# Supplementary material for: Diversity and determinants of recombination landscapes in flowering plants
Source: PLoS Genet. 2022 Aug 30;18(8):e1010141. doi: 10.1371/journal.pgen.1010141 (PMC9467342; doi:10.1371/journal.pgen.1010141)
Supplement: S1 Fig — The black vertical line is the centromere position estimated by cytological measures, when available in the literature. (PDF) [file pgen.1010141.s001.pdf]

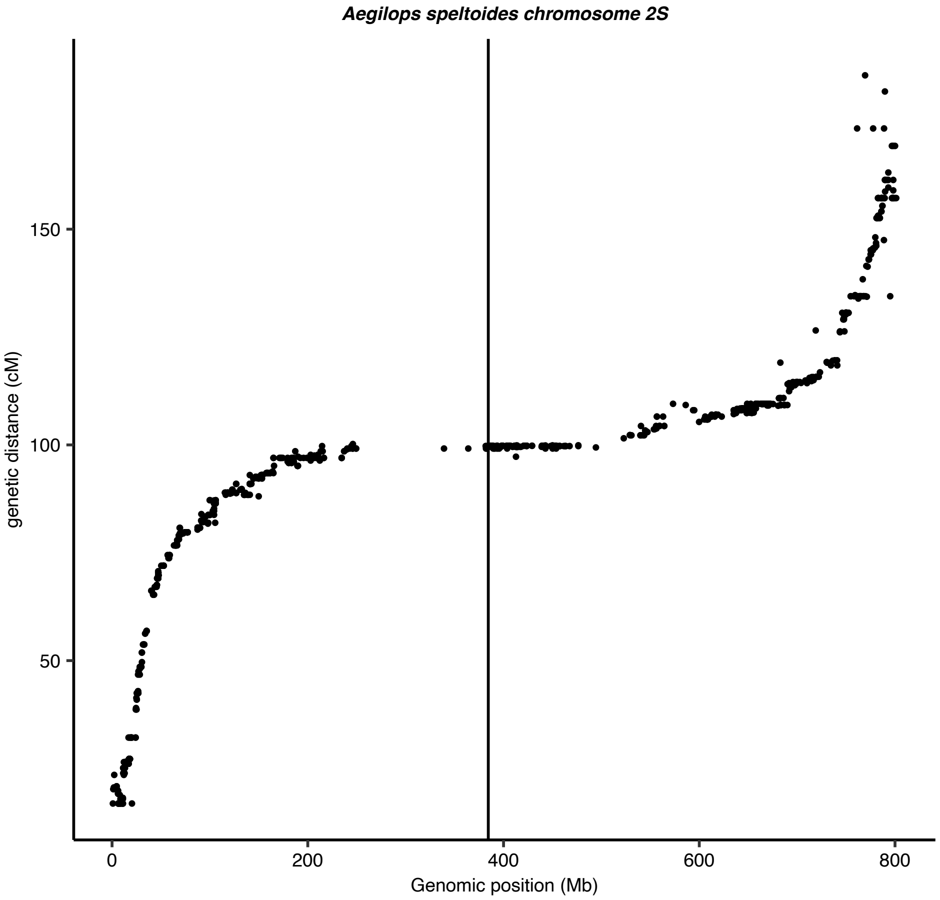
Aegilops speltoides chromosome 2S
genetic distance (cM)
150
100
50
0
200
400
600
800
Genomic position (Mb)

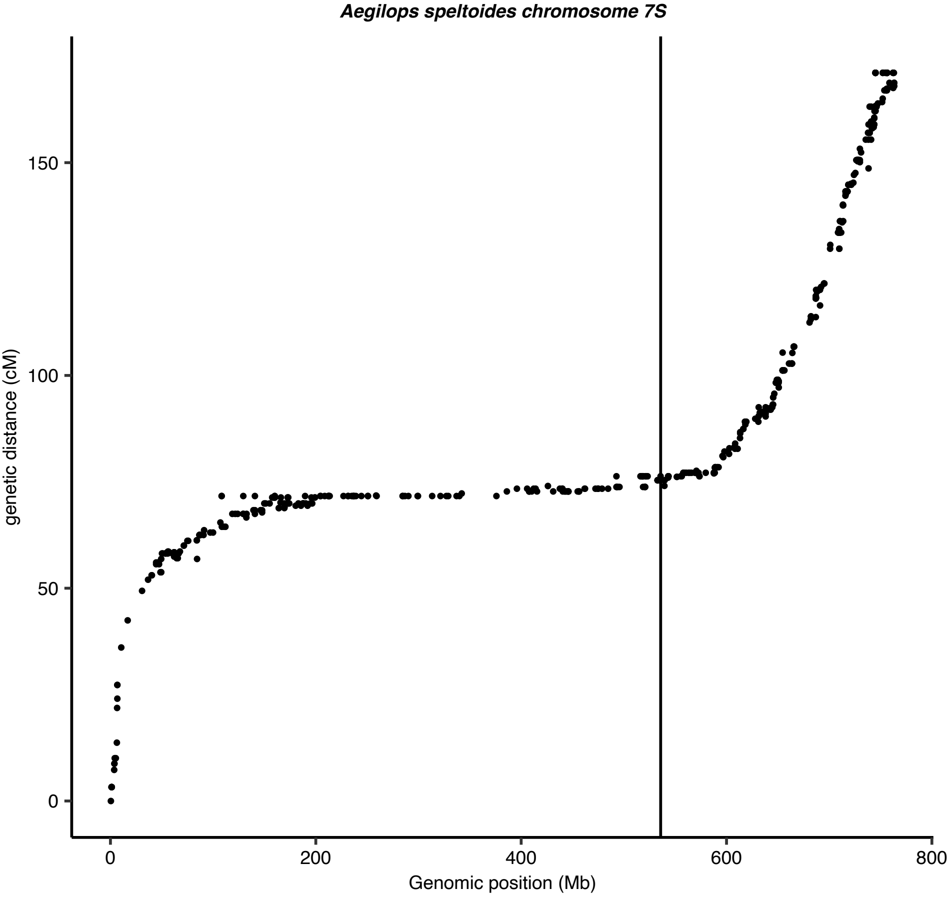
Aegilops speltoides chromosome 7S
genetic distance (cM)
150
100
50
0
0
200
400
600
800
Genomic position (Mb)

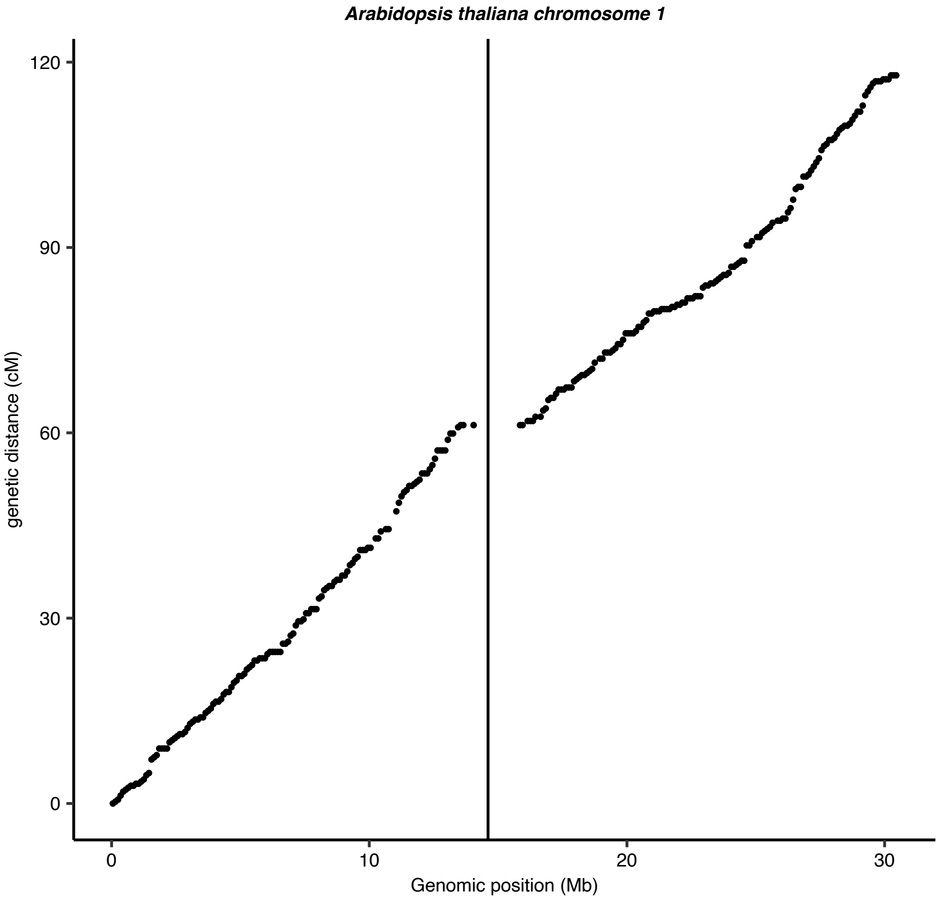
Arabidopsis thaliana chromosome 1
120
90
60
30
0
genetic distance (cM)
0
10
20
30
Genomic position (Mb)

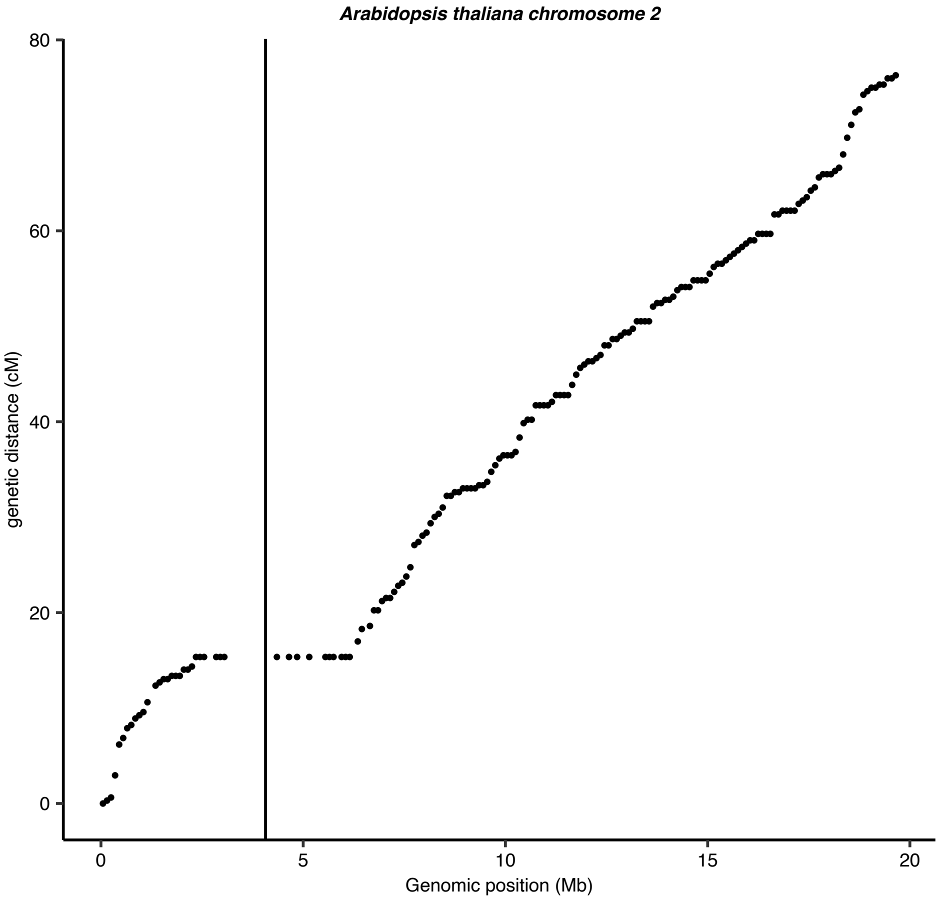
Arabidopsis thaliana chromosome 2
80
60
40
20
0
genetic distance (cM)
0
5
10
15
20
Genomic position (Mb)

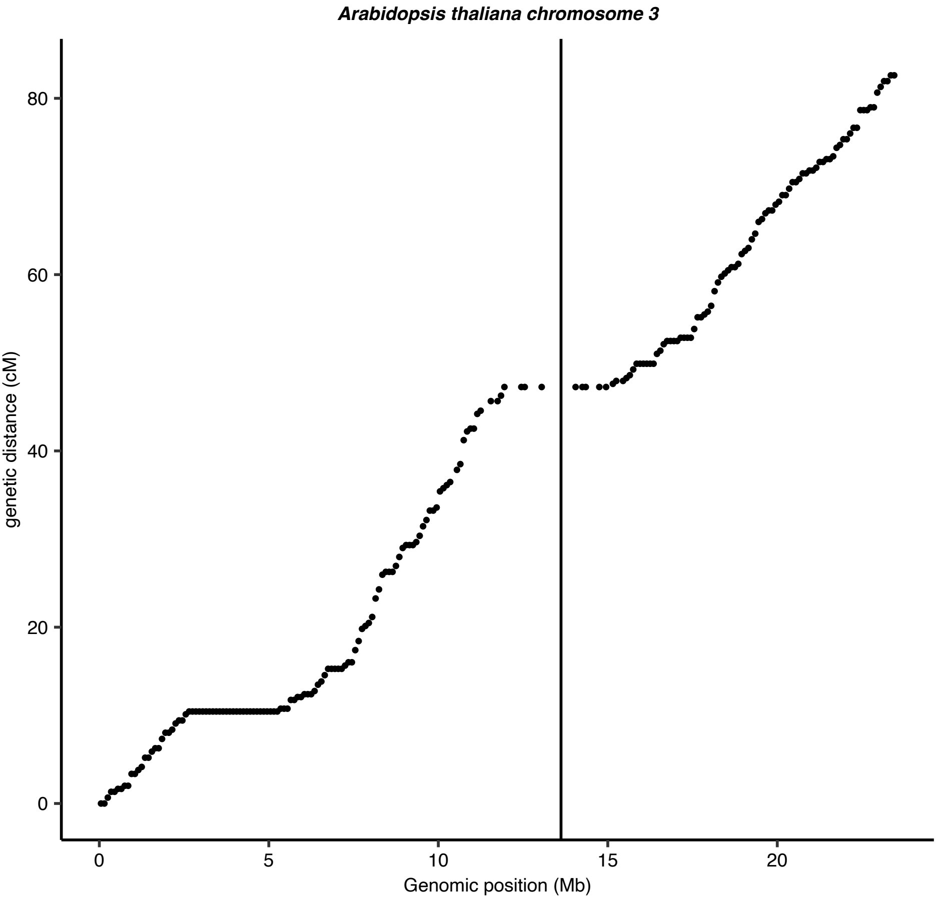

Arabidopsis thaliana chromosome 3
genetic distance (cM)
0
20
40
60
80
0
5
10
15
20
Genomic position (Mb)

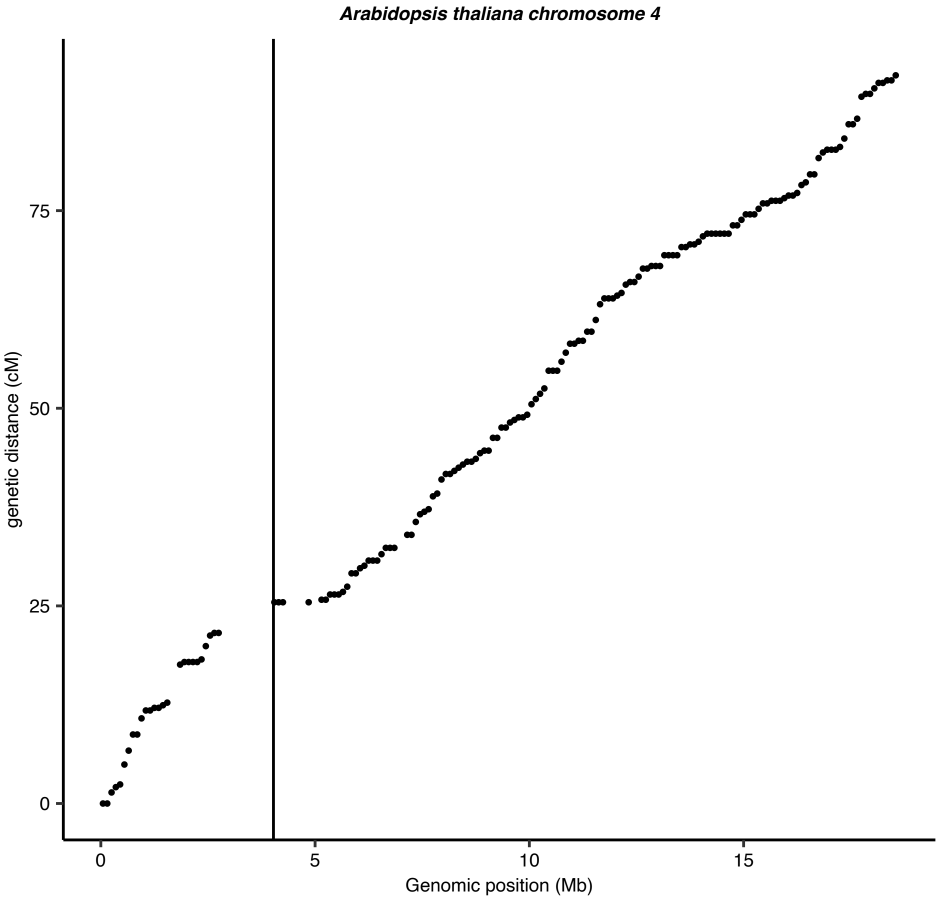
Arabidopsis thaliana chromosome 4
genetic distance (cM)
0
25
50
75
0
5
10
15
Genomic position (Mb)

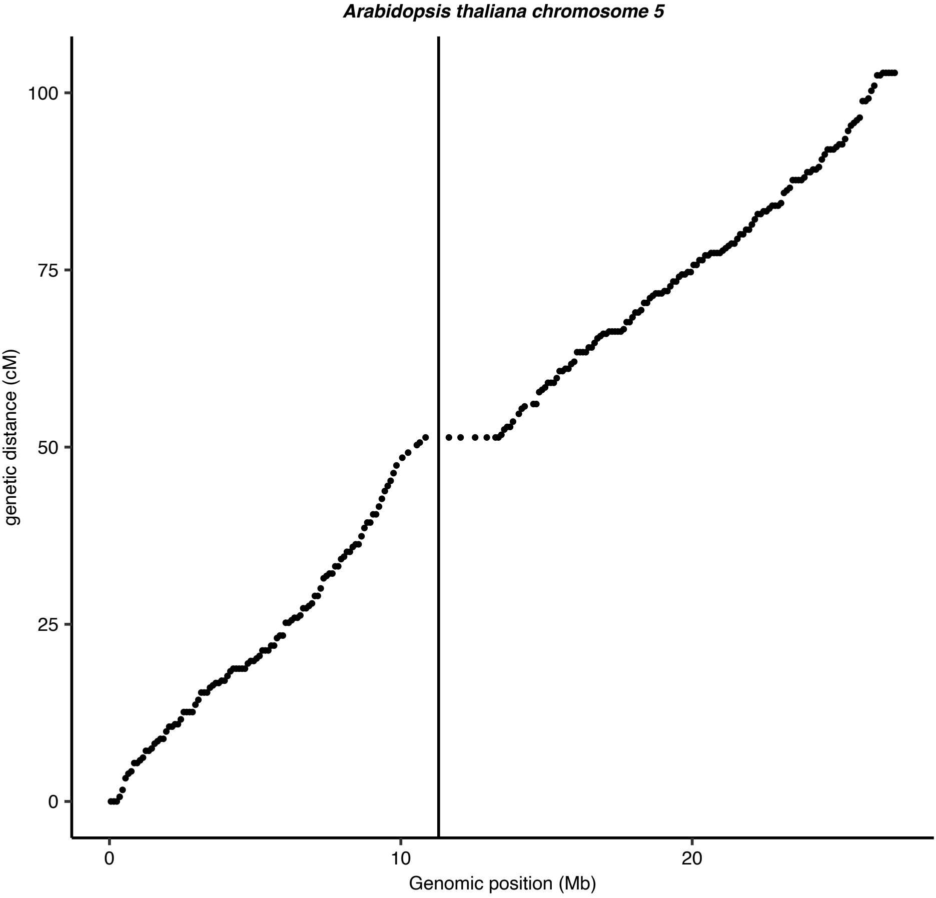
Arabidopsis thaliana chromosome 5
genetic distance (cM)
0
25
50
75
100
Genomic position (Mb)
0
10
20

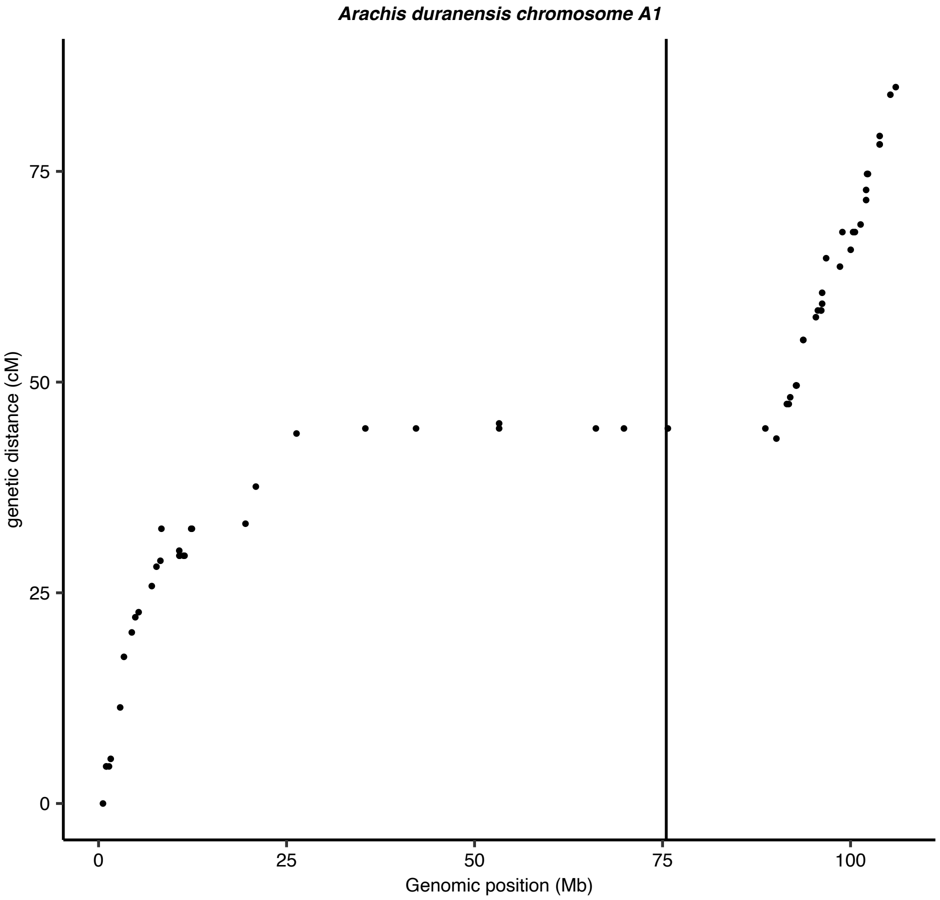
Arachis duranensis chromosome A1
genetic distance (cM)
0
25
50
75
Genomic position (Mb)
0
25
50
75
100

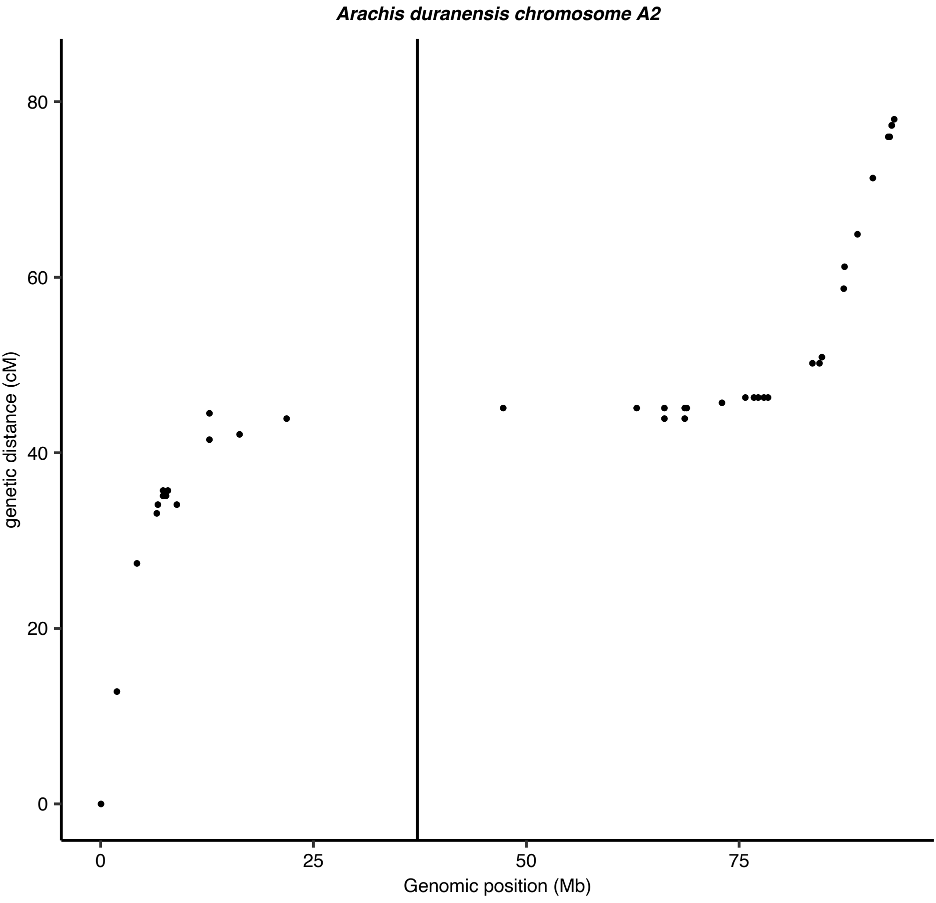
Arachis duranensis chromosome A2
genetic distance (cM)
0
20
40
60
80
Genomic position (Mb)
0
25
50
75

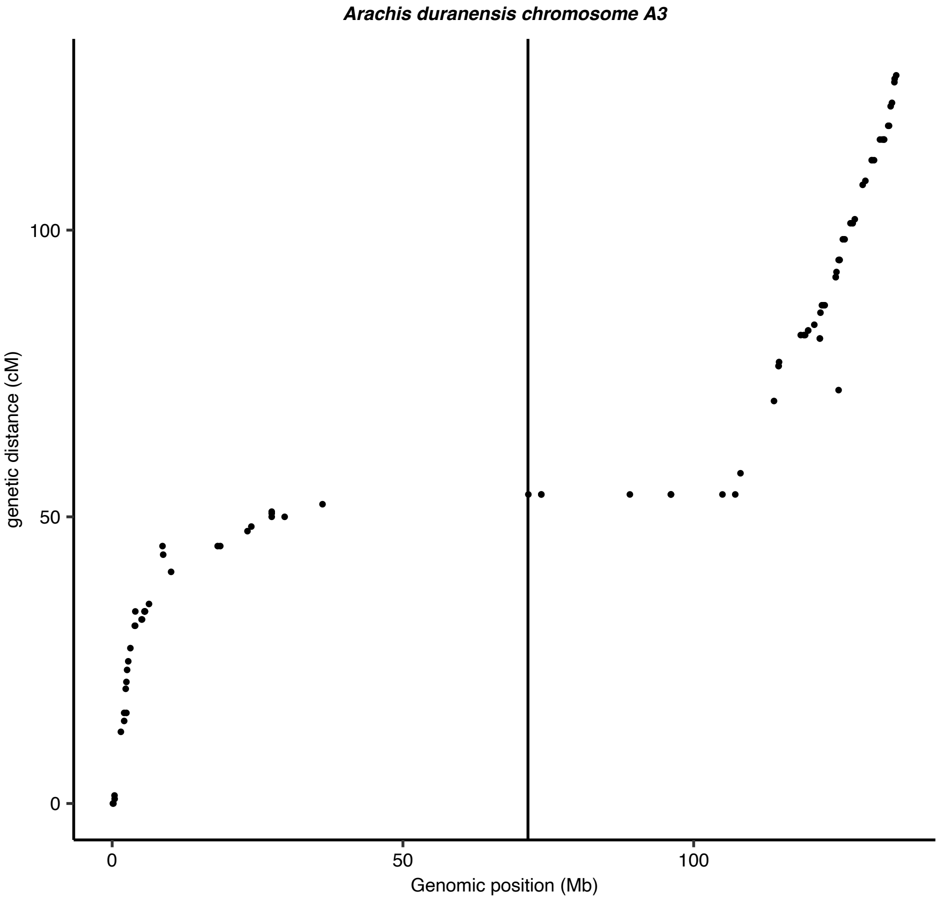
Arachis duranensis chromosome A3
genetic distance (cM)
100
50
0
Genomic position (Mb)
0
50
100

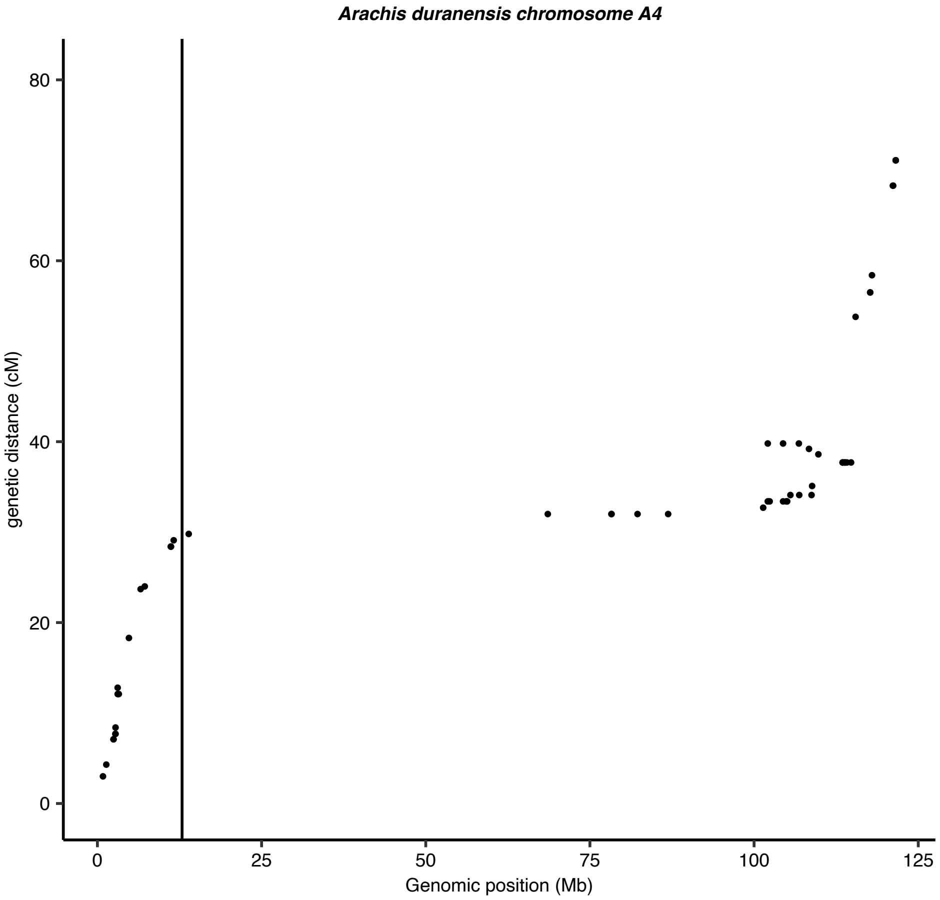
Arachis duranensis chromosome A4
genetic distance (cM)
0
20
40
60
80
Genomic position (Mb)
0
25
50
75
100
125

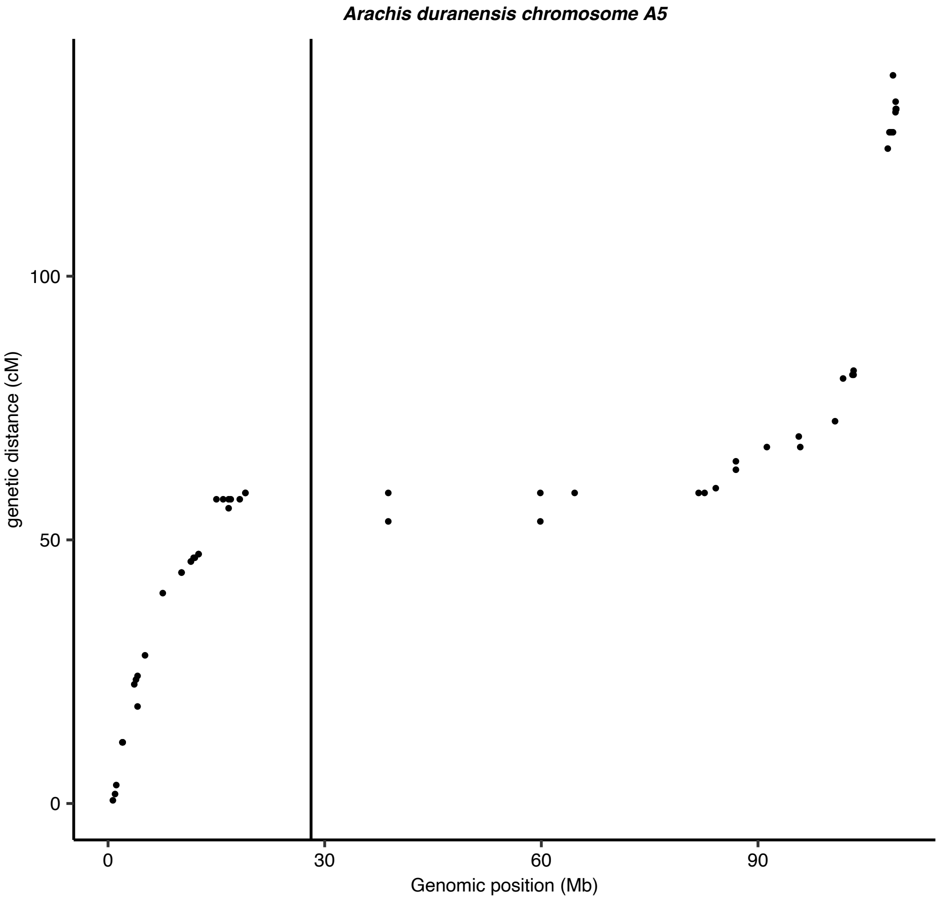
Arachis duranensis chromosome A5
genetic distance (cM)
100
50
0
0
30
60
90
Genomic position (Mb)

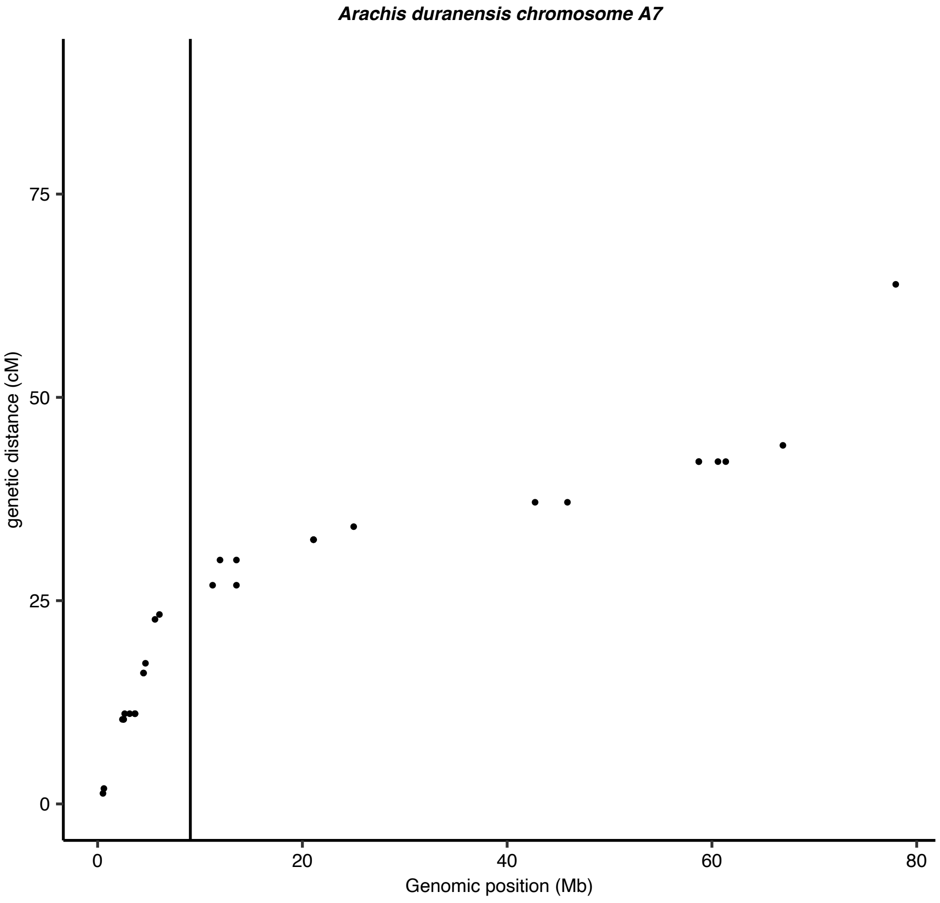
Arachis duranensis chromosome A7
genetic distance (cM)
75
50
25
0
0
20
40
60
80
Genomic position (Mb)

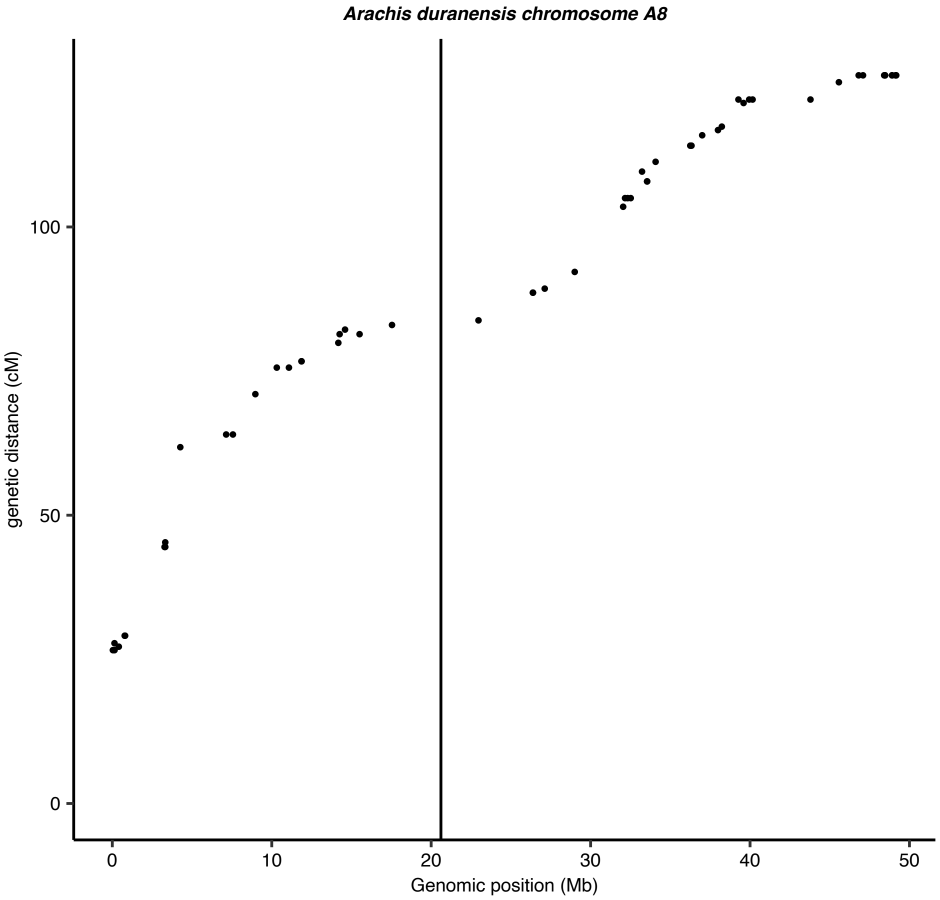
Arachis duranensis chromosome A8
genetic distance (cM)
0
50
100
Genomic position (Mb)
0
10
20
30
40
50

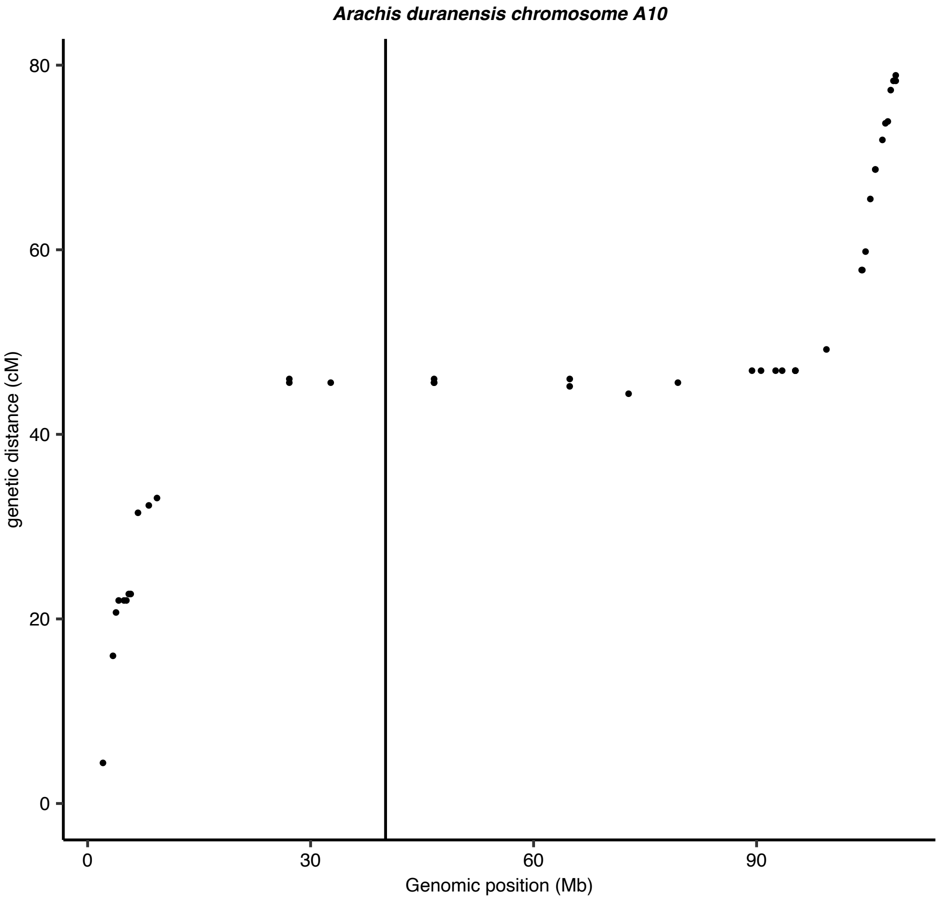
Arachis duranensis chromosome A10
80
60
40
20
0
genetic distance (cM)
0
30
60
90
Genomic position (Mb)

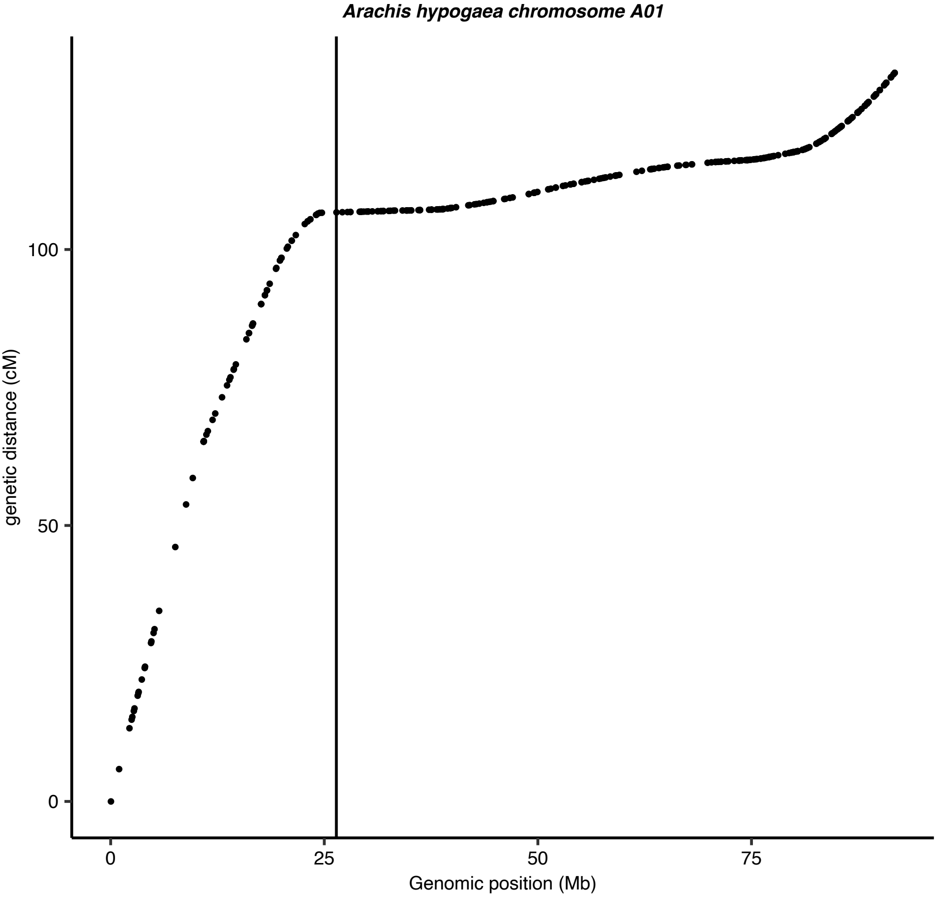
Arachis hypogaea chromosome A01
genetic distance (cM)
0
50
100
Genomic position (Mb)
0
25
50
75

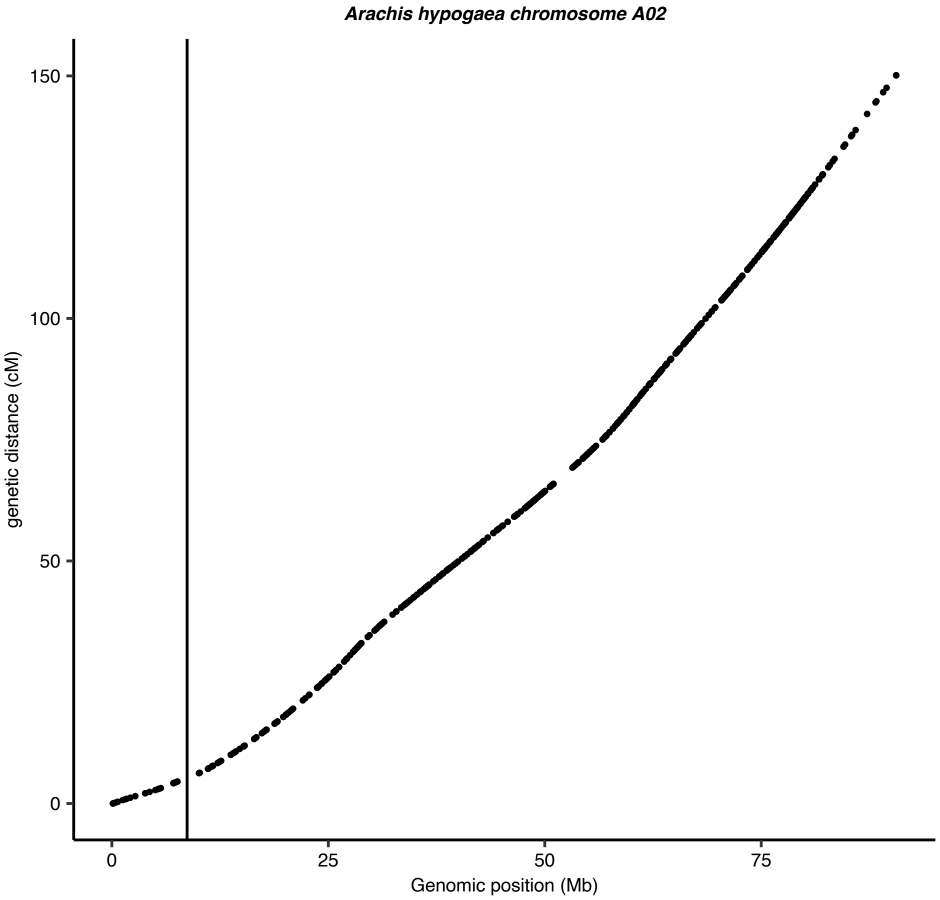
Arachis hypogaea chromosome A02
genetic distance (cM)
150
100
50
0
0
25
50
75
Genomic position (Mb)

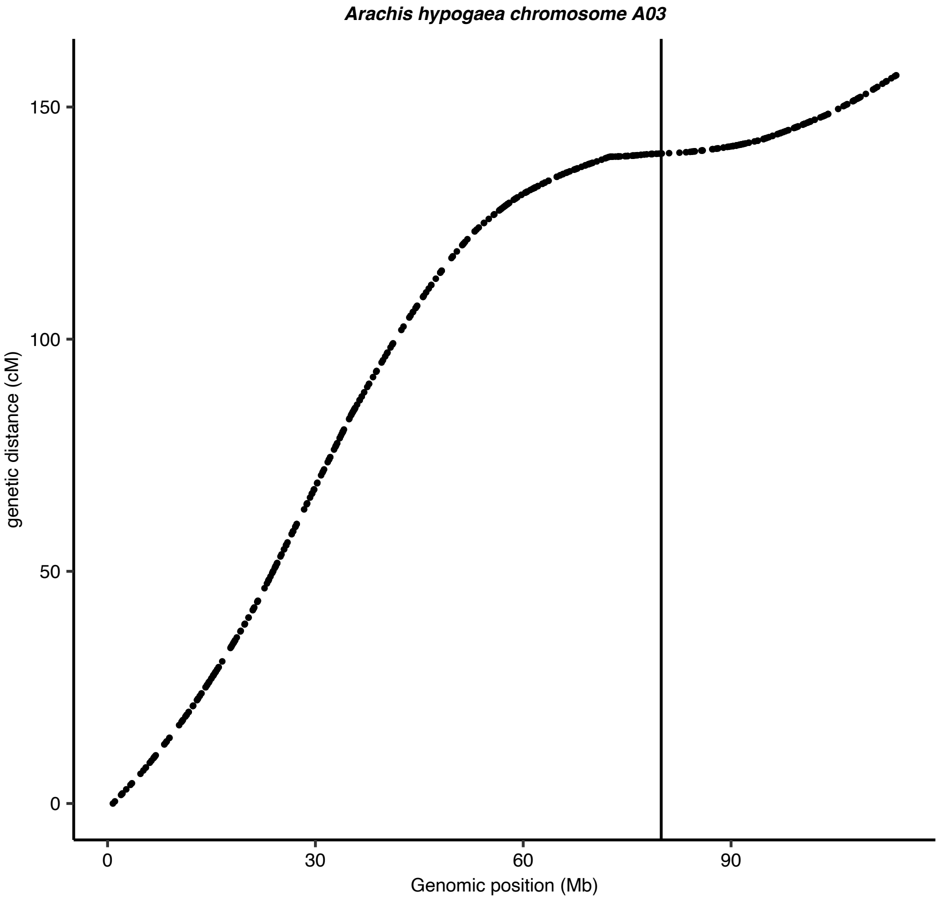
Arachis hypogaea chromosome A03
genetic distance (cM)
0
50
100
150
Genomic position (Mb)
0
30
60
90

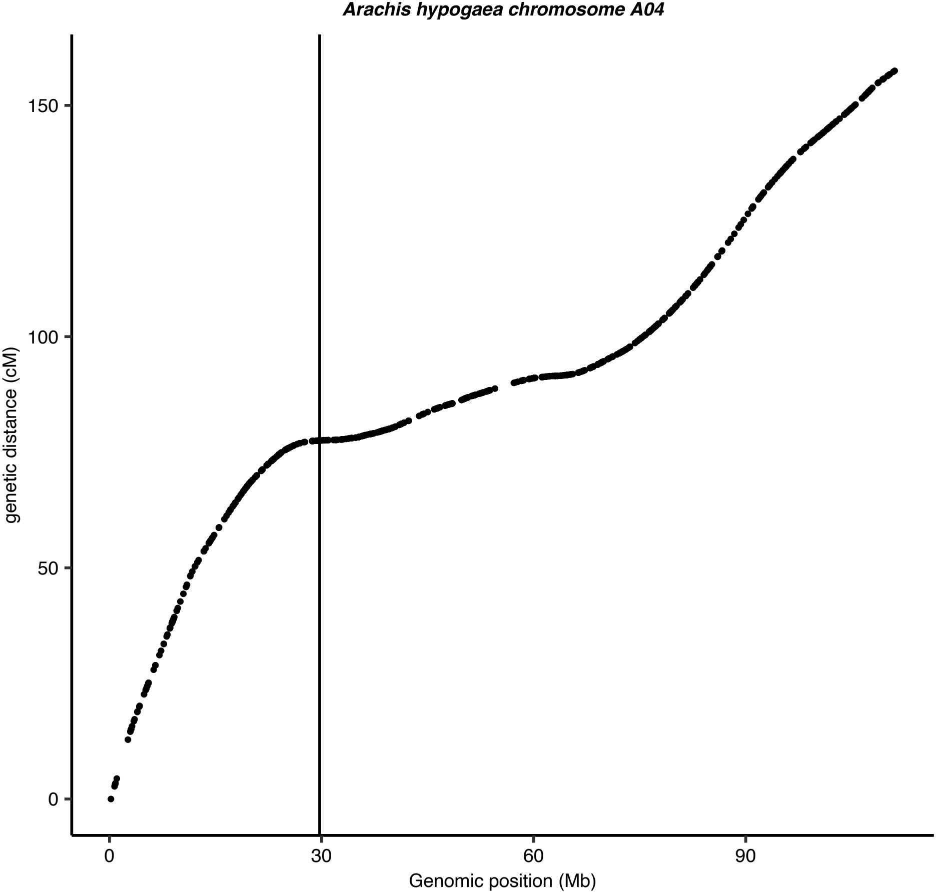
Arachis hypogaea chromosome A04
genetic distance (cM)
150
100
50
0
Genomic position (Mb)
0
30
60
90

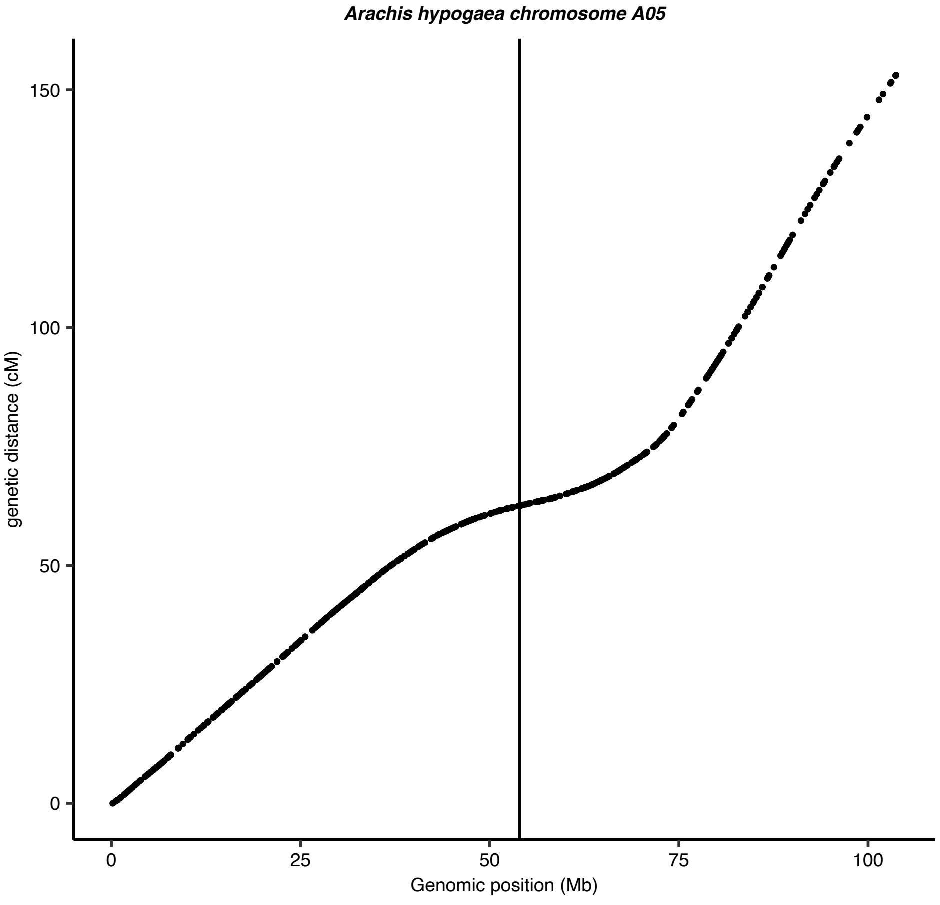
Arachis hypogaea chromosome A05
genetic distance (cM)
0
50
100
150
Genomic position (Mb)
0
25
50
75
100

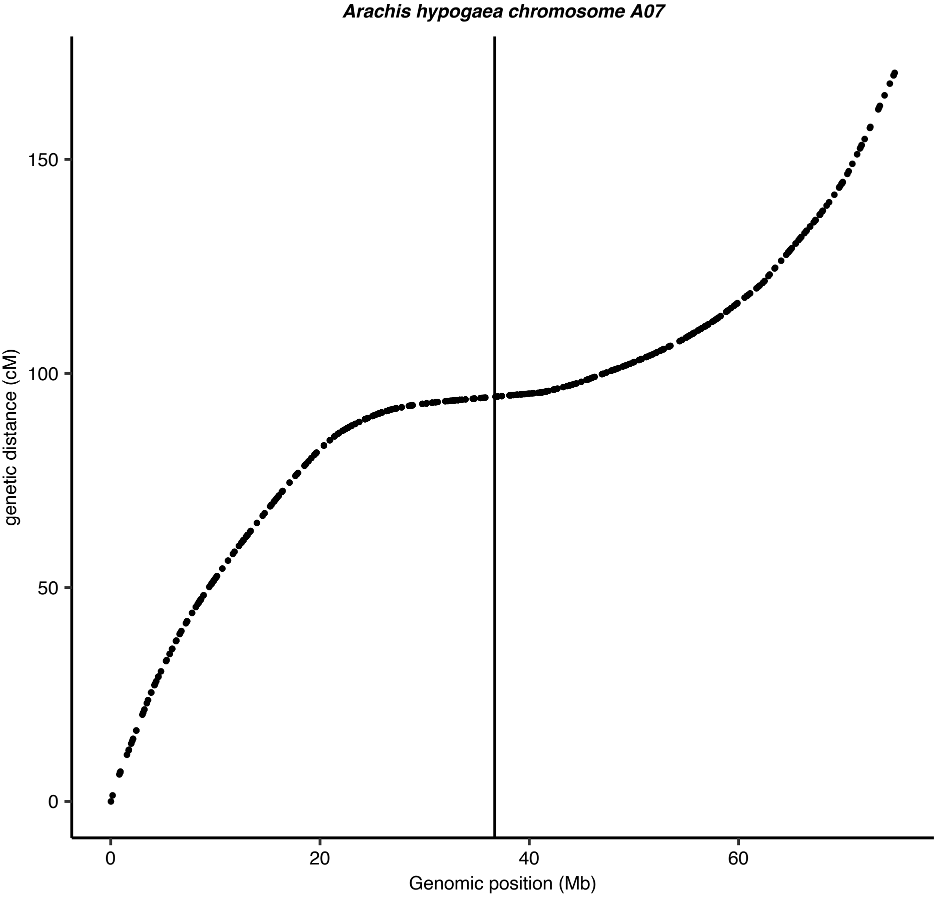
Arachis hypogaea chromosome A07
genetic distance (cM)
0
50
100
150
Genomic position (Mb)
0
20
40
60

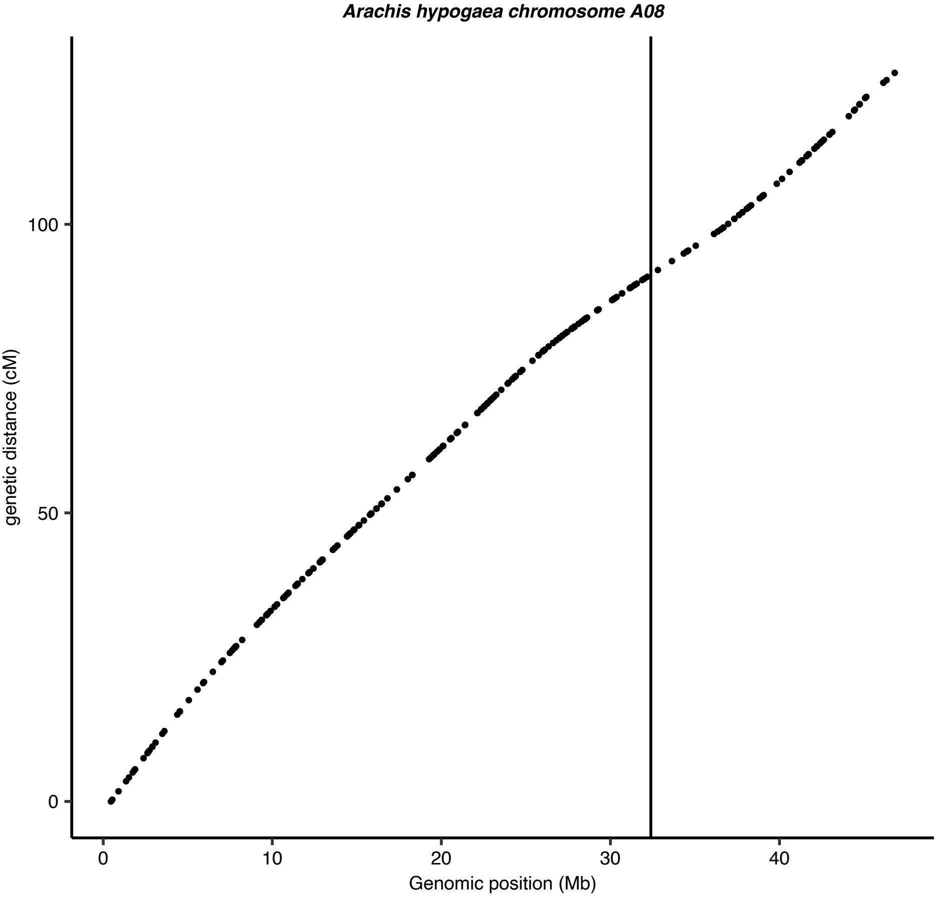
Arachis hypogaea chromosome A08
genetic distance (cM)
0
50
100
Genomic position (Mb)
0
10
20
30
40

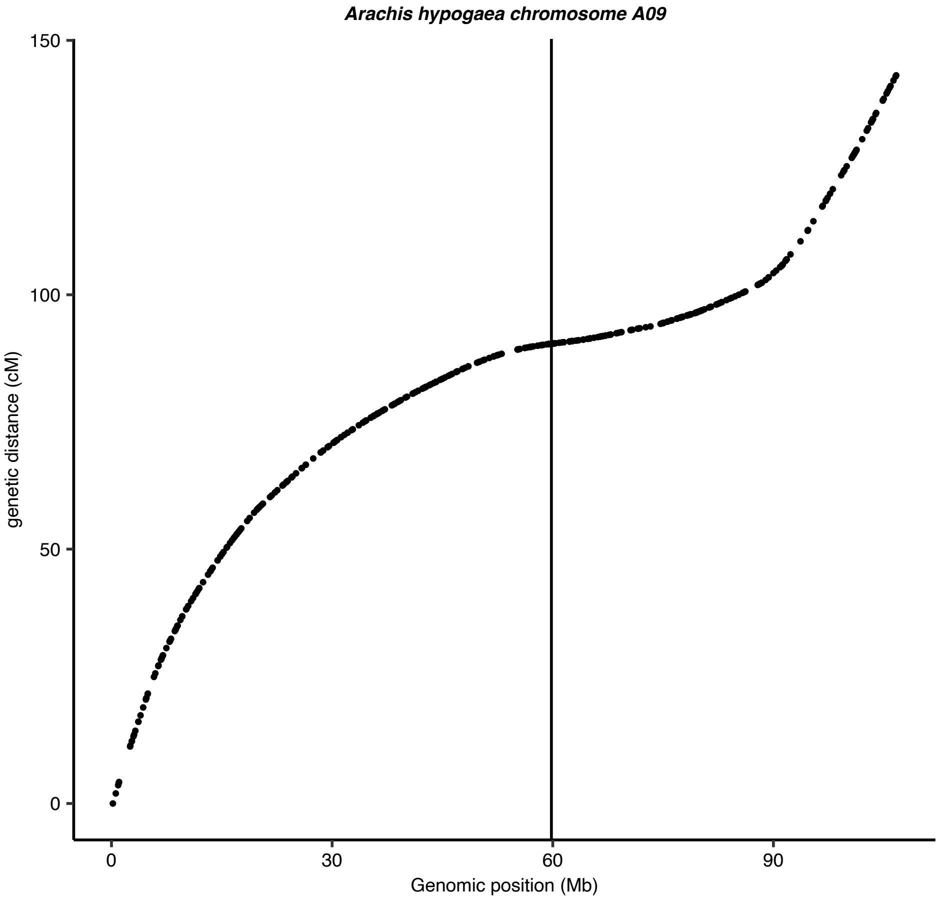
Arachis hypogaea chromosome A09
150
100
50
0
genetic distance (cM)
0
30
60
90
Genomic position (Mb)

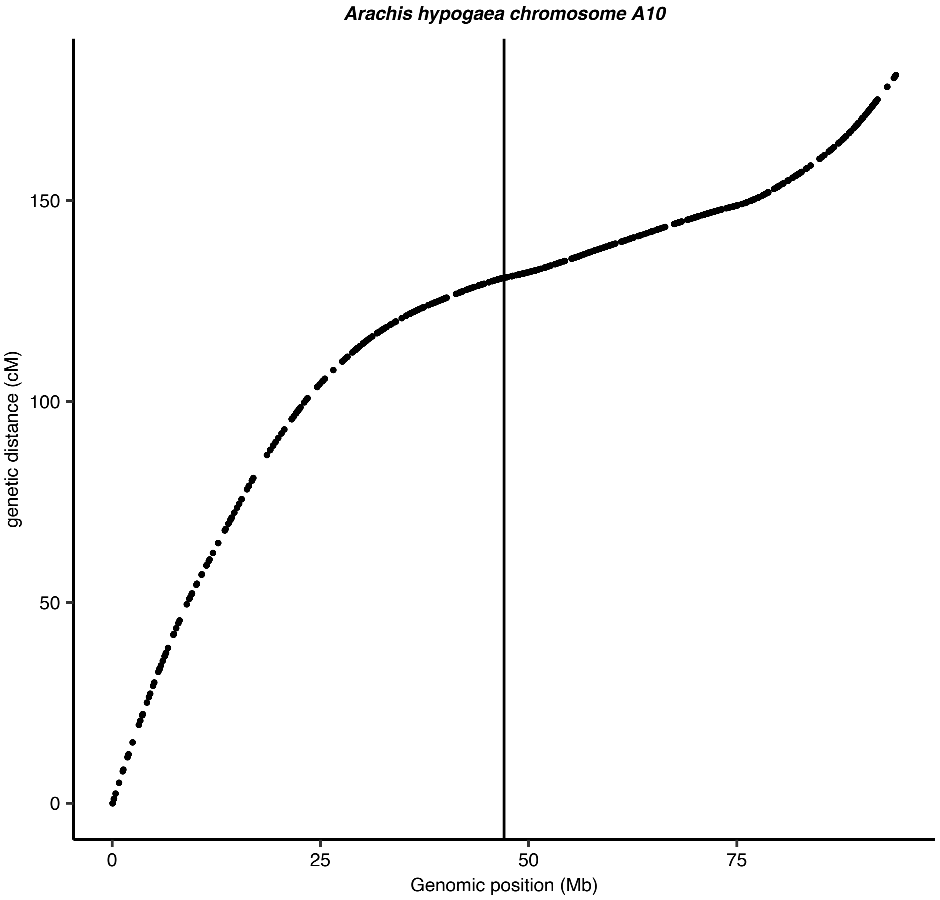
Arachis hypogaea chromosome A10
genetic distance (cM)
0
50
100
150
0
25
50
75
Genomic position (Mb)

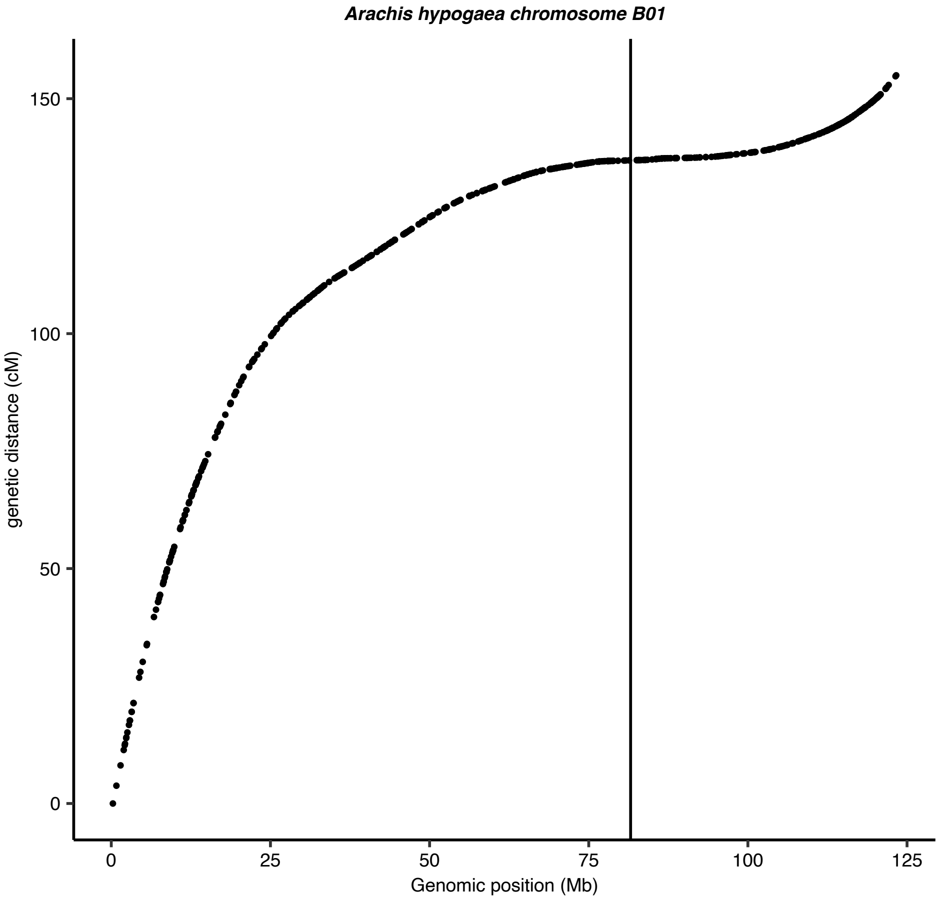
Arachis hypogaea chromosome B01
genetic distance (cM)
Genomic position (Mb)
0
50
100
150
0
25
50
75
100
125

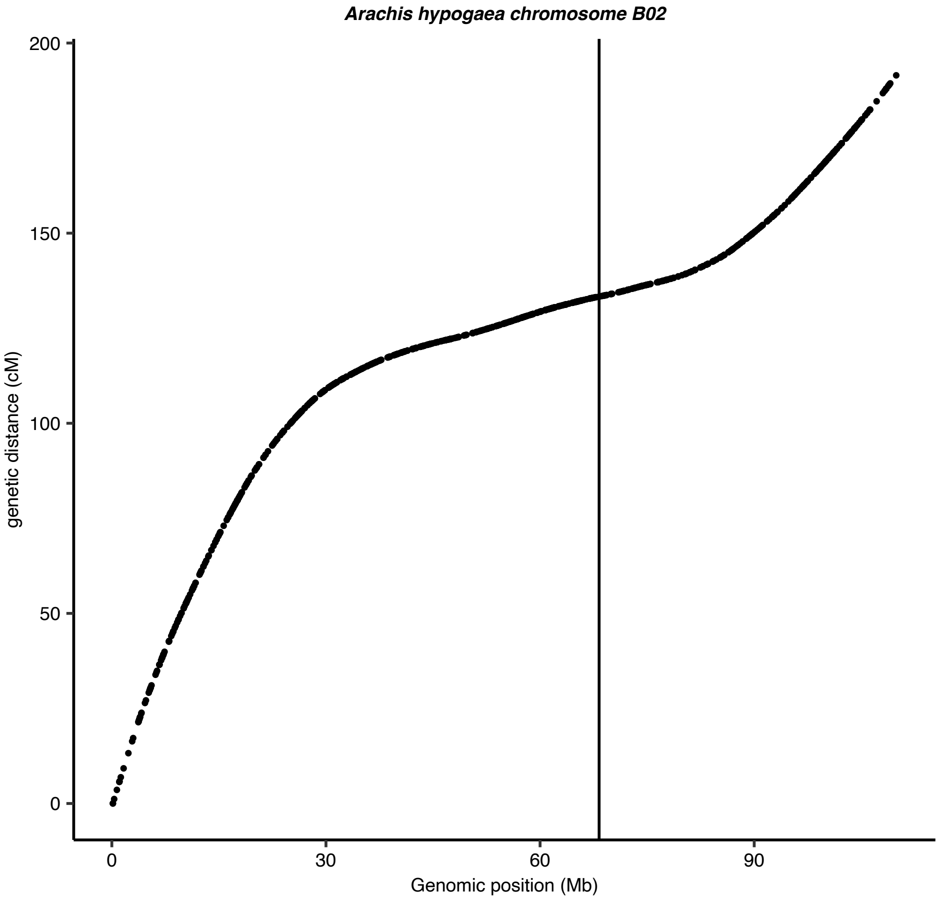
Arachis hypogaea chromosome B02
genetic distance (cM)
200
150
100
50
0
0
30
60
90
Genomic position (Mb)

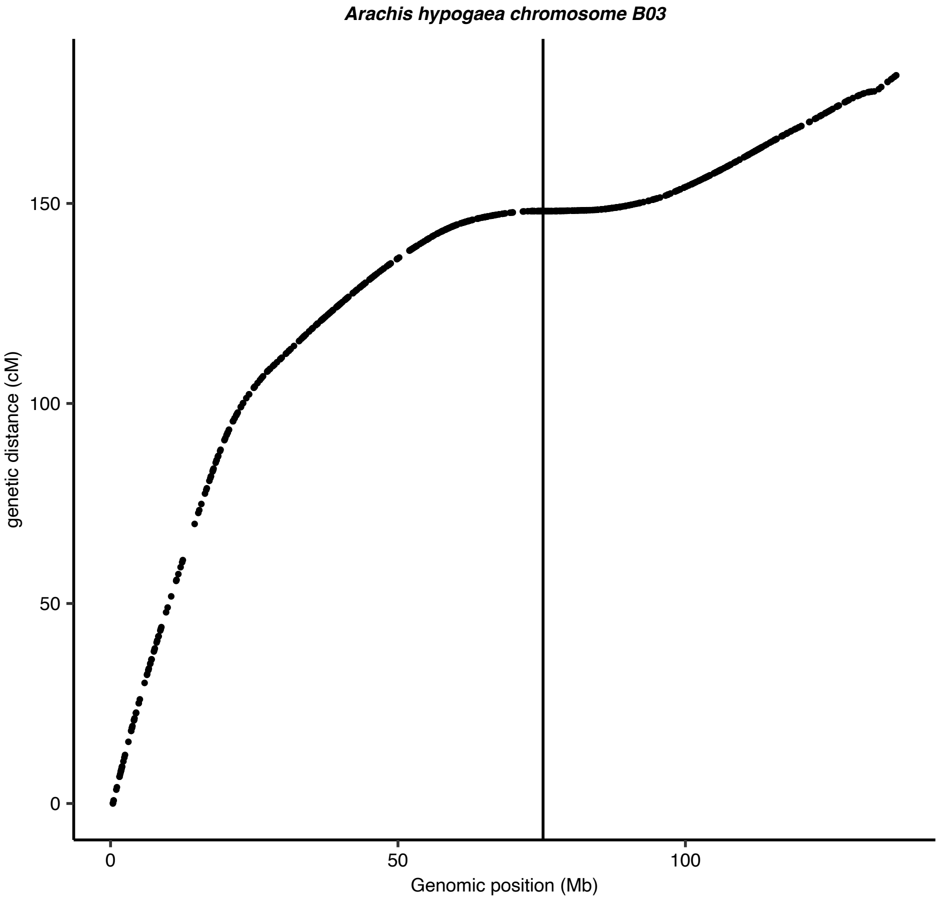
Arachis hypogaea chromosome B03
genetic distance (cM)
0
50
100
150
Genomic position (Mb)
0
50
100

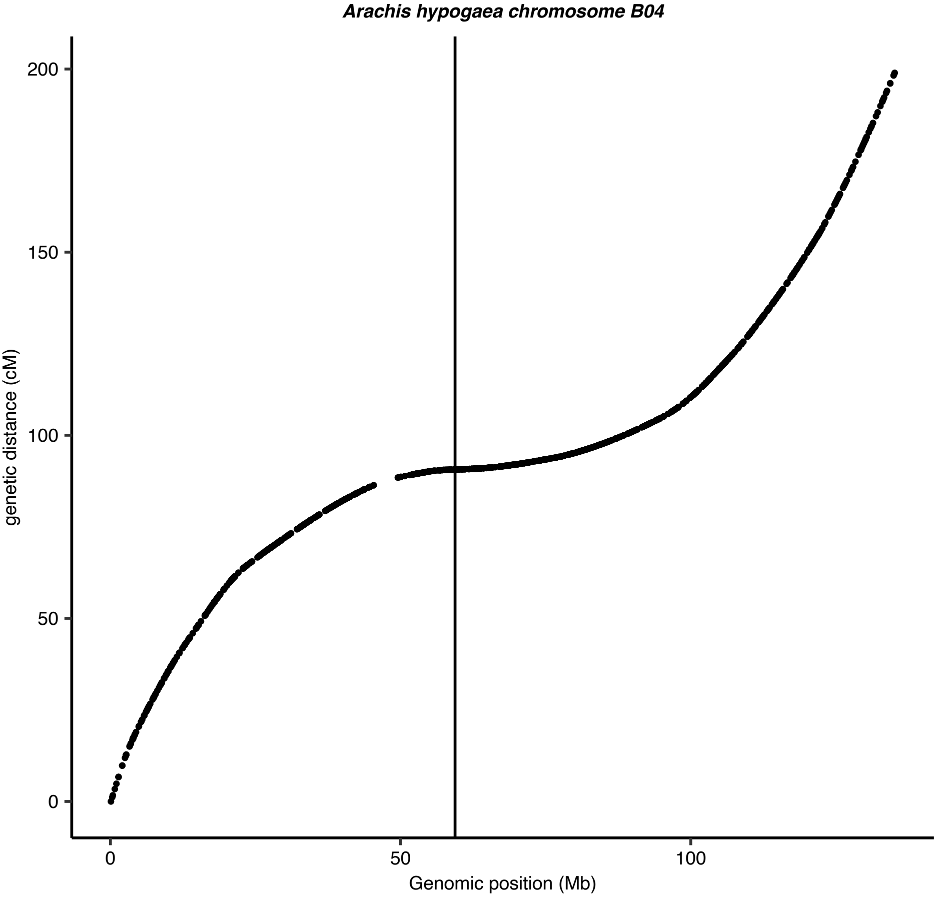

Arachis hypogaea chromosome B04
genetic distance (cM)
200
150
100
50
0
0
50
100
Genomic position (Mb)

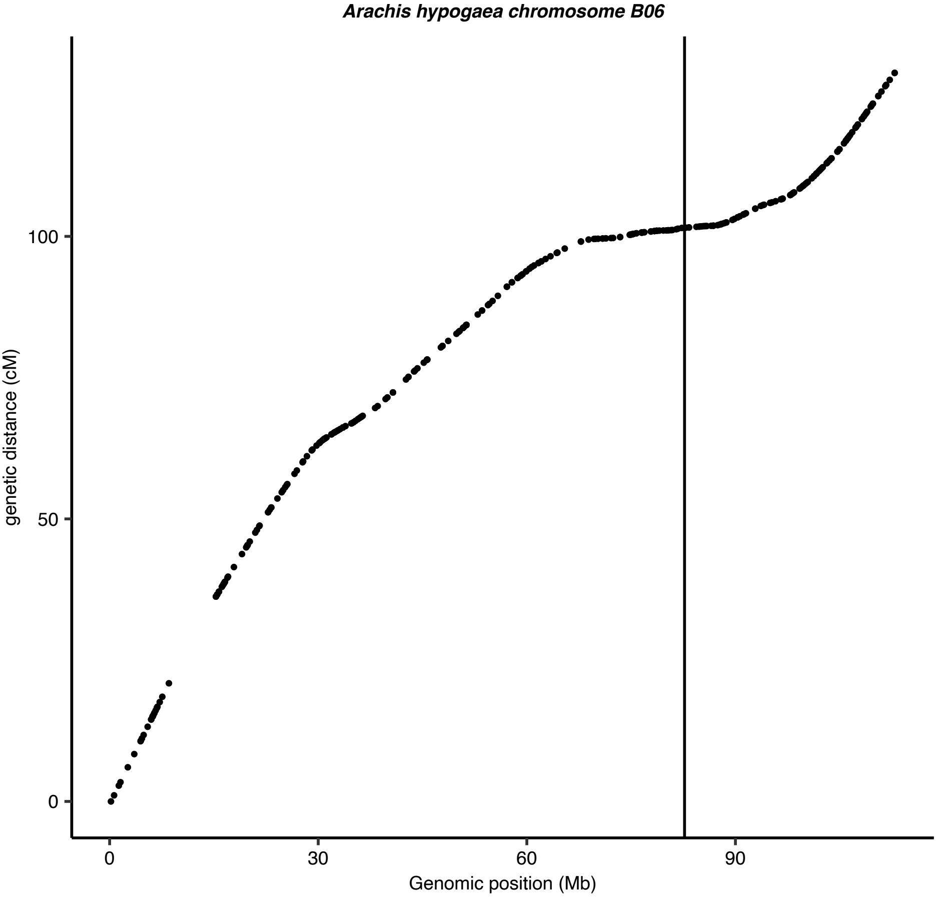
Arachis hypogaea chromosome B06
genetic distance (cM)
0
50
100
Genomic position (Mb)
0
30
60
90

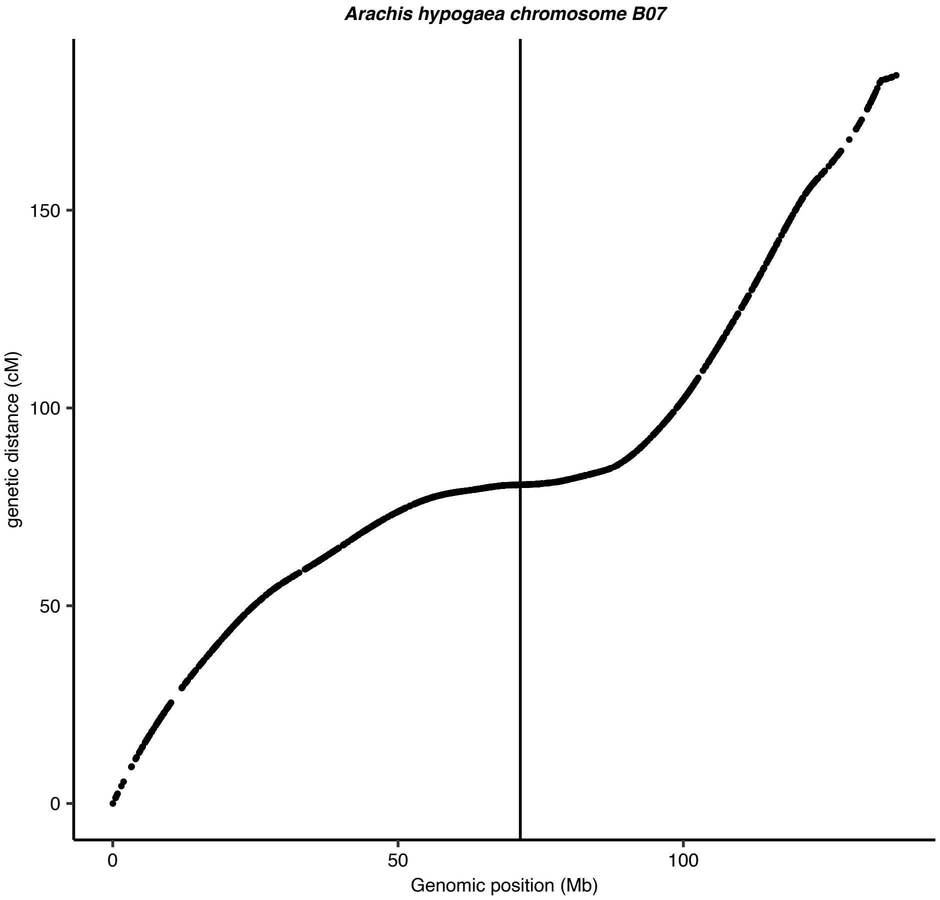
Arachis hypogaea chromosome B07
genetic distance (cM)
0
50
100
150
Genomic position (Mb)
0
50
100

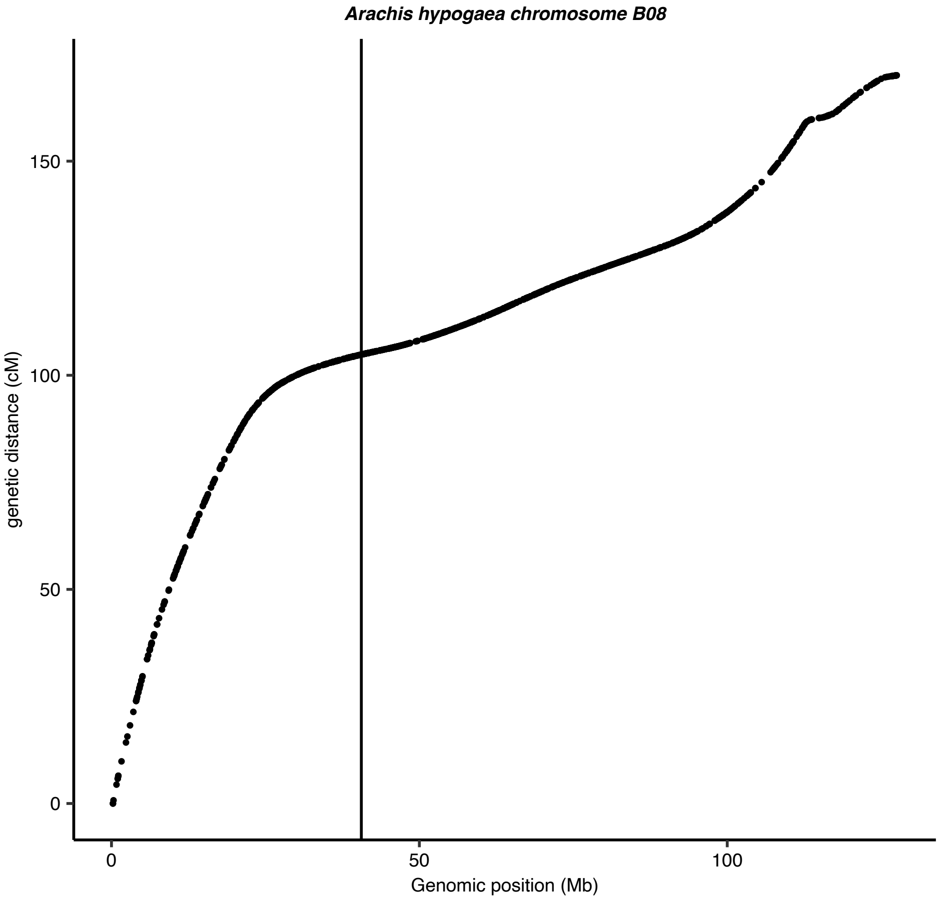
Arachis hypogaea chromosome B08
genetic distance (cM)
0
50
100
150
Genomic position (Mb)
0
50
100

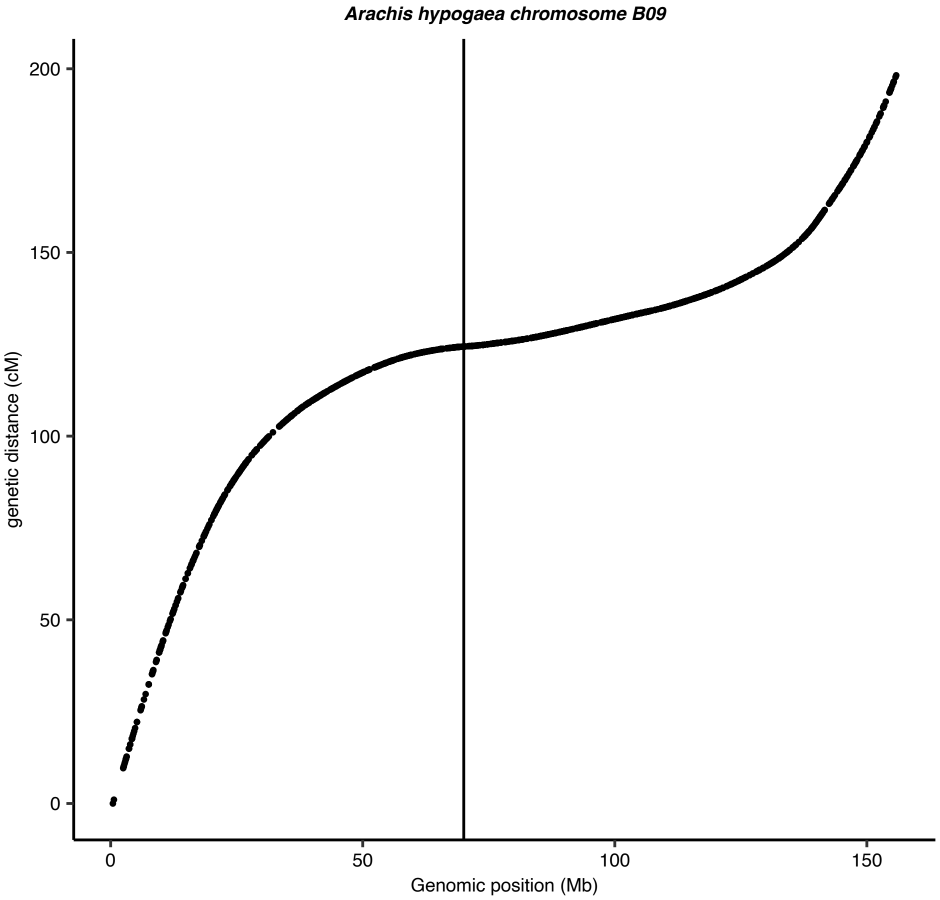

Arachis hypogaea chromosome B09
genetic distance (cM)
Genomic position (Mb)
0
50
100
150
200

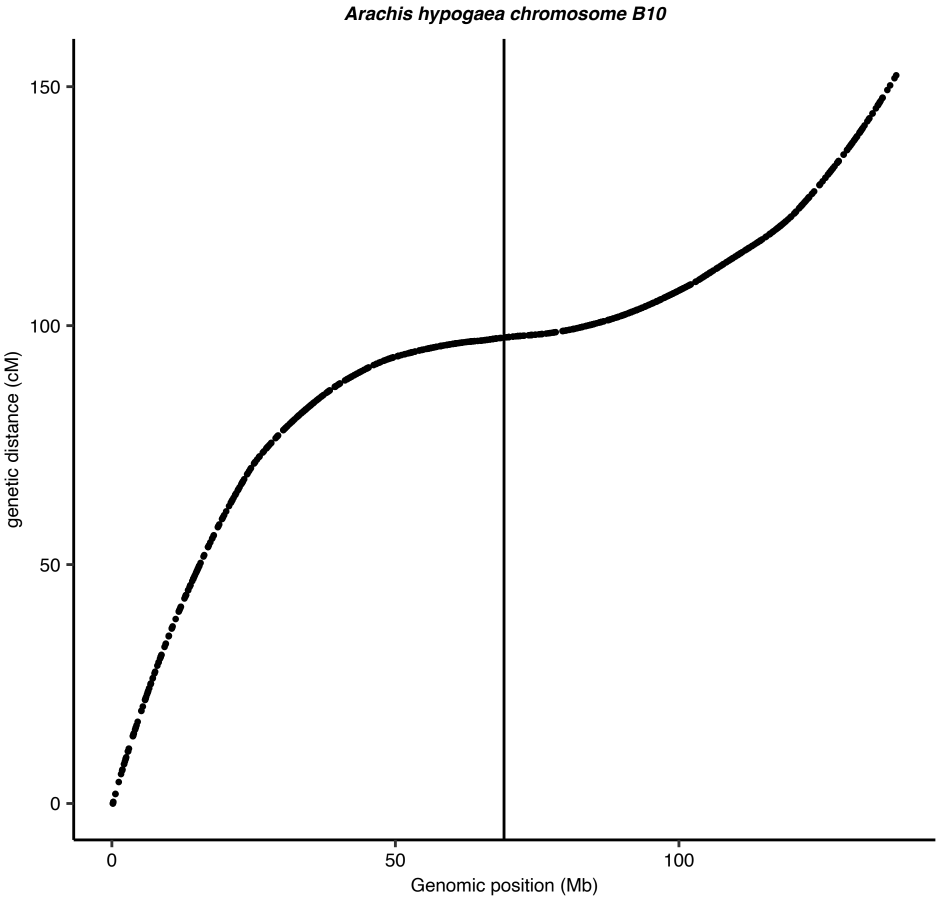
Arachis hypogaea chromosome B10
genetic distance (cM)
150
100
50
0
0
50
100
Genomic position (Mb)

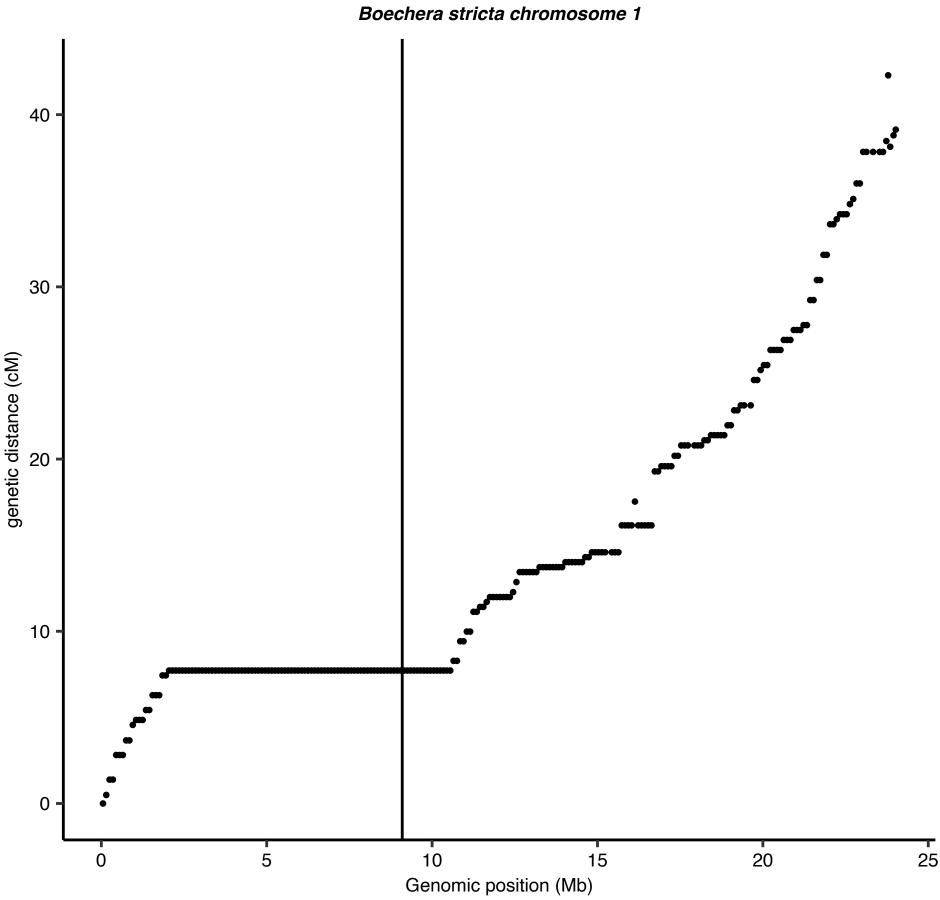
Boechera stricta chromosome 1
genetic distance (cM)
0
10
20
30
40
Genomic position (Mb)
0
5
10
15
20
25

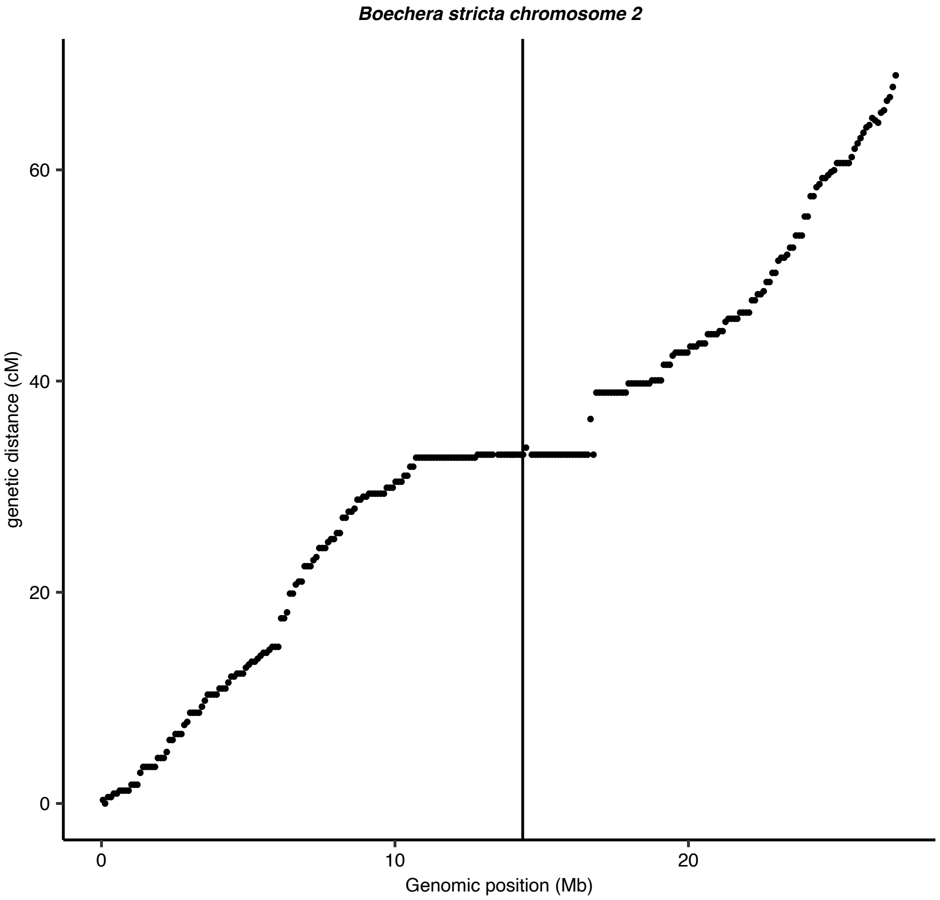
Boechera stricta chromosome 2
genetic distance (cM)
Genomic position (Mb)
0
20
40
60
0
10
20

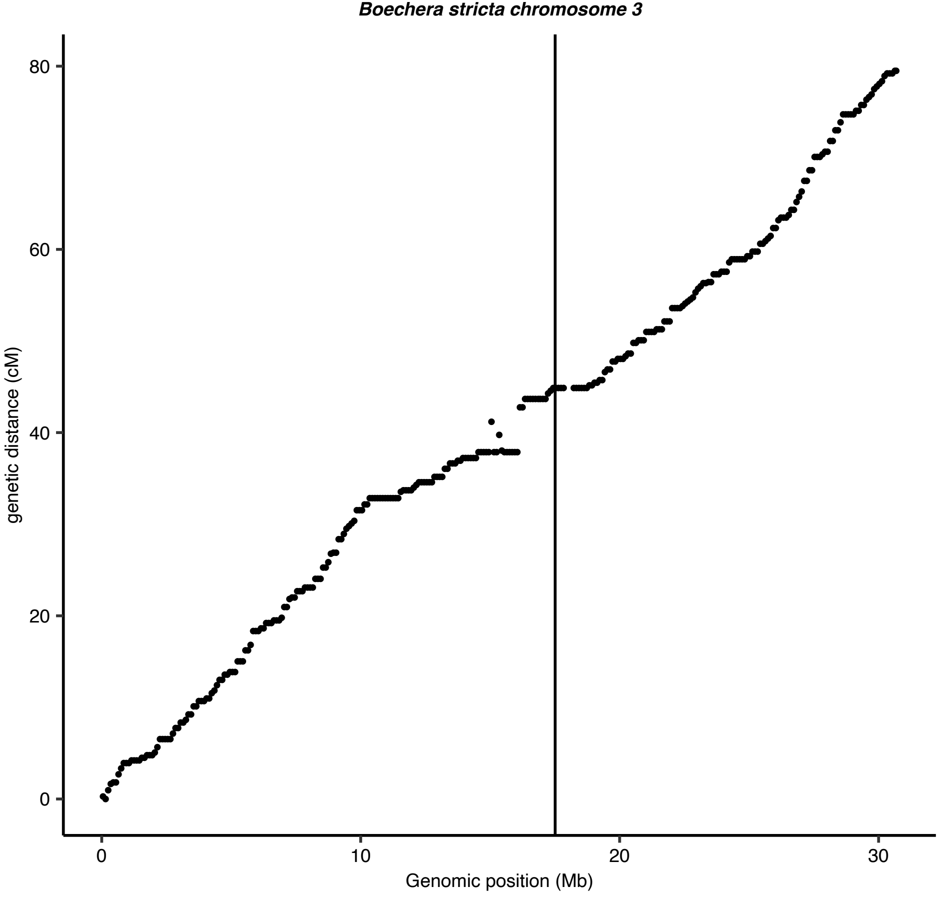
Boechera stricta chromosome 3
genetic distance (cM)
Genomic position (Mb)
0
20
40
60
80
0
10
20
30

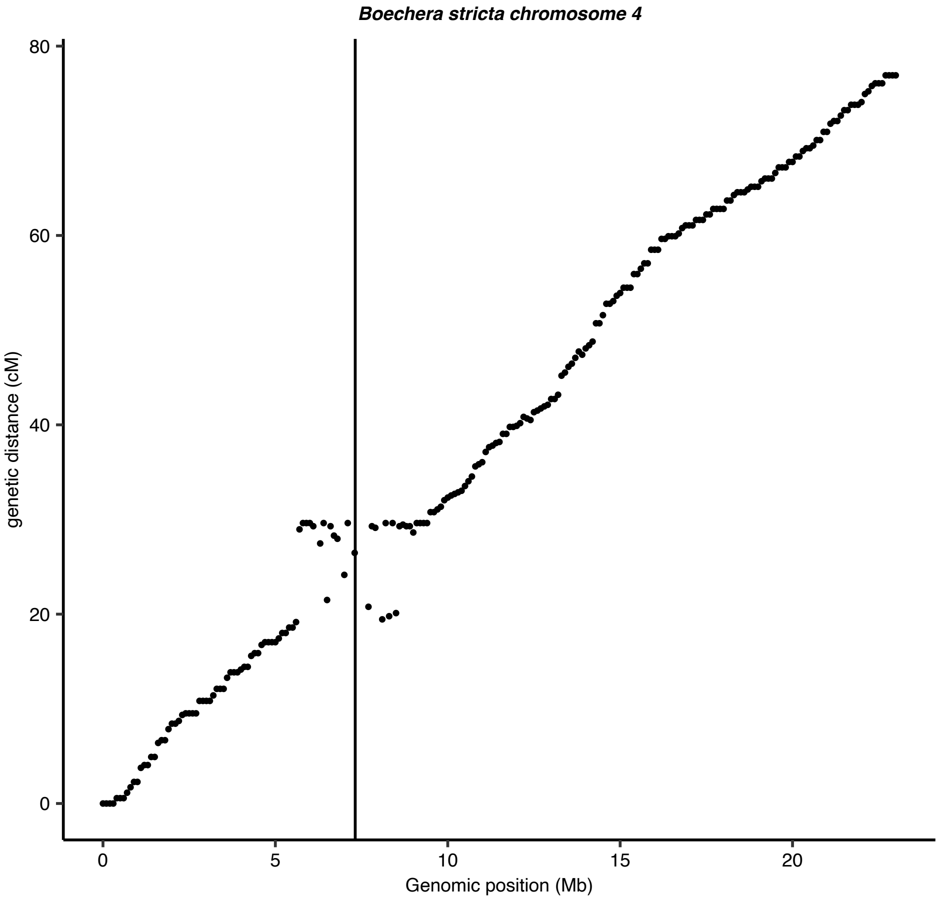
Boechera stricta chromosome 4
genetic distance (cM)
0
20
40
60
80
Genomic position (Mb)
0
5
10
15
20

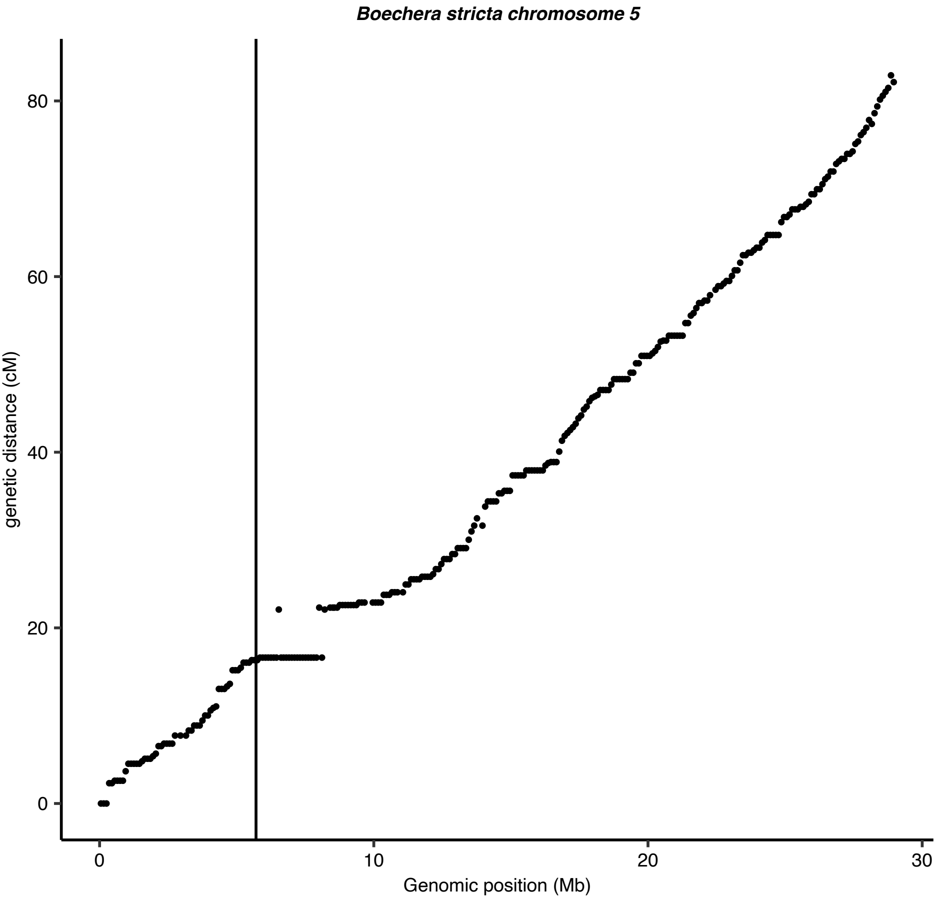
Boechera stricta chromosome 5
genetic distance (cM)
0
20
40
60
80
Genomic position (Mb)
0
10
20
30

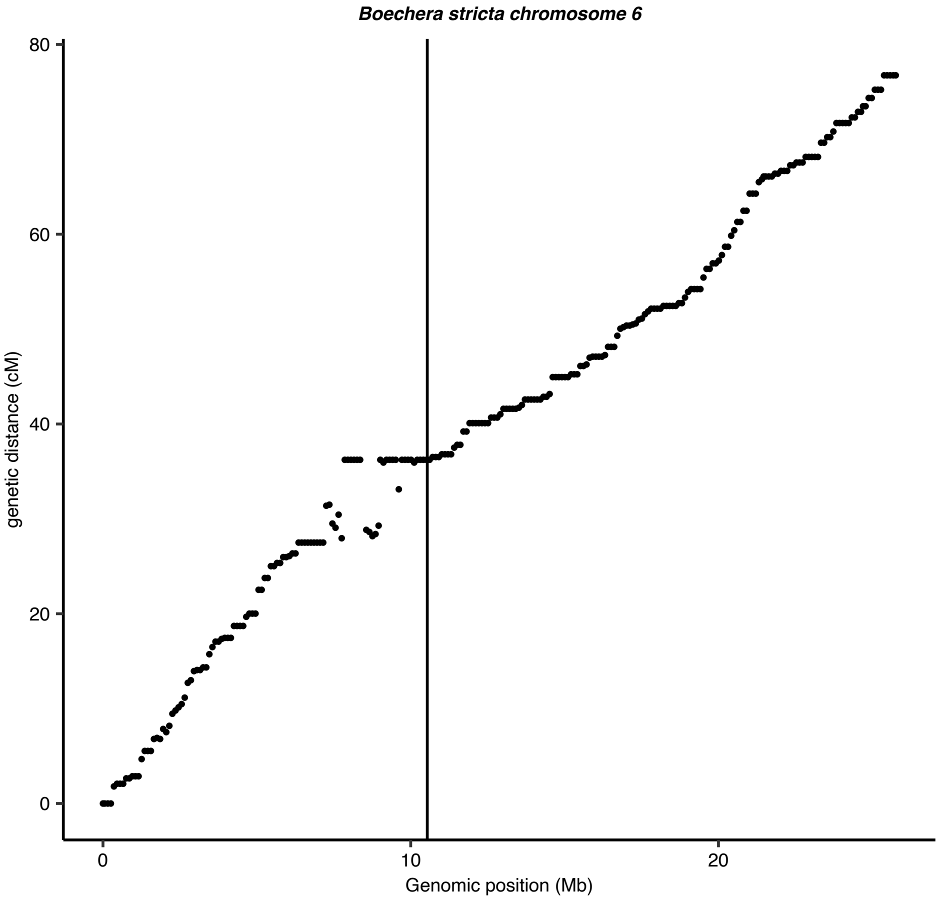
Boechera stricta chromosome 6
80
60
40
20
0
genetic distance (cM)
0
10
20
Genomic position (Mb)

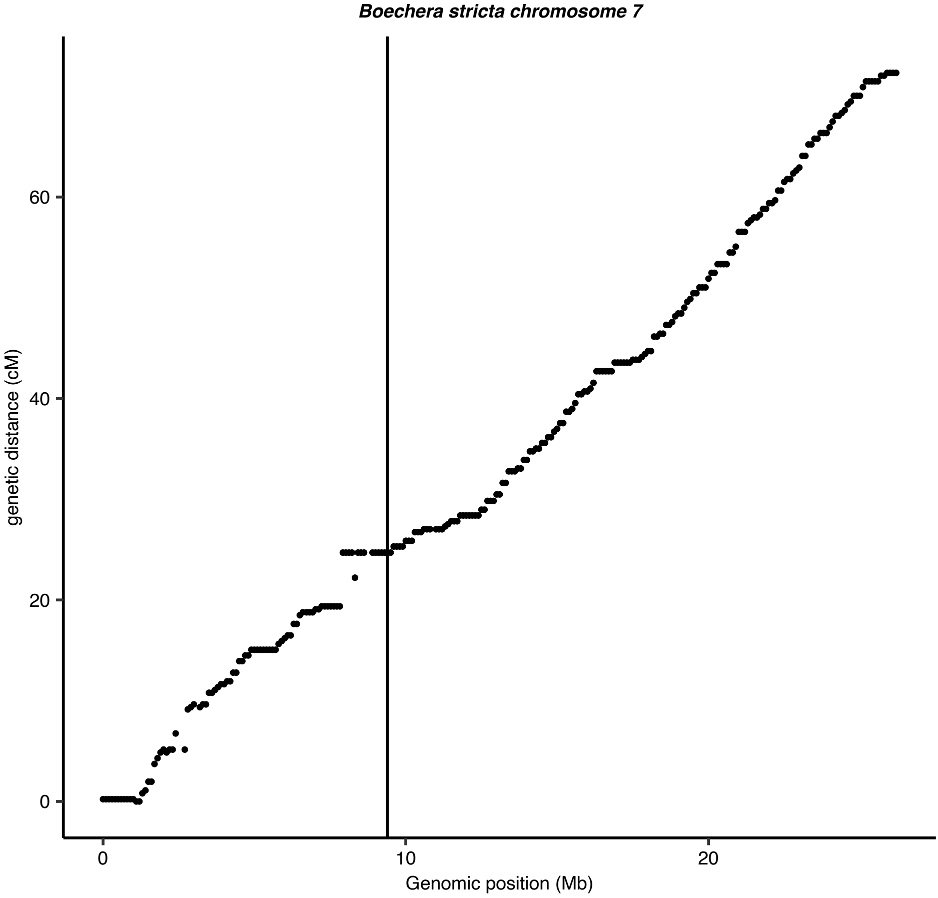
Boechera stricta chromosome 7
genetic distance (cM)
0
20
40
60
0
10
20
Genomic position (Mb)

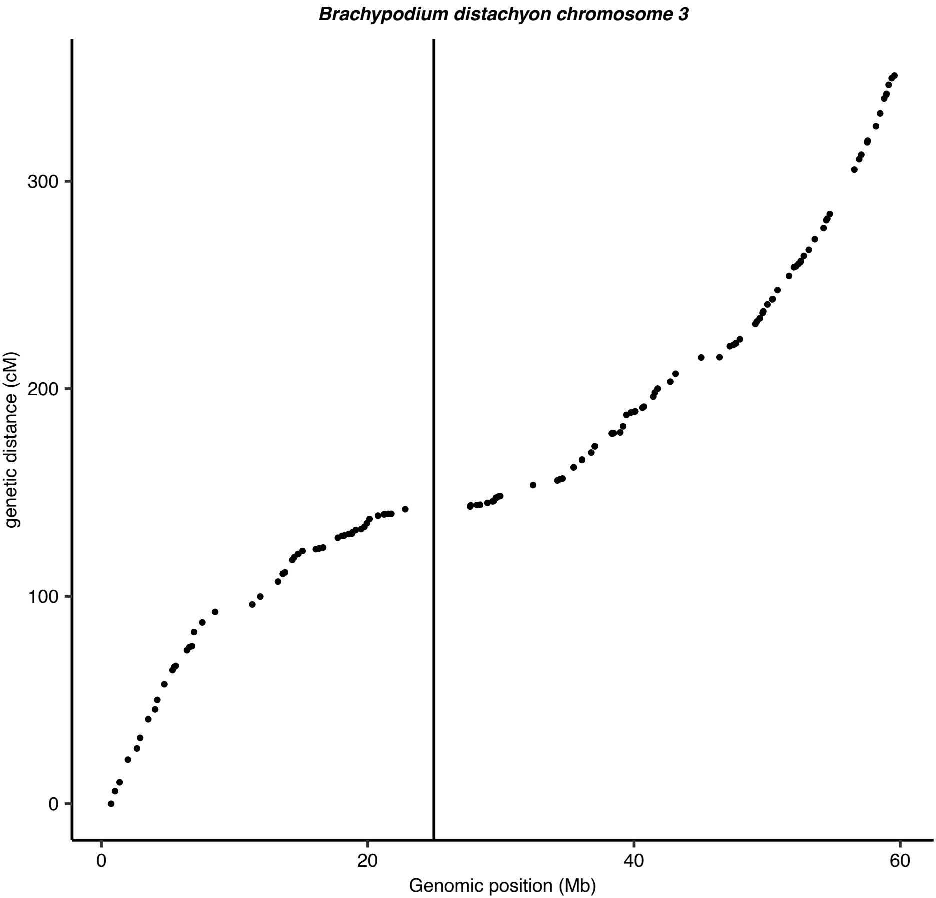
Brachypodium distachyon chromosome 3
genetic distance (cM)
0
100
200
300
Genomic position (Mb)
0
20
40
60

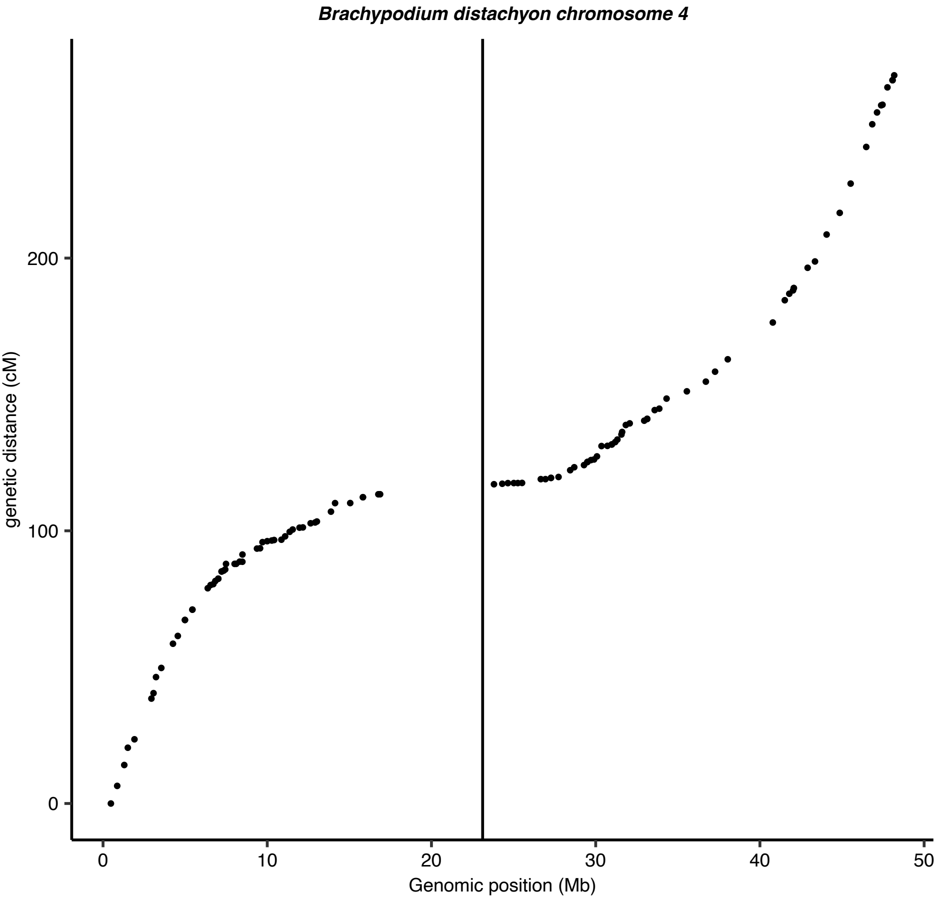
Brachypodium distachyon chromosome 4
genetic distance (cM)
0
100
200
Genomic position (Mb)
0
10
20
30
40
50

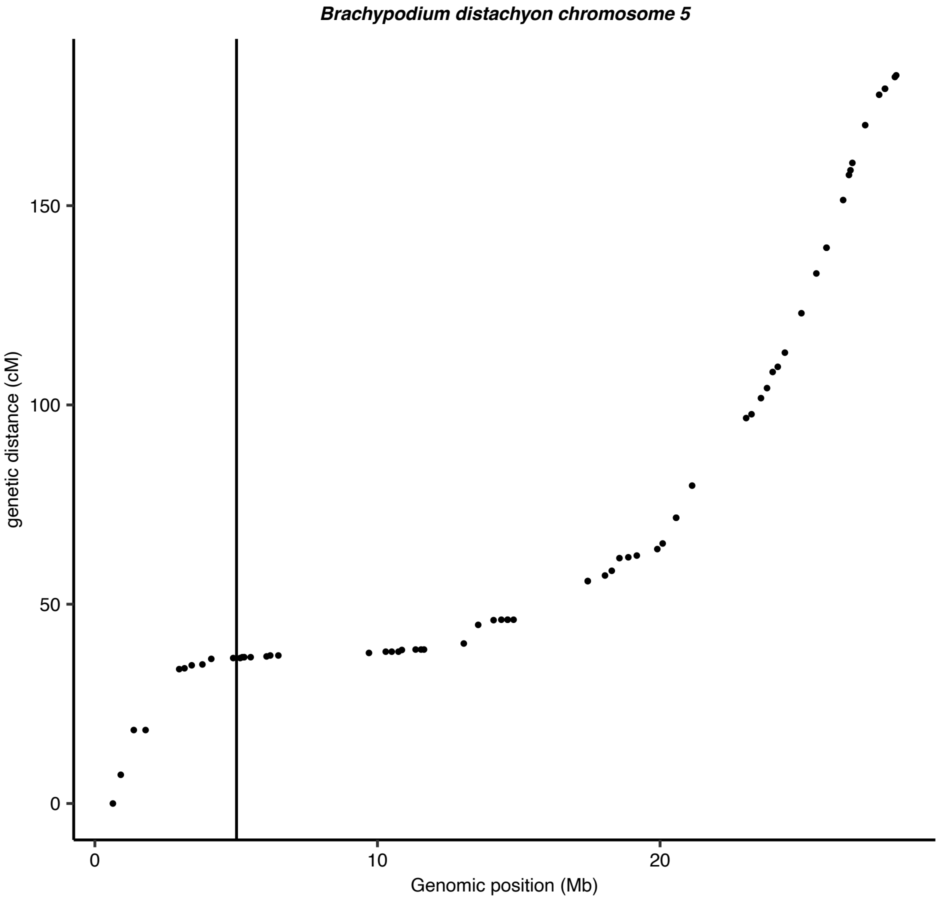
Brachypodium distachyon chromosome 5
genetic distance (cM)
0
50
100
150
0
10
20
Genomic position (Mb)

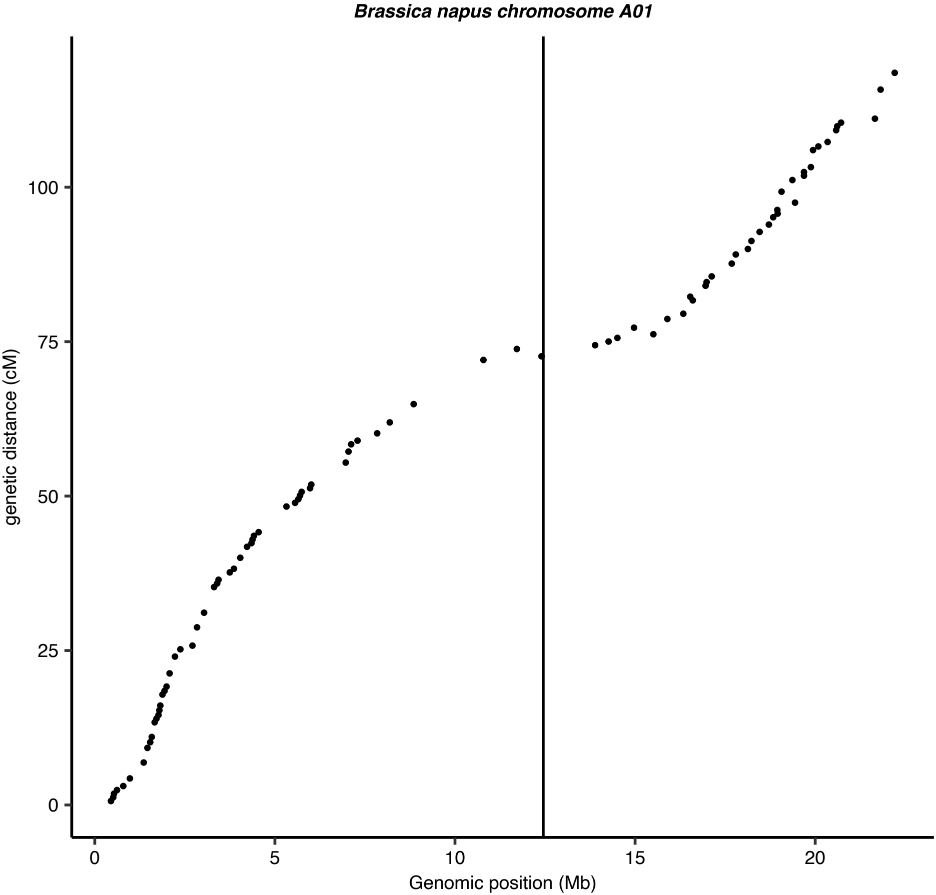

Brassica napus chromosome A01
genetic distance (cM)
0
25
50
75
100
Genomic position (Mb)
0
5
10
15
20

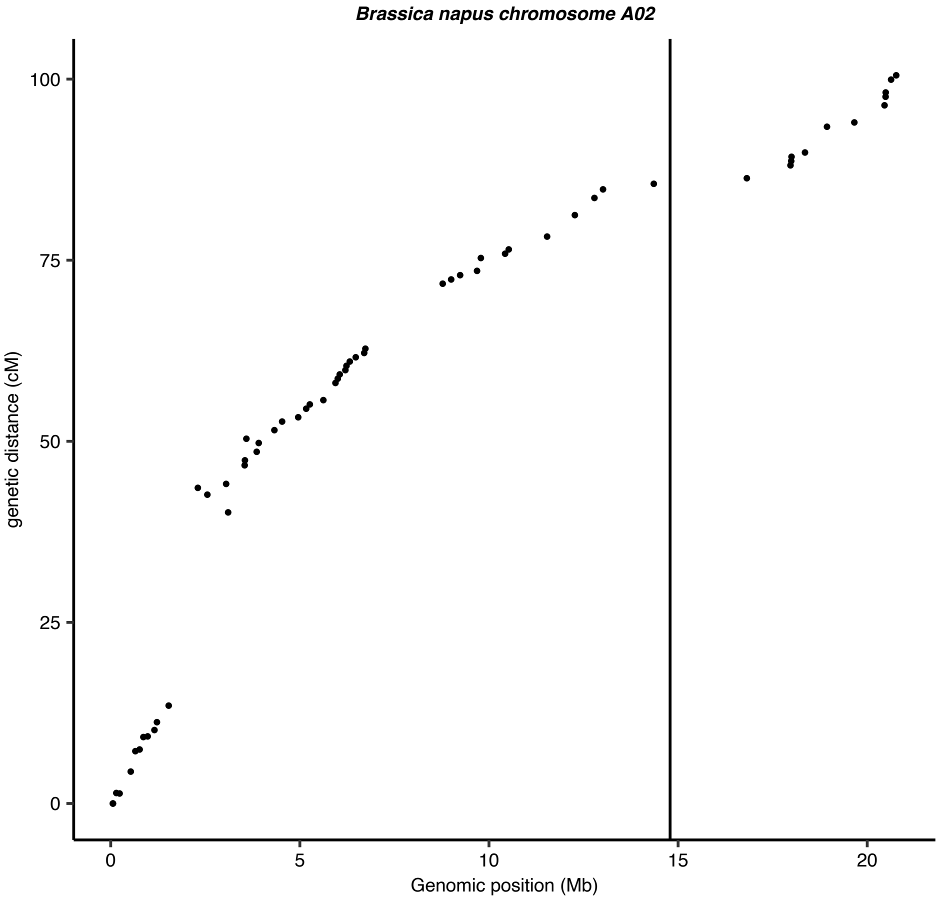
Brassica napus chromosome A02
genetic distance (cM)
Genomic position (Mb)
0
25
50
75
100
0
5
10
15
20

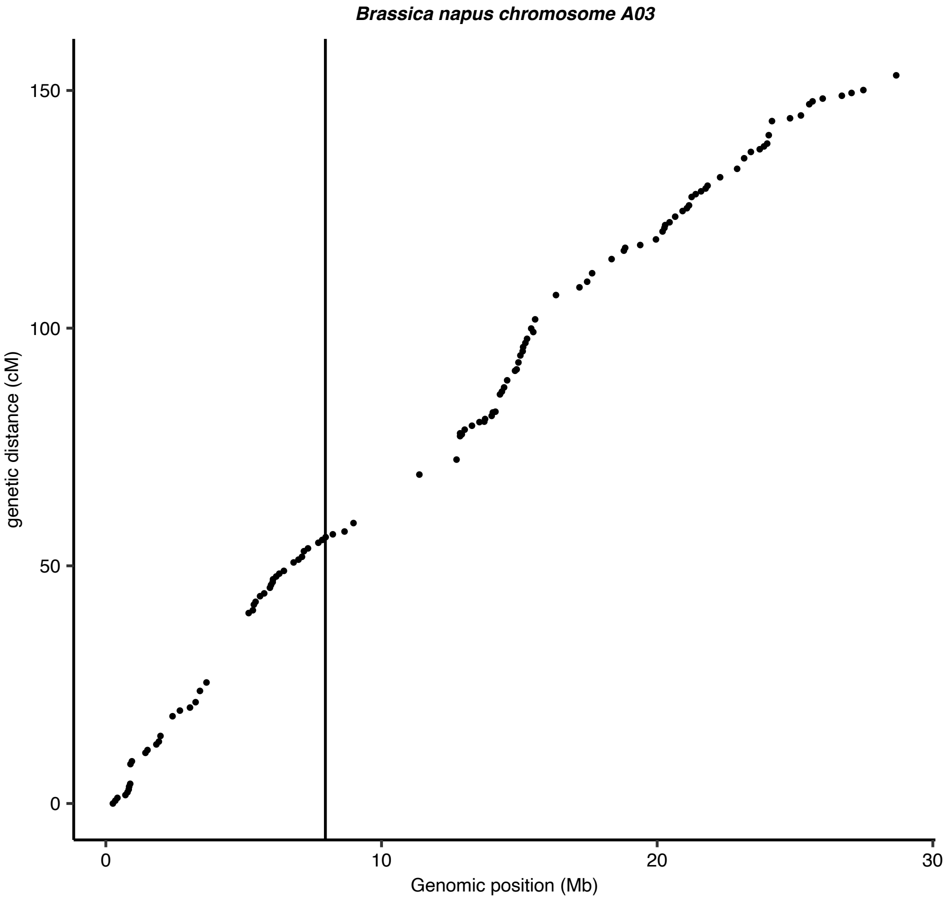
Brassica napus chromosome A03
genetic distance (cM)
150
100
50
0
Genomic position (Mb)
0
10
20
30

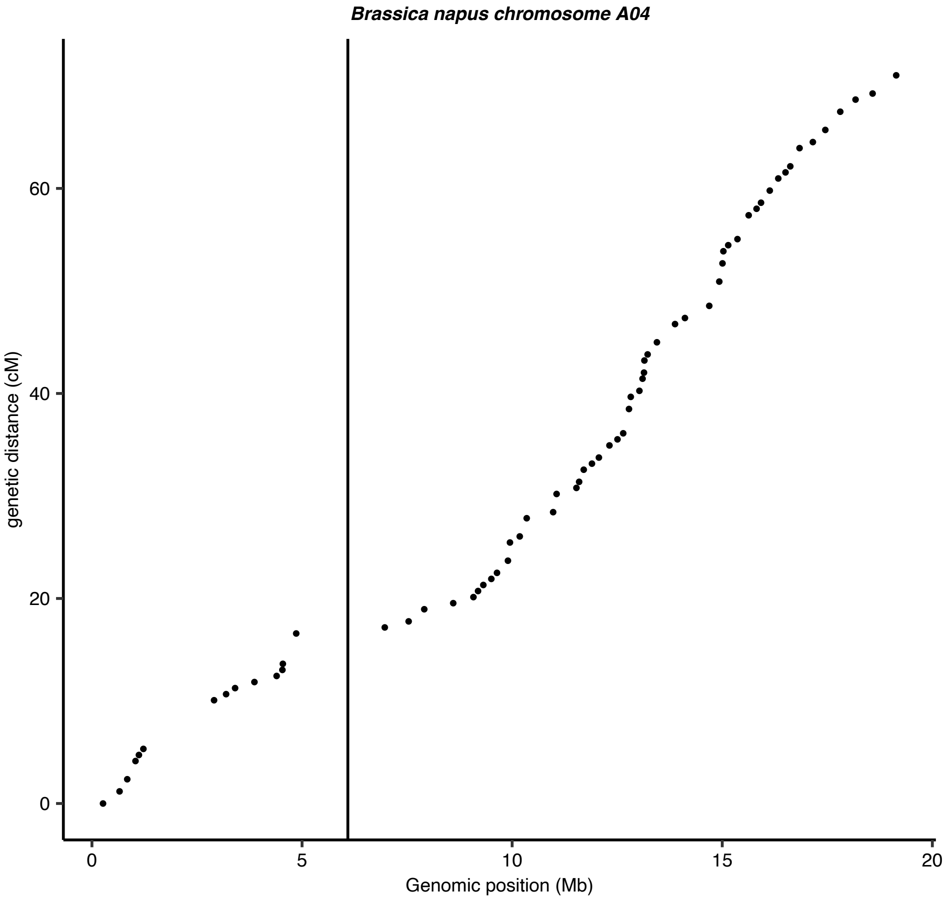
Brassica napus chromosome A04
genetic distance (cM)
0
20
40
60
0
5
10
15
20
Genomic position (Mb)

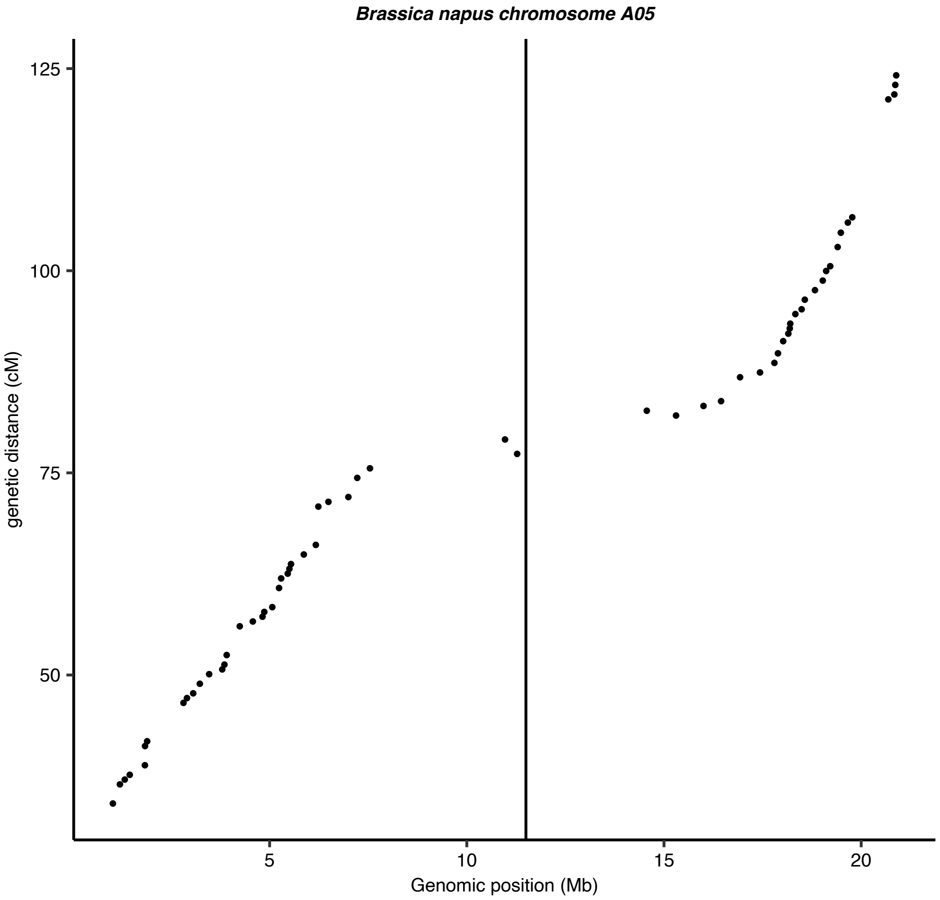
Brassica napus chromosome A05
genetic distance (cM)
125
100
75
50
5
10
15
20
Genomic position (Mb)

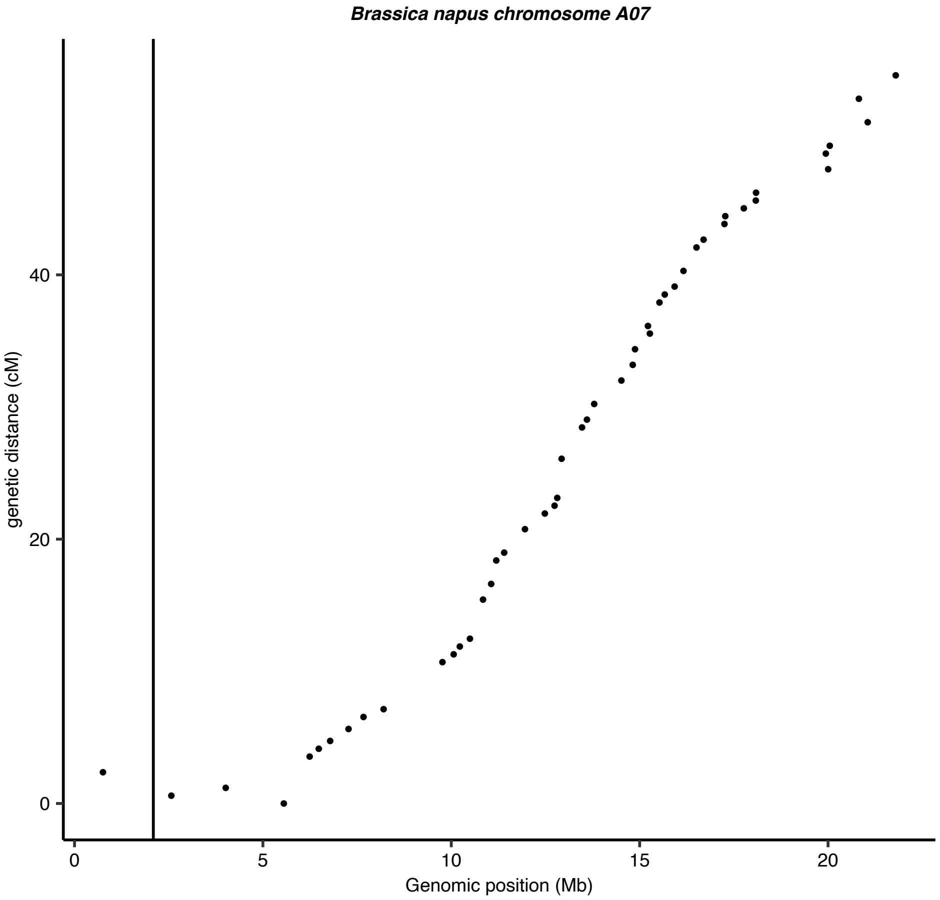
Brassica napus chromosome A07
genetic distance (cM)
0
20
40
Genomic position (Mb)
0
5
10
15
20

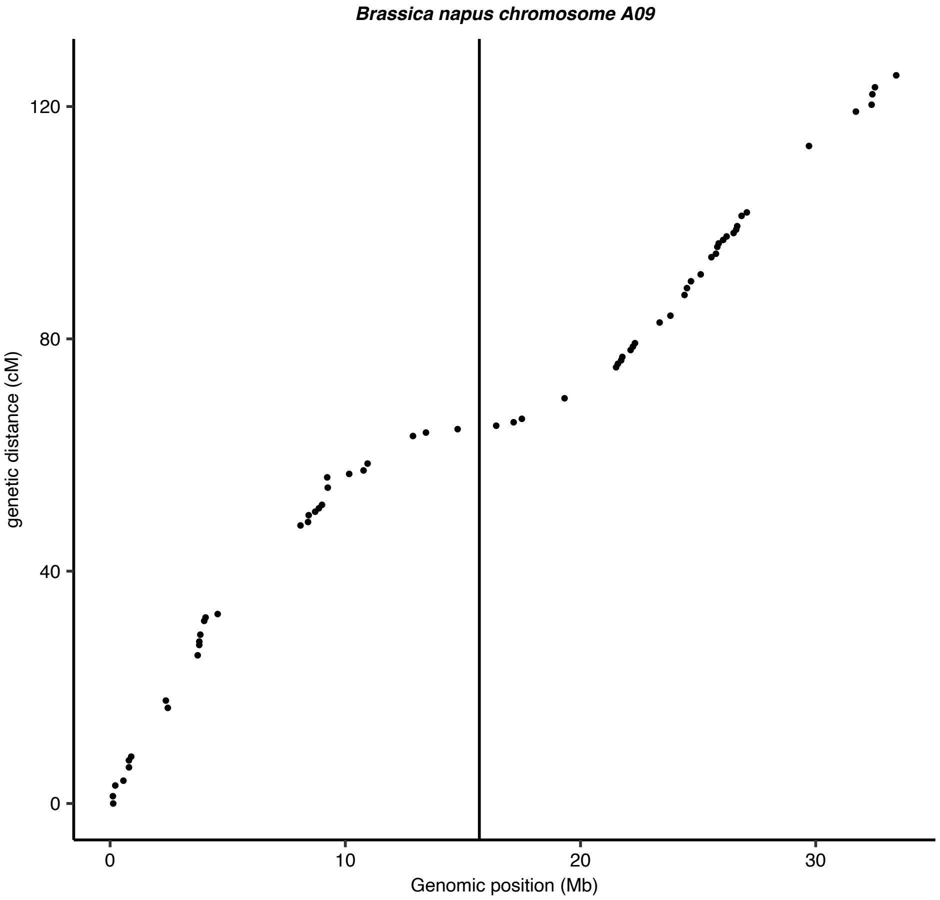
Brassica napus chromosome A09
genetic distance (cM)
Genomic position (Mb)
0
40
80
120
0
10
20
30

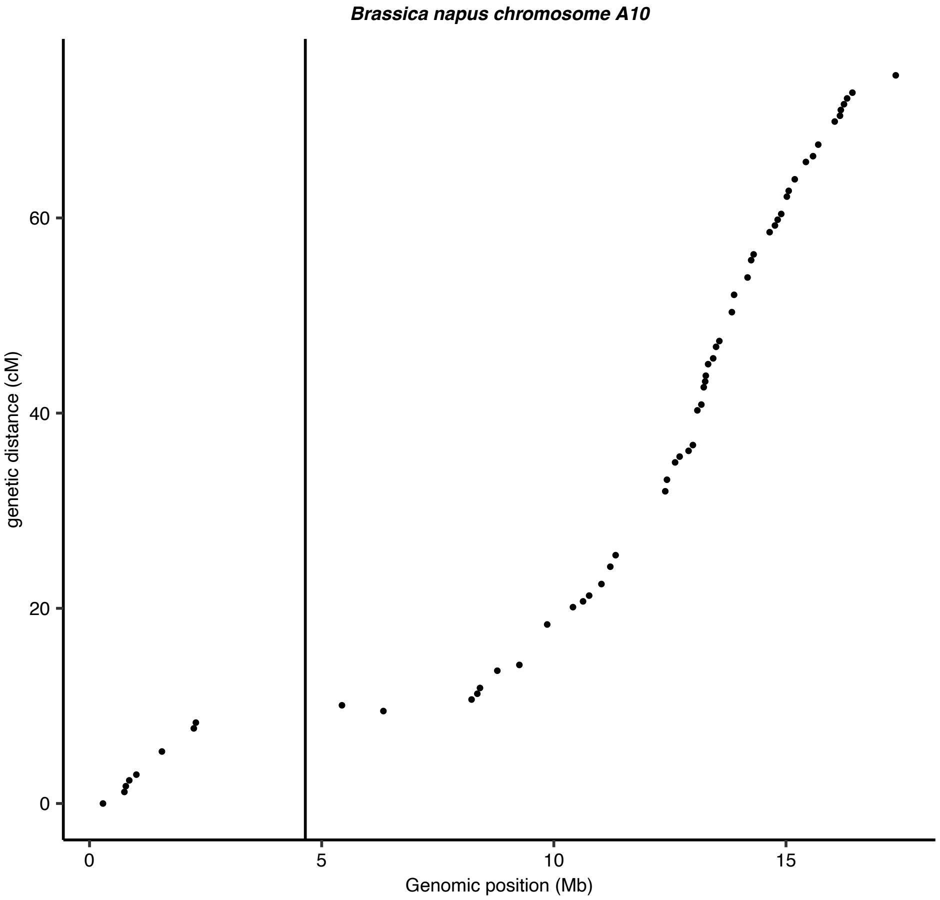
Brassica napus chromosome A10
genetic distance (cM)
Genomic position (Mb)
0
20
40
60
0
5
10
15

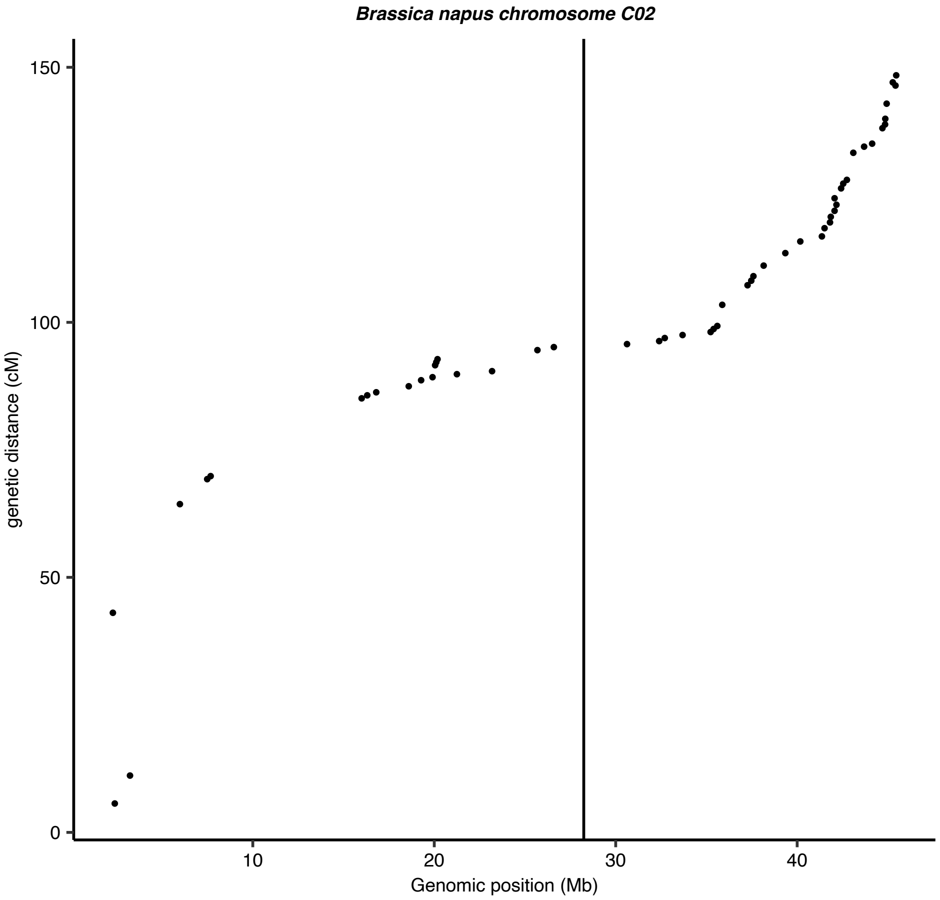

Brassica napus chromosome C02
150
100
50
0
10
20
30
40
Genomic position (Mb)
genetic distance (cM)

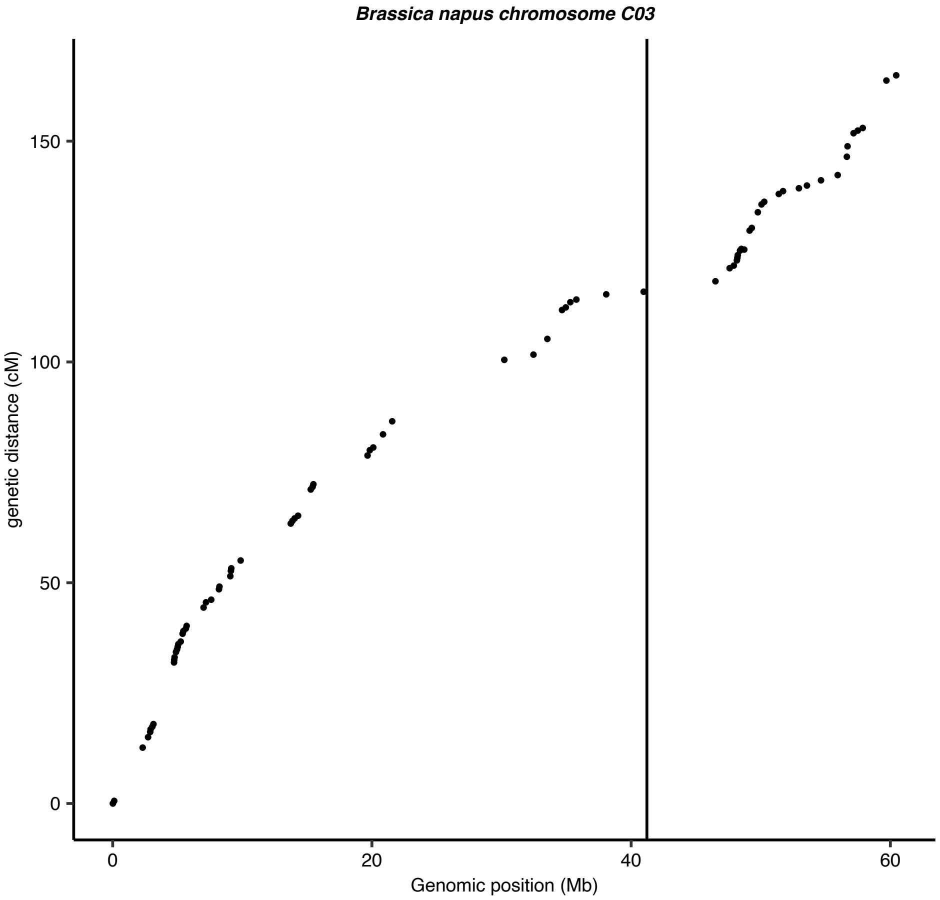
Brassica napus chromosome C03
genetic distance (cM)
0
50
100
150
Genomic position (Mb)
0
20
40
60

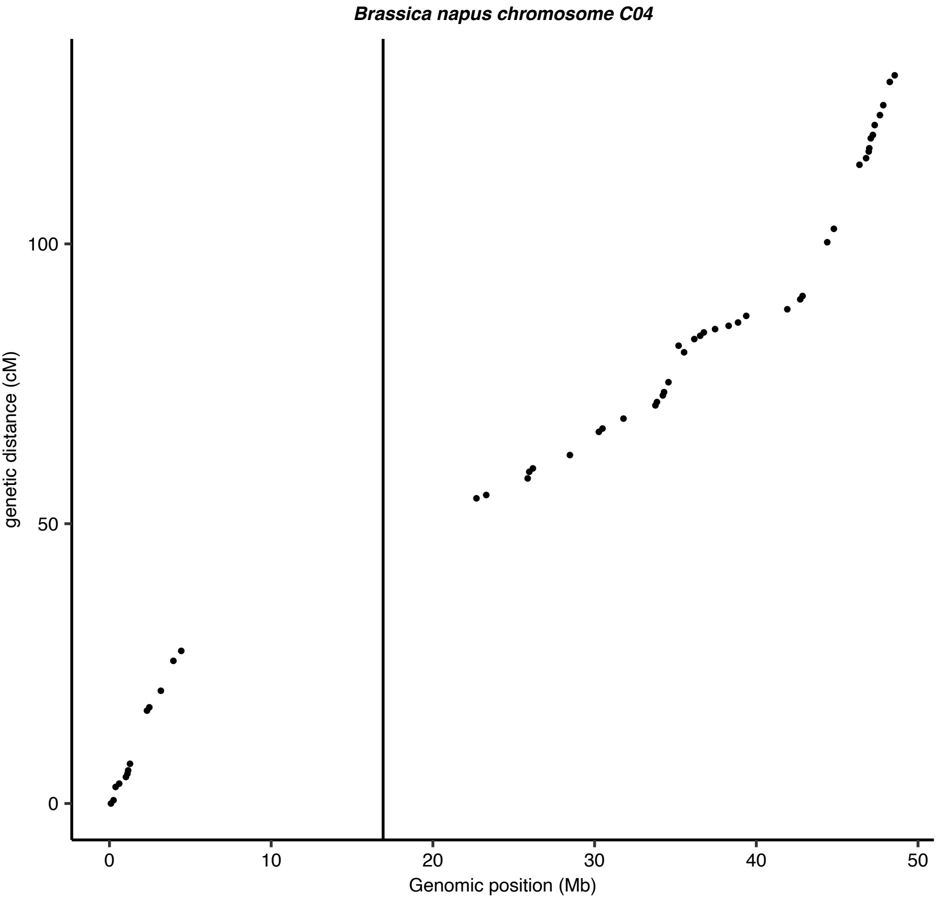
Brassica napus chromosome C04
genetic distance (cM)
0
50
100
Genomic position (Mb)
0
10
20
30
40
50

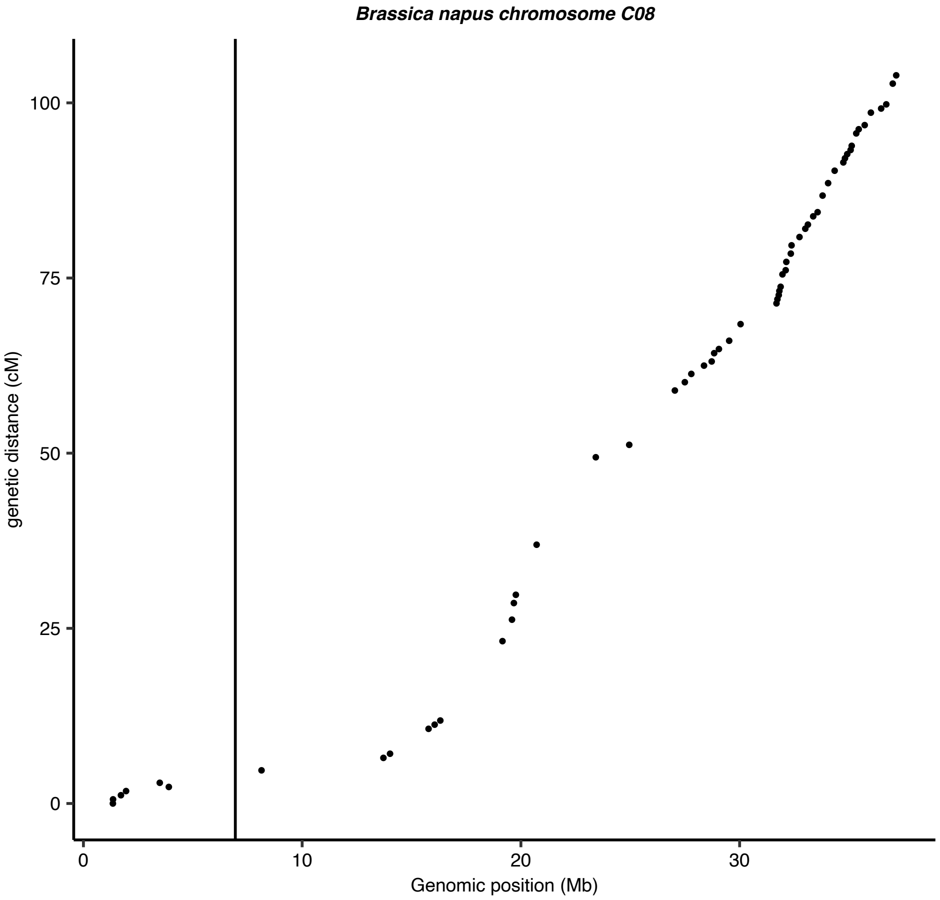
Brassica napus chromosome C08
genetic distance (cM)
100
75
50
25
0
0
10
20
30
Genomic position (Mb)

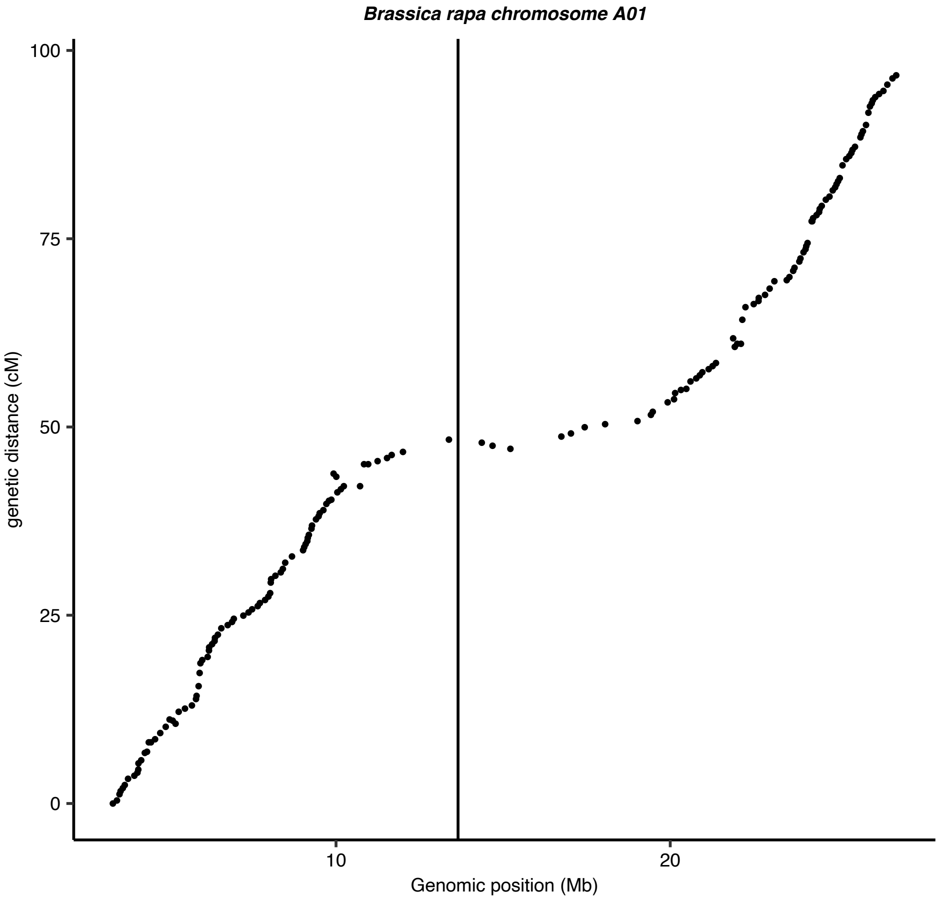

Brassica rapa chromosome A01
genetic distance (cM)
100
75
50
25
0
10
20
Genomic position (Mb)

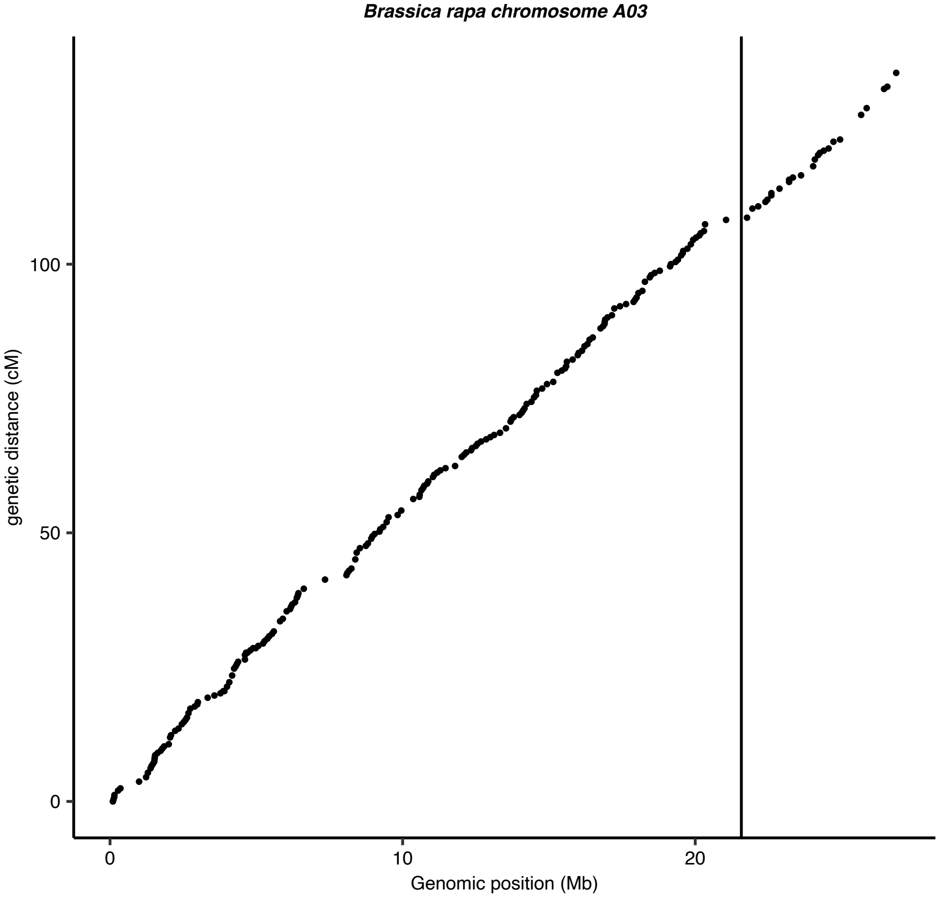
Brassica rapa chromosome A03
genetic distance (cM)
0
50
100
Genomic position (Mb)
0
10
20

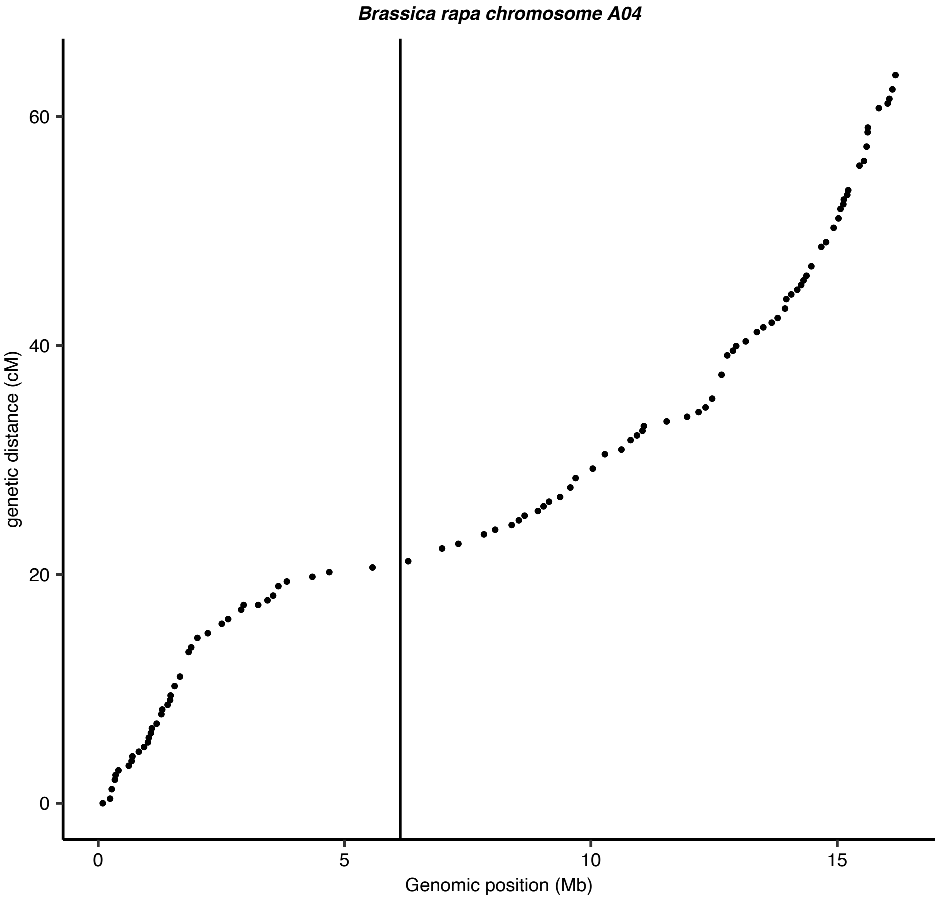
Brassica rapa chromosome A04
genetic distance (cM)
0
20
40
60
0
5
10
15
Genomic position (Mb)

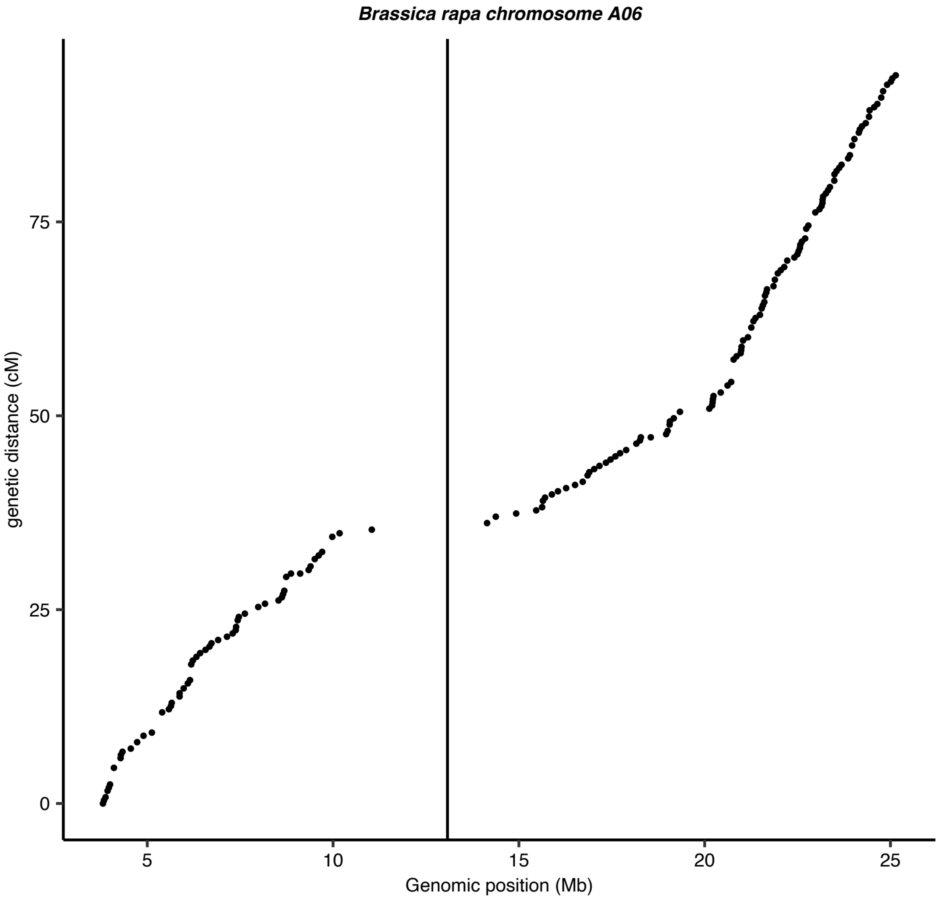

Brassica rapa chromosome A06
genetic distance (cM)
0
25
50
75
Genomic position (Mb)
5
10
15
20
25

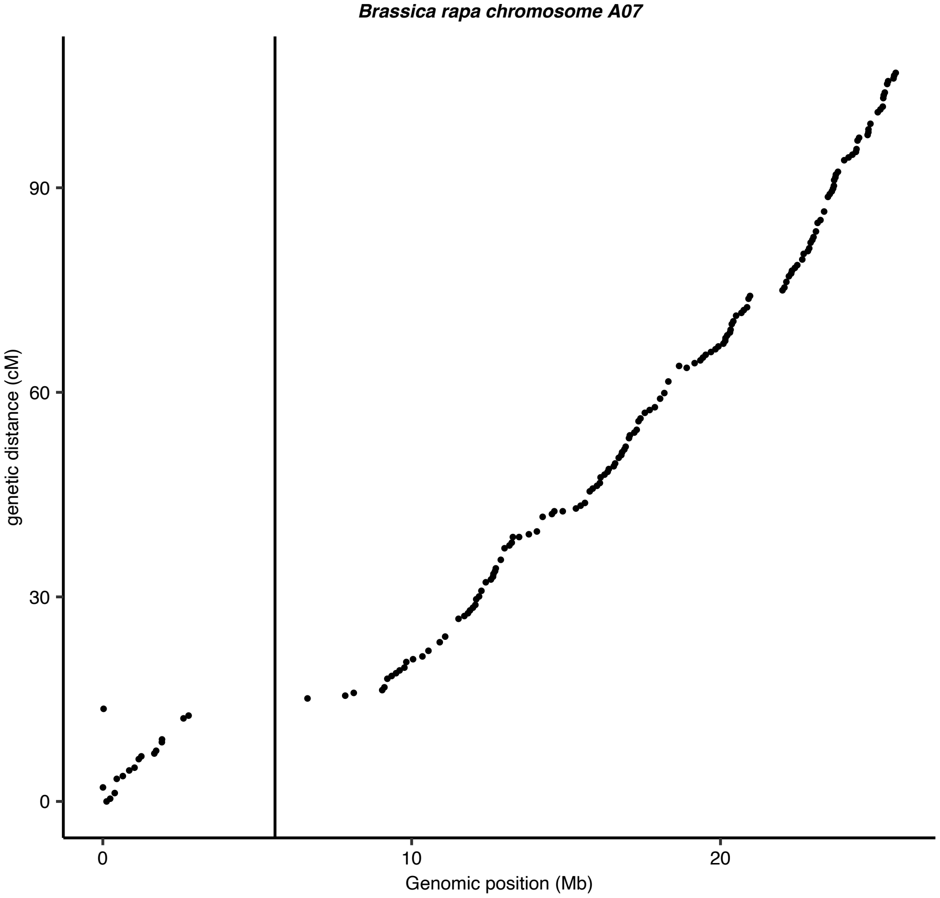

Brassica rapa chromosome A07
genetic distance (cM)
0
30
60
90
Genomic position (Mb)
0
10
20

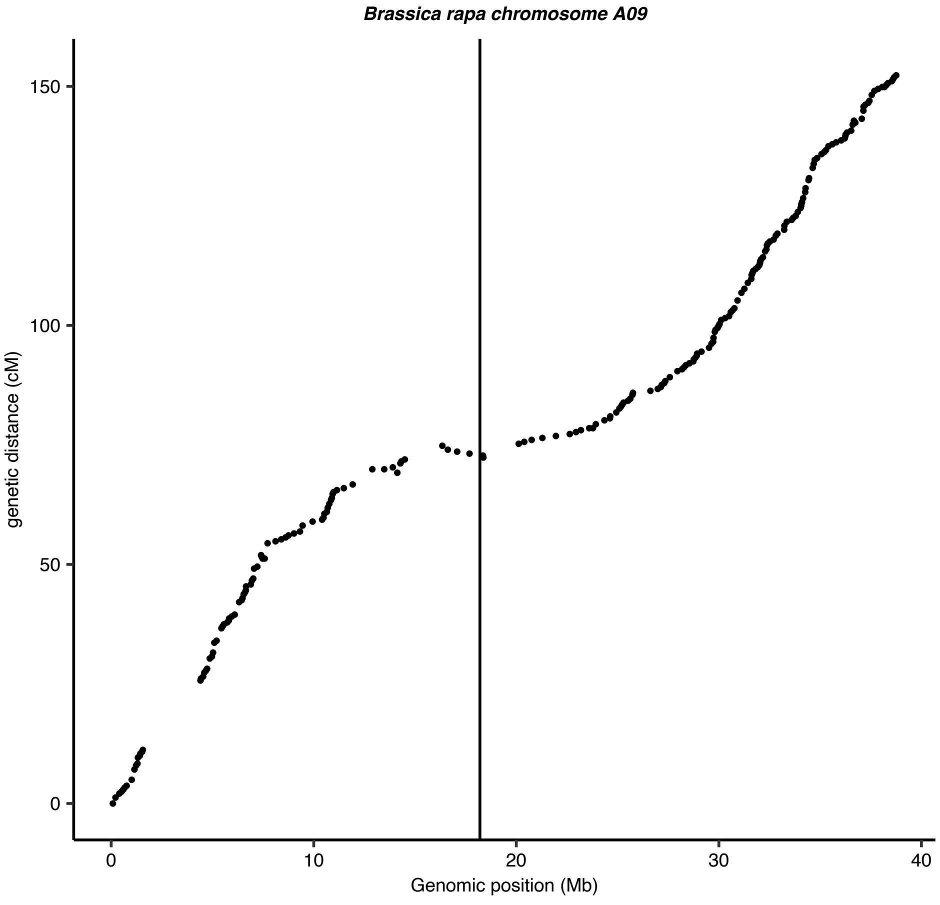
Brassica rapa chromosome A09
genetic distance (cM)
Genomic position (Mb)
0
50
100
150
0
10
20
30
40

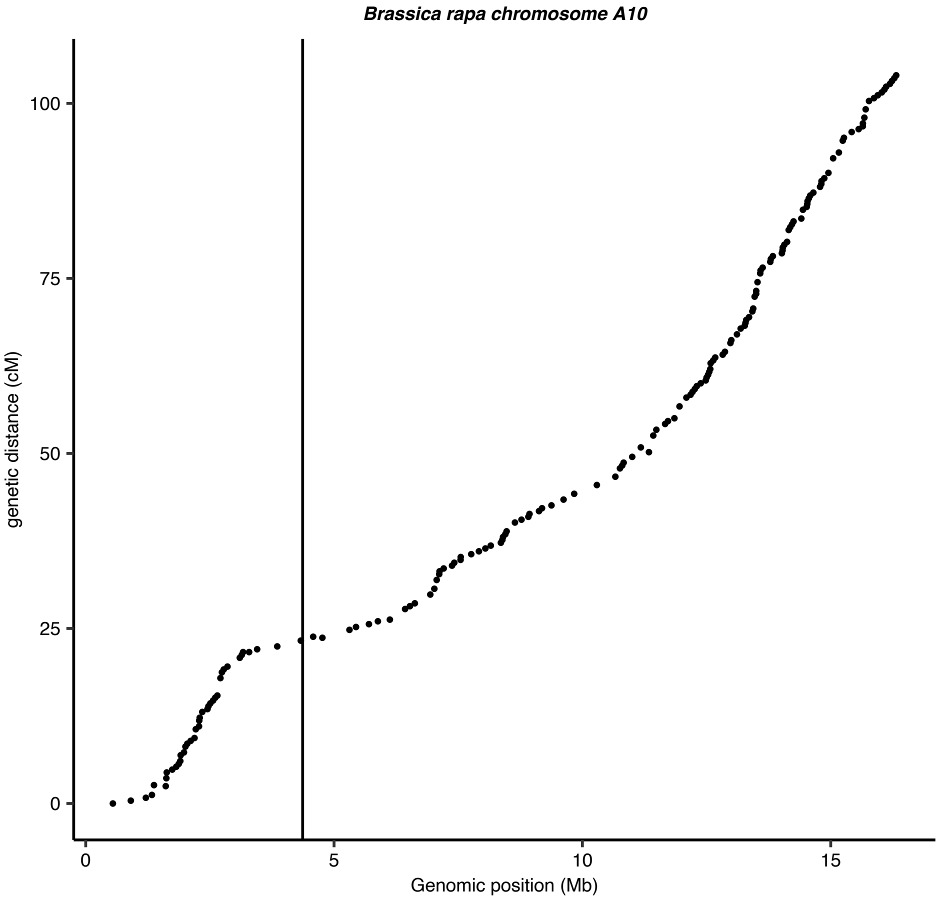
Brassica rapa chromosome A10
genetic distance (cM)
0
25
50
75
100
Genomic position (Mb)
0
5
10
15

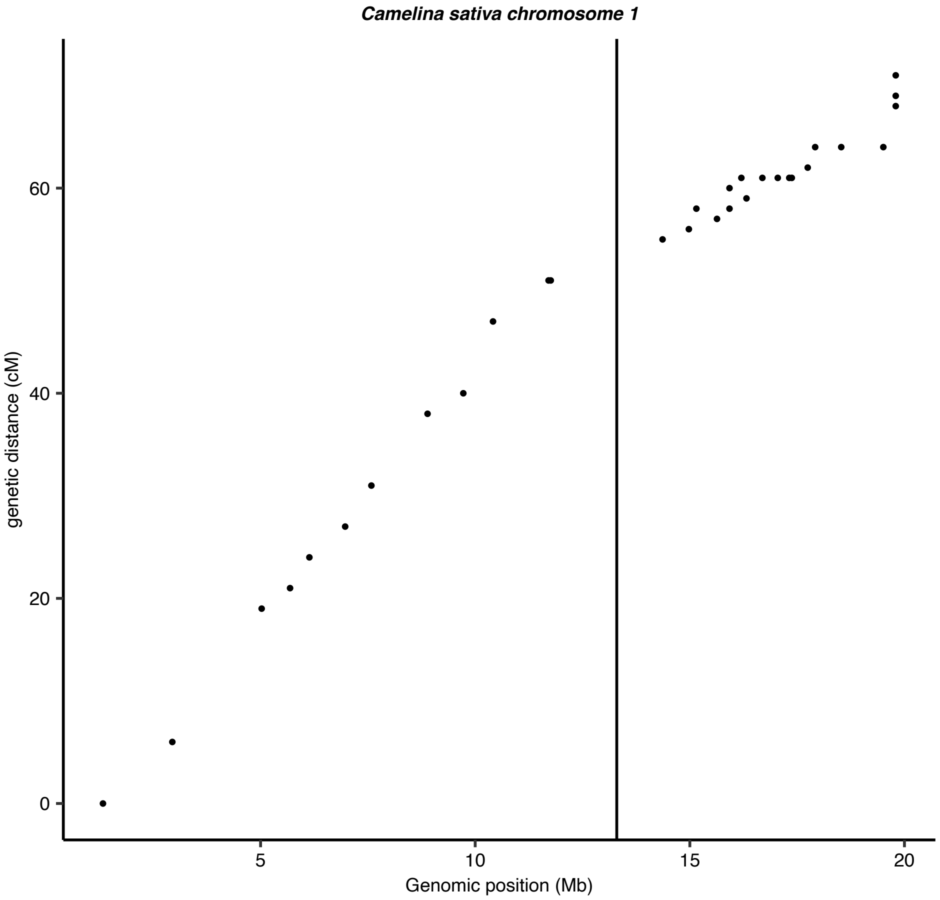
Camelina sativa chromosome 1
genetic distance (cM)
0
20
40
60
Genomic position (Mb)
5
10
15
20

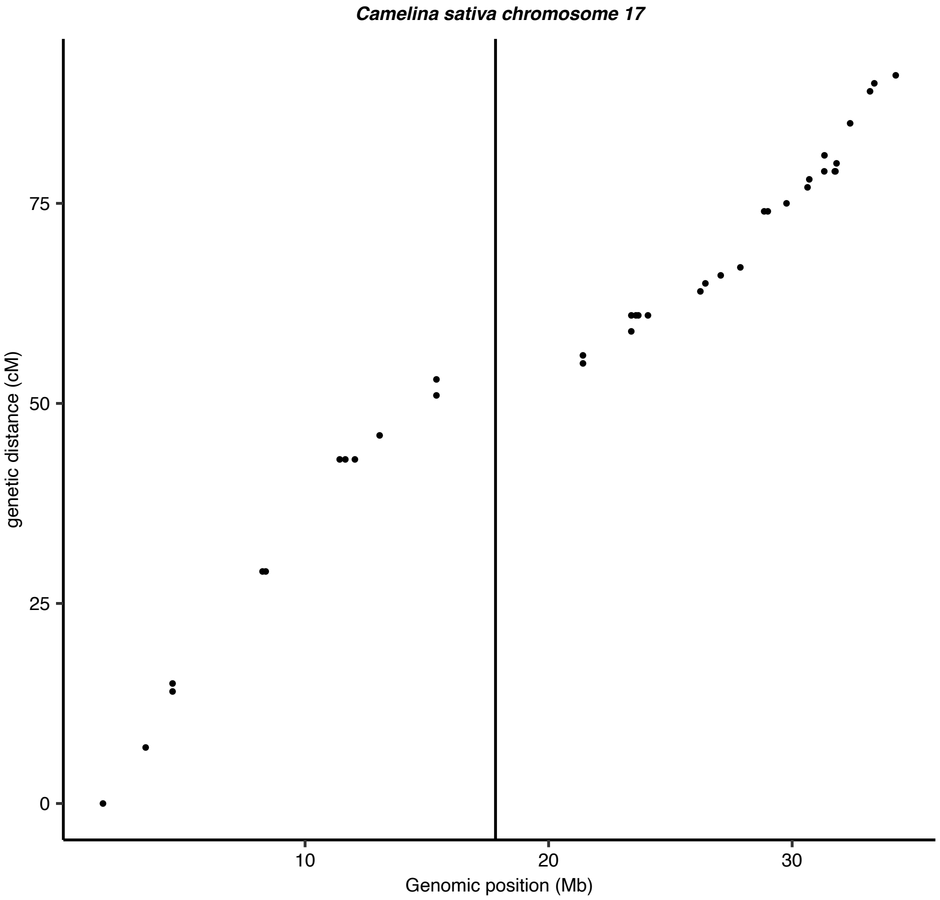
Camelina sativa chromosome 17
genetic distance (cM)
0
25
50
75
10
20
30
Genomic position (Mb)

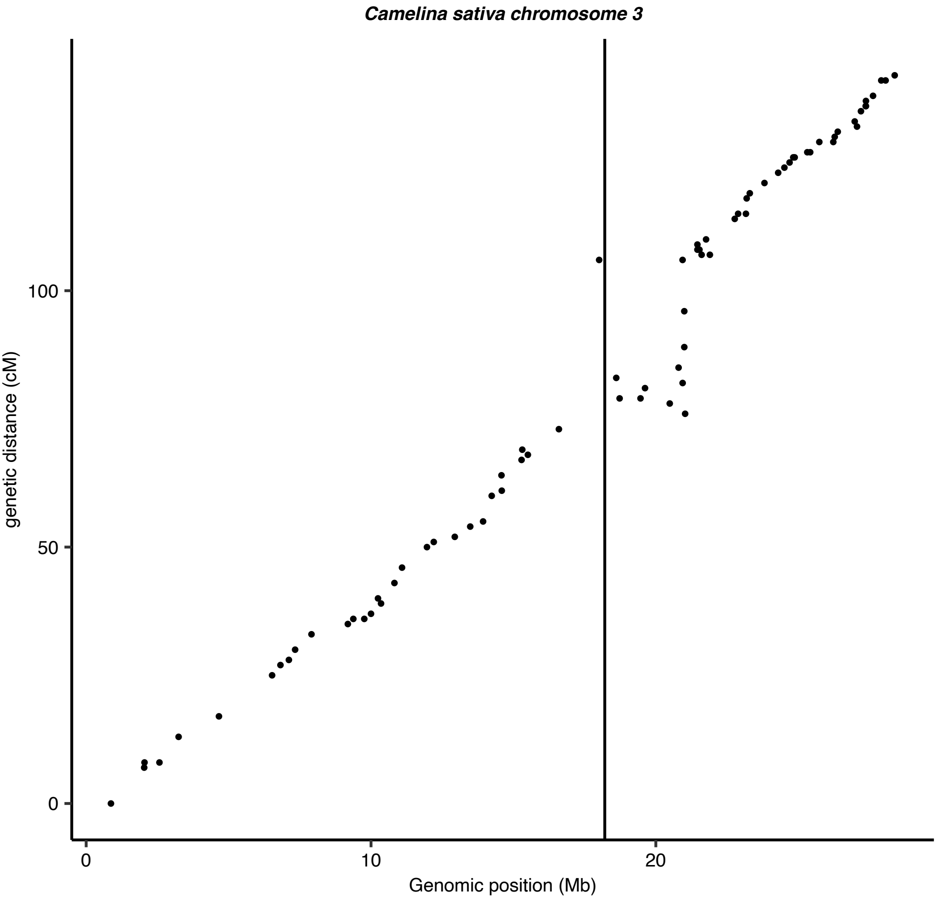
Camelina sativa chromosome 3
genetic distance (cM)
100
50
0
Genomic position (Mb)
0
10
20

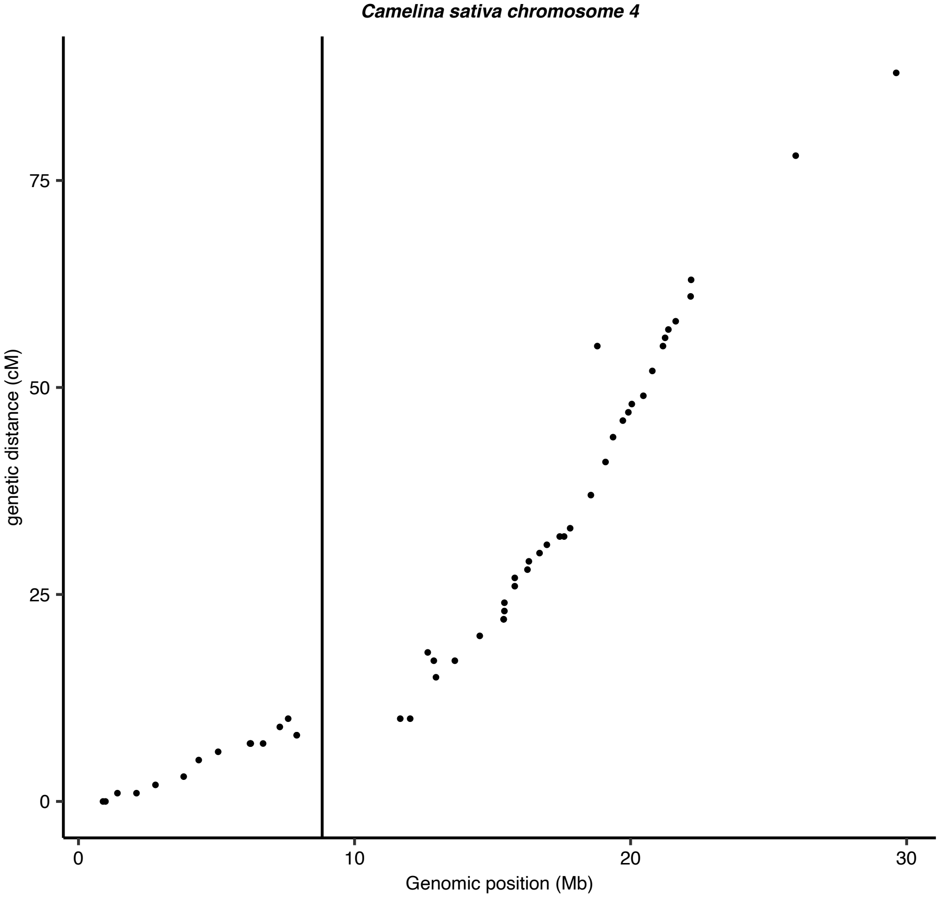
Camelina sativa chromosome 4
genetic distance (cM)
0
25
50
75
Genomic position (Mb)
0
10
20
30

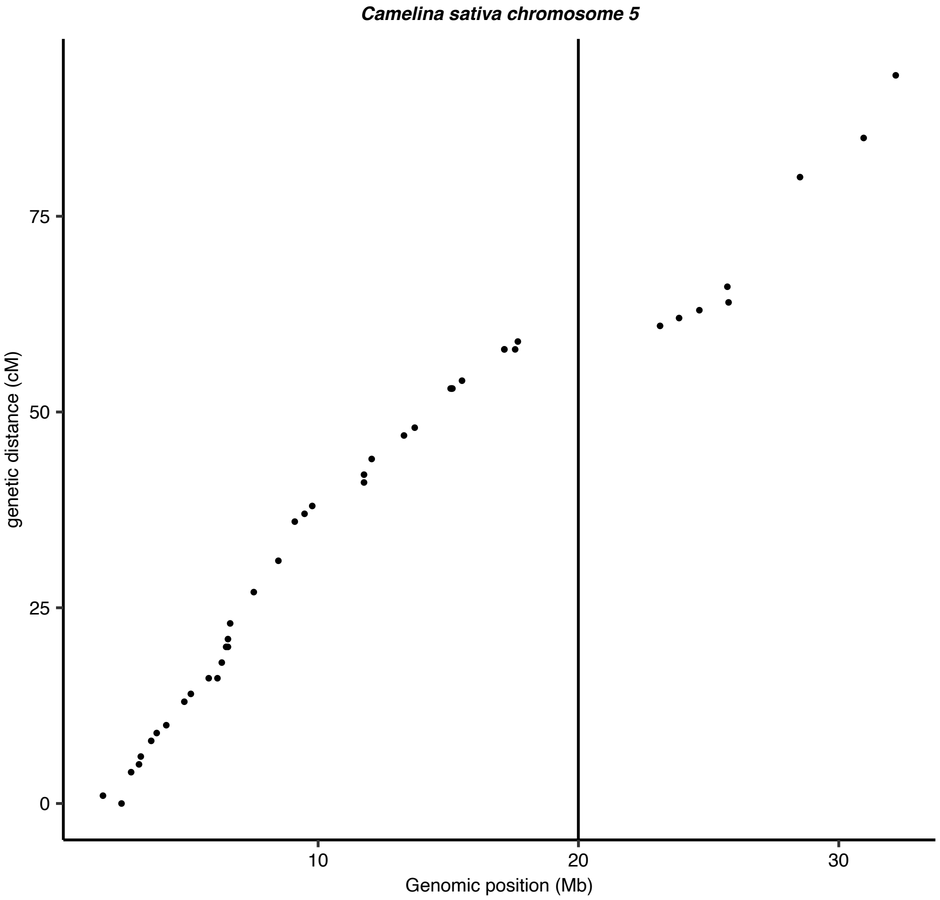
Camelina sativa chromosome 5
genetic distance (cM)
0
25
50
75
Genomic position (Mb)
10
20
30

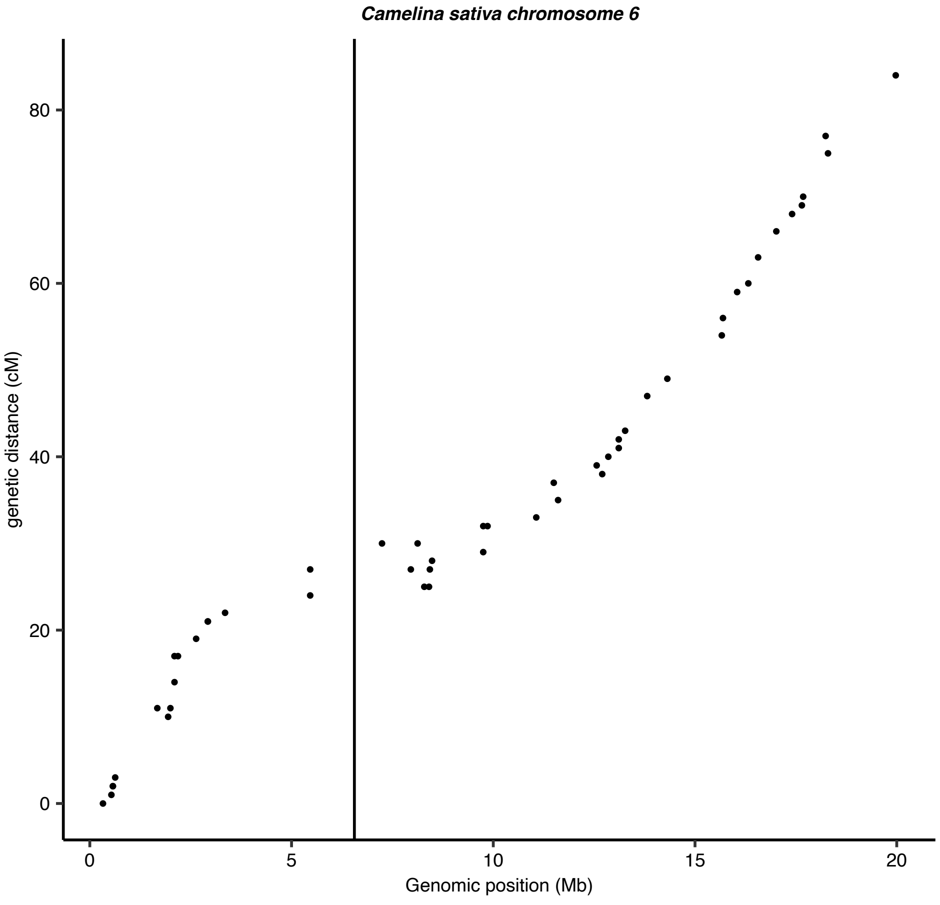

Camelina sativa chromosome 6
genetic distance (cM)
0
20
40
60
80
Genomic position (Mb)
0
5
10
15
20

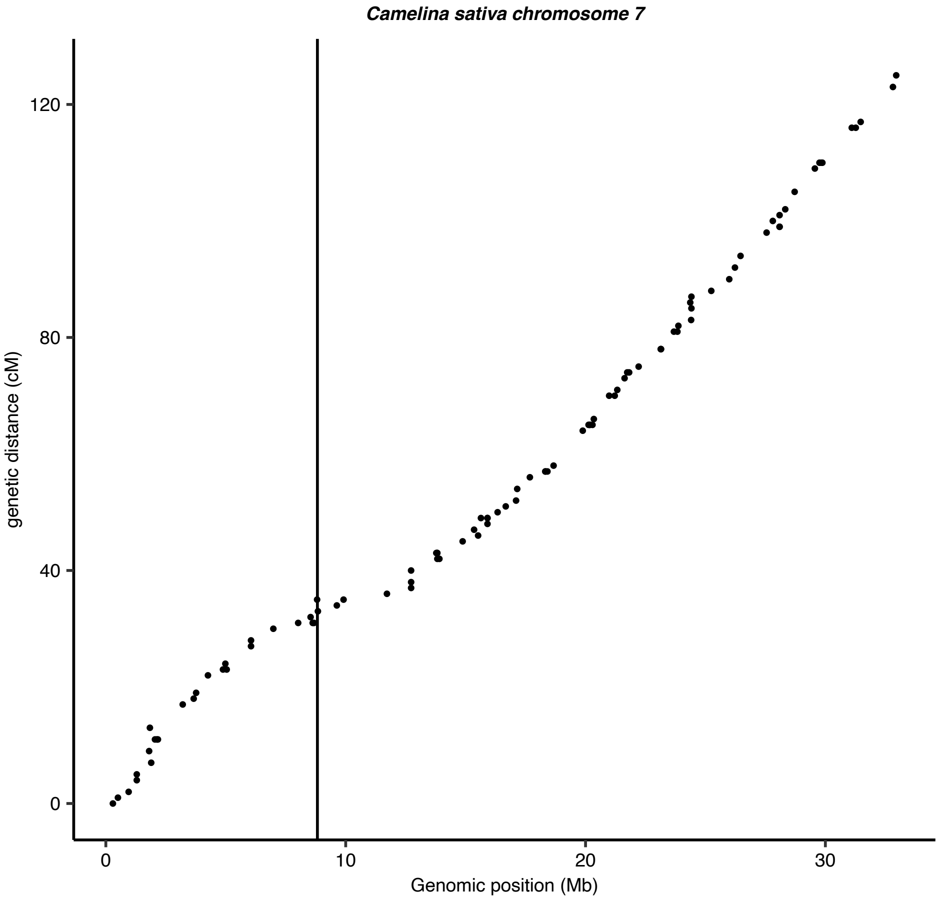
Camelina sativa chromosome 7
genetic distance (cM)
0
40
80
120
Genomic position (Mb)
0
10
20
30

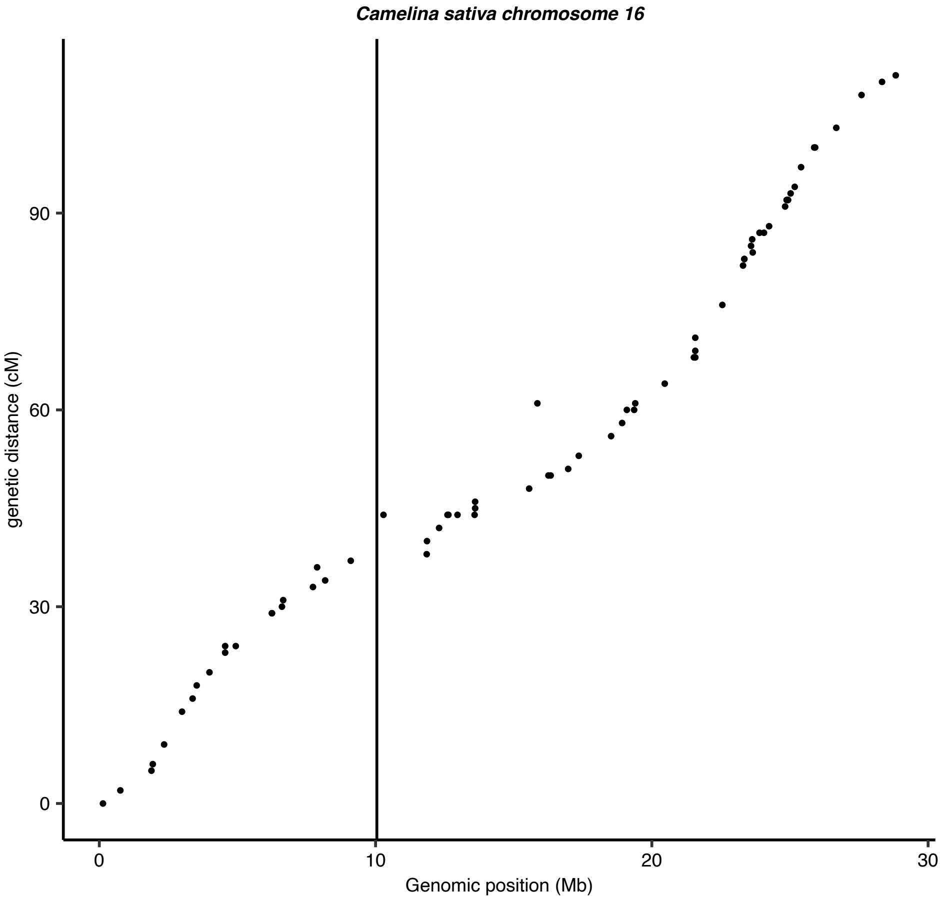
Camelina sativa chromosome 16
genetic distance (cM)
0
30
60
90
Genomic position (Mb)
0
10
20
30

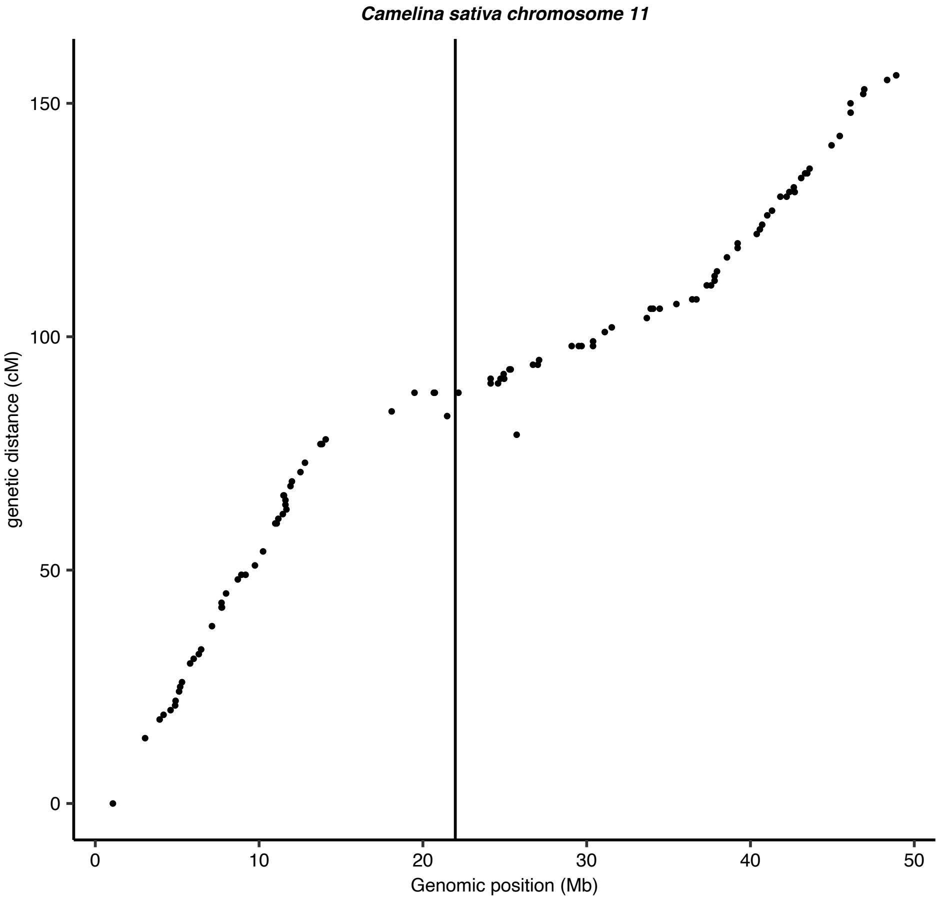
Camelina sativa chromosome 11
genetic distance (cM)
0
50
100
150
0
10
20
30
40
50
Genomic position (Mb)

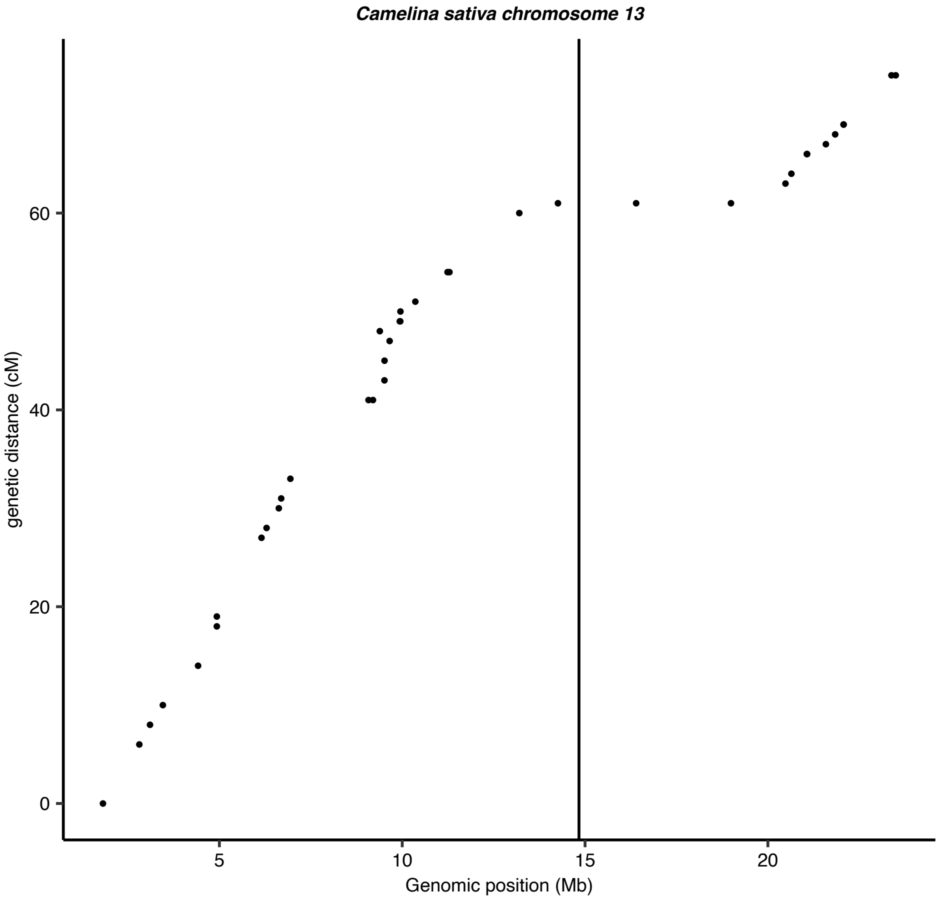
Camelina sativa chromosome 13
genetic distance (cM)
0
20
40
60
Genomic position (Mb)
5
10
15
20

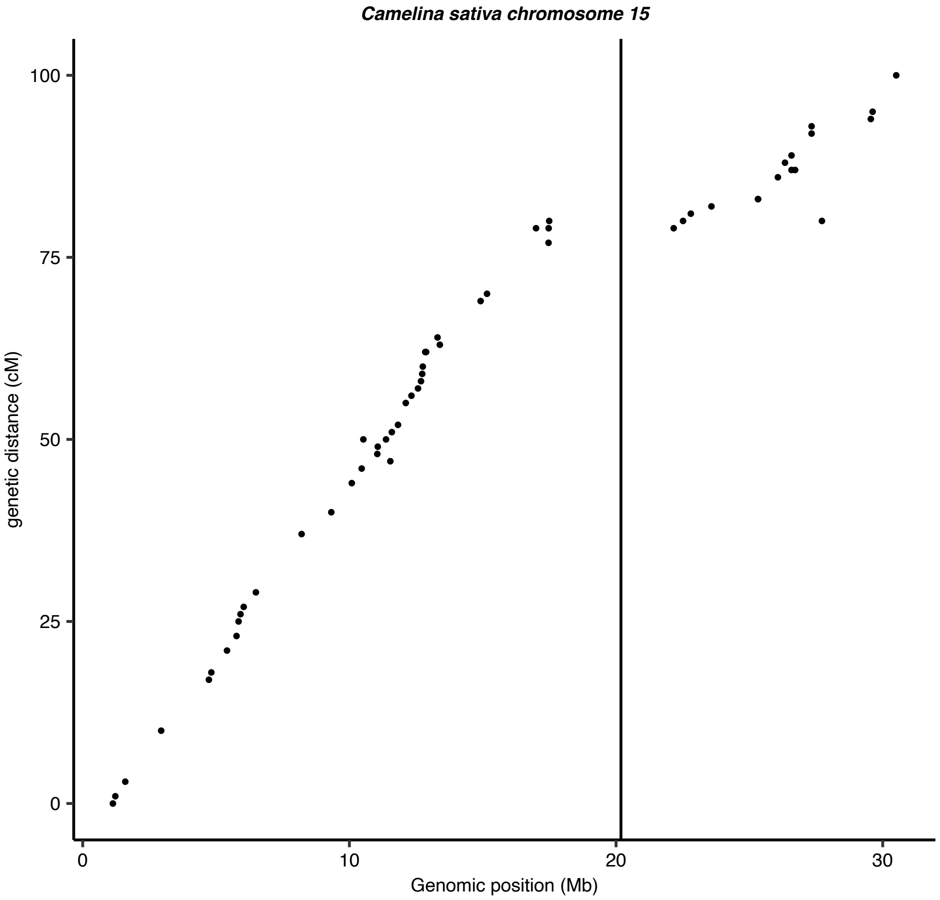
Camelina sativa chromosome 15
genetic distance (cM)
0
25
50
75
100
Genomic position (Mb)
0
10
20
30

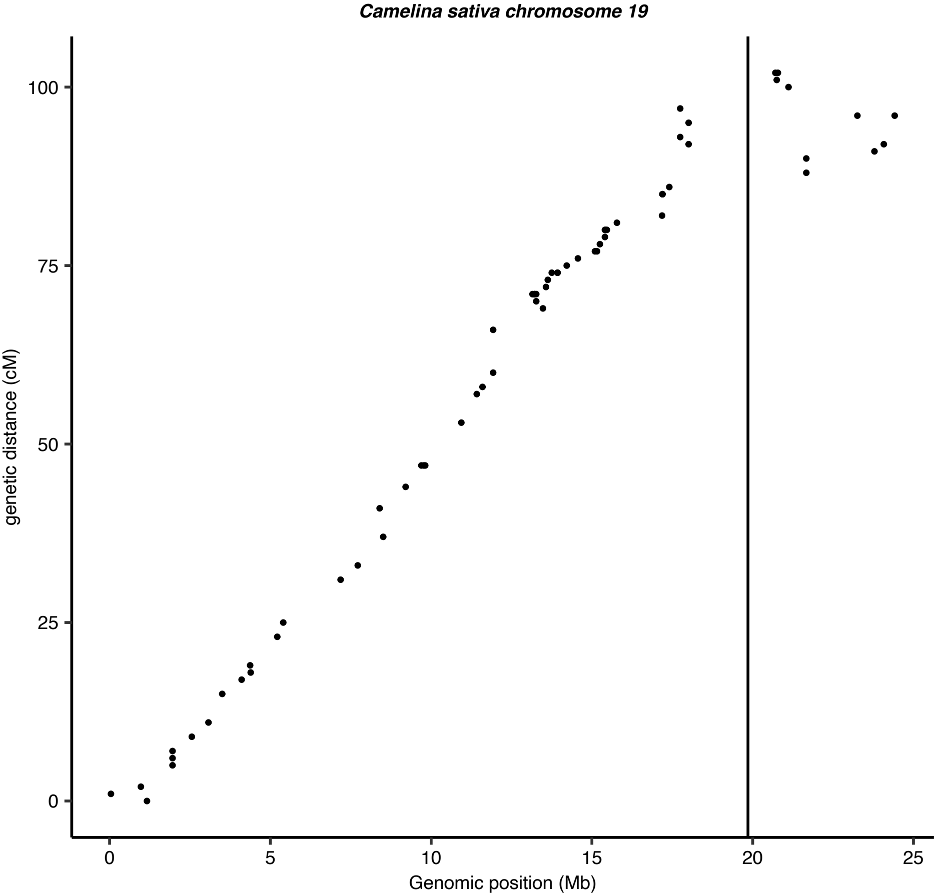

Camelina sativa chromosome 19
genetic distance (cM)
Genomic position (Mb)
0
25
50
75
100
0
5
10
15
20
25

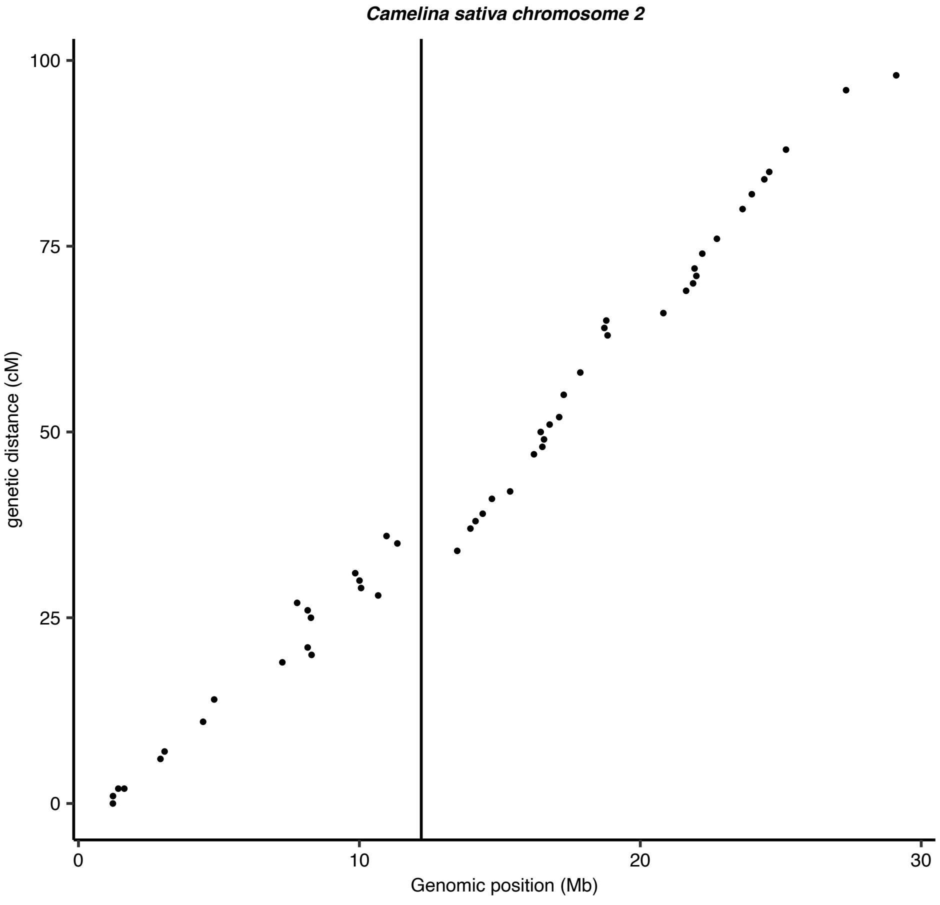
Camelina sativa chromosome 2
genetic distance (cM)
100
75
50
25
0
Genomic position (Mb)
0
10
20
30

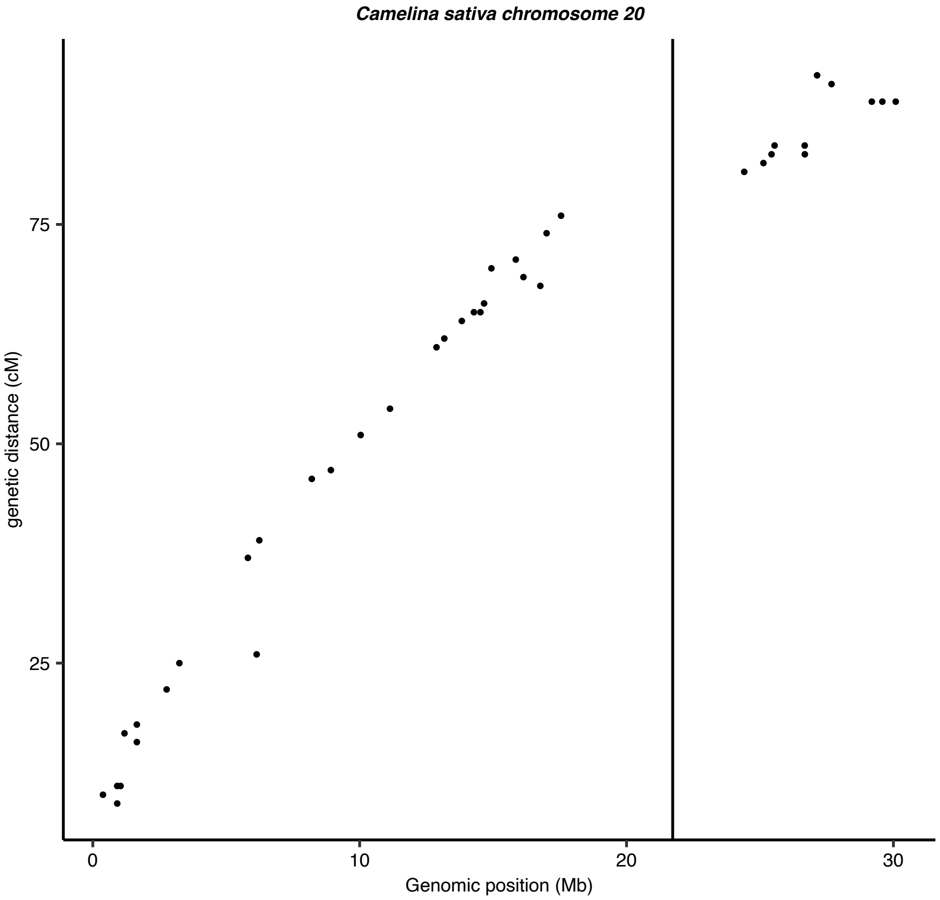
Camelina sativa chromosome 20
genetic distance (cM)
75
50
25
0
10
20
30
Genomic position (Mb)

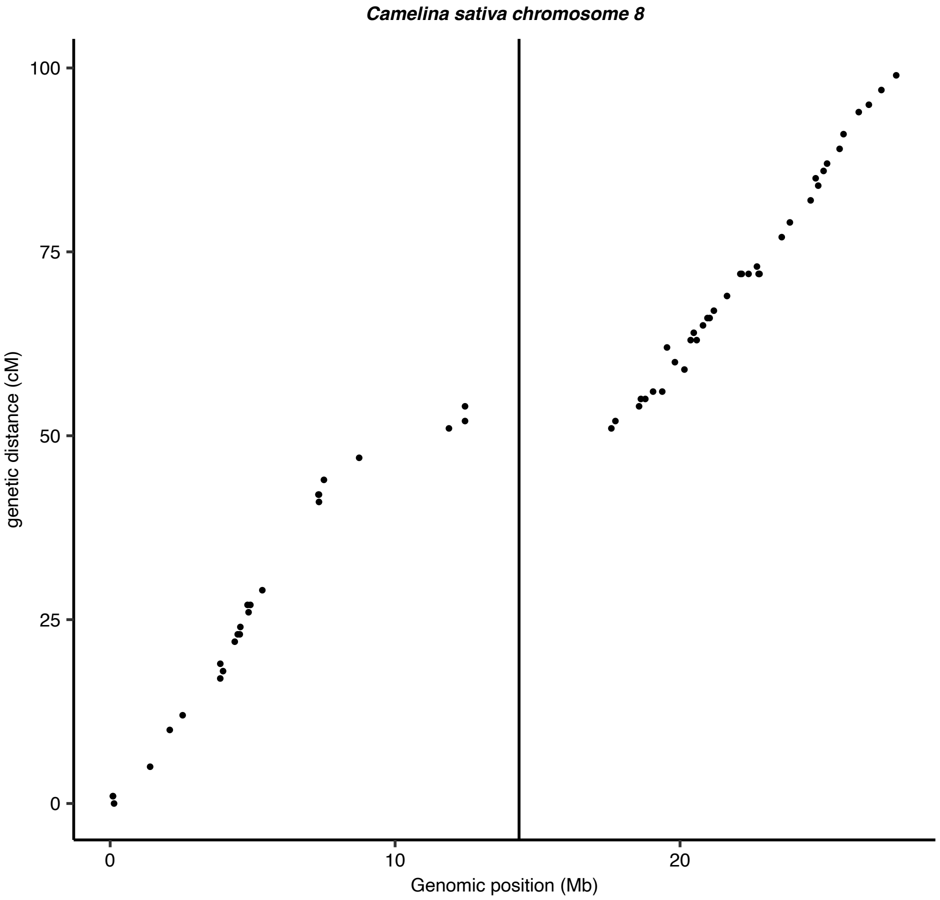
Camelina sativa chromosome 8
genetic distance (cM)
100
75
50
25
0
0
10
20
Genomic position (Mb)

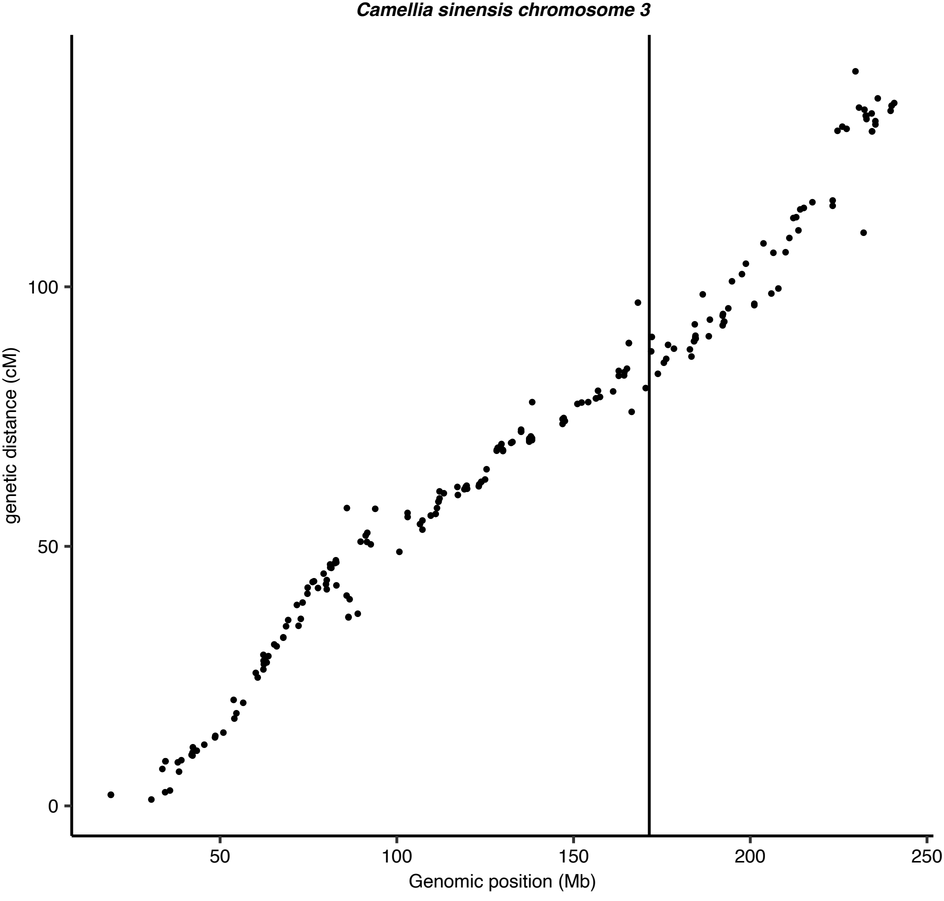
Camellia sinensis chromosome 3
genetic distance (cM)
0
50
100
Genomic position (Mb)
50
100
150
200
250

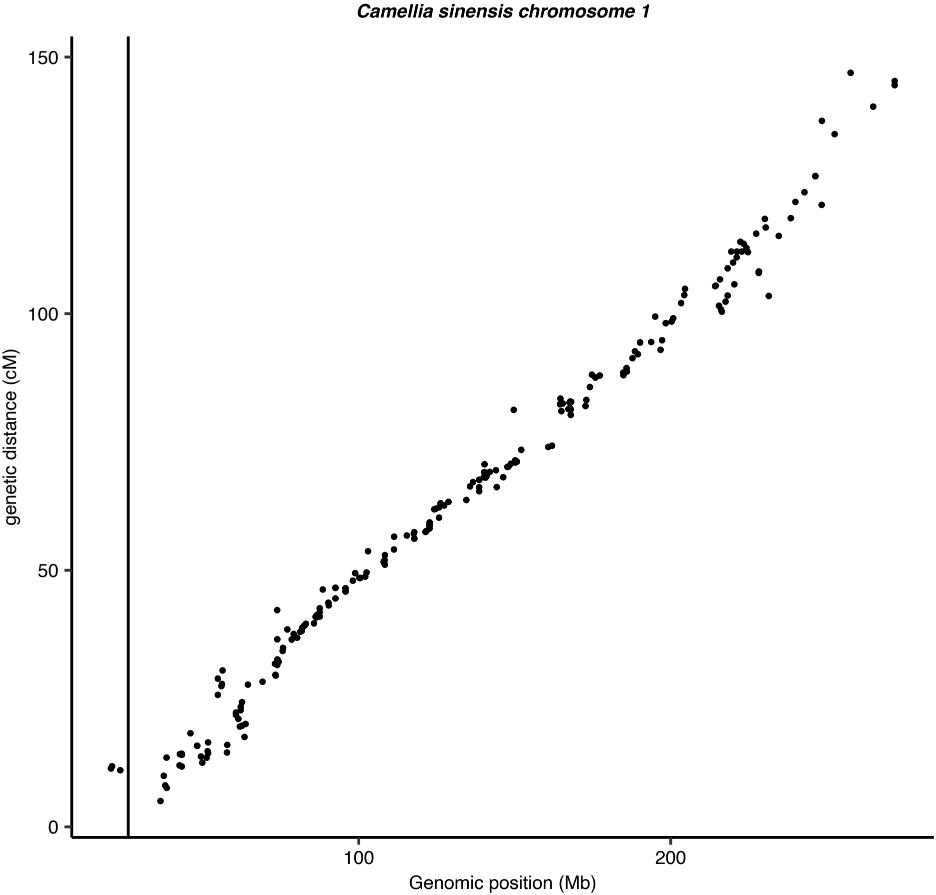
Camellia sinensis chromosome 1
150
100
50
0
100
200
Genomic position (Mb)
genetic distance (cM)

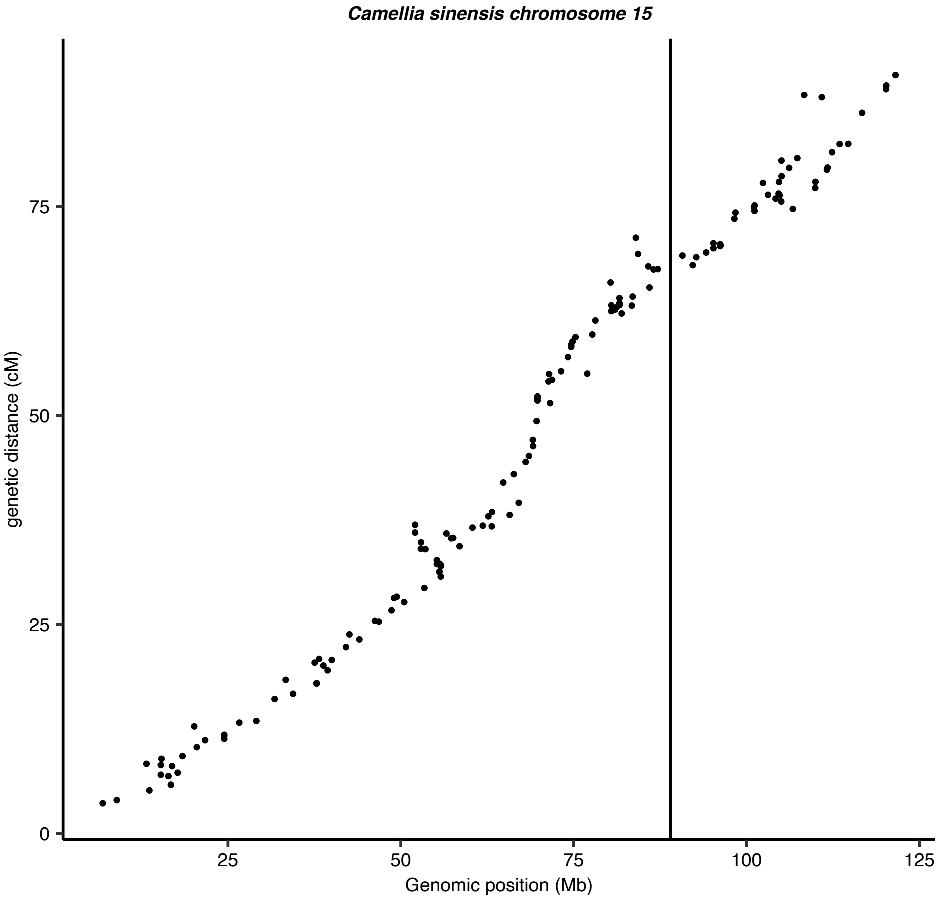

Camellia sinensis chromosome 15
genetic distance (cM)
0
25
50
75
Genomic position (Mb)
25
50
75
100
125

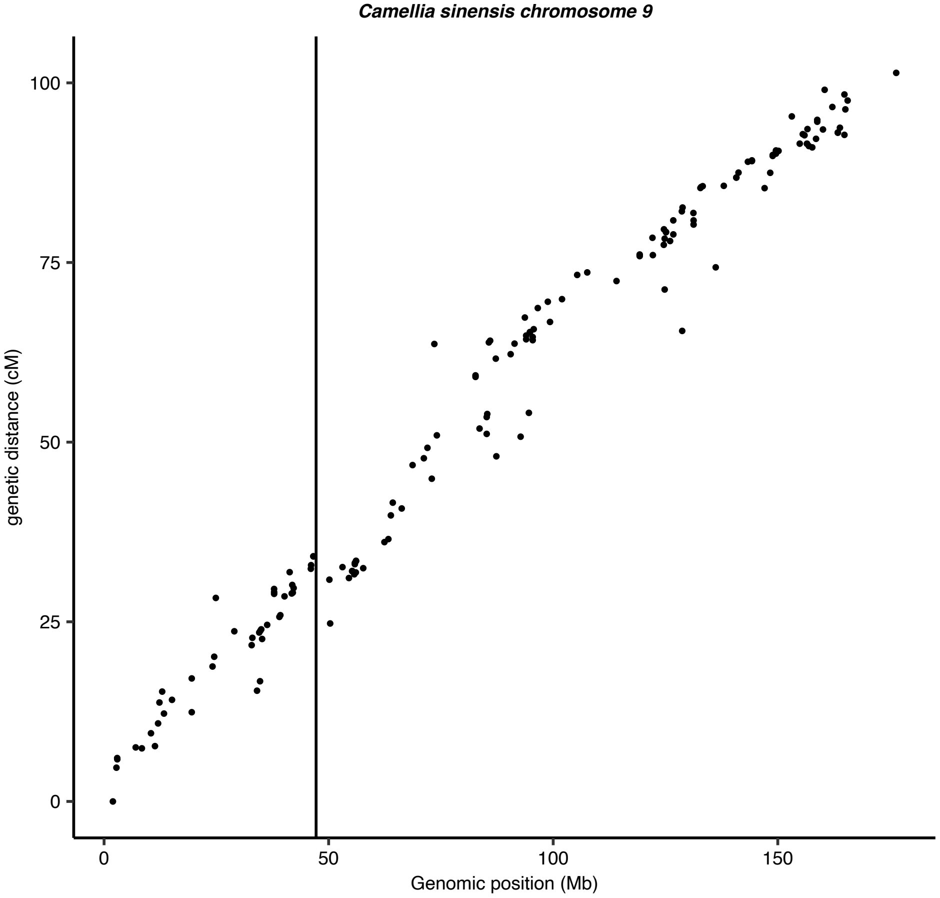

Camellia sinensis chromosome 9
genetic distance (cM)
0
25
50
75
100
Genomic position (Mb)
0
50
100
150

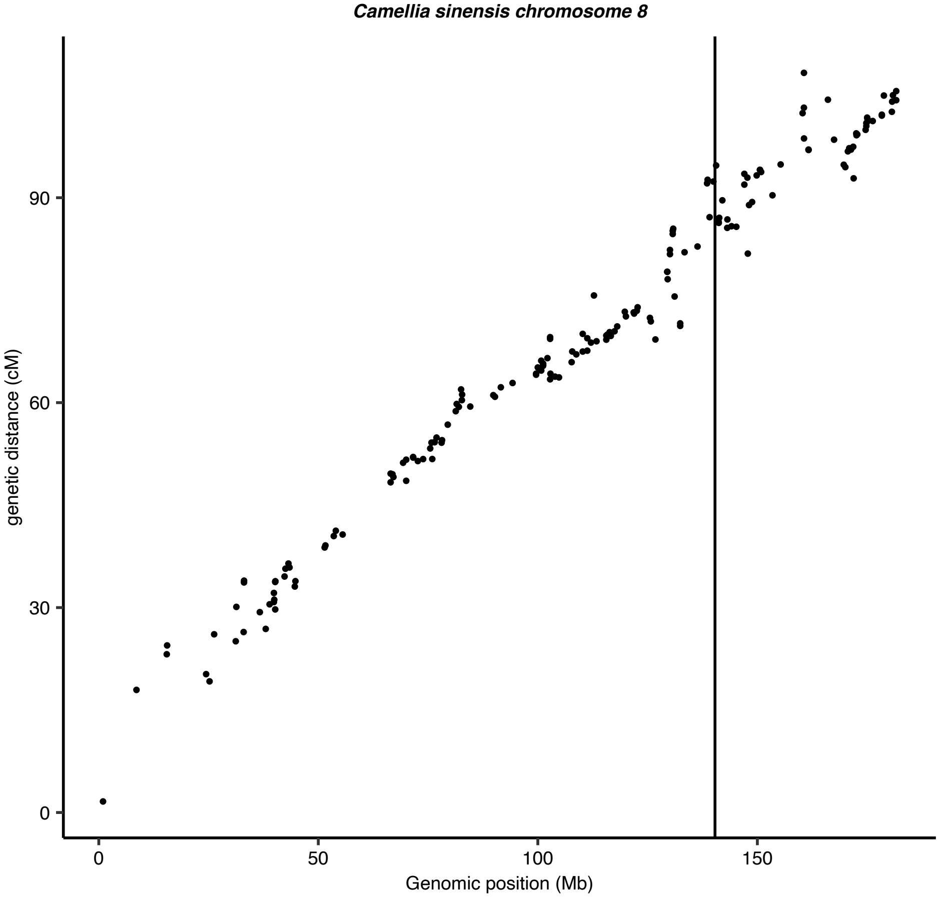
Camellia sinensis chromosome 8
genetic distance (cM)
0
30
60
90
Genomic position (Mb)
0
50
100
150

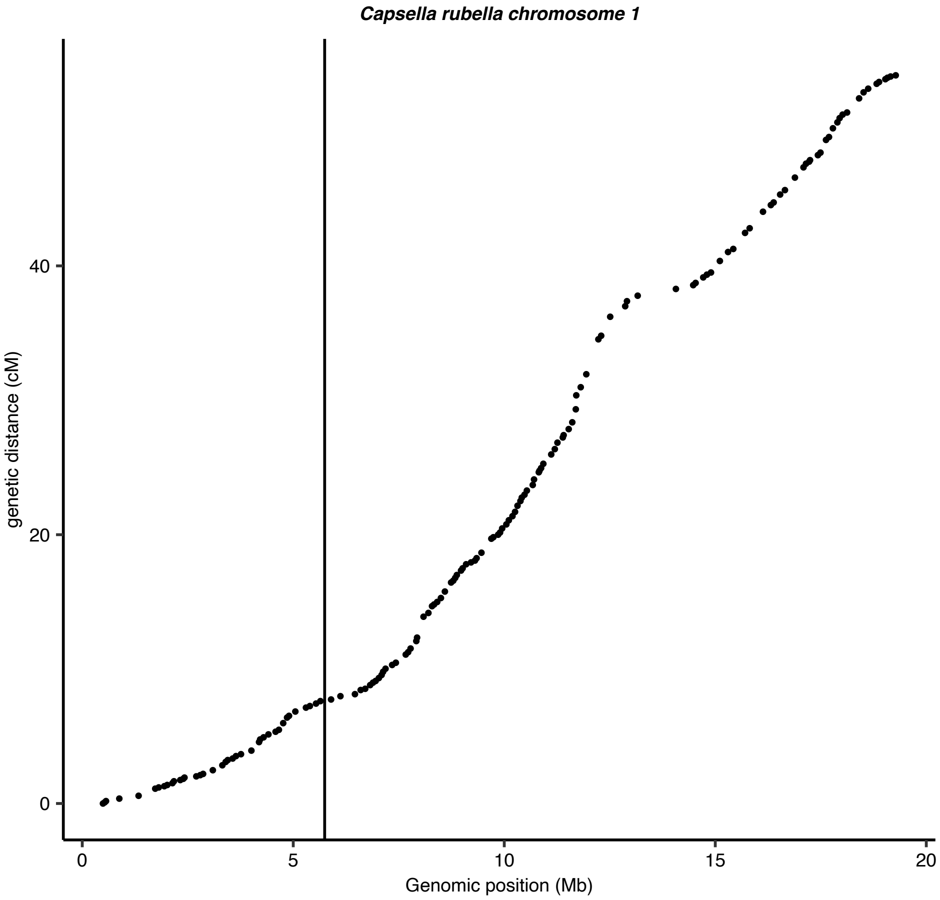
Capsella rubella chromosome 1
genetic distance (cM)
0
20
40
Genomic position (Mb)
0
5
10
15
20

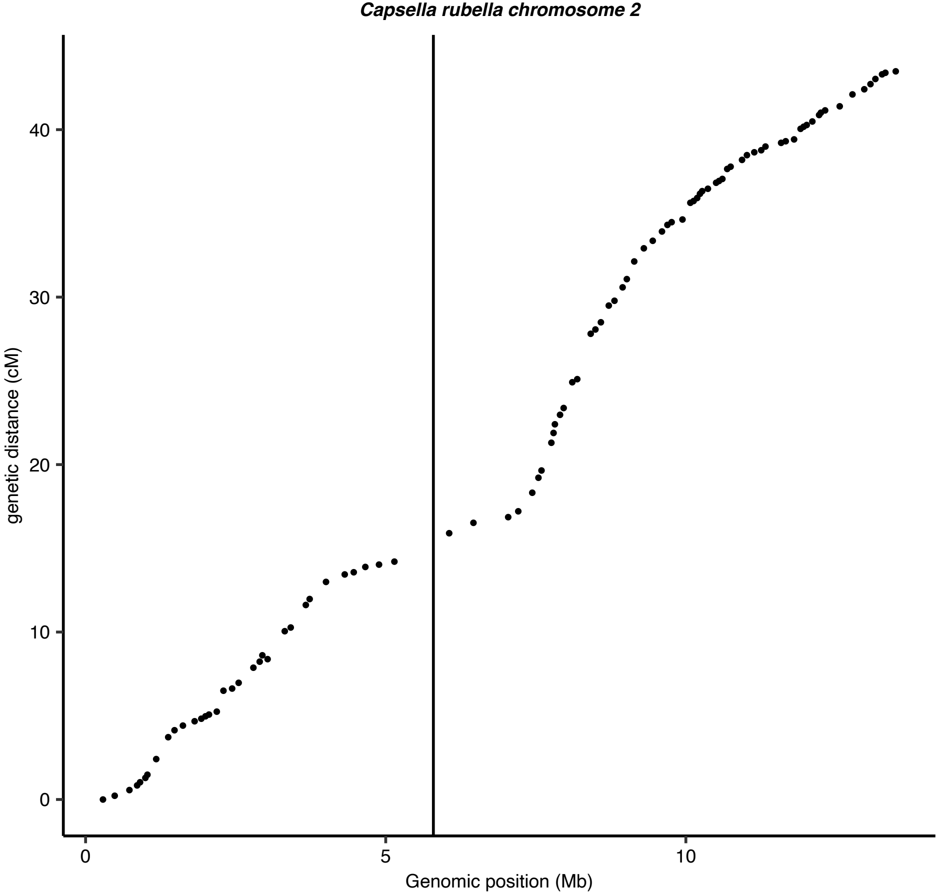
Capsella rubella chromosome 2
genetic distance (cM)
0
10
20
30
40
Genomic position (Mb)
0
5
10

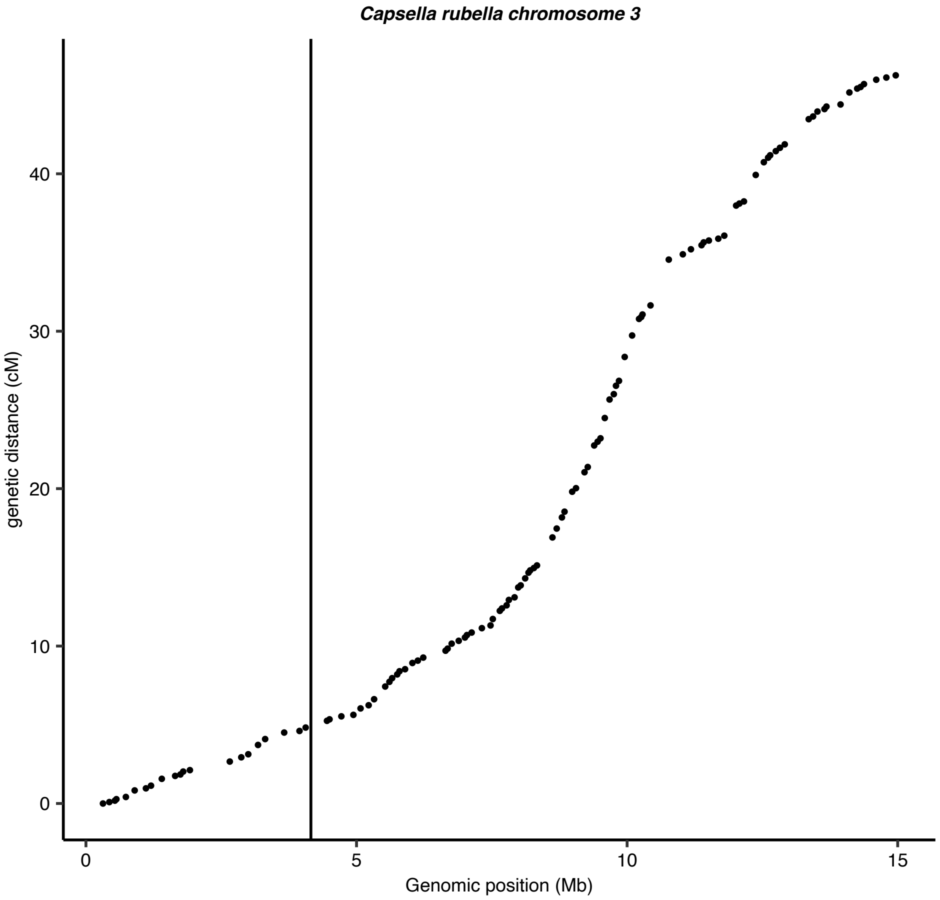
Capsella rubella chromosome 3
genetic distance (cM)
0
10
20
30
40
Genomic position (Mb)
0
5
10
15

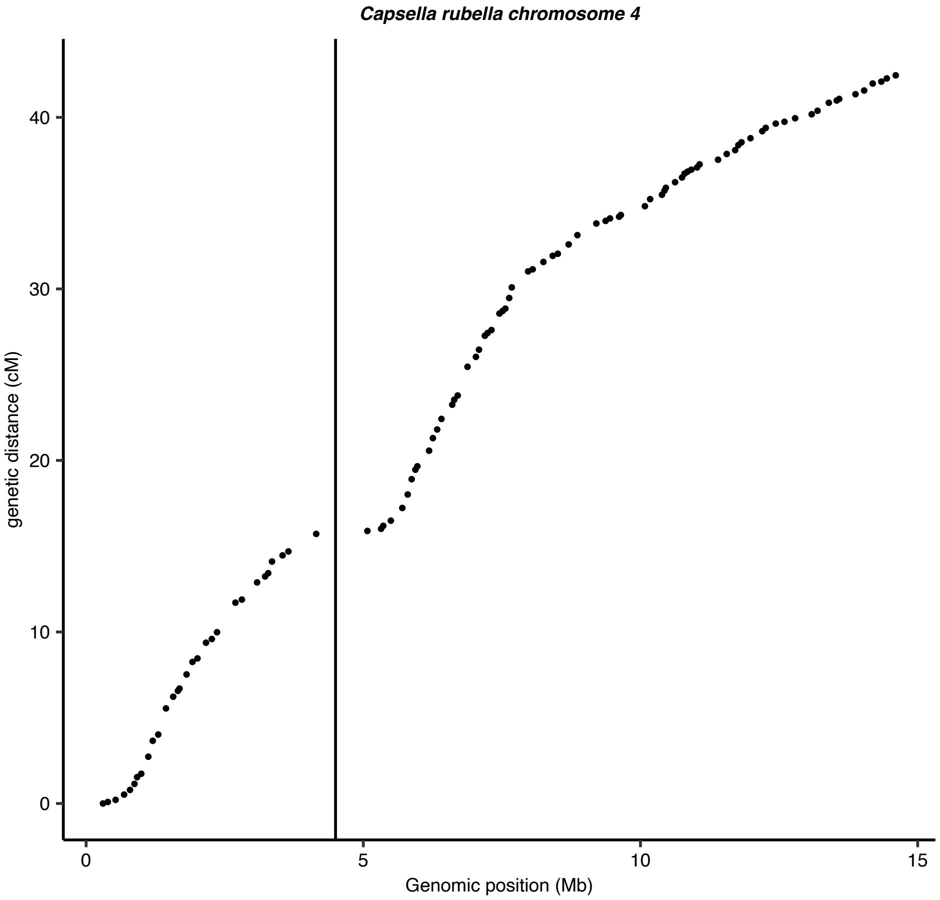
Capsella rubella chromosome 4
genetic distance (cM)
0
10
20
30
40
Genomic position (Mb)
0
5
10
15

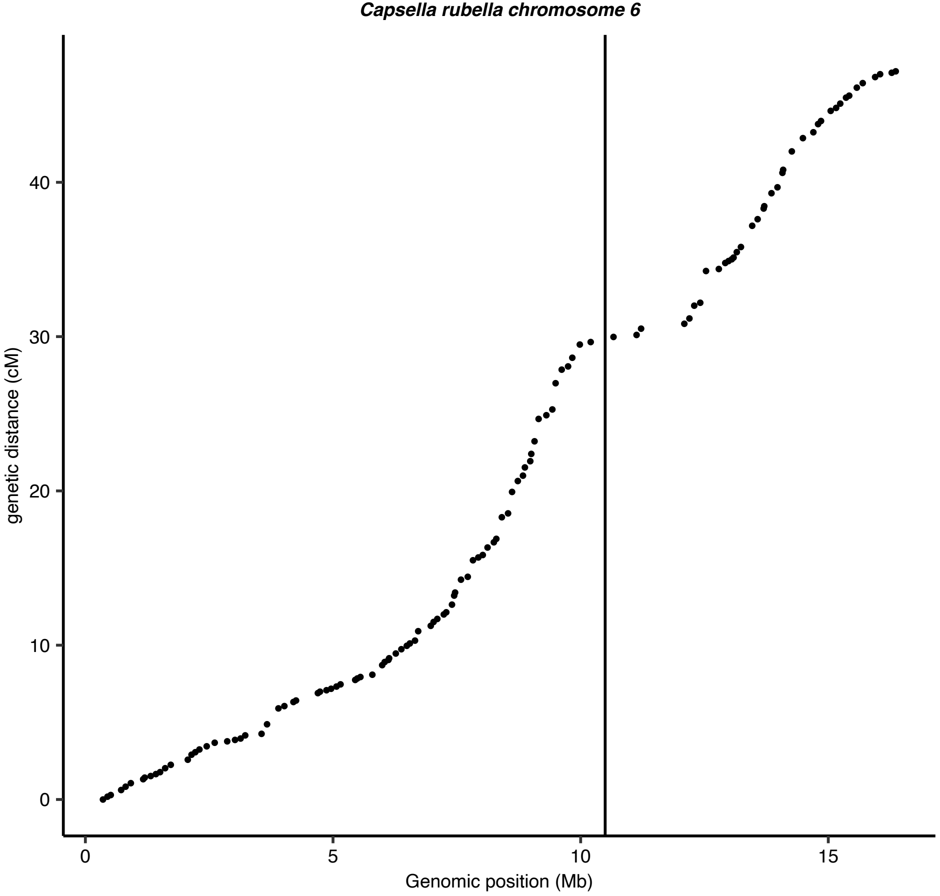

Capsella rubella chromosome 6
genetic distance (cM)
0
10
20
30
40
Genomic position (Mb)
0
5
10
15

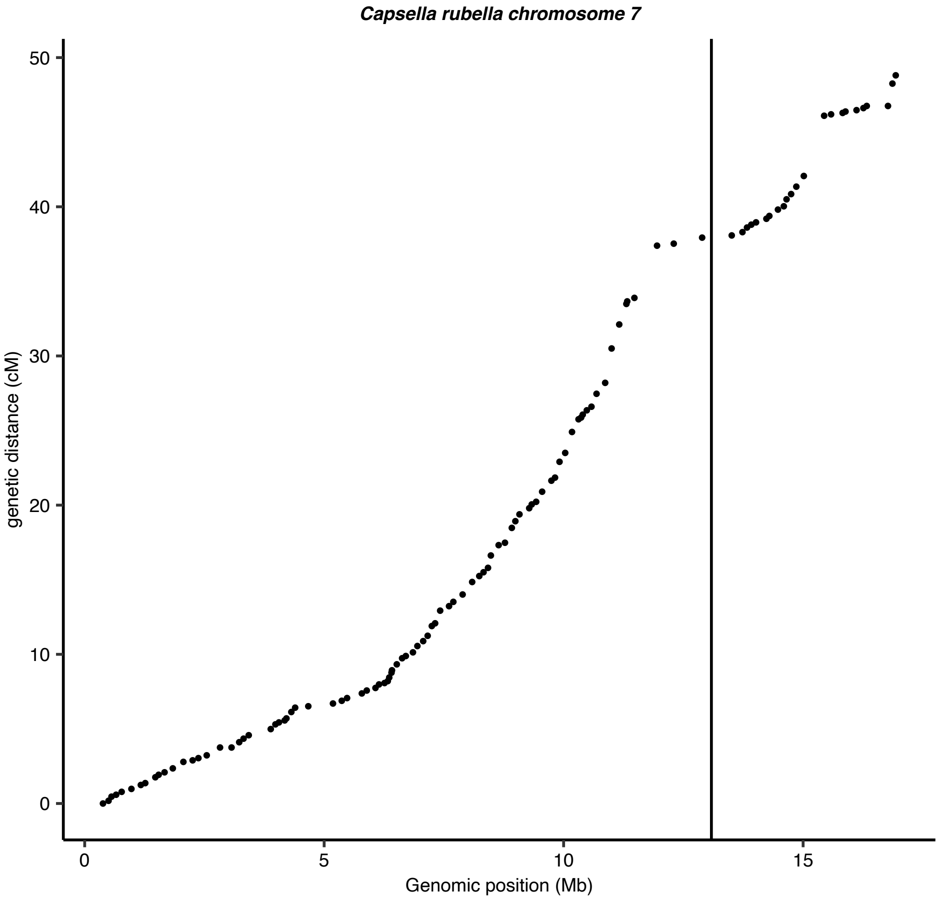
Capsella rubella chromosome 7
genetic distance (cM)
Genomic position (Mb)
0
10
20
30
40
50
0
5
10
15

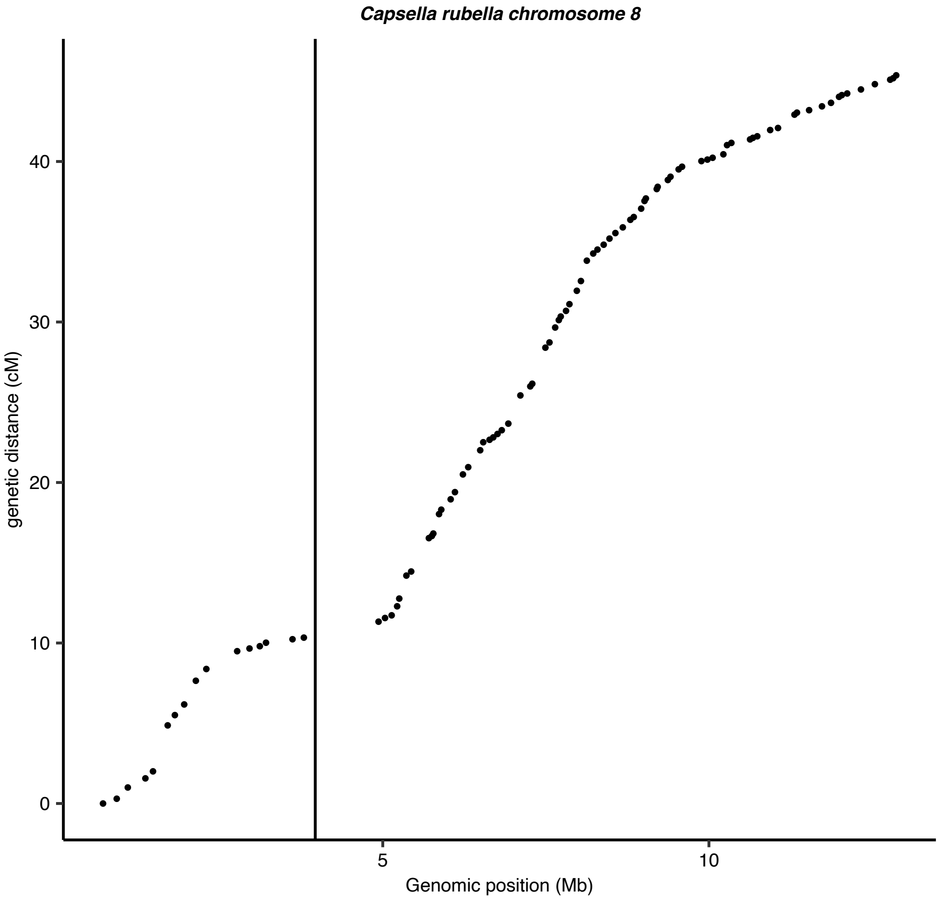
Capsella rubella chromosome 8
genetic distance (cM)
0
10
20
30
40
Genomic position (Mb)
5
10

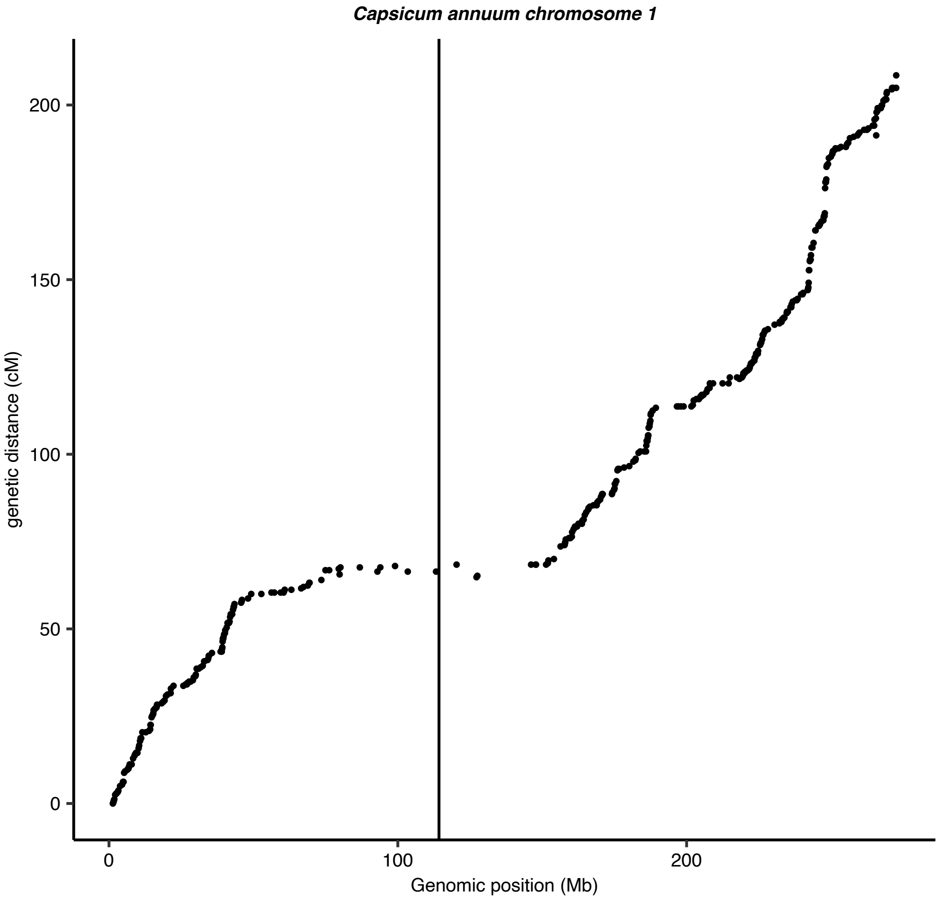
Capsicum annuum chromosome 1
genetic distance (cM)
Genomic position (Mb)
0
50
100
150
200
0
100
200

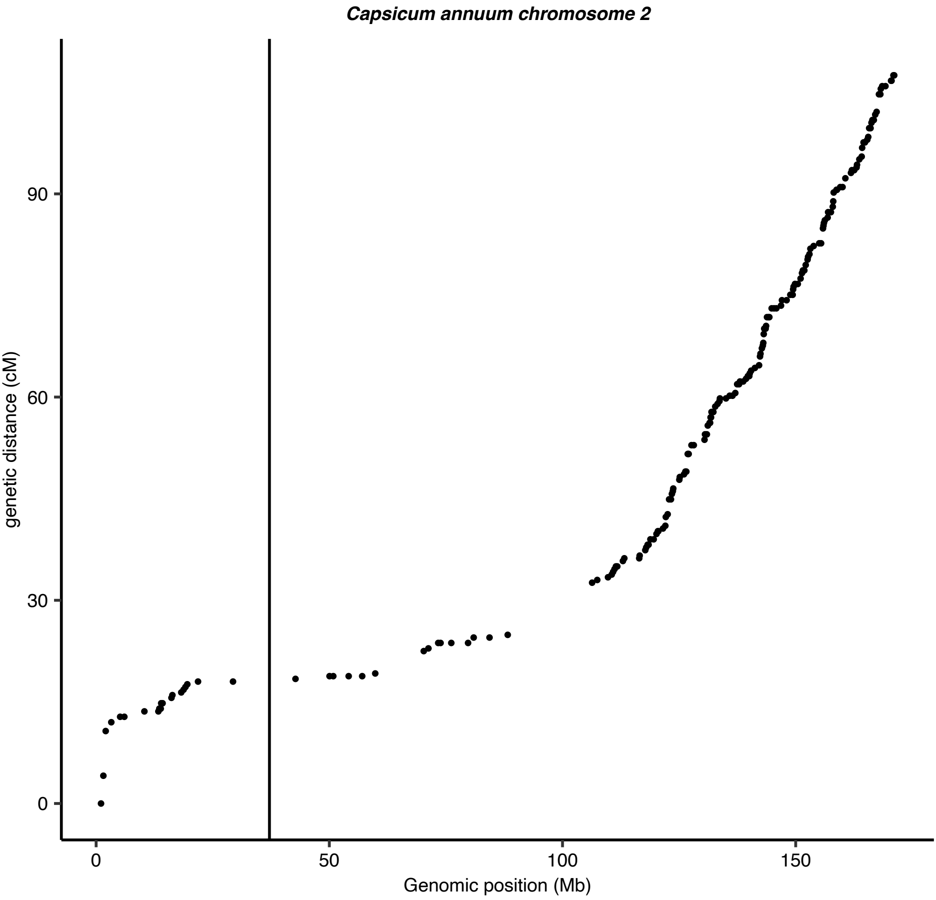
Capsicum annuum chromosome 2
genetic distance (cM)
0
30
60
90
Genomic position (Mb)
0
50
100
150

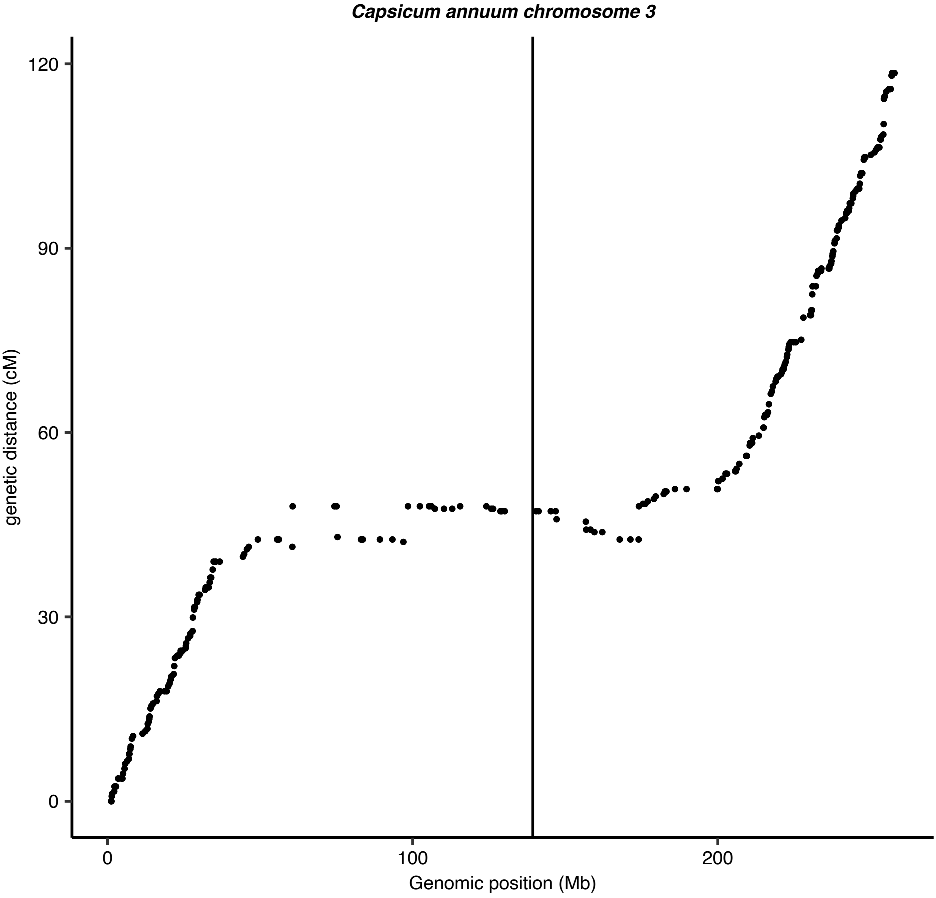
Capsicum annuum chromosome 3
genetic distance (cM)
120
90
60
30
0
0
100
200
Genomic position (Mb)

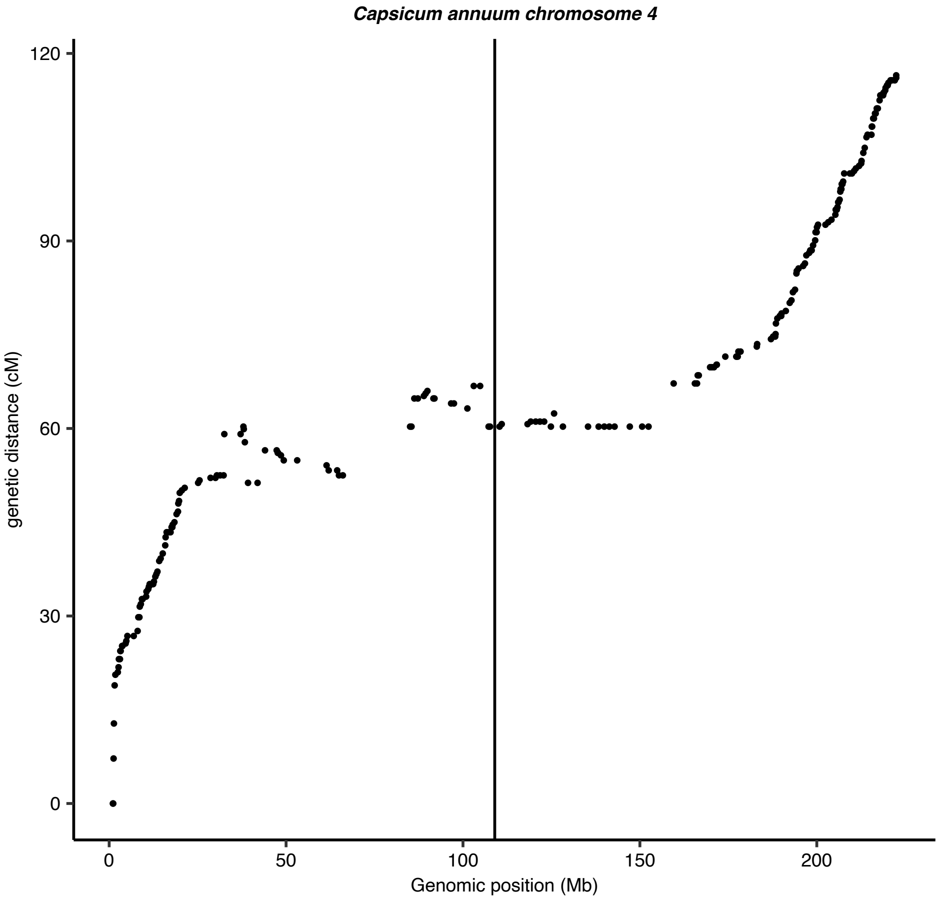
Capsicum annuum chromosome 4
genetic distance (cM)
120
90
60
30
0
0
50
100
150
200
Genomic position (Mb)

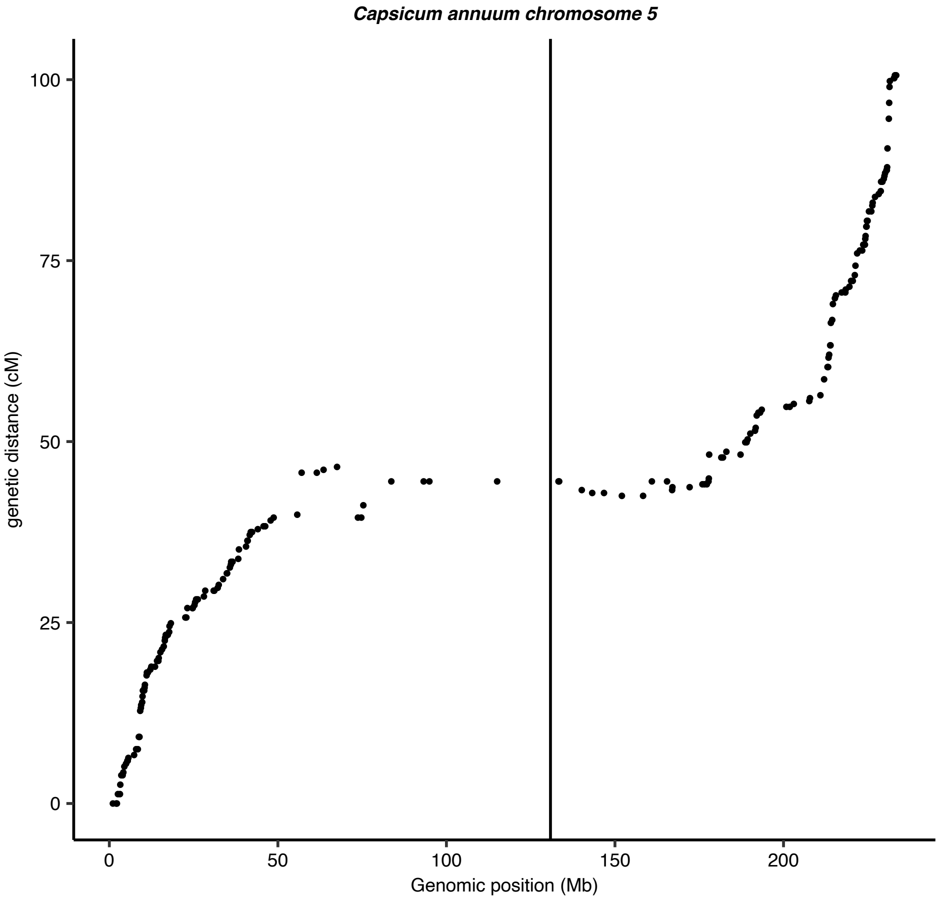
Capsicum annuum chromosome 5
genetic distance (cM)
Genomic position (Mb)
0
25
50
75
100
0
50
100
150
200

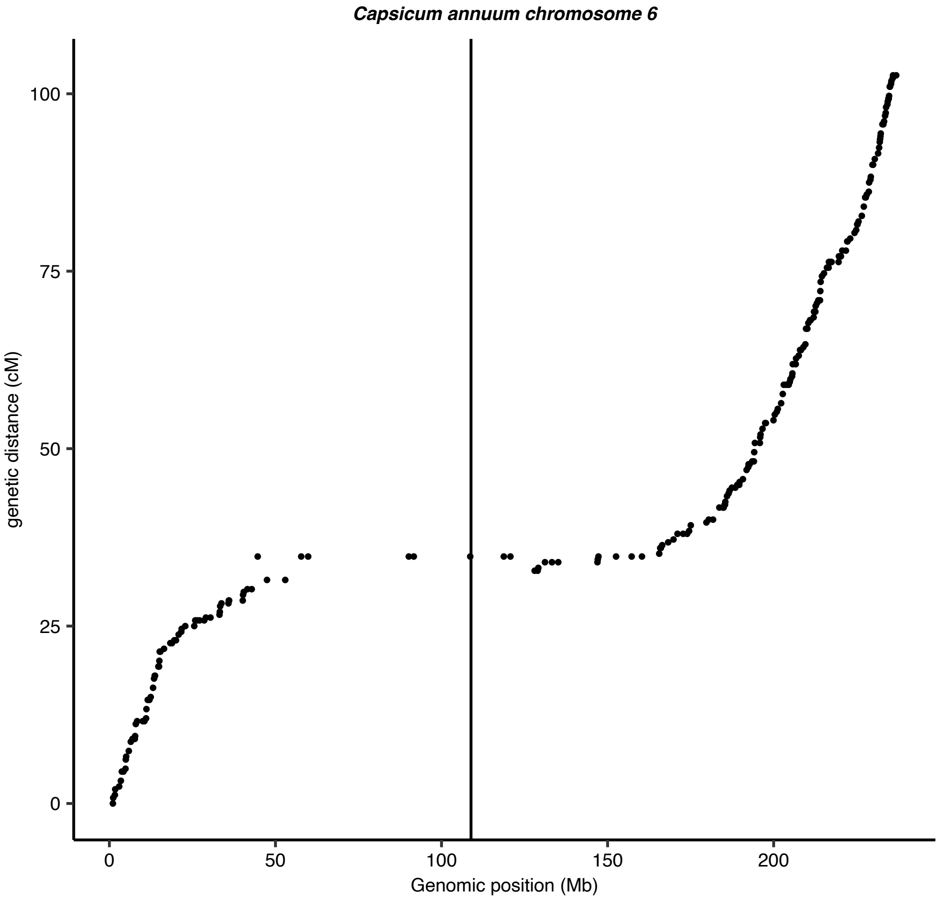
Capsicum annuum chromosome 6
genetic distance (cM)
100
75
50
25
0
0
50
100
150
200
Genomic position (Mb)

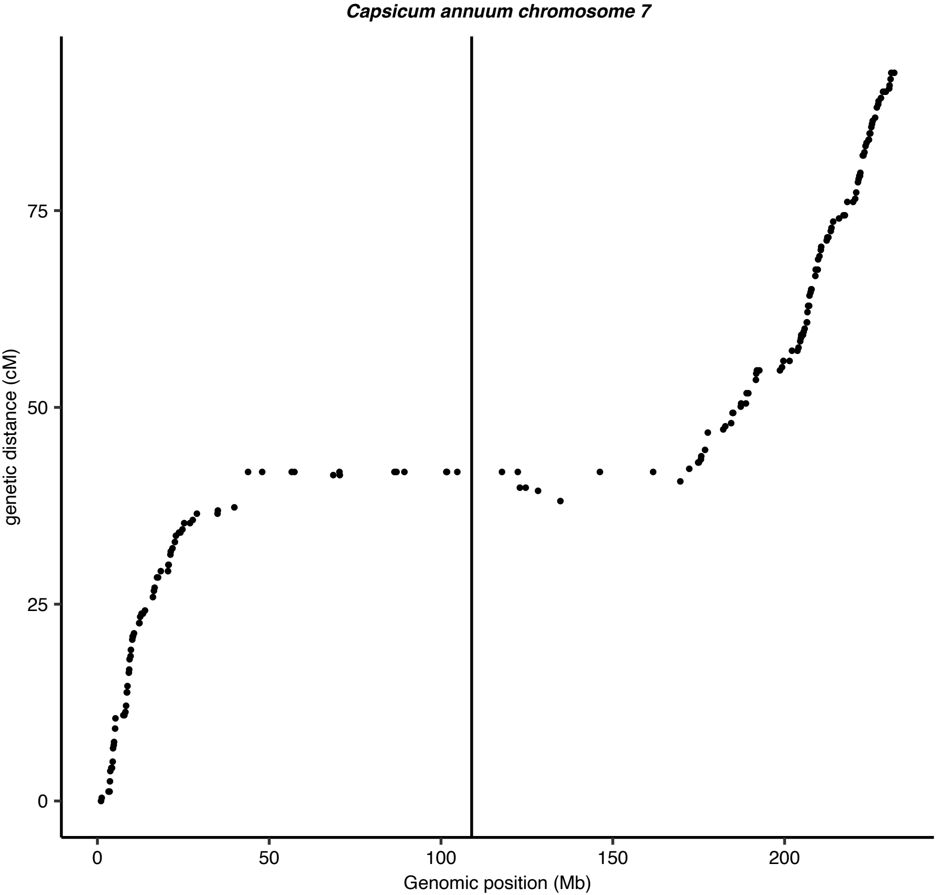
Capsicum annuum chromosome 7
genetic distance (cM)
Genomic position (Mb)
0
25
50
75
0
50
100
150
200

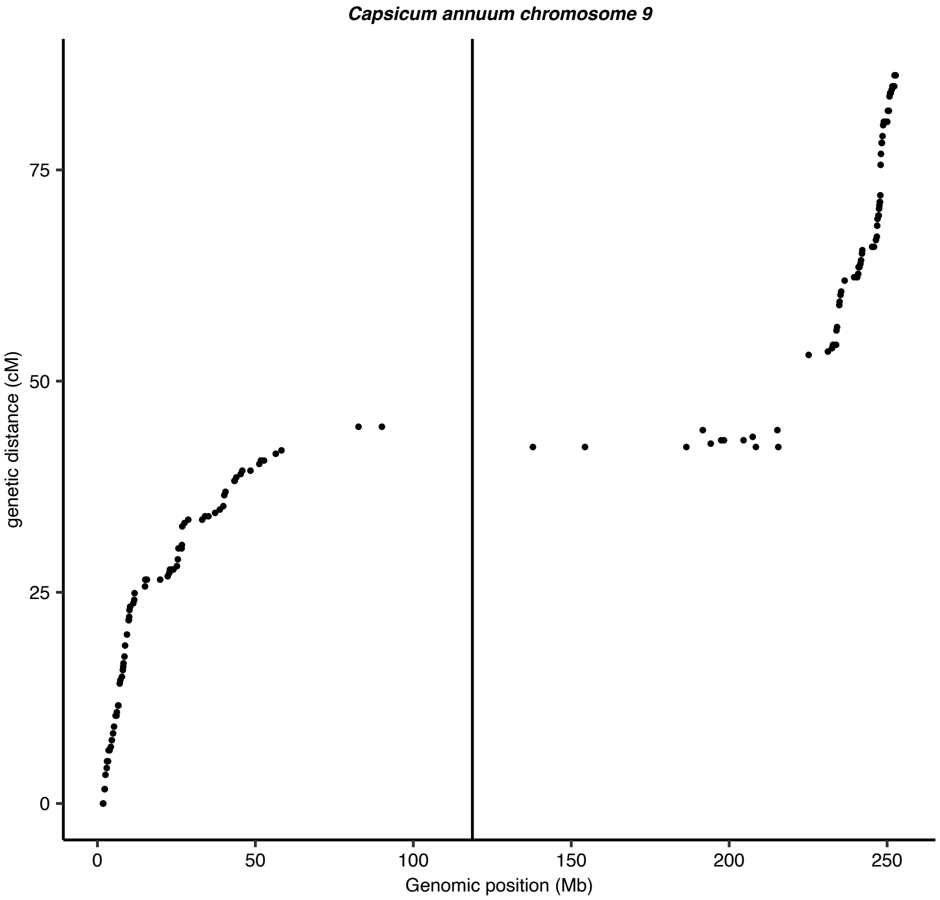
Capsicum annuum chromosome 9
genetic distance (cM)
0
25
50
75
Genomic position (Mb)
0
50
100
150
200
250

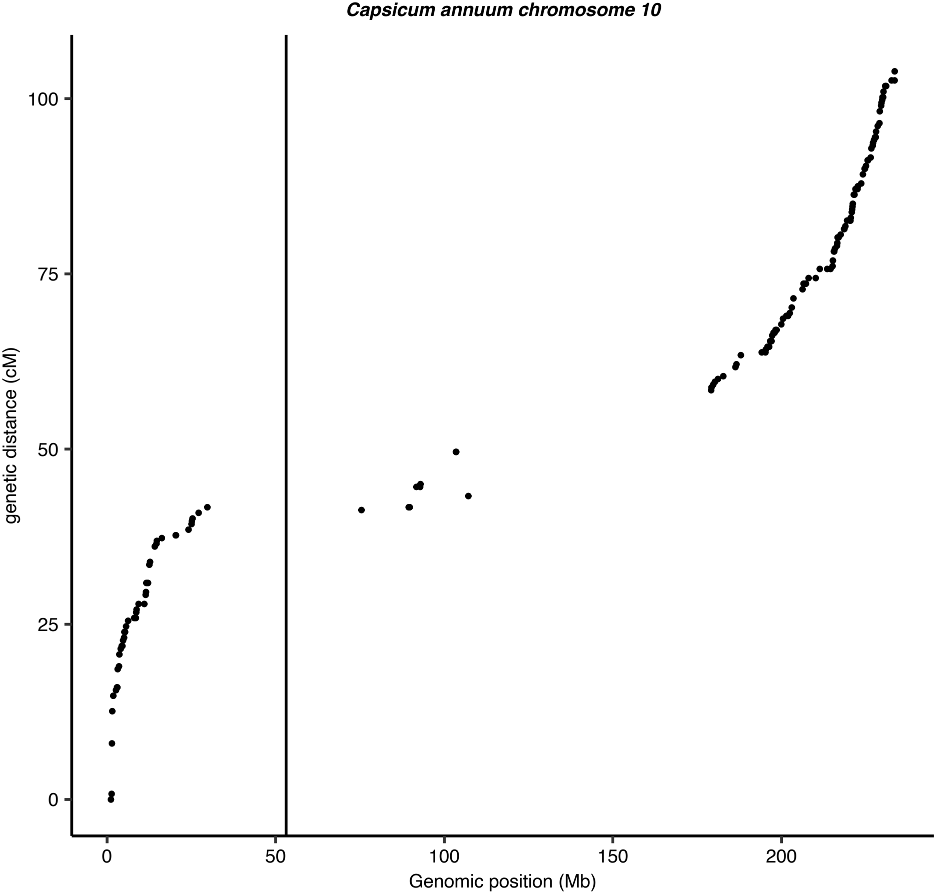
Capsicum annuum chromosome 10
genetic distance (cM)
0
25
50
75
100
0
50
100
150
200
Genomic position (Mb)

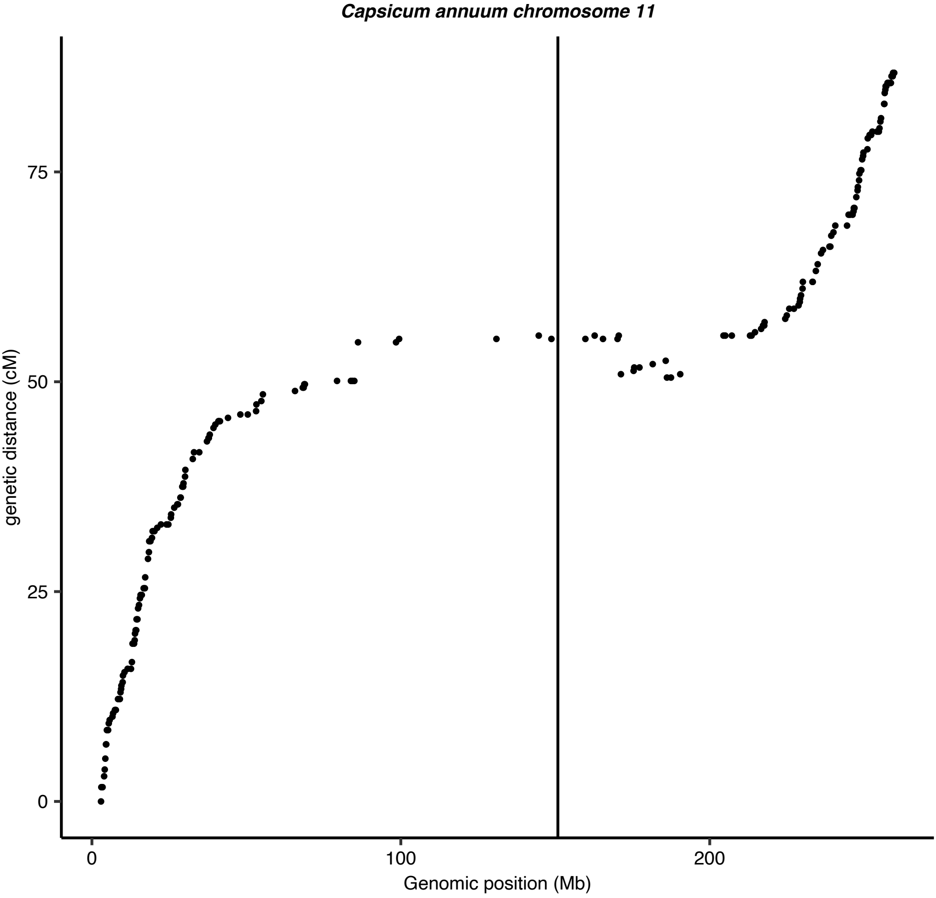
Capsicum annuum chromosome 11
genetic distance (cM)
0
25
50
75
Genomic position (Mb)
0
100
200

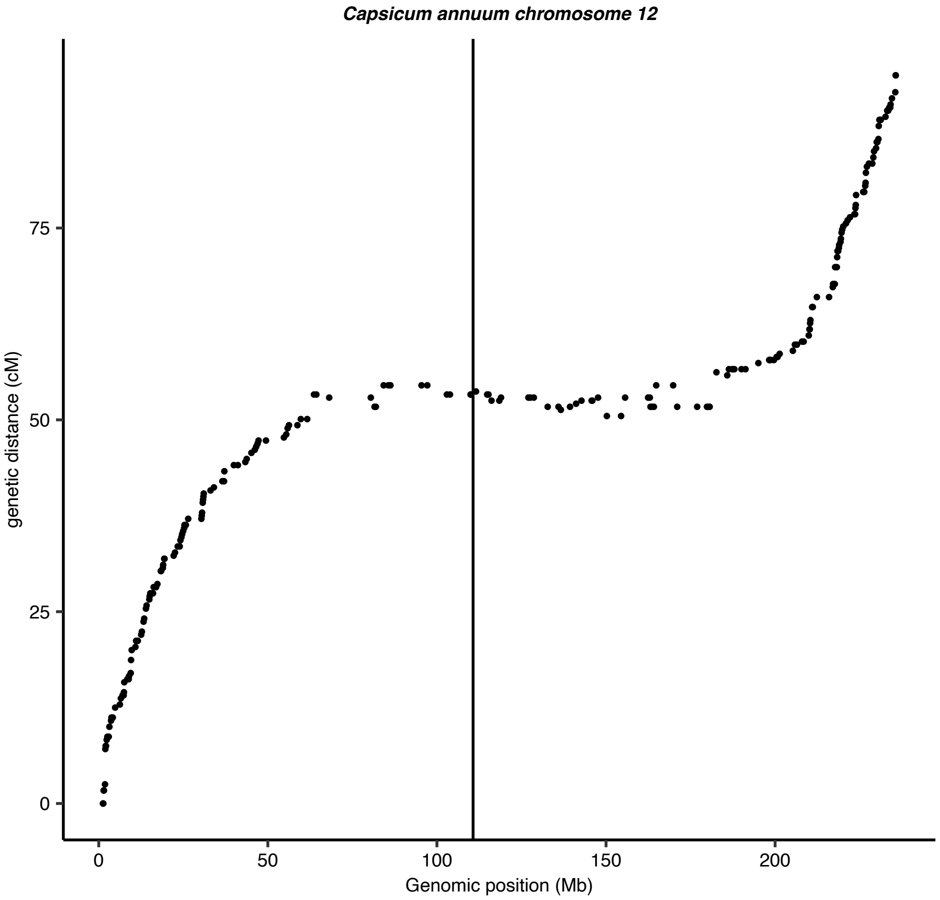
Capsicum annuum chromosome 12
genetic distance (cM)
Genomic position (Mb)
0
25
50
75
0
50
100
150
200

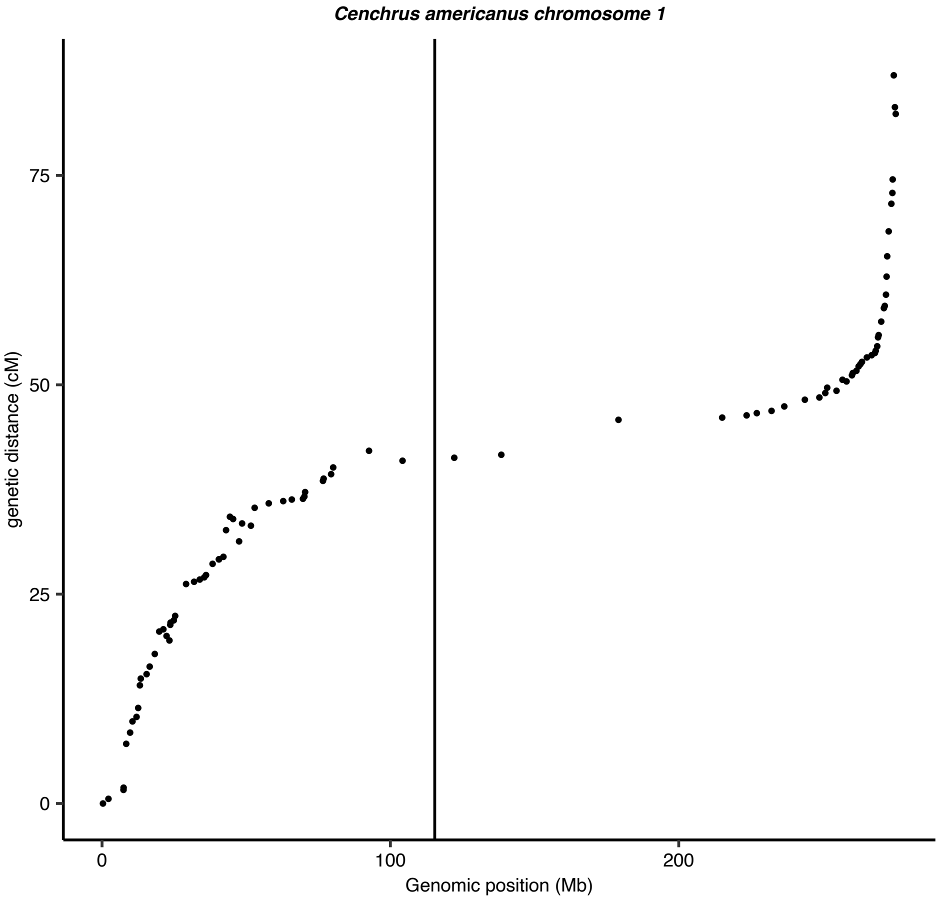
Cenchrus americanus chromosome 1
genetic distance (cM)
0
25
50
75
0
100
200
Genomic position (Mb)

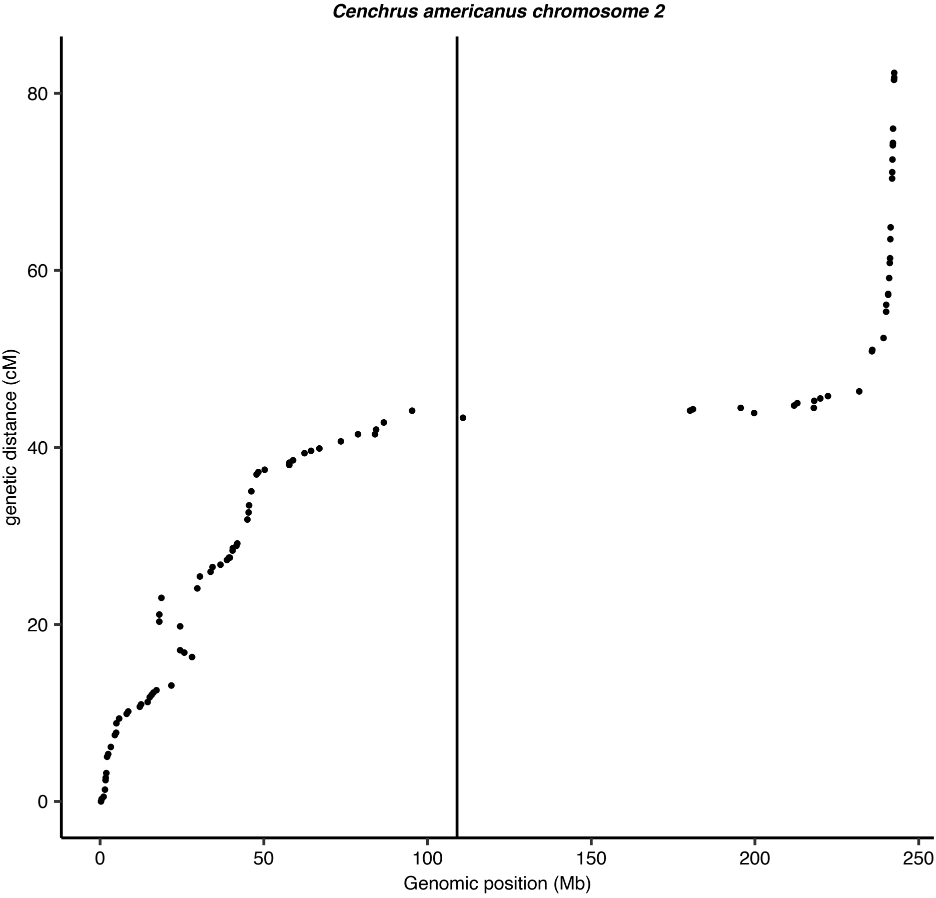

Cenchrus americanus chromosome 2
genetic distance (cM)
0
20
40
60
80
Genomic position (Mb)
0
50
100
150
200
250

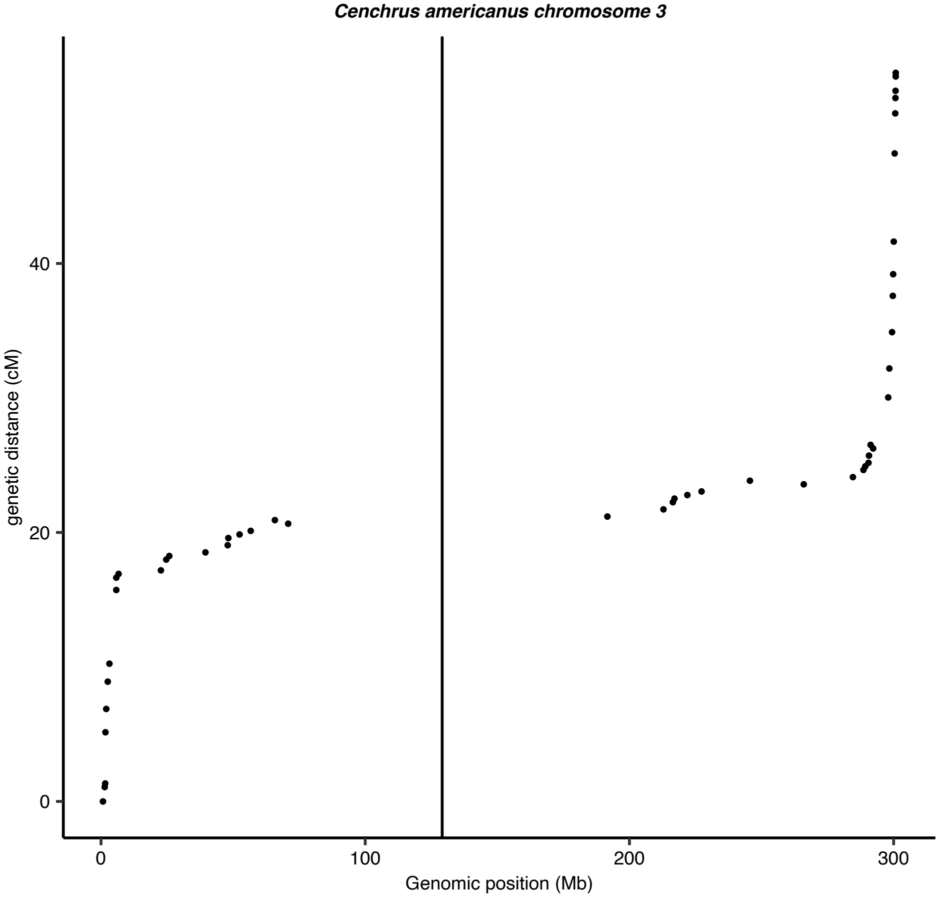
Cenchrus americanus chromosome 3
genetic distance (cM)
0
20
40
0
100
200
300
Genomic position (Mb)

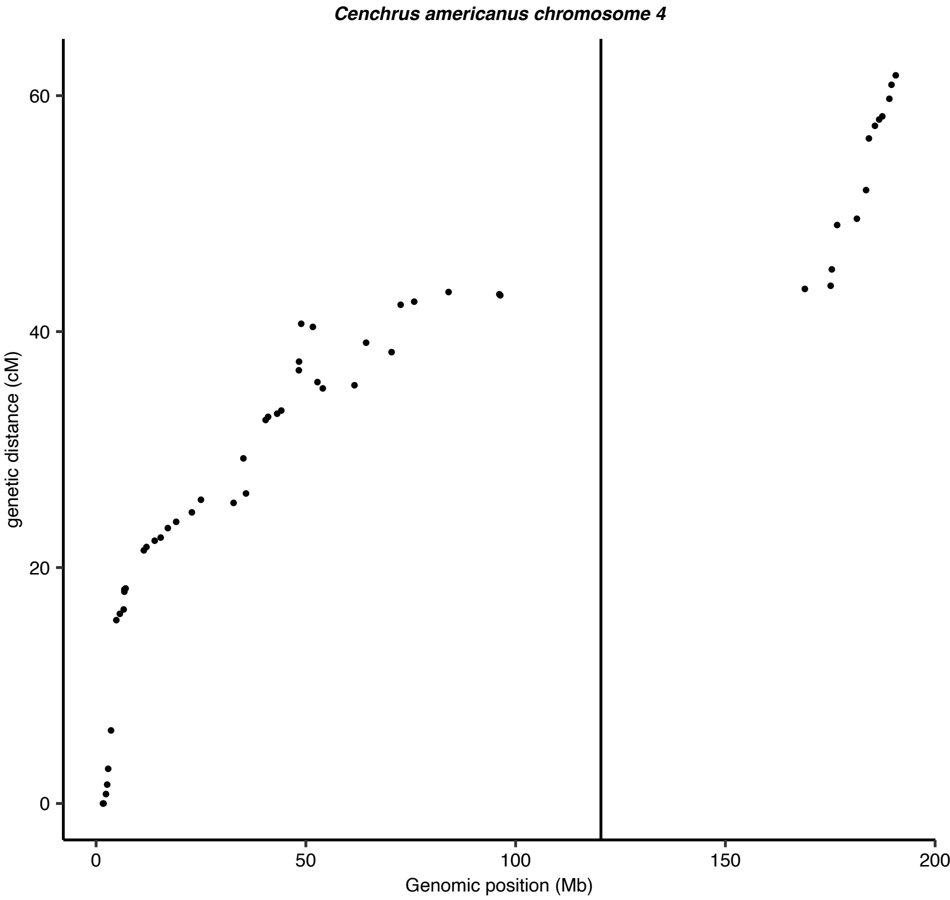
Cenchrus americanus chromosome 4
genetic distance (cM)
0
20
40
60
0
50
100
150
200
Genomic position (Mb)

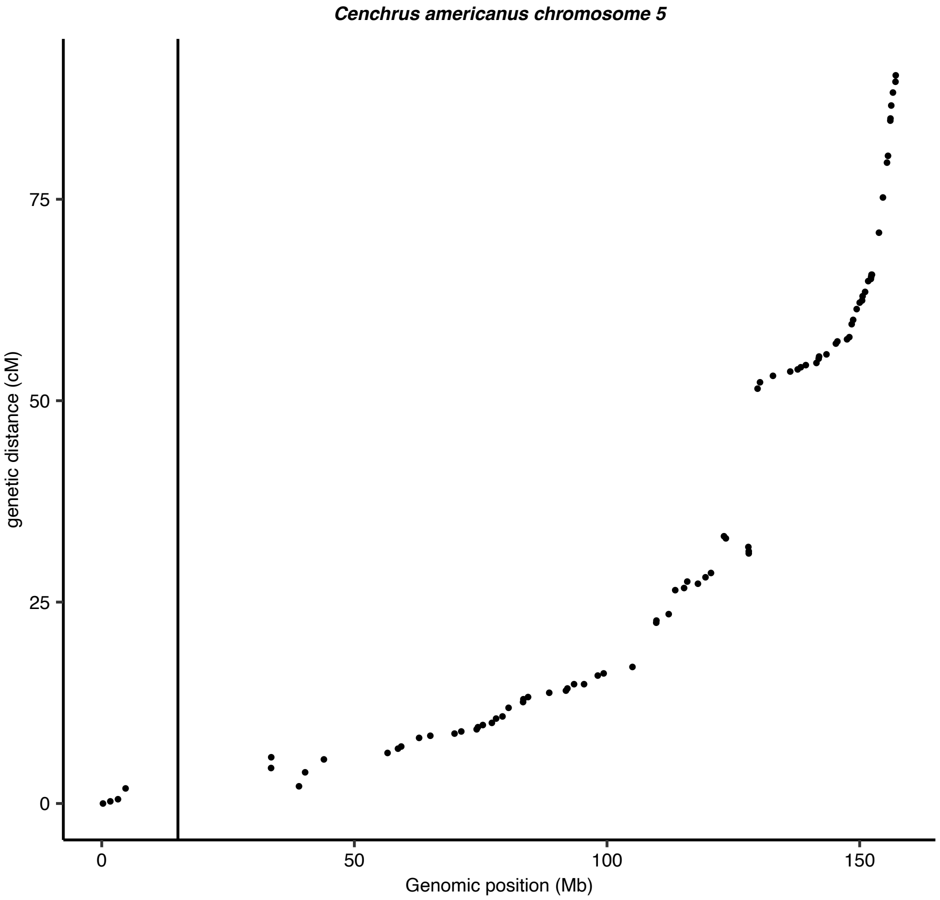
Cenchrus americanus chromosome 5
genetic distance (cM)
0
25
50
75
0
50
100
150
Genomic position (Mb)

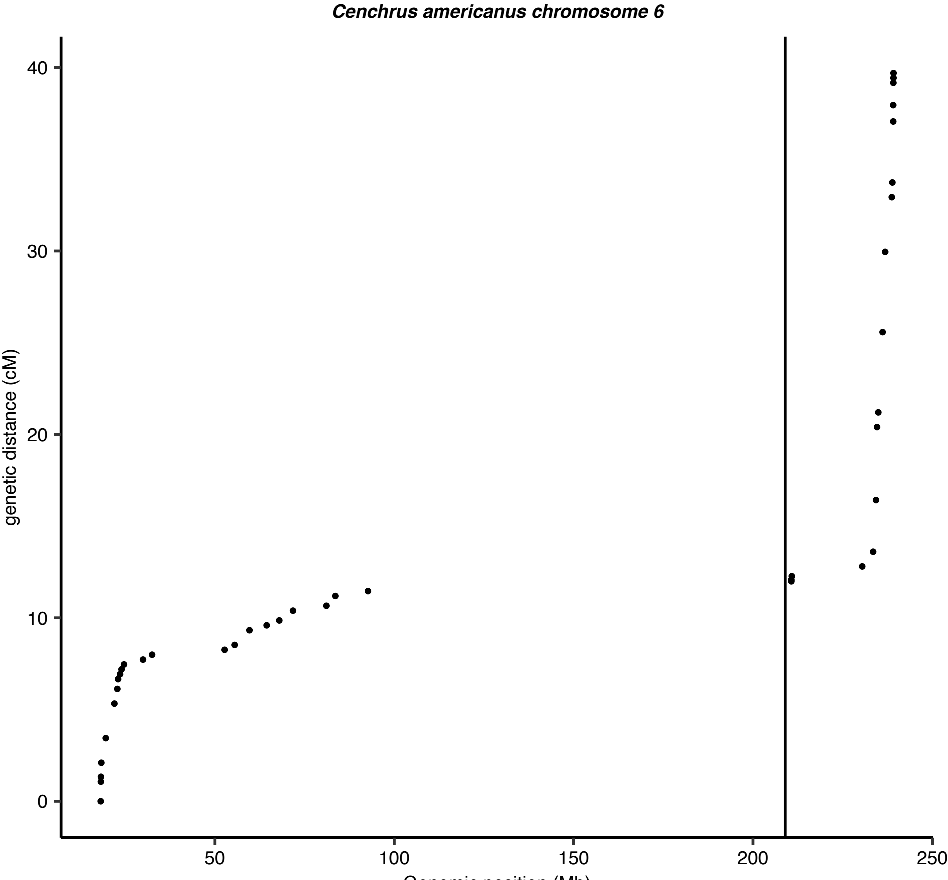

Cenchrus americanus chromosome 6
40
30
20
10
0
genetic distance (cM)
50
100
150
200
250
Genomic position (Mb)

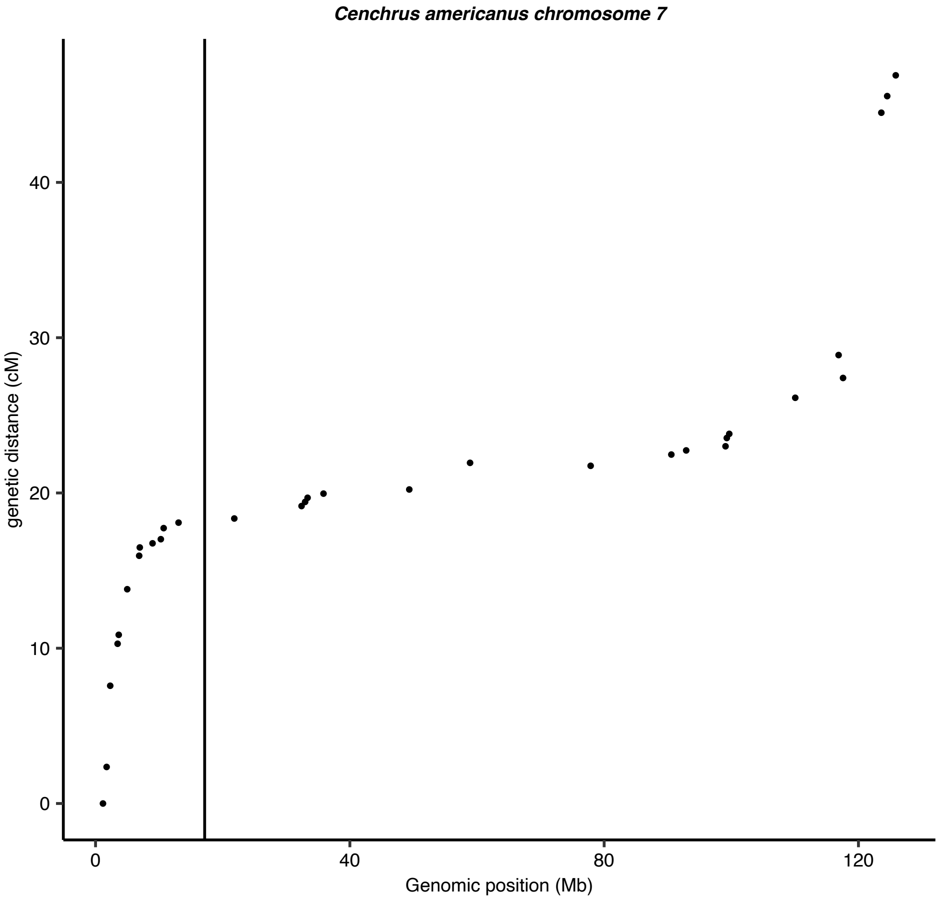
Cenchrus americanus chromosome 7
genetic distance (cM)
0
10
20
30
40
Genomic position (Mb)
0
40
80
120

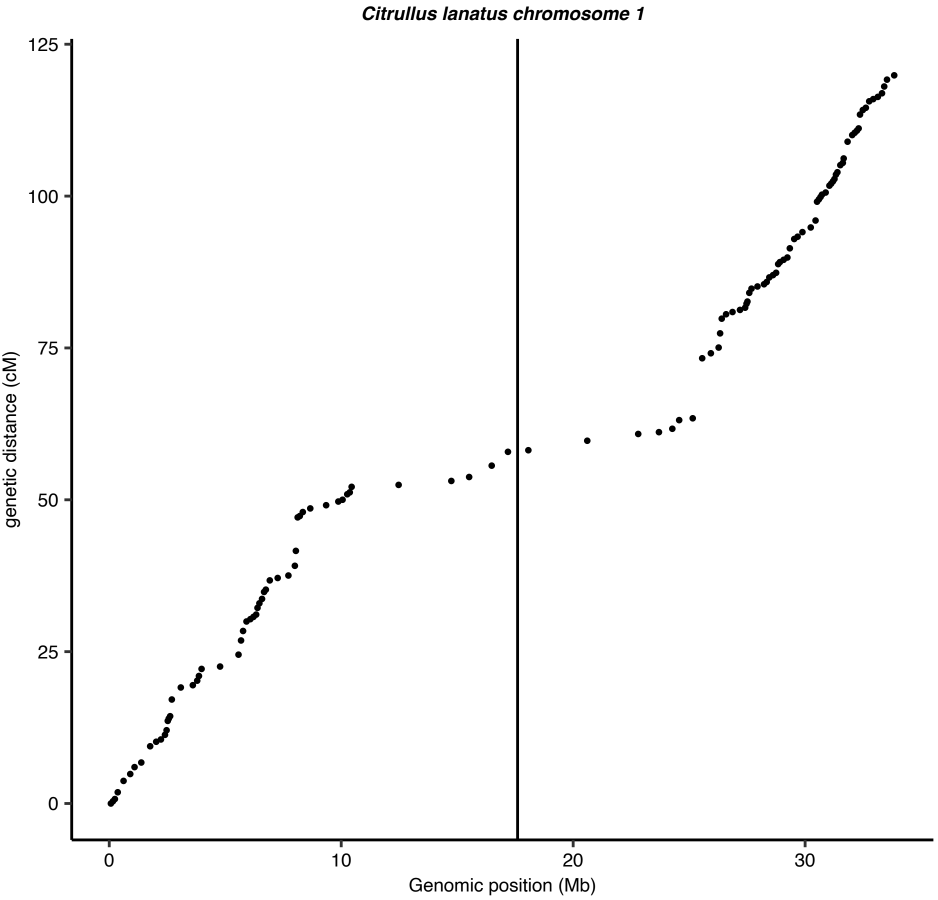
Citrullus lanatus chromosome 1
genetic distance (cM)
125
100
75
50
25
0
0
10
20
30
Genomic position (Mb)

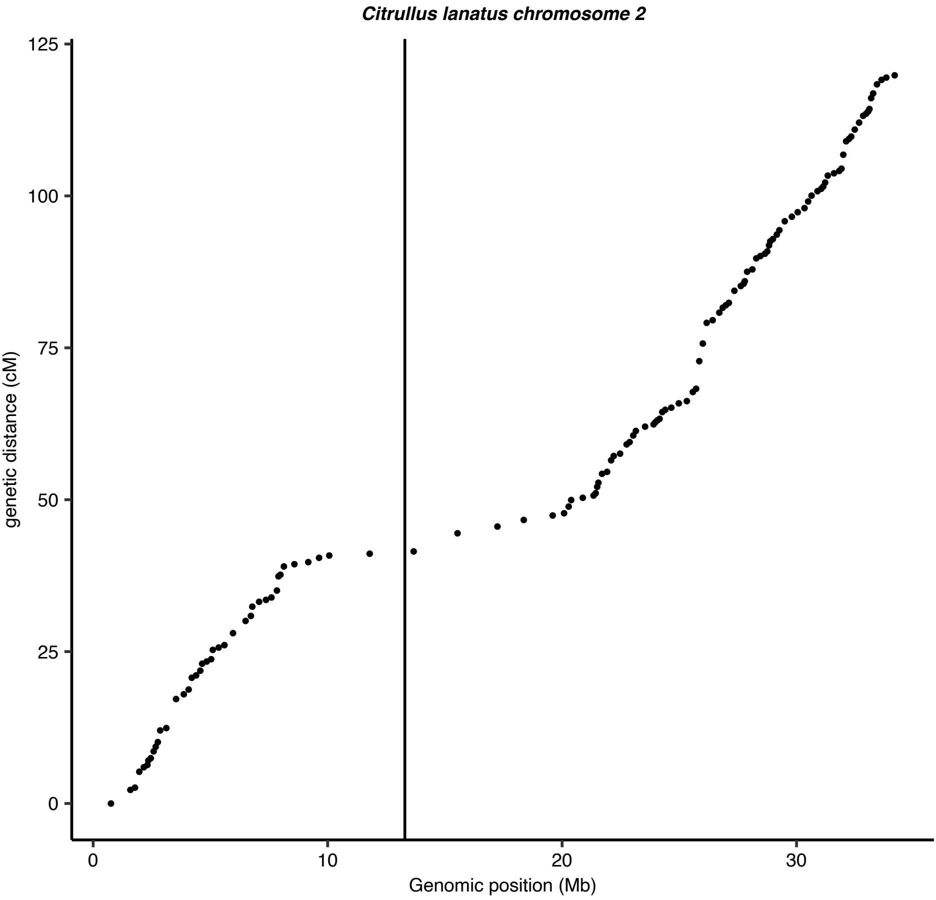
Citrullus lanatus chromosome 2
genetic distance (cM)
0
25
50
75
100
125
Genomic position (Mb)
0
10
20
30

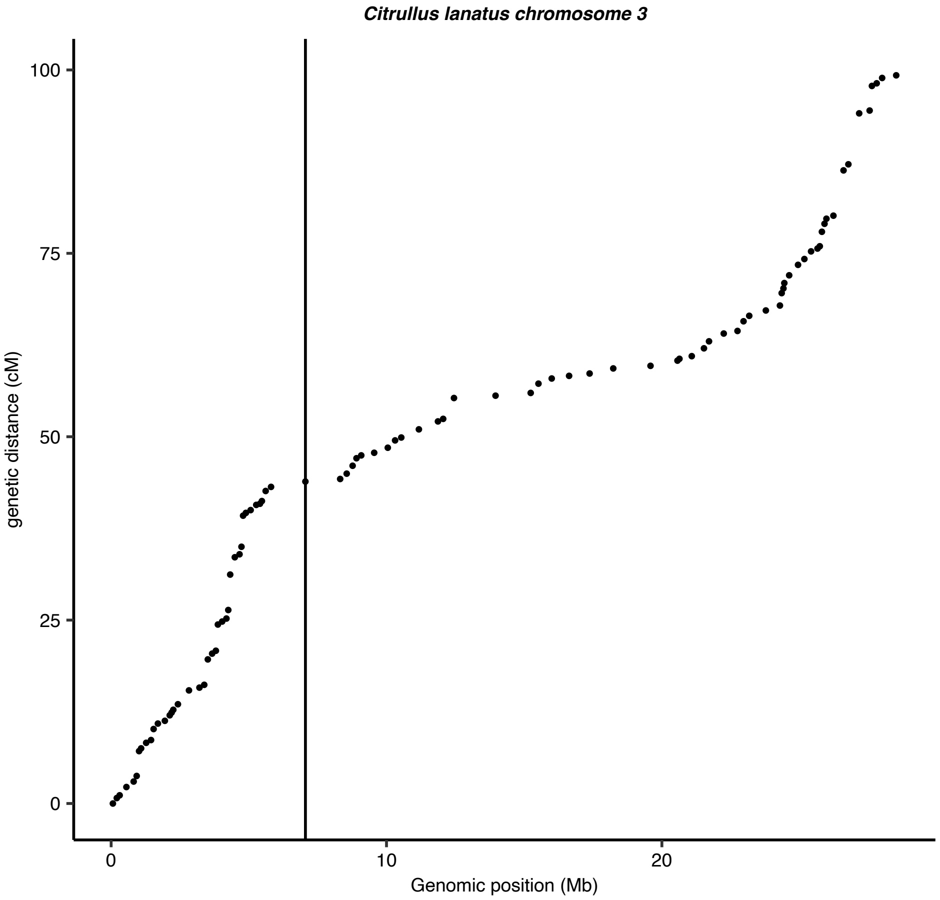
Citrullus lanatus chromosome 3
genetic distance (cM)
100
75
50
25
0
0
10
20
Genomic position (Mb)

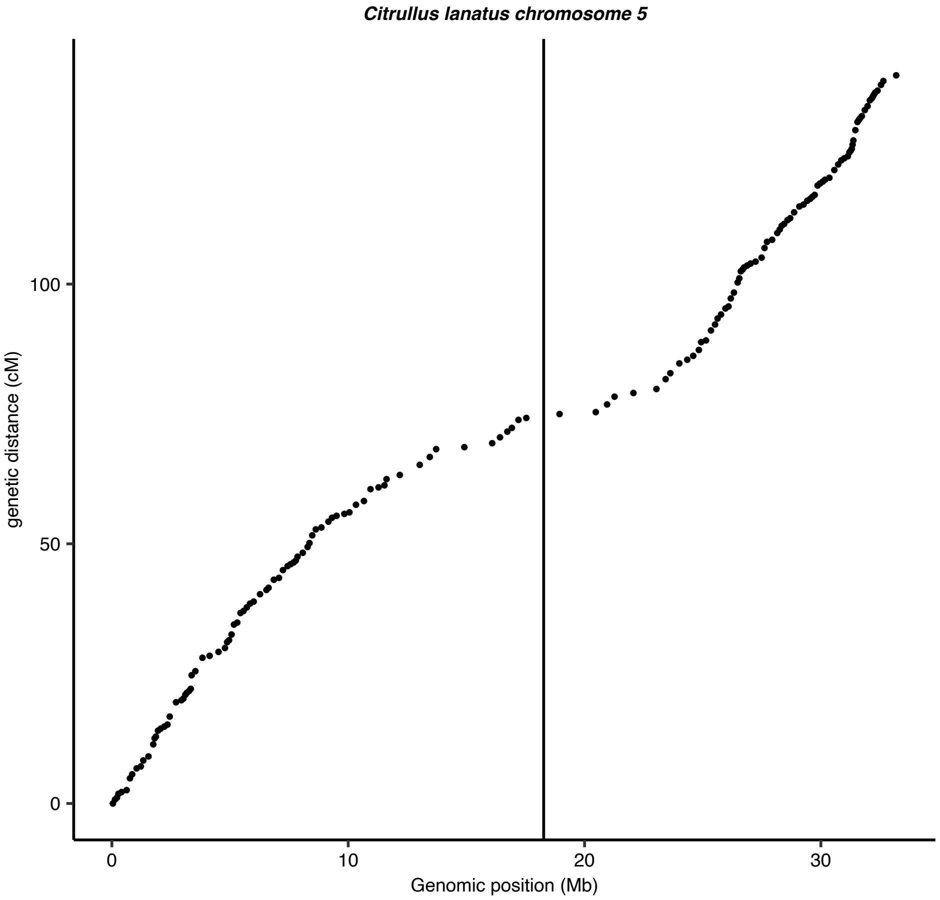
Citrullus lanatus chromosome 5
genetic distance (cM)
100
50
0
0
10
20
30
Genomic position (Mb)

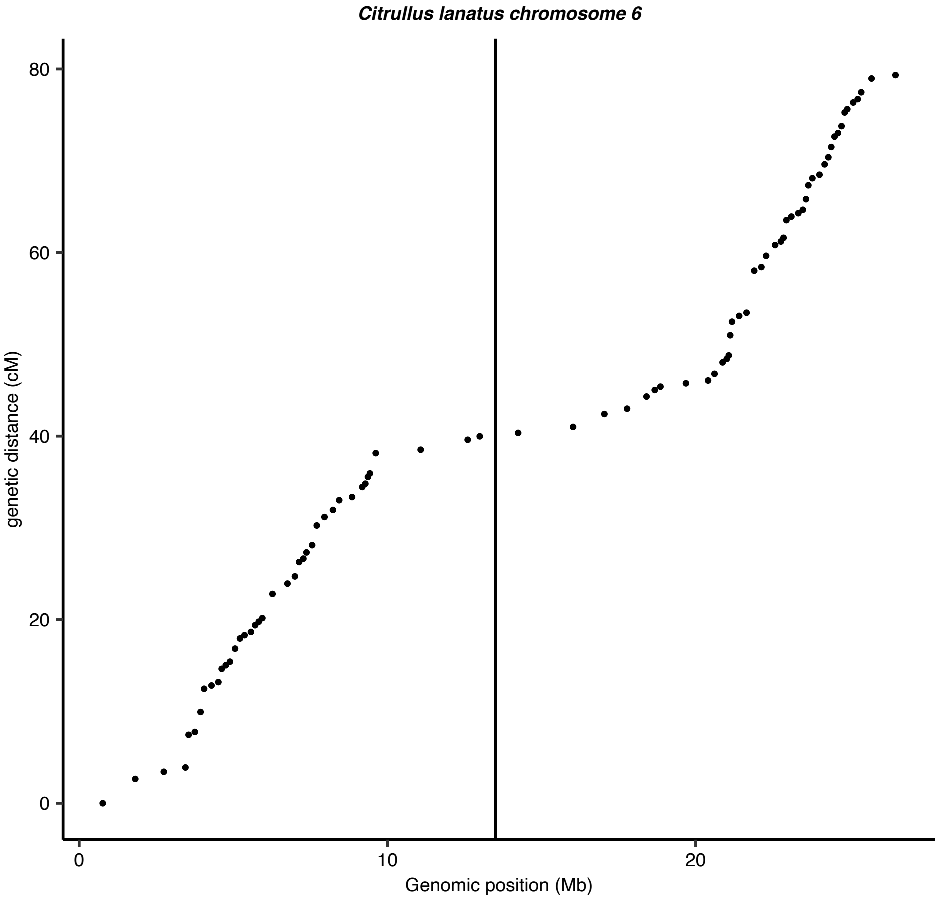
Citrullus lanatus chromosome 6
genetic distance (cM)
Genomic position (Mb)
0
20
40
60
80
0
10
20

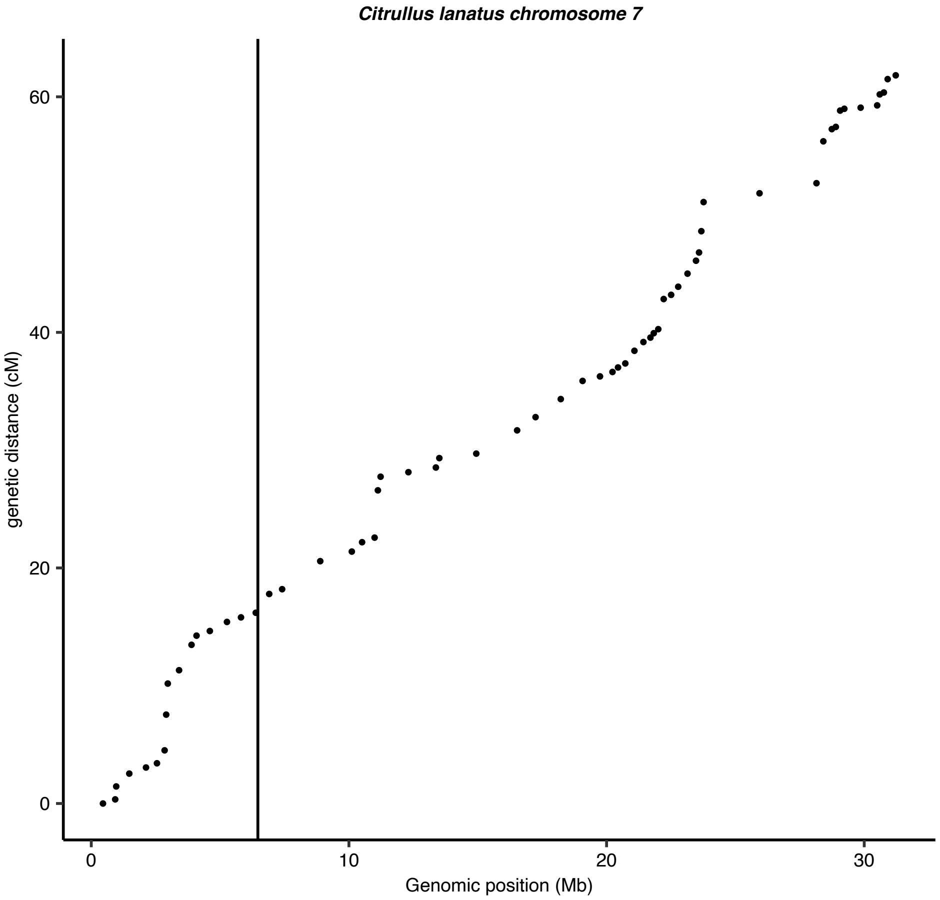
Citrullus lanatus chromosome 7
genetic distance (cM)
60
40
20
0
0
10
20
30
Genomic position (Mb)

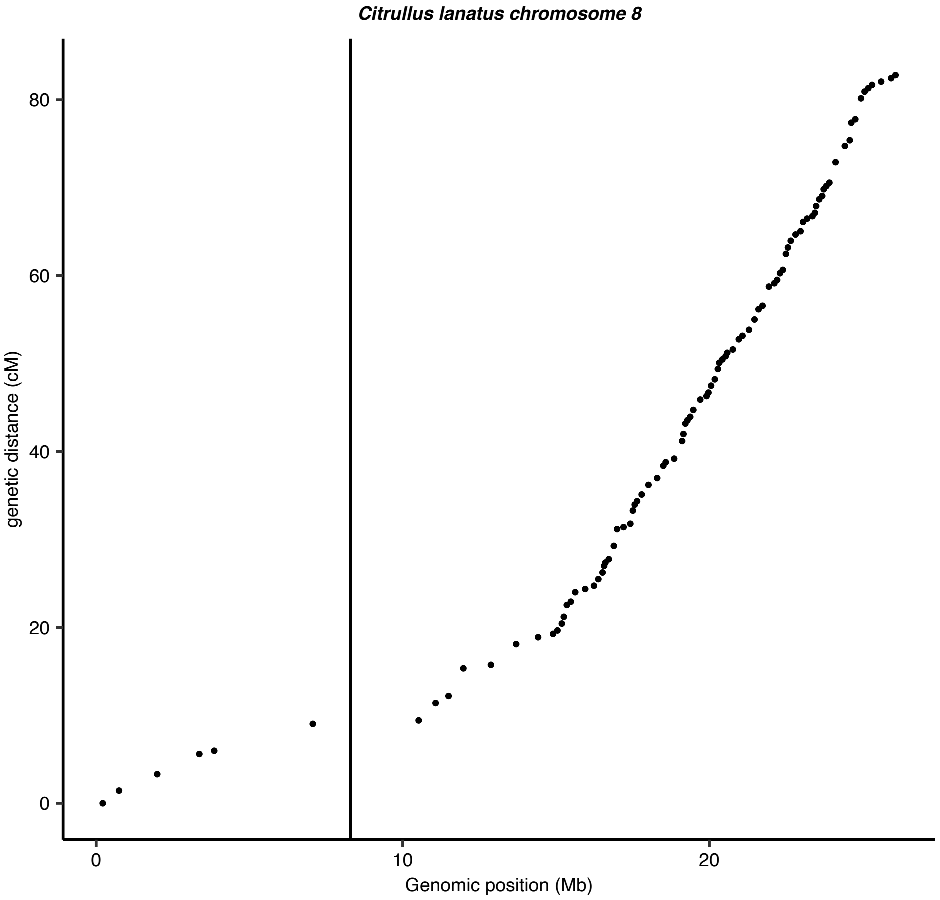
Citrullus lanatus chromosome 8
genetic distance (cM)
Genomic position (Mb)
0
20
40
60
80
0
10
20

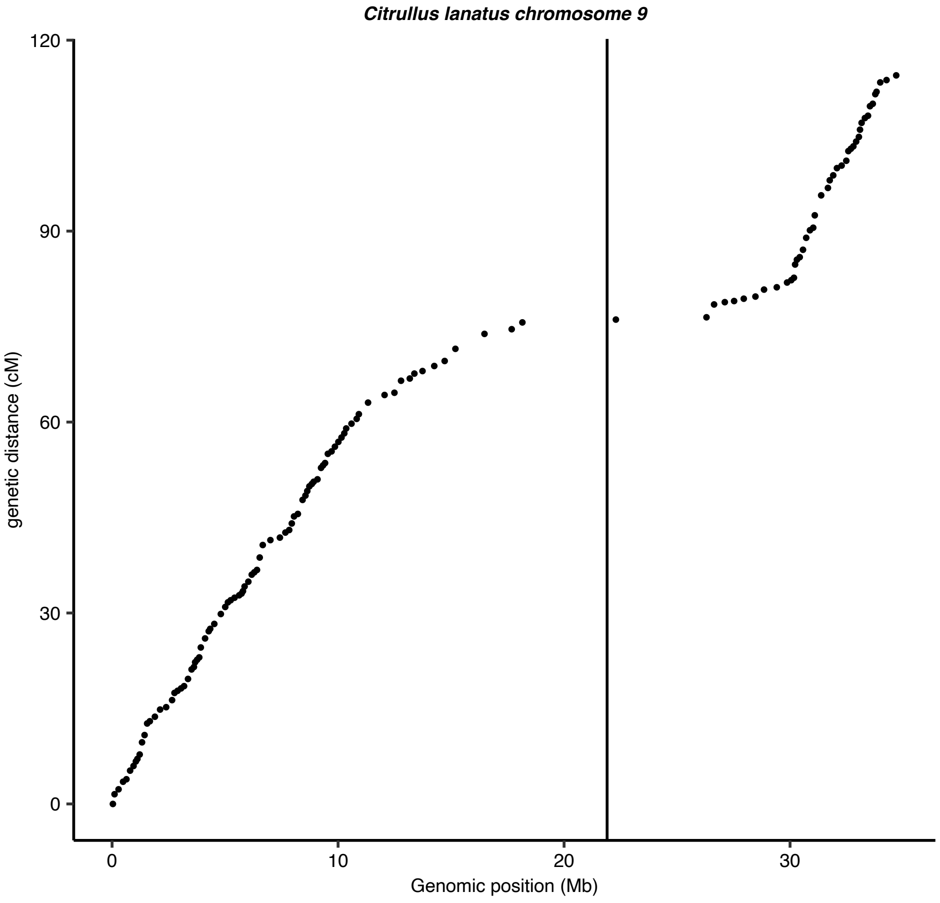
Citrullus lanatus chromosome 9
genetic distance (cM)
Genomic position (Mb)
0
30
60
90
120
0
10
20
30

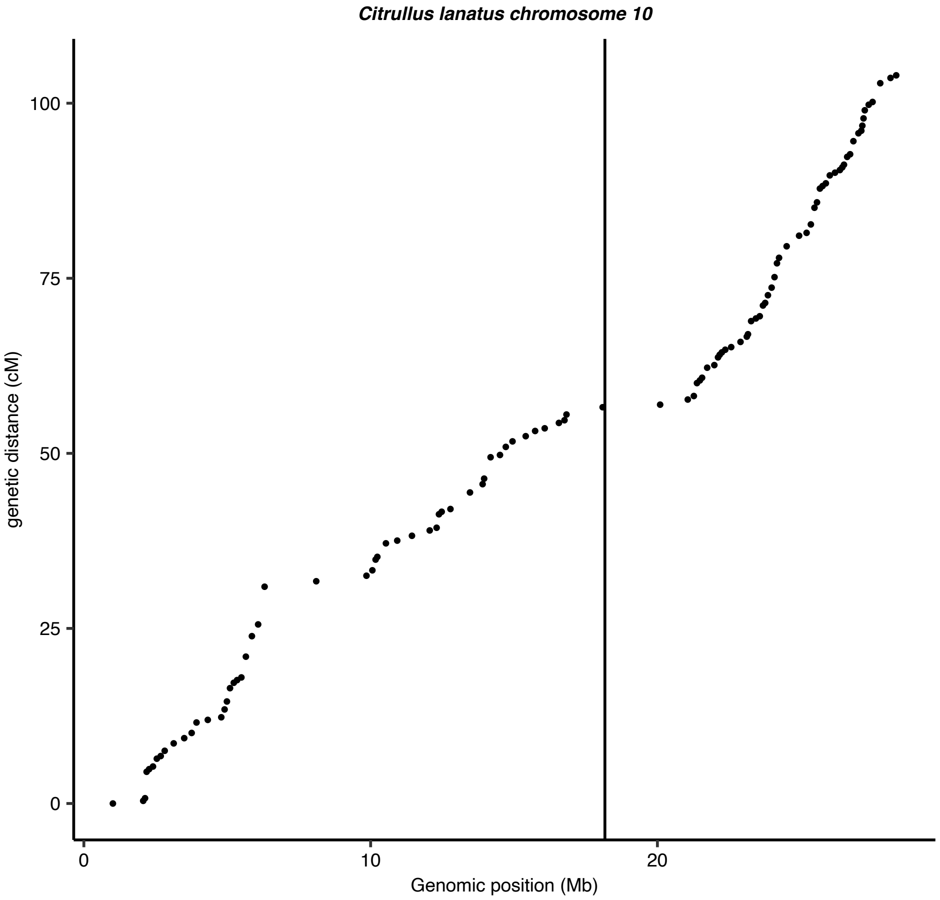
Citrullus lanatus chromosome 10
genetic distance (cM)
0
25
50
75
100
Genomic position (Mb)
0
10
20

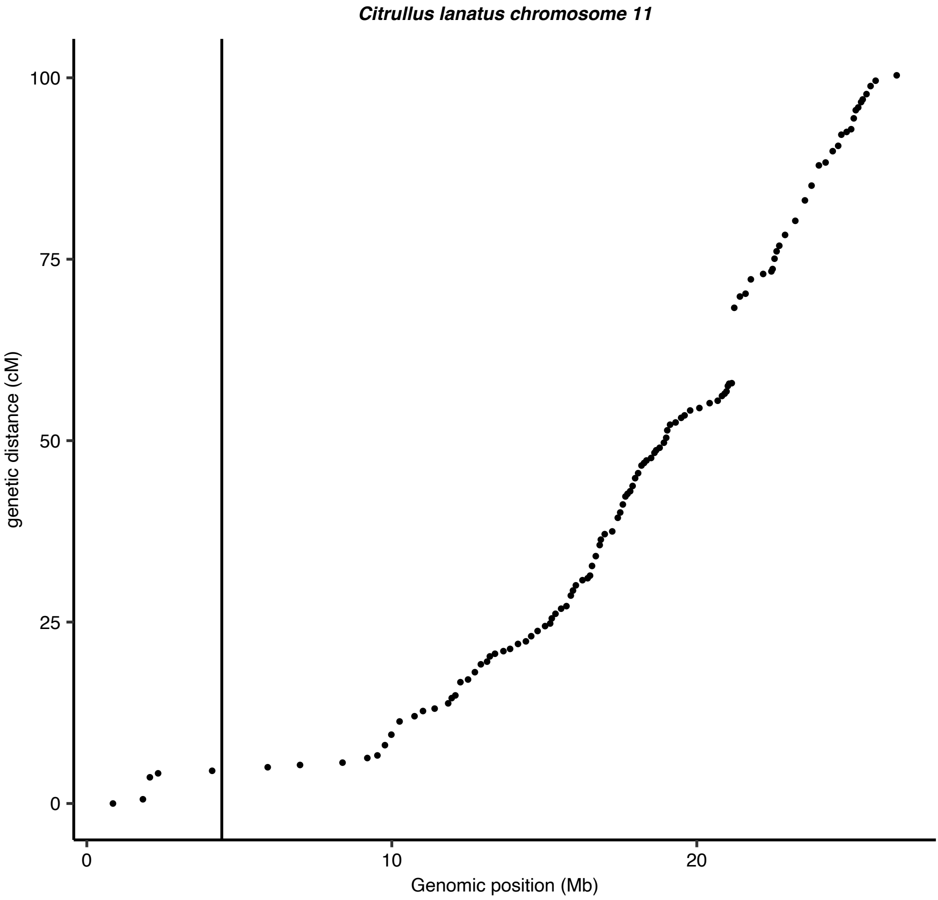
Citrullus lanatus chromosome 11
genetic distance (cM)
0
25
50
75
100
Genomic position (Mb)
0
10
20

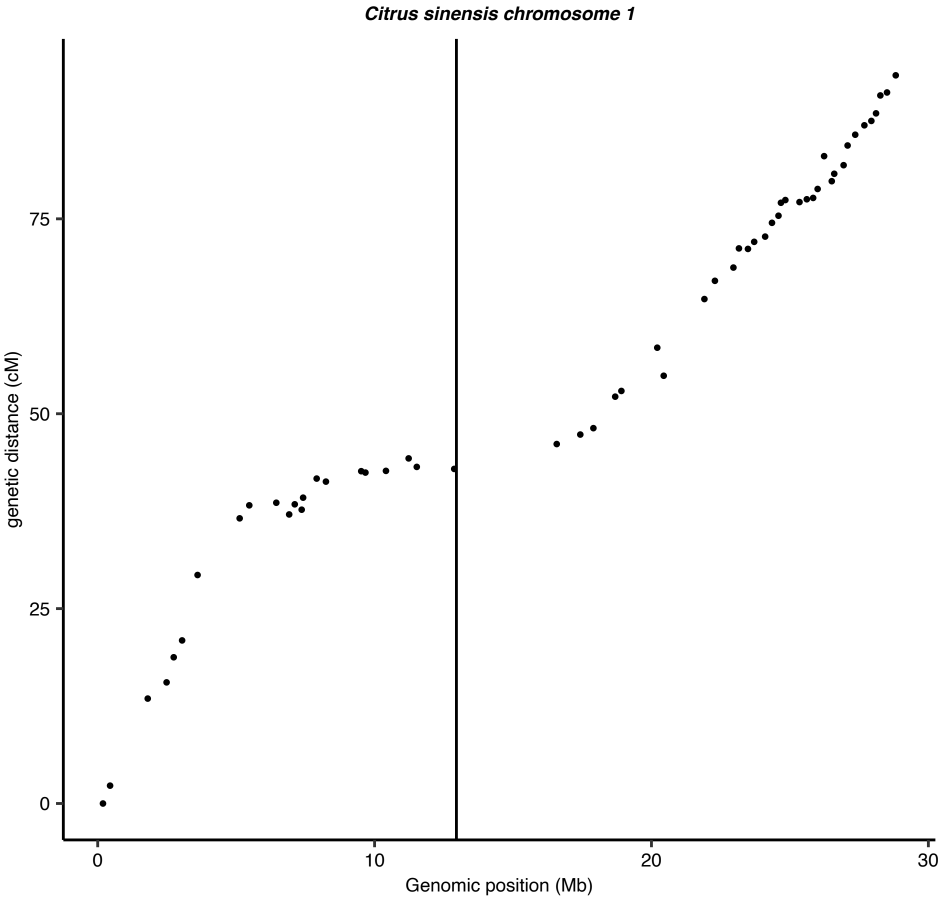
Citrus sinensis chromosome 1
genetic distance (cM)
75
50
25
0
0
10
20
30
Genomic position (Mb)

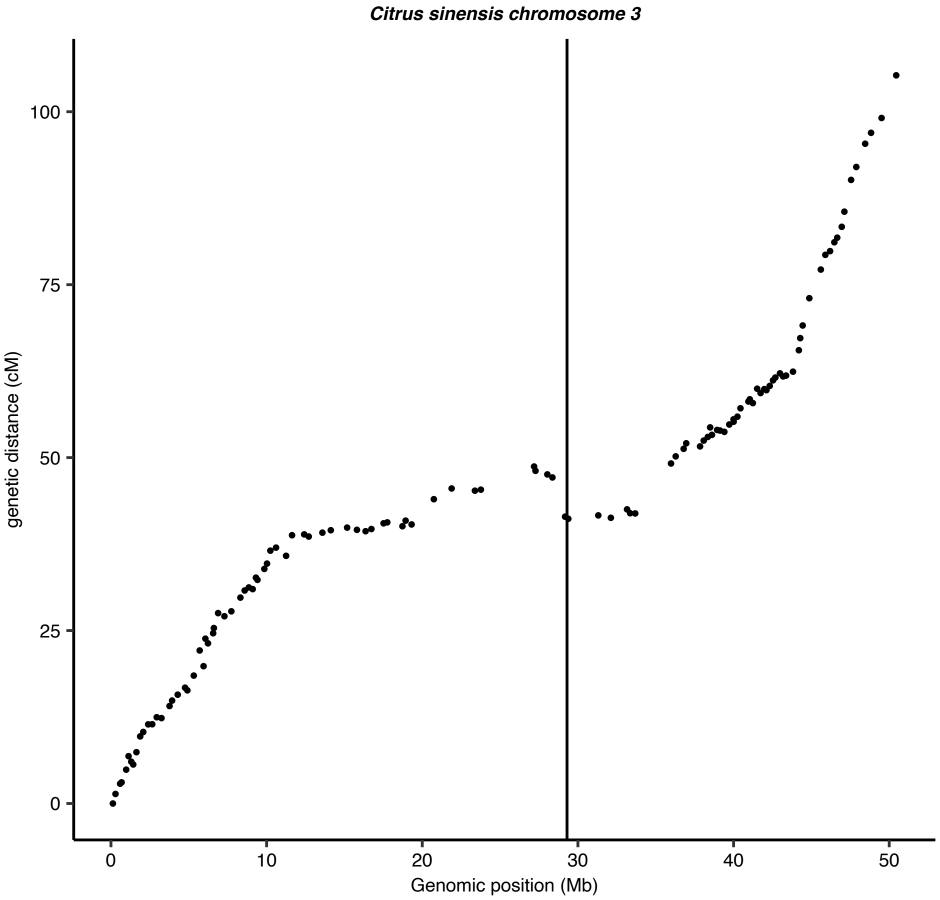
Citrus sinensis chromosome 3
genetic distance (cM)
Genomic position (Mb)
0
25
50
75
100
0
10
20
30
40
50

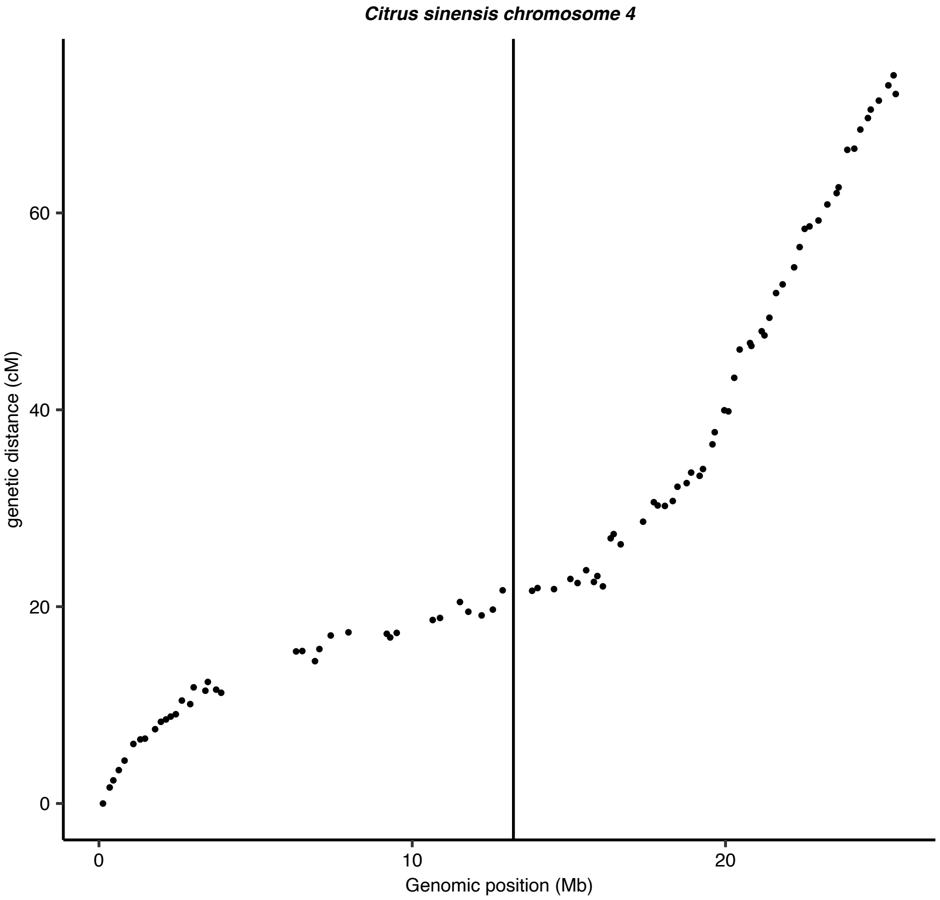
Citrus sinensis chromosome 4
genetic distance (cM)
0
20
40
60
Genomic position (Mb)
0
10
20

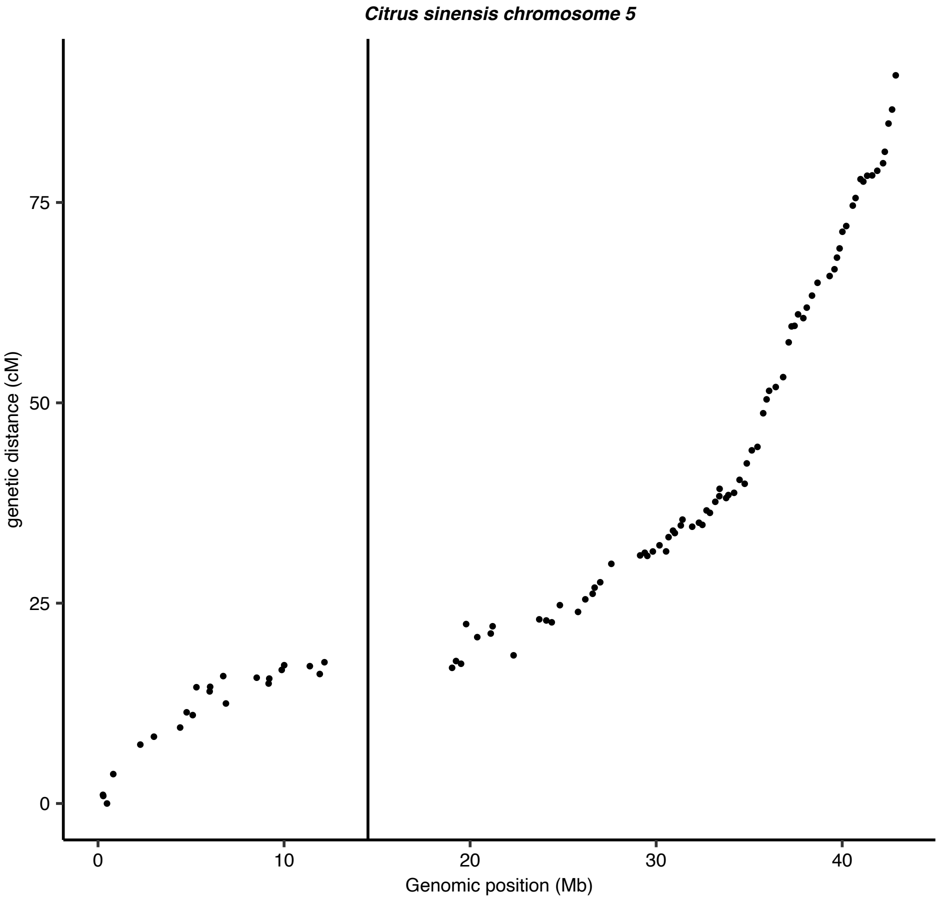
Citrus sinensis chromosome 5
genetic distance (cM)
75
50
25
0
0
10
20
30
40
Genomic position (Mb)

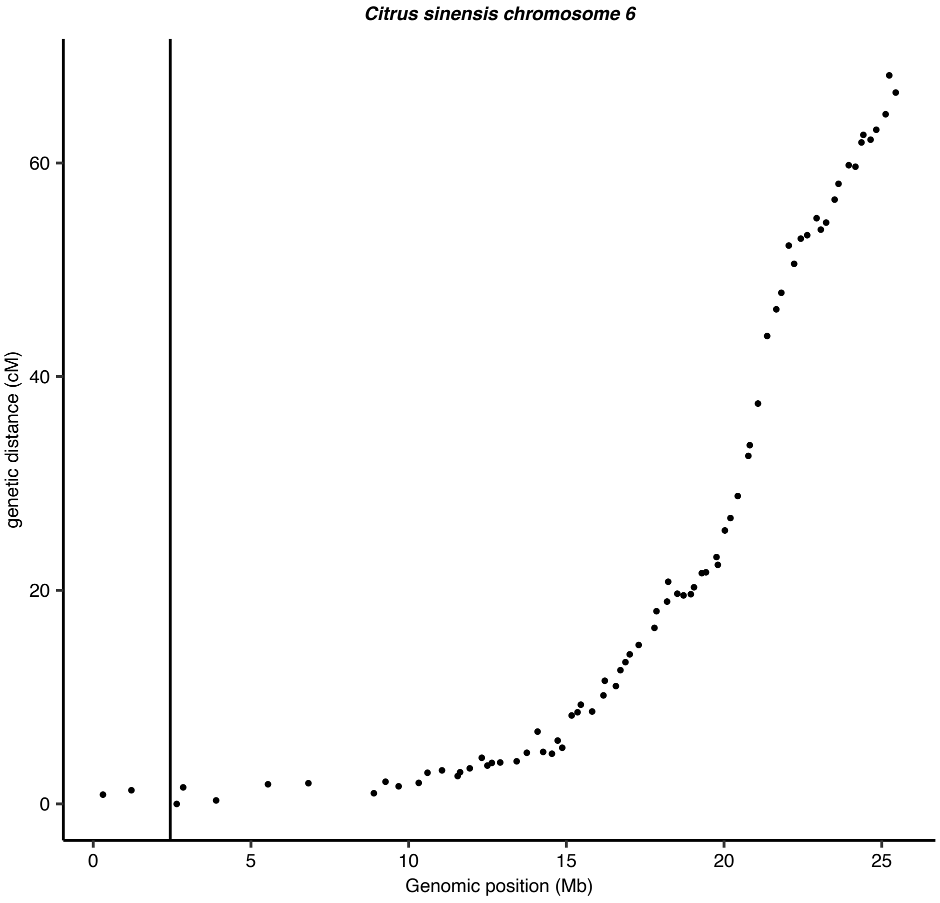
Citrus sinensis chromosome 6
genetic distance (cM)
0
20
40
60
0
5
10
15
20
25
Genomic position (Mb)

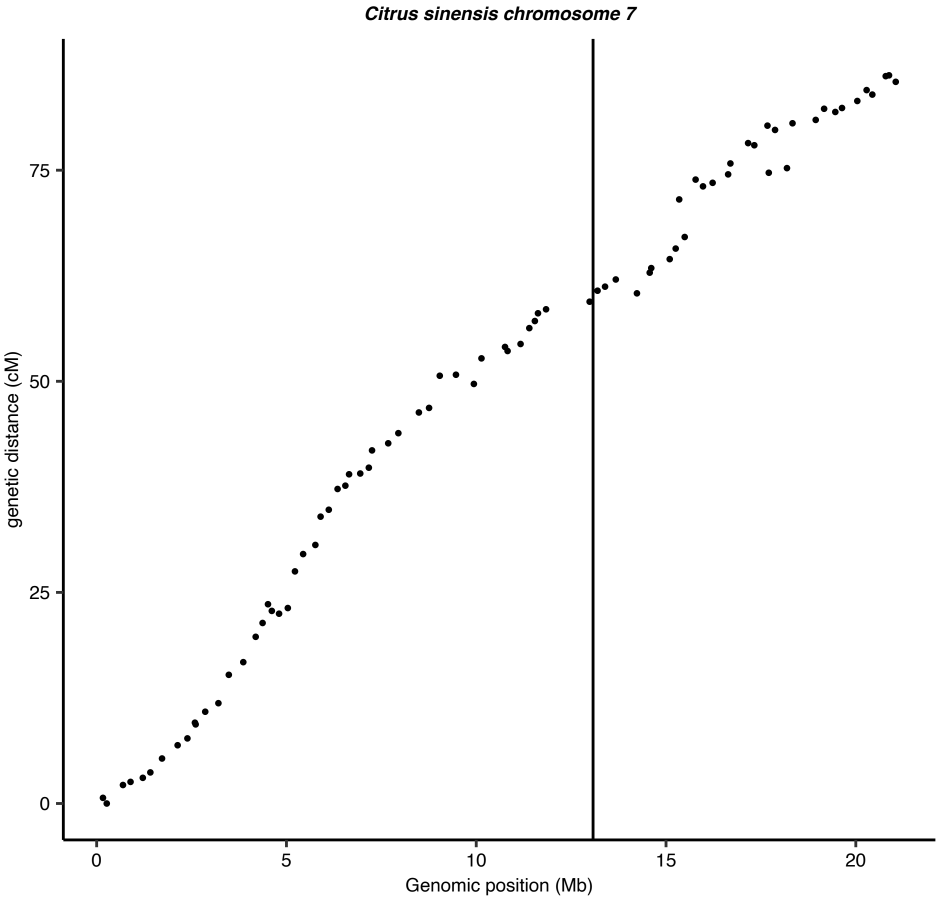
Citrus sinensis chromosome 7
genetic distance (cM)
0
25
50
75
Genomic position (Mb)
0
5
10
15
20

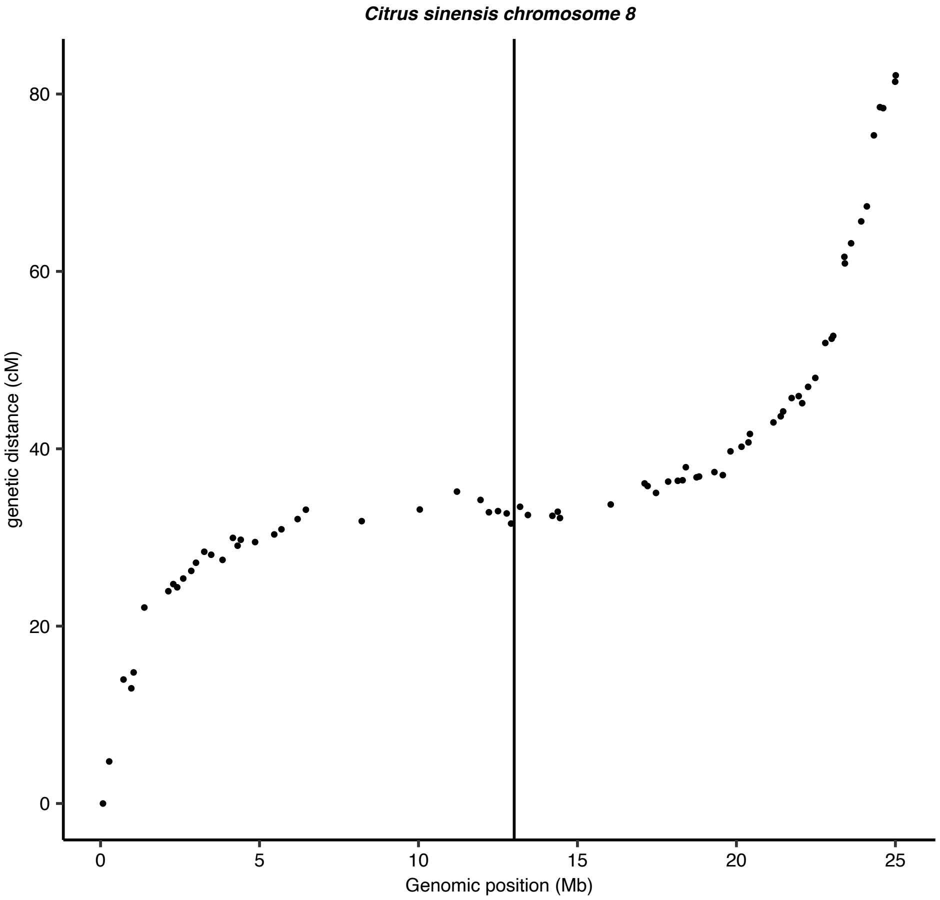
Citrus sinensis chromosome 8
genetic distance (cM)
Genomic position (Mb)
0
20
40
60
80
0
5
10
15
20
25

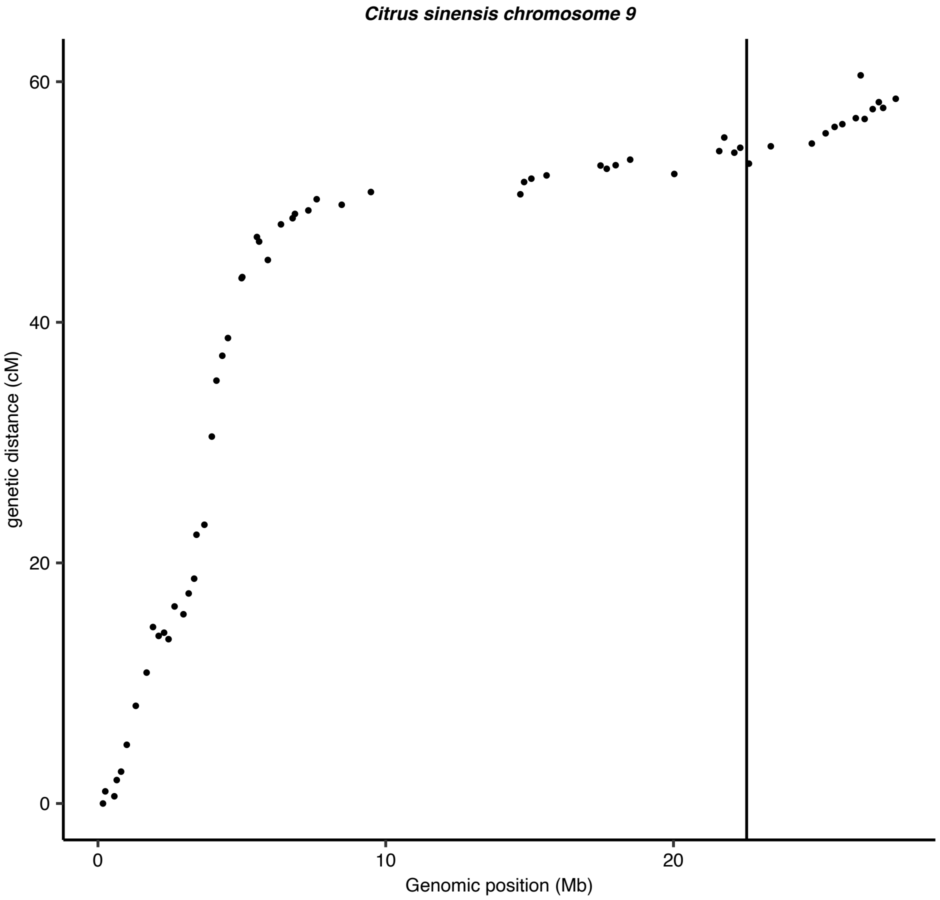
Citrus sinensis chromosome 9
60
40
20
0
0
10
20
genetic distance (cM)
Genomic position (Mb)

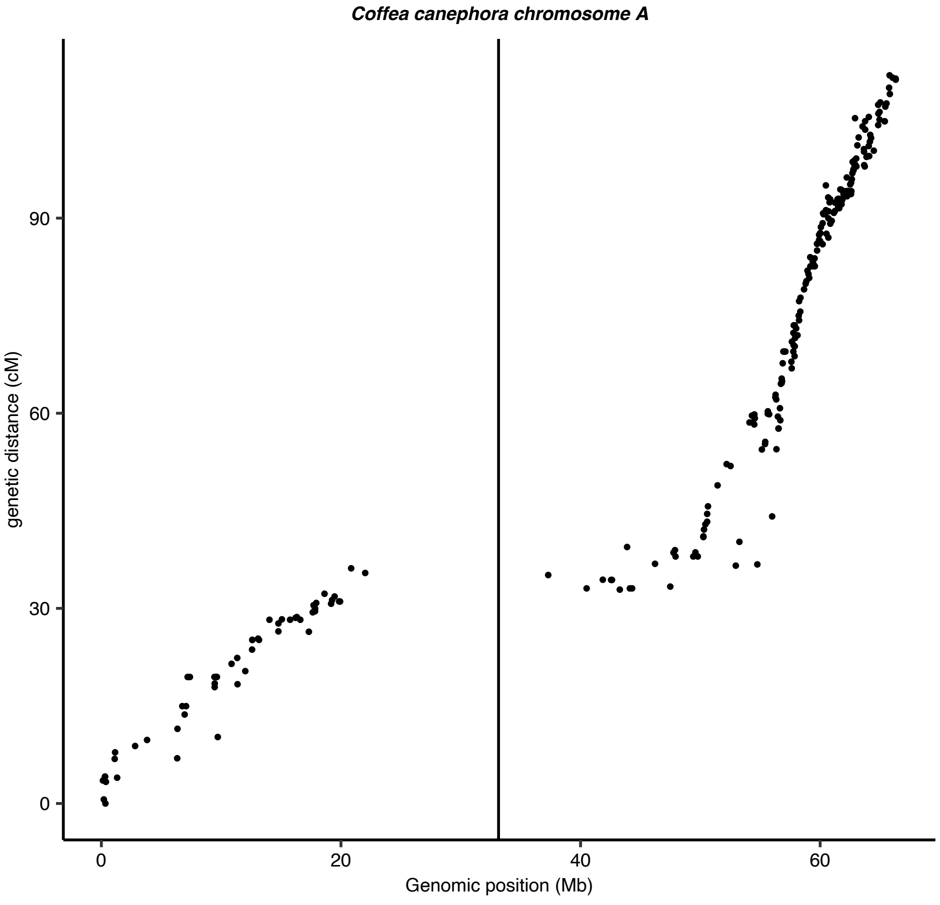
Coffea canephora chromosome A
genetic distance (cM)
0
30
60
90
Genomic position (Mb)
0
20
40
60

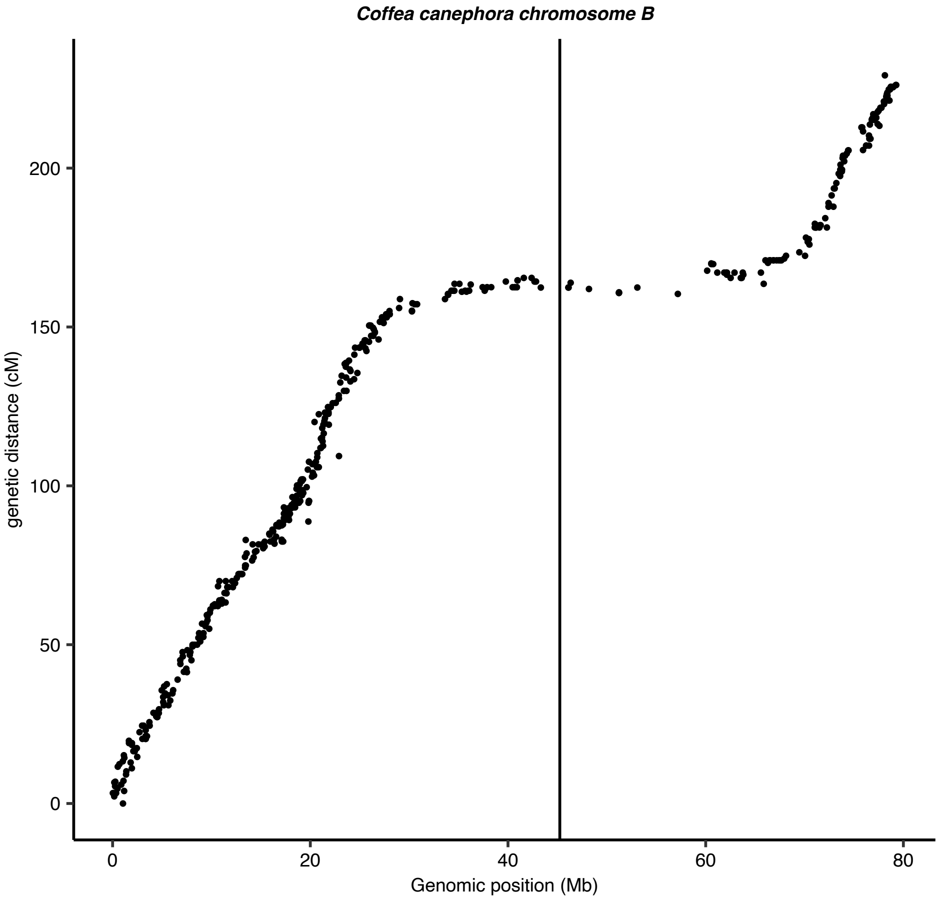
Coffea canephora chromosome B
genetic distance (cM)
Genomic position (Mb)
0
50
100
150
200
0
20
40
60
80

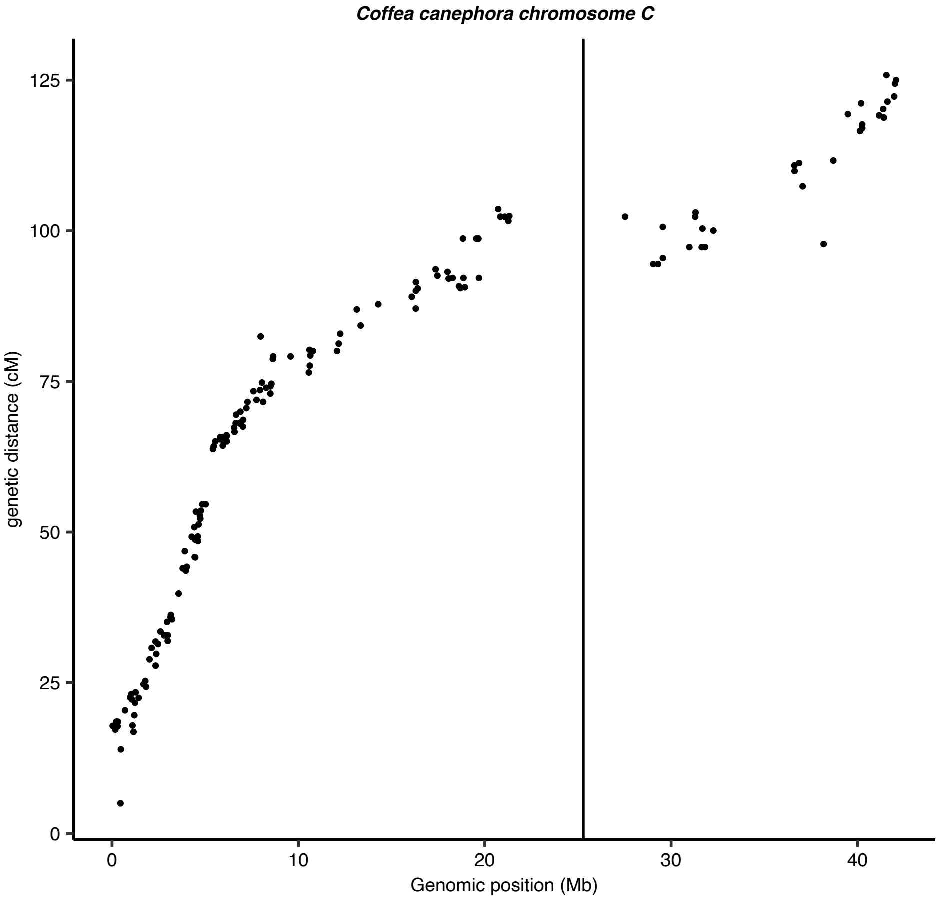
Coffea canephora chromosome C
genetic distance (cM)
125
100
75
50
25
0
0
10
20
30
40
Genomic position (Mb)

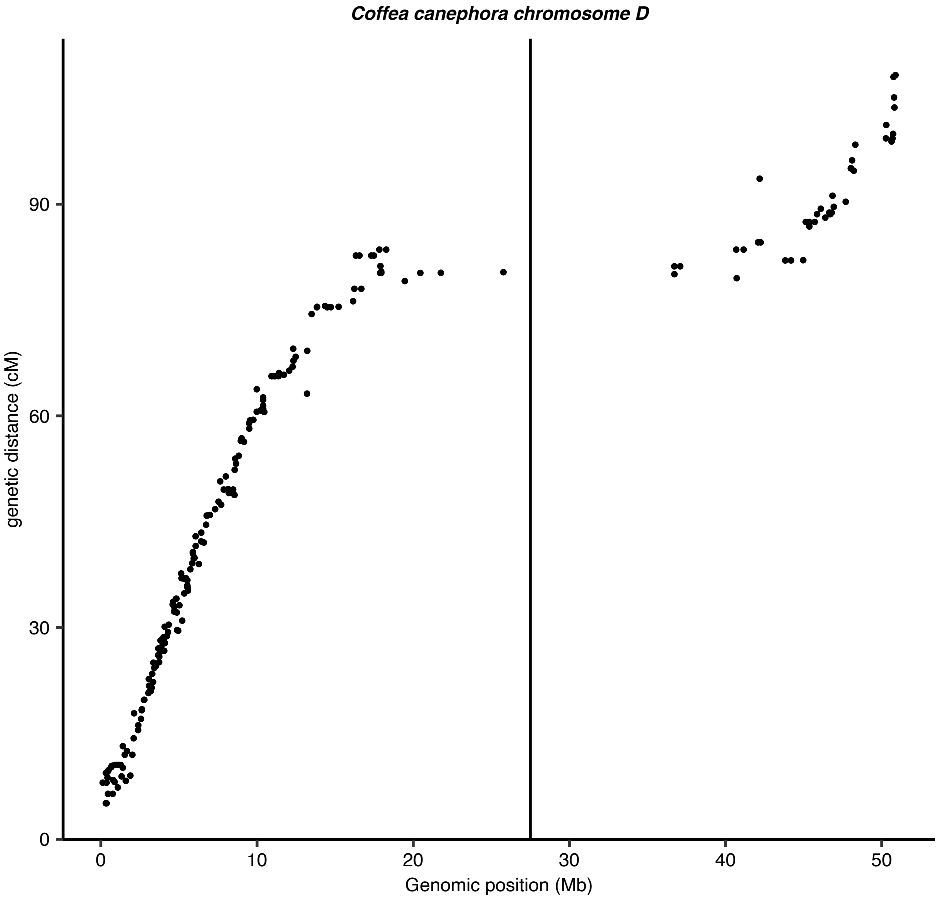
Coffea canephora chromosome D
genetic distance (cM)
0
30
60
90
Genomic position (Mb)
0
10
20
30
40
50

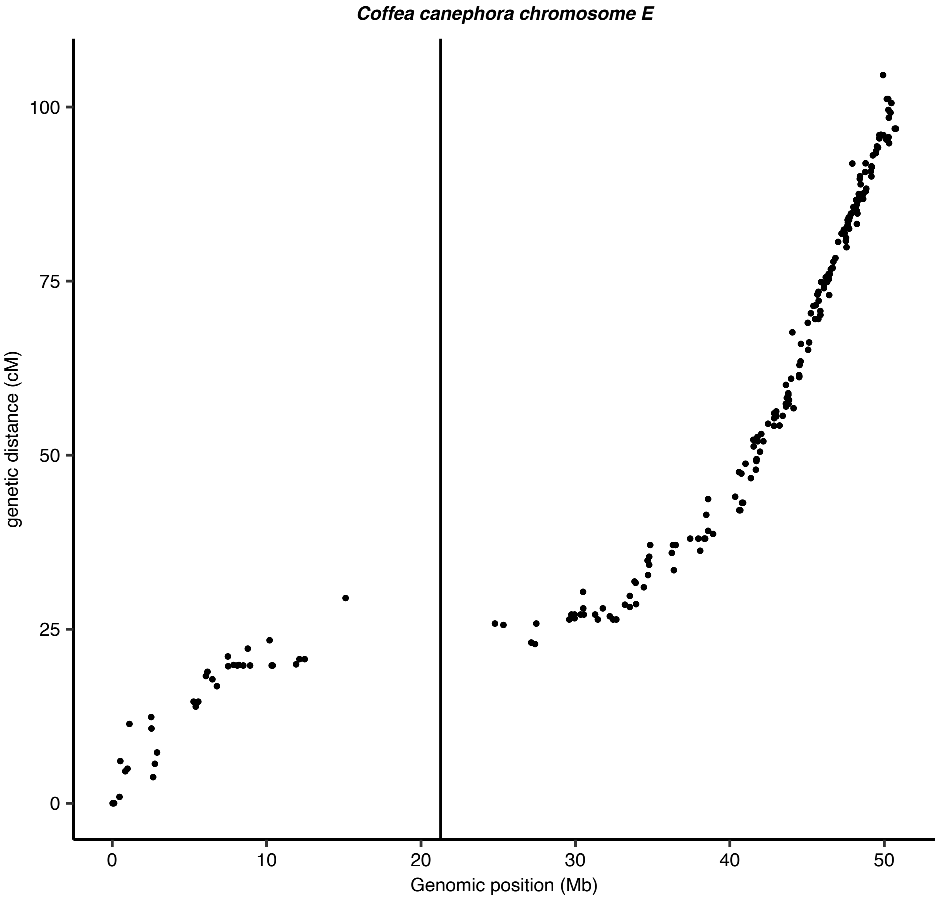
Coffea canephora chromosome E
genetic distance (cM)
Genomic position (Mb)
0
25
50
75
100
0
10
20
30
40
50

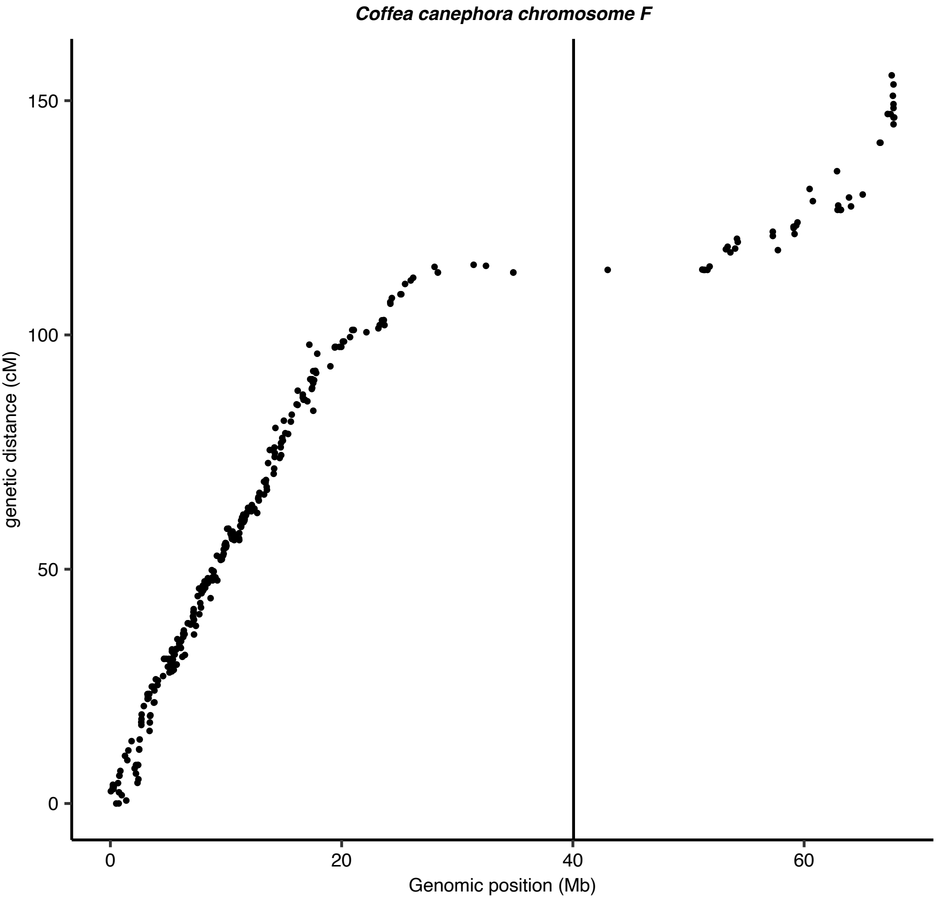
Coffea canephora chromosome F
genetic distance (cM)
150
100
50
0
0
20
40
60
Genomic position (Mb)

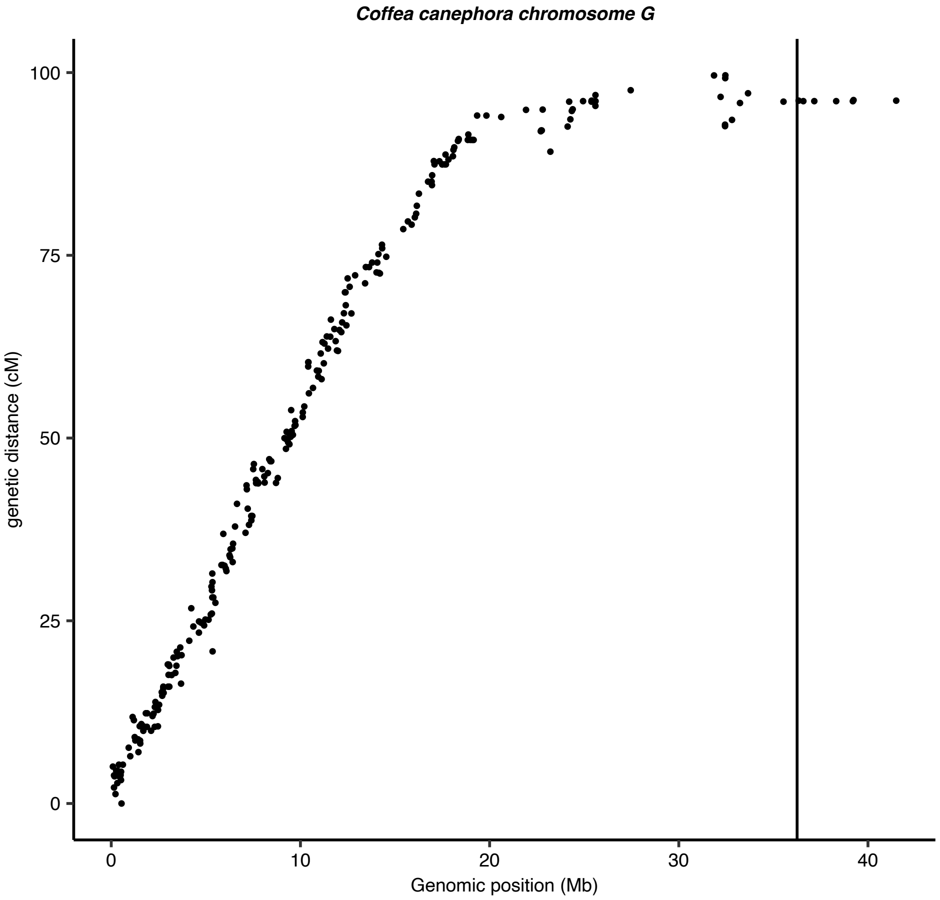
Coffea canephora chromosome G
genetic distance (cM)
Genomic position (Mb)
0
25
50
75
100
0
10
20
30
40

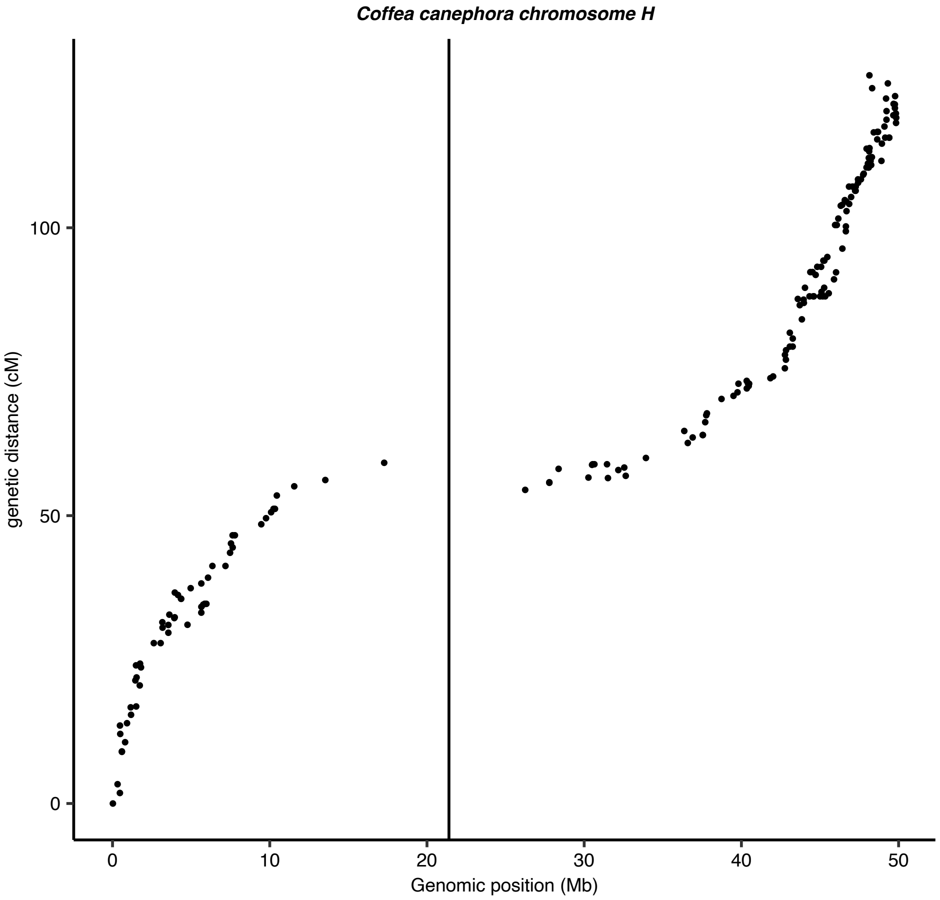
Coffea canephora chromosome H
genetic distance (cM)
0
50
100
Genomic position (Mb)
0
10
20
30
40
50

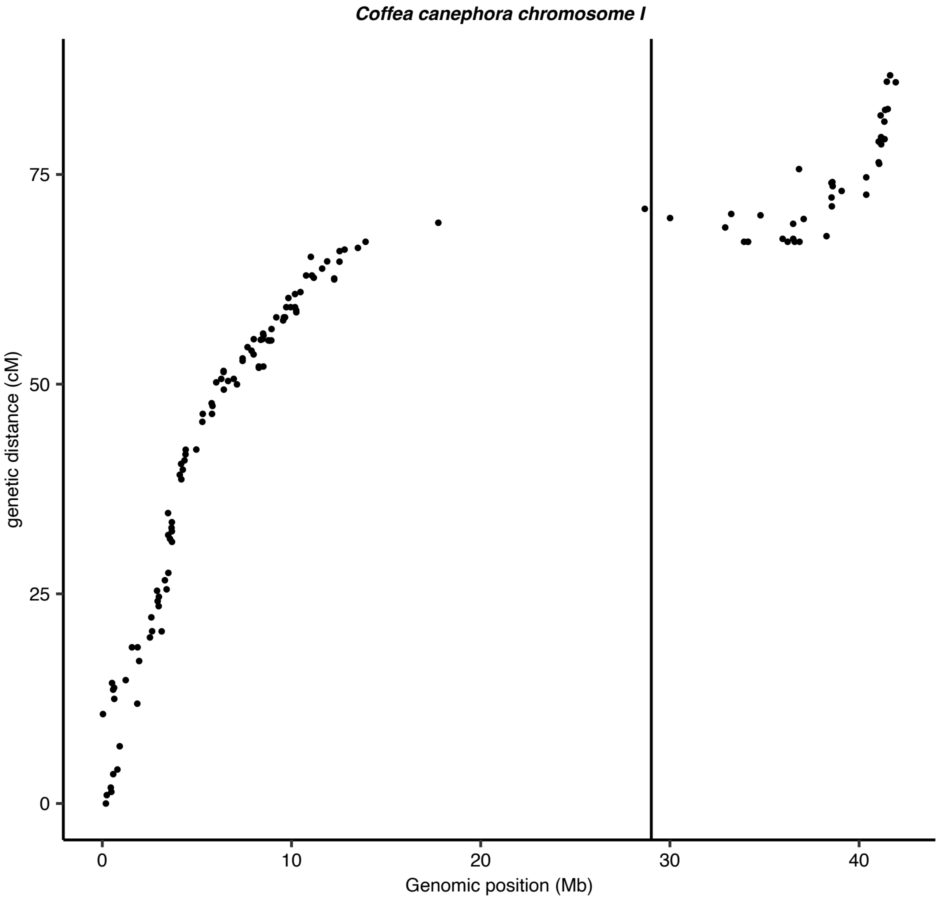
Coffea canephora chromosome I
genetic distance (cM)
0
25
50
75
0
10
20
30
40
Genomic position (Mb)

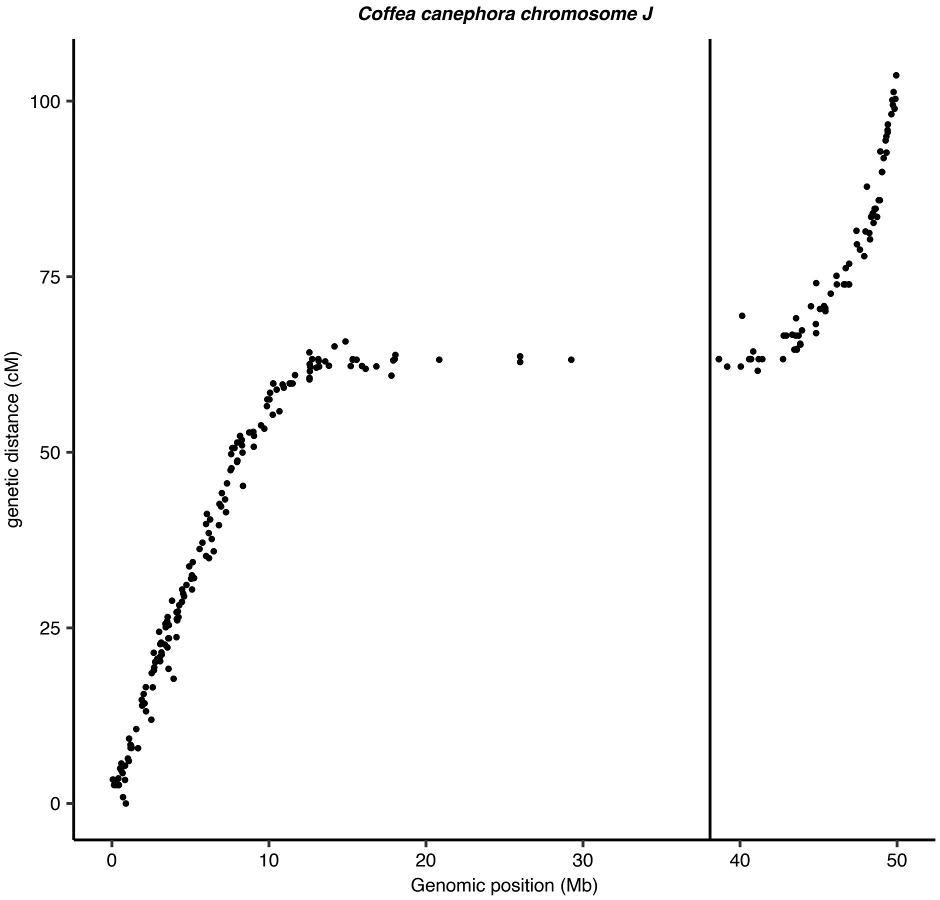
Coffea canephora chromosome J
genetic distance (cM)
Genomic position (Mb)
0
25
50
75
100
0
10
20
30
40
50

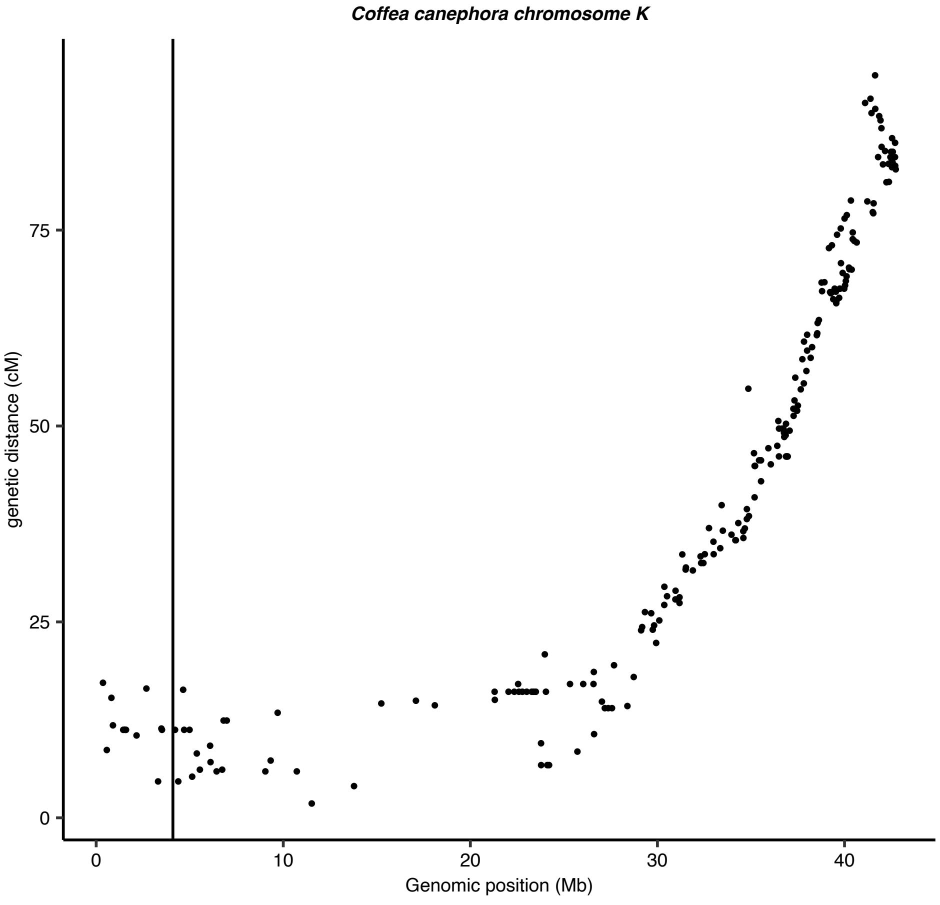
Coffea canephora chromosome K
genetic distance (cM)
0
25
50
75
Genomic position (Mb)
0
10
20
30
40

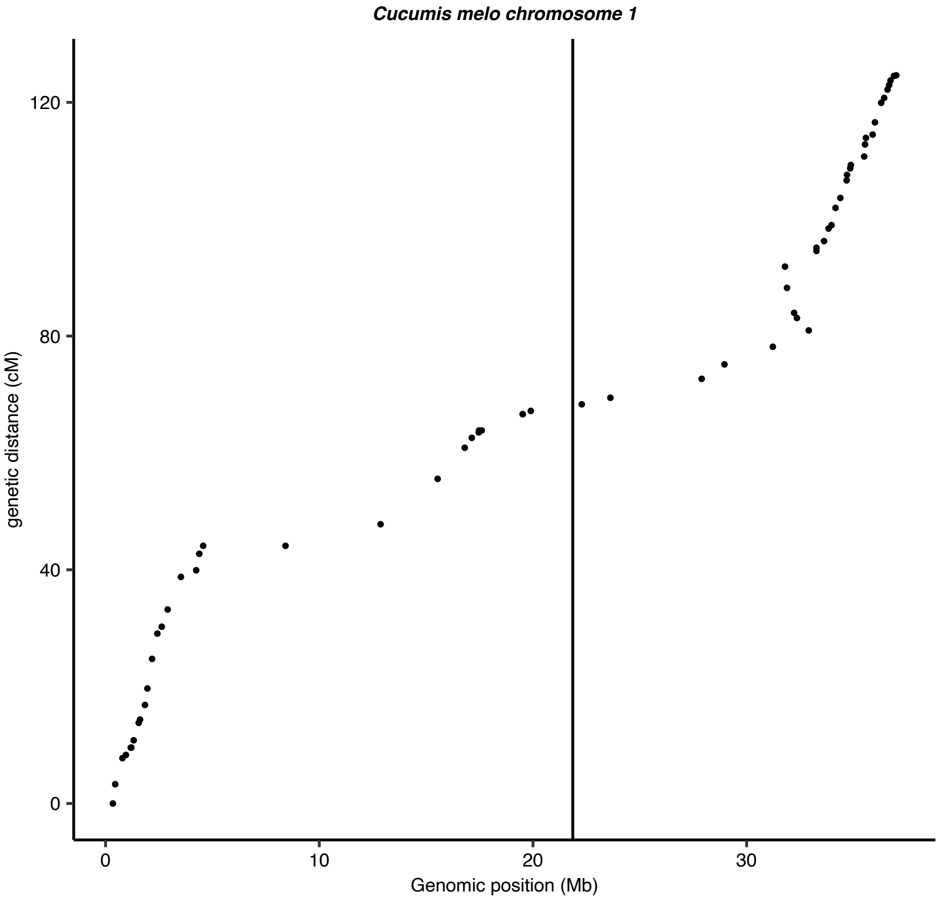
Cucumis melo chromosome 1
genetic distance (cM)
0
40
80
120
0
10
20
30
Genomic position (Mb)

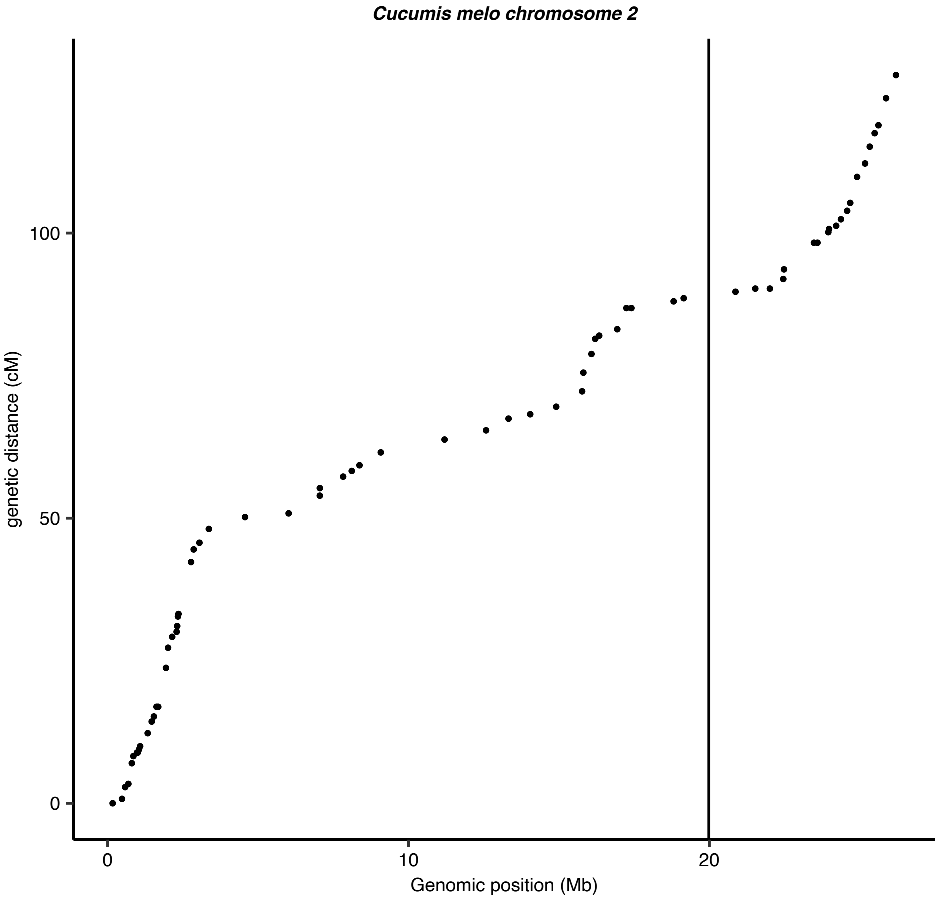
Cucumis melo chromosome 2
genetic distance (cM)
0
50
100
0
10
20
Genomic position (Mb)

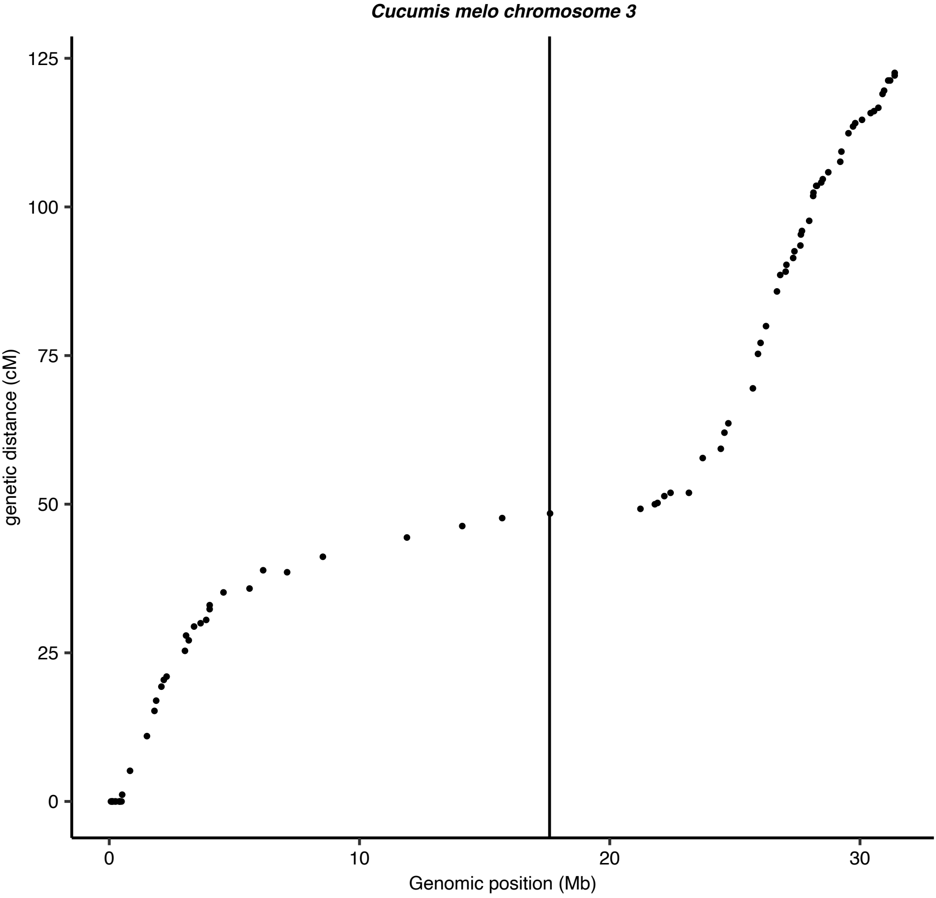
Cucumis melo chromosome 3
genetic distance (cM)
Genomic position (Mb)
0
25
50
75
100
125
0
10
20
30

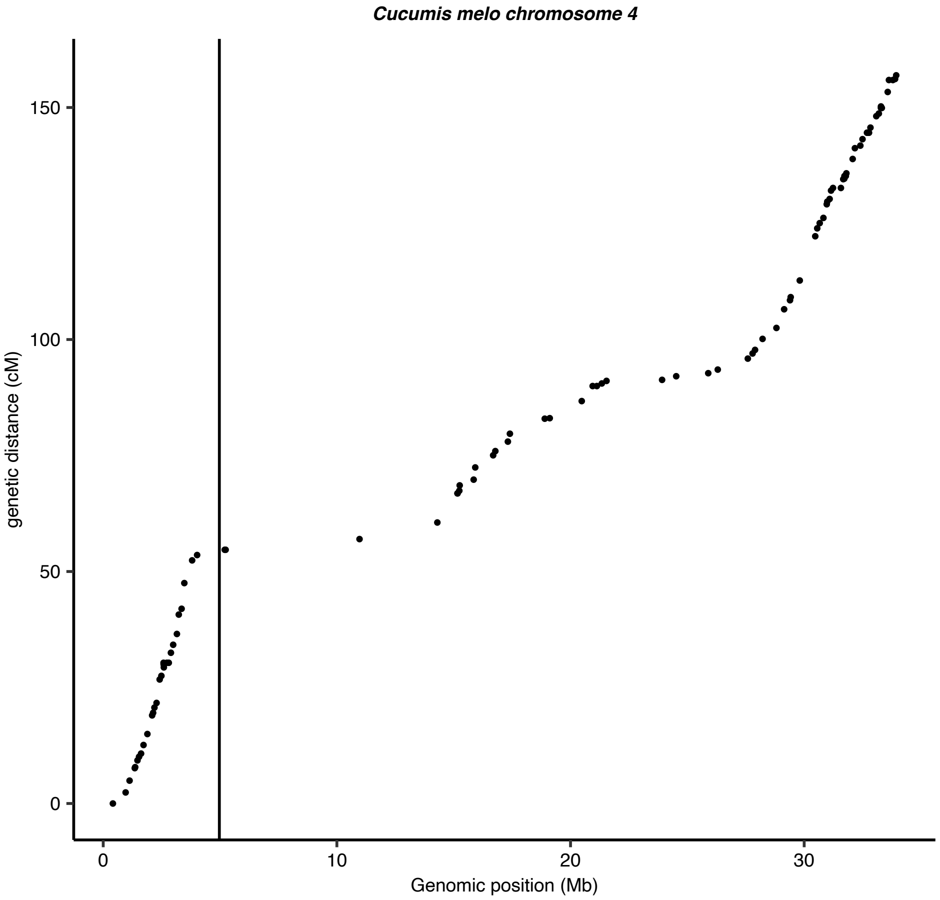
Cucumis melo chromosome 4
genetic distance (cM)
Genomic position (Mb)
0
50
100
150
0
10
20
30

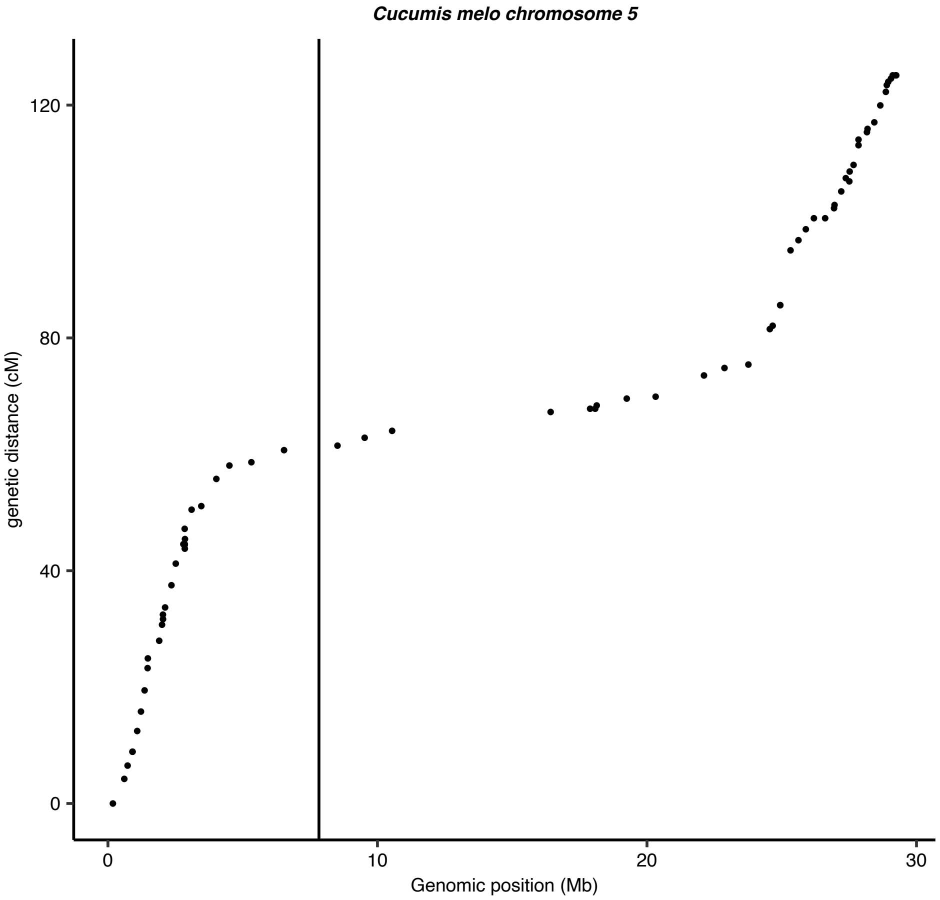
Cucumis melo chromosome 5
genetic distance (cM)
0
40
80
120
Genomic position (Mb)
0
10
20
30

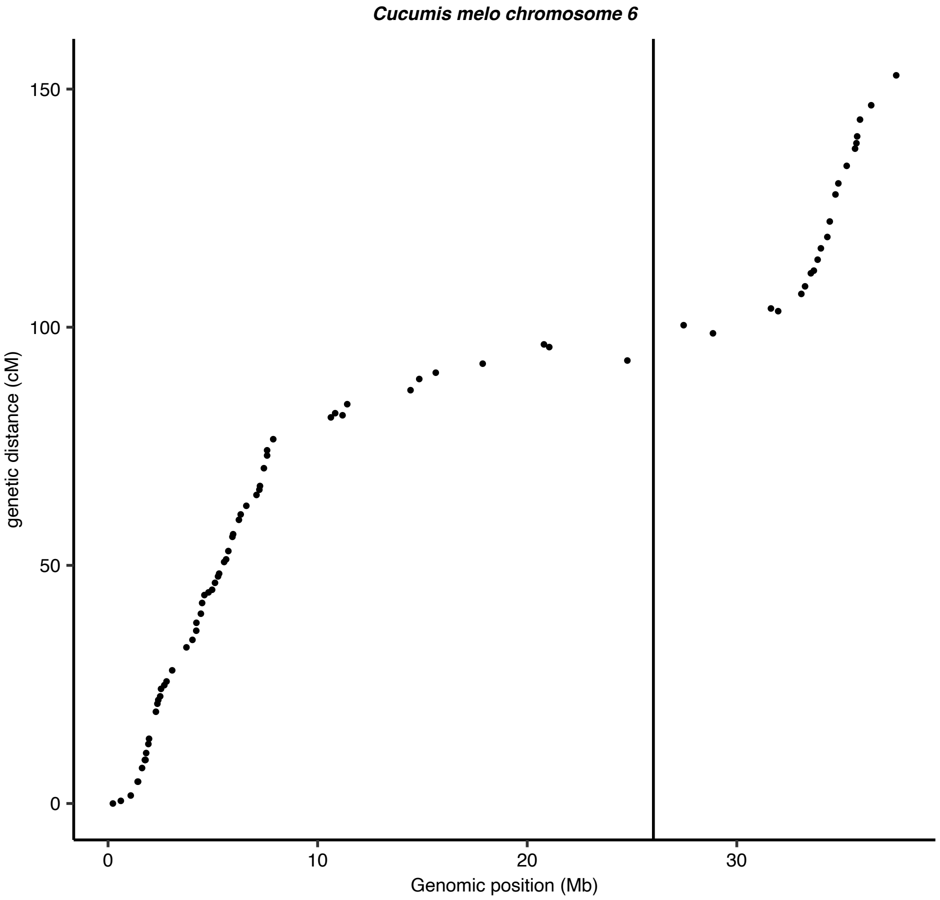
Cucumis melo chromosome 6
150
100
50
0
genetic distance (cM)
0
10
20
30
Genomic position (Mb)

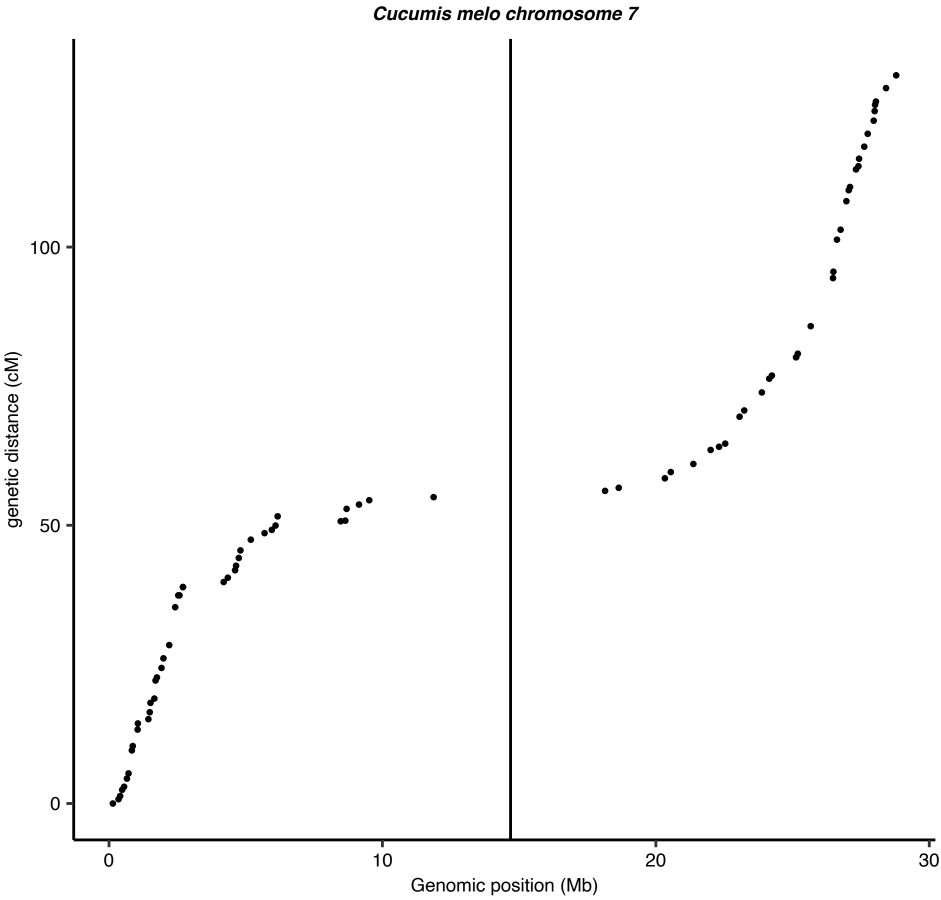
Cucumis melo chromosome 7
genetic distance (cM)
0
50
100
Genomic position (Mb)
0
10
20
30

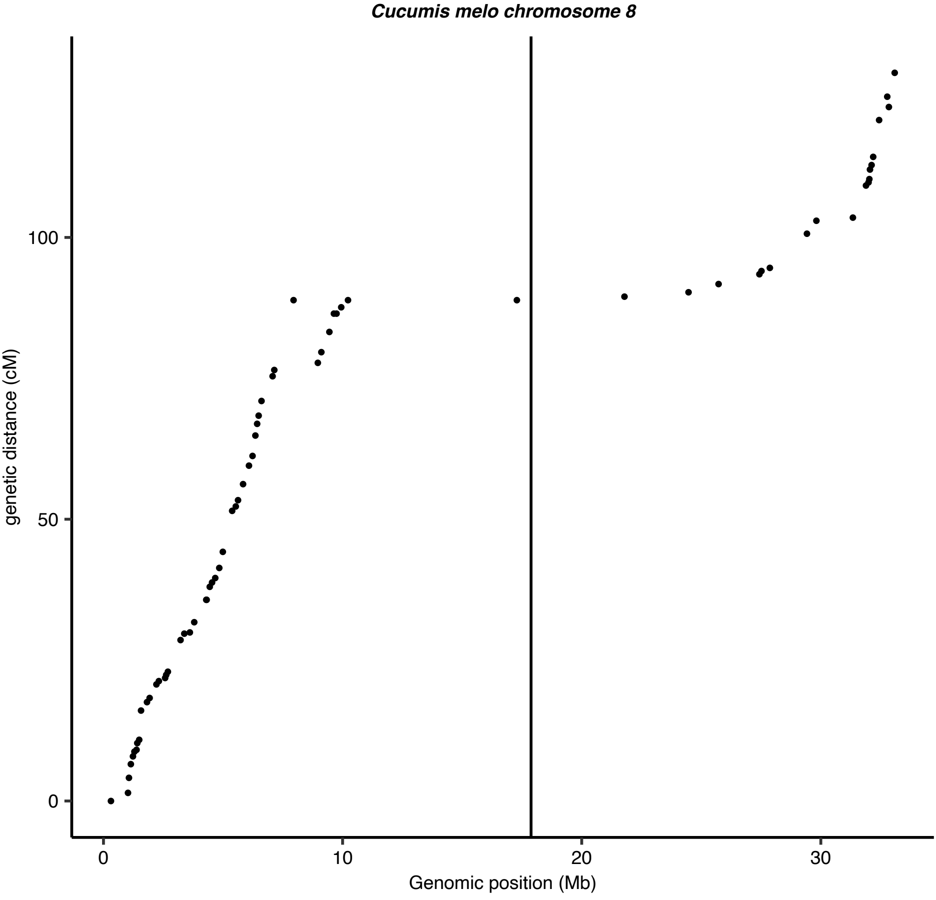
Cucumis melo chromosome 8
genetic distance (cM)
0
50
100
0
10
20
30
Genomic position (Mb)

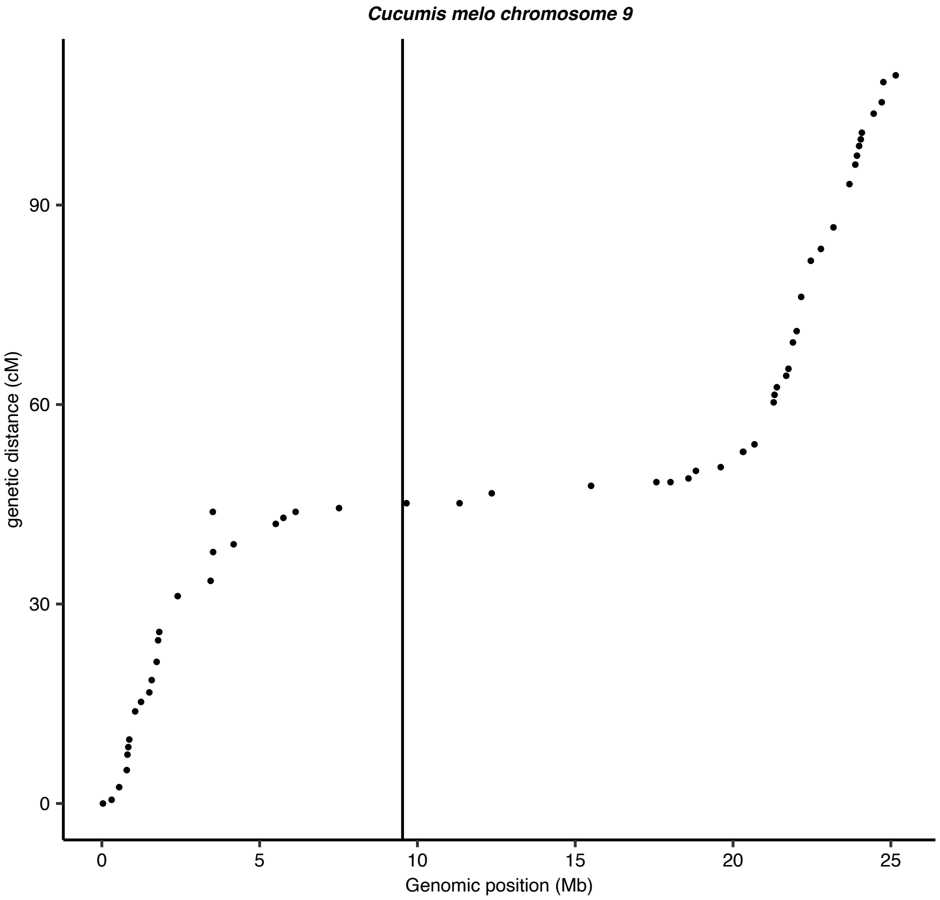
Cucumis melo chromosome 9
genetic distance (cM)
0
30
60
90
0
5
10
15
20
25
Genomic position (Mb)

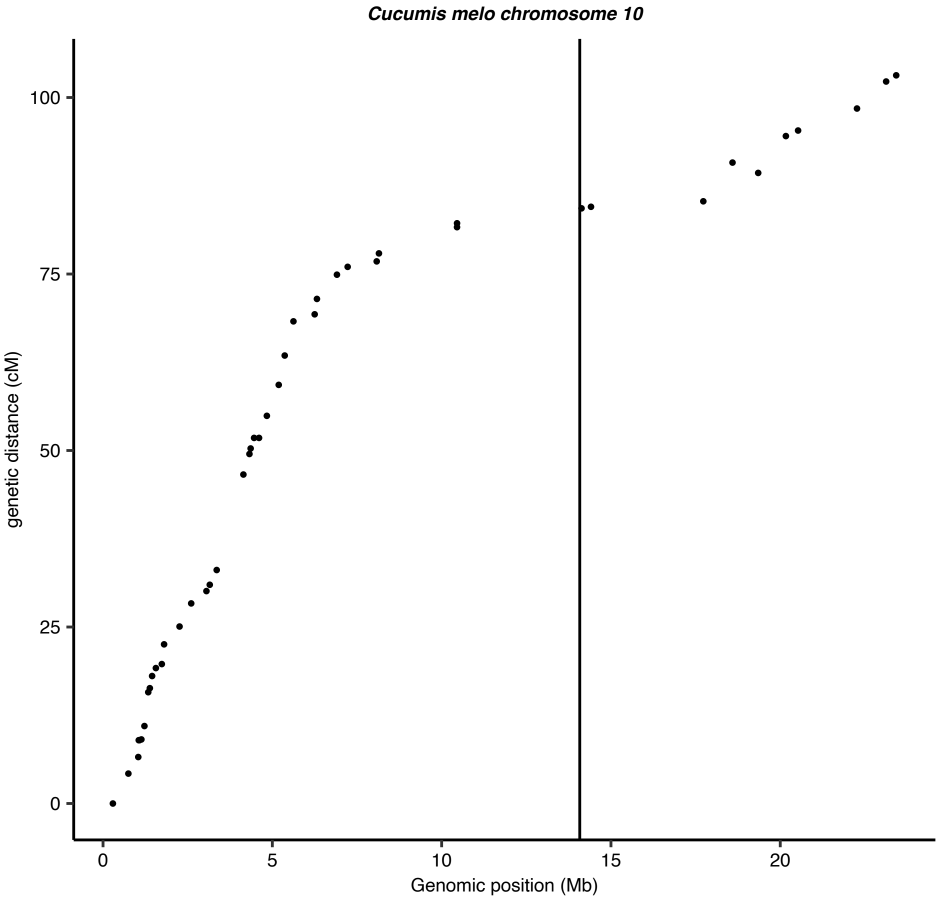
Cucumis melo chromosome 10
genetic distance (cM)
0
25
50
75
100
0
5
10
15
20
Genomic position (Mb)

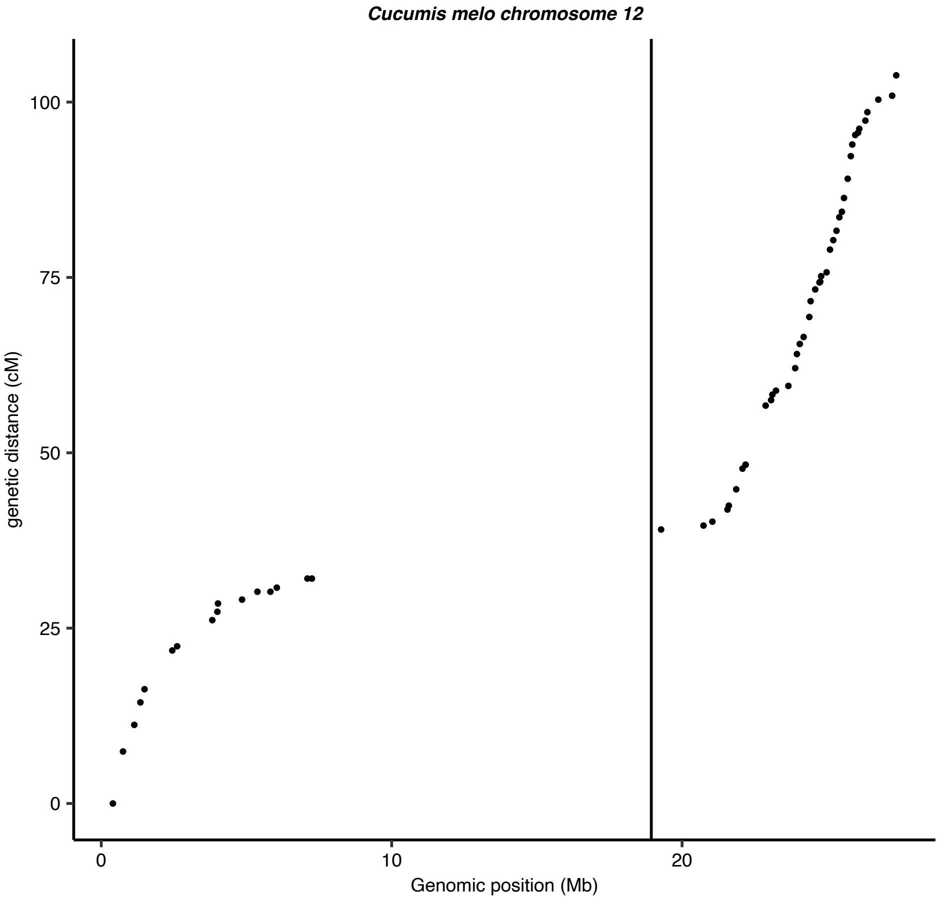
Cucumis melo chromosome 12
genetic distance (cM)
Genomic position (Mb)
0
25
50
75
100
0
10
20

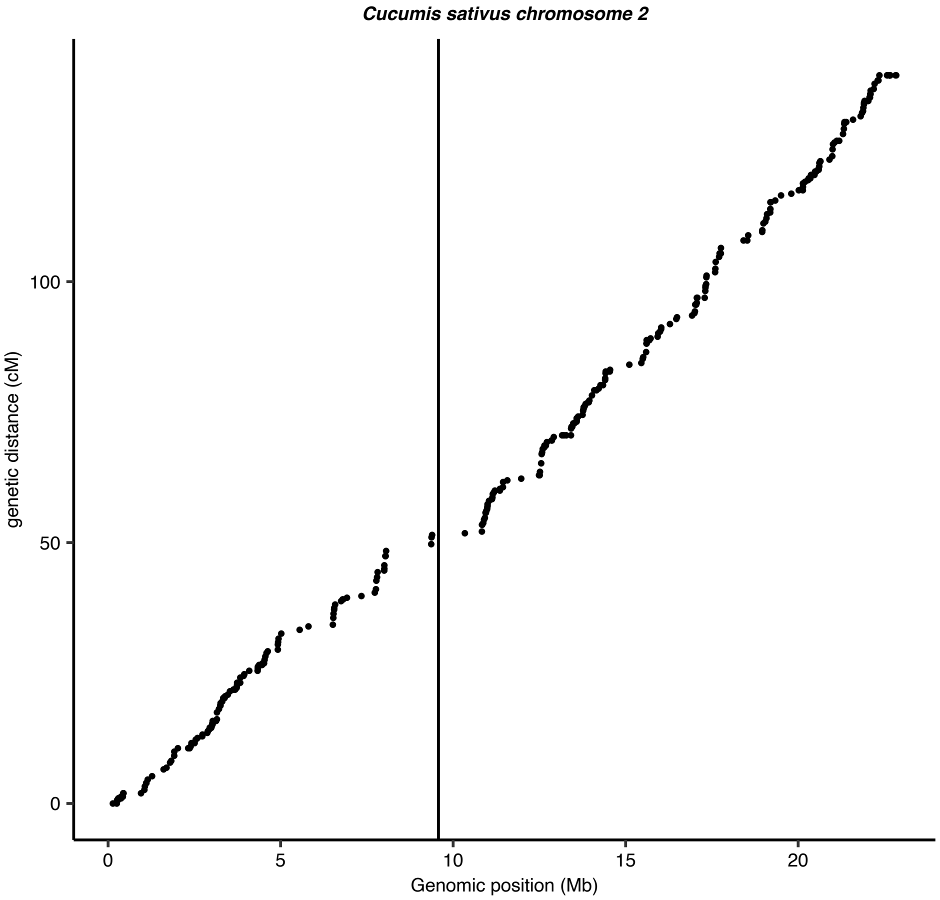
Cucumis sativus chromosome 2
genetic distance (cM)
0
50
100
Genomic position (Mb)
0
5
10
15
20

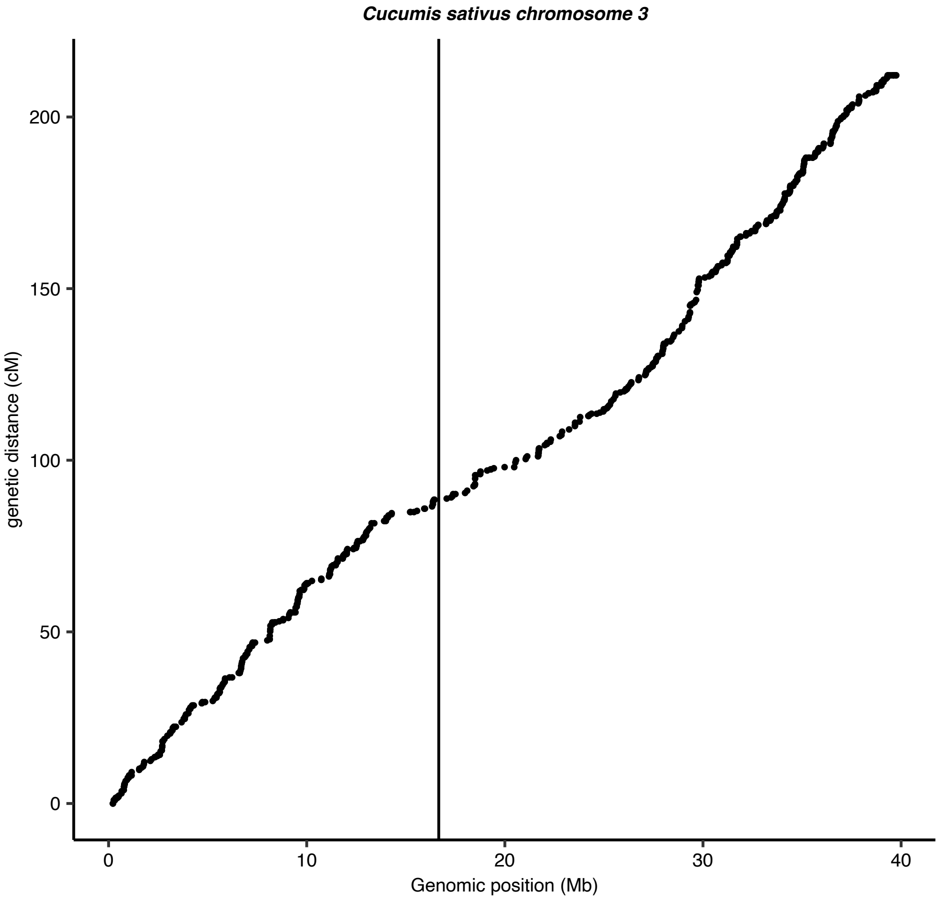
Cucumis sativus chromosome 3
genetic distance (cM)
0
50
100
150
200
Genomic position (Mb)
0
10
20
30
40

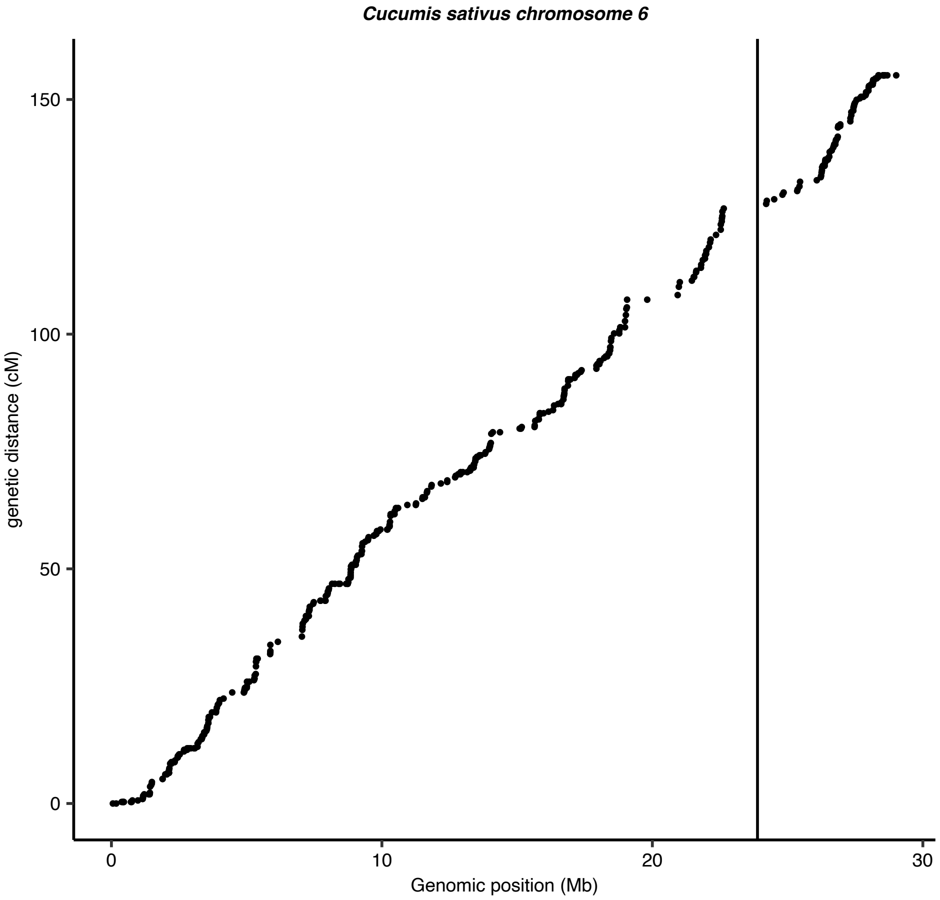
Cucumis sativus chromosome 6
genetic distance (cM)
Genomic position (Mb)
0
50
100
150
0
10
20
30

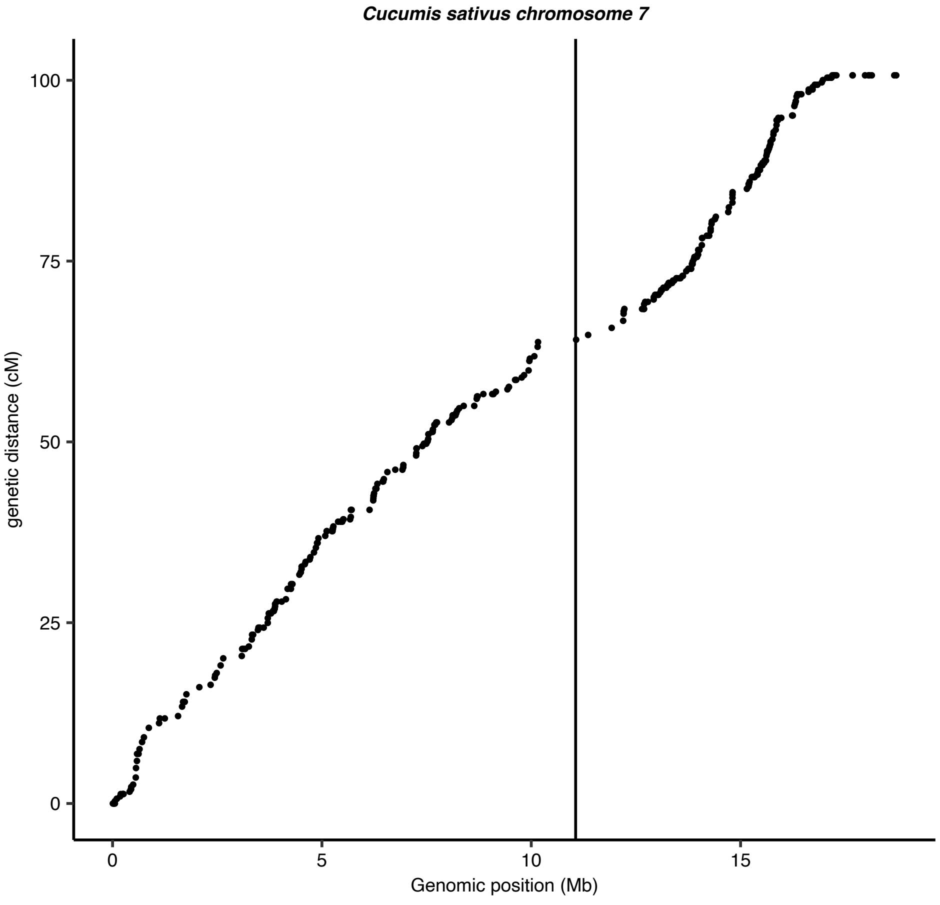
Cucumis sativus chromosome 7
genetic distance (cM)
Genomic position (Mb)
0
25
50
75
100
0
5
10
15

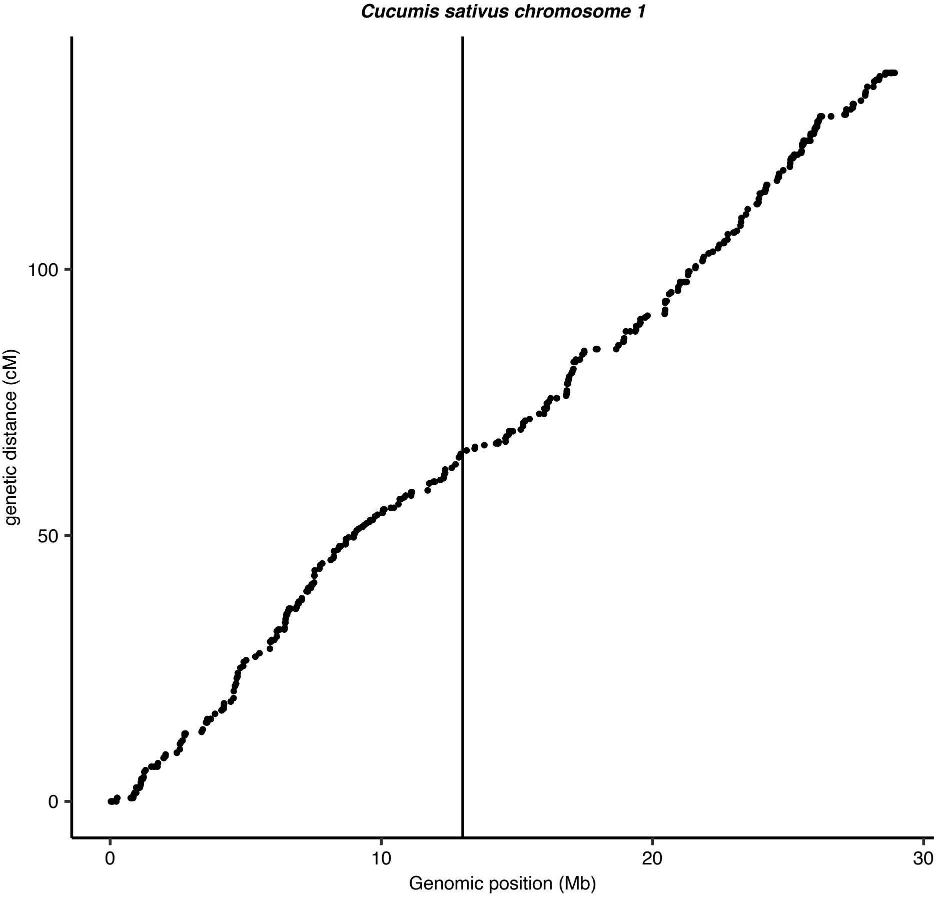
Cucumis sativus chromosome 1
genetic distance (cM)
100
50
0
0
10
20
30
Genomic position (Mb)

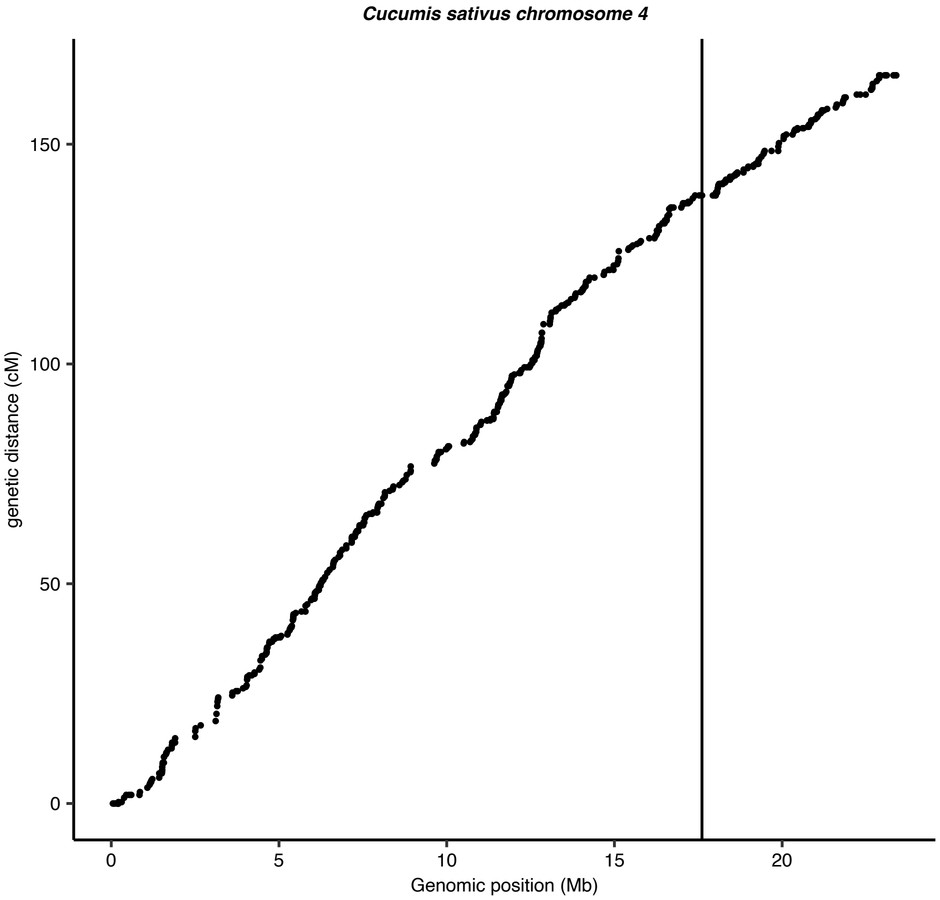
Cucumis sativus chromosome 4
genetic distance (cM)
150
100
50
0
0
5
10
15
20
Genomic position (Mb)

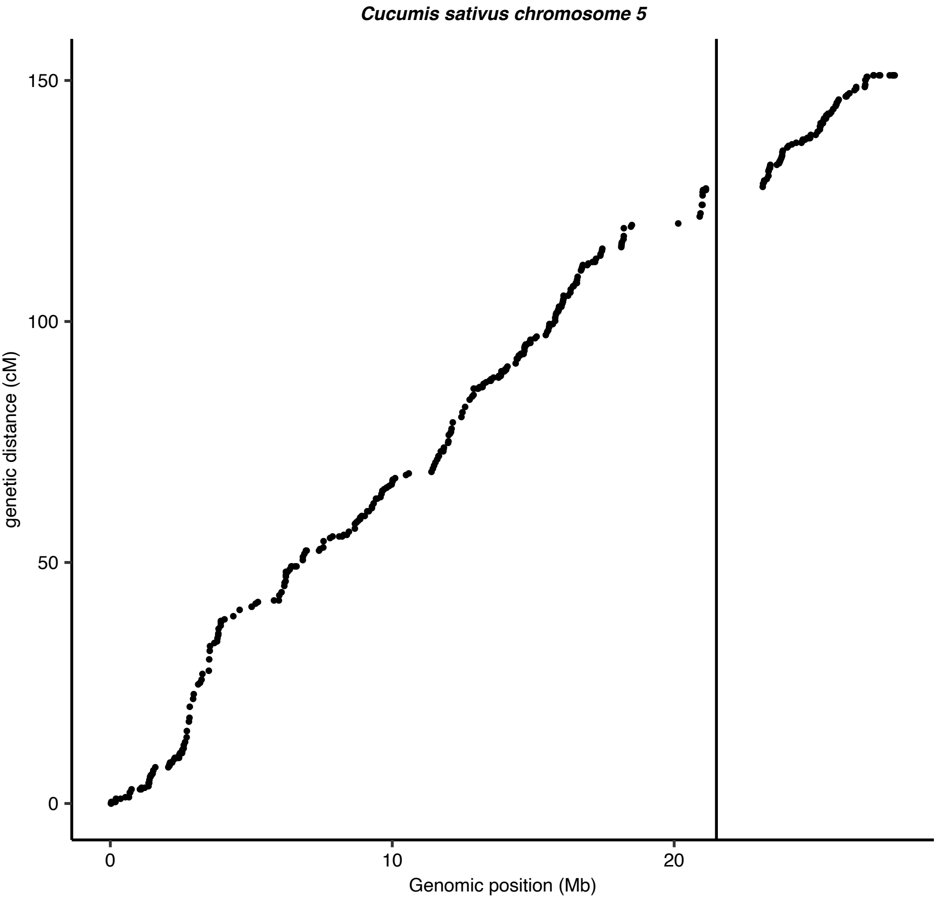
Cucumis sativus chromosome 5
genetic distance (cM)
150
100
50
0
0
10
20
Genomic position (Mb)

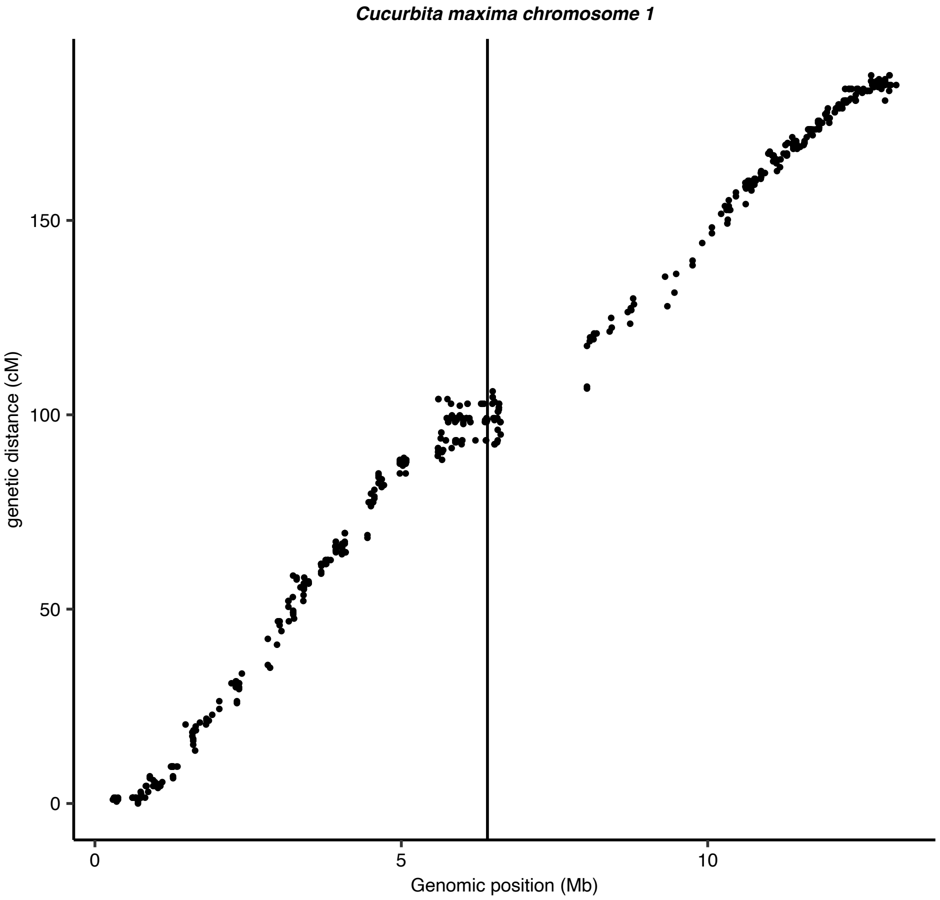
Cucurbita maxima chromosome 1
genetic distance (cM)
0
50
100
150
Genomic position (Mb)
0
5
10

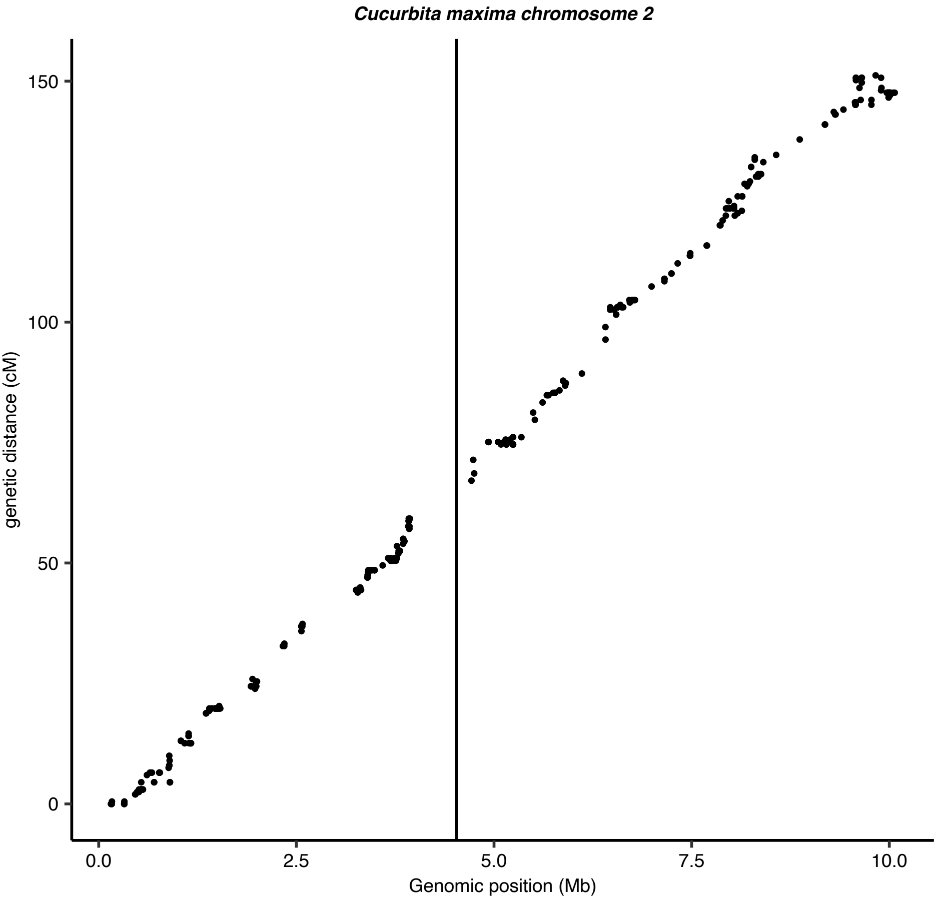
Cucurbita maxima chromosome 2
genetic distance (cM)
150
100
50
0
0.0
2.5
5.0
7.5
10.0
Genomic position (Mb)

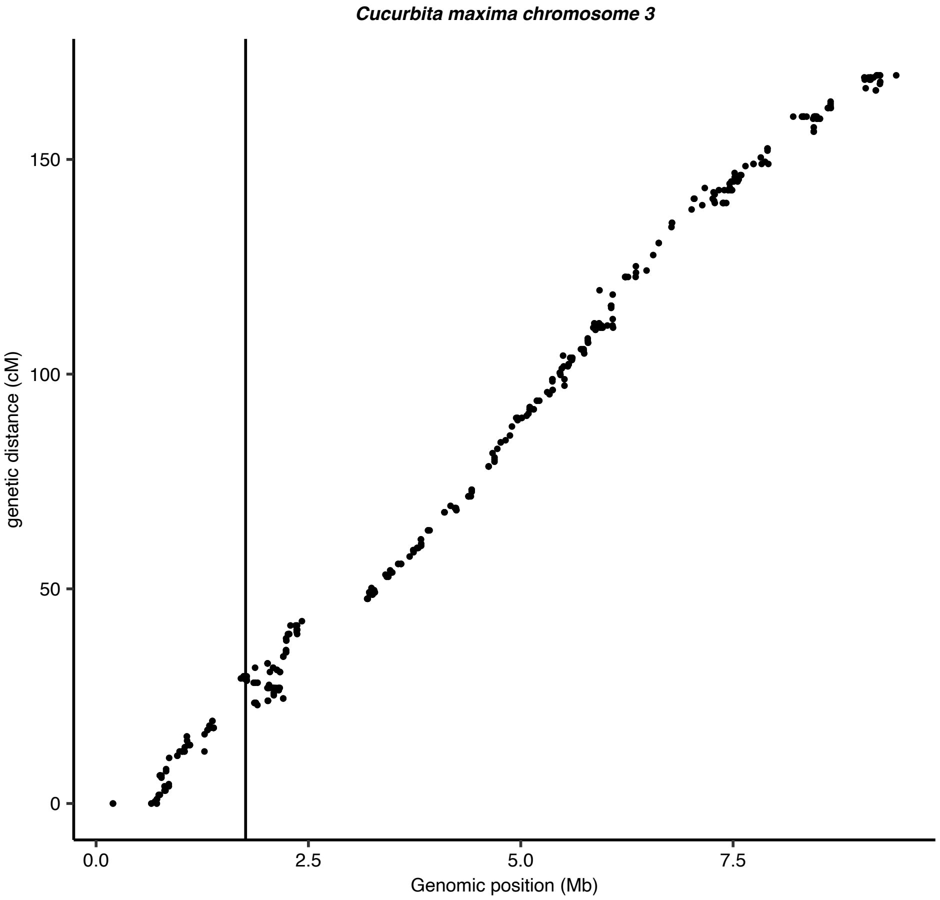
Cucurbita maxima chromosome 3
genetic distance (cM)
0
50
100
150
0.0
2.5
5.0
7.5
Genomic position (Mb)

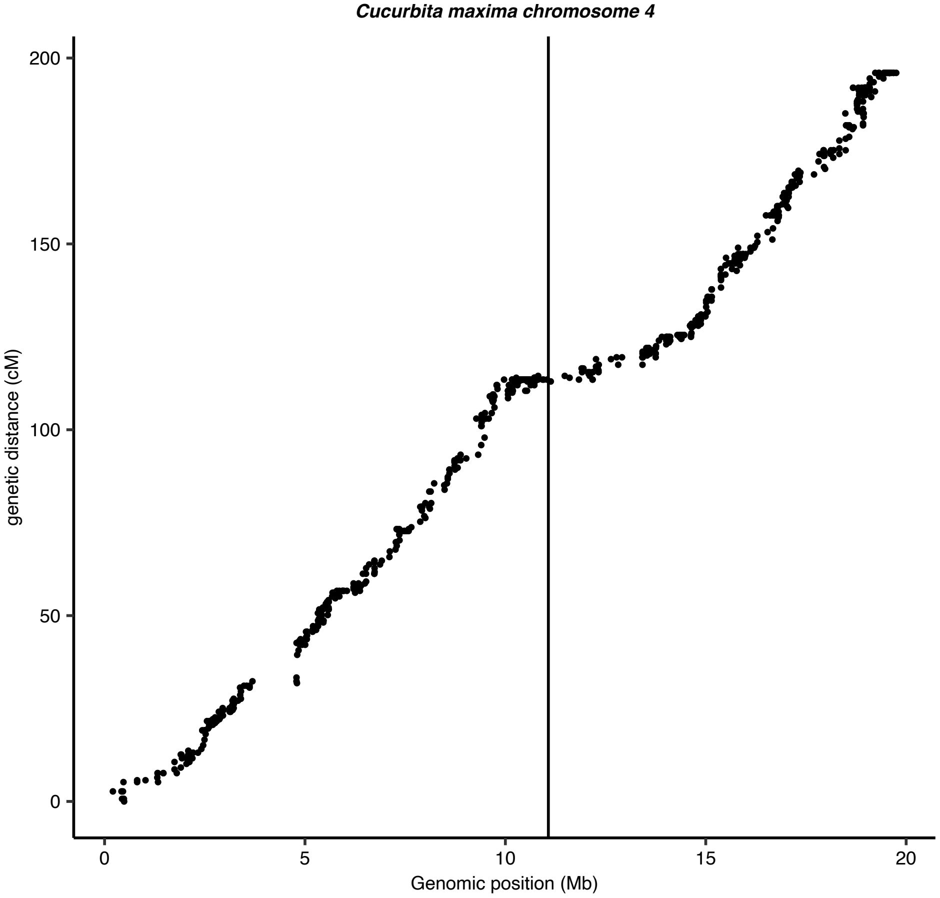
Cucurbita maxima chromosome 4
genetic distance (cM)
200
150
100
50
0
0
5
10
15
20
Genomic position (Mb)

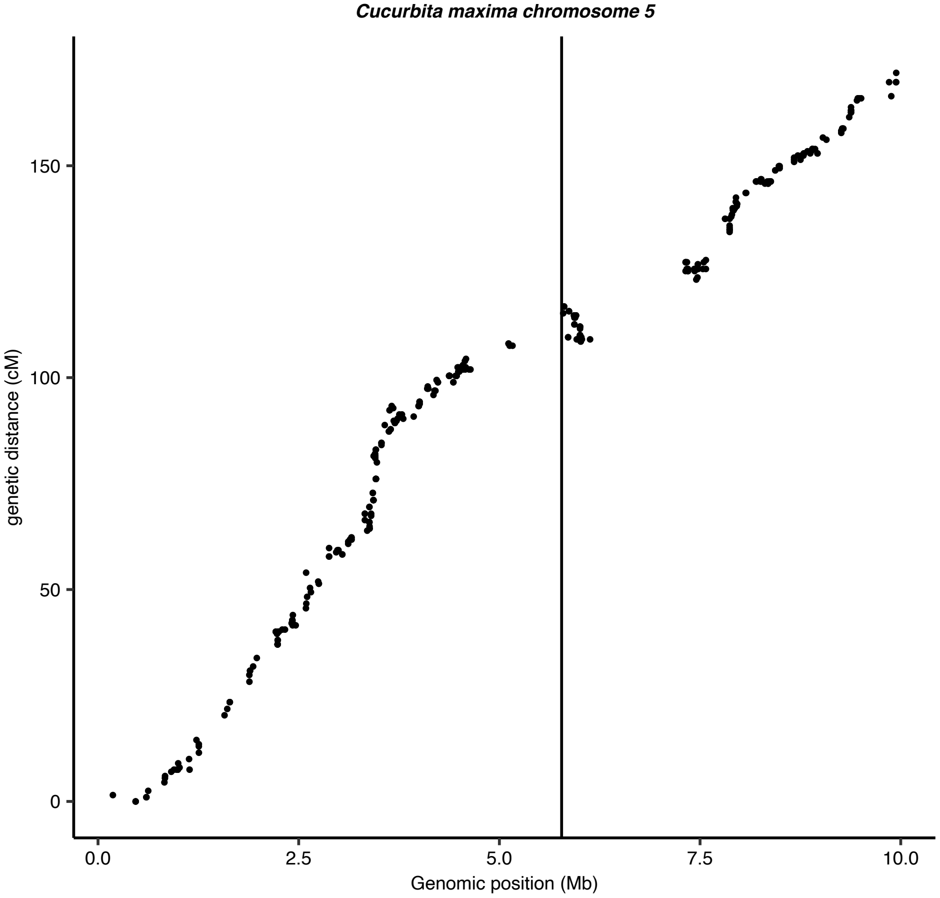
Cucurbita maxima chromosome 5
genetic distance (cM)
150
100
50
0
0.0
2.5
5.0
7.5
10.0
Genomic position (Mb)

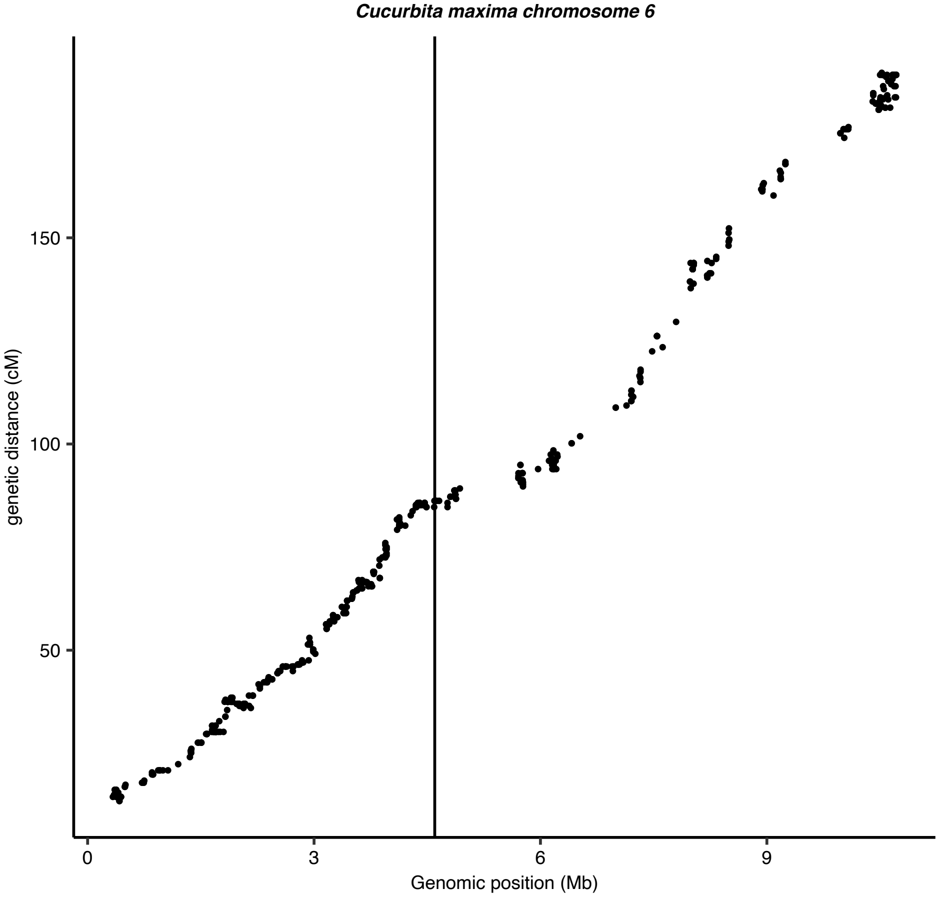

Cucurbita maxima chromosome 6
genetic distance (cM)
150
100
50
0
3
6
9
Genomic position (Mb)

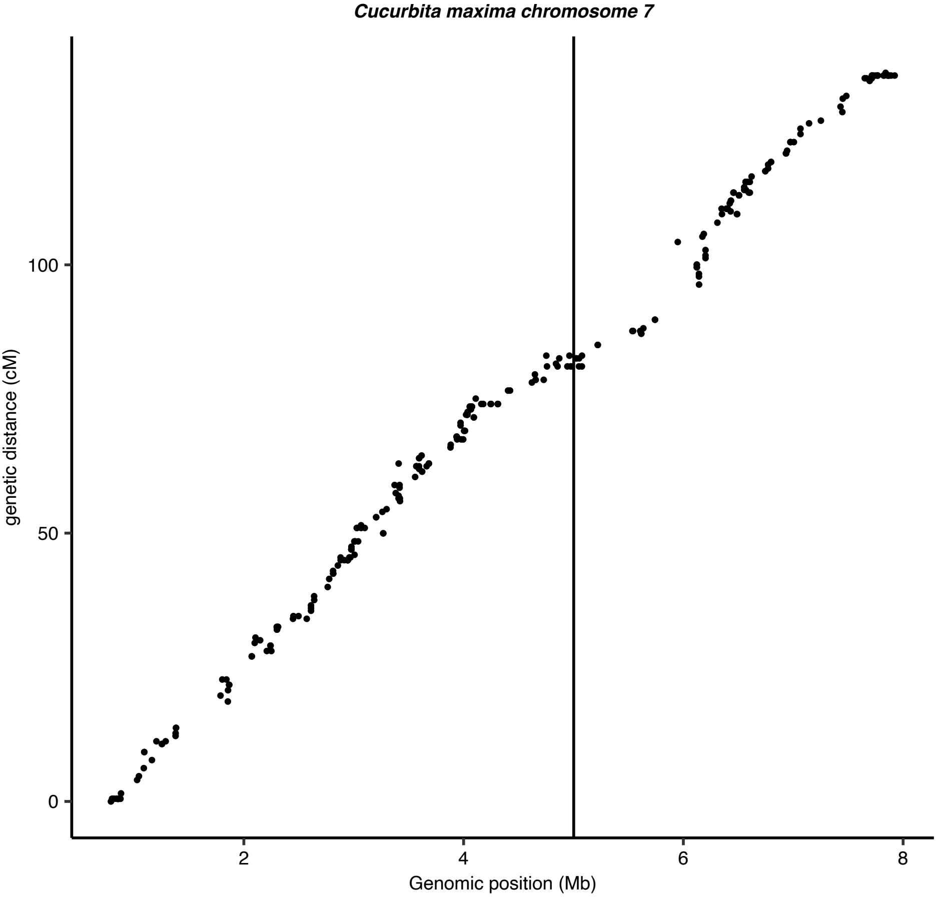
Cucurbita maxima chromosome 7
genetic distance (cM)
0
50
100
Genomic position (Mb)
2
4
6
8

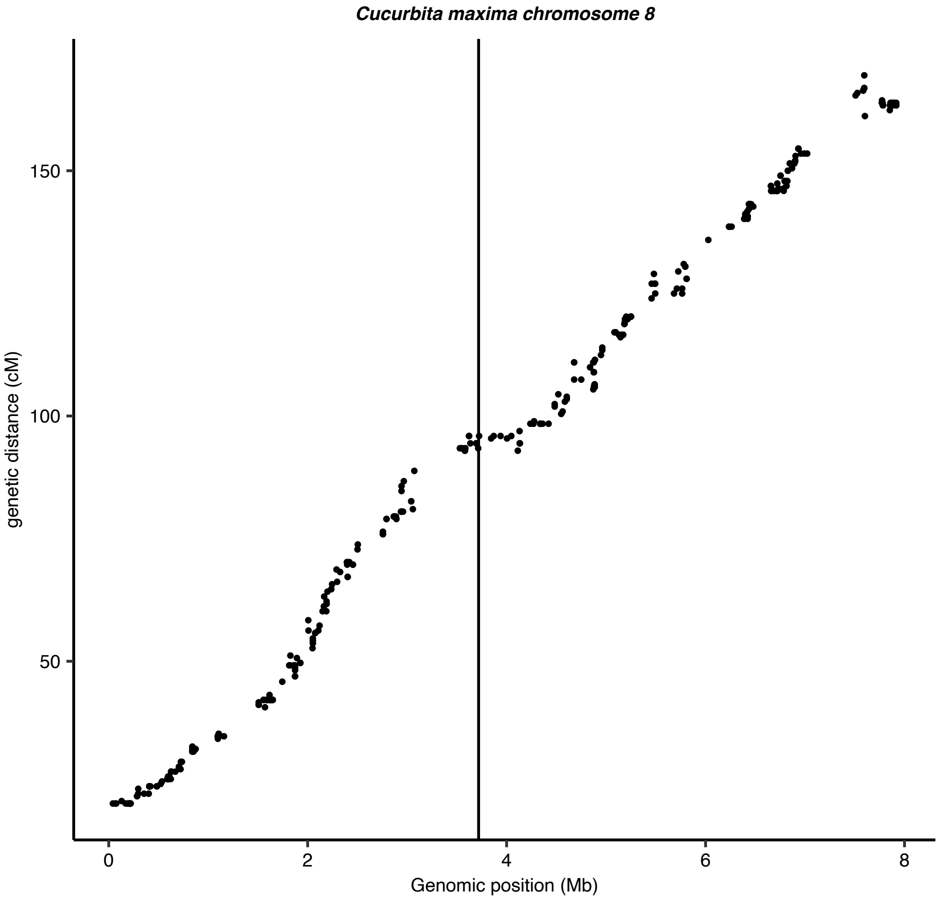
Cucurbita maxima chromosome 8
genetic distance (cM)
150
100
50
0
2
4
6
8
Genomic position (Mb)

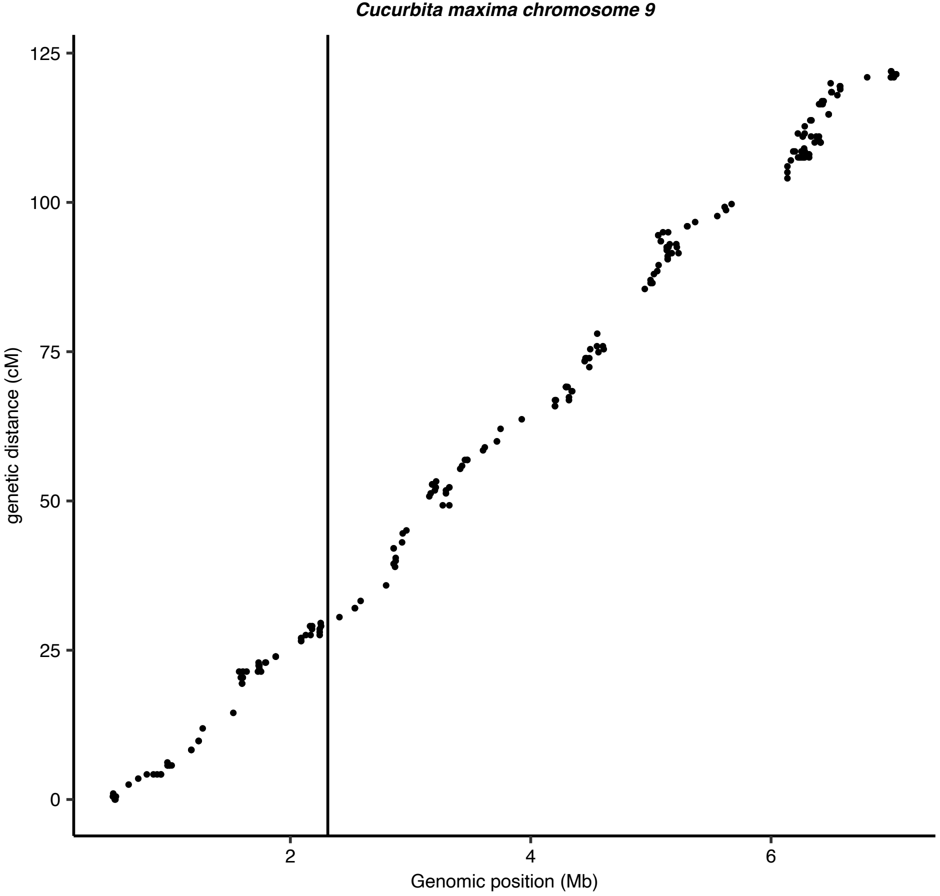
Cucurbita maxima chromosome 9
genetic distance (cM)
125
100
75
50
25
0
2
4
6
Genomic position (Mb)

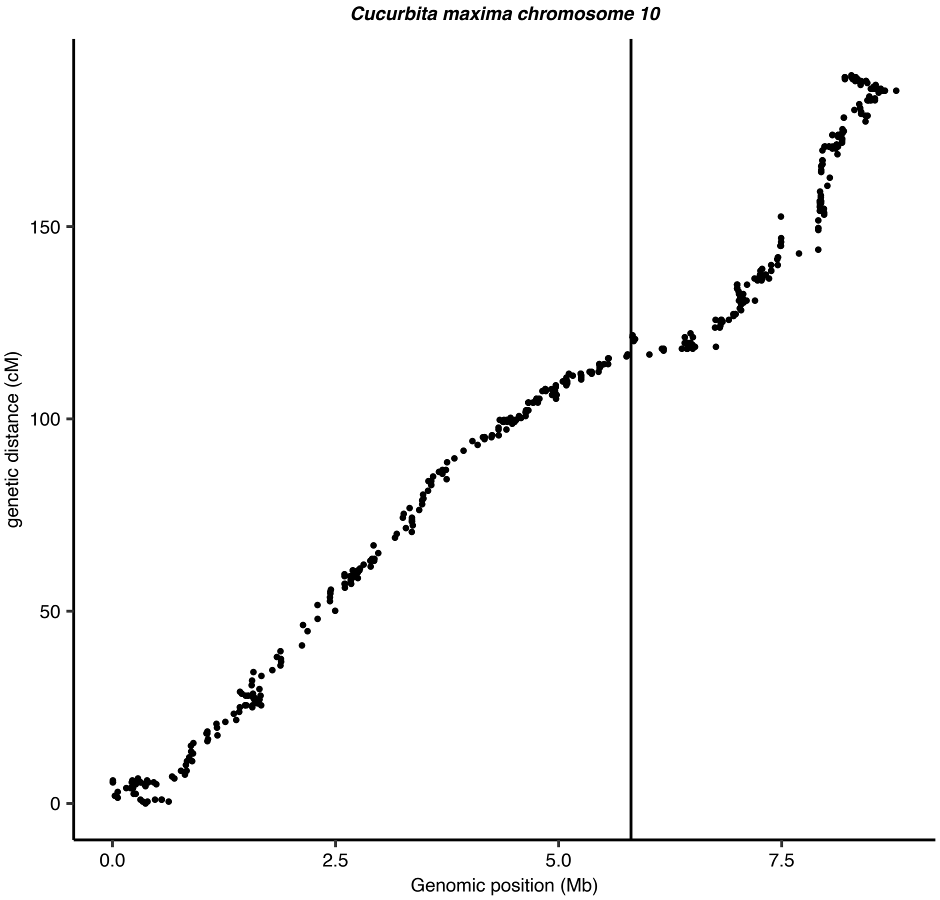
Cucurbita maxima chromosome 10
genetic distance (cM)
0
50
100
150
0.0
2.5
5.0
7.5
Genomic position (Mb)

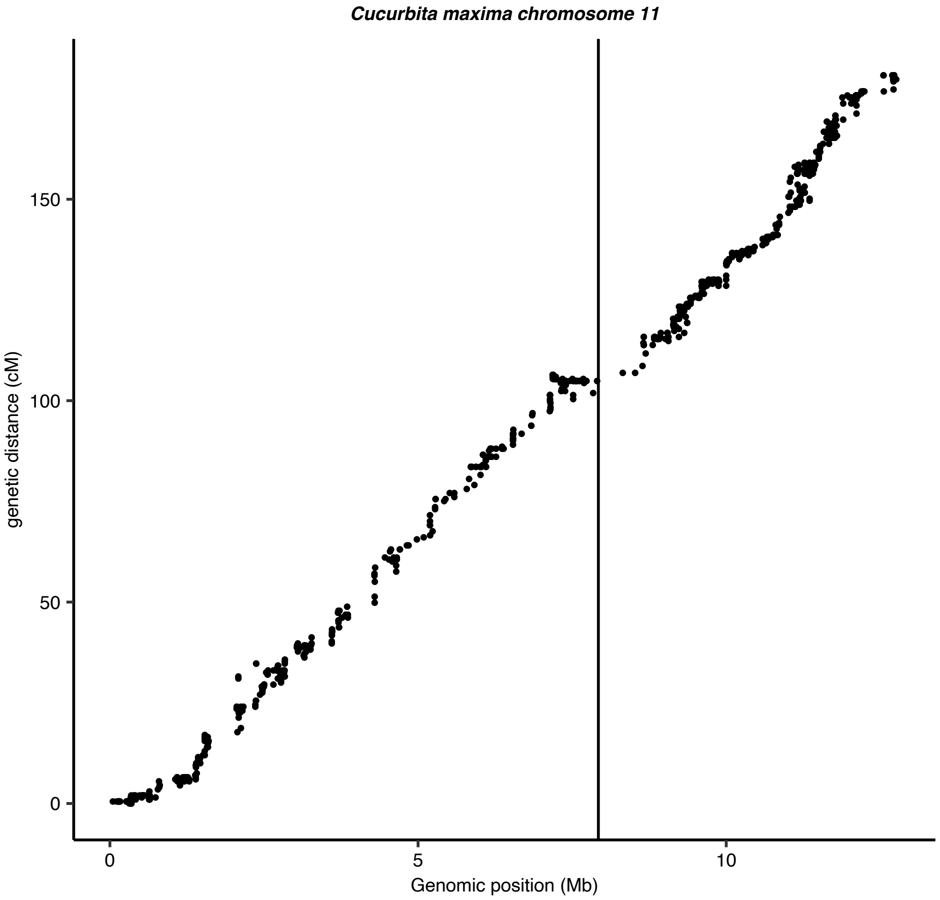
Cucurbita maxima chromosome 11
genetic distance (cM)
150
100
50
0
0
5
10
Genomic position (Mb)

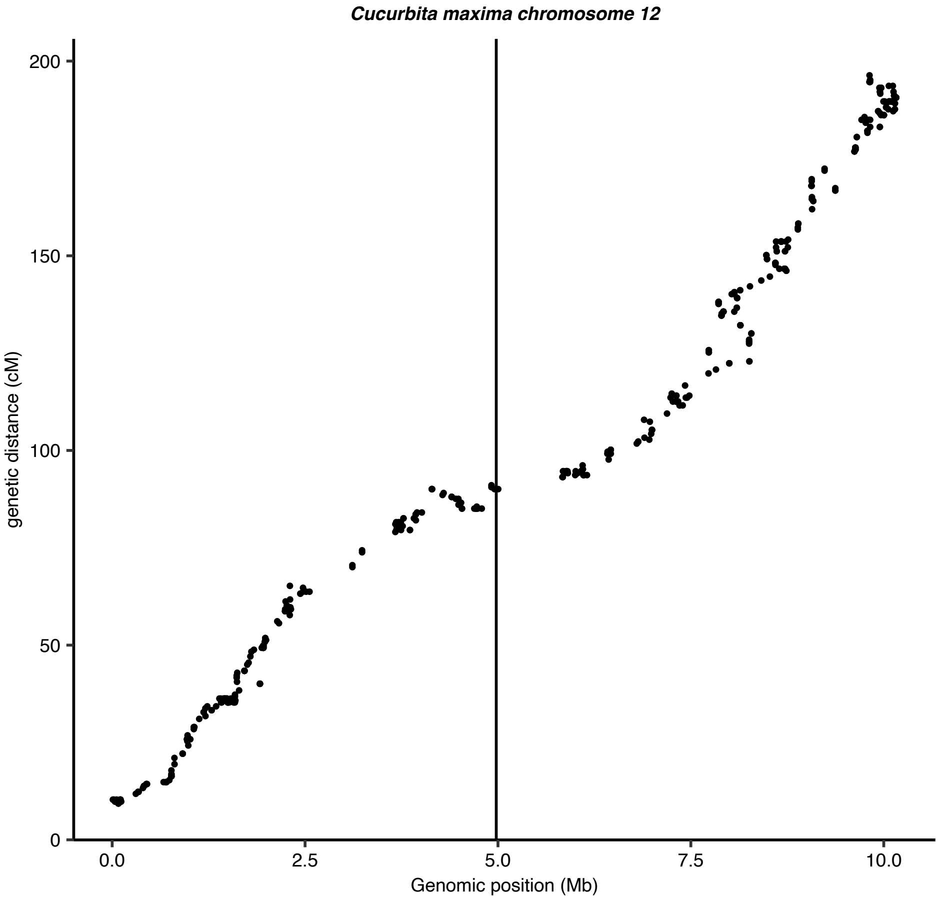
Cucurbita maxima chromosome 12
genetic distance (cM)
200
150
100
50
0
0.0
2.5
5.0
7.5
10.0
Genomic position (Mb)

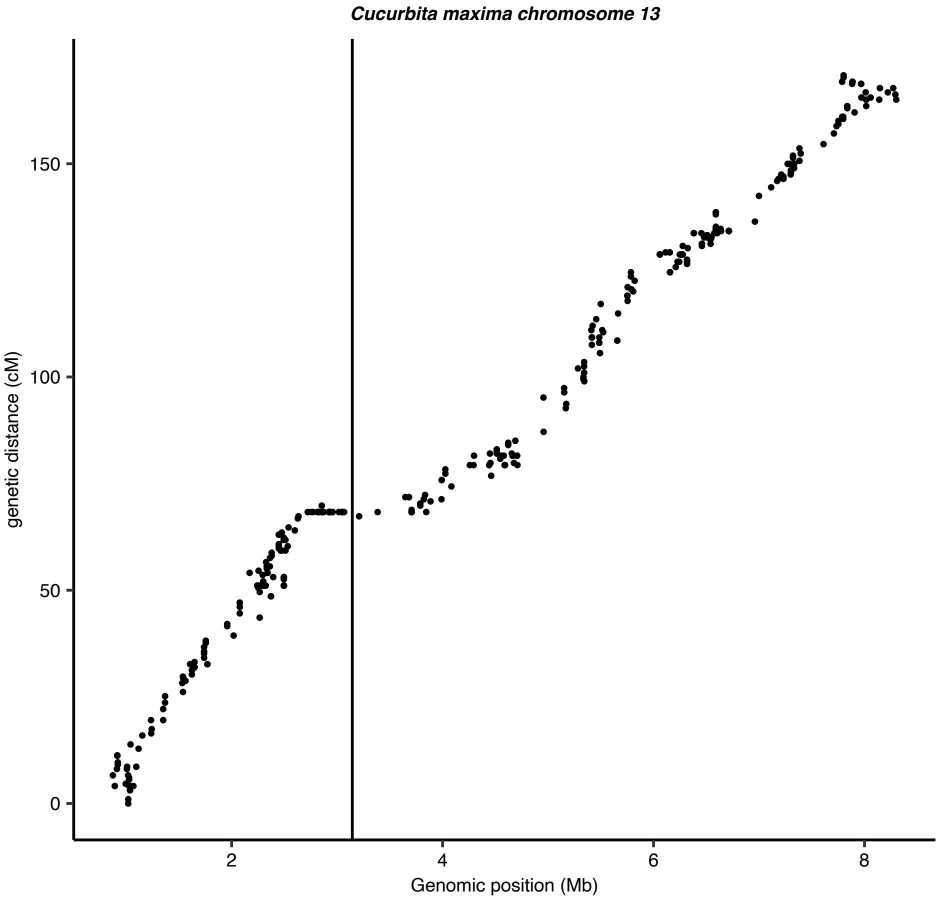
Cucurbita maxima chromosome 13
genetic distance (cM)
0
50
100
150
2
4
6
8
Genomic position (Mb)

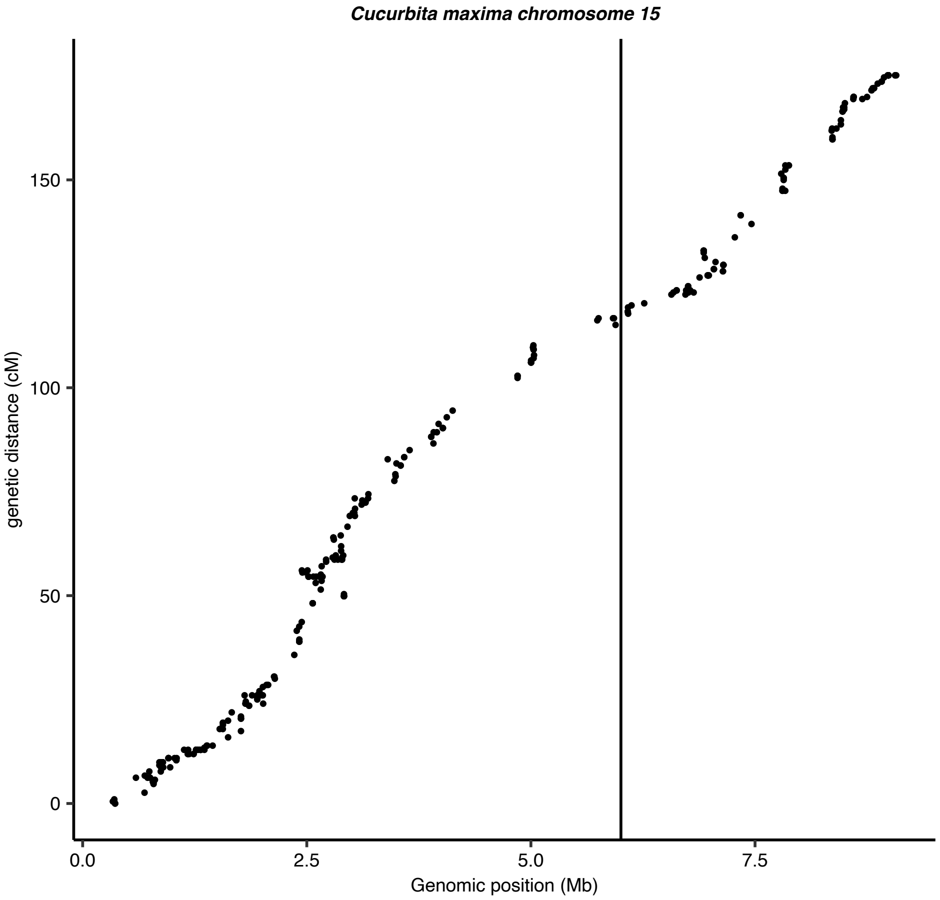
Cucurbita maxima chromosome 15
genetic distance (cM)
0
50
100
150
Genomic position (Mb)
0.0
2.5
5.0
7.5

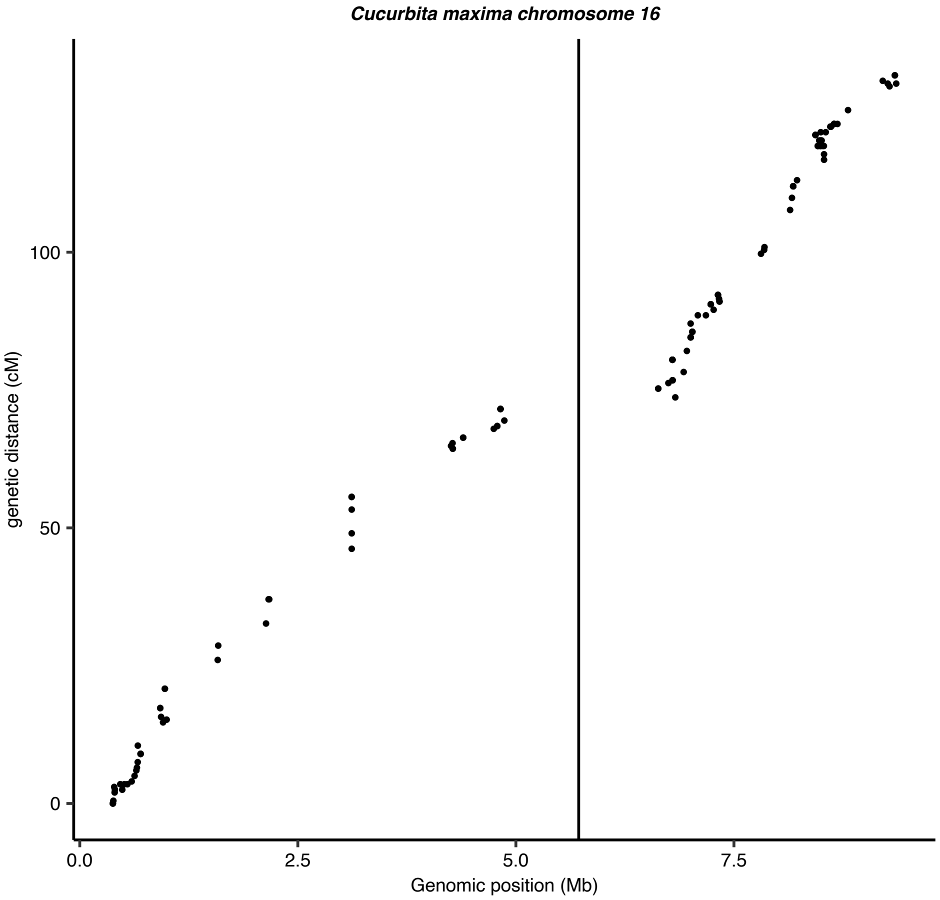
Cucurbita maxima chromosome 16
genetic distance (cM)
0
50
100
0.0
2.5
5.0
7.5
Genomic position (Mb)

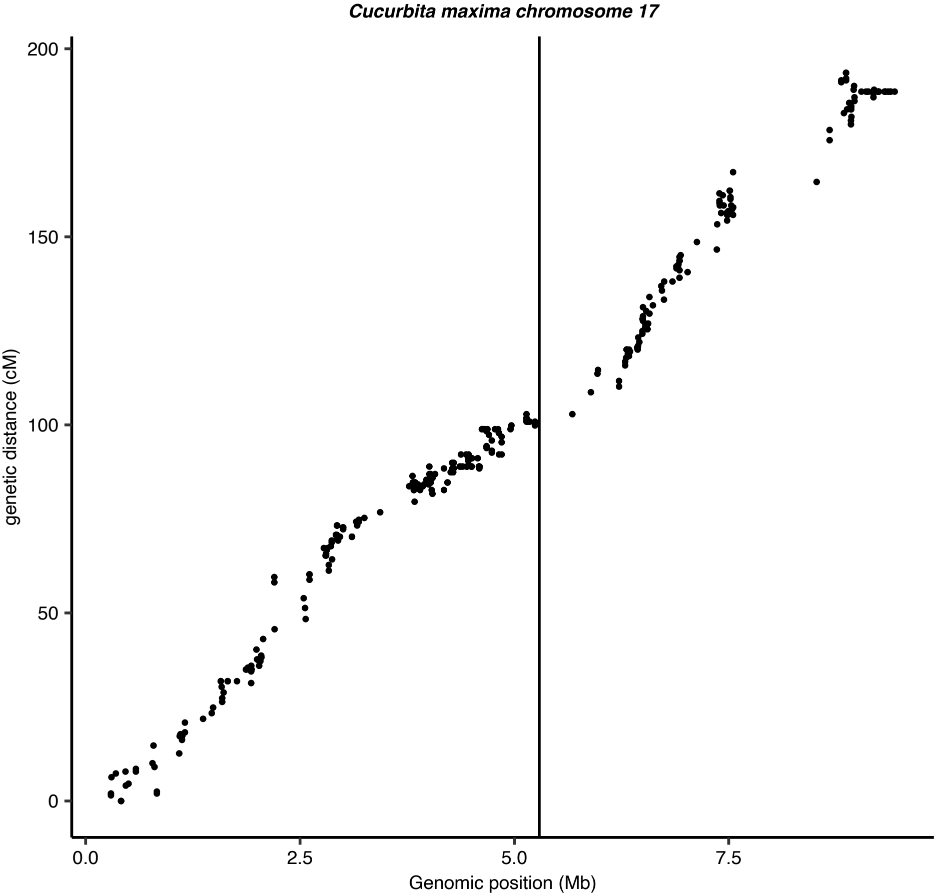
Cucurbita maxima chromosome 17
genetic distance (cM)
200
150
100
50
0
0.0
2.5
5.0
7.5
Genomic position (Mb)

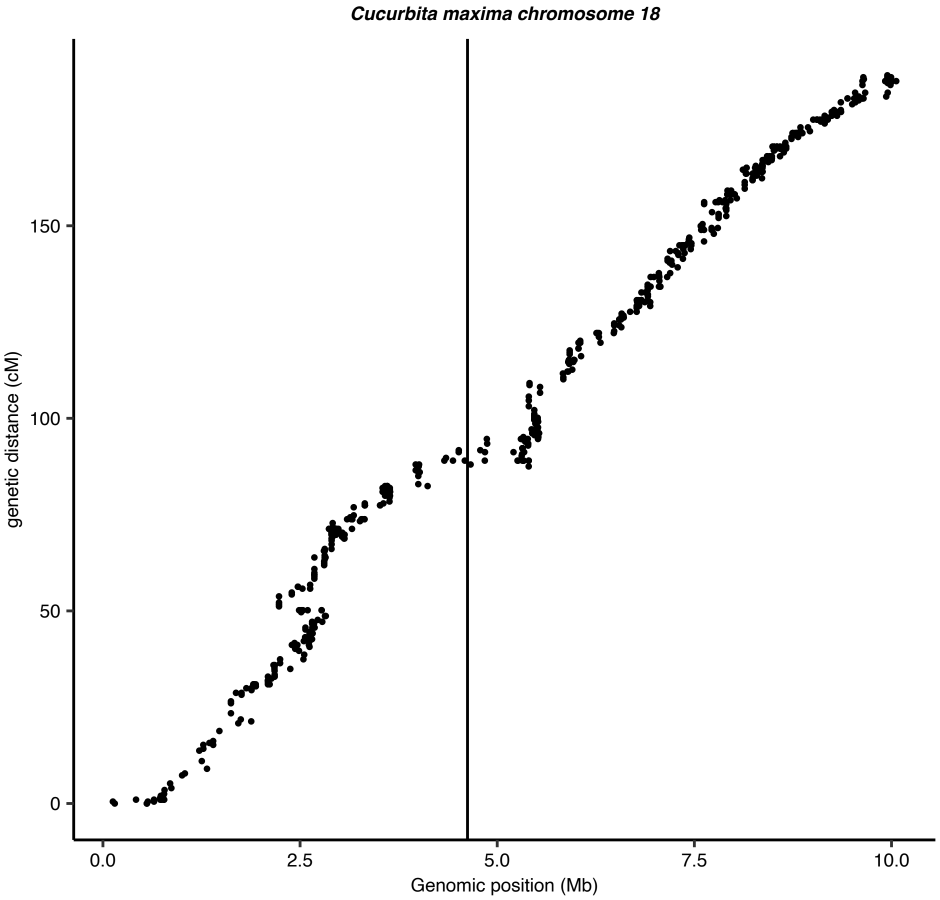
Cucurbita maxima chromosome 18
genetic distance (cM)
0
50
100
150
0.0
2.5
5.0
7.5
10.0
Genomic position (Mb)

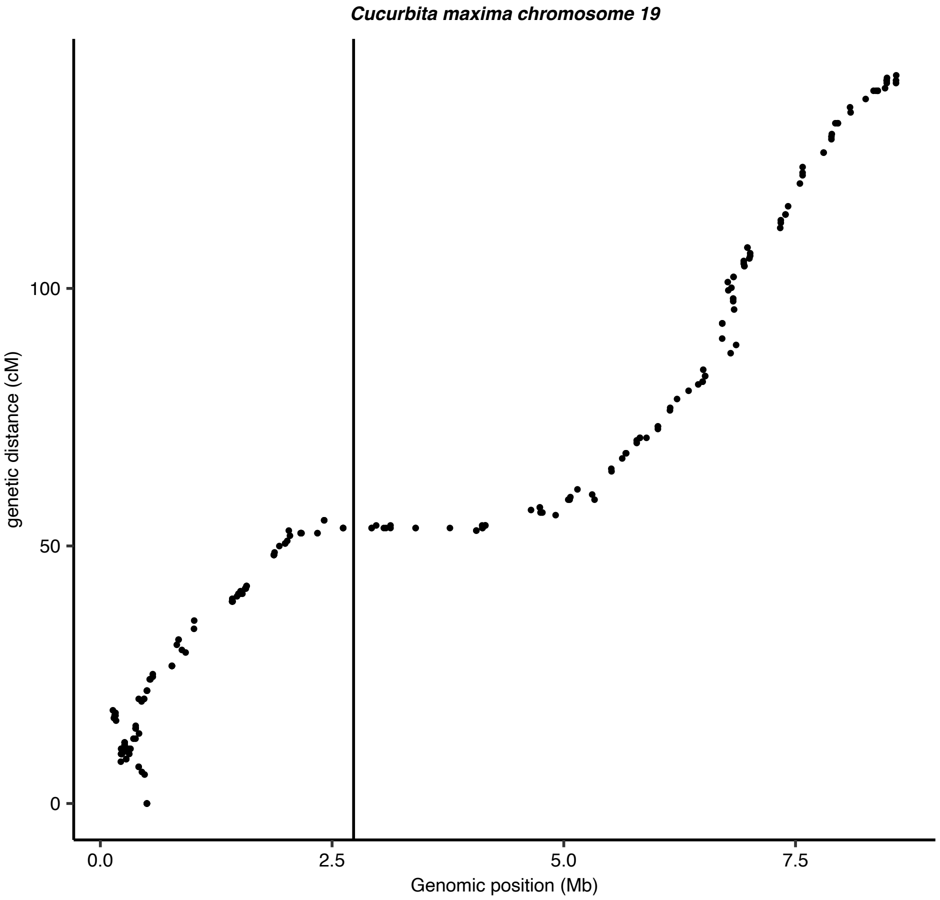
Cucurbita maxima chromosome 19
genetic distance (cM)
100
50
0
0.0
2.5
5.0
7.5
Genomic position (Mb)

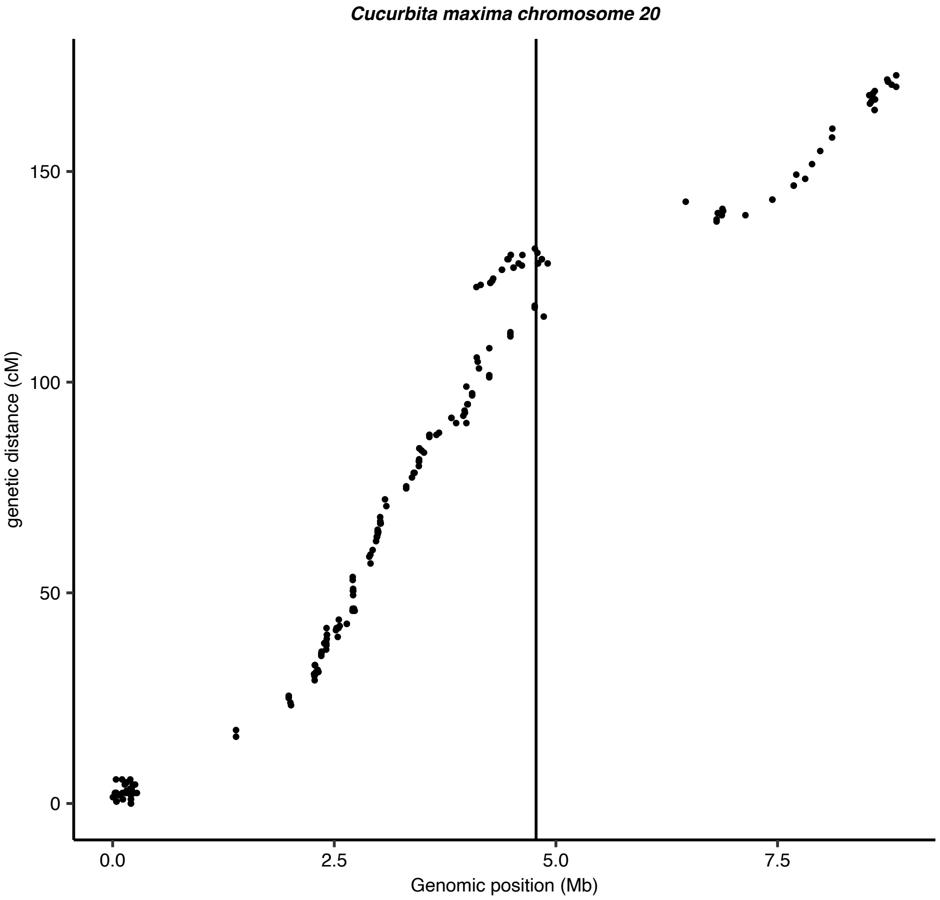
Cucurbita maxima chromosome 20
genetic distance (cM)
Genomic position (Mb)
0
50
100
150
0.0
2.5
5.0
7.5

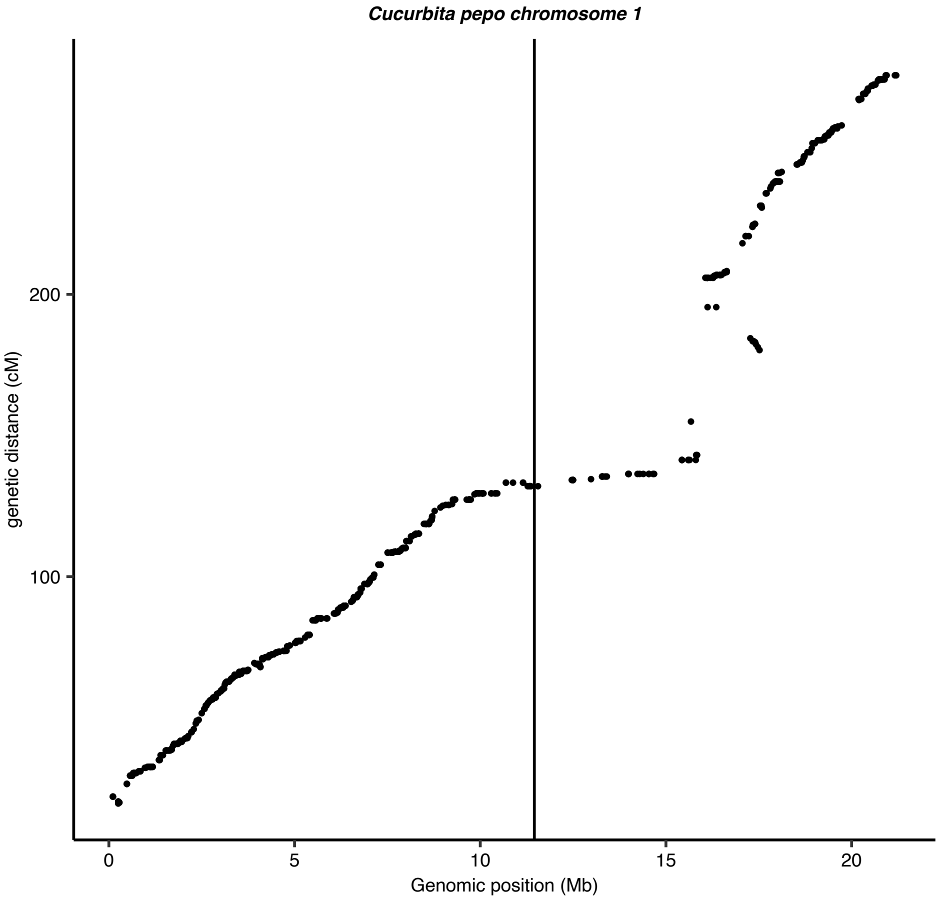
Cucurbita pepo chromosome 1
genetic distance (cM)
200
100
0
5
10
15
20
Genomic position (Mb)

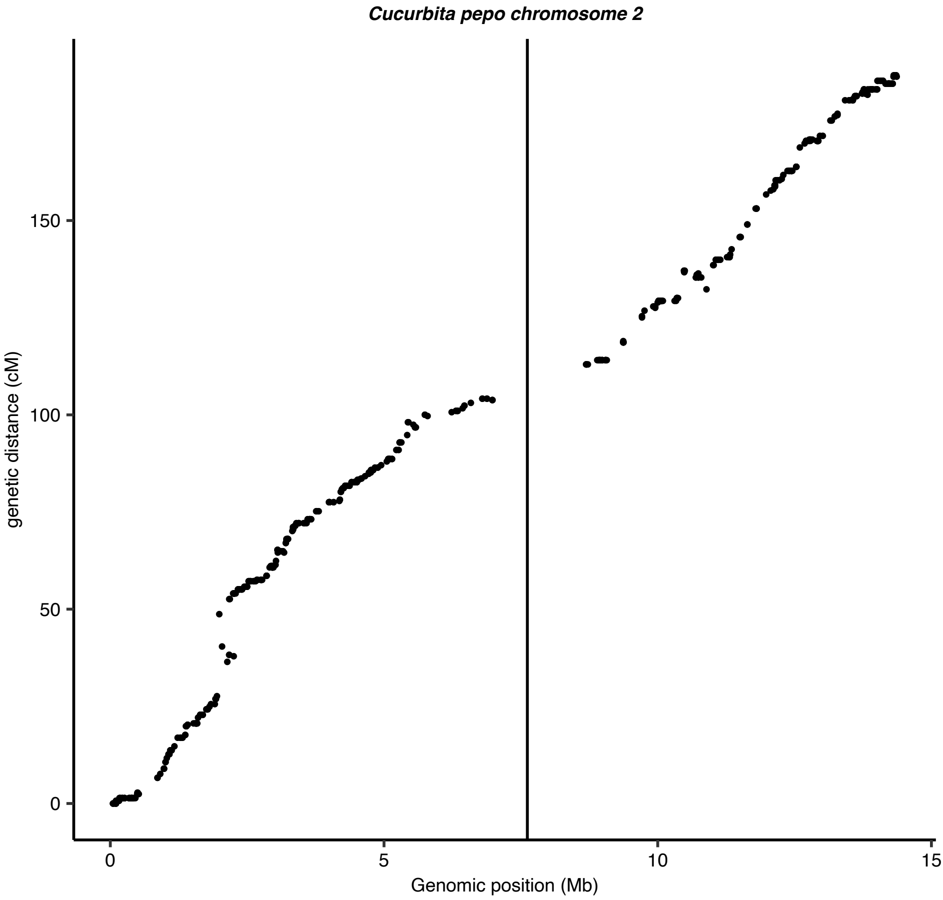
Cucurbita pepo chromosome 2
genetic distance (cM)
0
50
100
150
0
5
10
15
Genomic position (Mb)

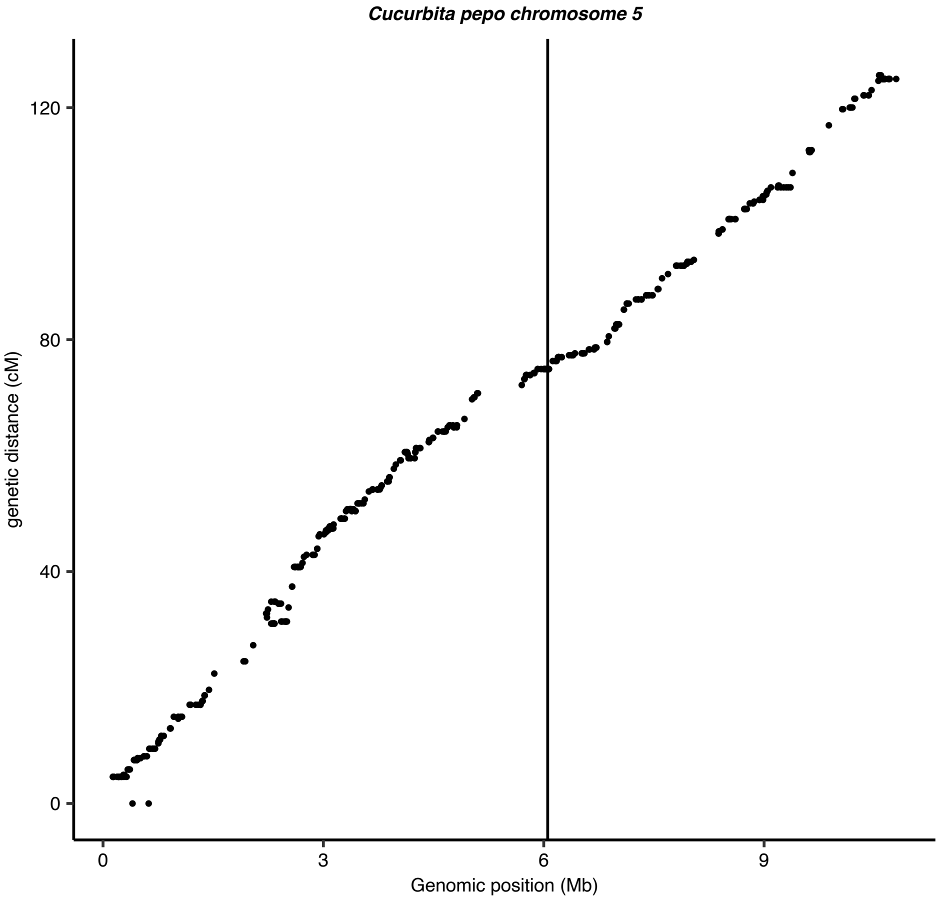
Cucurbita pepo chromosome 5
genetic distance (cM)
120
80
40
0
0
3
6
9
Genomic position (Mb)

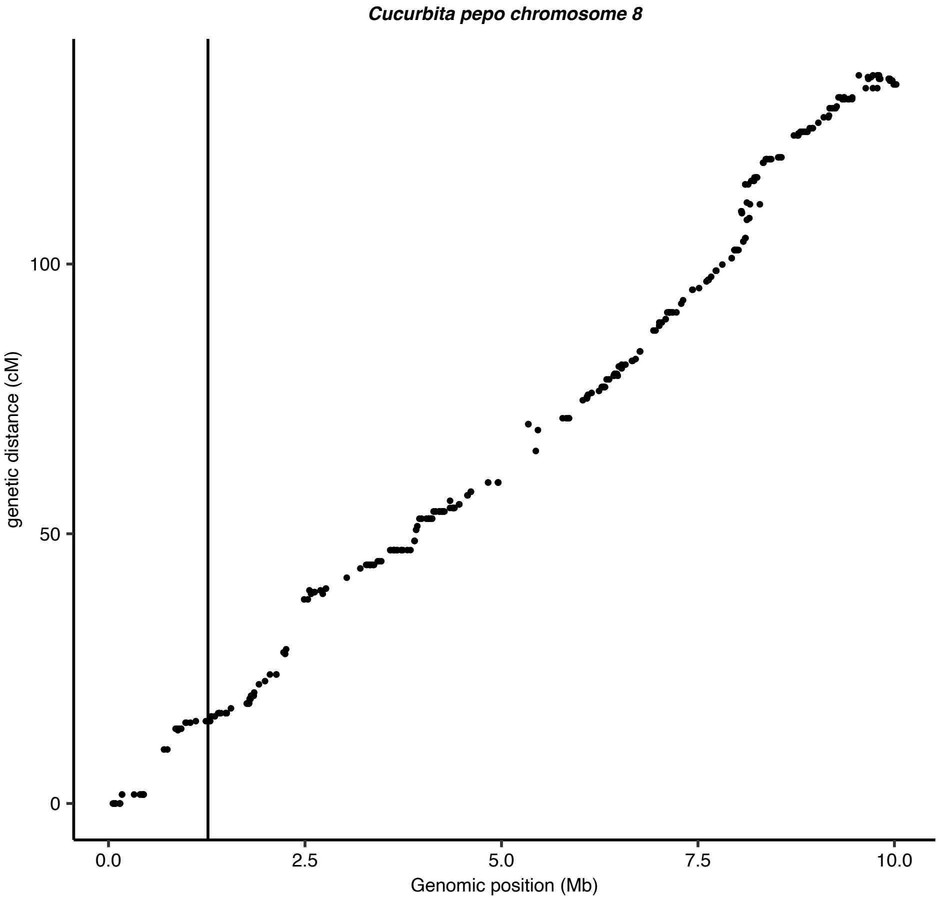

Cucurbita pepo chromosome 8
genetic distance (cM)
0
50
100
0.0
2.5
5.0
7.5
10.0
Genomic position (Mb)

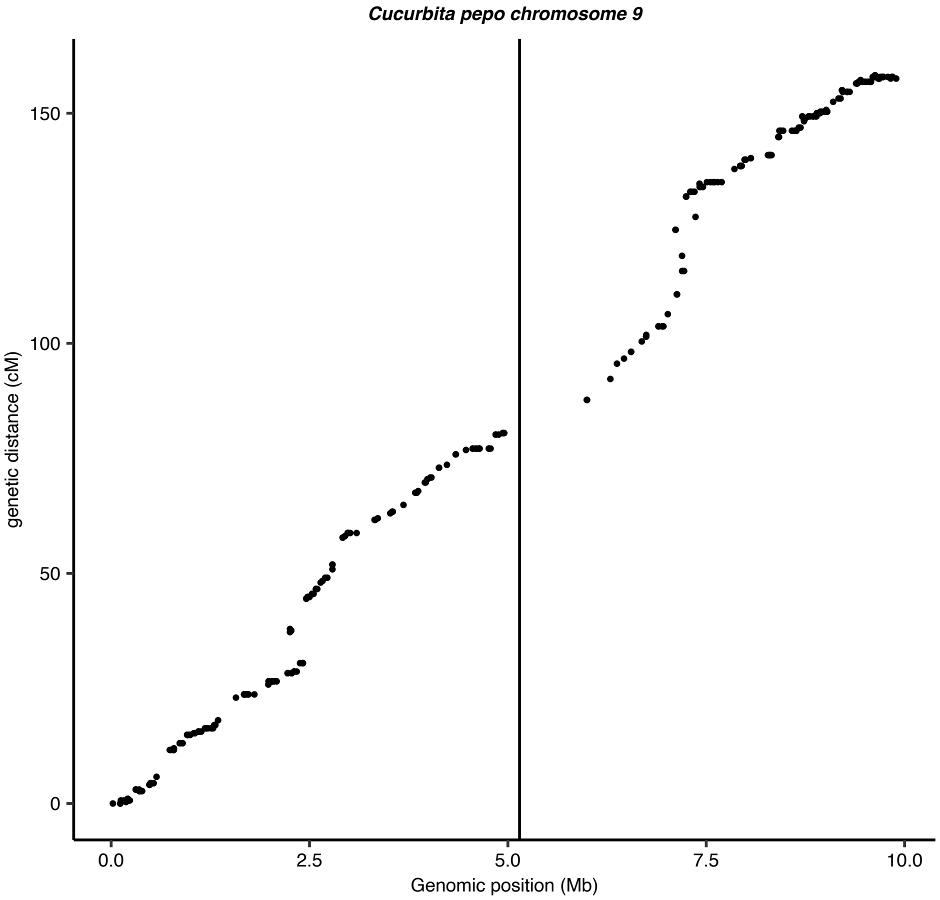

Cucurbita pepo chromosome 9
genetic distance (cM)
0
50
100
150
0.0
2.5
5.0
7.5
10.0
Genomic position (Mb)

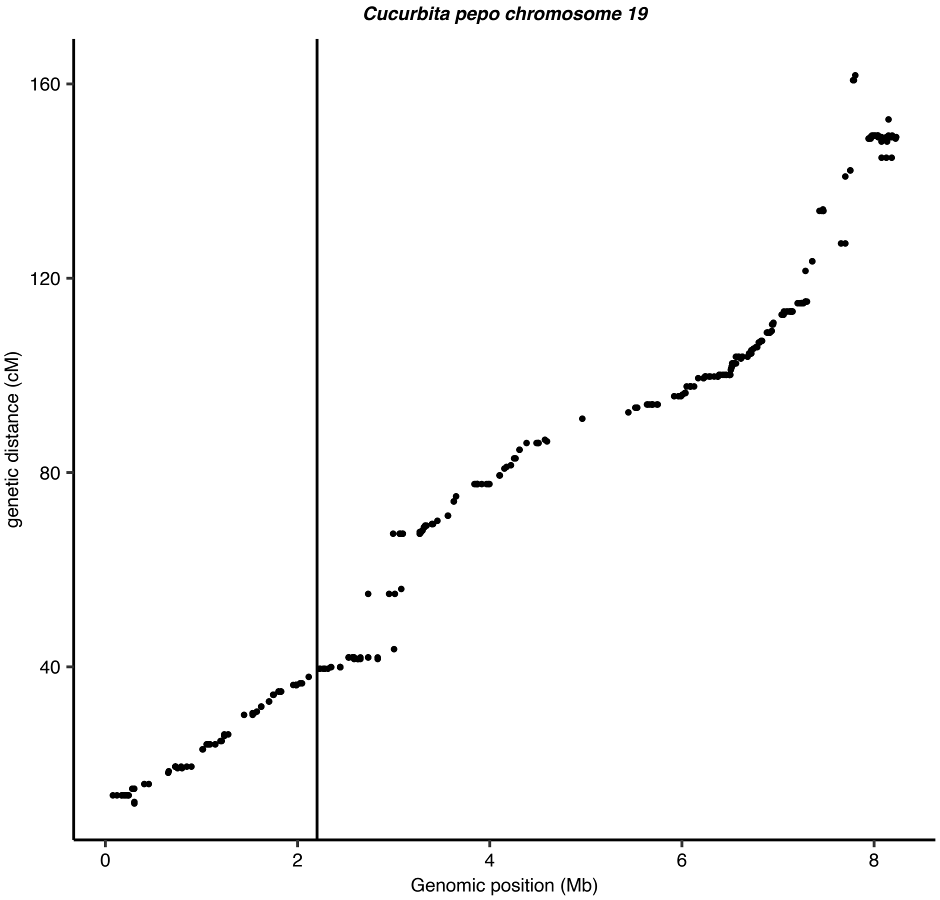

Cucurbita pepo chromosome 19
genetic distance (cM)
Genomic position (Mb)
160
120
80
40
0
2
4
6
8

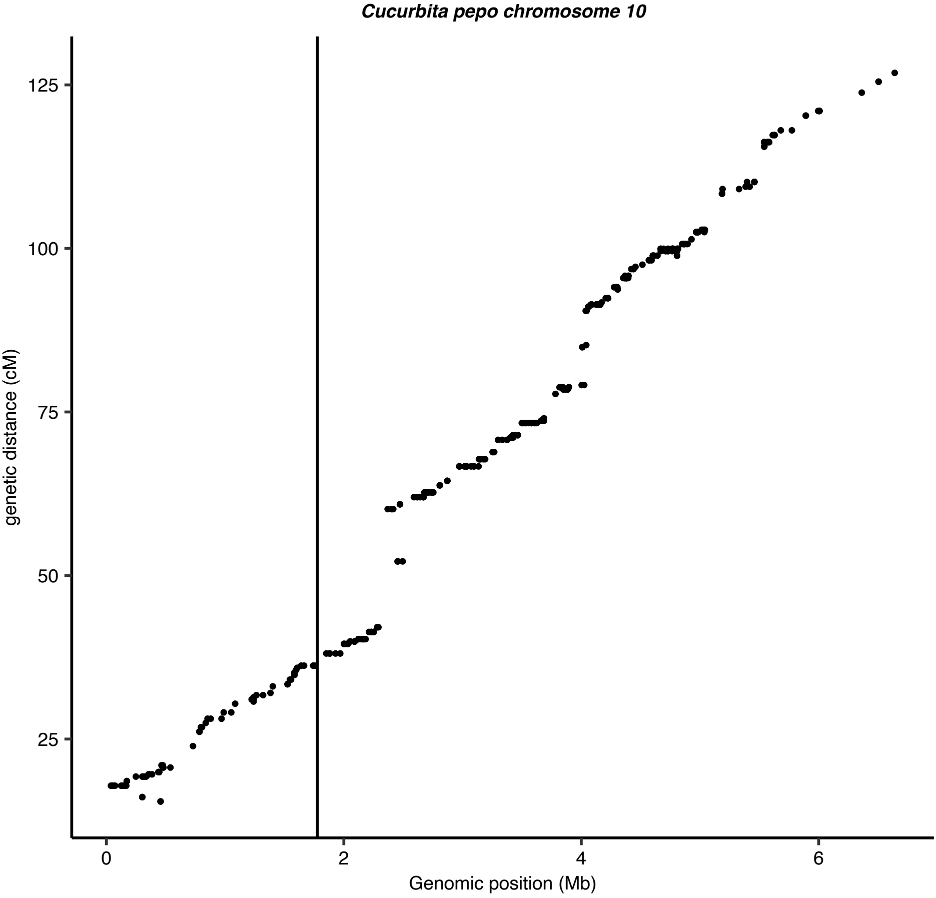
Cucurbita pepo chromosome 10
genetic distance (cM)
125
100
75
50
25
0
2
4
6
Genomic position (Mb)

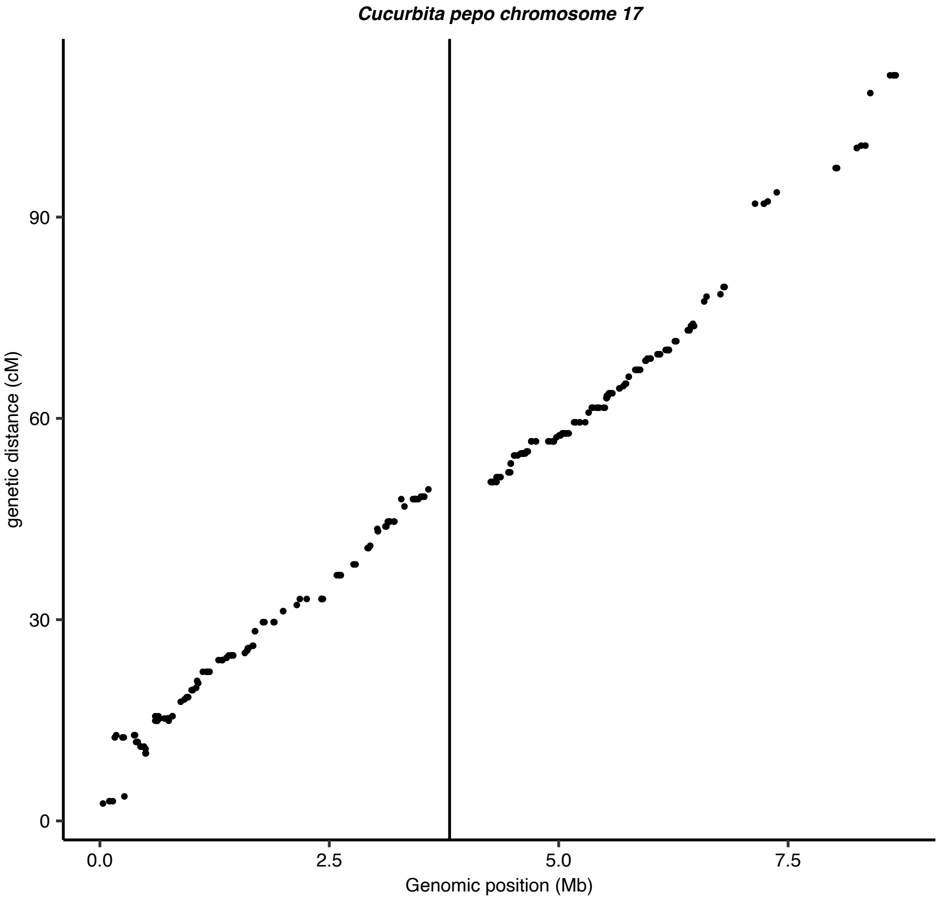
Cucurbita pepo chromosome 17
genetic distance (cM)
0
30
60
90
0.0
2.5
5.0
7.5
Genomic position (Mb)

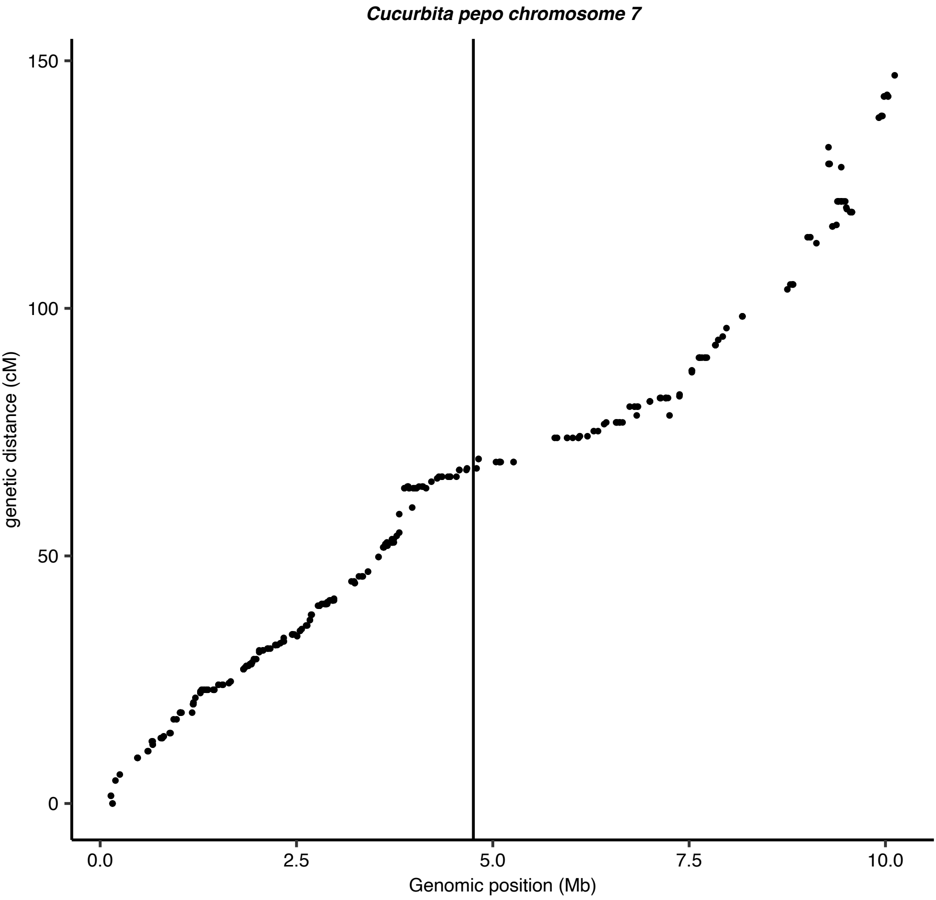
Cucurbita pepo chromosome 7
genetic distance (cM)
0
50
100
150
Genomic position (Mb)
0.0
2.5
5.0
7.5
10.0

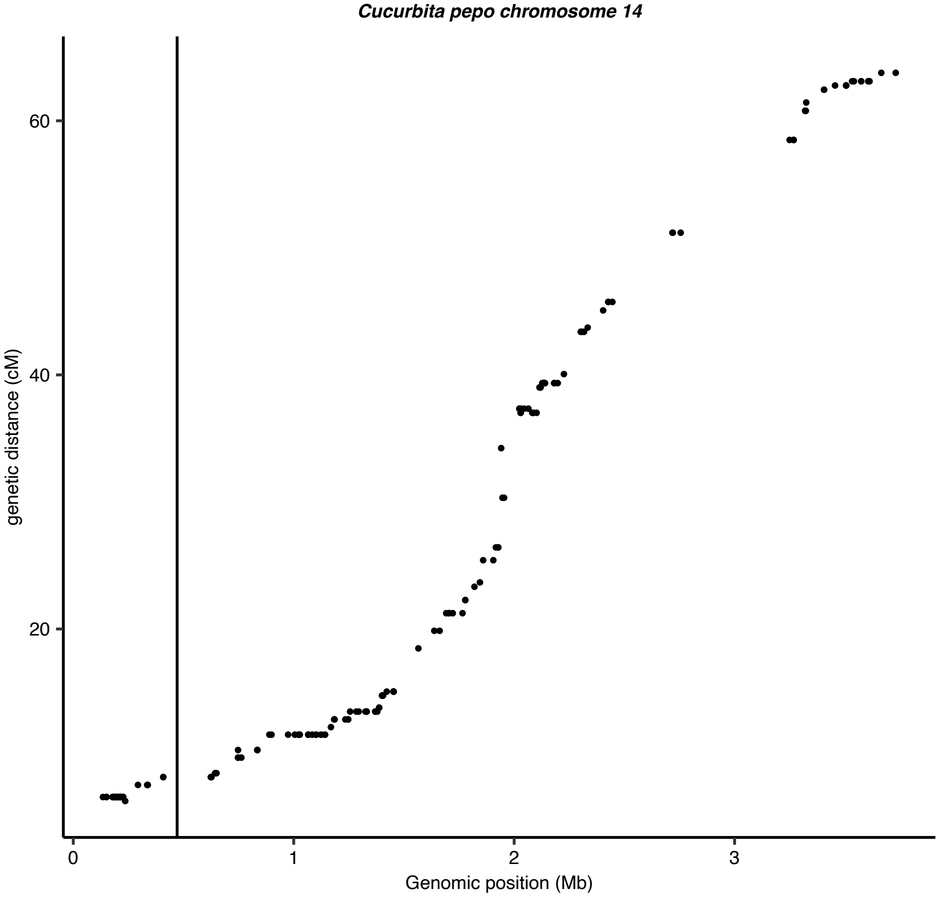
Cucurbita pepo chromosome 14
genetic distance (cM)
60
40
20
0
1
2
3
Genomic position (Mb)

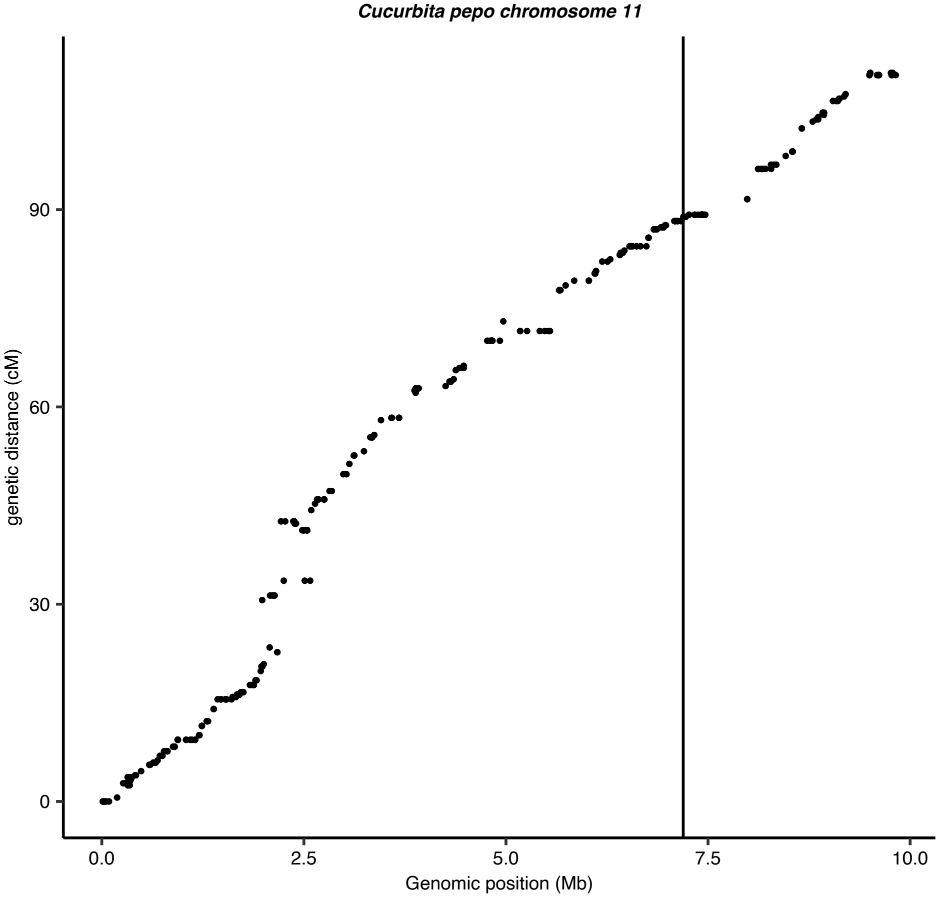
Cucurbita pepo chromosome 11
genetic distance (cM)
0
30
60
90
0.0
2.5
5.0
7.5
10.0
Genomic position (Mb)

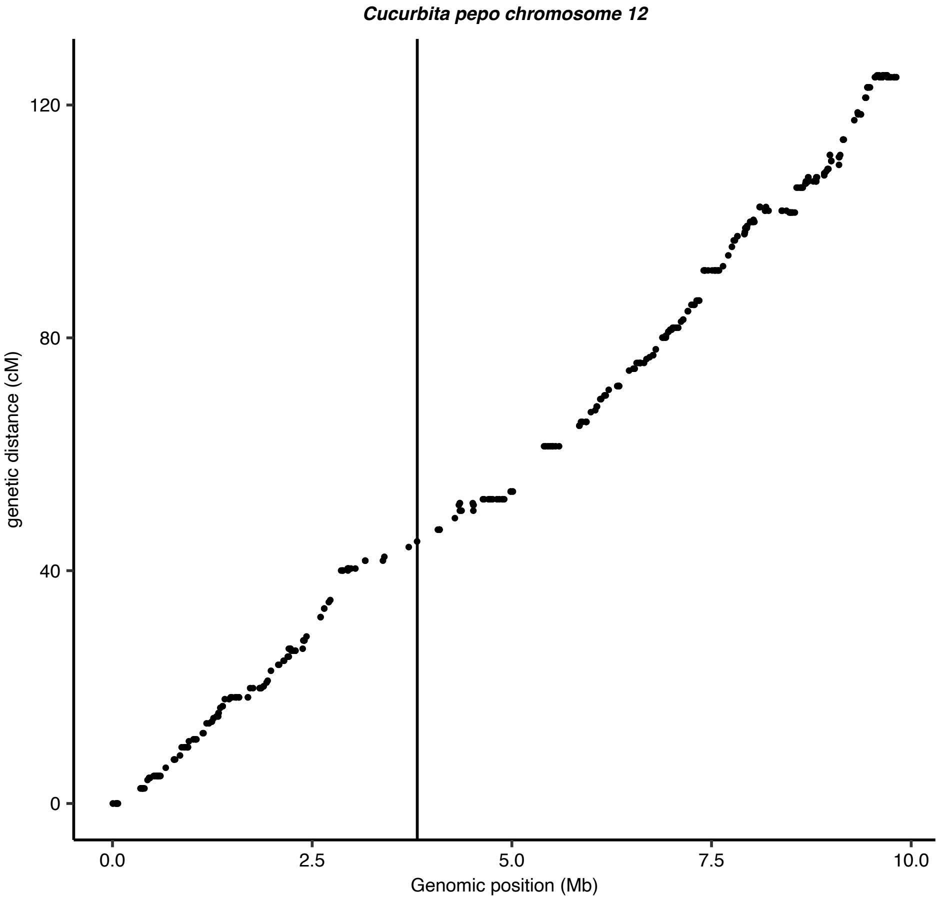
Cucurbita pepo chromosome 12
genetic distance (cM)
0
40
80
120
Genomic position (Mb)
0.0
2.5
5.0
7.5
10.0

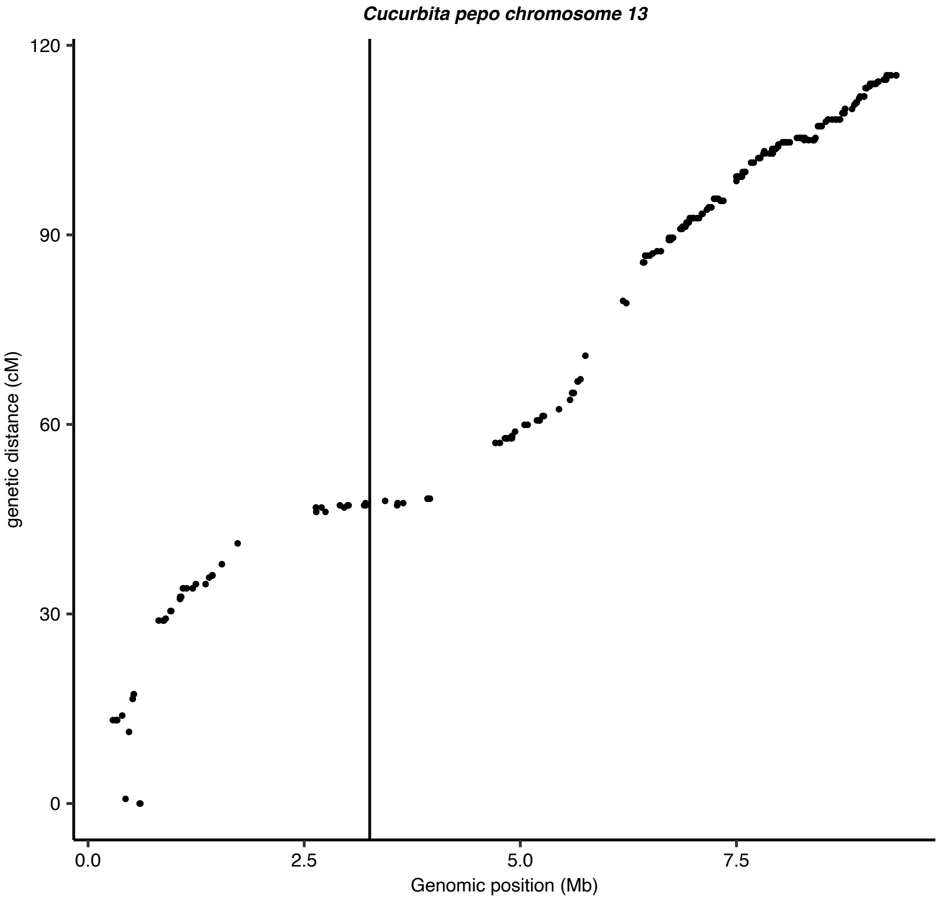
Cucurbita pepo chromosome 13
genetic distance (cM)
Genomic position (Mb)
0
30
60
90
120
0.0
2.5
5.0
7.5

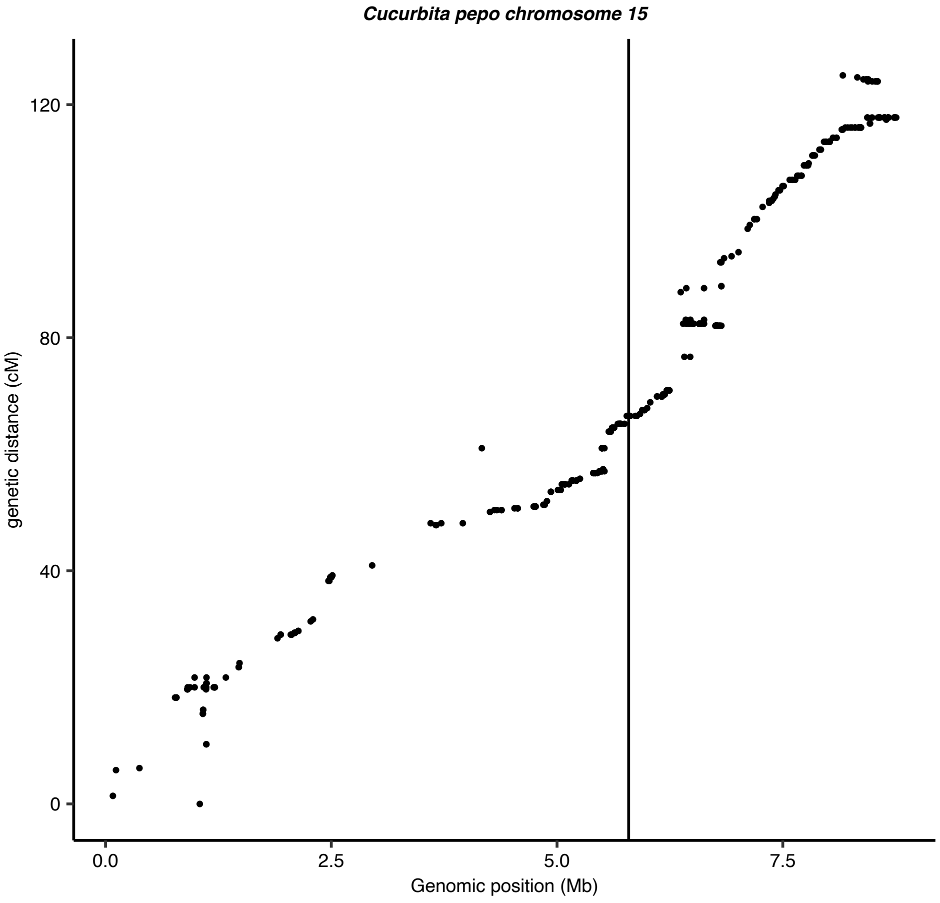
Cucurbita pepo chromosome 15
genetic distance (cM)
0
40
80
120
Genomic position (Mb)
0.0
2.5
5.0
7.5

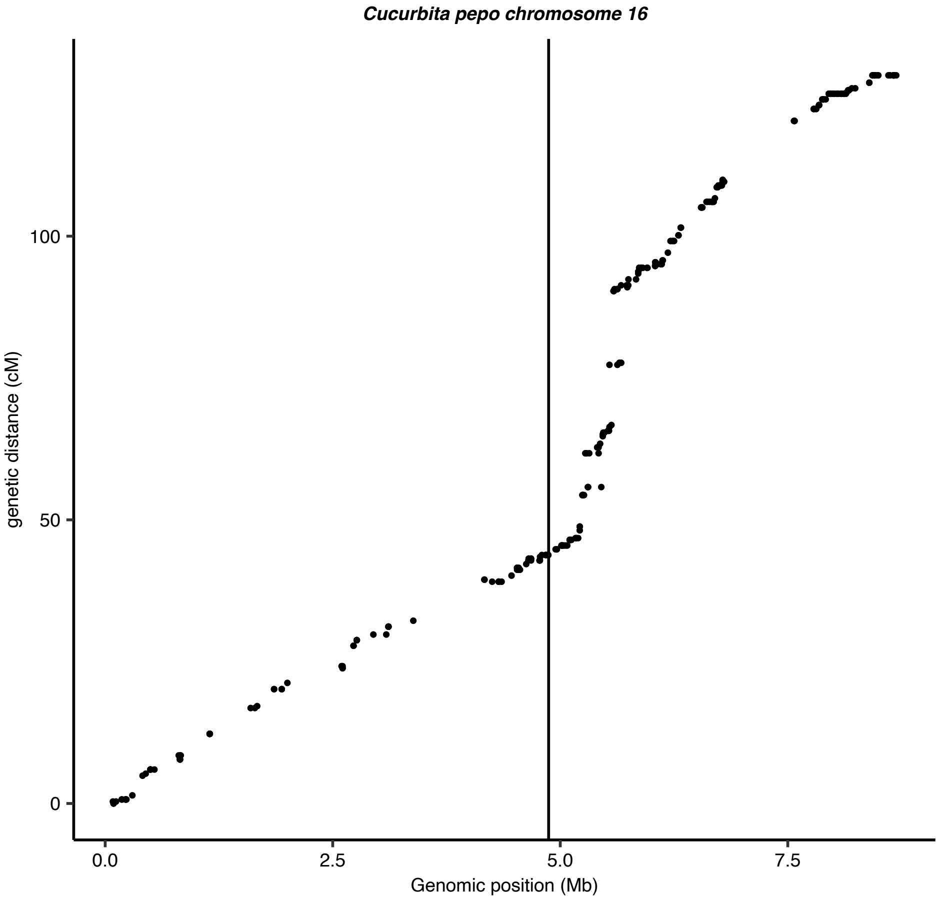
Cucurbita pepo chromosome 16
genetic distance (cM)
0
50
100
Genomic position (Mb)
0.0
2.5
5.0
7.5

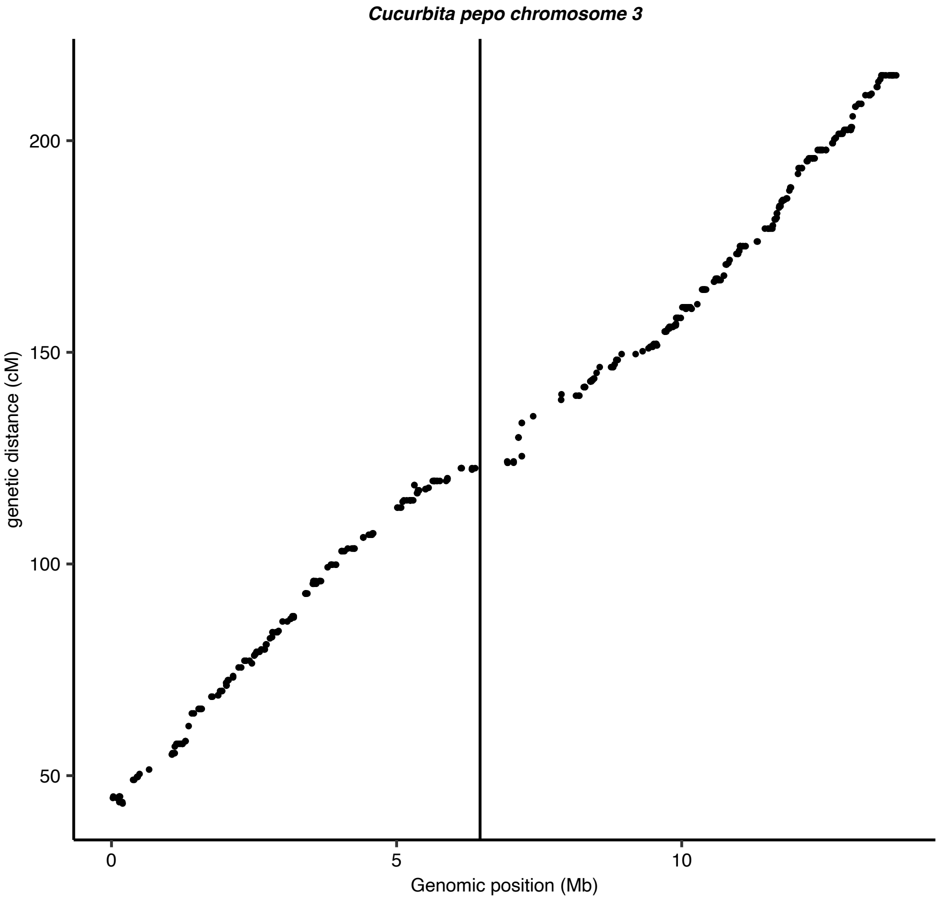
Cucurbita pepo chromosome 3
genetic distance (cM)
200
150
100
50
0
5
10
Genomic position (Mb)

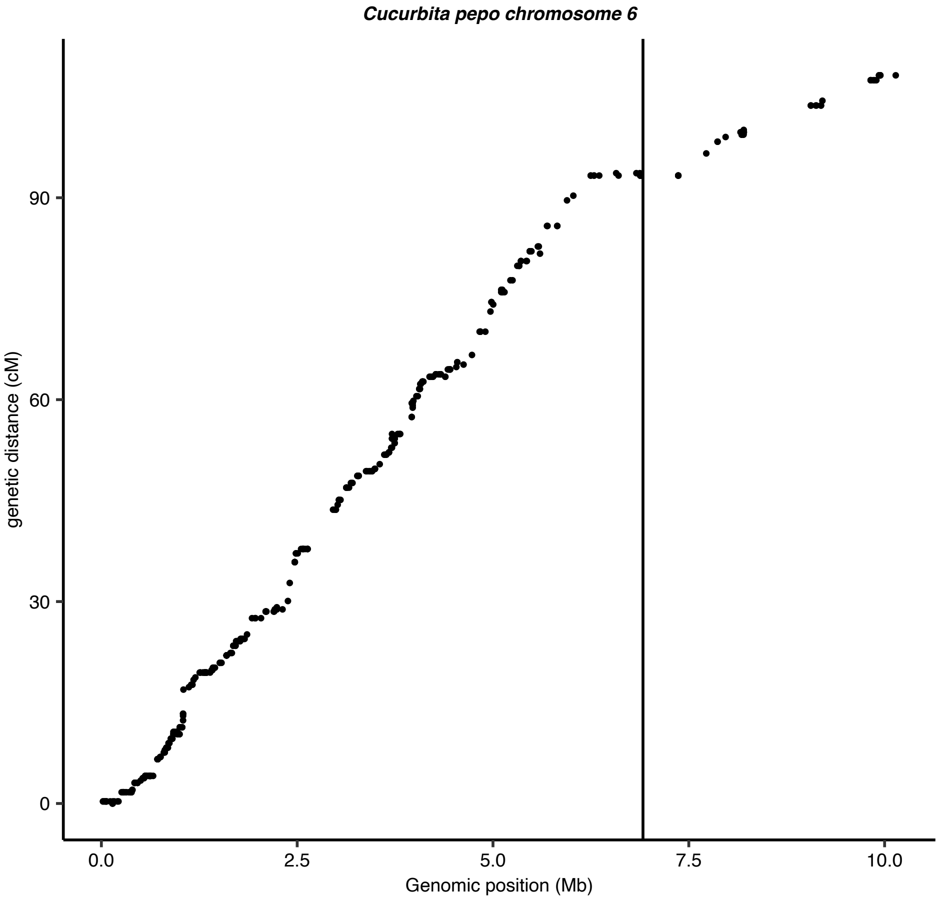
Cucurbita pepo chromosome 6
genetic distance (cM)
0
30
60
90
0.0
2.5
5.0
7.5
10.0
Genomic position (Mb)

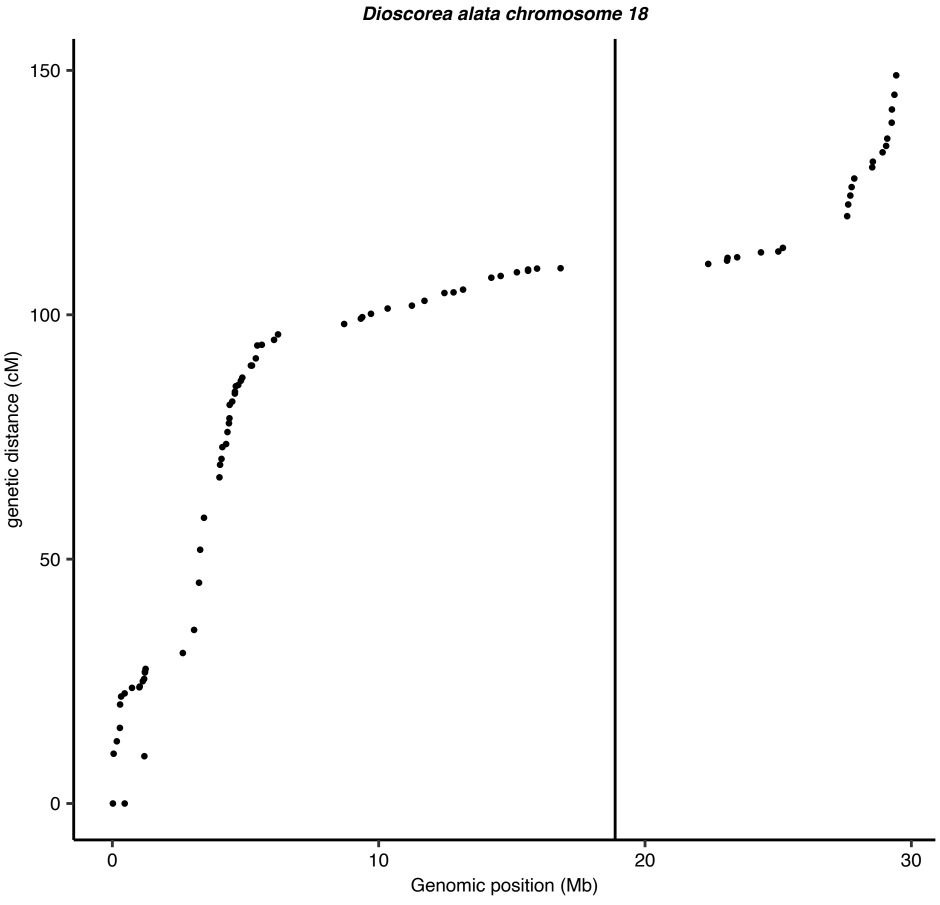
Dioscorea alata chromosome 18
genetic distance (cM)
0
50
100
150
Genomic position (Mb)
0
10
20
30

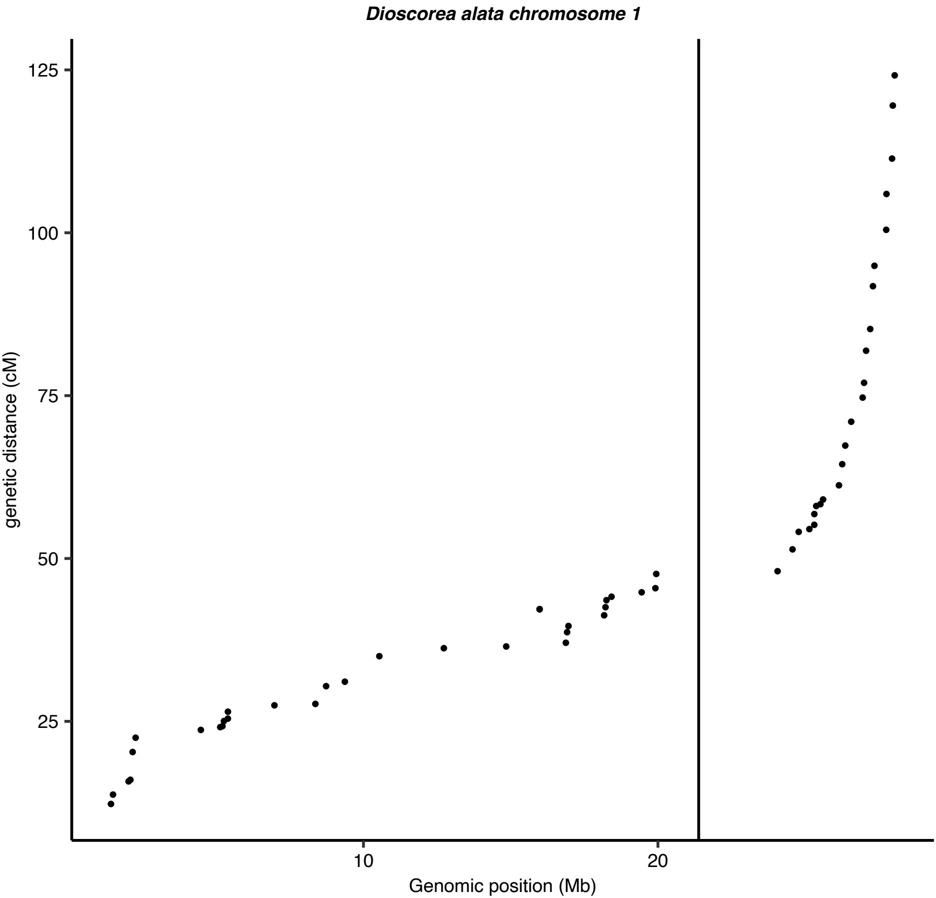
Dioscorea alata chromosome 1
genetic distance (cM)
125
100
75
50
25
10
20
Genomic position (Mb)

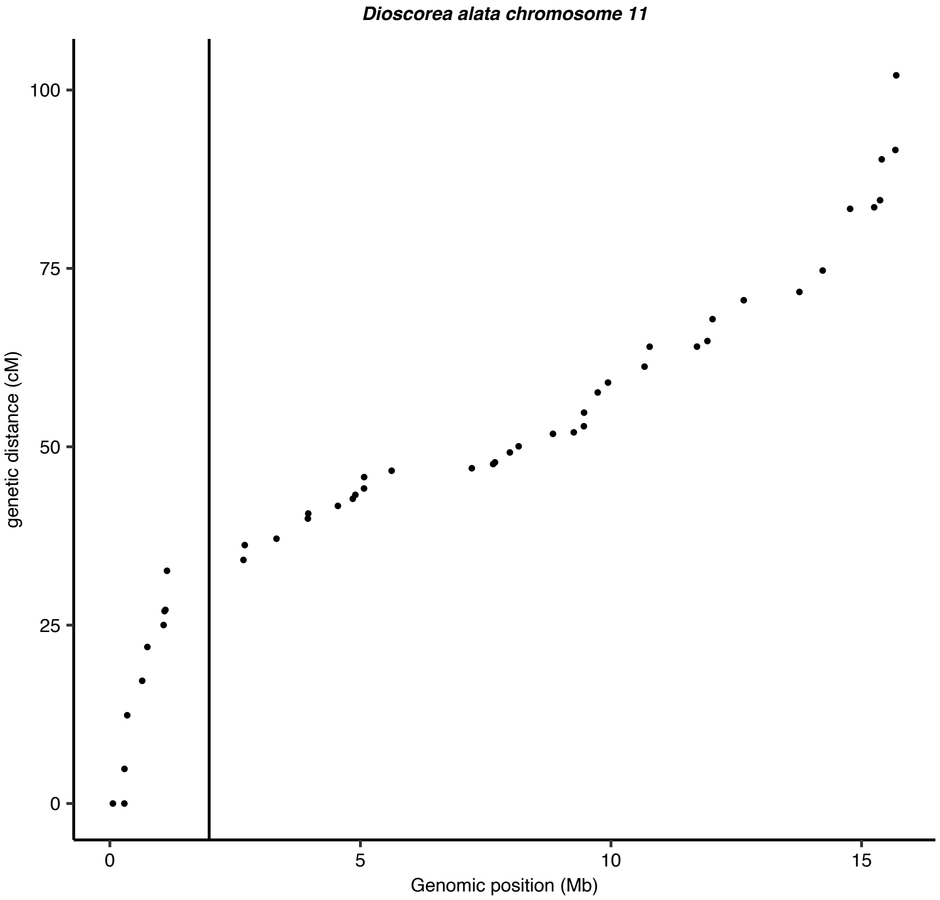
Dioscorea alata chromosome 11
genetic distance (cM)
Genomic position (Mb)
0
25
50
75
100
0
5
10
15

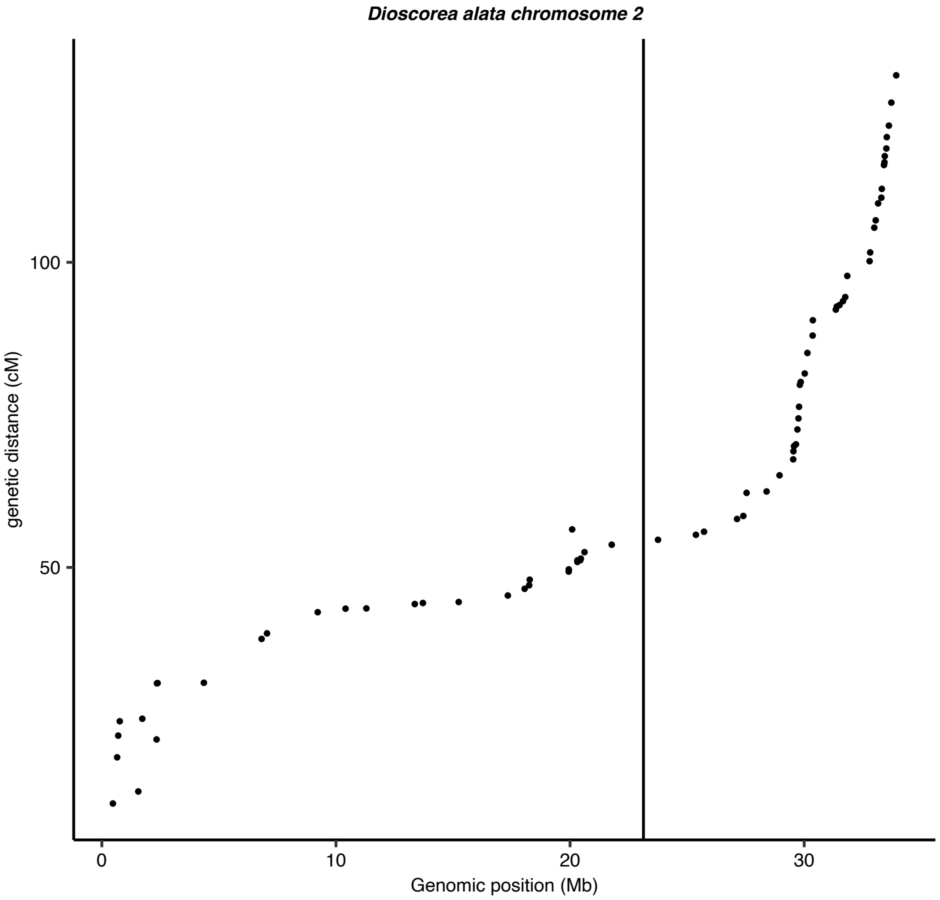
Dioscorea alata chromosome 2
genetic distance (cM)
100
50
0
10
20
30
Genomic position (Mb)

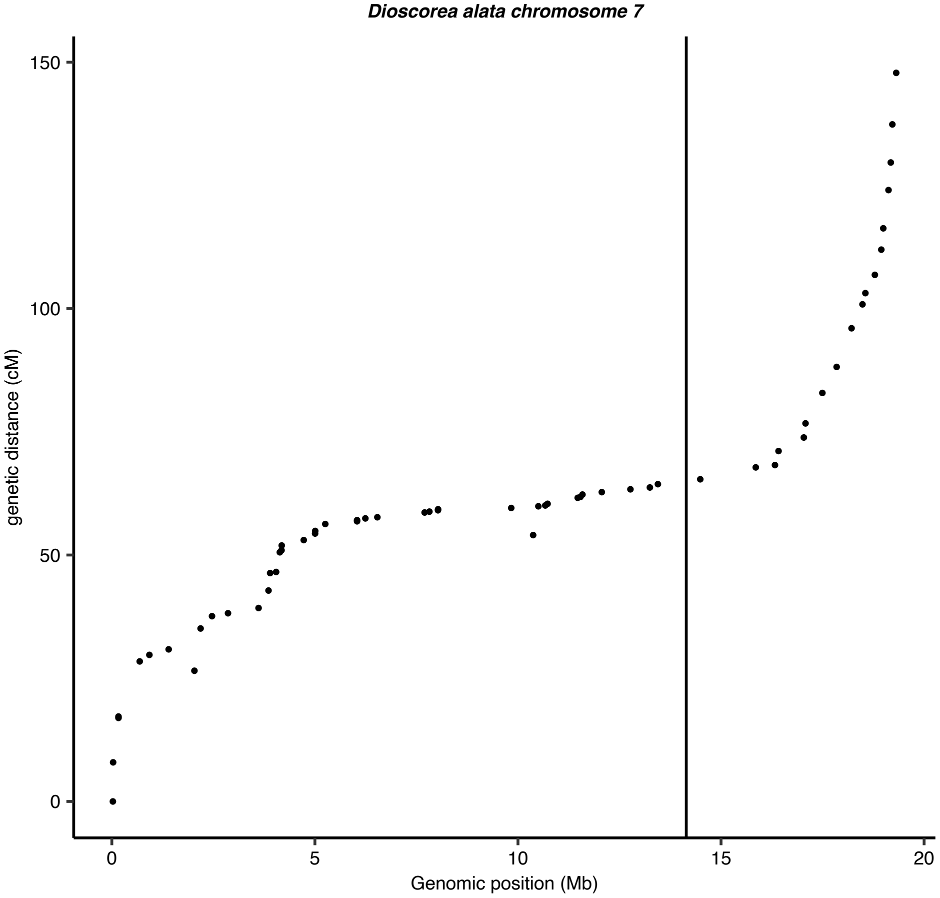
Dioscorea alata chromosome 7
150
100
50
0
genetic distance (cM)
0
5
10
15
20
Genomic position (Mb)

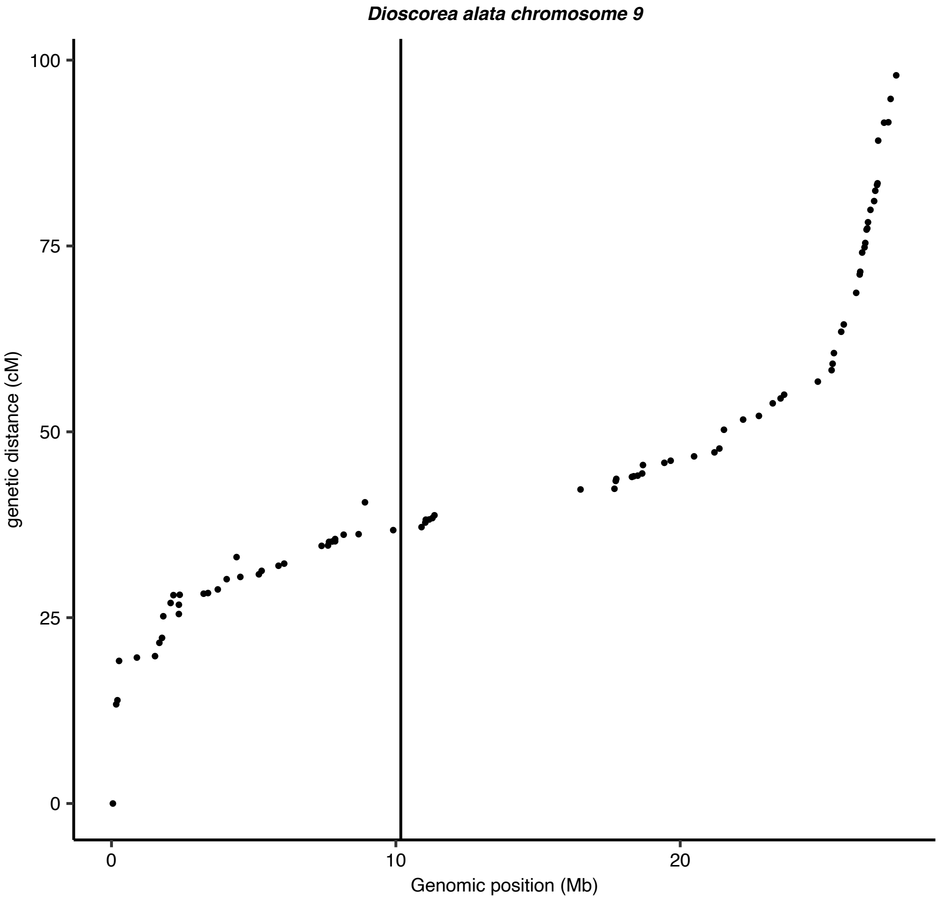
Dioscorea alata chromosome 9
genetic distance (cM)
Genomic position (Mb)
0
25
50
75
100
0
10
20

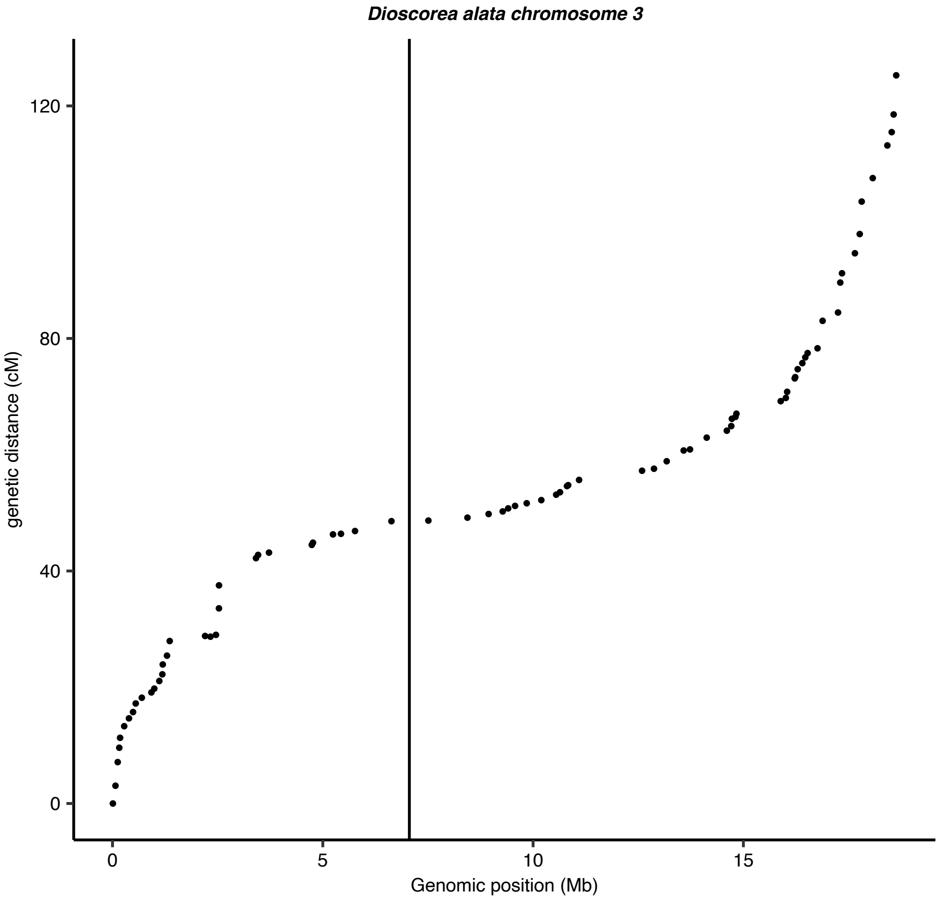
Dioscorea alata chromosome 3
genetic distance (cM)
0
40
80
120
Genomic position (Mb)
0
5
10
15

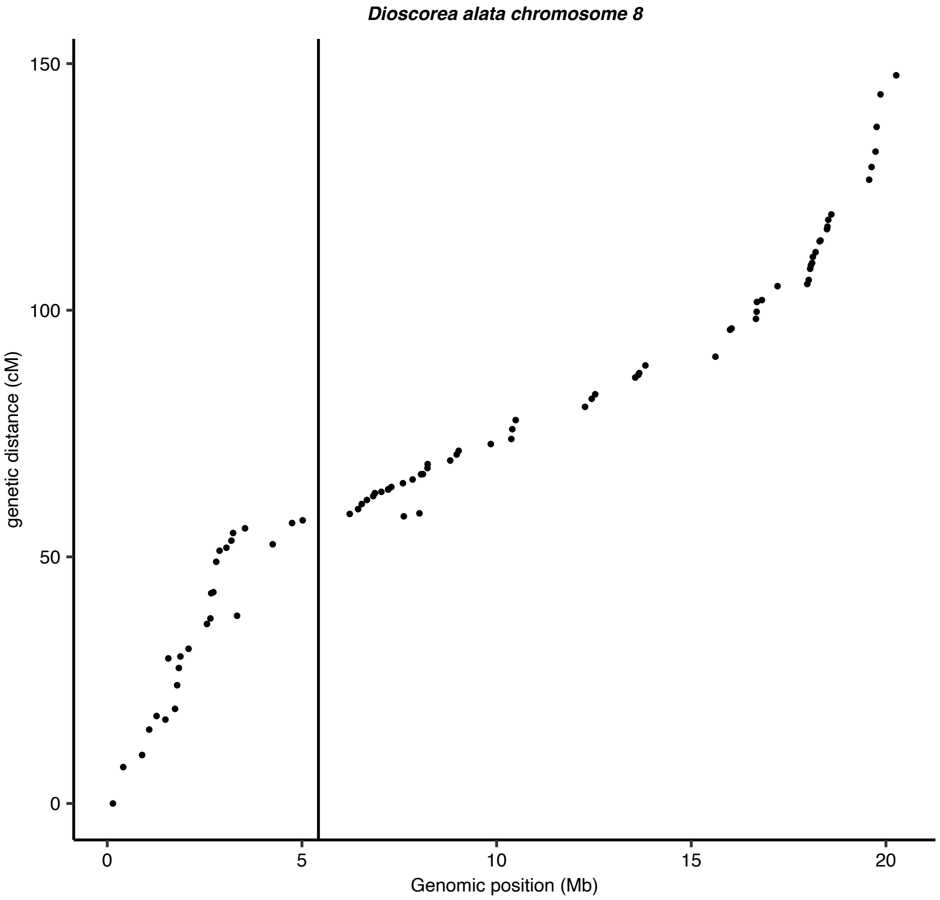
Dioscorea alata chromosome 8
genetic distance (cM)
0
50
100
150
Genomic position (Mb)
0
5
10
15
20

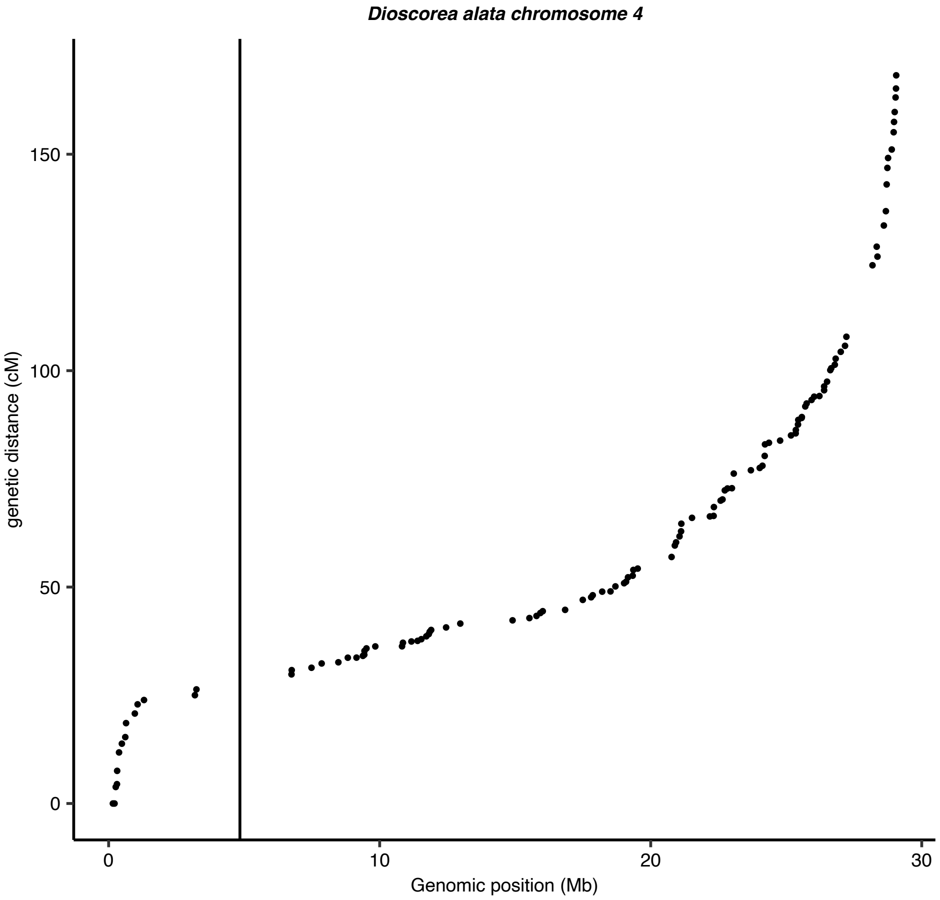

Dioscorea alata chromosome 4
genetic distance (cM)
150
100
50
0
0
10
20
30
Genomic position (Mb)

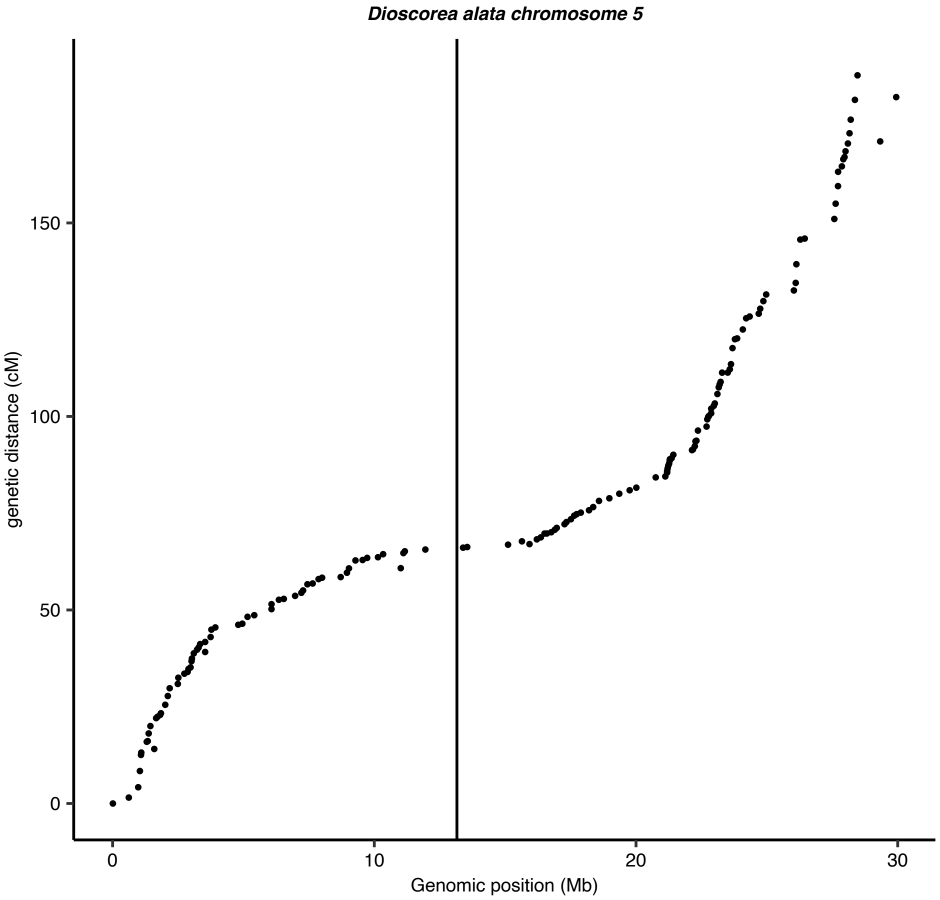
Dioscorea alata chromosome 5
genetic distance (cM)
150
100
50
0
0
10
20
30
Genomic position (Mb)

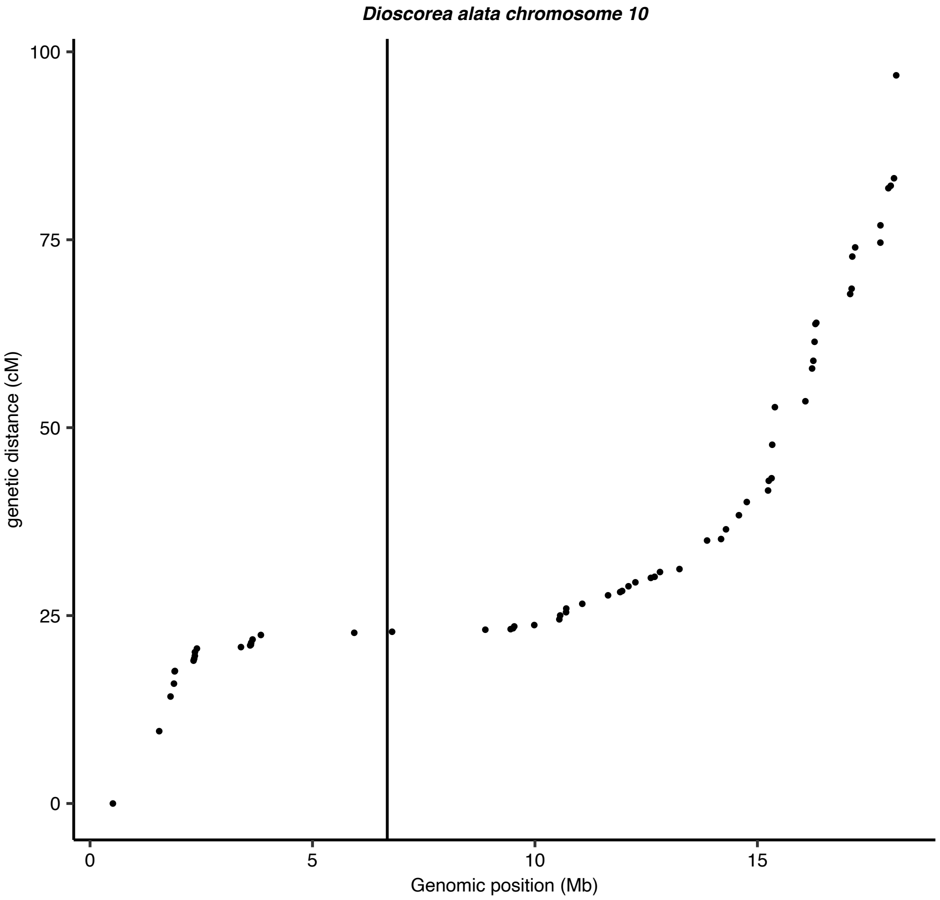

Dioscorea alata chromosome 10
genetic distance (cM)
100
75
50
25
0
0
5
10
15
Genomic position (Mb)

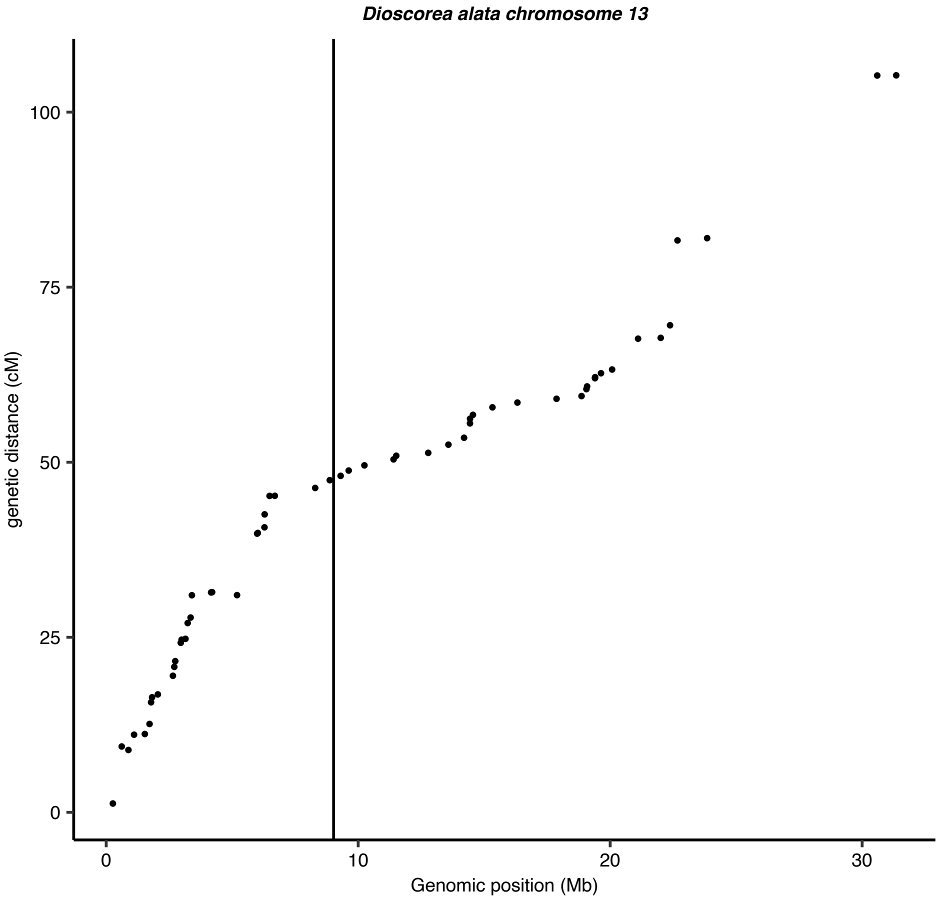
Dioscorea alata chromosome 13
genetic distance (cM)
Genomic position (Mb)
0
25
50
75
100
0
10
20
30

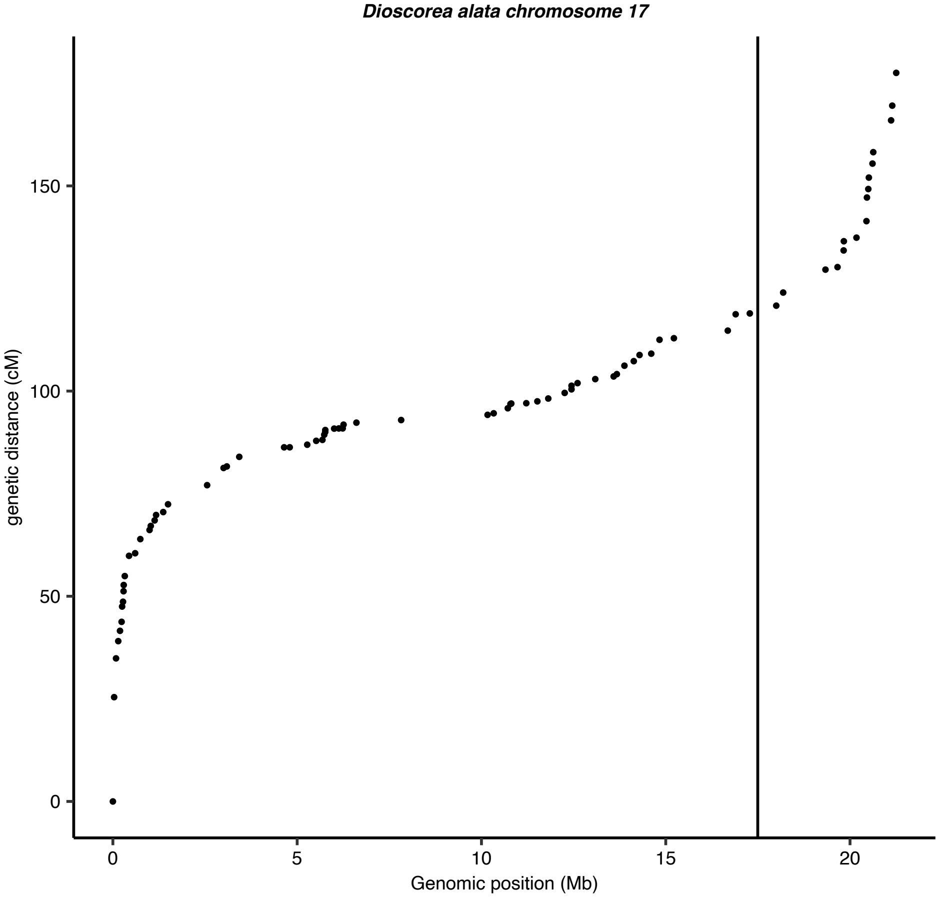
Dioscorea alata chromosome 17
genetic distance (cM)
0
50
100
150
0
5
10
15
20
Genomic position (Mb)

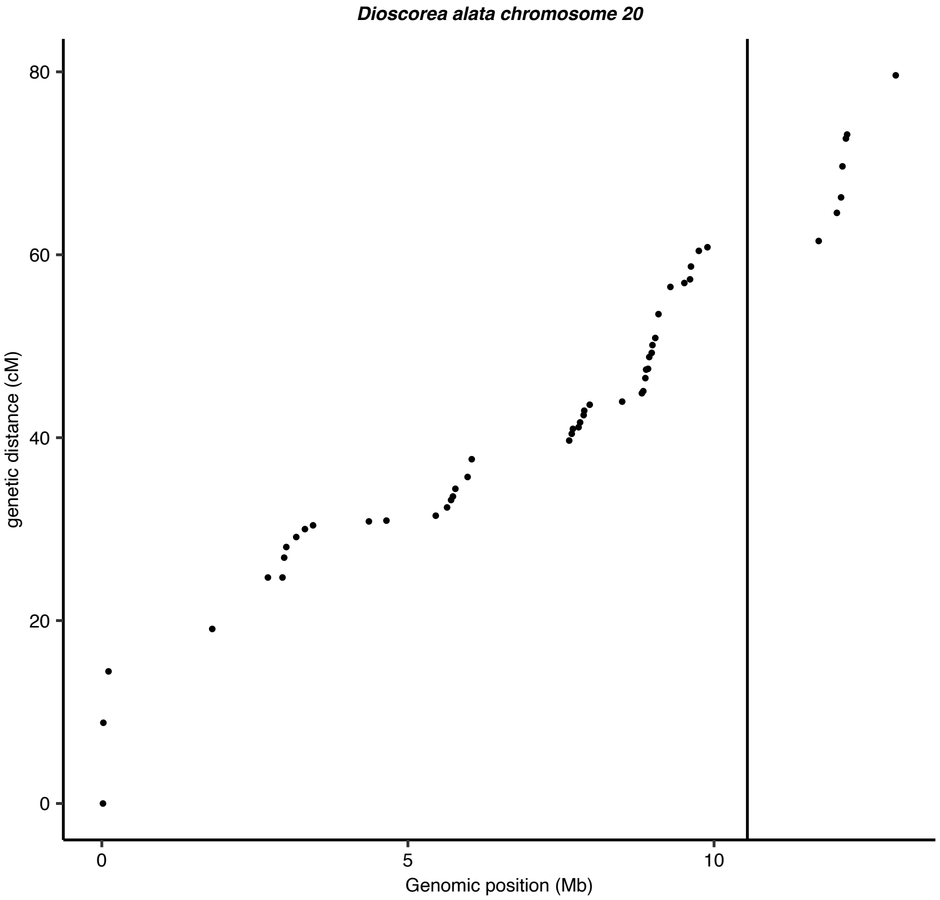
Dioscorea alata chromosome 20
genetic distance (cM)
0
20
40
60
80
Genomic position (Mb)
0
5
10

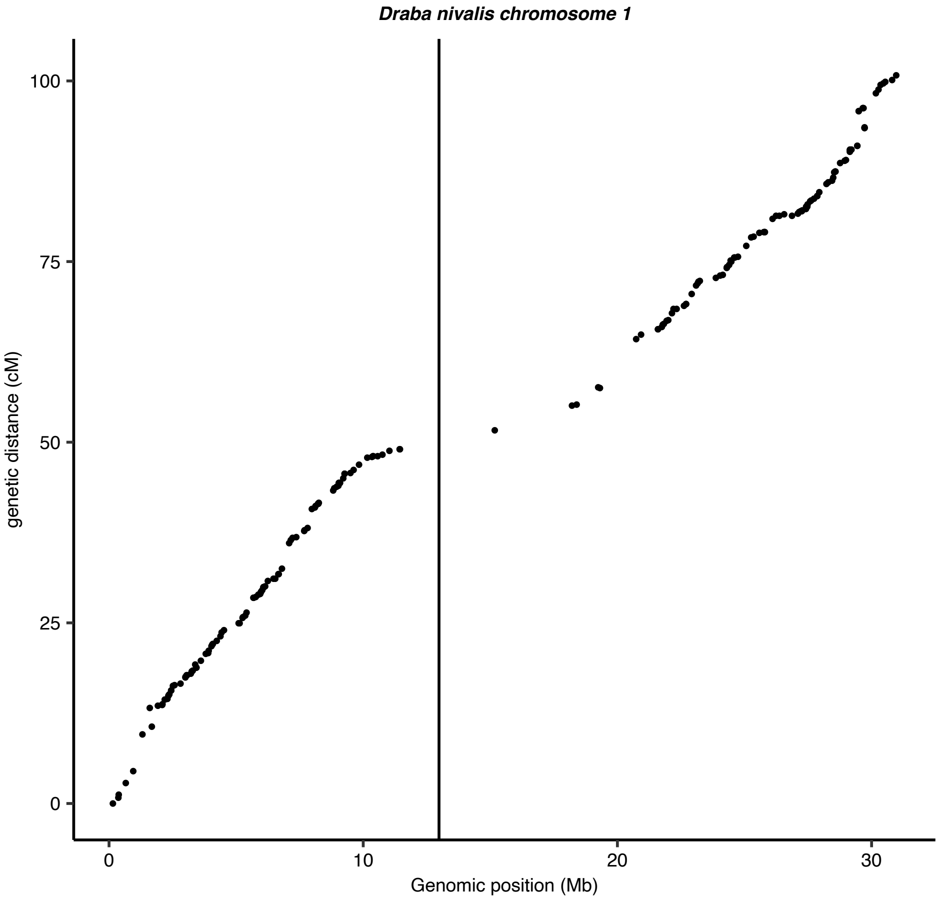
Draba nivalis chromosome 1
genetic distance (cM)
Genomic position (Mb)
0
25
50
75
100
0
10
20
30

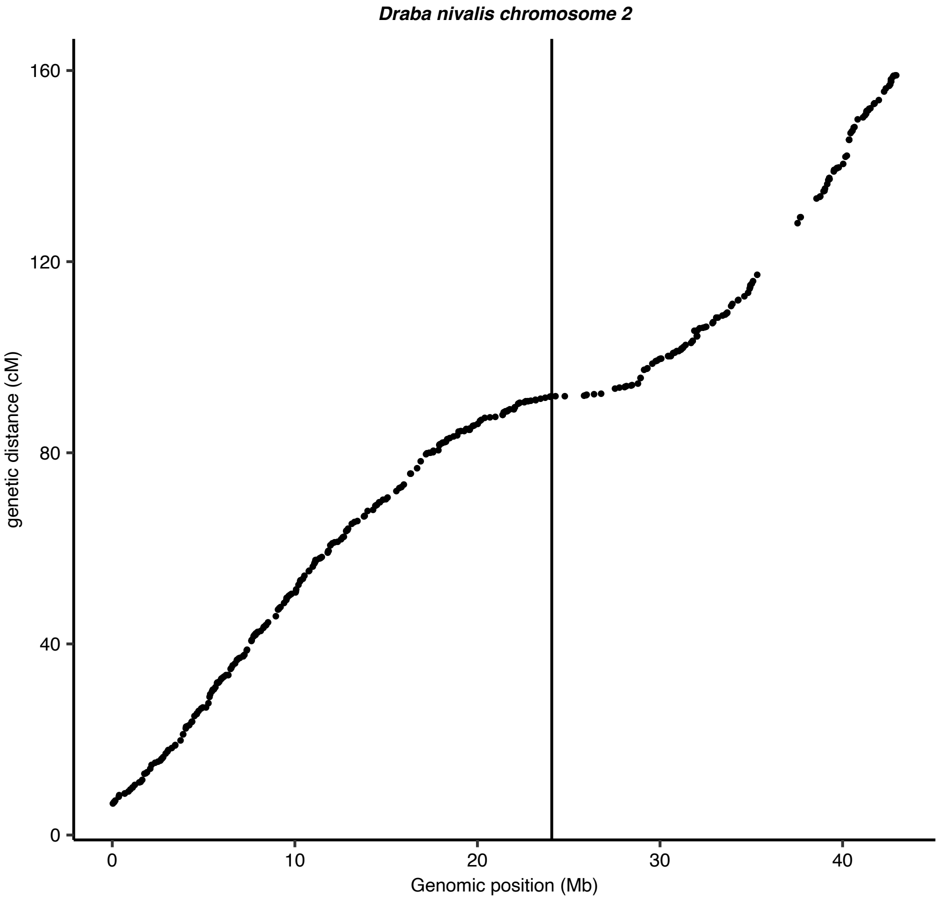
Draba nivalis chromosome 2
genetic distance (cM)
160
120
80
40
0
0
10
20
30
40
Genomic position (Mb)

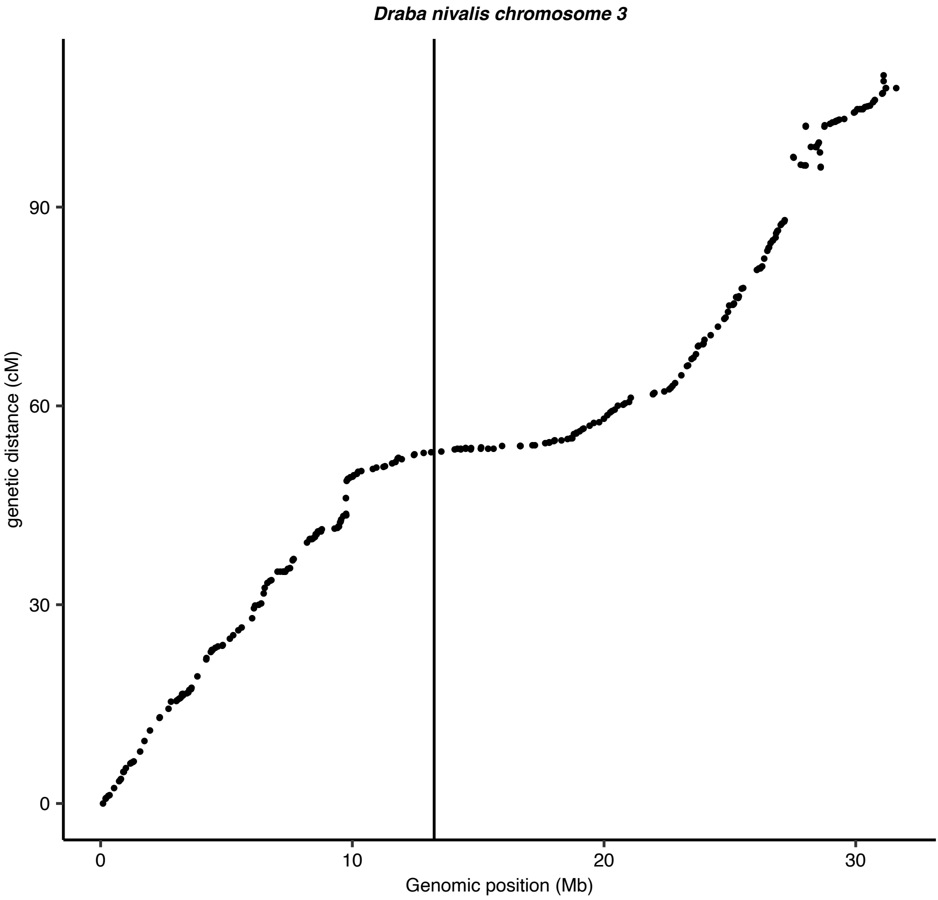
Draba nivalis chromosome 3
genetic distance (cM)
0
30
60
90
Genomic position (Mb)
0
10
20
30

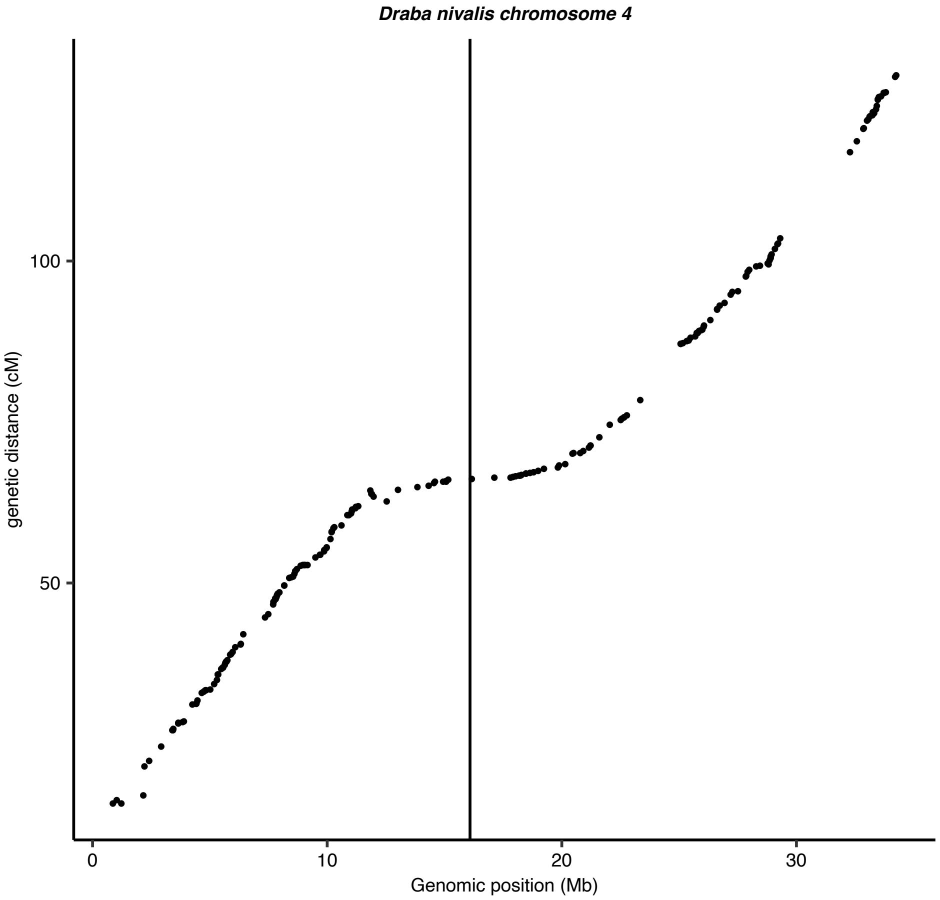
Draba nivalis chromosome 4
genetic distance (cM)
100
50
0
10
20
30
Genomic position (Mb)

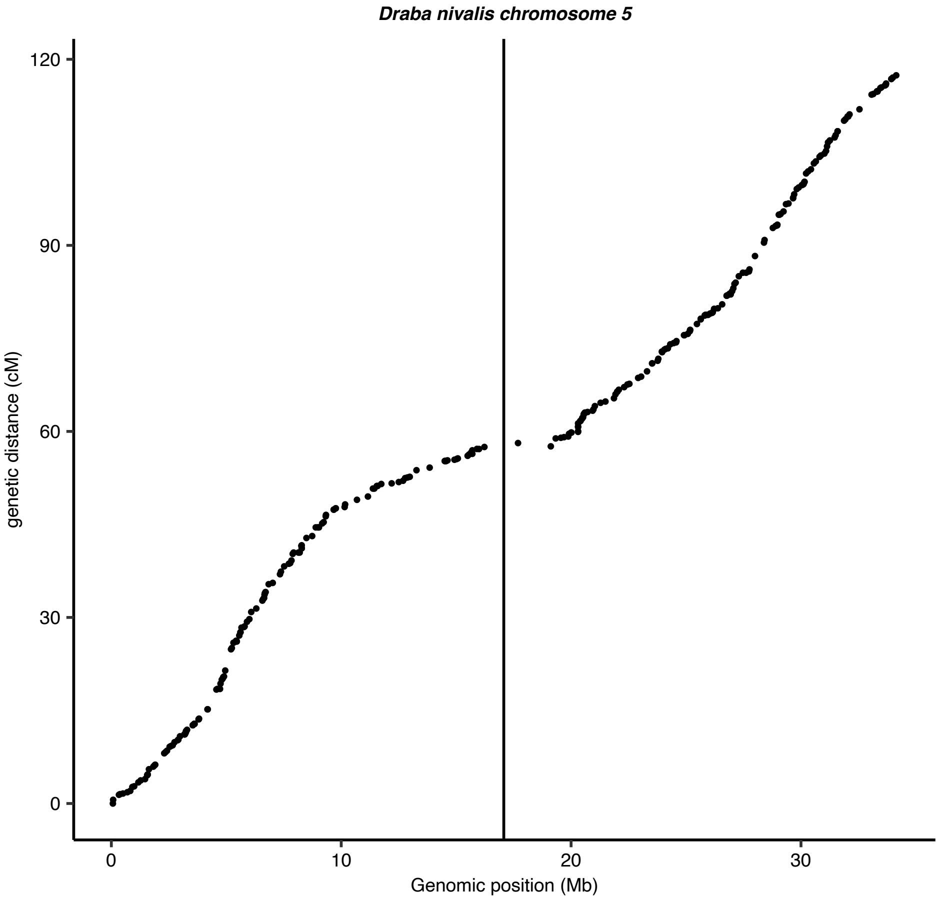
Draba nivalis chromosome 5
genetic distance (cM)
120
90
60
30
0
0
10
20
30
Genomic position (Mb)

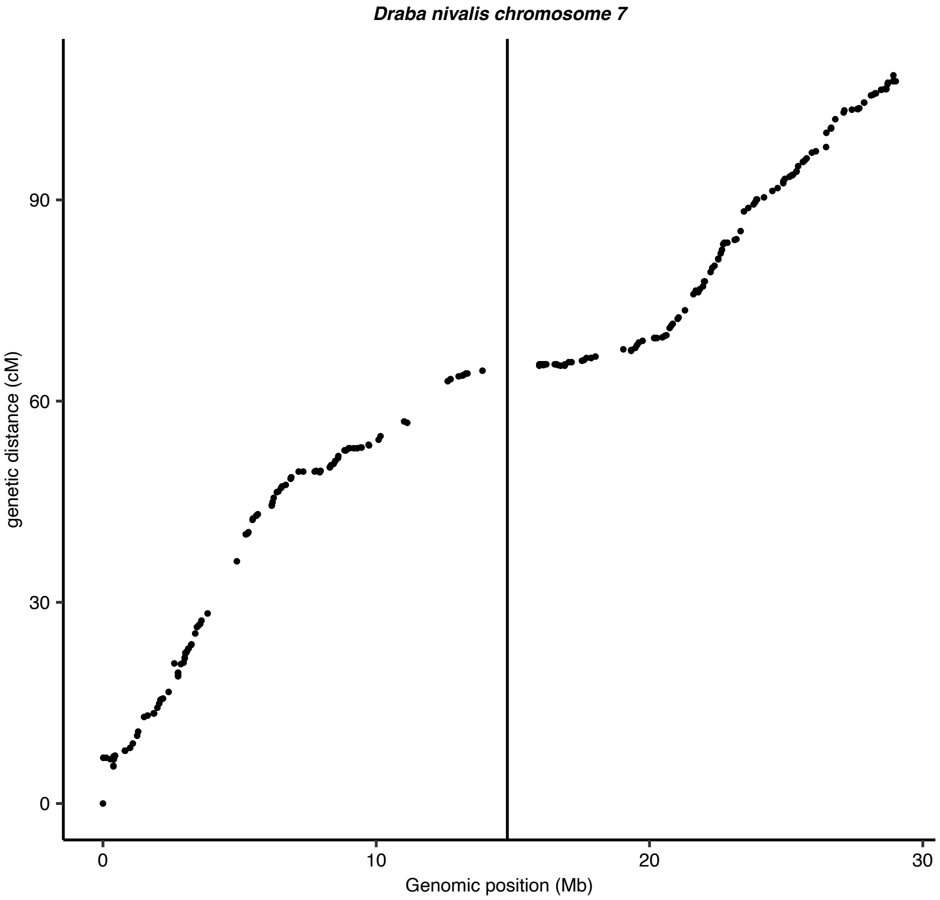
Draba nivalis chromosome 7
genetic distance (cM)
0
30
60
90
Genomic position (Mb)
0
10
20
30

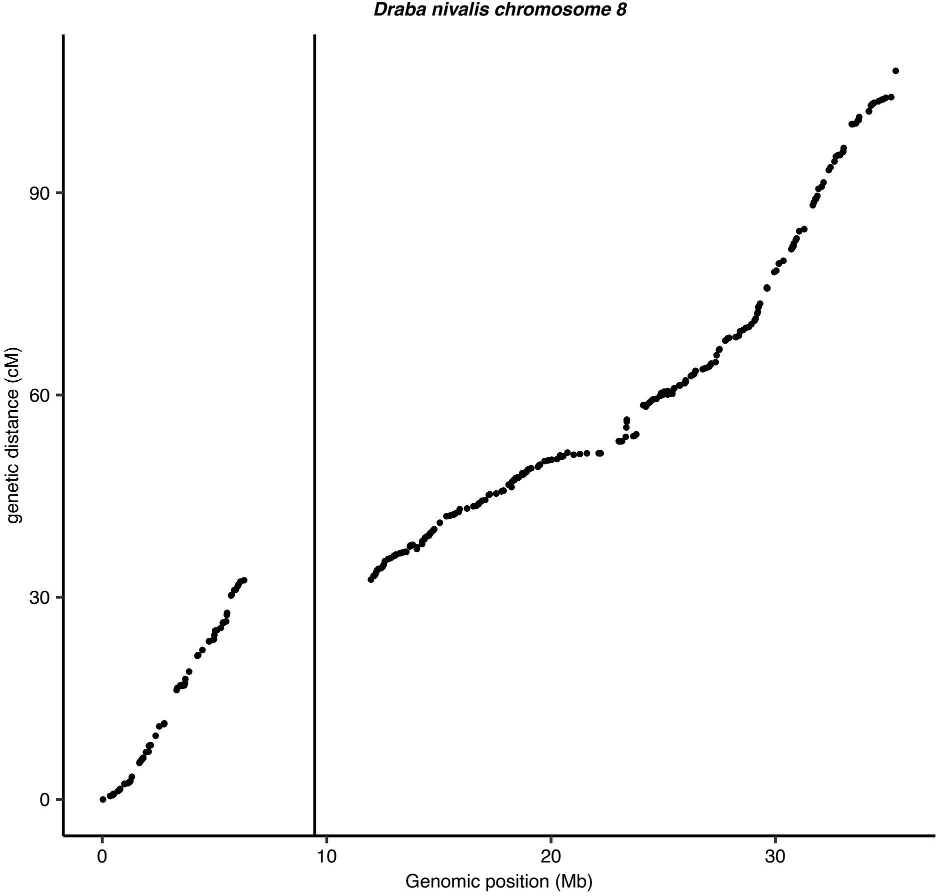
Draba nivalis chromosome 8
genetic distance (cM)
0
30
60
90
Genomic position (Mb)
0
10
20
30

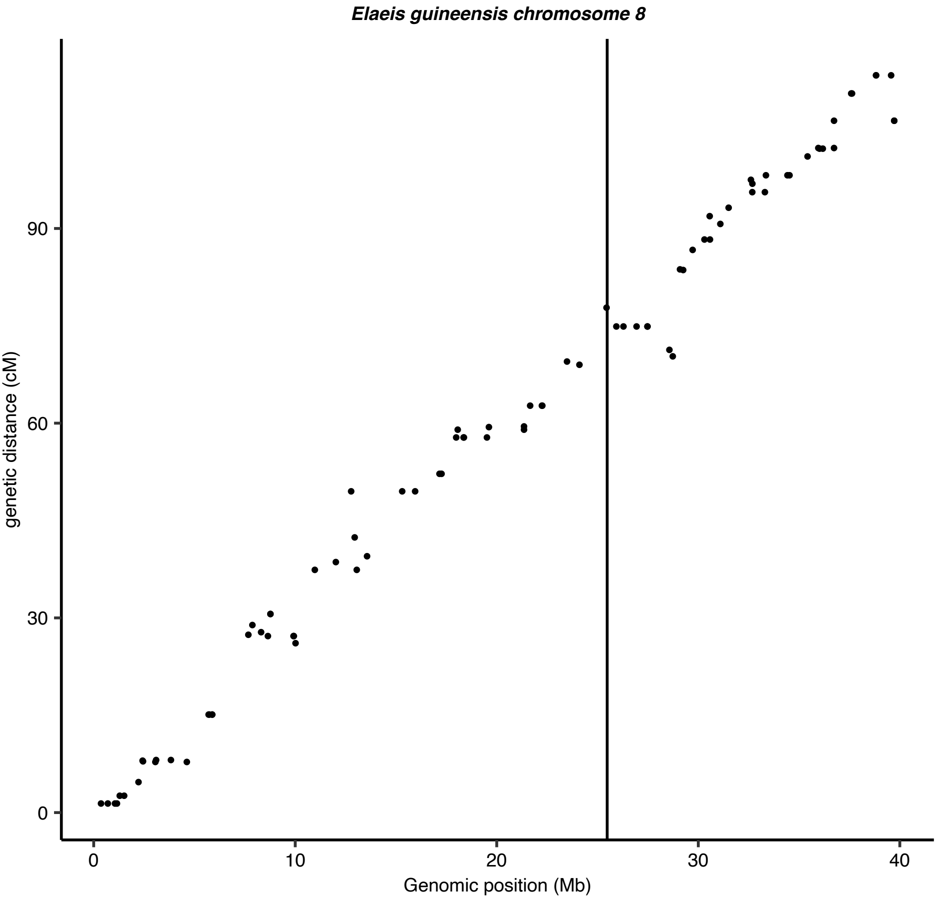
Elaeis guineensis chromosome 8
genetic distance (cM)
0
30
60
90
Genomic position (Mb)
0
10
20
30
40

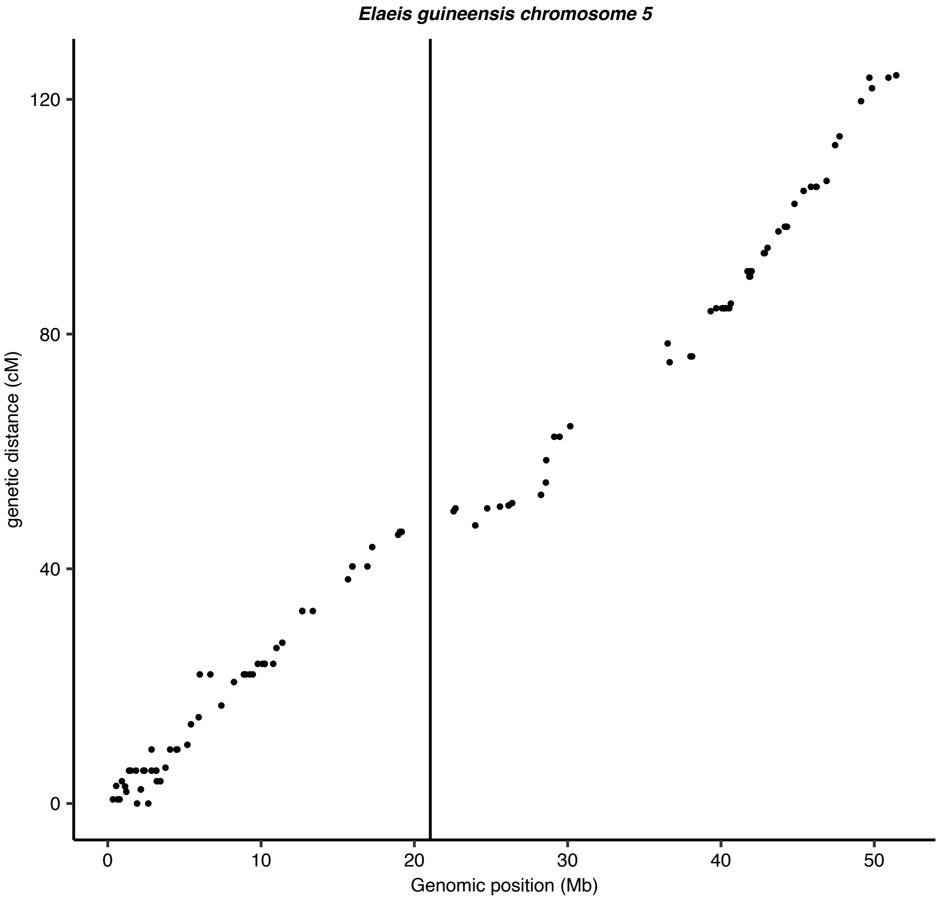
Elaeis guineensis chromosome 5
genetic distance (cM)
Genomic position (Mb)
0
40
80
120
0
10
20
30
40
50

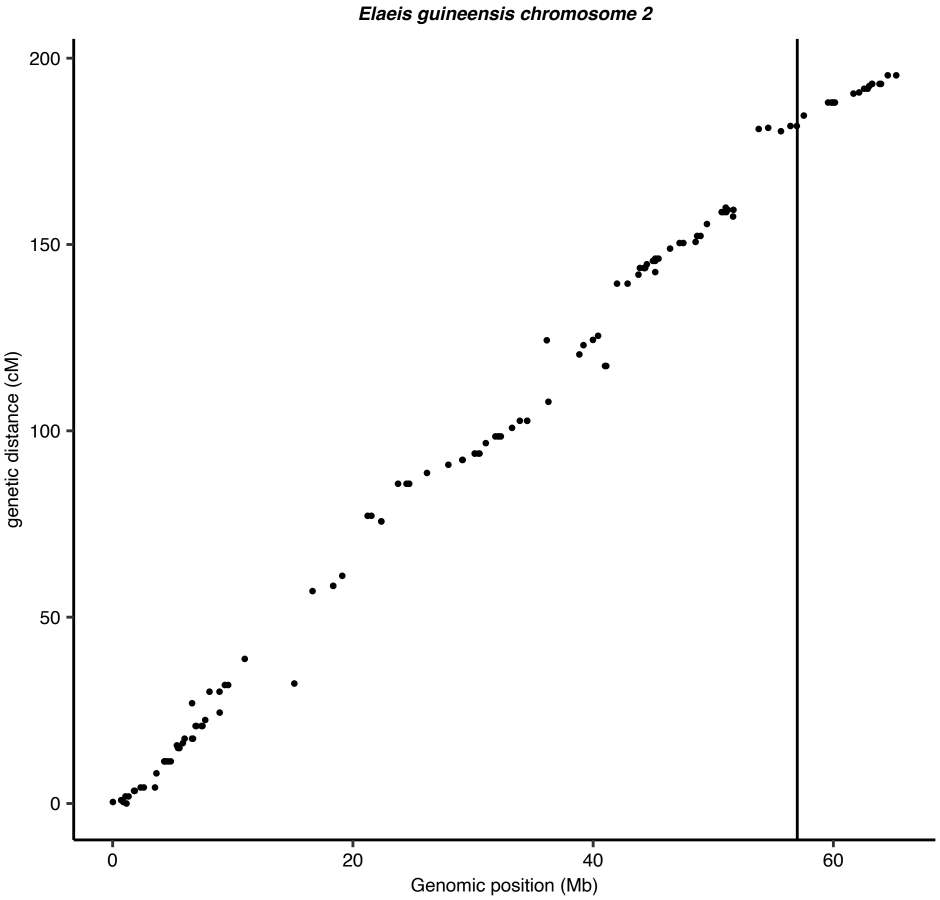
Elaeis guineensis chromosome 2
genetic distance (cM)
200
150
100
50
0
0
20
40
60
Genomic position (Mb)

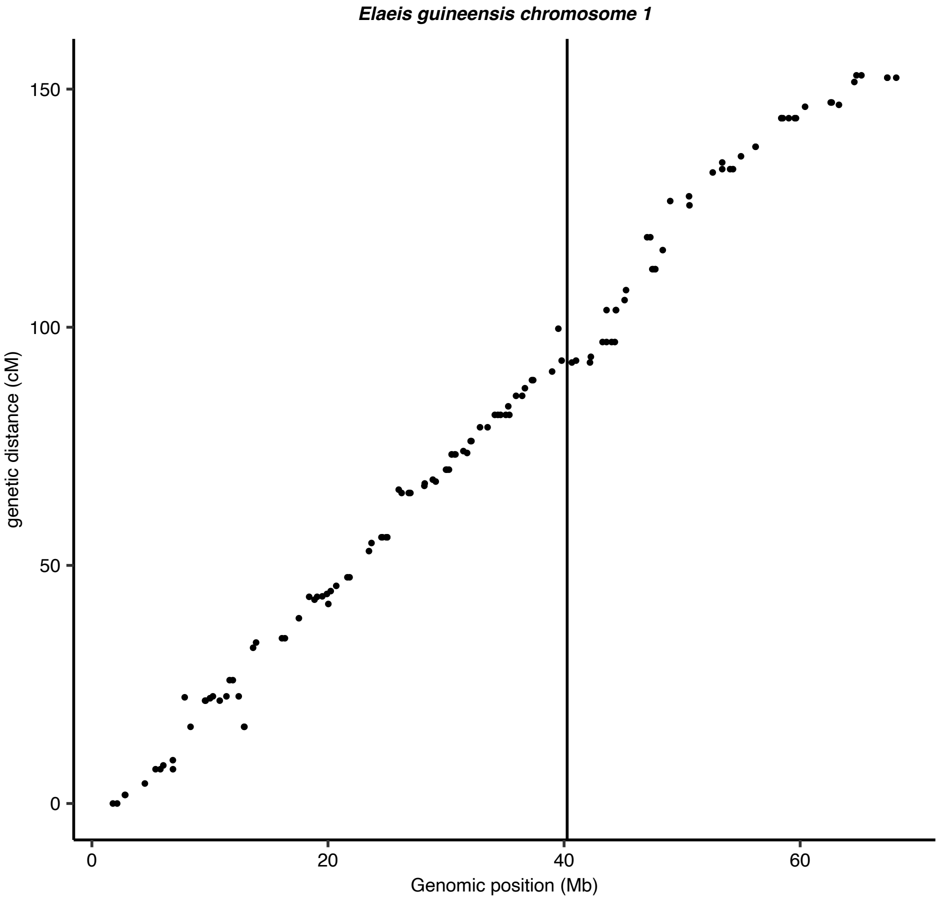
Elaeis guineensis chromosome 1
150
100
50
0
genetic distance (cM)
0
20
40
60
Genomic position (Mb)

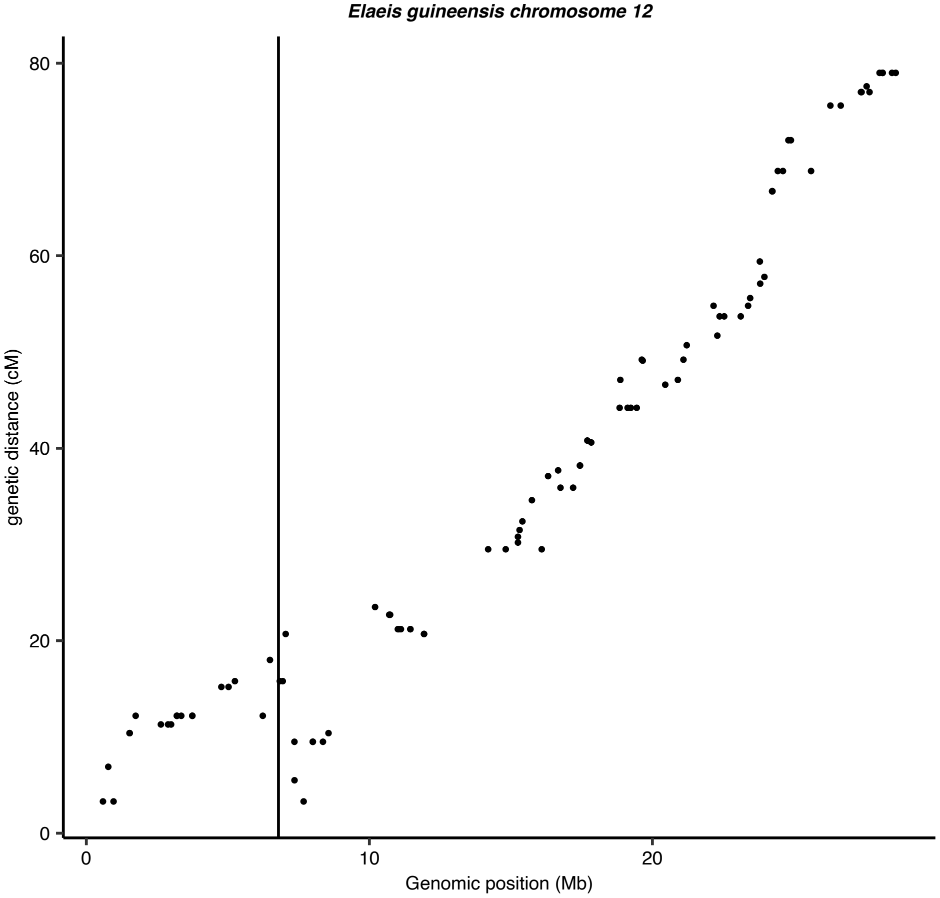
Elaeis guineensis chromosome 12
genetic distance (cM)
Genomic position (Mb)
0
20
40
60
80
0
10
20

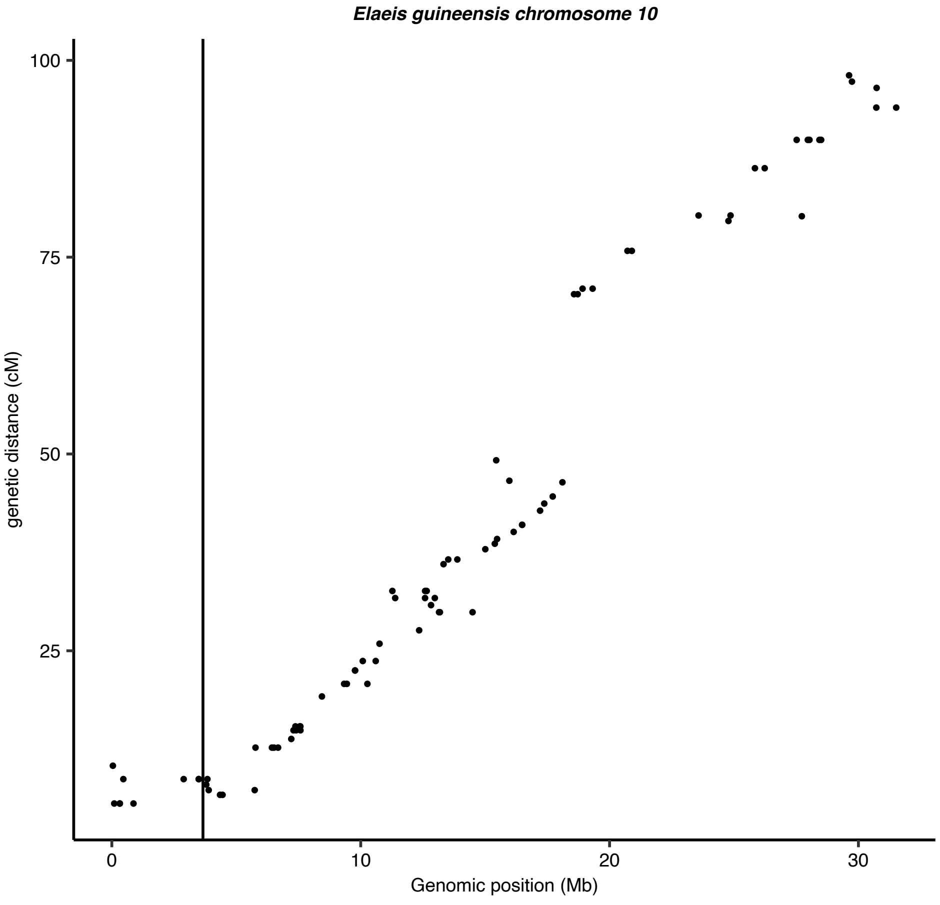
Elaeis guineensis chromosome 10
100
75
50
25
genetic distance (cM)
0
10
20
30
Genomic position (Mb)

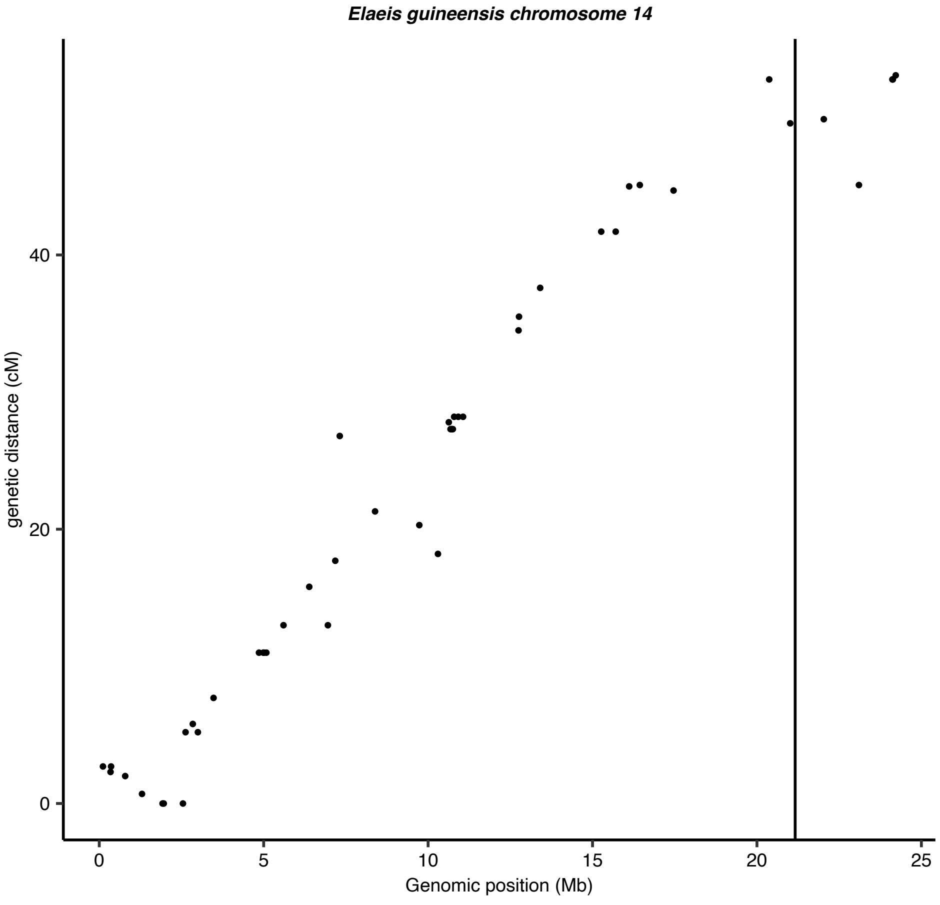
Elaeis guineensis chromosome 14
genetic distance (cM)
0
20
40
Genomic position (Mb)
0
5
10
15
20
25

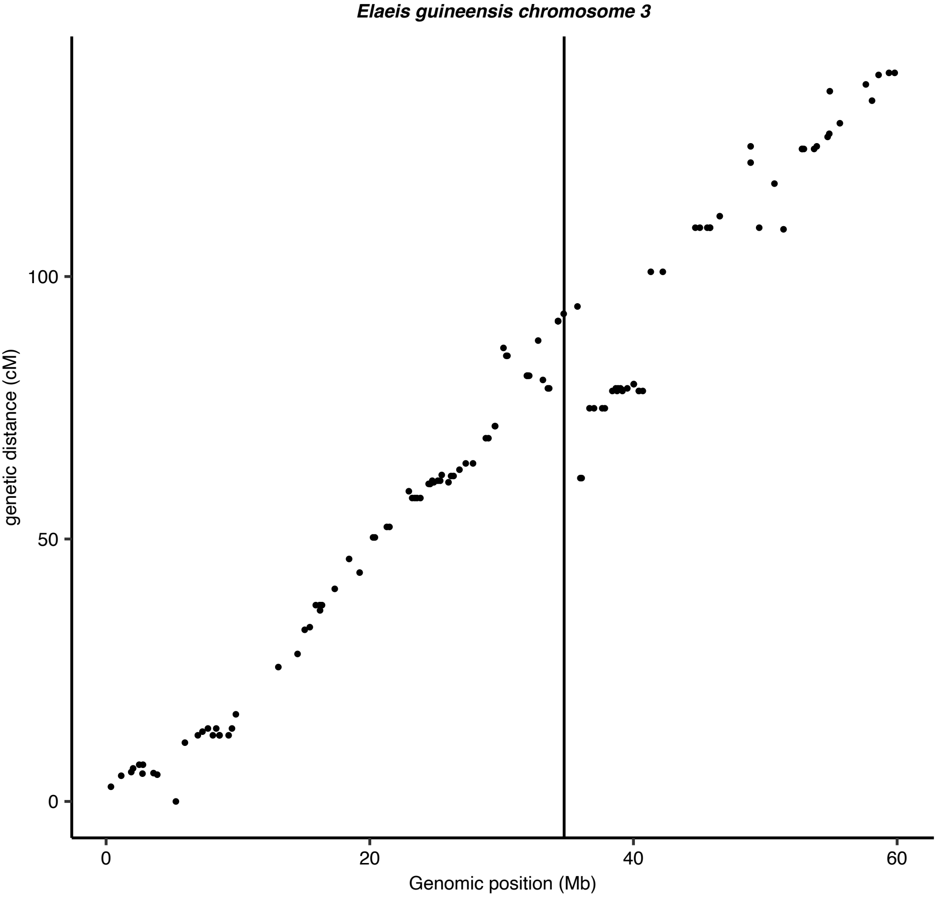
Elaeis guineensis chromosome 3
genetic distance (cM)
0
50
100
Genomic position (Mb)
0
20
40
60

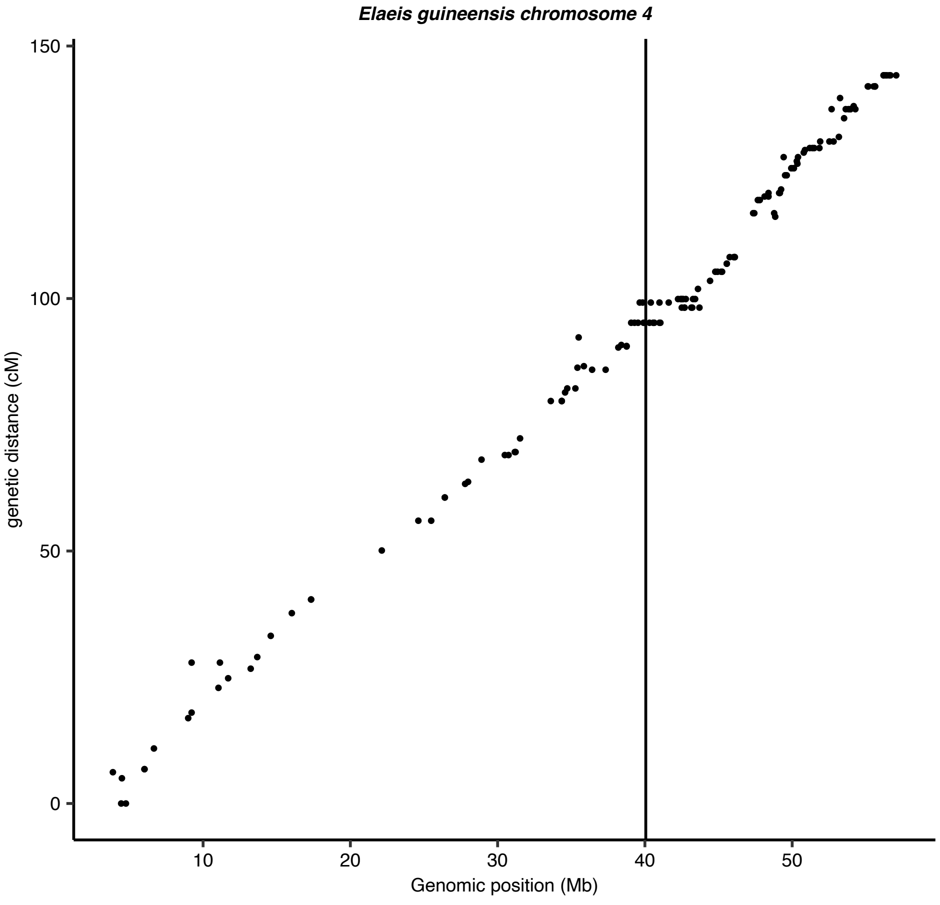
Elaeis guineensis chromosome 4
150
100
50
0
genetic distance (cM)
10
20
30
40
50
Genomic position (Mb)

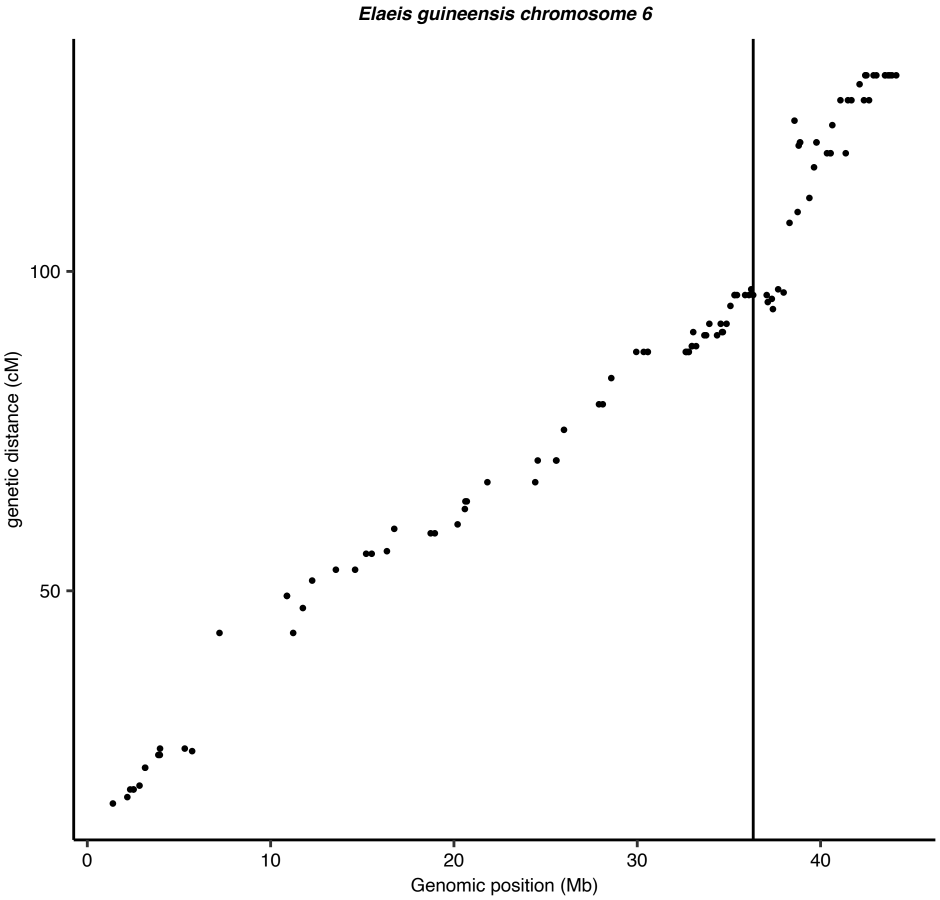
Elaeis guineensis chromosome 6
genetic distance (cM)
100
50
0
10
20
30
40
Genomic position (Mb)

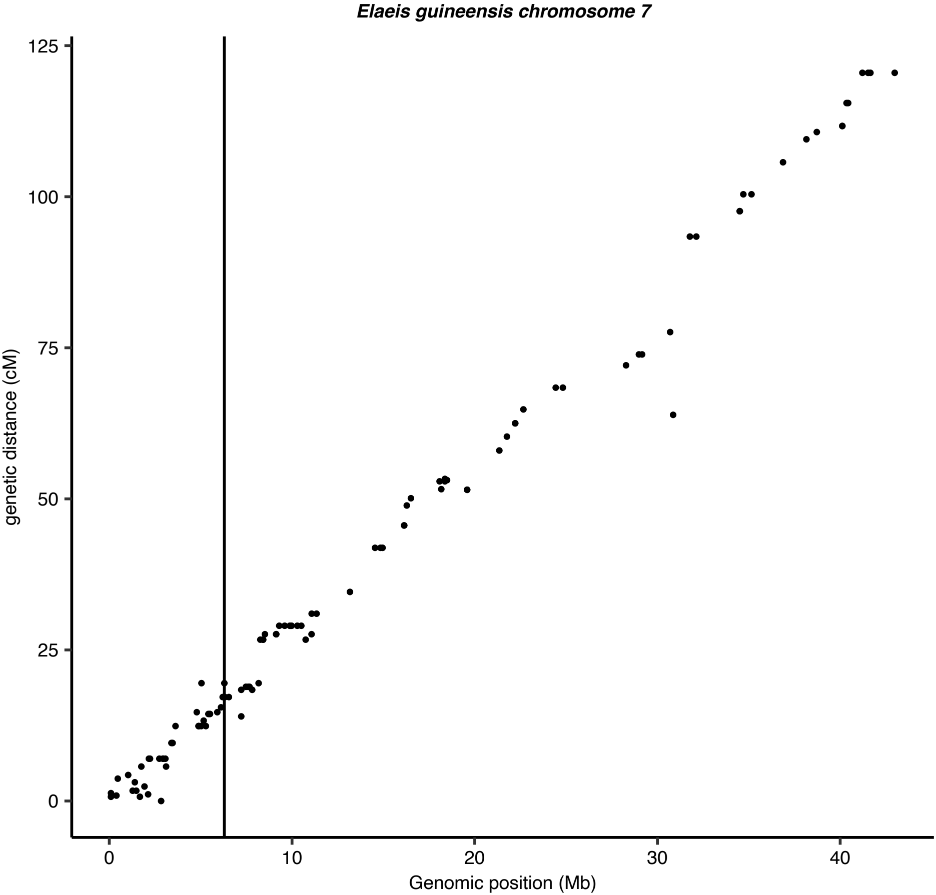
Elaeis guineensis chromosome 7
genetic distance (cM)
0
25
50
75
100
125
Genomic position (Mb)
0
10
20
30
40

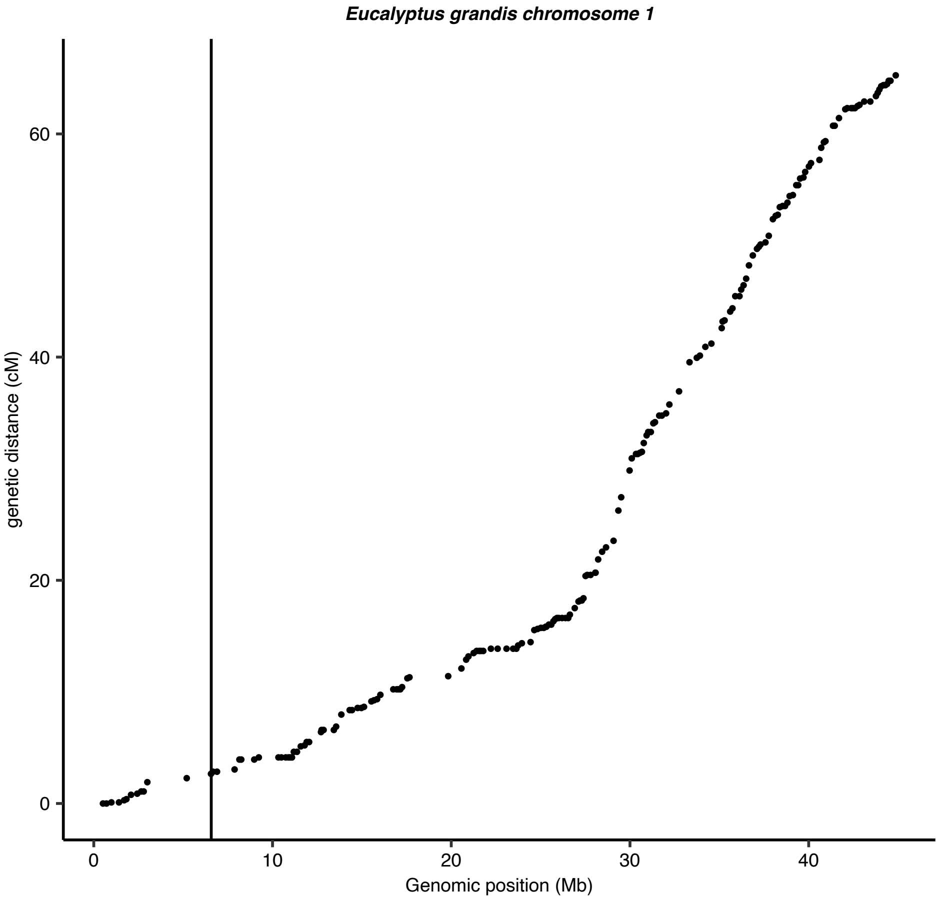
Eucalyptus grandis chromosome 1
genetic distance (cM)
0
20
40
60
Genomic position (Mb)
0
10
20
30
40

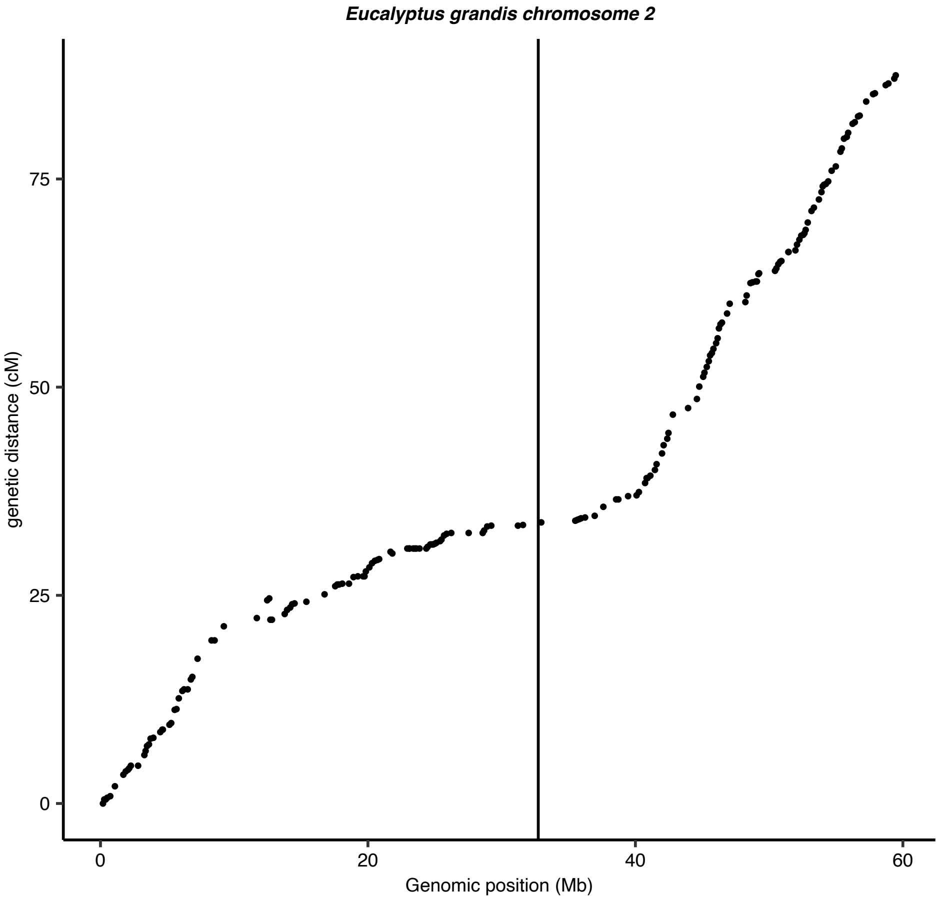

Eucalyptus grandis chromosome 2
genetic distance (cM)
0
25
50
75
Genomic position (Mb)
0
20
40
60

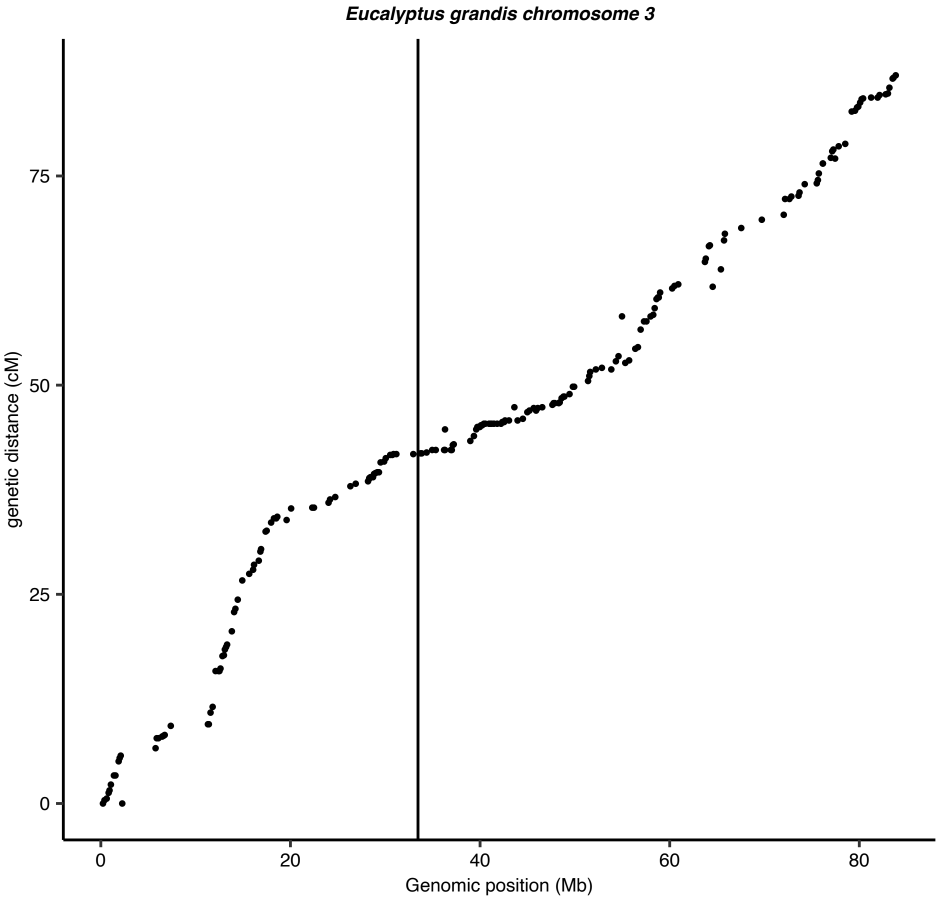
Eucalyptus grandis chromosome 3
genetic distance (cM)
0
25
50
75
Genomic position (Mb)
0
20
40
60
80

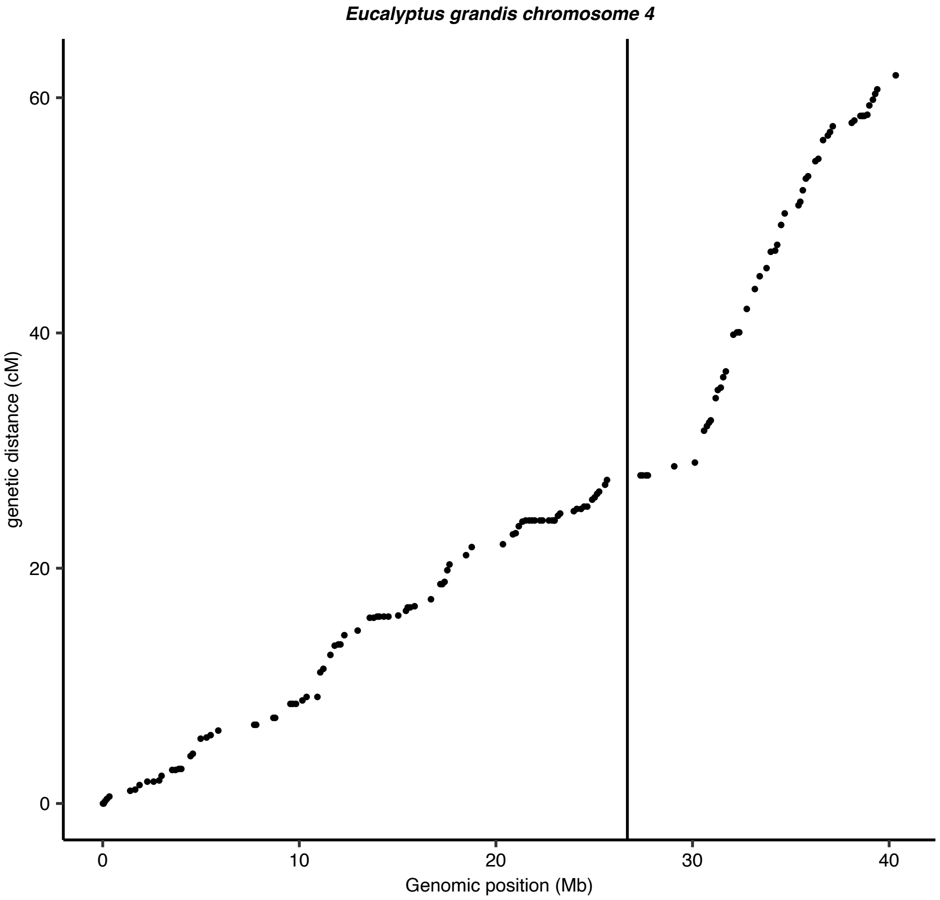
Eucalyptus grandis chromosome 4
genetic distance (cM)
Genomic position (Mb)
0
20
40
60
0
10
20
30
40

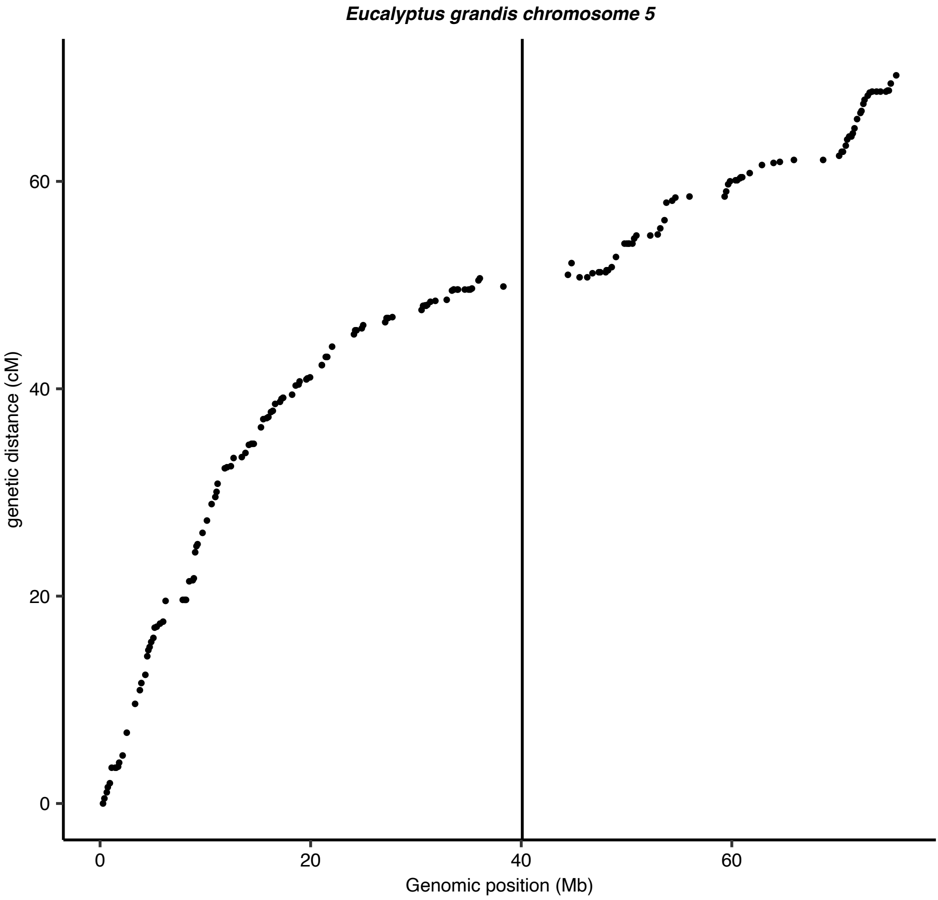
Eucalyptus grandis chromosome 5
genetic distance (cM)
Genomic position (Mb)
0
20
40
60
0
20
40
60

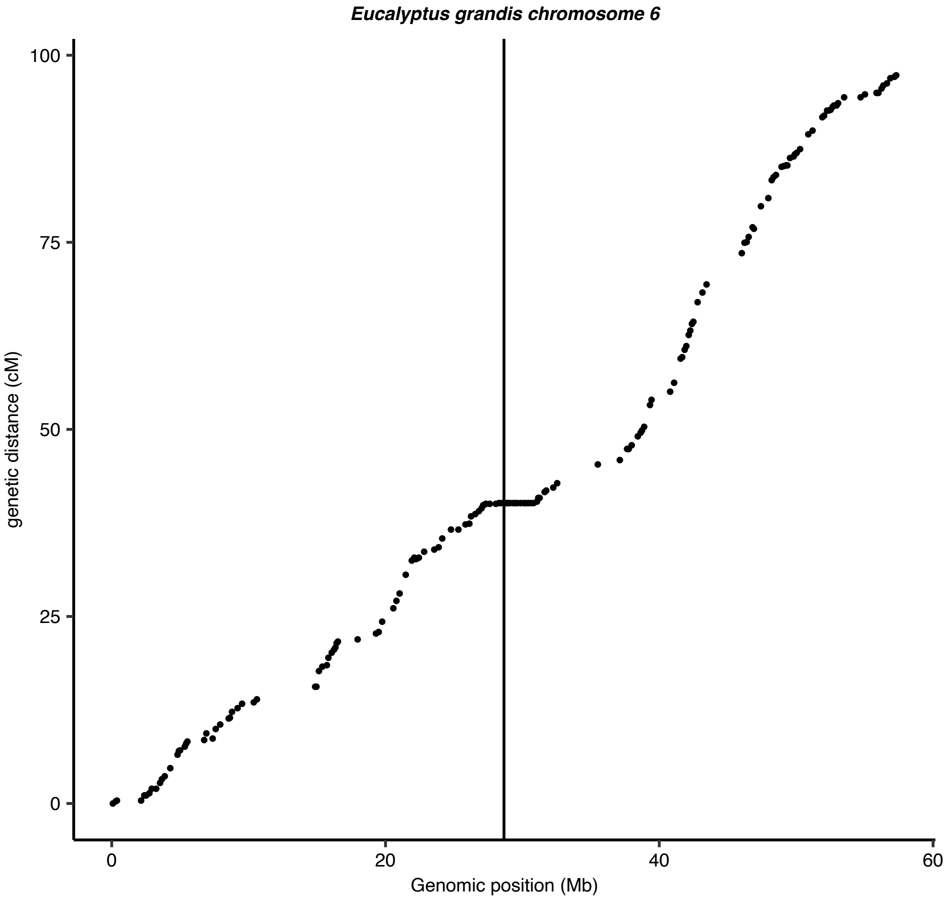

Eucalyptus grandis chromosome 6
genetic distance (cM)
Genomic position (Mb)
0
25
50
75
100
0
20
40
60

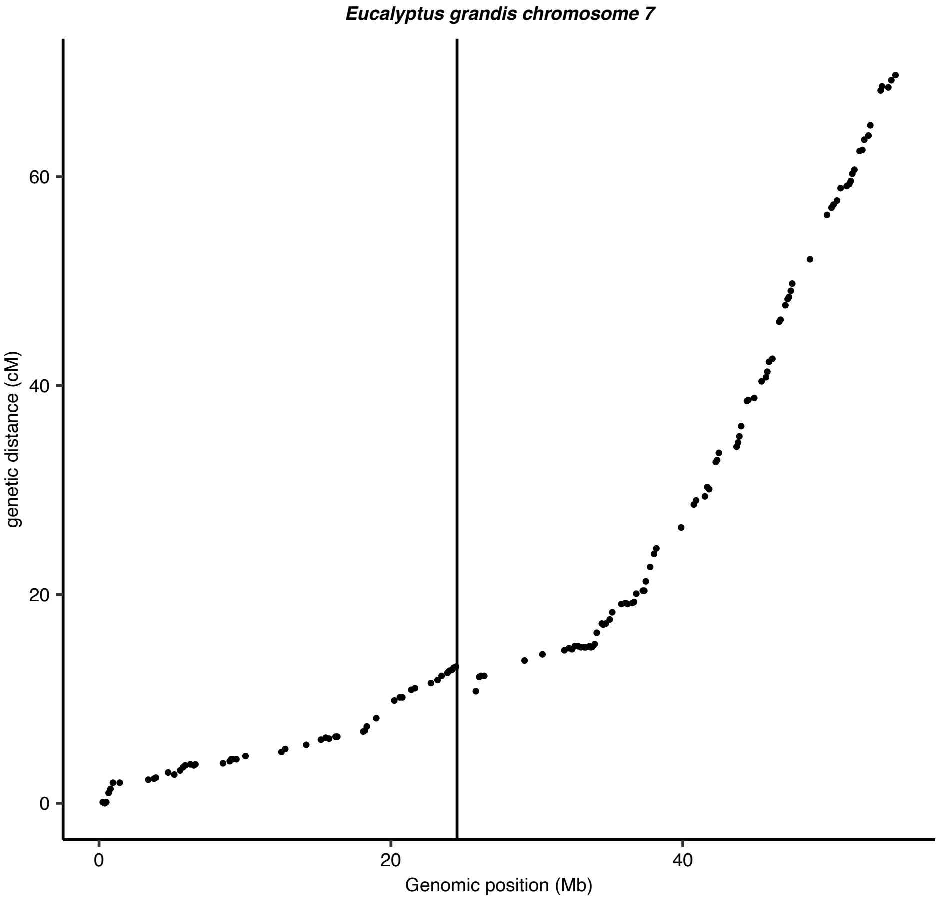
Eucalyptus grandis chromosome 7
genetic distance (cM)
0
20
40
60
Genomic position (Mb)
0
20
40

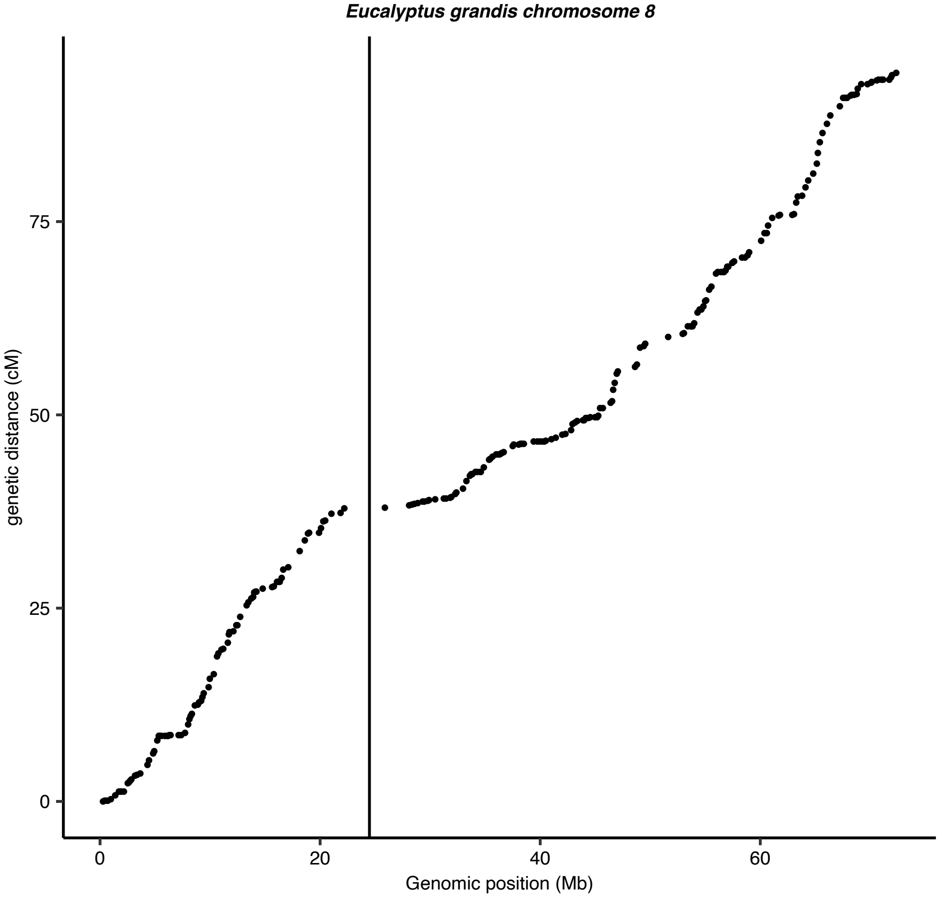

Eucalyptus grandis chromosome 8
genetic distance (cM)
Genomic position (Mb)
0
25
50
75
0
20
40
60

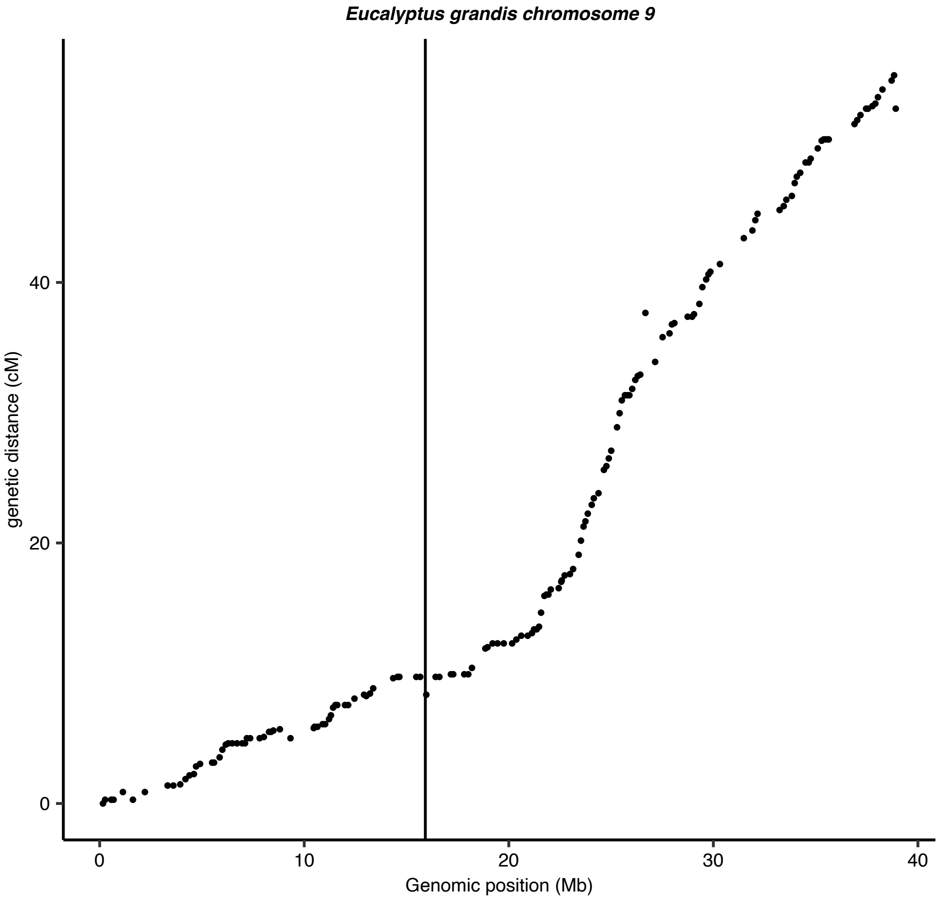
Eucalyptus grandis chromosome 9
genetic distance (cM)
Genomic position (Mb)
0
20
40
0
10
20
30
40

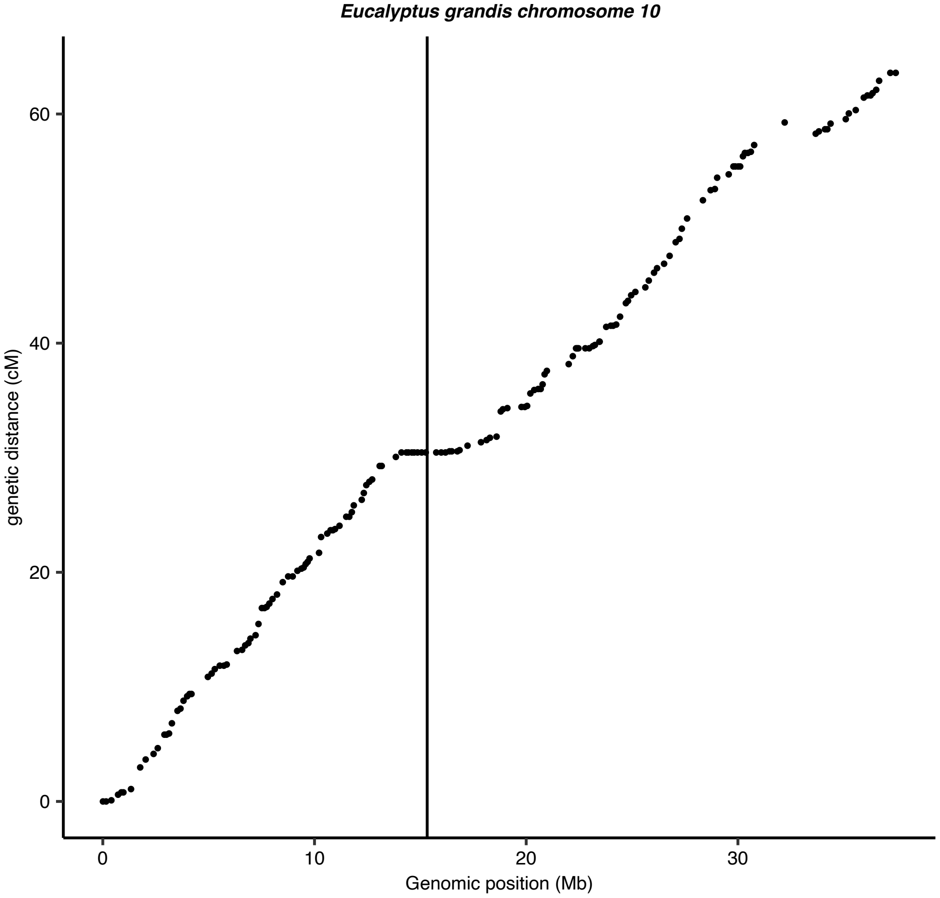
Eucalyptus grandis chromosome 10
genetic distance (cM)
0
20
40
60
0
10
20
30
Genomic position (Mb)

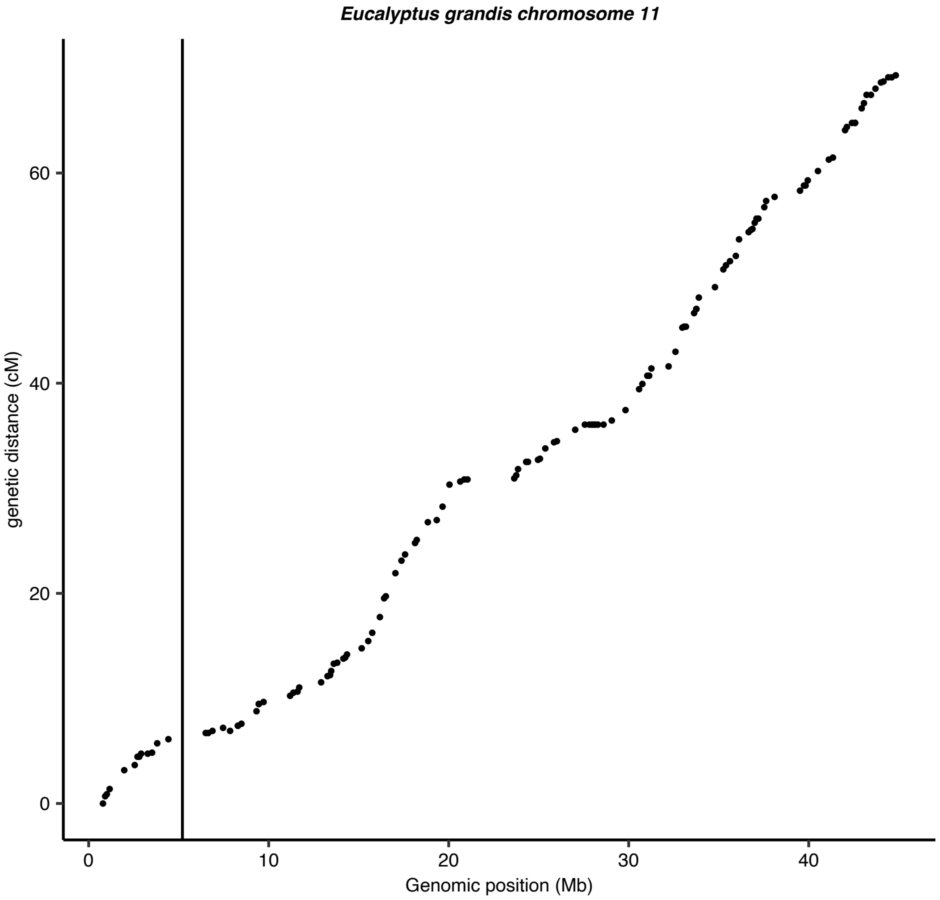
Eucalyptus grandis chromosome 11
genetic distance (cM)
0
20
40
60
Genomic position (Mb)
0
10
20
30
40

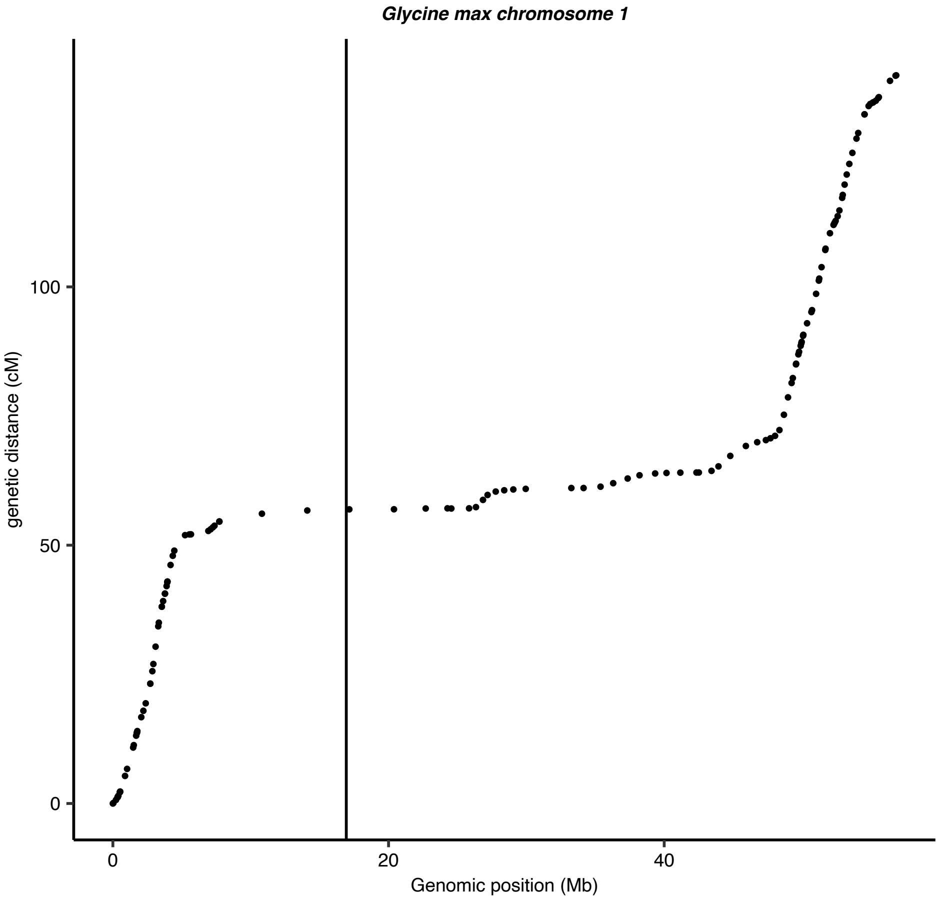
Glycine max chromosome 1
genetic distance (cM)
0
50
100
Genomic position (Mb)
0
20
40

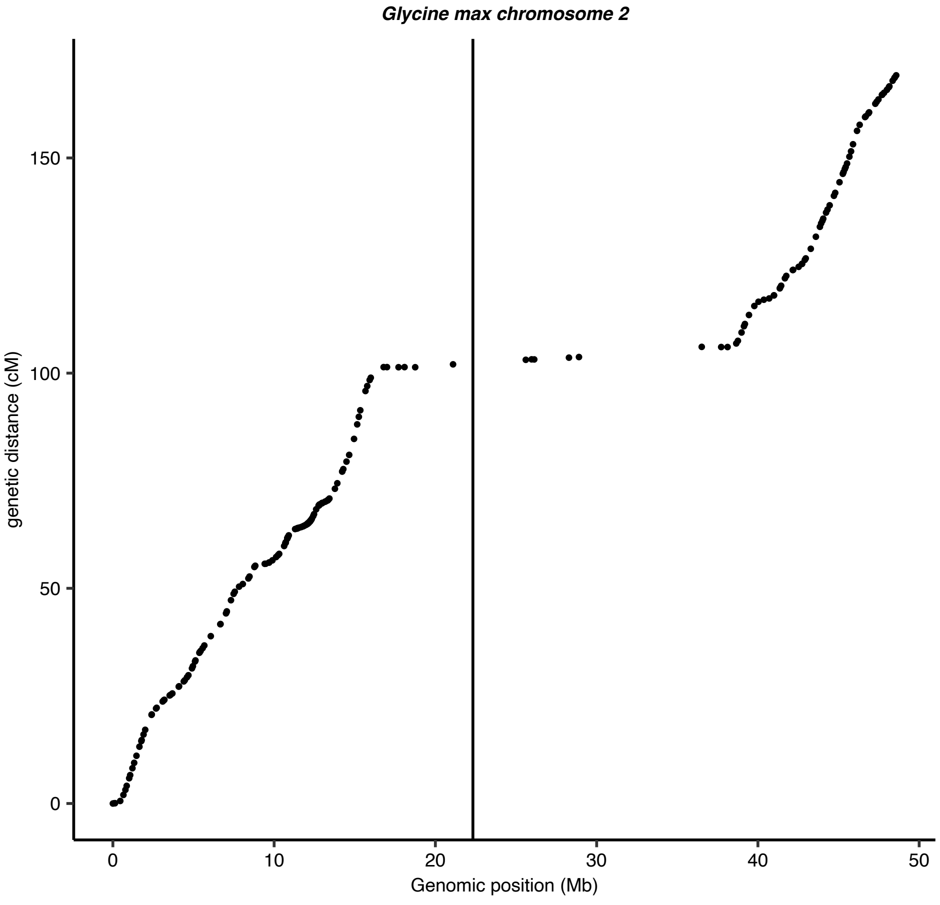
Glycine max chromosome 2
genetic distance (cM)
0
50
100
150
Genomic position (Mb)
0
10
20
30
40
50

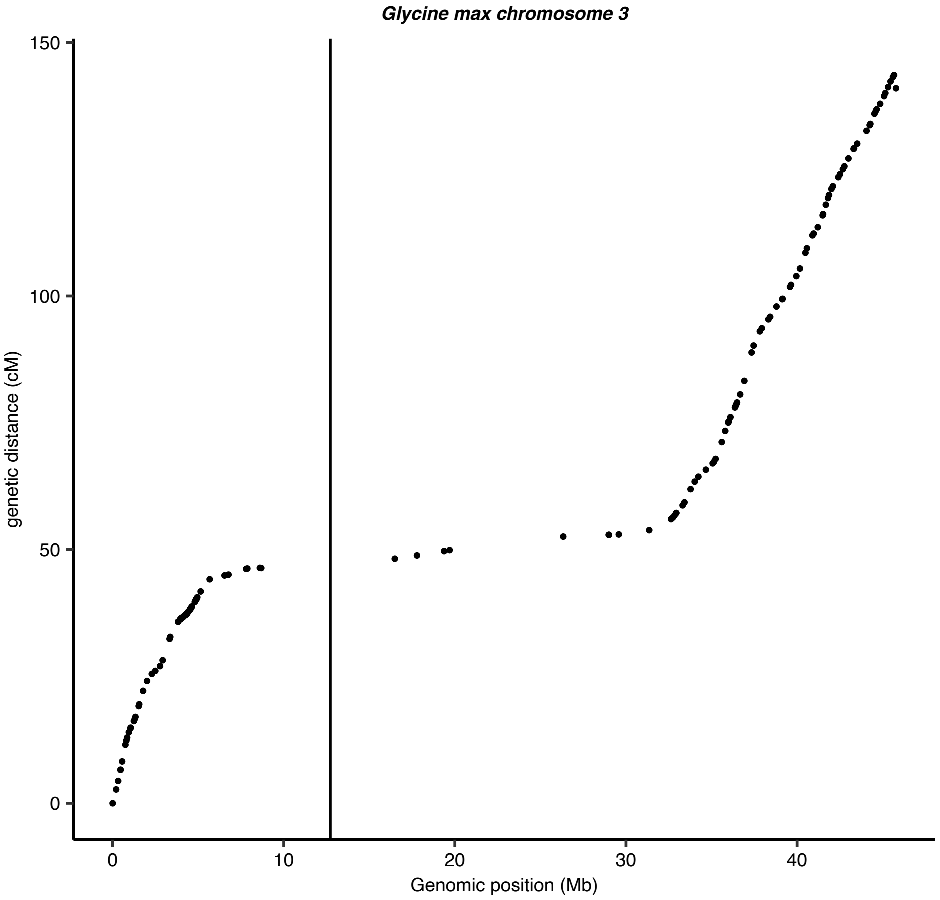
Glycine max chromosome 3
genetic distance (cM)
Genomic position (Mb)
0
50
100
150
0
10
20
30
40

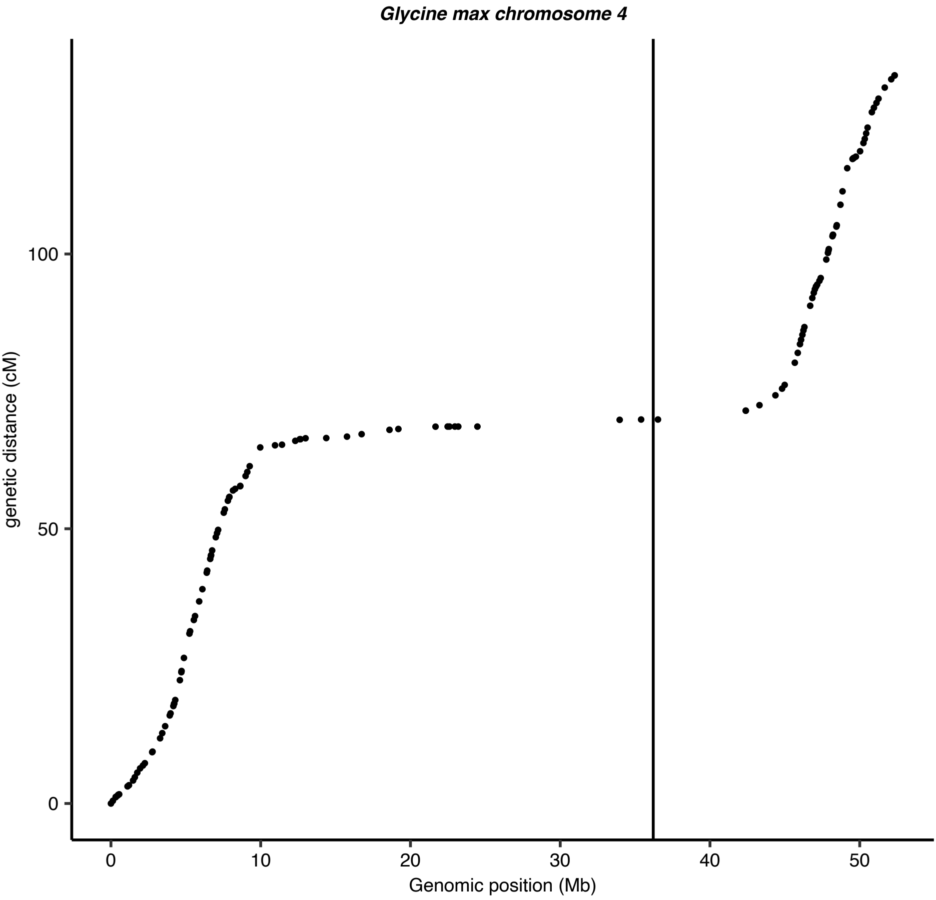
Glycine max chromosome 4
genetic distance (cM)
0
50
100
Genomic position (Mb)
0
10
20
30
40
50

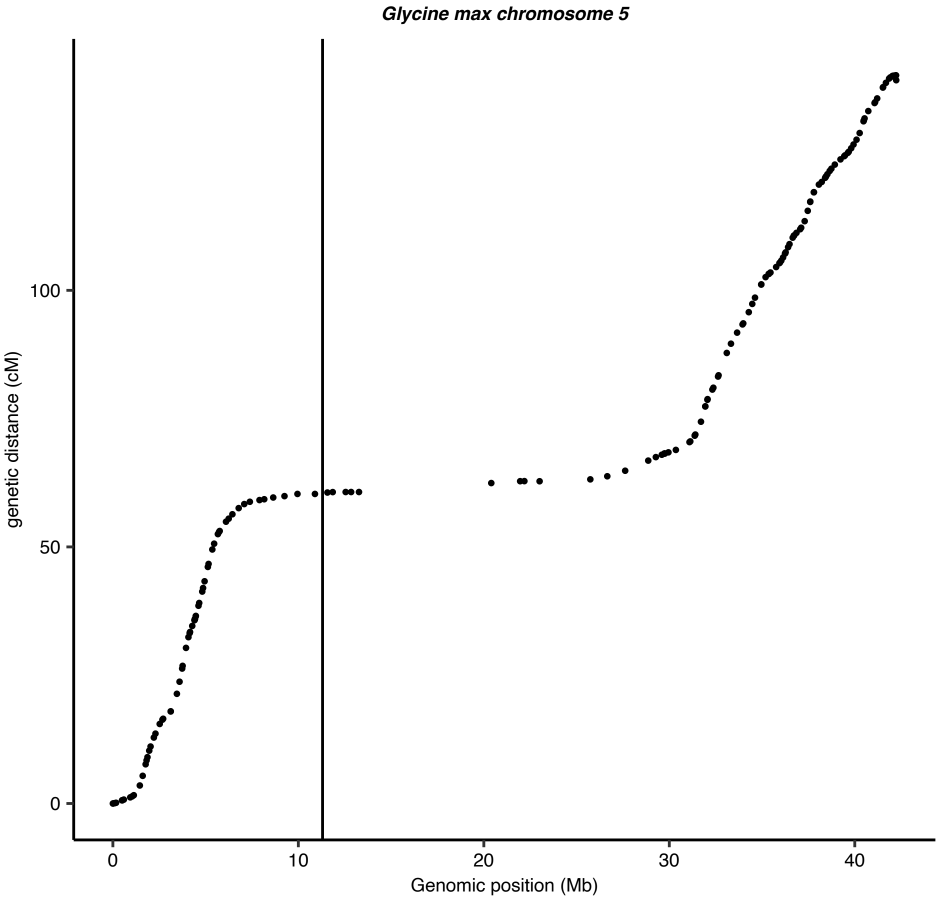
Glycine max chromosome 5
genetic distance (cM)
Genomic position (Mb)
0
50
100
0
10
20
30
40

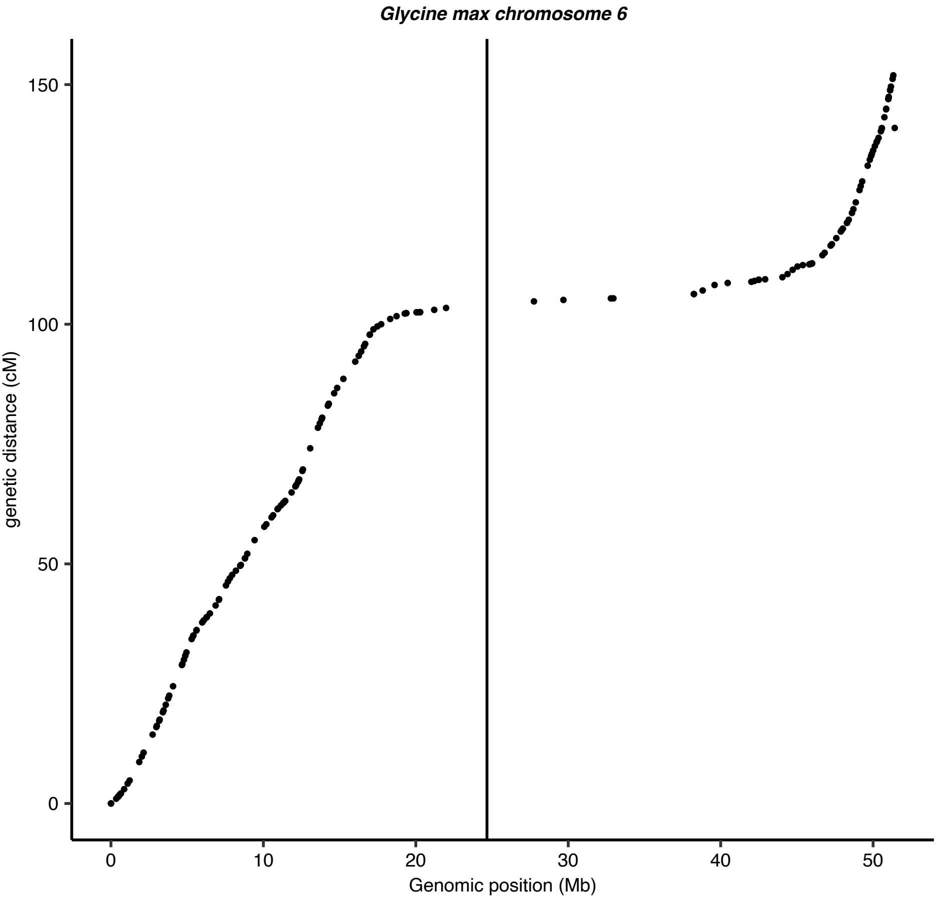
Glycine max chromosome 6
genetic distance (cM)
0
50
100
150
Genomic position (Mb)
0
10
20
30
40
50

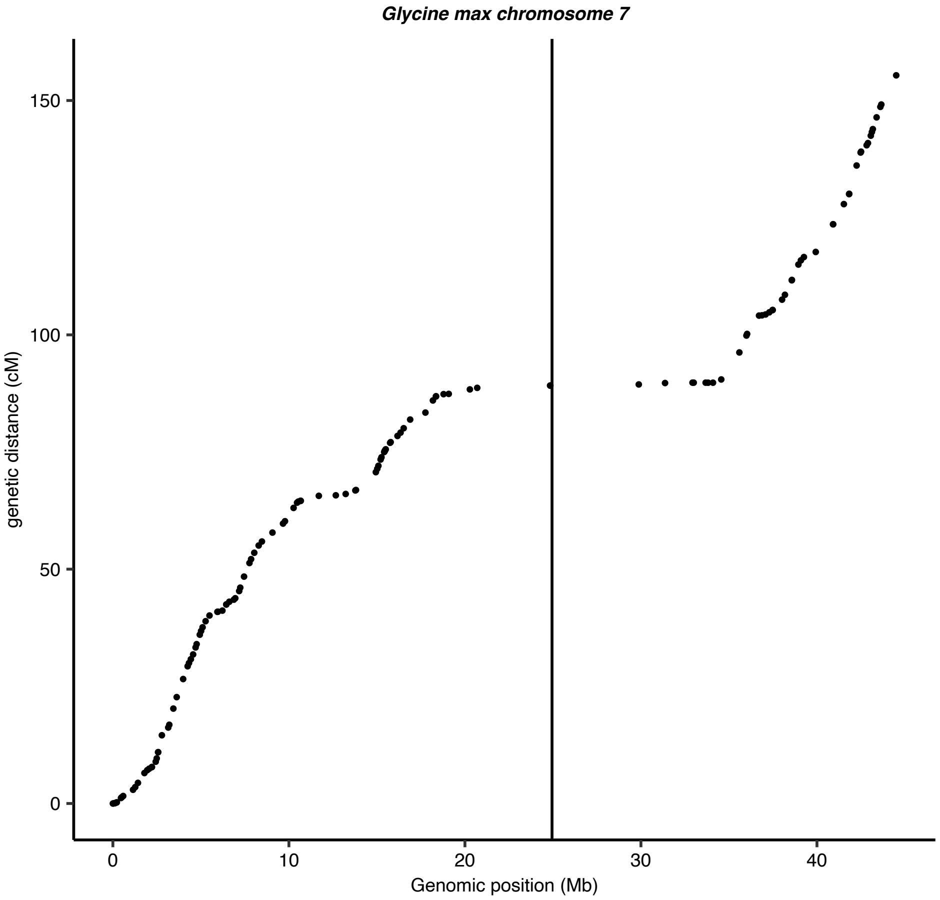
Glycine max chromosome 7
genetic distance (cM)
0
50
100
150
Genomic position (Mb)
0
10
20
30
40

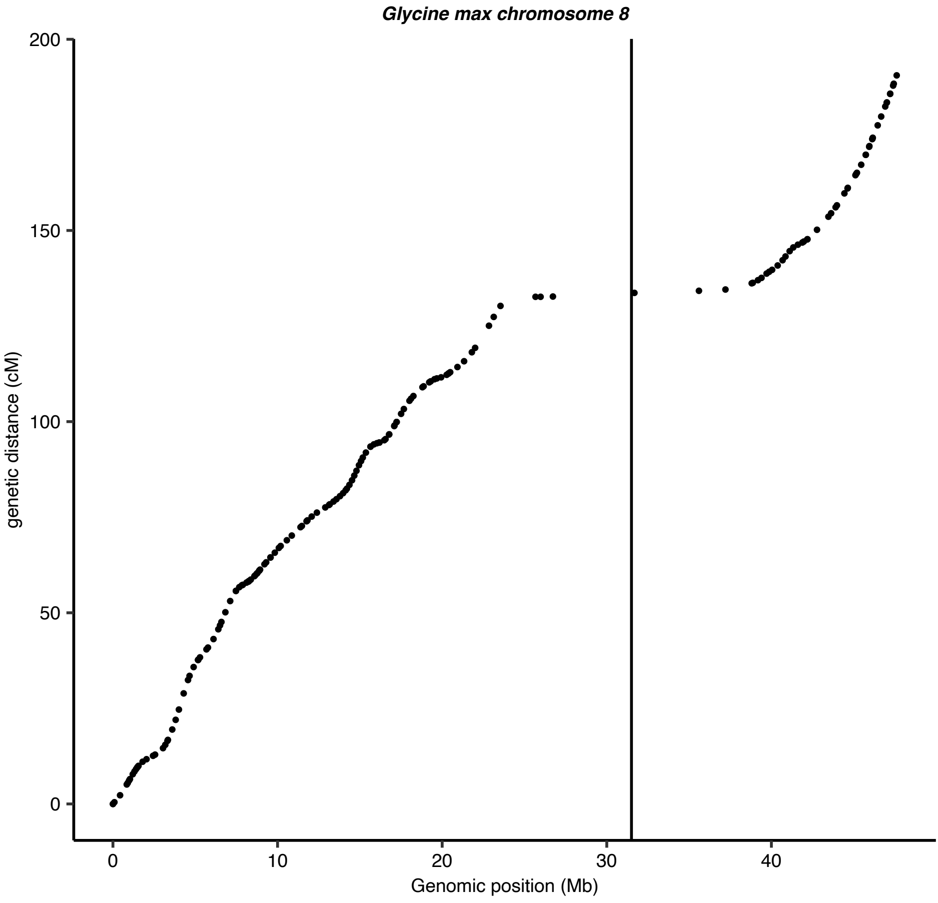

Glycine max chromosome 8
genetic distance (cM)
0
50
100
150
200
0
10
20
30
40
Genomic position (Mb)

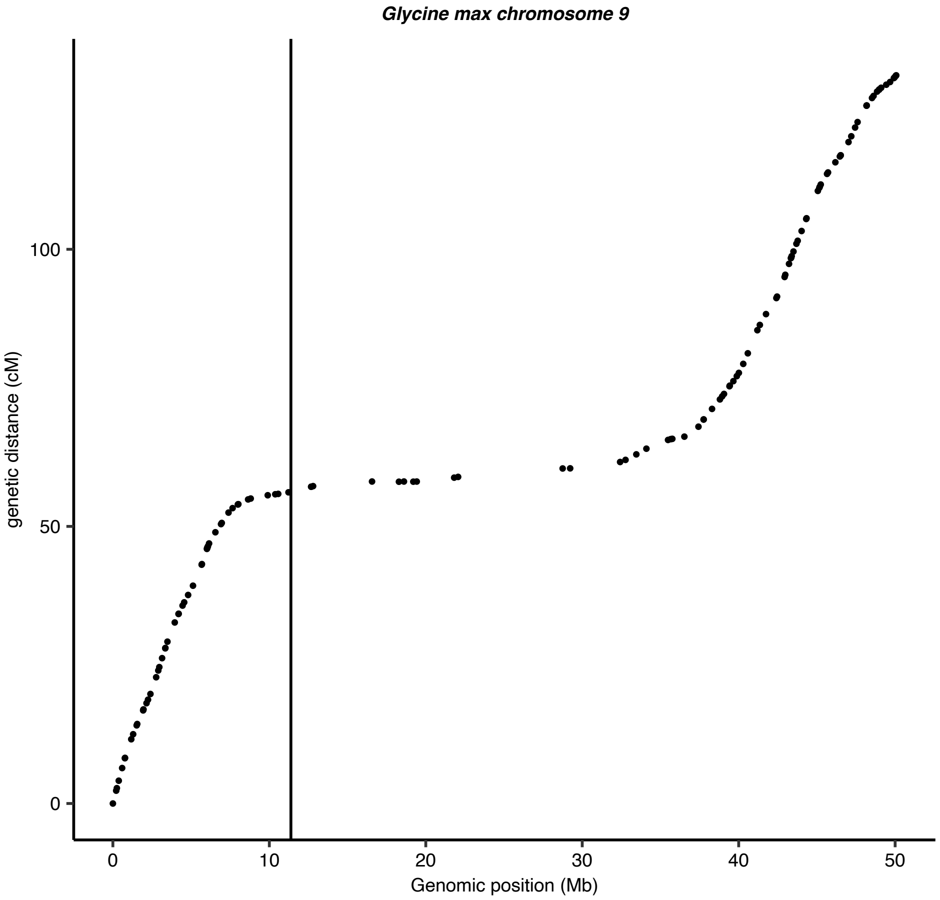
Glycine max chromosome 9
genetic distance (cM)
Genomic position (Mb)
0
50
100
0
10
20
30
40
50

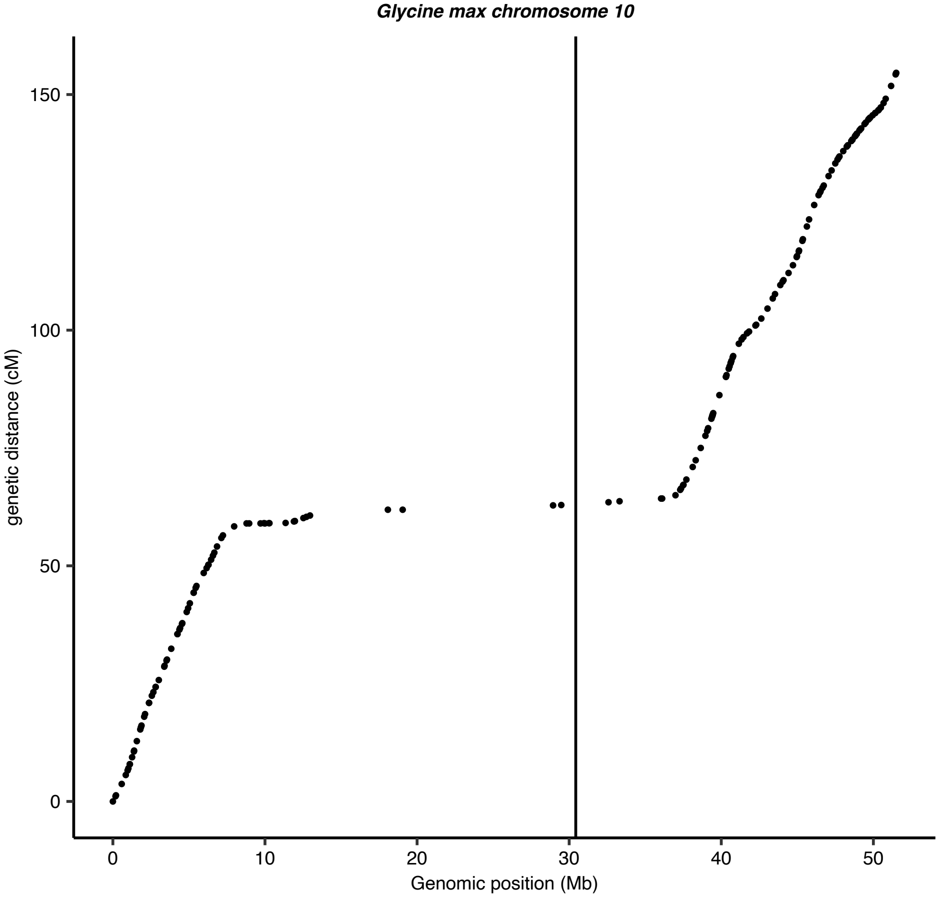
Glycine max chromosome 10
genetic distance (cM)
0
50
100
150
Genomic position (Mb)
0
10
20
30
40
50

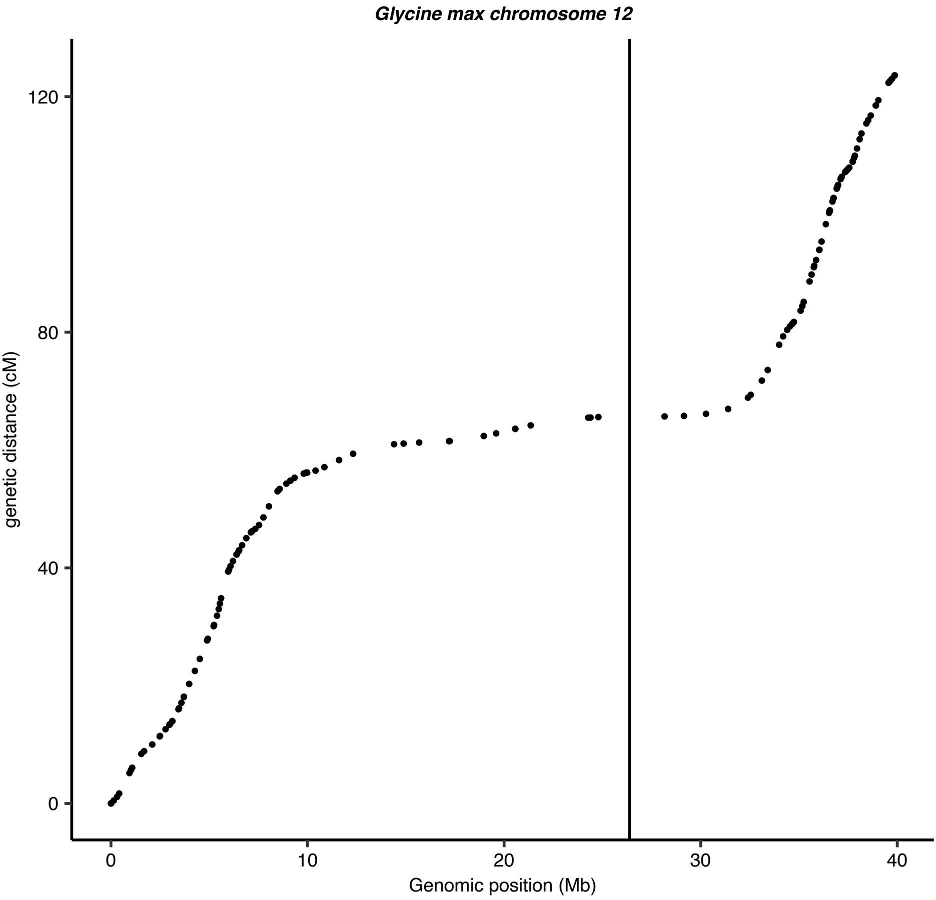
Glycine max chromosome 12
120
80
40
0
genetic distance (cM)
0
10
20
30
40
Genomic position (Mb)

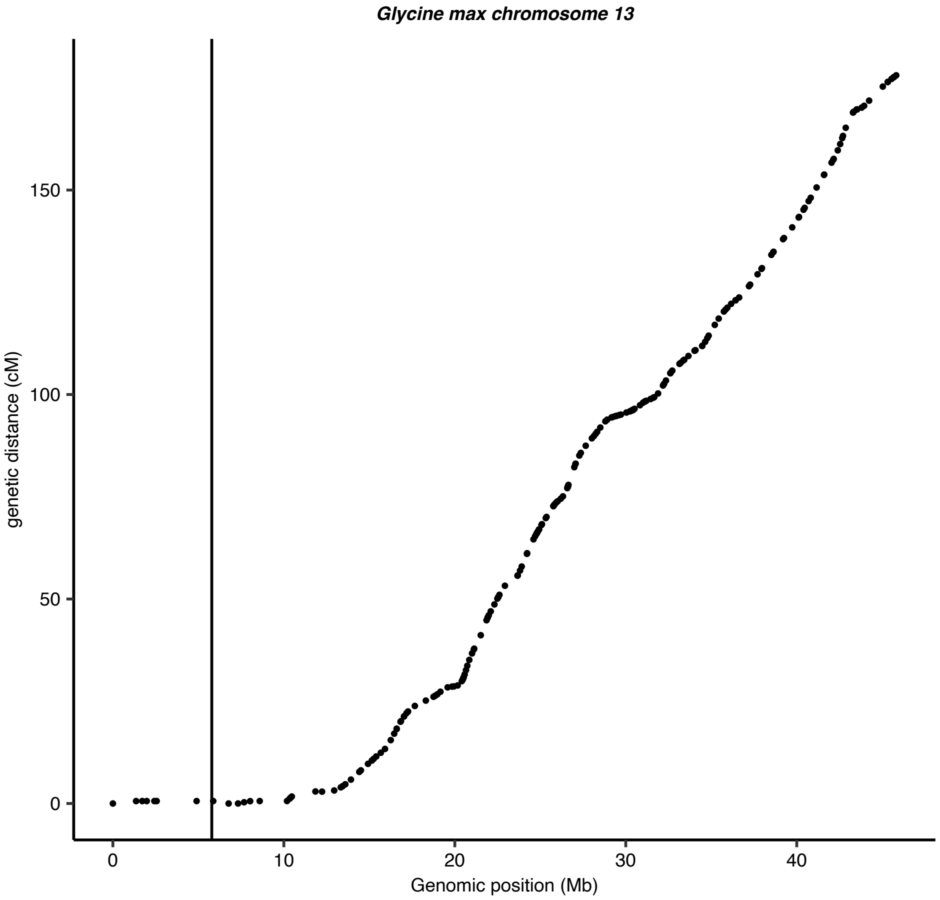
Glycine max chromosome 13
genetic distance (cM)
0
50
100
150
0
10
20
30
40
Genomic position (Mb)

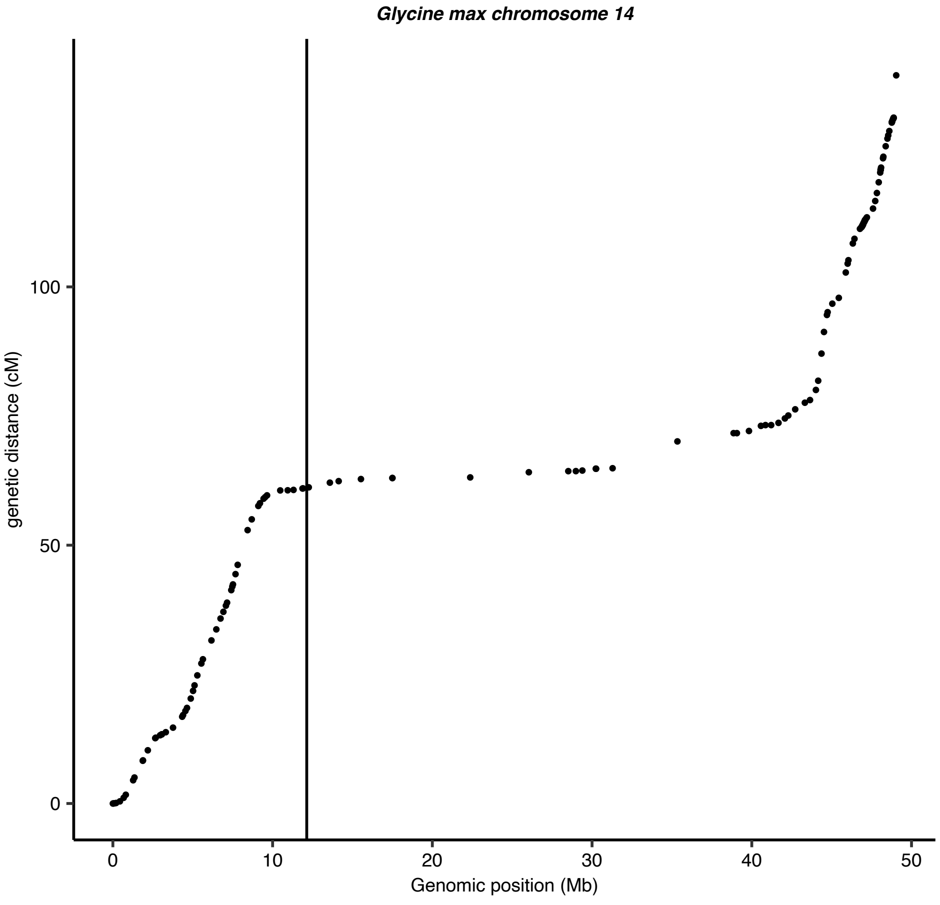
Glycine max chromosome 14
genetic distance (cM)
0
50
100
Genomic position (Mb)
0
10
20
30
40
50

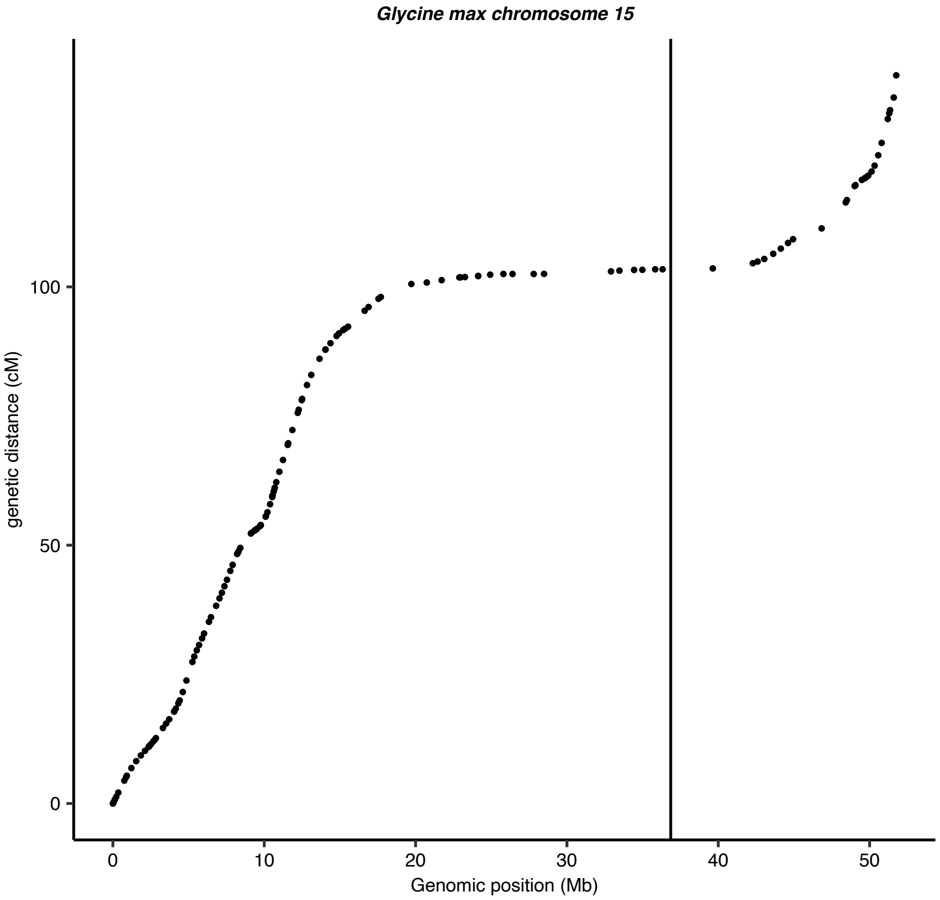
Glycine max chromosome 15
genetic distance (cM)
Genomic position (Mb)
0
50
100
0
10
20
30
40
50

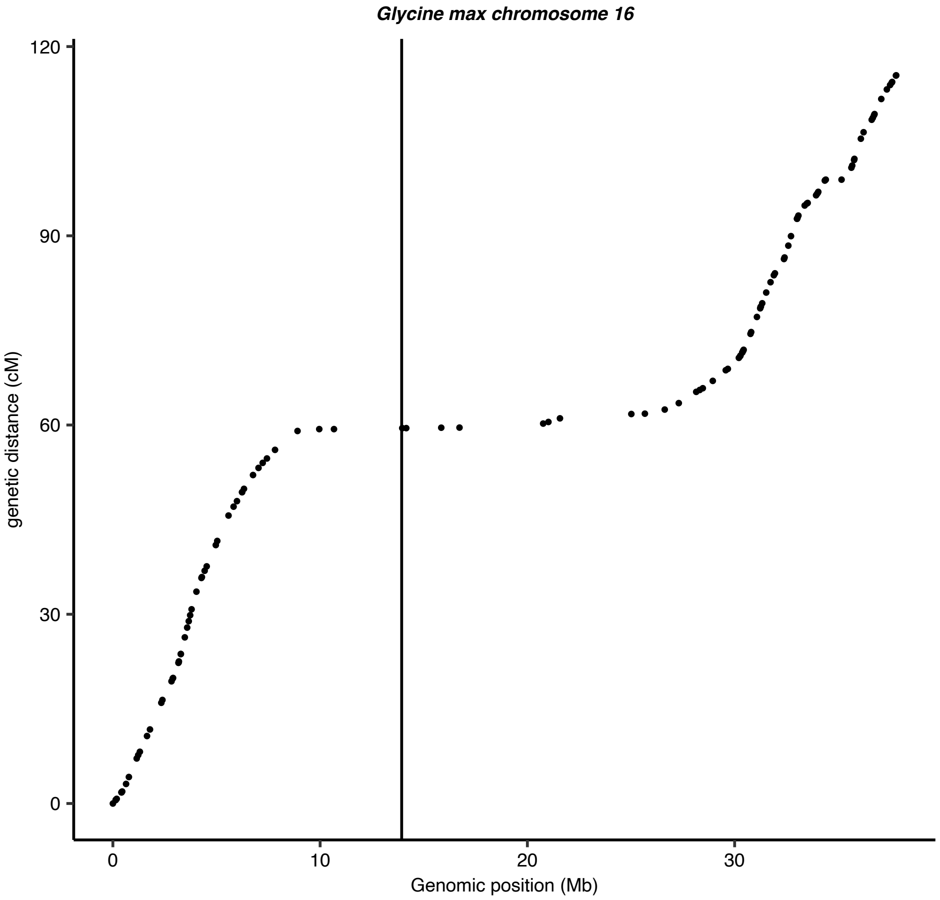
Glycine max chromosome 16
genetic distance (cM)
Genomic position (Mb)
0
30
60
90
120
0
10
20
30

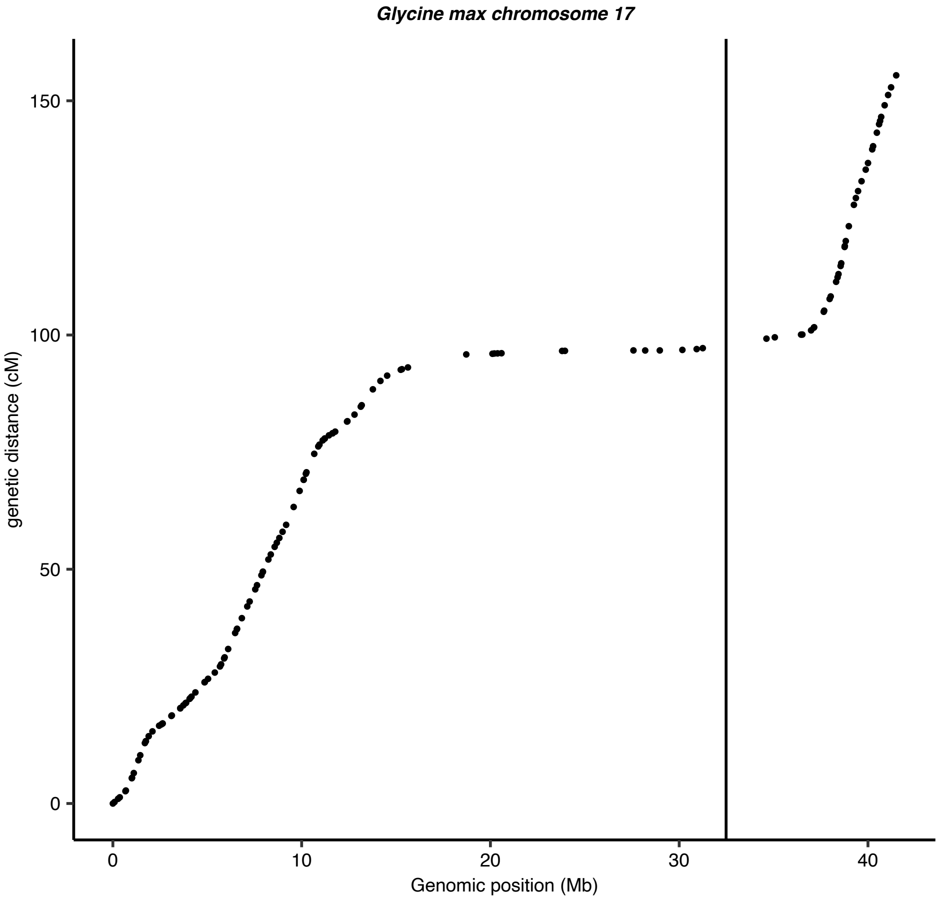
Glycine max chromosome 17
genetic distance (cM)
0
50
100
150
Genomic position (Mb)
0
10
20
30
40

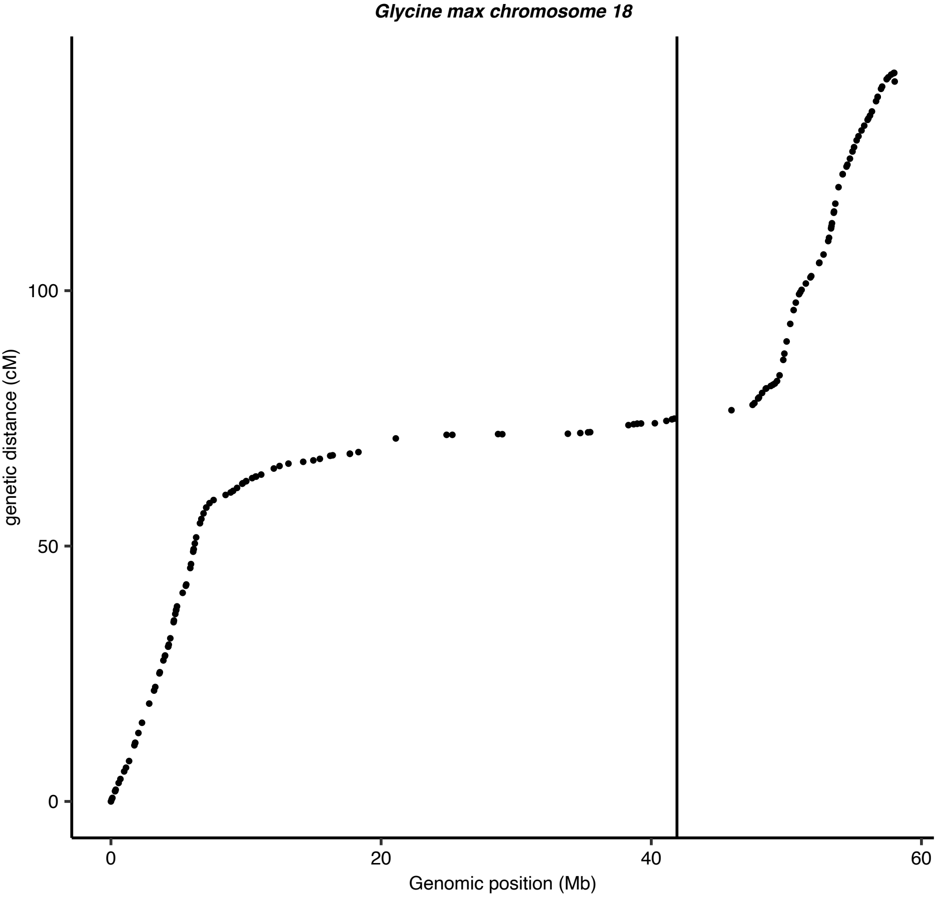
Glycine max chromosome 18
genetic distance (cM)
100
50
0
0
20
40
60
Genomic position (Mb)

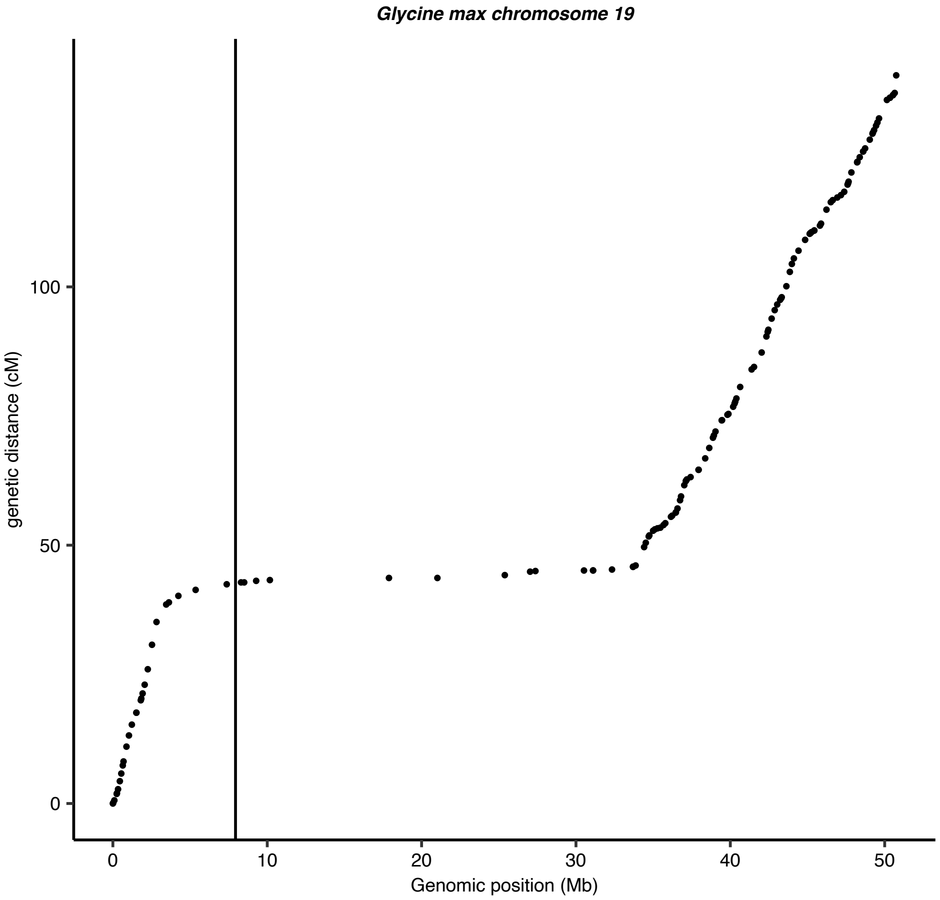
Glycine max chromosome 19
genetic distance (cM)
0
50
100
Genomic position (Mb)
0
10
20
30
40
50

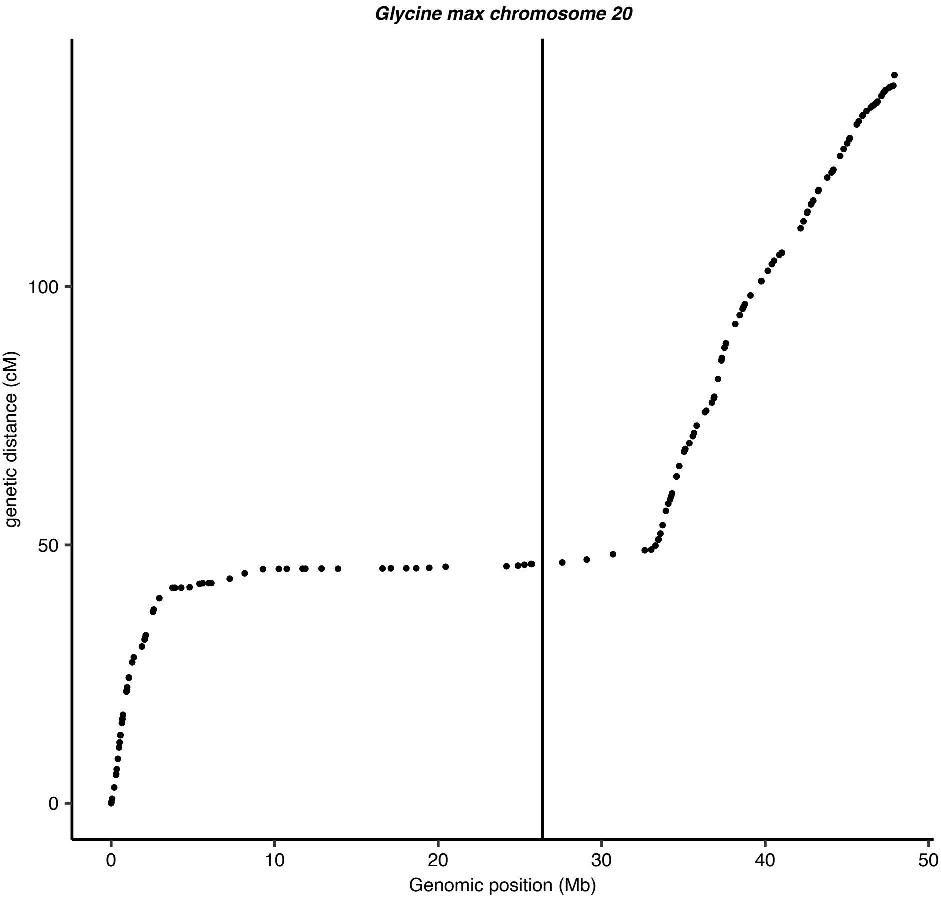

Glycine max chromosome 20
genetic distance (cM)
0
50
100
0
10
20
30
40
50
Genomic position (Mb)

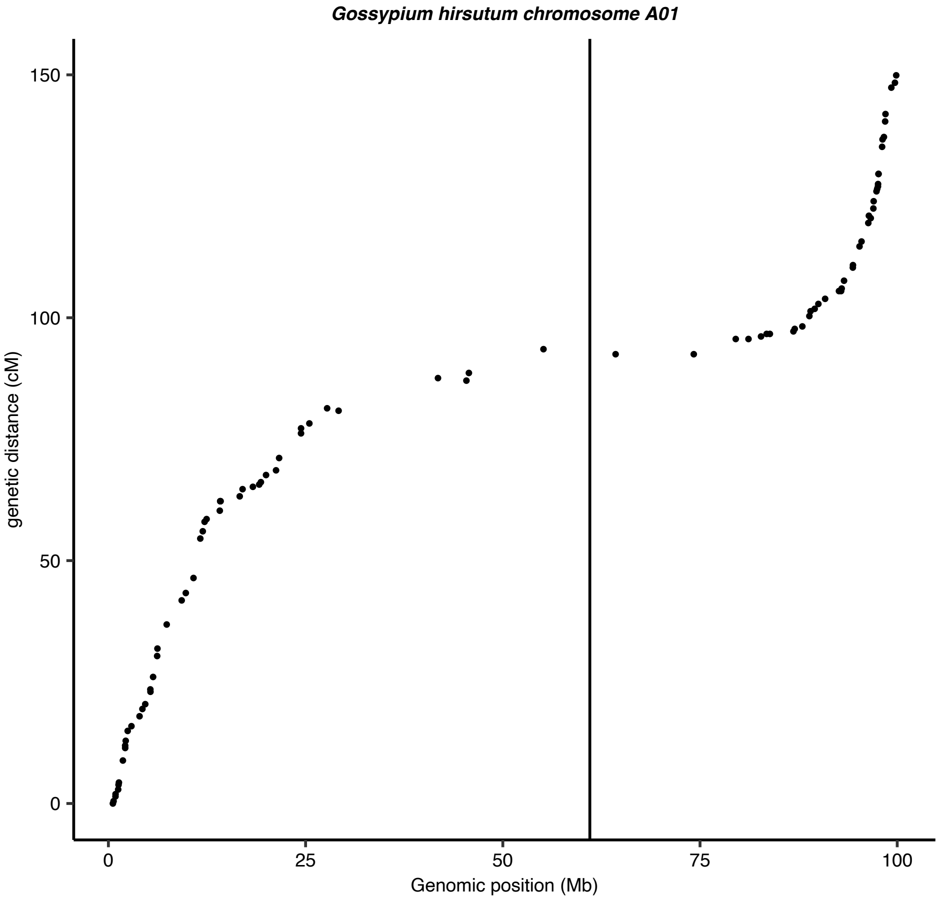
Gossypium hirsutum chromosome A01
genetic distance (cM)
Genomic position (Mb)
0
50
100
150
0
25
50
75
100

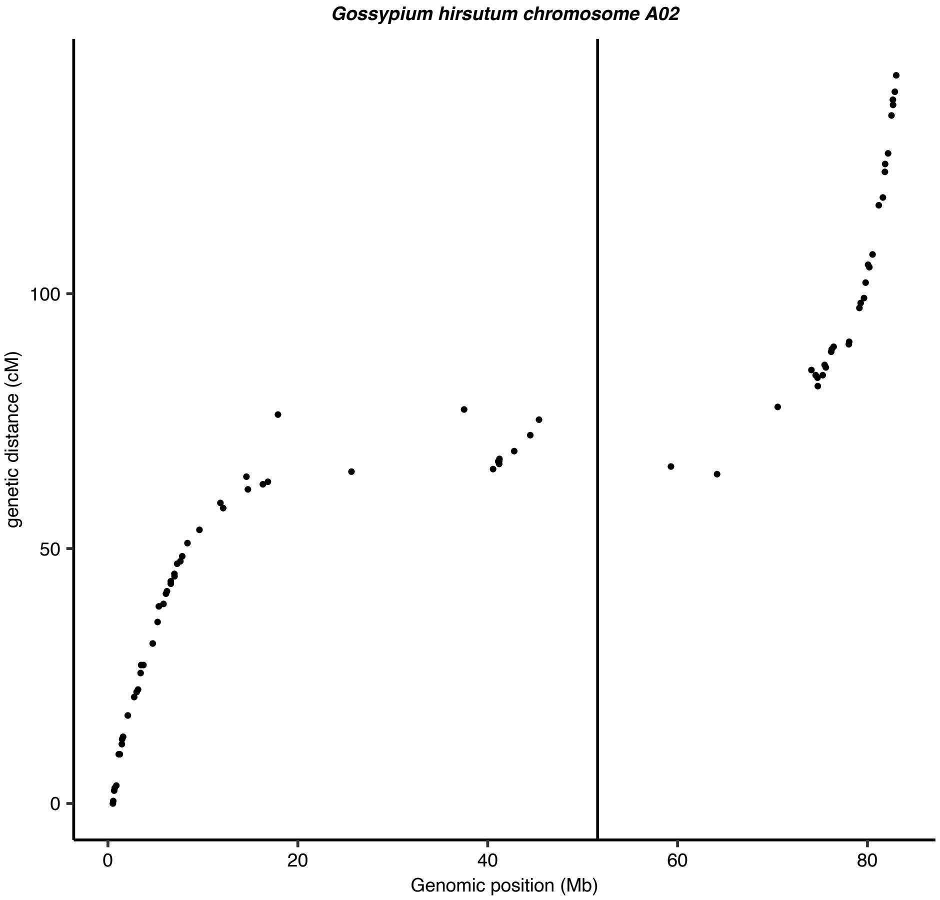
Gossypium hirsutum chromosome A02
genetic distance (cM)
100
50
0
0
20
40
60
80
Genomic position (Mb)

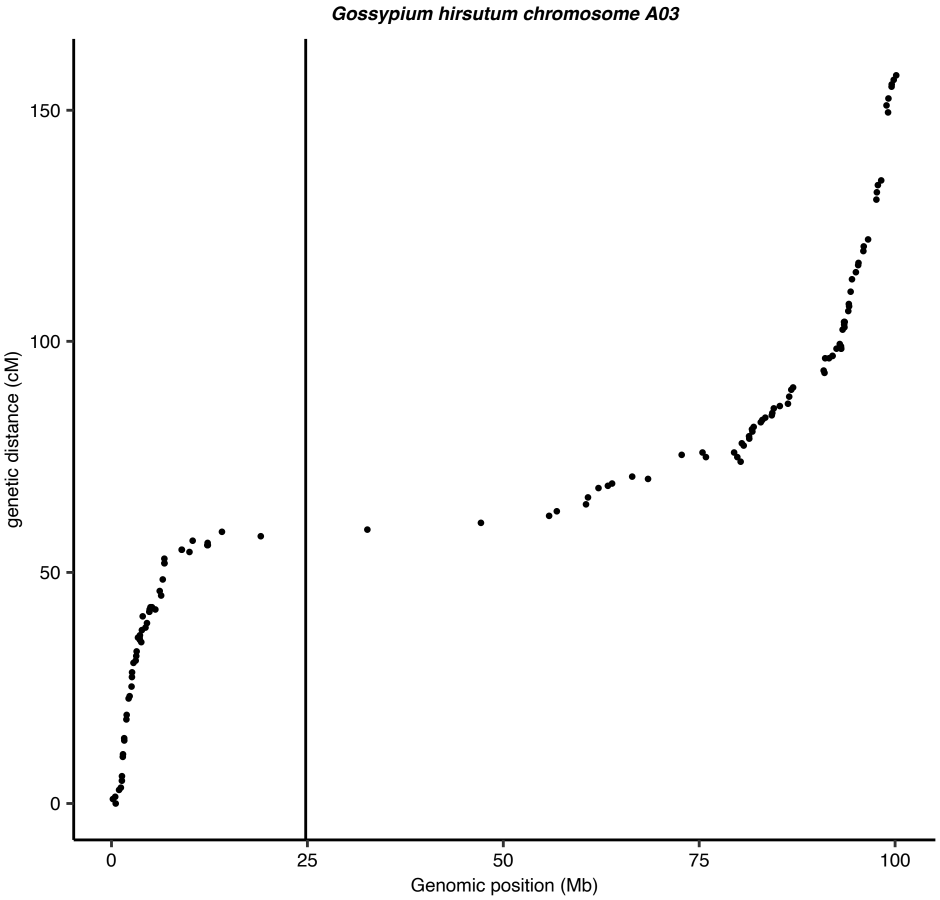
Gossypium hirsutum chromosome A03
genetic distance (cM)
150
100
50
0
0
25
50
75
100
Genomic position (Mb)

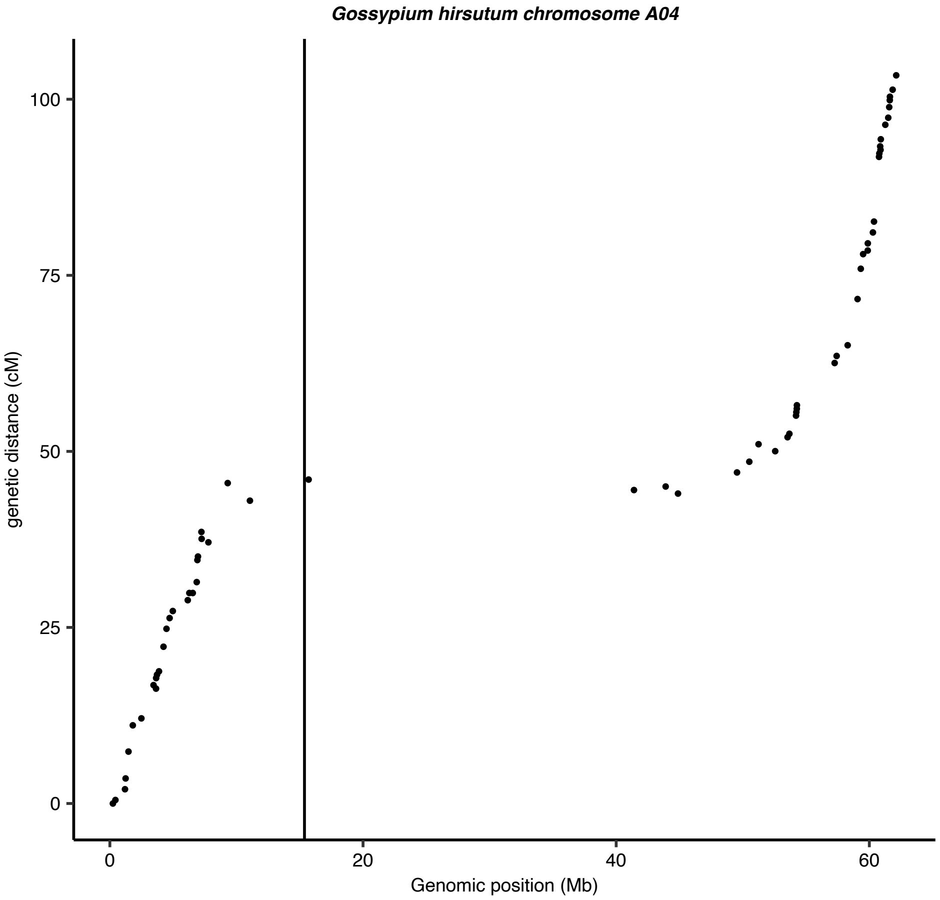
Gossypium hirsutum chromosome A04
genetic distance (cM)
100
75
50
25
0
0
20
40
60
Genomic position (Mb)

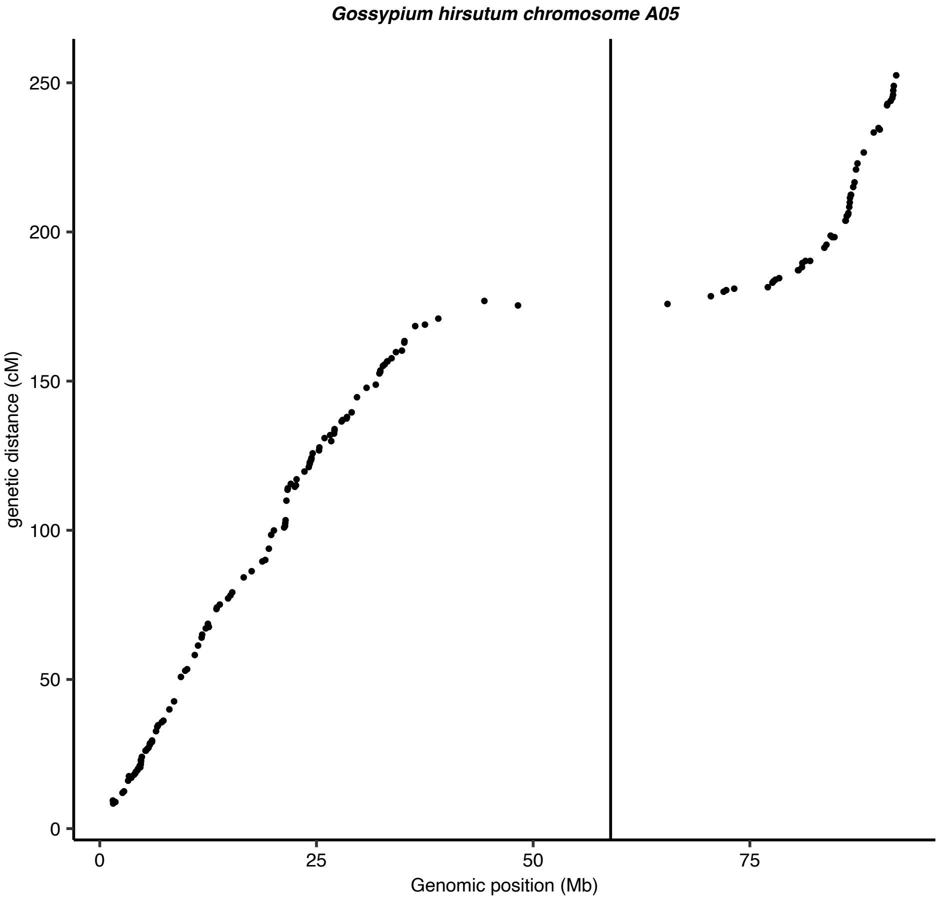
Gossypium hirsutum chromosome A05
genetic distance (cM)
0
50
100
150
200
250
Genomic position (Mb)
0
25
50
75

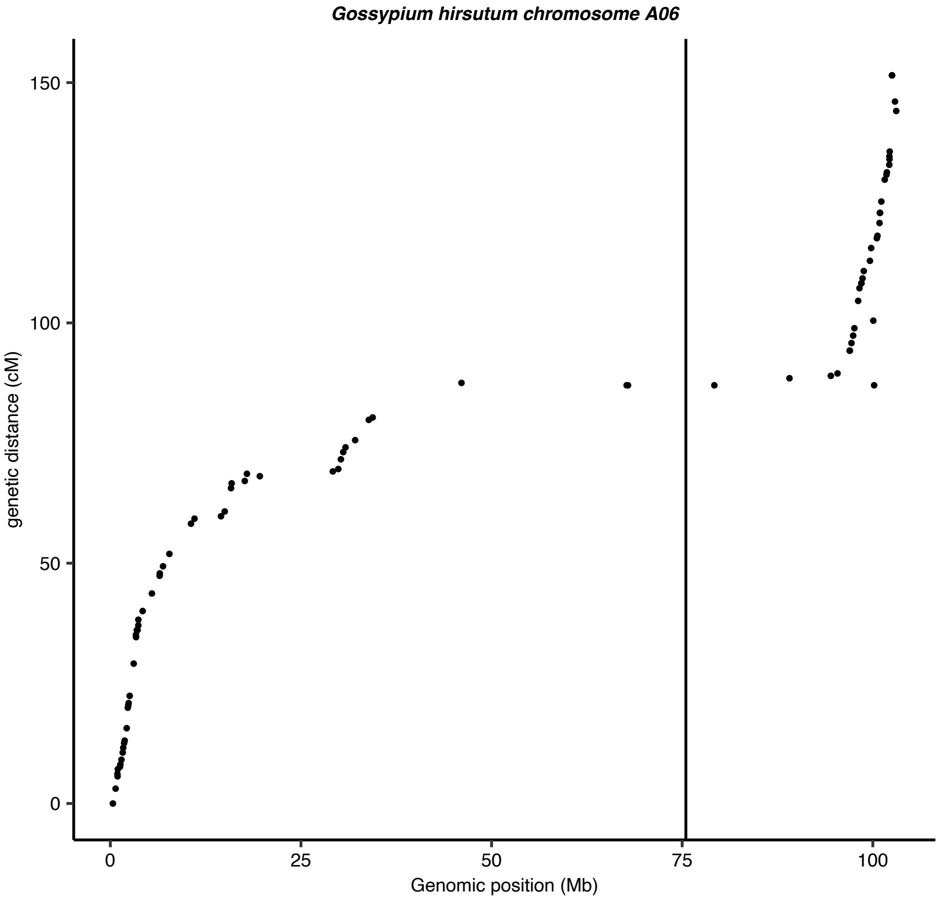
Gossypium hirsutum chromosome A06
genetic distance (cM)
150
100
50
0
0
25
50
75
100
Genomic position (Mb)

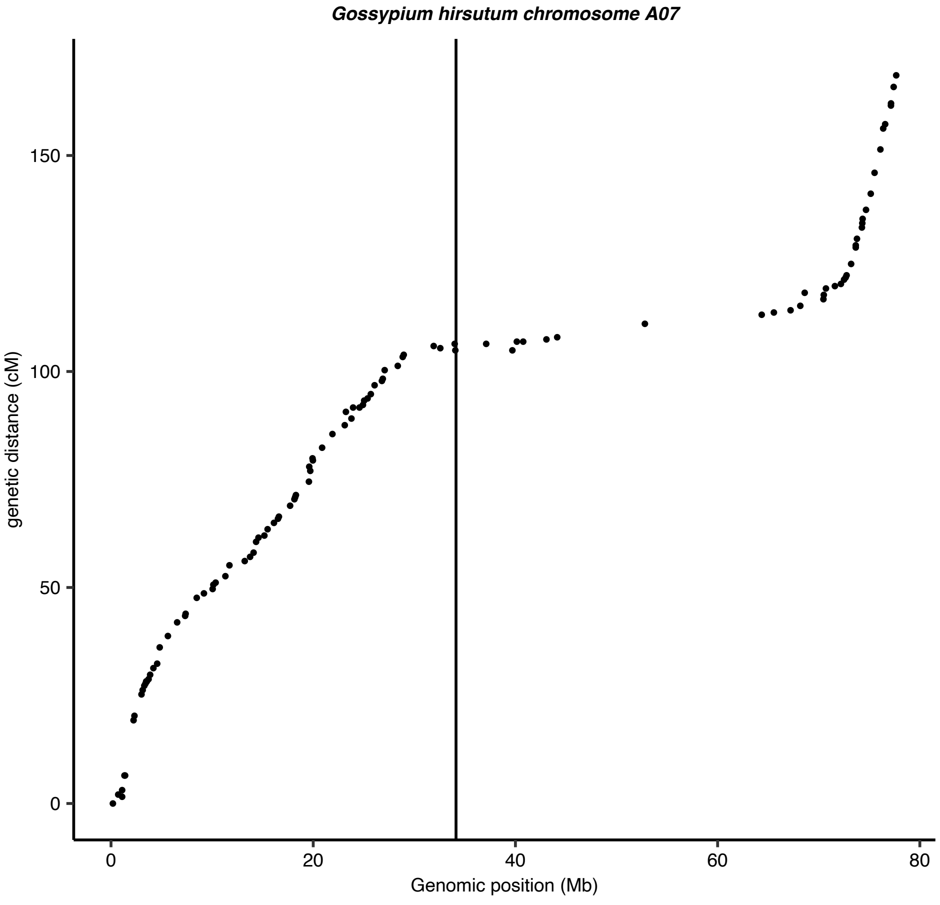
Gossypium hirsutum chromosome A07
genetic distance (cM)
150
100
50
0
0
20
40
60
80
Genomic position (Mb)

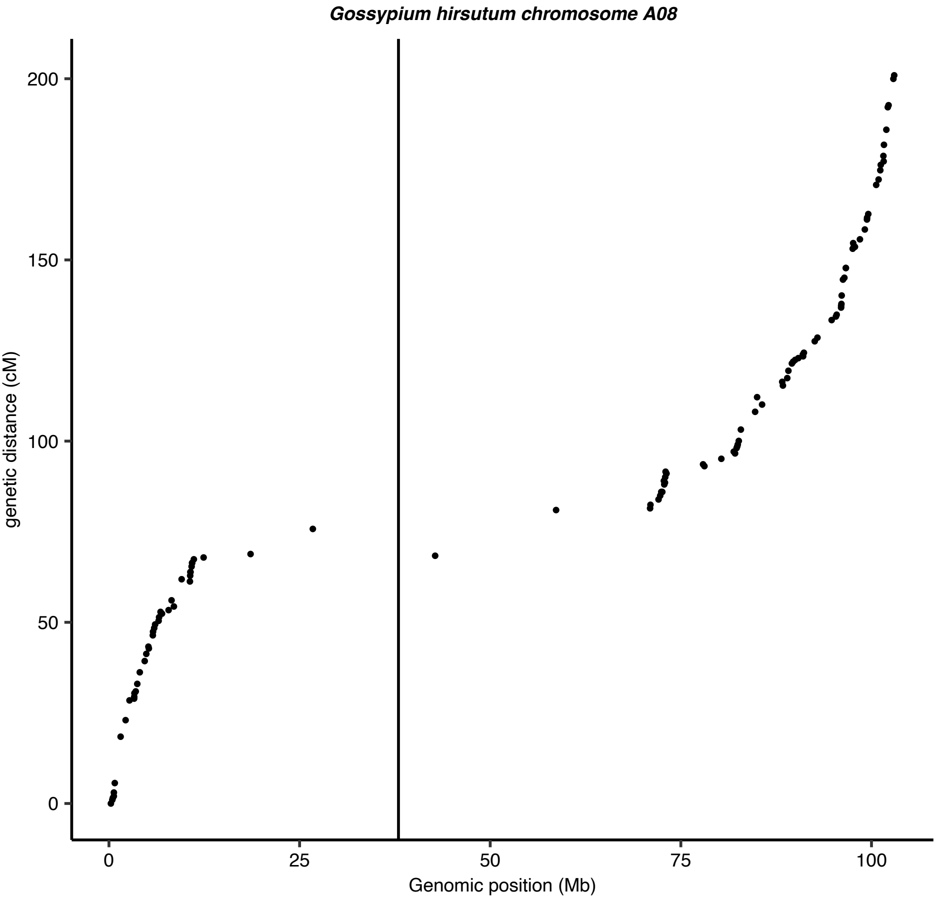
Gossypium hirsutum chromosome A08
genetic distance (cM)
Genomic position (Mb)
0
50
100
150
200
0
25
50
75
100

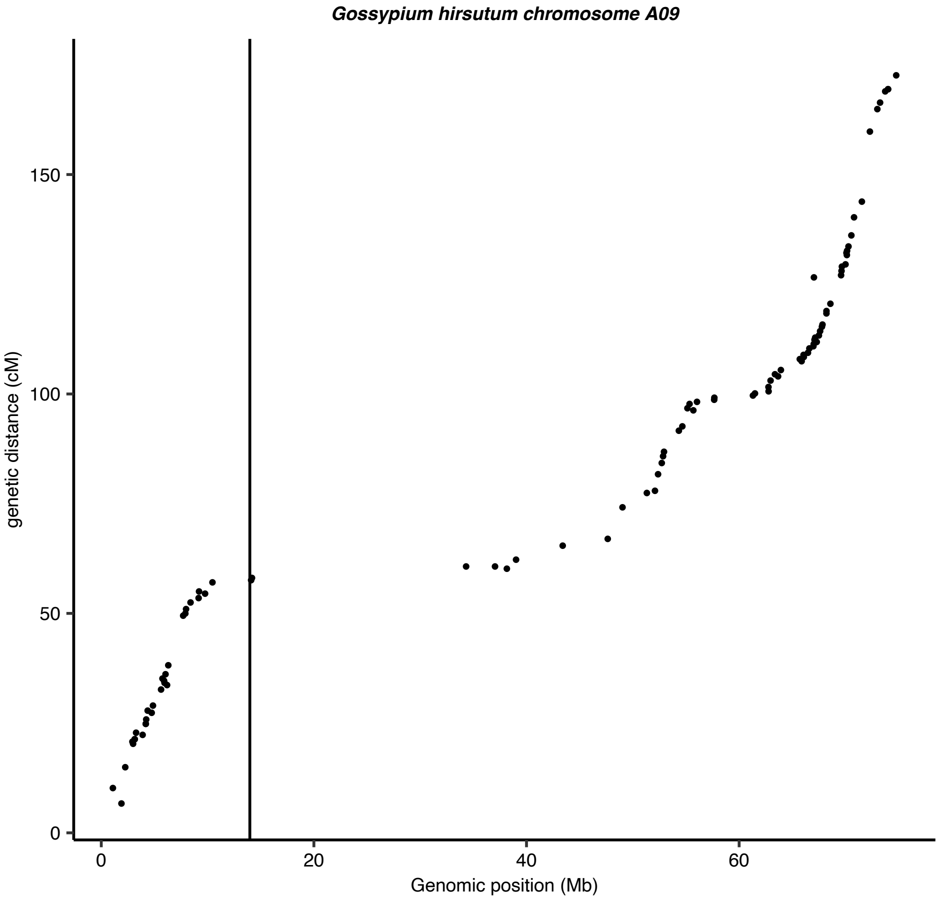
Gossypium hirsutum chromosome A09
genetic distance (cM)
0
50
100
150
0
20
40
60
Genomic position (Mb)

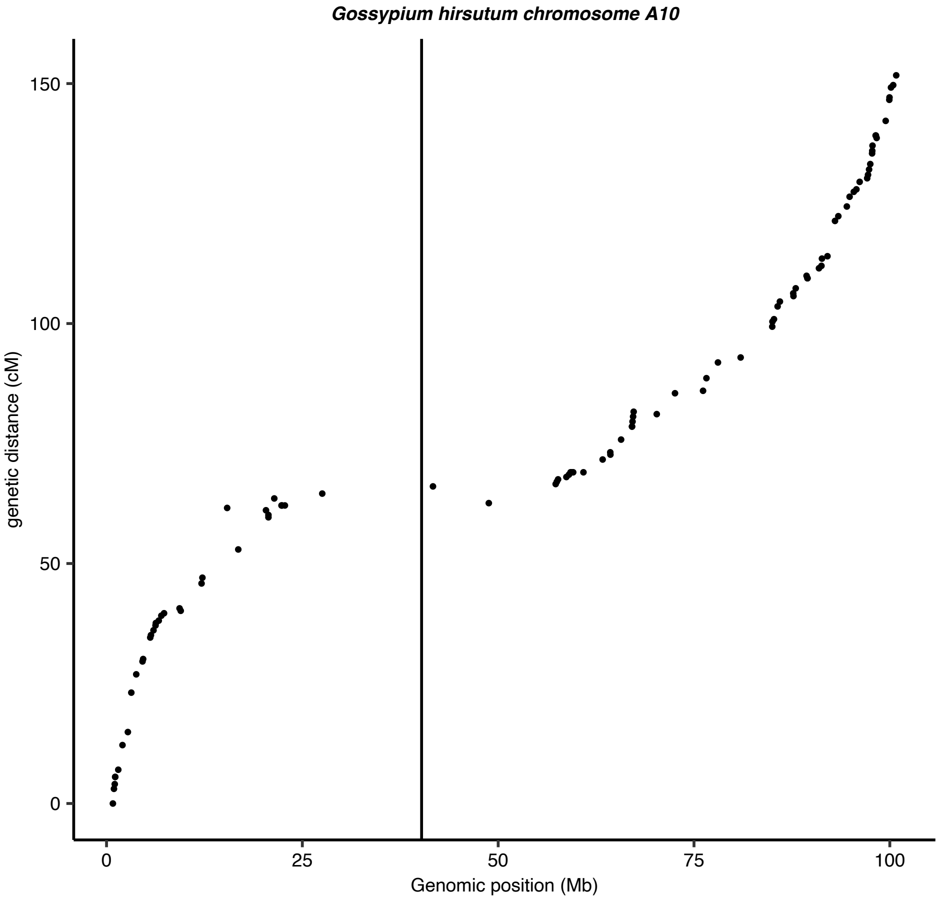
Gossypium hirsutum chromosome A10
150
100
50
0
genetic distance (cM)
0
25
50
75
100
Genomic position (Mb)

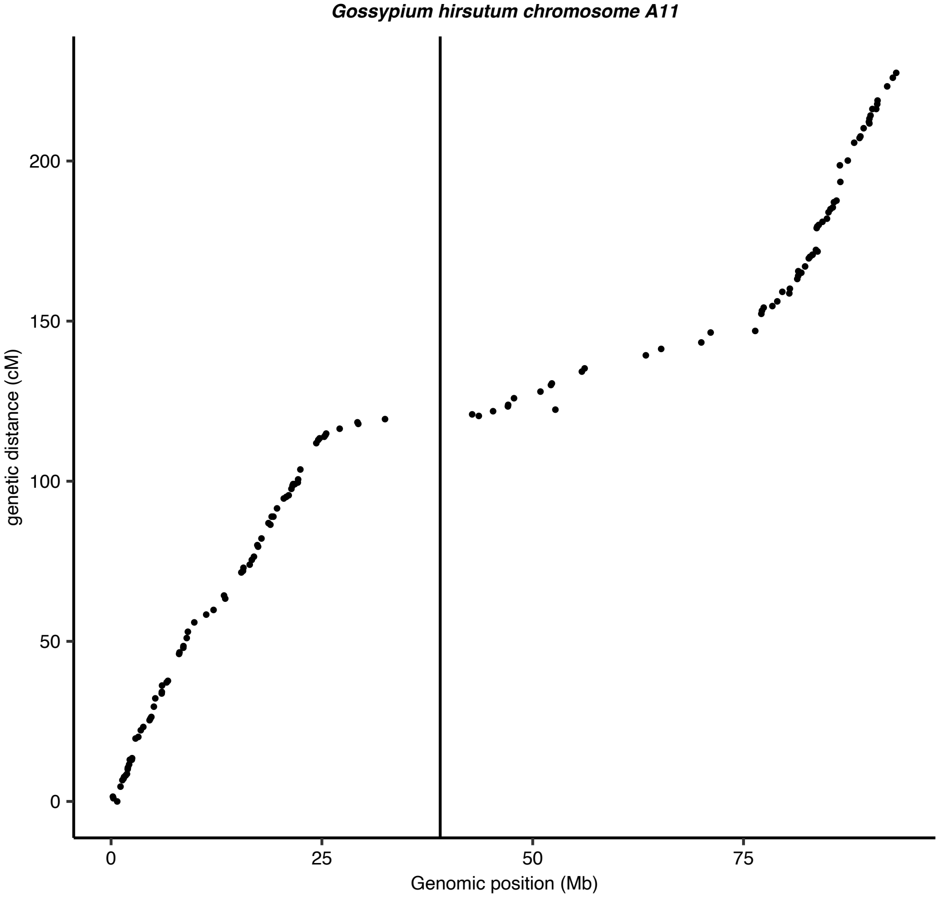
Gossypium hirsutum chromosome A11
genetic distance (cM)
0
50
100
150
200
Genomic position (Mb)
0
25
50
75

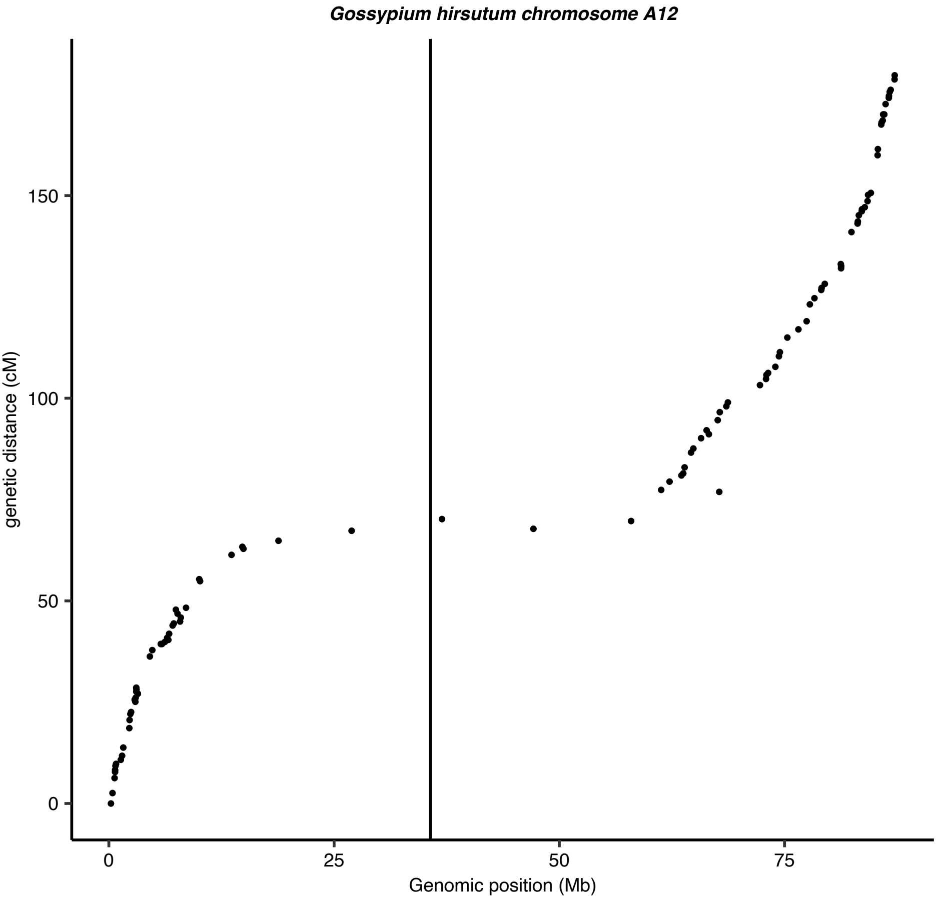
Gossypium hirsutum chromosome A12
genetic distance (cM)
0
50
100
150
Genomic position (Mb)
0
25
50
75

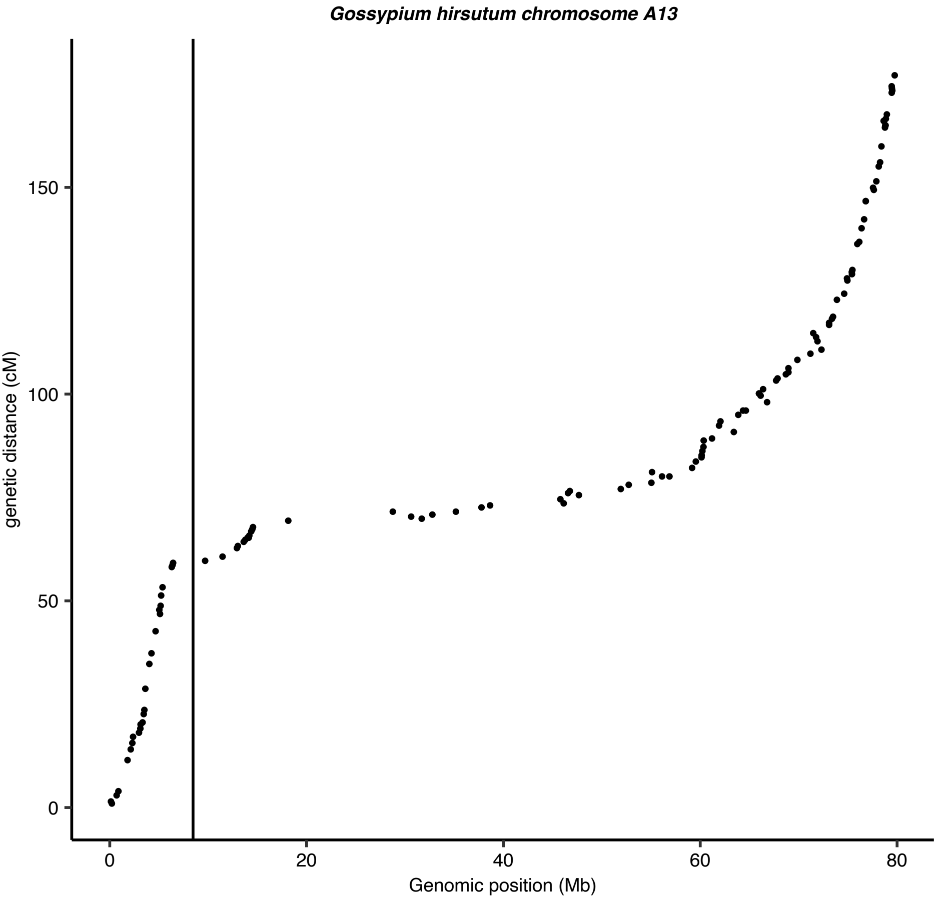
Gossypium hirsutum chromosome A13
genetic distance (cM)
150
100
50
0
0
20
40
60
80
Genomic position (Mb)

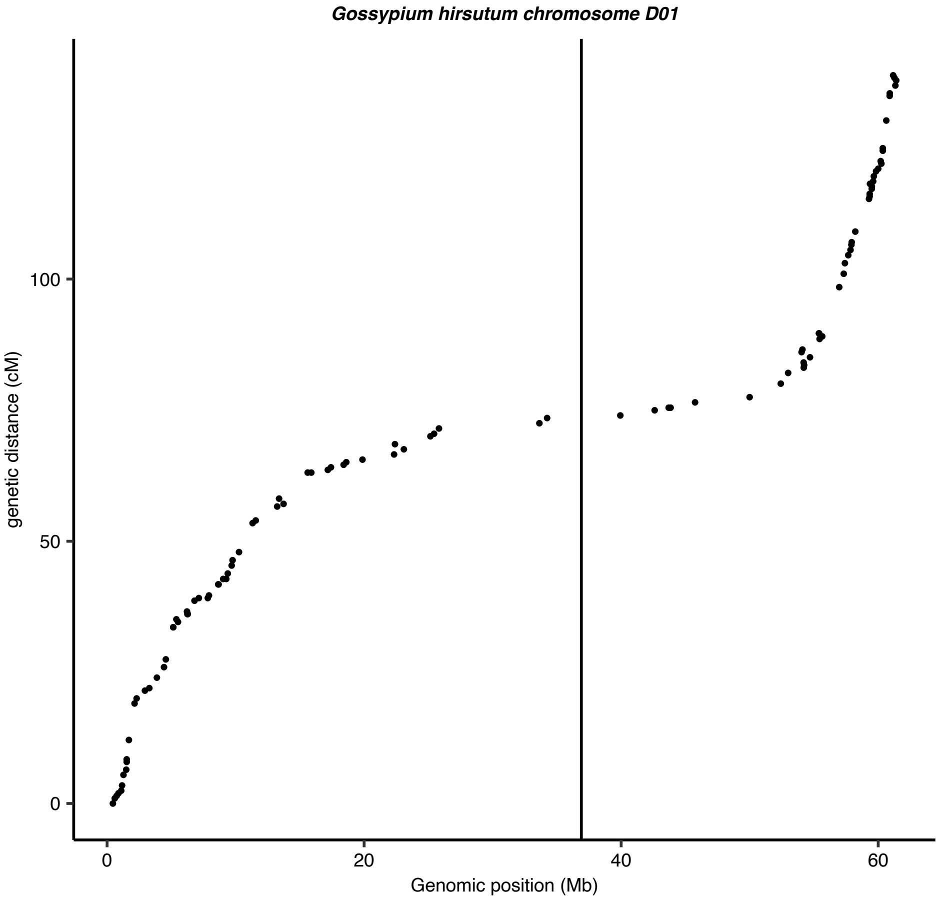
Gossypium hirsutum chromosome D01
genetic distance (cM)
100
50
0
0
20
40
60
Genomic position (Mb)

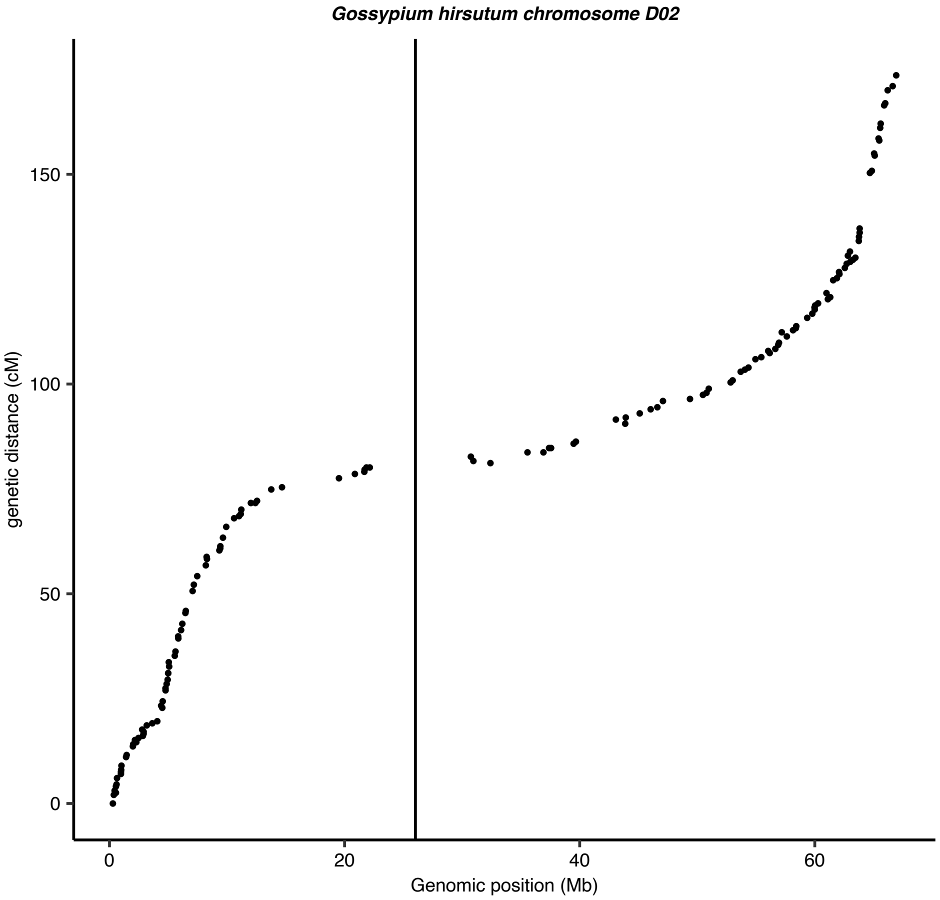
Gossypium hirsutum chromosome D02
genetic distance (cM)
150
100
50
0
0
20
40
60
Genomic position (Mb)

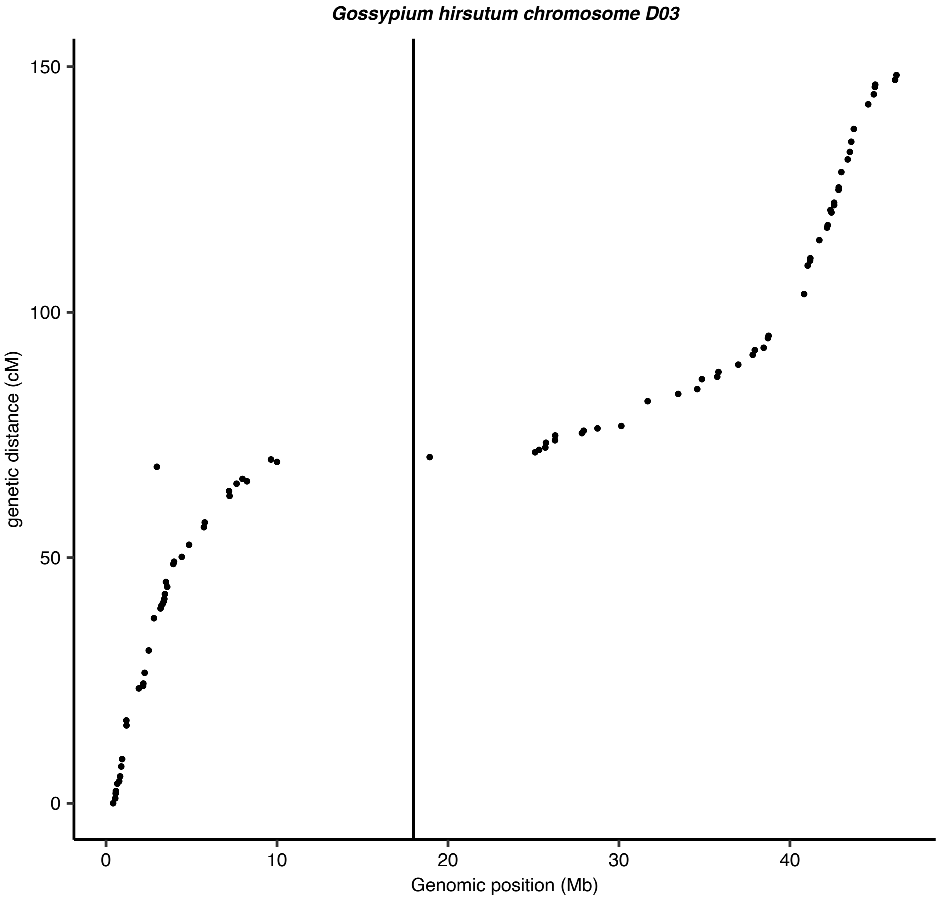
Gossypium hirsutum chromosome D03
genetic distance (cM)
150
100
50
0
0
10
20
30
40
Genomic position (Mb)

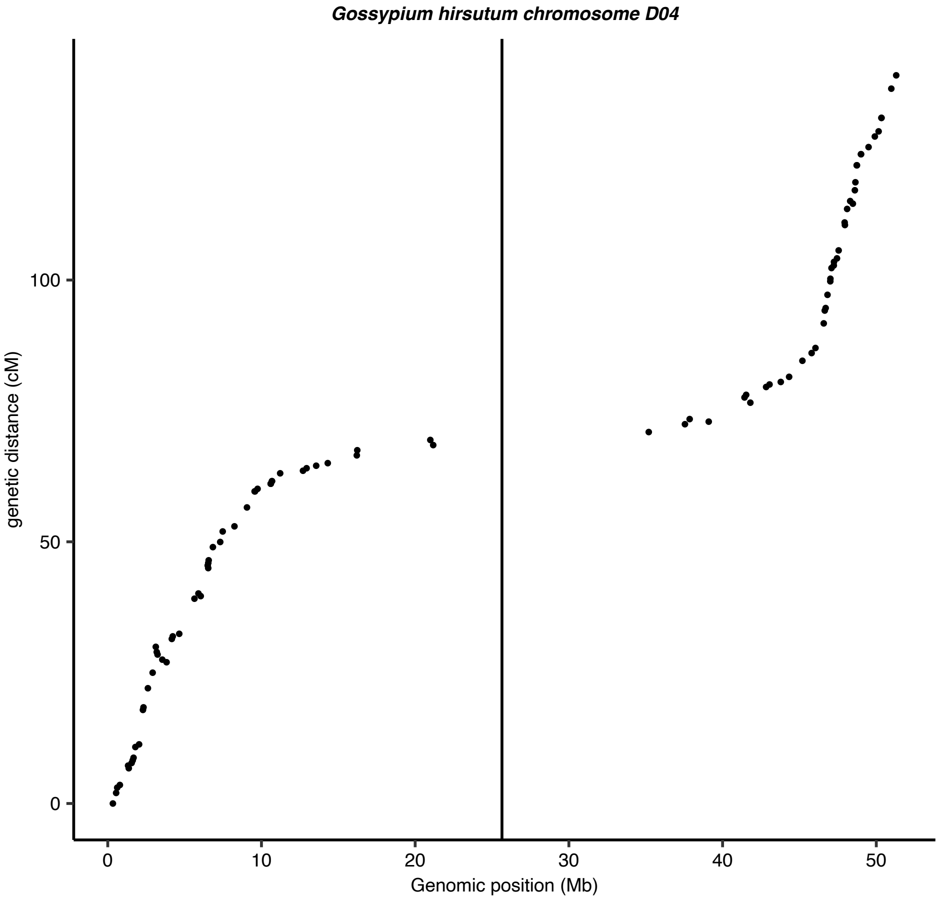
Gossypium hirsutum chromosome D04
genetic distance (cM)
0
50
100
Genomic position (Mb)
0
10
20
30
40
50

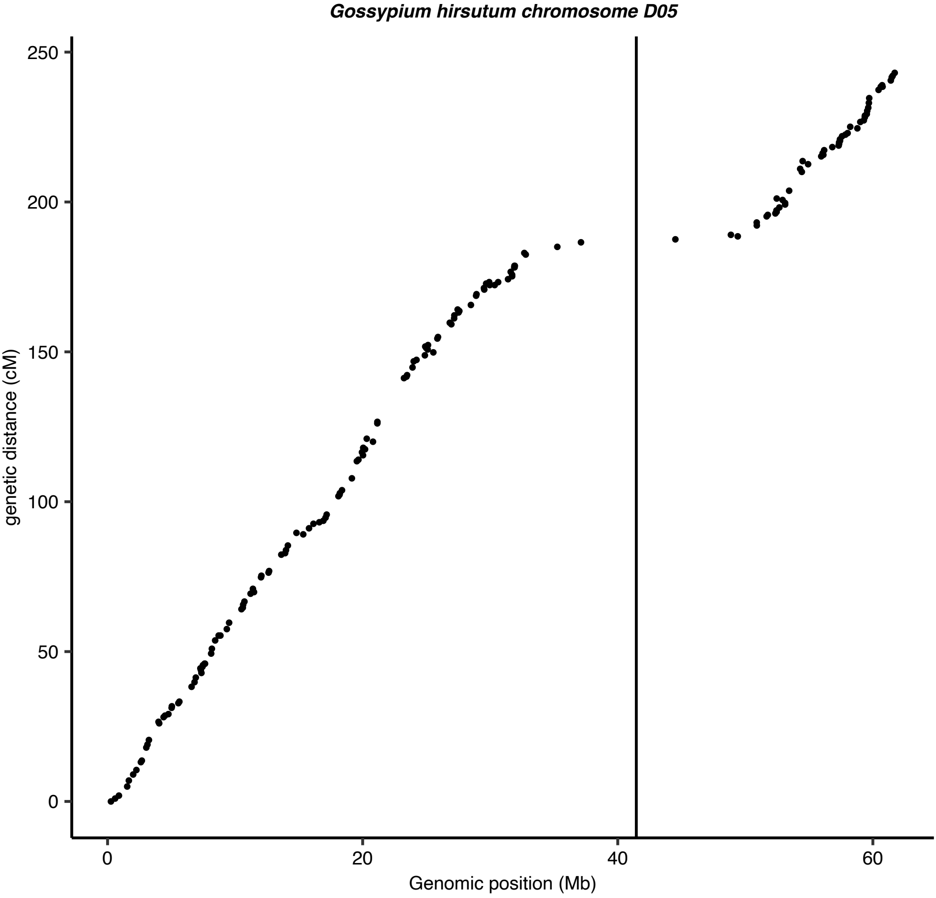
Gossypium hirsutum chromosome D05
250
200
150
100
50
0
genetic distance (cM)
0
20
40
60
Genomic position (Mb)

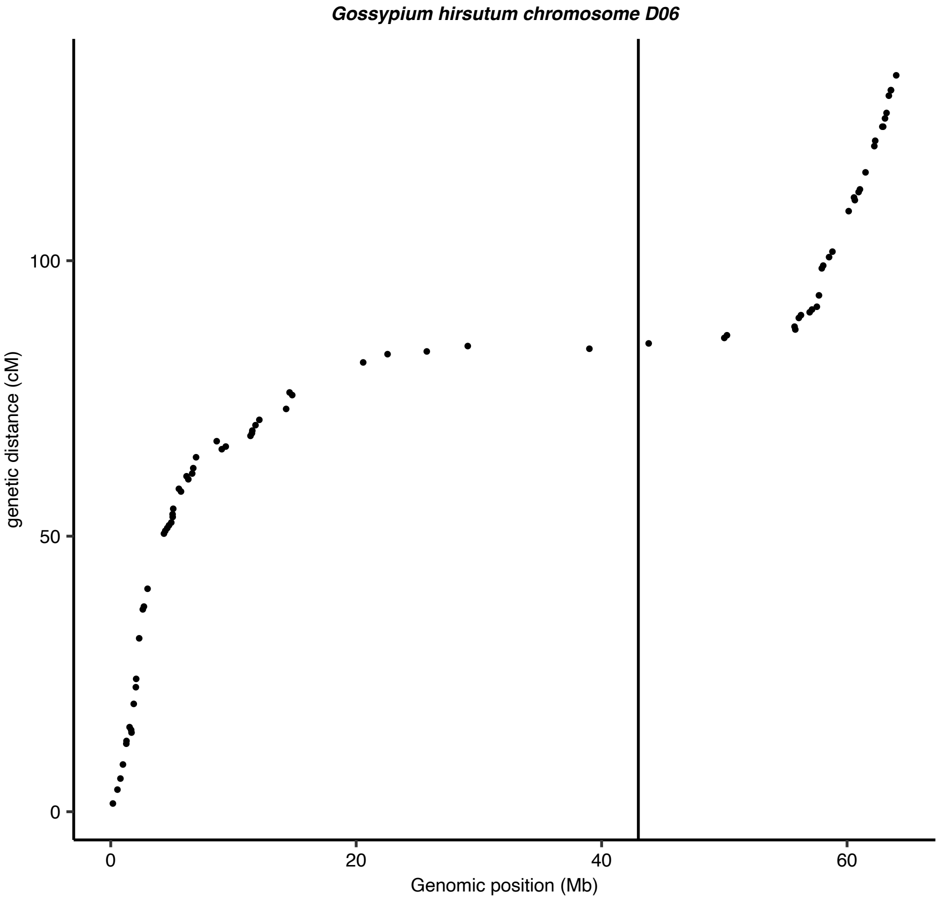
Gossypium hirsutum chromosome D06
genetic distance (cM)
100
50
0
Genomic position (Mb)
0
20
40
60

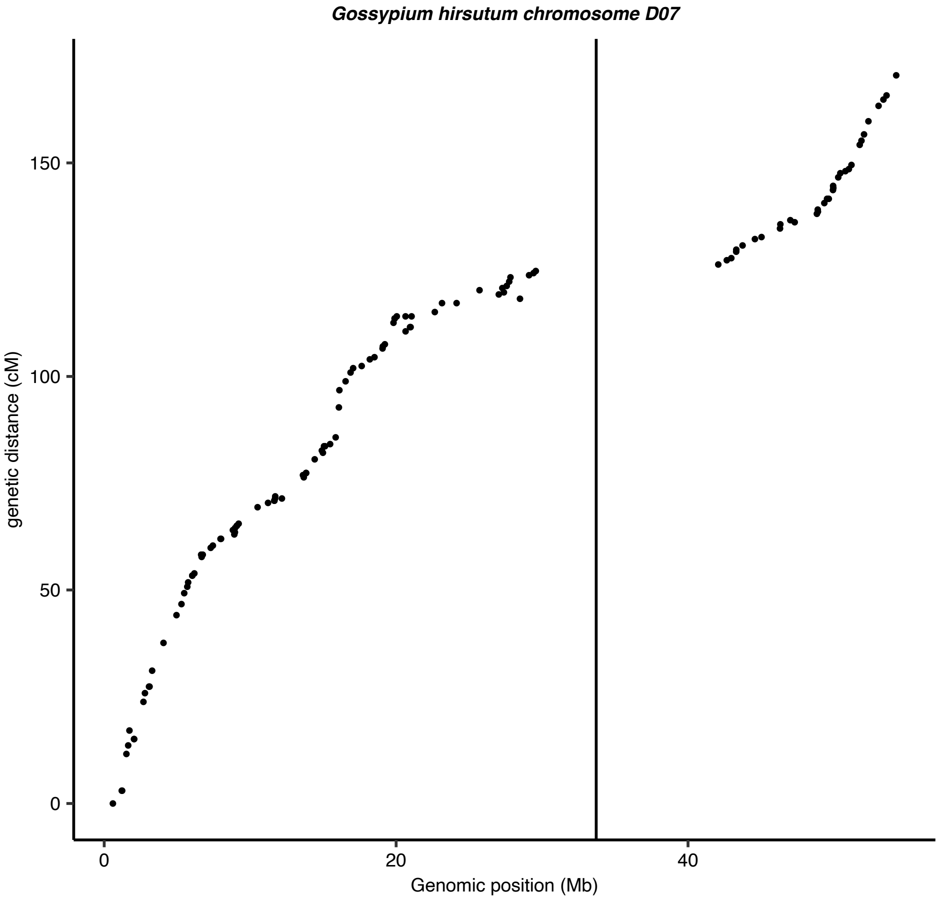
Gossypium hirsutum chromosome D07
genetic distance (cM)
150
100
50
0
0
20
40
Genomic position (Mb)

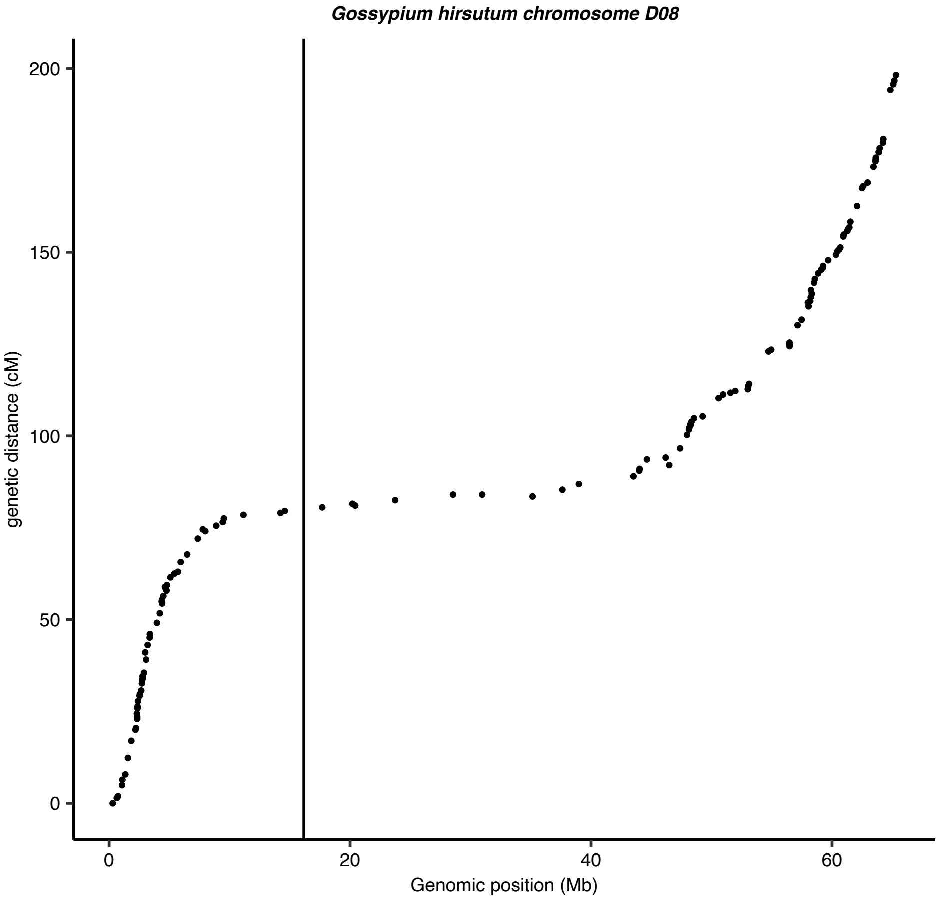
Gossypium hirsutum chromosome D08
genetic distance (cM)
200
150
100
50
0
0
20
40
60
Genomic position (Mb)

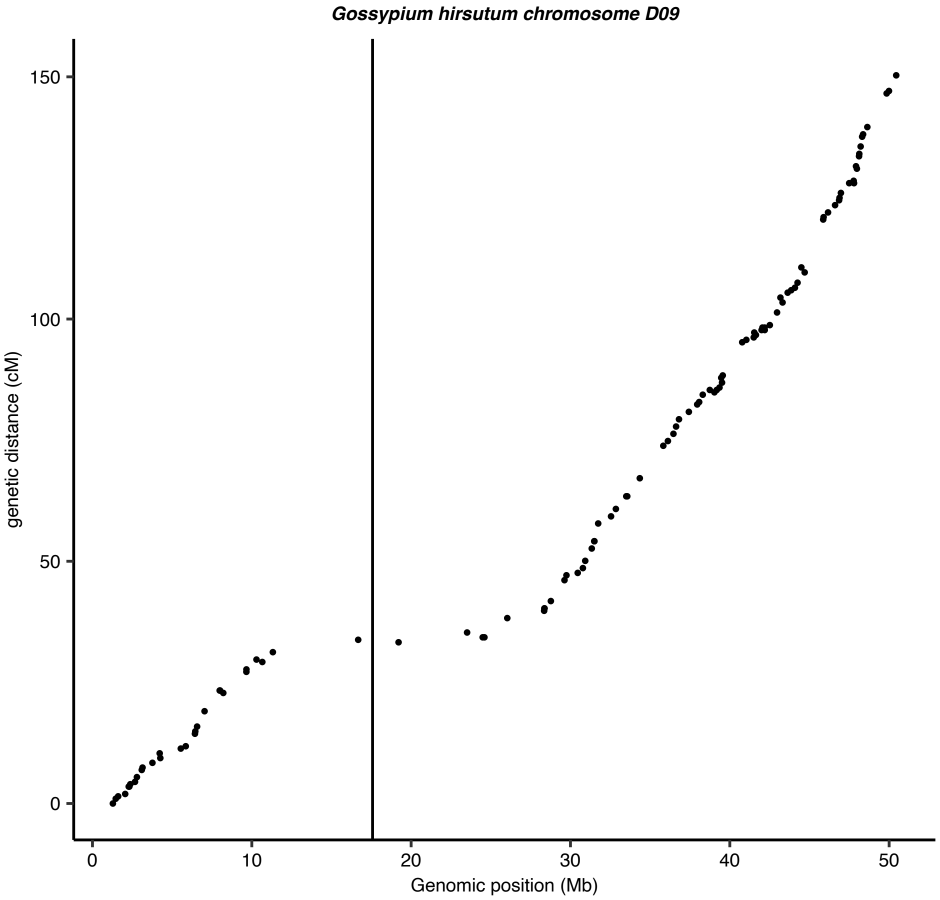
Gossypium hirsutum chromosome D09
genetic distance (cM)
Genomic position (Mb)
0
50
100
150
0
10
20
30
40
50

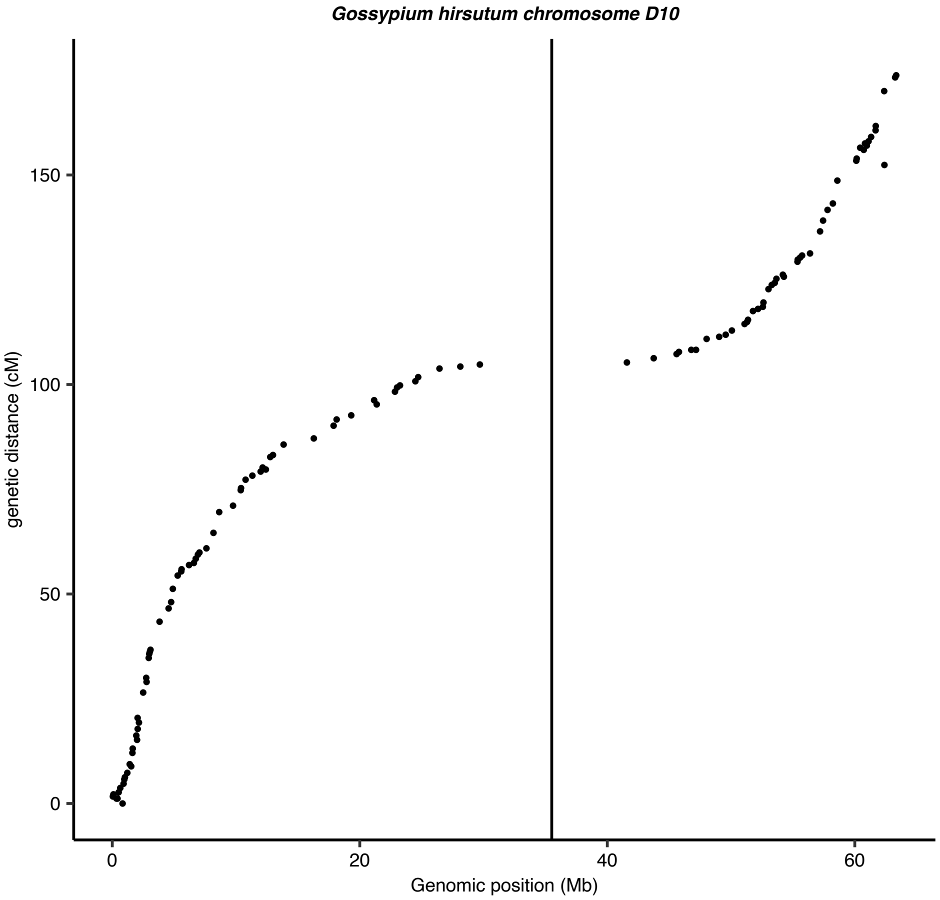
Gossypium hirsutum chromosome D10
genetic distance (cM)
0
50
100
150
0
20
40
60
Genomic position (Mb)

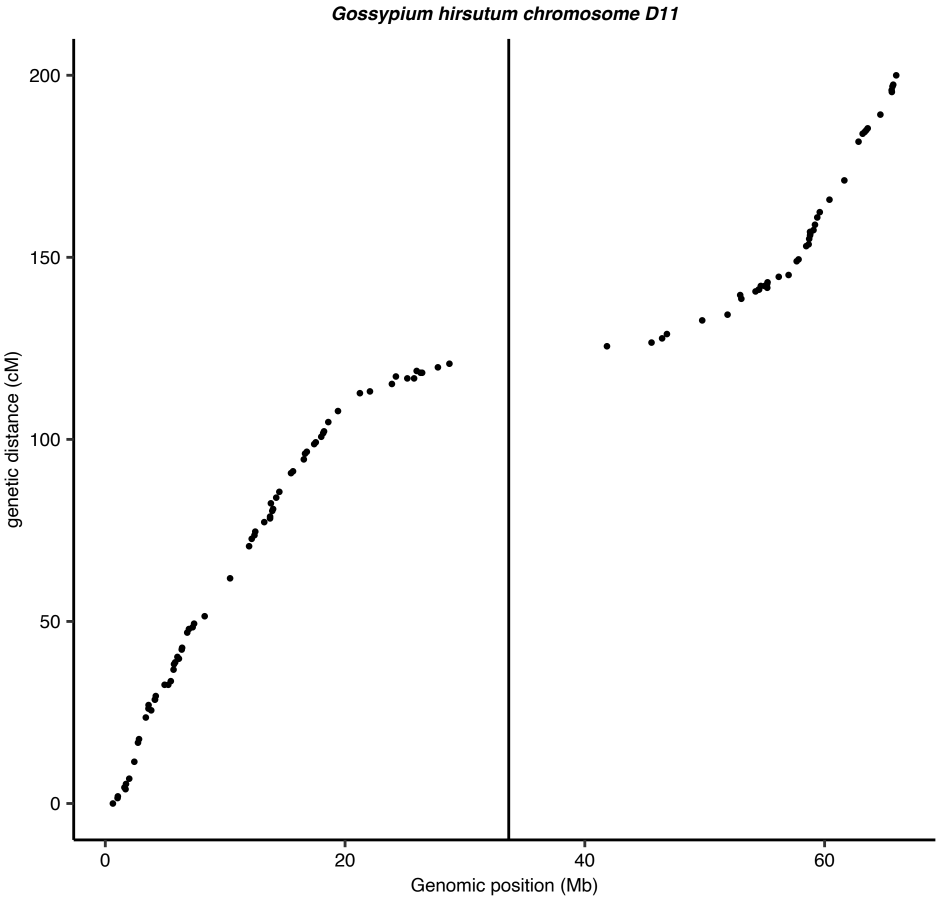
Gossypium hirsutum chromosome D11
genetic distance (cM)
0
50
100
150
200
Genomic position (Mb)
0
20
40
60

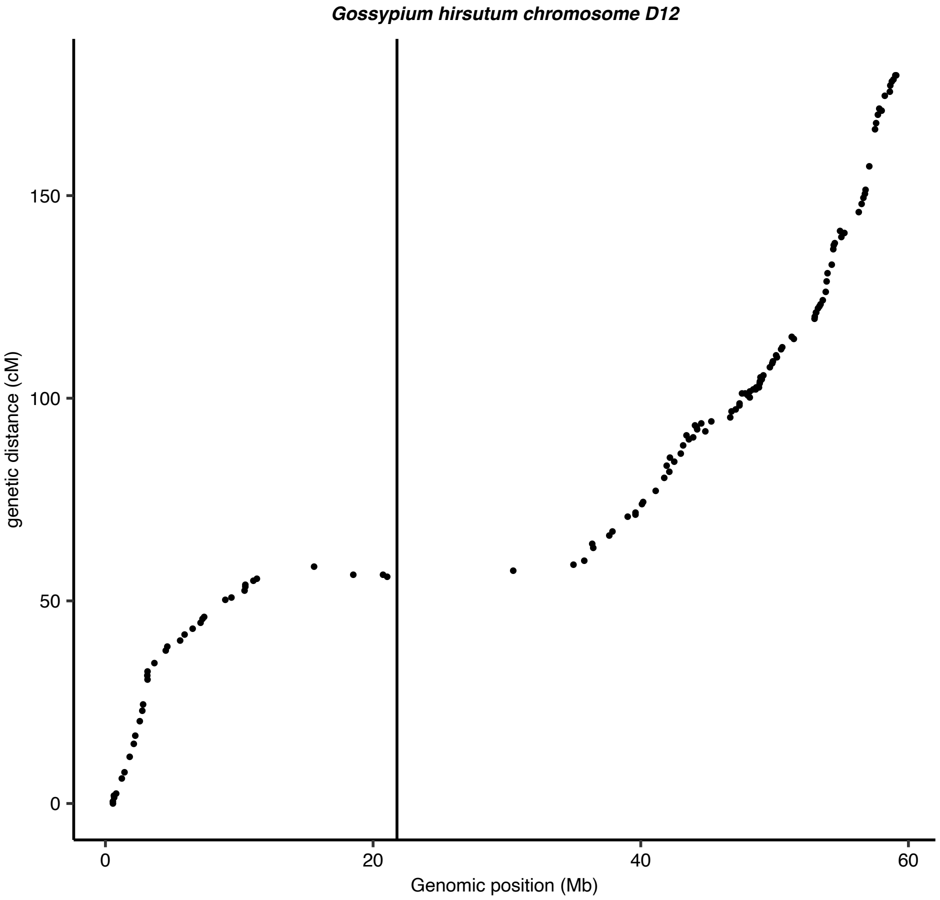
Gossypium hirsutum chromosome D12
genetic distance (cM)
Genomic position (Mb)
0
50
100
150
0
20
40
60

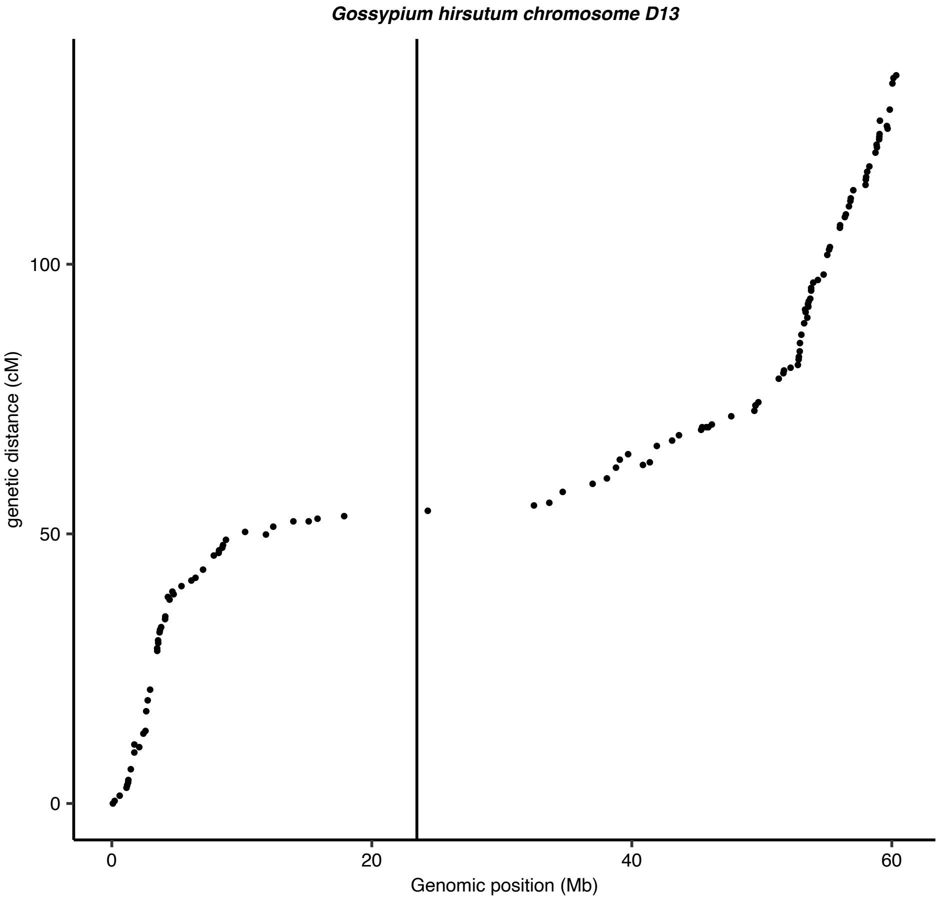
Gossypium hirsutum chromosome D13
genetic distance (cM)
100
50
0
0
20
40
60
Genomic position (Mb)

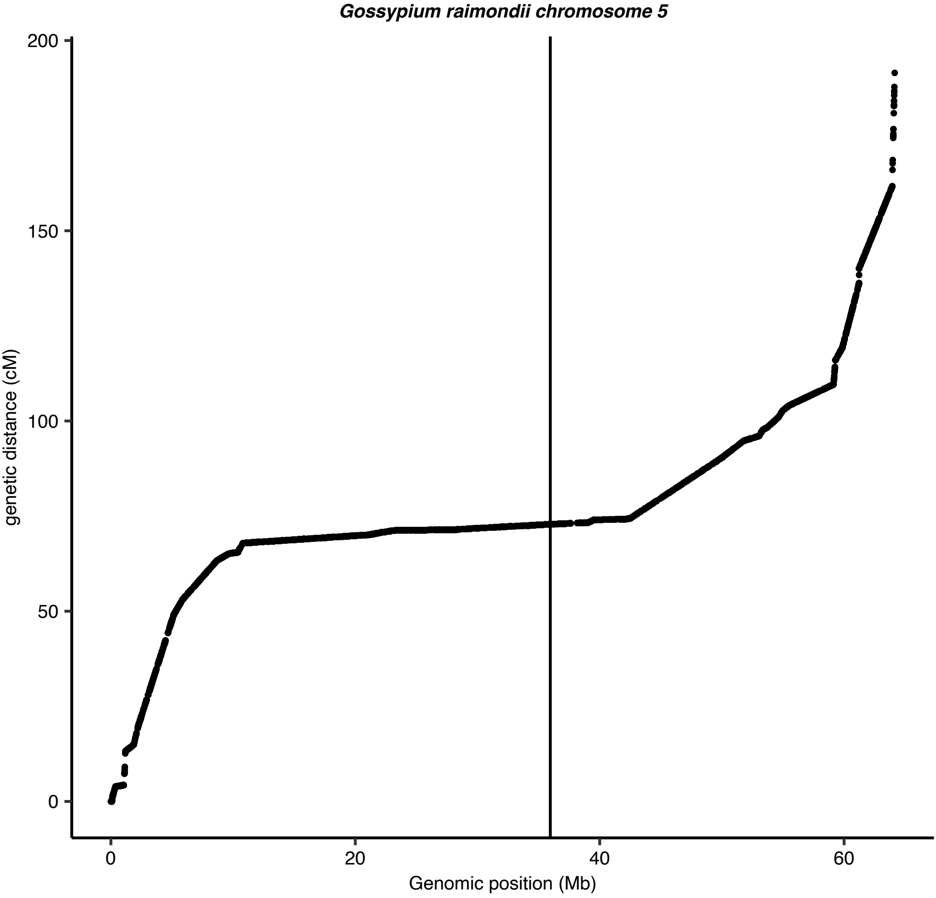
Gossypium raimondii chromosome 5
genetic distance (cM)
Genomic position (Mb)
0
50
100
150
200
0
20
40
60

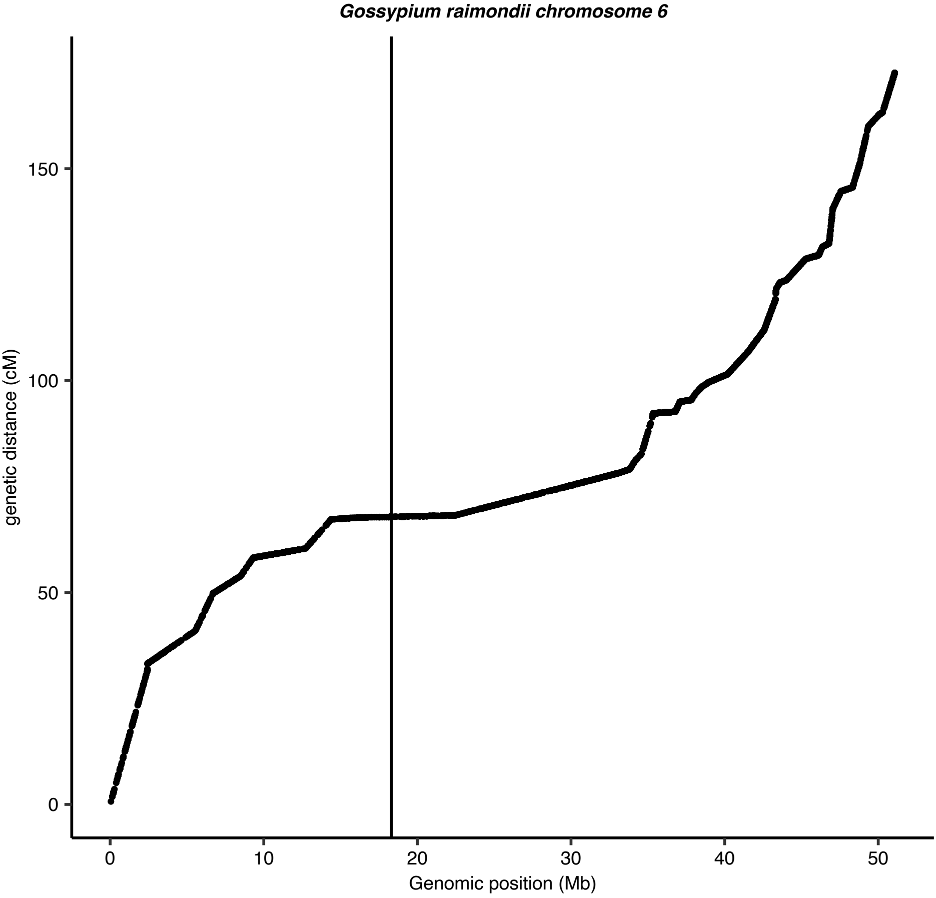

Gossypium raimondii chromosome 6
genetic distance (cM)
150
100
50
0
0
10
20
30
40
50
Genomic position (Mb)

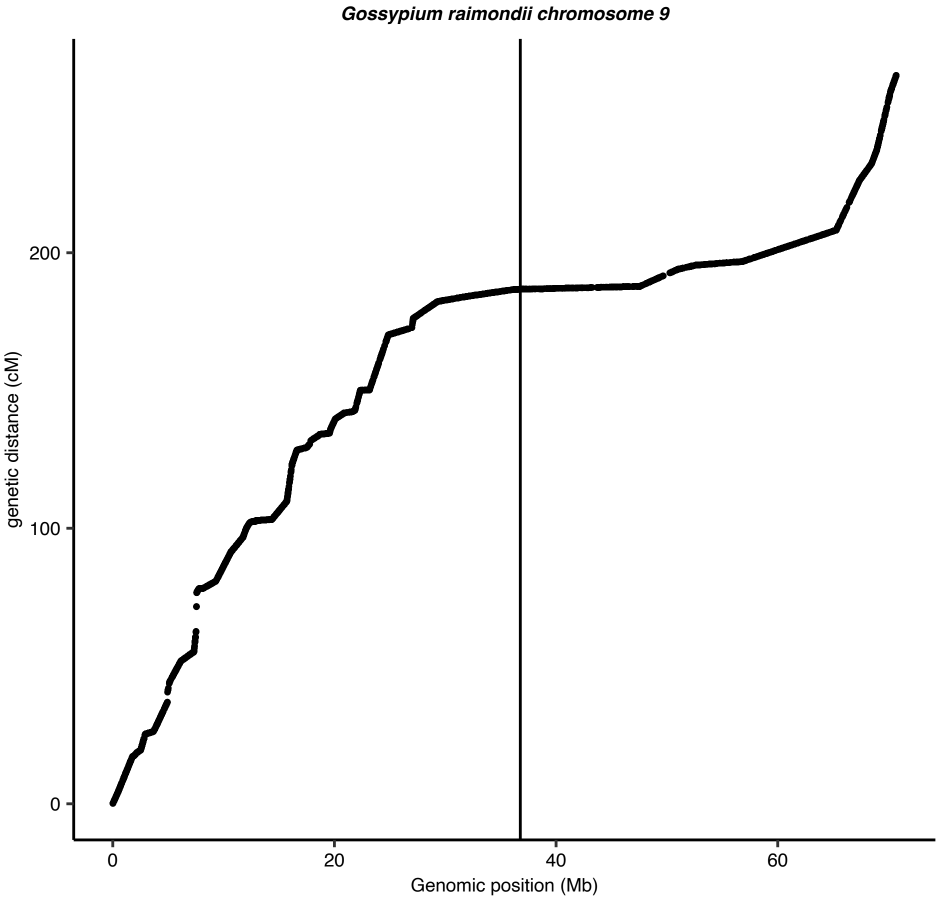
Gossypium raimondii chromosome 9
genetic distance (cM)
0
100
200
0
20
40
60
Genomic position (Mb)

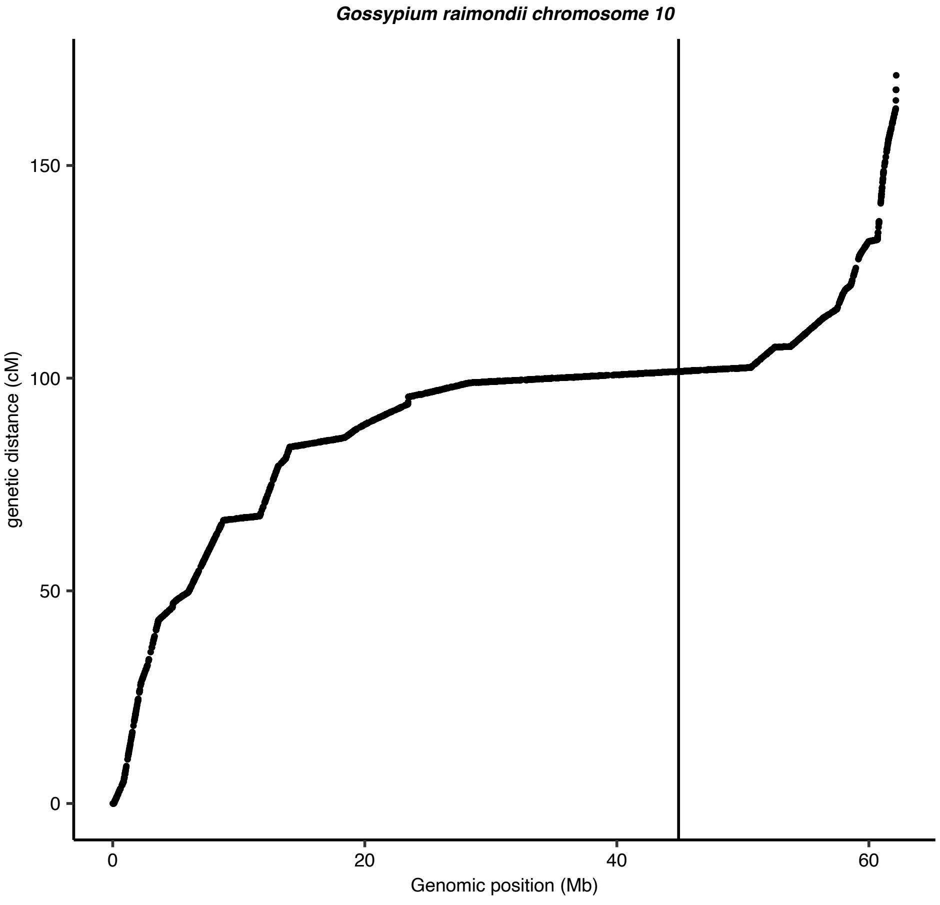
Gossypium raimondii chromosome 10
genetic distance (cM)
150
100
50
0
0
20
40
60
Genomic position (Mb)

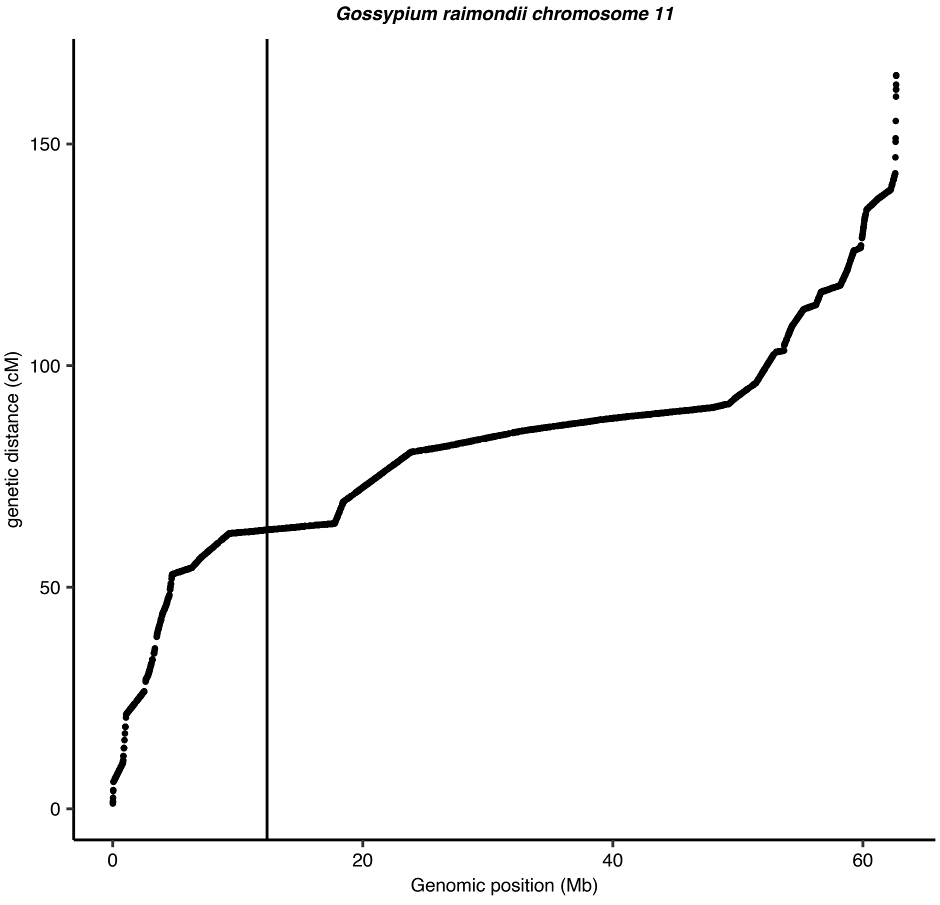
Gossypium raimondii chromosome 11
genetic distance (cM)
150
100
50
0
0
20
40
60
Genomic position (Mb)

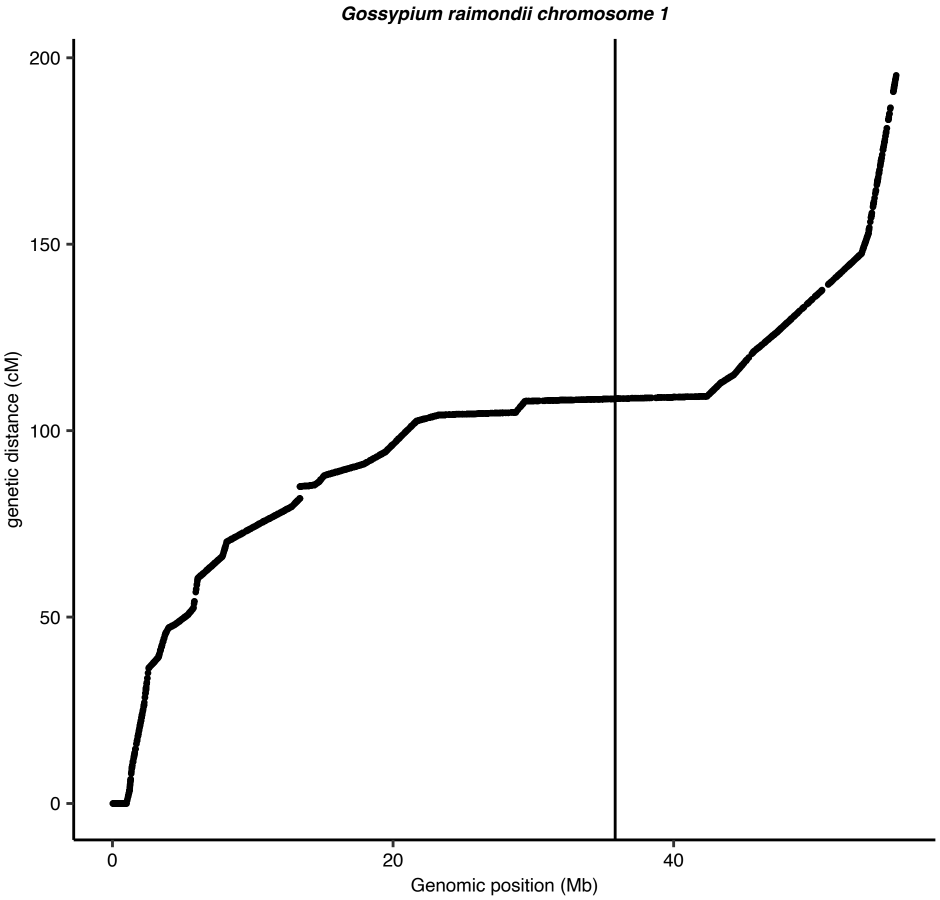
Gossypium raimondii chromosome 1
genetic distance (cM)
0
50
100
150
200
Genomic position (Mb)
0
20
40

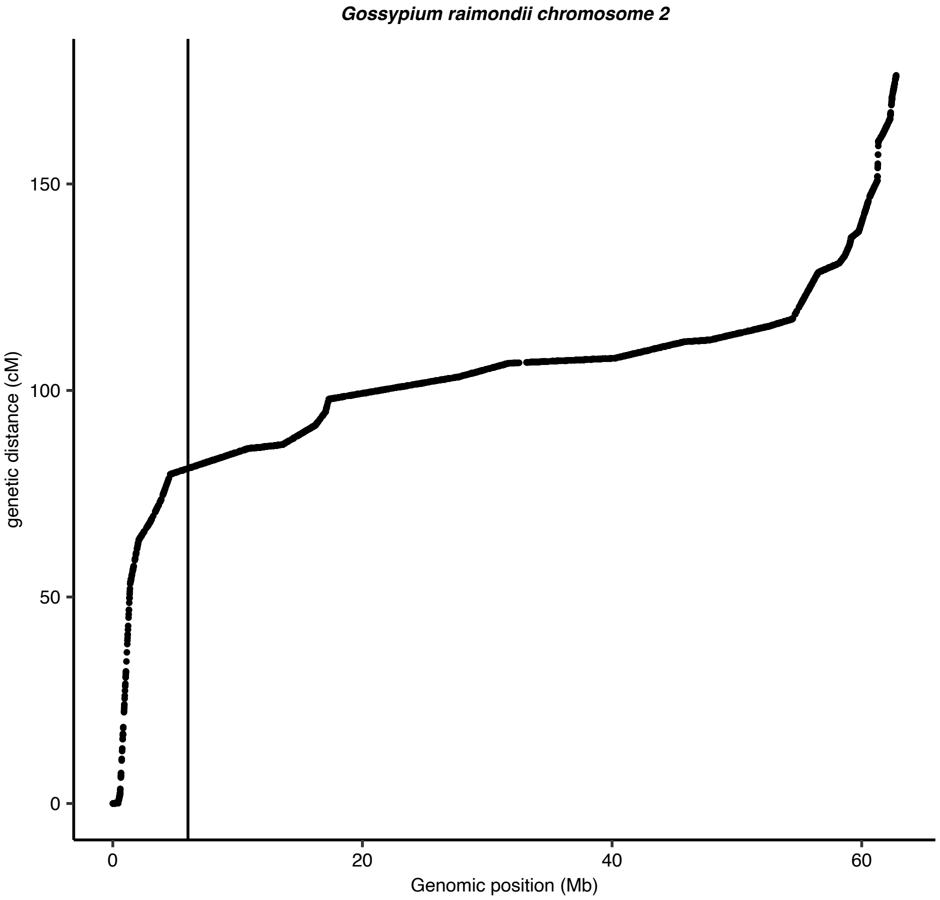
Gossypium raimondii chromosome 2
genetic distance (cM)
150
100
50
0
0
20
40
60
Genomic position (Mb)

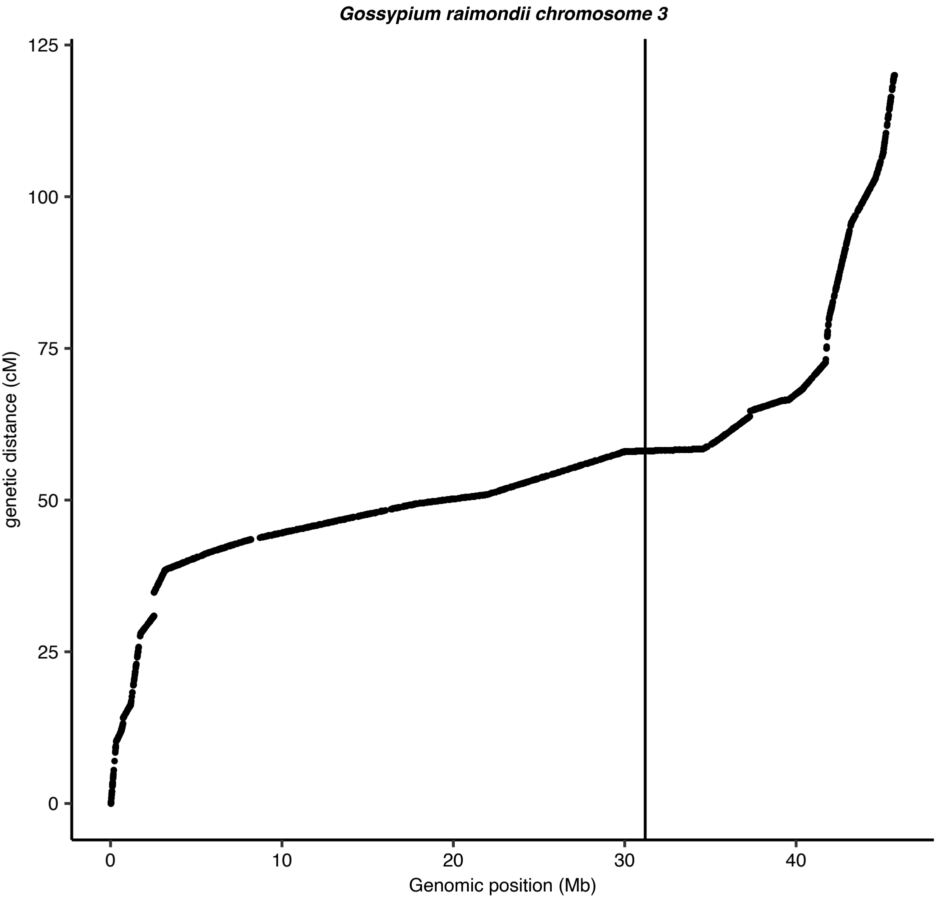
Gossypium raimondii chromosome 3
genetic distance (cM)
Genomic position (Mb)
0
25
50
75
100
125
0
10
20
30
40

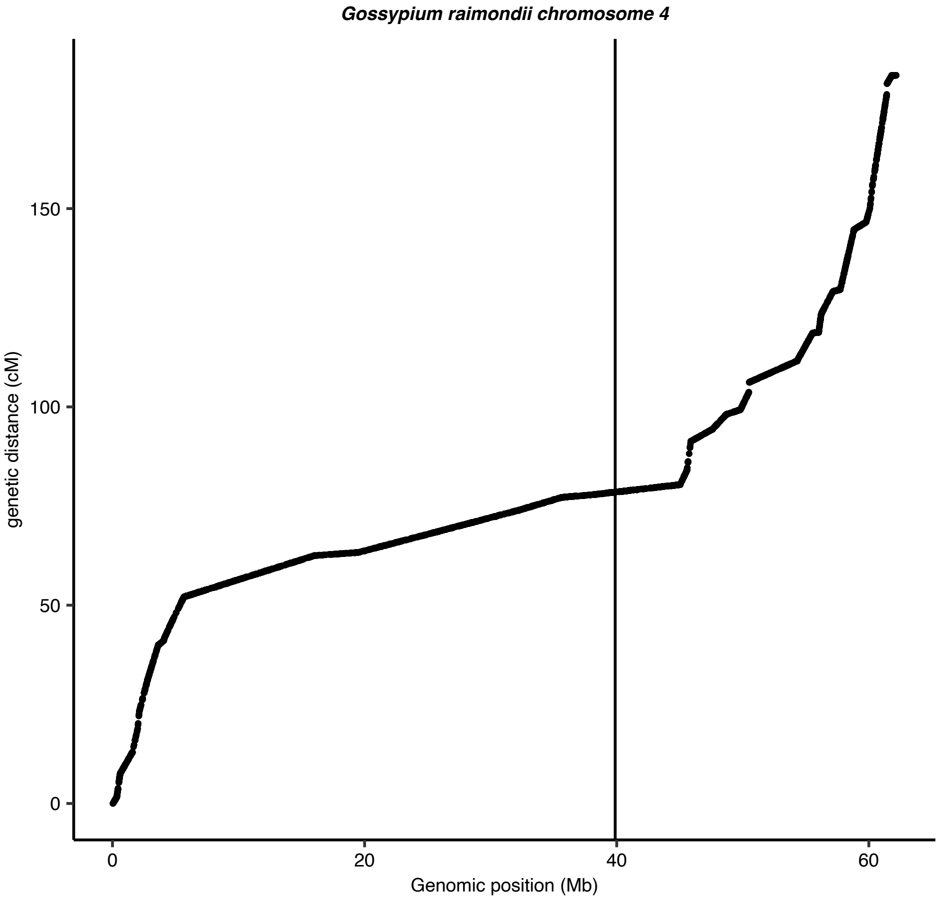
Gossypium raimondii chromosome 4
genetic distance (cM)
0
50
100
150
Genomic position (Mb)
0
20
40
60

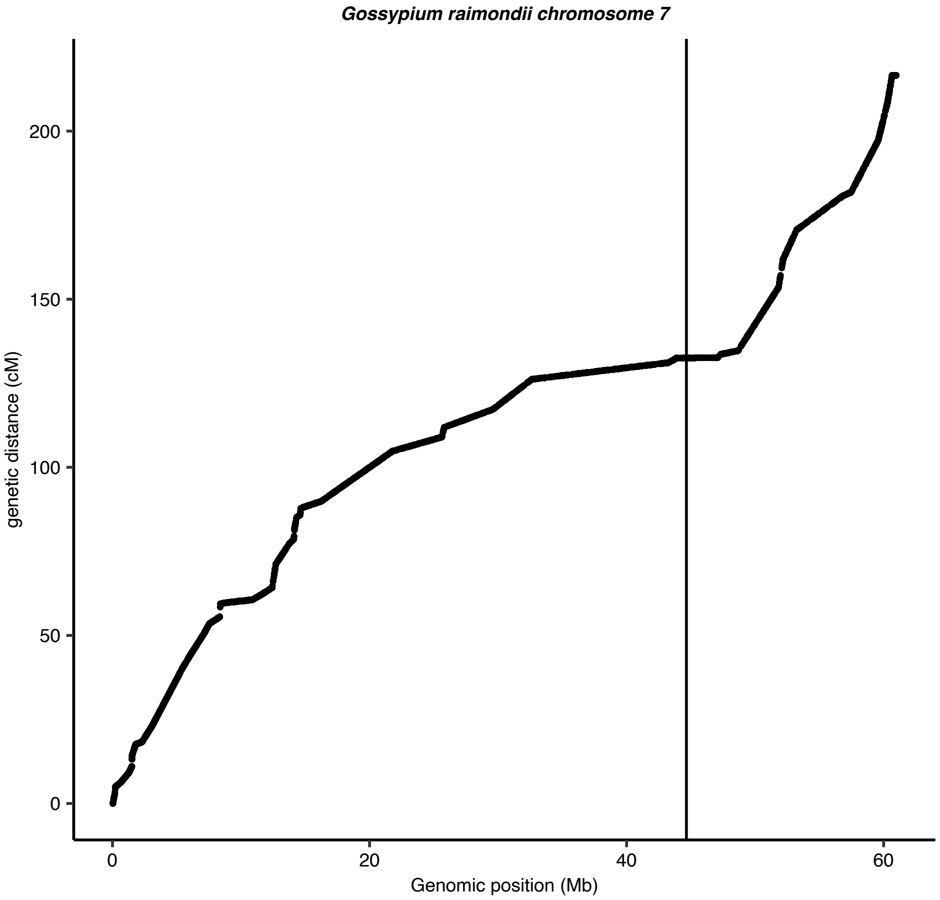
Gossypium raimondii chromosome 7
genetic distance (cM)
0
50
100
150
200
Genomic position (Mb)
0
20
40
60

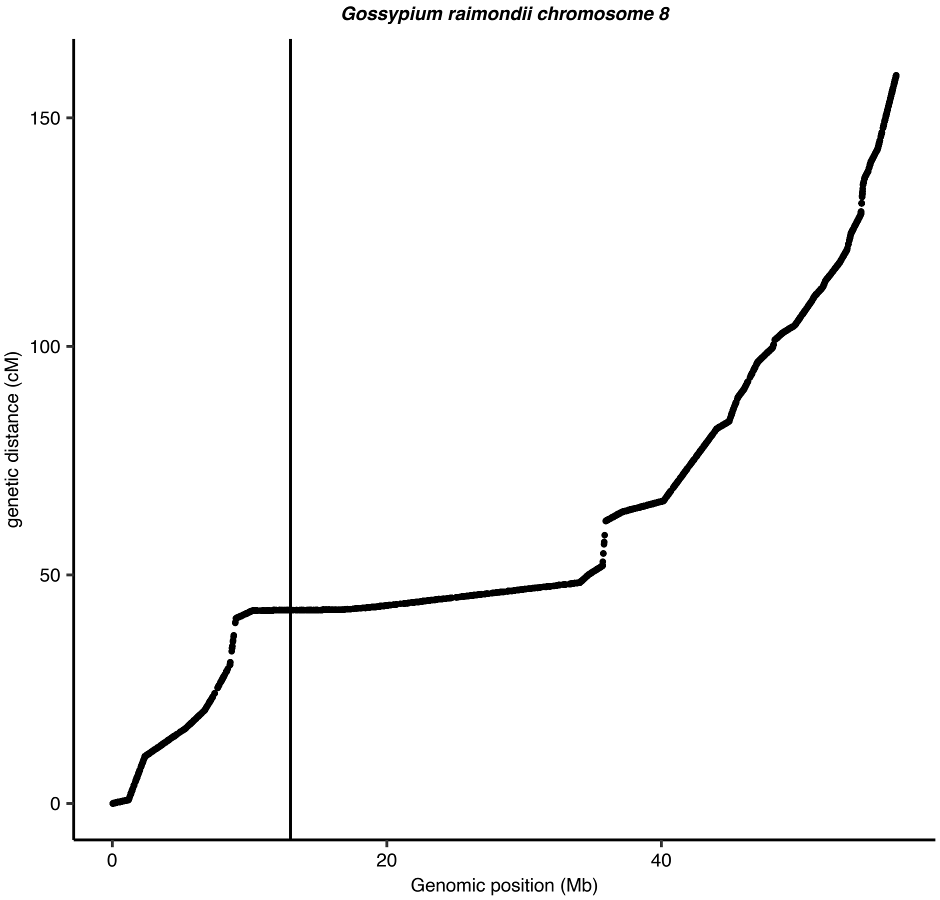
Gossypium raimondii chromosome 8
150
100
50
0
0
20
40
genetic distance (cM)
Genomic position (Mb)

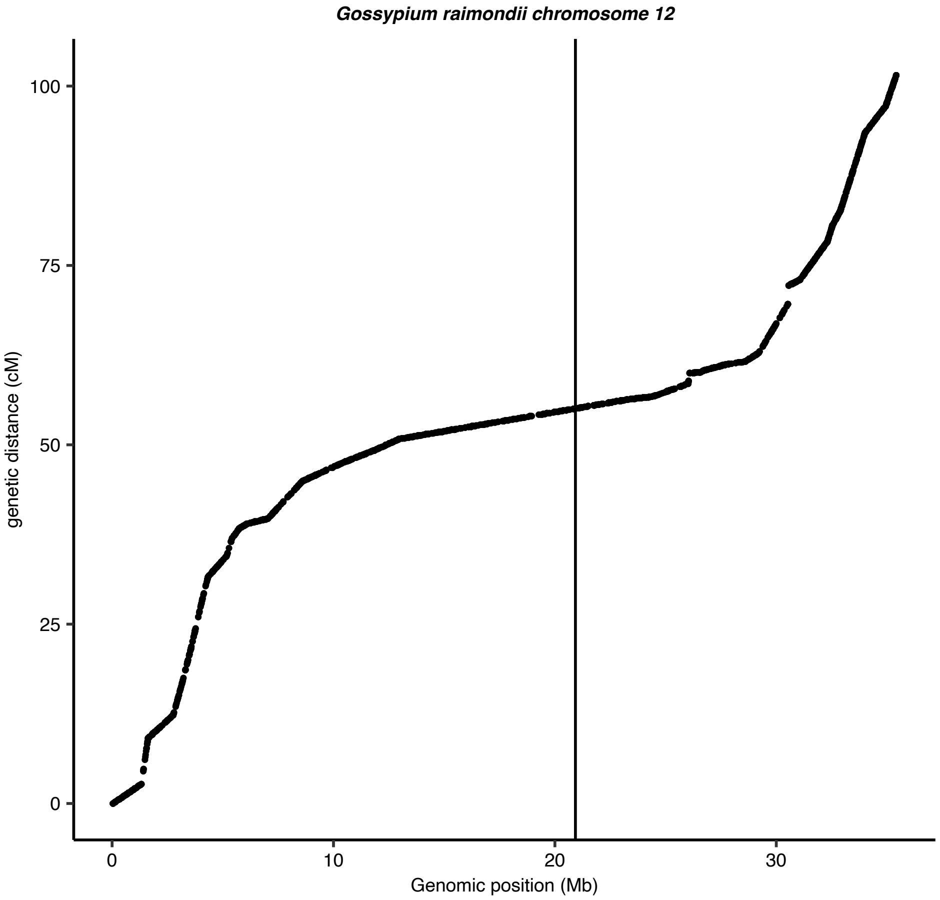
Gossypium raimondii chromosome 12
genetic distance (cM)
0
25
50
75
100
0
10
20
30
Genomic position (Mb)

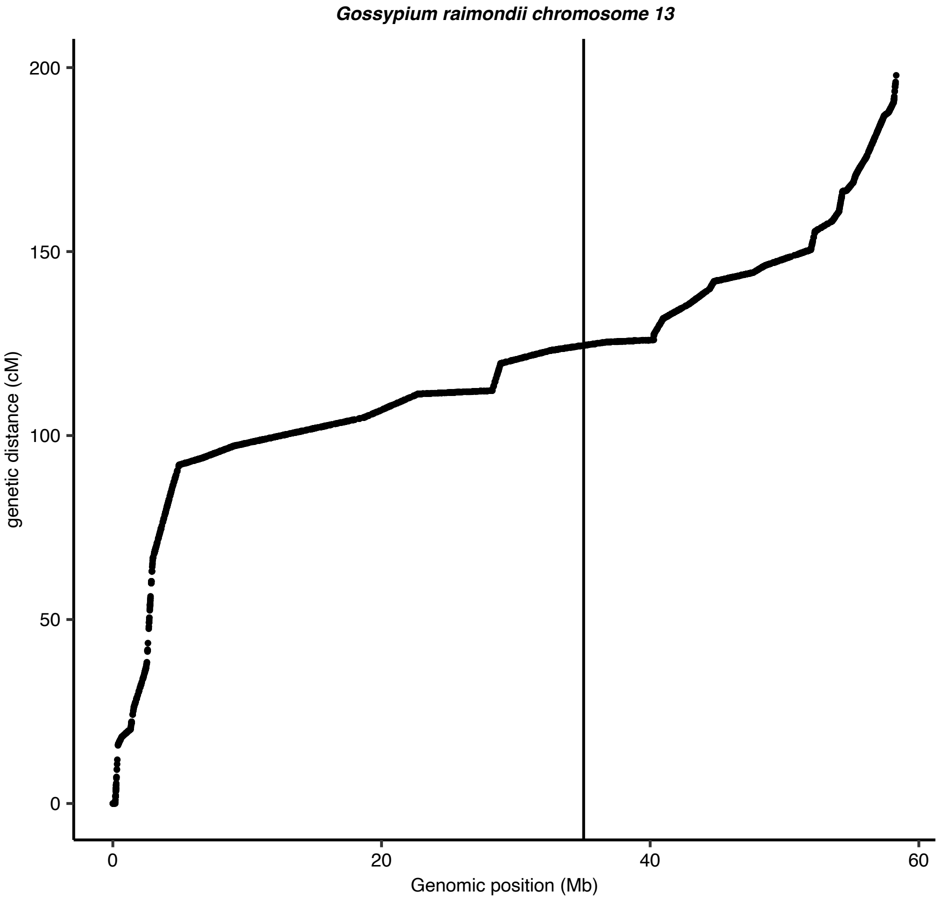
Gossypium raimondii chromosome 13
genetic distance (cM)
200
150
100
50
0
0
20
40
60
Genomic position (Mb)

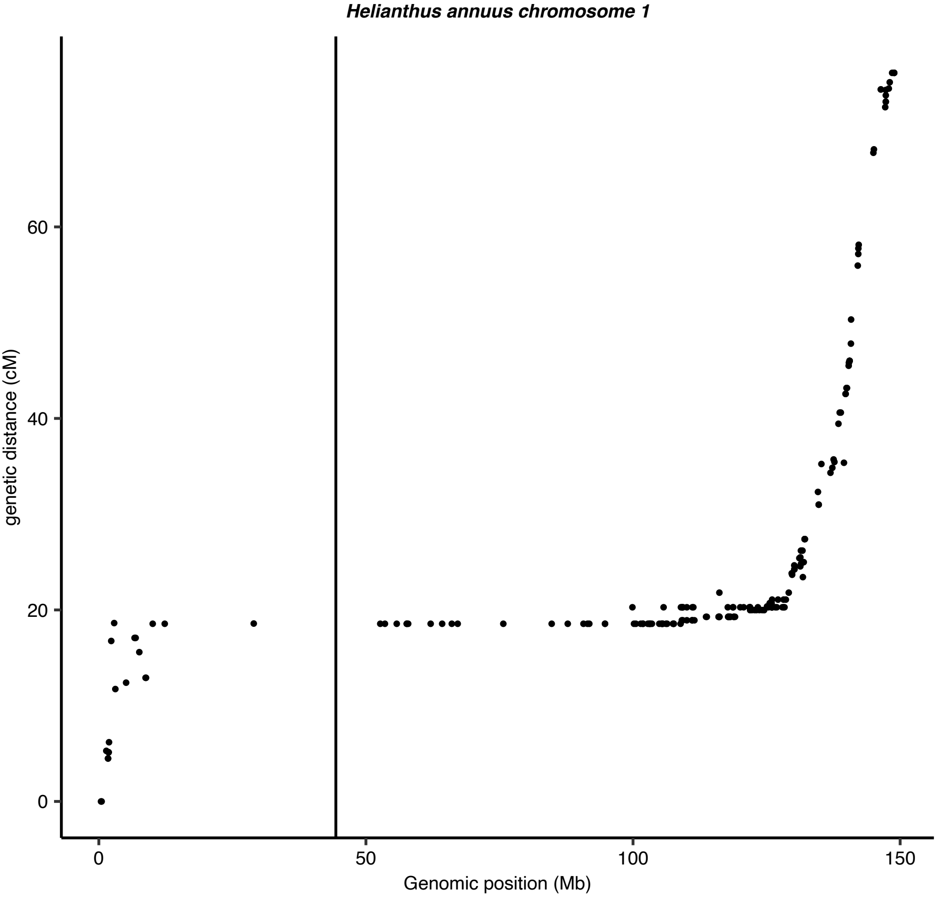

Helianthus annuus chromosome 1
genetic distance (cM)
0
20
40
60
0
50
100
150
Genomic position (Mb)

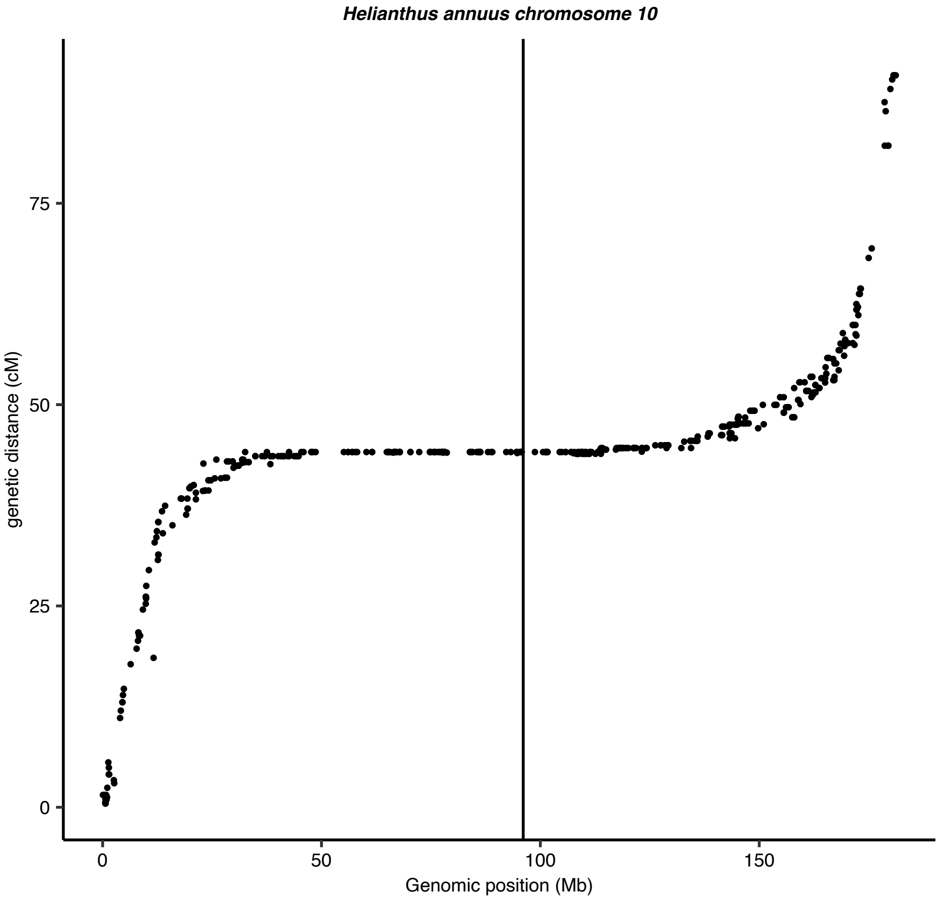
Helianthus annuus chromosome 10
genetic distance (cM)
0
25
50
75
Genomic position (Mb)
0
50
100
150

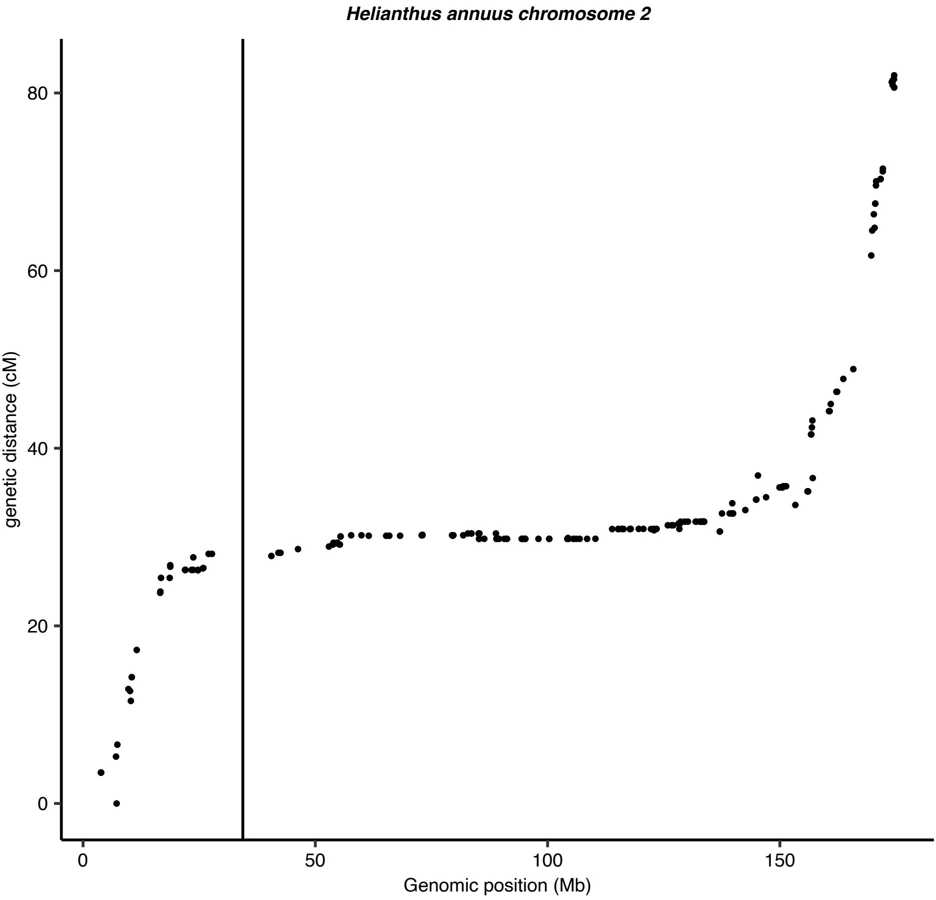
Helianthus annuus chromosome 2
genetic distance (cM)
0
20
40
60
80
Genomic position (Mb)
0
50
100
150

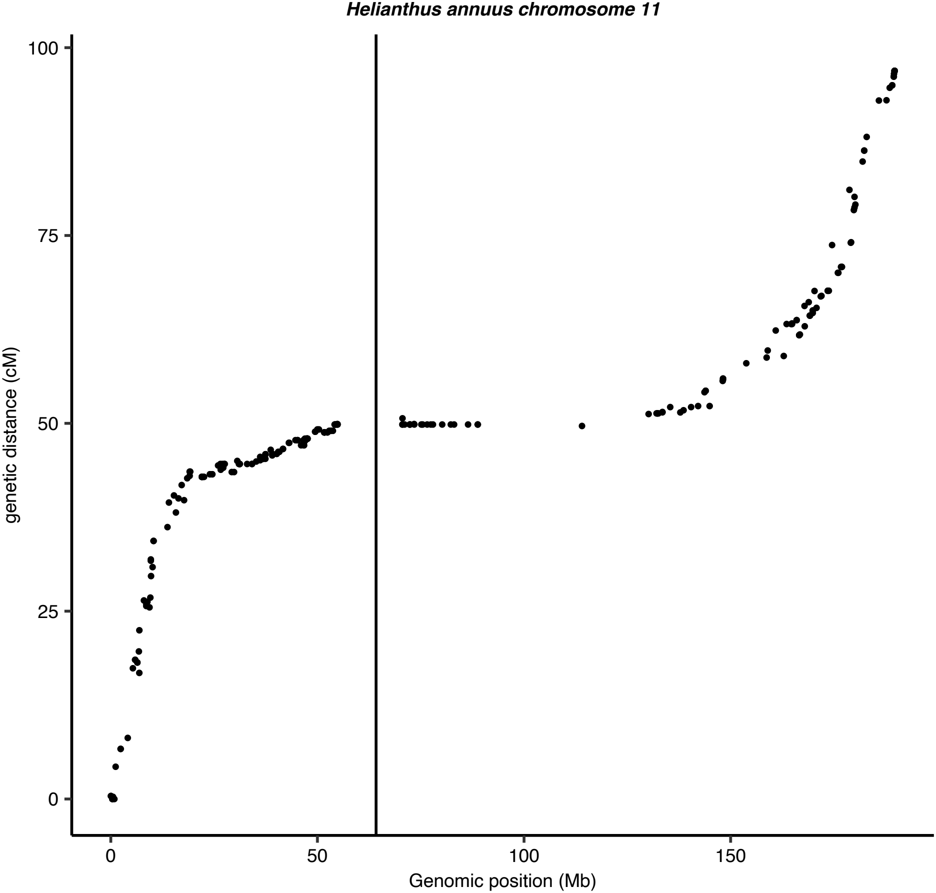
Helianthus annuus chromosome 11
genetic distance (cM)
Genomic position (Mb)
0
25
50
75
100
0
50
100
150

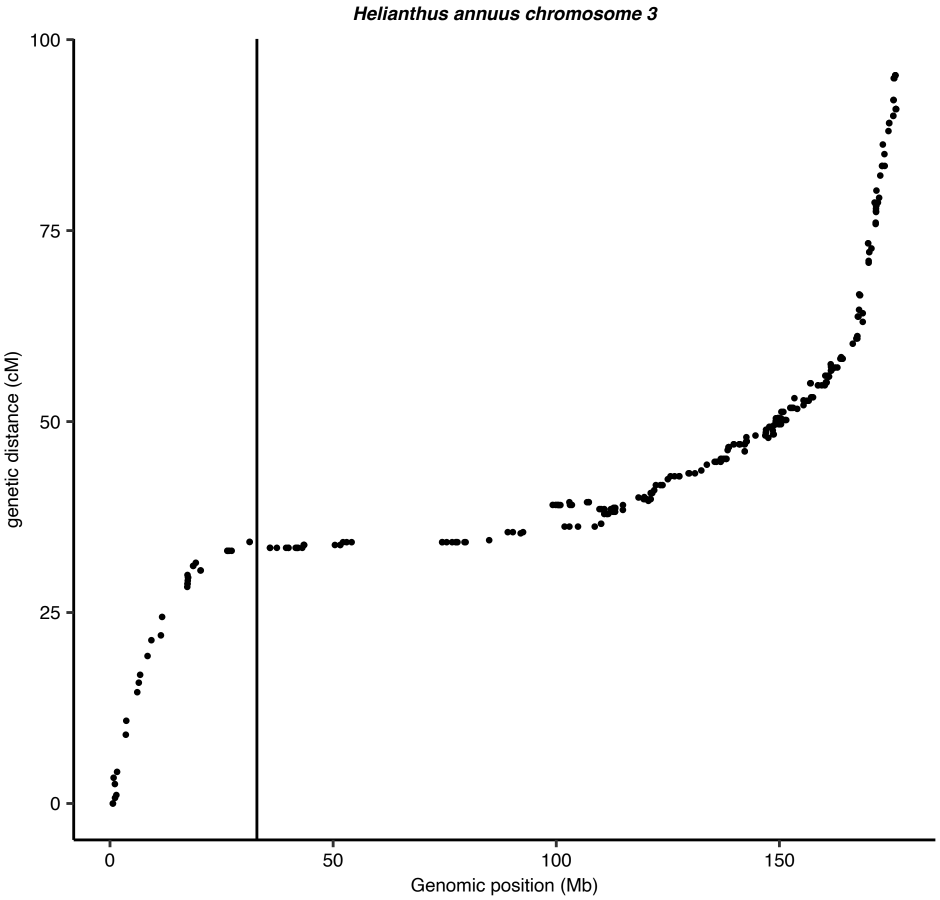
Helianthus annuus chromosome 3
100
75
50
25
0
genetic distance (cM)
0
50
100
150
Genomic position (Mb)

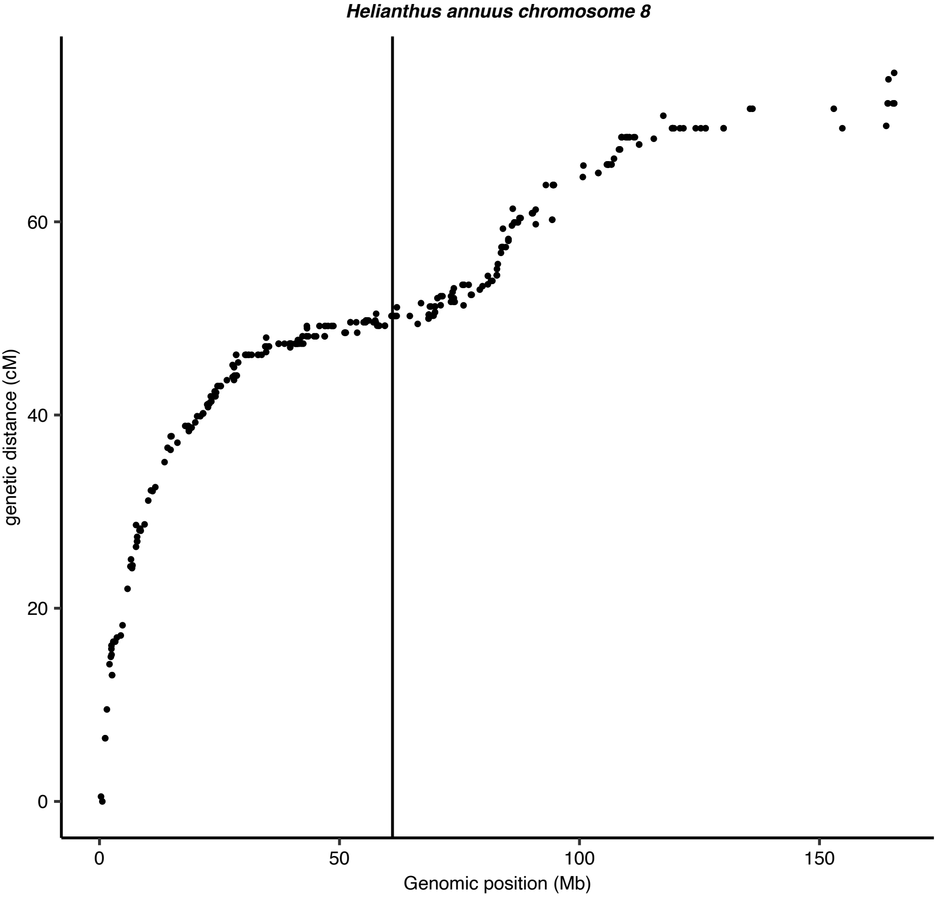
Helianthus annuus chromosome 8
genetic distance (cM)
0
20
40
60
Genomic position (Mb)
0
50
100
150

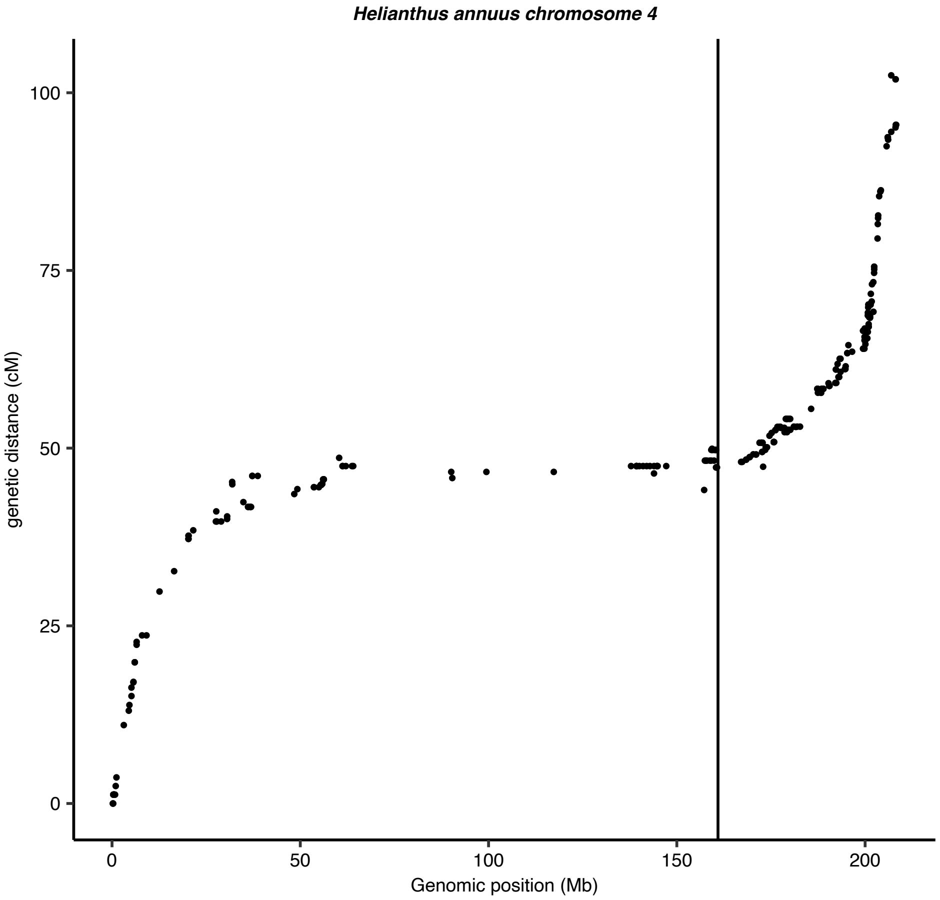

Helianthus annuus chromosome 4
genetic distance (cM)
100
75
50
25
0
0
50
100
150
200
Genomic position (Mb)

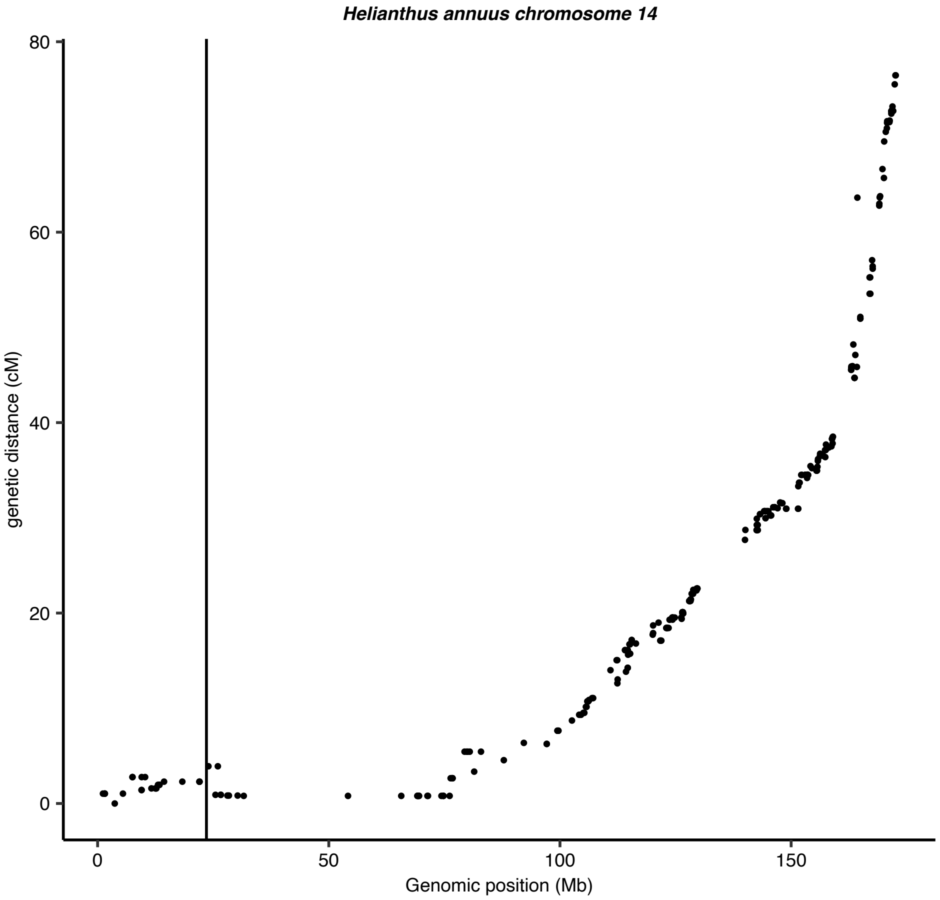
Helianthus annuus chromosome 14
genetic distance (cM)
Genomic position (Mb)
0
20
40
60
80
0
50
100
150

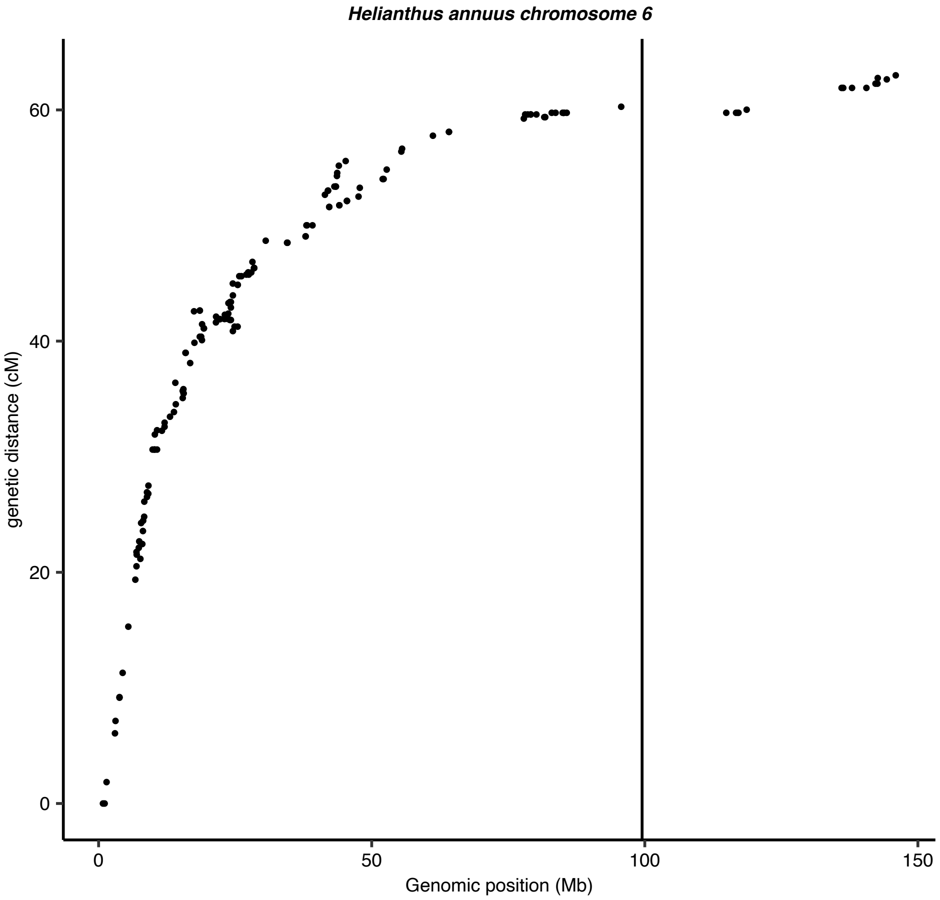
Helianthus annuus chromosome 6
genetic distance (cM)
0
20
40
60
Genomic position (Mb)
0
50
100
150

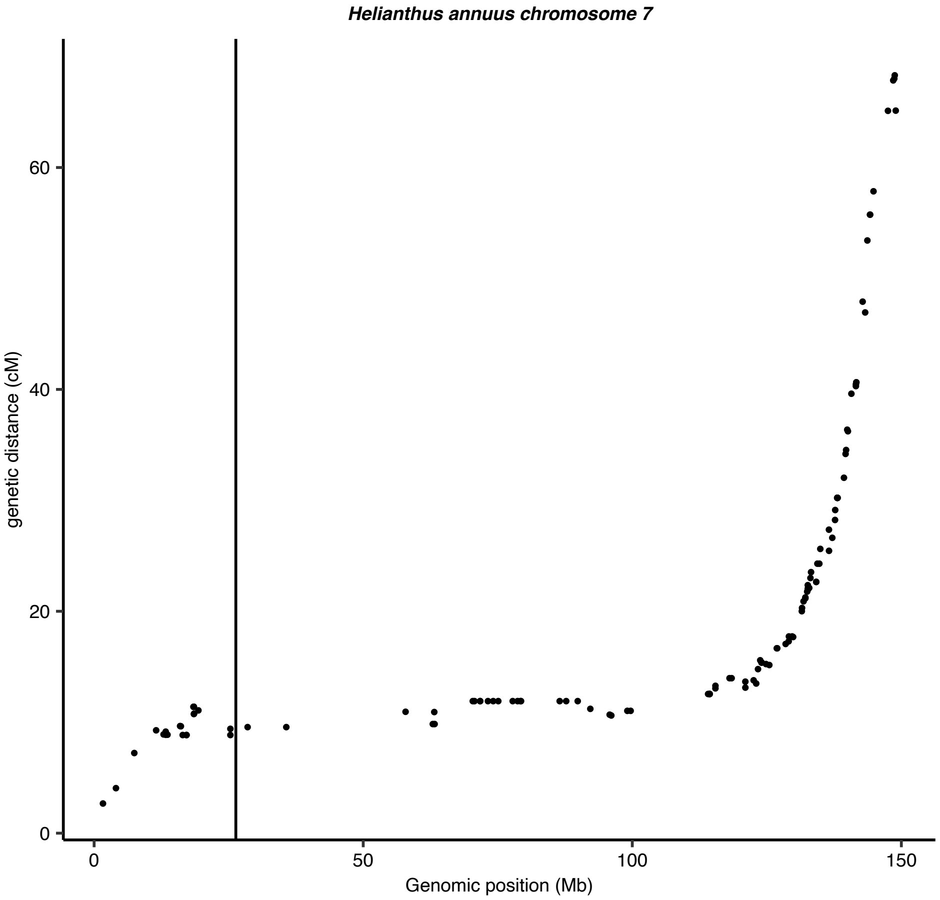

Helianthus annuus chromosome 7
genetic distance (cM)
0
20
40
60
Genomic position (Mb)
0
50
100
150

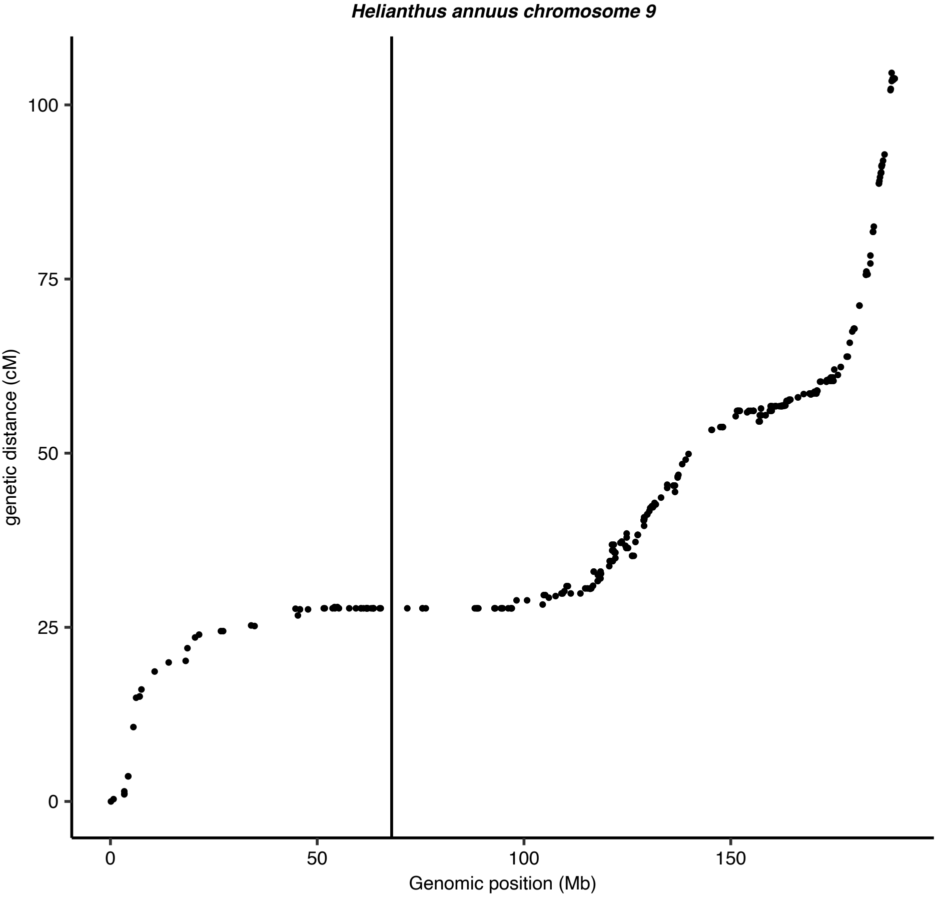
Helianthus annuus chromosome 9
genetic distance (cM)
100
75
50
25
0
0
50
100
150
Genomic position (Mb)

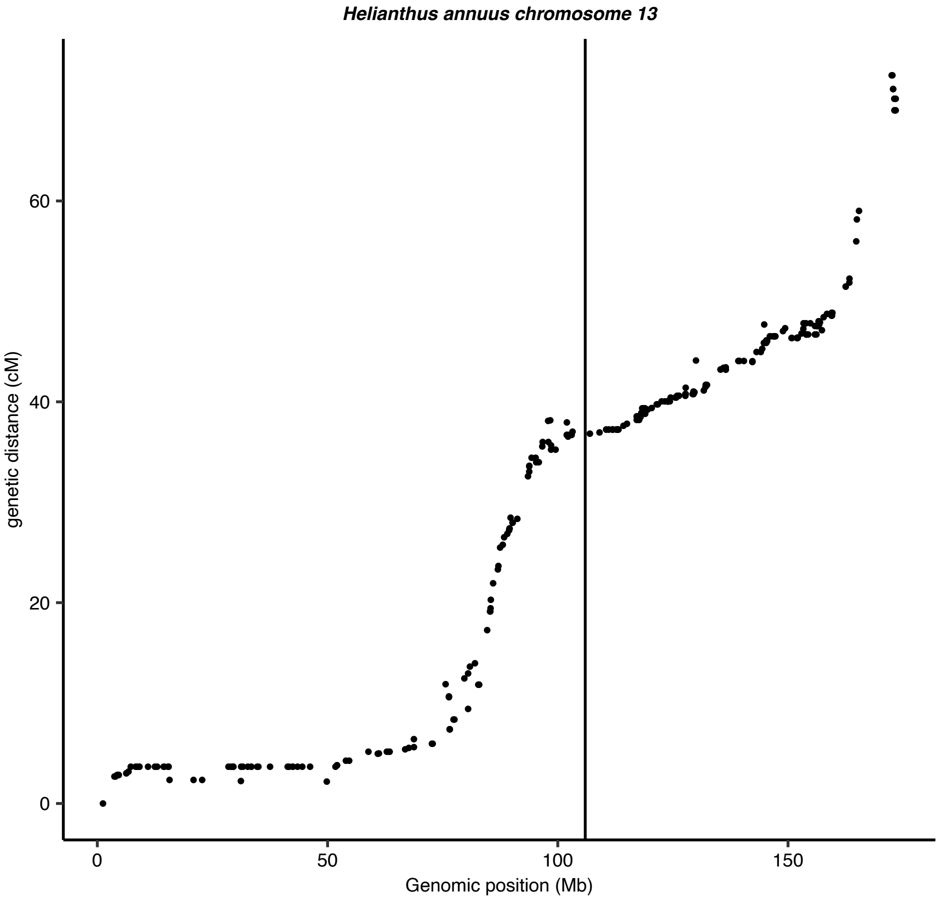
Helianthus annuus chromosome 13
genetic distance (cM)
0
20
40
60
Genomic position (Mb)
0
50
100
150

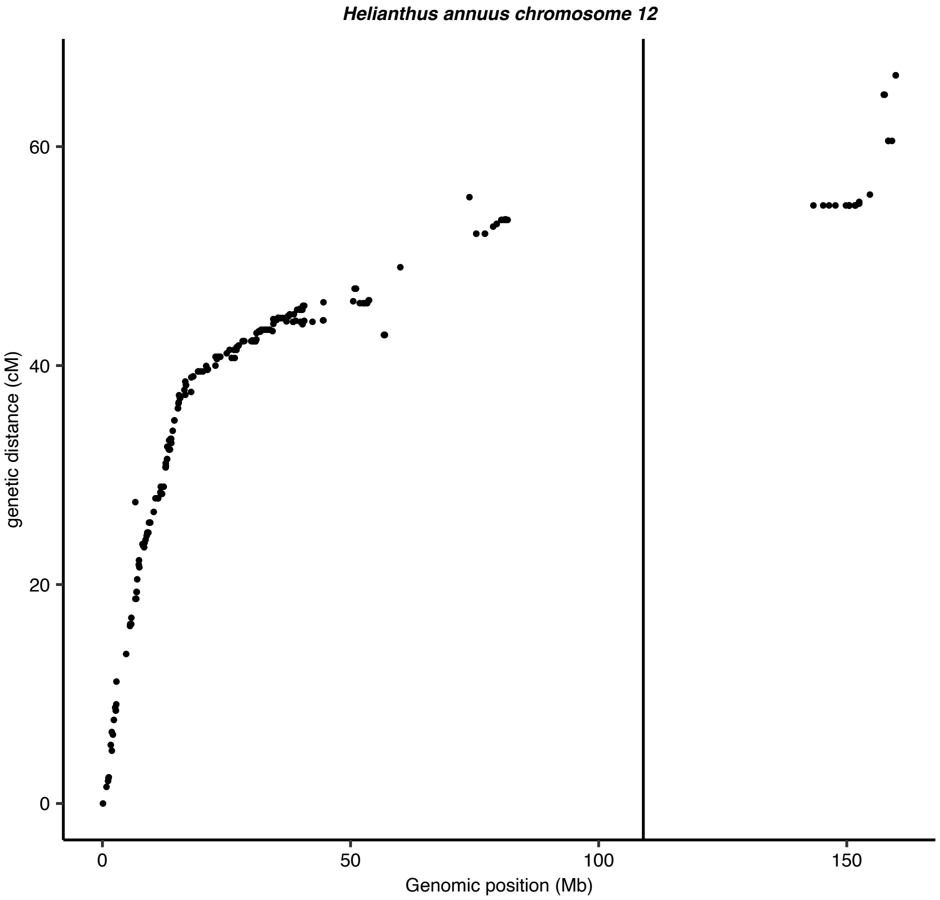
Helianthus annuus chromosome 12
genetic distance (cM)
0
20
40
60
Genomic position (Mb)
0
50
100
150

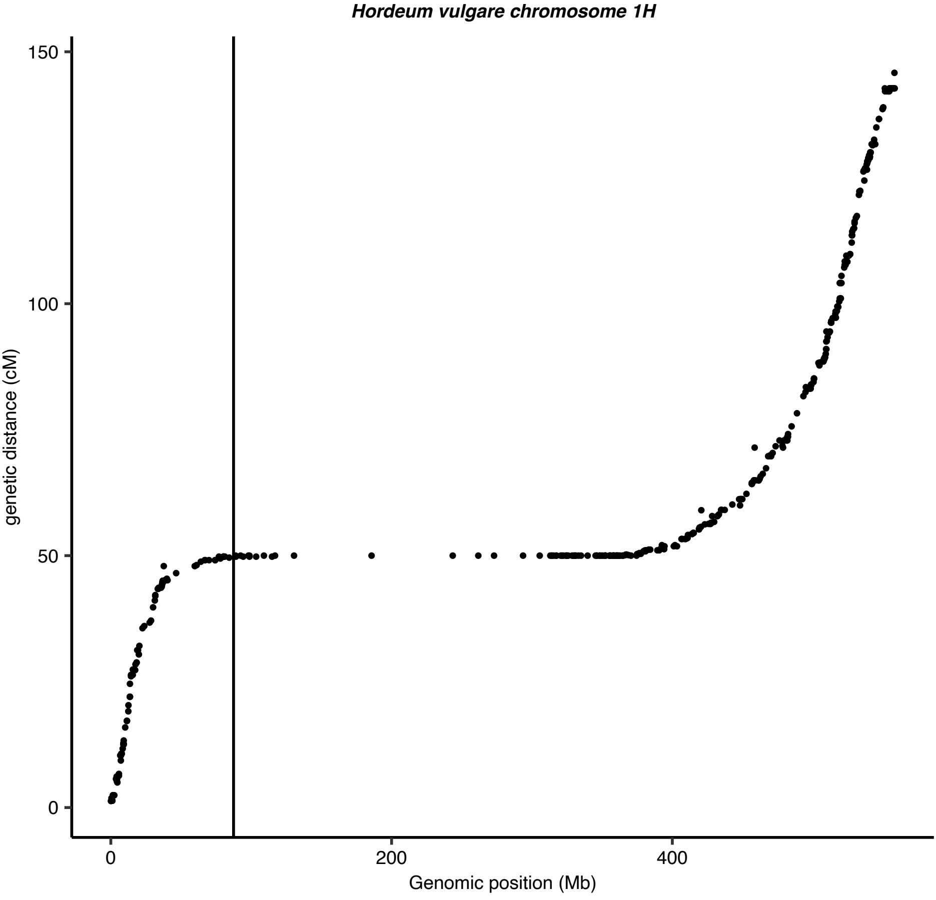
Hordeum vulgare chromosome 1H
150
100
50
0
genetic distance (cM)
0
200
400
Genomic position (Mb)

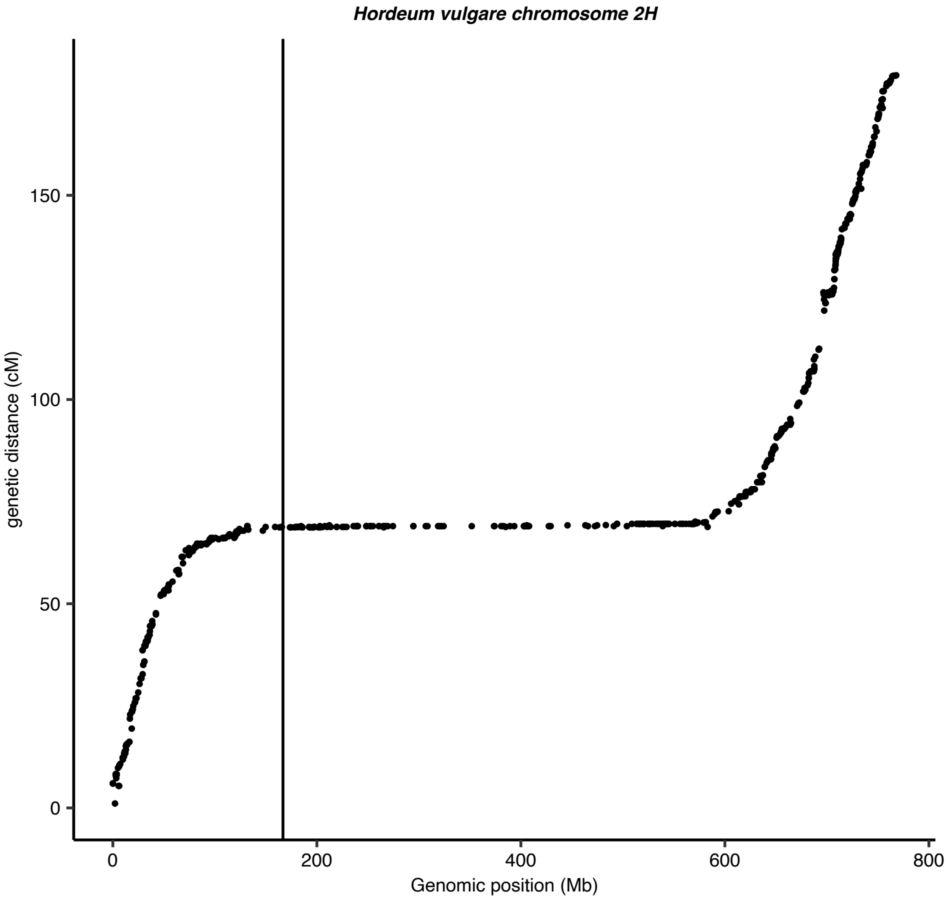

Hordeum vulgare chromosome 2H
genetic distance (cM)
0
50
100
150
0
200
400
600
800
Genomic position (Mb)

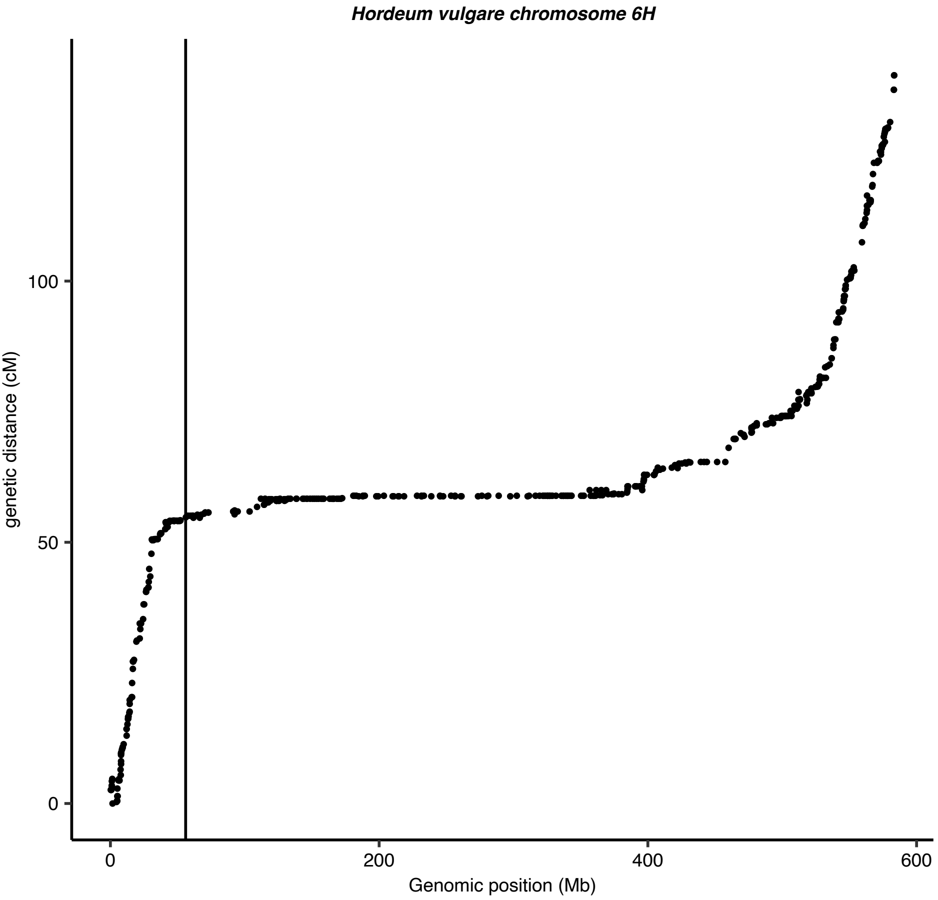
Hordeum vulgare chromosome 6H
genetic distance (cM)
0
50
100
0
200
400
600
Genomic position (Mb)

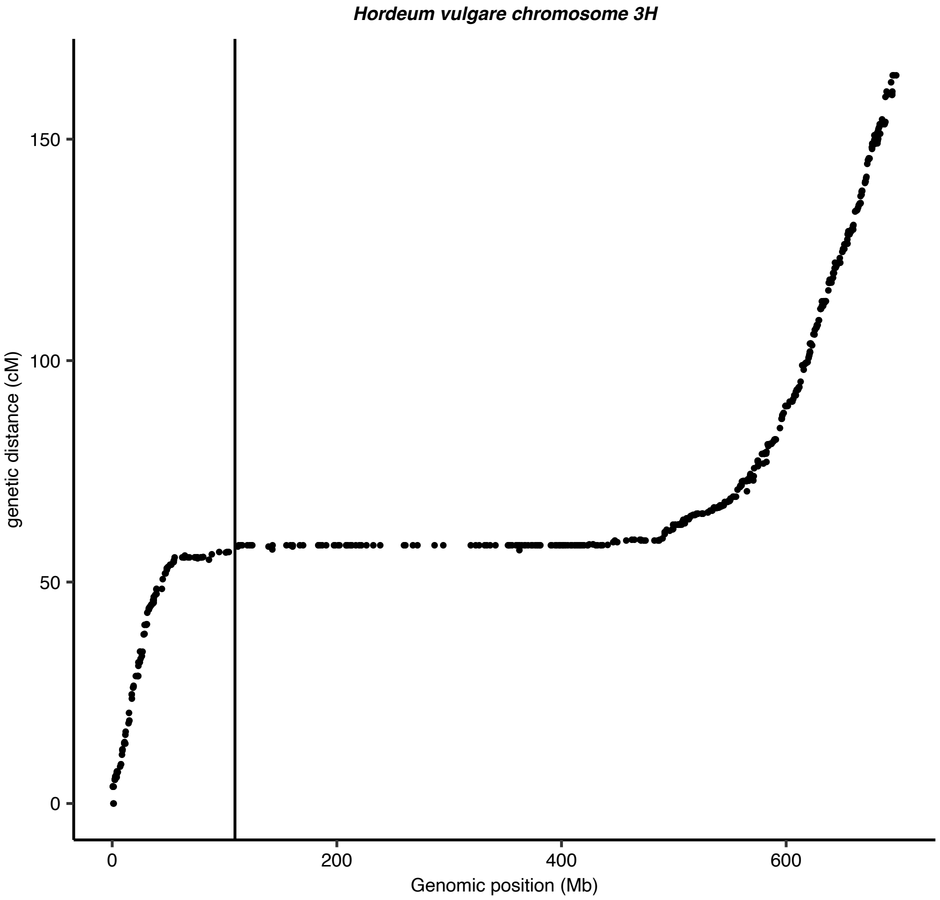
Hordeum vulgare chromosome 3H
genetic distance (cM)
0
50
100
150
Genomic position (Mb)
0
200
400
600

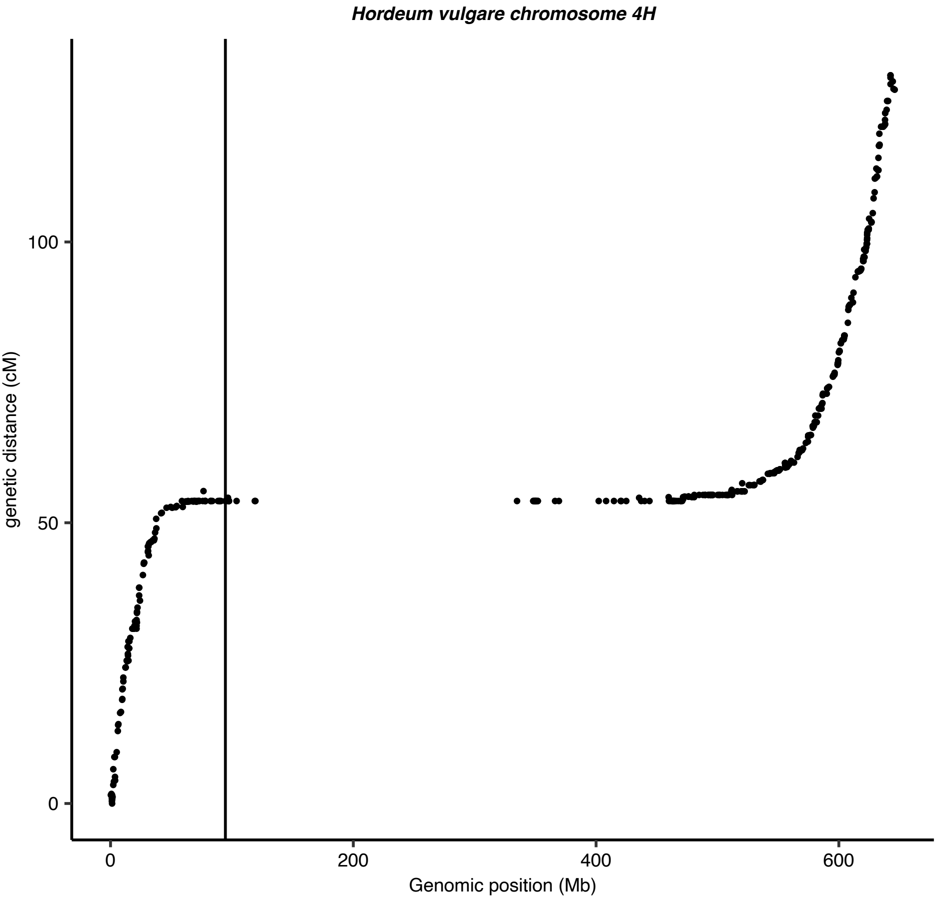
Hordeum vulgare chromosome 4H
genetic distance (cM)
100
50
0
0
200
400
600
Genomic position (Mb)

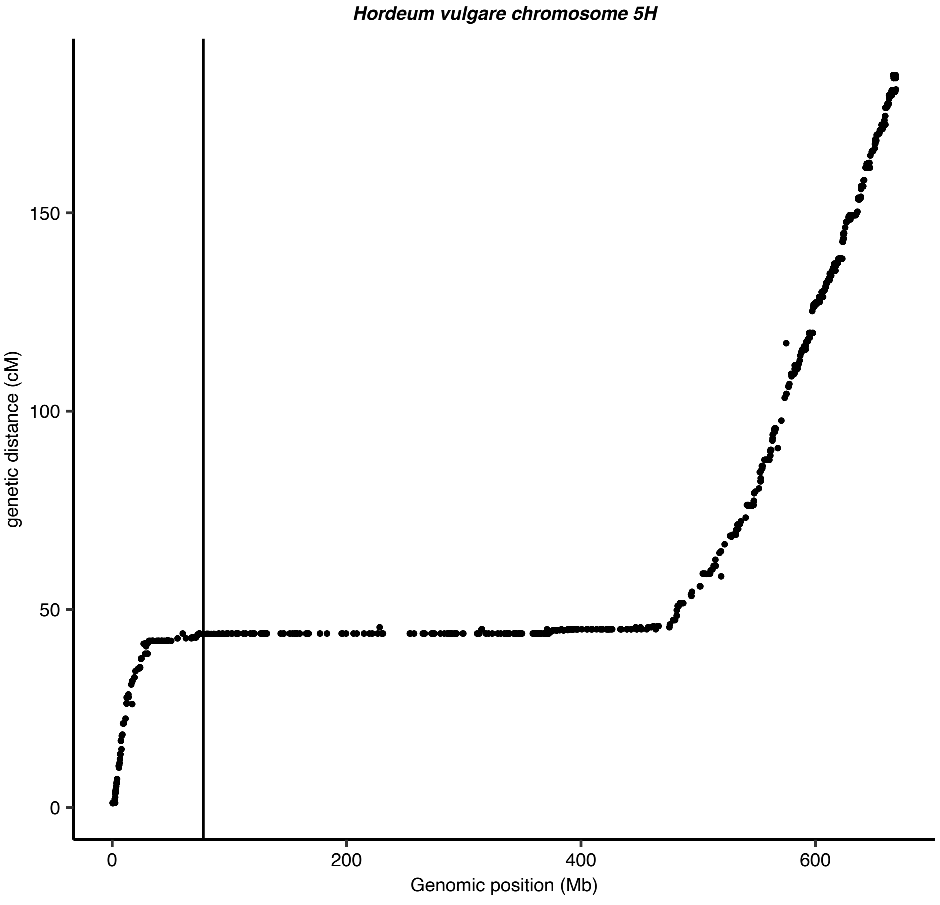
Hordeum vulgare chromosome 5H
genetic distance (cM)
0
50
100
150
Genomic position (Mb)
0
200
400
600

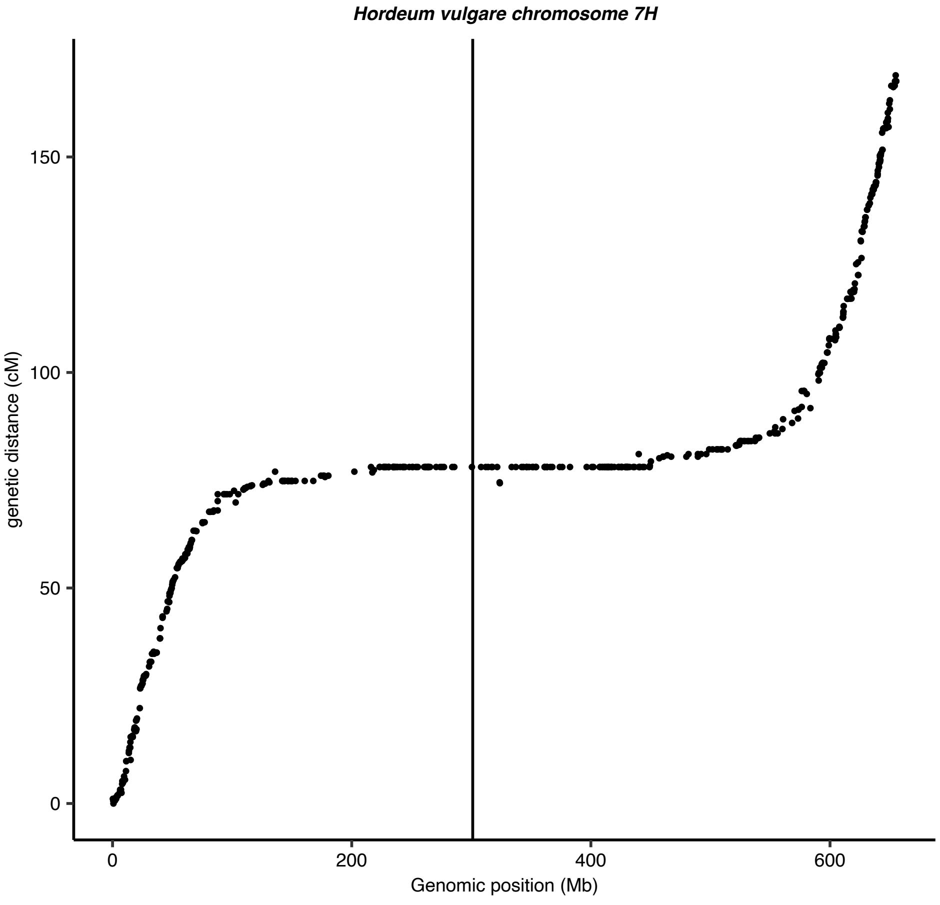
Hordeum vulgare chromosome 7H
genetic distance (cM)
0
50
100
150
Genomic position (Mb)
0
200
400
600

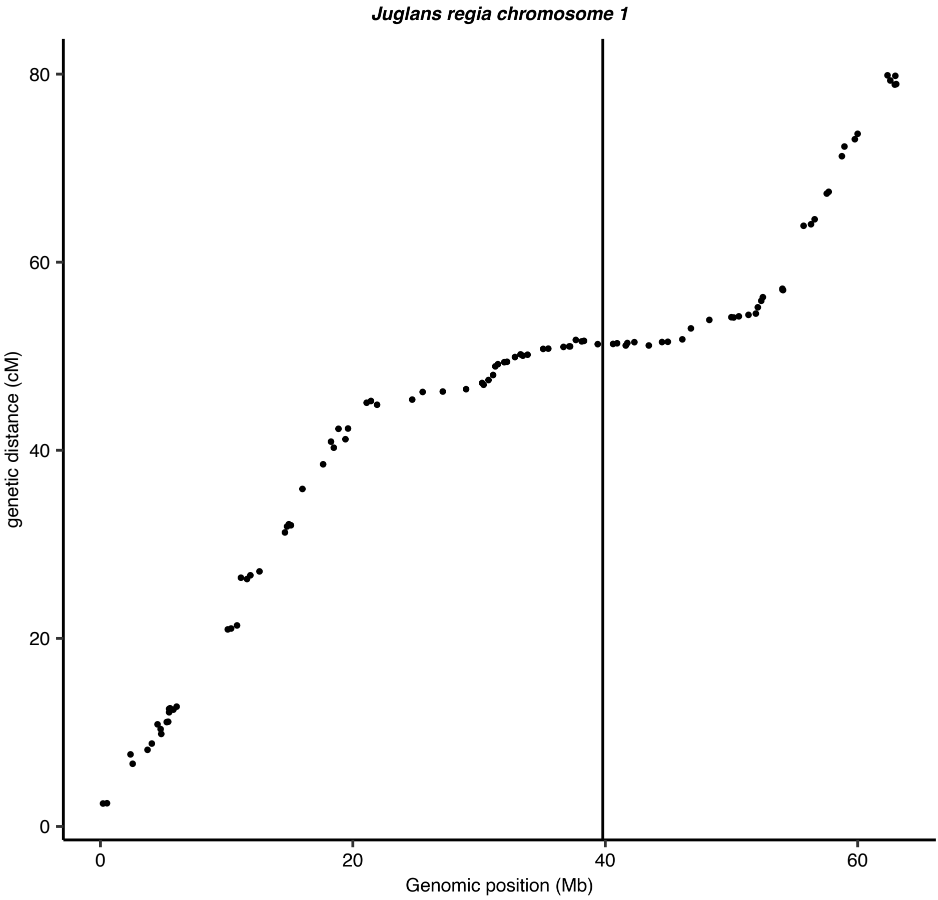
Juglans regia chromosome 1
genetic distance (cM)
Genomic position (Mb)
0
20
40
60
80
0
20
40
60

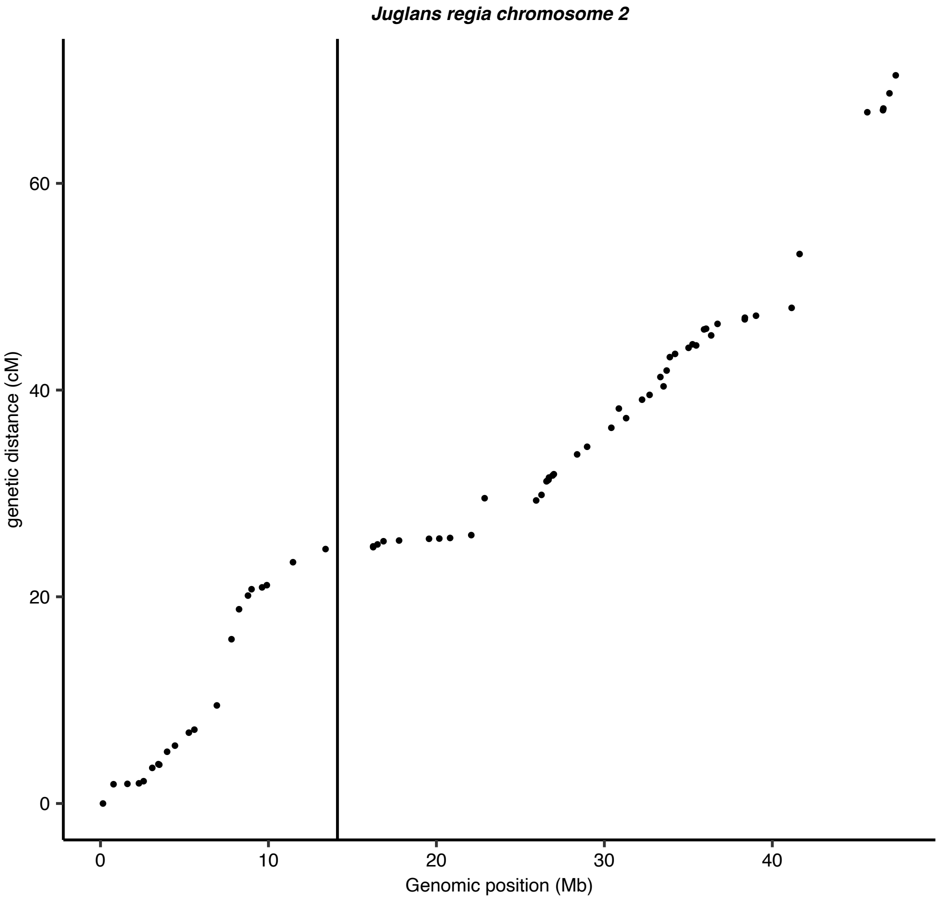
Juglans regia chromosome 2
genetic distance (cM)
0
20
40
60
0
10
20
30
40
Genomic position (Mb)

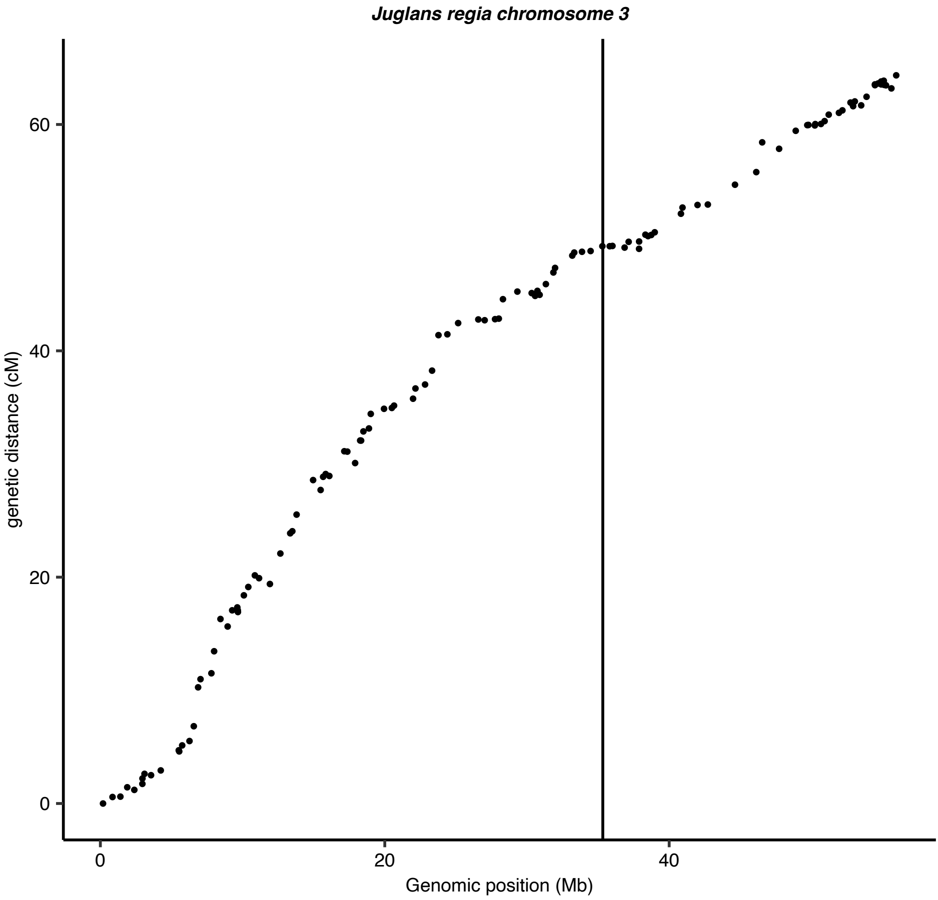
Juglans regia chromosome 3
genetic distance (cM)
60
40
20
0
0
20
40
Genomic position (Mb)

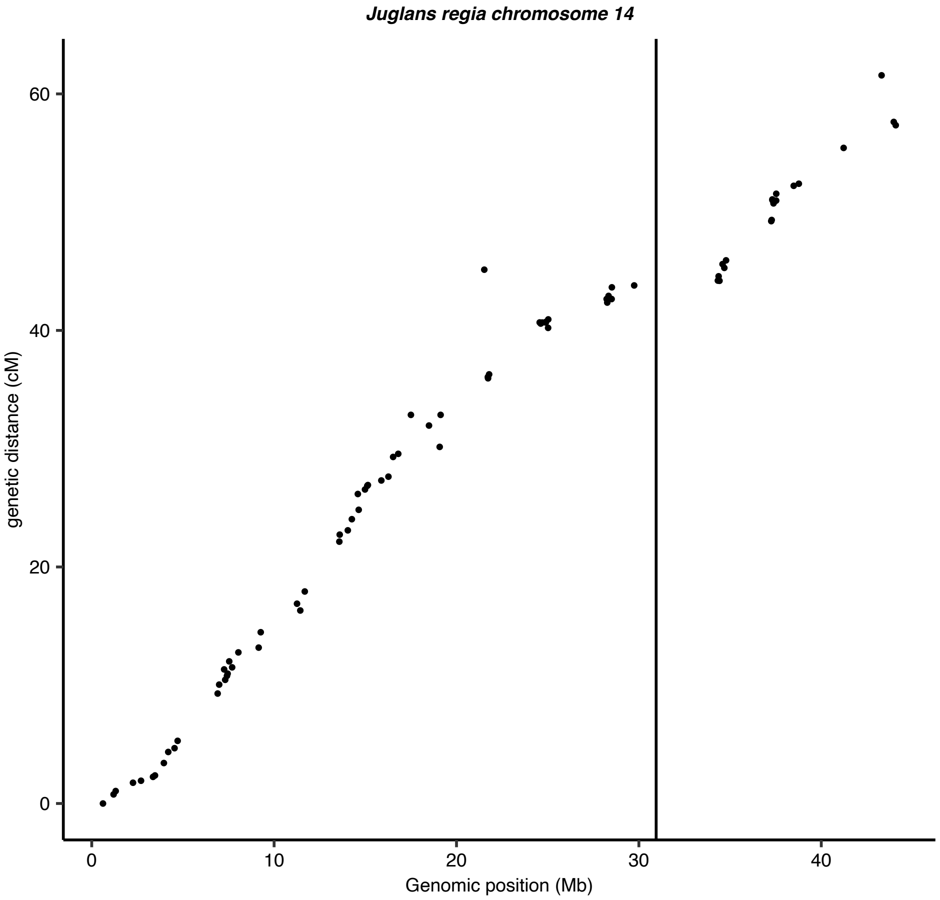
Juglans regia chromosome 14
genetic distance (cM)
Genomic position (Mb)
0
20
40
60
0
10
20
30
40

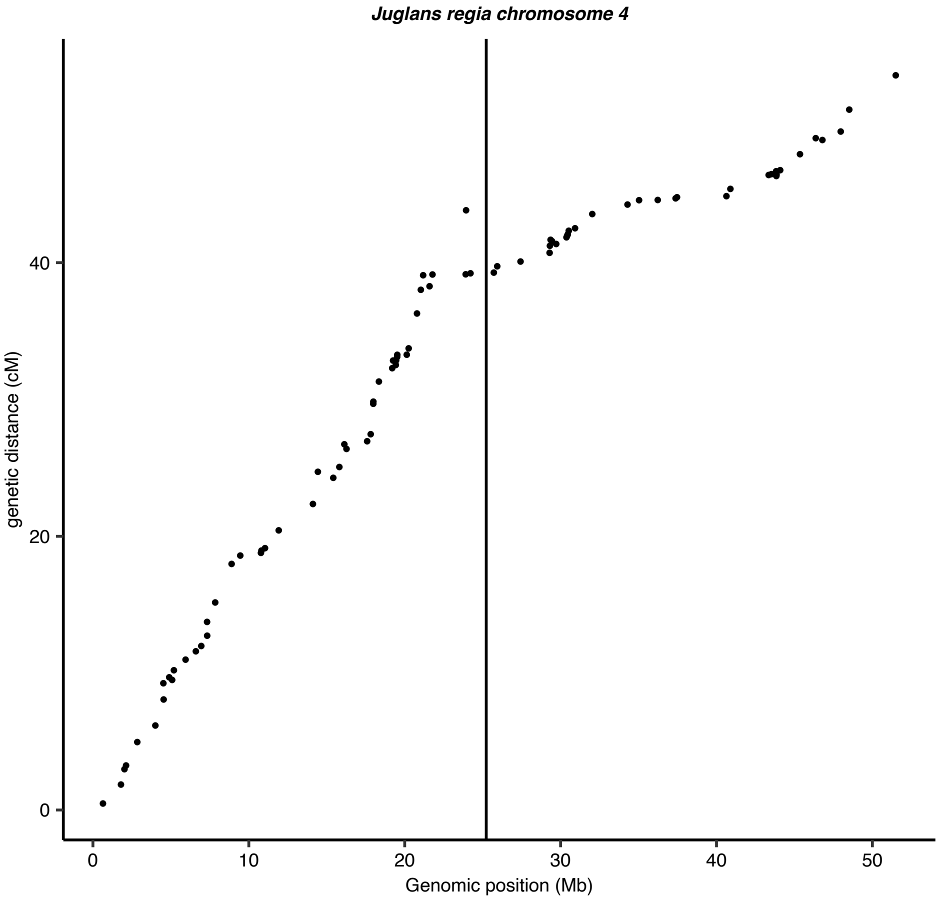

Juglans regia chromosome 4
genetic distance (cM)
0
20
40
Genomic position (Mb)
0
10
20
30
40
50

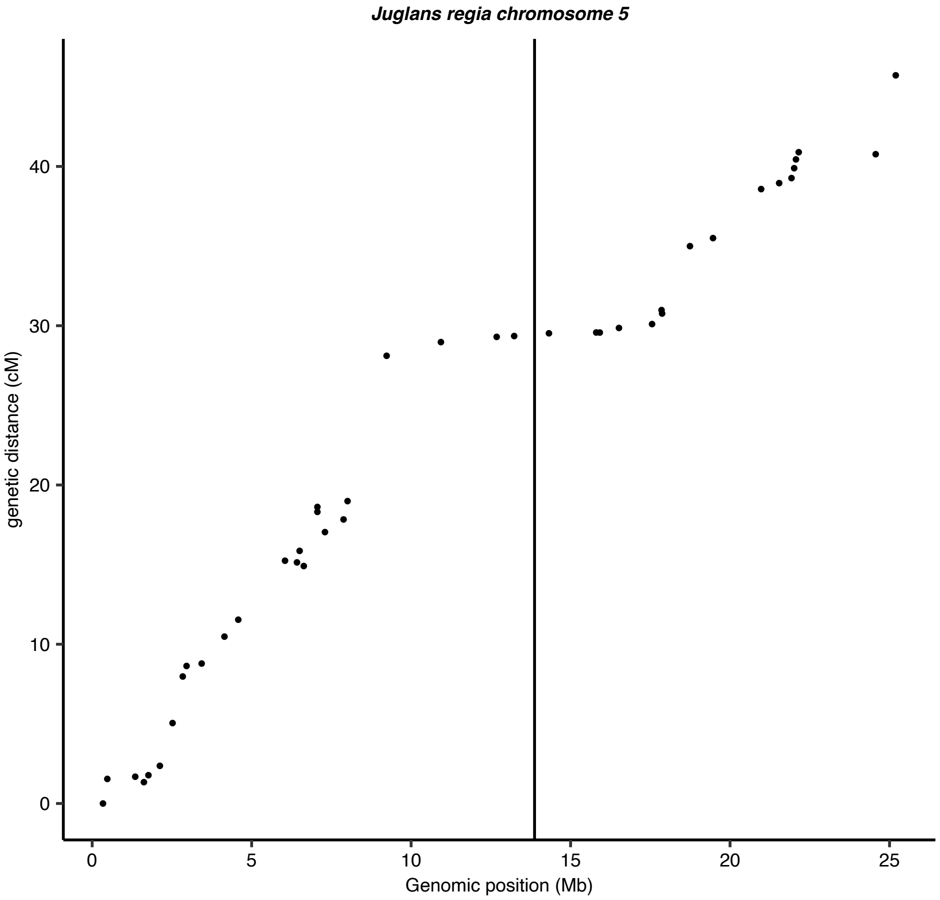
Juglans regia chromosome 5
genetic distance (cM)
0
10
20
30
40
Genomic position (Mb)
0
5
10
15
20
25

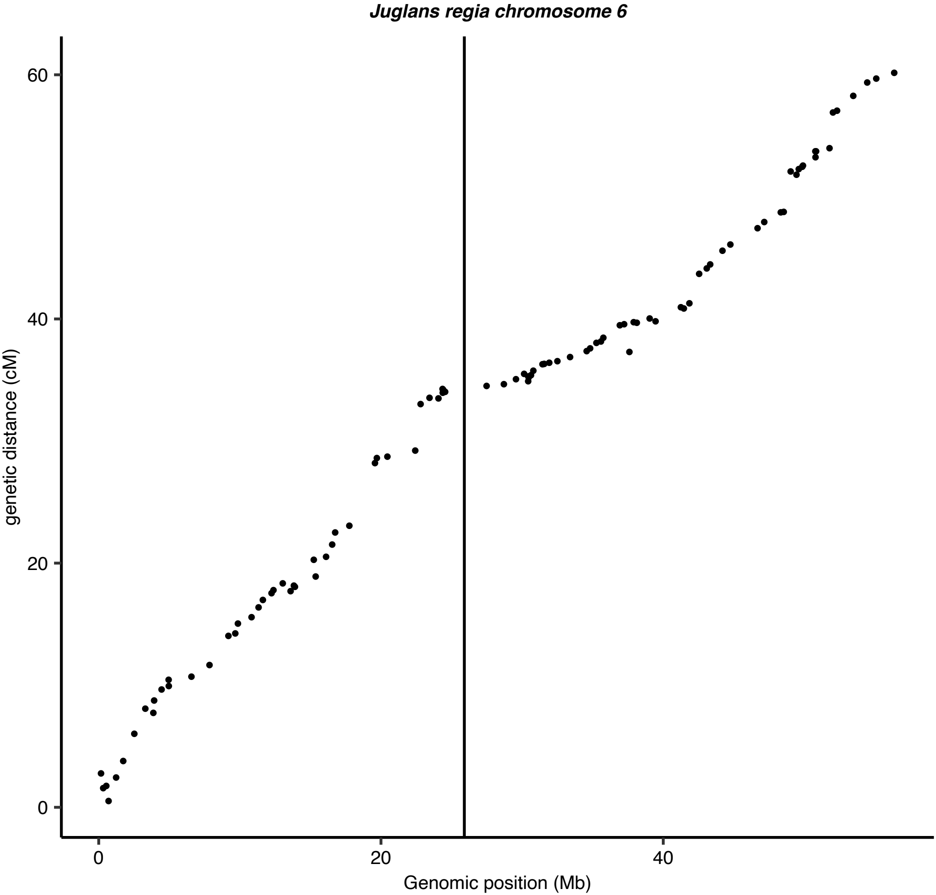
Juglans regia chromosome 6
genetic distance (cM)
60
40
20
0
0
20
40
Genomic position (Mb)

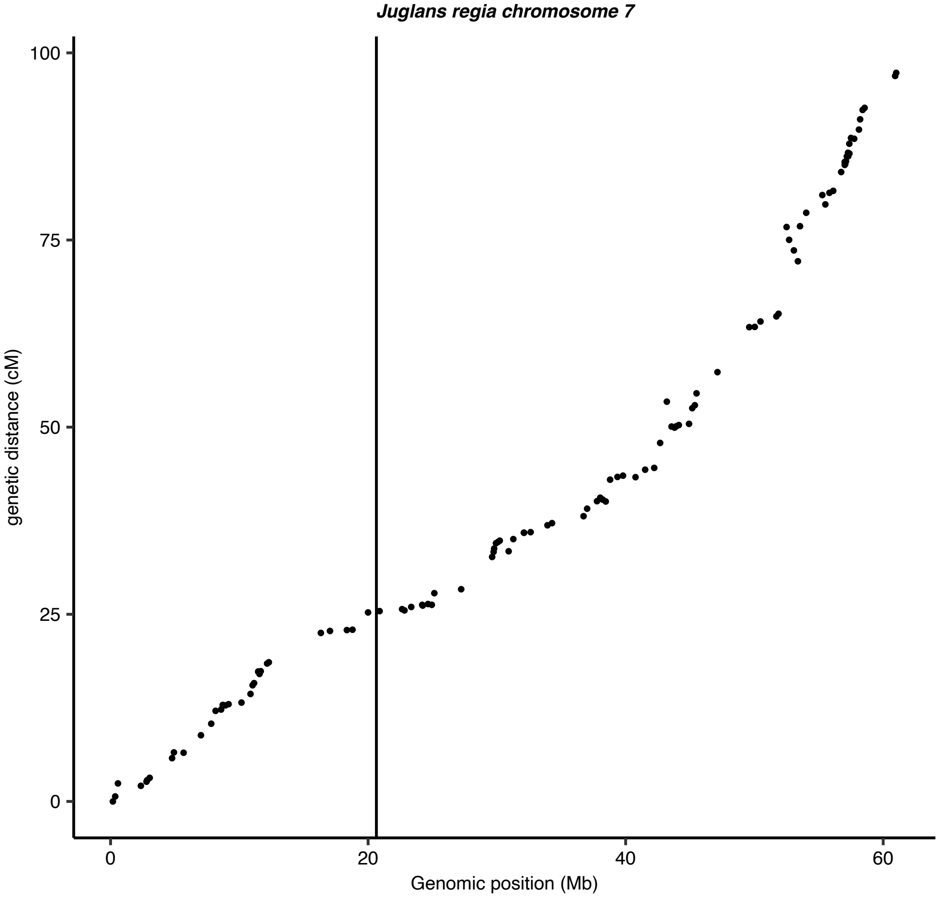
Juglans regia chromosome 7
genetic distance (cM)
Genomic position (Mb)
0
25
50
75
100
0
20
40
60

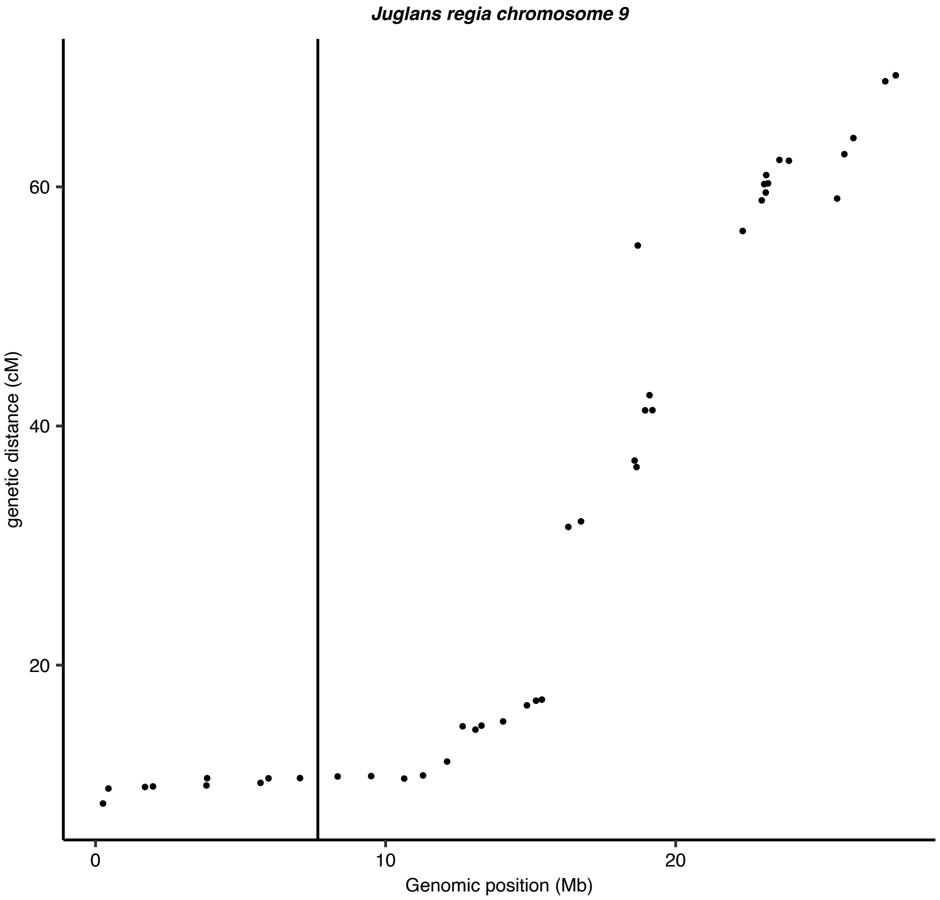
Juglans regia chromosome 9
genetic distance (cM)
20
40
60
0
10
20
Genomic position (Mb)

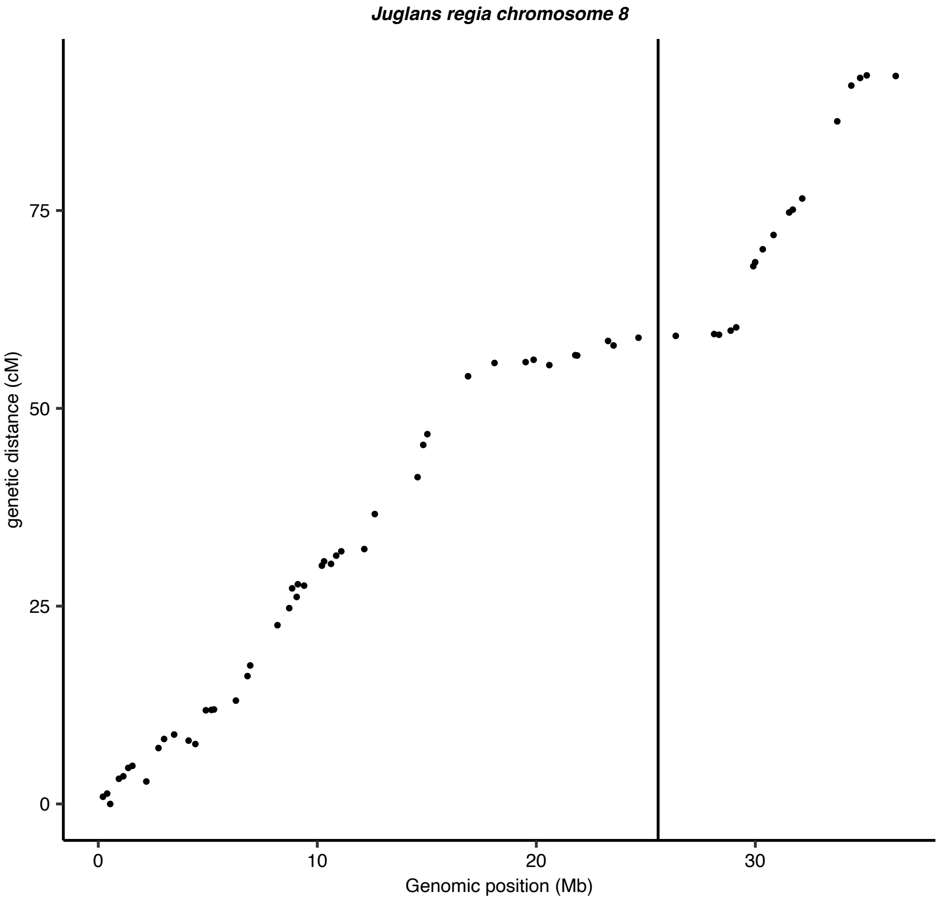

Juglans regia chromosome 8
genetic distance (cM)
0
25
50
75
0
10
20
30
Genomic position (Mb)

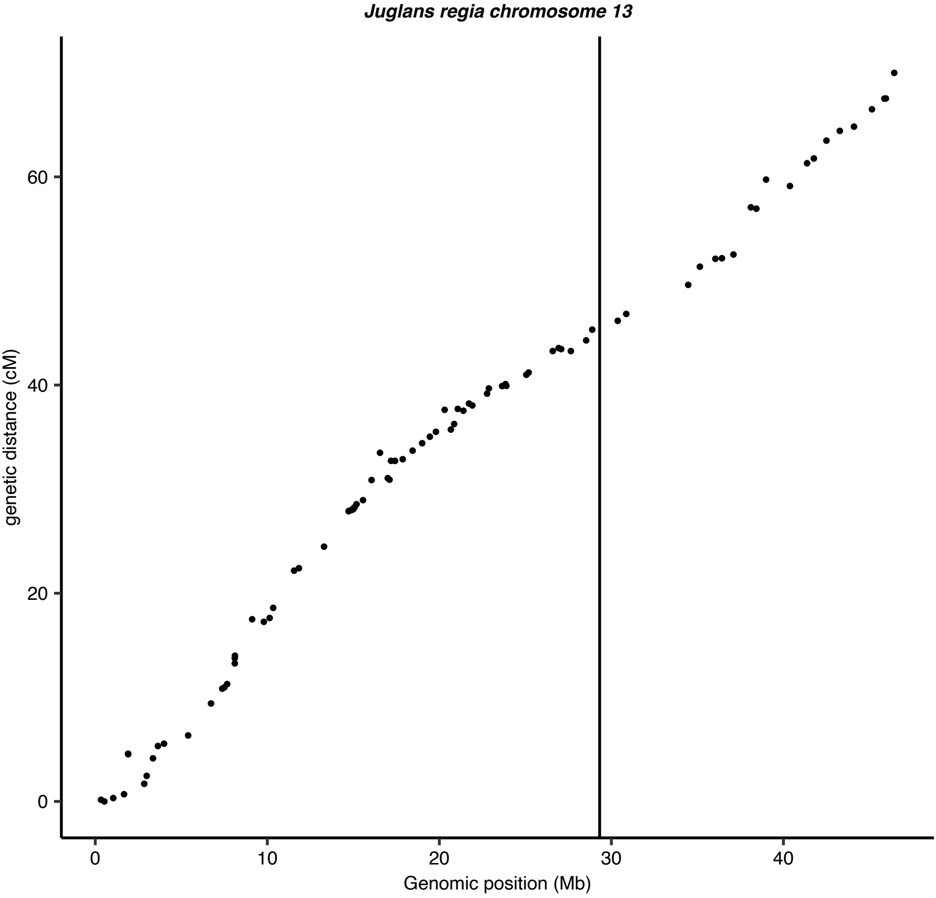
Juglans regia chromosome 13
genetic distance (cM)
Genomic position (Mb)
0
20
40
60
0
10
20
30
40

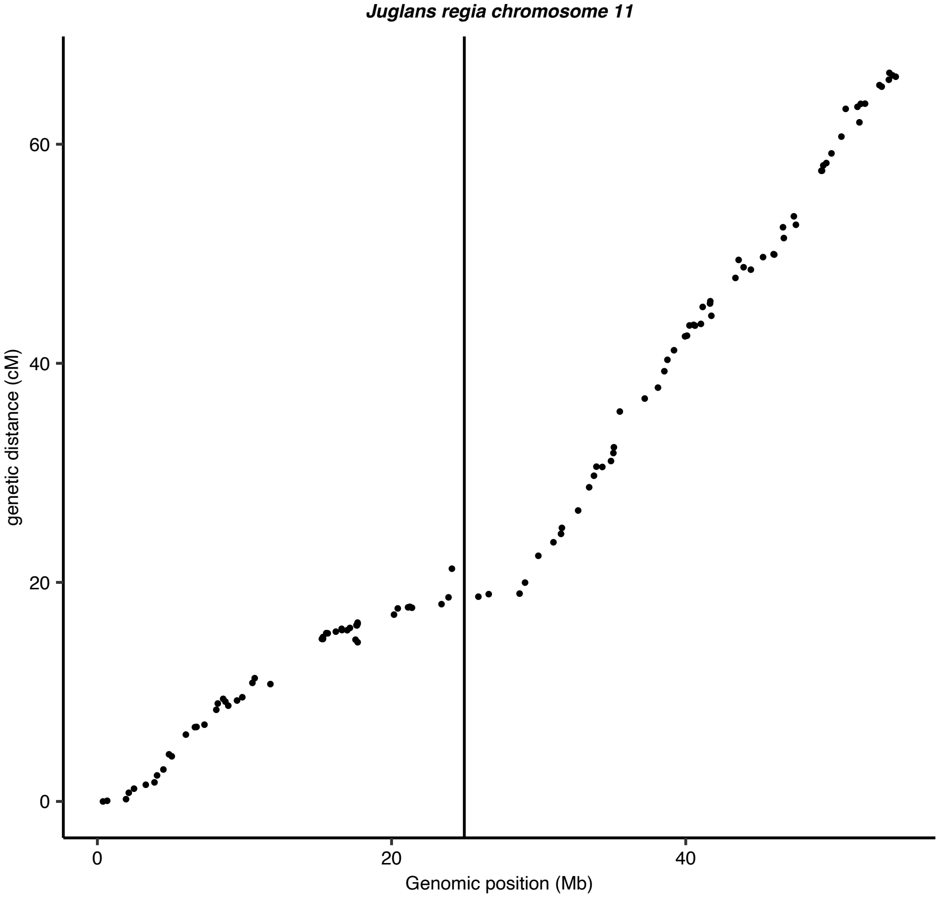
Juglans regia chromosome 11
genetic distance (cM)
Genomic position (Mb)
0
20
40
60
0
20
40

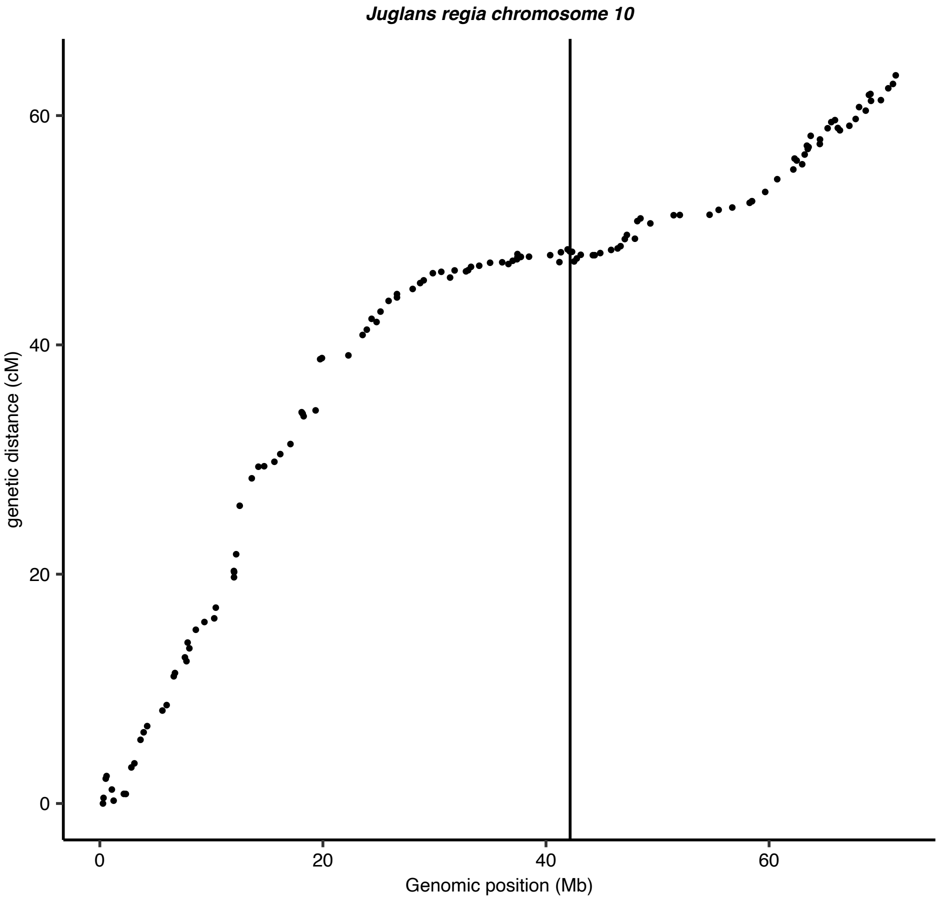
Juglans regia chromosome 10
genetic distance (cM)
0
20
40
60
Genomic position (Mb)
0
20
40
60

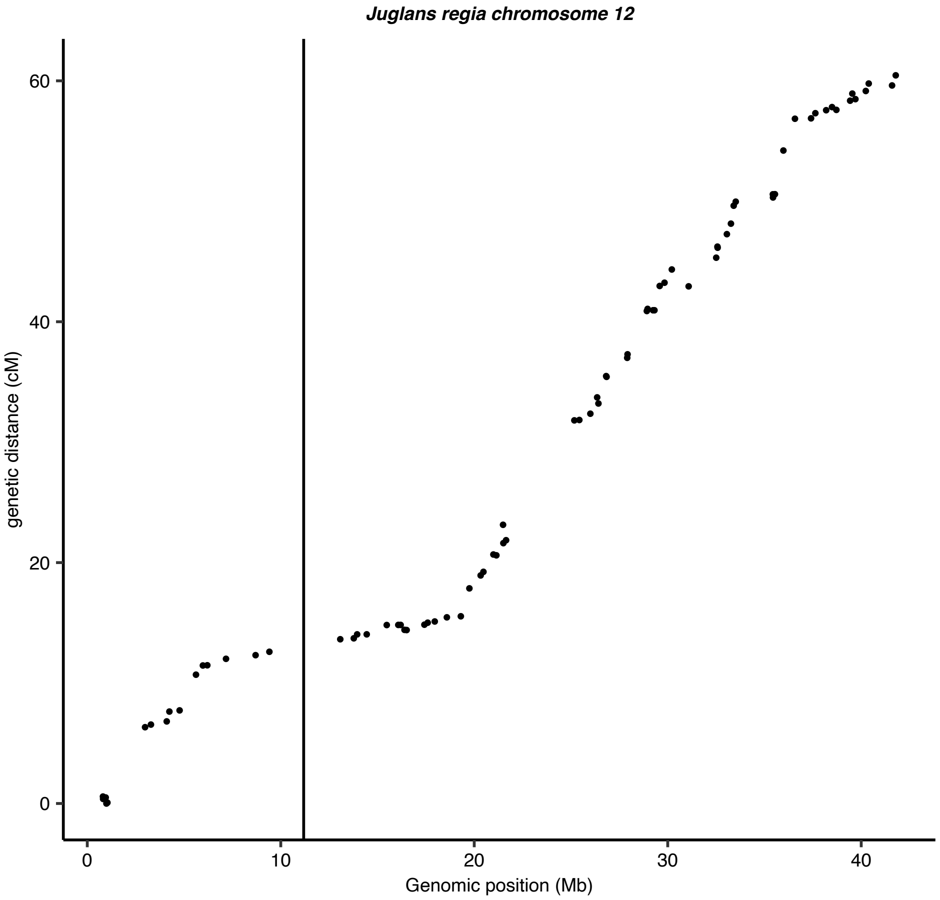
Juglans regia chromosome 12
genetic distance (cM)
0
20
40
60
Genomic position (Mb)
0
10
20
30
40

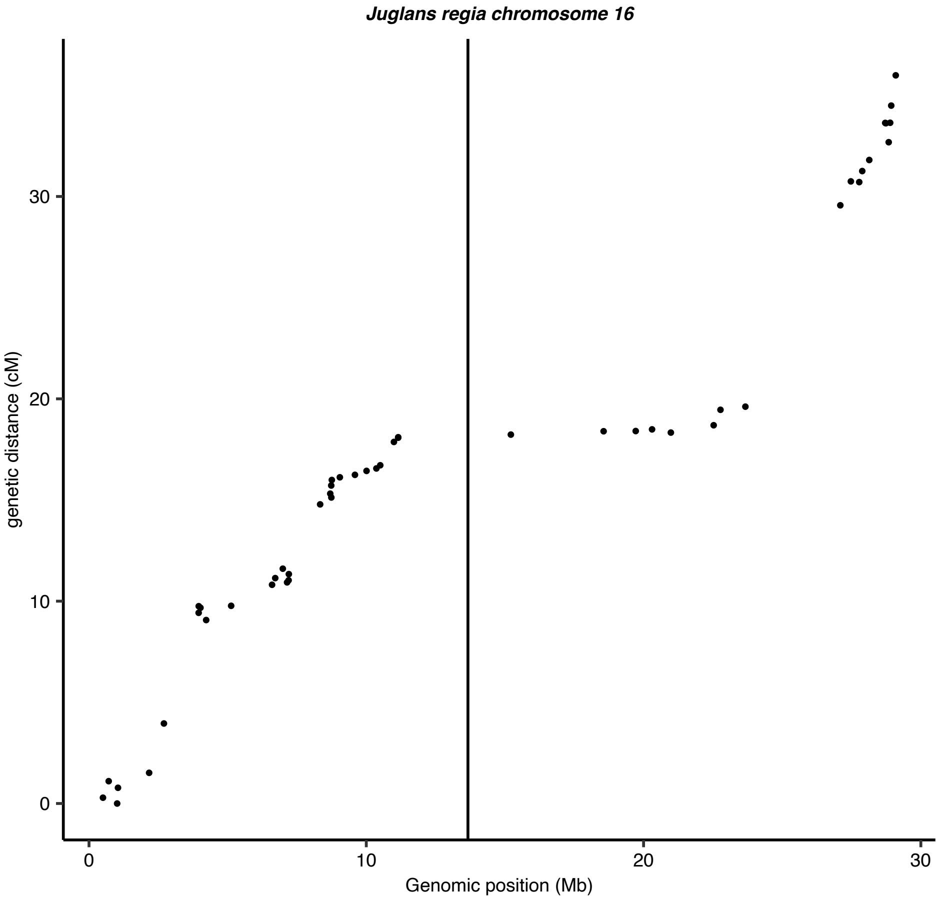
Juglans regia chromosome 16
genetic distance (cM)
0
10
20
30
Genomic position (Mb)
0
10
20
30

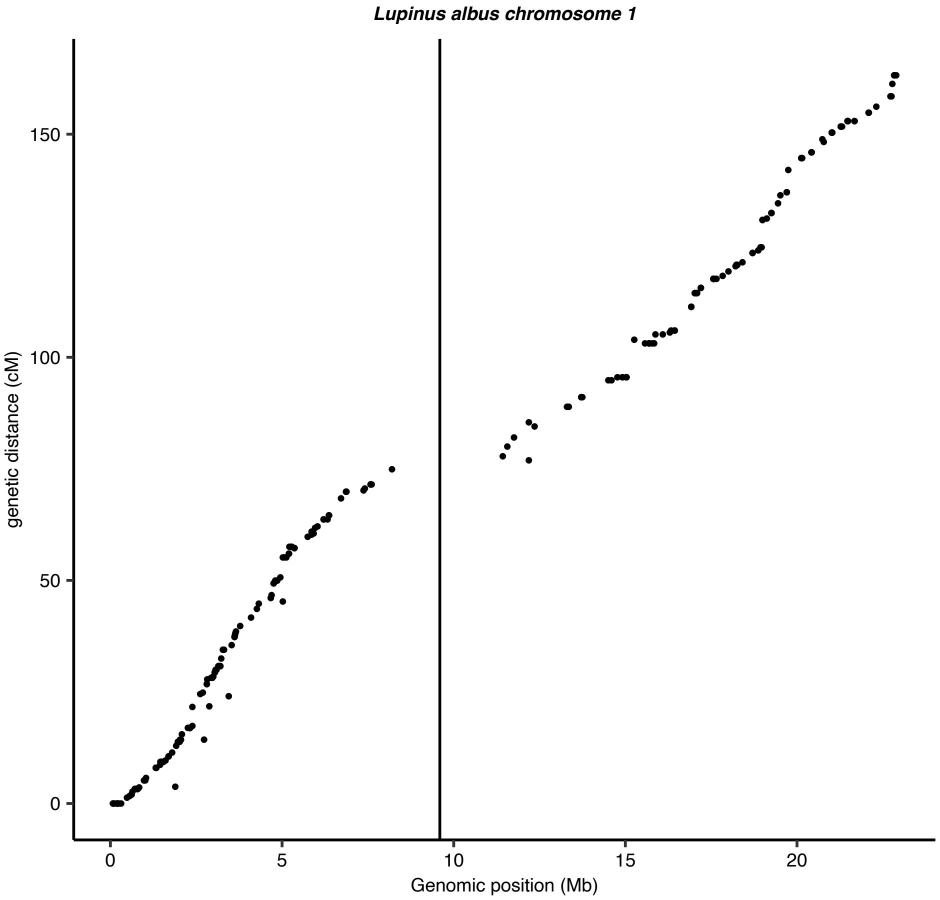
Lupinus albus chromosome 1
genetic distance (cM)
0
50
100
150
Genomic position (Mb)
0
5
10
15
20

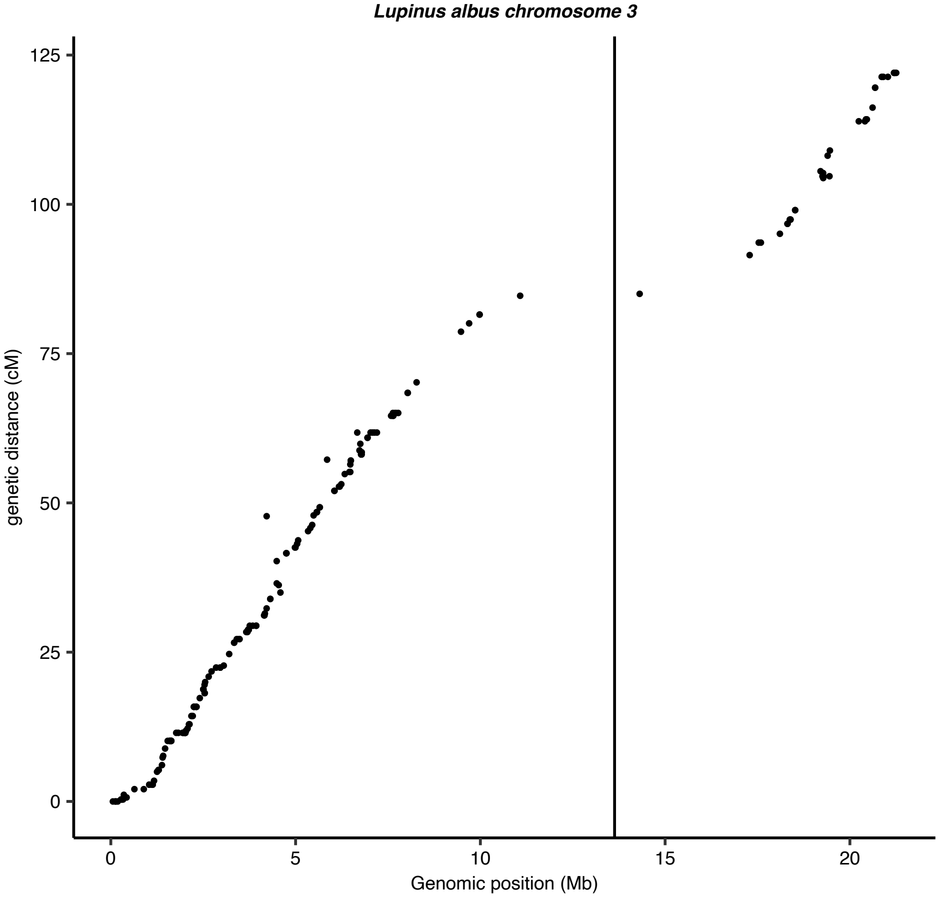
Lupinus albus chromosome 3
genetic distance (cM)
Genomic position (Mb)
0
25
50
75
100
125
0
5
10
15
20

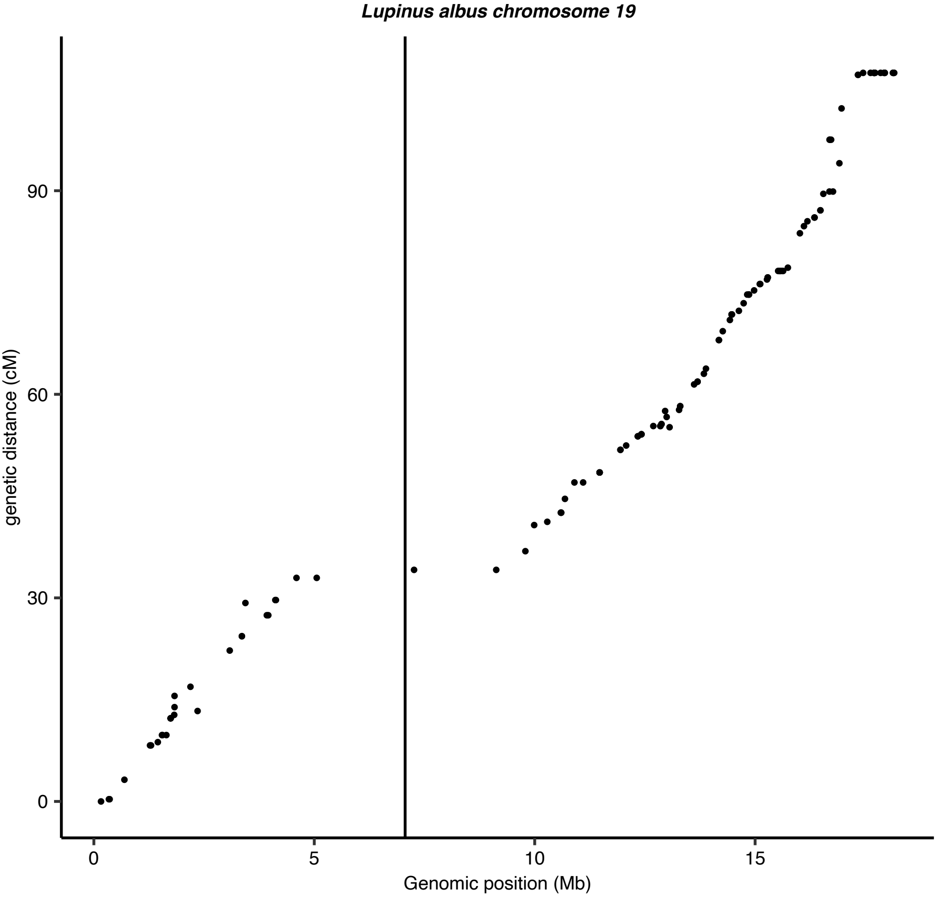
Lupinus albus chromosome 19
genetic distance (cM)
0
30
60
90
0
5
10
15
Genomic position (Mb)

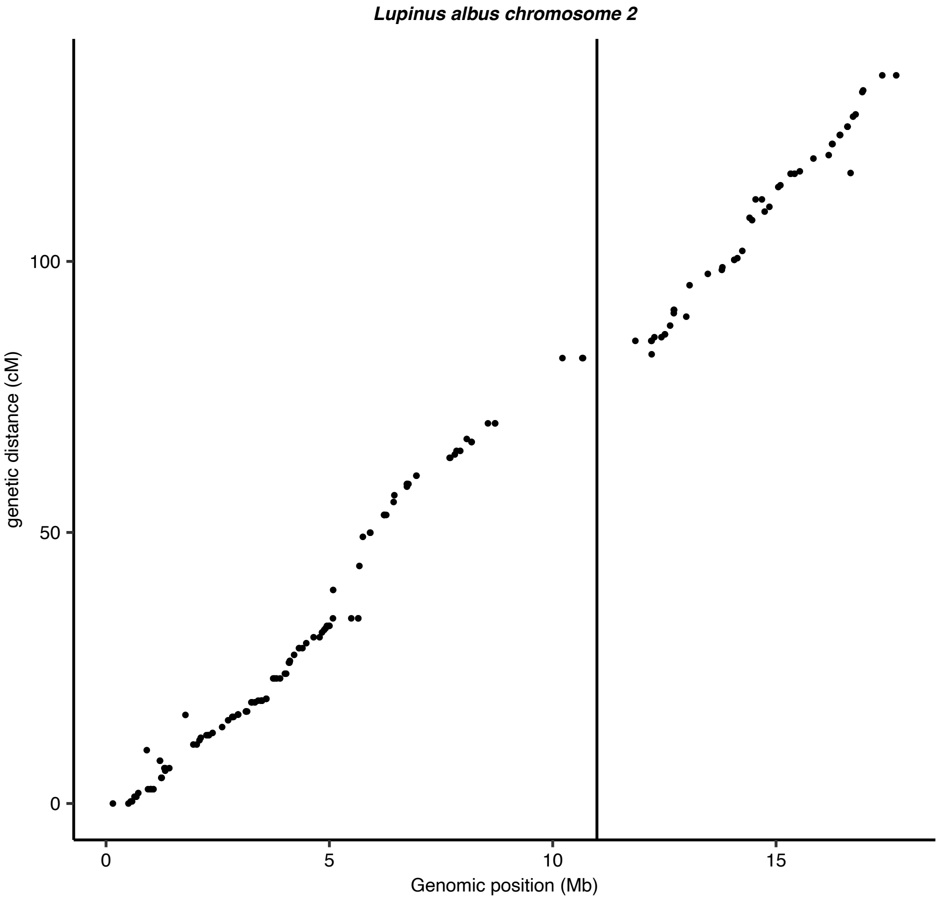
Lupinus albus chromosome 2
genetic distance (cM)
0
50
100
0
5
10
15
Genomic position (Mb)

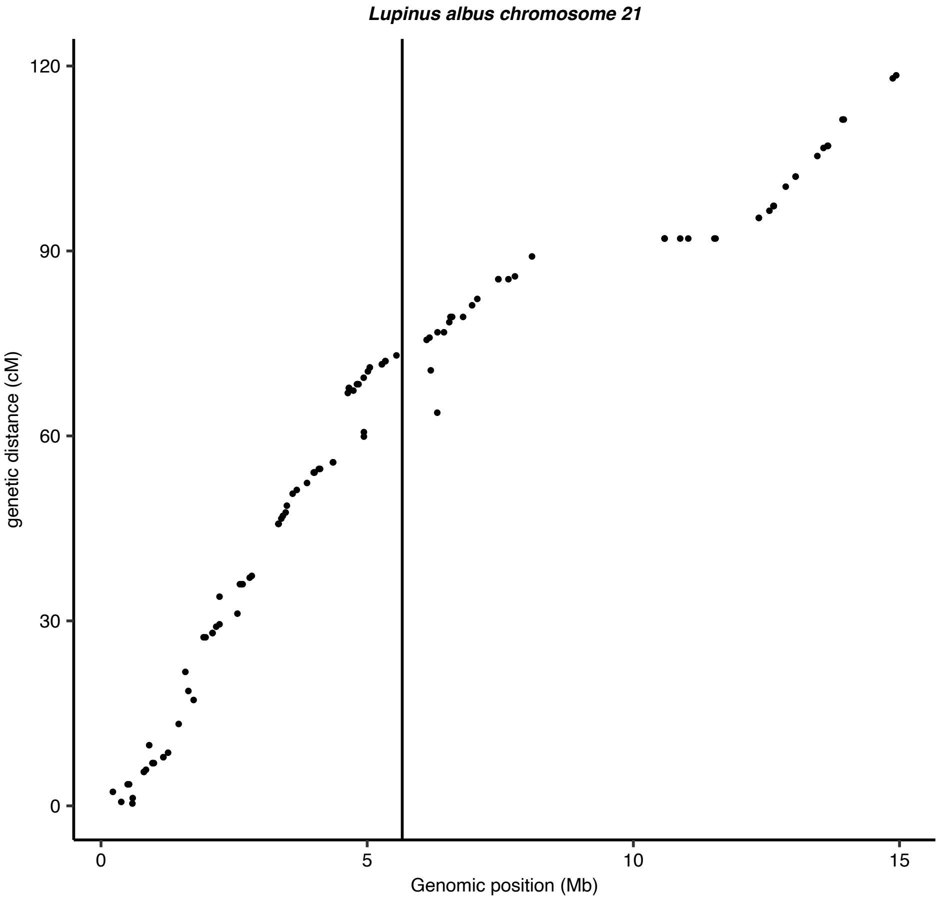
Lupinus albus chromosome 21
genetic distance (cM)
0
30
60
90
120
Genomic position (Mb)
0
5
10
15

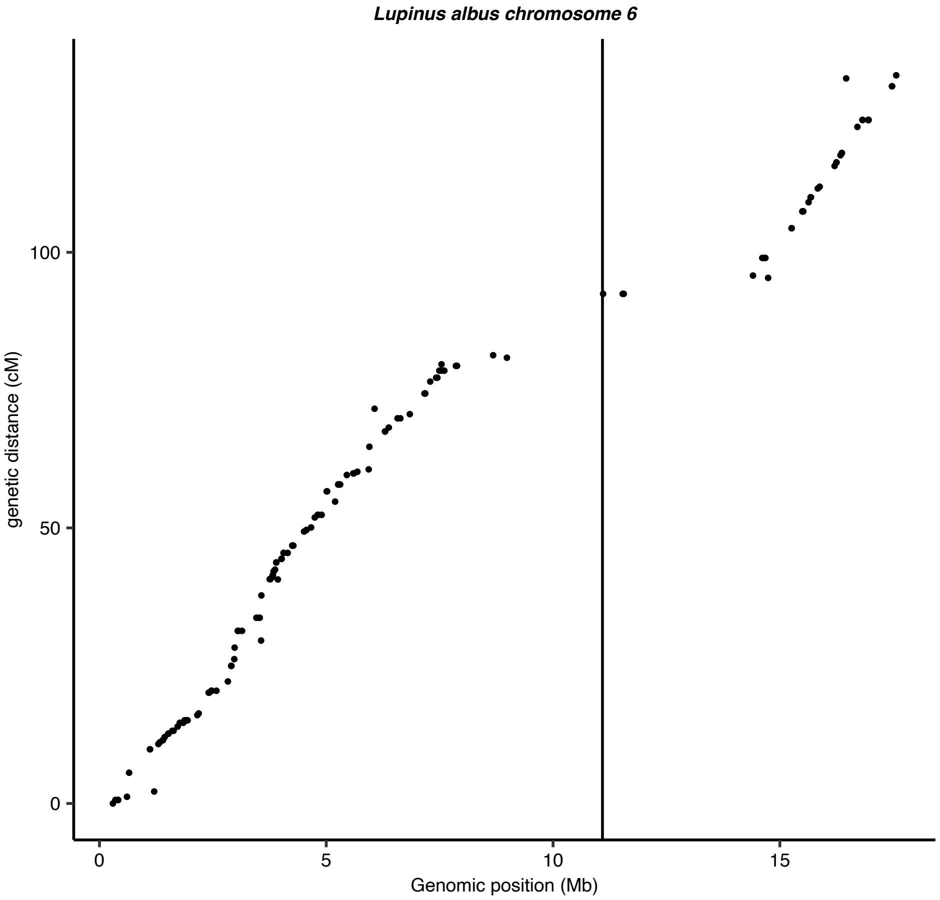
Lupinus albus chromosome 6
genetic distance (cM)
0
50
100
Genomic position (Mb)
0
5
10
15

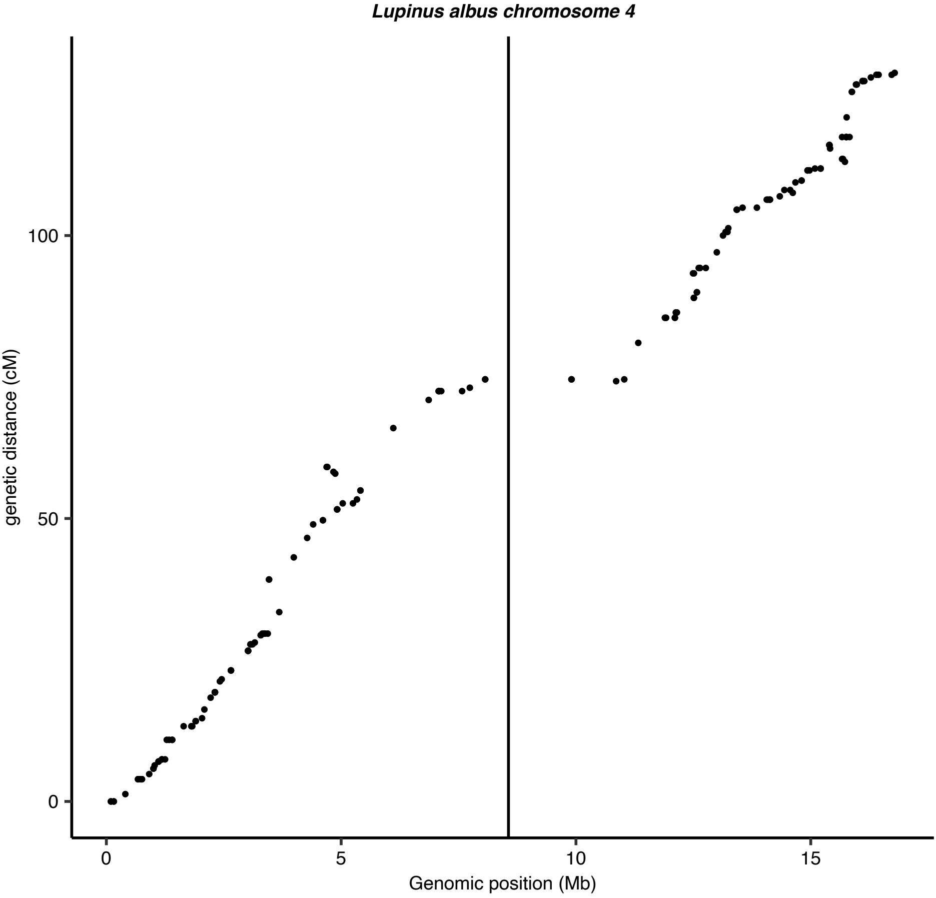
Lupinus albus chromosome 4
genetic distance (cM)
0
50
100
Genomic position (Mb)
0
5
10
15

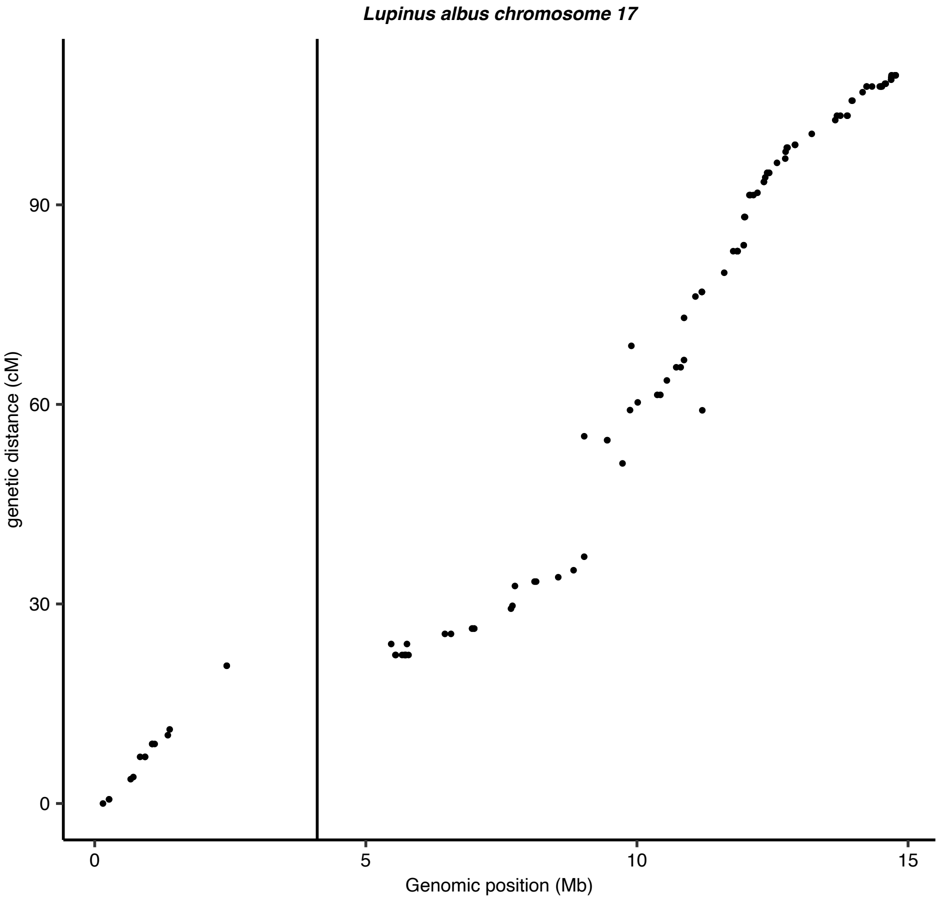
Lupinus albus chromosome 17
genetic distance (cM)
0
30
60
90
Genomic position (Mb)
0
5
10
15

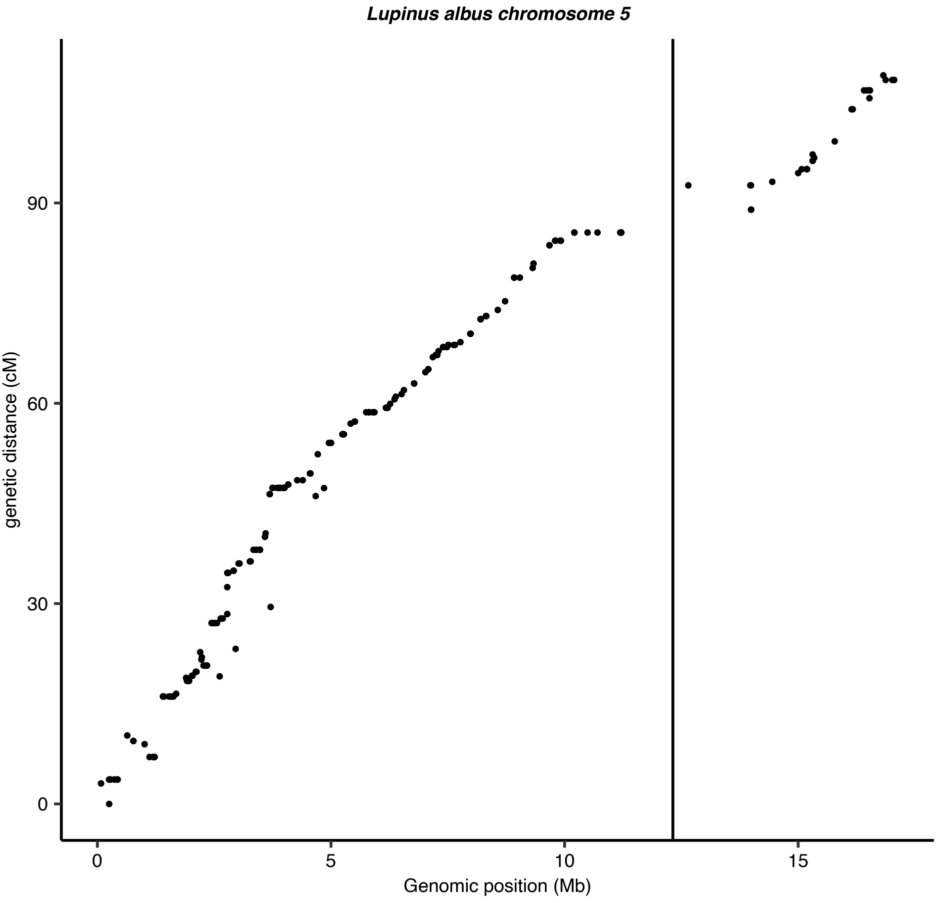
Lupinus albus chromosome 5
genetic distance (cM)
0
30
60
90
0
5
10
15
Genomic position (Mb)

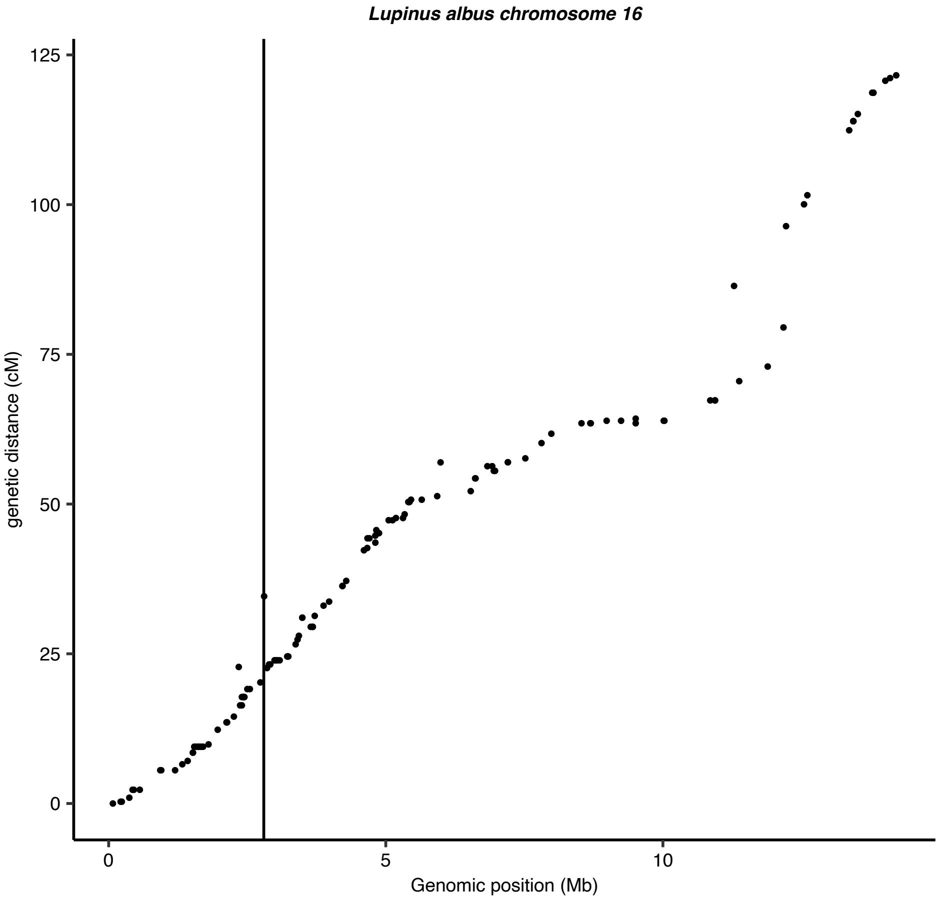
Lupinus albus chromosome 16
genetic distance (cM)
Genomic position (Mb)
0
25
50
75
100
125
0
5
10

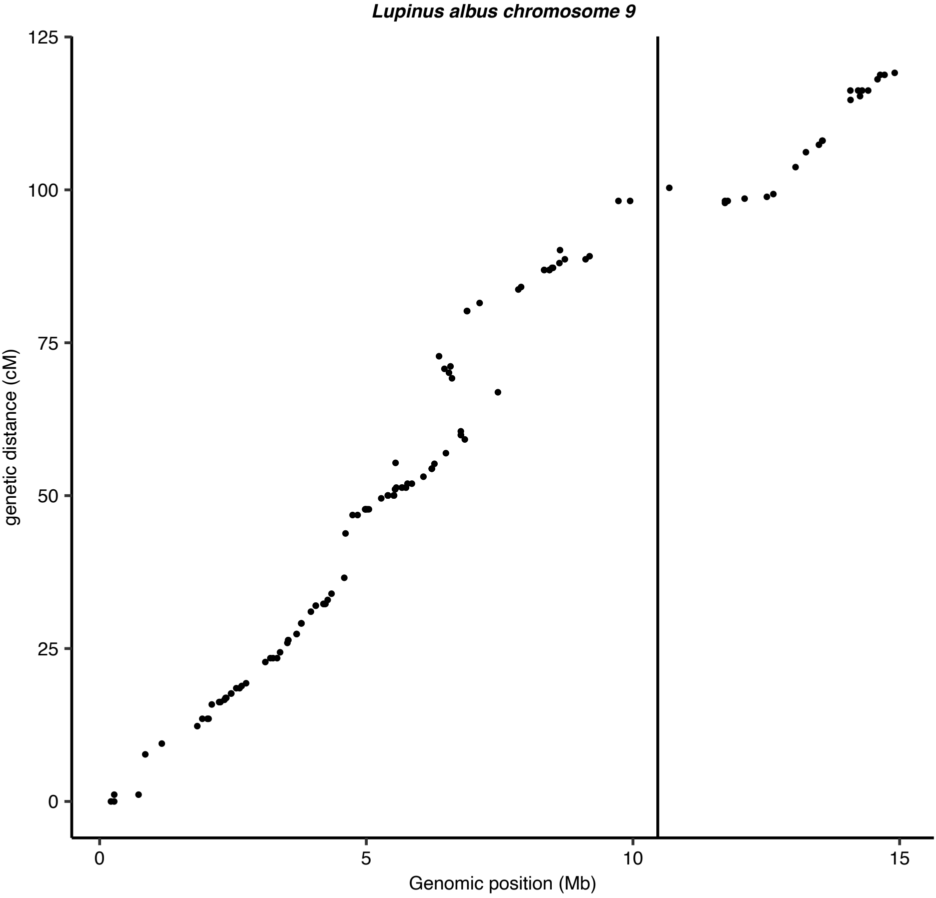
Lupinus albus chromosome 9
genetic distance (cM)
125
100
75
50
25
0
Genomic position (Mb)
0
5
10
15

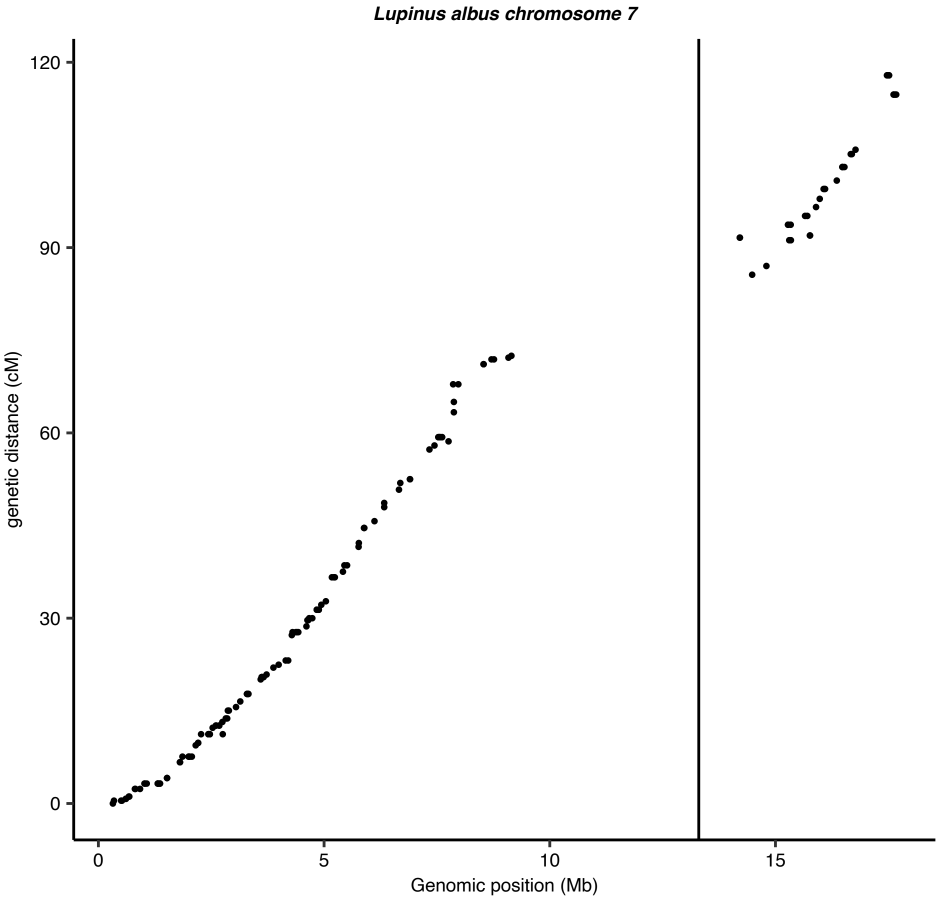
Lupinus albus chromosome 7
genetic distance (cM)
120
90
60
30
0
0
5
10
15
Genomic position (Mb)

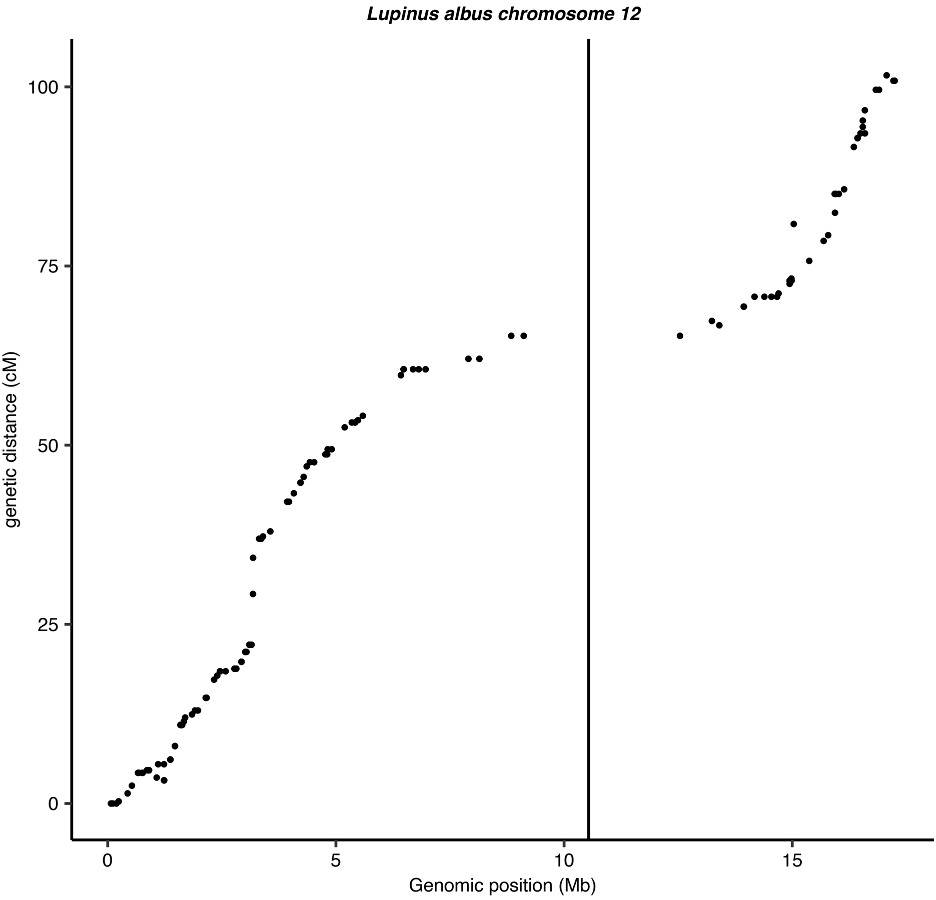
Lupinus albus chromosome 12
genetic distance (cM)
100
75
50
25
0
0
5
10
15
Genomic position (Mb)

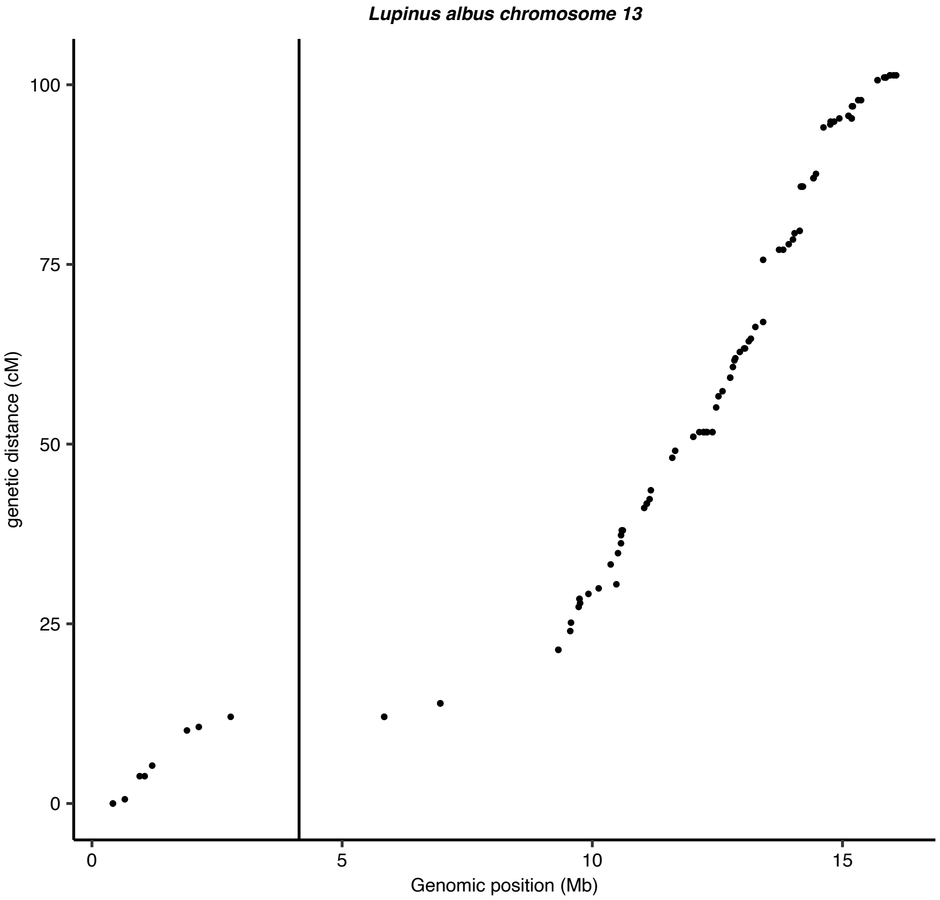
Lupinus albus chromosome 13
genetic distance (cM)
100
75
50
25
0
0
5
10
15
Genomic position (Mb)

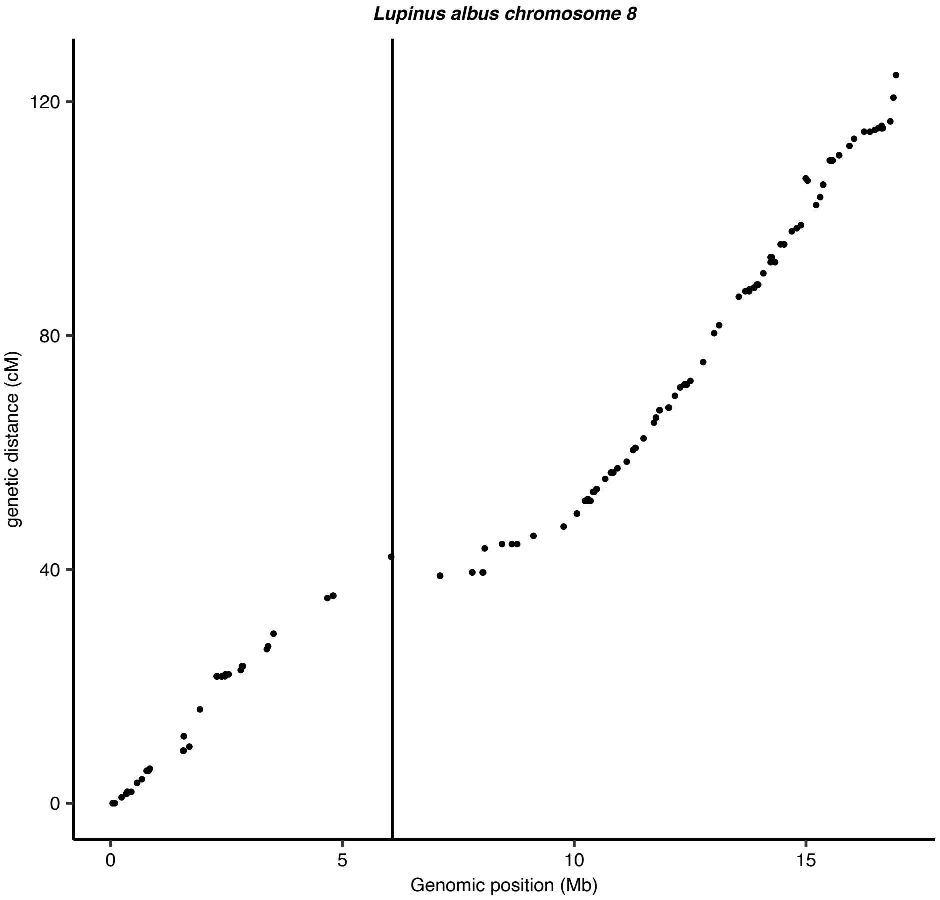

Lupinus albus chromosome 8
genetic distance (cM)
0
40
80
120
Genomic position (Mb)
0
5
10
15

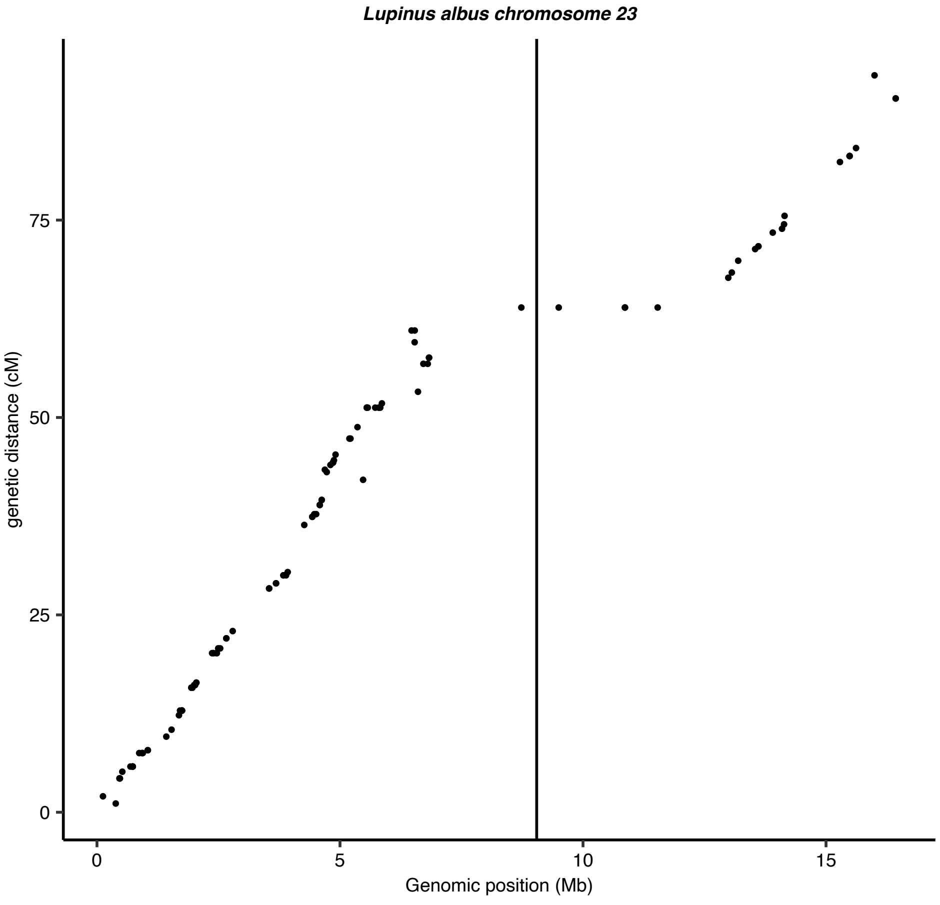
Lupinus albus chromosome 23
genetic distance (cM)
0
25
50
75
0
5
10
15
Genomic position (Mb)

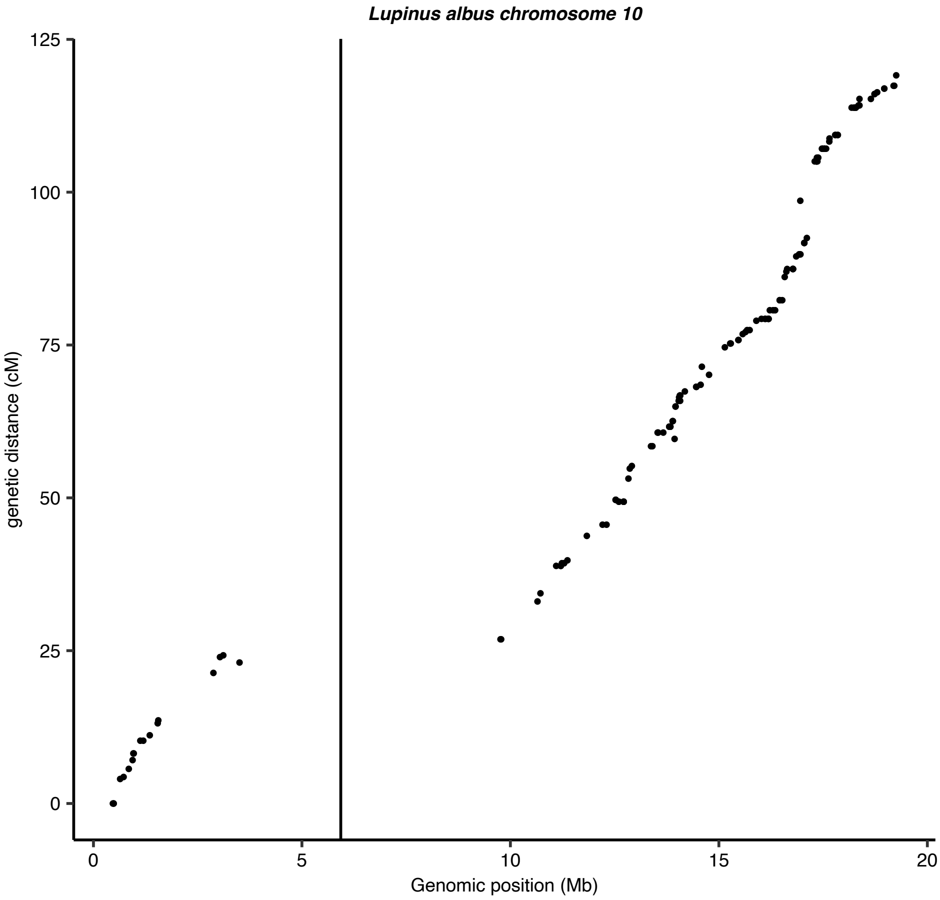
Lupinus albus chromosome 10
genetic distance (cM)
125
100
75
50
25
0
Genomic position (Mb)
0
5
10
15
20

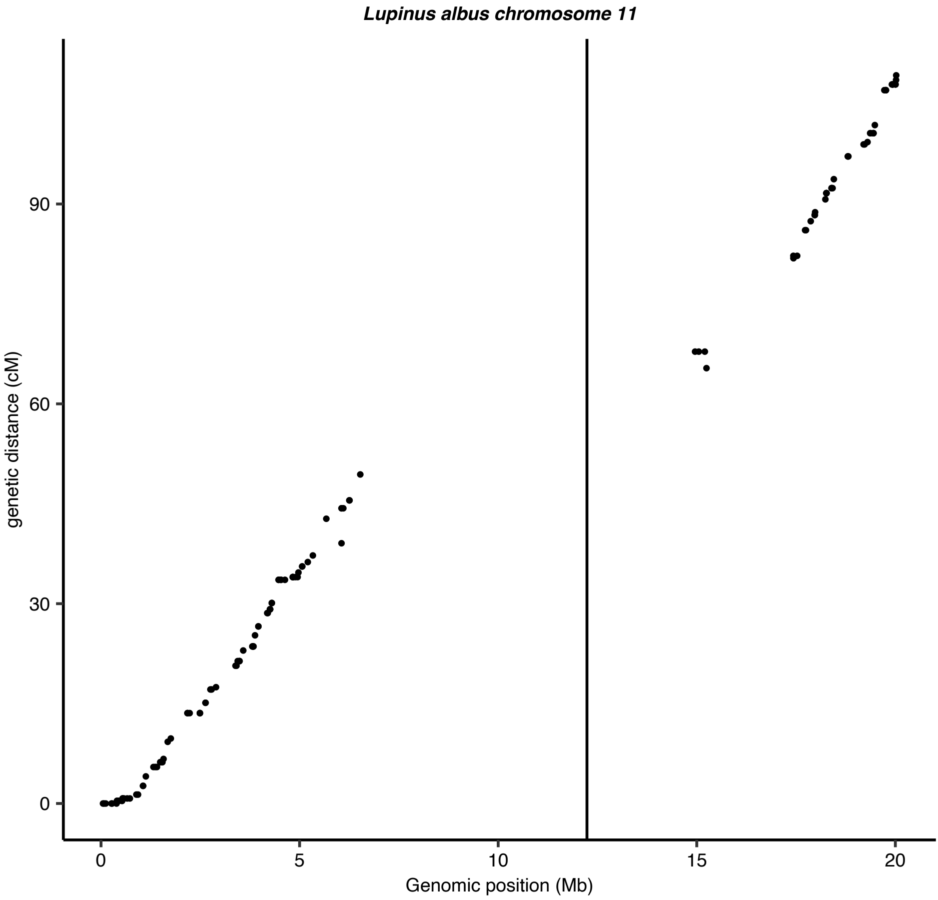
Lupinus albus chromosome 11
genetic distance (cM)
0
30
60
90
Genomic position (Mb)
0
5
10
15
20

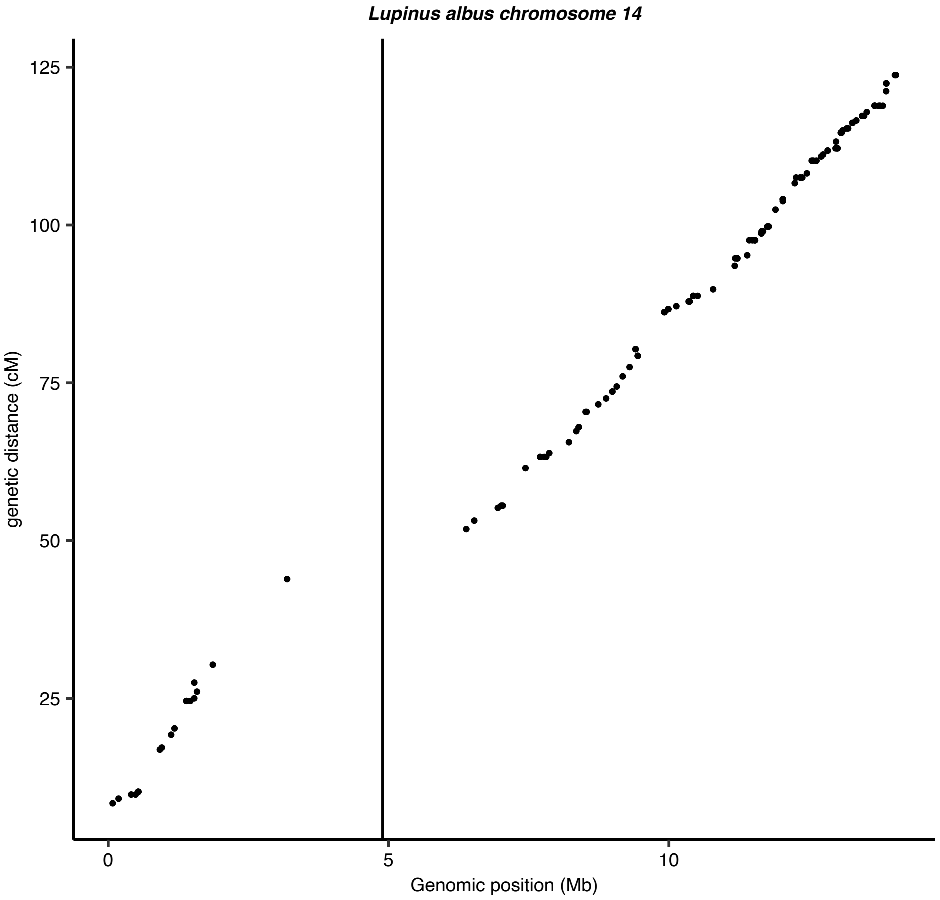
Lupinus albus chromosome 14
genetic distance (cM)
125
100
75
50
25
0
5
10
Genomic position (Mb)

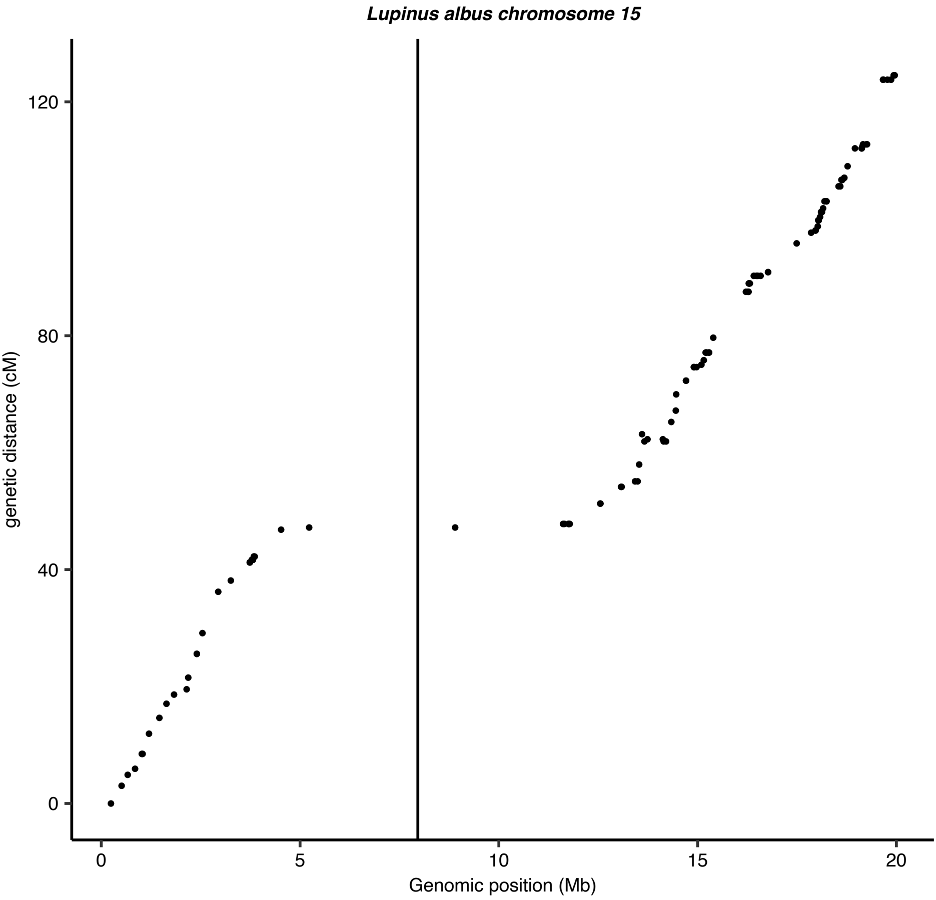
Lupinus albus chromosome 15
genetic distance (cM)
0
40
80
120
Genomic position (Mb)
0
5
10
15
20

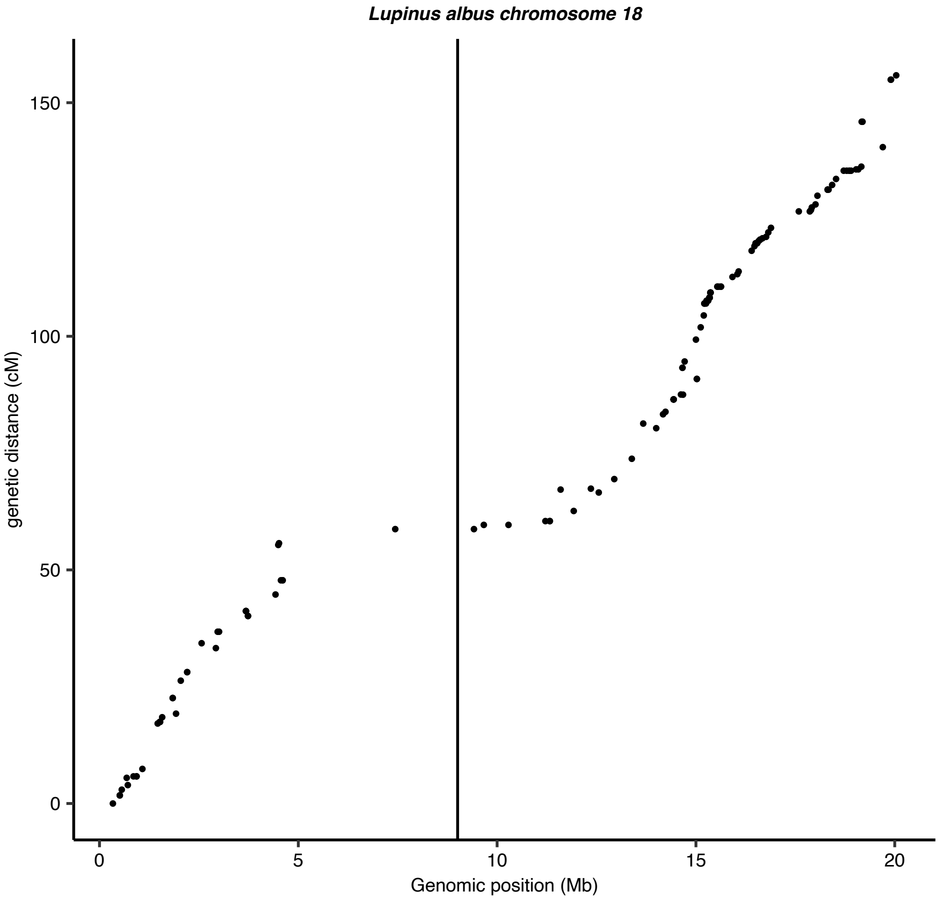

Lupinus albus chromosome 18
genetic distance (cM)
0
50
100
150
0
5
10
15
20
Genomic position (Mb)

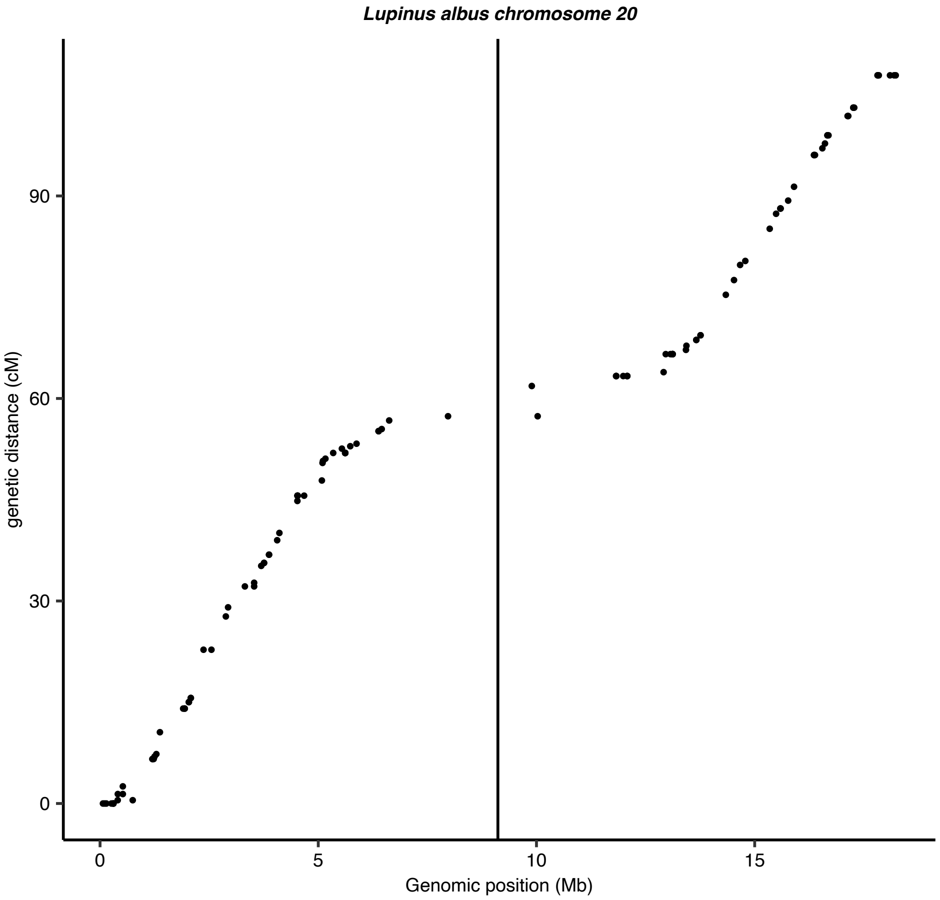
Lupinus albus chromosome 20
genetic distance (cM)
0
30
60
90
Genomic position (Mb)
0
5
10
15

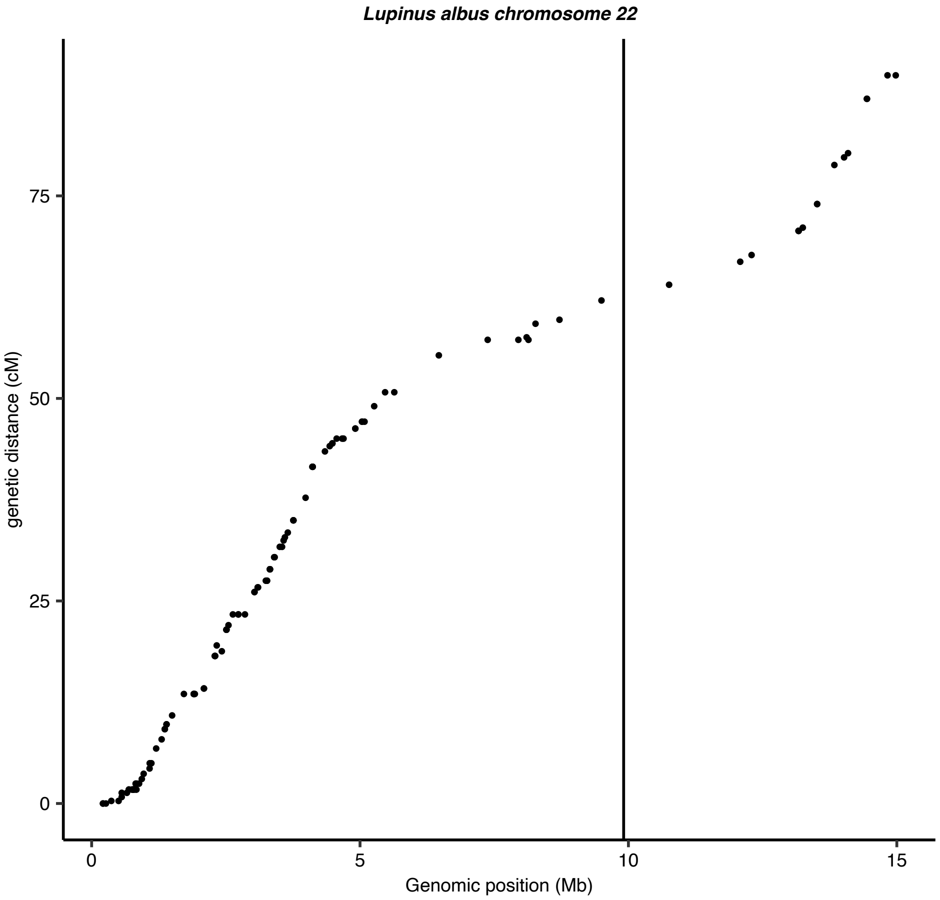
Lupinus albus chromosome 22
genetic distance (cM)
0
25
50
75
Genomic position (Mb)
0
5
10
15

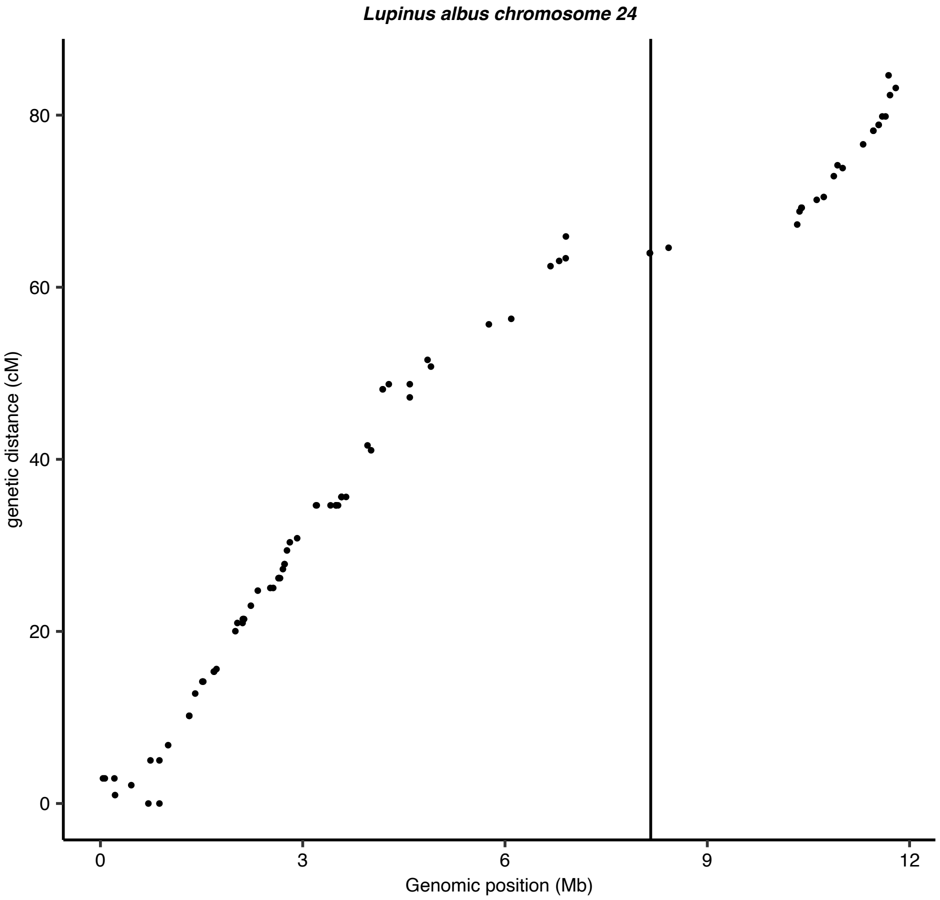
Lupinus albus chromosome 24
genetic distance (cM)
0
20
40
60
80
Genomic position (Mb)
0
3
6
9
12

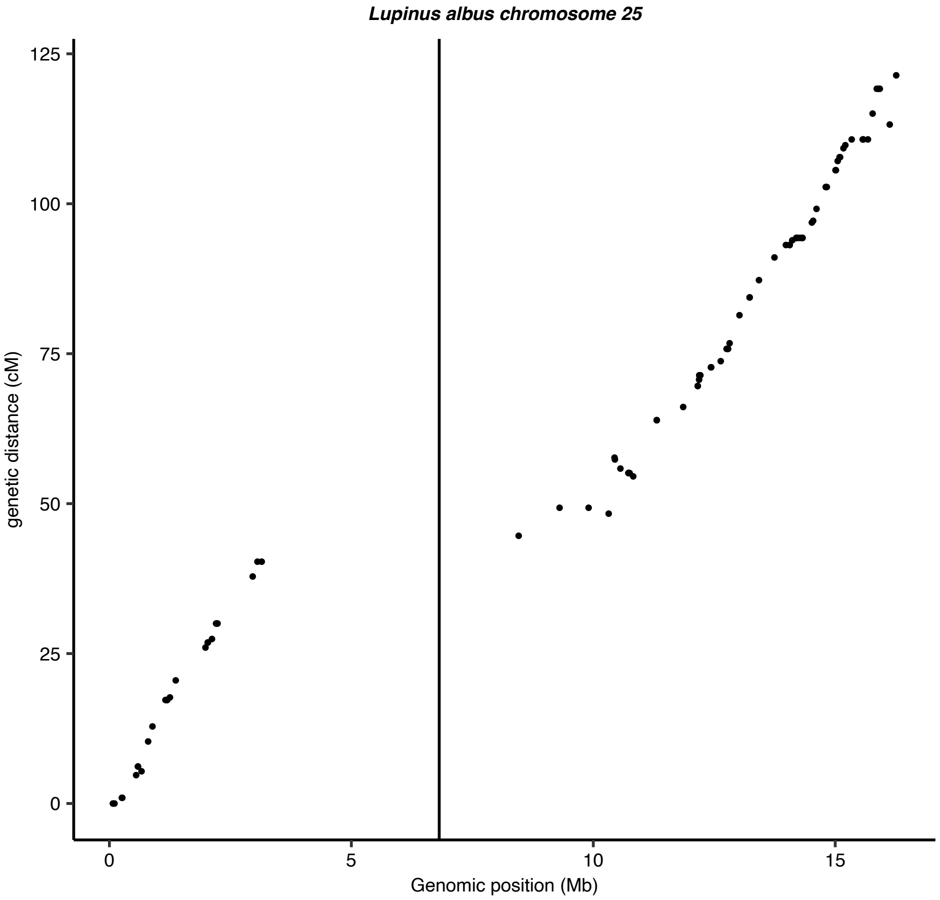
Lupinus albus chromosome 25
125
100
75
50
25
0
genetic distance (cM)
0
5
10
15
Genomic position (Mb)

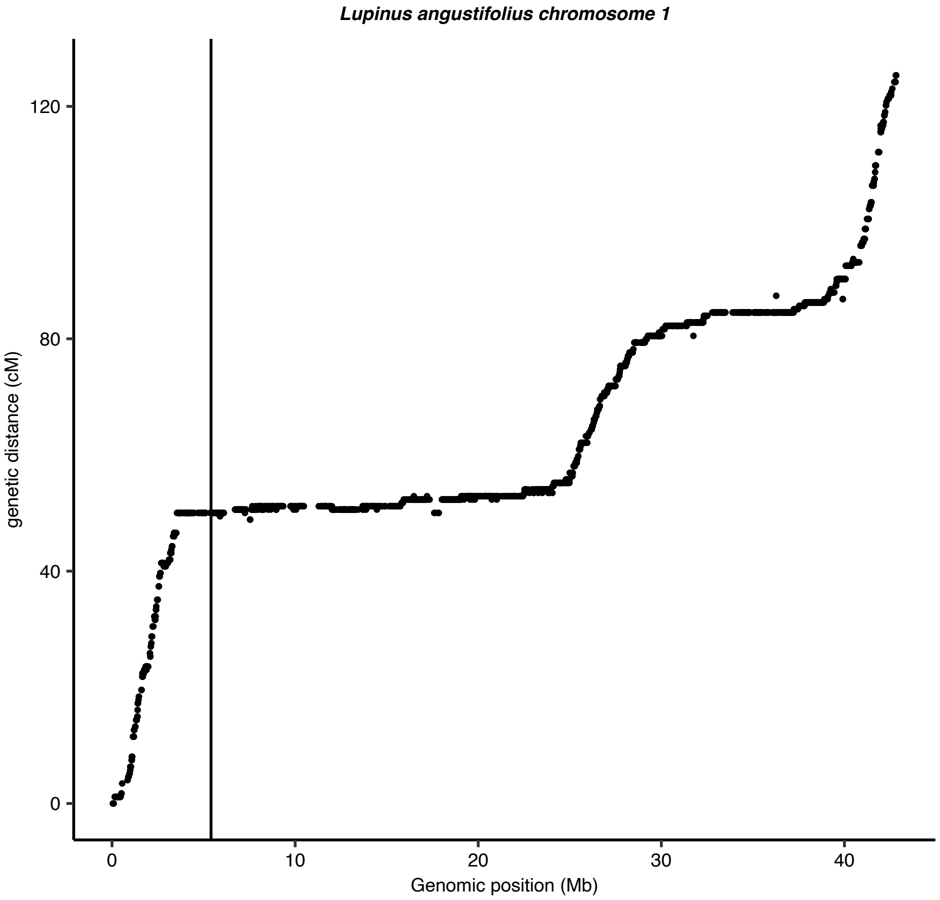
Lupinus angustifolius chromosome 1
genetic distance (cM)
0
40
80
120
Genomic position (Mb)
0
10
20
30
40

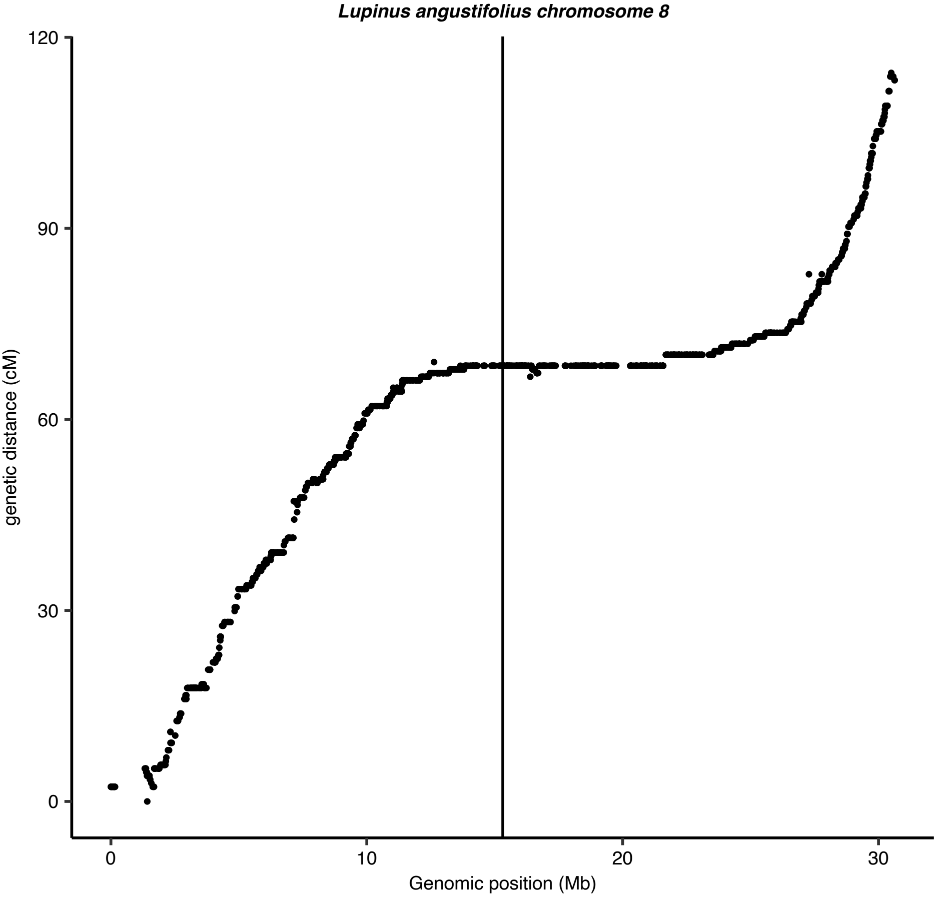
Lupinus angustifolius chromosome 8
120
90
60
30
0
genetic distance (cM)
0
10
20
30
Genomic position (Mb)

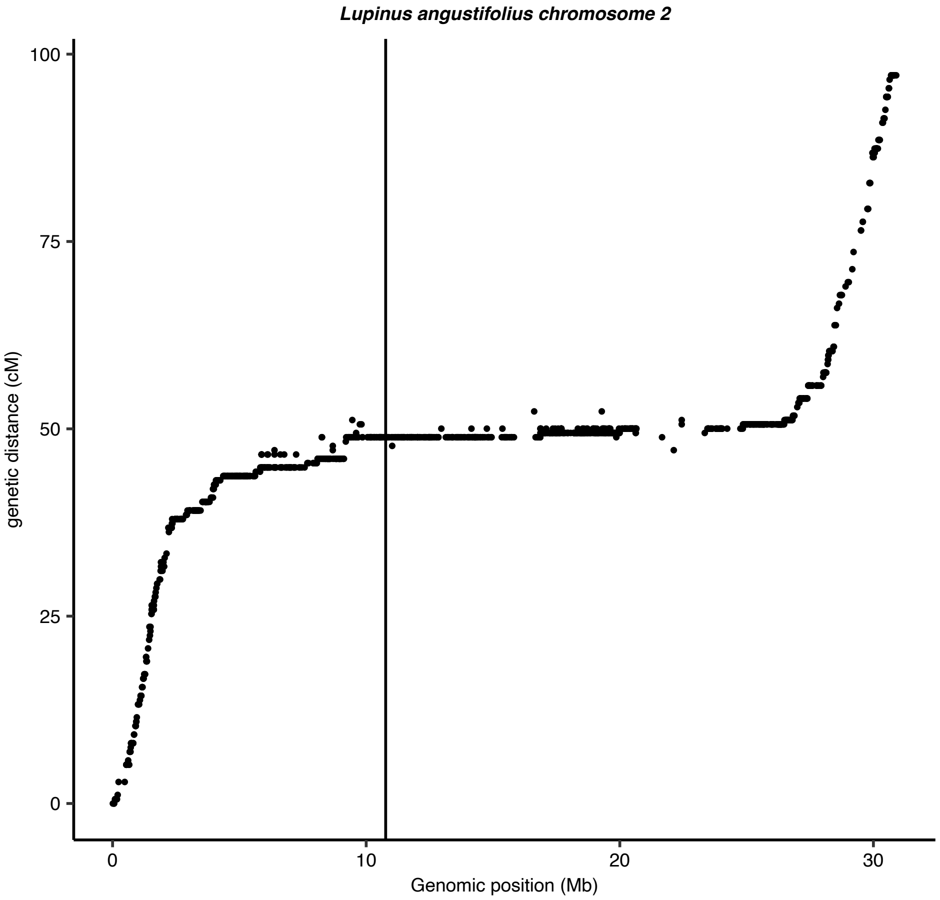
Lupinus angustifolius chromosome 2
genetic distance (cM)
Genomic position (Mb)
0
25
50
75
100
0
10
20
30

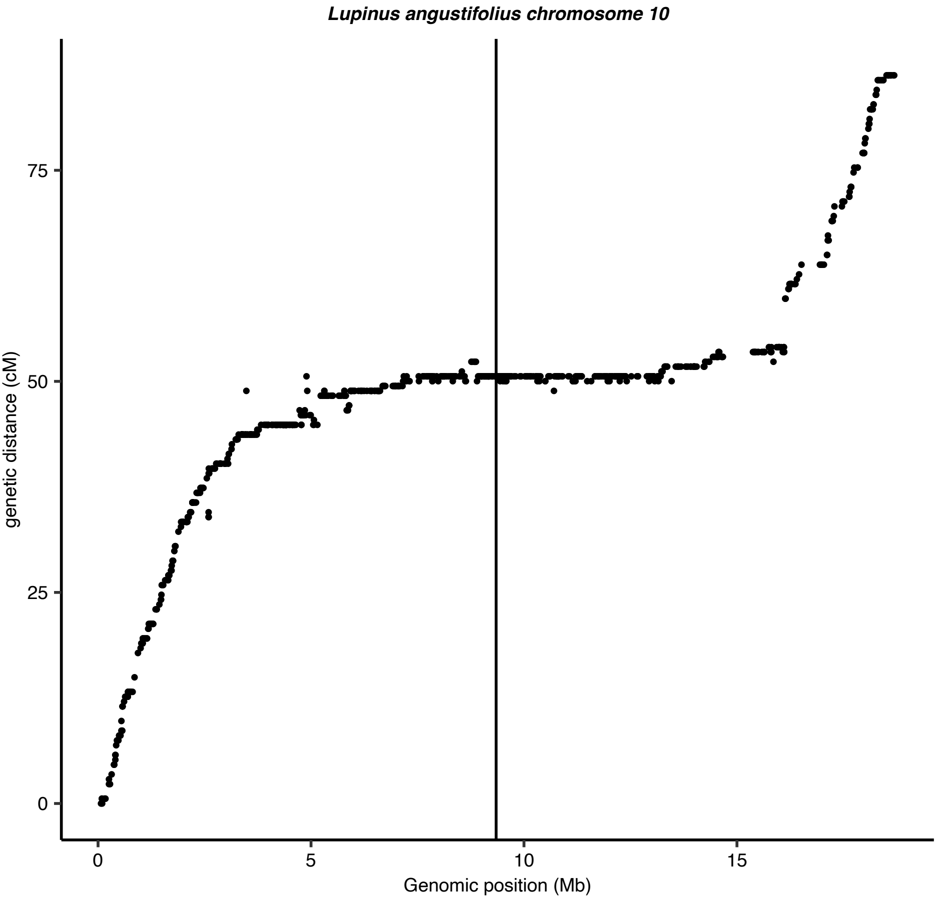
Lupinus angustifolius chromosome 10
genetic distance (cM)
0
25
50
75
Genomic position (Mb)
0
5
10
15

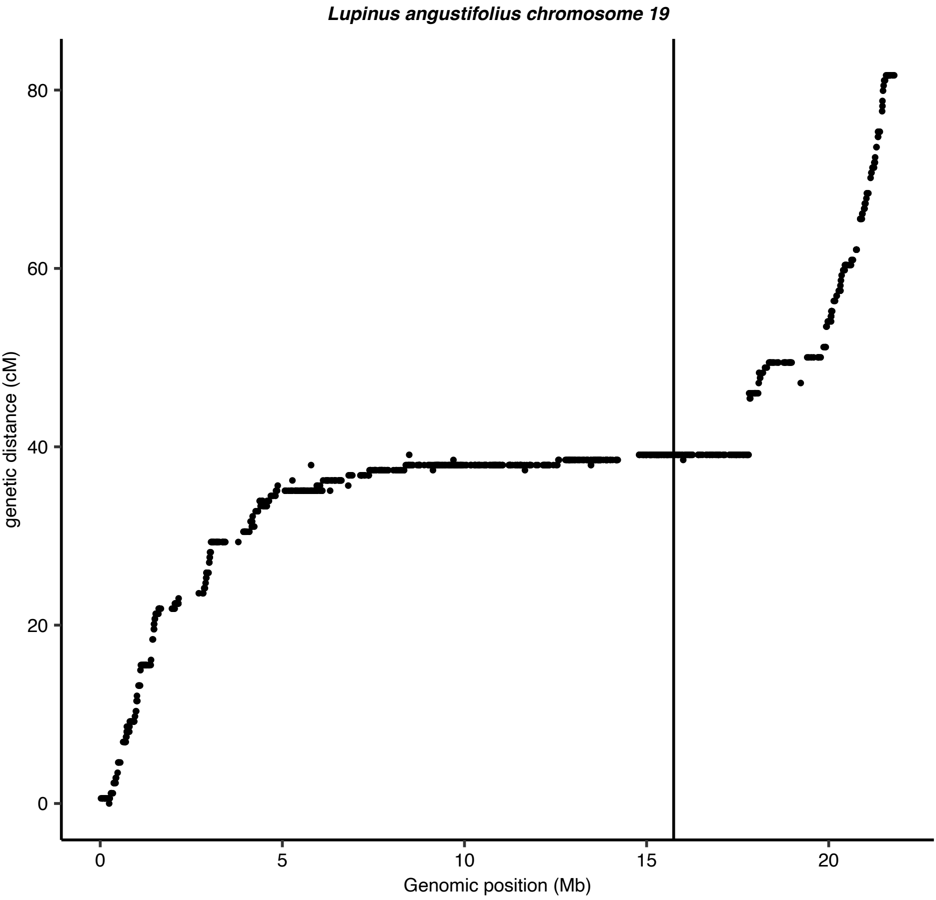
Lupinus angustifolius chromosome 19
genetic distance (cM)
0
20
40
60
80
0
5
10
15
20
Genomic position (Mb)

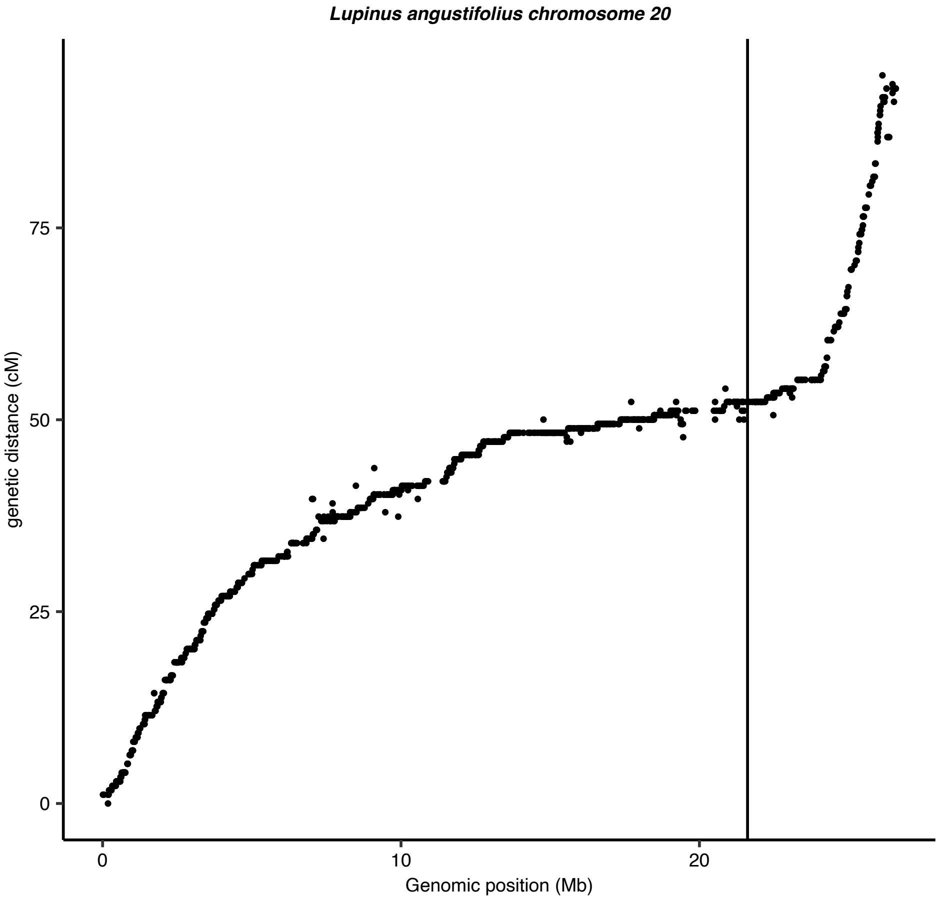
Lupinus angustifolius chromosome 20
genetic distance (cM)
0
25
50
75
Genomic position (Mb)
0
10
20

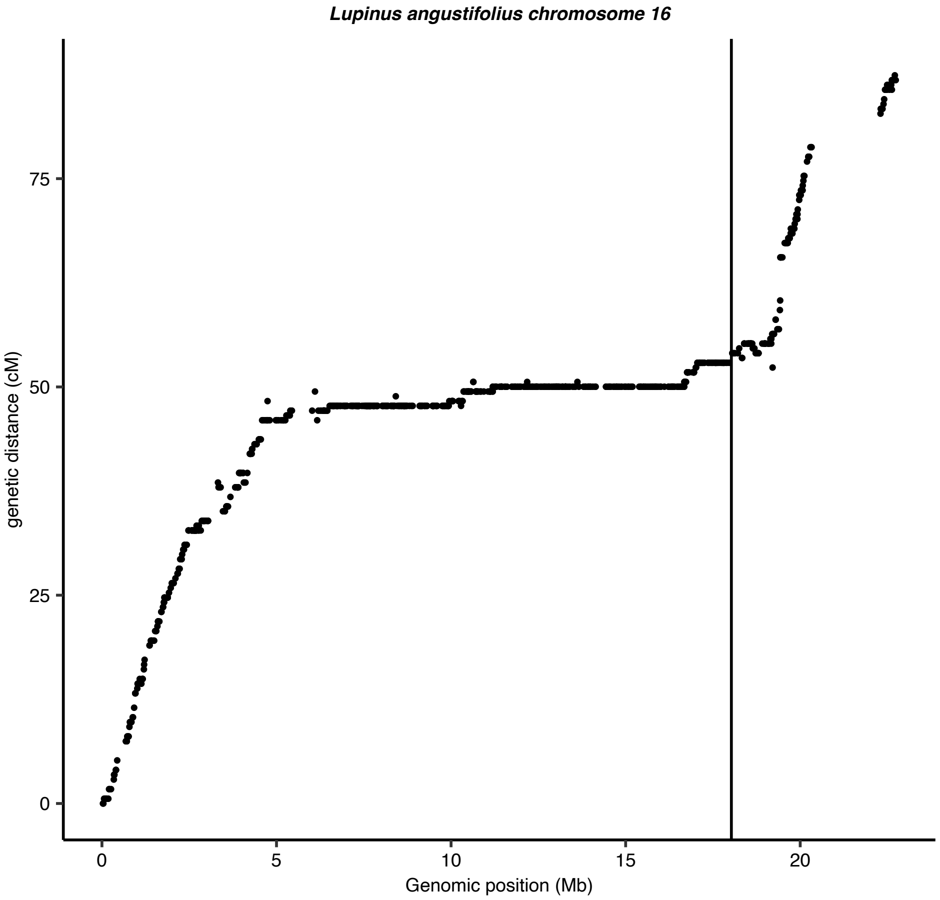
Lupinus angustifolius chromosome 16
genetic distance (cM)
0
25
50
75
Genomic position (Mb)
0
5
10
15
20

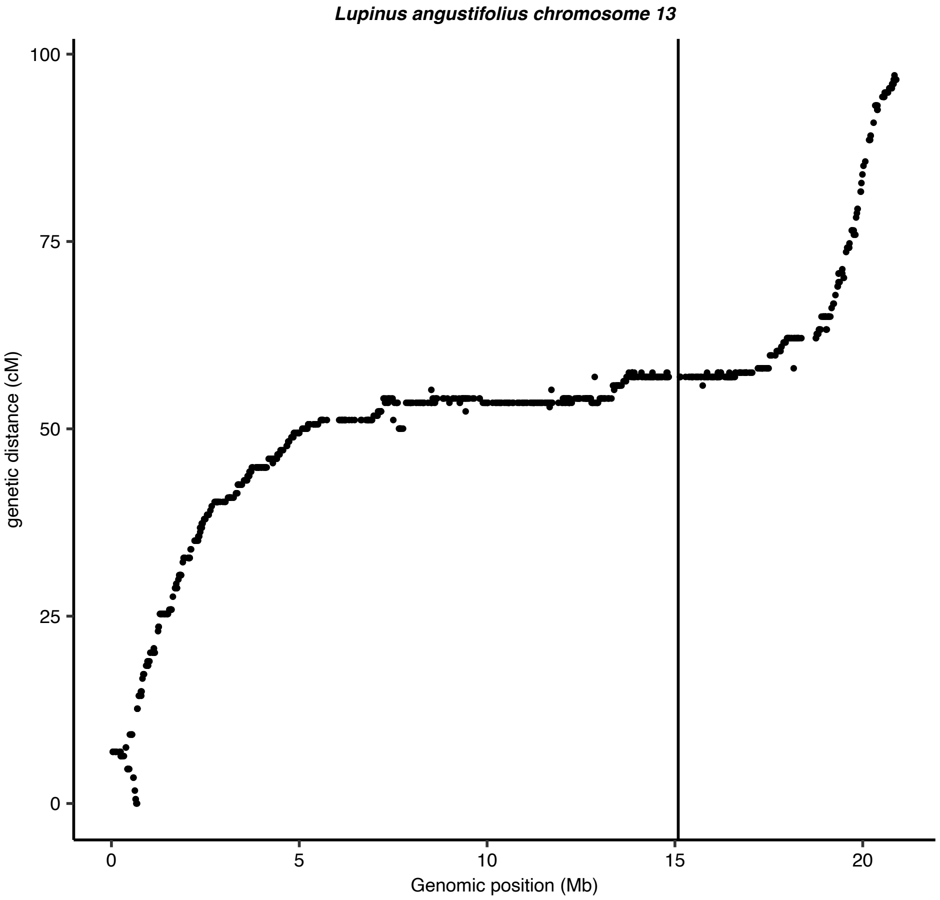
Lupinus angustifolius chromosome 13
genetic distance (cM)
Genomic position (Mb)
0
25
50
75
100
0
5
10
15
20

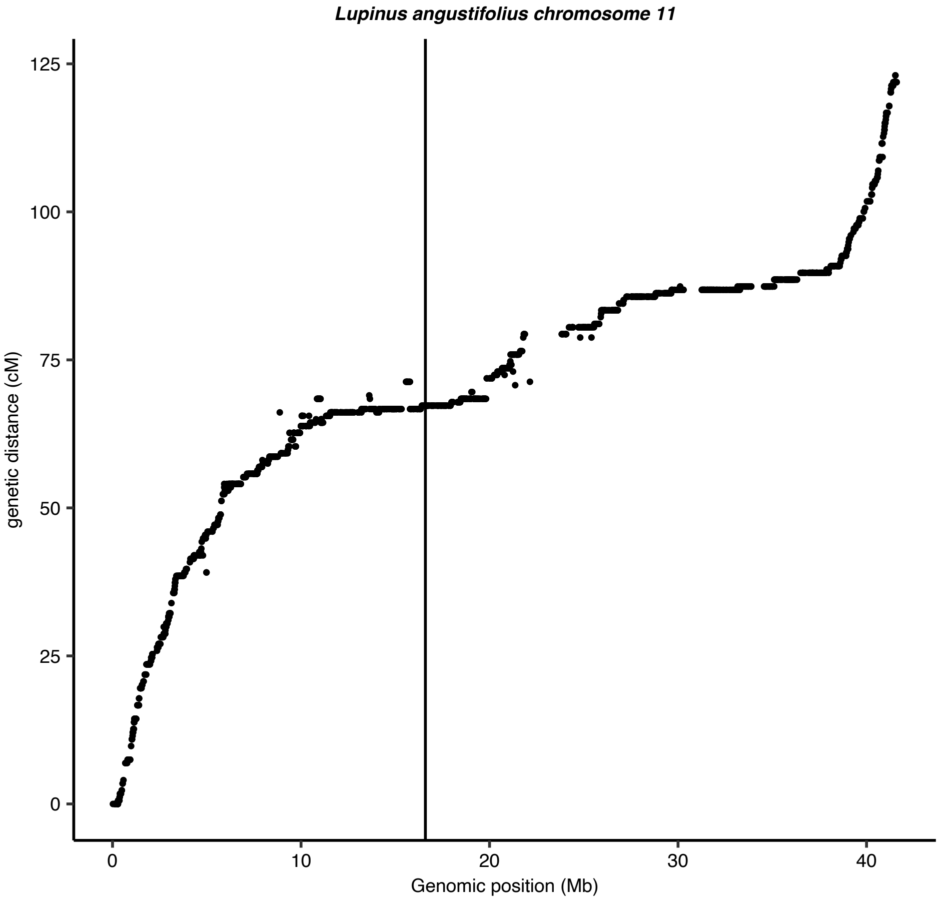
Lupinus angustifolius chromosome 11
125
100
75
50
25
0
genetic distance (cM)
0
10
20
30
40
Genomic position (Mb)

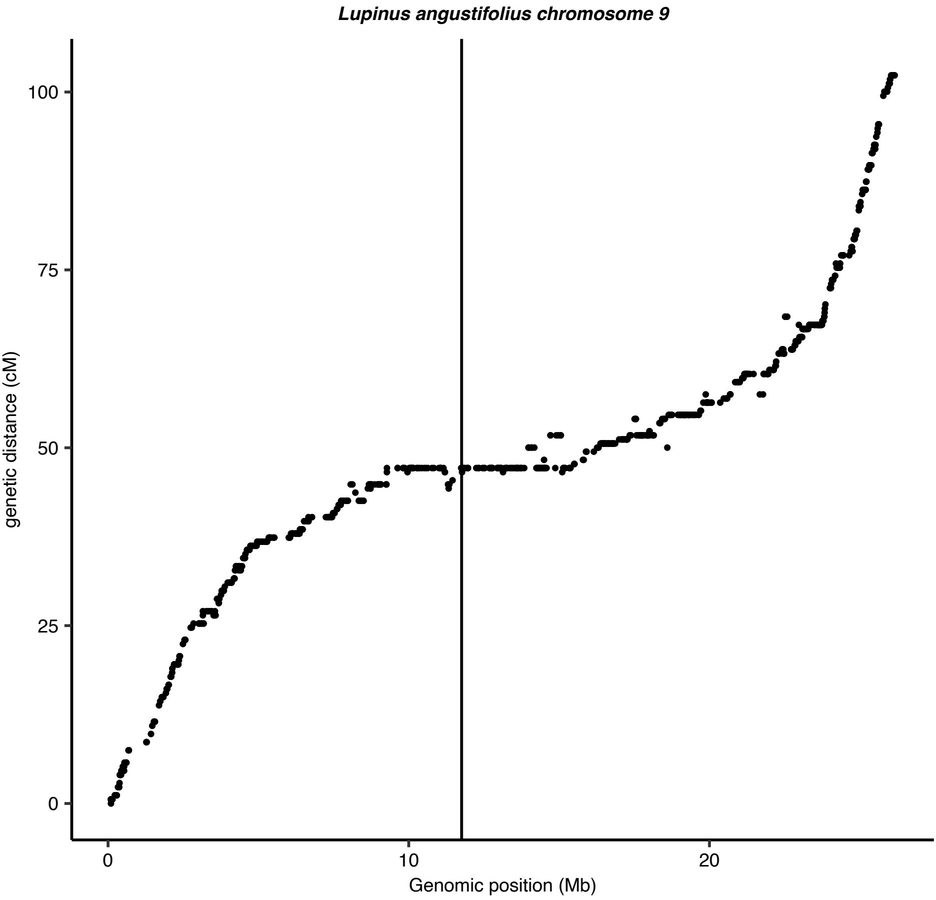
Lupinus angustifolius chromosome 9
genetic distance (cM)
100
75
50
25
0
0
10
20
Genomic position (Mb)

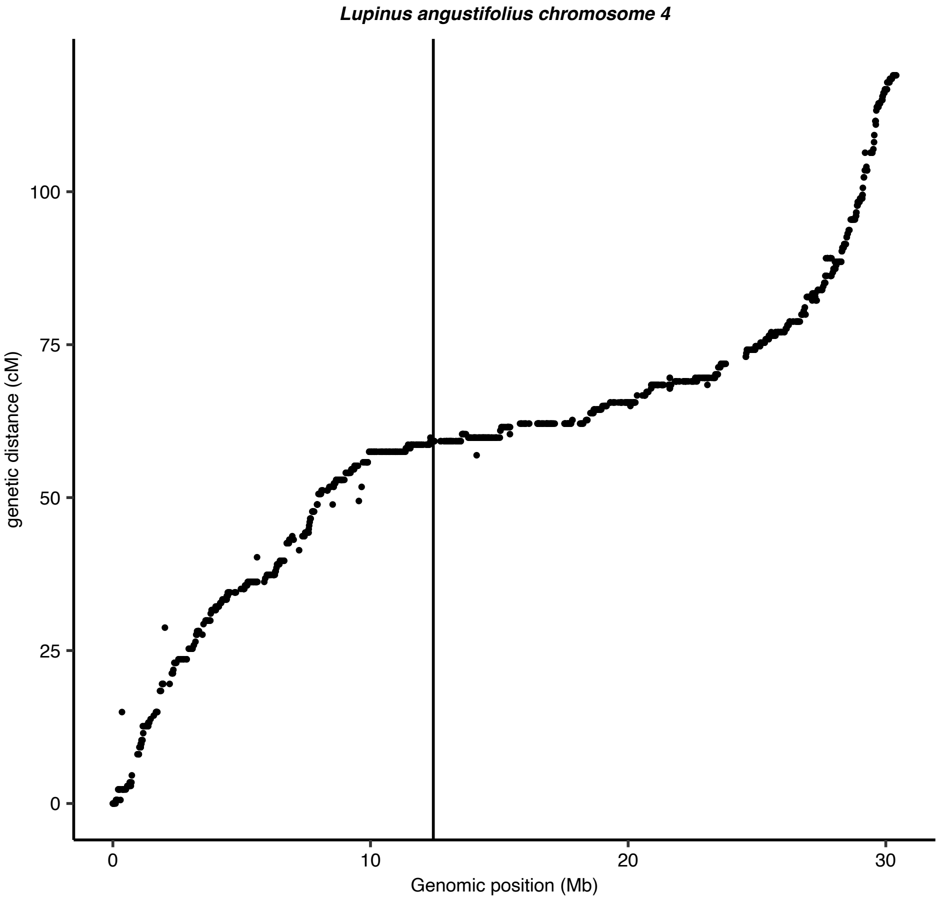
Lupinus angustifolius chromosome 4
genetic distance (cM)
0
25
50
75
100
Genomic position (Mb)
0
10
20
30

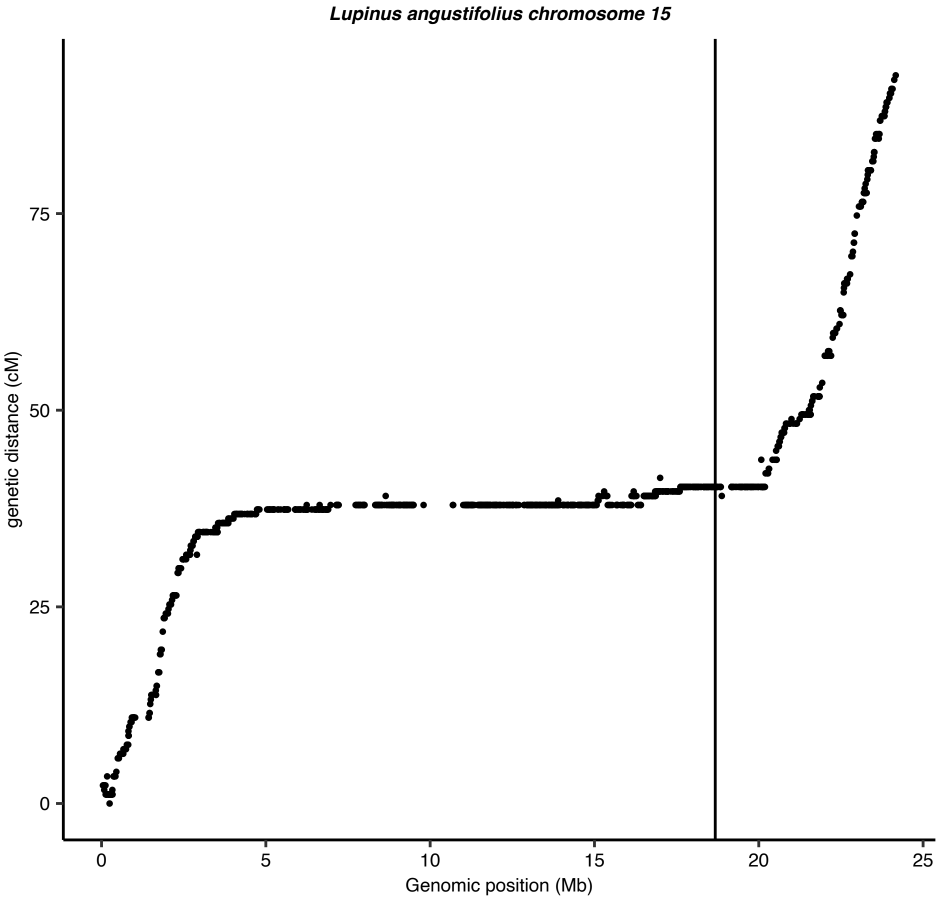
Lupinus angustifolius chromosome 15
genetic distance (cM)
0
25
50
75
Genomic position (Mb)
0
5
10
15
20
25

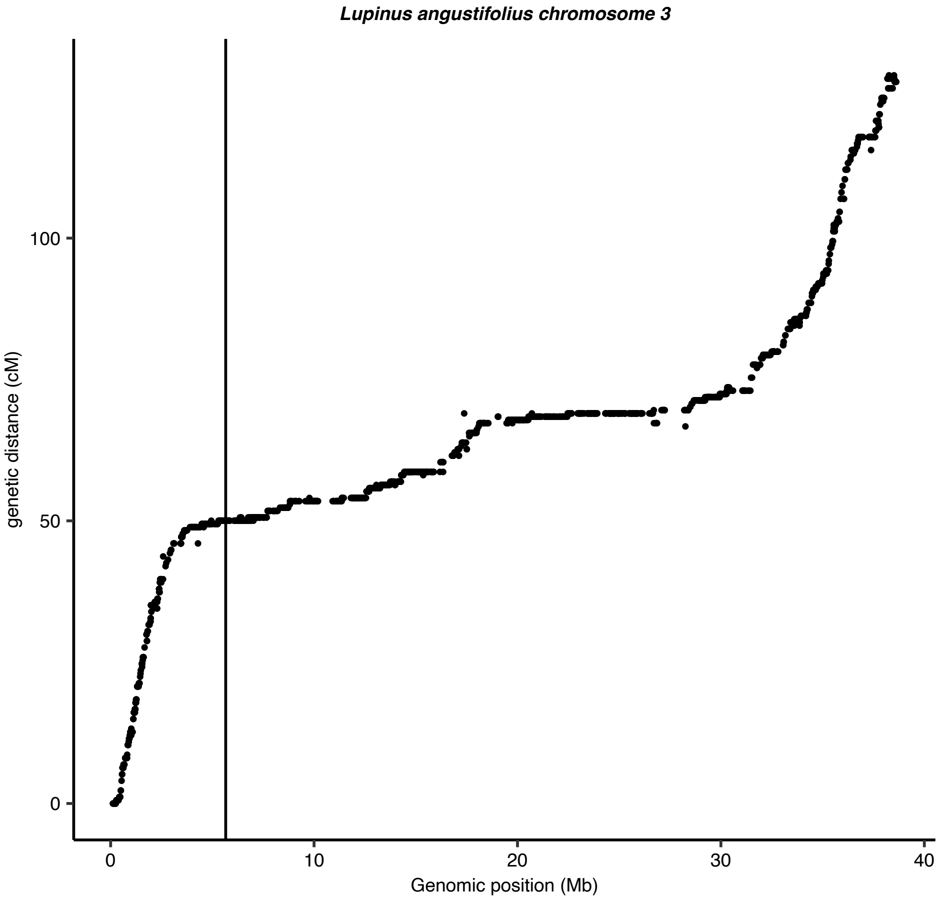
Lupinus angustifolius chromosome 3
genetic distance (cM)
0
50
100
Genomic position (Mb)
0
10
20
30
40

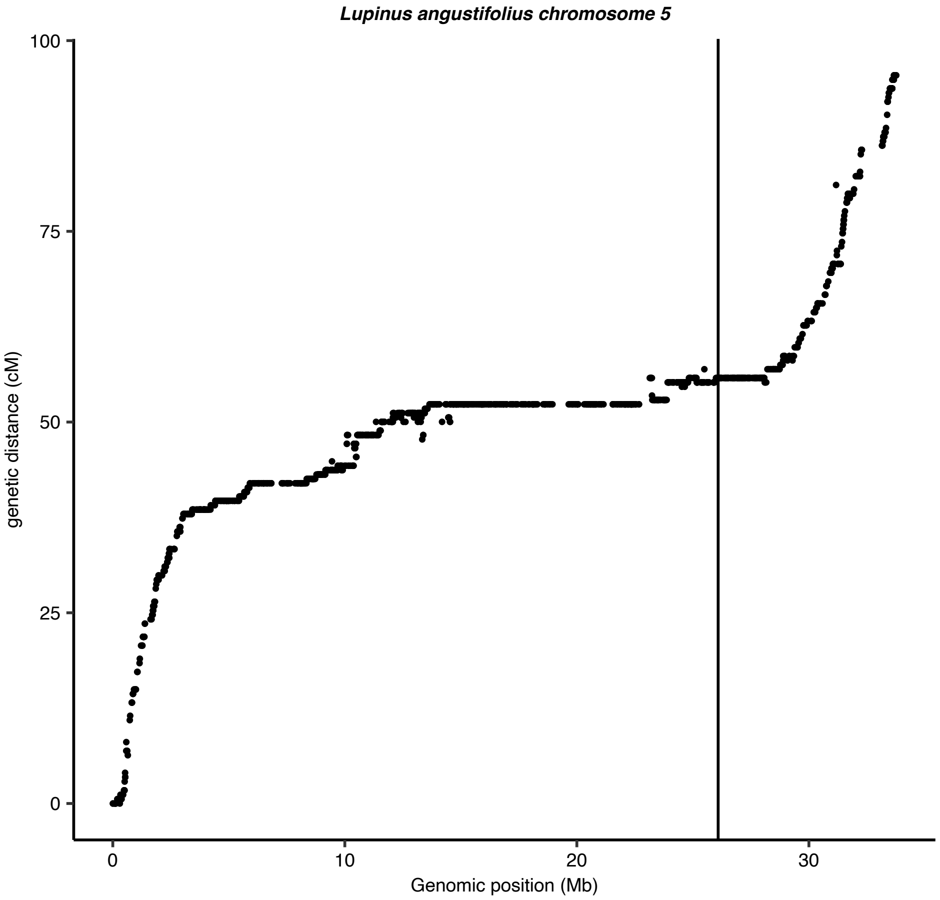
Lupinus angustifolius chromosome 5
100
75
50
25
0
genetic distance (cM)
0
10
20
30
Genomic position (Mb)

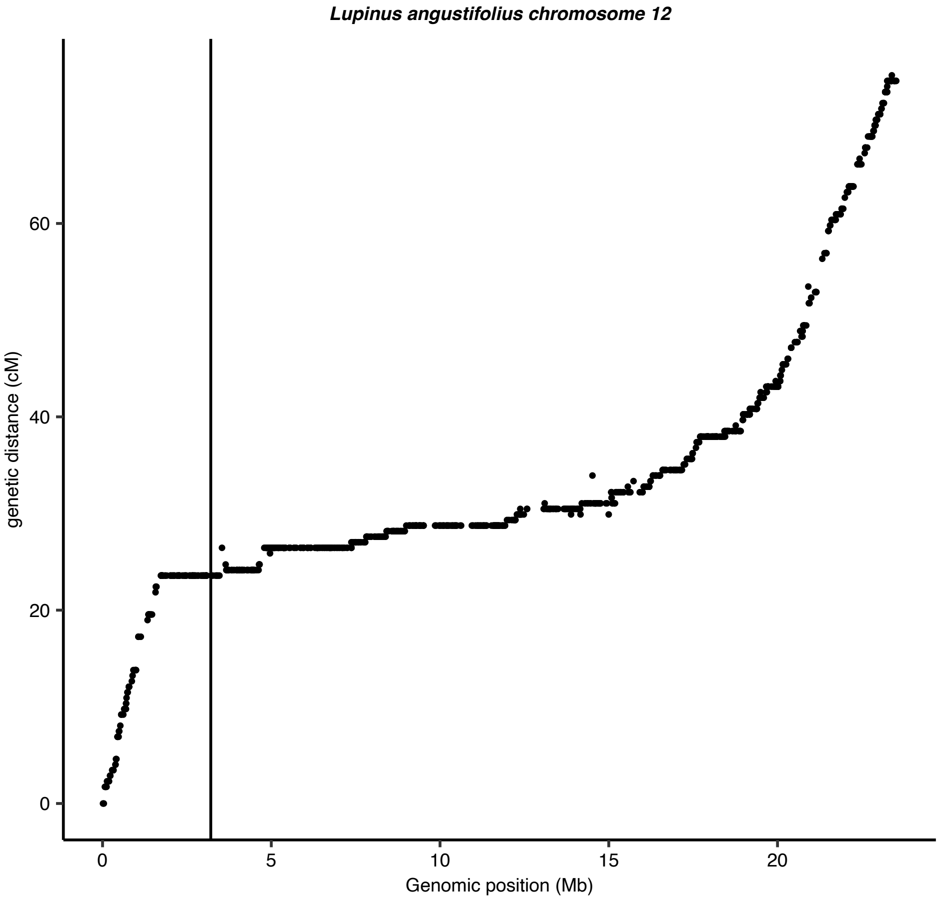
Lupinus angustifolius chromosome 12
genetic distance (cM)
0
20
40
60
Genomic position (Mb)
0
5
10
15
20

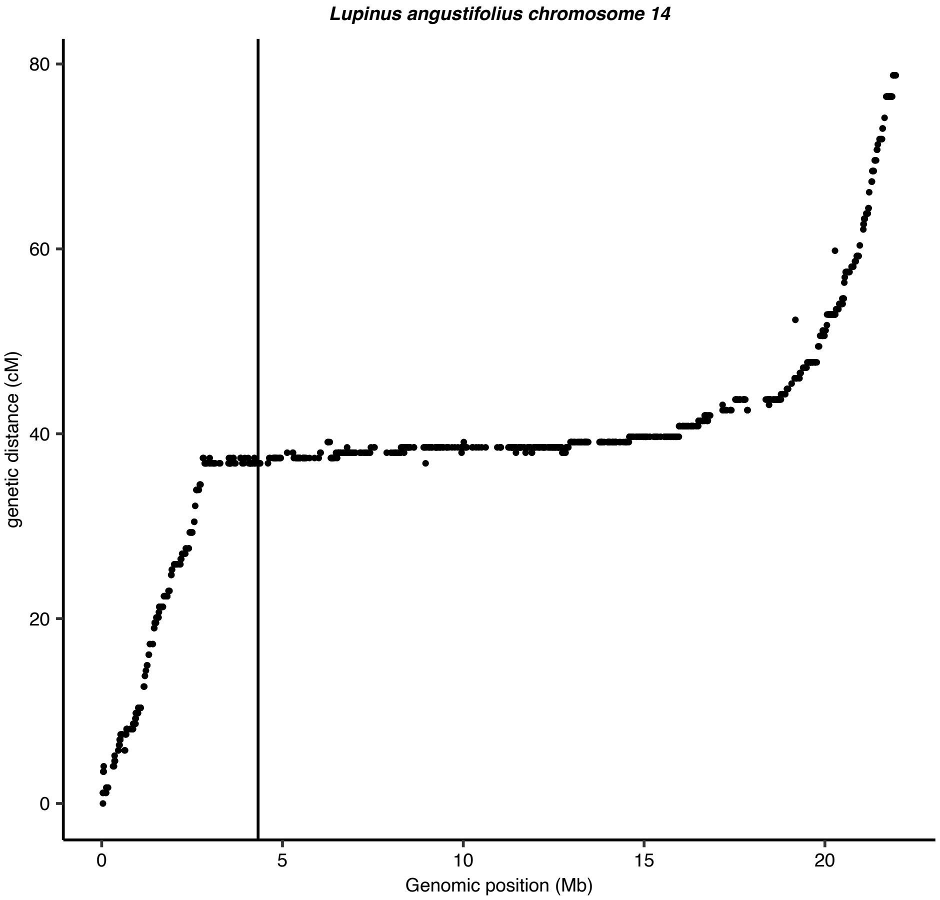
Lupinus angustifolius chromosome 14
genetic distance (cM)
0
20
40
60
80
Genomic position (Mb)
0
5
10
15
20

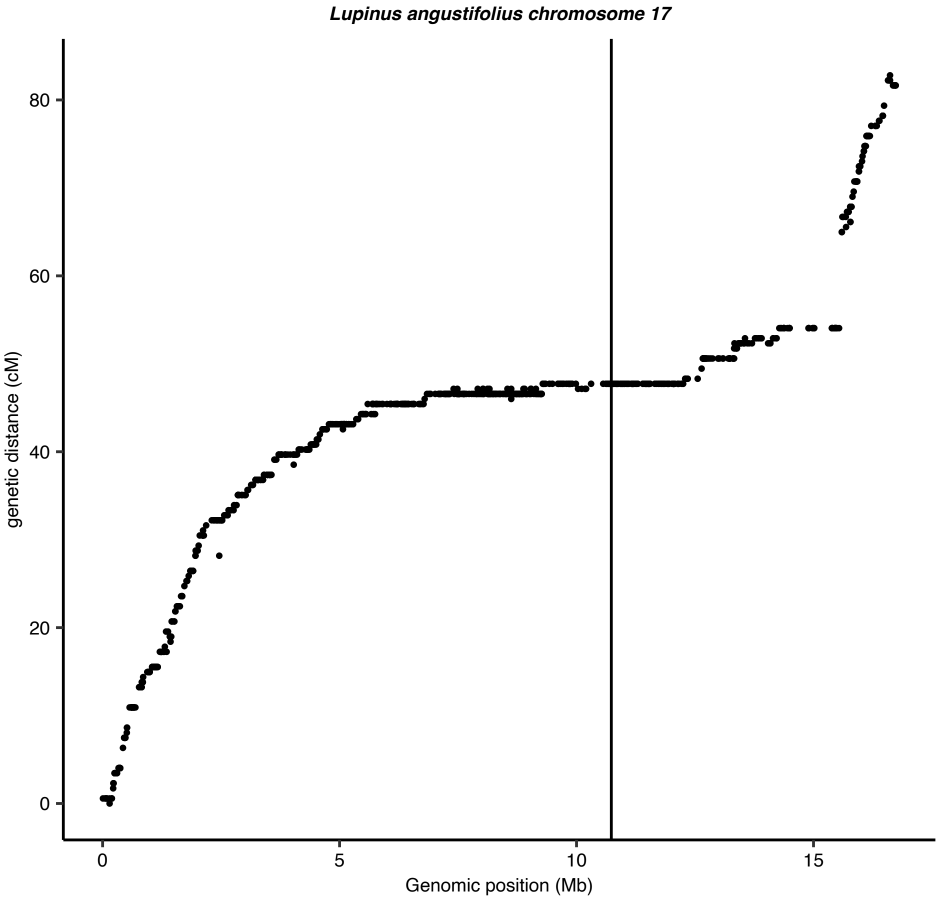
Lupinus angustifolius chromosome 17
genetic distance (cM)
0
20
40
60
80
Genomic position (Mb)
0
5
10
15

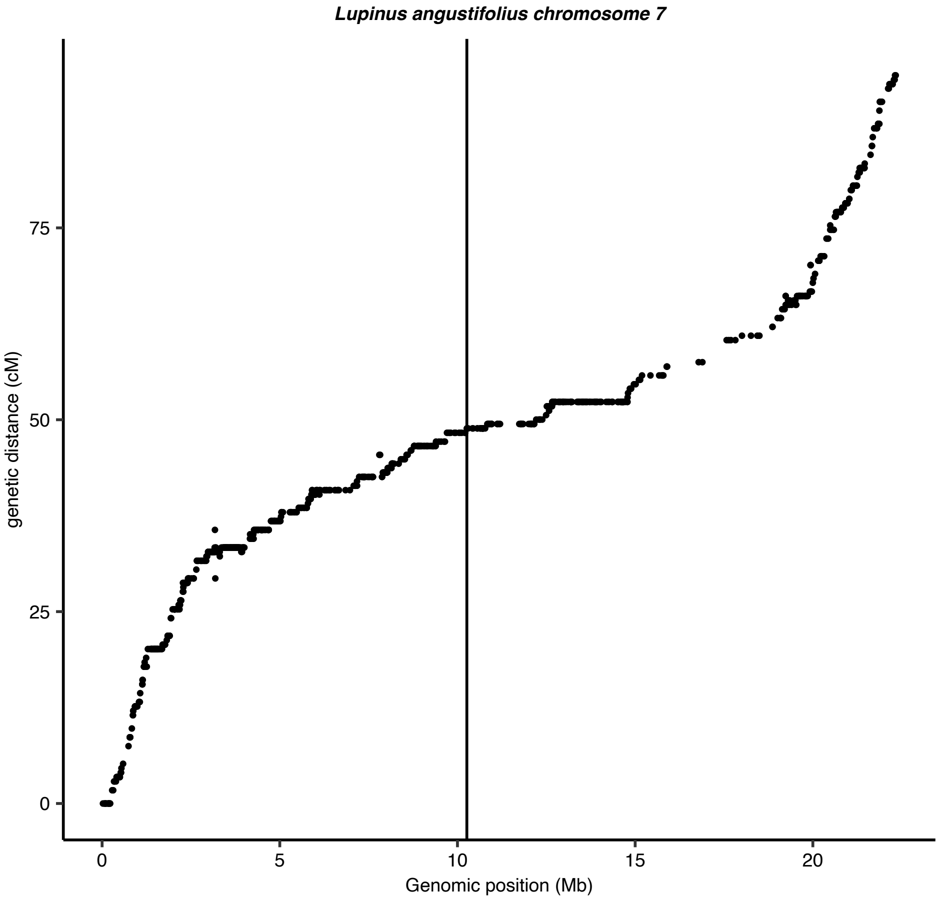

Lupinus angustifolius chromosome 7
genetic distance (cM)
0
25
50
75
Genomic position (Mb)
0
5
10
15
20

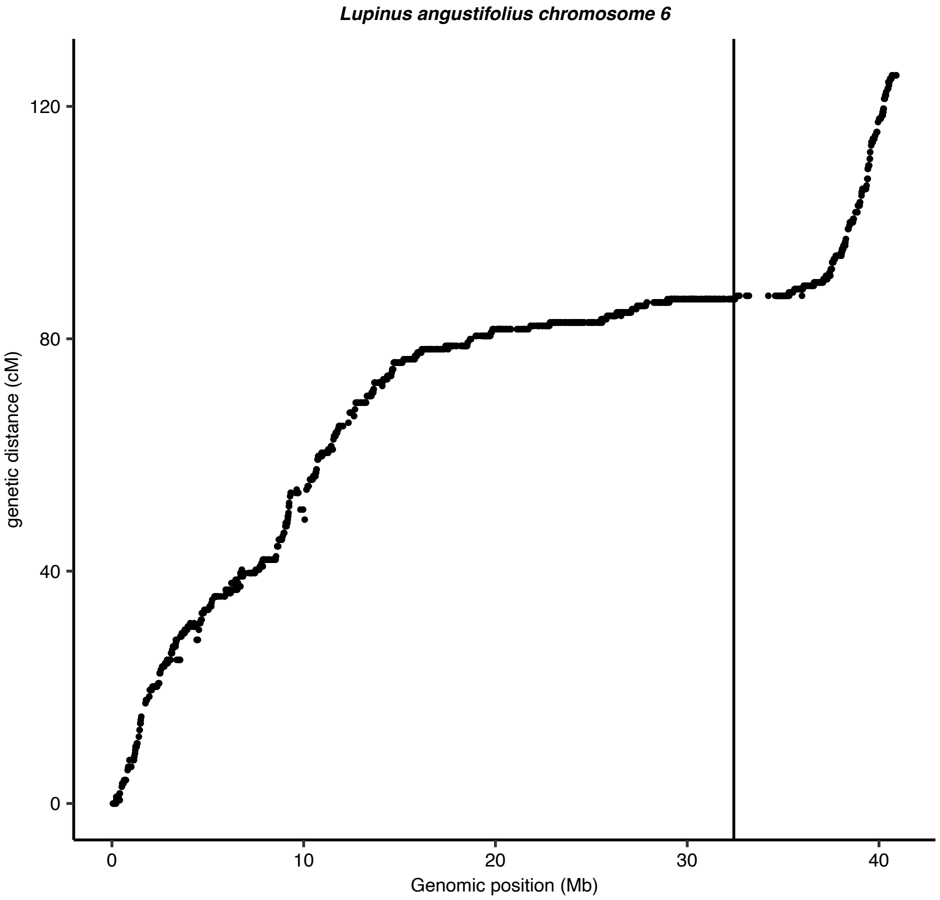
Lupinus angustifolius chromosome 6
genetic distance (cM)
Genomic position (Mb)
0
40
80
120
0
10
20
30
40

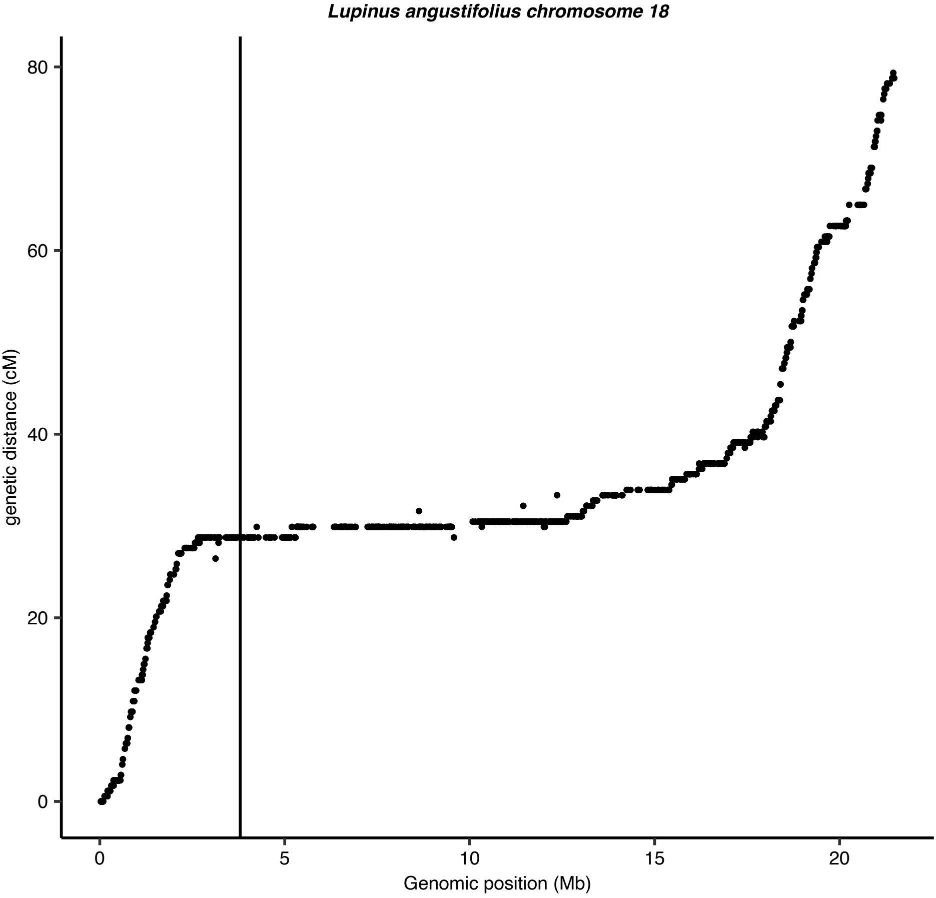
Lupinus angustifolius chromosome 18
genetic distance (cM)
Genomic position (Mb)
0
20
40
60
80
0
5
10
15
20

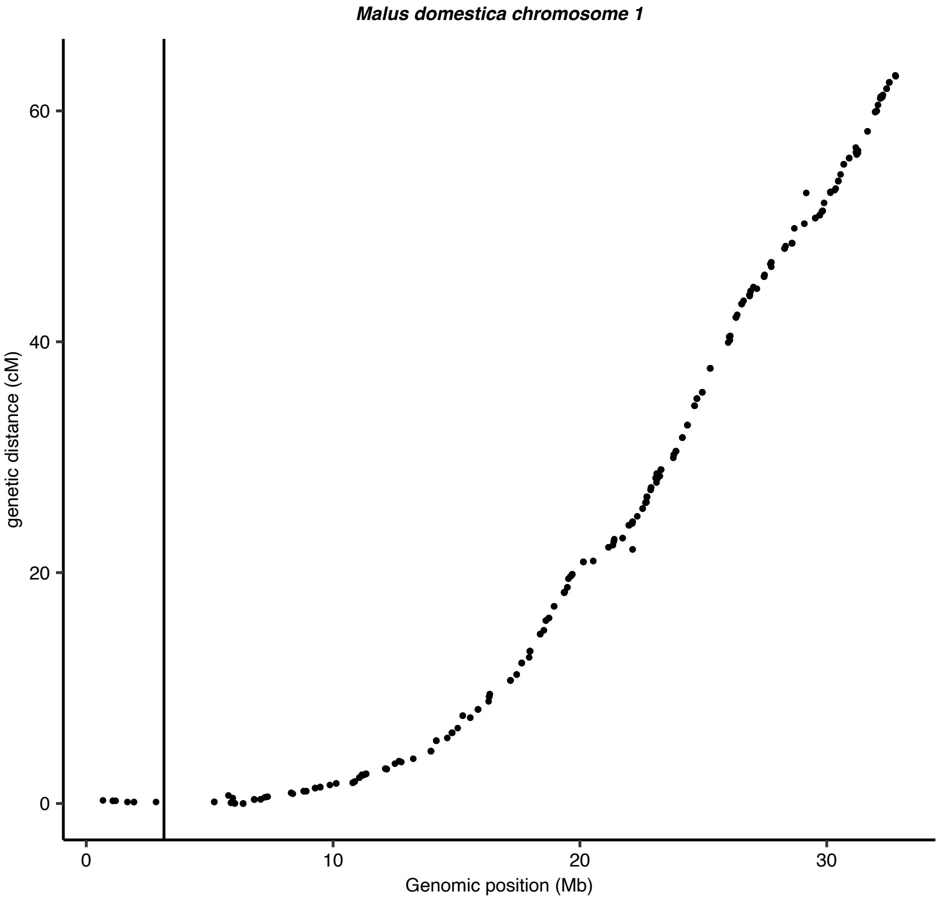
Malus domestica chromosome 1
genetic distance (cM)
Genomic position (Mb)
0
20
40
60
0
10
20
30

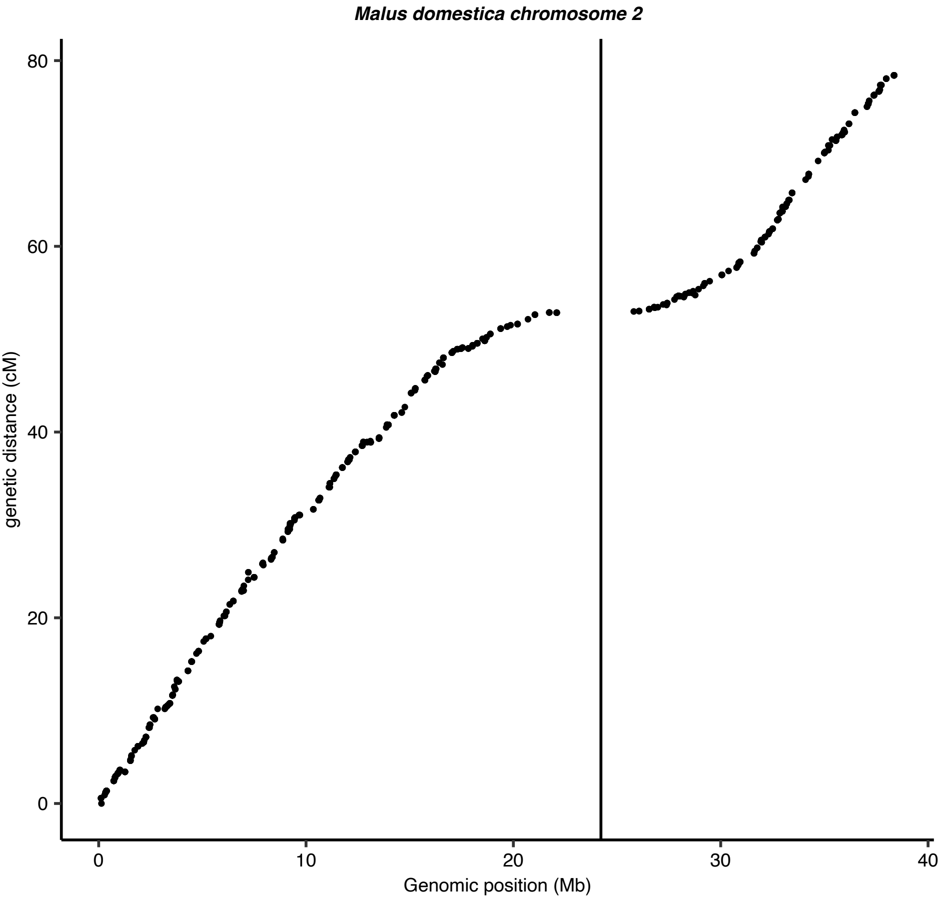
Malus domestica chromosome 2
genetic distance (cM)
0
20
40
60
80
Genomic position (Mb)
0
10
20
30
40

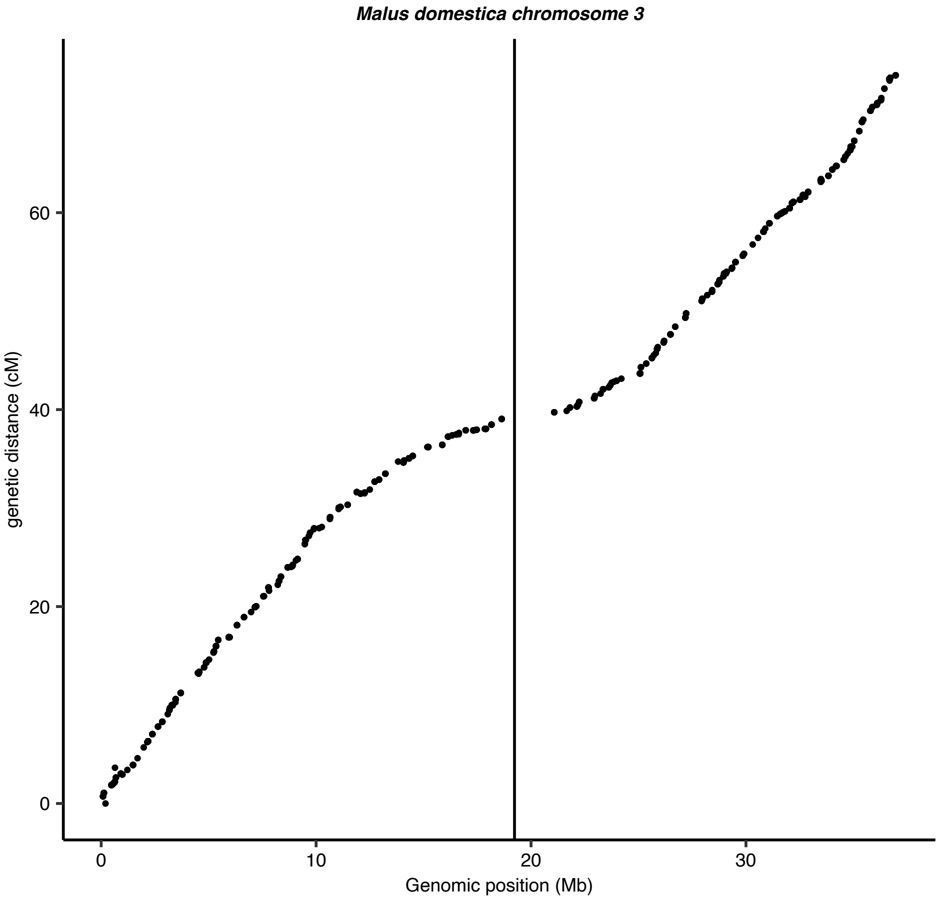
Malus domestica chromosome 3
genetic distance (cM)
Genomic position (Mb)
0
20
40
60
0
10
20
30

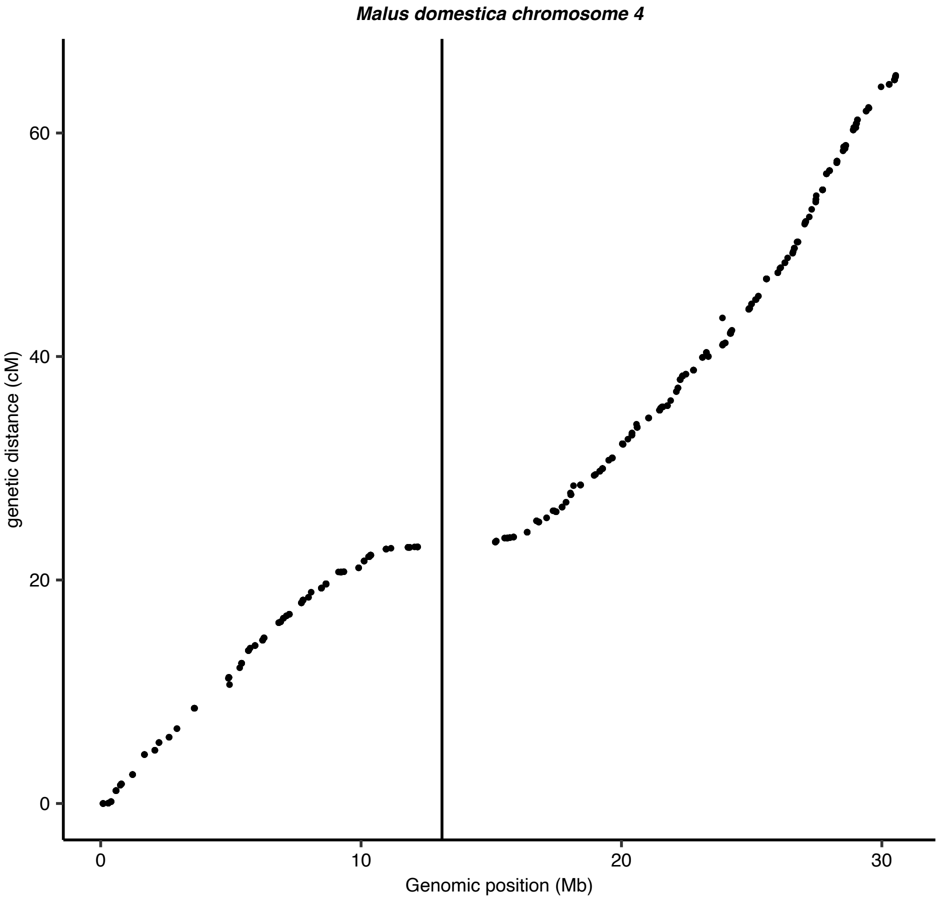
Malus domestica chromosome 4
genetic distance (cM)
0
20
40
60
Genomic position (Mb)
0
10
20
30

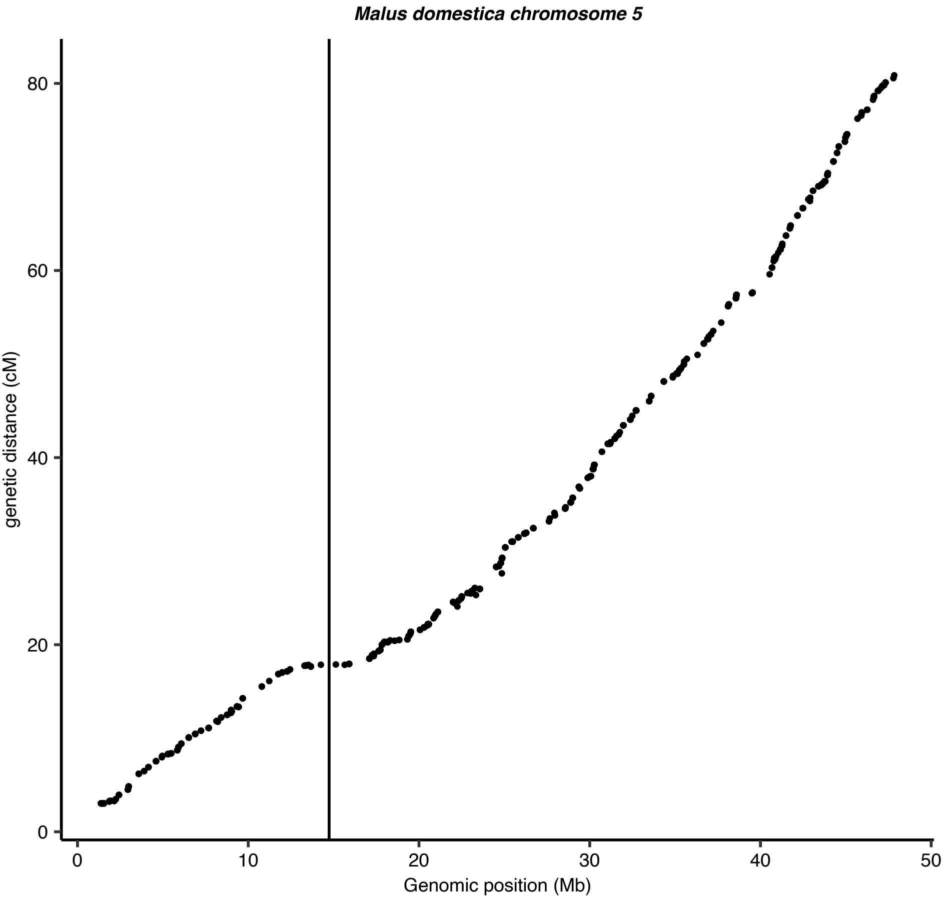
Malus domestica chromosome 5
genetic distance (cM)
Genomic position (Mb)
0
20
40
60
80
0
10
20
30
40
50

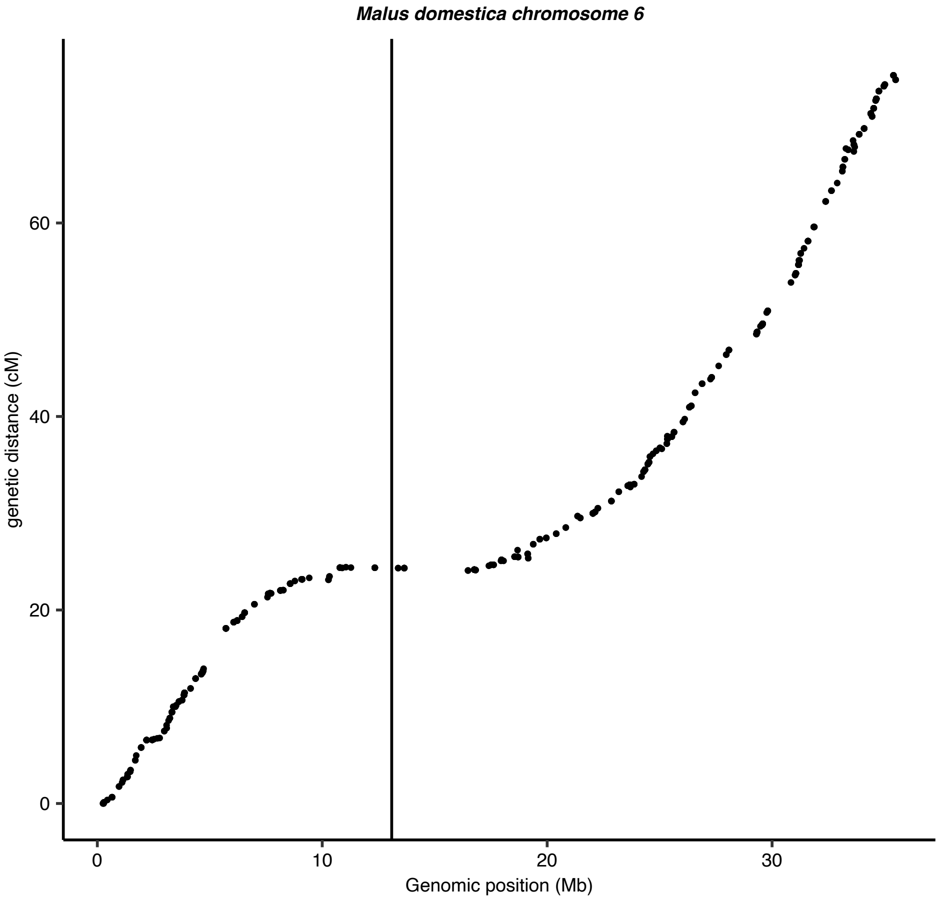
Malus domestica chromosome 6
genetic distance (cM)
Genomic position (Mb)
0
20
40
60
0
10
20
30

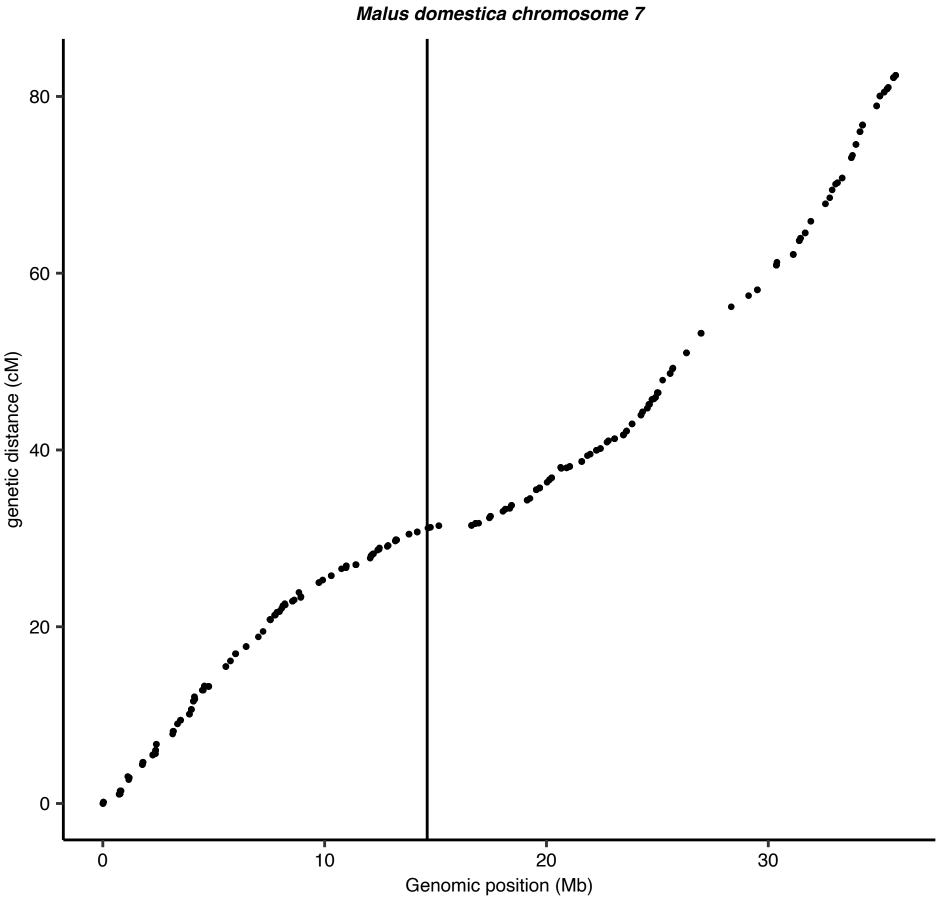
Malus domestica chromosome 7
genetic distance (cM)
Genomic position (Mb)
0
20
40
60
80
0
10
20
30

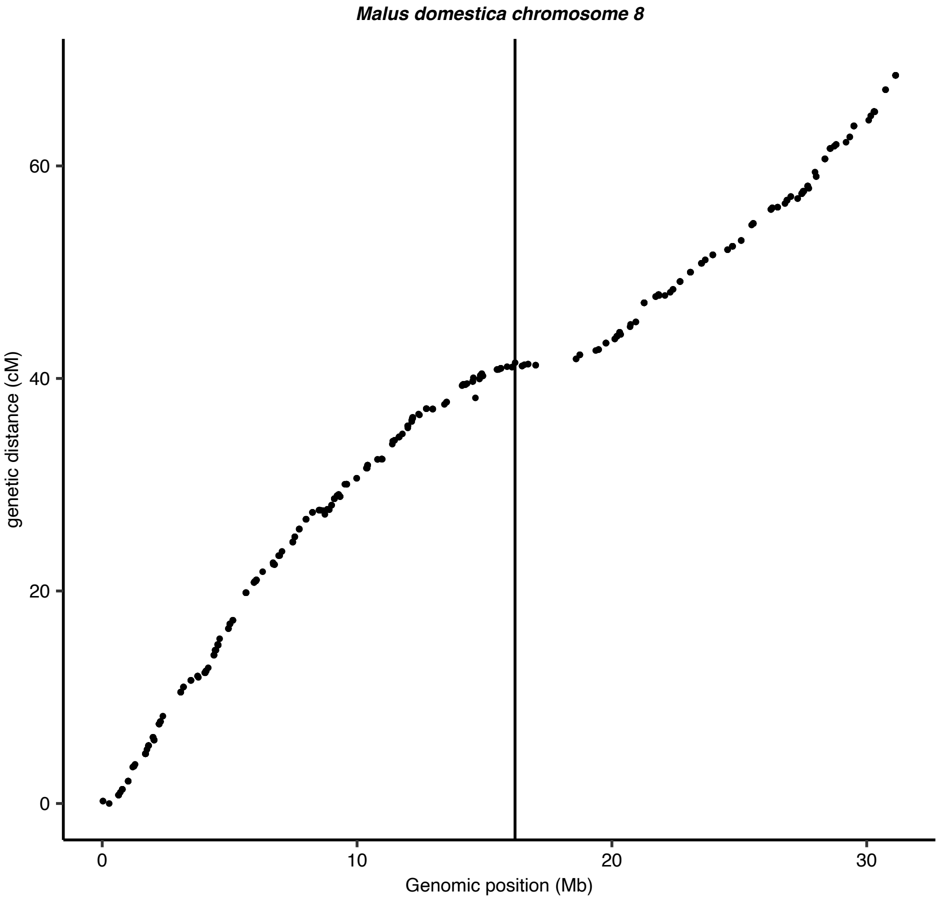
Malus domestica chromosome 8
genetic distance (cM)
0
20
40
60
0
10
20
30
Genomic position (Mb)

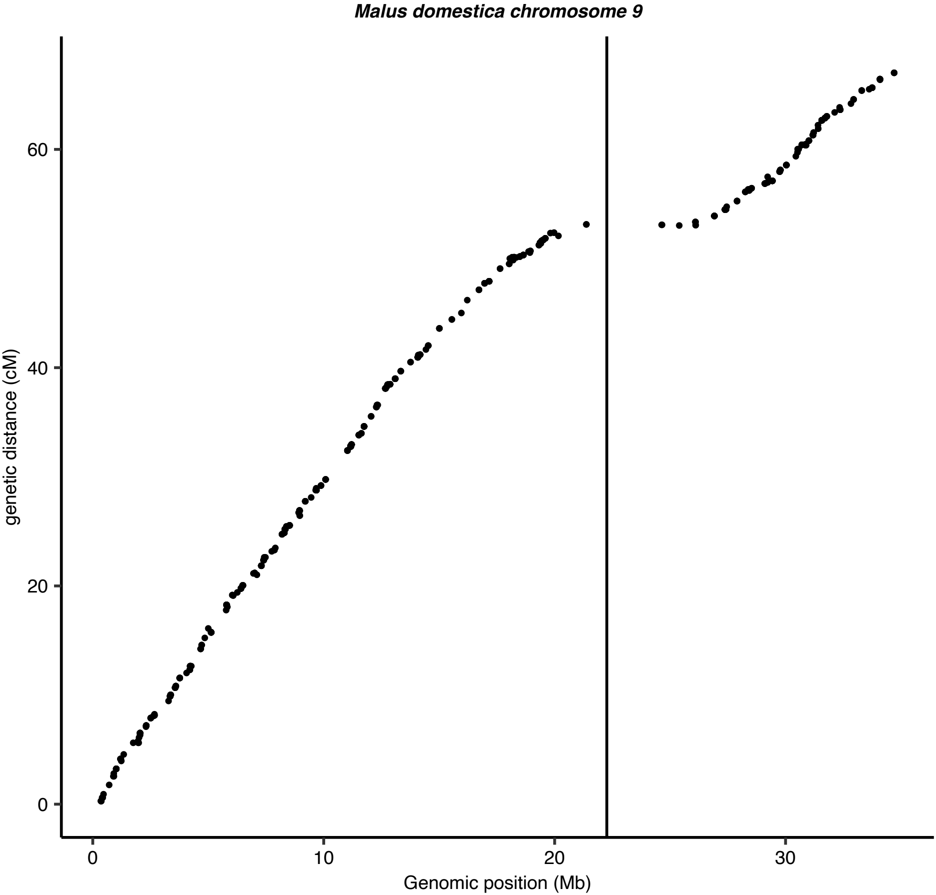
Malus domestica chromosome 9
genetic distance (cM)
0
20
40
60
0
10
20
30
Genomic position (Mb)

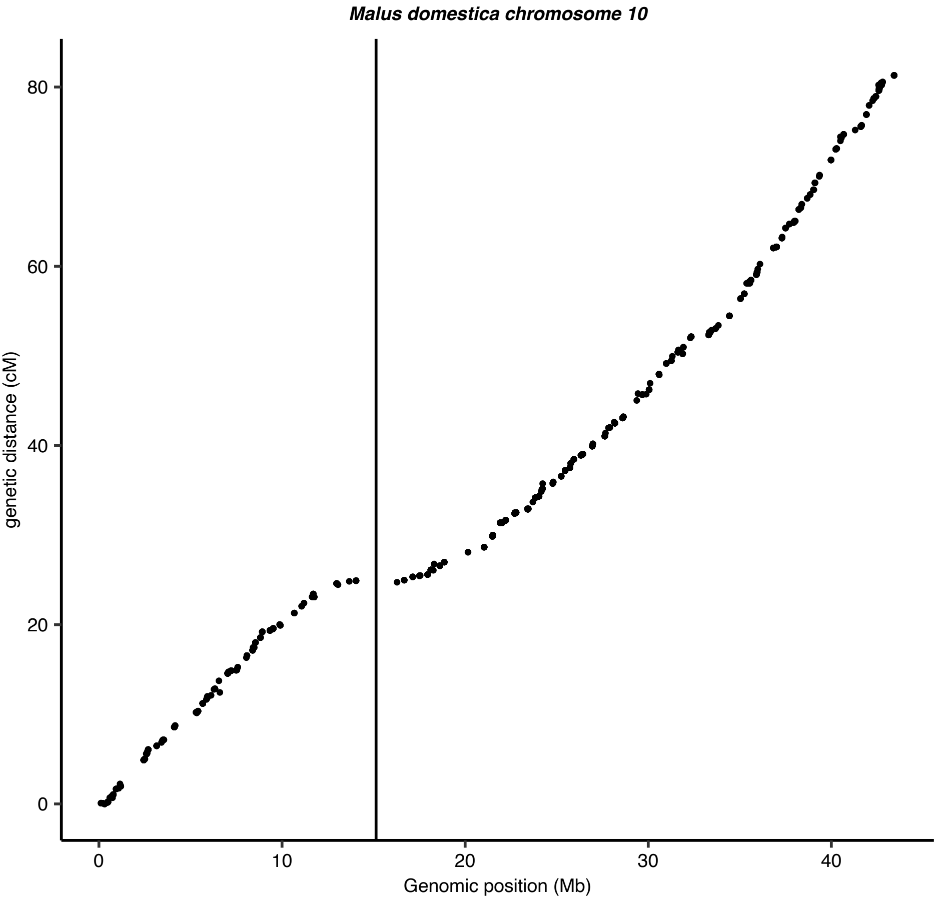
Malus domestica chromosome 10
genetic distance (cM)
Genomic position (Mb)
0
20
40
60
80
0
10
20
30
40

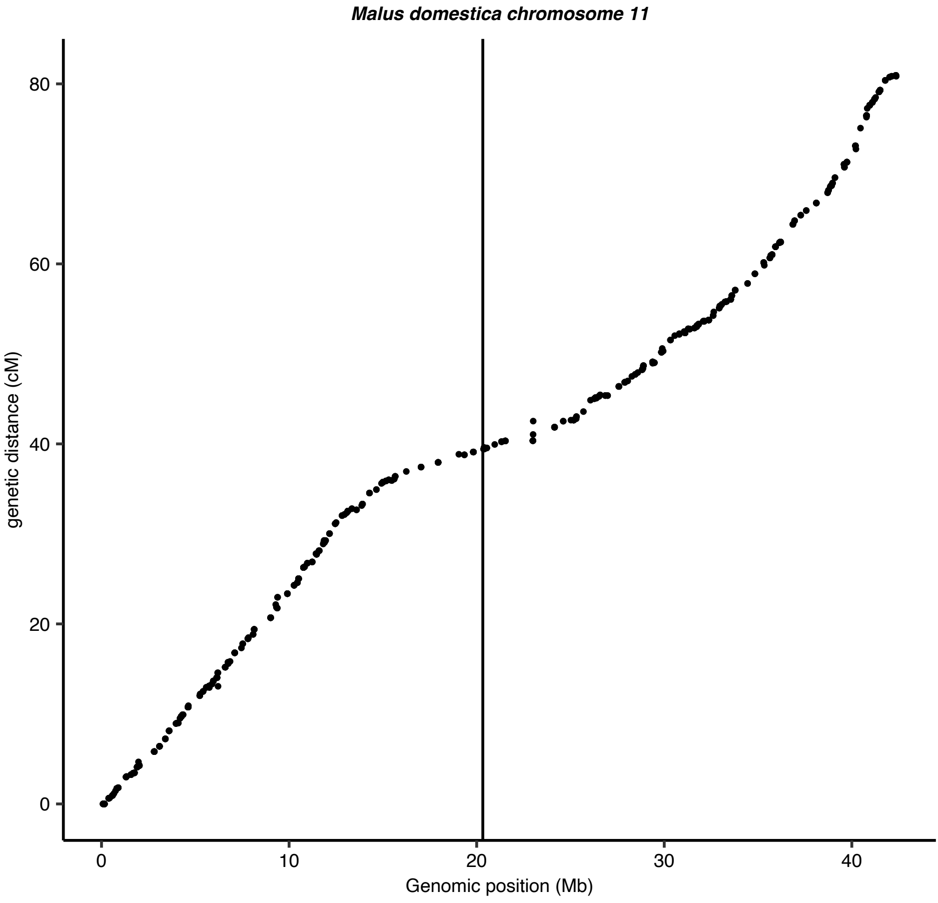
Malus domestica chromosome 11
genetic distance (cM)
Genomic position (Mb)
0
20
40
60
80
0
10
20
30
40

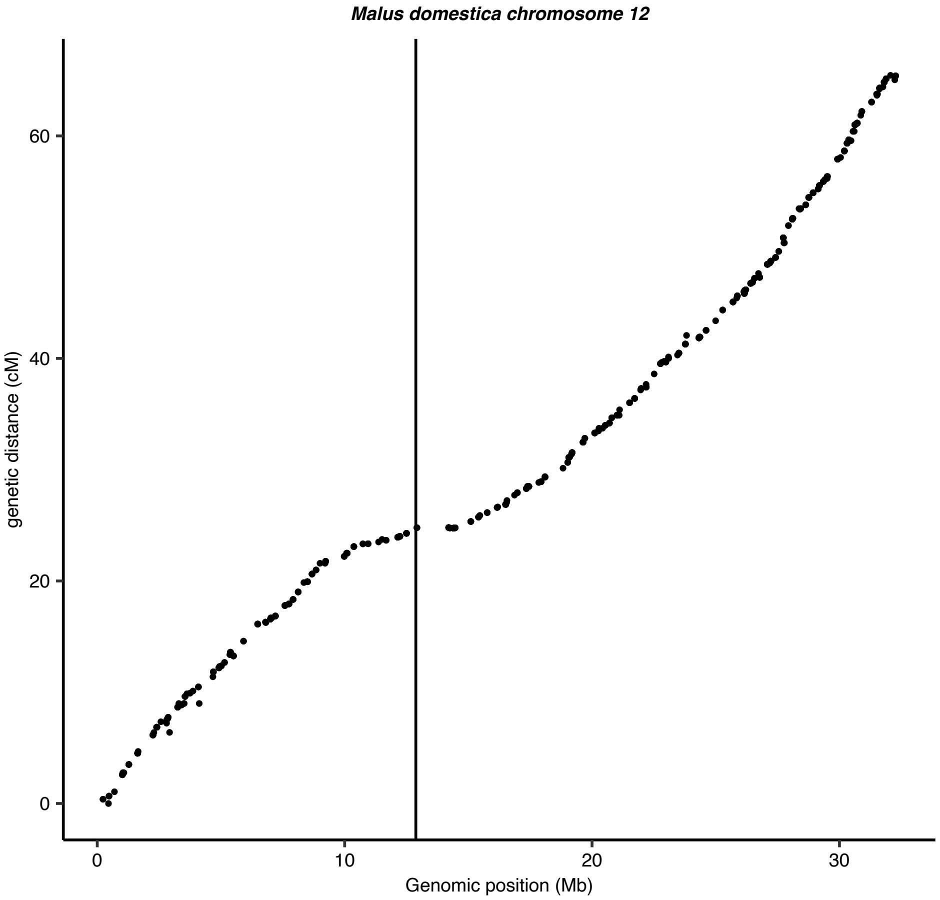
Malus domestica chromosome 12
genetic distance (cM)
0
20
40
60
0
10
20
30
Genomic position (Mb)

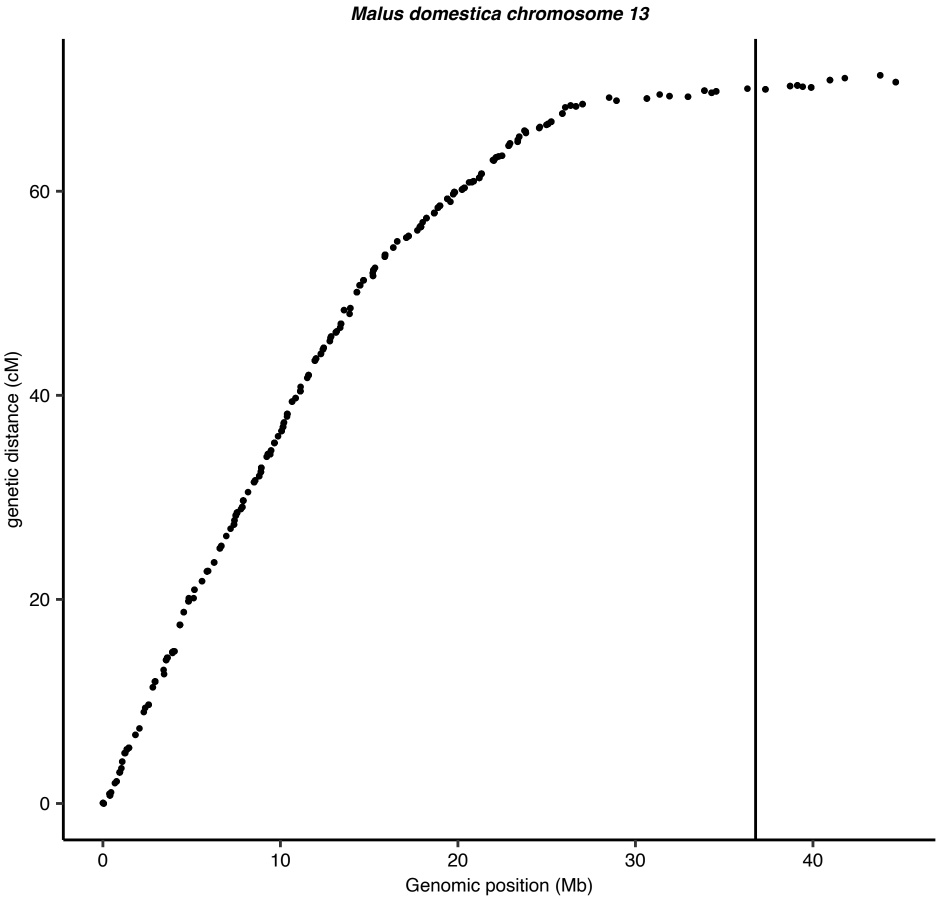
Malus domestica chromosome 13
genetic distance (cM)
0
20
40
60
Genomic position (Mb)
0
10
20
30
40

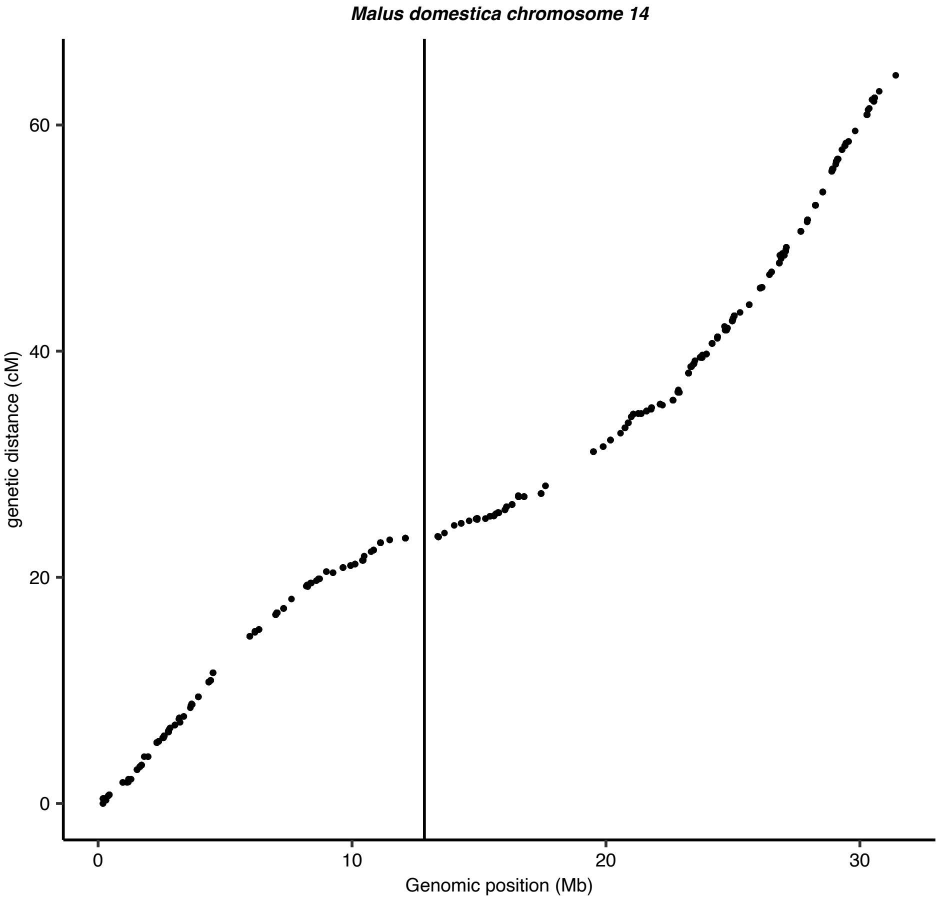
Malus domestica chromosome 14
genetic distance (cM)
Genomic position (Mb)
0
20
40
60
0
10
20
30

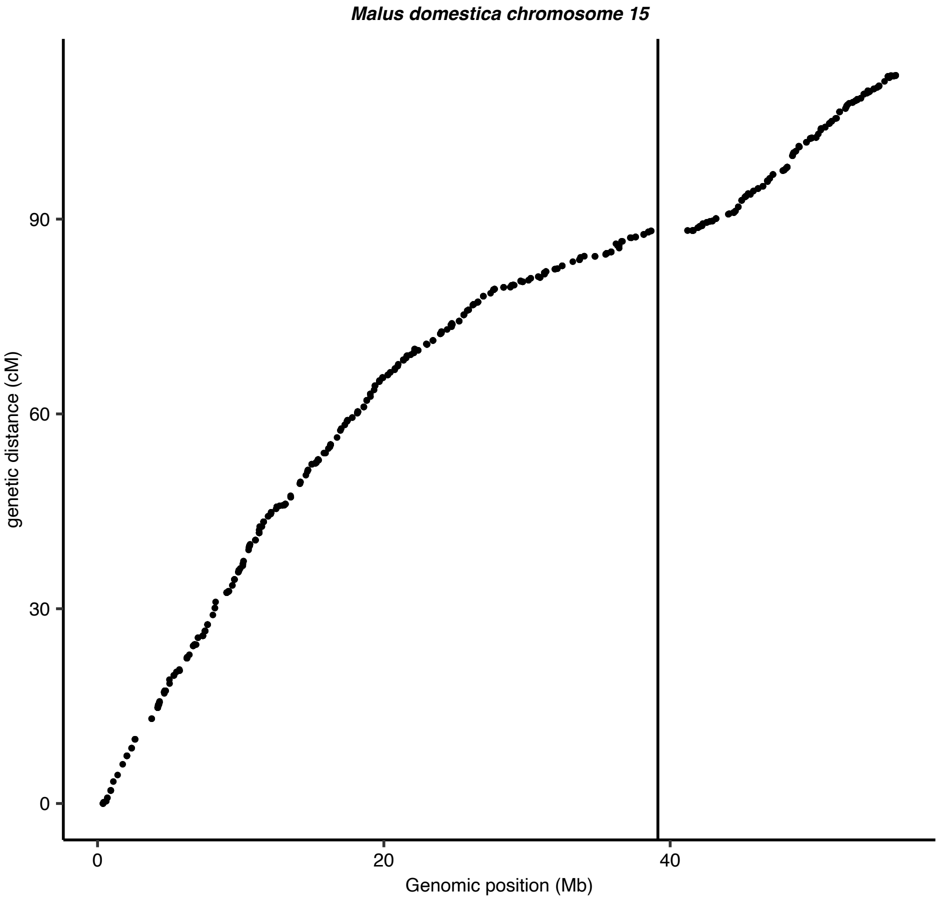
Malus domestica chromosome 15
genetic distance (cM)
0
30
60
90
Genomic position (Mb)
0
20
40

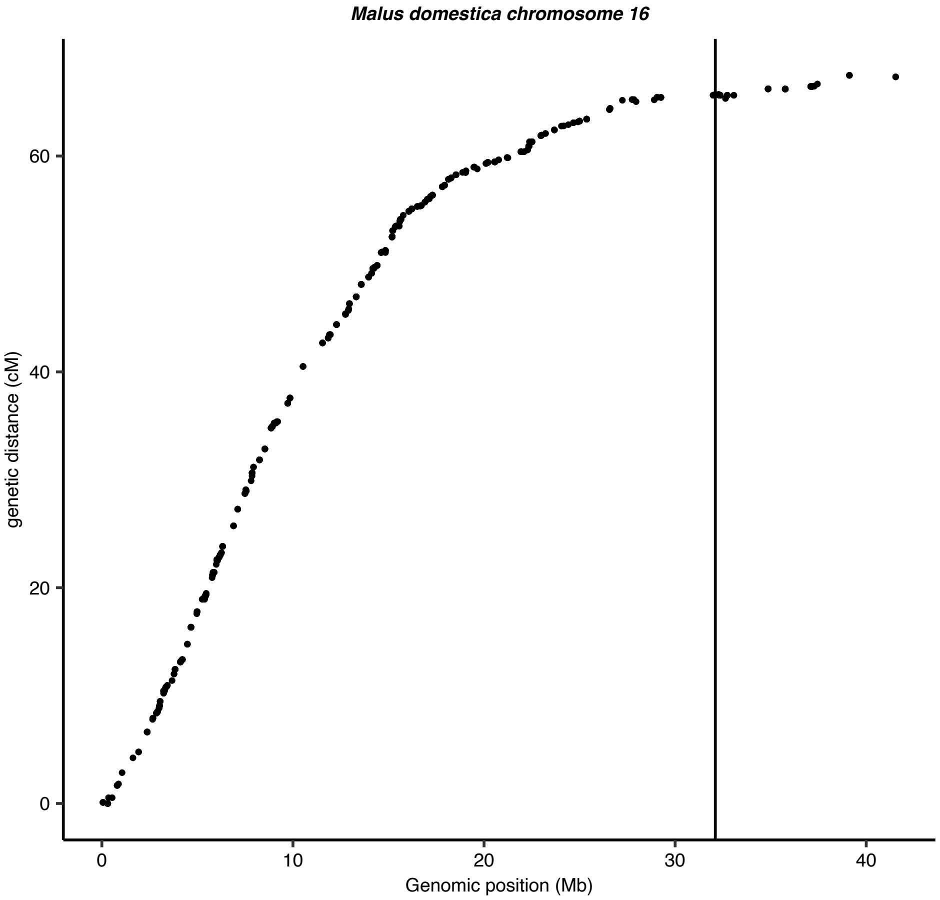
Malus domestica chromosome 16
genetic distance (cM)
60
40
20
0
0
10
20
30
40
Genomic position (Mb)

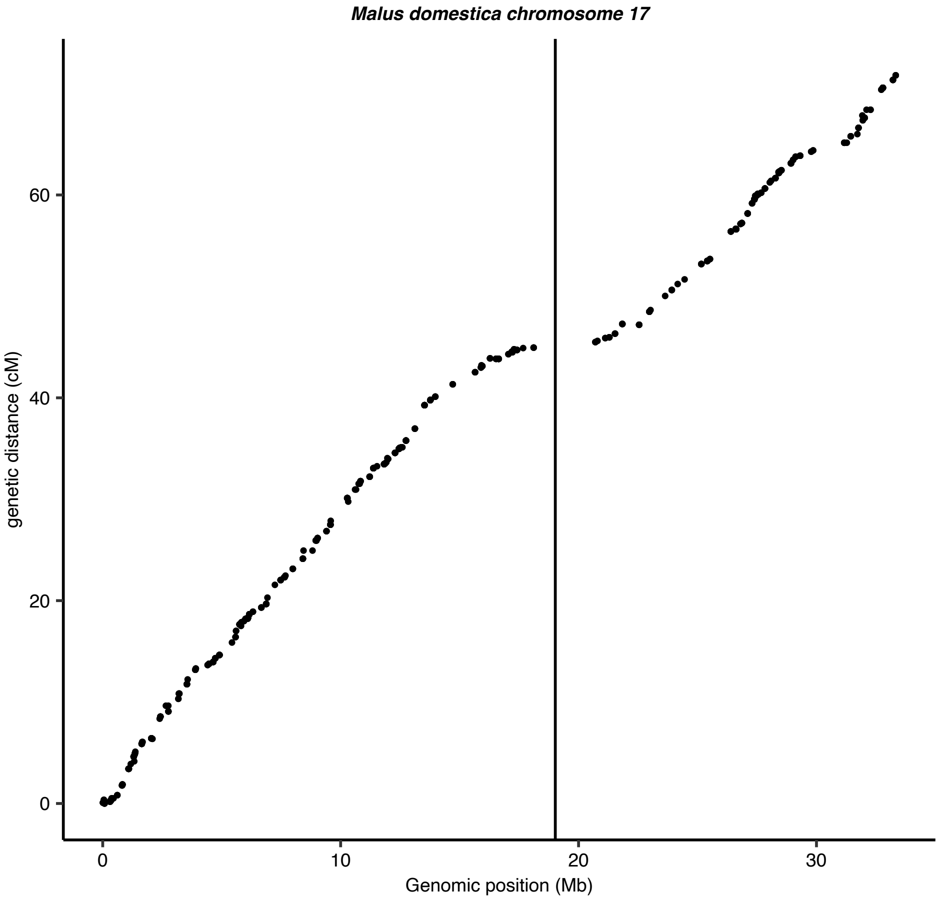
Malus domestica chromosome 17
genetic distance (cM)
Genomic position (Mb)
0
20
40
60
0
10
20
30

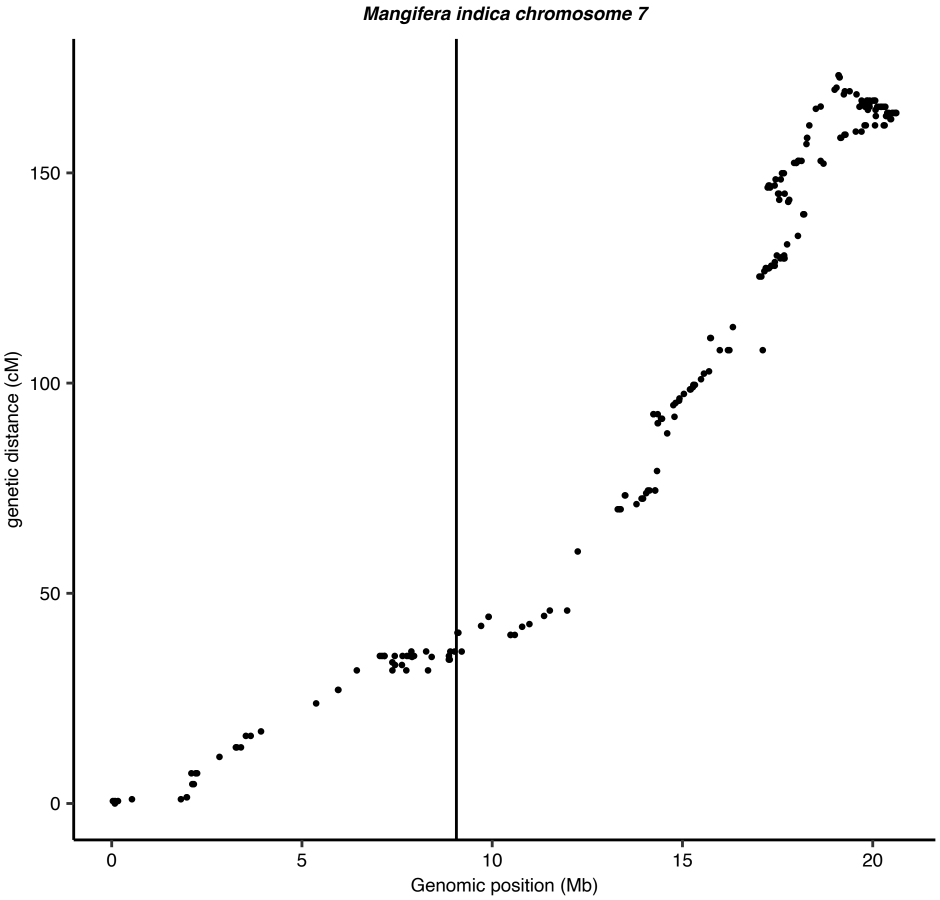
Mangifera indica chromosome 7
genetic distance (cM)
0
50
100
150
0
5
10
15
20
Genomic position (Mb)

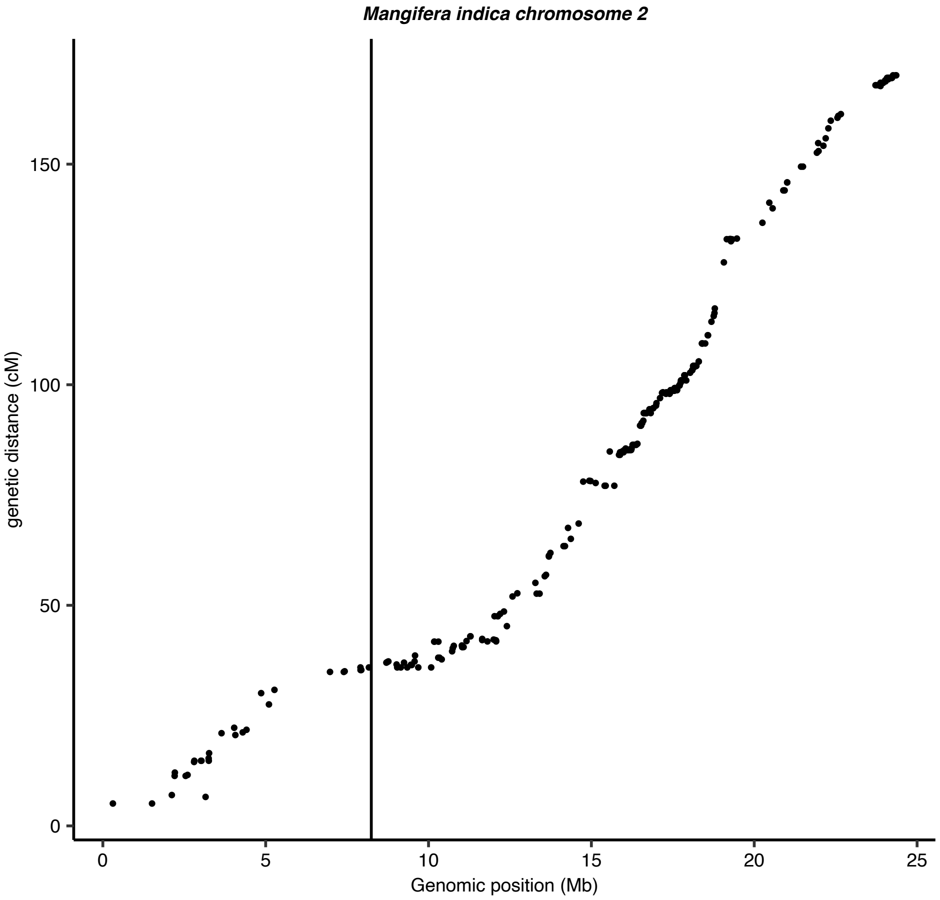
Mangifera indica chromosome 2
genetic distance (cM)
0
50
100
150
Genomic position (Mb)
0
5
10
15
20
25

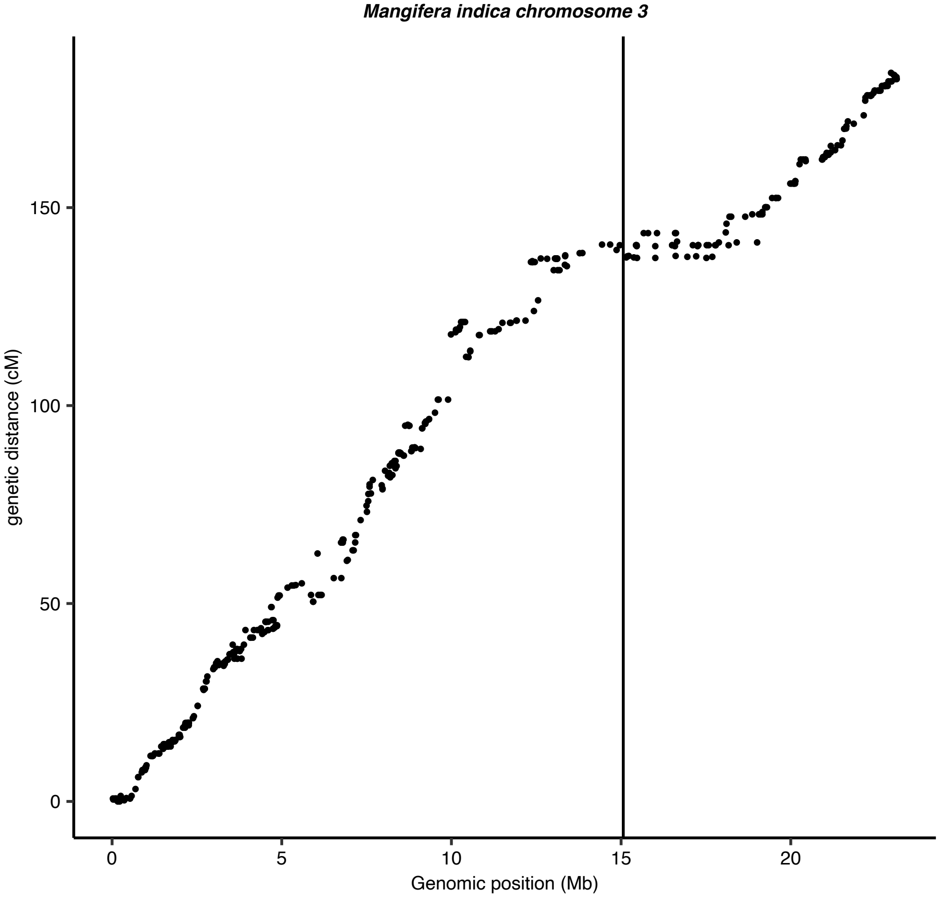
Mangifera indica chromosome 3
genetic distance (cM)
0
50
100
150
Genomic position (Mb)
0
5
10
15
20

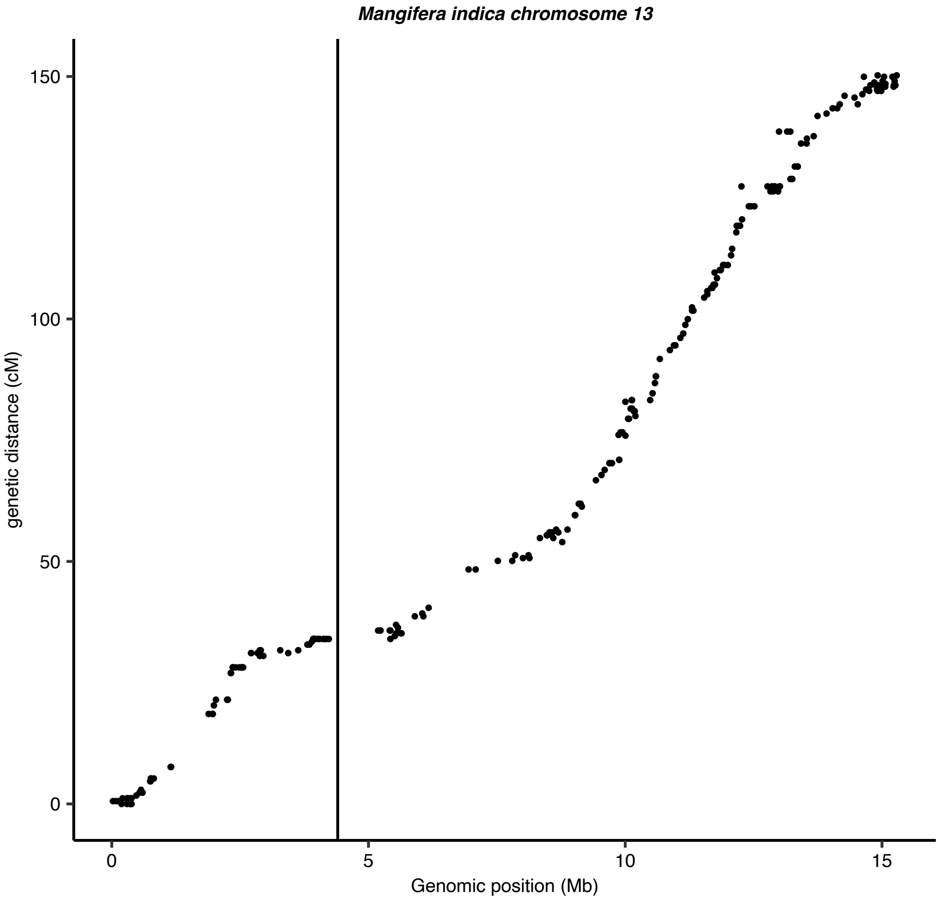
Mangifera indica chromosome 13
genetic distance (cM)
150
100
50
0
0
5
10
15
Genomic position (Mb)

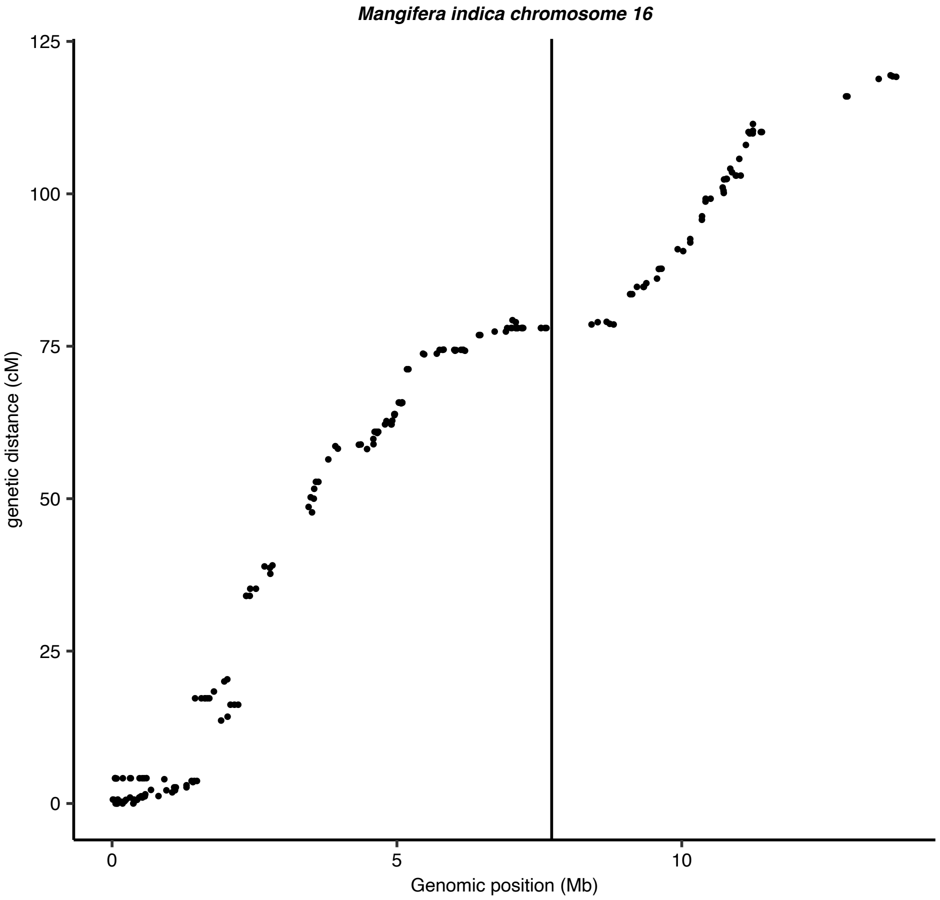
Mangifera indica chromosome 16
125
100
75
50
25
0
genetic distance (cM)
0
5
10
Genomic position (Mb)

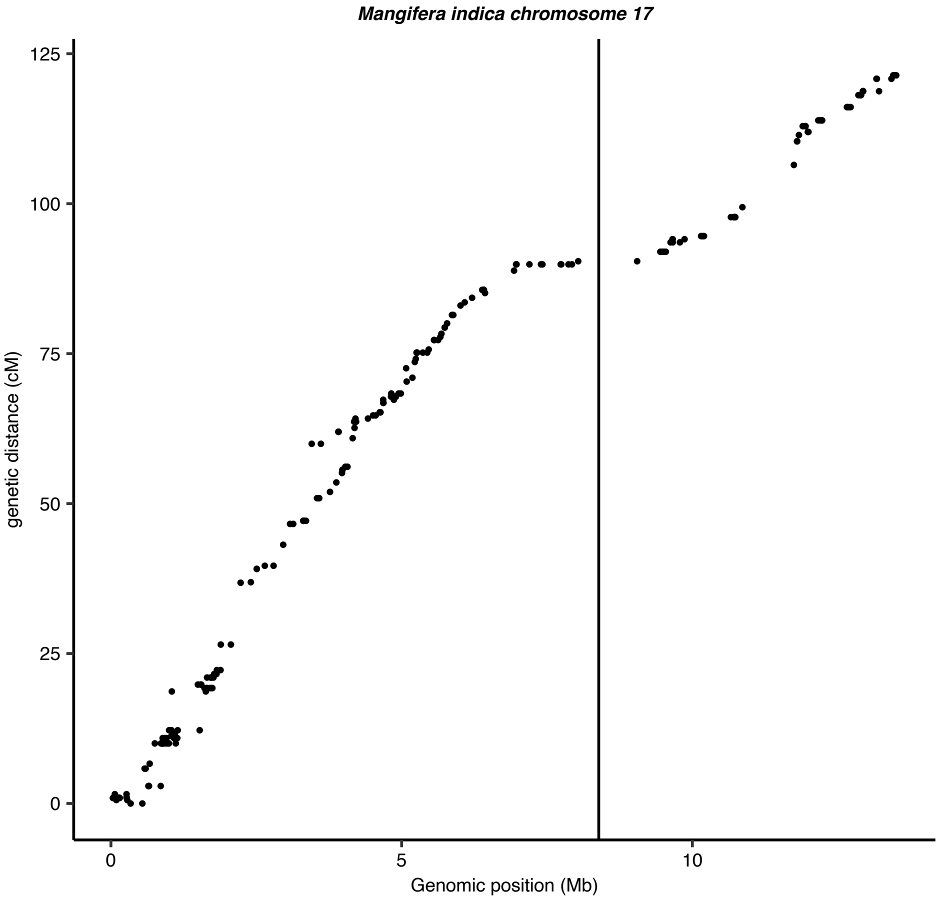
Mangifera indica chromosome 17
genetic distance (cM)
125
100
75
50
25
0
0
5
10
Genomic position (Mb)

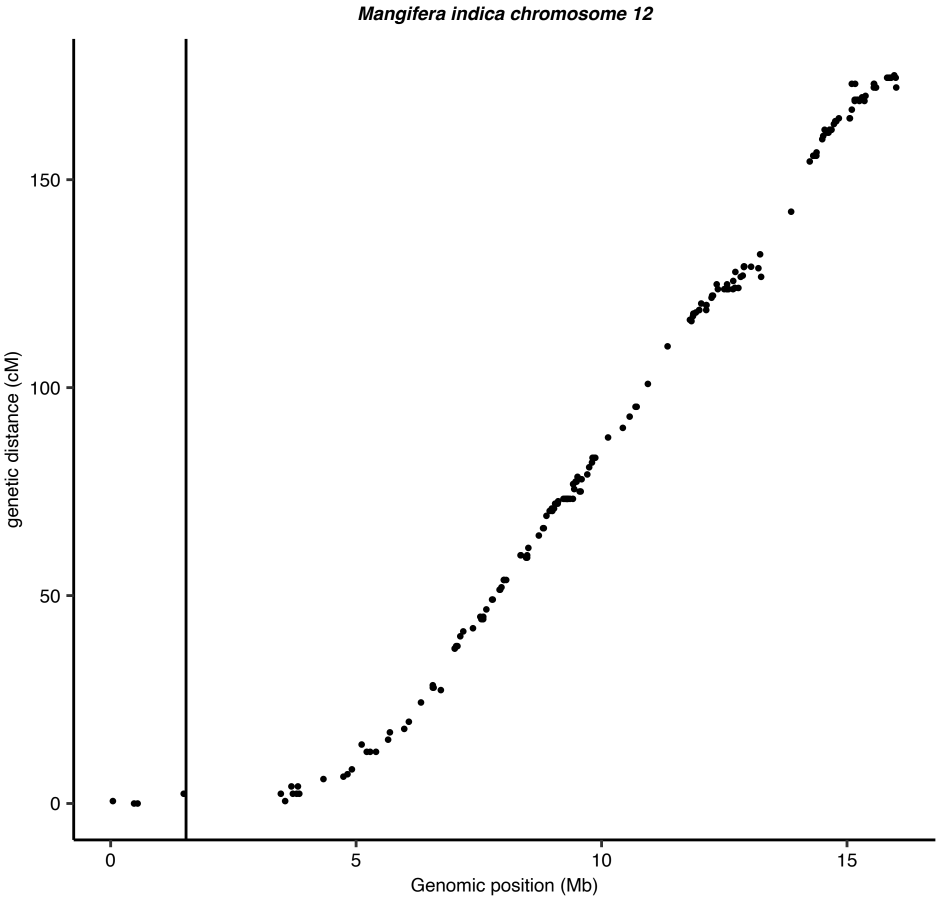
Mangifera indica chromosome 12
genetic distance (cM)
0
50
100
150
0
5
10
15
Genomic position (Mb)

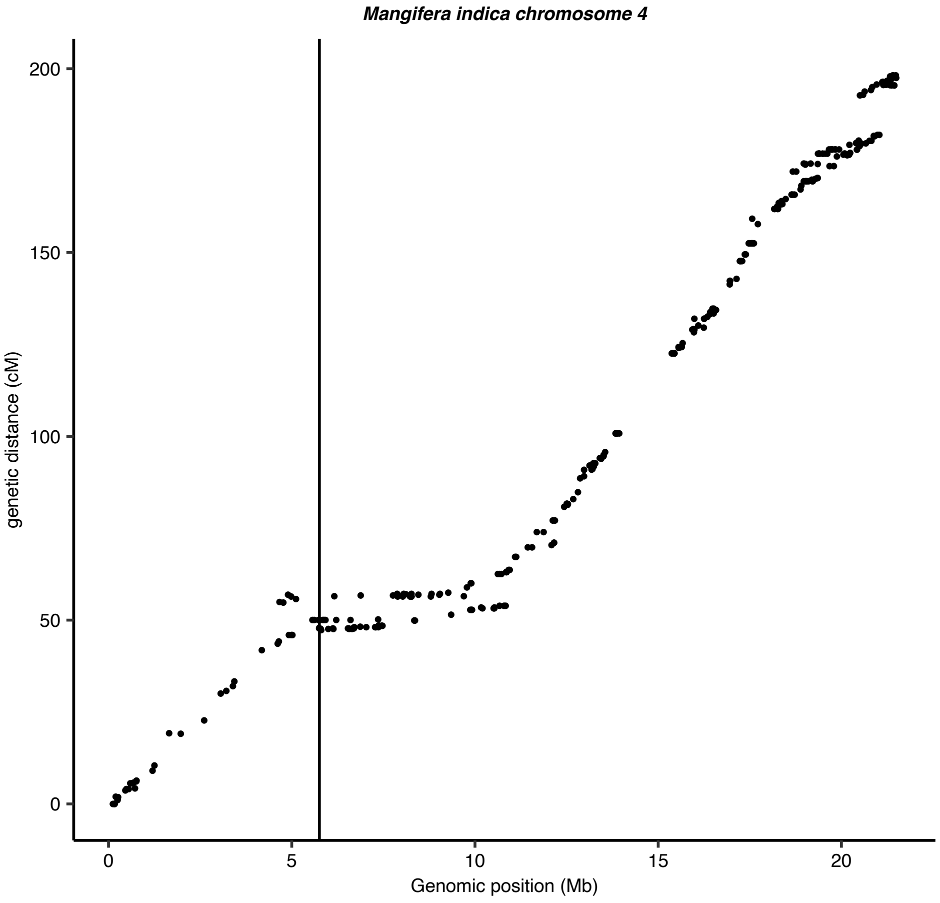
Mangifera indica chromosome 4
genetic distance (cM)
Genomic position (Mb)
0
50
100
150
200
0
5
10
15
20

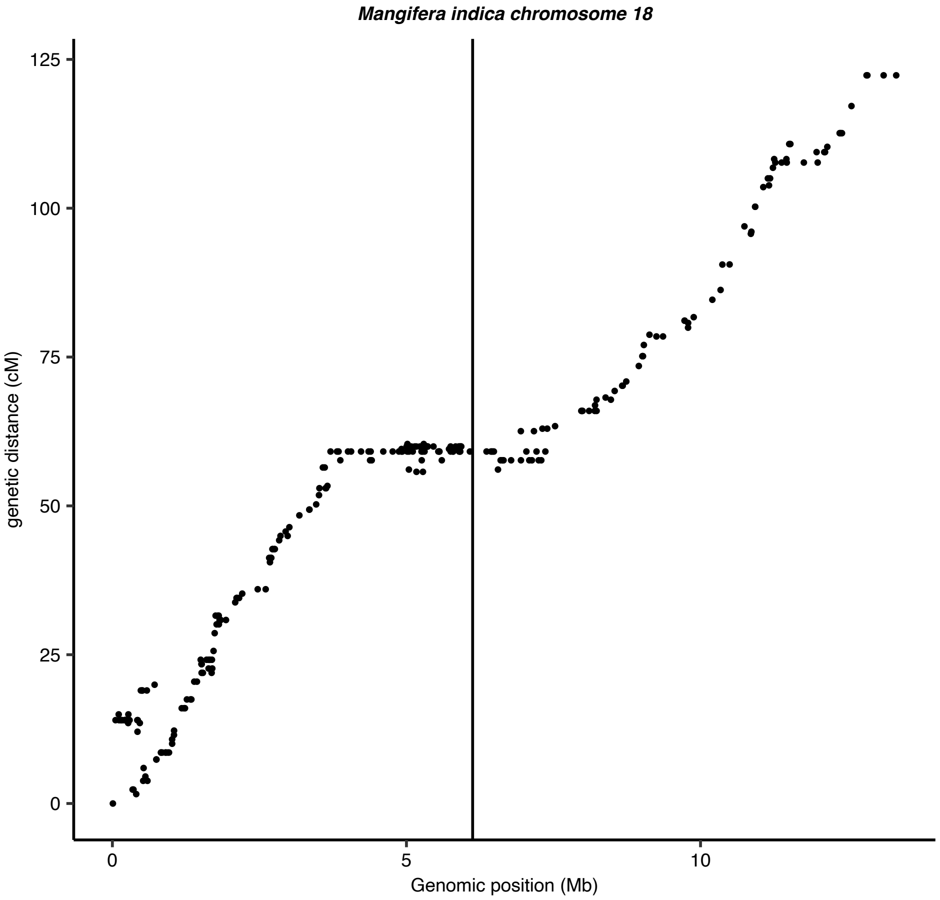
Mangifera indica chromosome 18
genetic distance (cM)
125
100
75
50
25
0
0
5
10
Genomic position (Mb)

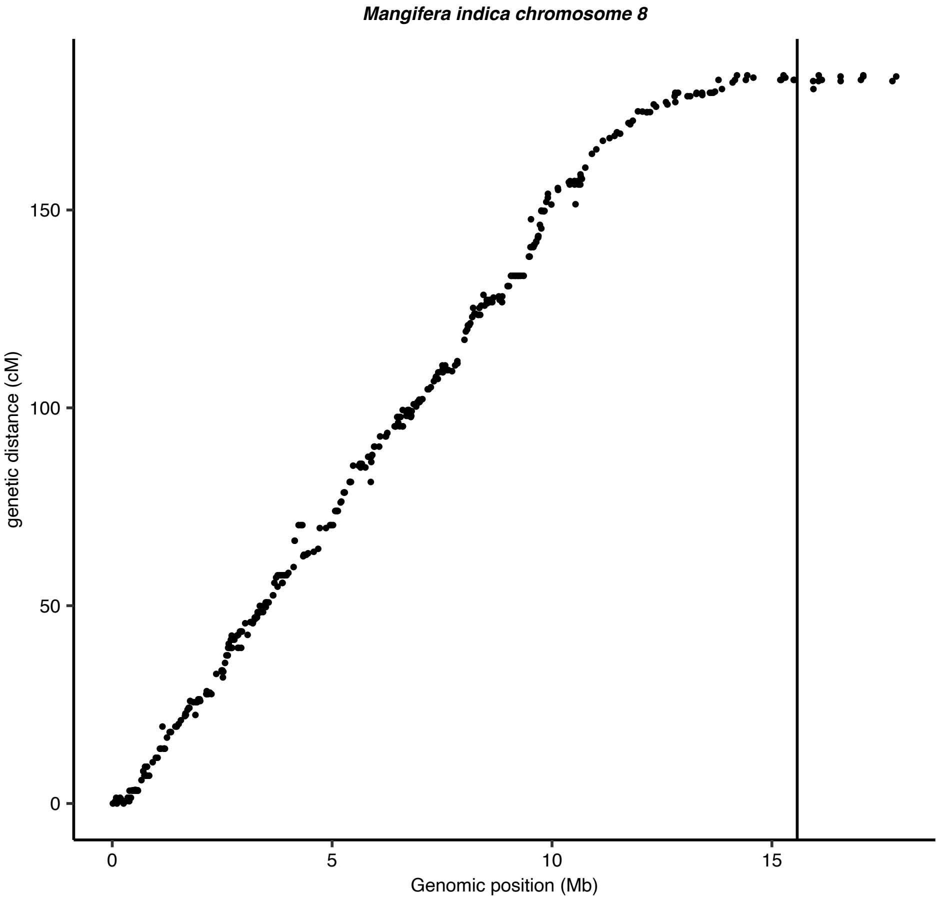
Mangifera indica chromosome 8
genetic distance (cM)
0
50
100
150
Genomic position (Mb)
0
5
10
15

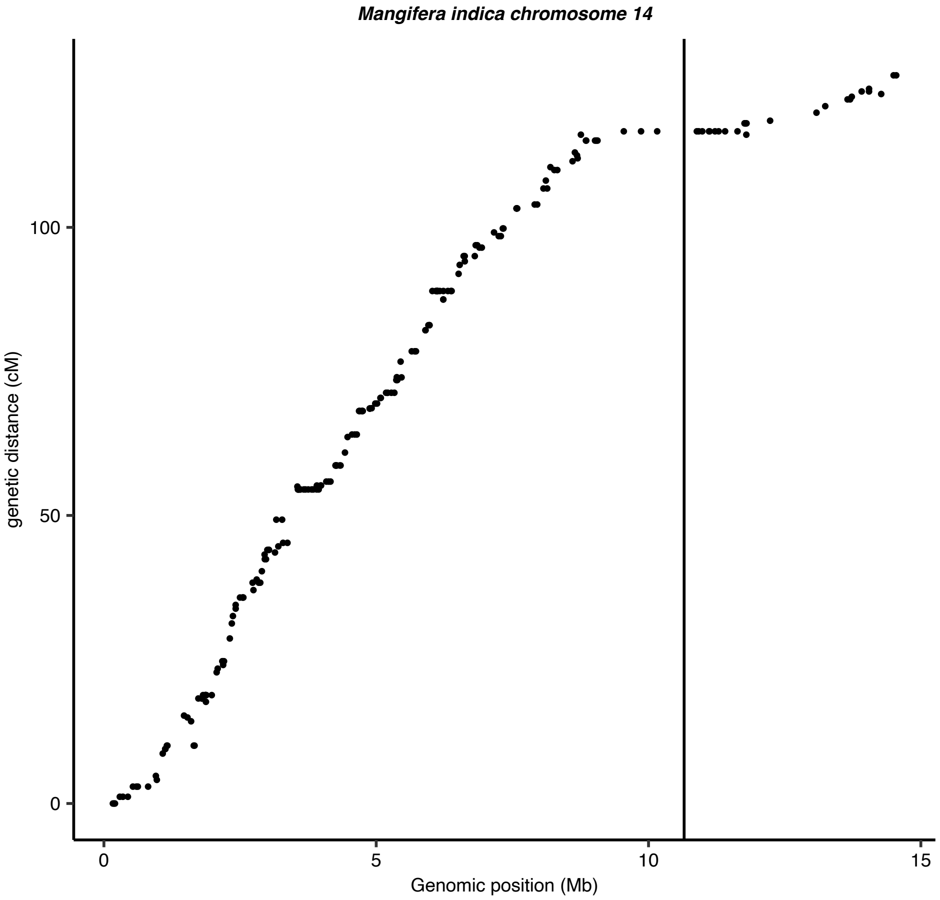
Mangifera indica chromosome 14
genetic distance (cM)
100
50
0
0
5
10
15
Genomic position (Mb)

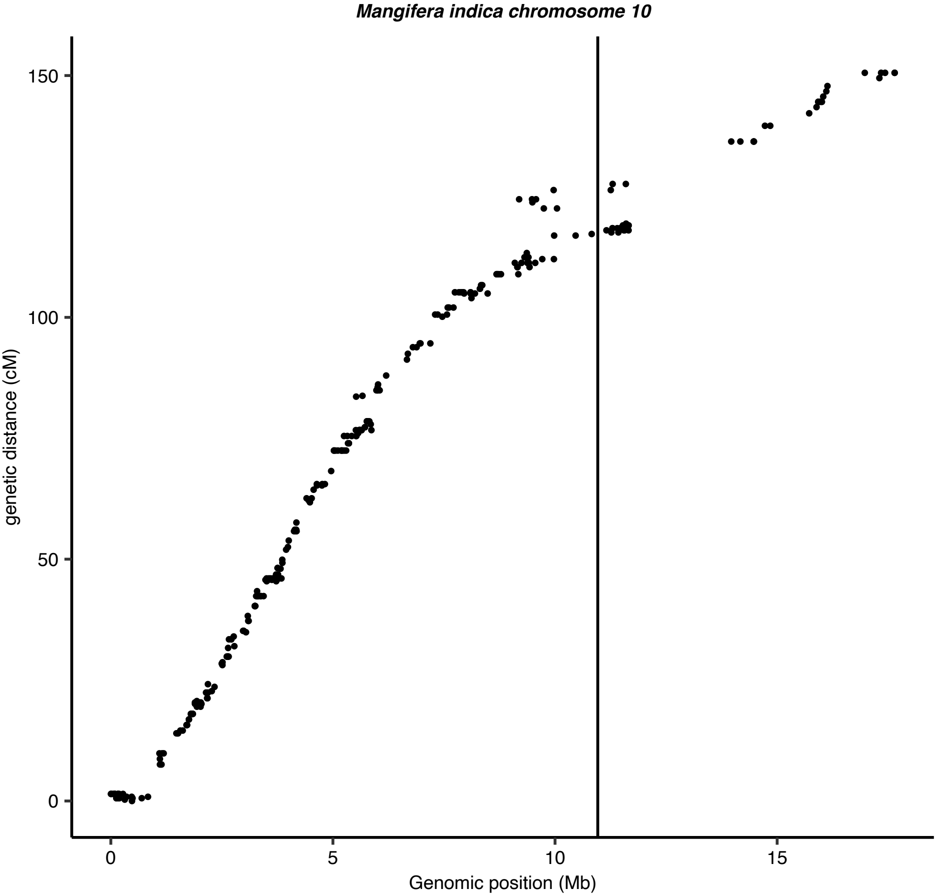
Mangifera indica chromosome 10
genetic distance (cM)
0
50
100
150
Genomic position (Mb)
0
5
10
15

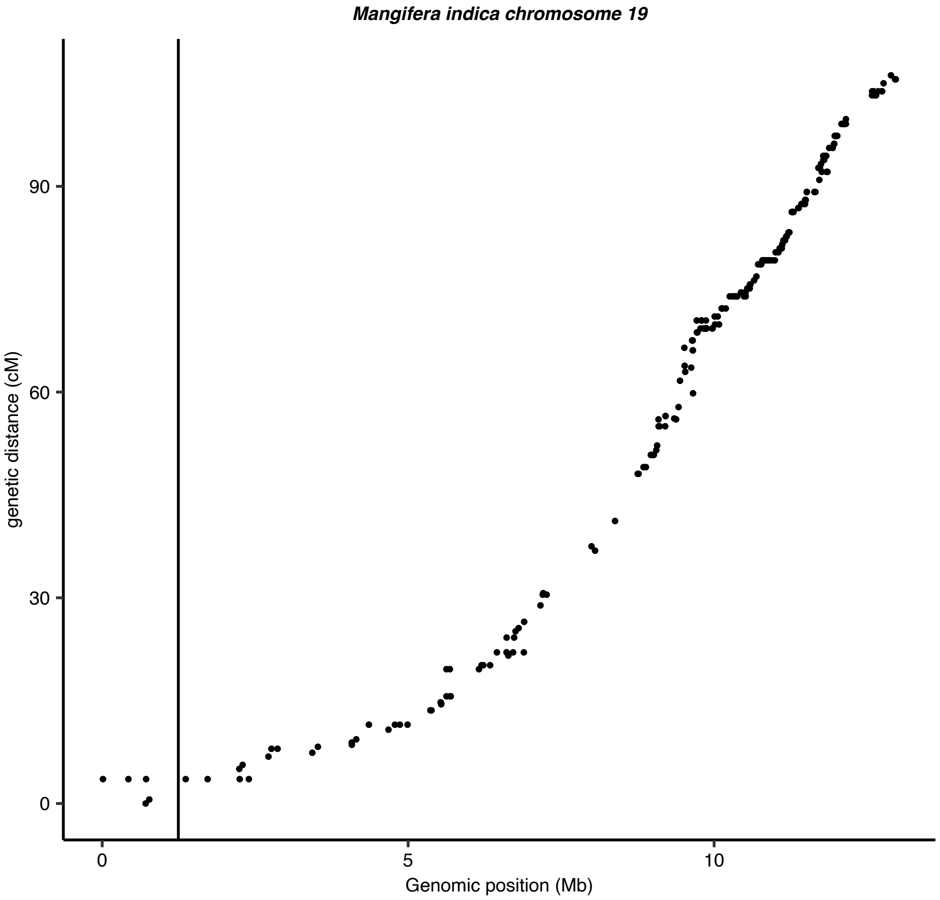
Mangifera indica chromosome 19
genetic distance (cM)
0
30
60
90
Genomic position (Mb)
0
5
10

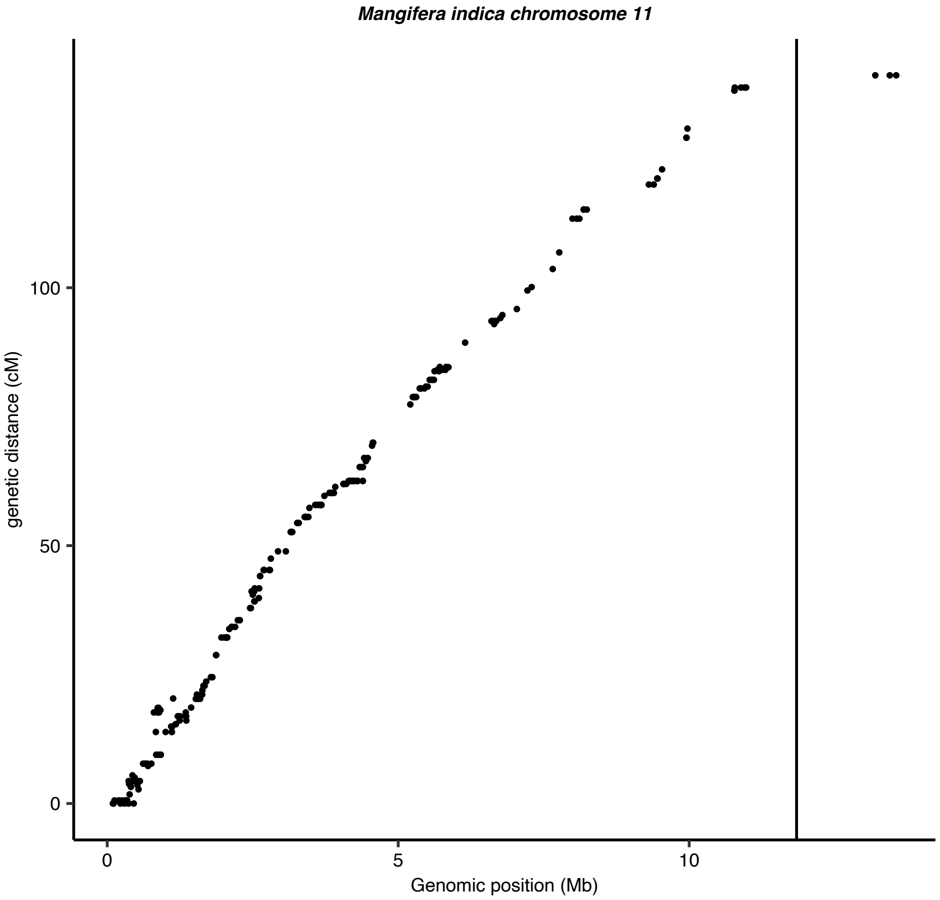
Mangifera indica chromosome 11
genetic distance (cM)
100
50
0
0
5
10
Genomic position (Mb)

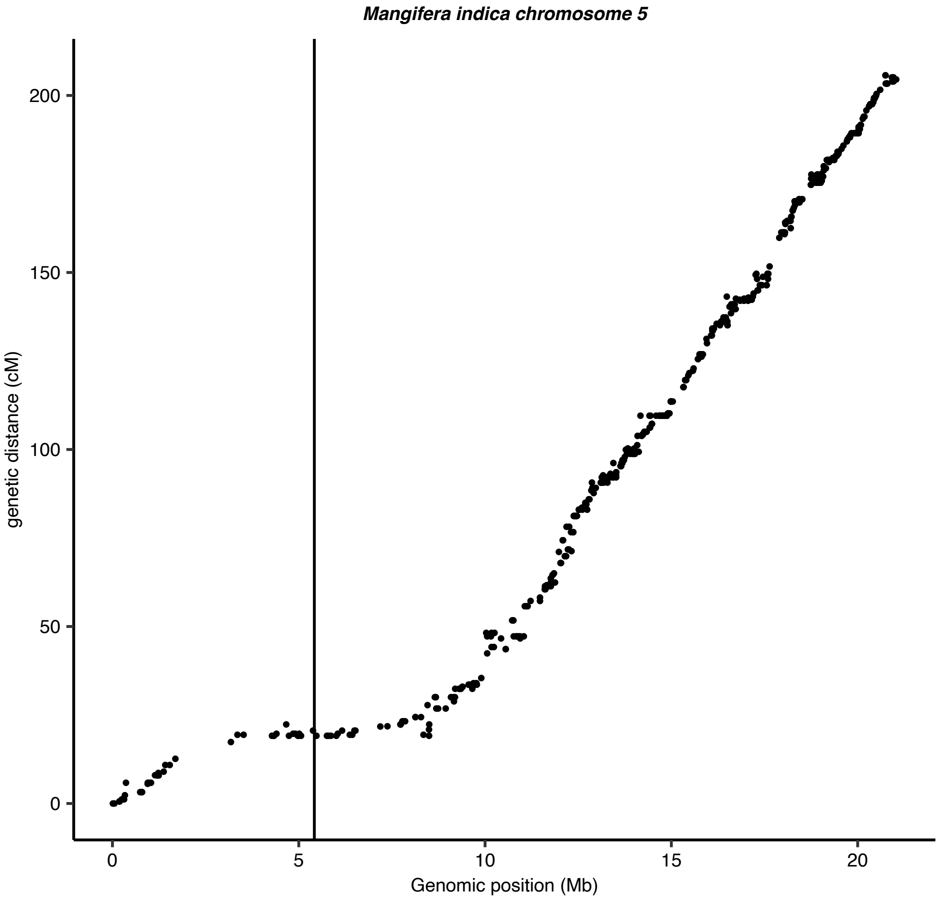
Mangifera indica chromosome 5
genetic distance (cM)
0
50
100
150
200
Genomic position (Mb)
0
5
10
15
20

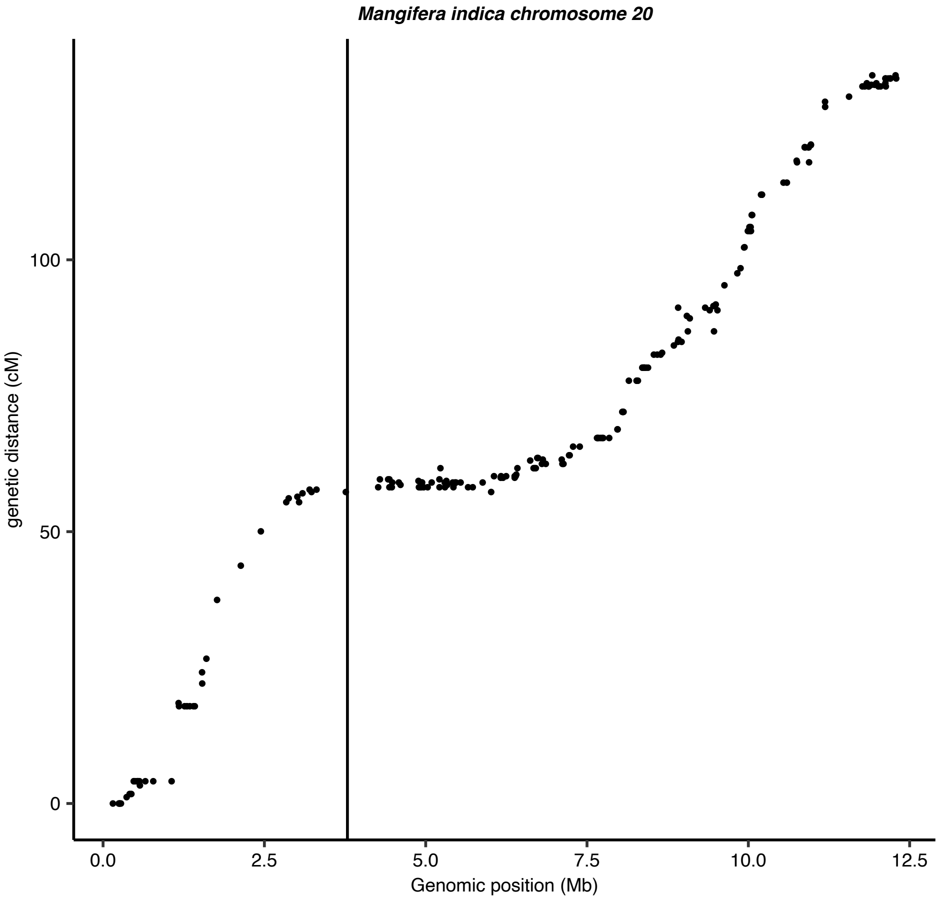
Mangifera indica chromosome 20
genetic distance (cM)
Genomic position (Mb)
0
50
100
0.0
2.5
5.0
7.5
10.0
12.5

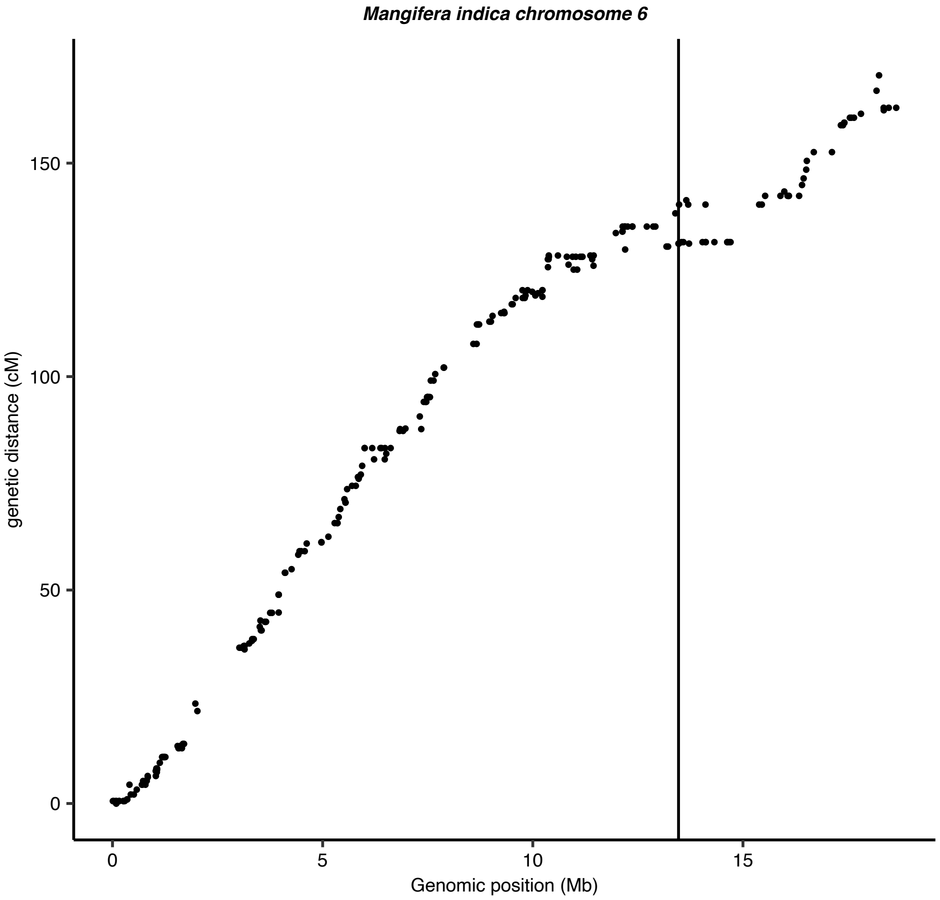
Mangifera indica chromosome 6
genetic distance (cM)
0
50
100
150
0
5
10
15
Genomic position (Mb)

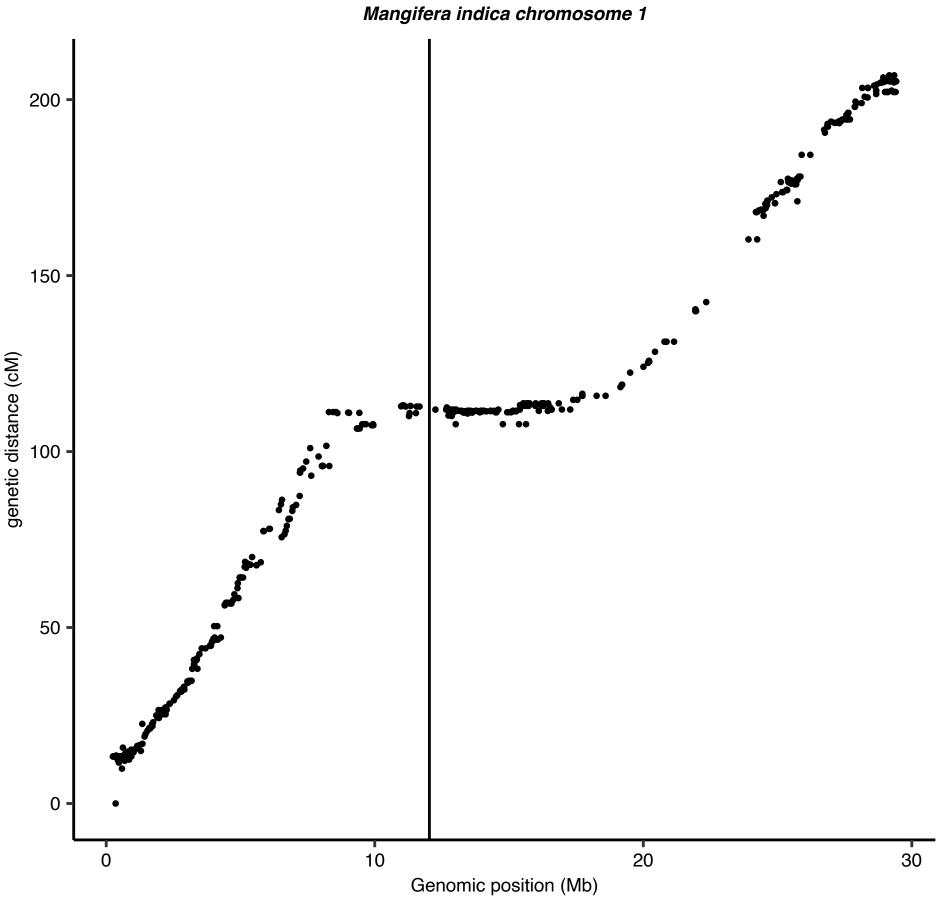
Mangifera indica chromosome 1
genetic distance (cM)
0
50
100
150
200
Genomic position (Mb)
0
10
20
30

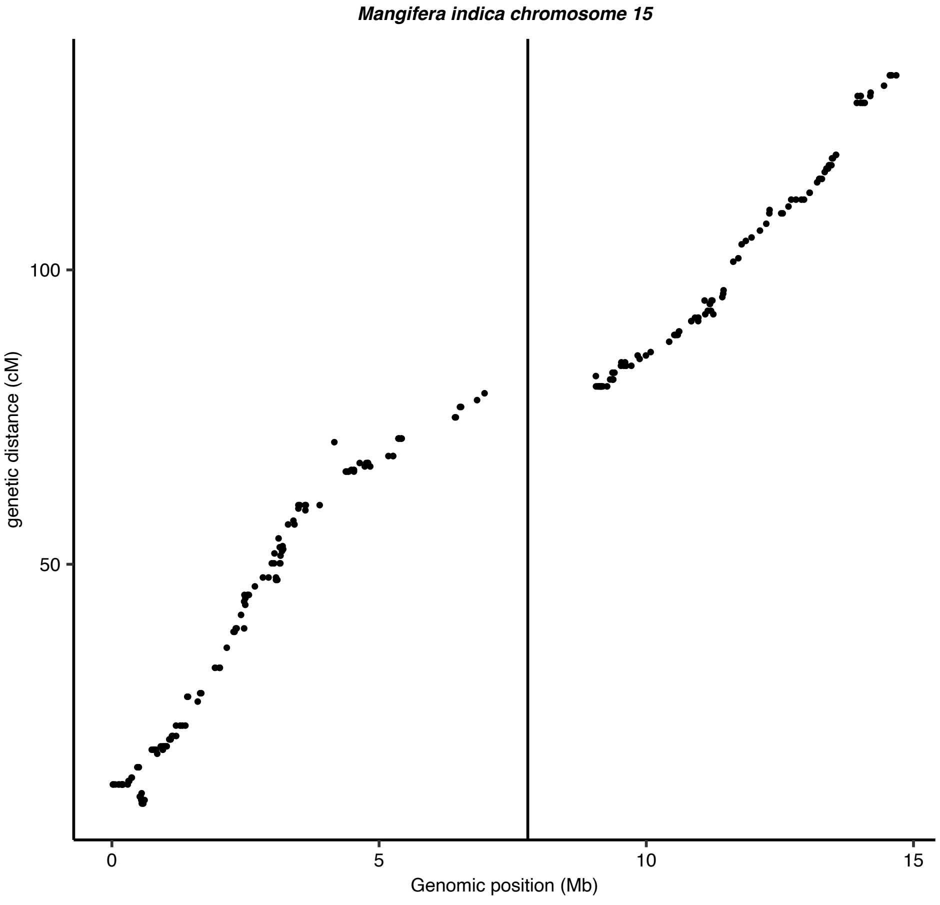

Mangifera indica chromosome 15
genetic distance (cM)
100
50
0
5
10
15
Genomic position (Mb)

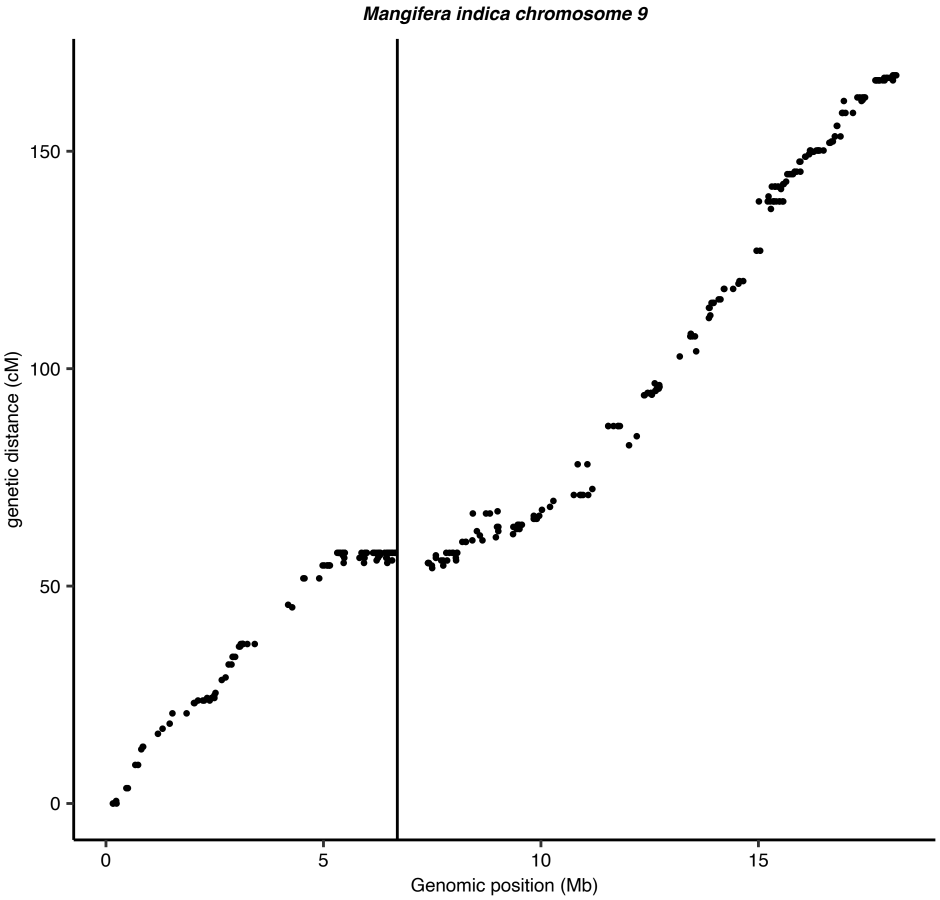
Mangifera indica chromosome 9
genetic distance (cM)
Genomic position (Mb)
0
50
100
150
0
5
10
15

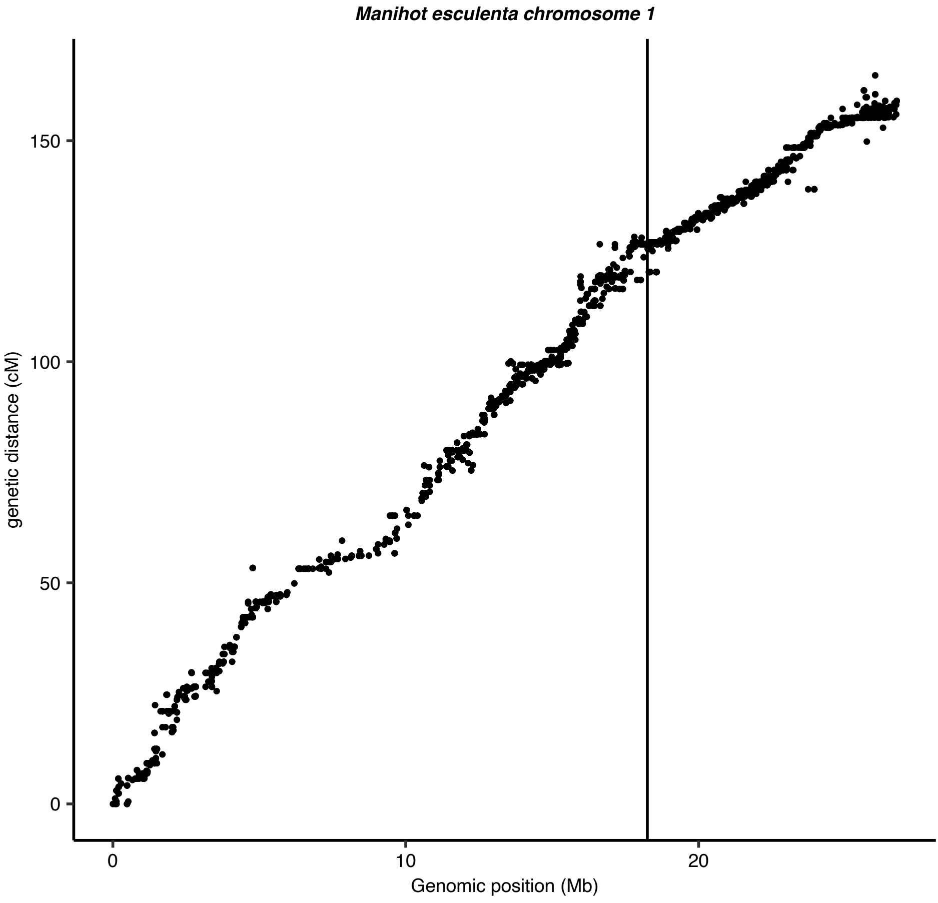
Manihot esculenta chromosome 1
genetic distance (cM)
150
100
50
0
0
10
20
Genomic position (Mb)

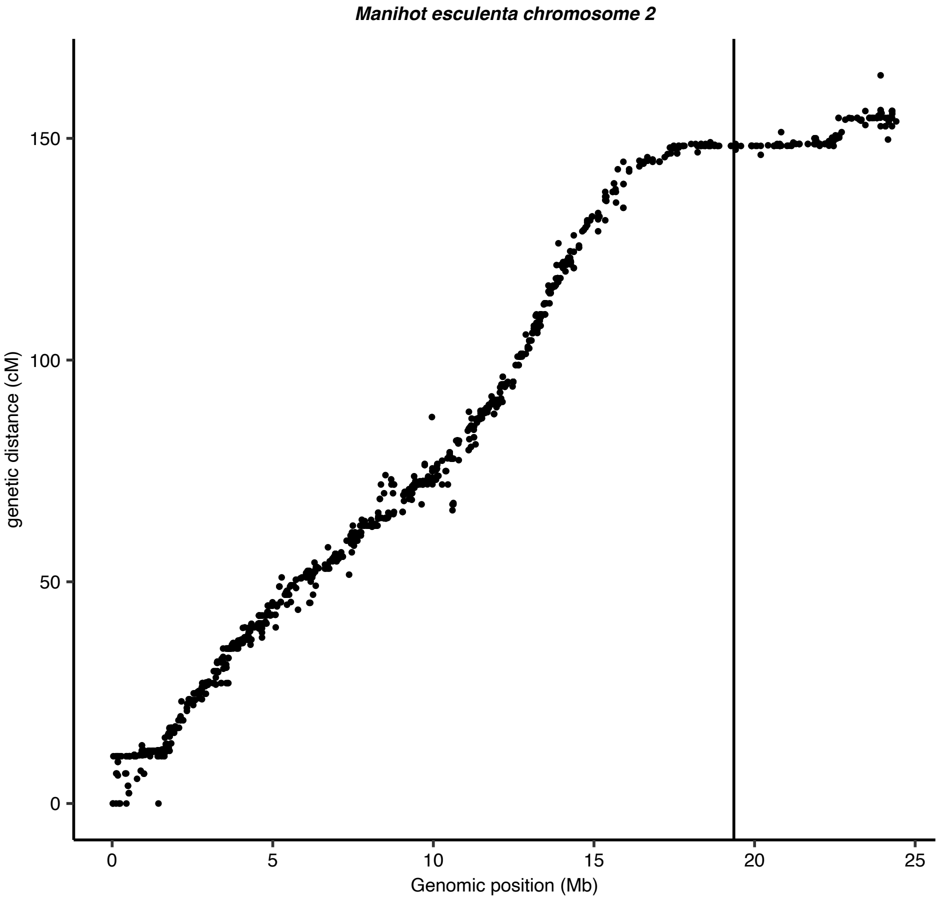
Manihot esculenta chromosome 2
genetic distance (cM)
Genomic position (Mb)
0
50
100
150
0
5
10
15
20
25

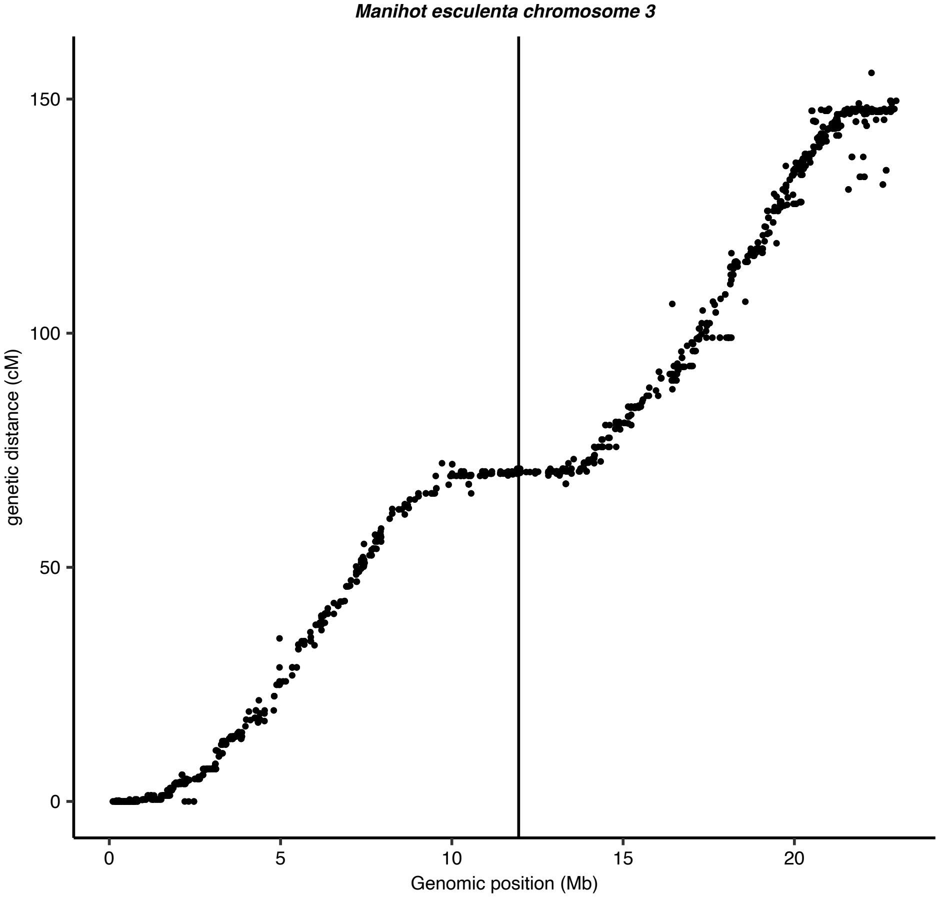
Manihot esculenta chromosome 3
genetic distance (cM)
0
50
100
150
Genomic position (Mb)
0
5
10
15
20

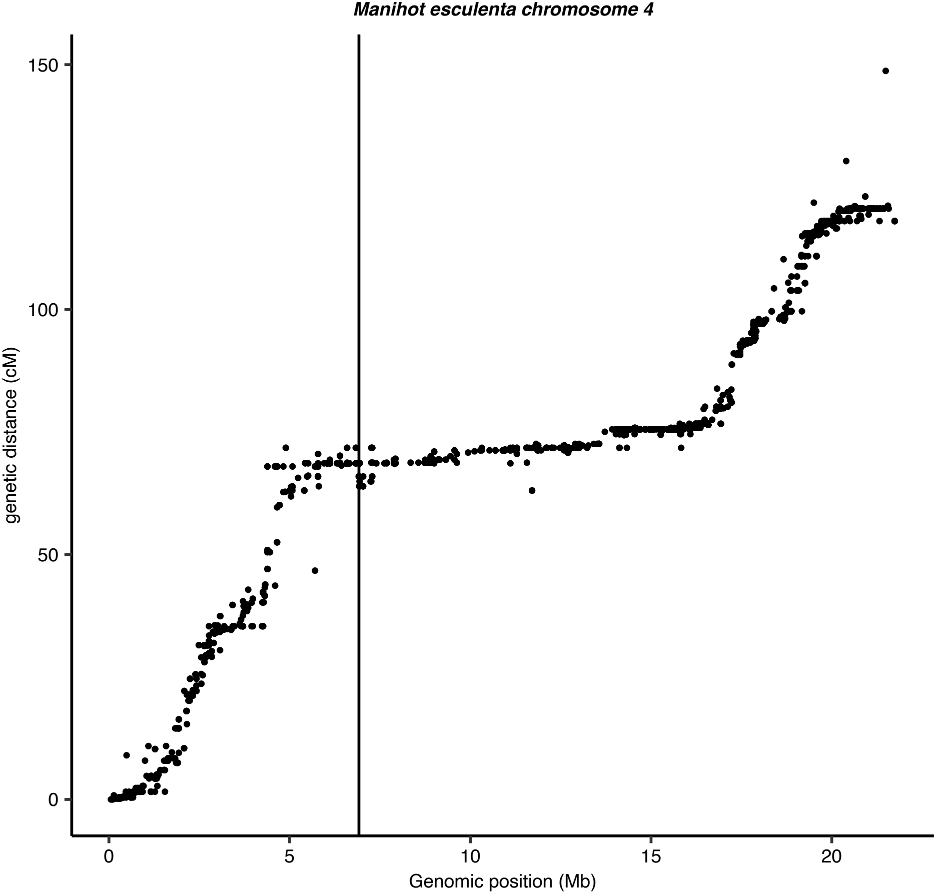
Manihot esculenta chromosome 4
genetic distance (cM)
150
100
50
0
0
5
10
15
20
Genomic position (Mb)

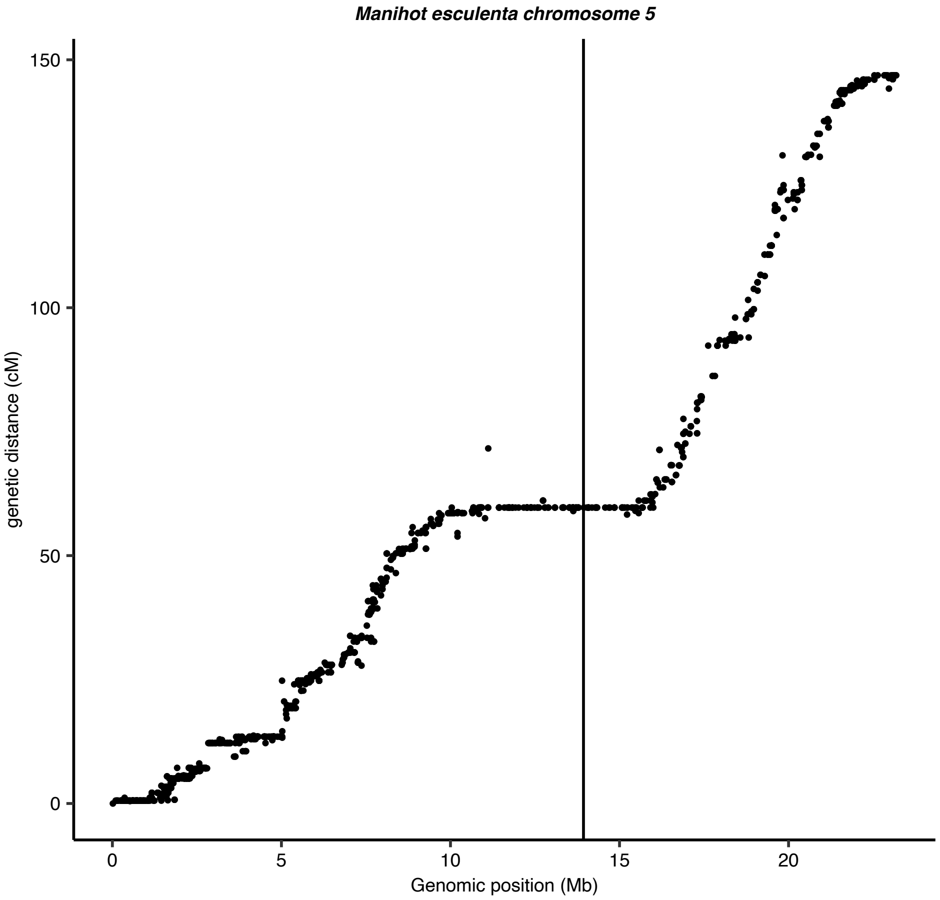
Manihot esculenta chromosome 5
genetic distance (cM)
150
100
50
0
0
5
10
15
20
Genomic position (Mb)

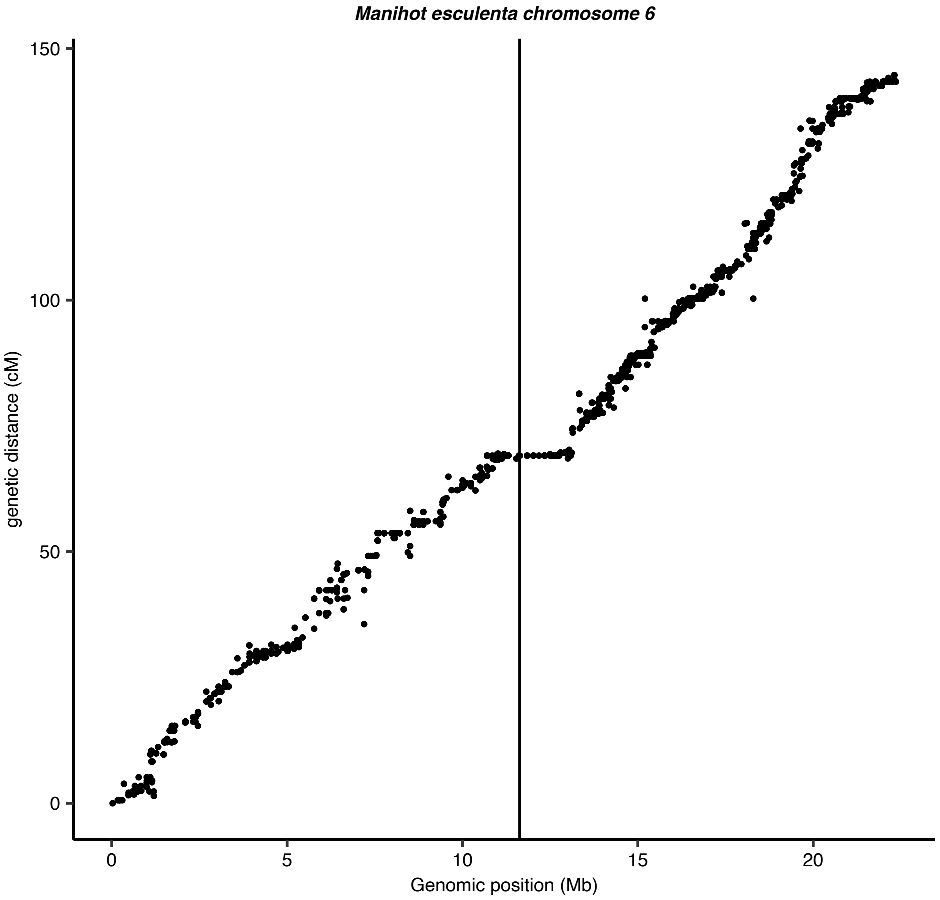
Manihot esculenta chromosome 6
150
100
50
0
genetic distance (cM)
0
5
10
15
20
Genomic position (Mb)

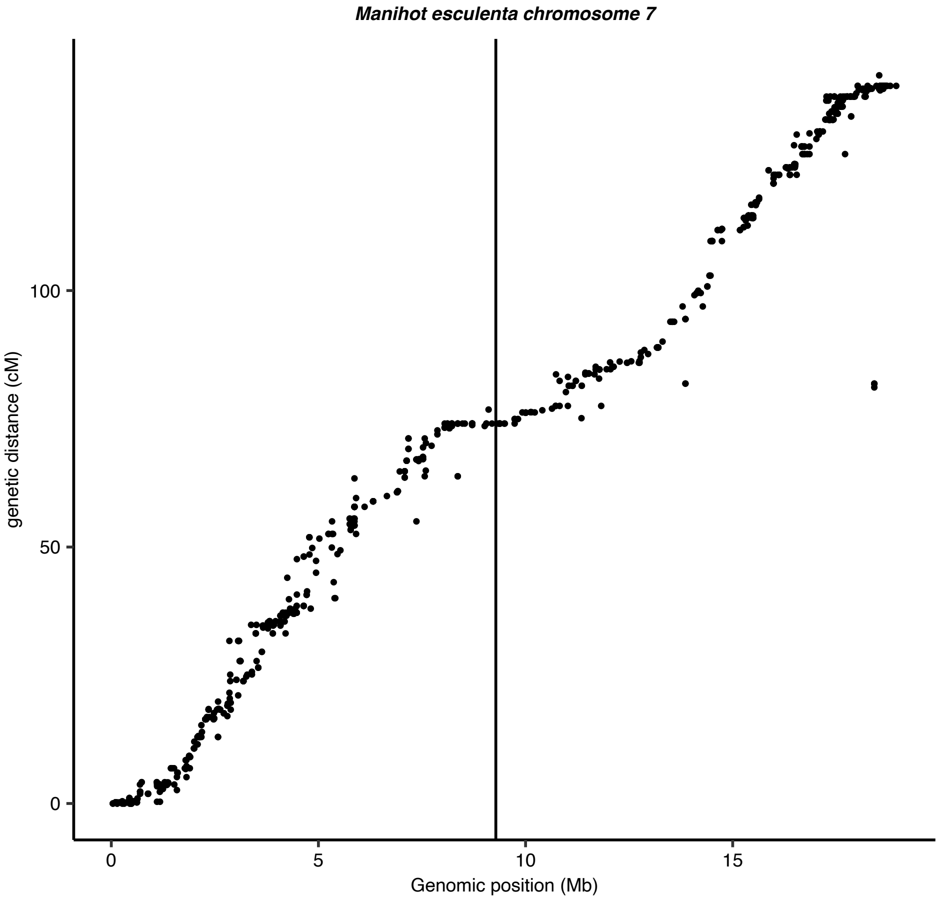
Manihot esculenta chromosome 7
genetic distance (cM)
0
50
100
Genomic position (Mb)
0
5
10
15

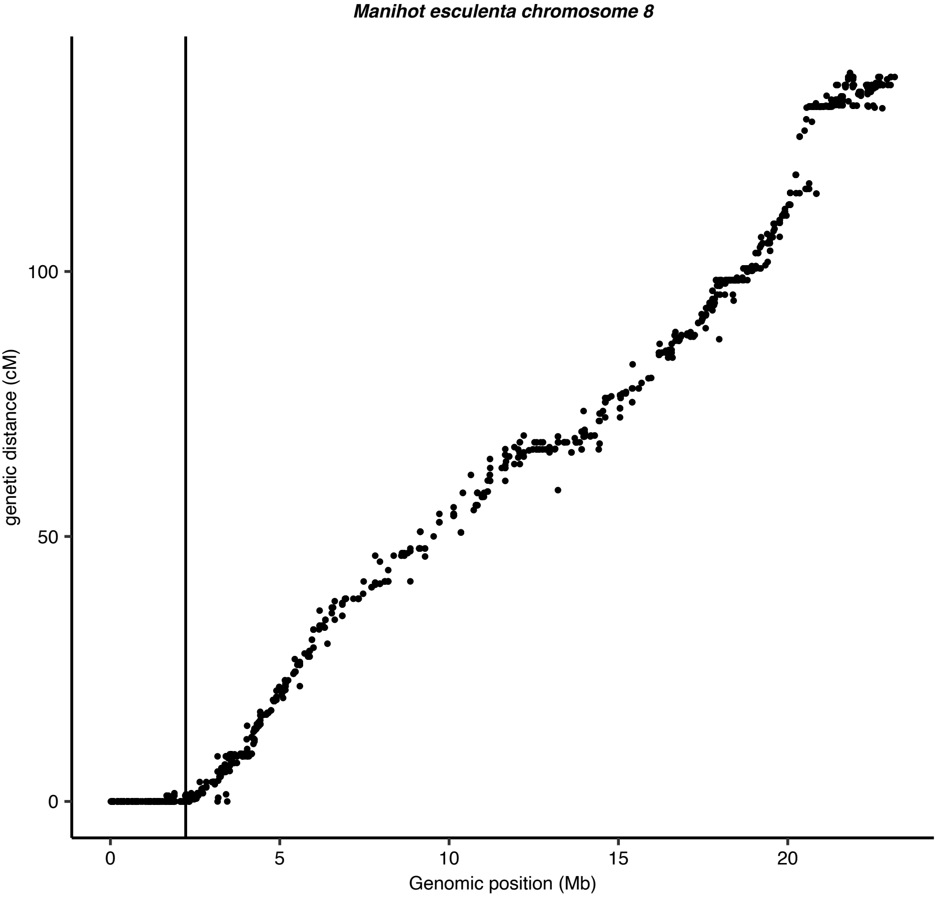
Manihot esculenta chromosome 8
genetic distance (cM)
0
50
100
Genomic position (Mb)
0
5
10
15
20

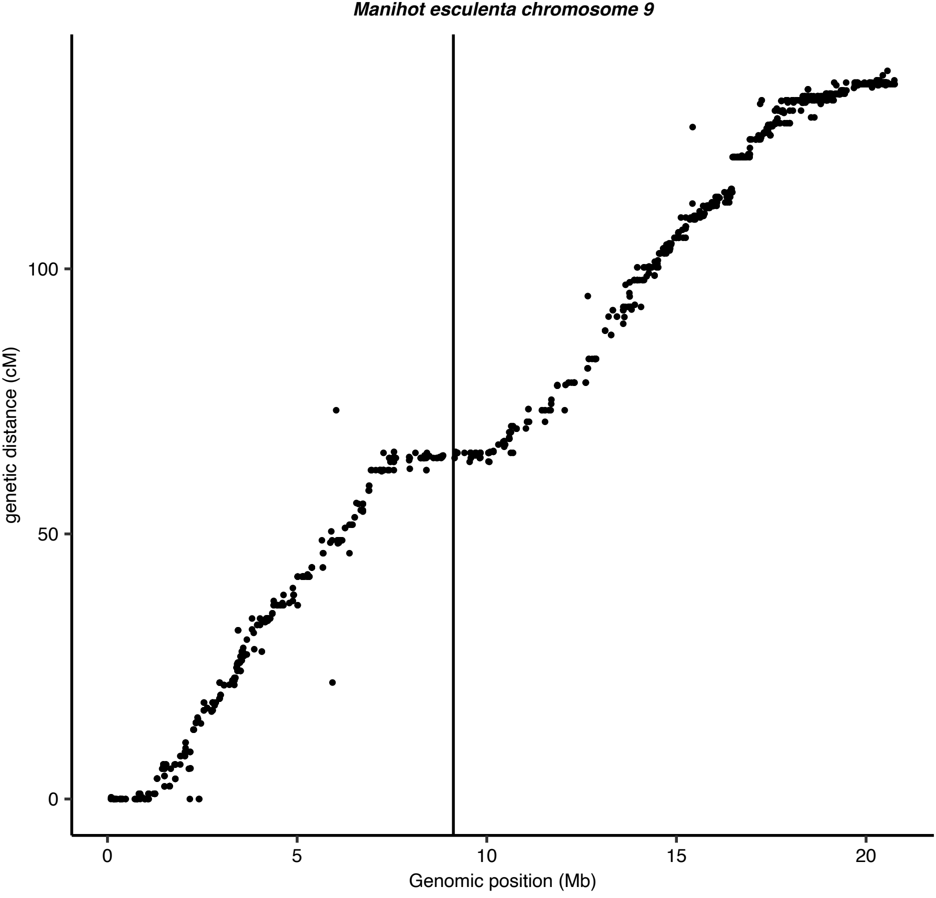
Manihot esculenta chromosome 9
genetic distance (cM)
Genomic position (Mb)
0
50
100
0
5
10
15
20

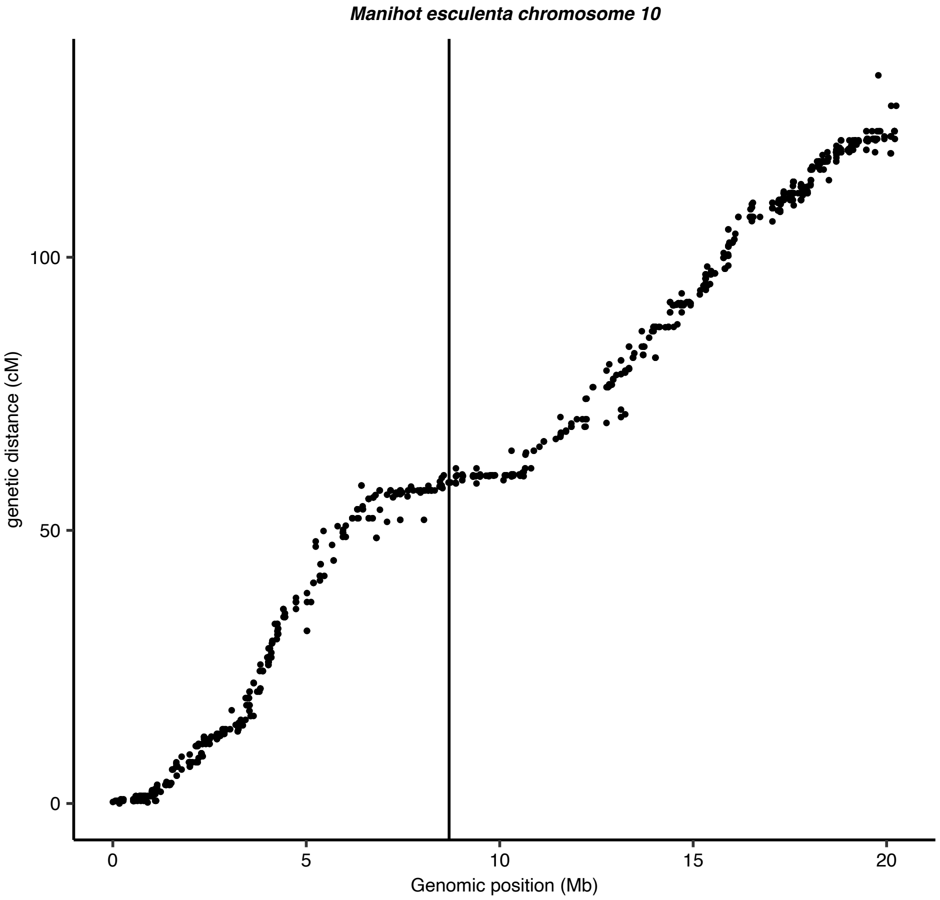
Manihot esculenta chromosome 10
genetic distance (cM)
0
50
100
Genomic position (Mb)
0
5
10
15
20

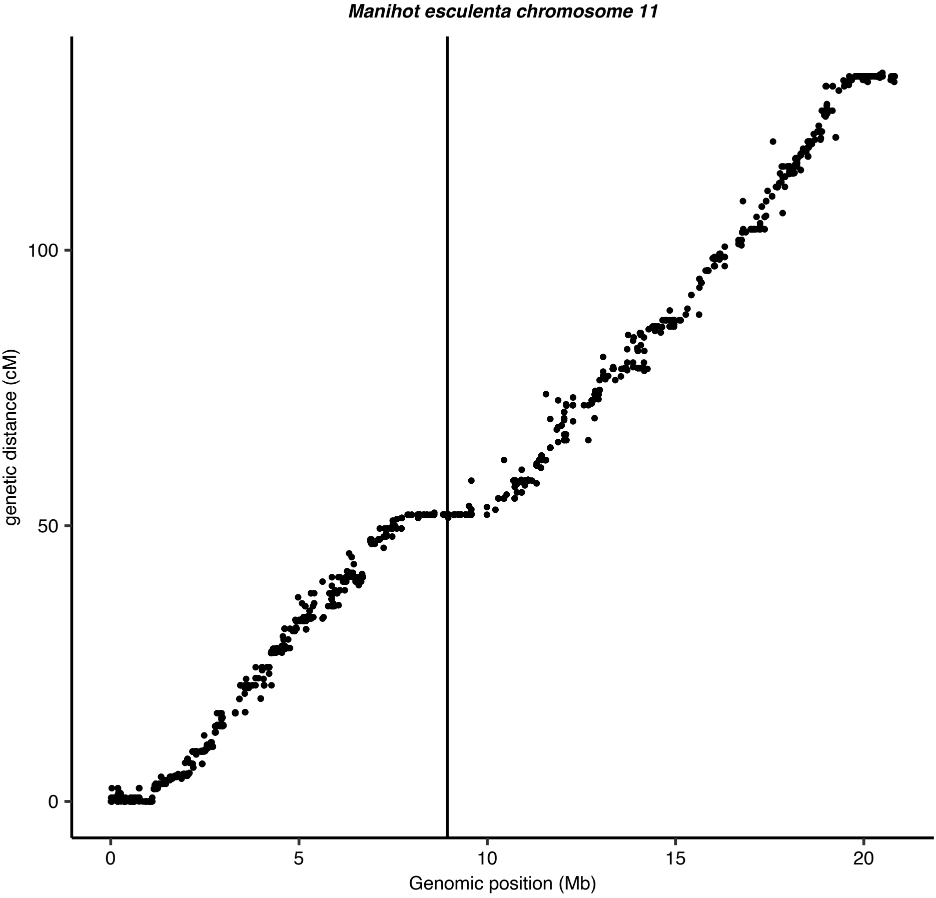
Manihot esculenta chromosome 11
genetic distance (cM)
0
50
100
Genomic position (Mb)
0
5
10
15
20

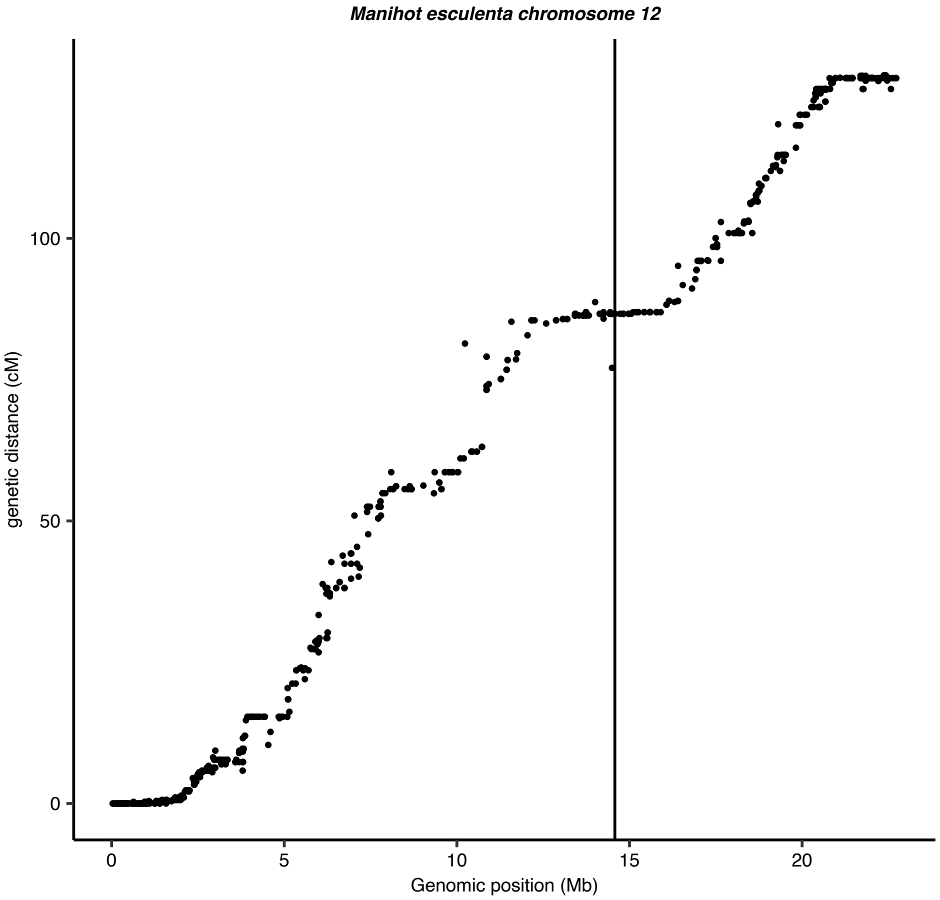
Manihot esculenta chromosome 12
genetic distance (cM)
100
50
0
0
5
10
15
20
Genomic position (Mb)

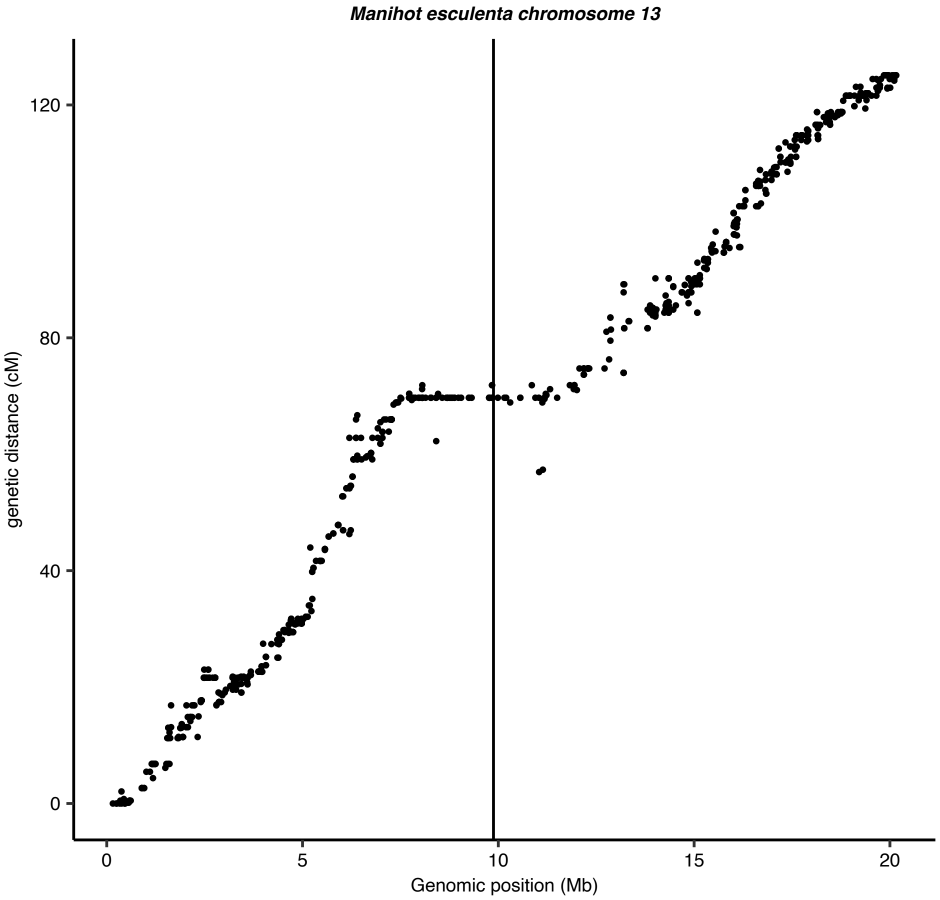
Manihot esculenta chromosome 13
genetic distance (cM)
Genomic position (Mb)
0
40
80
120
0
5
10
15
20

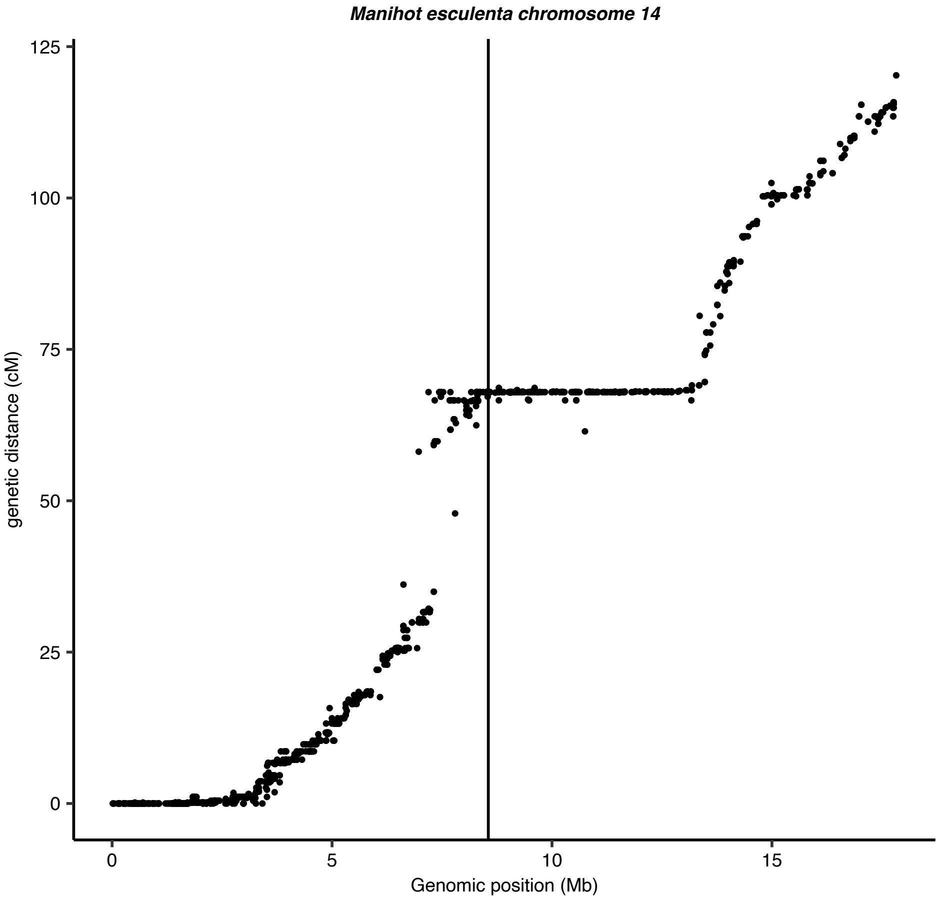
Manihot esculenta chromosome 14
genetic distance (cM)
Genomic position (Mb)
0
25
50
75
100
125
0
5
10
15

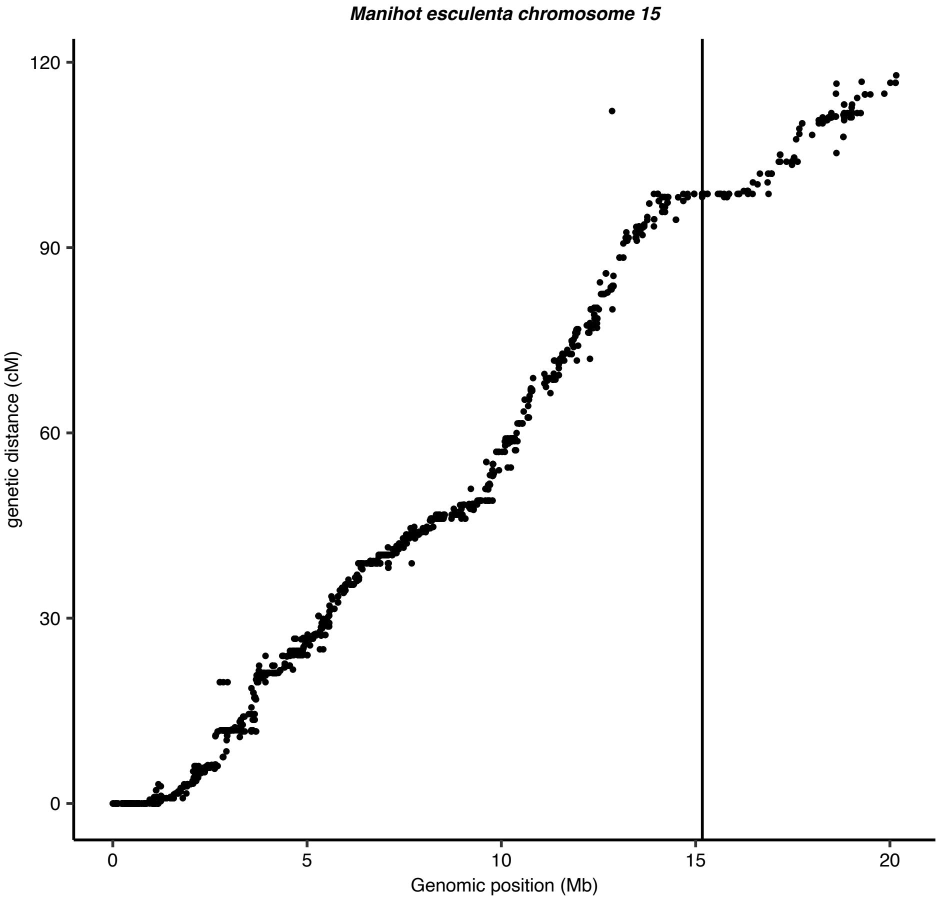
Manihot esculenta chromosome 15
genetic distance (cM)
Genomic position (Mb)
0
30
60
90
120
0
5
10
15
20

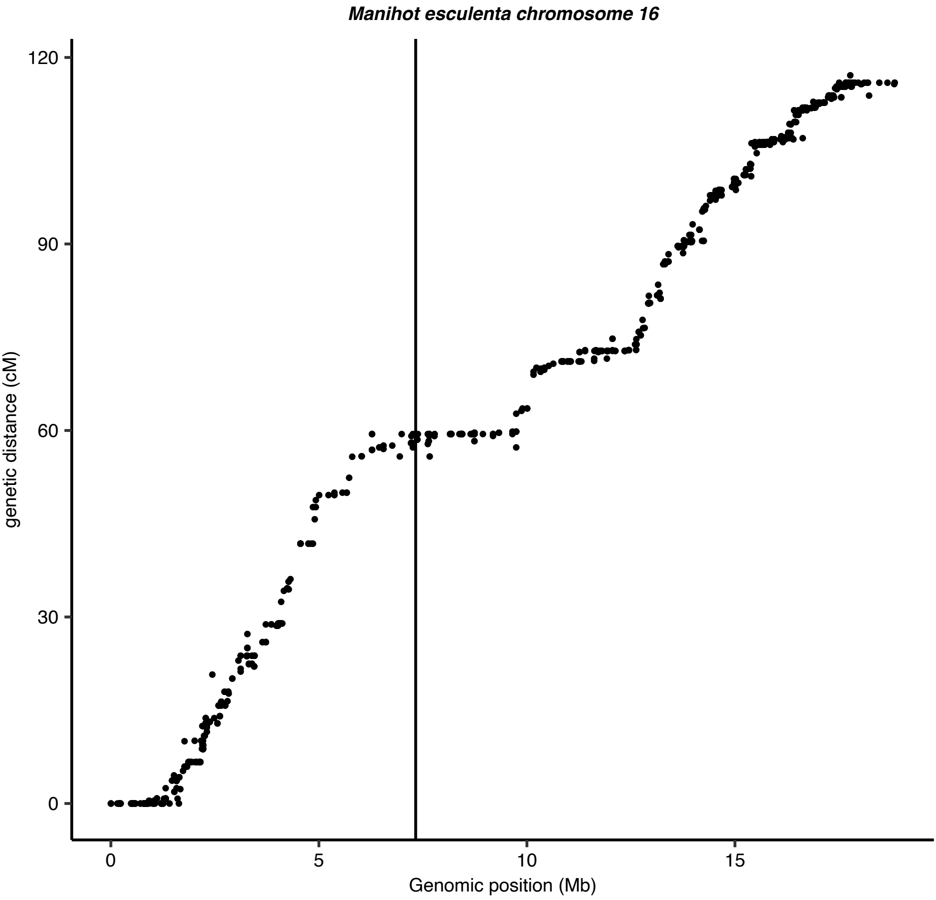
Manihot esculenta chromosome 16
genetic distance (cM)
120
90
60
30
0
Genomic position (Mb)
0
5
10
15

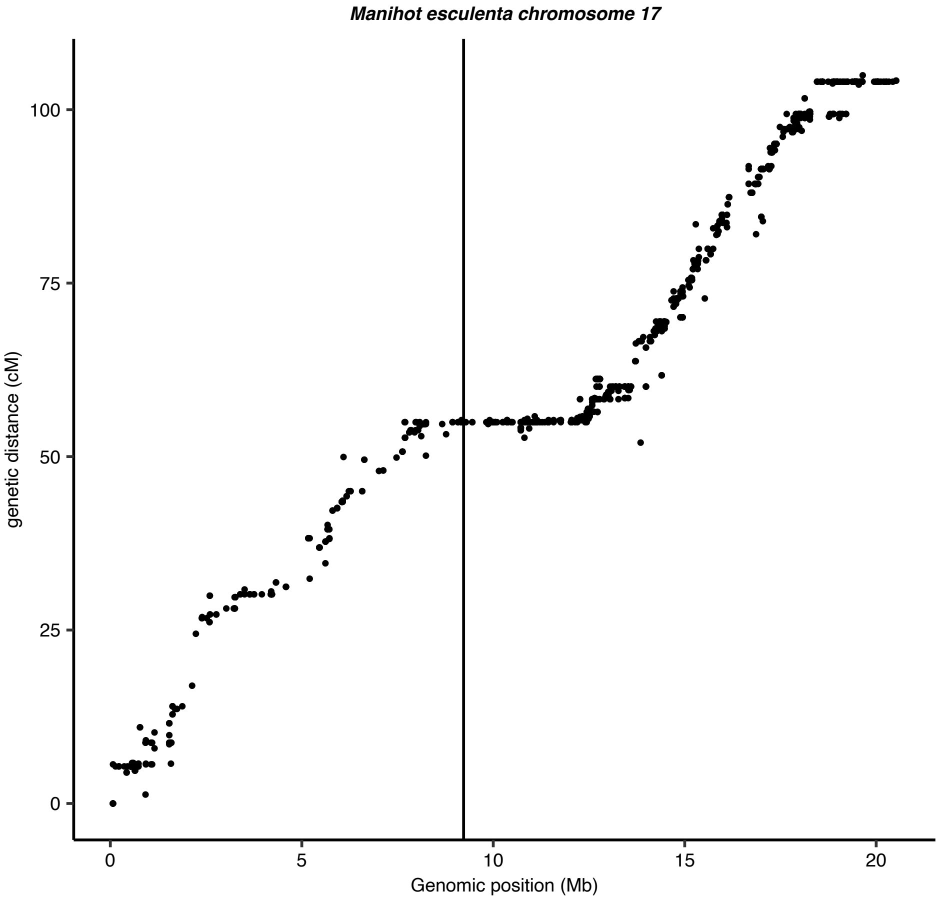
Manihot esculenta chromosome 17
genetic distance (cM)
0
25
50
75
100
Genomic position (Mb)
0
5
10
15
20

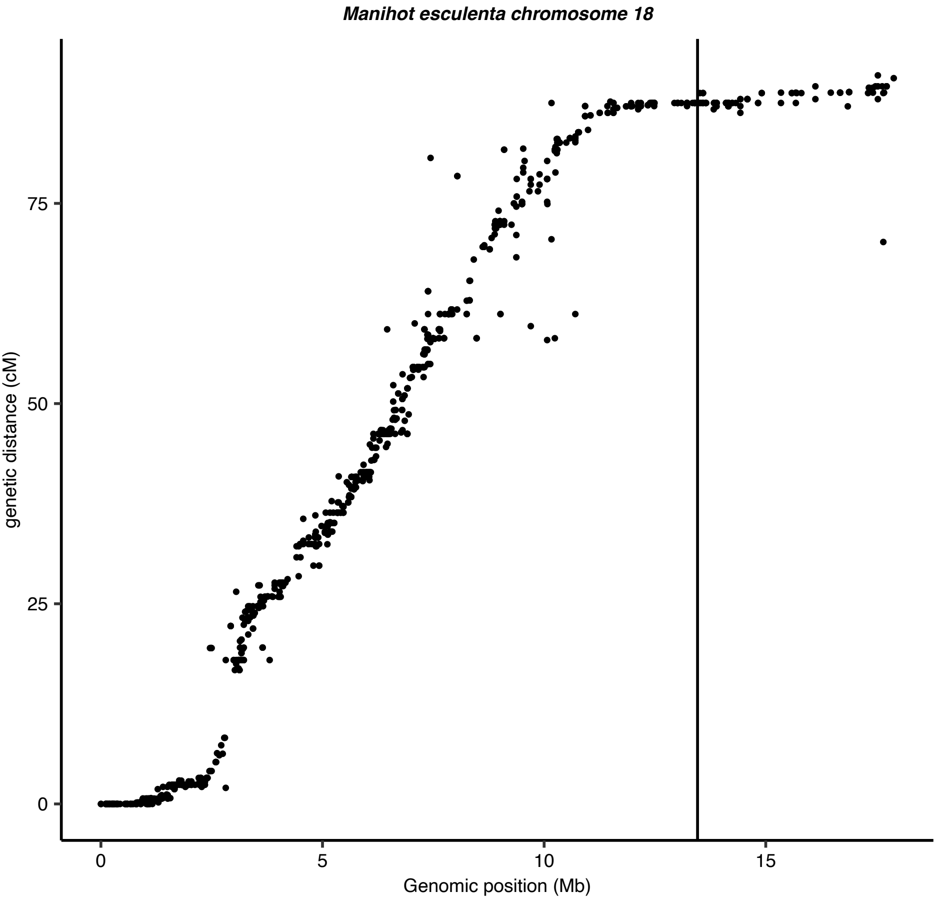
Manihot esculenta chromosome 18
genetic distance (cM)
0
25
50
75
Genomic position (Mb)
0
5
10
15

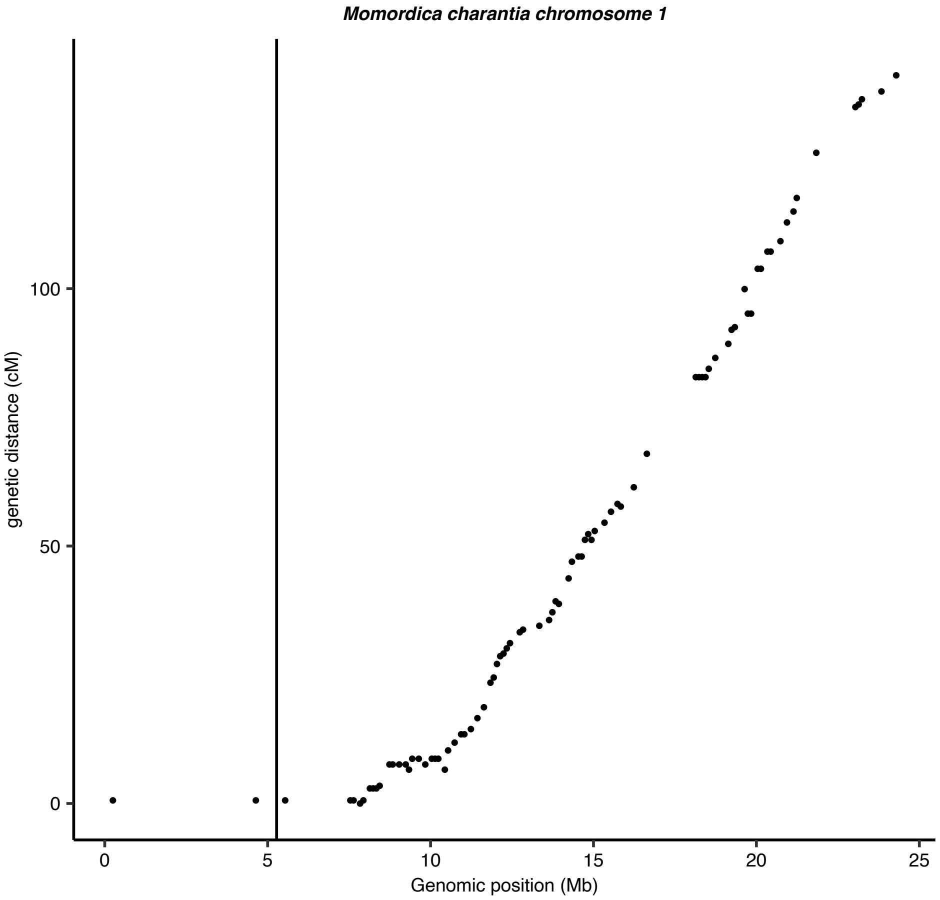
Momordica charantia chromosome 1
genetic distance (cM)
100
50
0
0
5
10
15
20
25
Genomic position (Mb)

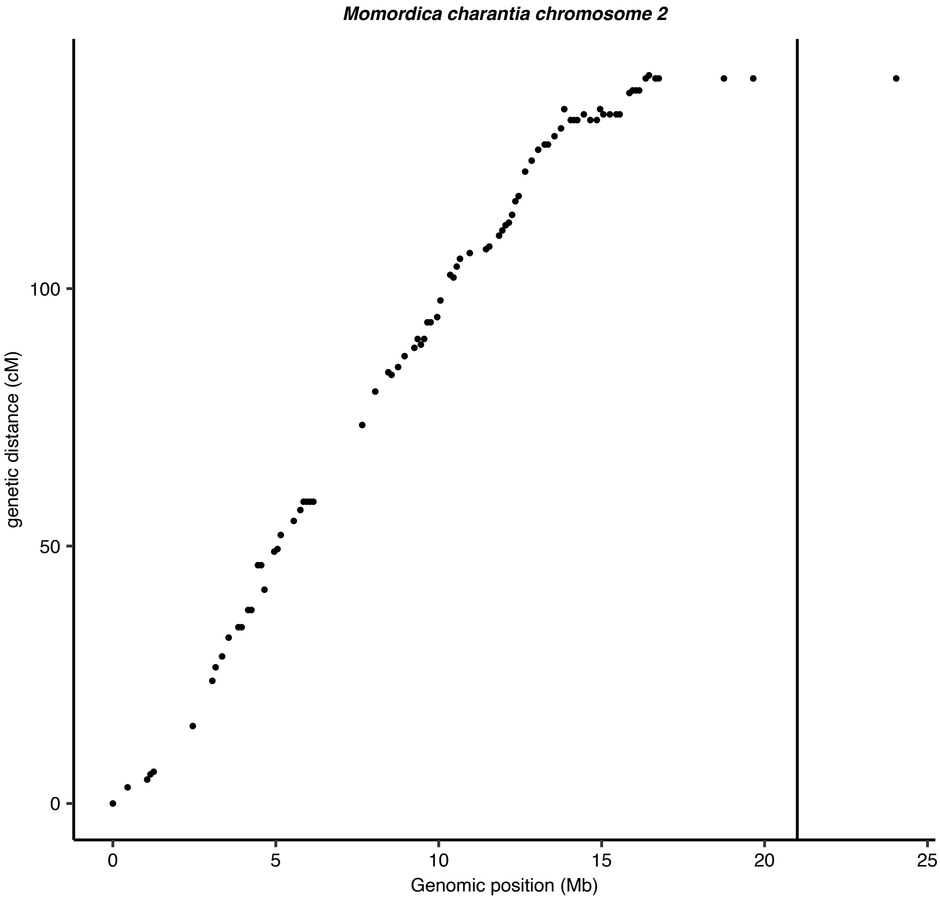
Momordica charantia chromosome 2
genetic distance (cM)
100
50
0
0
5
10
15
20
25
Genomic position (Mb)

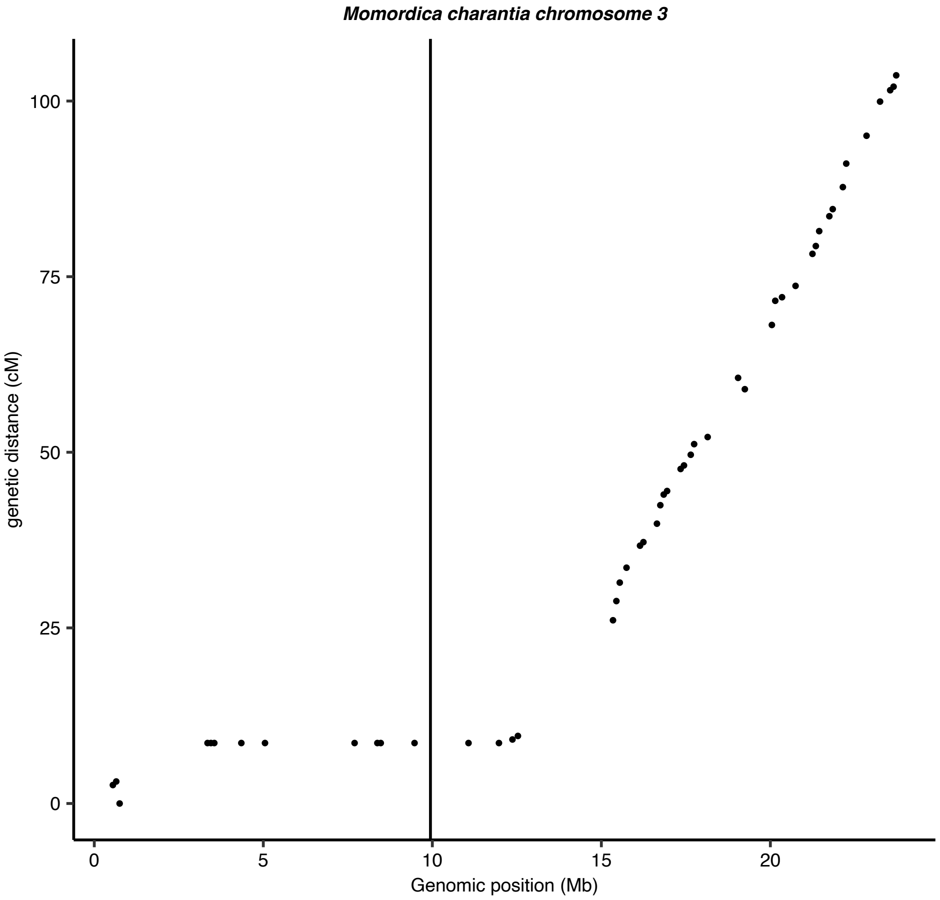
Momordica charantia chromosome 3
genetic distance (cM)
0
25
50
75
100
0
5
10
15
20
Genomic position (Mb)

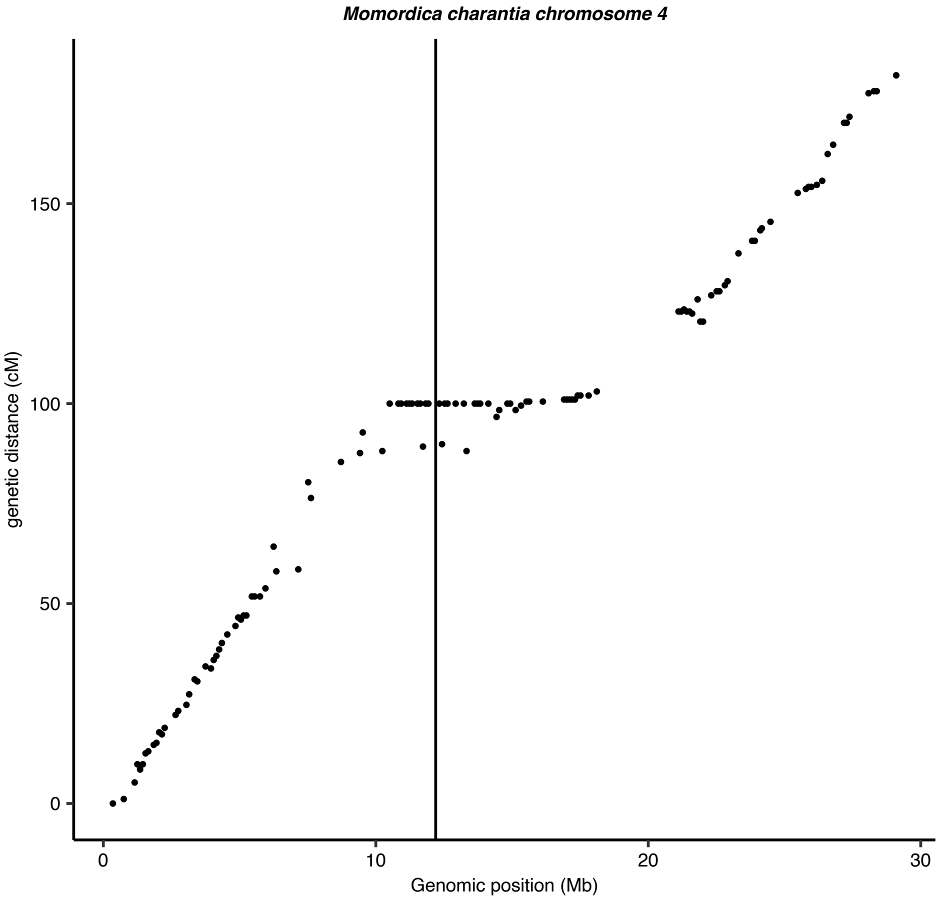
Momordica charantia chromosome 4
genetic distance (cM)
0
50
100
150
Genomic position (Mb)
0
10
20
30

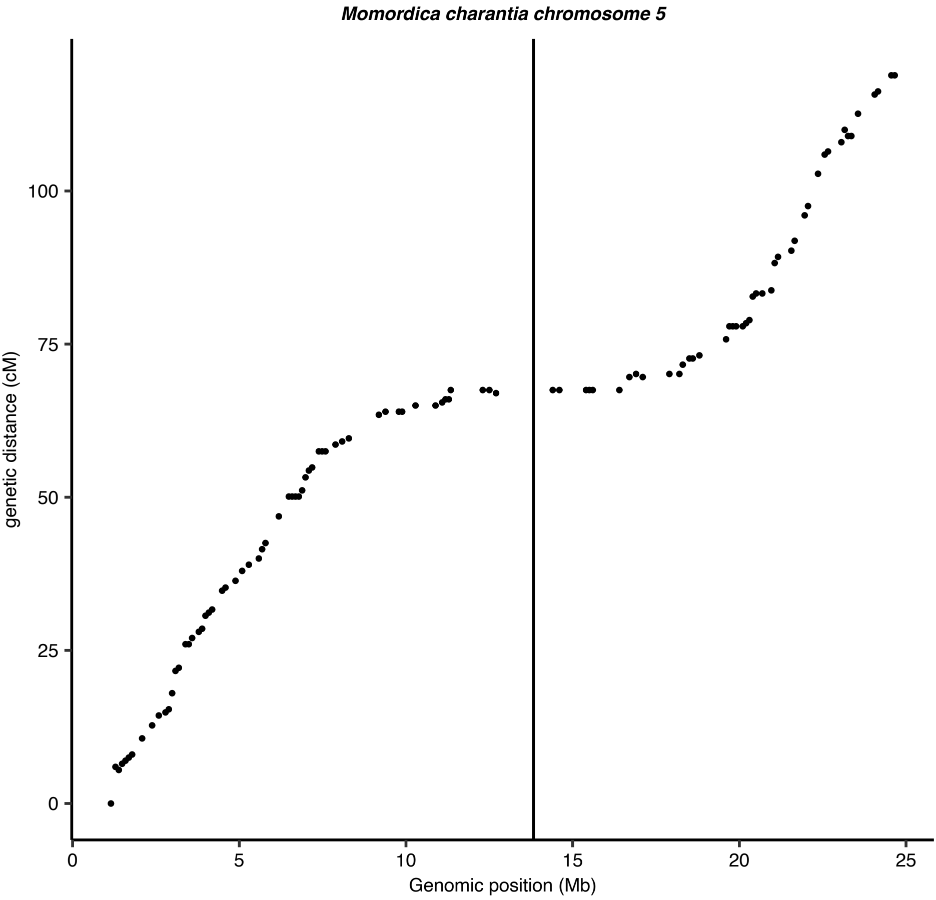

Momordica charantia chromosome 5
genetic distance (cM)
0
25
50
75
100
Genomic position (Mb)
0
5
10
15
20
25

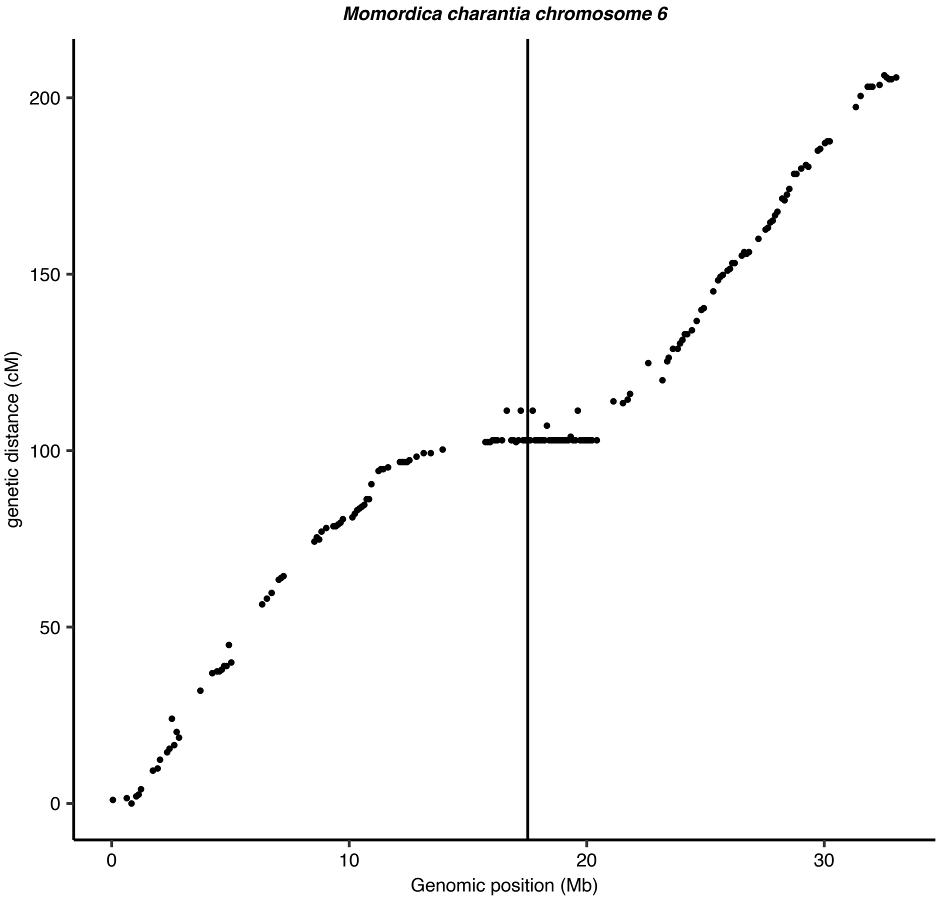
Momordica charantia chromosome 6
genetic distance (cM)
0
50
100
150
200
Genomic position (Mb)
0
10
20
30

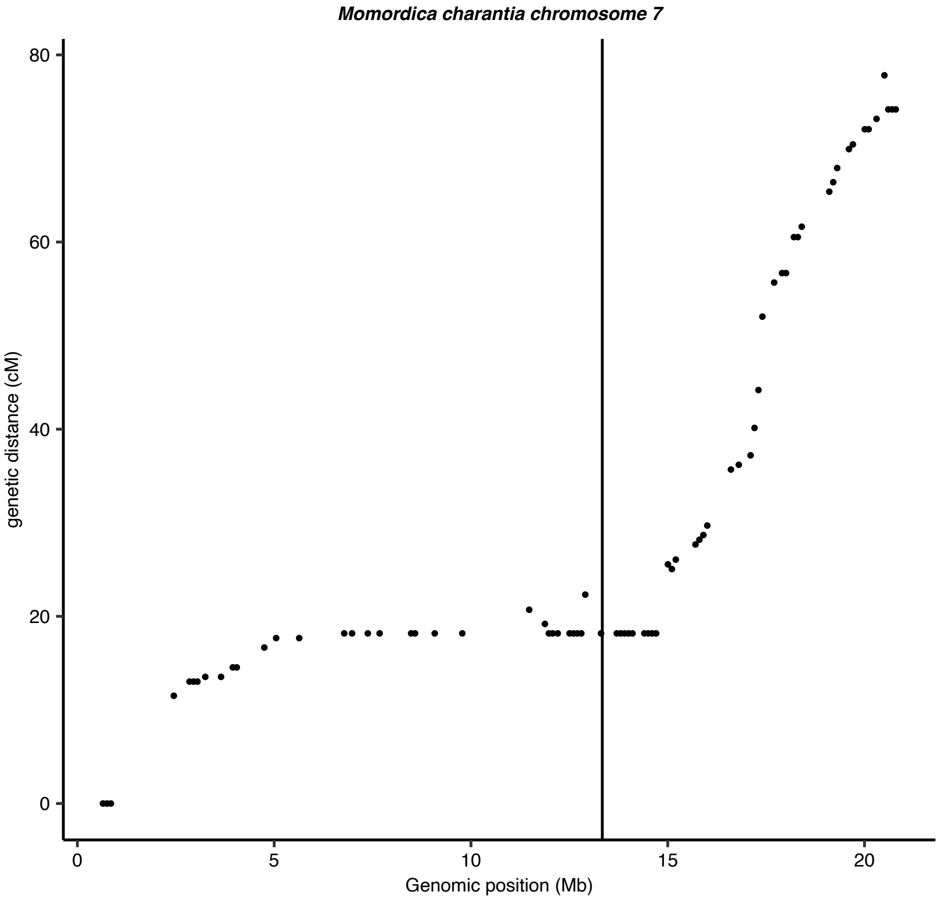
Momordica charantia chromosome 7
genetic distance (cM)
Genomic position (Mb)
0
20
40
60
80
0
5
10
15
20

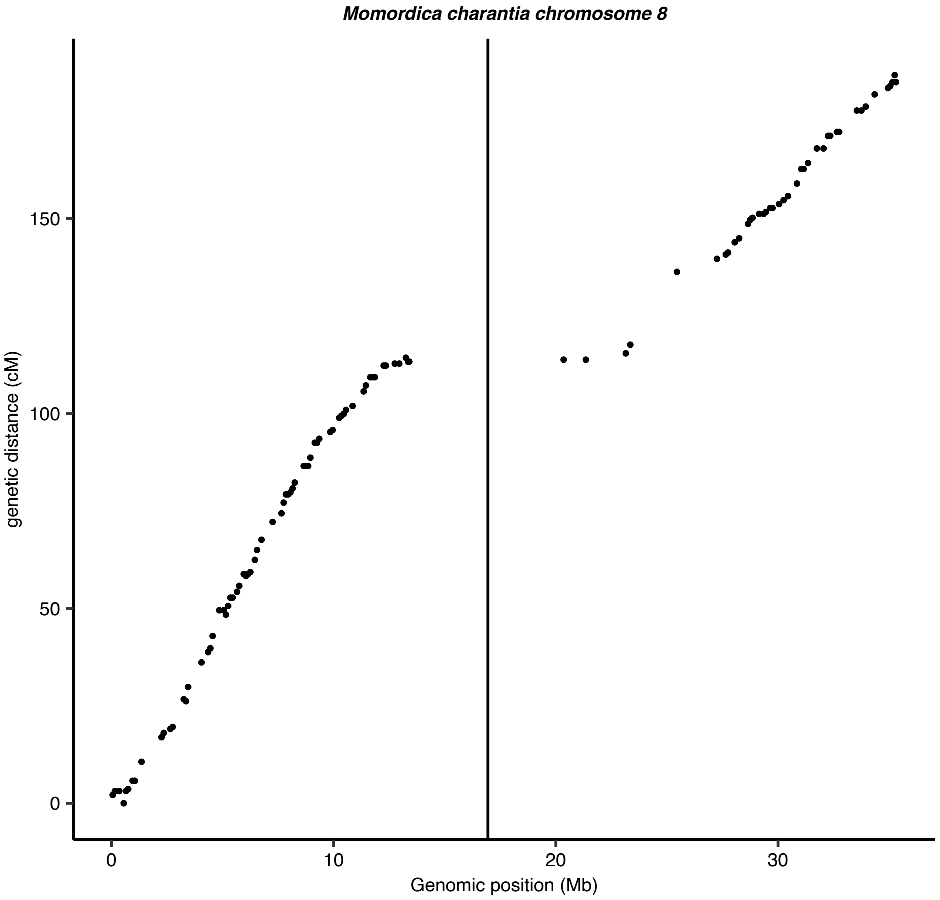
Momordica charantia chromosome 8
genetic distance (cM)
150
100
50
0
0
10
20
30
Genomic position (Mb)

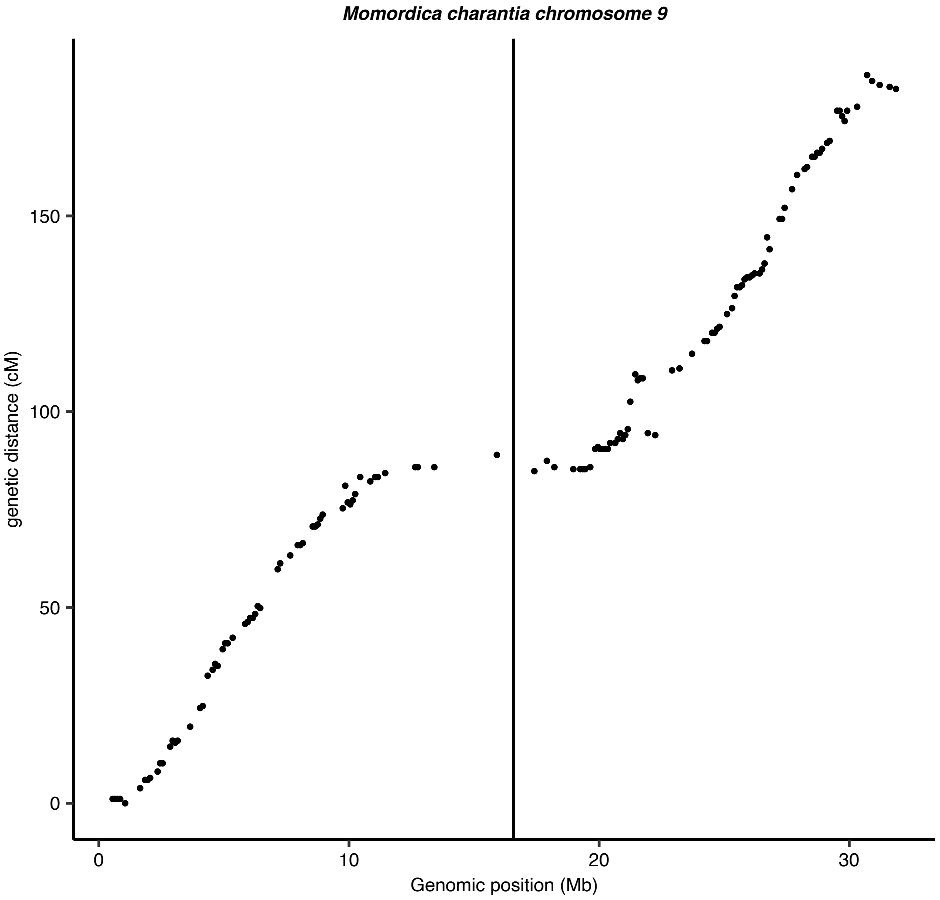

Momordica charantia chromosome 9
genetic distance (cM)
0
50
100
150
Genomic position (Mb)
0
10
20
30

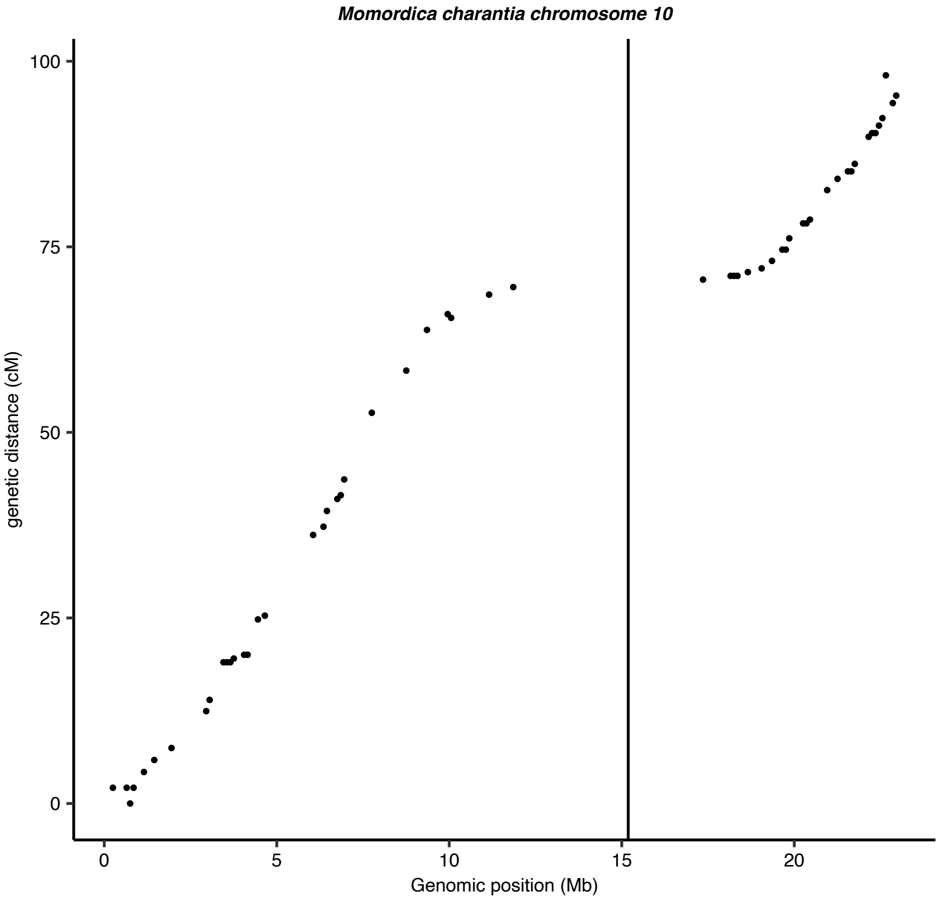
Momordica charantia chromosome 10
genetic distance (cM)
Genomic position (Mb)
0
25
50
75
100
0
5
10
15
20

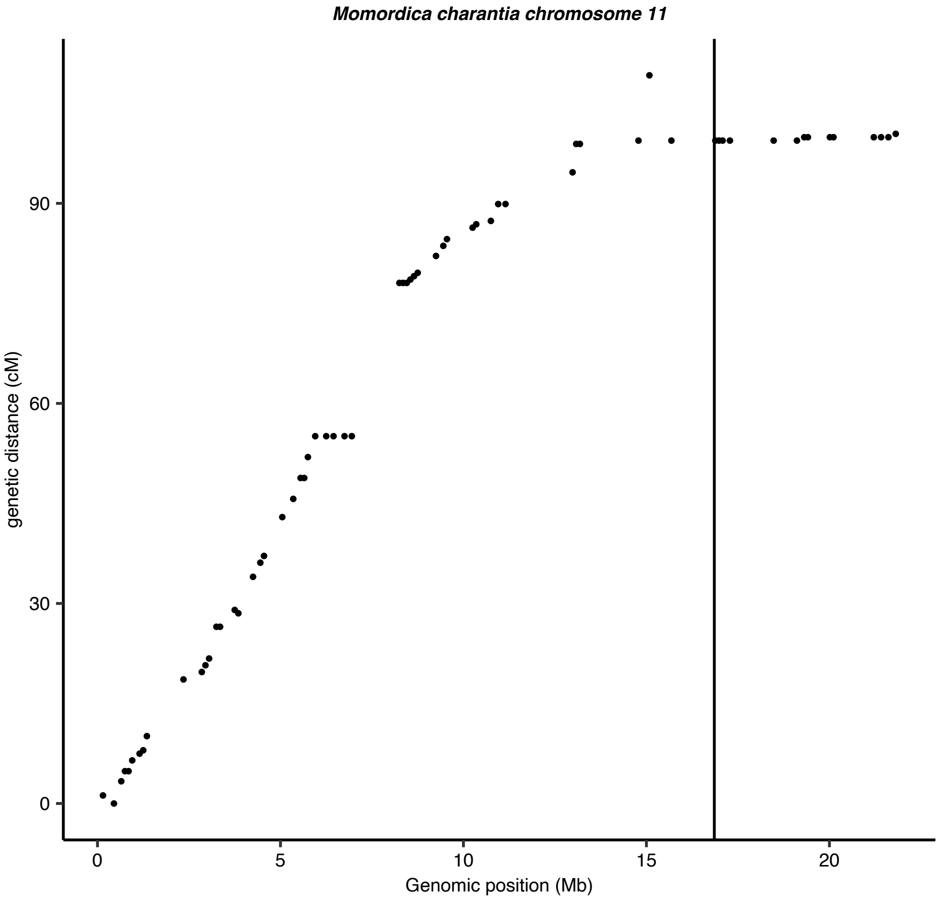

Momordica charantia chromosome 11
genetic distance (cM)
0
30
60
90
Genomic position (Mb)
0
5
10
15
20

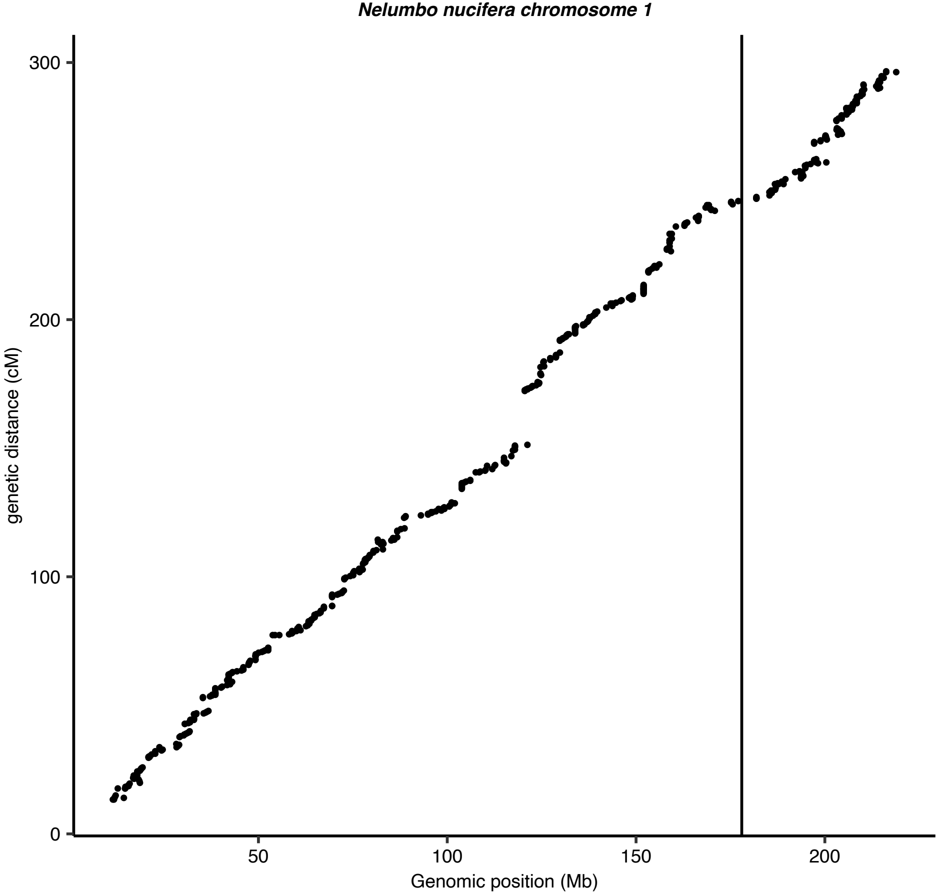
Nelumbo nucifera chromosome 1
300
200
100
0
50
100
150
200
genetic distance (cM)
Genomic position (Mb)

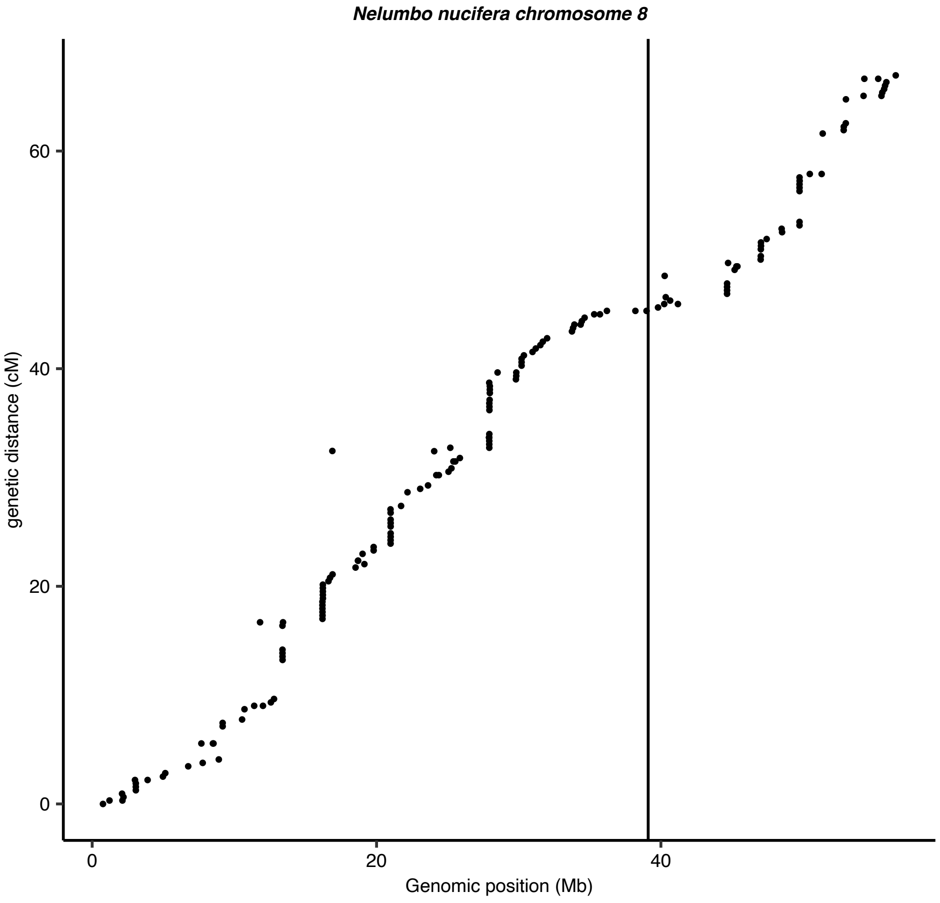

Nelumbo nucifera chromosome 8
genetic distance (cM)
0
20
40
60
Genomic position (Mb)
0
20
40

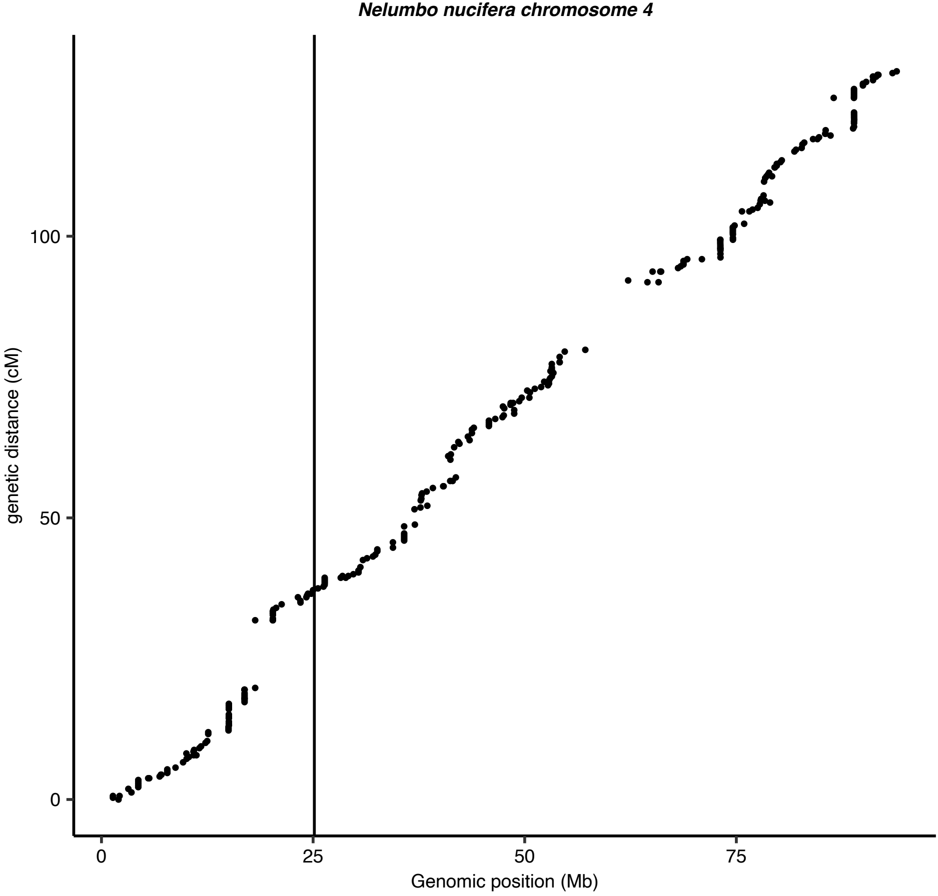
Nelumbo nucifera chromosome 4
genetic distance (cM)
Genomic position (Mb)
0
50
100
0
25
50
75

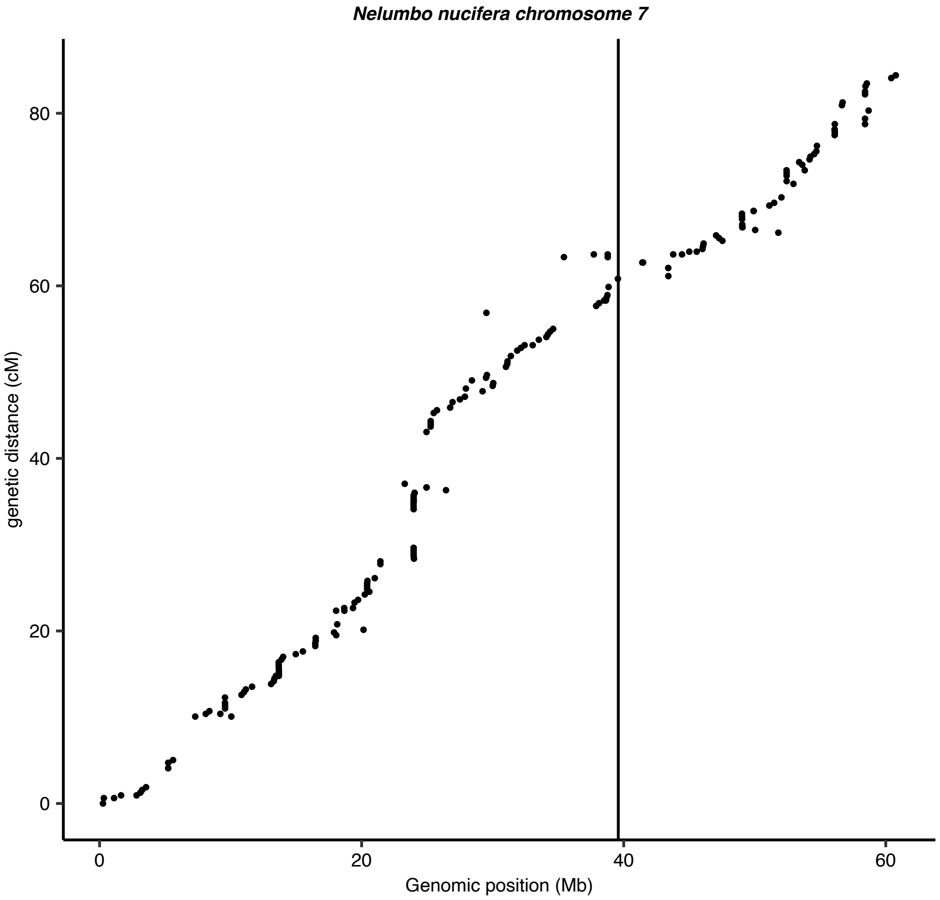
Nelumbo nucifera chromosome 7
genetic distance (cM)
0
20
40
60
80
Genomic position (Mb)
0
20
40
60

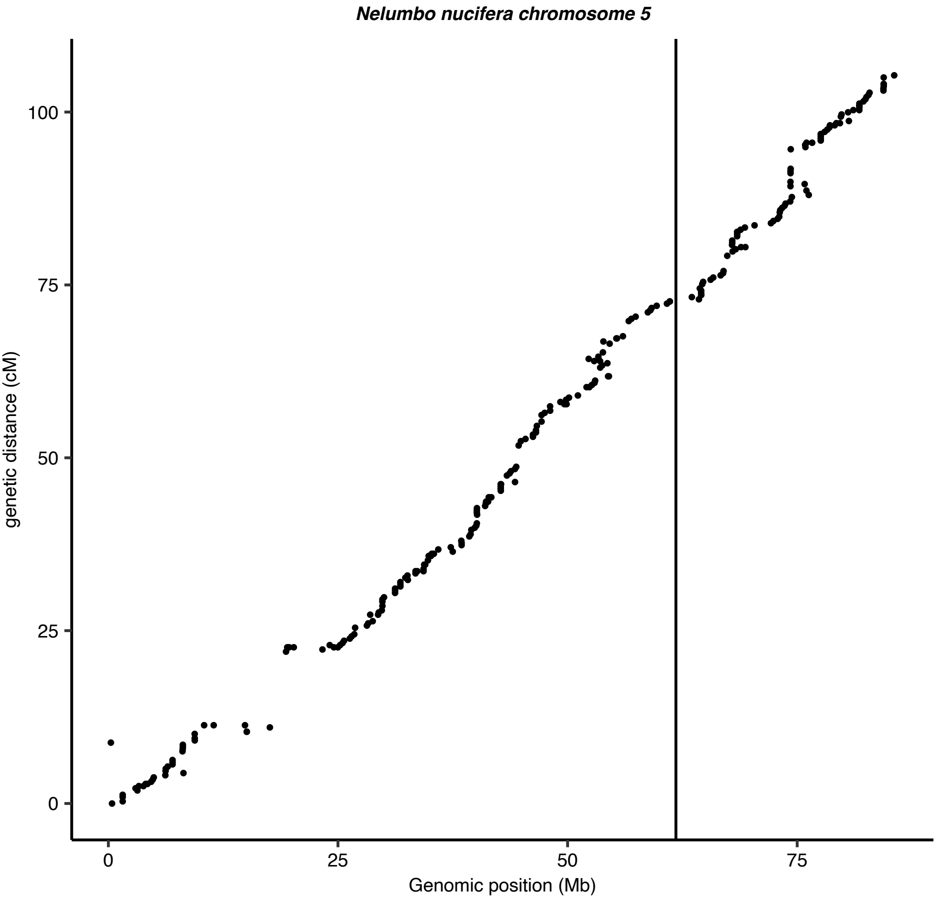

Nelumbo nucifera chromosome 5
genetic distance (cM)
0
25
50
75
100
Genomic position (Mb)
0
25
50
75

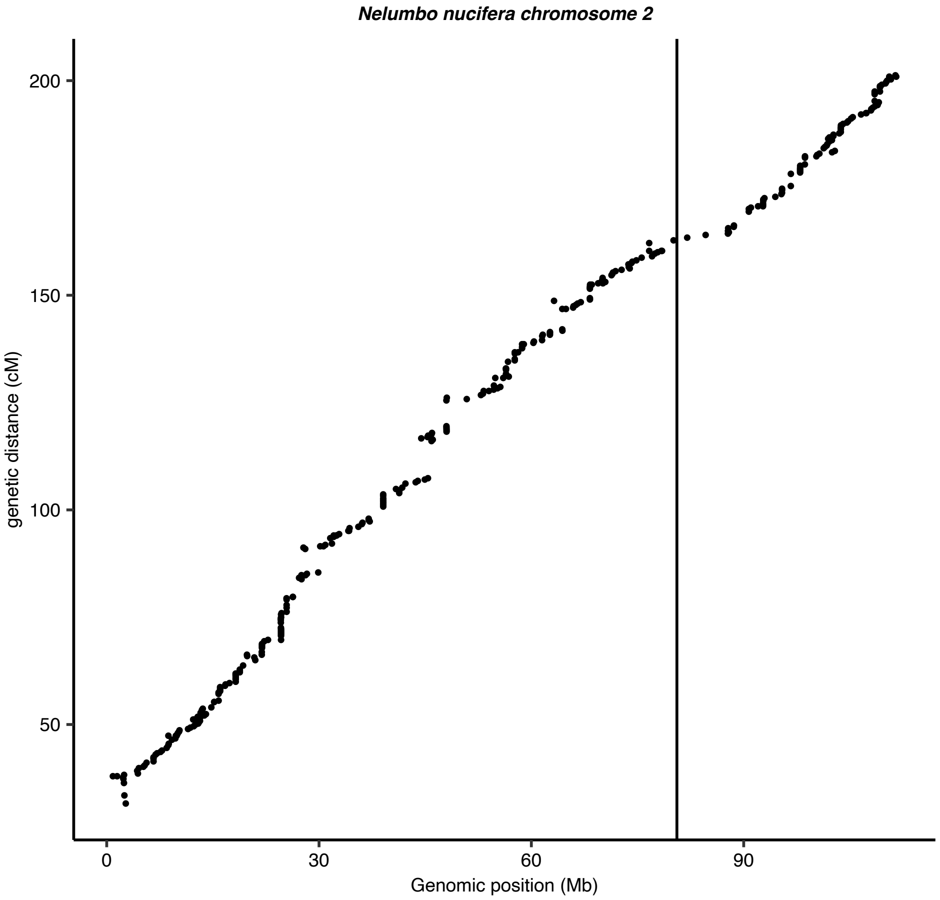
Nelumbo nucifera chromosome 2
200
150
100
50
genetic distance (cM)
0
30
60
90
Genomic position (Mb)

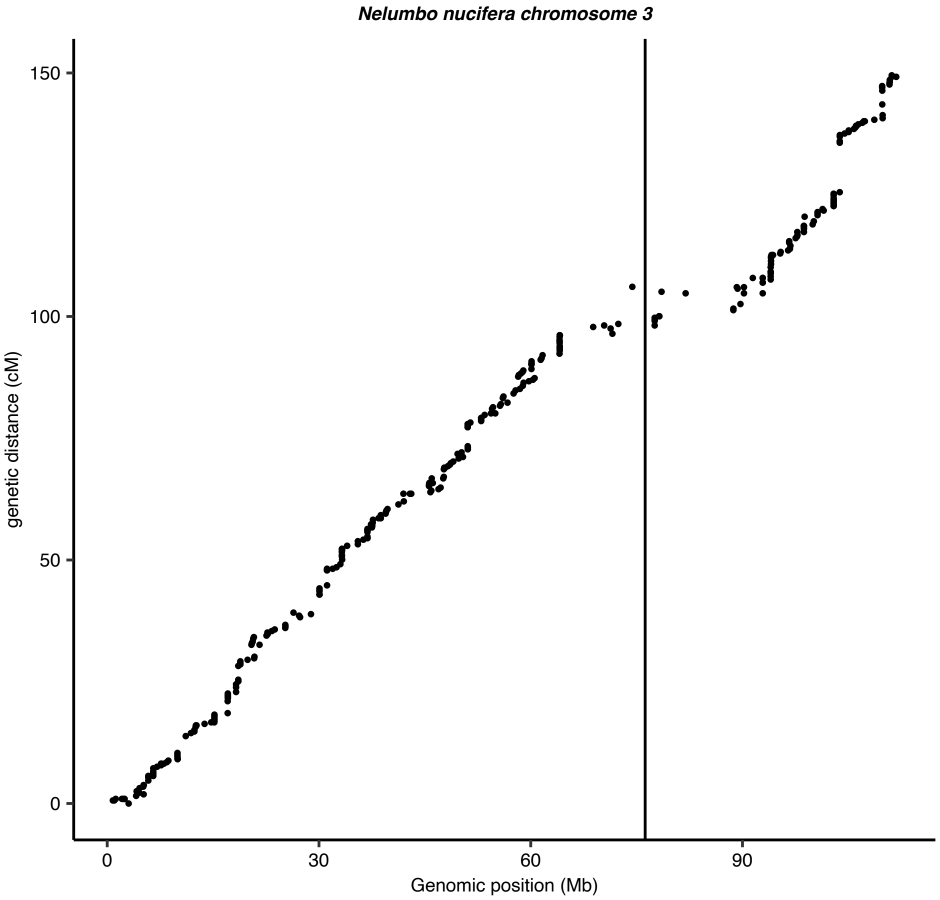
Nelumbo nucifera chromosome 3
genetic distance (cM)
150
100
50
0
0
30
60
90
Genomic position (Mb)

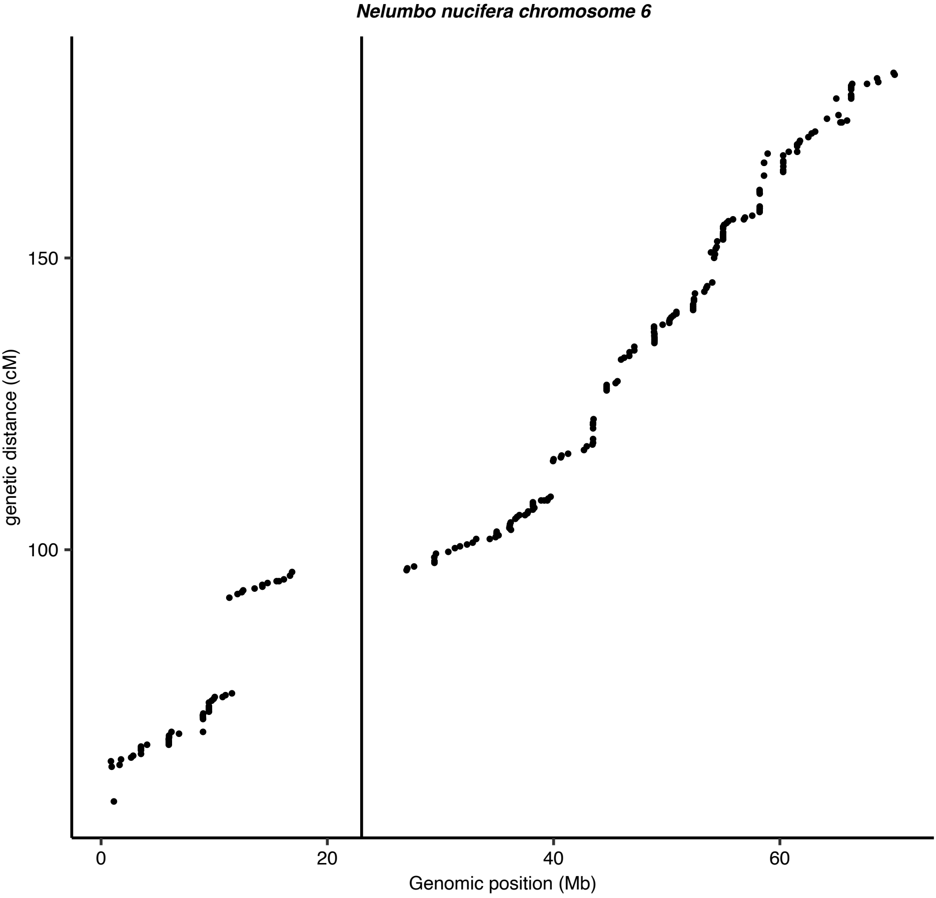
Nelumbo nucifera chromosome 6
genetic distance (cM)
150
100
0
20
40
60
Genomic position (Mb)

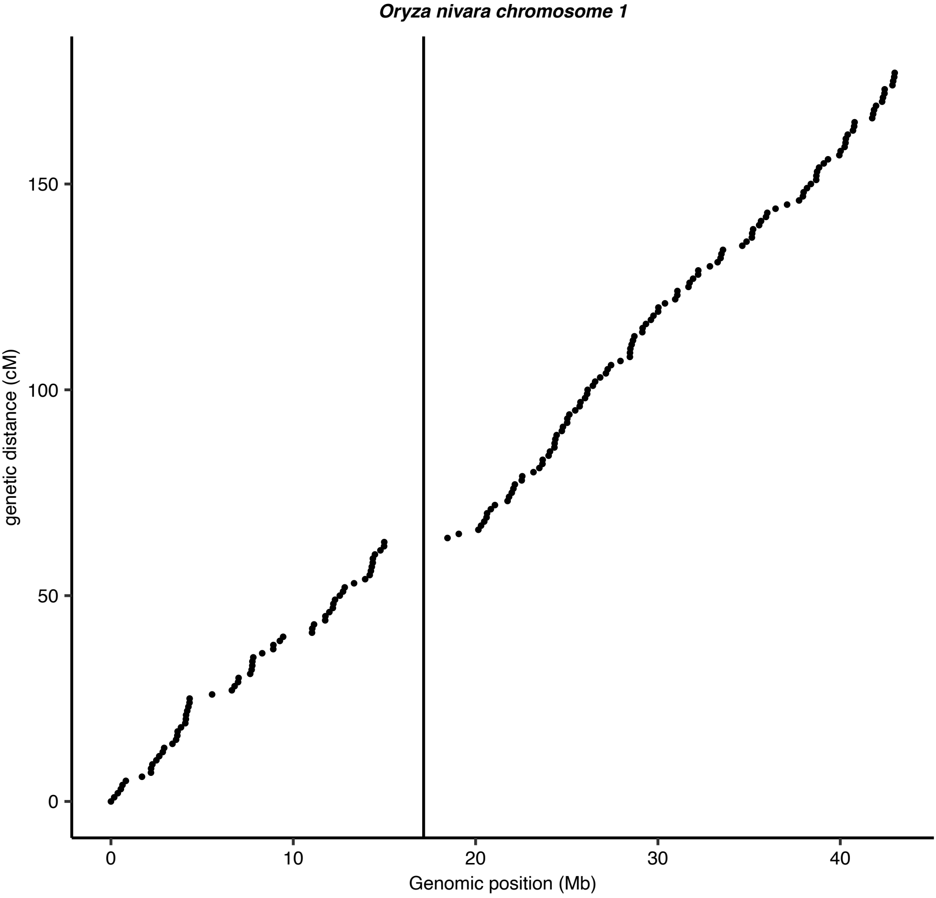
Oryza nivara chromosome 1
genetic distance (cM)
0
50
100
150
Genomic position (Mb)
0
10
20
30
40

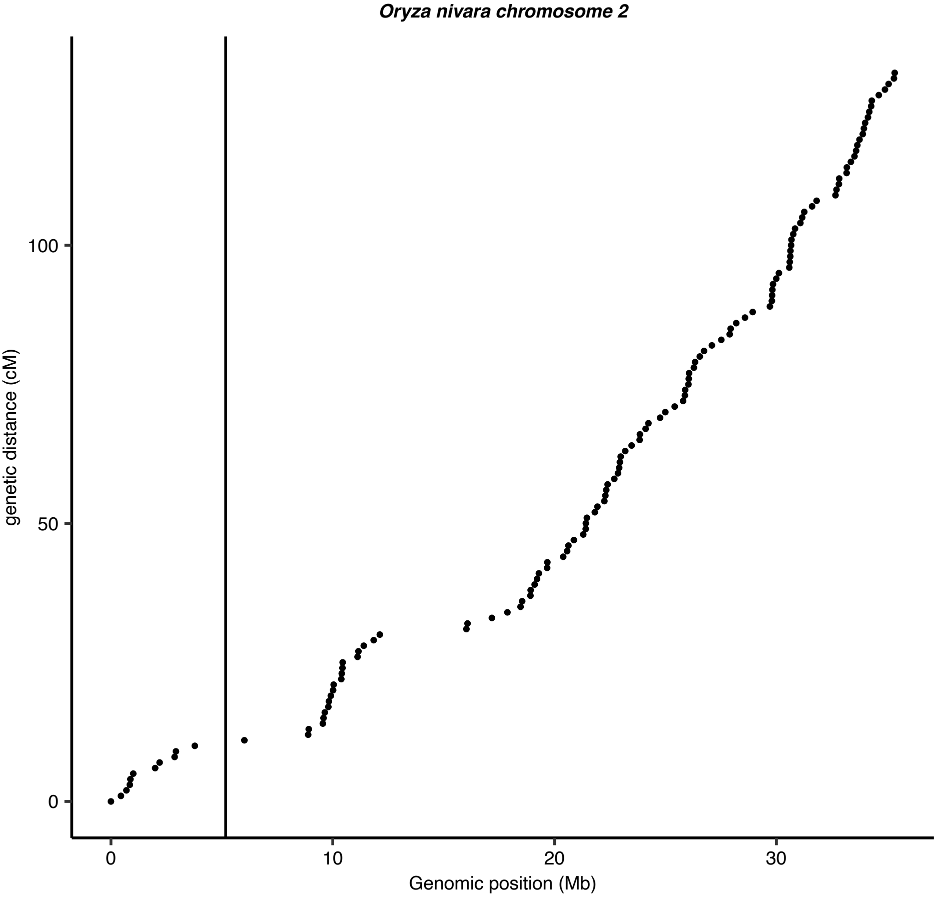
Oryza nivara chromosome 2
genetic distance (cM)
0
50
100
Genomic position (Mb)
0
10
20
30

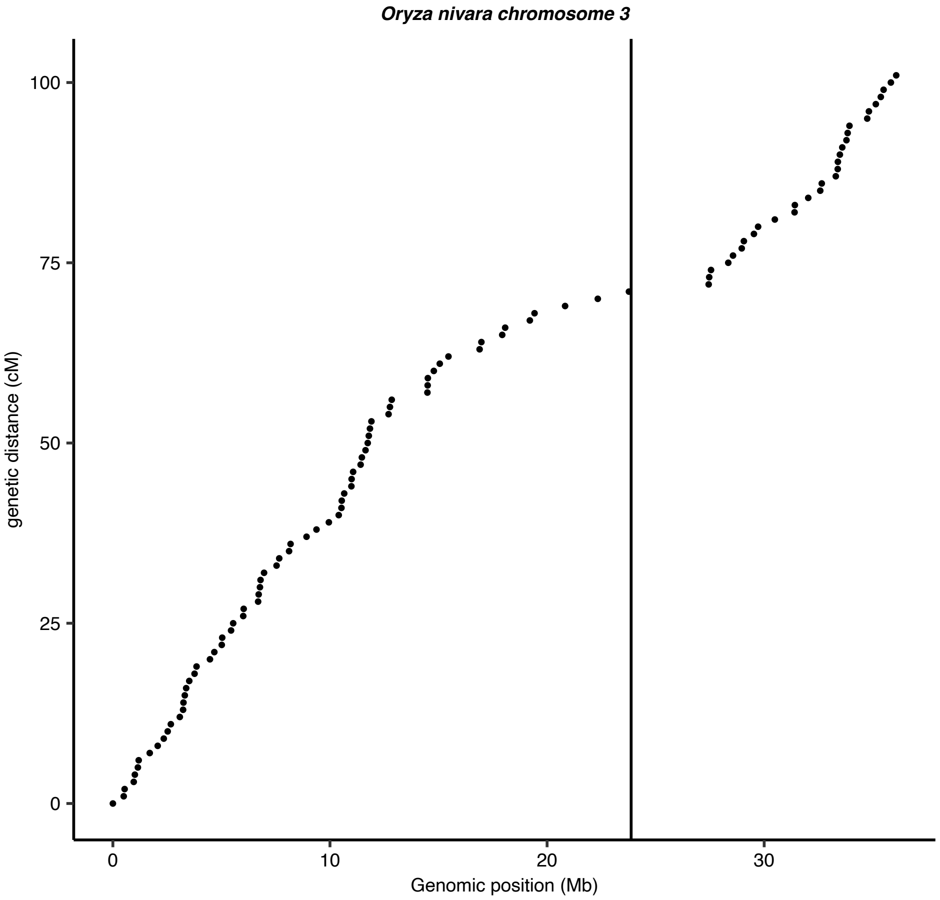
Oryza nivara chromosome 3
genetic distance (cM)
Genomic position (Mb)
0
25
50
75
100
0
10
20
30

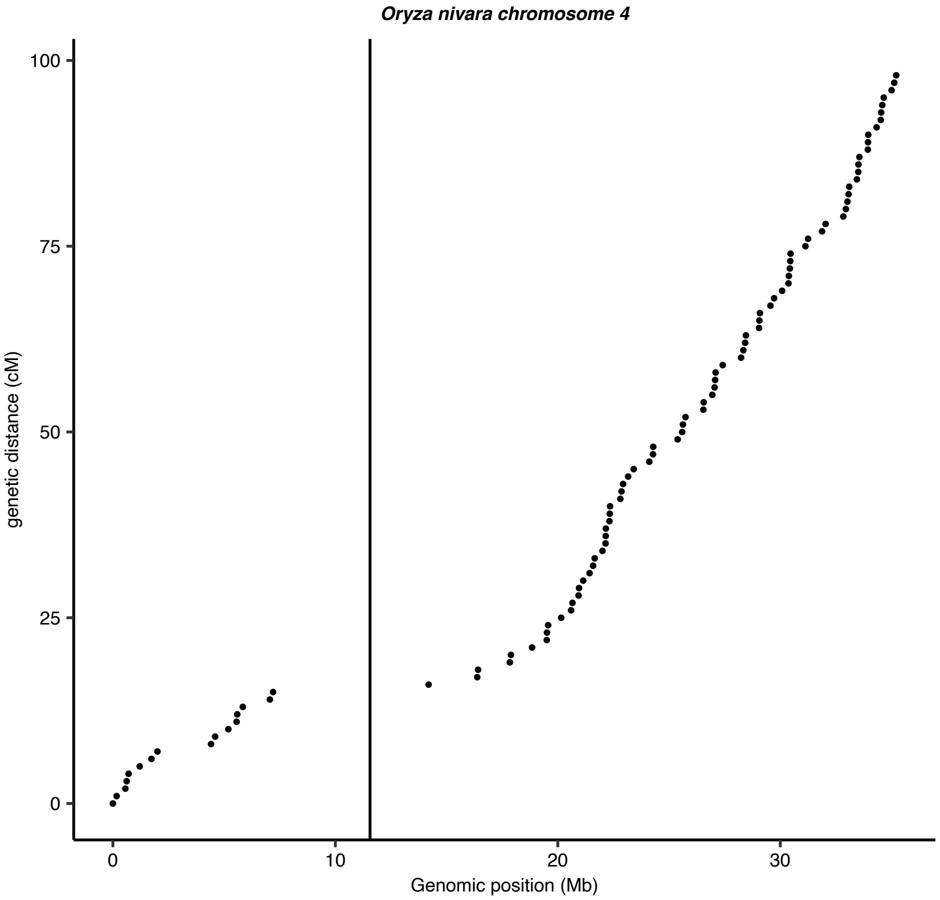
Oryza nivara chromosome 4
genetic distance (cM)
Genomic position (Mb)
0
25
50
75
100
0
10
20
30

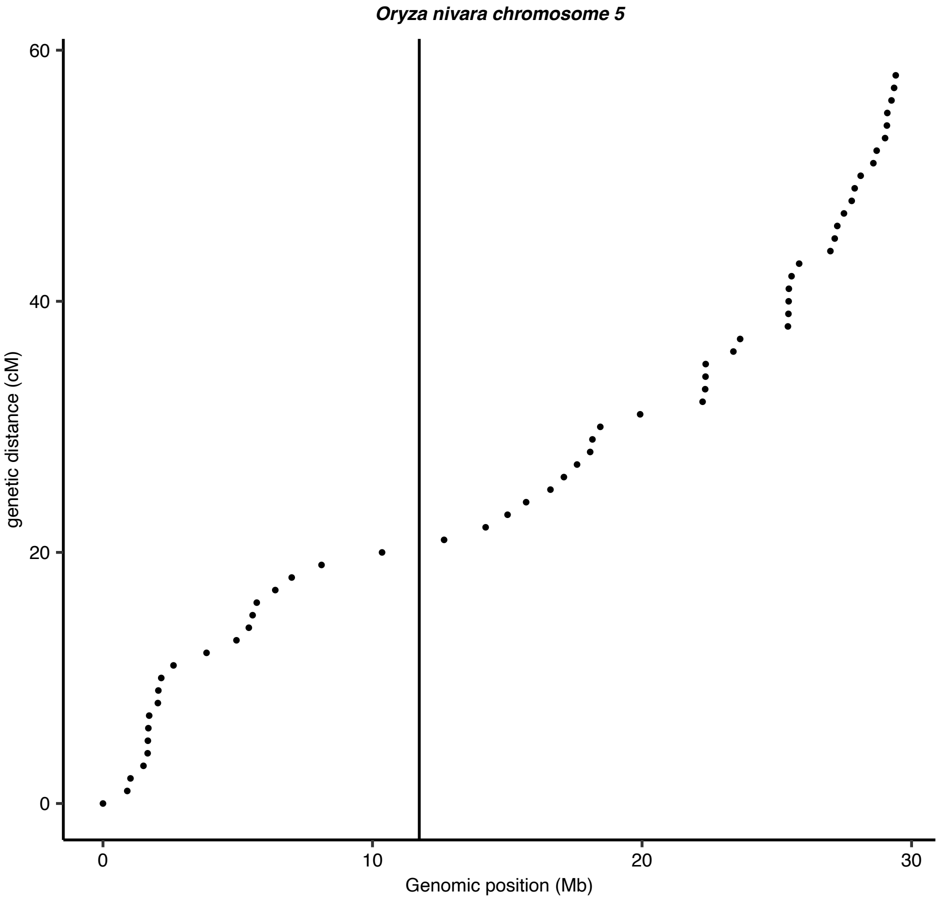
Oryza nivara chromosome 5
genetic distance (cM)
60
40
20
0
0
10
20
30
Genomic position (Mb)

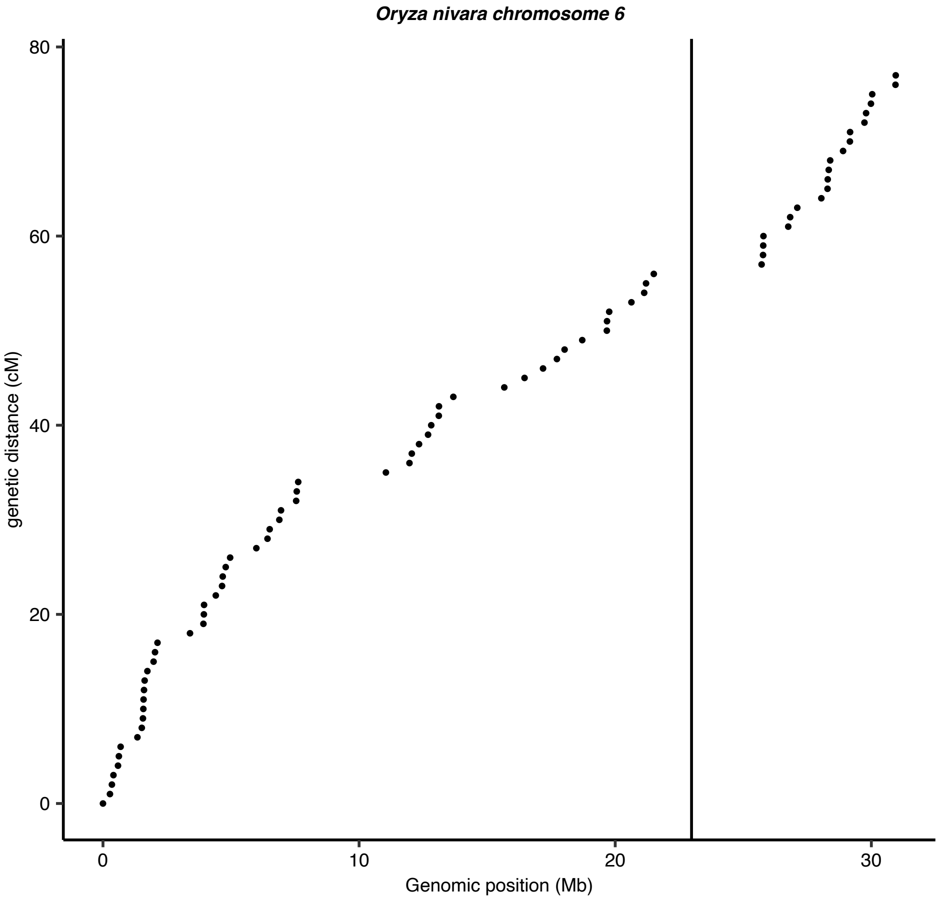
Oryza nivara chromosome 6
genetic distance (cM)
Genomic position (Mb)
0
20
40
60
80
0
10
20
30

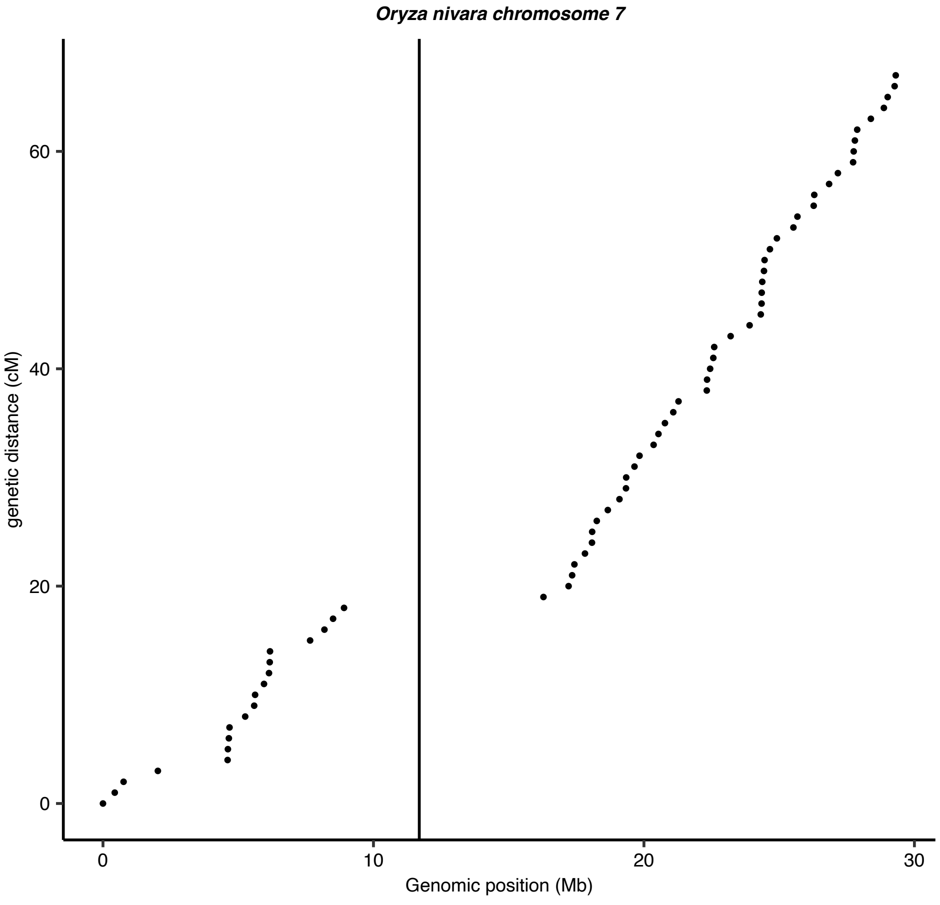
Oryza nivara chromosome 7
genetic distance (cM)
0
20
40
60
Genomic position (Mb)
0
10
20
30

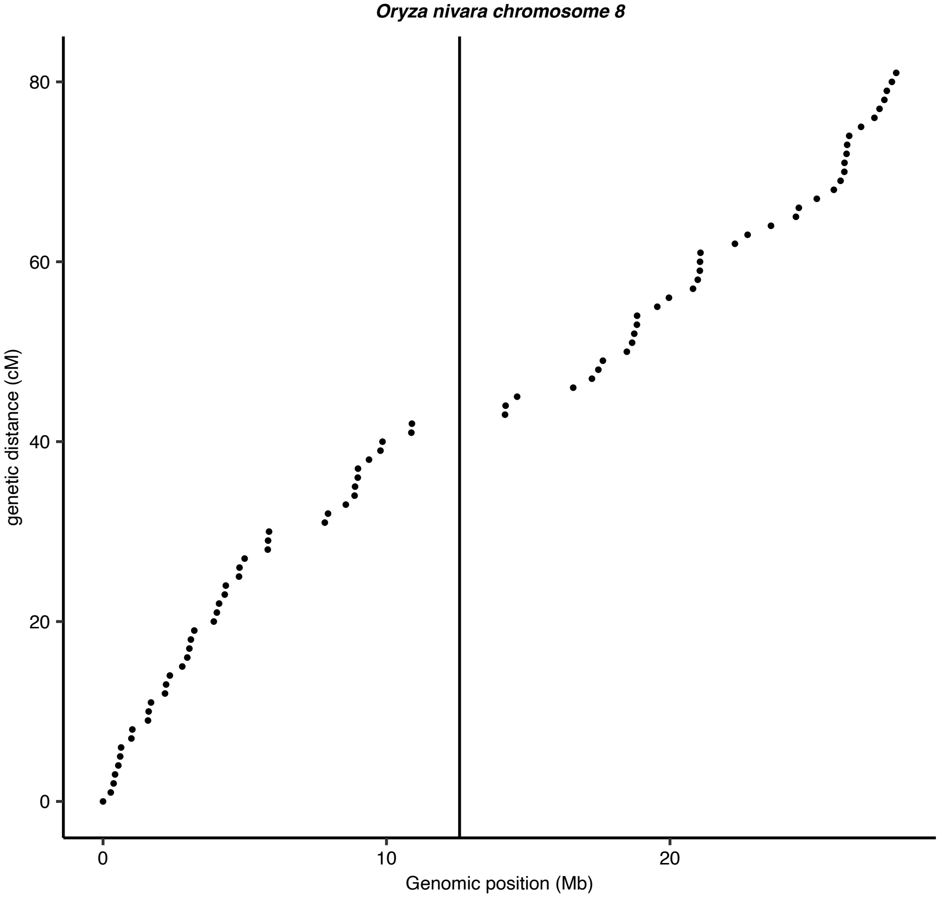
Oryza nivara chromosome 8
genetic distance (cM)
Genomic position (Mb)
0
20
40
60
80
0
10
20

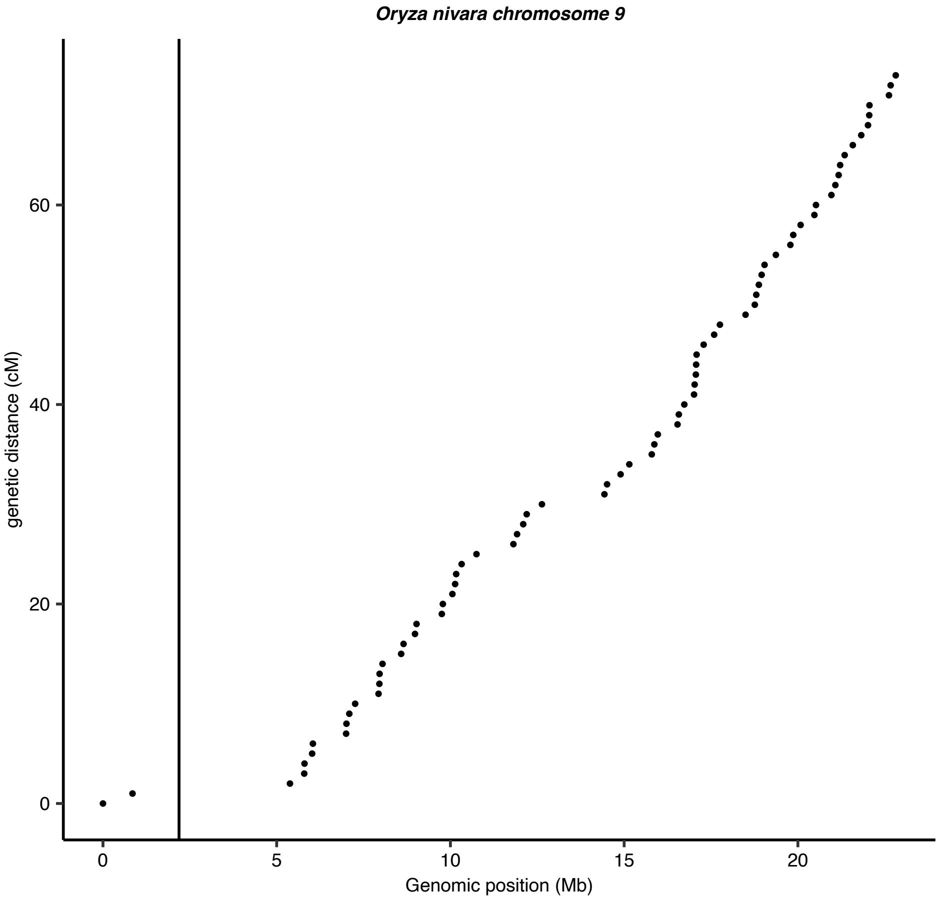
Oryza nivara chromosome 9
genetic distance (cM)
0
20
40
60
0
5
10
15
20
Genomic position (Mb)

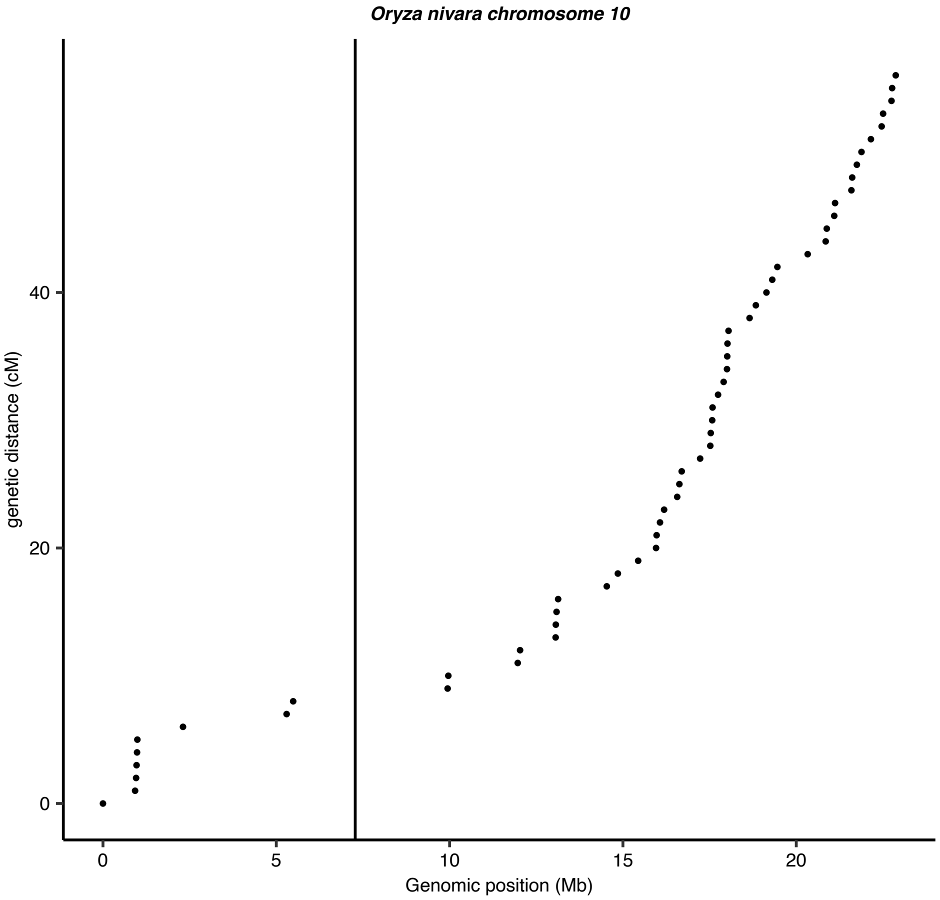
Oryza nivara chromosome 10
genetic distance (cM)
0
20
40
Genomic position (Mb)
0
5
10
15
20

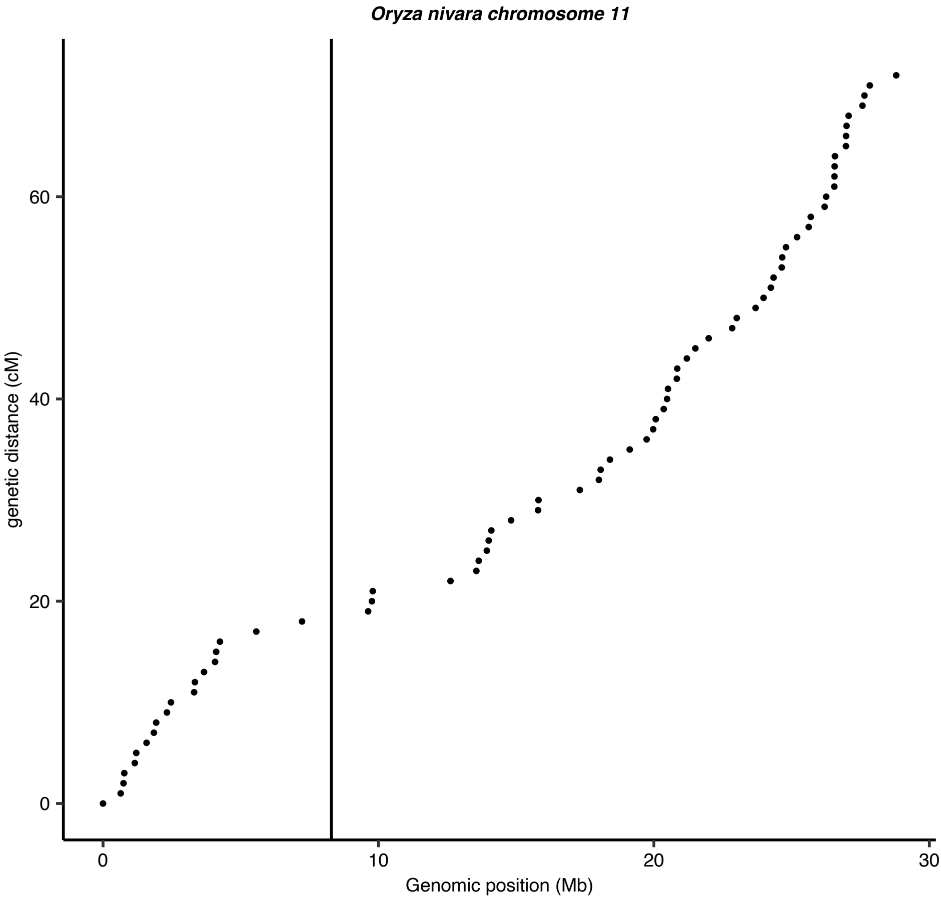
Oryza nivara chromosome 11
genetic distance (cM)
0
20
40
60
0
10
20
30
Genomic position (Mb)

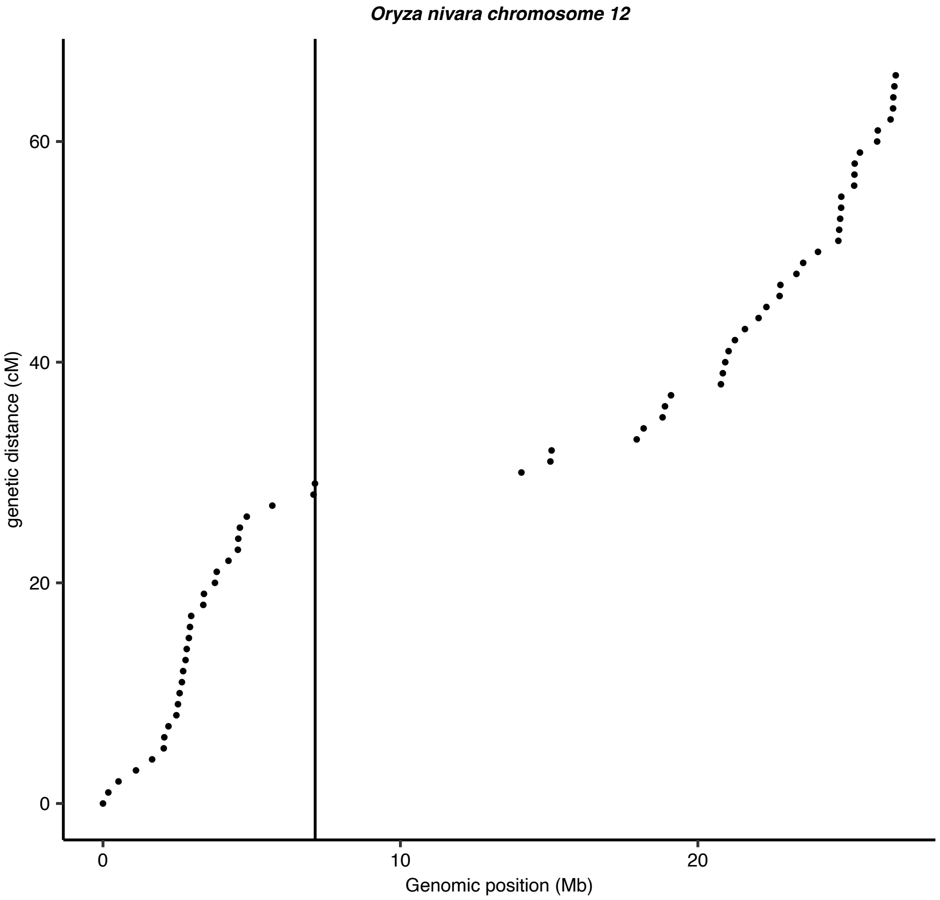
Oryza nivara chromosome 12
genetic distance (cM)
0
20
40
60
0
10
20
Genomic position (Mb)

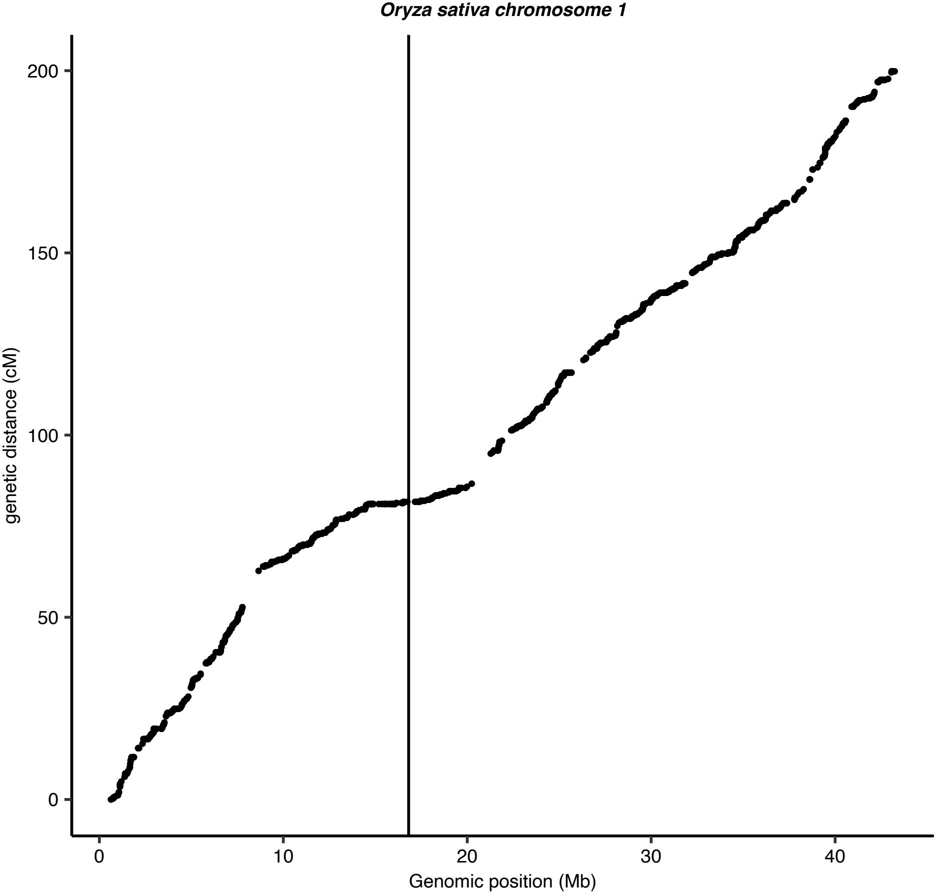
Oryza sativa chromosome 1
genetic distance (cM)
0
50
100
150
200
Genomic position (Mb)
0
10
20
30
40

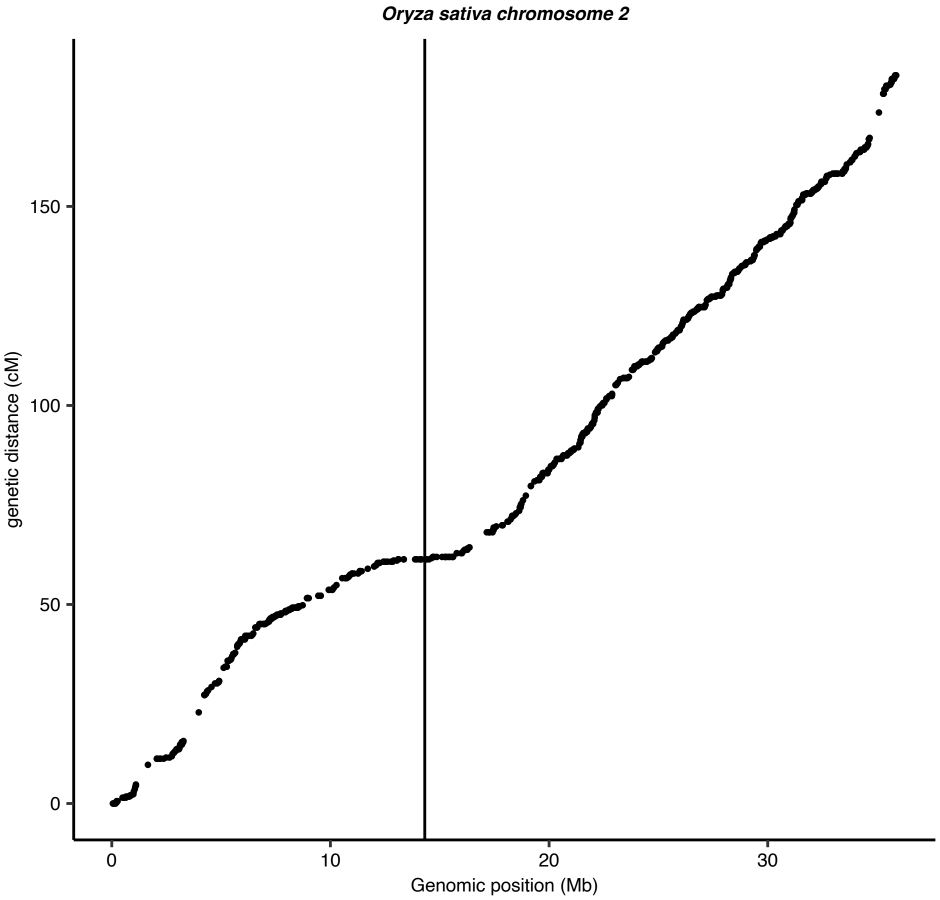
Oryza sativa chromosome 2
genetic distance (cM)
0
50
100
150
Genomic position (Mb)
0
10
20
30

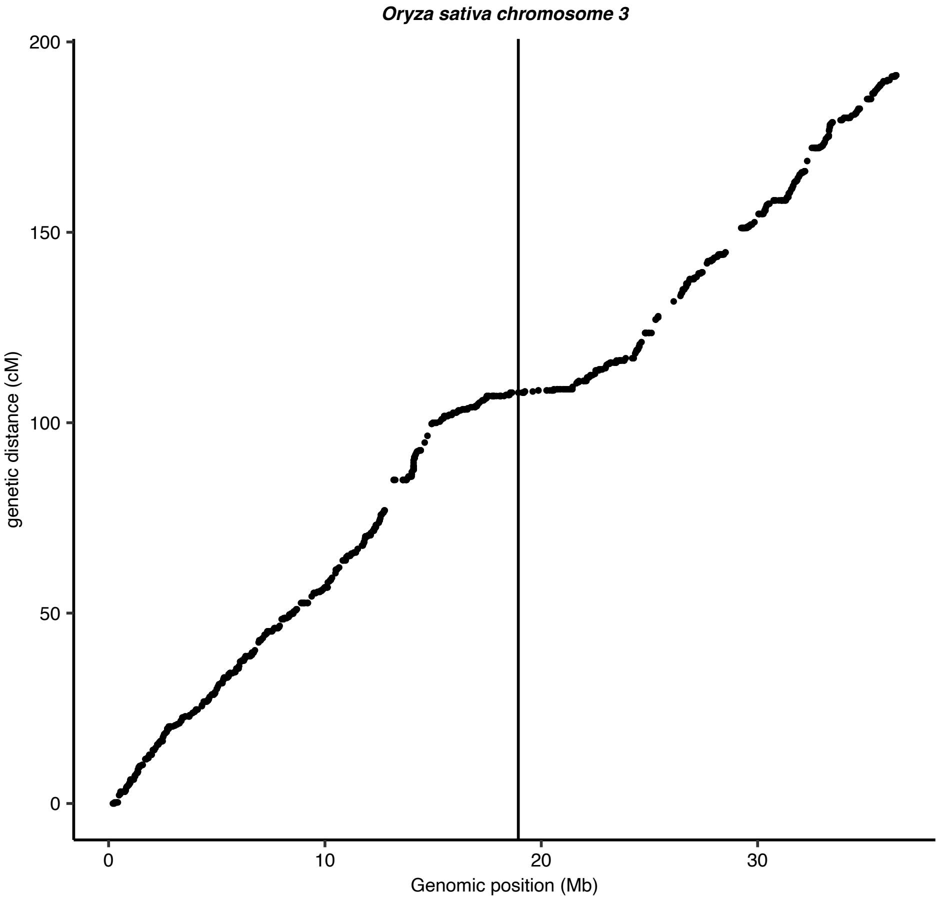
Oryza sativa chromosome 3
200
150
100
50
0
genetic distance (cM)
0
10
20
30
Genomic position (Mb)

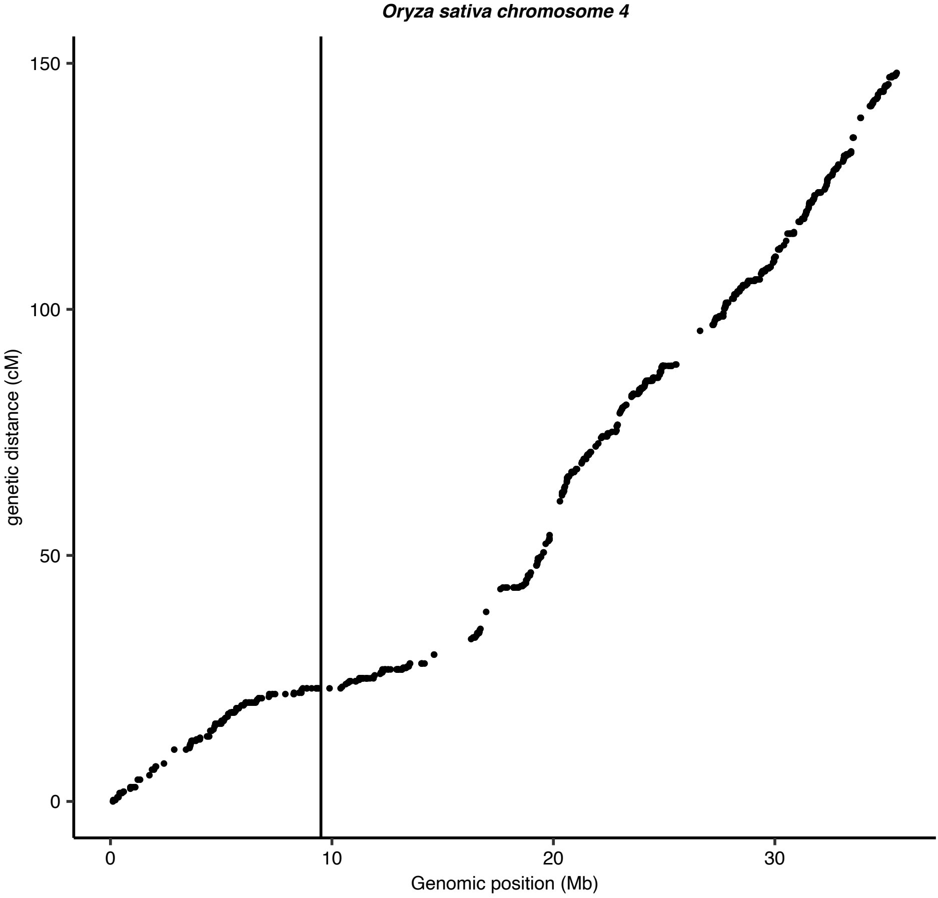
Oryza sativa chromosome 4
genetic distance (cM)
150
100
50
0
0
10
20
30
Genomic position (Mb)

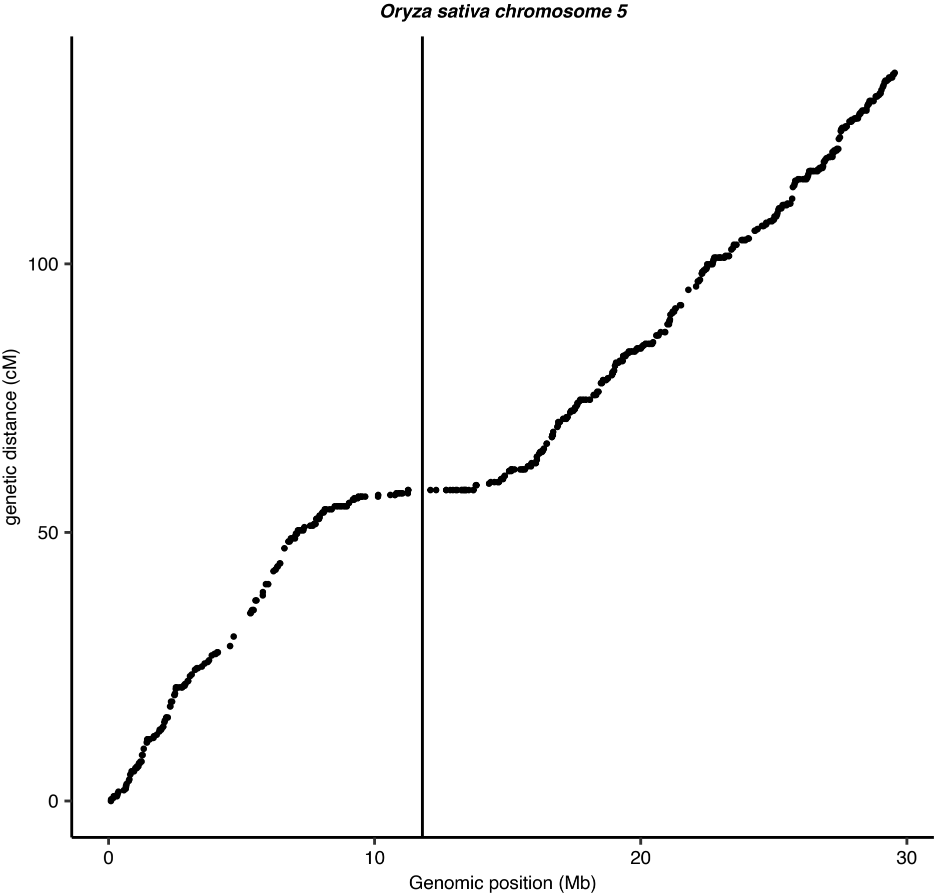
Oryza sativa chromosome 5
genetic distance (cM)
0
50
100
0
10
20
30
Genomic position (Mb)

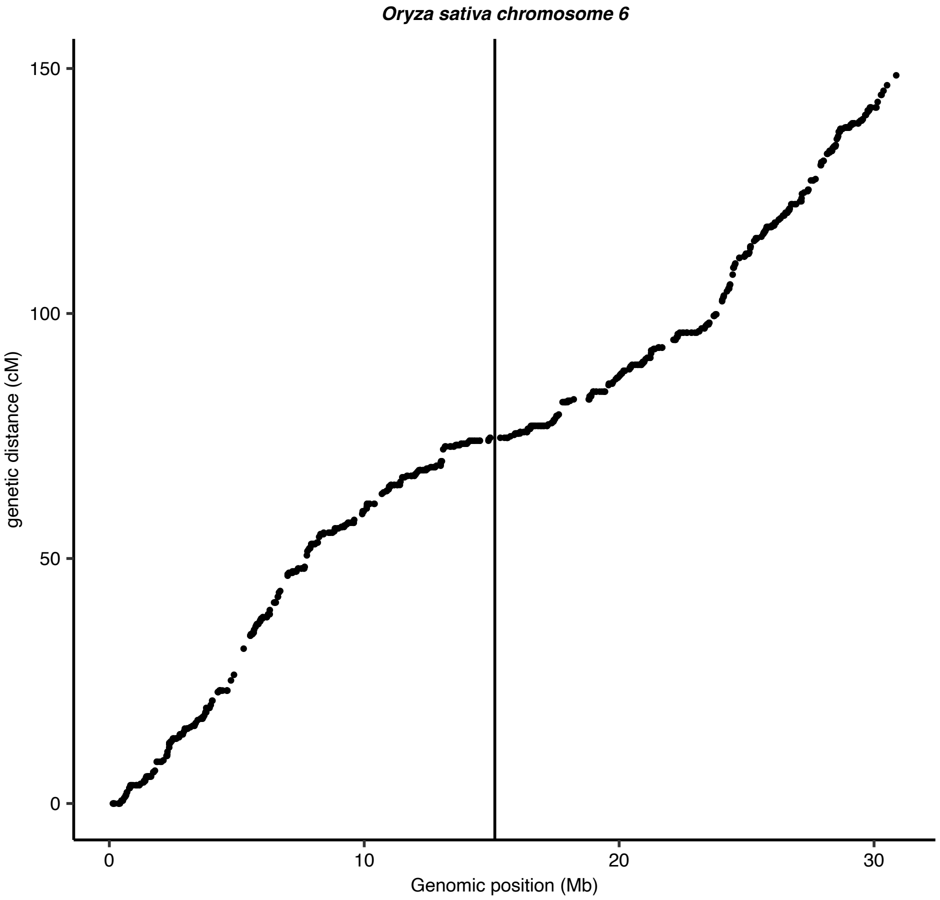
Oryza sativa chromosome 6
genetic distance (cM)
Genomic position (Mb)
0
50
100
150
0
10
20
30

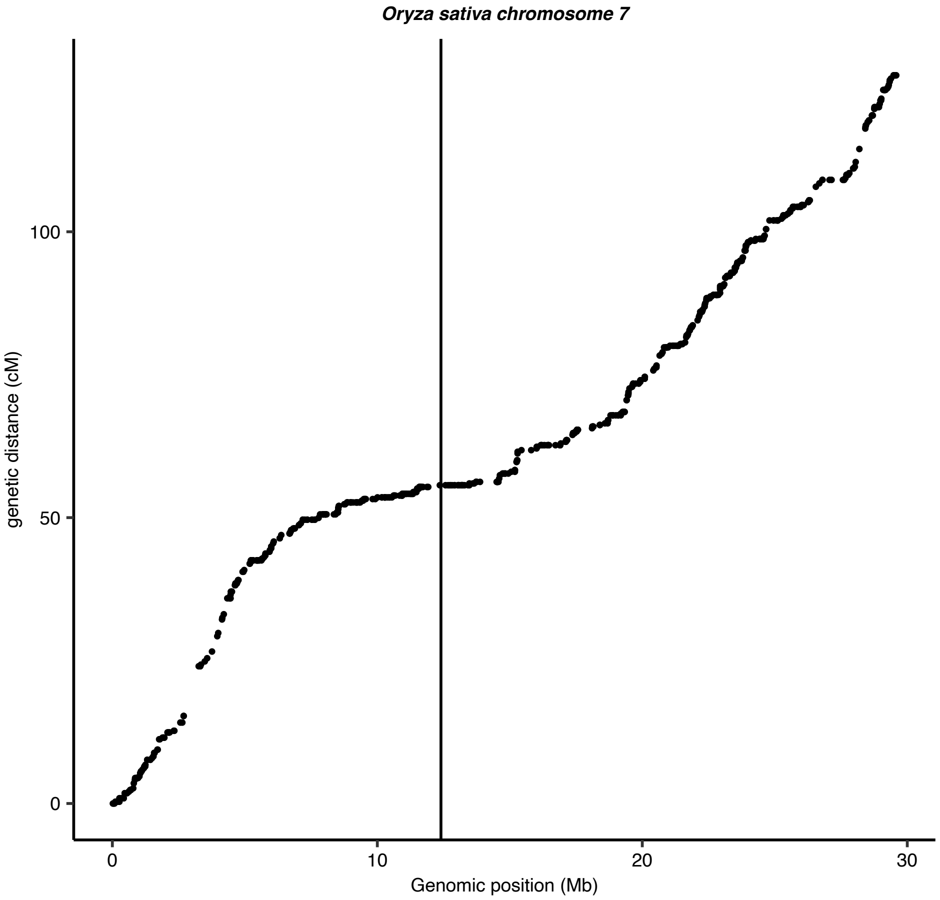
Oryza sativa chromosome 7
genetic distance (cM)
0
50
100
0
10
20
30
Genomic position (Mb)

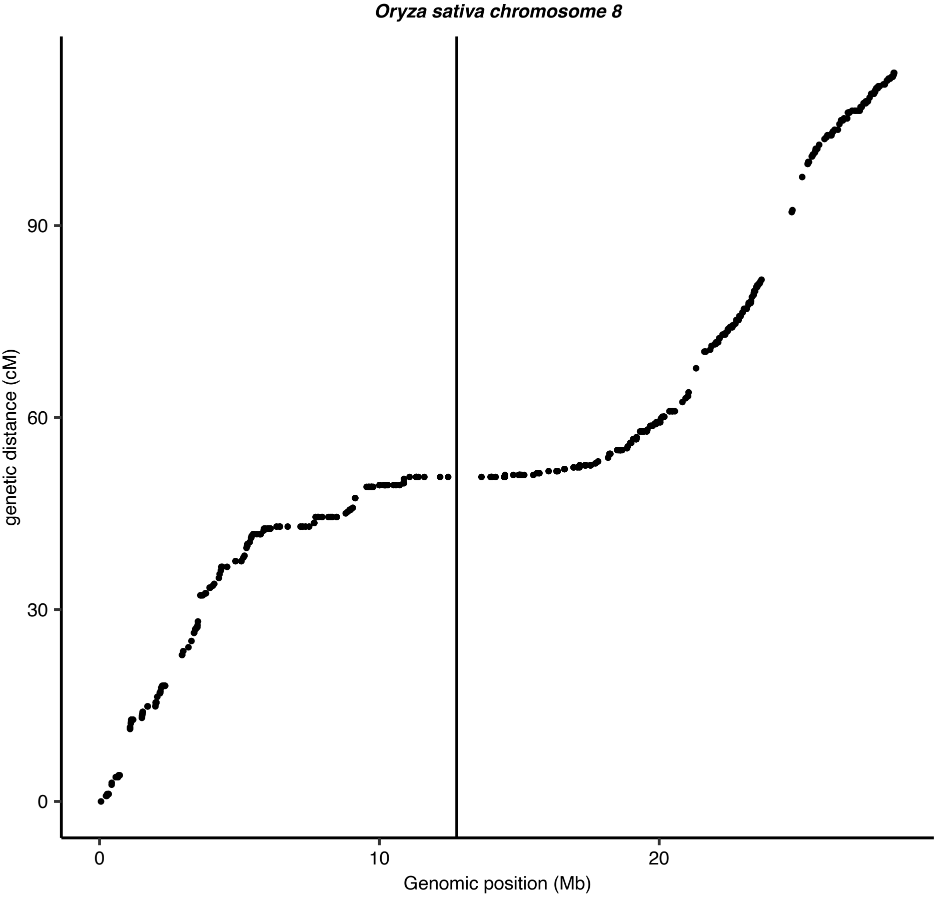
Oryza sativa chromosome 8
90
60
30
0
genetic distance (cM)
0
10
20
Genomic position (Mb)

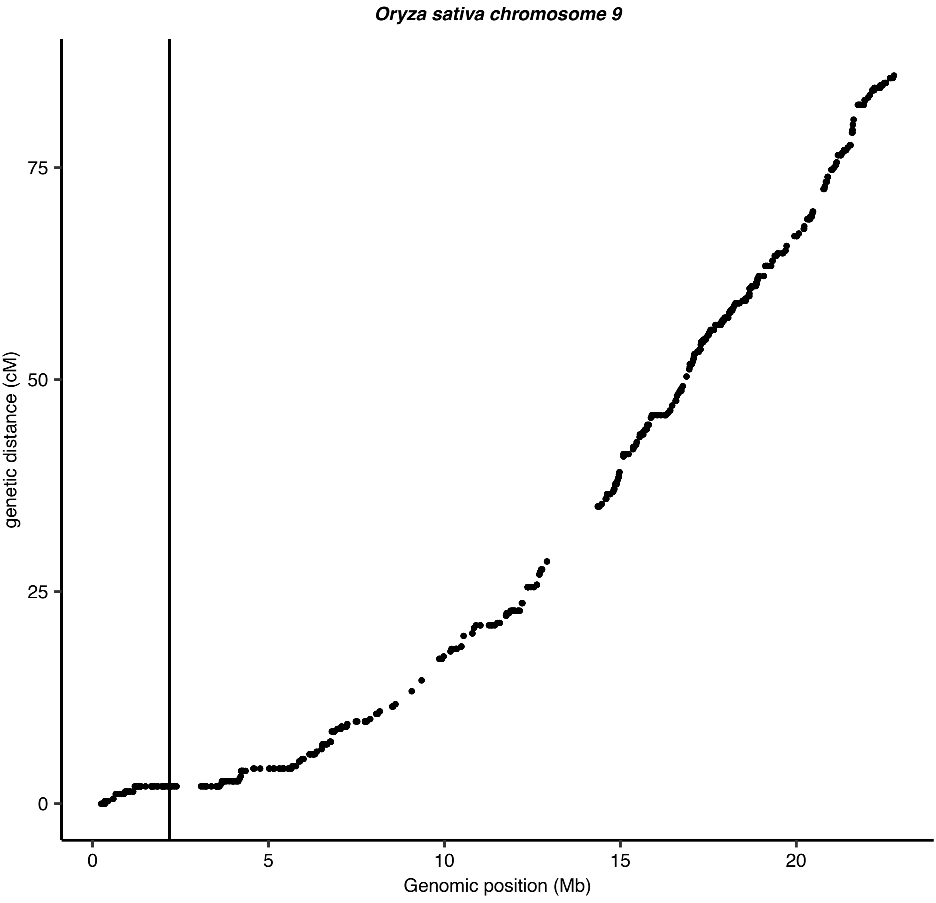

Oryza sativa chromosome 9
genetic distance (cM)
0
25
50
75
Genomic position (Mb)
0
5
10
15
20

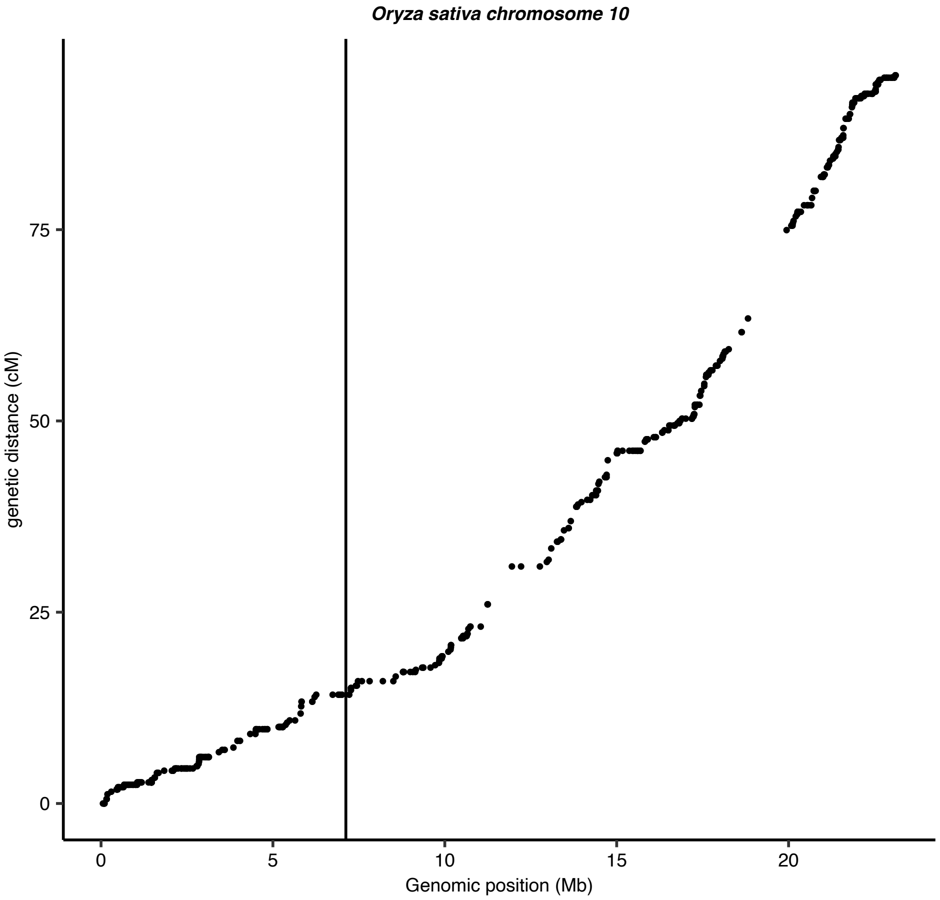
Oryza sativa chromosome 10
genetic distance (cM)
0
25
50
75
0
5
10
15
20
Genomic position (Mb)

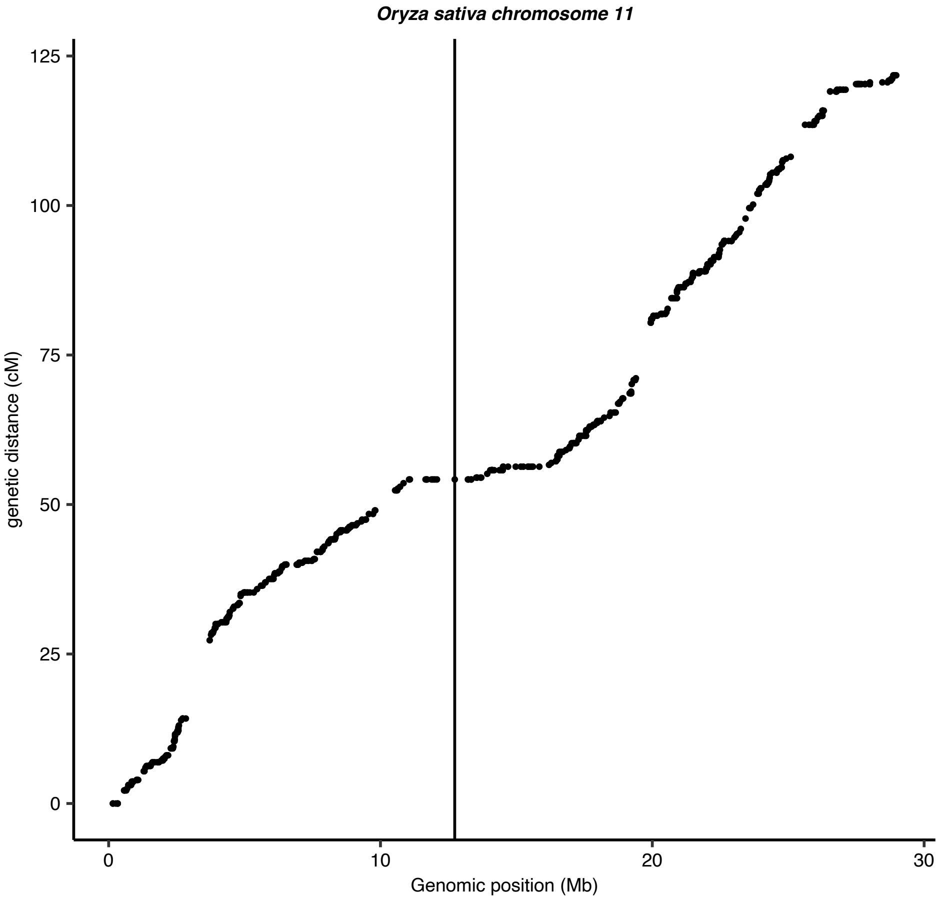

Oryza sativa chromosome 11
genetic distance (cM)
125
100
75
50
25
0
0
10
20
30
Genomic position (Mb)

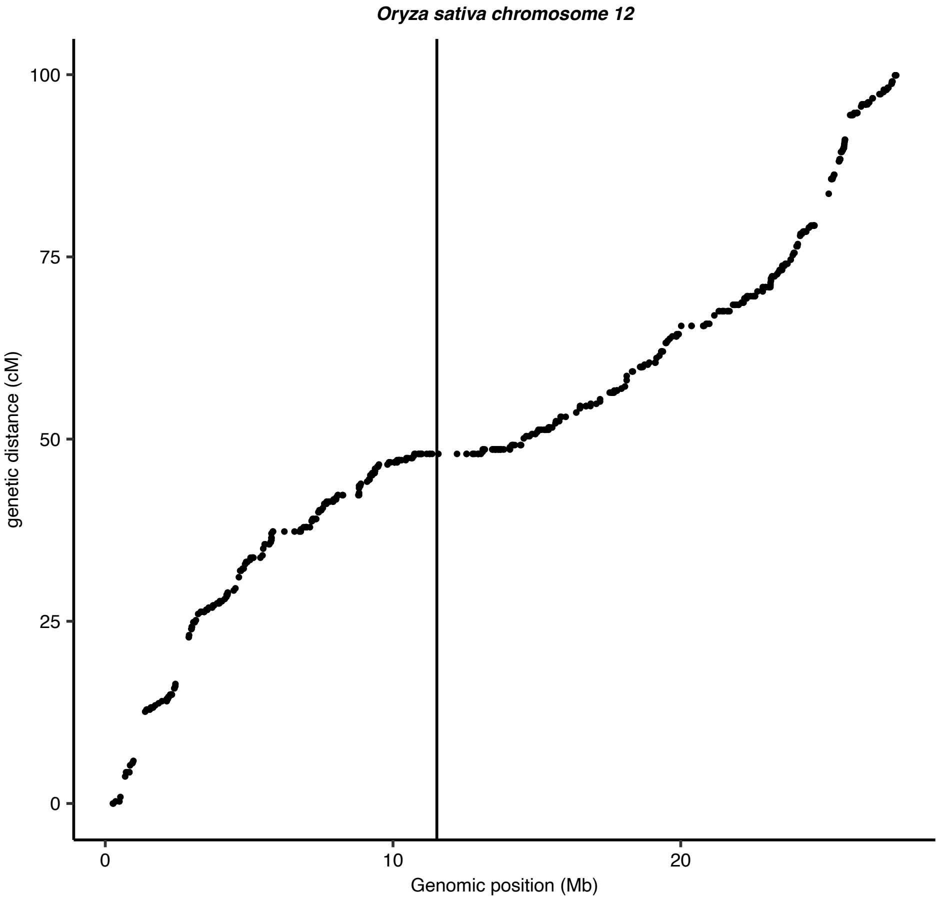
Oryza sativa chromosome 12
genetic distance (cM)
100
75
50
25
0
0
10
20
Genomic position (Mb)

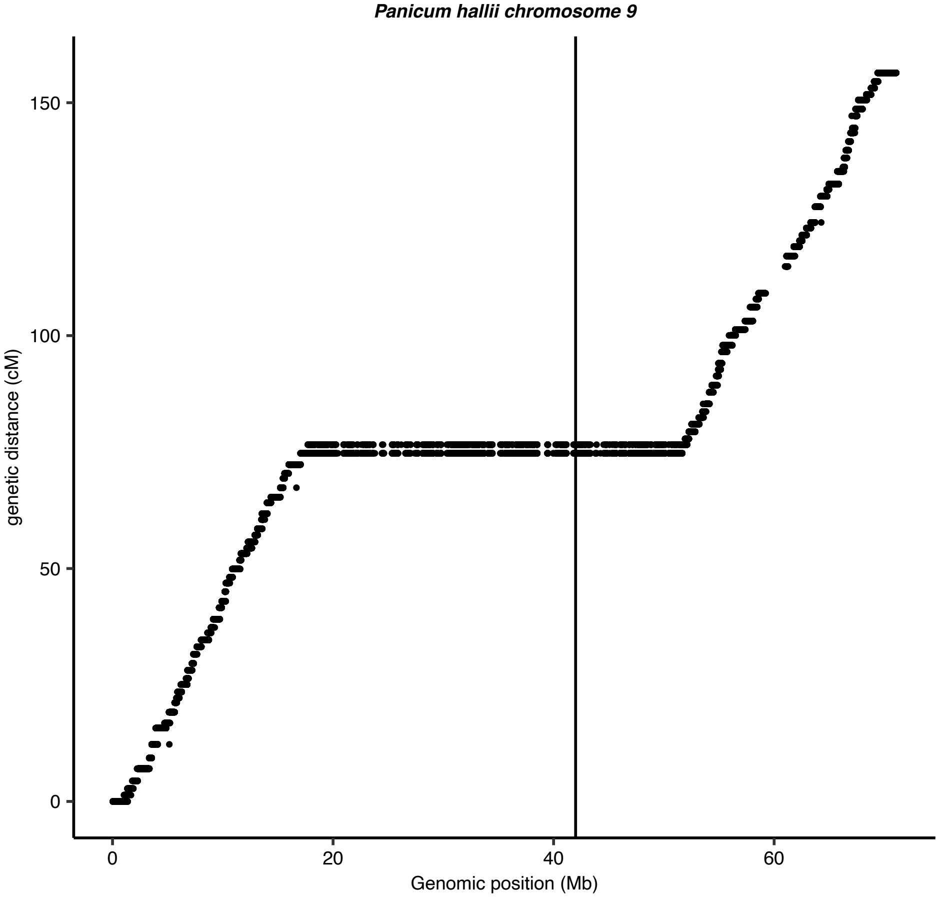
Panicum hallii chromosome 9
genetic distance (cM)
150
100
50
0
0
20
40
60
Genomic position (Mb)

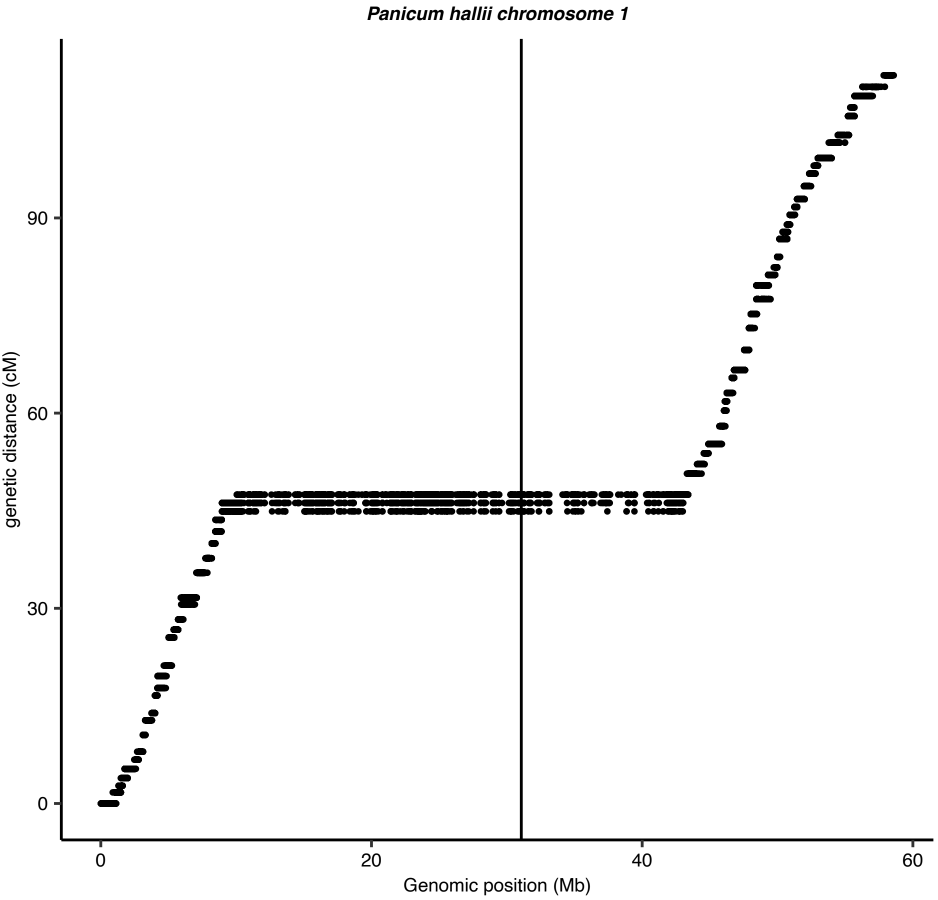
Panicum hallii chromosome 1
genetic distance (cM)
0
30
60
90
Genomic position (Mb)
0
20
40
60

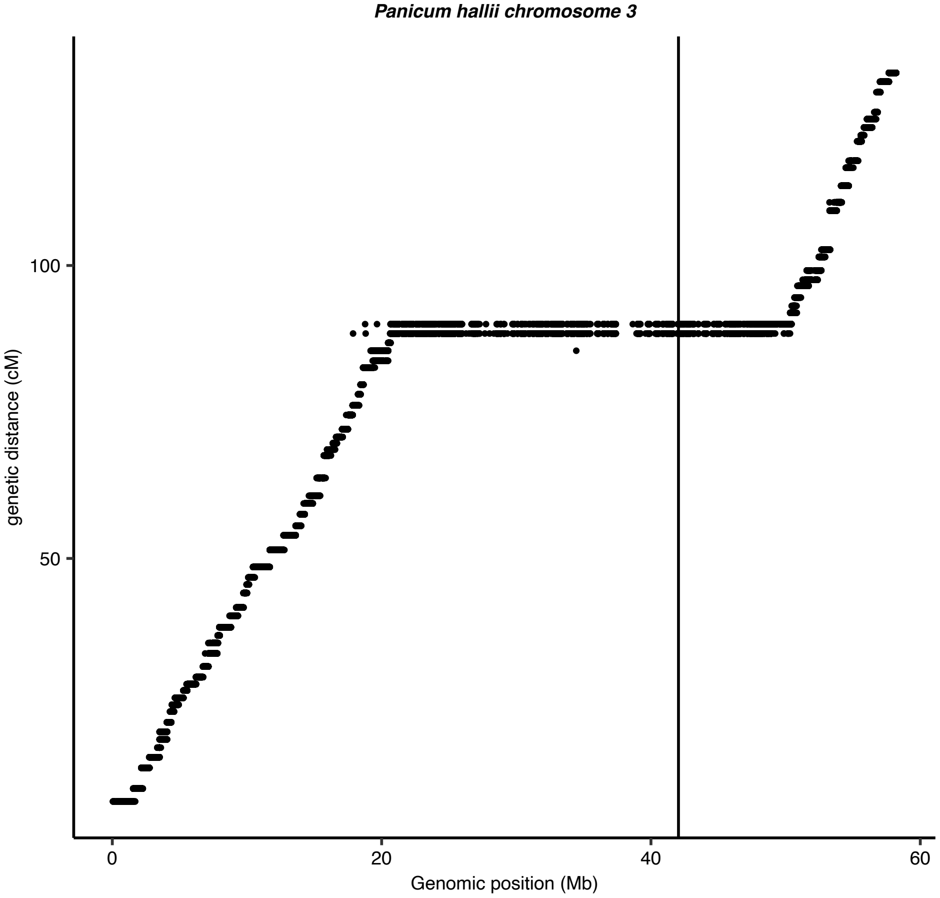

Panicum hallii chromosome 3
genetic distance (cM)
100
50
0
20
40
60
Genomic position (Mb)

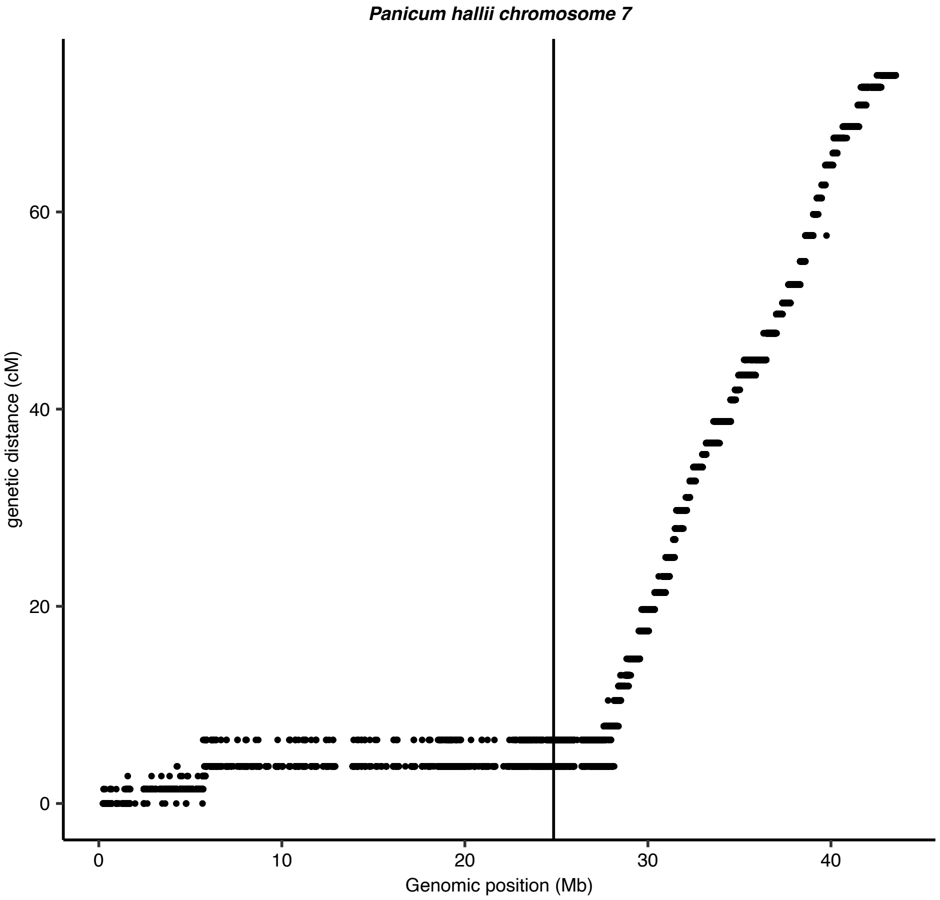
Panicum hallii chromosome 7
genetic distance (cM)
0
20
40
60
Genomic position (Mb)
0
10
20
30
40

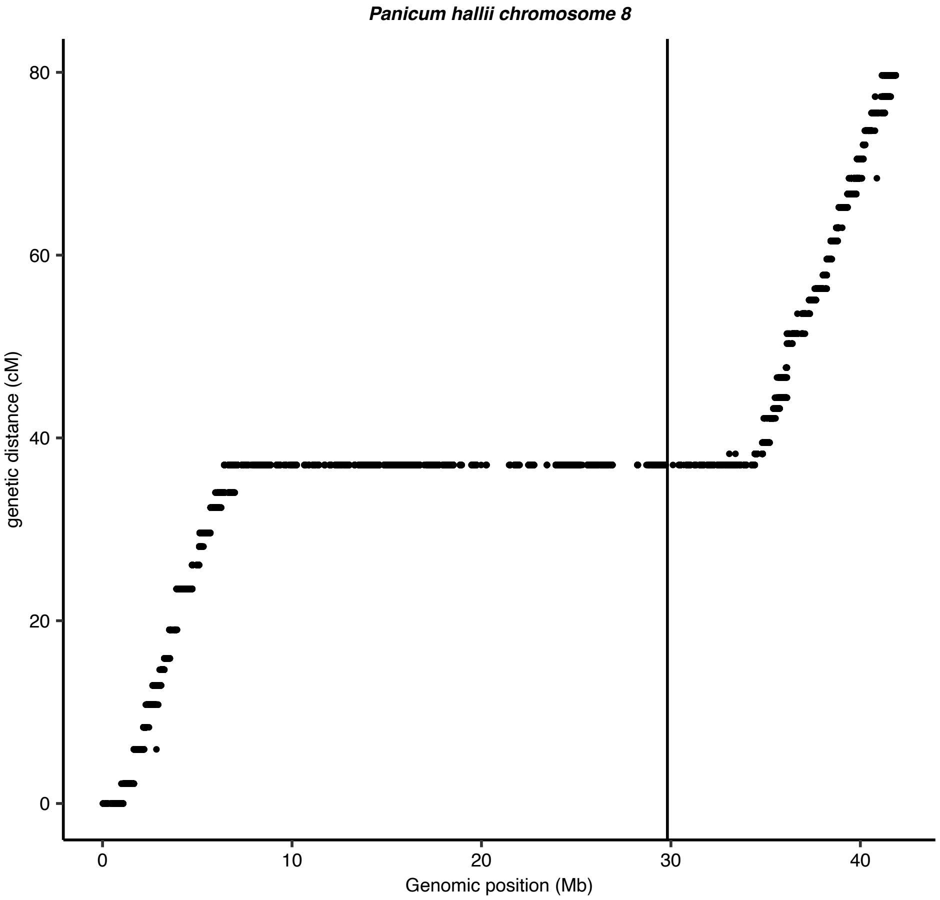
Panicum hallii chromosome 8
genetic distance (cM)
0
20
40
60
80
Genomic position (Mb)
0
10
20
30
40

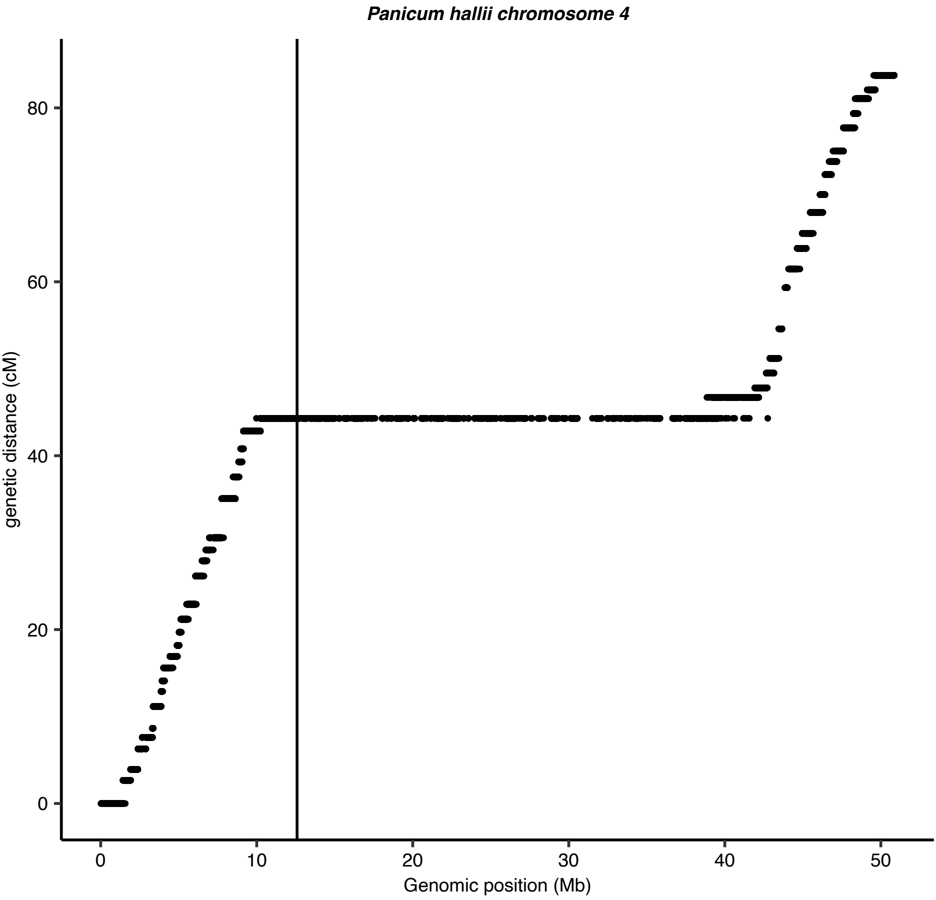
Panicum hallii chromosome 4
genetic distance (cM)
Genomic position (Mb)
0
20
40
60
80
0
10
20
30
40
50

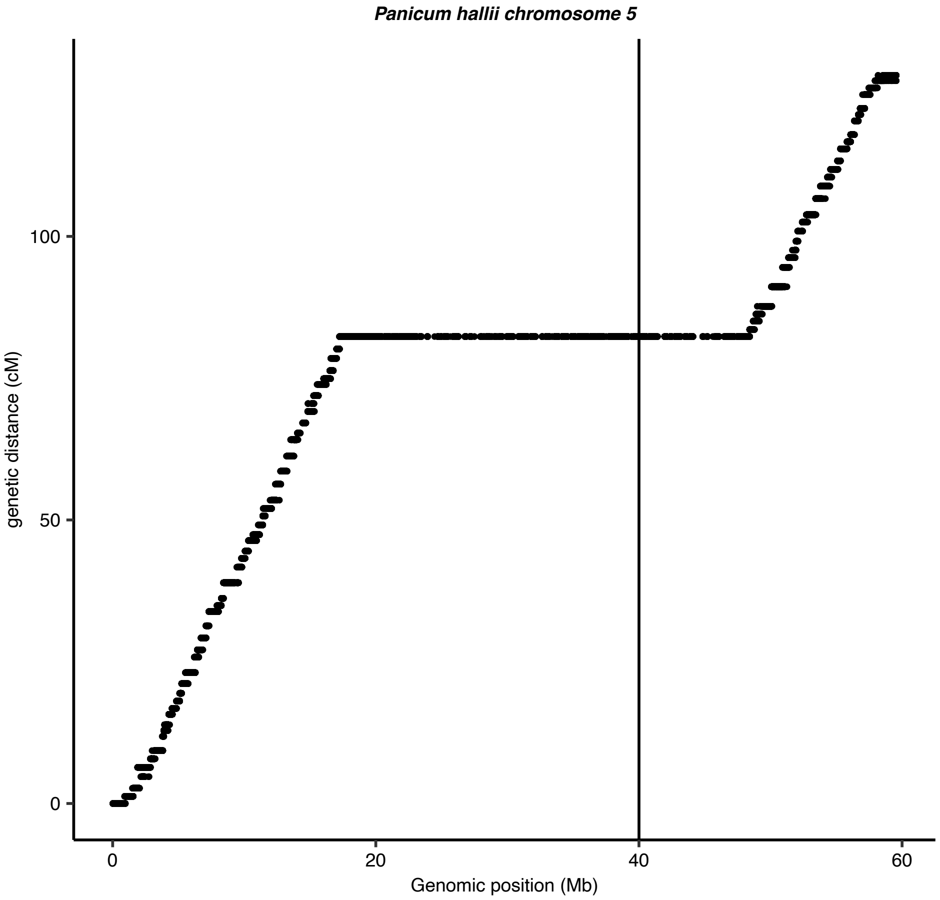
Panicum hallii chromosome 5
genetic distance (cM)
0
50
100
Genomic position (Mb)
0
20
40
60

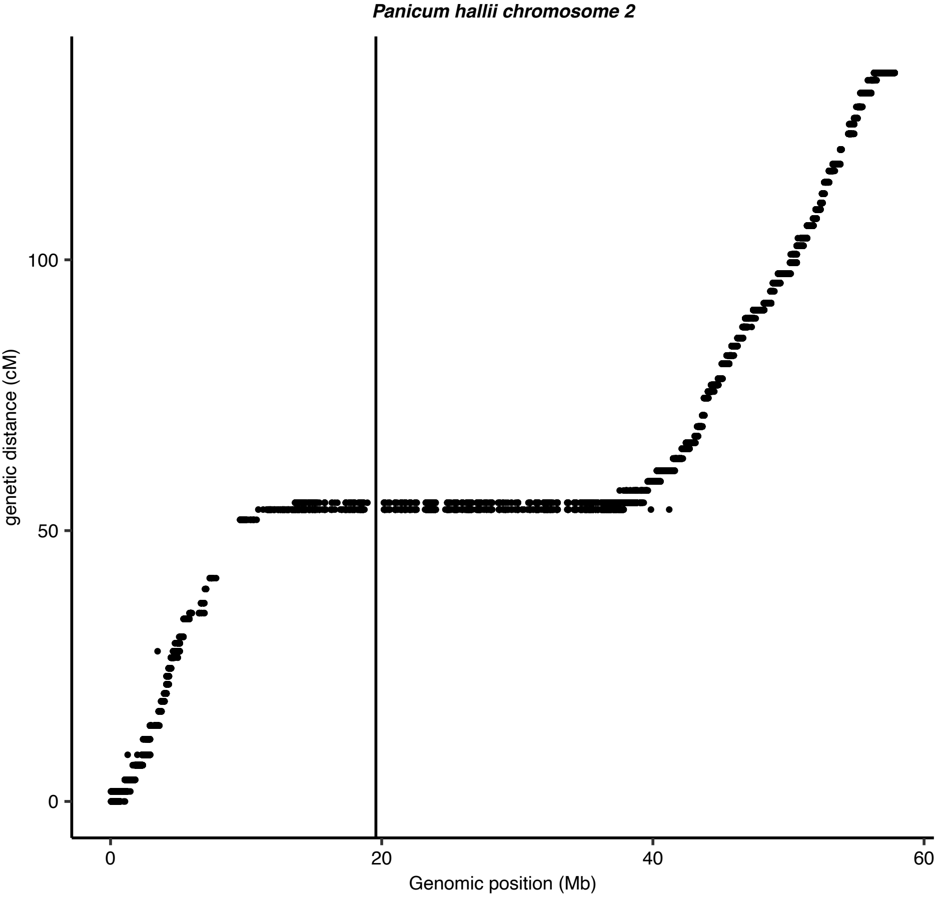
Panicum hallii chromosome 2
genetic distance (cM)
100
50
0
0
20
40
60
Genomic position (Mb)

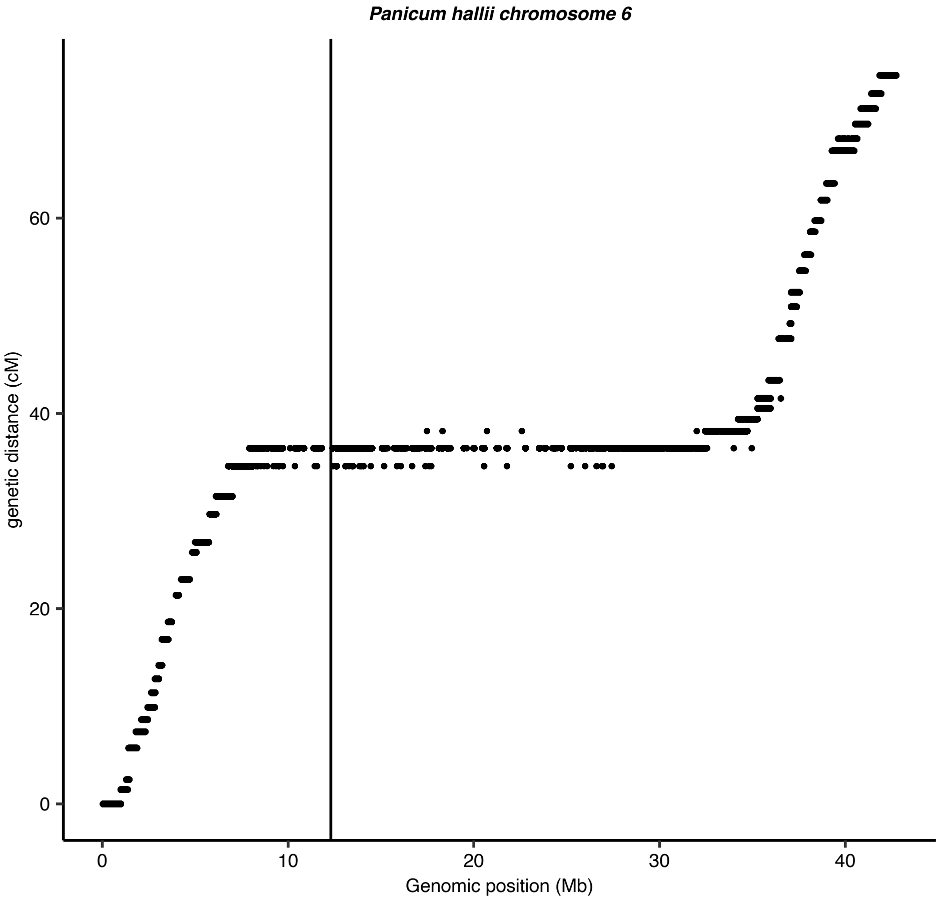
Panicum hallii chromosome 6
genetic distance (cM)
60
40
20
0
0
10
20
30
40
Genomic position (Mb)

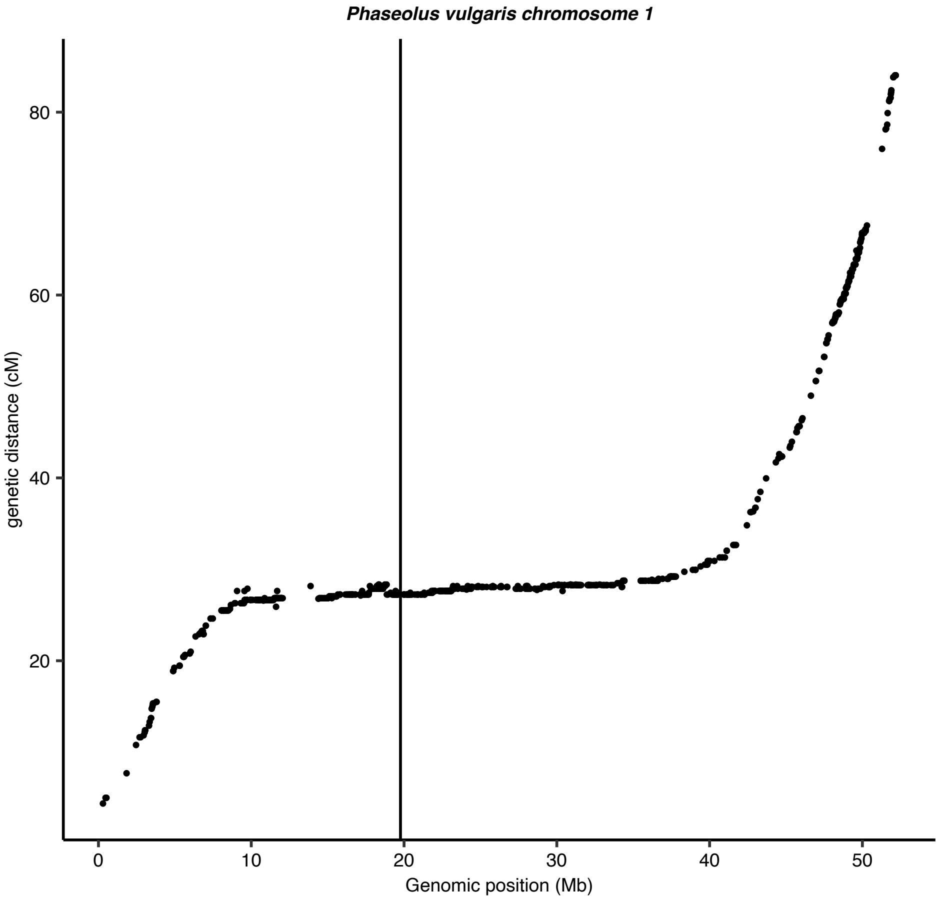
Phaseolus vulgaris chromosome 1
genetic distance (cM)
Genomic position (Mb)
0
10
20
30
40
50
20
40
60
80

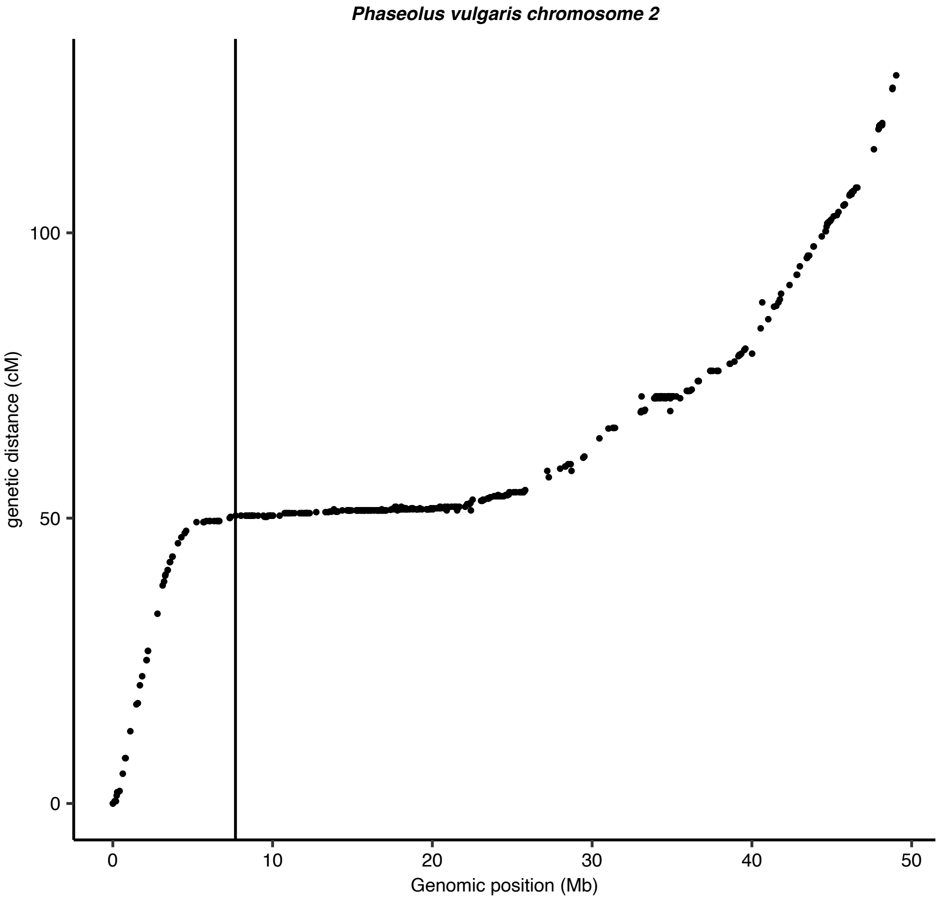
Phaseolus vulgaris chromosome 2
genetic distance (cM)
0
50
100
Genomic position (Mb)
0
10
20
30
40
50

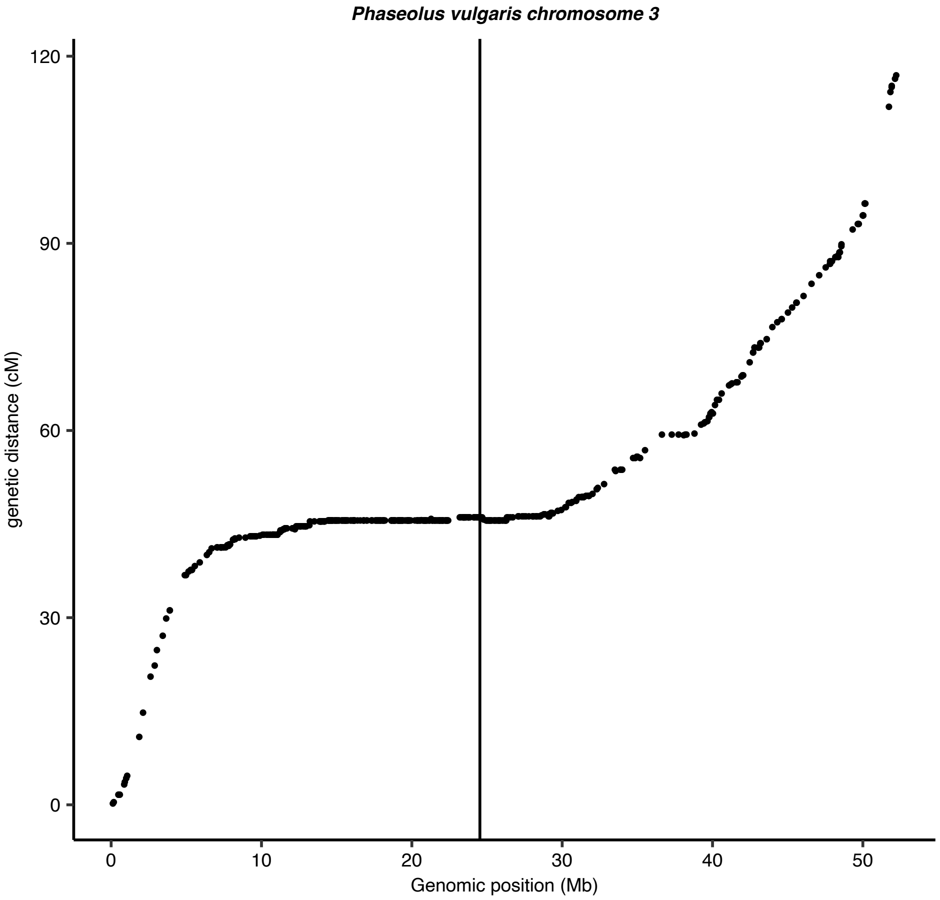
Phaseolus vulgaris chromosome 3
genetic distance (cM)
0
30
60
90
120
Genomic position (Mb)
0
10
20
30
40
50

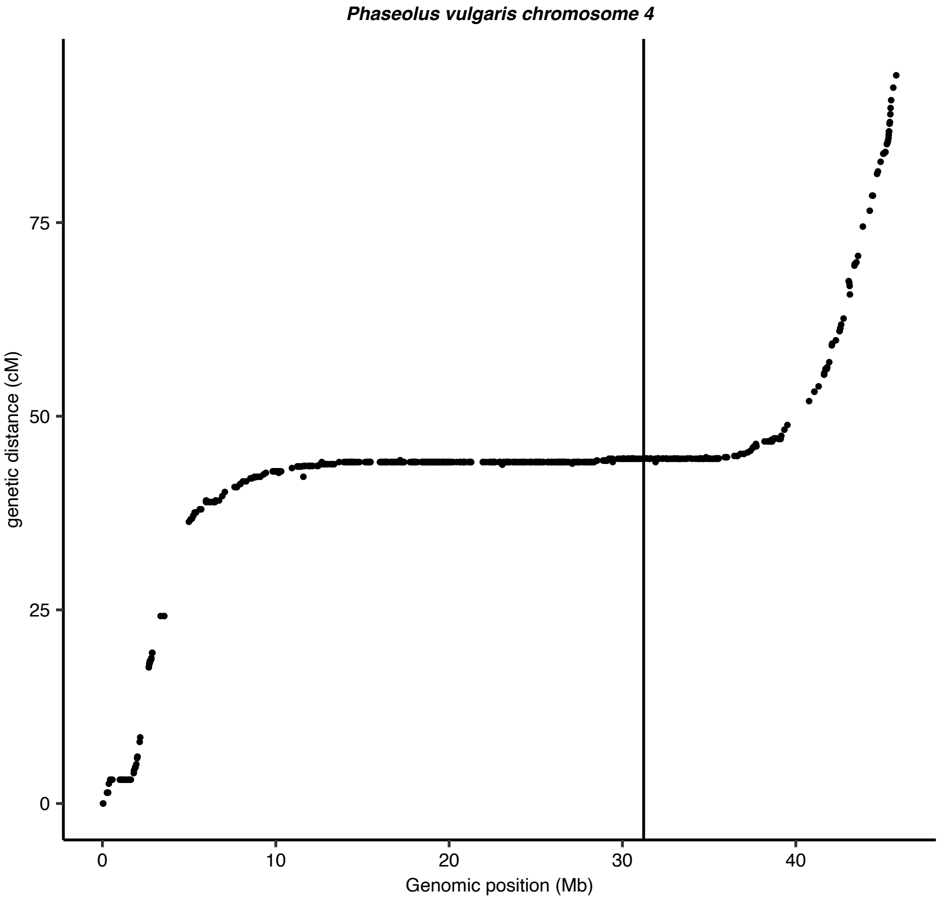
Phaseolus vulgaris chromosome 4
genetic distance (cM)
0
25
50
75
Genomic position (Mb)
0
10
20
30
40

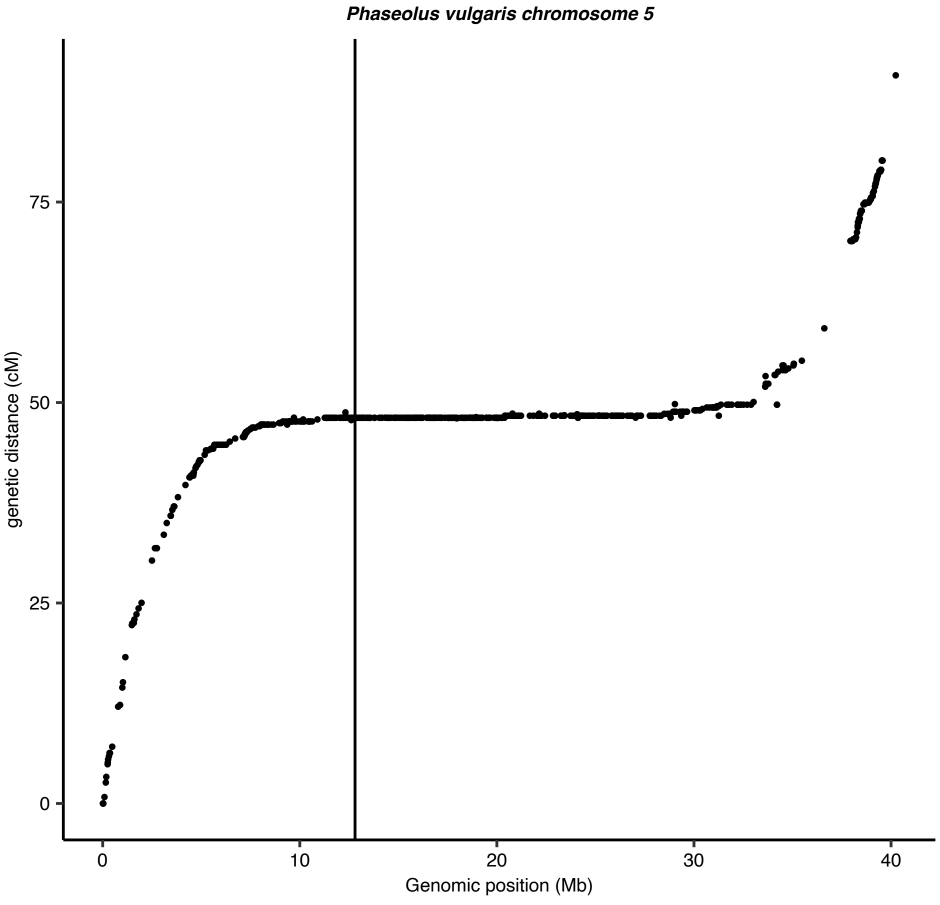

Phaseolus vulgaris chromosome 5
genetic distance (cM)
0
25
50
75
0
10
20
30
40
Genomic position (Mb)

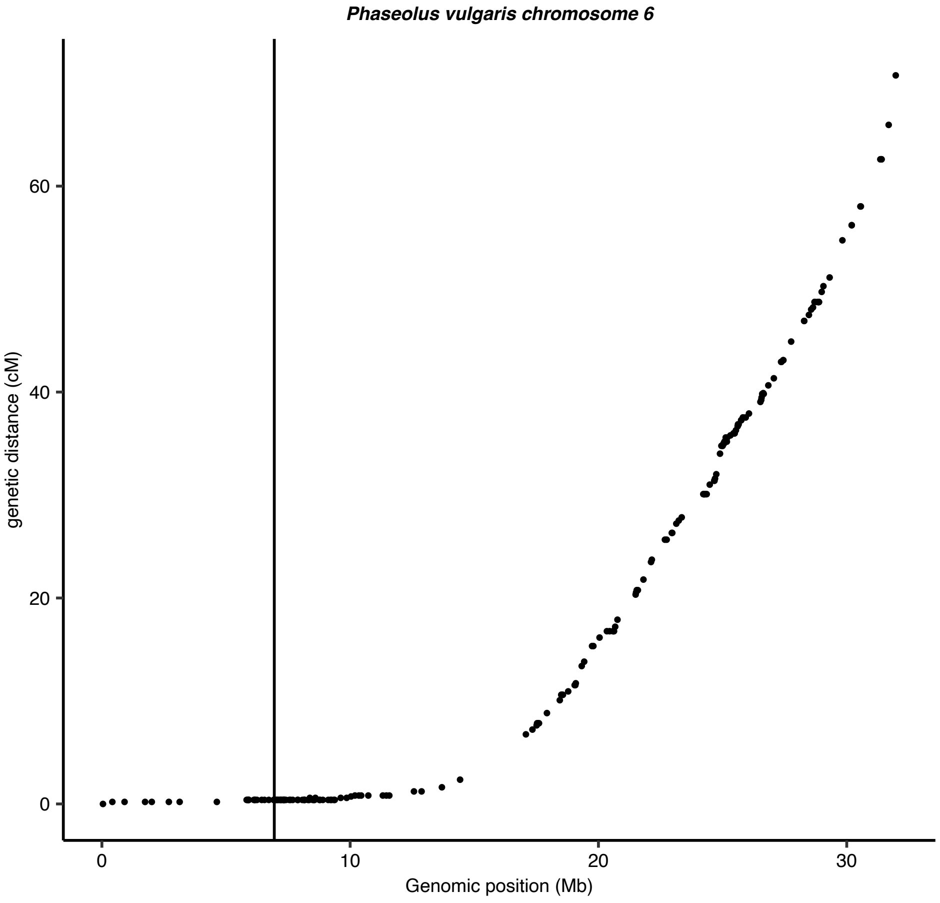
Phaseolus vulgaris chromosome 6
genetic distance (cM)
0
20
40
60
0
10
20
30
Genomic position (Mb)

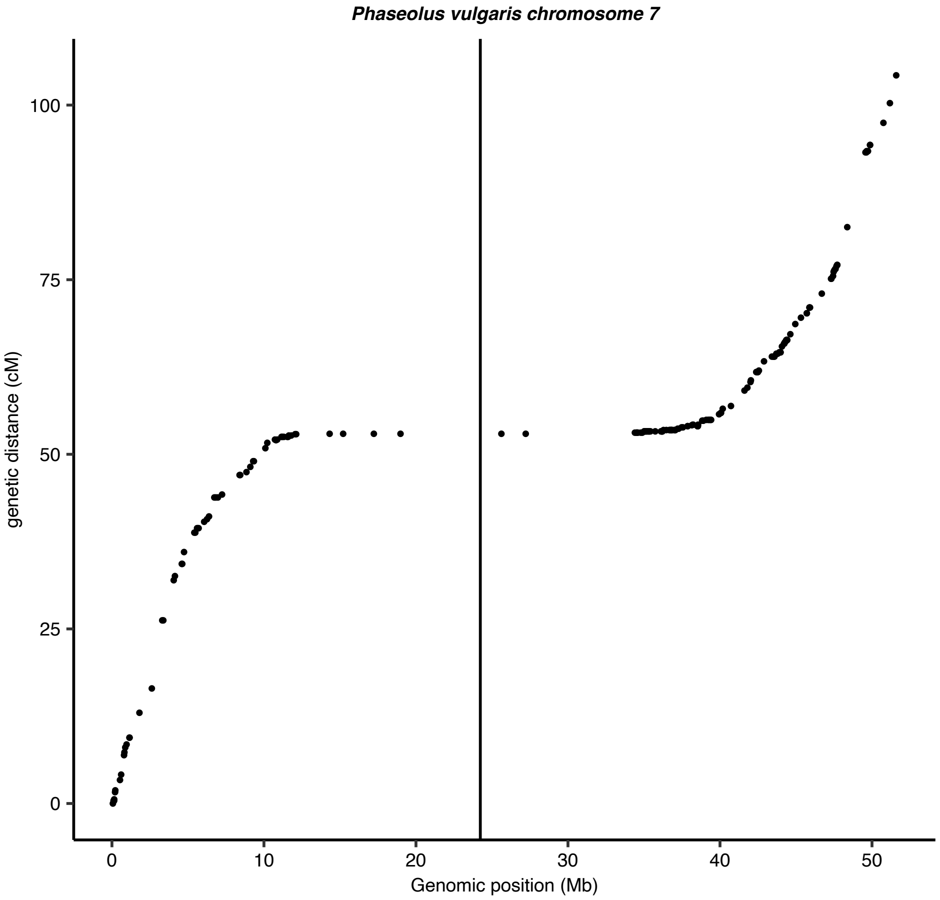
Phaseolus vulgaris chromosome 7
genetic distance (cM)
0
25
50
75
100
Genomic position (Mb)
0
10
20
30
40
50

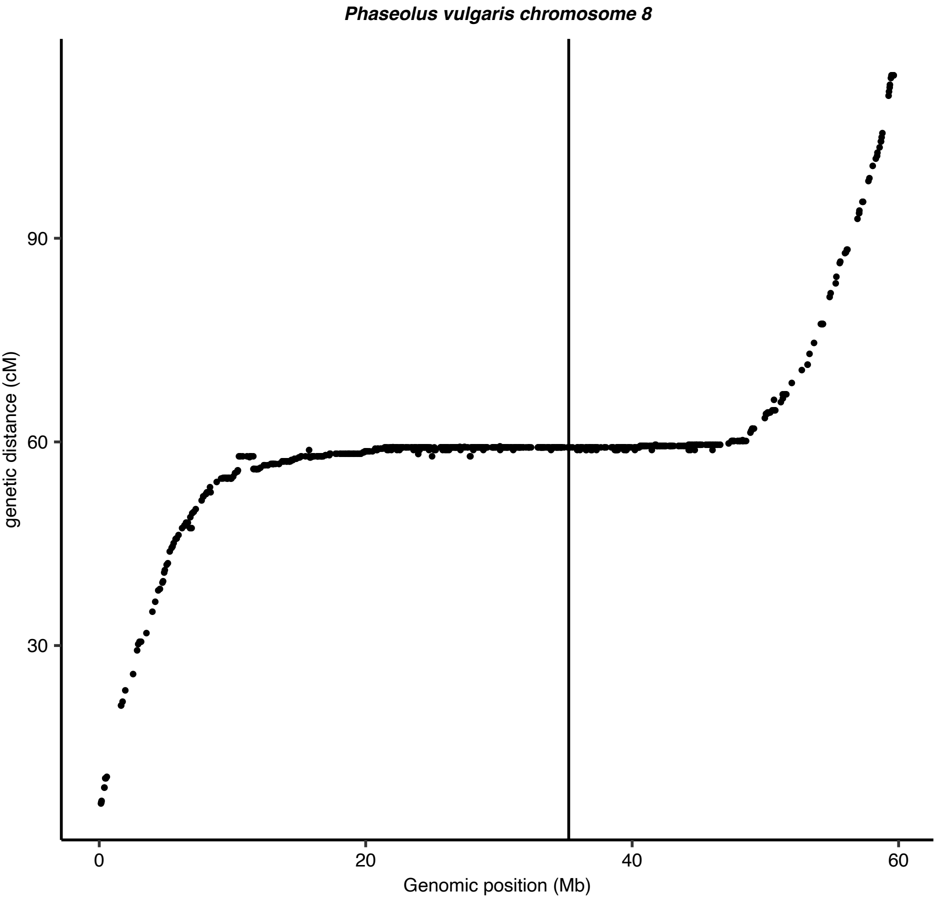
Phaseolus vulgaris chromosome 8
genetic distance (cM)
90
60
30
0
20
40
60
Genomic position (Mb)

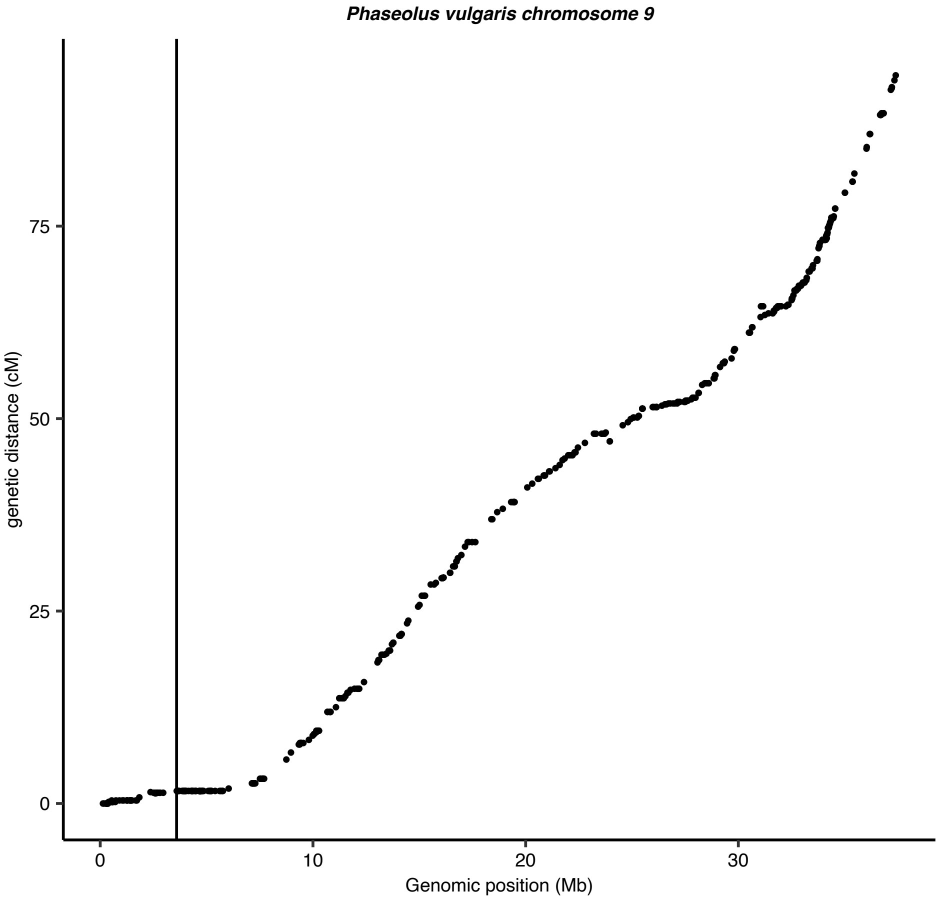

Phaseolus vulgaris chromosome 9
genetic distance (cM)
75
50
25
0
0
10
20
30
Genomic position (Mb)

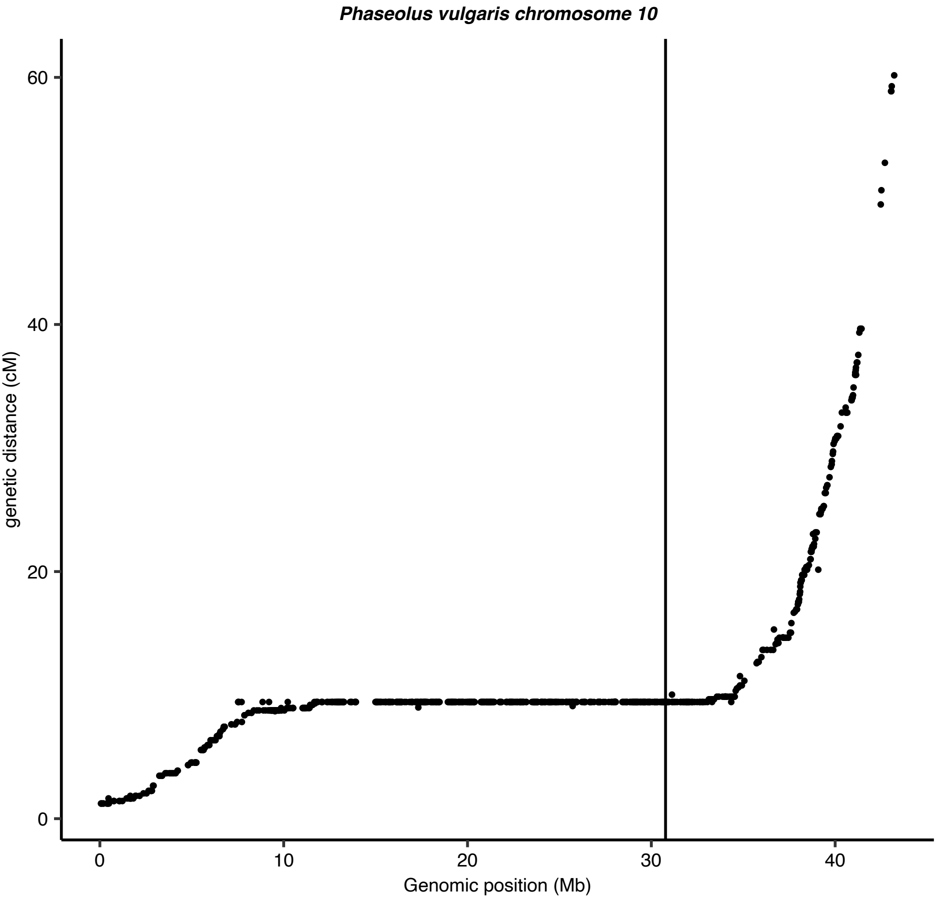
Phaseolus vulgaris chromosome 10
genetic distance (cM)
0
20
40
60
Genomic position (Mb)
0
10
20
30
40

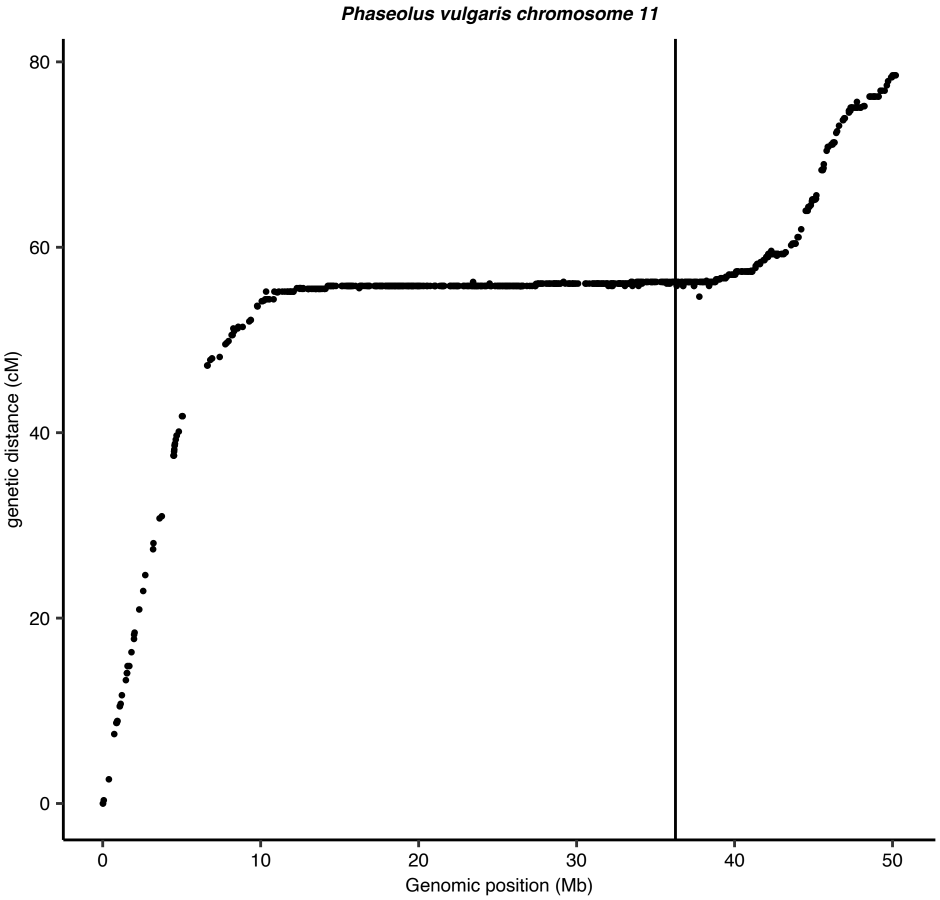
Phaseolus vulgaris chromosome 11
genetic distance (cM)
0
20
40
60
80
Genomic position (Mb)
0
10
20
30
40
50

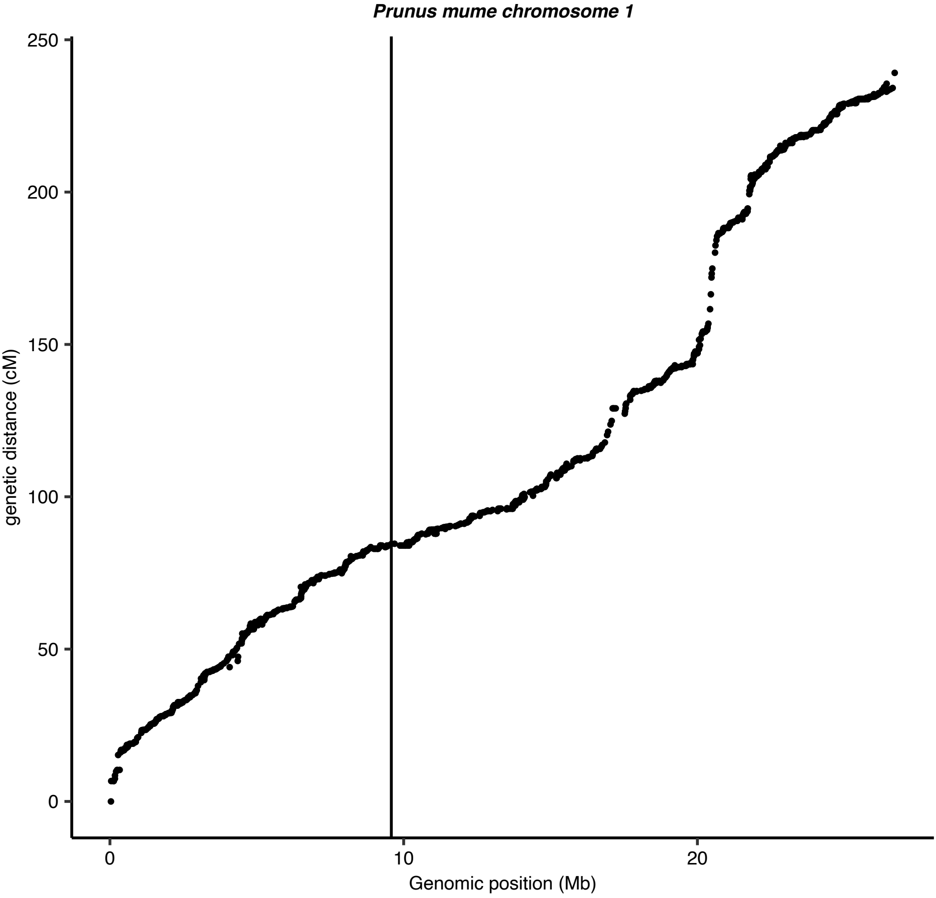
Prunus mume chromosome 1
genetic distance (cM)
250
200
150
100
50
0
0
10
20
Genomic position (Mb)

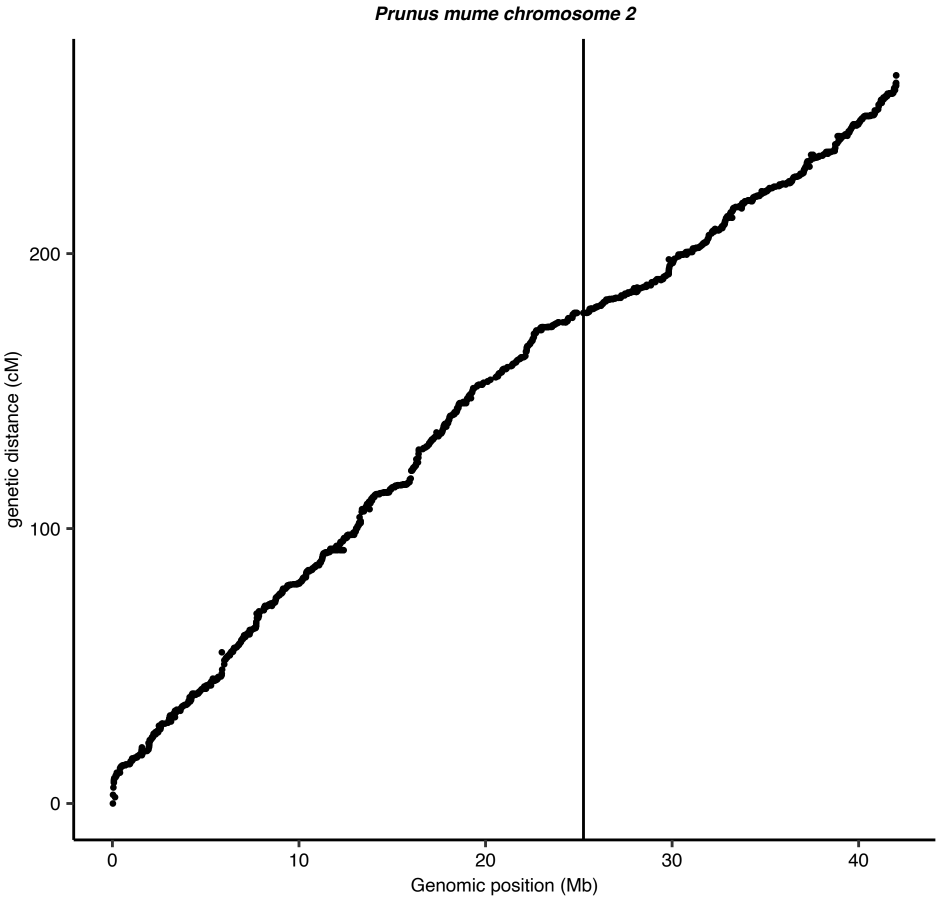
Prunus mume chromosome 2
genetic distance (cM)
0
100
200
Genomic position (Mb)
0
10
20
30
40

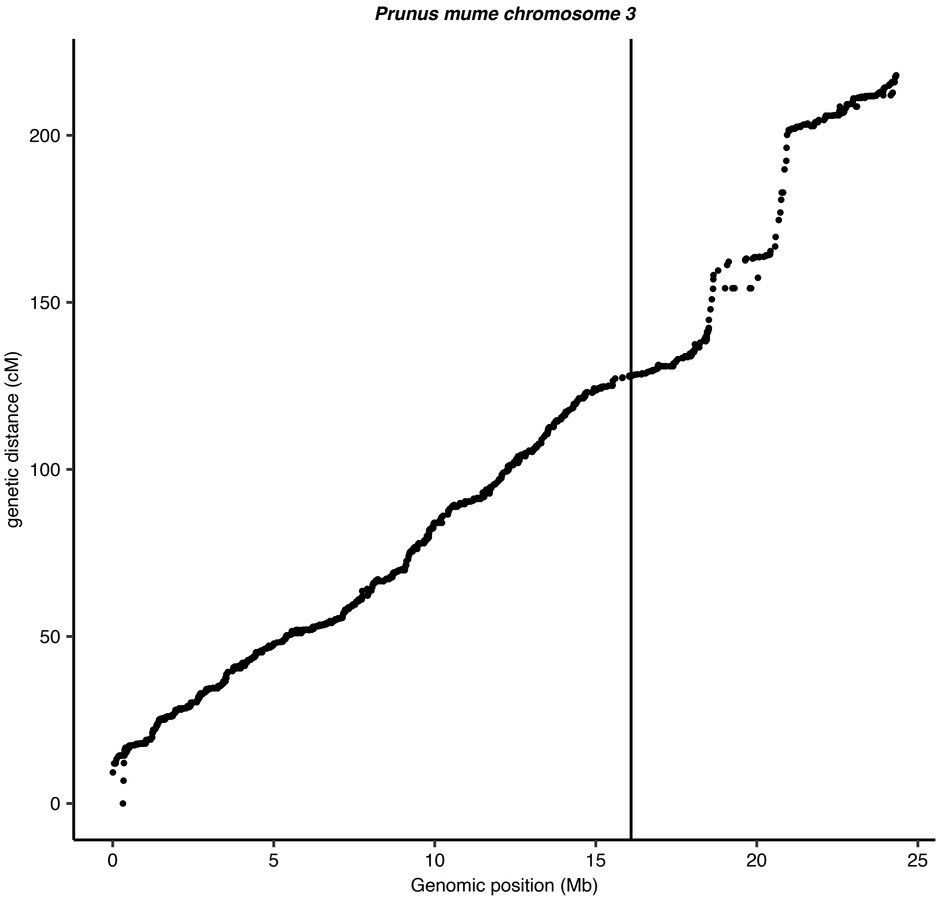

Prunus mume chromosome 3
genetic distance (cM)
0
50
100
150
200
Genomic position (Mb)
0
5
10
15
20
25

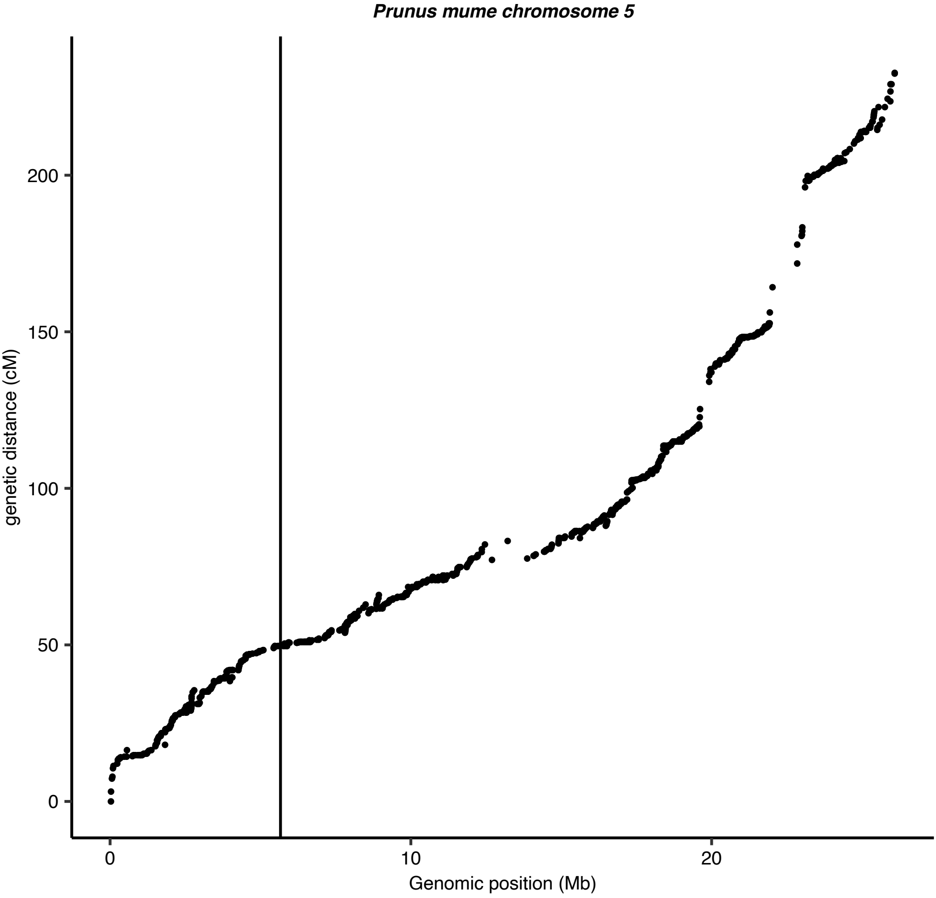
Prunus mume chromosome 5
genetic distance (cM)
0
50
100
150
200
Genomic position (Mb)
0
10
20

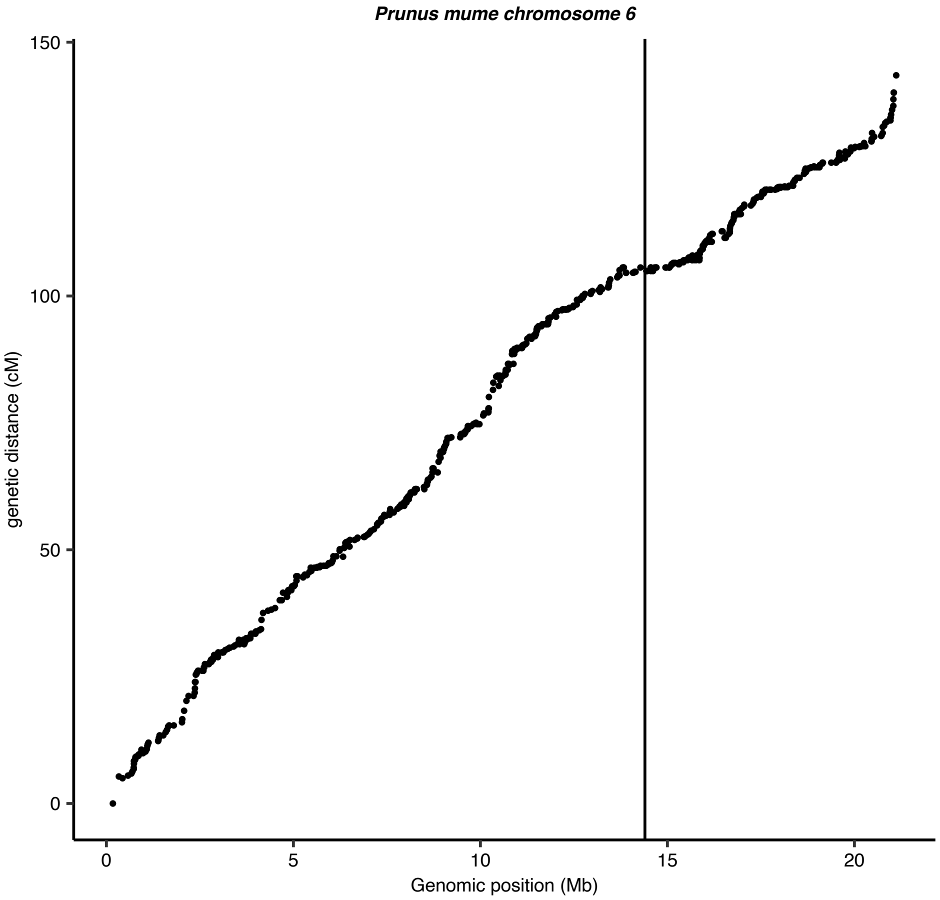

Prunus mume chromosome 6
genetic distance (cM)
150
100
50
0
Genomic position (Mb)
0
5
10
15
20

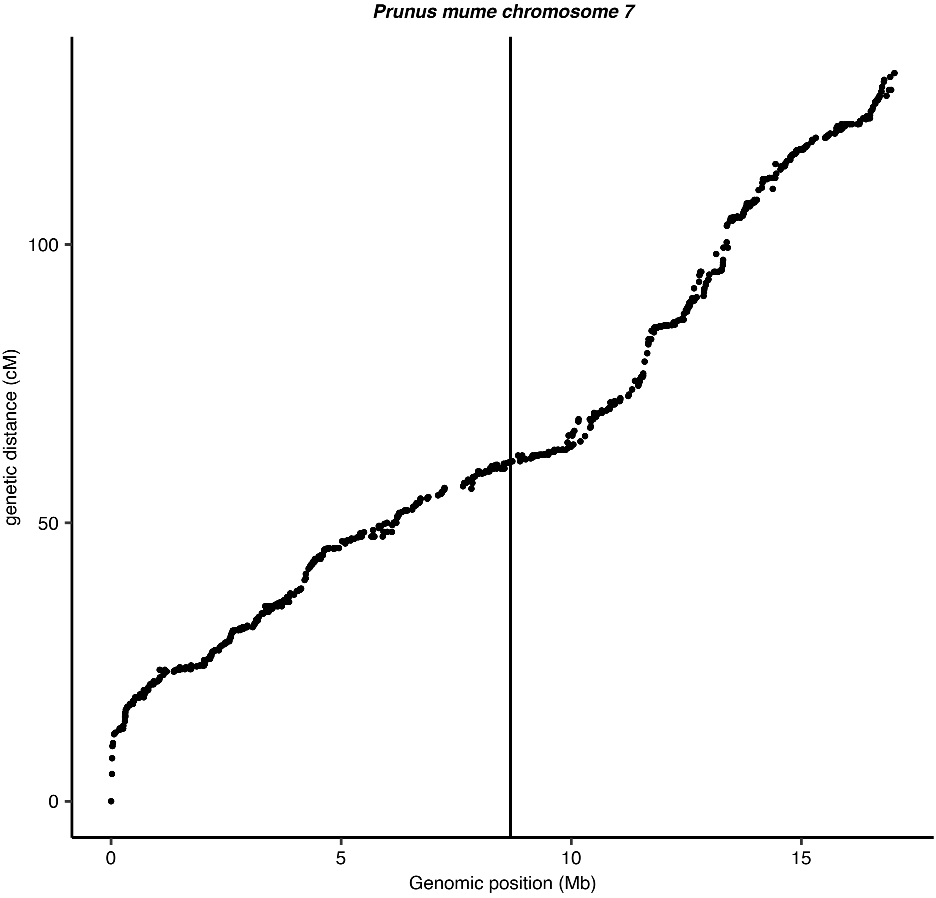
Prunus mume chromosome 7
genetic distance (cM)
0
50
100
Genomic position (Mb)
0
5
10
15

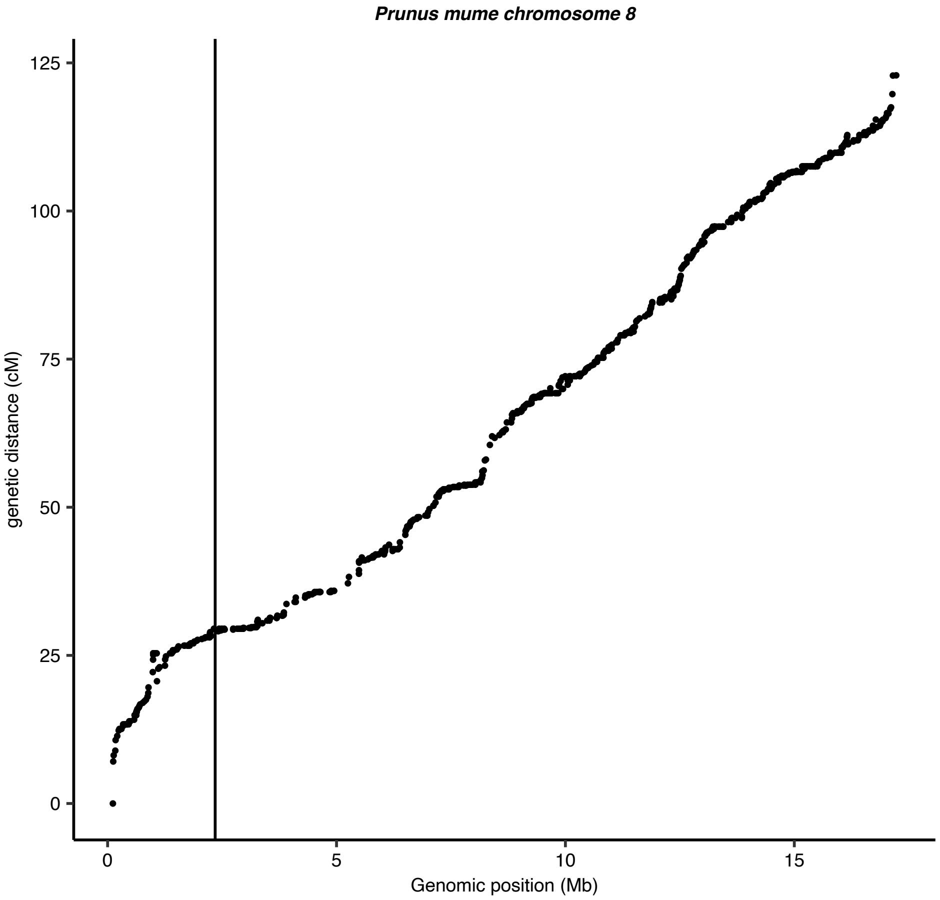
Prunus mume chromosome 8
genetic distance (cM)
0
25
50
75
100
125
Genomic position (Mb)
0
5
10
15

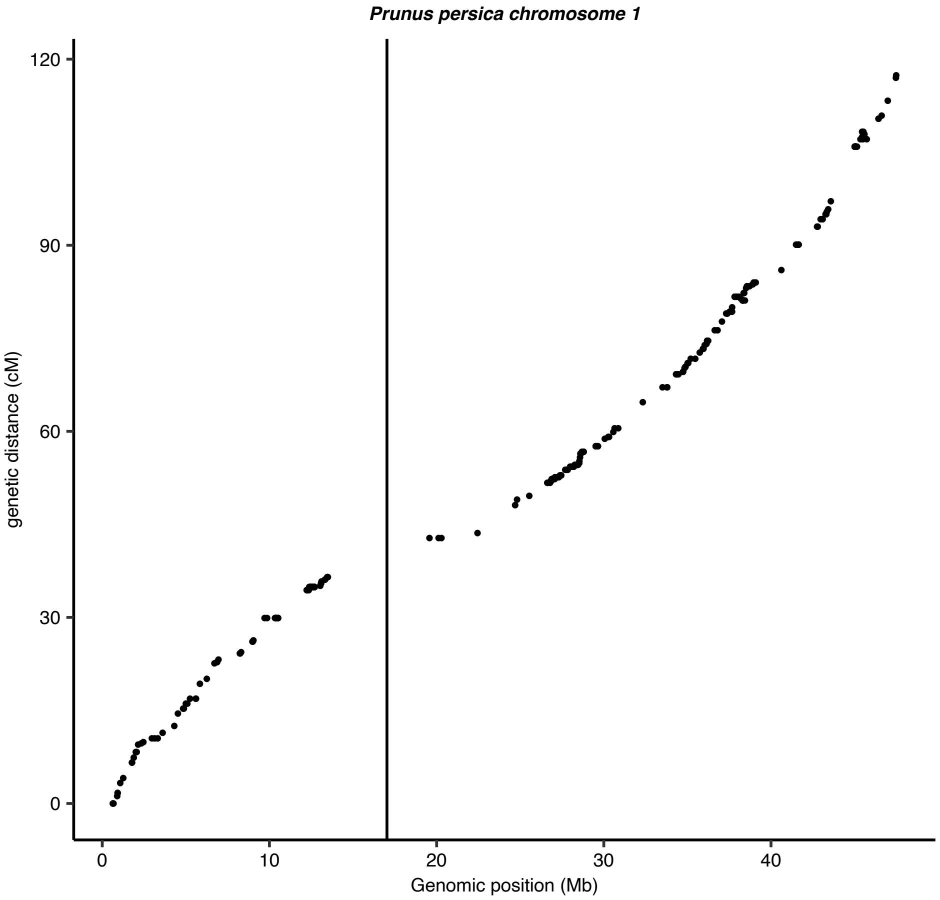
Prunus persica chromosome 1
120
90
60
30
0
0
10
20
30
40
Genomic position (Mb)
genetic distance (cM)

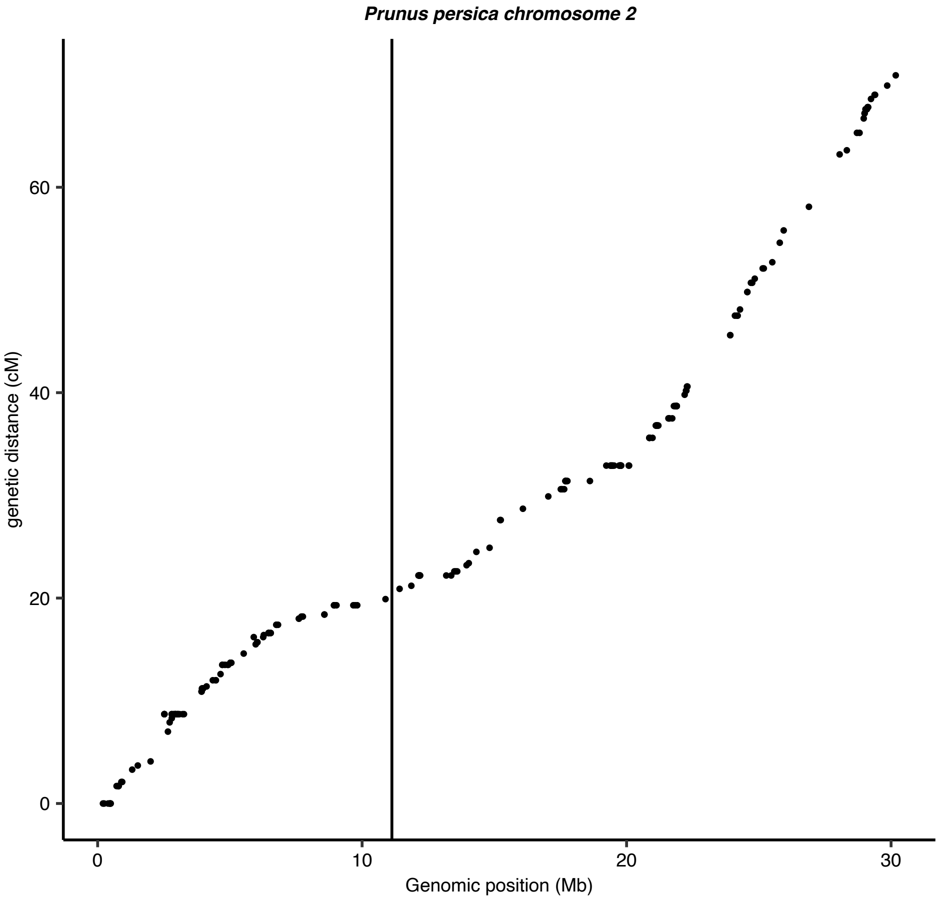
Prunus persica chromosome 2
genetic distance (cM)
0
20
40
60
Genomic position (Mb)
0
10
20
30

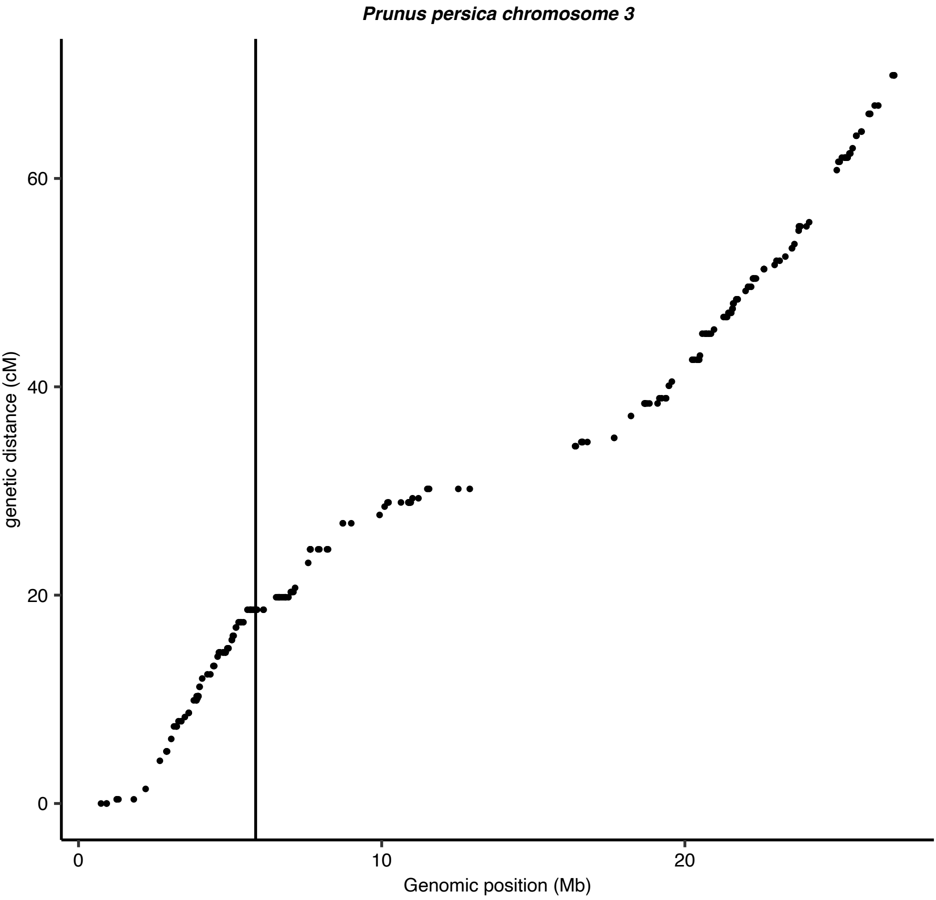
Prunus persica chromosome 3
genetic distance (cM)
0
20
40
60
0
10
20
Genomic position (Mb)

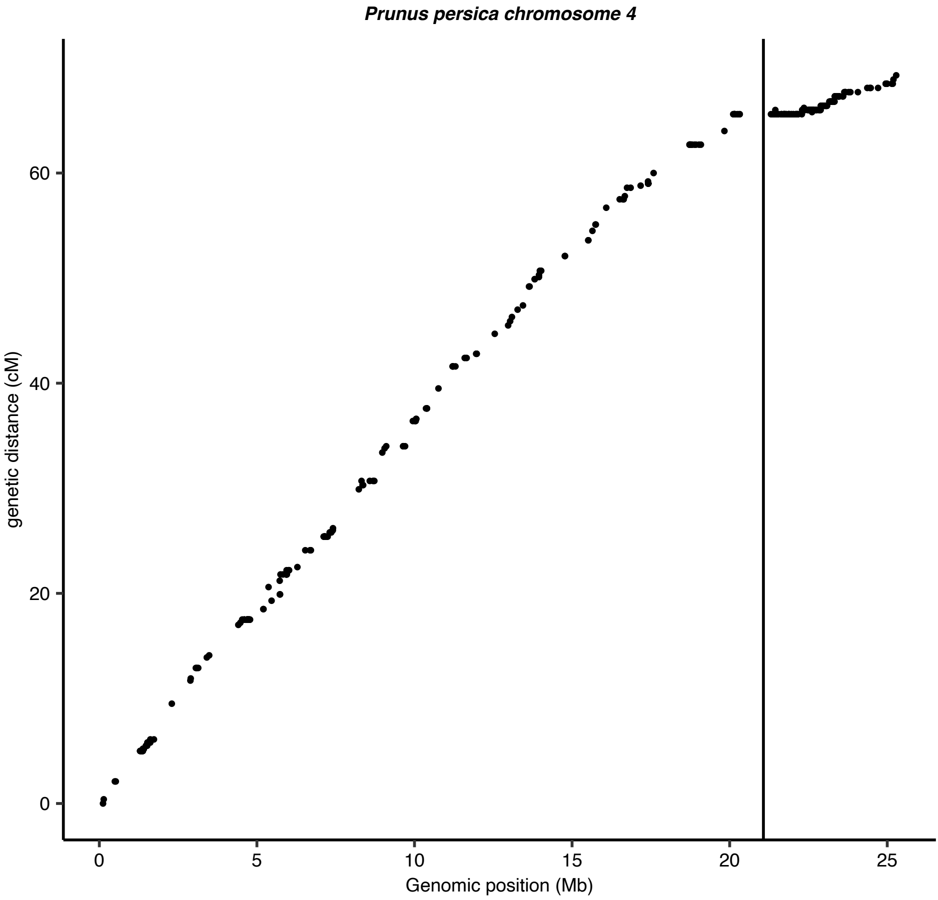
Prunus persica chromosome 4
genetic distance (cM)
0
20
40
60
Genomic position (Mb)
0
5
10
15
20
25

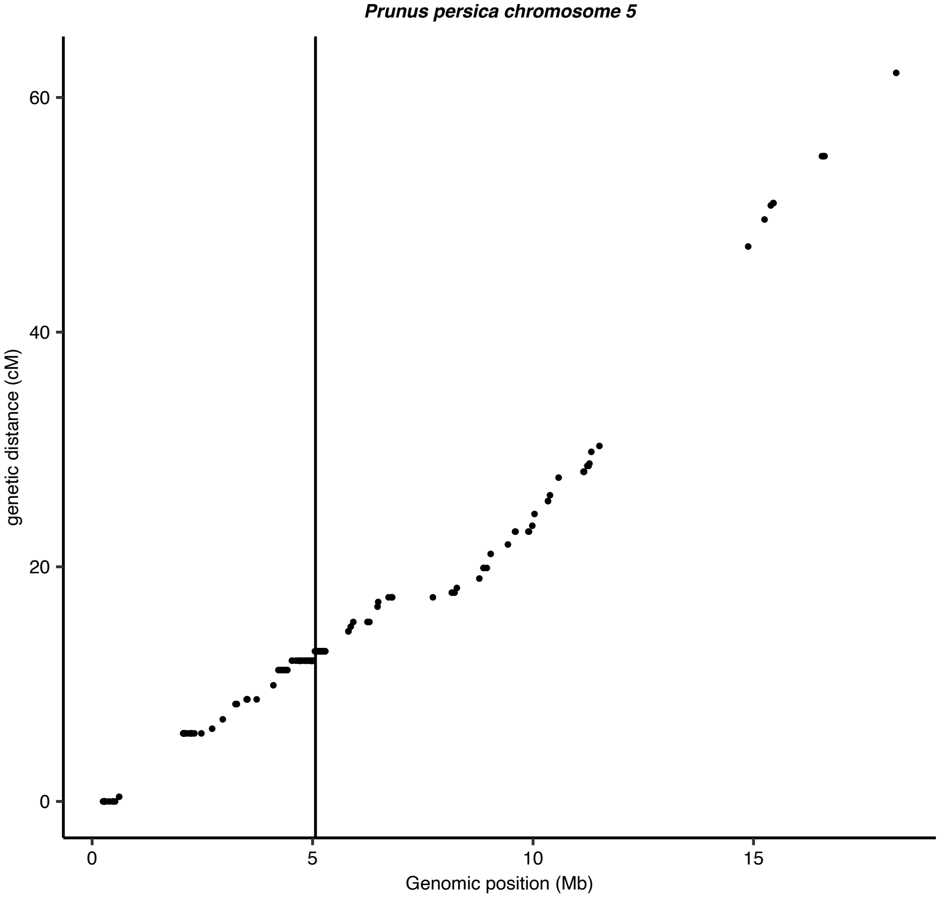
Prunus persica chromosome 5
genetic distance (cM)
60
40
20
0
0
5
10
15
Genomic position (Mb)

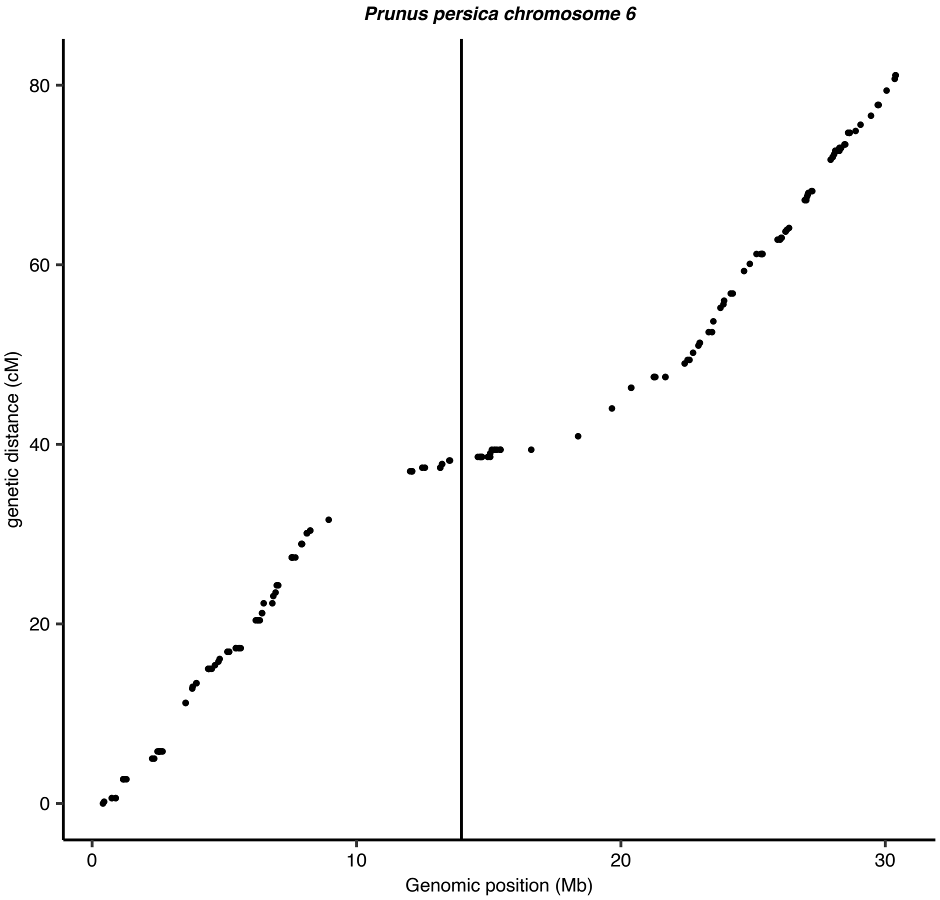
Prunus persica chromosome 6
genetic distance (cM)
0
20
40
60
80
Genomic position (Mb)
0
10
20
30

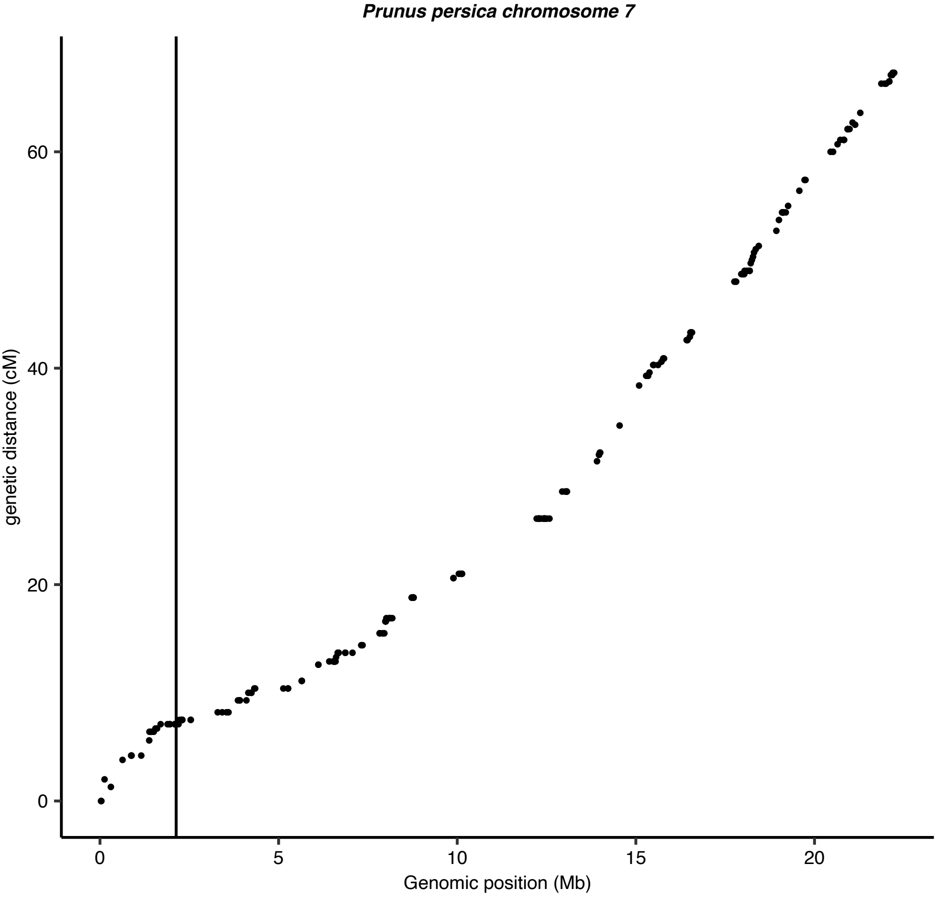
Prunus persica chromosome 7
genetic distance (cM)
0
20
40
60
Genomic position (Mb)
0
5
10
15
20

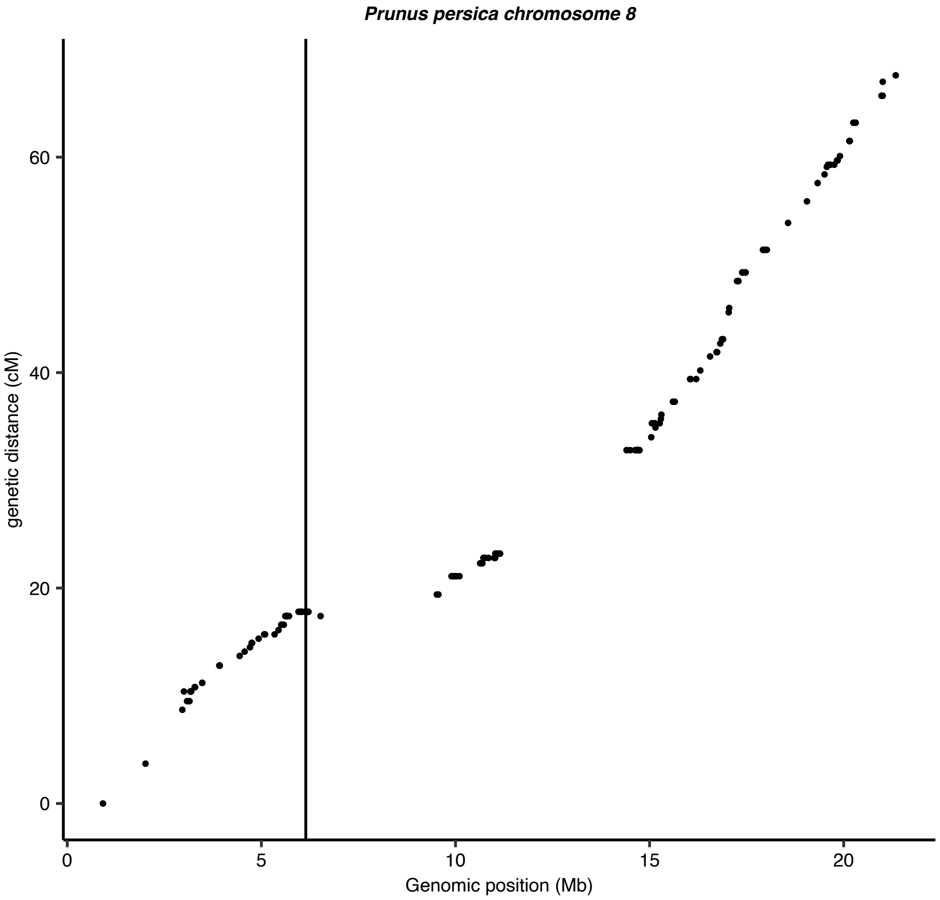
Prunus persica chromosome 8
genetic distance (cM)
Genomic position (Mb)
0
20
40
60
0
5
10
15
20

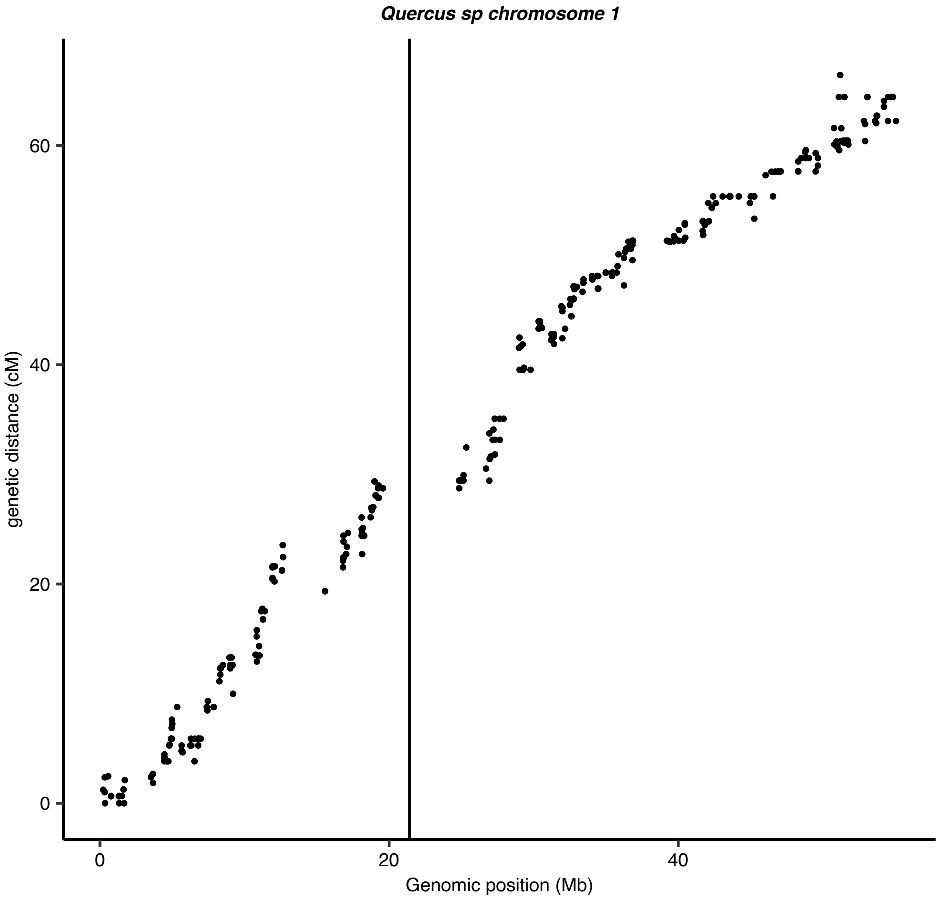
Quercus sp chromosome 1
genetic distance (cM)
Genomic position (Mb)
0
20
40
60
0
20
40

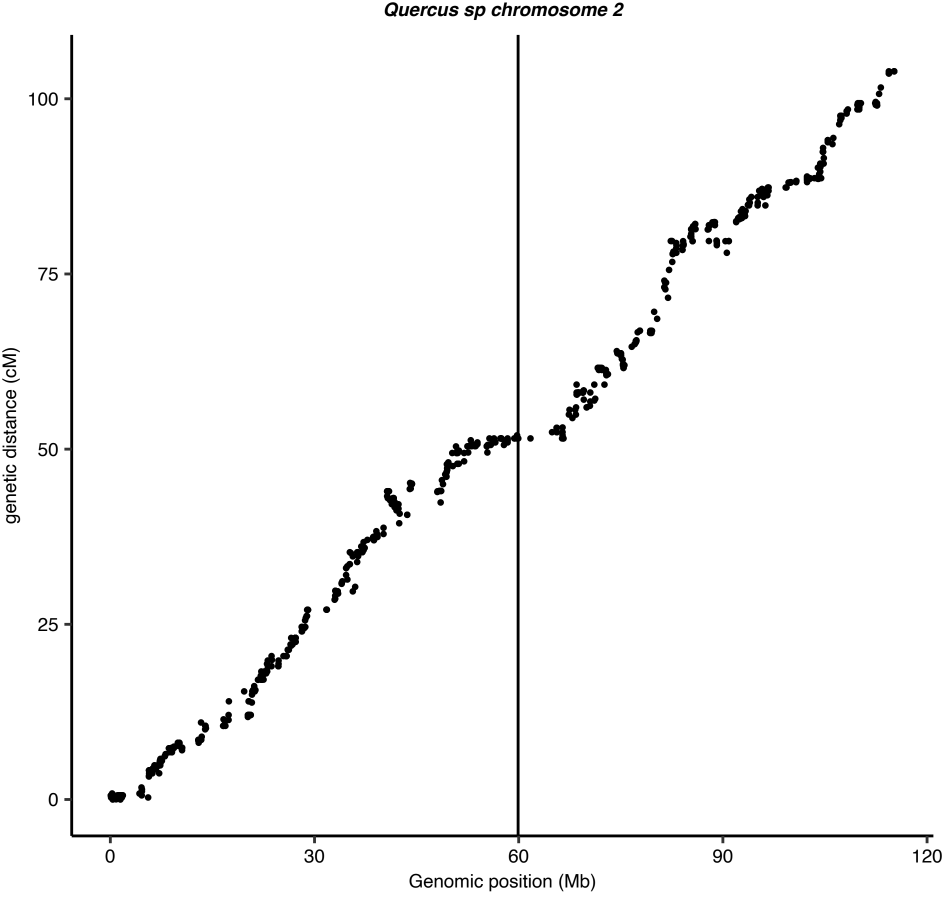
Quercus sp chromosome 2
genetic distance (cM)
0
25
50
75
100
Genomic position (Mb)
0
30
60
90
120

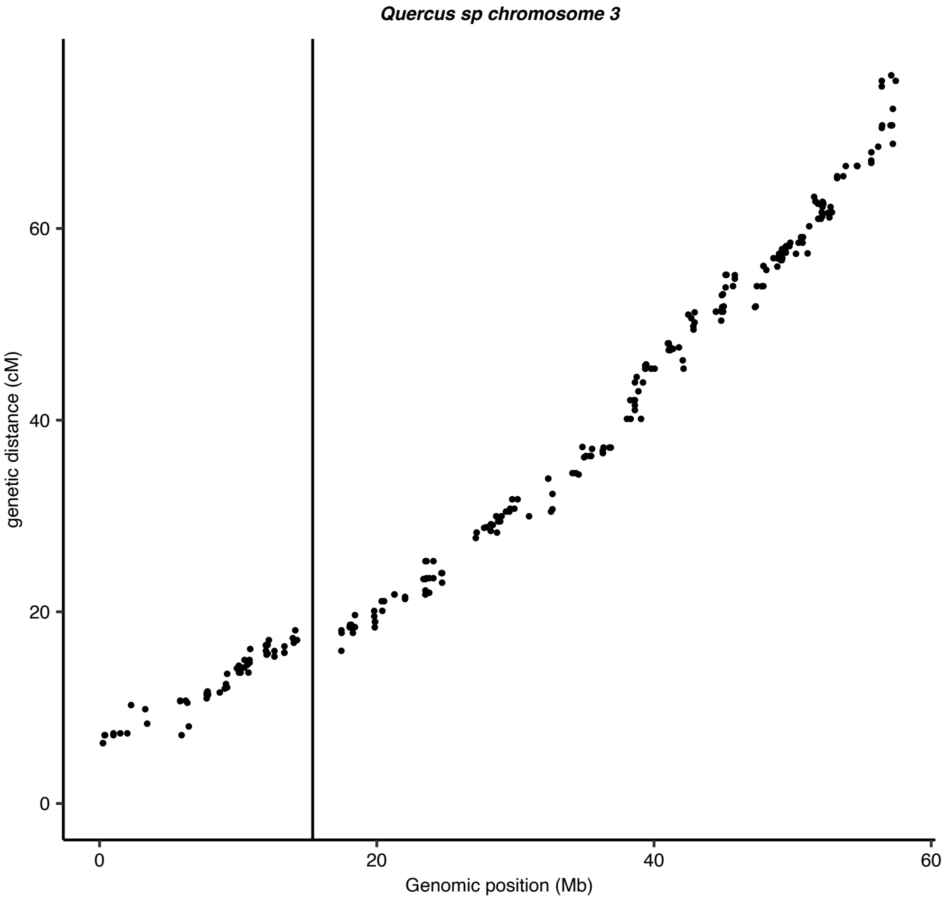
Quercus sp chromosome 3
genetic distance (cM)
Genomic position (Mb)
0
20
40
60
0
20
40
60

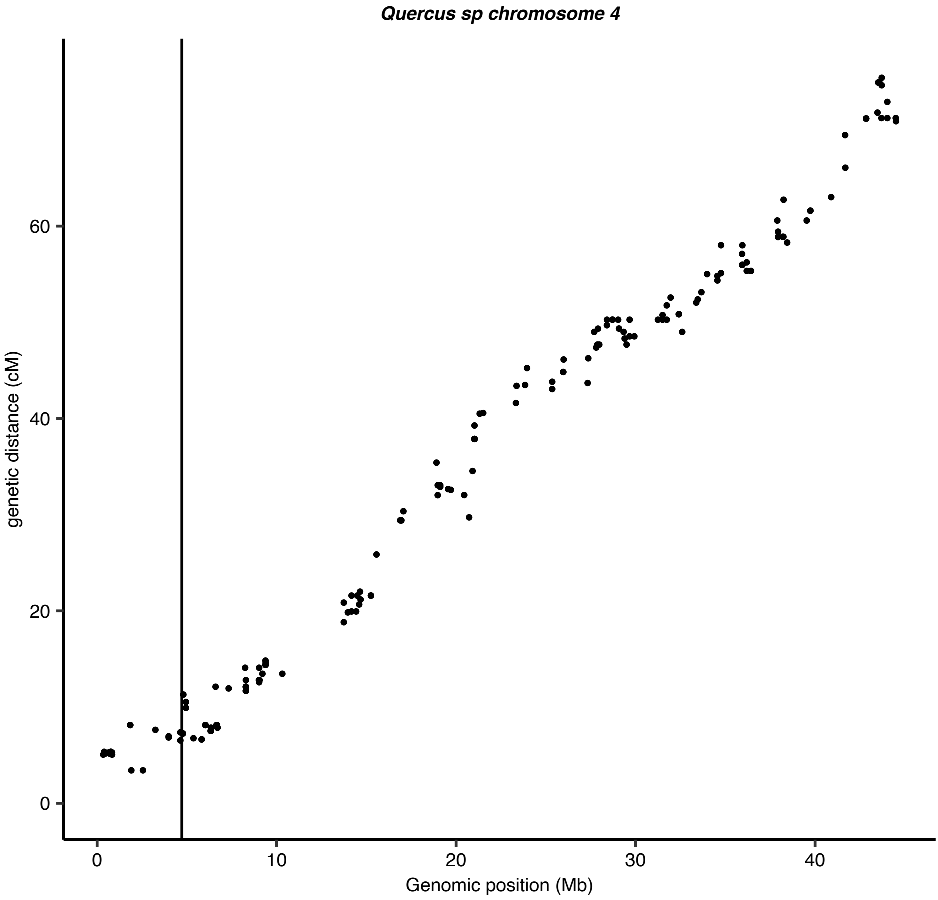
Quercus sp chromosome 4
genetic distance (cM)
0
20
40
60
Genomic position (Mb)
0
10
20
30
40

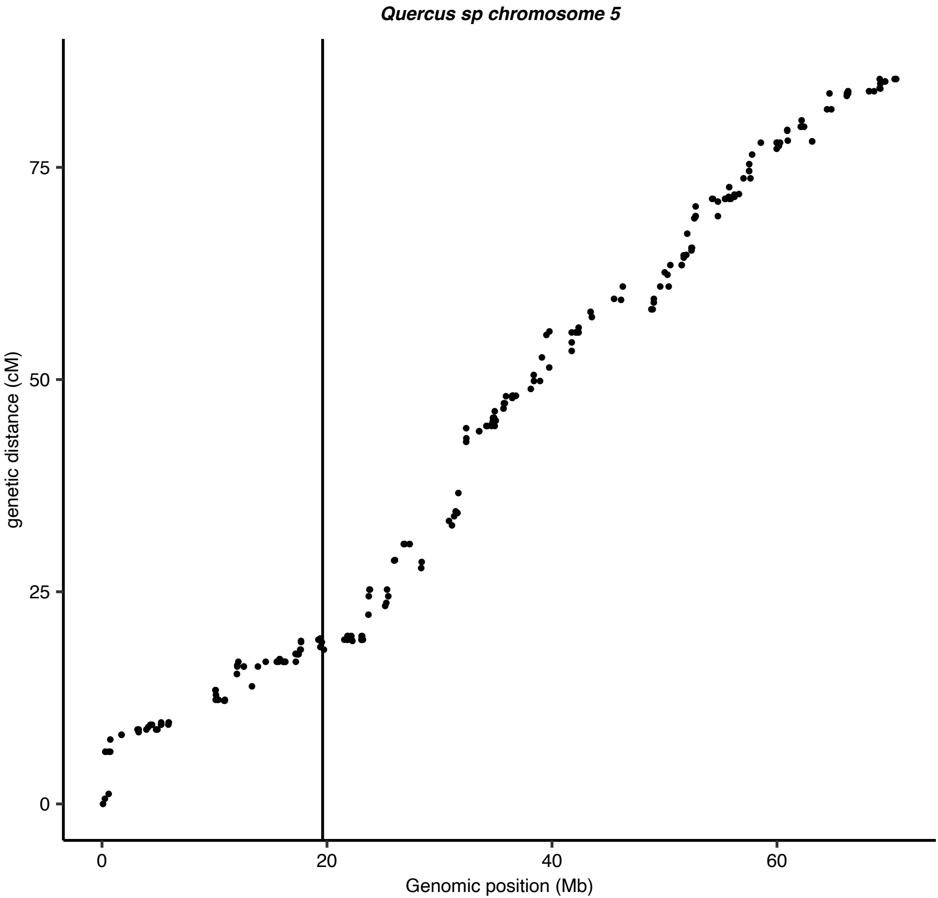
Quercus sp chromosome 5
genetic distance (cM)
Genomic position (Mb)
0
25
50
75
0
20
40
60

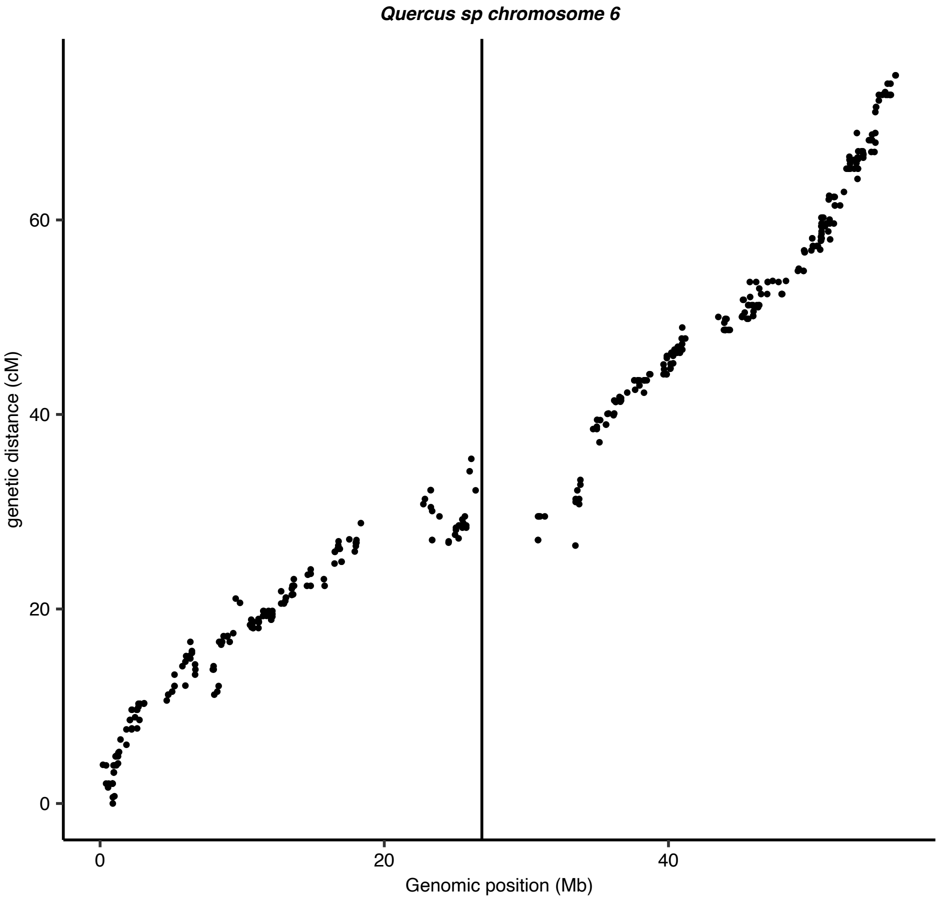
Quercus sp chromosome 6
genetic distance (cM)
Genomic position (Mb)
0
20
40
60
0
20
40

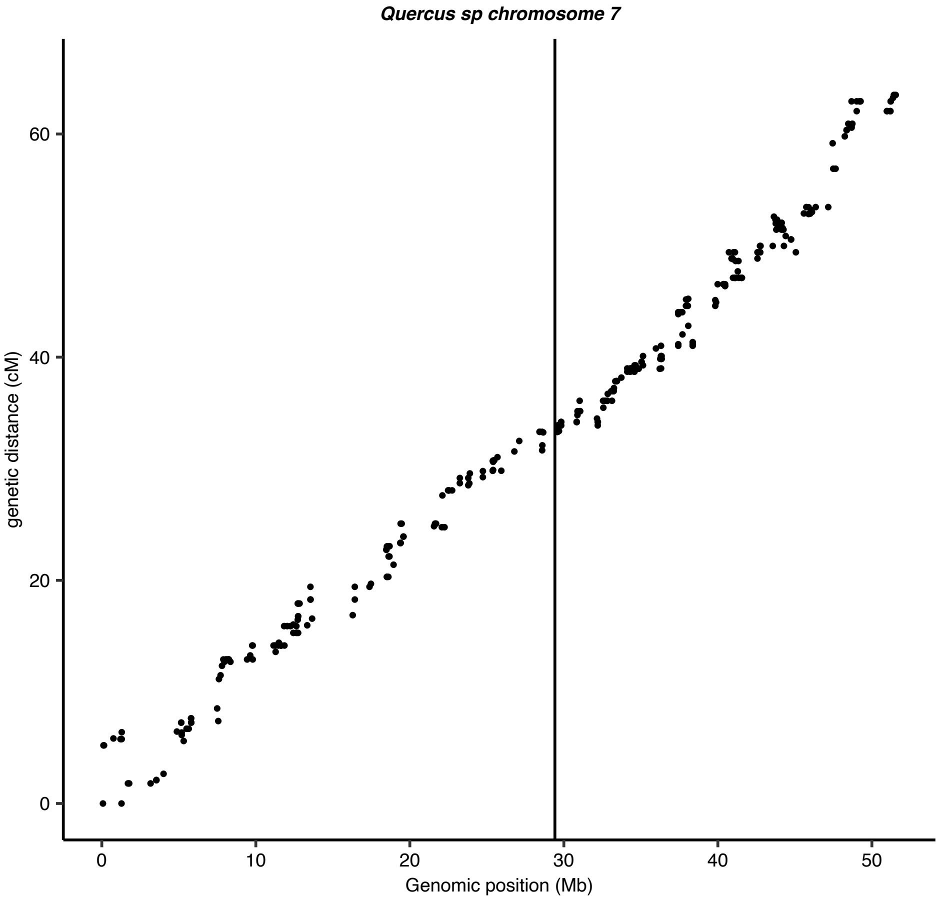
Quercus sp chromosome 7
genetic distance (cM)
Genomic position (Mb)
0
20
40
60
0
10
20
30
40
50

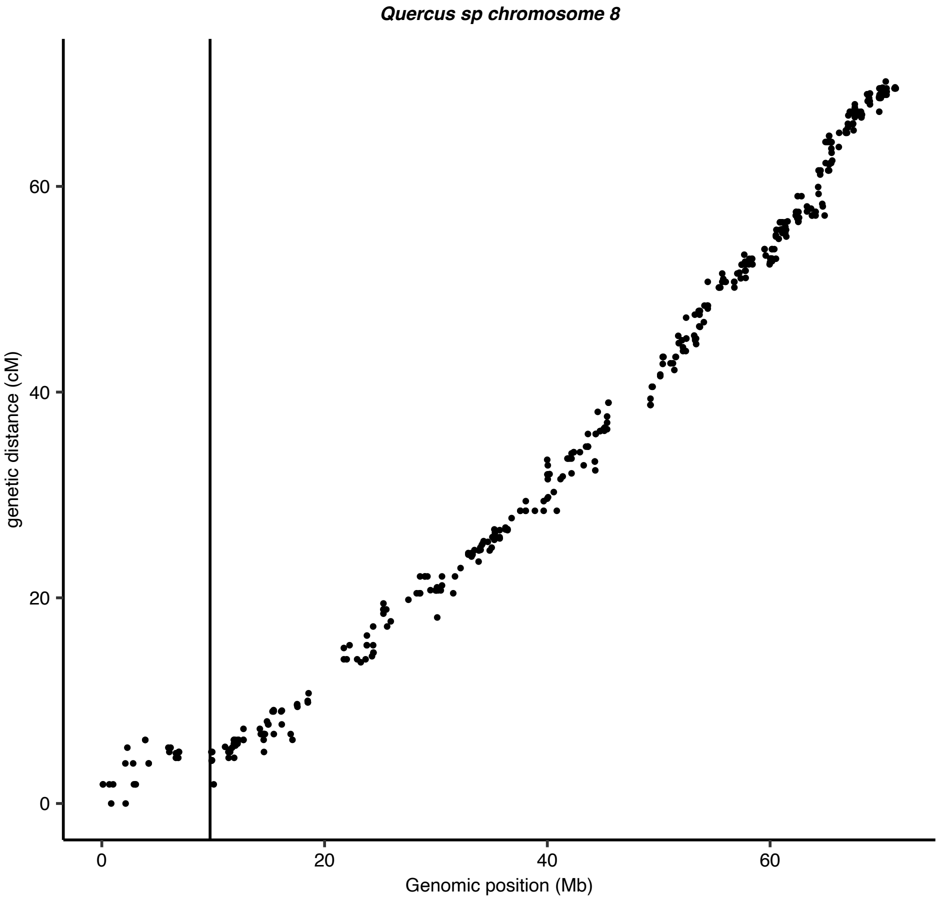
Quercus sp chromosome 8
genetic distance (cM)
0
20
40
60
Genomic position (Mb)
0
20
40
60

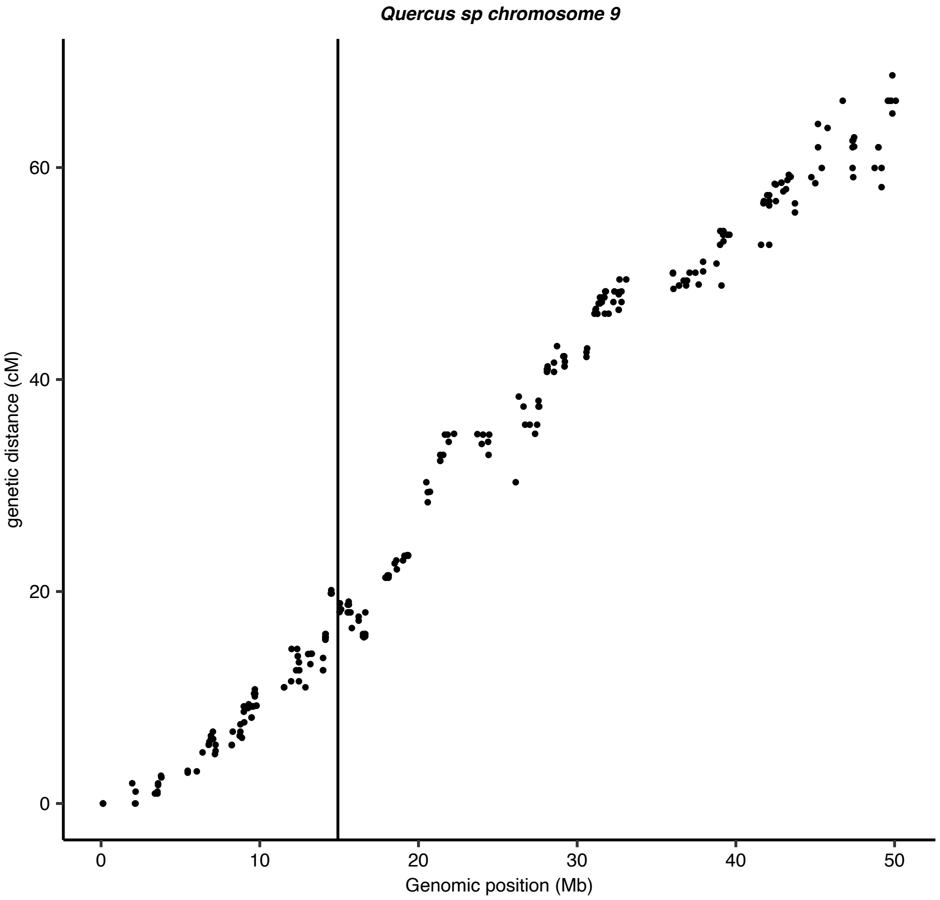
Quercus sp chromosome 9
genetic distance (cM)
0
20
40
60
Genomic position (Mb)
0
10
20
30
40
50

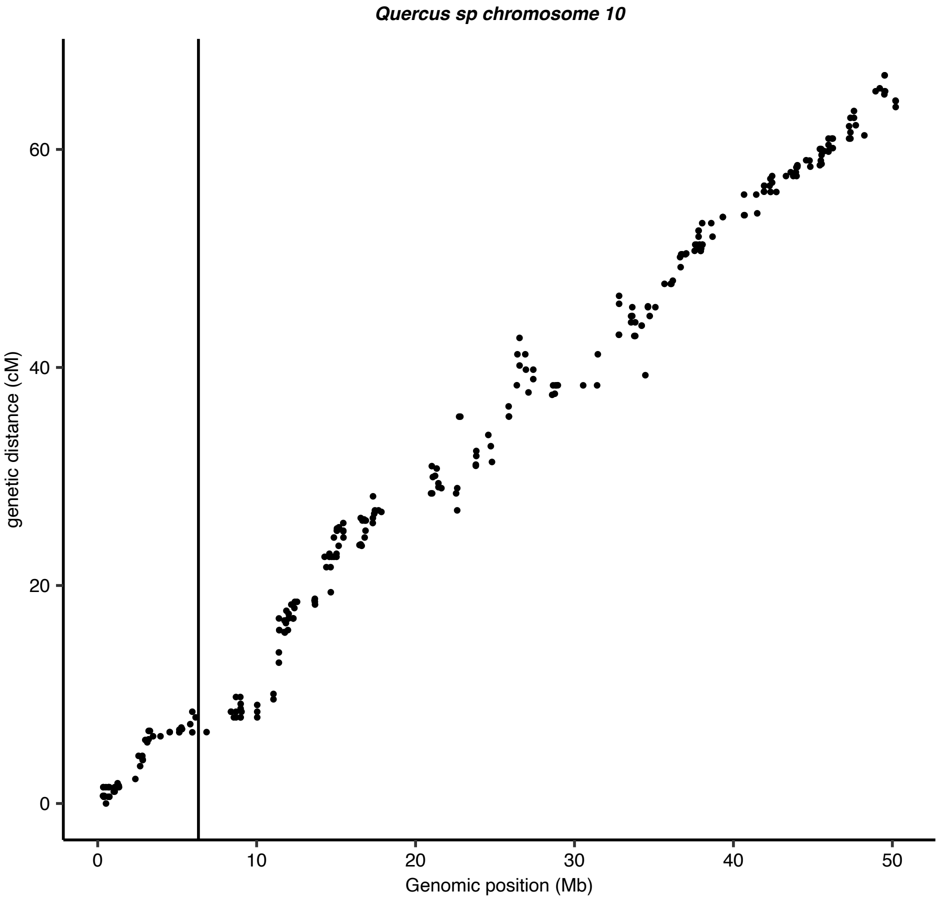
Quercus sp chromosome 10
genetic distance (cM)
Genomic position (Mb)
0
20
40
60
0
10
20
30
40
50

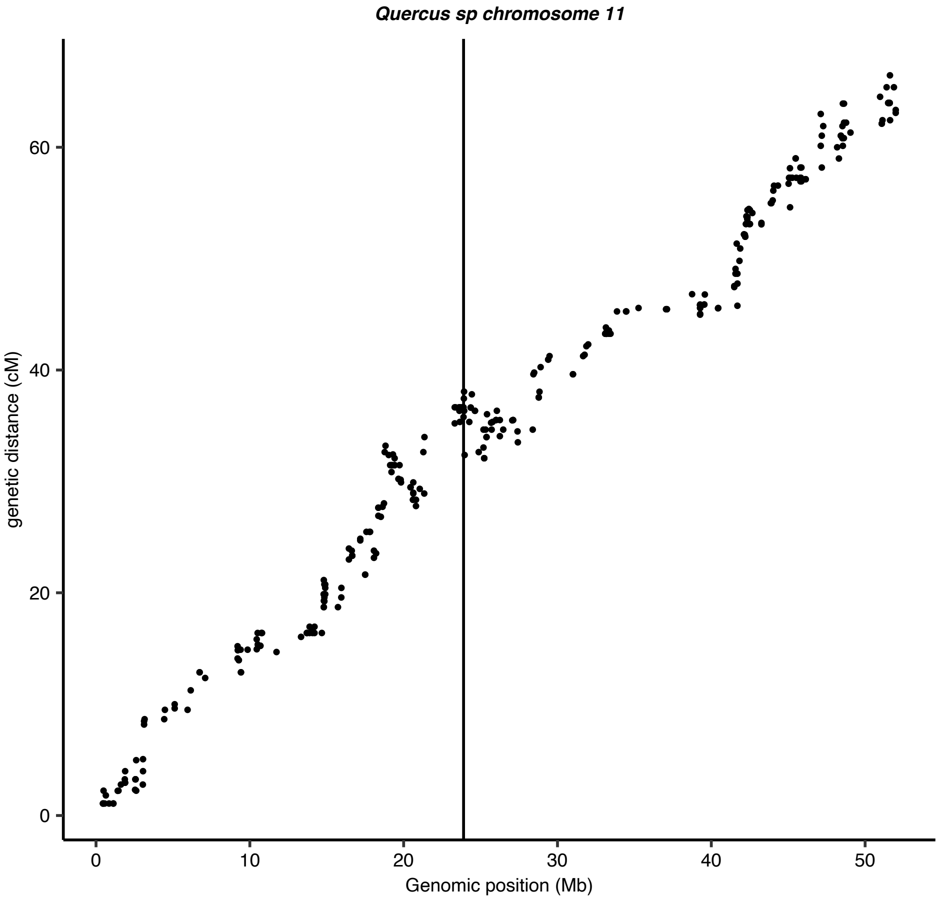
Quercus sp chromosome 11
genetic distance (cM)
0
20
40
60
Genomic position (Mb)
0
10
20
30
40
50

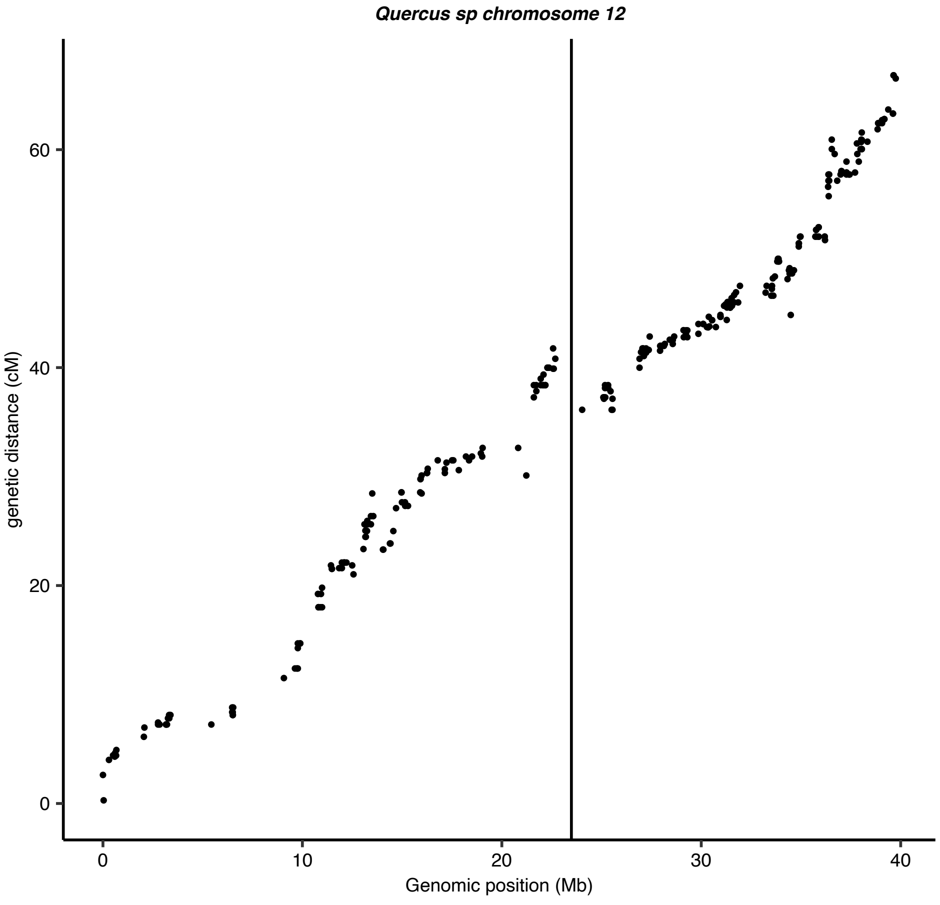
Quercus sp chromosome 12
genetic distance (cM)
0
20
40
60
0
10
20
30
40
Genomic position (Mb)

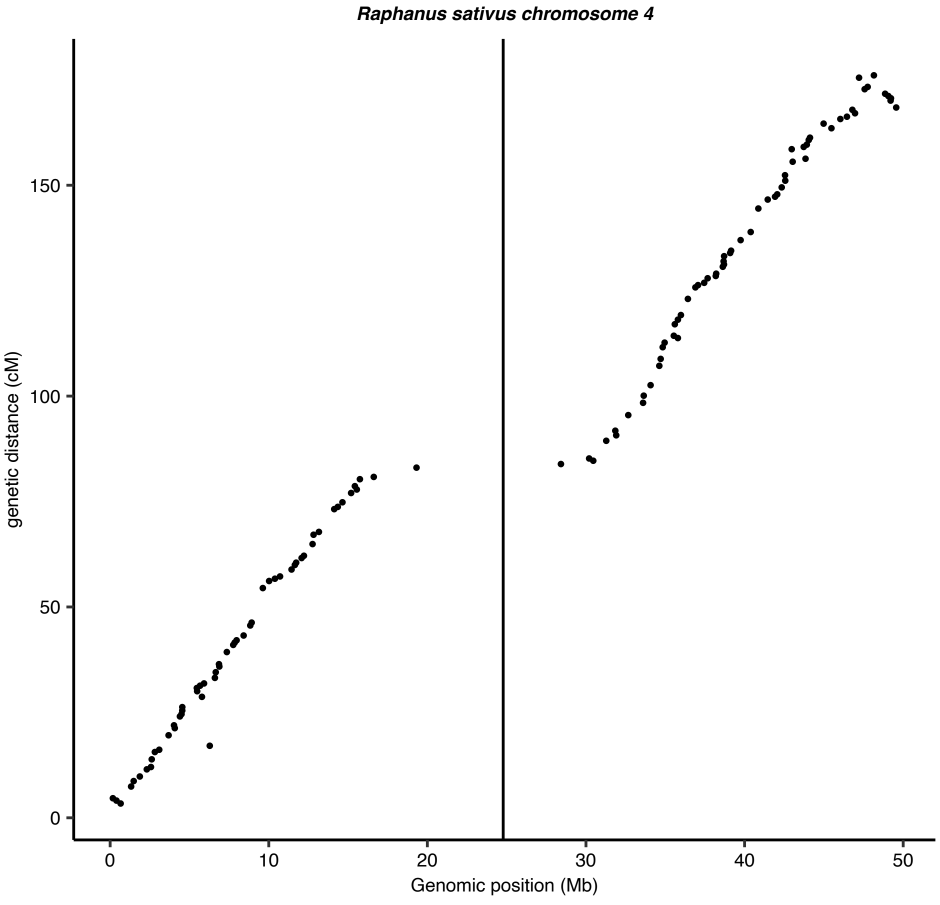
Raphanus sativus chromosome 4
genetic distance (cM)
0
50
100
150
Genomic position (Mb)
0
10
20
30
40
50

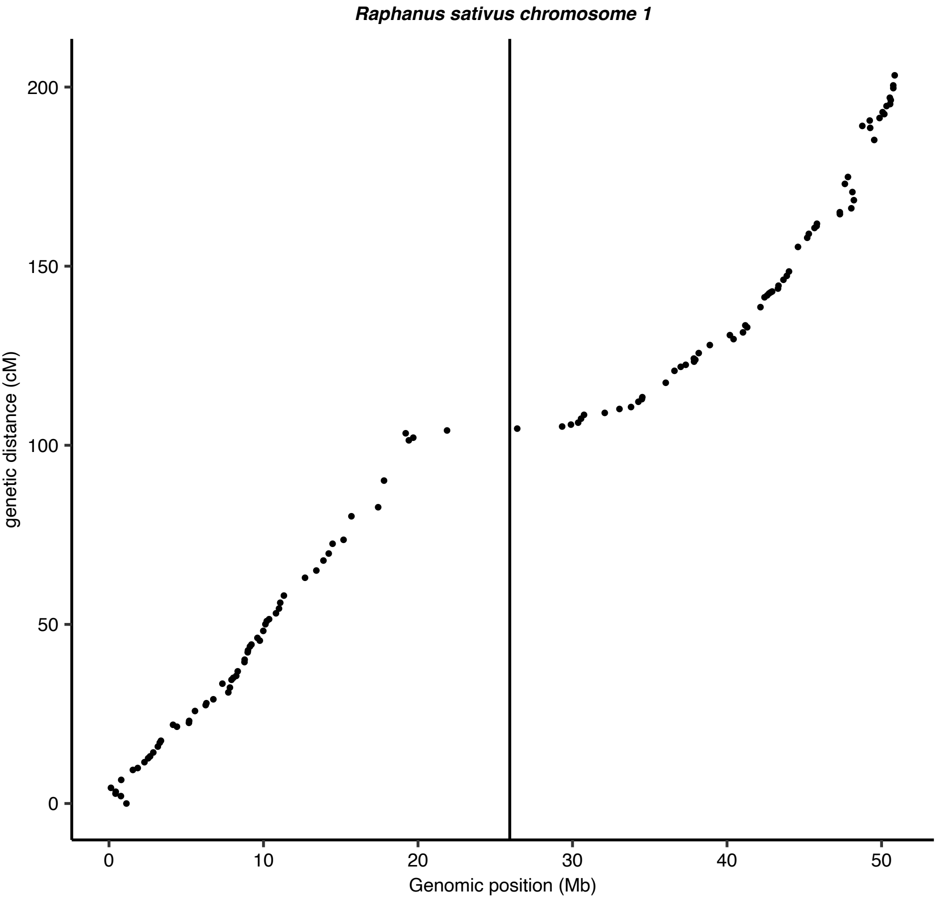
Raphanus sativus chromosome 1
genetic distance (cM)
200
150
100
50
0
0
10
20
30
40
50
Genomic position (Mb)

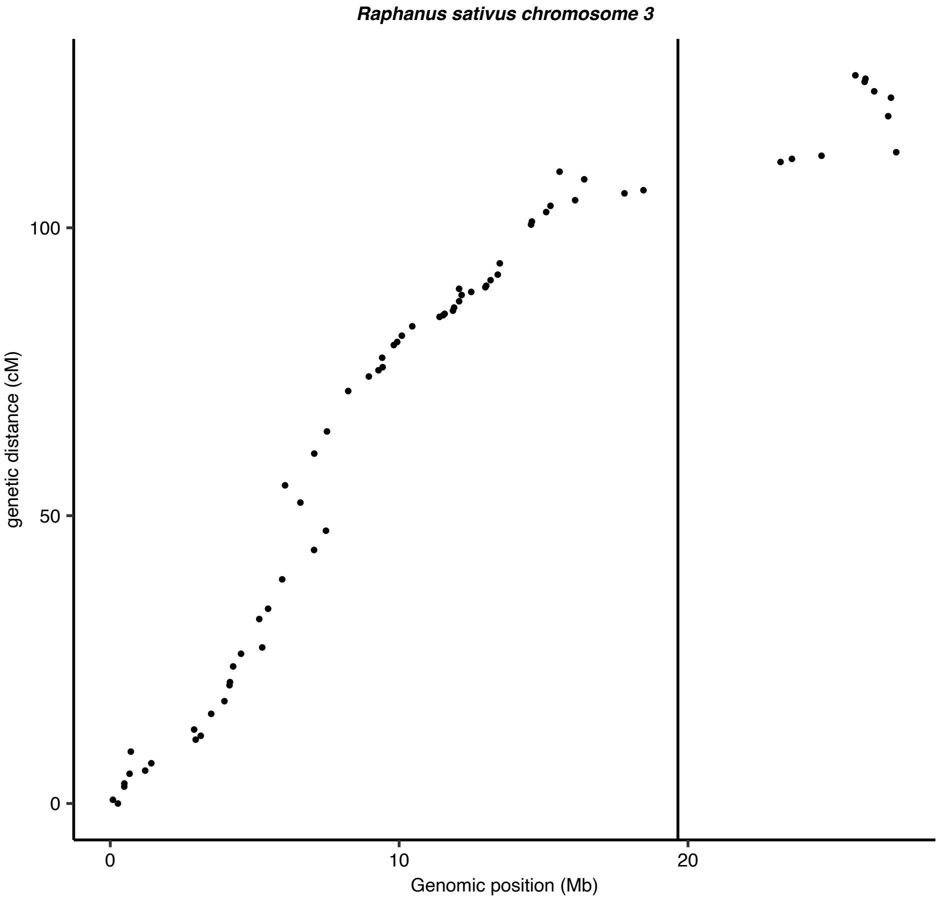
Raphanus sativus chromosome 3
genetic distance (cM)
100
50
0
0
10
20
Genomic position (Mb)

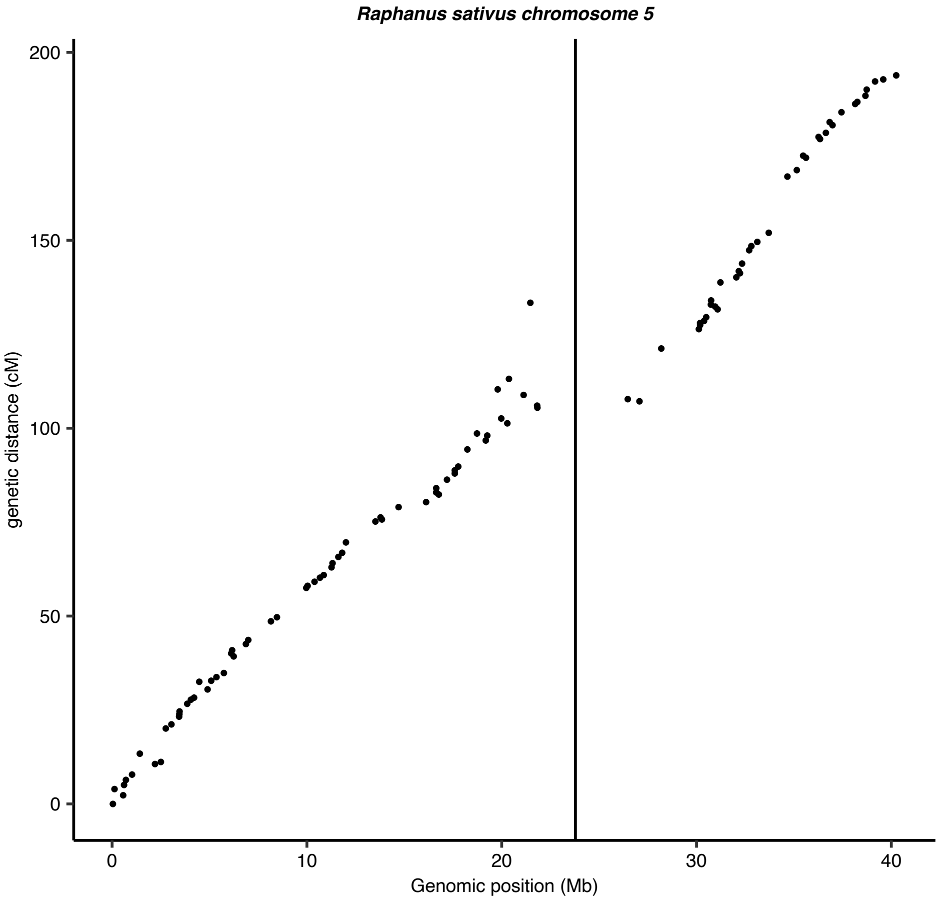
Raphanus sativus chromosome 5
genetic distance (cM)
Genomic position (Mb)
0
50
100
150
200
0
10
20
30
40

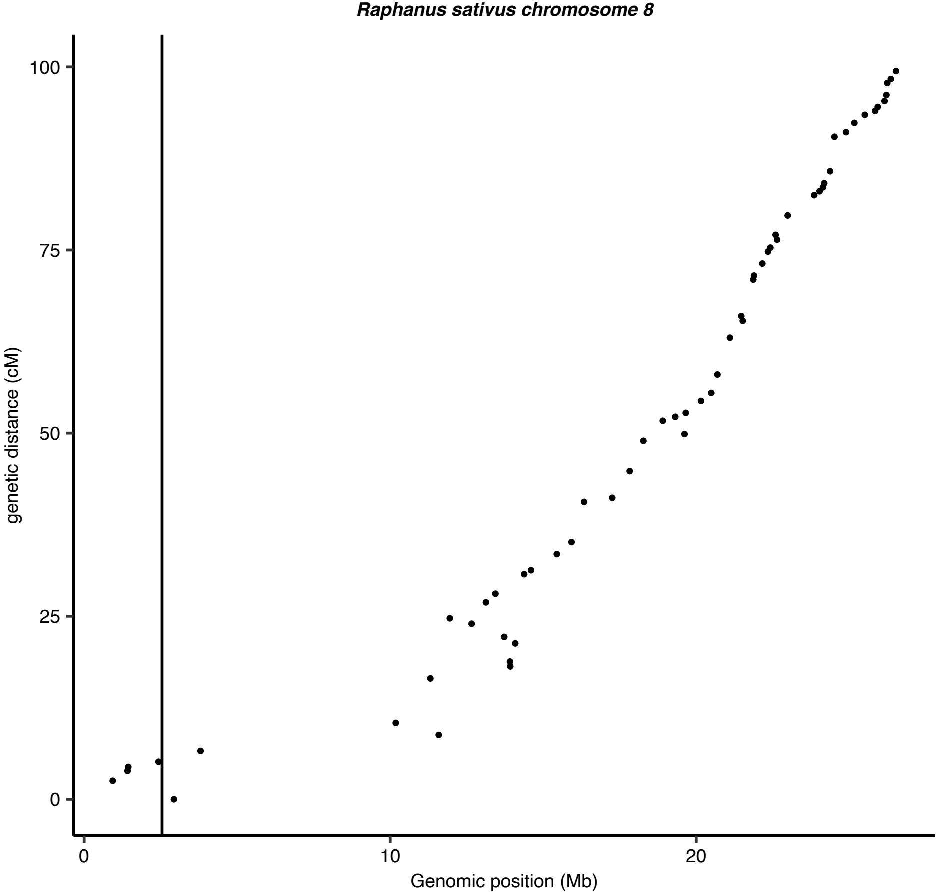
Raphanus sativus chromosome 8
genetic distance (cM)
0
25
50
75
100
0
10
20
Genomic position (Mb)

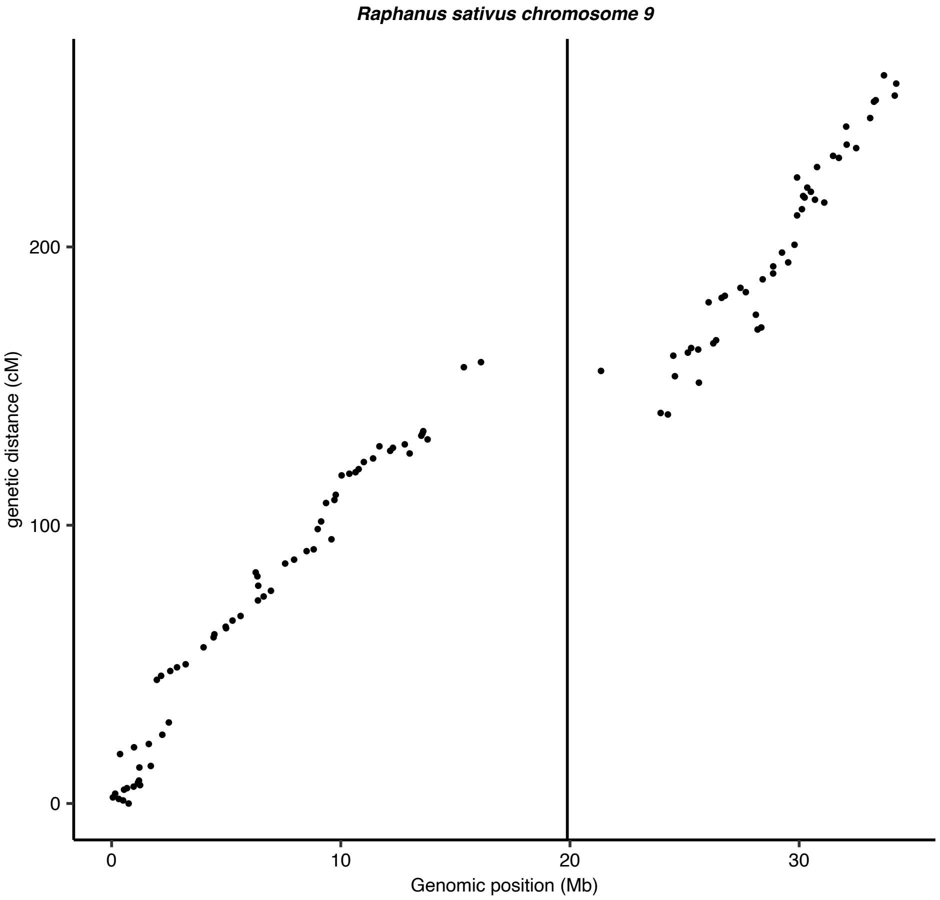
Raphanus sativus chromosome 9
genetic distance (cM)
0
100
200
Genomic position (Mb)
0
10
20
30

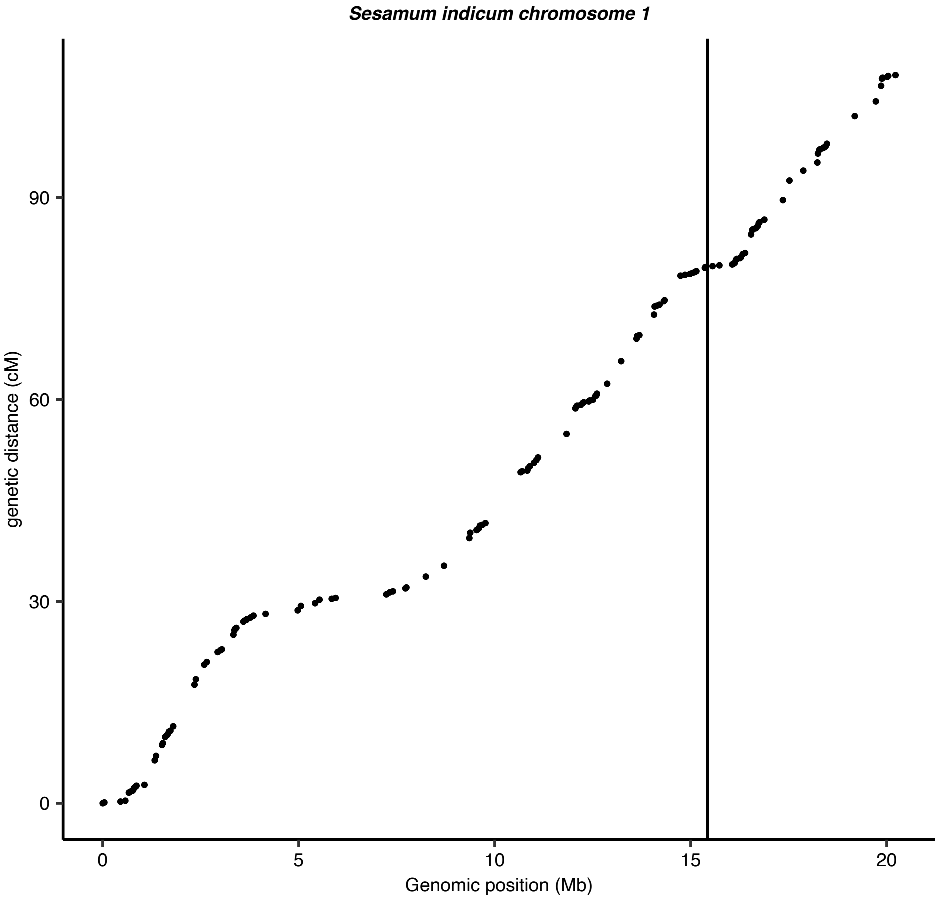
Sesamum indicum chromosome 1
genetic distance (cM)
0
30
60
90
Genomic position (Mb)
0
5
10
15
20

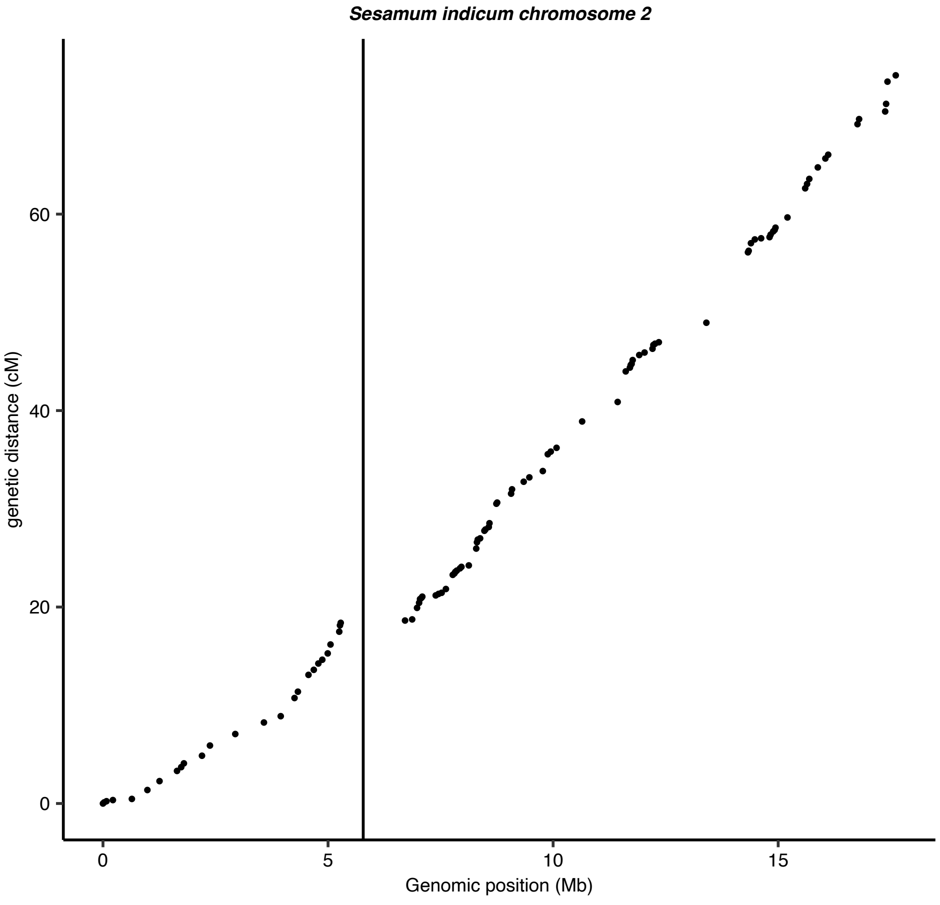
Sesamum indicum chromosome 2
genetic distance (cM)
0
20
40
60
Genomic position (Mb)
0
5
10
15

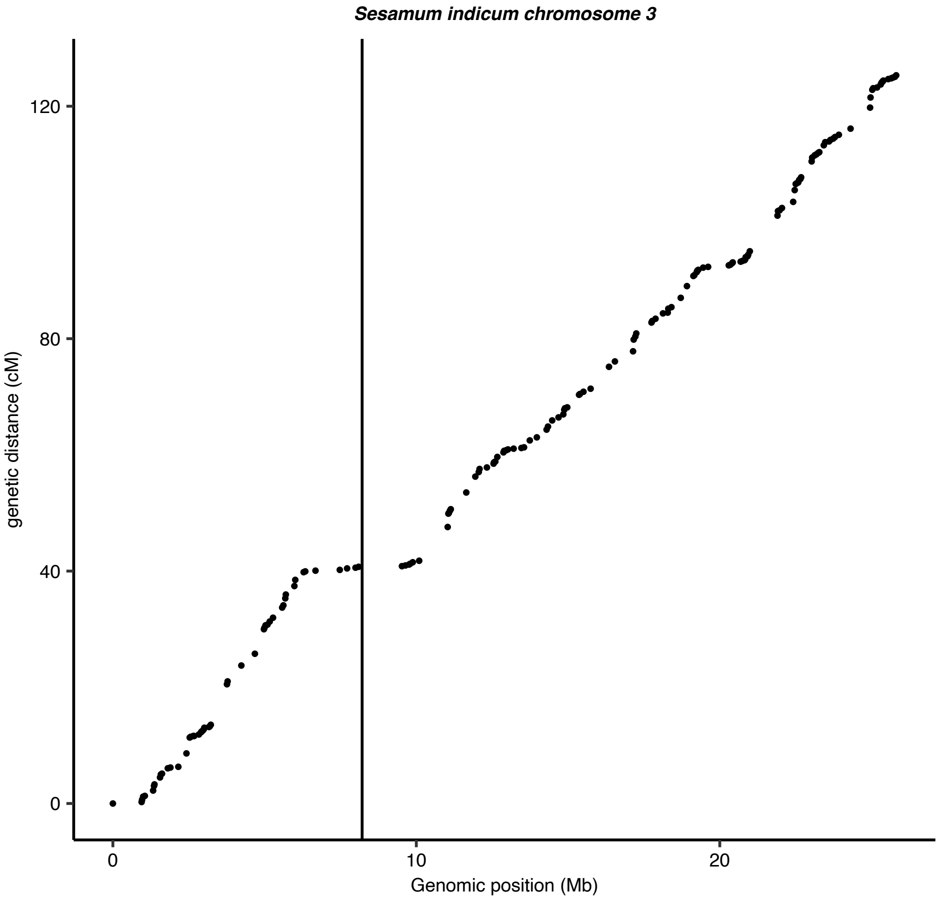
Sesamum indicum chromosome 3
genetic distance (cM)
120
80
40
0
0
10
20
Genomic position (Mb)

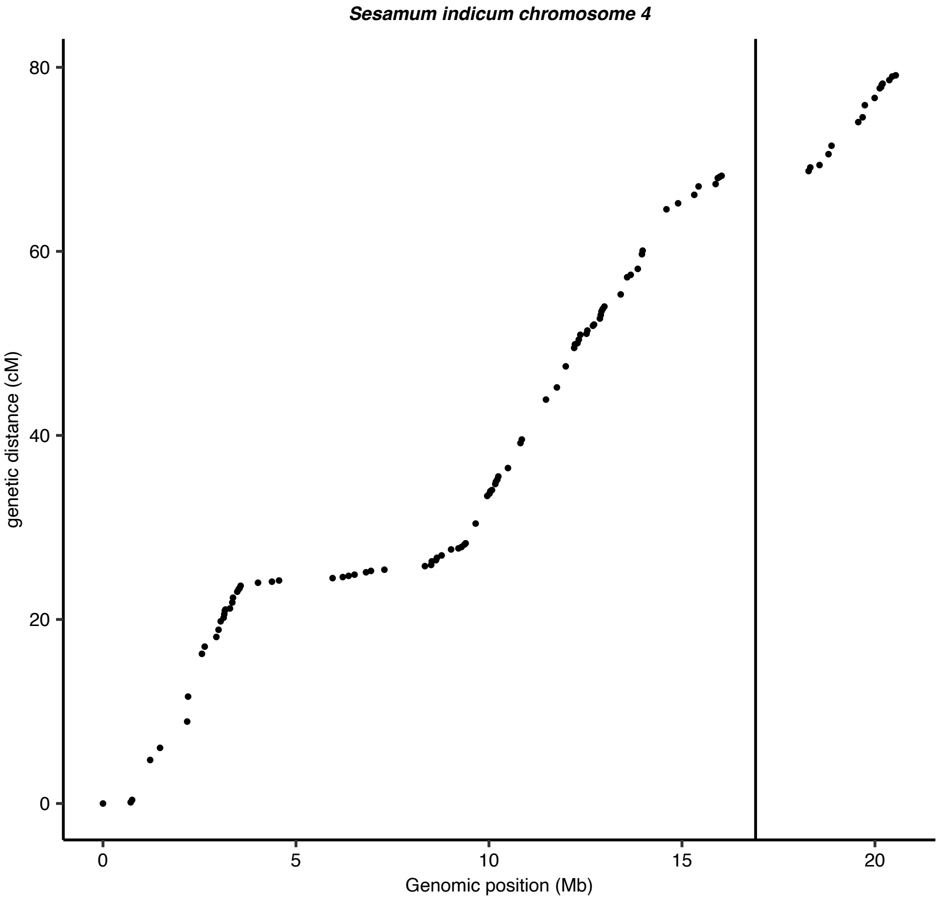
Sesamum indicum chromosome 4
genetic distance (cM)
0
20
40
60
80
Genomic position (Mb)
0
5
10
15
20

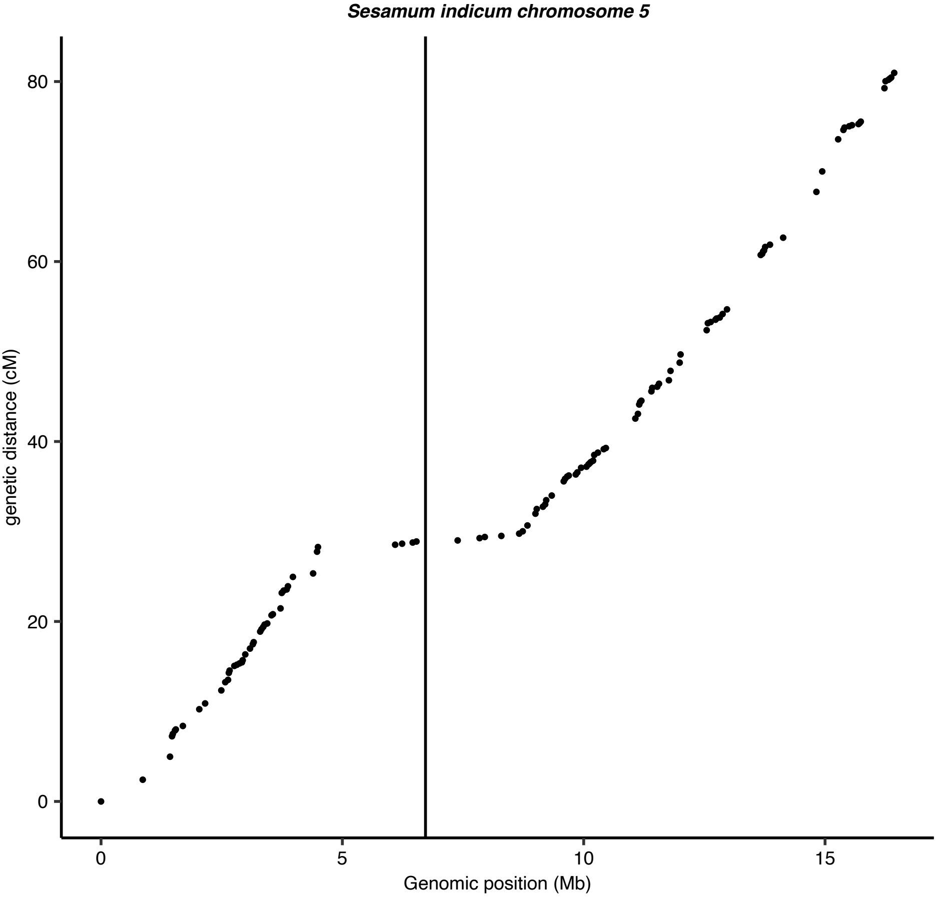
Sesamum indicum chromosome 5
genetic distance (cM)
80
60
40
20
0
0
5
10
15
Genomic position (Mb)

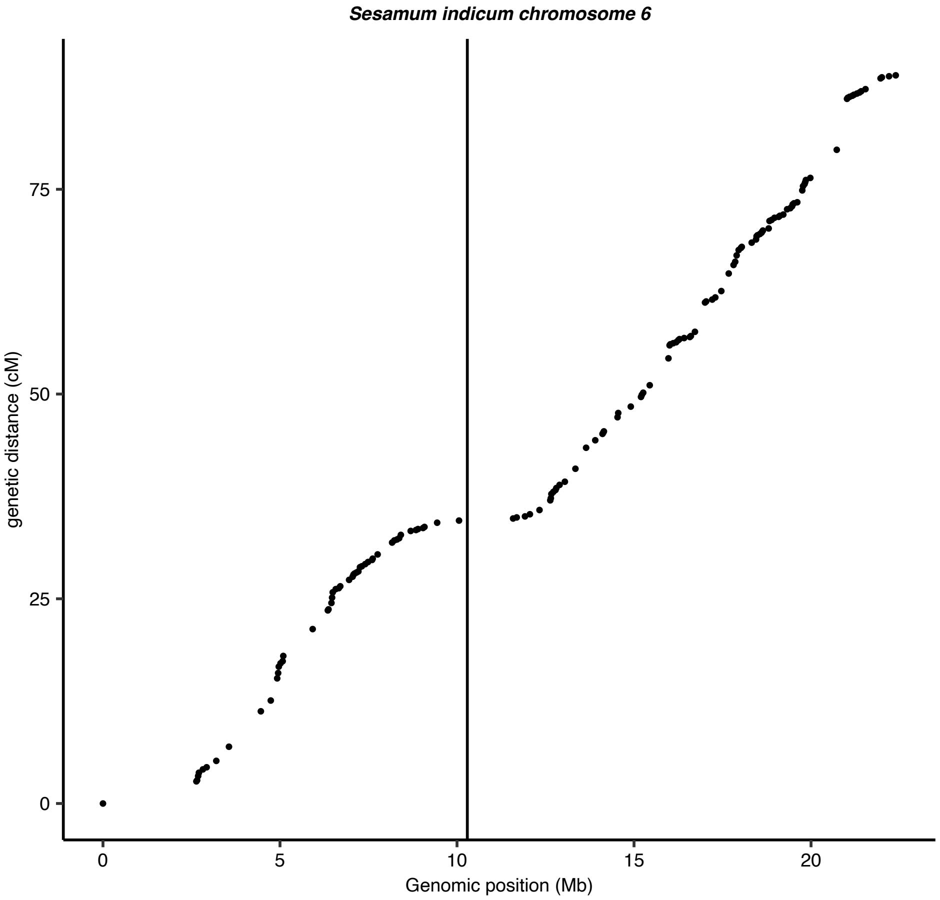
Sesamum indicum chromosome 6
genetic distance (cM)
0
25
50
75
Genomic position (Mb)
0
5
10
15
20

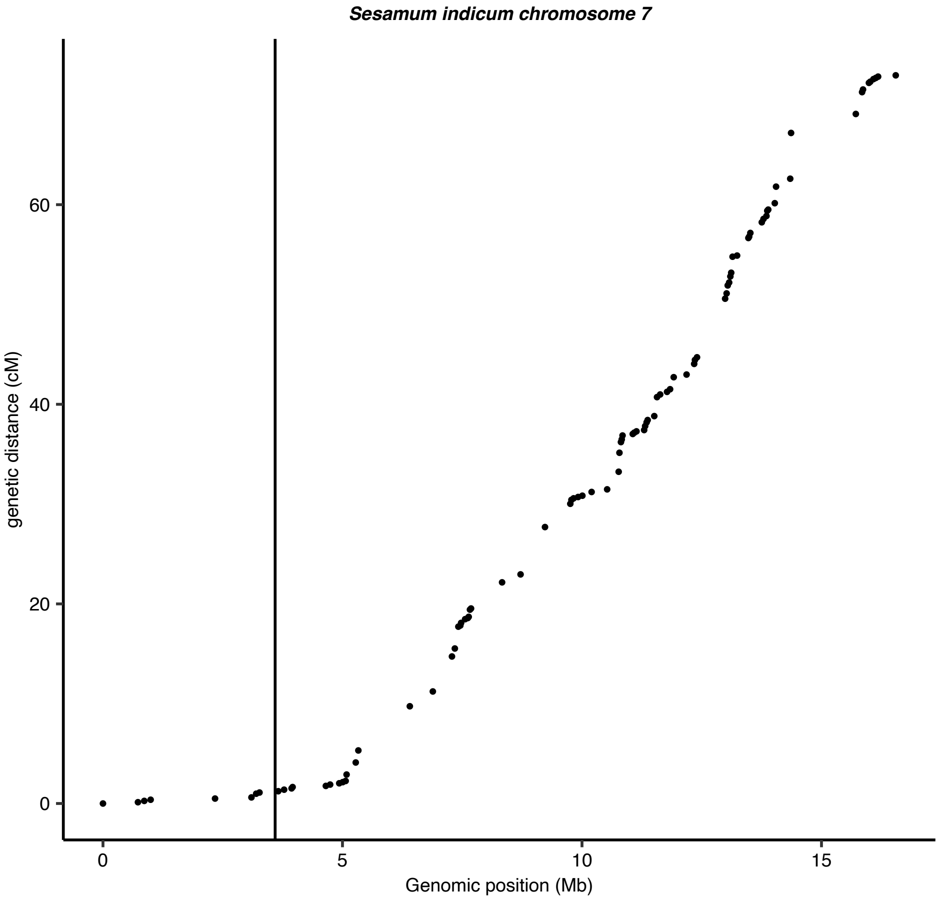
Sesamum indicum chromosome 7
genetic distance (cM)
Genomic position (Mb)
0
20
40
60
0
5
10
15

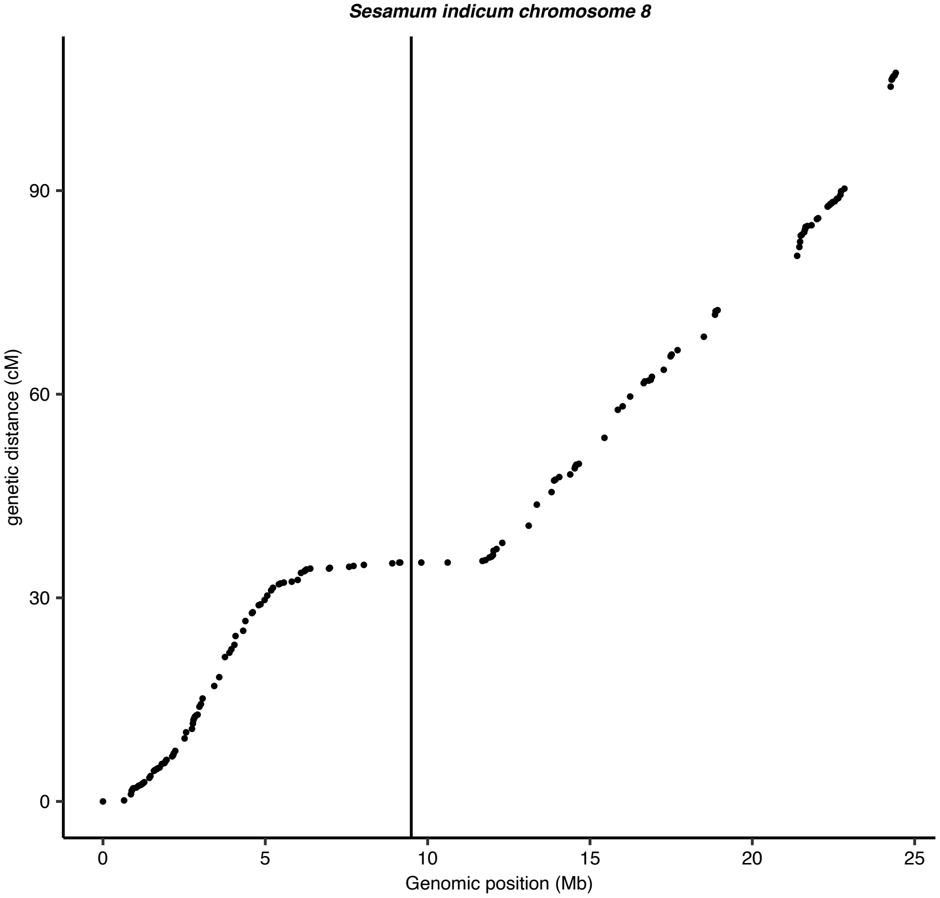

Sesamum indicum chromosome 8
genetic distance (cM)
0
30
60
90
0
5
10
15
20
25
Genomic position (Mb)

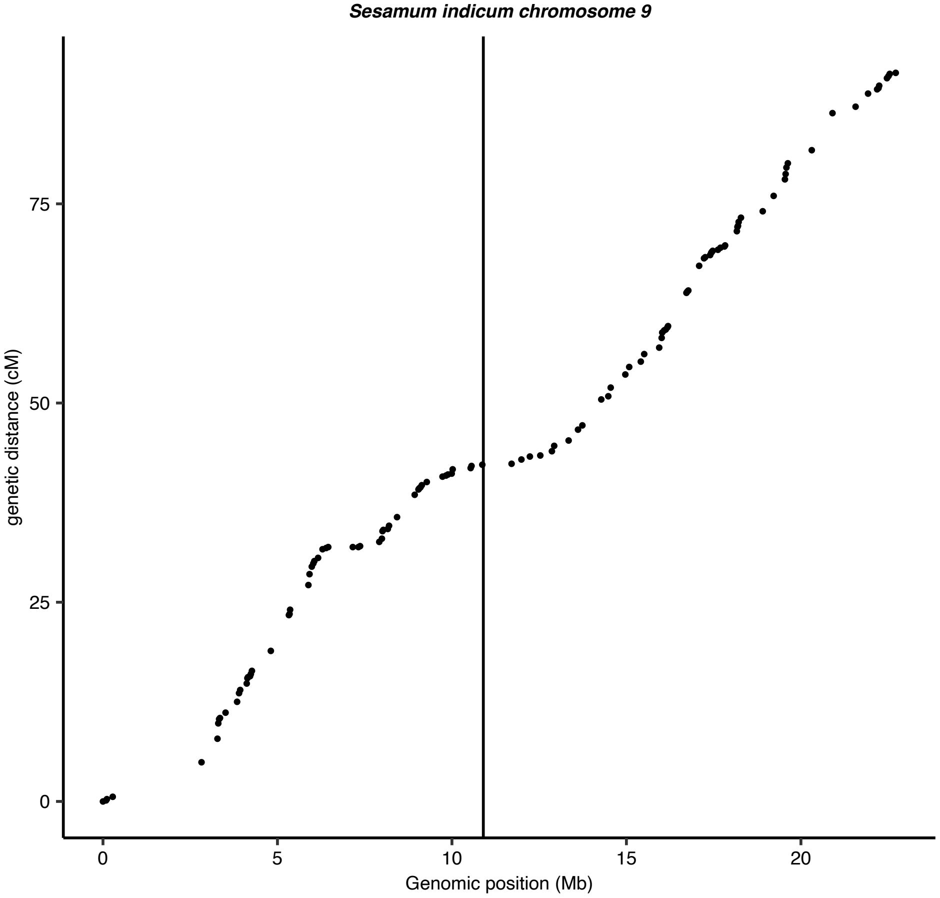
Sesamum indicum chromosome 9
genetic distance (cM)
0
25
50
75
Genomic position (Mb)
0
5
10
15
20

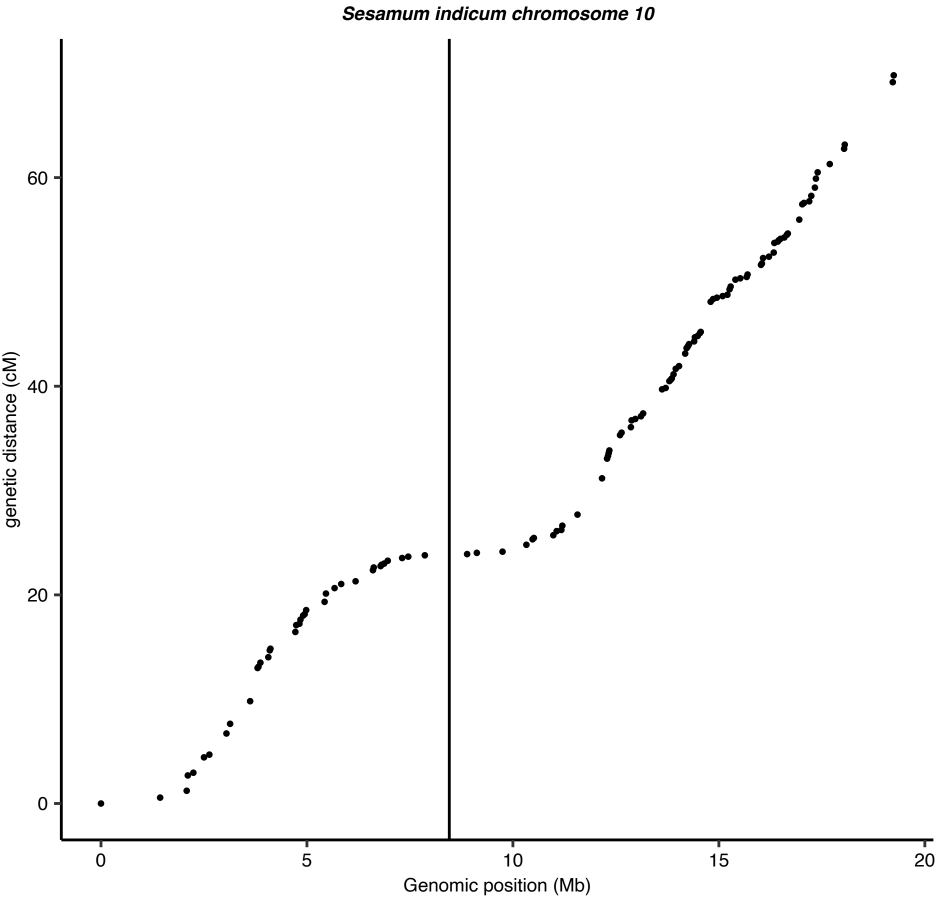
Sesamum indicum chromosome 10
genetic distance (cM)
0
20
40
60
Genomic position (Mb)
0
5
10
15
20

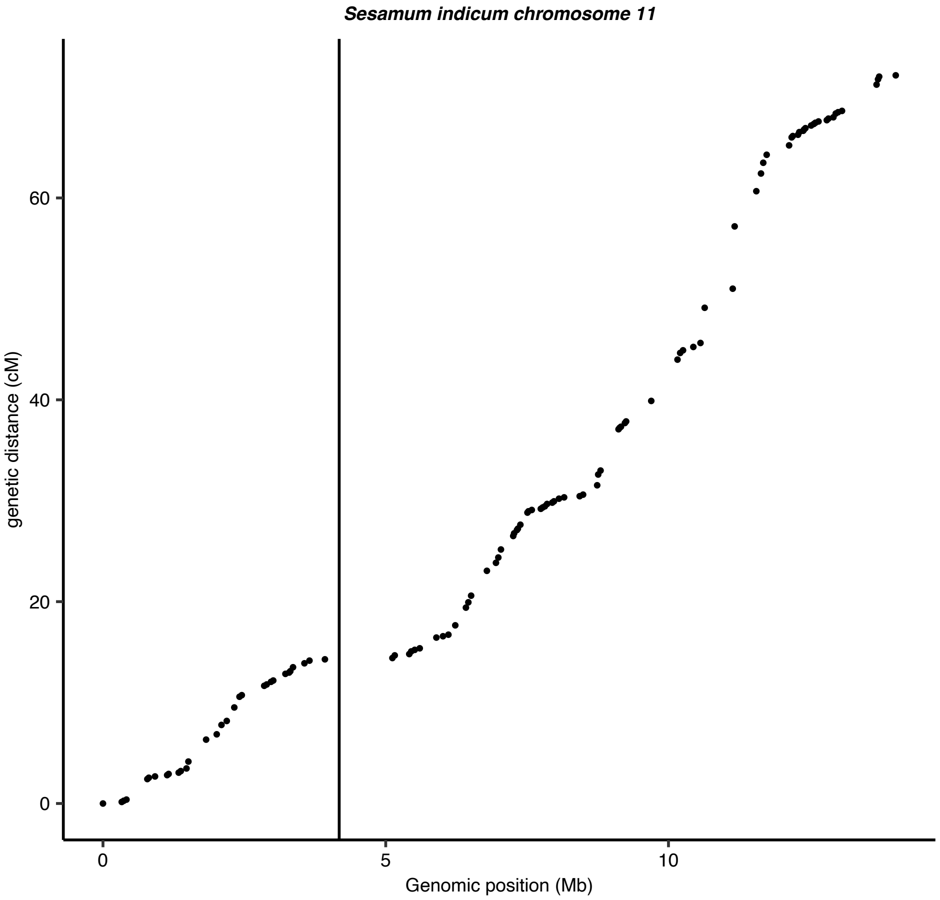
Sesamum indicum chromosome 11
genetic distance (cM)
0
20
40
60
0
5
10
Genomic position (Mb)

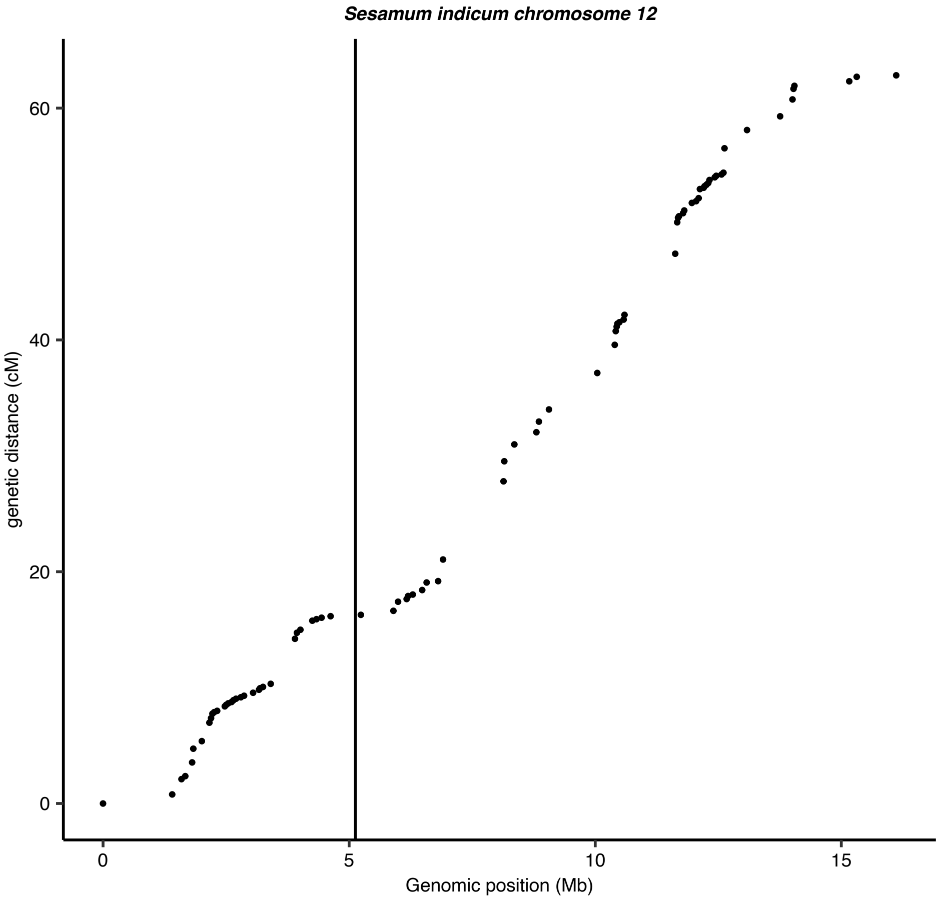
Sesamum indicum chromosome 12
genetic distance (cM)
0
20
40
60
Genomic position (Mb)
0
5
10
15

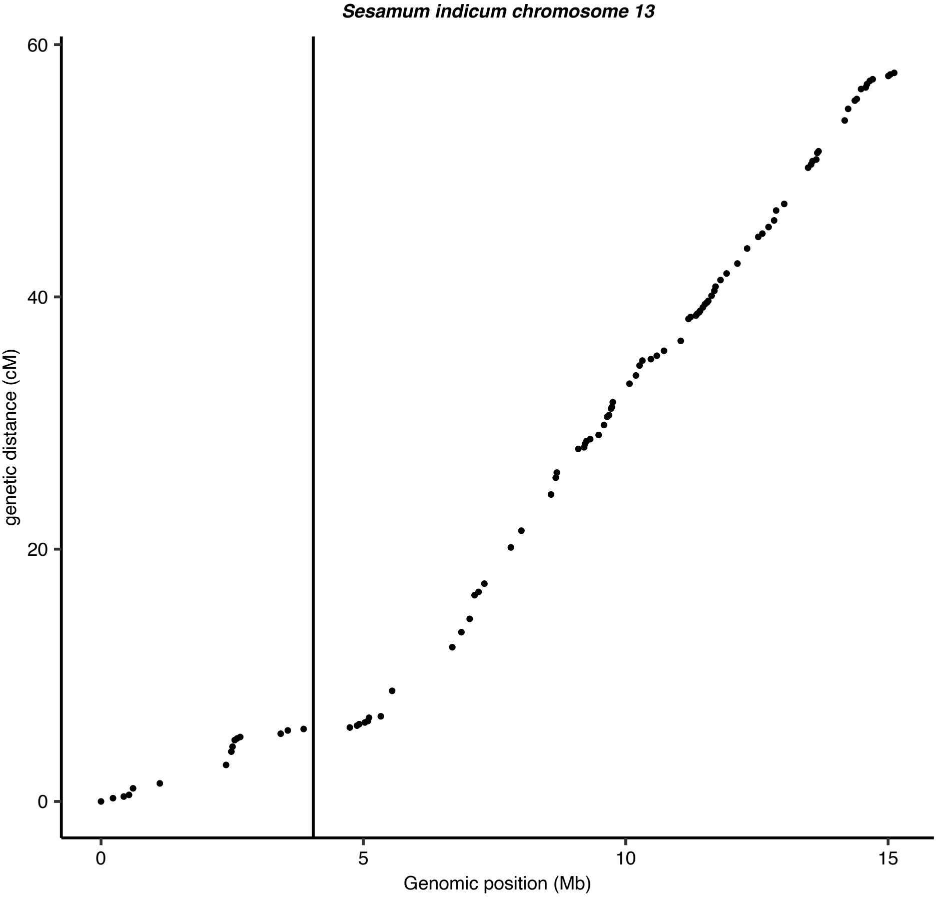
Sesamum indicum chromosome 13
genetic distance (cM)
Genomic position (Mb)
0
20
40
60
0
5
10
15

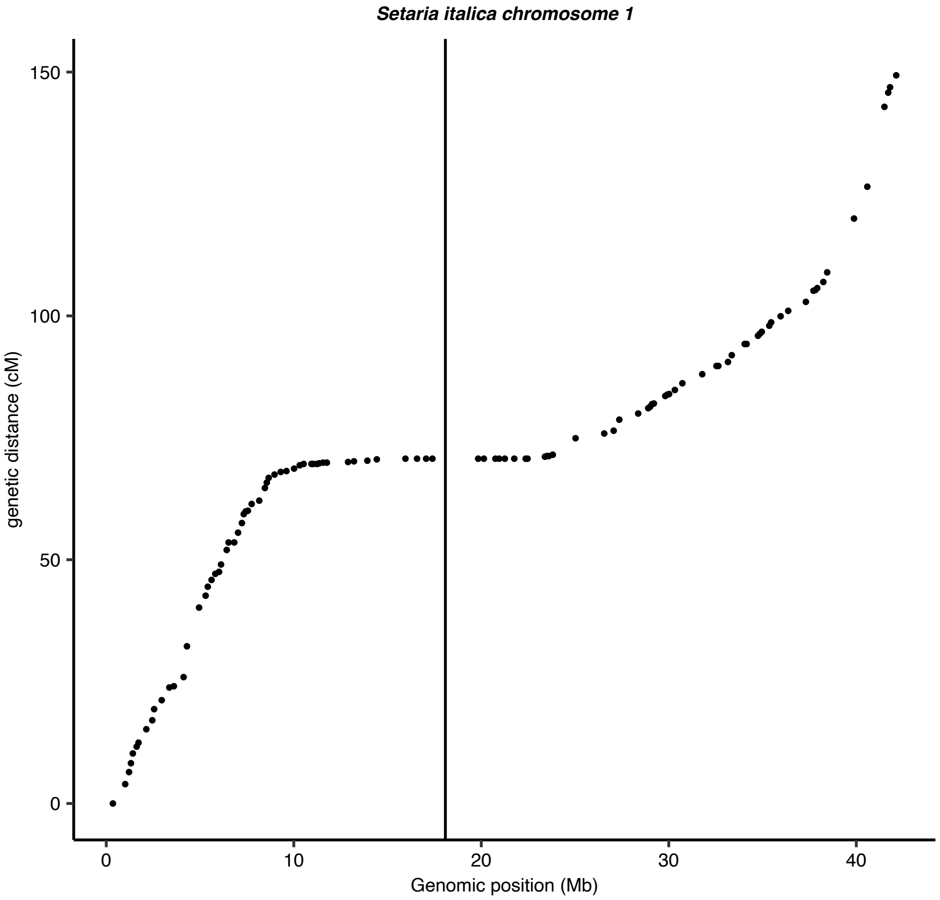
Setaria italica chromosome 1
genetic distance (cM)
Genomic position (Mb)
0
50
100
150
0
10
20
30
40

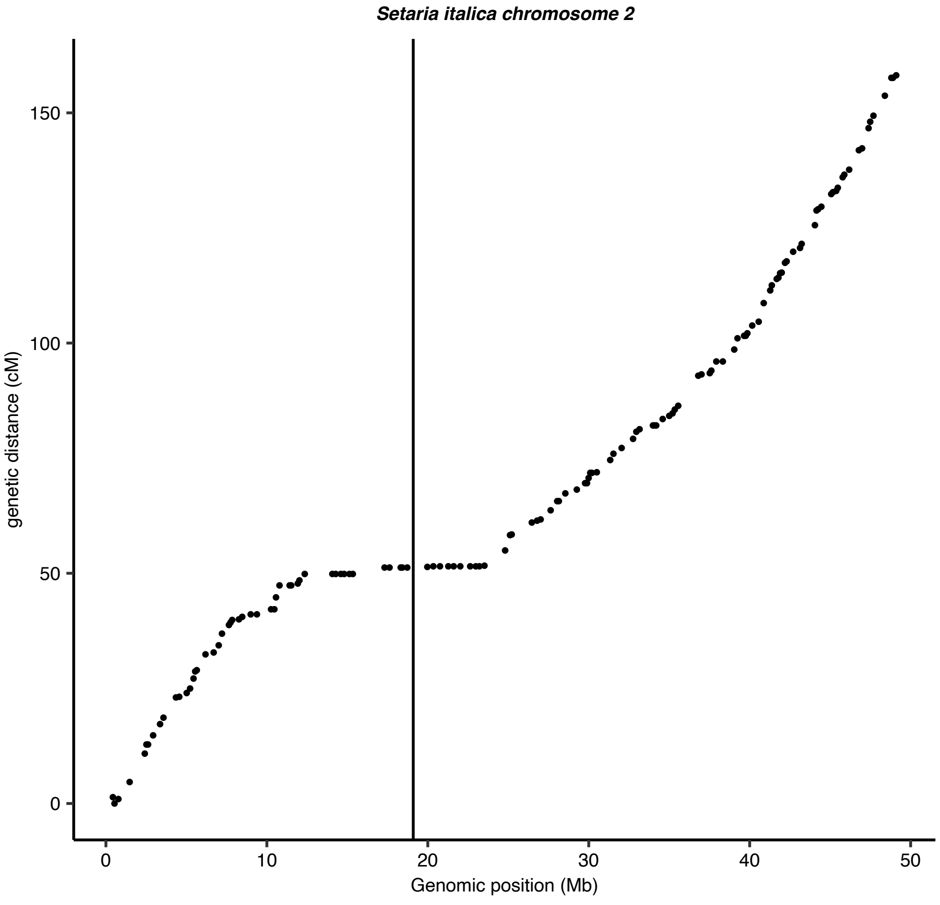
Setaria italica chromosome 2
genetic distance (cM)
Genomic position (Mb)
0
50
100
150
0
10
20
30
40
50

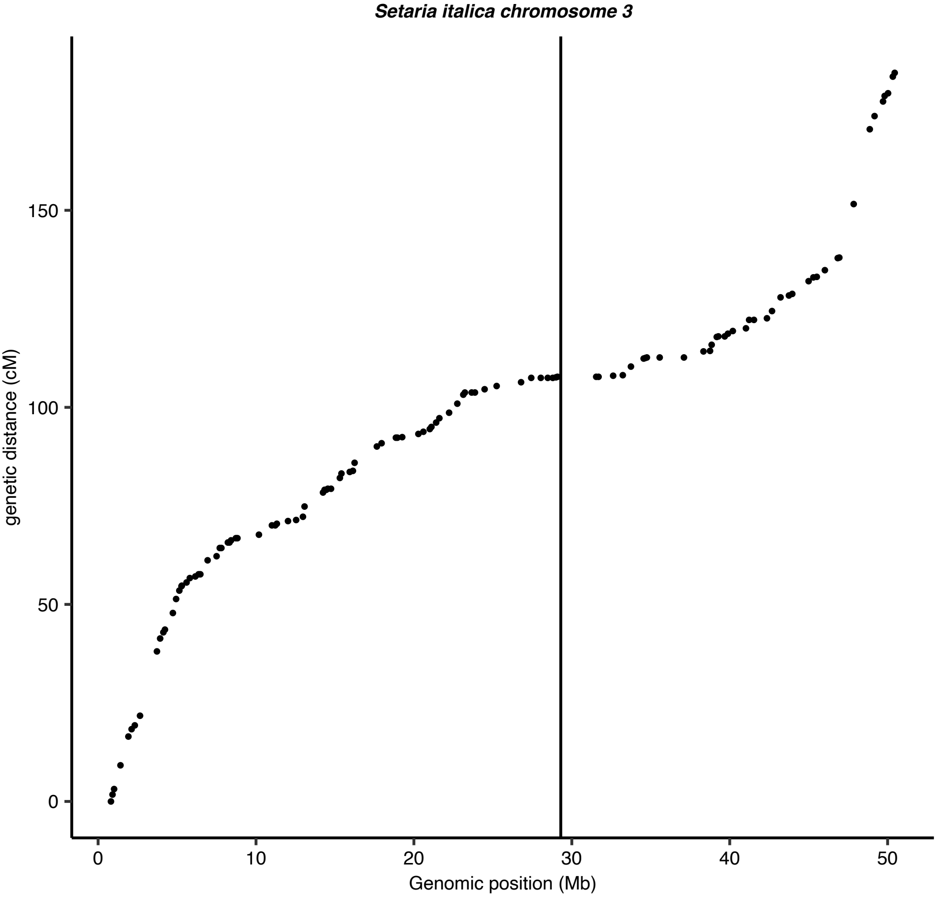
Setaria italica chromosome 3
genetic distance (cM)
150
100
50
0
0
10
20
30
40
50
Genomic position (Mb)

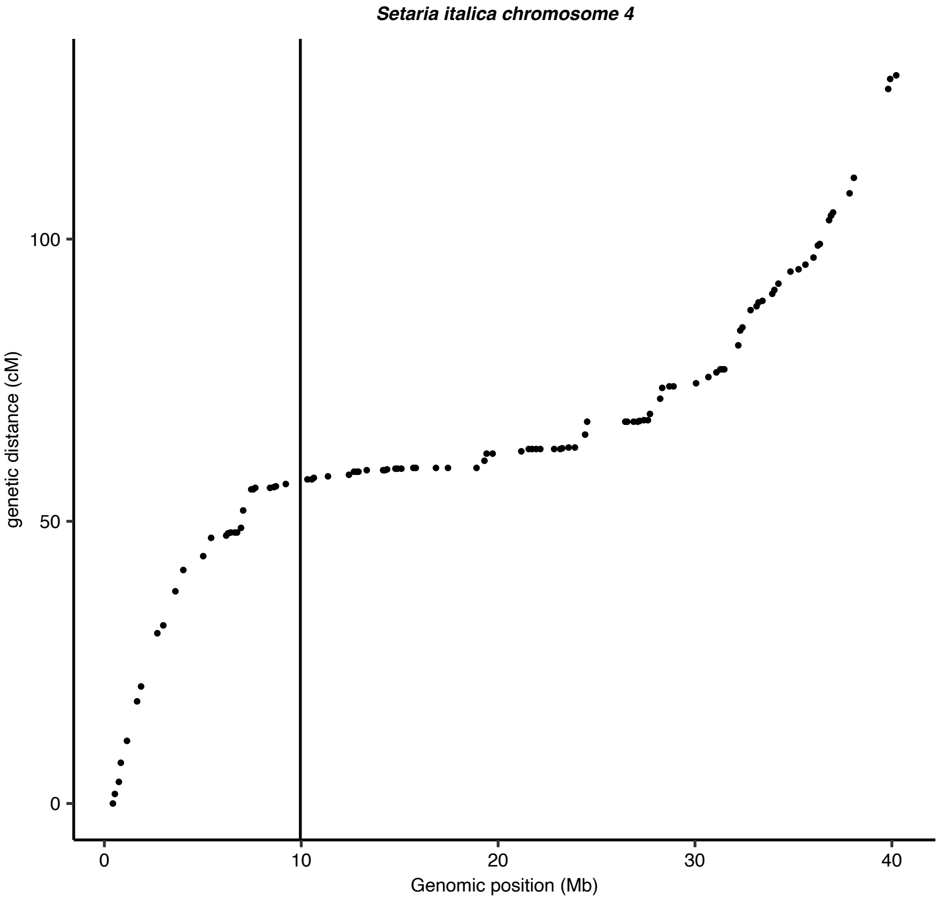
Setaria italica chromosome 4
genetic distance (cM)
Genomic position (Mb)
0
50
100
0
10
20
30
40

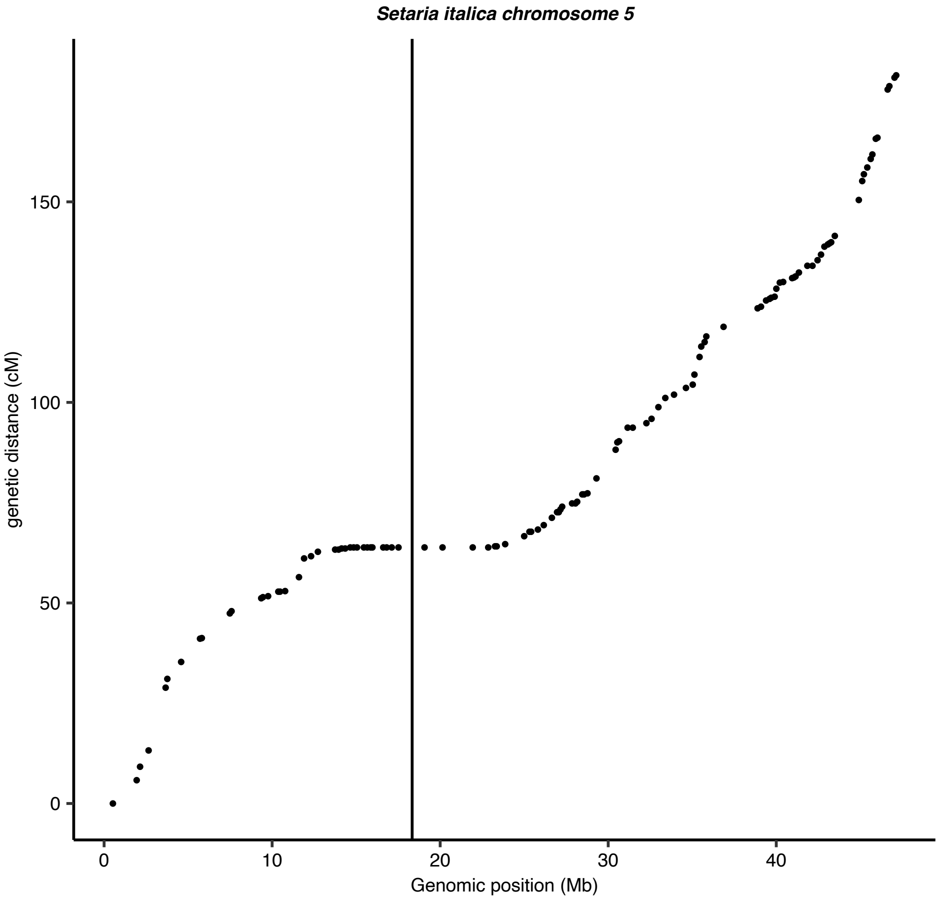
Setaria italica chromosome 5
genetic distance (cM)
0
50
100
150
Genomic position (Mb)
0
10
20
30
40

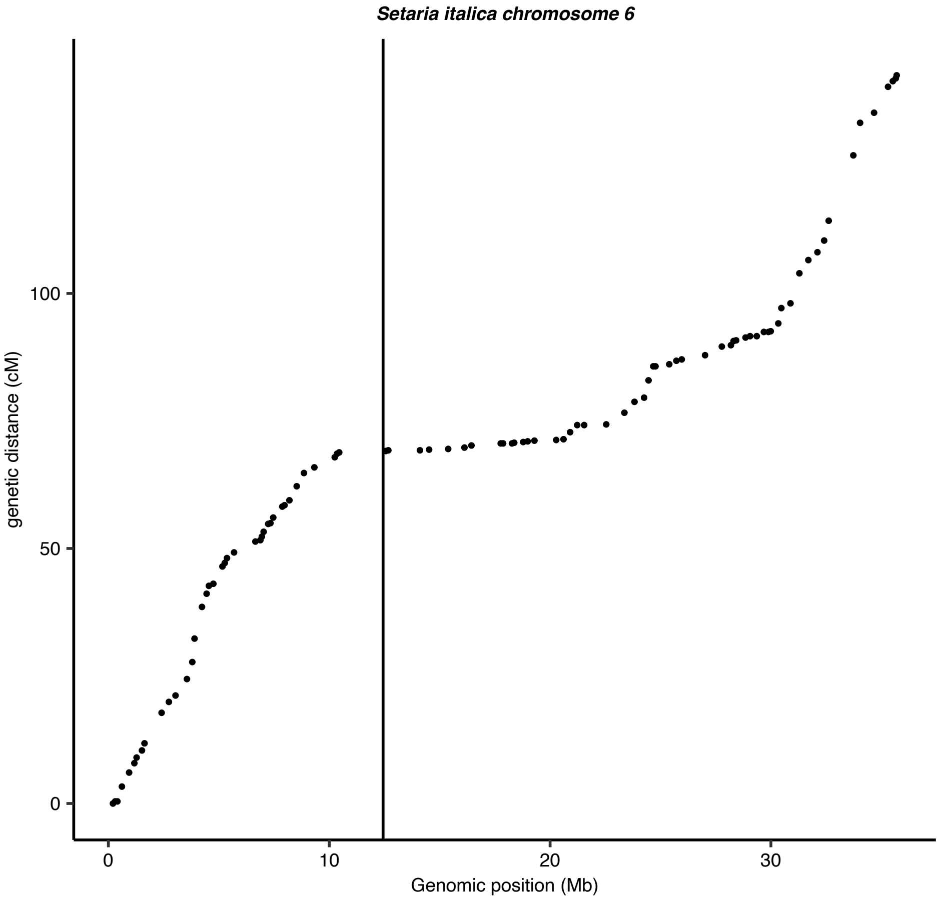
Setaria italica chromosome 6
genetic distance (cM)
Genomic position (Mb)
0
50
100
0
10
20
30

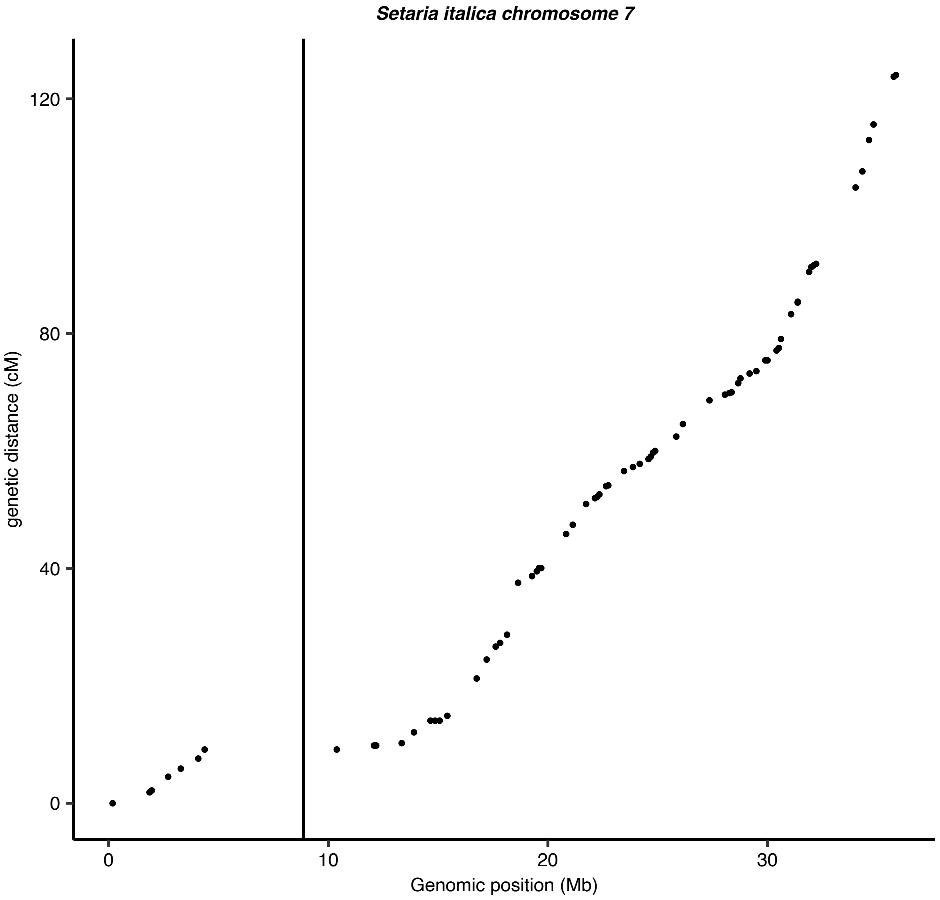
Setaria italica chromosome 7
genetic distance (cM)
Genomic position (Mb)
0
40
80
120
0
10
20
30

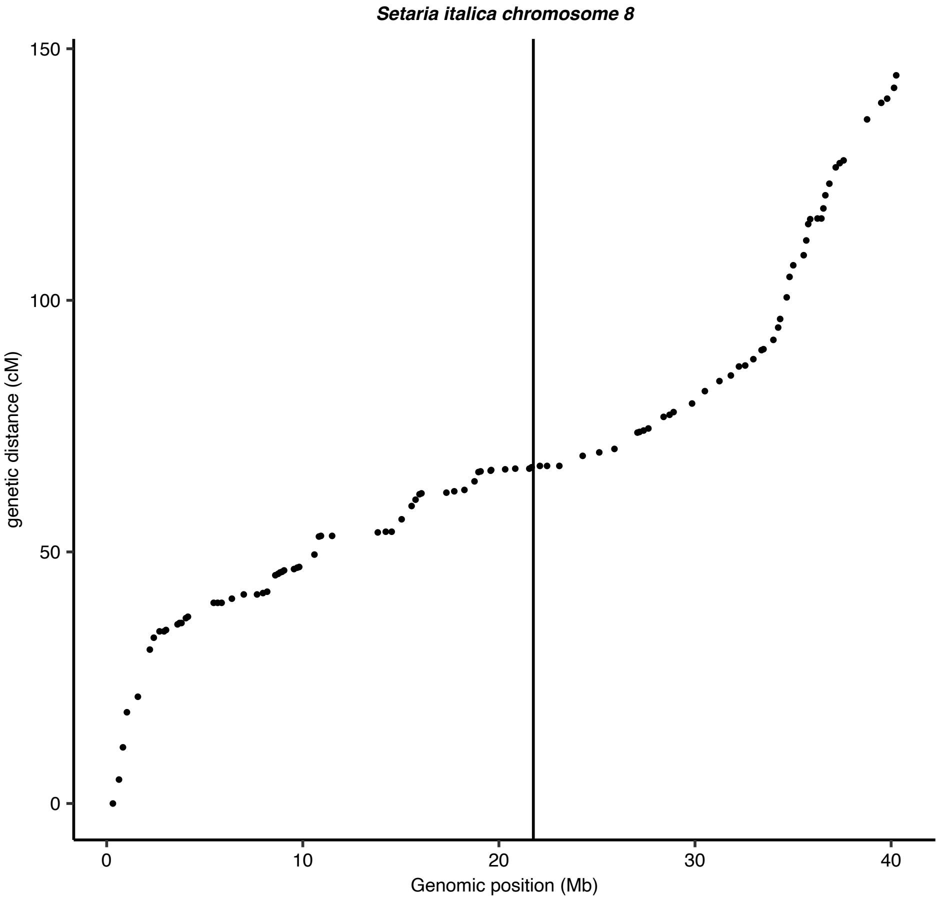

Setaria italica chromosome 8
genetic distance (cM)
Genomic position (Mb)
0
50
100
150
0
10
20
30
40

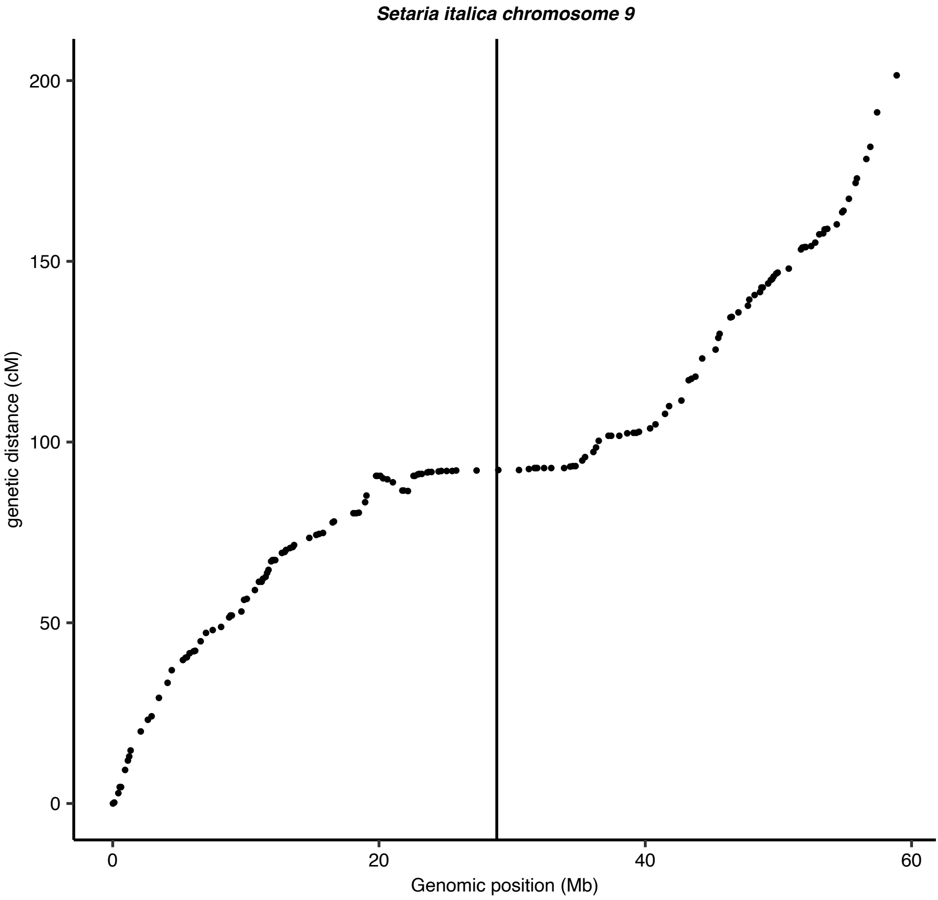
Setaria italica chromosome 9
genetic distance (cM)
Genomic position (Mb)
0
50
100
150
200
0
20
40
60

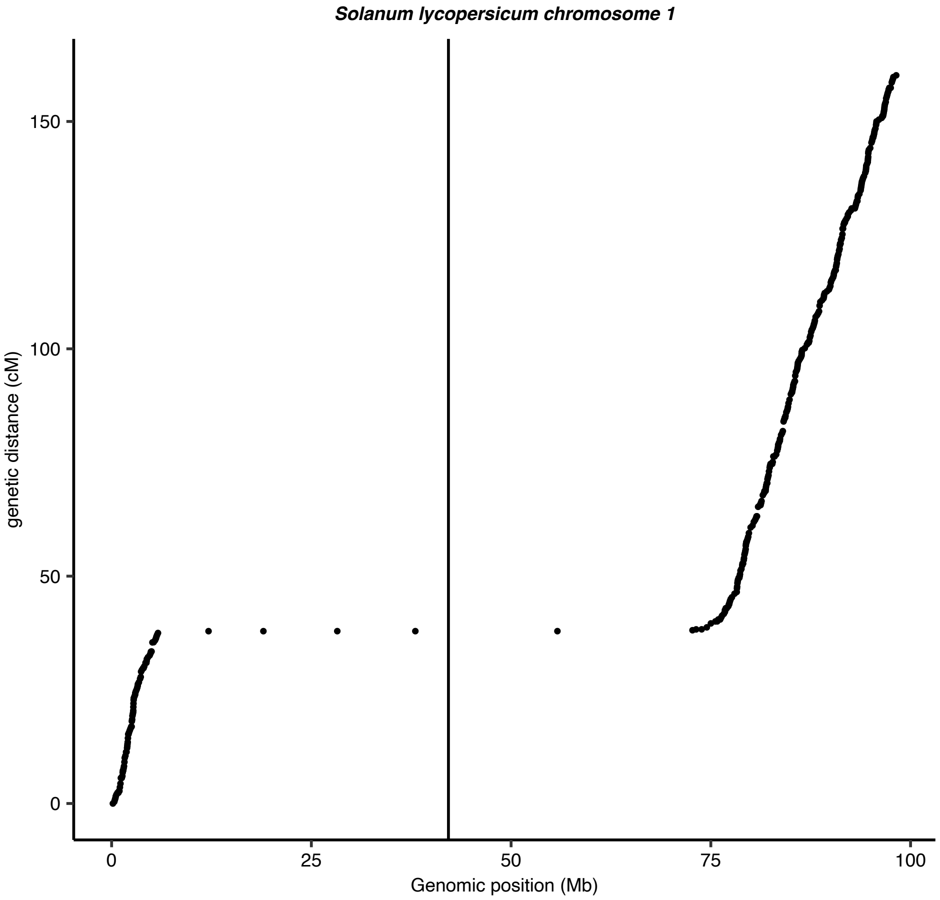

Solanum lycopersicum chromosome 1
genetic distance (cM)
0
50
100
150
Genomic position (Mb)
0
25
50
75
100

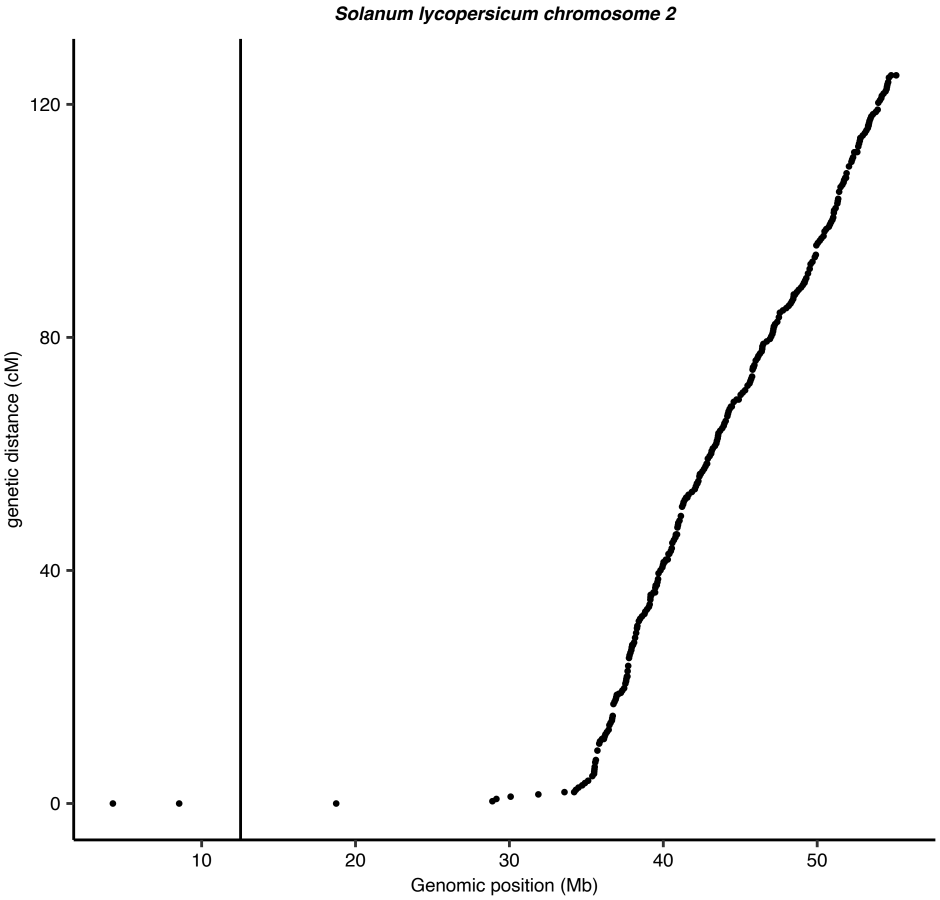
Solanum lycopersicum chromosome 2
genetic distance (cM)
0
40
80
120
Genomic position (Mb)
10
20
30
40
50

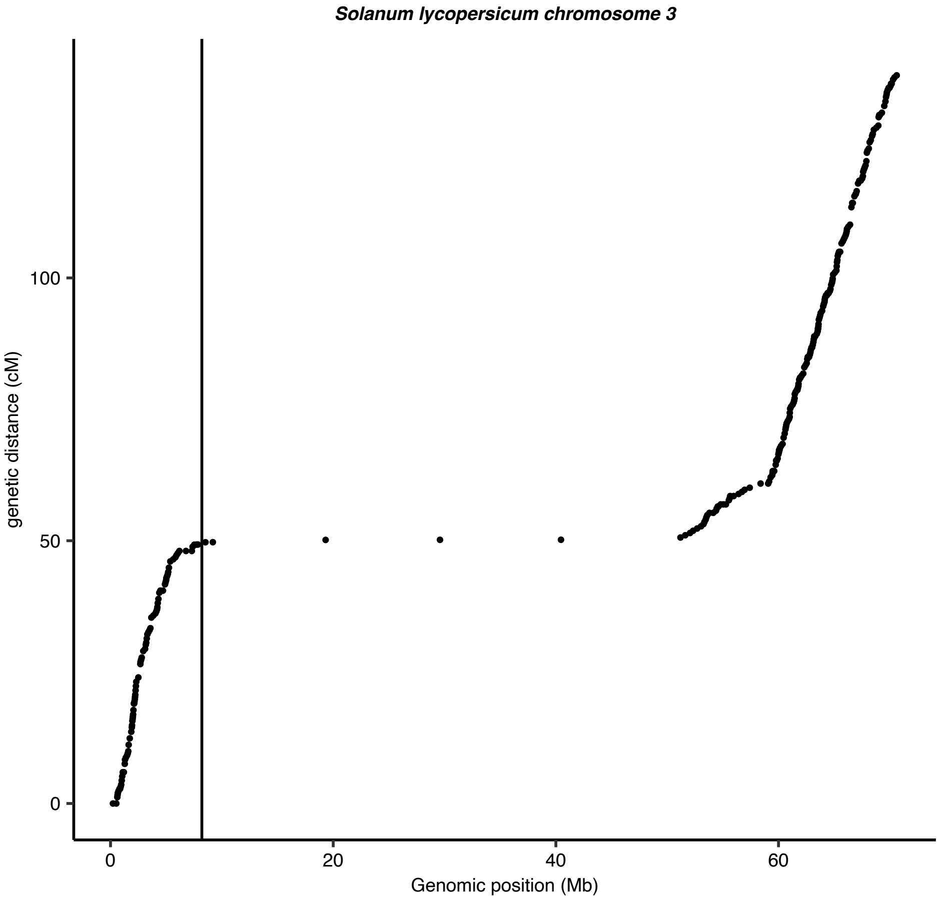
Solanum lycopersicum chromosome 3
genetic distance (cM)
100
50
0
0
20
40
60
Genomic position (Mb)

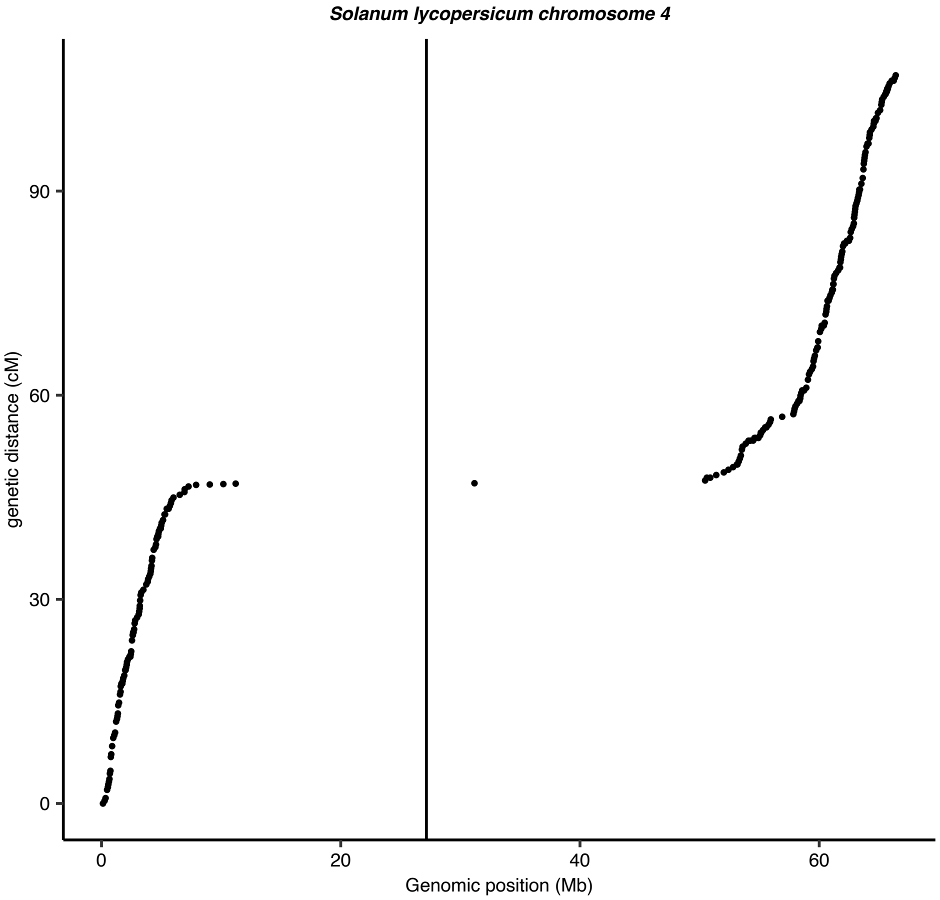
Solanum lycopersicum chromosome 4
genetic distance (cM)
Genomic position (Mb)
0
30
60
90
0
20
40
60

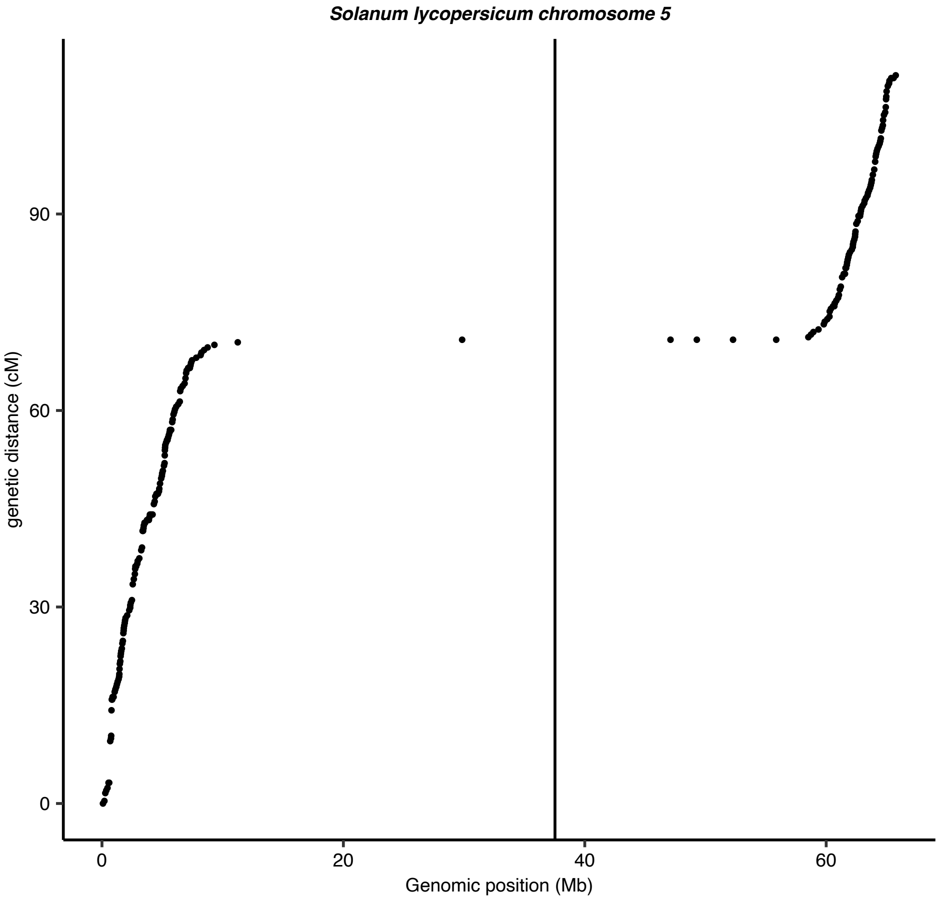
Solanum lycopersicum chromosome 5
genetic distance (cM)
90
60
30
0
0
20
40
60
Genomic position (Mb)

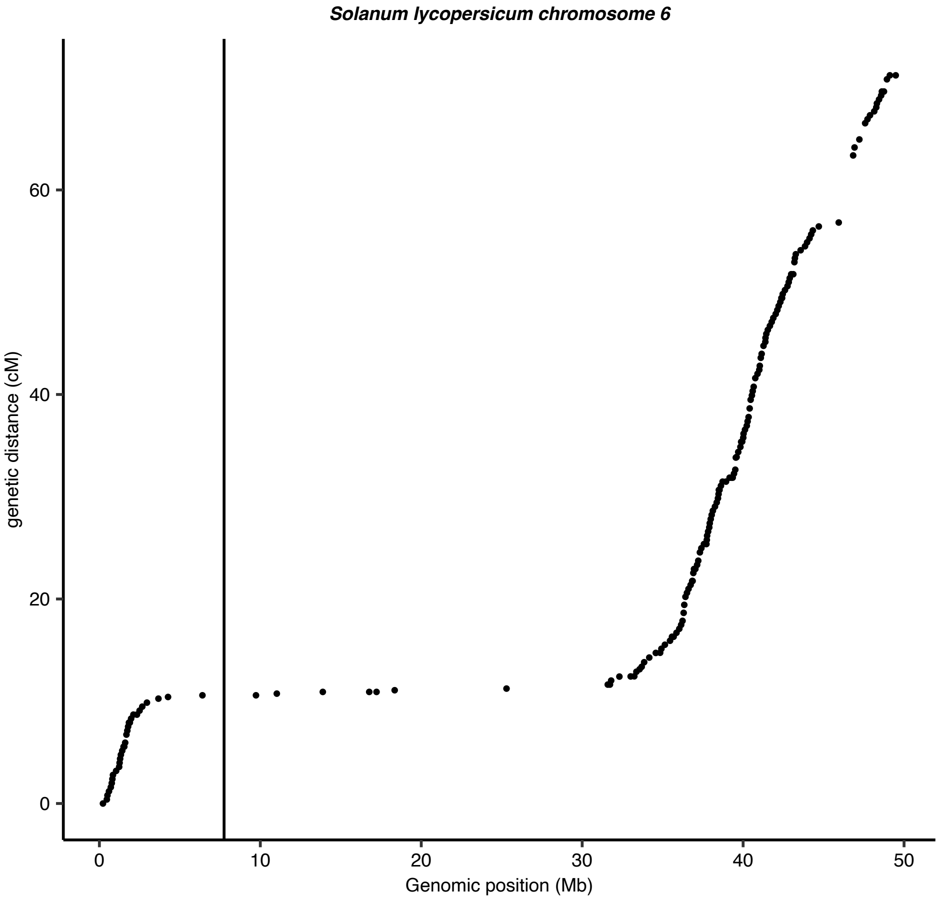
Solanum lycopersicum chromosome 6
genetic distance (cM)
0
20
40
60
0
10
20
30
40
50
Genomic position (Mb)

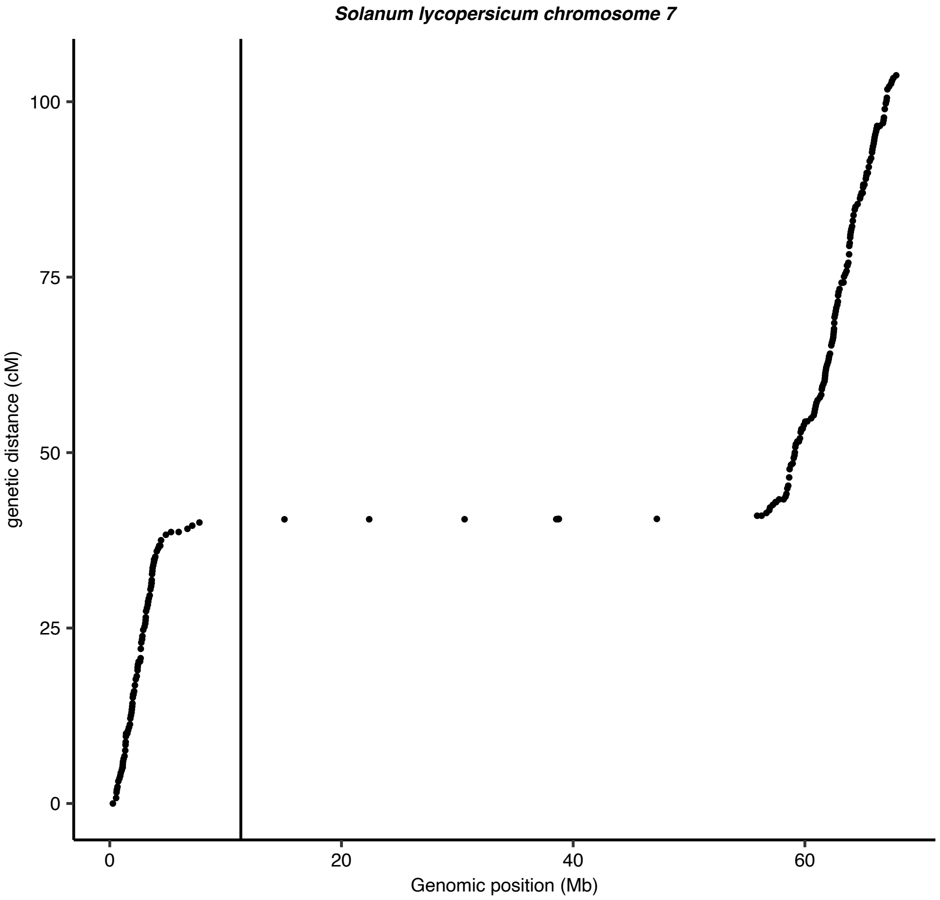
Solanum lycopersicum chromosome 7
genetic distance (cM)
Genomic position (Mb)
0
25
50
75
100
0
20
40
60

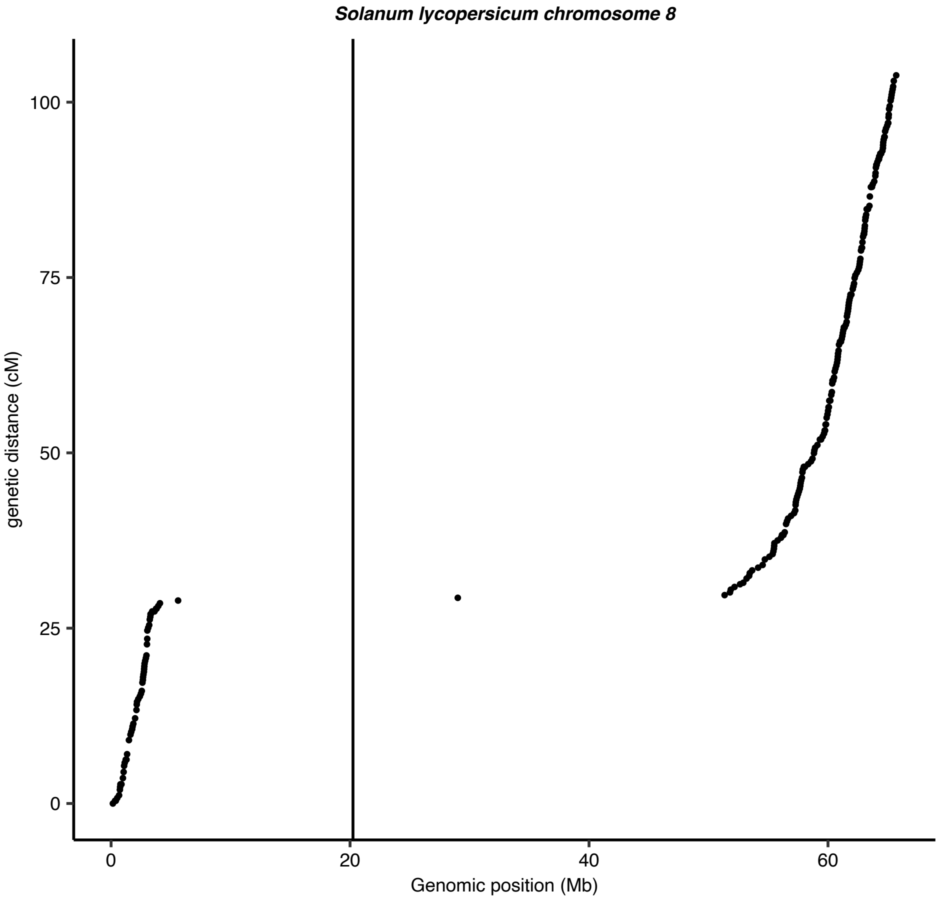
Solanum lycopersicum chromosome 8
genetic distance (cM)
0
25
50
75
100
Genomic position (Mb)
0
20
40
60

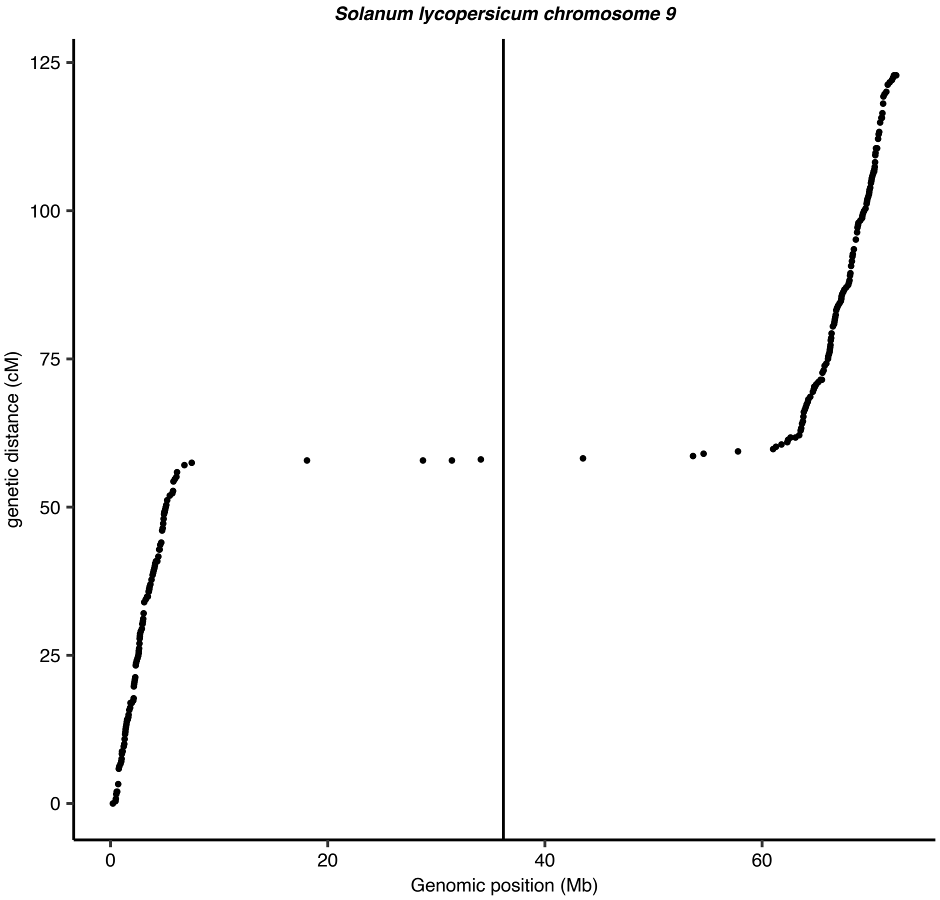
Solanum lycopersicum chromosome 9
genetic distance (cM)
0
25
50
75
100
125
0
20
40
60
Genomic position (Mb)

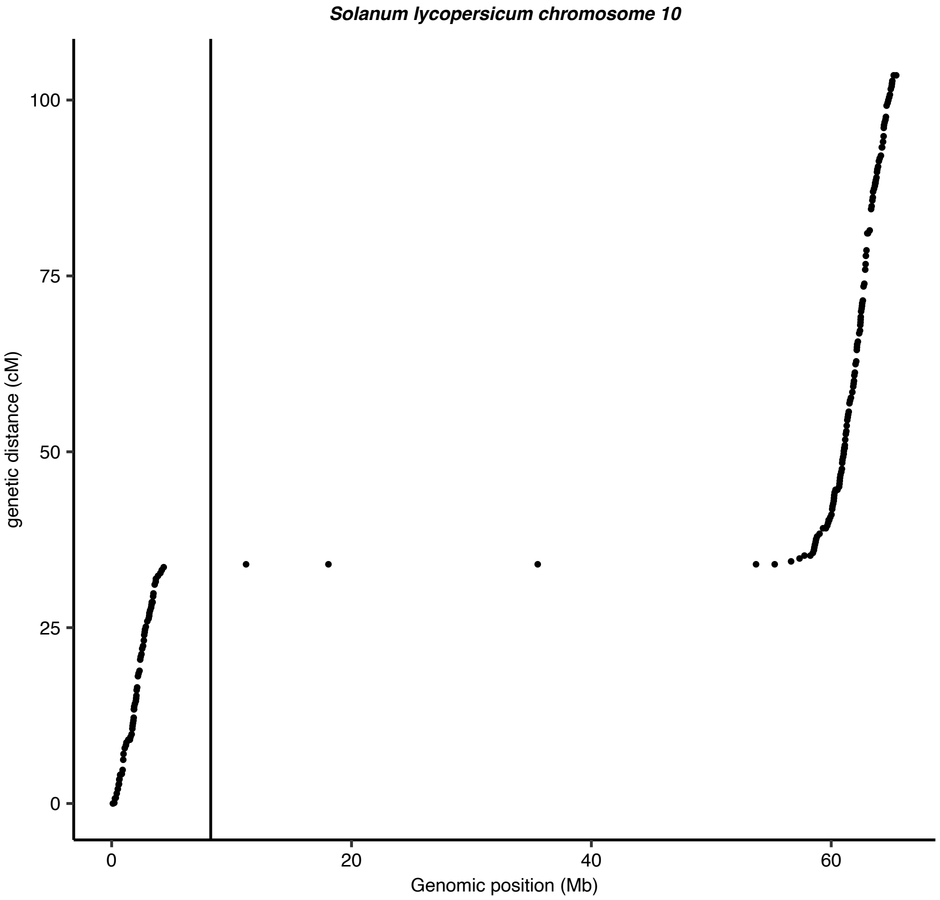
Solanum lycopersicum chromosome 10
genetic distance (cM)
100
75
50
25
0
0
20
40
60
Genomic position (Mb)

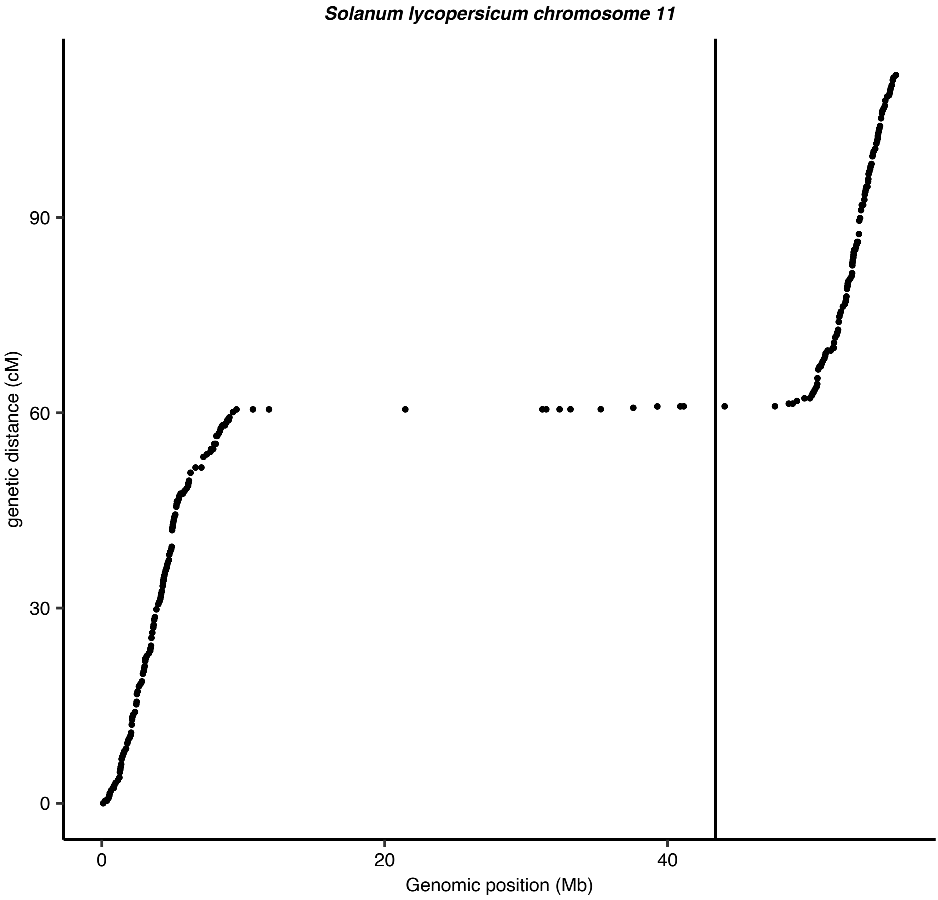
Solanum lycopersicum chromosome 11
genetic distance (cM)
0
30
60
90
Genomic position (Mb)
0
20
40

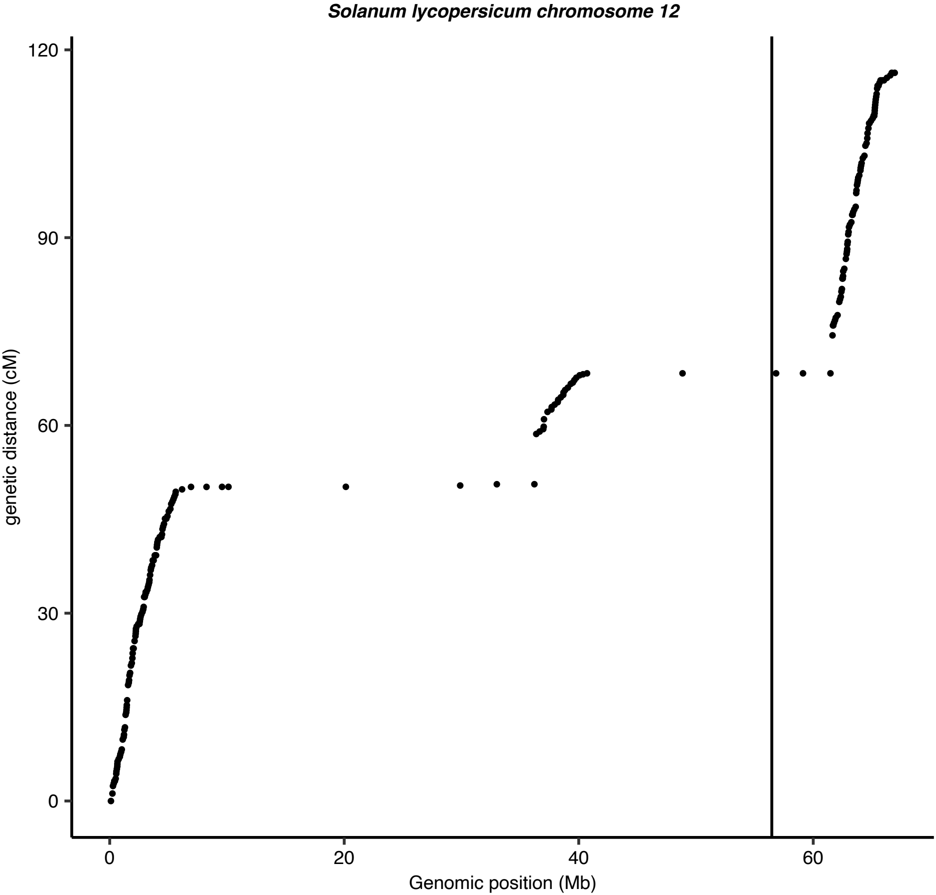

Solanum lycopersicum chromosome 12
genetic distance (cM)
Genomic position (Mb)
0
30
60
90
120
0
20
40
60

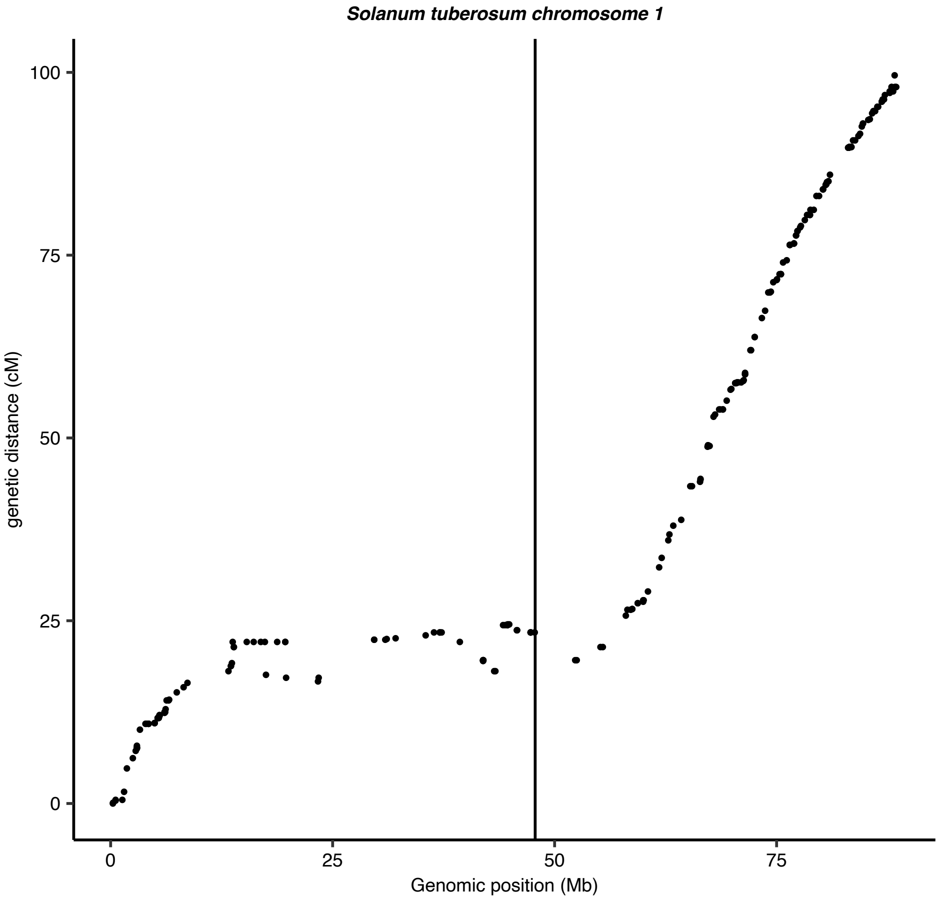
Solanum tuberosum chromosome 1
genetic distance (cM)
Genomic position (Mb)
0
25
50
75
100
0
25
50
75

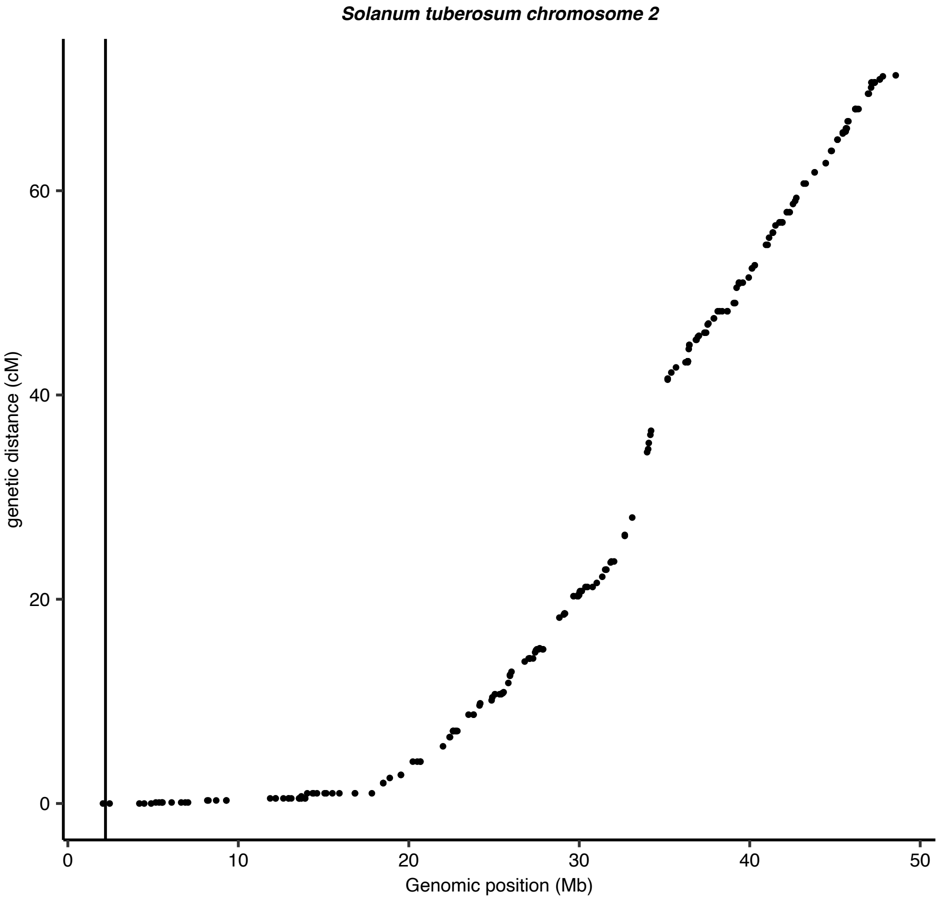
Solanum tuberosum chromosome 2
genetic distance (cM)
Genomic position (Mb)
0
20
40
60
0
10
20
30
40
50

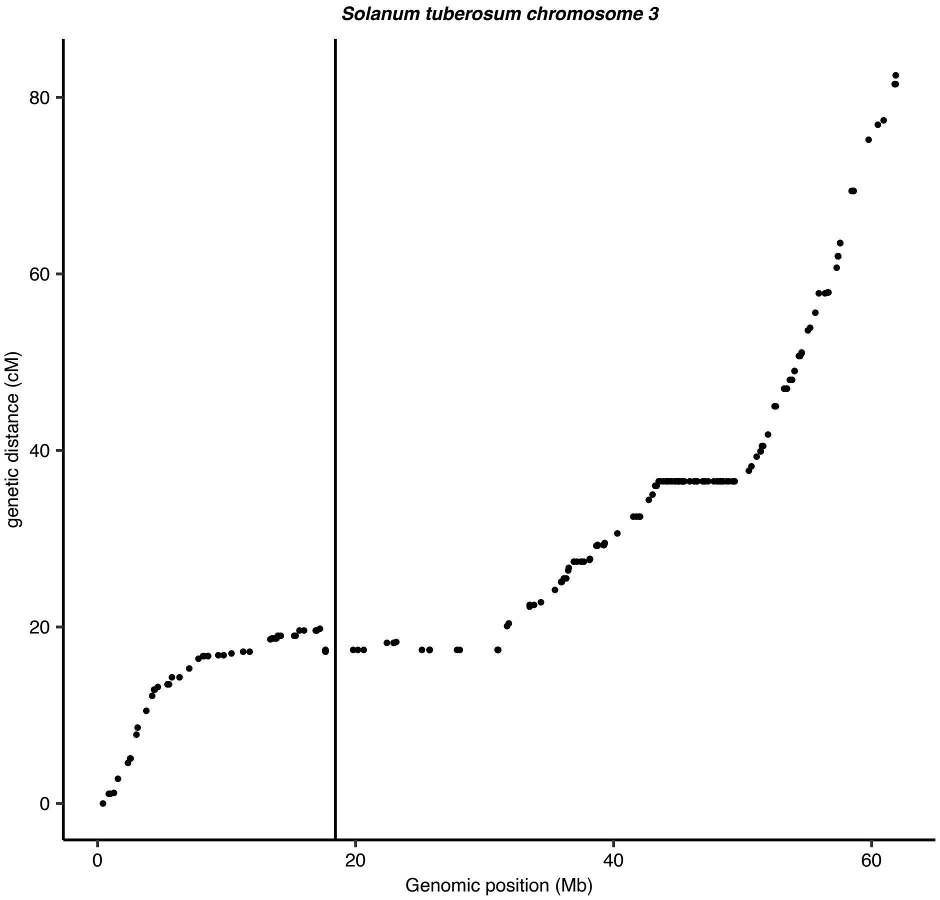
Solanum tuberosum chromosome 3
genetic distance (cM)
Genomic position (Mb)
0
20
40
60
80
0
20
40
60

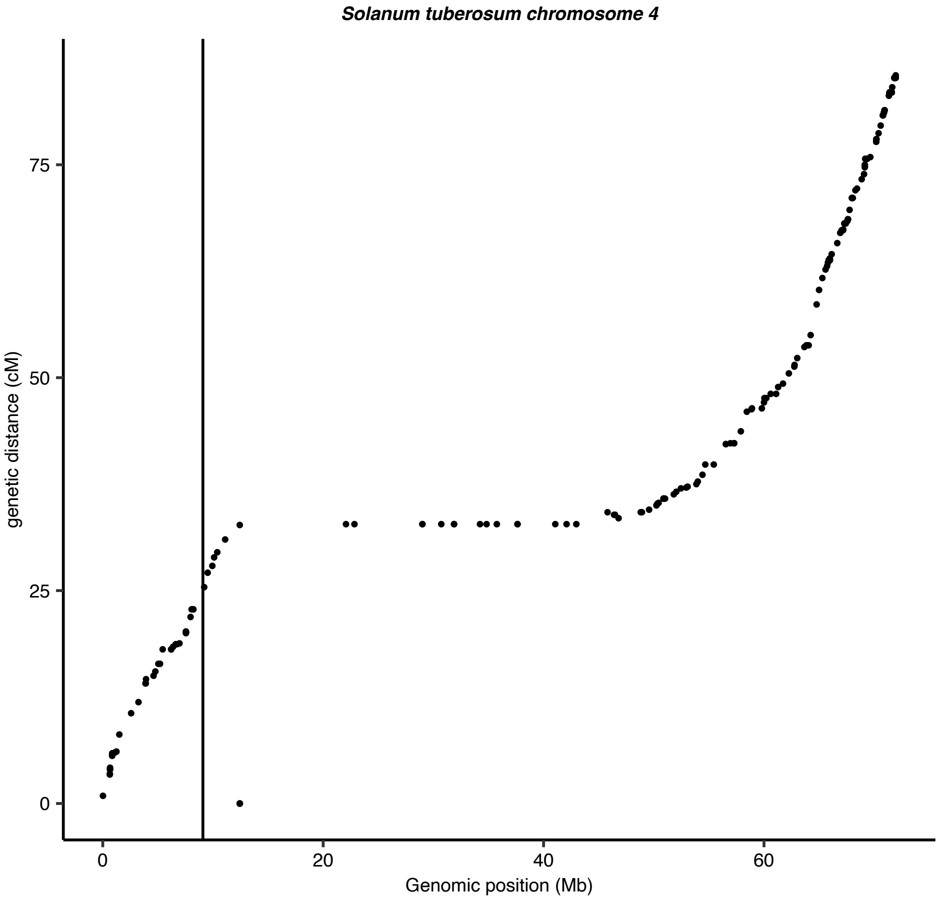
Solanum tuberosum chromosome 4
genetic distance (cM)
Genomic position (Mb)
0
25
50
75
0
20
40
60

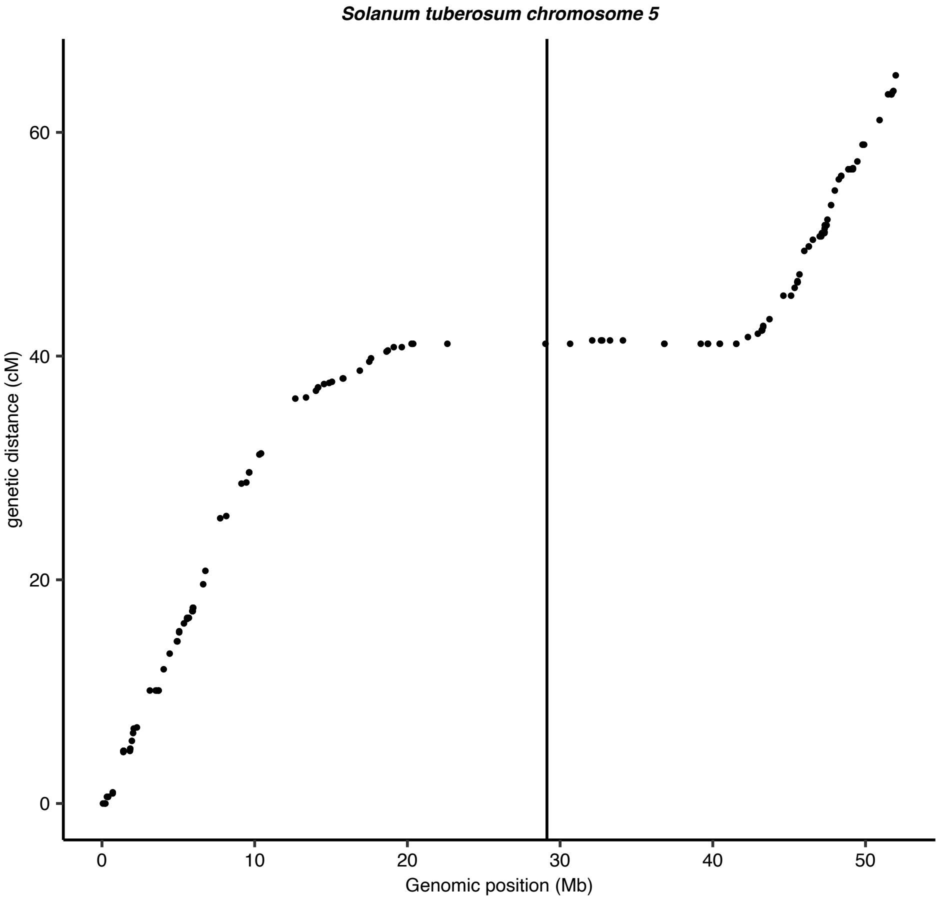
Solanum tuberosum chromosome 5
genetic distance (cM)
0
20
40
60
Genomic position (Mb)
0
10
20
30
40
50

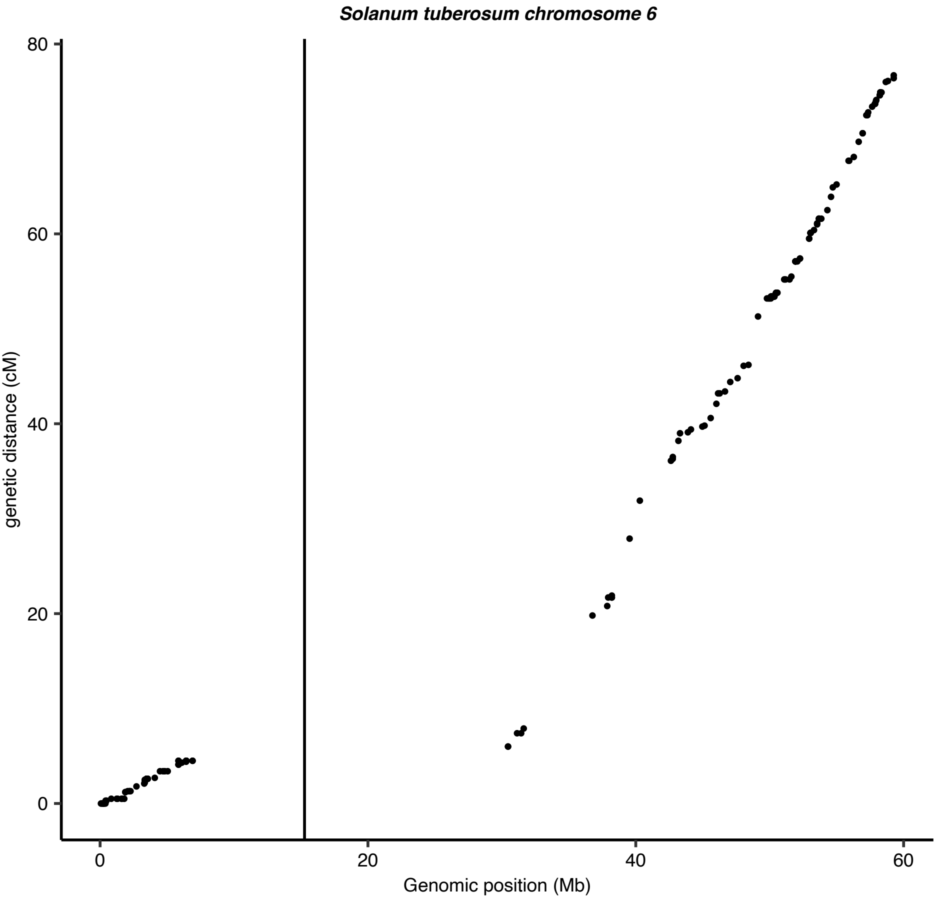
Solanum tuberosum chromosome 6
genetic distance (cM)
Genomic position (Mb)
0
20
40
60
80
0
20
40
60

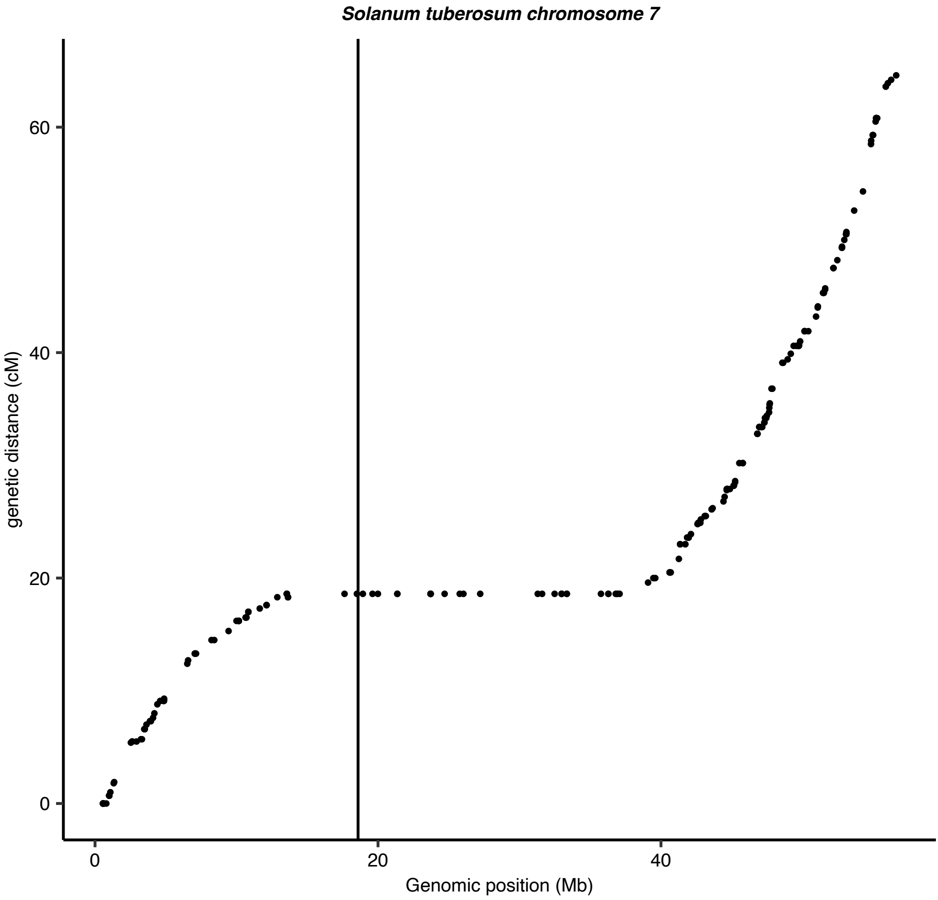
Solanum tuberosum chromosome 7
genetic distance (cM)
Genomic position (Mb)
0
20
40
60
0
20
40

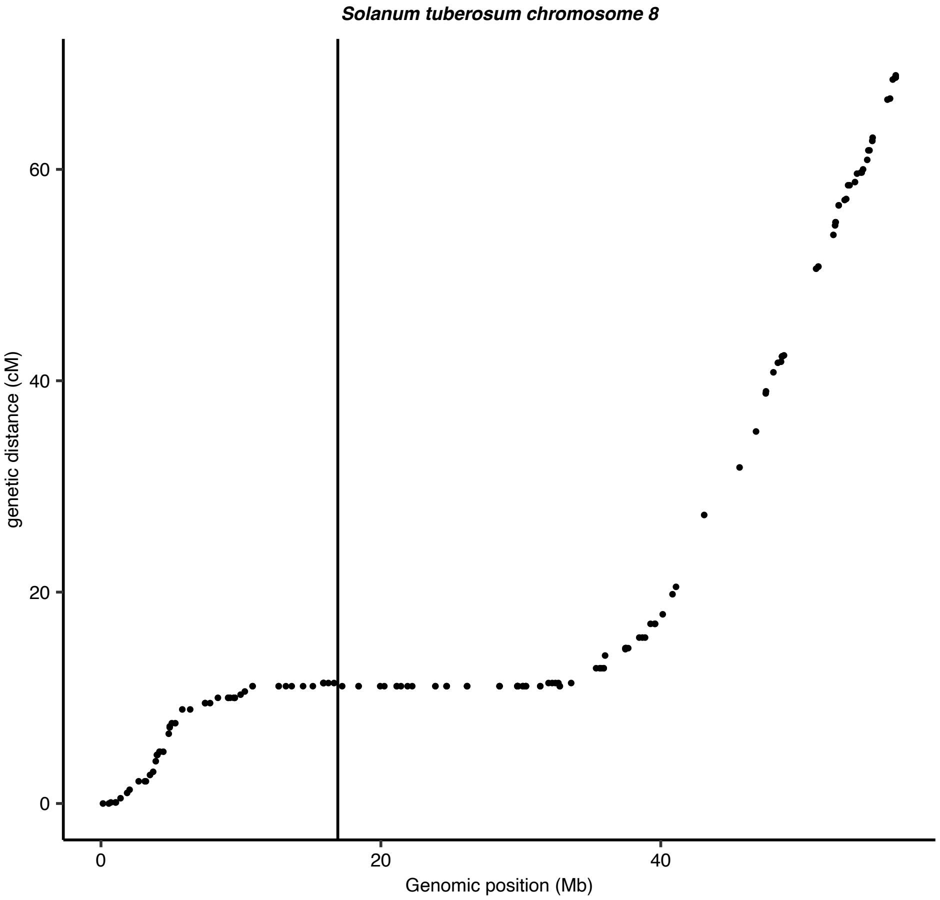
Solanum tuberosum chromosome 8
genetic distance (cM)
60
40
20
0
0
20
40
Genomic position (Mb)

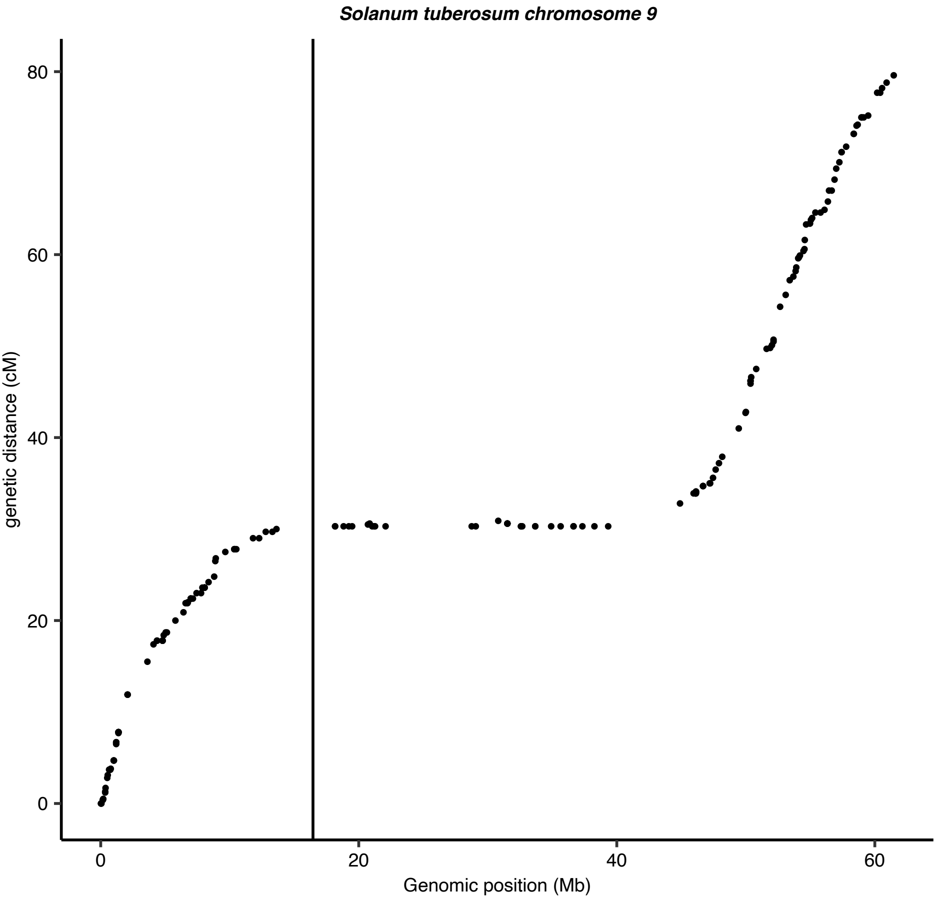
Solanum tuberosum chromosome 9
genetic distance (cM)
Genomic position (Mb)
0
20
40
60
80
0
20
40
60

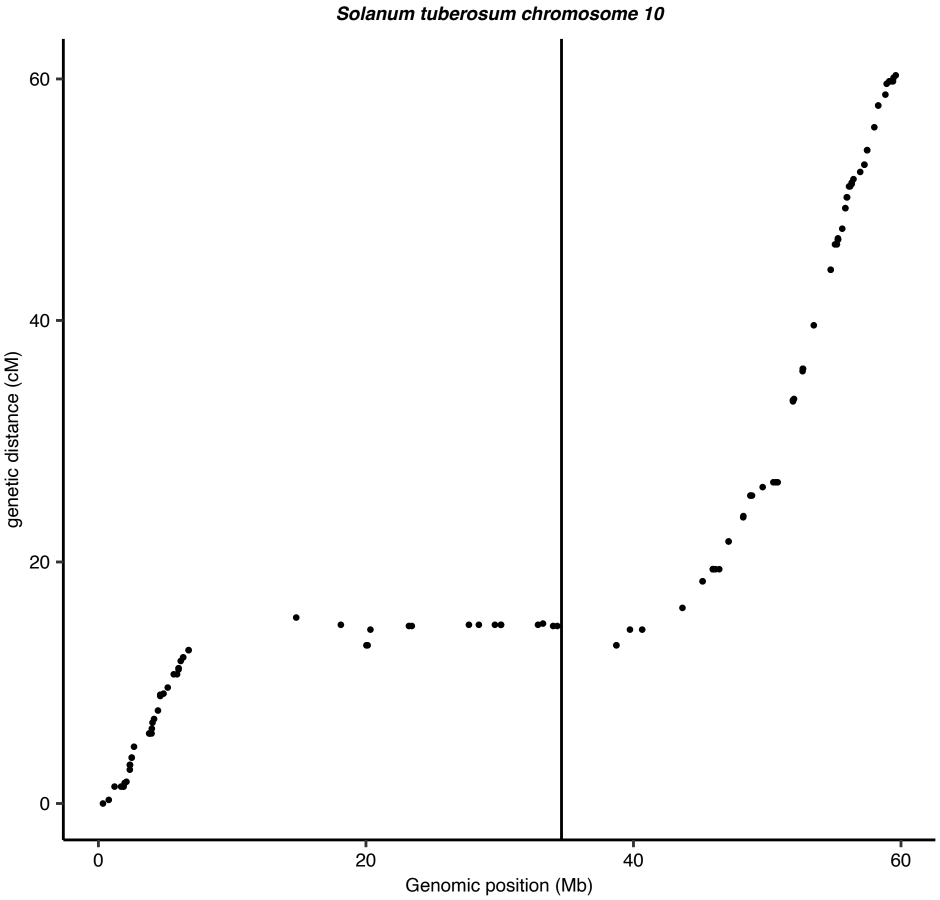

Solanum tuberosum chromosome 10
genetic distance (cM)
Genomic position (Mb)
0
20
40
60
0
20
40
60

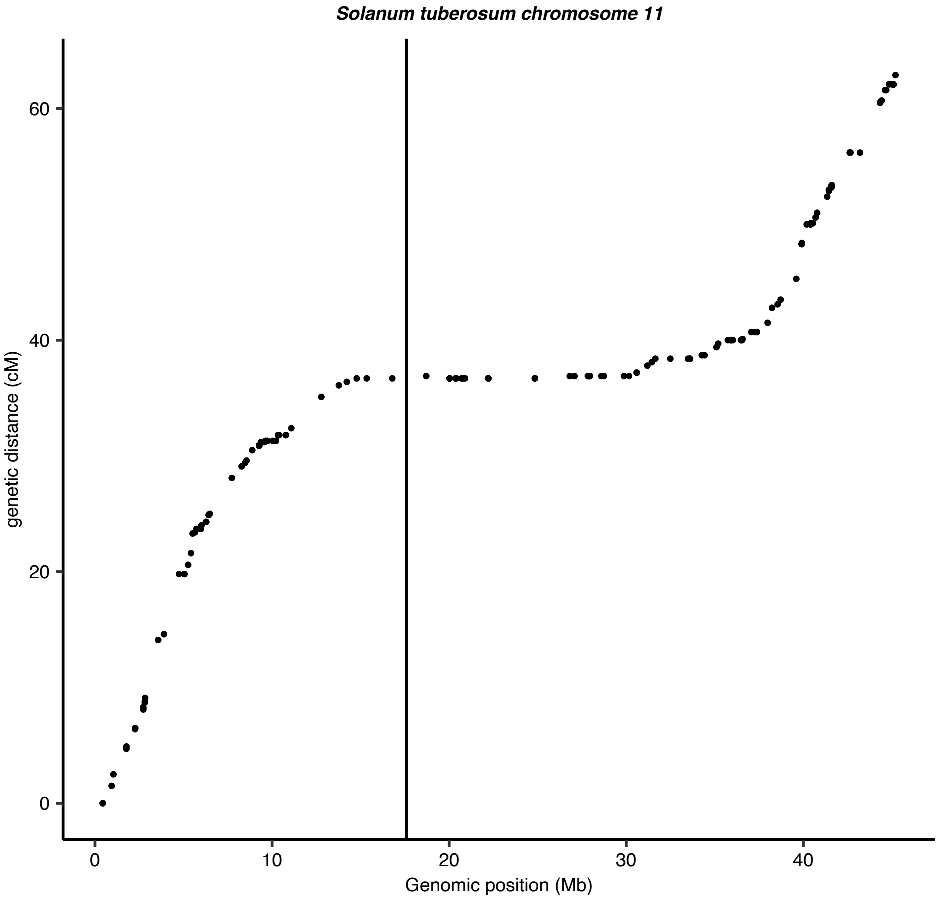
Solanum tuberosum chromosome 11
genetic distance (cM)
Genomic position (Mb)
0
20
40
60
0
10
20
30
40

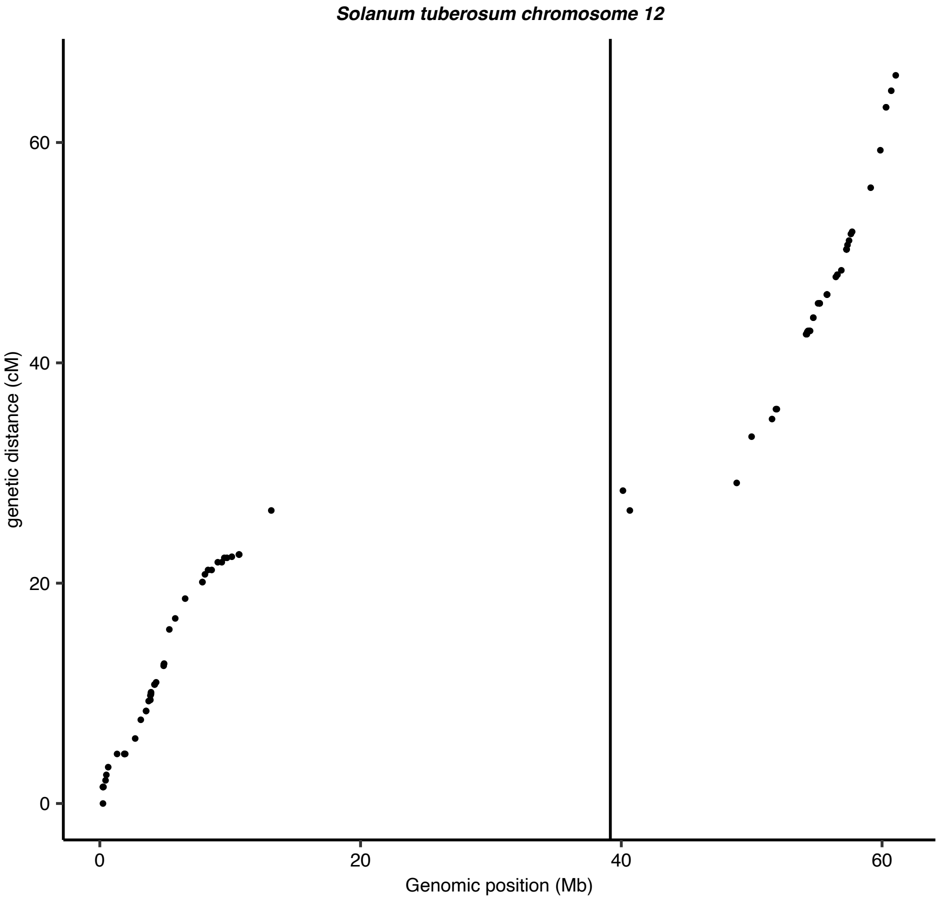
Solanum tuberosum chromosome 12
genetic distance (cM)
0
20
40
60
Genomic position (Mb)
0
20
40
60

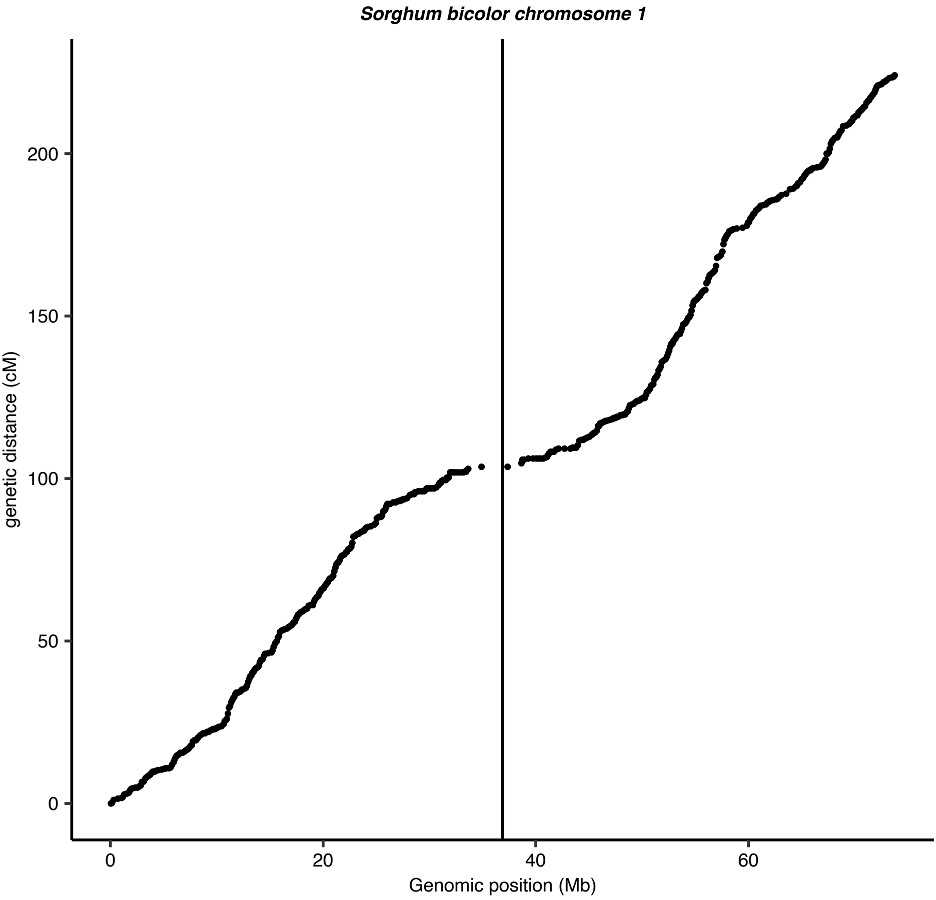
Sorghum bicolor chromosome 1
genetic distance (cM)
0
50
100
150
200
Genomic position (Mb)
0
20
40
60

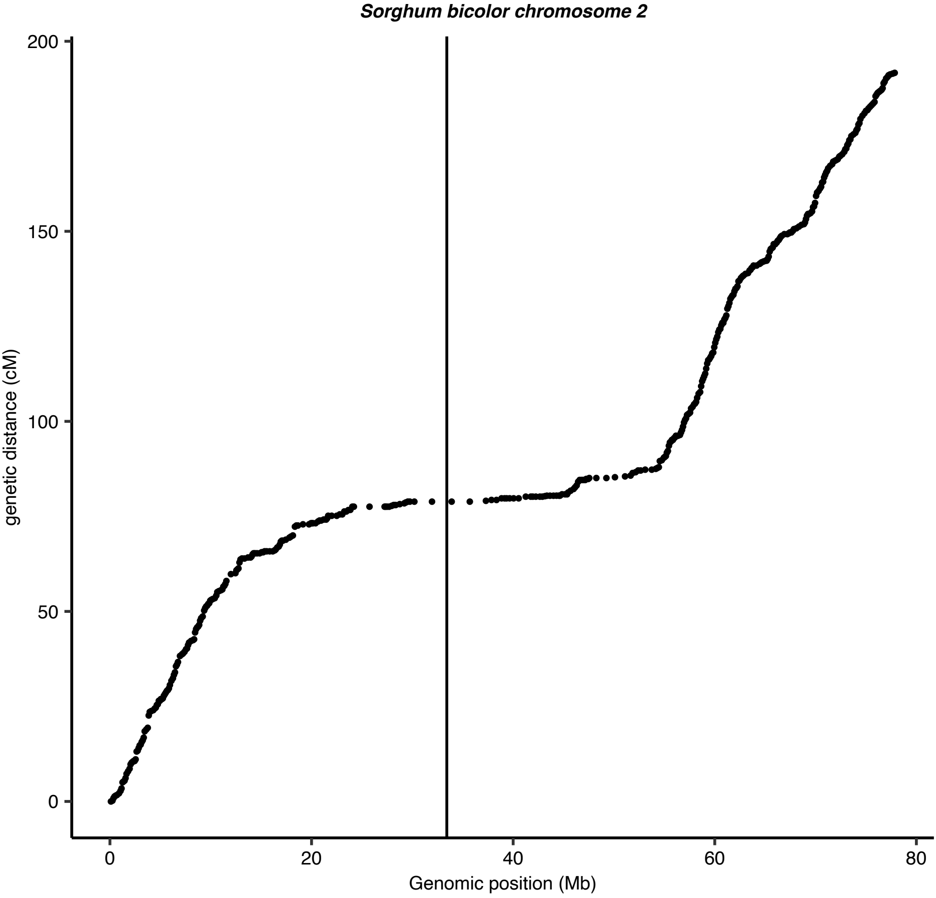
Sorghum bicolor chromosome 2
genetic distance (cM)
200
150
100
50
0
0
20
40
60
80
Genomic position (Mb)

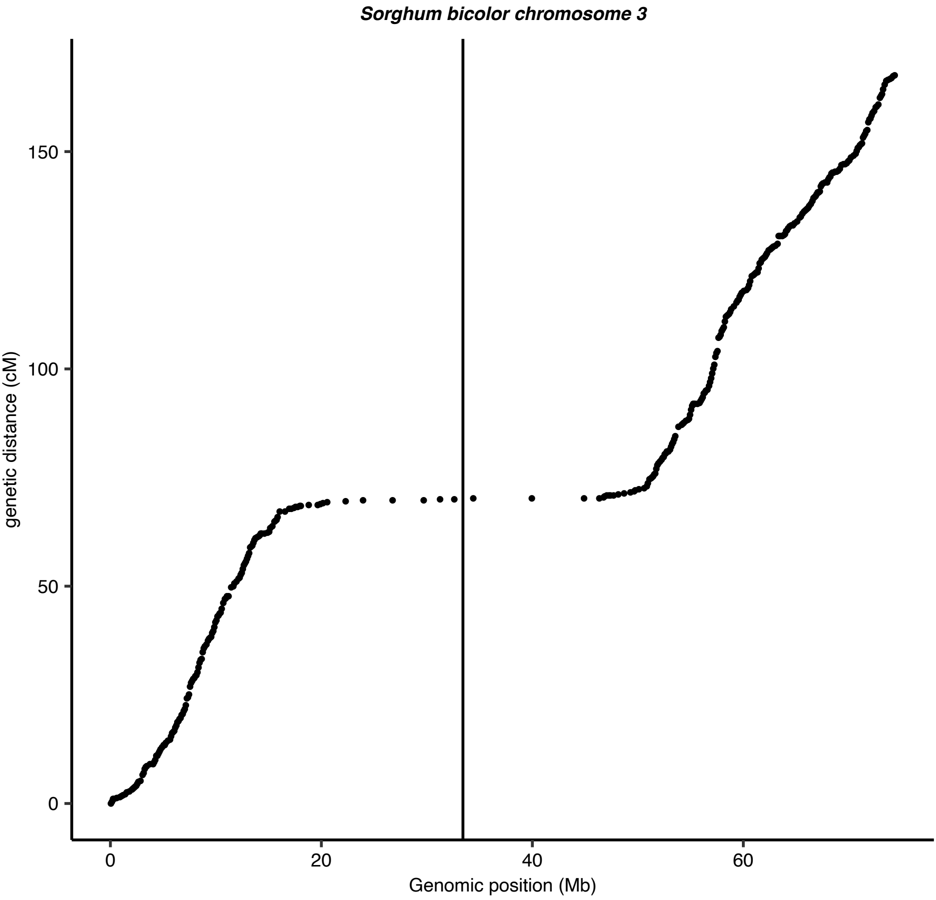
Sorghum bicolor chromosome 3
genetic distance (cM)
0
50
100
150
Genomic position (Mb)
0
20
40
60

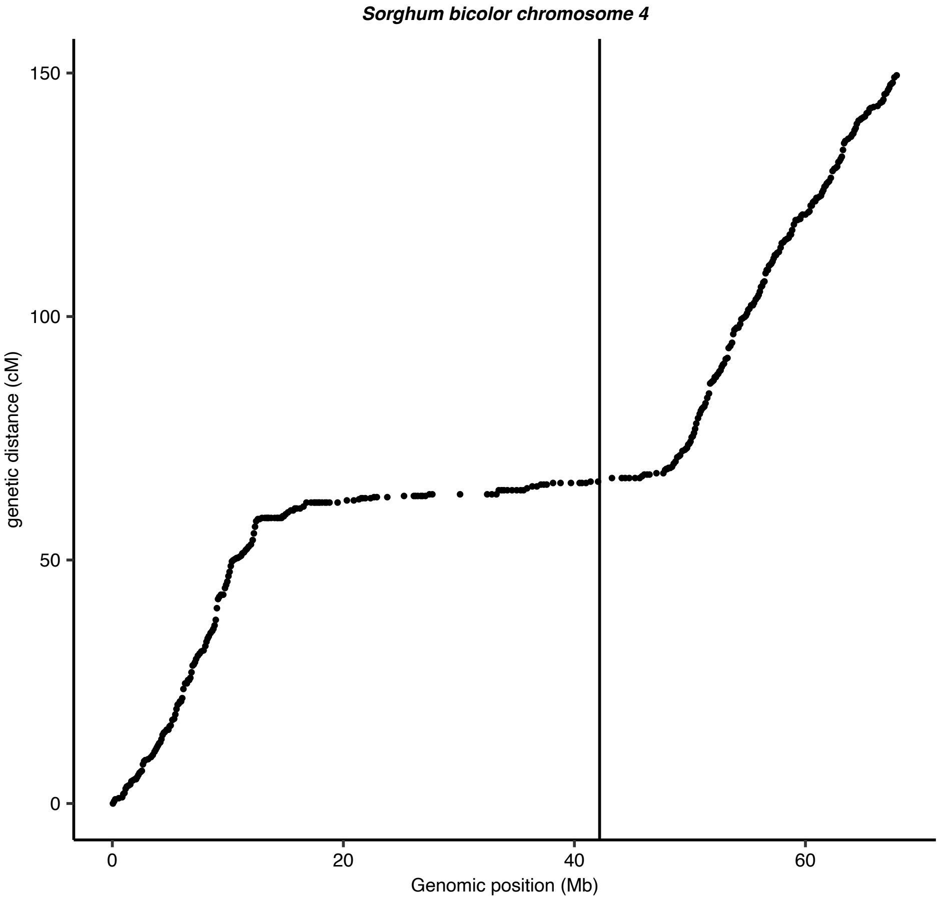

Sorghum bicolor chromosome 4
genetic distance (cM)
150
100
50
0
0
20
40
60
Genomic position (Mb)

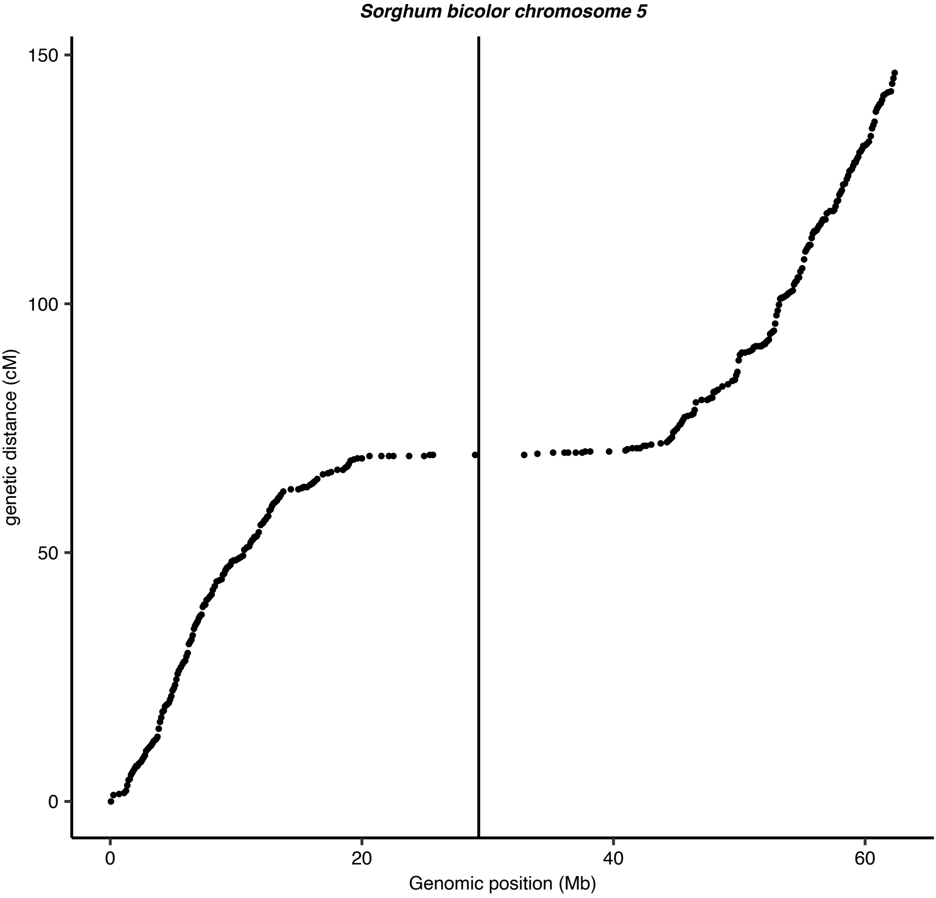
Sorghum bicolor chromosome 5
genetic distance (cM)
150
100
50
0
0
20
40
60
Genomic position (Mb)

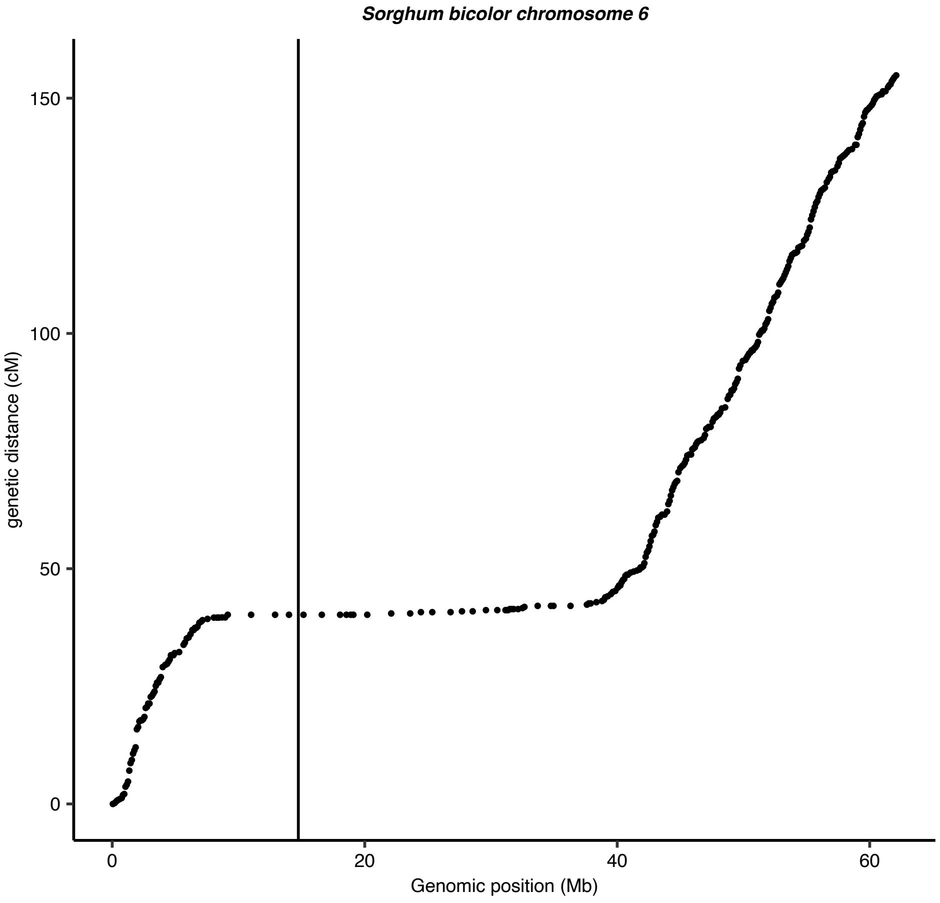
Sorghum bicolor chromosome 6
genetic distance (cM)
0
50
100
150
Genomic position (Mb)
0
20
40
60

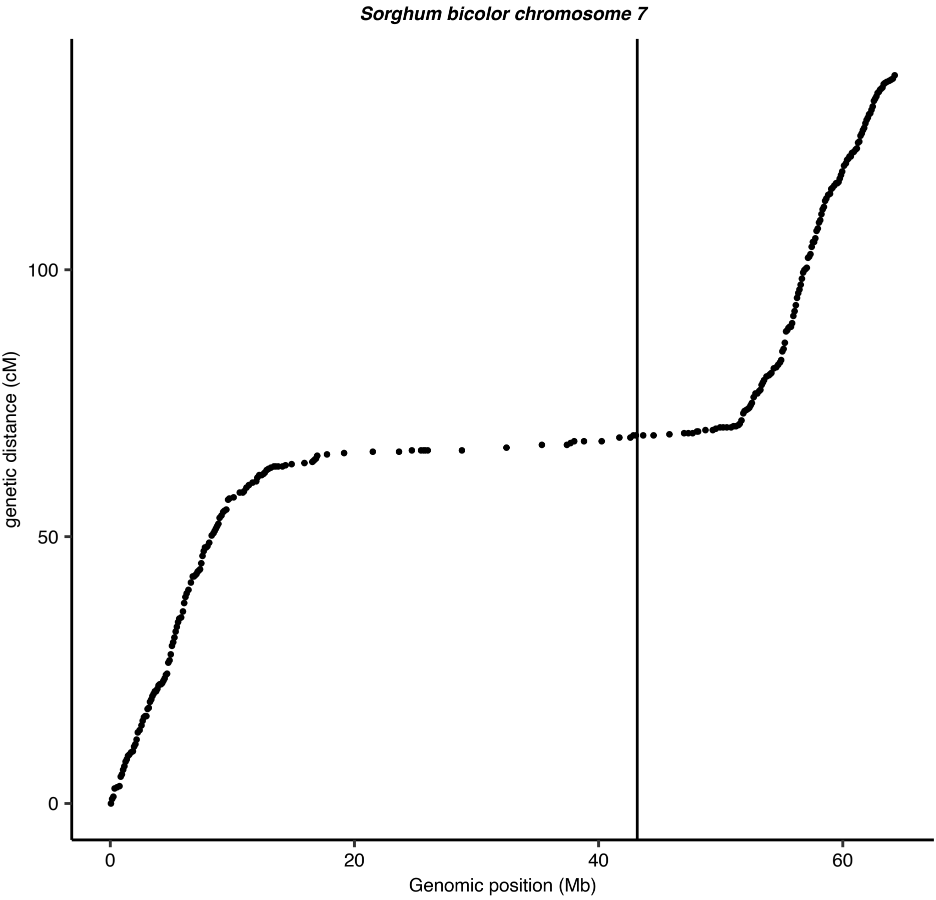

Sorghum bicolor chromosome 7
genetic distance (cM)
0
50
100
0
20
40
60
Genomic position (Mb)

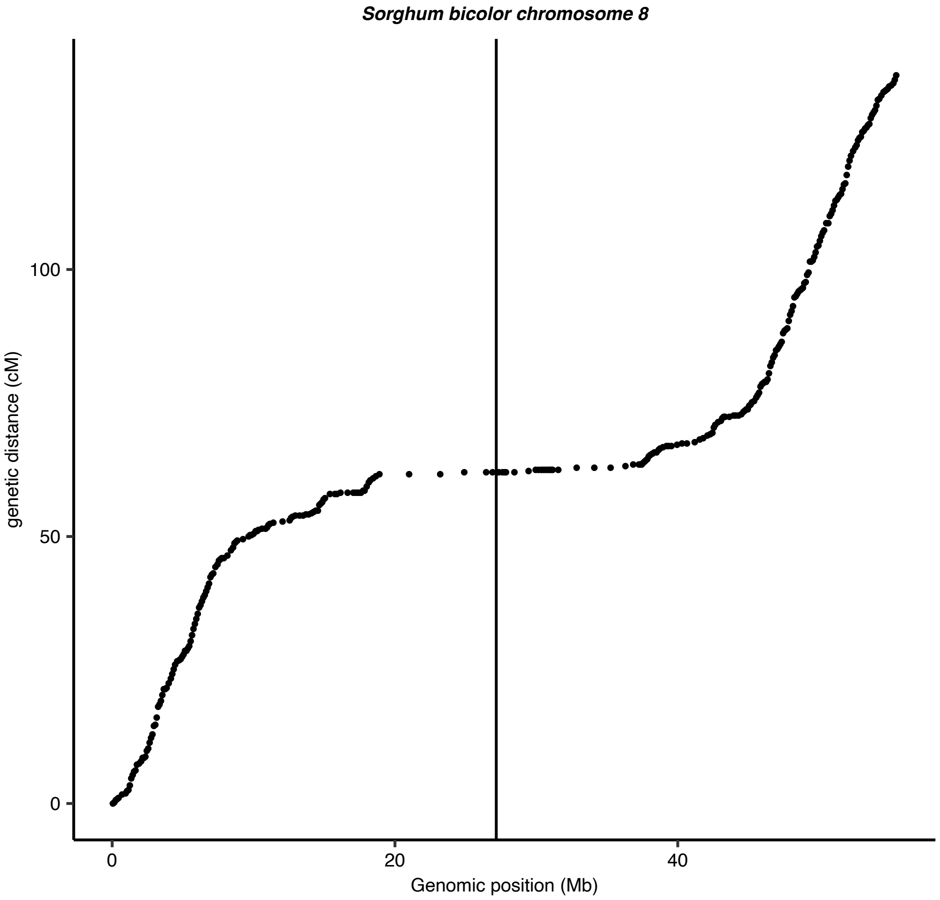
Sorghum bicolor chromosome 8
genetic distance (cM)
Genomic position (Mb)
0
50
100
0
20
40

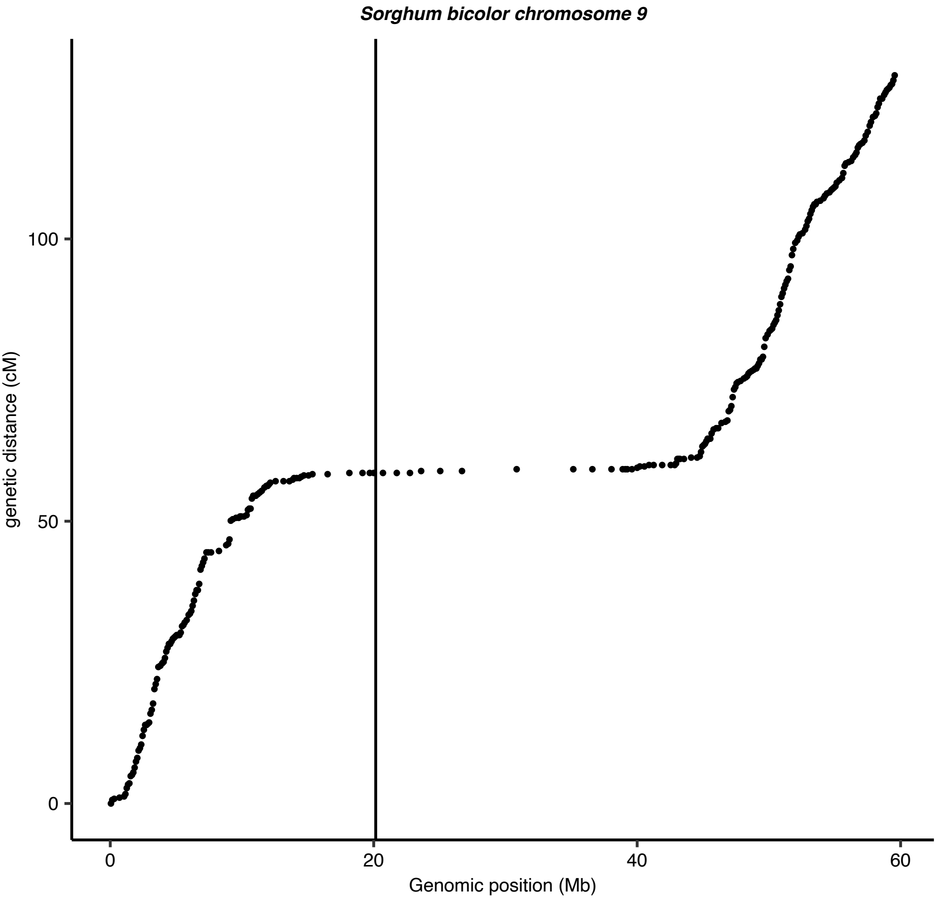

Sorghum bicolor chromosome 9
genetic distance (cM)
100
50
0
0
20
40
60
Genomic position (Mb)

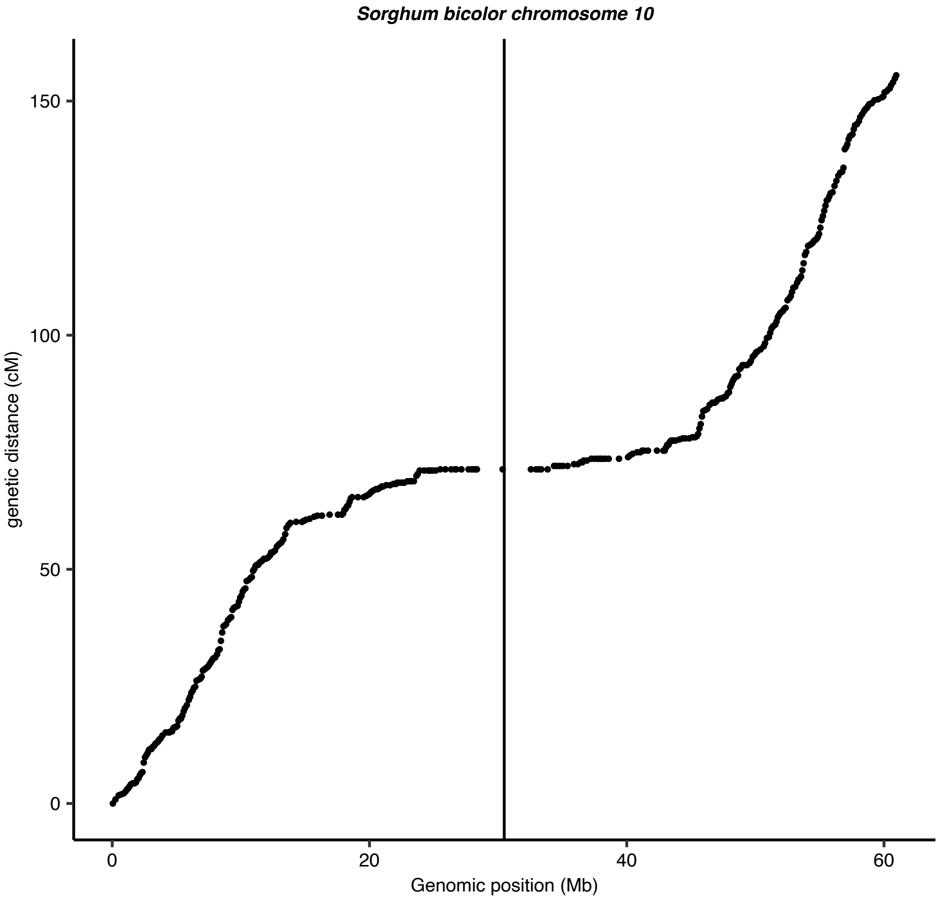
Sorghum bicolor chromosome 10
genetic distance (cM)
0
50
100
150
Genomic position (Mb)
0
20
40
60

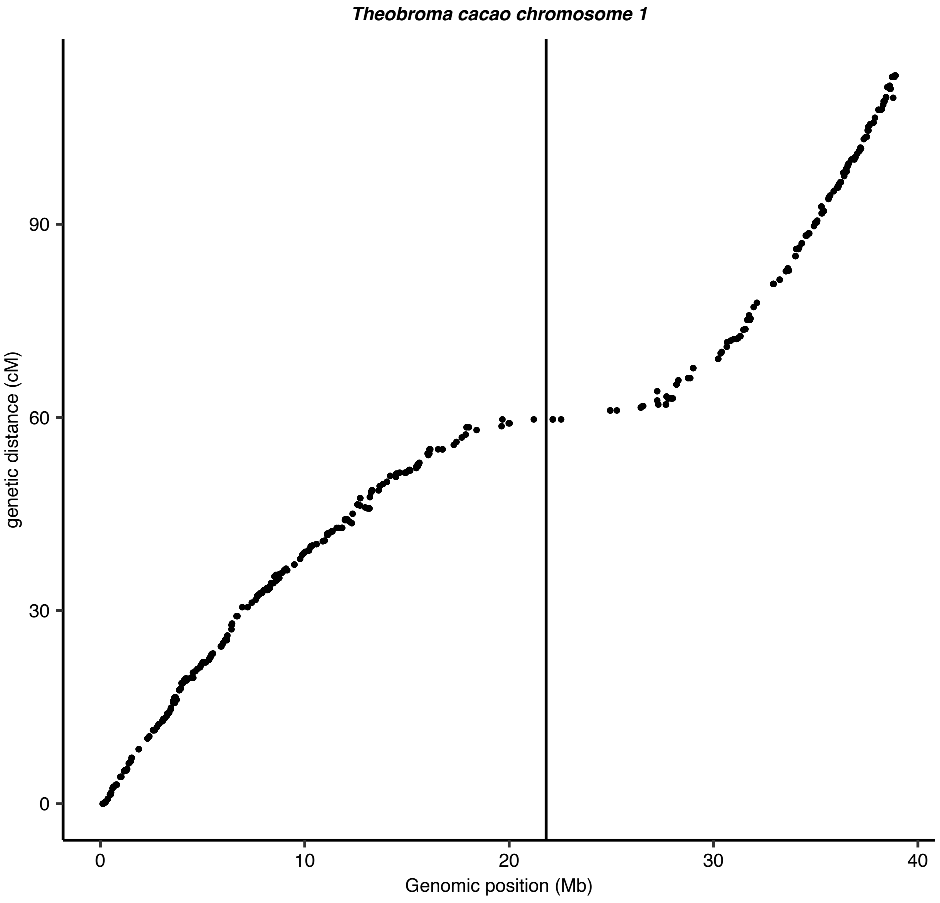
Theobroma cacao chromosome 1
genetic distance (cM)
Genomic position (Mb)
0
30
60
90
0
10
20
30
40

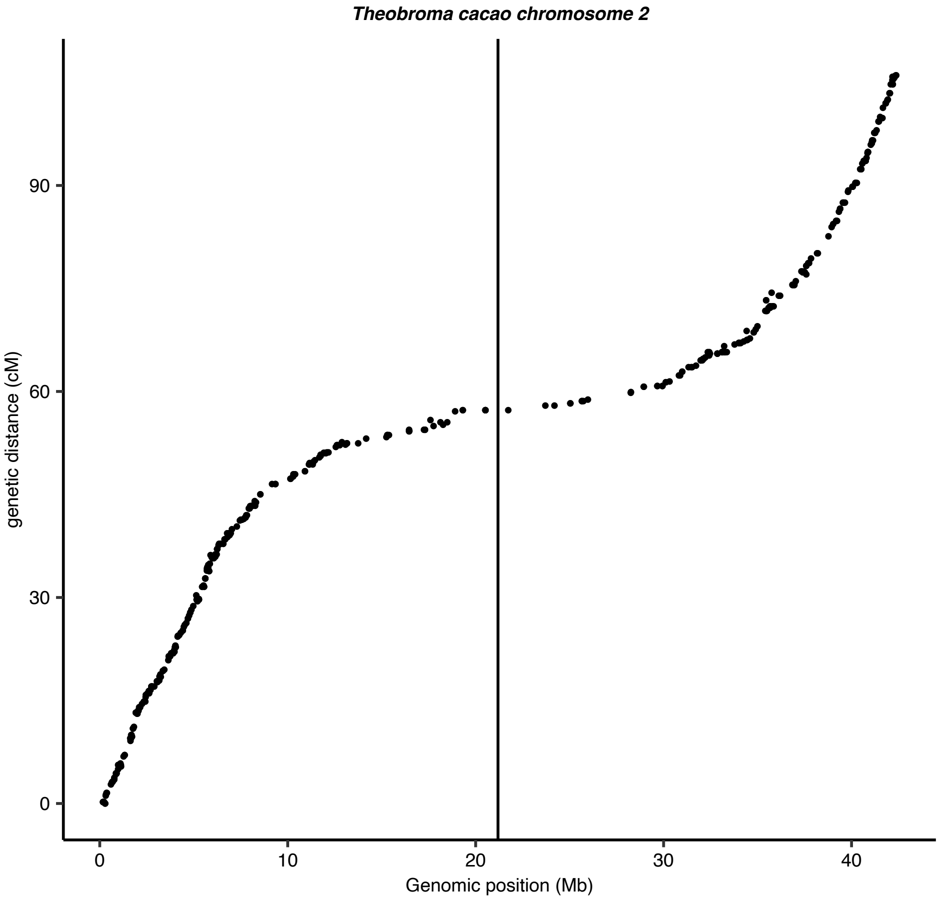
Theobroma cacao chromosome 2
genetic distance (cM)
0
30
60
90
Genomic position (Mb)
0
10
20
30
40

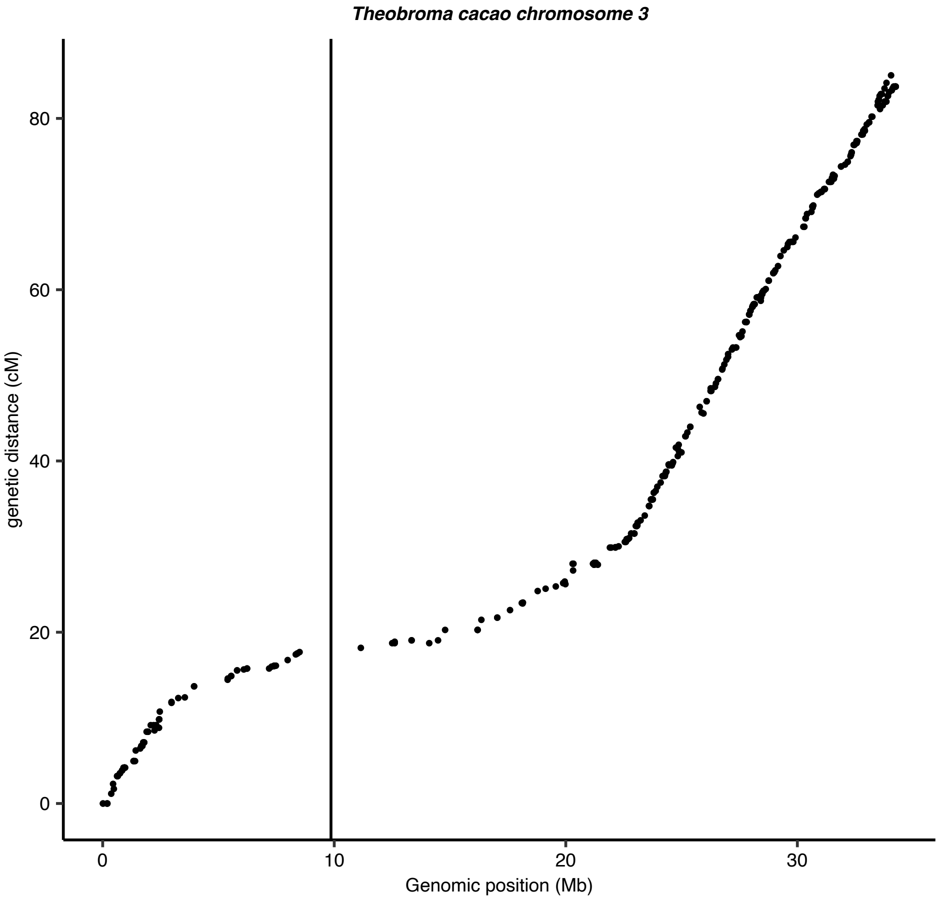
Theobroma cacao chromosome 3
genetic distance (cM)
Genomic position (Mb)
0
20
40
60
80
0
10
20
30

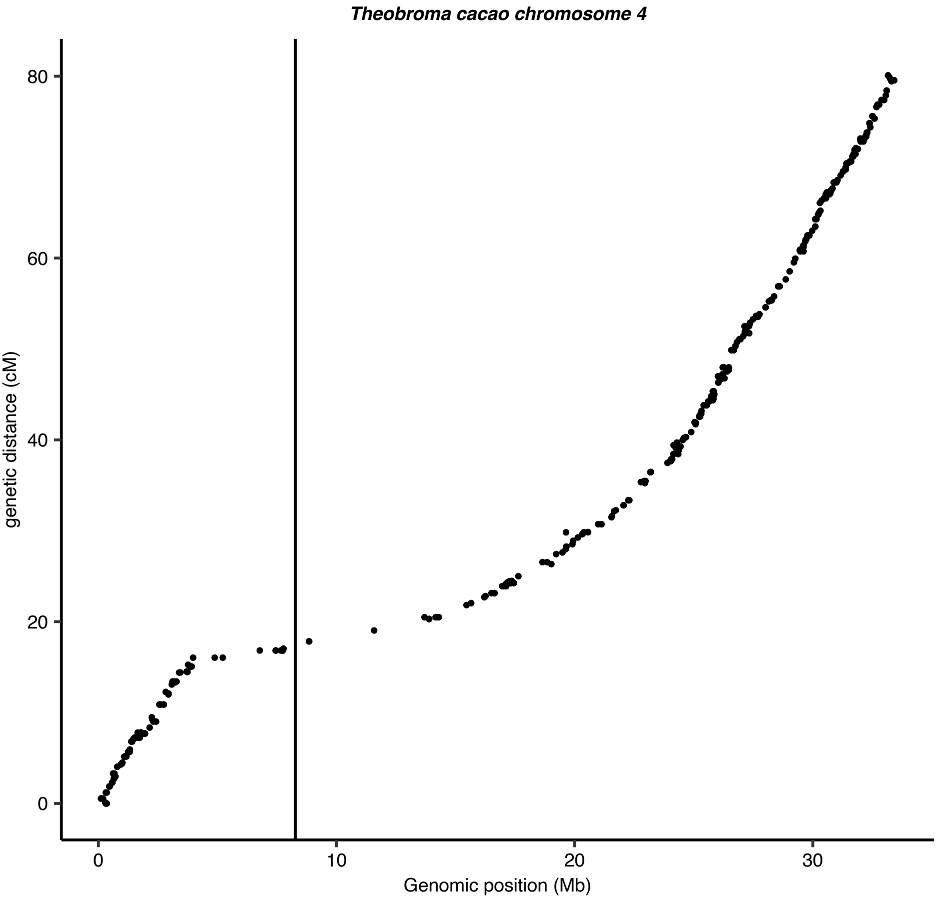
Theobroma cacao chromosome 4
genetic distance (cM)
0
20
40
60
80
Genomic position (Mb)
0
10
20
30

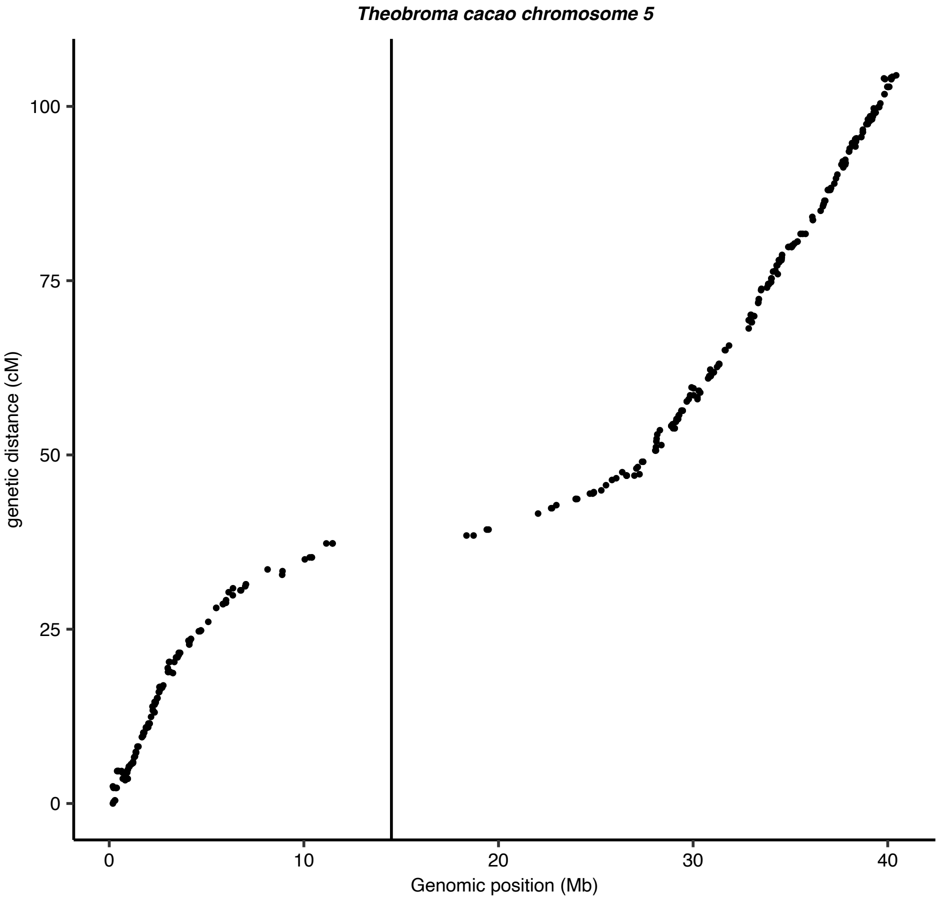
Theobroma cacao chromosome 5
genetic distance (cM)
0
25
50
75
100
Genomic position (Mb)
0
10
20
30
40

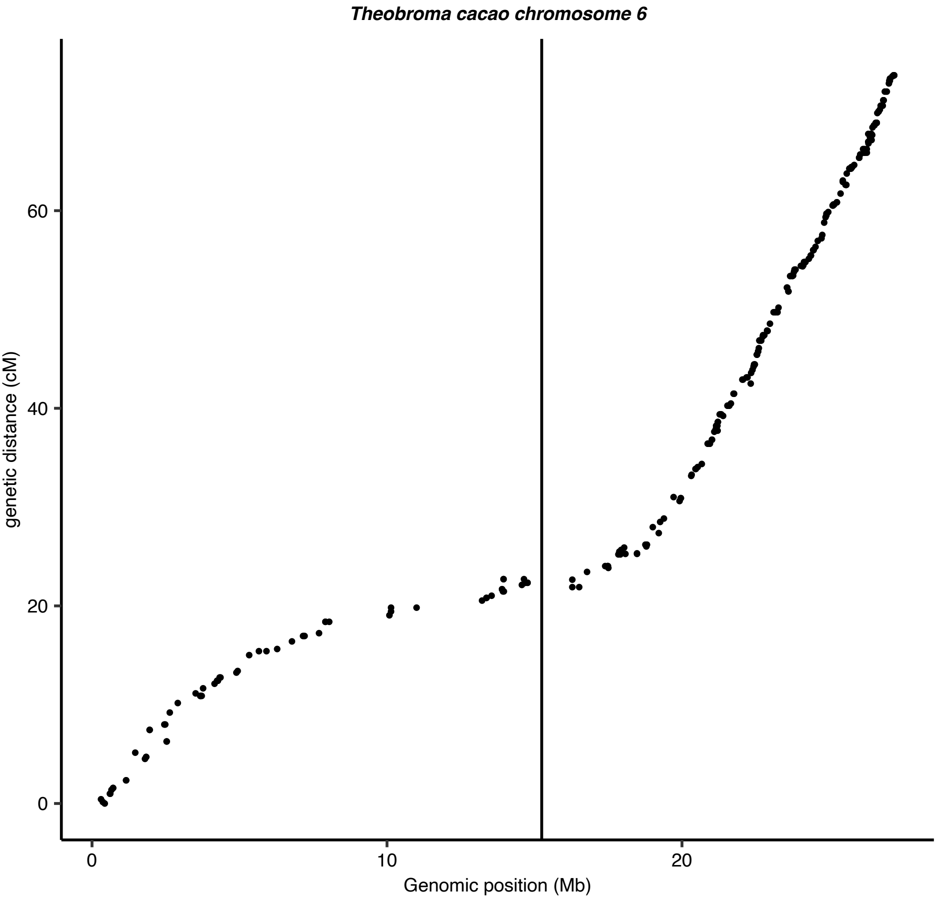
Theobroma cacao chromosome 6
genetic distance (cM)
0
20
40
60
0
10
20
Genomic position (Mb)

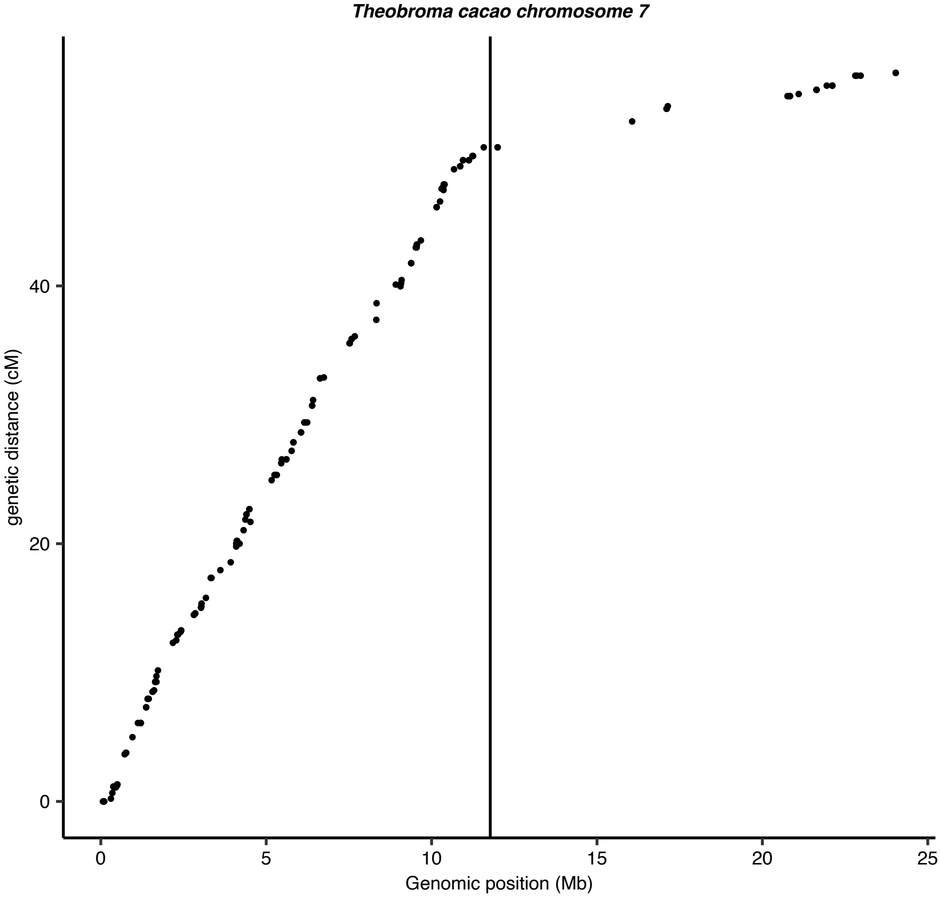
Theobroma cacao chromosome 7
genetic distance (cM)
0
20
40
Genomic position (Mb)
0
5
10
15
20
25

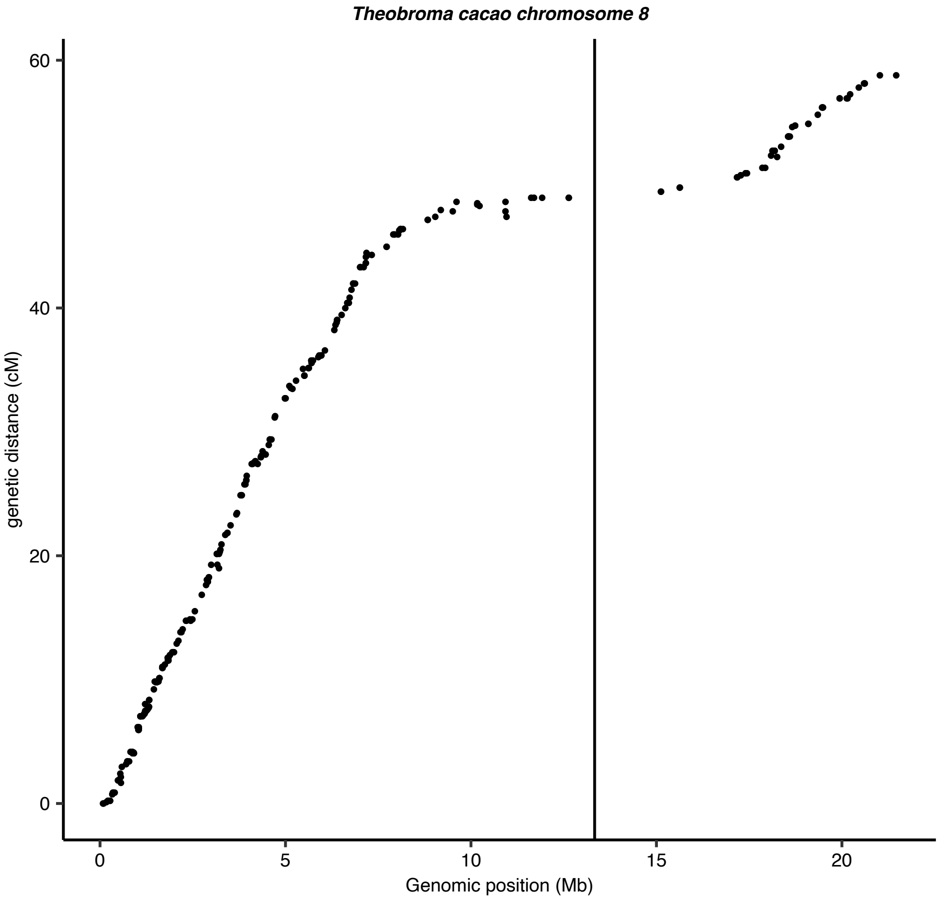
Theobroma cacao chromosome 8
60
40
20
0
genetic distance (cM)
0
5
10
15
20
Genomic position (Mb)

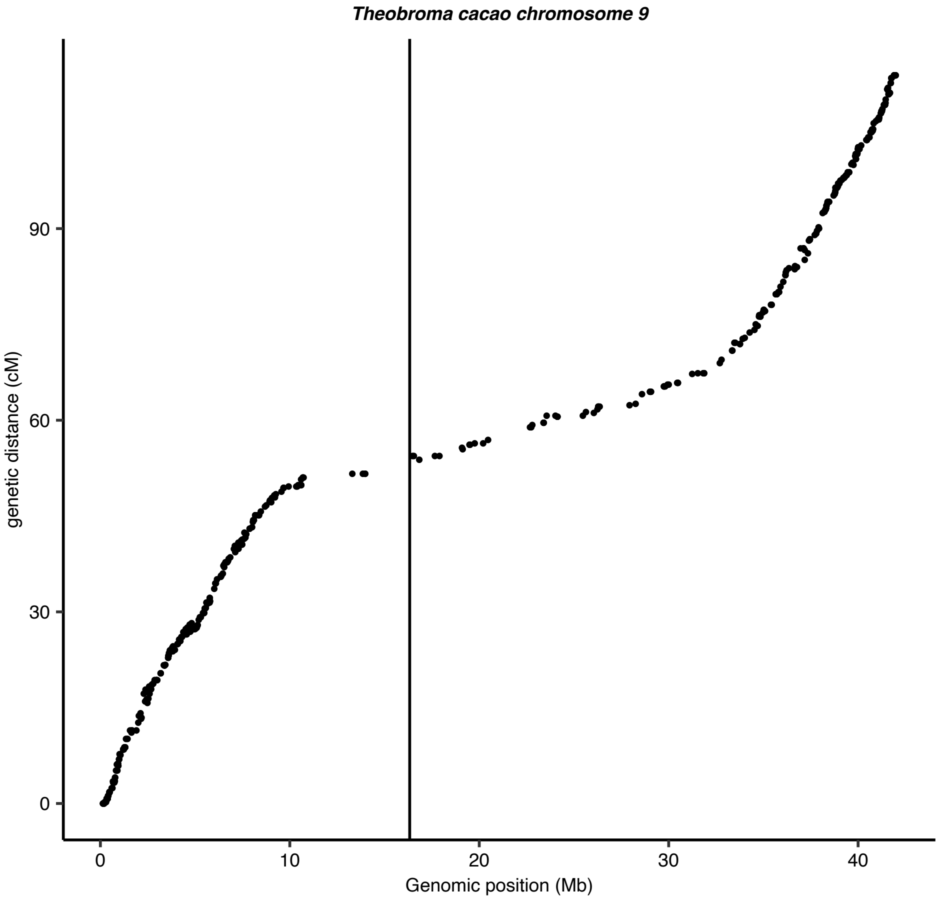
Theobroma cacao chromosome 9
genetic distance (cM)
Genomic position (Mb)
0
30
60
90
0
10
20
30
40

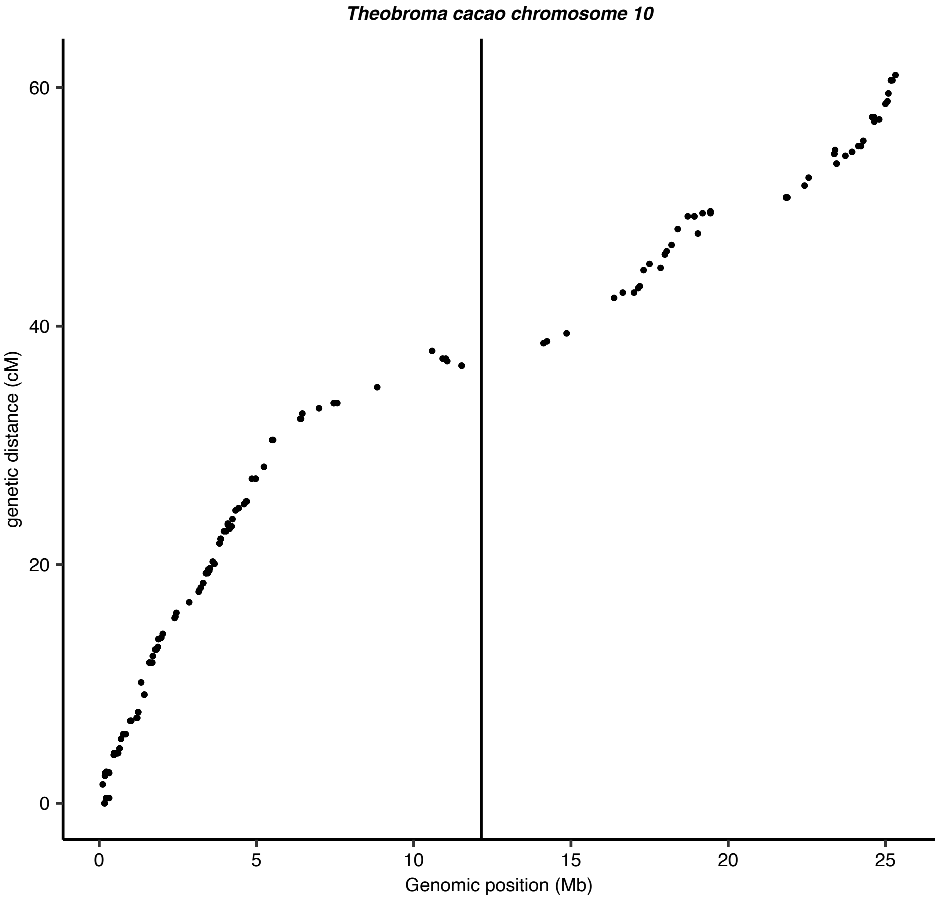
Theobroma cacao chromosome 10
genetic distance (cM)
Genomic position (Mb)
0
20
40
60
0
5
10
15
20
25

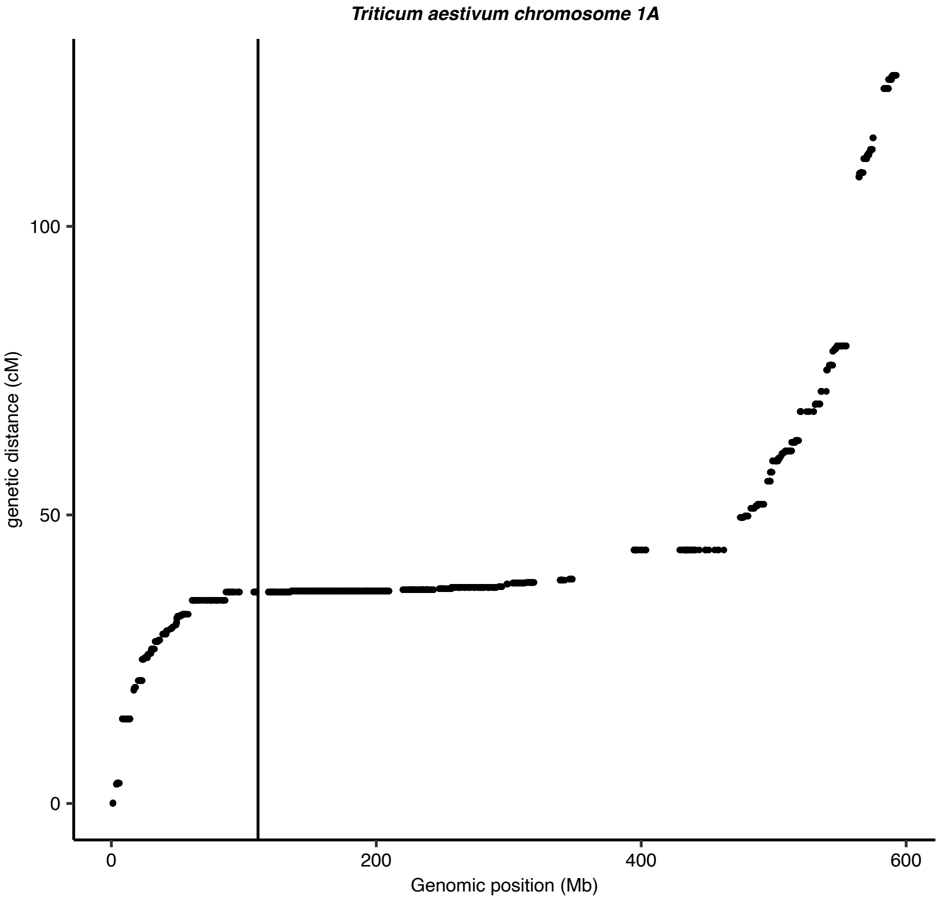

Triticum aestivum chromosome 1A
genetic distance (cM)
100
50
0
0
200
400
600
Genomic position (Mb)

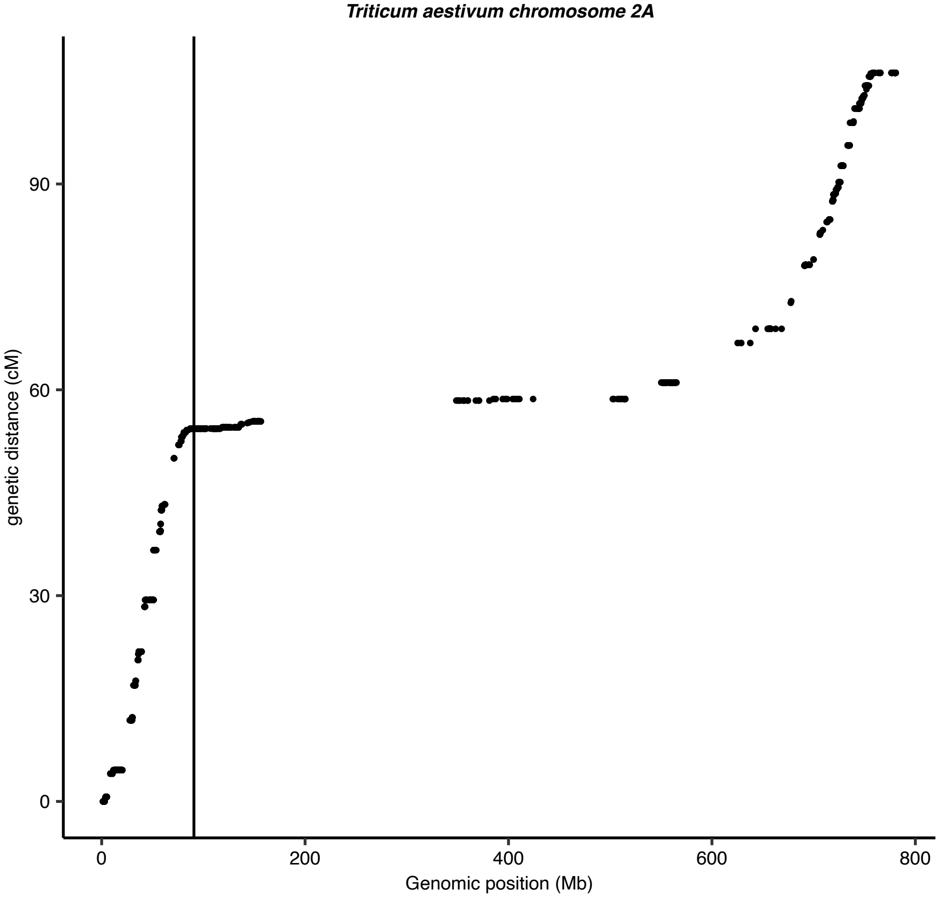
Triticum aestivum chromosome 2A
genetic distance (cM)
0
30
60
90
Genomic position (Mb)
0
200
400
600
800

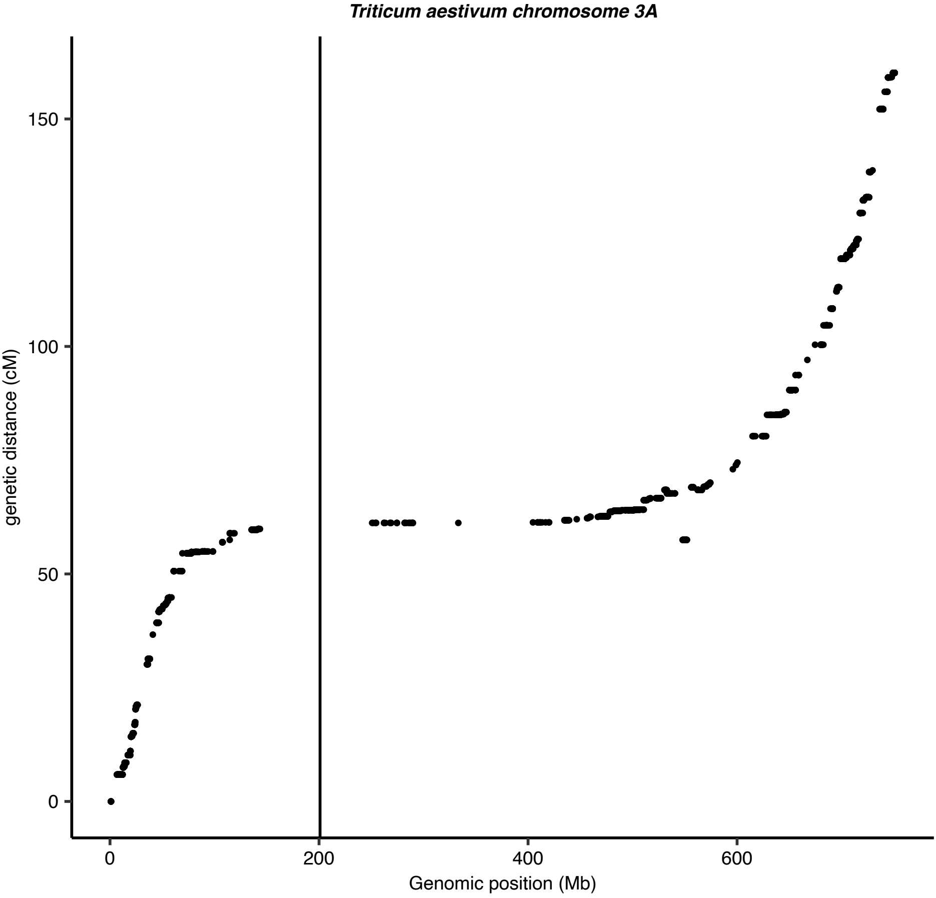
Triticum aestivum chromosome 3A
genetic distance (cM)
Genomic position (Mb)
0
50
100
150
0
200
400
600

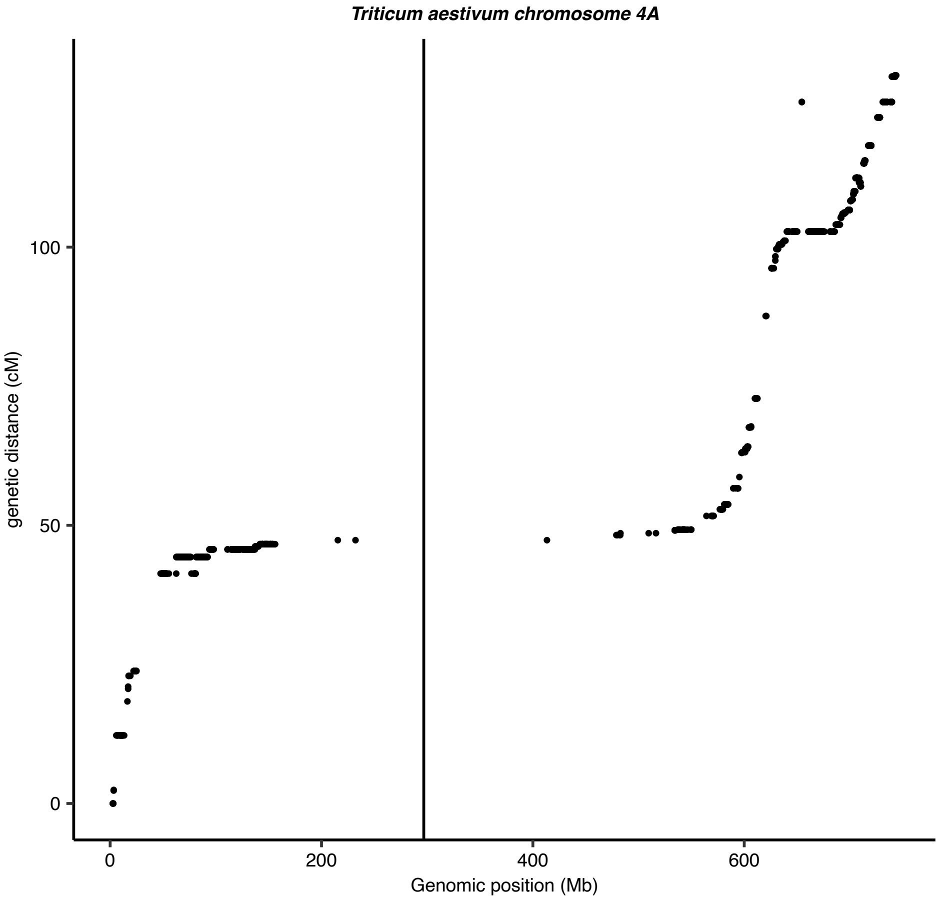
Triticum aestivum chromosome 4A
genetic distance (cM)
100
50
0
0
200
400
600
Genomic position (Mb)

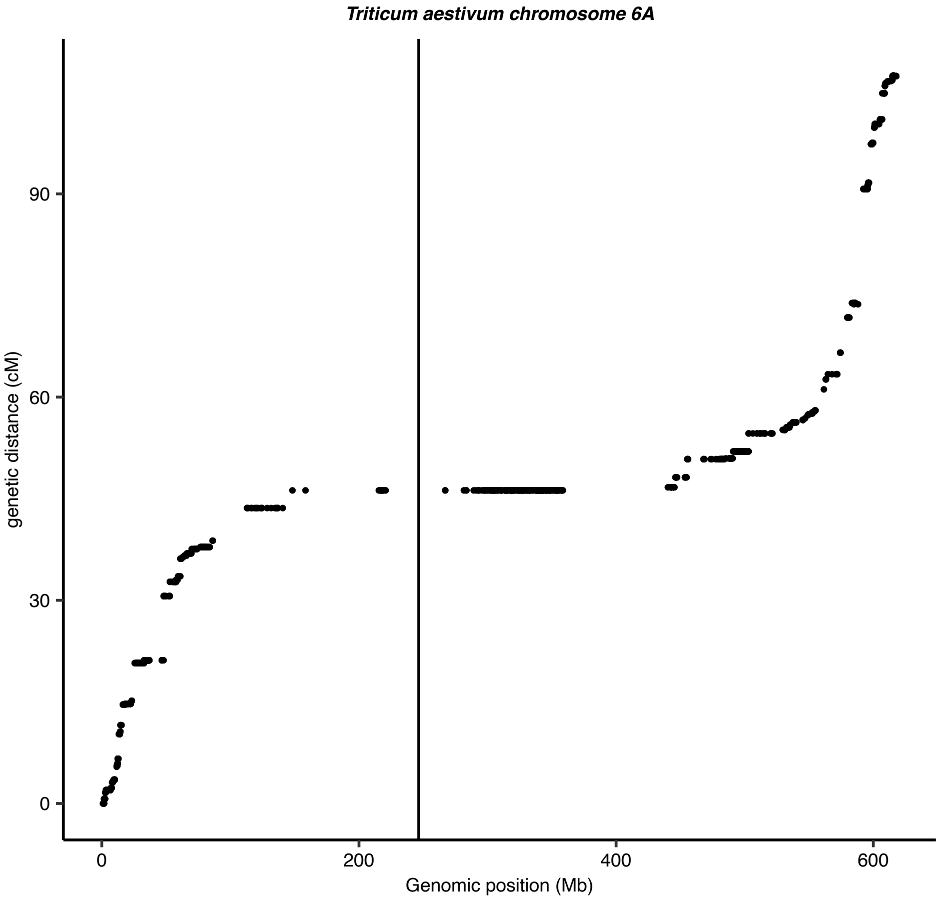
Triticum aestivum chromosome 6A
genetic distance (cM)
90
60
30
0
0
200
400
600
Genomic position (Mb)

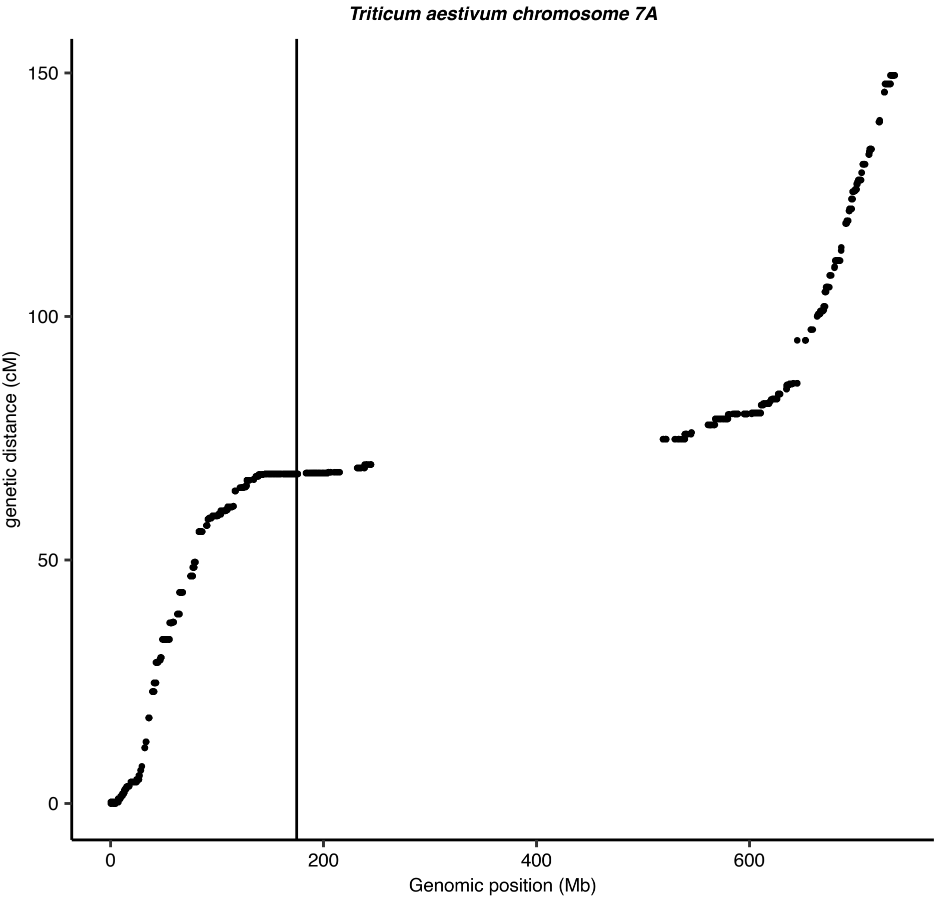
Triticum aestivum chromosome 7A
genetic distance (cM)
150
100
50
0
0
200
400
600
Genomic position (Mb)

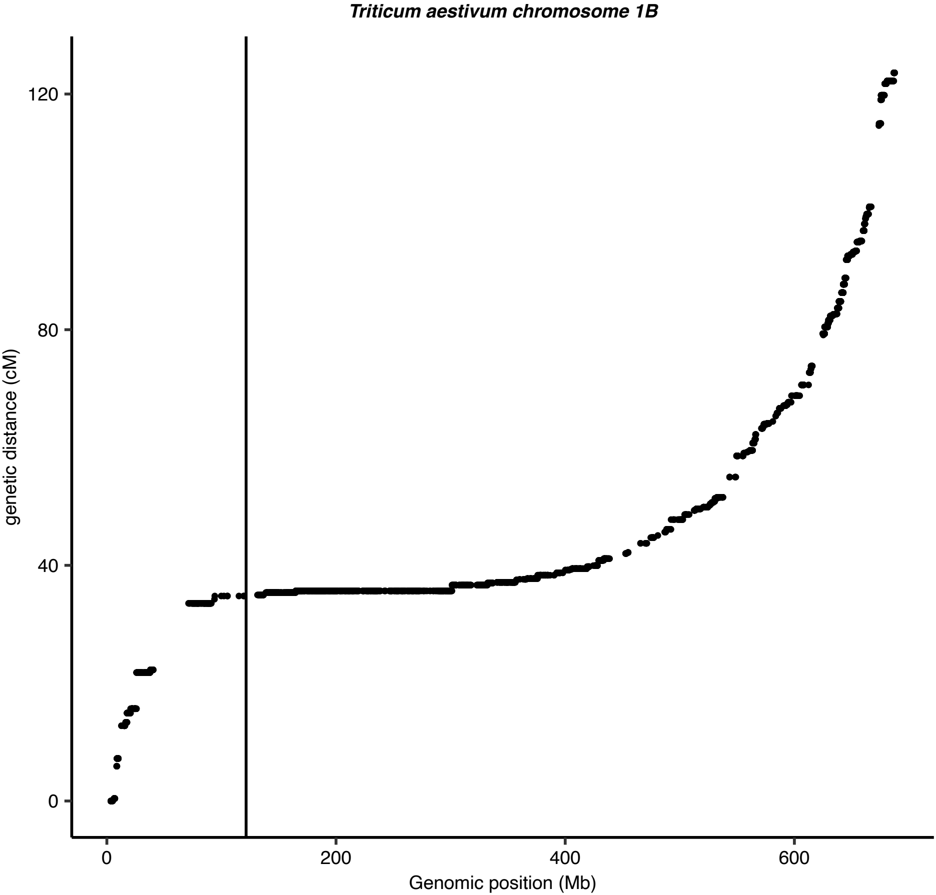
Triticum aestivum chromosome 1B
genetic distance (cM)
Genomic position (Mb)
0
40
80
120
0
200
400
600

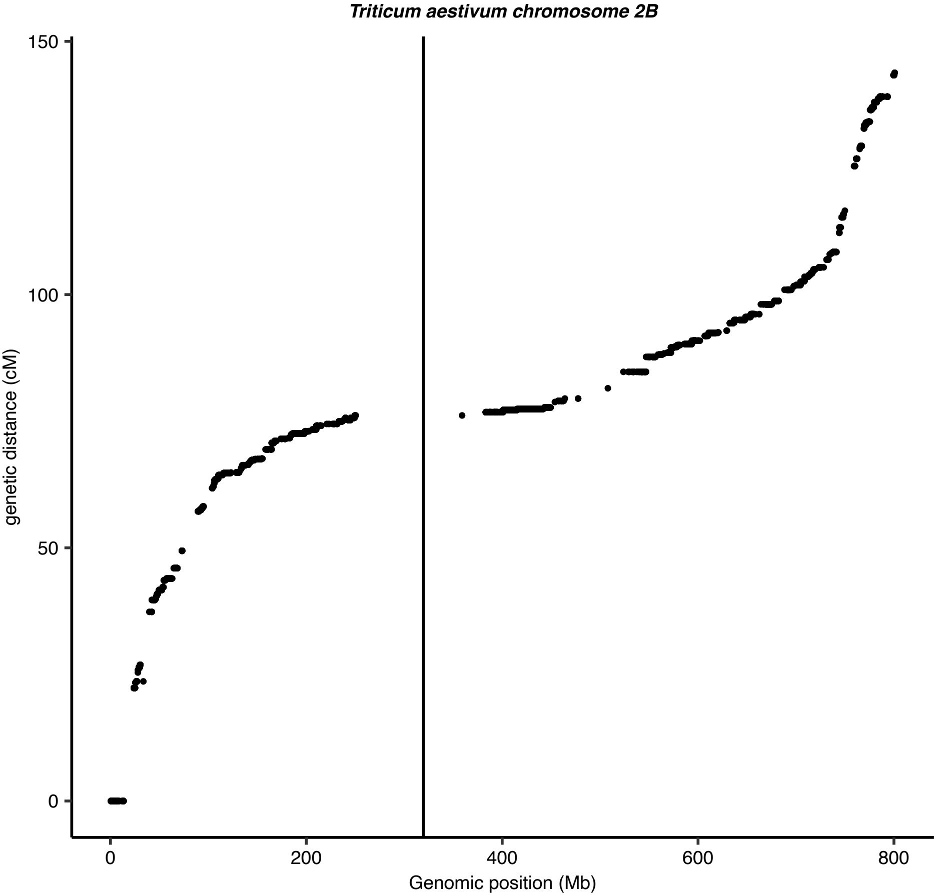
Triticum aestivum chromosome 2B
genetic distance (cM)
0
50
100
150
Genomic position (Mb)
0
200
400
600
800

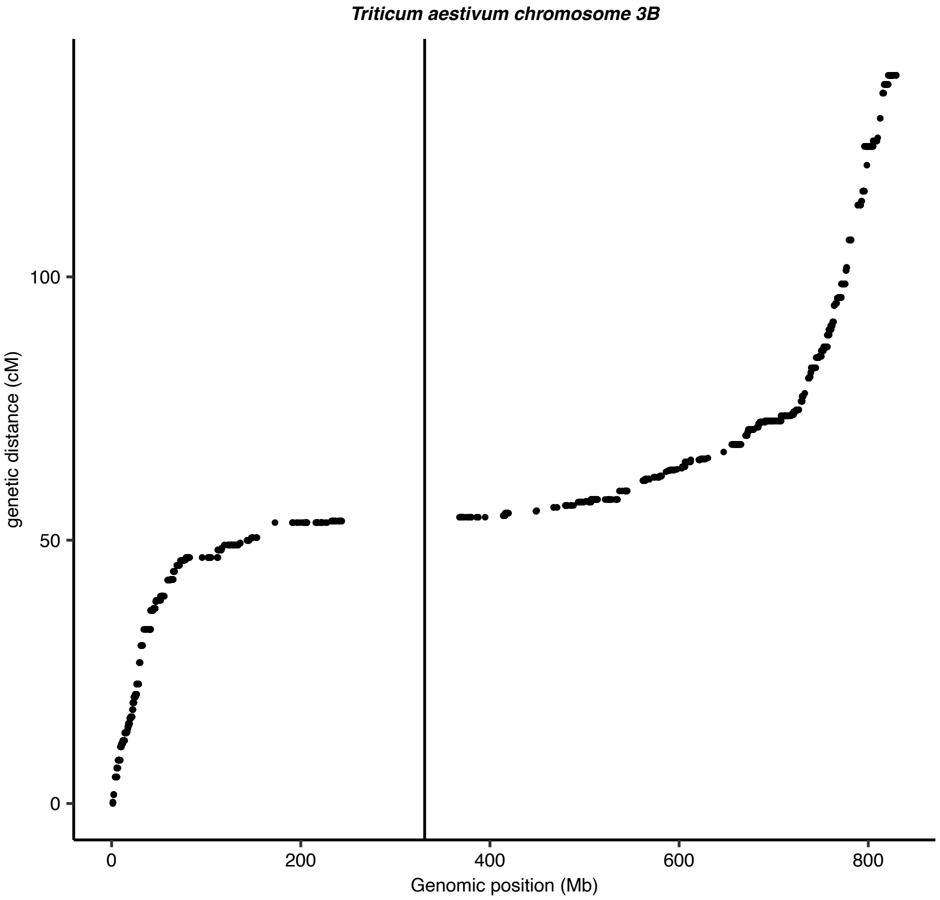
Triticum aestivum chromosome 3B
genetic distance (cM)
100
50
0
0
200
400
600
800
Genomic position (Mb)

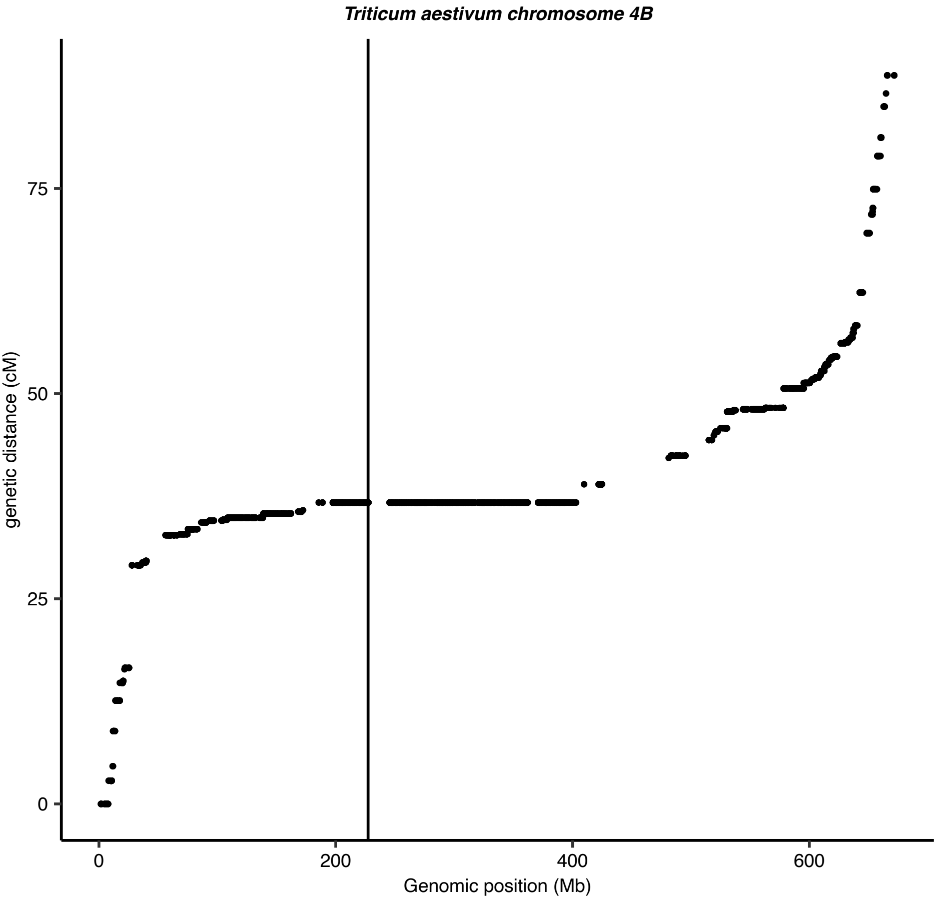
Triticum aestivum chromosome 4B
genetic distance (cM)
0
25
50
75
Genomic position (Mb)
0
200
400
600

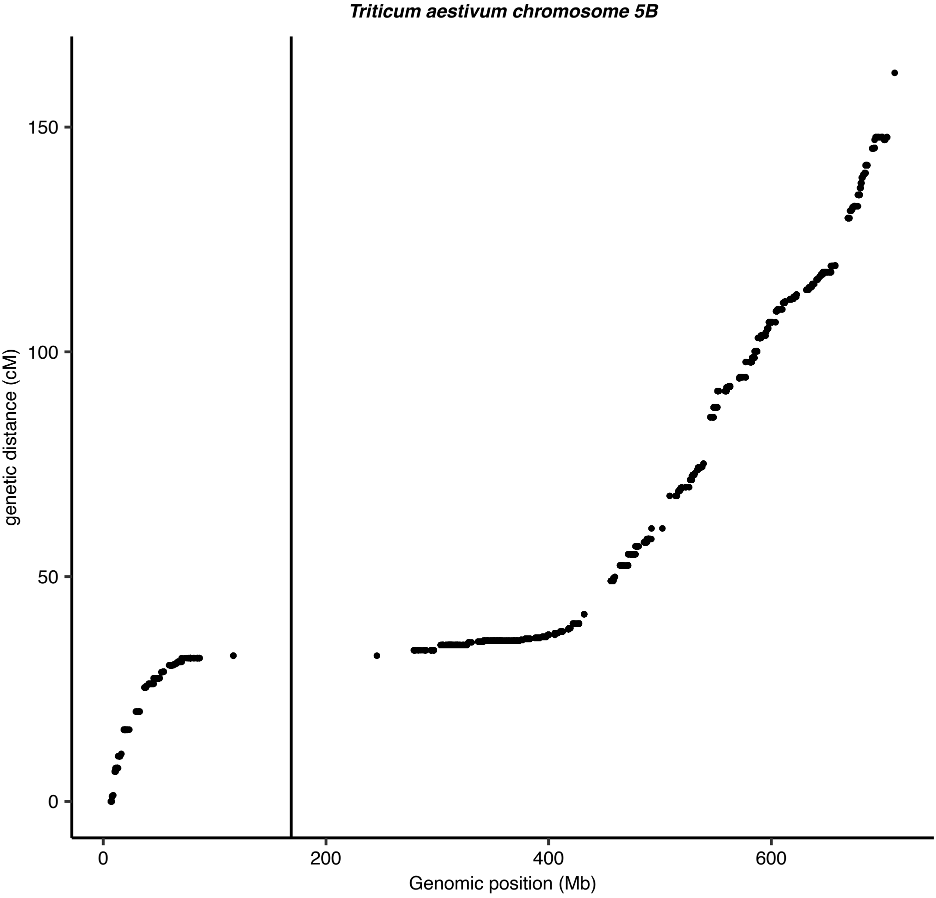
Triticum aestivum chromosome 5B
genetic distance (cM)
150
100
50
0
0
200
400
600
Genomic position (Mb)

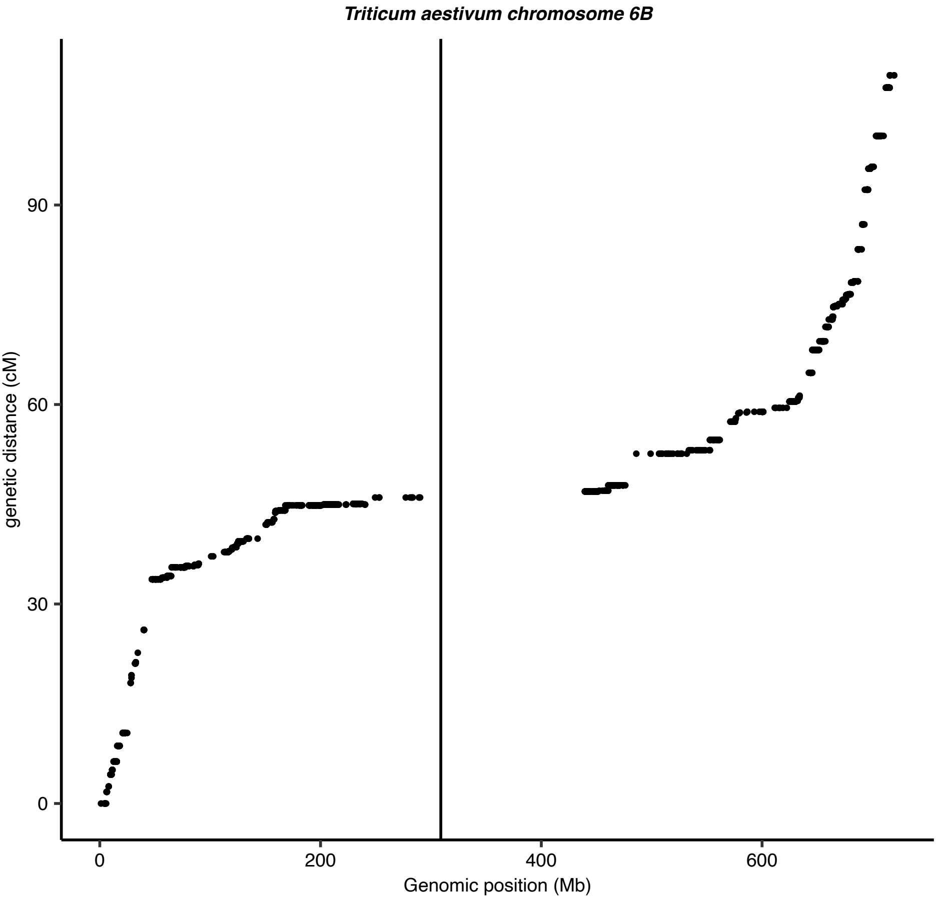
Triticum aestivum chromosome 6B
genetic distance (cM)
90
60
30
0
0
200
400
600
Genomic position (Mb)

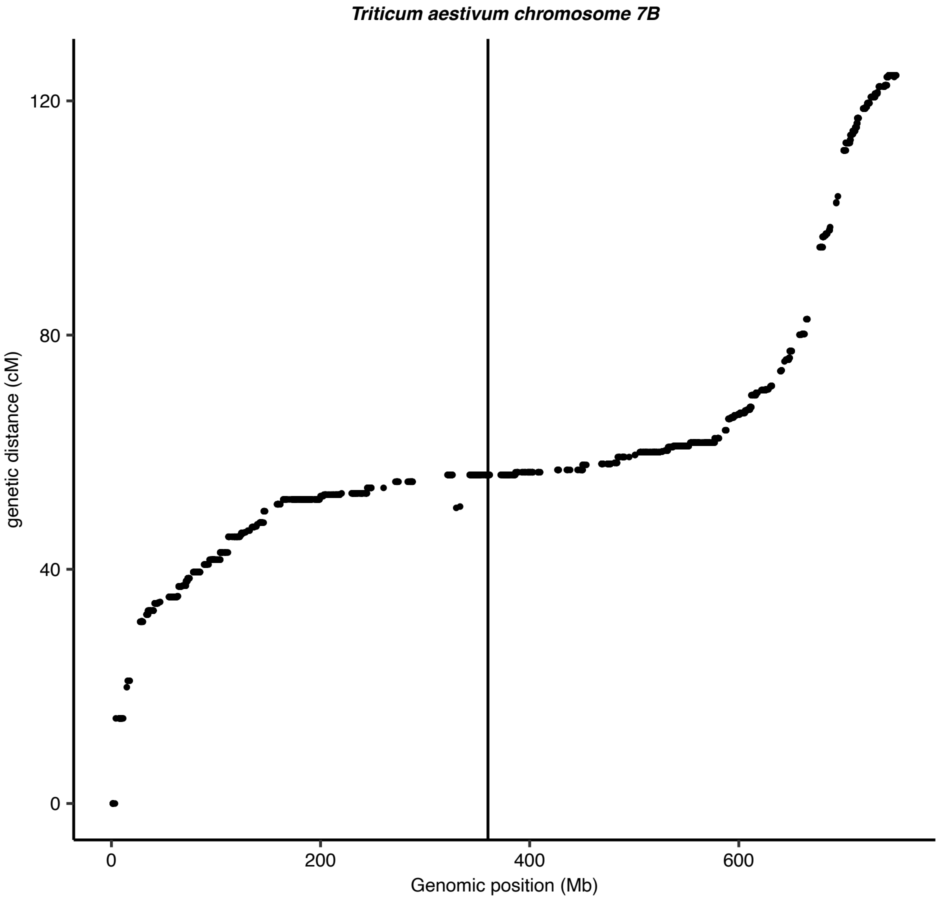
Triticum aestivum chromosome 7B
genetic distance (cM)
0
40
80
120
Genomic position (Mb)
0
200
400
600

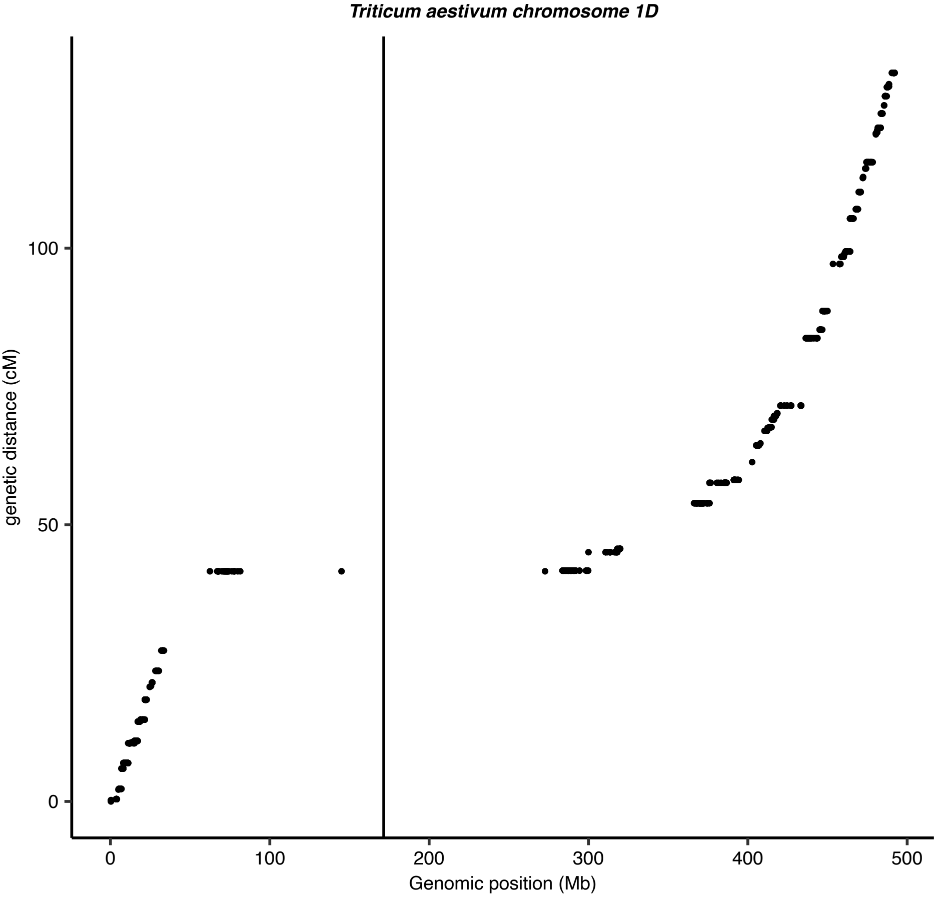
Triticum aestivum chromosome 1D
genetic distance (cM)
0
50
100
Genomic position (Mb)
0
100
200
300
400
500

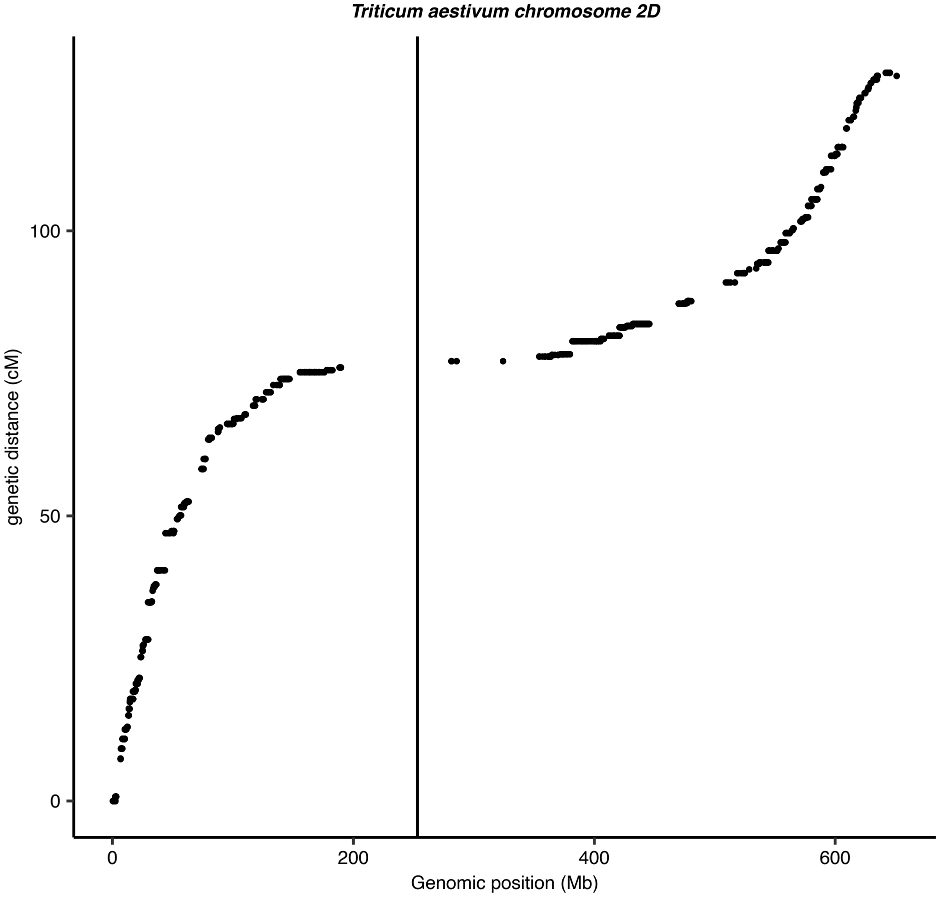
Triticum aestivum chromosome 2D
genetic distance (cM)
100
50
0
0
200
400
600
Genomic position (Mb)

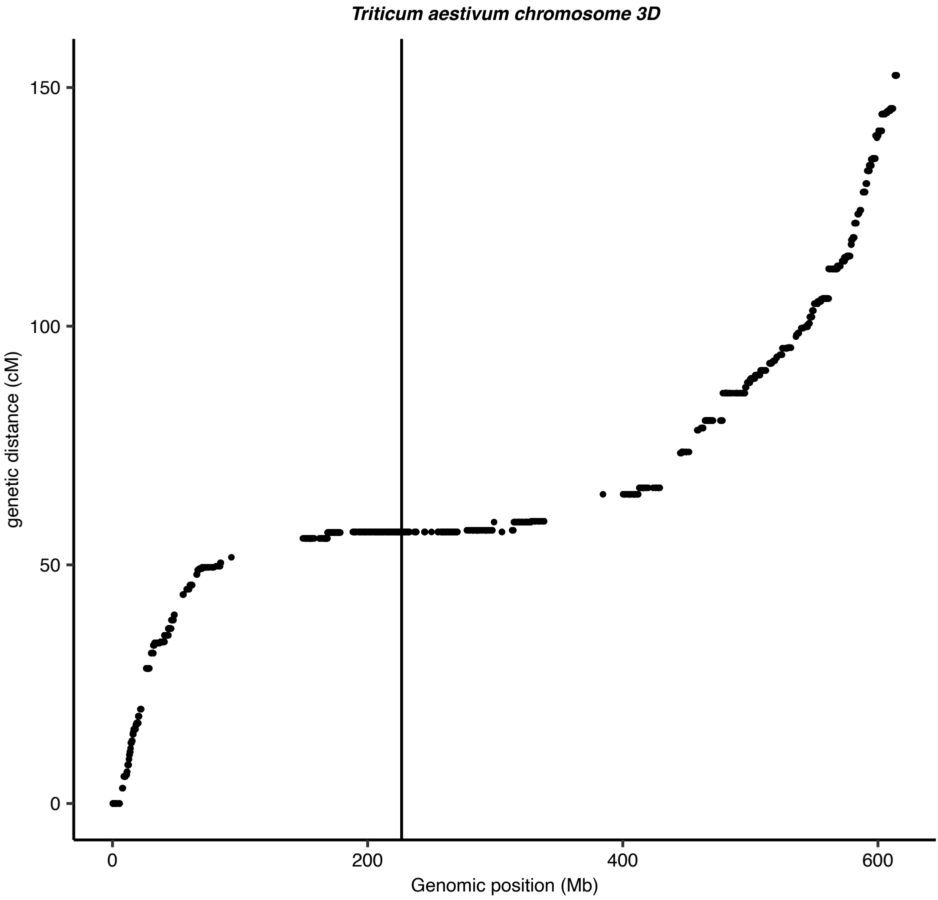
Triticum aestivum chromosome 3D
genetic distance (cM)
150
100
50
0
0
200
400
600
Genomic position (Mb)

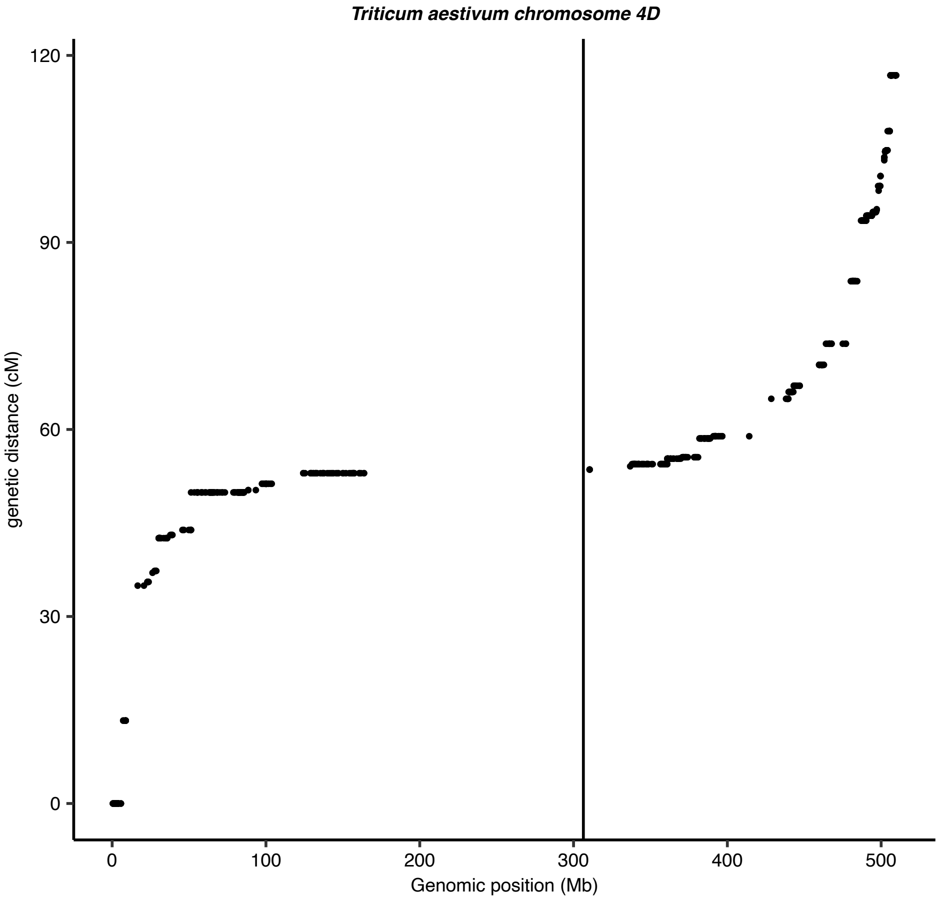
Triticum aestivum chromosome 4D
genetic distance (cM)
120
90
60
30
0
0
100
200
300
400
500
Genomic position (Mb)

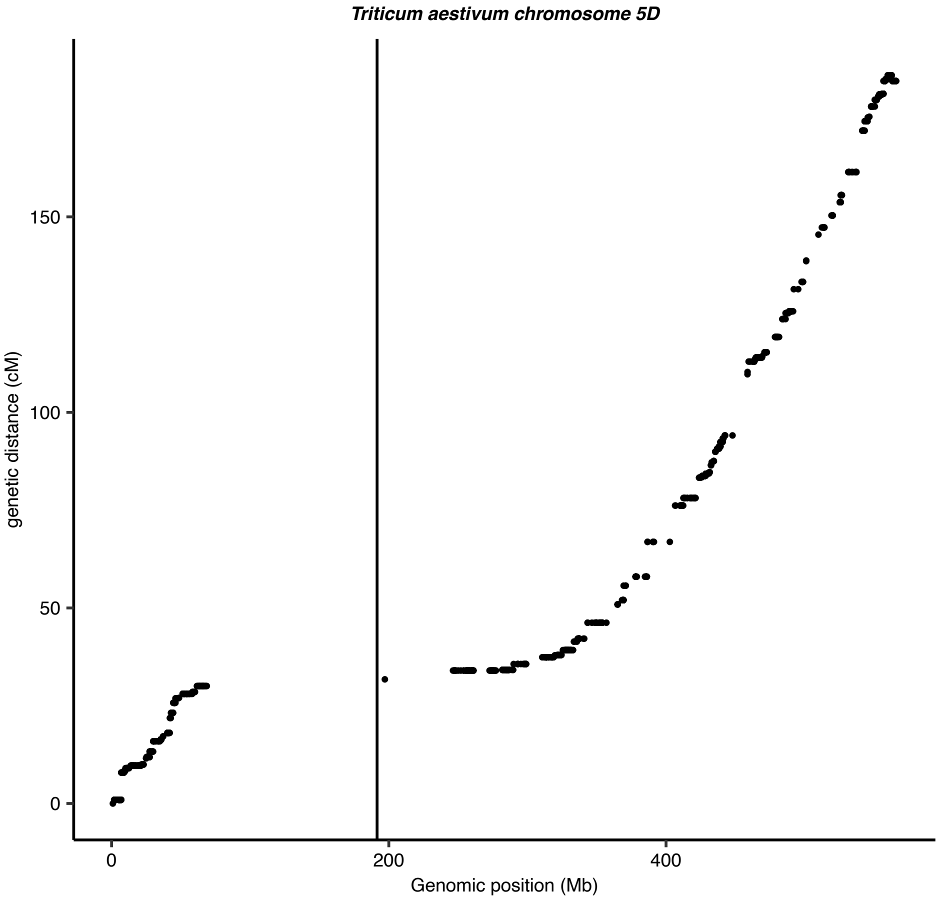
Triticum aestivum chromosome 5D
genetic distance (cM)
0
50
100
150
0
200
400
Genomic position (Mb)

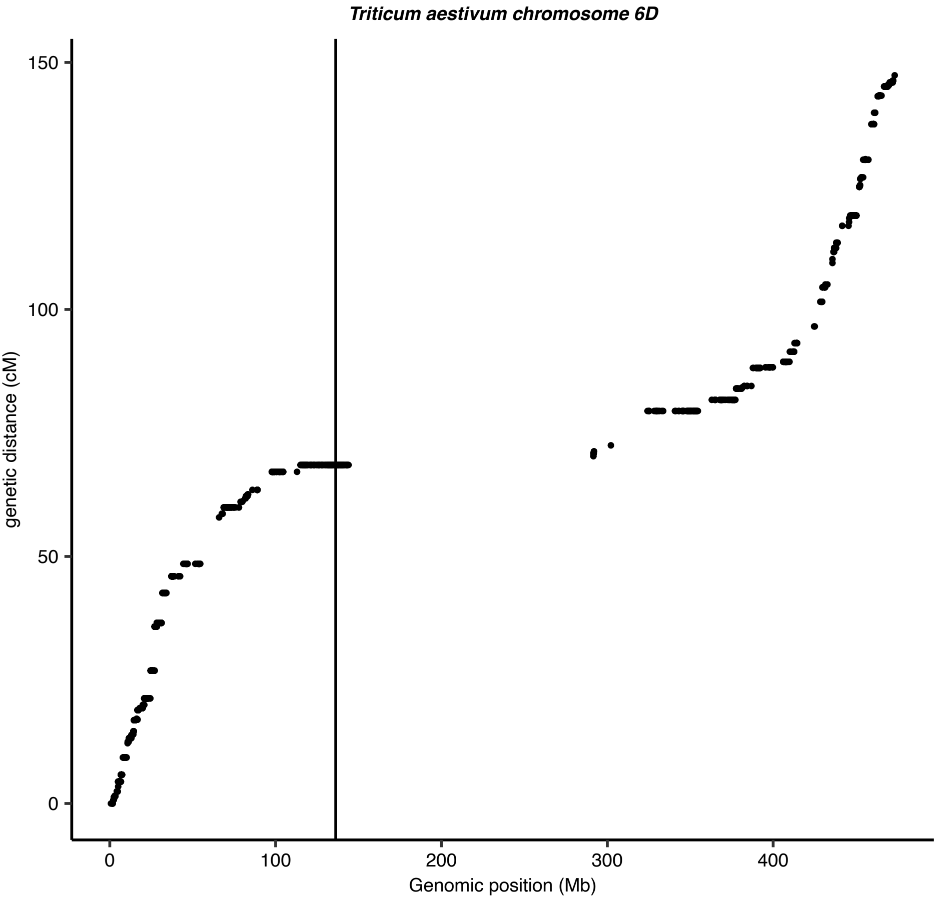
Triticum aestivum chromosome 6D
genetic distance (cM)
150
100
50
0
0
100
200
300
400
Genomic position (Mb)

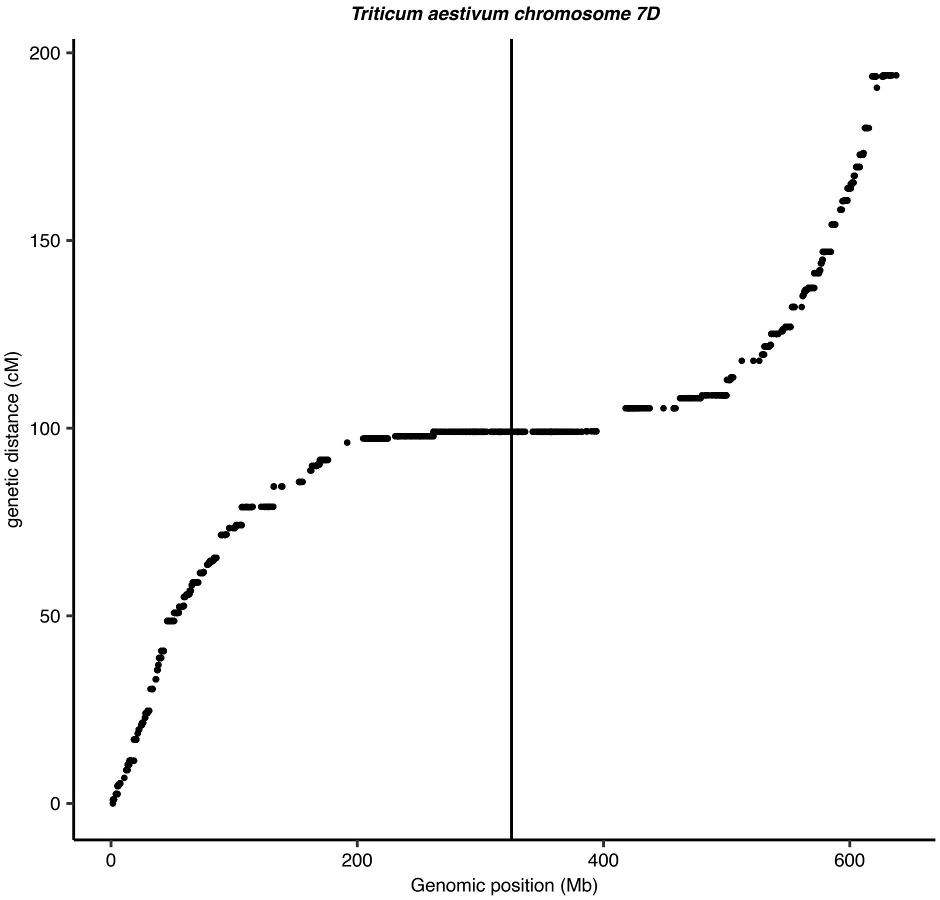
Triticum aestivum chromosome 7D
genetic distance (cM)
Genomic position (Mb)
0
50
100
150
200
0
200
400
600

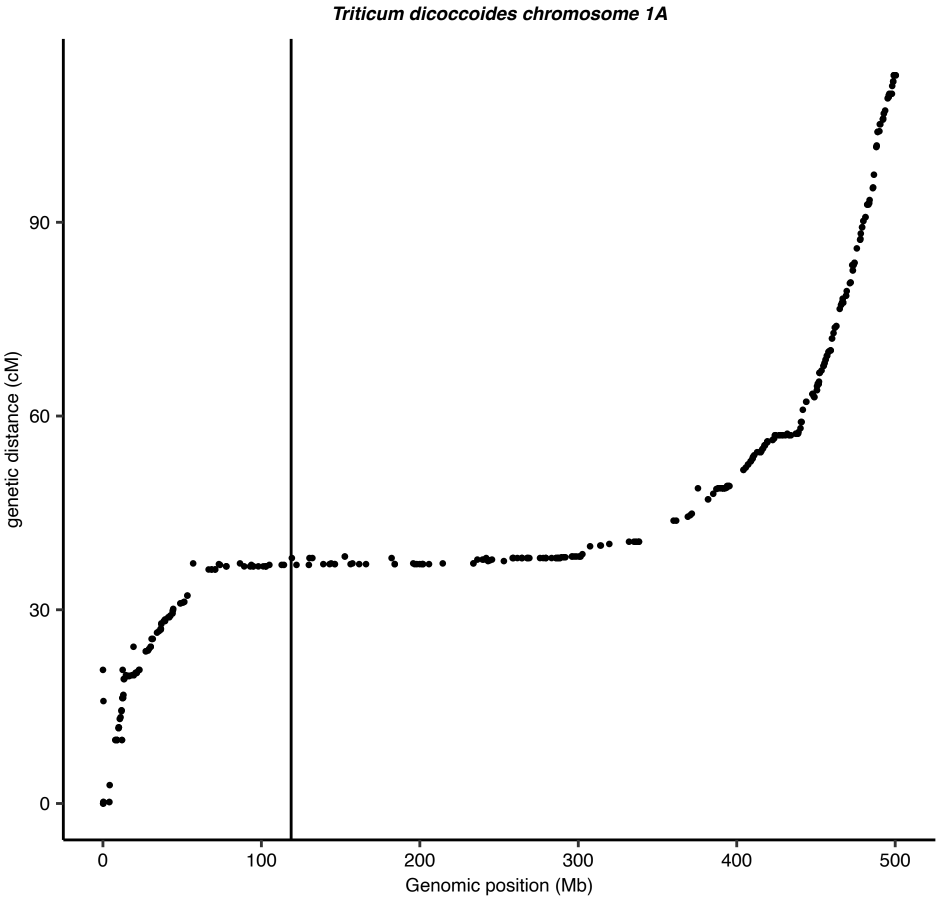
Triticum dicoccoides chromosome 1A
genetic distance (cM)
0
30
60
90
Genomic position (Mb)
0
100
200
300
400
500

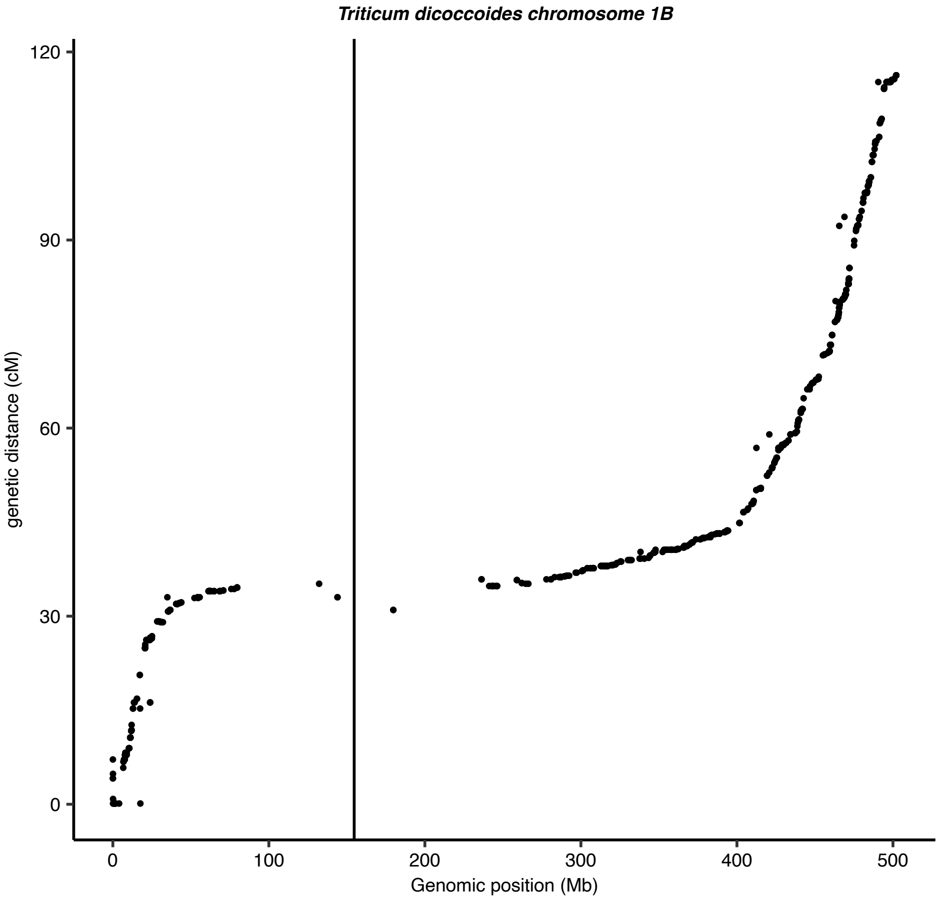
Triticum dicoccoides chromosome 1B
genetic distance (cM)
Genomic position (Mb)
0
30
60
90
120
0
100
200
300
400
500

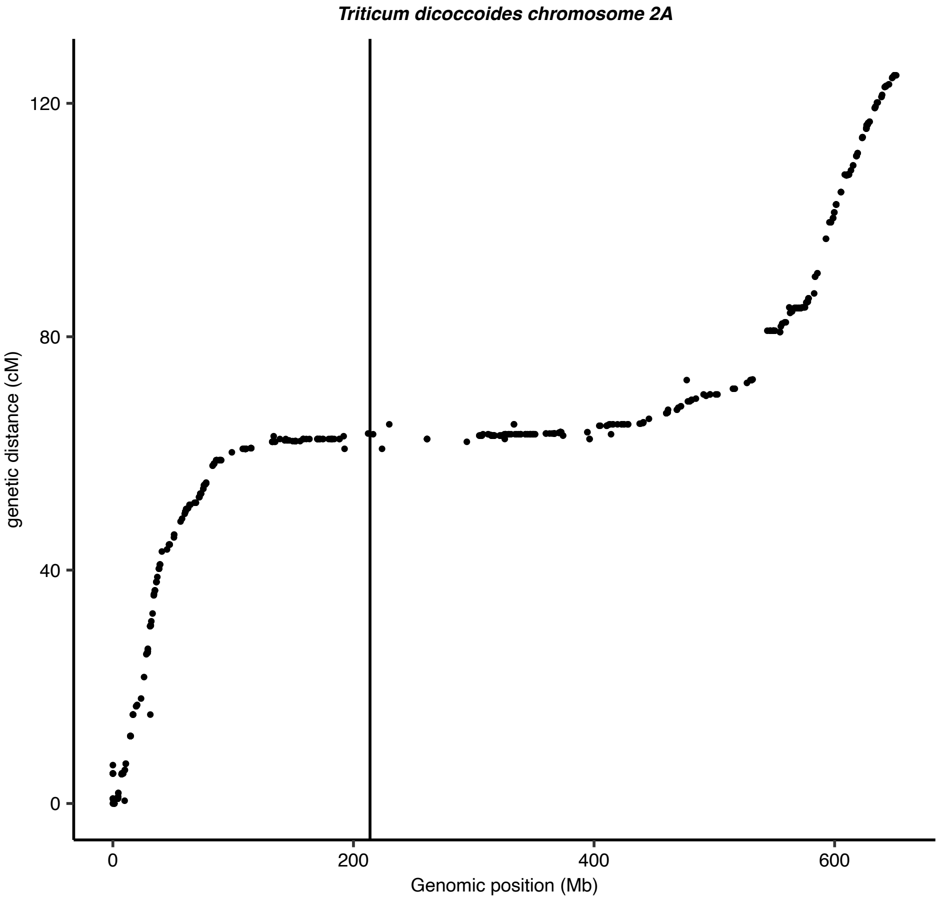
Triticum dicoccoides chromosome 2A
genetic distance (cM)
0
40
80
120
0
200
400
600
Genomic position (Mb)

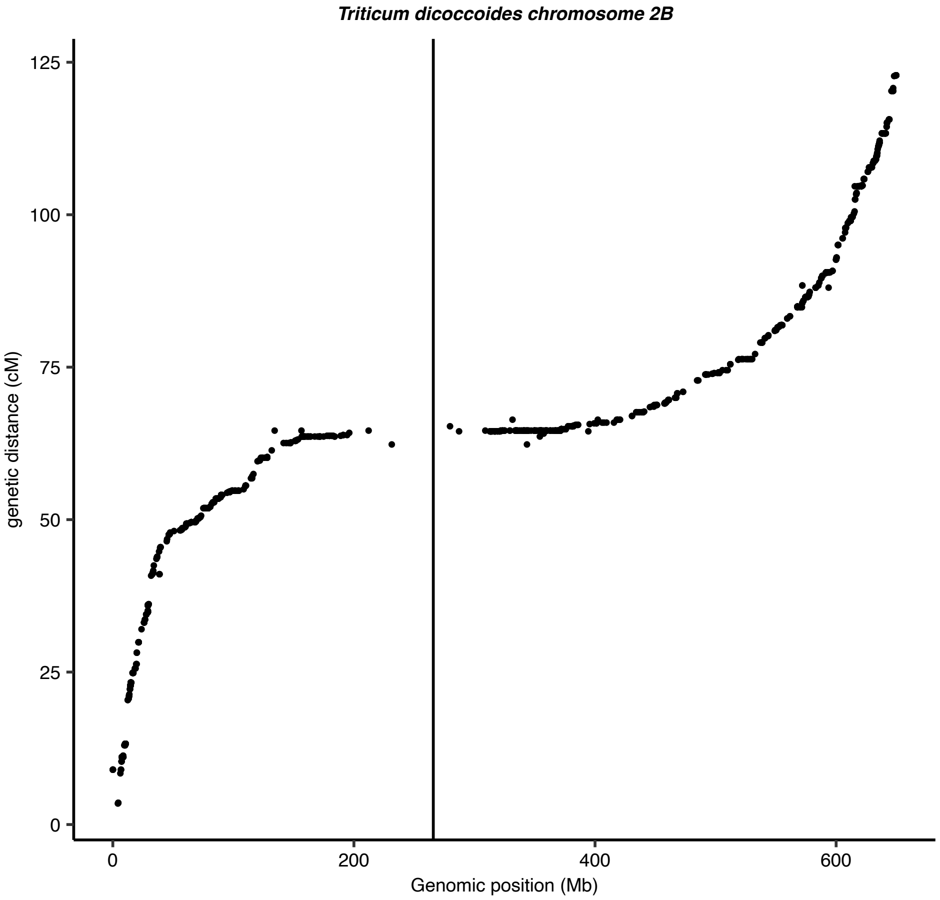
Triticum dicoccoides chromosome 2B
genetic distance (cM)
Genomic position (Mb)
0
25
50
75
100
125
0
200
400
600

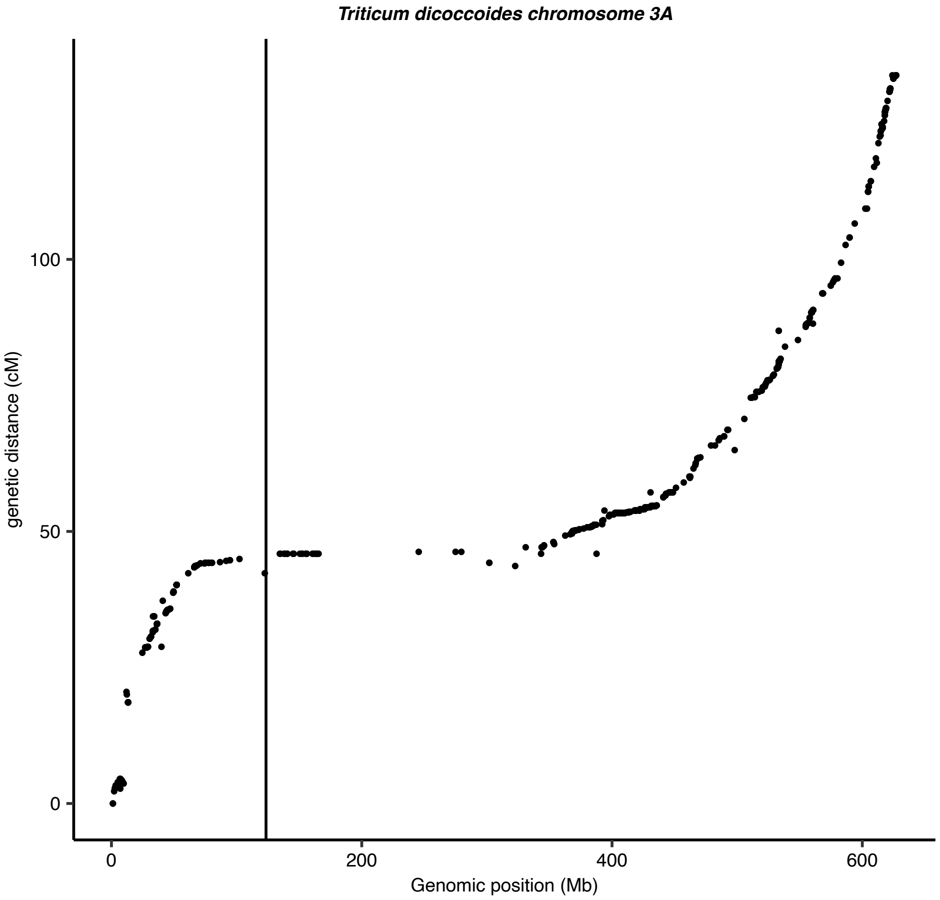
Triticum dicoccoides chromosome 3A
genetic distance (cM)
100
50
0
0
200
400
600
Genomic position (Mb)

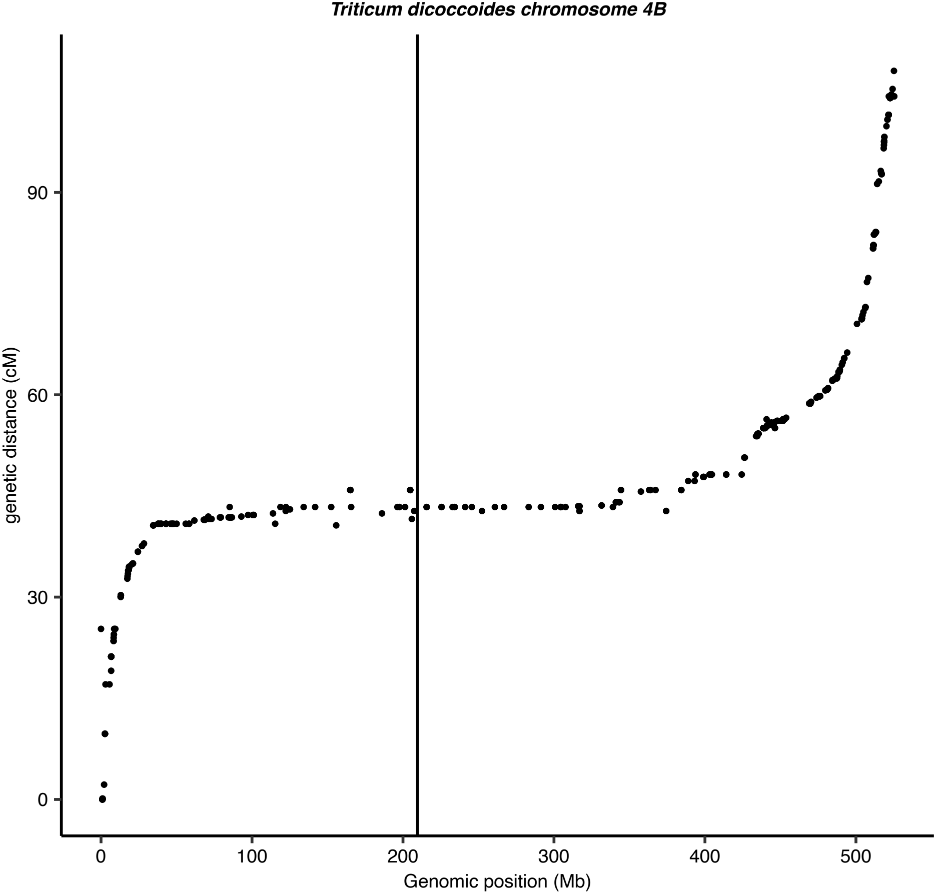
Triticum dicoccoides chromosome 4B
genetic distance (cM)
Genomic position (Mb)
0
30
60
90
0
100
200
300
400
500

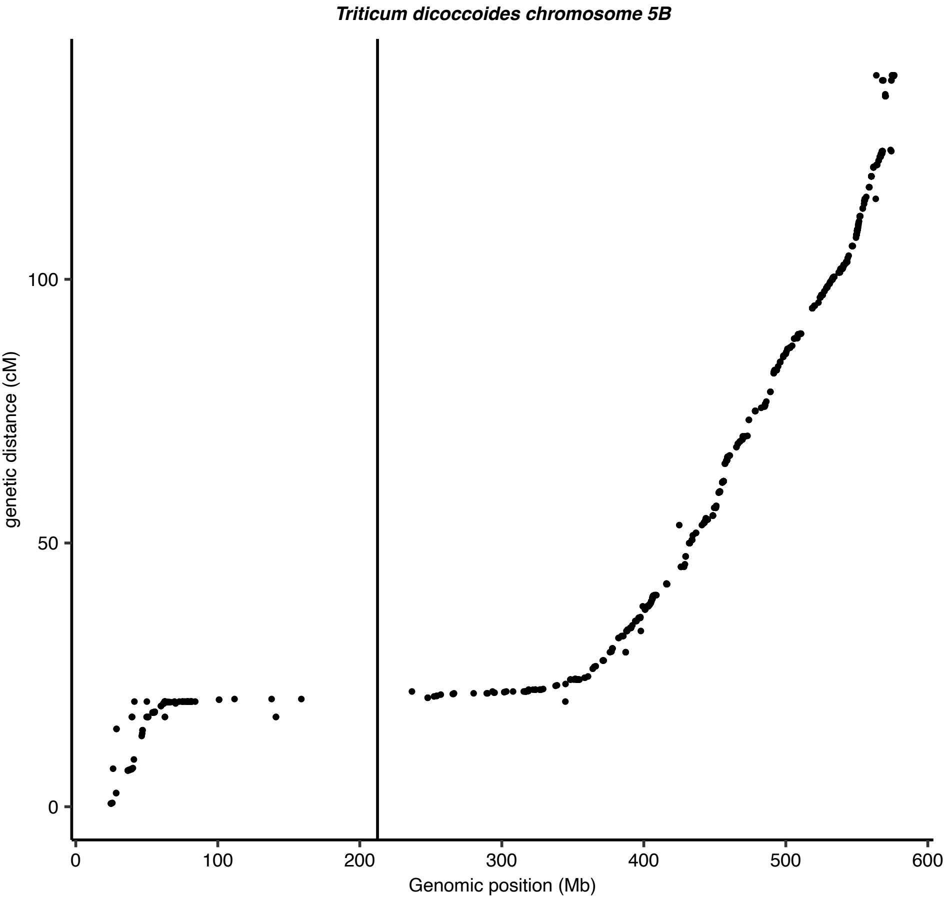
Triticum dicoccoides chromosome 5B
genetic distance (cM)
0
50
100
Genomic position (Mb)
0
100
200
300
400
500
600

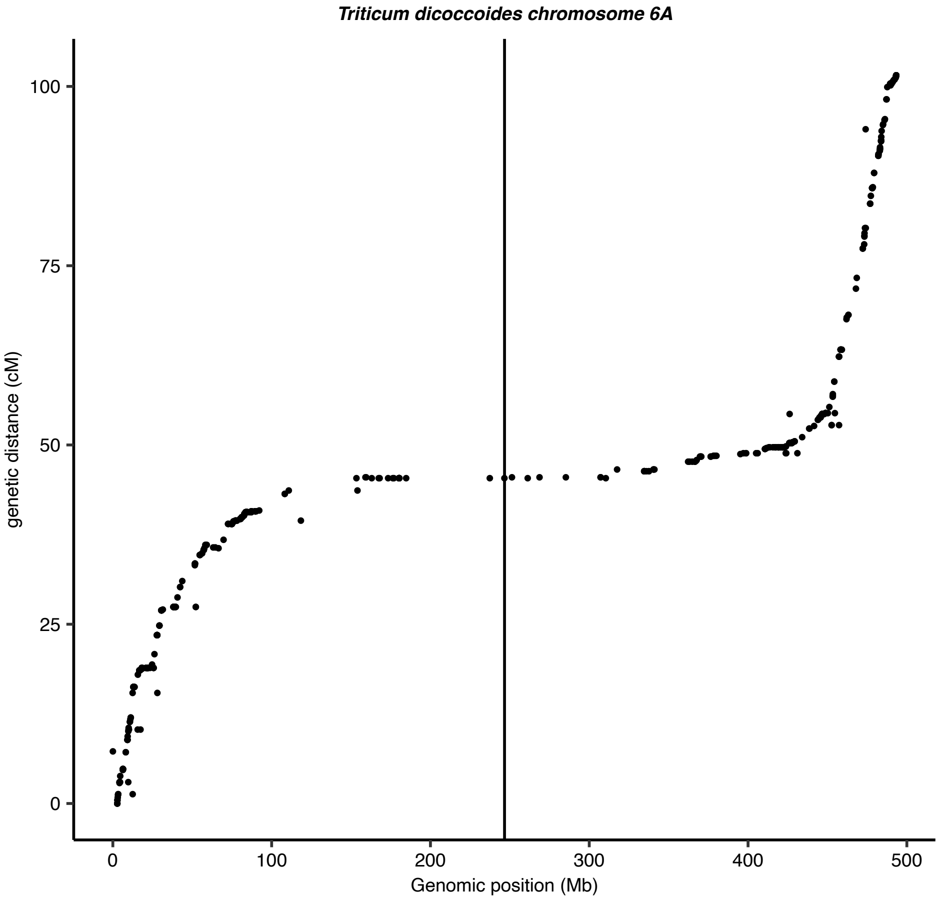
Triticum dicoccoides chromosome 6A
genetic distance (cM)
0
25
50
75
100
Genomic position (Mb)
0
100
200
300
400
500

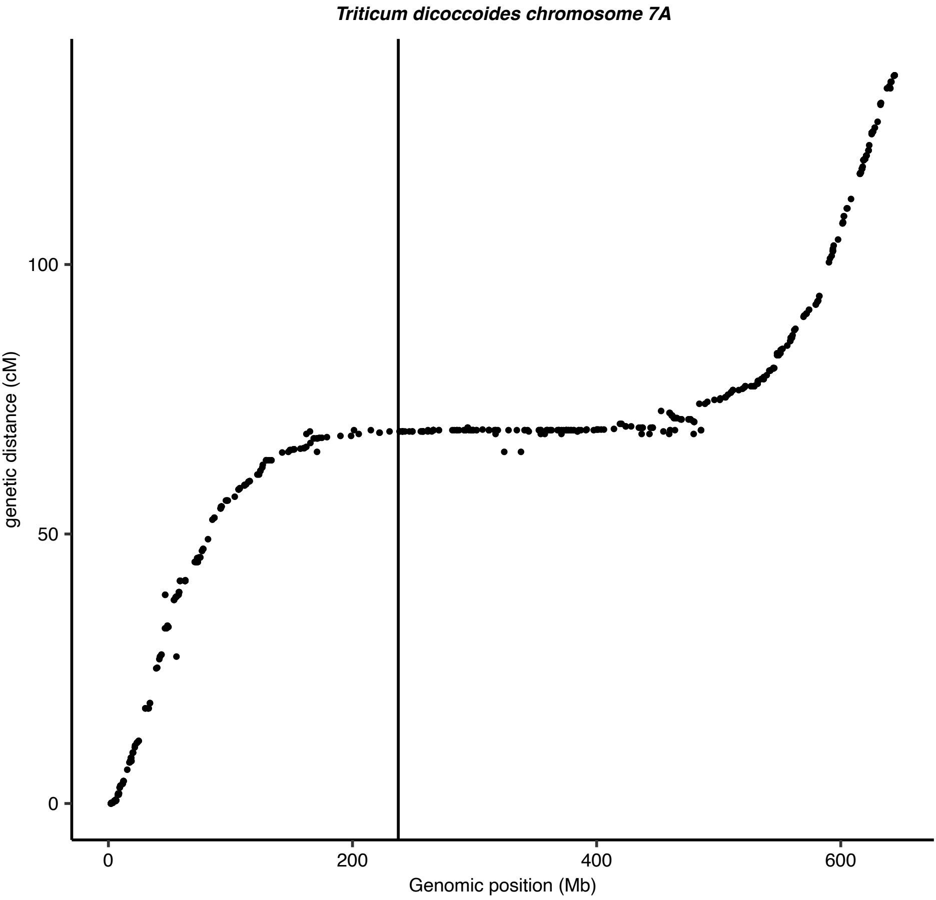
Triticum dicoccoides chromosome 7A
genetic distance (cM)
0
50
100
Genomic position (Mb)
0
200
400
600

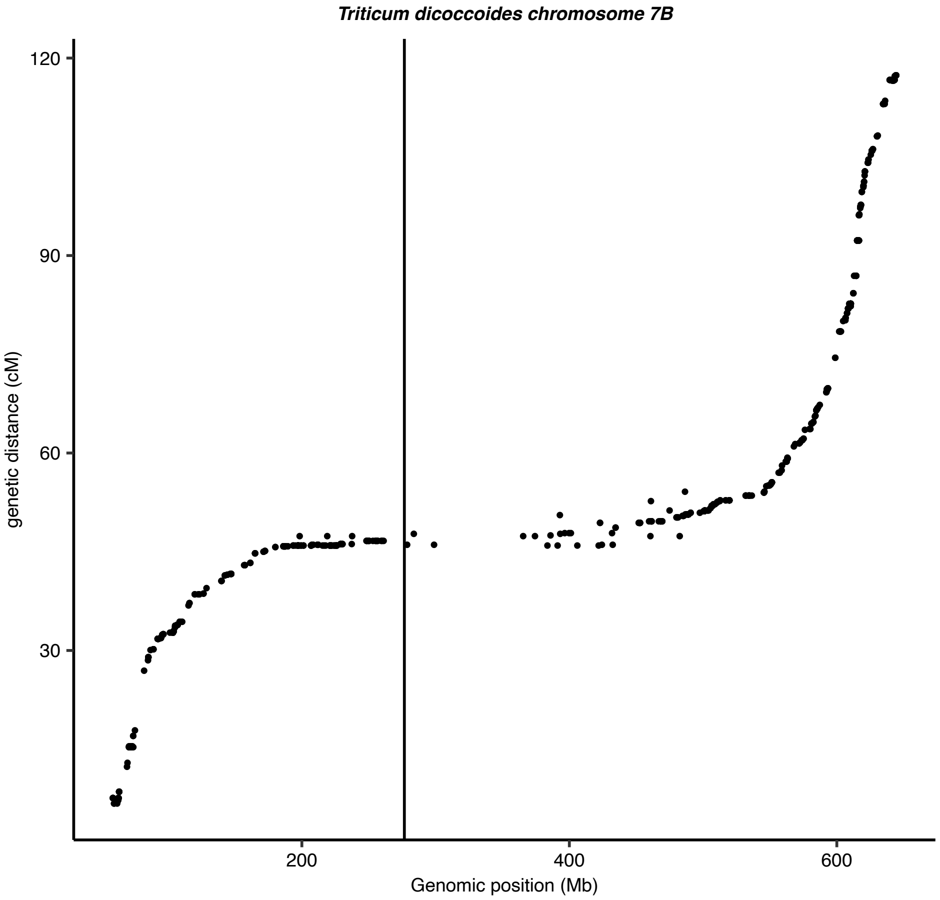
Triticum dicoccoides chromosome 7B
120
90
60
30
genetic distance (cM)
200
400
600
Genomic position (Mb)

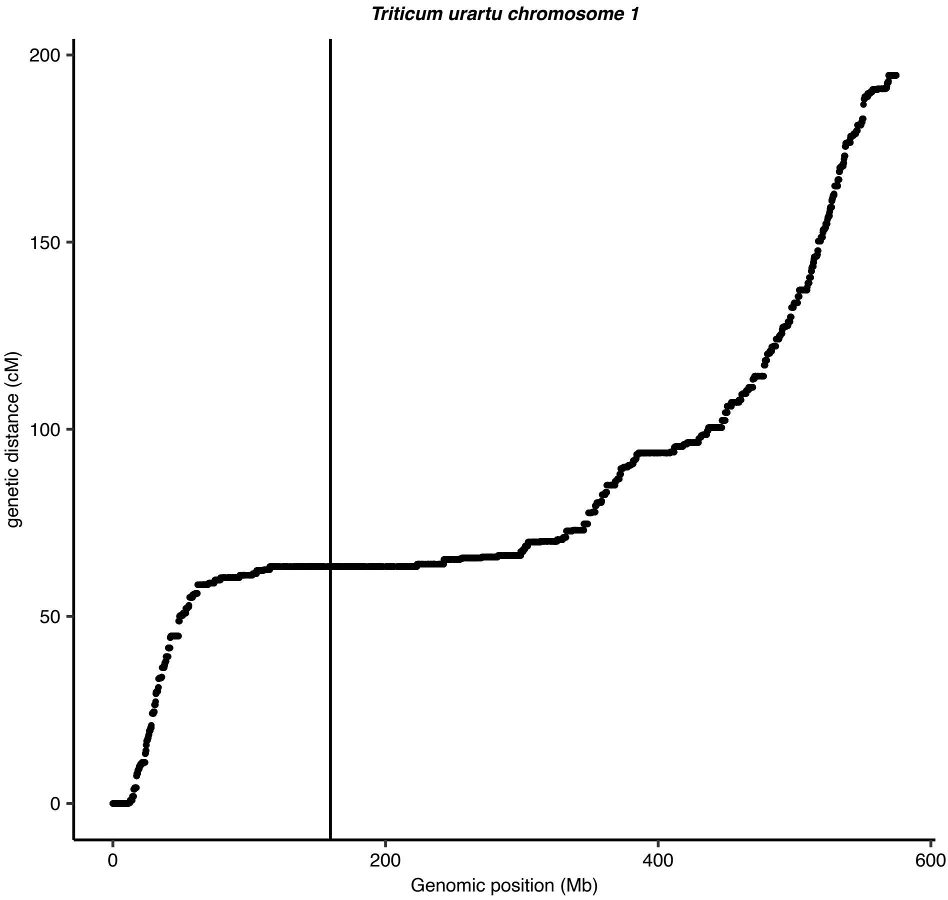
Triticum urartu chromosome 1
genetic distance (cM)
200
150
100
50
0
0
200
400
600
Genomic position (Mb)

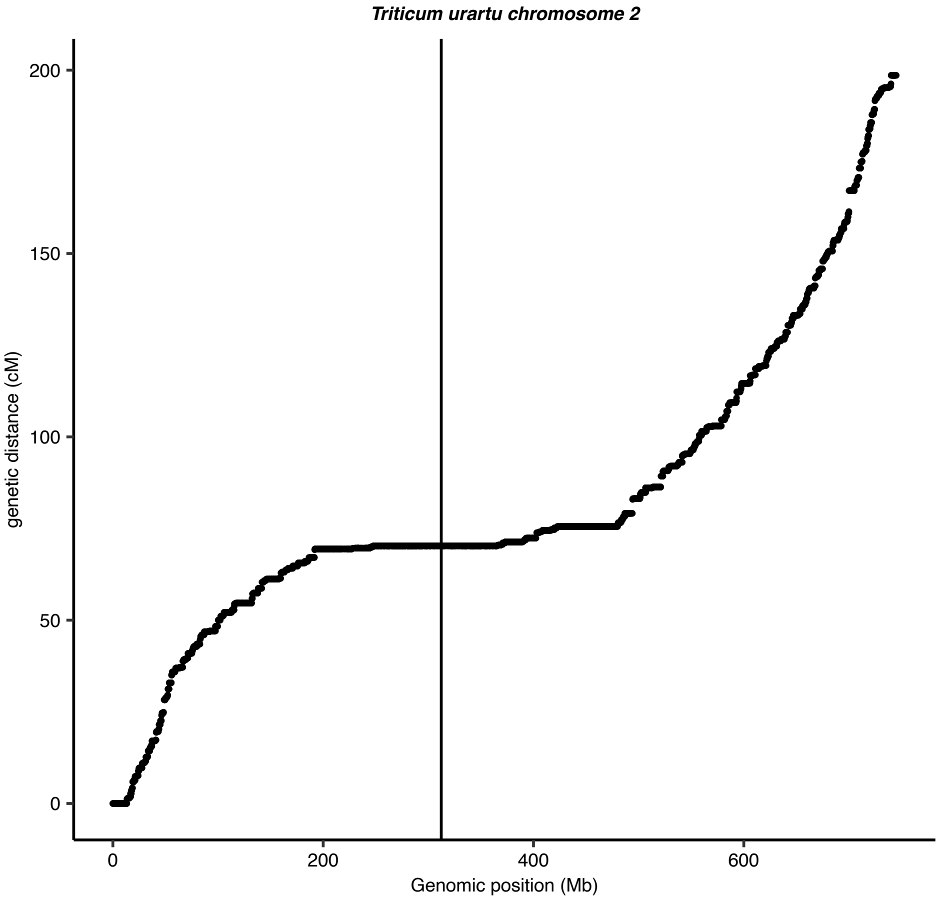
Triticum urartu chromosome 2
genetic distance (cM)
200
150
100
50
0
0
200
400
600
Genomic position (Mb)

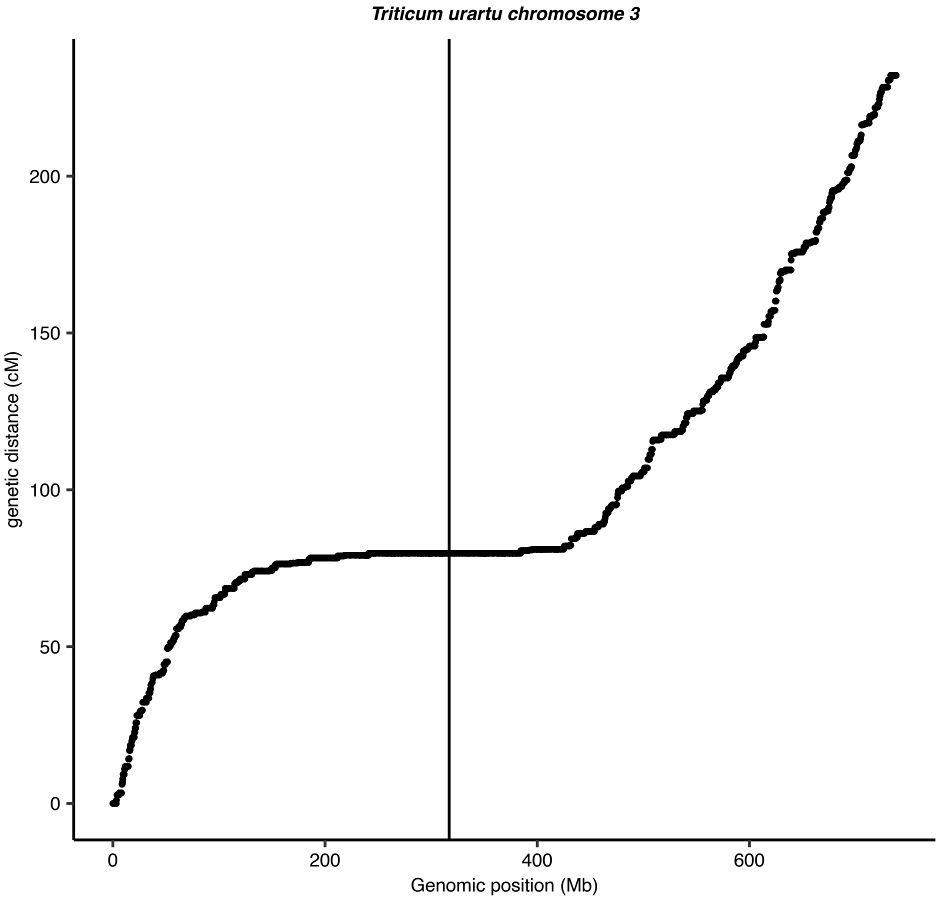
Triticum urartu chromosome 3
genetic distance (cM)
Genomic position (Mb)
0
50
100
150
200
0
200
400
600

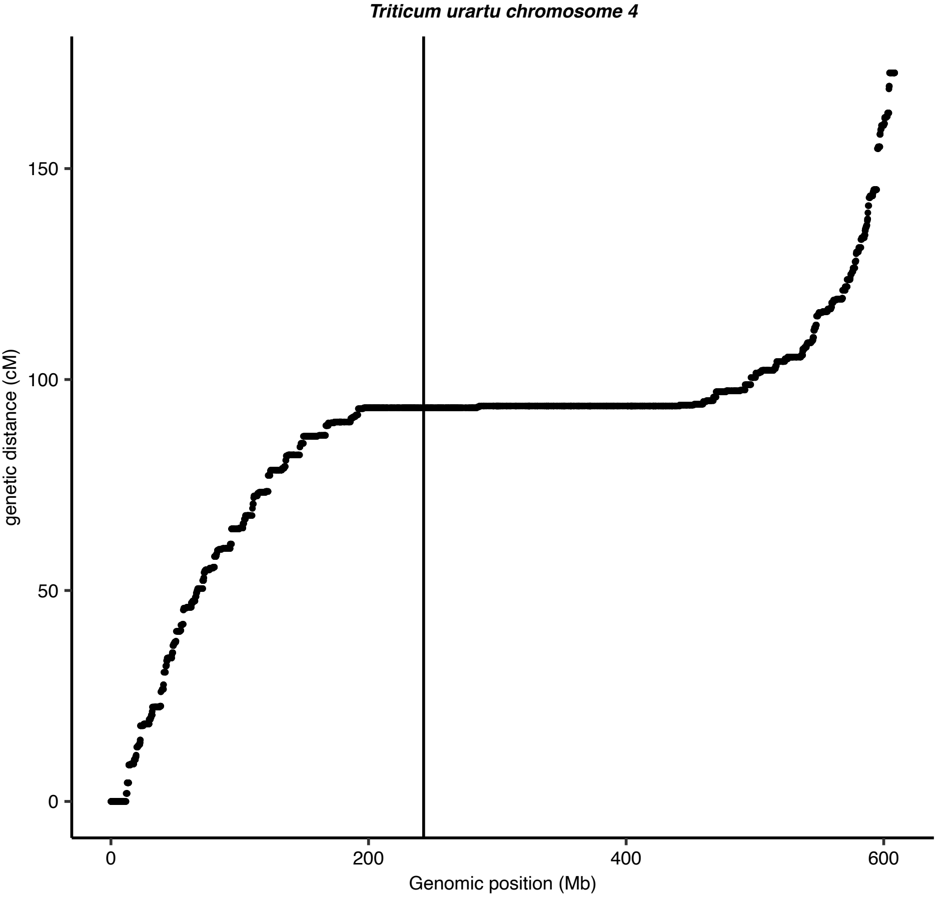
Triticum urartu chromosome 4
genetic distance (cM)
150
100
50
0
0
200
400
600
Genomic position (Mb)

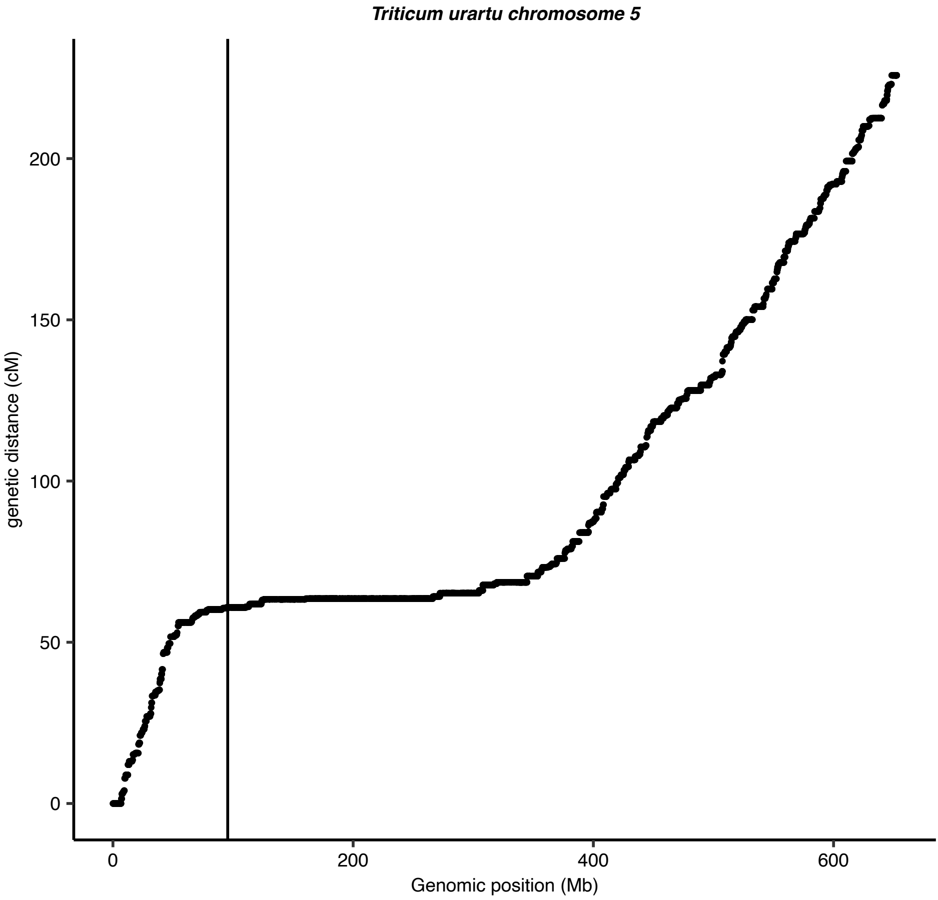
Triticum urartu chromosome 5
genetic distance (cM)
0
50
100
150
200
Genomic position (Mb)
0
200
400
600

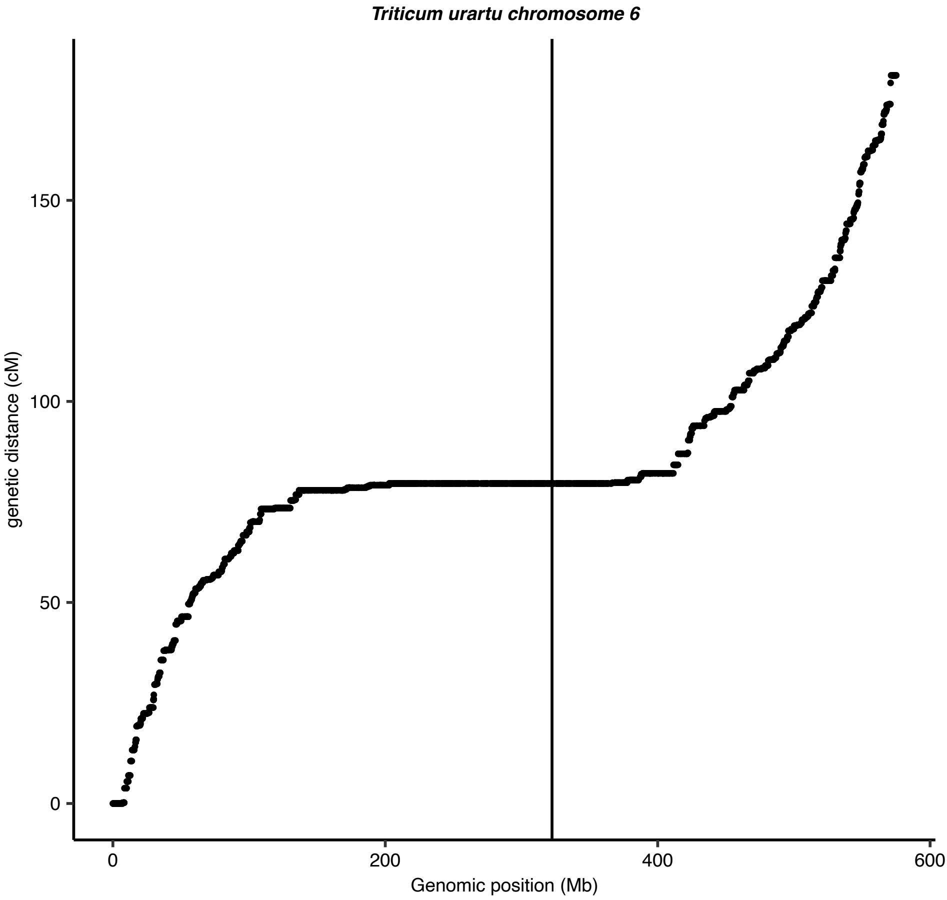

Triticum urartu chromosome 6
genetic distance (cM)
150
100
50
0
0
200
400
600
Genomic position (Mb)

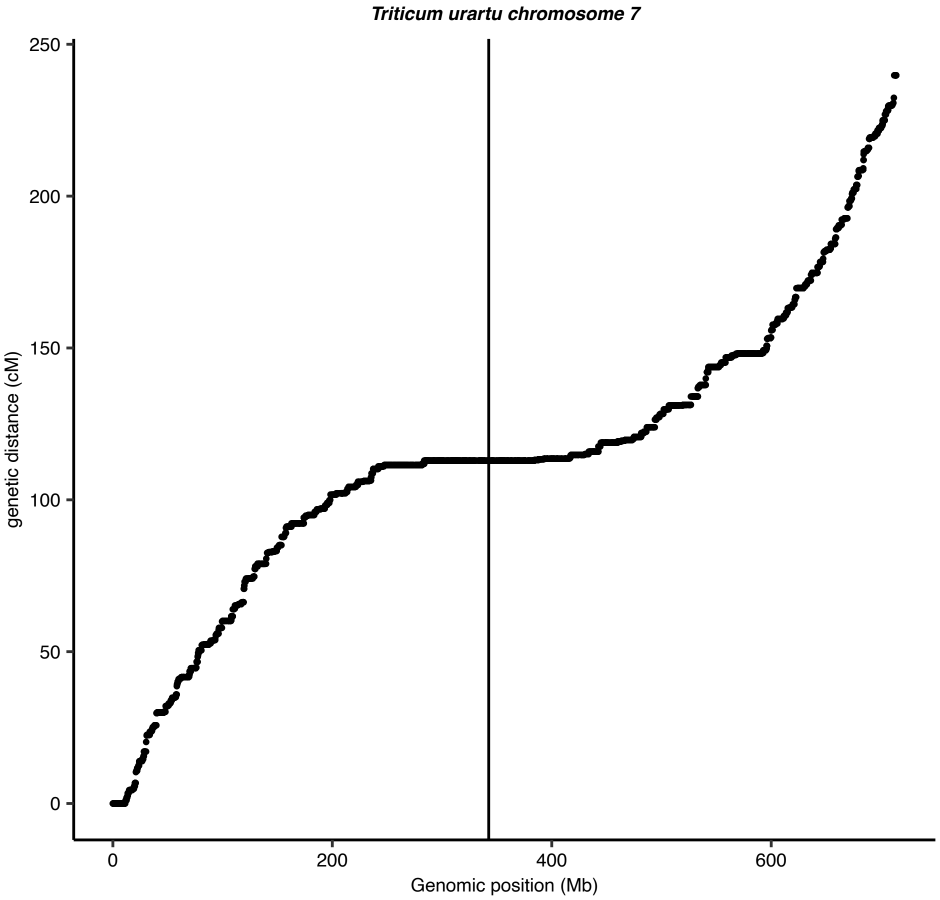
Triticum urartu chromosome 7
genetic distance (cM)
Genomic position (Mb)
0
50
100
150
200
250
0
200
400
600

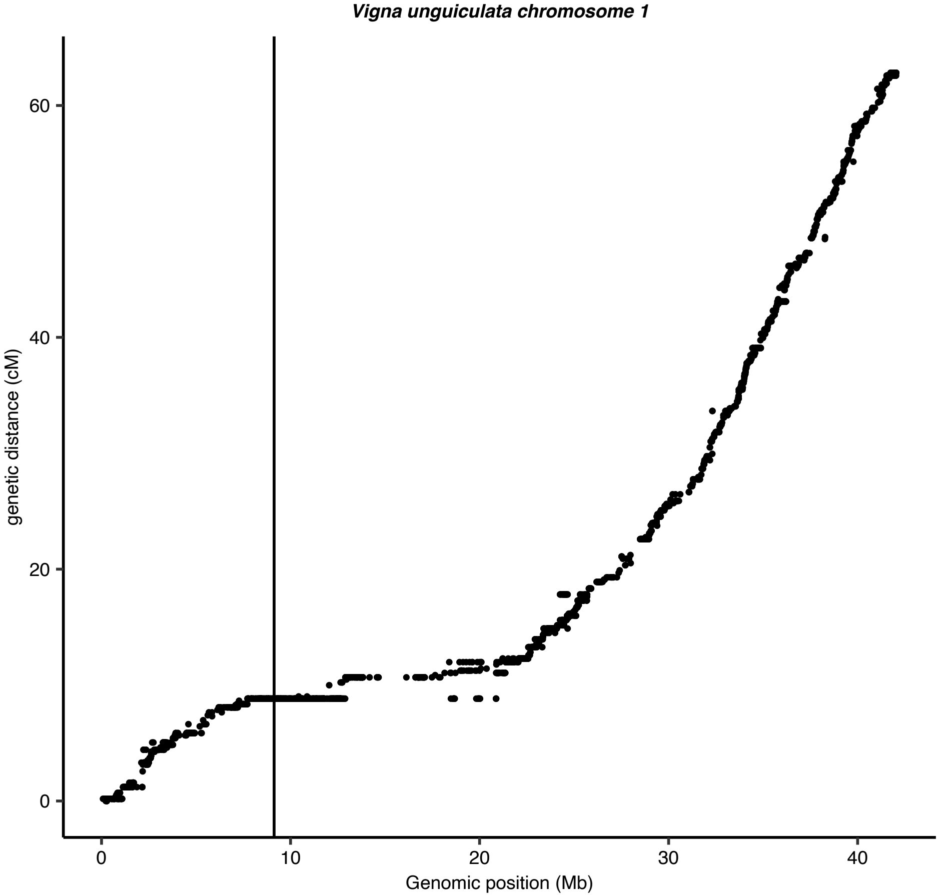
Vigna unguiculata chromosome 1
genetic distance (cM)
Genomic position (Mb)
0
20
40
60
0
10
20
30
40

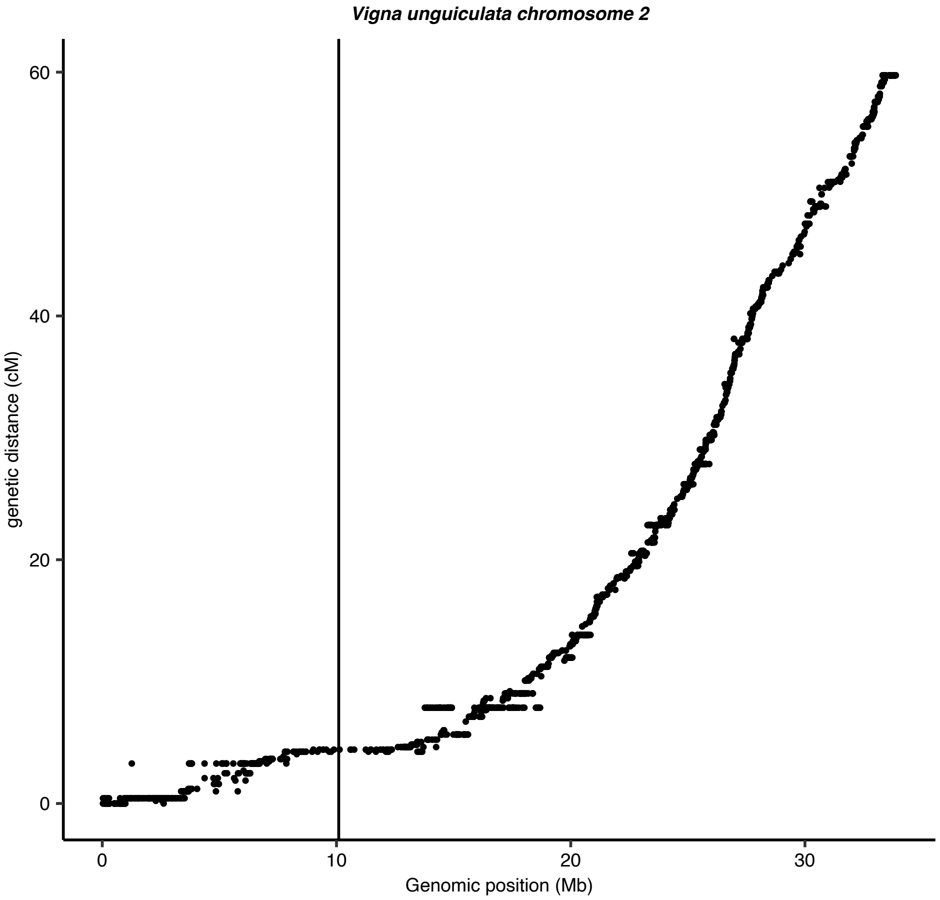
Vigna unguiculata chromosome 2
genetic distance (cM)
60
40
20
0
0
10
20
30
Genomic position (Mb)

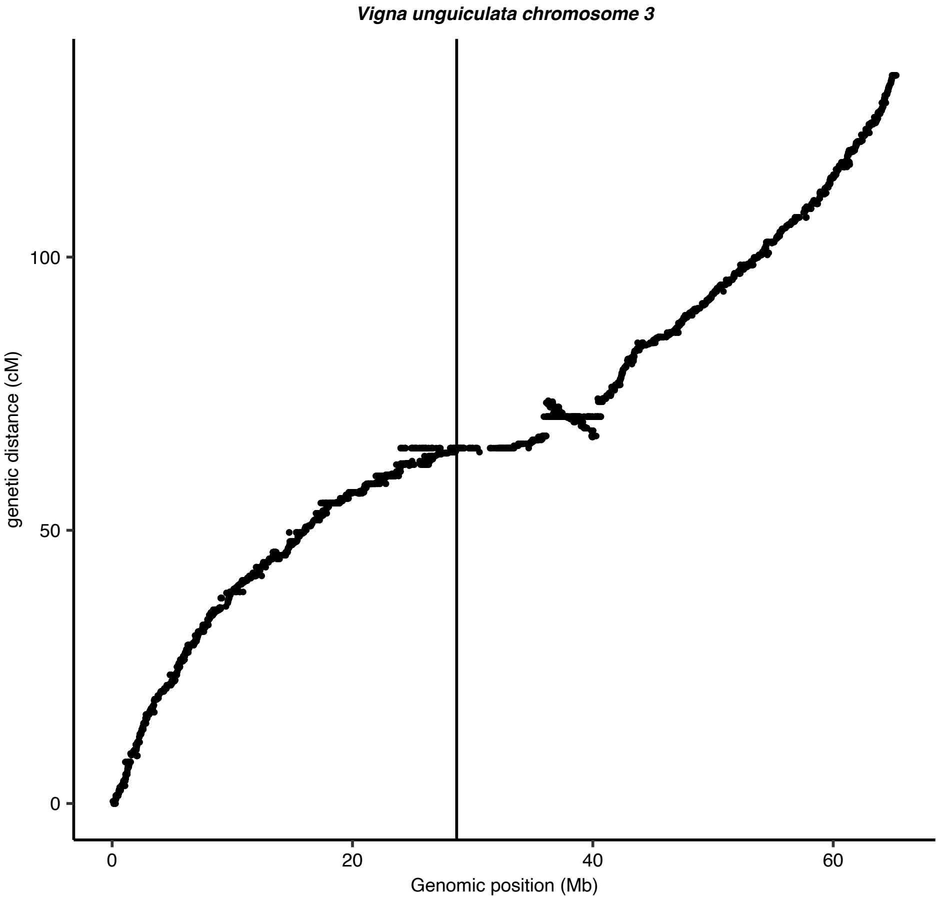
Vigna unguiculata chromosome 3
genetic distance (cM)
0
50
100
Genomic position (Mb)
0
20
40
60

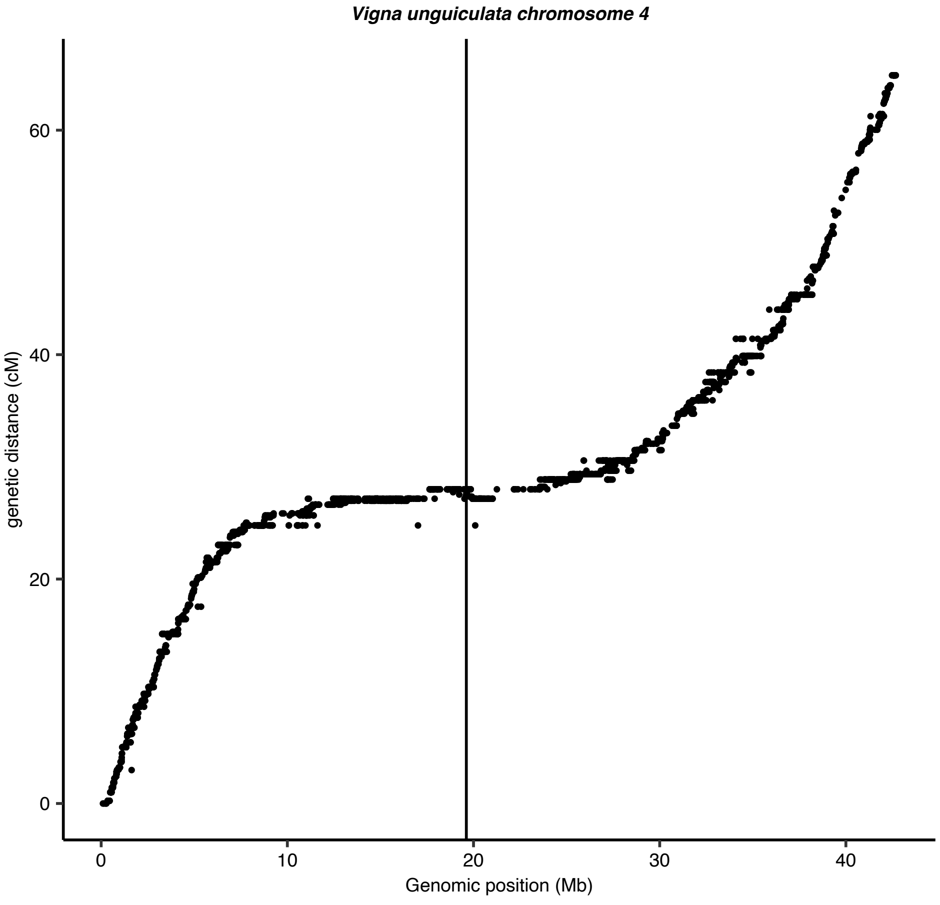
Vigna unguiculata chromosome 4
genetic distance (cM)
0
20
40
60
Genomic position (Mb)
0
10
20
30
40

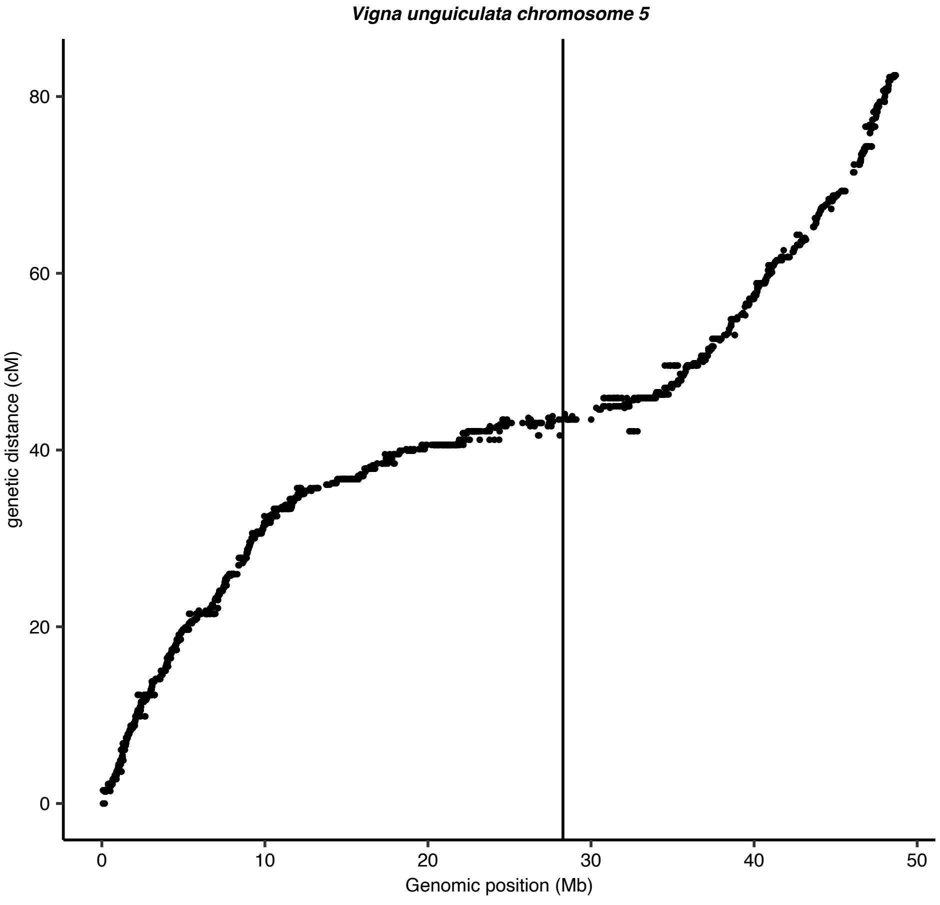
Vigna unguiculata chromosome 5
genetic distance (cM)
0
20
40
60
80
Genomic position (Mb)
0
10
20
30
40
50

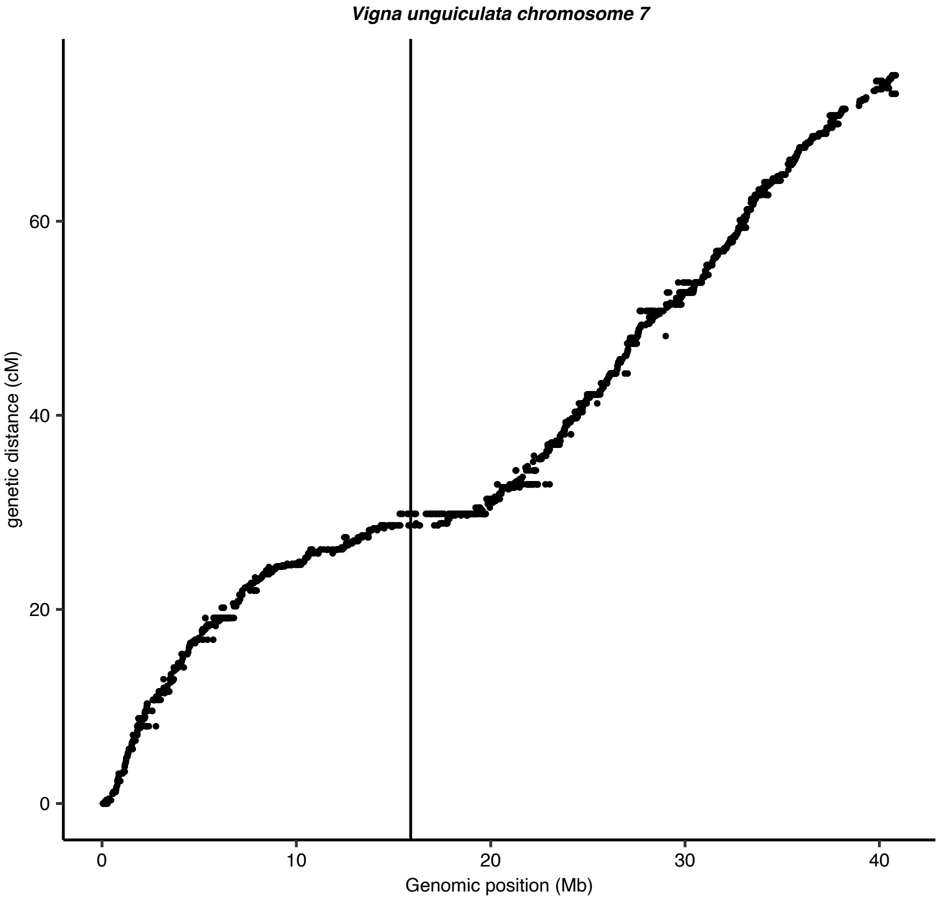
Vigna unguiculata chromosome 7
genetic distance (cM)
0
20
40
60
Genomic position (Mb)
0
10
20
30
40

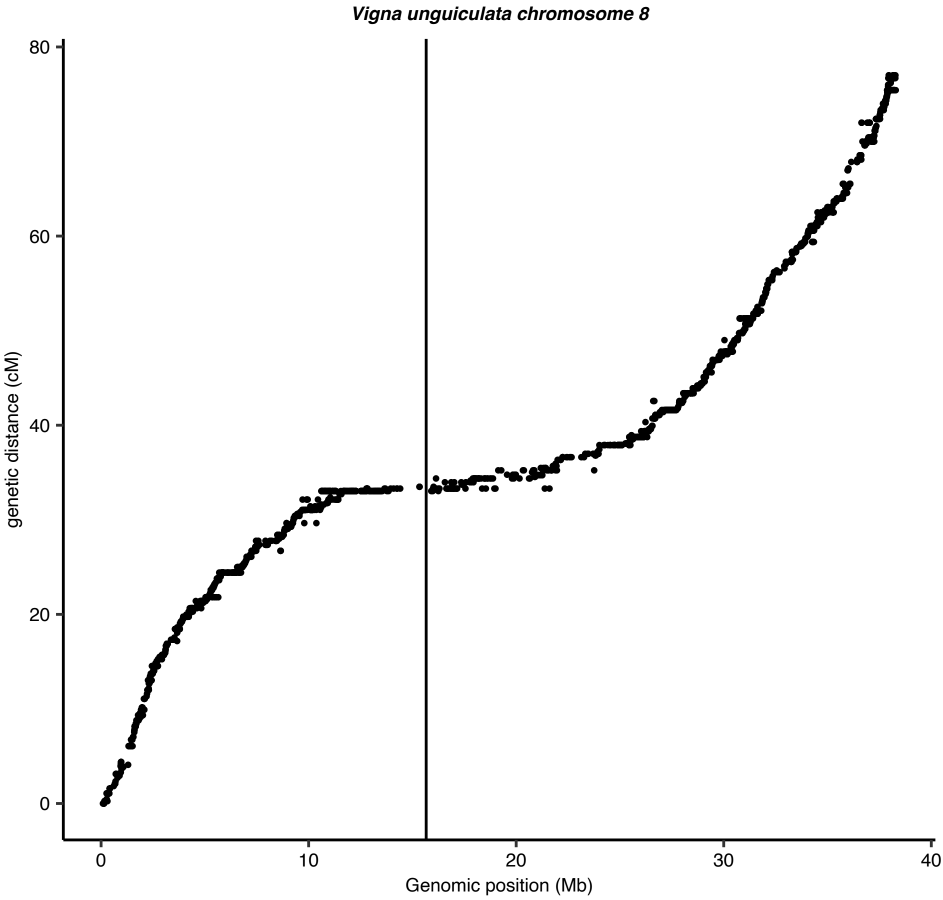
Vigna unguiculata chromosome 8
genetic distance (cM)
Genomic position (Mb)
0
20
40
60
80
0
10
20
30
40

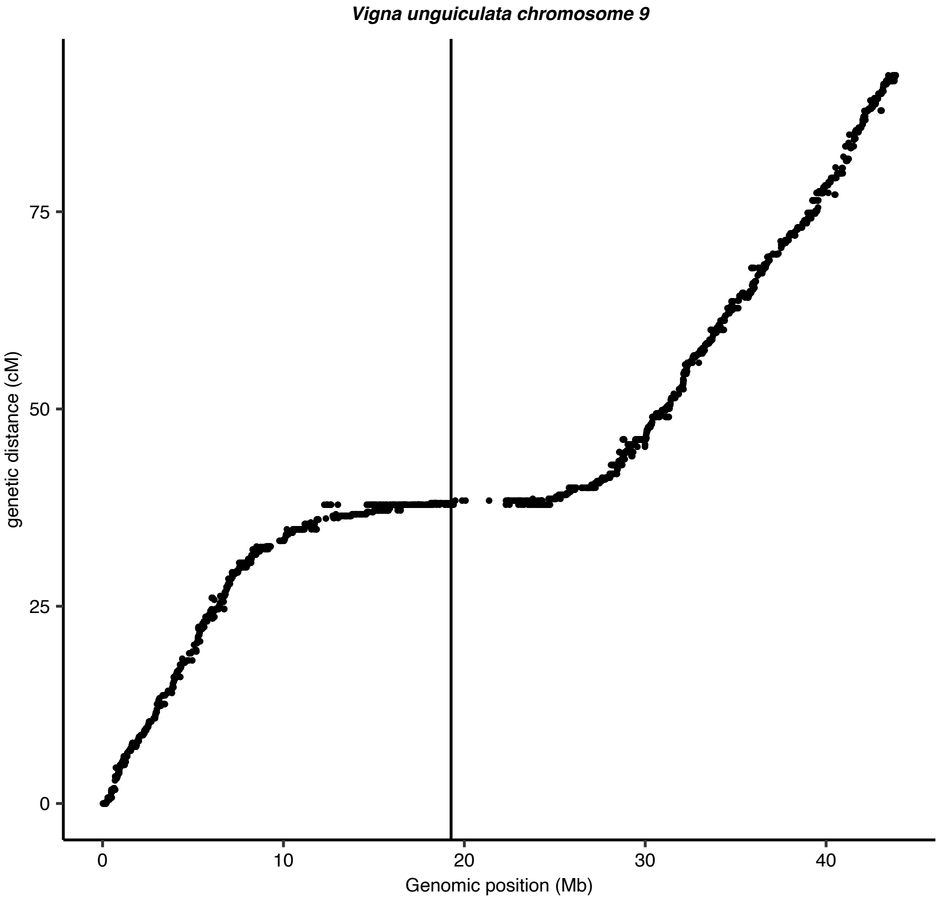
Vigna unguiculata chromosome 9
genetic distance (cM)
0
25
50
75
Genomic position (Mb)
0
10
20
30
40

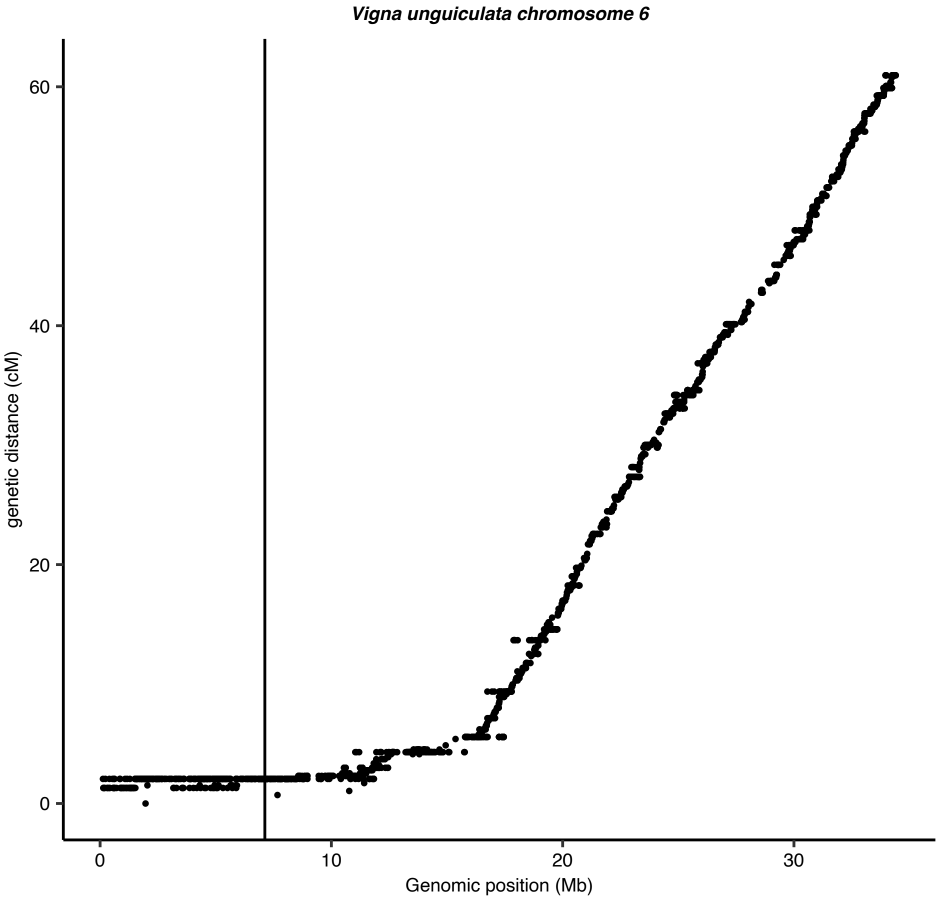
Vigna unguiculata chromosome 6
genetic distance (cM)
60
40
20
0
0
10
20
30
Genomic position (Mb)

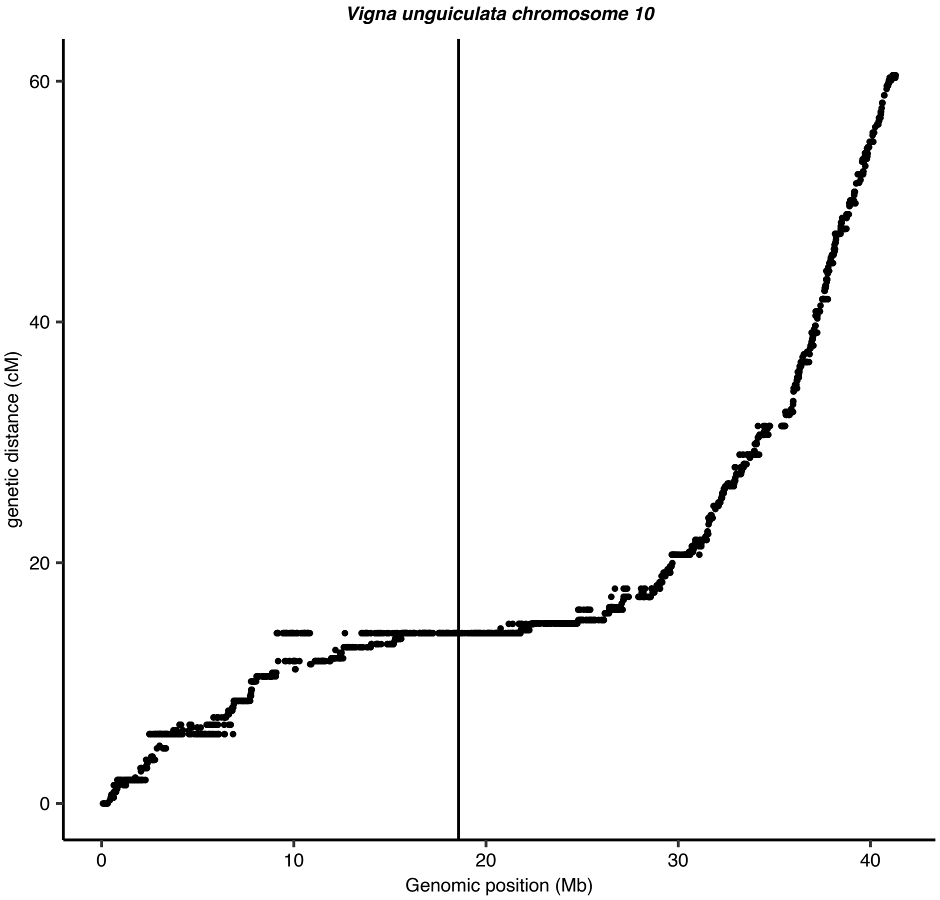
Vigna unguiculata chromosome 10
genetic distance (cM)
Genomic position (Mb)
0
20
40
60
0
10
20
30
40

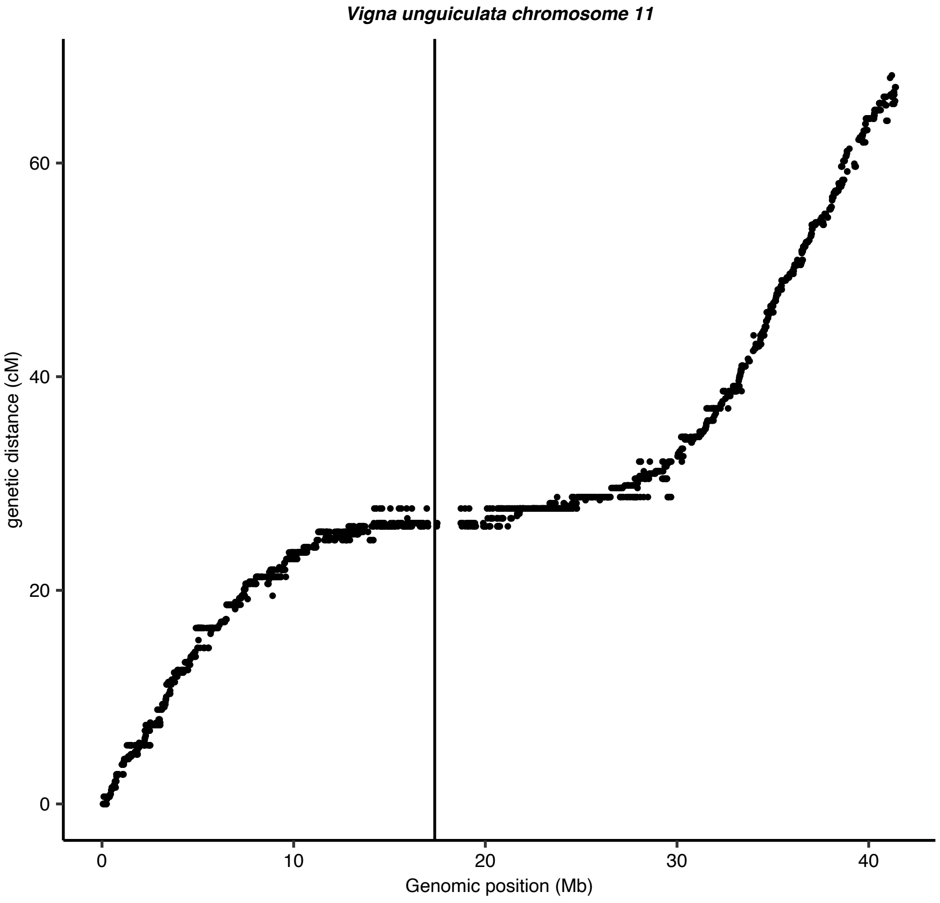
Vigna unguiculata chromosome 11
genetic distance (cM)
0
20
40
60
Genomic position (Mb)
0
10
20
30
40

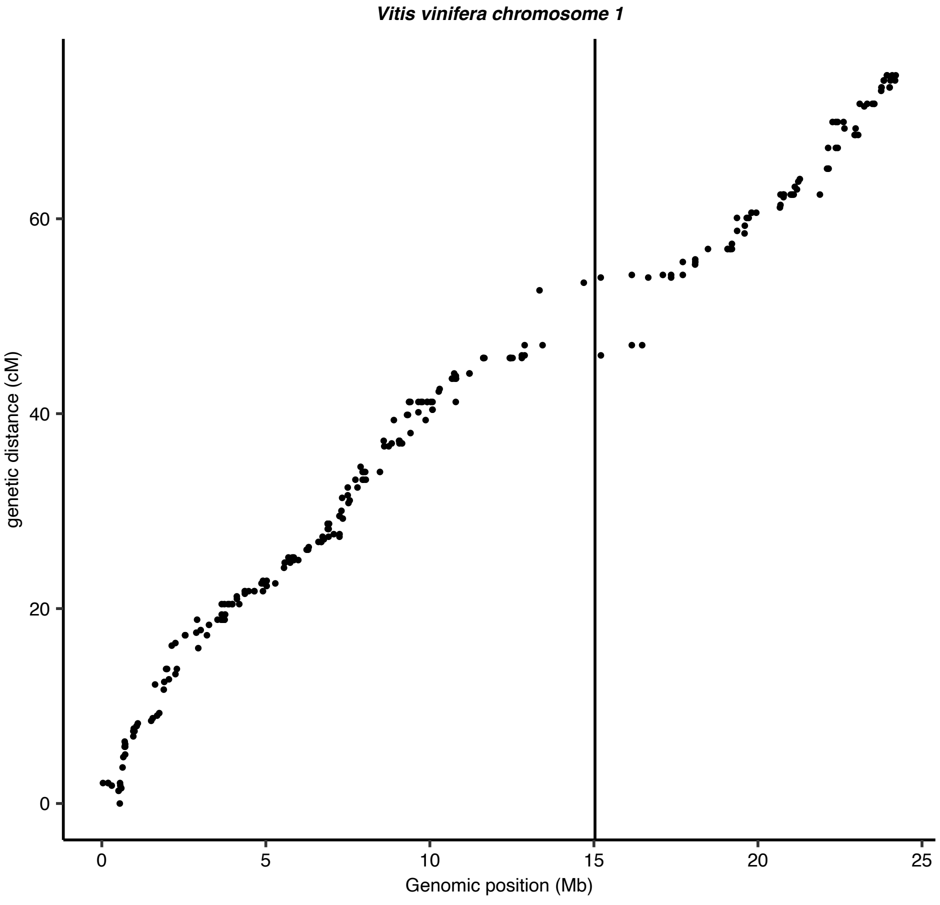
Vitis vinifera chromosome 1
genetic distance (cM)
0
20
40
60
Genomic position (Mb)
0
5
10
15
20
25

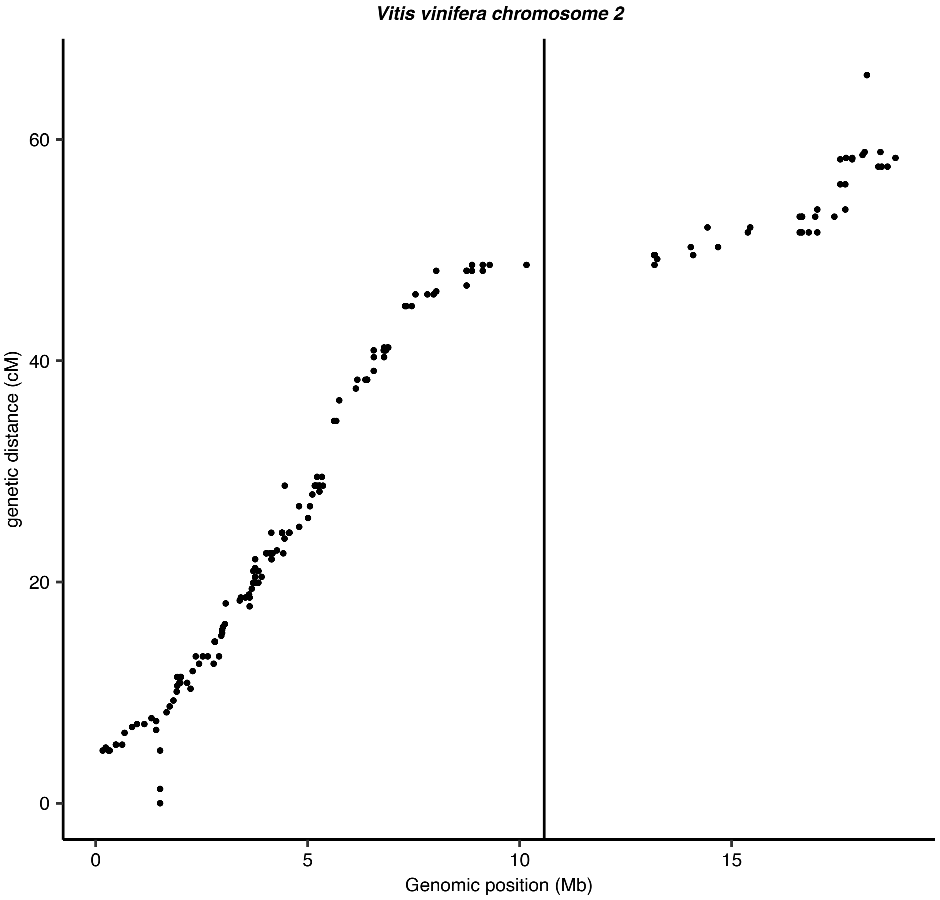
Vitis vinifera chromosome 2
genetic distance (cM)
0
20
40
60
Genomic position (Mb)
0
5
10
15

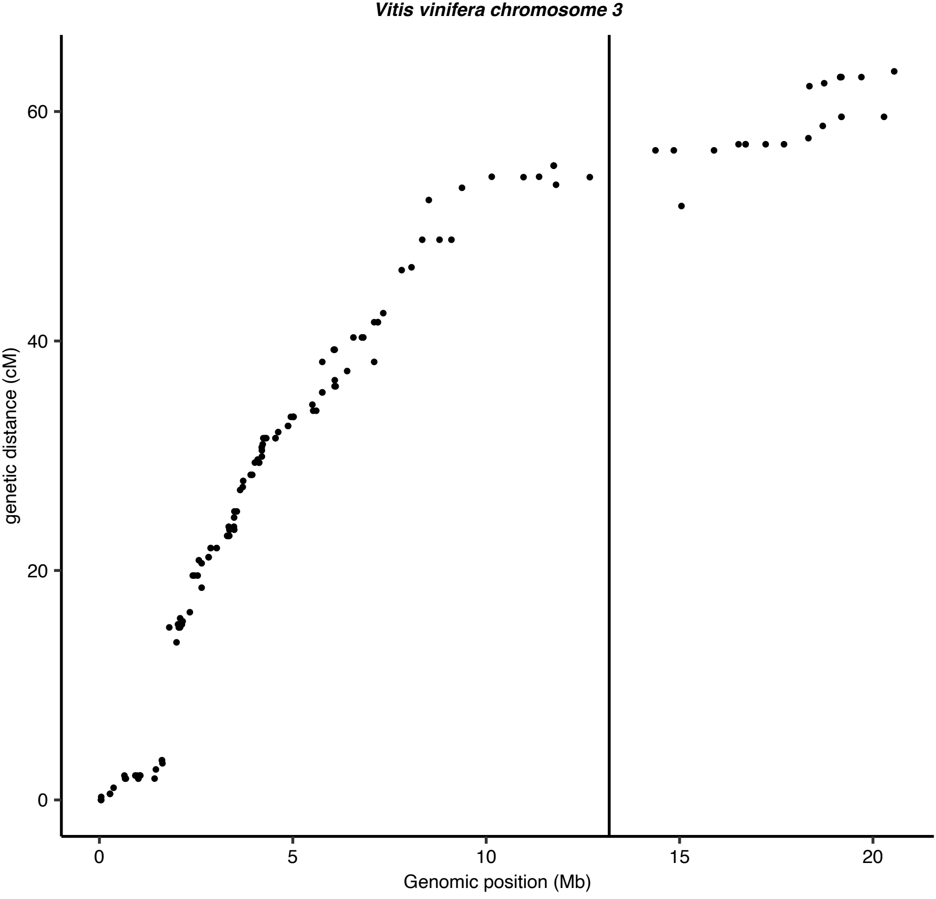
Vitis vinifera chromosome 3
genetic distance (cM)
Genomic position (Mb)
0
20
40
60
0
5
10
15
20

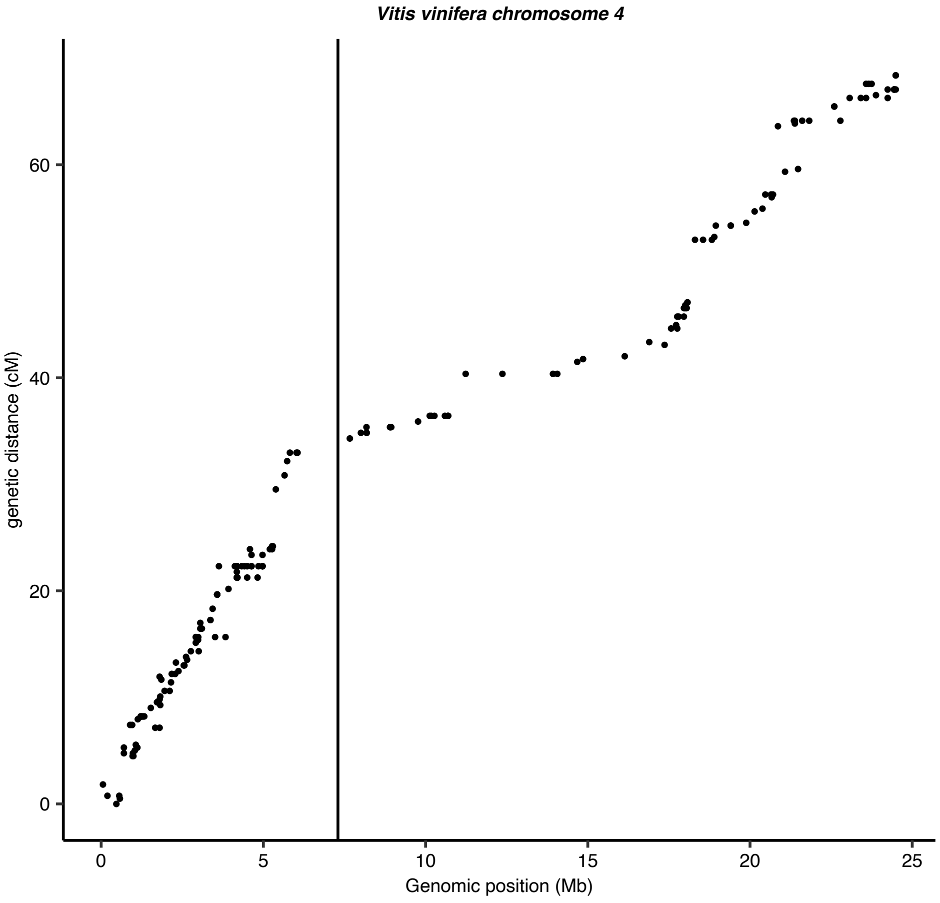
Vitis vinifera chromosome 4
genetic distance (cM)
0
20
40
60
Genomic position (Mb)
0
5
10
15
20
25

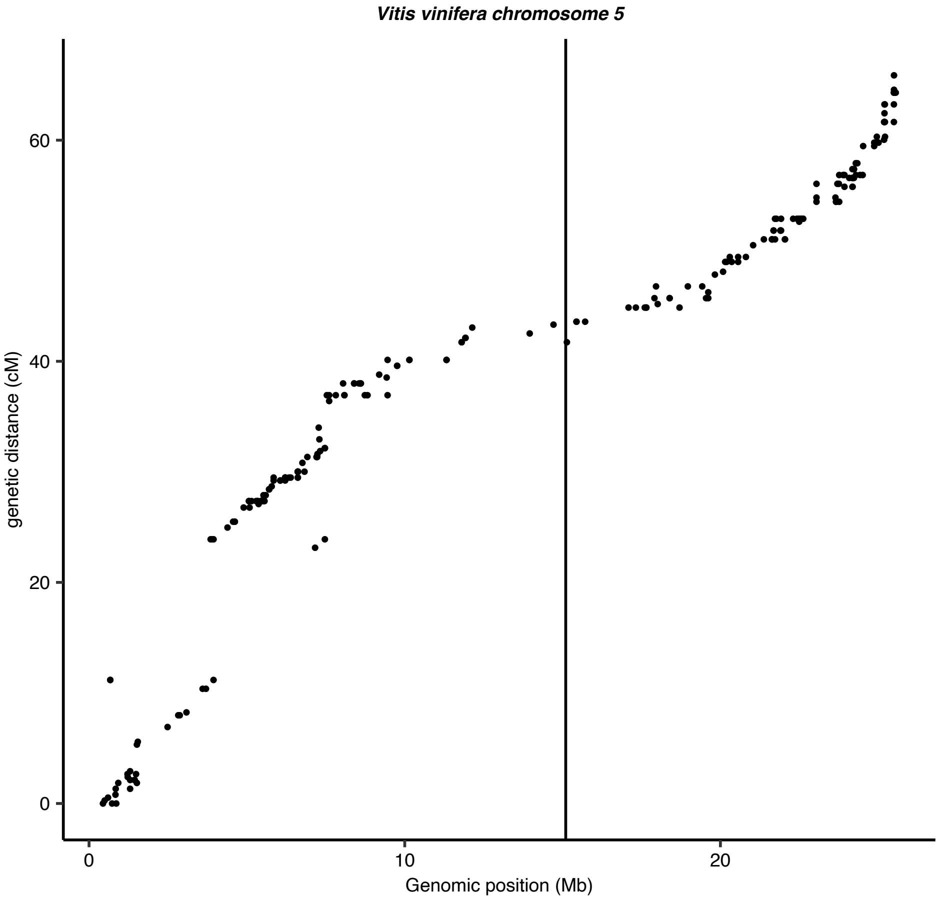
Vitis vinifera chromosome 5
genetic distance (cM)
Genomic position (Mb)
0
20
40
60
0
10
20

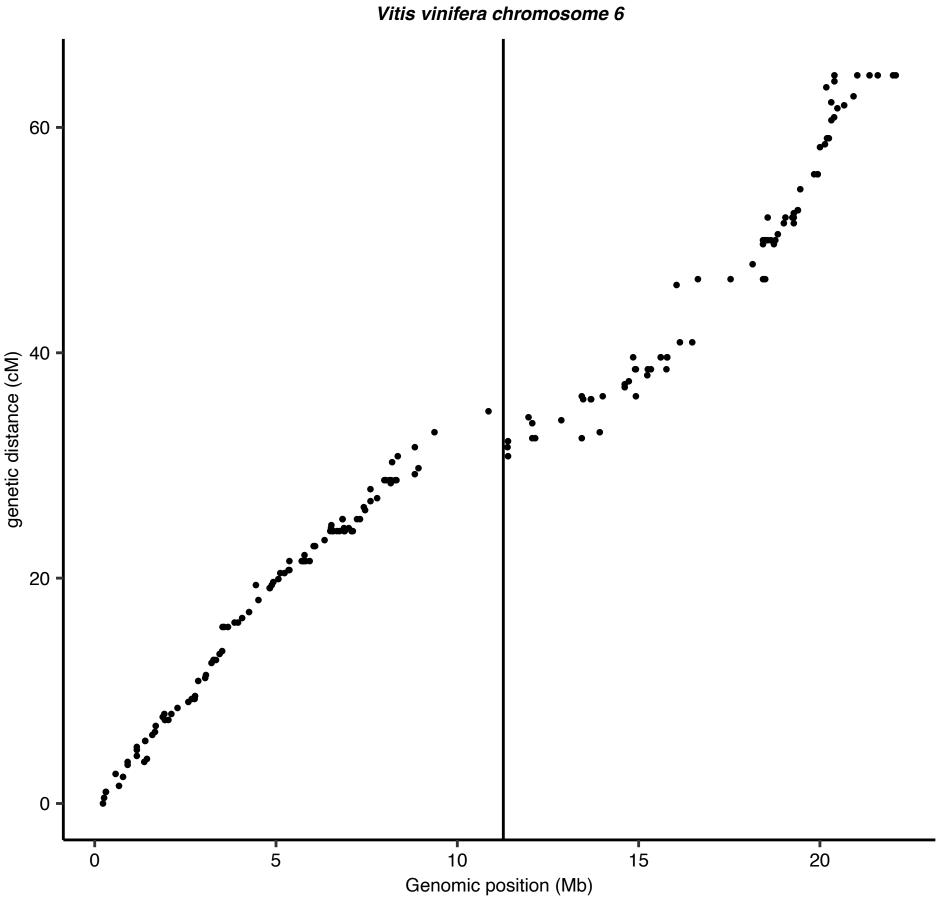
Vitis vinifera chromosome 6
genetic distance (cM)
0
20
40
60
0
5
10
15
20
Genomic position (Mb)

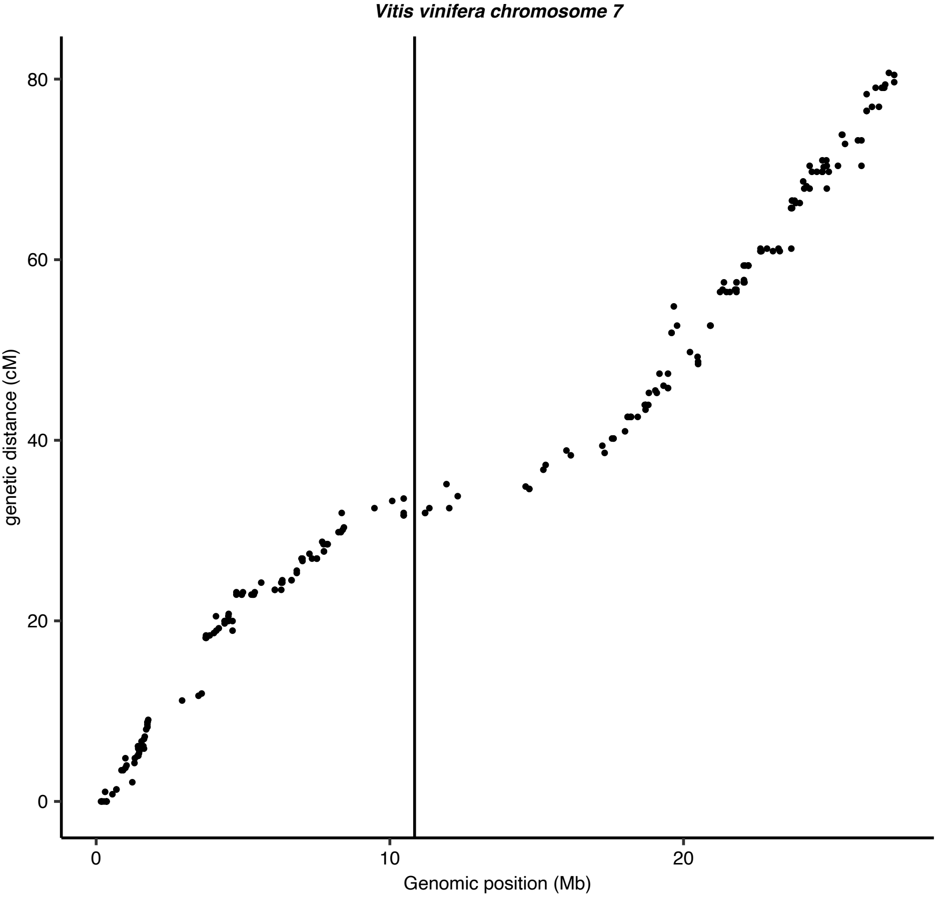
Vitis vinifera chromosome 7
genetic distance (cM)
0
20
40
60
80
Genomic position (Mb)
0
10
20

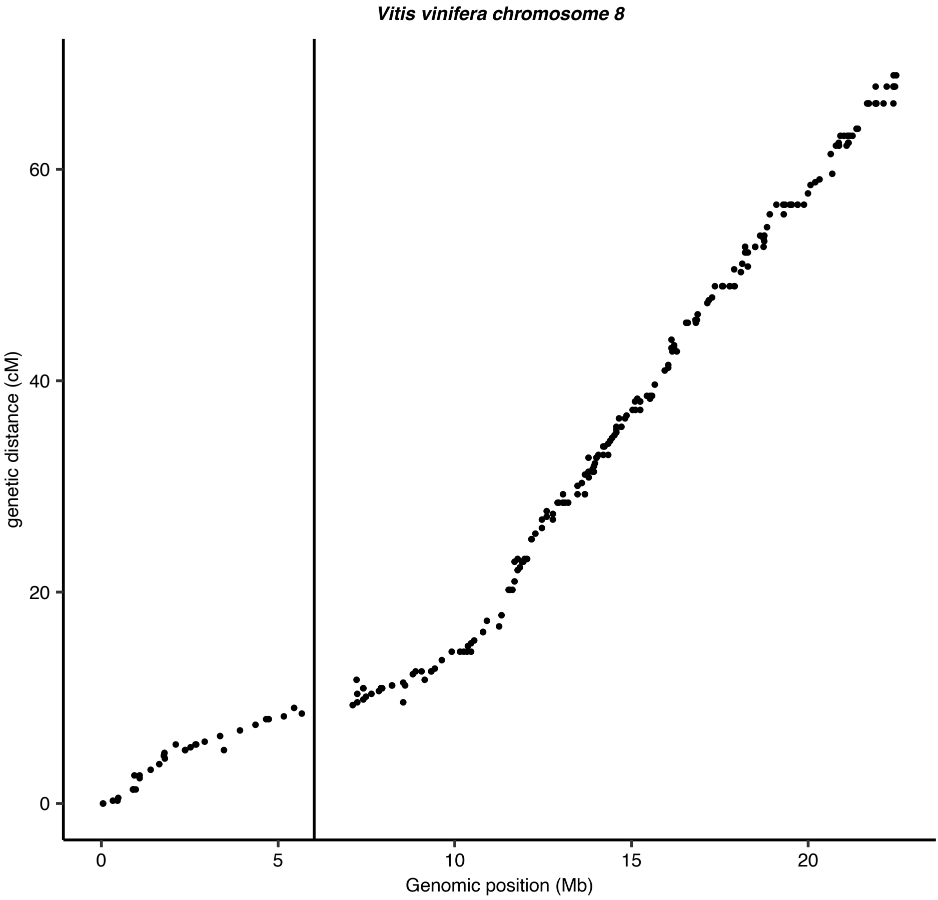
Vitis vinifera chromosome 8
genetic distance (cM)
0
20
40
60
Genomic position (Mb)
0
5
10
15
20

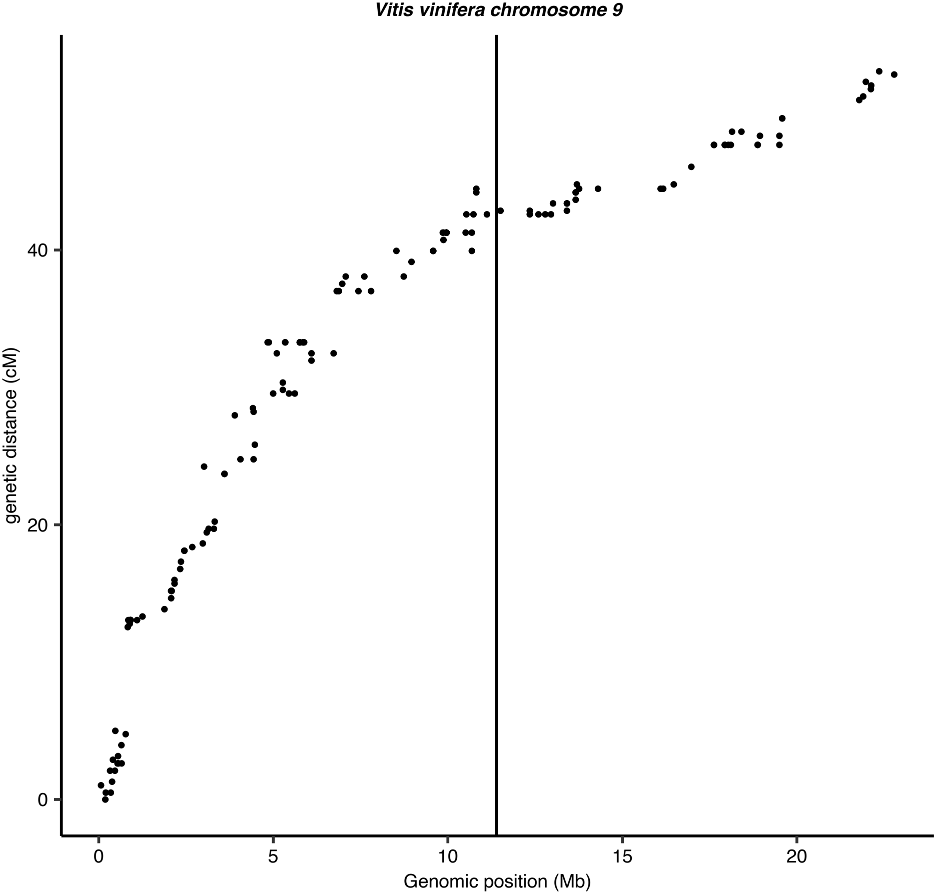
Vitis vinifera chromosome 9
genetic distance (cM)
0
20
40
Genomic position (Mb)
0
5
10
15
20

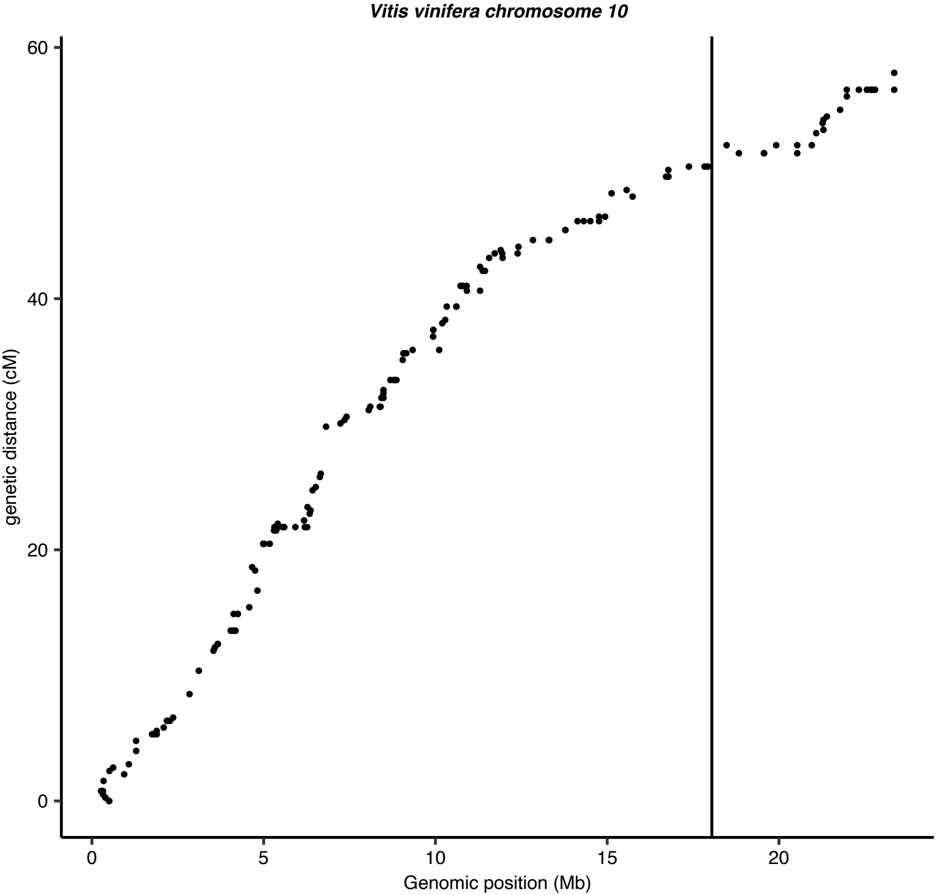
Vitis vinifera chromosome 10
genetic distance (cM)
Genomic position (Mb)
0
20
40
60
0
5
10
15
20

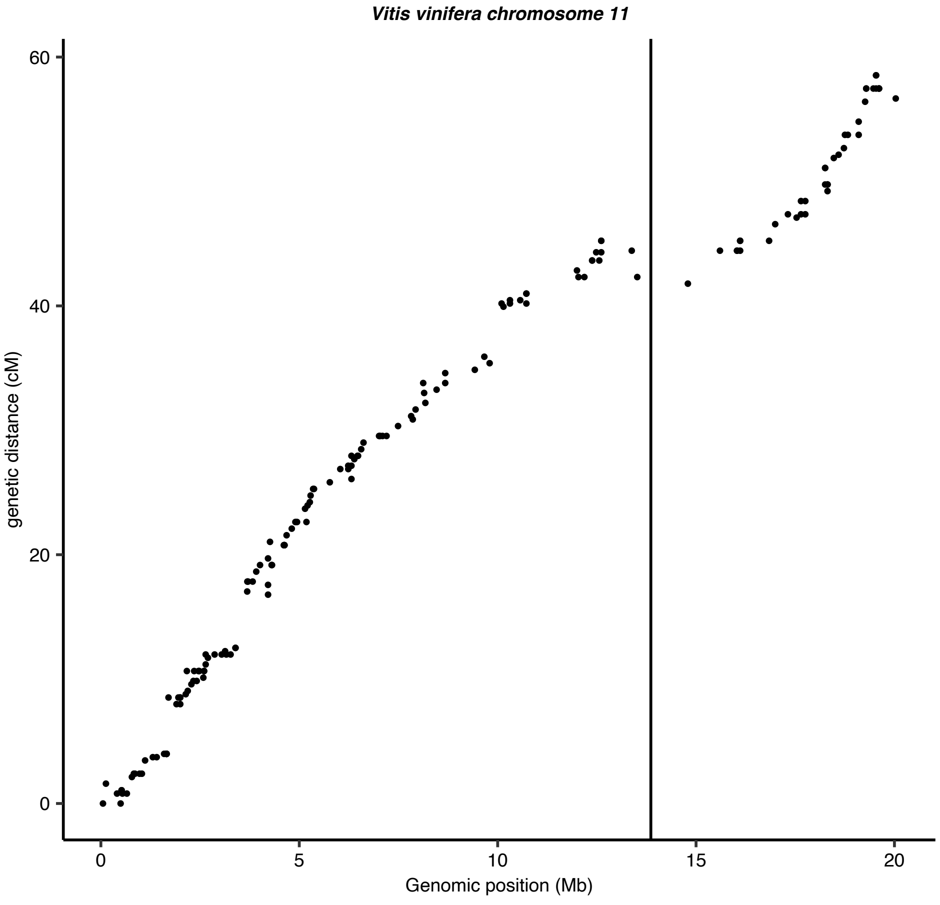
Vitis vinifera chromosome 11
genetic distance (cM)
0
20
40
60
Genomic position (Mb)
0
5
10
15
20

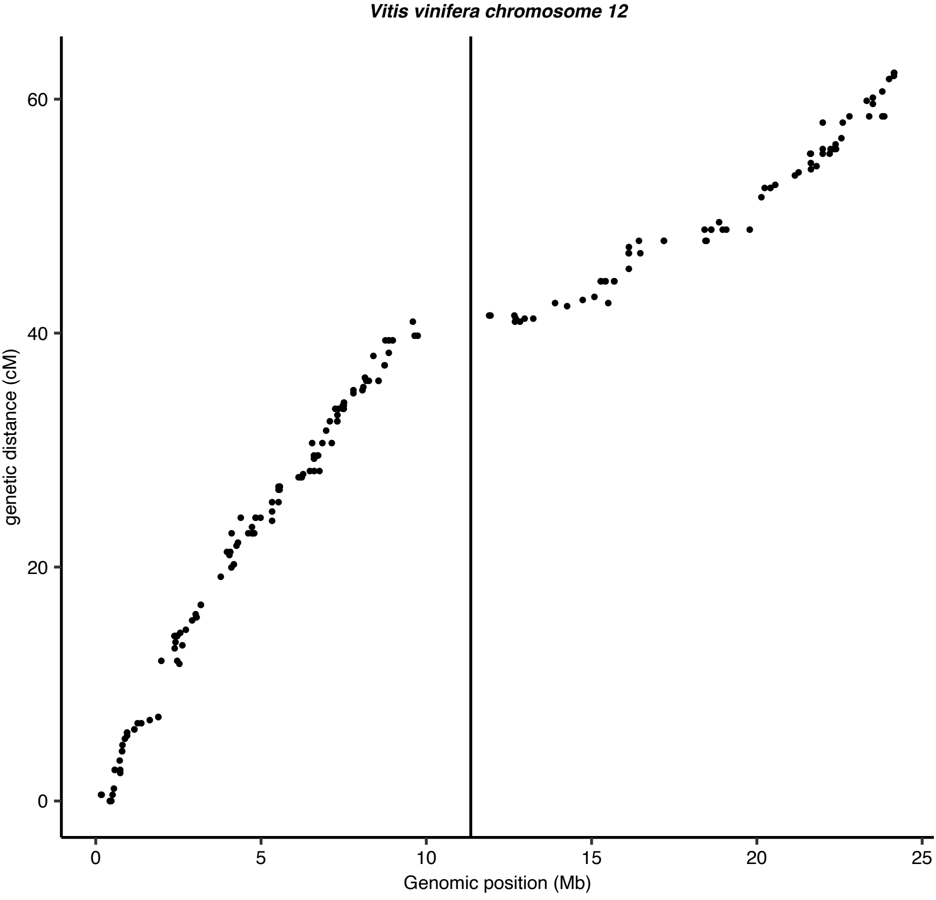
Vitis vinifera chromosome 12
genetic distance (cM)
0
20
40
60
Genomic position (Mb)
0
5
10
15
20
25

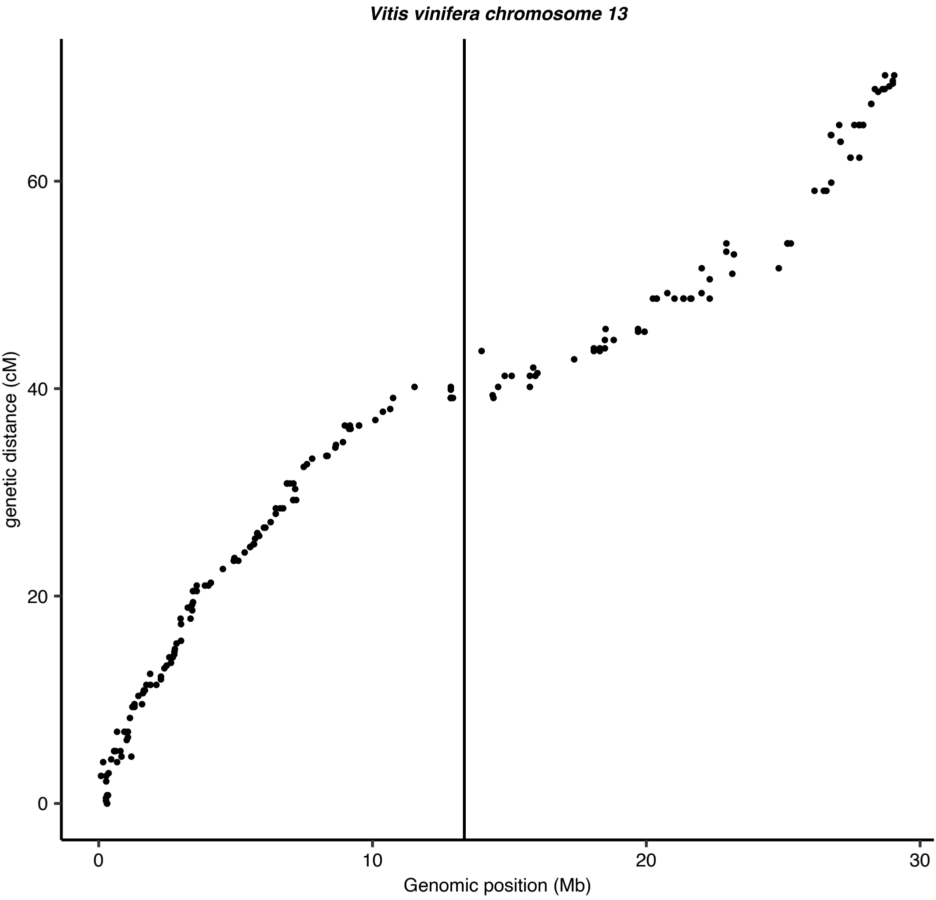
Vitis vinifera chromosome 13
genetic distance (cM)
0
20
40
60
0
10
20
30
Genomic position (Mb)

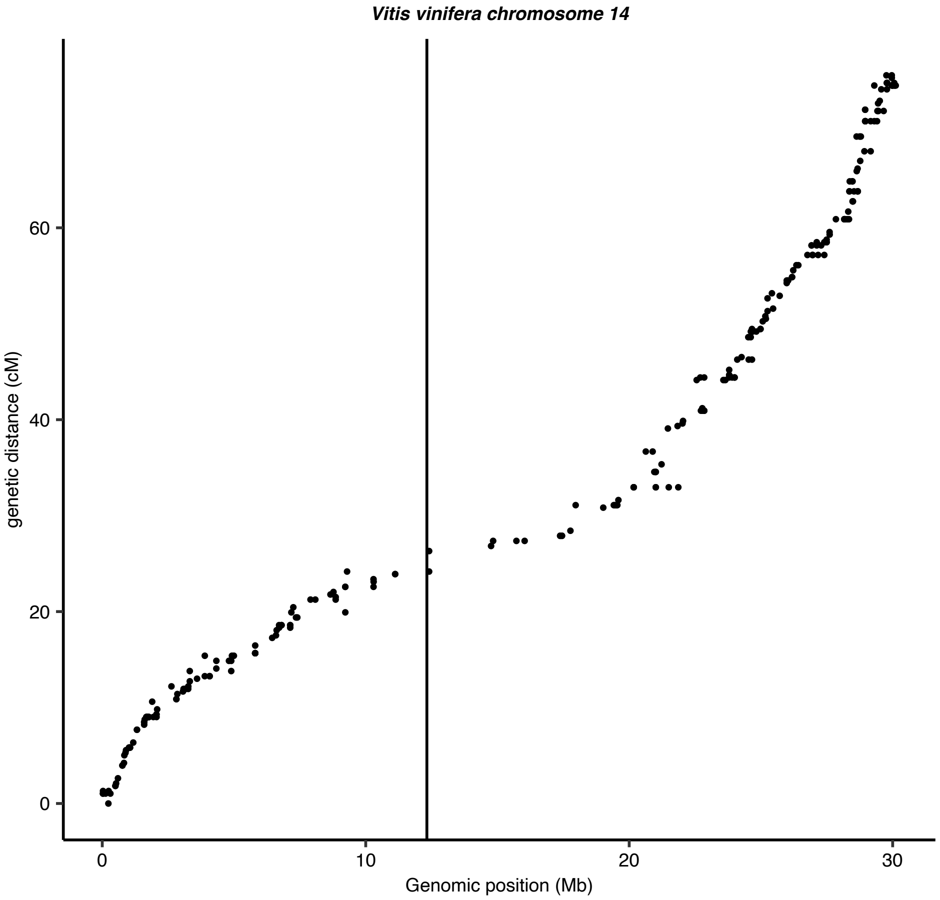
Vitis vinifera chromosome 14
genetic distance (cM)
0
20
40
60
Genomic position (Mb)
0
10
20
30

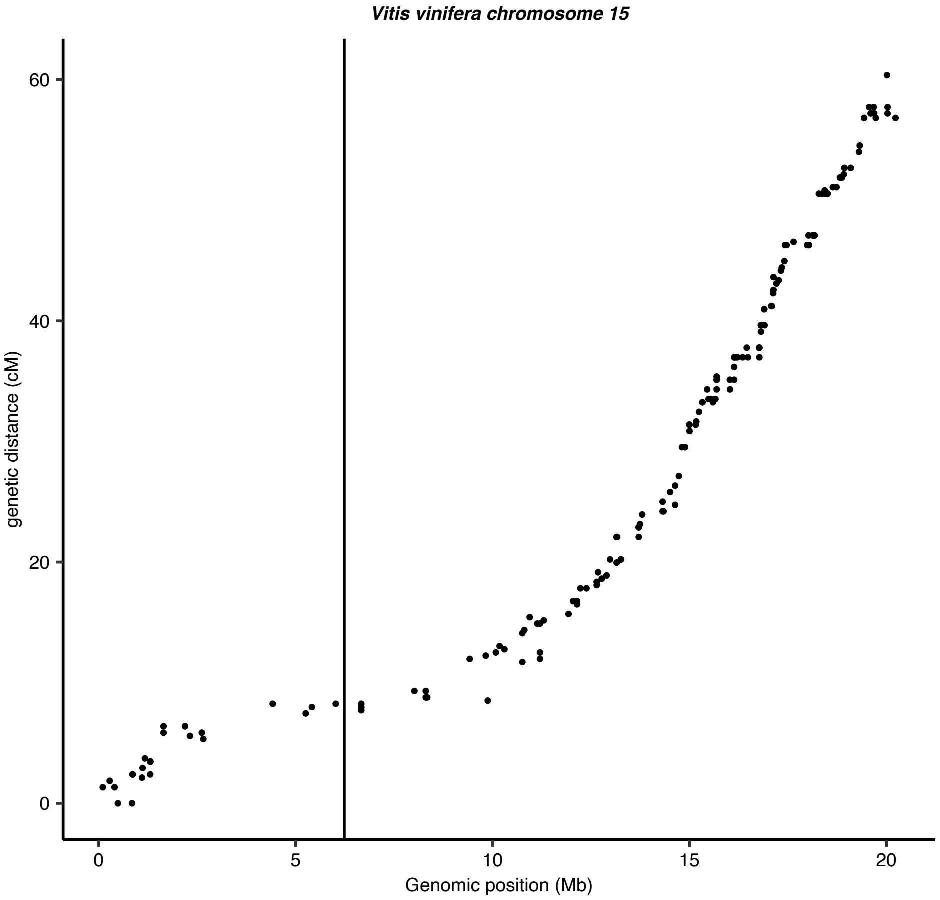
Vitis vinifera chromosome 15
genetic distance (cM)
0
20
40
60
Genomic position (Mb)
0
5
10
15
20

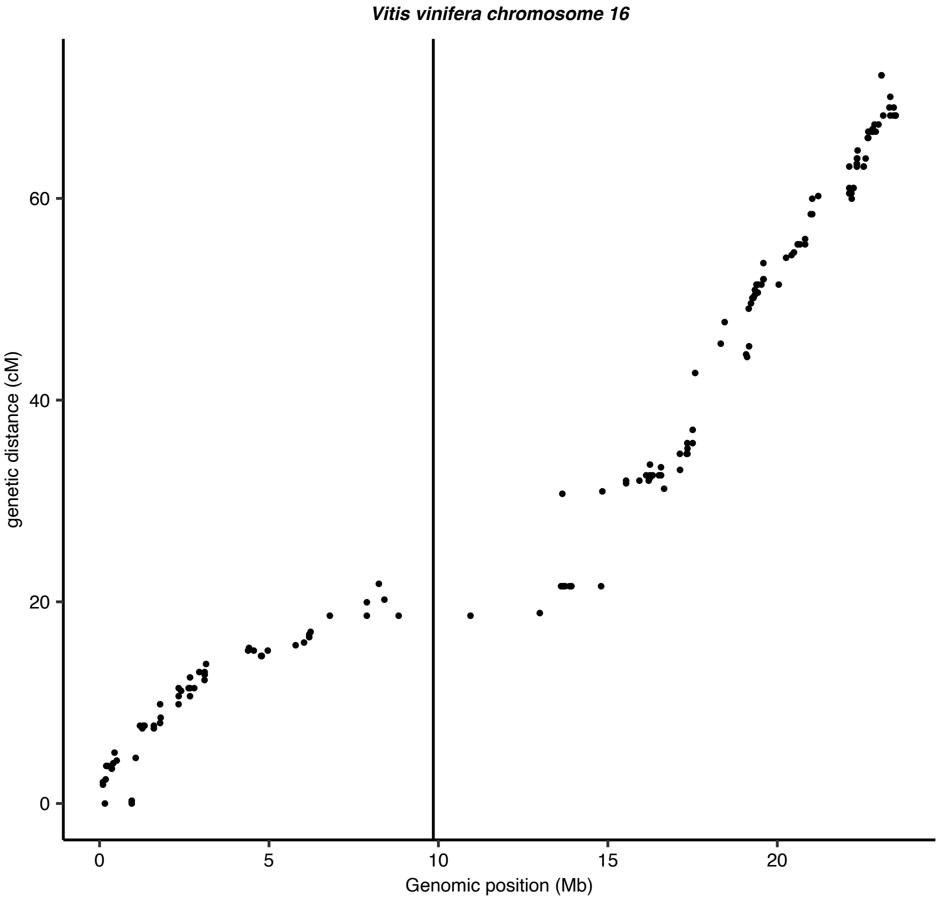
Vitis vinifera chromosome 16
genetic distance (cM)
0
20
40
60
Genomic position (Mb)
0
5
10
15
20

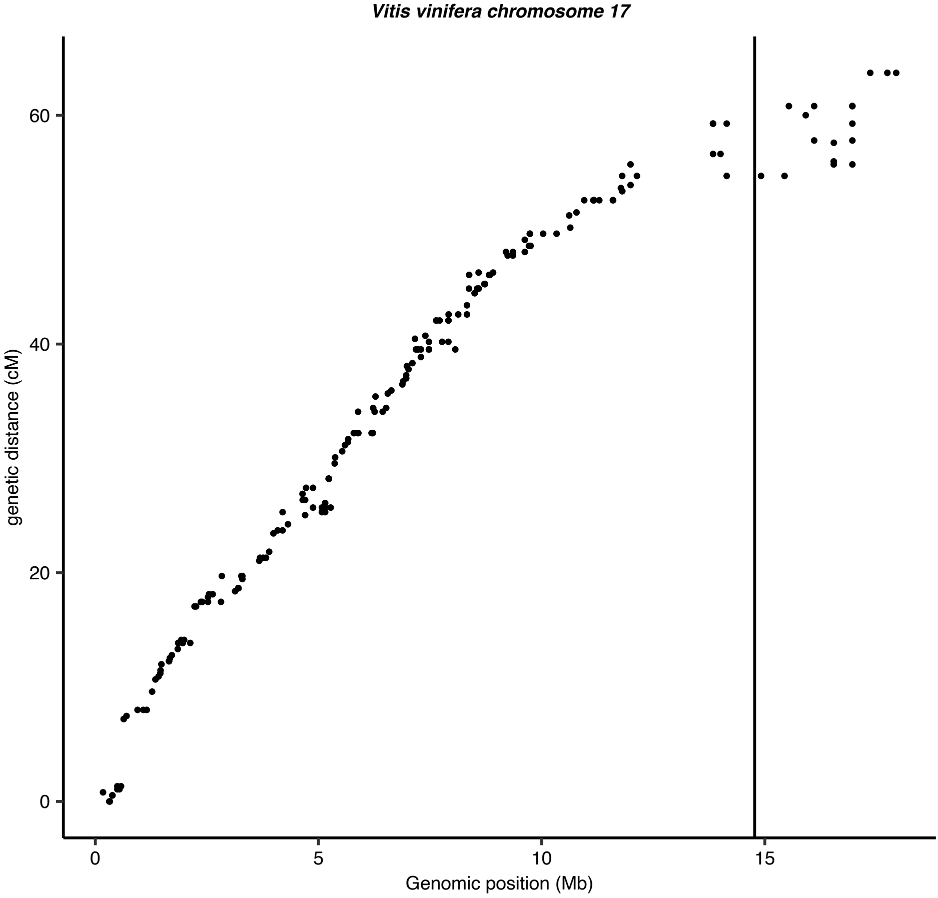
Vitis vinifera chromosome 17
genetic distance (cM)
Genomic position (Mb)
0
20
40
60
0
5
10
15

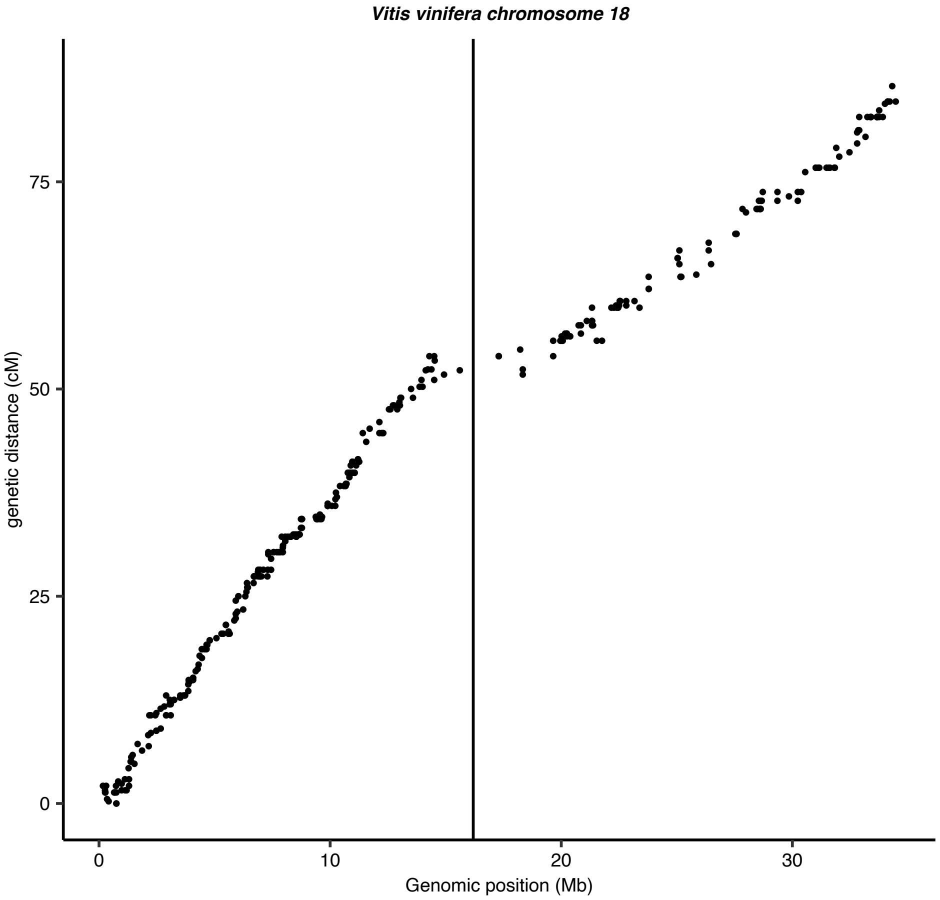
Vitis vinifera chromosome 18
genetic distance (cM)
0
25
50
75
Genomic position (Mb)
0
10
20
30

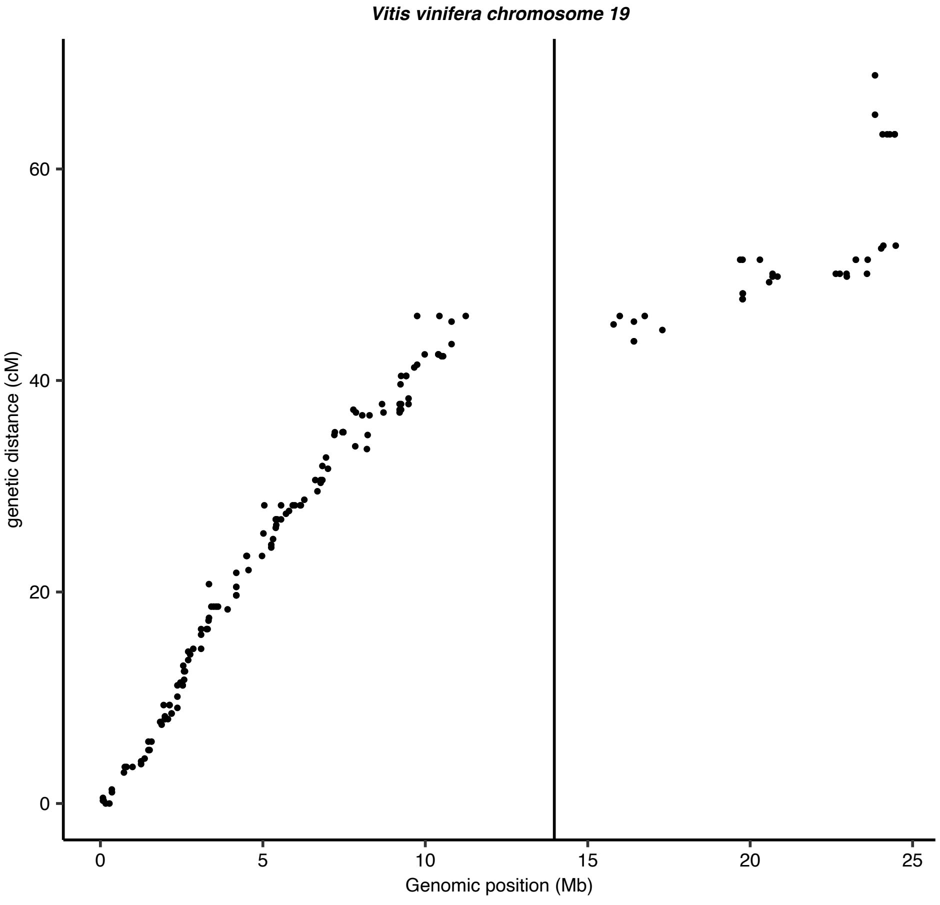
Vitis vinifera chromosome 19
genetic distance (cM)
0
20
40
60
0
5
10
15
20
25
Genomic position (Mb)

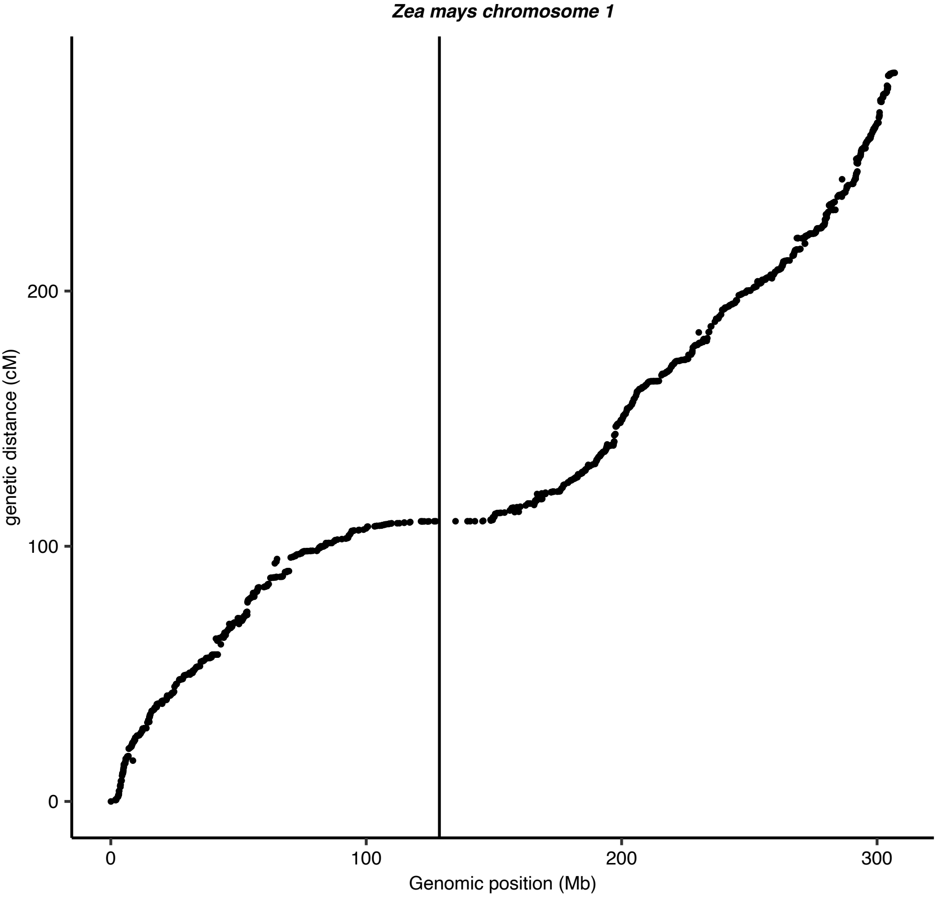
Zea mays chromosome 1
genetic distance (cM)
Genomic position (Mb)
0
100
200
0
100
200
300

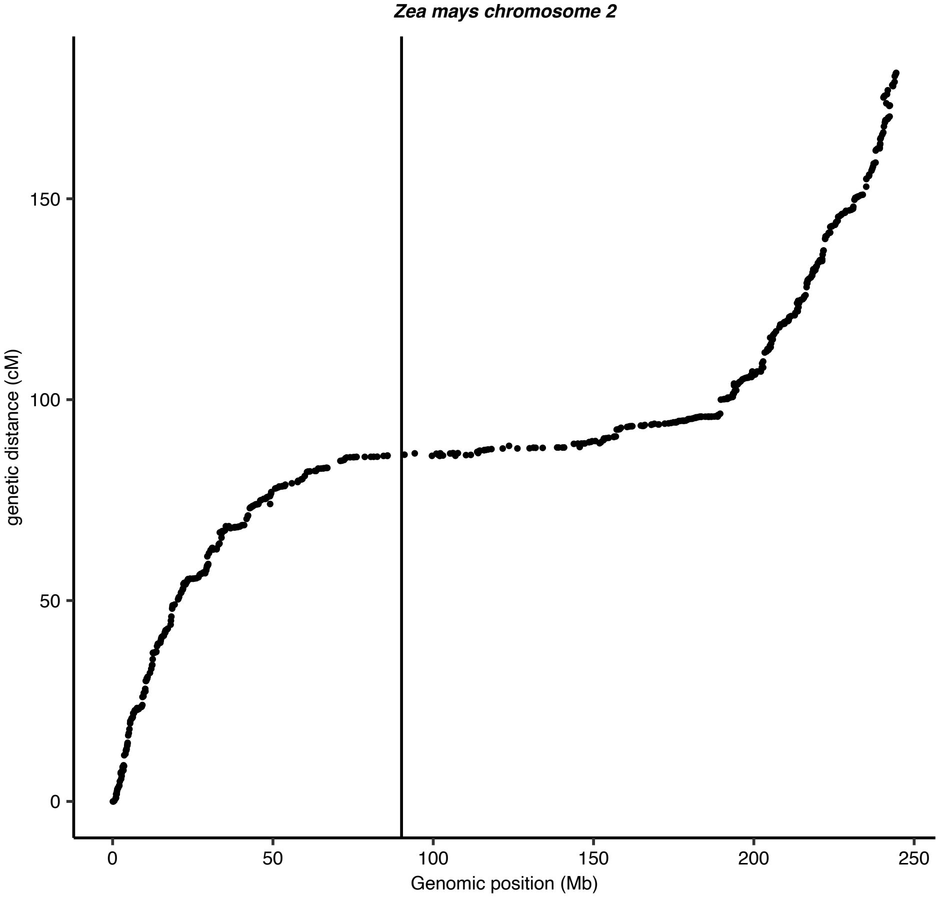
Zea mays chromosome 2
genetic distance (cM)
Genomic position (Mb)
0
50
100
150
200
250

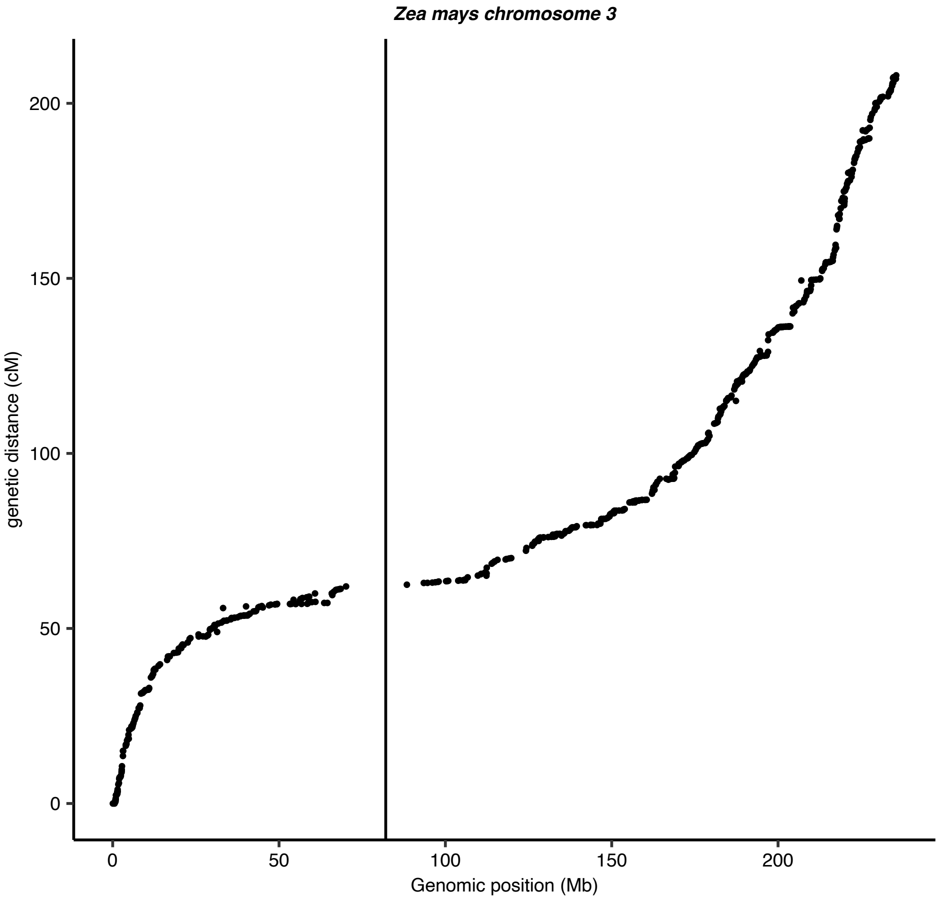
Zea mays chromosome 3
genetic distance (cM)
0
50
100
150
200
Genomic position (Mb)
0
50
100
150
200

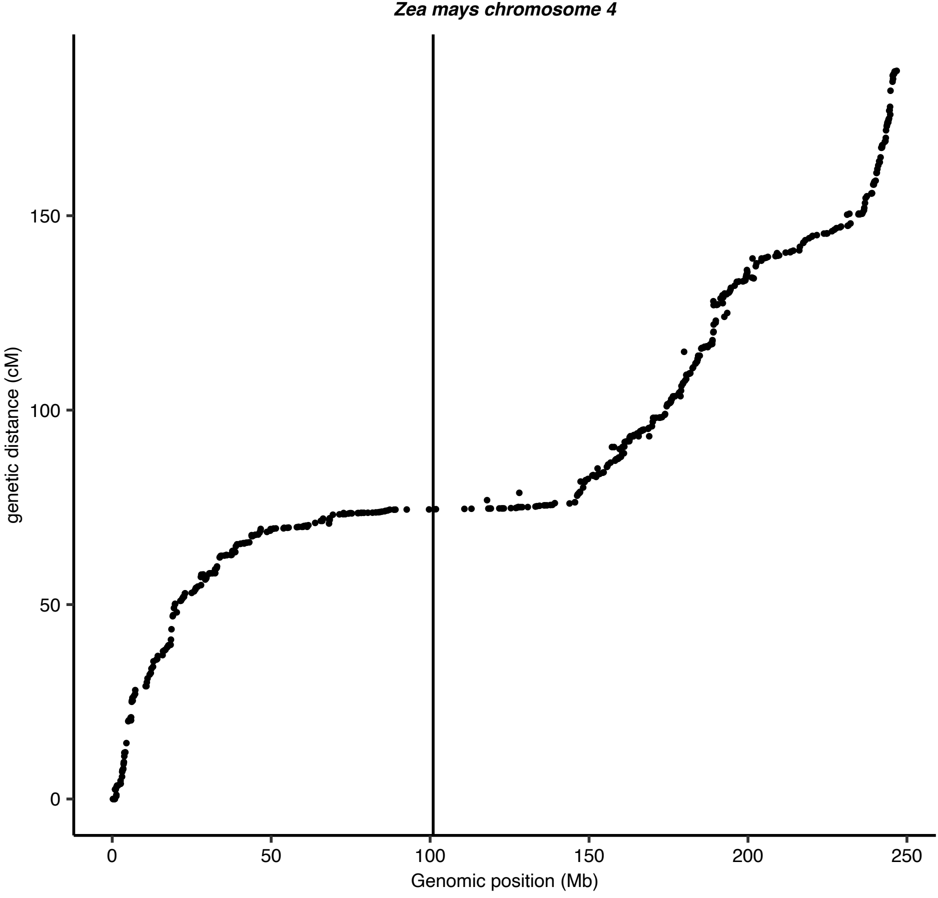
Zea mays chromosome 4
genetic distance (cM)
0
50
100
150
Genomic position (Mb)
0
50
100
150
200
250

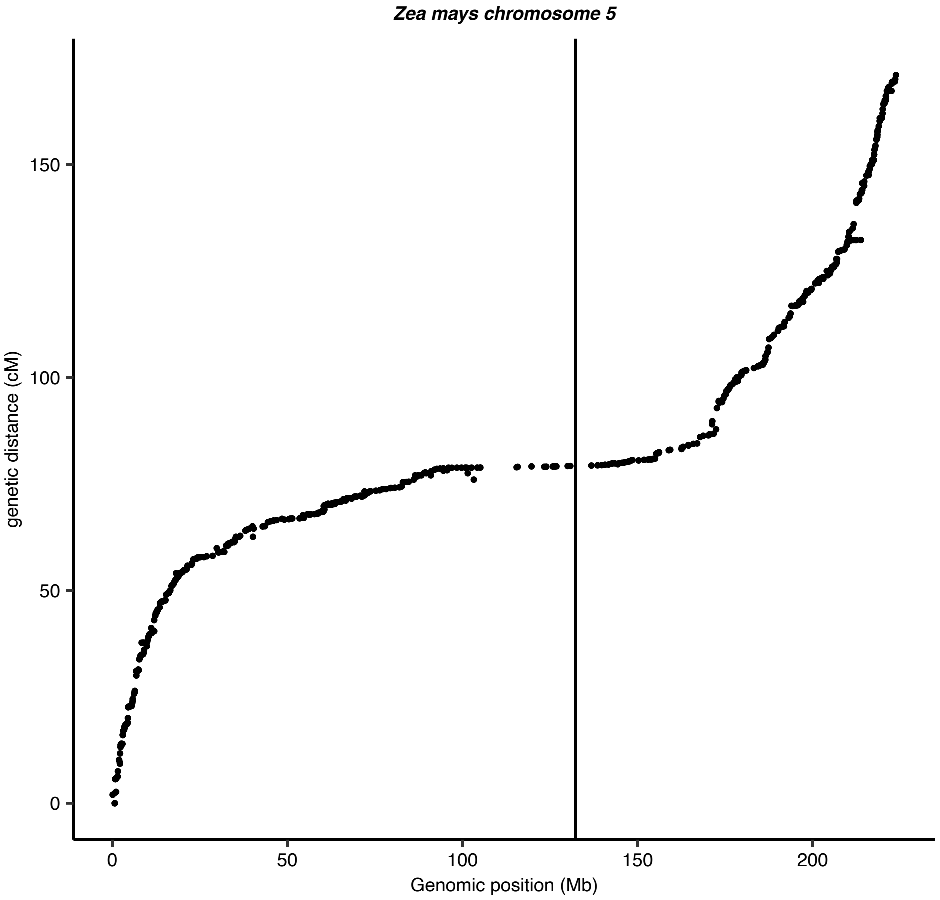
Zea mays chromosome 5
genetic distance (cM)
0
50
100
150
Genomic position (Mb)
0
50
100
150
200

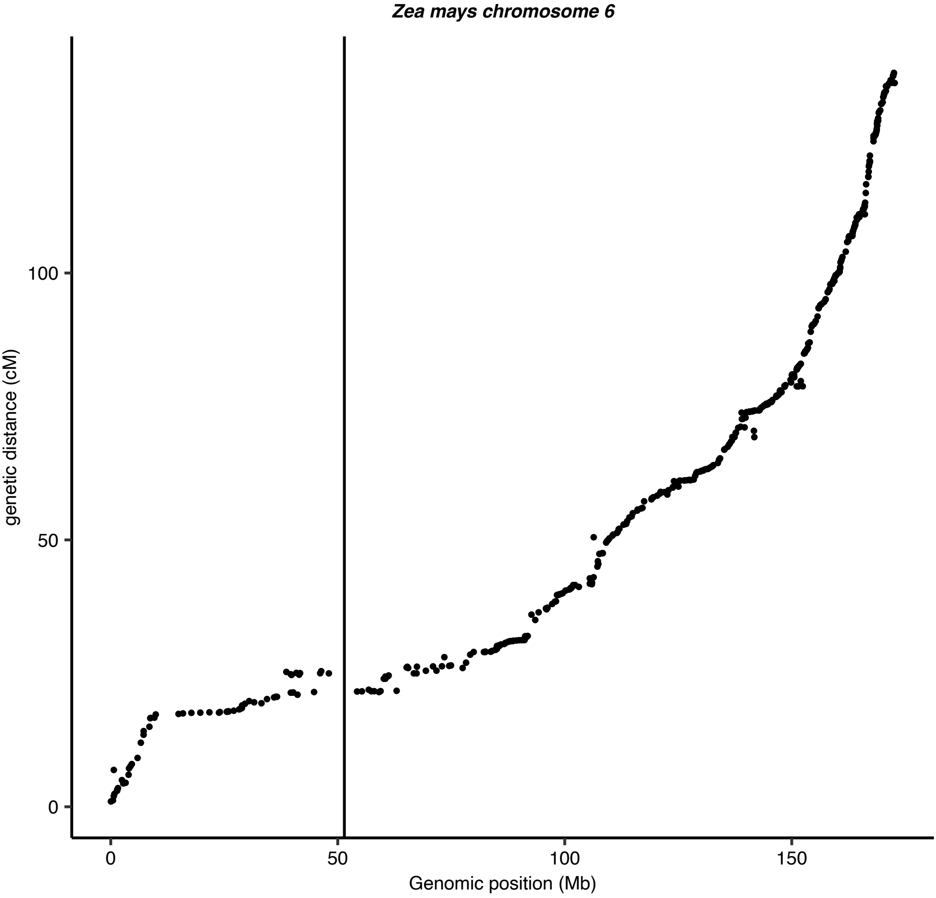
Zea mays chromosome 6
genetic distance (cM)
100
50
0
0
50
100
150
Genomic position (Mb)

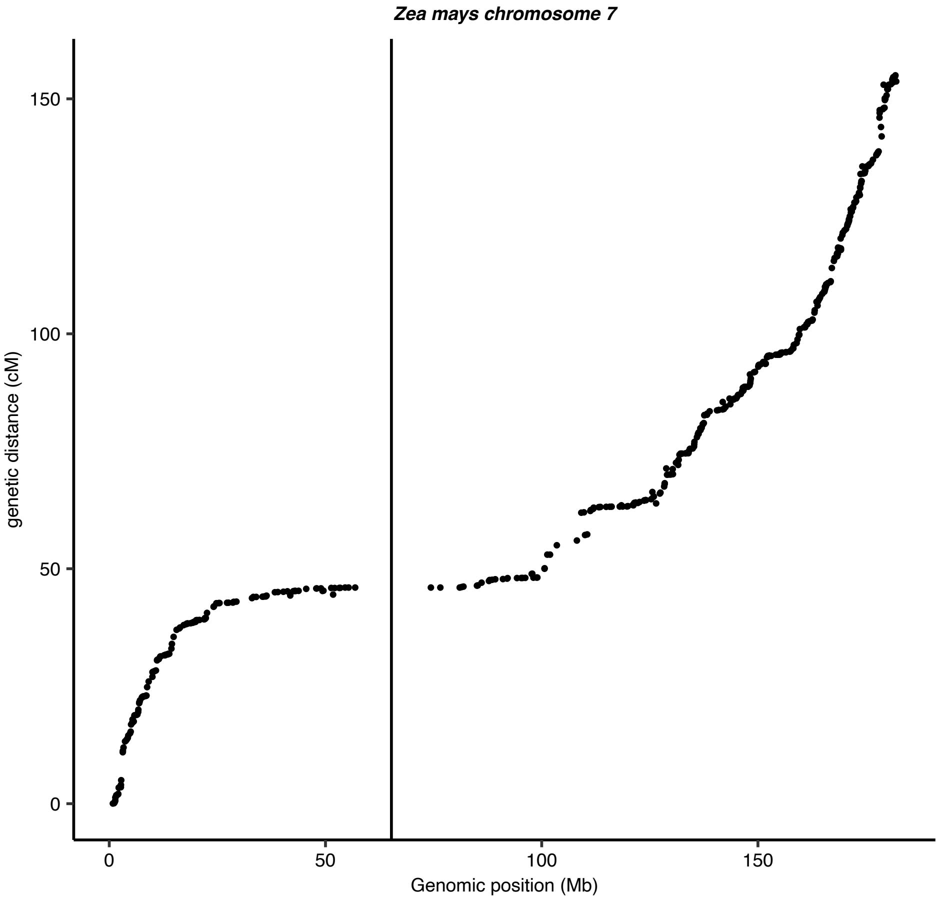
Zea mays chromosome 7
genetic distance (cM)
150
100
50
0
0
50
100
150
Genomic position (Mb)

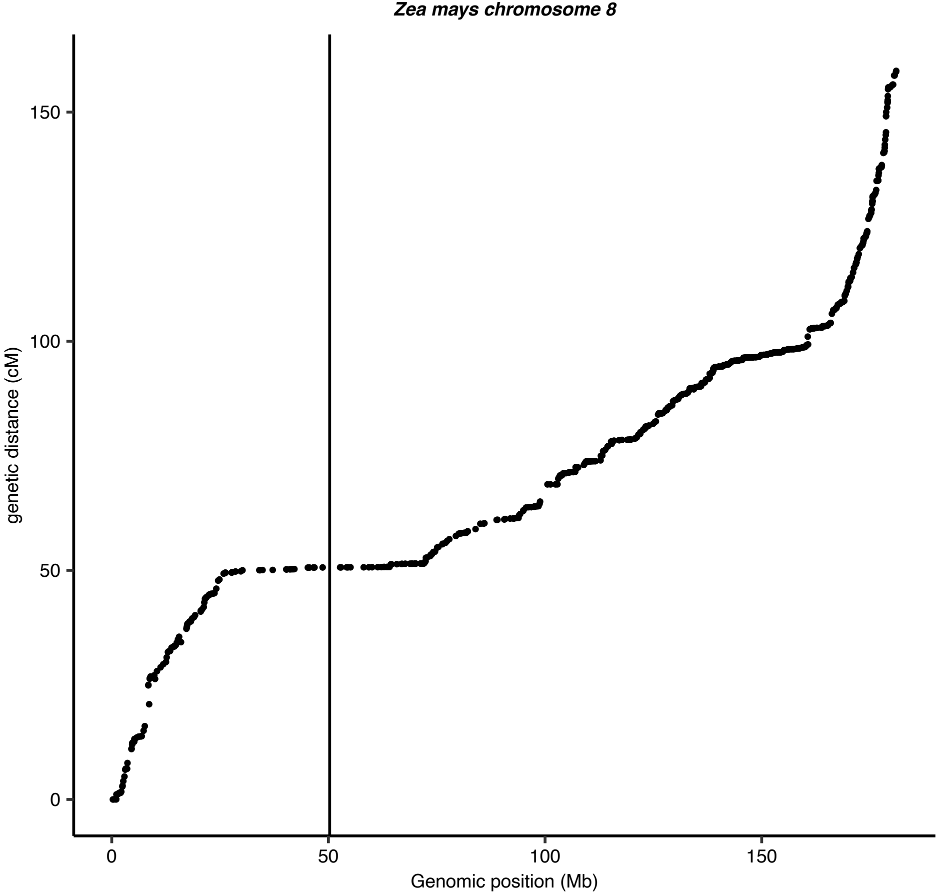

Zea mays chromosome 8
genetic distance (cM)
150
100
50
0
0
50
100
150
Genomic position (Mb)

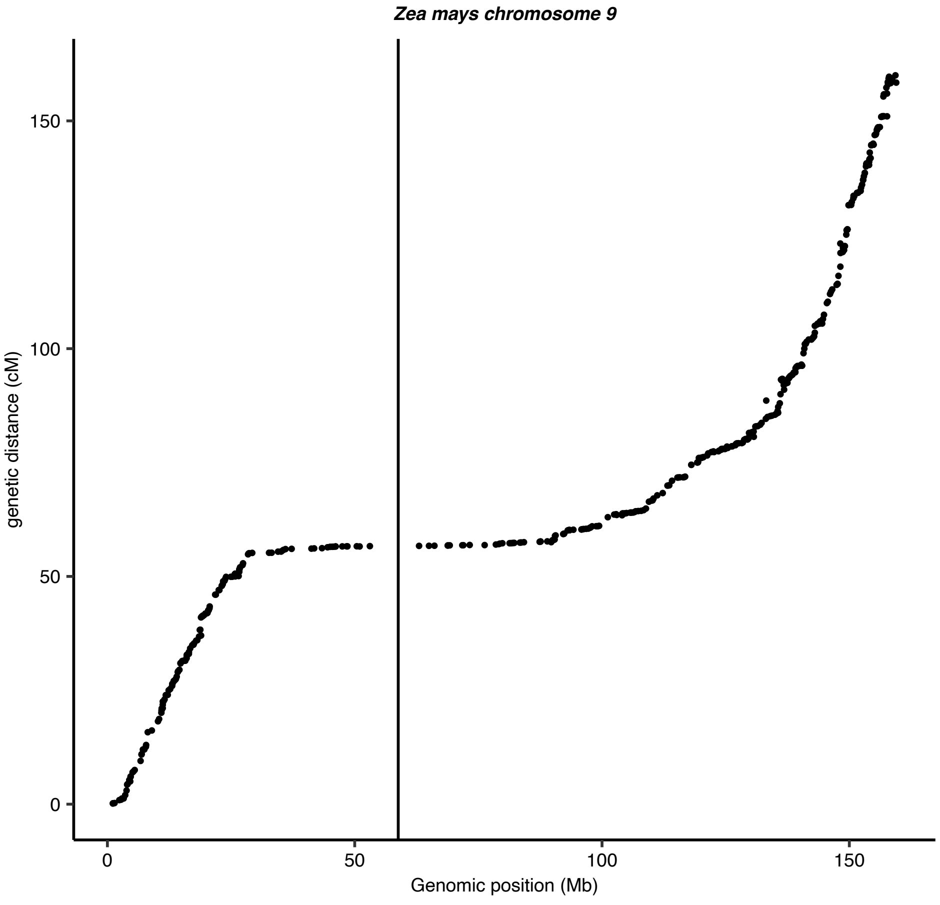
Zea mays chromosome 9
genetic distance (cM)
Genomic position (Mb)
0
50
100
150
0
50
100
150

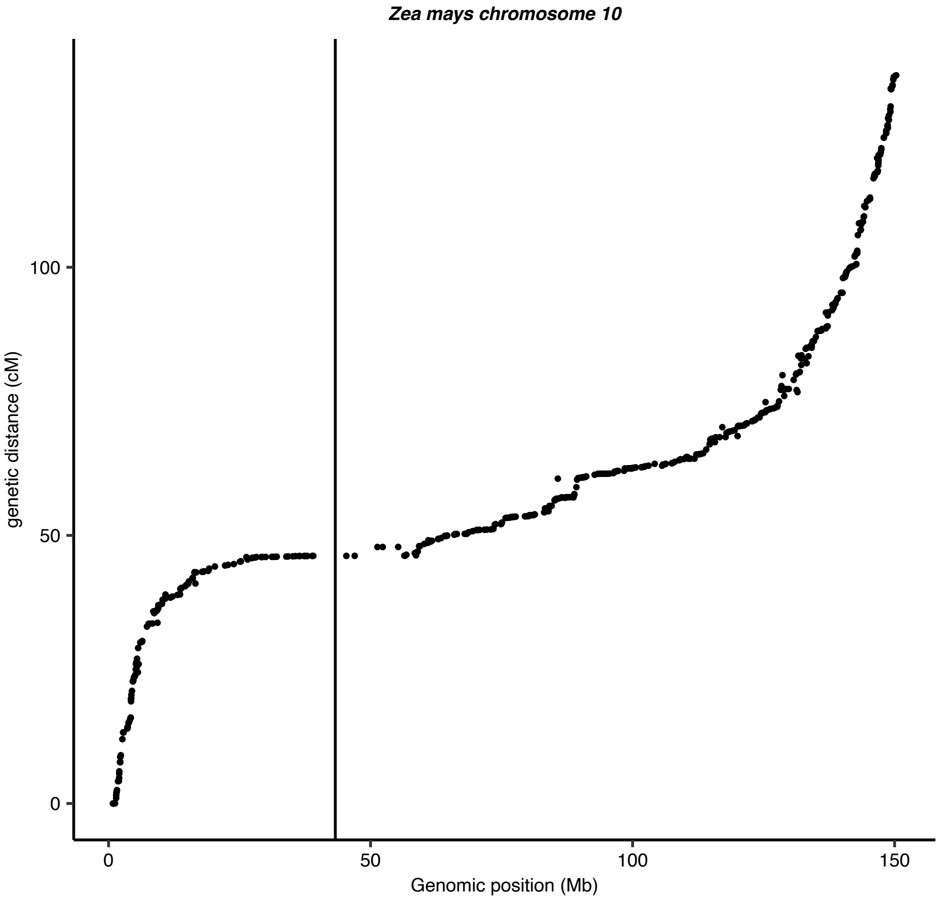
Zea mays chromosome 10
genetic distance (cM)
0
50
100
Genomic position (Mb)
0
50
100
150
