# Supplementary figures and images for: Diversity and determinants of recombination landscapes in flowering plants
Source: PLoS Genet. 2022 Aug 30;18(8):e1010141. doi: 10.1371/journal.pgen.1010141 (PMC9467342; doi:10.1371/journal.pgen.1010141)

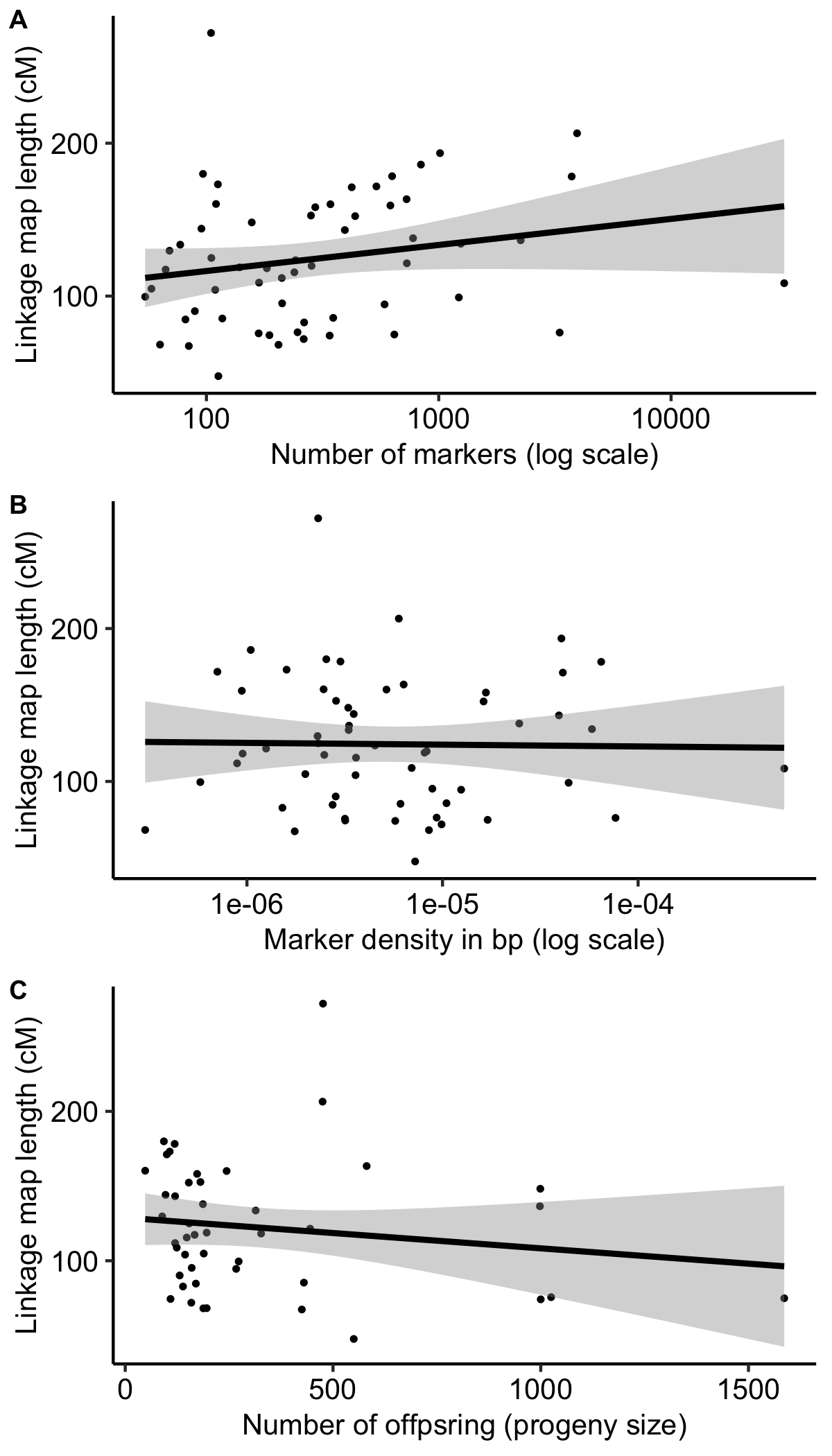

Supplement: S3 Fig — The averaged linkage map length (total linkage map length divided by the number of chromosomes, cM) is not correlated with (A) the number of markers (linkage map length ~ log10(number of markers), adjusted R2 = 0.04, p = 0.11), (B) marker density (linkage map length ~ marker density, adjusted R2 = -0.018, p = 0.90) and (C) the progeny size (linkage map length ~ progeny size, adjusted R2 = 0.022, p < 0.32). Regression lines with 95% parametric confidence interval estimated with ggplot2. (TIF) [file pgen.1010141.s003.tif]

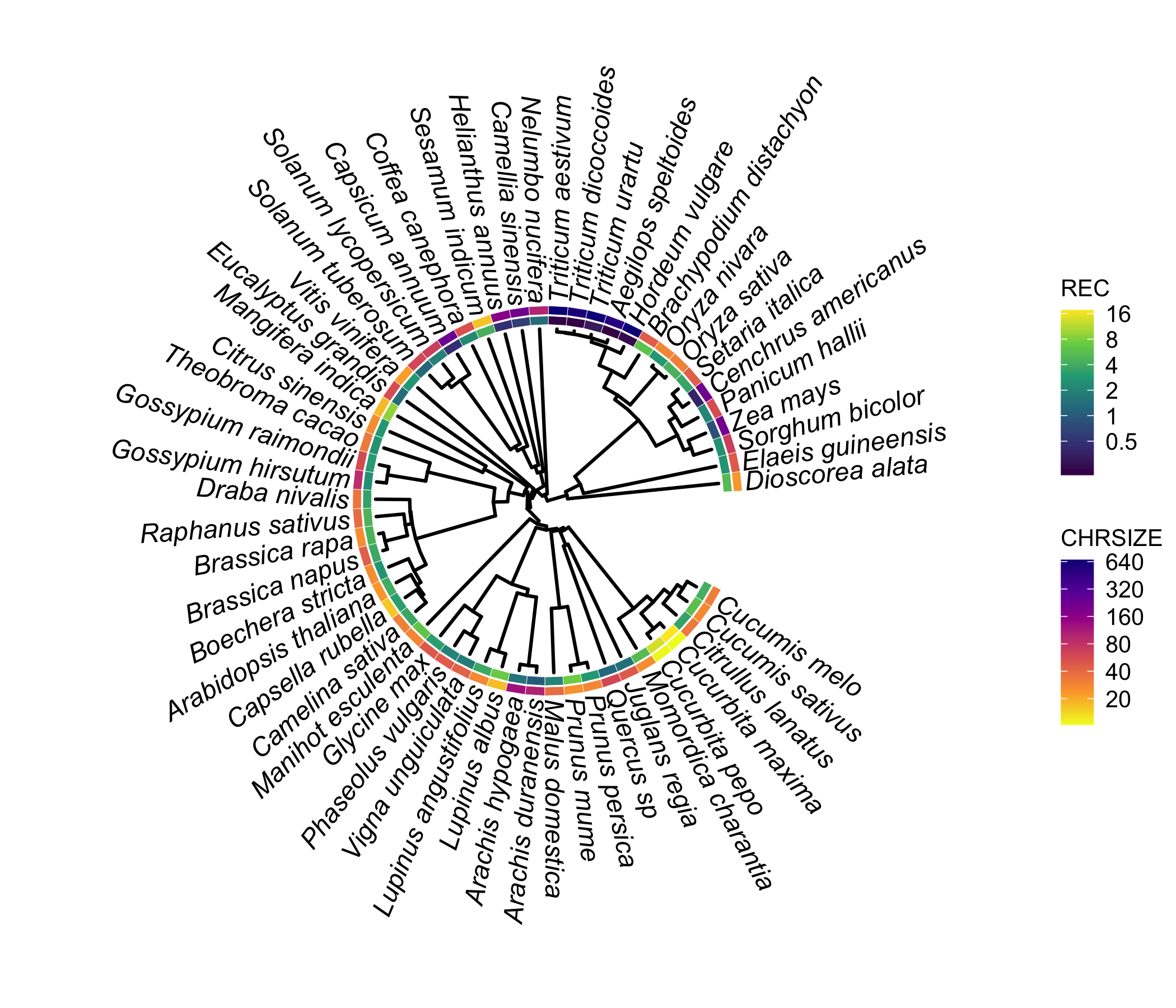

Supplement: S4 Fig — The supertree was retrieved from the publicly available phylogeny constructed by Smith and Brown (Smith & Brown, 2018). (TIF) [file pgen.1010141.s004.tif]

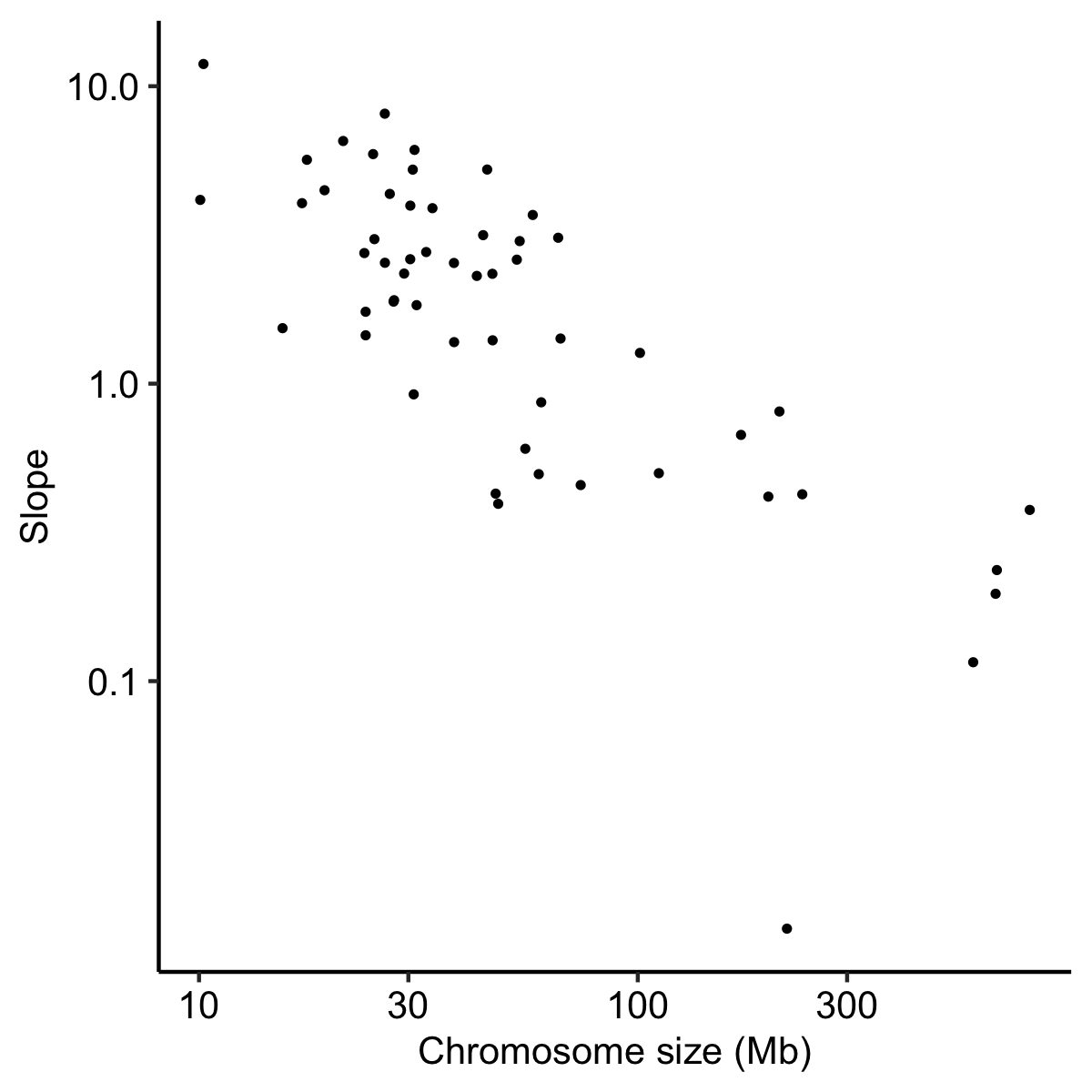

Supplement: S5 Fig — (TIF) [file pgen.1010141.s005.tif]

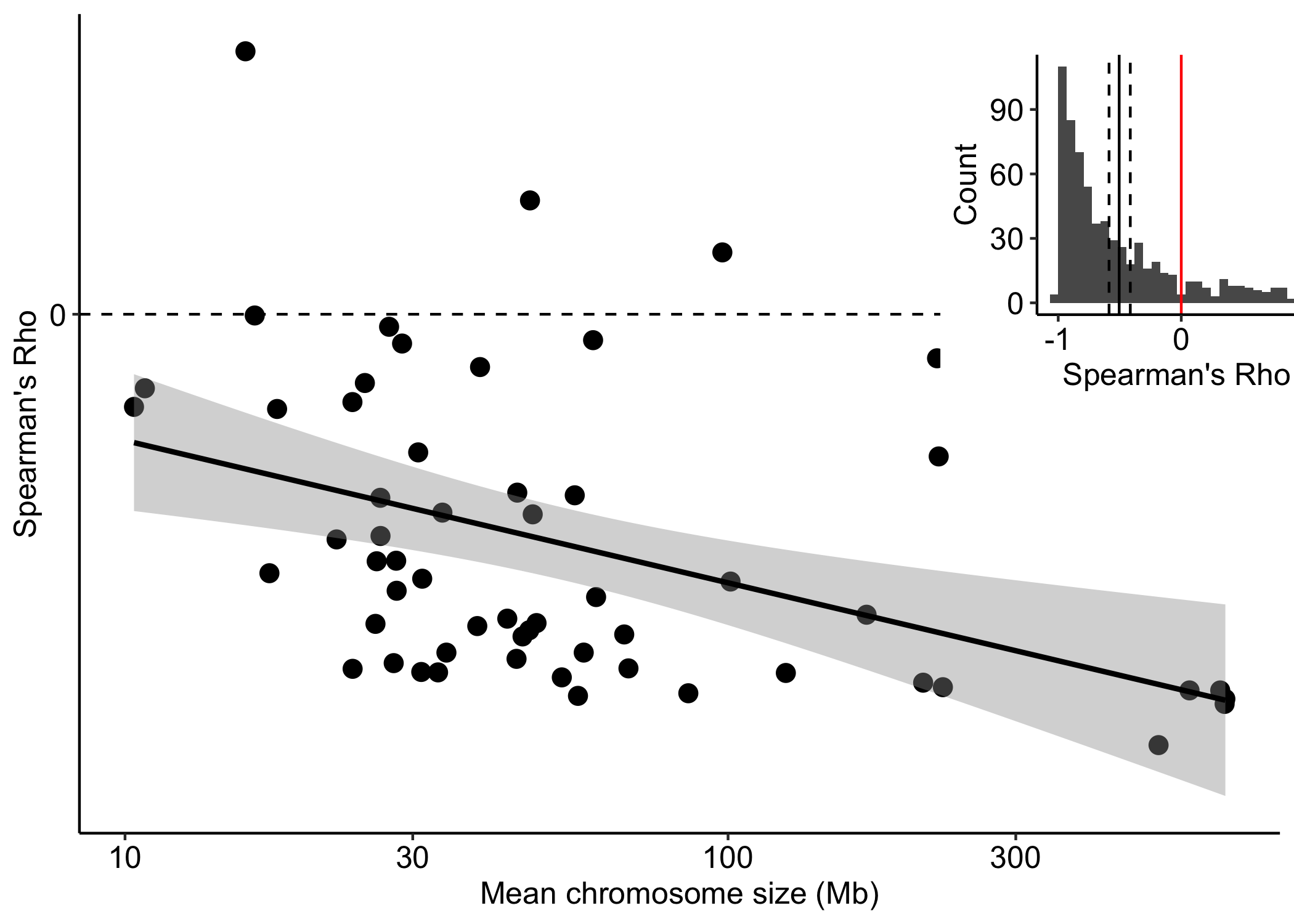

Supplement: S6 Fig — The linear regression line and its parametric 95% confidence interval were estimated in ggplot2. The inset presents the distribution of Spearman’s Rho coefficients for chromosomes (n = 665 chromosomes). The mean correlation and its 95% confidence interval (black solid and dashed lines) were estimated by 1,000 bootstraps. The red vertical line is for a null correlation. (TIF) [file pgen.1010141.s006.tif]

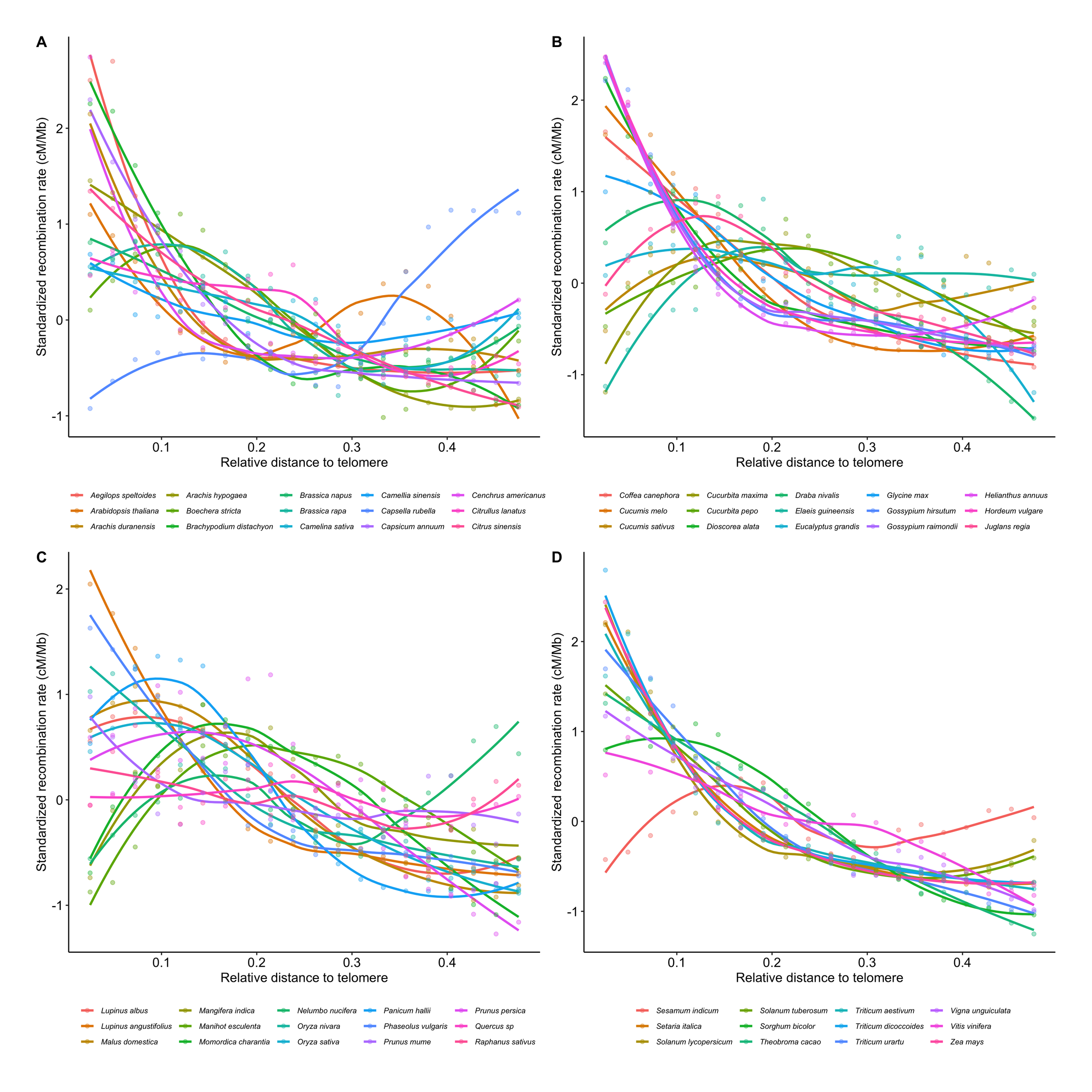

Supplement: S7 Fig — Chromosomes were split in halves, a relative distance of 0.5 being the centre of the chromosome, and only one side was randomly sampled to avoid averaging patterns. Then, chromosomes were pooled per species. Each colour is a species. A loess regression was estimated for each species. Species presented in four plots for clarity. (TIF) [file pgen.1010141.s007.tif]

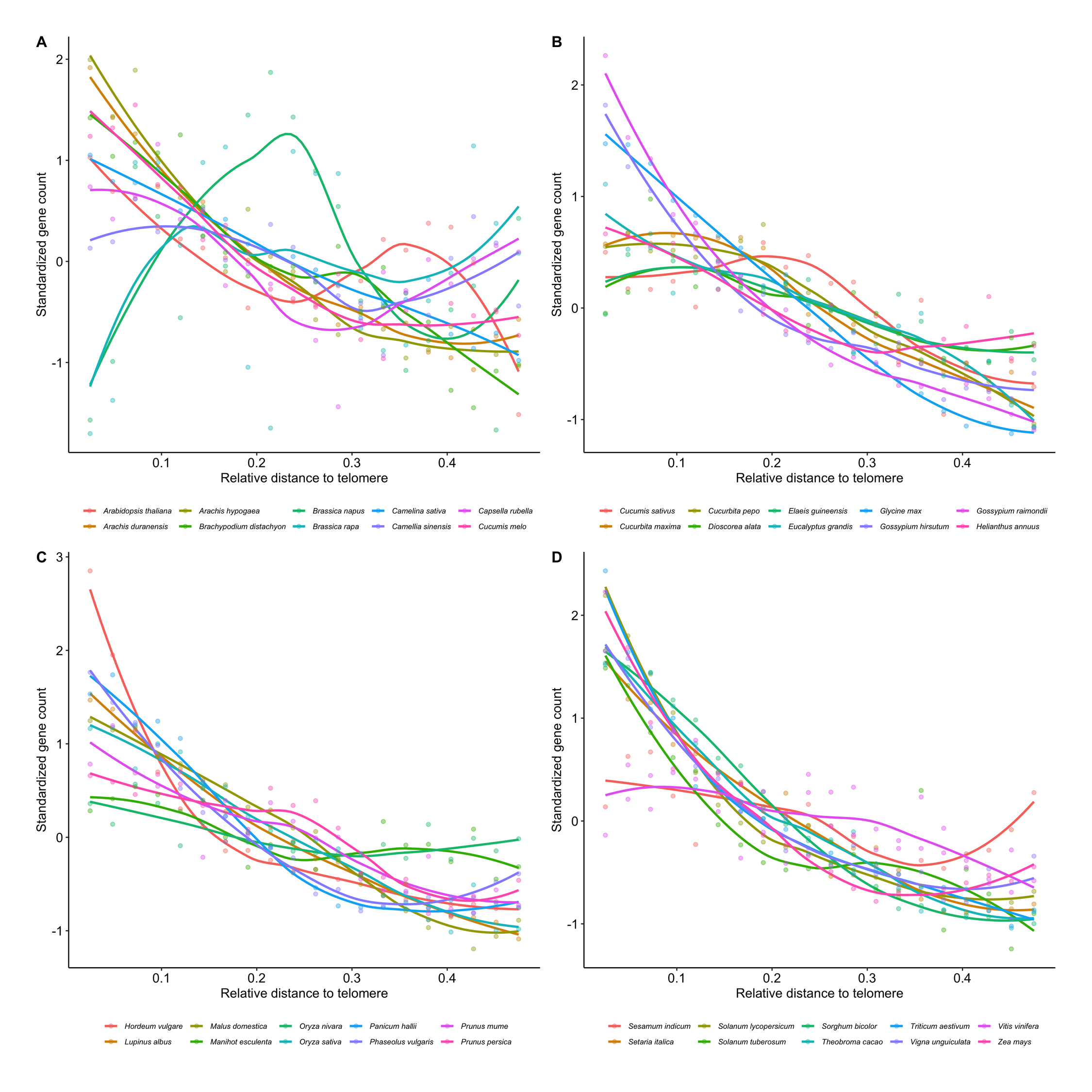

Supplement: S8 Fig — Chromosomes were split in halves, a relative distance of 0.5 being the centre of the chromosome, and only one side was randomly sampled to avoid averaging patterns. Then, chromosomes were pooled per species. Each colour is a species. A loess regression was estimated for each species. Species presented in four plots for clarity. (TIF) [file pgen.1010141.s008.tif]

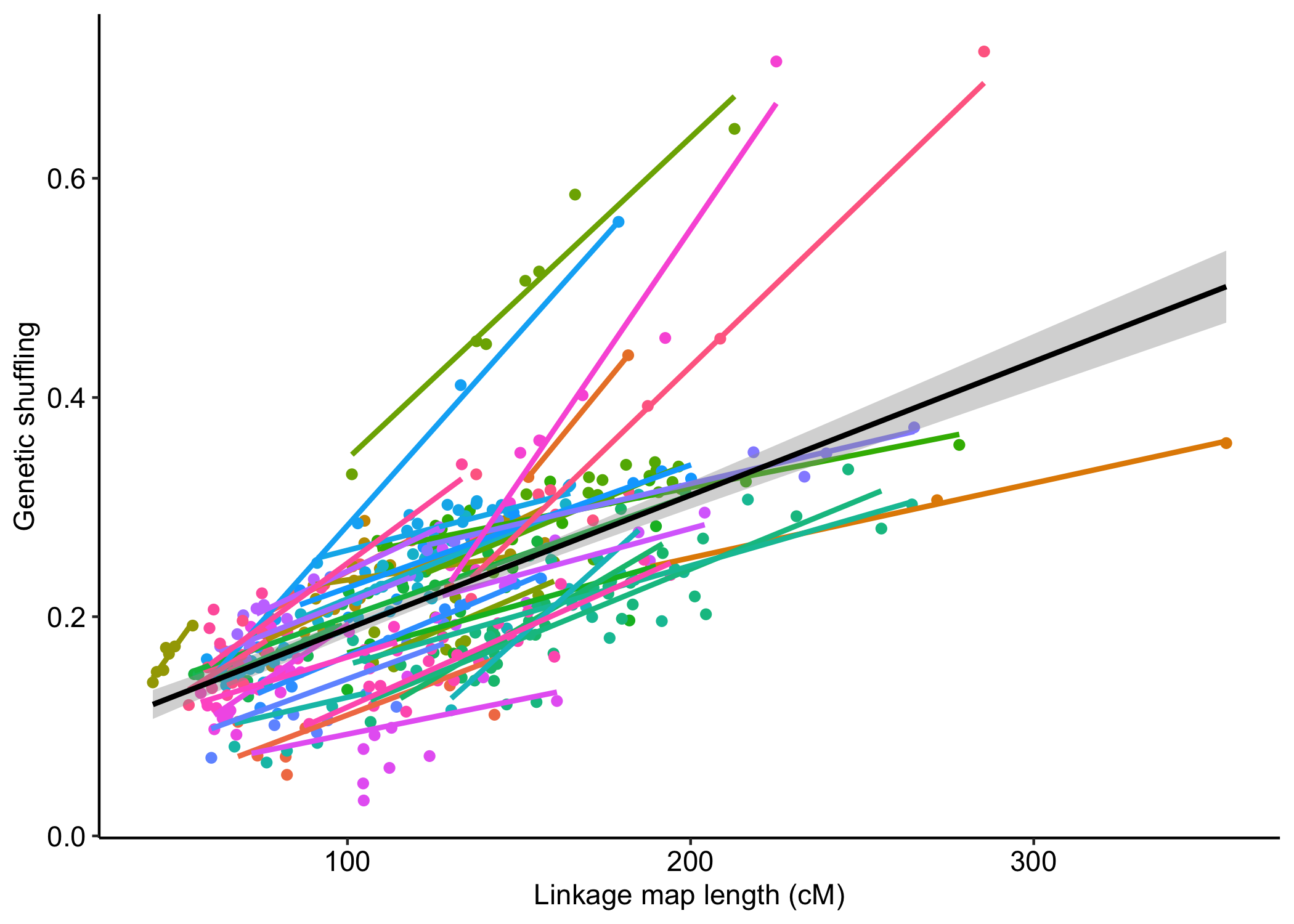

Supplement: S9 Fig — Linear mixed regression with a species random effect and its 95% confidence interval estimated by ggplot2 (black line and grey ribbon). Each colour is a species. A linear regression was estimated for each species. (TIF) [file pgen.1010141.s009.tif]

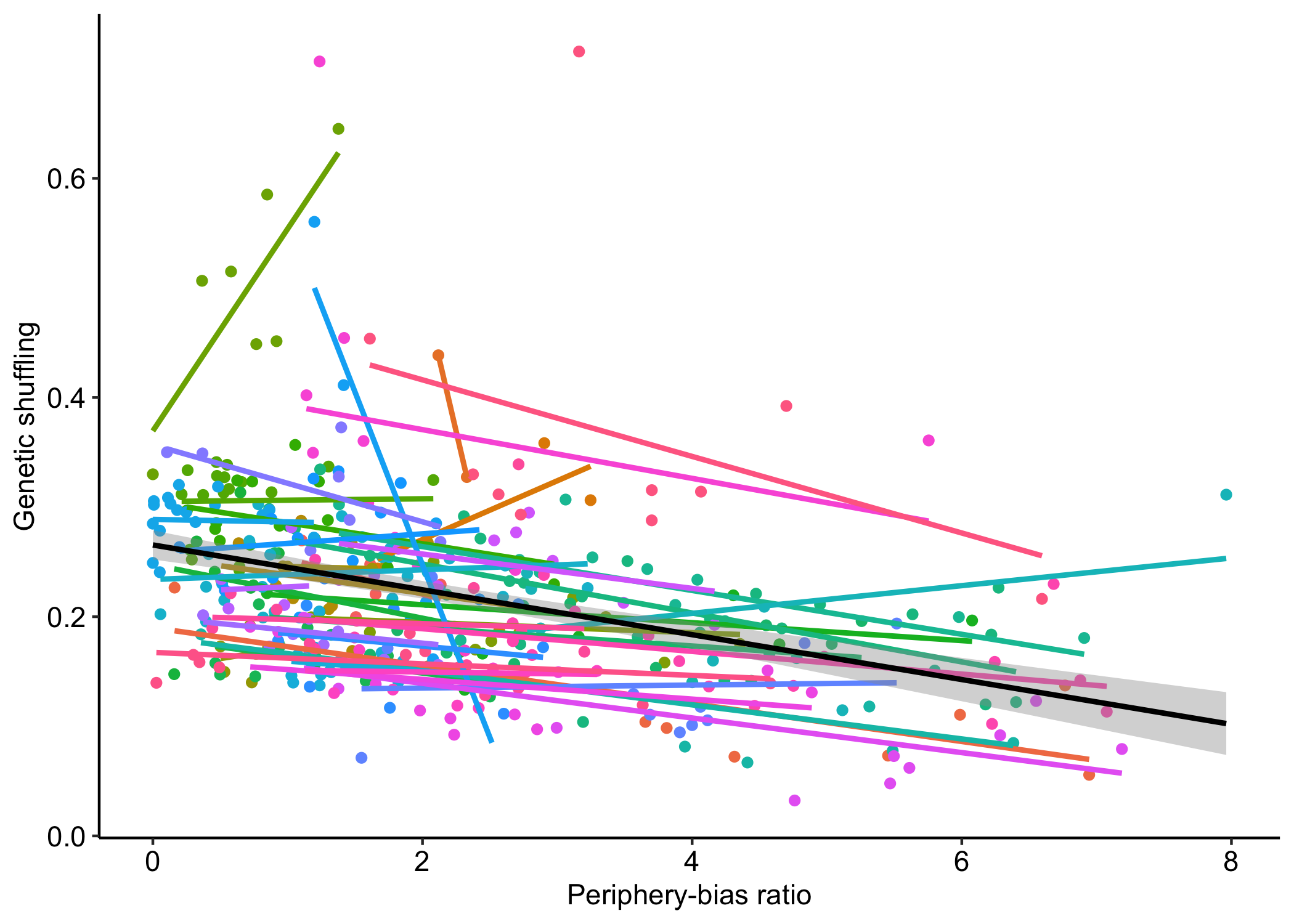

Supplement: S10 Fig — Each colour is a species. A linear regression was estimated for each species. (TIF) [file pgen.1010141.s010.tif]

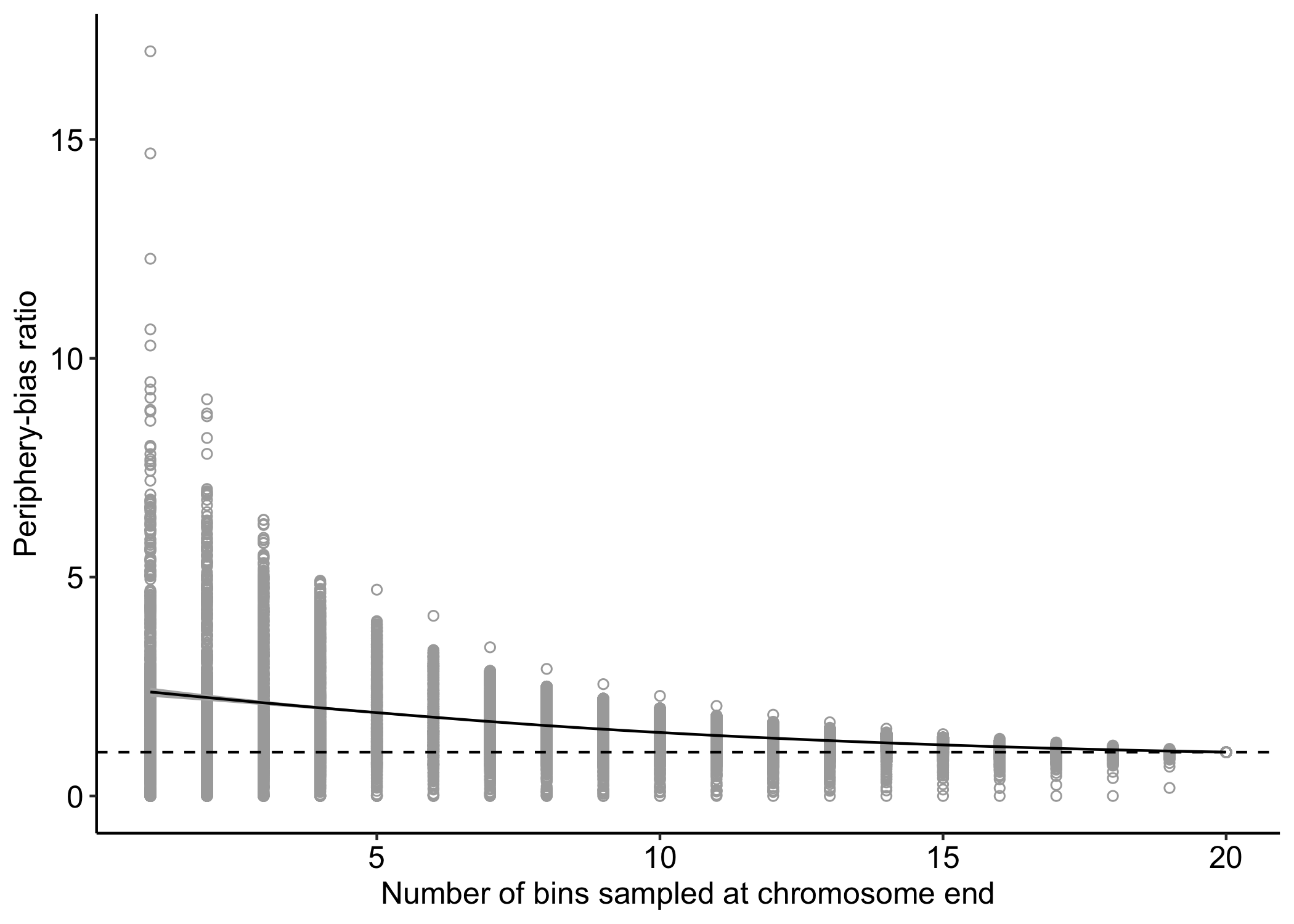

Supplement: S13 Fig — The periphery-bias ratio was estimated for different numbers of bins sampled and always divided by the mean chromosomal recombination rate. Linear regression (black line) shows a decrease of the periphery-bias ratio as the number of bins increases, towards a ratio value of 1 (dashed line). (TIF) [file pgen.1010141.s013.tif]
