# Supplementary material for: Diversity and determinants of recombination landscapes in flowering plants
Source: PLoS Genet. 2022 Aug 30;18(8):e1010141. doi: 10.1371/journal.pgen.1010141 (PMC9467342; doi:10.1371/journal.pgen.1010141)
Supplement: S11 Fig — Recombination rate (cM/Mb) estimated in windows of 100kb. Loess regression of gene count along the chromosome in blue line with parametric confidence interval at 95% in grey. (PDF) [file pgen.1010141.s011.pdf]

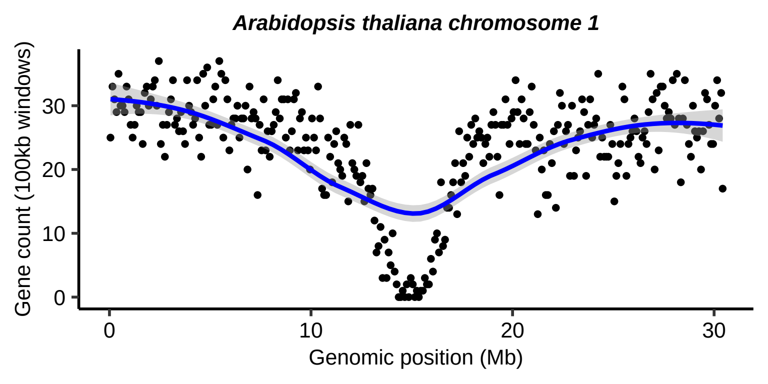

Arabidopsis thaliana chromosome 1
Gene count (100kb windows)
0
10
20
30
Genomic position (Mb)

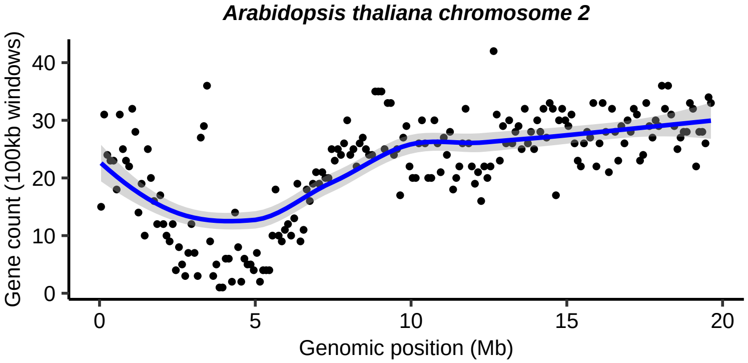

Arabidopsis thaliana chromosome 2
Gene count (100kb windows)
0
10
20
30
40
0
5
10
15
20
Genomic position (Mb)

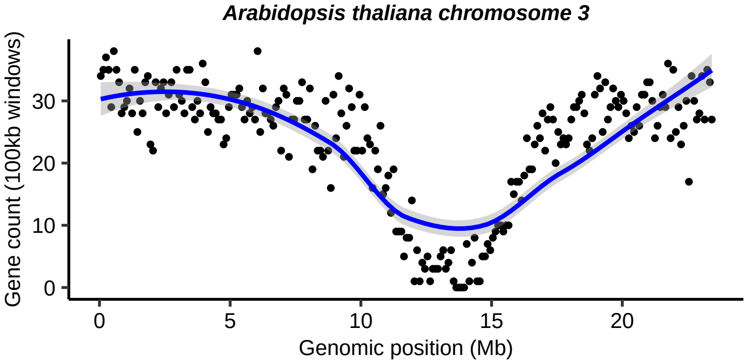

Arabidopsis thaliana chromosome 3
Gene count (100kb windows)
0
10
20
30
0
5
10
15
20
Genomic position (Mb)

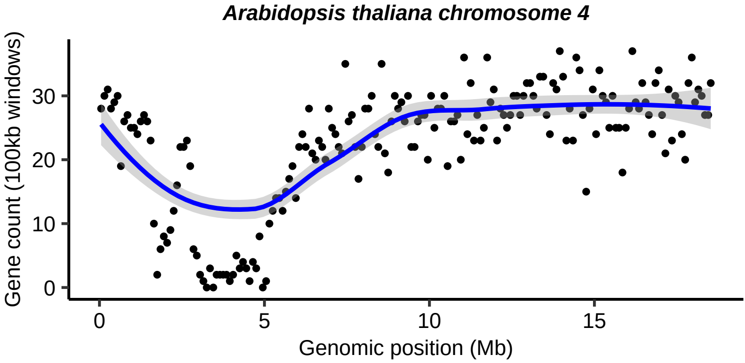

Arabidopsis thaliana chromosome 4
Gene count (100kb windows)
0
10
20
30
0
5
10
15
Genomic position (Mb)

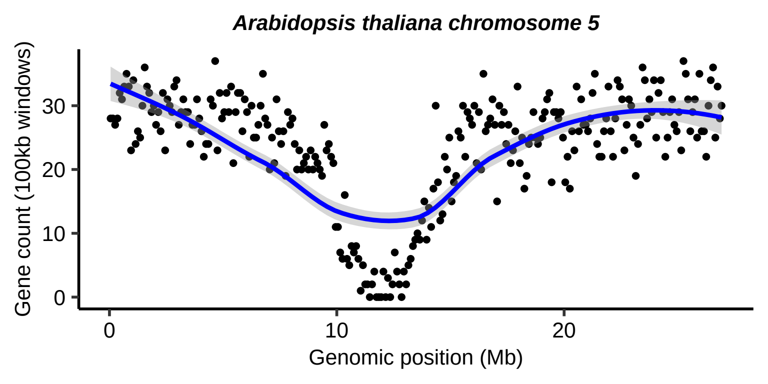
Arabidopsis thaliana chromosome 5
Gene count (100kb windows)
0
10
20
30
0
10
20
Genomic position (Mb)

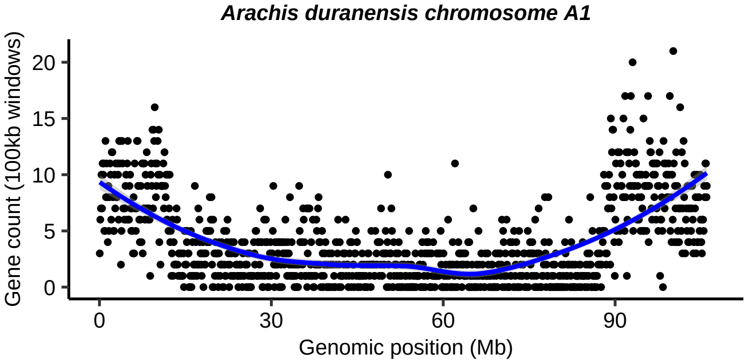

Arachis duranensis chromosome A1
Gene count (100kb windows)
0
5
10
15
20
0
30
60
90
Genomic position (Mb)

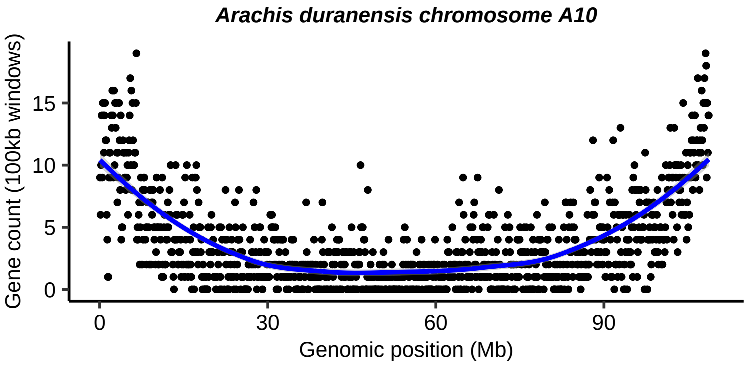

Arachis duranensis chromosome A10
Gene count (100kb windows)
0
5
10
15
0
30
60
90
Genomic position (Mb)

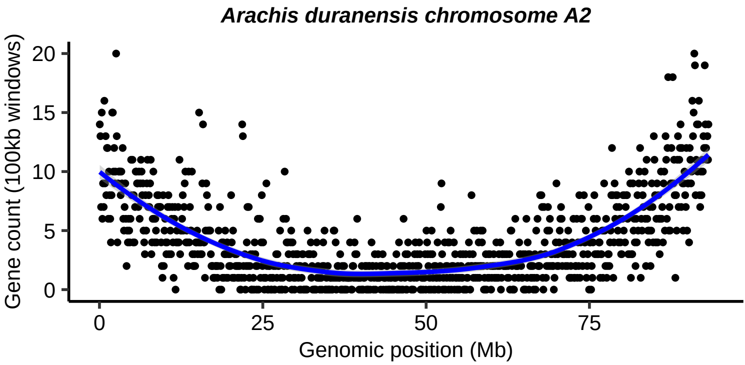

Arachis duranensis chromosome A2
Gene count (100kb windows)
0
5
10
15
20
0
25
50
75
Genomic position (Mb)

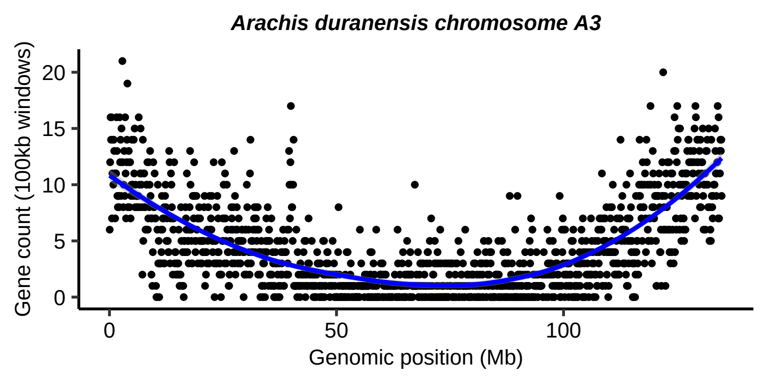
Arachis duranensis chromosome A3
Gene count (100kb windows)
0
5
10
15
20
0
50
100
Genomic position (Mb)

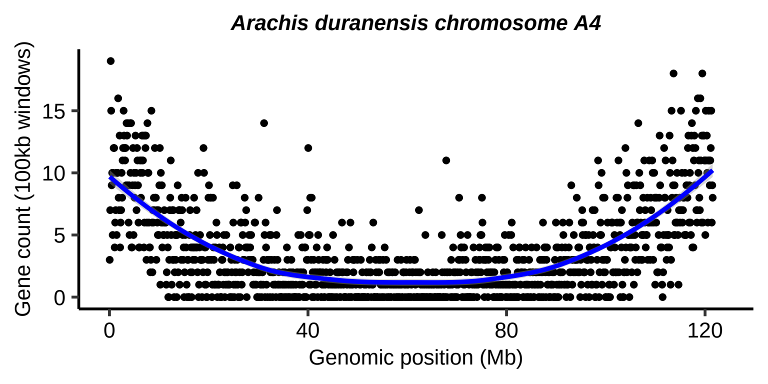

Arachis duranensis chromosome A4
Gene count (100kb windows)
0
5
10
15
0
40
80
120
Genomic position (Mb)

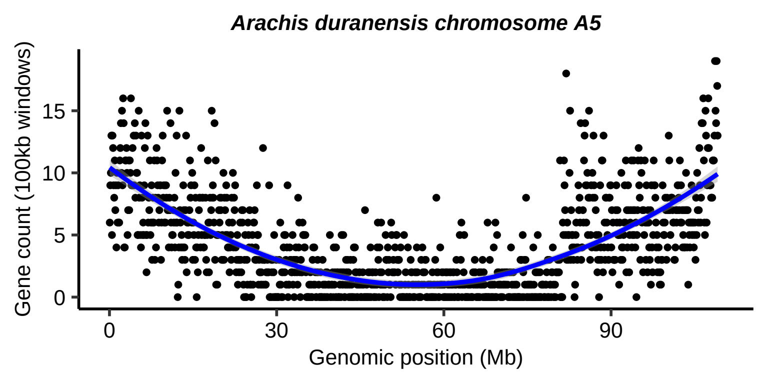

Arachis duranensis chromosome A5
Gene count (100kb windows)
0
5
10
15
0
30
60
90
Genomic position (Mb)

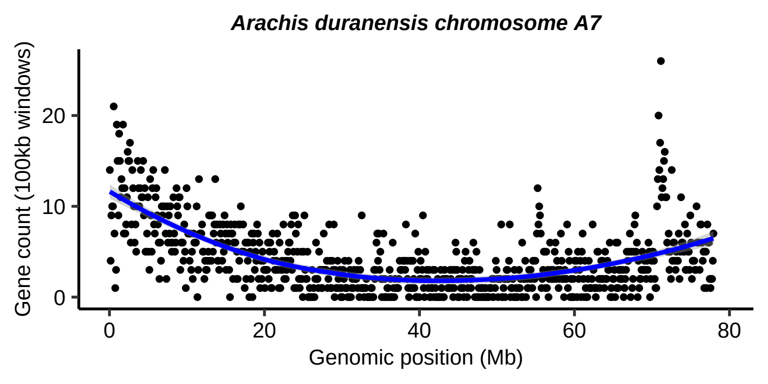

Arachis duranensis chromosome A7
Gene count (100kb windows)
0
10
20
0
20
40
60
80
Genomic position (Mb)

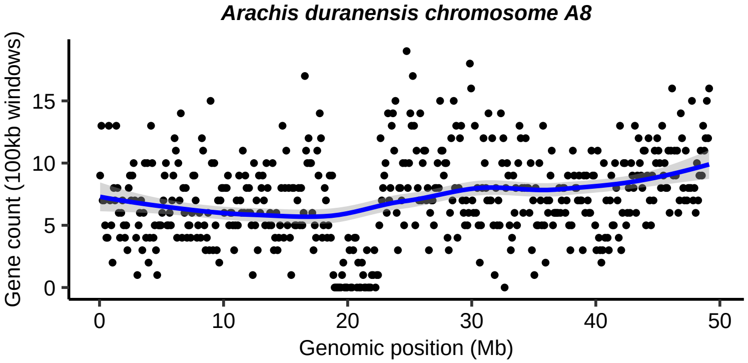

Arachis duranensis chromosome A8
Gene count (100kb windows)
0
5
10
15
0
10
20
30
40
50
Genomic position (Mb)

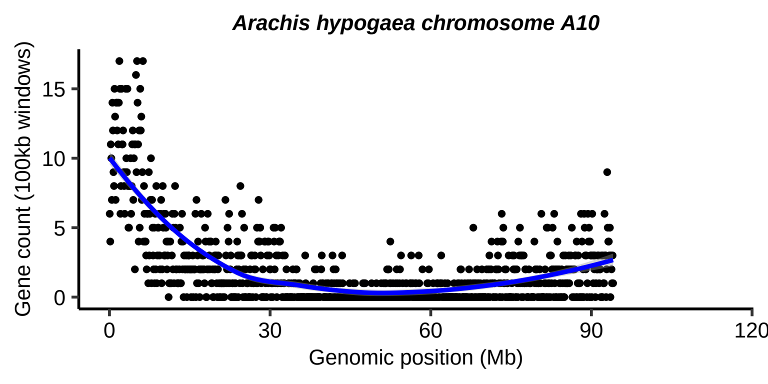
Arachis hypogaea chromosome A10
Gene count (100kb windows)
0
5
10
15
0
30
60
90
120
Genomic position (Mb)

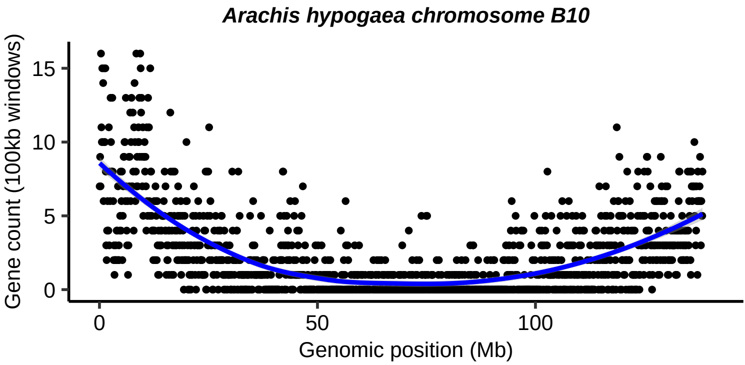

Arachis hypogaea chromosome B10
Gene count (100kb windows)
0
5
10
15
0
50
100
Genomic position (Mb)

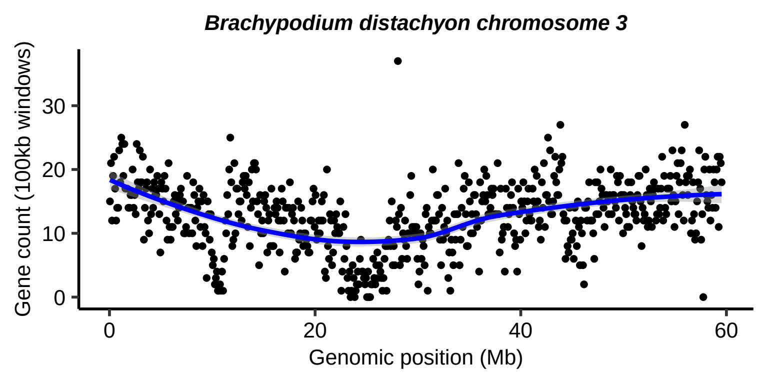
Brachypodium distachyon chromosome 3
Gene count (100kb windows)
0
10
20
30
0
20
40
60
Genomic position (Mb)

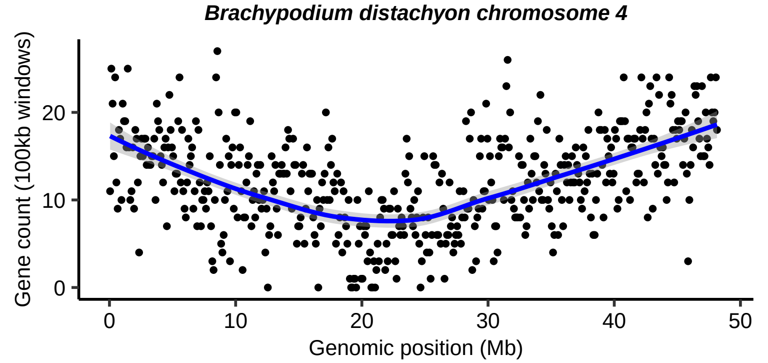

Brachypodium distachyon chromosome 4
Gene count (100kb windows)
20
10
0
0
10
20
30
40
50
Genomic position (Mb)

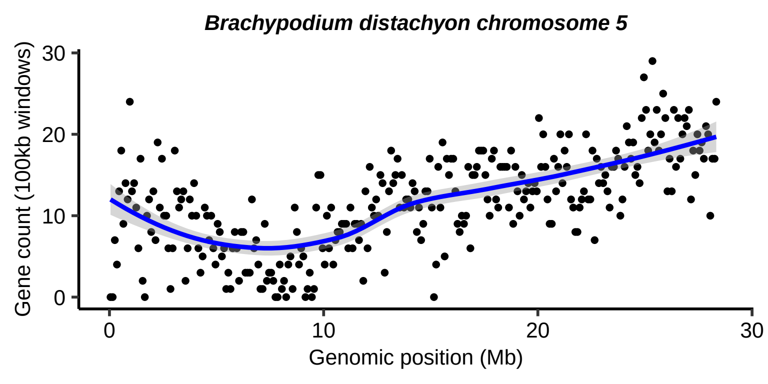

Brachypodium distachyon chromosome 5
Gene count (100kb windows)
0
10
20
30
0
10
20
30
Genomic position (Mb)

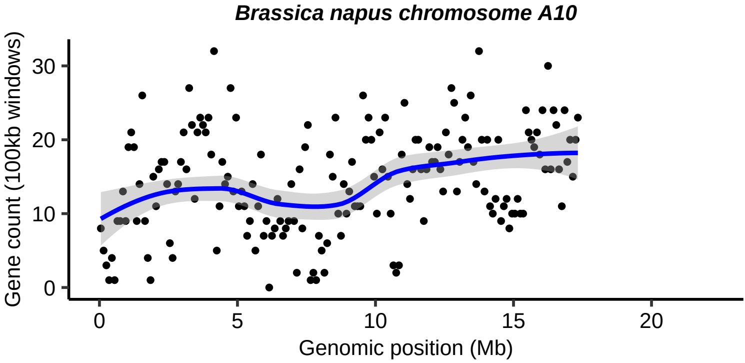

Brassica napus chromosome A10
Gene count (100kb windows)
30
20
10
0
0
5
10
15
20
Genomic position (Mb)

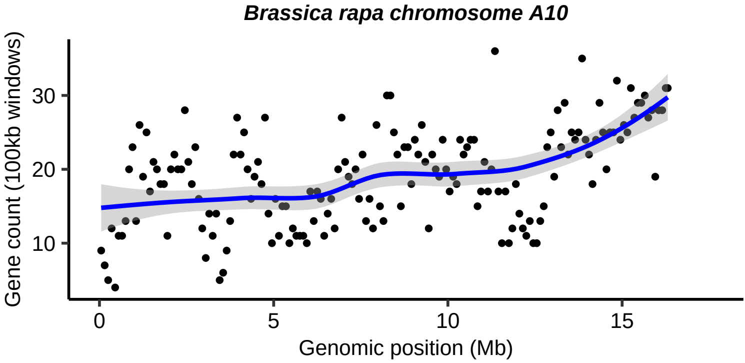

Brassica rapa chromosome A10
Gene count (100kb windows)
30
20
10
0
5
10
15
Genomic position (Mb)

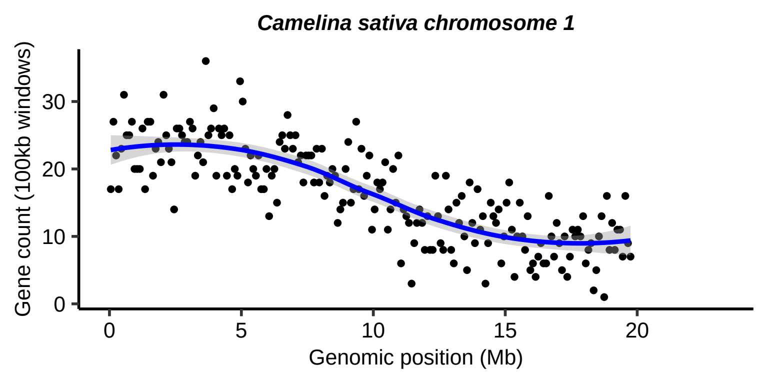

Camelina sativa chromosome 1
Gene count (100kb windows)
0
10
20
30
0
5
10
15
20
Genomic position (Mb)

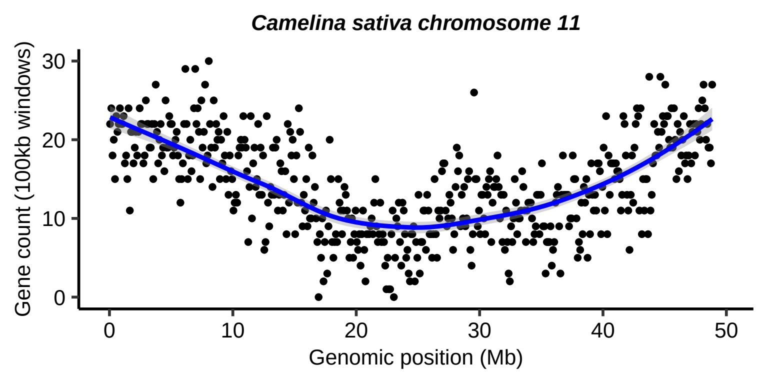

Camelina sativa chromosome 11
Gene count (100kb windows)
0
10
20
30
0
10
20
30
40
50
Genomic position (Mb)

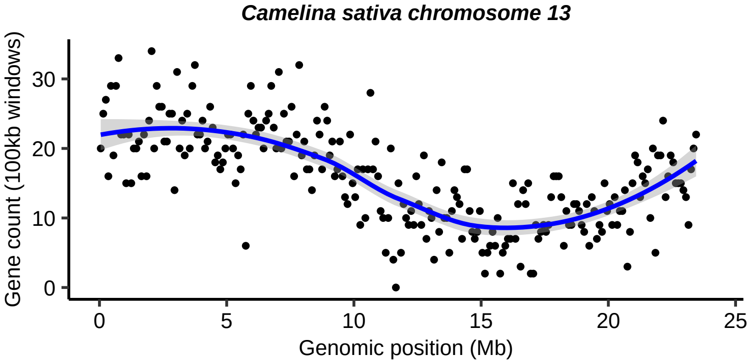
Camelina sativa chromosome 13
Gene count (100kb windows)
0
10
20
30
0
5
10
15
20
25
Genomic position (Mb)

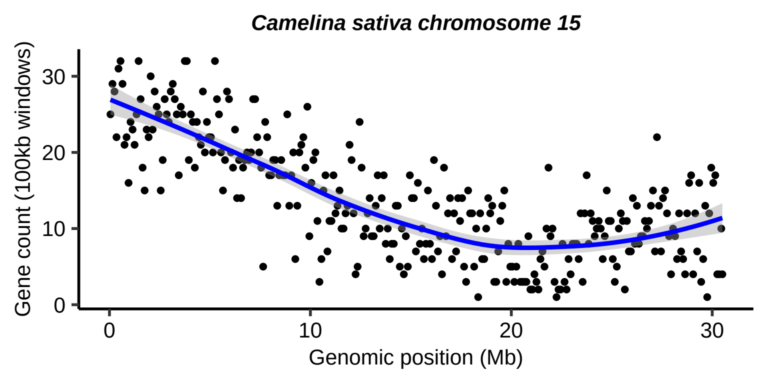

Camelina sativa chromosome 15
Gene count (100kb windows)
0
10
20
30
Genomic position (Mb)

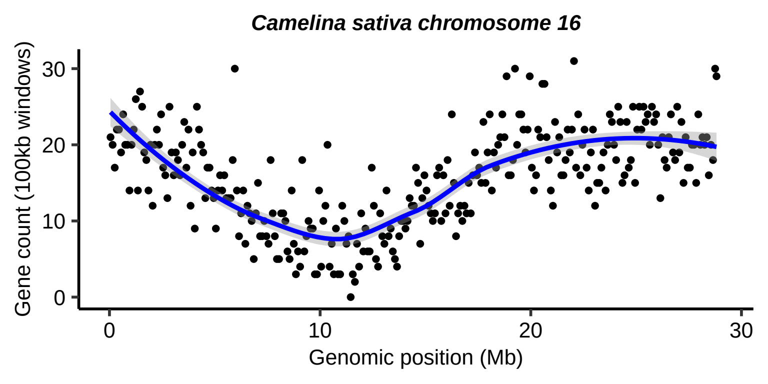

Camelina sativa chromosome 16
Gene count (100kb windows)
0
10
20
30
Genomic position (Mb)

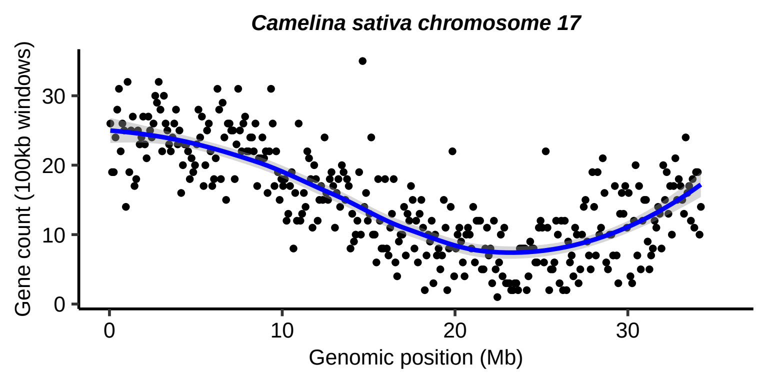

Camelina sativa chromosome 17
Gene count (100kb windows)
0
10
20
30
0
10
20
30
Genomic position (Mb)

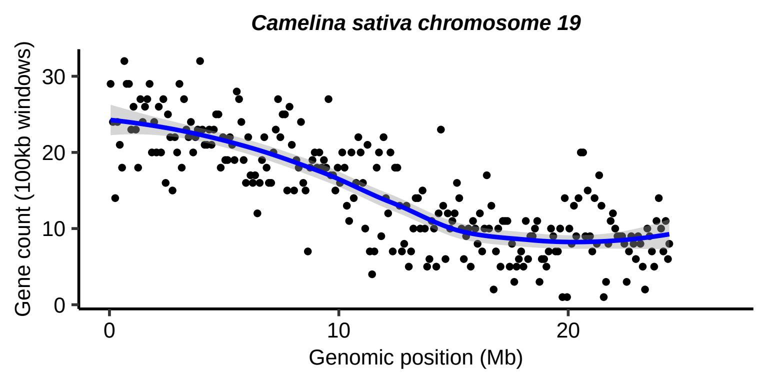
Camelina sativa chromosome 19
Gene count (100kb windows)
30
20
10
0
0
10
20
Genomic position (Mb)

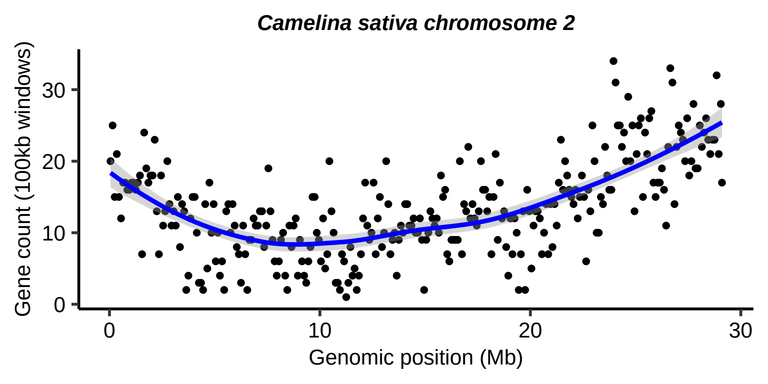

Camelina sativa chromosome 2
Gene count (100kb windows)
0
10
20
30
0
10
20
30
Genomic position (Mb)

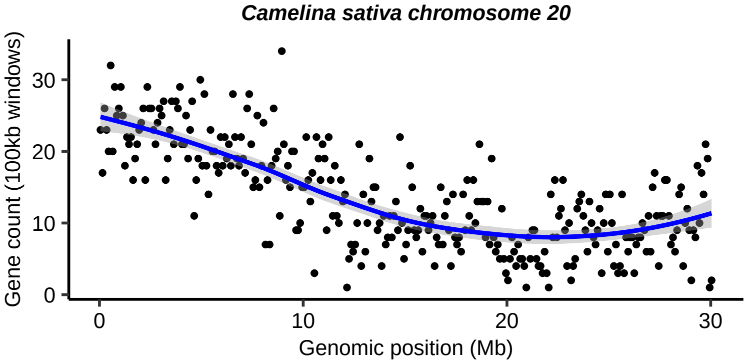

Camelina sativa chromosome 20
Gene count (100kb windows)
0
10
20
30
Genomic position (Mb)

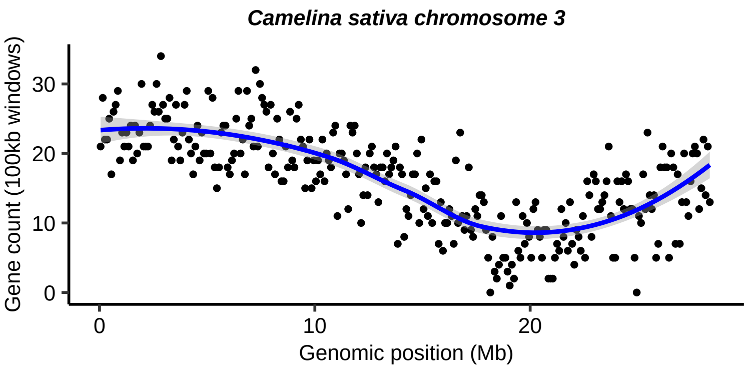

Camelina sativa chromosome 3
Gene count (100kb windows)
0
10
20
30
Genomic position (Mb)

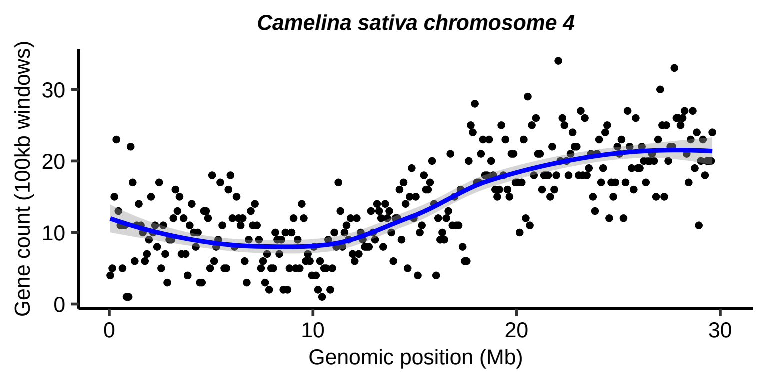

Camelina sativa chromosome 4
Gene count (100kb windows)
0
10
20
30
Genomic position (Mb)

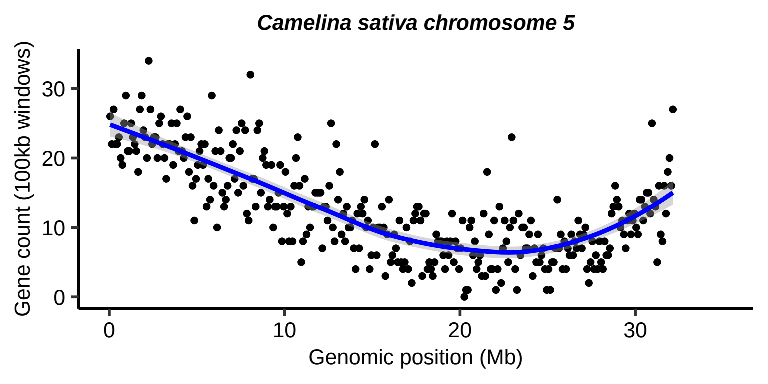

Camelina sativa chromosome 5
Gene count (100kb windows)
0
10
20
30
Genomic position (Mb)

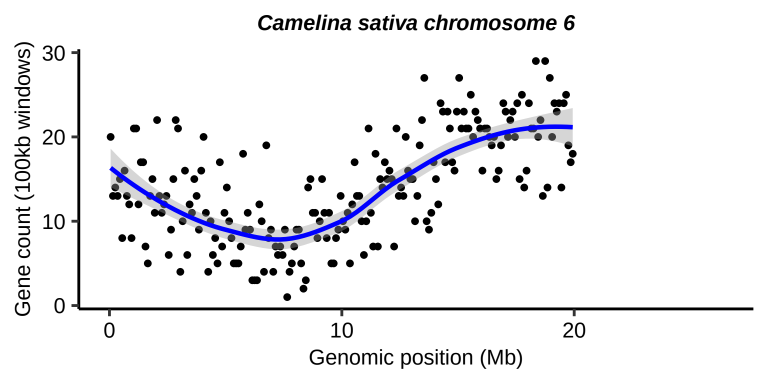
Camelina sativa chromosome 6
Gene count (100kb windows)
30
20
10
0
0
10
20
Genomic position (Mb)

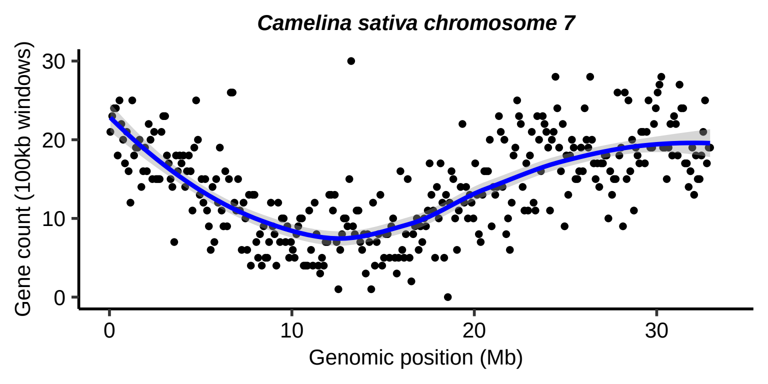
Camelina sativa chromosome 7
Gene count (100kb windows)
0
10
20
30
Genomic position (Mb)
0
10
20
30

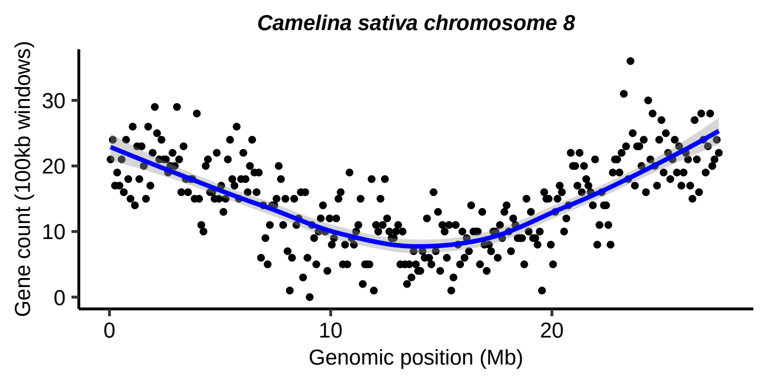

Camelina sativa chromosome 8
Gene count (100kb windows)
0
10
20
30
Genomic position (Mb)

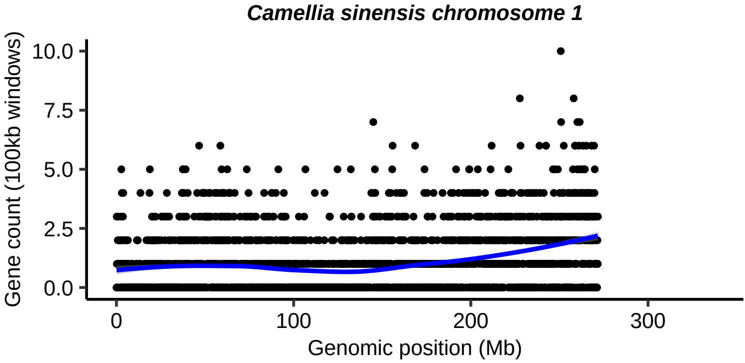
Camellia sinensis chromosome 1
Gene count (100kb windows)
10.0
7.5
5.0
2.5
0.0
0
100
200
300
Genomic position (Mb)

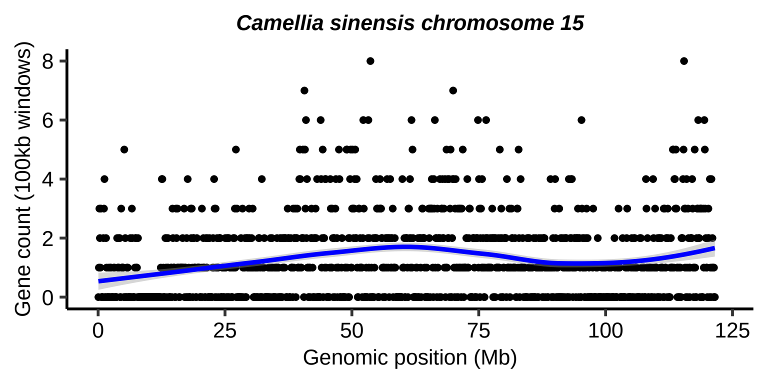

Camellia sinensis chromosome 15
Gene count (100kb windows)
0
2
4
6
8
0
25
50
75
100
125
Genomic position (Mb)

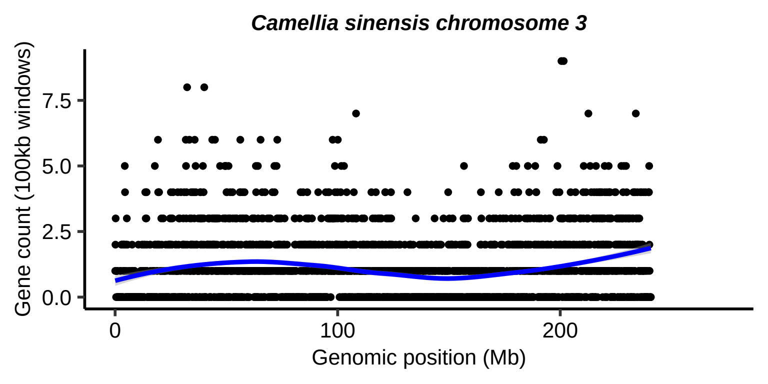

Camellia sinensis chromosome 3
Gene count (100kb windows)
7.5
5.0
2.5
0.0
0
100
200
Genomic position (Mb)

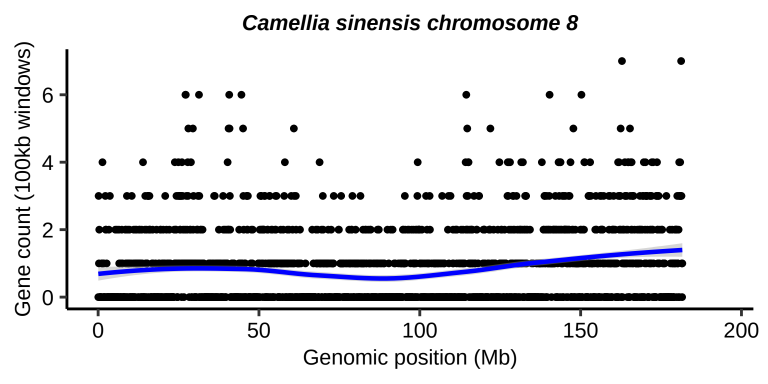

Camellia sinensis chromosome 8
Gene count (100kb windows)
0
2
4
6
0
50
100
150
200
Genomic position (Mb)

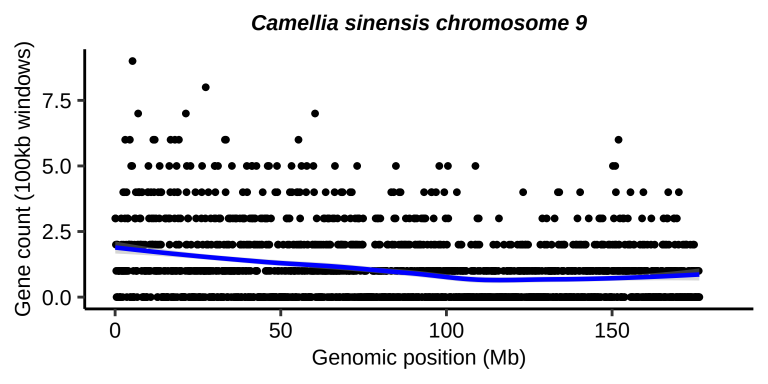
Camellia sinensis chromosome 9
Gene count (100kb windows)
0.0
2.5
5.0
7.5
0
50
100
150
Genomic position (Mb)

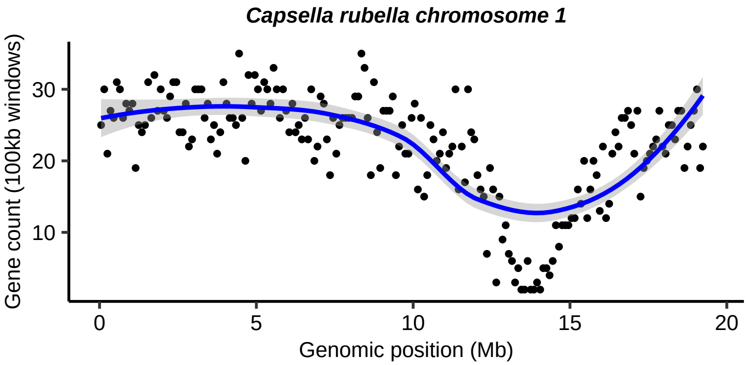
Capsella rubella chromosome 1
Gene count (100kb windows)
30
20
10
0
5
10
15
20
Genomic position (Mb)

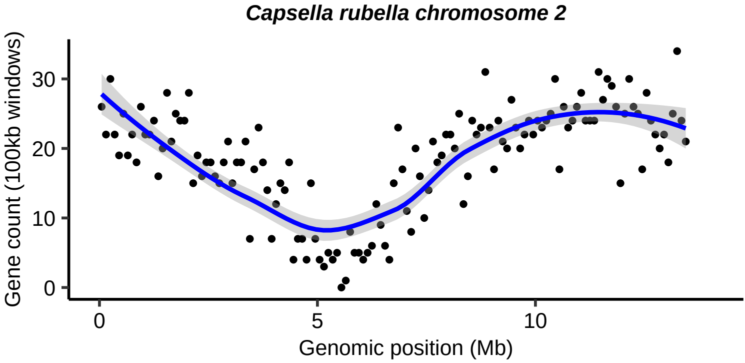

Capsella rubella chromosome 2
Gene count (100kb windows)
0
10
20
30
0
5
10
Genomic position (Mb)

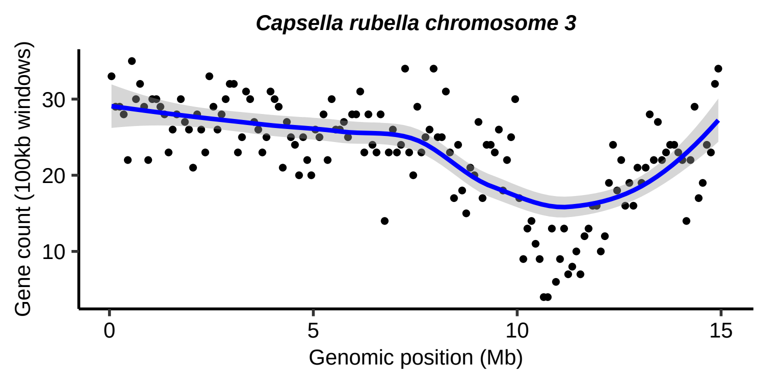

Capsella rubella chromosome 3
Gene count (100kb windows)
30
20
10
0
5
10
15
Genomic position (Mb)

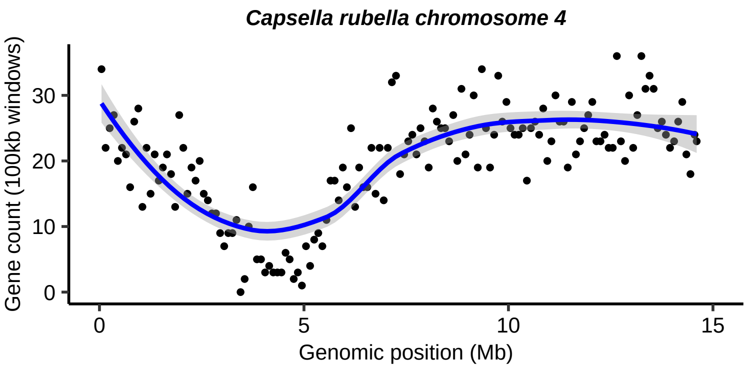

Capsella rubella chromosome 4
Gene count (100kb windows)
30
20
10
0
0
5
10
15
Genomic position (Mb)

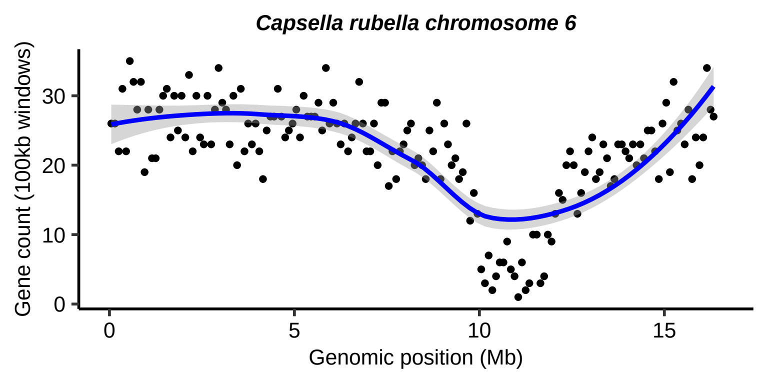
Capsella rubella chromosome 6
Gene count (100kb windows)
0
10
20
30
0
5
10
15
Genomic position (Mb)

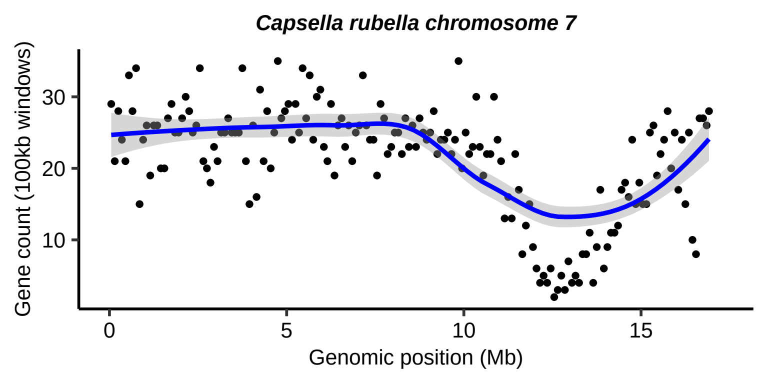

Capsella rubella chromosome 7
Gene count (100kb windows)
30
20
10
0
5
10
15
Genomic position (Mb)

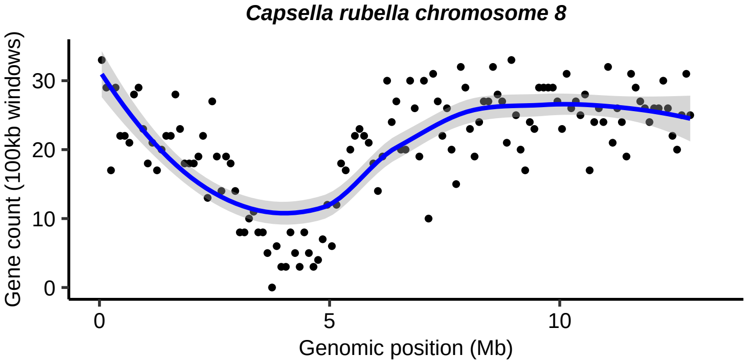

Capsella rubella chromosome 8
Gene count (100kb windows)
0
10
20
30
0
5
10
Genomic position (Mb)

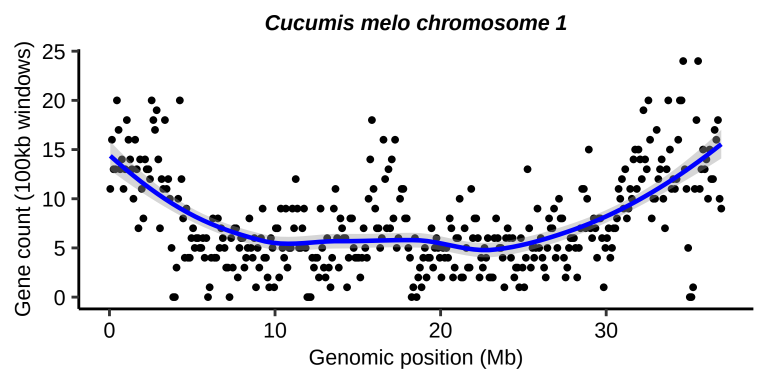

Cucumis melo chromosome 1
Gene count (100kb windows)
0
5
10
15
20
25
0
10
20
30
Genomic position (Mb)

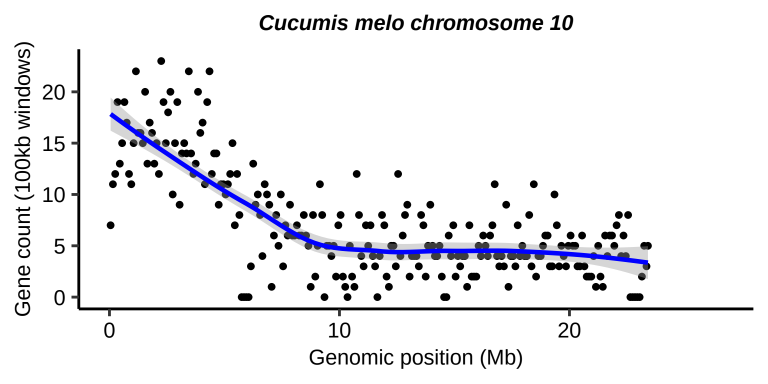

Cucumis melo chromosome 10
Gene count (100kb windows)
0
5
10
15
20
0
10
20
Genomic position (Mb)

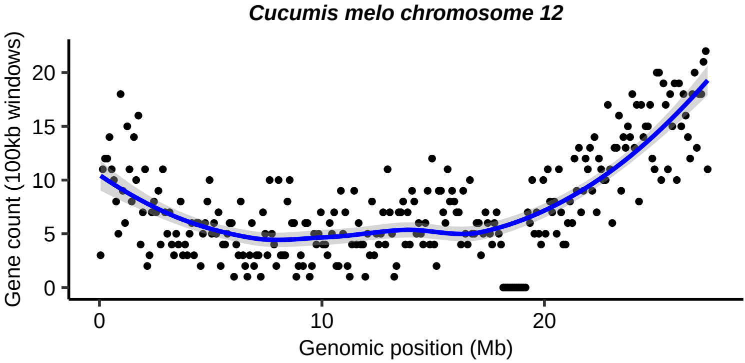

Cucumis melo chromosome 12
Gene count (100kb windows)
0
5
10
15
20
0
10
20
Genomic position (Mb)

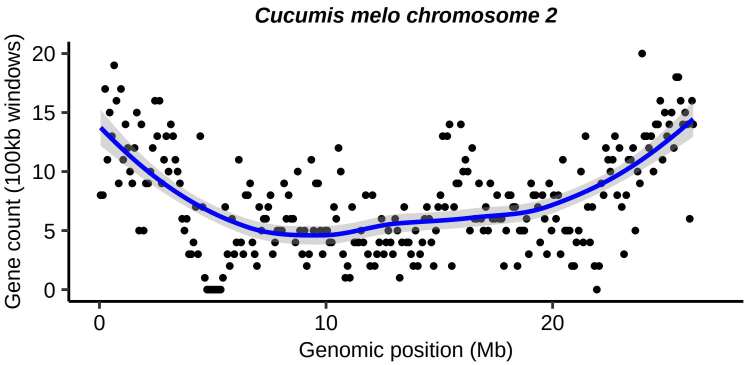

Cucumis melo chromosome 2
Gene count (100kb windows)
0
5
10
15
20
0
10
20
Genomic position (Mb)

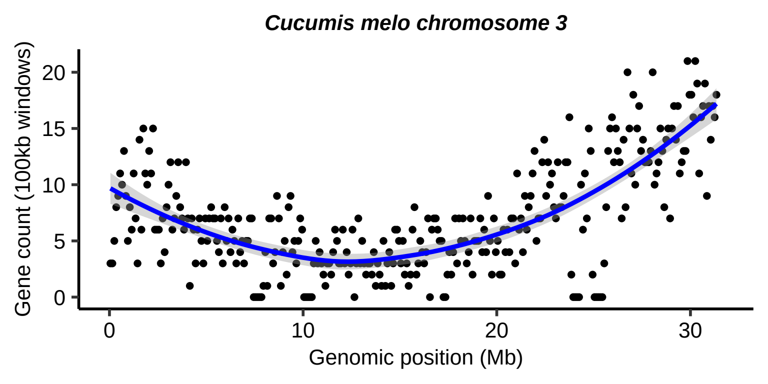
Cucumis melo chromosome 3
Gene count (100kb windows)
0
5
10
15
20
0
10
20
30
Genomic position (Mb)

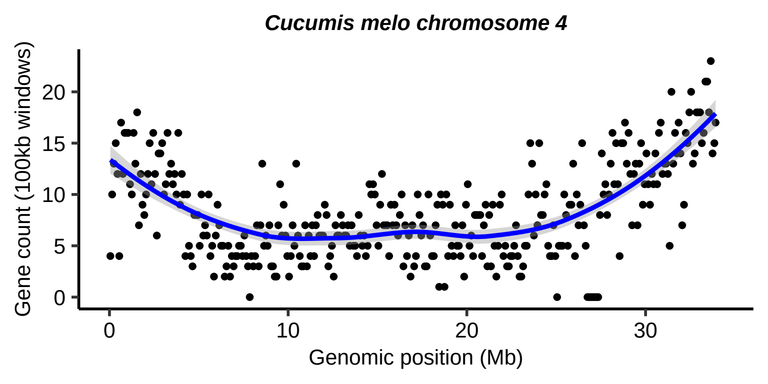

Cucumis melo chromosome 4
Gene count (100kb windows)
0
5
10
15
20
0
10
20
30
Genomic position (Mb)

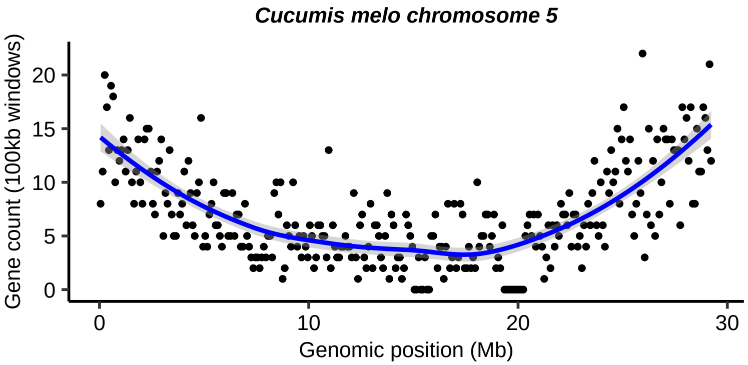

Cucumis melo chromosome 5
Gene count (100kb windows)
0
5
10
15
20
0
10
20
30
Genomic position (Mb)

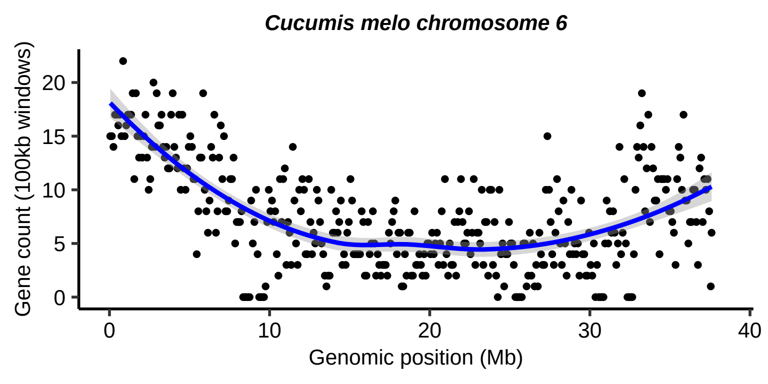

Cucumis melo chromosome 6
Gene count (100kb windows)
0
5
10
15
20
0
10
20
30
40
Genomic position (Mb)

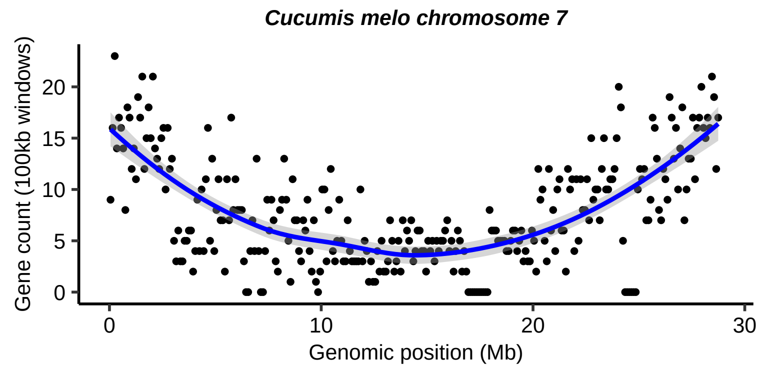
Cucumis melo chromosome 7
Gene count (100kb windows)
0
5
10
15
20
0
10
20
30
Genomic position (Mb)

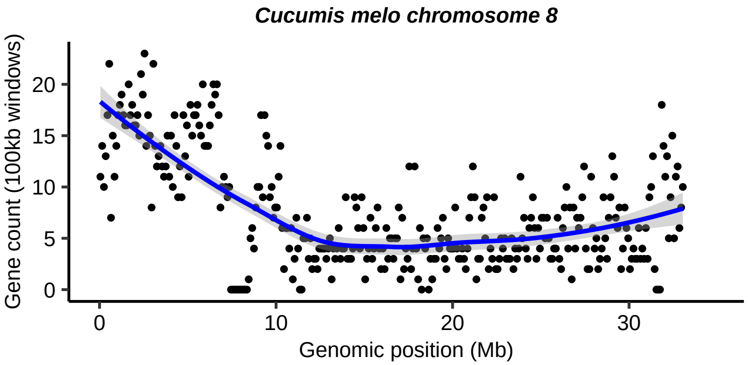

Cucumis melo chromosome 8
Gene count (100kb windows)
0
5
10
15
20
0
10
20
30
Genomic position (Mb)

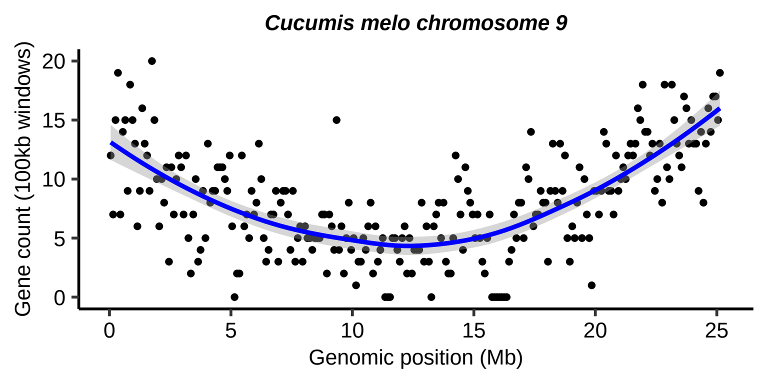

Cucumis melo chromosome 9
Gene count (100kb windows)
20
15
10
5
0
0
5
10
15
20
25
Genomic position (Mb)

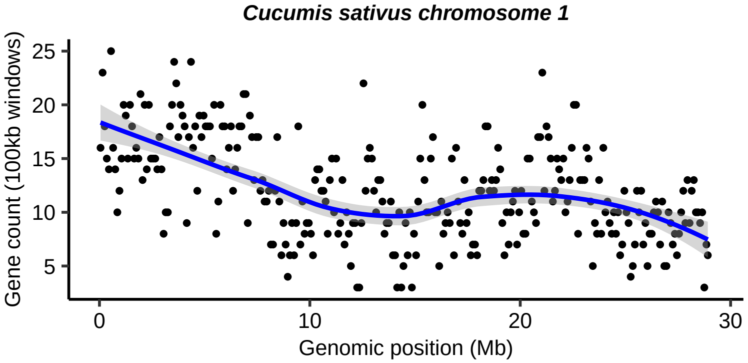

Cucumis sativus chromosome 1
Gene count (100kb windows)
25
20
15
10
5
0
10
20
30
Genomic position (Mb)

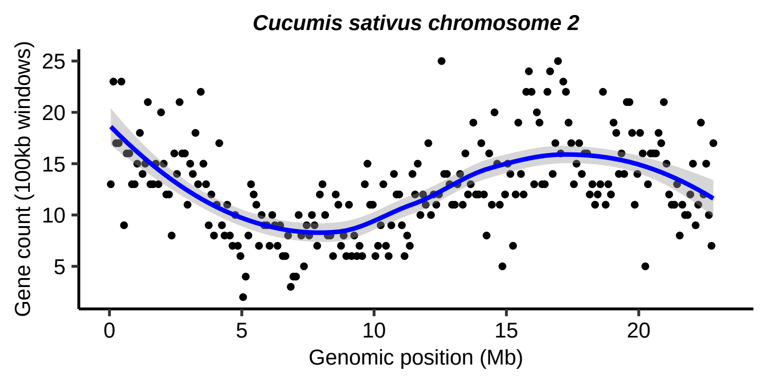

Cucumis sativus chromosome 2
Gene count (100kb windows)
25
20
15
10
5
0
5
10
15
20
Genomic position (Mb)

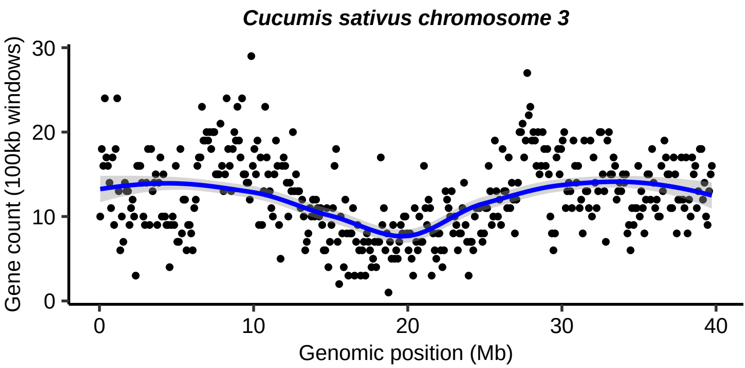

Cucumis sativus chromosome 3
Gene count (100kb windows)
30
20
10
0
0
10
20
30
40
Genomic position (Mb)

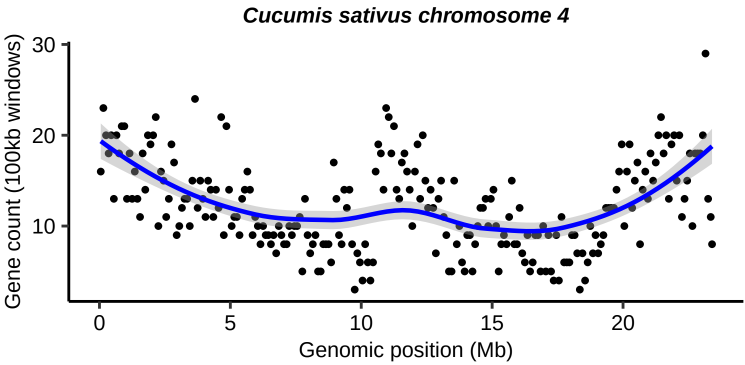
Cucumis sativus chromosome 4
Gene count (100kb windows)
30
20
10
0
5
10
15
20
Genomic position (Mb)

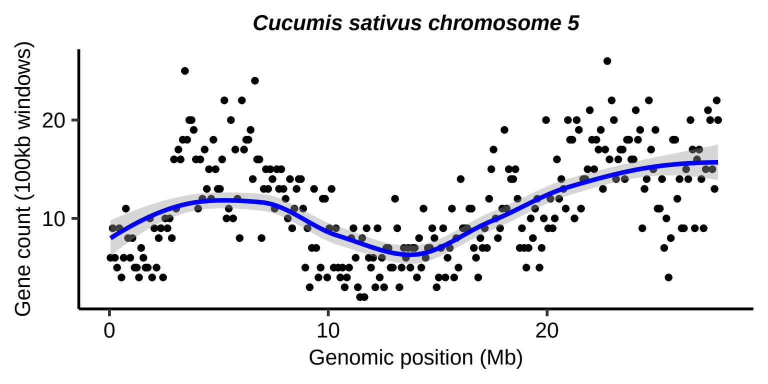

Cucumis sativus chromosome 5
Gene count (100kb windows)
10
20
0
10
20
Genomic position (Mb)

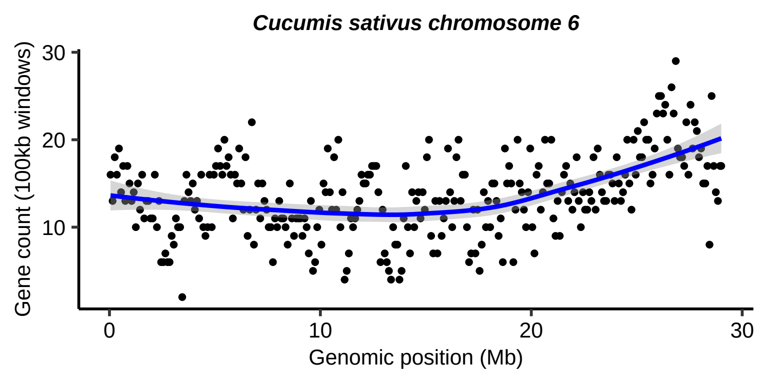
Cucumis sativus chromosome 6
Gene count (100kb windows)
30
20
10
0
10
20
30
Genomic position (Mb)

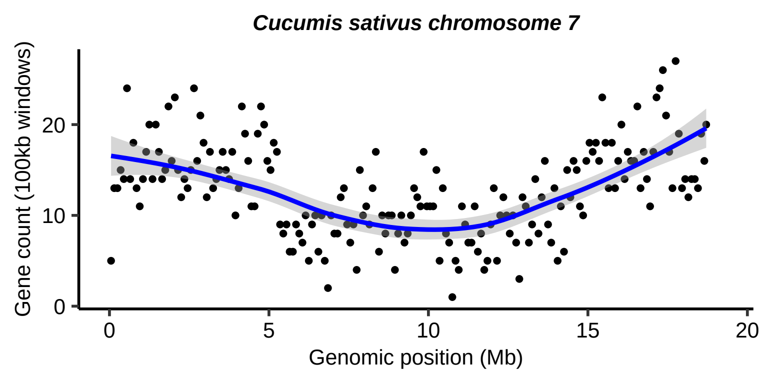

Cucumis sativus chromosome 7
Gene count (100kb windows)
0
10
20
0
5
10
15
20
Genomic position (Mb)

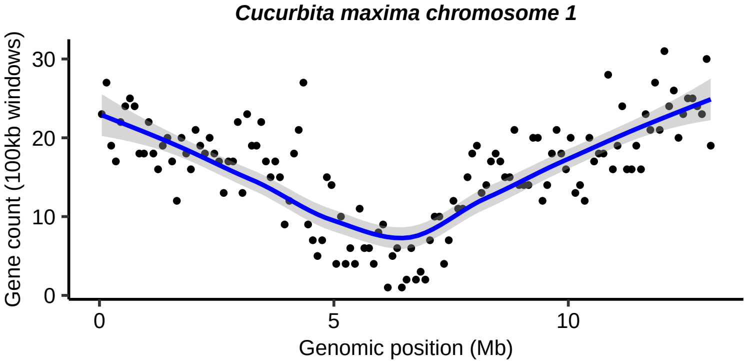

Cucurbita maxima chromosome 1
Gene count (100kb windows)
0
10
20
30
0
5
10
Genomic position (Mb)

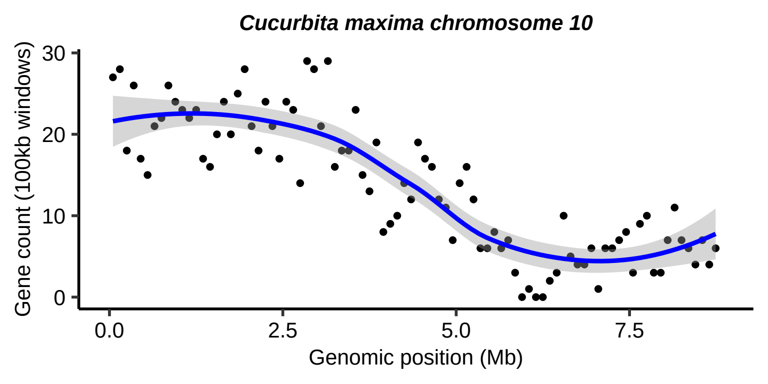

Cucurbita maxima chromosome 10
Gene count (100kb windows)
30
20
10
0
0.0
2.5
5.0
7.5
Genomic position (Mb)

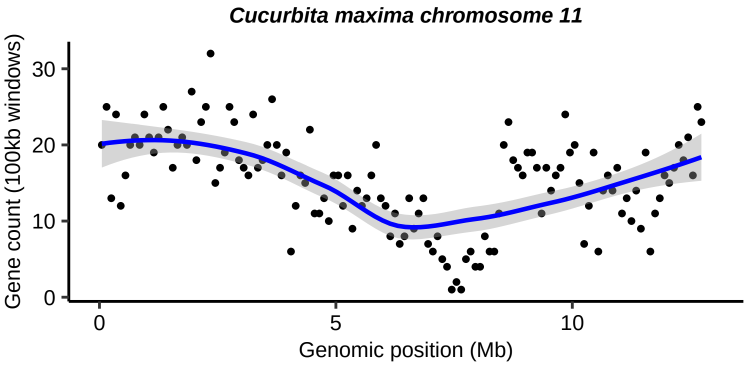

Cucurbita maxima chromosome 11
Gene count (100kb windows)
0
10
20
30
0
5
10
Genomic position (Mb)

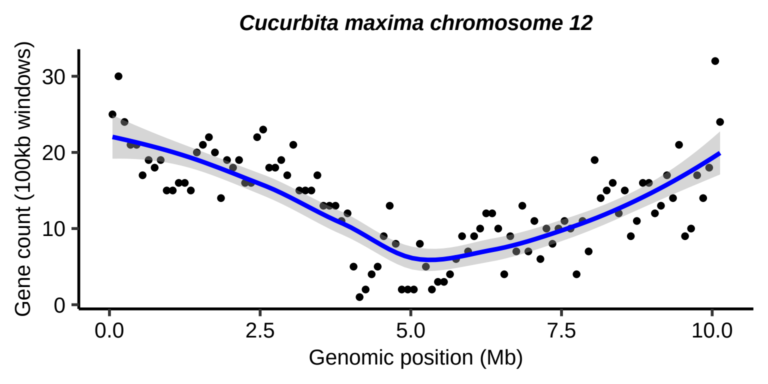

Cucurbita maxima chromosome 12
Gene count (100kb windows)
0
10
20
30
0.0
2.5
5.0
7.5
10.0
Genomic position (Mb)

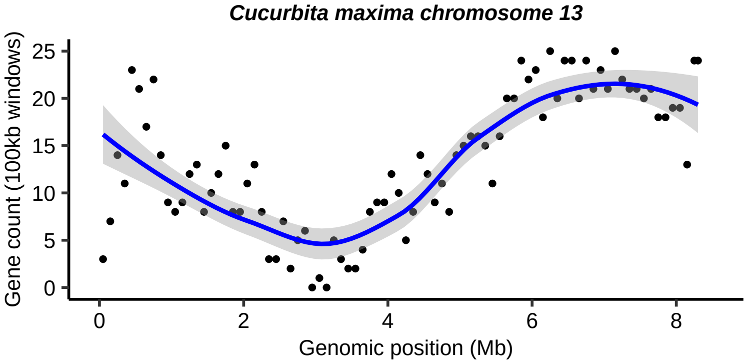

Cucurbita maxima chromosome 13
Gene count (100kb windows)
0
5
10
15
20
25
0
2
4
6
8
Genomic position (Mb)

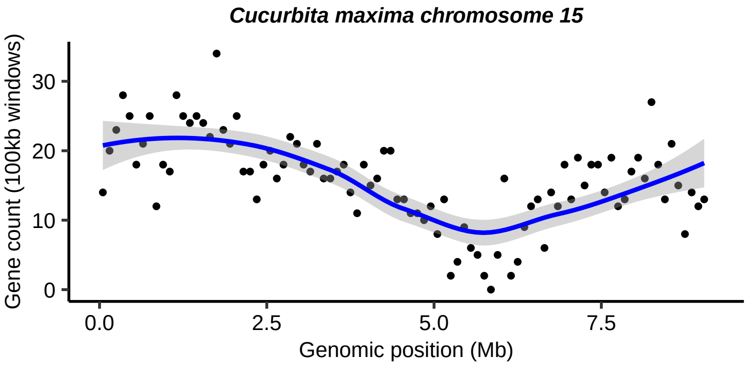

Cucurbita maxima chromosome 15
Gene count (100kb windows)
0
10
20
30
0.0
2.5
5.0
7.5
Genomic position (Mb)

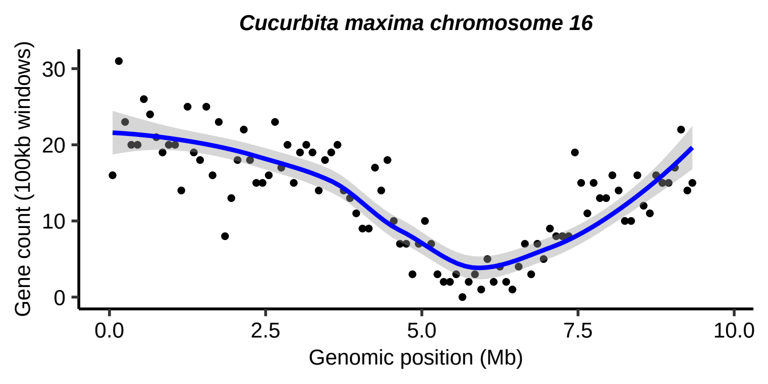
Cucurbita maxima chromosome 16
Gene count (100kb windows)
0
10
20
30
0.0
2.5
5.0
7.5
10.0
Genomic position (Mb)

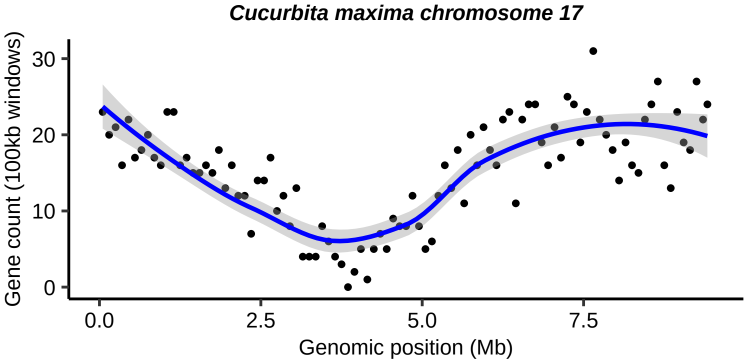
Cucurbita maxima chromosome 17
Gene count (100kb windows)
0
10
20
30
0.0
2.5
5.0
7.5
Genomic position (Mb)

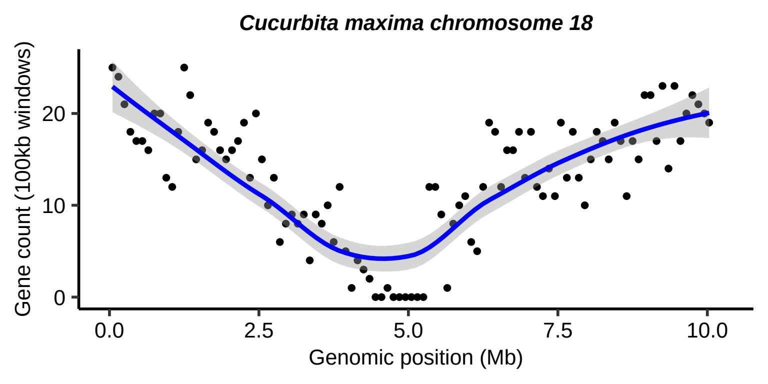
Cucurbita maxima chromosome 18
Gene count (100kb windows)
0
10
20
0.0
2.5
5.0
7.5
10.0
Genomic position (Mb)

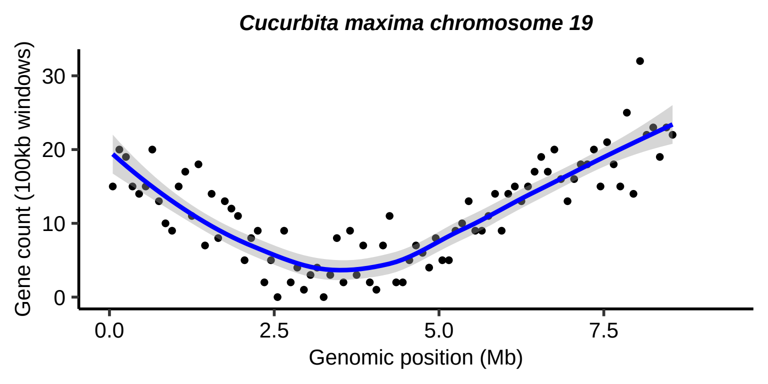

Cucurbita maxima chromosome 19
Gene count (100kb windows)
30
20
10
0
0.0
2.5
5.0
7.5
Genomic position (Mb)

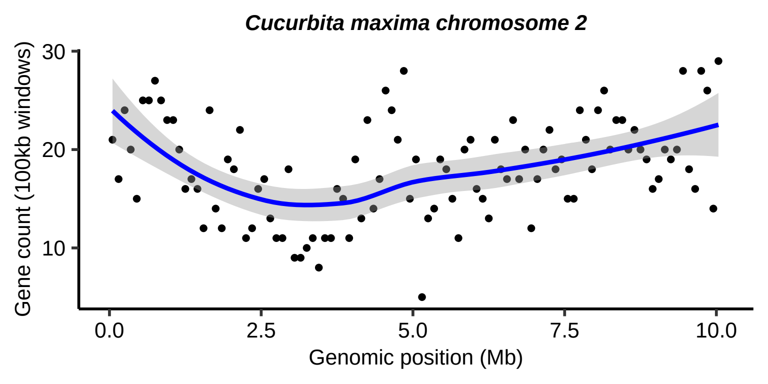

Cucurbita maxima chromosome 2
Gene count (100kb windows)
30
20
10
0.0
2.5
5.0
7.5
10.0
Genomic position (Mb)

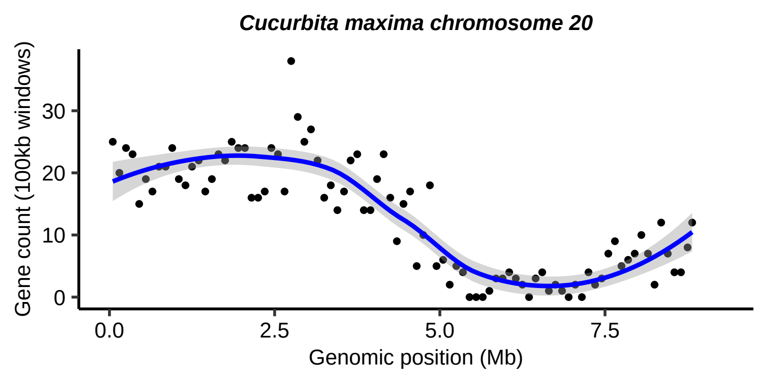

Cucurbita maxima chromosome 20
Gene count (100kb windows)
0
10
20
30
0.0
2.5
5.0
7.5
Genomic position (Mb)

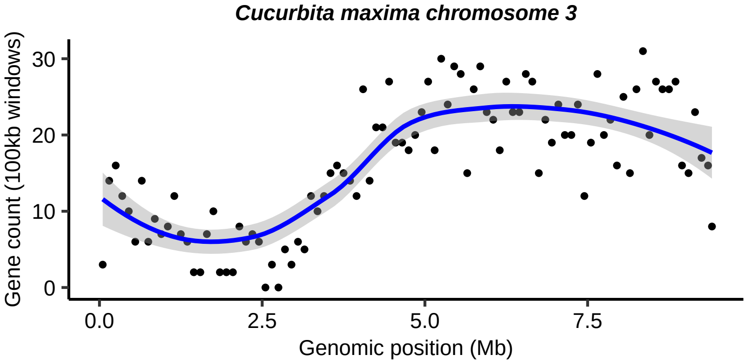

Cucurbita maxima chromosome 3
Gene count (100kb windows)
30
20
10
0
0.0
2.5
5.0
7.5
Genomic position (Mb)

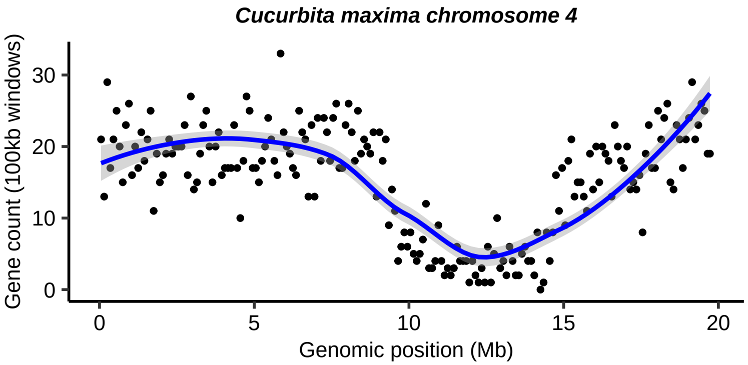

Cucurbita maxima chromosome 4
Gene count (100kb windows)
0
10
20
30
0
5
10
15
20
Genomic position (Mb)

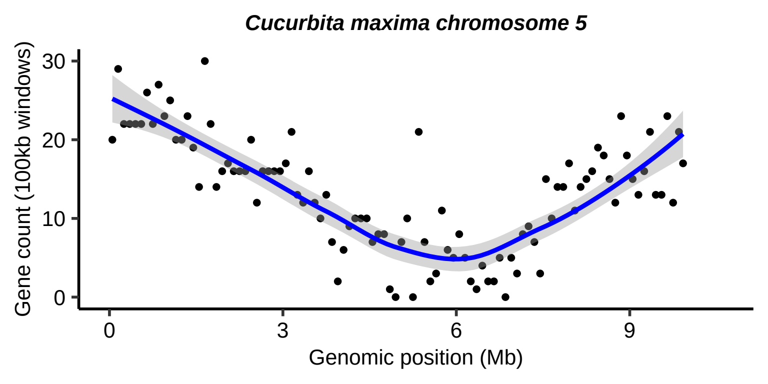

Cucurbita maxima chromosome 5
Gene count (100kb windows)
0
10
20
30
0
3
6
9
Genomic position (Mb)

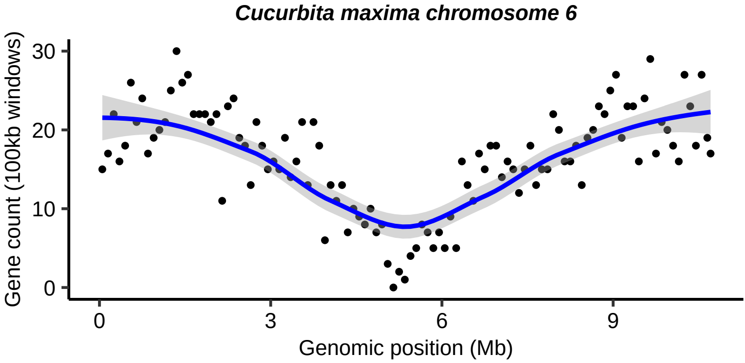
Cucurbita maxima chromosome 6
Gene count (100kb windows)
30
20
10
0
0
3
6
9
Genomic position (Mb)

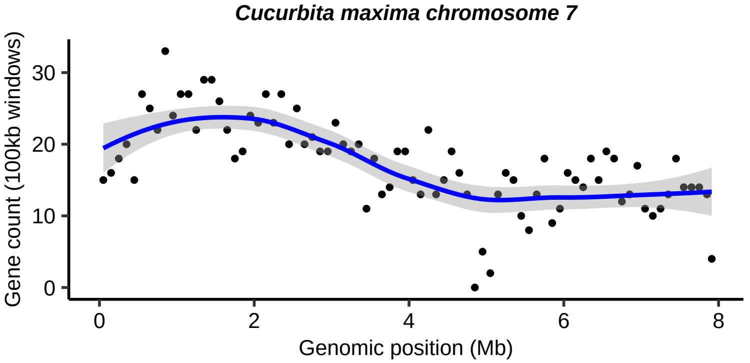

Cucurbita maxima chromosome 7
Gene count (100kb windows)
0
10
20
30
0
2
4
6
8
Genomic position (Mb)

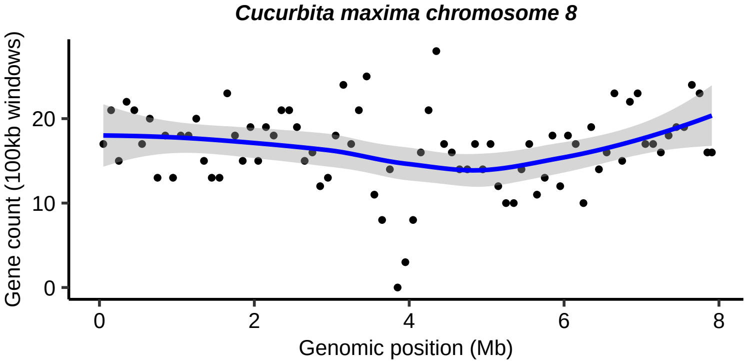

Cucurbita maxima chromosome 8
Gene count (100kb windows)
0
10
20
0
2
4
6
8
Genomic position (Mb)

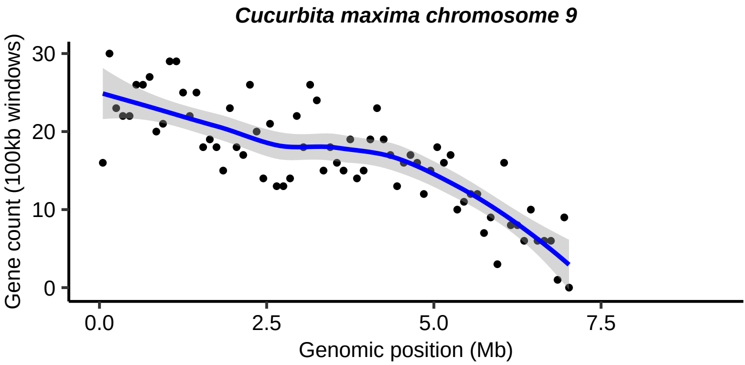

Cucurbita maxima chromosome 9
Gene count (100kb windows)
30
20
10
0
0.0
2.5
5.0
7.5
Genomic position (Mb)

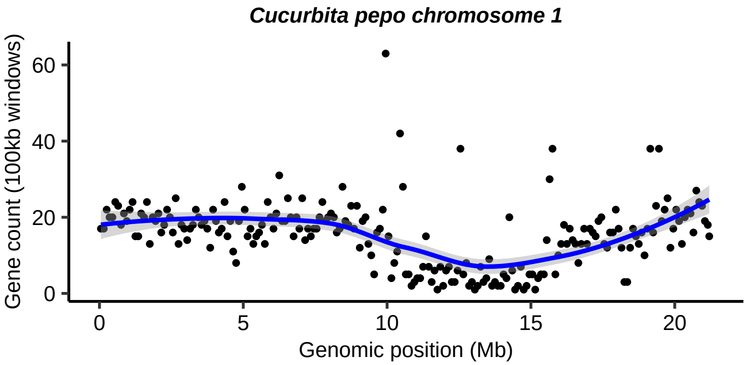

Cucurbita pepo chromosome 1
Gene count (100kb windows)
0
20
40
60
0
5
10
15
20
Genomic position (Mb)

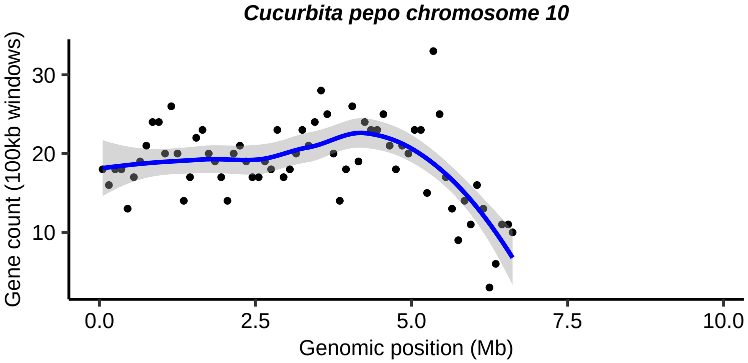

Cucurbita pepo chromosome 10
Gene count (100kb windows)
30
20
10
0.0
2.5
5.0
7.5
10.0
Genomic position (Mb)

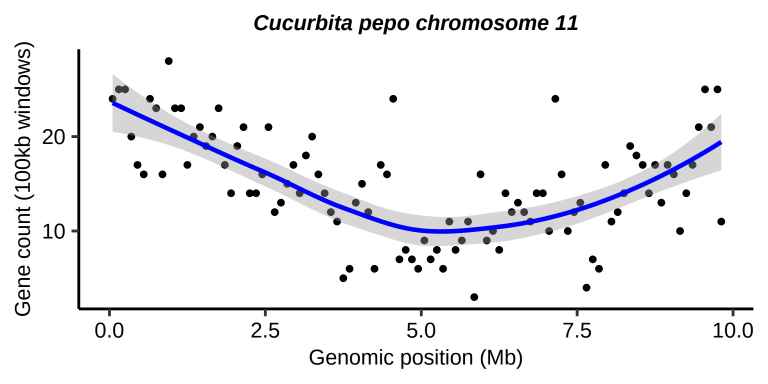
Cucurbita pepo chromosome 11
Gene count (100kb windows)
20
10
0.0
2.5
5.0
7.5
10.0
Genomic position (Mb)

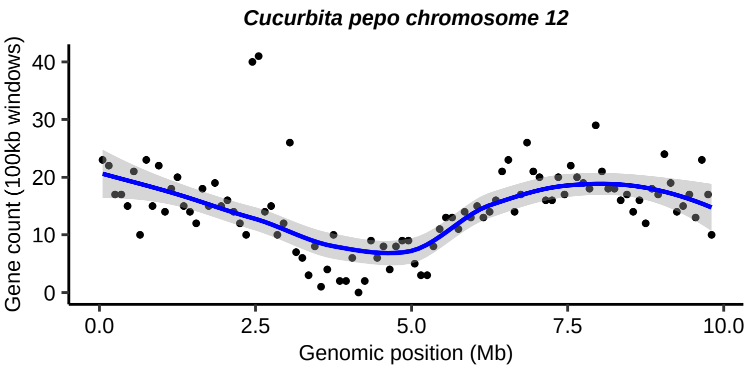

Cucurbita pepo chromosome 12
Gene count (100kb windows)
0
10
20
30
40
0.0
2.5
5.0
7.5
10.0
Genomic position (Mb)

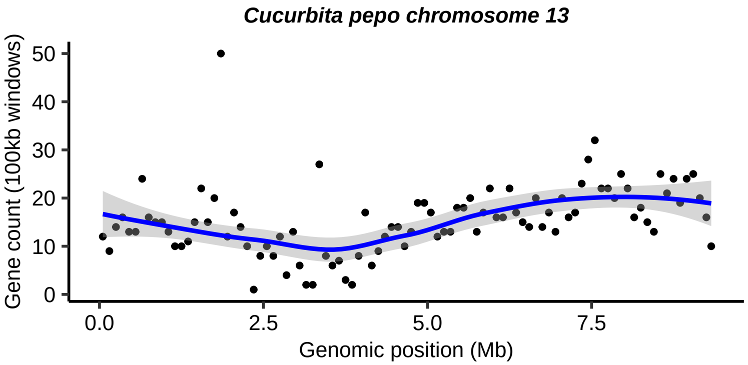

Cucurbita pepo chromosome 13
Gene count (100kb windows)
50
40
30
20
10
0
0.0
2.5
5.0
7.5
Genomic position (Mb)

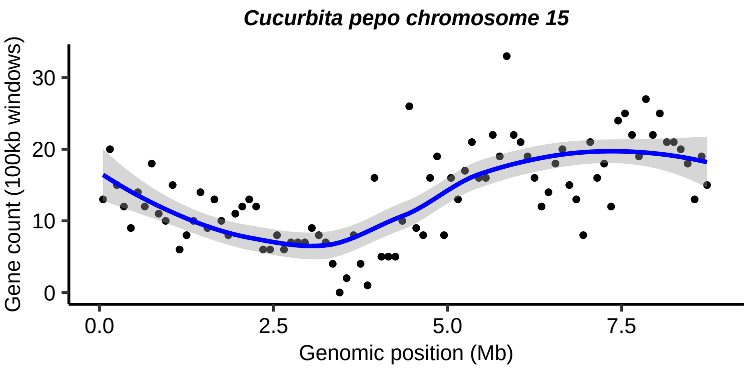
Cucurbita pepo chromosome 15
Gene count (100kb windows)
0
10
20
30
0.0
2.5
5.0
7.5
Genomic position (Mb)

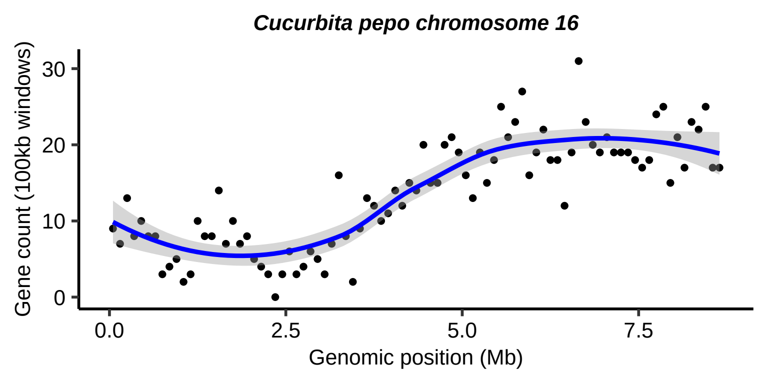

Cucurbita pepo chromosome 16
Gene count (100kb windows)
0
10
20
30
0.0
2.5
5.0
7.5
Genomic position (Mb)

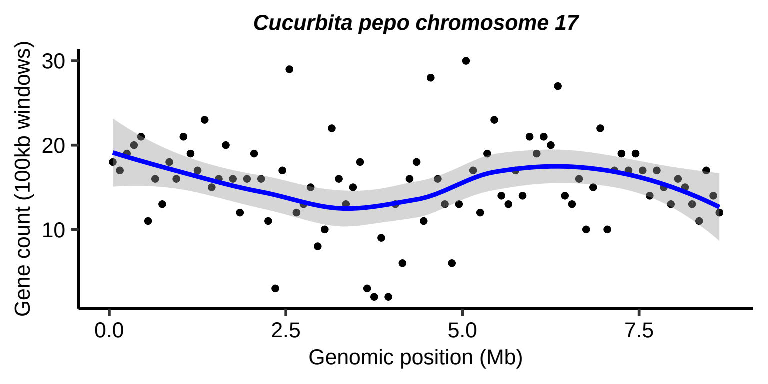

Cucurbita pepo chromosome 17
Gene count (100kb windows)
30
20
10
0.0
2.5
5.0
7.5
Genomic position (Mb)

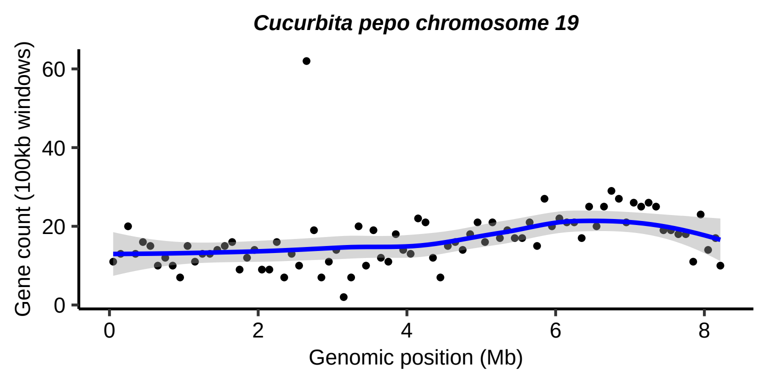

Cucurbita pepo chromosome 19
Gene count (100kb windows)
0
20
40
60
0
2
4
6
8
Genomic position (Mb)

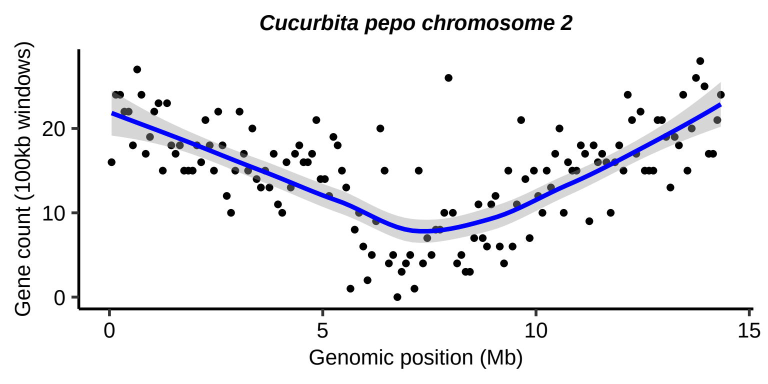

Cucurbita pepo chromosome 2
Gene count (100kb windows)
0
10
20
0
5
10
15
Genomic position (Mb)

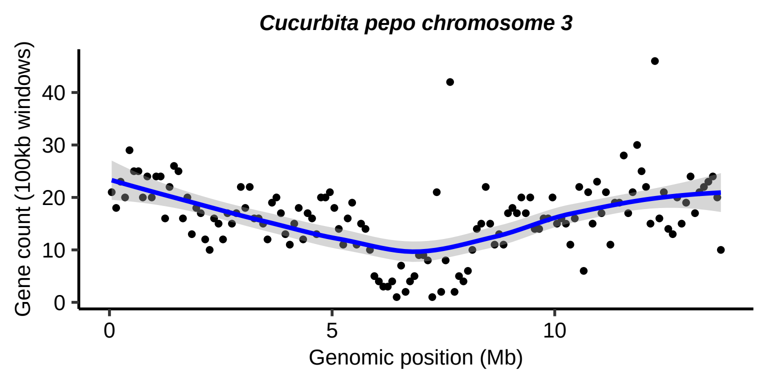
Cucurbita pepo chromosome 3
Gene count (100kb windows)
Genomic position (Mb)
0
10
20
30
40
0
5
10

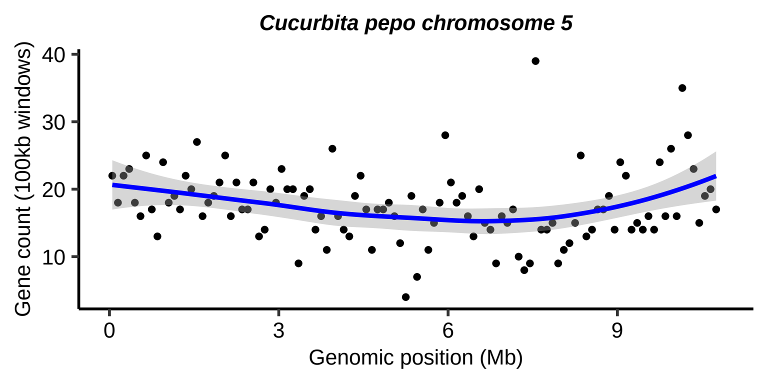
Cucurbita pepo chromosome 5
Gene count (100kb windows)
40
30
20
10
0
3
6
9
Genomic position (Mb)

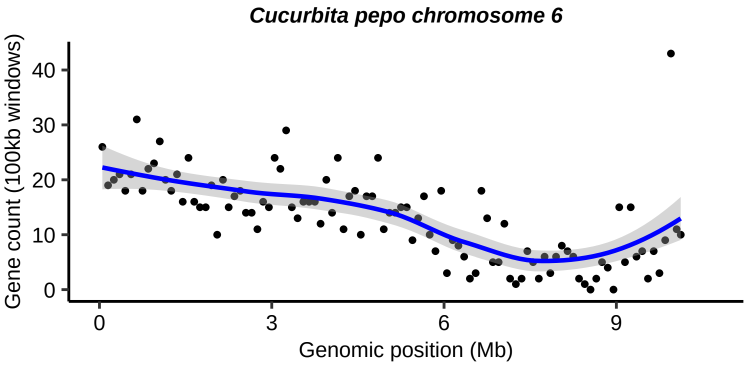

Cucurbita pepo chromosome 6
Gene count (100kb windows)
0
10
20
30
40
0
3
6
9
Genomic position (Mb)

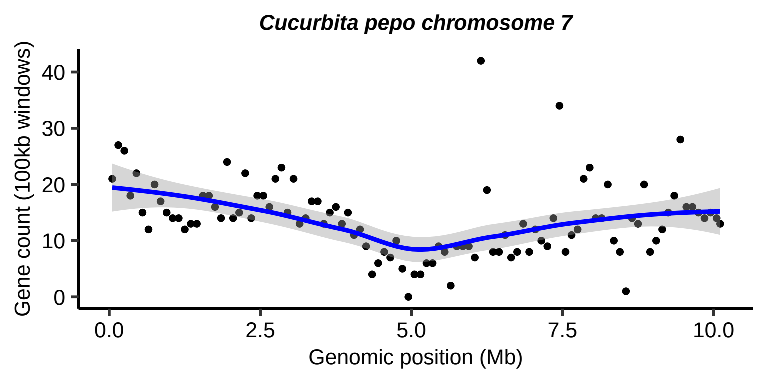
Cucurbita pepo chromosome 7
Gene count (100kb windows)
Genomic position (Mb)
0
10
20
30
40
0.0
2.5
5.0
7.5
10.0

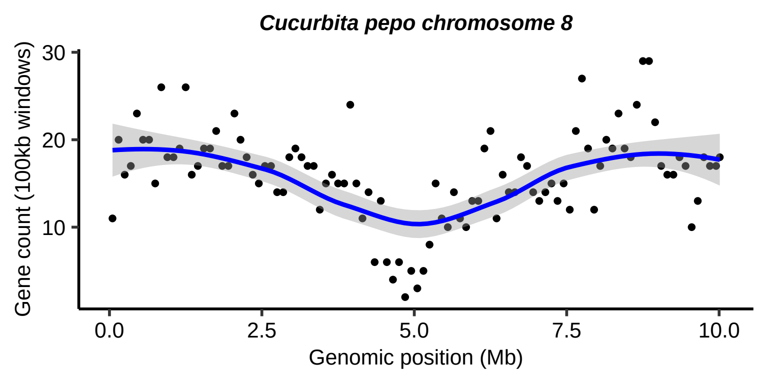
Cucurbita pepo chromosome 8
Gene count (100kb windows)
30
20
10
0.0
2.5
5.0
7.5
10.0
Genomic position (Mb)

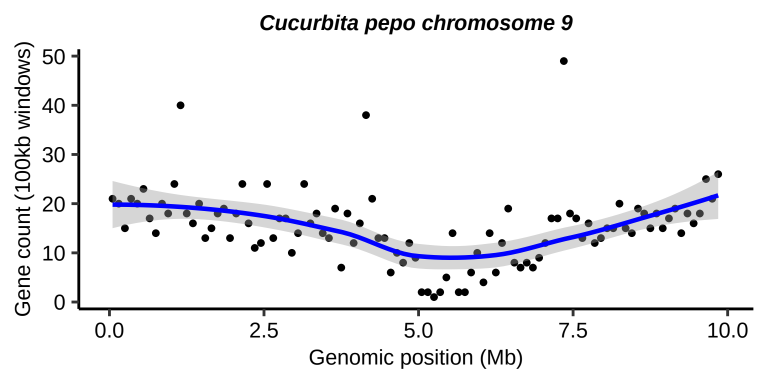

Cucurbita pepo chromosome 9
Gene count (100kb windows)
0
10
20
30
40
50
0.0
2.5
5.0
7.5
10.0
Genomic position (Mb)

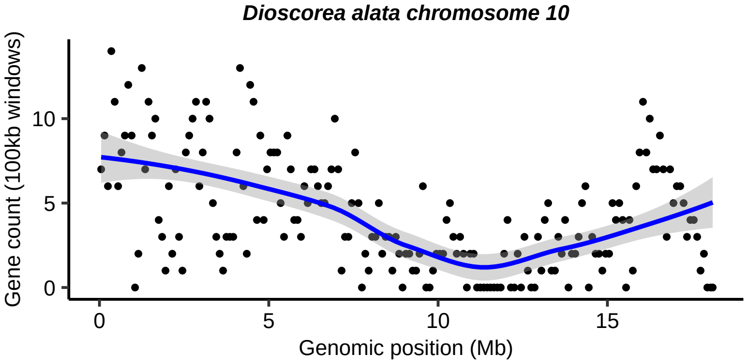

Dioscorea alata chromosome 10
Gene count (100kb windows)
0
5
10
0
5
10
15
Genomic position (Mb)

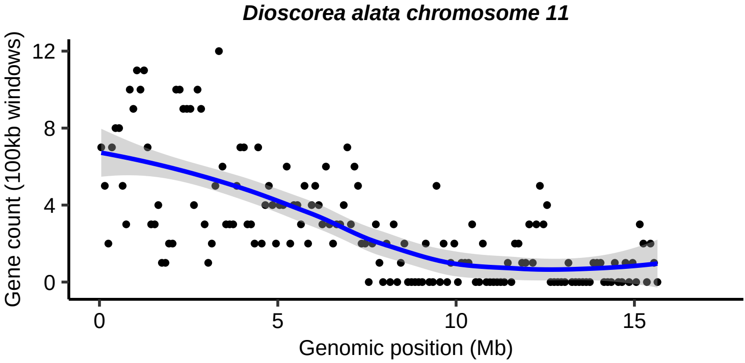

Dioscorea alata chromosome 11
Gene count (100kb windows)
0
4
8
12
0
5
10
15
Genomic position (Mb)

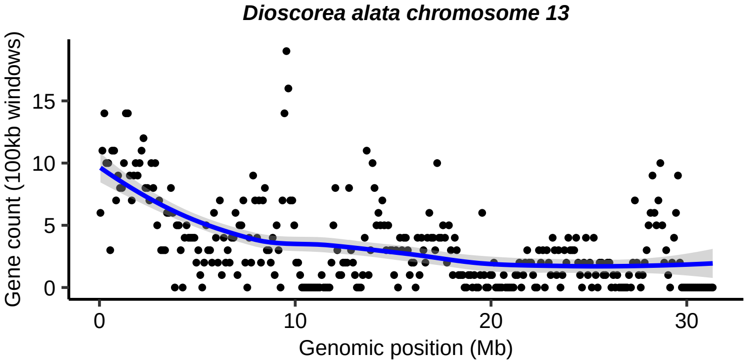
Dioscorea alata chromosome 13
Gene count (100kb windows)
0
5
10
15
0
10
20
30
Genomic position (Mb)

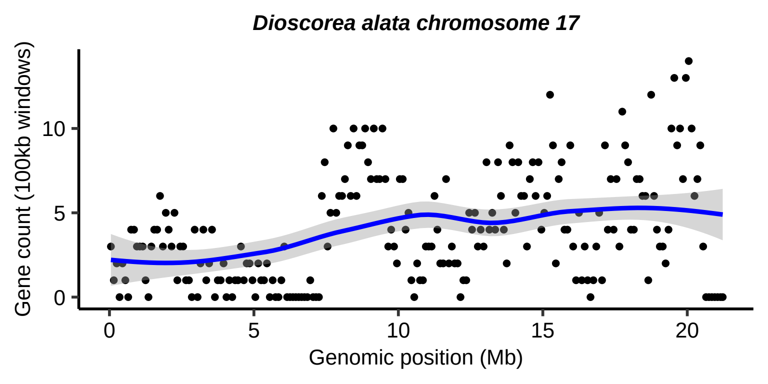

Dioscorea alata chromosome 17
Gene count (100kb windows)
0
5
10
0
5
10
15
20
Genomic position (Mb)

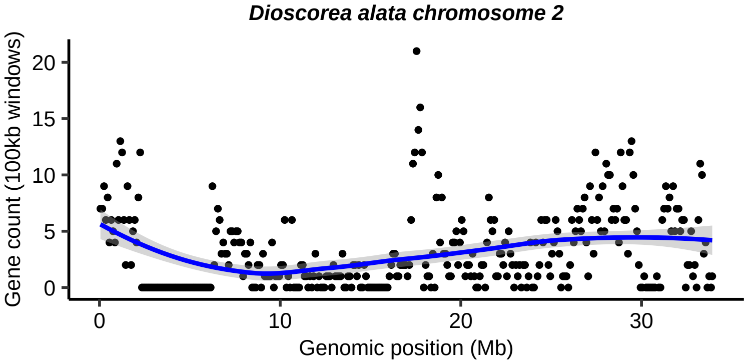

Dioscorea alata chromosome 2
Gene count (100kb windows)
20
15
10
5
0
0
10
20
30
Genomic position (Mb)

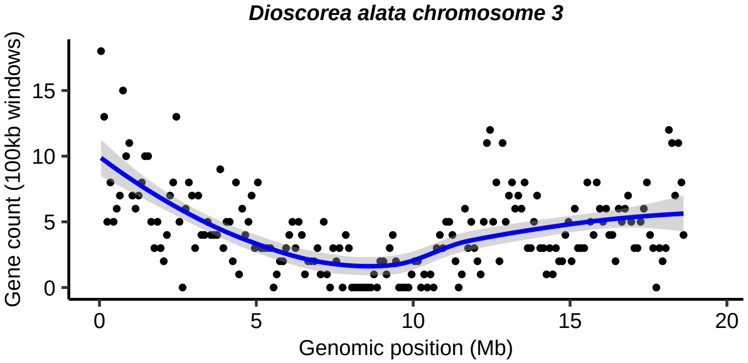

Dioscorea alata chromosome 3
Gene count (100kb windows)
0
5
10
15
0
5
10
15
20
Genomic position (Mb)

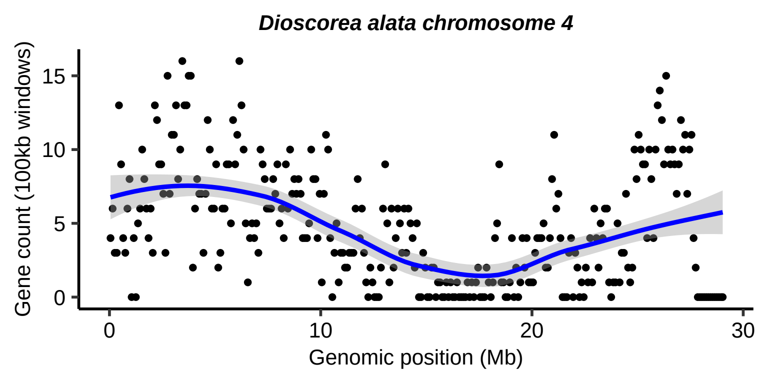

Dioscorea alata chromosome 4
Gene count (100kb windows)
0
5
10
15
0
10
20
30
Genomic position (Mb)

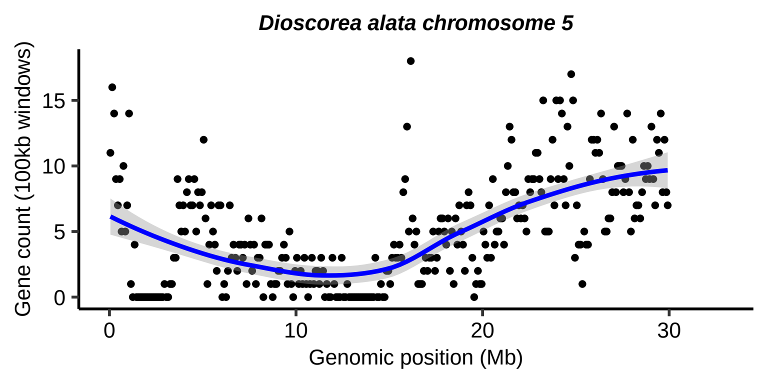

Dioscorea alata chromosome 5
Gene count (100kb windows)
15
10
5
0
0
10
20
30
Genomic position (Mb)

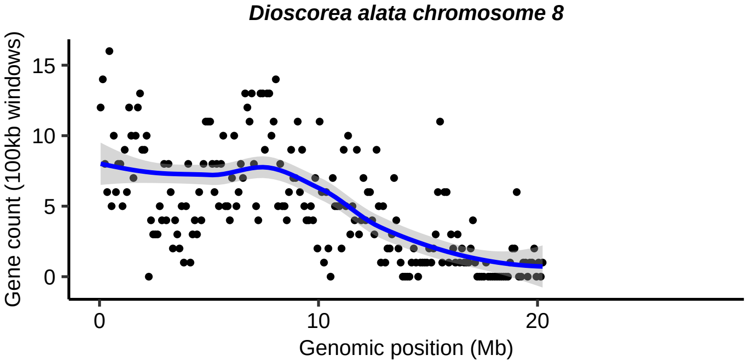

Dioscorea alata chromosome 8
Gene count (100kb windows)
0
5
10
15
0
10
20
Genomic position (Mb)

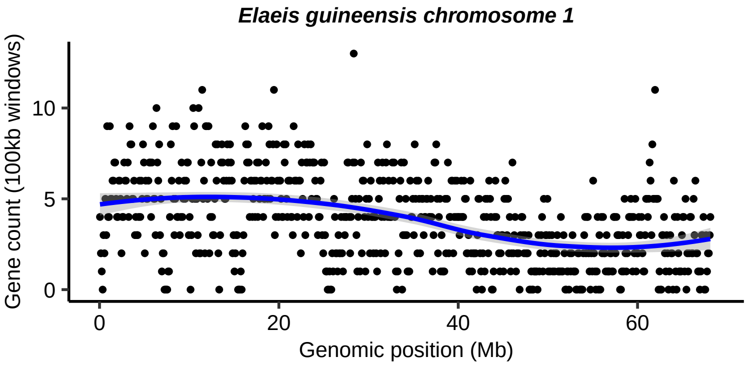

Elaeis guineensis chromosome 1
Gene count (100kb windows)
10
5
0
0
20
40
60
Genomic position (Mb)

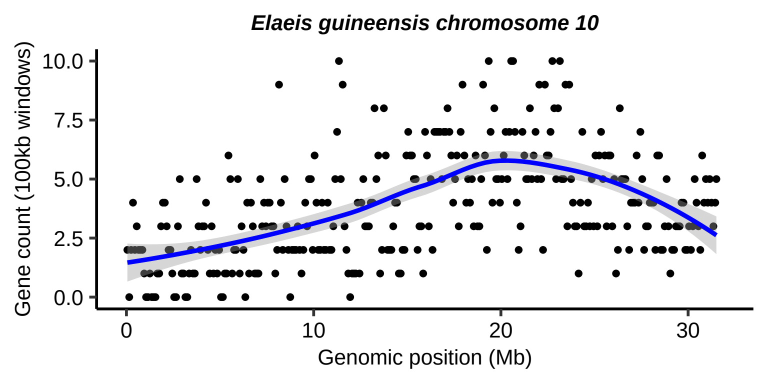

Elaeis guineensis chromosome 10
Gene count (100kb windows)
10.0
7.5
5.0
2.5
0.0
0
10
20
30
Genomic position (Mb)

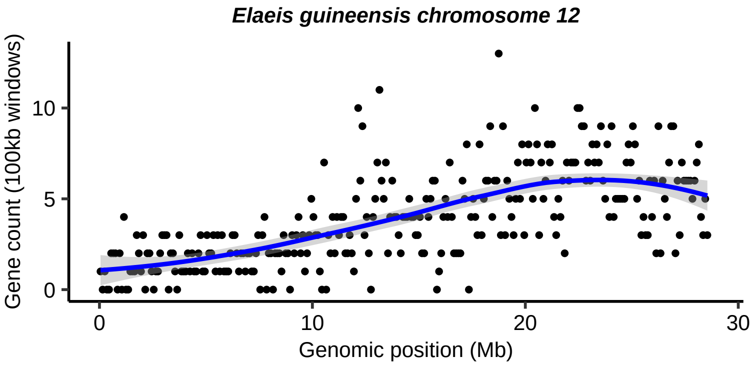

Elaeis guineensis chromosome 12
Gene count (100kb windows)
10
5
0
0
10
20
30
Genomic position (Mb)

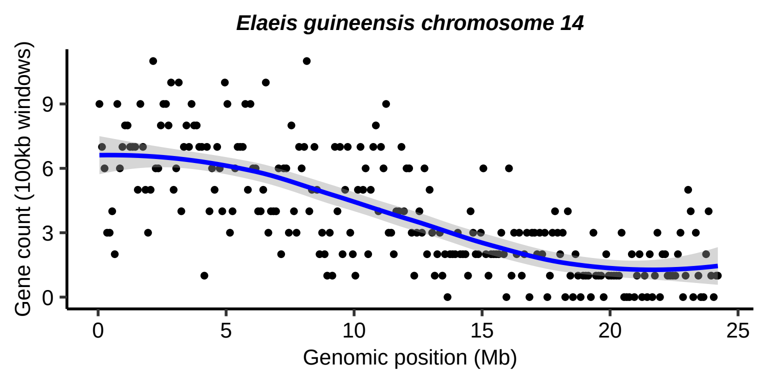

Elaeis guineensis chromosome 14
Gene count (100kb windows)
0
3
6
9
0
5
10
15
20
25
Genomic position (Mb)

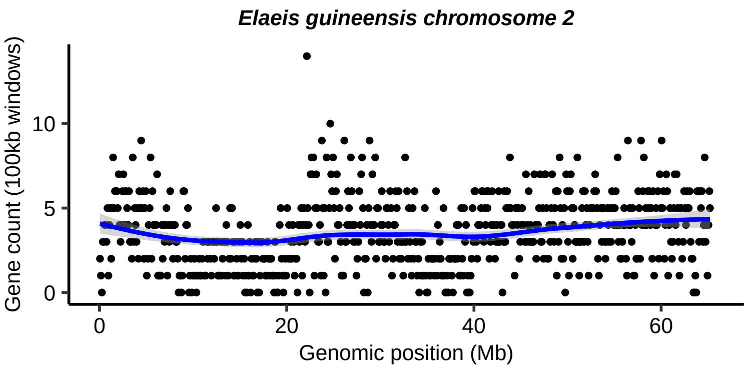

Elaeis guineensis chromosome 2
Gene count (100kb windows)
0
5
10
0
20
40
60
Genomic position (Mb)

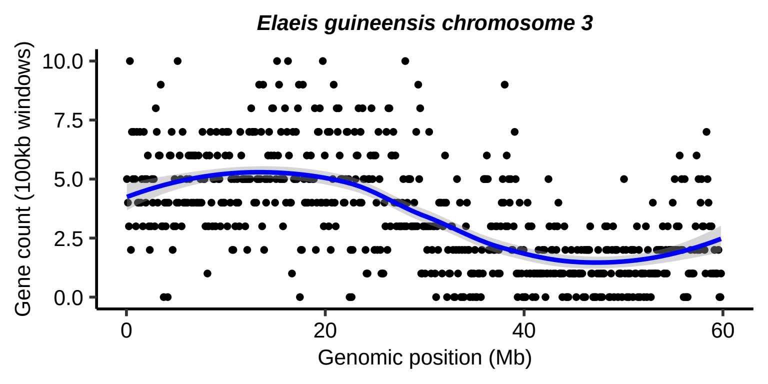

Elaeis guineensis chromosome 3
Gene count (100kb windows)
10.0
7.5
5.0
2.5
0.0
0
20
40
60
Genomic position (Mb)

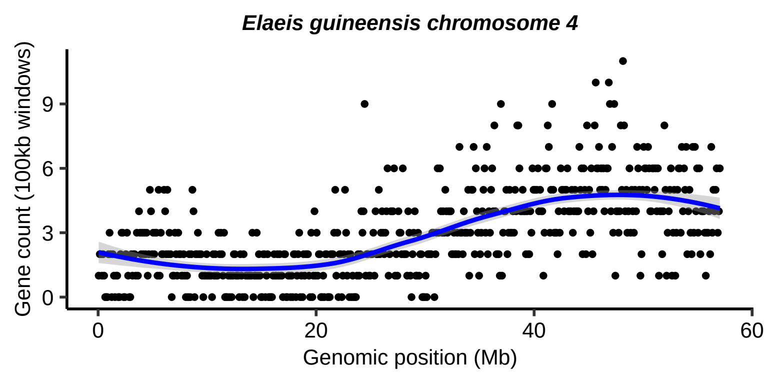

Elaeis guineensis chromosome 4
Gene count (100kb windows)
0
3
6
9
0
20
40
60
Genomic position (Mb)

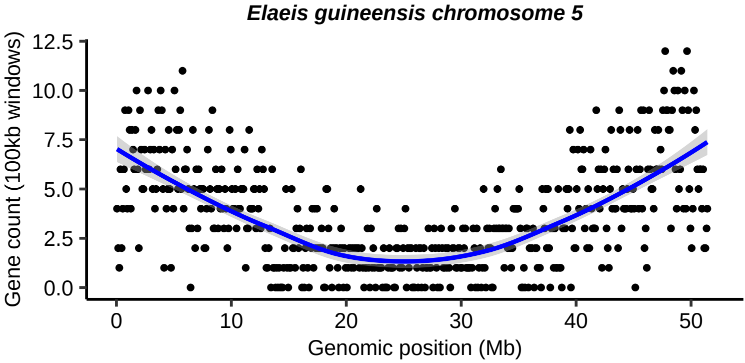

Elaeis guineensis chromosome 5
Gene count (100kb windows)
12.5
10.0
7.5
5.0
2.5
0.0
0
10
20
30
40
50
Genomic position (Mb)

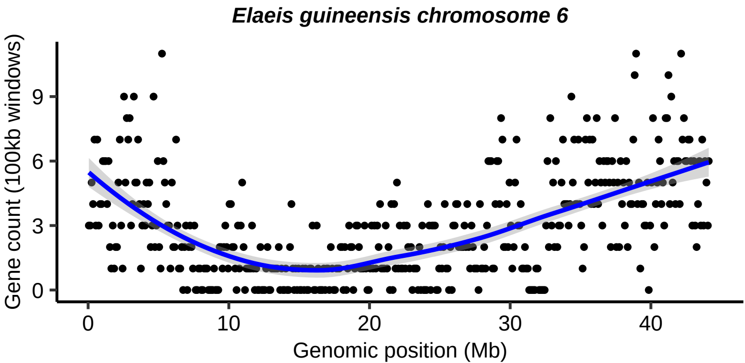

Elaeis guineensis chromosome 6
Gene count (100kb windows)
0
3
6
9
0
10
20
30
40
Genomic position (Mb)

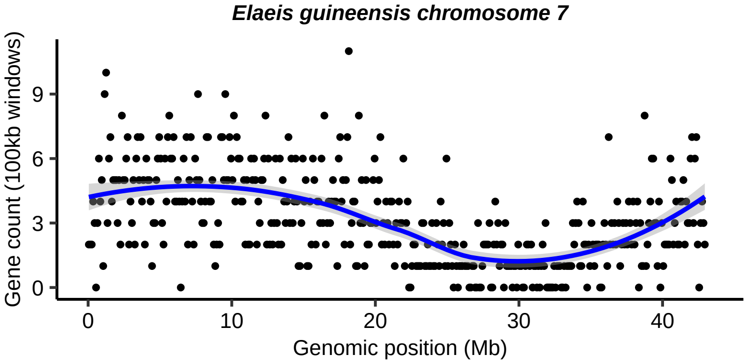

Elaeis guineensis chromosome 7
Gene count (100kb windows)
0
3
6
9
0
10
20
30
40
Genomic position (Mb)

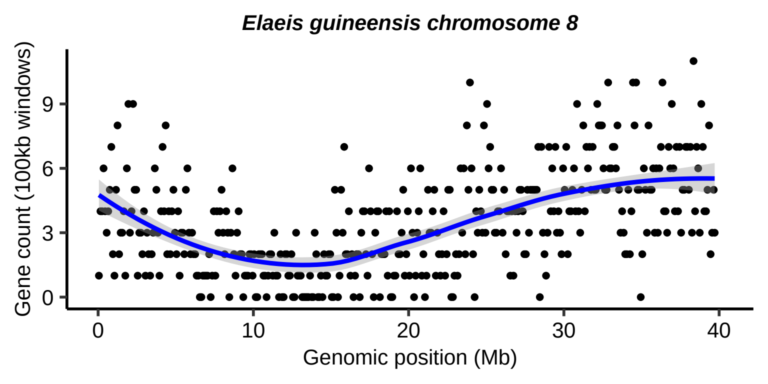

Elaeis guineensis chromosome 8
Gene count (100kb windows)
0
3
6
9
0
10
20
30
40
Genomic position (Mb)

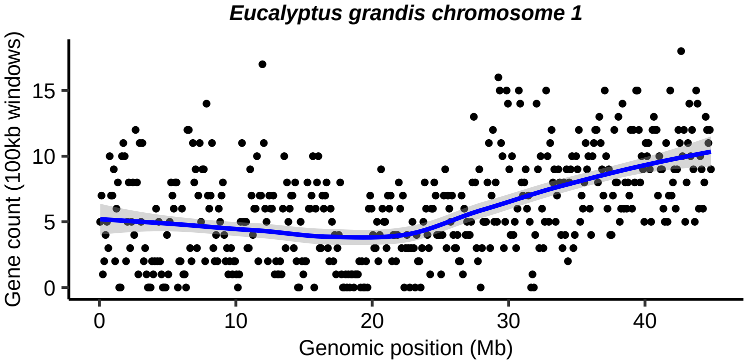

Eucalyptus grandis chromosome 1
Gene count (100kb windows)
0
5
10
15
0
10
20
30
40
Genomic position (Mb)

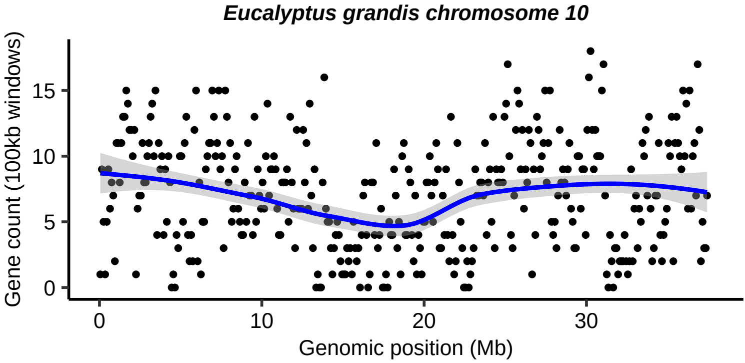

Eucalyptus grandis chromosome 10
Gene count (100kb windows)
0
5
10
15
0
10
20
30
Genomic position (Mb)

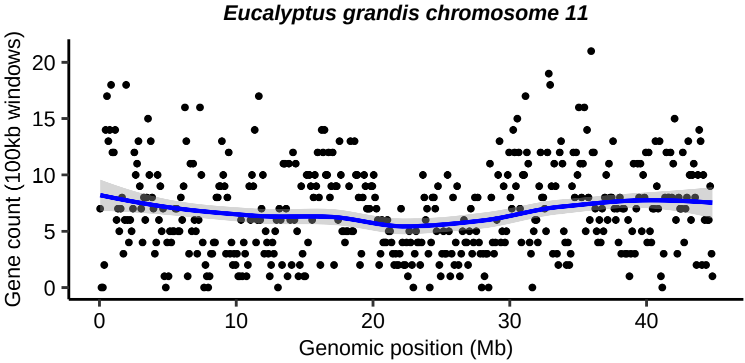

Eucalyptus grandis chromosome 11
Gene count (100kb windows)
0
5
10
15
20
0
10
20
30
40
Genomic position (Mb)

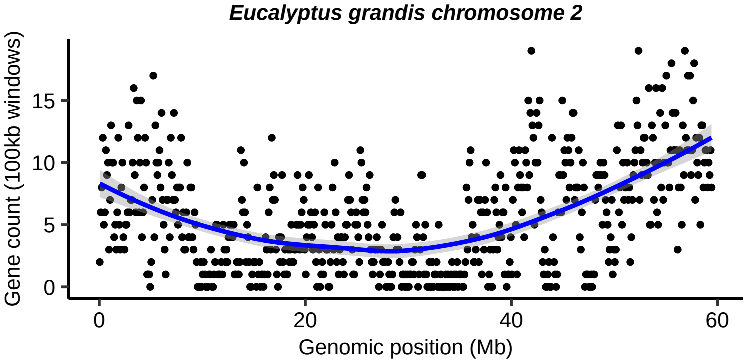
Eucalyptus grandis chromosome 2
Gene count (100kb windows)
0
5
10
15
0
20
40
60
Genomic position (Mb)

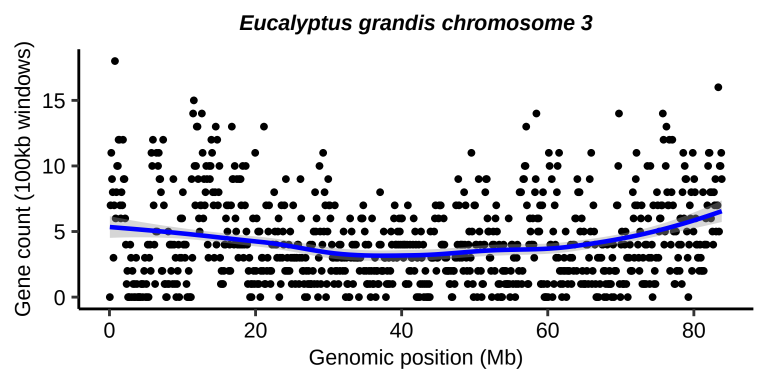
Eucalyptus grandis chromosome 3
Gene count (100kb windows)
0
5
10
15
0
20
40
60
80
Genomic position (Mb)

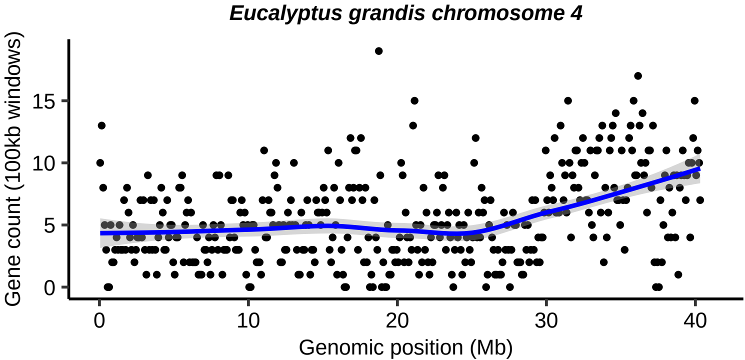

Eucalyptus grandis chromosome 4
Gene count (100kb windows)
0
5
10
15
0
10
20
30
40
Genomic position (Mb)

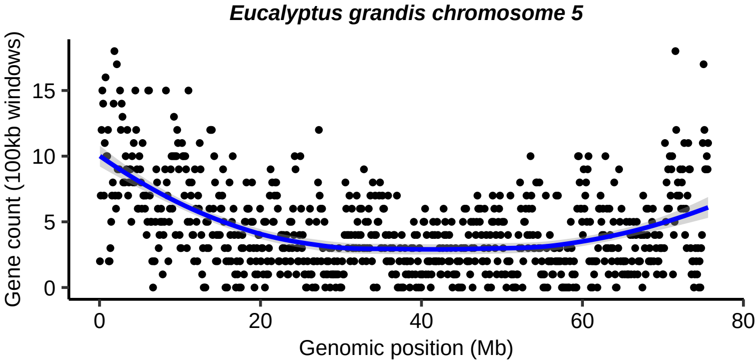

Eucalyptus grandis chromosome 5
Gene count (100kb windows)
0
5
10
15
0
20
40
60
80
Genomic position (Mb)

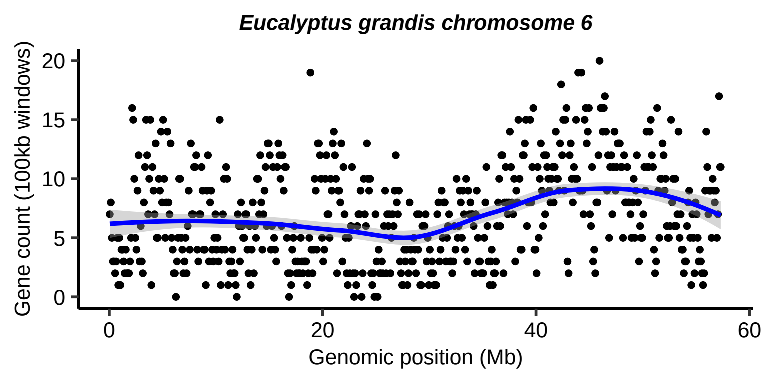
Eucalyptus grandis chromosome 6
Gene count (100kb windows)
20
15
10
5
0
0
20
40
60
Genomic position (Mb)

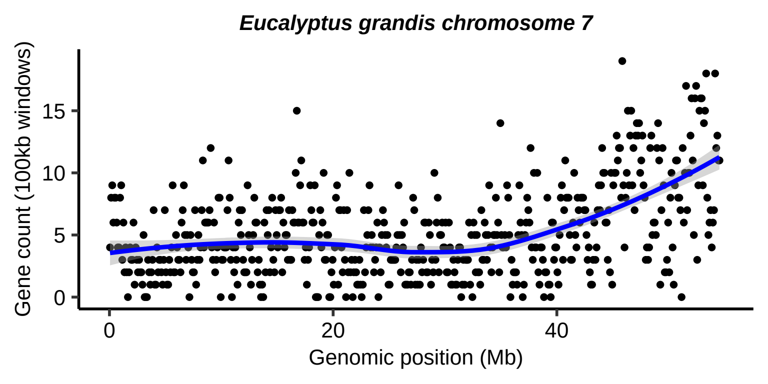

Eucalyptus grandis chromosome 7
Gene count (100kb windows)
15
10
5
0
0
20
40
Genomic position (Mb)

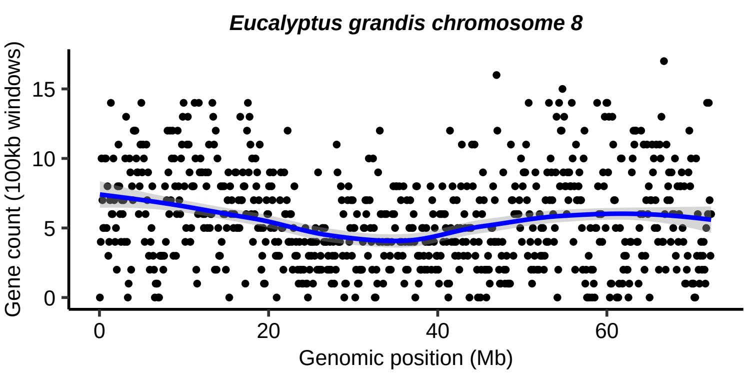
Eucalyptus grandis chromosome 8
Gene count (100kb windows)
Genomic position (Mb)
0
5
10
15
0
20
40
60

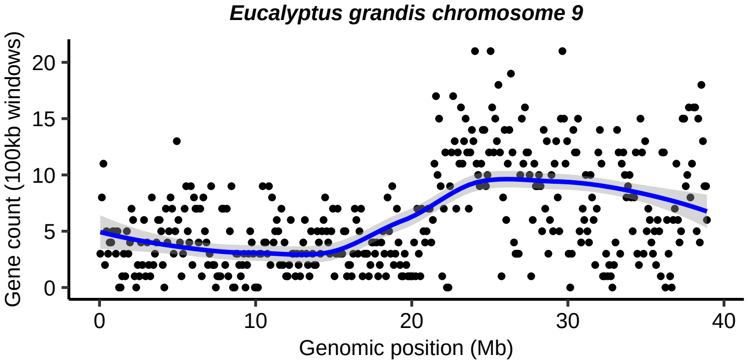

Eucalyptus grandis chromosome 9
Gene count (100kb windows)
0
5
10
15
20
0
10
20
30
40
Genomic position (Mb)

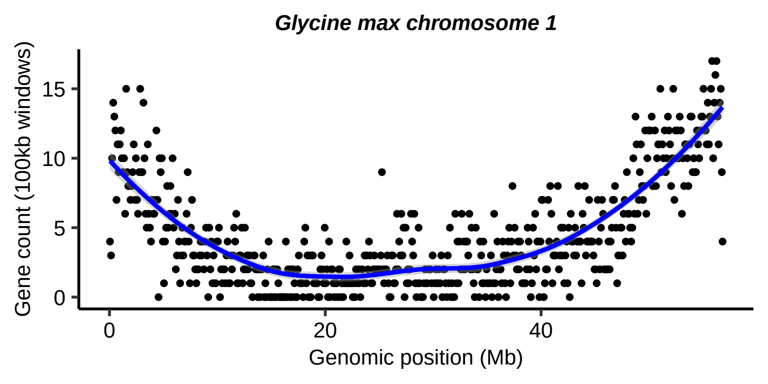

Glycine max chromosome 1
Gene count (100kb windows)
0
5
10
15
0
20
40
Genomic position (Mb)

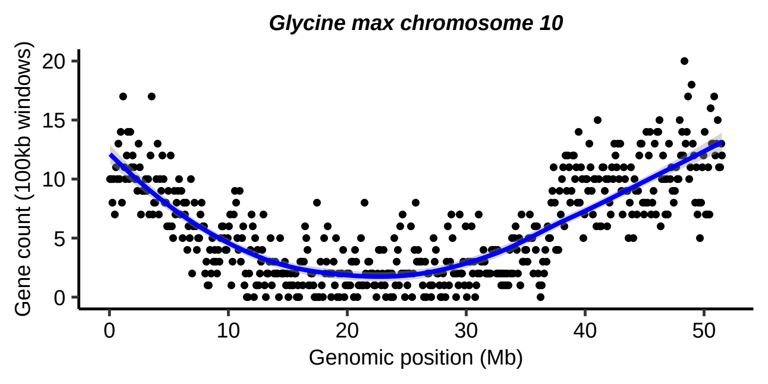

Glycine max chromosome 10
Gene count (100kb windows)
0
5
10
15
20
0
10
20
30
40
50
Genomic position (Mb)

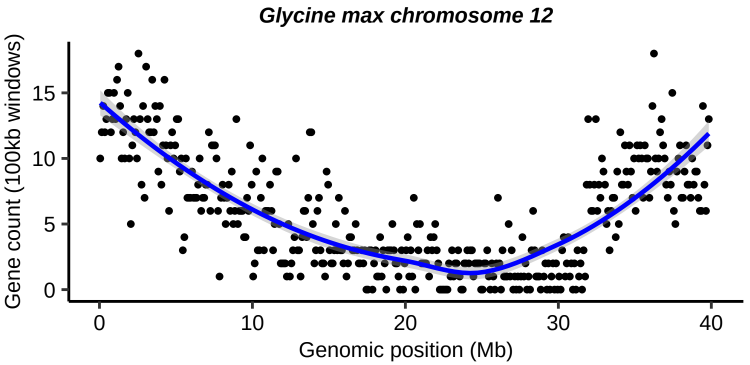

Glycine max chromosome 12
Gene count (100kb windows)
0
5
10
15
0
10
20
30
40
Genomic position (Mb)

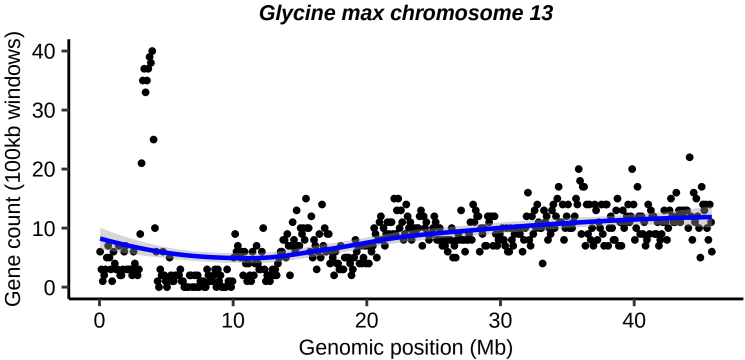

Glycine max chromosome 13
Gene count (100kb windows)
0
10
20
30
40
Genomic position (Mb)

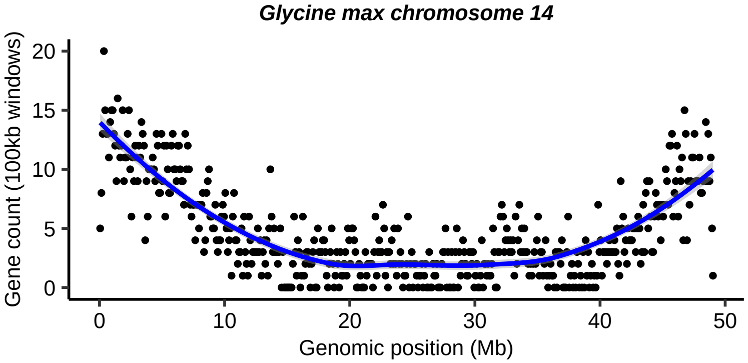

Glycine max chromosome 14
Gene count (100kb windows)
20
15
10
5
0
0
10
20
30
40
50
Genomic position (Mb)

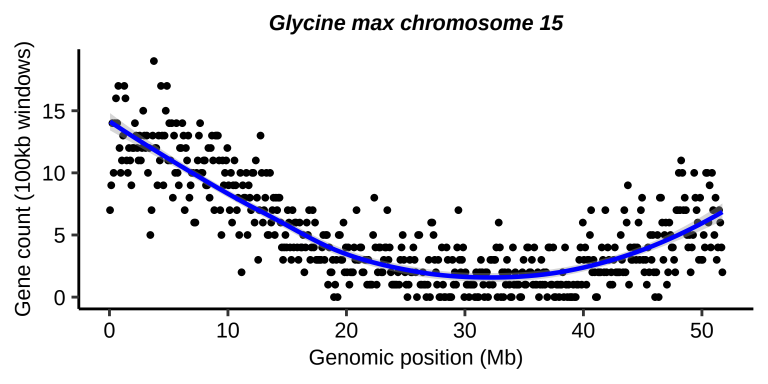

Glycine max chromosome 15
Gene count (100kb windows)
15
10
5
0
0
10
20
30
40
50
Genomic position (Mb)

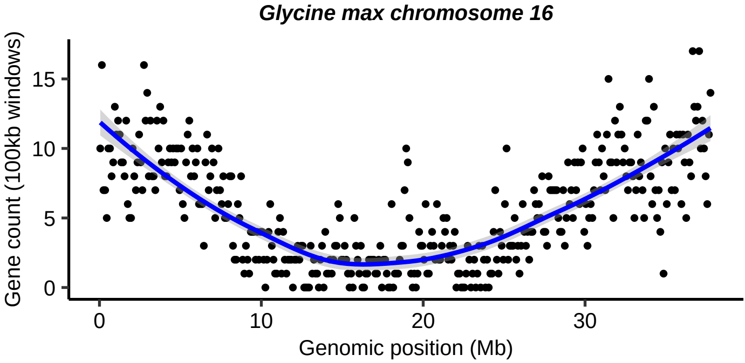

Glycine max chromosome 16
Gene count (100kb windows)
15
10
5
0
0
10
20
30
Genomic position (Mb)

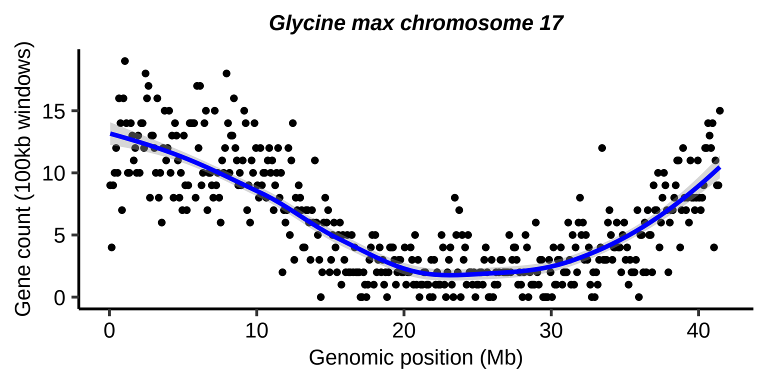

Glycine max chromosome 17
Gene count (100kb windows)
0
5
10
15
0
10
20
30
40
Genomic position (Mb)

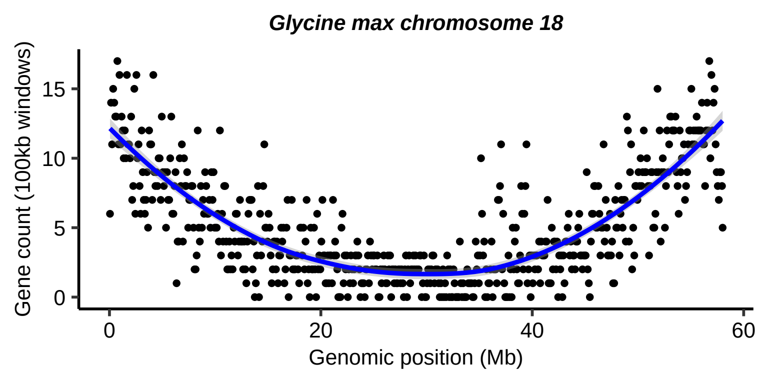

Glycine max chromosome 18
Gene count (100kb windows)
0
5
10
15
0
20
40
60
Genomic position (Mb)

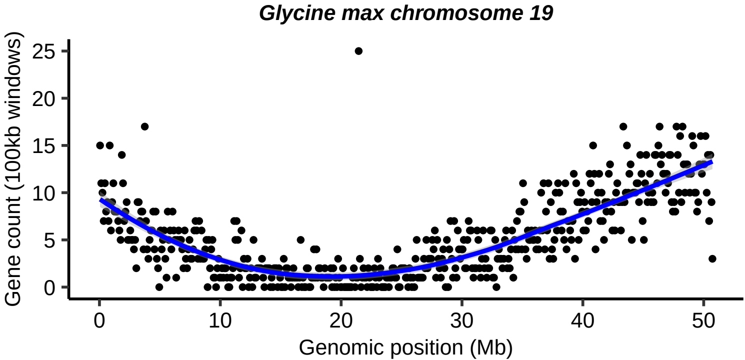

Glycine max chromosome 19
Gene count (100kb windows)
25
20
15
10
5
0
0
10
20
30
40
50
Genomic position (Mb)

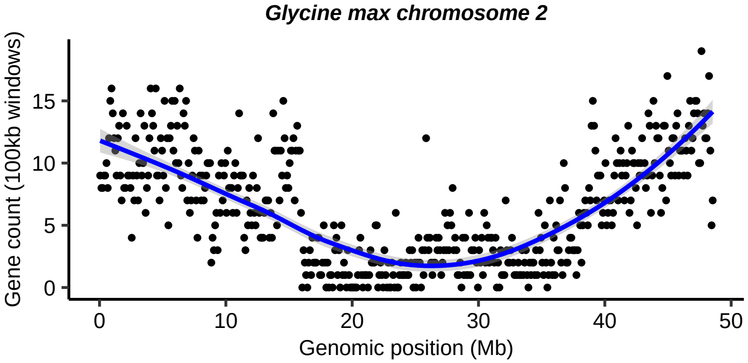

Glycine max chromosome 2
Gene count (100kb windows)
0
5
10
15
0
10
20
30
40
50
Genomic position (Mb)

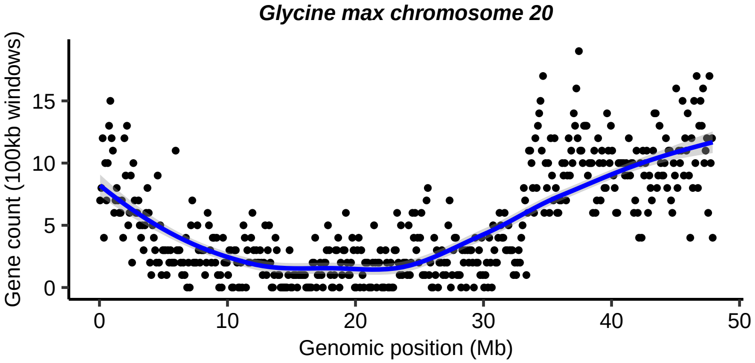

Glycine max chromosome 20
Gene count (100kb windows)
Genomic position (Mb)
0
5
10
15
0
10
20
30
40
50

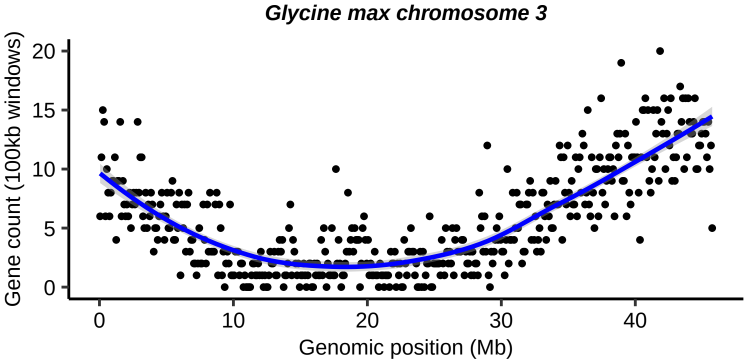
Glycine max chromosome 3
Gene count (100kb windows)
20
15
10
5
0
0
10
20
30
40
Genomic position (Mb)

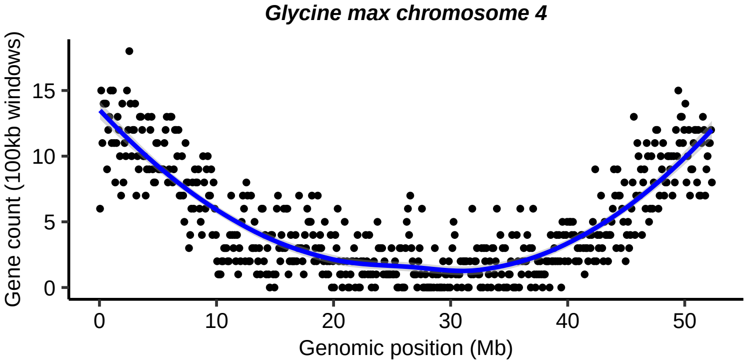

Glycine max chromosome 4
Gene count (100kb windows)
0
5
10
15
0
10
20
30
40
50
Genomic position (Mb)

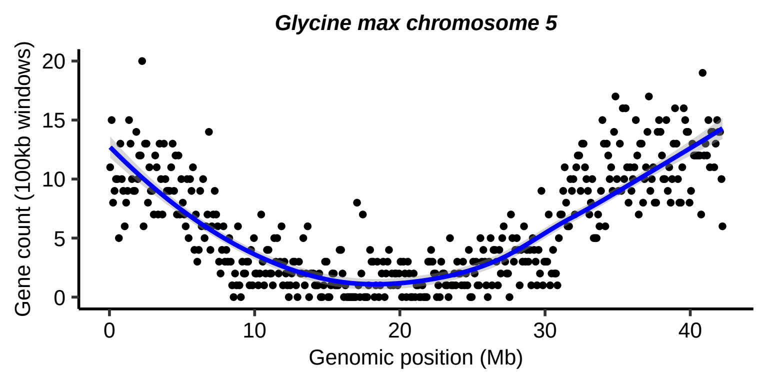
Glycine max chromosome 5
Gene count (100kb windows)
0
5
10
15
20
0
10
20
30
40
Genomic position (Mb)

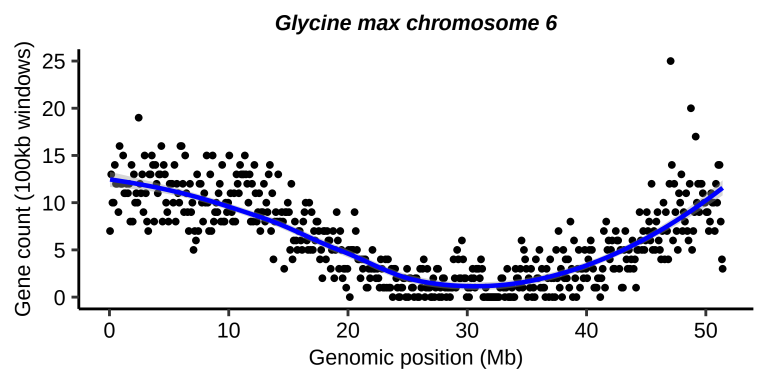

Glycine max chromosome 6
Gene count (100kb windows)
0
5
10
15
20
25
0
10
20
30
40
50
Genomic position (Mb)

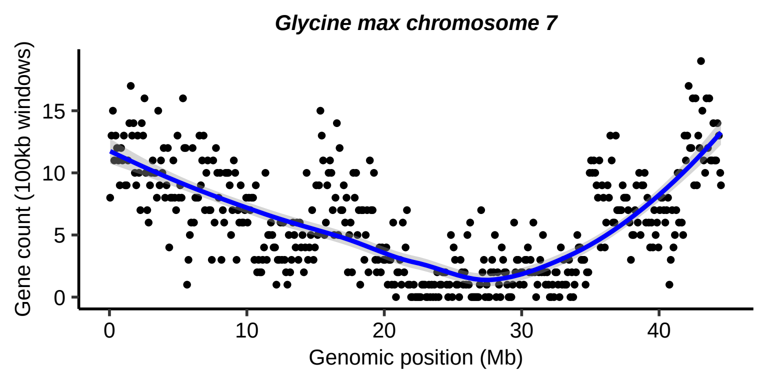

Glycine max chromosome 7
Gene count (100kb windows)
0
5
10
15
0
10
20
30
40
Genomic position (Mb)

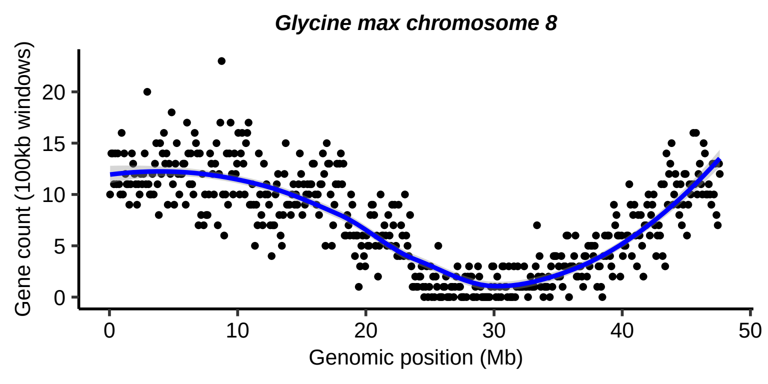

Glycine max chromosome 8
Gene count (100kb windows)
0
5
10
15
20
0
10
20
30
40
50
Genomic position (Mb)

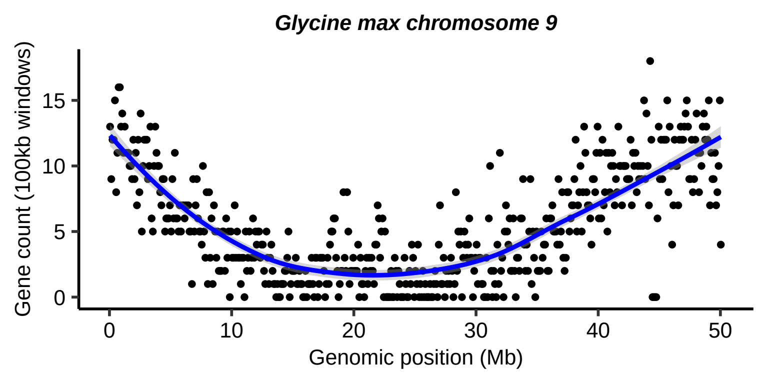
Glycine max chromosome 9
Gene count (100kb windows)
0
5
10
15
0
10
20
30
40
50
Genomic position (Mb)

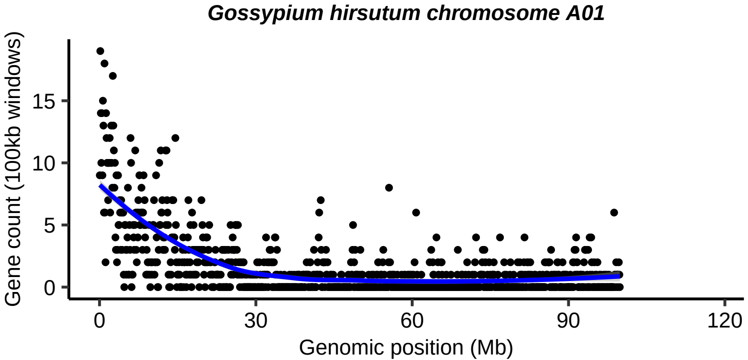

Gossypium hirsutum chromosome A01
Gene count (100kb windows)
15
10
5
0
0
30
60
90
120
Genomic position (Mb)

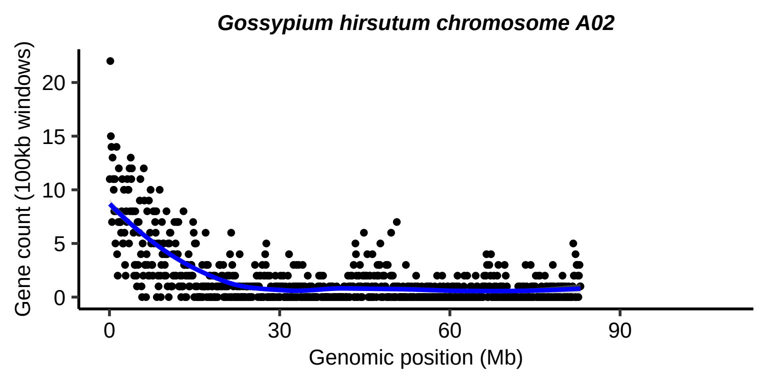
Gossypium hirsutum chromosome A02
Gene count (100kb windows)
0
5
10
15
20
0
30
60
90
Genomic position (Mb)

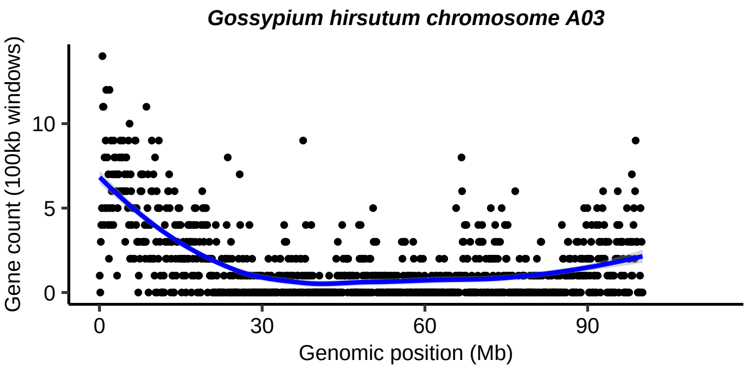

Gossypium hirsutum chromosome A03
Gene count (100kb windows)
0
5
10
0
30
60
90
Genomic position (Mb)

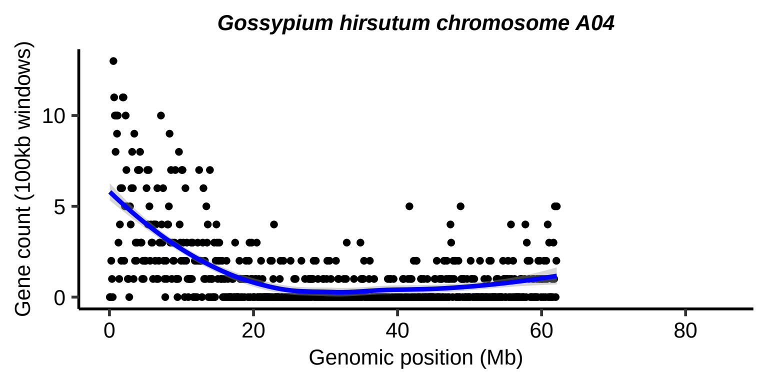
Gossypium hirsutum chromosome A04
Gene count (100kb windows)
0
5
10
0
20
40
60
80
Genomic position (Mb)

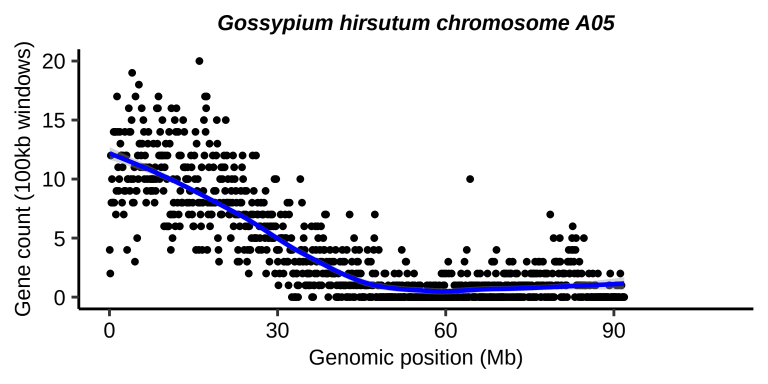

Gossypium hirsutum chromosome A05
Gene count (100kb windows)
0
5
10
15
20
0
30
60
90
Genomic position (Mb)

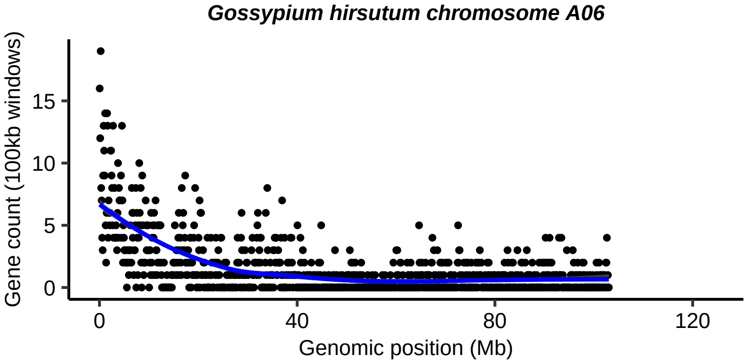

Gossypium hirsutum chromosome A06
Gene count (100kb windows)
0
5
10
15
0
40
80
120
Genomic position (Mb)

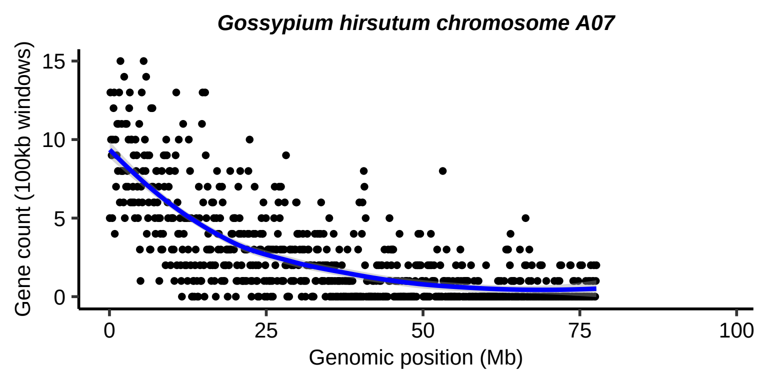
Gossypium hirsutum chromosome A07
Gene count (100kb windows)
15
10
5
0
0
25
50
75
100
Genomic position (Mb)

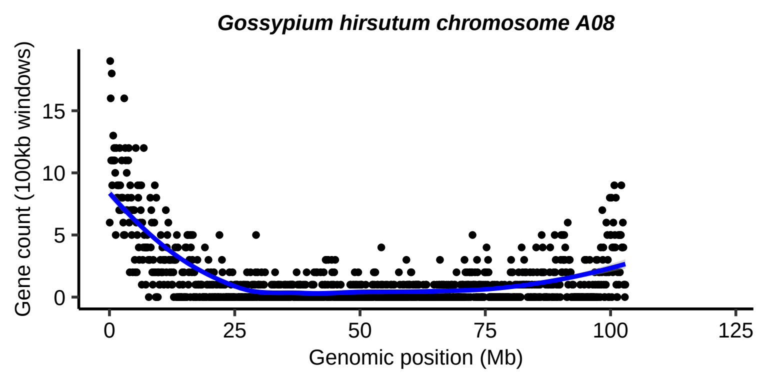
Gossypium hirsutum chromosome A08
Gene count (100kb windows)
15
10
5
0
0
25
50
75
100
125
Genomic position (Mb)

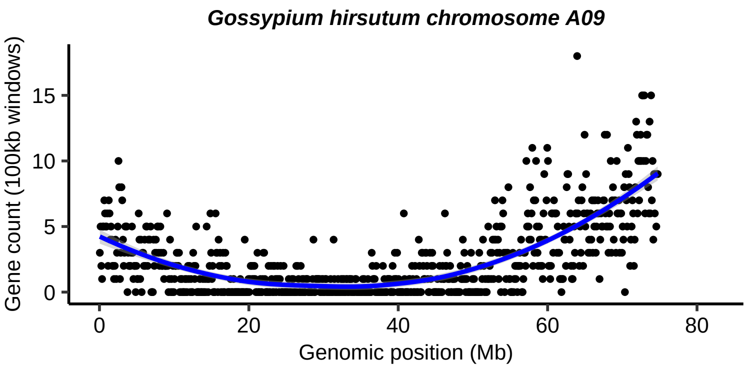

Gossypium hirsutum chromosome A09
Gene count (100kb windows)
15
10
5
0
0
20
40
60
80
Genomic position (Mb)

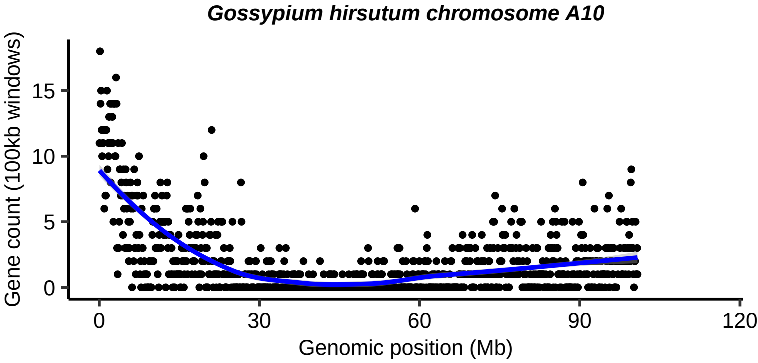

Gossypium hirsutum chromosome A10
Gene count (100kb windows)
0
5
10
15
0
30
60
90
120
Genomic position (Mb)

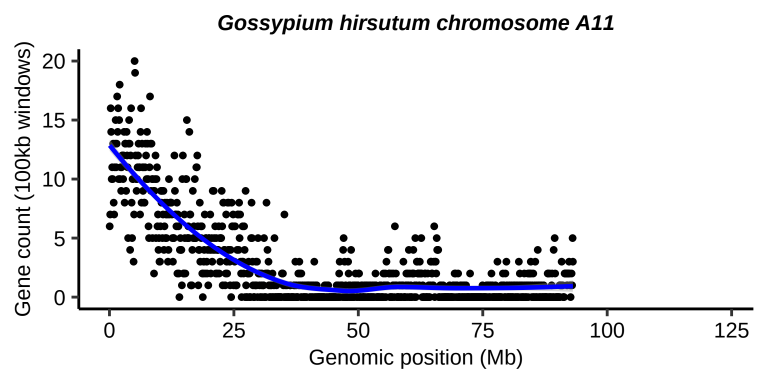

Gossypium hirsutum chromosome A11
Gene count (100kb windows)
20
15
10
5
0
0
25
50
75
100
125
Genomic position (Mb)

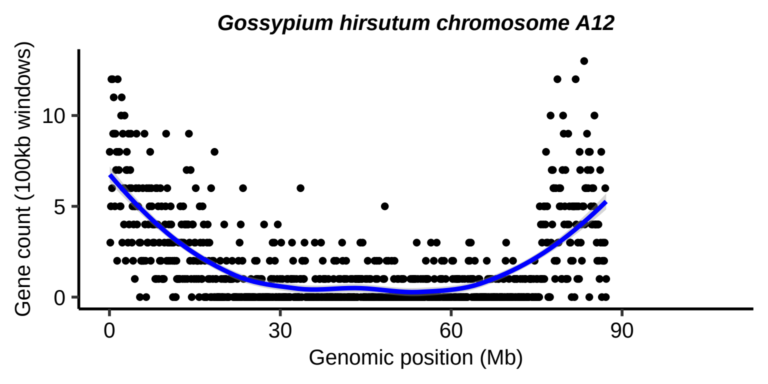

Gossypium hirsutum chromosome A12
Gene count (100kb windows)
0
5
10
0
30
60
90
Genomic position (Mb)

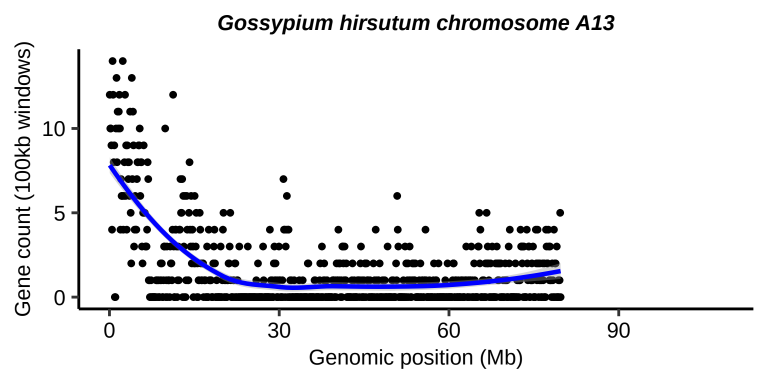
Gossypium hirsutum chromosome A13
Gene count (100kb windows)
0
5
10
0
30
60
90
Genomic position (Mb)

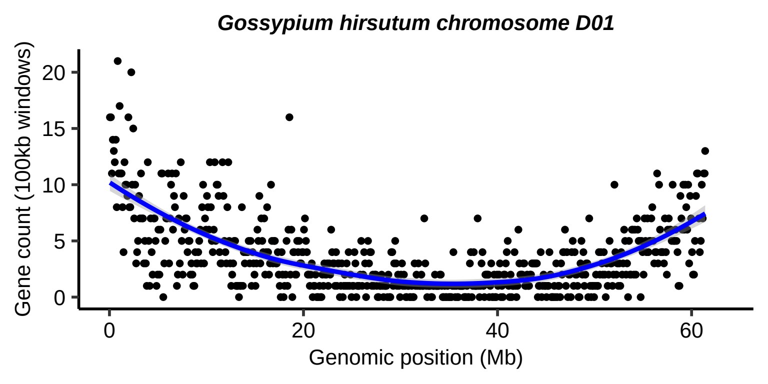

Gossypium hirsutum chromosome D01
Gene count (100kb windows)
0
5
10
15
20
0
20
40
60
Genomic position (Mb)

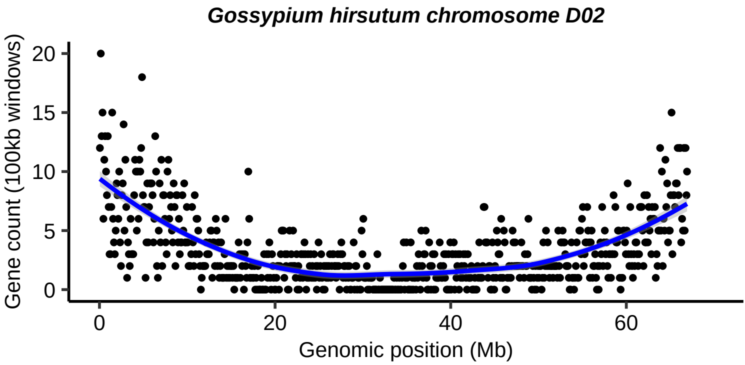

Gossypium hirsutum chromosome D02
Gene count (100kb windows)
20
15
10
5
0
0
20
40
60
Genomic position (Mb)

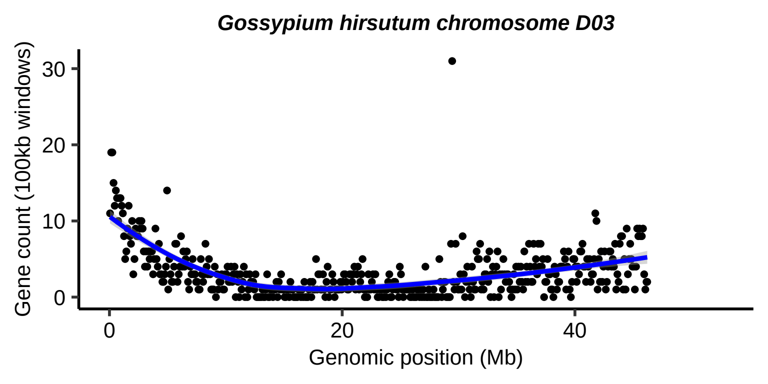
Gossypium hirsutum chromosome D03
Gene count (100kb windows)
0
10
20
30
0
20
40
Genomic position (Mb)

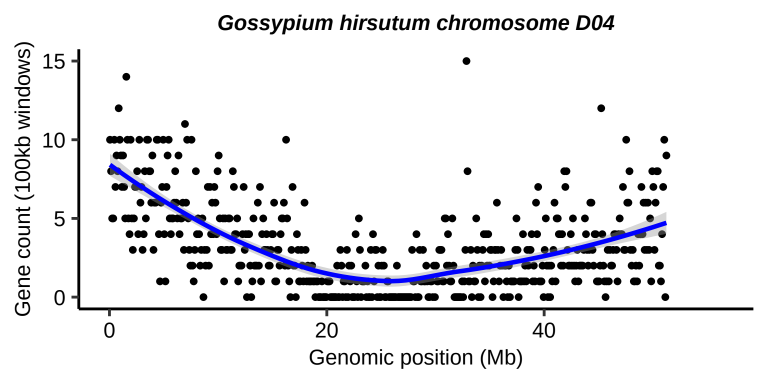

Gossypium hirsutum chromosome D04
Gene count (100kb windows)
15
10
5
0
0
20
40
Genomic position (Mb)

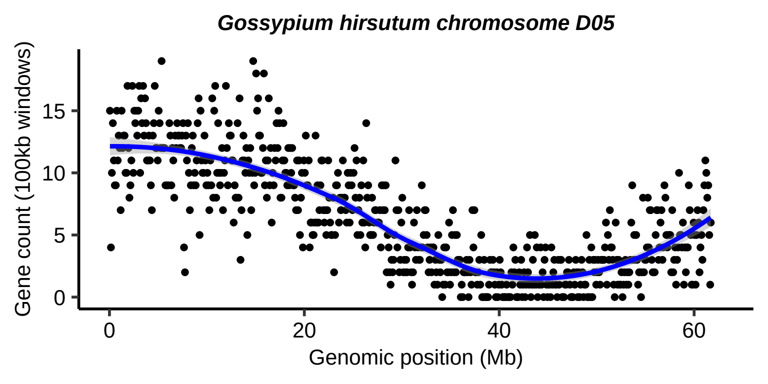
Gossypium hirsutum chromosome D05
Gene count (100kb windows)
0
5
10
15
0
20
40
60
Genomic position (Mb)

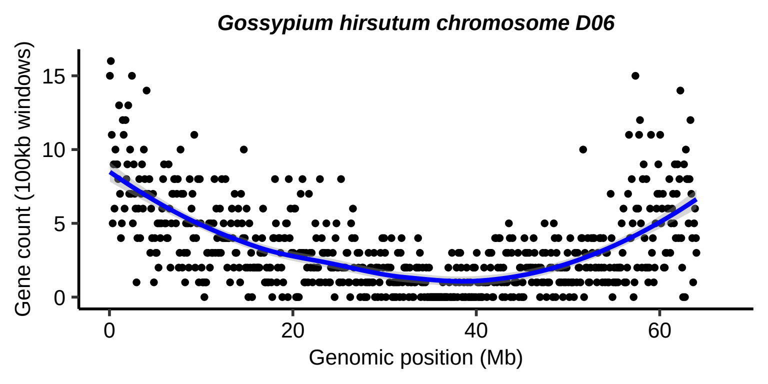
Gossypium hirsutum chromosome D06
Gene count (100kb windows)
0
5
10
15
0
20
40
60
Genomic position (Mb)

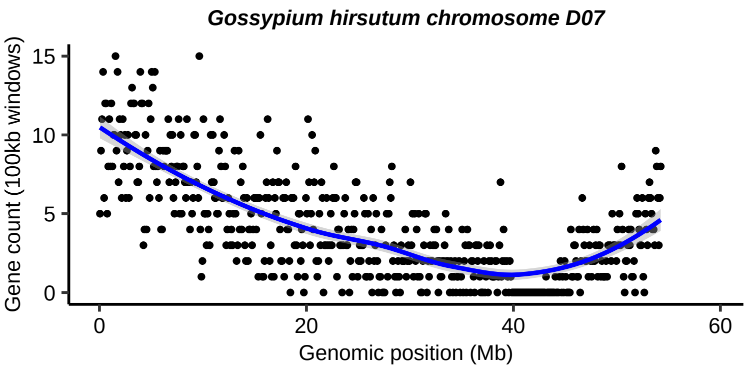

Gossypium hirsutum chromosome D07
Gene count (100kb windows)
Genomic position (Mb)
0
5
10
15
0
20
40
60

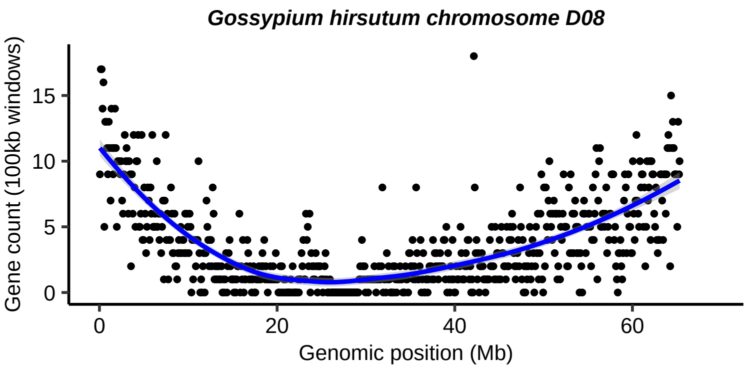
Gossypium hirsutum chromosome D08
Gene count (100kb windows)
0
5
10
15
0
20
40
60
Genomic position (Mb)

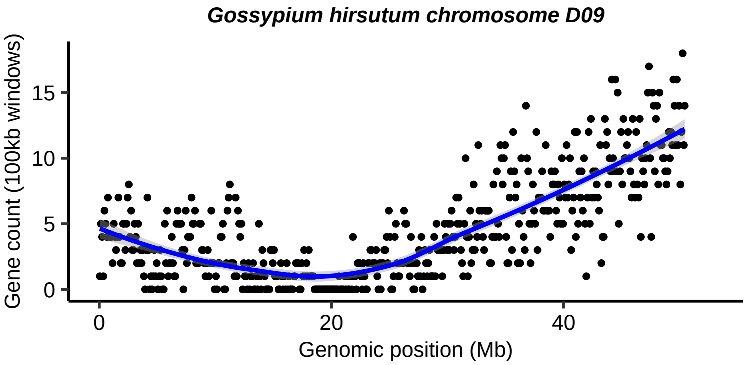

Gossypium hirsutum chromosome D09
Gene count (100kb windows)
0
5
10
15
0
20
40
Genomic position (Mb)

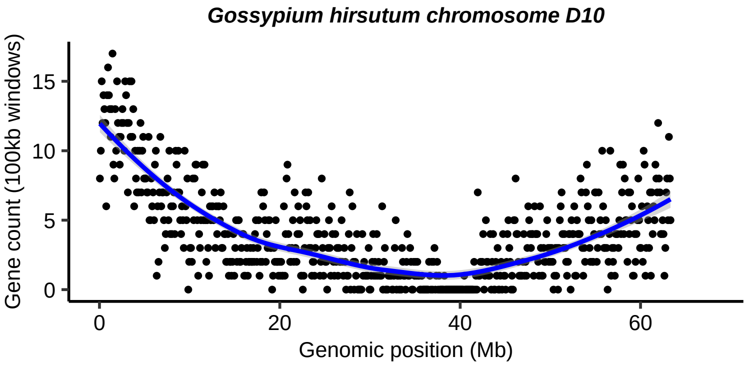
Gossypium hirsutum chromosome D10
Gene count (100kb windows)
15
10
5
0
0
20
40
60
Genomic position (Mb)

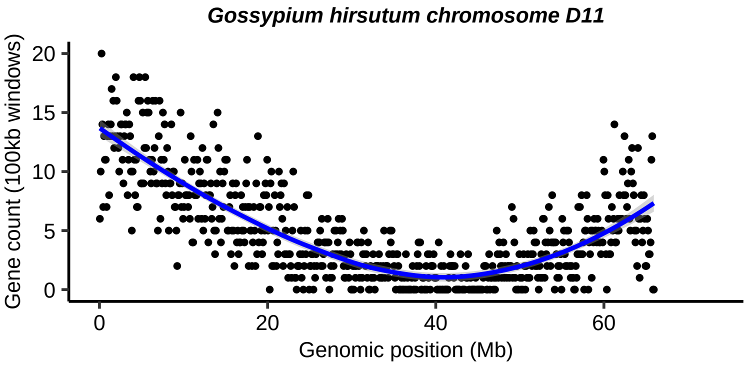
Gossypium hirsutum chromosome D11
Gene count (100kb windows)
0
5
10
15
20
0
20
40
60
Genomic position (Mb)

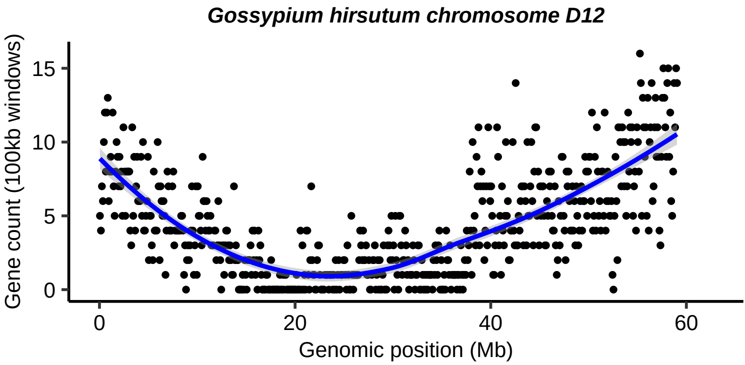
Gossypium hirsutum chromosome D12
Gene count (100kb windows)
15
10
5
0
0
20
40
60
Genomic position (Mb)

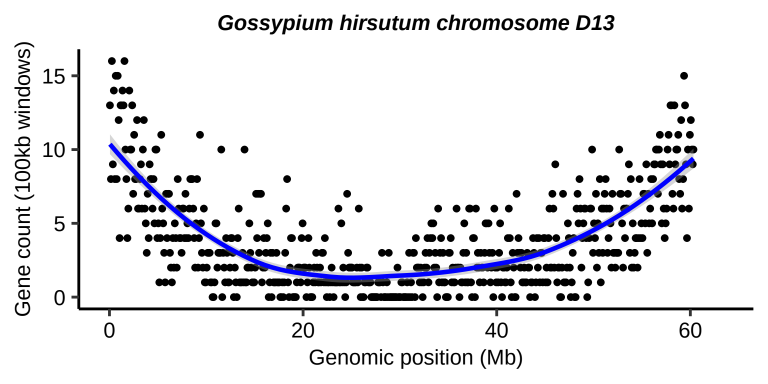
Gossypium hirsutum chromosome D13
Gene count (100kb windows)
0
5
10
15
0
20
40
60
Genomic position (Mb)

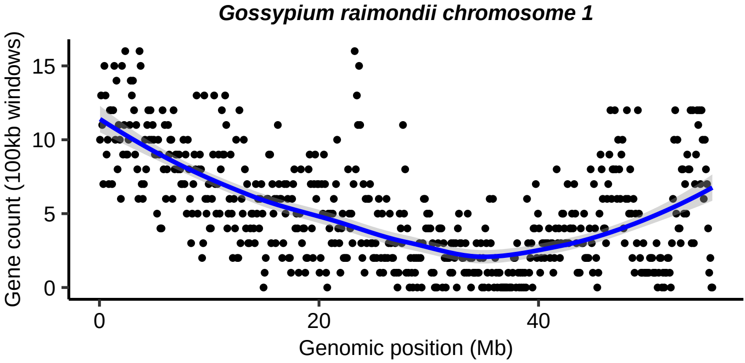

Gossypium raimondii chromosome 1
Gene count (100kb windows)
0
5
10
15
0
20
40
Genomic position (Mb)

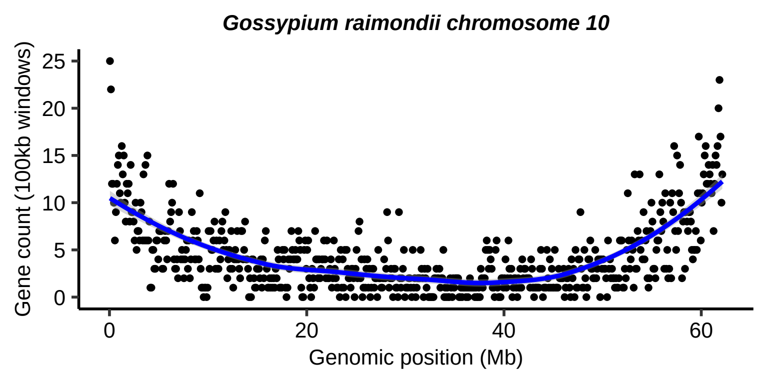

Gossypium raimondii chromosome 10
Gene count (100kb windows)
25
20
15
10
5
0
0
20
40
60
Genomic position (Mb)

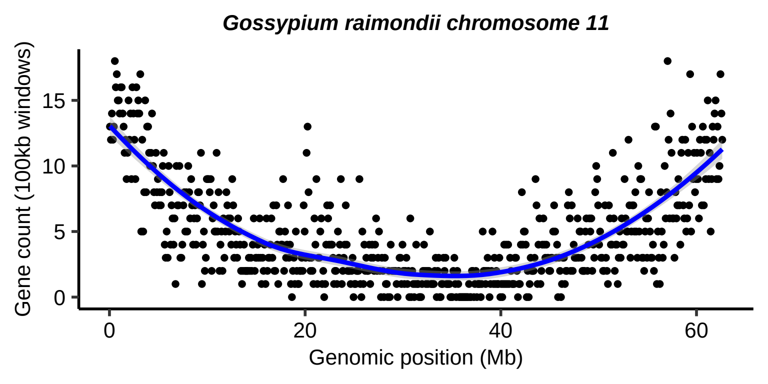
Gossypium raimondii chromosome 11
Gene count (100kb windows)
0
5
10
15
0
20
40
60
Genomic position (Mb)

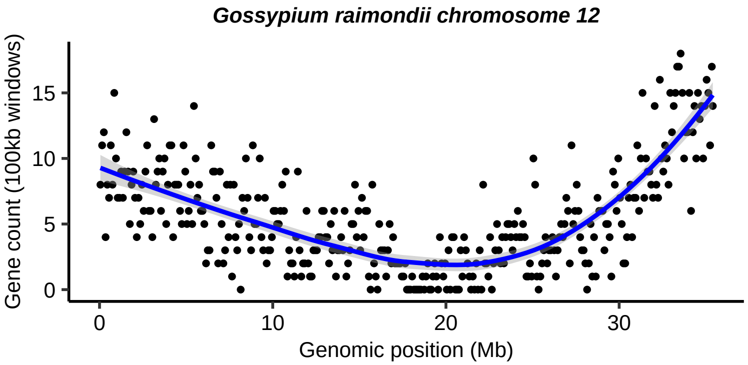

Gossypium raimondii chromosome 12
Gene count (100kb windows)
0
5
10
15
0
10
20
30
Genomic position (Mb)

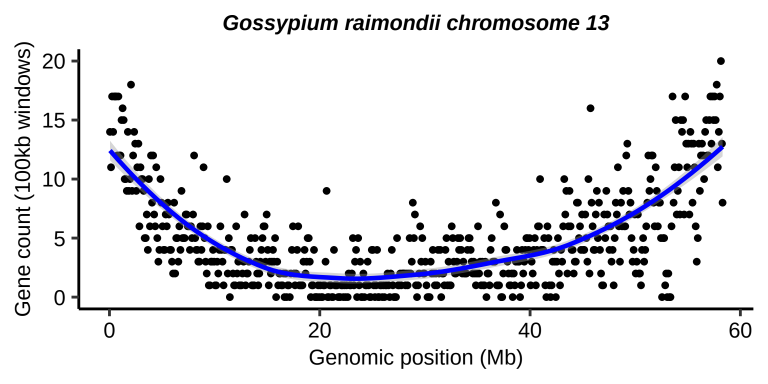
Gossypium raimondii chromosome 13
Gene count (100kb windows)
Genomic position (Mb)
0
5
10
15
20
0
20
40
60

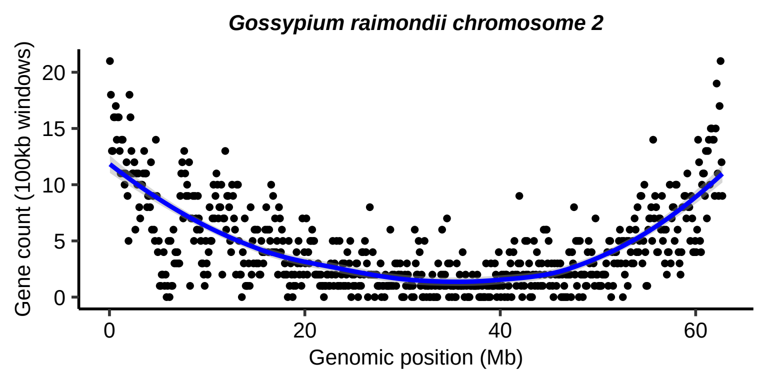

Gossypium raimondii chromosome 2
Gene count (100kb windows)
0
5
10
15
20
0
20
40
60
Genomic position (Mb)

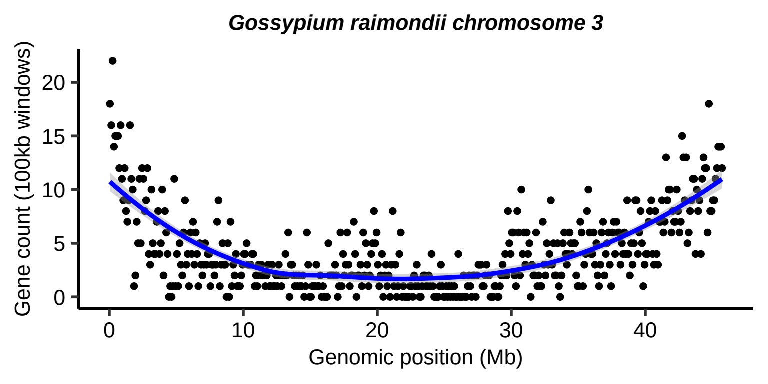

Gossypium raimondii chromosome 3
Gene count (100kb windows)
0
5
10
15
20
0
10
20
30
40
Genomic position (Mb)

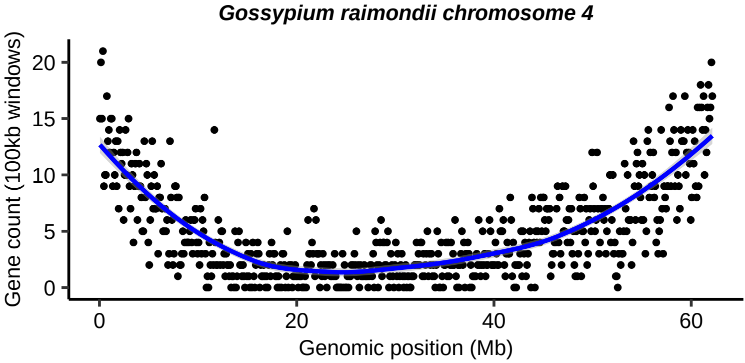

Gossypium raimondii chromosome 4
Gene count (100kb windows)
0
5
10
15
20
0
20
40
60
Genomic position (Mb)

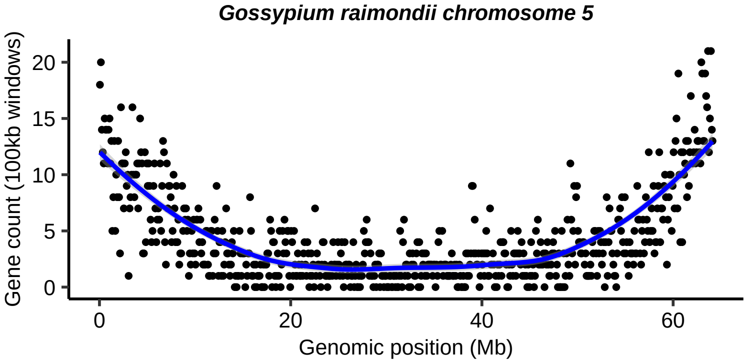

Gossypium raimondii chromosome 5
Gene count (100kb windows)
20
15
10
5
0
0
20
40
60
Genomic position (Mb)

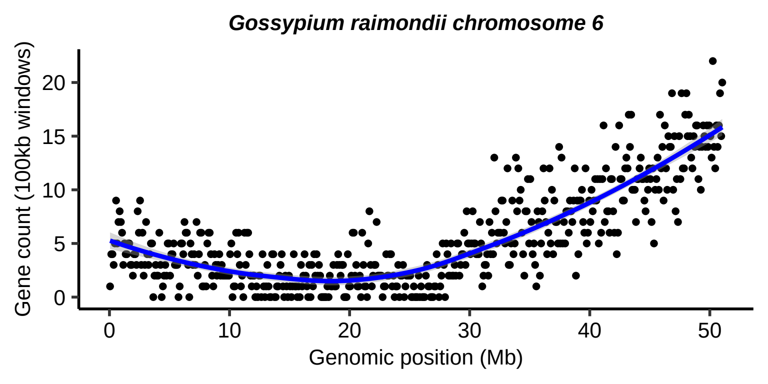

Gossypium raimondii chromosome 6
Gene count (100kb windows)
0
5
10
15
20
0
10
20
30
40
50
Genomic position (Mb)

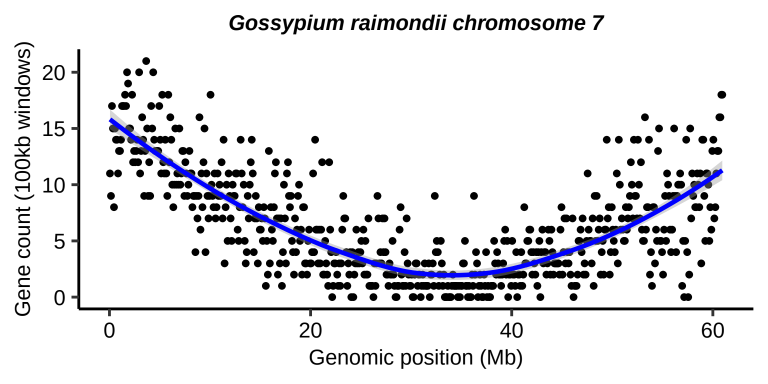
Gossypium raimondii chromosome 7
Gene count (100kb windows)
0
5
10
15
20
0
20
40
60
Genomic position (Mb)

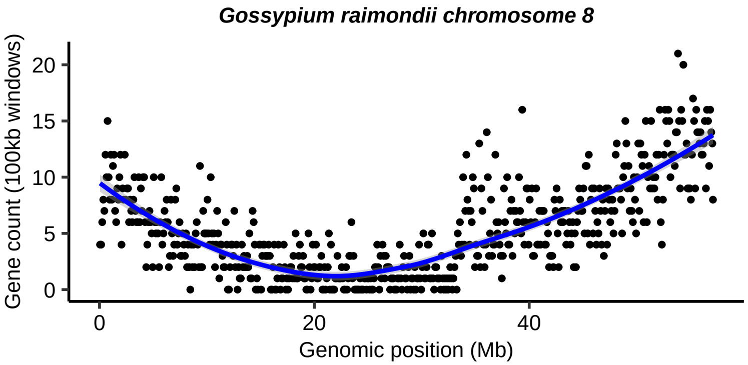

Gossypium raimondii chromosome 8
Gene count (100kb windows)
0
5
10
15
20
0
20
40
Genomic position (Mb)

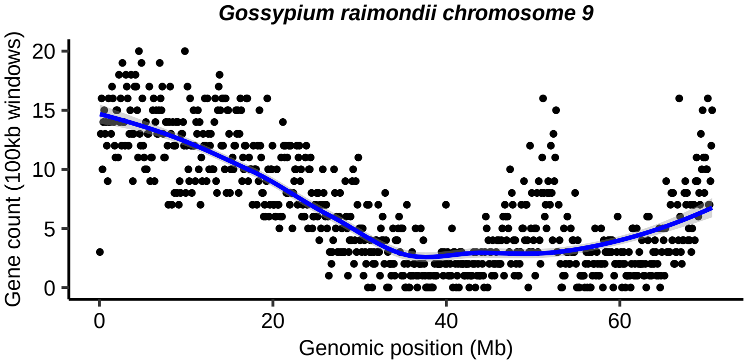

Gossypium raimondii chromosome 9
Gene count (100kb windows)
0
5
10
15
20
0
20
40
60
Genomic position (Mb)

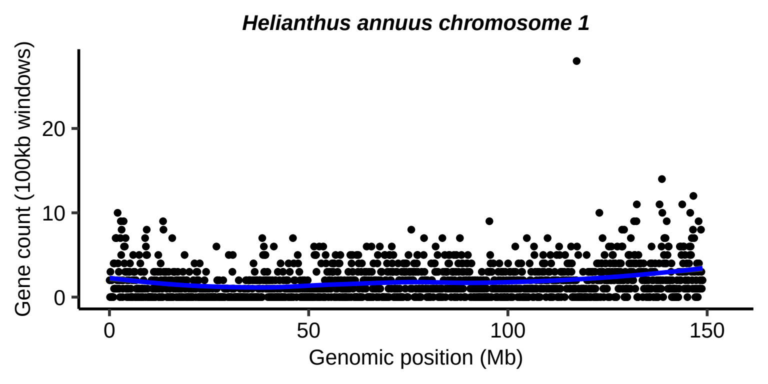

Helianthus annuus chromosome 1
Gene count (100kb windows)
0
10
20
0
50
100
150
Genomic position (Mb)

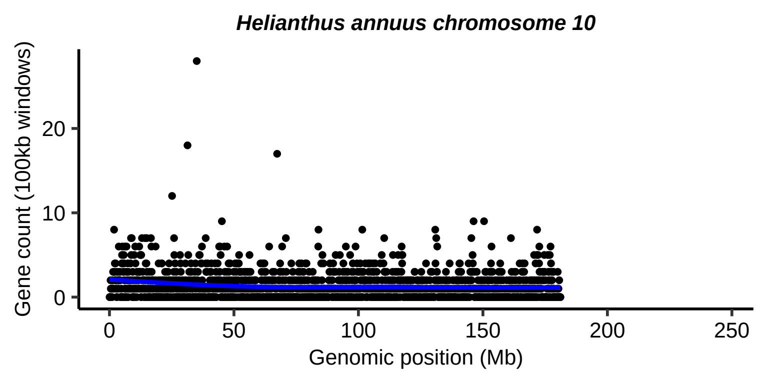

Helianthus annuus chromosome 10
Gene count (100kb windows)
0
10
20
0
50
100
150
200
250
Genomic position (Mb)

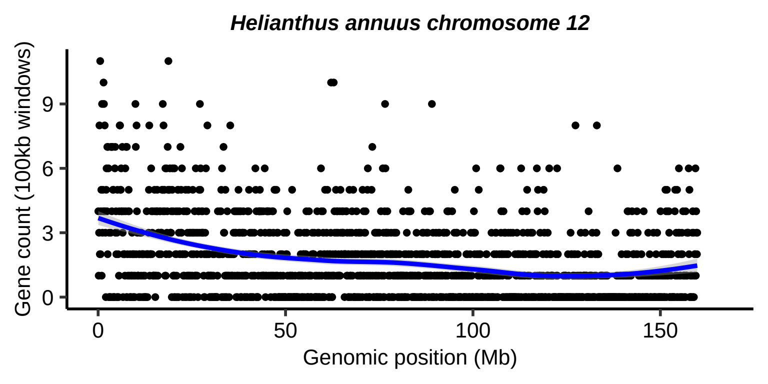
Helianthus annuus chromosome 12
Gene count (100kb windows)
0
3
6
9
0
50
100
150
Genomic position (Mb)

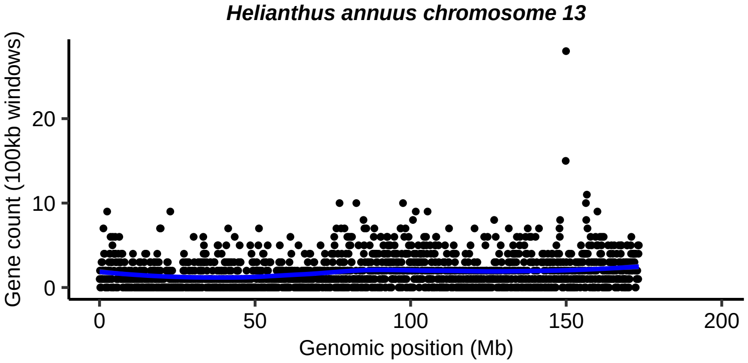

Helianthus annuus chromosome 13
Gene count (100kb windows)
20
10
0
0
50
100
150
200
Genomic position (Mb)

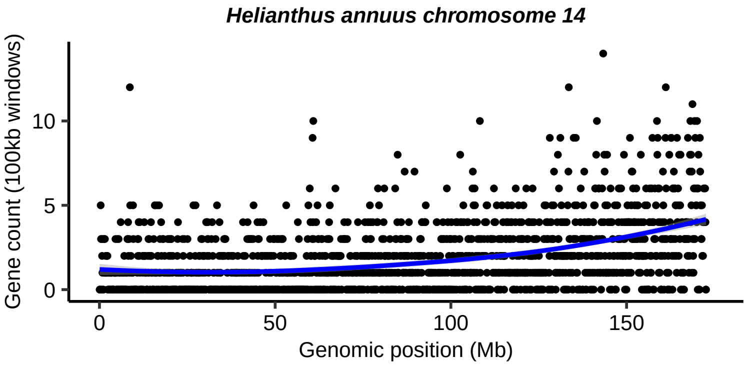
Helianthus annuus chromosome 14
Gene count (100kb windows)
0
5
10
0
50
100
150
Genomic position (Mb)

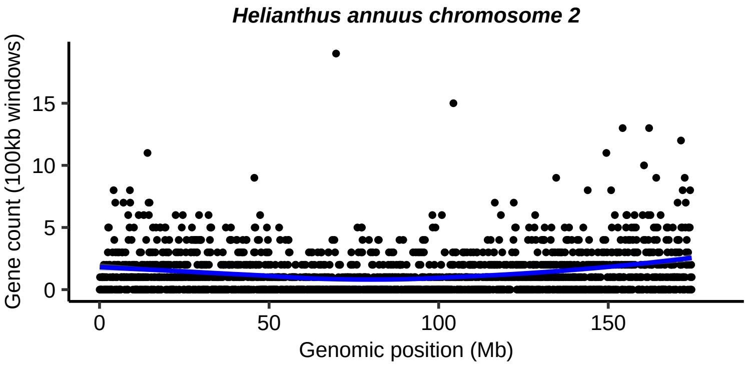

Helianthus annuus chromosome 2
Gene count (100kb windows)
0
5
10
15
0
50
100
150
Genomic position (Mb)

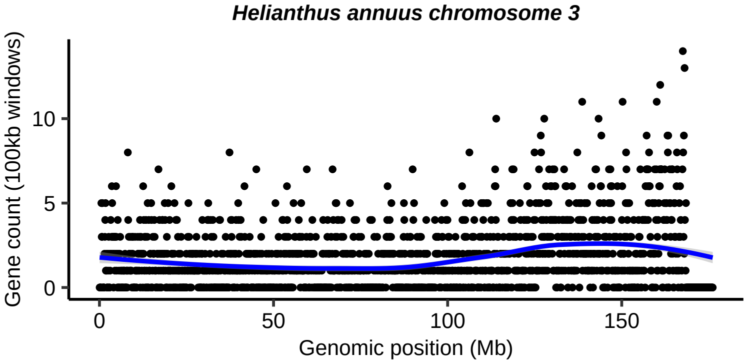
Helianthus annuus chromosome 3
Gene count (100kb windows)
0
5
10
0
50
100
150
Genomic position (Mb)

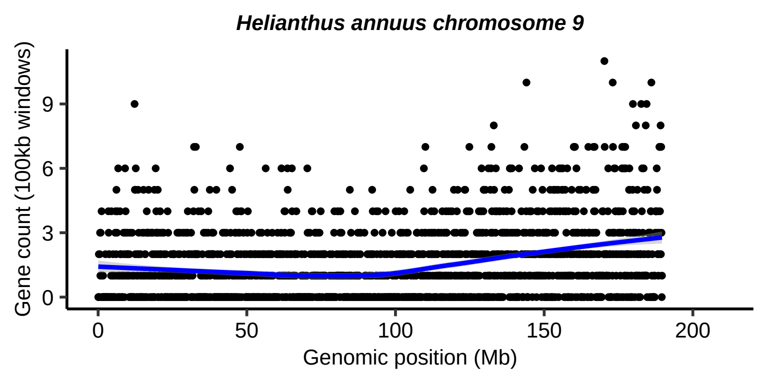

Helianthus annuus chromosome 9
Gene count (100kb windows)
0
3
6
9
0
50
100
150
200
Genomic position (Mb)

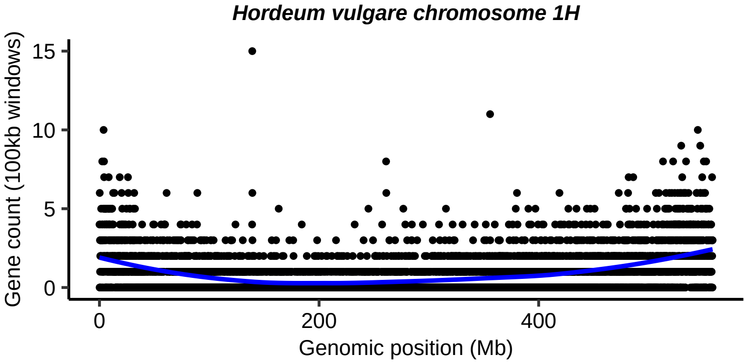

Hordeum vulgare chromosome 1H
Gene count (100kb windows)
15
10
5
0
0
200
400
Genomic position (Mb)

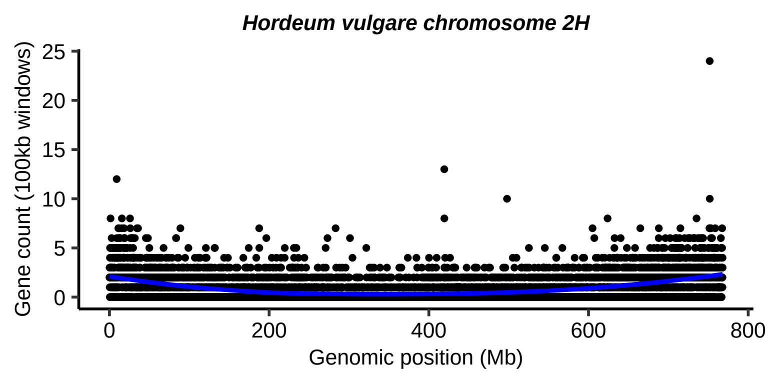

Hordeum vulgare chromosome 2H
Gene count (100kb windows)
25
20
15
10
5
0
0
200
400
600
800
Genomic position (Mb)

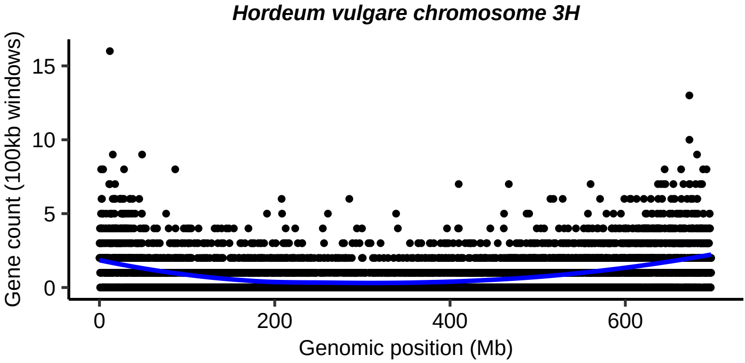

Hordeum vulgare chromosome 3H
Gene count (100kb windows)
15
10
5
0
0
200
400
600
Genomic position (Mb)

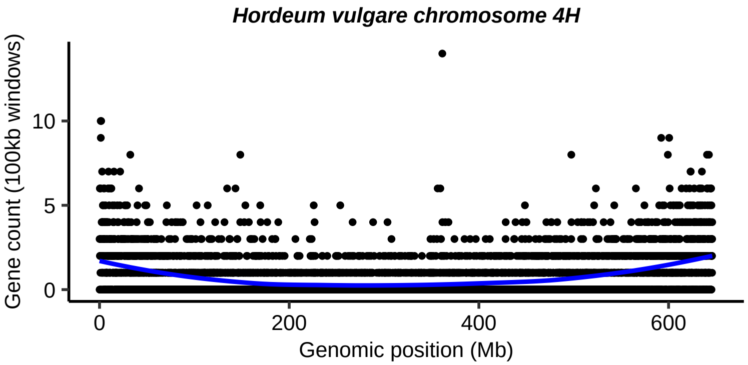

Hordeum vulgare chromosome 4H
Gene count (100kb windows)
10
5
0
0
200
400
600
Genomic position (Mb)

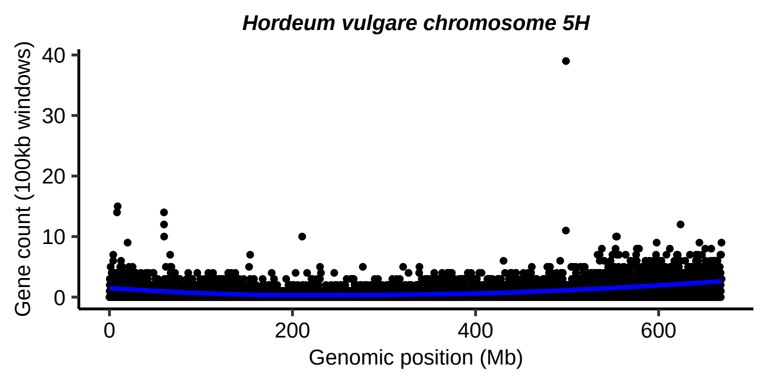

Hordeum vulgare chromosome 5H
Gene count (100kb windows)
0
10
20
30
40
0
200
400
600
Genomic position (Mb)

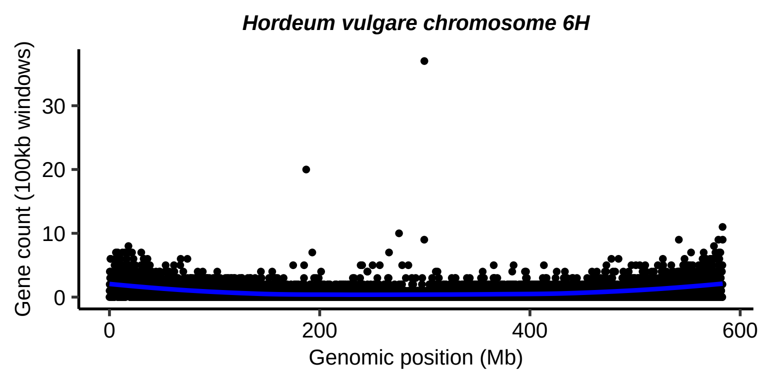

Hordeum vulgare chromosome 6H
Gene count (100kb windows)
0
10
20
30
0
200
400
600
Genomic position (Mb)

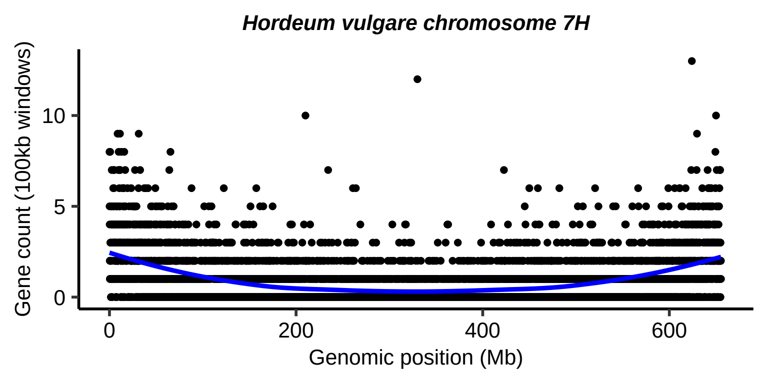
Hordeum vulgare chromosome 7H
Gene count (100kb windows)
0
5
10
0
200
400
600
Genomic position (Mb)

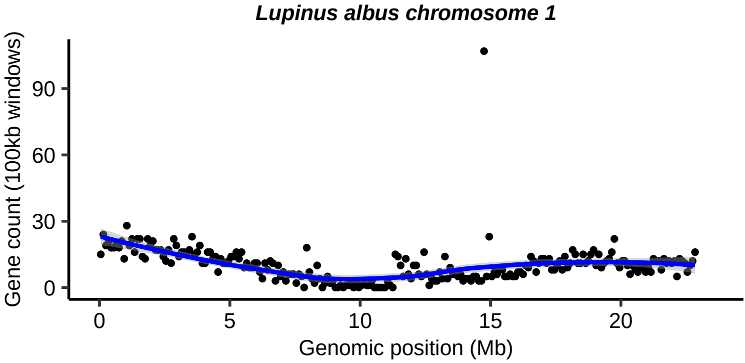

Lupinus albus chromosome 1
Gene count (100kb windows)
0
30
60
90
0
5
10
15
20
Genomic position (Mb)

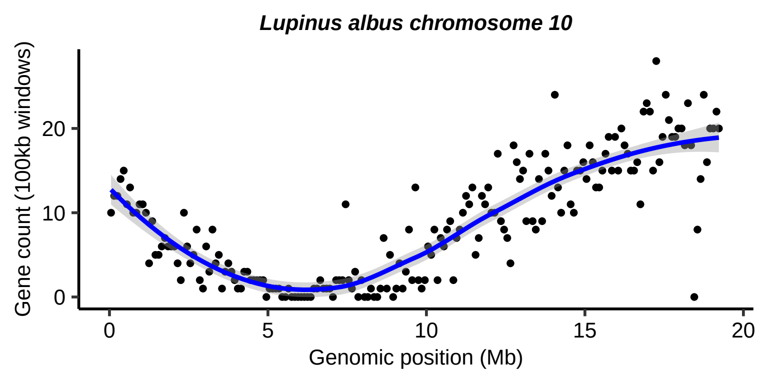

Lupinus albus chromosome 10
Gene count (100kb windows)
0
10
20
0
5
10
15
20
Genomic position (Mb)

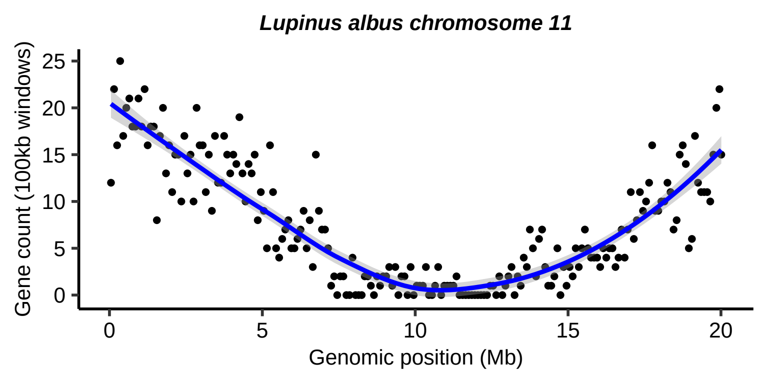

Lupinus albus chromosome 11
Gene count (100kb windows)
25
20
15
10
5
0
0
5
10
15
20
Genomic position (Mb)

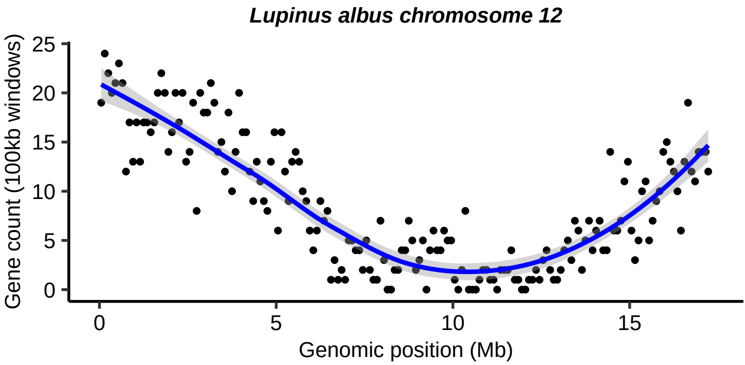

Lupinus albus chromosome 12
Gene count (100kb windows)
25
20
15
10
5
0
0
5
10
15
Genomic position (Mb)

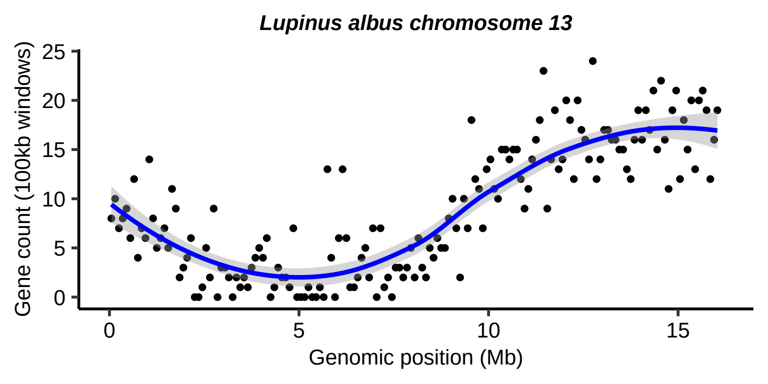

Lupinus albus chromosome 13
Gene count (100kb windows)
0
5
10
15
20
25
0
5
10
15
Genomic position (Mb)

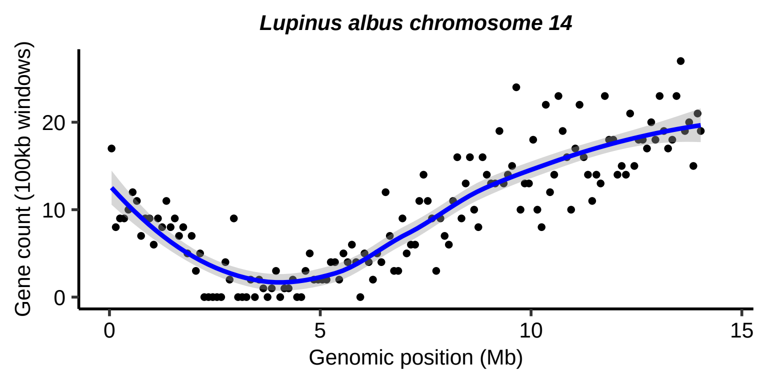
Lupinus albus chromosome 14
Gene count (100kb windows)
0
10
20
0
5
10
15
Genomic position (Mb)

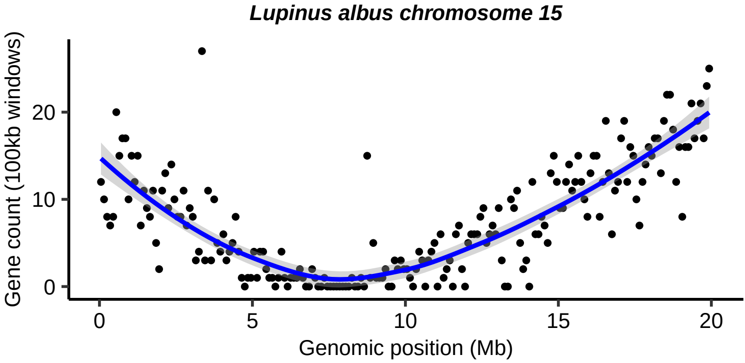
Lupinus albus chromosome 15
Gene count (100kb windows)
0
10
20
0
5
10
15
20
Genomic position (Mb)

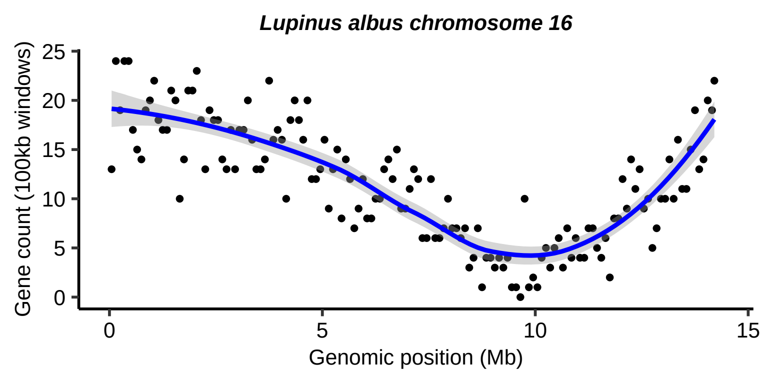

Lupinus albus chromosome 16
Gene count (100kb windows)
0
5
10
15
20
25
0
5
10
15
Genomic position (Mb)

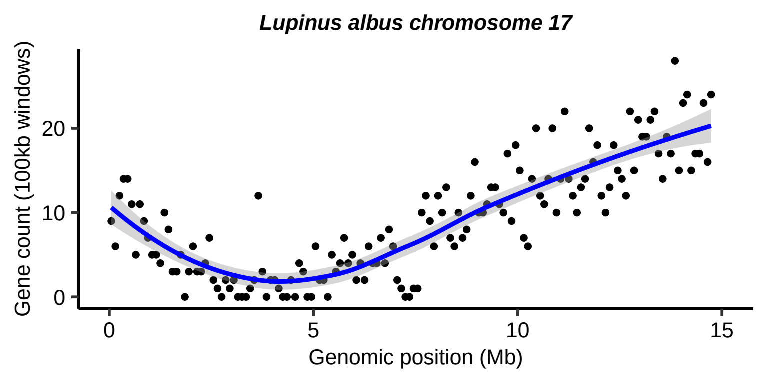

Lupinus albus chromosome 17
Gene count (100kb windows)
0
10
20
0
5
10
15
Genomic position (Mb)

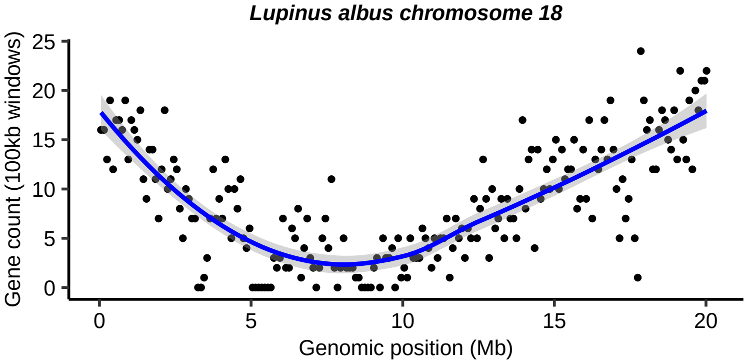
Lupinus albus chromosome 18
Gene count (100kb windows)
0
5
10
15
20
25
Genomic position (Mb)

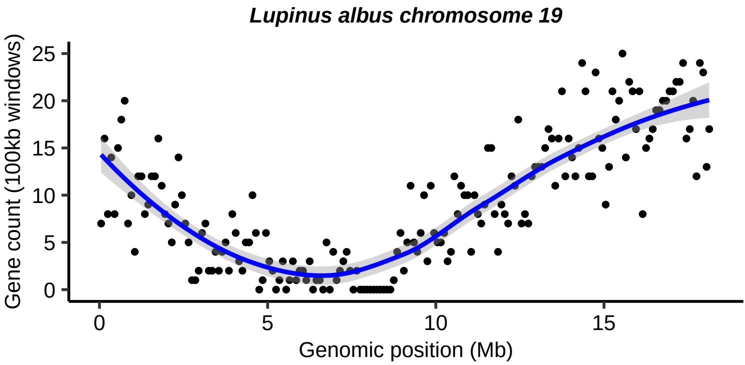

Lupinus albus chromosome 19
Gene count (100kb windows)
0
5
10
15
20
25
0
5
10
15
Genomic position (Mb)

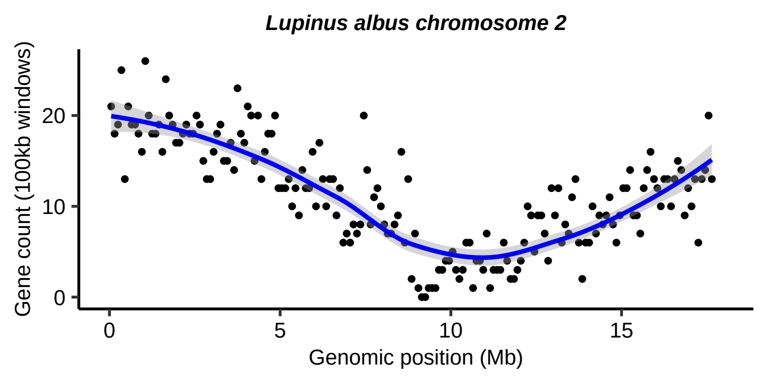
Lupinus albus chromosome 2
Gene count (100kb windows)
0
10
20
0
5
10
15
Genomic position (Mb)

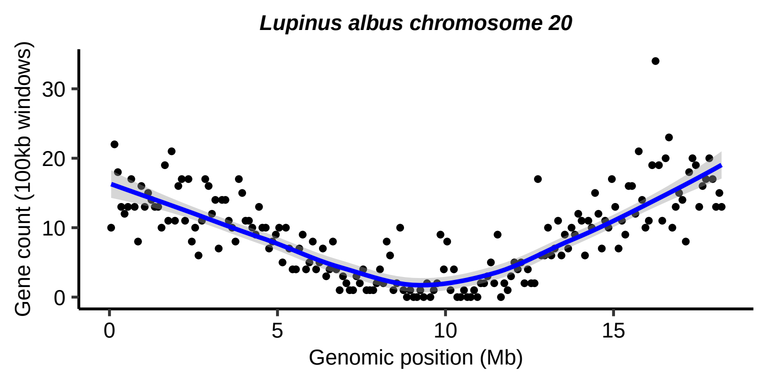

Lupinus albus chromosome 20
Gene count (100kb windows)
0
10
20
30
0
5
10
15
Genomic position (Mb)

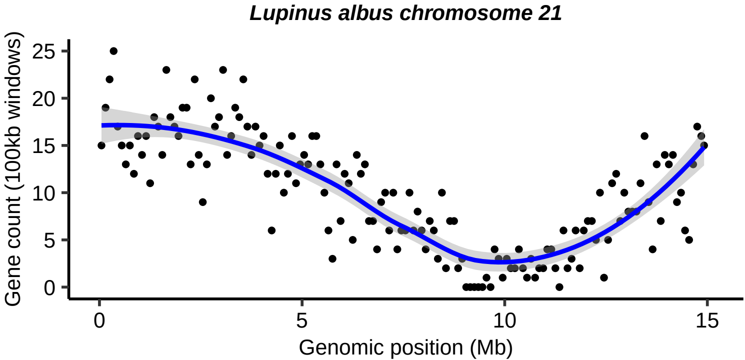

Lupinus albus chromosome 21
Gene count (100kb windows)
25
20
15
10
5
0
0
5
10
15
Genomic position (Mb)

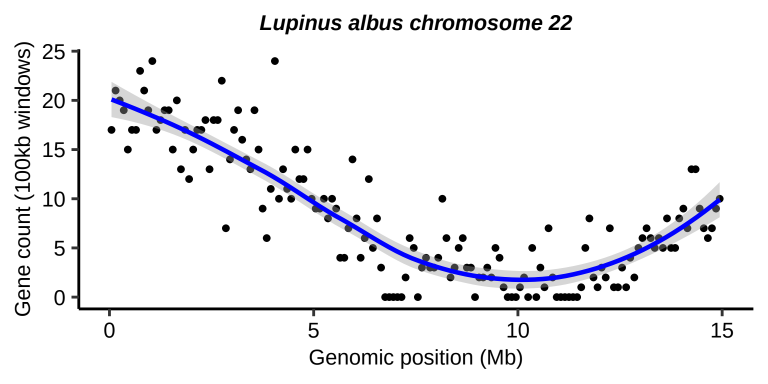

Lupinus albus chromosome 22
Gene count (100kb windows)
25
20
15
10
5
0
0
5
10
15
Genomic position (Mb)

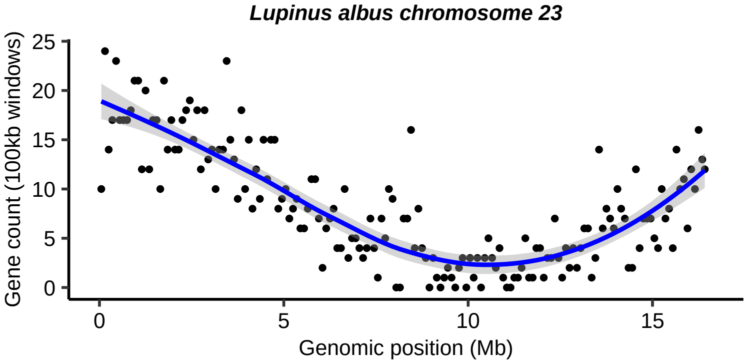

Lupinus albus chromosome 23
Gene count (100kb windows)
25
20
15
10
5
0
0
5
10
15
Genomic position (Mb)

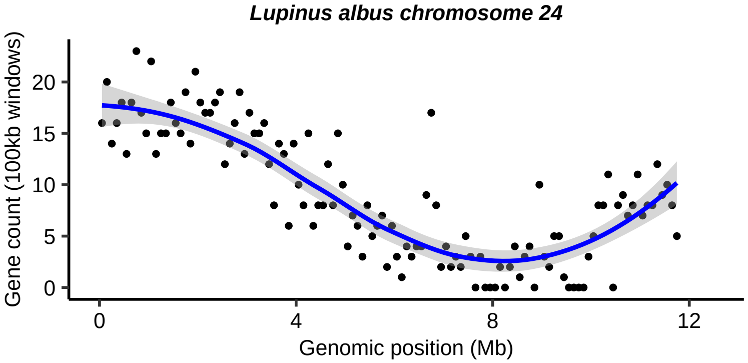

Lupinus albus chromosome 24
Gene count (100kb windows)
20
15
10
5
0
0
4
8
12
Genomic position (Mb)

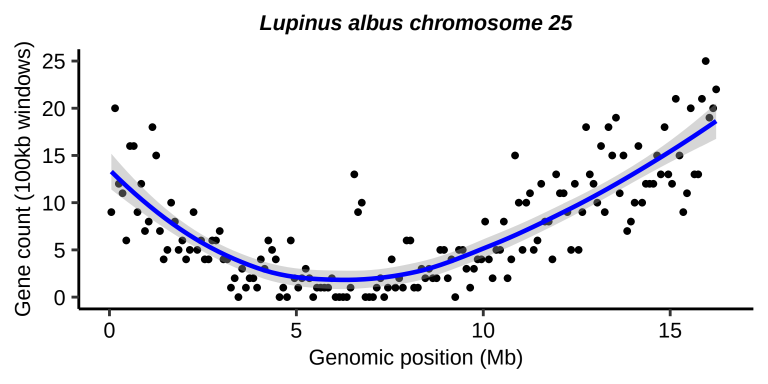
Lupinus albus chromosome 25
Gene count (100kb windows)
0
5
10
15
20
25
0
5
10
15
Genomic position (Mb)

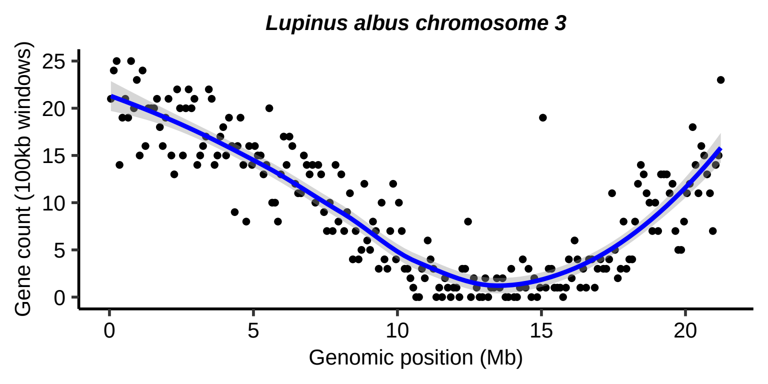

Lupinus albus chromosome 3
Gene count (100kb windows)
25
20
15
10
5
0
0
5
10
15
20
Genomic position (Mb)

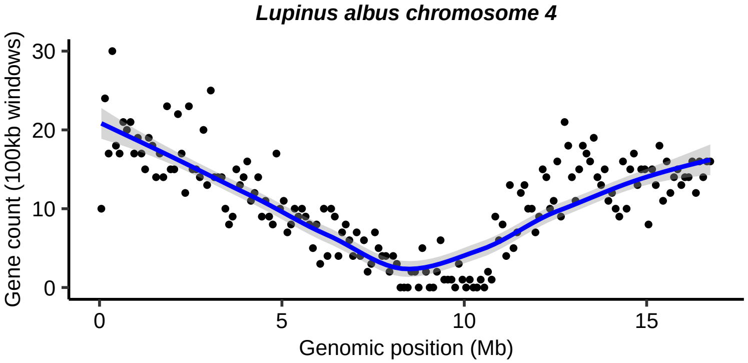

Lupinus albus chromosome 4
Gene count (100kb windows)
30
20
10
0
0
5
10
15
Genomic position (Mb)

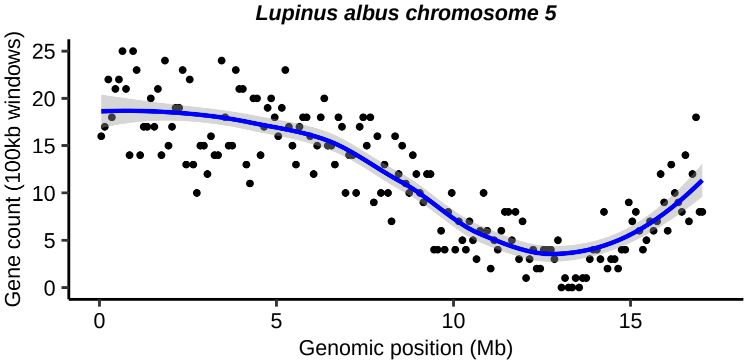

Lupinus albus chromosome 5
Gene count (100kb windows)
0
5
10
15
20
25
0
5
10
15
Genomic position (Mb)

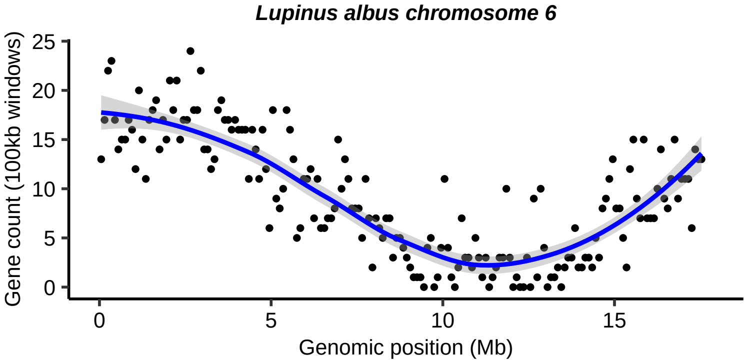

Lupinus albus chromosome 6
Gene count (100kb windows)
25
20
15
10
5
0
0
5
10
15
Genomic position (Mb)

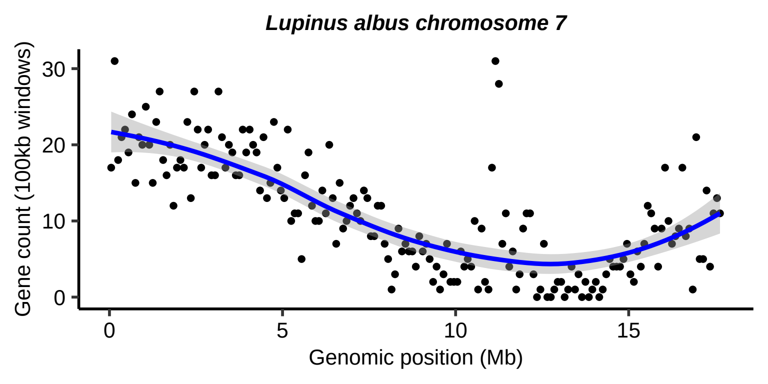

Lupinus albus chromosome 7
Gene count (100kb windows)
0
10
20
30
0
5
10
15
Genomic position (Mb)

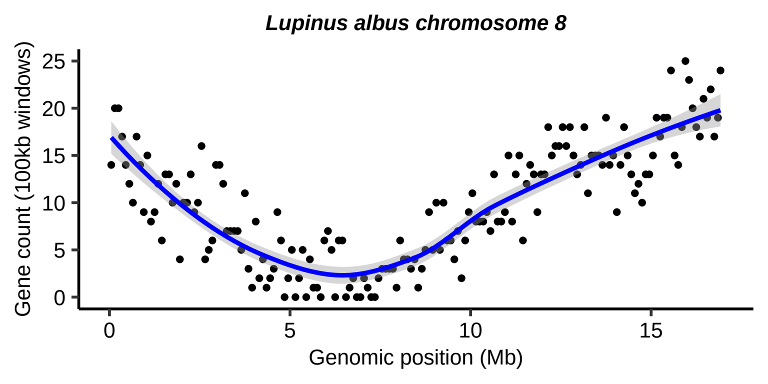

Lupinus albus chromosome 8
Gene count (100kb windows)
0
5
10
15
20
25
0
5
10
15
Genomic position (Mb)

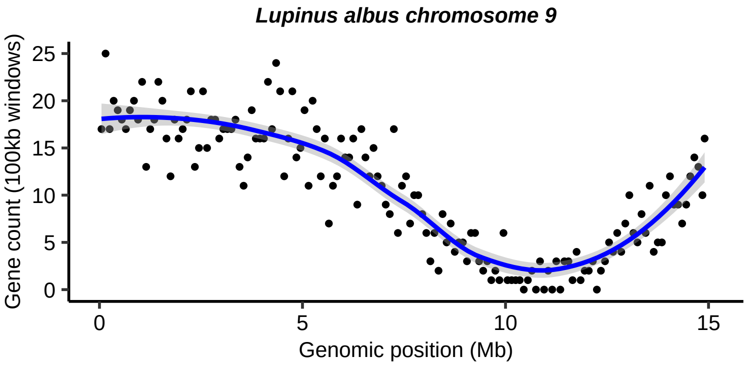
Lupinus albus chromosome 9
Gene count (100kb windows)
0
5
10
15
20
25
0
5
10
15
Genomic position (Mb)

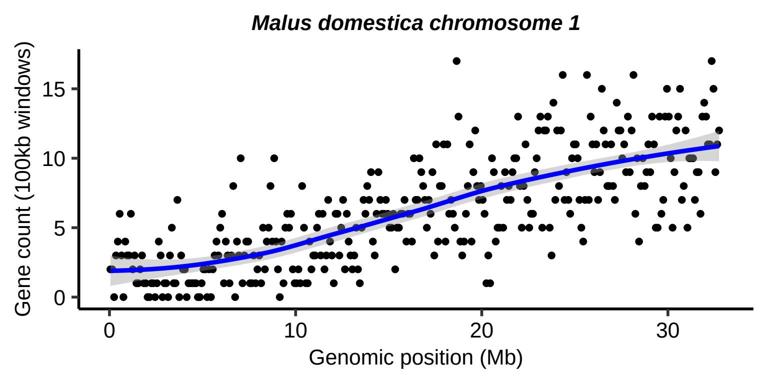

Malus domestica chromosome 1
Gene count (100kb windows)
0
5
10
15
0
10
20
30
Genomic position (Mb)

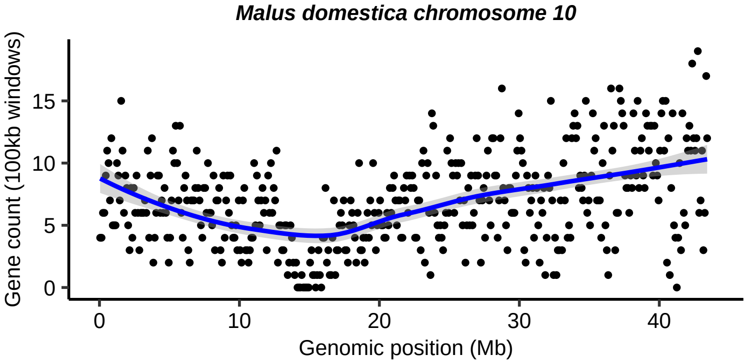

Malus domestica chromosome 10
Gene count (100kb windows)
0
5
10
15
0
10
20
30
40
Genomic position (Mb)

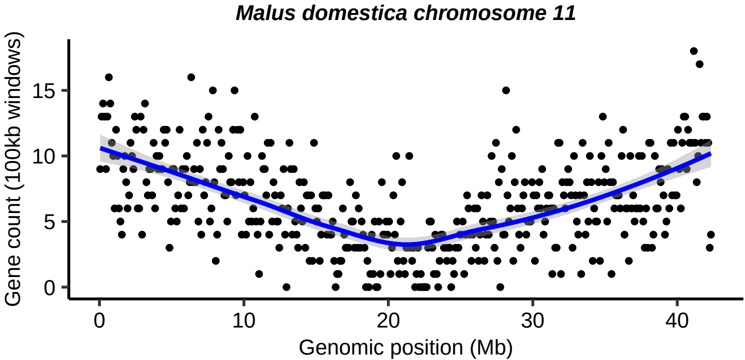

Malus domestica chromosome 11
Gene count (100kb windows)
0
5
10
15
0
10
20
30
40
Genomic position (Mb)

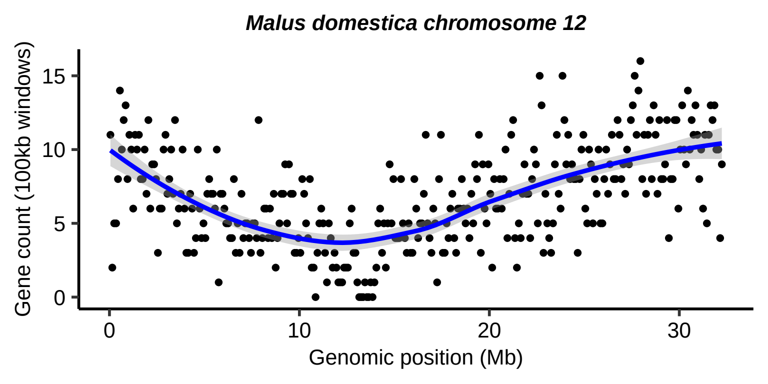

Malus domestica chromosome 12
Gene count (100kb windows)
15
10
5
0
0
10
20
30
Genomic position (Mb)

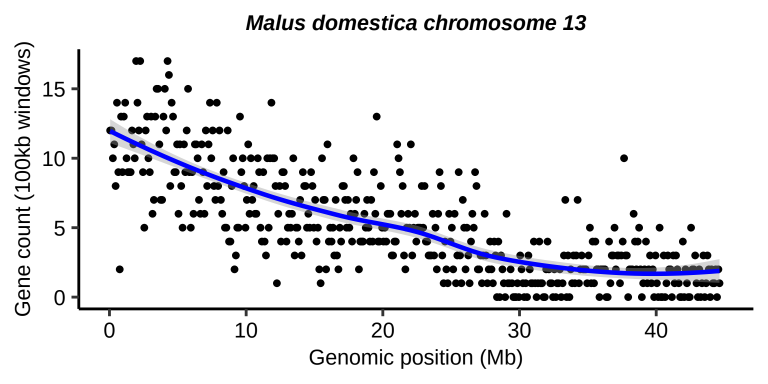
Malus domestica chromosome 13
Gene count (100kb windows)
Genomic position (Mb)
0
5
10
15
0
10
20
30
40

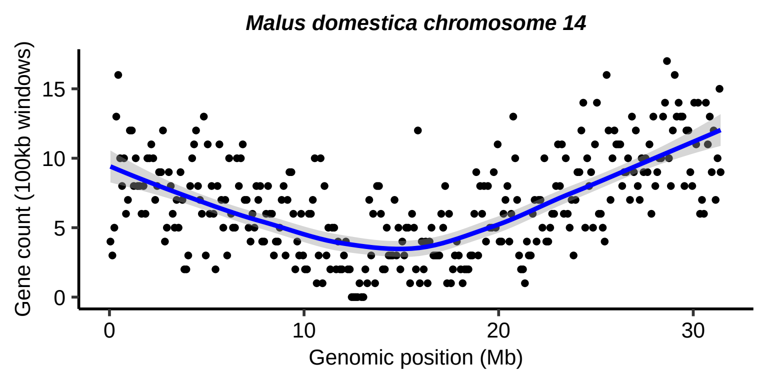

Malus domestica chromosome 14
Gene count (100kb windows)
0
5
10
15
0
10
20
30
Genomic position (Mb)

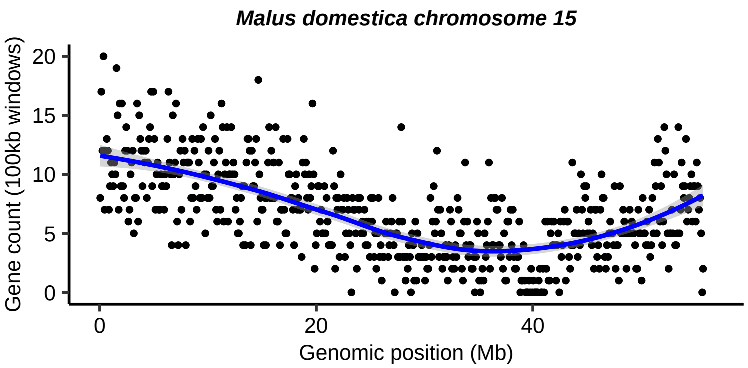

Malus domestica chromosome 15
Gene count (100kb windows)
20
15
10
5
0
0
20
40
Genomic position (Mb)

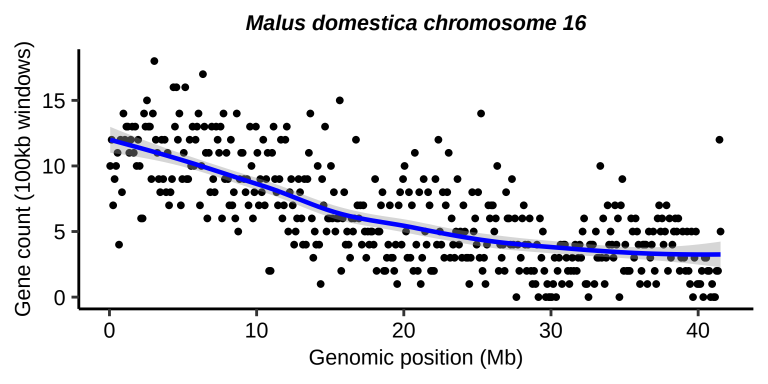
Malus domestica chromosome 16
Gene count (100kb windows)
0
5
10
15
0
10
20
30
40
Genomic position (Mb)

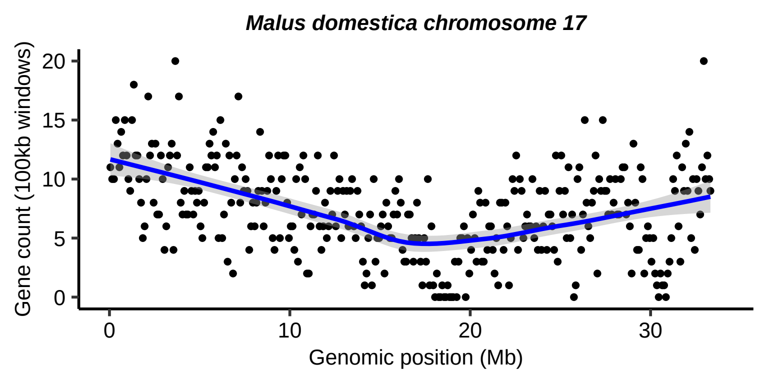

Malus domestica chromosome 17
Gene count (100kb windows)
0
5
10
15
20
0
10
20
30
Genomic position (Mb)

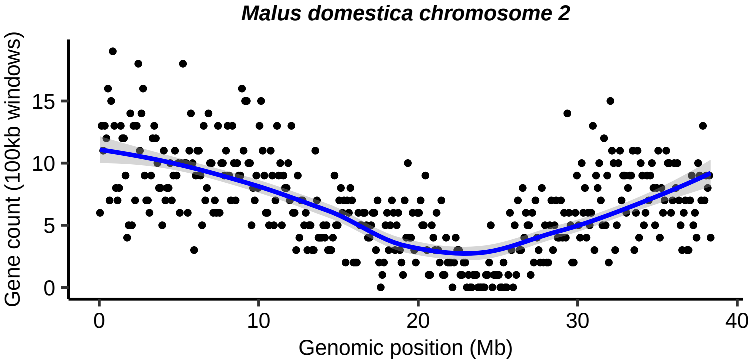

Malus domestica chromosome 2
Gene count (100kb windows)
15
10
5
0
0
10
20
30
40
Genomic position (Mb)

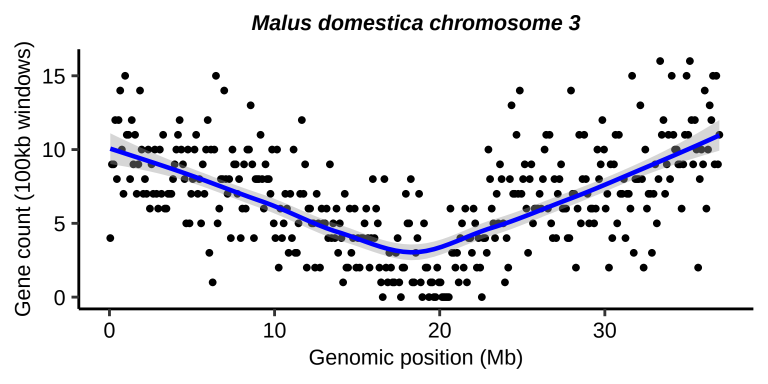

Malus domestica chromosome 3
Gene count (100kb windows)
0
5
10
15
0
10
20
30
Genomic position (Mb)

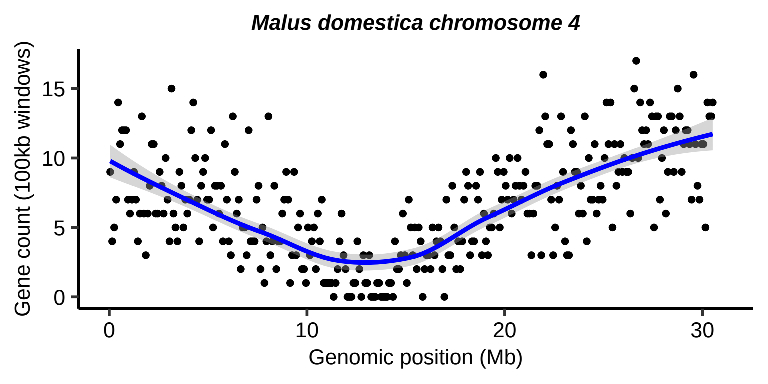

Malus domestica chromosome 4
Gene count (100kb windows)
0
5
10
15
0
10
20
30
Genomic position (Mb)

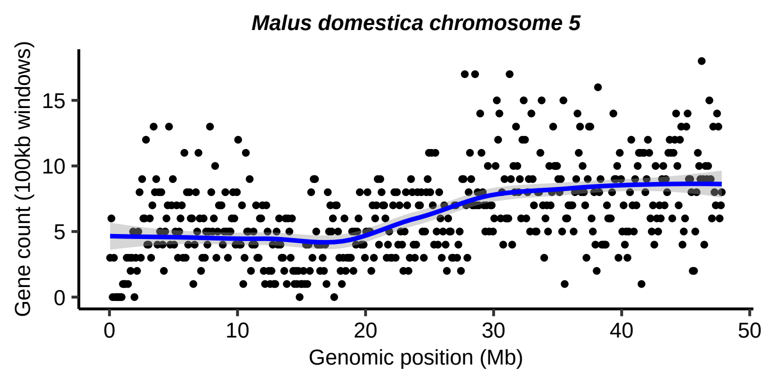
Malus domestica chromosome 5
Gene count (100kb windows)
0
5
10
15
0
10
20
30
40
50
Genomic position (Mb)

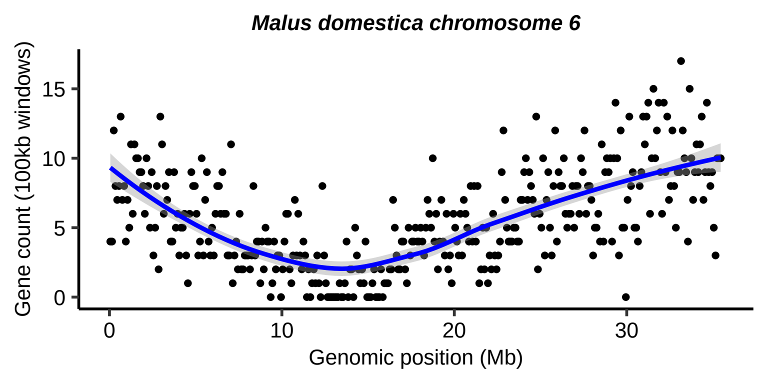

Malus domestica chromosome 6
Gene count (100kb windows)
15
10
5
0
0
10
20
30
Genomic position (Mb)

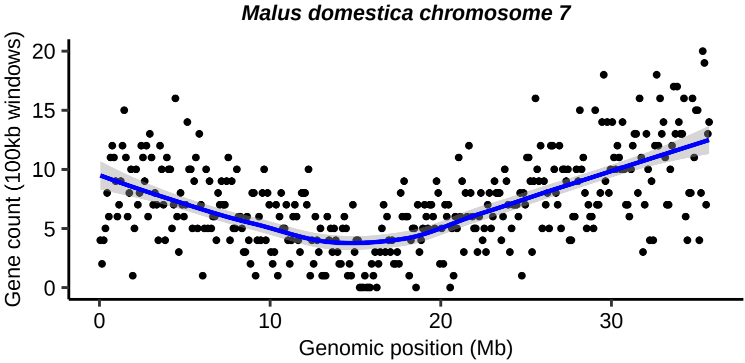

Malus domestica chromosome 7
Gene count (100kb windows)
20
15
10
5
0
0
10
20
30
Genomic position (Mb)

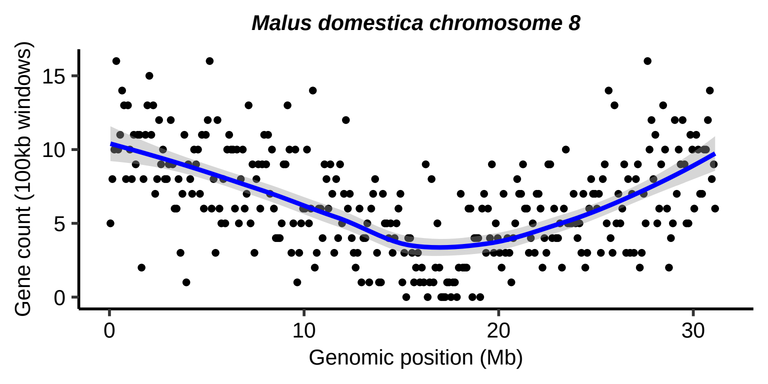

Malus domestica chromosome 8
Gene count (100kb windows)
0
5
10
15
0
10
20
30
Genomic position (Mb)

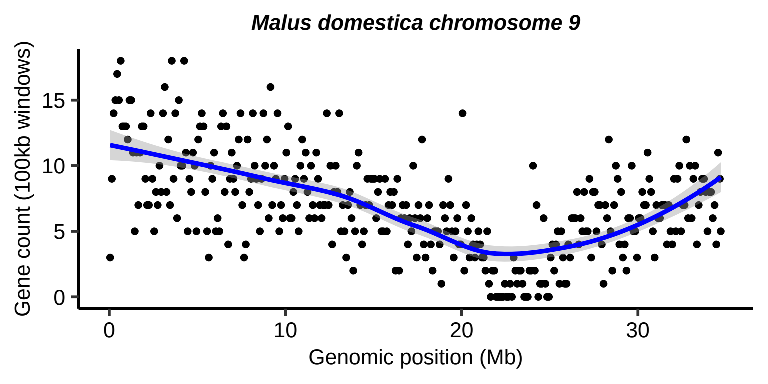

Malus domestica chromosome 9
Gene count (100kb windows)
0
5
10
15
0
10
20
30
Genomic position (Mb)

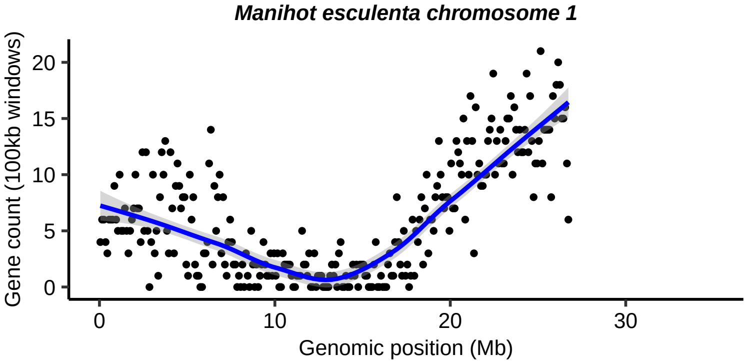
Manihot esculenta chromosome 1
Gene count (100kb windows)
0
5
10
15
20
0
10
20
30
Genomic position (Mb)

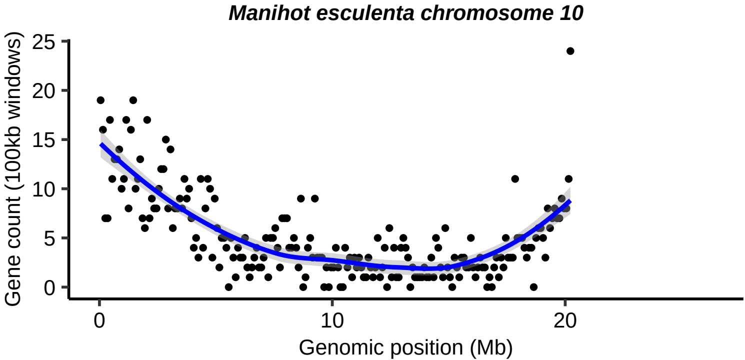

Manihot esculenta chromosome 10
Gene count (100kb windows)
25
20
15
10
5
0
0
10
20
Genomic position (Mb)

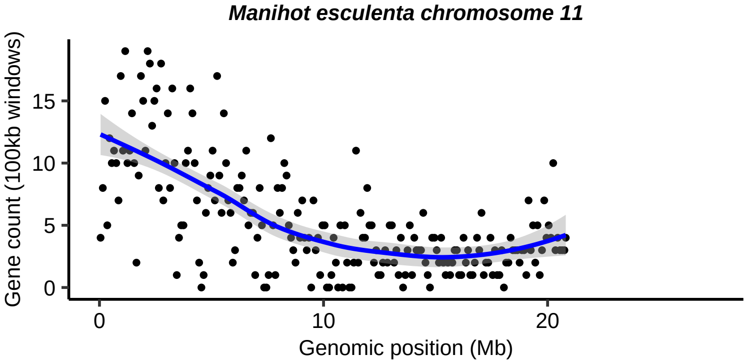

Manihot esculenta chromosome 11
Gene count (100kb windows)
0
5
10
15
0
10
20
Genomic position (Mb)

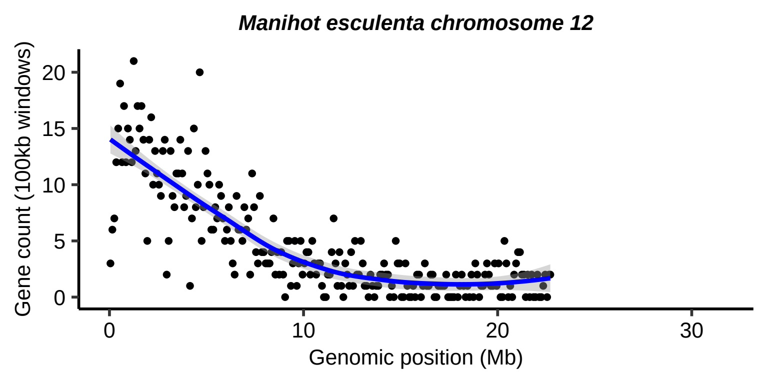

Manihot esculenta chromosome 12
Gene count (100kb windows)
0
5
10
15
20
0
10
20
30
Genomic position (Mb)

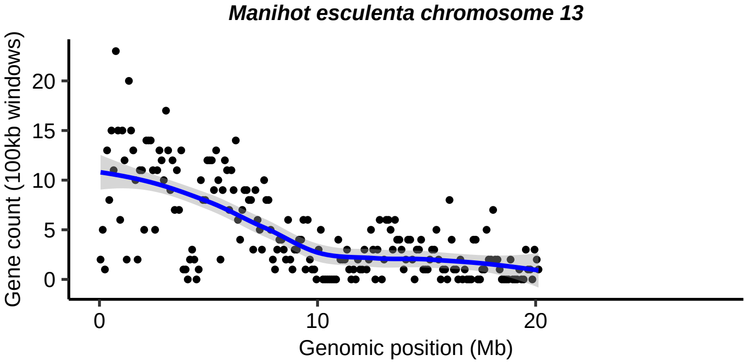

Manihot esculenta chromosome 13
Gene count (100kb windows)
0
5
10
15
20
0
10
20
Genomic position (Mb)

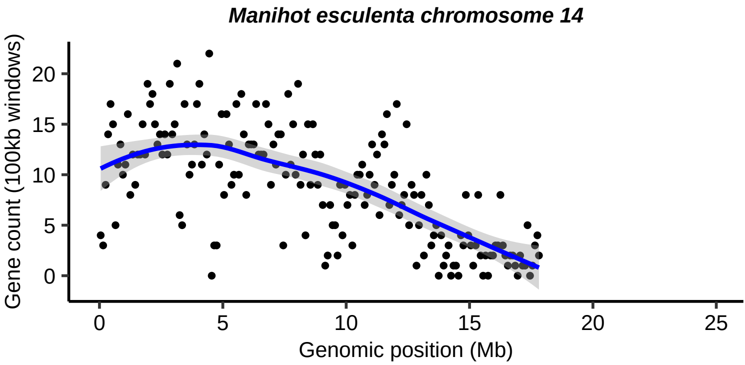

Manihot esculenta chromosome 14
Gene count (100kb windows)
0
5
10
15
20
0
5
10
15
20
25
Genomic position (Mb)

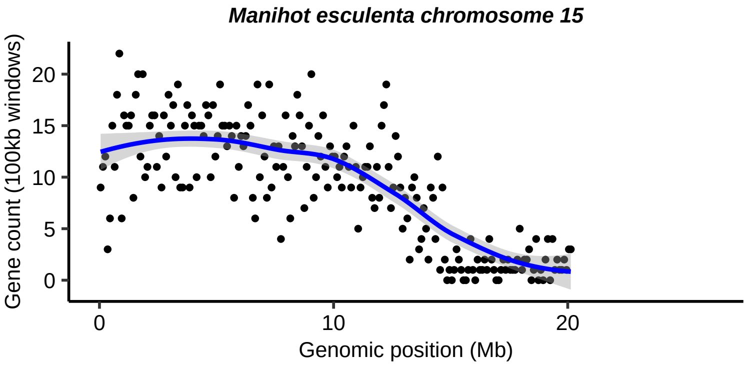
Manihot esculenta chromosome 15
Gene count (100kb windows)
0
5
10
15
20
0
10
20
Genomic position (Mb)

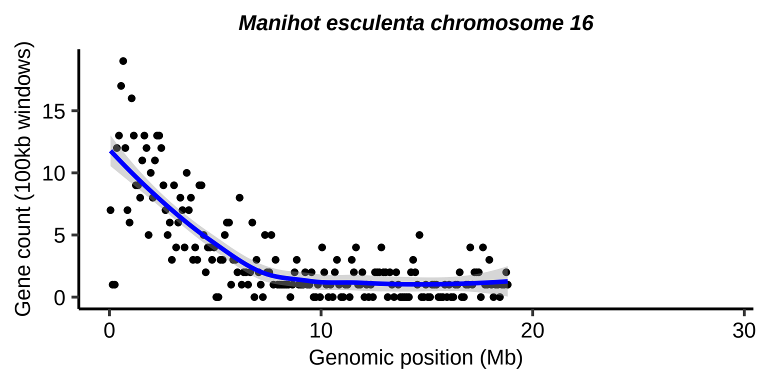

Manihot esculenta chromosome 16
Gene count (100kb windows)
0
5
10
15
0
10
20
30
Genomic position (Mb)

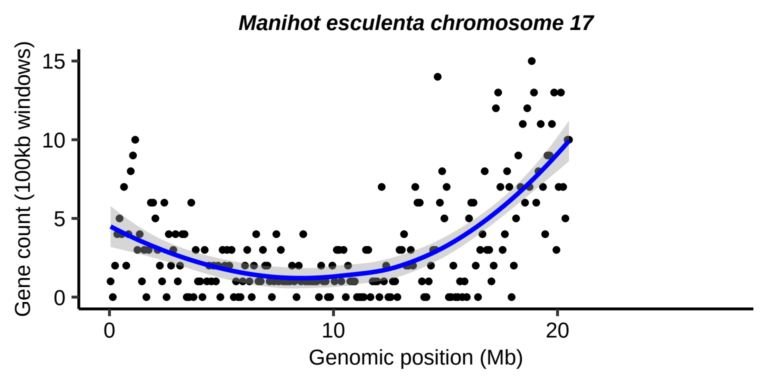
Manihot esculenta chromosome 17
Gene count (100kb windows)
15
10
5
0
0
10
20
Genomic position (Mb)

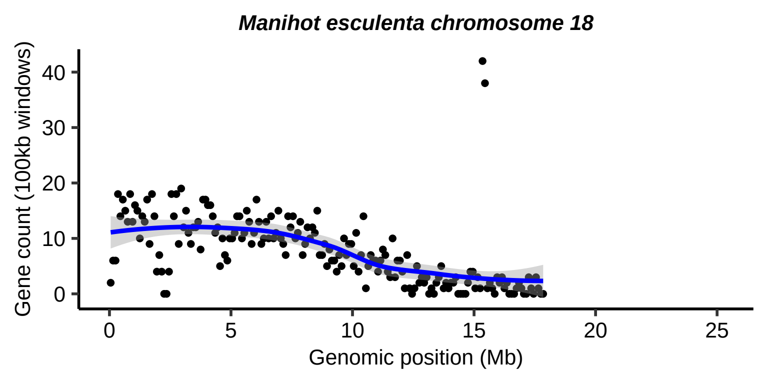
Manihot esculenta chromosome 18
Gene count (100kb windows)
40
30
20
10
0
0
5
10
15
20
25
Genomic position (Mb)

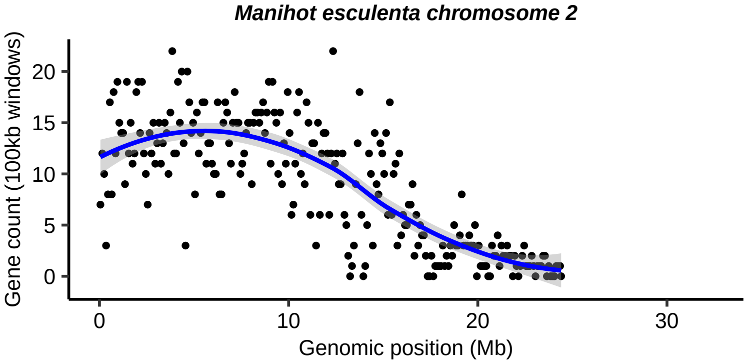
Manihot esculenta chromosome 2
Gene count (100kb windows)
0
5
10
15
20
0
10
20
30
Genomic position (Mb)

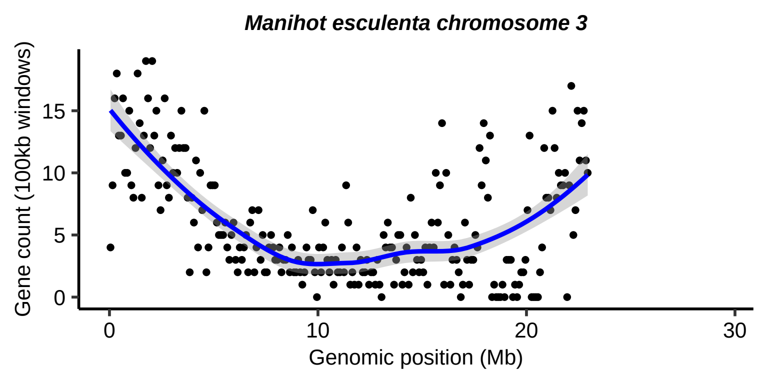

Manihot esculenta chromosome 3
Gene count (100kb windows)
0
5
10
15
0
10
20
30
Genomic position (Mb)

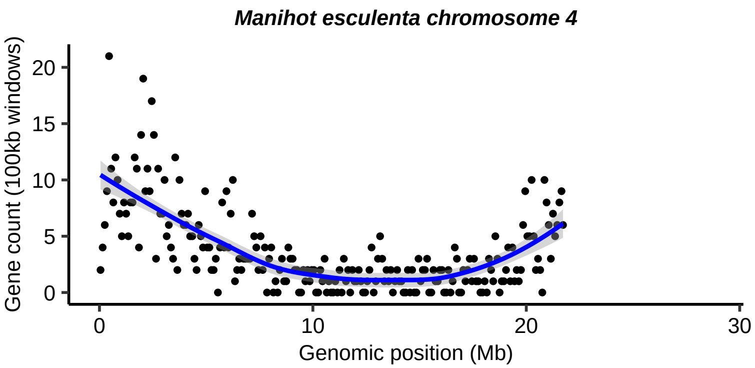

Manihot esculenta chromosome 4
Gene count (100kb windows)
0
5
10
15
20
0
10
20
30
Genomic position (Mb)

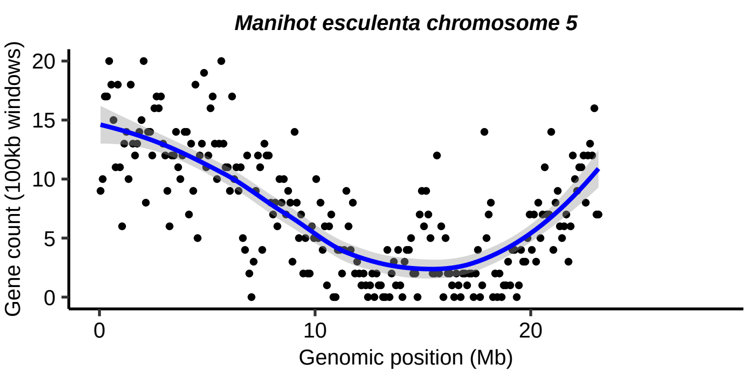
Manihot esculenta chromosome 5
Gene count (100kb windows)
0
5
10
15
20
0
10
20
Genomic position (Mb)

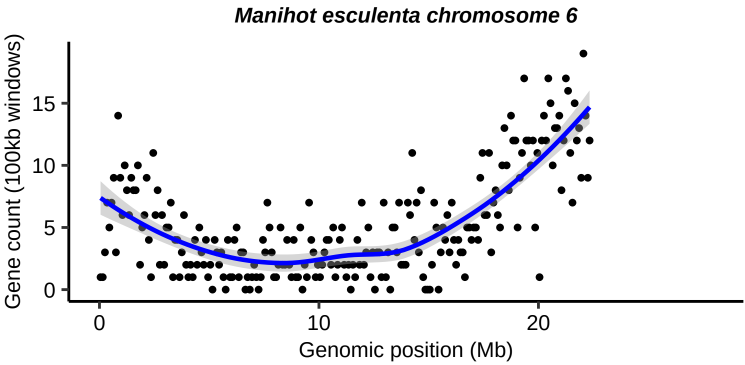

Manihot esculenta chromosome 6
Gene count (100kb windows)
0
5
10
15
0
10
20
Genomic position (Mb)

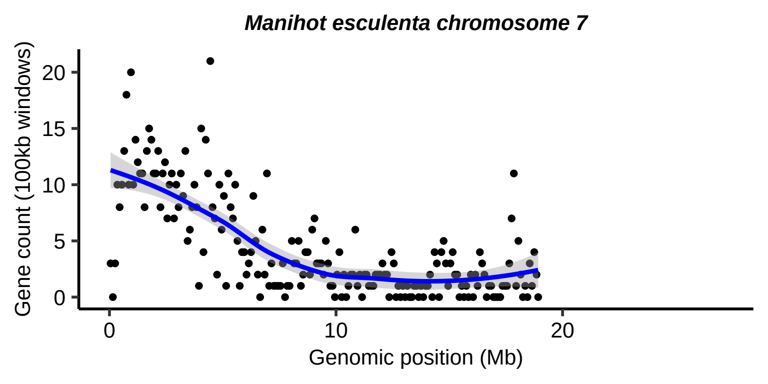

Manihot esculenta chromosome 7
Gene count (100kb windows)
20
15
10
5
0
0
10
20
Genomic position (Mb)

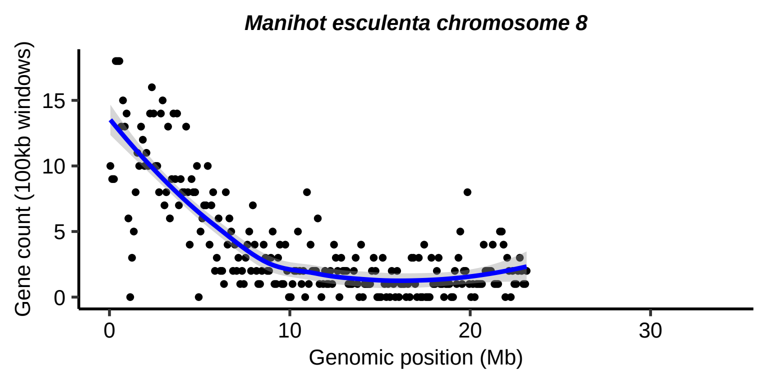

Manihot esculenta chromosome 8
Gene count (100kb windows)
0
5
10
15
0
10
20
30
Genomic position (Mb)

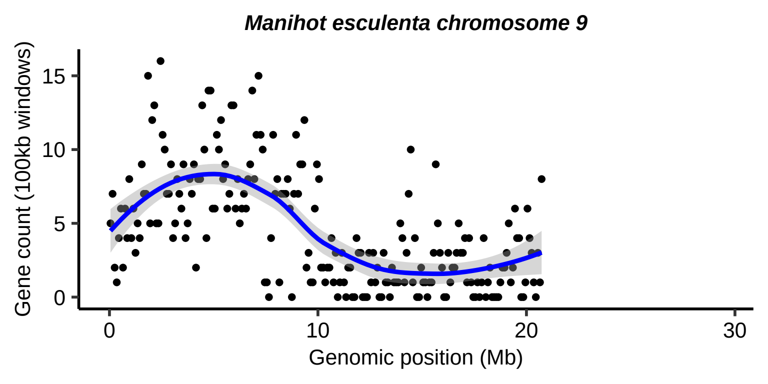

Manihot esculenta chromosome 9
Gene count (100kb windows)
15
10
5
0
0
10
20
30
Genomic position (Mb)

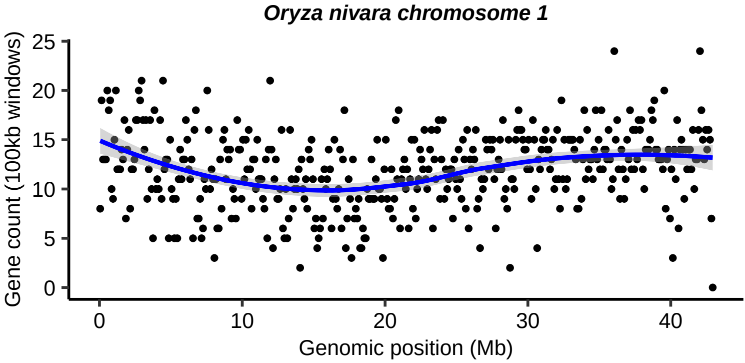

Oryza nivara chromosome 1
Gene count (100kb windows)
Genomic position (Mb)
0
5
10
15
20
25
0
10
20
30
40

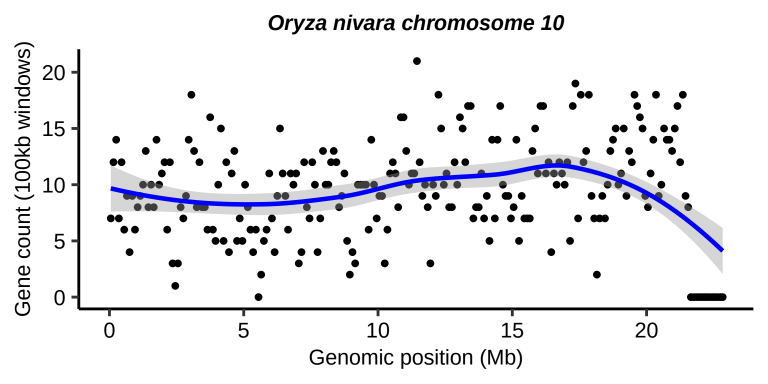

Oryza nivara chromosome 10
Gene count (100kb windows)
0
5
10
15
20
Genomic position (Mb)

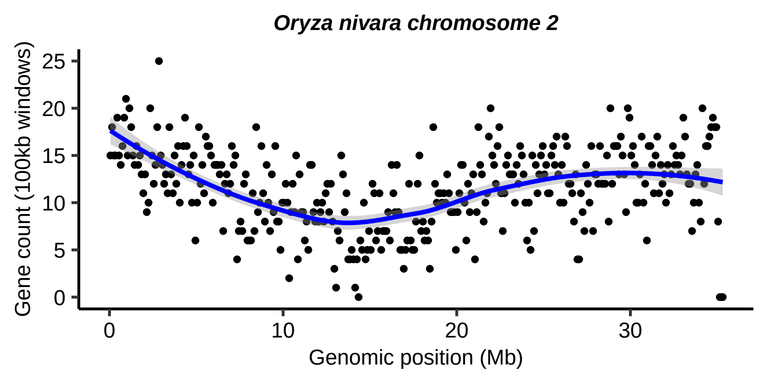
Oryza nivara chromosome 2
Gene count (100kb windows)
0
5
10
15
20
25
0
10
20
30
Genomic position (Mb)

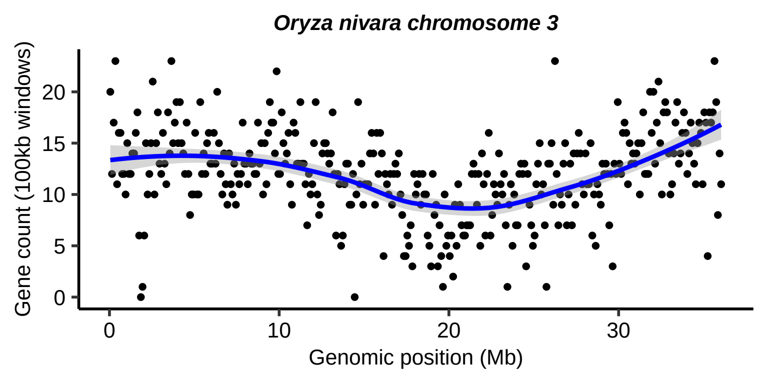

Oryza nivara chromosome 3
Gene count (100kb windows)
0
5
10
15
20
0
10
20
30
Genomic position (Mb)

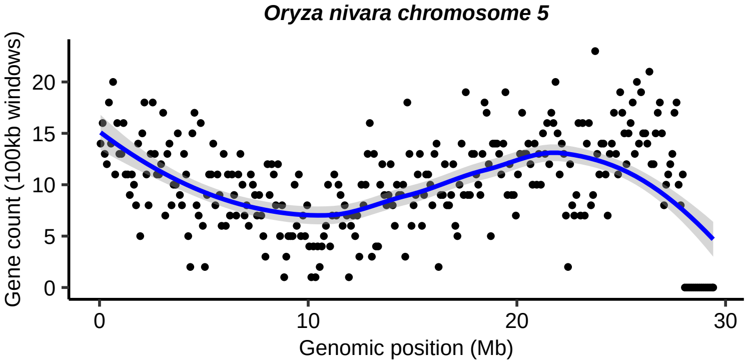

Oryza nivara chromosome 5
Gene count (100kb windows)
0
5
10
15
20
0
10
20
30
Genomic position (Mb)

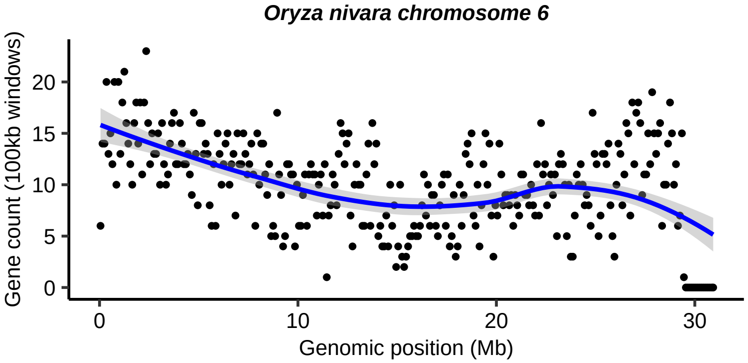

Oryza nivara chromosome 6
Gene count (100kb windows)
0
5
10
15
20
0
10
20
30
Genomic position (Mb)

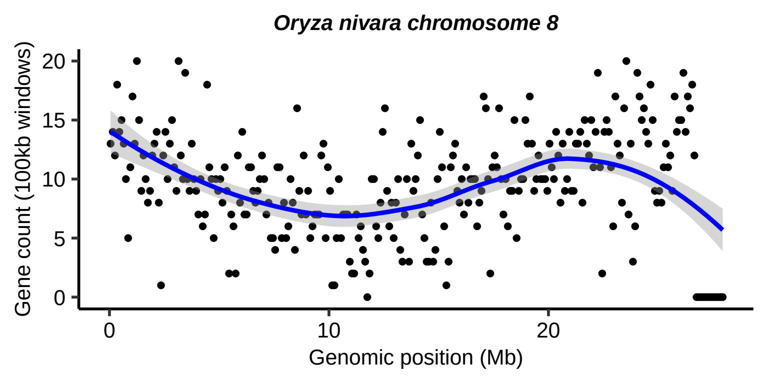

Oryza nivara chromosome 8
Gene count (100kb windows)
20
15
10
5
0
0
10
20
Genomic position (Mb)

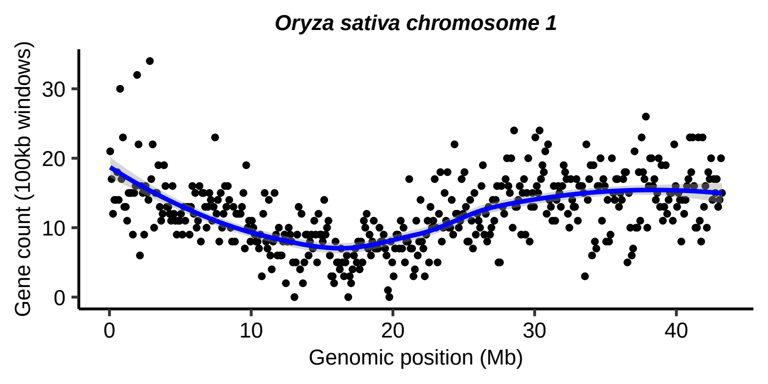

Oryza sativa chromosome 1
Gene count (100kb windows)
0
10
20
30
0
10
20
30
40
Genomic position (Mb)

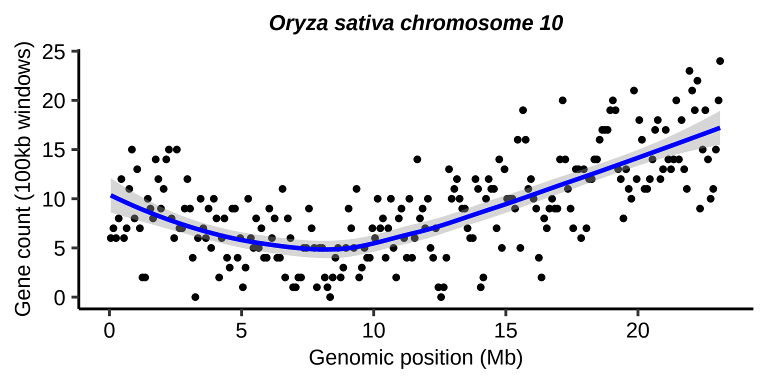

Oryza sativa chromosome 10
Gene count (100kb windows)
0
5
10
15
20
25
0
5
10
15
20
Genomic position (Mb)

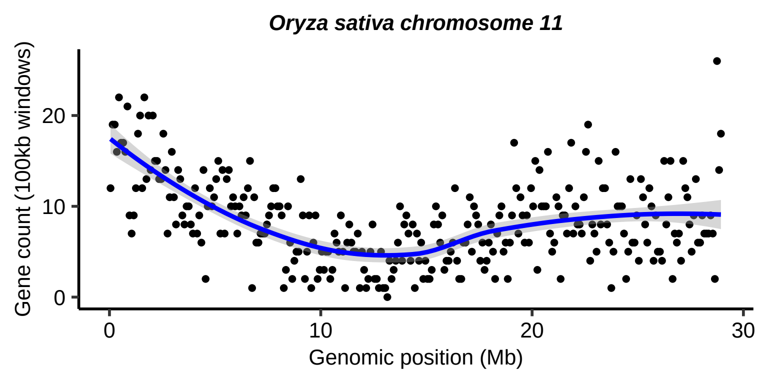

Oryza sativa chromosome 11
Gene count (100kb windows)
20
10
0
0
10
20
30
Genomic position (Mb)

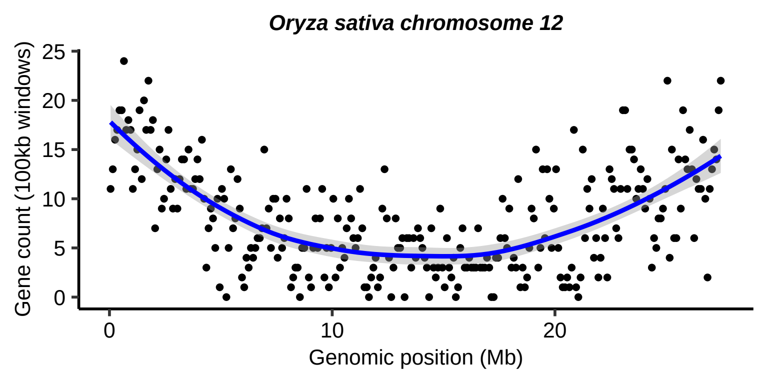

Oryza sativa chromosome 12
Gene count (100kb windows)
0
5
10
15
20
25
0
10
20
Genomic position (Mb)

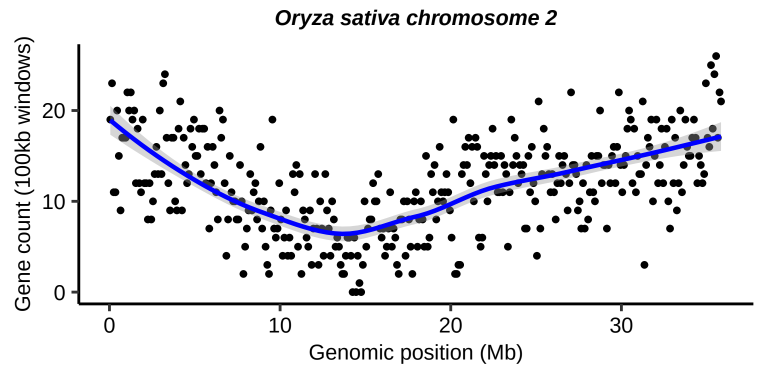

Oryza sativa chromosome 2
Gene count (100kb windows)
0
10
20
0
10
20
30
Genomic position (Mb)

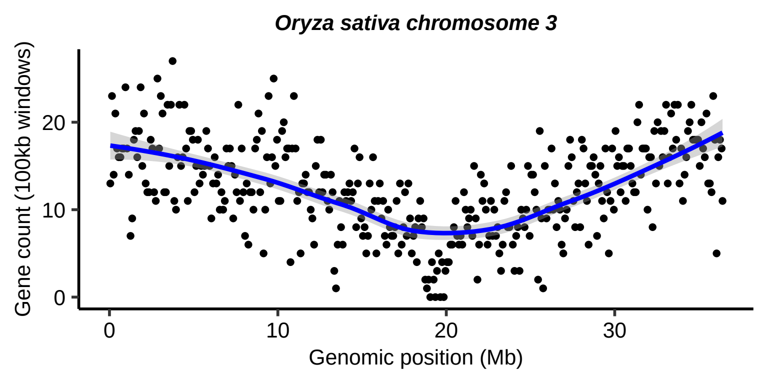
Oryza sativa chromosome 3
Gene count (100kb windows)
0
10
20
0
10
20
30
Genomic position (Mb)

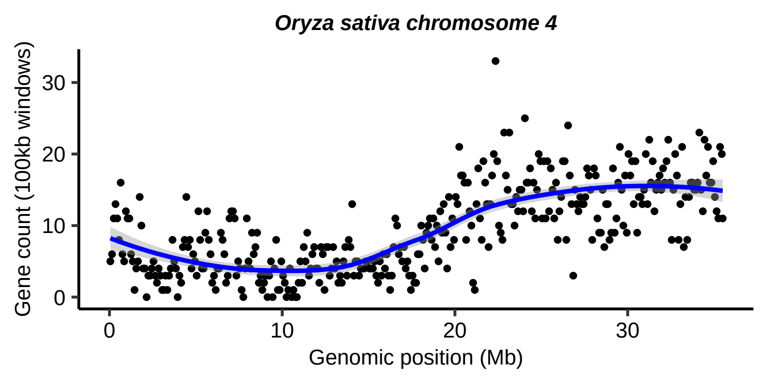

Oryza sativa chromosome 4
Gene count (100kb windows)
0
10
20
30
Genomic position (Mb)

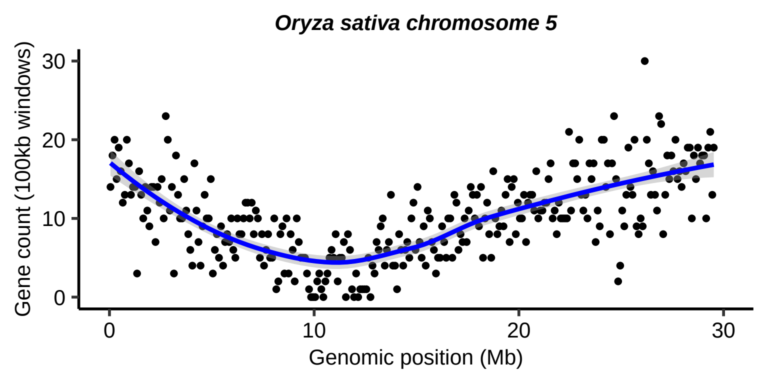

Oryza sativa chromosome 5
Gene count (100kb windows)
0
10
20
30
Genomic position (Mb)

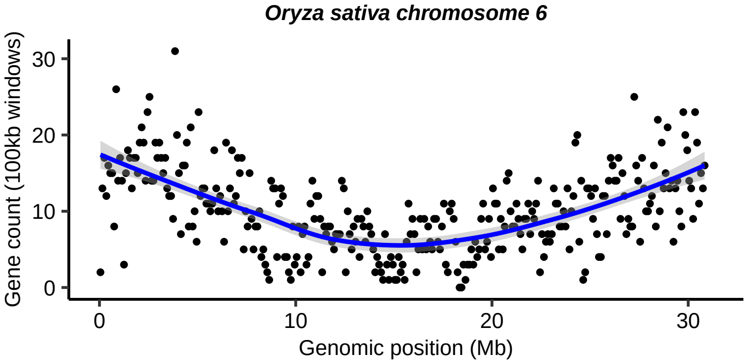
Oryza sativa chromosome 6
Gene count (100kb windows)
0
10
20
30
Genomic position (Mb)

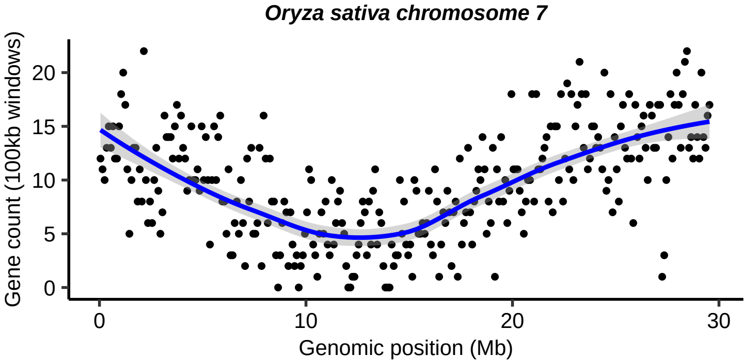

Oryza sativa chromosome 7
Gene count (100kb windows)
0
5
10
15
20
0
10
20
30
Genomic position (Mb)

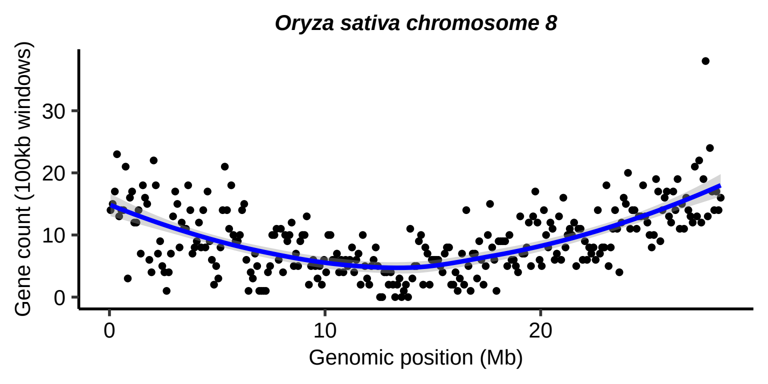

Oryza sativa chromosome 8
Gene count (100kb windows)
0
10
20
30
0
10
20
Genomic position (Mb)

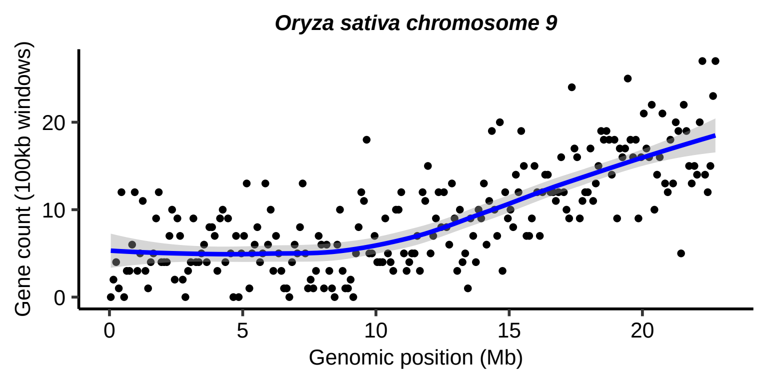

Oryza sativa chromosome 9
Gene count (100kb windows)
0
10
20
0
5
10
15
20
Genomic position (Mb)

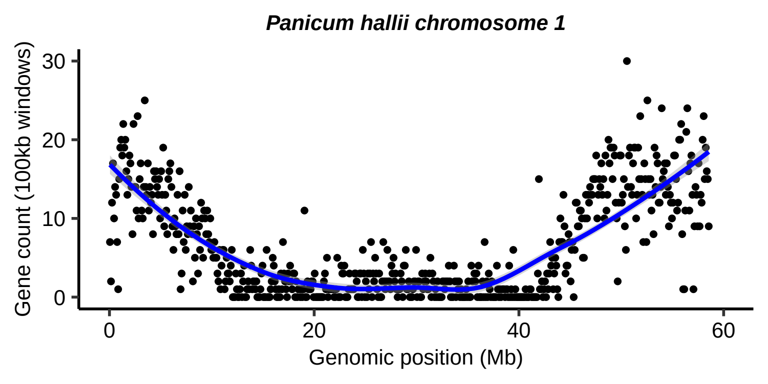

Panicum hallii chromosome 1
Gene count (100kb windows)
Genomic position (Mb)
0
10
20
30
0
20
40
60

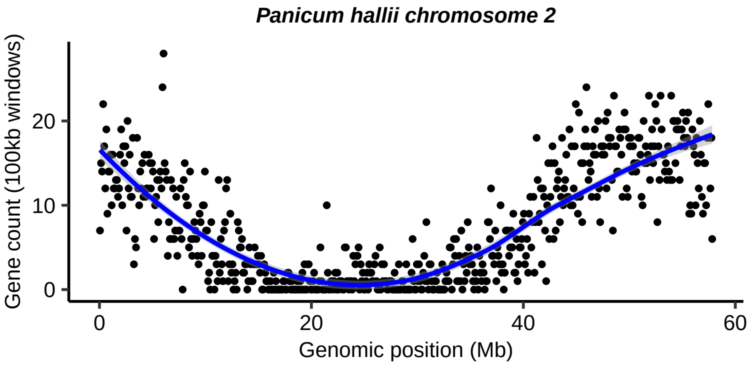

Panicum hallii chromosome 2
Gene count (100kb windows)
0
10
20
0
20
40
60
Genomic position (Mb)

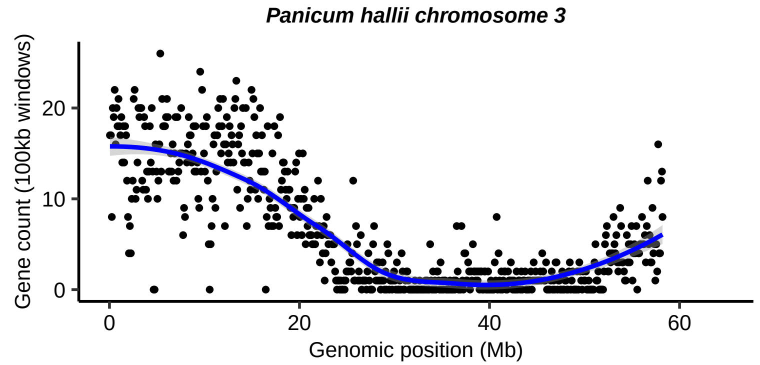

Panicum hallii chromosome 3
Gene count (100kb windows)
0
10
20
0
20
40
60
Genomic position (Mb)

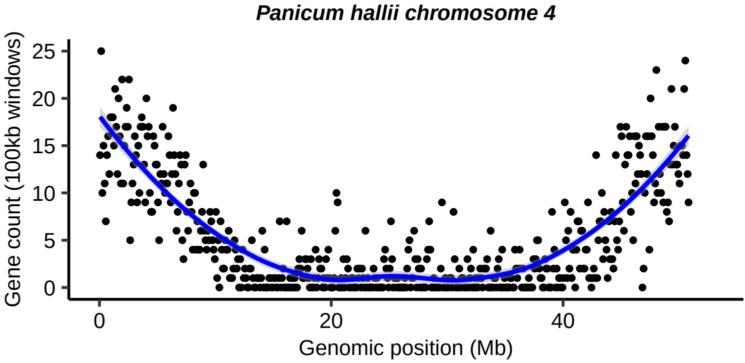
Panicum hallii chromosome 4
Gene count (100kb windows)
25
20
15
10
5
0
0
20
40
Genomic position (Mb)

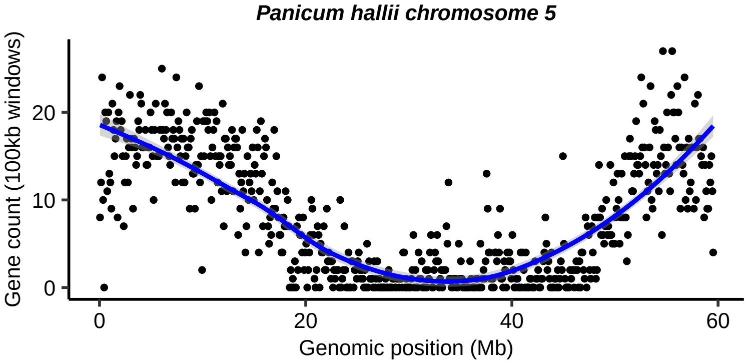

Panicum hallii chromosome 5
Gene count (100kb windows)
0
10
20
0
20
40
60
Genomic position (Mb)

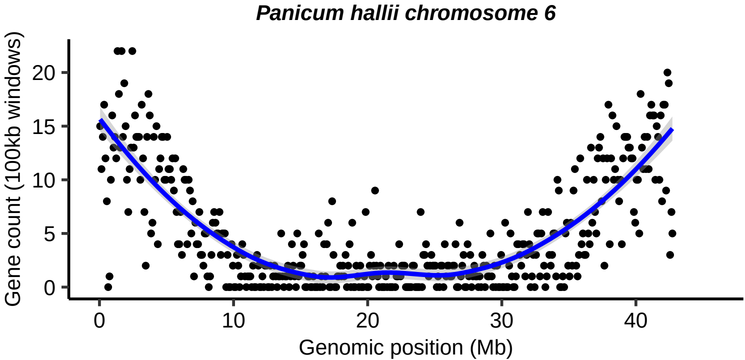
Panicum hallii chromosome 6
Gene count (100kb windows)
0
5
10
15
20
0
10
20
30
40
Genomic position (Mb)

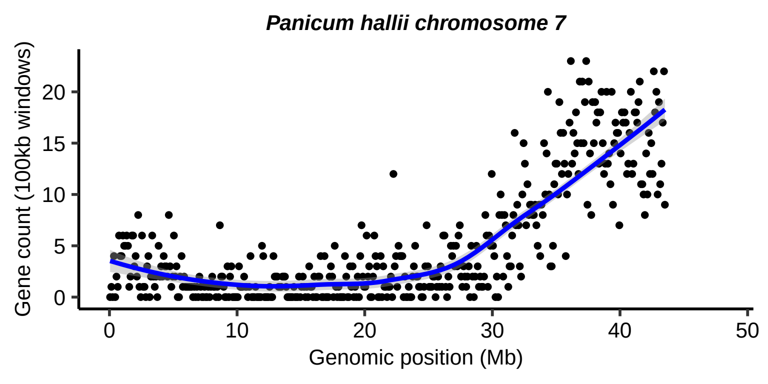

Panicum hallii chromosome 7
Gene count (100kb windows)
0
5
10
15
20
0
10
20
30
40
50
Genomic position (Mb)

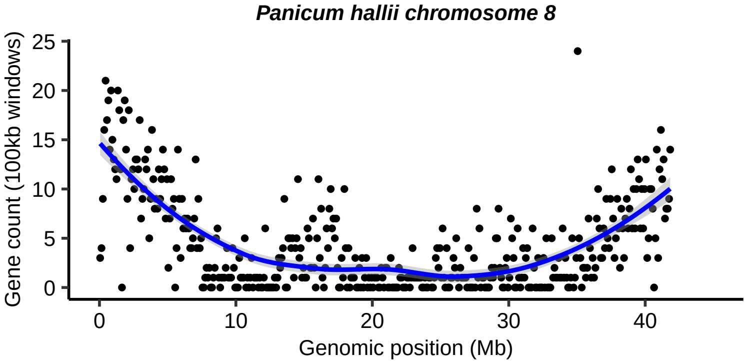
Panicum hallii chromosome 8
Gene count (100kb windows)
Genomic position (Mb)
0
5
10
15
20
25
0
10
20
30
40

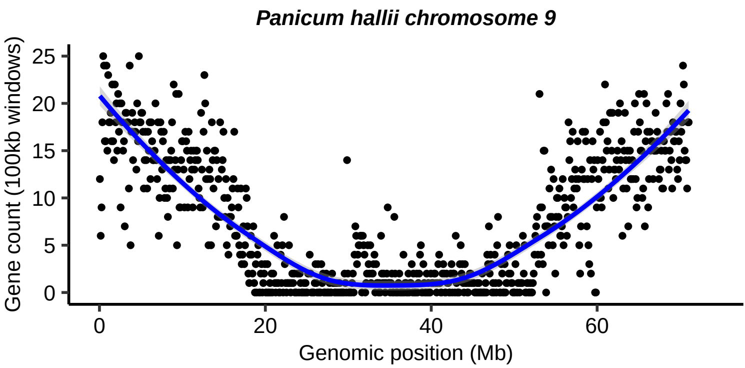

Panicum hallii chromosome 9
Gene count (100kb windows)
25
20
15
10
5
0
0
20
40
60
Genomic position (Mb)

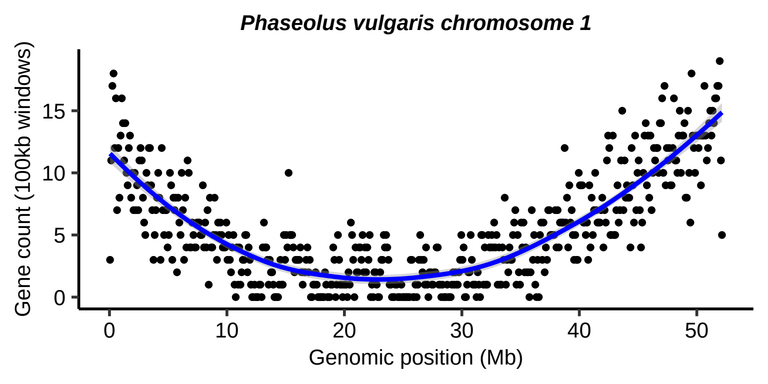
Phaseolus vulgaris chromosome 1
Gene count (100kb windows)
0
5
10
15
0
10
20
30
40
50
Genomic position (Mb)

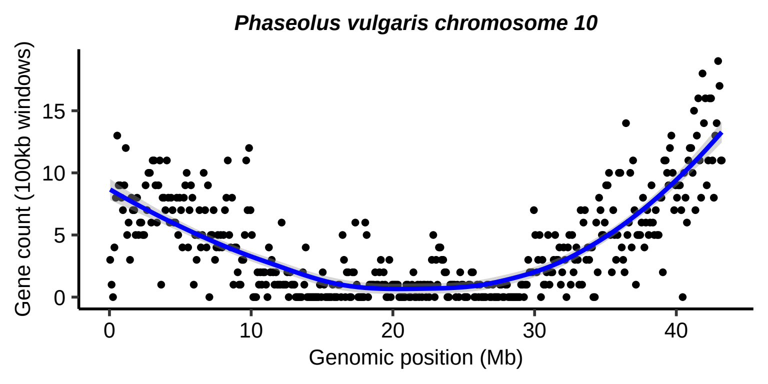

Phaseolus vulgaris chromosome 10
Gene count (100kb windows)
0
5
10
15
0
10
20
30
40
Genomic position (Mb)

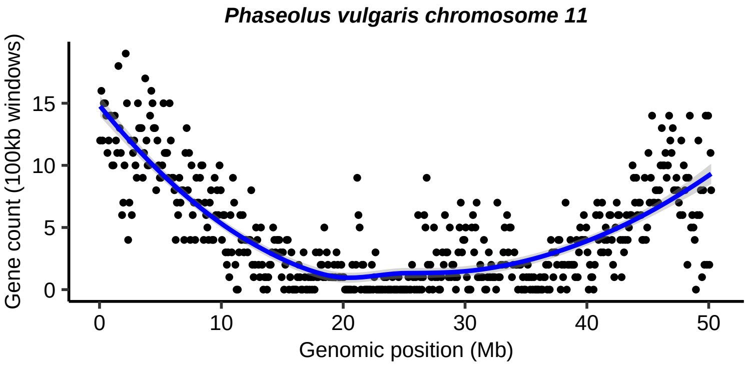

Phaseolus vulgaris chromosome 11
Gene count (100kb windows)
0
5
10
15
0
10
20
30
40
50
Genomic position (Mb)

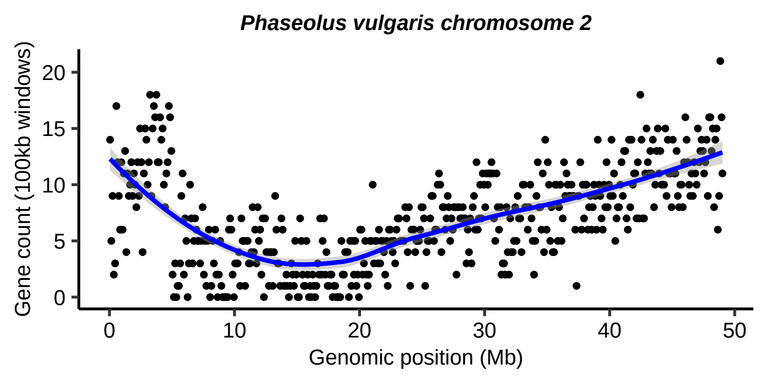
Phaseolus vulgaris chromosome 2
Gene count (100kb windows)
0
5
10
15
20
0
10
20
30
40
50
Genomic position (Mb)

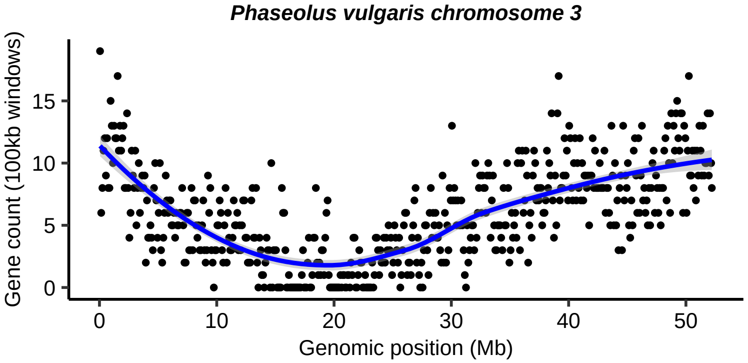

Phaseolus vulgaris chromosome 3
Gene count (100kb windows)
0
5
10
15
0
10
20
30
40
50
Genomic position (Mb)

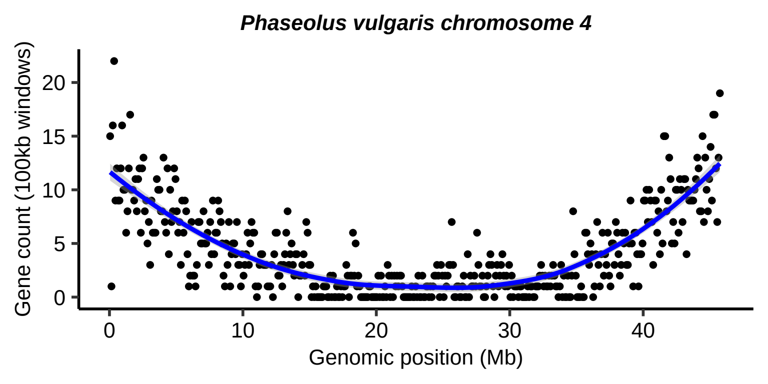
Phaseolus vulgaris chromosome 4
Gene count (100kb windows)
0
5
10
15
20
0
10
20
30
40
Genomic position (Mb)

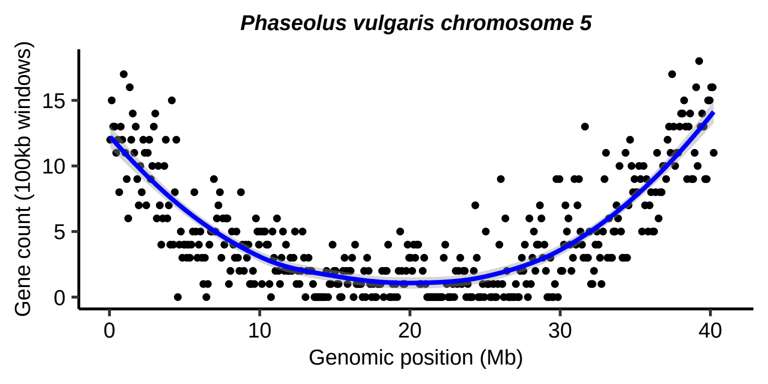

Phaseolus vulgaris chromosome 5
Gene count (100kb windows)
0
5
10
15
0
10
20
30
40
Genomic position (Mb)

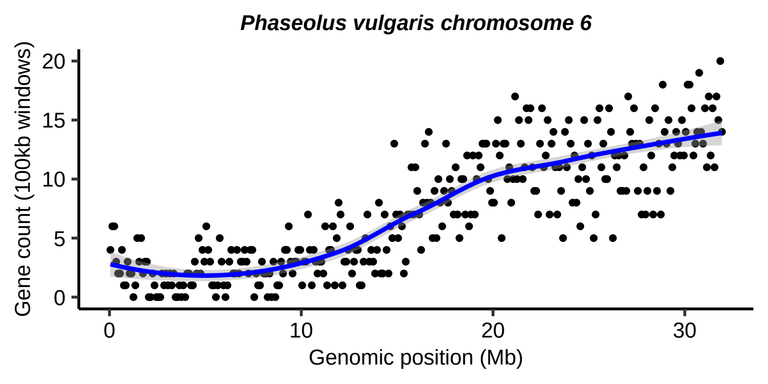

Phaseolus vulgaris chromosome 6
Gene count (100kb windows)
0
5
10
15
20
0
10
20
30
Genomic position (Mb)

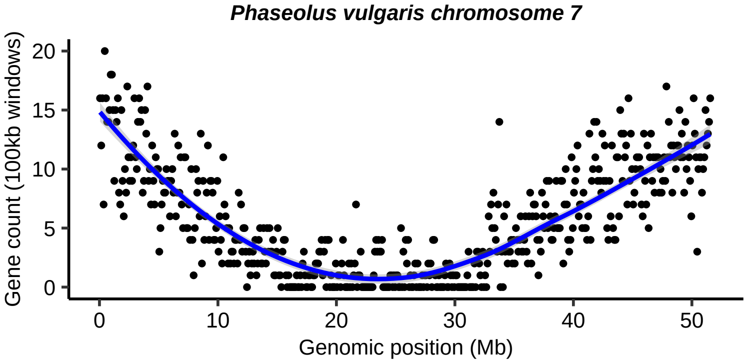

Phaseolus vulgaris chromosome 7
Gene count (100kb windows)
0
5
10
15
20
0
10
20
30
40
50
Genomic position (Mb)

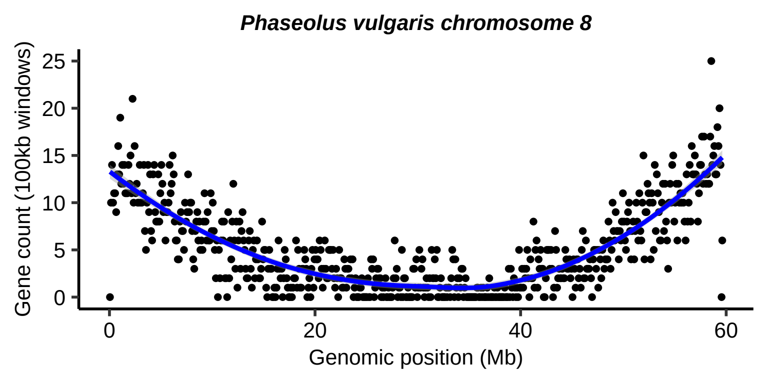

Phaseolus vulgaris chromosome 8
Gene count (100kb windows)
0
5
10
15
20
25
0
20
40
60
Genomic position (Mb)

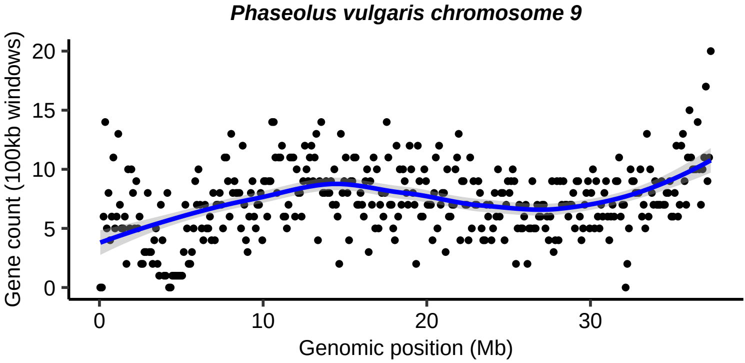

Phaseolus vulgaris chromosome 9
Gene count (100kb windows)
20
15
10
5
0
0
10
20
30
Genomic position (Mb)

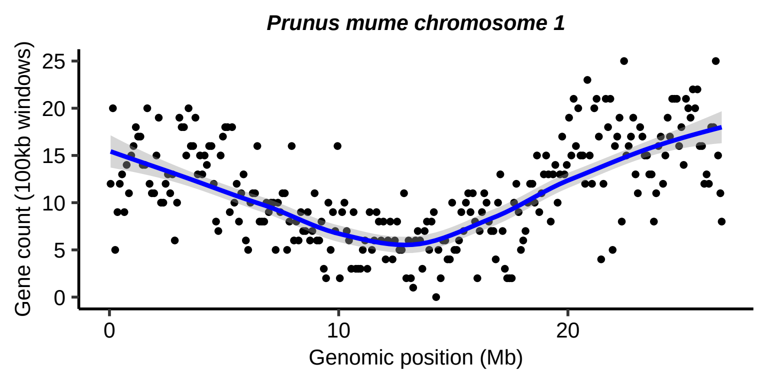

Prunus mume chromosome 1
Gene count (100kb windows)
25
20
15
10
5
0
0
10
20
Genomic position (Mb)

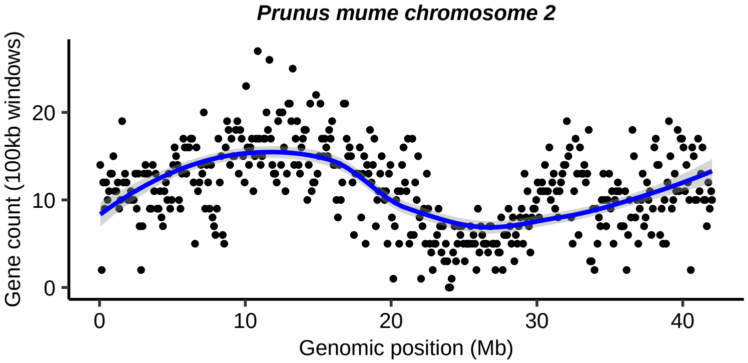

Prunus mume chromosome 2
Gene count (100kb windows)
0
10
20
0
10
20
30
40
Genomic position (Mb)

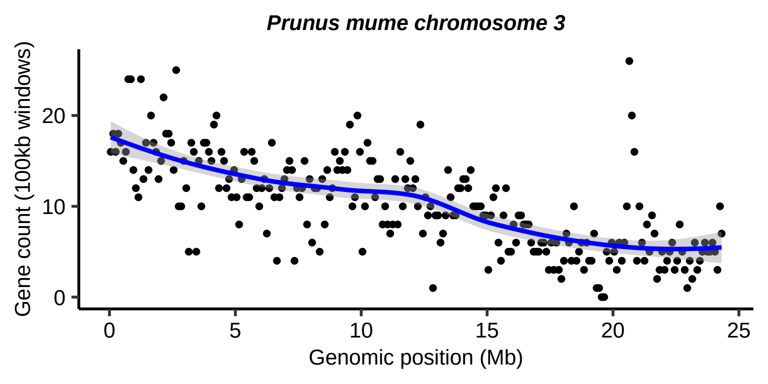

Prunus mume chromosome 3
Gene count (100kb windows)
0
10
20
0
5
10
15
20
25
Genomic position (Mb)

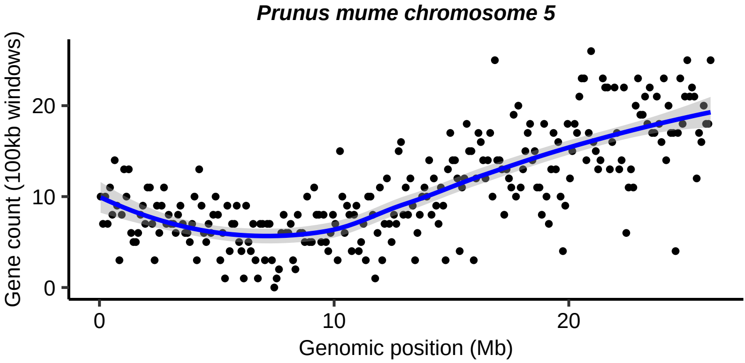

Prunus mume chromosome 5
Gene count (100kb windows)
0
10
20
0
10
20
Genomic position (Mb)

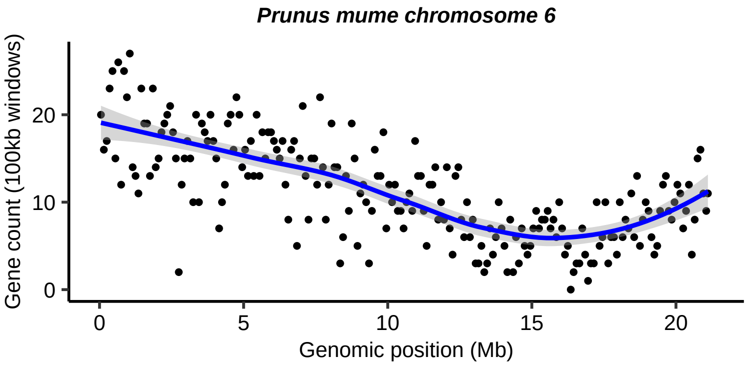

Prunus mume chromosome 6
Gene count (100kb windows)
0
10
20
0
5
10
15
20
Genomic position (Mb)

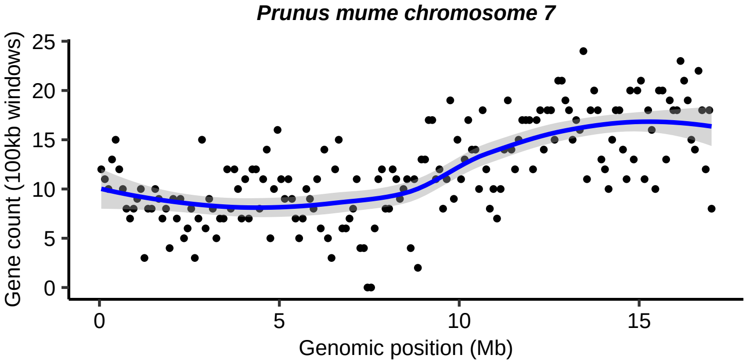
Prunus mume chromosome 7
Gene count (100kb windows)
25
20
15
10
5
0
0
5
10
15
Genomic position (Mb)

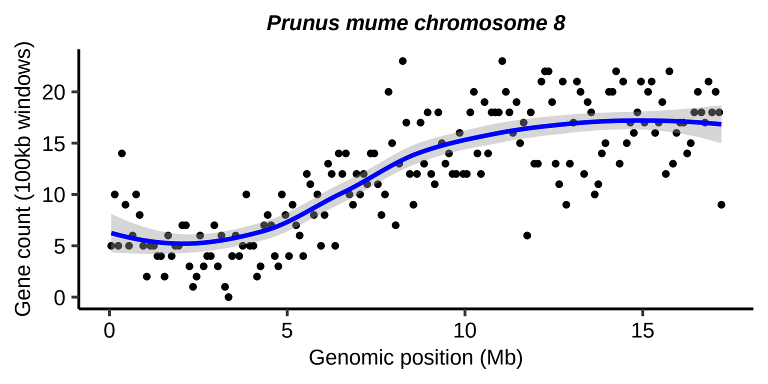

Prunus mume chromosome 8
Gene count (100kb windows)
0
5
10
15
20
0
5
10
15
Genomic position (Mb)

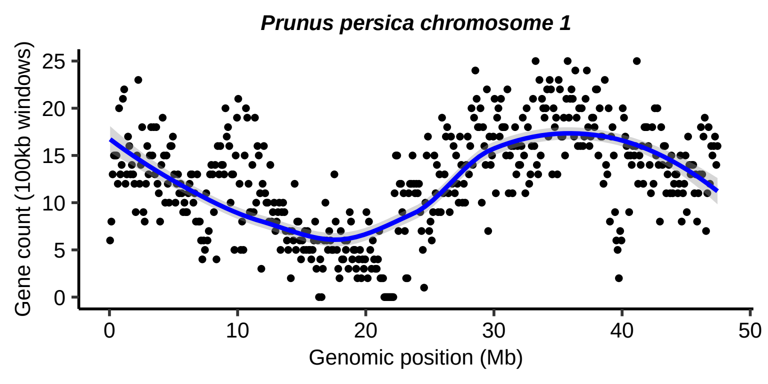
Prunus persica chromosome 1
Gene count (100kb windows)
25
20
15
10
5
0
0
10
20
30
40
50
Genomic position (Mb)

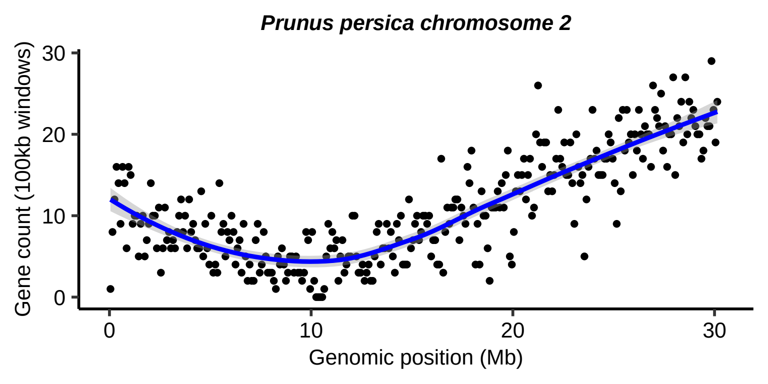
Prunus persica chromosome 2
Gene count (100kb windows)
0
10
20
30
Genomic position (Mb)

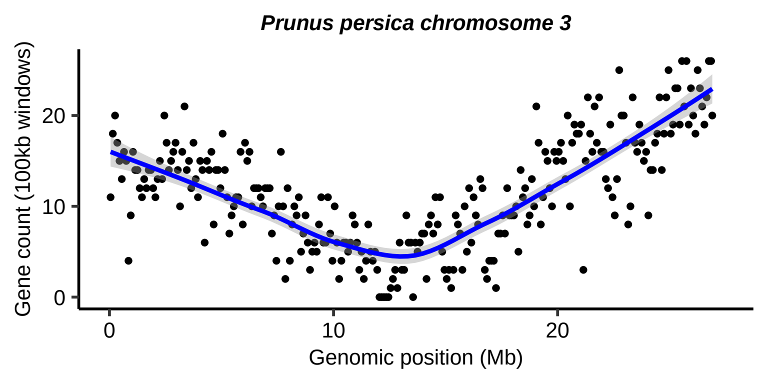
Prunus persica chromosome 3
Gene count (100kb windows)
0
10
20
0
10
20
Genomic position (Mb)

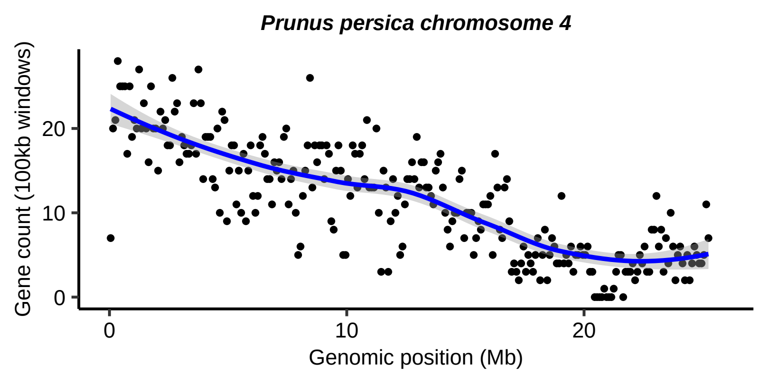
Prunus persica chromosome 4
Gene count (100kb windows)
20
10
0
0
10
20
Genomic position (Mb)

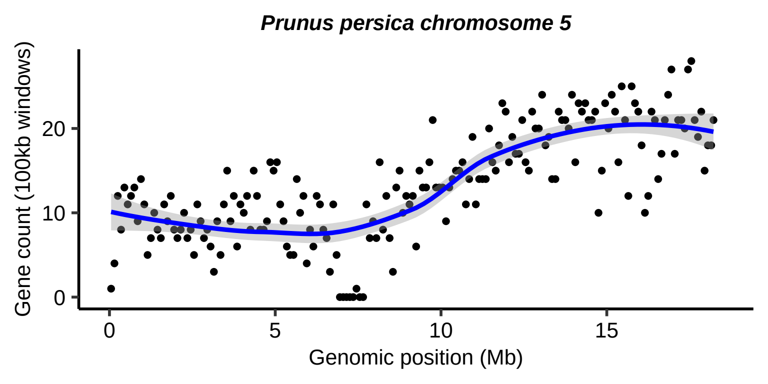

Prunus persica chromosome 5
Gene count (100kb windows)
0
10
20
0
5
10
15
Genomic position (Mb)

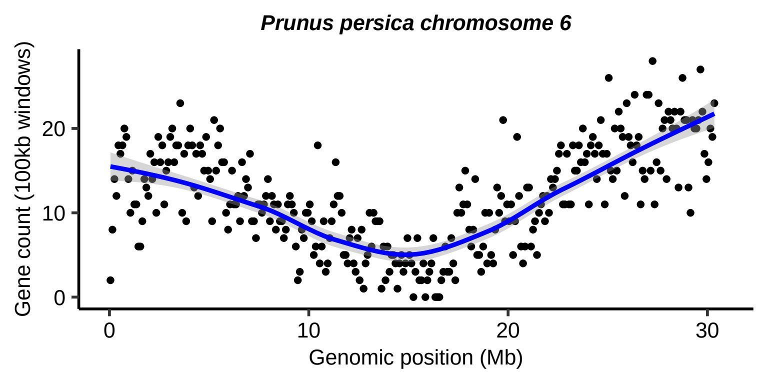

Prunus persica chromosome 6
Gene count (100kb windows)
0
10
20
0
10
20
30
Genomic position (Mb)

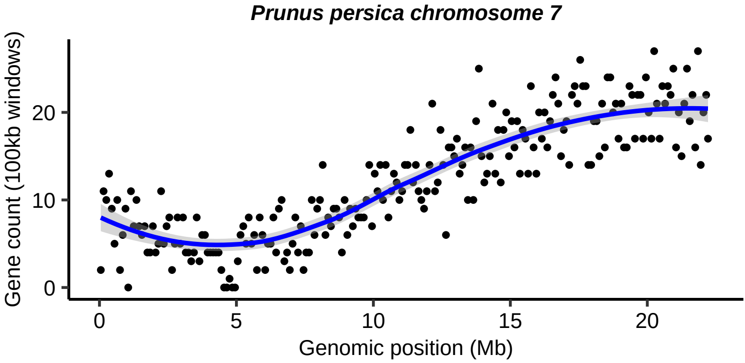

Prunus persica chromosome 7
Gene count (100kb windows)
20
10
0
0
5
10
15
20
Genomic position (Mb)

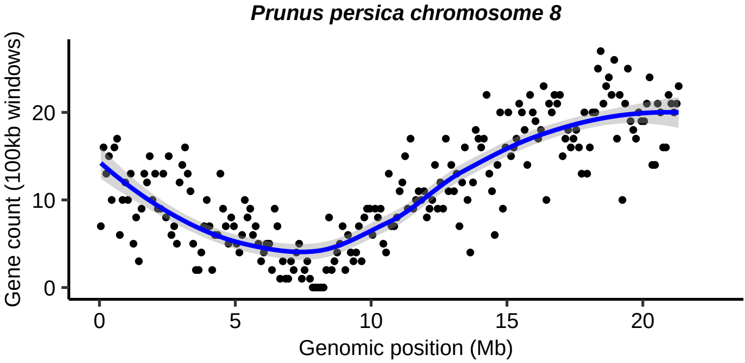
Prunus persica chromosome 8
Gene count (100kb windows)
0
10
20
0
5
10
15
20
Genomic position (Mb)

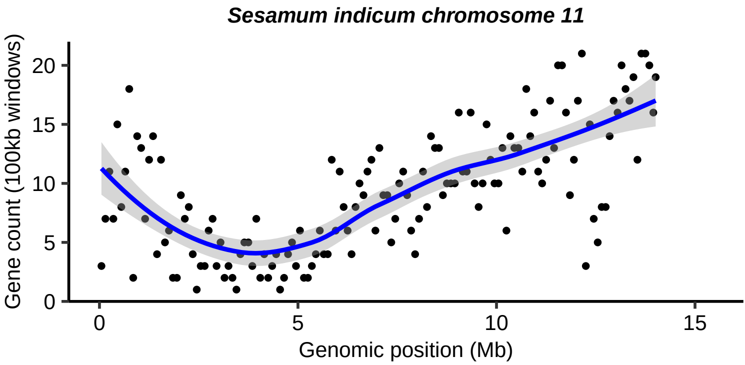
Sesamum indicum chromosome 11
Gene count (100kb windows)
0
5
10
15
20
0
5
10
15
Genomic position (Mb)

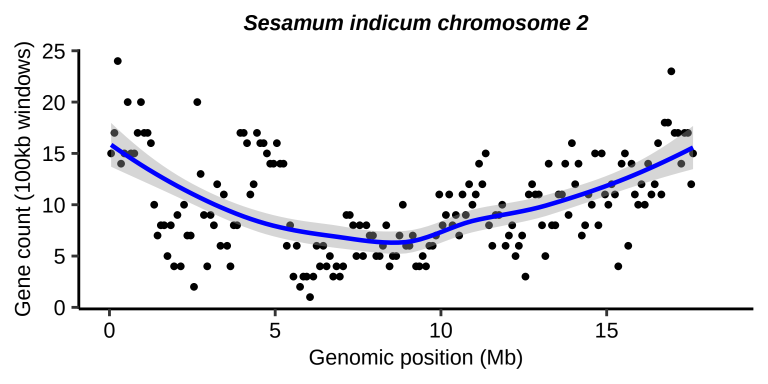

Sesamum indicum chromosome 2
Gene count (100kb windows)
25
20
15
10
5
0
0
5
10
15
Genomic position (Mb)

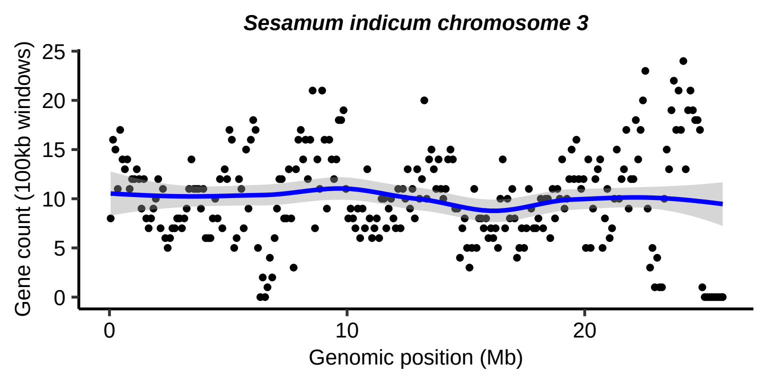
Sesamum indicum chromosome 3
Gene count (100kb windows)
25
20
15
10
5
0
0
10
20
Genomic position (Mb)

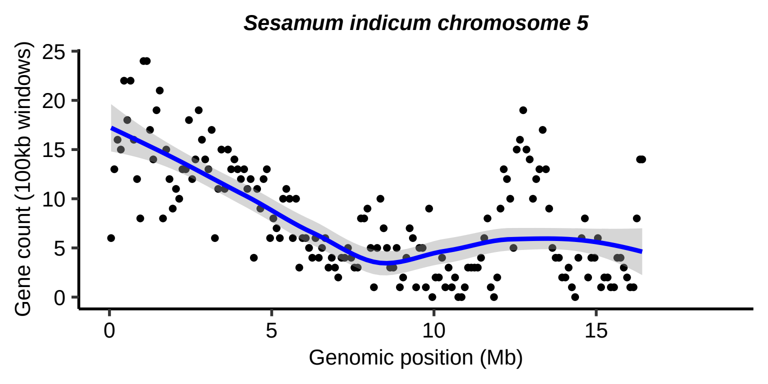

Sesamum indicum chromosome 5
Gene count (100kb windows)
0
5
10
15
20
25
0
5
10
15
Genomic position (Mb)

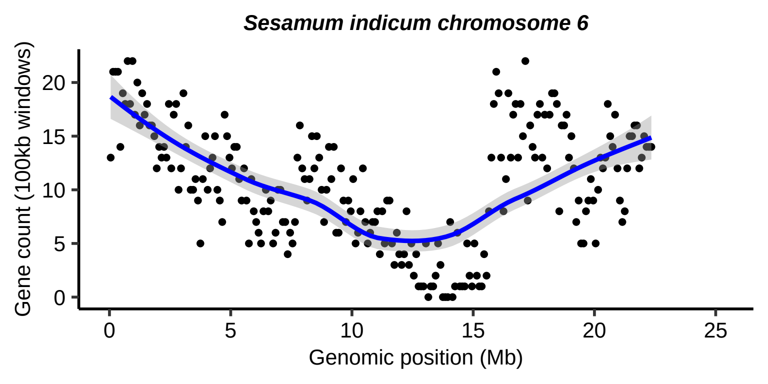

Sesamum indicum chromosome 6
Gene count (100kb windows)
0
5
10
15
20
25
Genomic position (Mb)

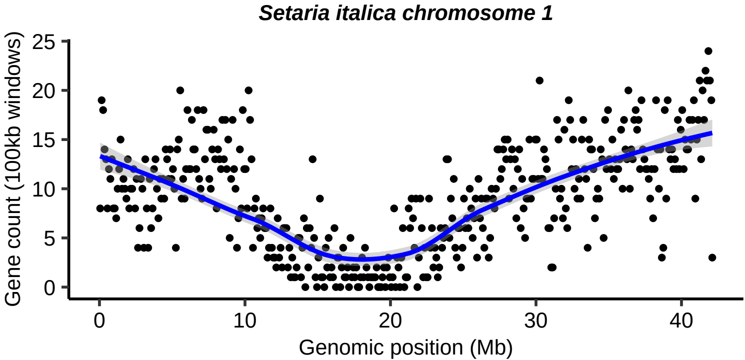
Setaria italica chromosome 1
Gene count (100kb windows)
0
5
10
15
20
25
0
10
20
30
40
Genomic position (Mb)

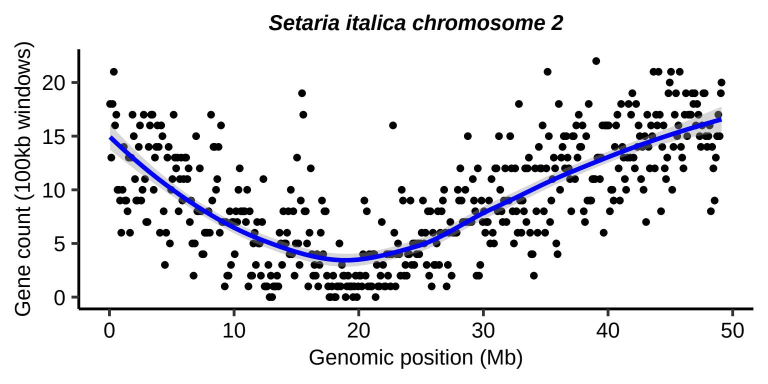
Setaria italica chromosome 2
Gene count (100kb windows)
0
5
10
15
20
0
10
20
30
40
50
Genomic position (Mb)

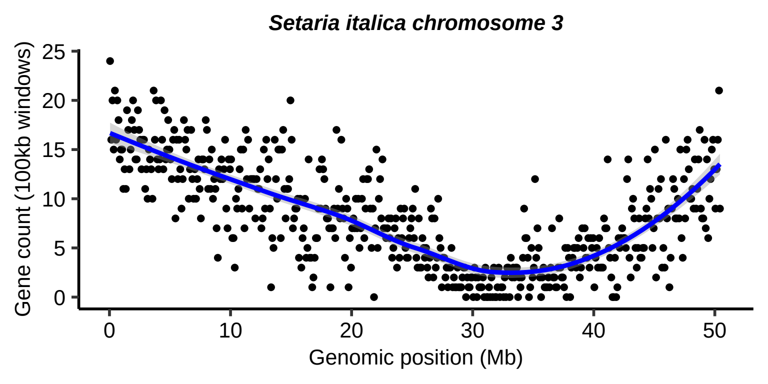

Setaria italica chromosome 3
Gene count (100kb windows)
25
20
15
10
5
0
0
10
20
30
40
50
Genomic position (Mb)

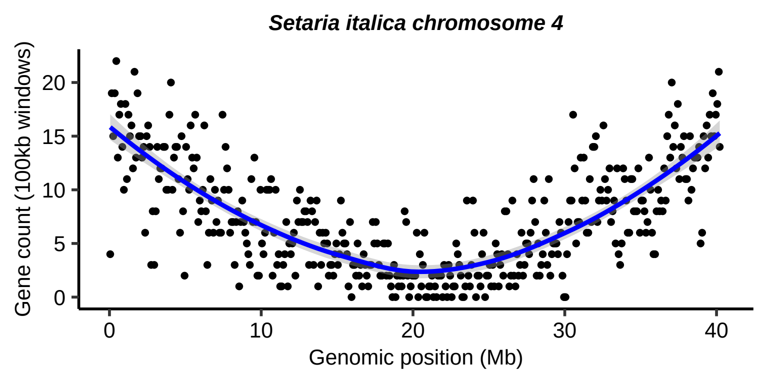

Setaria italica chromosome 4
Gene count (100kb windows)
0
5
10
15
20
0
10
20
30
40
Genomic position (Mb)

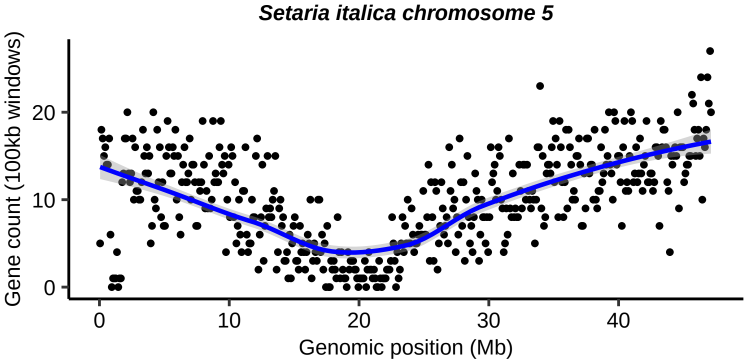

Setaria italica chromosome 5
Gene count (100kb windows)
0
10
20
0
10
20
30
40
Genomic position (Mb)

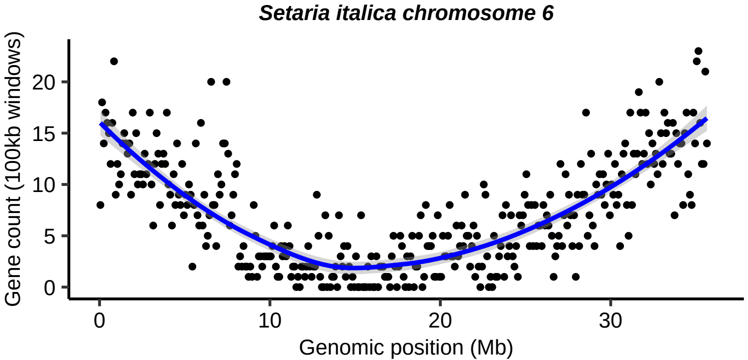

Setaria italica chromosome 6
Gene count (100kb windows)
0
5
10
15
20
0
10
20
30
Genomic position (Mb)

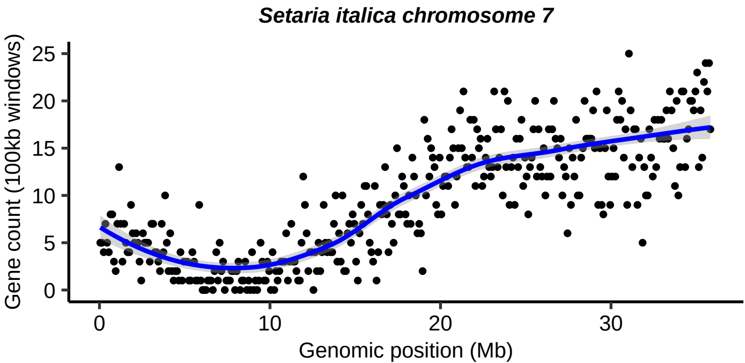
Setaria italica chromosome 7
Gene count (100kb windows)
0
5
10
15
20
25
0
10
20
30
Genomic position (Mb)

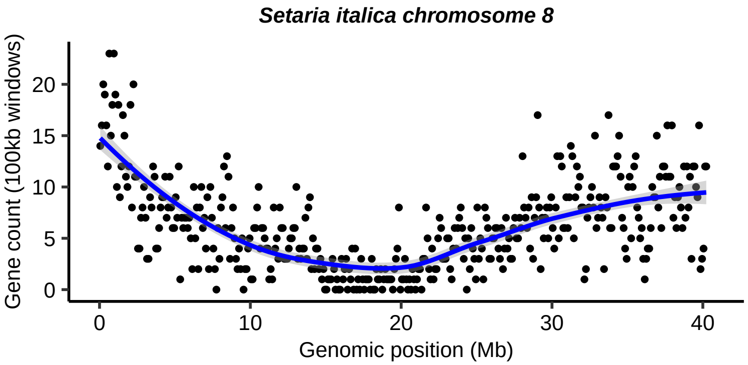

Setaria italica chromosome 8
Gene count (100kb windows)
0
5
10
15
20
0
10
20
30
40
Genomic position (Mb)

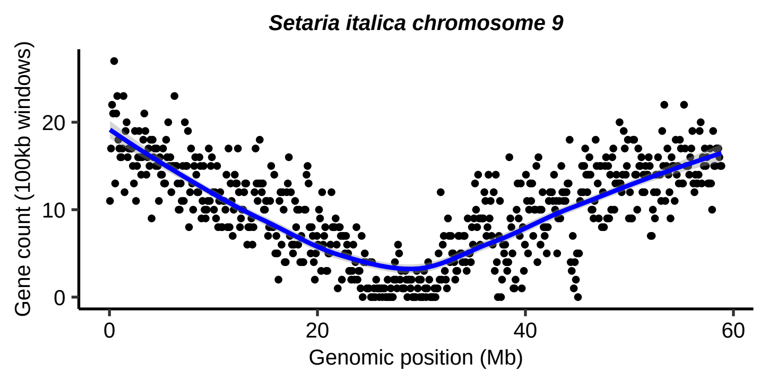

Setaria italica chromosome 9
Gene count (100kb windows)
0
10
20
0
20
40
60
Genomic position (Mb)

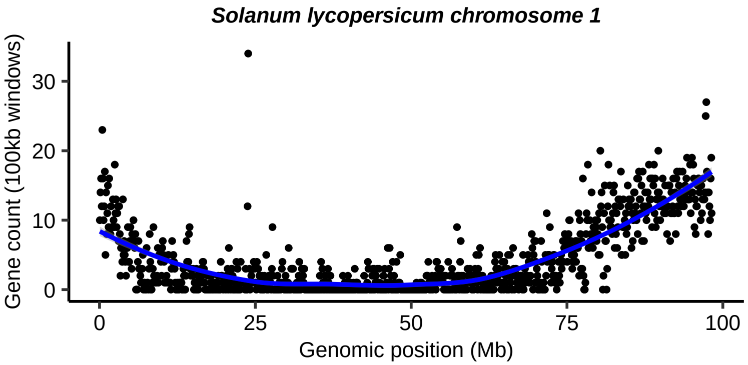
Solanum lycopersicum chromosome 1
Gene count (100kb windows)
0
10
20
30
0
25
50
75
100
Genomic position (Mb)

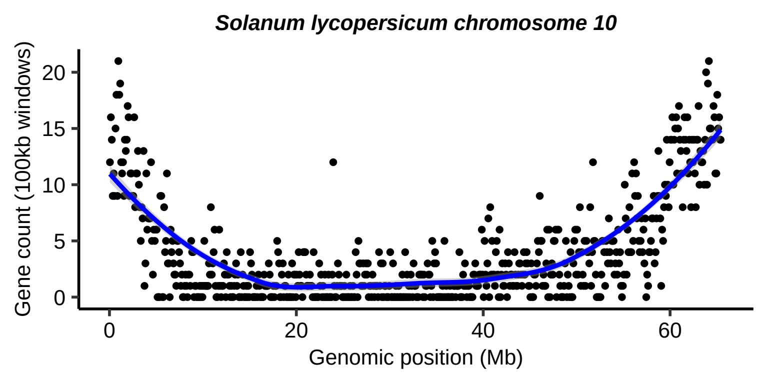

Solanum lycopersicum chromosome 10
Gene count (100kb windows)
0
5
10
15
20
0
20
40
60
Genomic position (Mb)

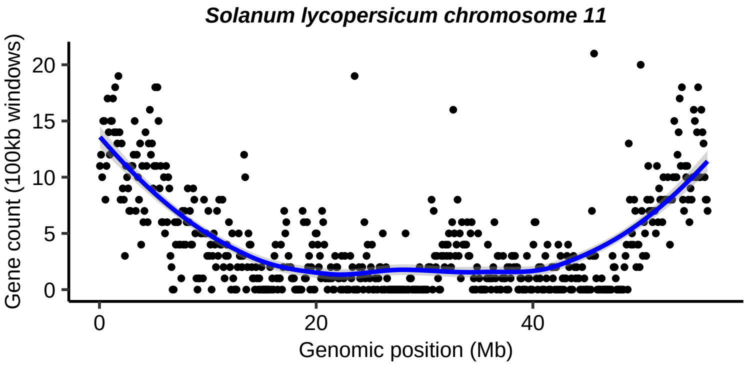
Solanum lycopersicum chromosome 11
Gene count (100kb windows)
0
5
10
15
20
0
20
40
Genomic position (Mb)

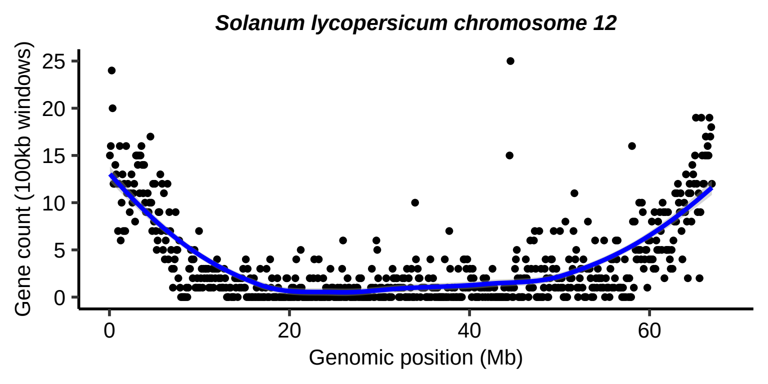

Solanum lycopersicum chromosome 12
Gene count (100kb windows)
25
20
15
10
5
0
0
20
40
60
Genomic position (Mb)

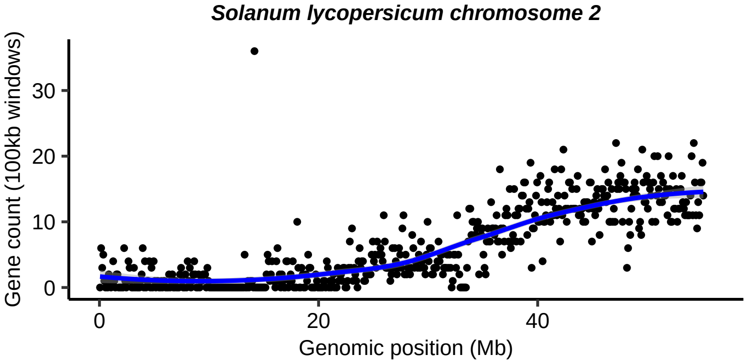

Solanum lycopersicum chromosome 2
Gene count (100kb windows)
0
10
20
30
0
20
40
Genomic position (Mb)

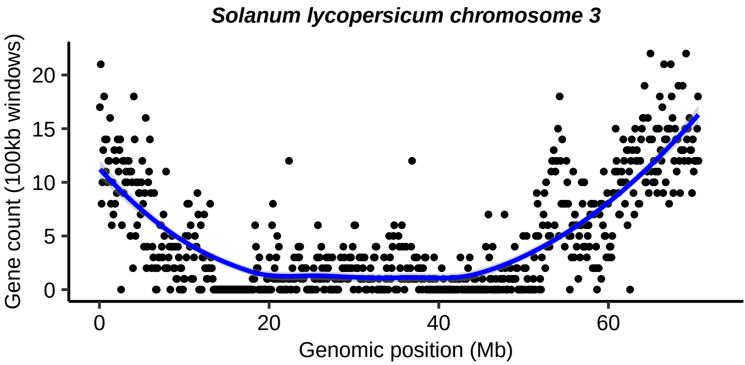

Solanum lycopersicum chromosome 3
Gene count (100kb windows)
20
15
10
5
0
0
20
40
60
Genomic position (Mb)

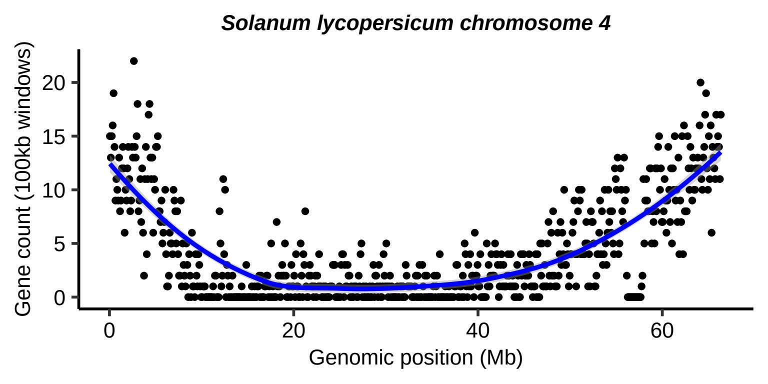

Solanum lycopersicum chromosome 4
Gene count (100kb windows)
0
5
10
15
20
0
20
40
60
Genomic position (Mb)

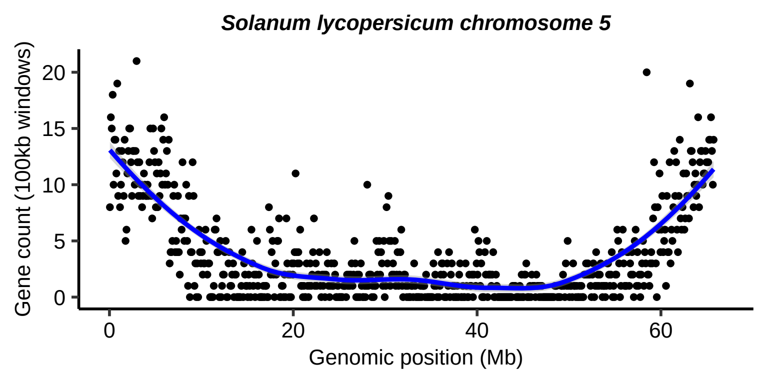

Solanum lycopersicum chromosome 5
Gene count (100kb windows)
0
5
10
15
20
0
20
40
60
Genomic position (Mb)

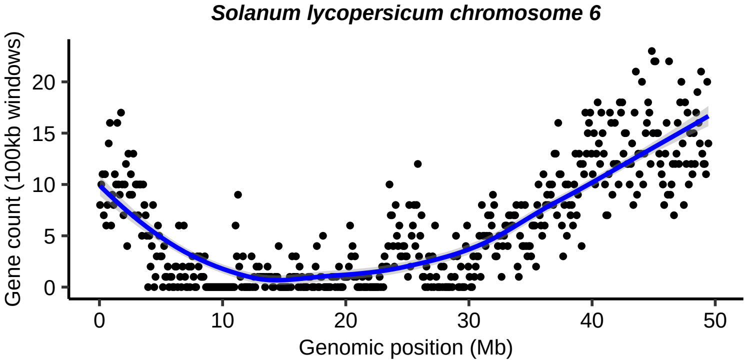
Solanum lycopersicum chromosome 6
Gene count (100kb windows)
0
5
10
15
20
0
10
20
30
40
50
Genomic position (Mb)

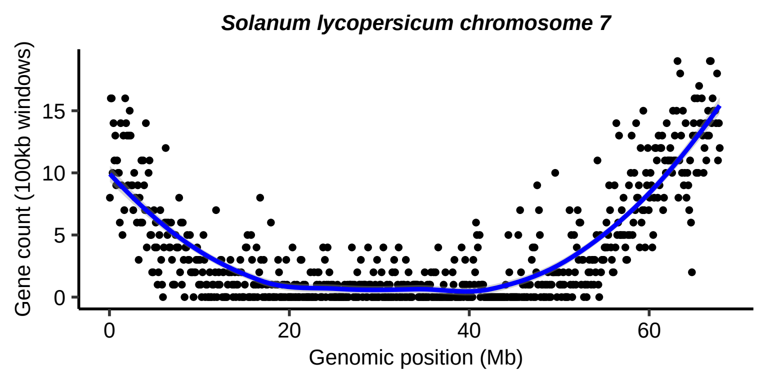
Solanum lycopersicum chromosome 7
Gene count (100kb windows)
0
5
10
15
0
20
40
60
Genomic position (Mb)

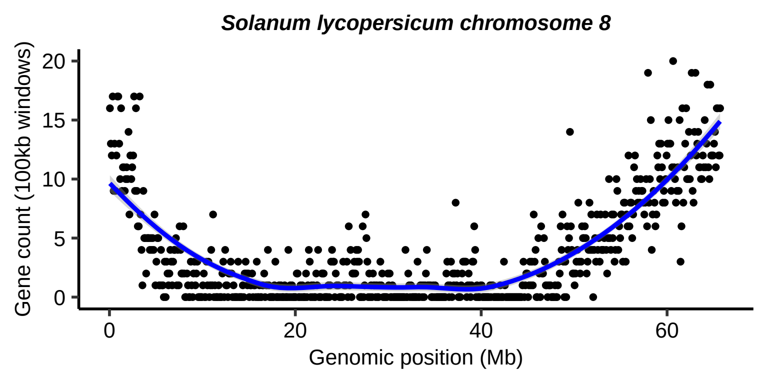

Solanum lycopersicum chromosome 8
Gene count (100kb windows)
0
5
10
15
20
0
20
40
60
Genomic position (Mb)

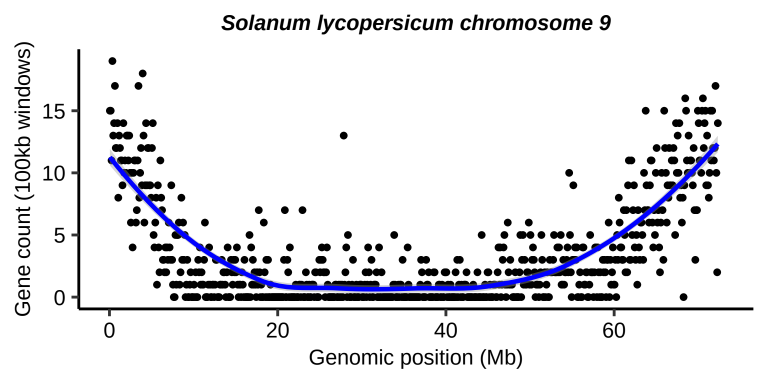

Solanum lycopersicum chromosome 9
Gene count (100kb windows)
0
5
10
15
0
20
40
60
Genomic position (Mb)

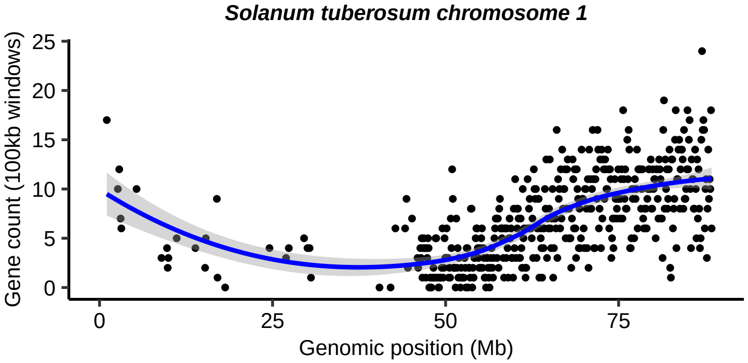

Solanum tuberosum chromosome 1
Gene count (100kb windows)
0
5
10
15
20
25
0
25
50
75
Genomic position (Mb)

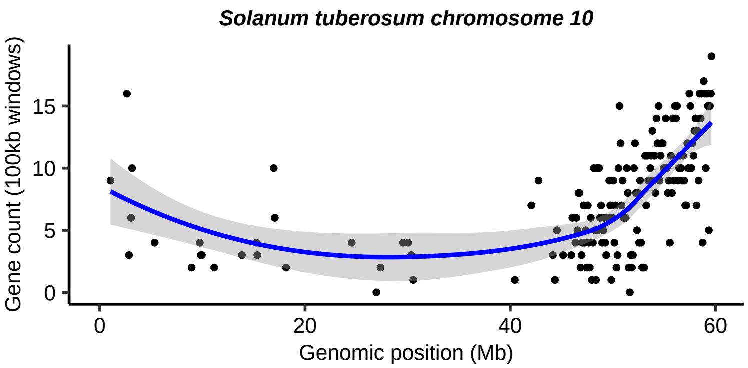

Solanum tuberosum chromosome 10
Gene count (100kb windows)
0
5
10
15
0
20
40
60
Genomic position (Mb)

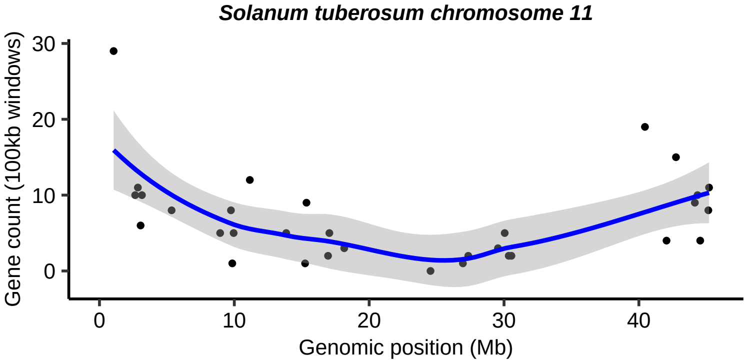

Solanum tuberosum chromosome 11
Gene count (100kb windows)
30
20
10
0
0
10
20
30
40
Genomic position (Mb)

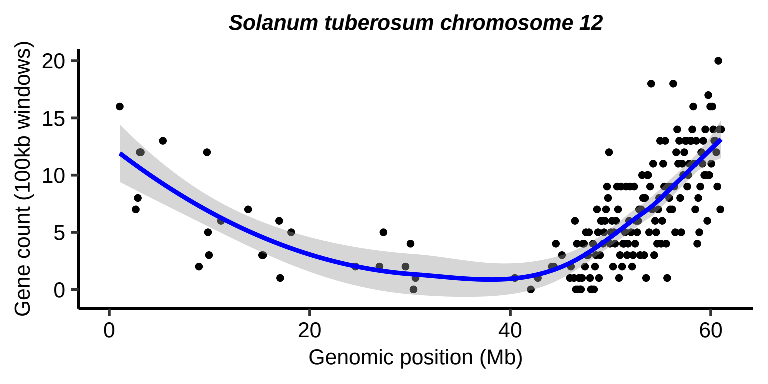
Solanum tuberosum chromosome 12
Gene count (100kb windows)
20
15
10
5
0
0
20
40
60
Genomic position (Mb)

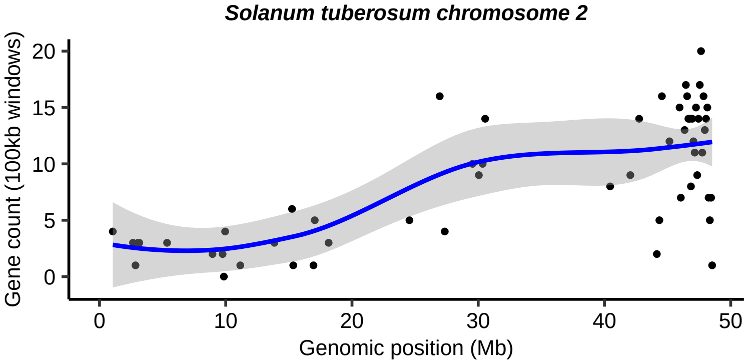
Solanum tuberosum chromosome 2
Gene count (100kb windows)
0
5
10
15
20
0
10
20
30
40
50
Genomic position (Mb)

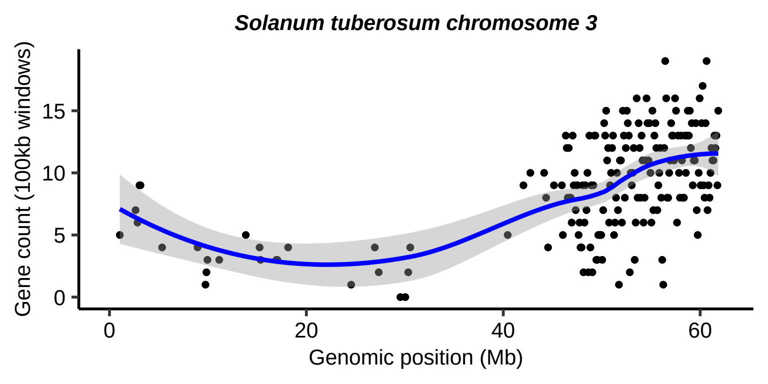

Solanum tuberosum chromosome 3
Gene count (100kb windows)
0
5
10
15
0
20
40
60
Genomic position (Mb)

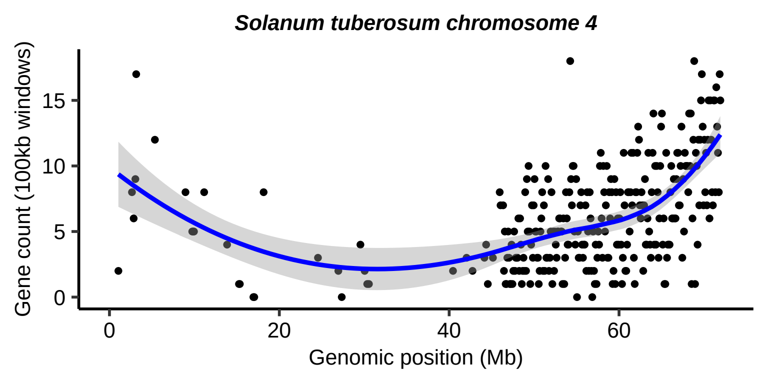

Solanum tuberosum chromosome 4
Gene count (100kb windows)
0
5
10
15
0
20
40
60
Genomic position (Mb)

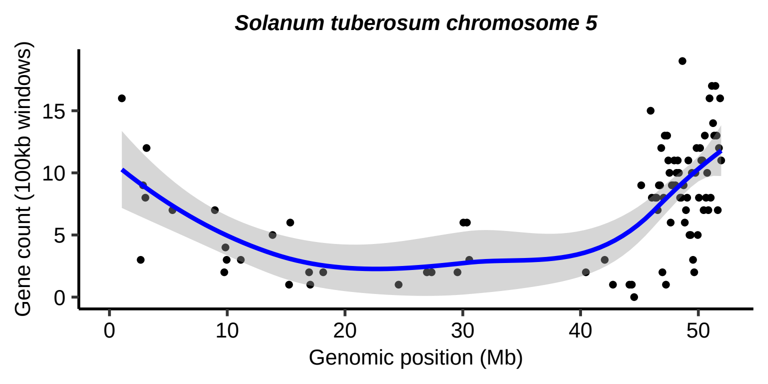

Solanum tuberosum chromosome 5
Gene count (100kb windows)
0
5
10
15
0
10
20
30
40
50
Genomic position (Mb)

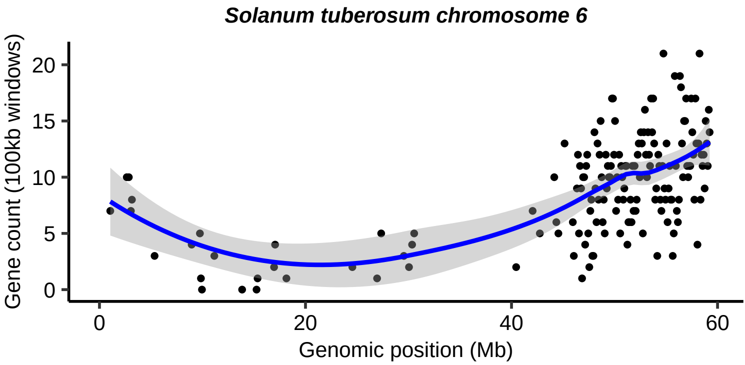

Solanum tuberosum chromosome 6
Gene count (100kb windows)
0
5
10
15
20
0
20
40
60
Genomic position (Mb)

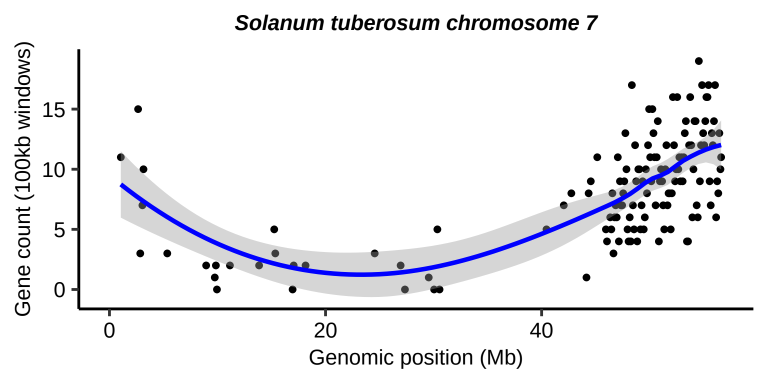

Solanum tuberosum chromosome 7
Gene count (100kb windows)
15
10
5
0
0
20
40
Genomic position (Mb)

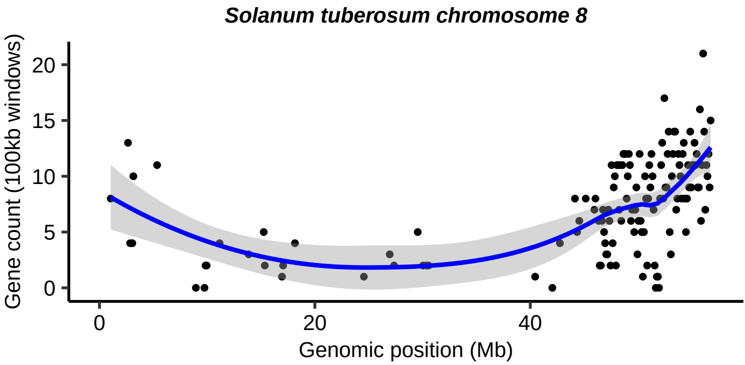

Solanum tuberosum chromosome 8
Gene count (100kb windows)
0
5
10
15
20
0
20
40
Genomic position (Mb)

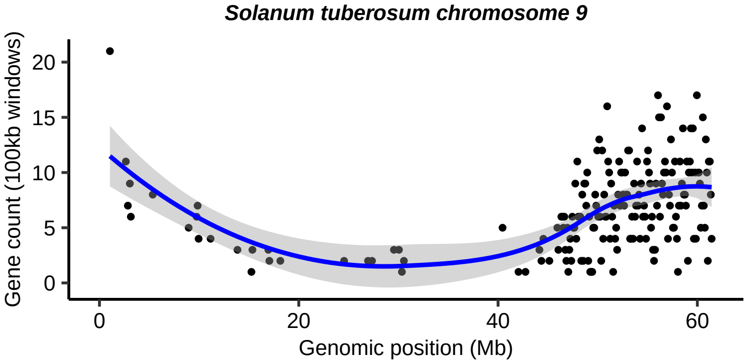

Solanum tuberosum chromosome 9
Gene count (100kb windows)
0
5
10
15
20
0
20
40
60
Genomic position (Mb)

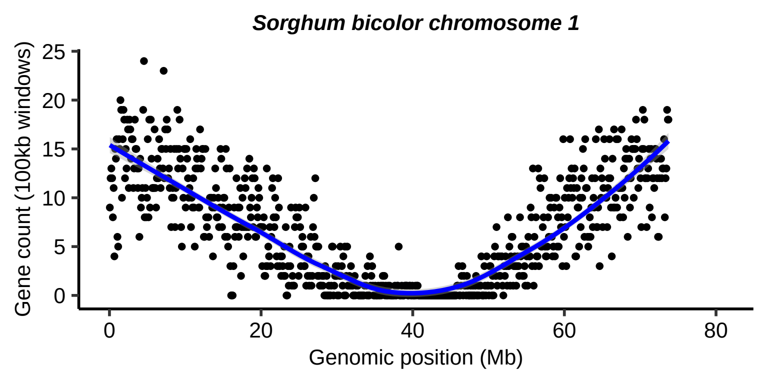
Sorghum bicolor chromosome 1
Gene count (100kb windows)
25
20
15
10
5
0
0
20
40
60
80
Genomic position (Mb)

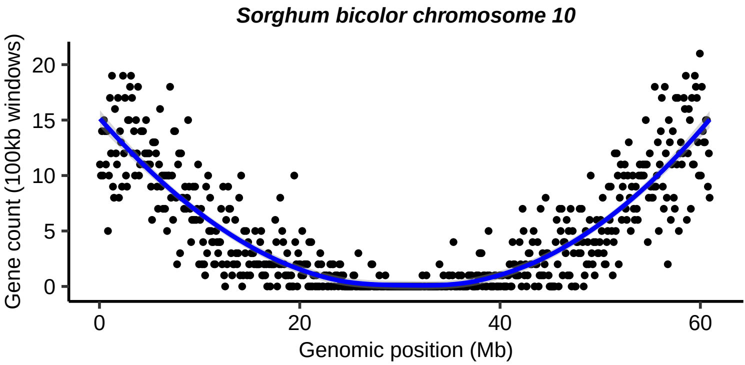

Sorghum bicolor chromosome 10
Gene count (100kb windows)
0
5
10
15
20
0
20
40
60
Genomic position (Mb)

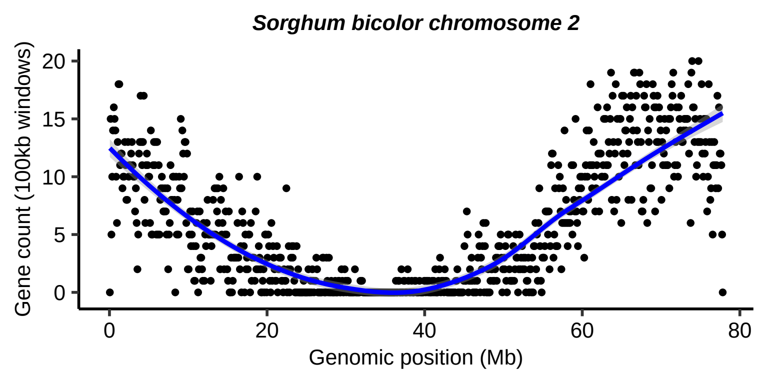

Sorghum bicolor chromosome 2
Gene count (100kb windows)
20
15
10
5
0
0
20
40
60
80
Genomic position (Mb)

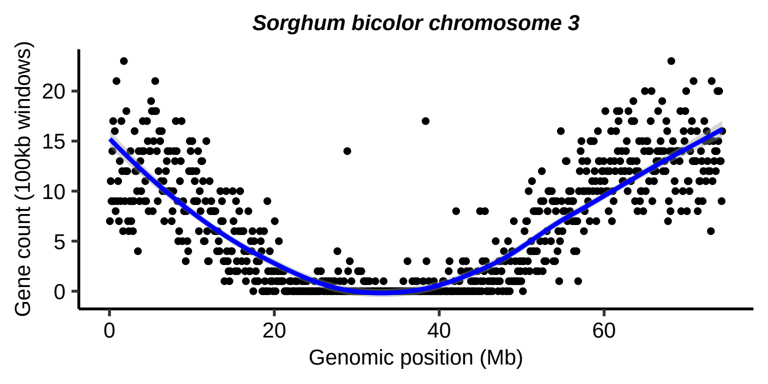
Sorghum bicolor chromosome 3
Gene count (100kb windows)
0
5
10
15
20
0
20
40
60
Genomic position (Mb)

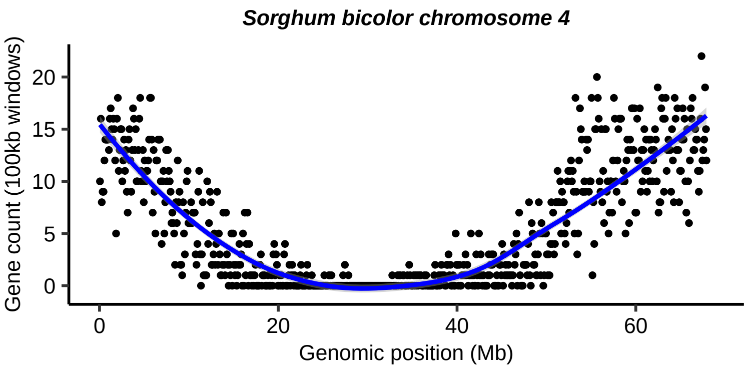
Sorghum bicolor chromosome 4
Gene count (100kb windows)
0
5
10
15
20
0
20
40
60
Genomic position (Mb)

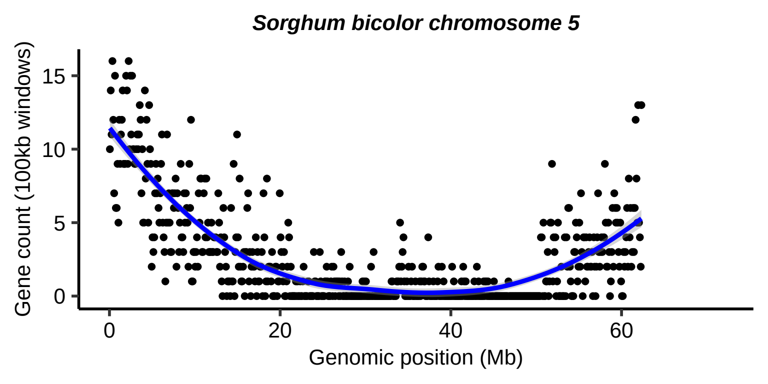
Sorghum bicolor chromosome 5
Gene count (100kb windows)
0
5
10
15
0
20
40
60
Genomic position (Mb)

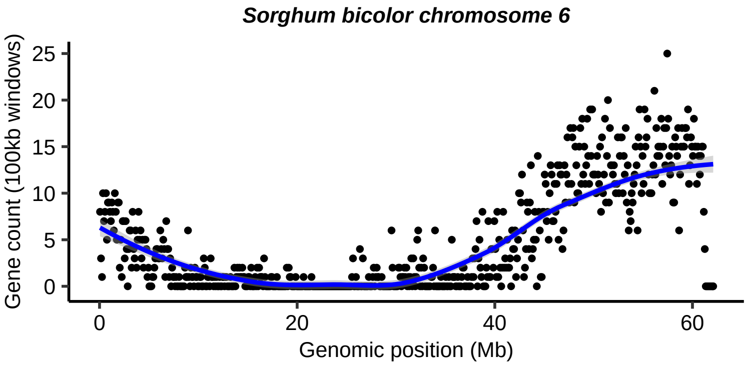

Sorghum bicolor chromosome 6
Gene count (100kb windows)
0
5
10
15
20
25
0
20
40
60
Genomic position (Mb)

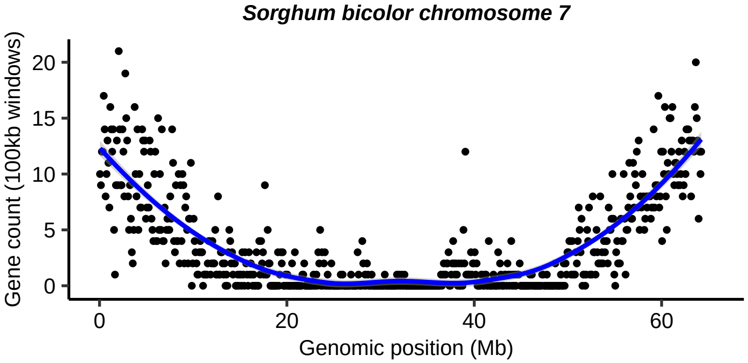

Sorghum bicolor chromosome 7
Gene count (100kb windows)
0
5
10
15
20
0
20
40
60
Genomic position (Mb)

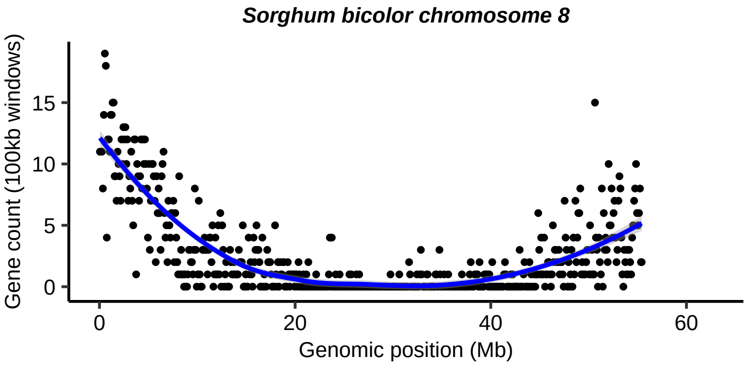

Sorghum bicolor chromosome 8
Gene count (100kb windows)
15
10
5
0
0
20
40
60
Genomic position (Mb)

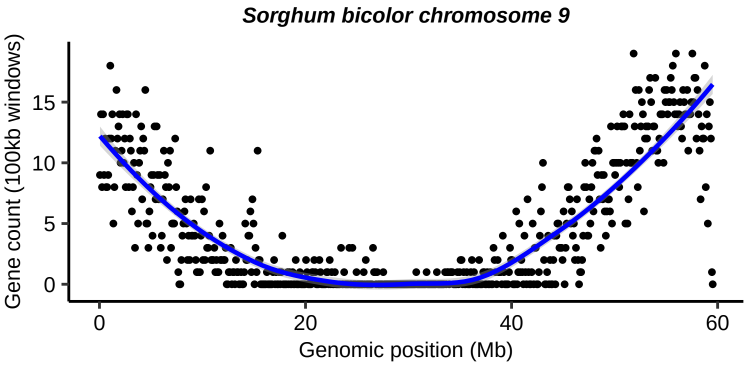

Sorghum bicolor chromosome 9
Gene count (100kb windows)
0
5
10
15
0
20
40
60
Genomic position (Mb)

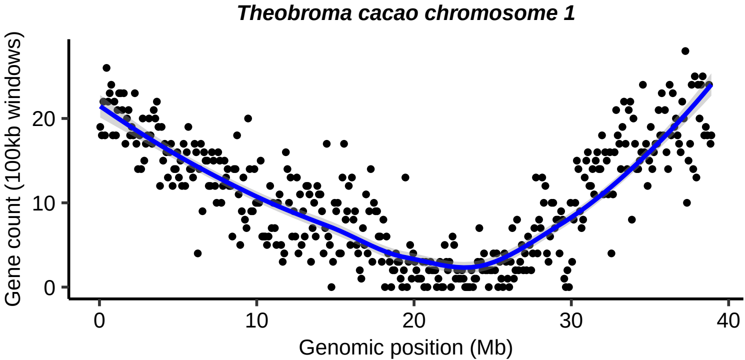

Theobroma cacao chromosome 1
Gene count (100kb windows)
0
10
20
0
10
20
30
40
Genomic position (Mb)

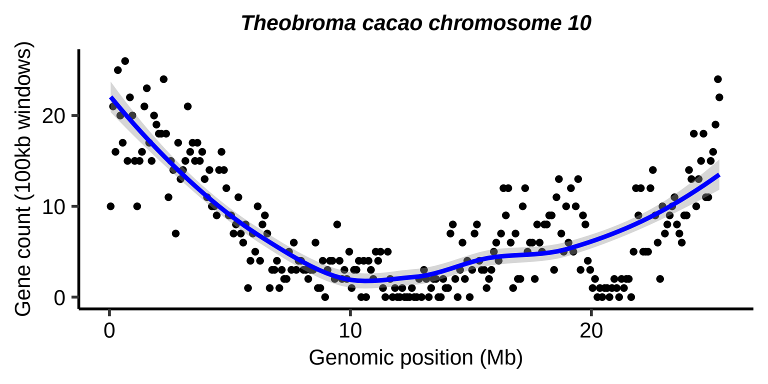
Theobroma cacao chromosome 10
Gene count (100kb windows)
0
10
20
Genomic position (Mb)

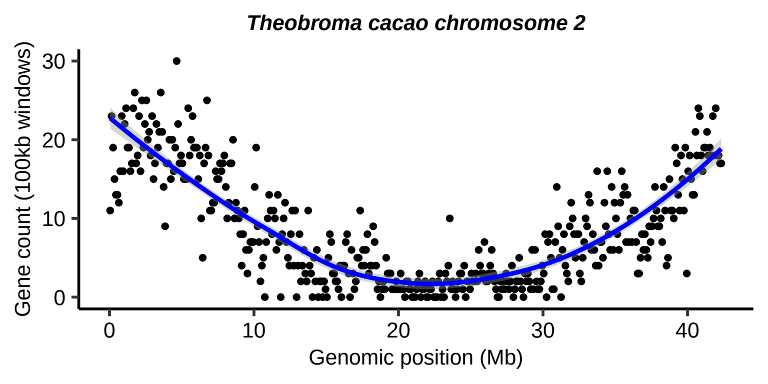

Theobroma cacao chromosome 2
Gene count (100kb windows)
30
20
10
0
0
10
20
30
40
Genomic position (Mb)

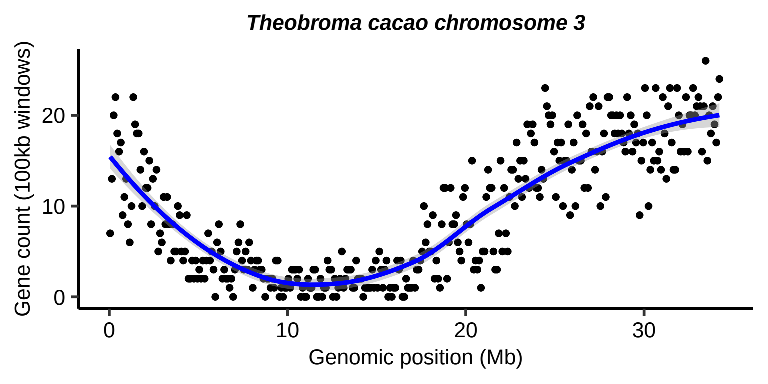

Theobroma cacao chromosome 3
Gene count (100kb windows)
0
10
20
0
10
20
30
Genomic position (Mb)

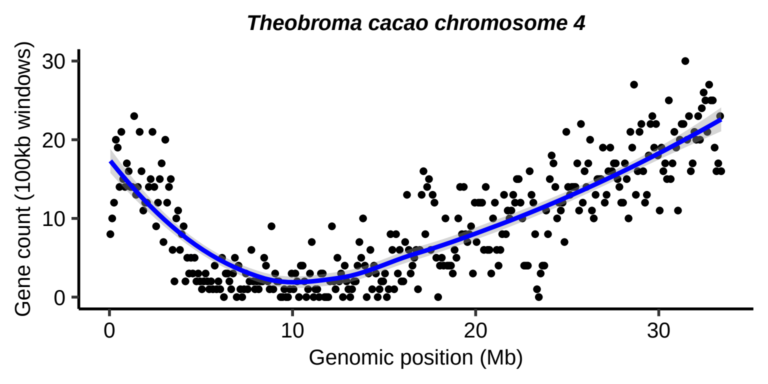
Theobroma cacao chromosome 4
Gene count (100kb windows)
30
20
10
0
0
10
20
30
Genomic position (Mb)

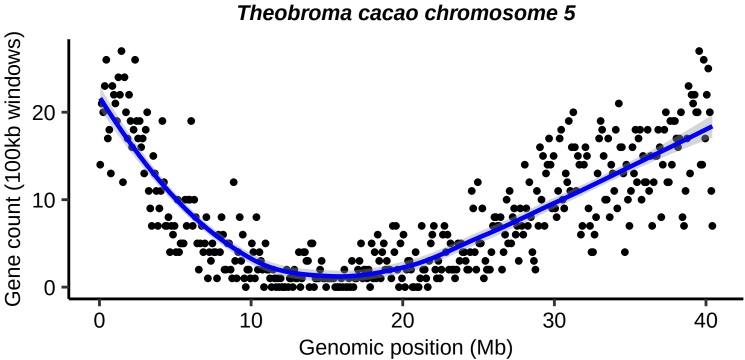

Theobroma cacao chromosome 5
Gene count (100kb windows)
0
10
20
0
10
20
30
40
Genomic position (Mb)

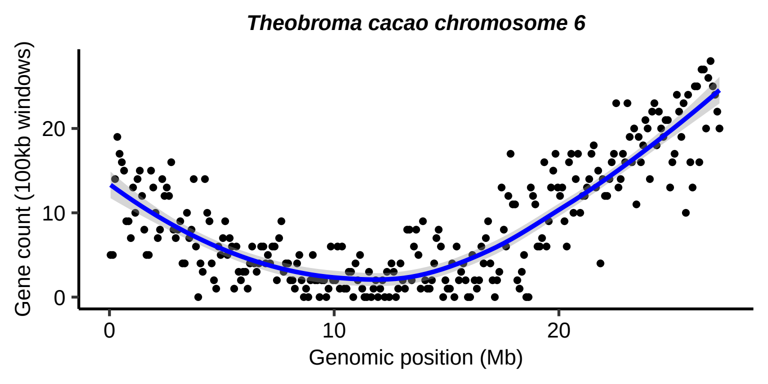
Theobroma cacao chromosome 6
Gene count (100kb windows)
0
10
20
Genomic position (Mb)

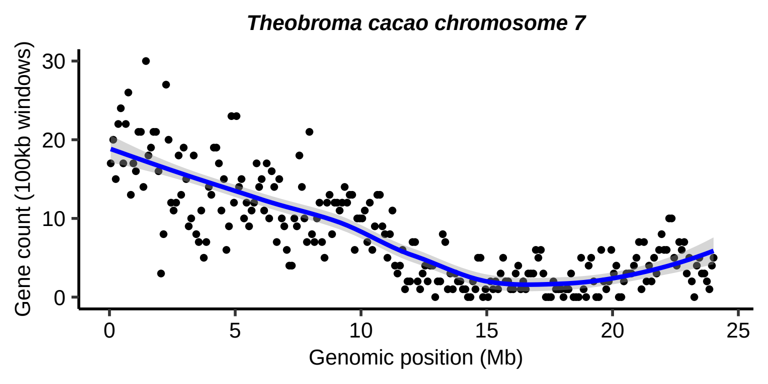

Theobroma cacao chromosome 7
Gene count (100kb windows)
30
20
10
0
0
5
10
15
20
25
Genomic position (Mb)

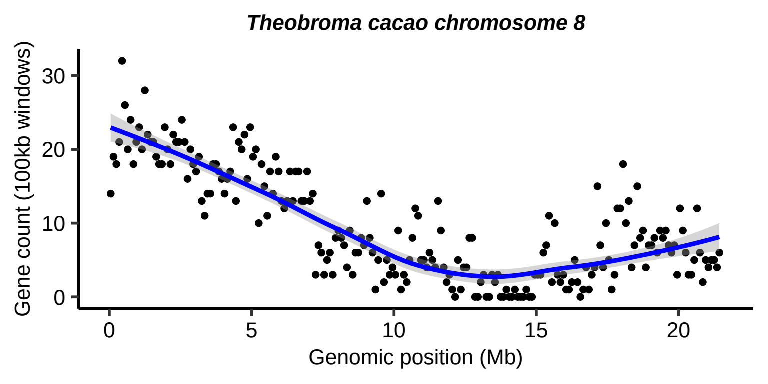
Theobroma cacao chromosome 8
Gene count (100kb windows)
30
20
10
0
0
5
10
15
20
Genomic position (Mb)

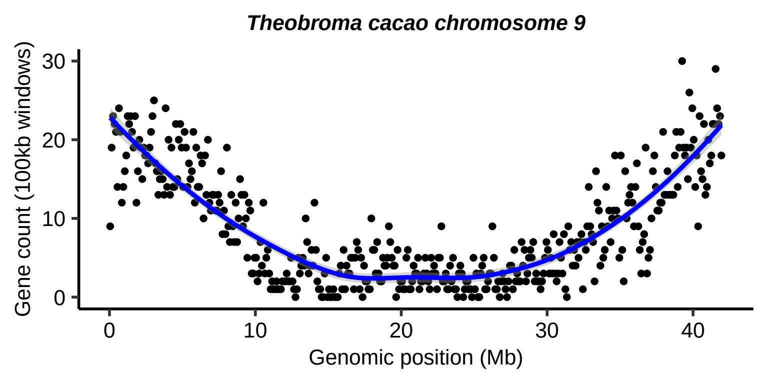

Theobroma cacao chromosome 9
Gene count (100kb windows)
30
20
10
0
0
10
20
30
40
Genomic position (Mb)

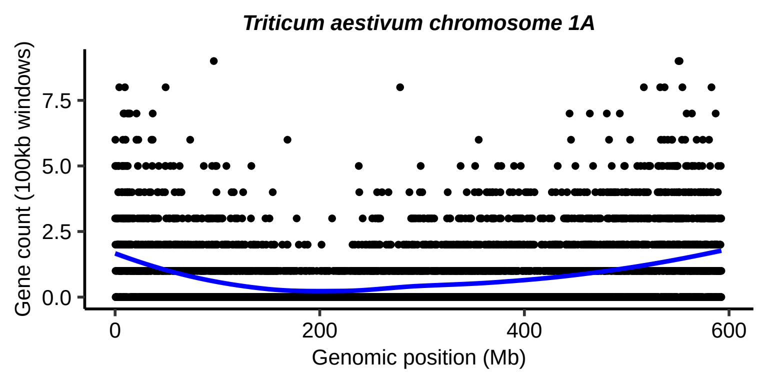
Triticum aestivum chromosome 1A
Gene count (100kb windows)
7.5
5.0
2.5
0.0
0
200
400
600
Genomic position (Mb)

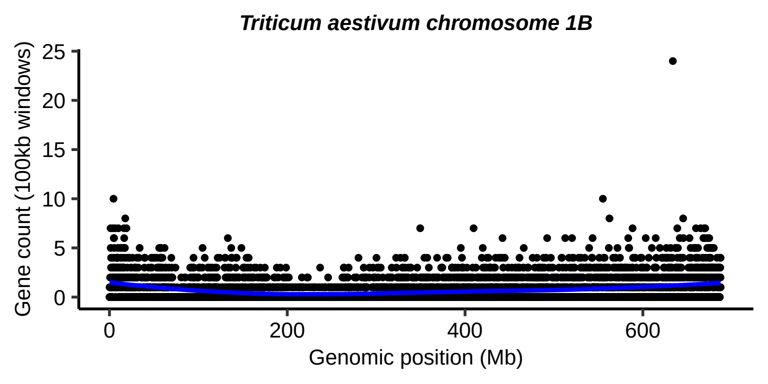
Triticum aestivum chromosome 1B
Gene count (100kb windows)
0
5
10
15
20
25
0
200
400
600
Genomic position (Mb)

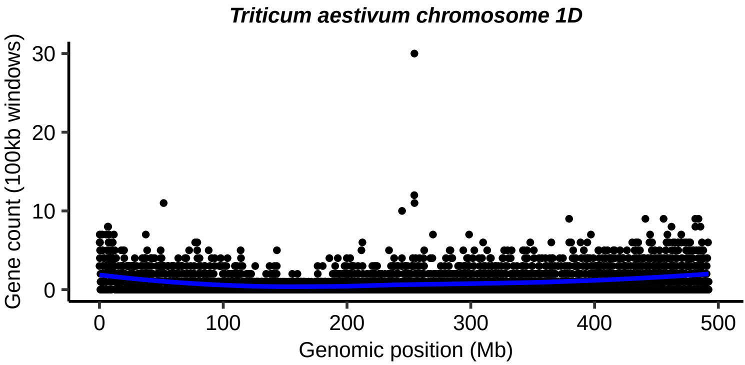

Triticum aestivum chromosome 1D
Gene count (100kb windows)
30
20
10
0
0
100
200
300
400
500
Genomic position (Mb)

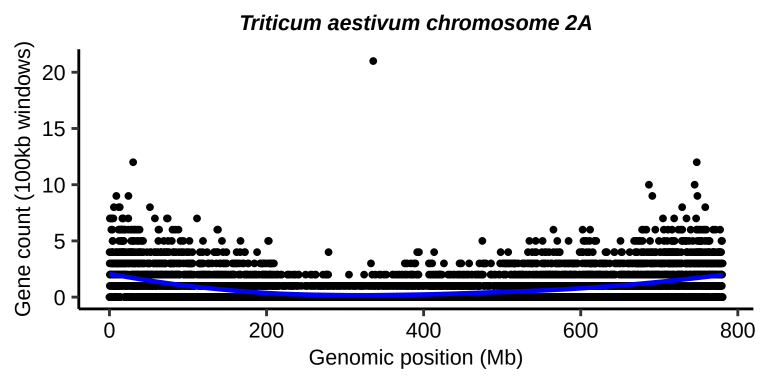

Triticum aestivum chromosome 2A
Gene count (100kb windows)
0
5
10
15
20
0
200
400
600
800
Genomic position (Mb)

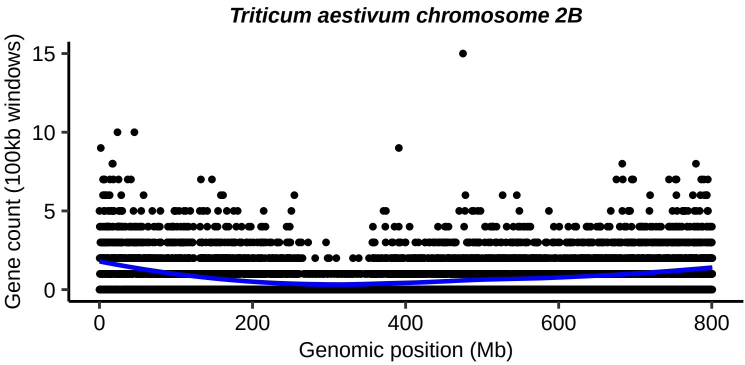

Triticum aestivum chromosome 2B
Gene count (100kb windows)
15
10
5
0
0
200
400
600
800
Genomic position (Mb)

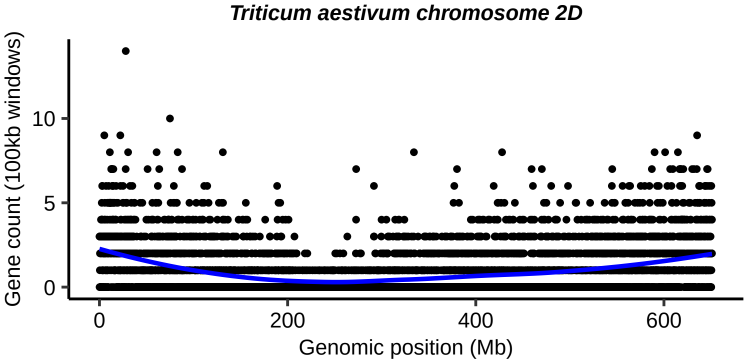

Triticum aestivum chromosome 2D
Gene count (100kb windows)
10
5
0
0
200
400
600
Genomic position (Mb)

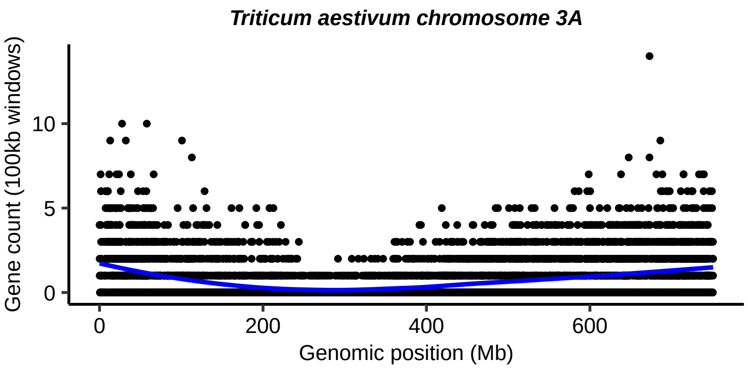
Triticum aestivum chromosome 3A
Gene count (100kb windows)
0
5
10
0
200
400
600
Genomic position (Mb)

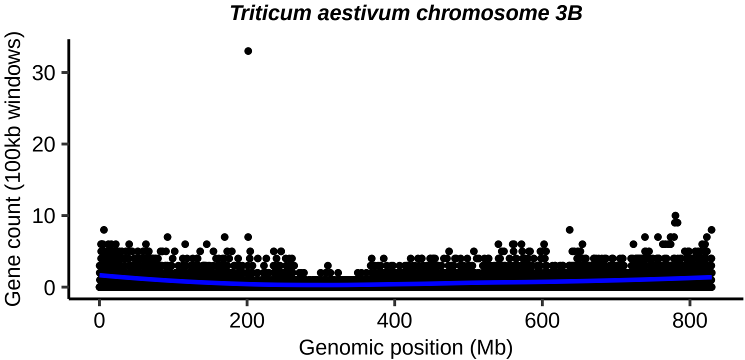

Triticum aestivum chromosome 3B
Gene count (100kb windows)
30
20
10
0
0
200
400
600
800
Genomic position (Mb)

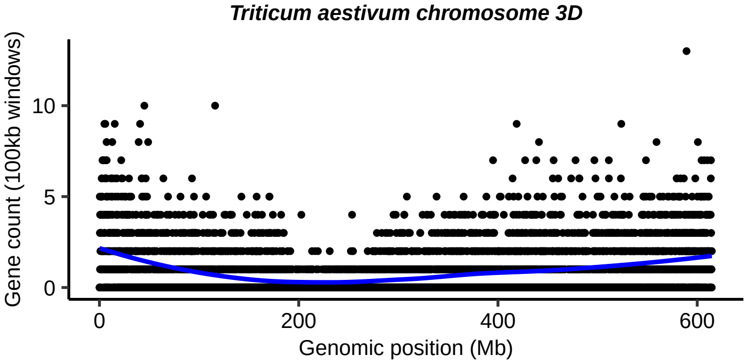

Triticum aestivum chromosome 3D
Gene count (100kb windows)
0
5
10
0
200
400
600
Genomic position (Mb)

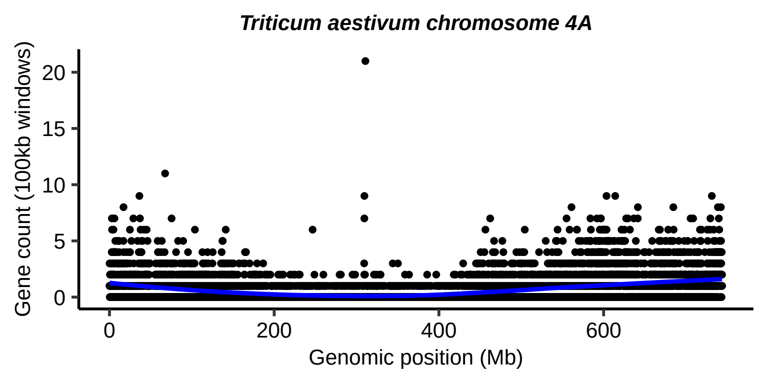

Triticum aestivum chromosome 4A
Gene count (100kb windows)
0
5
10
15
20
0
200
400
600
Genomic position (Mb)

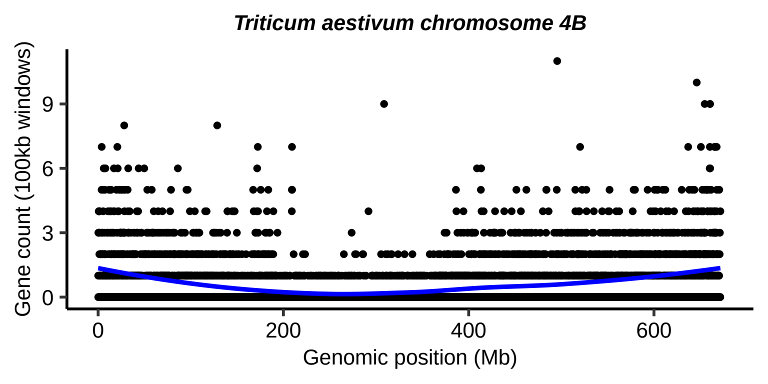
Triticum aestivum chromosome 4B
Gene count (100kb windows)
0
3
6
9
0
200
400
600
Genomic position (Mb)

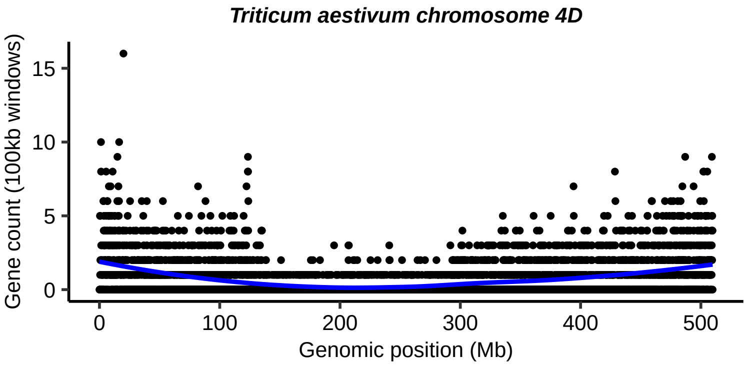
Triticum aestivum chromosome 4D
Gene count (100kb windows)
0
5
10
15
0
100
200
300
400
500
Genomic position (Mb)

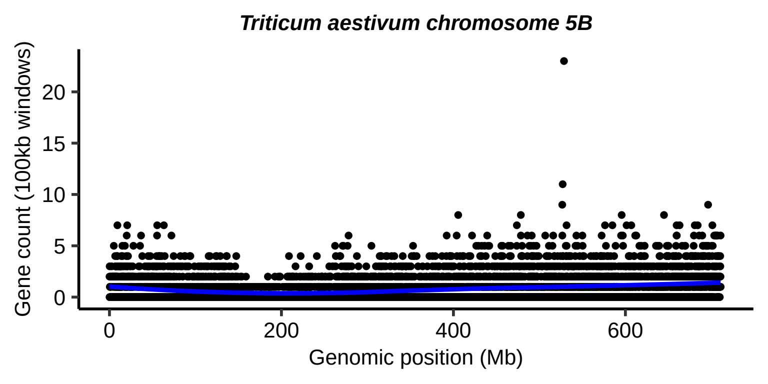
Triticum aestivum chromosome 5B
Gene count (100kb windows)
0
5
10
15
20
0
200
400
600
Genomic position (Mb)

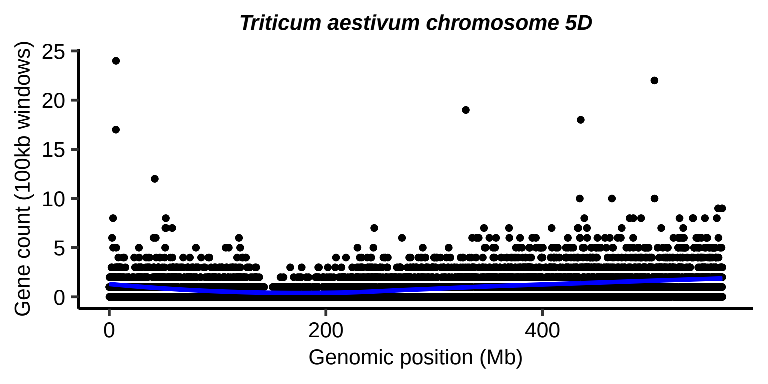

Triticum aestivum chromosome 5D
Gene count (100kb windows)
25
20
15
10
5
0
0
200
400
Genomic position (Mb)

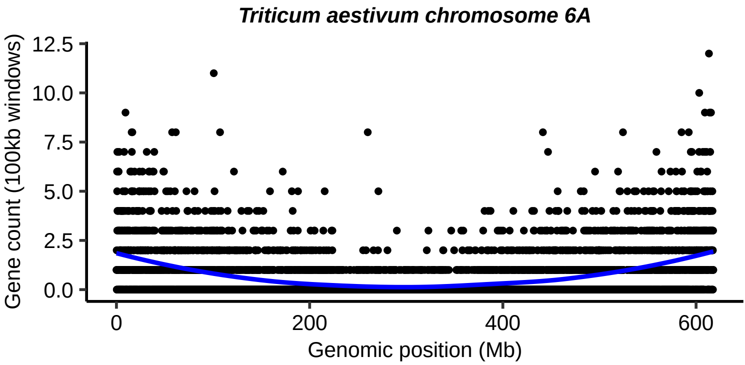

Triticum aestivum chromosome 6A
Gene count (100kb windows)
12.5
10.0
7.5
5.0
2.5
0.0
0
200
400
600
Genomic position (Mb)

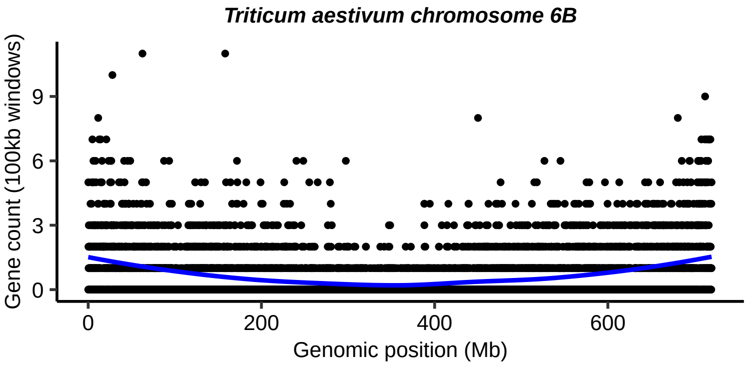

Triticum aestivum chromosome 6B
Gene count (100kb windows)
0
3
6
9
0
200
400
600
Genomic position (Mb)

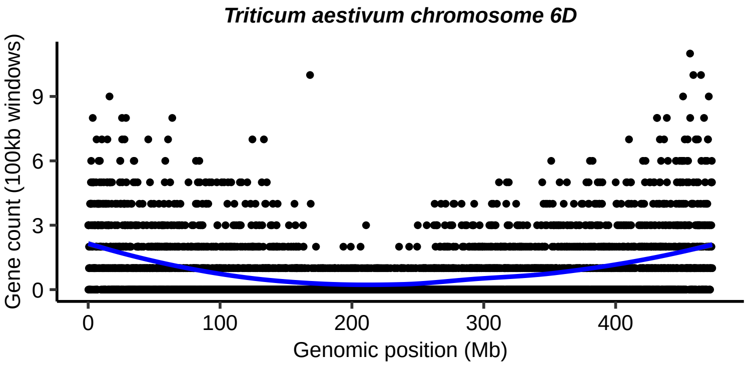

Triticum aestivum chromosome 6D
Gene count (100kb windows)
0
3
6
9
0
100
200
300
400
Genomic position (Mb)

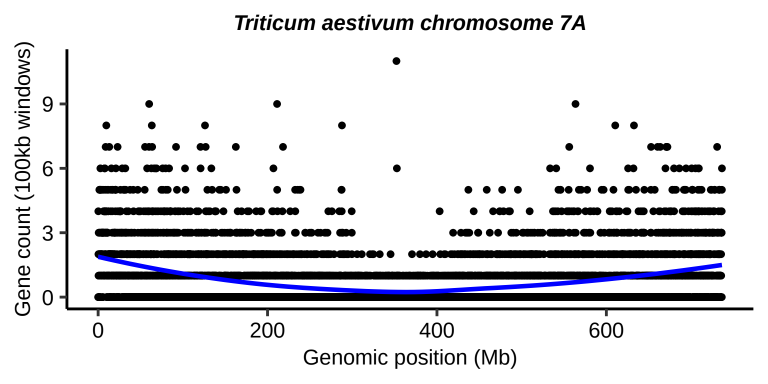
Triticum aestivum chromosome 7A
Gene count (100kb windows)
0
3
6
9
0
200
400
600
Genomic position (Mb)

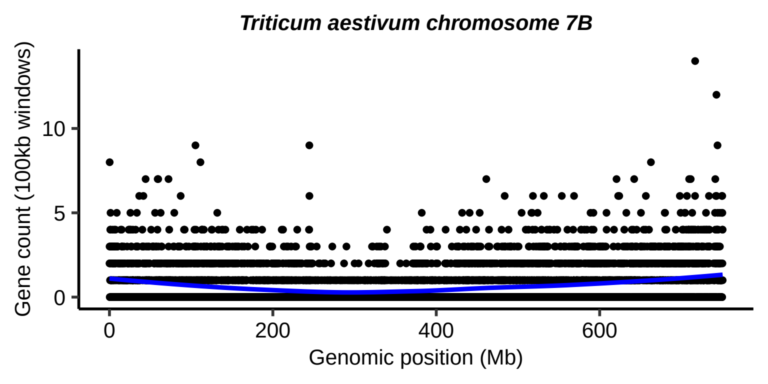

Triticum aestivum chromosome 7B
Gene count (100kb windows)
0
5
10
0
200
400
600
Genomic position (Mb)

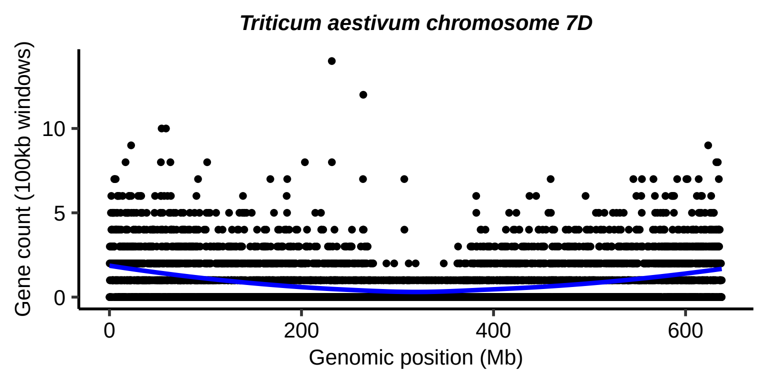

Triticum aestivum chromosome 7D
Gene count (100kb windows)
0
5
10
0
200
400
600
Genomic position (Mb)

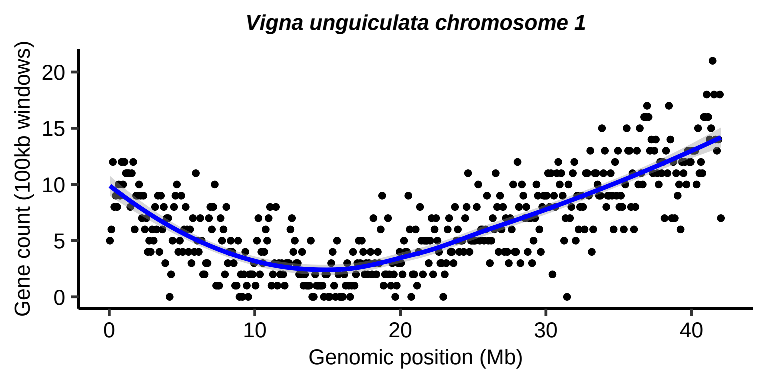

Vigna unguiculata chromosome 1
Gene count (100kb windows)
0
5
10
15
20
0
10
20
30
40
Genomic position (Mb)

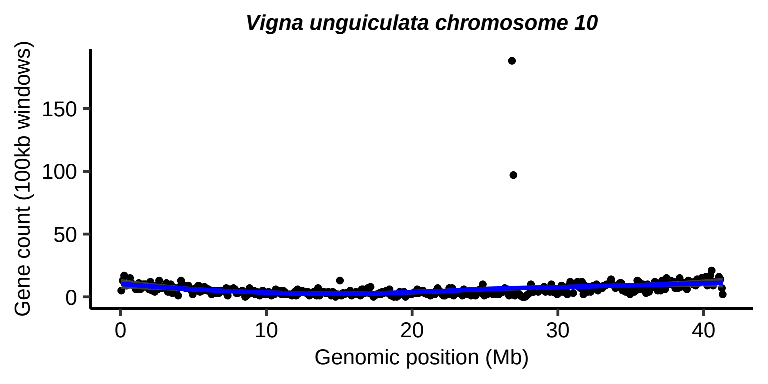

Vigna unguiculata chromosome 10
Gene count (100kb windows)
150
100
50
0
0
10
20
30
40
Genomic position (Mb)

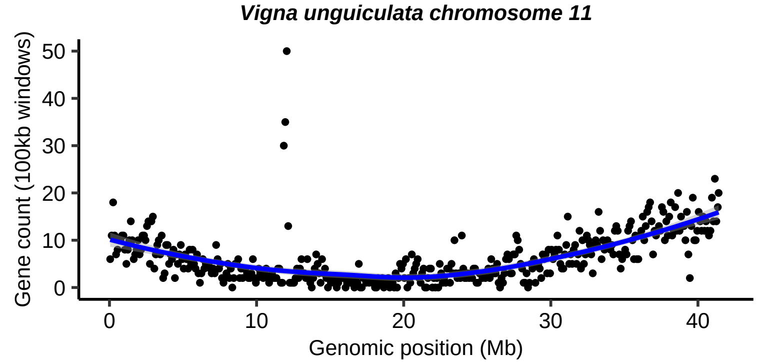

Vigna unguiculata chromosome 11
Gene count (100kb windows)
50
40
30
20
10
0
0
10
20
30
40
Genomic position (Mb)

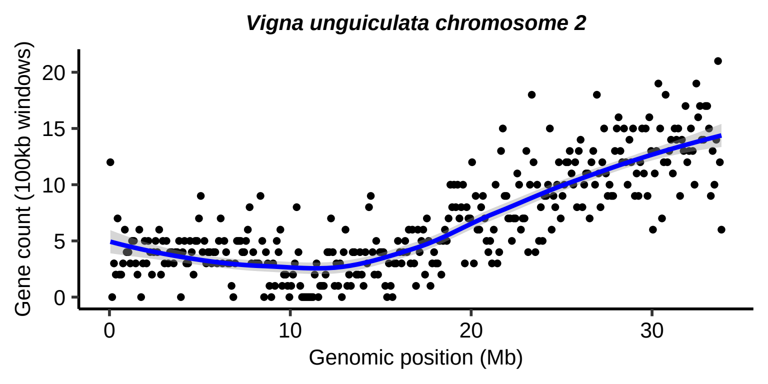
Vigna unguiculata chromosome 2
Gene count (100kb windows)
Genomic position (Mb)
0
5
10
15
20
0
10
20
30

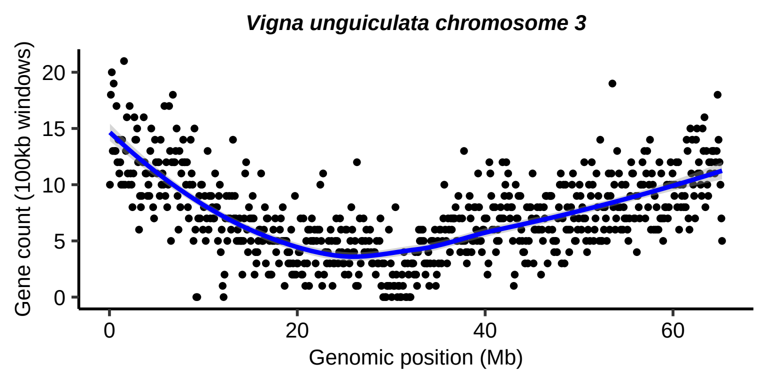

Vigna unguiculata chromosome 3
Gene count (100kb windows)
0
5
10
15
20
0
20
40
60
Genomic position (Mb)

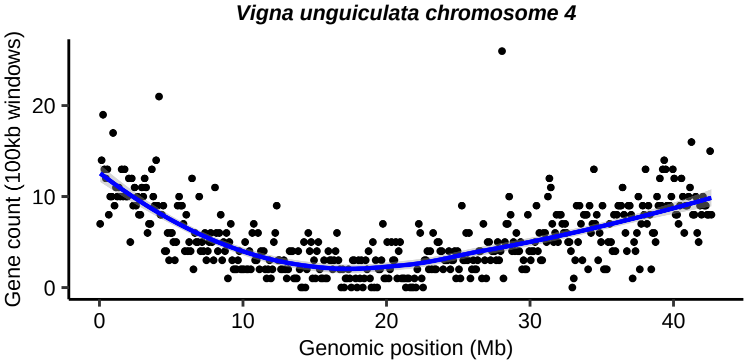
Vigna unguiculata chromosome 4
Gene count (100kb windows)
0
10
20
0
10
20
30
40
Genomic position (Mb)

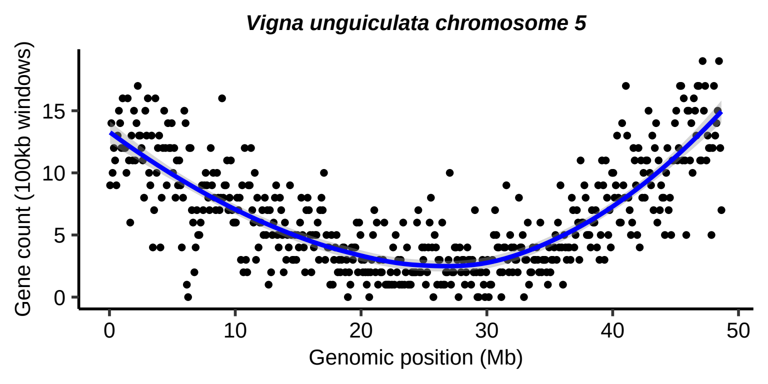

Vigna unguiculata chromosome 5
Gene count (100kb windows)
0
5
10
15
0
10
20
30
40
50
Genomic position (Mb)

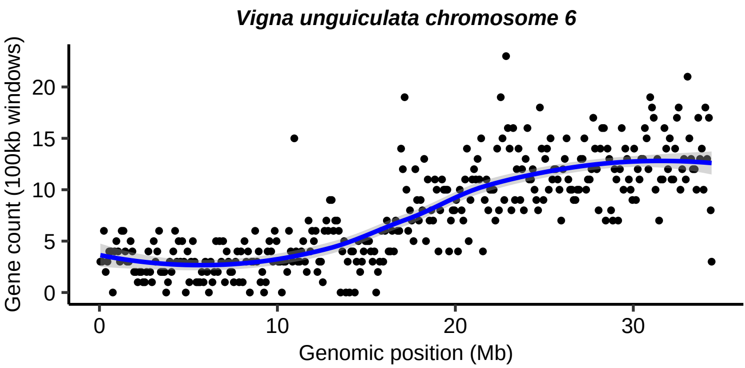

Vigna unguiculata chromosome 6
Gene count (100kb windows)
0
5
10
15
20
0
10
20
30
Genomic position (Mb)

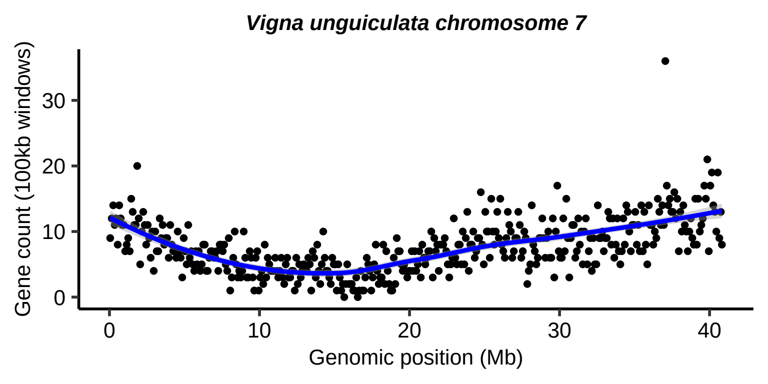

Vigna unguiculata chromosome 7
Gene count (100kb windows)
0
10
20
30
0
10
20
30
40
Genomic position (Mb)

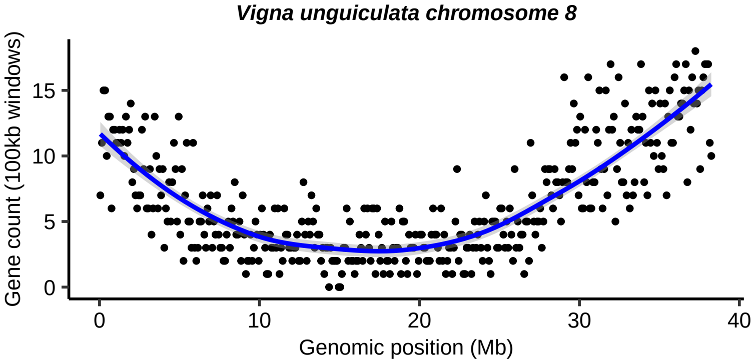

Vigna unguiculata chromosome 8
Gene count (100kb windows)
0
5
10
15
0
10
20
30
40
Genomic position (Mb)

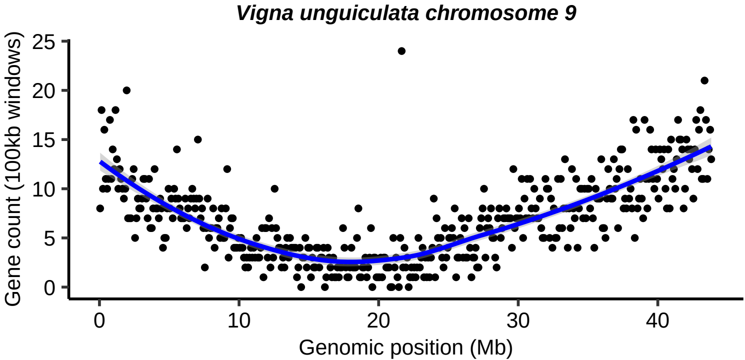

Vigna unguiculata chromosome 9
Gene count (100kb windows)
25
20
15
10
5
0
0
10
20
30
40
Genomic position (Mb)

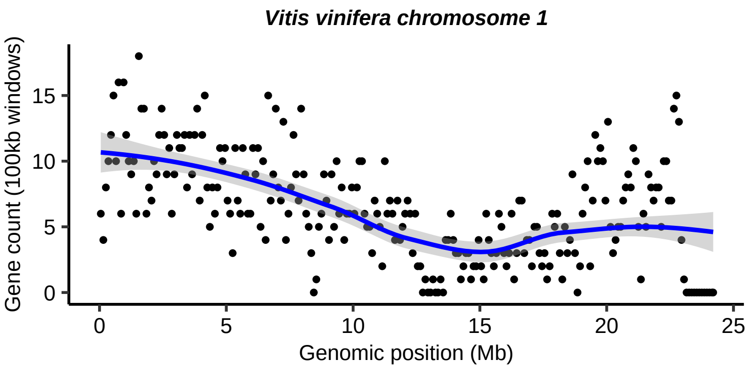

Vitis vinifera chromosome 1
Gene count (100kb windows)
0
5
10
15
0
5
10
15
20
25
Genomic position (Mb)

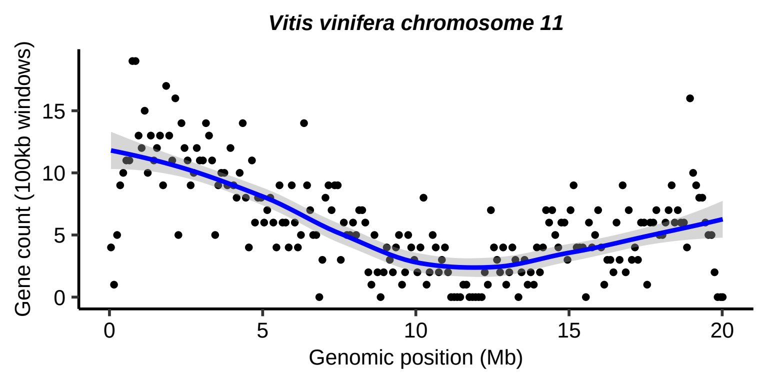
Vitis vinifera chromosome 11
Gene count (100kb windows)
0
5
10
15
20
Genomic position (Mb)

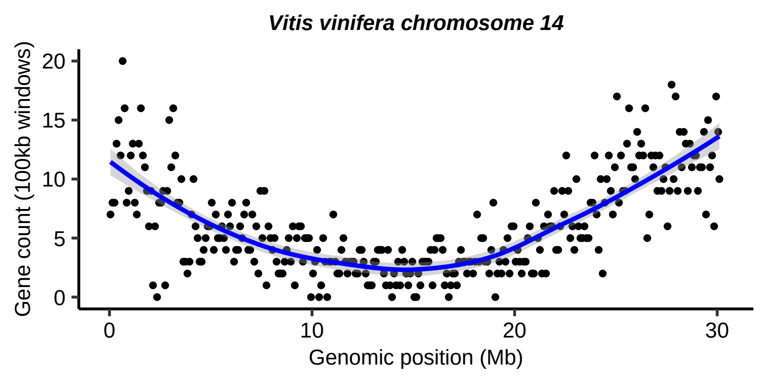

Vitis vinifera chromosome 14
Gene count (100kb windows)
0
5
10
15
20
0
10
20
30
Genomic position (Mb)

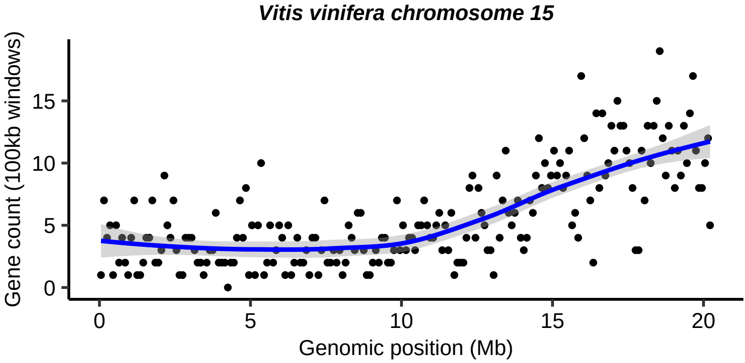

Vitis vinifera chromosome 15
Gene count (100kb windows)
0
5
10
15
0
5
10
15
20
Genomic position (Mb)

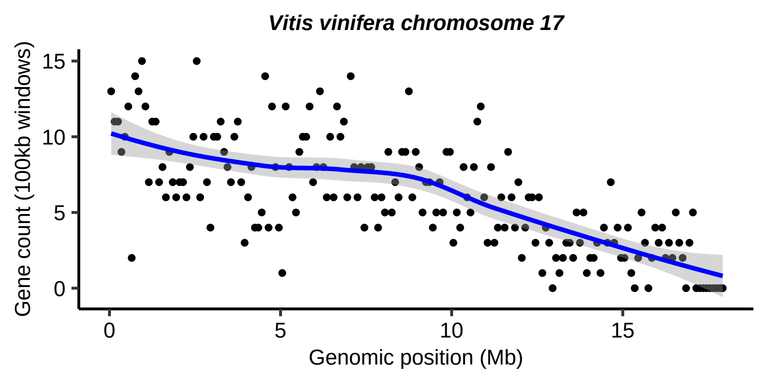

Vitis vinifera chromosome 17
Gene count (100kb windows)
0
5
10
15
Genomic position (Mb)

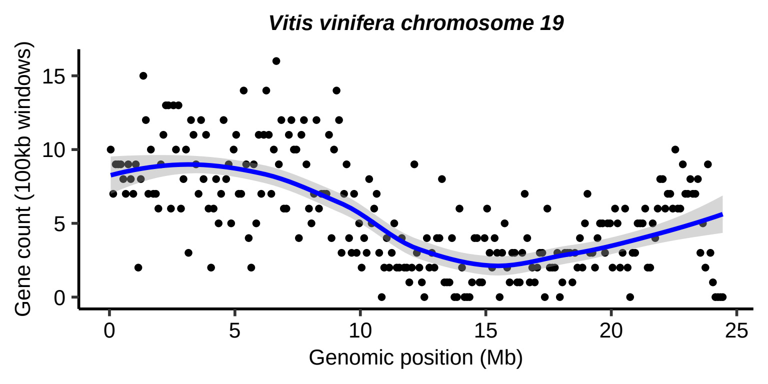
Vitis vinifera chromosome 19
Gene count (100kb windows)
0
5
10
15
0
5
10
15
20
25
Genomic position (Mb)

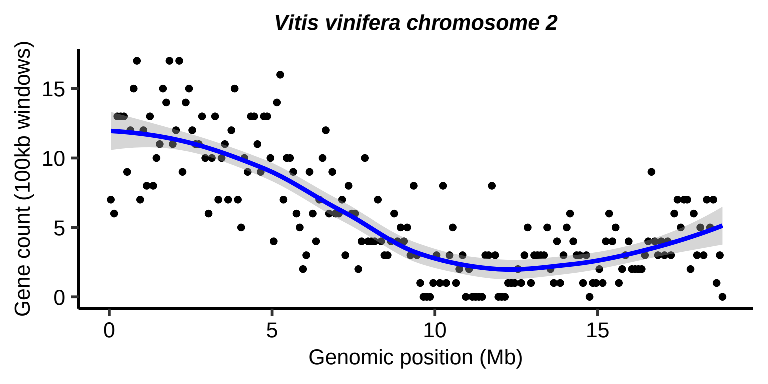
Vitis vinifera chromosome 2
Gene count (100kb windows)
0
5
10
15
0
5
10
15
Genomic position (Mb)

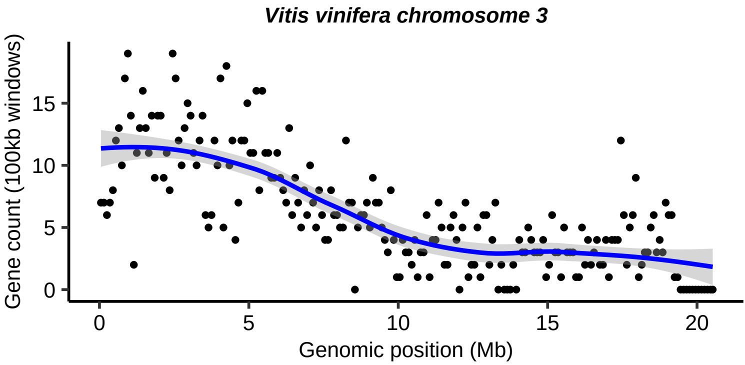

Vitis vinifera chromosome 3
Gene count (100kb windows)
0
5
10
15
0
5
10
15
20
Genomic position (Mb)

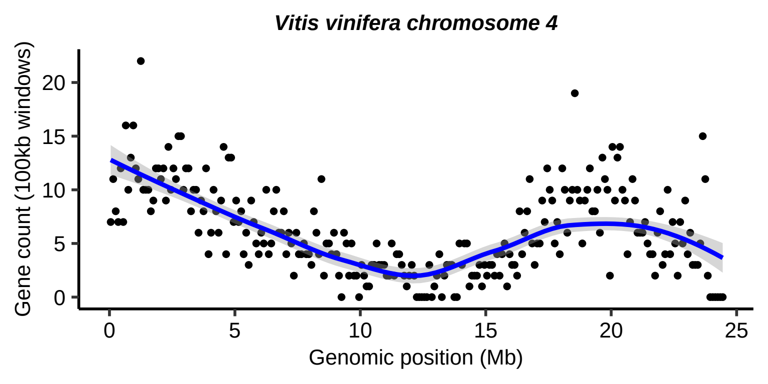

Vitis vinifera chromosome 4
Gene count (100kb windows)
0
5
10
15
20
0
5
10
15
20
25
Genomic position (Mb)

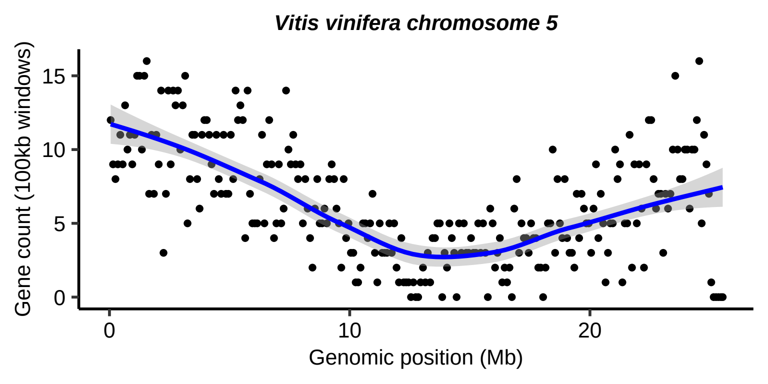

Vitis vinifera chromosome 5
Gene count (100kb windows)
0
5
10
15
0
10
20
Genomic position (Mb)

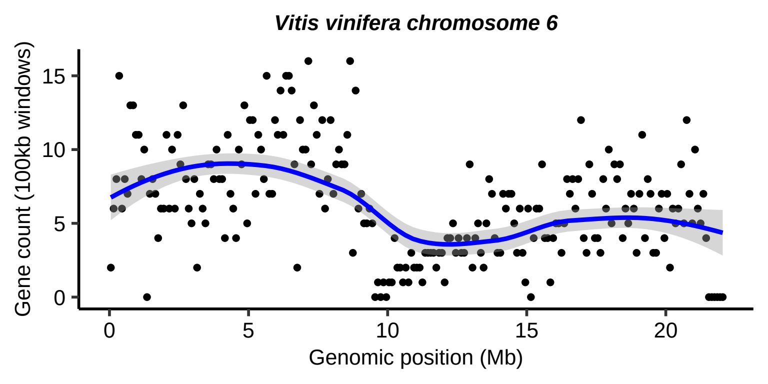
Vitis vinifera chromosome 6
Gene count (100kb windows)
0
5
10
15
0
5
10
15
20
Genomic position (Mb)

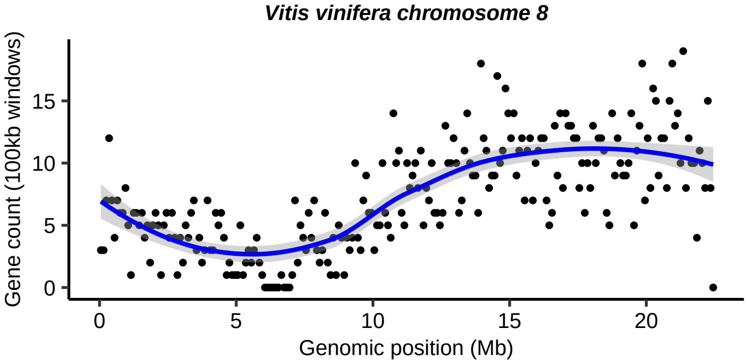

Vitis vinifera chromosome 8
Gene count (100kb windows)
0
5
10
15
0
5
10
15
20
Genomic position (Mb)

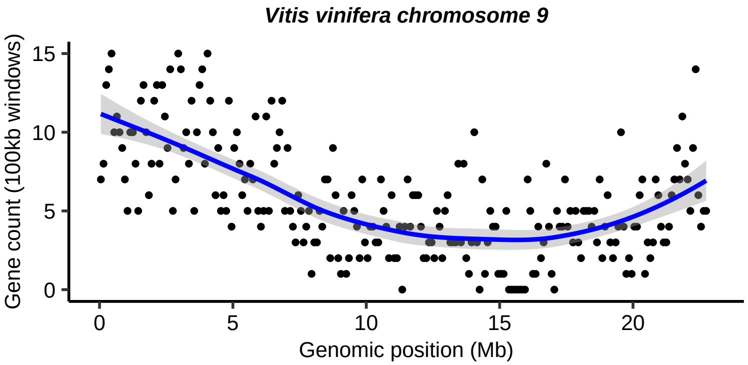

Vitis vinifera chromosome 9
Gene count (100kb windows)
0
5
10
15
0
5
10
15
20
Genomic position (Mb)

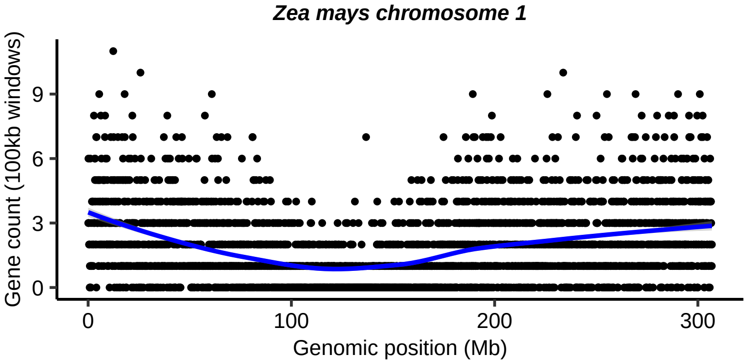

Zea mays chromosome 1
Gene count (100kb windows)
0
3
6
9
0
100
200
300
Genomic position (Mb)

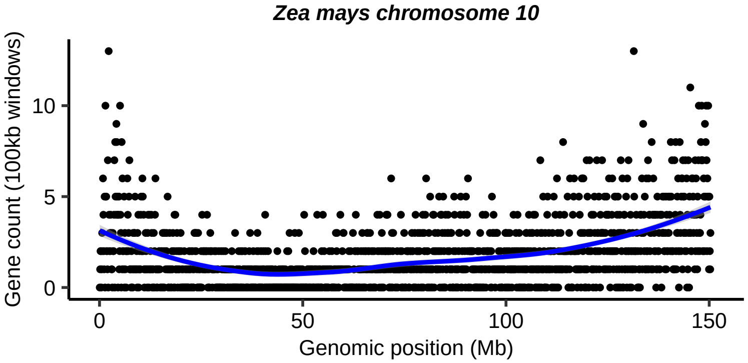

Zea mays chromosome 10
Gene count (100kb windows)
0
5
10
0
50
100
150
Genomic position (Mb)

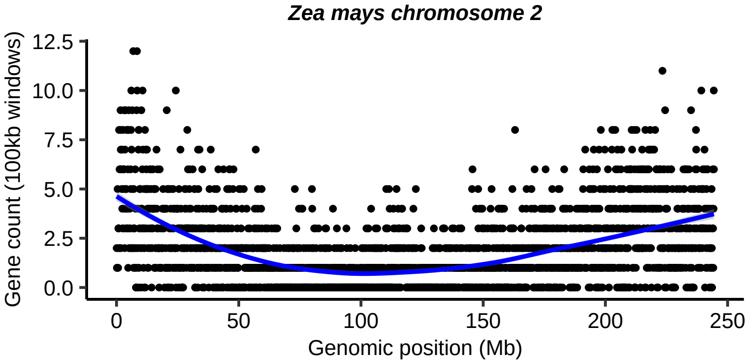

Zea mays chromosome 2
Gene count (100kb windows)
0.0
2.5
5.0
7.5
10.0
12.5
0
50
100
150
200
250
Genomic position (Mb)

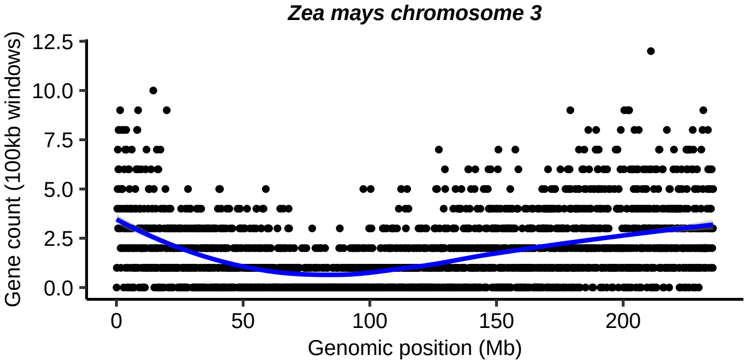

Zea mays chromosome 3
Gene count (100kb windows)
12.5
10.0
7.5
5.0
2.5
0.0
0
50
100
150
200
Genomic position (Mb)

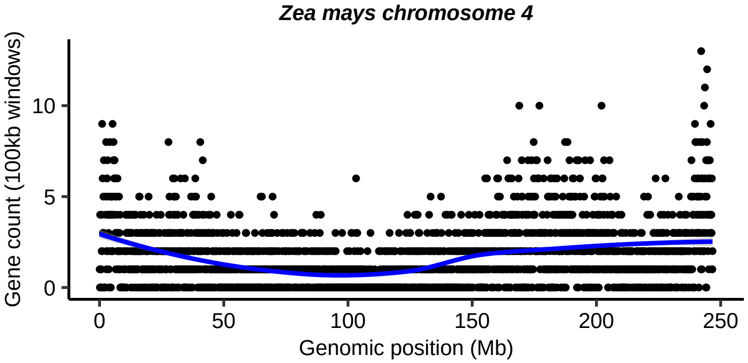

Zea mays chromosome 4
Gene count (100kb windows)
0
5
10
0
50
100
150
200
250
Genomic position (Mb)

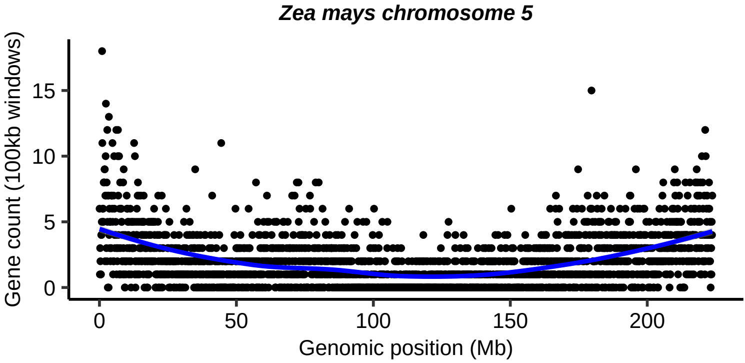

Zea mays chromosome 5
Gene count (100kb windows)
0
5
10
15
0
50
100
150
200
Genomic position (Mb)

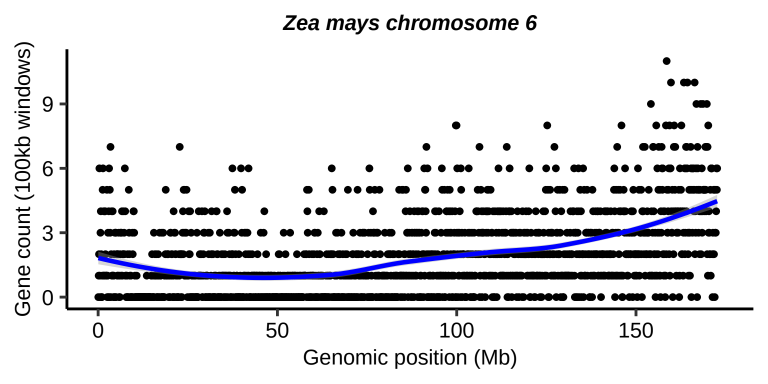
Zea mays chromosome 6
Gene count (100kb windows)
0
3
6
9
0
50
100
150
Genomic position (Mb)

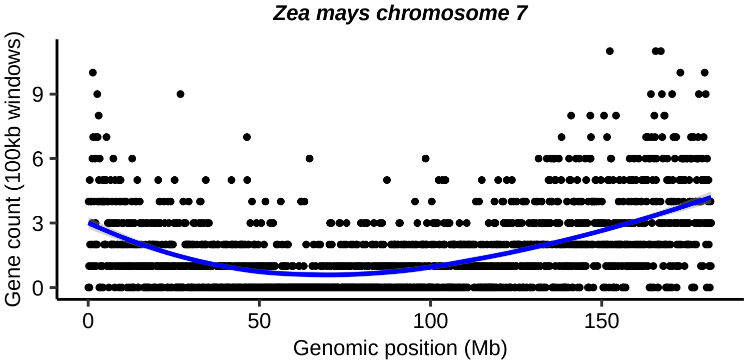

Zea mays chromosome 7
Gene count (100kb windows)
0
3
6
9
0
50
100
150
Genomic position (Mb)

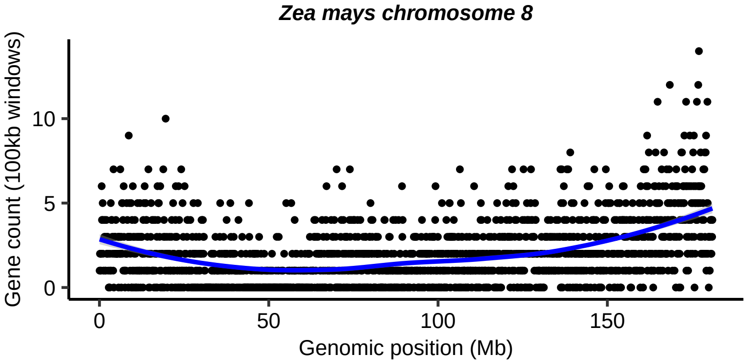

Zea mays chromosome 8
Gene count (100kb windows)
0
5
10
0
50
100
150
Genomic position (Mb)

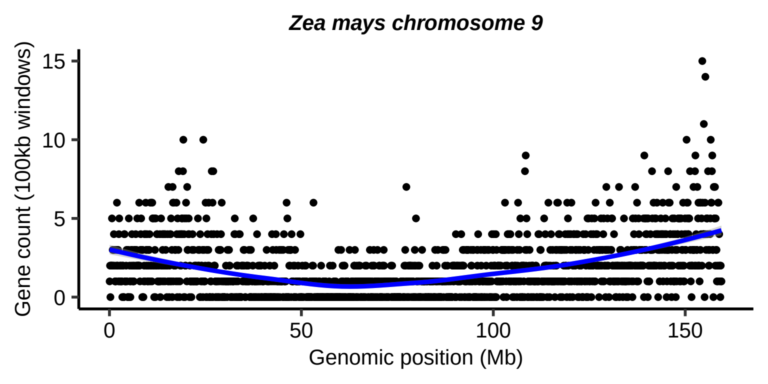
Zea mays chromosome 9
Gene count (100kb windows)
15
10
5
0
0
50
100
150
Genomic position (Mb)
