# Supplementary material for: Diversity and determinants of recombination landscapes in flowering plants
Source: PLoS Genet. 2022 Aug 30;18(8):e1010141. doi: 10.1371/journal.pgen.1010141 (PMC9467342; doi:10.1371/journal.pgen.1010141)
Supplement: S12 Fig — The black dashed line is a theoretical uniform distribution of markers. The black vertical line is the centromere position estimated by cytological measures, when available in the literature. (PDF) [file pgen.1010141.s012.pdf]

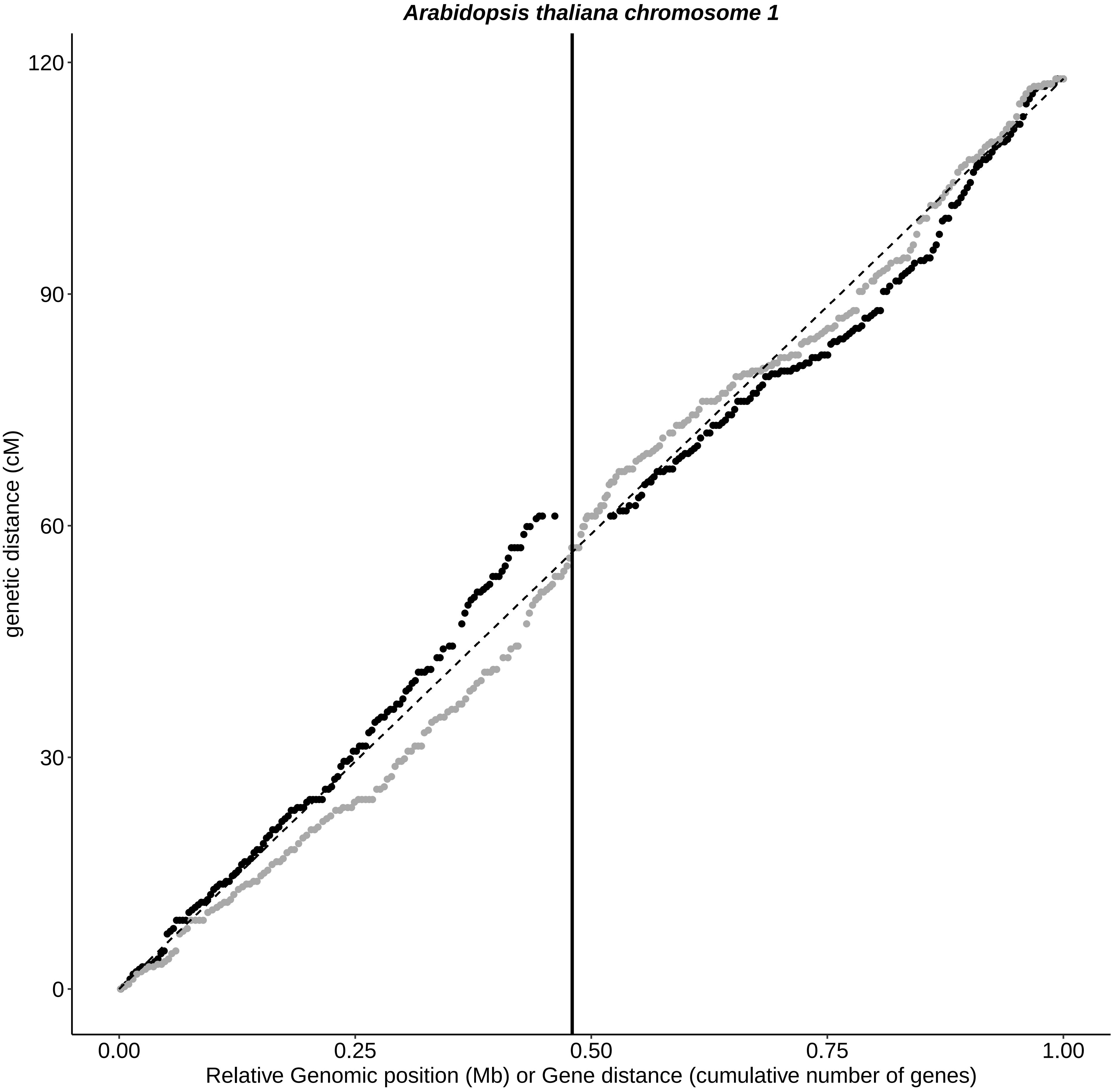
Arabidopsis thaliana chromosome 1
genetic distance (cM)
120
90
60
30
0
0.00
0.25
0.50
0.75
1.00
Relative Genomic position (Mb) or Gene distance (cumulative number of genes)

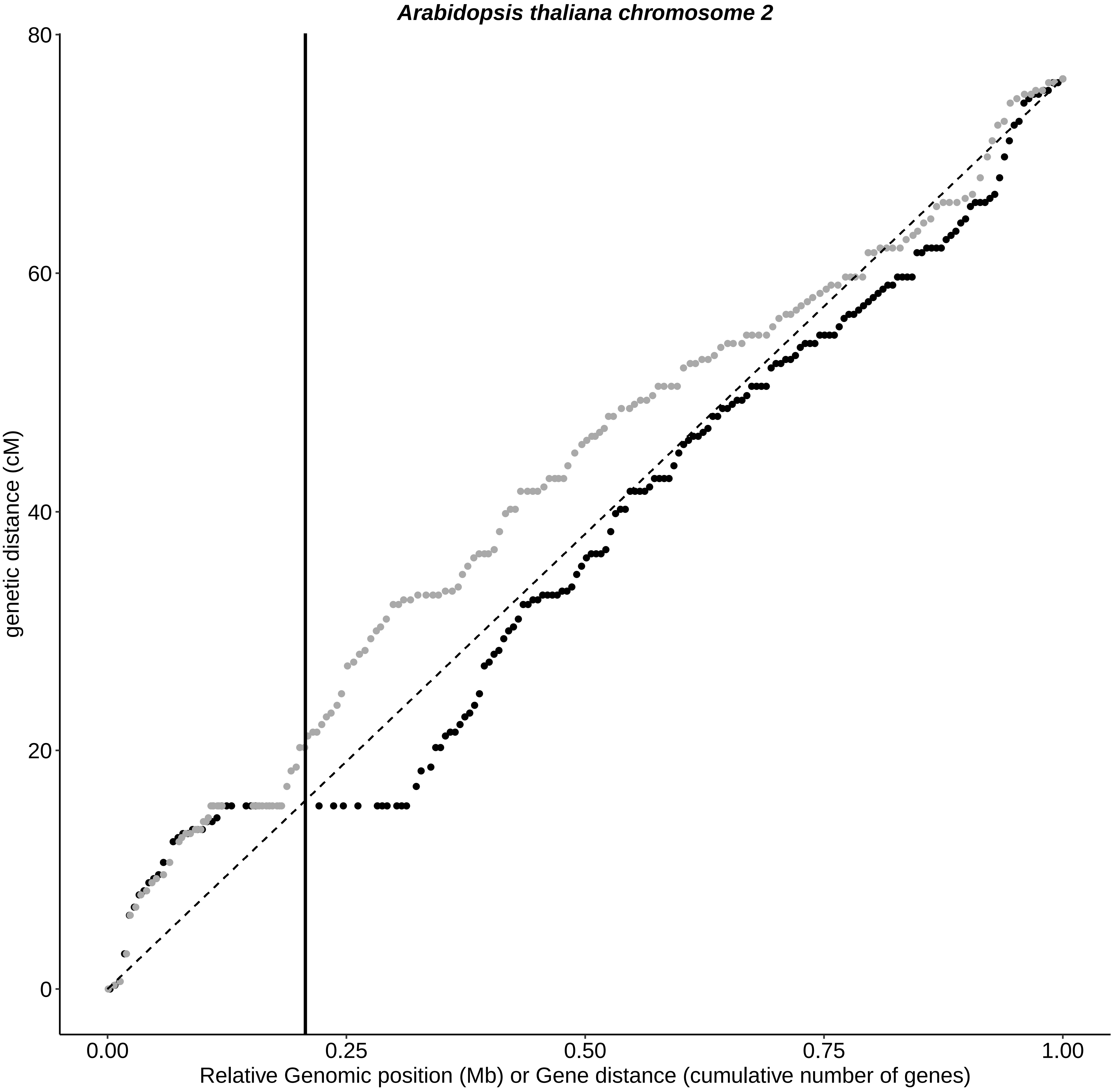
Arabidopsis thaliana chromosome 2
genetic distance (cM)
0
20
40
60
80
0.00
0.25
0.50
0.75
1.00
Relative Genomic position (Mb) or Gene distance (cumulative number of genes)

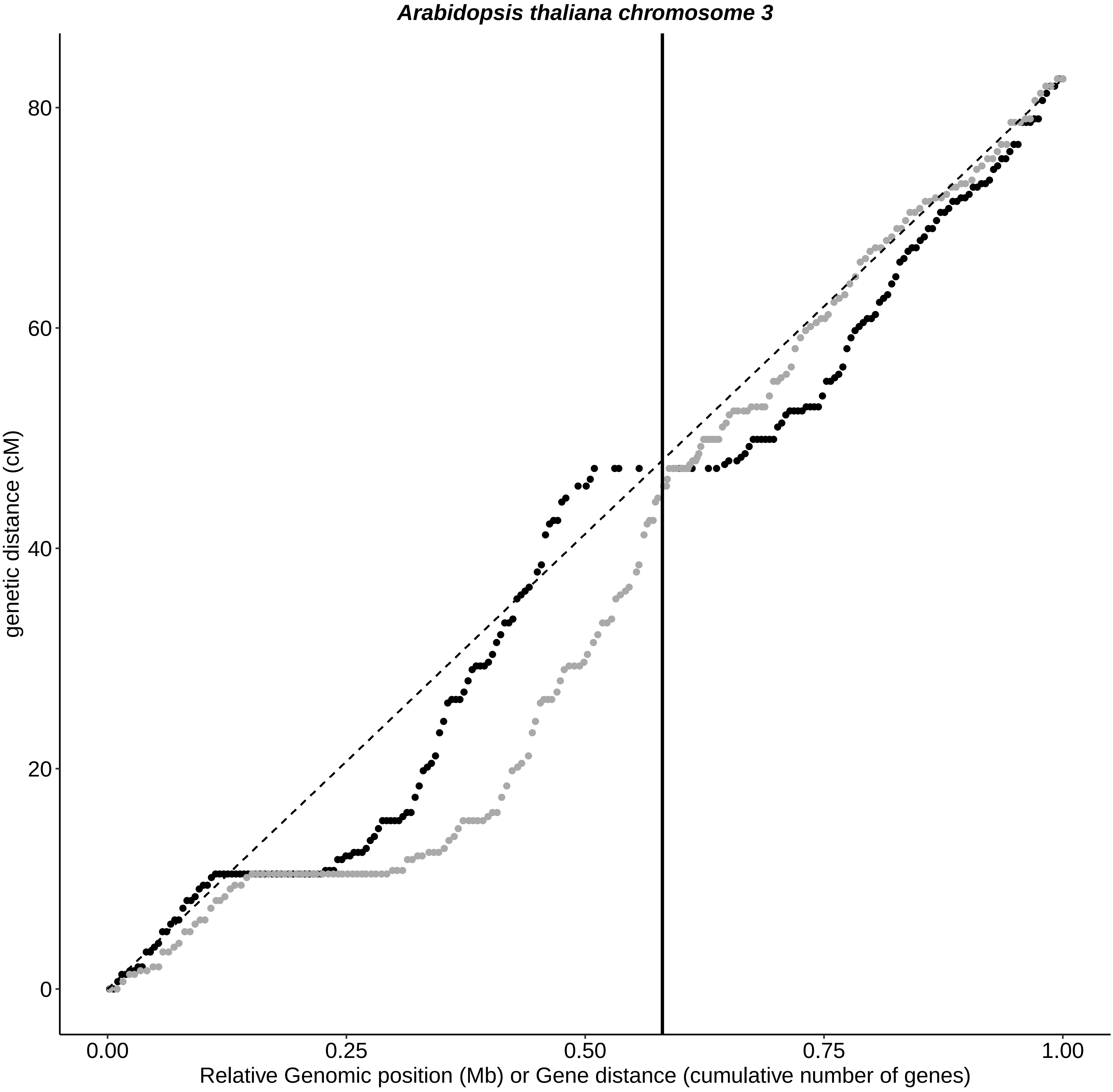
Arabidopsis thaliana chromosome 3
genetic distance (cM)
0
20
40
60
80
0.00
0.25
0.50
0.75
1.00
Relative Genomic position (Mb) or Gene distance (cumulative number of genes)

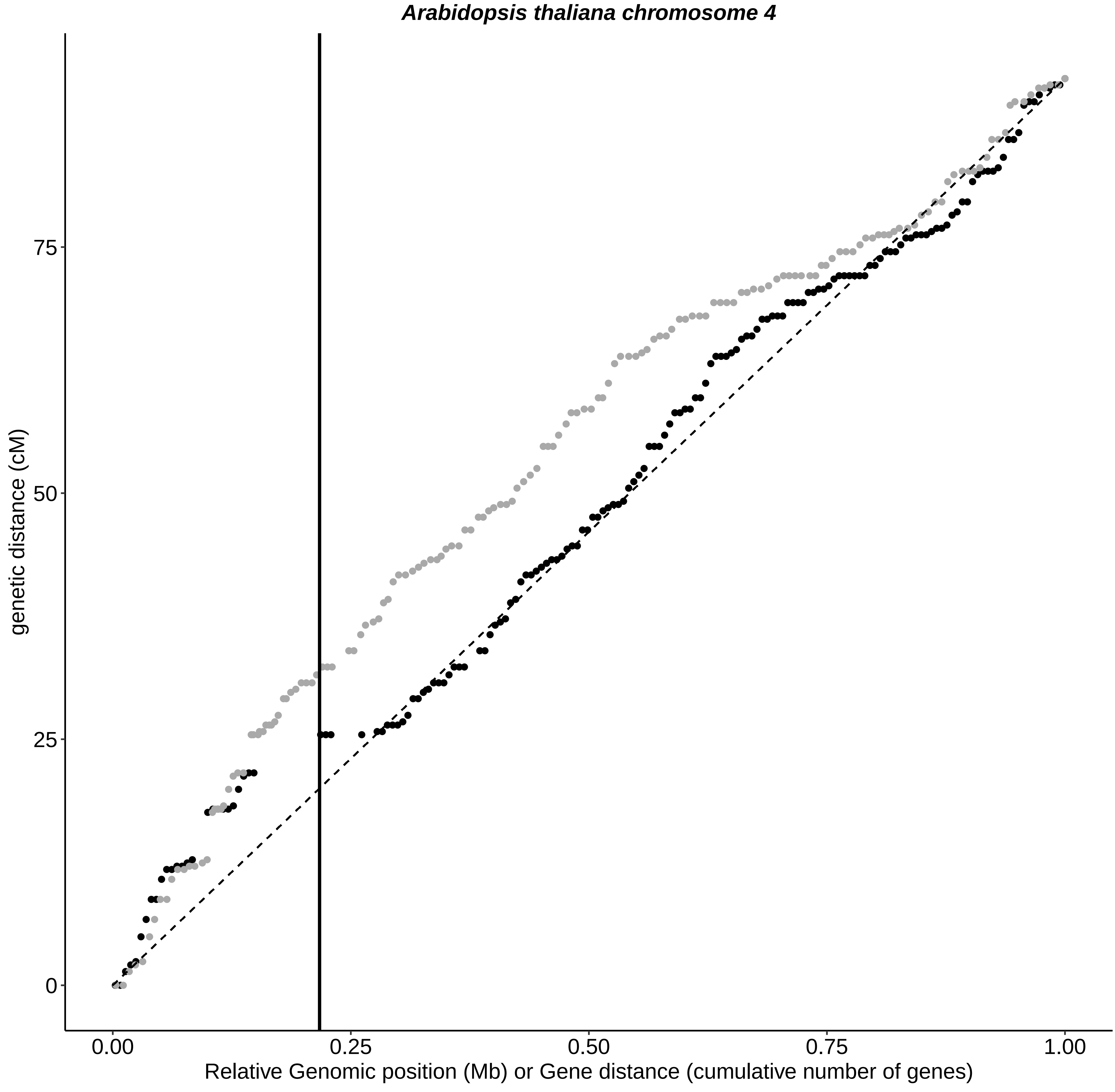
Arabidopsis thaliana chromosome 4
genetic distance (cM)
0
25
50
75
0.00
0.25
0.50
0.75
1.00
Relative Genomic position (Mb) or Gene distance (cumulative number of genes)

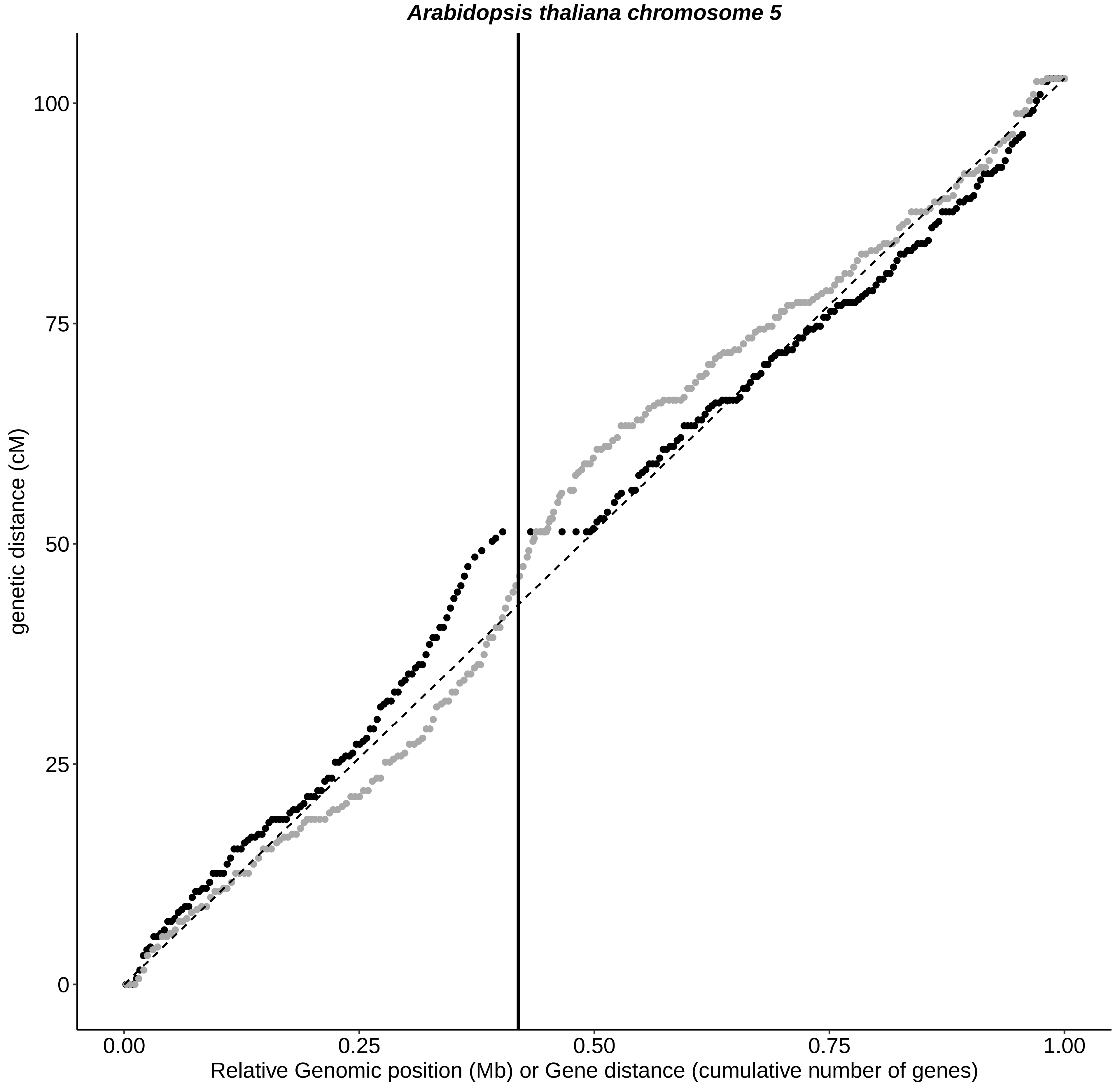
Arabidopsis thaliana chromosome 5
genetic distance (cM)
0
25
50
75
100
0.00
0.25
0.50
0.75
1.00
Relative Genomic position (Mb) or Gene distance (cumulative number of genes)

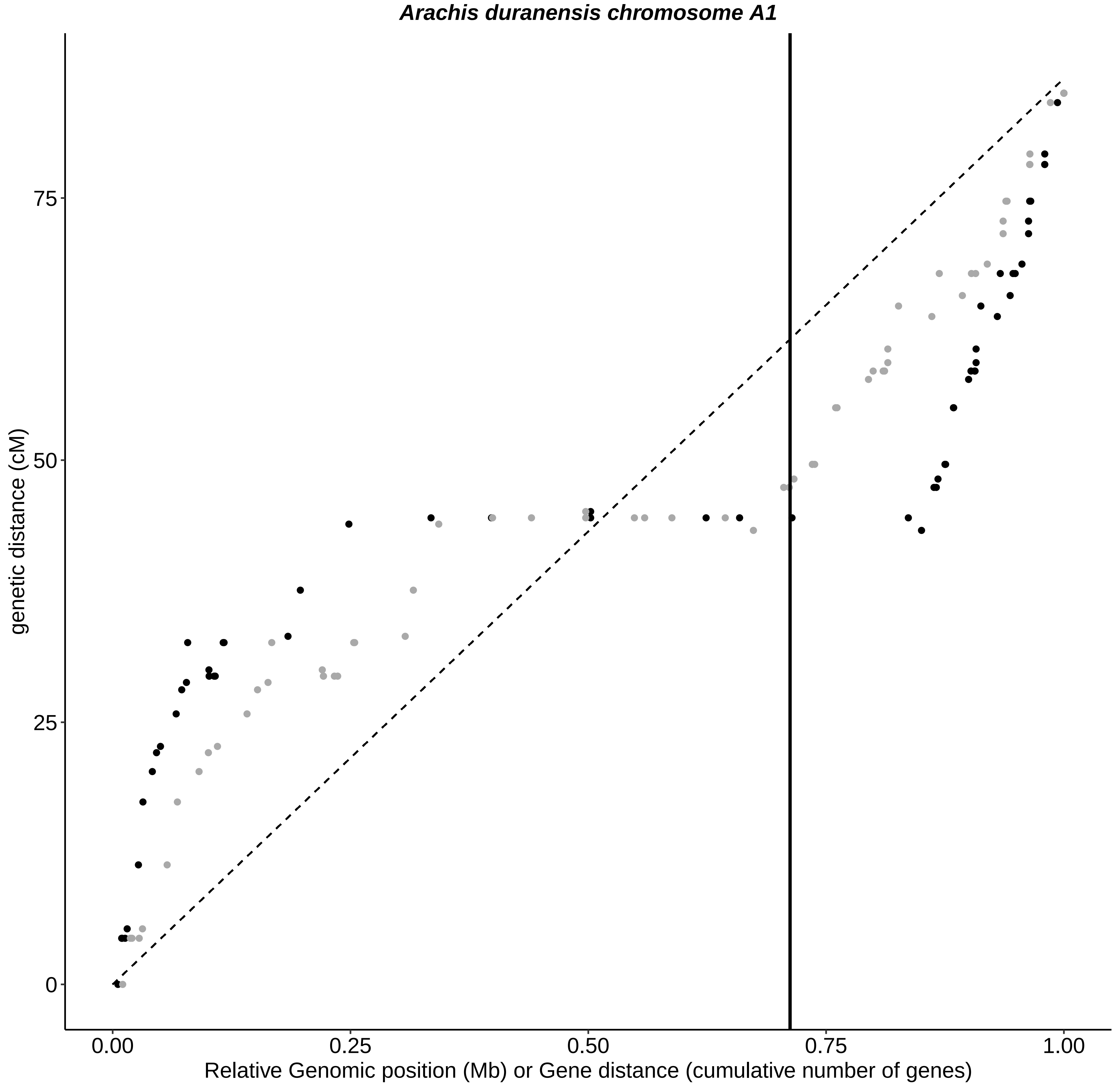
Arachis duranensis chromosome A1
genetic distance (cM)
0
25
50
75
0.00
0.25
0.50
0.75
1.00
Relative Genomic position (Mb) or Gene distance (cumulative number of genes)

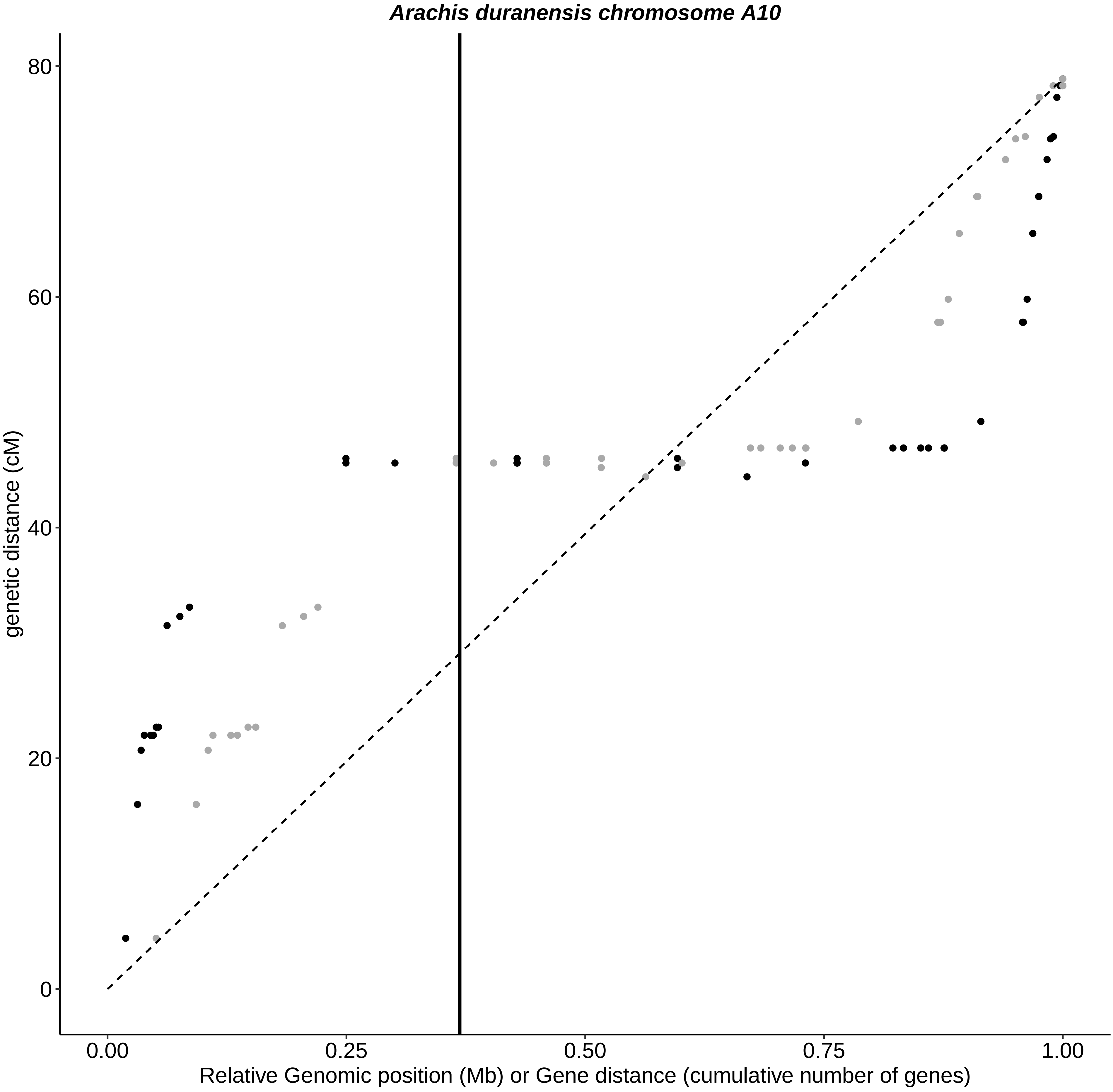
Arachis duranensis chromosome A10
genetic distance (cM)
0
20
40
60
80
0.00
0.25
0.50
0.75
1.00
Relative Genomic position (Mb) or Gene distance (cumulative number of genes)

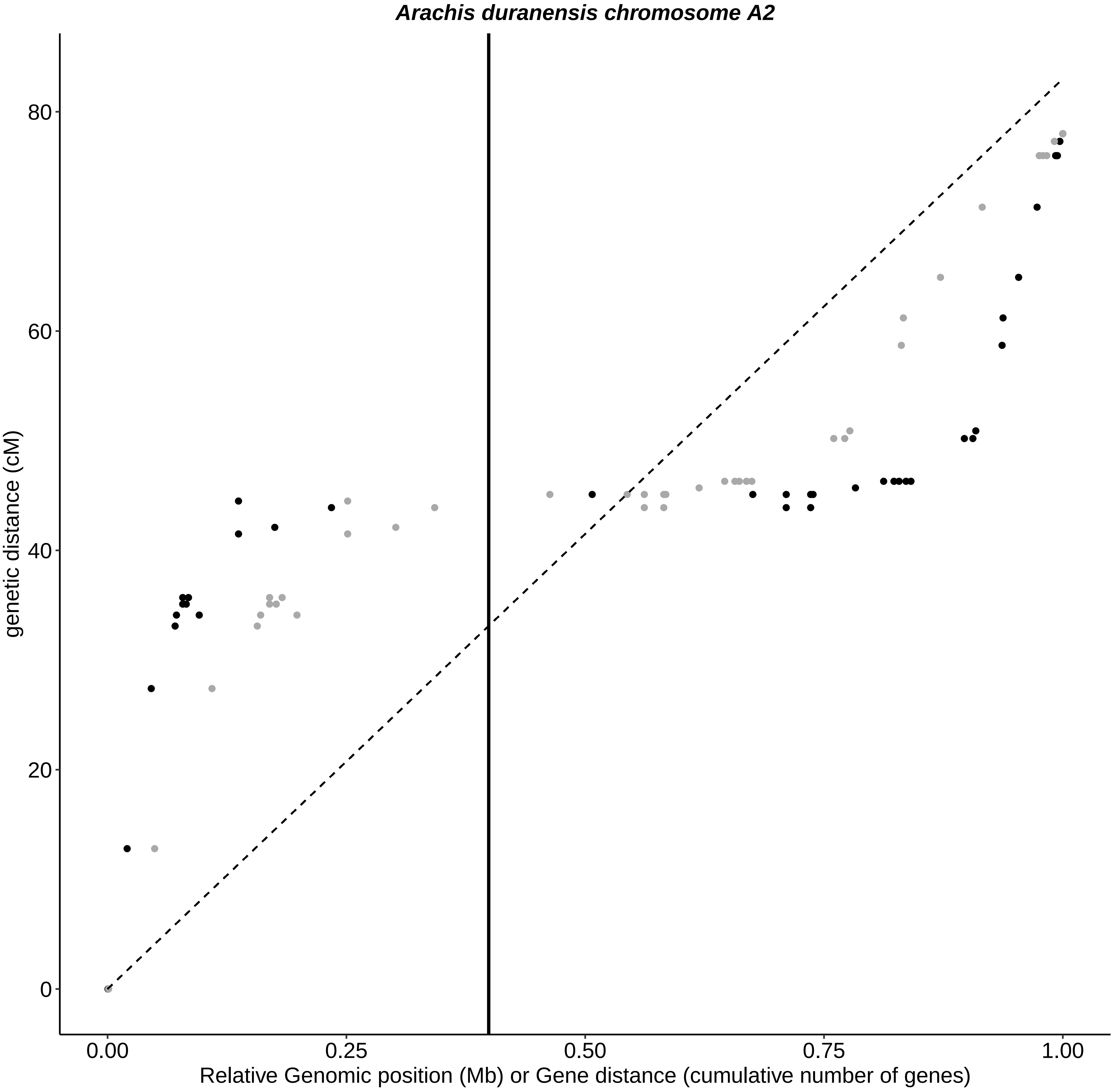
Arachis duranensis chromosome A2
genetic distance (cM)
80
60
40
20
0
0.00
0.25
0.50
0.75
1.00
Relative Genomic position (Mb) or Gene distance (cumulative number of genes)

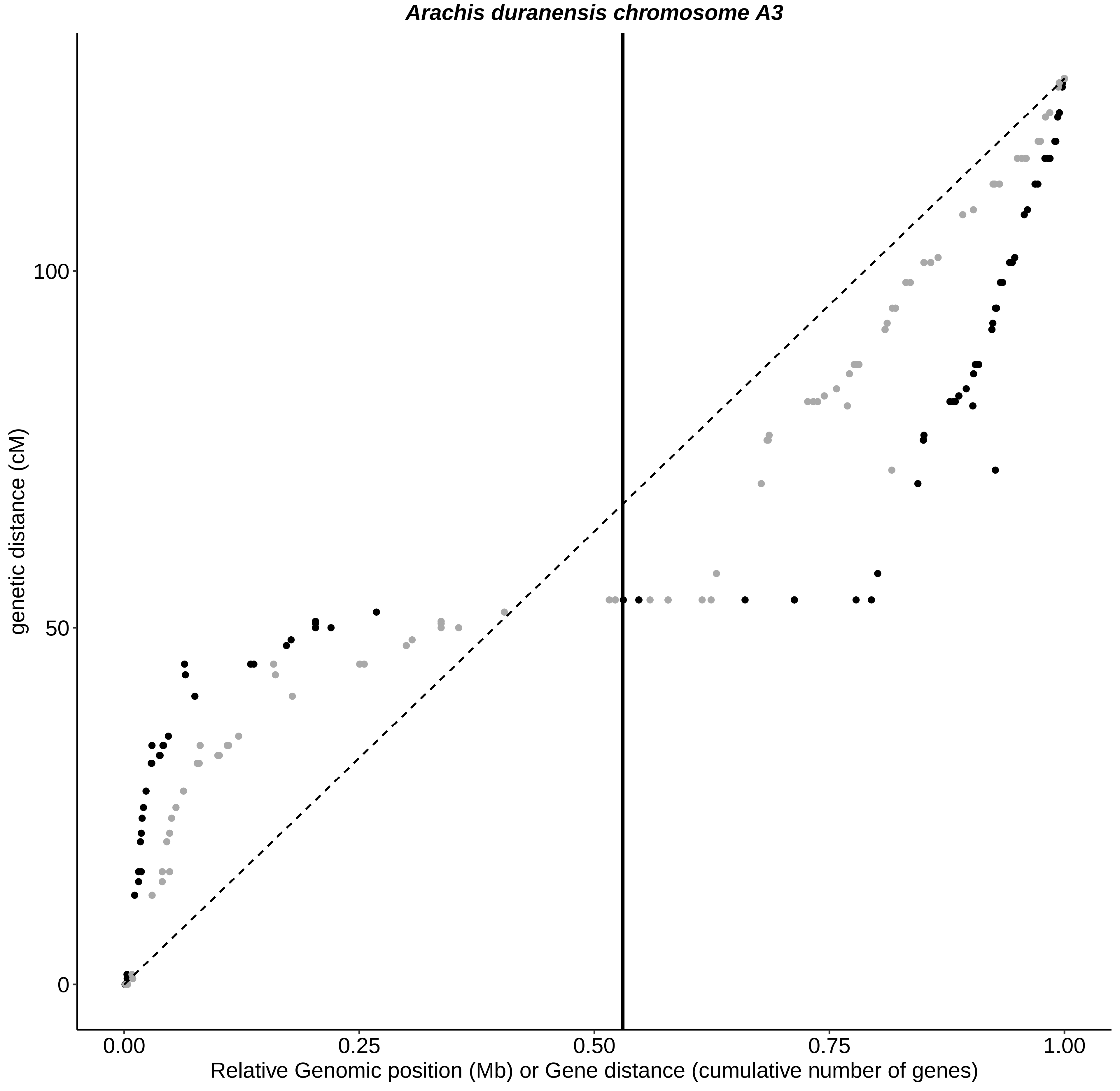
Arachis duranensis chromosome A3
genetic distance (cM)
100
50
0
0.00
0.25
0.50
0.75
1.00
Relative Genomic position (Mb) or Gene distance (cumulative number of genes)

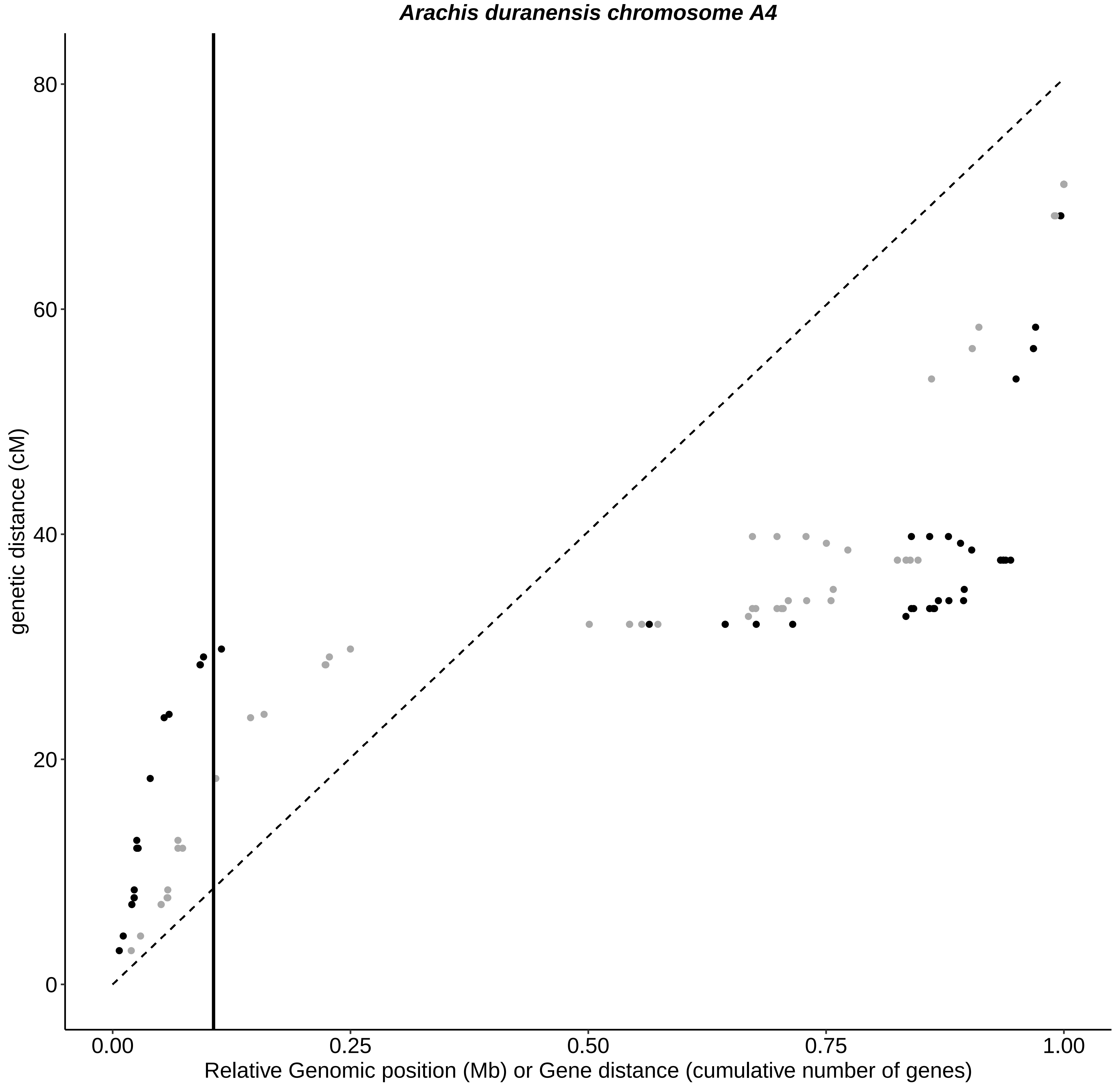
Arachis duranensis chromosome A4
genetic distance (cM)
0
20
40
60
80
0.00
0.25
0.50
0.75
1.00
Relative Genomic position (Mb) or Gene distance (cumulative number of genes)

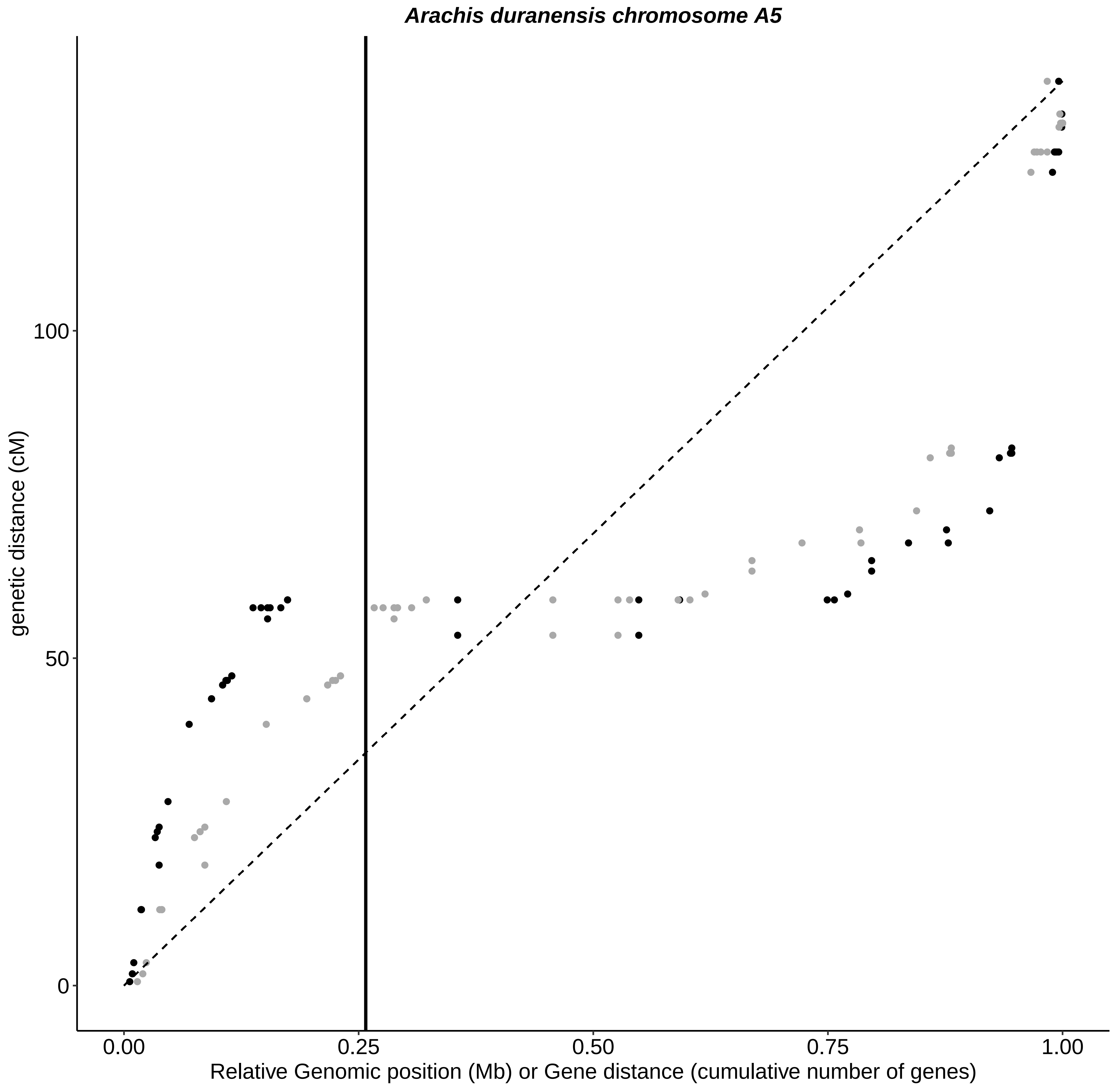
Arachis duranensis chromosome A5
genetic distance (cM)
100
50
0
0.00
0.25
0.50
0.75
1.00
Relative Genomic position (Mb) or Gene distance (cumulative number of genes)

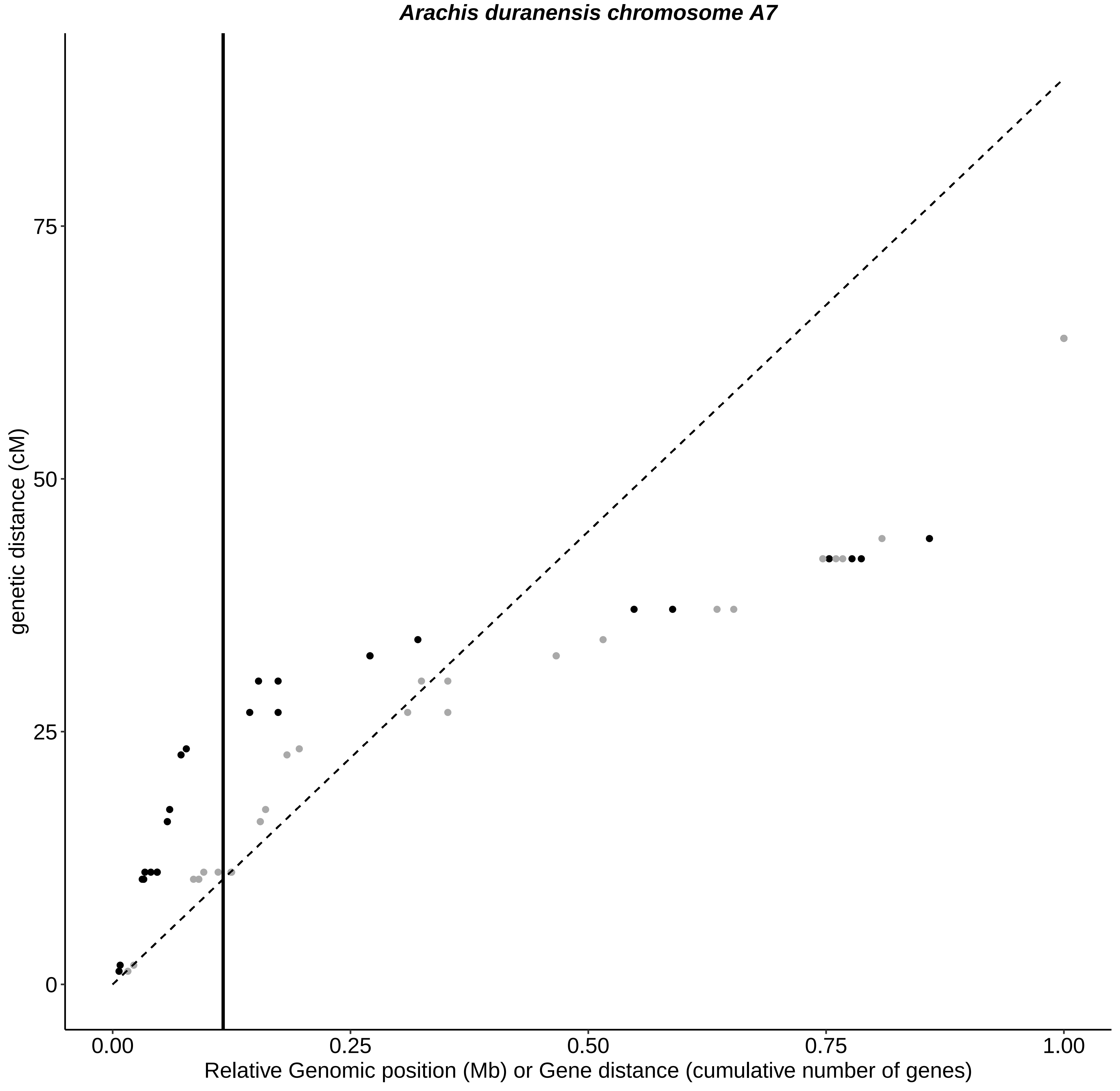
Arachis duranensis chromosome A7
genetic distance (cM)
0
25
50
75
0.00
0.25
0.50
0.75
1.00
Relative Genomic position (Mb) or Gene distance (cumulative number of genes)

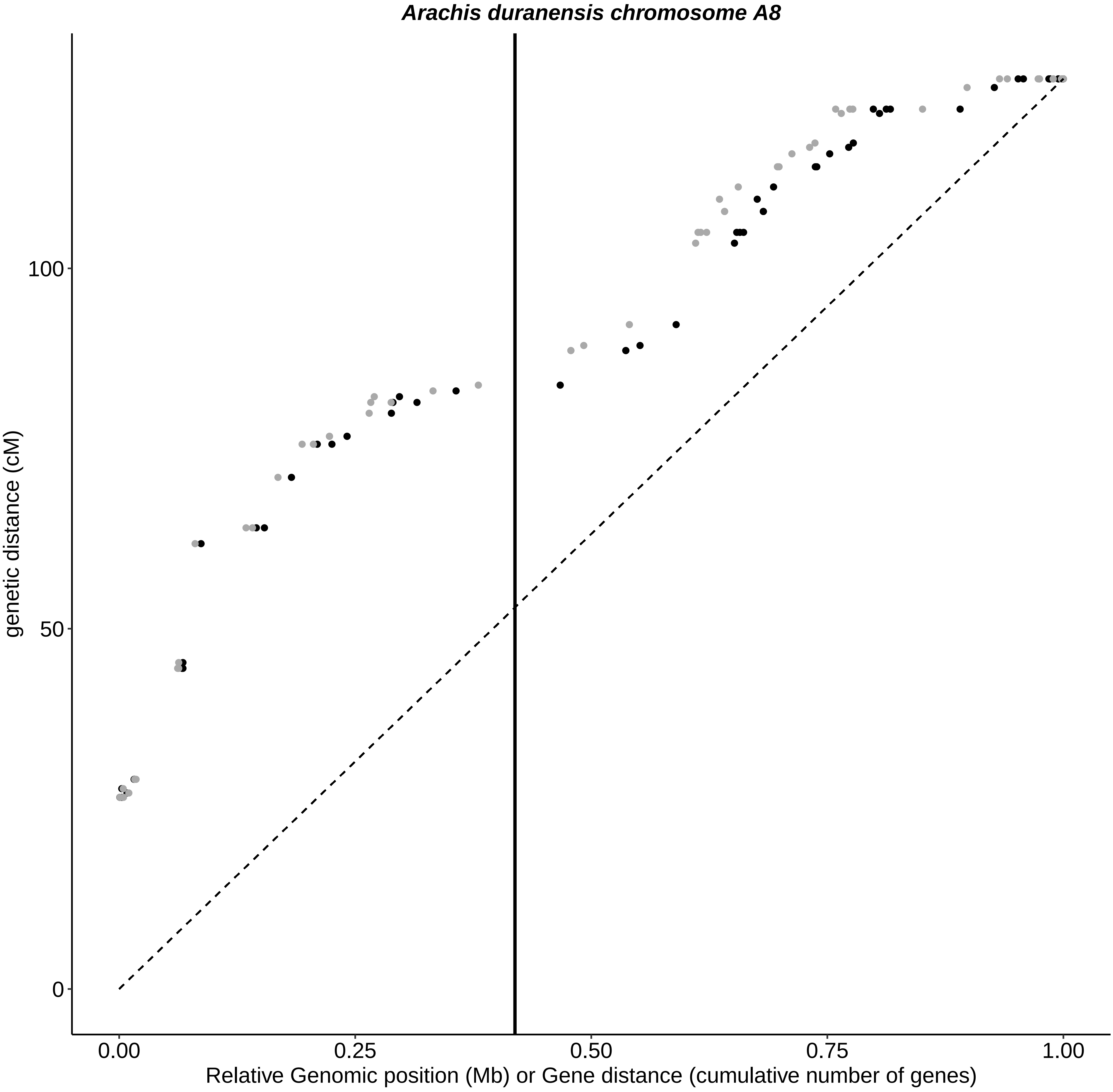
Arachis duranensis chromosome A8
genetic distance (cM)
100
50
0
0.00
0.25
0.50
0.75
1.00
Relative Genomic position (Mb) or Gene distance (cumulative number of genes)

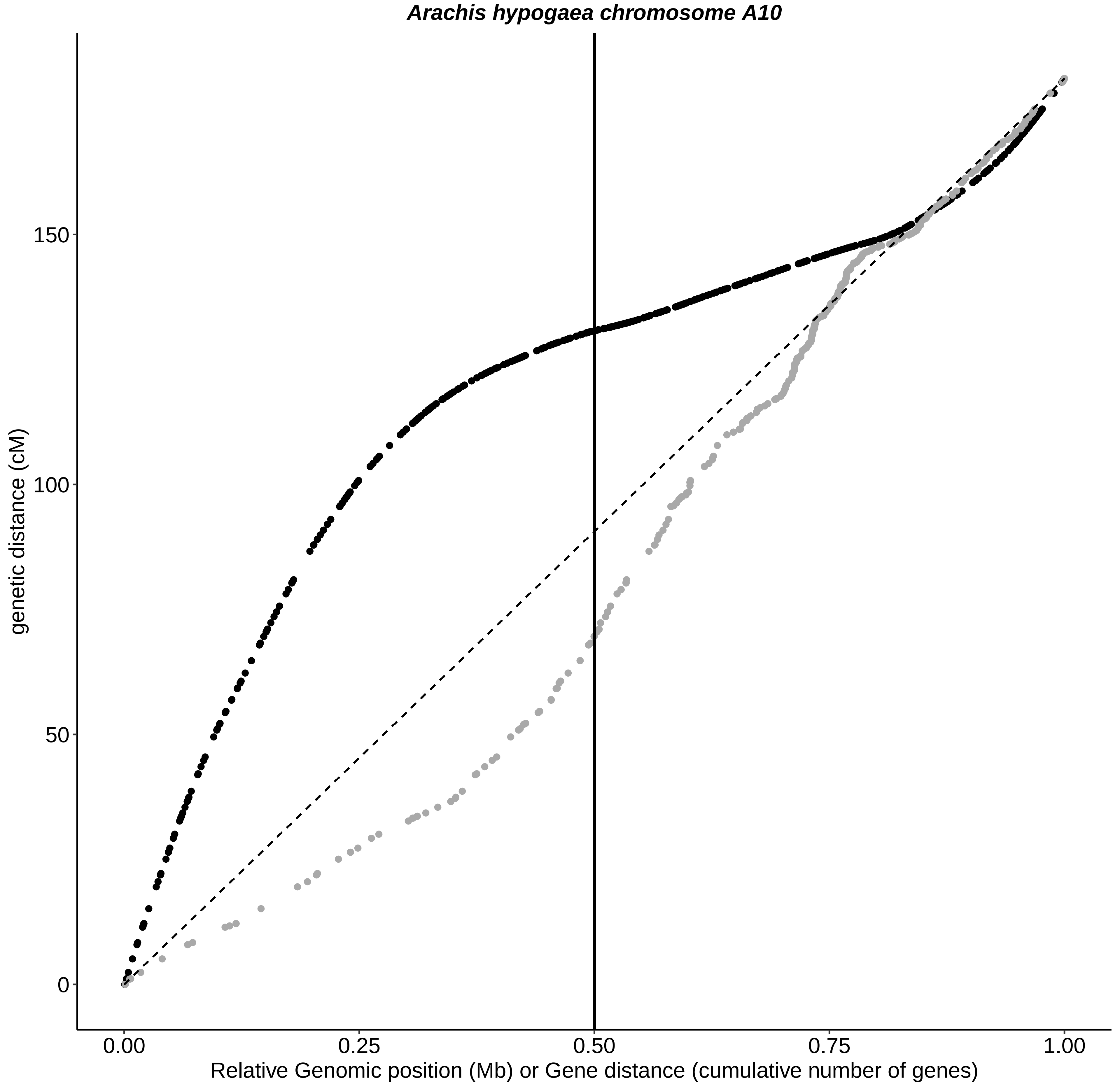
Arachis hypogaea chromosome A10
genetic distance (cM)
0
50
100
150
0.00
0.25
0.50
0.75
1.00
Relative Genomic position (Mb) or Gene distance (cumulative number of genes)

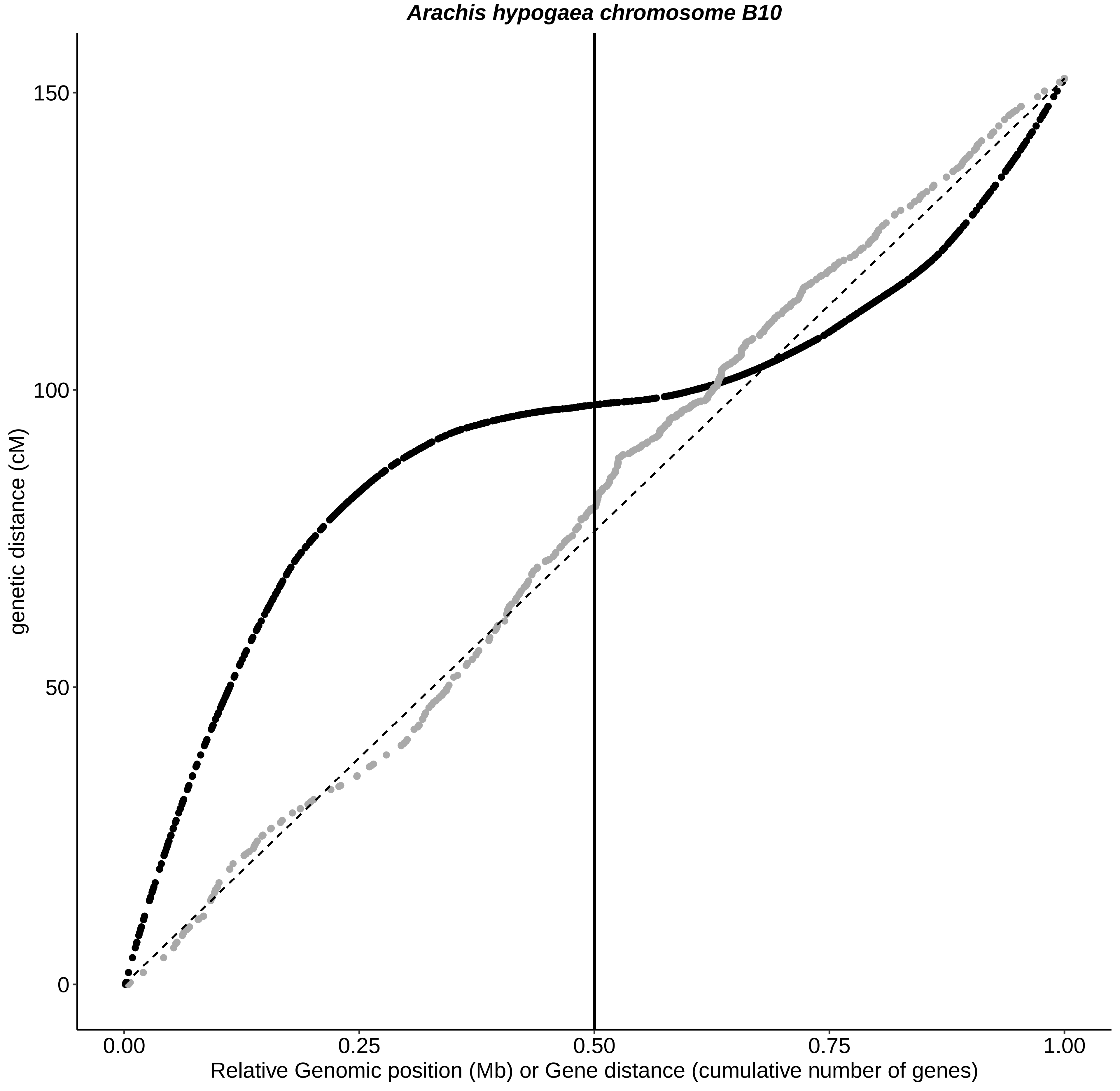
Arachis hypogaea chromosome B10
genetic distance (cM)
150
100
50
0
0.00
0.25
0.50
0.75
1.00
Relative Genomic position (Mb) or Gene distance (cumulative number of genes)

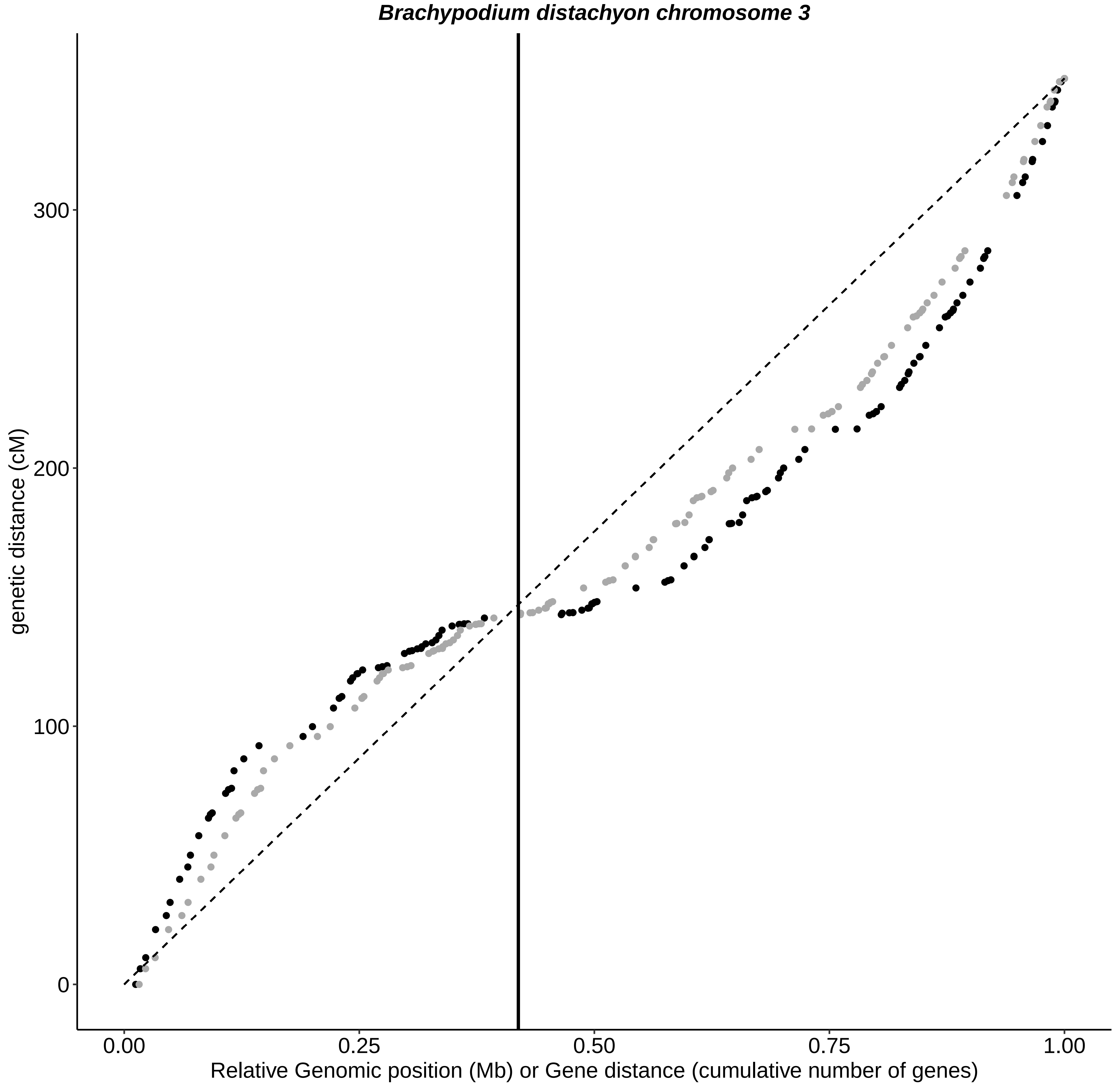
Brachypodium distachyon chromosome 3
genetic distance (cM)
0
100
200
300
0.00
0.25
0.50
0.75
1.00
Relative Genomic position (Mb) or Gene distance (cumulative number of genes)

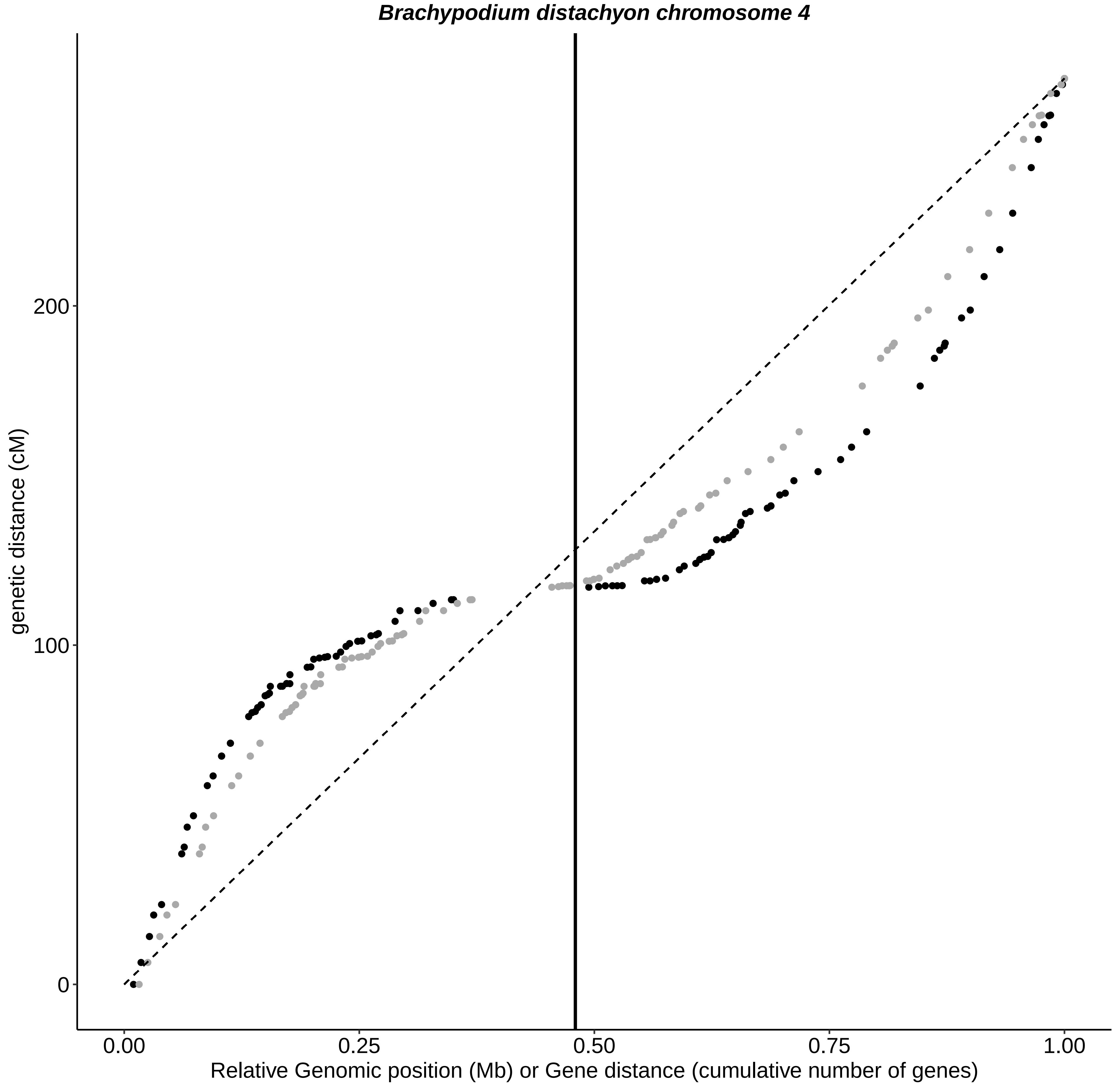
Brachypodium distachyon chromosome 4
genetic distance (cM)
0
100
200
0.00
0.25
0.50
0.75
1.00
Relative Genomic position (Mb) or Gene distance (cumulative number of genes)

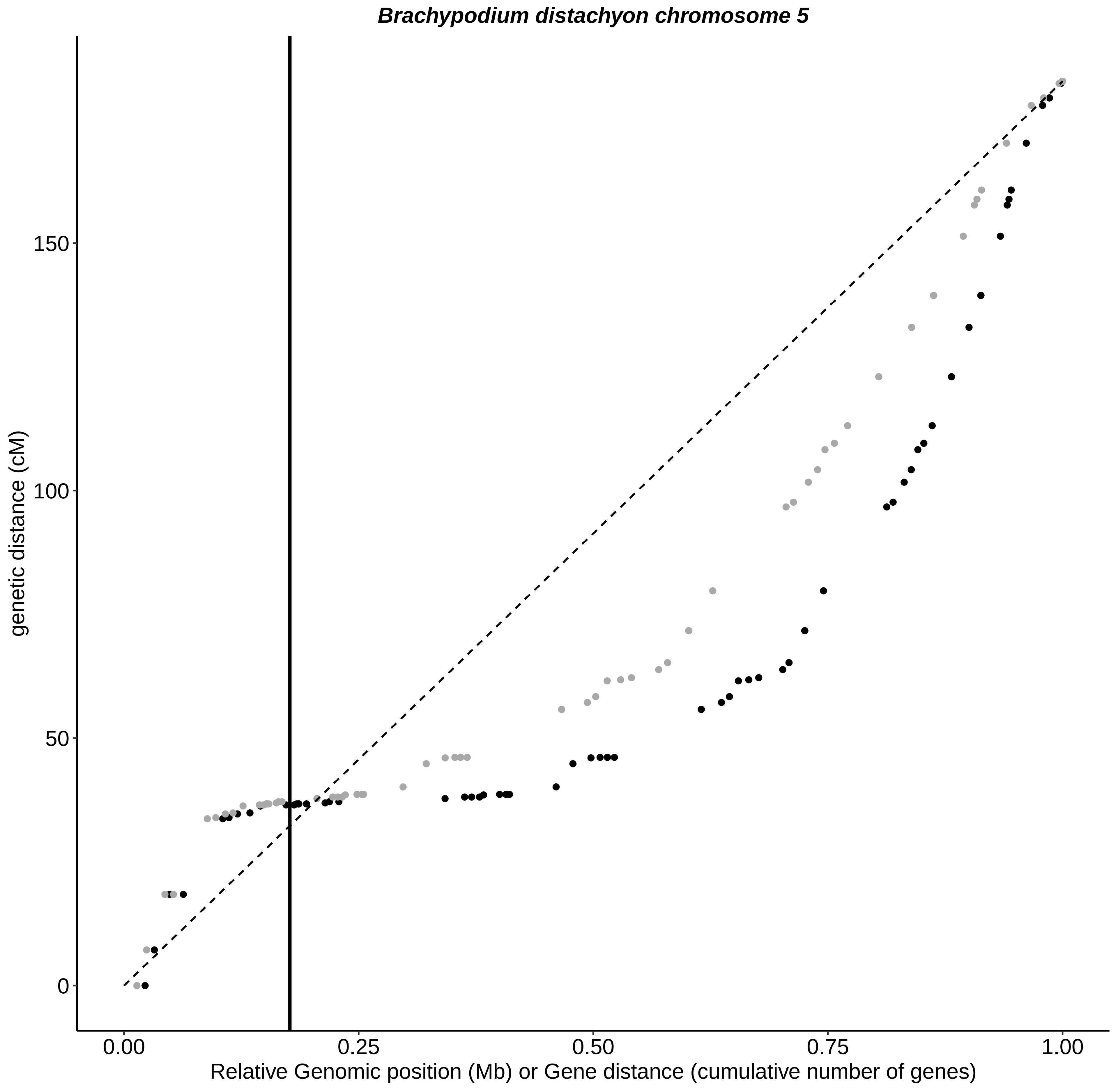
Brachypodium distachyon chromosome 5
genetic distance (cM)
0
50
100
150
0.00
0.25
0.50
0.75
1.00
Relative Genomic position (Mb) or Gene distance (cumulative number of genes)

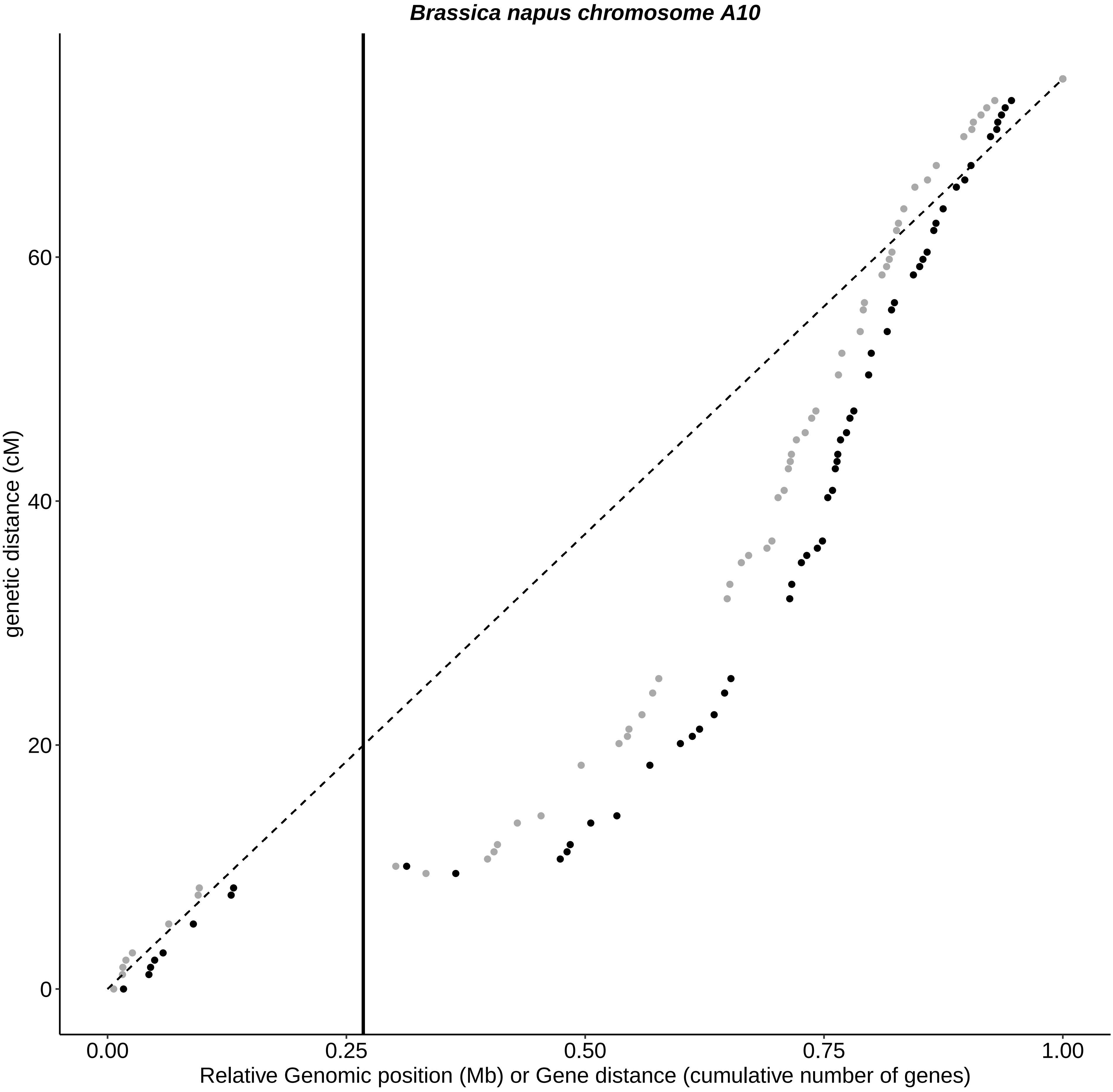
Brassica napus chromosome A10
genetic distance (cM)
0
20
40
60
0.00
0.25
0.50
0.75
1.00
Relative Genomic position (Mb) or Gene distance (cumulative number of genes)

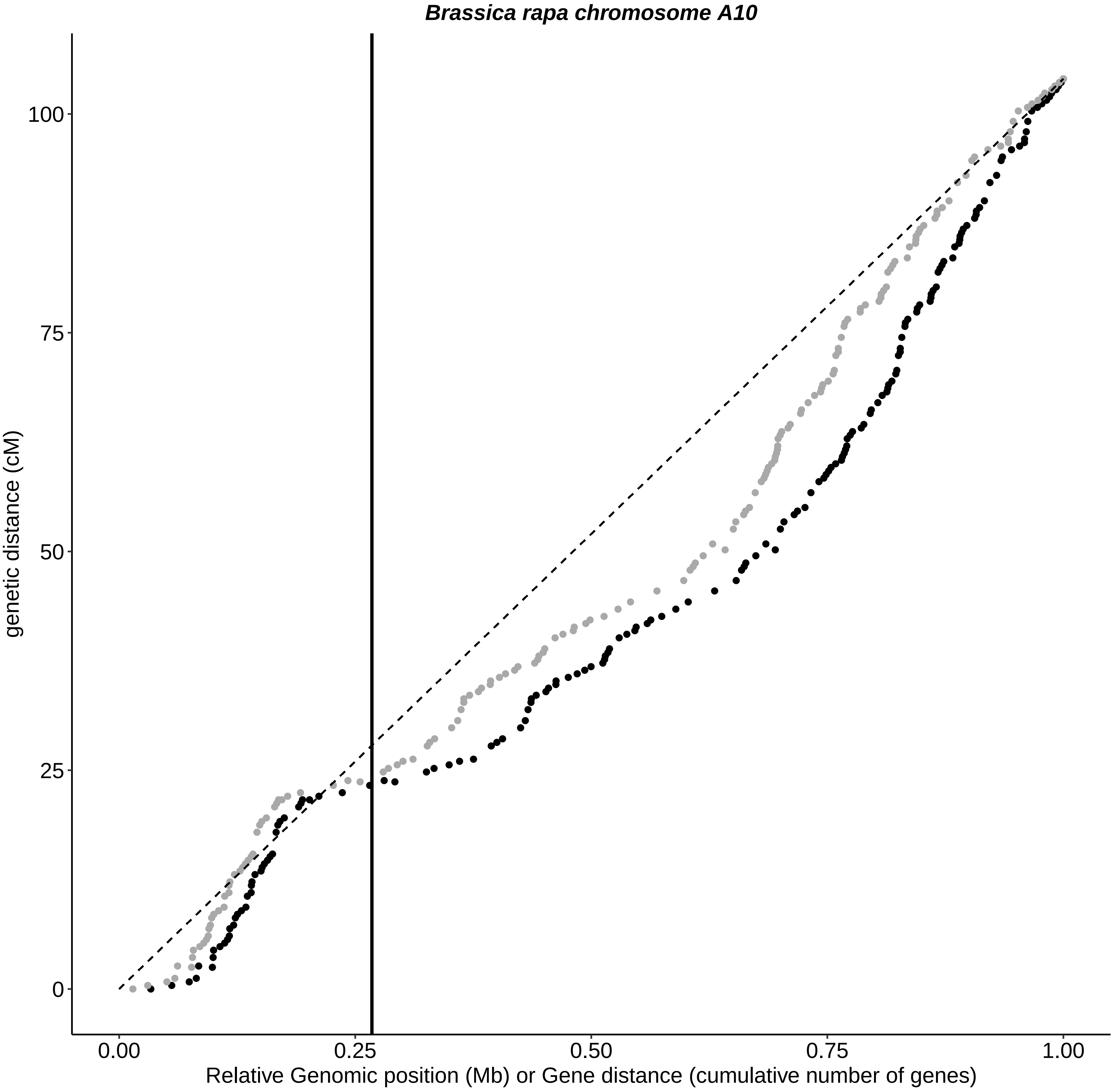
Brassica rapa chromosome A10
genetic distance (cM)
0
25
50
75
100
0.00
0.25
0.50
0.75
1.00
Relative Genomic position (Mb) or Gene distance (cumulative number of genes)

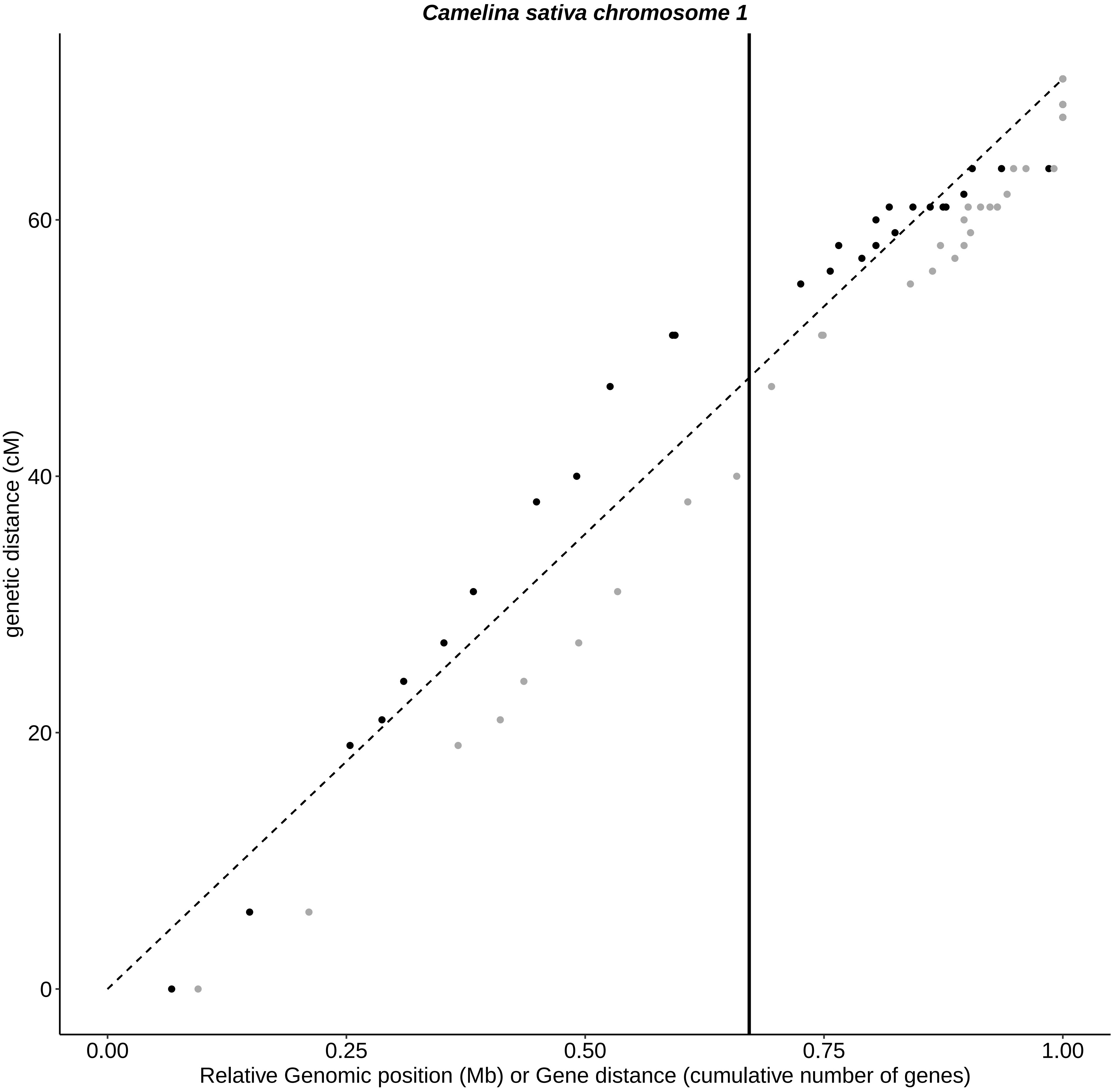
Camelina sativa chromosome 1
genetic distance (cM)
0
20
40
60
0.00
0.25
0.50
0.75
1.00
Relative Genomic position (Mb) or Gene distance (cumulative number of genes)

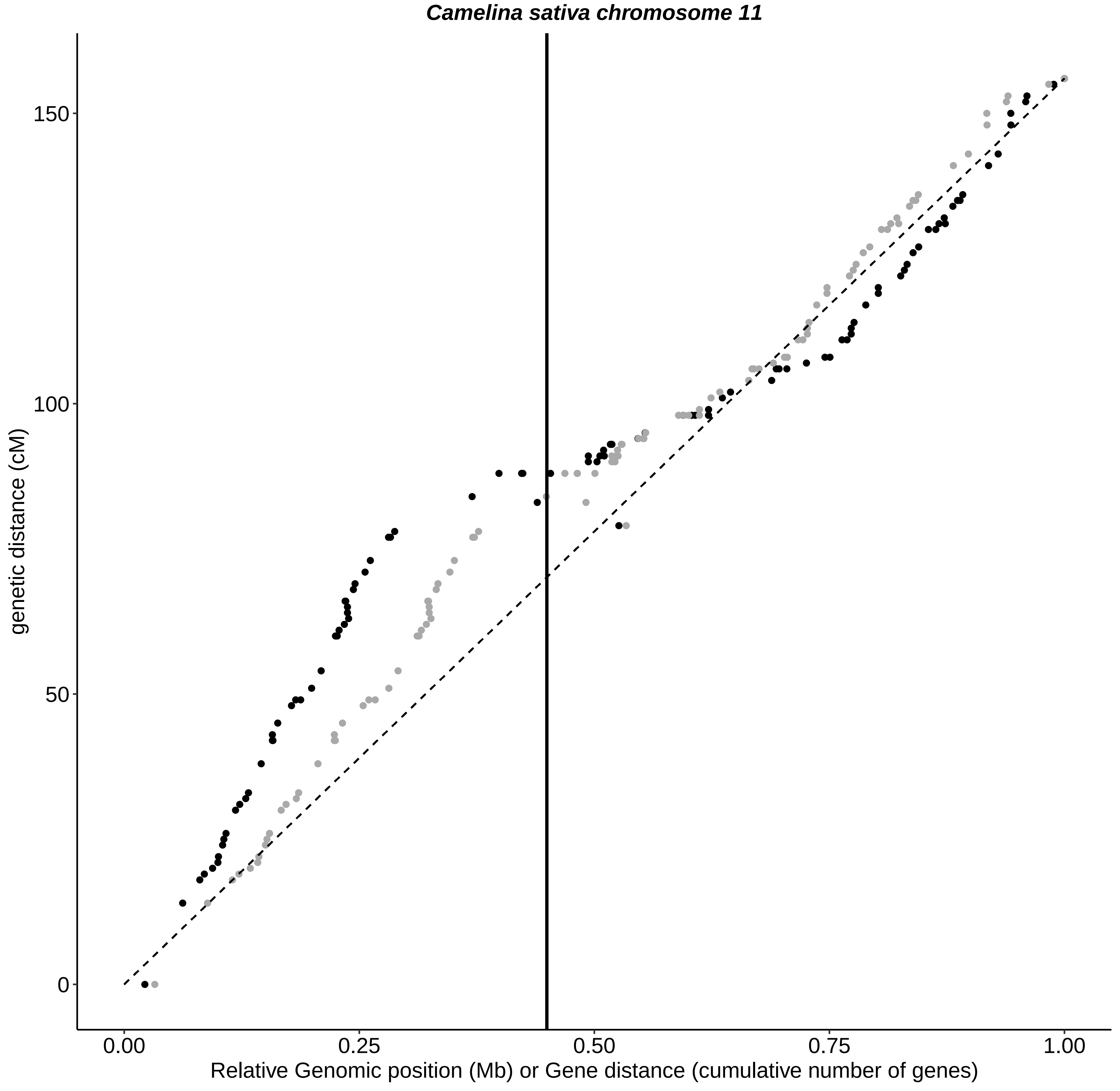
Camelina sativa chromosome 11
genetic distance (cM)
0
50
100
150
0.00
0.25
0.50
0.75
1.00
Relative Genomic position (Mb) or Gene distance (cumulative number of genes)

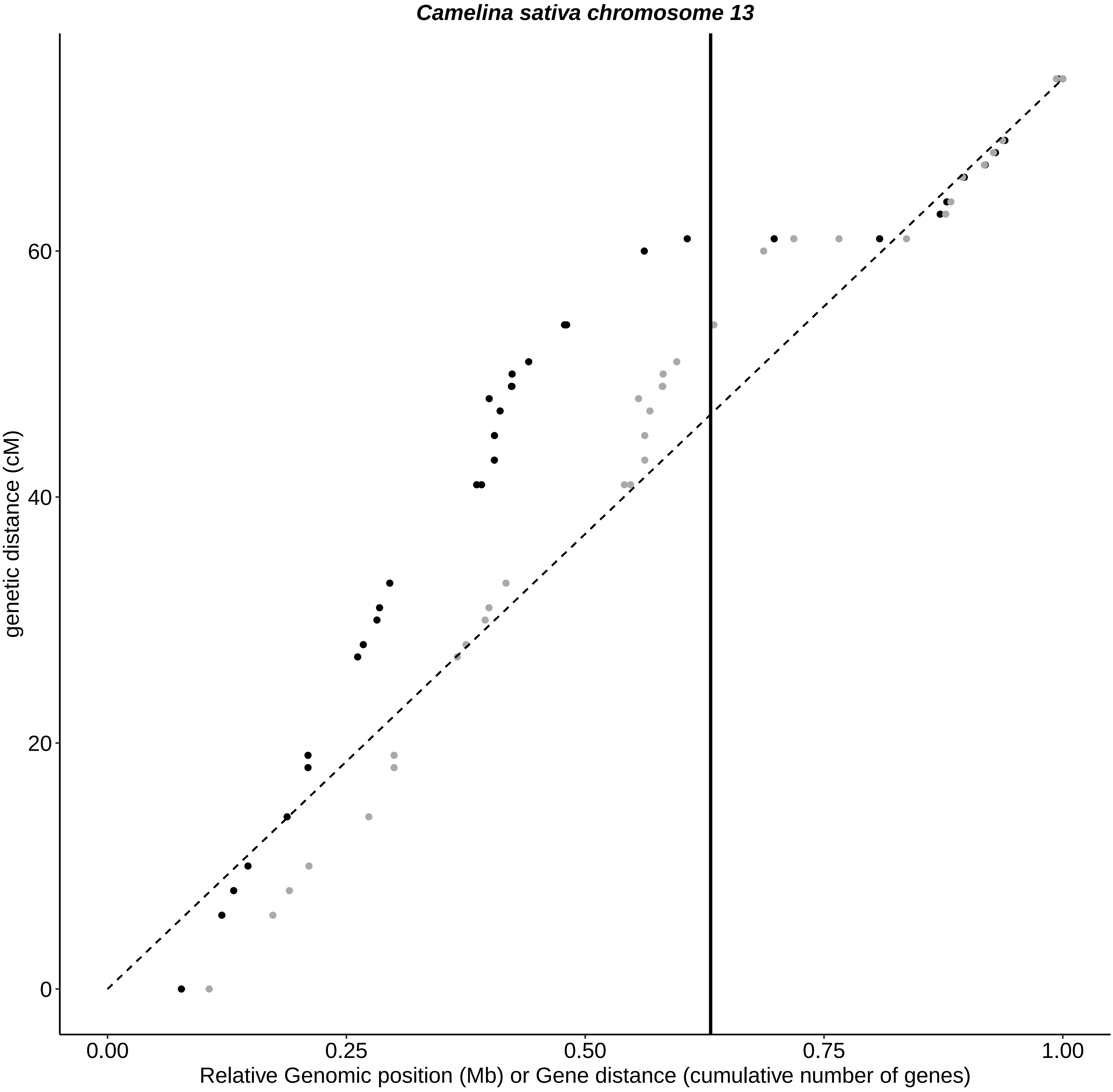
Camelina sativa chromosome 13
genetic distance (cM)
0
20
40
60
0.00
0.25
0.50
0.75
1.00
Relative Genomic position (Mb) or Gene distance (cumulative number of genes)

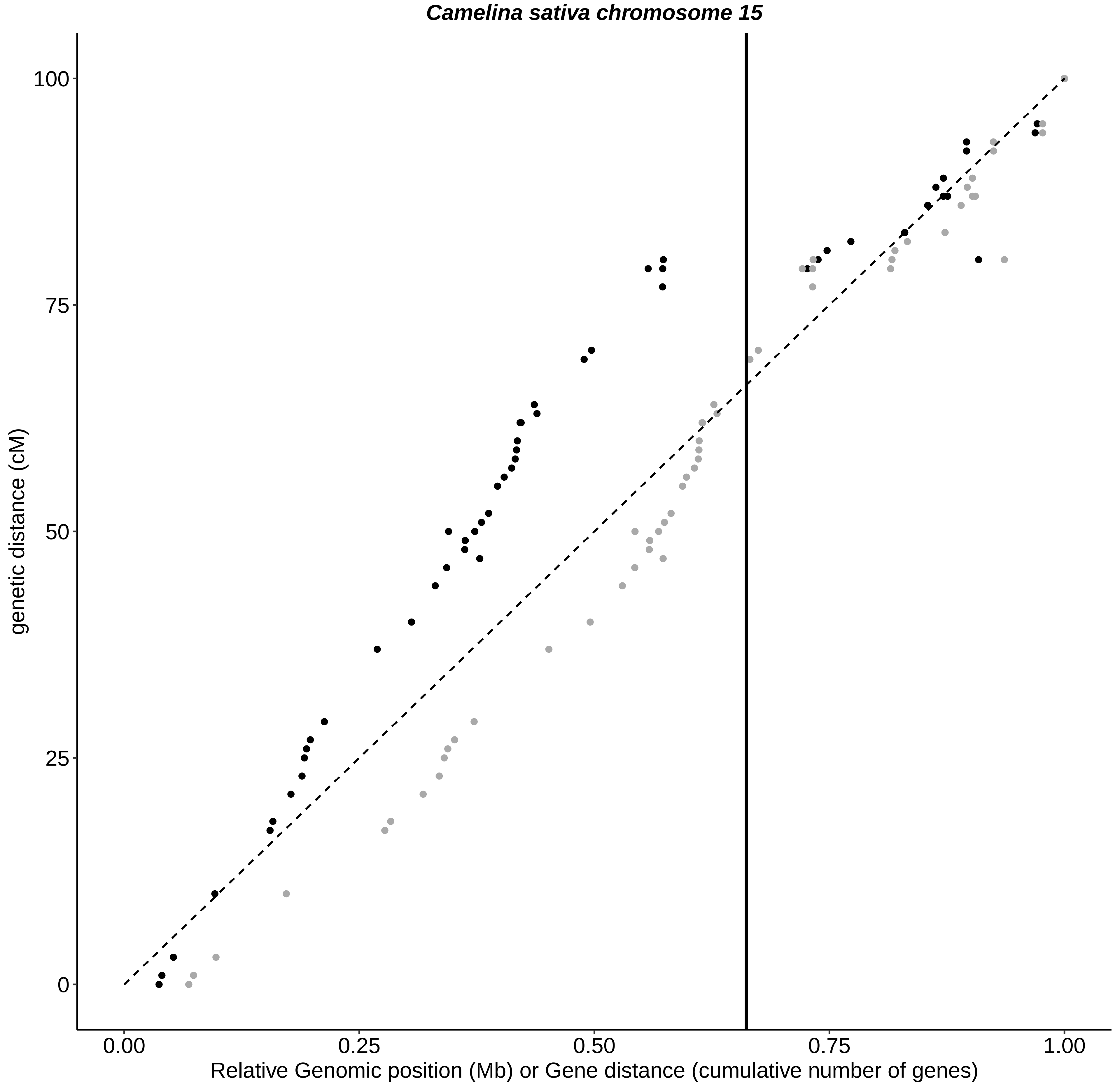
Camelina sativa chromosome 15
genetic distance (cM)
100
75
50
25
0
0.00
0.25
0.50
0.75
1.00
Relative Genomic position (Mb) or Gene distance (cumulative number of genes)

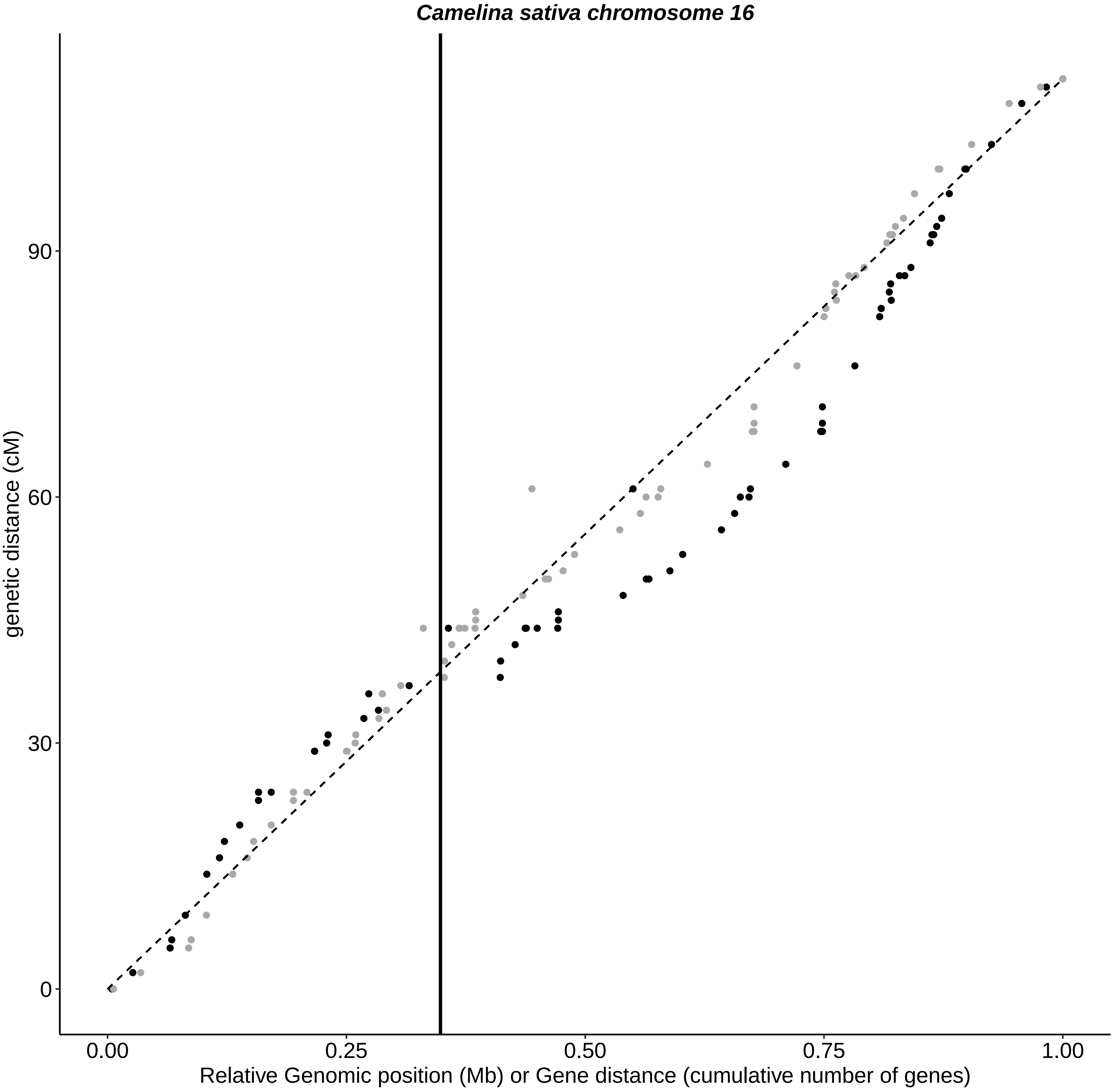
Camelina sativa chromosome 16
genetic distance (cM)
0
30
60
90
0.00
0.25
0.50
0.75
1.00
Relative Genomic position (Mb) or Gene distance (cumulative number of genes)

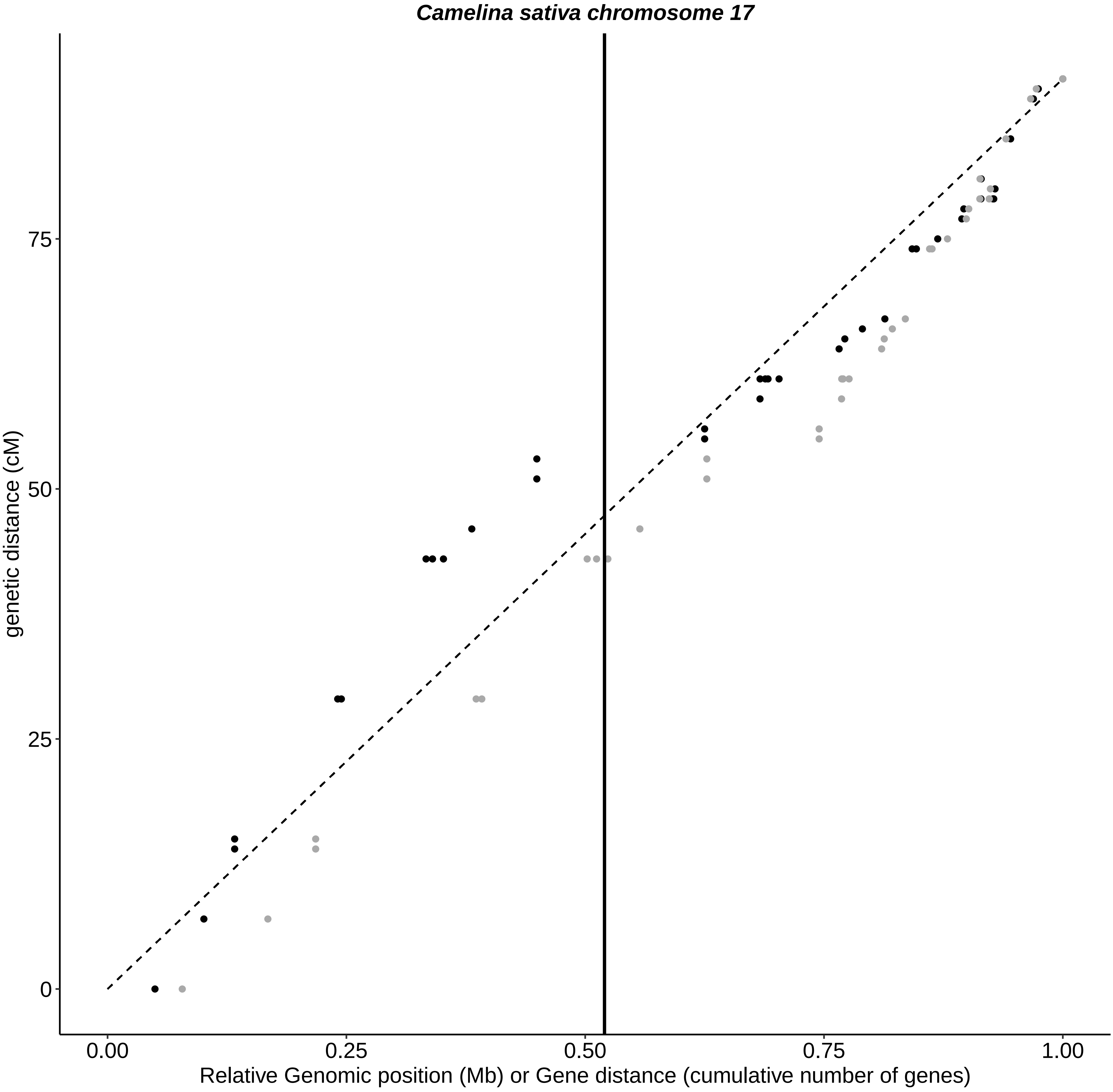
Camelina sativa chromosome 17
genetic distance (cM)
0
25
50
75
0.00
0.25
0.50
0.75
1.00
Relative Genomic position (Mb) or Gene distance (cumulative number of genes)

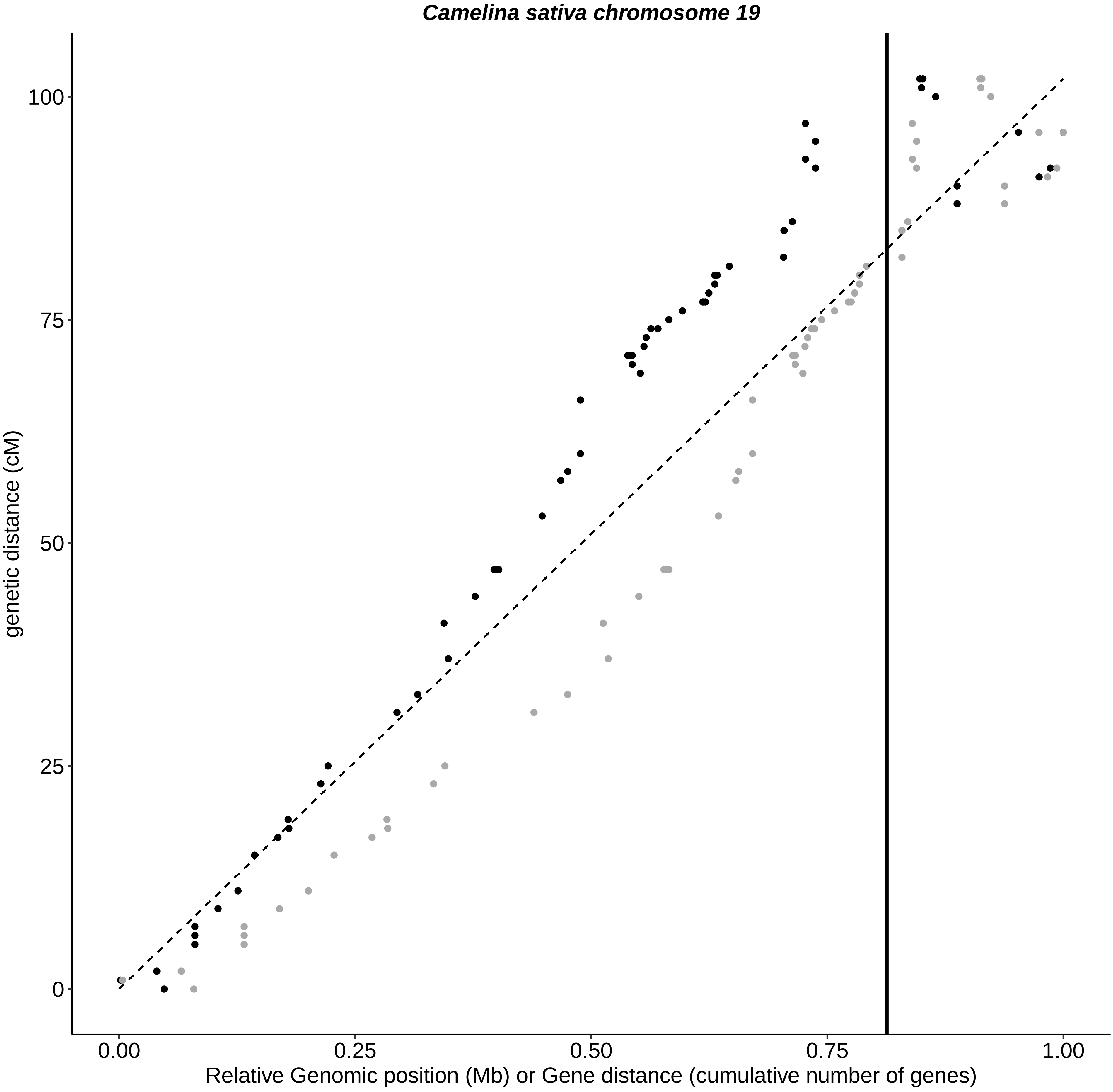
Camelina sativa chromosome 19
genetic distance (cM)
0
25
50
75
100
0.00
0.25
0.50
0.75
1.00
Relative Genomic position (Mb) or Gene distance (cumulative number of genes)

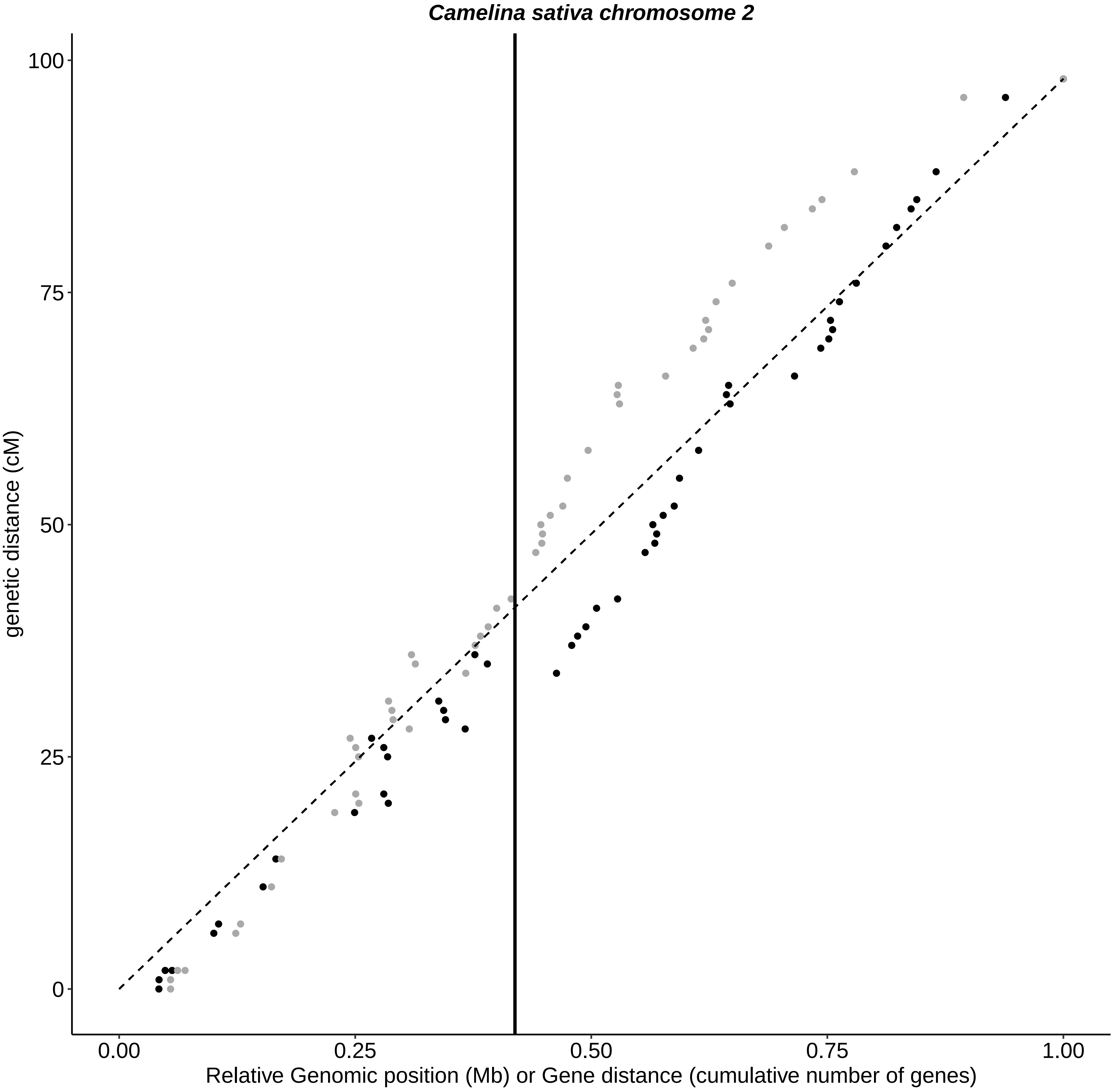

Camelina sativa chromosome 2
100
75
50
25
0
genetic distance (cM)
0.00
0.25
0.50
0.75
1.00
Relative Genomic position (Mb) or Gene distance (cumulative number of genes)

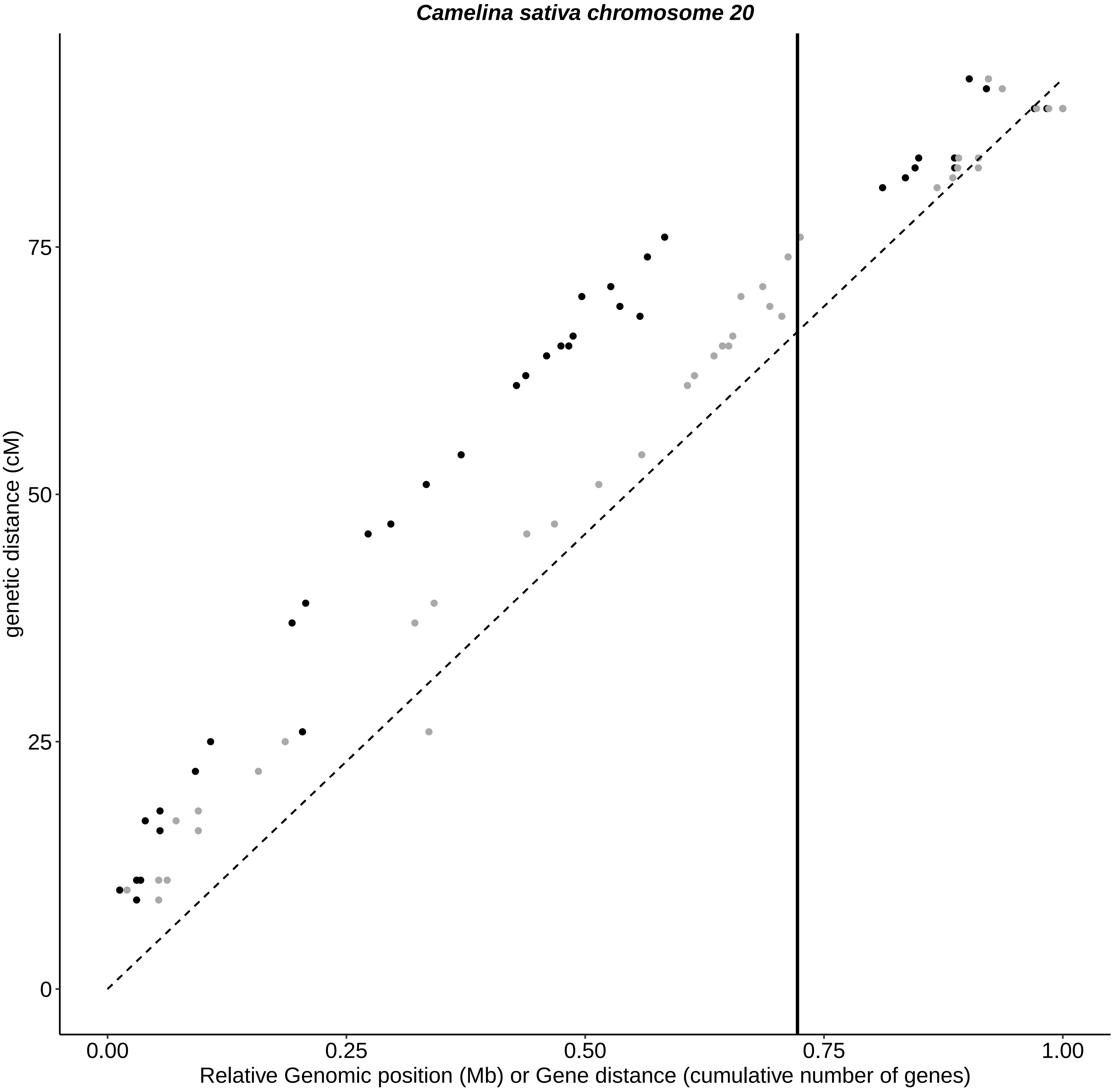
Camelina sativa chromosome 20
genetic distance (cM)
0
25
50
75
0.00
0.25
0.50
0.75
1.00
Relative Genomic position (Mb) or Gene distance (cumulative number of genes)

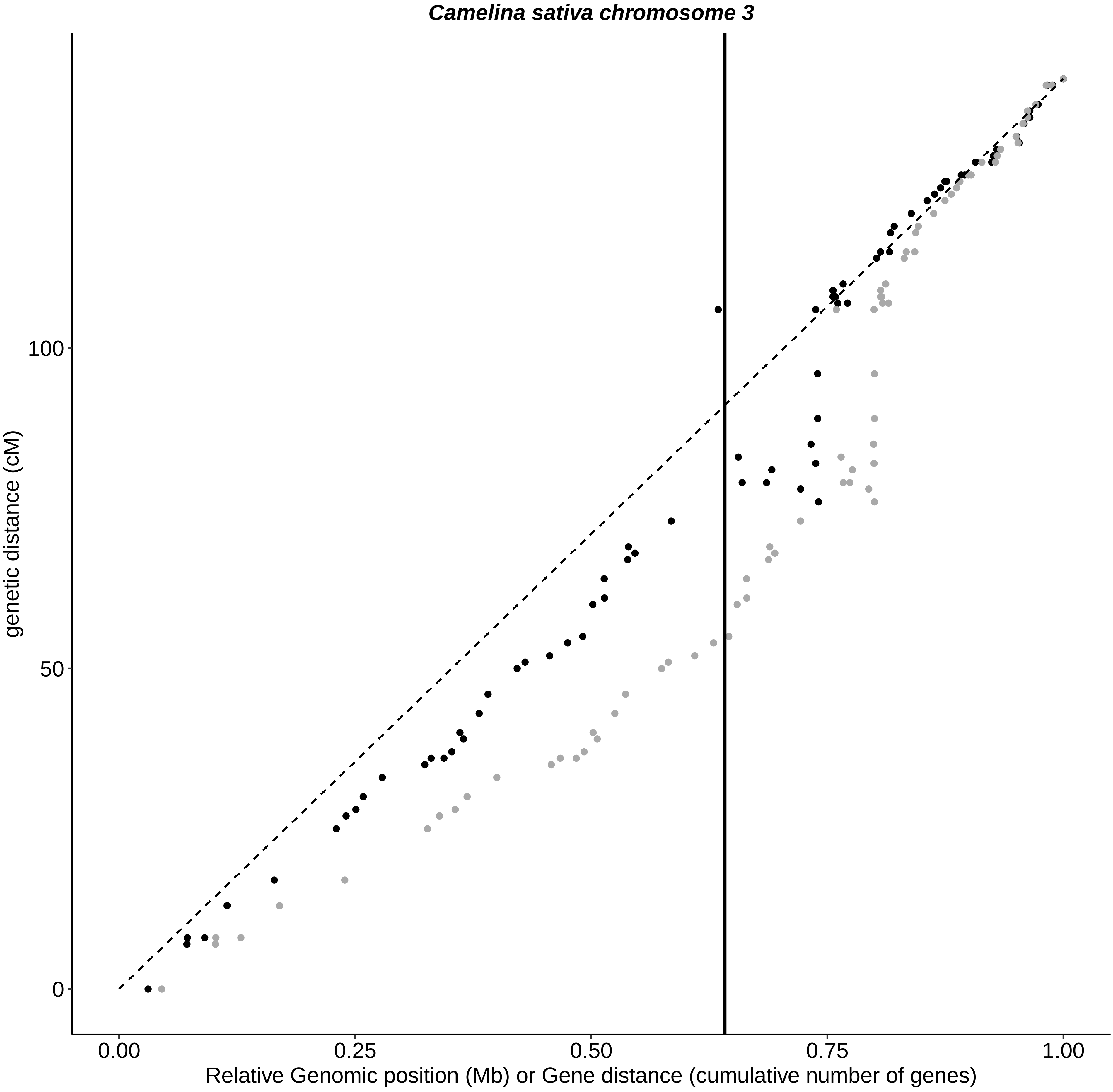
Camelina sativa chromosome 3
genetic distance (cM)
0
50
100
0.00
0.25
0.50
0.75
1.00
Relative Genomic position (Mb) or Gene distance (cumulative number of genes)

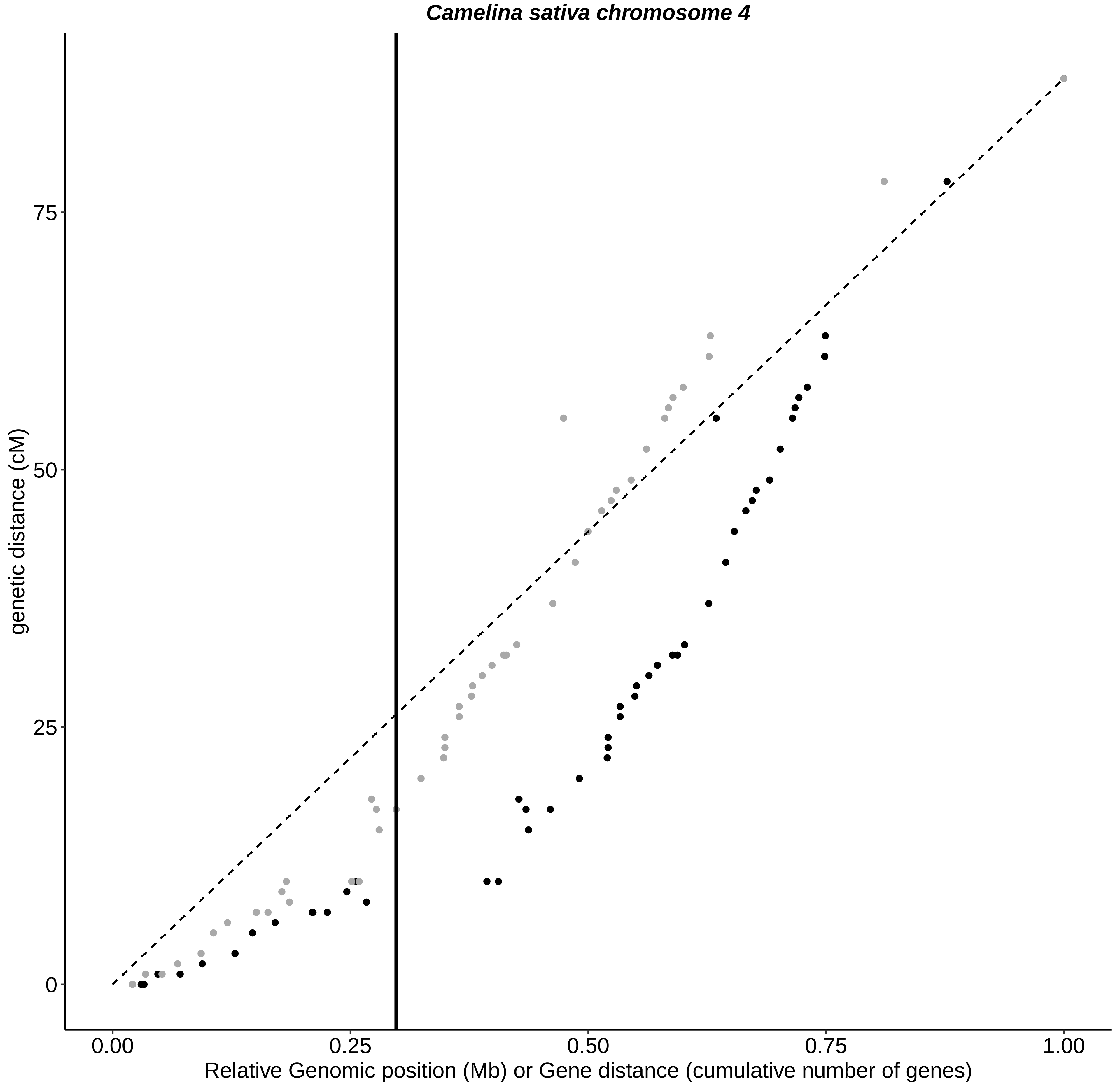
Camelina sativa chromosome 4
genetic distance (cM)
0
25
50
75
0.00
0.25
0.50
0.75
1.00
Relative Genomic position (Mb) or Gene distance (cumulative number of genes)

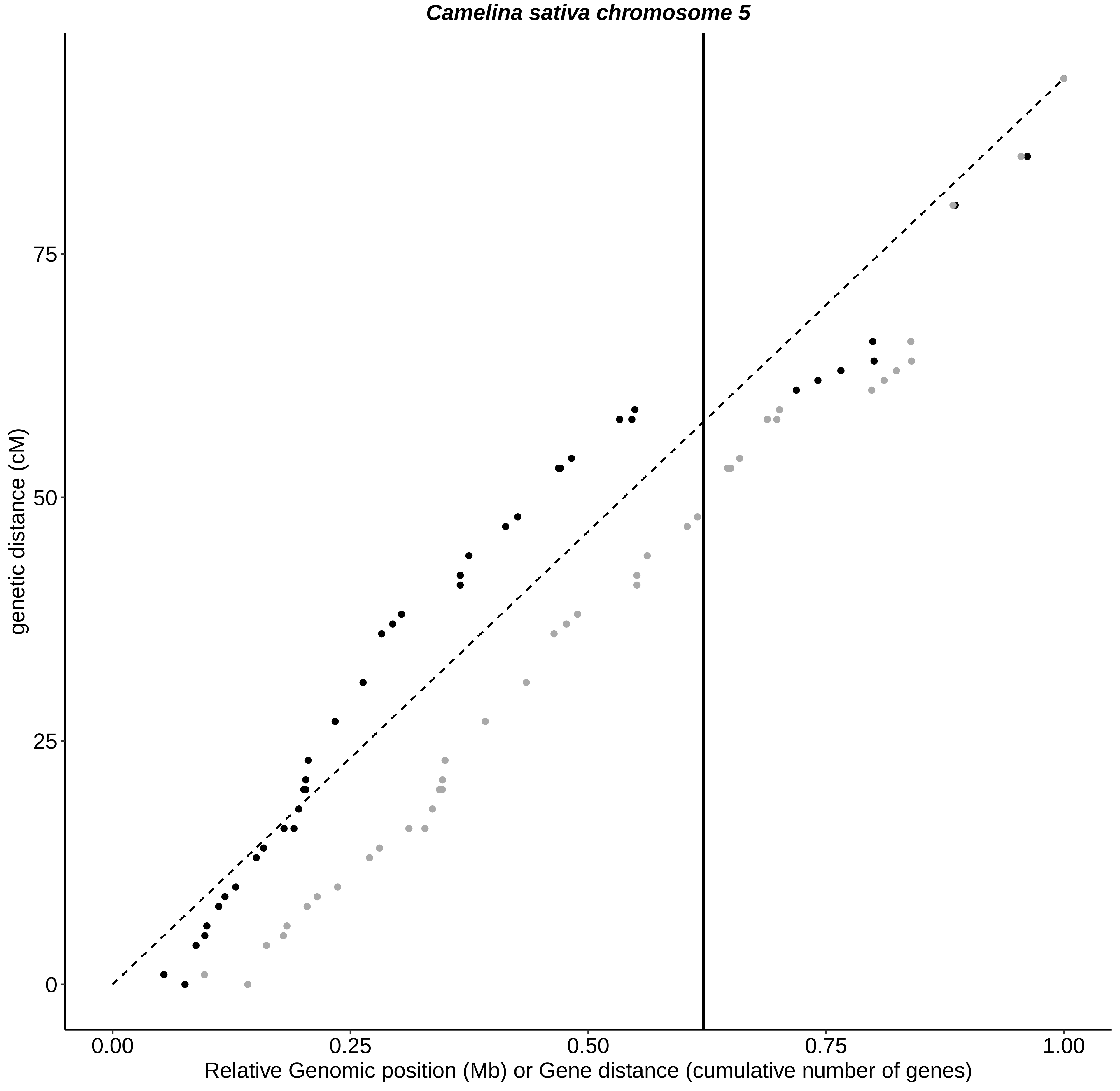
Camelina sativa chromosome 5
genetic distance (cM)
0
25
50
75
0.00
0.25
0.50
0.75
1.00
Relative Genomic position (Mb) or Gene distance (cumulative number of genes)

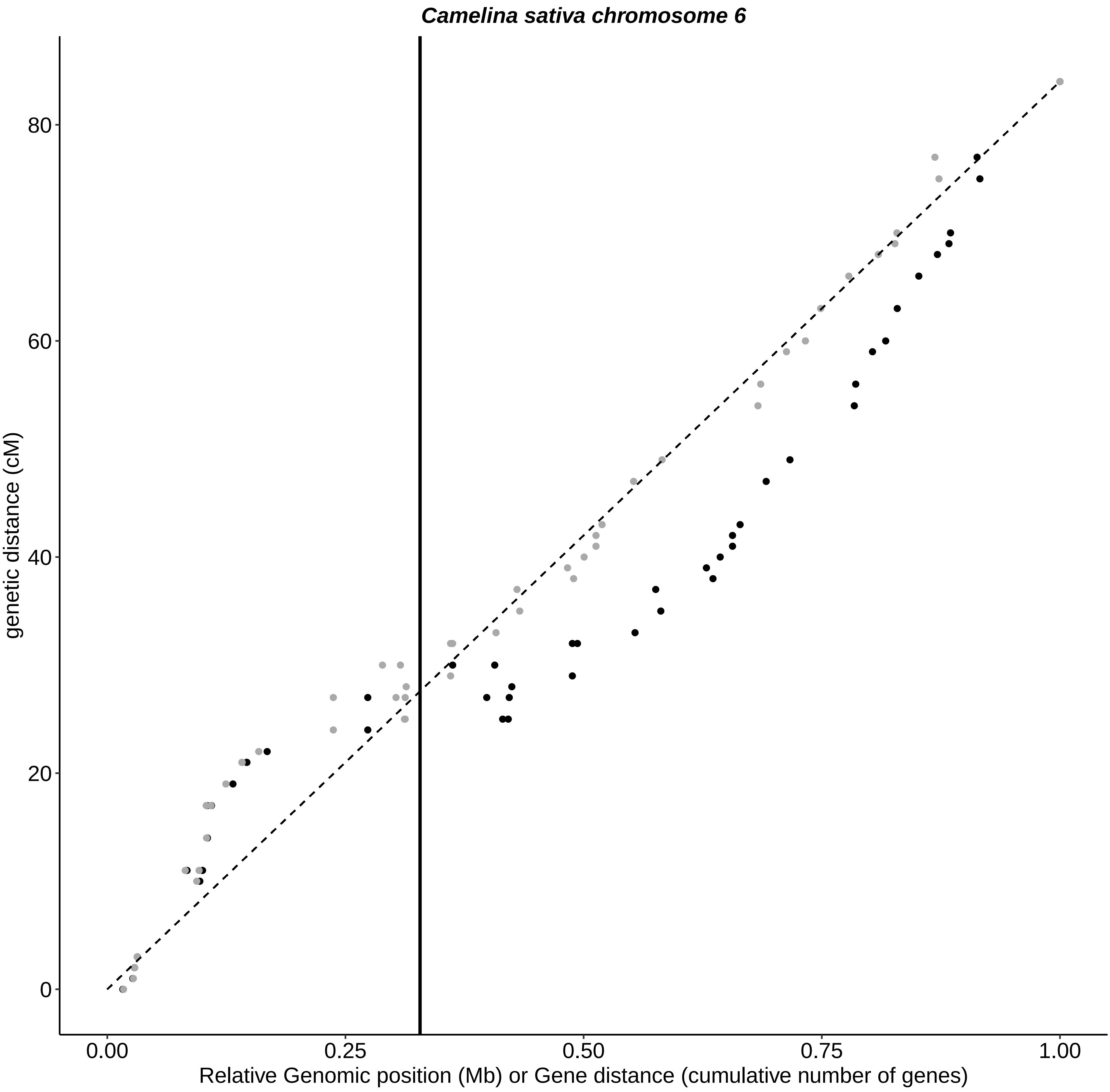
Camelina sativa chromosome 6
genetic distance (cM)
0
20
40
60
80
0.00
0.25
0.50
0.75
1.00
Relative Genomic position (Mb) or Gene distance (cumulative number of genes)

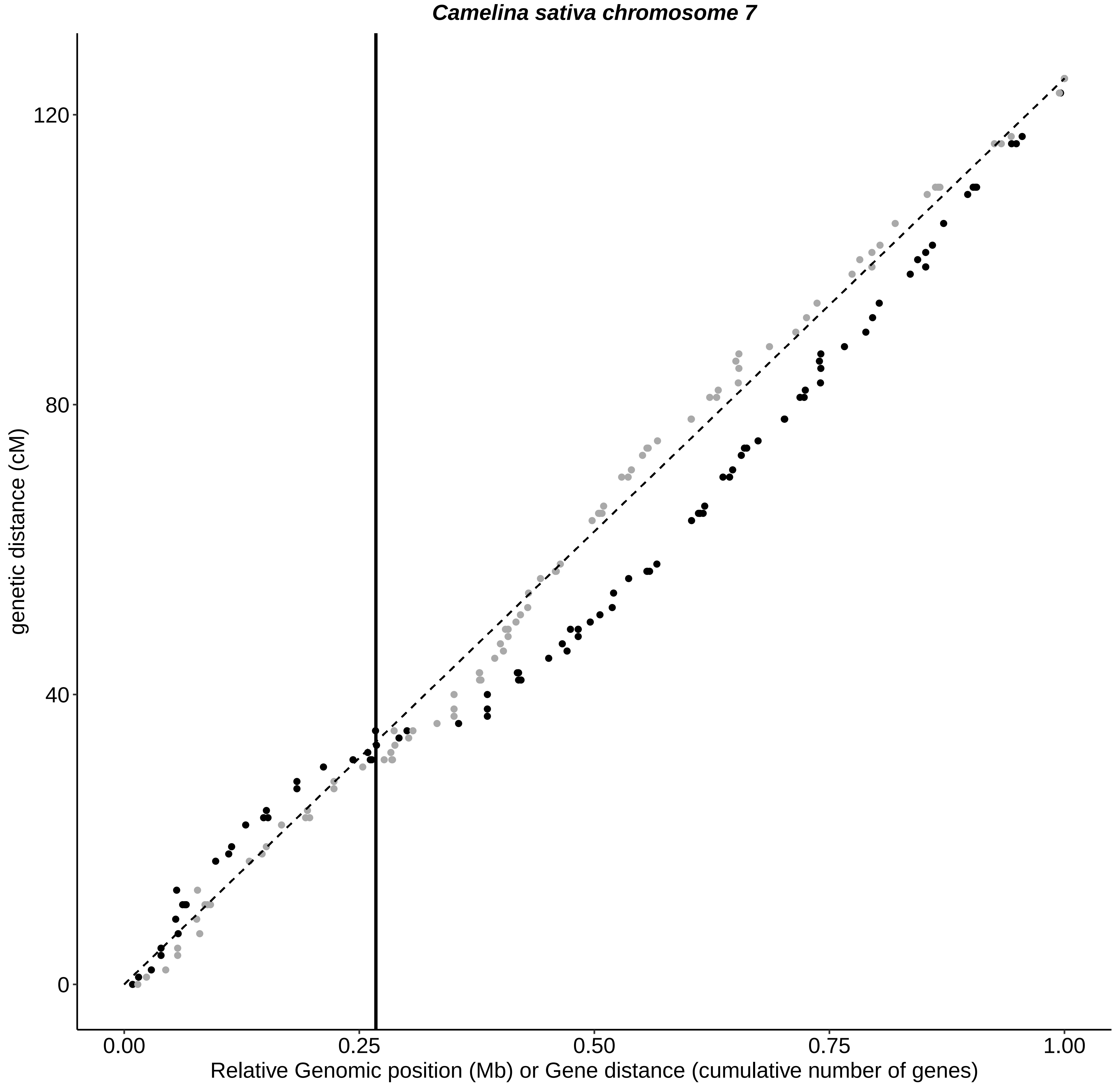
Camelina sativa chromosome 7
120
80
40
0
genetic distance (cM)
0.00
0.25
0.50
0.75
1.00
Relative Genomic position (Mb) or Gene distance (cumulative number of genes)

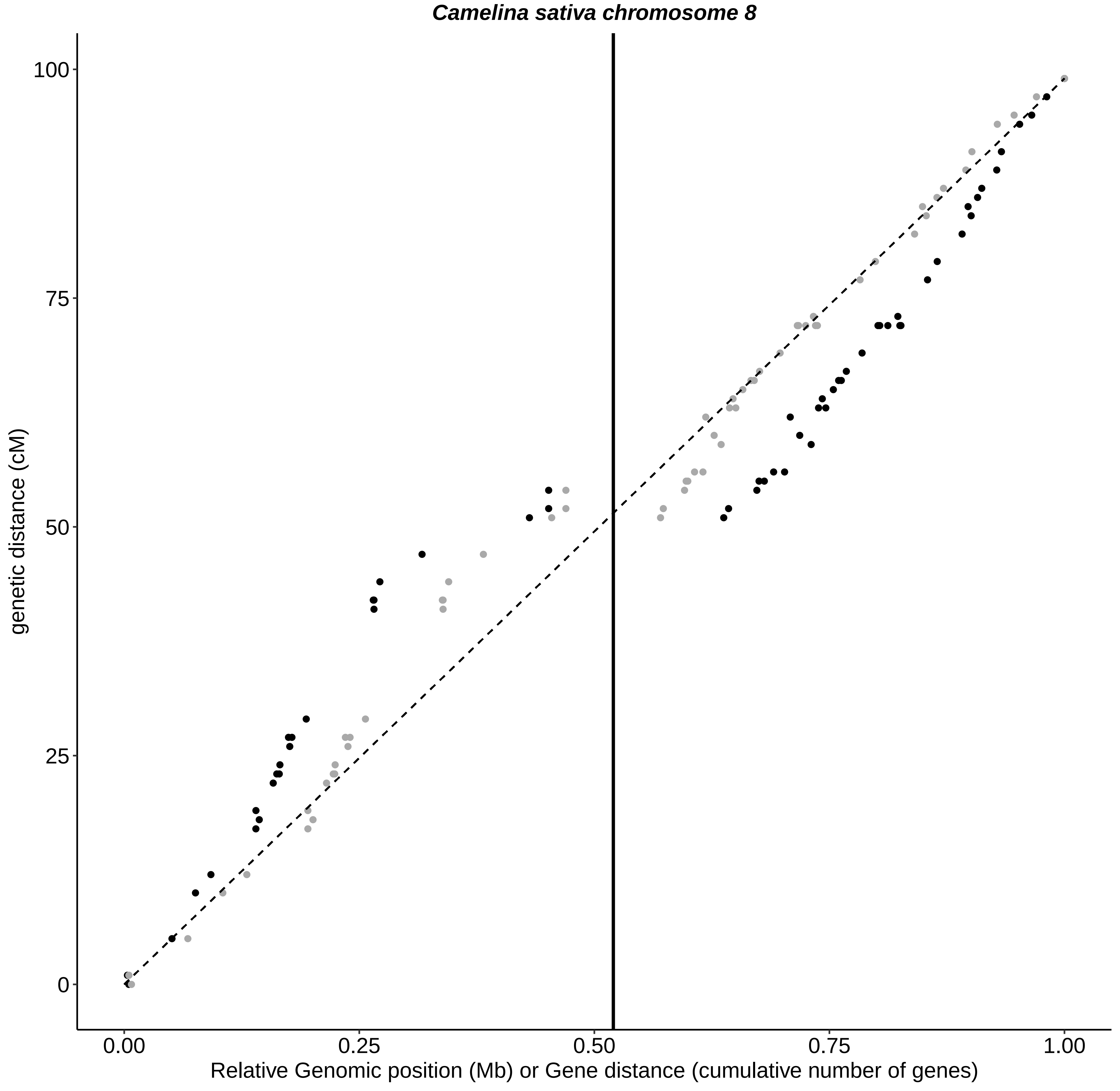
Camelina sativa chromosome 8
100
75
50
25
0
genetic distance (cM)
0.00
0.25
0.50
0.75
1.00
Relative Genomic position (Mb) or Gene distance (cumulative number of genes)

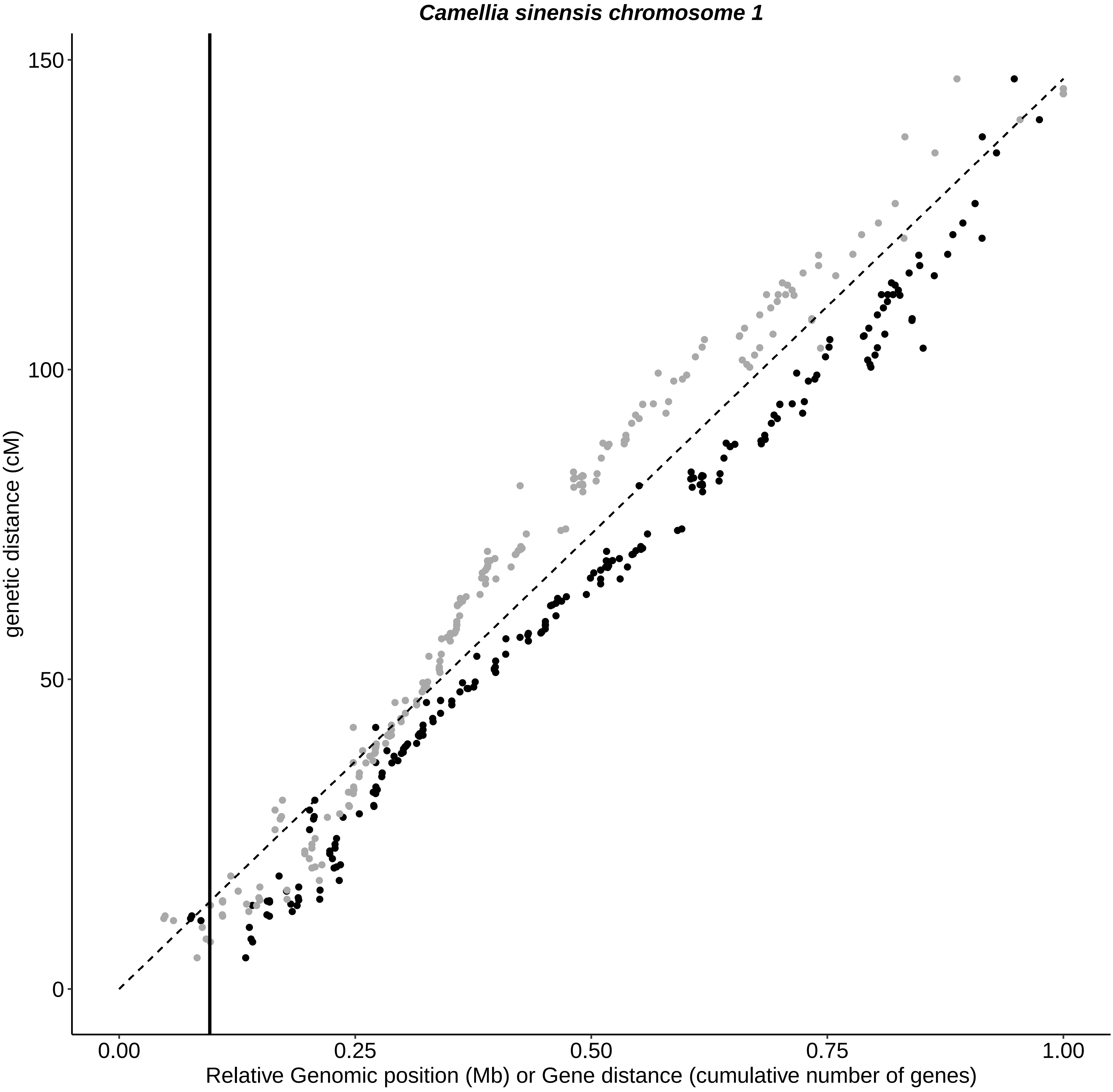
Camellia sinensis chromosome 1
genetic distance (cM)
150
100
50
0
0.00
0.25
0.50
0.75
1.00
Relative Genomic position (Mb) or Gene distance (cumulative number of genes)

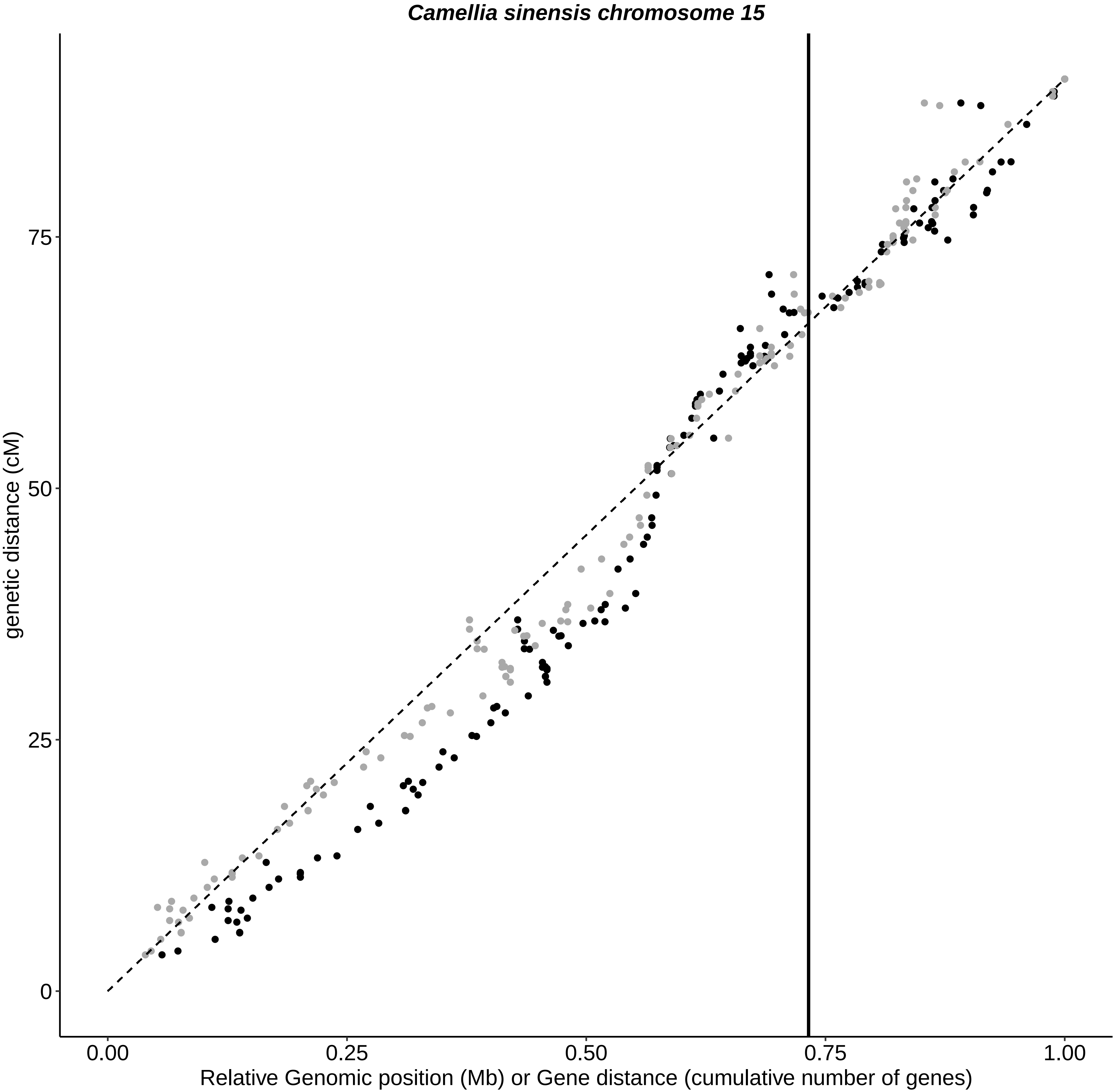
Camellia sinensis chromosome 15
genetic distance (cM)
0
25
50
75
0.00
0.25
0.50
0.75
1.00
Relative Genomic position (Mb) or Gene distance (cumulative number of genes)

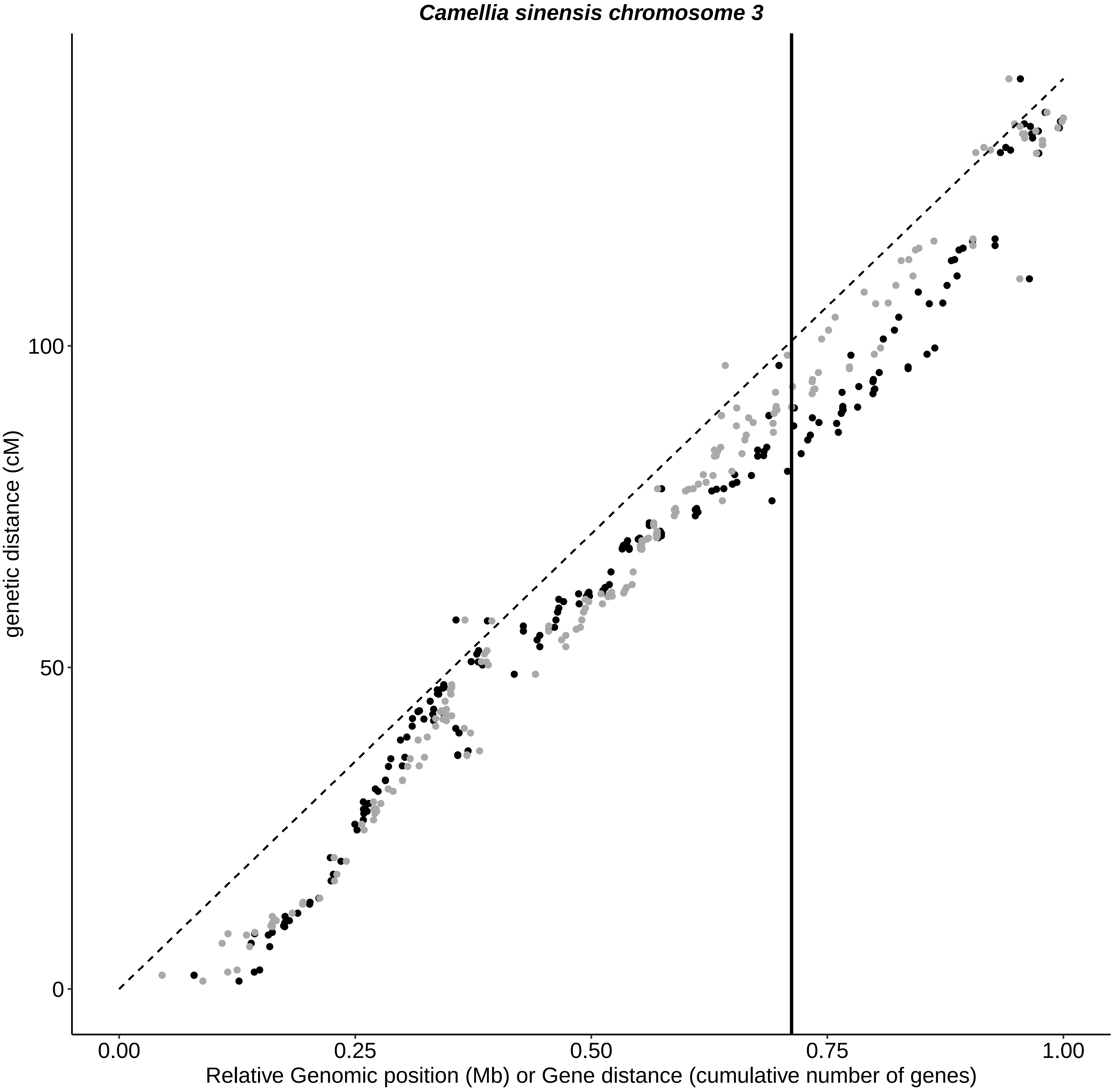
Camellia sinensis chromosome 3
genetic distance (cM)
0
50
100
0.00
0.25
0.50
0.75
1.00
Relative Genomic position (Mb) or Gene distance (cumulative number of genes)

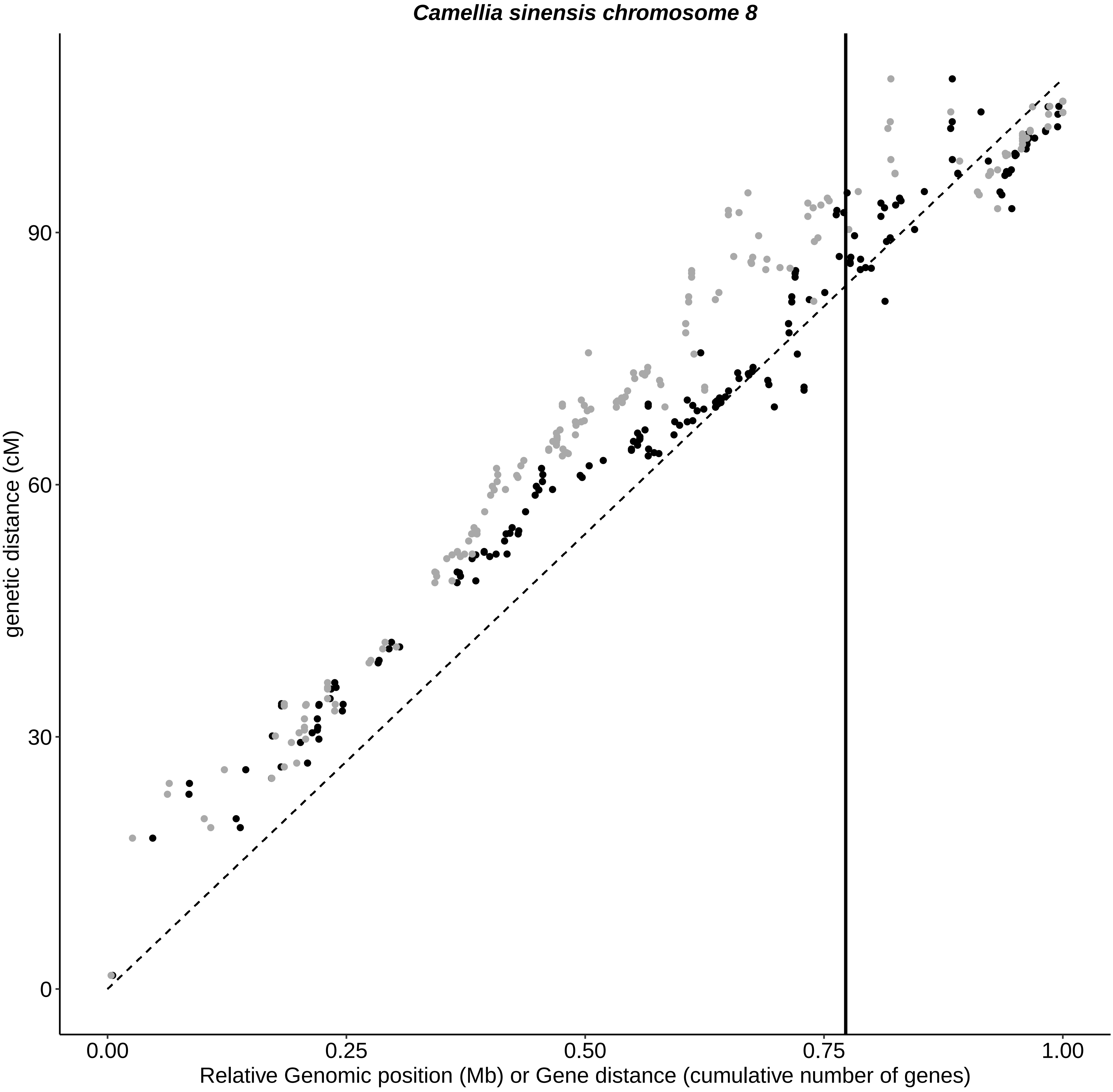

Camellia sinensis chromosome 8
genetic distance (cM)
0
30
60
90
0.00
0.25
0.50
0.75
1.00
Relative Genomic position (Mb) or Gene distance (cumulative number of genes)

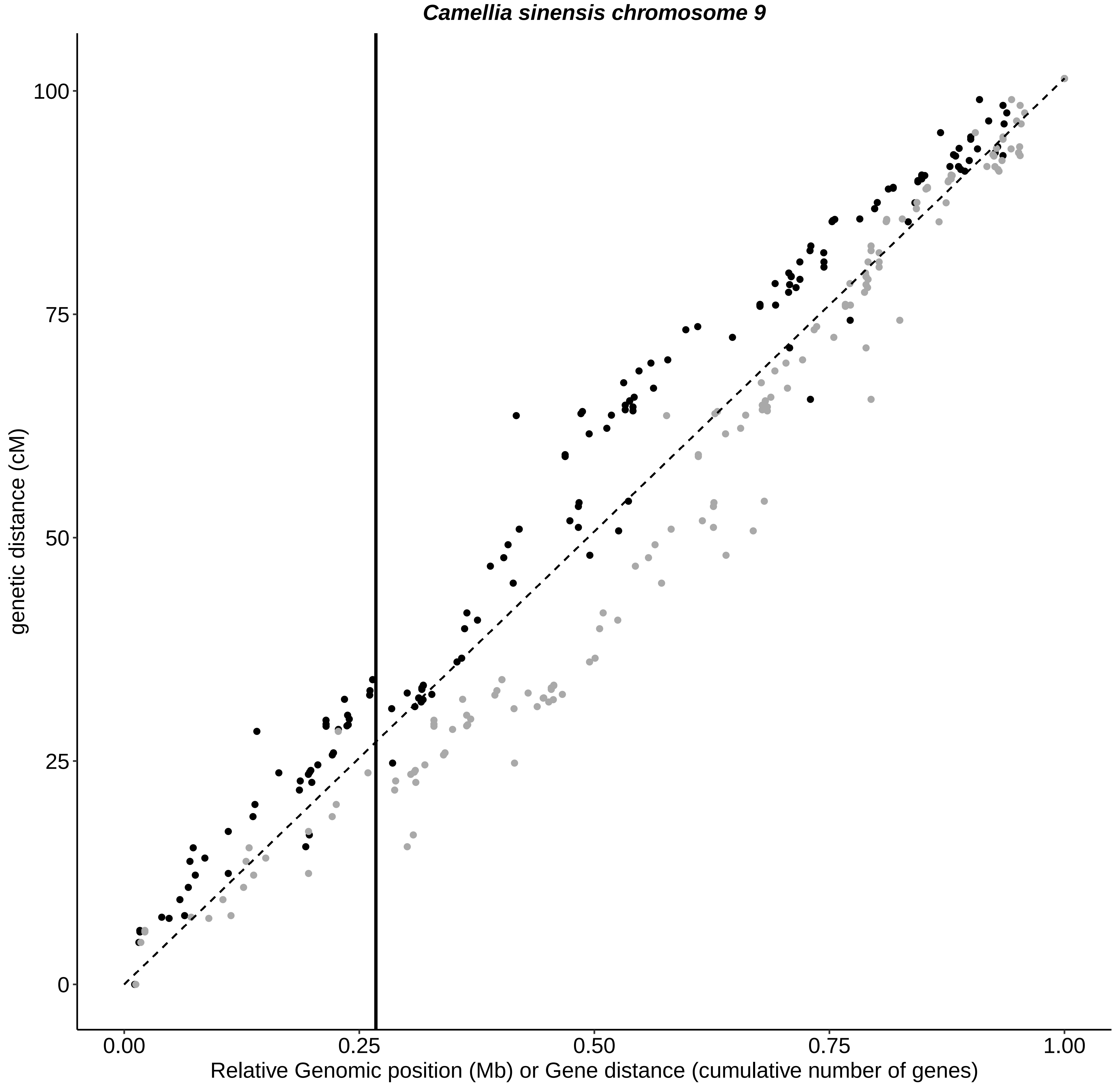
Camellia sinensis chromosome 9
genetic distance (cM)
100
75
50
25
0
0.00
0.25
0.50
0.75
1.00
Relative Genomic position (Mb) or Gene distance (cumulative number of genes)

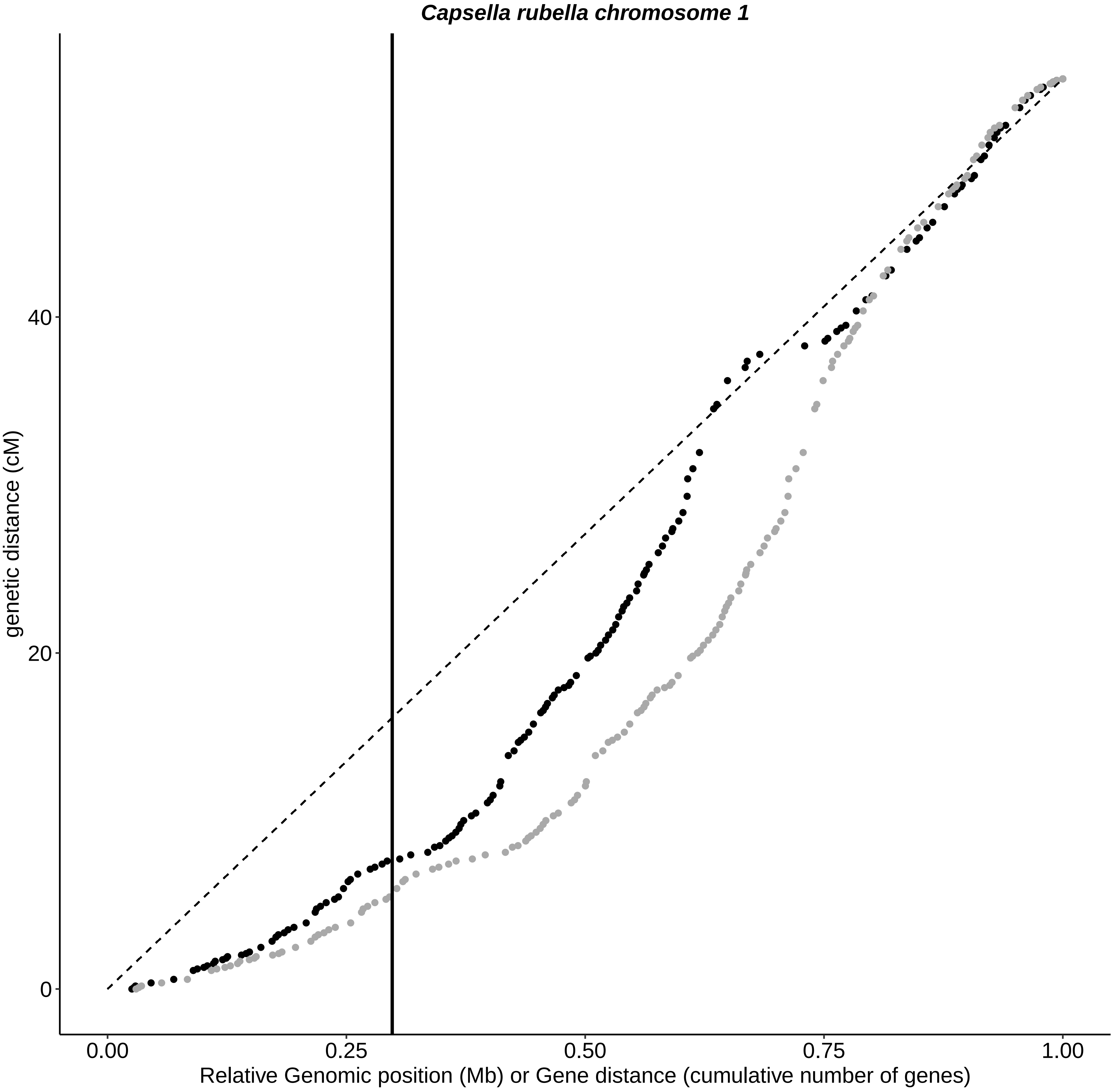
Capsella rubella chromosome 1
genetic distance (cM)
0
20
40
0.00
0.25
0.50
0.75
1.00
Relative Genomic position (Mb) or Gene distance (cumulative number of genes)

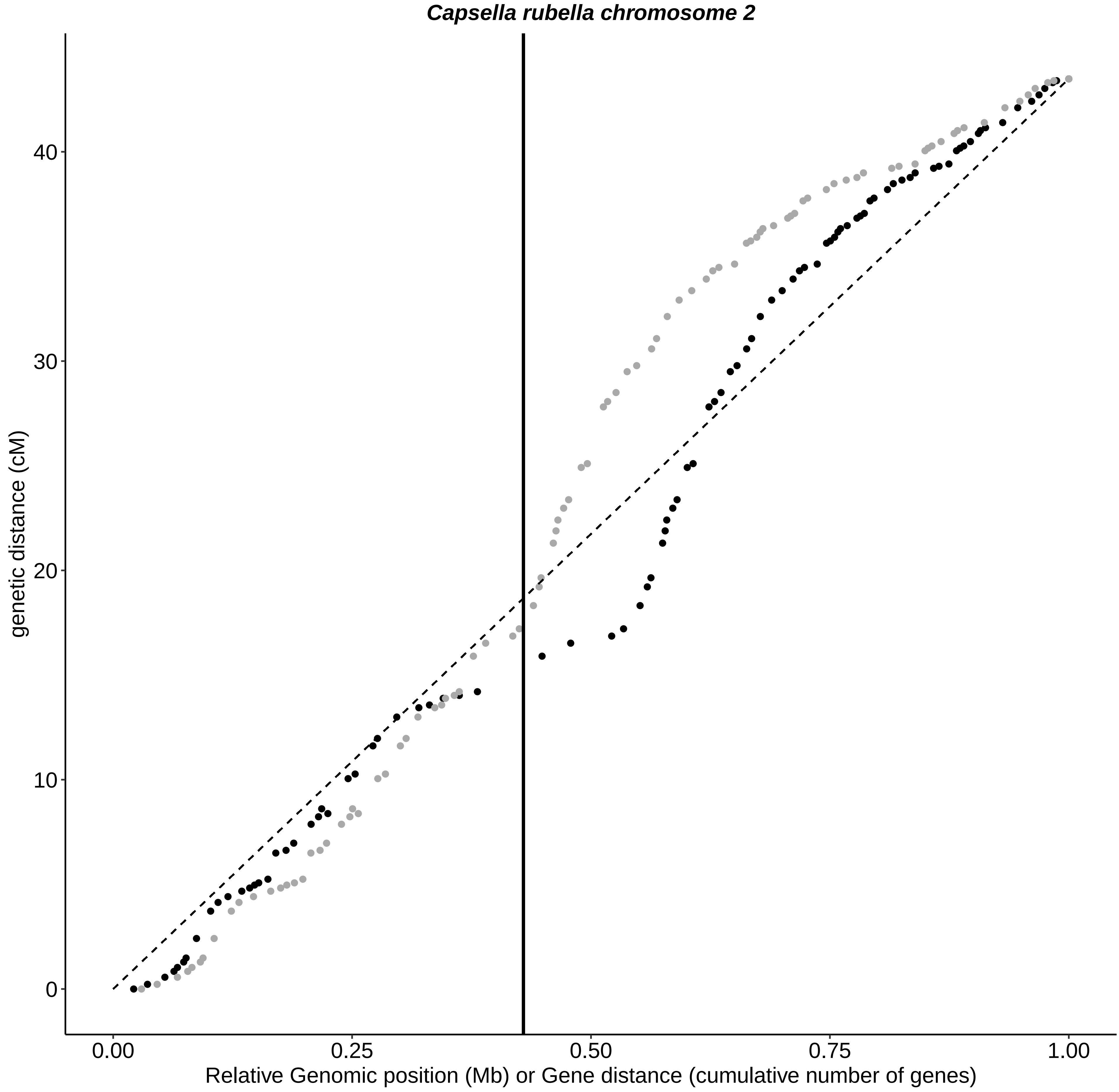
Capsella rubella chromosome 2
genetic distance (cM)
0
10
20
30
40
0.00
0.25
0.50
0.75
1.00
Relative Genomic position (Mb) or Gene distance (cumulative number of genes)

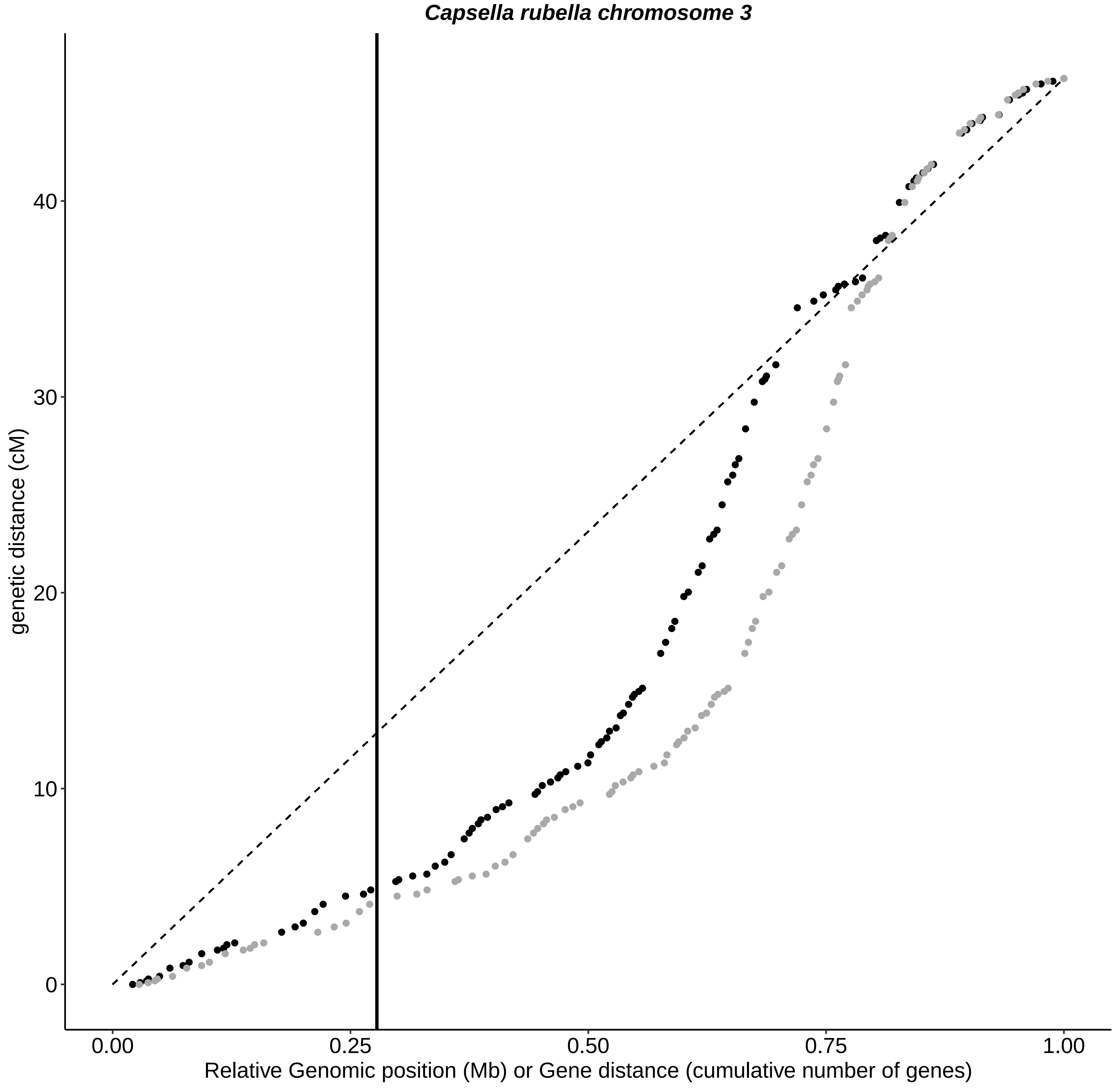
Capsella rubella chromosome 3
genetic distance (cM)
0
10
20
30
40
0.00
0.25
0.50
0.75
1.00
Relative Genomic position (Mb) or Gene distance (cumulative number of genes)

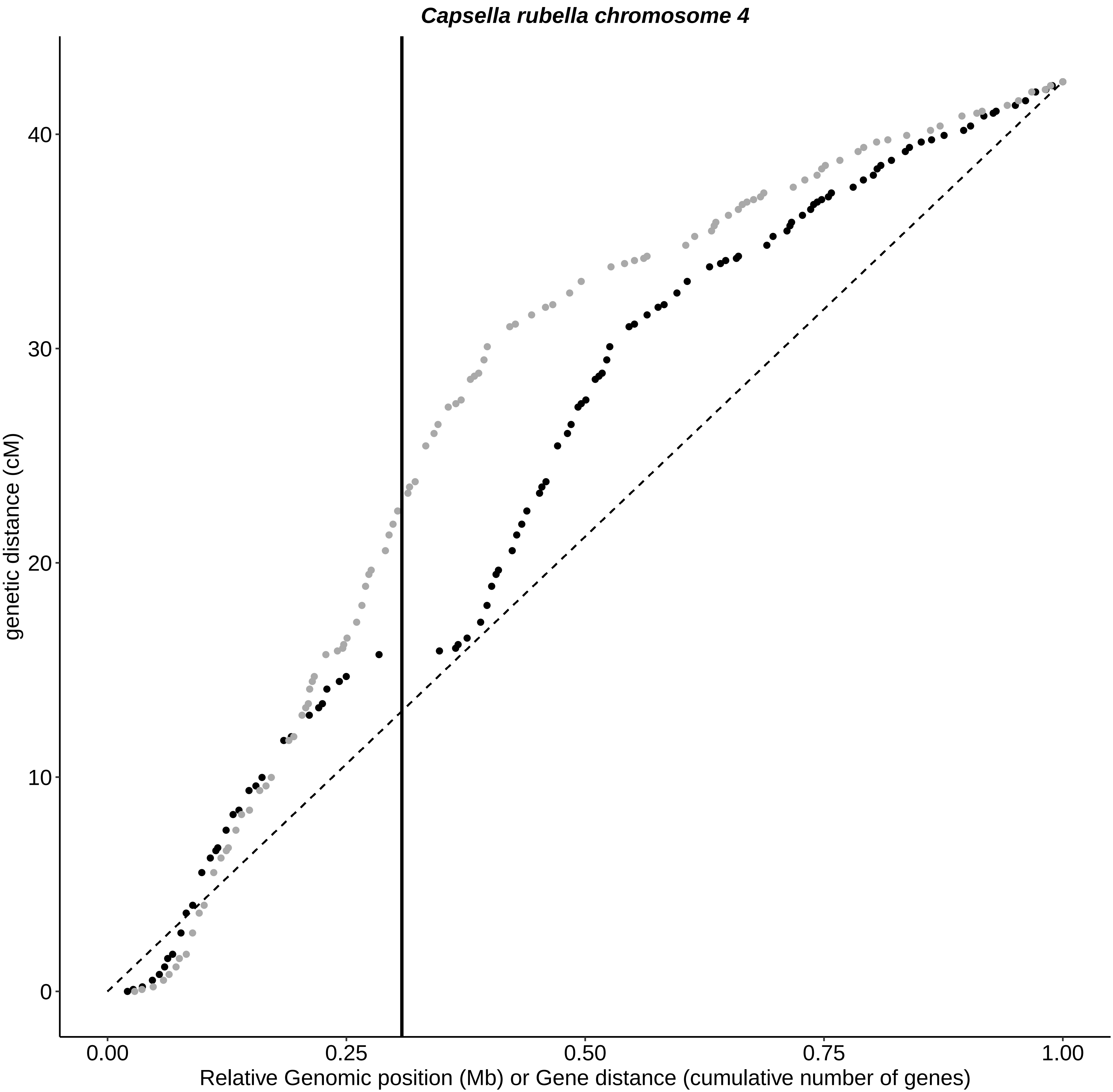
Capsella rubella chromosome 4
genetic distance (cM)
0
10
20
30
40
0.00
0.25
0.50
0.75
1.00
Relative Genomic position (Mb) or Gene distance (cumulative number of genes)

Capsella rubella chromosome 6

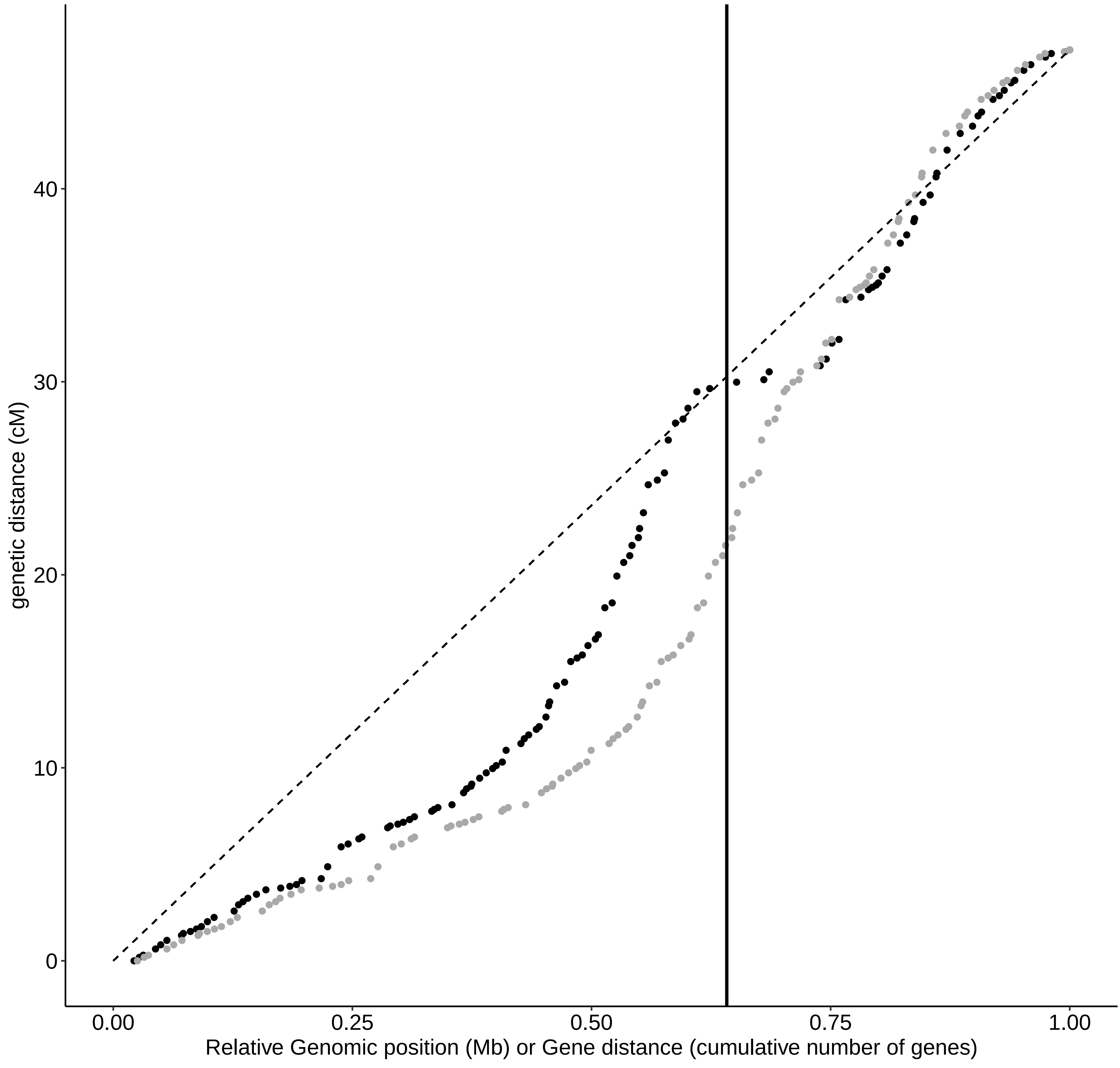

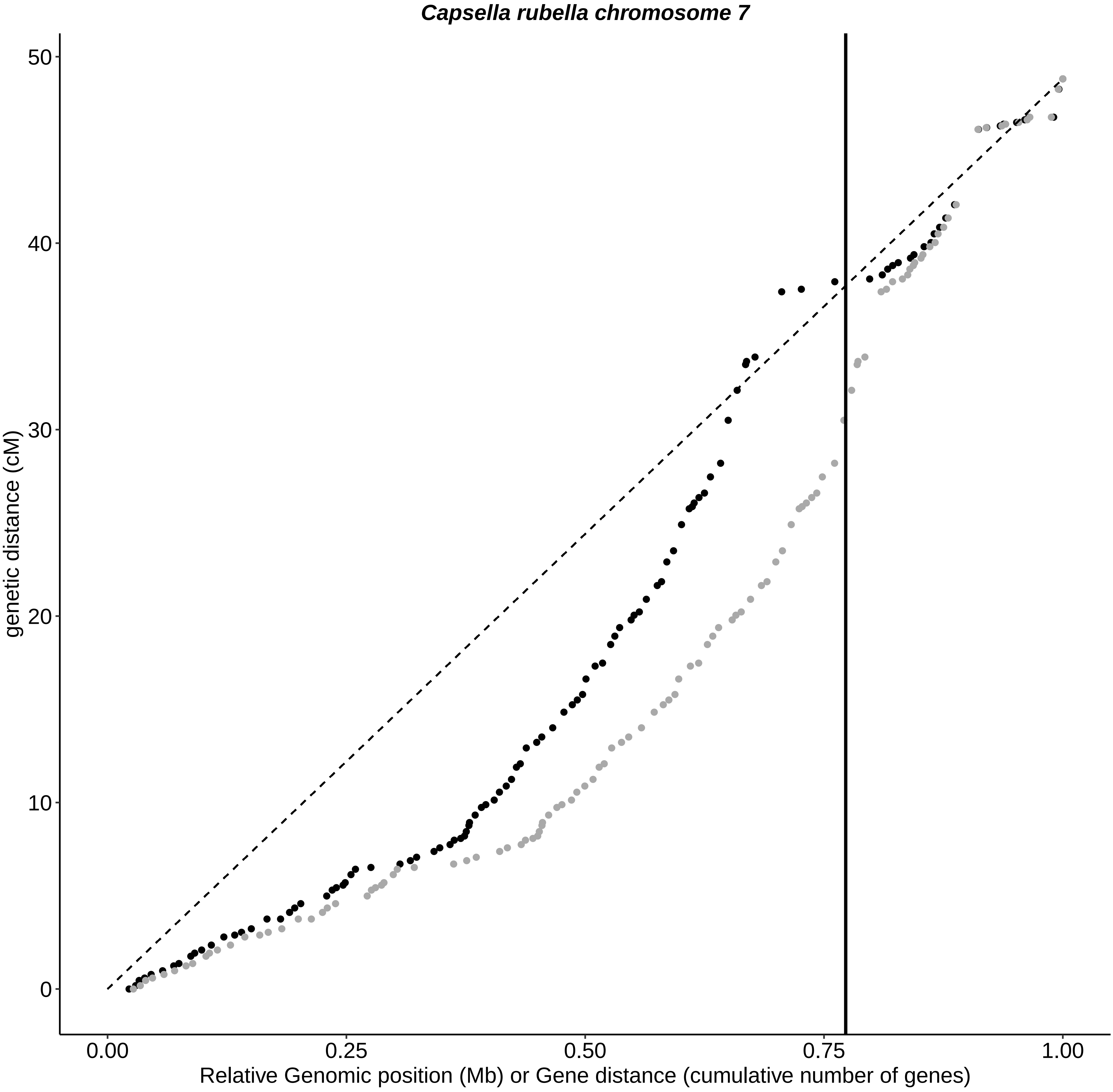
Capsella rubella chromosome 7
genetic distance (cM)
0
10
20
30
40
50
0.00
0.25
0.50
0.75
1.00
Relative Genomic position (Mb) or Gene distance (cumulative number of genes)

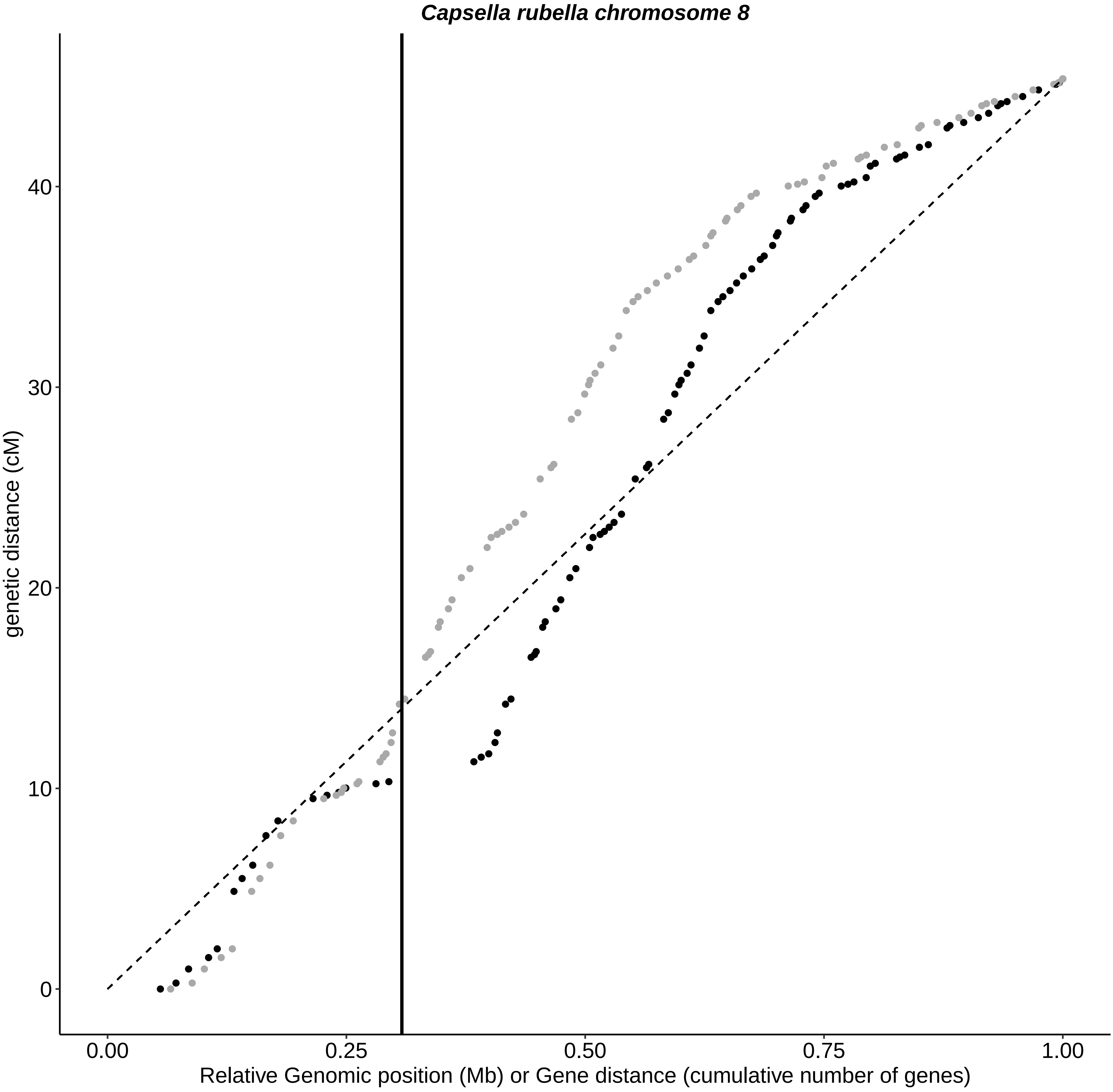
Capsella rubella chromosome 8
genetic distance (cM)
0
10
20
30
40
0.00
0.25
0.50
0.75
1.00
Relative Genomic position (Mb) or Gene distance (cumulative number of genes)

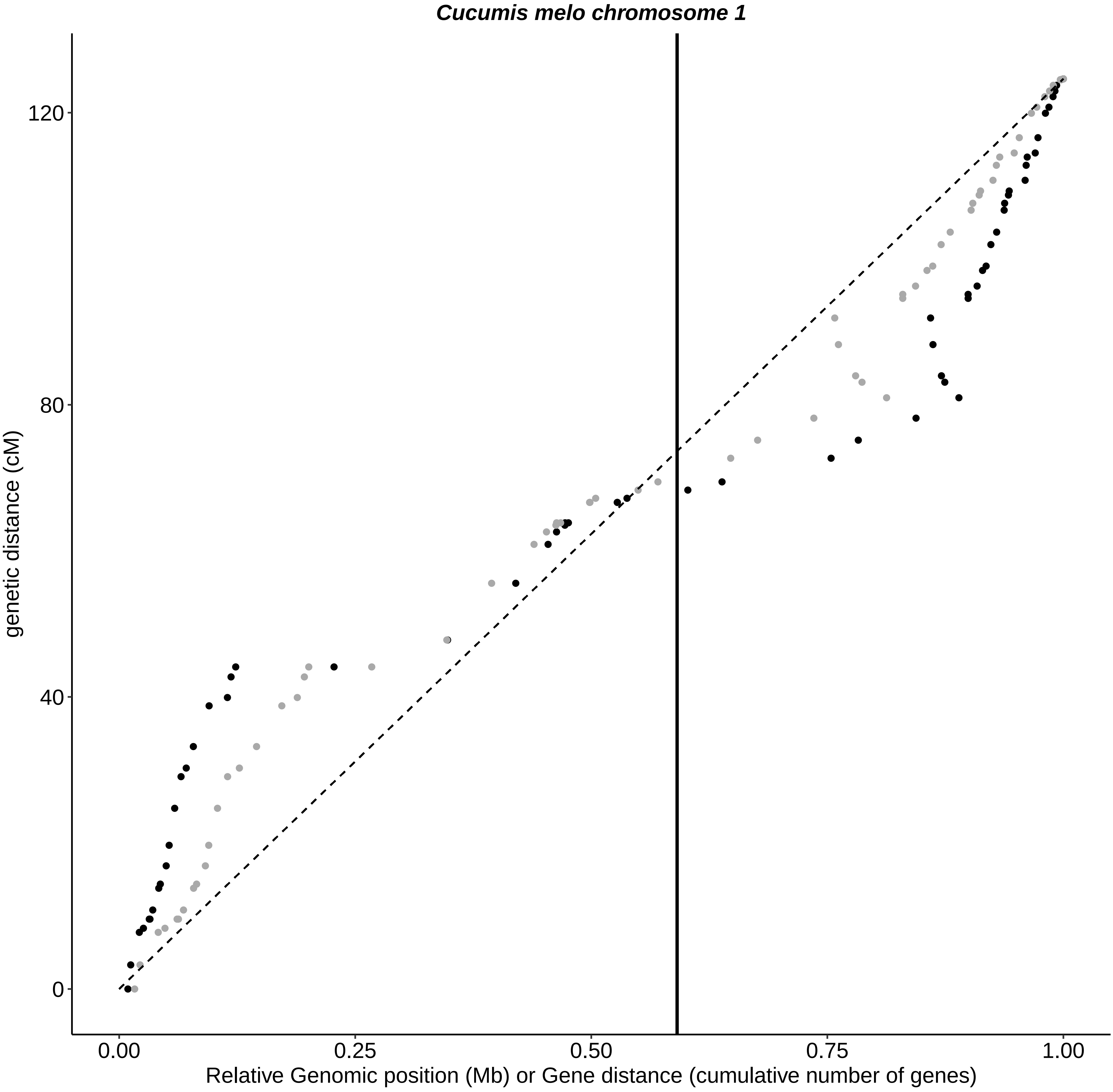
Cucumis melo chromosome 1
genetic distance (cM)
120
80
40
0
0.00
0.25
0.50
0.75
1.00
Relative Genomic position (Mb) or Gene distance (cumulative number of genes)

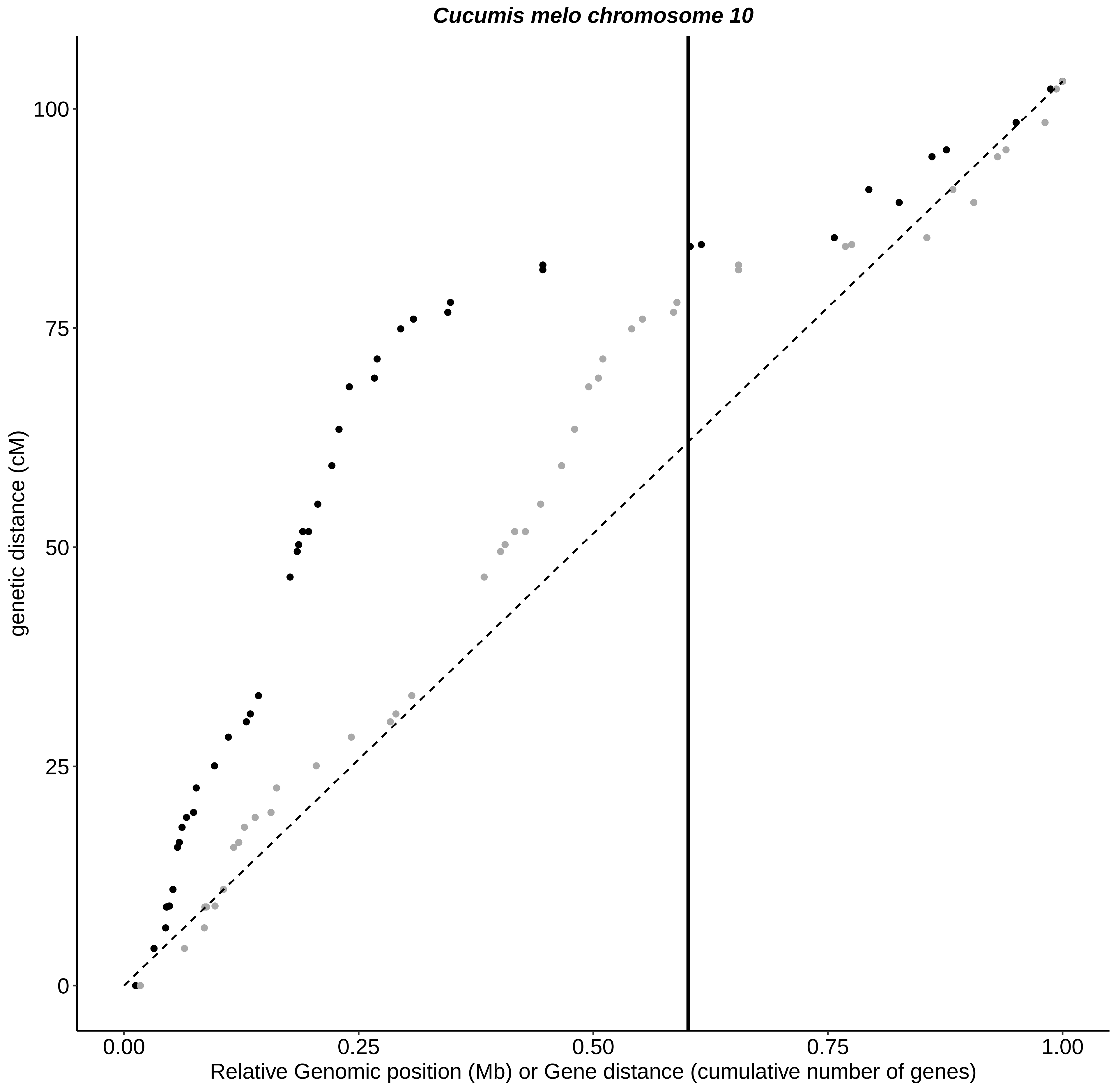
Cucumis melo chromosome 10
genetic distance (cM)
0
25
50
75
100
0.00
0.25
0.50
0.75
1.00
Relative Genomic position (Mb) or Gene distance (cumulative number of genes)

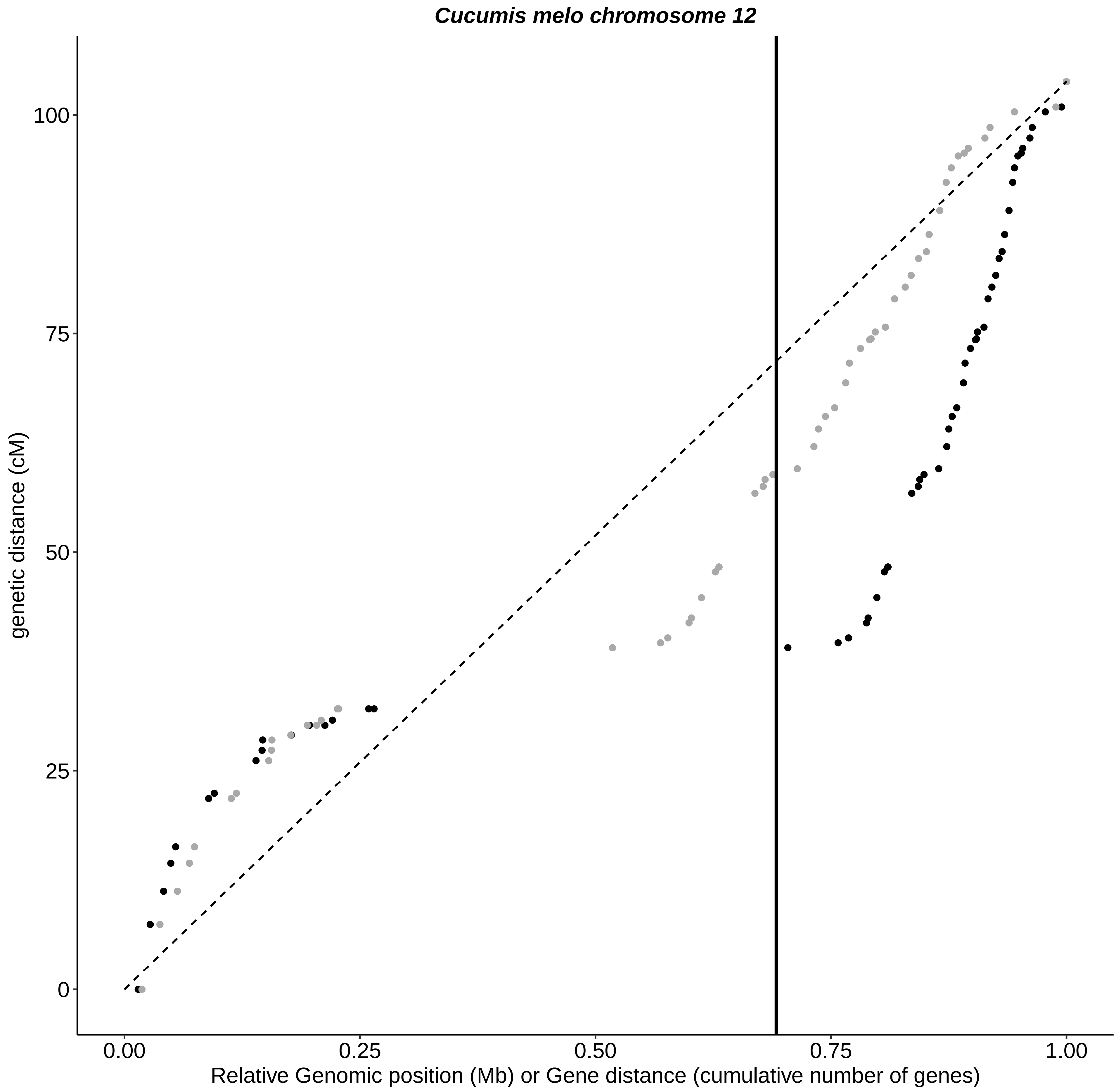
Cucumis melo chromosome 12
genetic distance (cM)
0
25
50
75
100
0.00
0.25
0.50
0.75
1.00
Relative Genomic position (Mb) or Gene distance (cumulative number of genes)

Cucumis melo chromosome 2

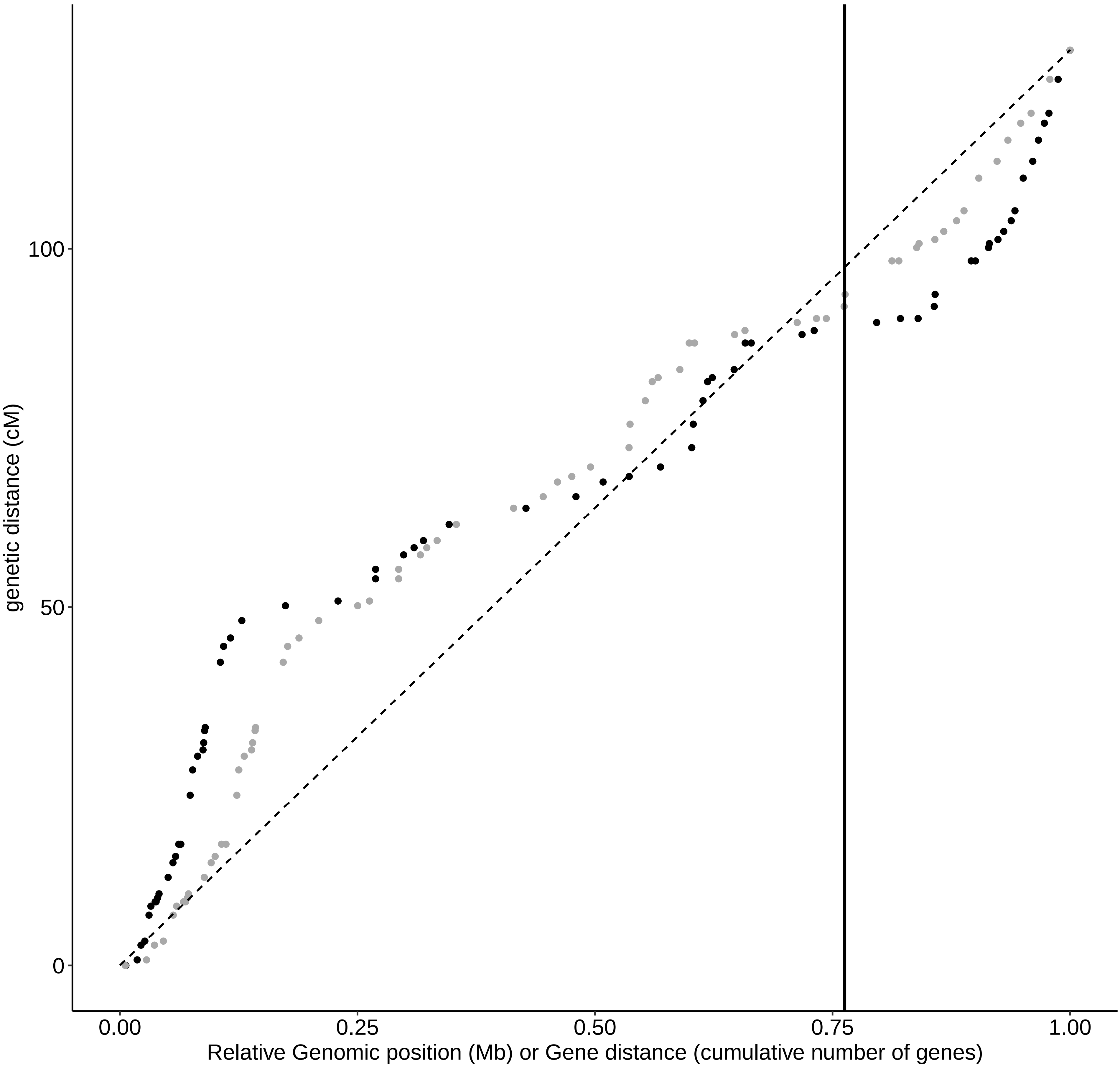

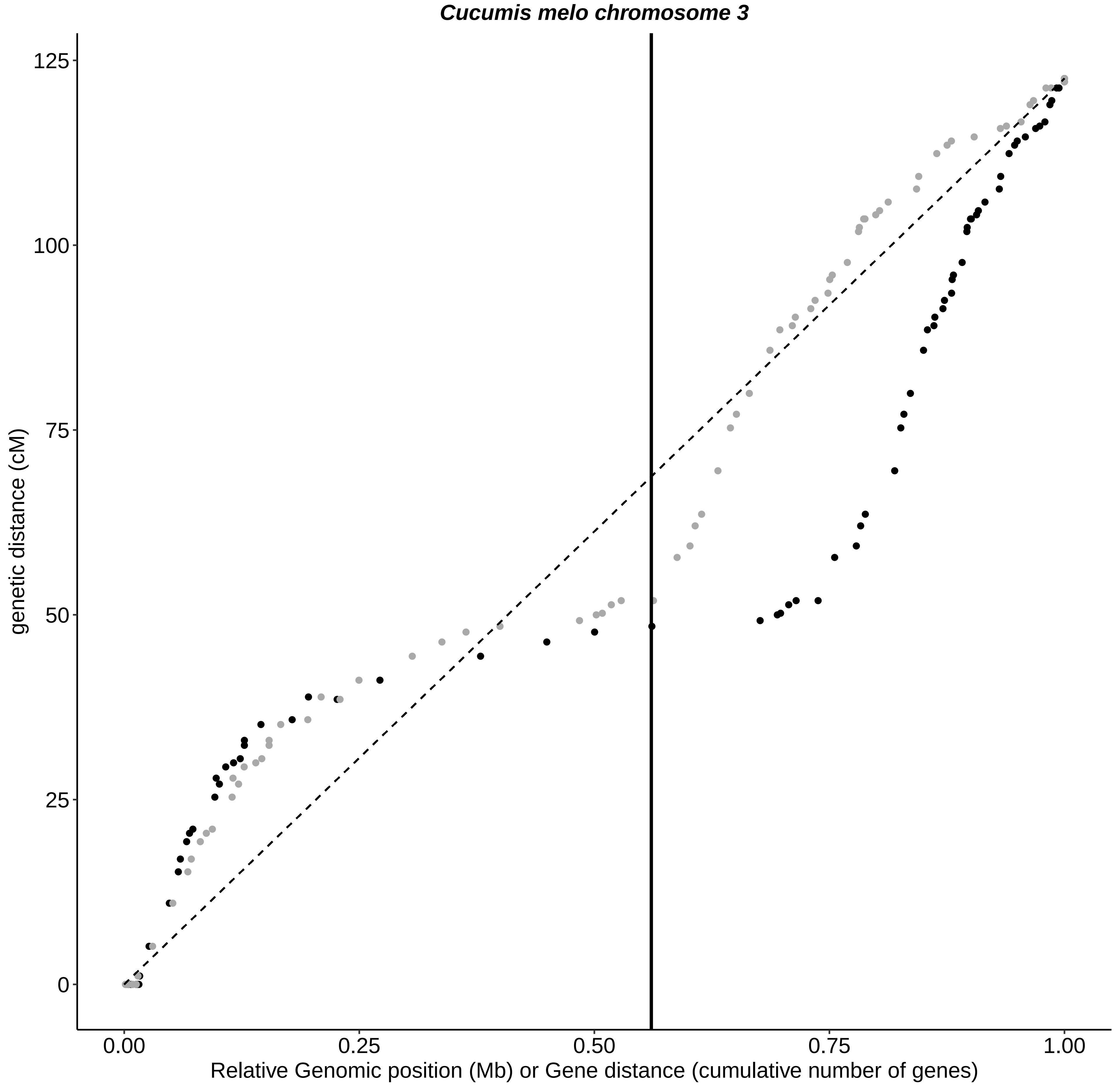
Cucumis melo chromosome 3
genetic distance (cM)
125
100
75
50
25
0
0.00
0.25
0.50
0.75
1.00
Relative Genomic position (Mb) or Gene distance (cumulative number of genes)

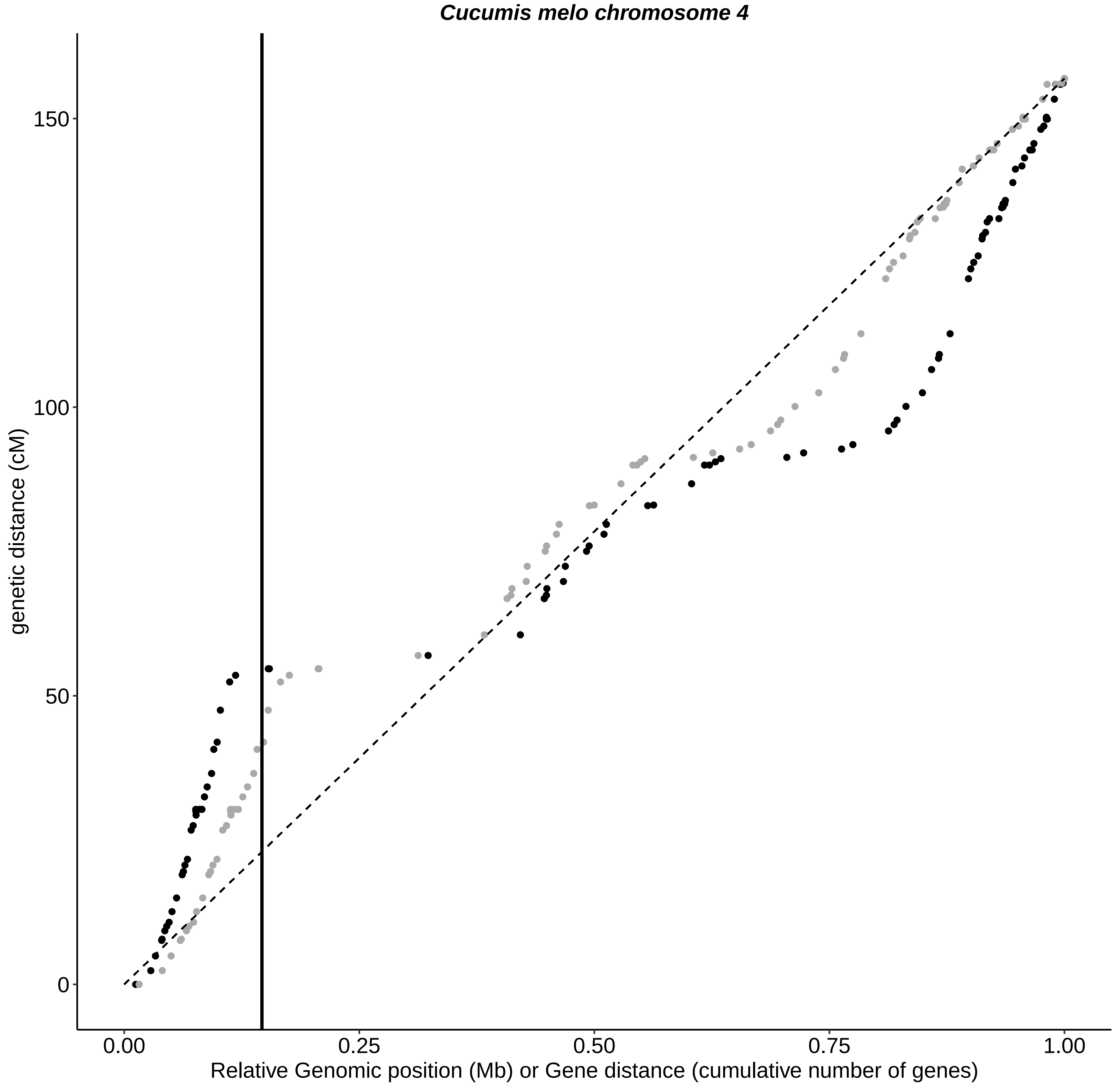
Cucumis melo chromosome 4
genetic distance (cM)
0
50
100
150
0.00
0.25
0.50
0.75
1.00
Relative Genomic position (Mb) or Gene distance (cumulative number of genes)

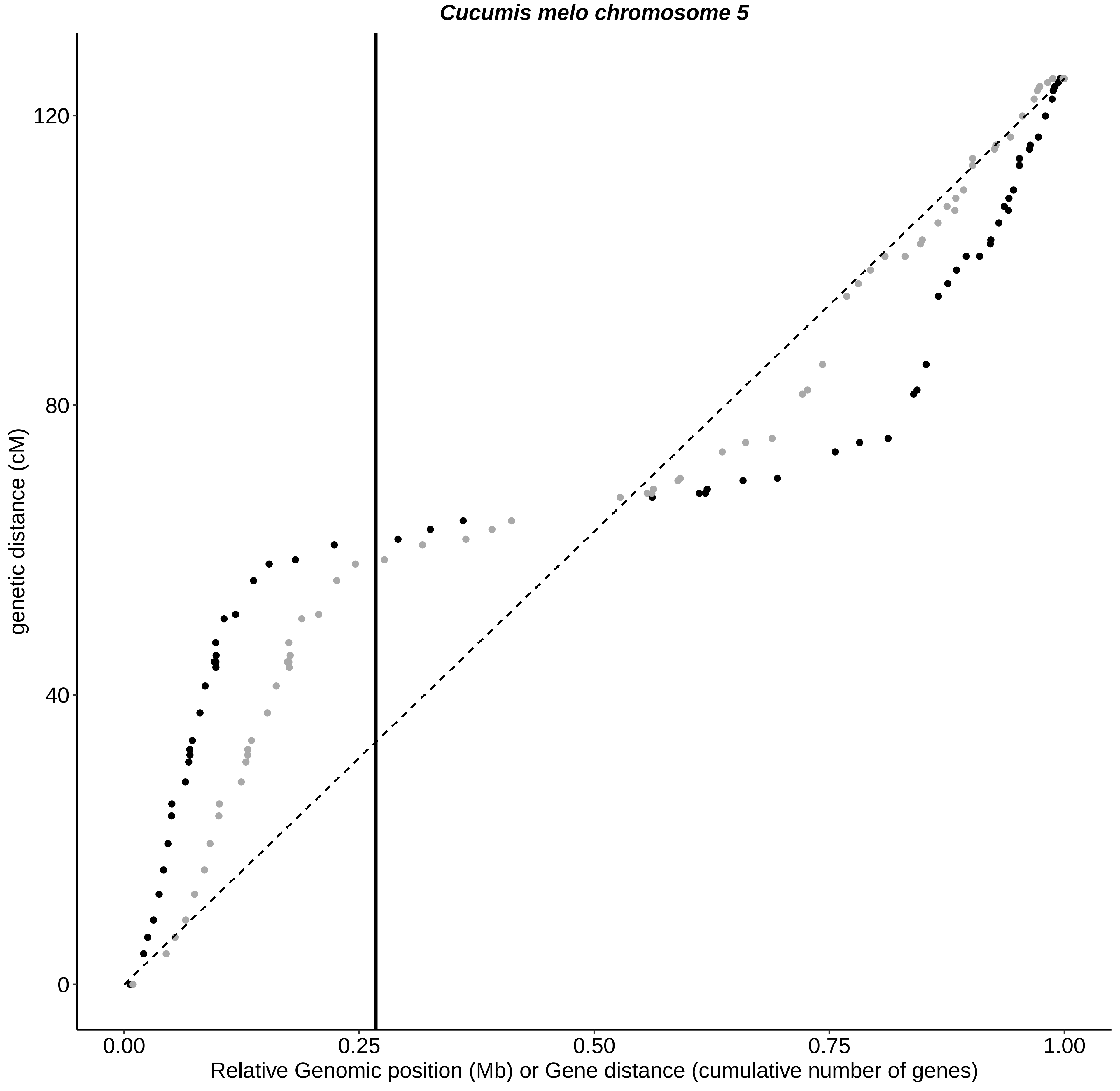
Cucumis melo chromosome 5
genetic distance (cM)
0
40
80
120
0.00
0.25
0.50
0.75
1.00
Relative Genomic position (Mb) or Gene distance (cumulative number of genes)

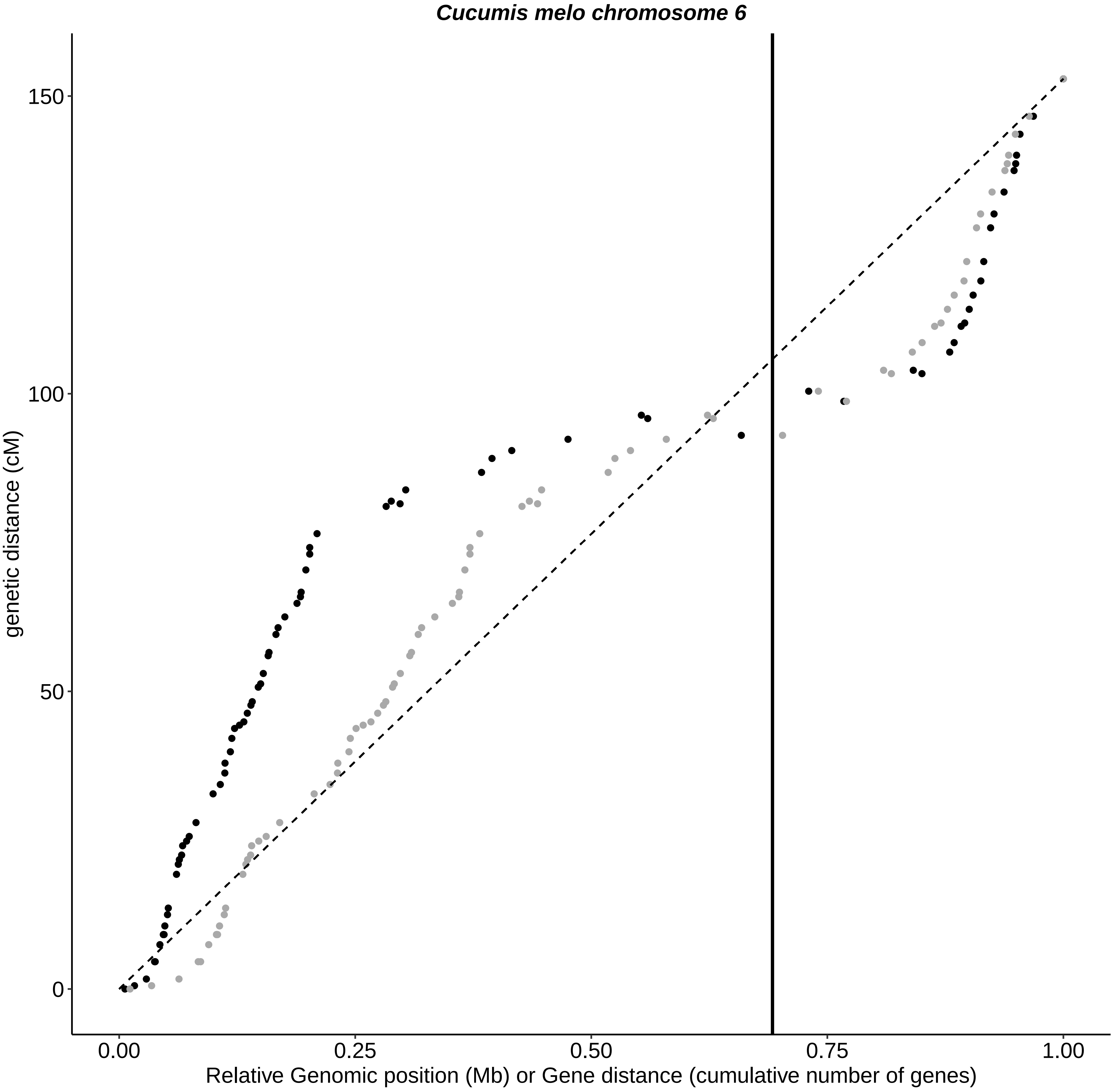
Cucumis melo chromosome 6
genetic distance (cM)
150
100
50
0
0.00
0.25
0.50
0.75
1.00
Relative Genomic position (Mb) or Gene distance (cumulative number of genes)

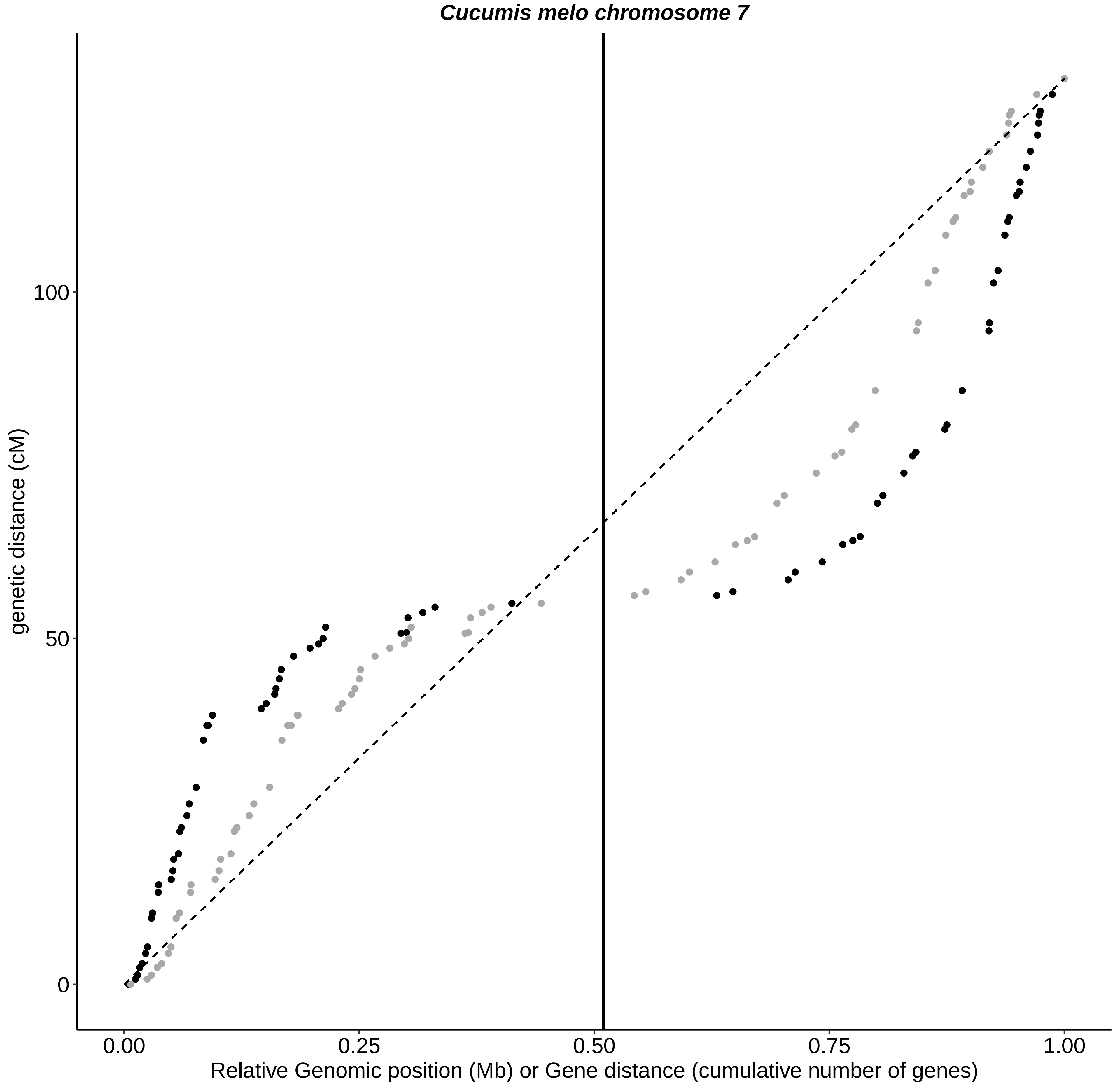
Cucumis melo chromosome 7
genetic distance (cM)
0
50
100
0.00
0.25
0.50
0.75
1.00
Relative Genomic position (Mb) or Gene distance (cumulative number of genes)

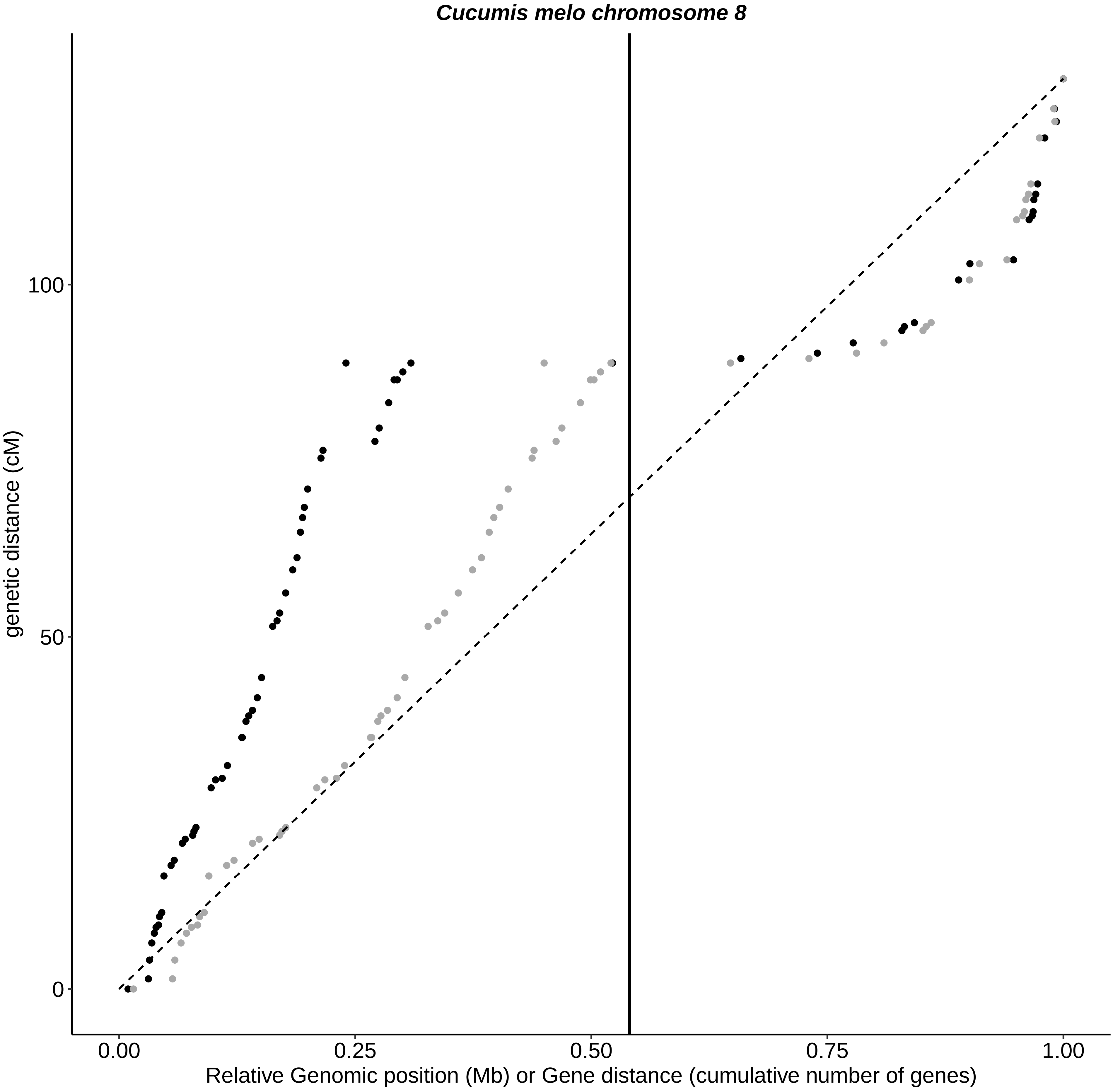
Cucumis melo chromosome 8
genetic distance (cM)
100
50
0
0.00
0.25
0.50
0.75
1.00
Relative Genomic position (Mb) or Gene distance (cumulative number of genes)

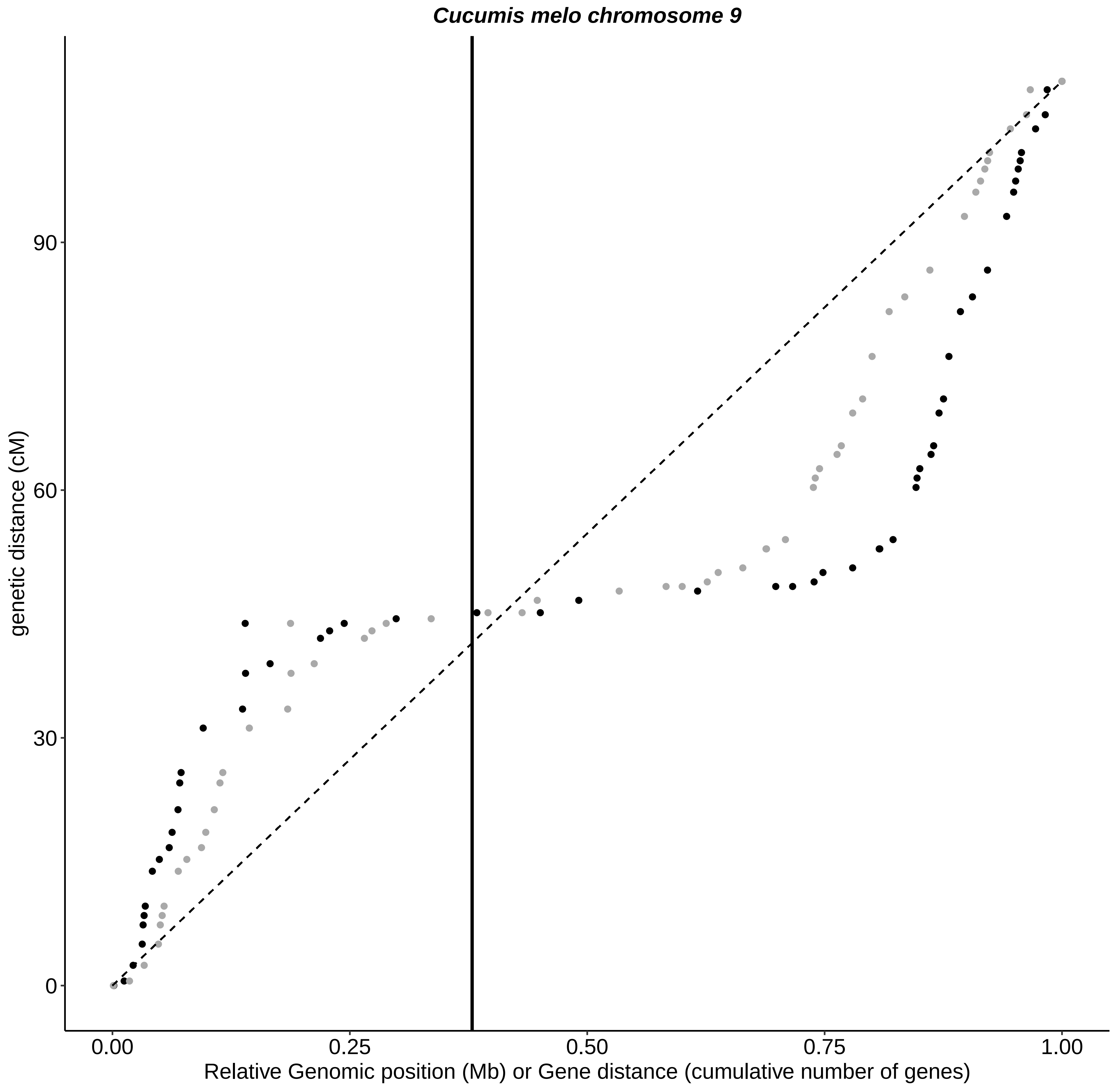
Cucumis melo chromosome 9
genetic distance (cM)
0
30
60
90
0.00
0.25
0.50
0.75
1.00
Relative Genomic position (Mb) or Gene distance (cumulative number of genes)

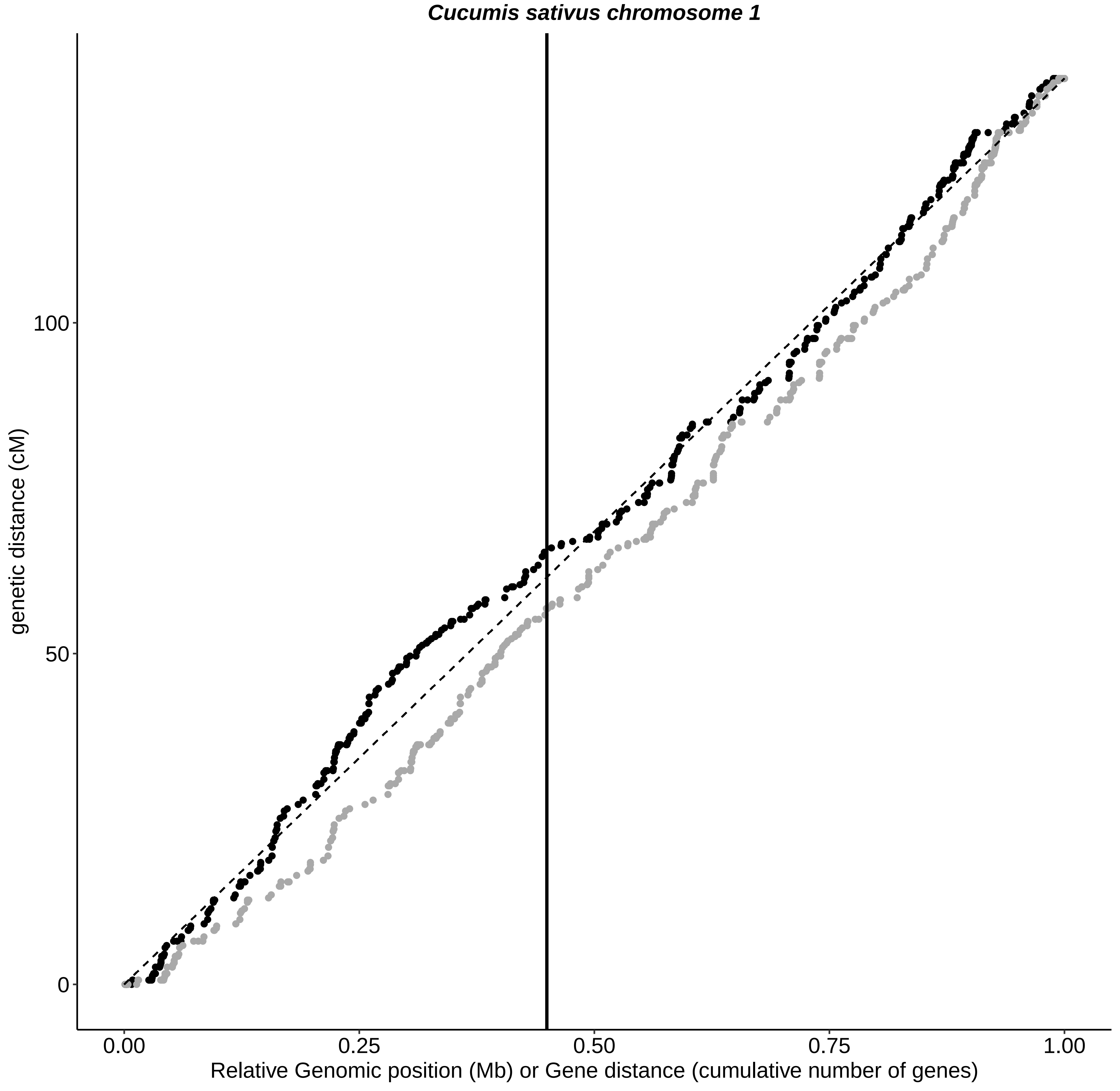
Cucumis sativus chromosome 1
genetic distance (cM)
0
50
100
0.00
0.25
0.50
0.75
1.00
Relative Genomic position (Mb) or Gene distance (cumulative number of genes)

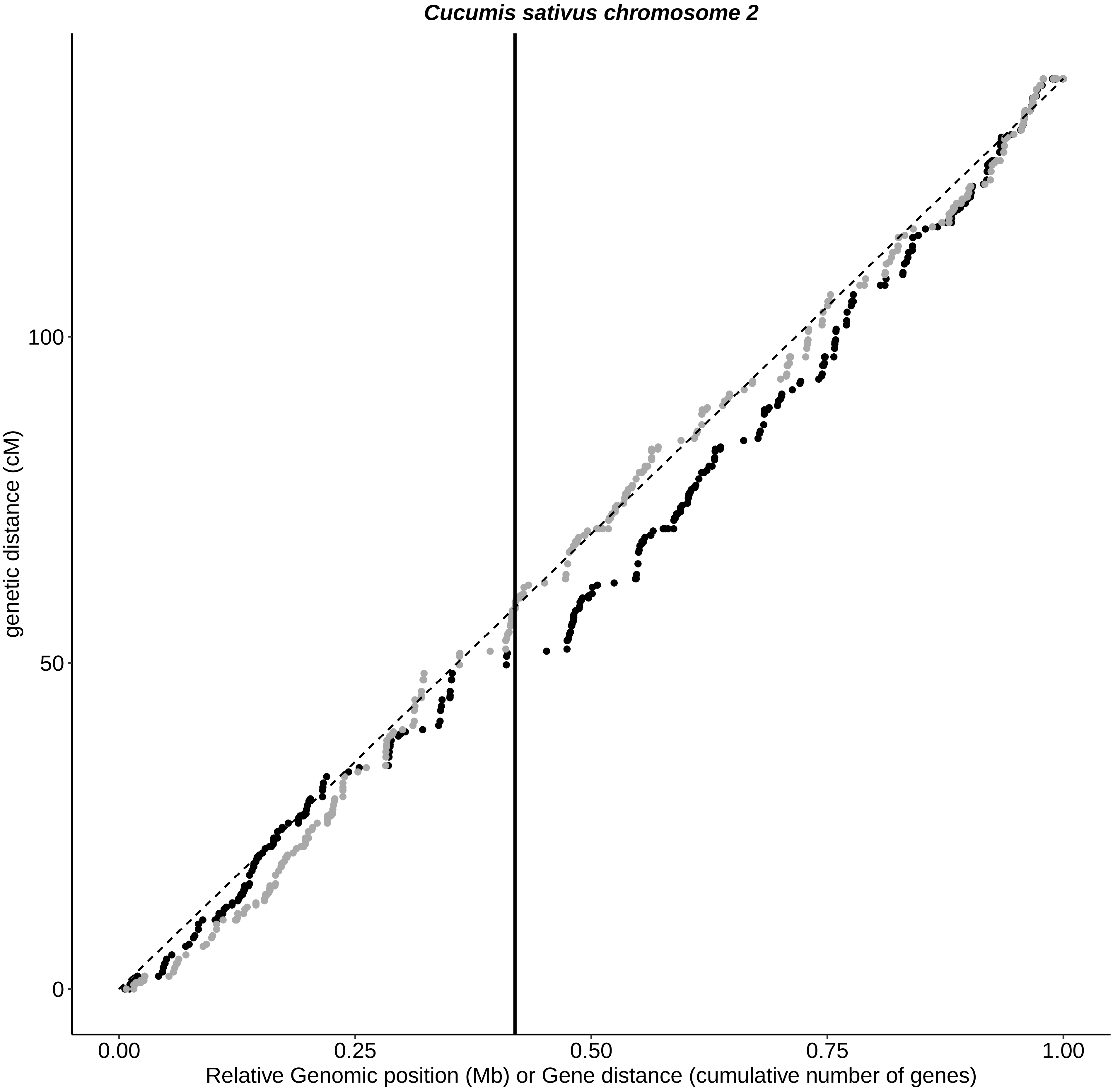
Cucumis sativus chromosome 2
genetic distance (cM)
0
50
100
0.00
0.25
0.50
0.75
1.00
Relative Genomic position (Mb) or Gene distance (cumulative number of genes)

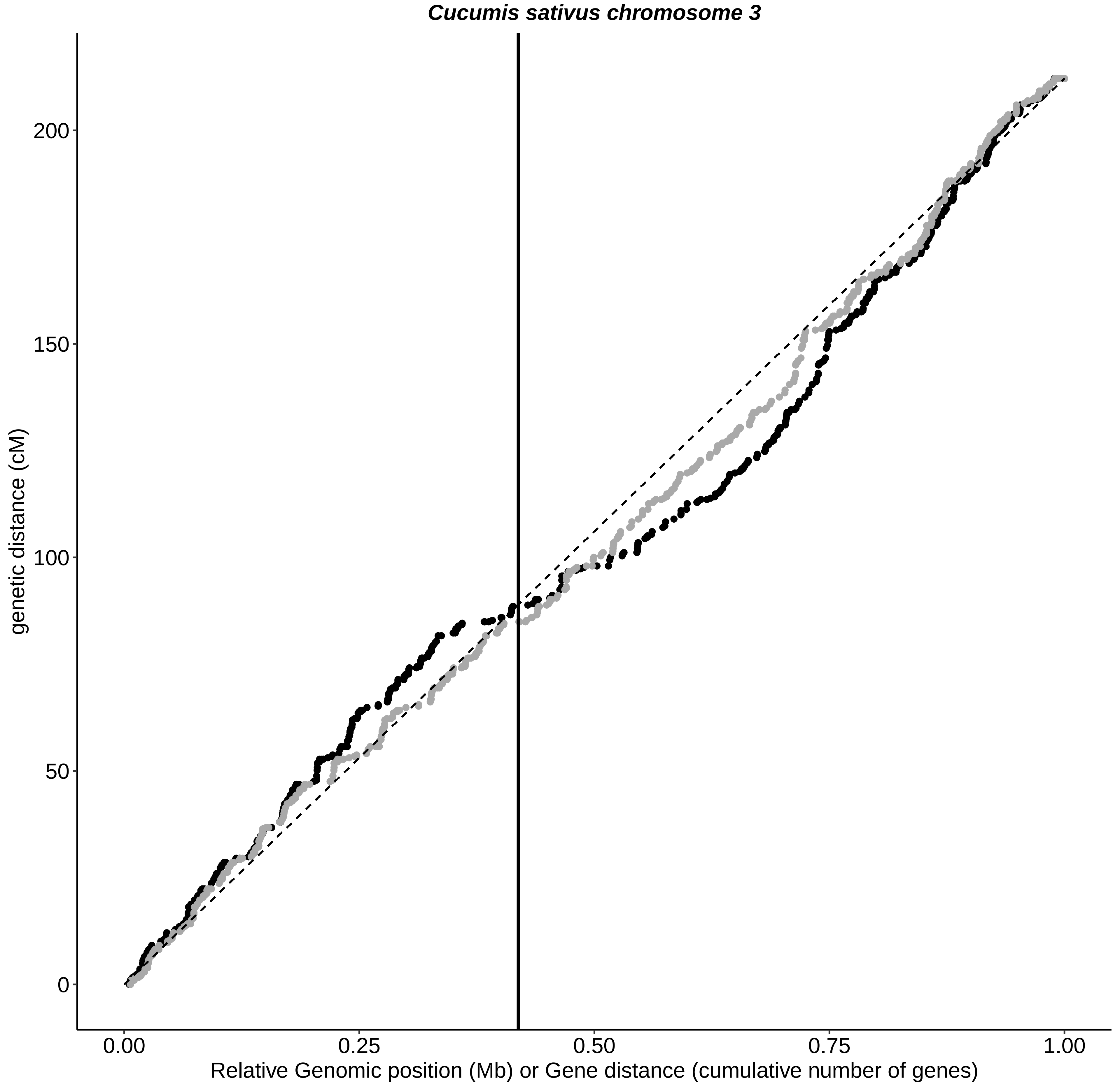
Cucumis sativus chromosome 3
genetic distance (cM)
0
50
100
150
200
0.00
0.25
0.50
0.75
1.00
Relative Genomic position (Mb) or Gene distance (cumulative number of genes)

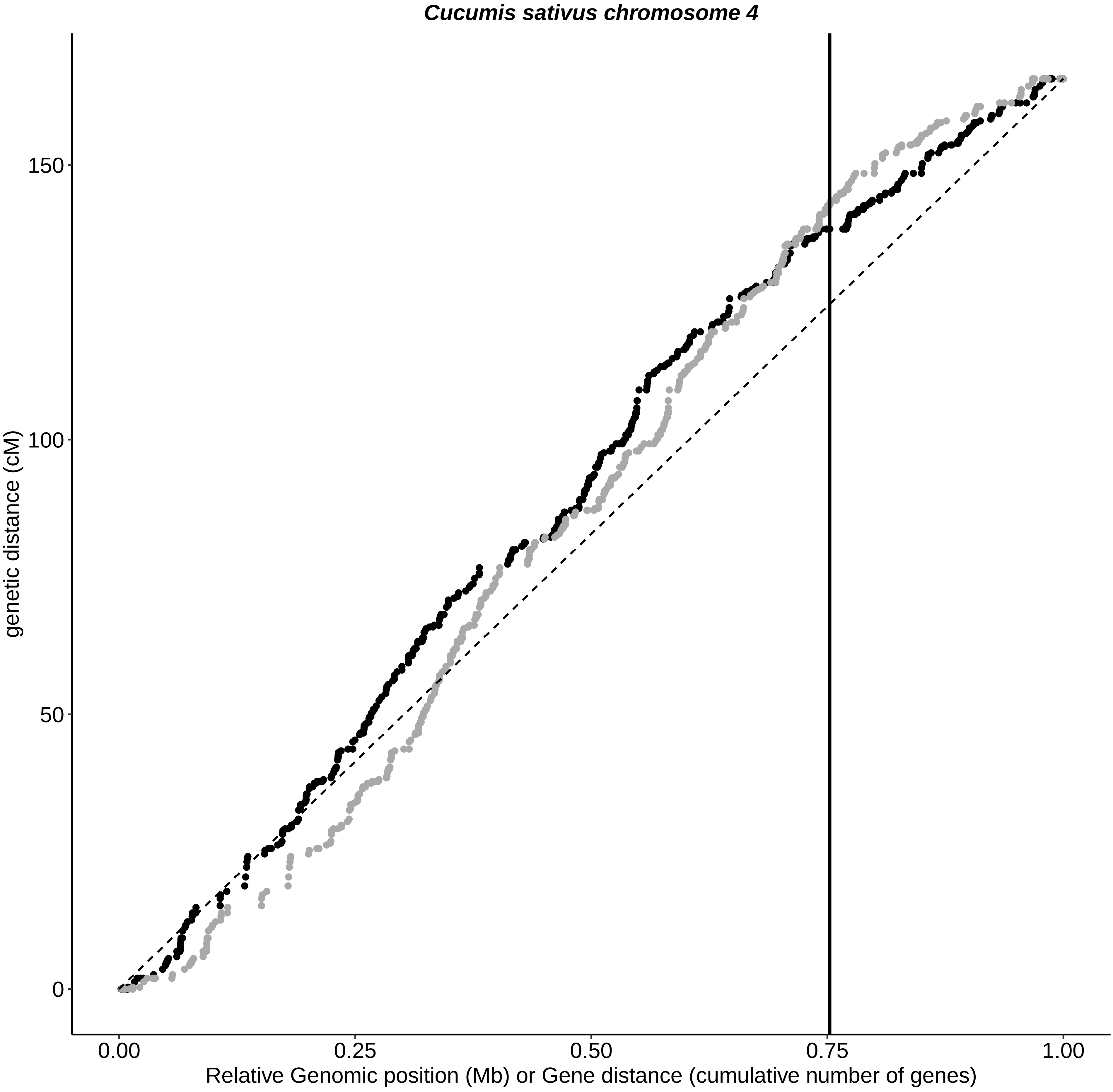
Cucumis sativus chromosome 4
genetic distance (cM)
150
100
50
0
0.00
0.25
0.50
0.75
1.00
Relative Genomic position (Mb) or Gene distance (cumulative number of genes)

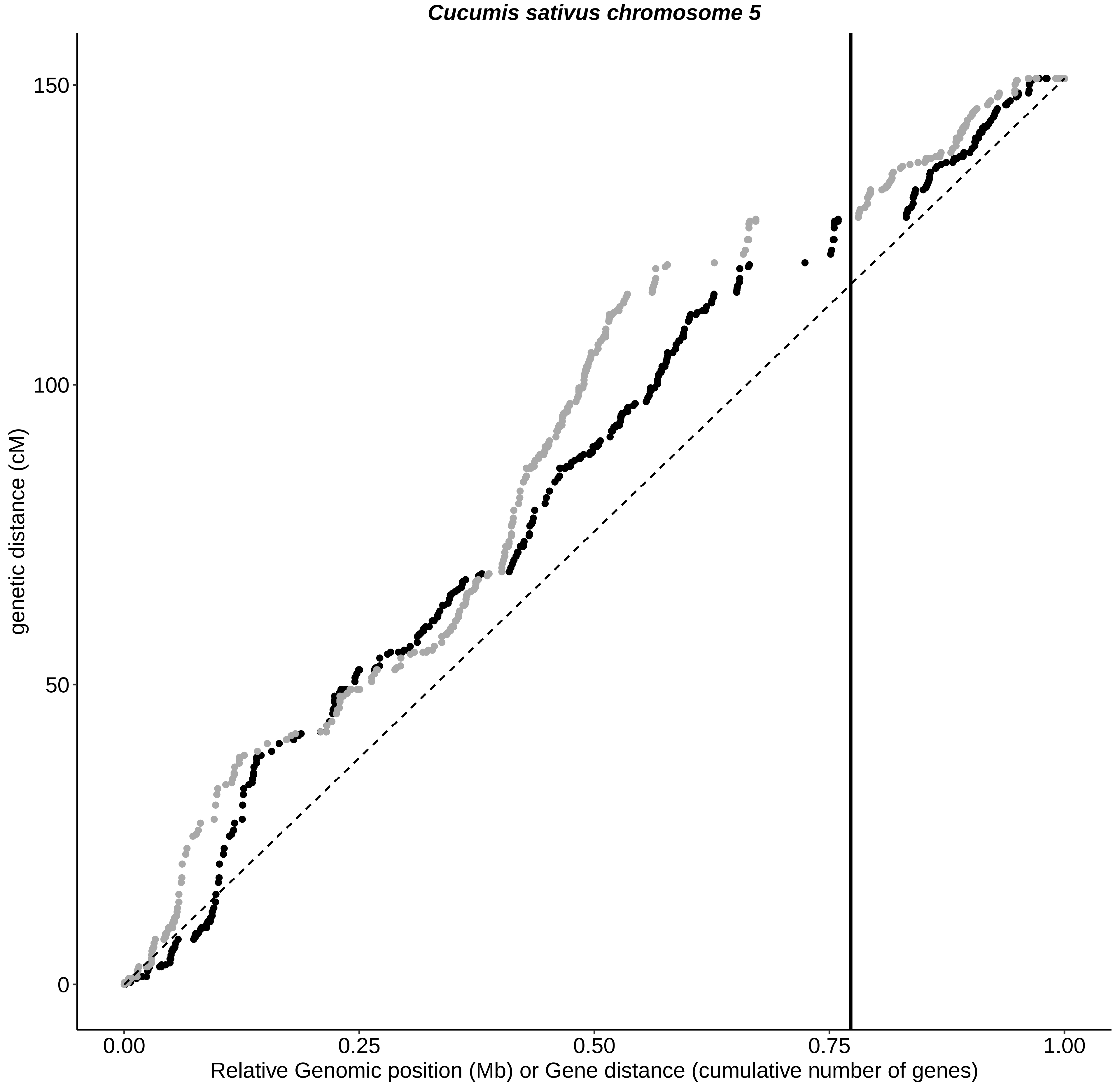
Cucumis sativus chromosome 5
genetic distance (cM)
150
100
50
0
0.00
0.25
0.50
0.75
1.00
Relative Genomic position (Mb) or Gene distance (cumulative number of genes)

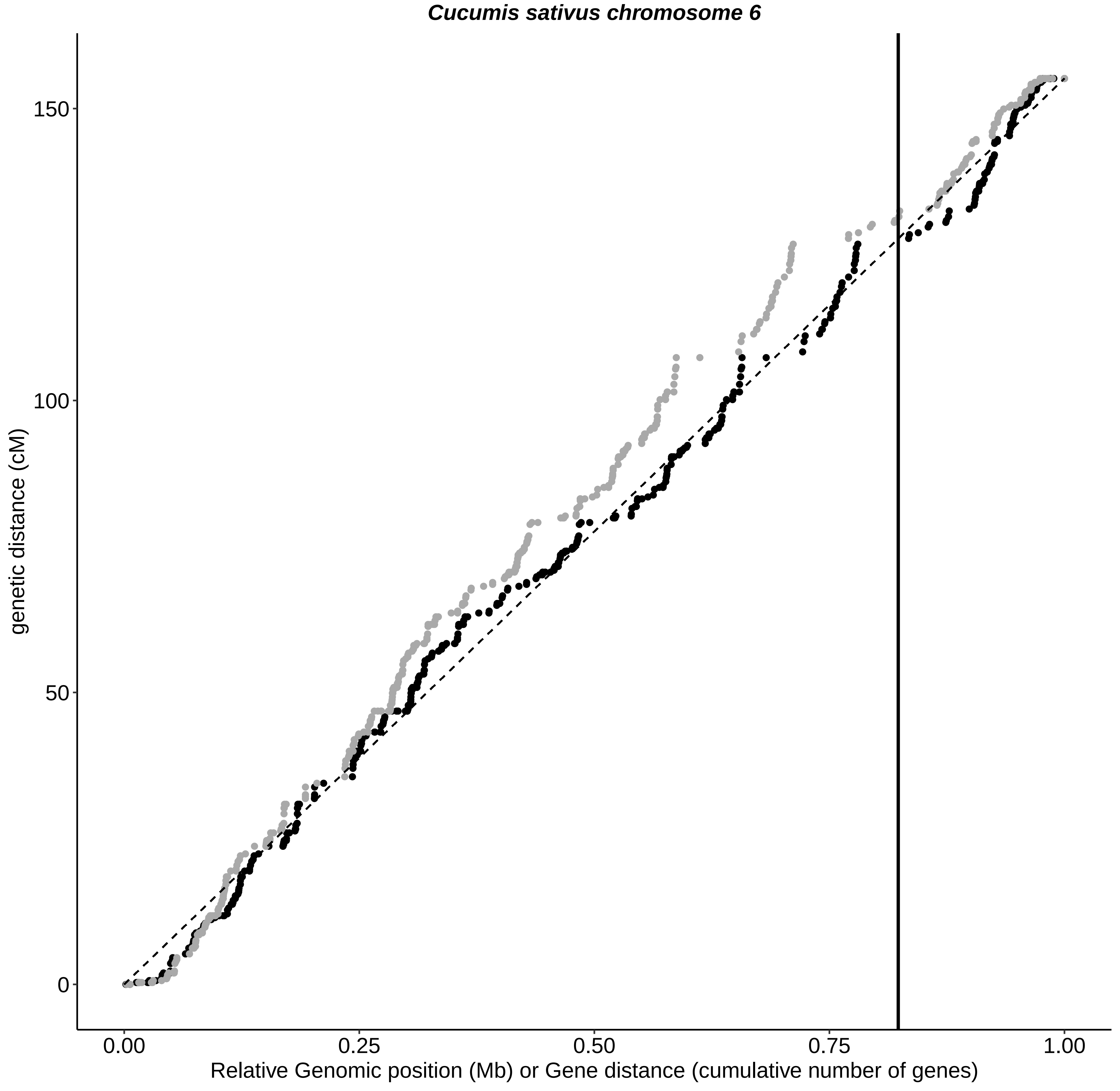
Cucumis sativus chromosome 6
genetic distance (cM)
0
50
100
150
0.00
0.25
0.50
0.75
1.00
Relative Genomic position (Mb) or Gene distance (cumulative number of genes)

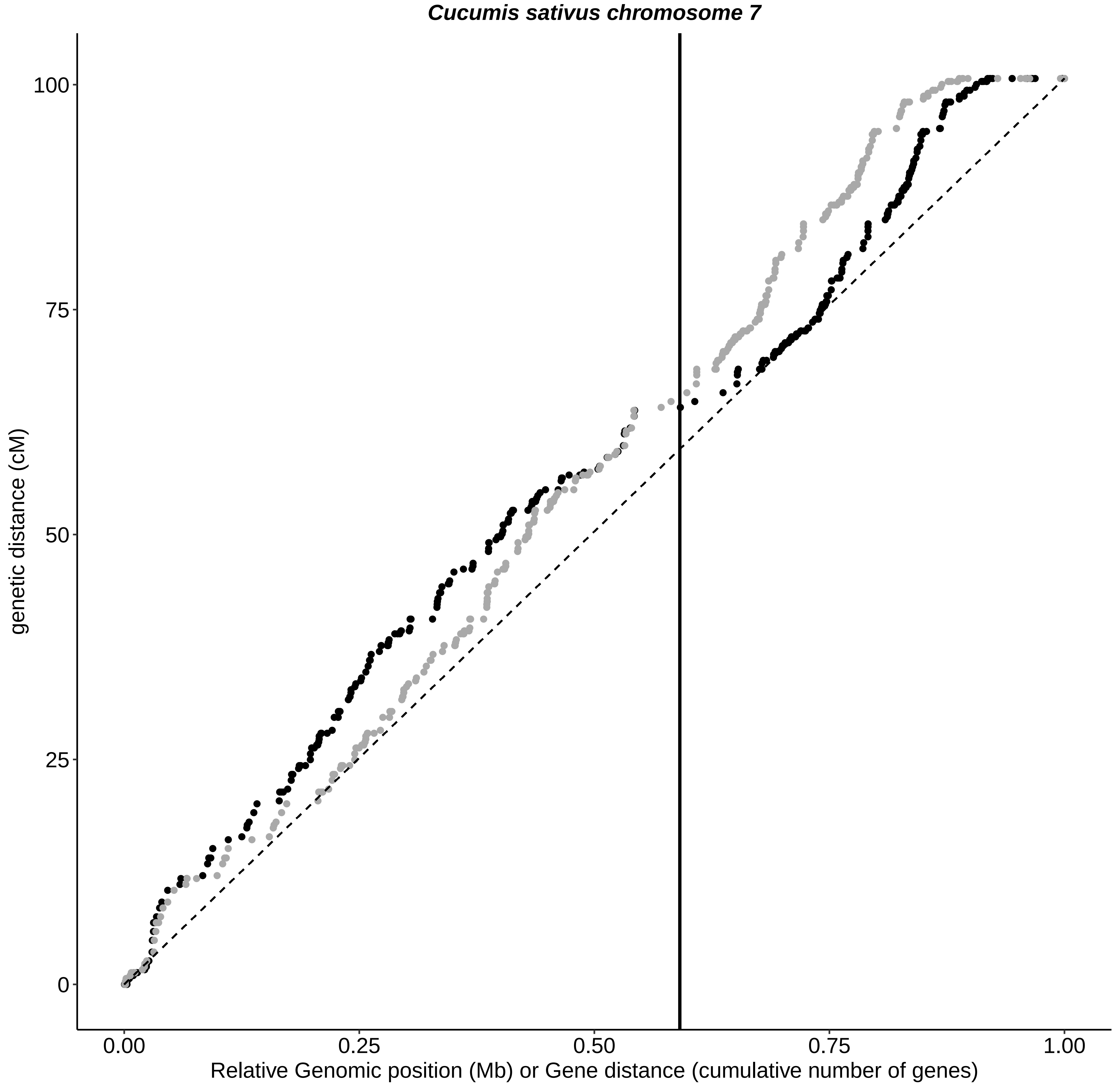
Cucumis sativus chromosome 7
genetic distance (cM)
0
25
50
75
100
0.00
0.25
0.50
0.75
1.00
Relative Genomic position (Mb) or Gene distance (cumulative number of genes)

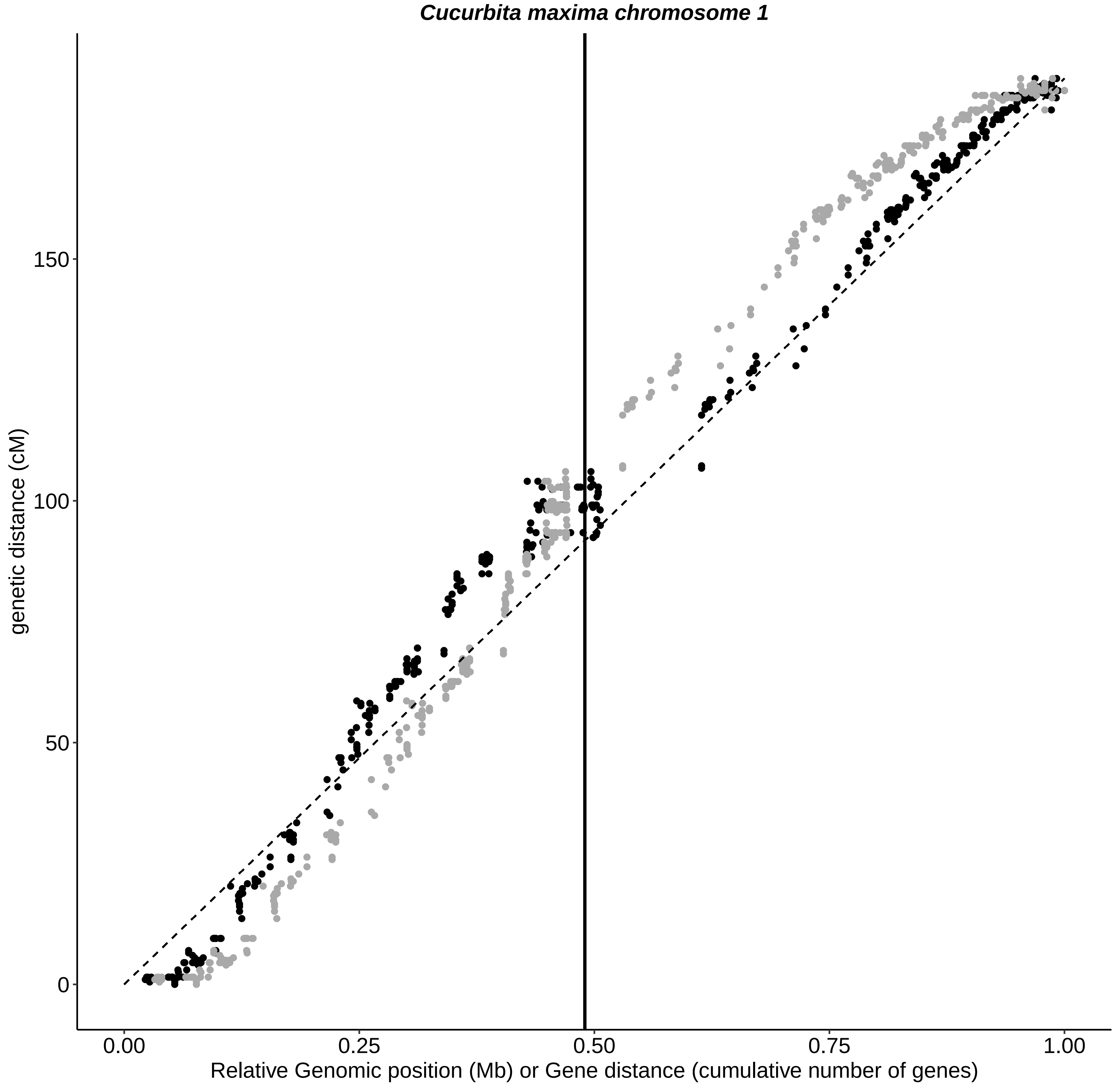
Cucurbita maxima chromosome 1
genetic distance (cM)
Relative Genomic position (Mb) or Gene distance (cumulative number of genes)
0
50
100
150
0.00
0.25
0.50
0.75
1.00

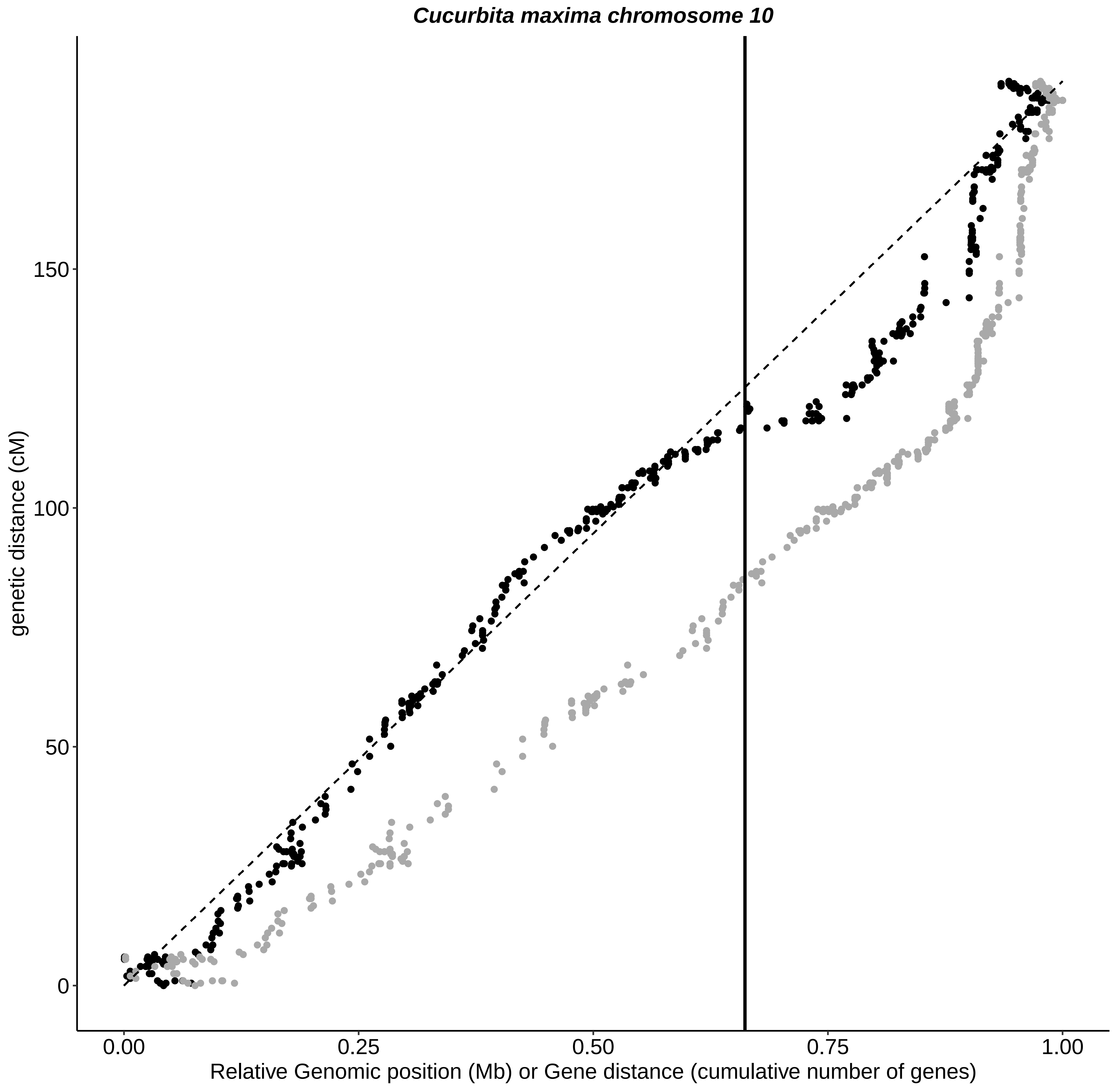
Cucurbita maxima chromosome 10
genetic distance (cM)
150
100
50
0
0.00
0.25
0.50
0.75
1.00
Relative Genomic position (Mb) or Gene distance (cumulative number of genes)

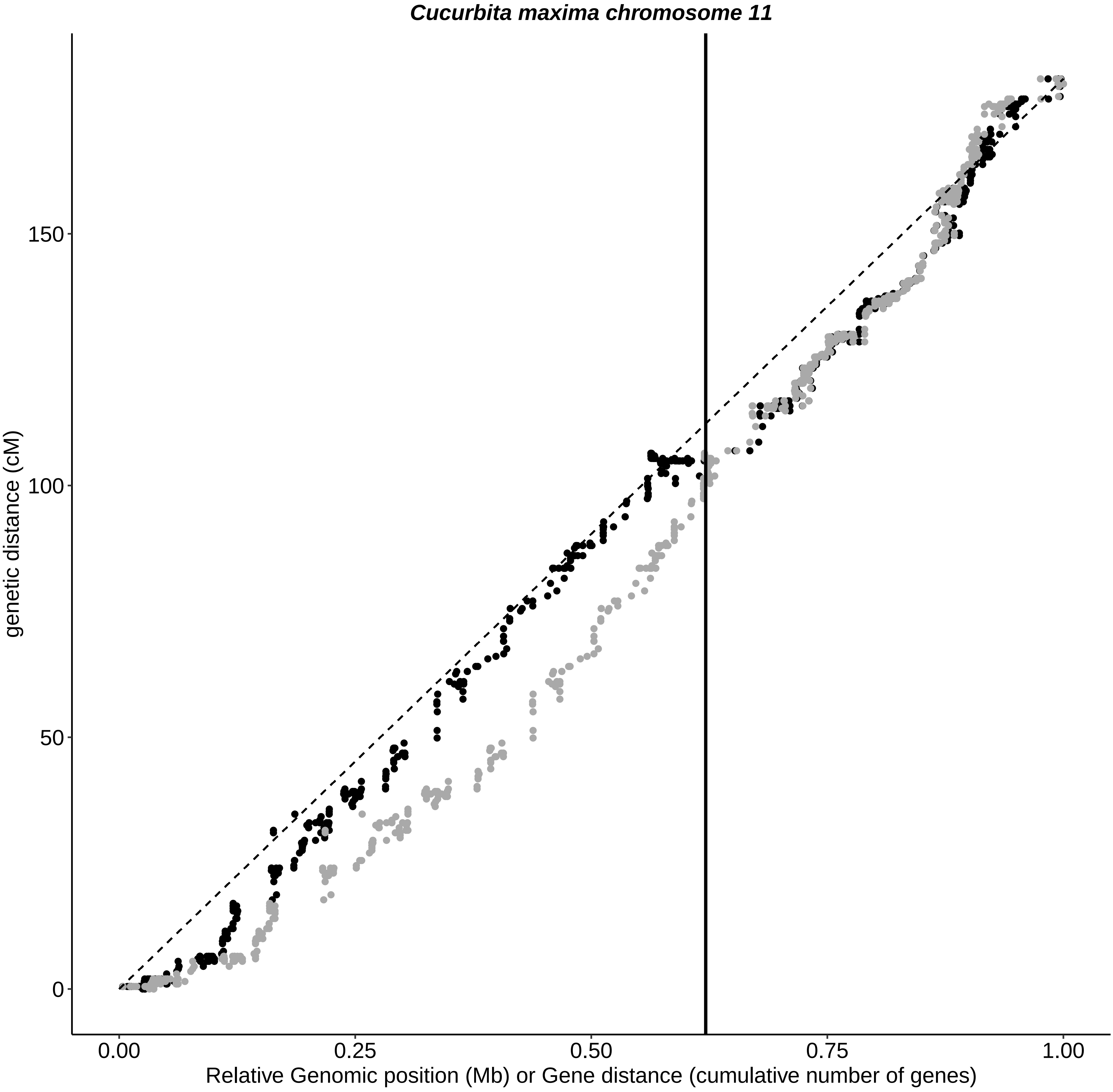
Cucurbita maxima chromosome 11
genetic distance (cM)
0
50
100
150
0.00
0.25
0.50
0.75
1.00
Relative Genomic position (Mb) or Gene distance (cumulative number of genes)

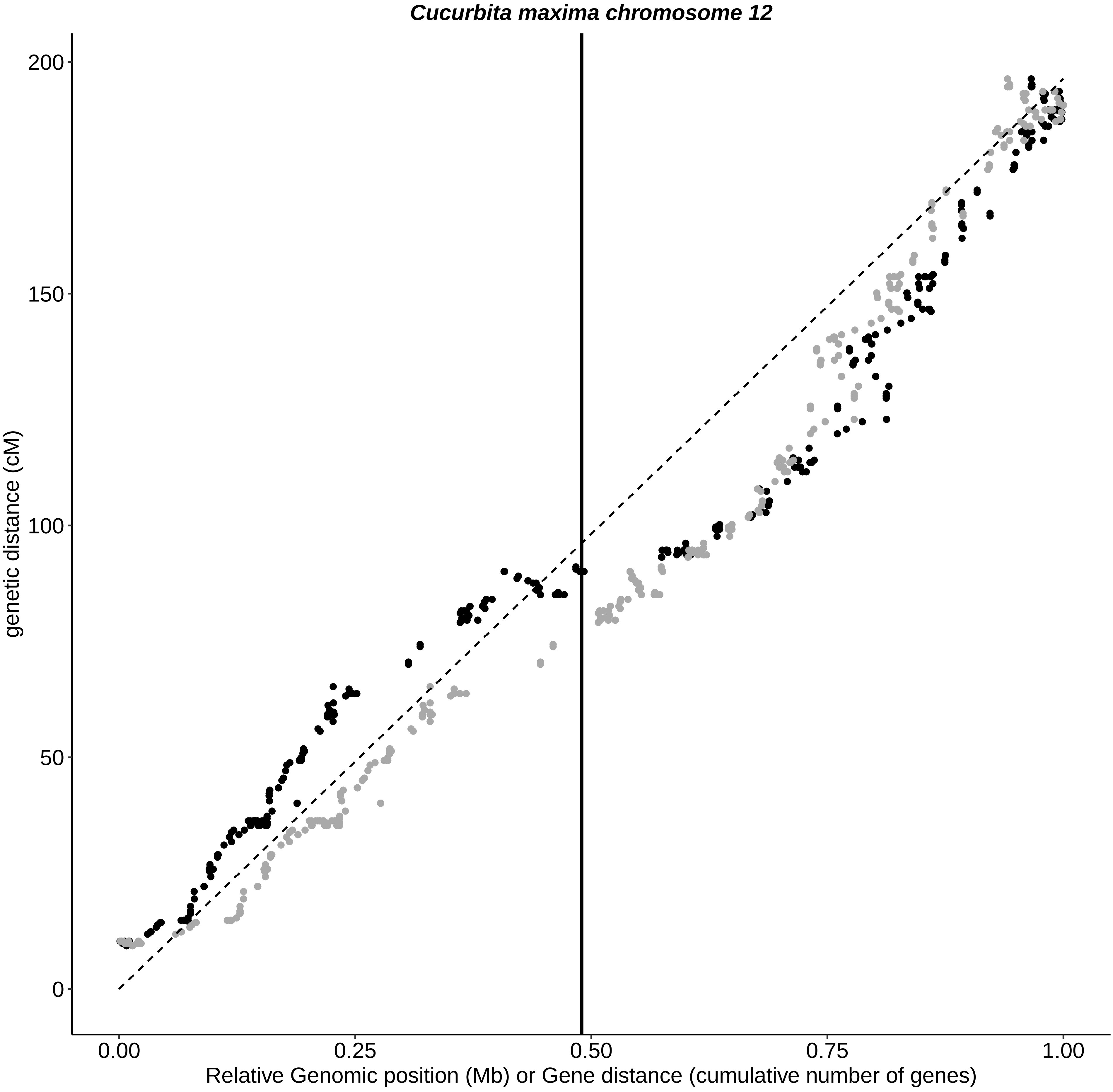
Cucurbita maxima chromosome 12
genetic distance (cM)
0
50
100
150
200
0.00
0.25
0.50
0.75
1.00
Relative Genomic position (Mb) or Gene distance (cumulative number of genes)

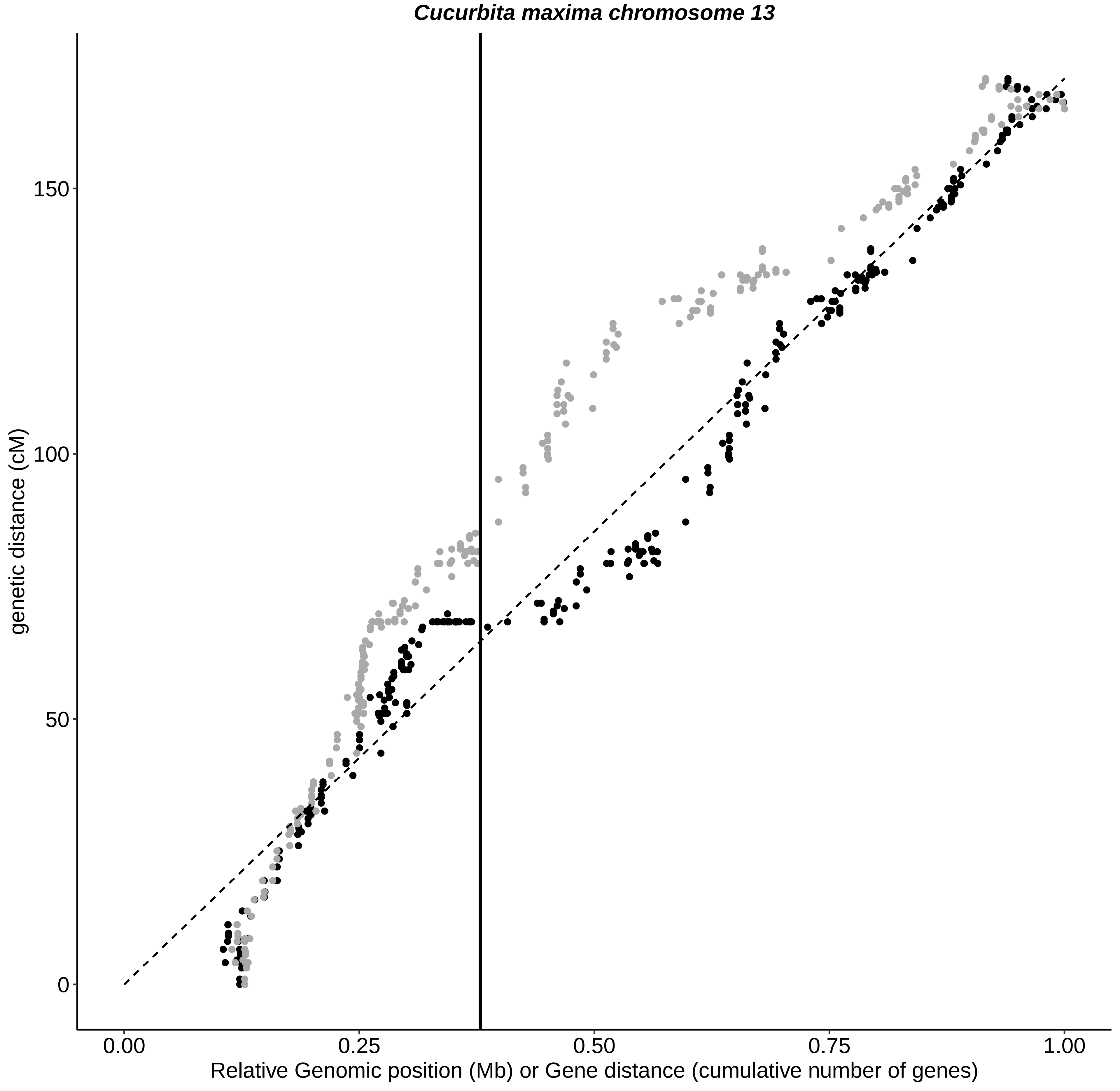
Cucurbita maxima chromosome 13
genetic distance (cM)
150
100
50
0
0.00
0.25
0.50
0.75
1.00
Relative Genomic position (Mb) or Gene distance (cumulative number of genes)

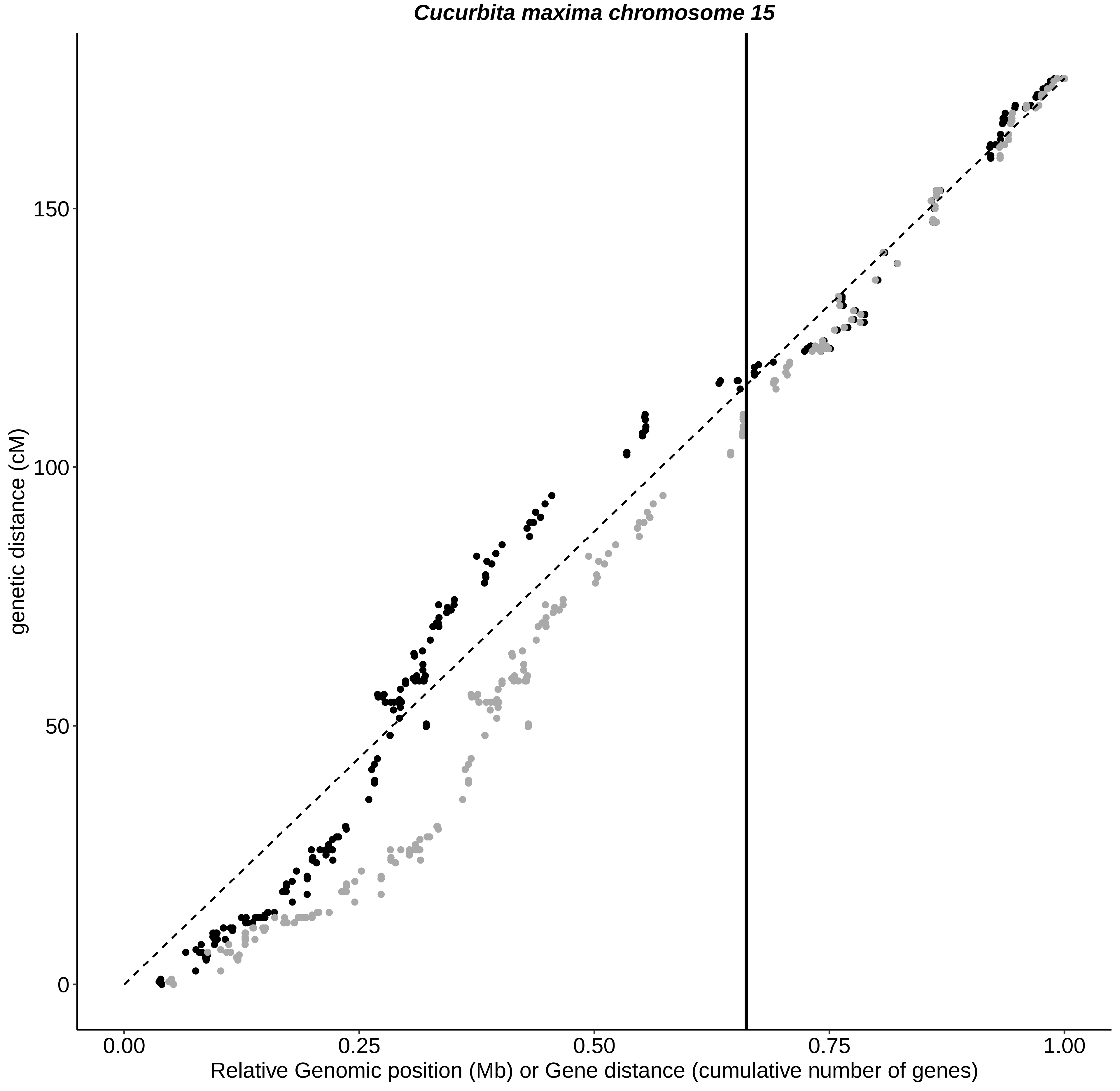
Cucurbita maxima chromosome 15
genetic distance (cM)
0
50
100
150
0.00
0.25
0.50
0.75
1.00
Relative Genomic position (Mb) or Gene distance (cumulative number of genes)

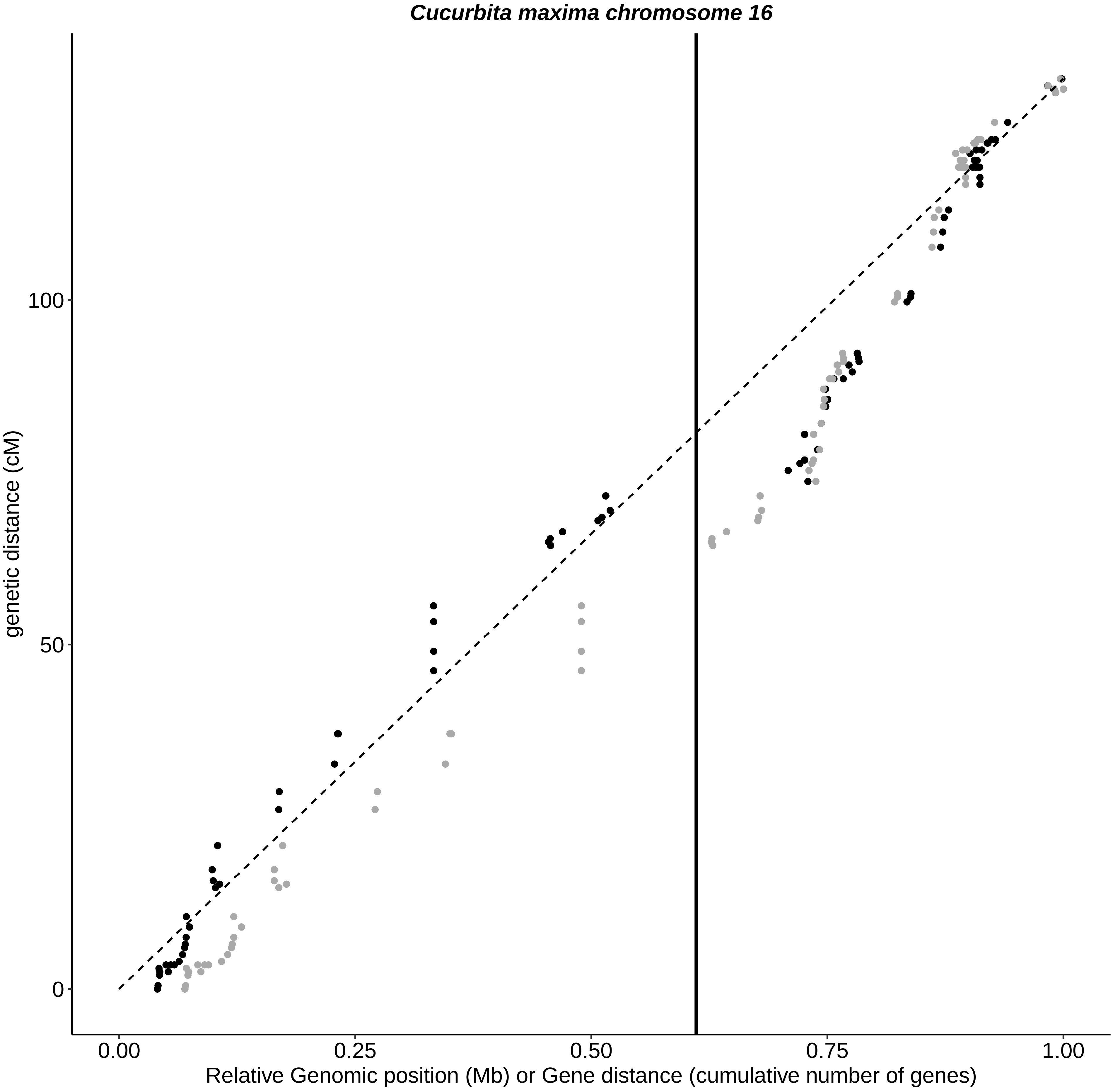
Cucurbita maxima chromosome 16
genetic distance (cM)
100
50
0
0.00
0.25
0.50
0.75
1.00
Relative Genomic position (Mb) or Gene distance (cumulative number of genes)

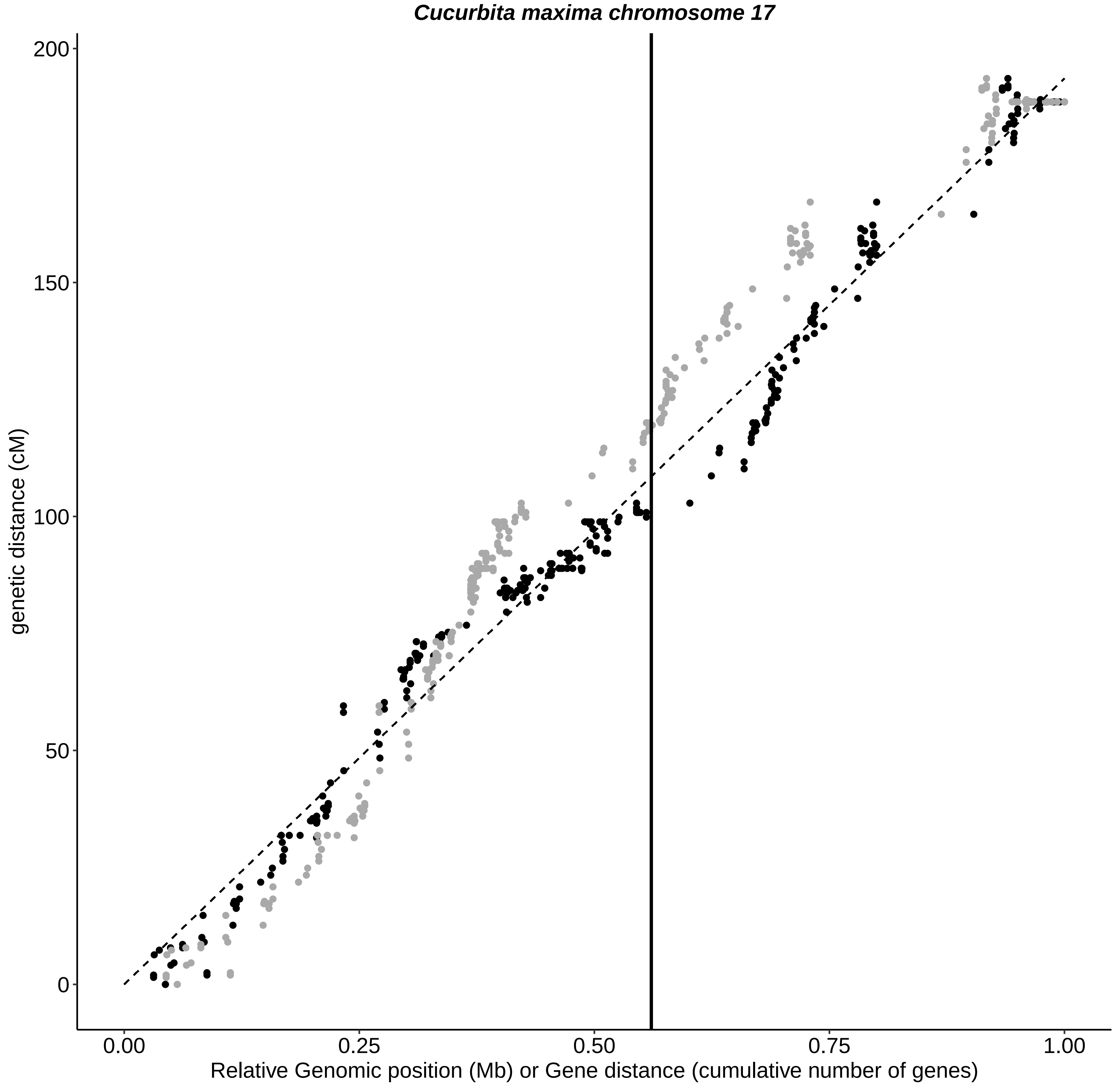

Cucurbita maxima chromosome 17
genetic distance (cM)
0
50
100
150
200
0.00
0.25
0.50
0.75
1.00
Relative Genomic position (Mb) or Gene distance (cumulative number of genes)

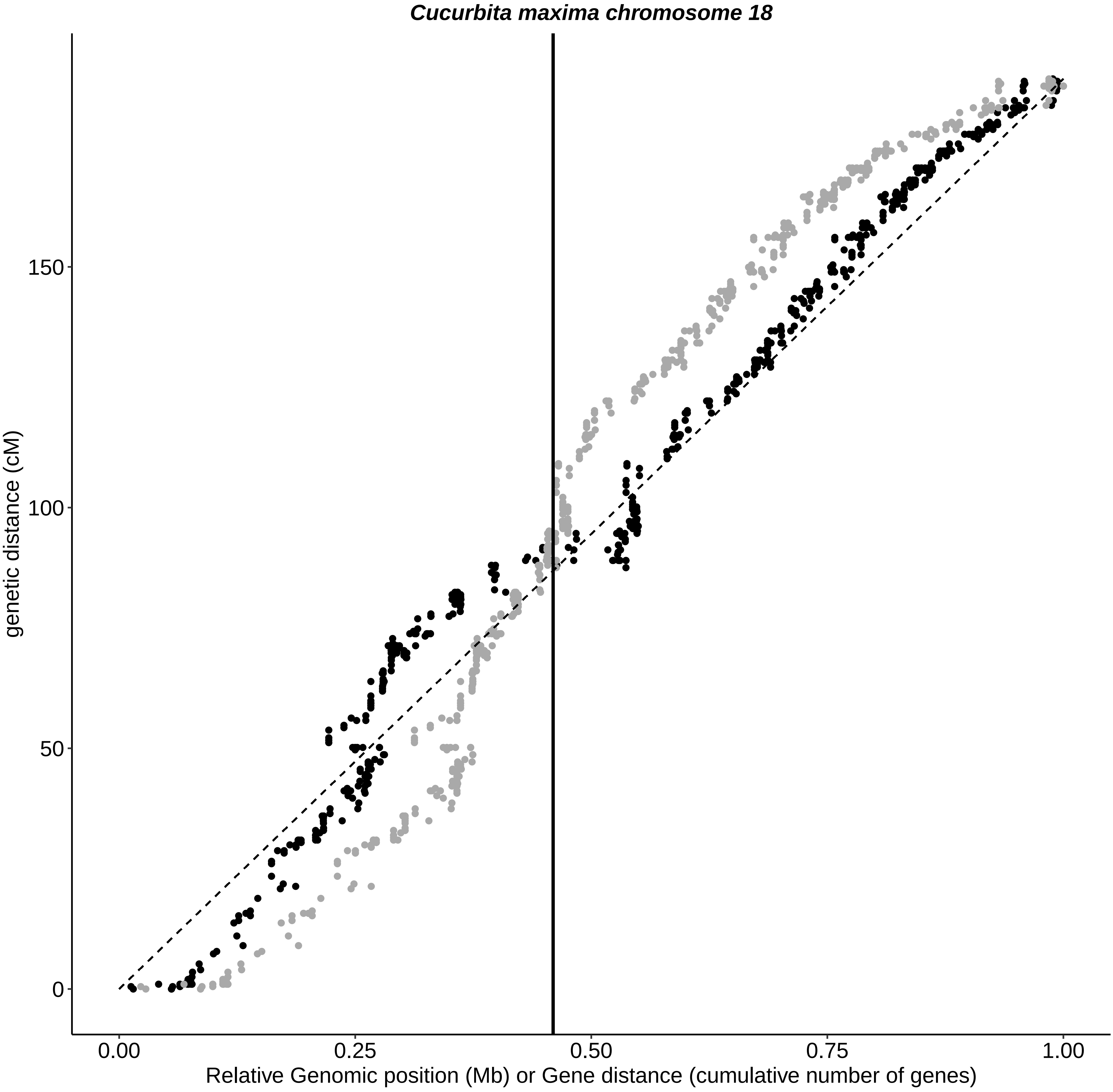
Cucurbita maxima chromosome 18
genetic distance (cM)
0
50
100
150
0.00
0.25
0.50
0.75
1.00
Relative Genomic position (Mb) or Gene distance (cumulative number of genes)

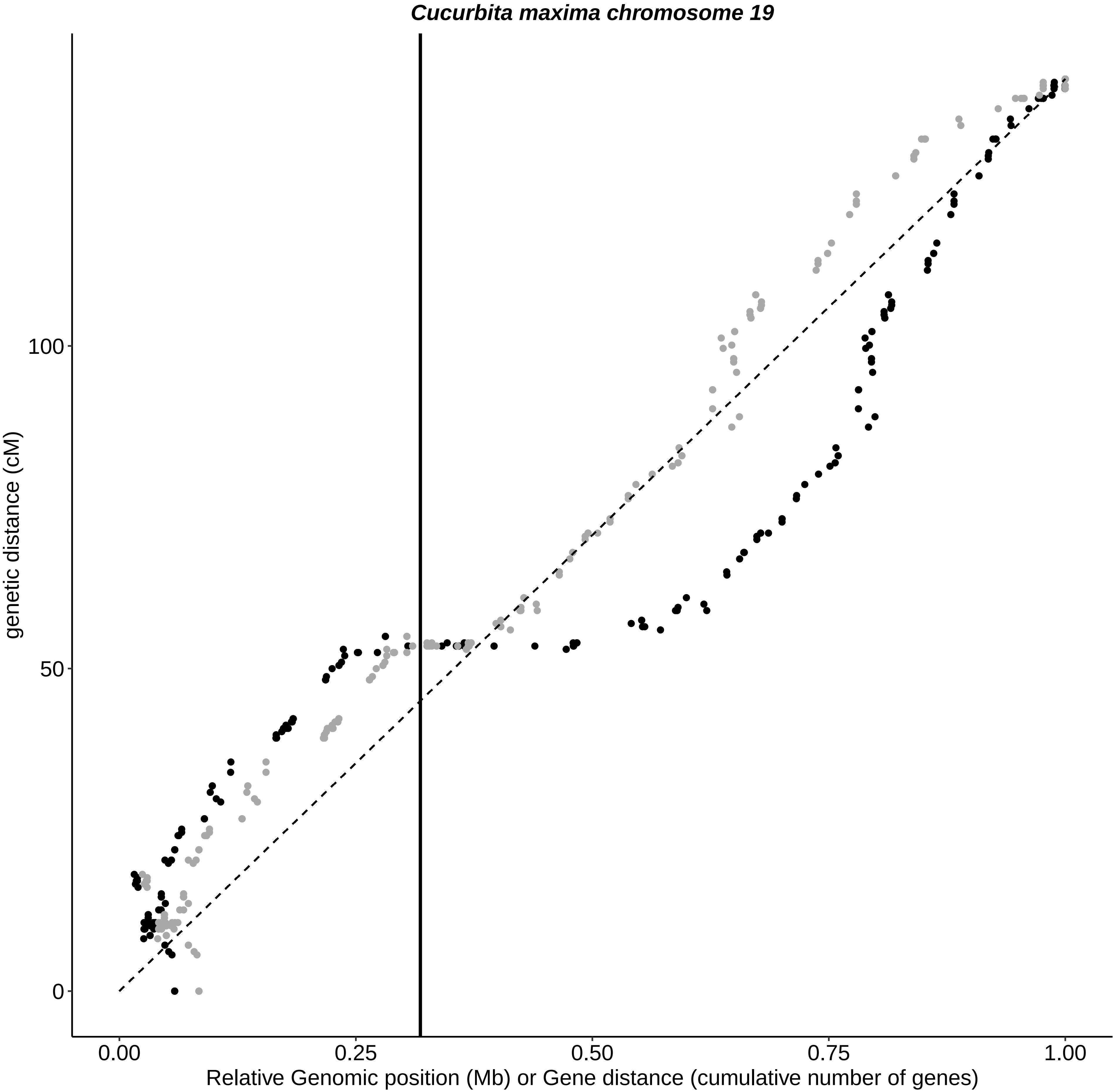
Cucurbita maxima chromosome 19
genetic distance (cM)
0
50
100
0.00
0.25
0.50
0.75
1.00
Relative Genomic position (Mb) or Gene distance (cumulative number of genes)

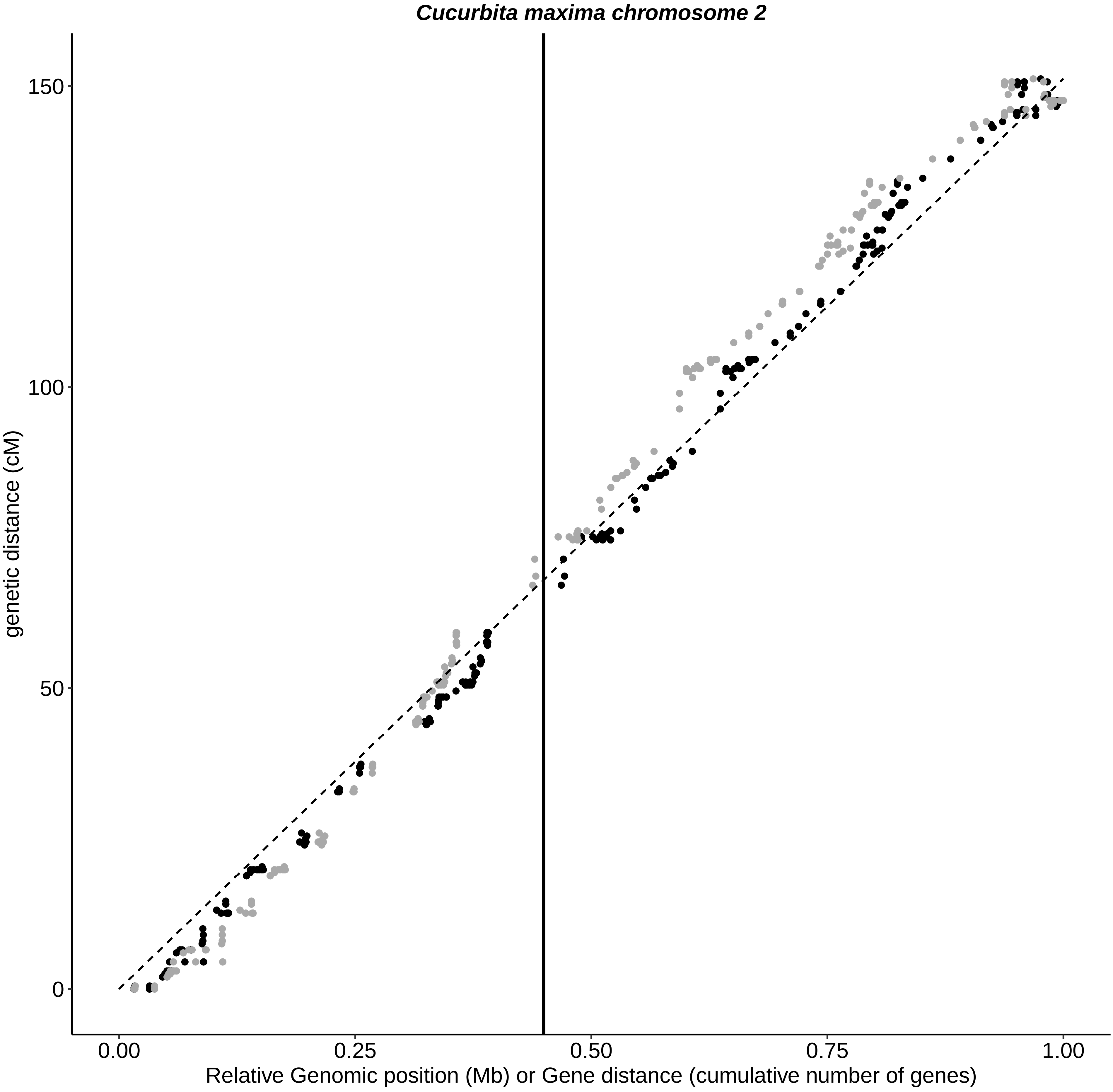
Cucurbita maxima chromosome 2
genetic distance (cM)
150
100
50
0
0.00
0.25
0.50
0.75
1.00
Relative Genomic position (Mb) or Gene distance (cumulative number of genes)

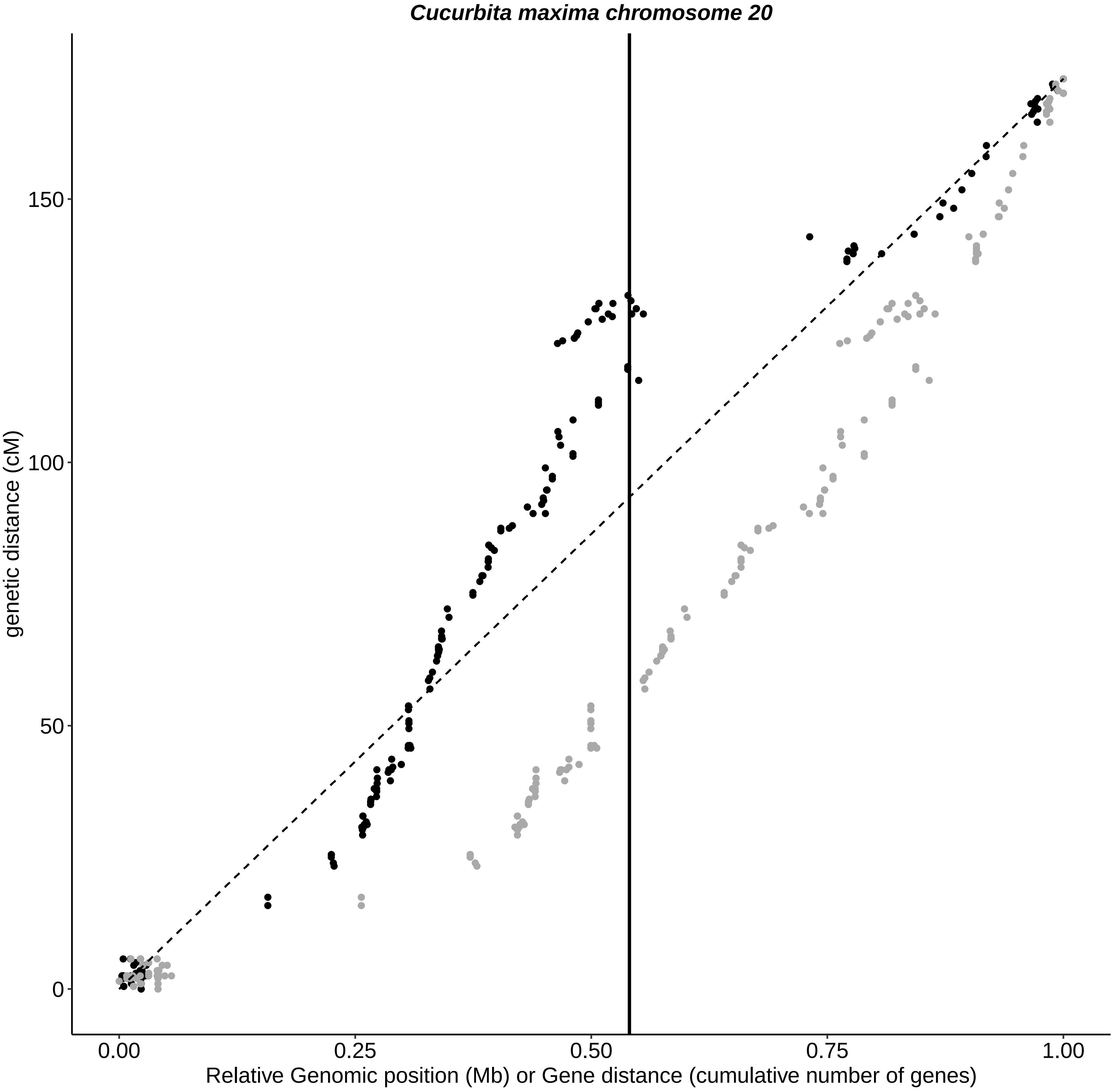

Cucurbita maxima chromosome 20
genetic distance (cM)
0
50
100
150
0.00
0.25
0.50
0.75
1.00
Relative Genomic position (Mb) or Gene distance (cumulative number of genes)

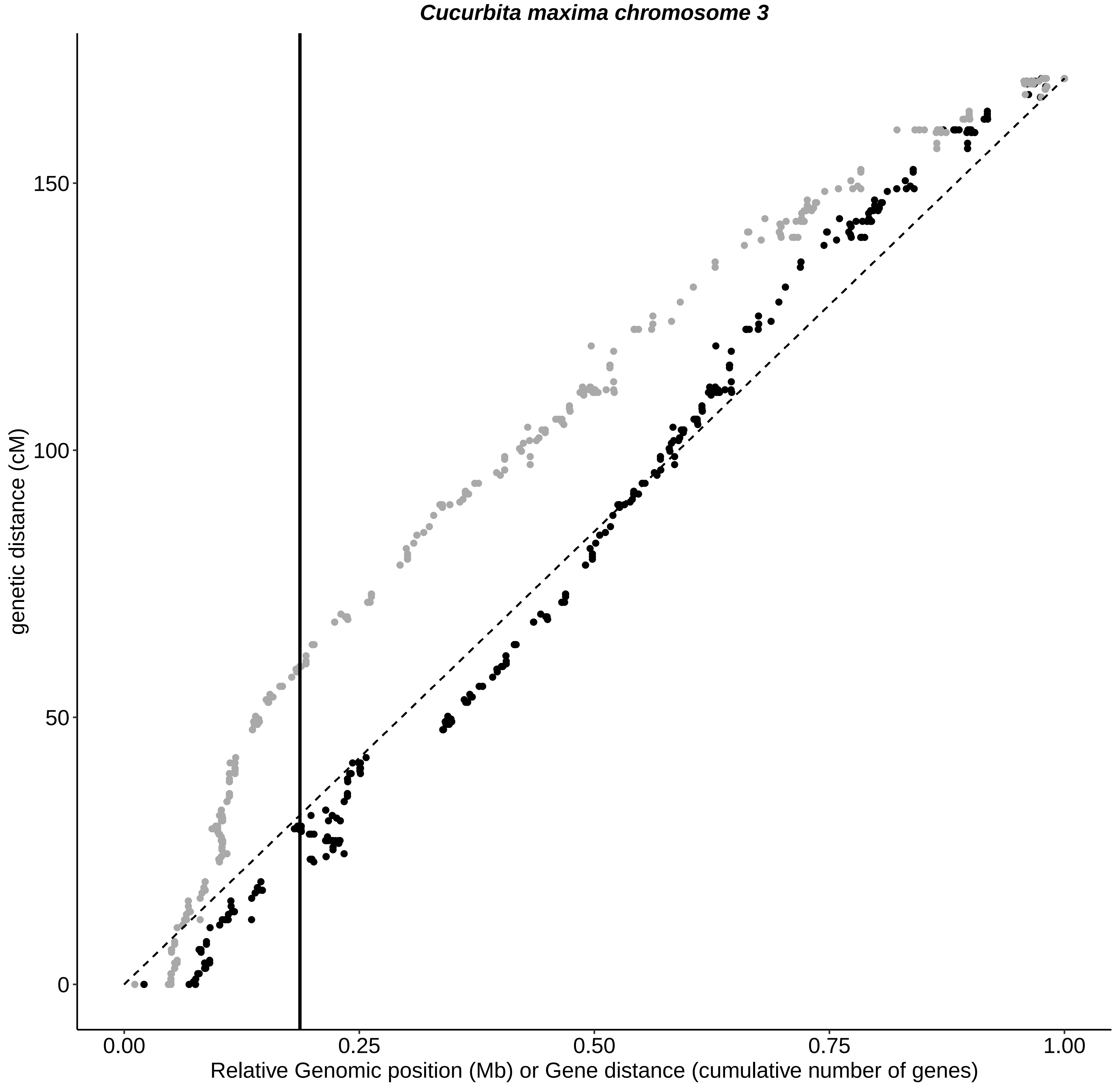
Cucurbita maxima chromosome 3
genetic distance (cM)
0
50
100
150
0.00
0.25
0.50
0.75
1.00
Relative Genomic position (Mb) or Gene distance (cumulative number of genes)

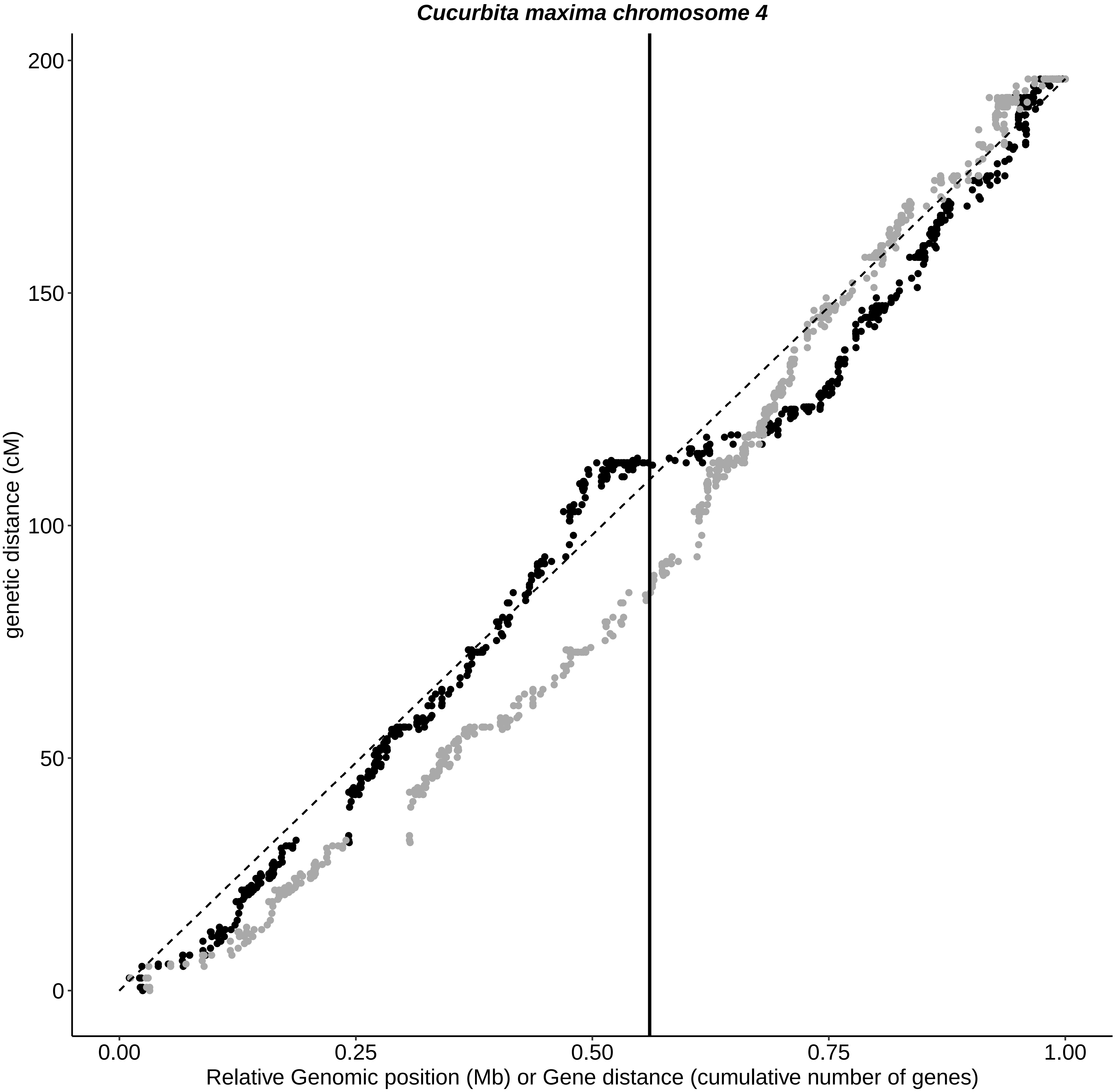
Cucurbita maxima chromosome 4
genetic distance (cM)
200
150
100
50
0
0.00
0.25
0.50
0.75
1.00
Relative Genomic position (Mb) or Gene distance (cumulative number of genes)

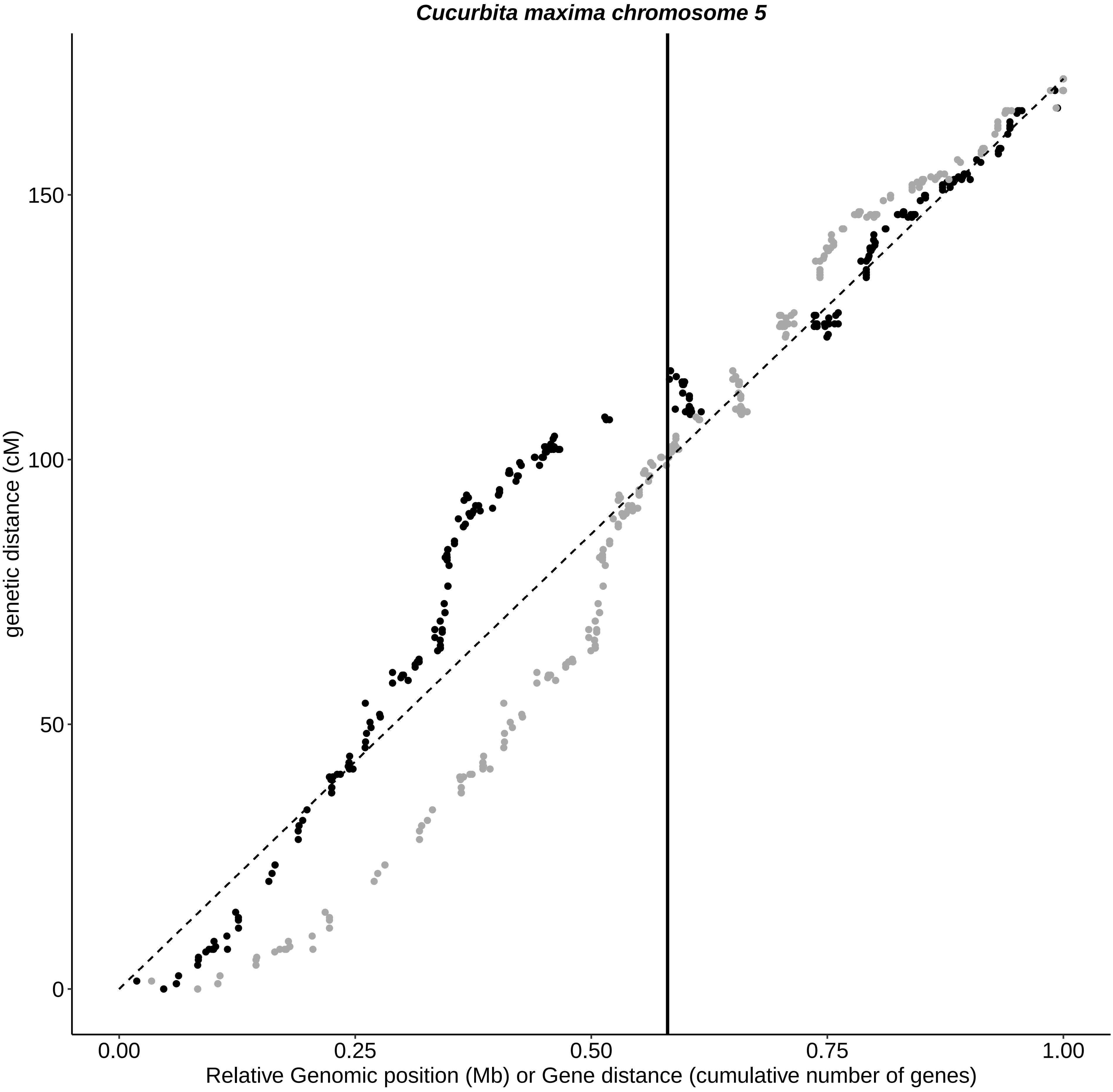
Cucurbita maxima chromosome 5
genetic distance (cM)
0
50
100
150
0.00
0.25
0.50
0.75
1.00
Relative Genomic position (Mb) or Gene distance (cumulative number of genes)

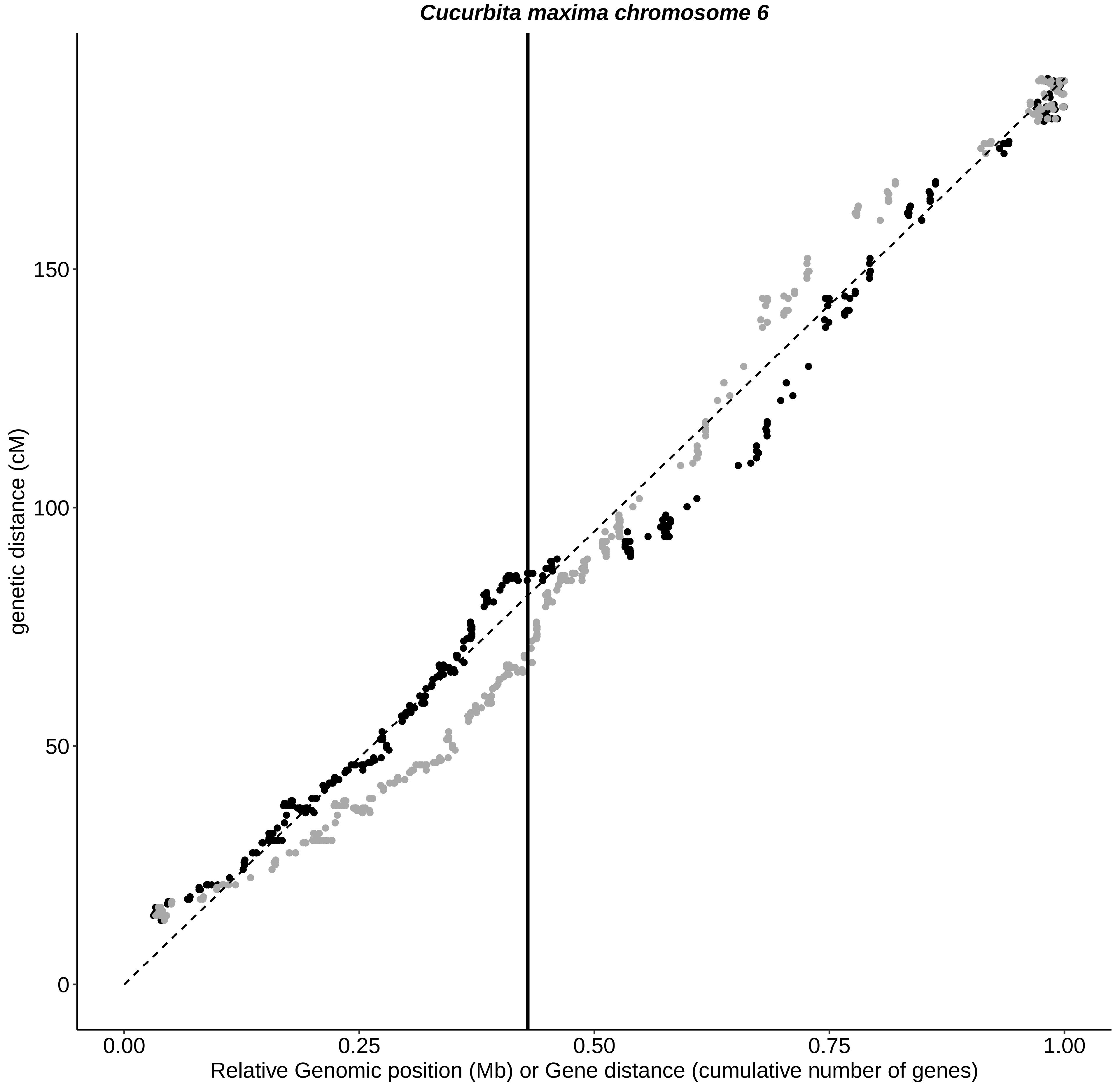
Cucurbita maxima chromosome 6
genetic distance (cM)
0
50
100
150
0.00
0.25
0.50
0.75
1.00
Relative Genomic position (Mb) or Gene distance (cumulative number of genes)

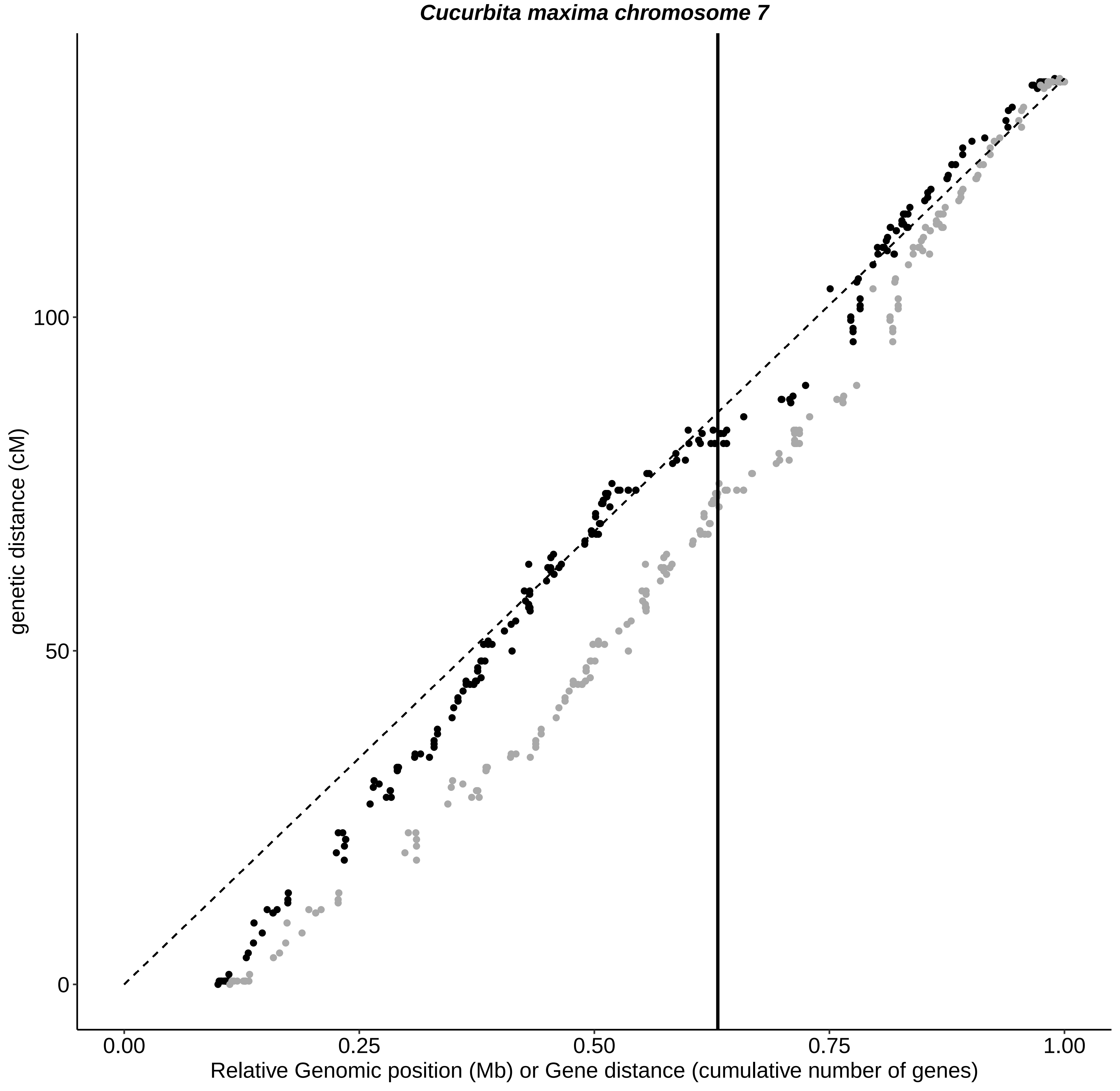
Cucurbita maxima chromosome 7
genetic distance (cM)
0
50
100
0.00
0.25
0.50
0.75
1.00
Relative Genomic position (Mb) or Gene distance (cumulative number of genes)

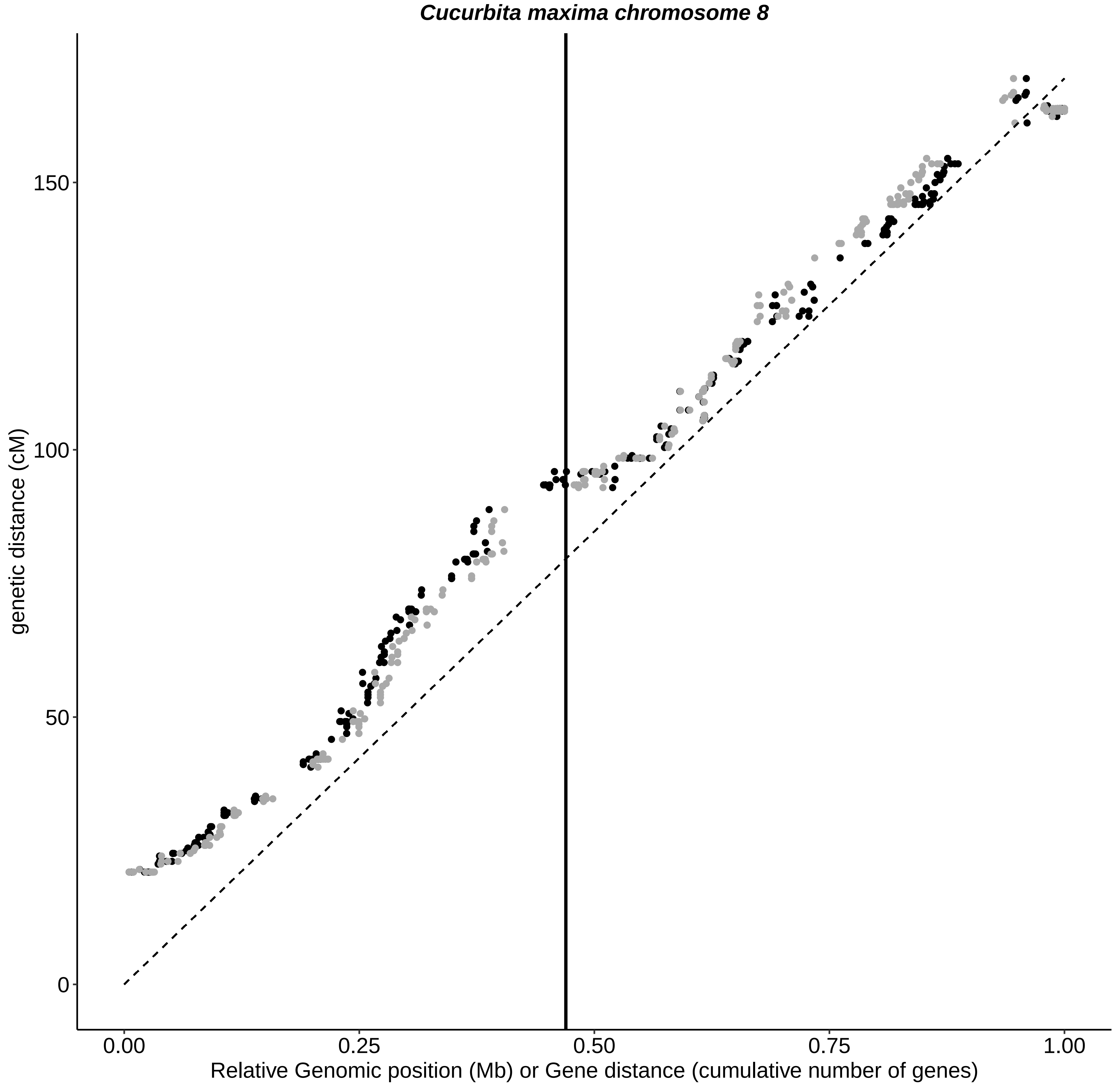
Cucurbita maxima chromosome 8
genetic distance (cM)
150
100
50
0
0.00
0.25
0.50
0.75
1.00
Relative Genomic position (Mb) or Gene distance (cumulative number of genes)

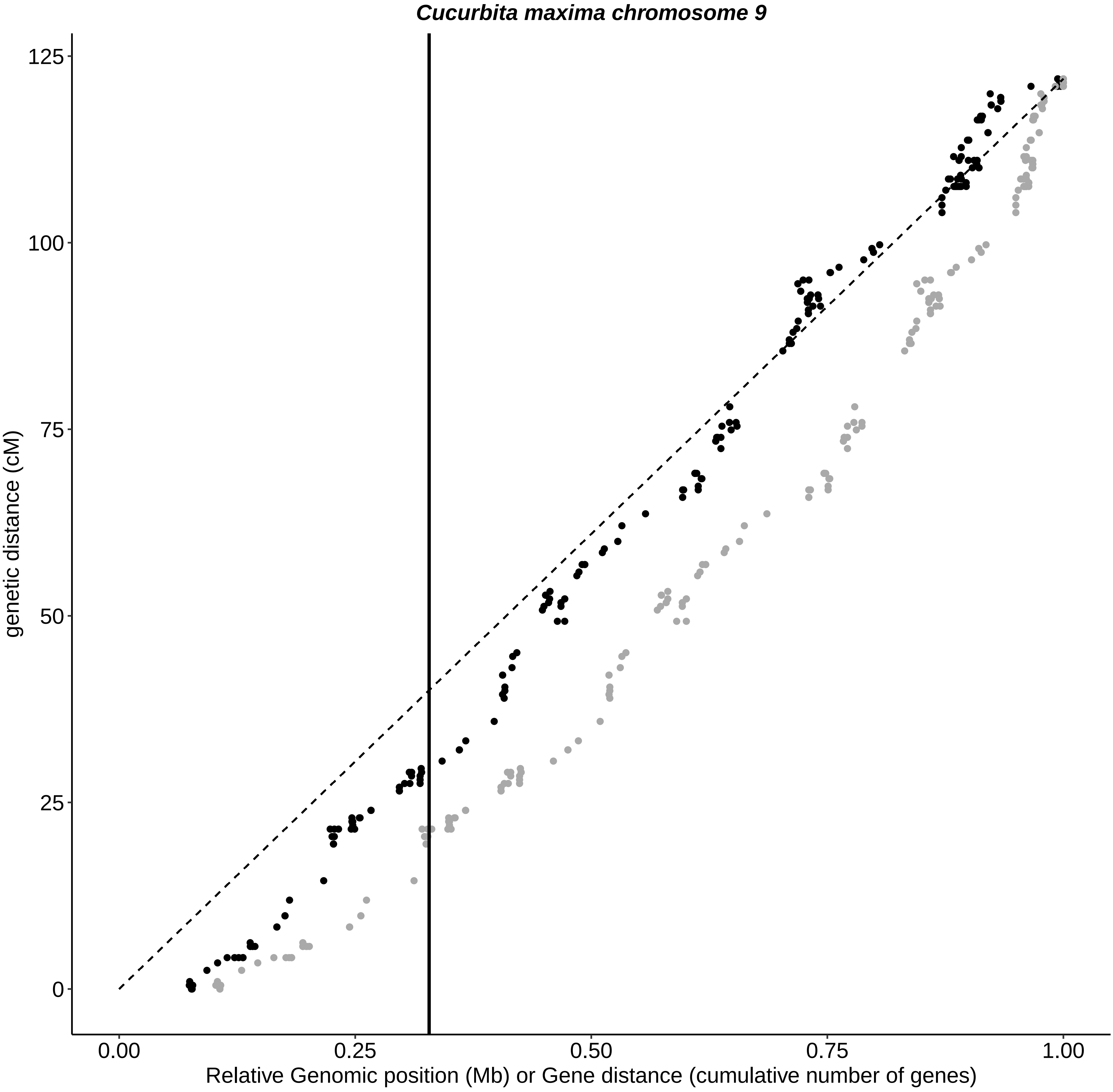
Cucurbita maxima chromosome 9
genetic distance (cM)
125
100
75
50
25
0
0.00
0.25
0.50
0.75
1.00
Relative Genomic position (Mb) or Gene distance (cumulative number of genes)

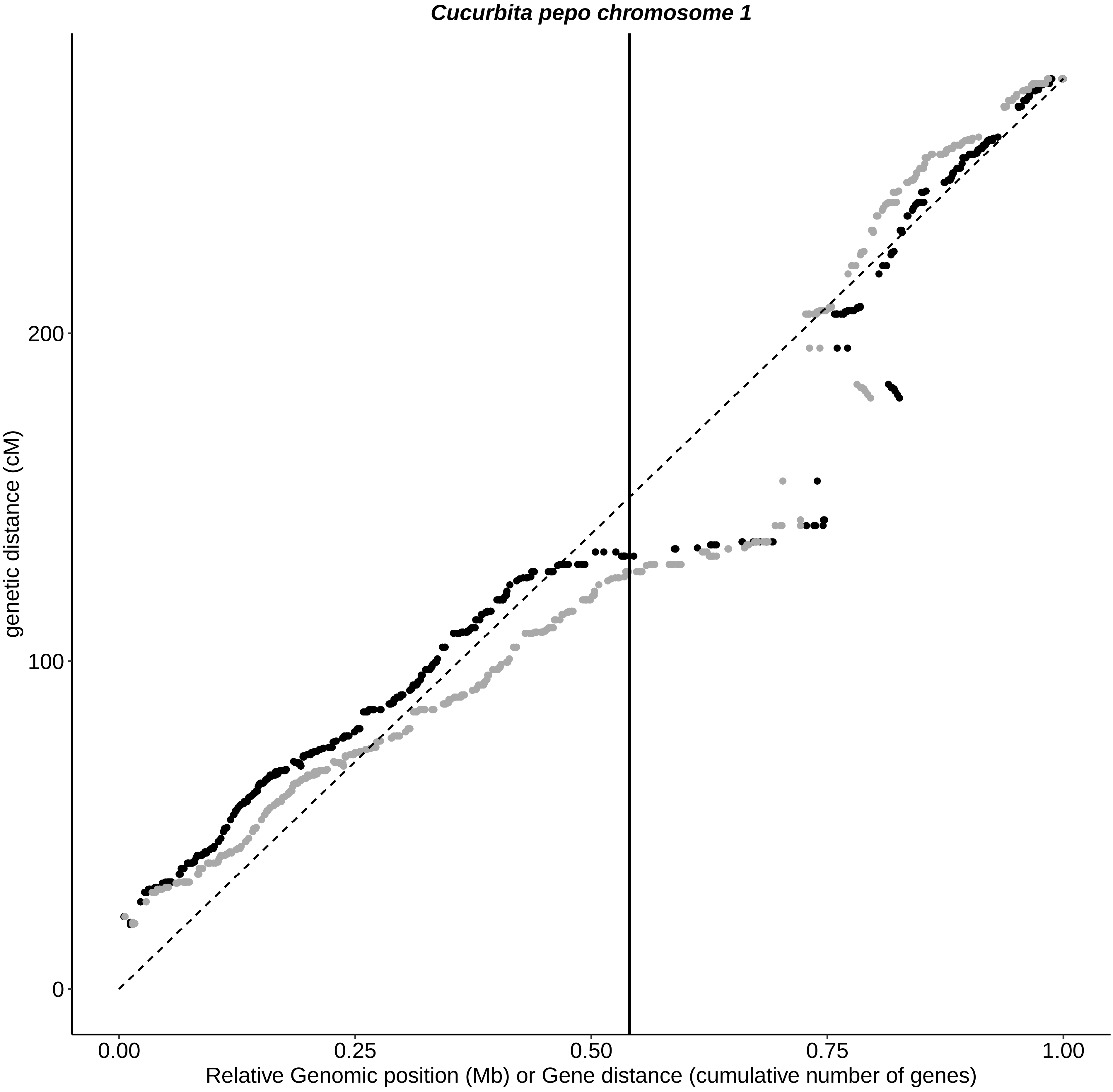
Cucurbita pepo chromosome 1
genetic distance (cM)
0
100
200
0.00
0.25
0.50
0.75
1.00
Relative Genomic position (Mb) or Gene distance (cumulative number of genes)

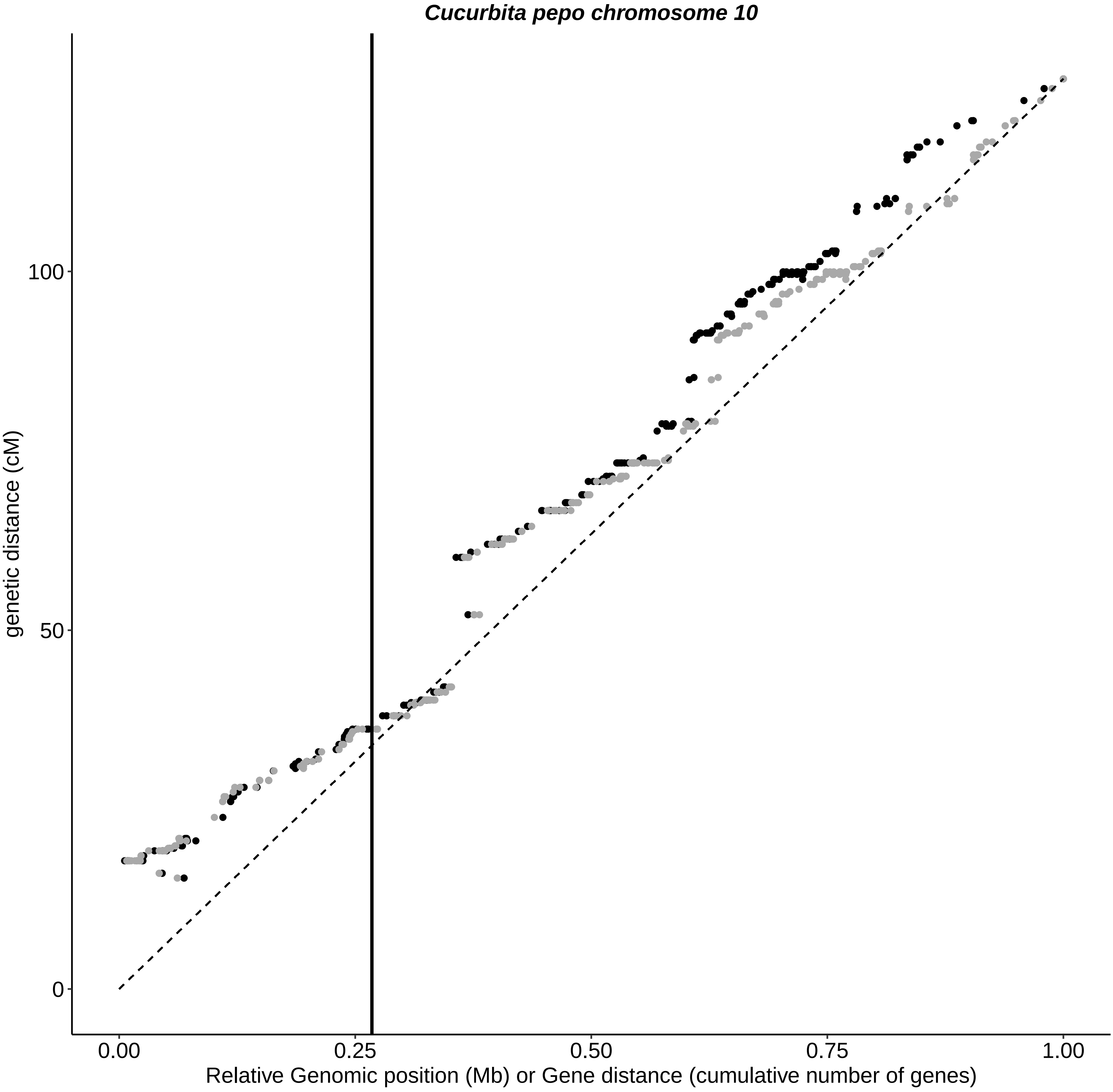

Cucurbita pepo chromosome 10
genetic distance (cM)
0
50
100
0.00
0.25
0.50
0.75
1.00
Relative Genomic position (Mb) or Gene distance (cumulative number of genes)

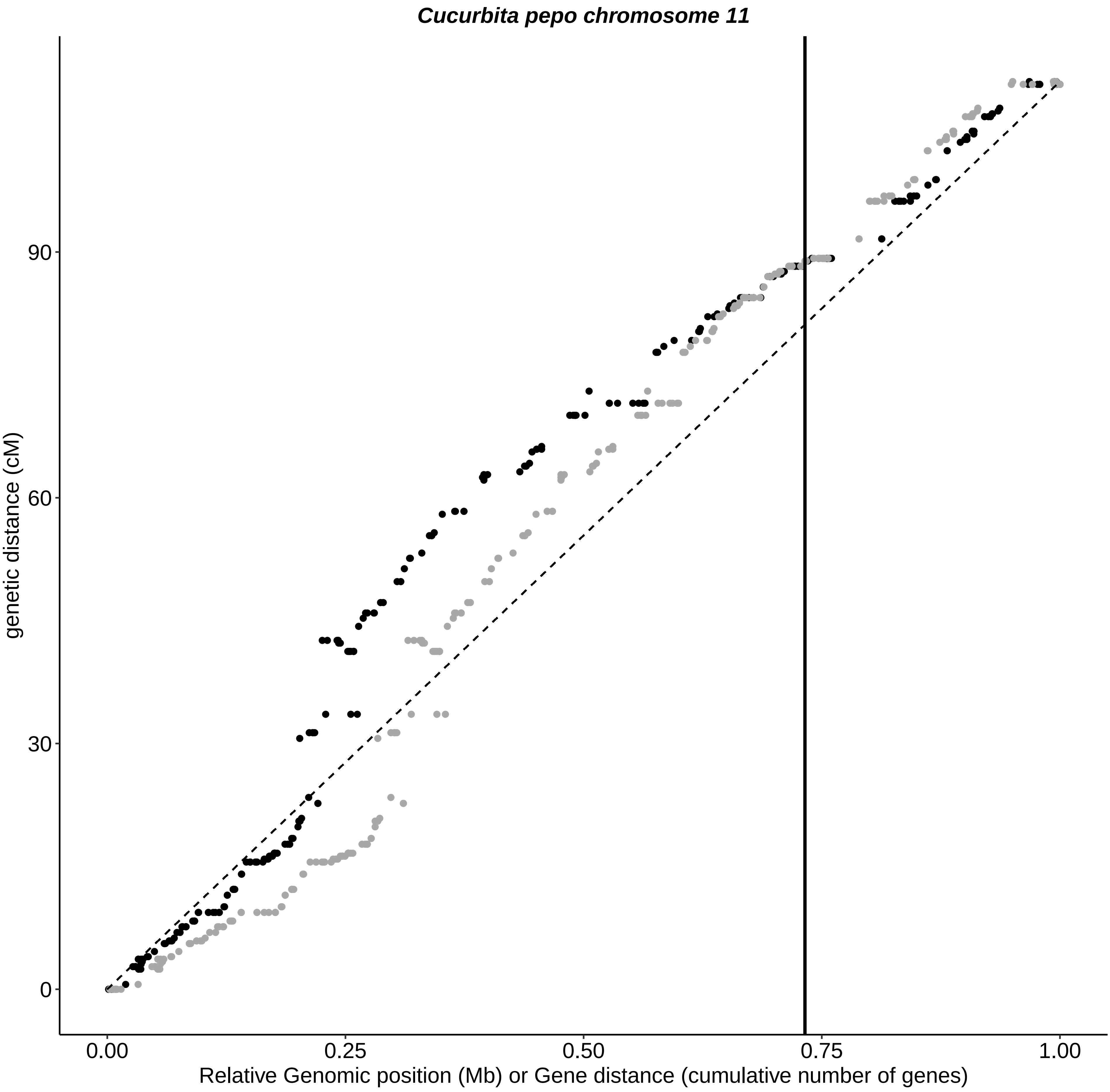

Cucurbita pepo chromosome 11
genetic distance (cM)
0
30
60
90
0.00
0.25
0.50
0.75
1.00
Relative Genomic position (Mb) or Gene distance (cumulative number of genes)

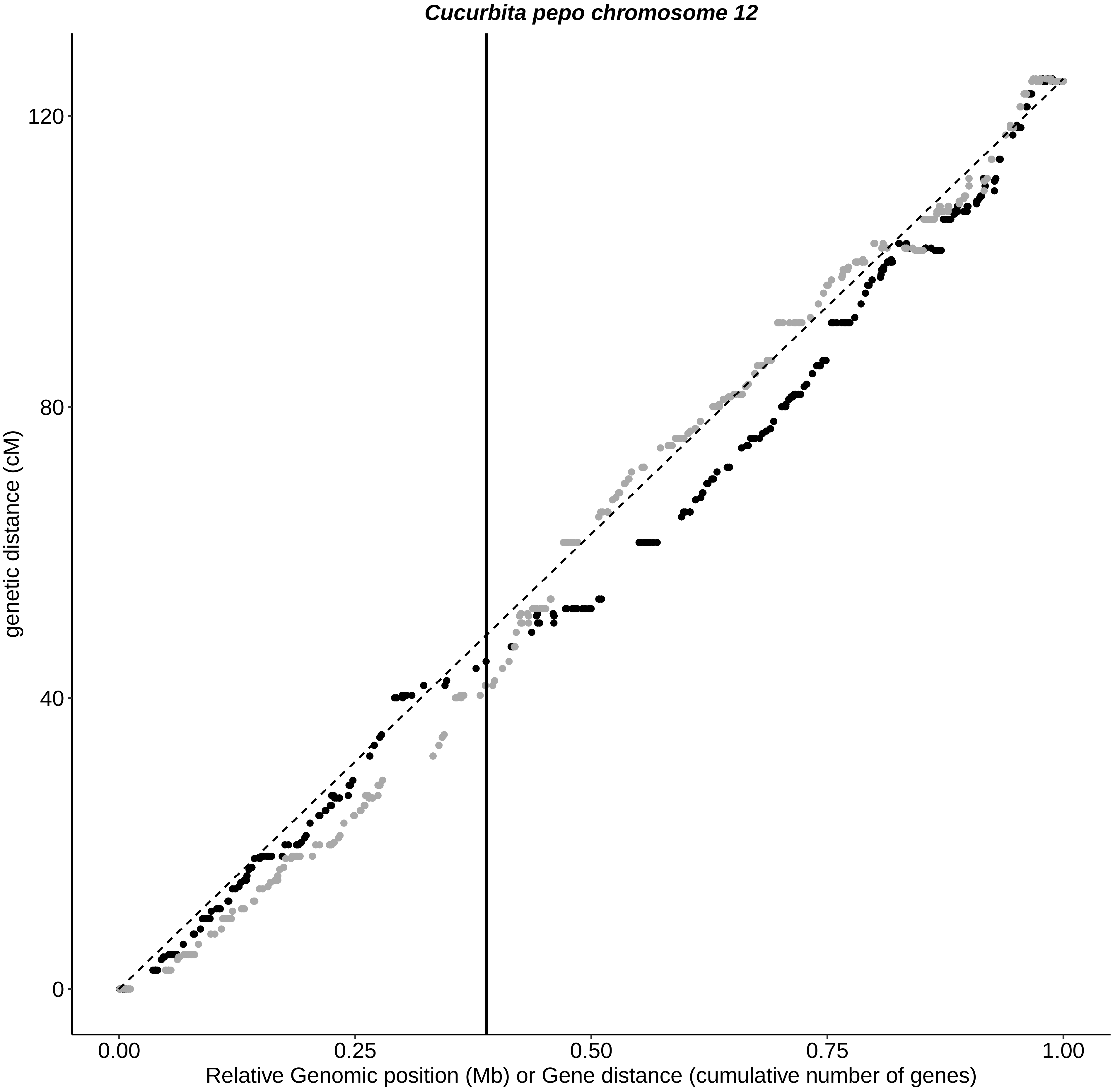
Cucurbita pepo chromosome 12
genetic distance (cM)
0
40
80
120
0.00
0.25
0.50
0.75
1.00
Relative Genomic position (Mb) or Gene distance (cumulative number of genes)

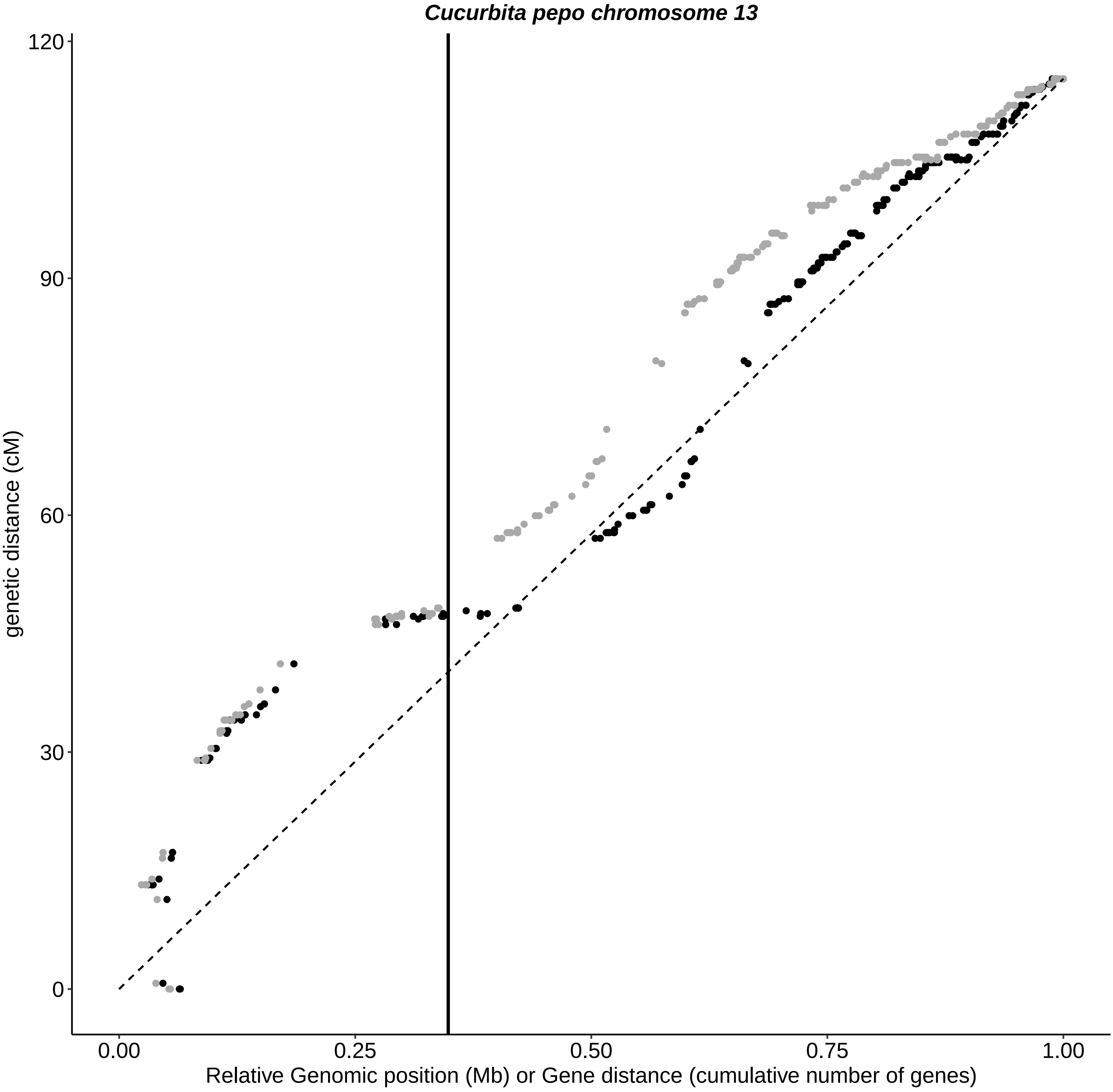
Cucurbita pepo chromosome 13
genetic distance (cM)
120
90
60
30
0
0.00
0.25
0.50
0.75
1.00
Relative Genomic position (Mb) or Gene distance (cumulative number of genes)

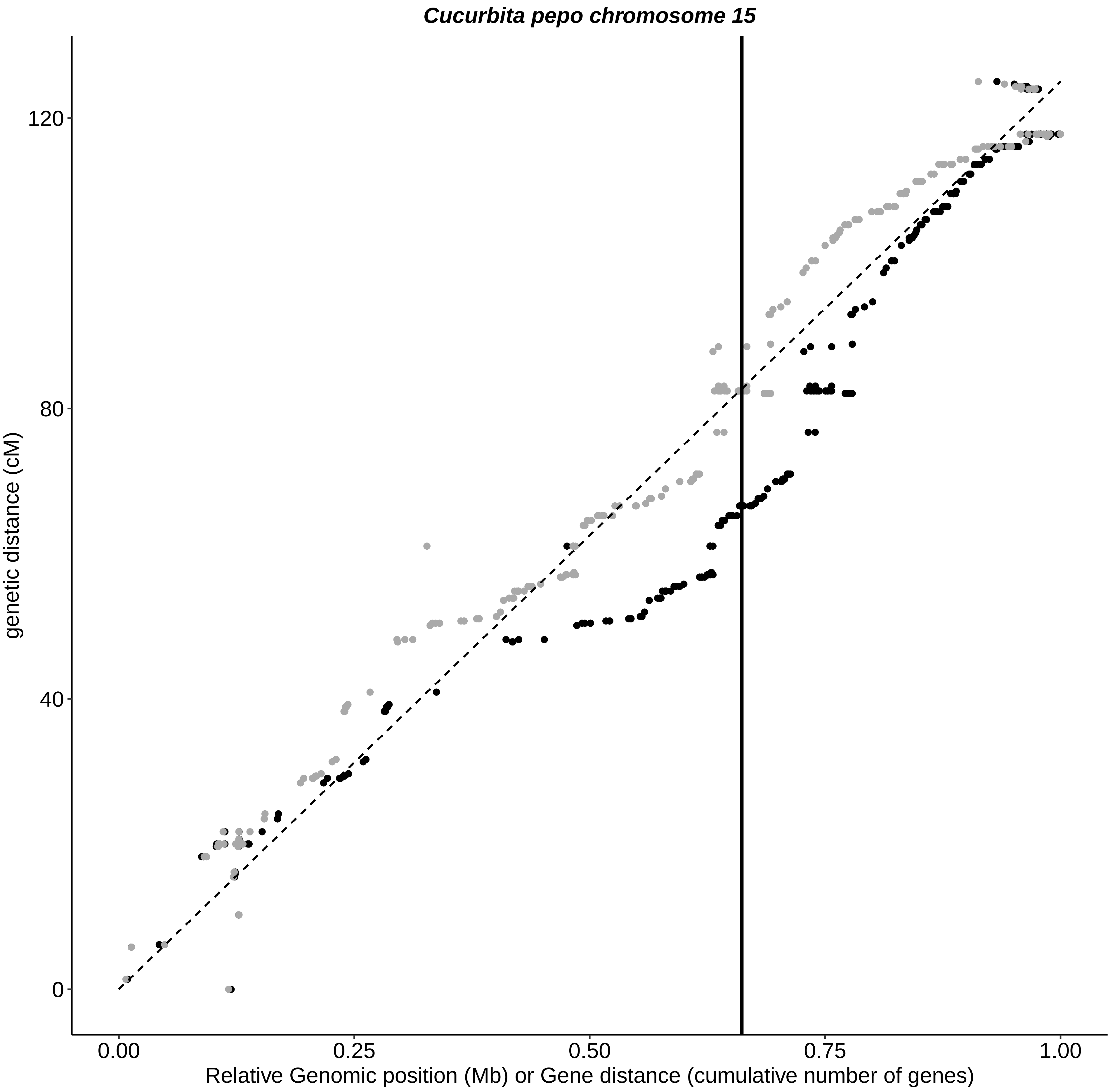

Cucurbita pepo chromosome 15
genetic distance (cM)
0
40
80
120
0.00
0.25
0.50
0.75
1.00
Relative Genomic position (Mb) or Gene distance (cumulative number of genes)

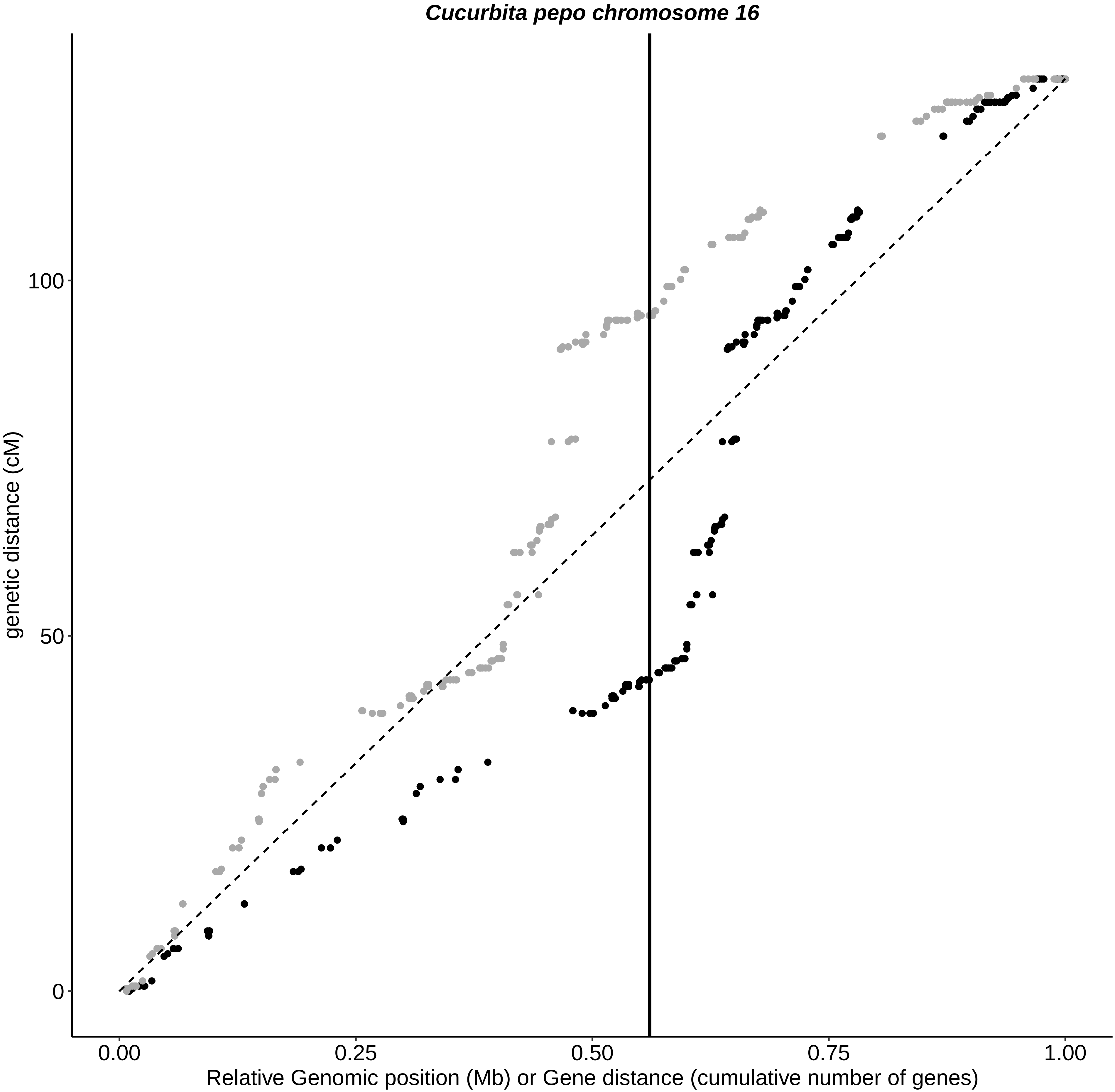
Cucurbita pepo chromosome 16
genetic distance (cM)
0
50
100
0.00
0.25
0.50
0.75
1.00
Relative Genomic position (Mb) or Gene distance (cumulative number of genes)

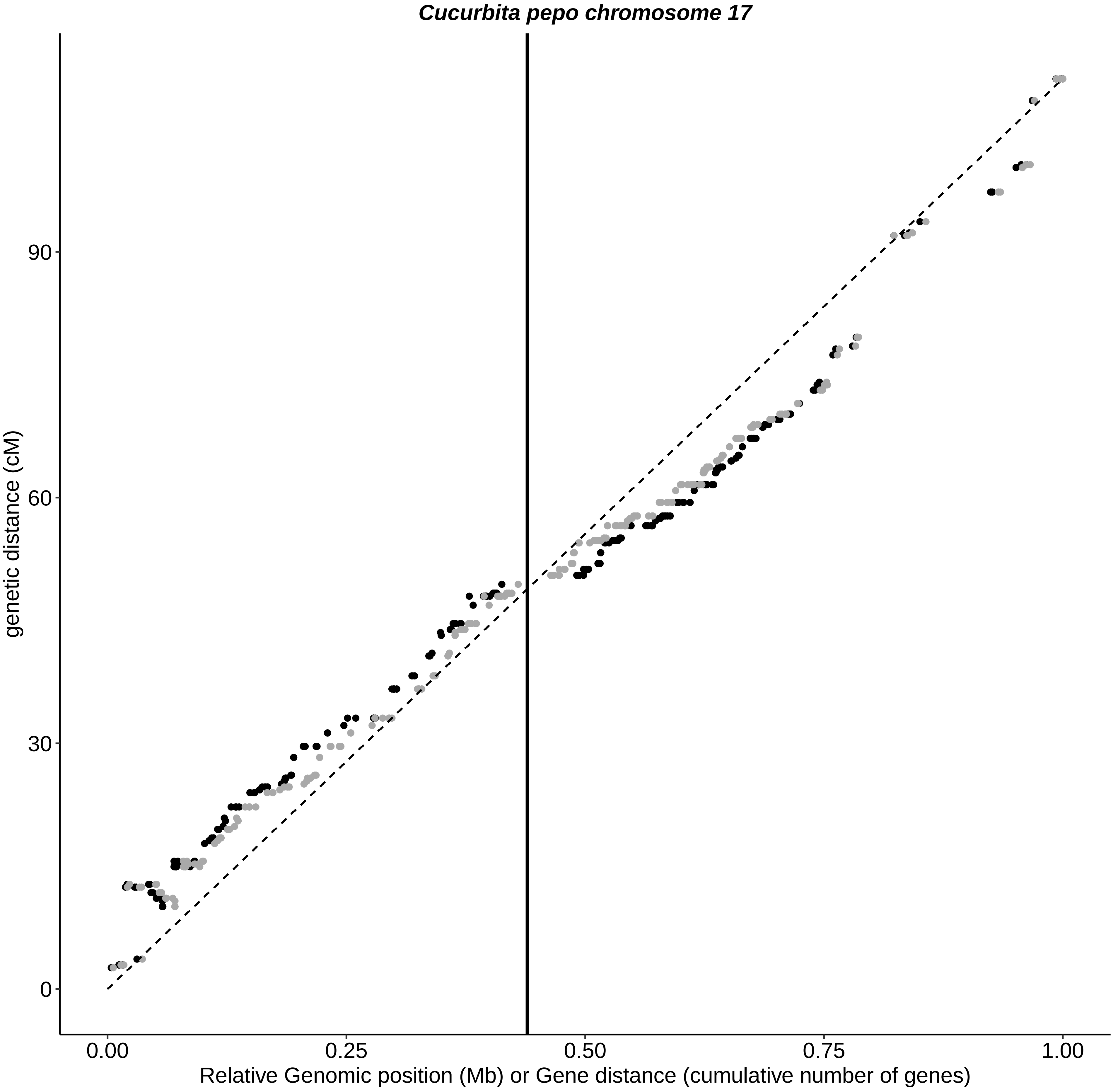
Cucurbita pepo chromosome 17
genetic distance (cM)
0
30
60
90
0.00
0.25
0.50
0.75
1.00
Relative Genomic position (Mb) or Gene distance (cumulative number of genes)

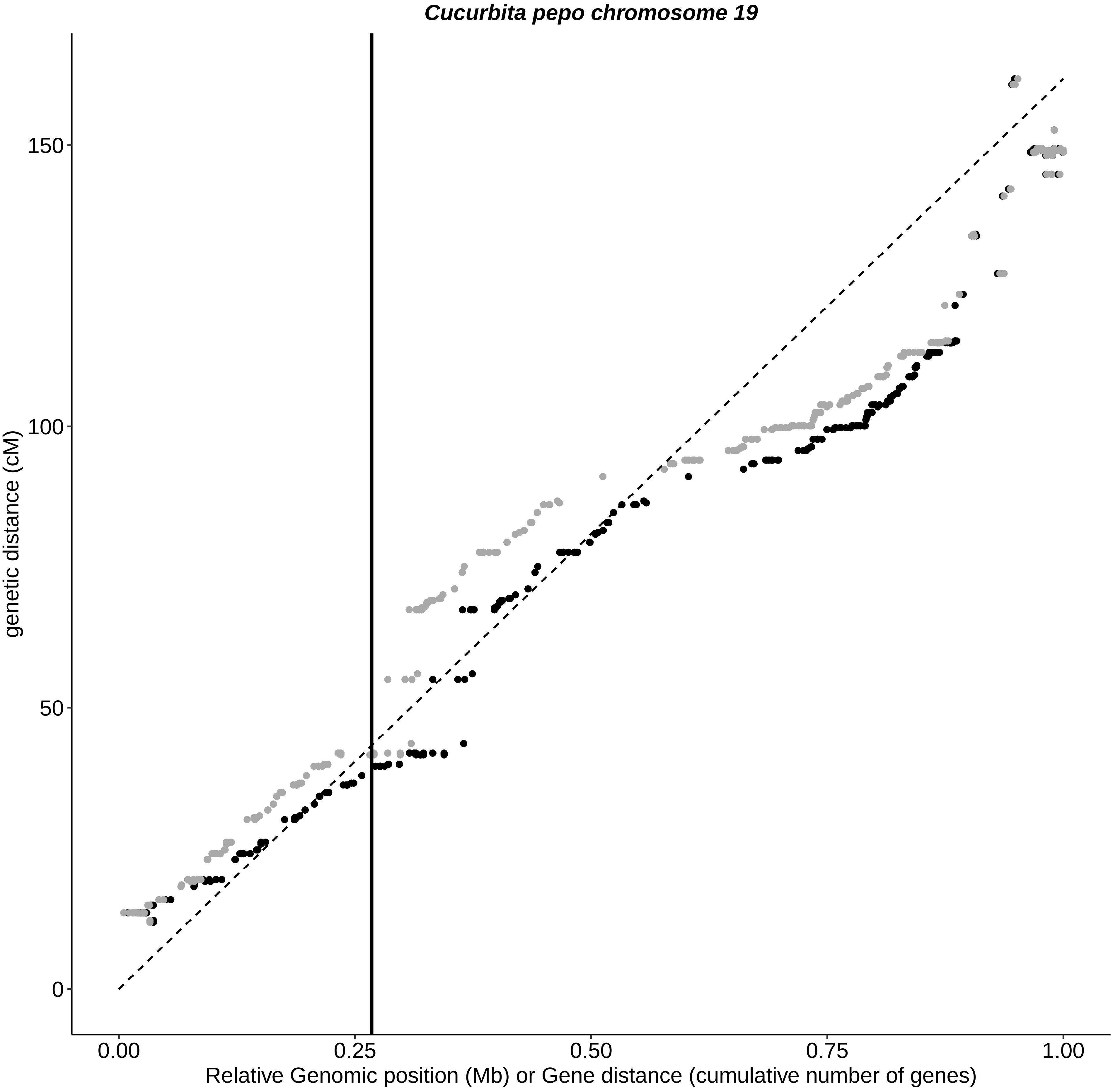

Cucurbita pepo chromosome 19
genetic distance (cM)
150
100
50
0
0.00
0.25
0.50
0.75
1.00
Relative Genomic position (Mb) or Gene distance (cumulative number of genes)

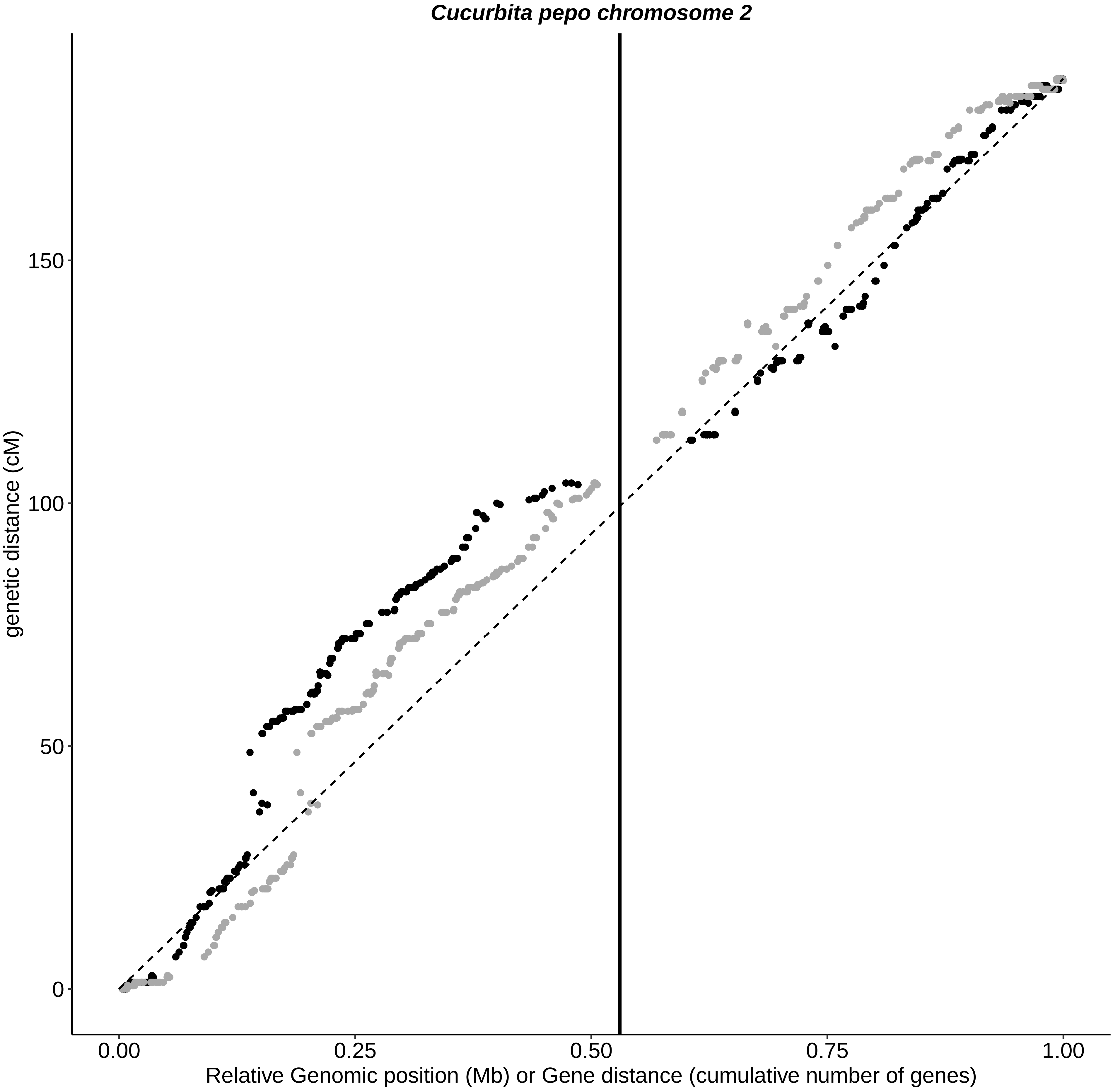
Cucurbita pepo chromosome 2
genetic distance (cM)
0
50
100
150
0.00
0.25
0.50
0.75
1.00
Relative Genomic position (Mb) or Gene distance (cumulative number of genes)

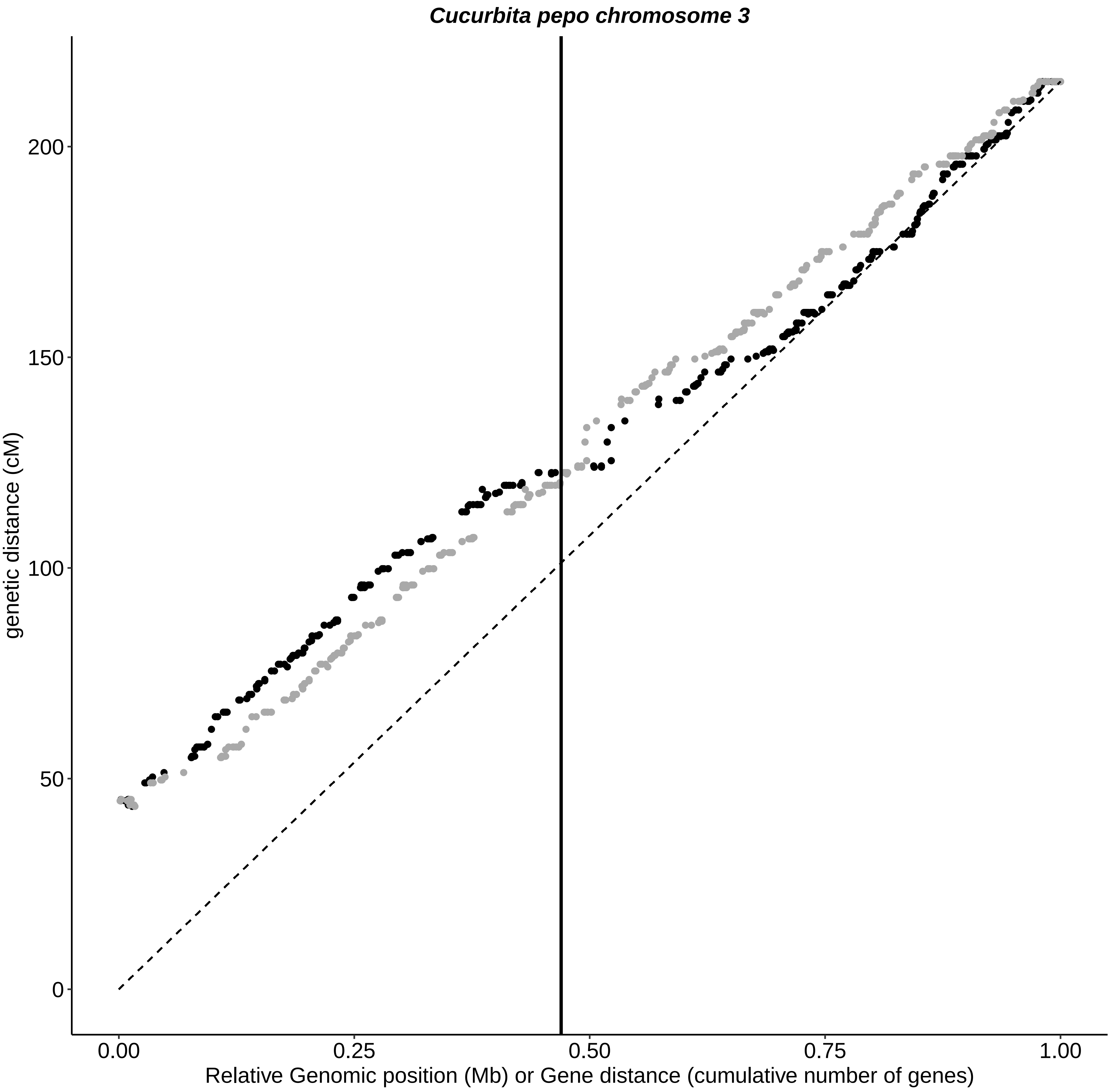
Cucurbita pepo chromosome 3
genetic distance (cM)
0
50
100
150
200
0.00
0.25
0.50
0.75
1.00
Relative Genomic position (Mb) or Gene distance (cumulative number of genes)

Cucurbita pepo chromosome 5

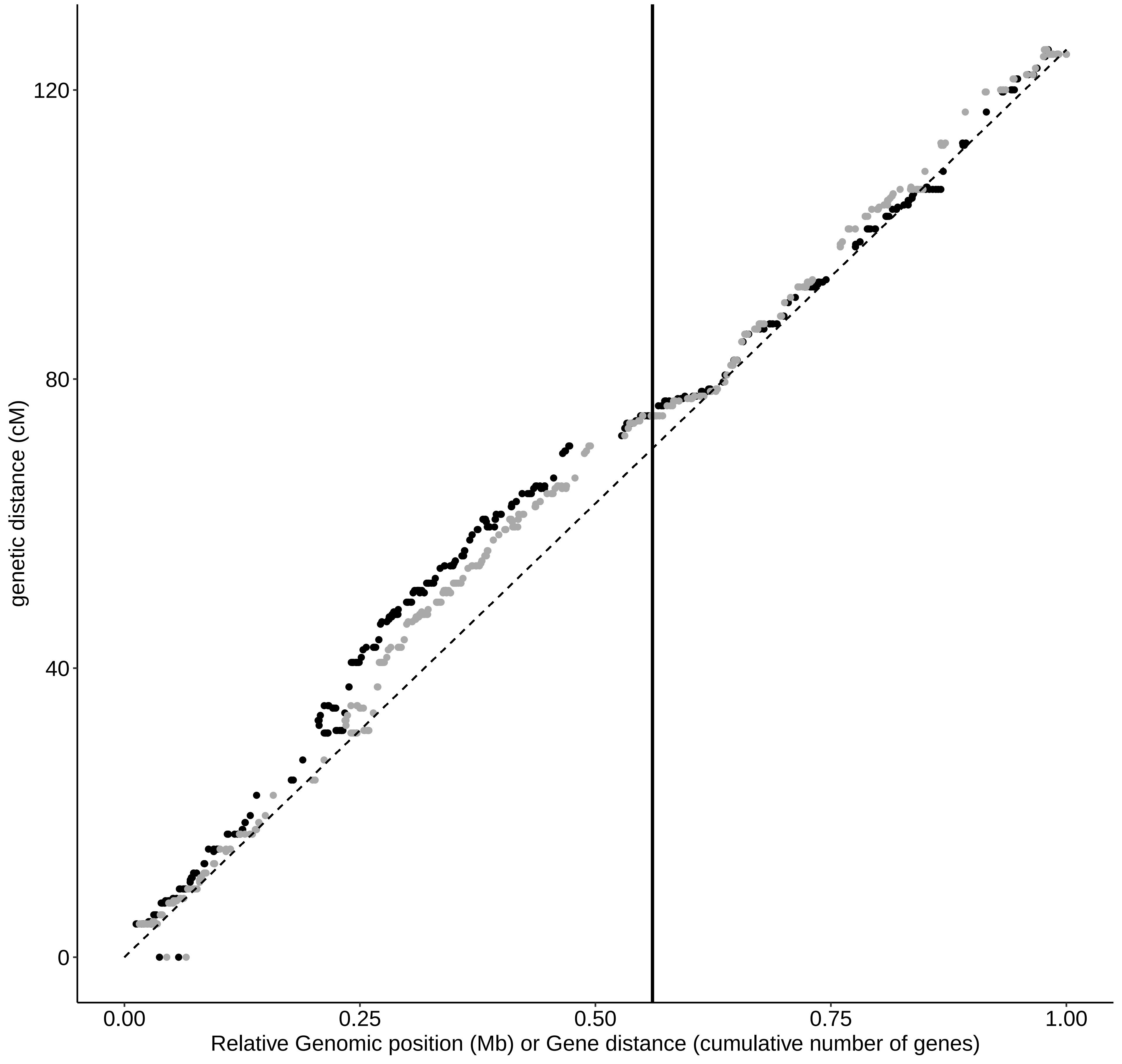

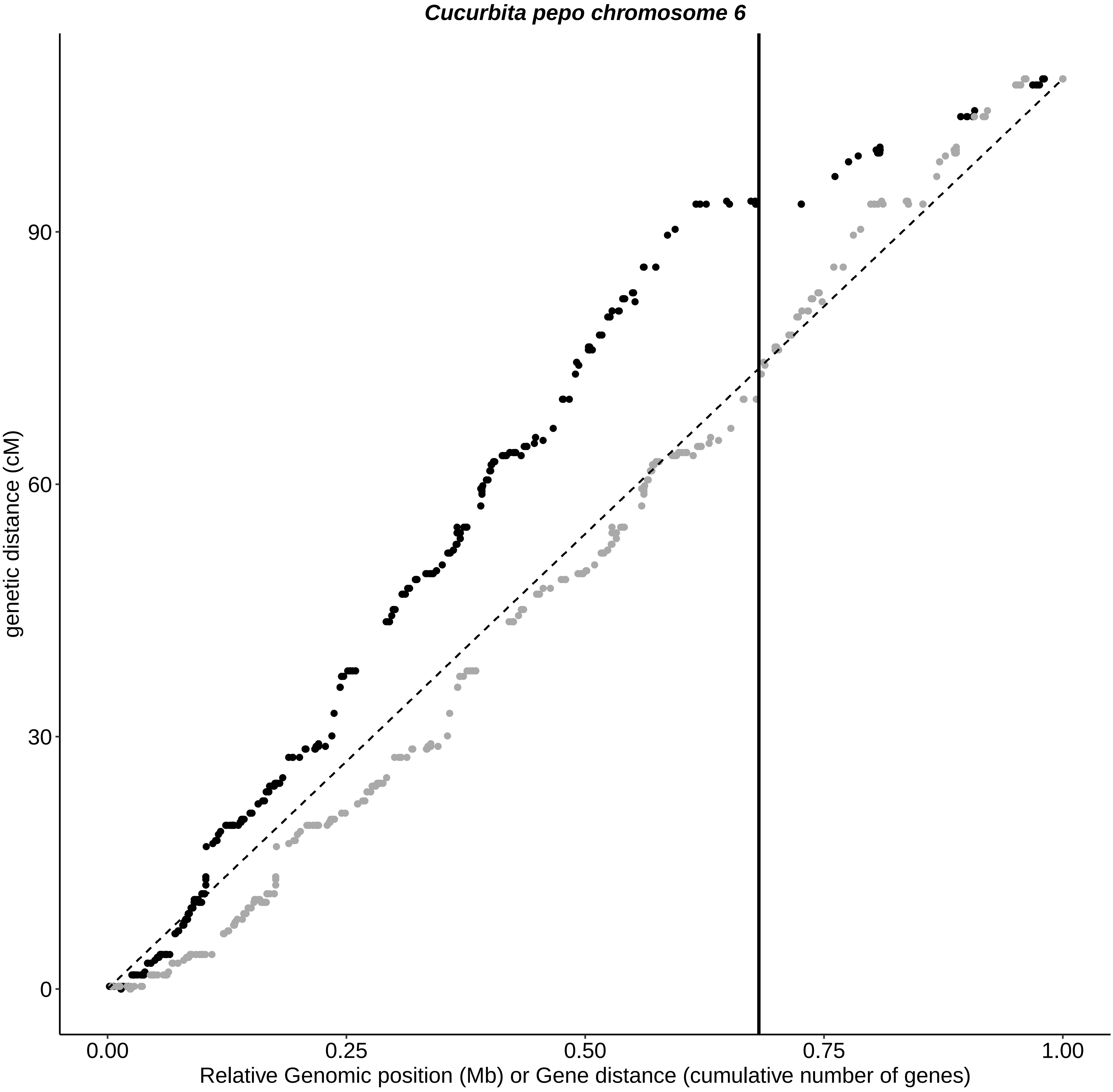
Cucurbita pepo chromosome 6
genetic distance (cM)
0
30
60
90
0.00
0.25
0.50
0.75
1.00
Relative Genomic position (Mb) or Gene distance (cumulative number of genes)

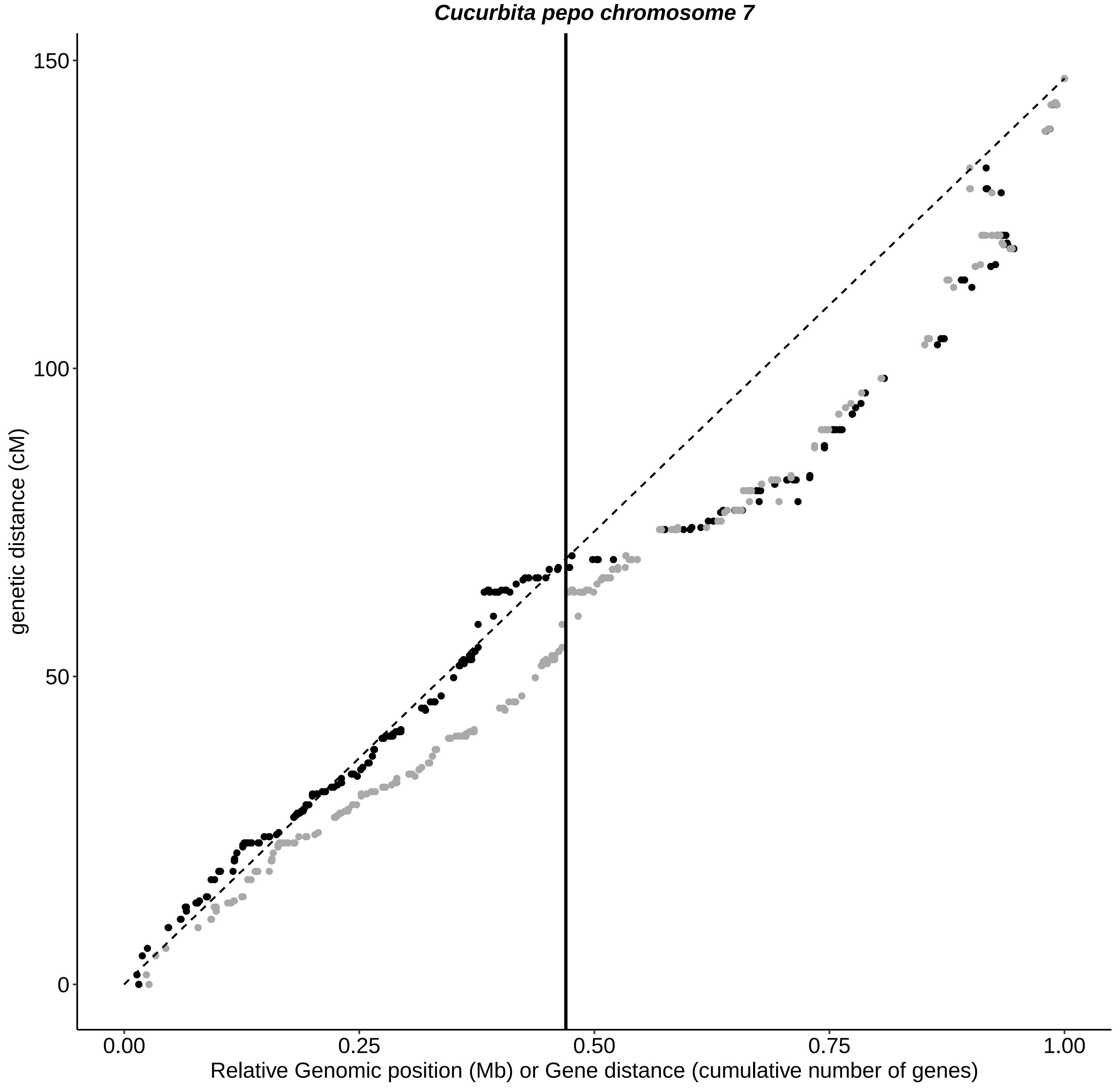

Cucurbita pepo chromosome 7
genetic distance (cM)
150
100
50
0
0.00
0.25
0.50
0.75
1.00
Relative Genomic position (Mb) or Gene distance (cumulative number of genes)

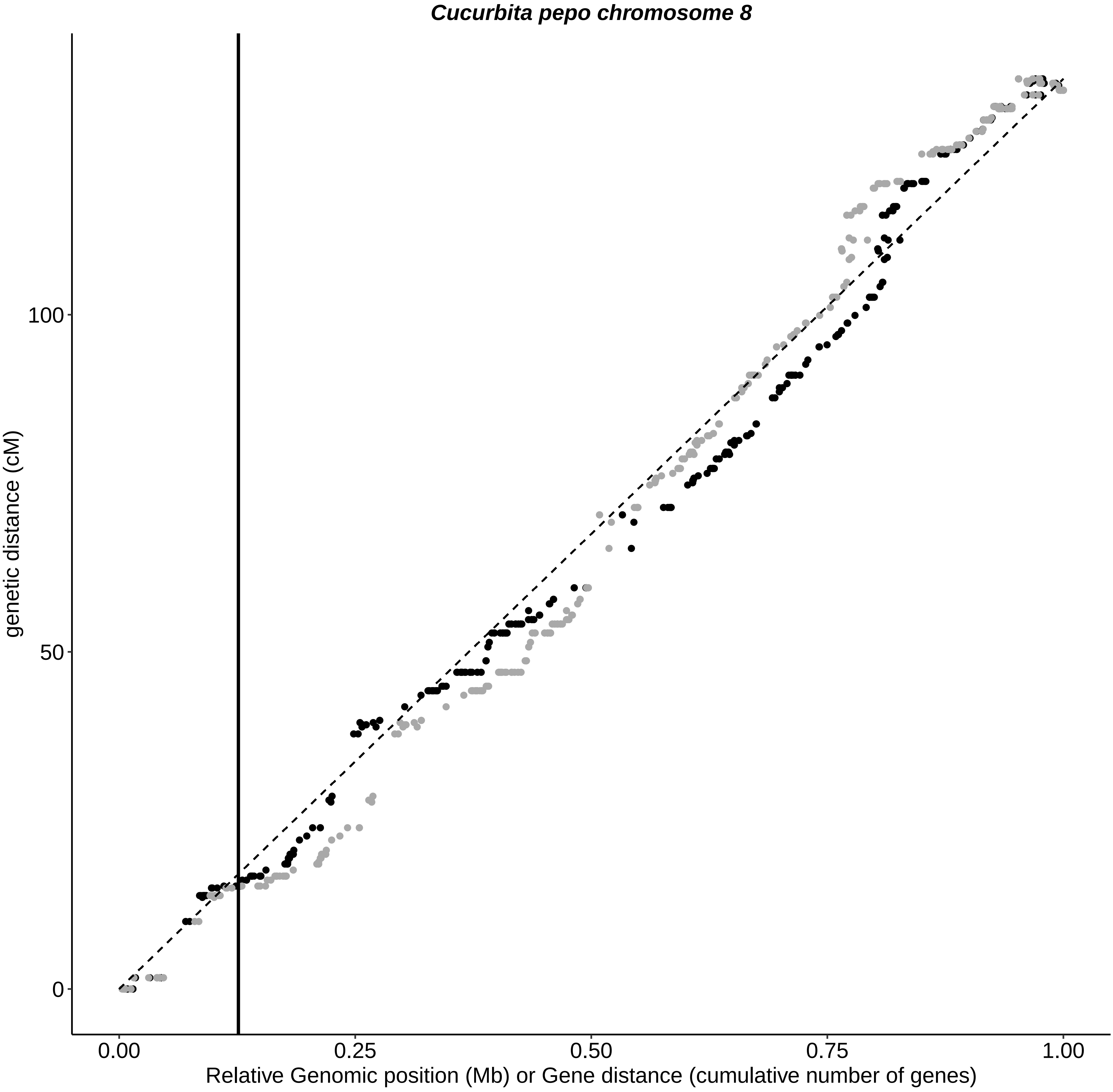
Cucurbita pepo chromosome 8
genetic distance (cM)
100
50
0
0.00
0.25
0.50
0.75
1.00
Relative Genomic position (Mb) or Gene distance (cumulative number of genes)

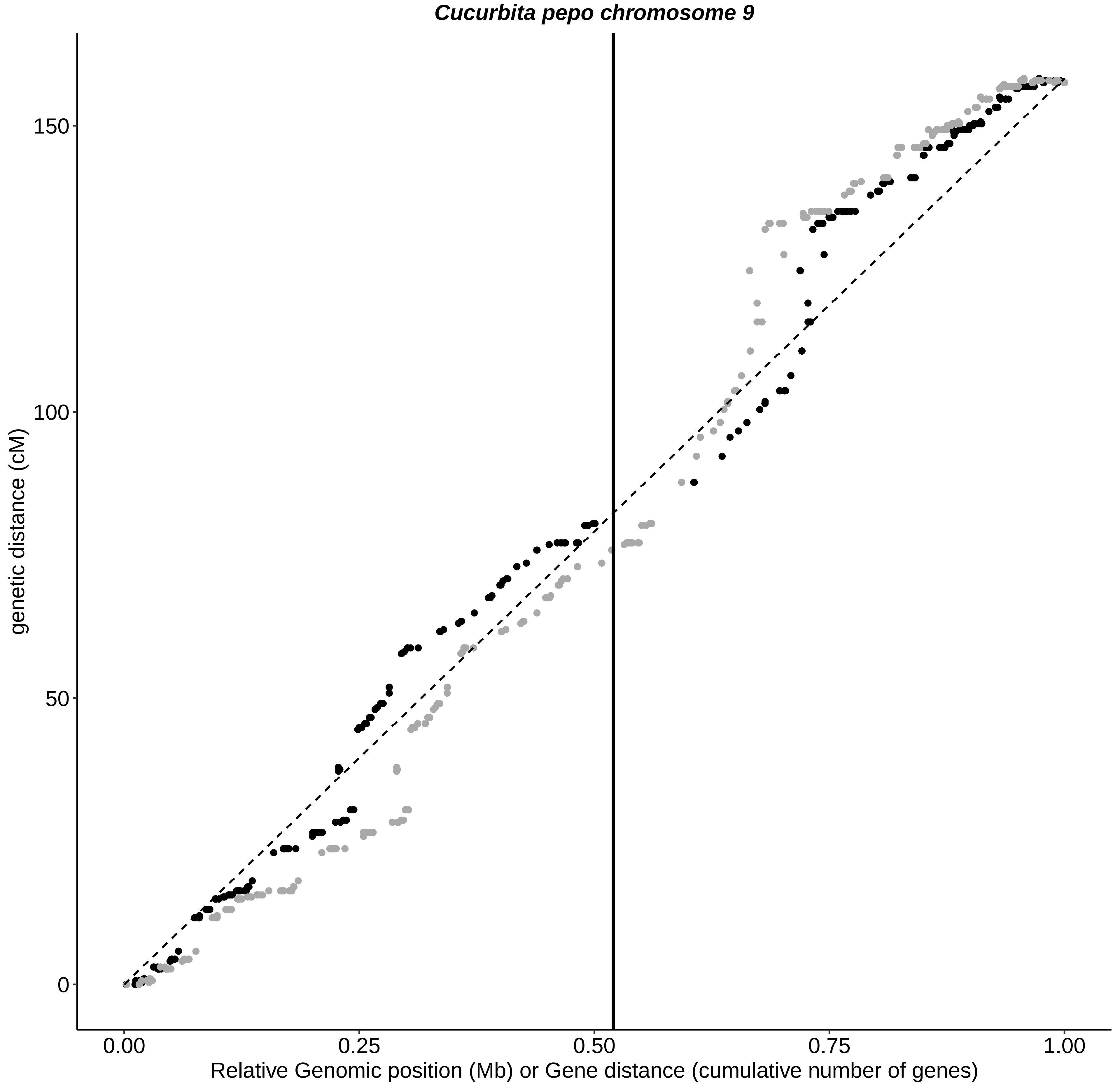
Cucurbita pepo chromosome 9
genetic distance (cM)
150
100
50
0
0.00
0.25
0.50
0.75
1.00
Relative Genomic position (Mb) or Gene distance (cumulative number of genes)

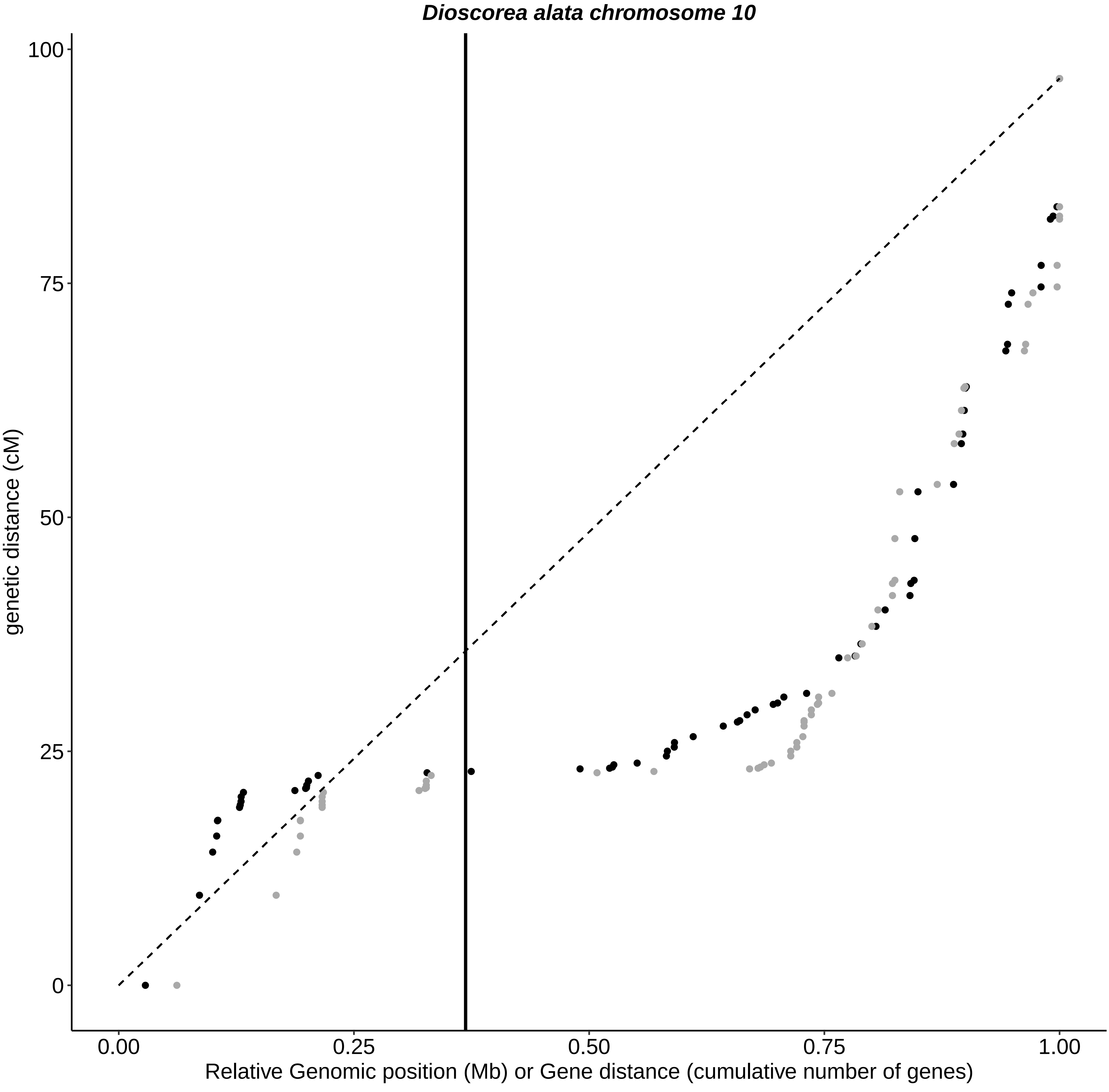
Dioscorea alata chromosome 10
genetic distance (cM)
0
25
50
75
100
0.00
0.25
0.50
0.75
1.00
Relative Genomic position (Mb) or Gene distance (cumulative number of genes)

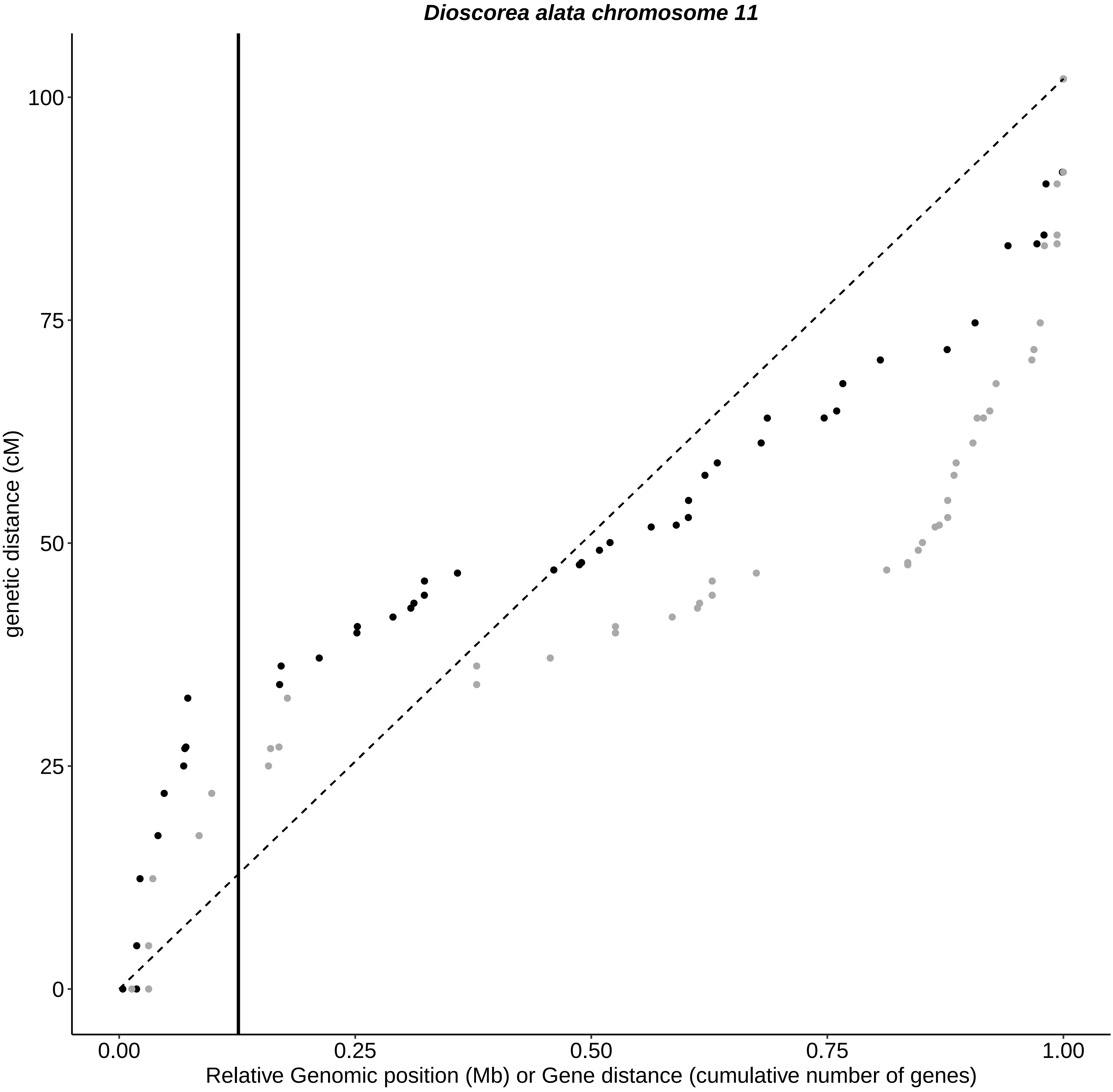
Dioscorea alata chromosome 11
genetic distance (cM)
0
25
50
75
100
0.00
0.25
0.50
0.75
1.00
Relative Genomic position (Mb) or Gene distance (cumulative number of genes)

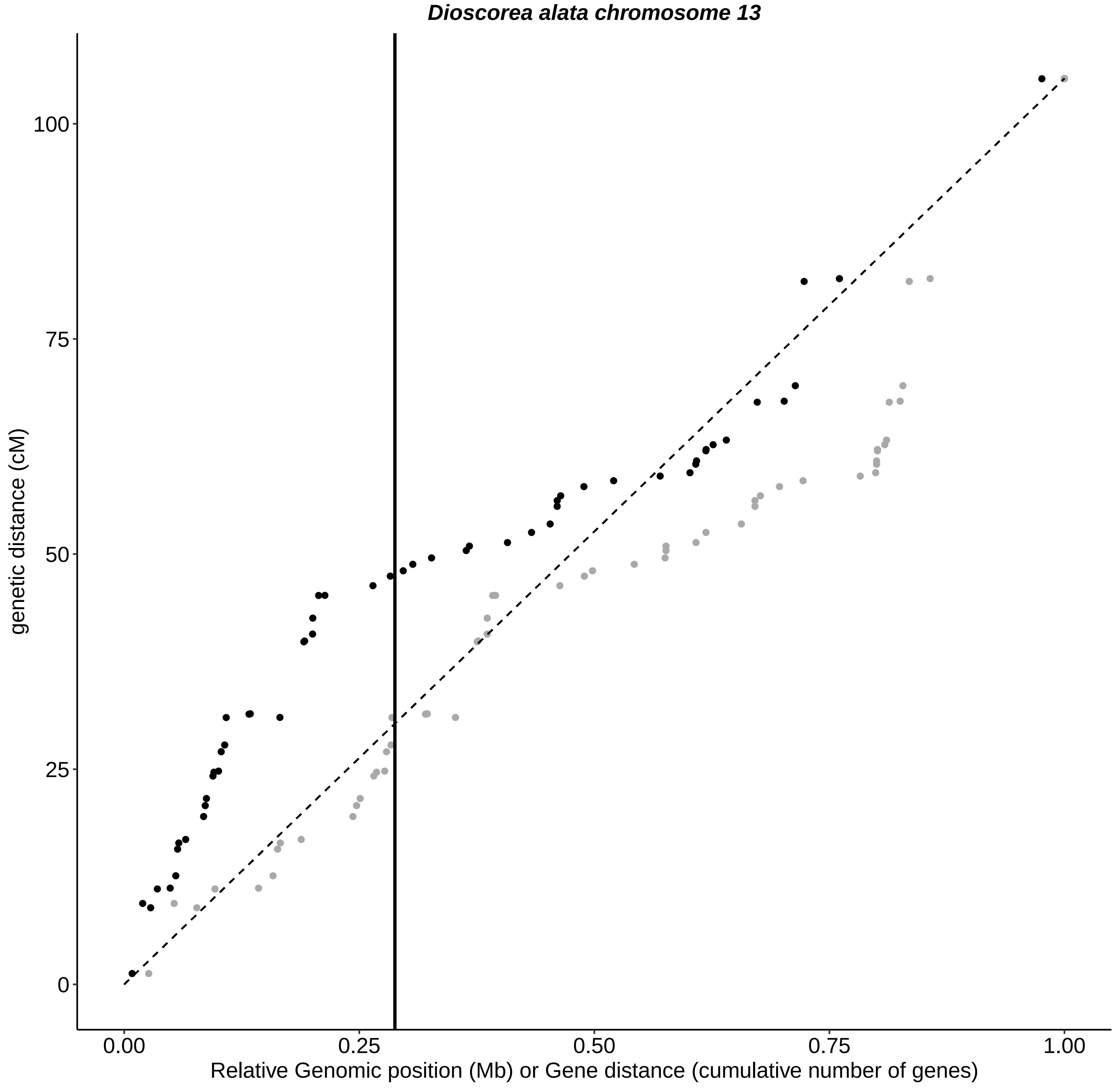
Dioscorea alata chromosome 13
genetic distance (cM)
100
75
50
25
0
0.00
0.25
0.50
0.75
1.00
Relative Genomic position (Mb) or Gene distance (cumulative number of genes)

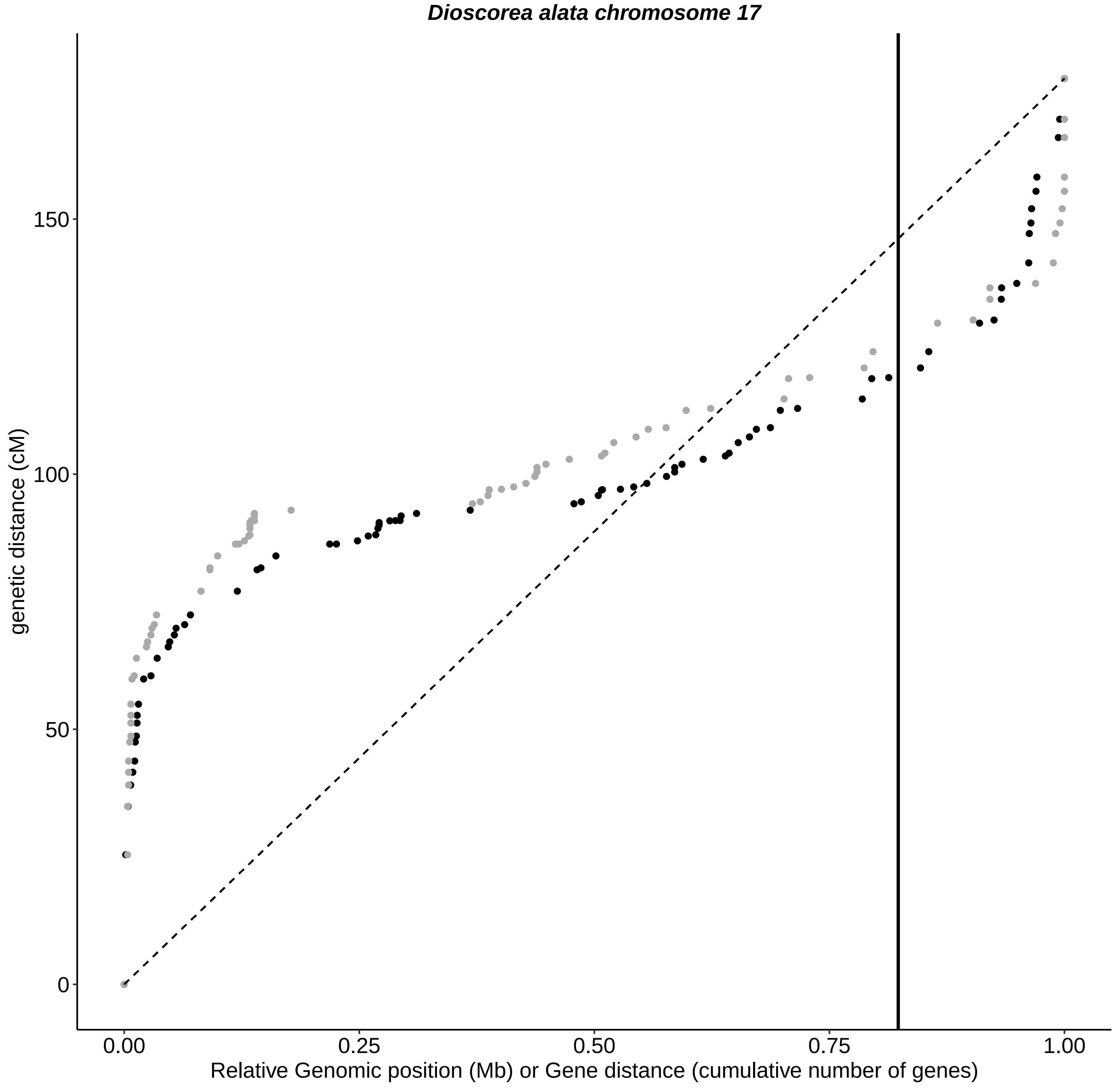
Dioscorea alata chromosome 17
genetic distance (cM)
0
50
100
150
0.00
0.25
0.50
0.75
1.00
Relative Genomic position (Mb) or Gene distance (cumulative number of genes)

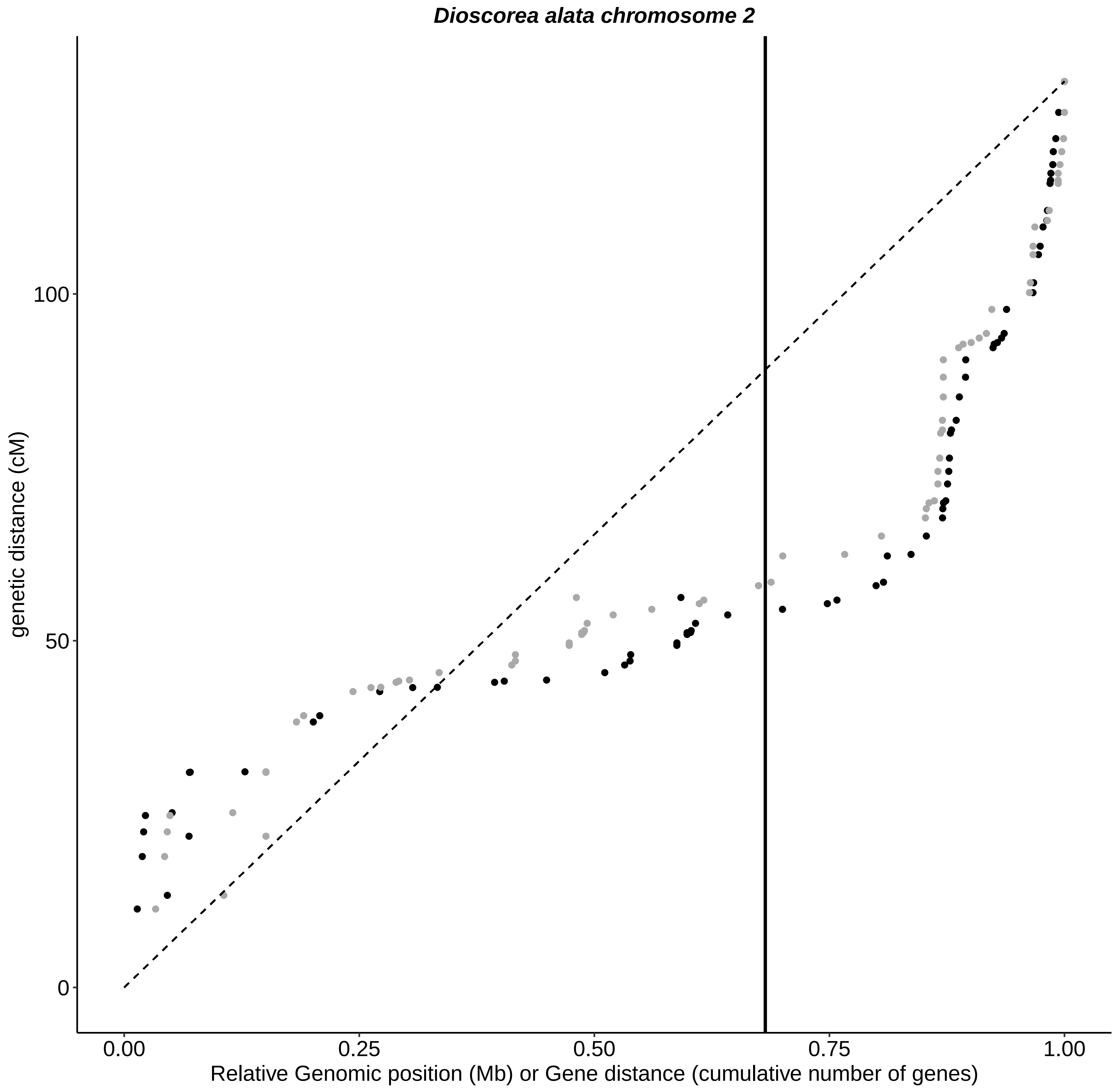
Dioscorea alata chromosome 2
genetic distance (cM)
0
50
100
0.00
0.25
0.50
0.75
1.00
Relative Genomic position (Mb) or Gene distance (cumulative number of genes)

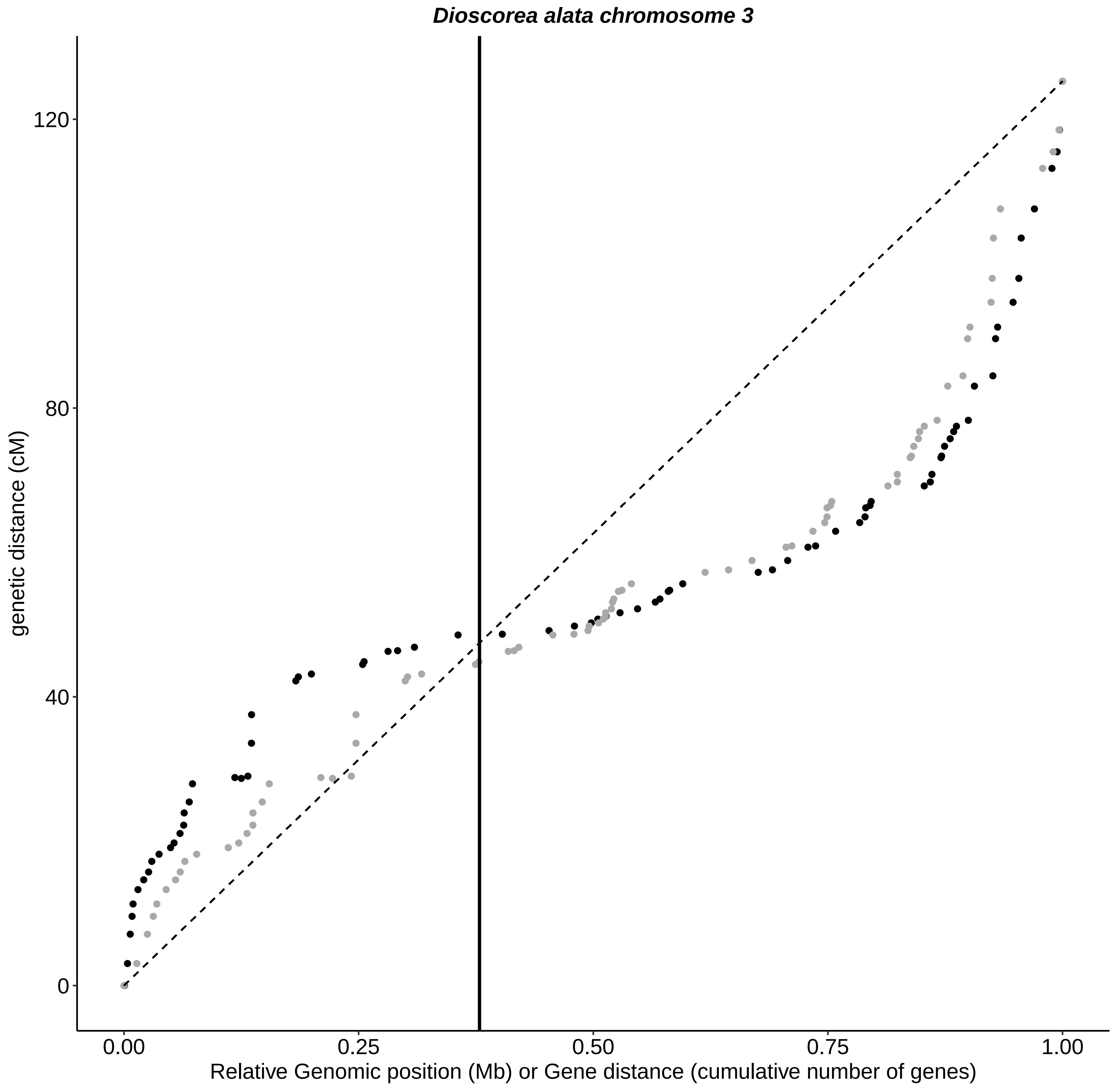
Dioscorea alata chromosome 3
genetic distance (cM)
0
40
80
120
0.00
0.25
0.50
0.75
1.00
Relative Genomic position (Mb) or Gene distance (cumulative number of genes)

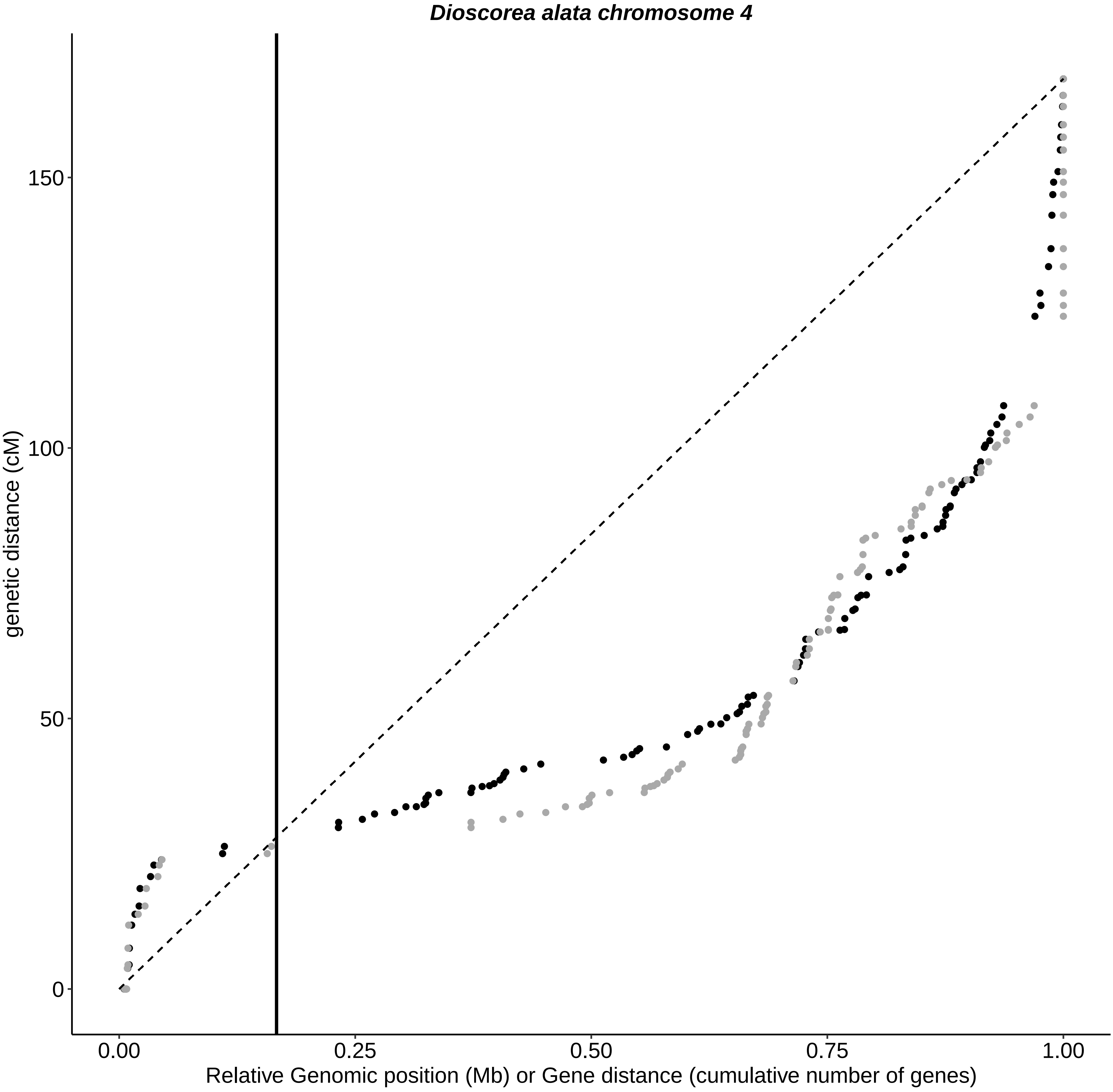
Dioscorea alata chromosome 4
genetic distance (cM)
0
50
100
150
0.00
0.25
0.50
0.75
1.00
Relative Genomic position (Mb) or Gene distance (cumulative number of genes)

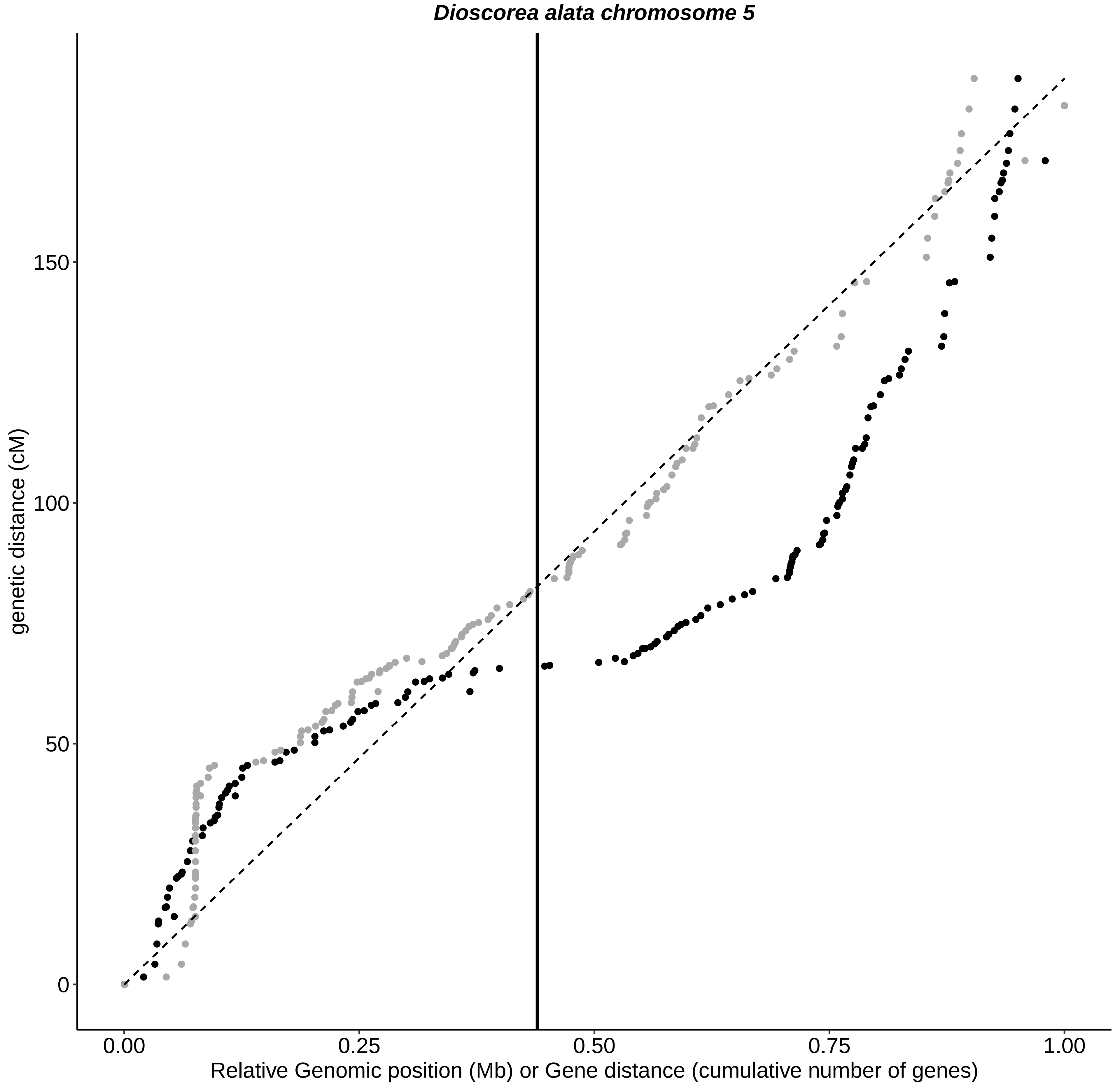
Dioscorea alata chromosome 5
genetic distance (cM)
0
50
100
150
0.00
0.25
0.50
0.75
1.00
Relative Genomic position (Mb) or Gene distance (cumulative number of genes)

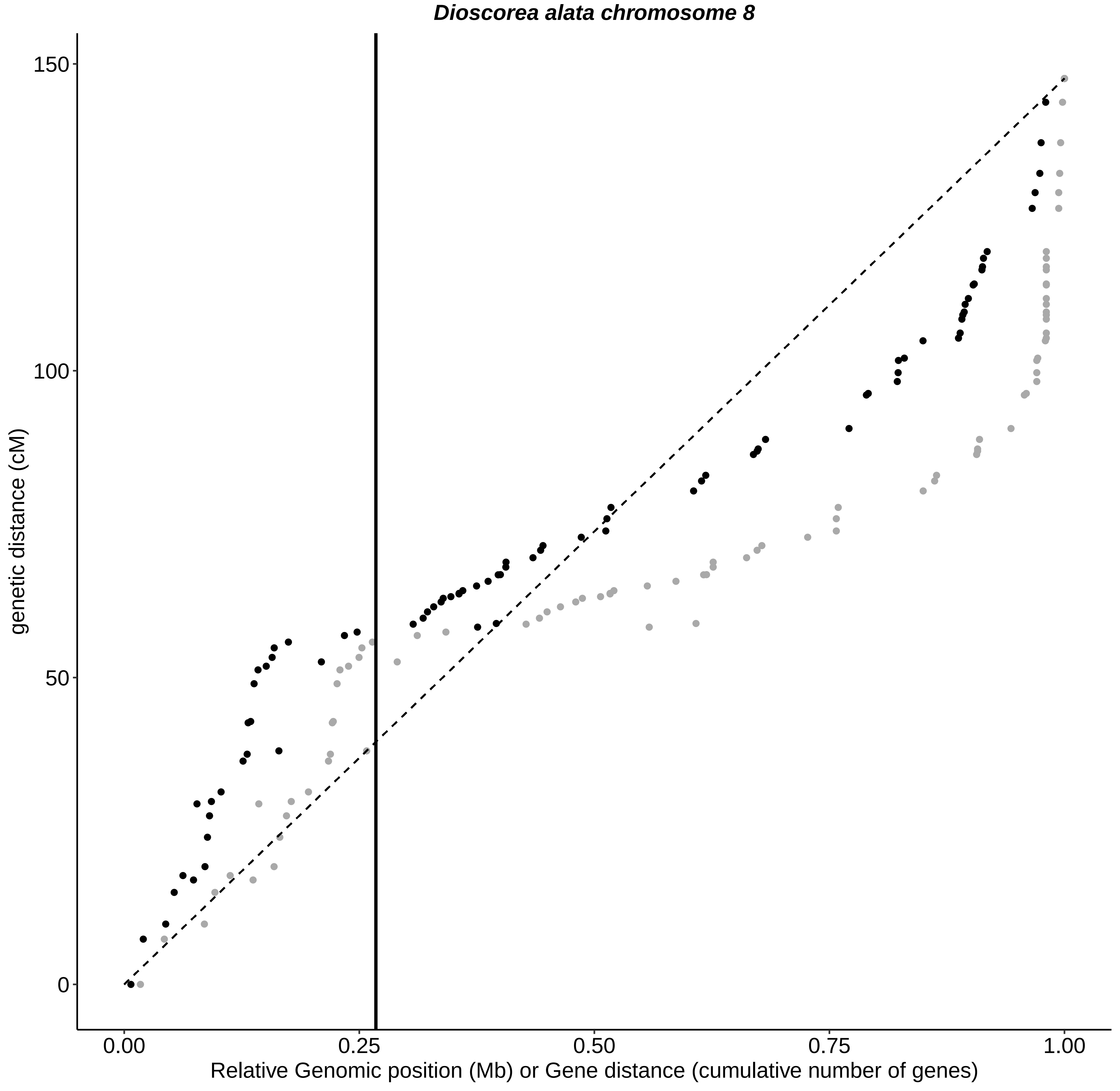
Dioscorea alata chromosome 8
genetic distance (cM)
150
100
50
0
0.00
0.25
0.50
0.75
1.00
Relative Genomic position (Mb) or Gene distance (cumulative number of genes)

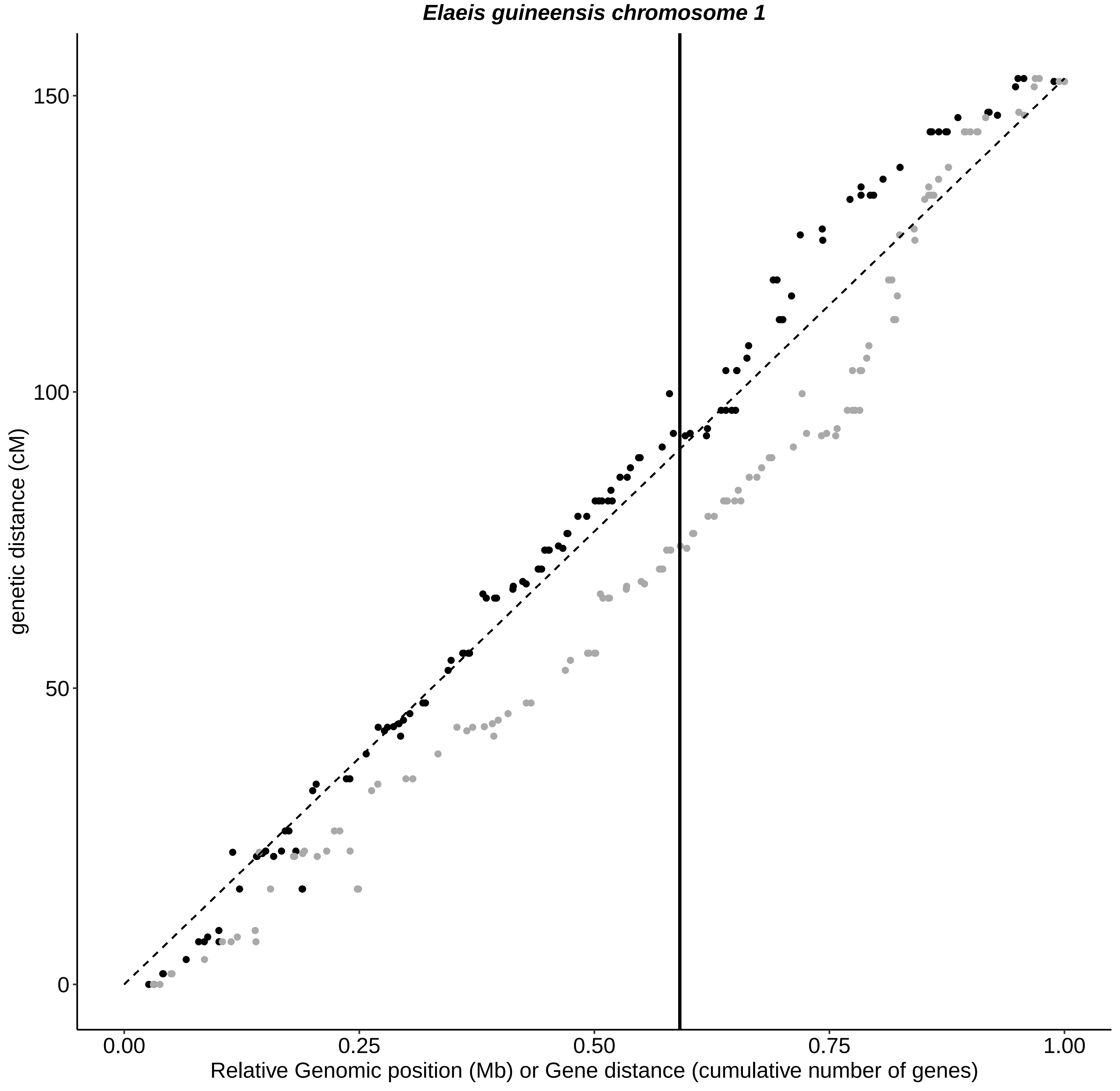
Elaeis guineensis chromosome 1
genetic distance (cM)
150
100
50
0
0.00
0.25
0.50
0.75
1.00
Relative Genomic position (Mb) or Gene distance (cumulative number of genes)

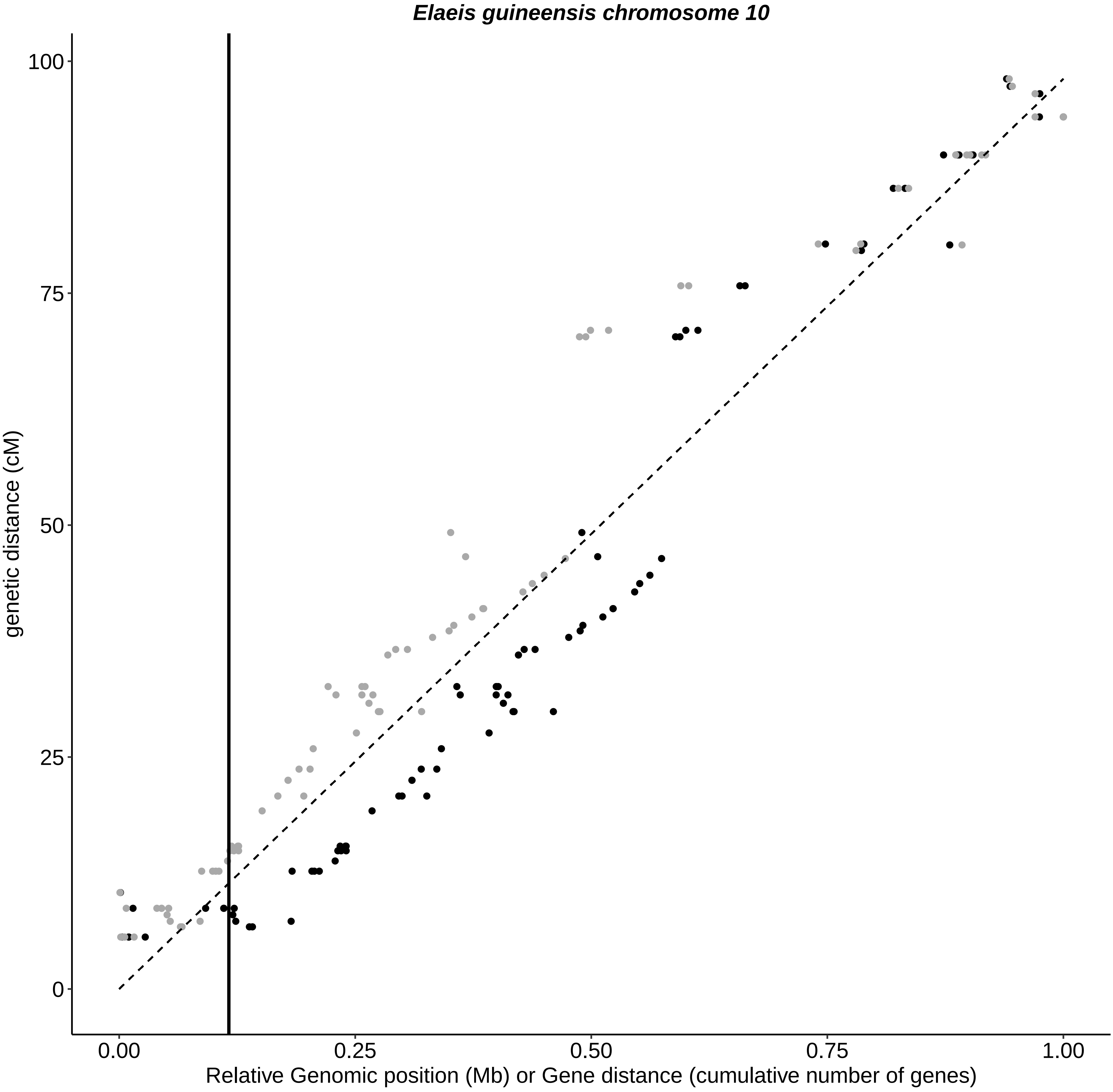

Elaeis guineensis chromosome 10
genetic distance (cM)
0
25
50
75
100
0.00
0.25
0.50
0.75
1.00
Relative Genomic position (Mb) or Gene distance (cumulative number of genes)

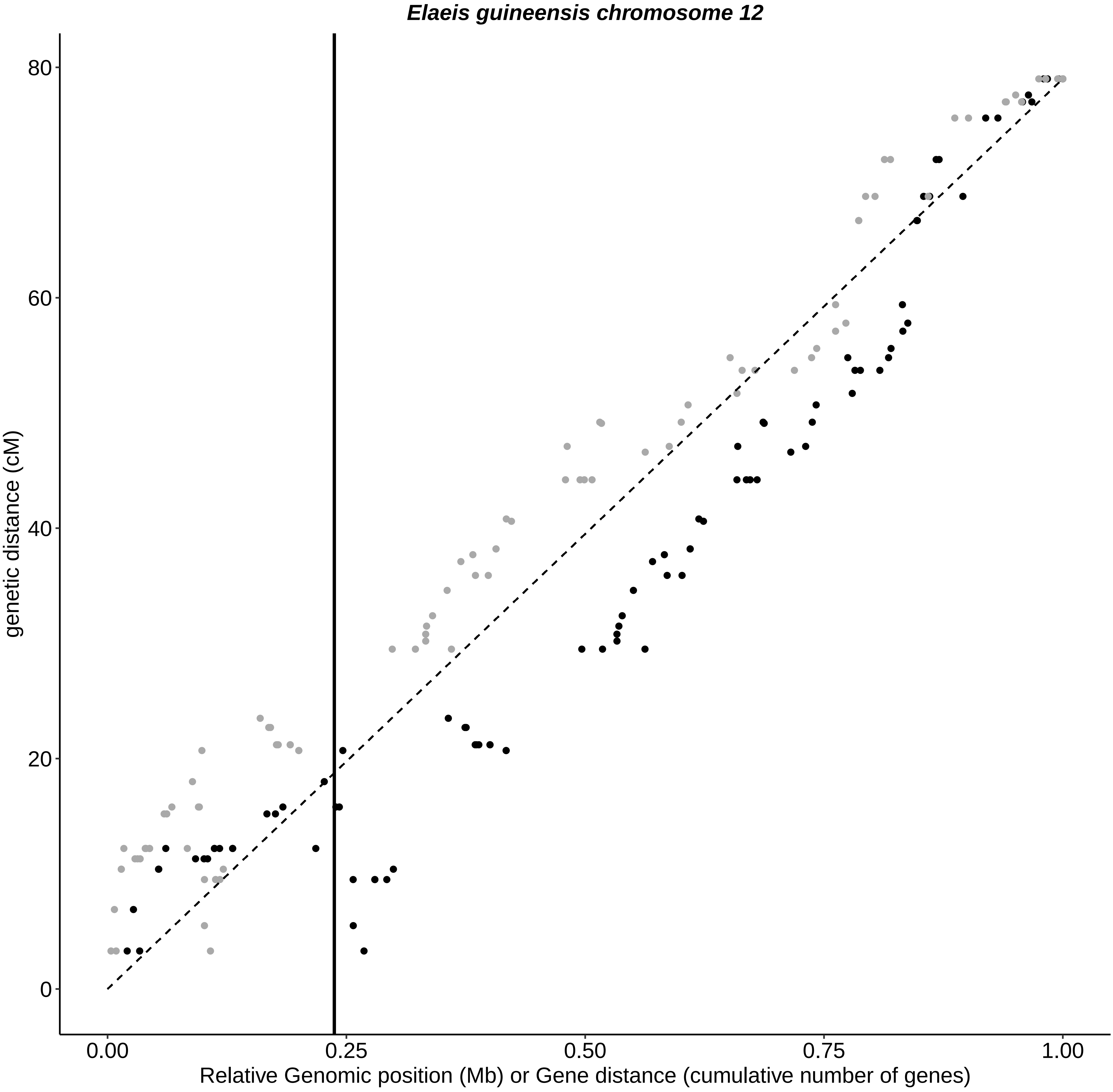
Elaeis guineensis chromosome 12
genetic distance (cM)
0
20
40
60
80
0.00
0.25
0.50
0.75
1.00
Relative Genomic position (Mb) or Gene distance (cumulative number of genes)

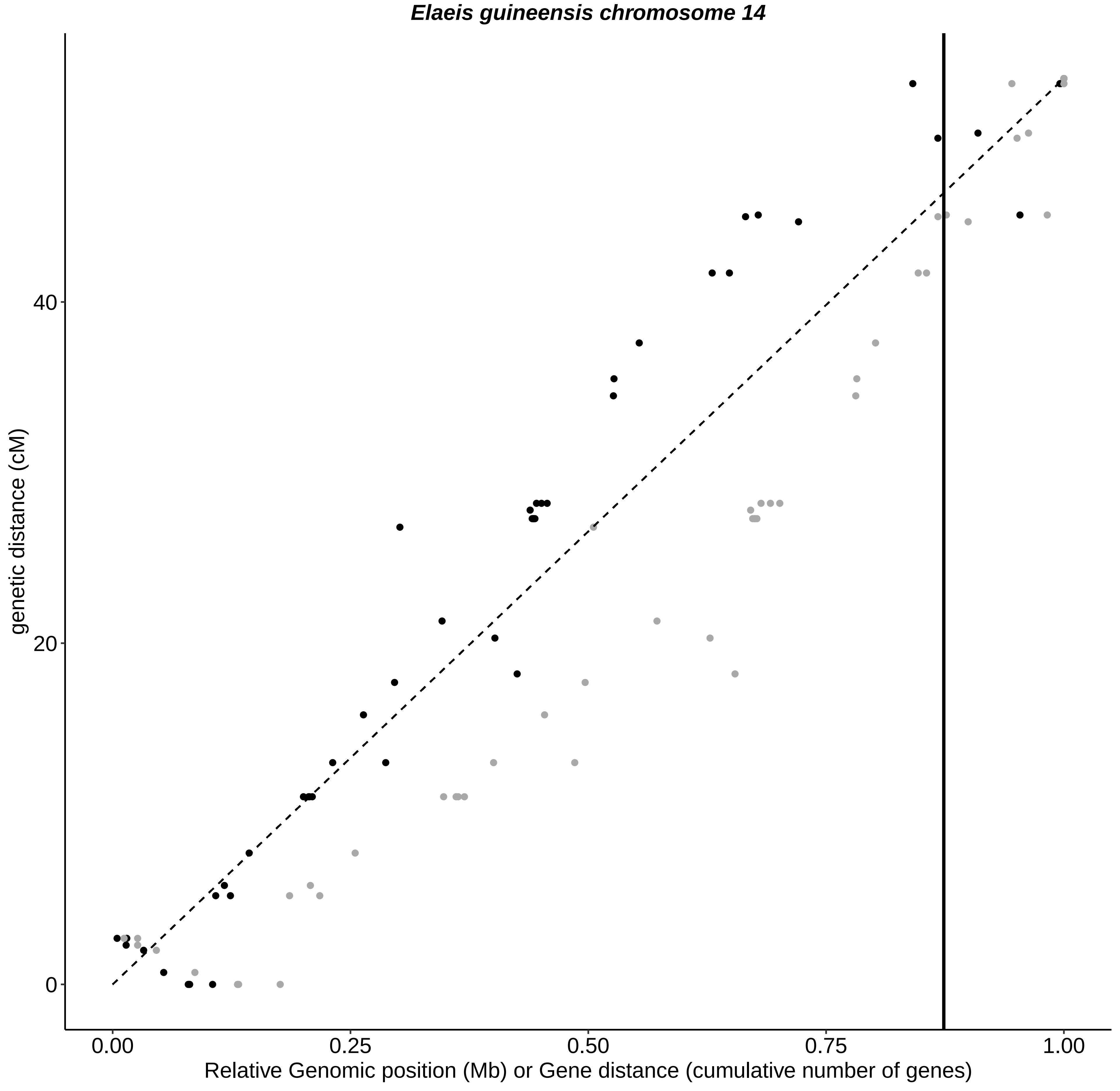
Elaeis guineensis chromosome 14
genetic distance (cM)
0
20
40
0.00
0.25
0.50
0.75
1.00
Relative Genomic position (Mb) or Gene distance (cumulative number of genes)

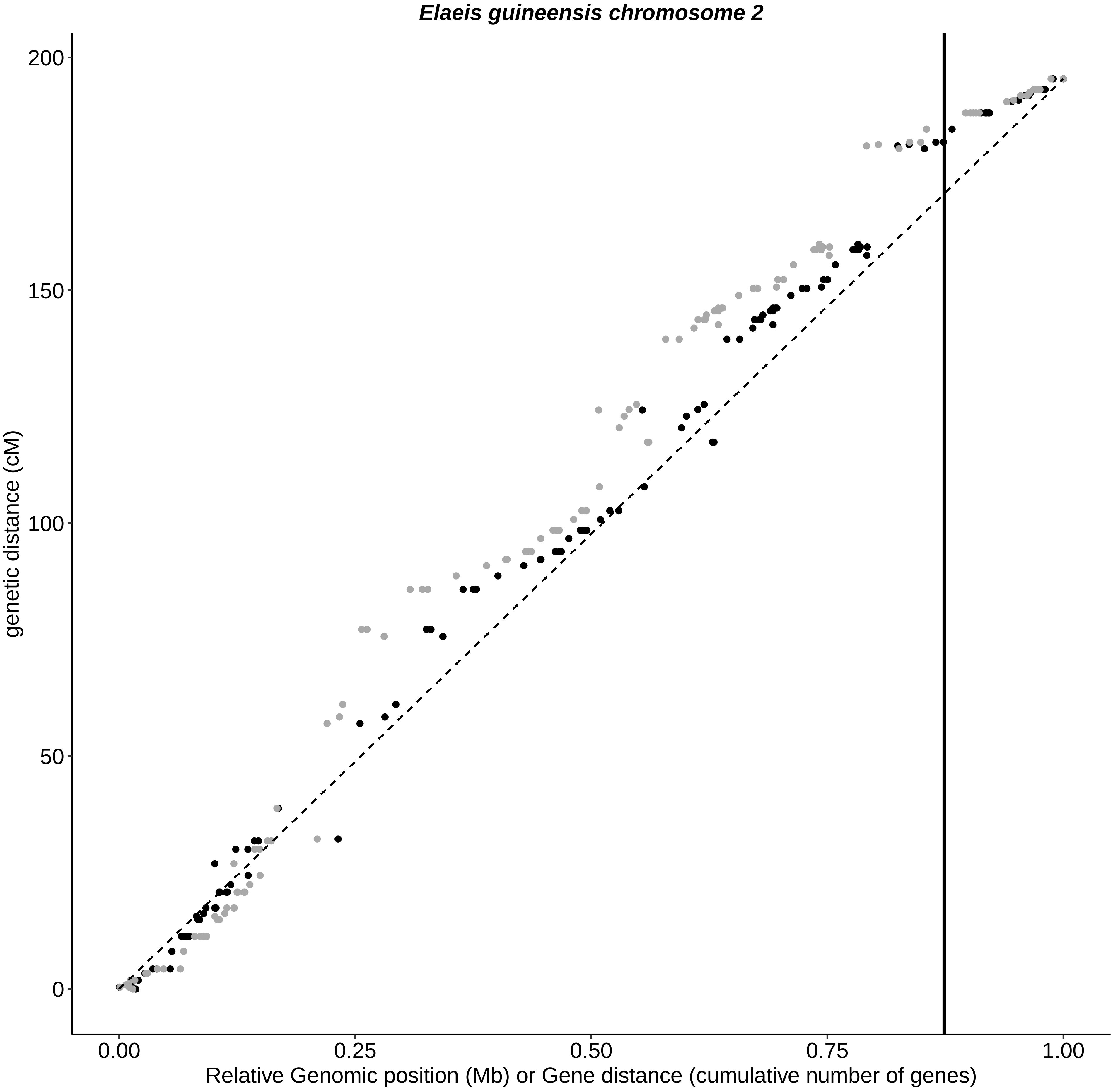
Elaeis guineensis chromosome 2
genetic distance (cM)
0
50
100
150
200
0.00
0.25
0.50
0.75
1.00
Relative Genomic position (Mb) or Gene distance (cumulative number of genes)

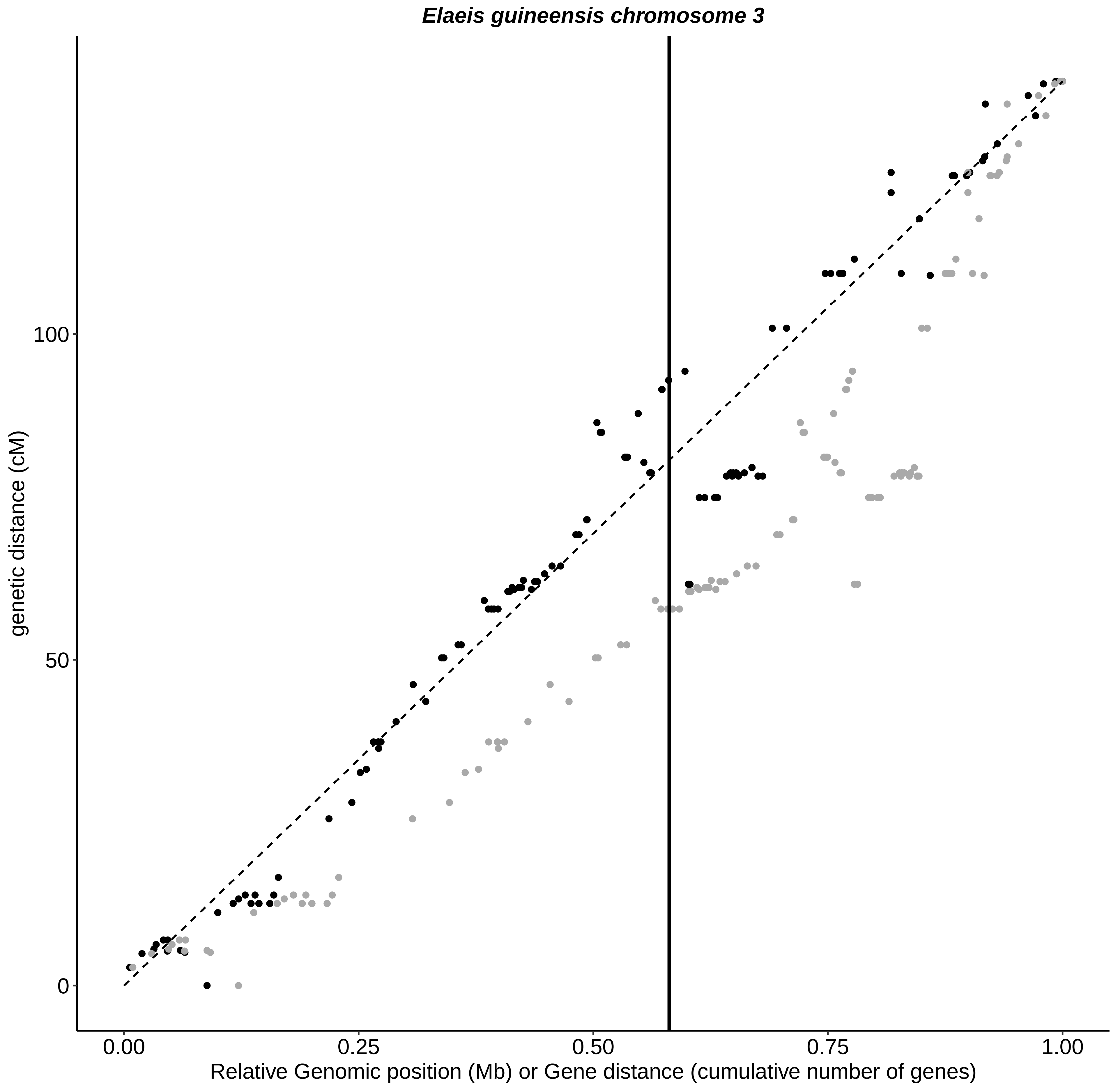

Elaeis guineensis chromosome 3
genetic distance (cM)
0
50
100
0.00
0.25
0.50
0.75
1.00
Relative Genomic position (Mb) or Gene distance (cumulative number of genes)

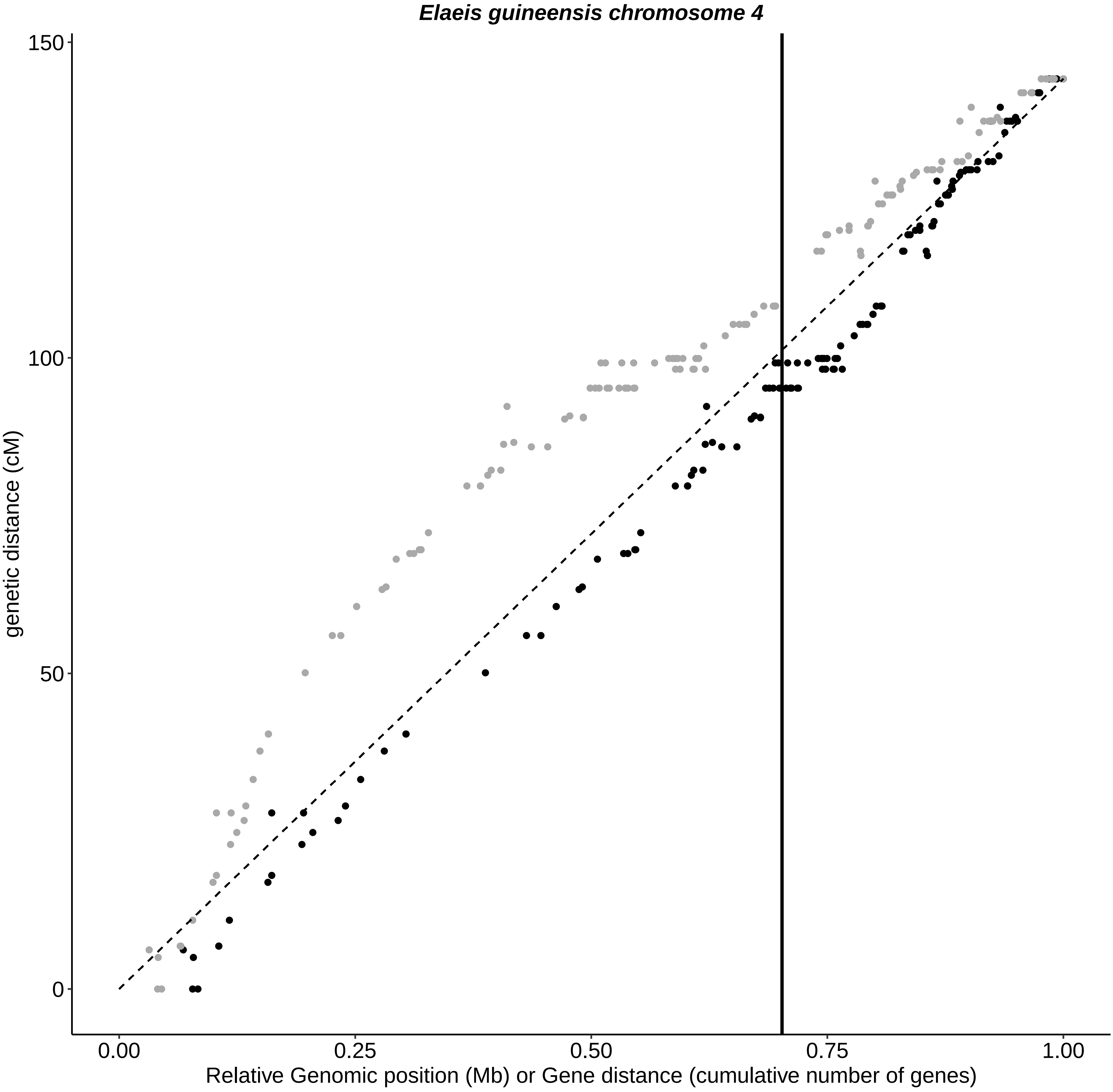
Elaeis guineensis chromosome 4
150
100
50
0
genetic distance (cM)
0.00
0.25
0.50
0.75
1.00
Relative Genomic position (Mb) or Gene distance (cumulative number of genes)

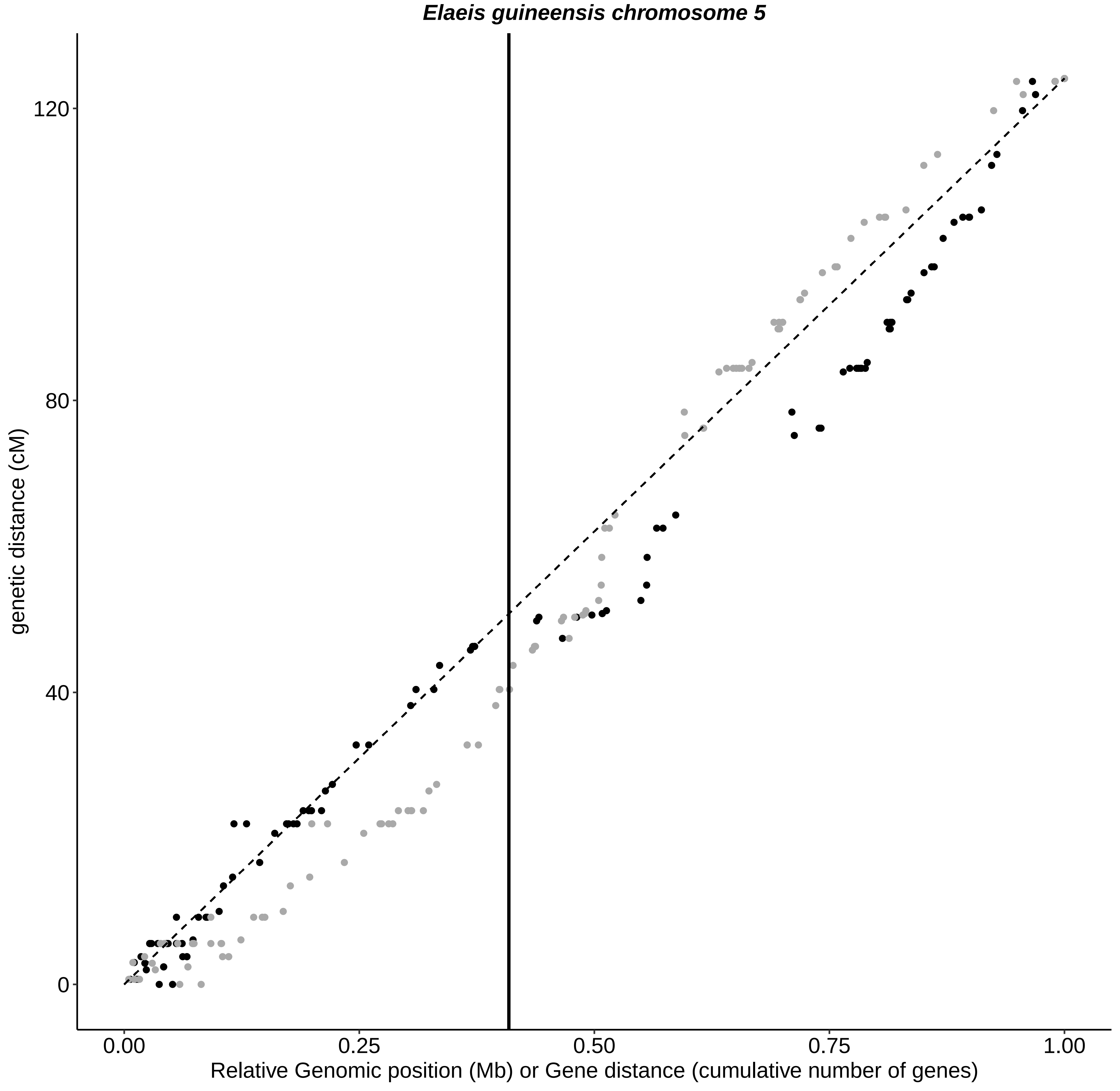
Elaeis guineensis chromosome 5
genetic distance (cM)
0
40
80
120
0.00
0.25
0.50
0.75
1.00
Relative Genomic position (Mb) or Gene distance (cumulative number of genes)

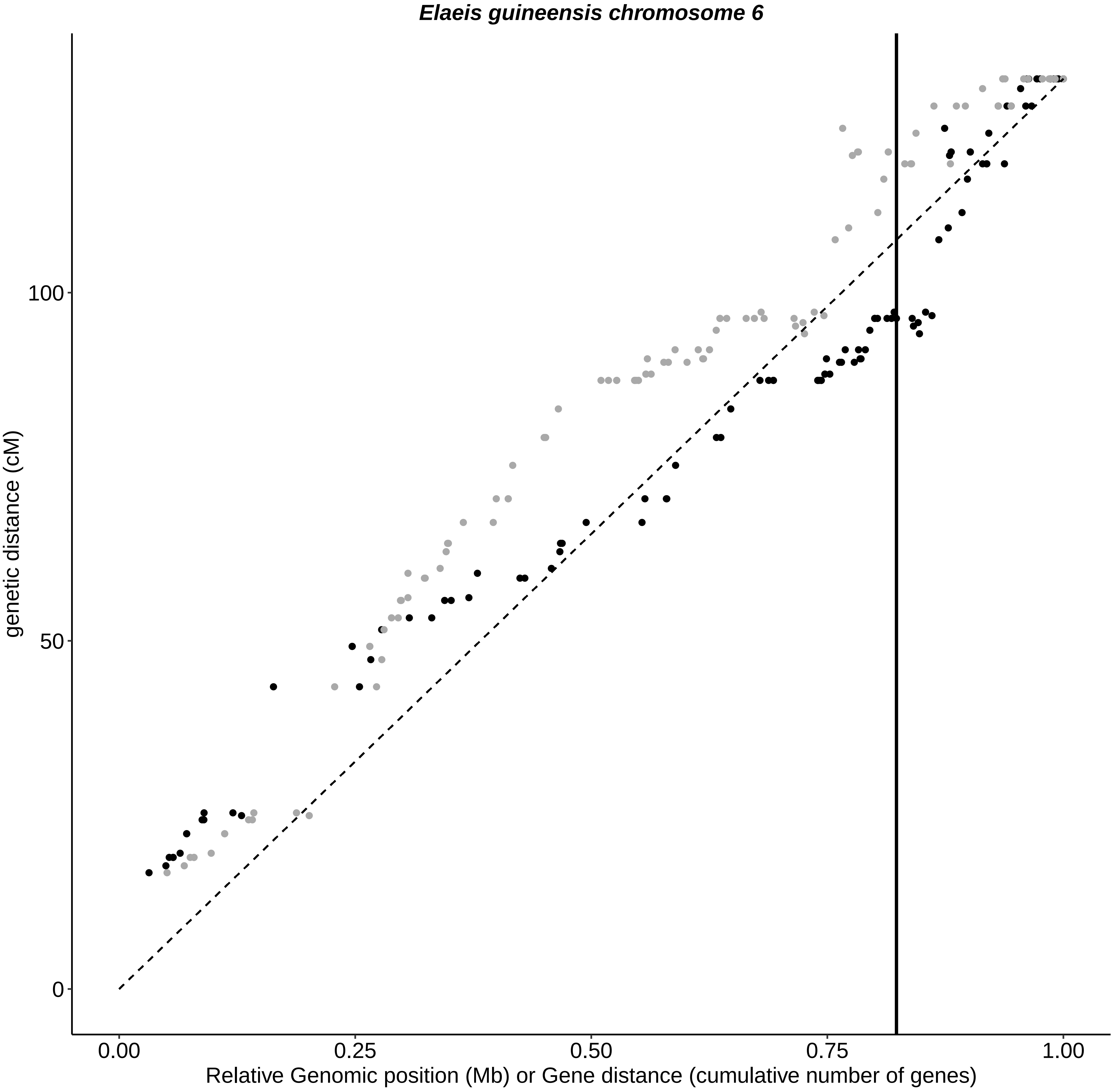
Elaeis guineensis chromosome 6
genetic distance (cM)
100
50
0
0.00
0.25
0.50
0.75
1.00
Relative Genomic position (Mb) or Gene distance (cumulative number of genes)

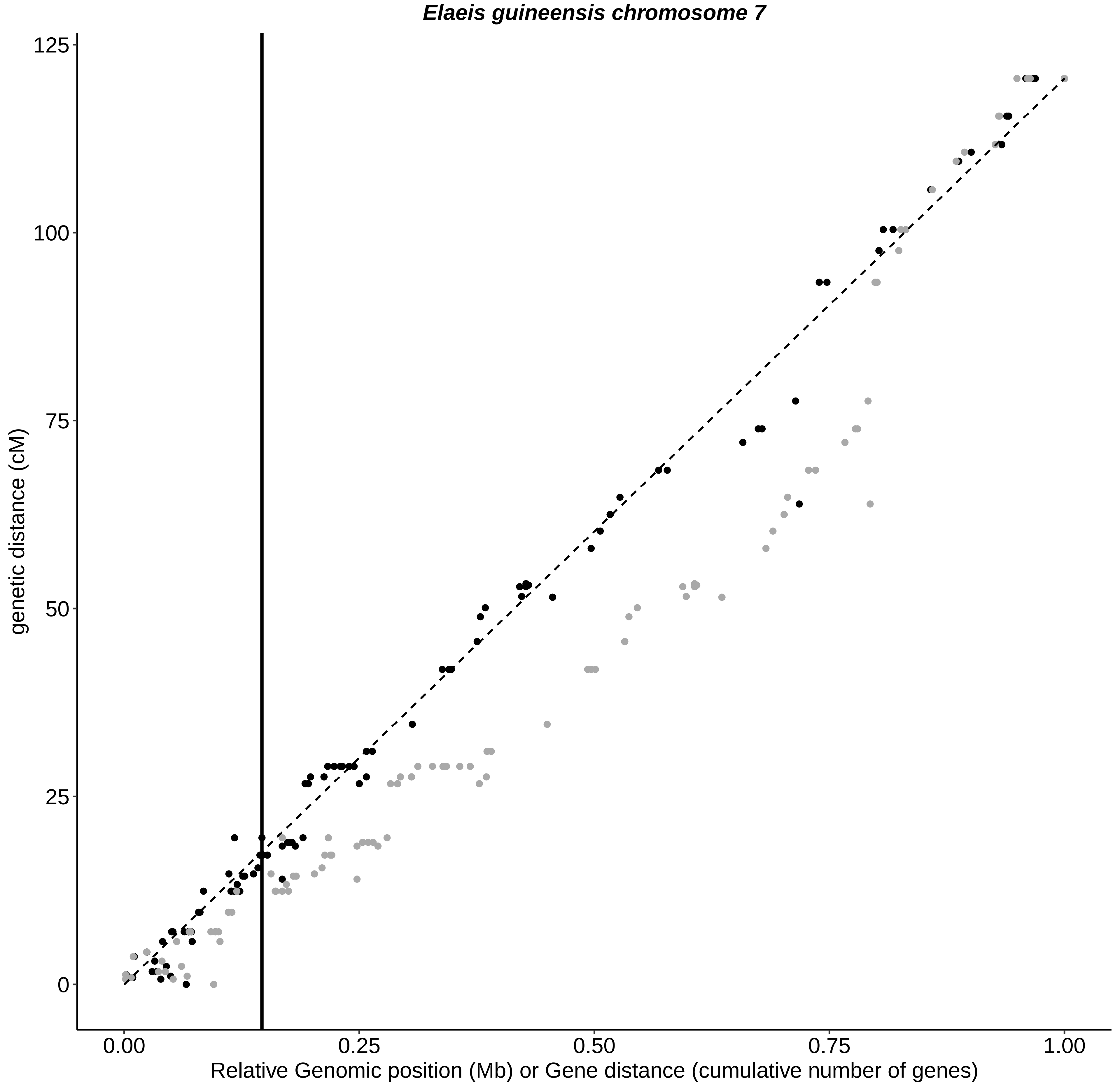
Elaeis guineensis chromosome 7
125
100
75
50
25
0
genetic distance (cM)
0.00
0.25
0.50
0.75
1.00
Relative Genomic position (Mb) or Gene distance (cumulative number of genes)

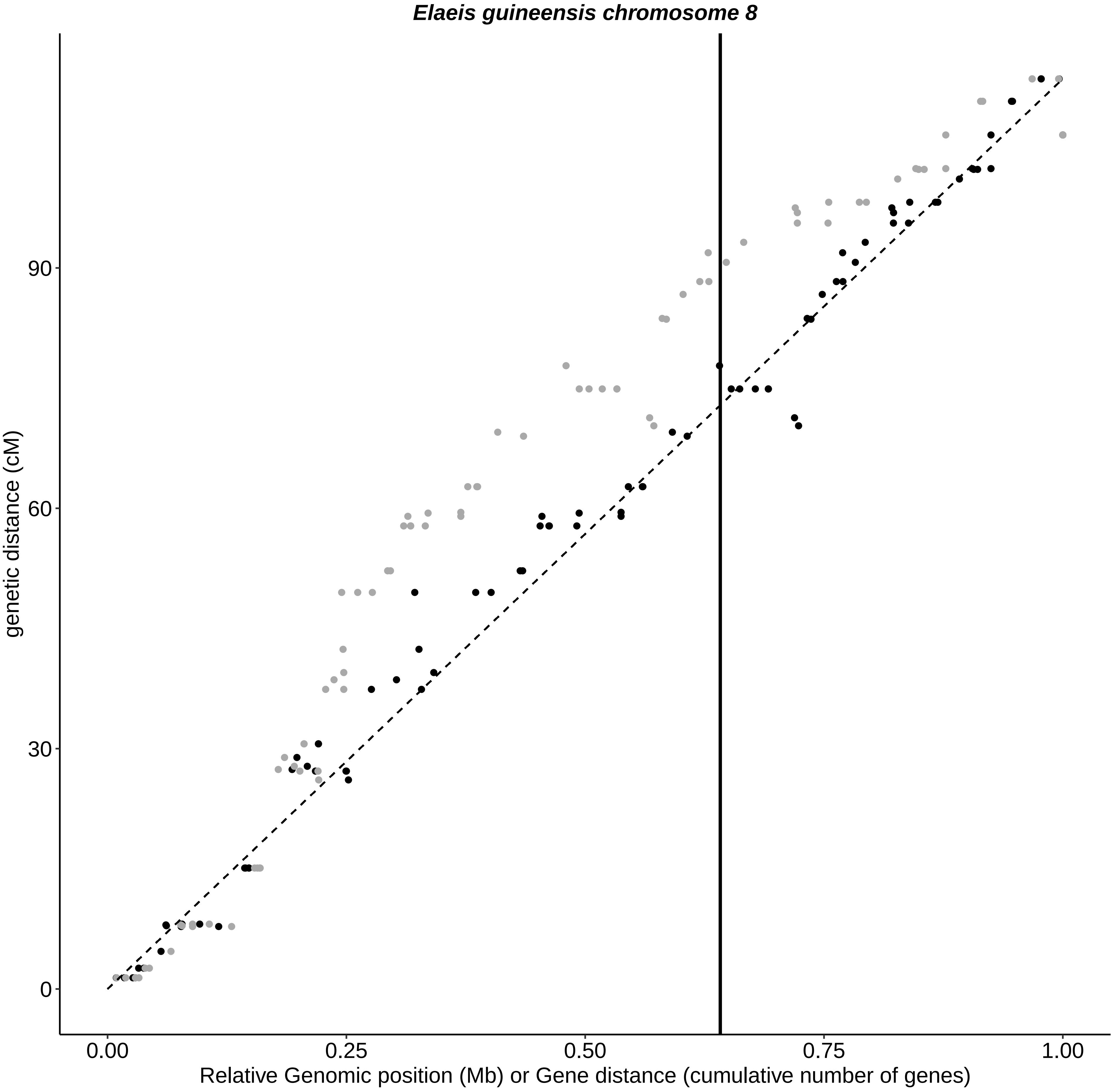
Elaeis guineensis chromosome 8
genetic distance (cM)
0
30
60
90
0.00
0.25
0.50
0.75
1.00
Relative Genomic position (Mb) or Gene distance (cumulative number of genes)

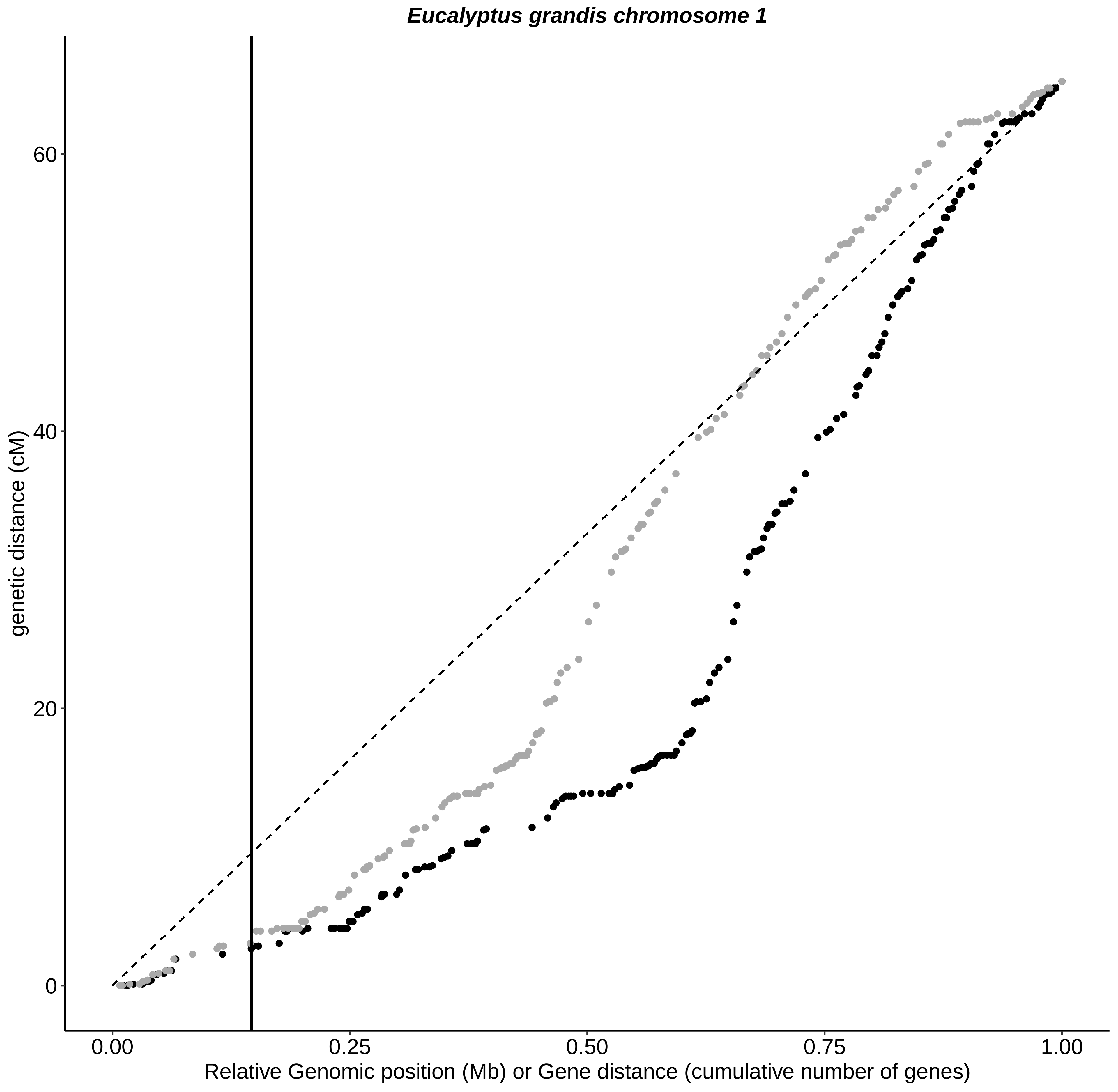
Eucalyptus grandis chromosome 1
genetic distance (cM)
0
20
40
60
0.00
0.25
0.50
0.75
1.00
Relative Genomic position (Mb) or Gene distance (cumulative number of genes)

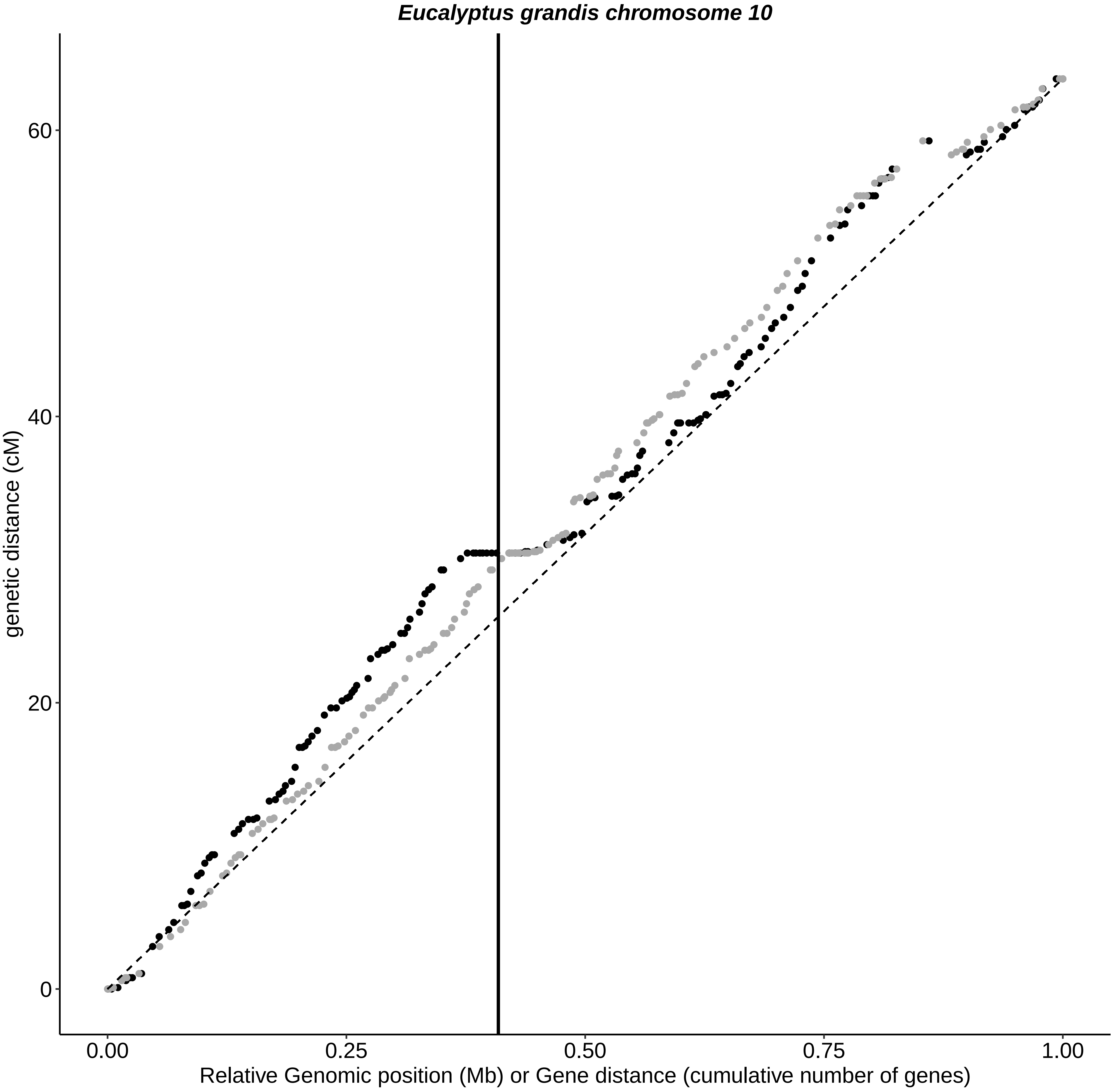

Eucalyptus grandis chromosome 10
genetic distance (cM)
0
20
40
60
0.00
0.25
0.50
0.75
1.00
Relative Genomic position (Mb) or Gene distance (cumulative number of genes)

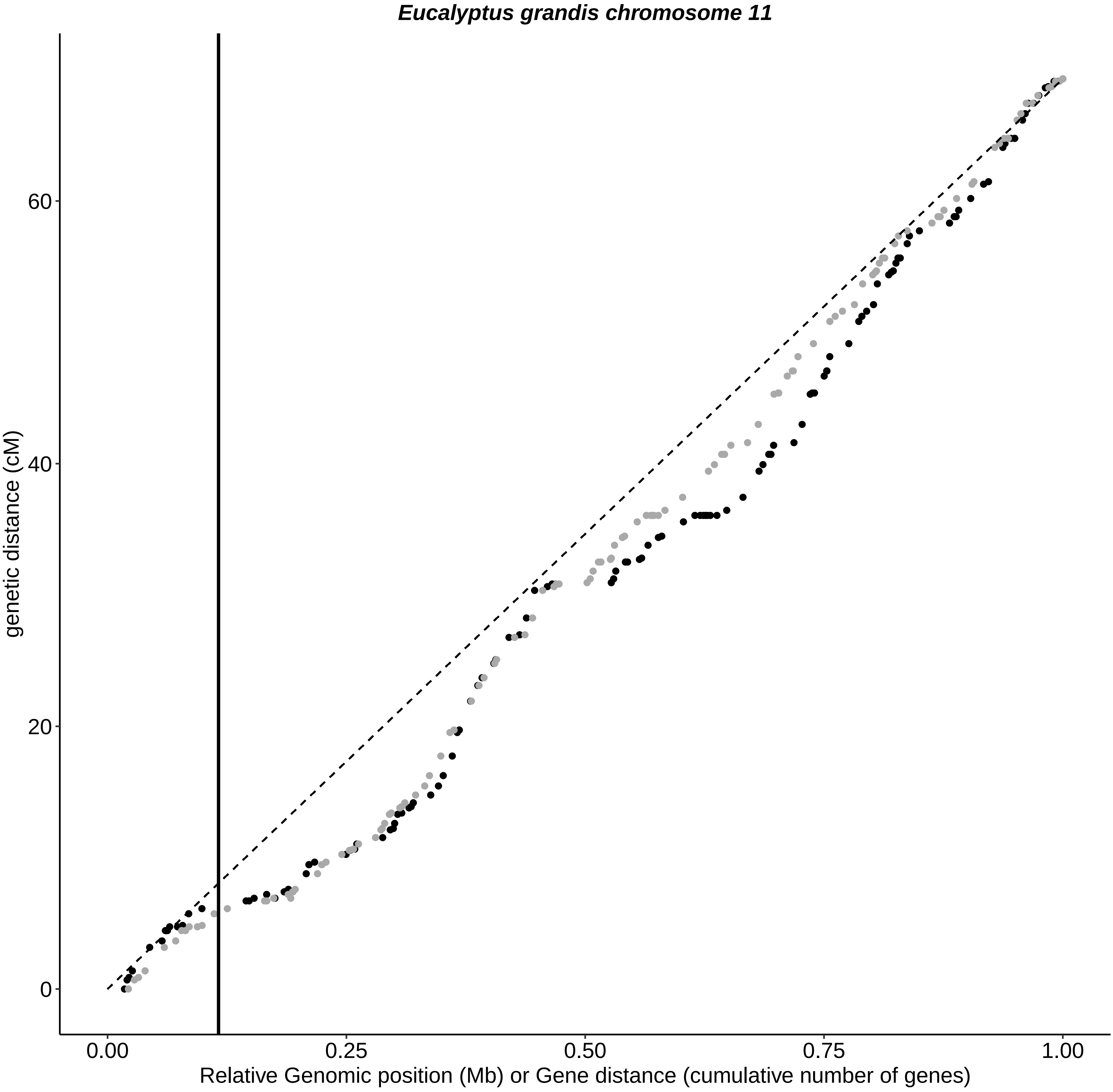
Eucalyptus grandis chromosome 11
genetic distance (cM)
0
20
40
60
0.00
0.25
0.50
0.75
1.00
Relative Genomic position (Mb) or Gene distance (cumulative number of genes)

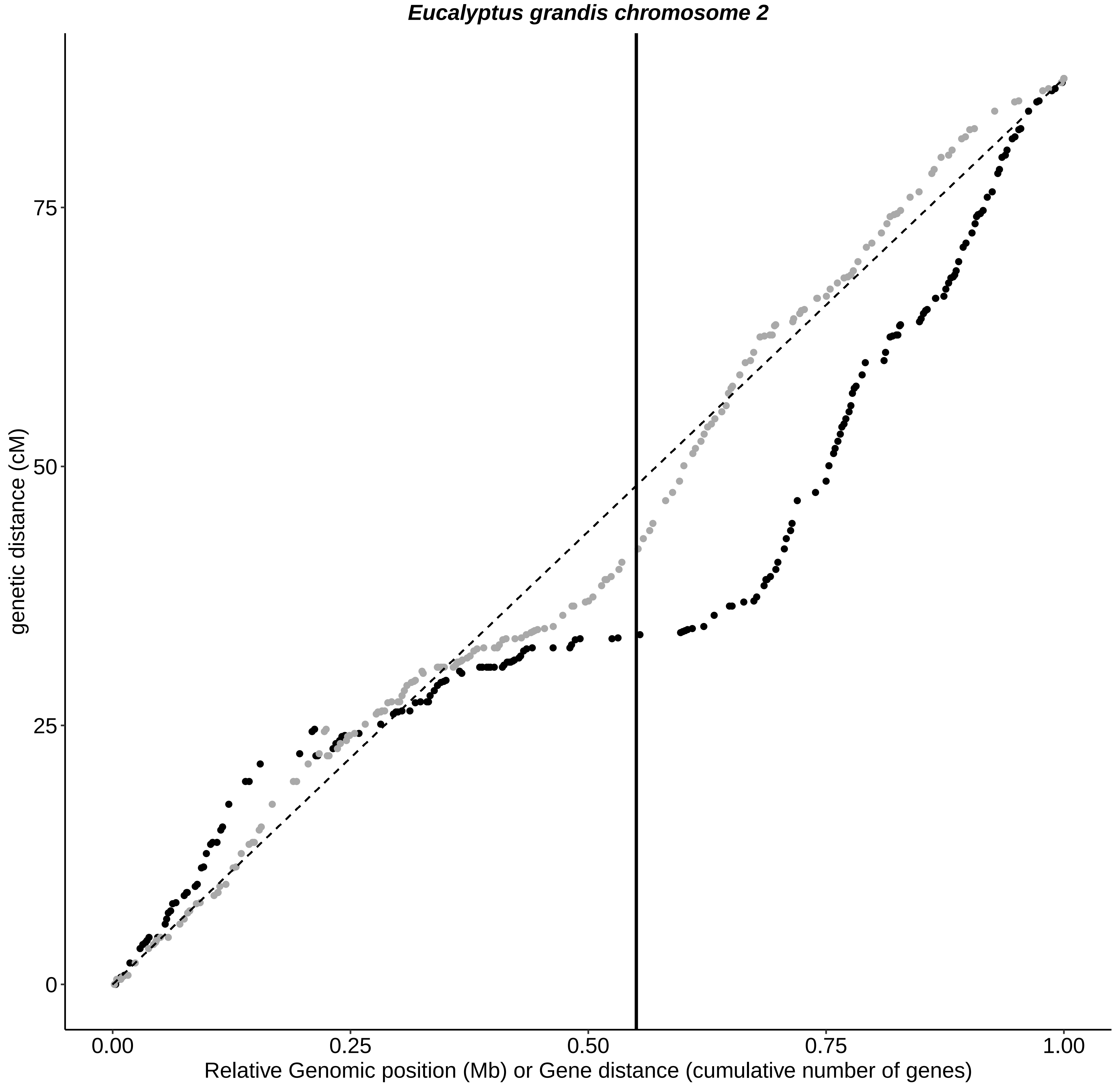
Eucalyptus grandis chromosome 2
genetic distance (cM)
0
25
50
75
0.00
0.25
0.50
0.75
1.00
Relative Genomic position (Mb) or Gene distance (cumulative number of genes)

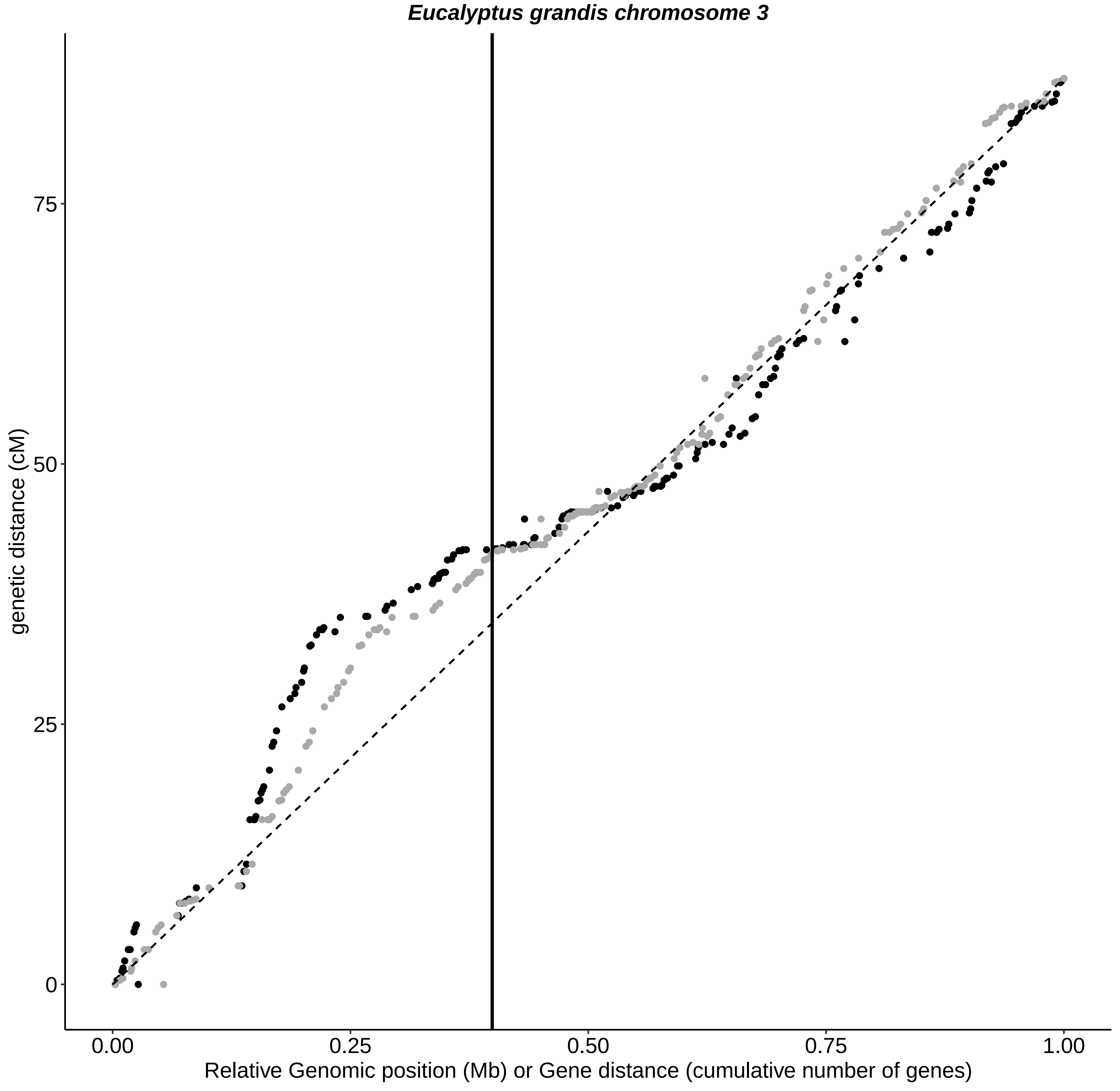
Eucalyptus grandis chromosome 3
genetic distance (cM)
0
25
50
75
0.00
0.25
0.50
0.75
1.00
Relative Genomic position (Mb) or Gene distance (cumulative number of genes)

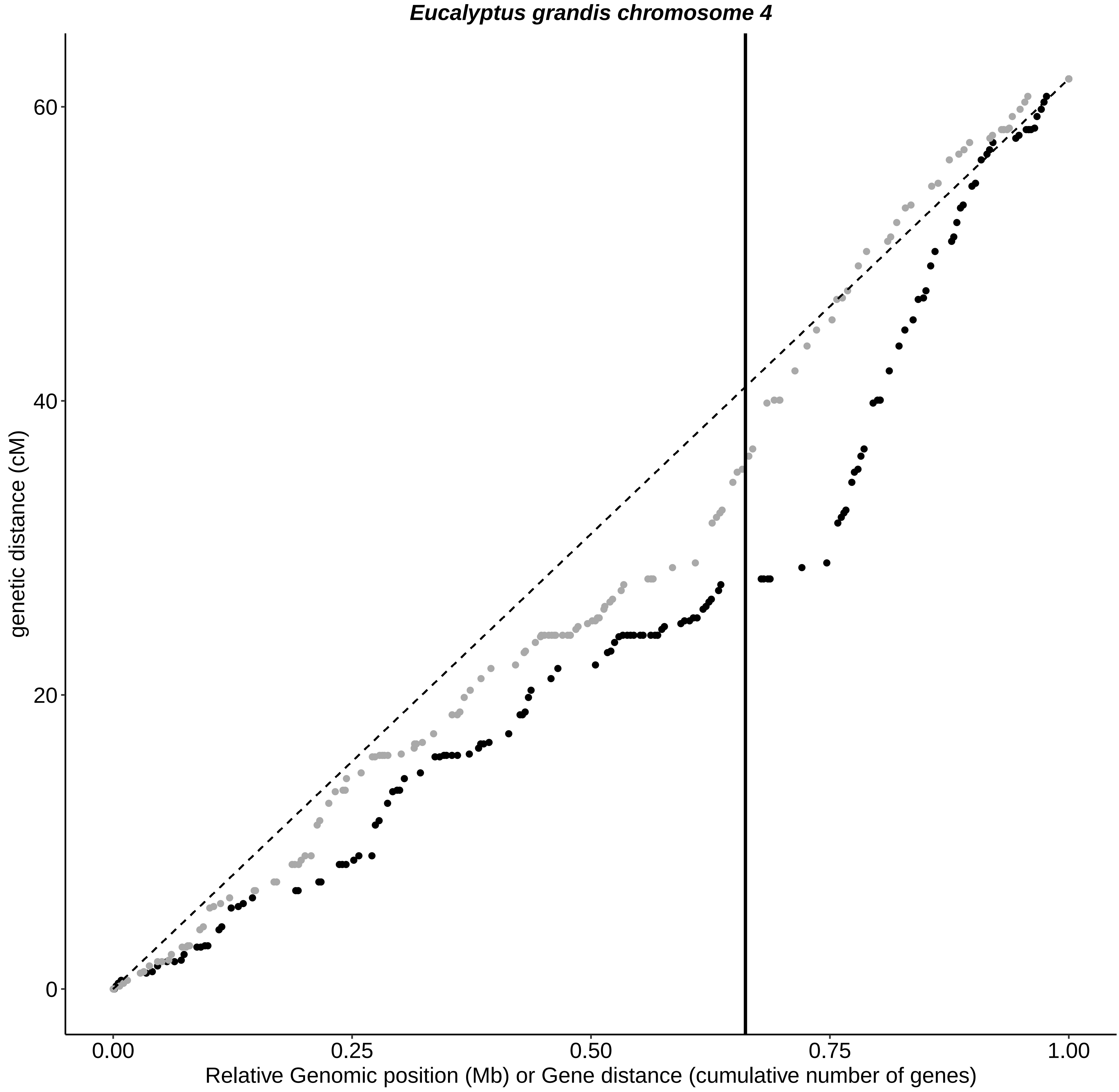
Eucalyptus grandis chromosome 4
genetic distance (cM)
0
20
40
60
0.00
0.25
0.50
0.75
1.00
Relative Genomic position (Mb) or Gene distance (cumulative number of genes)

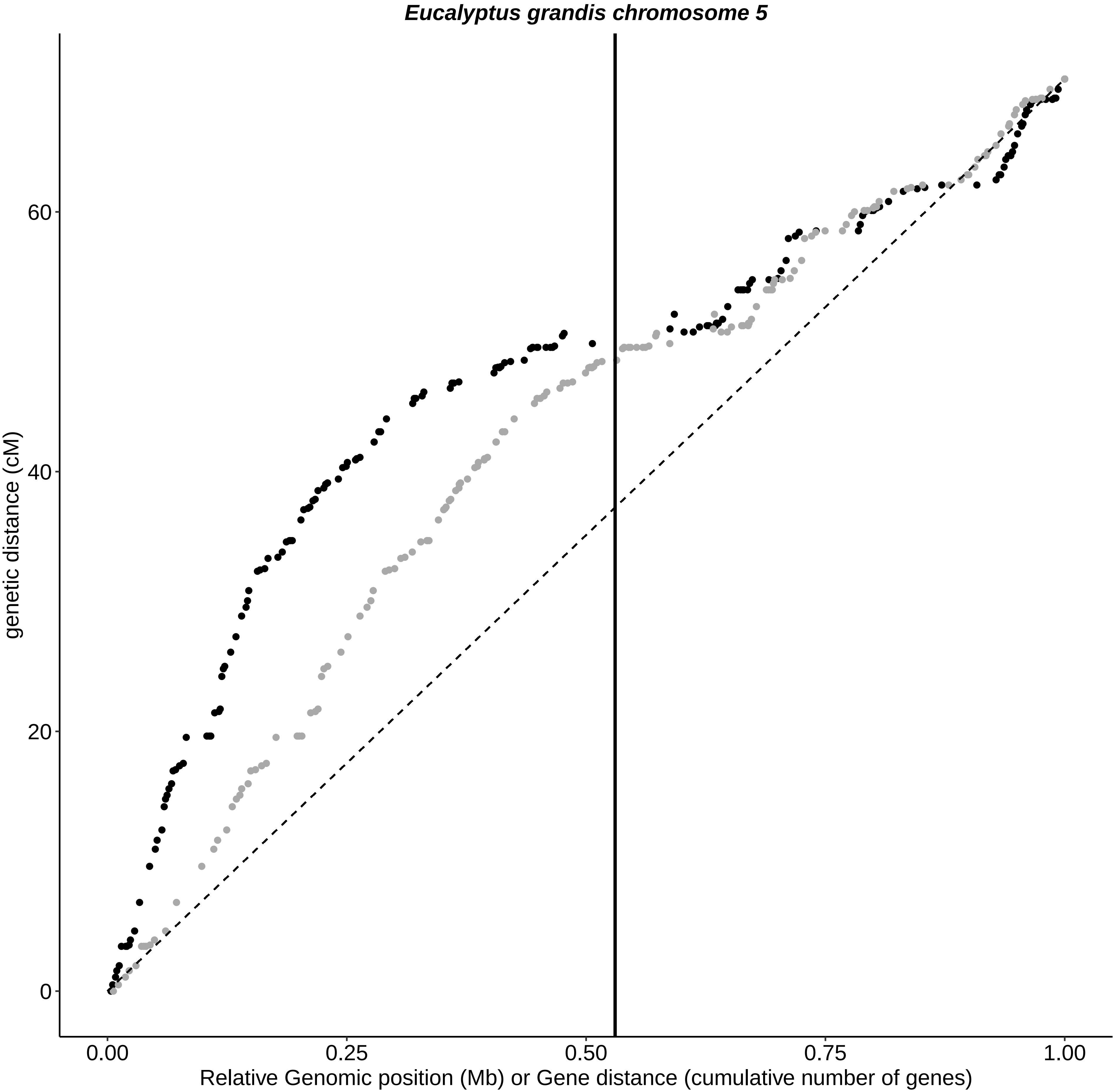
Eucalyptus grandis chromosome 5
genetic distance (cM)
0
20
40
60
0.00
0.25
0.50
0.75
1.00
Relative Genomic position (Mb) or Gene distance (cumulative number of genes)

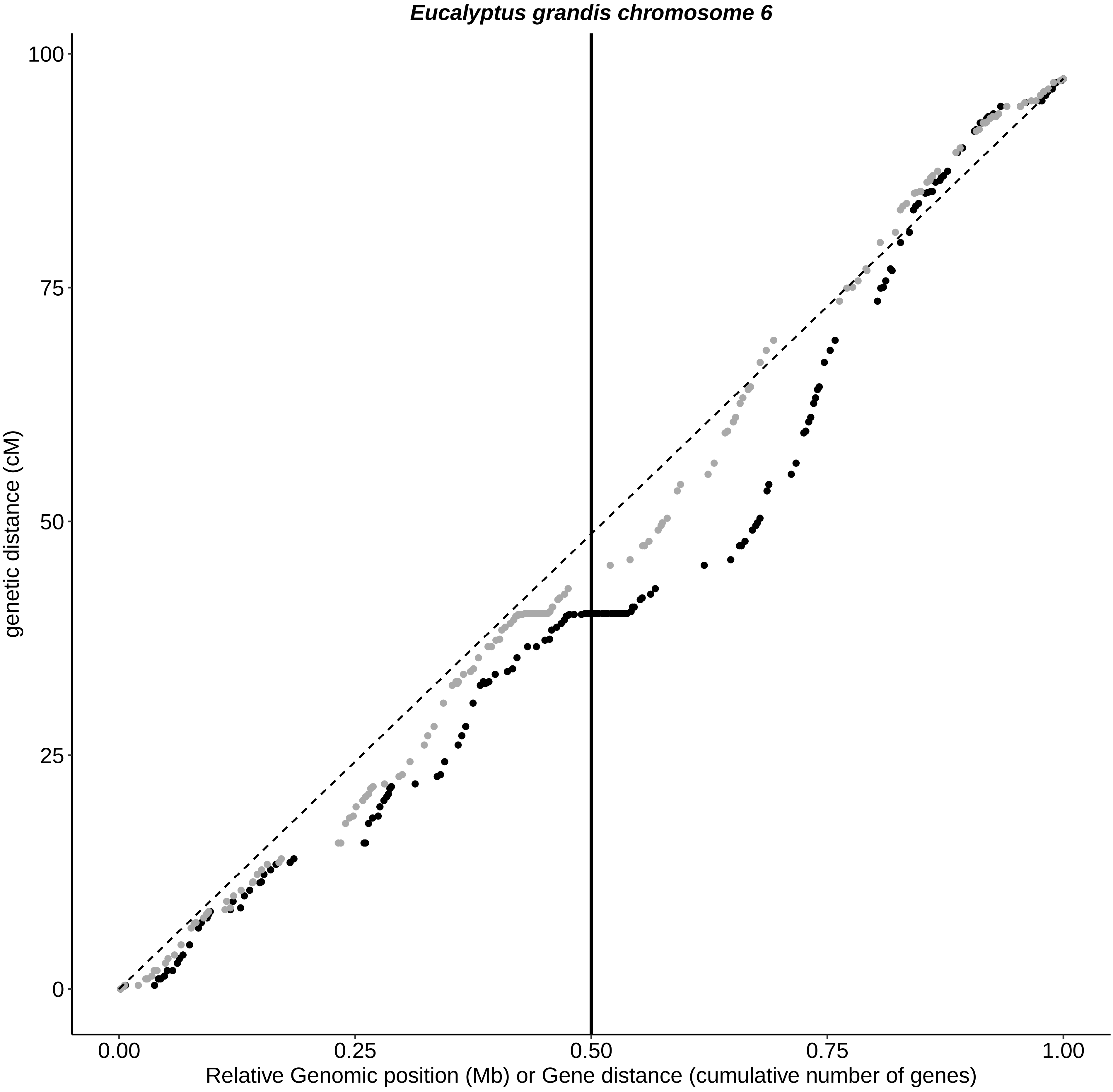
Eucalyptus grandis chromosome 6
genetic distance (cM)
100
75
50
25
0
0.00
0.25
0.50
0.75
1.00
Relative Genomic position (Mb) or Gene distance (cumulative number of genes)

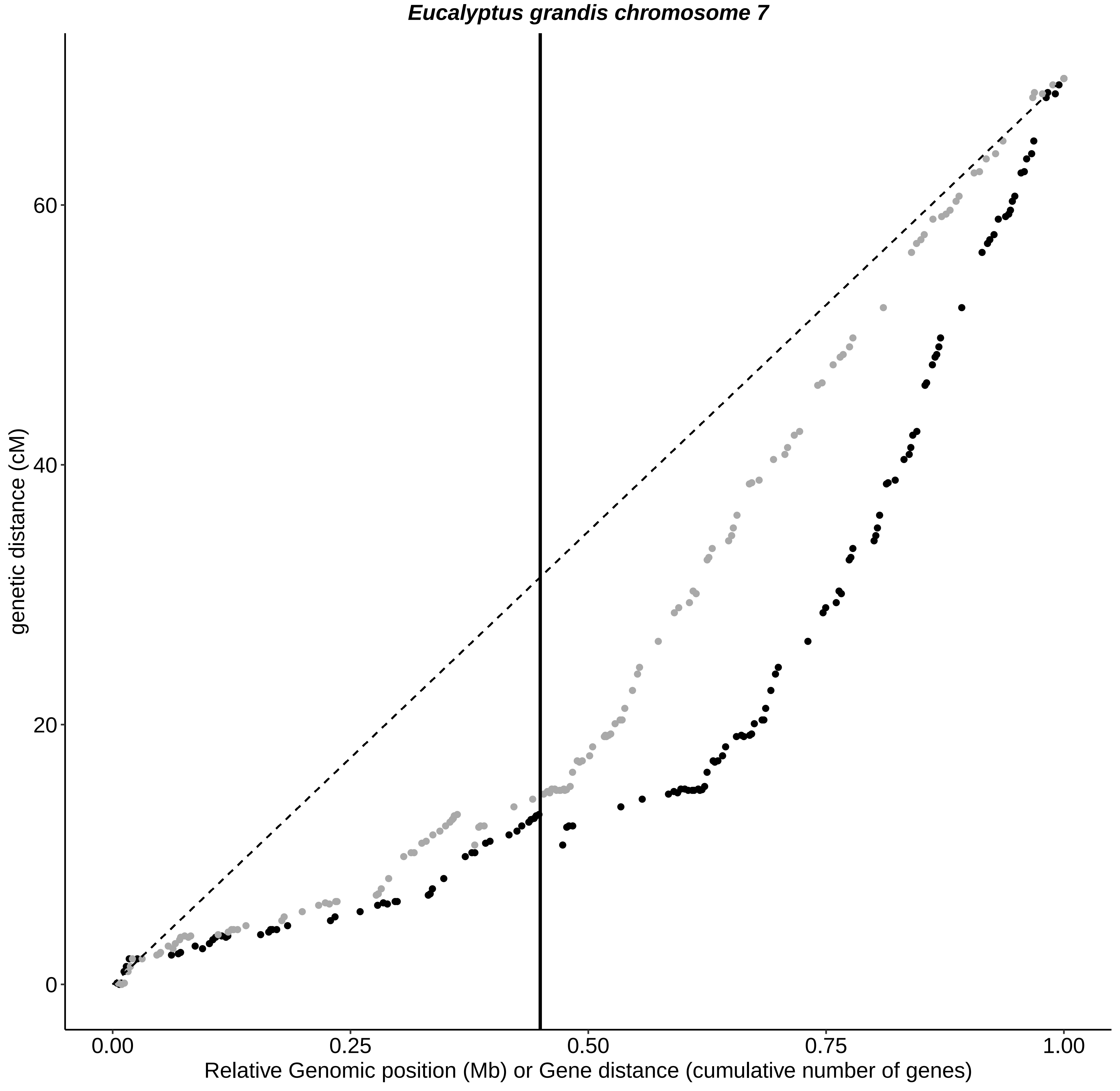
Eucalyptus grandis chromosome 7
genetic distance (cM)
0
20
40
60
0.00
0.25
0.50
0.75
1.00
Relative Genomic position (Mb) or Gene distance (cumulative number of genes)

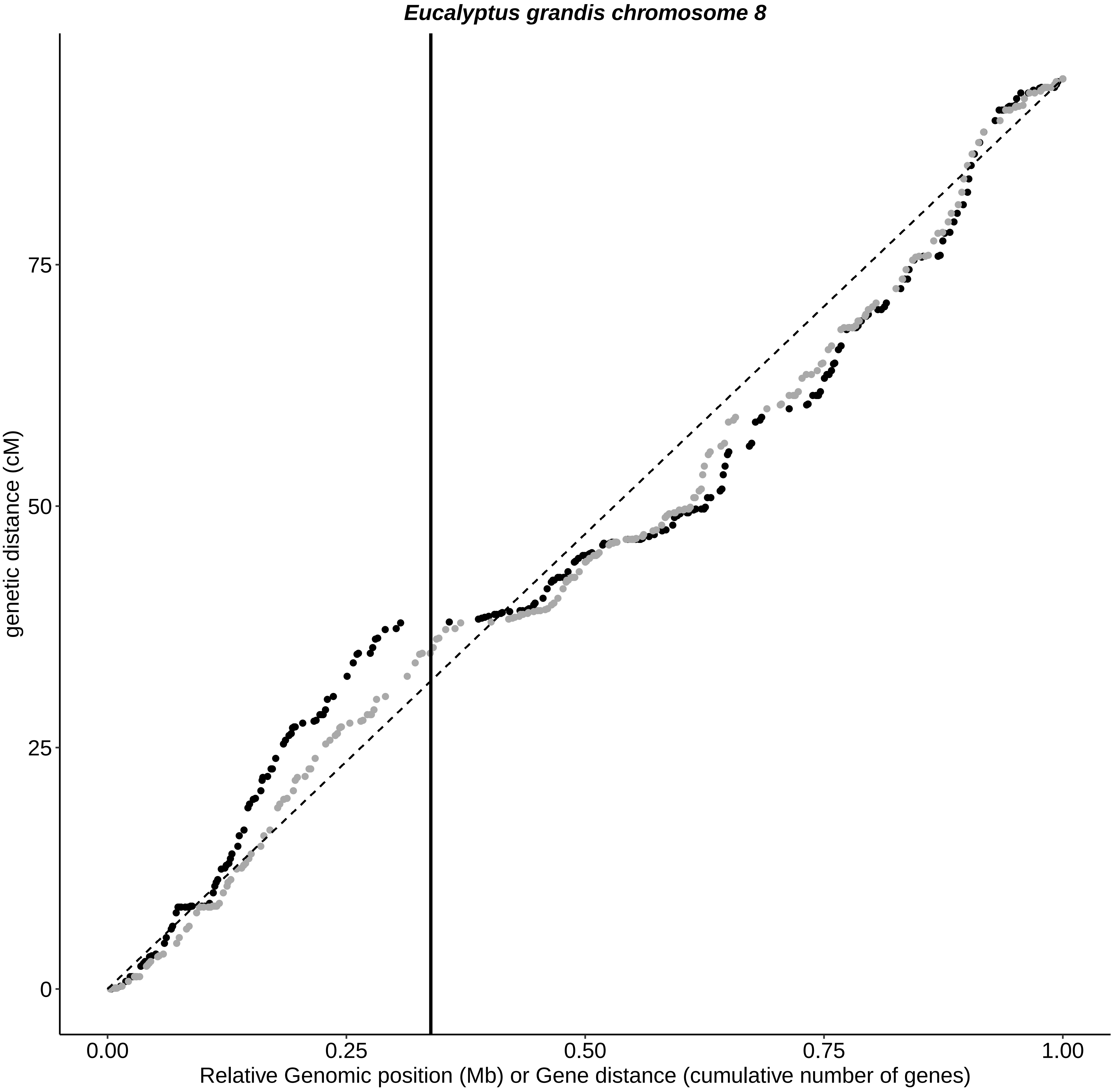
Eucalyptus grandis chromosome 8
genetic distance (cM)
0
25
50
75
0.00
0.25
0.50
0.75
1.00
Relative Genomic position (Mb) or Gene distance (cumulative number of genes)

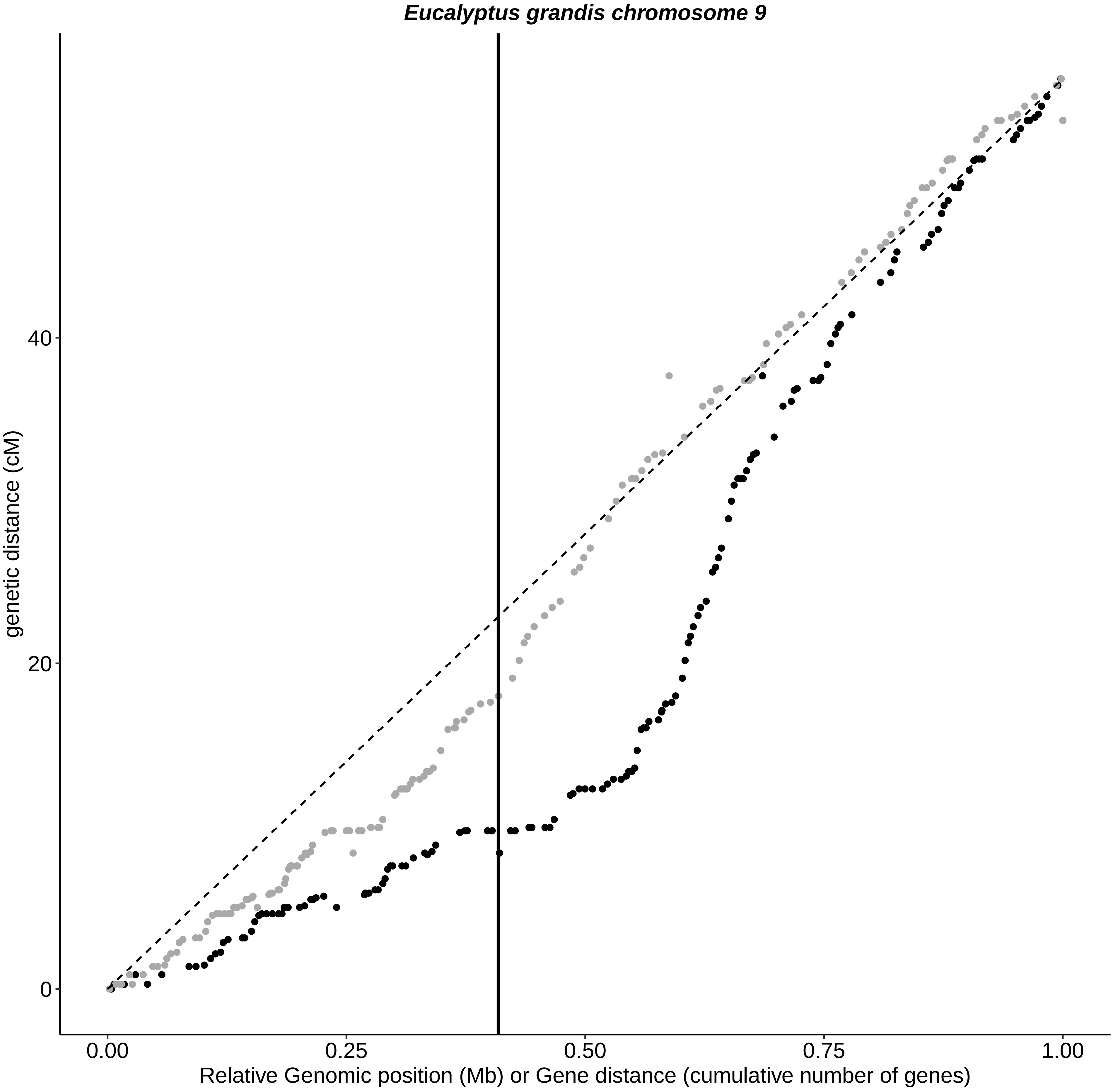
Eucalyptus grandis chromosome 9
genetic distance (cM)
0
20
40
0.00
0.25
0.50
0.75
1.00
Relative Genomic position (Mb) or Gene distance (cumulative number of genes)

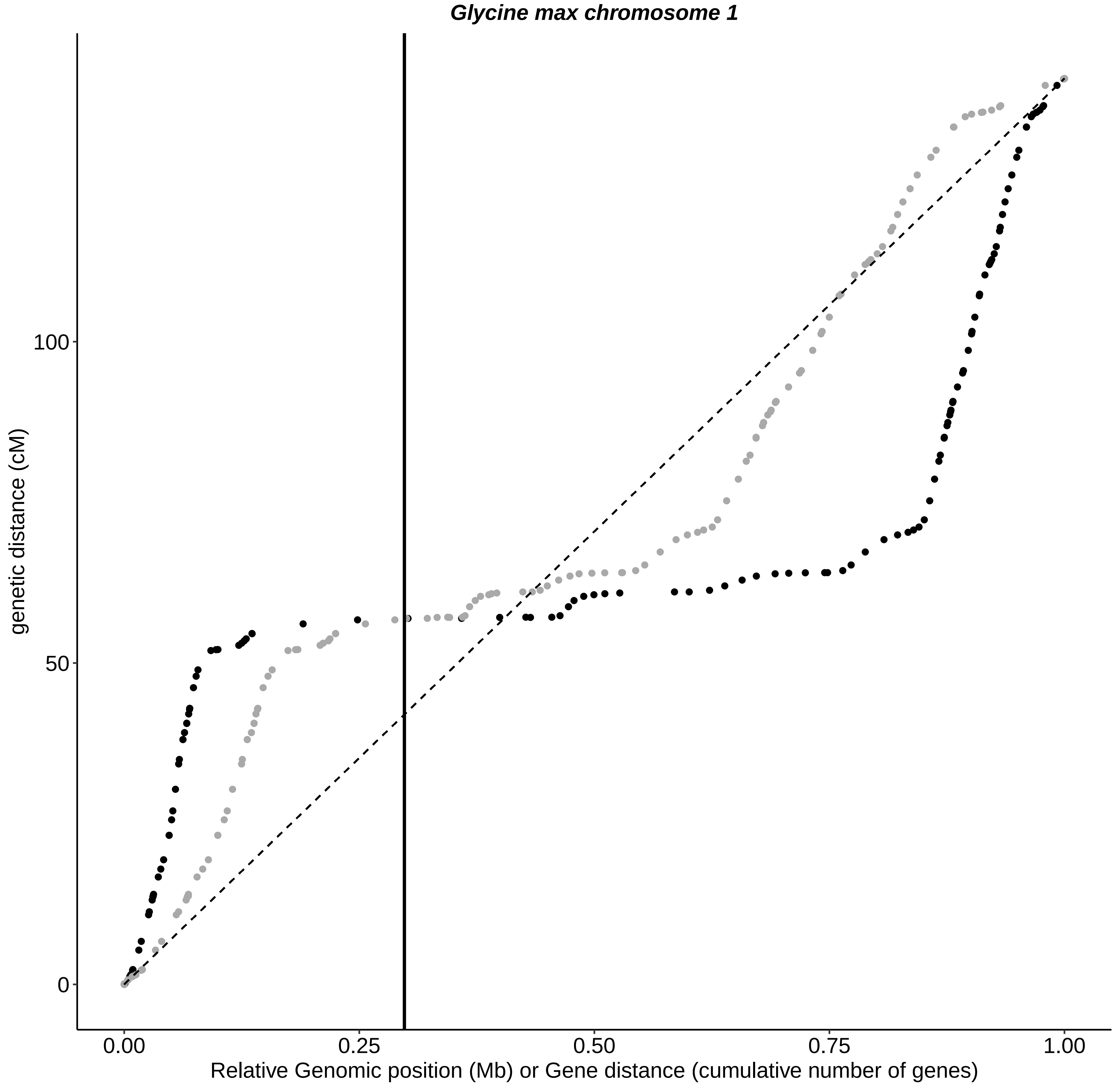
Glycine max chromosome 1
genetic distance (cM)
0
50
100
0.00
0.25
0.50
0.75
1.00
Relative Genomic position (Mb) or Gene distance (cumulative number of genes)

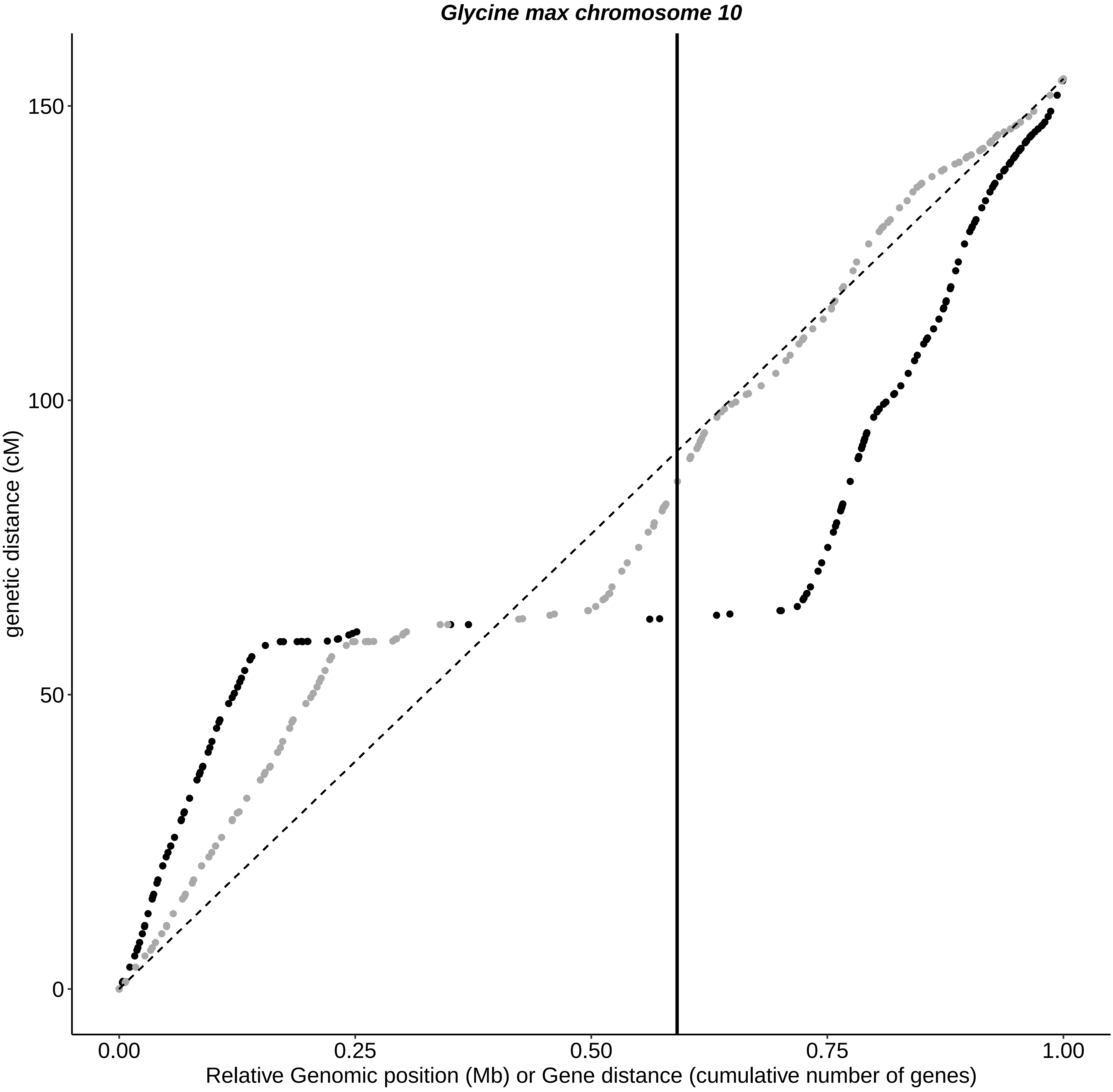
Glycine max chromosome 10
genetic distance (cM)
0
50
100
150
0.00
0.25
0.50
0.75
1.00
Relative Genomic position (Mb) or Gene distance (cumulative number of genes)

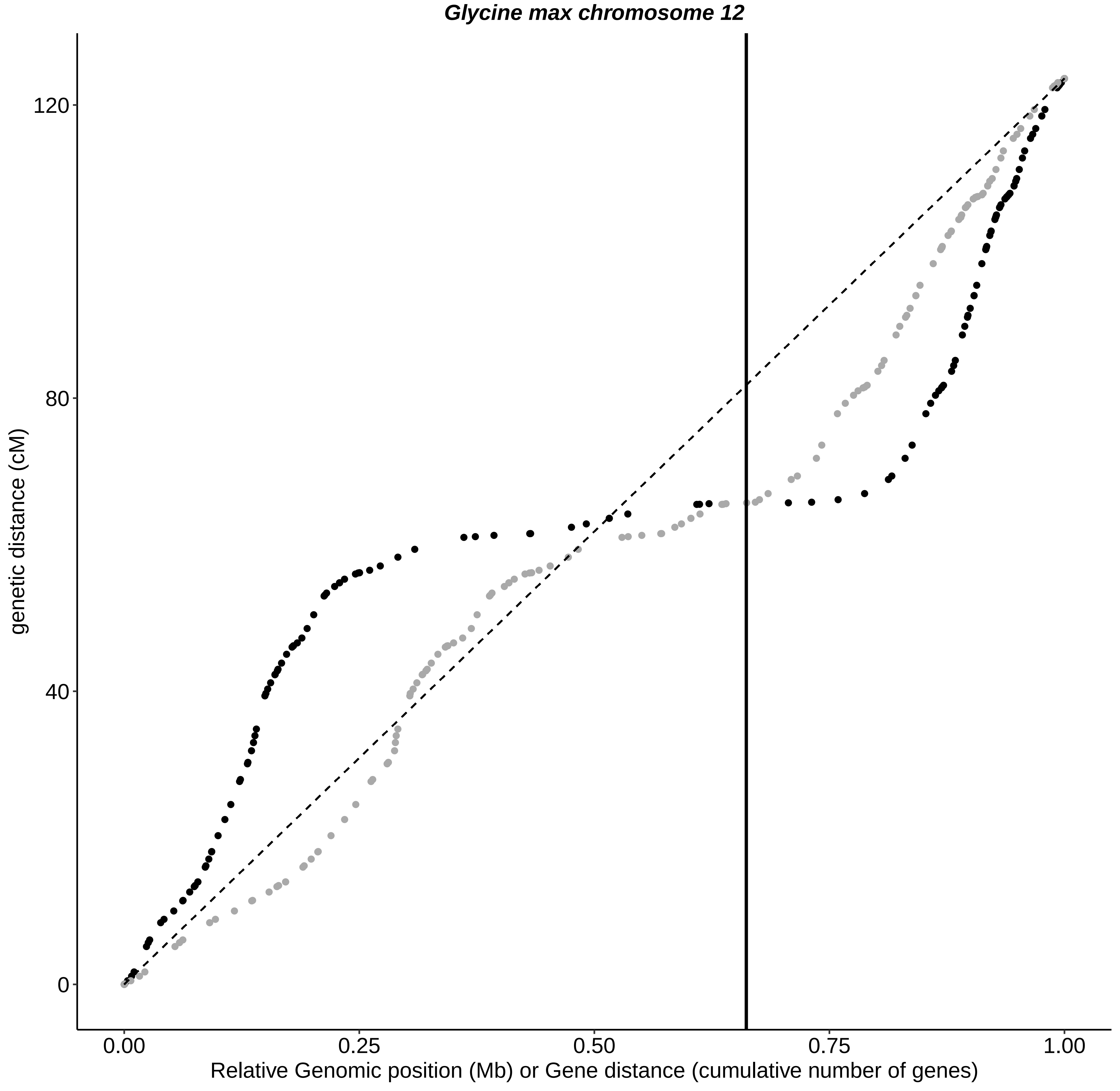
Glycine max chromosome 12
genetic distance (cM)
0
40
80
120
0.00
0.25
0.50
0.75
1.00
Relative Genomic position (Mb) or Gene distance (cumulative number of genes)

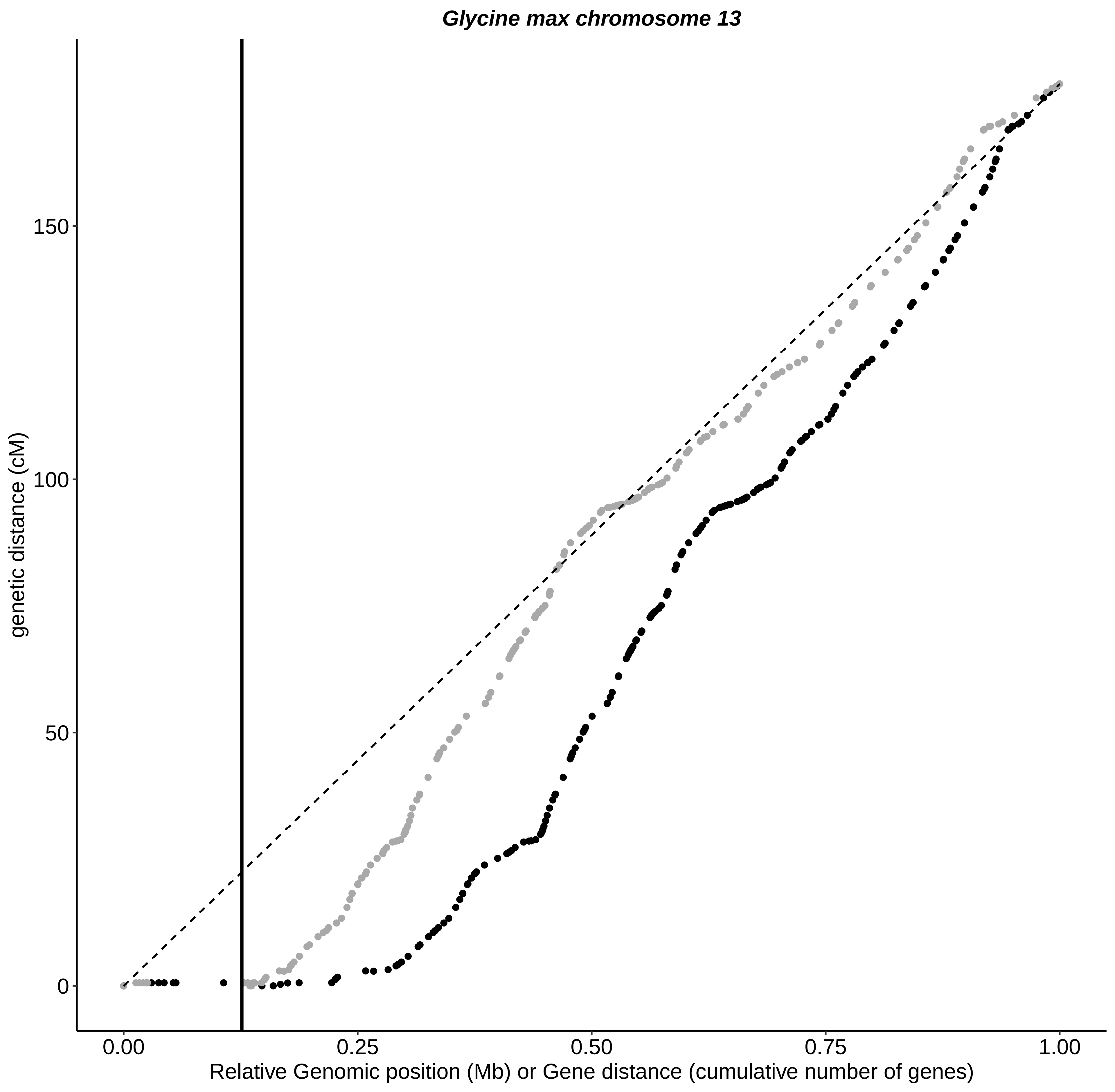
Glycine max chromosome 13
genetic distance (cM)
150
100
50
0
0.00
0.25
0.50
0.75
1.00
Relative Genomic position (Mb) or Gene distance (cumulative number of genes)

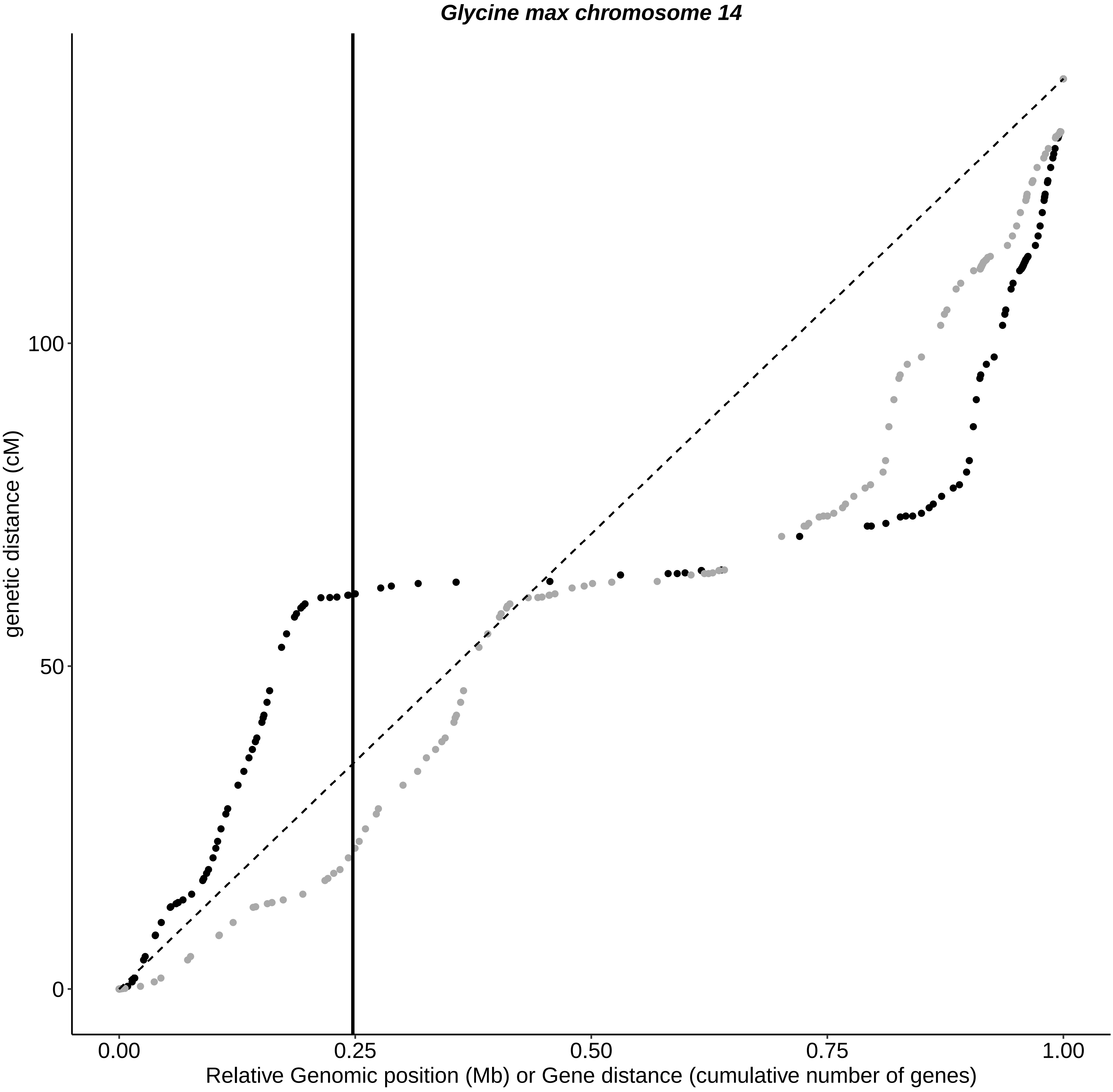
Glycine max chromosome 14
genetic distance (cM)
0
50
100
0.00
0.25
0.50
0.75
1.00
Relative Genomic position (Mb) or Gene distance (cumulative number of genes)

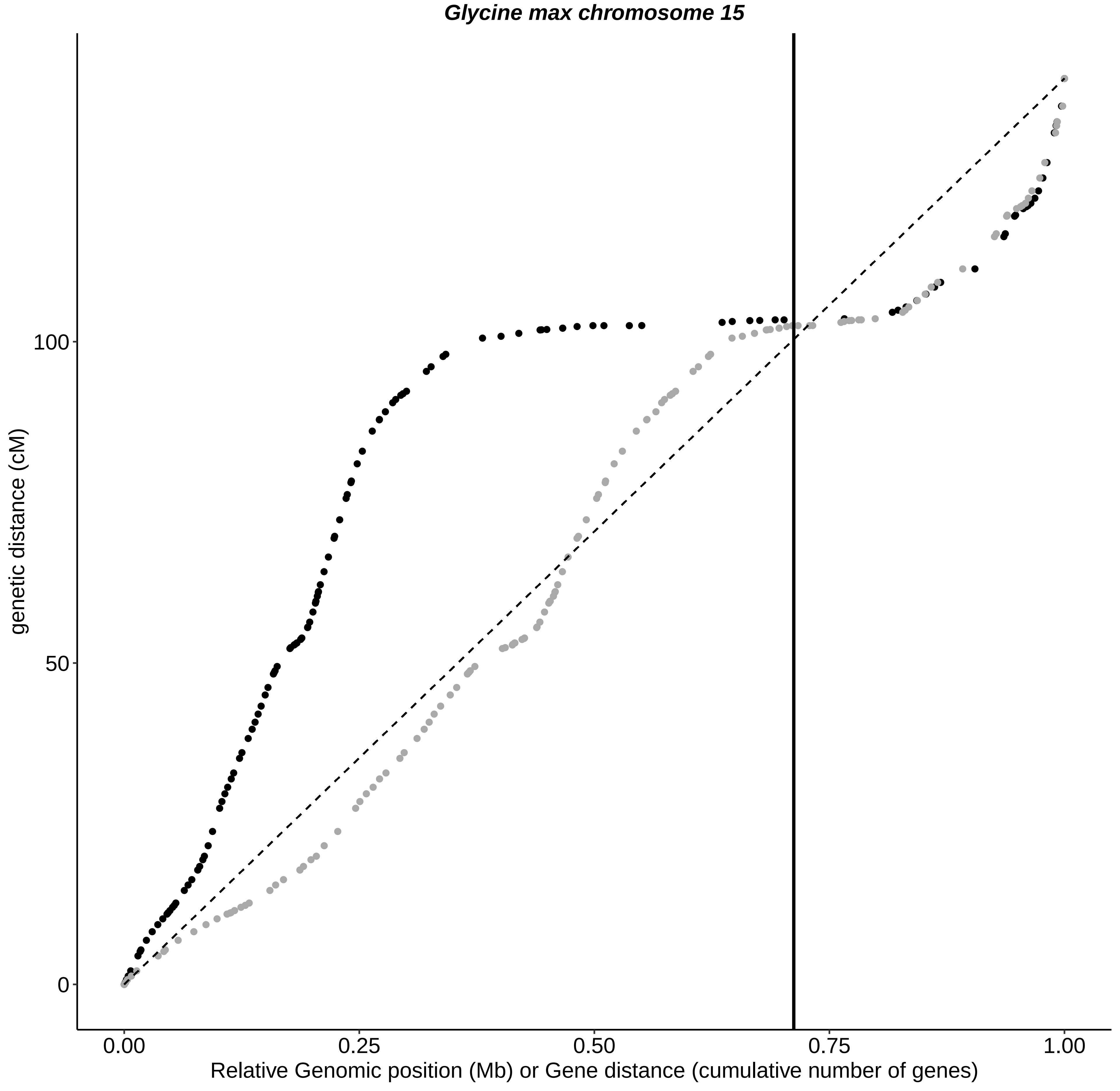

Glycine max chromosome 15
genetic distance (cM)
100
50
0
0.00
0.25
0.50
0.75
1.00
Relative Genomic position (Mb) or Gene distance (cumulative number of genes)

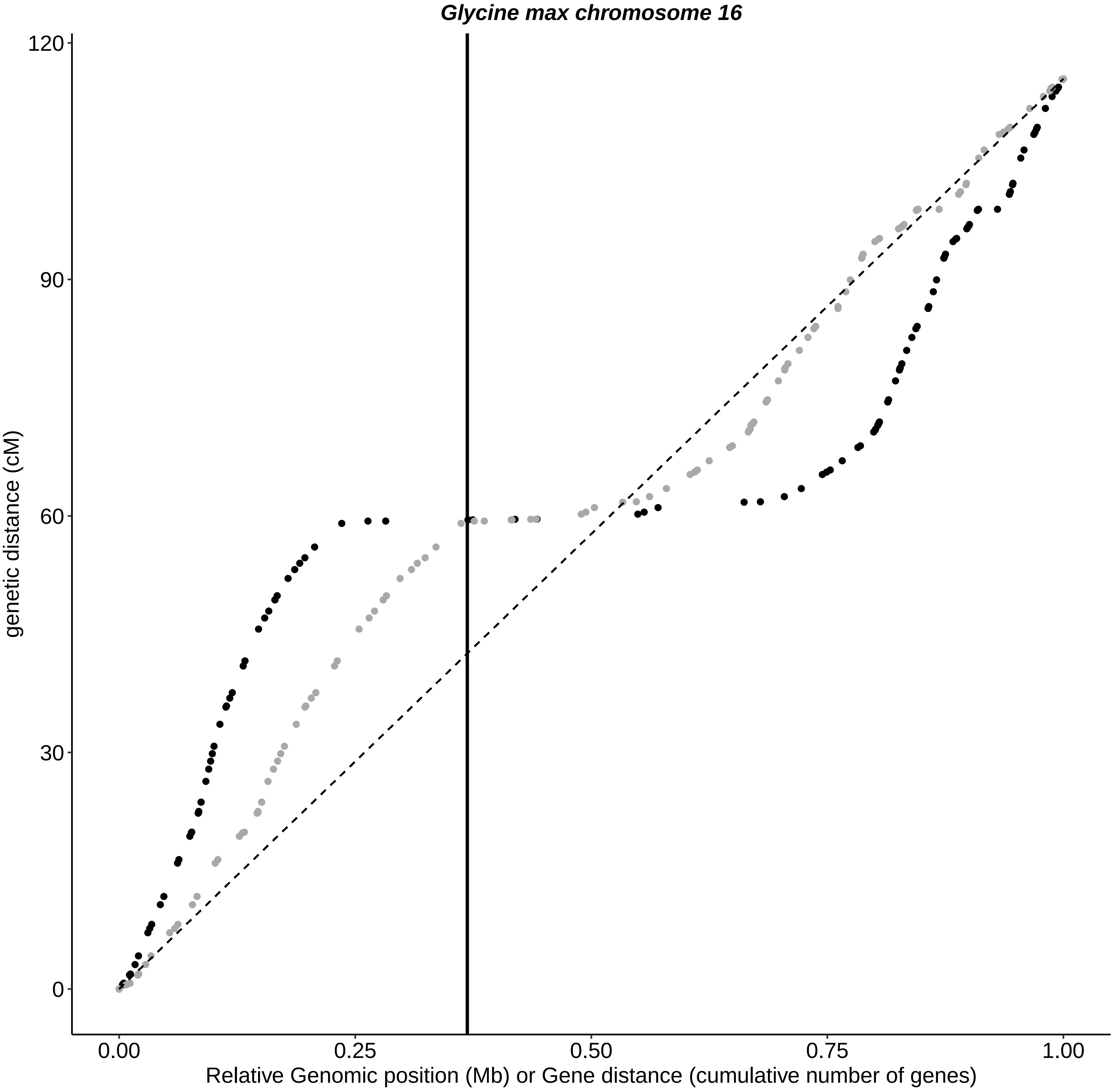
Glycine max chromosome 16
genetic distance (cM)
0
30
60
90
120
0.00
0.25
0.50
0.75
1.00
Relative Genomic position (Mb) or Gene distance (cumulative number of genes)

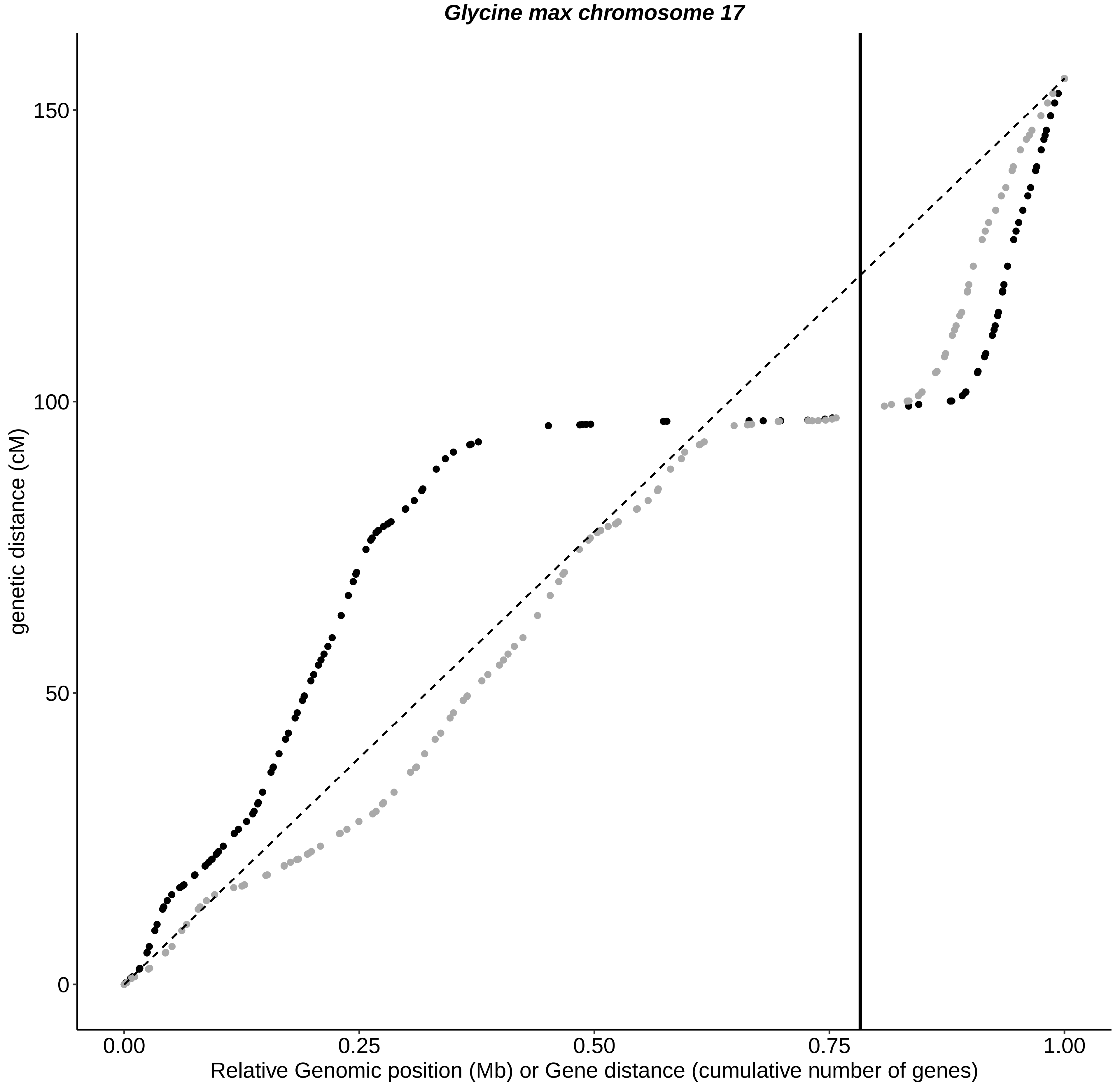
Glycine max chromosome 17
genetic distance (cM)
150
100
50
0
0.00
0.25
0.50
0.75
1.00
Relative Genomic position (Mb) or Gene distance (cumulative number of genes)

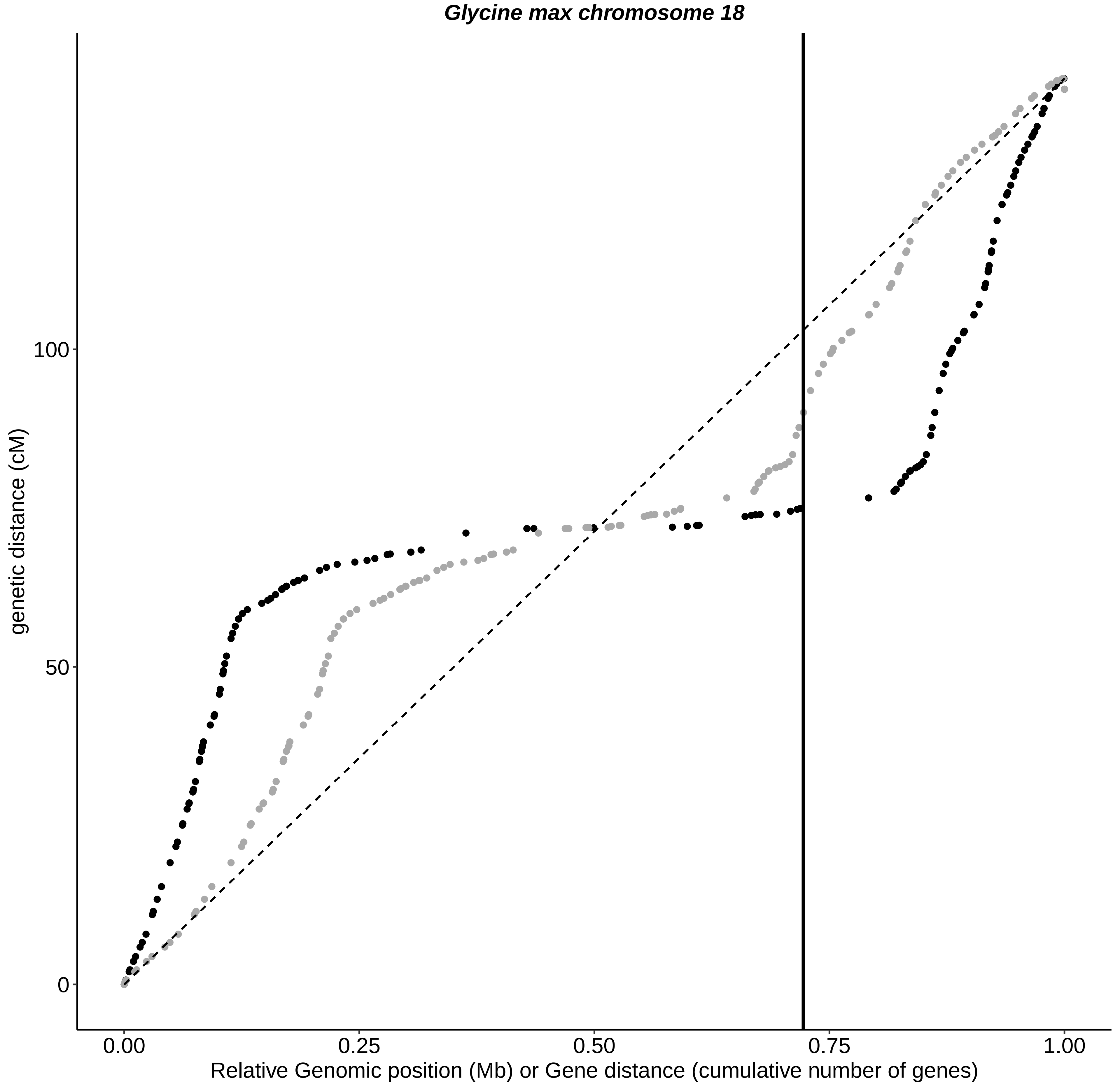
Glycine max chromosome 18
genetic distance (cM)
0
50
100
0.00
0.25
0.50
0.75
1.00
Relative Genomic position (Mb) or Gene distance (cumulative number of genes)

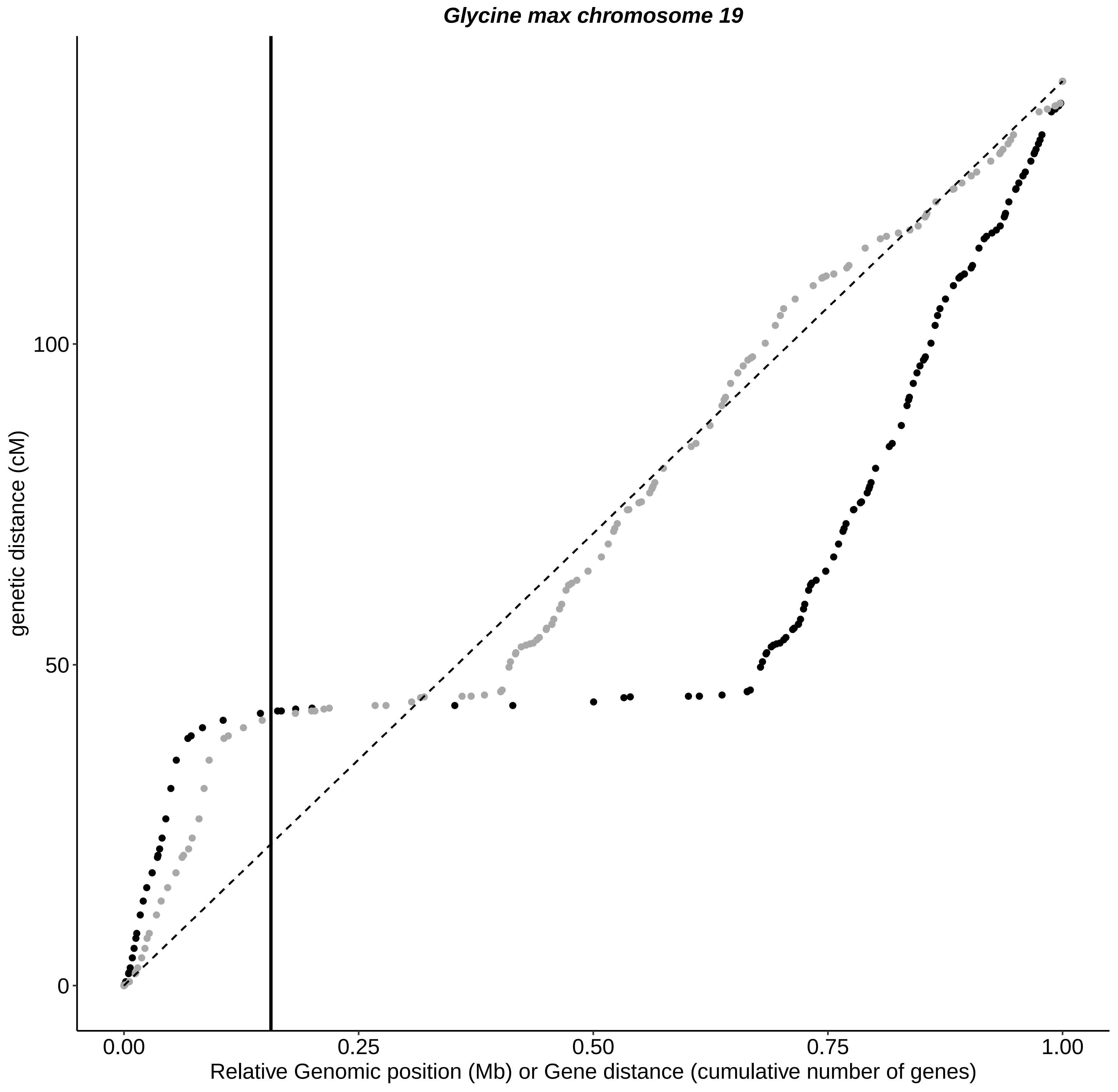
Glycine max chromosome 19
genetic distance (cM)
0
50
100
0.00
0.25
0.50
0.75
1.00
Relative Genomic position (Mb) or Gene distance (cumulative number of genes)

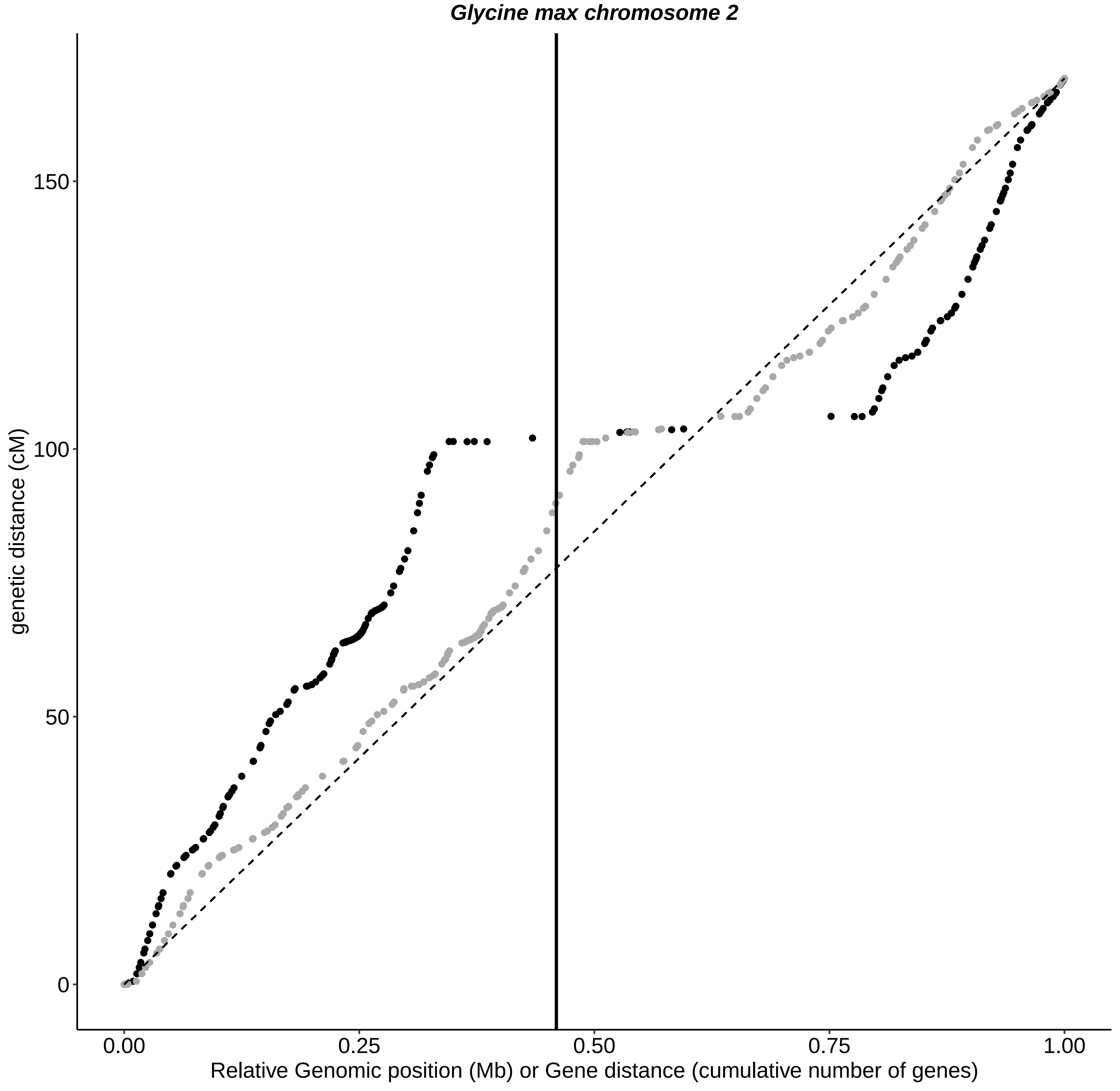
Glycine max chromosome 2
genetic distance (cM)
150
100
50
0
0.00
0.25
0.50
0.75
1.00
Relative Genomic position (Mb) or Gene distance (cumulative number of genes)

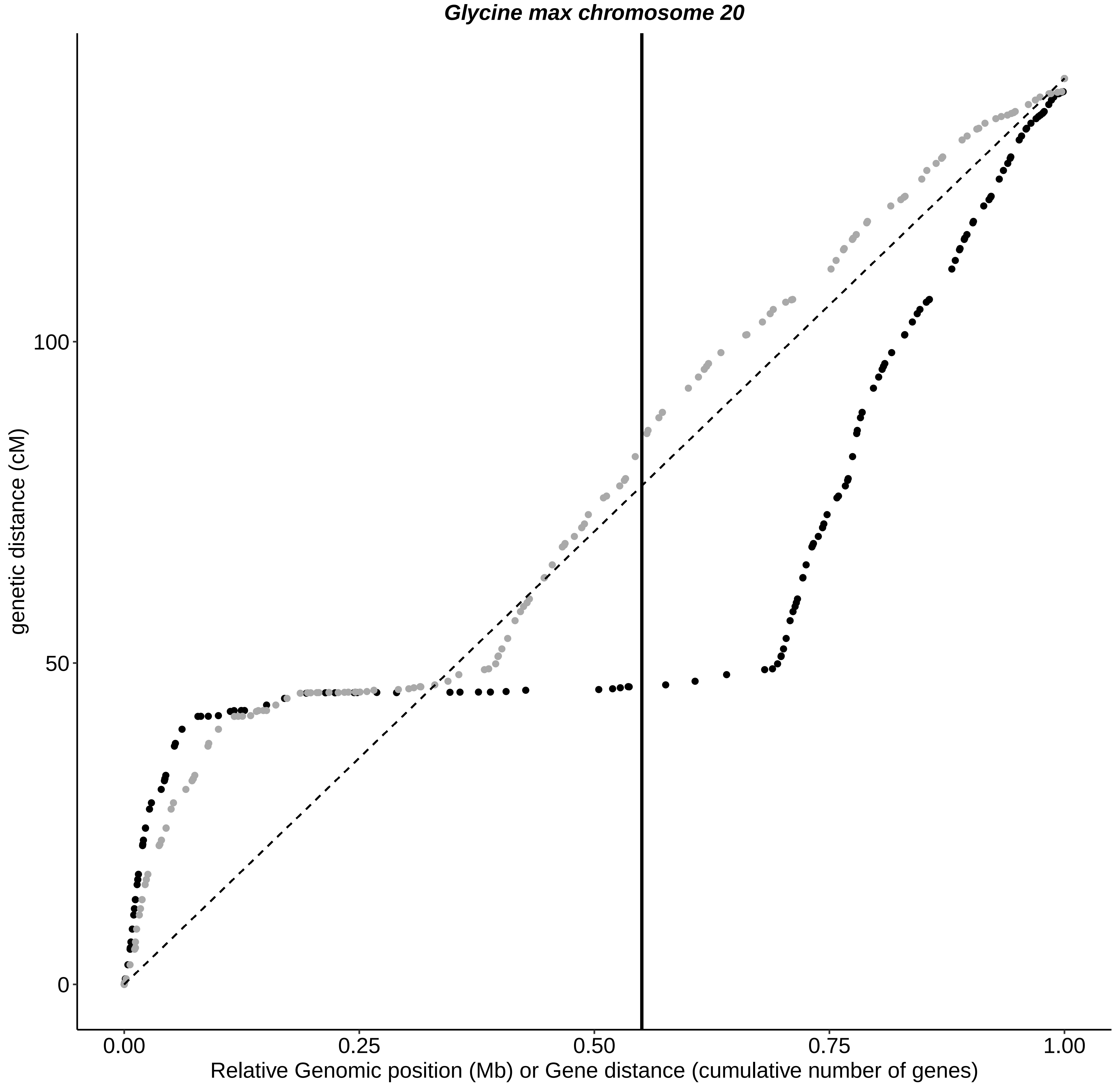
Glycine max chromosome 20
genetic distance (cM)
0
50
100
0.00
0.25
0.50
0.75
1.00
Relative Genomic position (Mb) or Gene distance (cumulative number of genes)

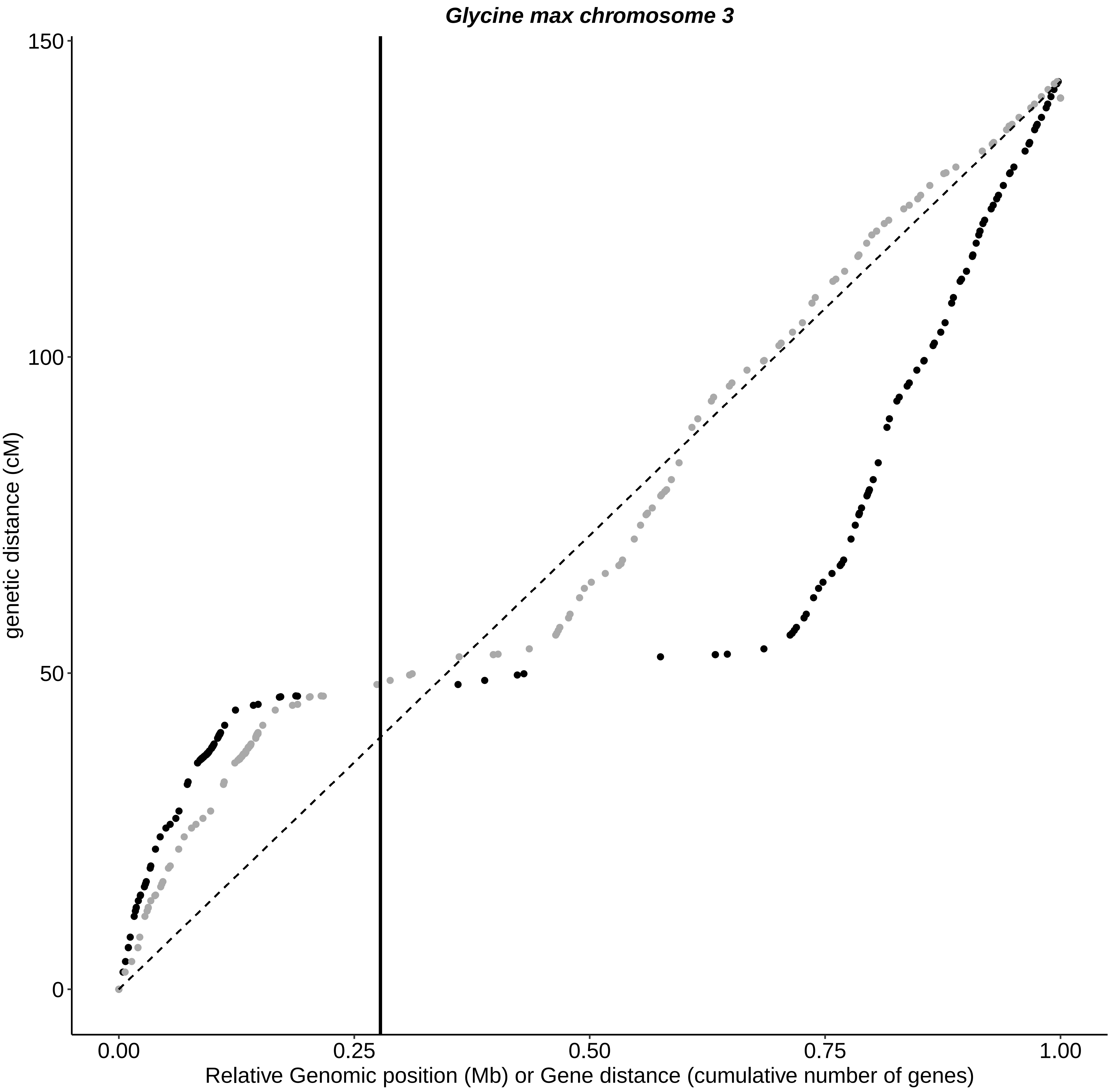
Glycine max chromosome 3
150
100
50
0
genetic distance (cM)
0.00
0.25
0.50
0.75
1.00
Relative Genomic position (Mb) or Gene distance (cumulative number of genes)

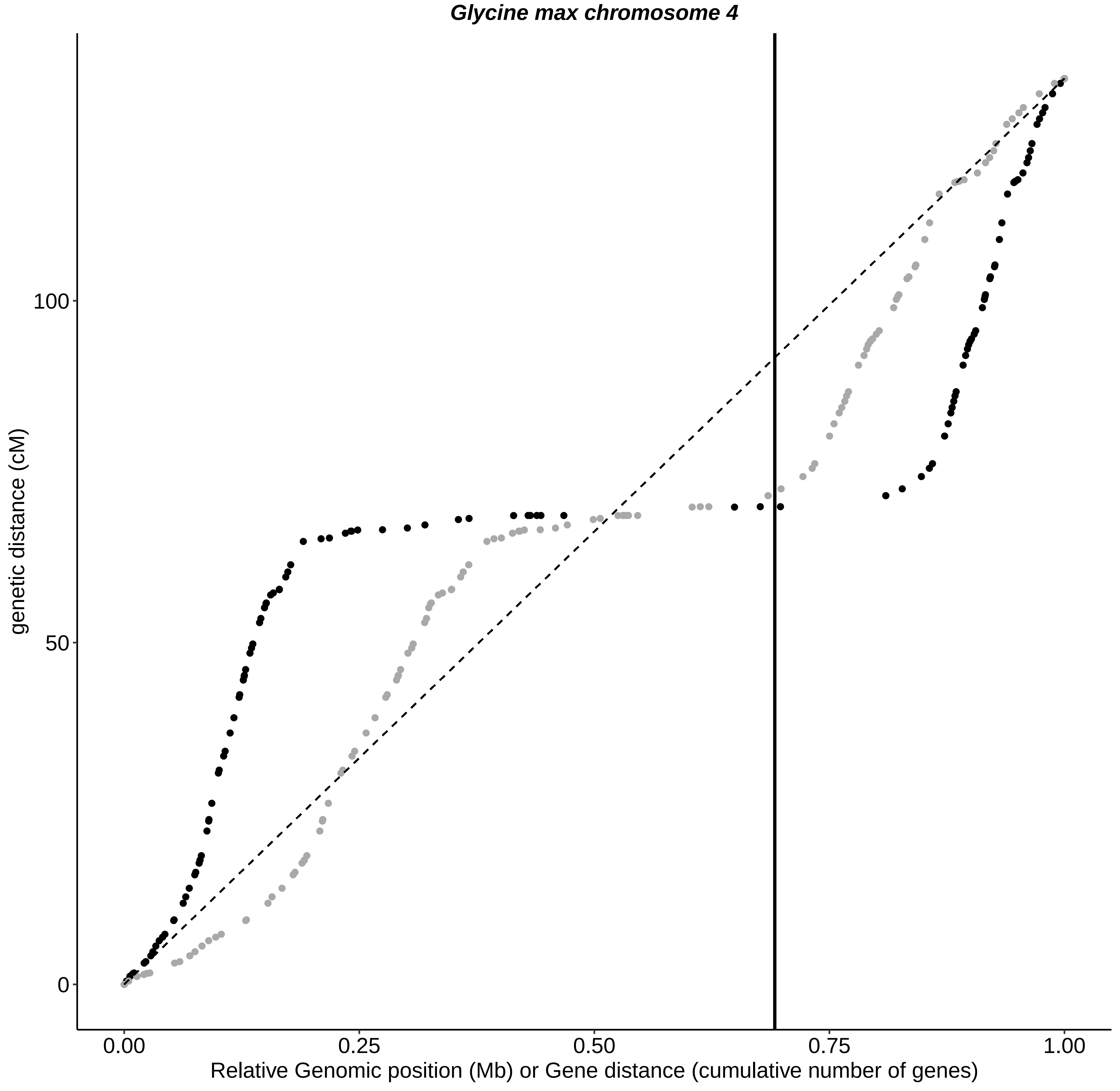
Glycine max chromosome 4
genetic distance (cM)
0
50
100
0.00
0.25
0.50
0.75
1.00
Relative Genomic position (Mb) or Gene distance (cumulative number of genes)

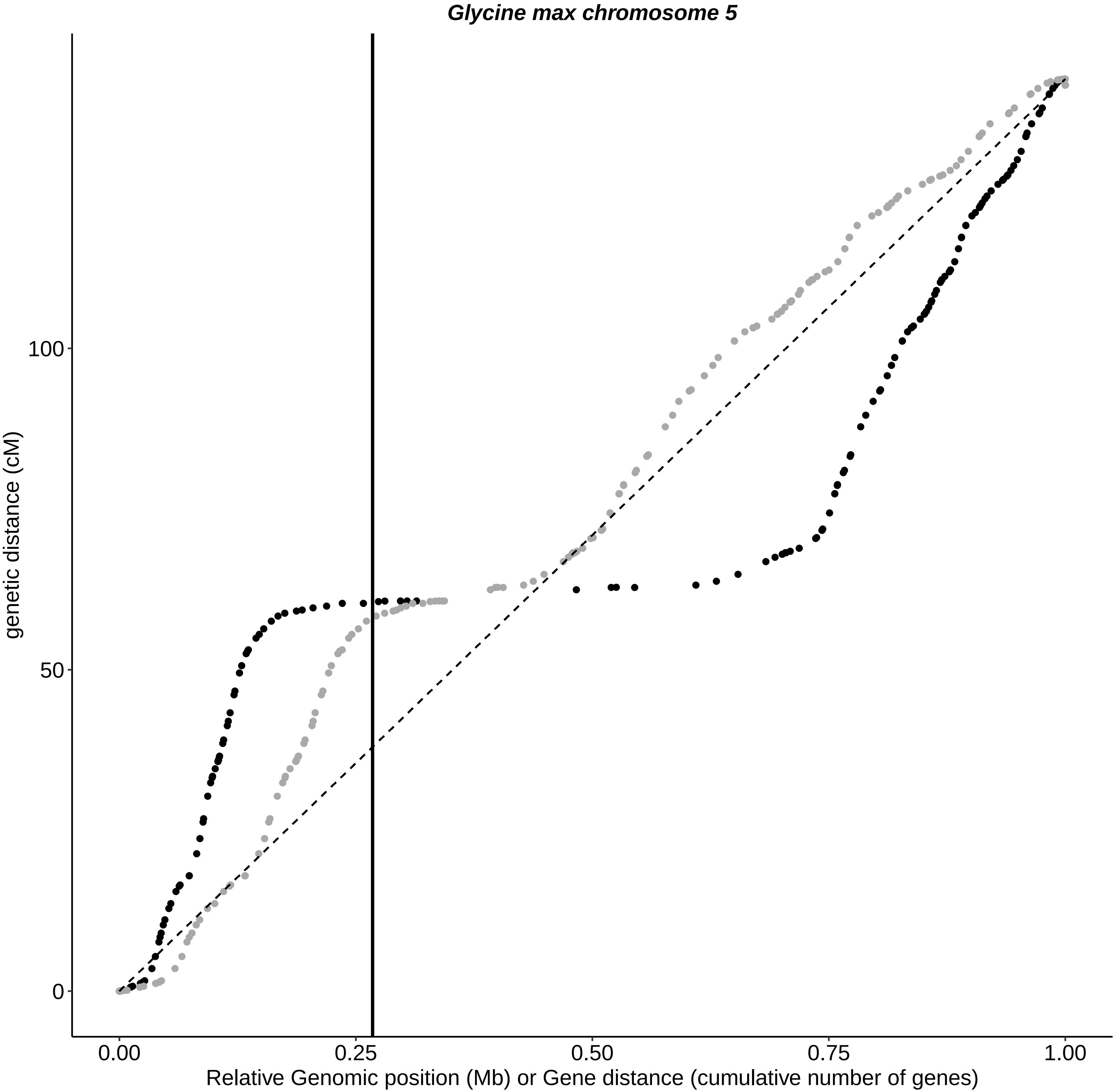
Glycine max chromosome 5
genetic distance (cM)
100
50
0
0.00
0.25
0.50
0.75
1.00
Relative Genomic position (Mb) or Gene distance (cumulative number of genes)

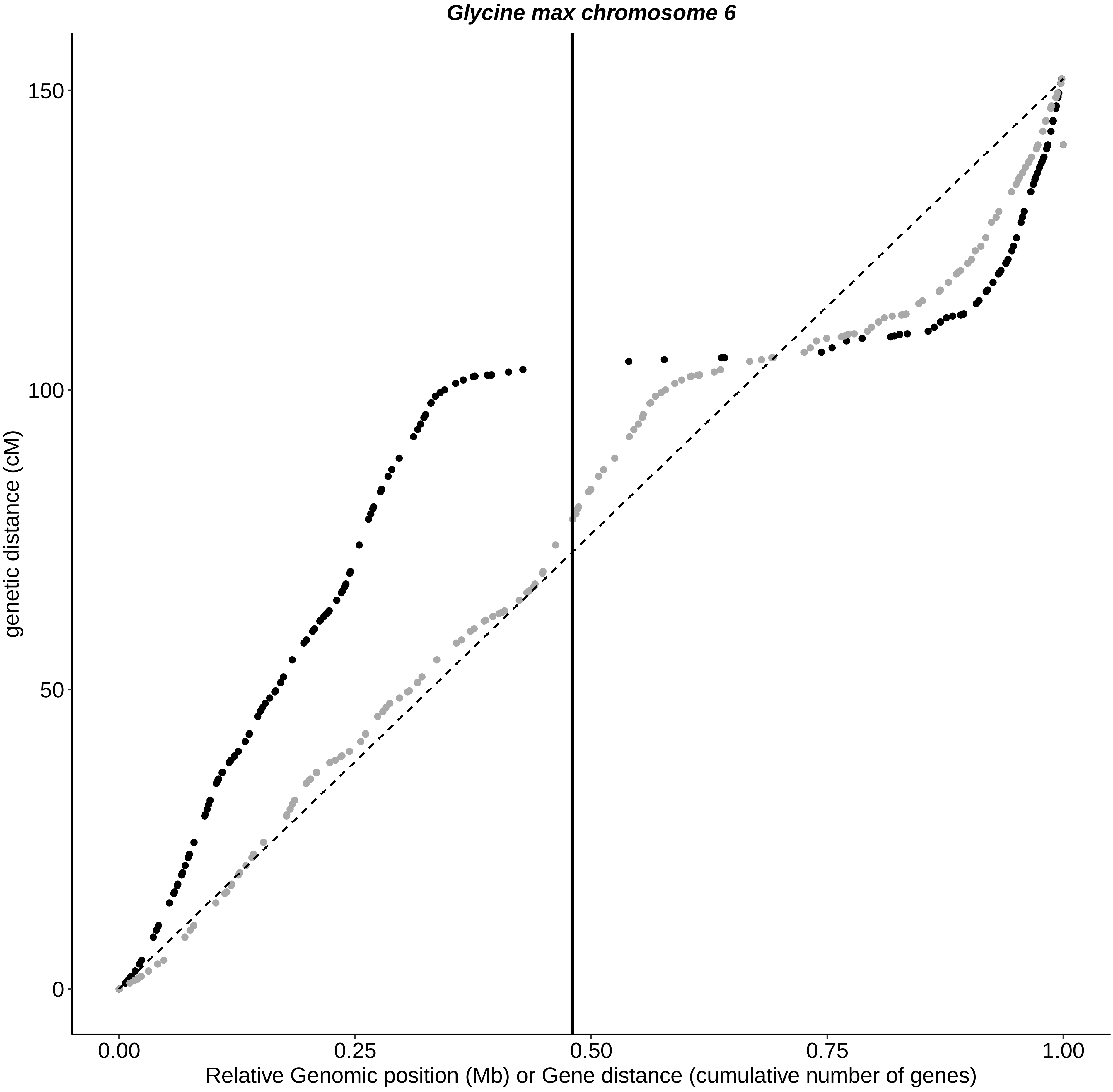

Glycine max chromosome 6
genetic distance (cM)
150
100
50
0
0.00
0.25
0.50
0.75
1.00
Relative Genomic position (Mb) or Gene distance (cumulative number of genes)

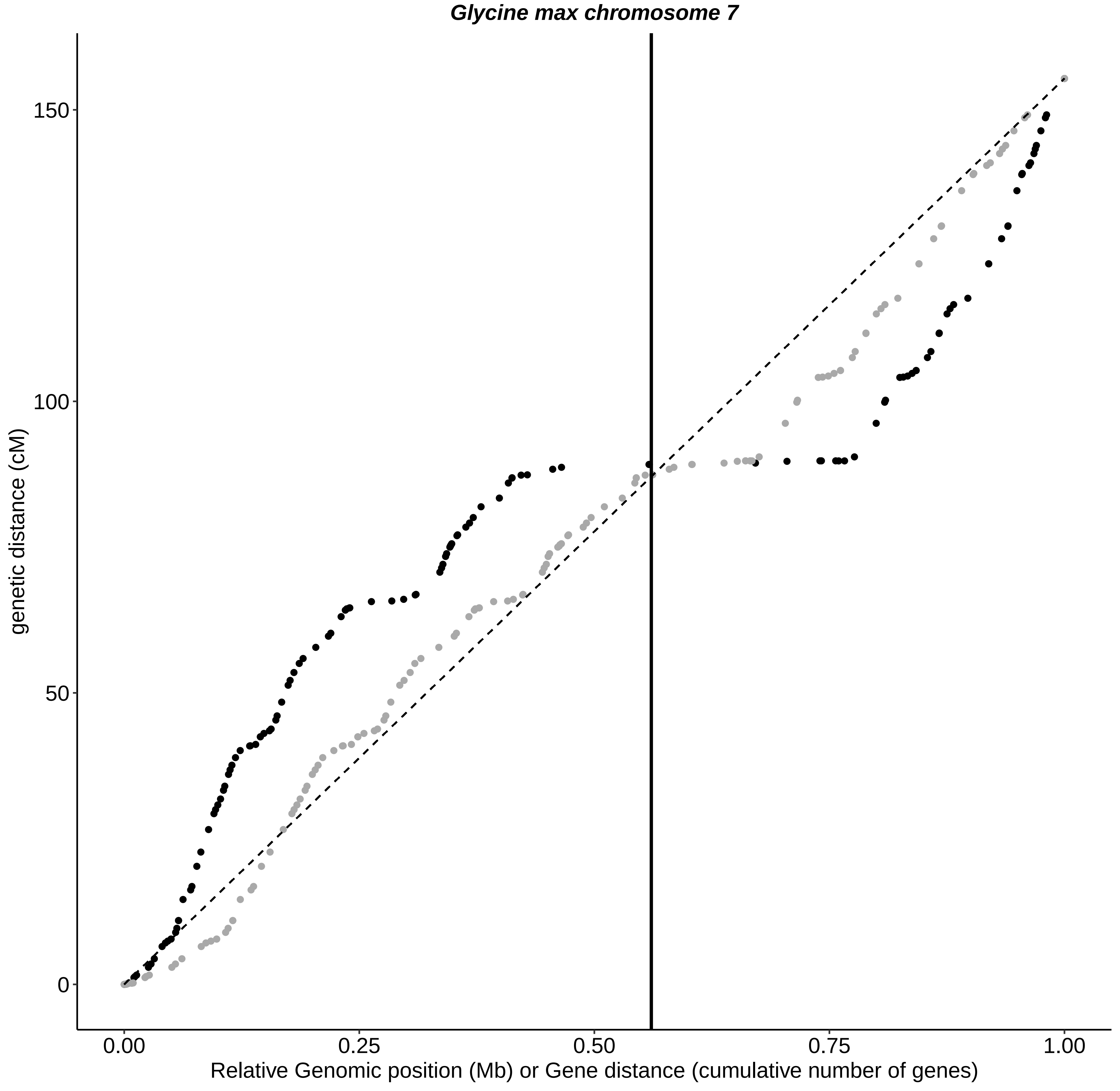
Glycine max chromosome 7
genetic distance (cM)
150
100
50
0
0.00
0.25
0.50
0.75
1.00
Relative Genomic position (Mb) or Gene distance (cumulative number of genes)

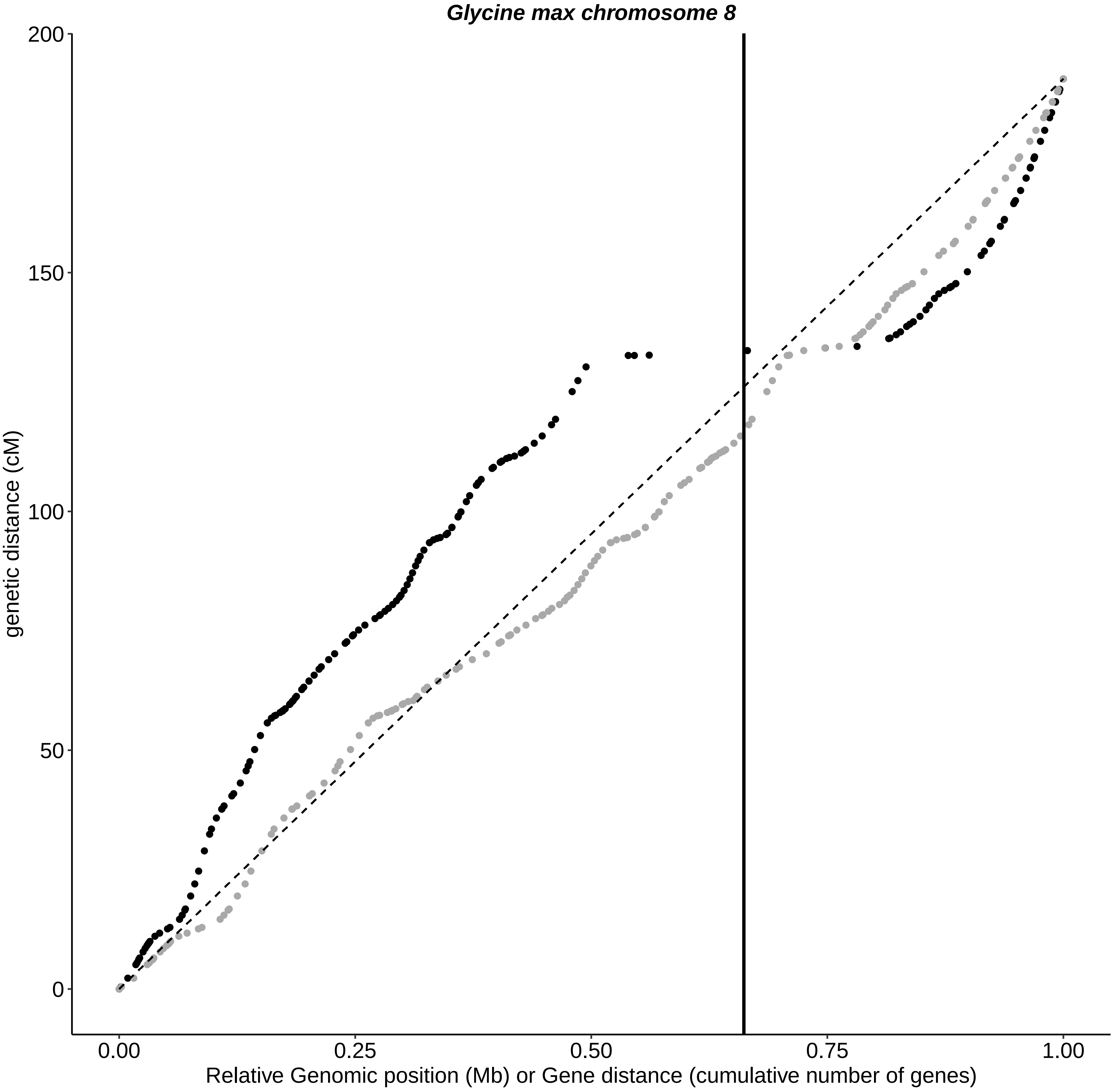

Glycine max chromosome 8
genetic distance (cM)
200
150
100
50
0
0.00
0.25
0.50
0.75
1.00
Relative Genomic position (Mb) or Gene distance (cumulative number of genes)

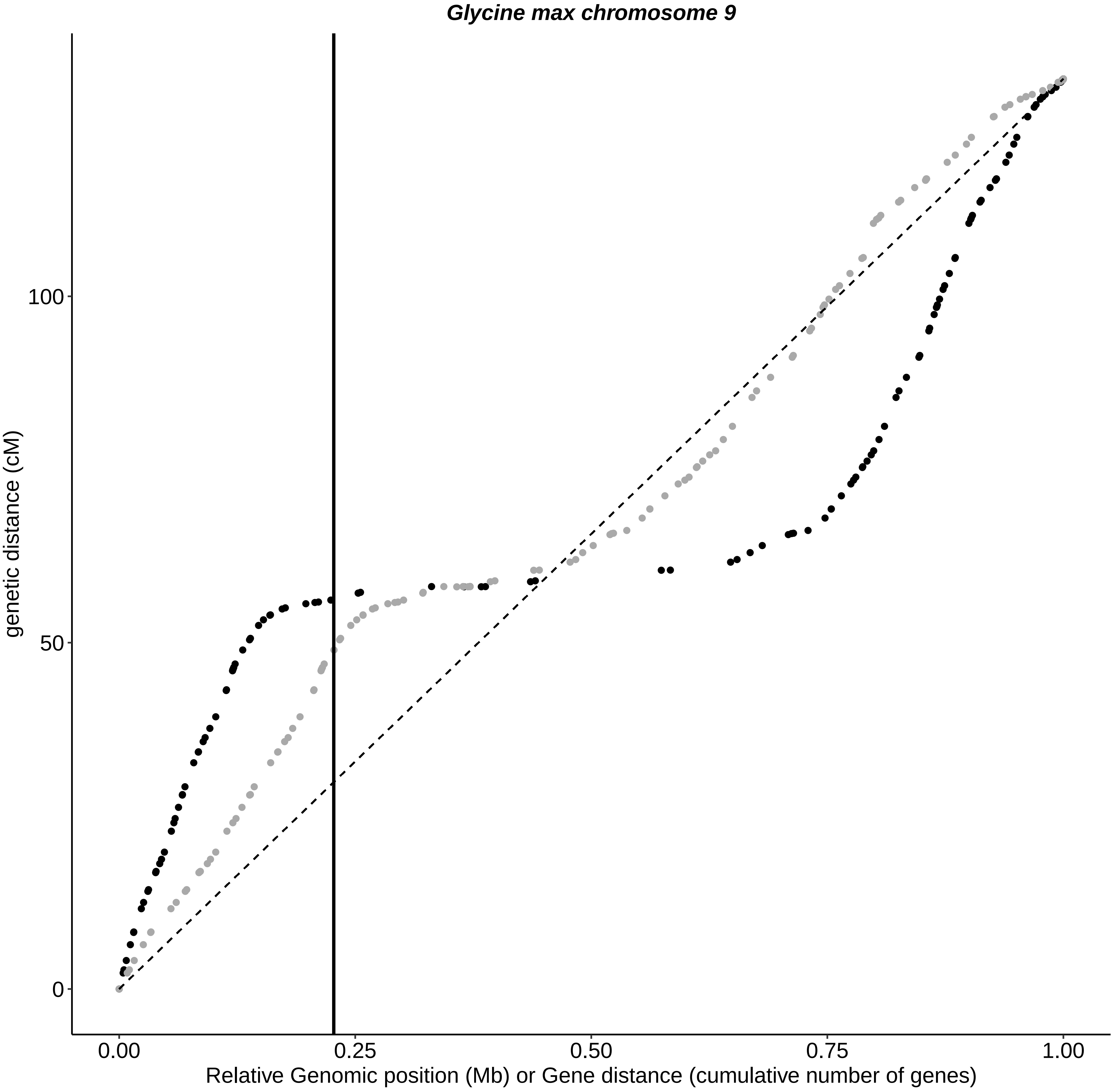

Glycine max chromosome 9
genetic distance (cM)
0
50
100
0.00
0.25
0.50
0.75
1.00
Relative Genomic position (Mb) or Gene distance (cumulative number of genes)

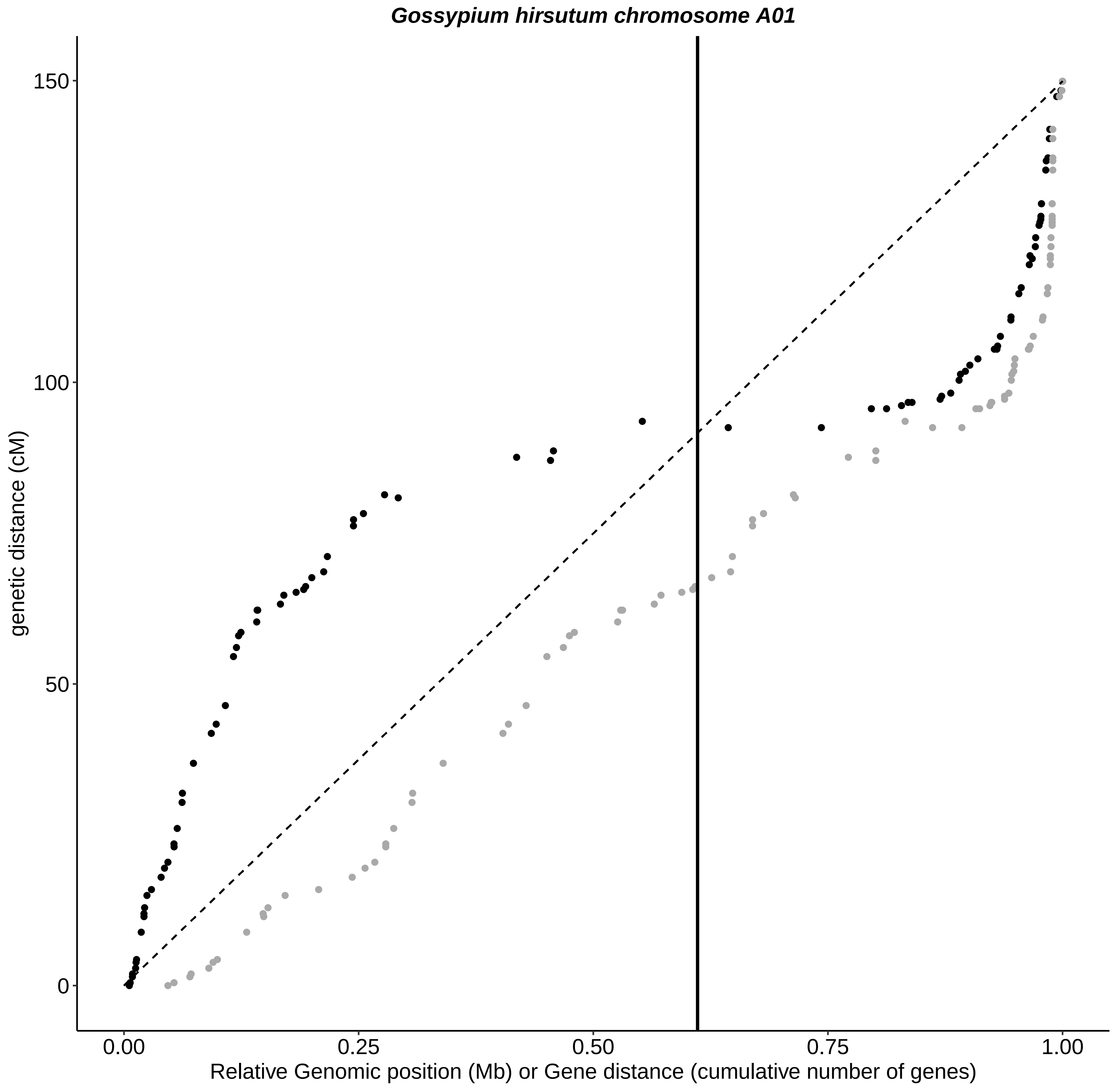
Gossypium hirsutum chromosome A01
genetic distance (cM)
Relative Genomic position (Mb) or Gene distance (cumulative number of genes)
150
100
50
0
0.00
0.25
0.50
0.75
1.00

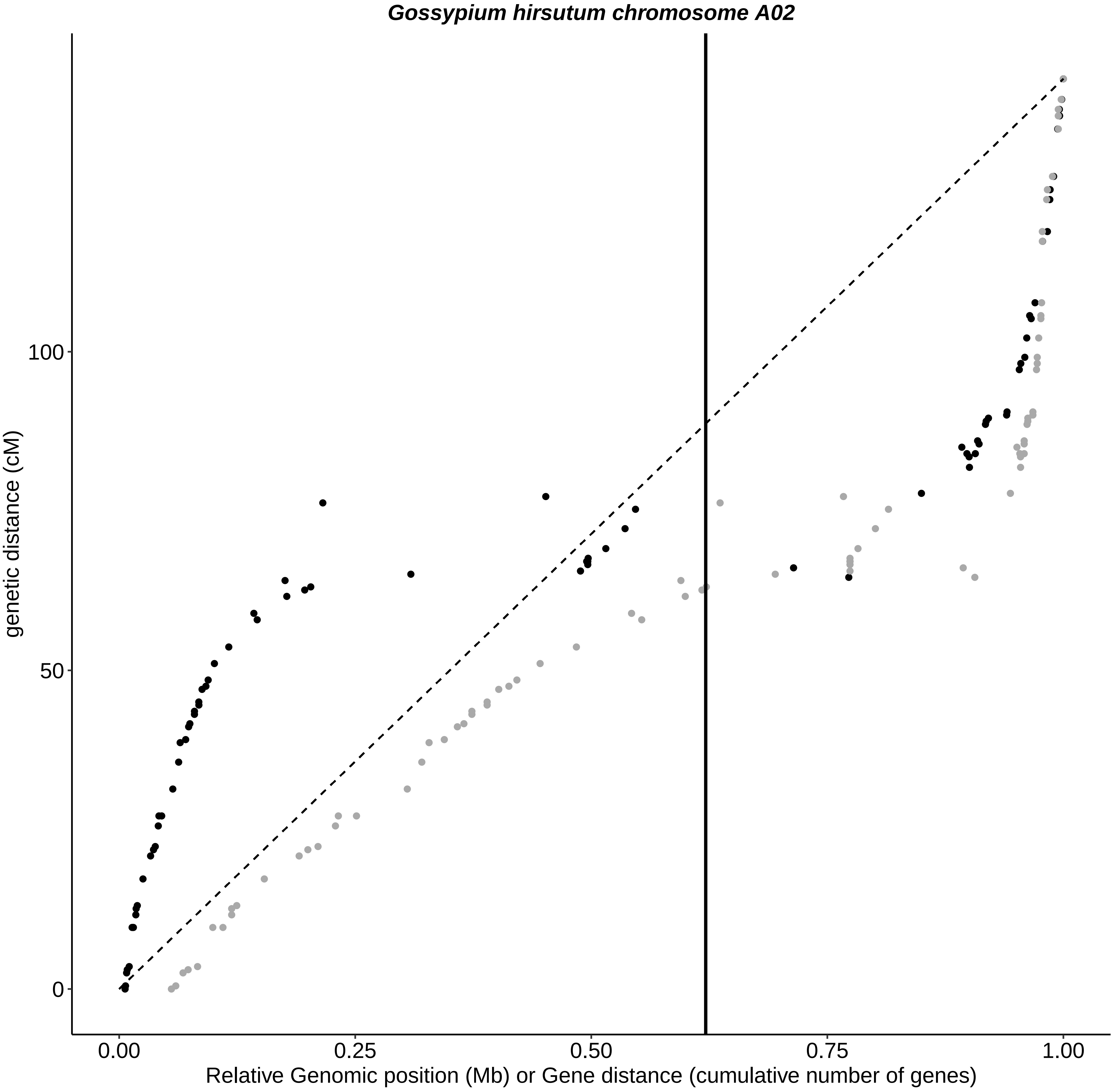
Gossypium hirsutum chromosome A02
genetic distance (cM)
100
50
0
0.00
0.25
0.50
0.75
1.00
Relative Genomic position (Mb) or Gene distance (cumulative number of genes)

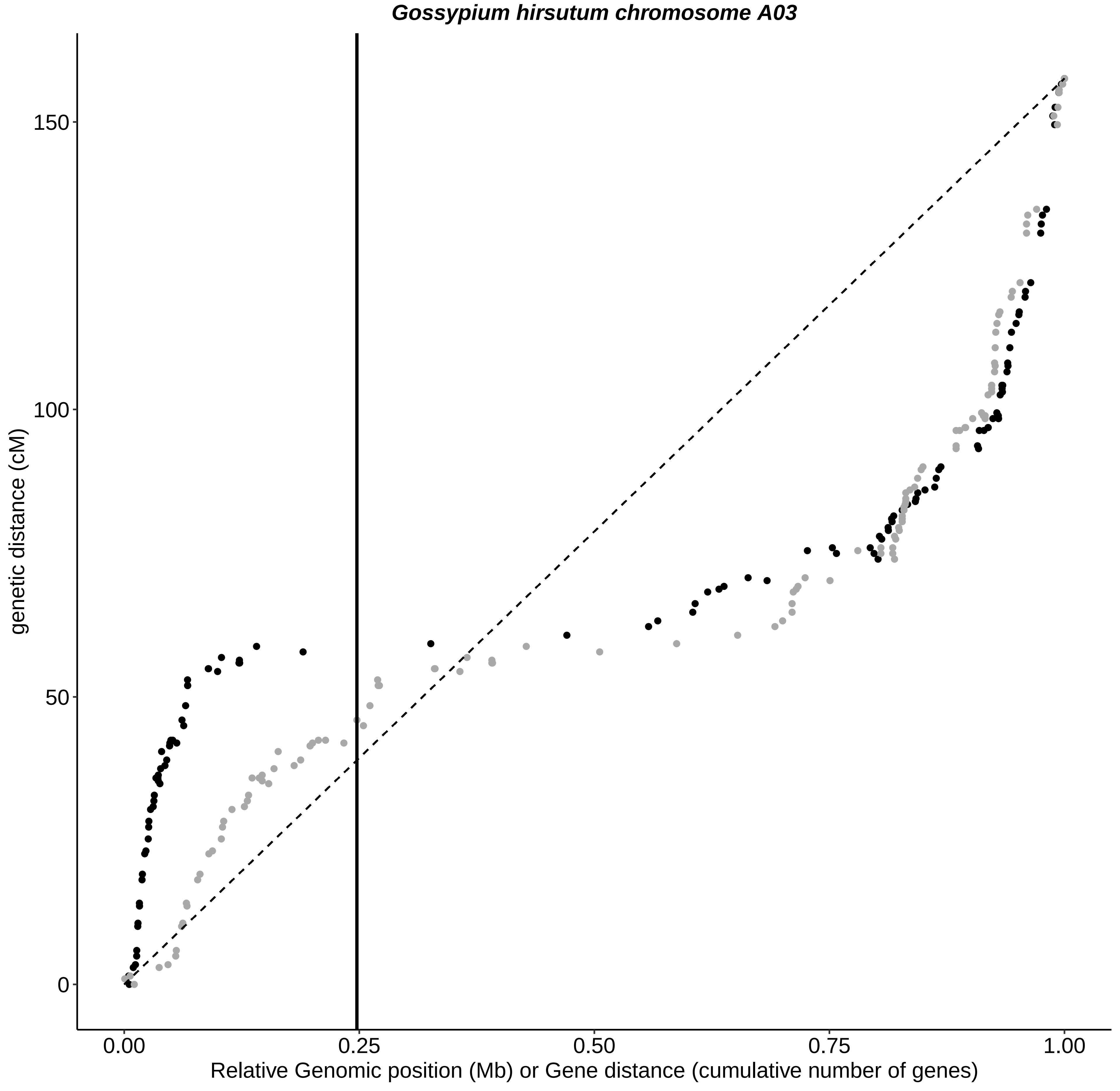
Gossypium hirsutum chromosome A03
genetic distance (cM)
0
50
100
150
0.00
0.25
0.50
0.75
1.00
Relative Genomic position (Mb) or Gene distance (cumulative number of genes)

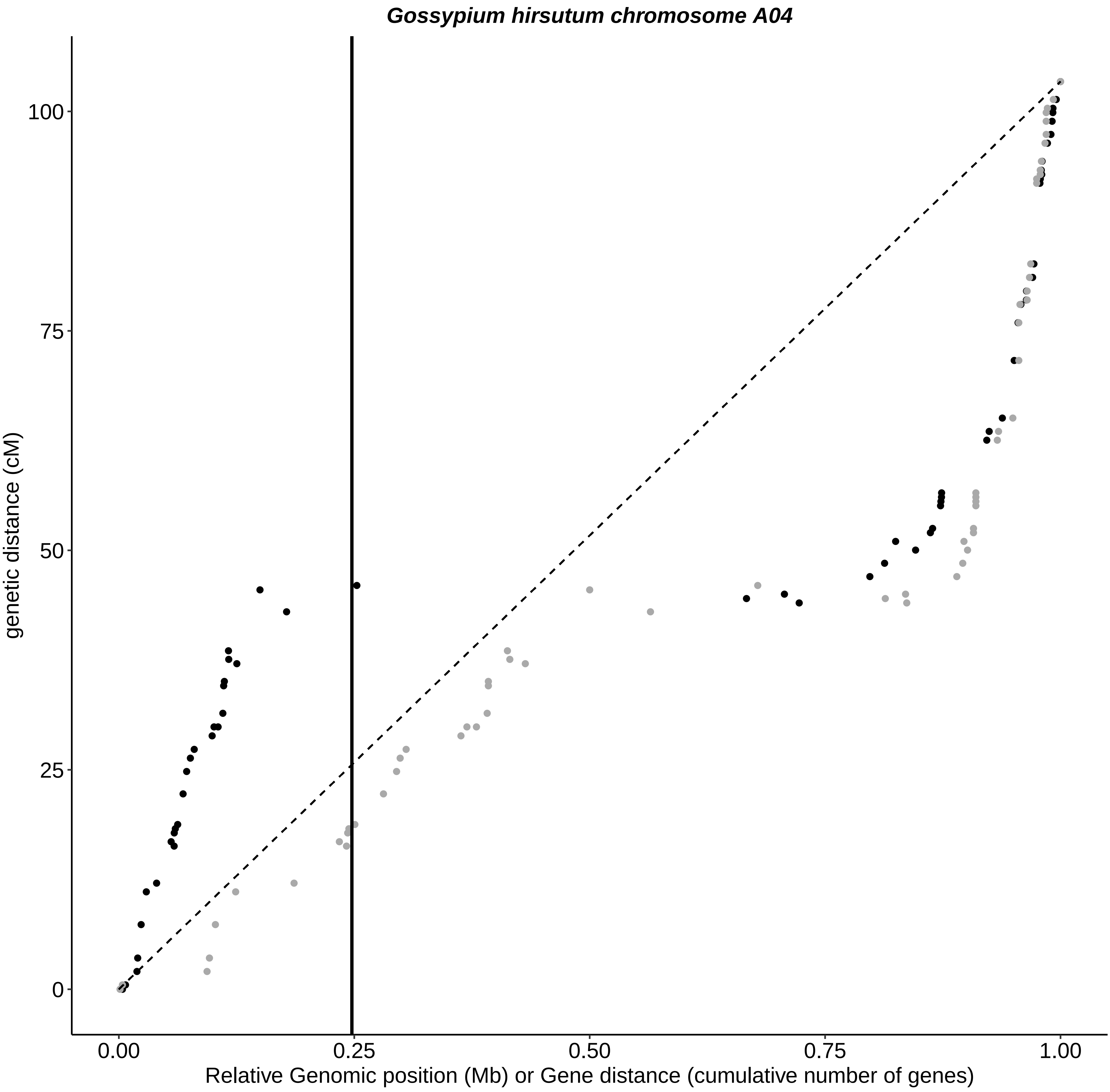
Gossypium hirsutum chromosome A04
genetic distance (cM)
0
25
50
75
100
0.00
0.25
0.50
0.75
1.00
Relative Genomic position (Mb) or Gene distance (cumulative number of genes)

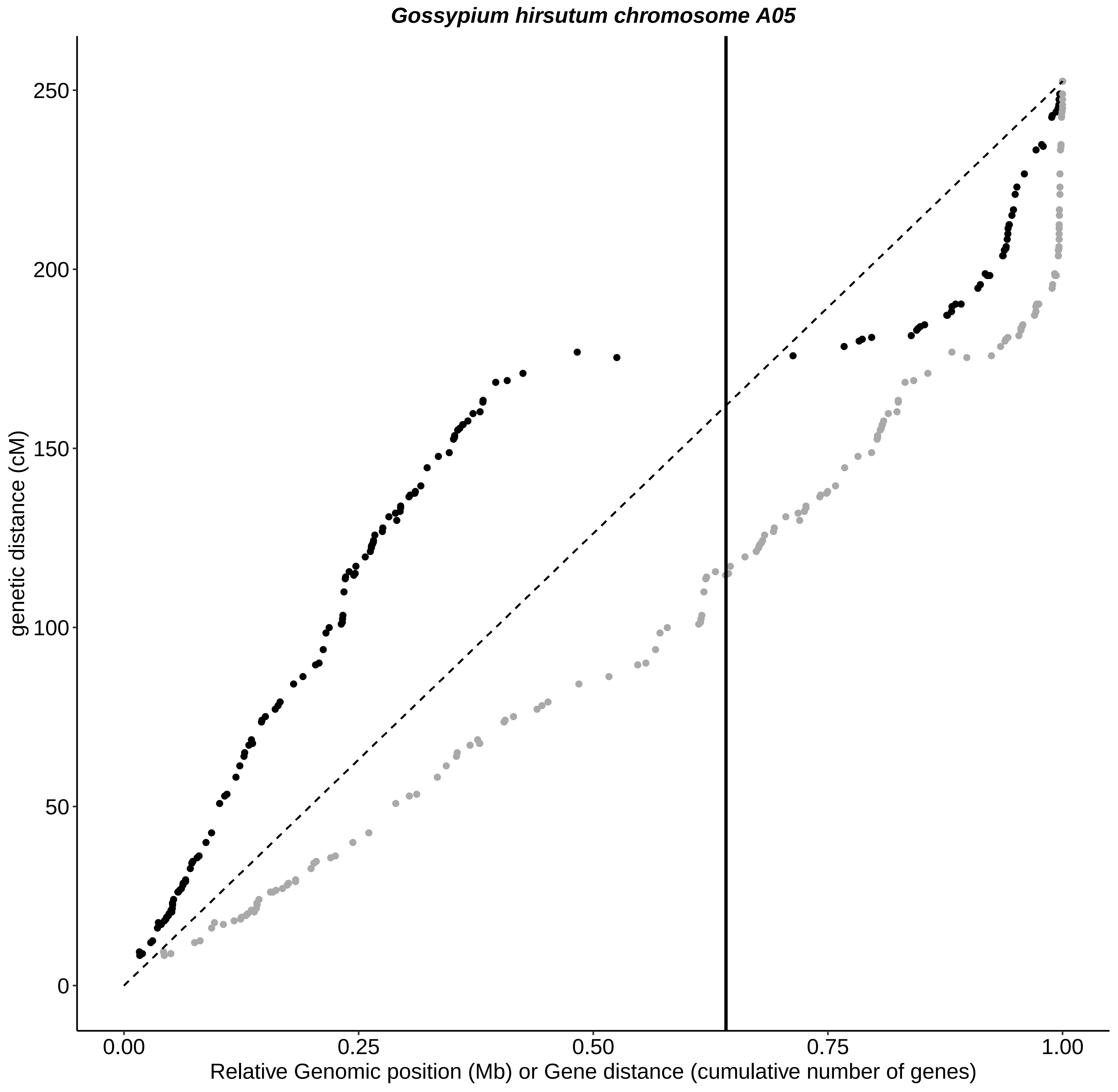
Gossypium hirsutum chromosome A05
genetic distance (cM)
0
50
100
150
200
250
0.00
0.25
0.50
0.75
1.00
Relative Genomic position (Mb) or Gene distance (cumulative number of genes)

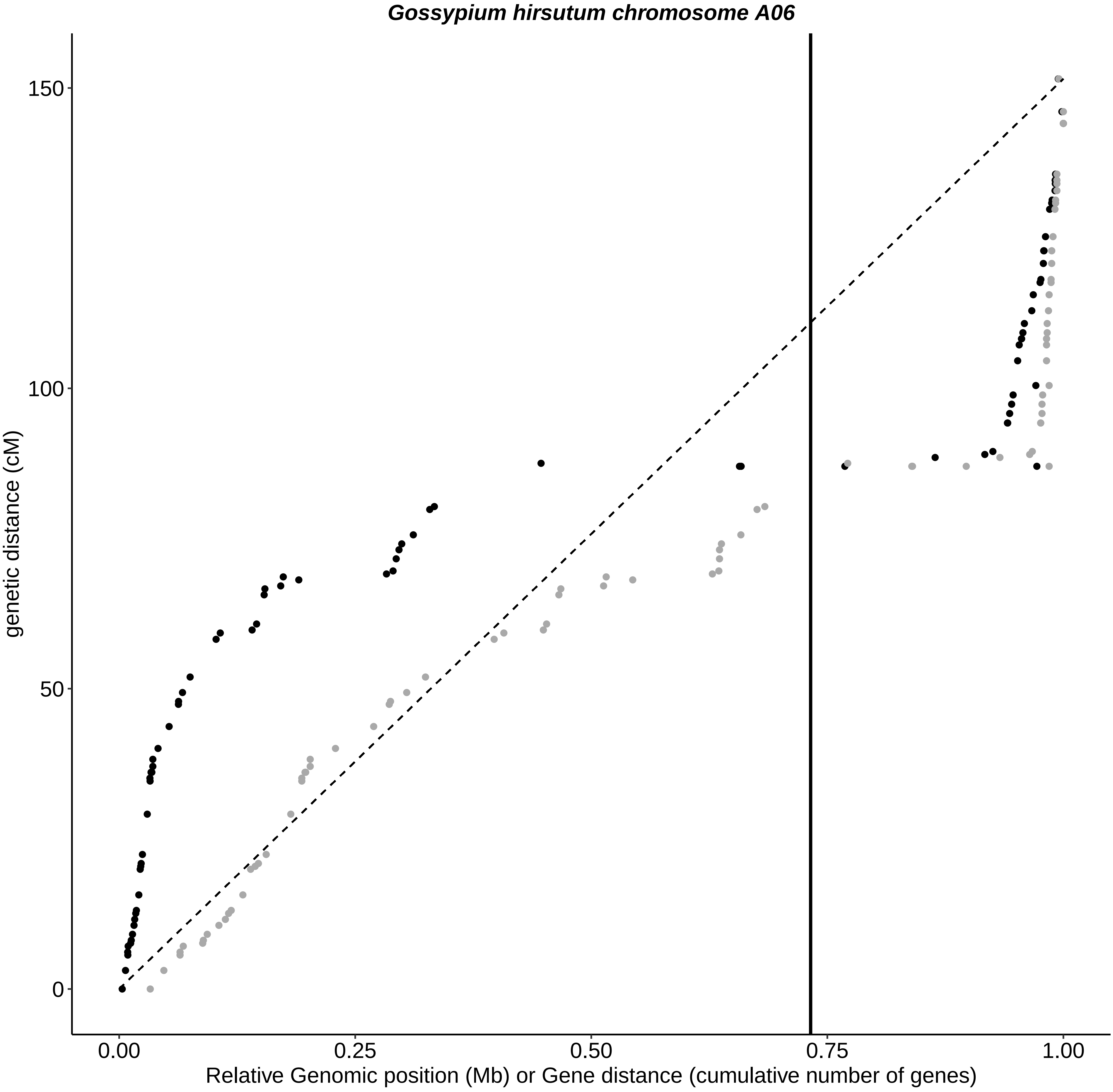
Gossypium hirsutum chromosome A06
150
100
50
0
genetic distance (cM)
0.00
0.25
0.50
0.75
1.00
Relative Genomic position (Mb) or Gene distance (cumulative number of genes)

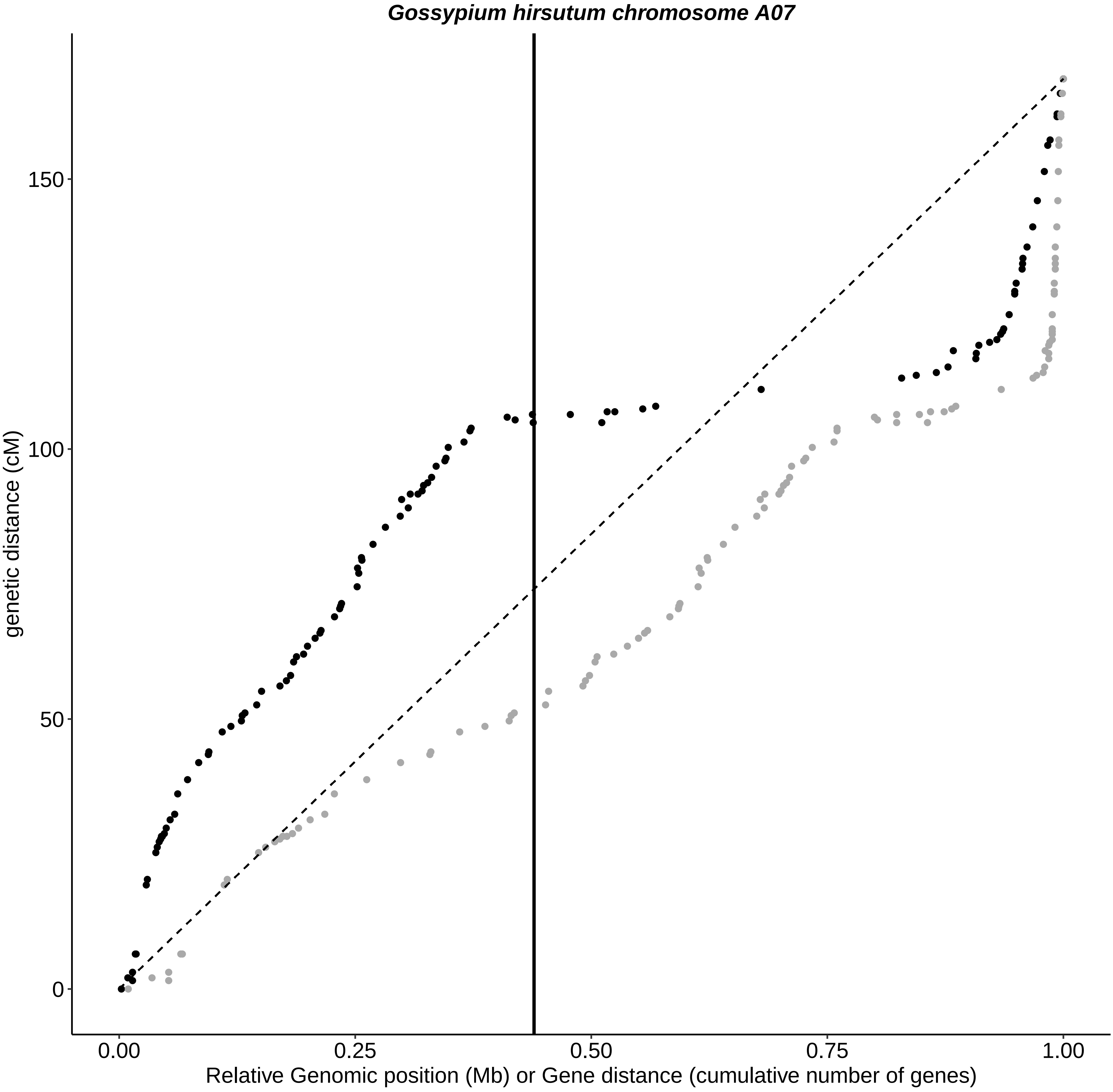
Gossypium hirsutum chromosome A07
genetic distance (cM)
150
100
50
0
0.00
0.25
0.50
0.75
1.00
Relative Genomic position (Mb) or Gene distance (cumulative number of genes)

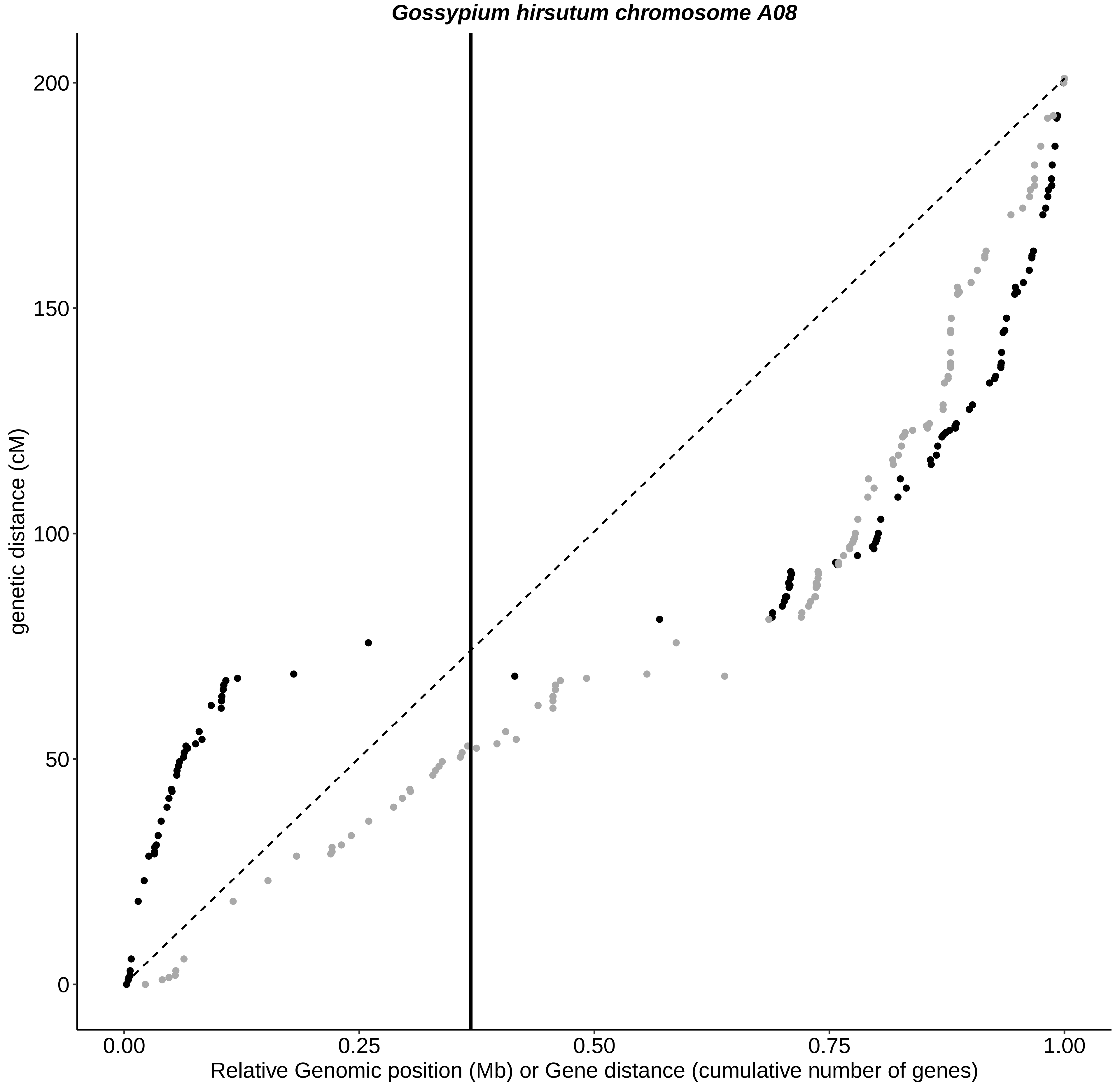
Gossypium hirsutum chromosome A08
genetic distance (cM)
200
150
100
50
0
0.00
0.25
0.50
0.75
1.00
Relative Genomic position (Mb) or Gene distance (cumulative number of genes)

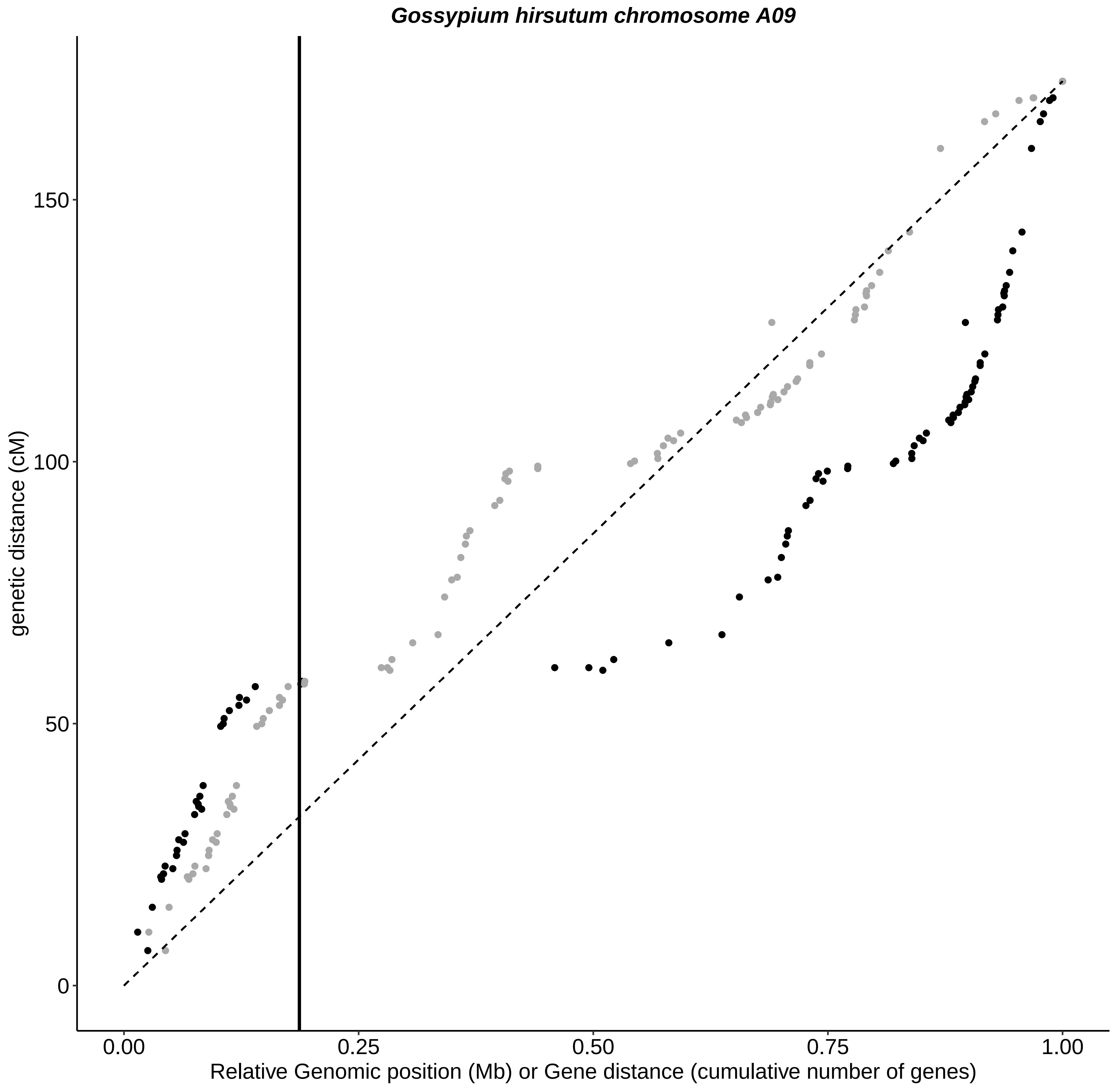
Gossypium hirsutum chromosome A09
genetic distance (cM)
150
100
50
0
0.00
0.25
0.50
0.75
1.00
Relative Genomic position (Mb) or Gene distance (cumulative number of genes)

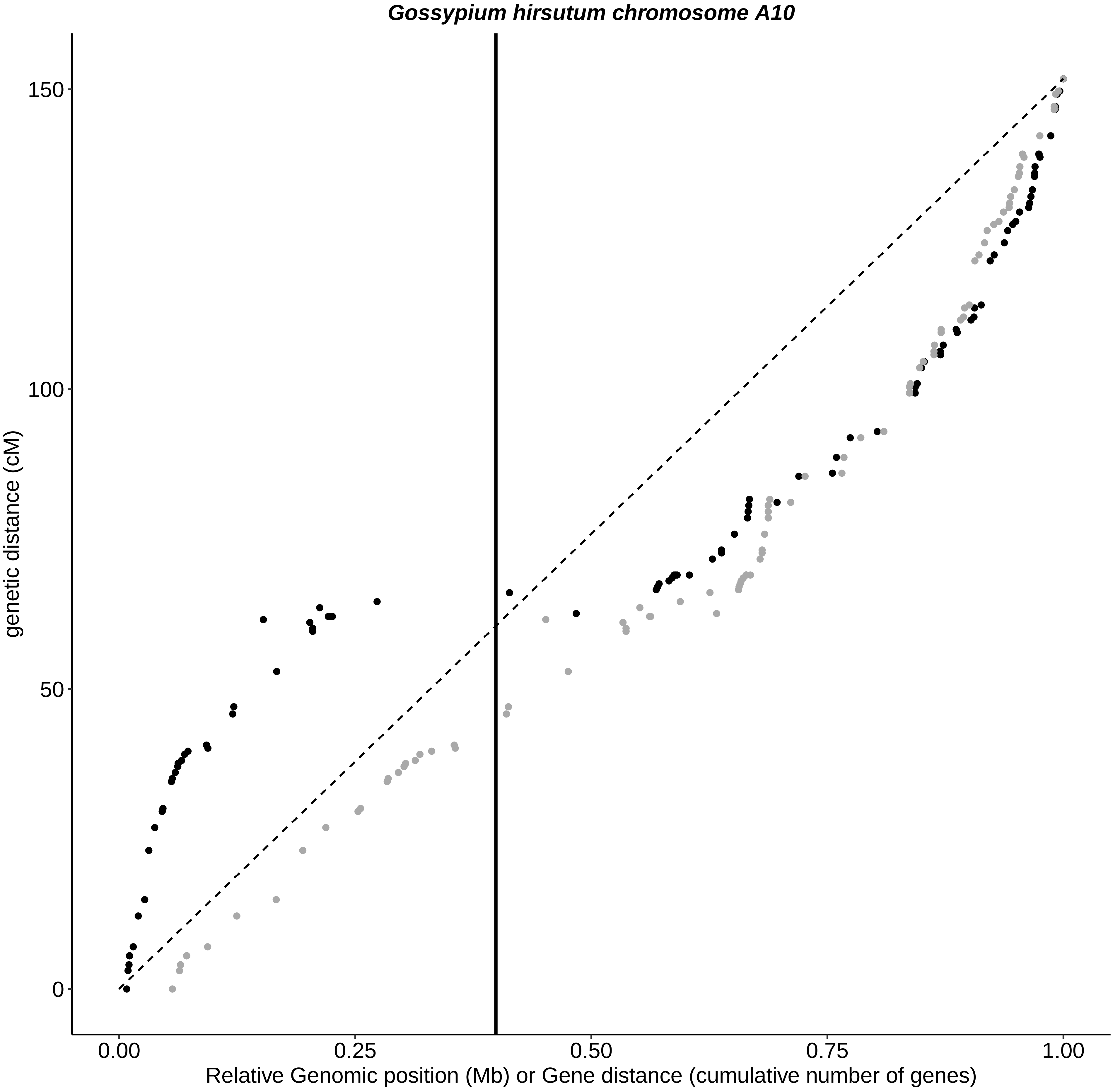
Gossypium hirsutum chromosome A10
genetic distance (cM)
Relative Genomic position (Mb) or Gene distance (cumulative number of genes)
0
50
100
150
0.00
0.25
0.50
0.75
1.00

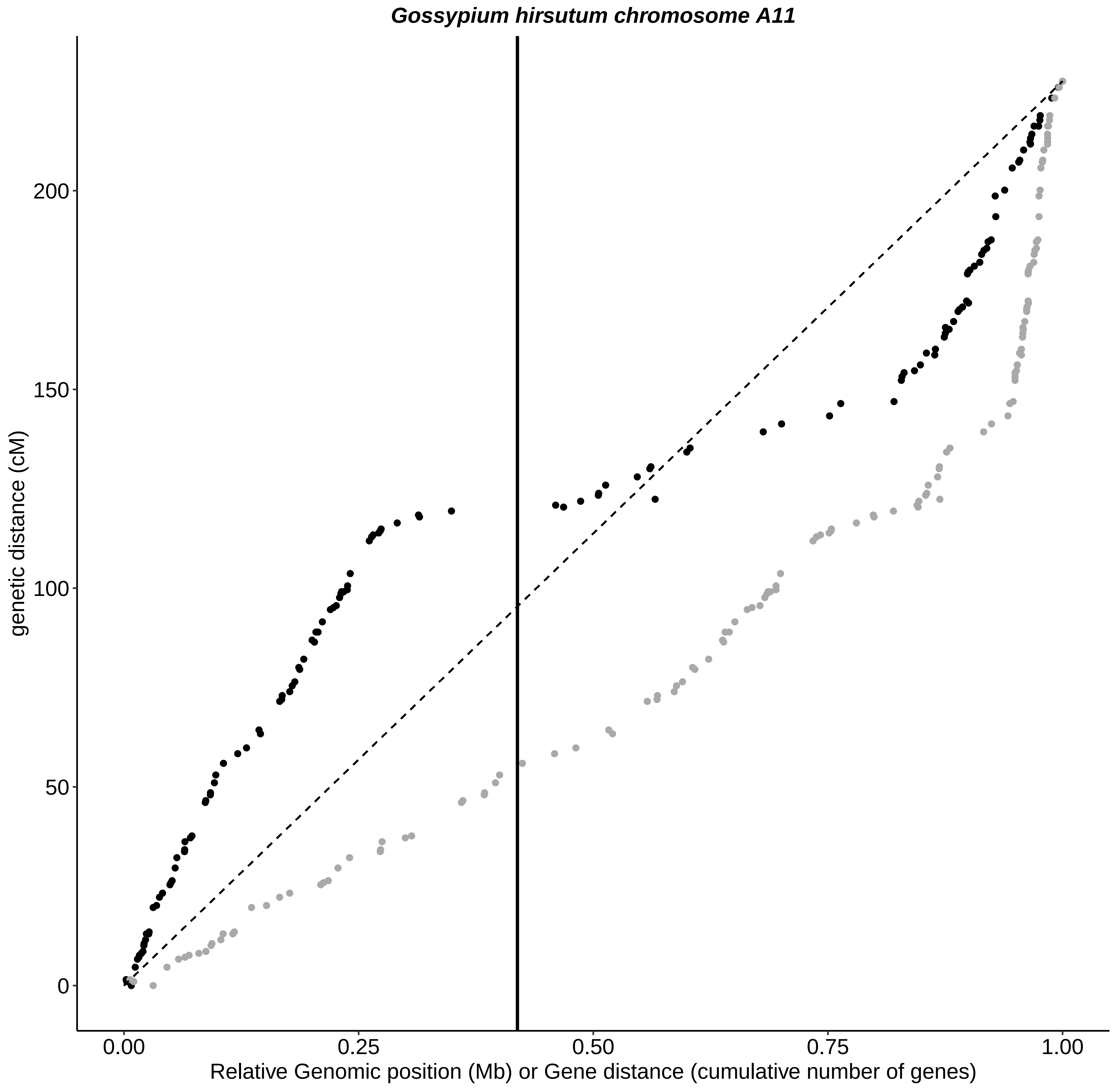
Gossypium hirsutum chromosome A11
genetic distance (cM)
0
50
100
150
200
0.00
0.25
0.50
0.75
1.00
Relative Genomic position (Mb) or Gene distance (cumulative number of genes)

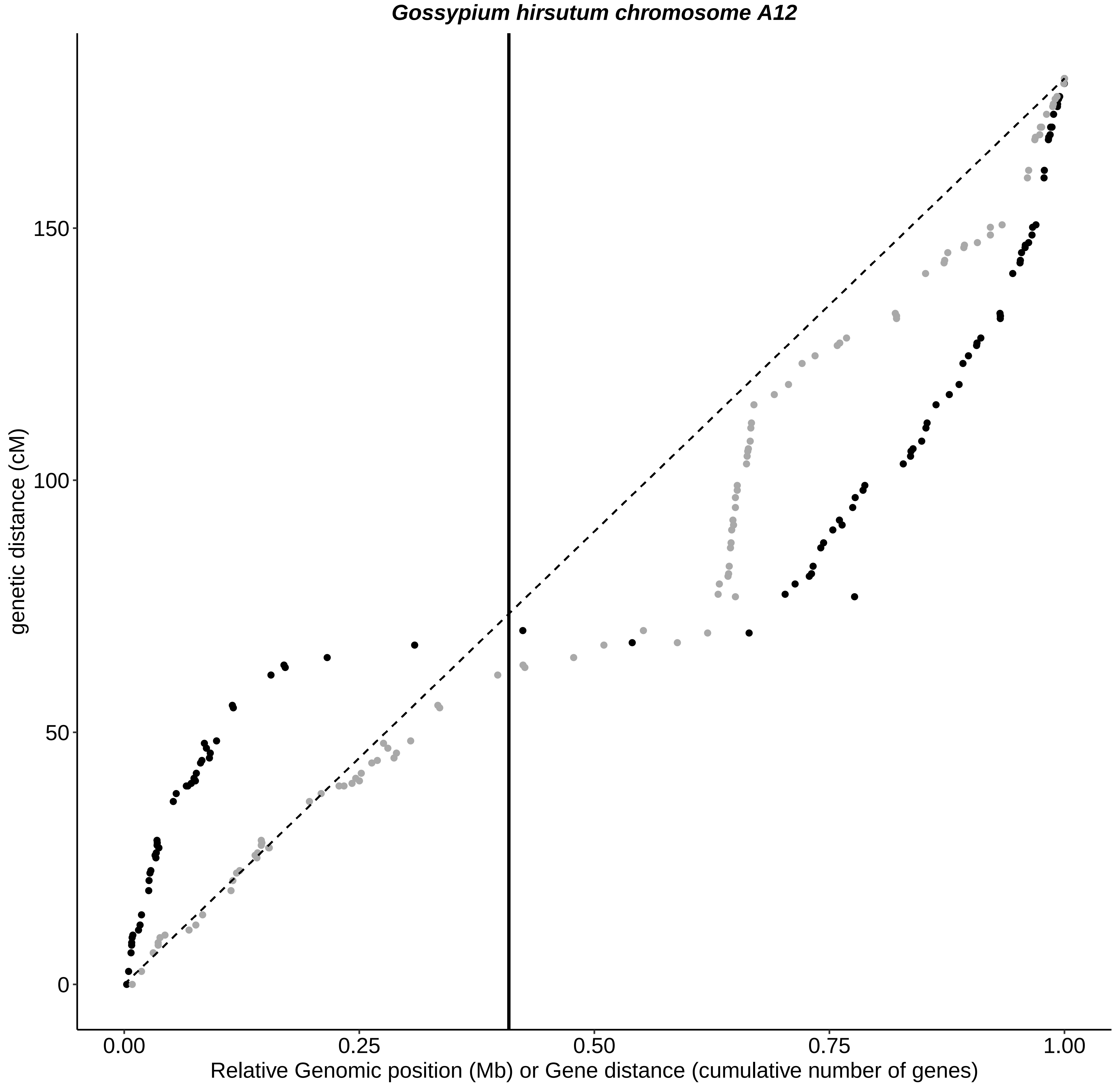
Gossypium hirsutum chromosome A12
genetic distance (cM)
0
50
100
150
0.00
0.25
0.50
0.75
1.00
Relative Genomic position (Mb) or Gene distance (cumulative number of genes)

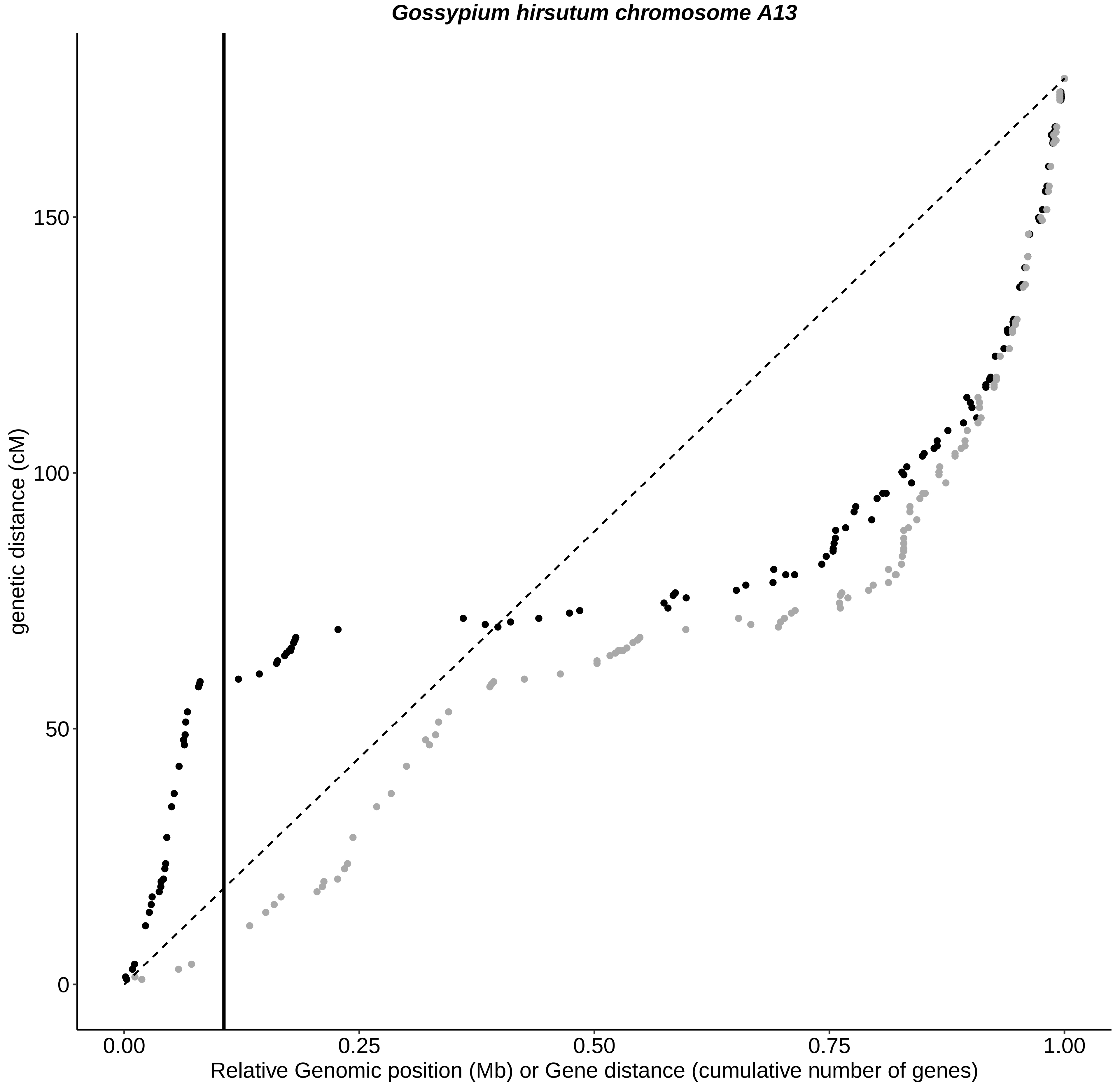
Gossypium hirsutum chromosome A13
genetic distance (cM)
150
100
50
0
0.00
0.25
0.50
0.75
1.00
Relative Genomic position (Mb) or Gene distance (cumulative number of genes)

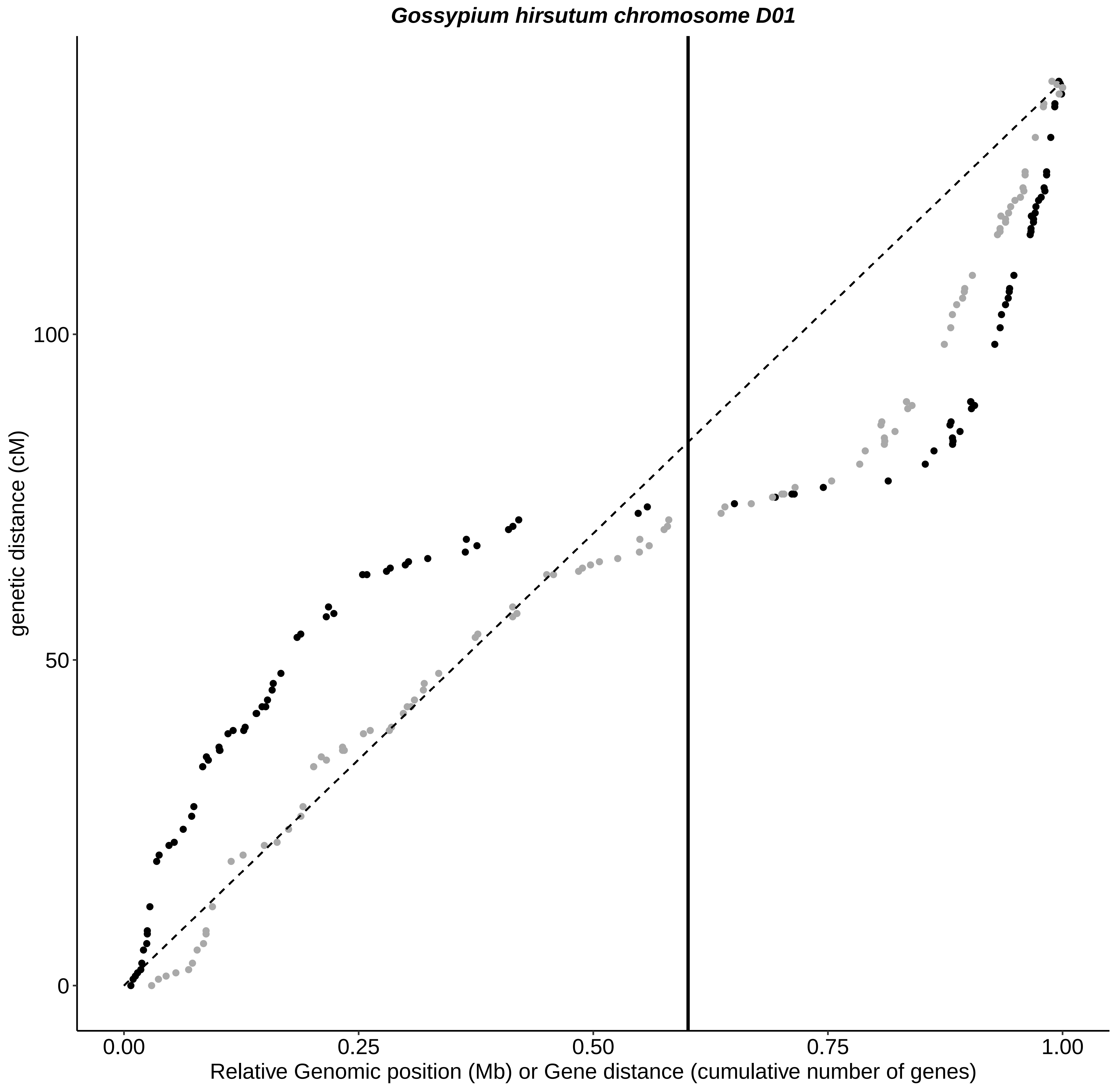
Gossypium hirsutum chromosome D01
genetic distance (cM)
100
50
0
0.00
0.25
0.50
0.75
1.00
Relative Genomic position (Mb) or Gene distance (cumulative number of genes)

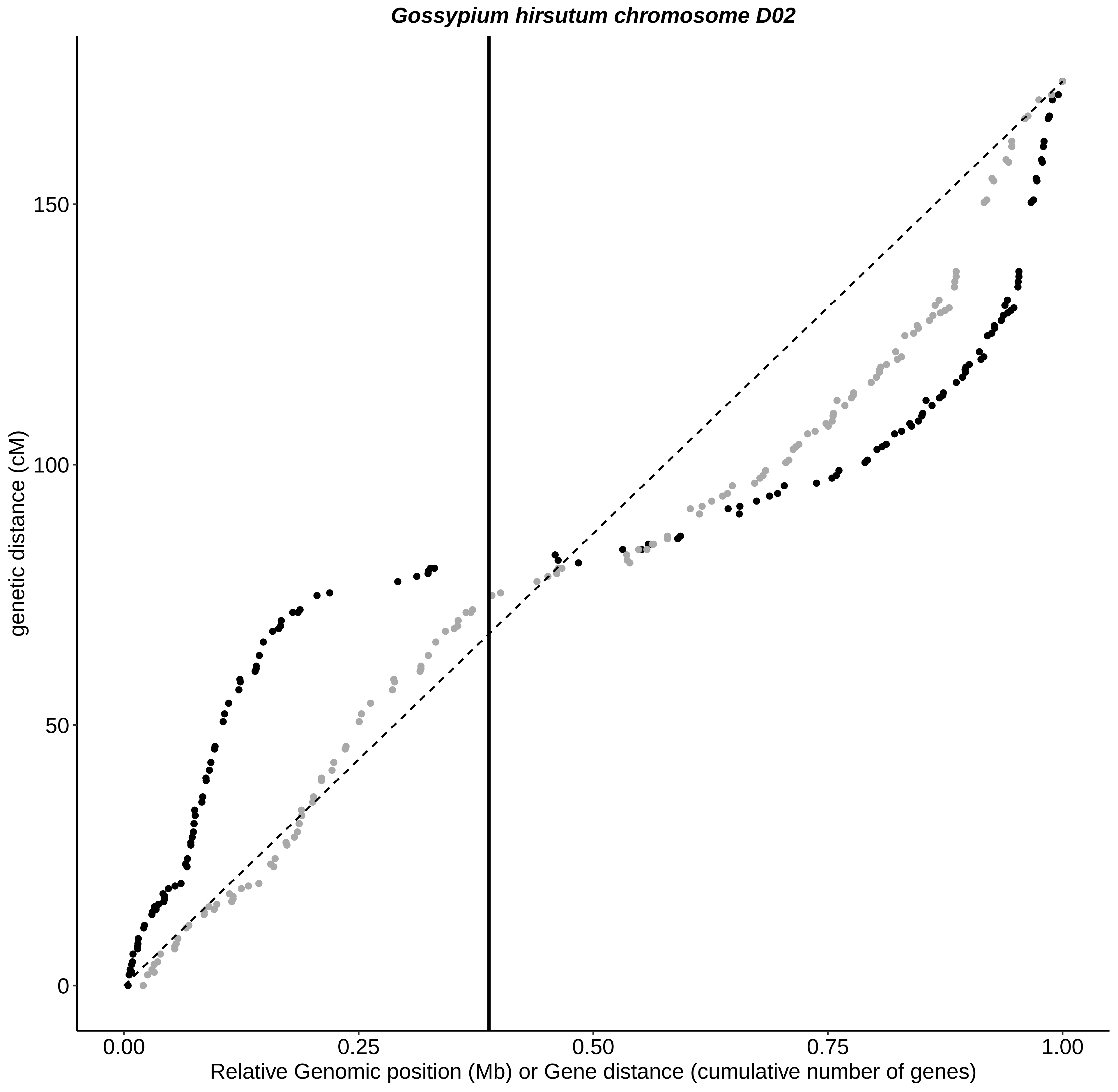
Gossypium hirsutum chromosome D02
genetic distance (cM)
0
50
100
150
0.00
0.25
0.50
0.75
1.00
Relative Genomic position (Mb) or Gene distance (cumulative number of genes)

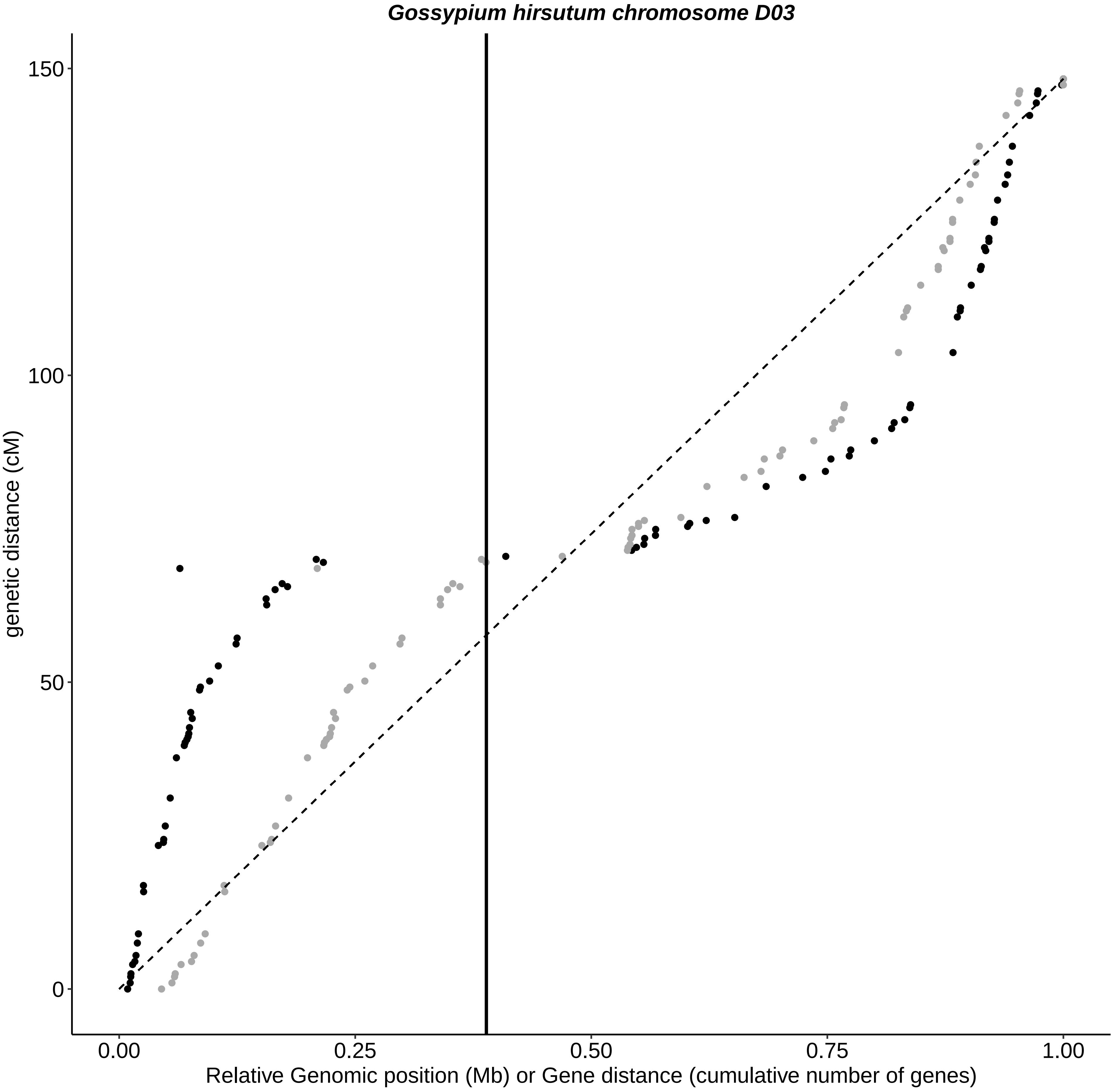
Gossypium hirsutum chromosome D03
genetic distance (cM)
150
100
50
0
0.00
0.25
0.50
0.75
1.00
Relative Genomic position (Mb) or Gene distance (cumulative number of genes)

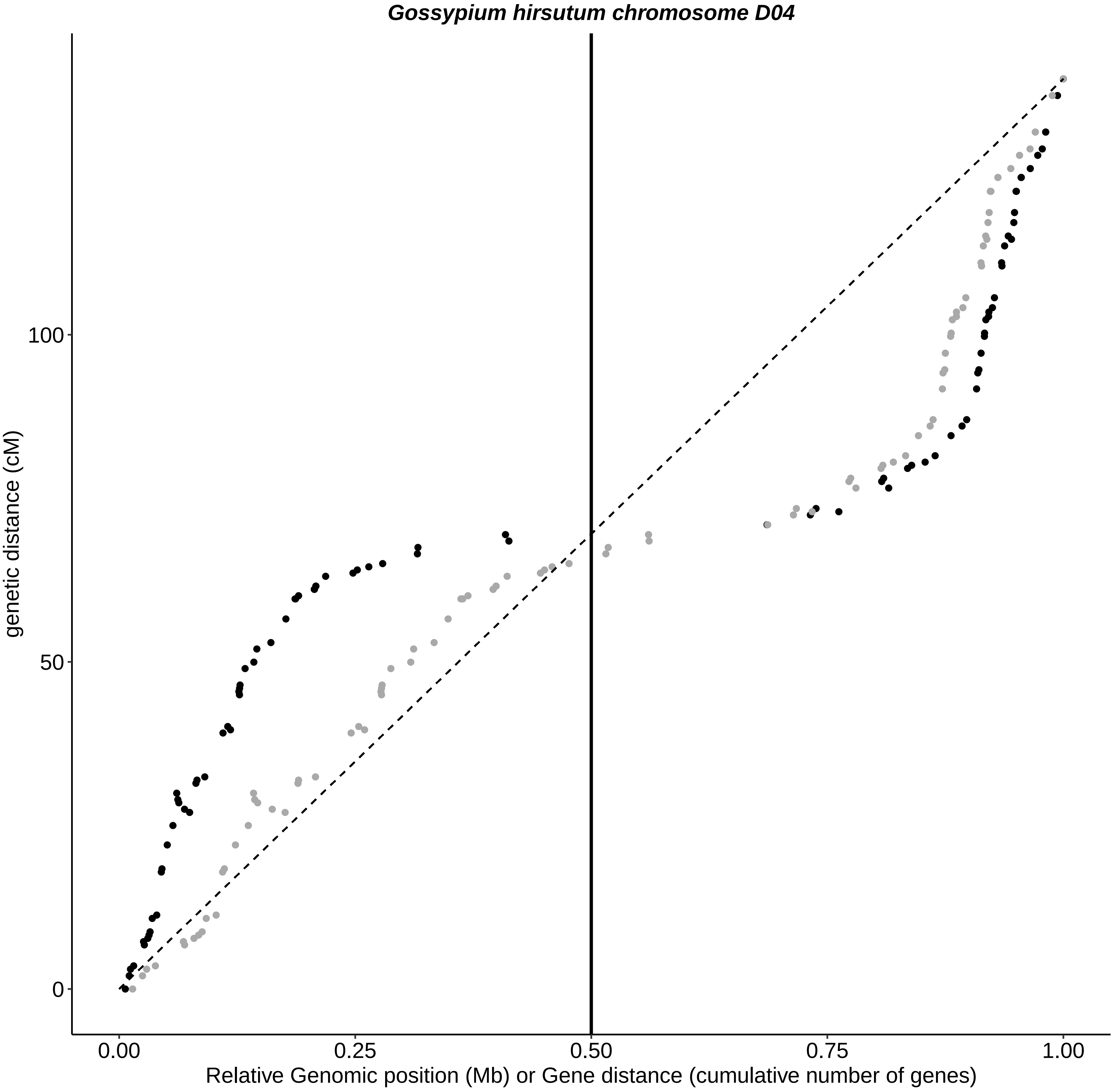

Gossypium hirsutum chromosome D04
genetic distance (cM)
100
50
0
0.00
0.25
0.50
0.75
1.00
Relative Genomic position (Mb) or Gene distance (cumulative number of genes)

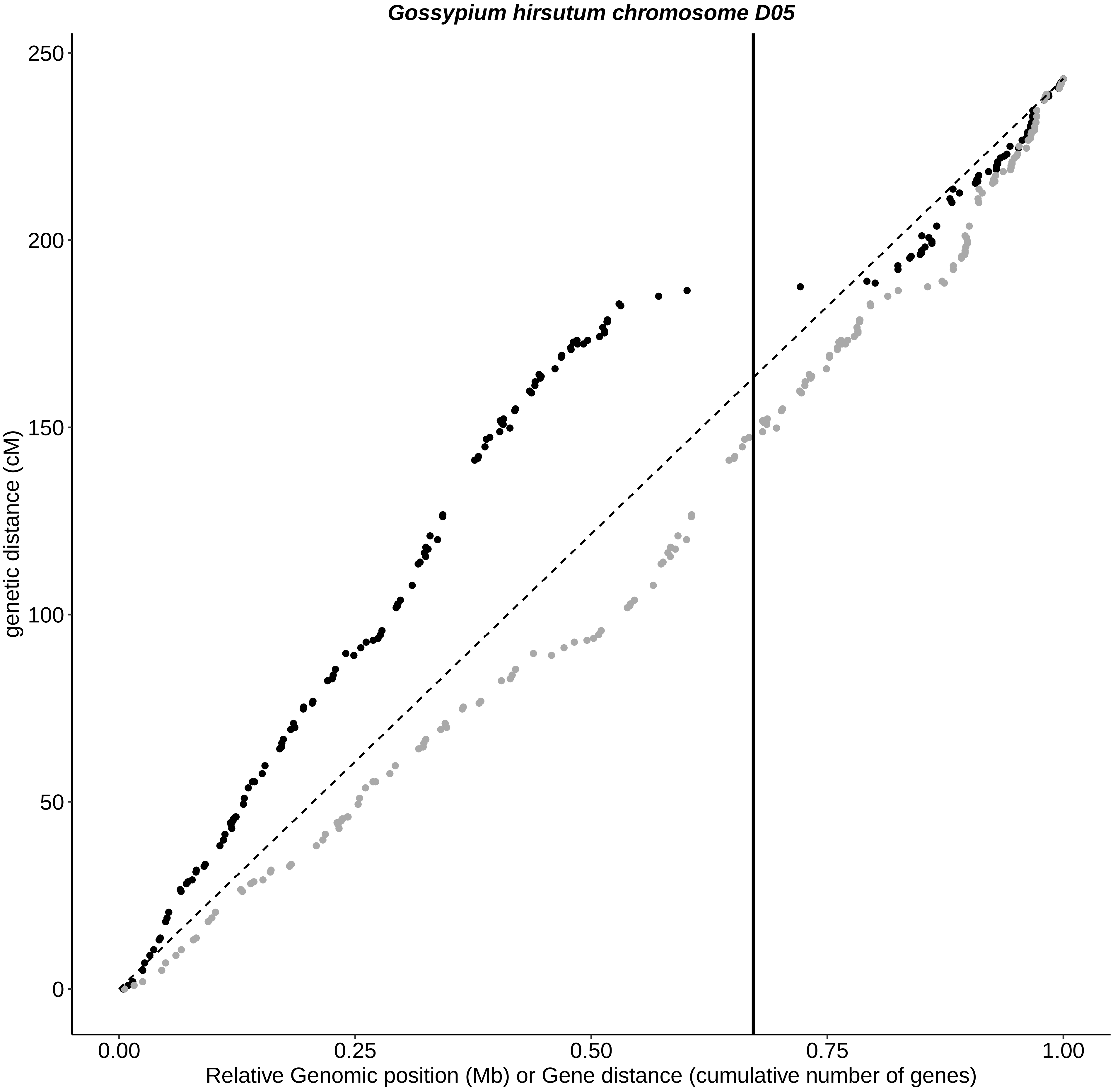
Gossypium hirsutum chromosome D05
genetic distance (cM)
250
200
150
100
50
0
0.00
0.25
0.50
0.75
1.00
Relative Genomic position (Mb) or Gene distance (cumulative number of genes)

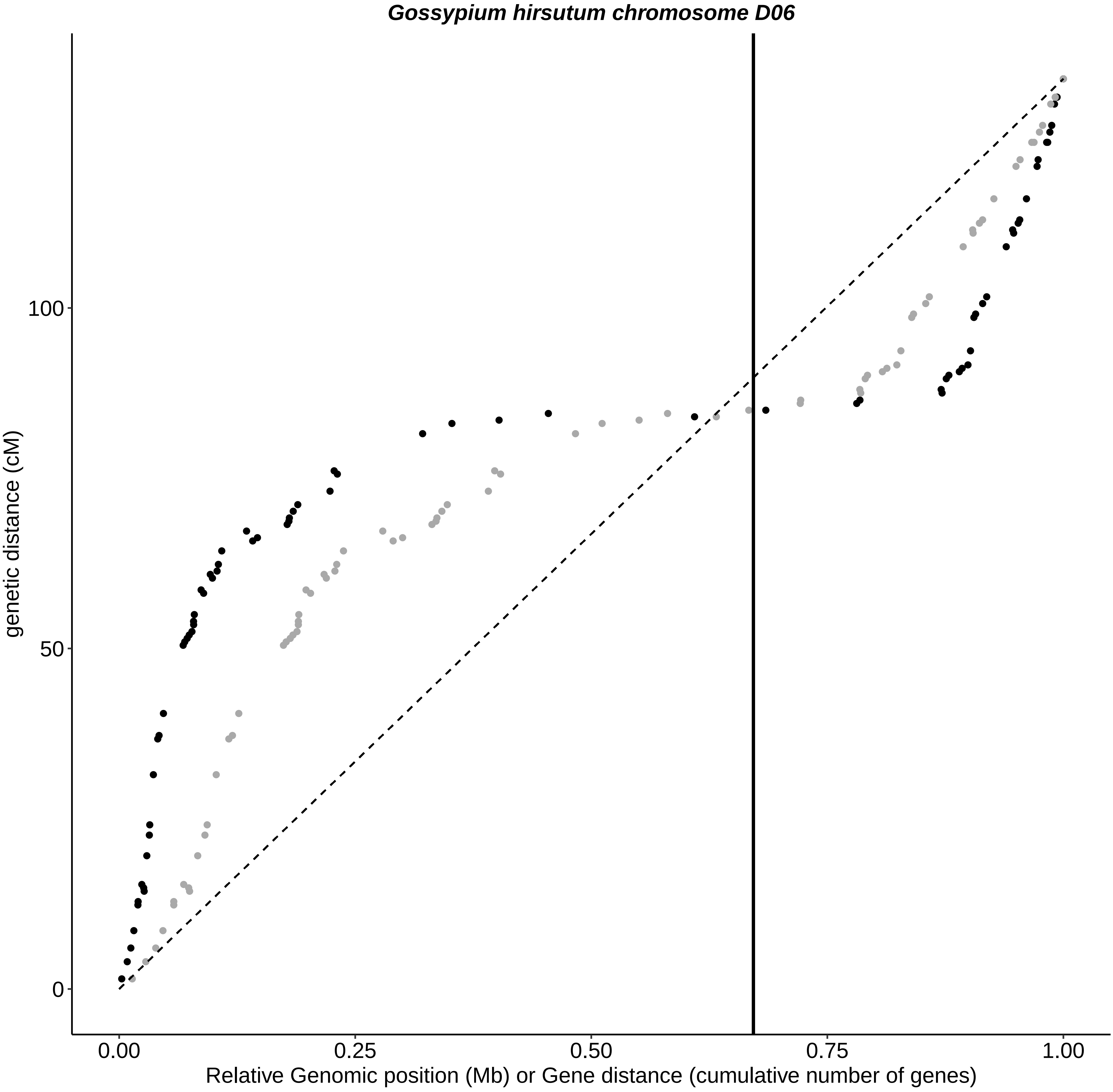

Gossypium hirsutum chromosome D06
genetic distance (cM)
0
50
100
0.00
0.25
0.50
0.75
1.00
Relative Genomic position (Mb) or Gene distance (cumulative number of genes)

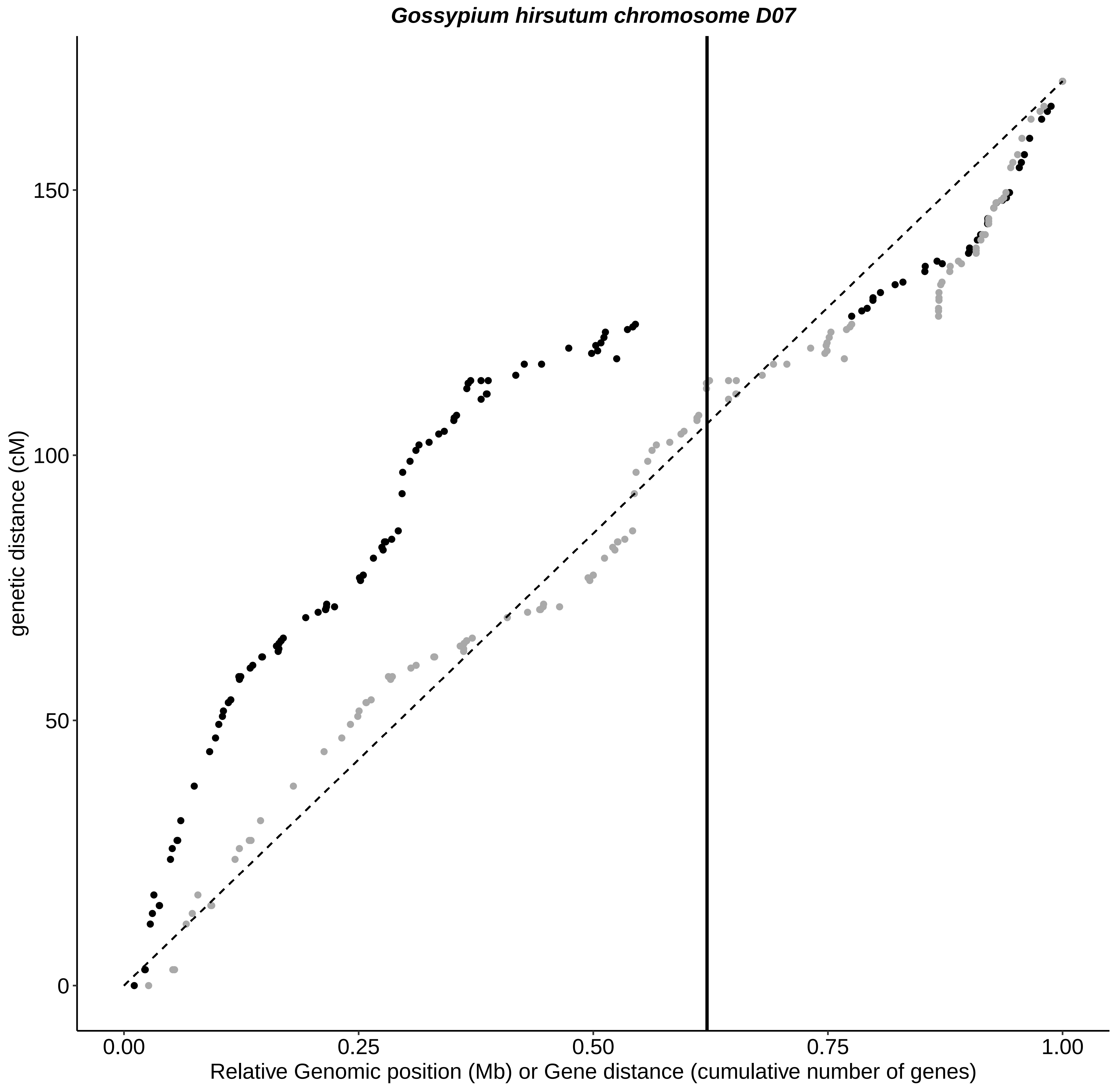
Gossypium hirsutum chromosome D07
genetic distance (cM)
150
100
50
0
0.00
0.25
0.50
0.75
1.00
Relative Genomic position (Mb) or Gene distance (cumulative number of genes)

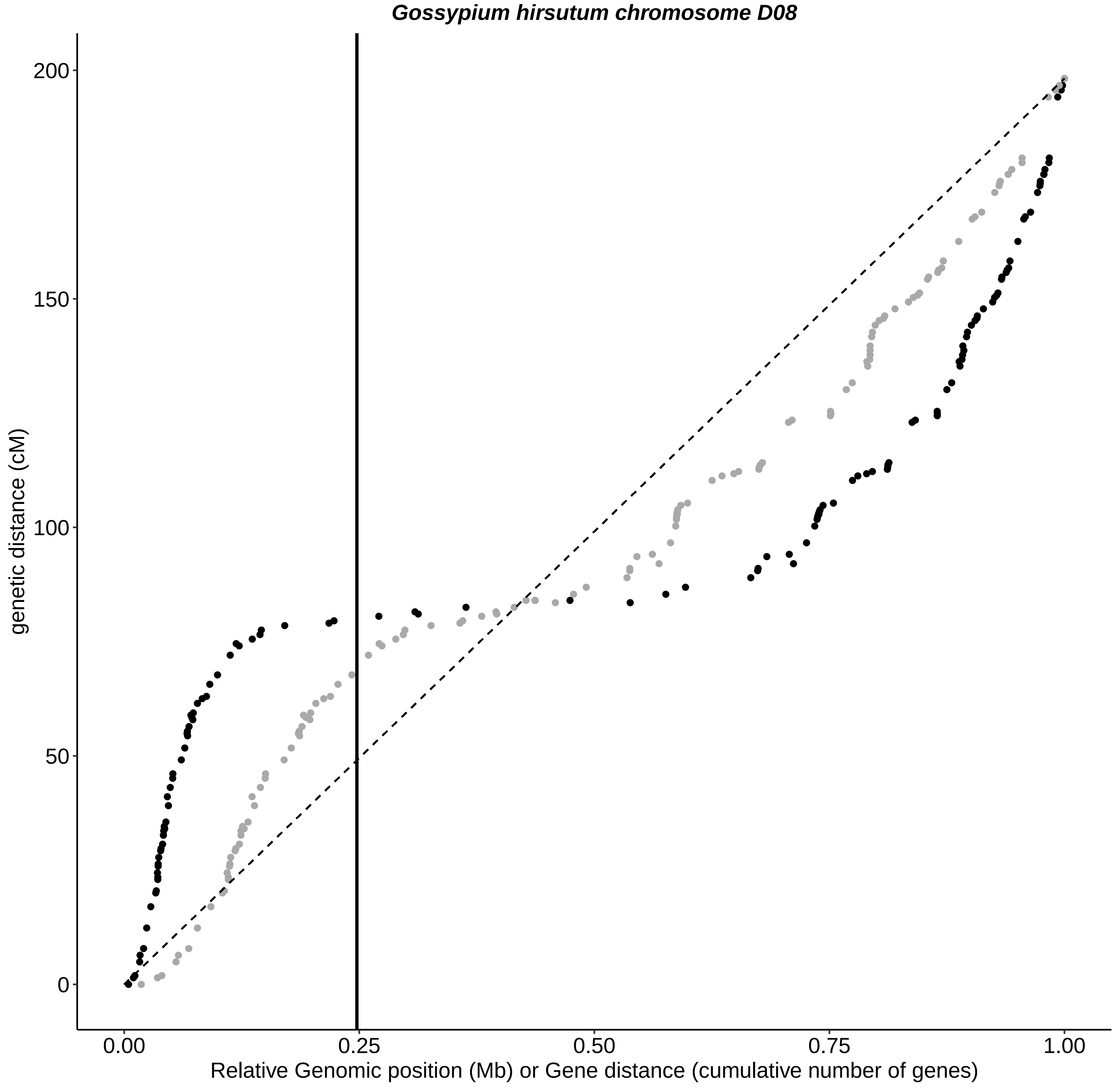
Gossypium hirsutum chromosome D08
genetic distance (cM)
0
50
100
150
200
0.00
0.25
0.50
0.75
1.00
Relative Genomic position (Mb) or Gene distance (cumulative number of genes)

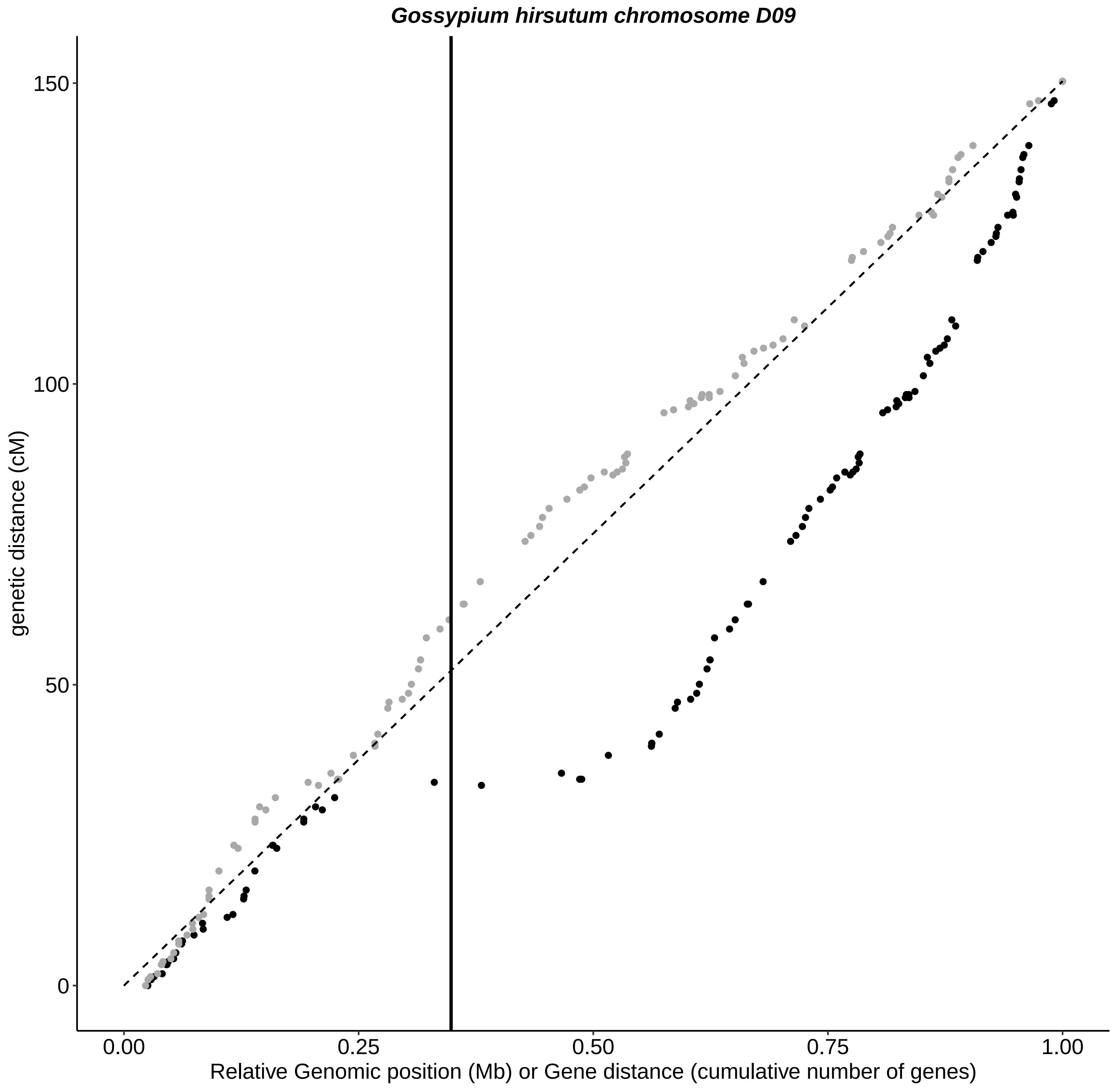
Gossypium hirsutum chromosome D09
genetic distance (cM)
150
100
50
0
0.00
0.25
0.50
0.75
1.00
Relative Genomic position (Mb) or Gene distance (cumulative number of genes)

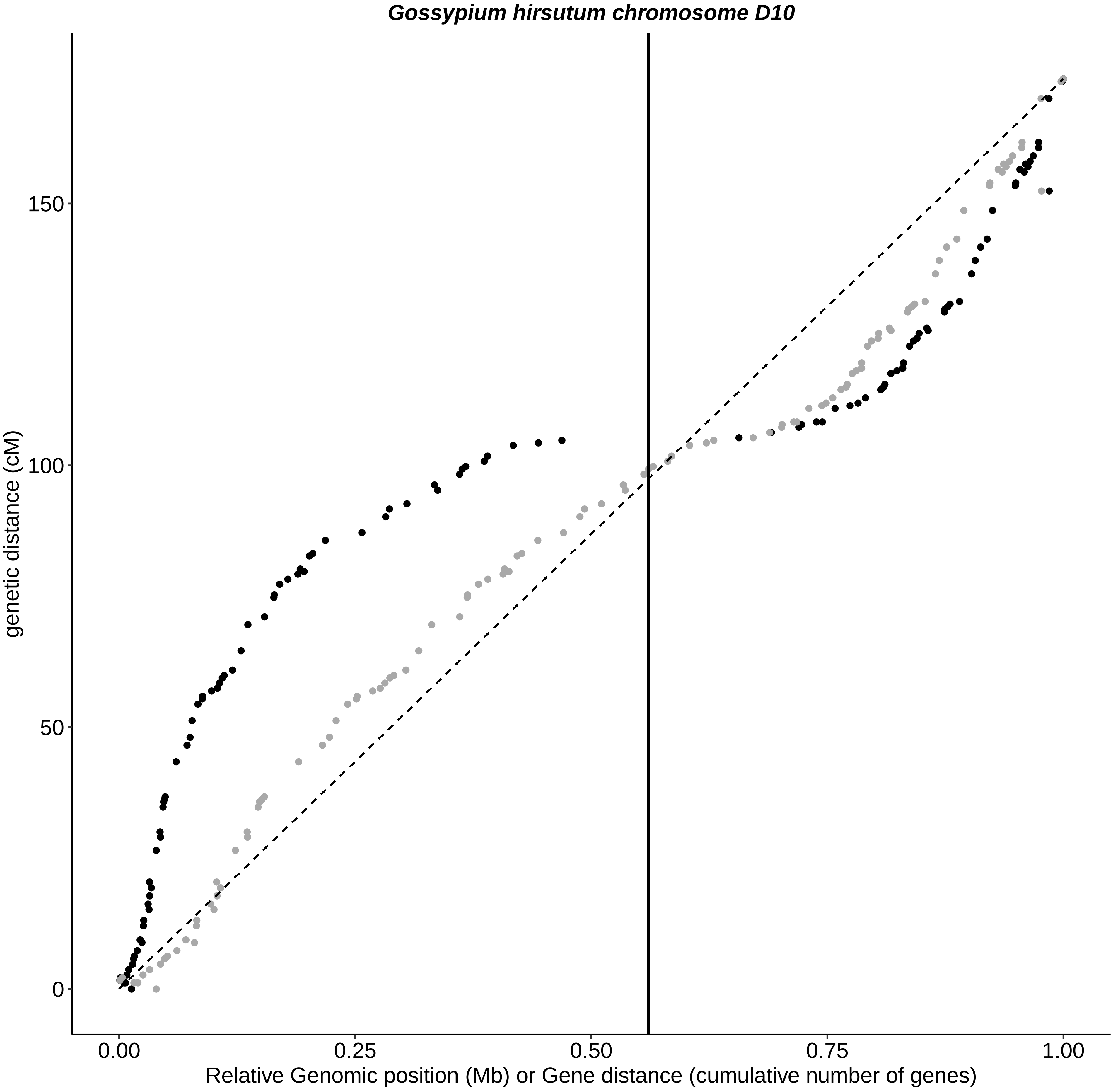
Gossypium hirsutum chromosome D10
genetic distance (cM)
0
50
100
150
0.00
0.25
0.50
0.75
1.00
Relative Genomic position (Mb) or Gene distance (cumulative number of genes)

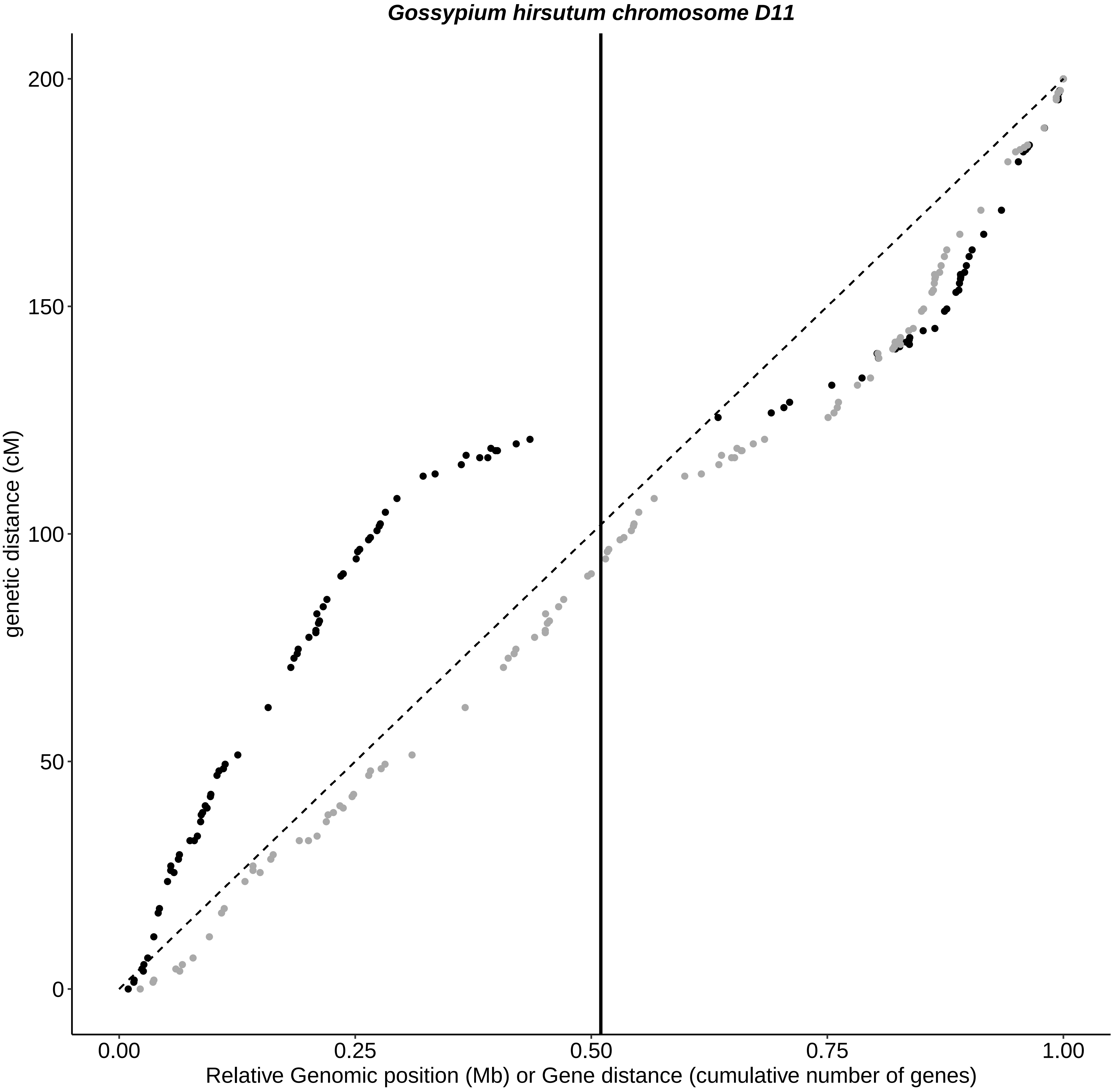
Gossypium hirsutum chromosome D11
genetic distance (cM)
200
150
100
50
0
0.00
0.25
0.50
0.75
1.00
Relative Genomic position (Mb) or Gene distance (cumulative number of genes)

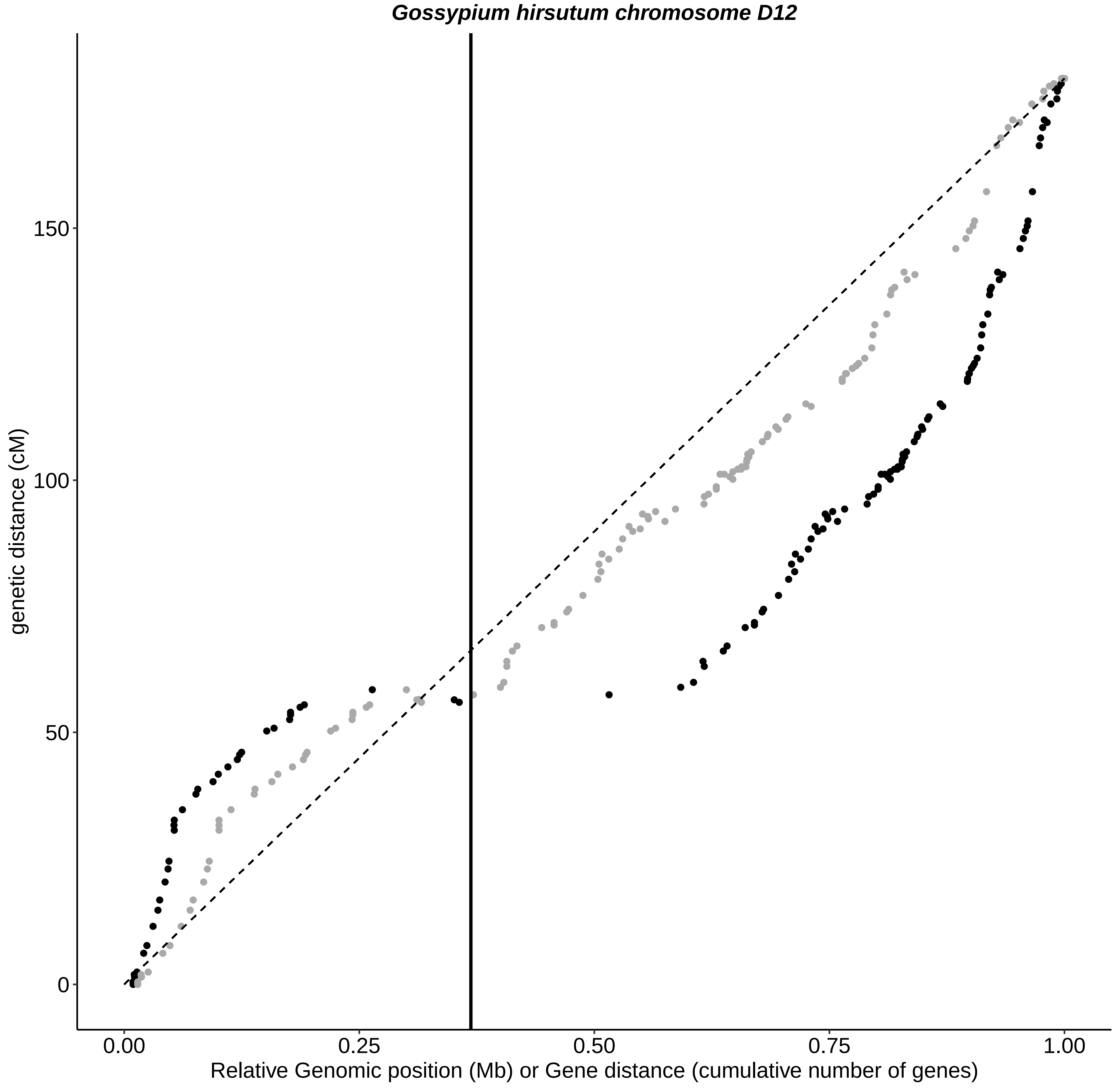

Gossypium hirsutum chromosome D12
genetic distance (cM)
0
50
100
150
0.00
0.25
0.50
0.75
1.00
Relative Genomic position (Mb) or Gene distance (cumulative number of genes)

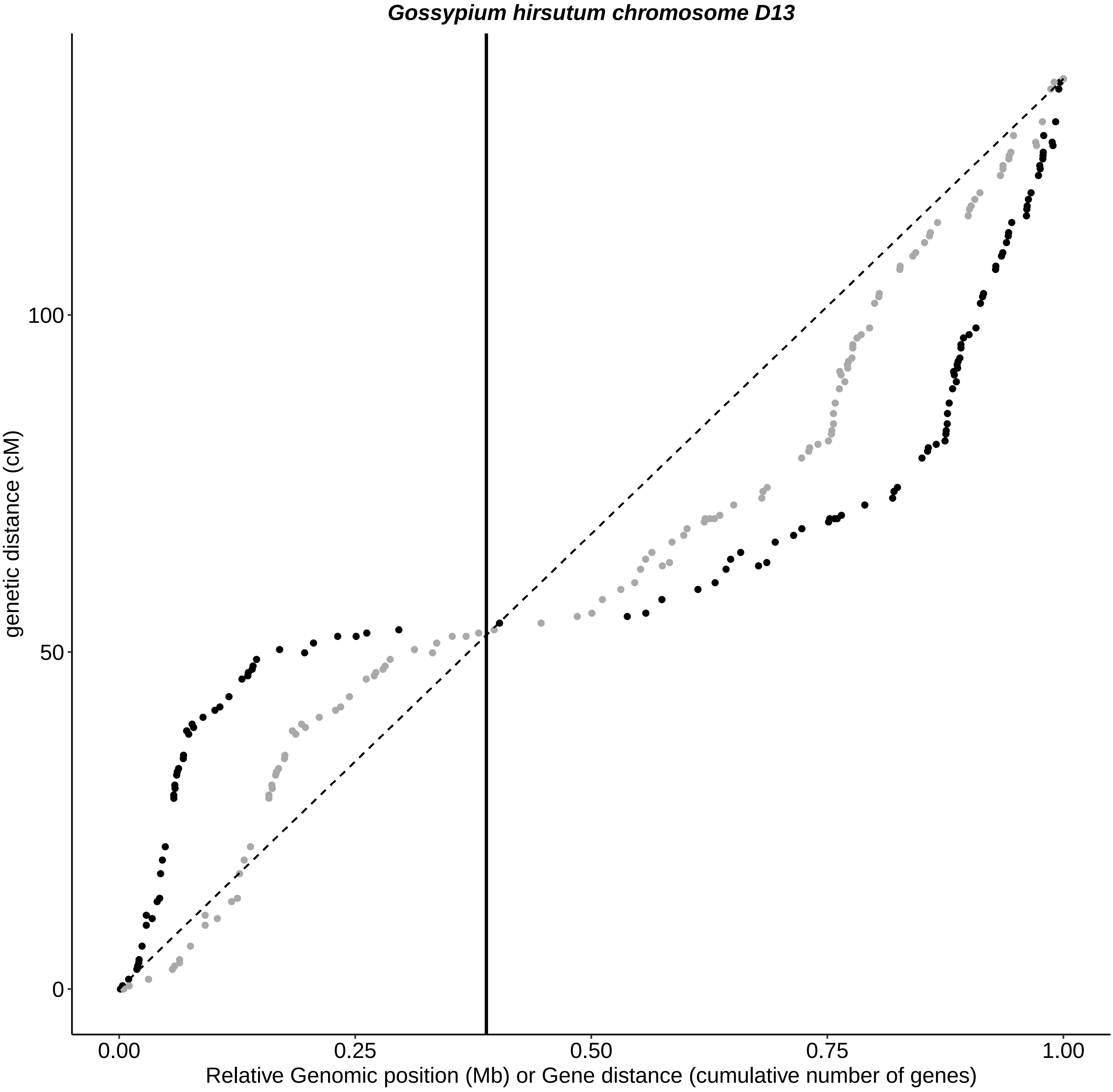
Gossypium hirsutum chromosome D13
genetic distance (cM)
100
50
0
0.00
0.25
0.50
0.75
1.00
Relative Genomic position (Mb) or Gene distance (cumulative number of genes)

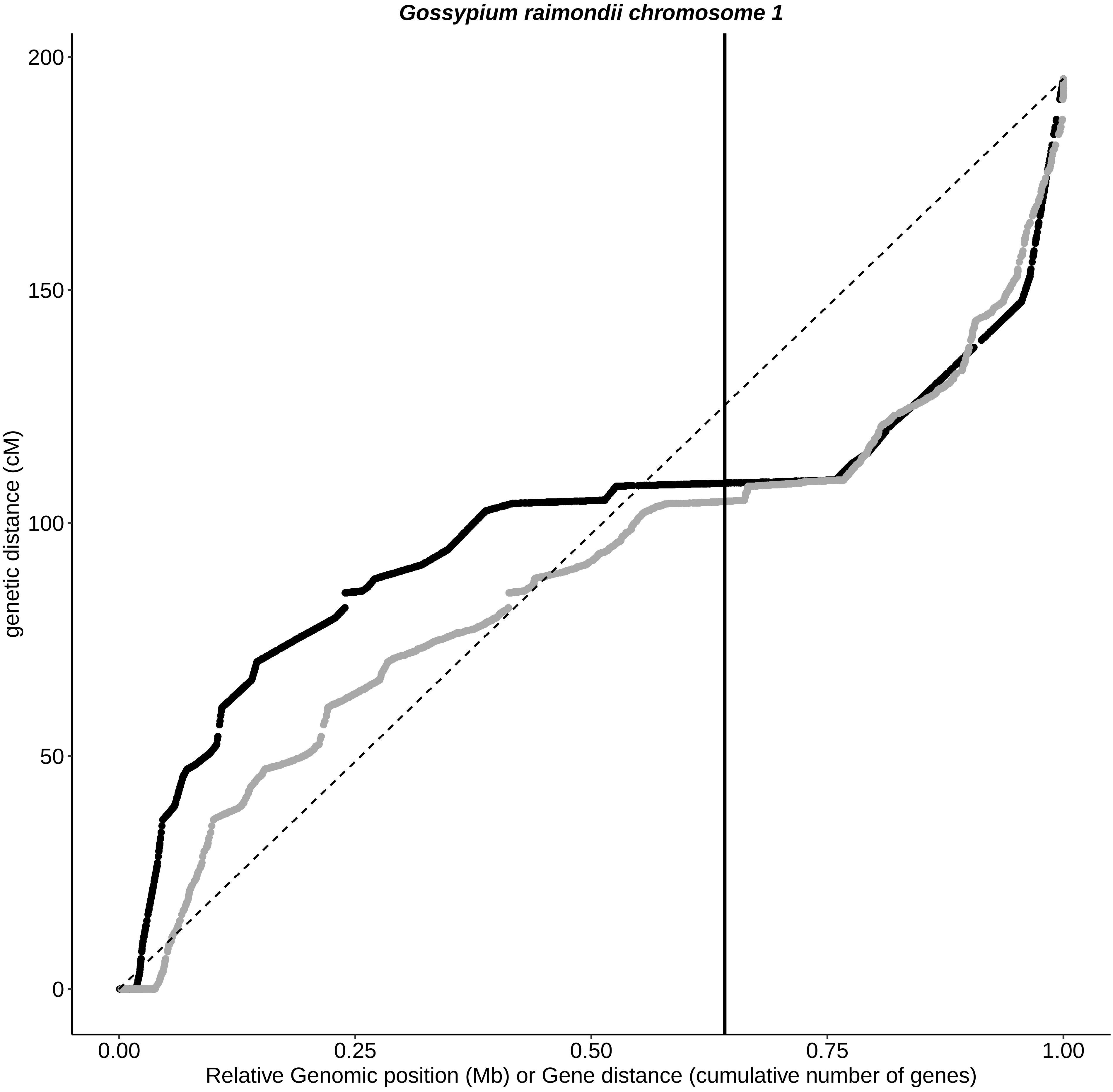
Gossypium raimondii chromosome 1
genetic distance (cM)
200
150
100
50
0
0.00
0.25
0.50
0.75
1.00
Relative Genomic position (Mb) or Gene distance (cumulative number of genes)

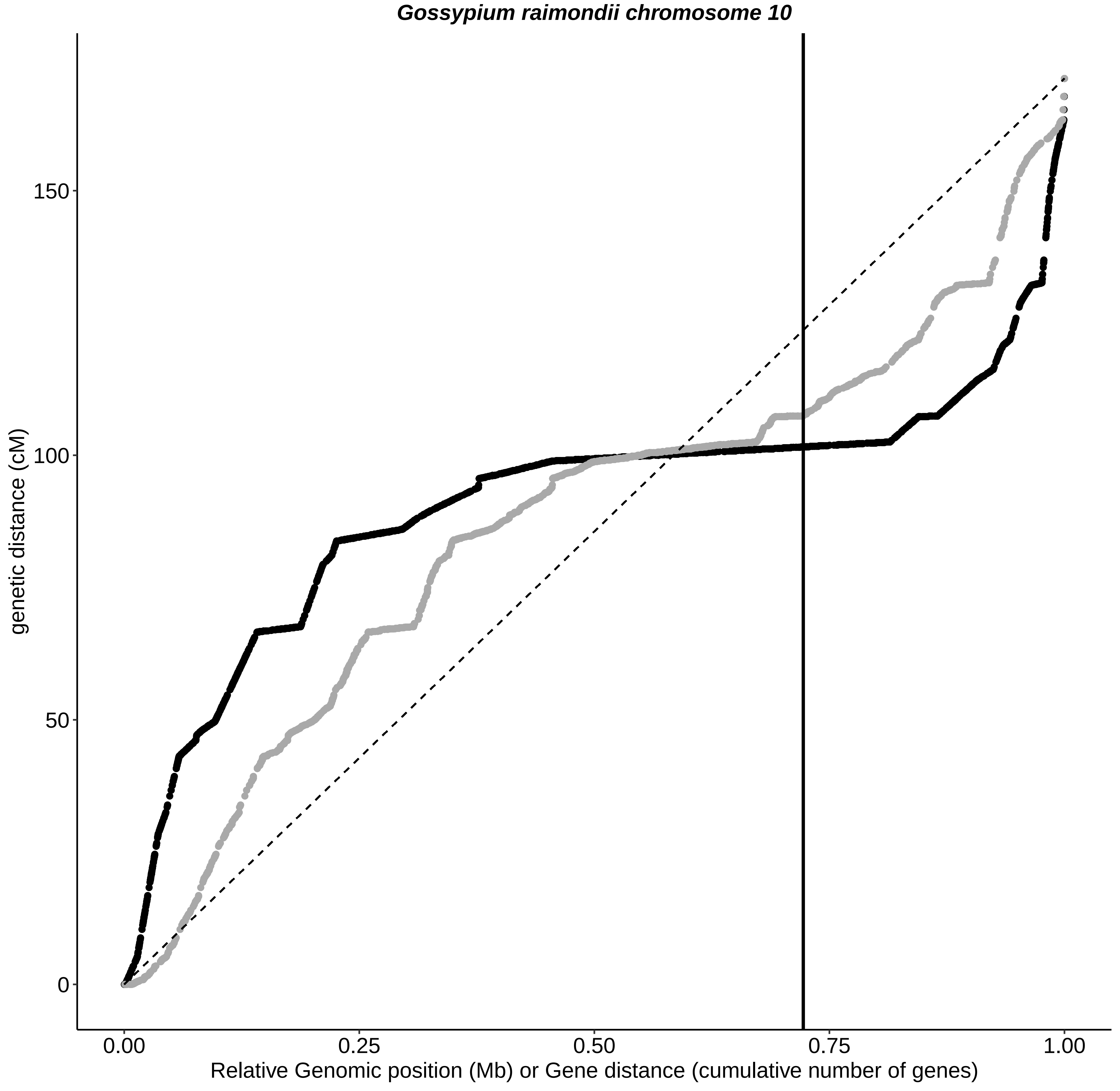
Gossypium raimondii chromosome 10
genetic distance (cM)
150
100
50
0
0.00
0.25
0.50
0.75
1.00
Relative Genomic position (Mb) or Gene distance (cumulative number of genes)

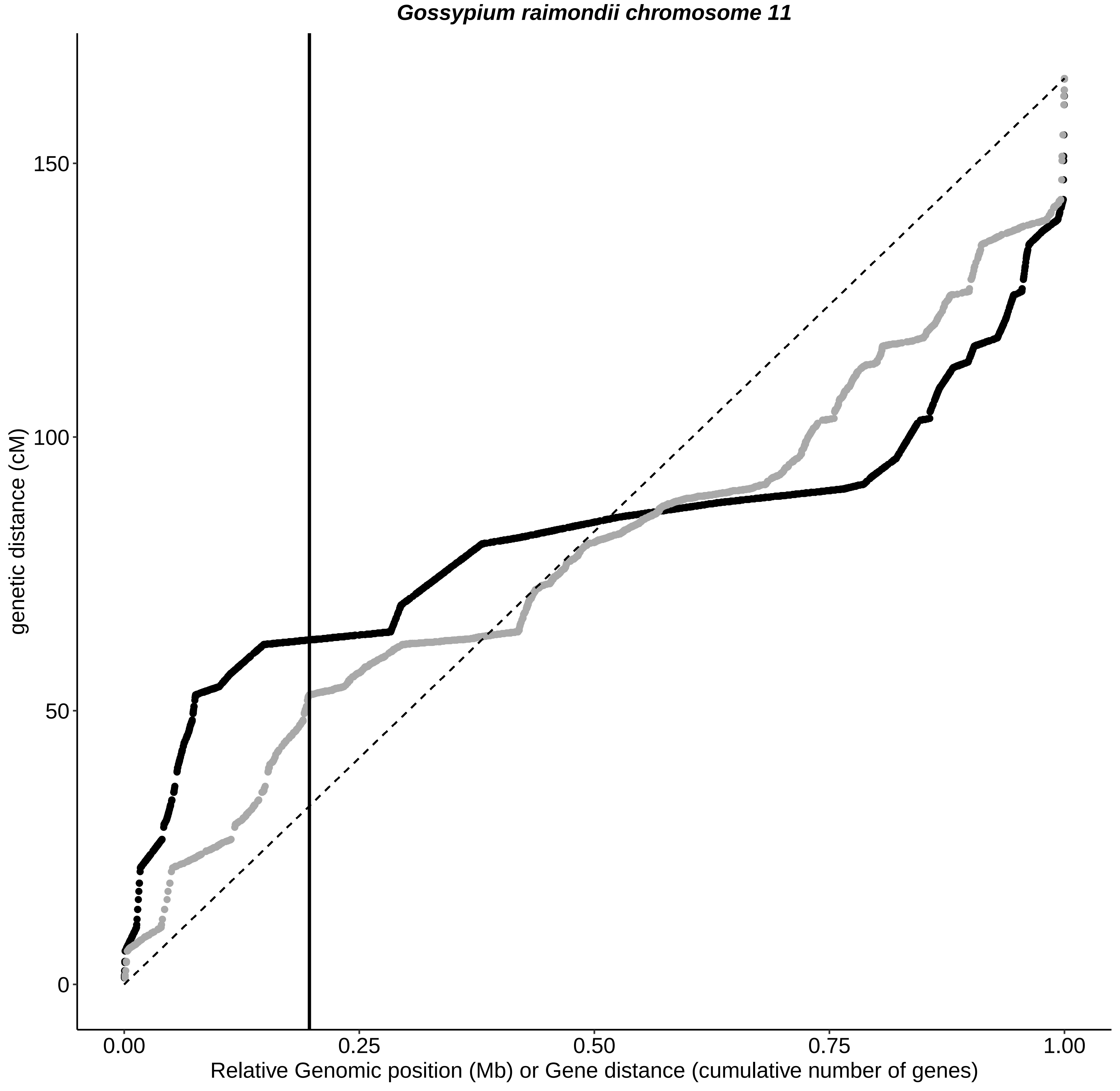
Gossypium raimondii chromosome 11
genetic distance (cM)
150
100
50
0
0.00
0.25
0.50
0.75
1.00
Relative Genomic position (Mb) or Gene distance (cumulative number of genes)

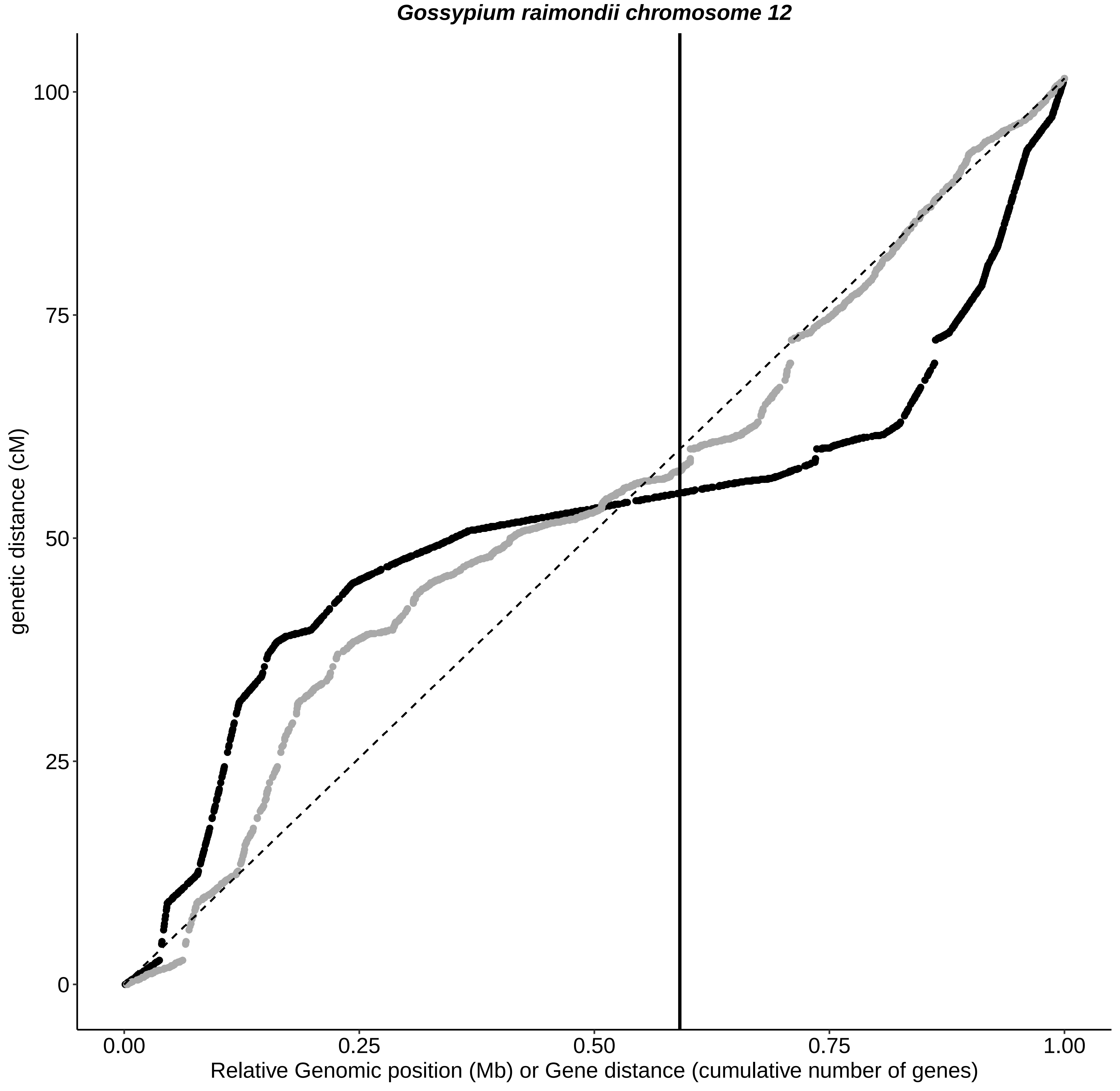
Gossypium raimondii chromosome 12
genetic distance (cM)
0
25
50
75
100
0.00
0.25
0.50
0.75
1.00
Relative Genomic position (Mb) or Gene distance (cumulative number of genes)

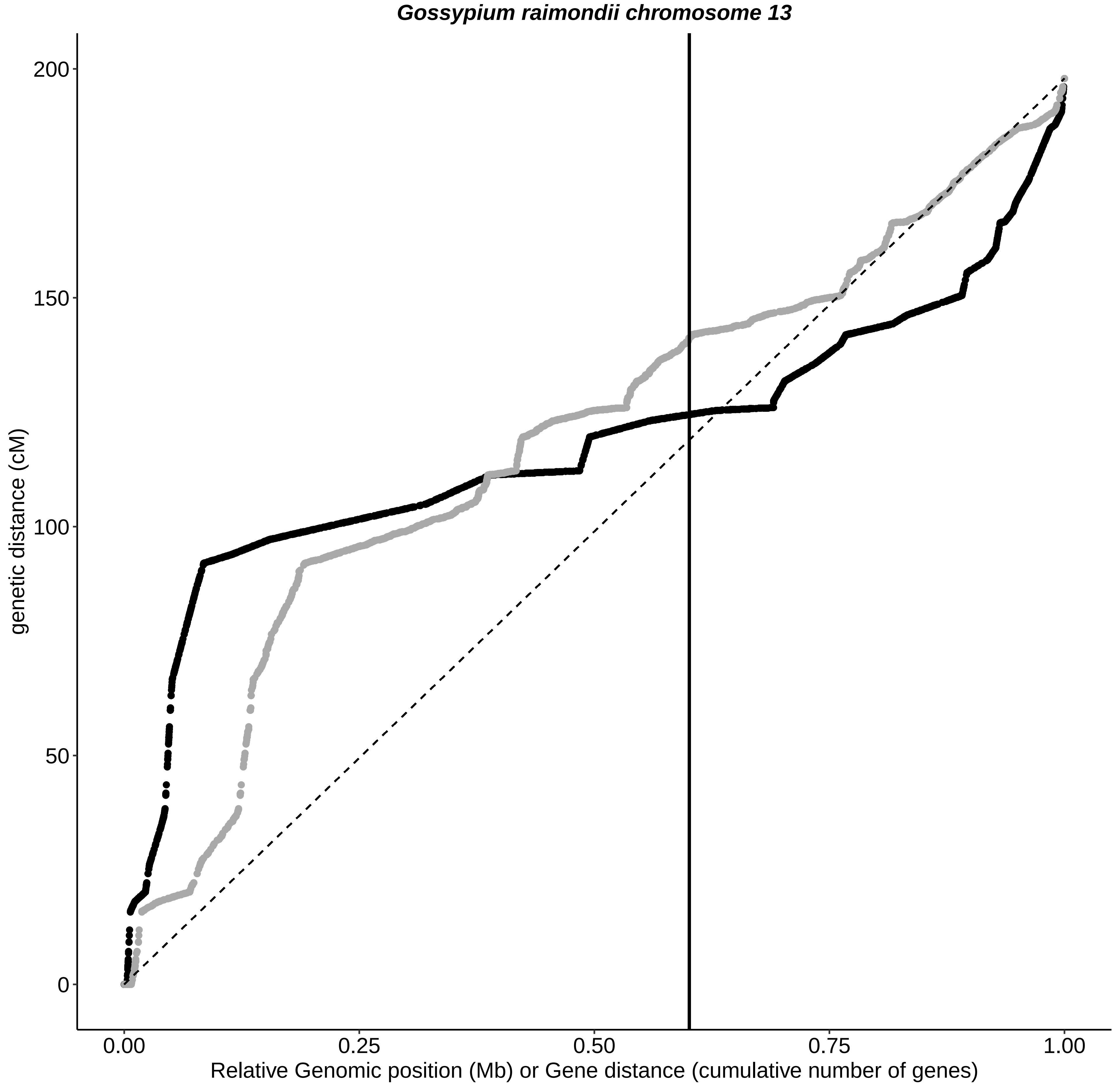
Gossypium raimondii chromosome 13
genetic distance (cM)
0
50
100
150
200
0.00
0.25
0.50
0.75
1.00
Relative Genomic position (Mb) or Gene distance (cumulative number of genes)

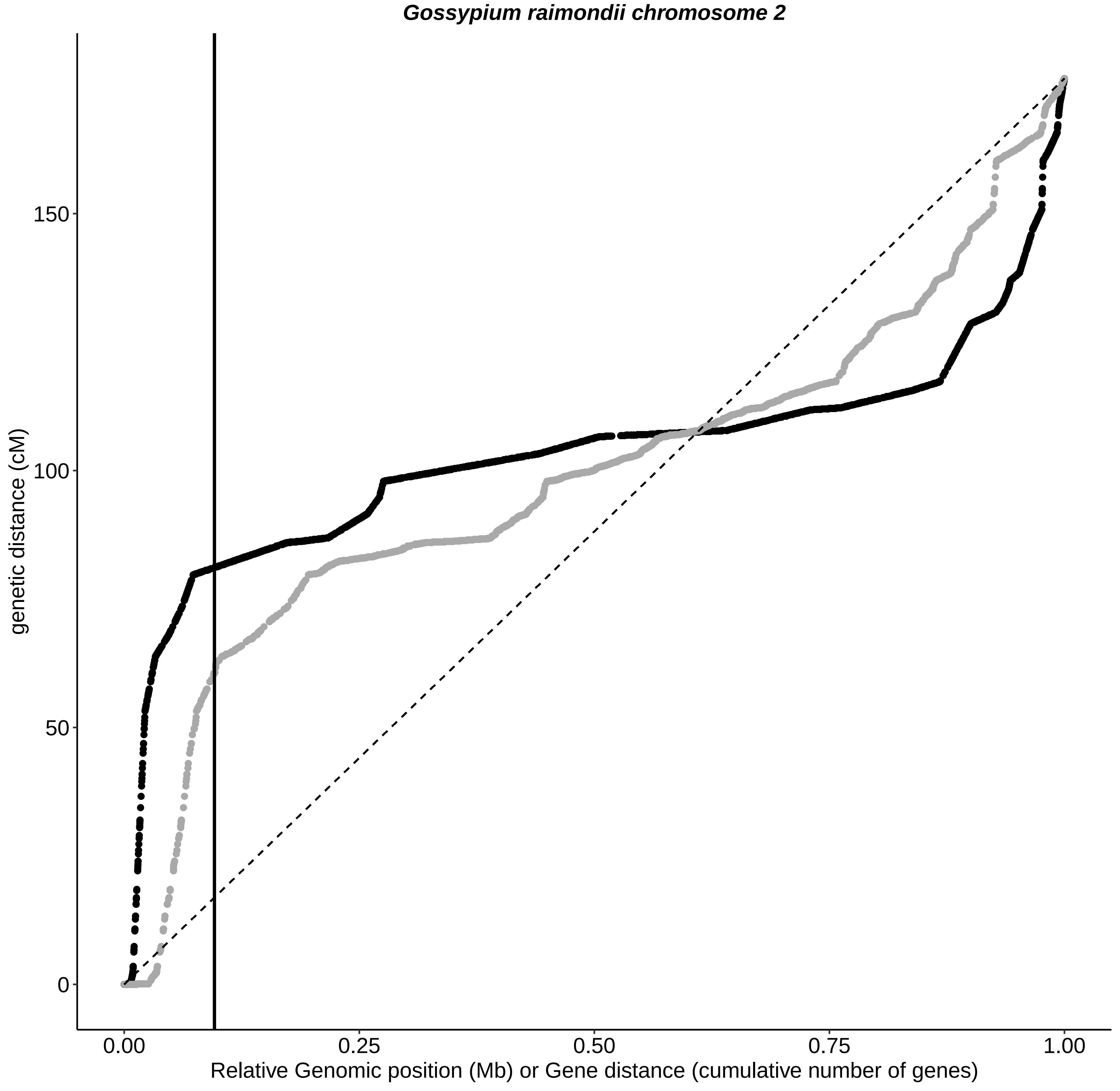
Gossypium raimondii chromosome 2
genetic distance (cM)
150
100
50
0
0.00
0.25
0.50
0.75
1.00
Relative Genomic position (Mb) or Gene distance (cumulative number of genes)

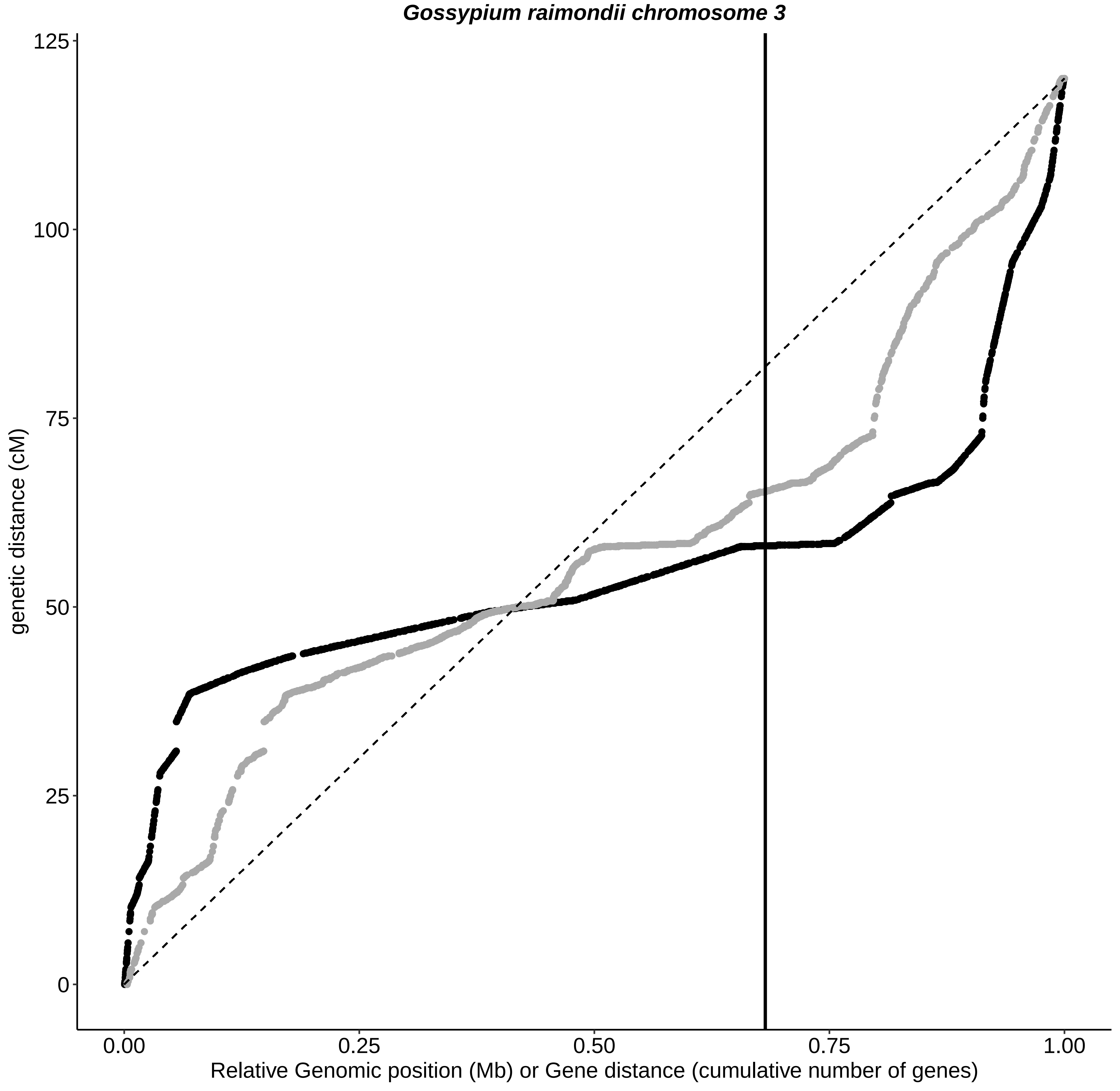
Gossypium raimondii chromosome 3
125
100
75
50
25
0
genetic distance (cM)
0.00
0.25
0.50
0.75
1.00
Relative Genomic position (Mb) or Gene distance (cumulative number of genes)

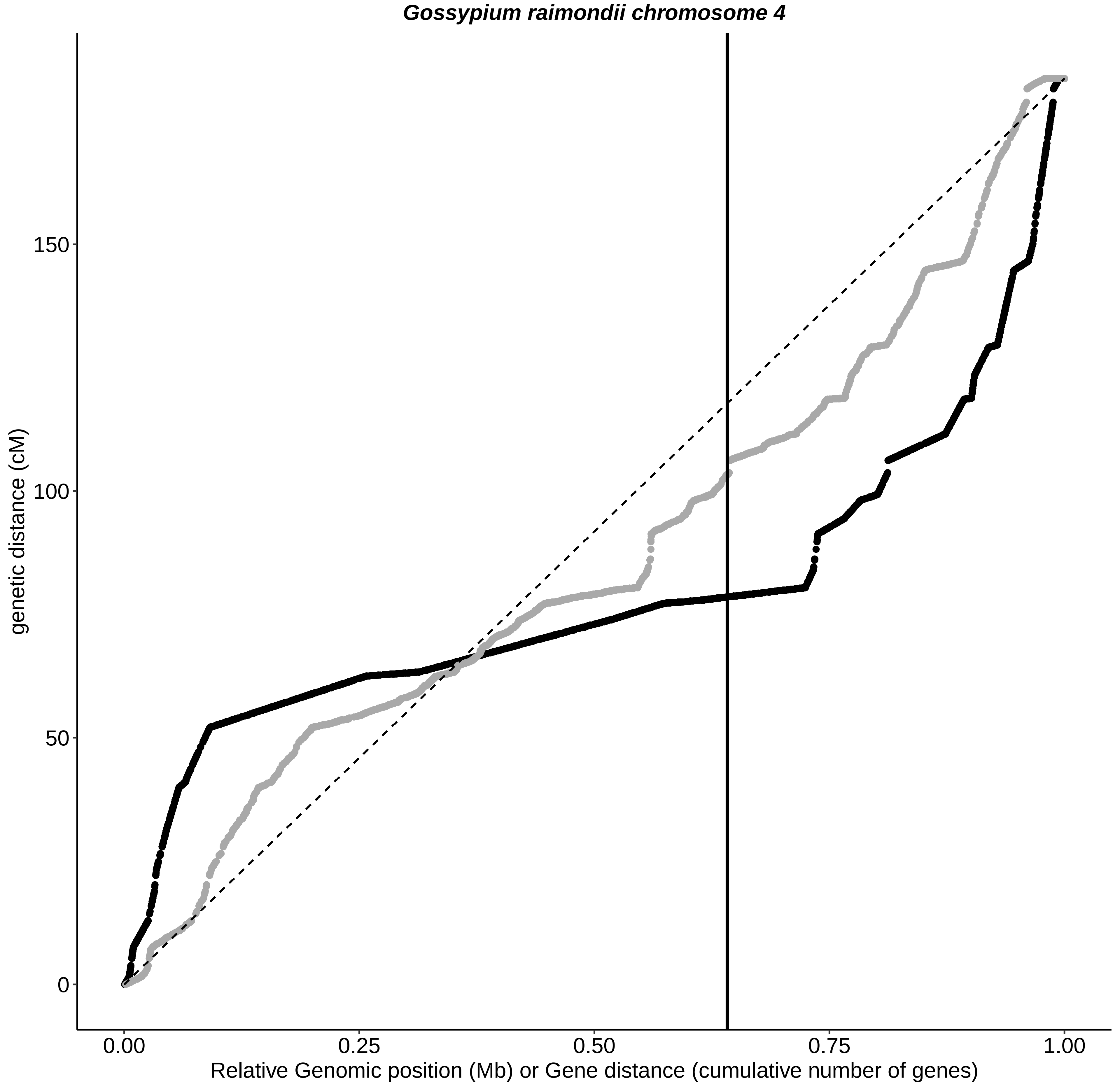
Gossypium raimondii chromosome 4
genetic distance (cM)
0
50
100
150
0.00
0.25
0.50
0.75
1.00
Relative Genomic position (Mb) or Gene distance (cumulative number of genes)

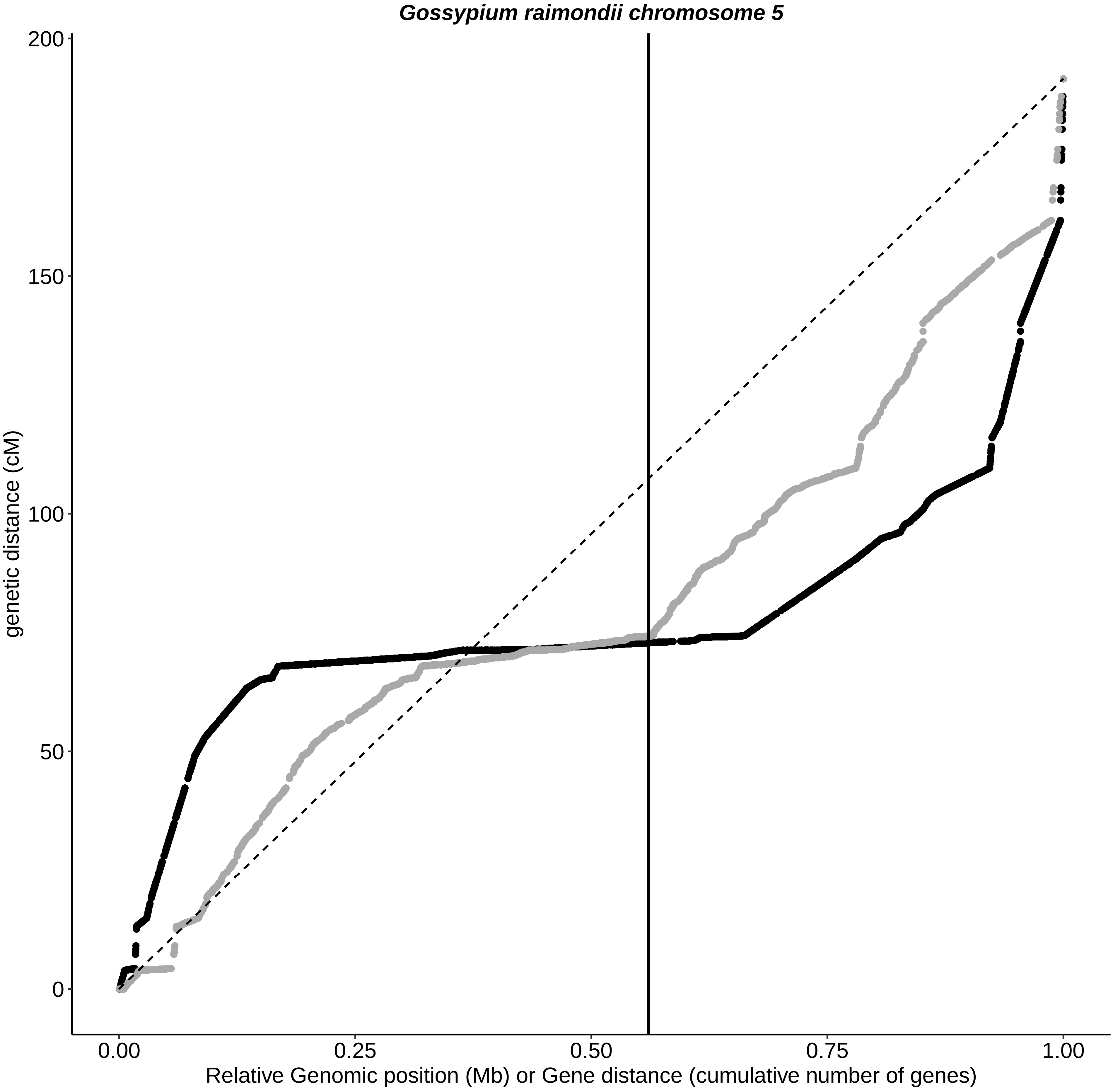
Gossypium raimondii chromosome 5
genetic distance (cM)
0
50
100
150
200
0.00
0.25
0.50
0.75
1.00
Relative Genomic position (Mb) or Gene distance (cumulative number of genes)

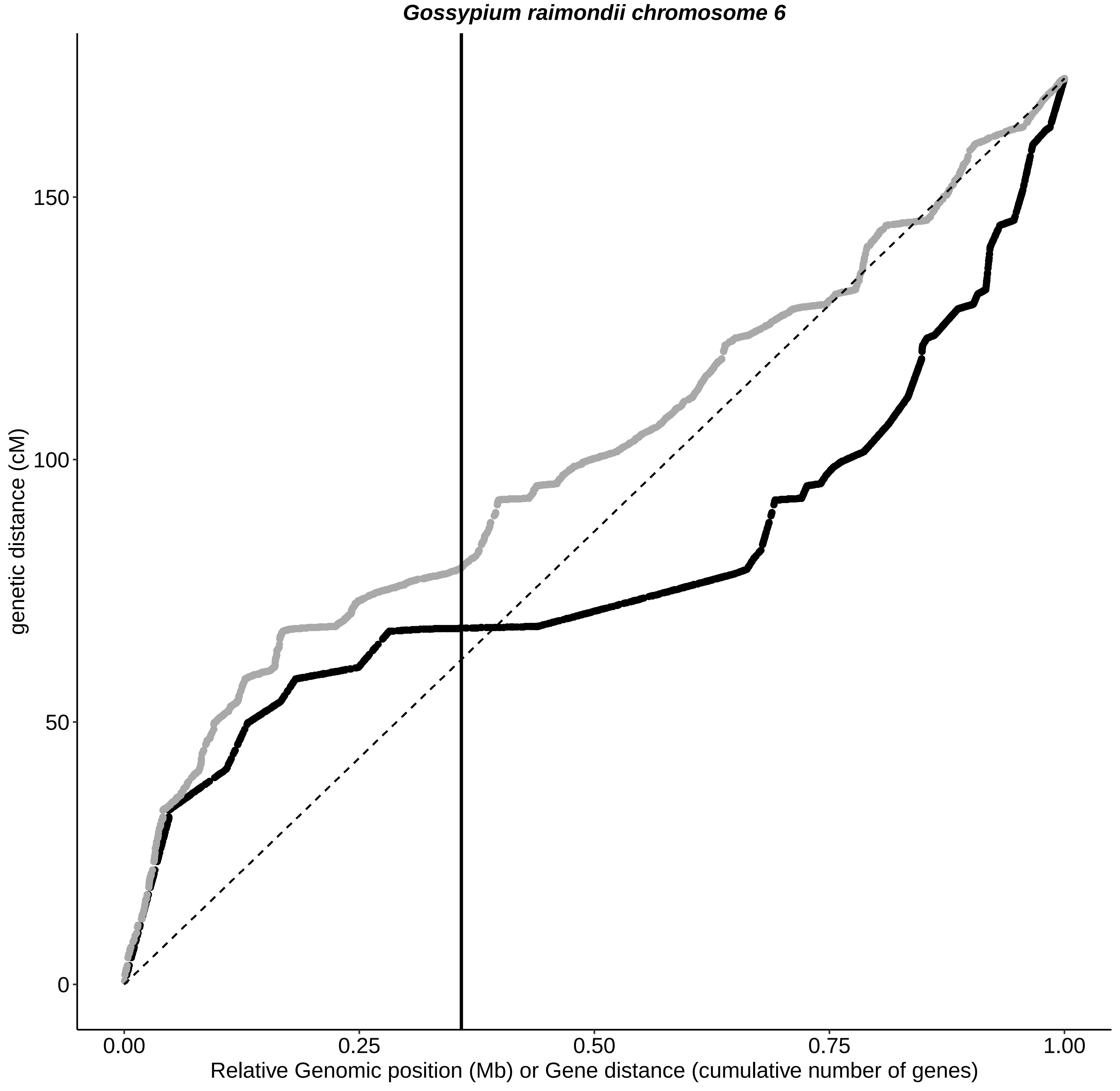

Gossypium raimondii chromosome 6
genetic distance (cM)
0
50
100
150
0.00
0.25
0.50
0.75
1.00
Relative Genomic position (Mb) or Gene distance (cumulative number of genes)

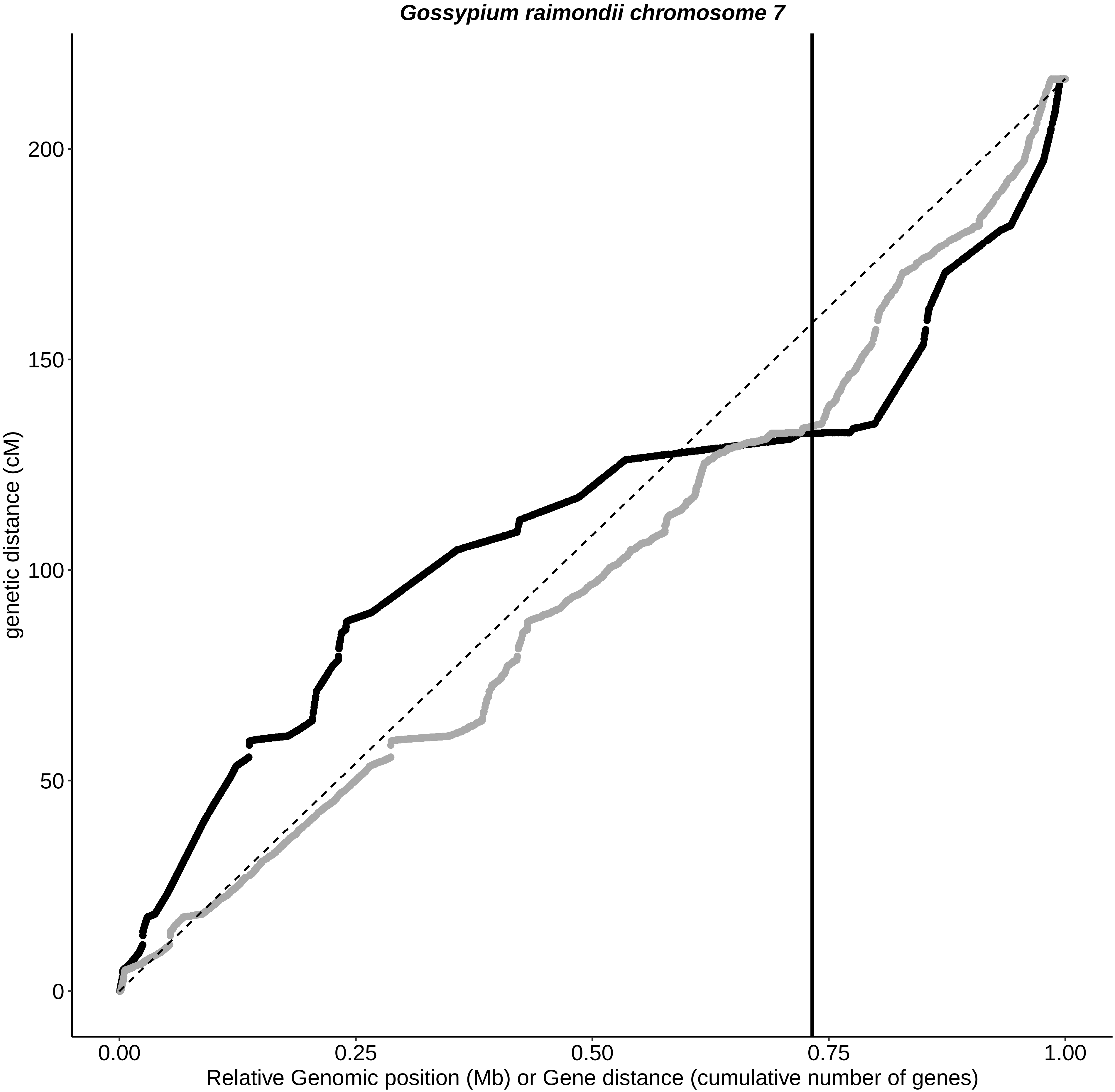
Gossypium raimondii chromosome 7
genetic distance (cM)
0
50
100
150
200
0.00
0.25
0.50
0.75
1.00
Relative Genomic position (Mb) or Gene distance (cumulative number of genes)

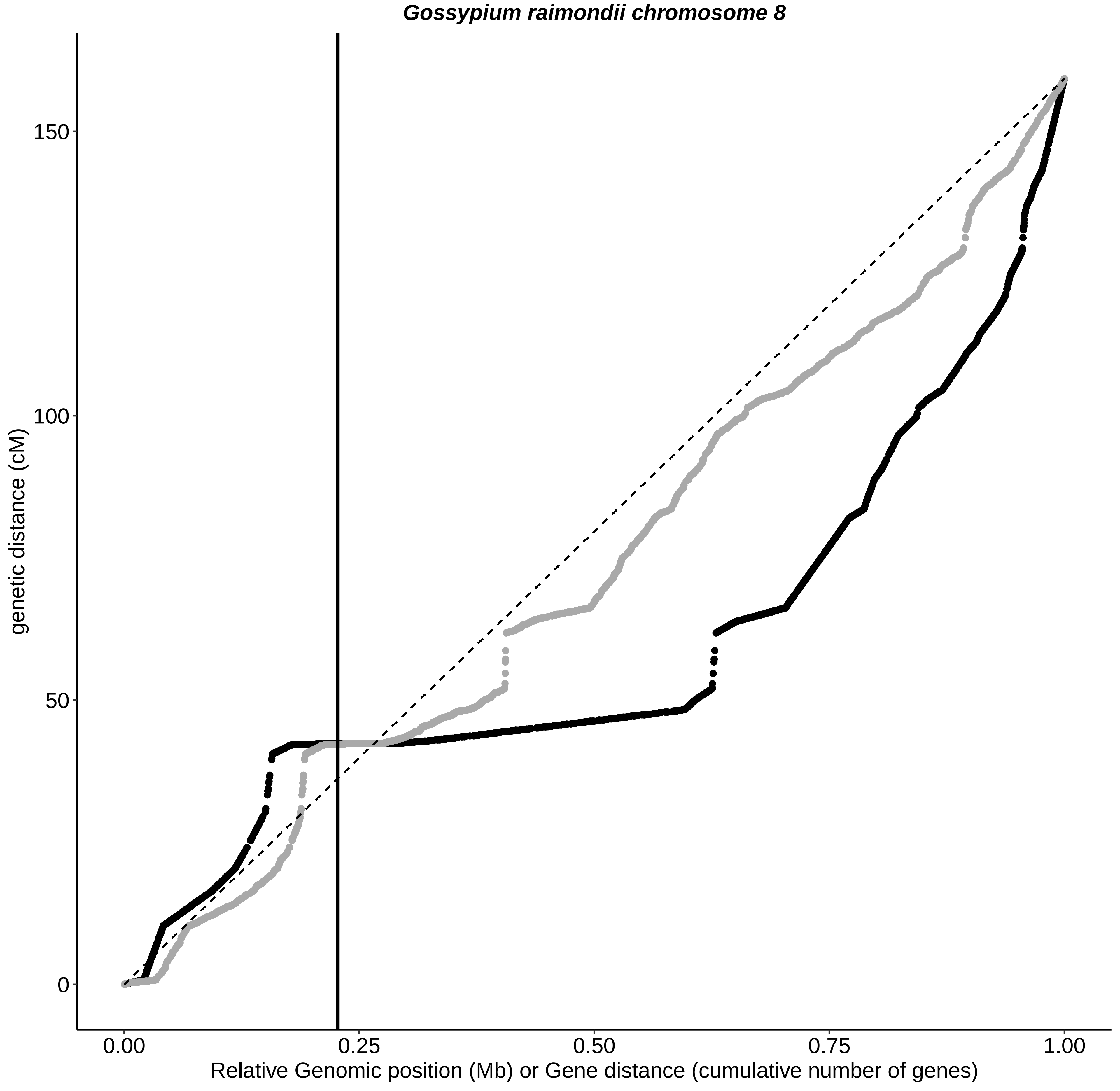
Gossypium raimondii chromosome 8
150
100
50
0
genetic distance (cM)
0.00
0.25
0.50
0.75
1.00
Relative Genomic position (Mb) or Gene distance (cumulative number of genes)

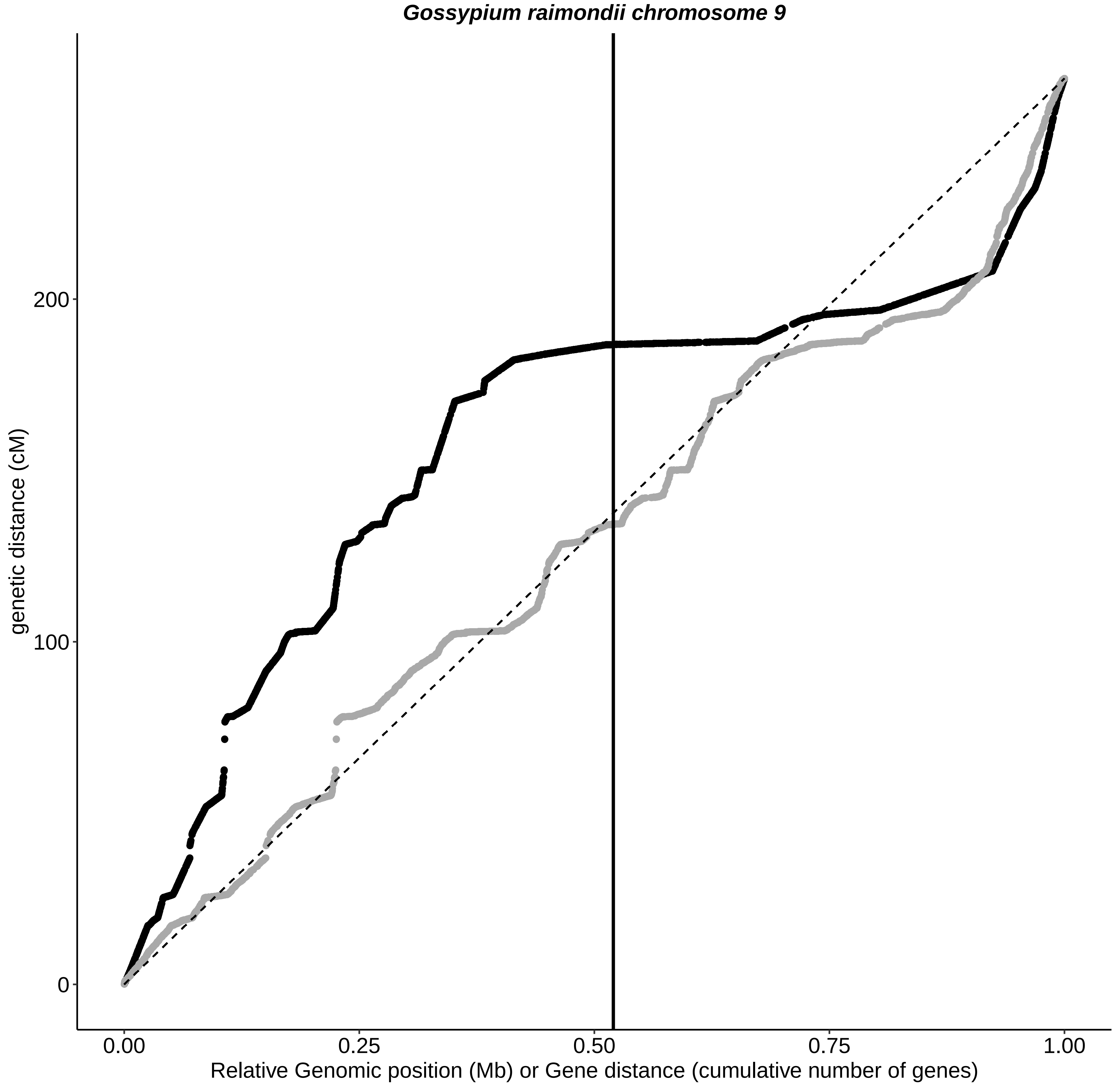
Gossypium raimondii chromosome 9
genetic distance (cM)
200
100
0
0.00
0.25
0.50
0.75
1.00
Relative Genomic position (Mb) or Gene distance (cumulative number of genes)

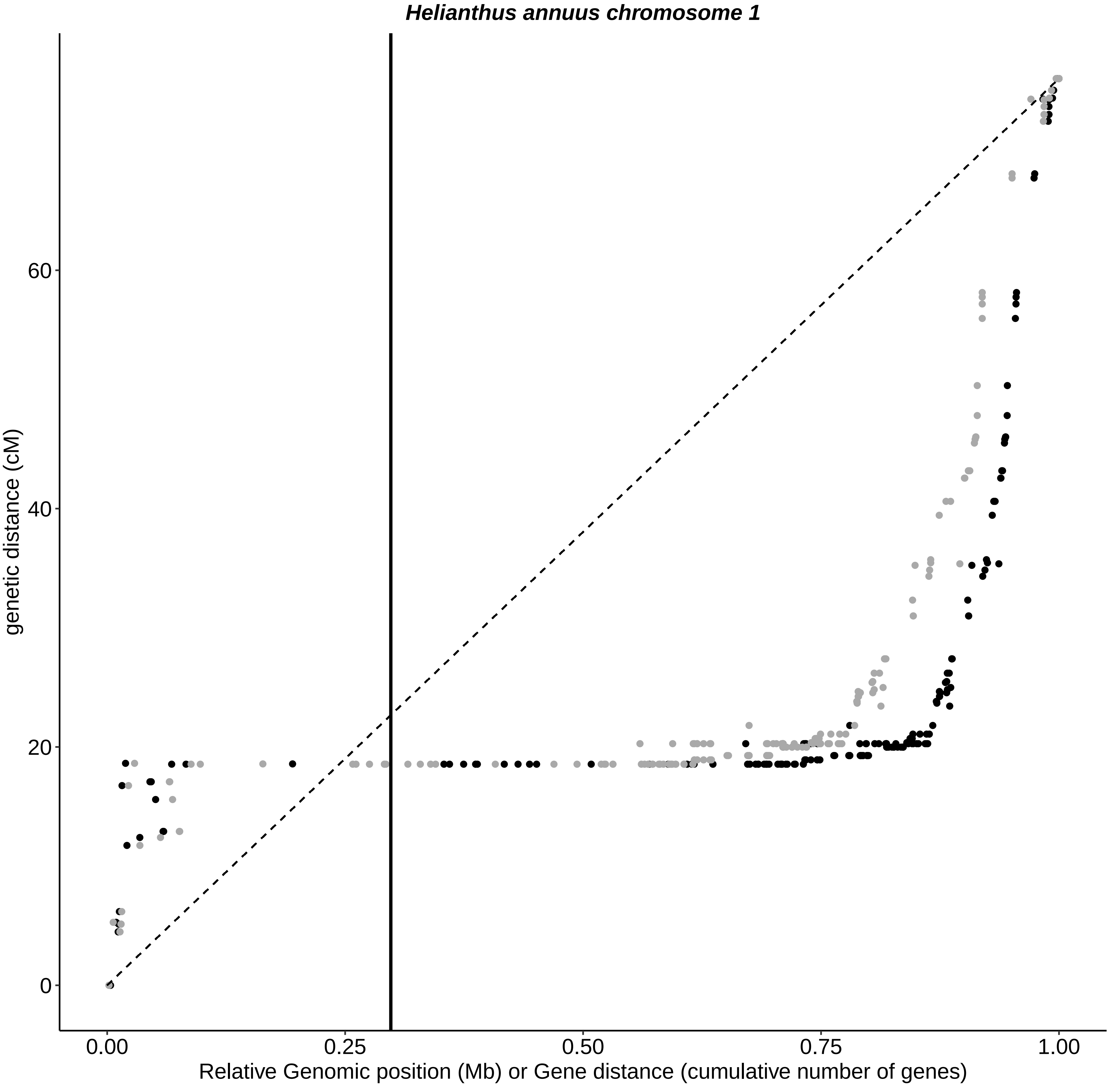

Helianthus annuus chromosome 1
genetic distance (cM)
0
20
40
60
0.00
0.25
0.50
0.75
1.00
Relative Genomic position (Mb) or Gene distance (cumulative number of genes)

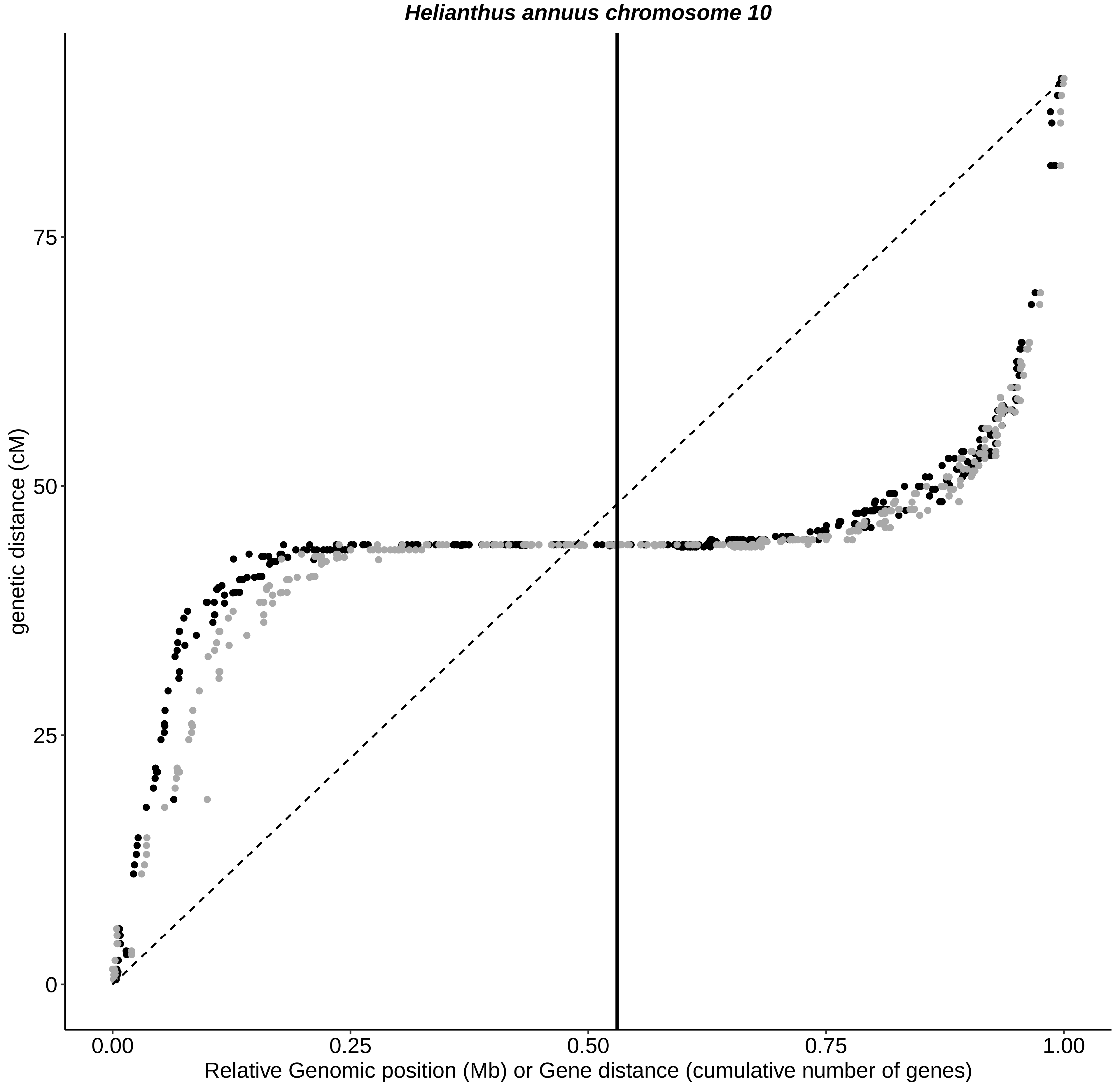

Helianthus annuus chromosome 10
genetic distance (cM)
0
25
50
75
0.00
0.25
0.50
0.75
1.00
Relative Genomic position (Mb) or Gene distance (cumulative number of genes)

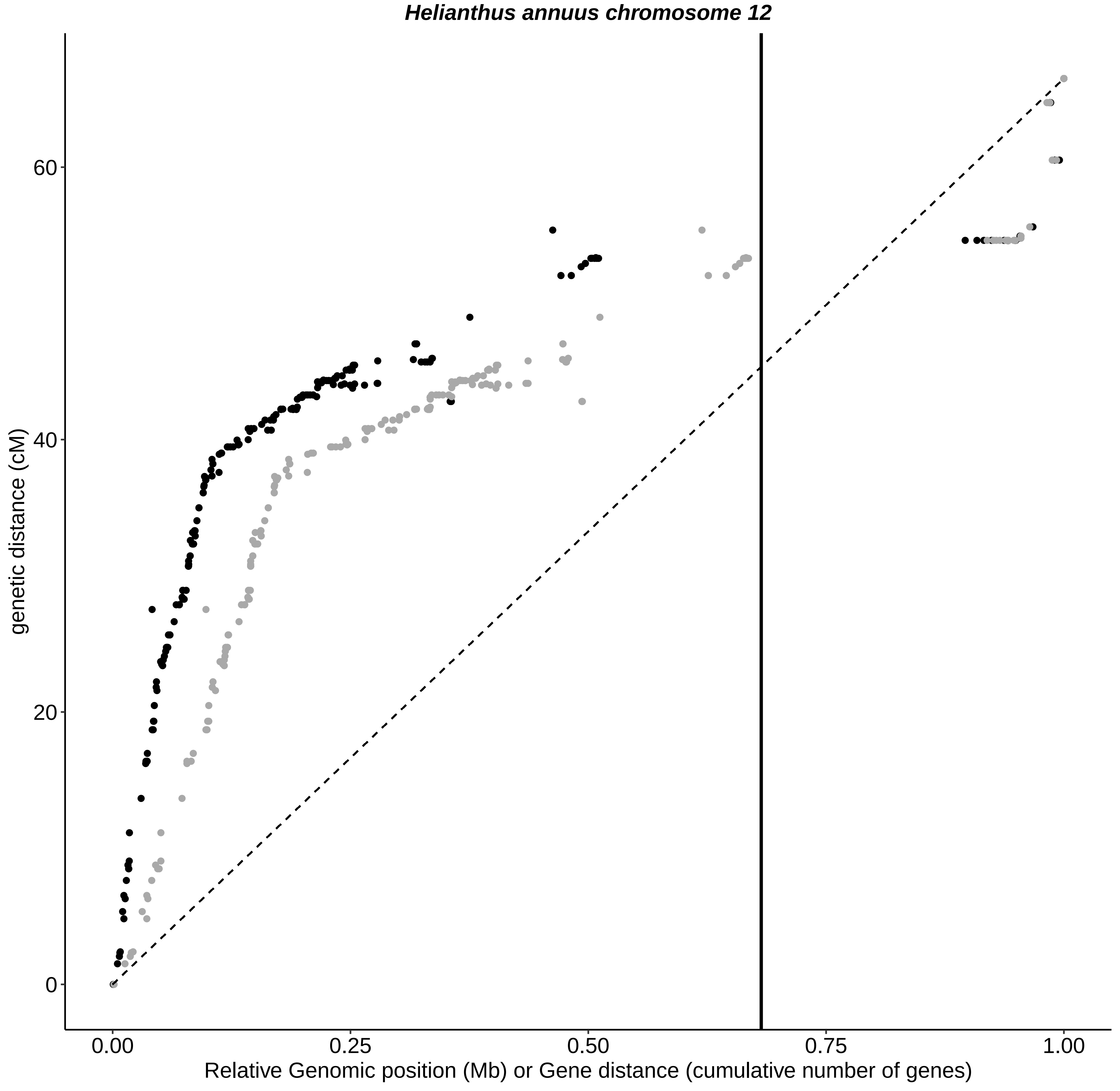
Helianthus annuus chromosome 12
genetic distance (cM)
0
20
40
60
0.00
0.25
0.50
0.75
1.00
Relative Genomic position (Mb) or Gene distance (cumulative number of genes)

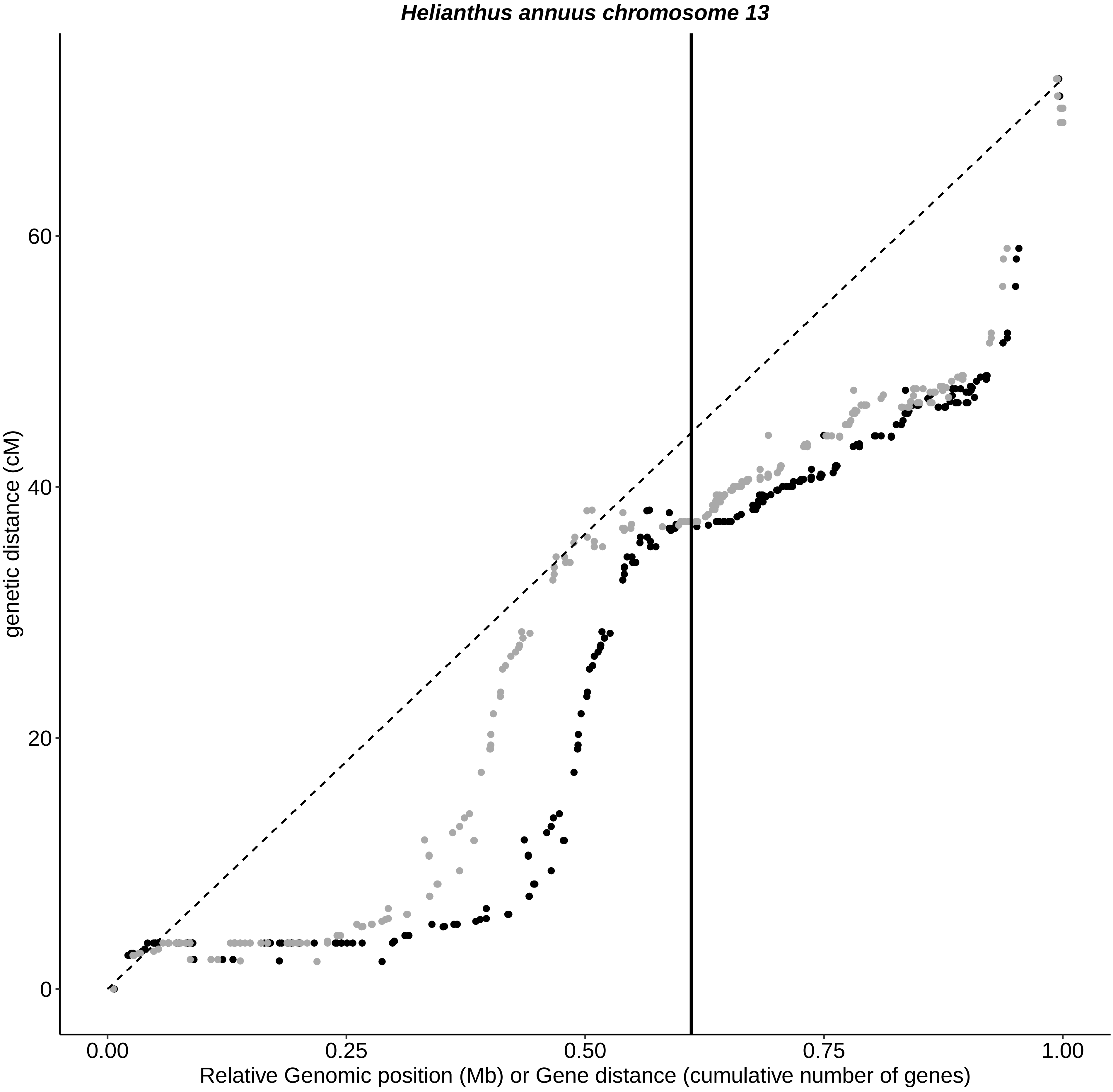
Helianthus annuus chromosome 13
genetic distance (cM)
0
20
40
60
0.00
0.25
0.50
0.75
1.00
Relative Genomic position (Mb) or Gene distance (cumulative number of genes)

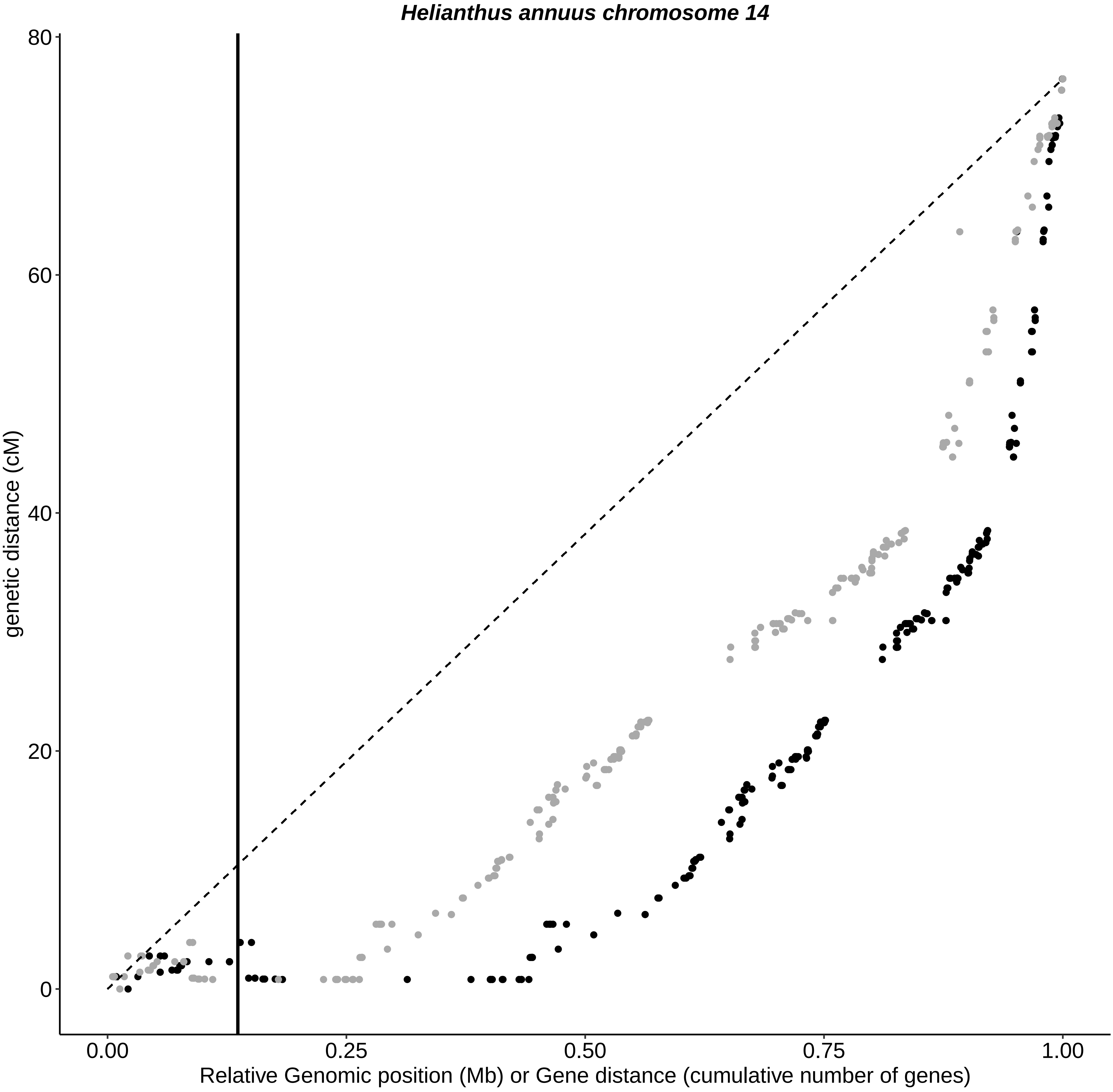
Helianthus annuus chromosome 14
genetic distance (cM)
0
20
40
60
80
0.00
0.25
0.50
0.75
1.00
Relative Genomic position (Mb) or Gene distance (cumulative number of genes)

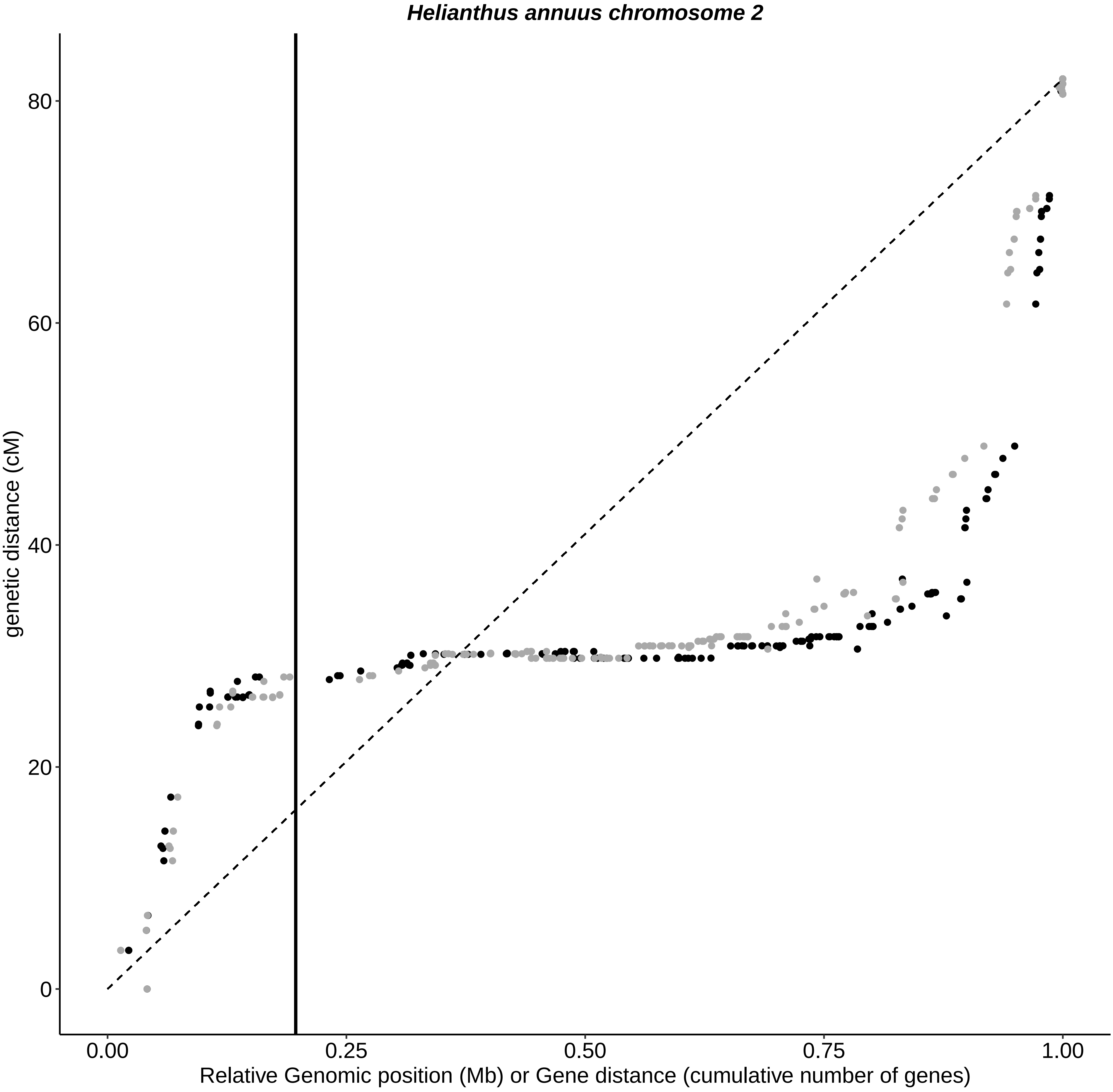
Helianthus annuus chromosome 2
genetic distance (cM)
0
20
40
60
80
0.00
0.25
0.50
0.75
1.00
Relative Genomic position (Mb) or Gene distance (cumulative number of genes)

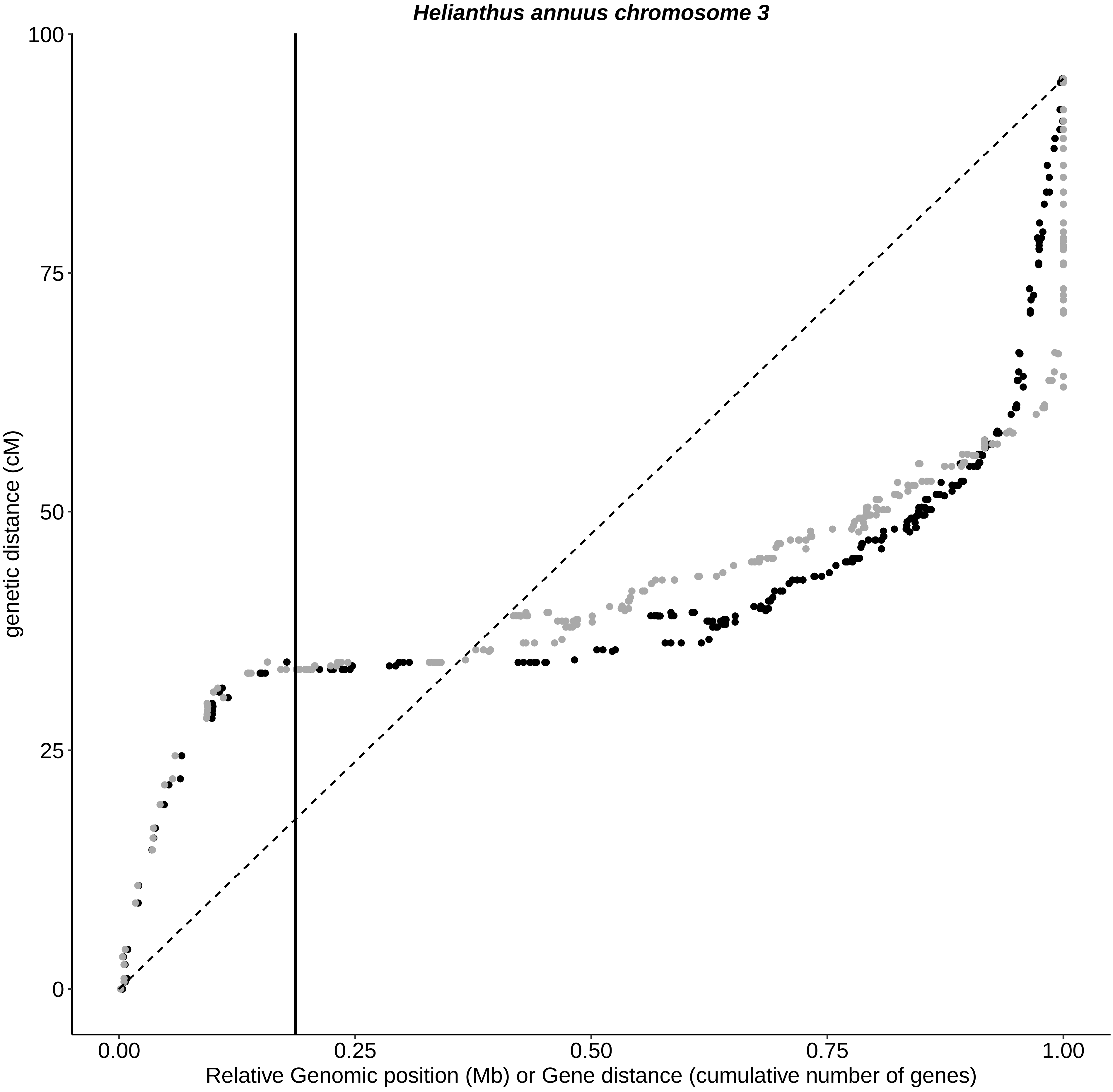
Helianthus annuus chromosome 3
100
75
50
25
0
genetic distance (cM)
0.00
0.25
0.50
0.75
1.00
Relative Genomic position (Mb) or Gene distance (cumulative number of genes)

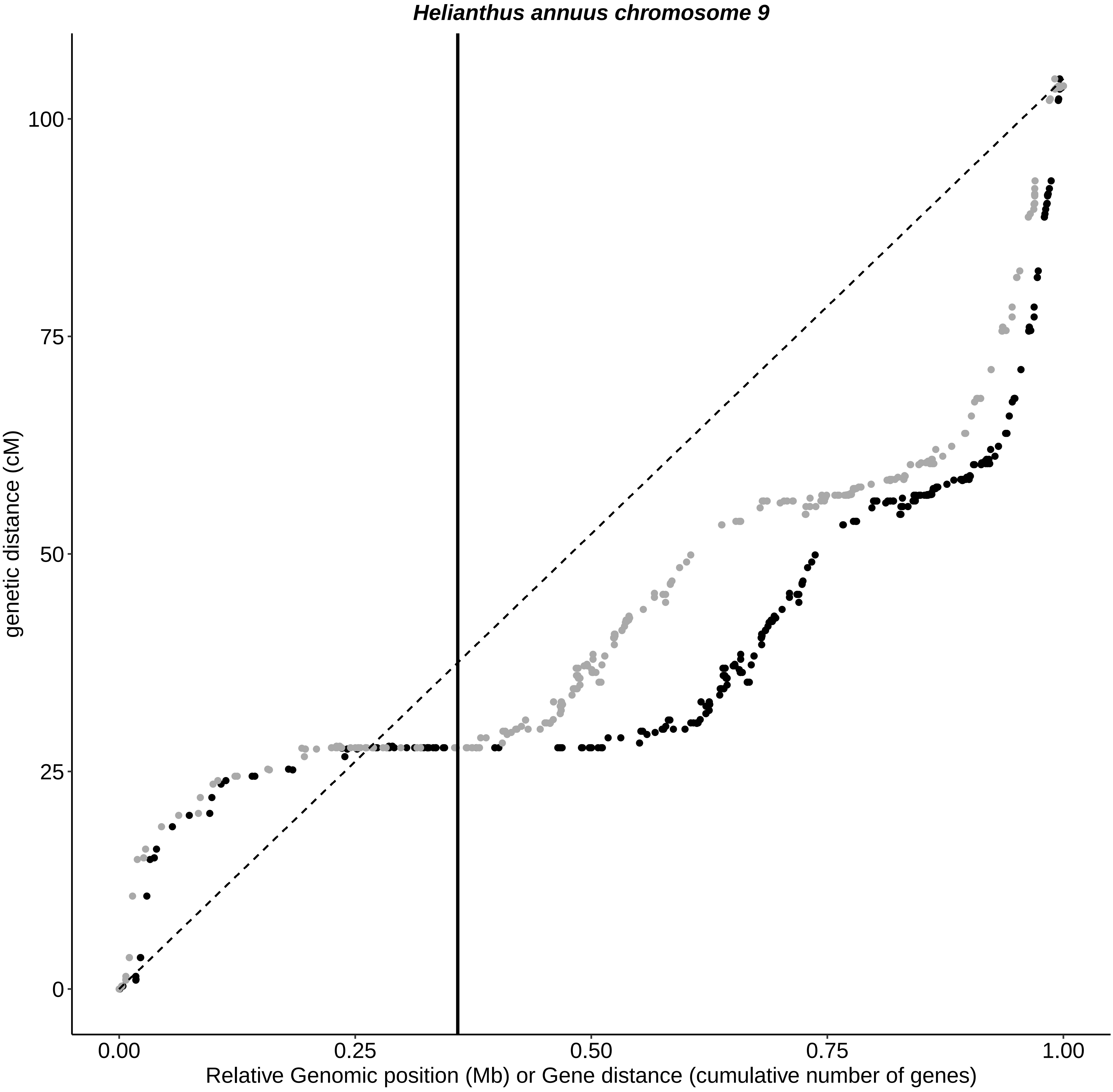
Helianthus annuus chromosome 9
genetic distance (cM)
0
25
50
75
100
0.00
0.25
0.50
0.75
1.00
Relative Genomic position (Mb) or Gene distance (cumulative number of genes)

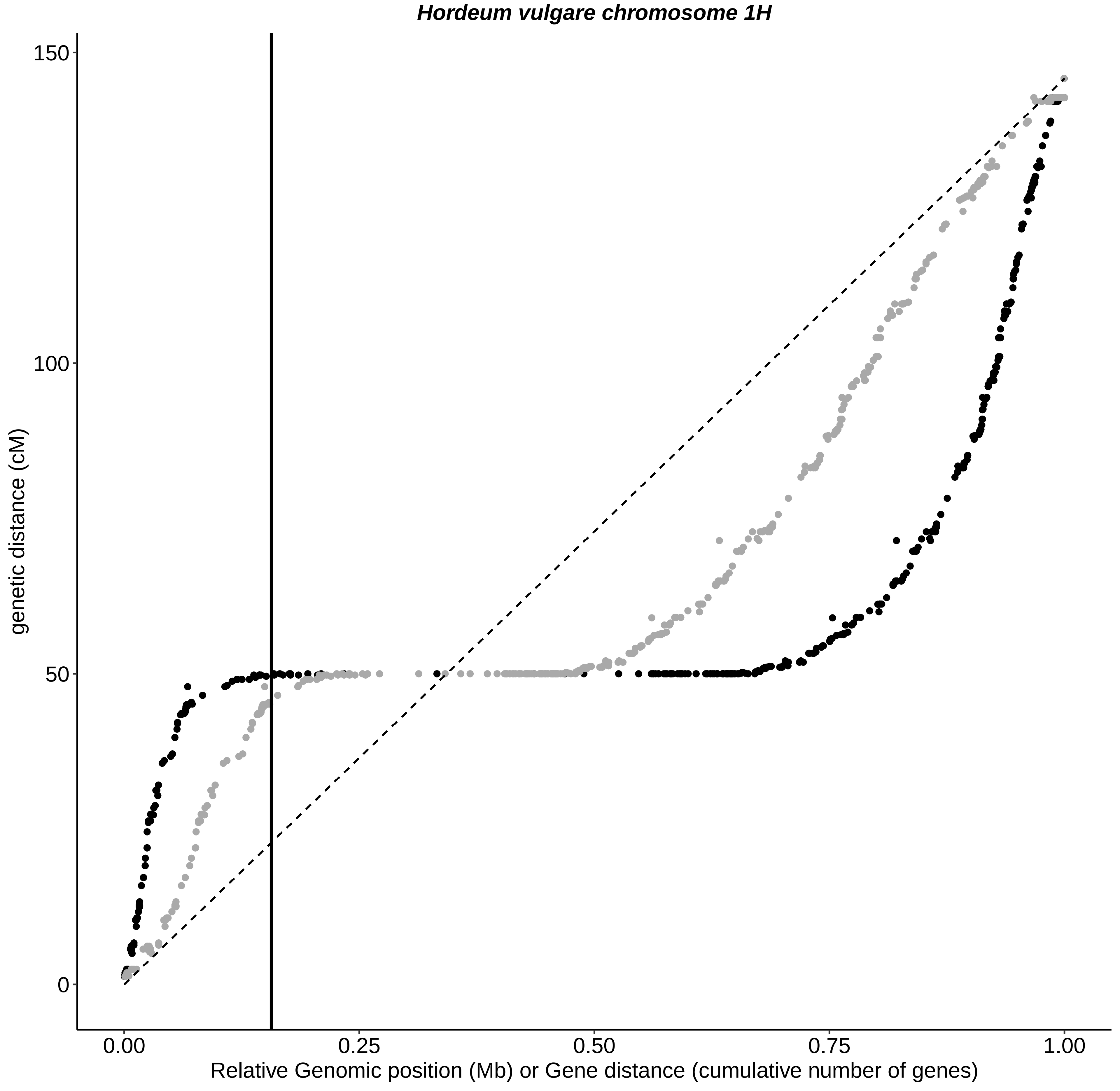
Hordeum vulgare chromosome 1H
genetic distance (cM)
150
100
50
0
0.00
0.25
0.50
0.75
1.00
Relative Genomic position (Mb) or Gene distance (cumulative number of genes)

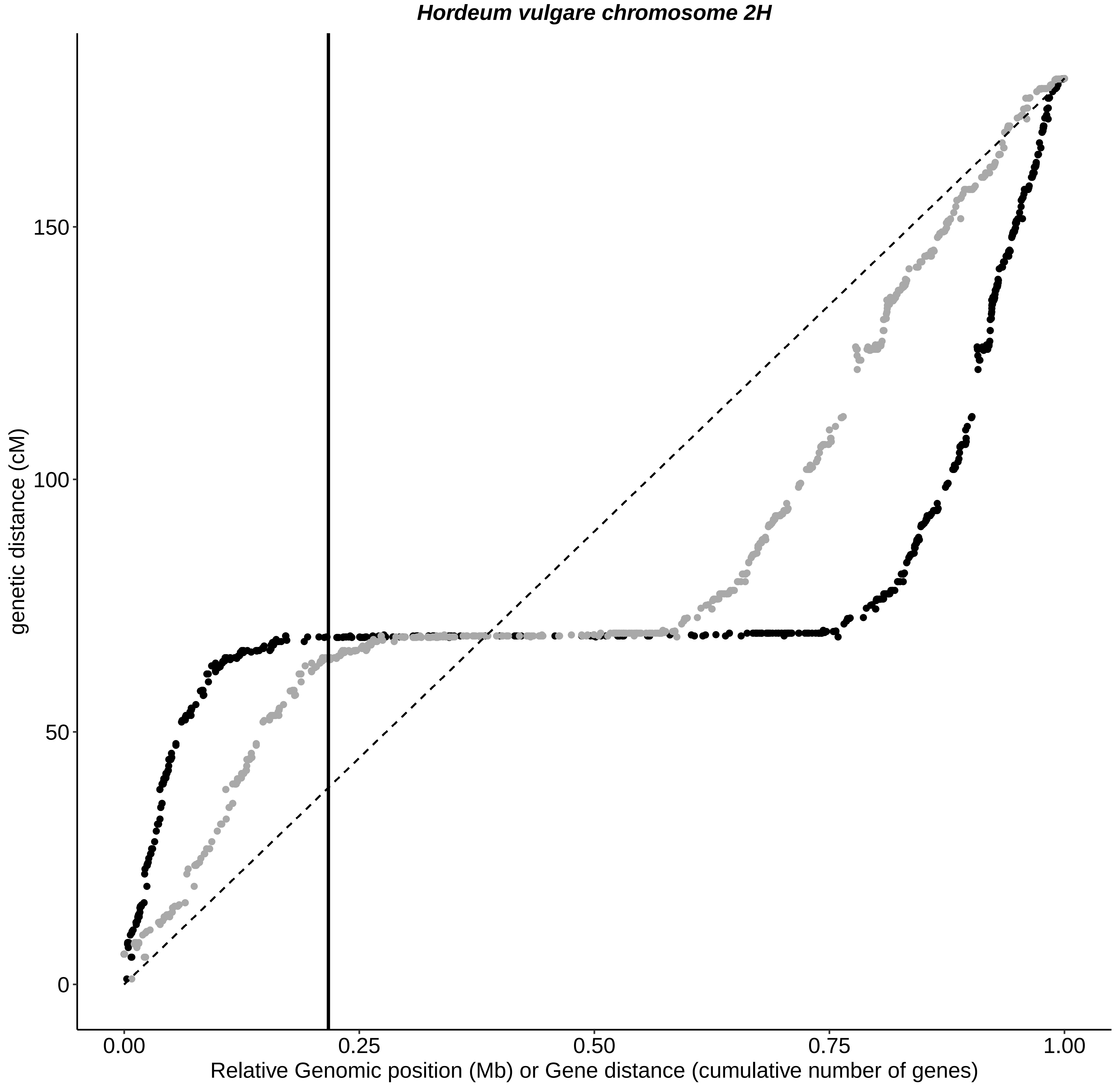
Hordeum vulgare chromosome 2H
genetic distance (cM)
0
50
100
150
0.00
0.25
0.50
0.75
1.00
Relative Genomic position (Mb) or Gene distance (cumulative number of genes)

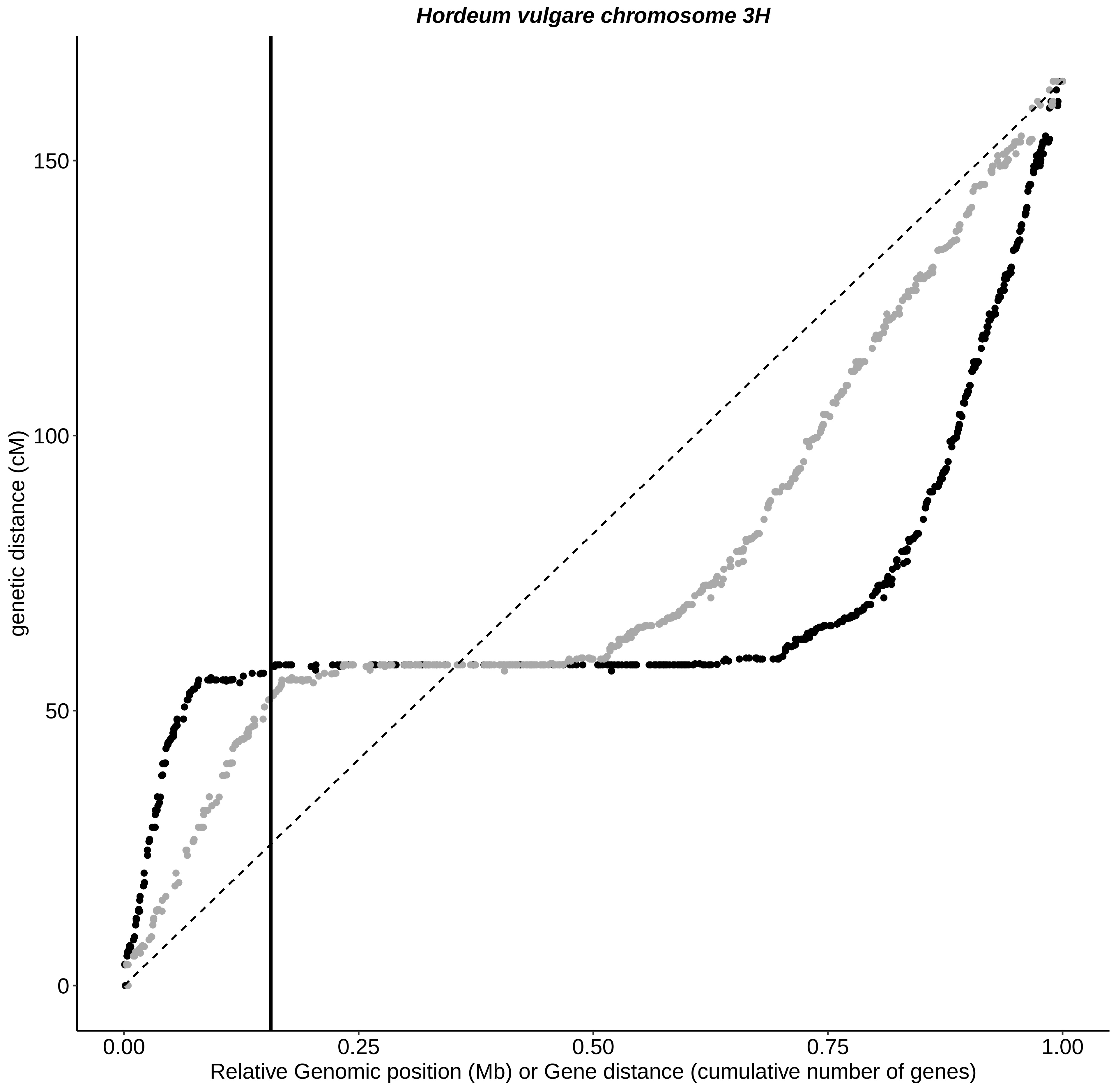
Hordeum vulgare chromosome 3H
genetic distance (cM)
150
100
50
0
0.00
0.25
0.50
0.75
1.00
Relative Genomic position (Mb) or Gene distance (cumulative number of genes)

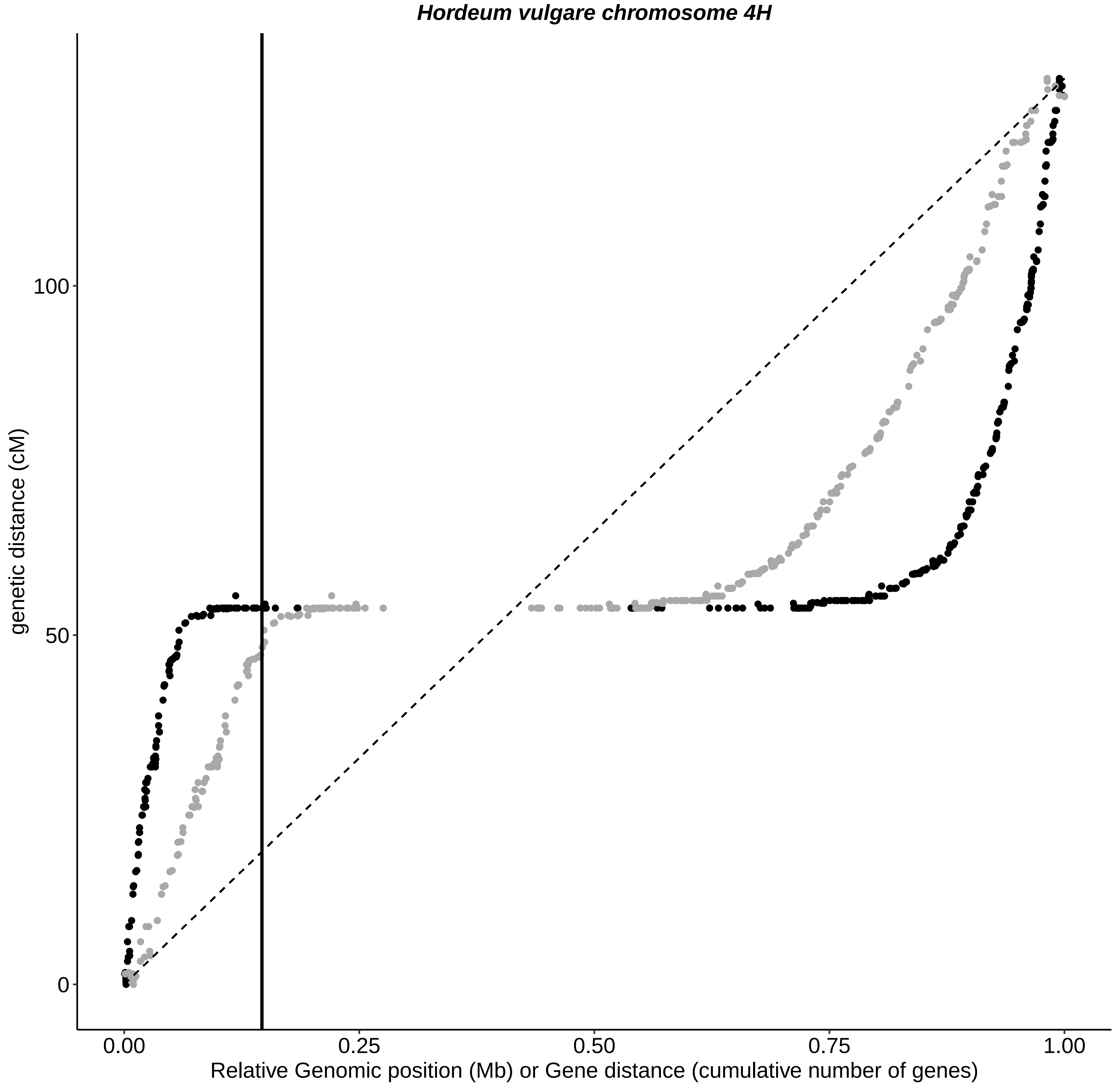
Hordeum vulgare chromosome 4H
genetic distance (cM)
0
50
100
0.00
0.25
0.50
0.75
1.00
Relative Genomic position (Mb) or Gene distance (cumulative number of genes)

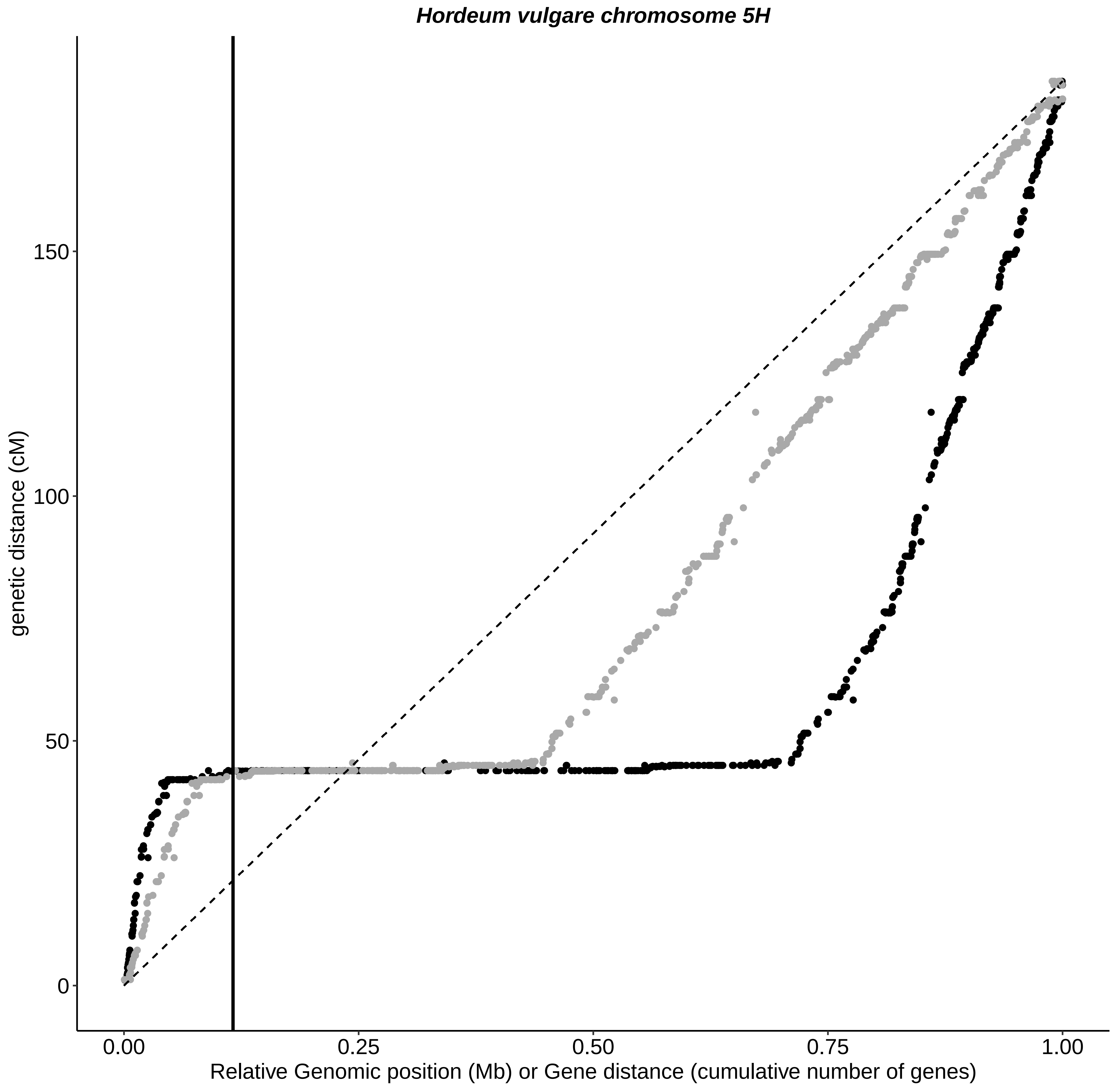
Hordeum vulgare chromosome 5H
genetic distance (cM)
150
100
50
0
0.00
0.25
0.50
0.75
1.00
Relative Genomic position (Mb) or Gene distance (cumulative number of genes)

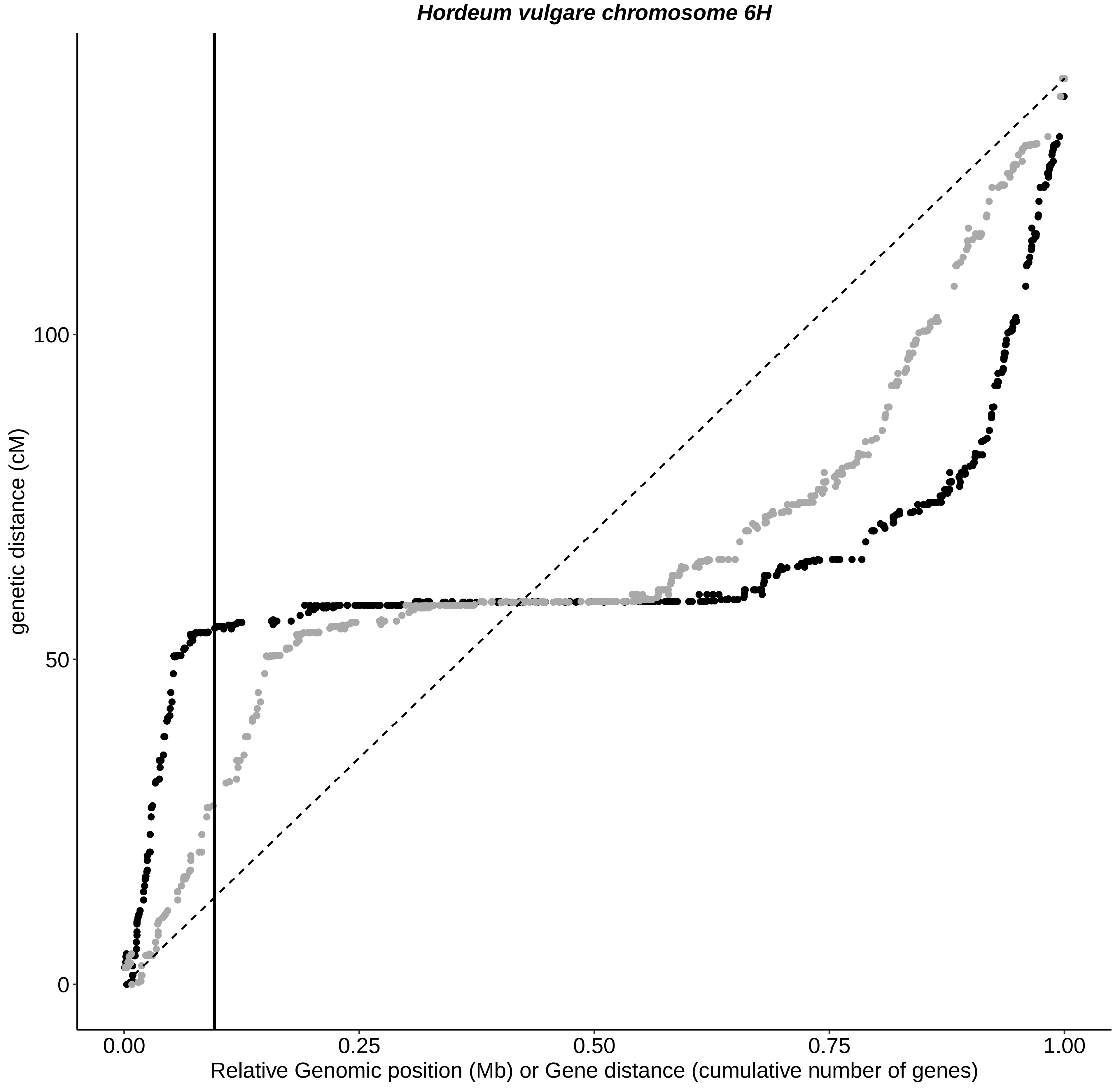
Hordeum vulgare chromosome 6H
genetic distance (cM)
0
50
100
0.00
0.25
0.50
0.75
1.00
Relative Genomic position (Mb) or Gene distance (cumulative number of genes)

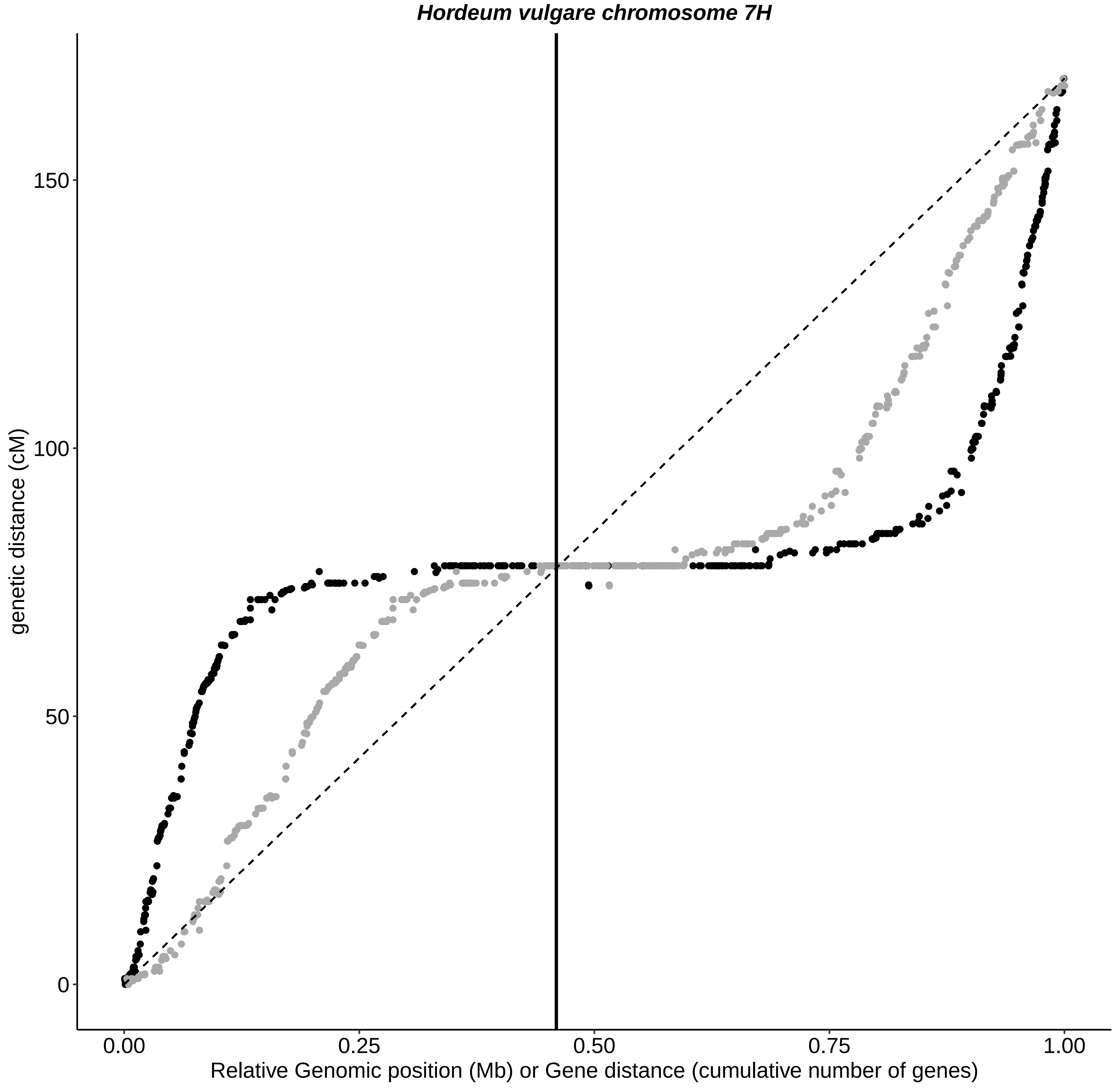
Hordeum vulgare chromosome 7H
genetic distance (cM)
0
50
100
150
0.00
0.25
0.50
0.75
1.00
Relative Genomic position (Mb) or Gene distance (cumulative number of genes)

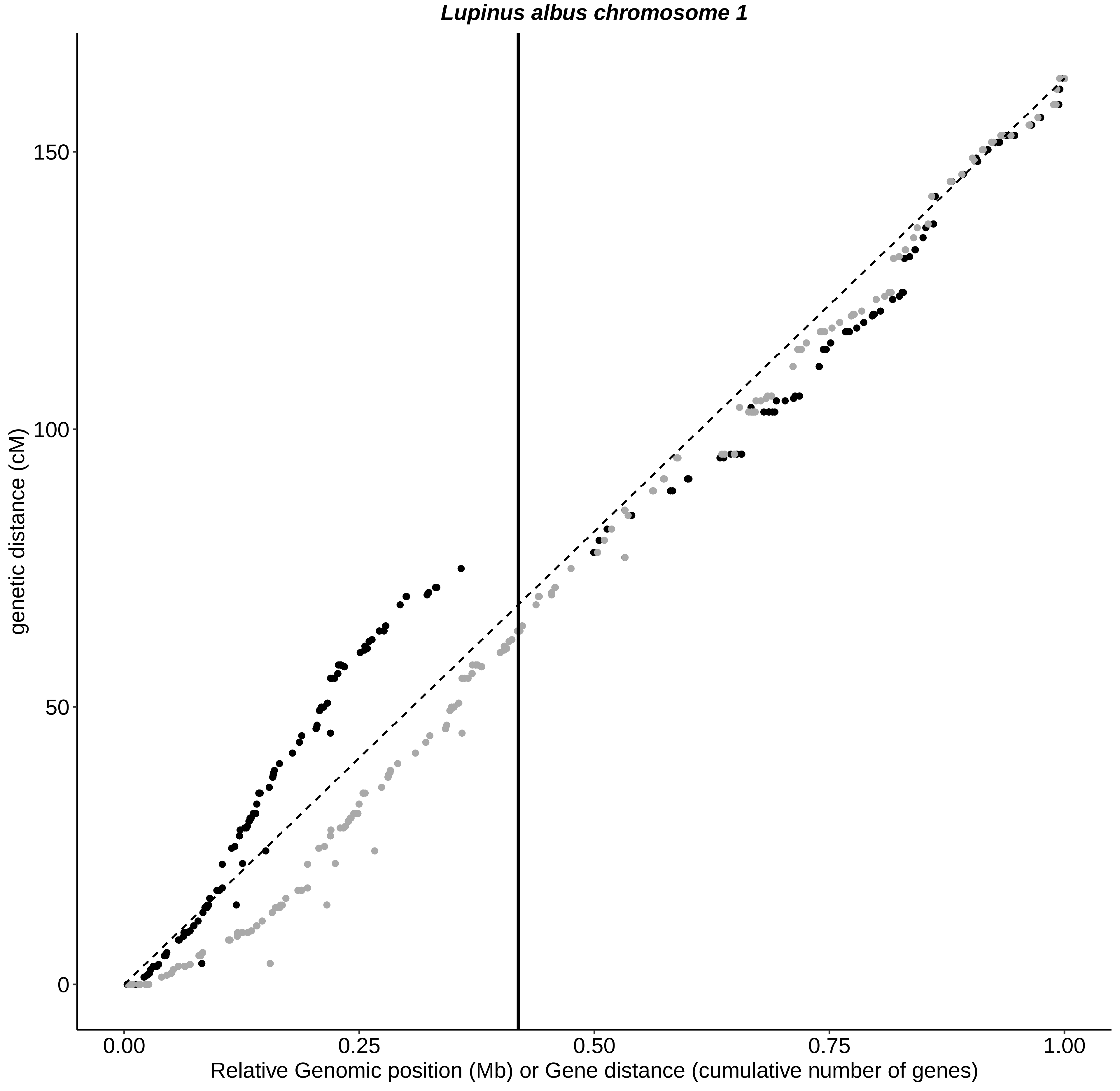
Lupinus albus chromosome 1
genetic distance (cM)
0
50
100
150
0.00
0.25
0.50
0.75
1.00
Relative Genomic position (Mb) or Gene distance (cumulative number of genes)

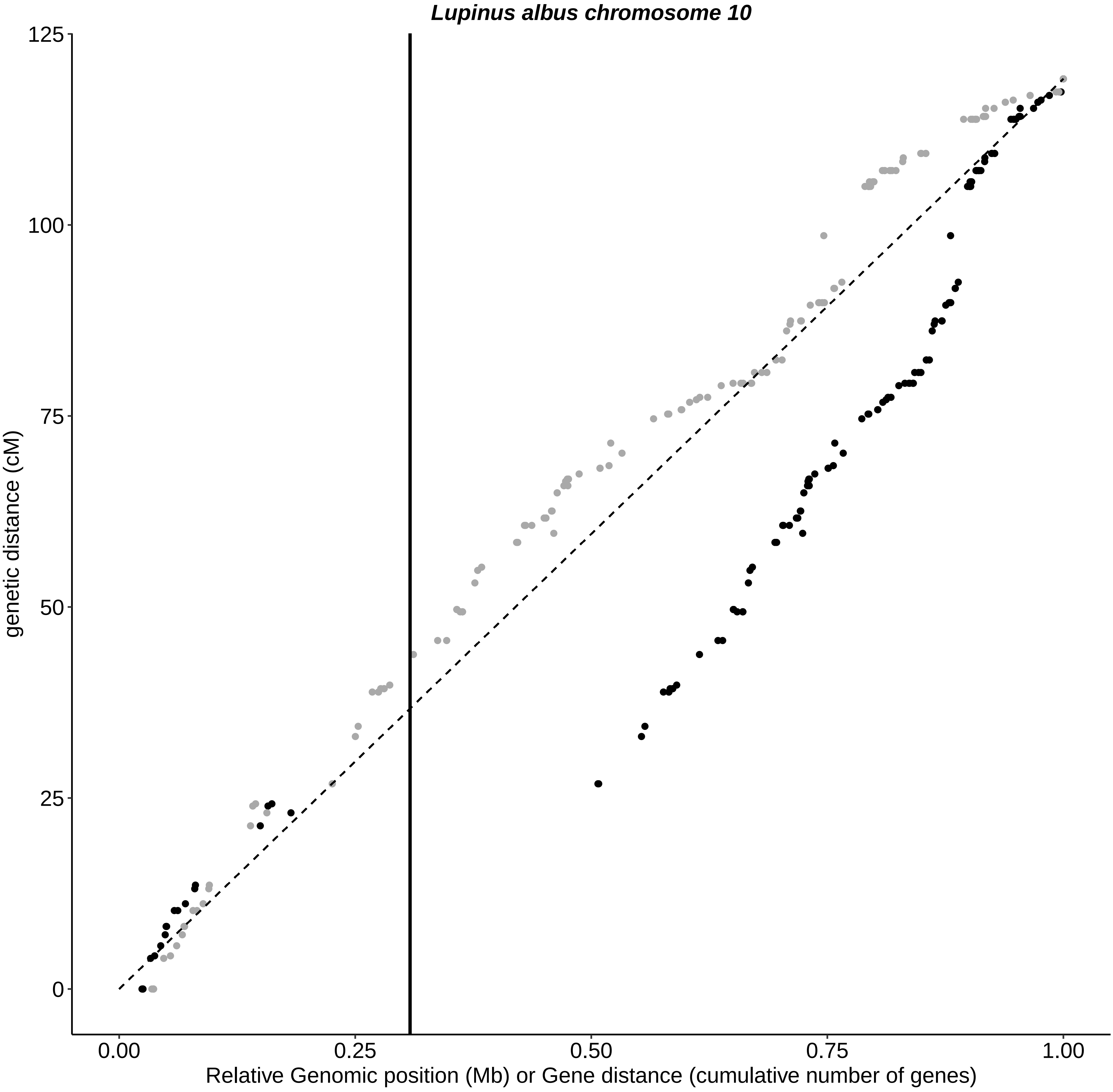
Lupinus albus chromosome 10
125
100
75
50
25
0
genetic distance (cM)
0.00
0.25
0.50
0.75
1.00
Relative Genomic position (Mb) or Gene distance (cumulative number of genes)

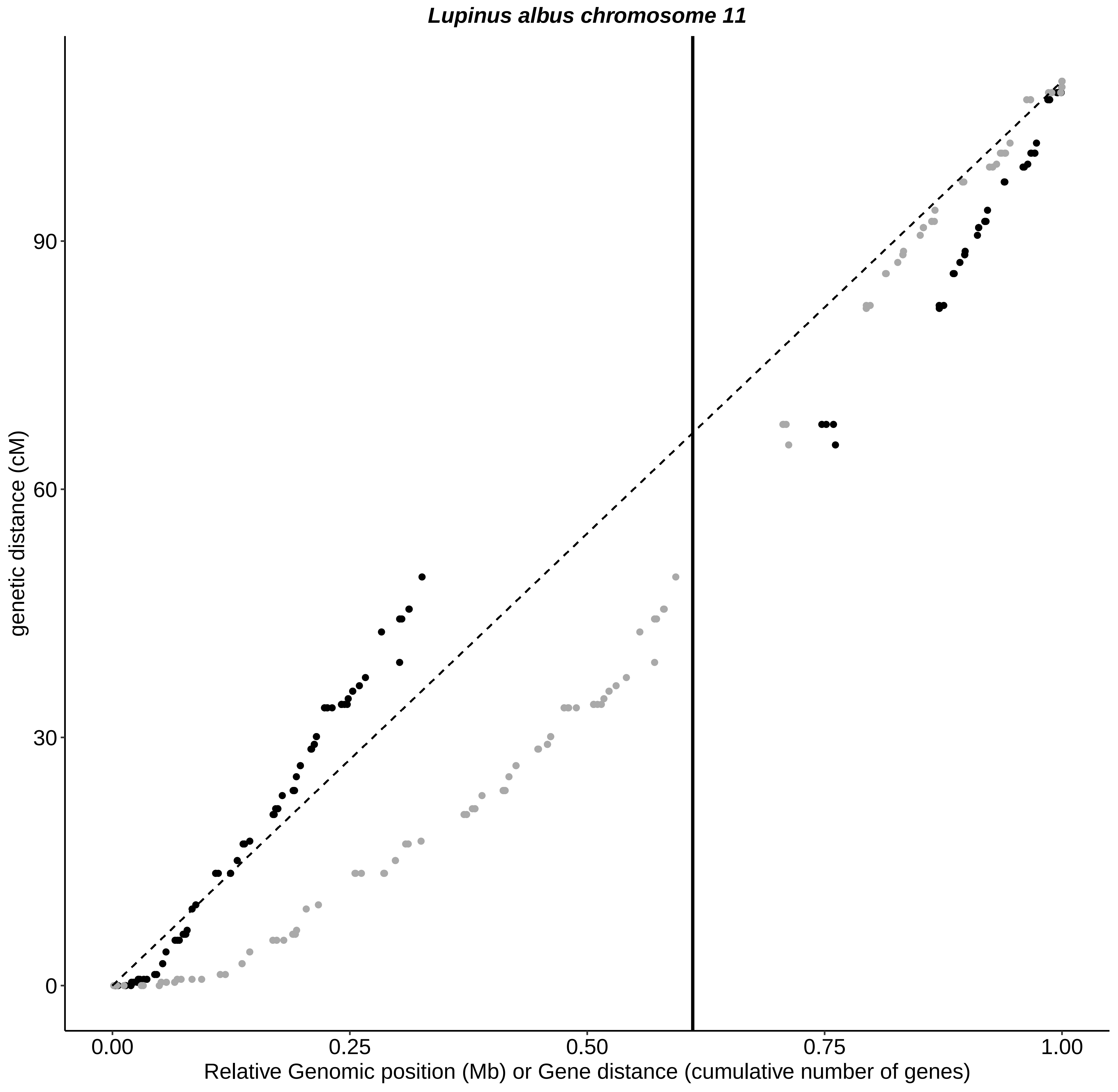
Lupinus albus chromosome 11
genetic distance (cM)
90
60
30
0
0.00
0.25
0.50
0.75
1.00
Relative Genomic position (Mb) or Gene distance (cumulative number of genes)

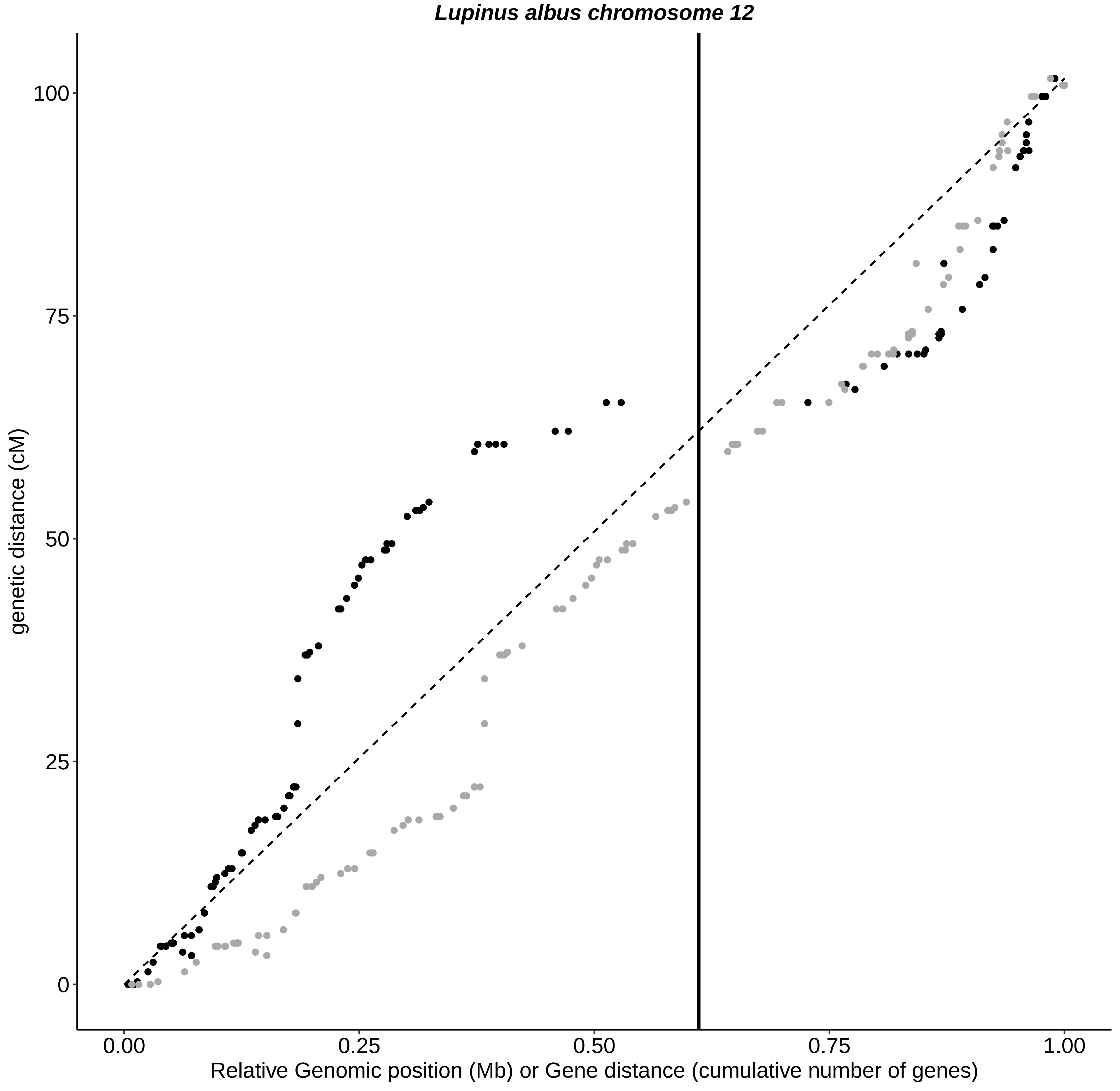
Lupinus albus chromosome 12
genetic distance (cM)
0
25
50
75
100
Relative Genomic position (Mb) or Gene distance (cumulative number of genes)
0.00
0.25
0.50
0.75
1.00

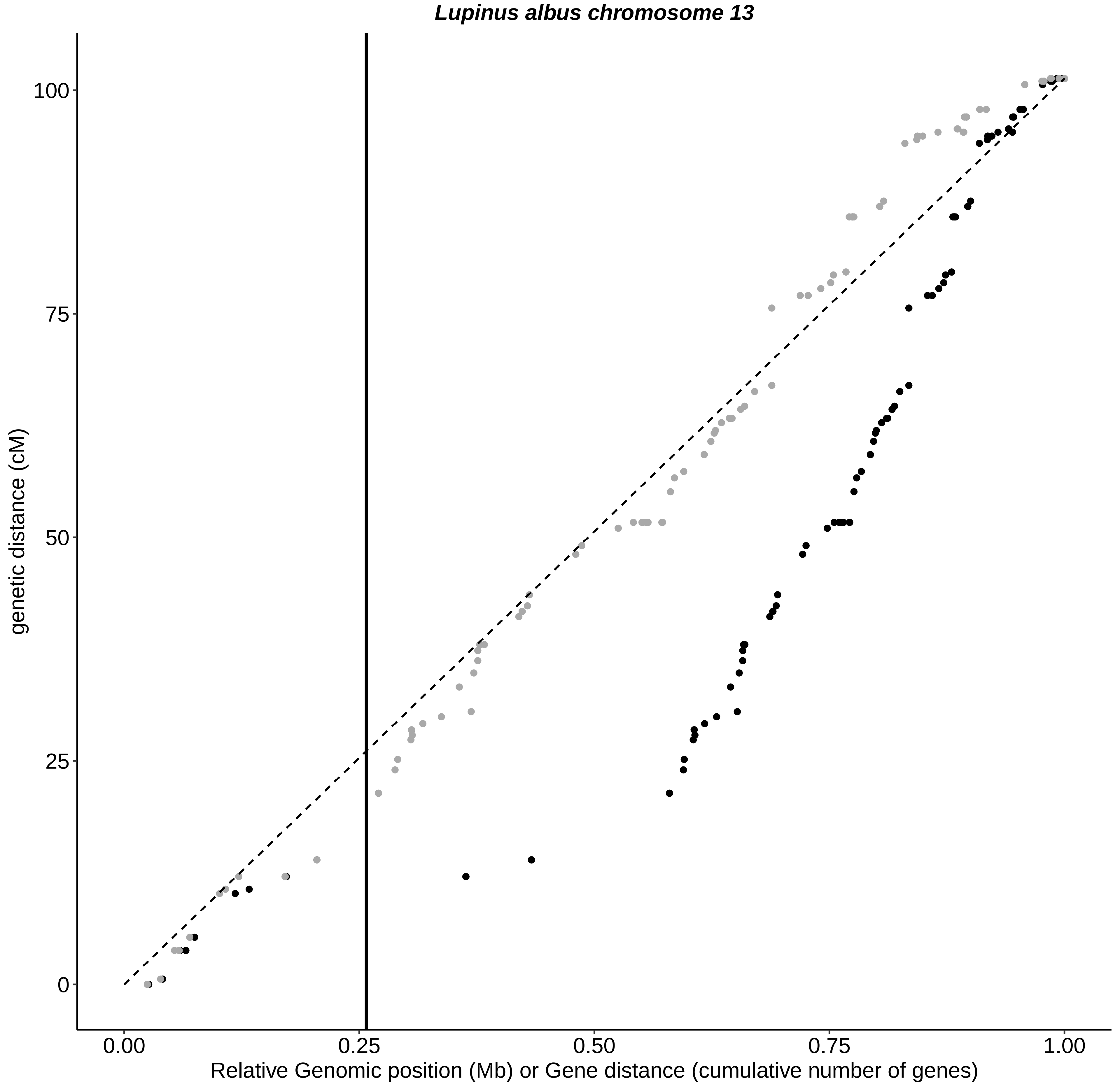
Lupinus albus chromosome 13
genetic distance (cM)
0
25
50
75
100
0.00
0.25
0.50
0.75
1.00
Relative Genomic position (Mb) or Gene distance (cumulative number of genes)

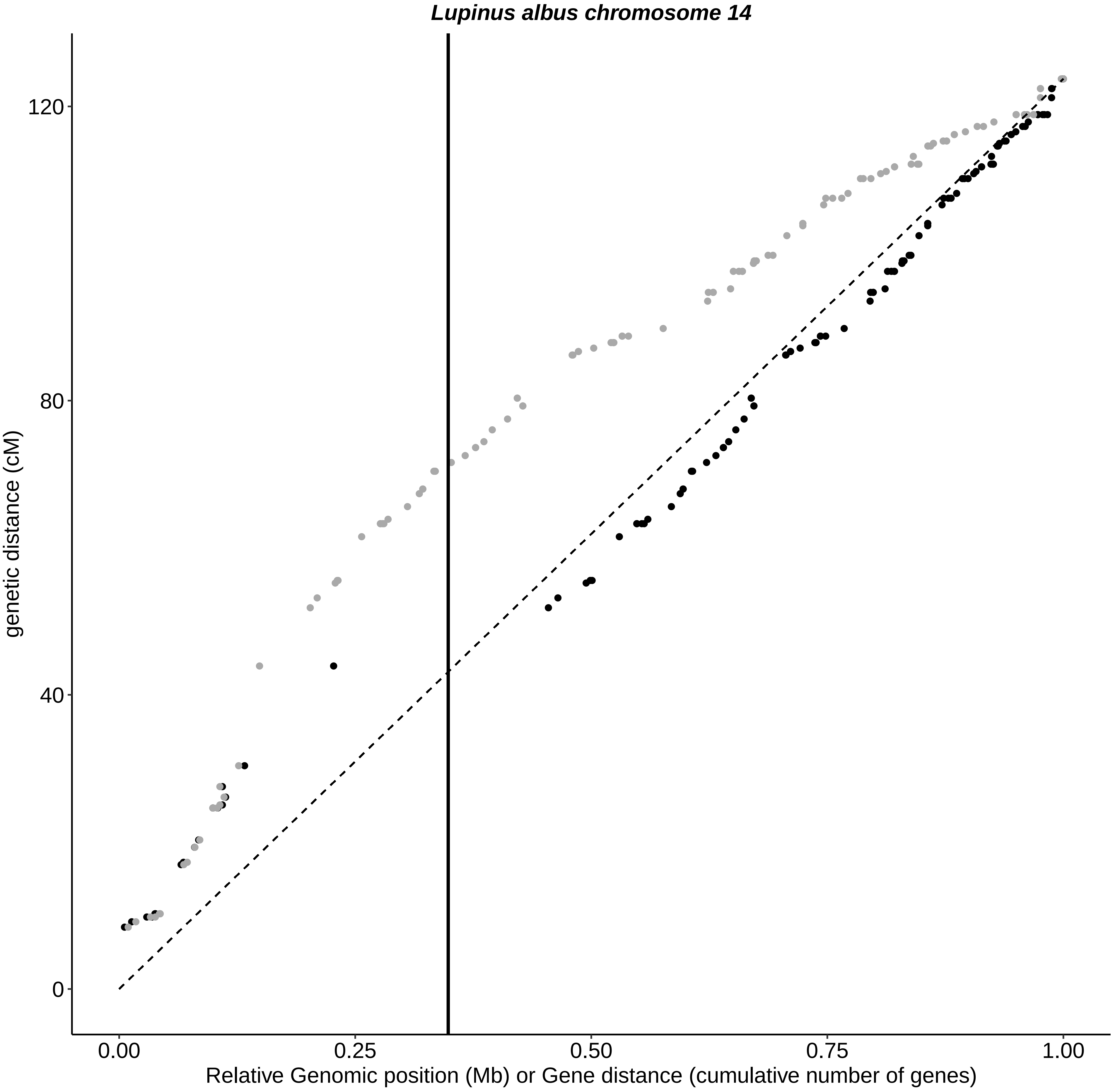
Lupinus albus chromosome 14
genetic distance (cM)
0
40
80
120
0.00
0.25
0.50
0.75
1.00
Relative Genomic position (Mb) or Gene distance (cumulative number of genes)

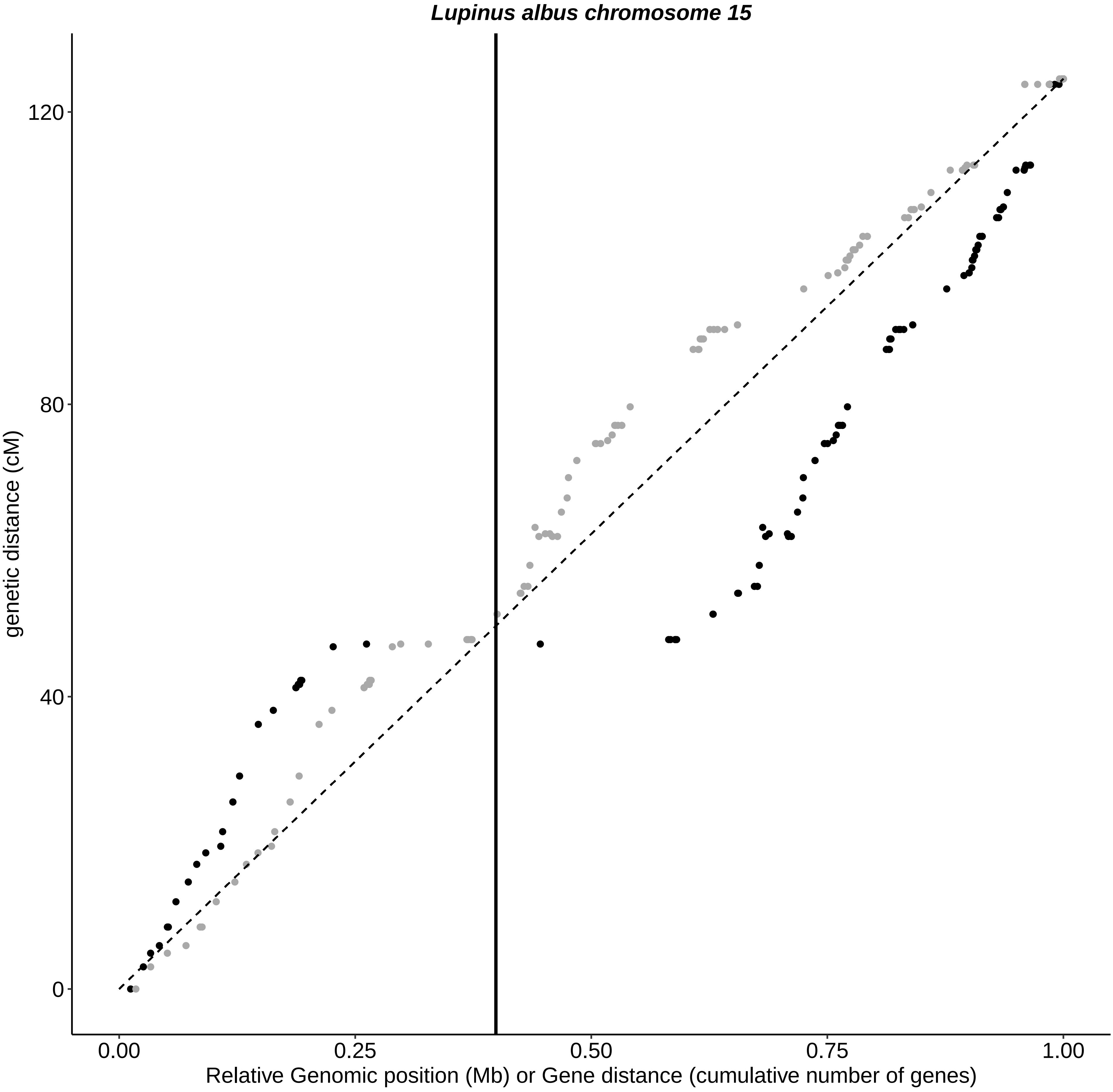
Lupinus albus chromosome 15
genetic distance (cM)
0
40
80
120
0.00
0.25
0.50
0.75
1.00
Relative Genomic position (Mb) or Gene distance (cumulative number of genes)

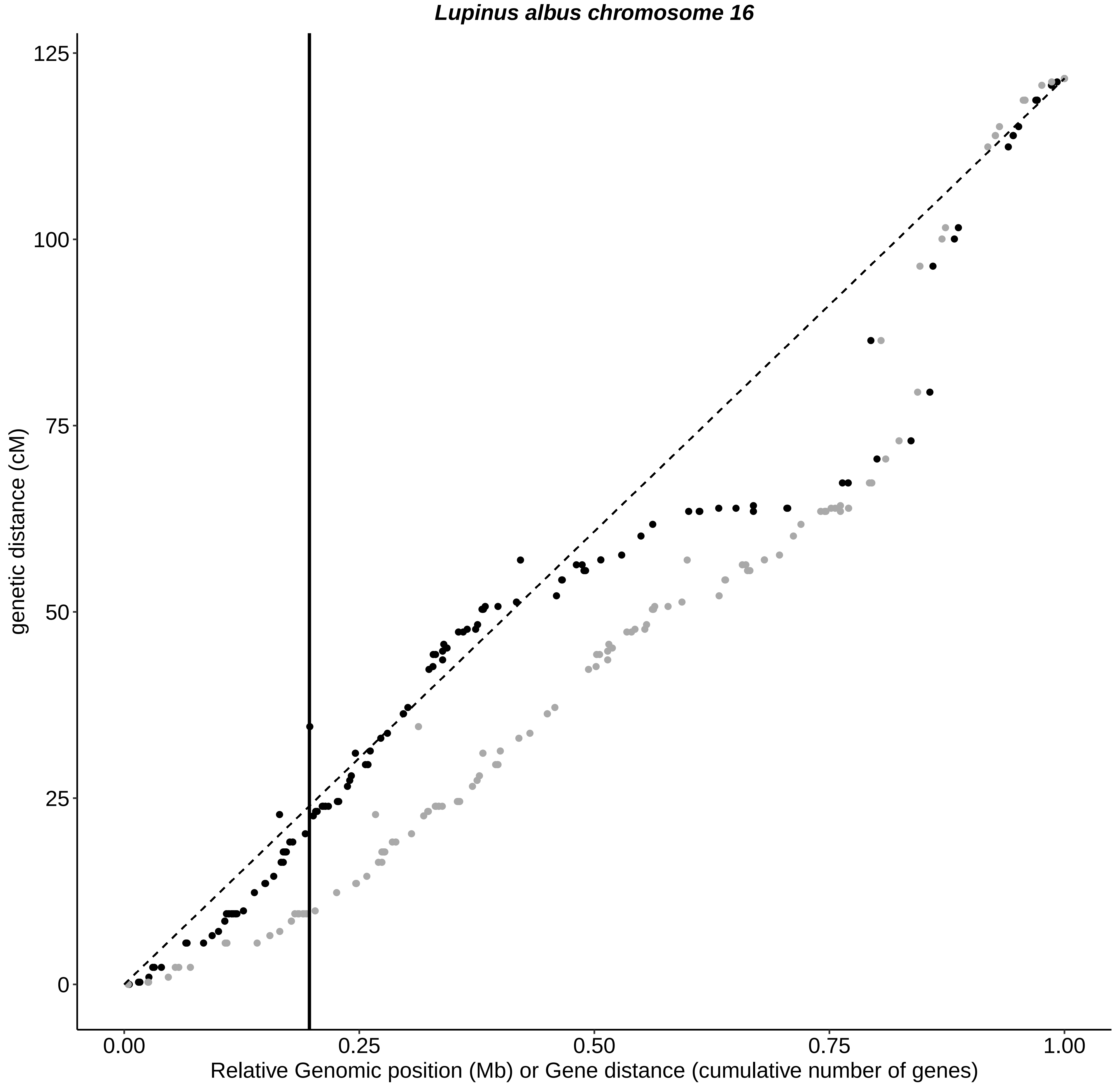
Lupinus albus chromosome 16
genetic distance (cM)
125
100
75
50
25
0
0.00
0.25
0.50
0.75
1.00
Relative Genomic position (Mb) or Gene distance (cumulative number of genes)

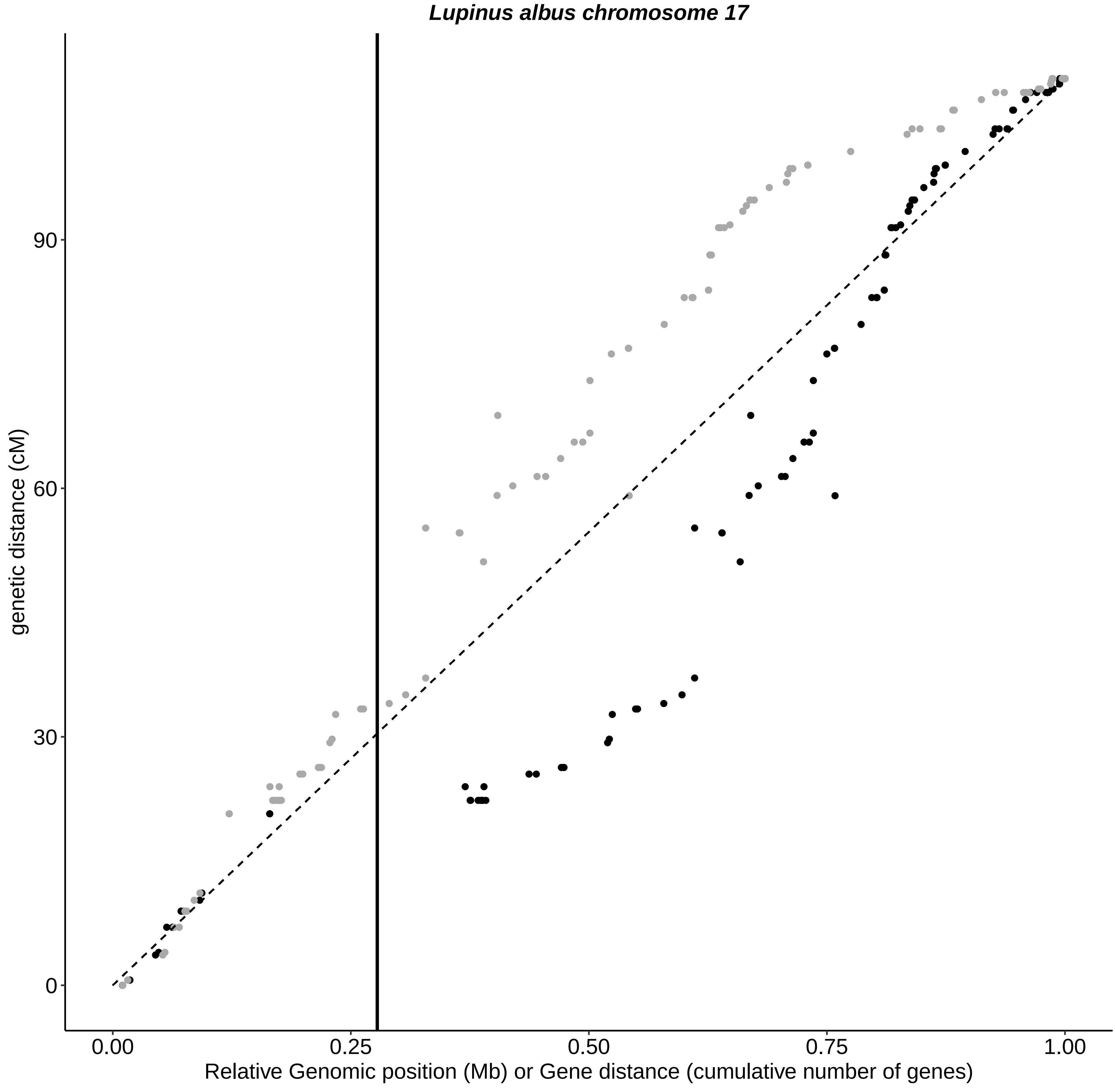

Lupinus albus chromosome 17
genetic distance (cM)
0
30
60
90
0.00
0.25
0.50
0.75
1.00
Relative Genomic position (Mb) or Gene distance (cumulative number of genes)

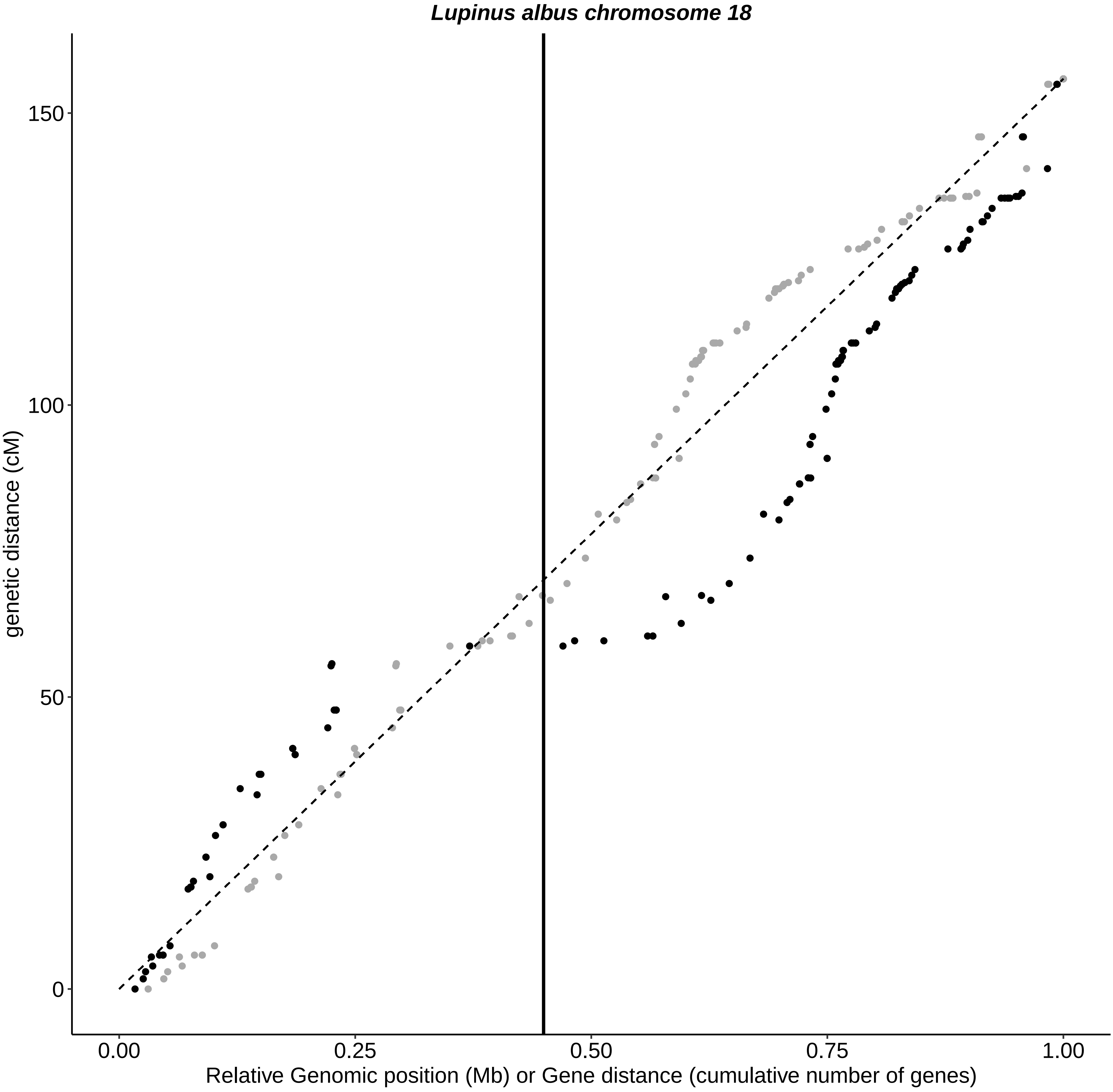
Lupinus albus chromosome 18
genetic distance (cM)
0
50
100
150
0.00
0.25
0.50
0.75
1.00
Relative Genomic position (Mb) or Gene distance (cumulative number of genes)

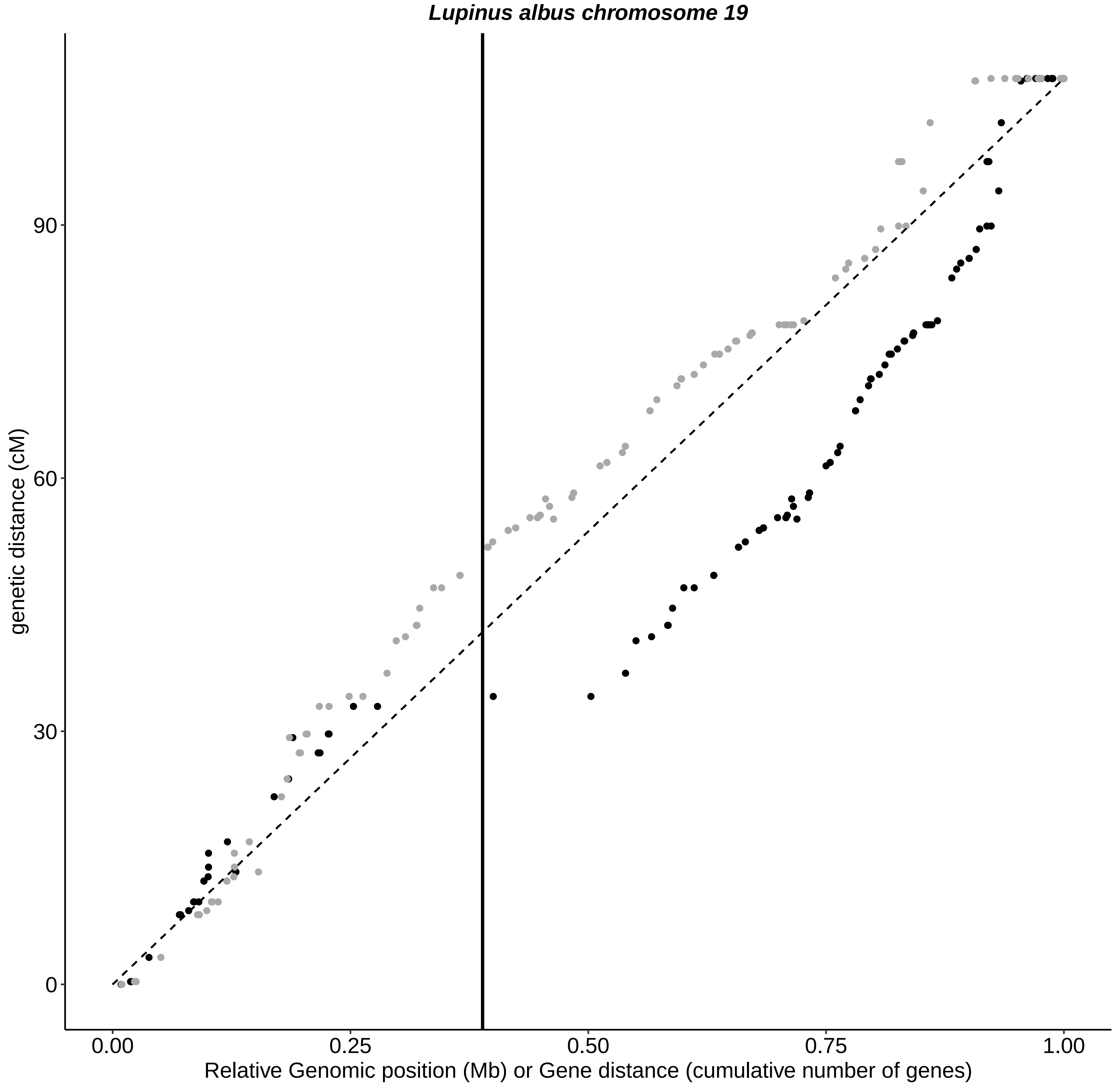
Lupinus albus chromosome 19
genetic distance (cM)
0
30
60
90
0.00
0.25
0.50
0.75
1.00
Relative Genomic position (Mb) or Gene distance (cumulative number of genes)

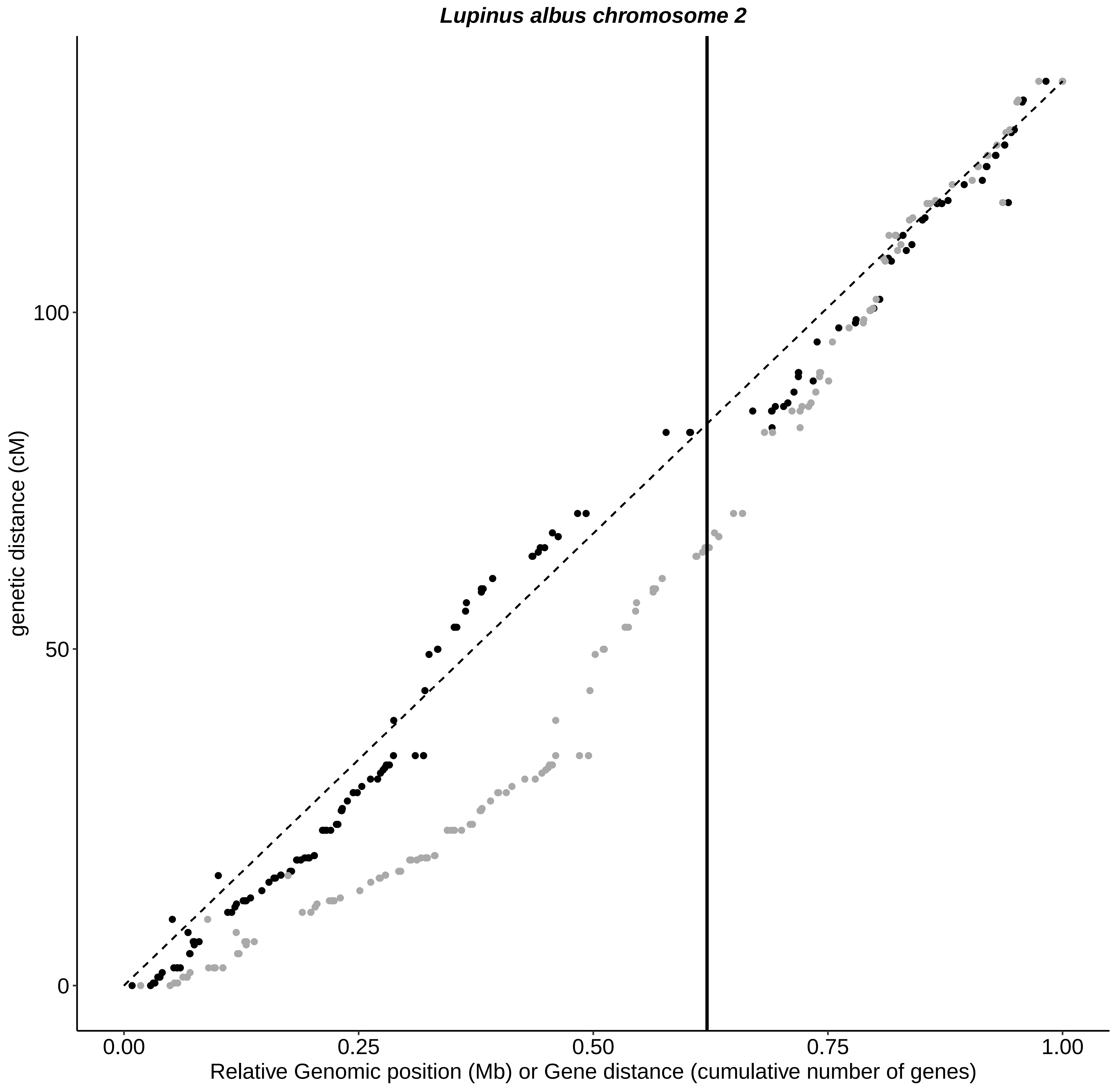
Lupinus albus chromosome 2
genetic distance (cM)
0
50
100
0.00
0.25
0.50
0.75
1.00
Relative Genomic position (Mb) or Gene distance (cumulative number of genes)

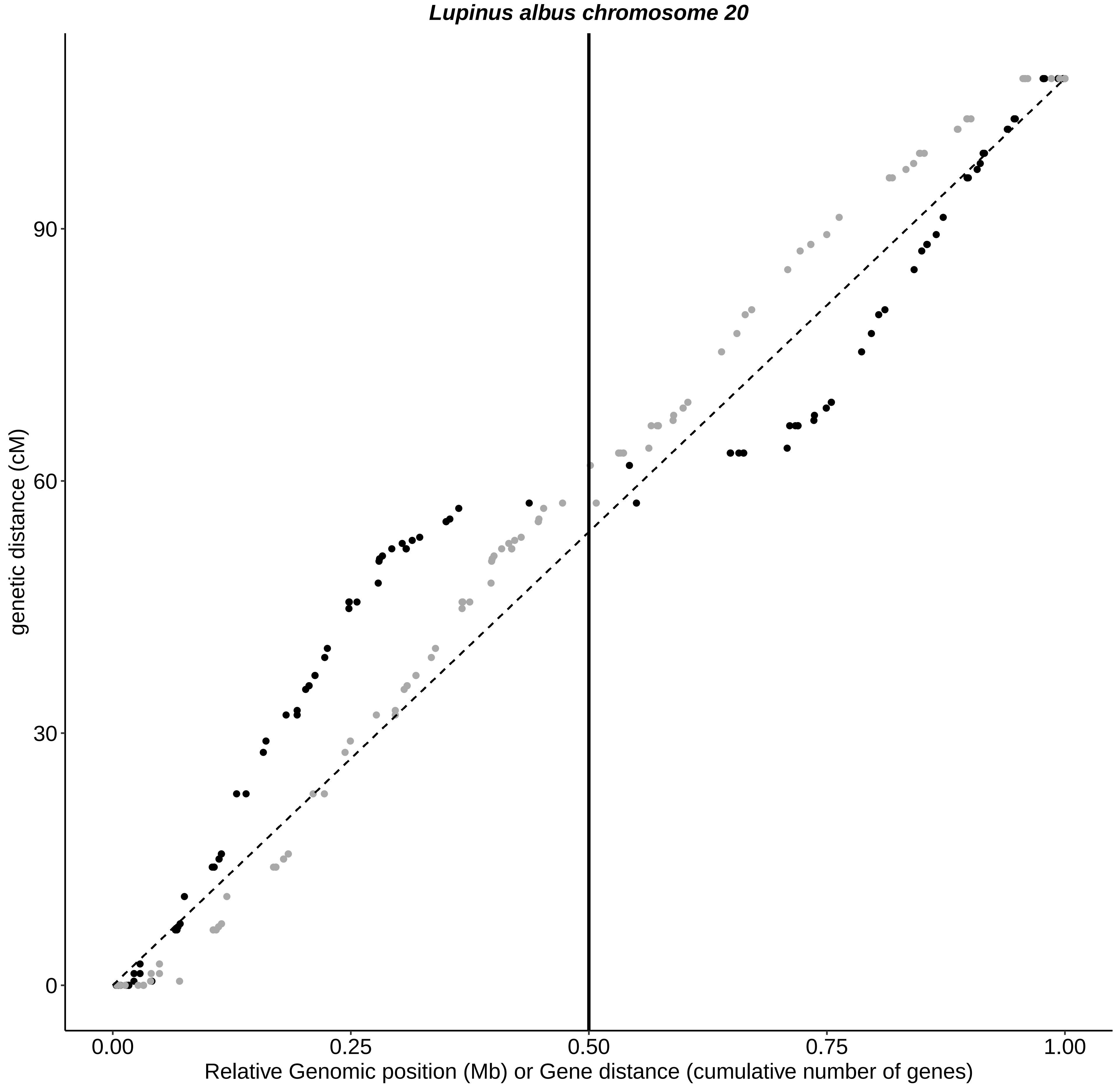
Lupinus albus chromosome 20
genetic distance (cM)
0
30
60
90
0.00
0.25
0.50
0.75
1.00
Relative Genomic position (Mb) or Gene distance (cumulative number of genes)

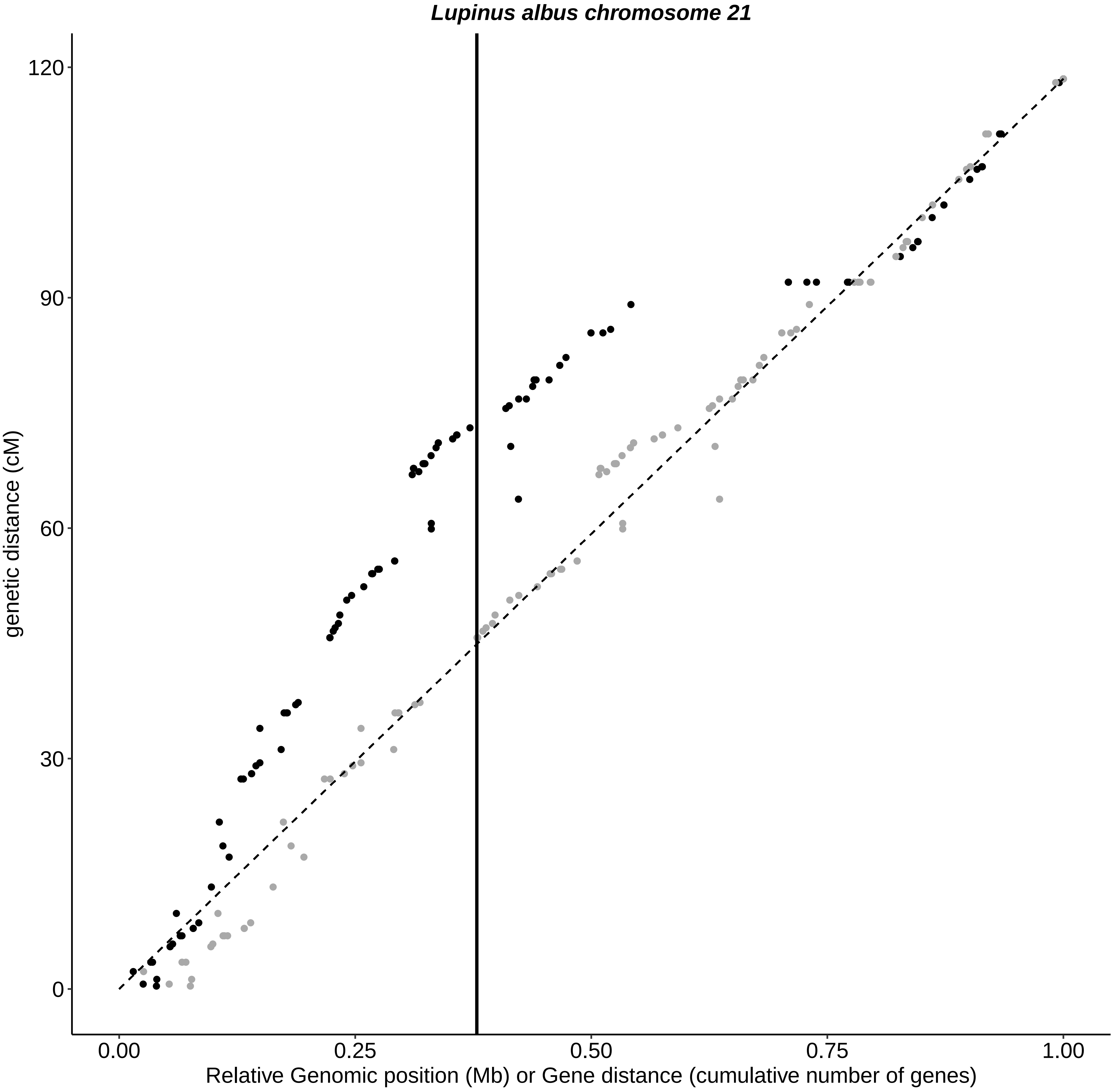
Lupinus albus chromosome 21
genetic distance (cM)
120
90
60
30
0
0.00
0.25
0.50
0.75
1.00
Relative Genomic position (Mb) or Gene distance (cumulative number of genes)

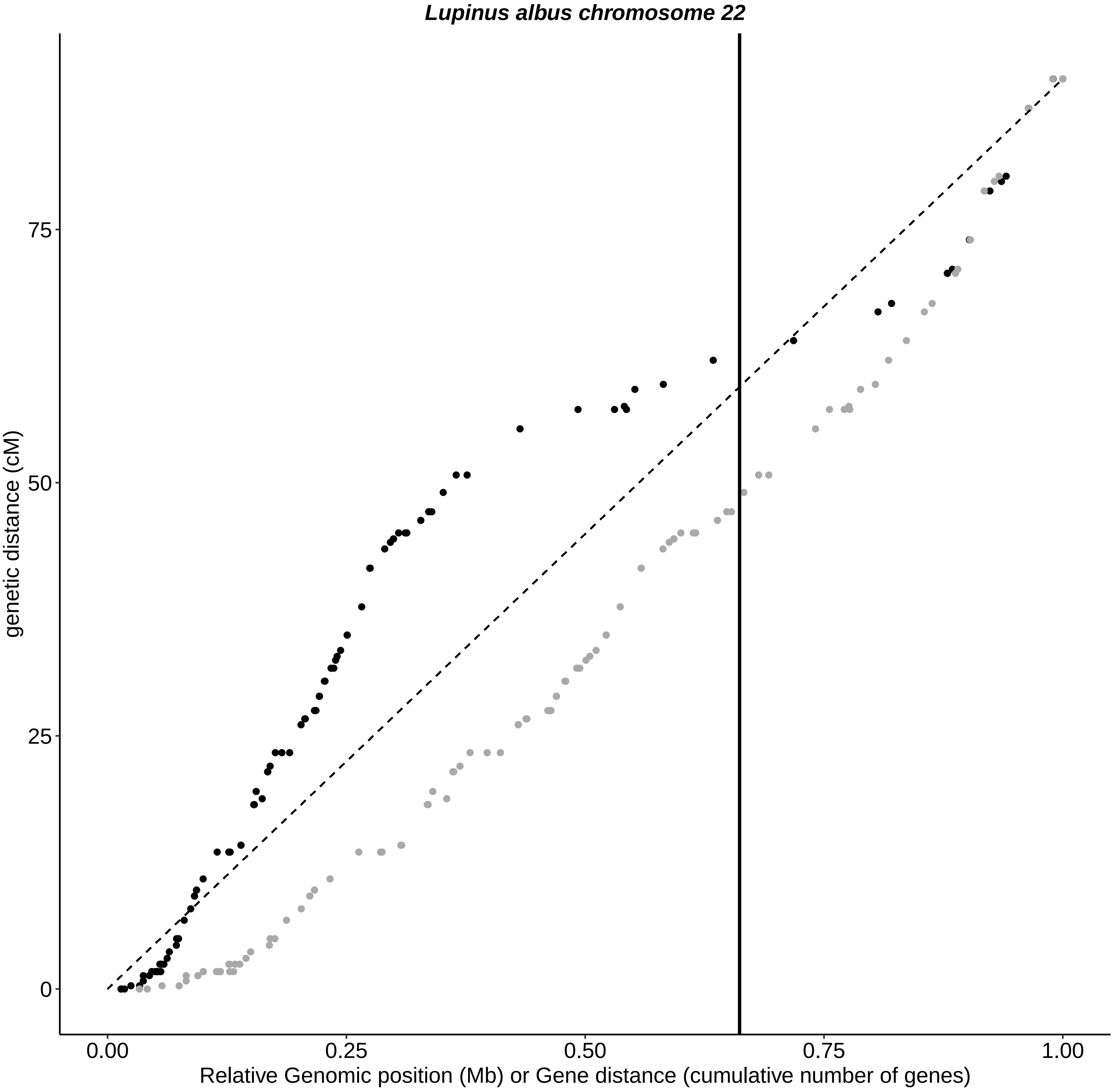
Lupinus albus chromosome 22
genetic distance (cM)
0
25
50
75
0.00
0.25
0.50
0.75
1.00
Relative Genomic position (Mb) or Gene distance (cumulative number of genes)

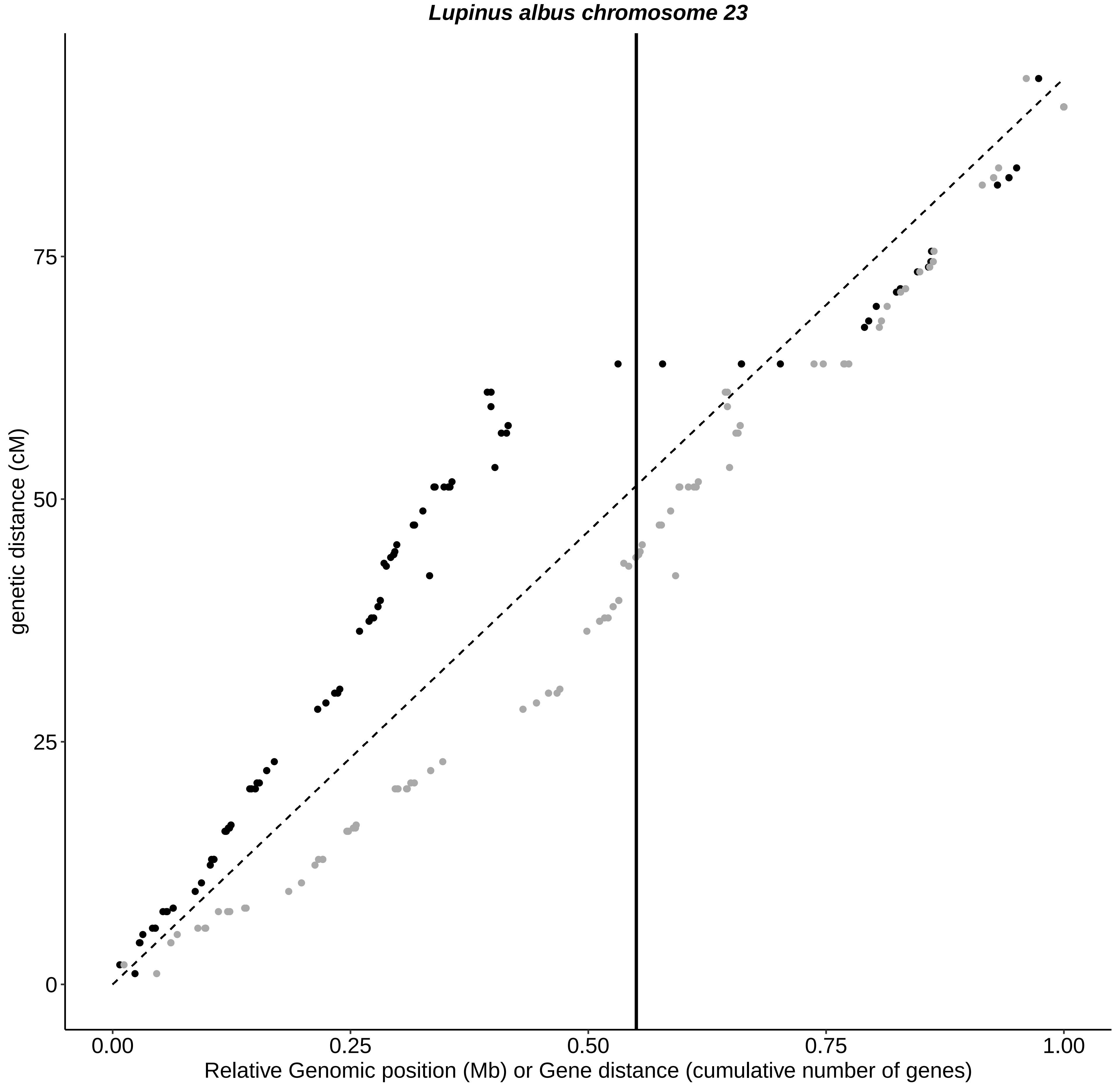
Lupinus albus chromosome 23
genetic distance (cM)
0
25
50
75
0.00
0.25
0.50
0.75
1.00
Relative Genomic position (Mb) or Gene distance (cumulative number of genes)

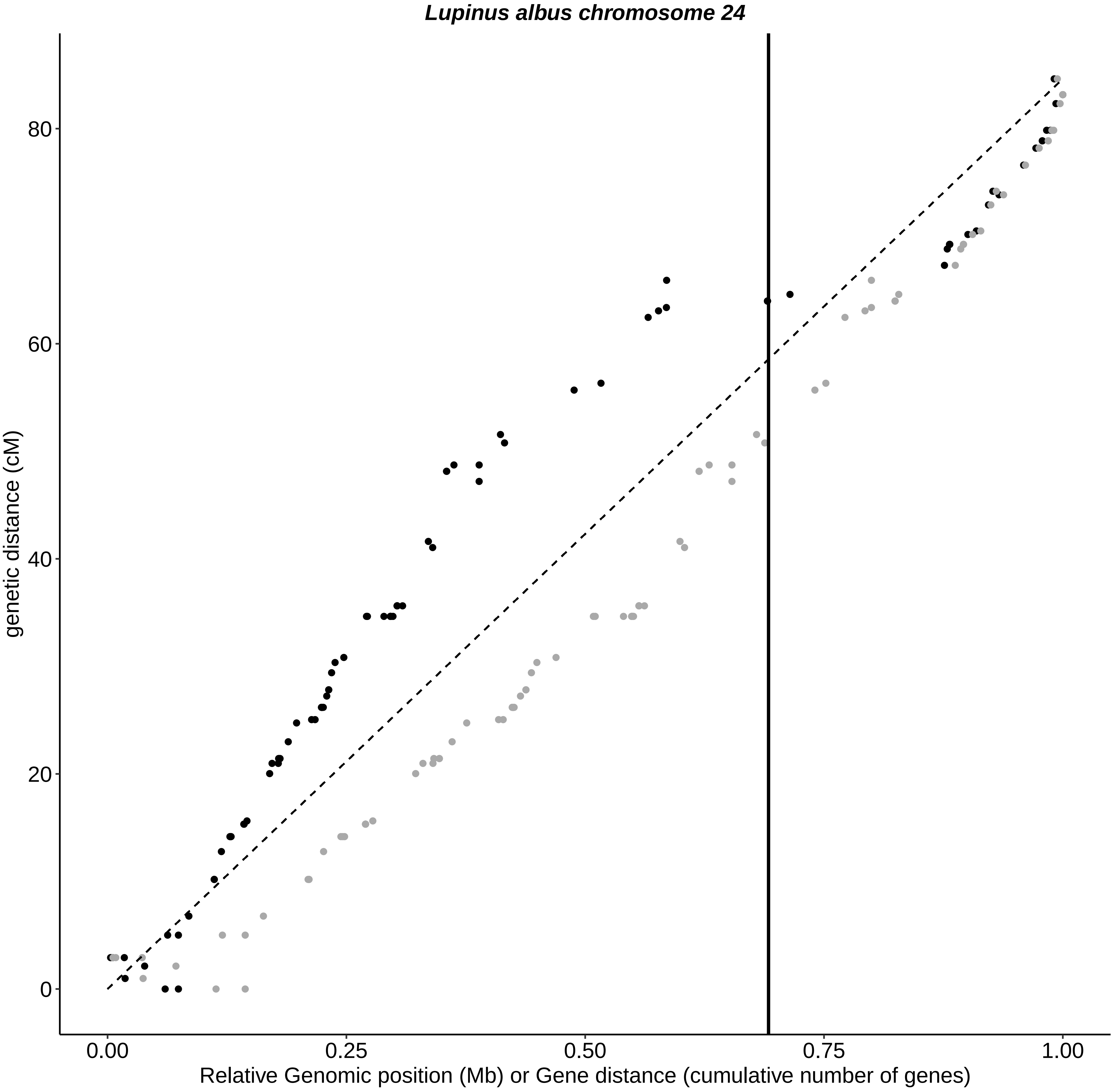
Lupinus albus chromosome 24
genetic distance (cM)
0
20
40
60
80
0.00
0.25
0.50
0.75
1.00
Relative Genomic position (Mb) or Gene distance (cumulative number of genes)

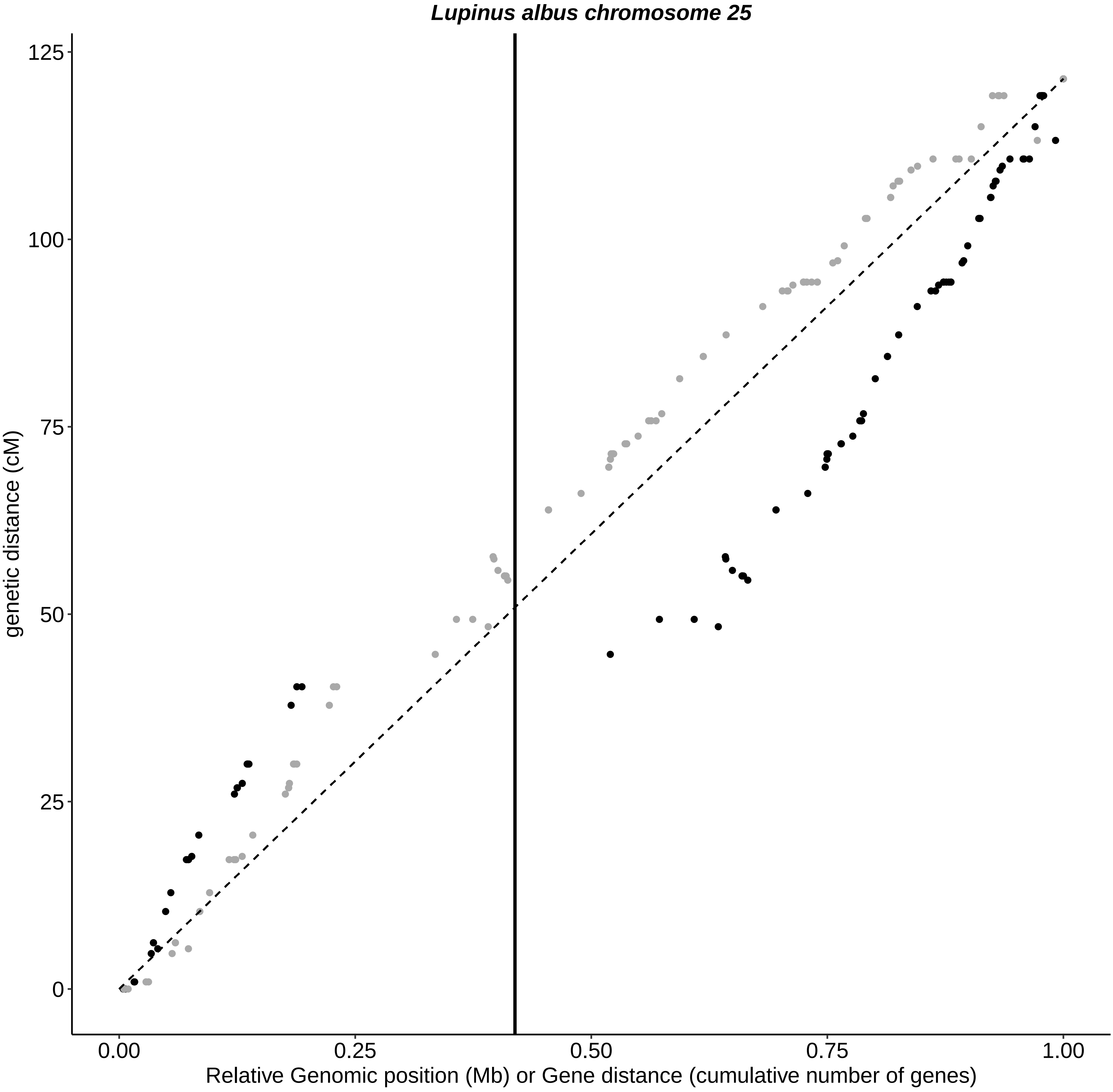
Lupinus albus chromosome 25
genetic distance (cM)
0
25
50
75
100
125
0.00
0.25
0.50
0.75
1.00
Relative Genomic position (Mb) or Gene distance (cumulative number of genes)

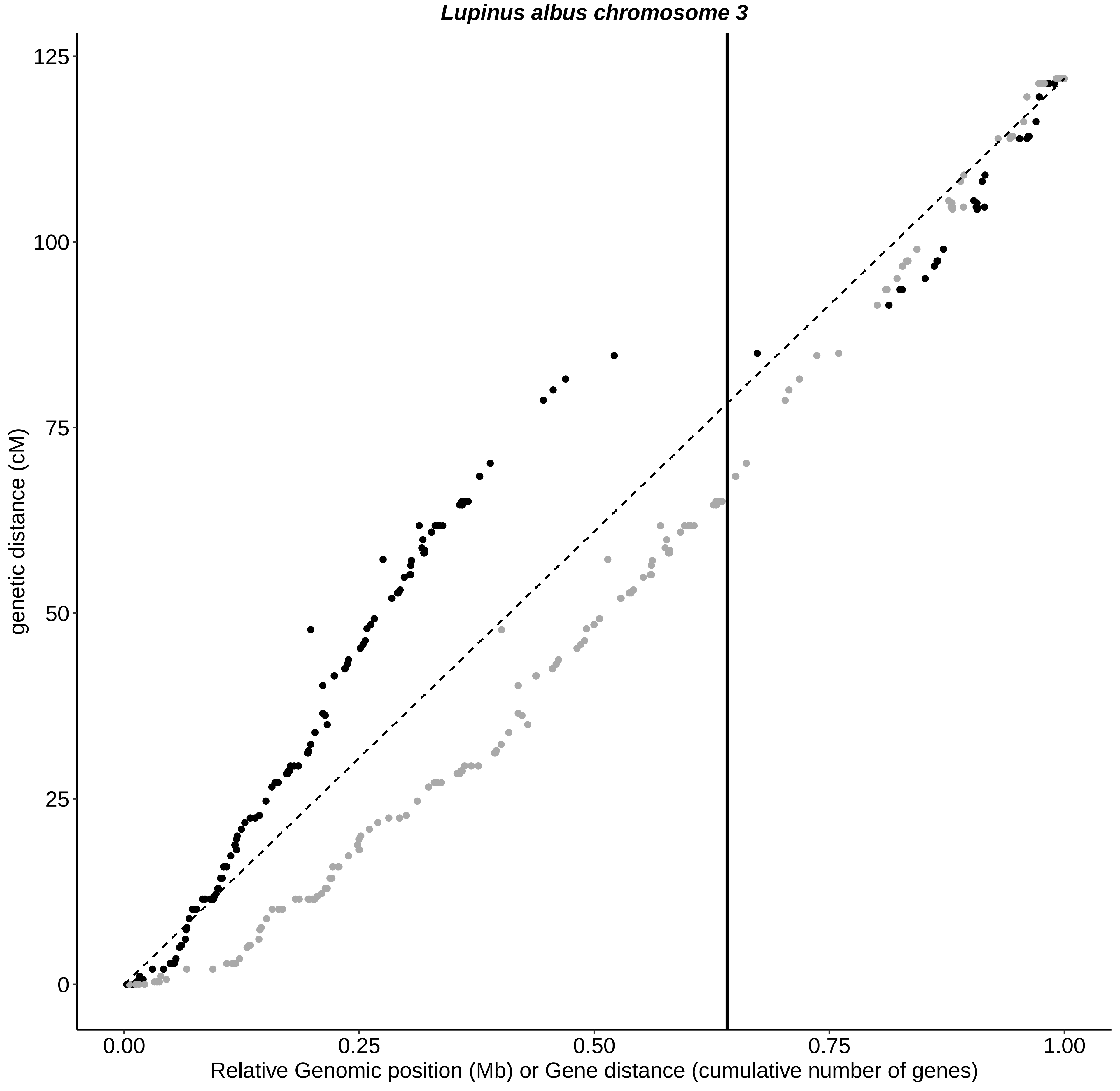
Lupinus albus chromosome 3
genetic distance (cM)
125
100
75
50
25
0
0.00
0.25
0.50
0.75
1.00
Relative Genomic position (Mb) or Gene distance (cumulative number of genes)

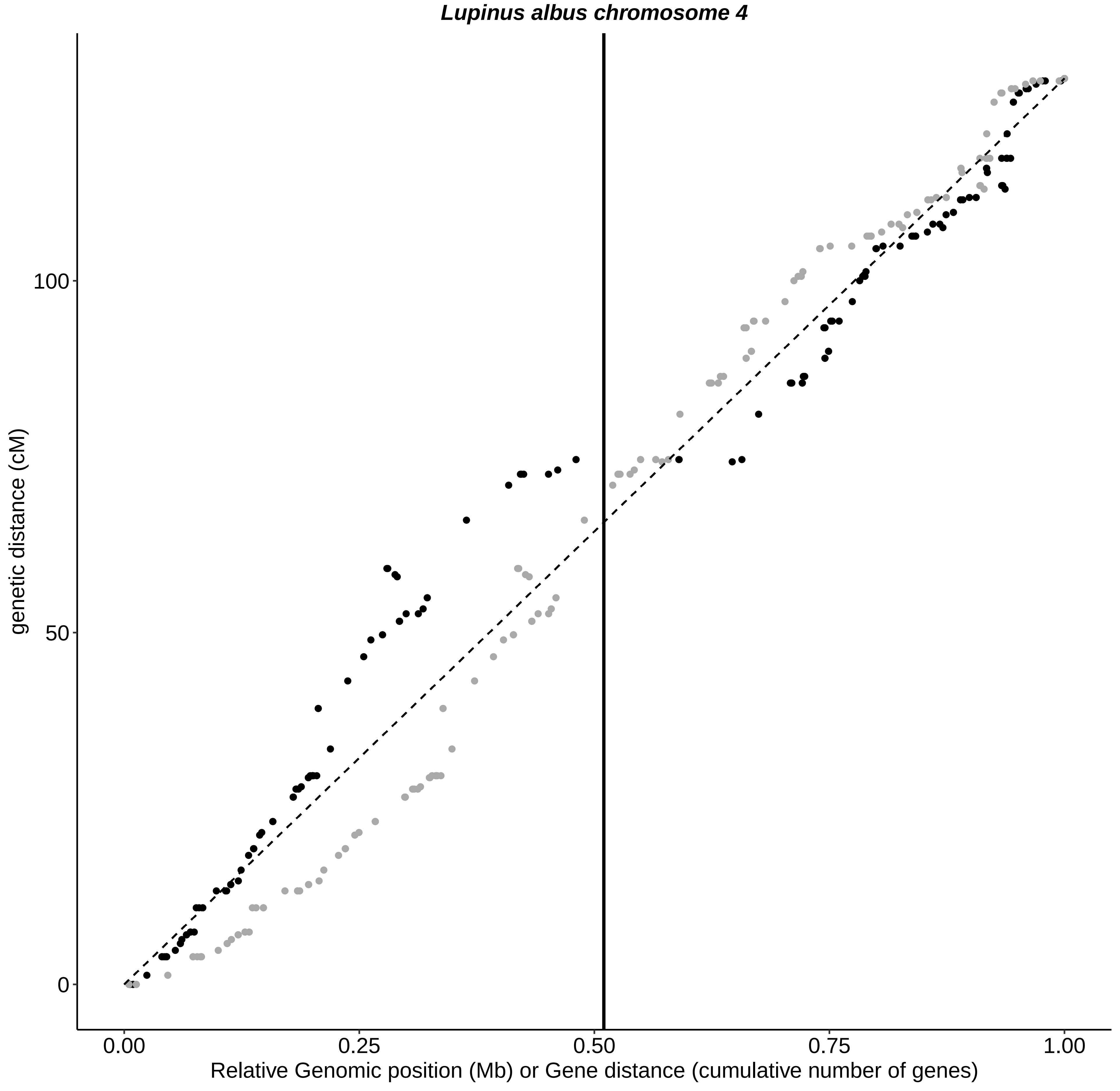

Lupinus albus chromosome 4
genetic distance (cM)
0
50
100
0.00
0.25
0.50
0.75
1.00
Relative Genomic position (Mb) or Gene distance (cumulative number of genes)

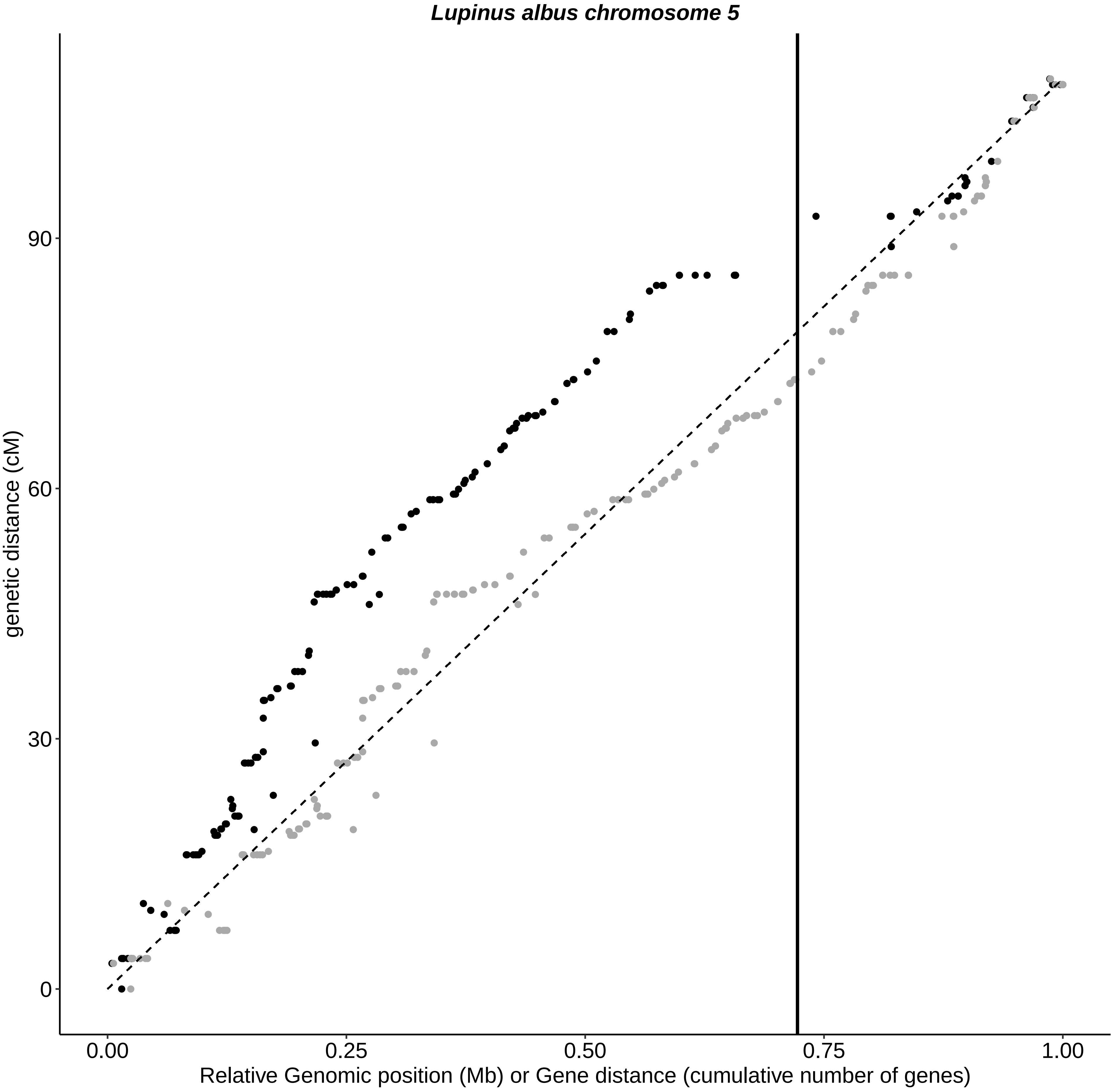
Lupinus albus chromosome 5
genetic distance (cM)
0
30
60
90
0.00
0.25
0.50
0.75
1.00
Relative Genomic position (Mb) or Gene distance (cumulative number of genes)

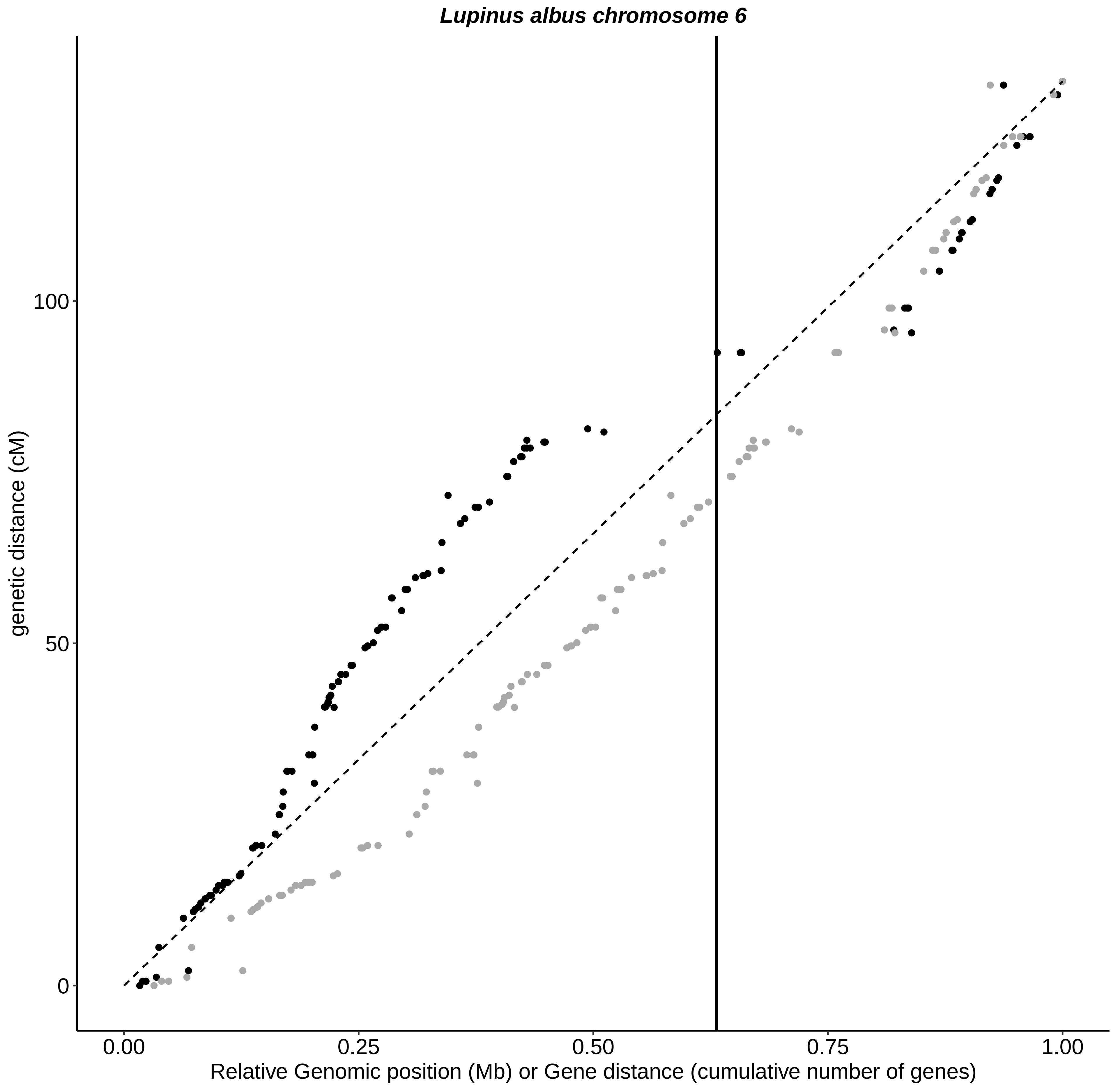
Lupinus albus chromosome 6
genetic distance (cM)
0
50
100
0.00
0.25
0.50
0.75
1.00
Relative Genomic position (Mb) or Gene distance (cumulative number of genes)

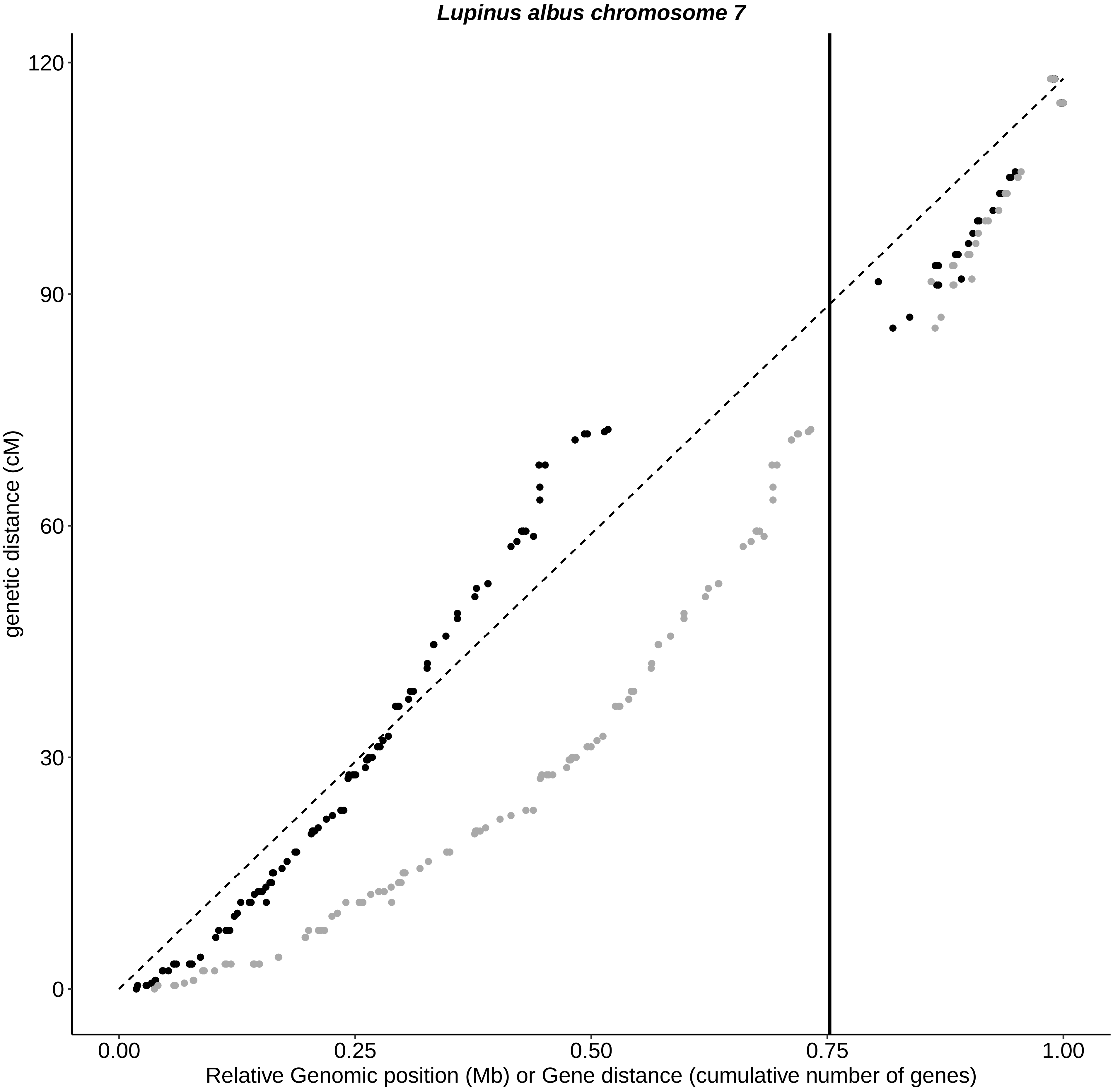
Lupinus albus chromosome 7
genetic distance (cM)
120
90
60
30
0
0.00
0.25
0.50
0.75
1.00
Relative Genomic position (Mb) or Gene distance (cumulative number of genes)

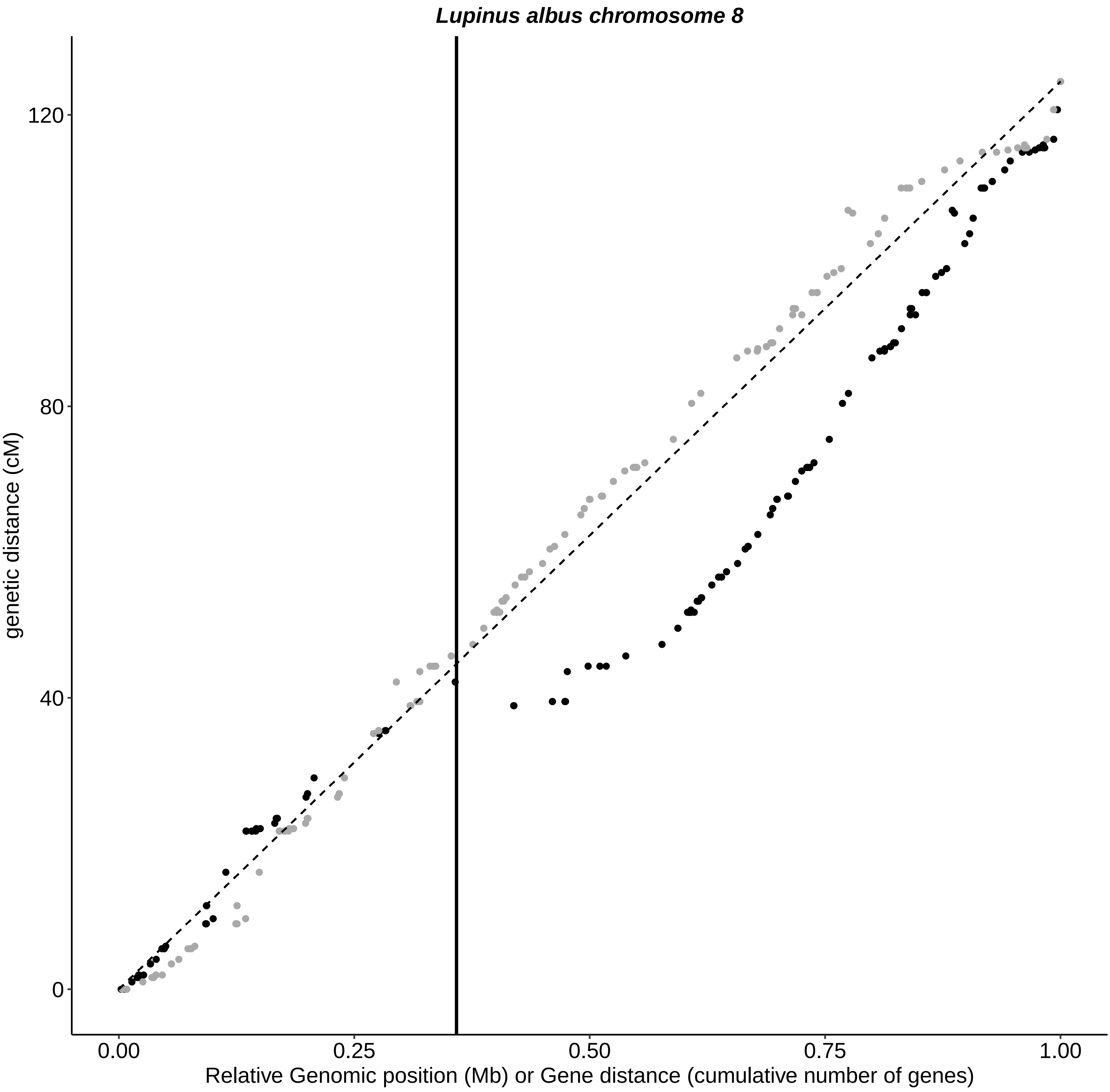
Lupinus albus chromosome 8
genetic distance (cM)
0
40
80
120
0.00
0.25
0.50
0.75
1.00
Relative Genomic position (Mb) or Gene distance (cumulative number of genes)

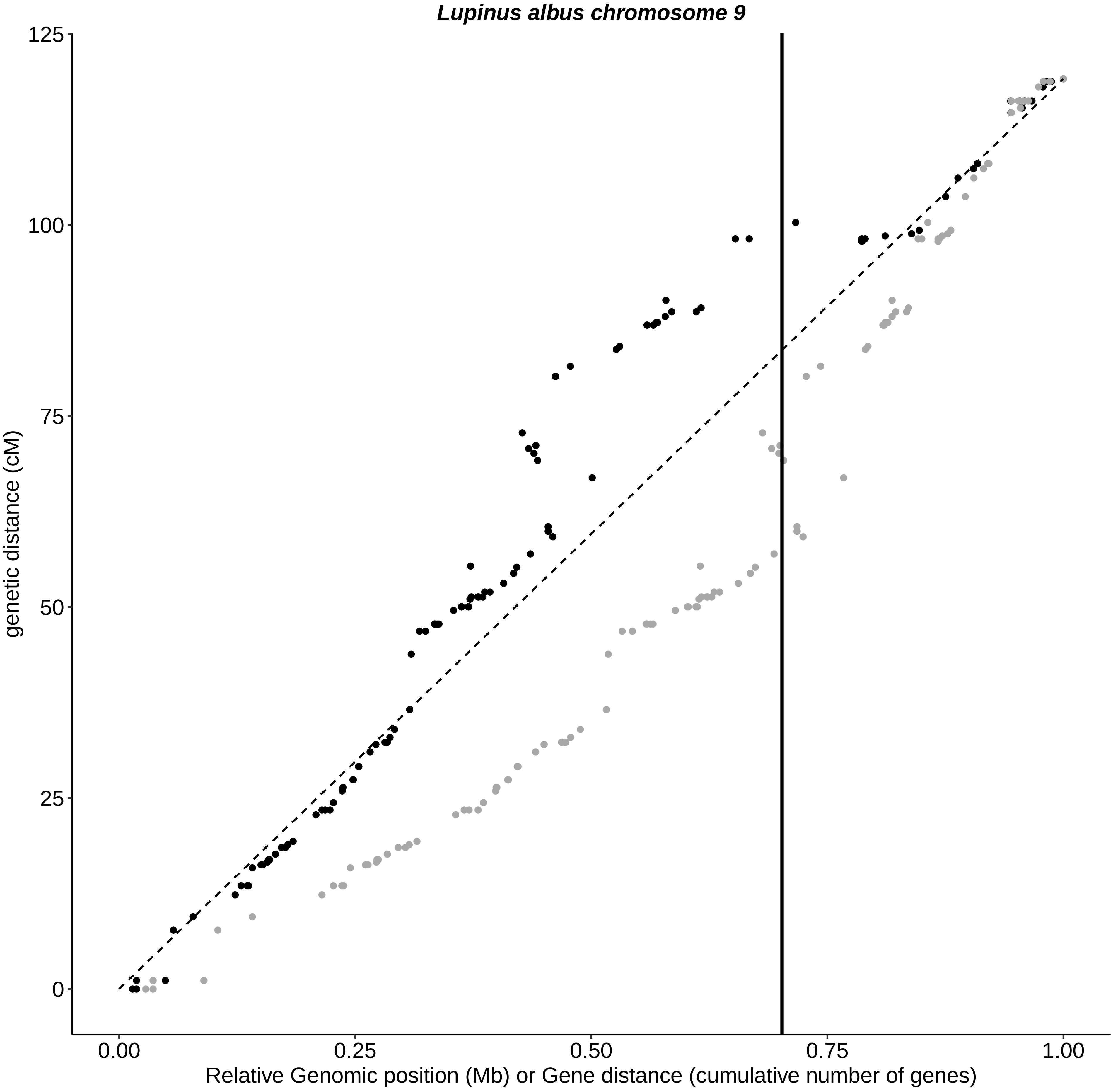
Lupinus albus chromosome 9
genetic distance (cM)
0
25
50
75
100
125
0.00
0.25
0.50
0.75
1.00
Relative Genomic position (Mb) or Gene distance (cumulative number of genes)

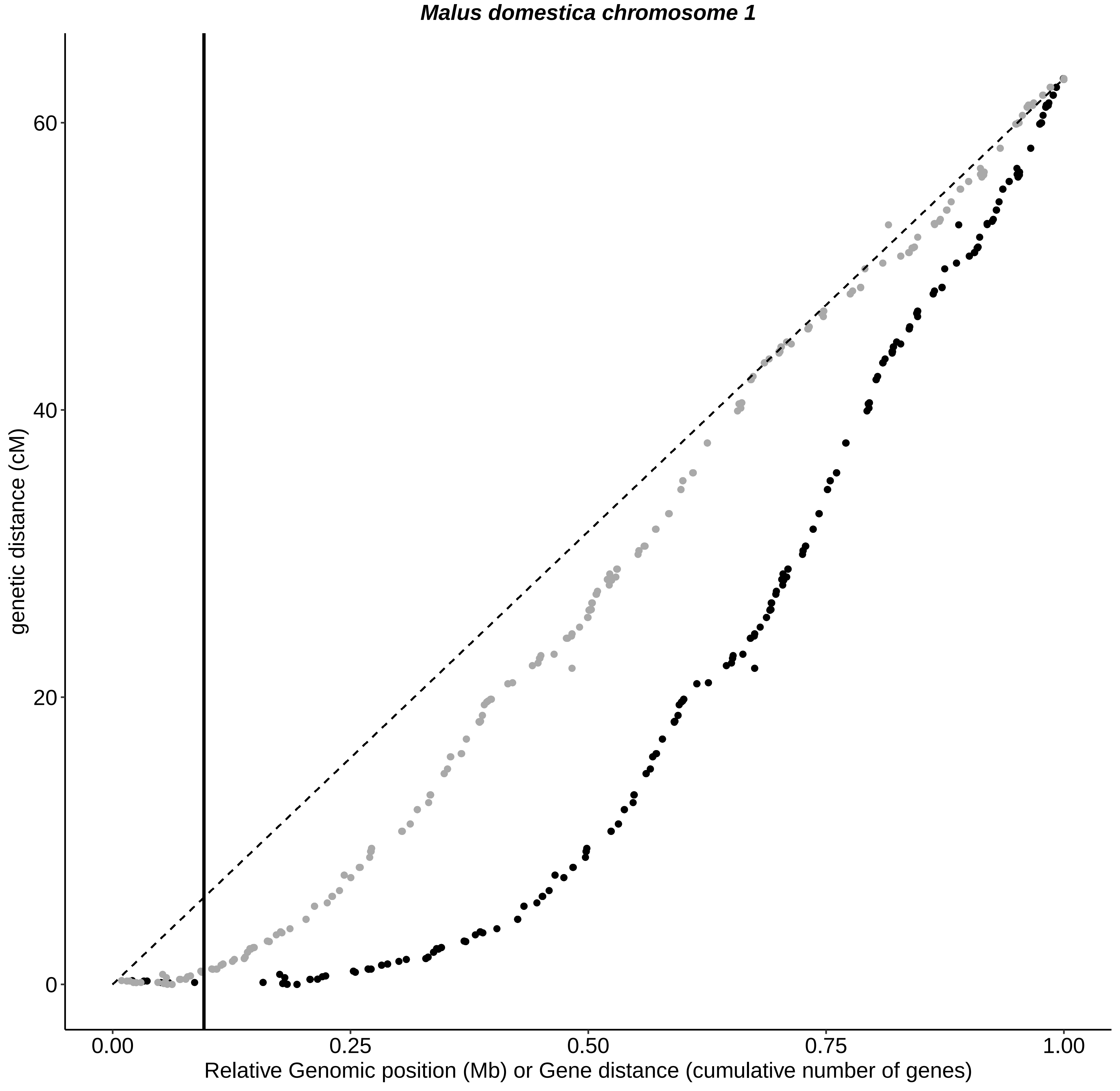
Malus domestica chromosome 1
genetic distance (cM)
0
20
40
60
0.00
0.25
0.50
0.75
1.00
Relative Genomic position (Mb) or Gene distance (cumulative number of genes)

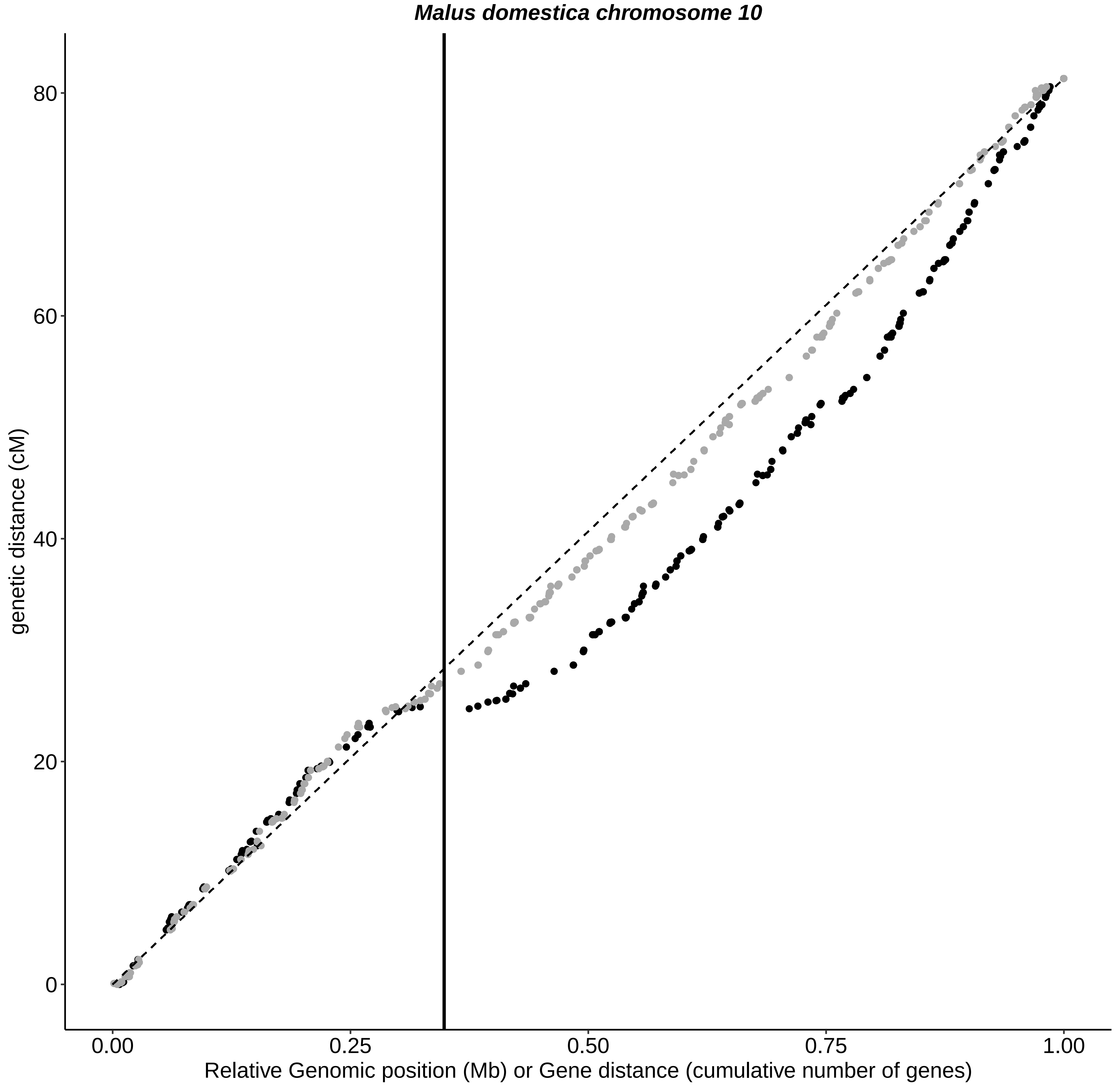
Malus domestica chromosome 10
genetic distance (cM)
0
20
40
60
80
0.00
0.25
0.50
0.75
1.00
Relative Genomic position (Mb) or Gene distance (cumulative number of genes)

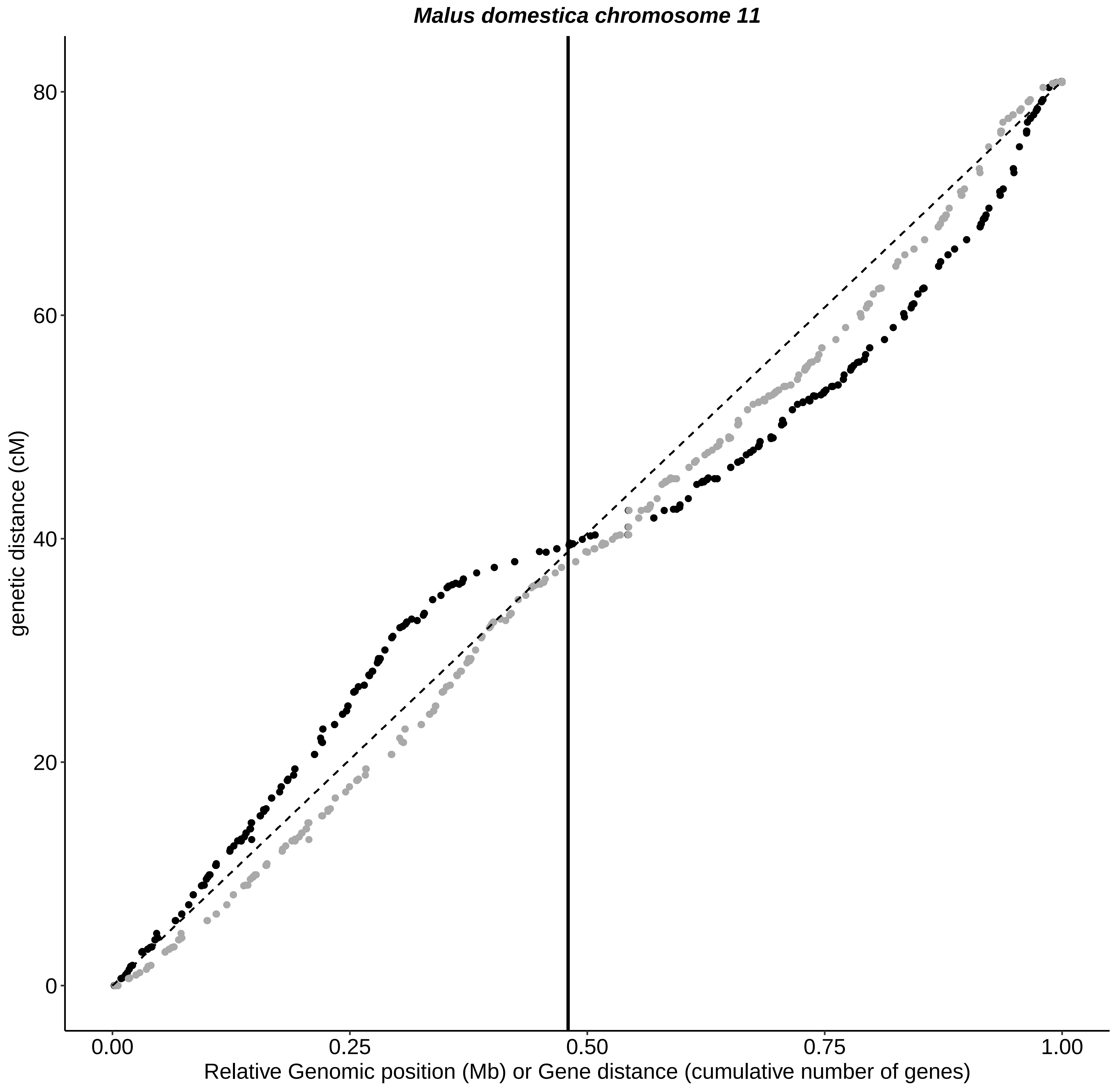
Malus domestica chromosome 11
genetic distance (cM)
0
20
40
60
80
0.00
0.25
0.50
0.75
1.00
Relative Genomic position (Mb) or Gene distance (cumulative number of genes)

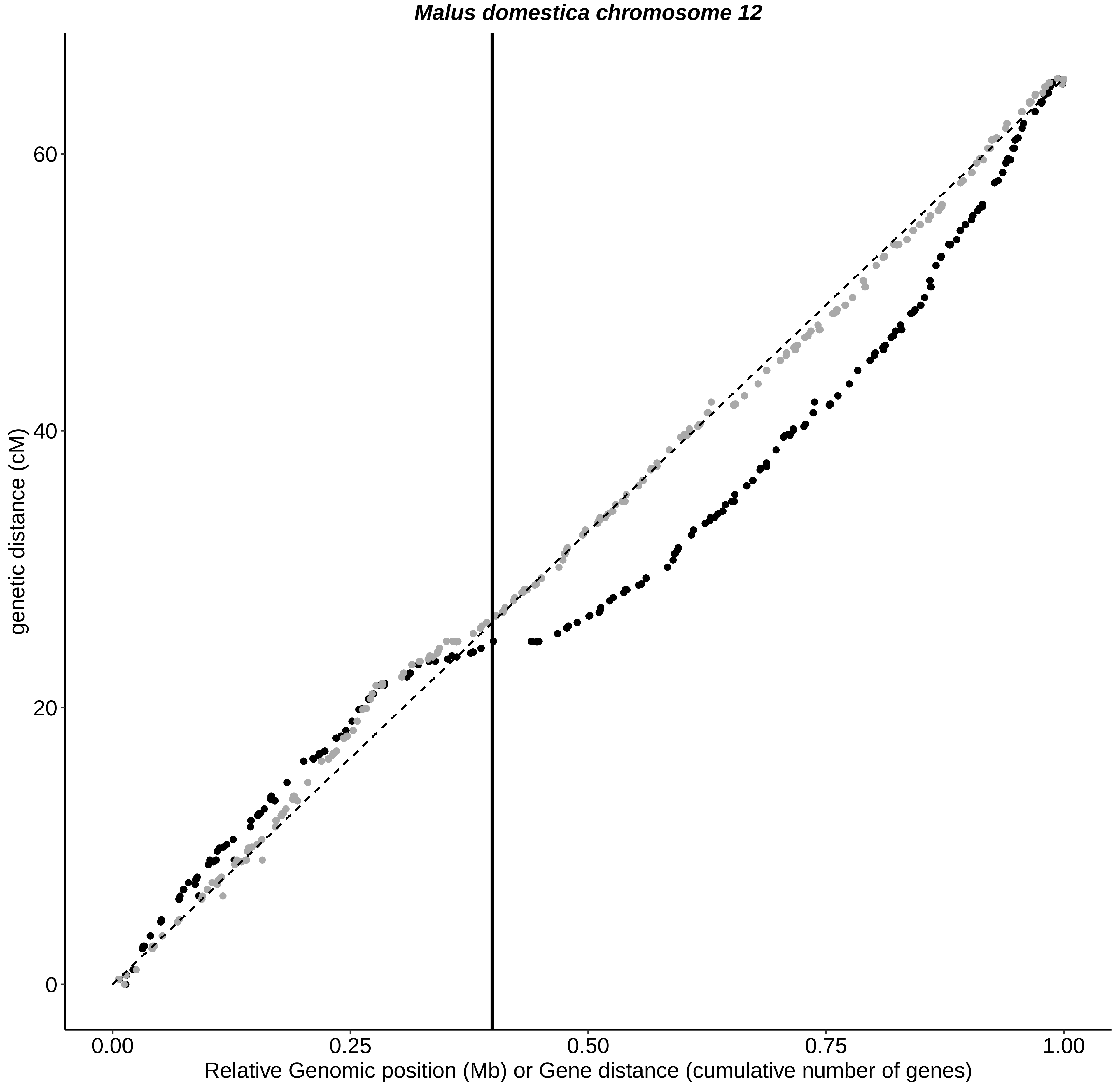
Malus domestica chromosome 12
genetic distance (cM)
0
20
40
60
0.00
0.25
0.50
0.75
1.00
Relative Genomic position (Mb) or Gene distance (cumulative number of genes)

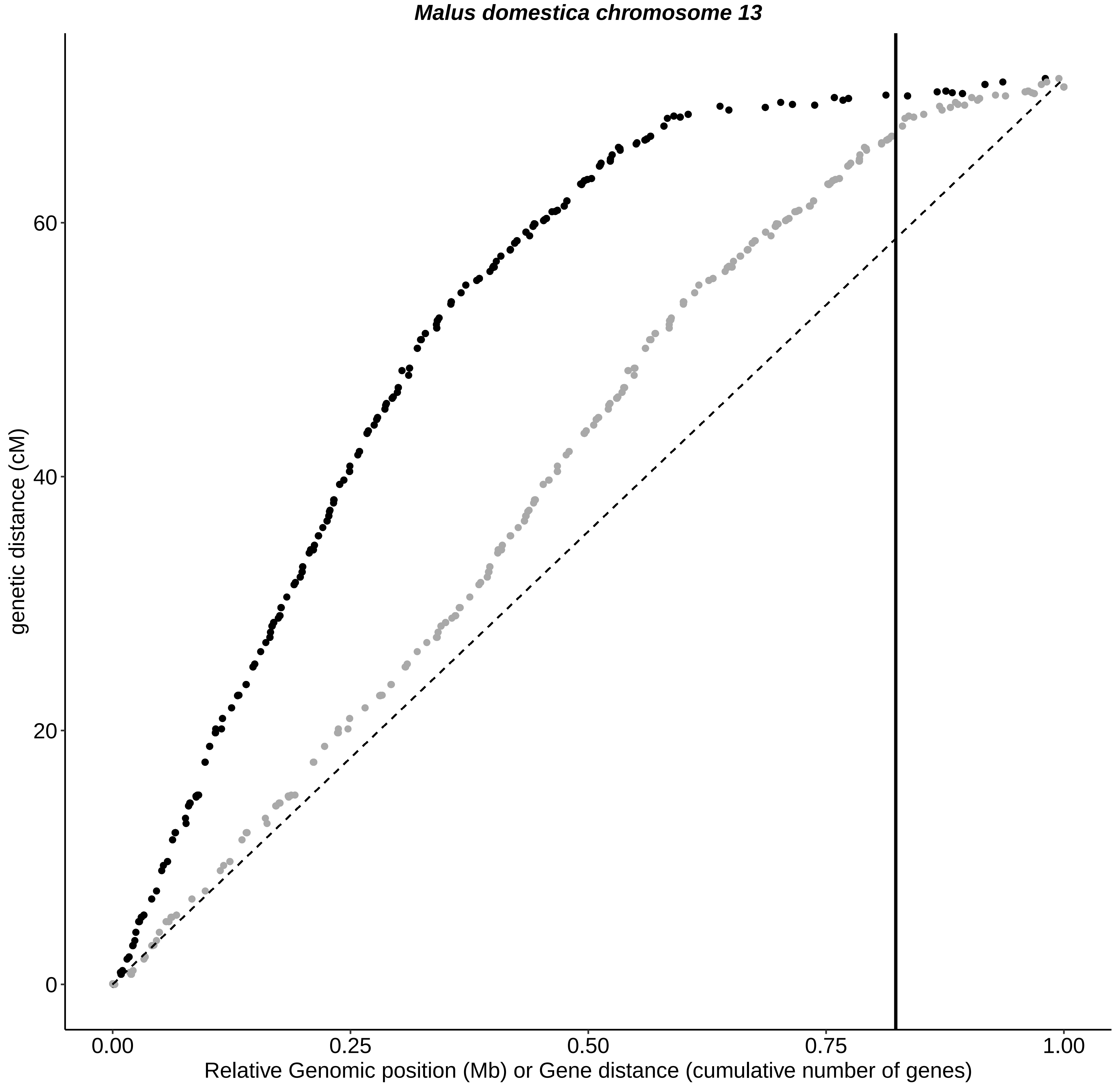
Malus domestica chromosome 13
genetic distance (cM)
0
20
40
60
0.00
0.25
0.50
0.75
1.00
Relative Genomic position (Mb) or Gene distance (cumulative number of genes)

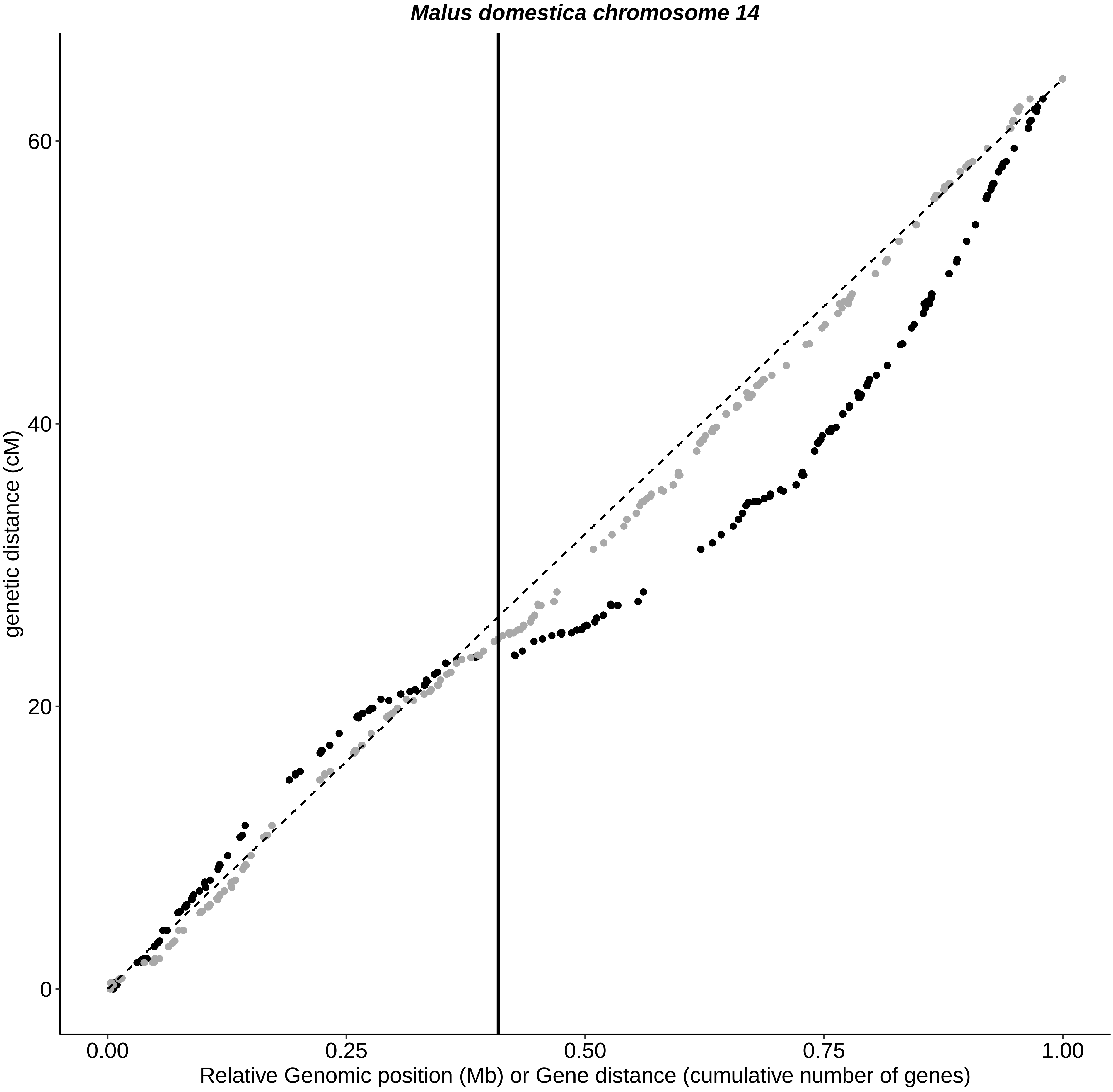
Malus domestica chromosome 14
genetic distance (cM)
0
20
40
60
0.00
0.25
0.50
0.75
1.00
Relative Genomic position (Mb) or Gene distance (cumulative number of genes)

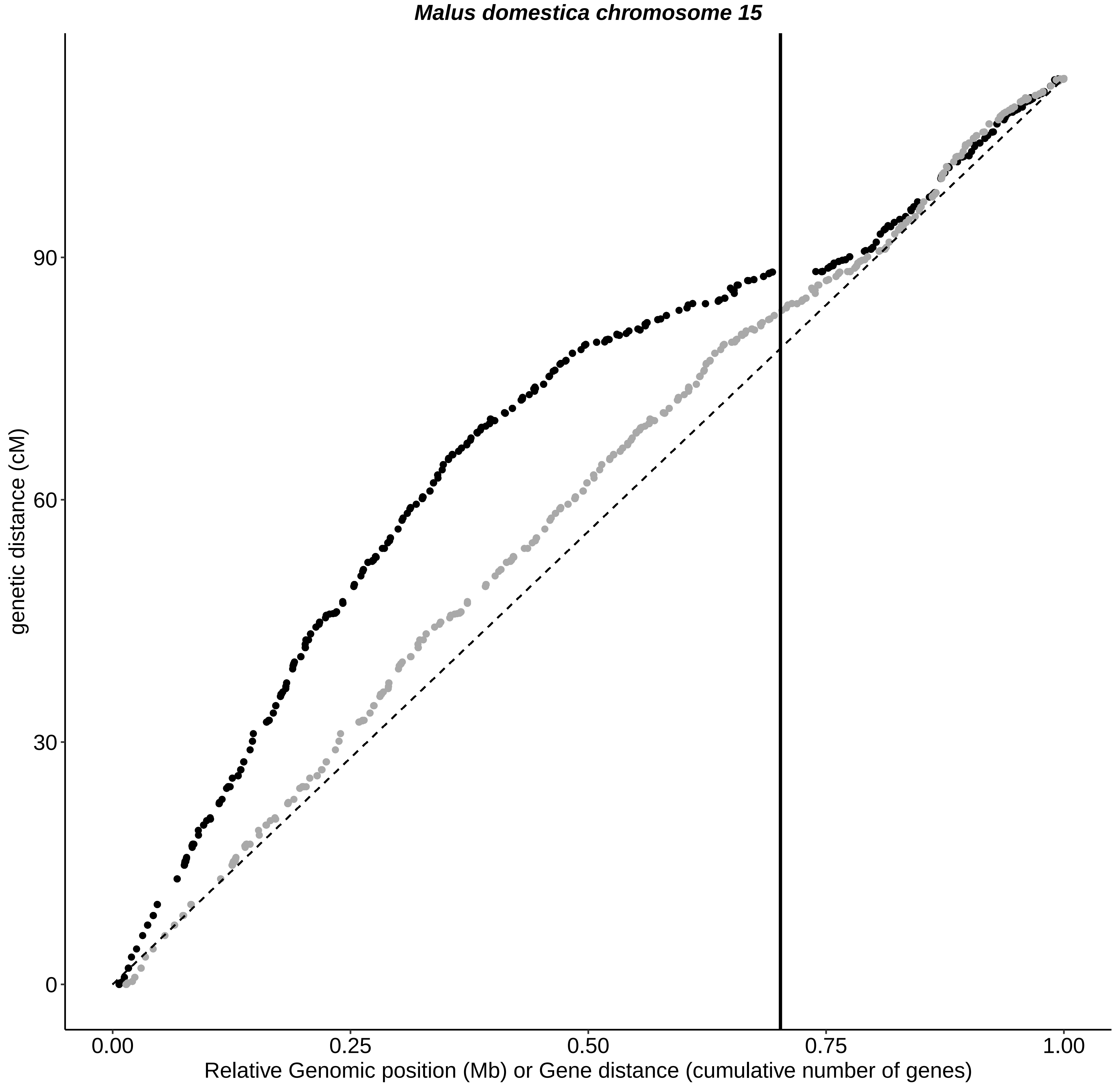
Malus domestica chromosome 15
genetic distance (cM)
0
30
60
90
0.00
0.25
0.50
0.75
1.00
Relative Genomic position (Mb) or Gene distance (cumulative number of genes)

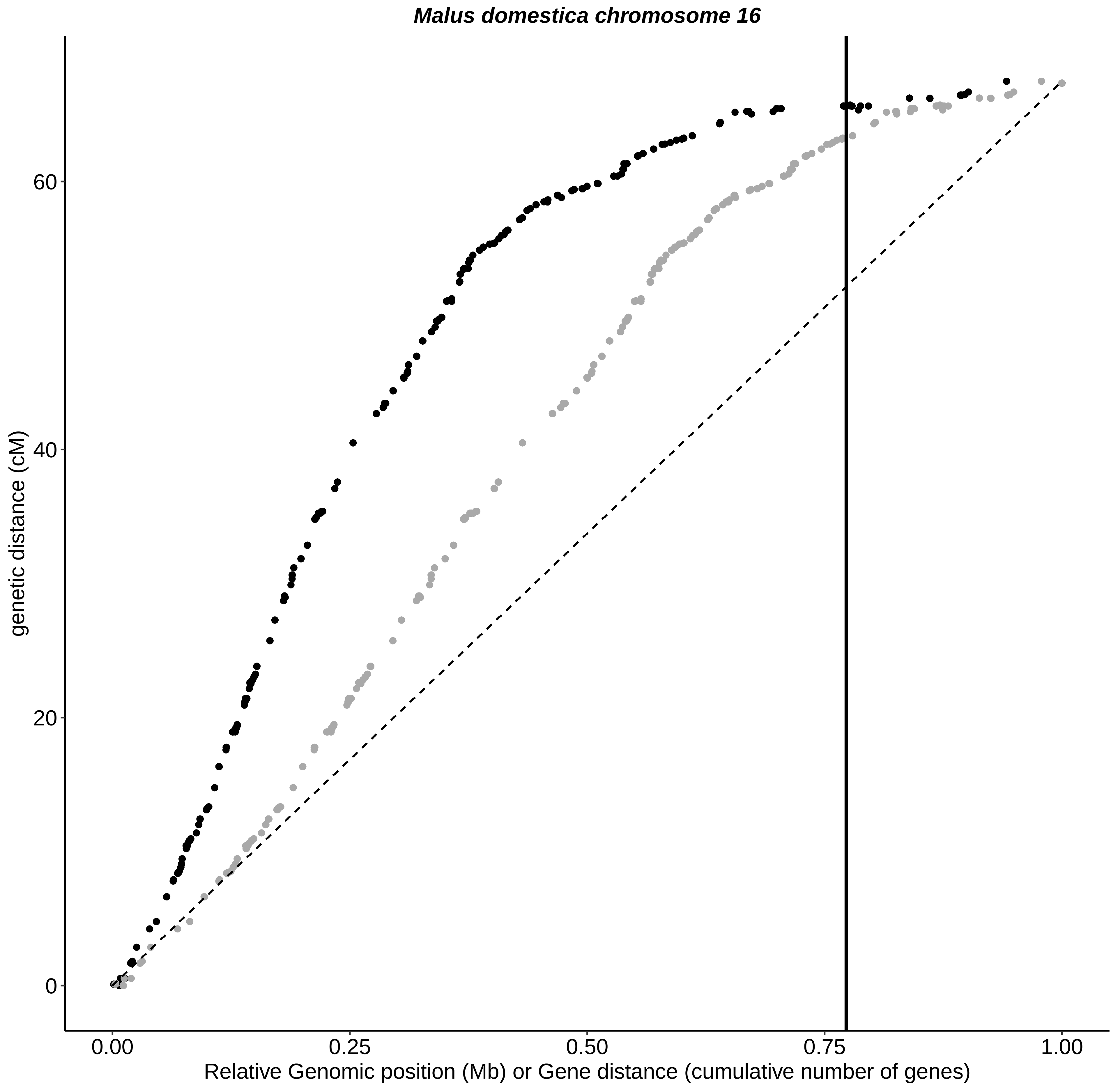
Malus domestica chromosome 16
genetic distance (cM)
0
20
40
60
0.00
0.25
0.50
0.75
1.00
Relative Genomic position (Mb) or Gene distance (cumulative number of genes)

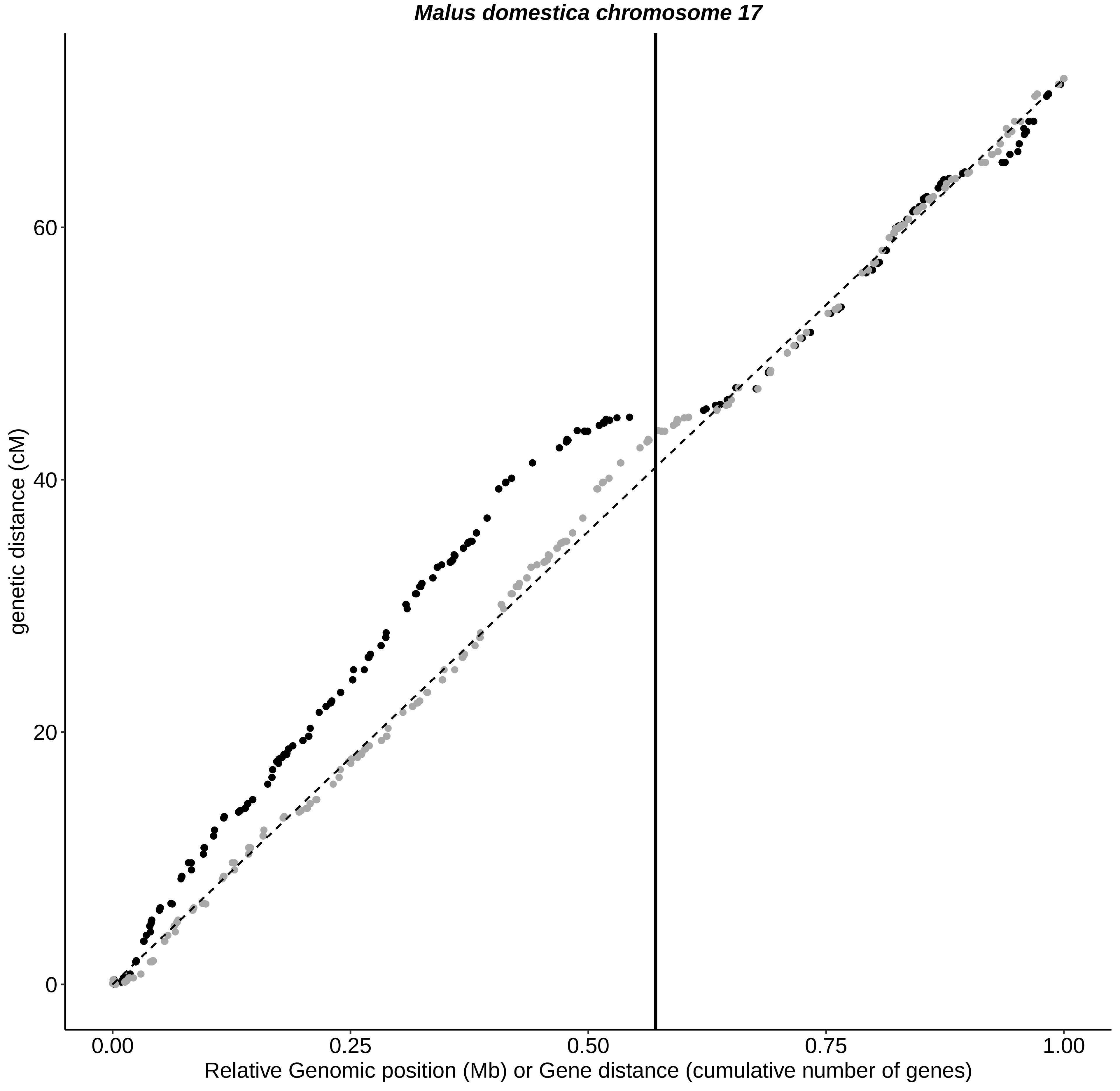
Malus domestica chromosome 17
genetic distance (cM)
0
20
40
60
0.00
0.25
0.50
0.75
1.00
Relative Genomic position (Mb) or Gene distance (cumulative number of genes)

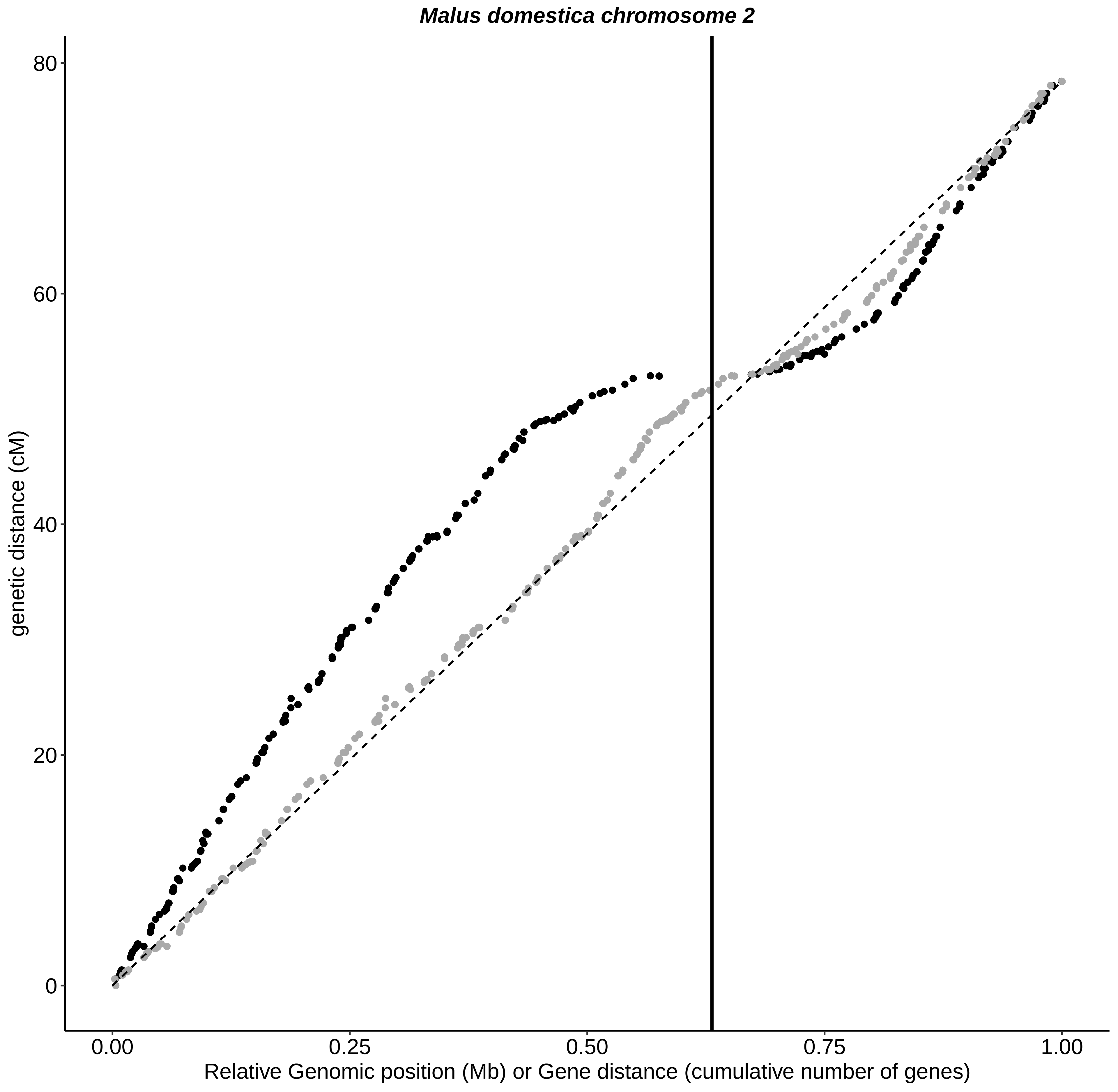
Malus domestica chromosome 2
80
60
40
20
0
genetic distance (cM)
0.00
0.25
0.50
0.75
1.00
Relative Genomic position (Mb) or Gene distance (cumulative number of genes)

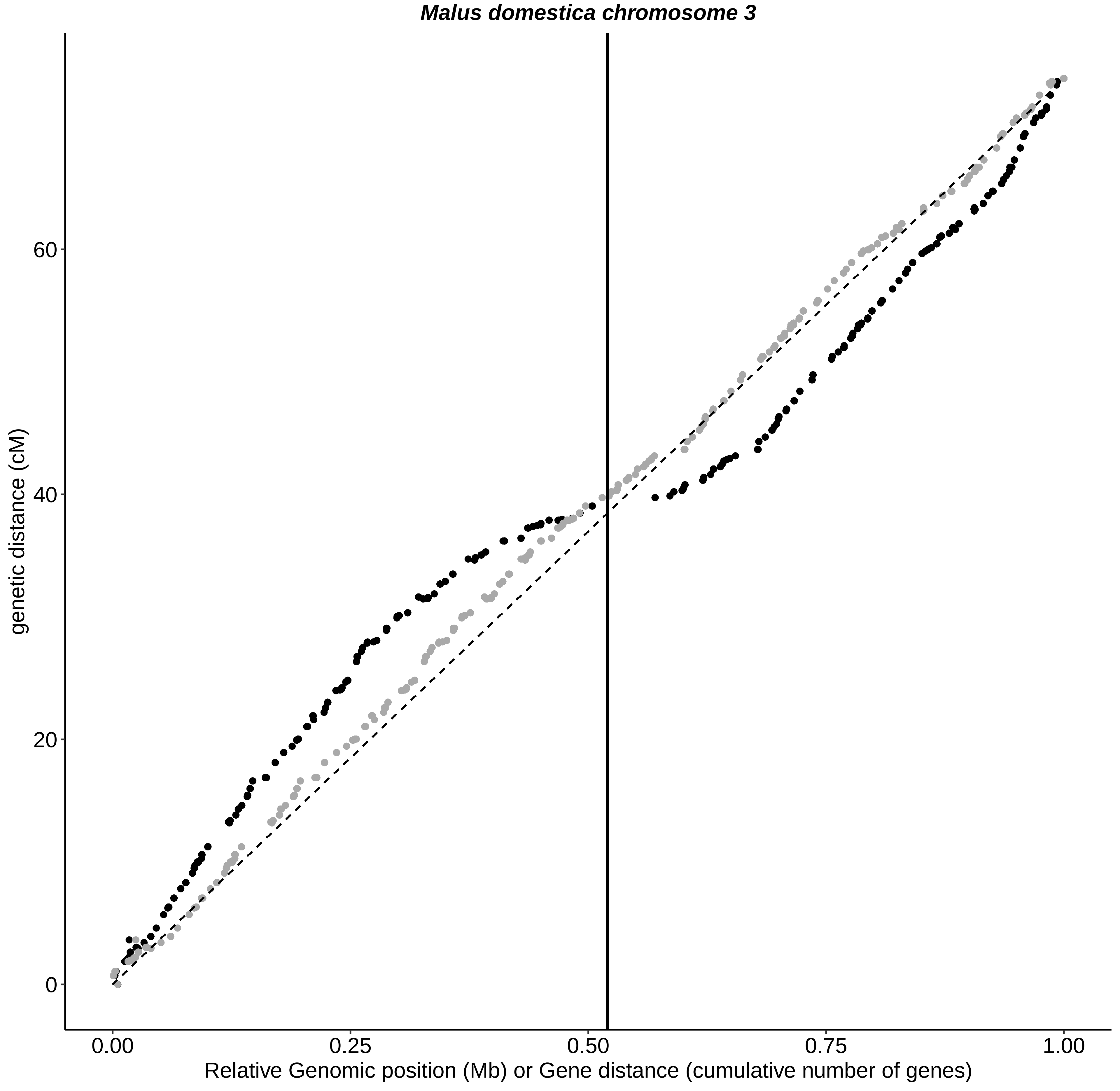
Malus domestica chromosome 3
genetic distance (cM)
0
20
40
60
0.00
0.25
0.50
0.75
1.00
Relative Genomic position (Mb) or Gene distance (cumulative number of genes)

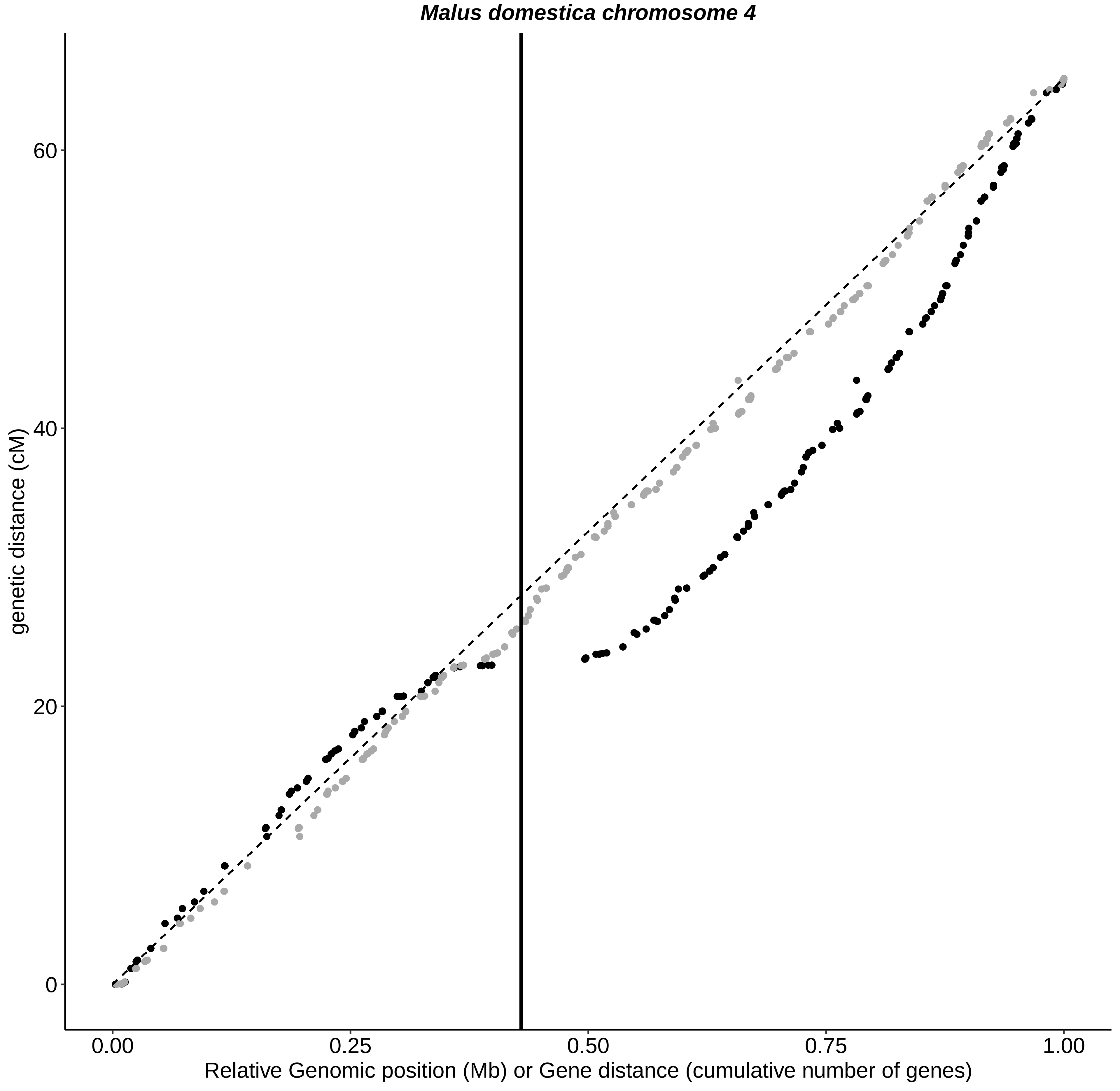

Malus domestica chromosome 4
genetic distance (cM)
0
20
40
60
0.00
0.25
0.50
0.75
1.00
Relative Genomic position (Mb) or Gene distance (cumulative number of genes)

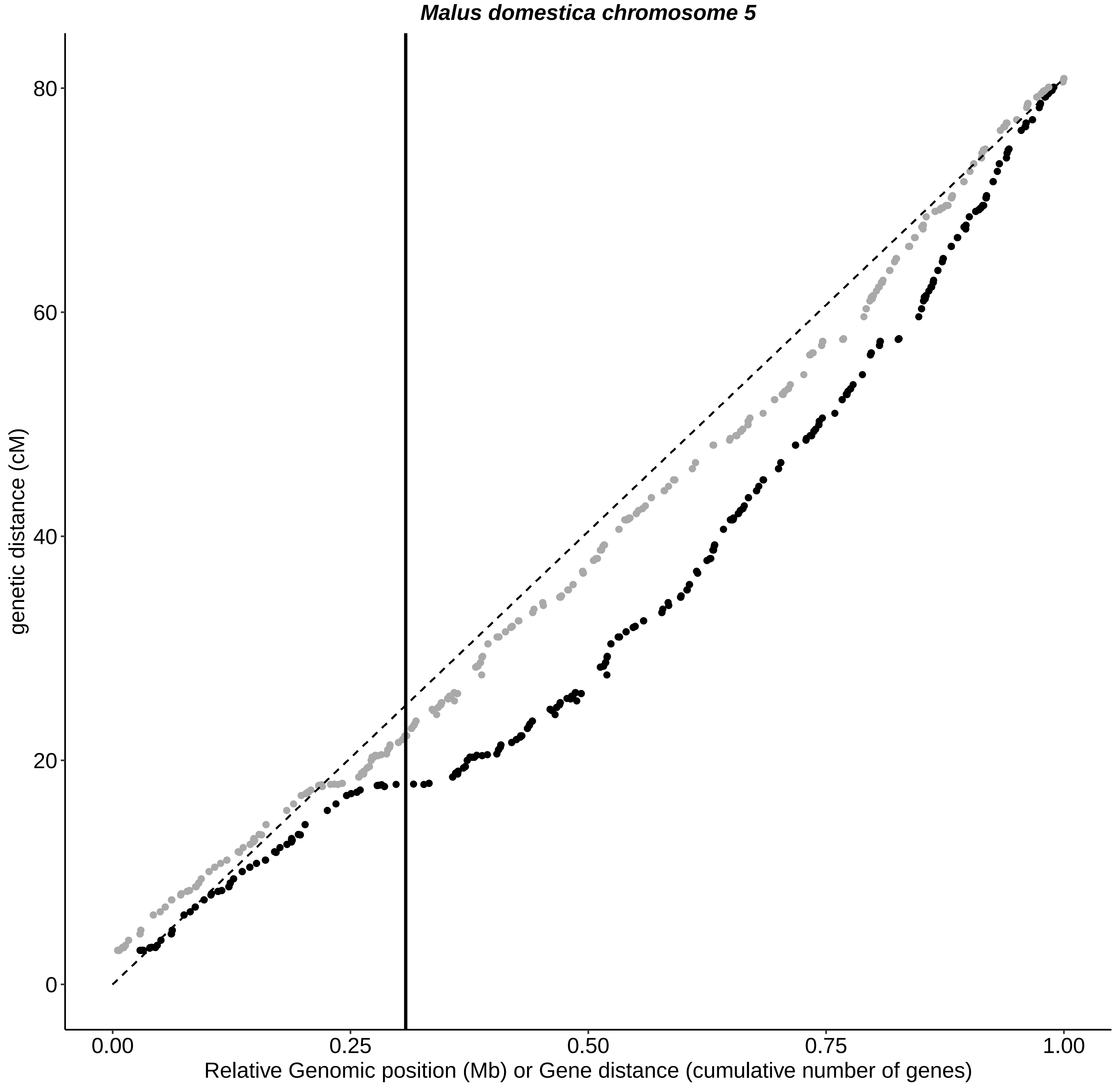
Malus domestica chromosome 5
genetic distance (cM)
0
20
40
60
80
0.00
0.25
0.50
0.75
1.00
Relative Genomic position (Mb) or Gene distance (cumulative number of genes)

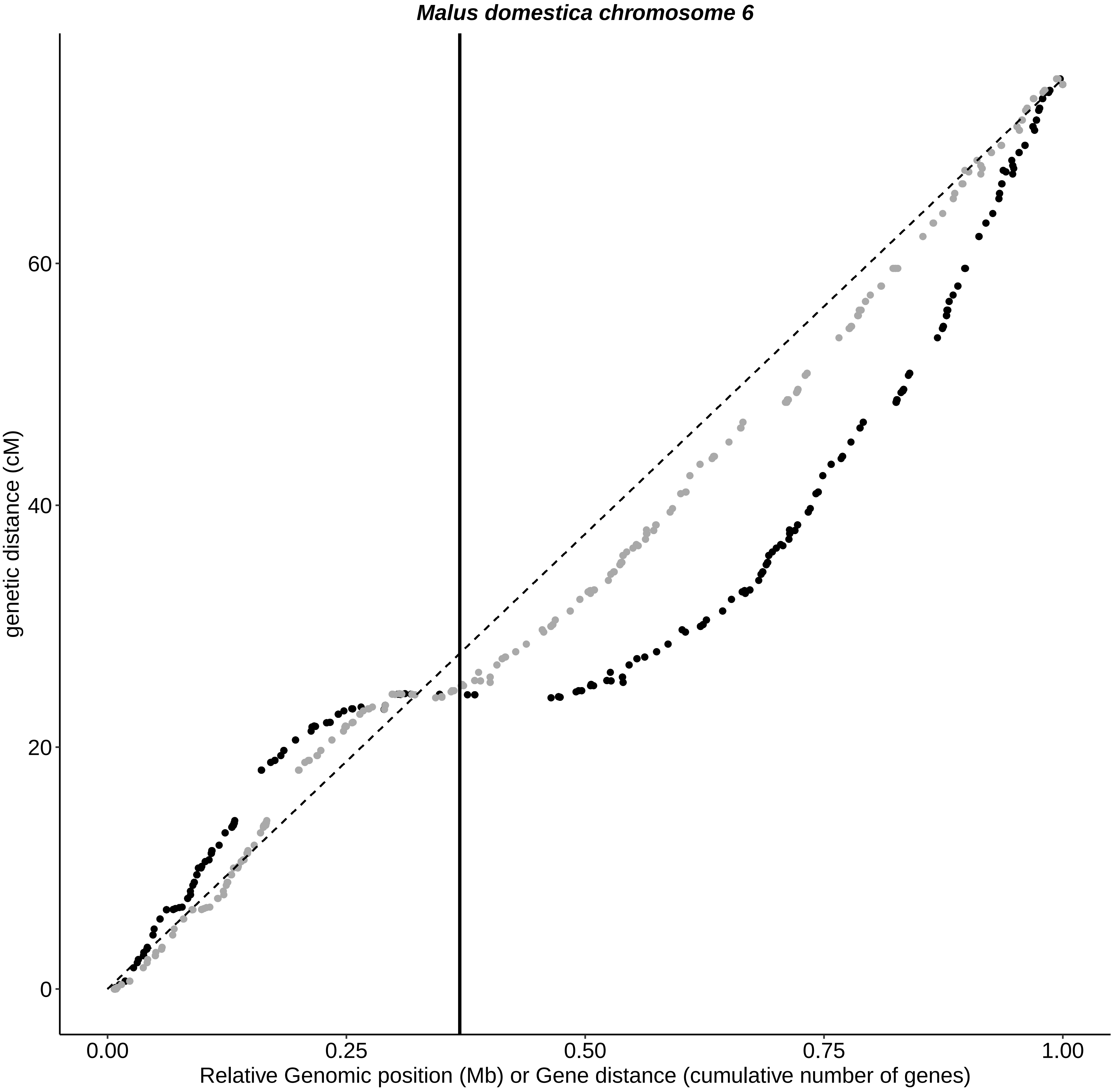
Malus domestica chromosome 6
genetic distance (cM)
0
20
40
60
0.00
0.25
0.50
0.75
1.00
Relative Genomic position (Mb) or Gene distance (cumulative number of genes)

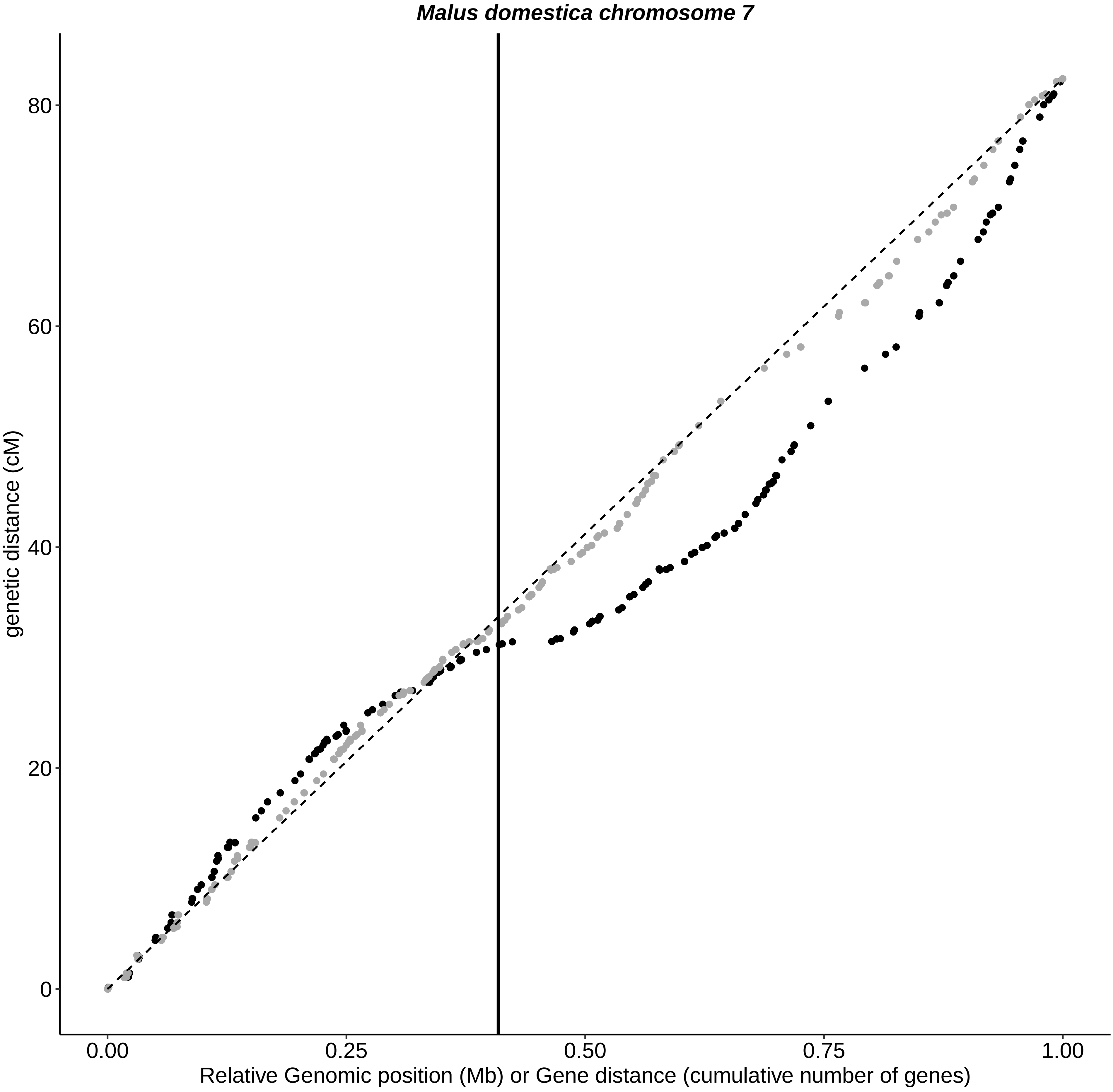
Malus domestica chromosome 7
genetic distance (cM)
0
20
40
60
80
0.00
0.25
0.50
0.75
1.00
Relative Genomic position (Mb) or Gene distance (cumulative number of genes)

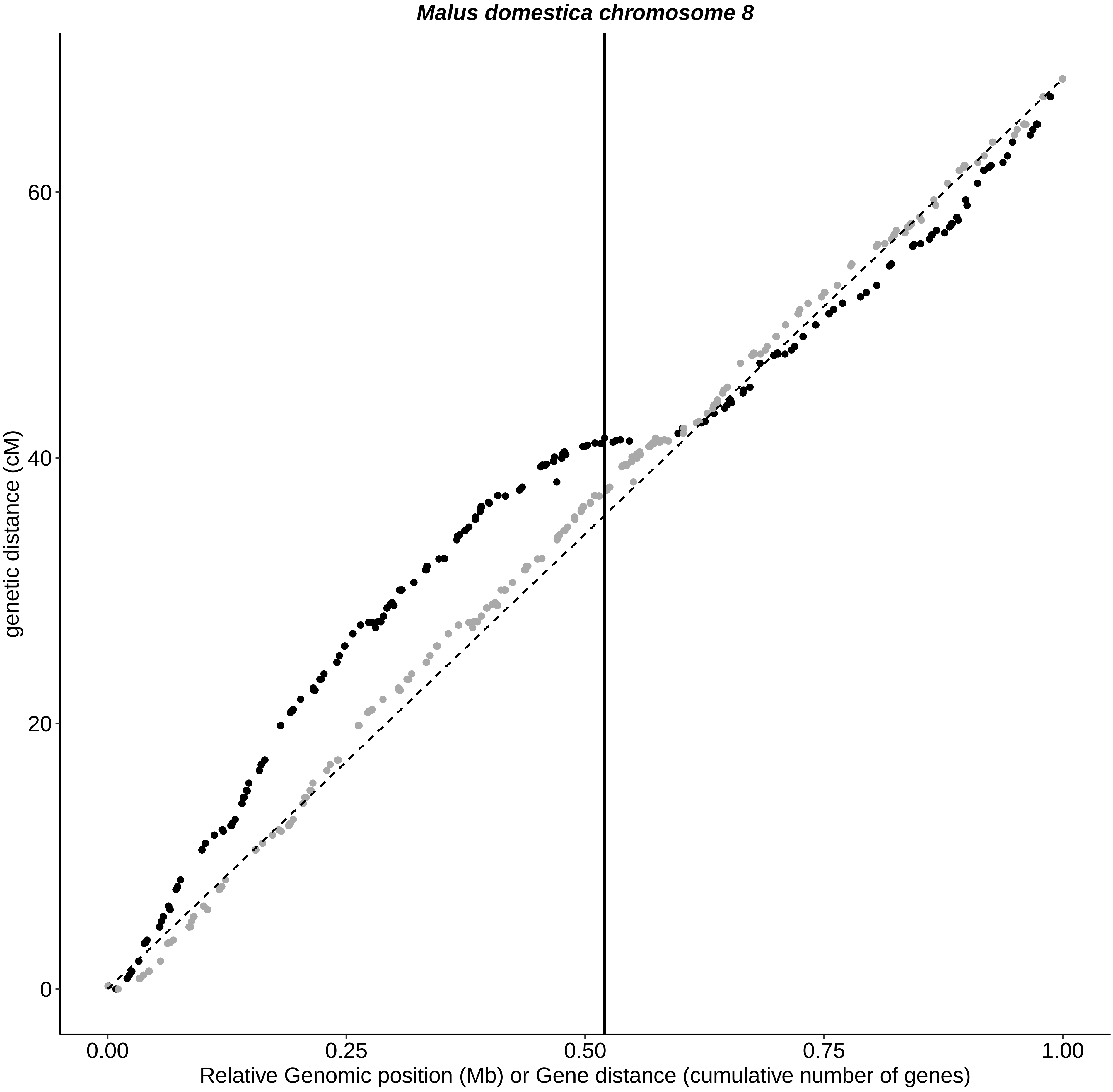
Malus domestica chromosome 8
genetic distance (cM)
0
20
40
60
0.00
0.25
0.50
0.75
1.00
Relative Genomic position (Mb) or Gene distance (cumulative number of genes)

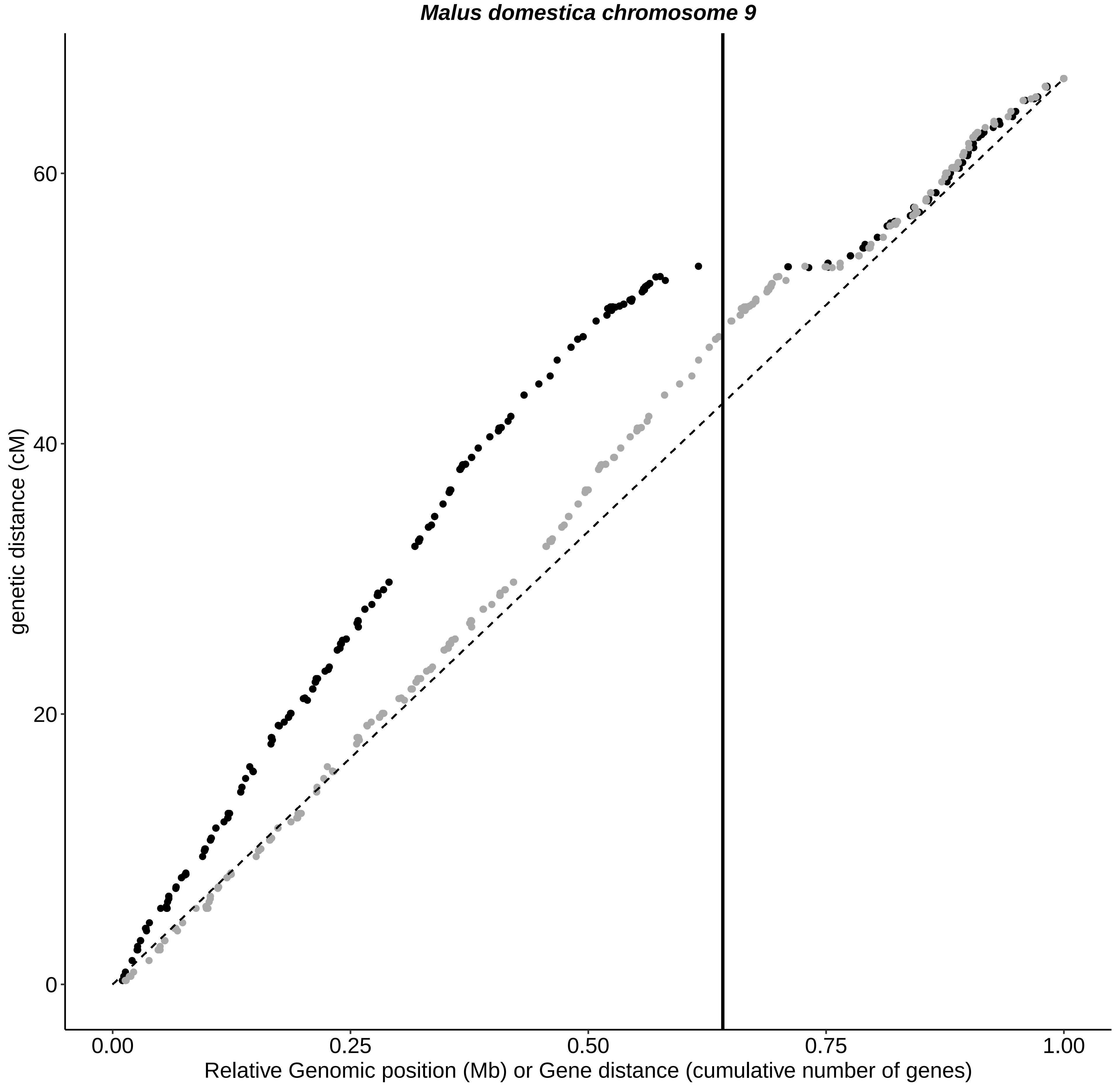
Malus domestica chromosome 9
genetic distance (cM)
0
20
40
60
0.00
0.25
0.50
0.75
1.00
Relative Genomic position (Mb) or Gene distance (cumulative number of genes)

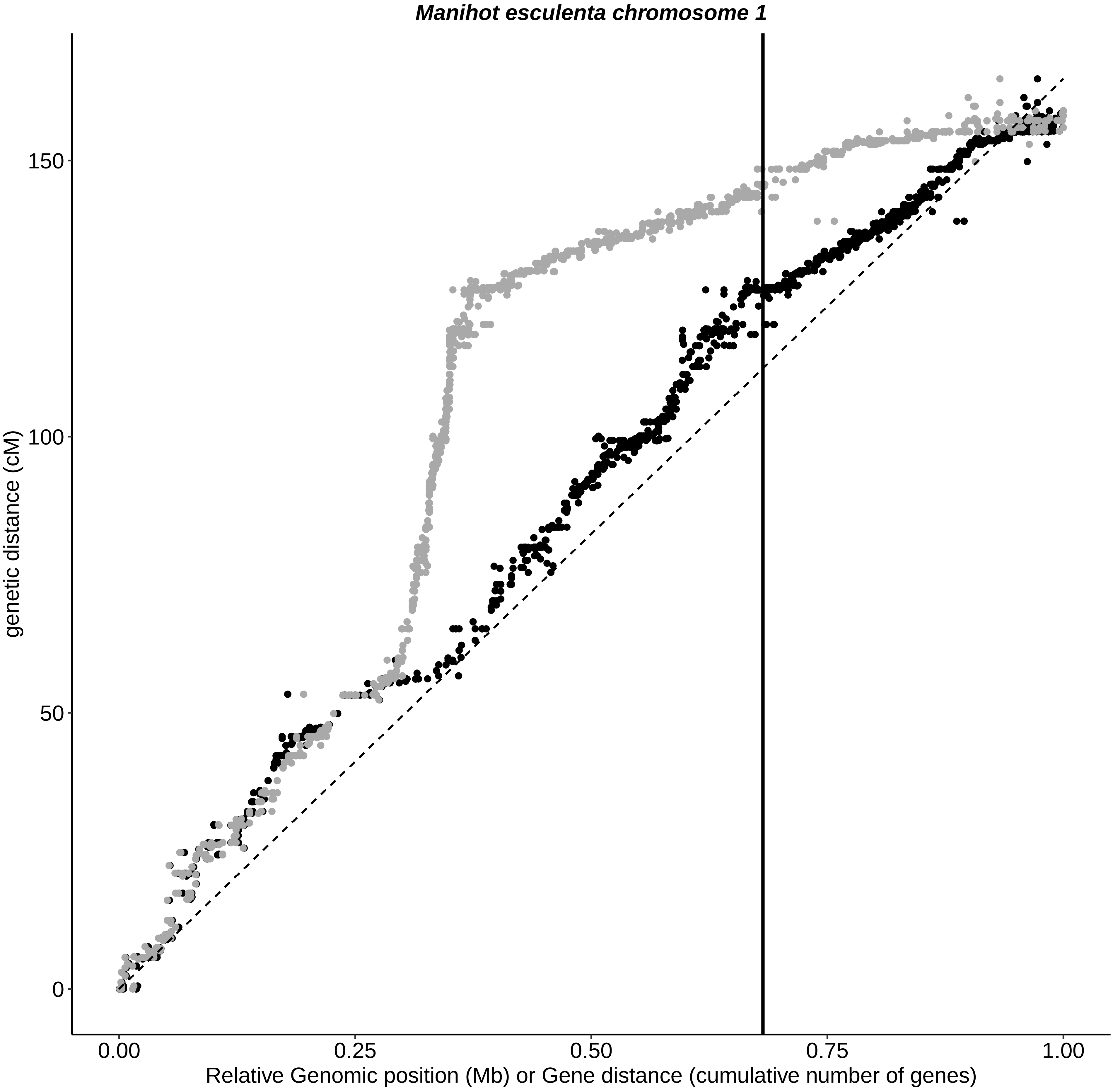
Manihot esculenta chromosome 1
genetic distance (cM)
0
50
100
150
0.00
0.25
0.50
0.75
1.00
Relative Genomic position (Mb) or Gene distance (cumulative number of genes)

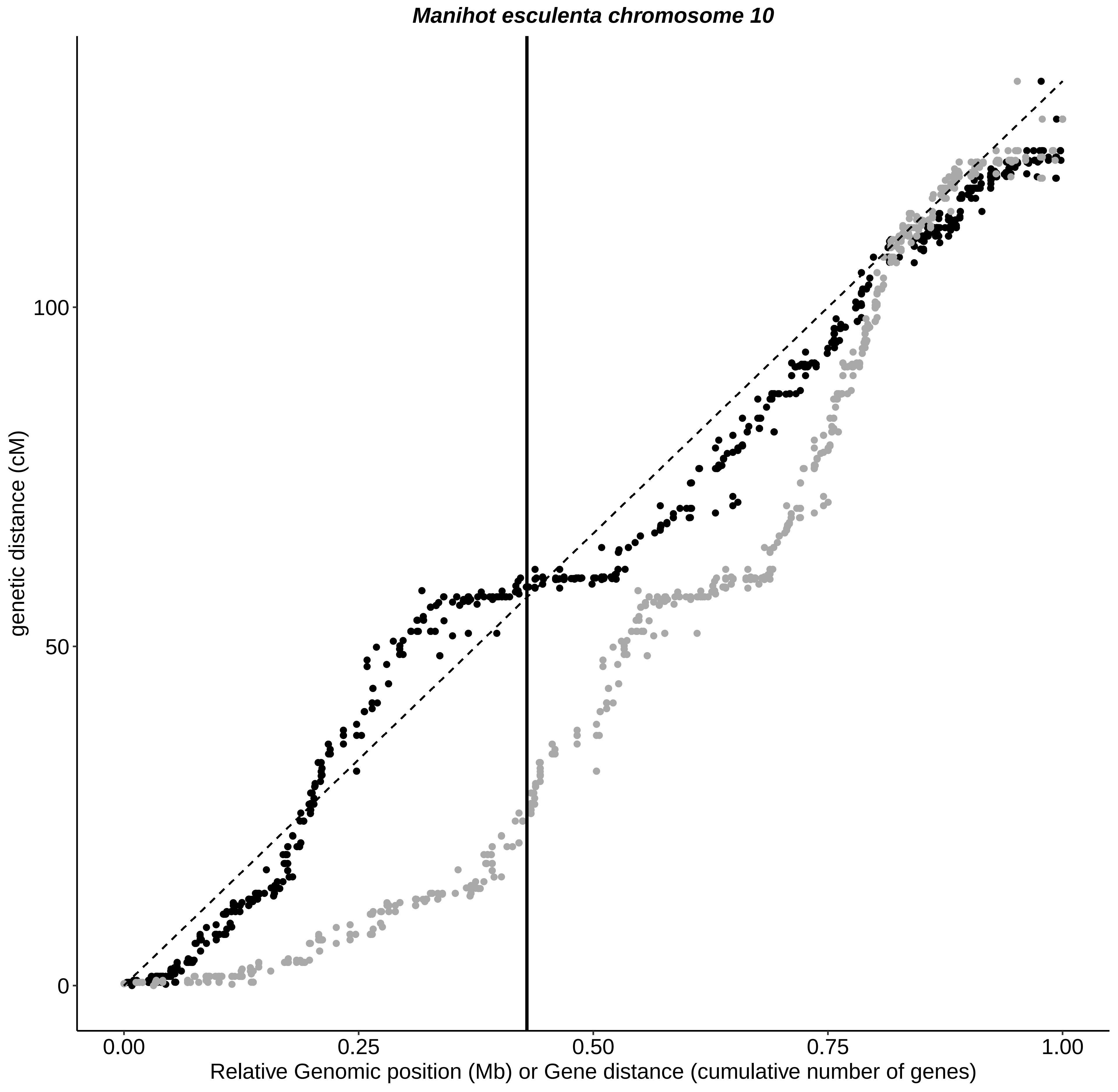
Manihot esculenta chromosome 10
genetic distance (cM)
0
50
100
0.00
0.25
0.50
0.75
1.00
Relative Genomic position (Mb) or Gene distance (cumulative number of genes)

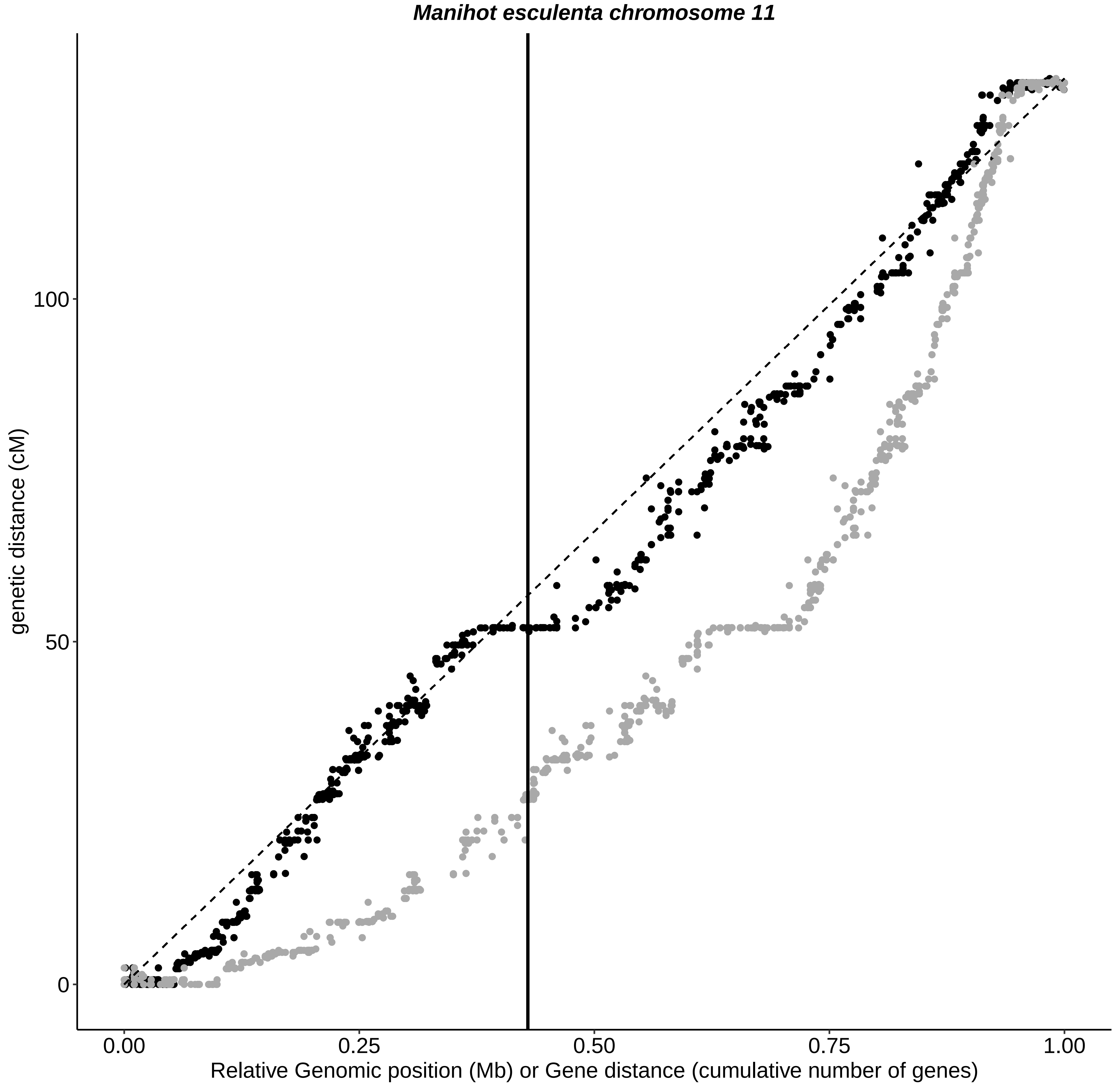

Manihot esculenta chromosome 11
genetic distance (cM)
100
50
0
0.00
0.25
0.50
0.75
1.00
Relative Genomic position (Mb) or Gene distance (cumulative number of genes)

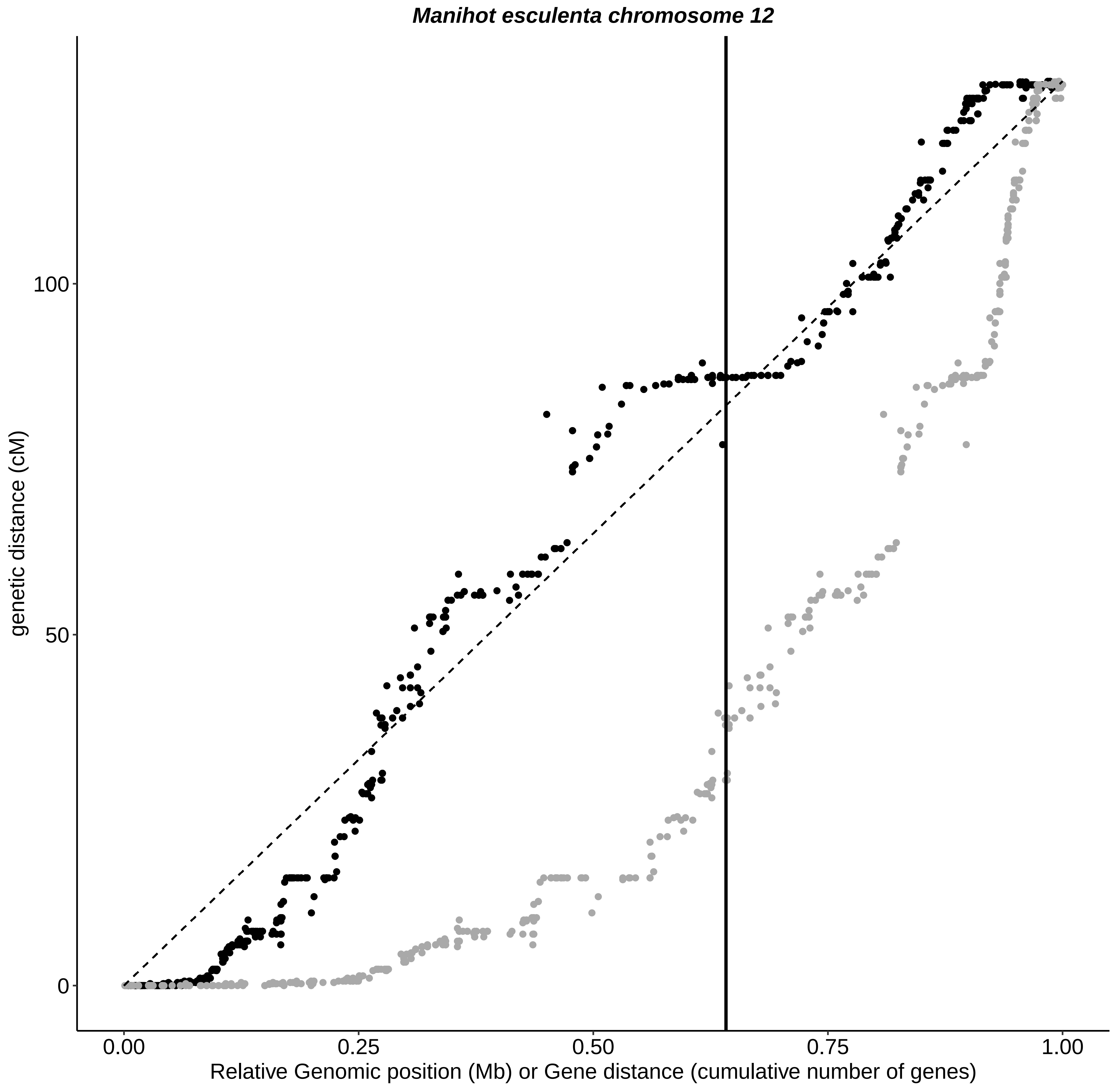

Manihot esculenta chromosome 12
genetic distance (cM)
0
50
100
0.00
0.25
0.50
0.75
1.00
Relative Genomic position (Mb) or Gene distance (cumulative number of genes)

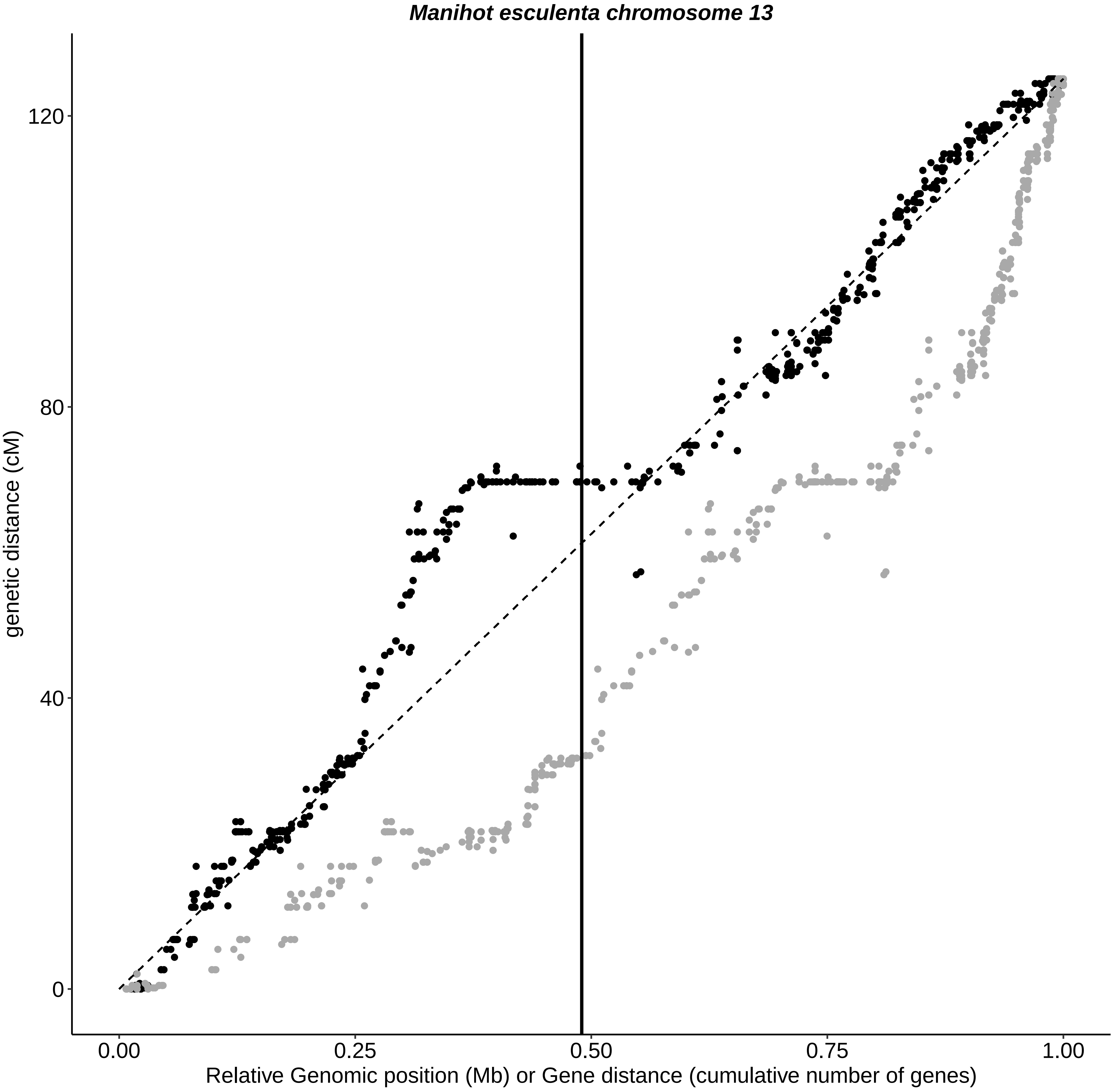
Manihot esculenta chromosome 13
genetic distance (cM)
0
40
80
120
0.00
0.25
0.50
0.75
1.00
Relative Genomic position (Mb) or Gene distance (cumulative number of genes)

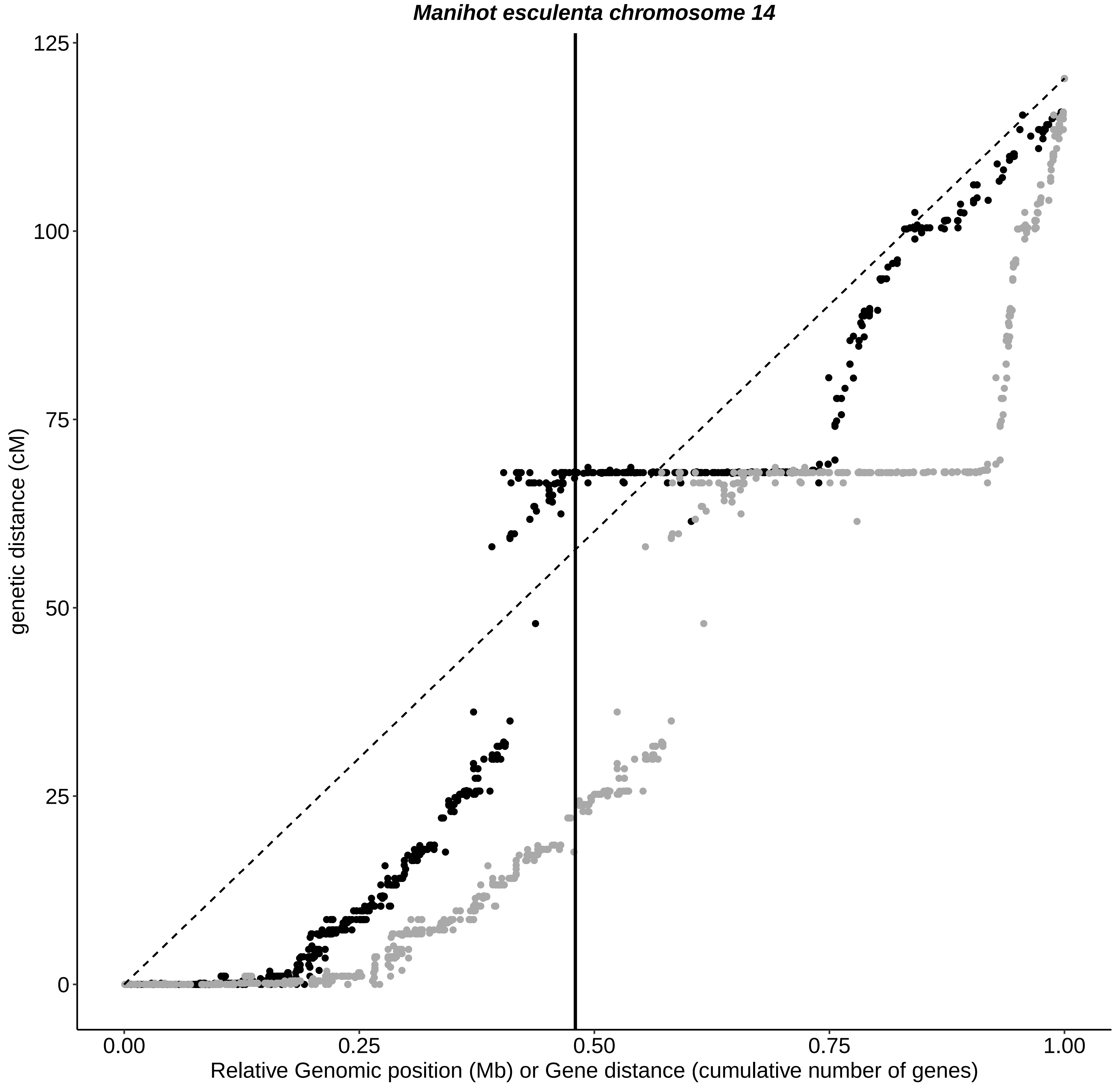

Manihot esculenta chromosome 14
genetic distance (cM)
125
100
75
50
25
0
0.00
0.25
0.50
0.75
1.00
Relative Genomic position (Mb) or Gene distance (cumulative number of genes)

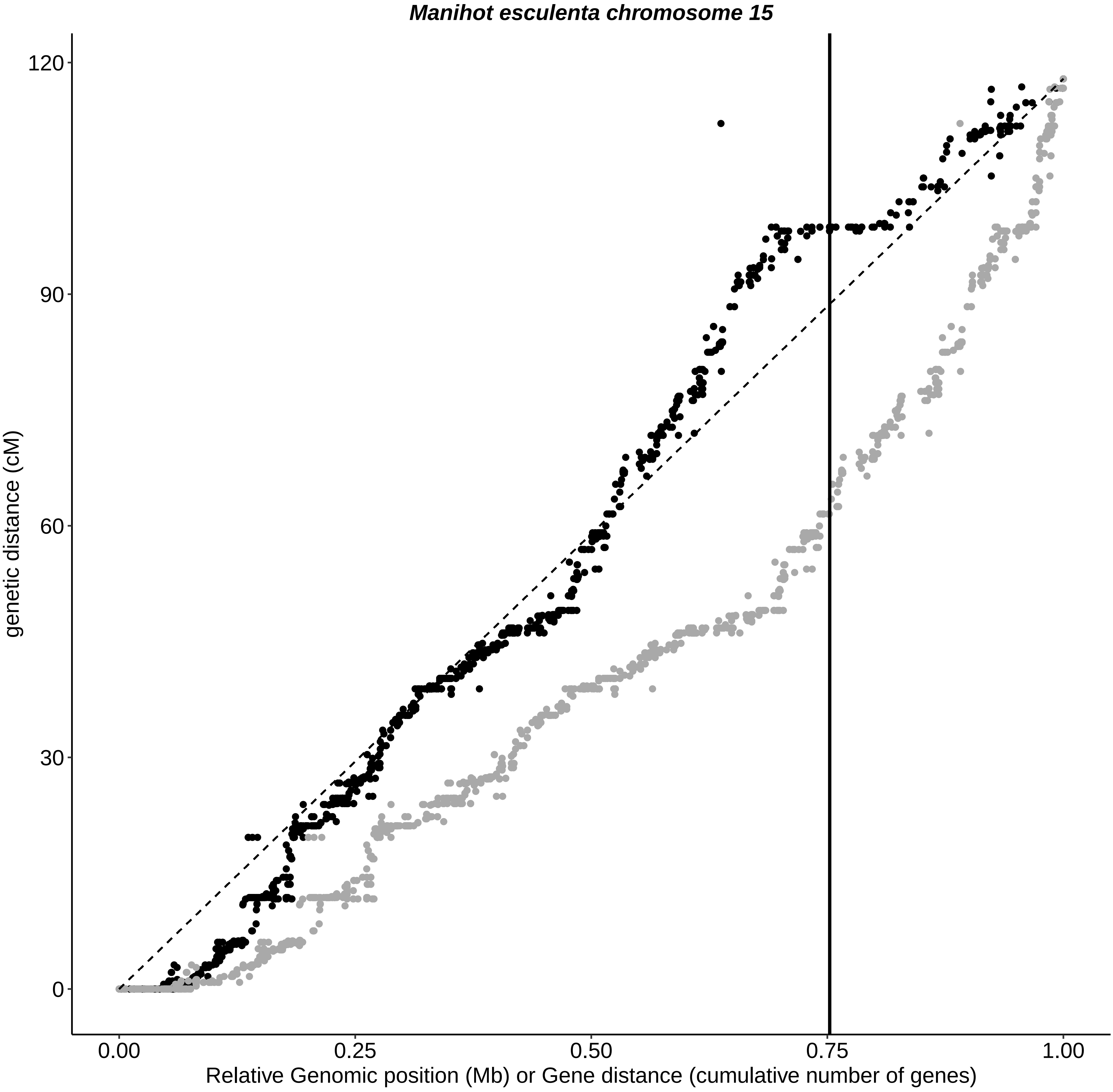
Manihot esculenta chromosome 15
genetic distance (cM)
120
90
60
30
0
0.00
0.25
0.50
0.75
1.00
Relative Genomic position (Mb) or Gene distance (cumulative number of genes)

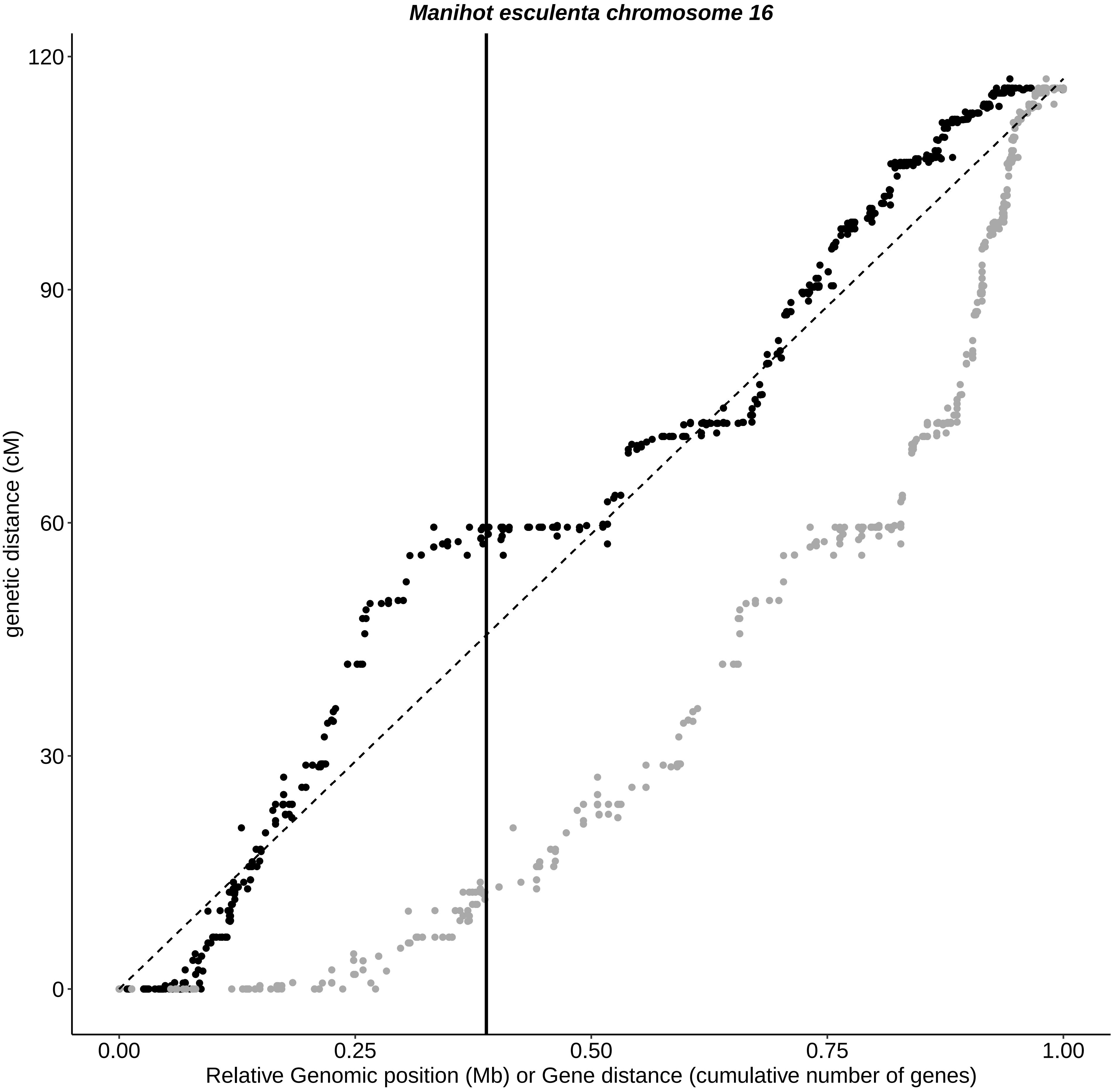
Manihot esculenta chromosome 16
genetic distance (cM)
0
30
60
90
120
0.00
0.25
0.50
0.75
1.00
Relative Genomic position (Mb) or Gene distance (cumulative number of genes)

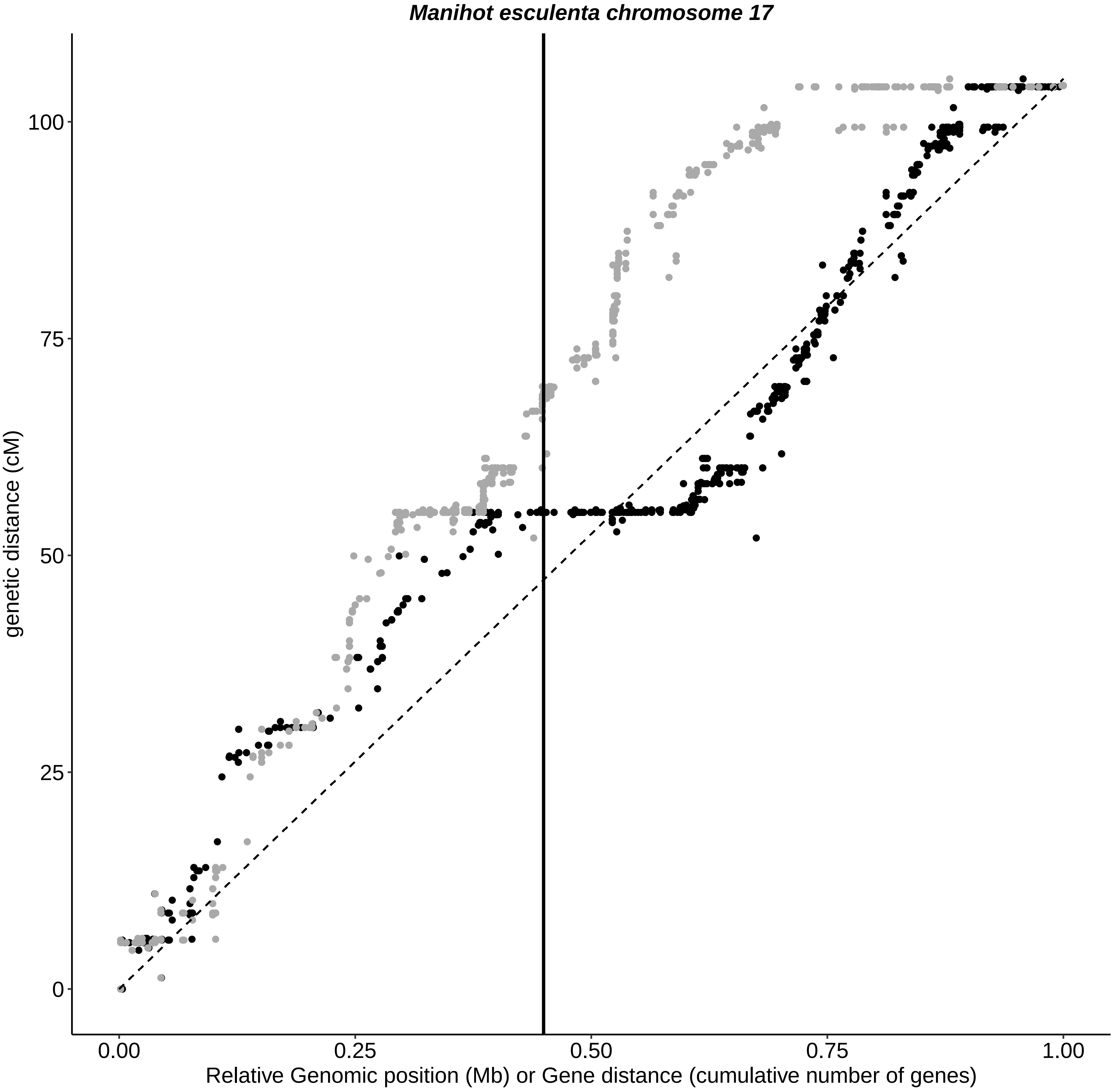

Manihot esculenta chromosome 17
genetic distance (cM)
0
25
50
75
100
0.00
0.25
0.50
0.75
1.00
Relative Genomic position (Mb) or Gene distance (cumulative number of genes)

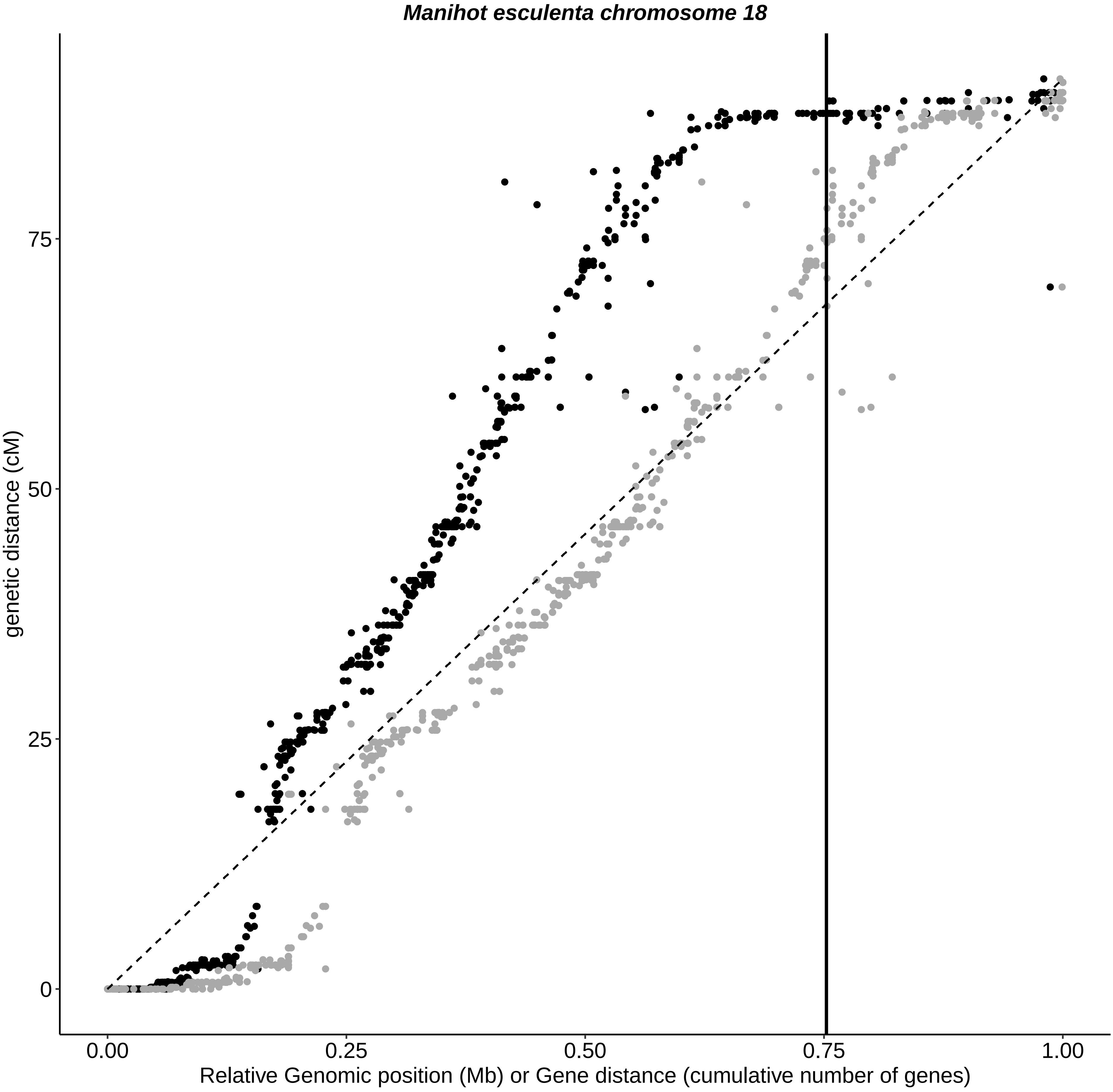
Manihot esculenta chromosome 18
genetic distance (cM)
0
25
50
75
0.00
0.25
0.50
0.75
1.00
Relative Genomic position (Mb) or Gene distance (cumulative number of genes)

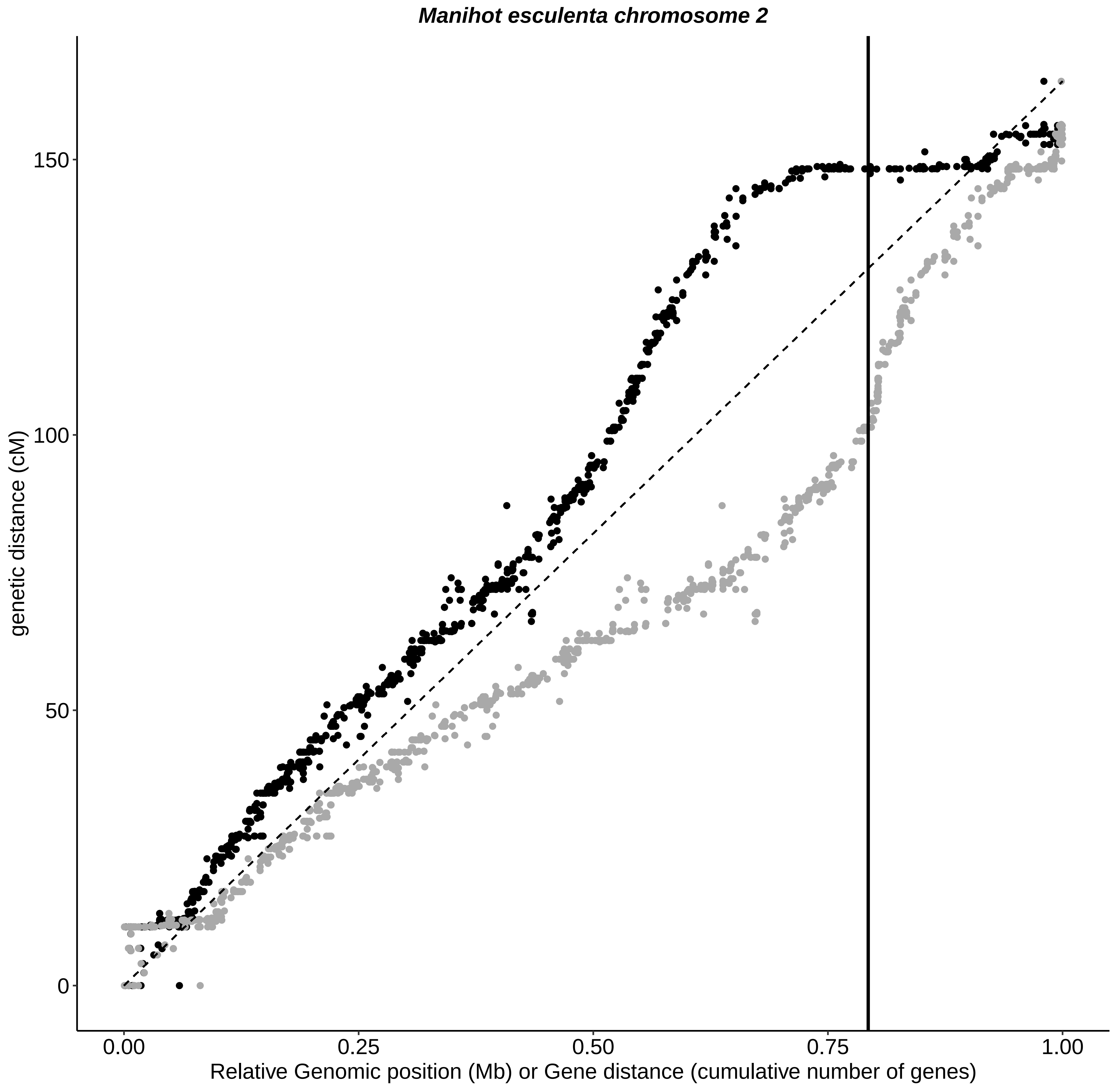
Manihot esculenta chromosome 2
genetic distance (cM)
150
100
50
0
0.00
0.25
0.50
0.75
1.00
Relative Genomic position (Mb) or Gene distance (cumulative number of genes)

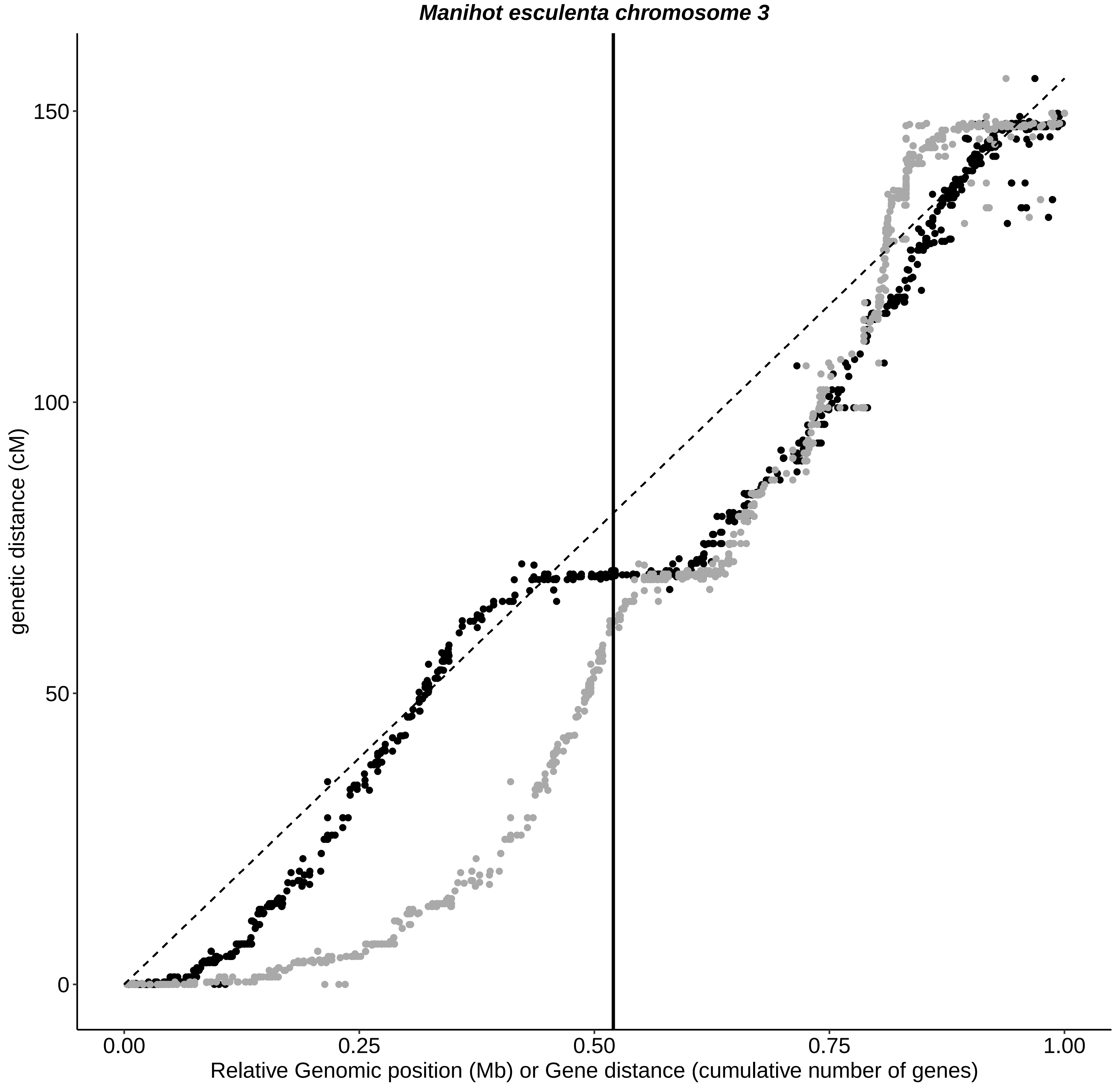
Manihot esculenta chromosome 3
genetic distance (cM)
150
100
50
0
0.00
0.25
0.50
0.75
1.00
Relative Genomic position (Mb) or Gene distance (cumulative number of genes)

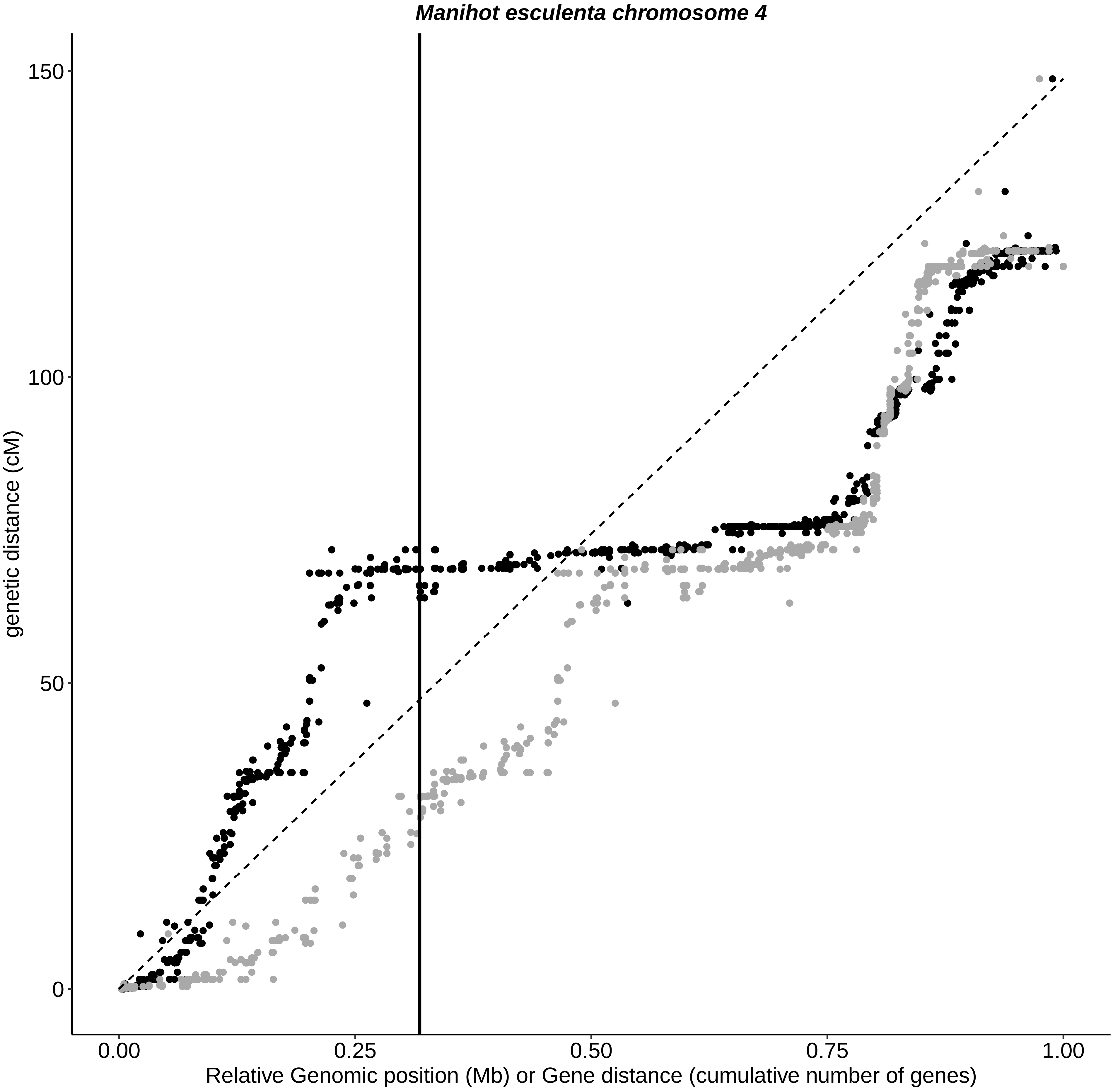
Manihot esculenta chromosome 4
genetic distance (cM)
150
100
50
0
0.00
0.25
0.50
0.75
1.00
Relative Genomic position (Mb) or Gene distance (cumulative number of genes)

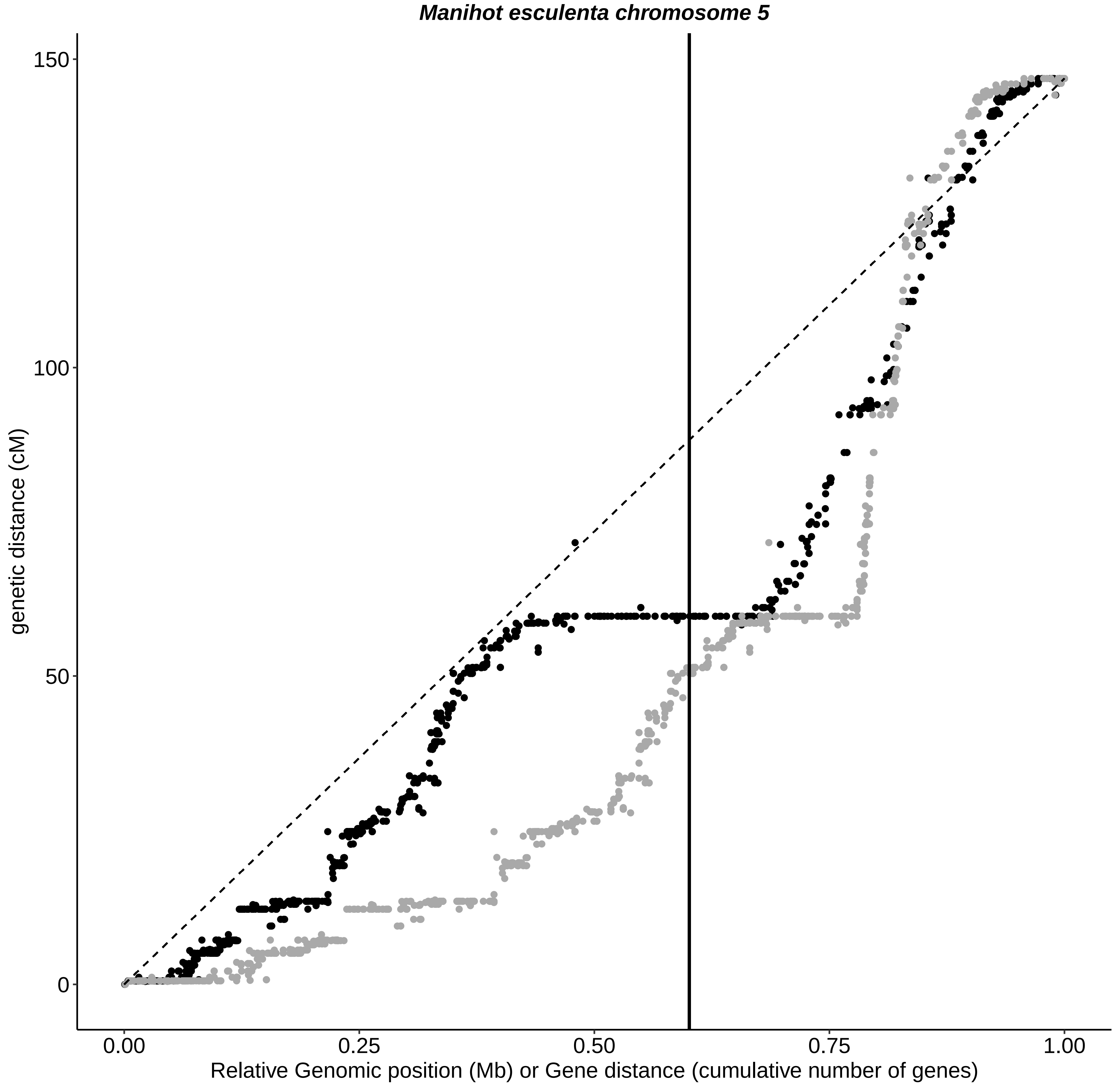
Manihot esculenta chromosome 5
genetic distance (cM)
150
100
50
0
0.00
0.25
0.50
0.75
1.00
Relative Genomic position (Mb) or Gene distance (cumulative number of genes)

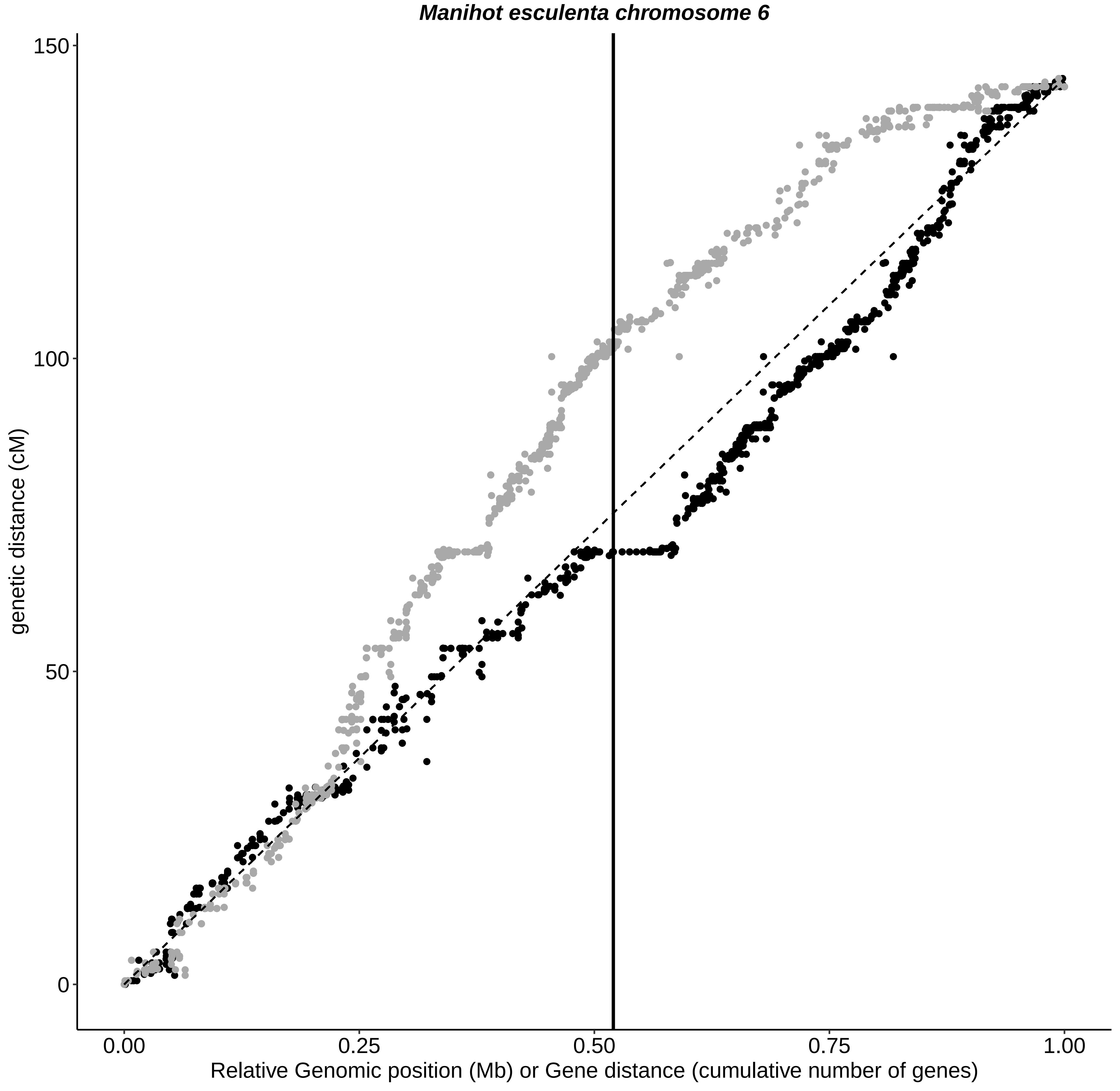
Manihot esculenta chromosome 6
genetic distance (cM)
150
100
50
0
0.00
0.25
0.50
0.75
1.00
Relative Genomic position (Mb) or Gene distance (cumulative number of genes)

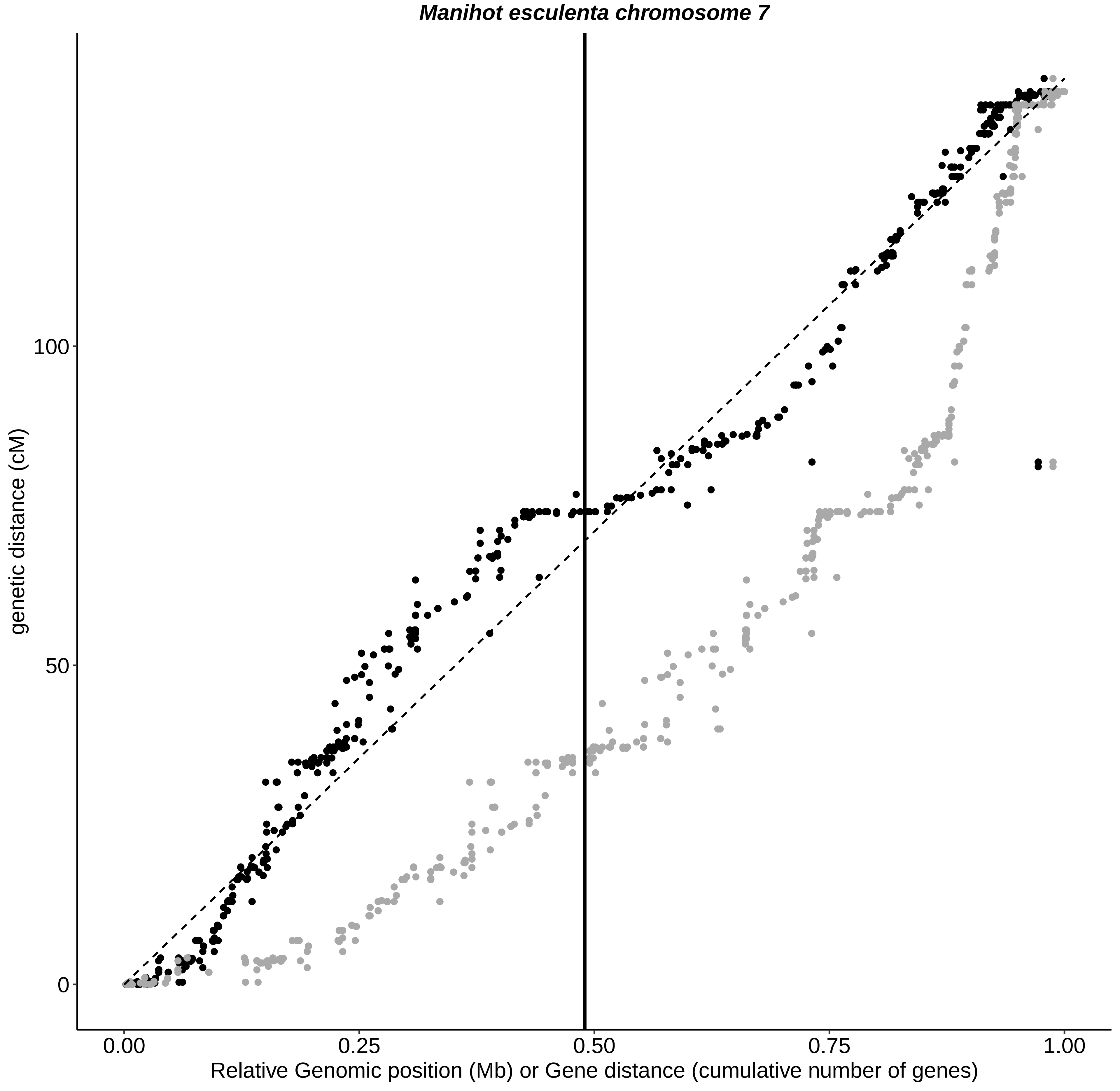
Manihot esculenta chromosome 7
genetic distance (cM)
100
50
0
0.00
0.25
0.50
0.75
1.00
Relative Genomic position (Mb) or Gene distance (cumulative number of genes)

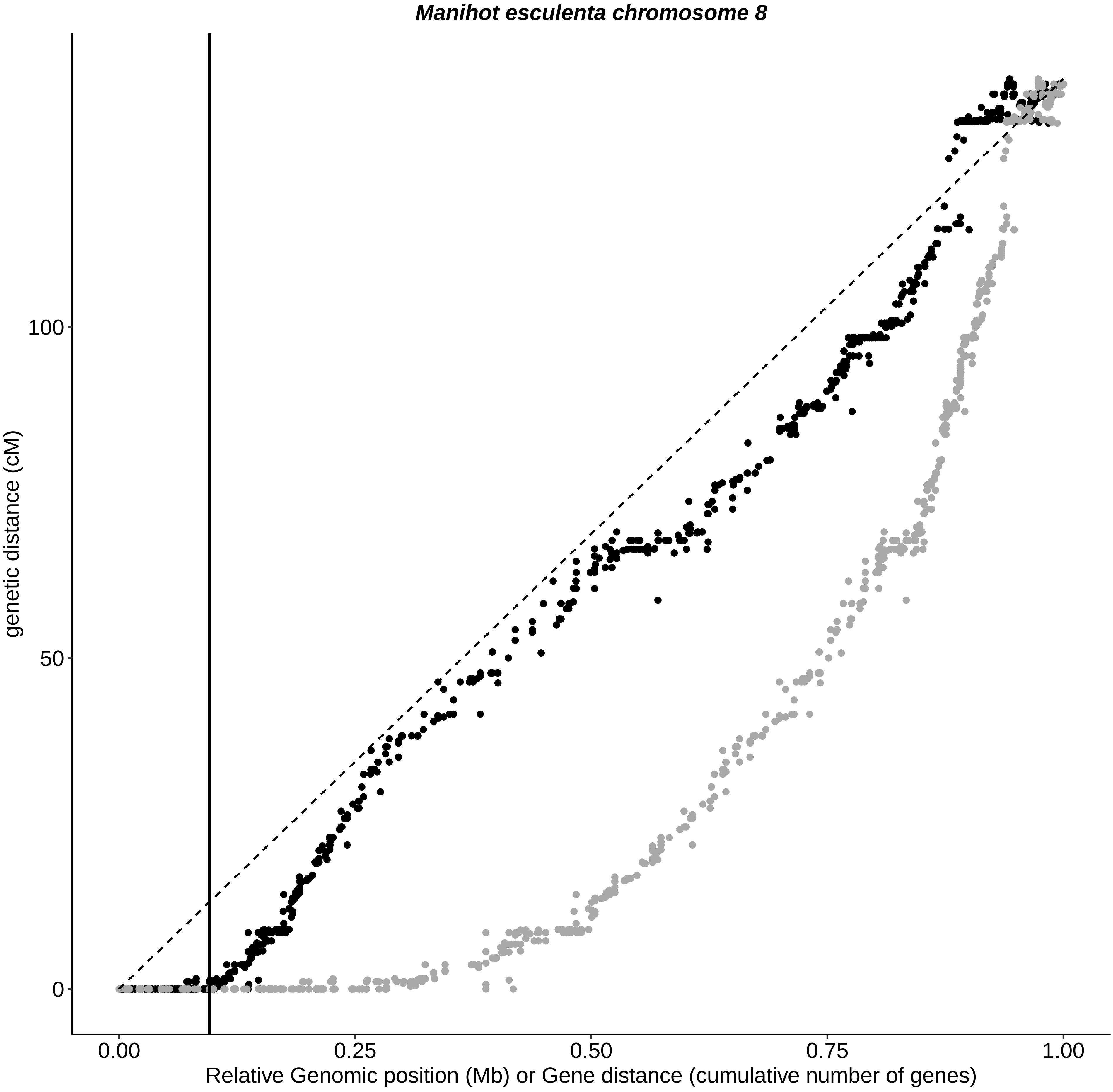
Manihot esculenta chromosome 8
genetic distance (cM)
100
50
0
0.00
0.25
0.50
0.75
1.00
Relative Genomic position (Mb) or Gene distance (cumulative number of genes)

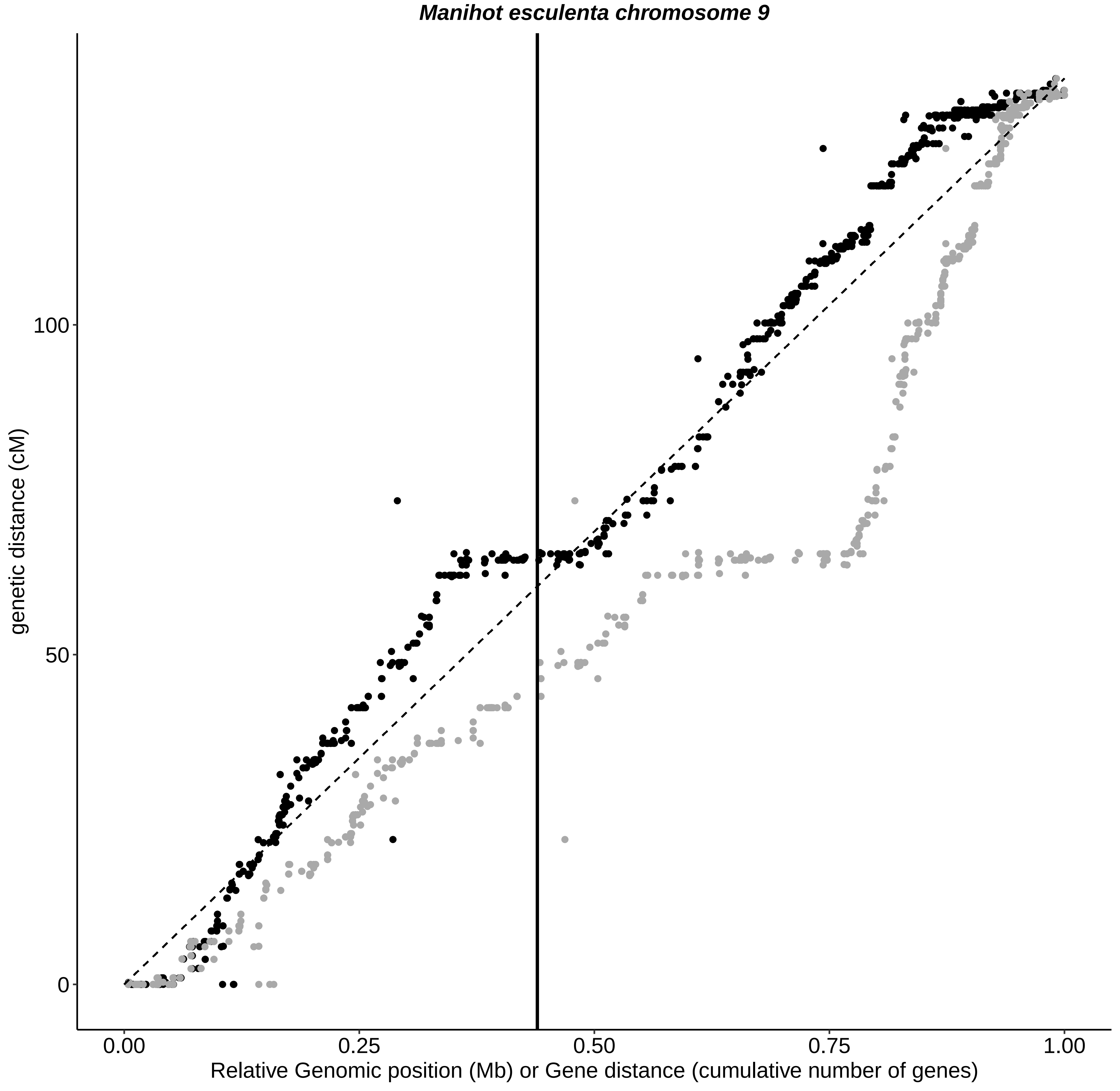
Manihot esculenta chromosome 9
genetic distance (cM)
100
50
0
0.00
0.25
0.50
0.75
1.00
Relative Genomic position (Mb) or Gene distance (cumulative number of genes)

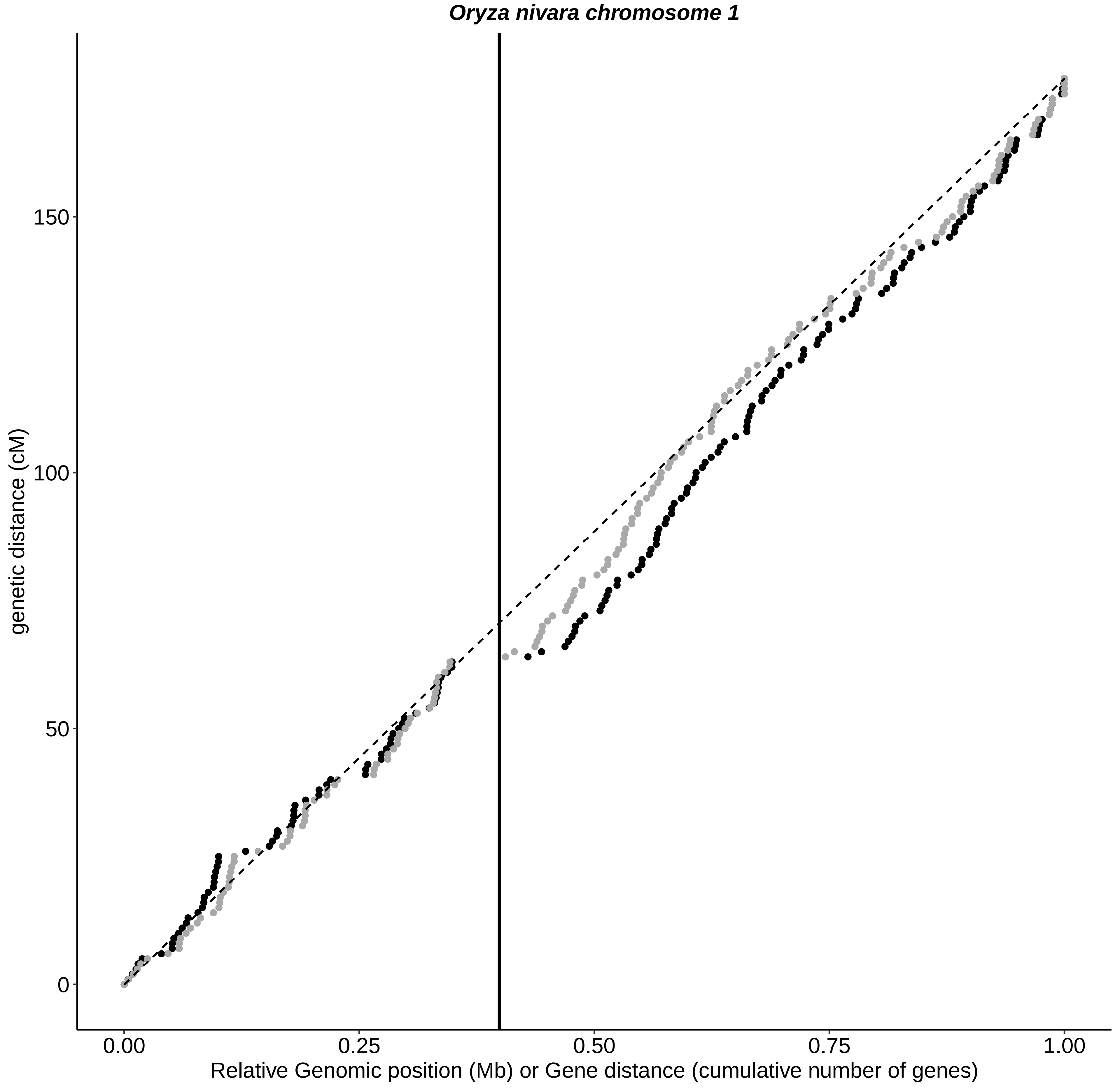
Oryza nivara chromosome 1
genetic distance (cM)
0
50
100
150
0.00
0.25
0.50
0.75
1.00
Relative Genomic position (Mb) or Gene distance (cumulative number of genes)

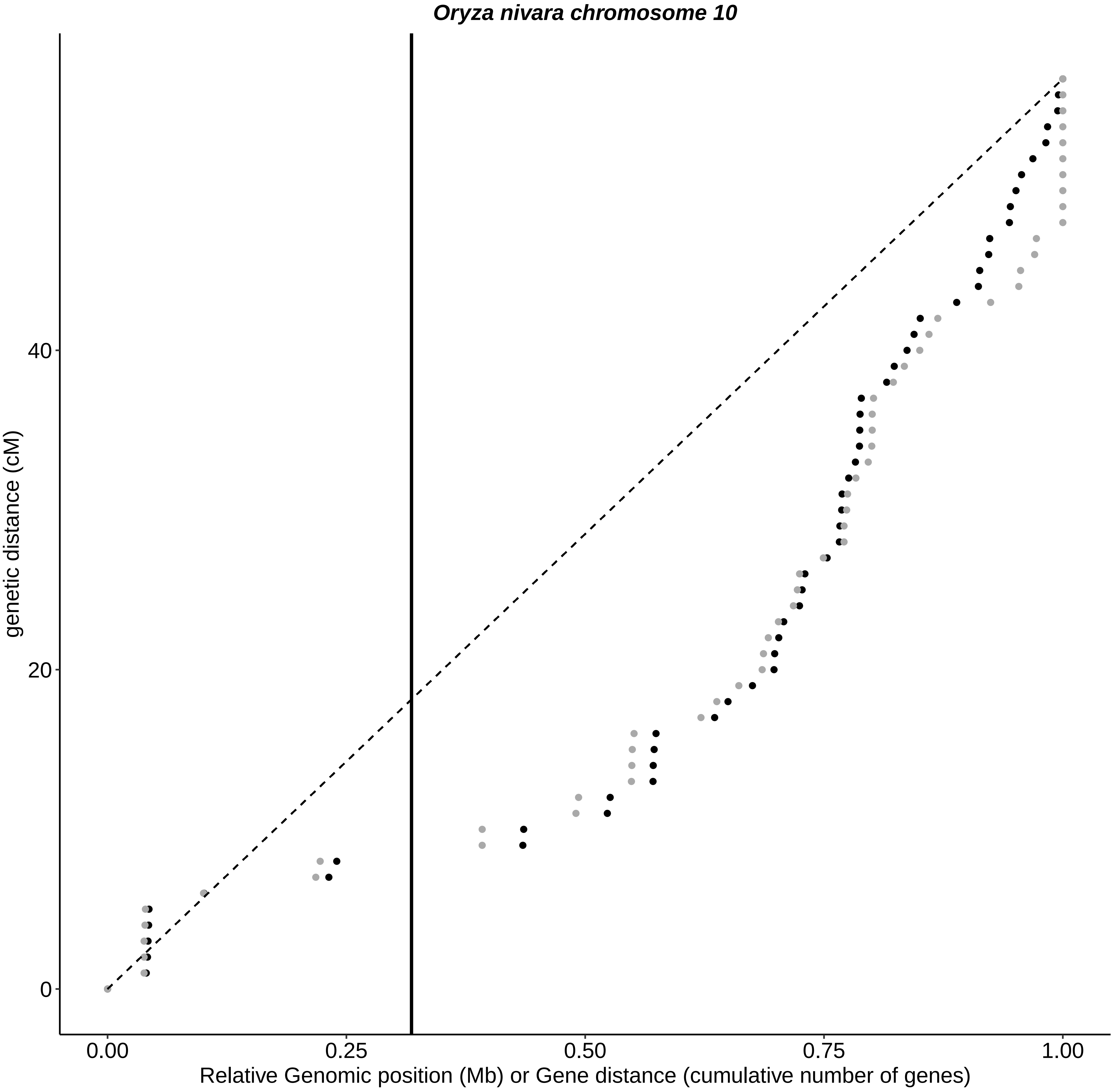
Oryza nivara chromosome 10
genetic distance (cM)
0
20
40
0.00
0.25
0.50
0.75
1.00
Relative Genomic position (Mb) or Gene distance (cumulative number of genes)

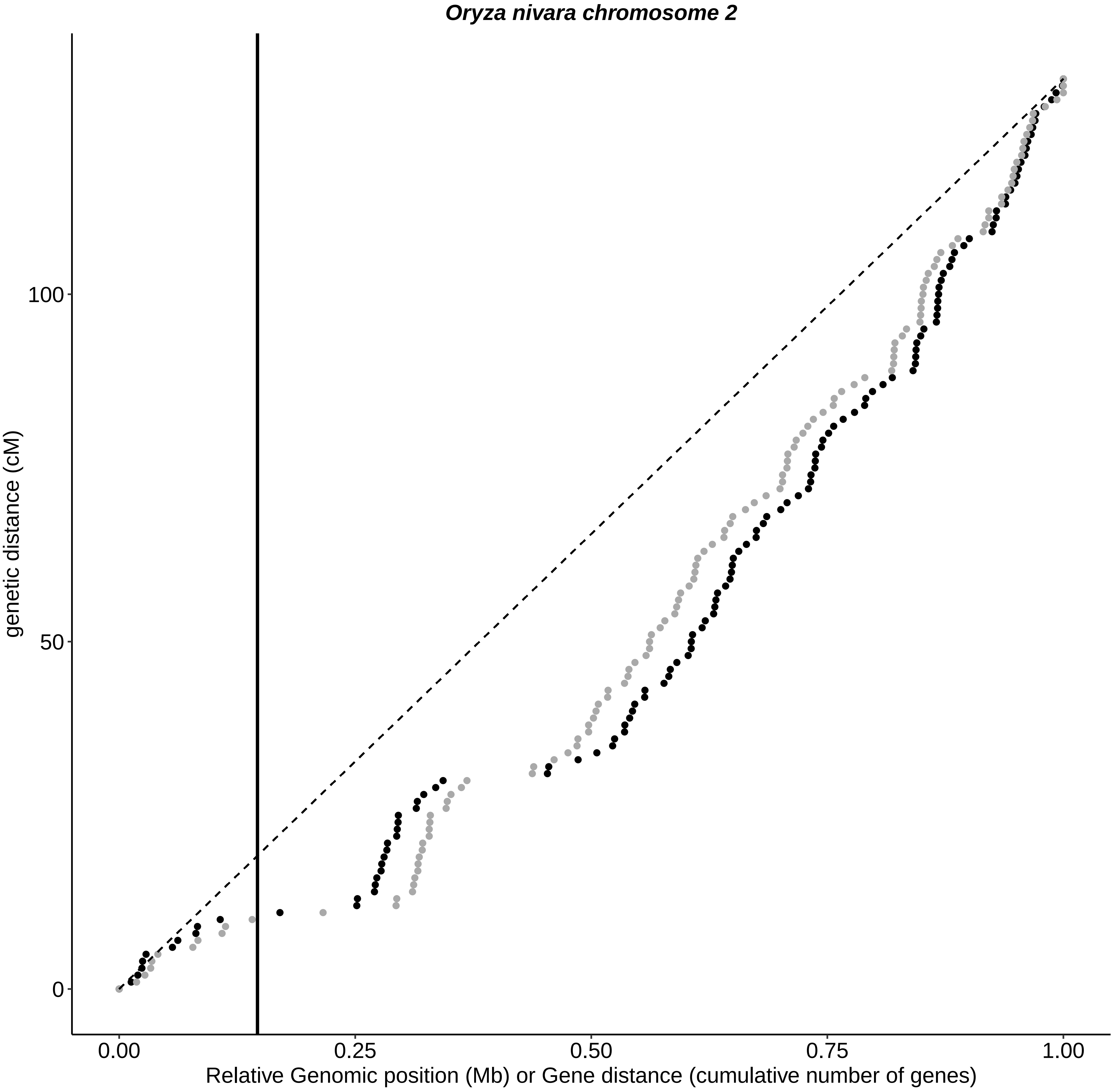
Oryza nivara chromosome 2
genetic distance (cM)
100
50
0
0.00
0.25
0.50
0.75
1.00
Relative Genomic position (Mb) or Gene distance (cumulative number of genes)

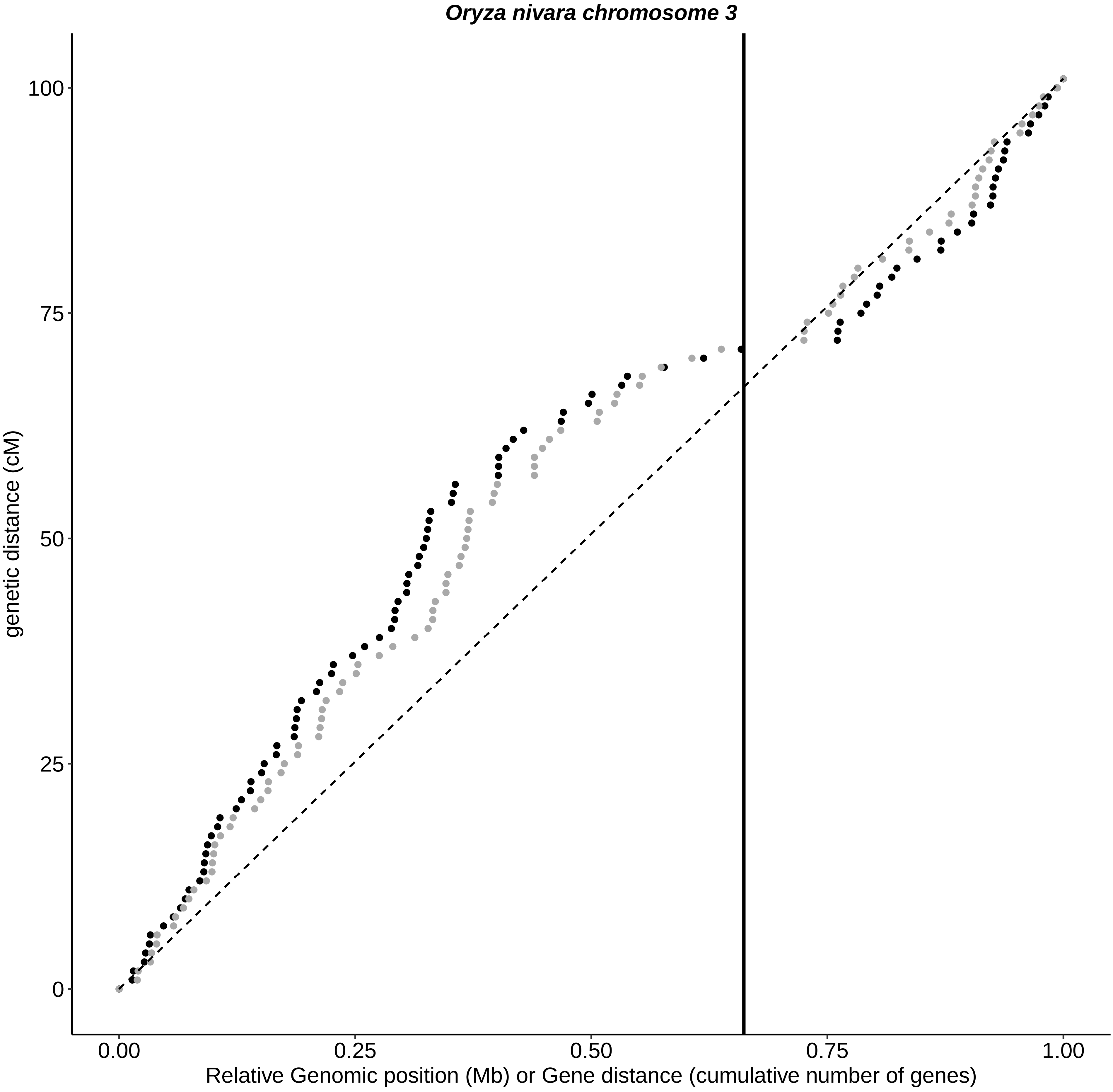
Oryza nivara chromosome 3
genetic distance (cM)
0
25
50
75
100
0.00
0.25
0.50
0.75
1.00
Relative Genomic position (Mb) or Gene distance (cumulative number of genes)

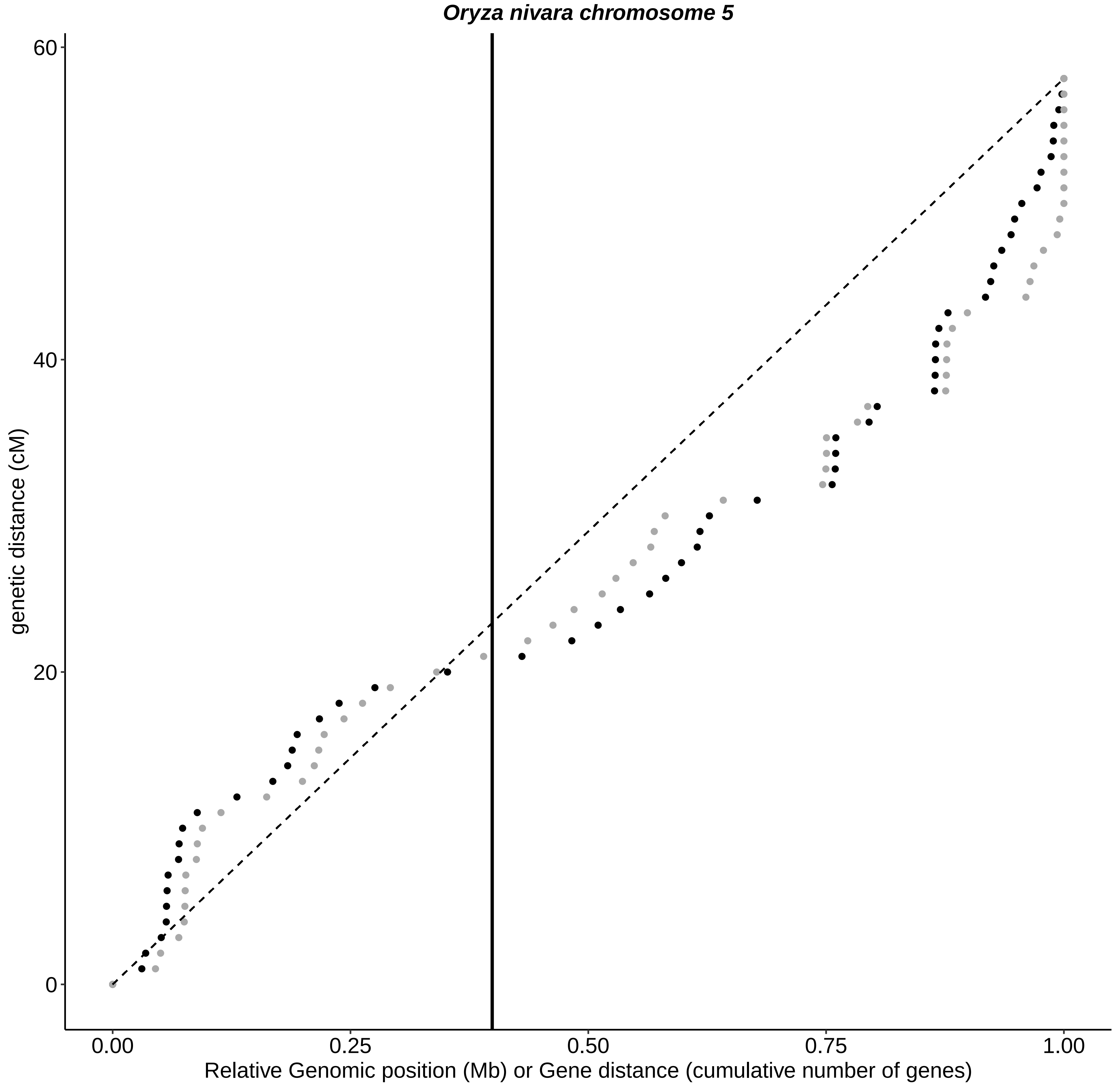
Oryza nivara chromosome 5
genetic distance (cM)
60
40
20
0
0.00
0.25
0.50
0.75
1.00
Relative Genomic position (Mb) or Gene distance (cumulative number of genes)

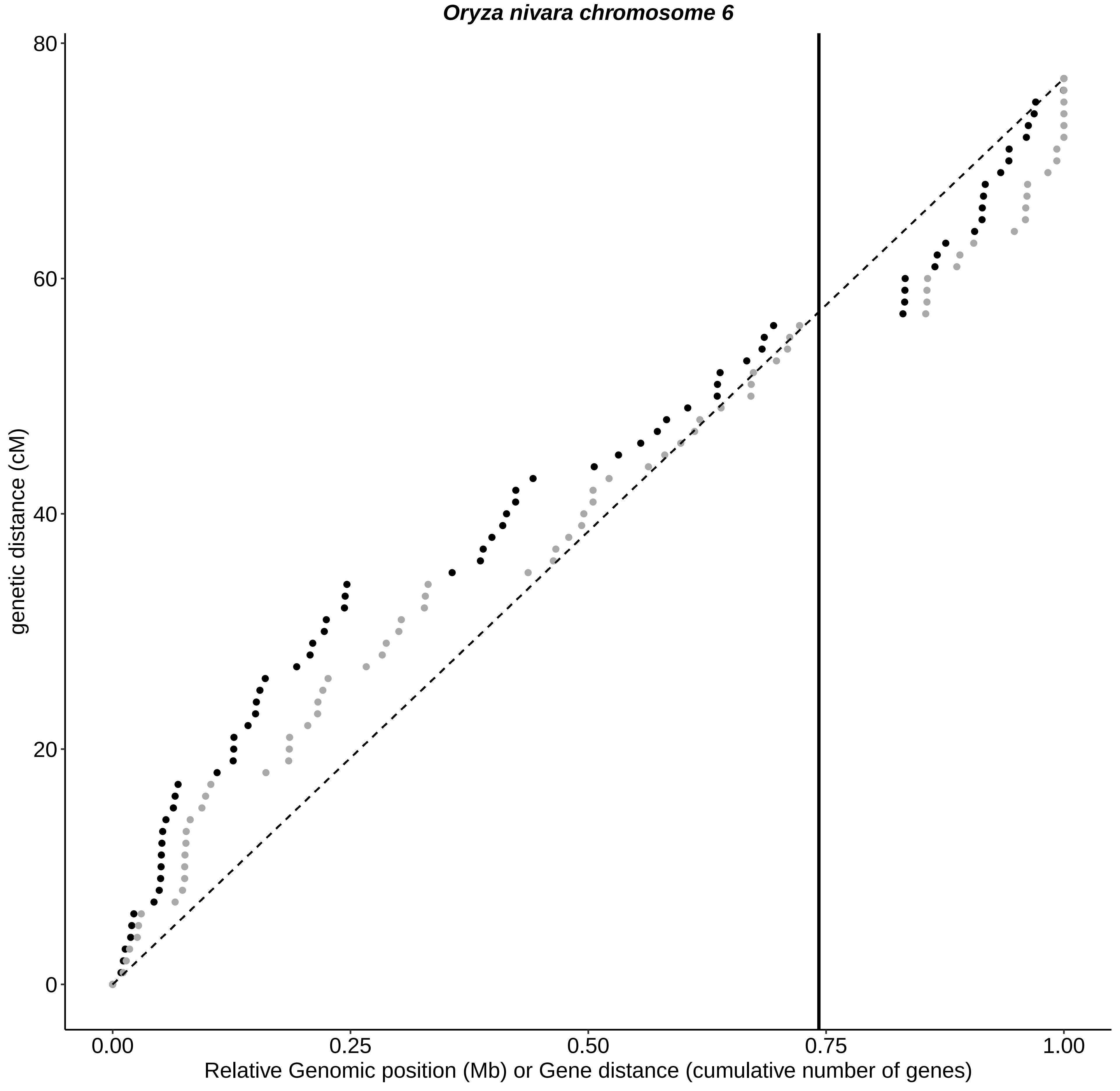
Oryza nivara chromosome 6
genetic distance (cM)
0
20
40
60
80
0.00
0.25
0.50
0.75
1.00
Relative Genomic position (Mb) or Gene distance (cumulative number of genes)

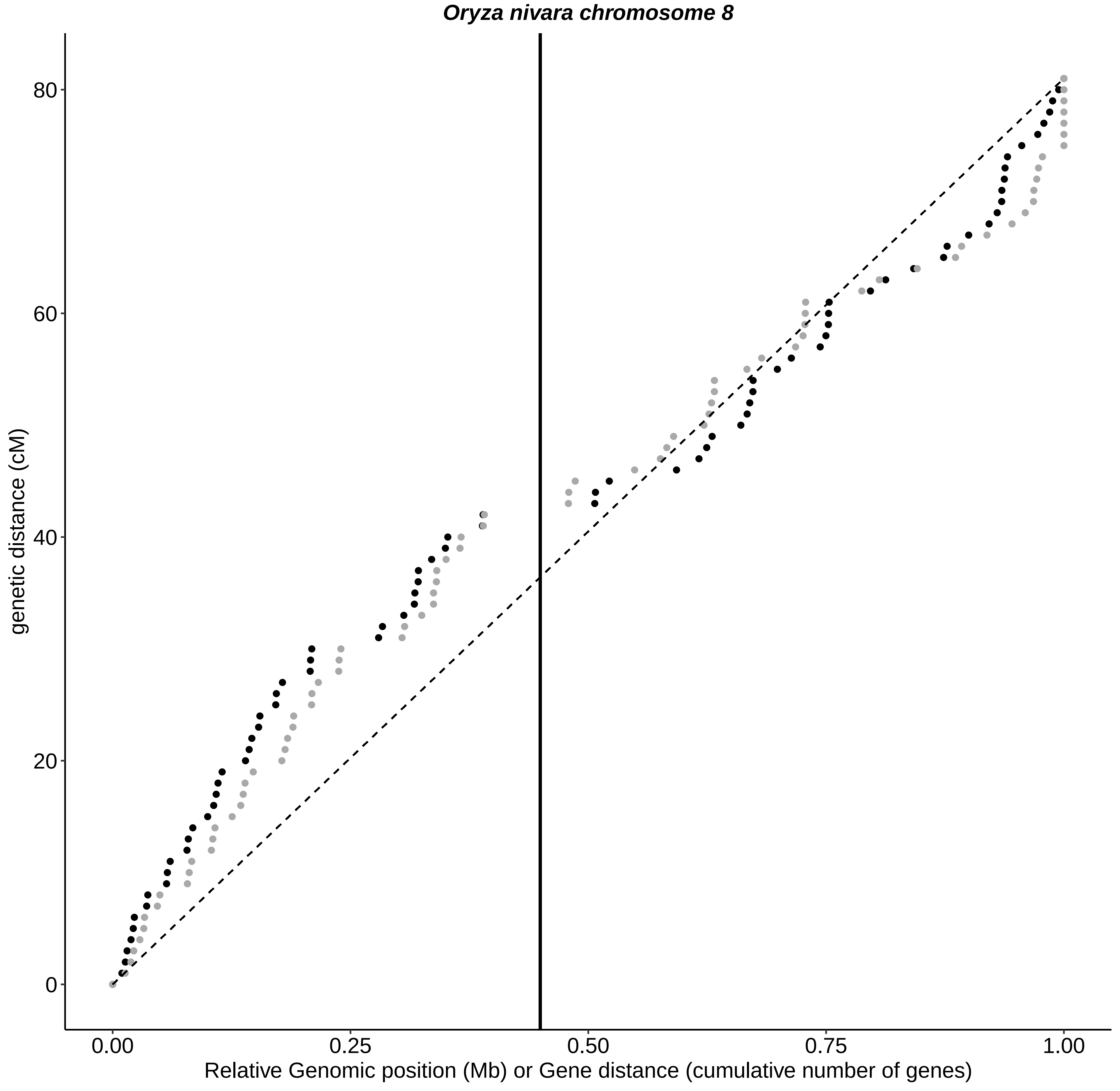
Oryza nivara chromosome 8
genetic distance (cM)
Relative Genomic position (Mb) or Gene distance (cumulative number of genes)
0
20
40
60
80
0.00
0.25
0.50
0.75
1.00

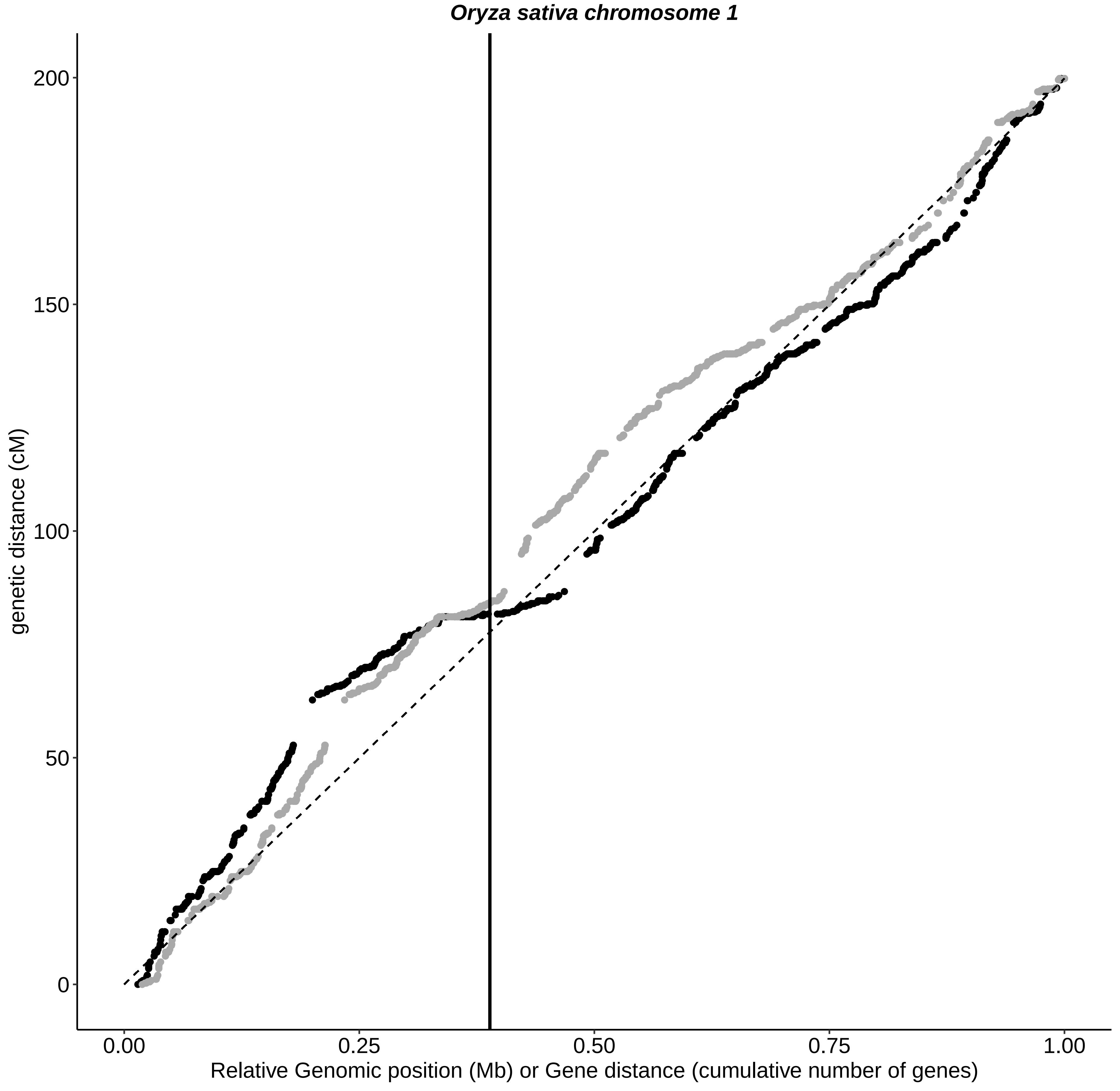
Oryza sativa chromosome 1
genetic distance (cM)
0
50
100
150
200
0.00
0.25
0.50
0.75
1.00
Relative Genomic position (Mb) or Gene distance (cumulative number of genes)

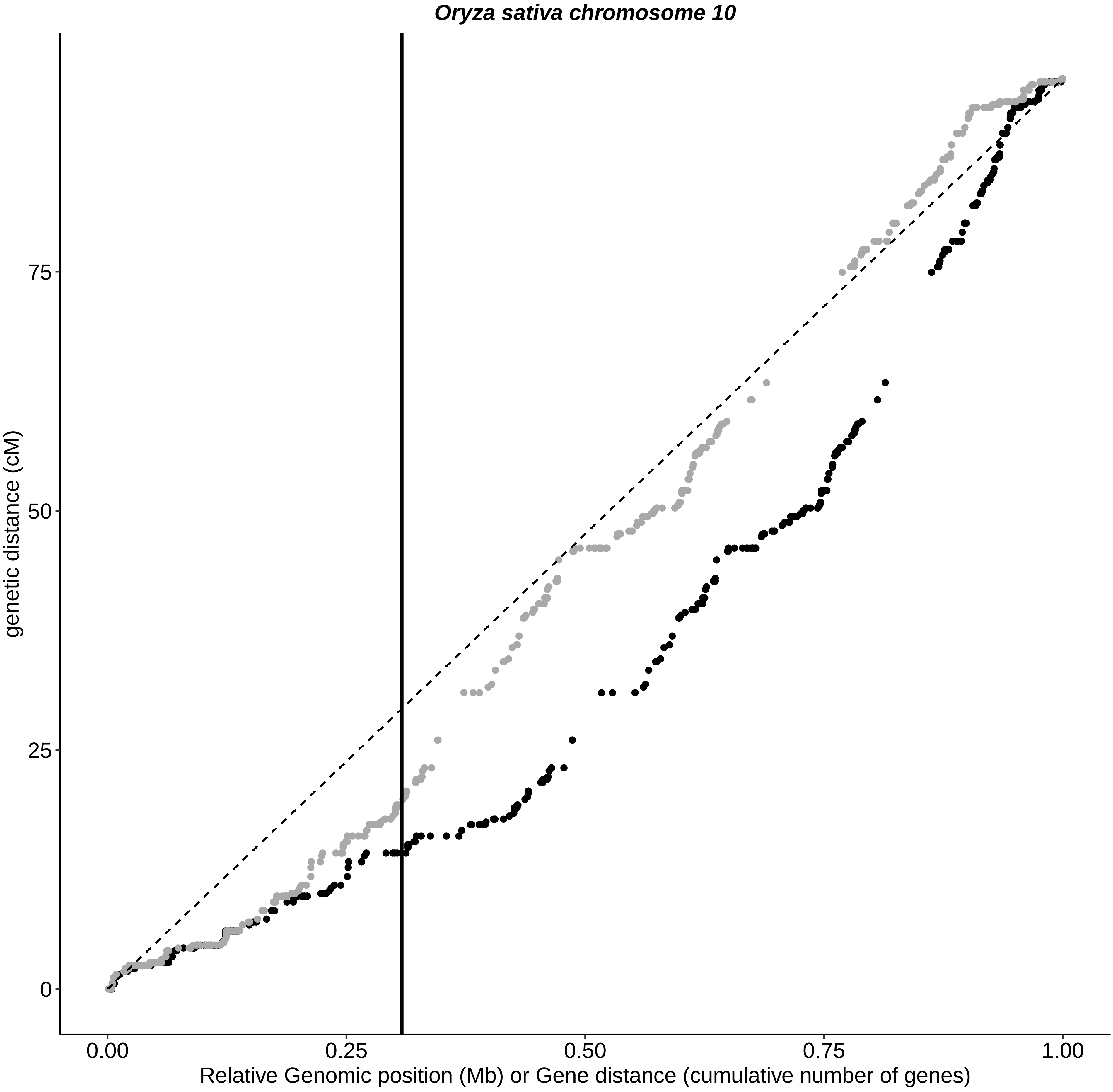
Oryza sativa chromosome 10
genetic distance (cM)
0
25
50
75
0.00
0.25
0.50
0.75
1.00
Relative Genomic position (Mb) or Gene distance (cumulative number of genes)

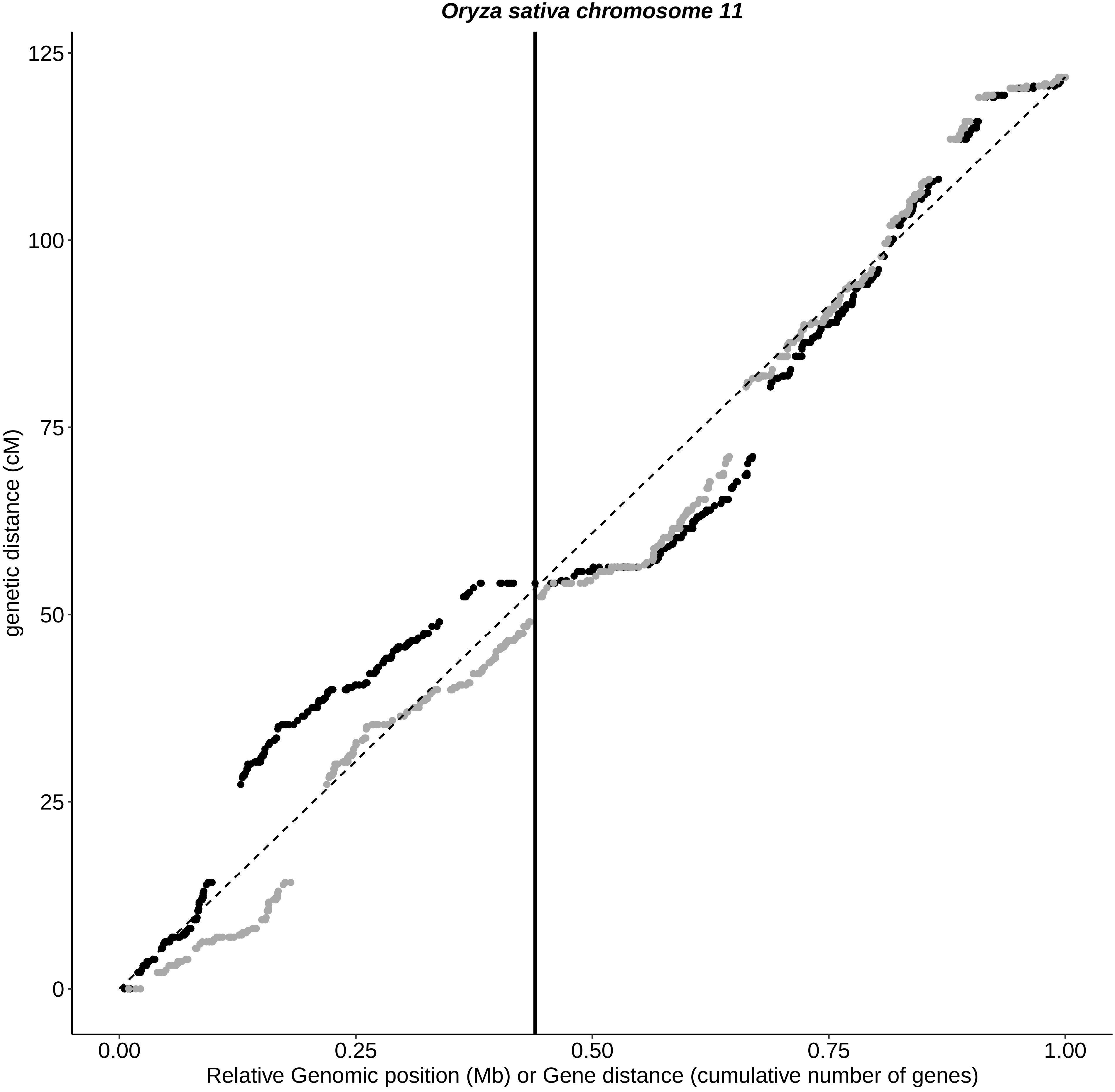
Oryza sativa chromosome 11
genetic distance (cM)
125
100
75
50
25
0
0.00
0.25
0.50
0.75
1.00
Relative Genomic position (Mb) or Gene distance (cumulative number of genes)

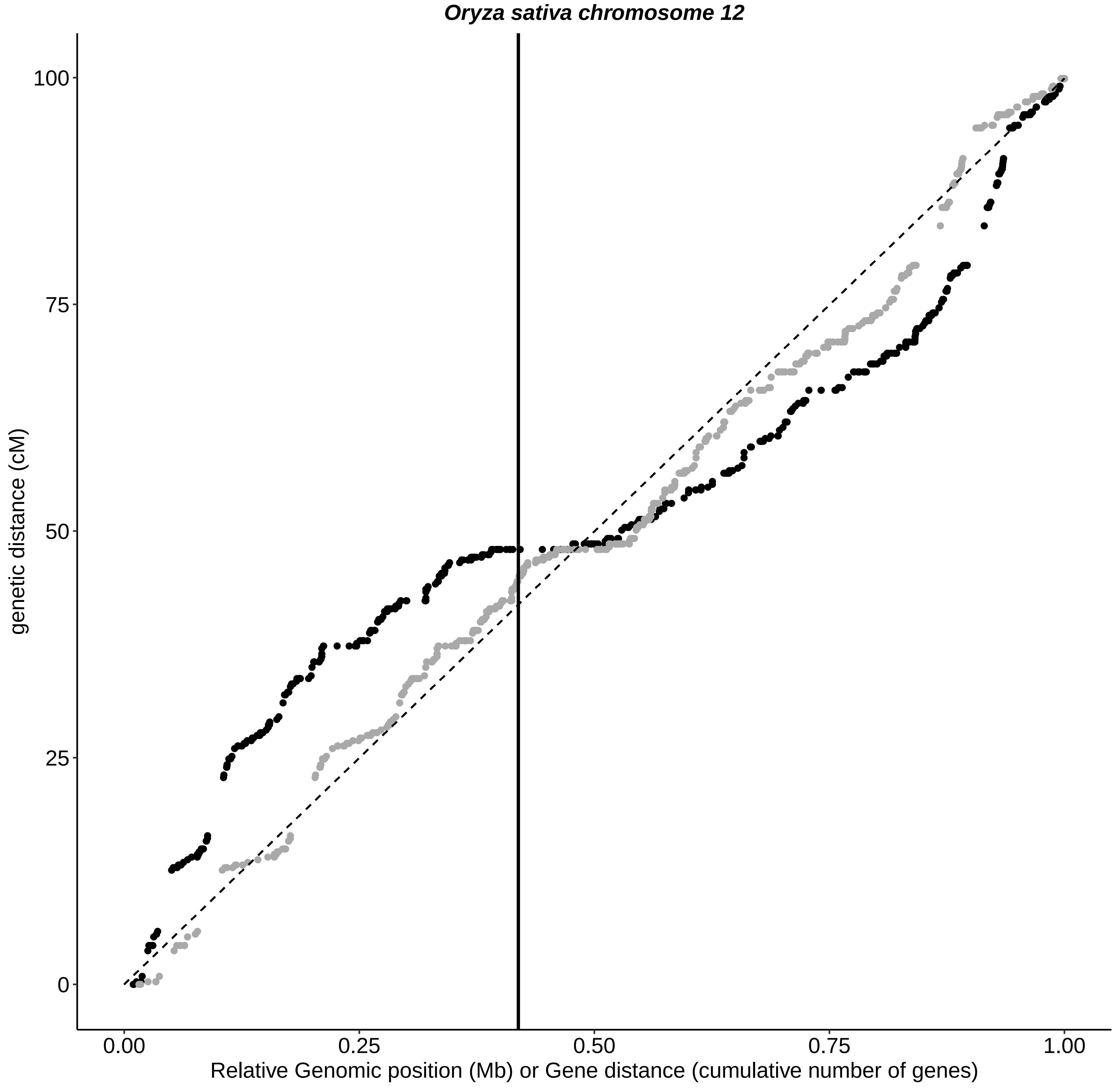

Oryza sativa chromosome 12
genetic distance (cM)
0
25
50
75
100
0.00
0.25
0.50
0.75
1.00
Relative Genomic position (Mb) or Gene distance (cumulative number of genes)

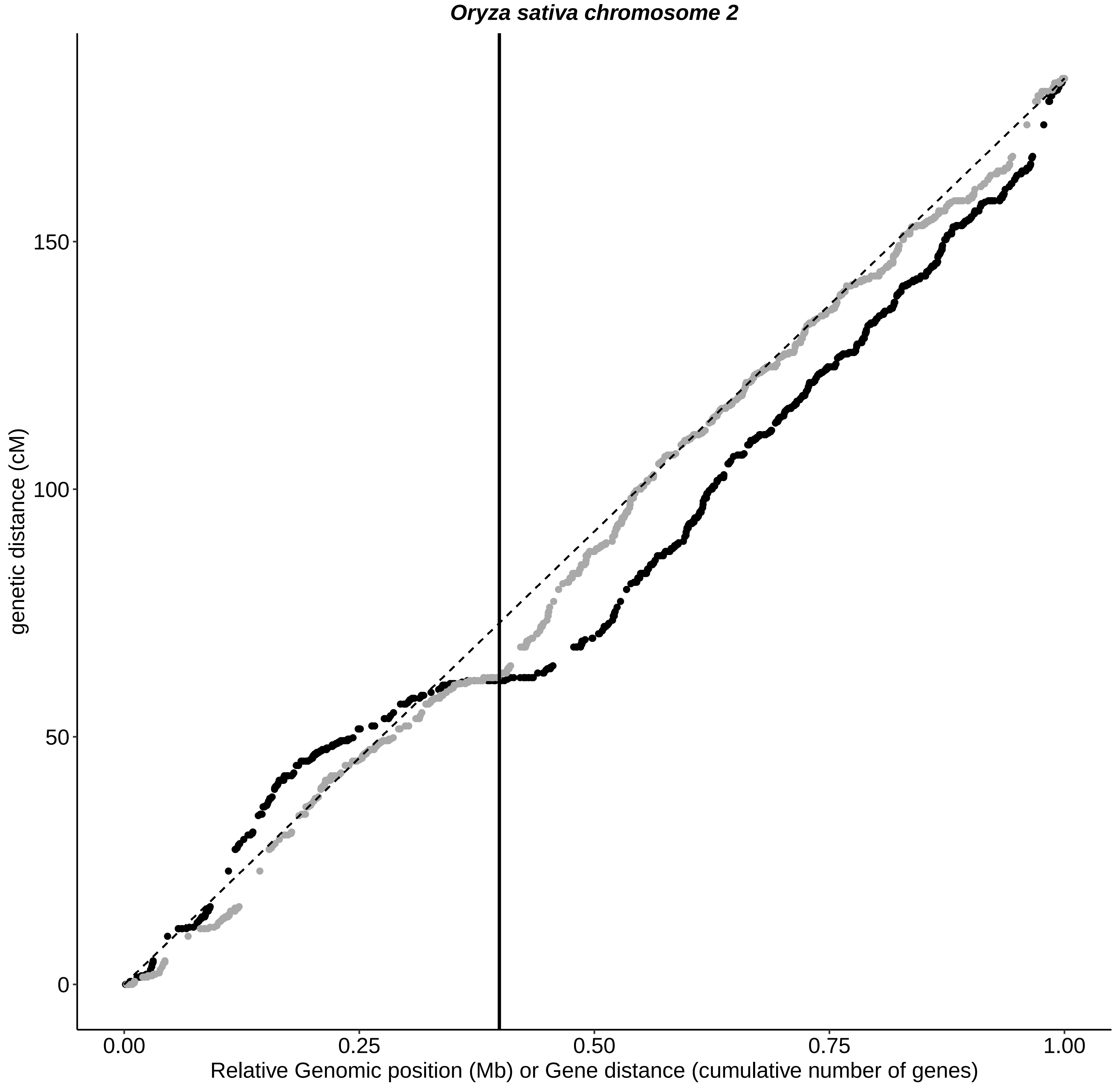

Oryza sativa chromosome 2
genetic distance (cM)
150
100
50
0
0.00
0.25
0.50
0.75
1.00
Relative Genomic position (Mb) or Gene distance (cumulative number of genes)

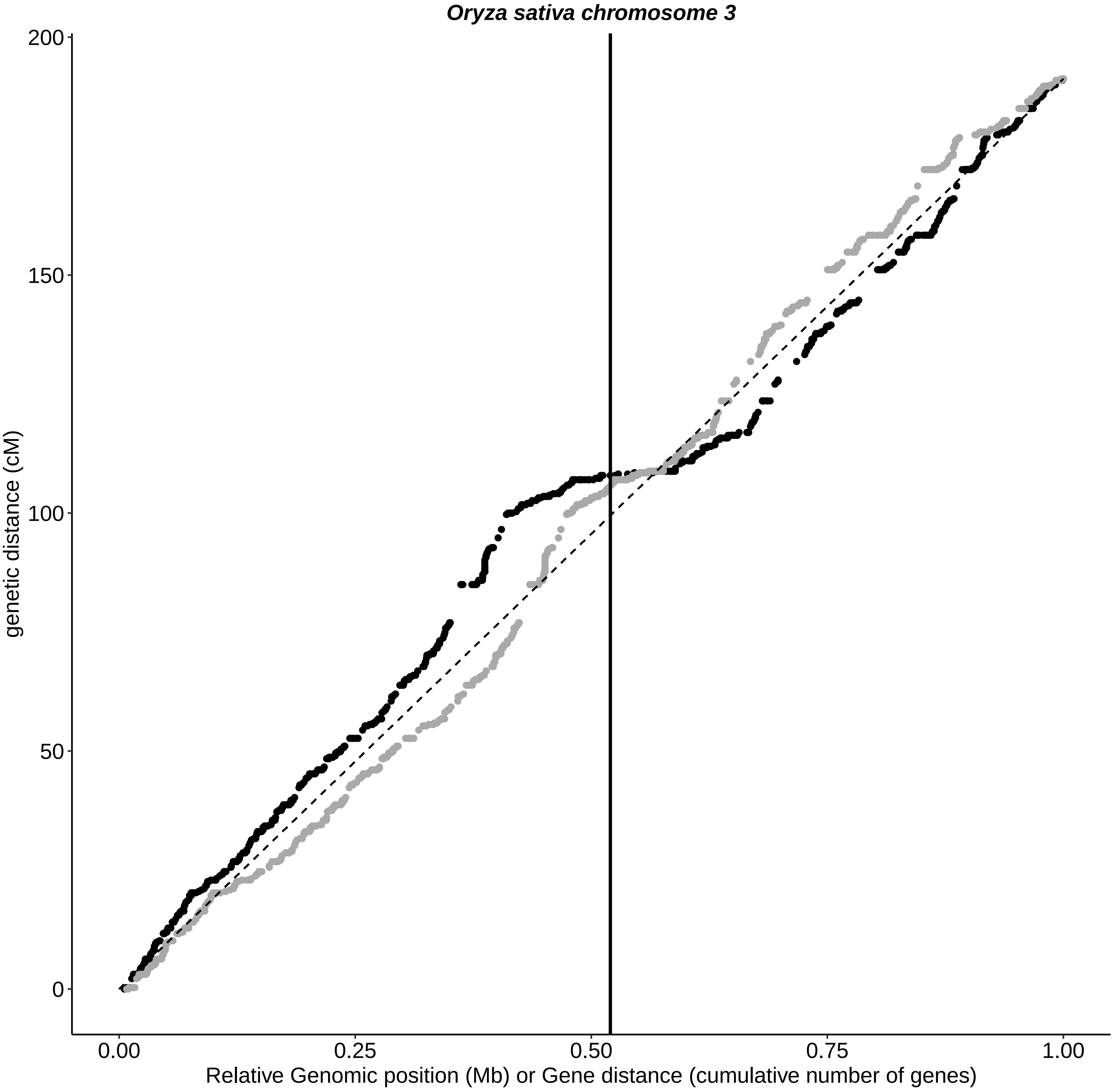
Oryza sativa chromosome 3
genetic distance (cM)
200
150
100
50
0
0.00
0.25
0.50
0.75
1.00
Relative Genomic position (Mb) or Gene distance (cumulative number of genes)

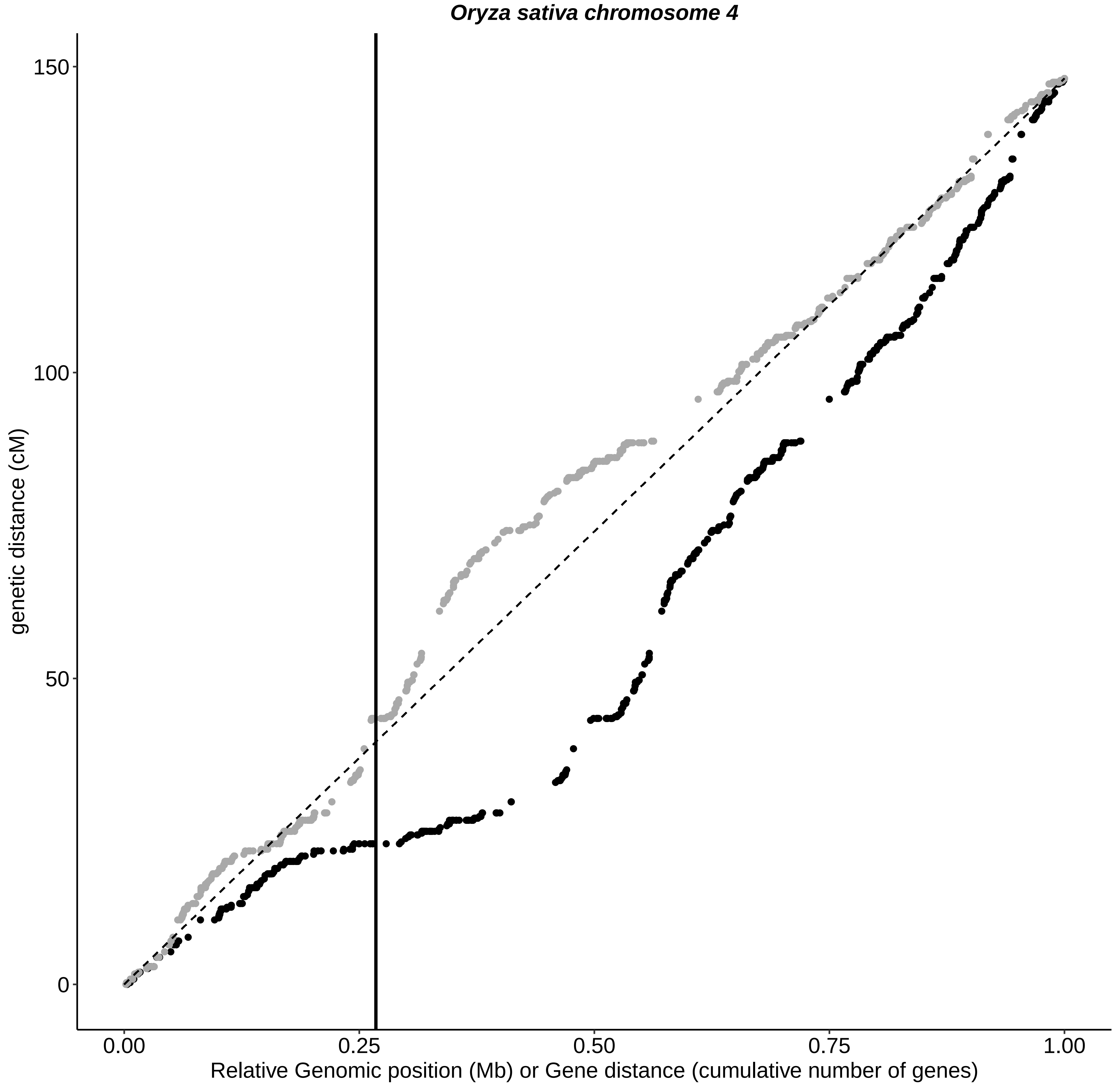
Oryza sativa chromosome 4
genetic distance (cM)
150
100
50
0
0.00
0.25
0.50
0.75
1.00
Relative Genomic position (Mb) or Gene distance (cumulative number of genes)

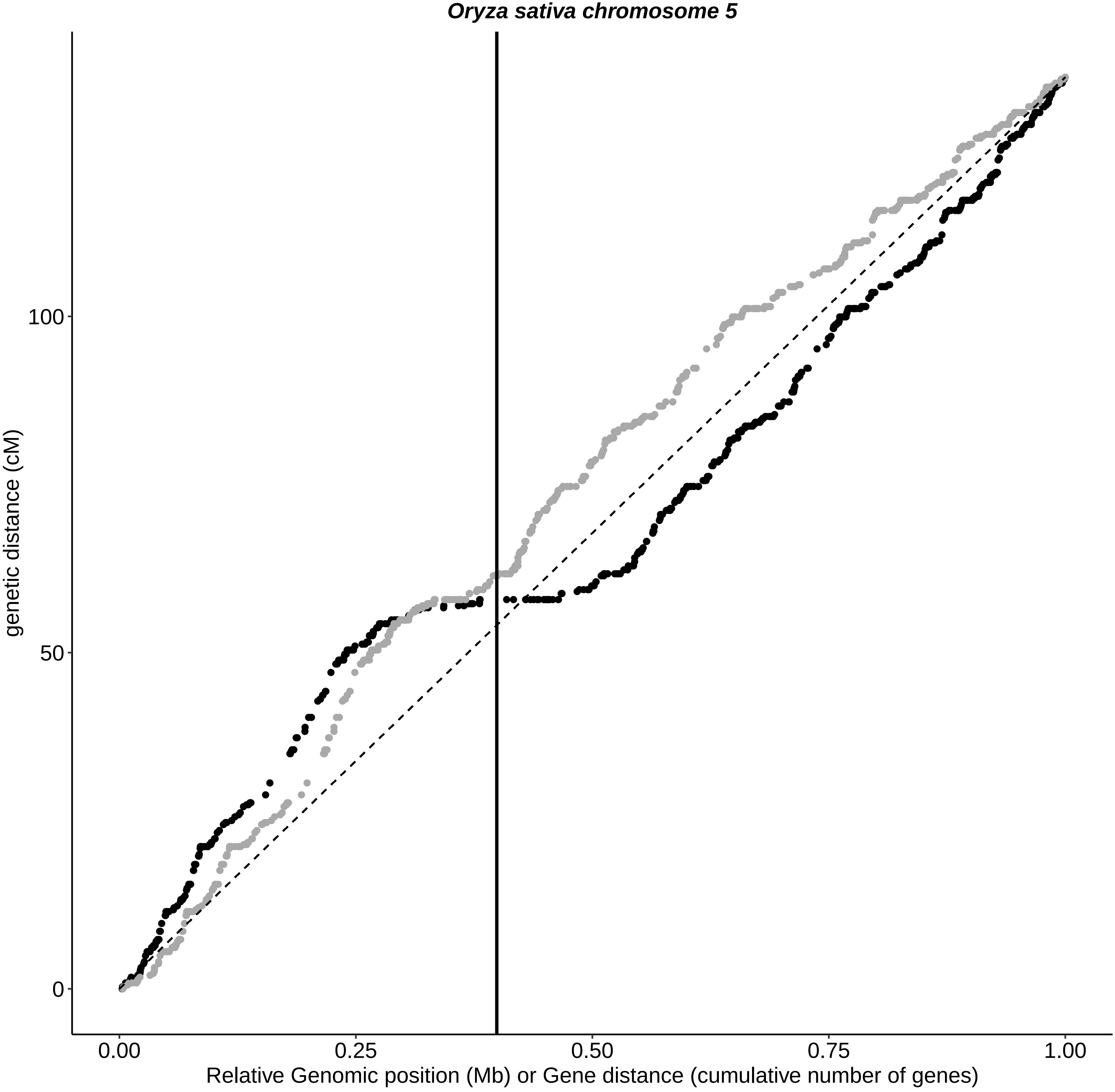
Oryza sativa chromosome 5
genetic distance (cM)
100
50
0
0.00
0.25
0.50
0.75
1.00
Relative Genomic position (Mb) or Gene distance (cumulative number of genes)

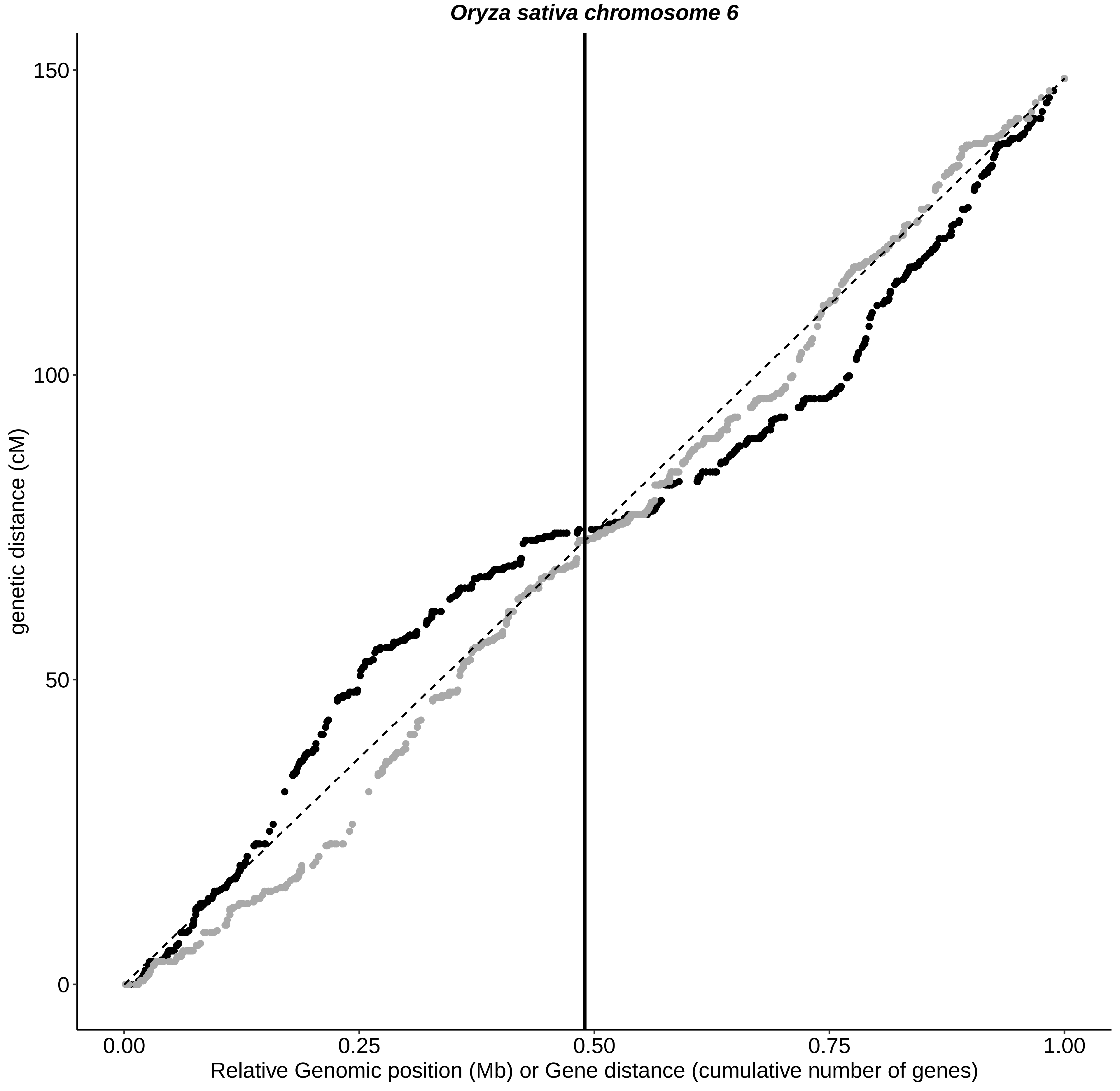

Oryza sativa chromosome 6
genetic distance (cM)
0
50
100
150
0.00
0.25
0.50
0.75
1.00
Relative Genomic position (Mb) or Gene distance (cumulative number of genes)

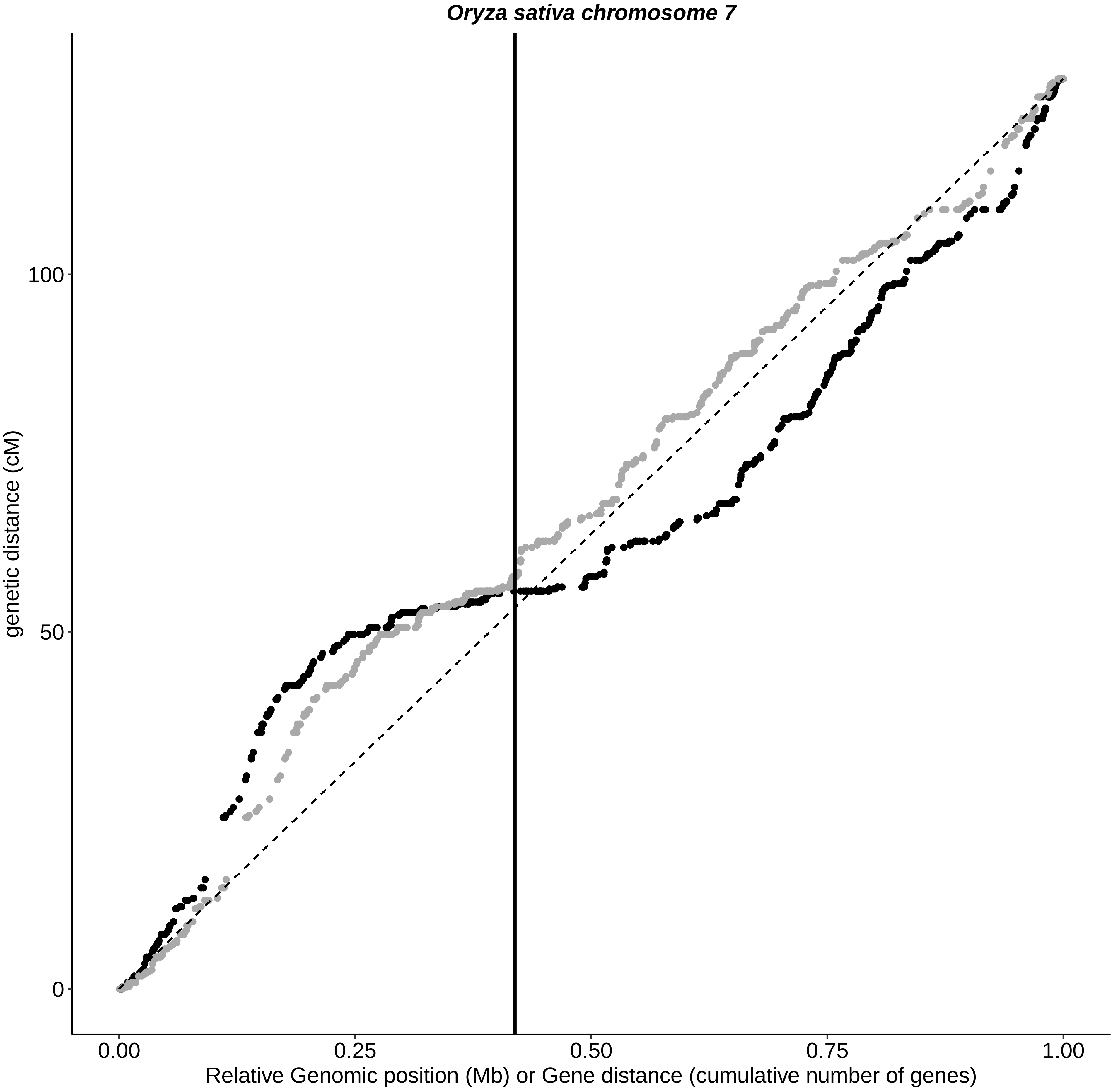
Oryza sativa chromosome 7
genetic distance (cM)
0
50
100
0.00
0.25
0.50
0.75
1.00
Relative Genomic position (Mb) or Gene distance (cumulative number of genes)

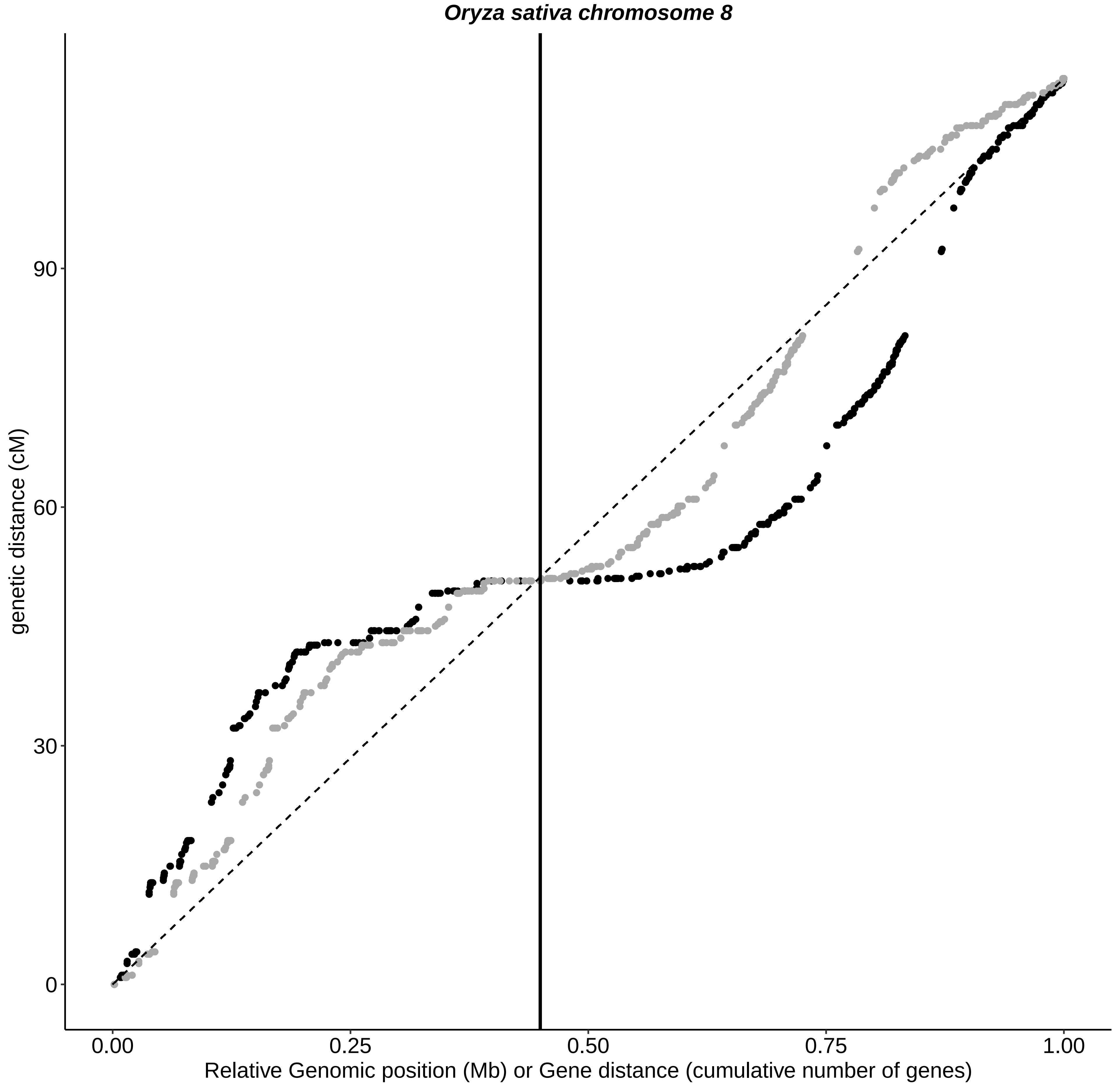
Oryza sativa chromosome 8
genetic distance (cM)
0
30
60
90
0.00
0.25
0.50
0.75
1.00
Relative Genomic position (Mb) or Gene distance (cumulative number of genes)

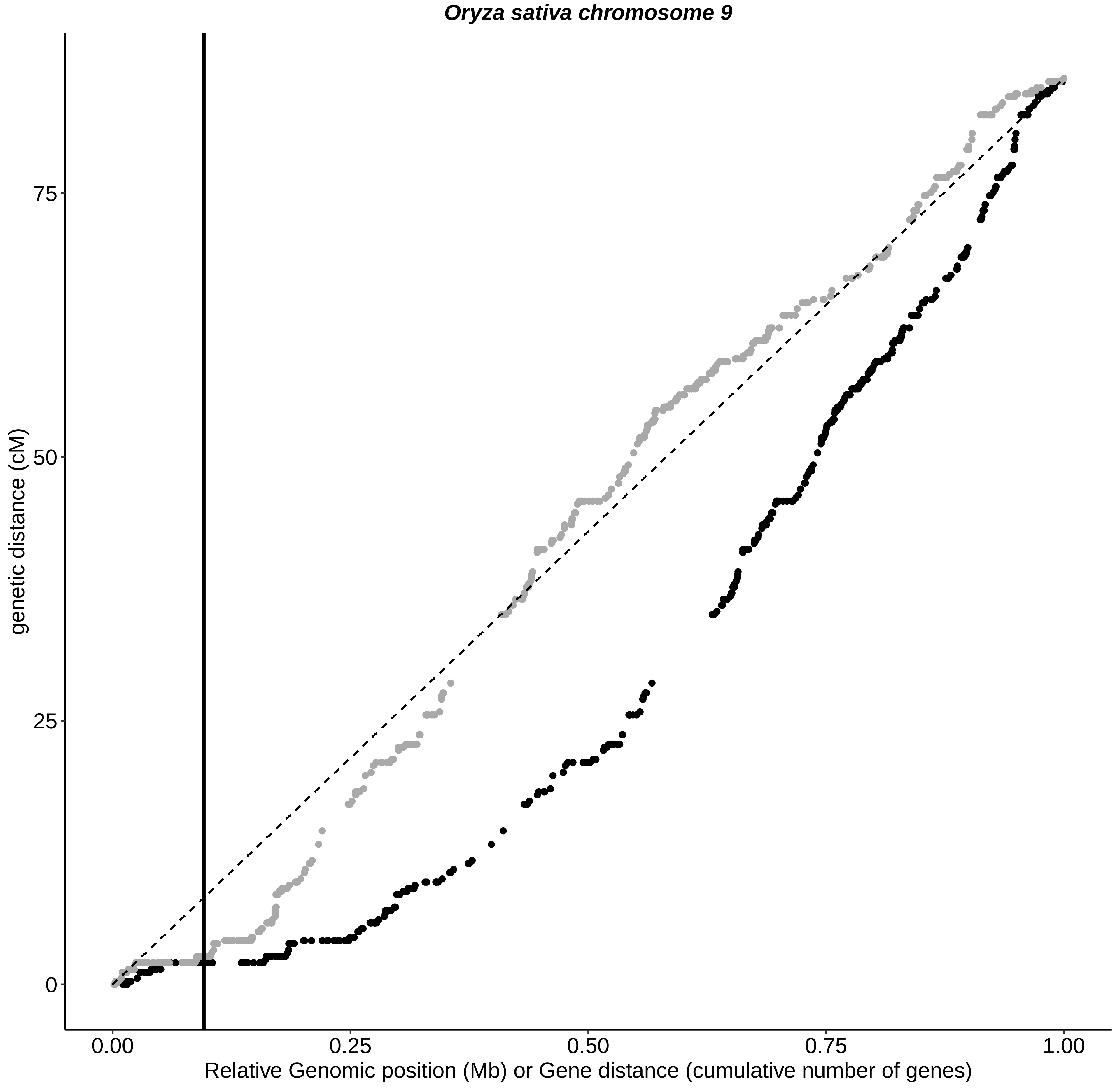
Oryza sativa chromosome 9
genetic distance (cM)
0
25
50
75
0.00
0.25
0.50
0.75
1.00
Relative Genomic position (Mb) or Gene distance (cumulative number of genes)

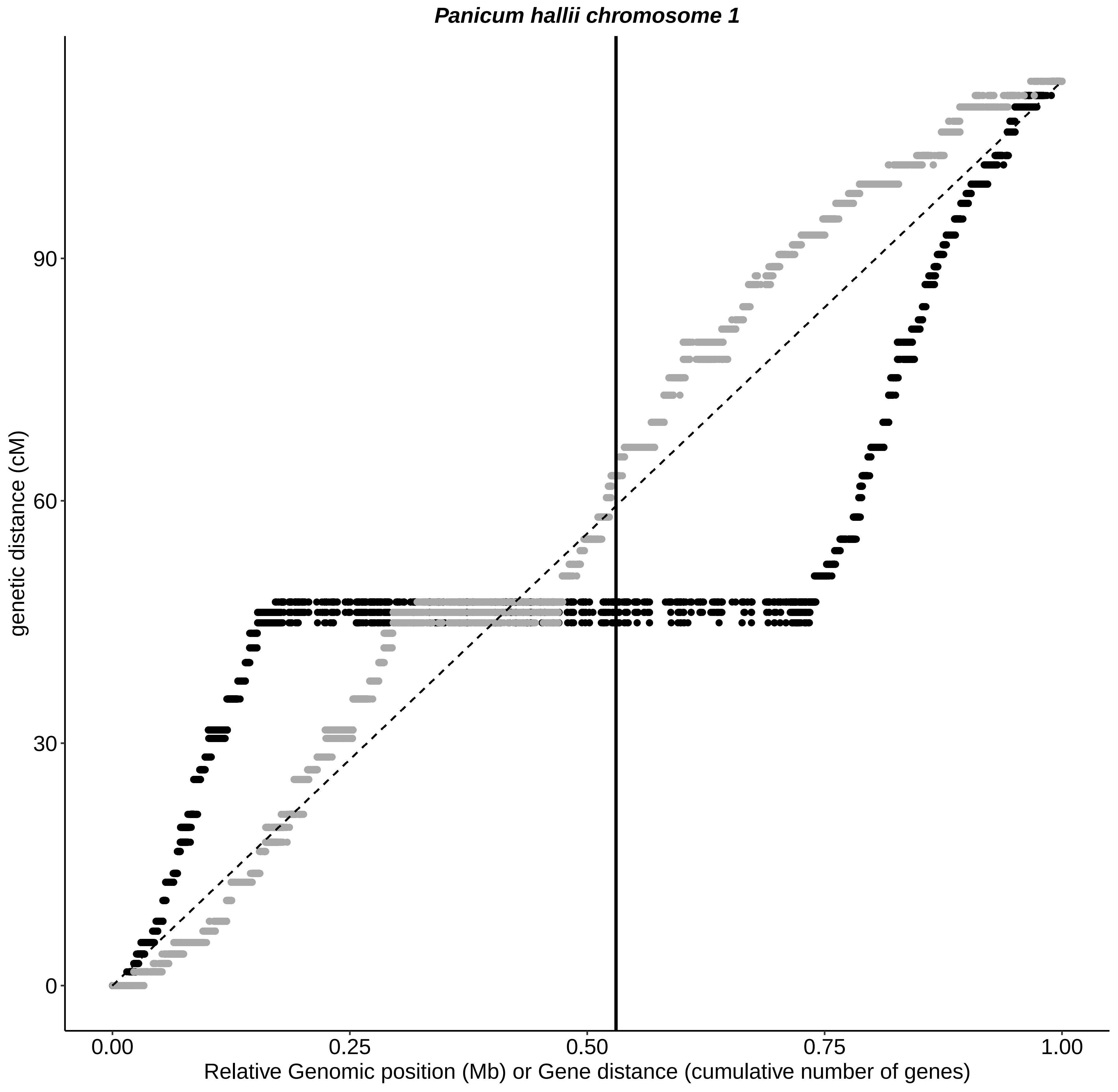
Panicum hallii chromosome 1
genetic distance (cM)
0
30
60
90
0.00
0.25
0.50
0.75
1.00
Relative Genomic position (Mb) or Gene distance (cumulative number of genes)

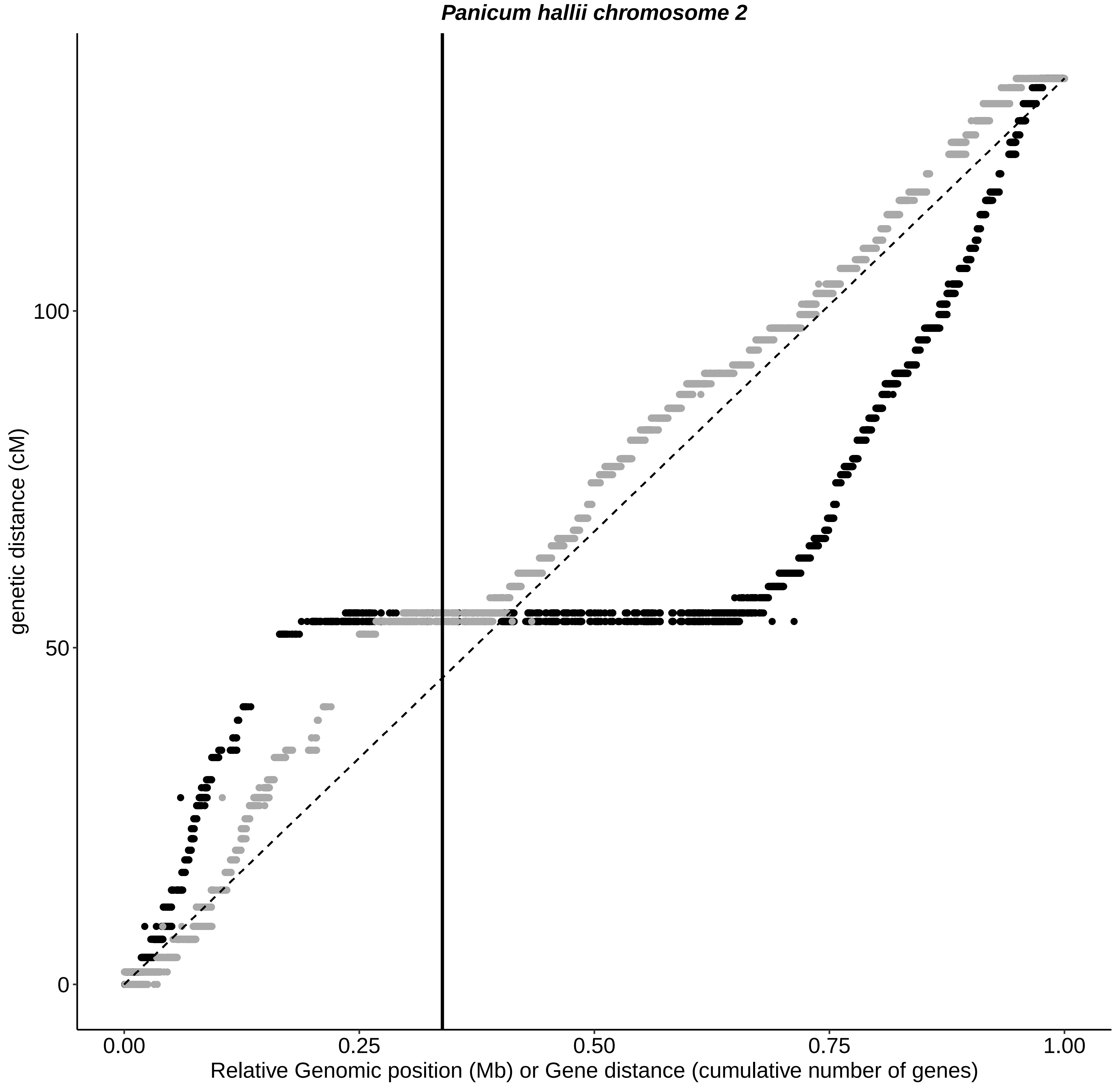

Panicum hallii chromosome 2
genetic distance (cM)
0
50
100
0.00
0.25
0.50
0.75
1.00
Relative Genomic position (Mb) or Gene distance (cumulative number of genes)

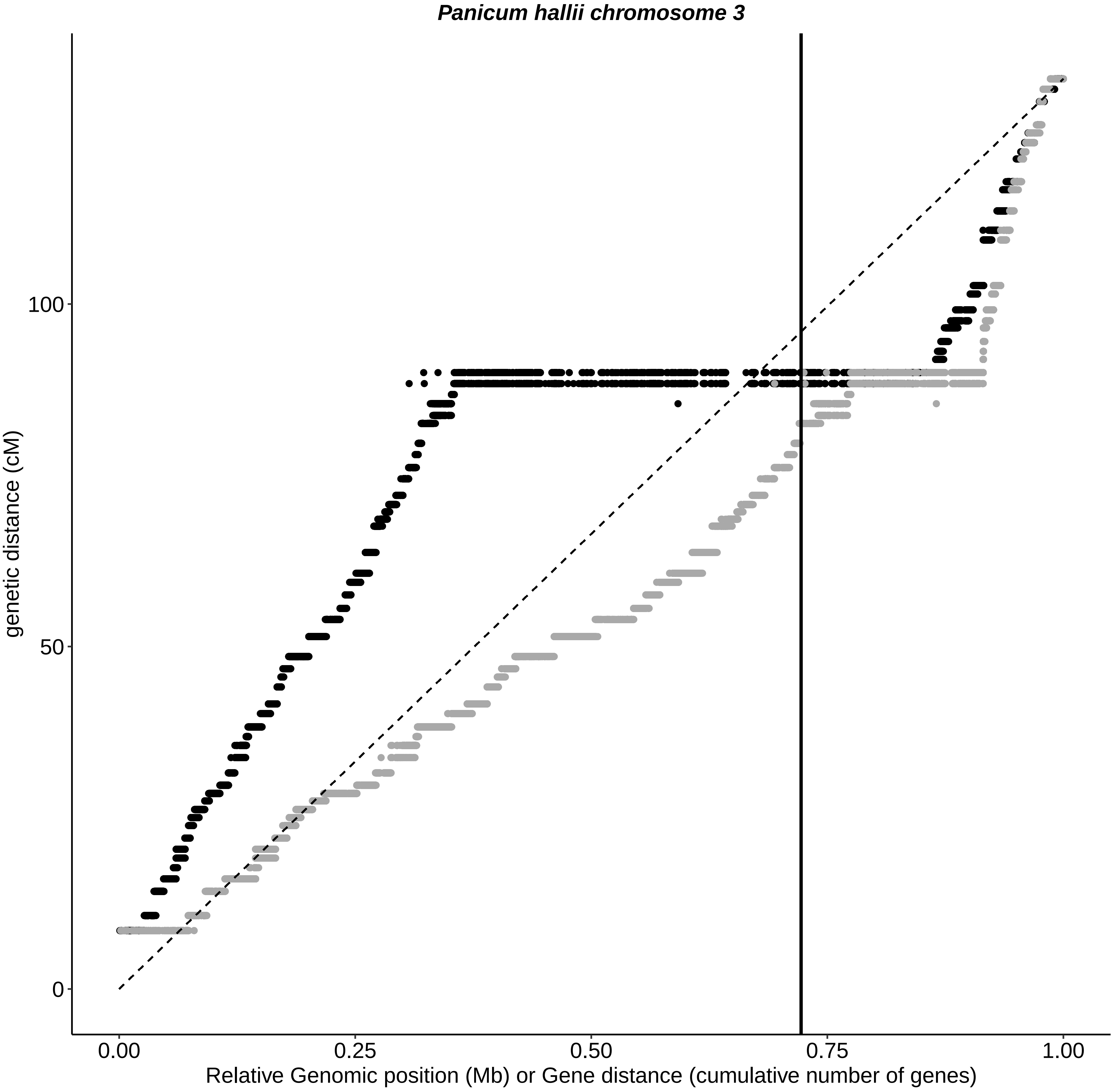
Panicum hallii chromosome 3
genetic distance (cM)
0
50
100
0.00
0.25
0.50
0.75
1.00
Relative Genomic position (Mb) or Gene distance (cumulative number of genes)

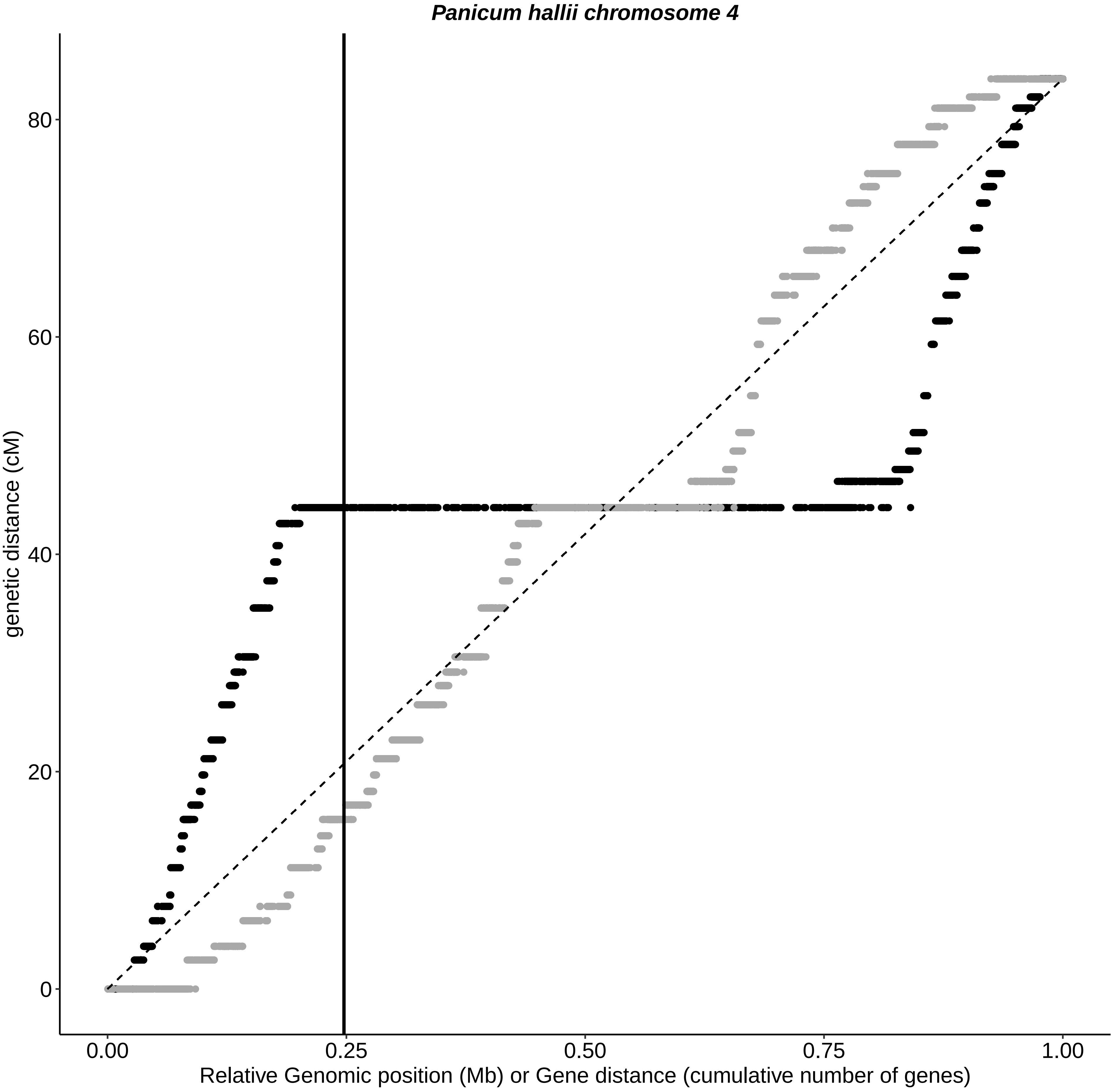
Panicum hallii chromosome 4
genetic distance (cM)
0
20
40
60
80
0.00
0.25
0.50
0.75
1.00
Relative Genomic position (Mb) or Gene distance (cumulative number of genes)

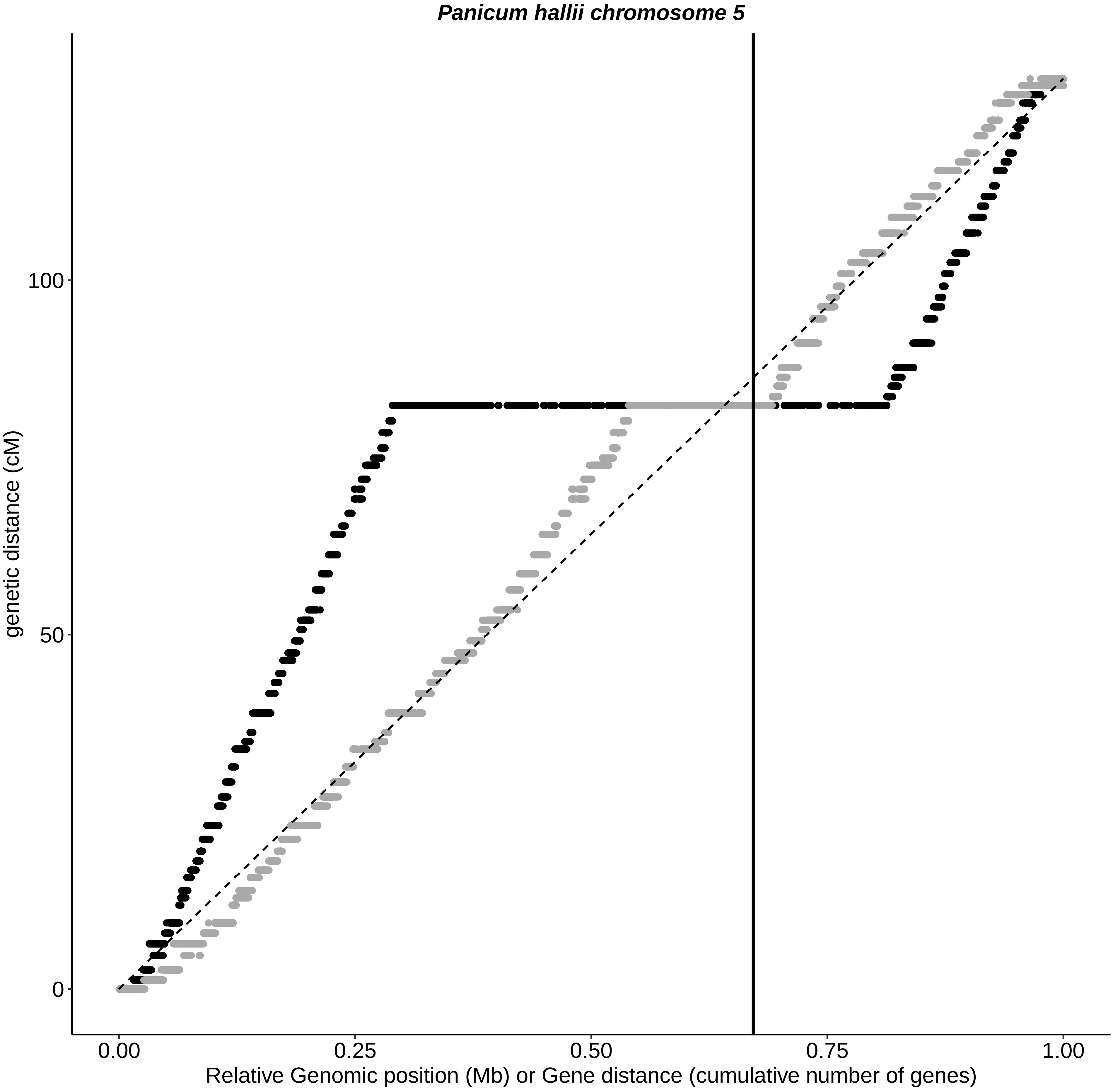
Panicum hallii chromosome 5
genetic distance (cM)
0
50
100
0.00
0.25
0.50
0.75
1.00
Relative Genomic position (Mb) or Gene distance (cumulative number of genes)

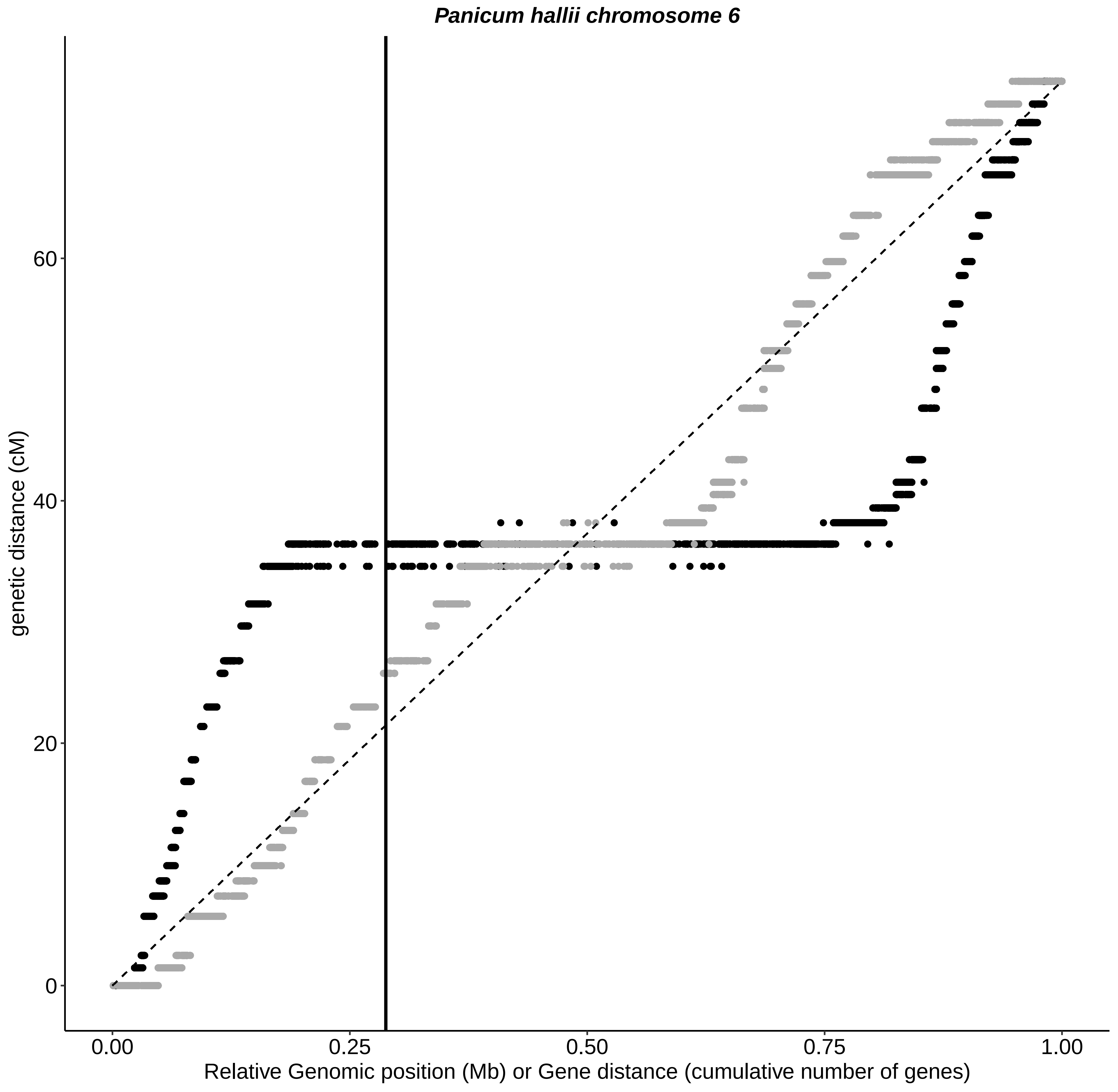
Panicum hallii chromosome 6
genetic distance (cM)
0
20
40
60
0.00
0.25
0.50
0.75
1.00
Relative Genomic position (Mb) or Gene distance (cumulative number of genes)

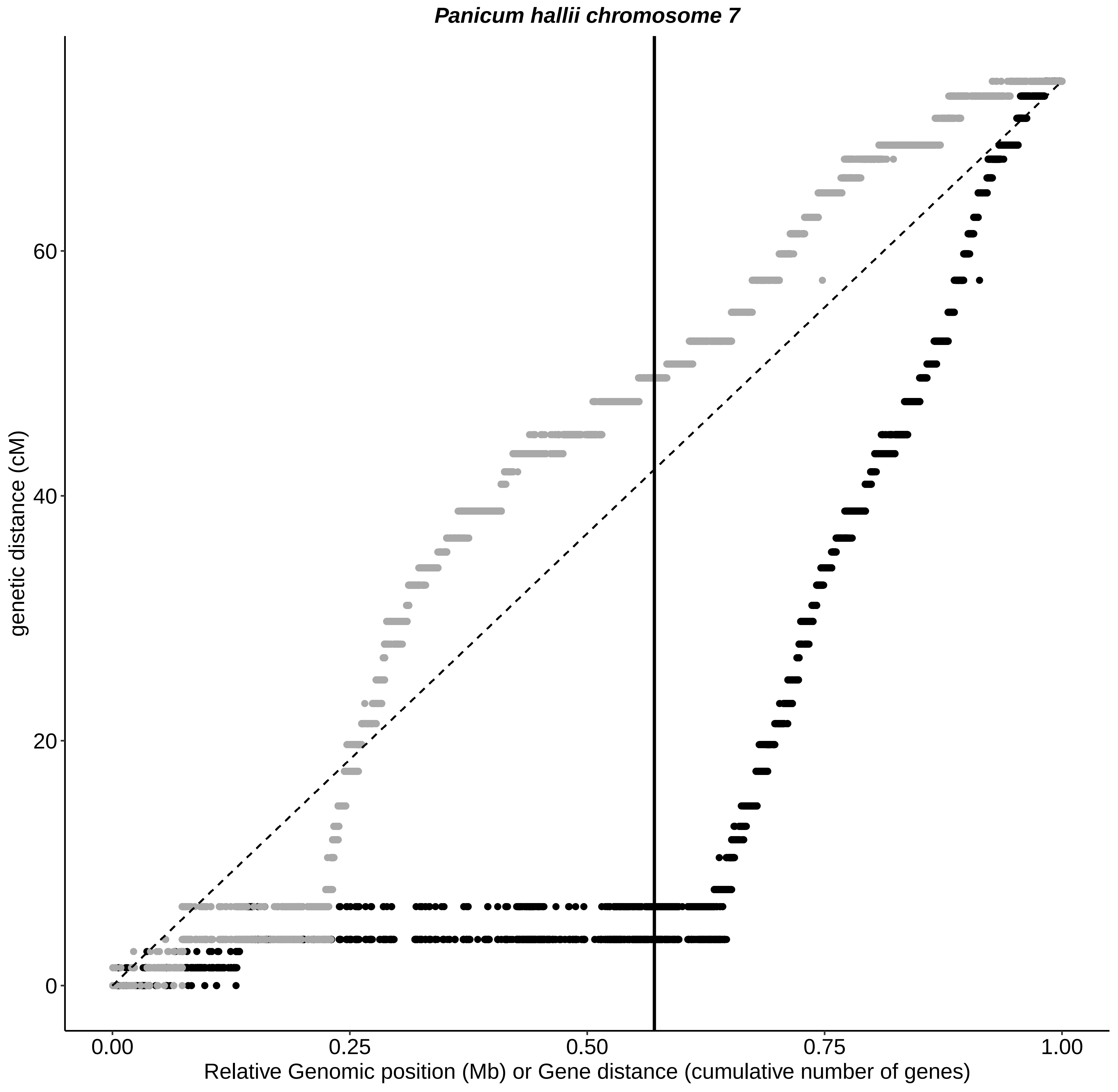
Panicum hallii chromosome 7
genetic distance (cM)
0
20
40
60
0.00
0.25
0.50
0.75
1.00
Relative Genomic position (Mb) or Gene distance (cumulative number of genes)

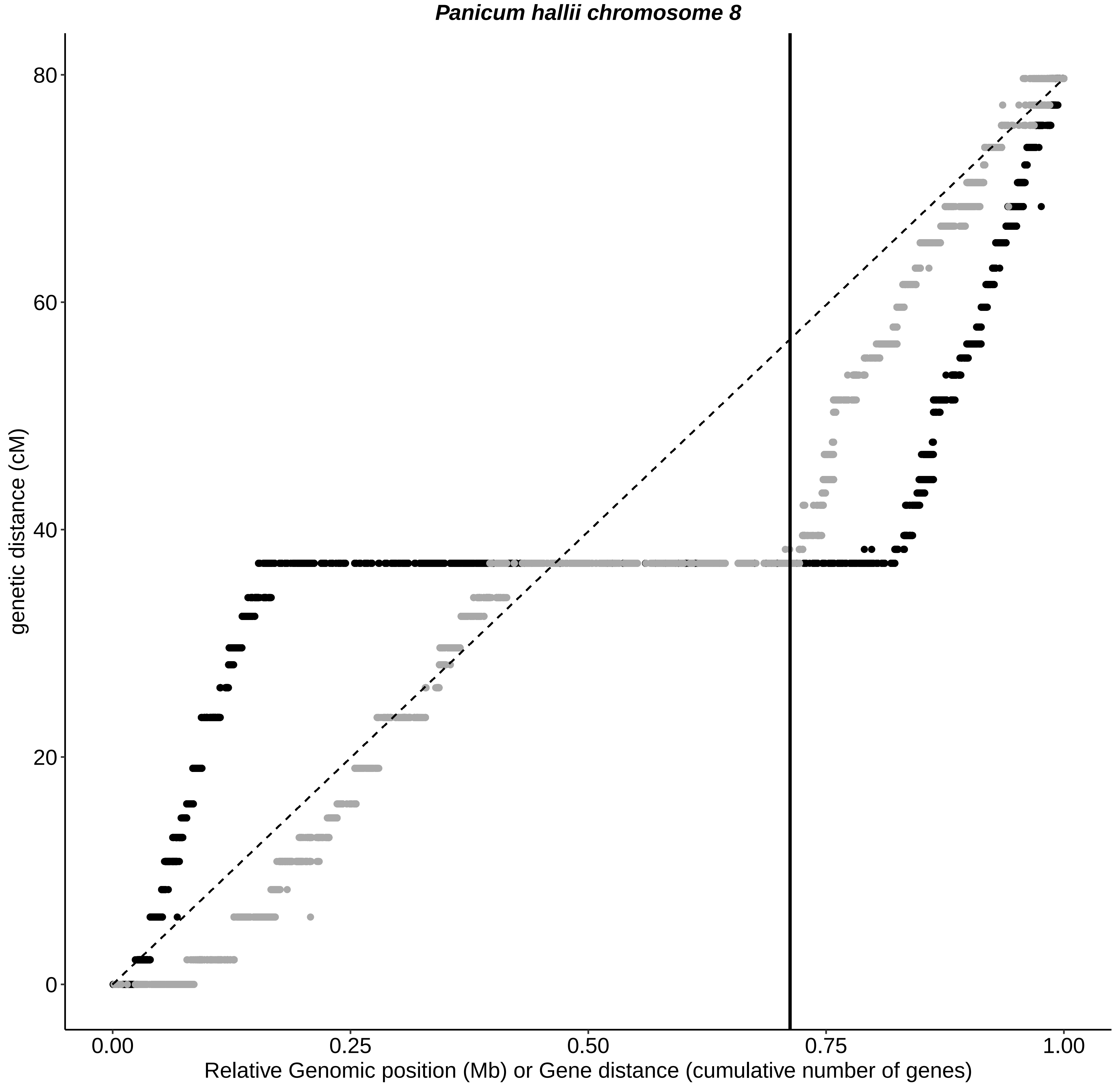
Panicum hallii chromosome 8
genetic distance (cM)
80
60
40
20
0
0.00
0.25
0.50
0.75
1.00
Relative Genomic position (Mb) or Gene distance (cumulative number of genes)

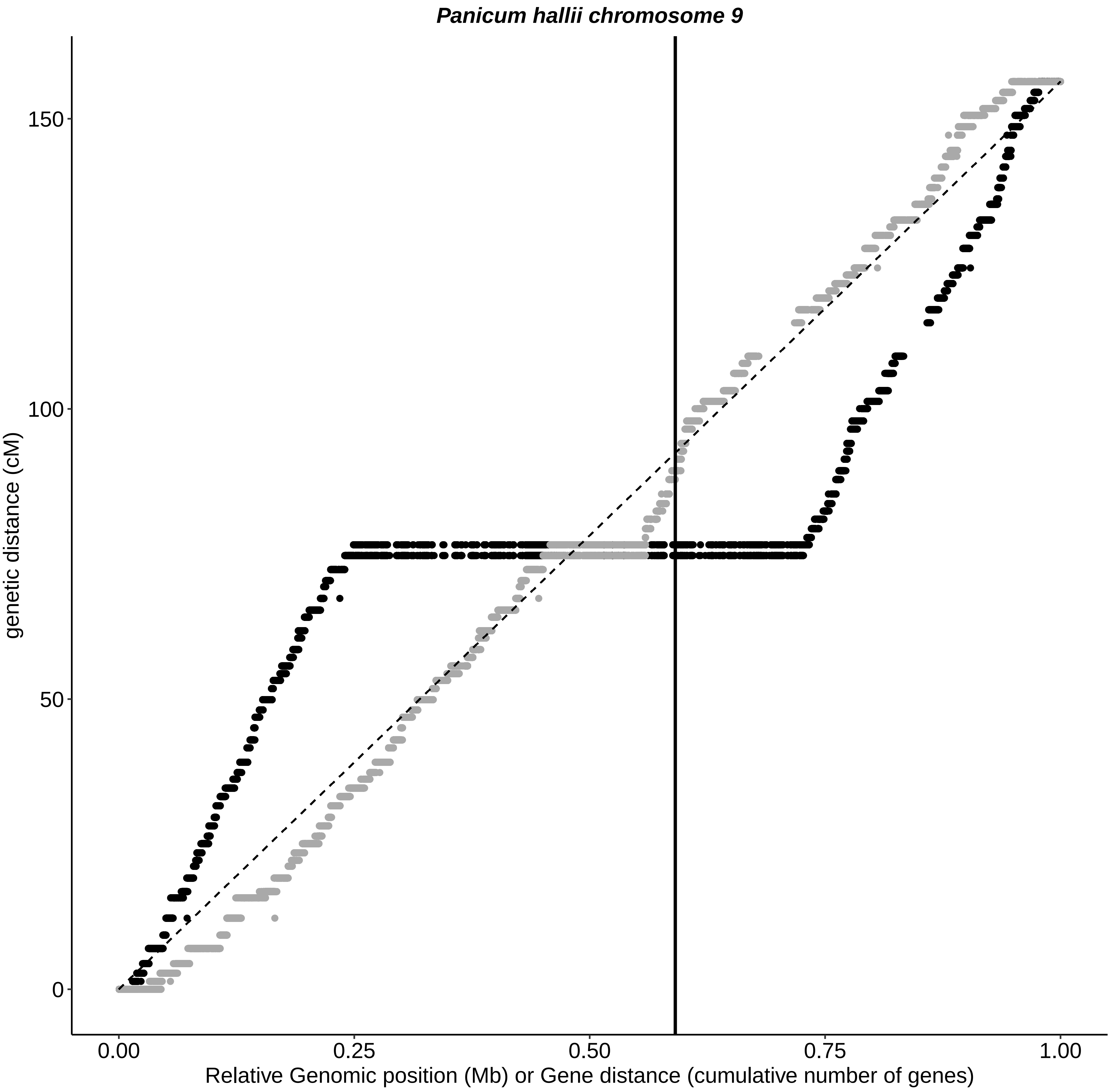
Panicum hallii chromosome 9
genetic distance (cM)
0
50
100
150
0.00
0.25
0.50
0.75
1.00
Relative Genomic position (Mb) or Gene distance (cumulative number of genes)

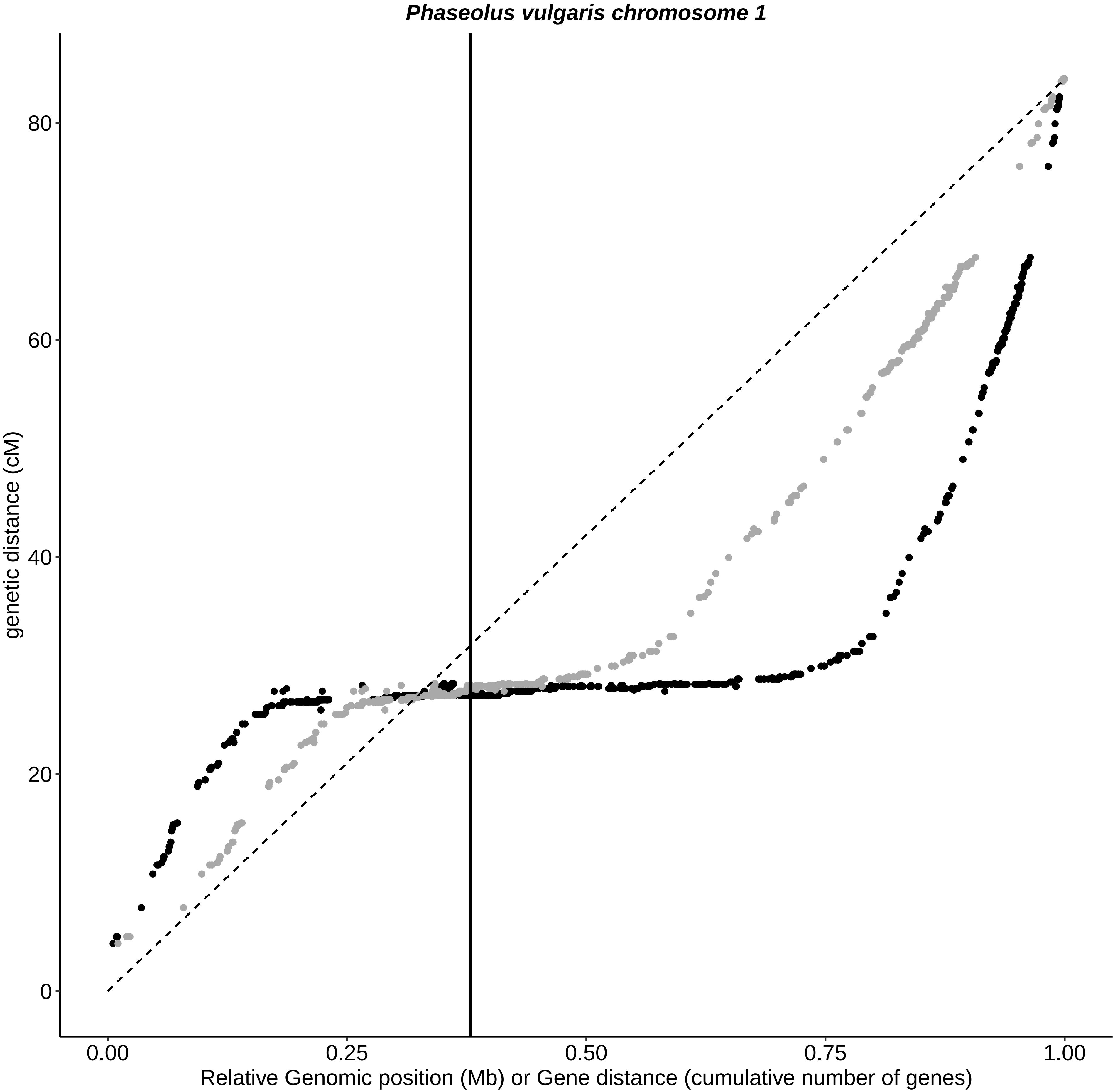
Phaseolus vulgaris chromosome 1
genetic distance (cM)
0
20
40
60
80
0.00
0.25
0.50
0.75
1.00
Relative Genomic position (Mb) or Gene distance (cumulative number of genes)

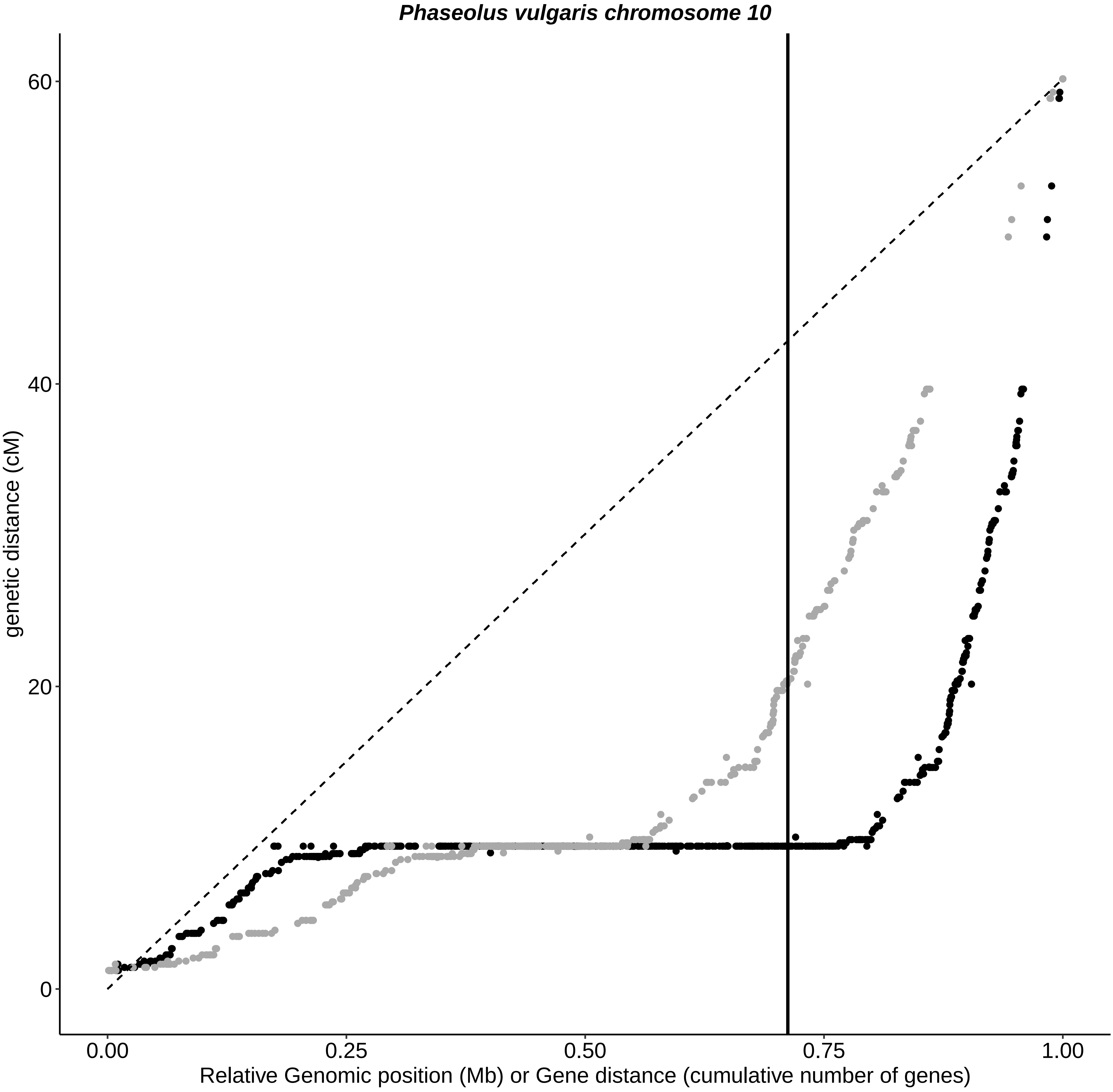
Phaseolus vulgaris chromosome 10
genetic distance (cM)
0
20
40
60
0.00
0.25
0.50
0.75
1.00
Relative Genomic position (Mb) or Gene distance (cumulative number of genes)

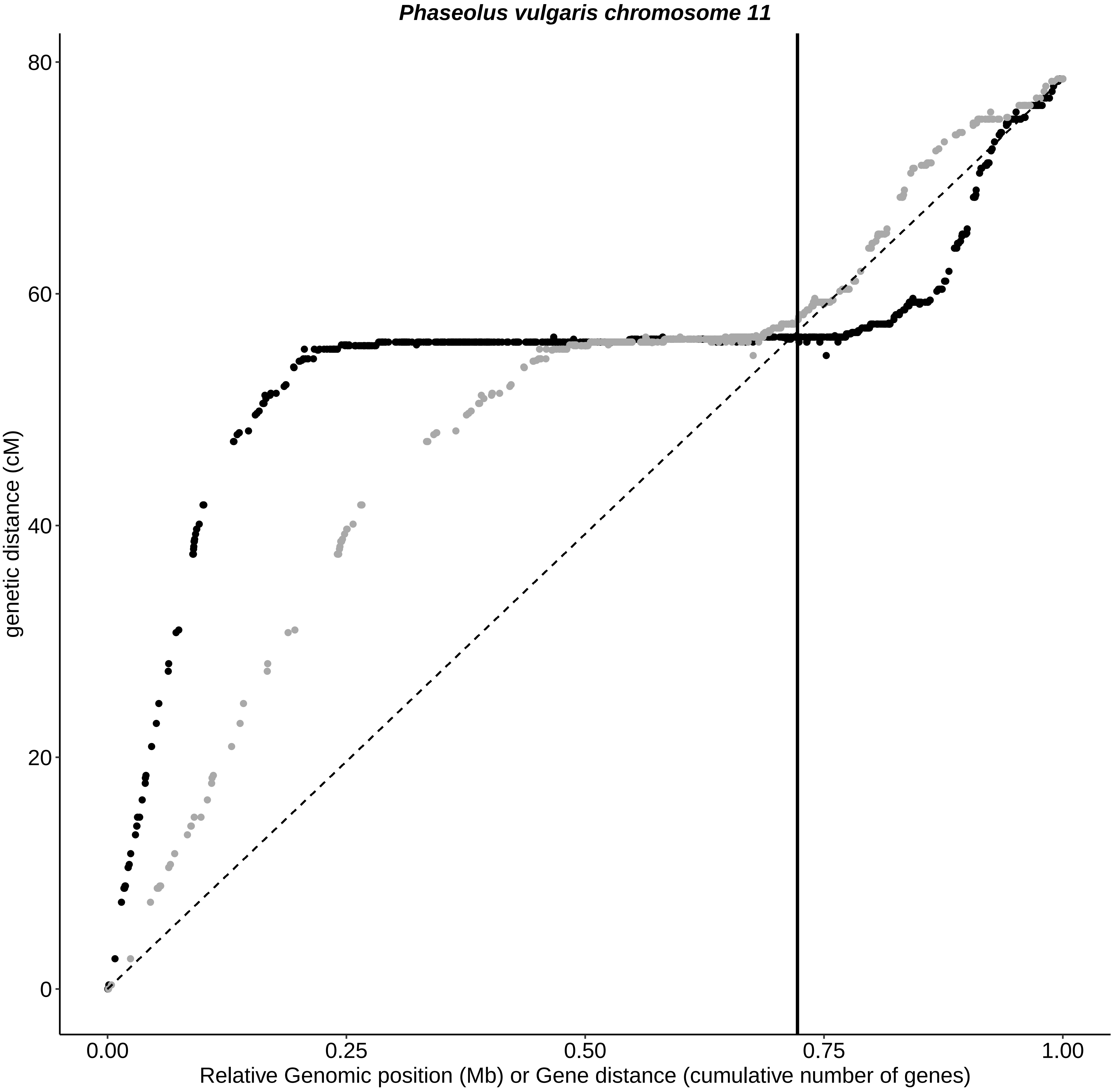
Phaseolus vulgaris chromosome 11
genetic distance (cM)
0
20
40
60
80
0.00
0.25
0.50
0.75
1.00
Relative Genomic position (Mb) or Gene distance (cumulative number of genes)

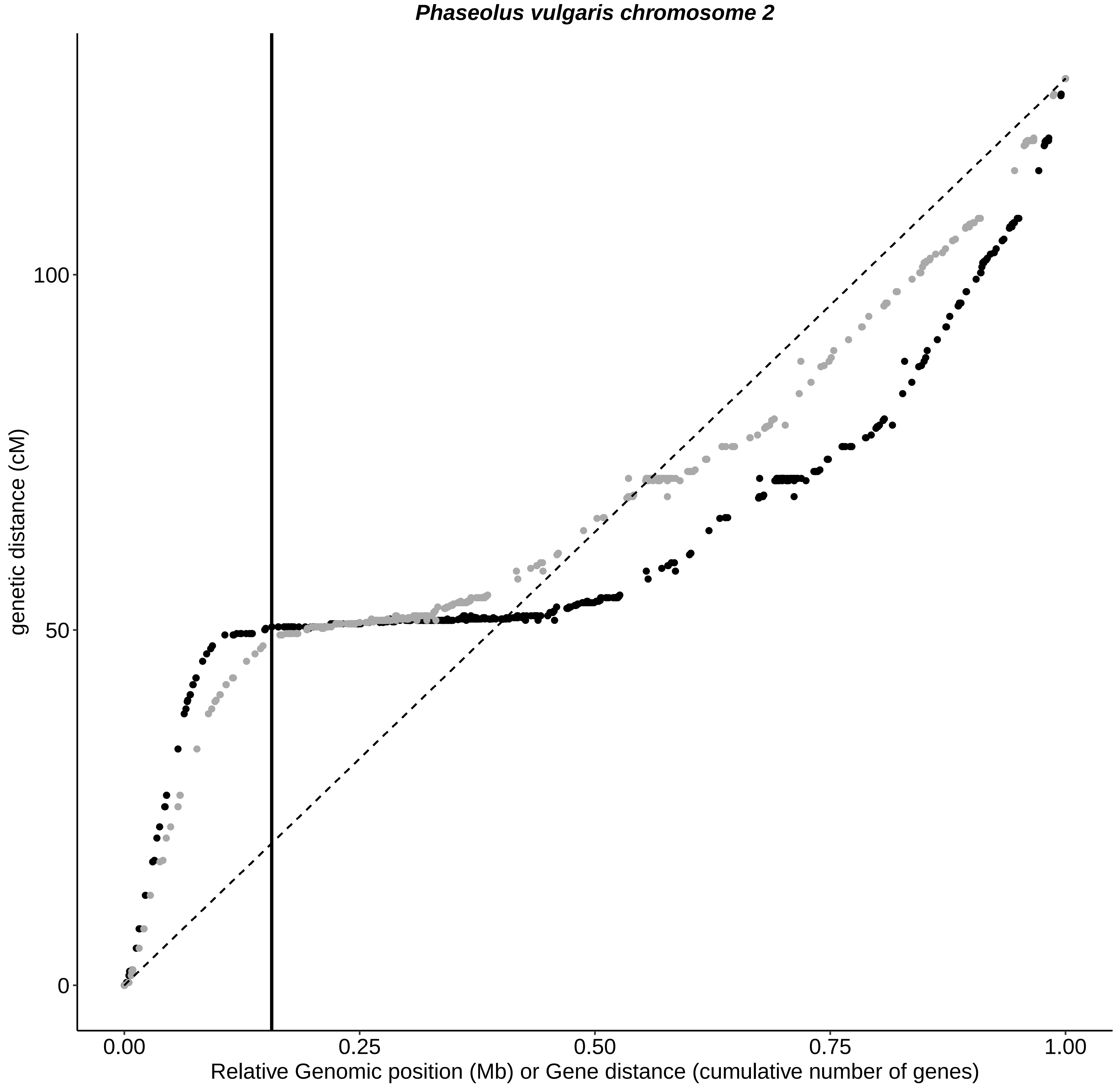
Phaseolus vulgaris chromosome 2
genetic distance (cM)
100
50
0
0.00
0.25
0.50
0.75
1.00
Relative Genomic position (Mb) or Gene distance (cumulative number of genes)

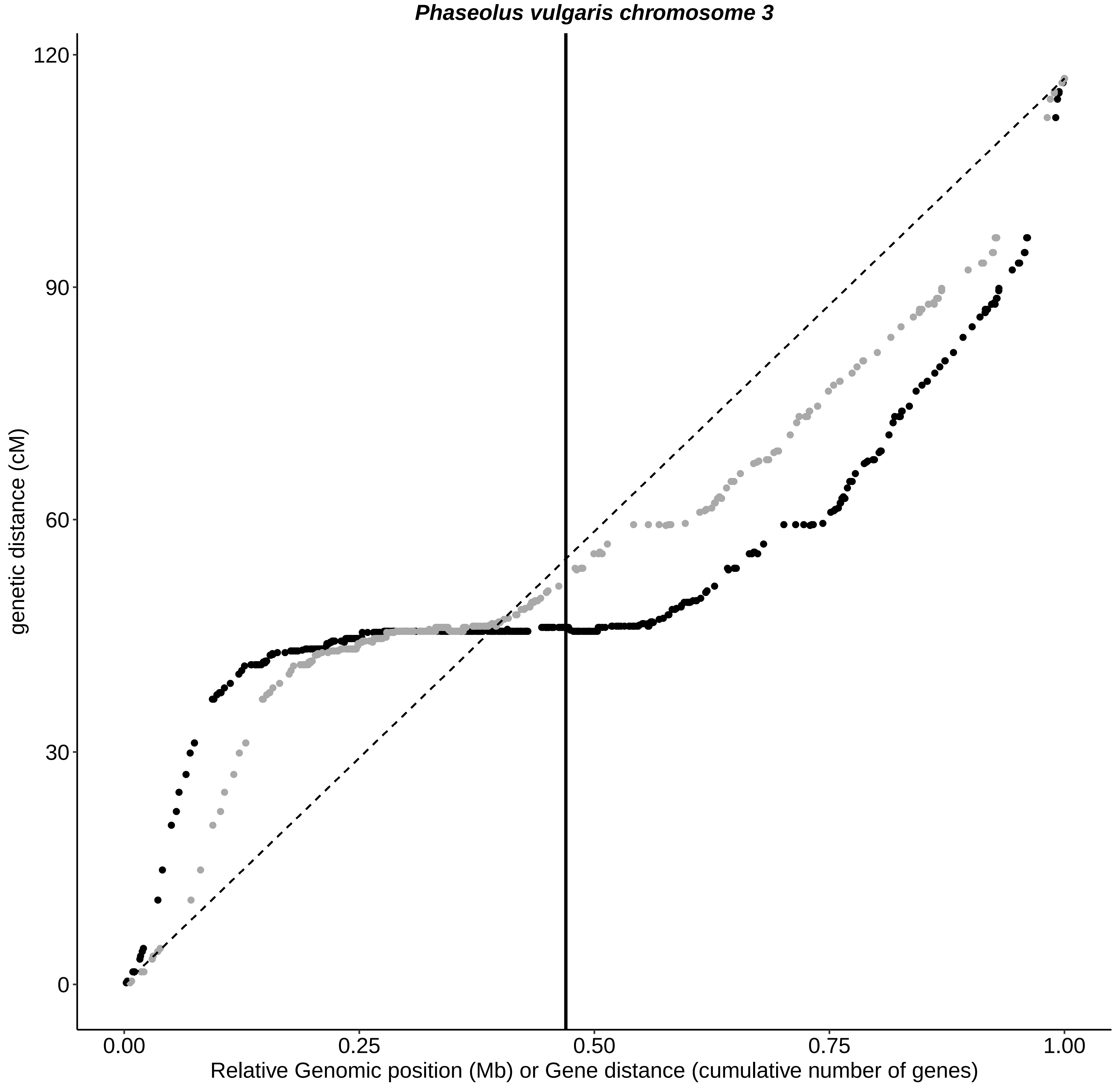
Phaseolus vulgaris chromosome 3
genetic distance (cM)
120
90
60
30
0
0.00
0.25
0.50
0.75
1.00
Relative Genomic position (Mb) or Gene distance (cumulative number of genes)

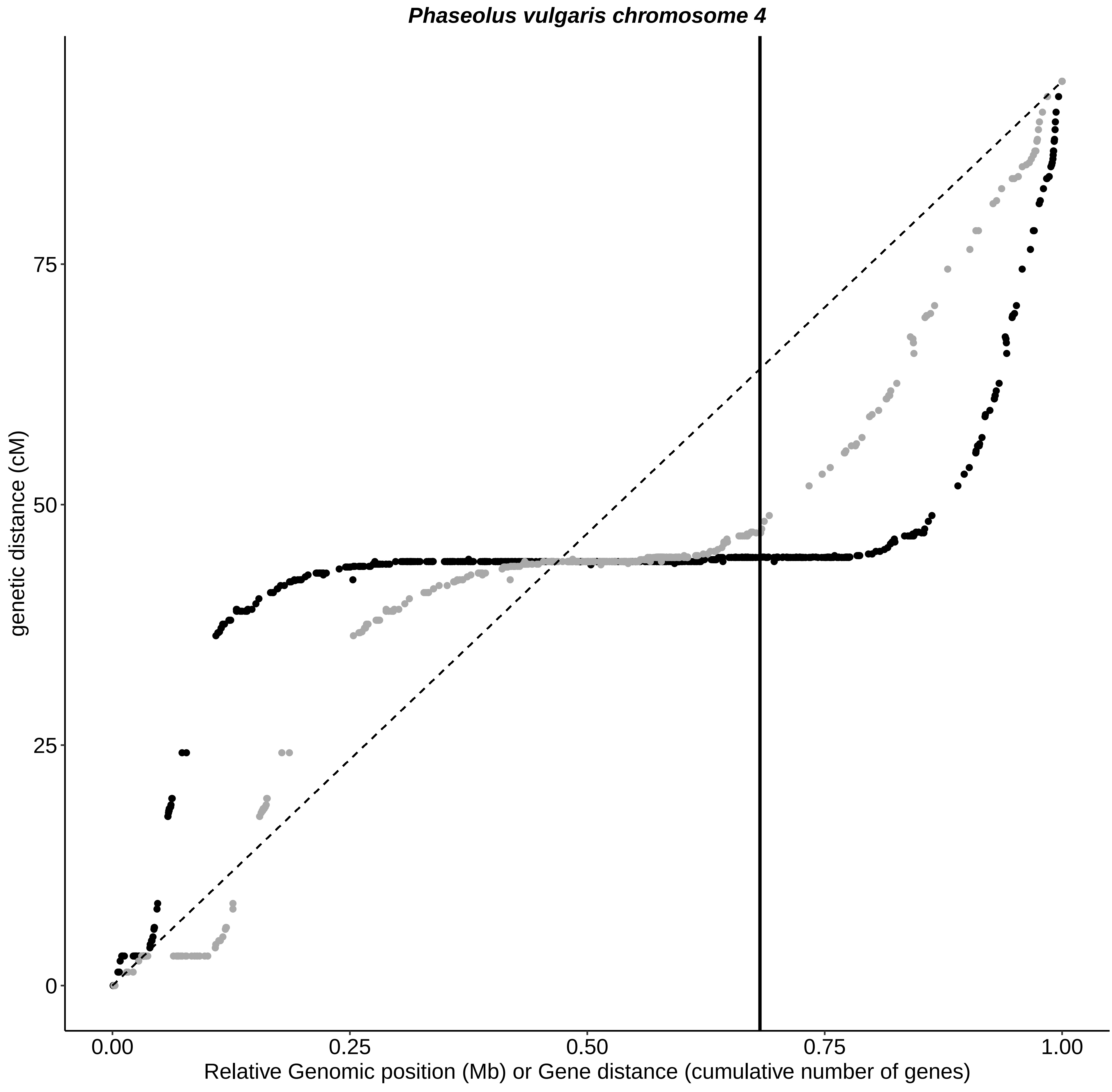

Phaseolus vulgaris chromosome 4
genetic distance (cM)
0
25
50
75
0.00
0.25
0.50
0.75
1.00
Relative Genomic position (Mb) or Gene distance (cumulative number of genes)

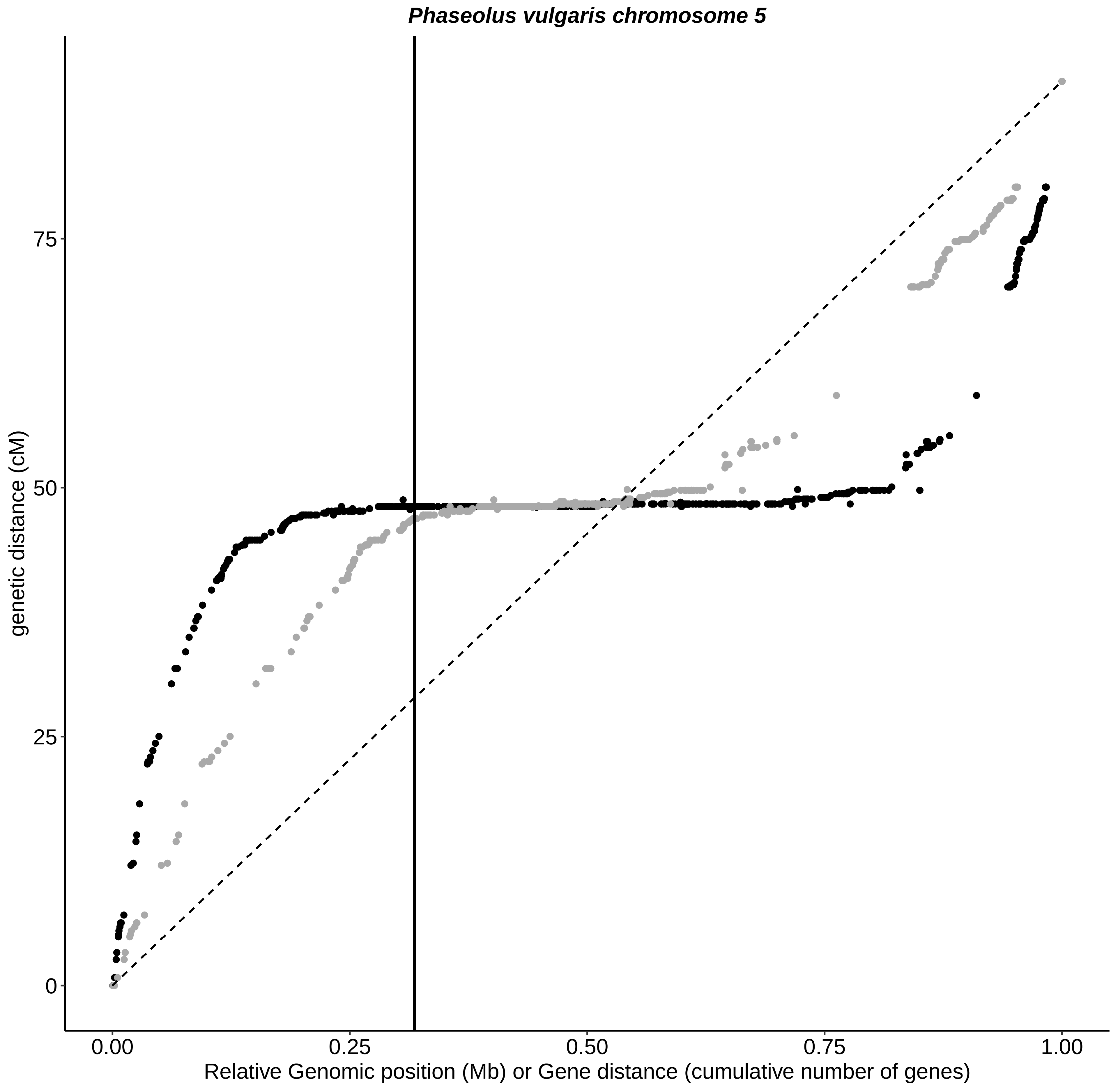

Phaseolus vulgaris chromosome 5
genetic distance (cM)
0
25
50
75
0.00
0.25
0.50
0.75
1.00
Relative Genomic position (Mb) or Gene distance (cumulative number of genes)

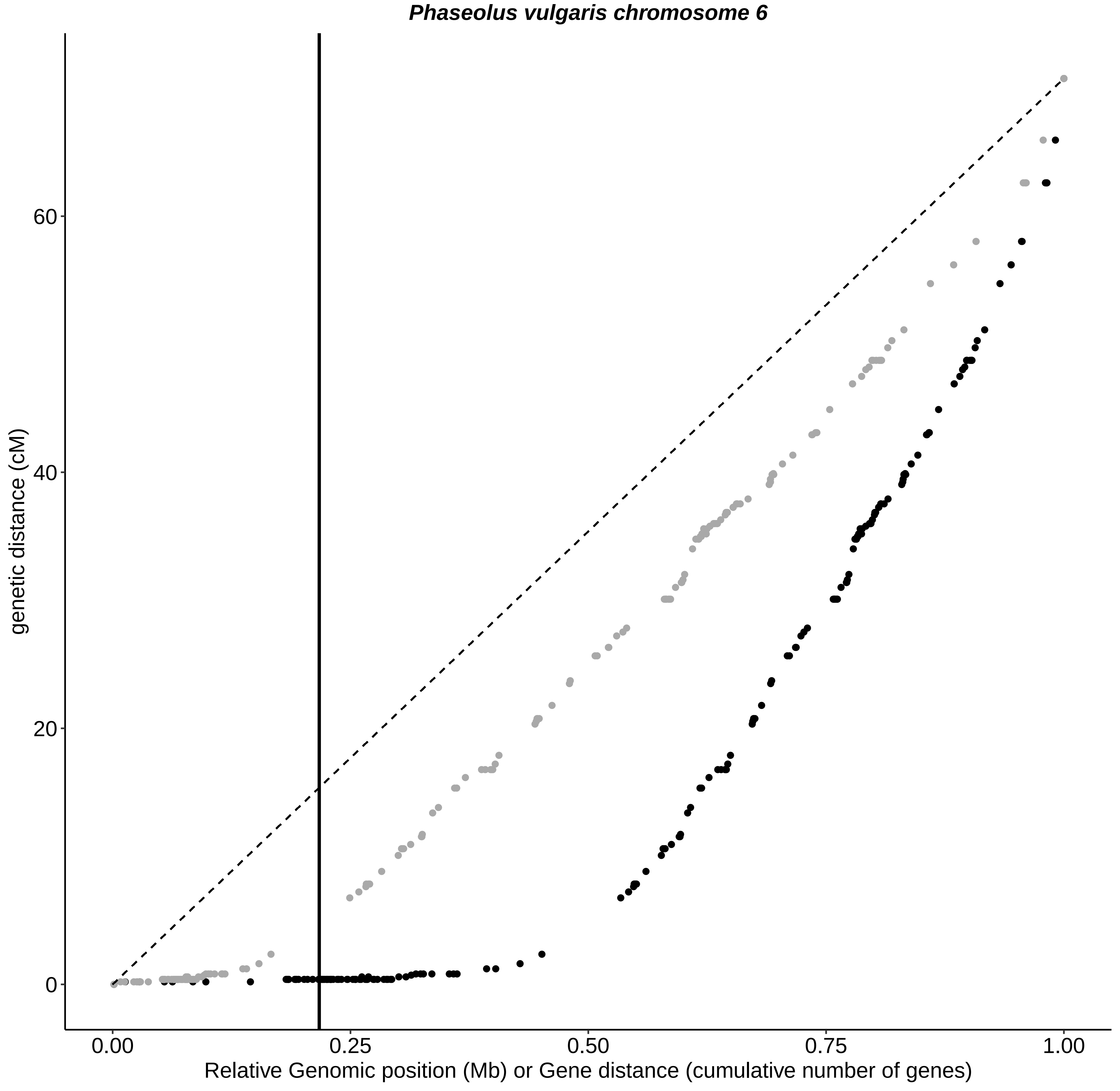
Phaseolus vulgaris chromosome 6
genetic distance (cM)
0
20
40
60
0.00
0.25
0.50
0.75
1.00
Relative Genomic position (Mb) or Gene distance (cumulative number of genes)

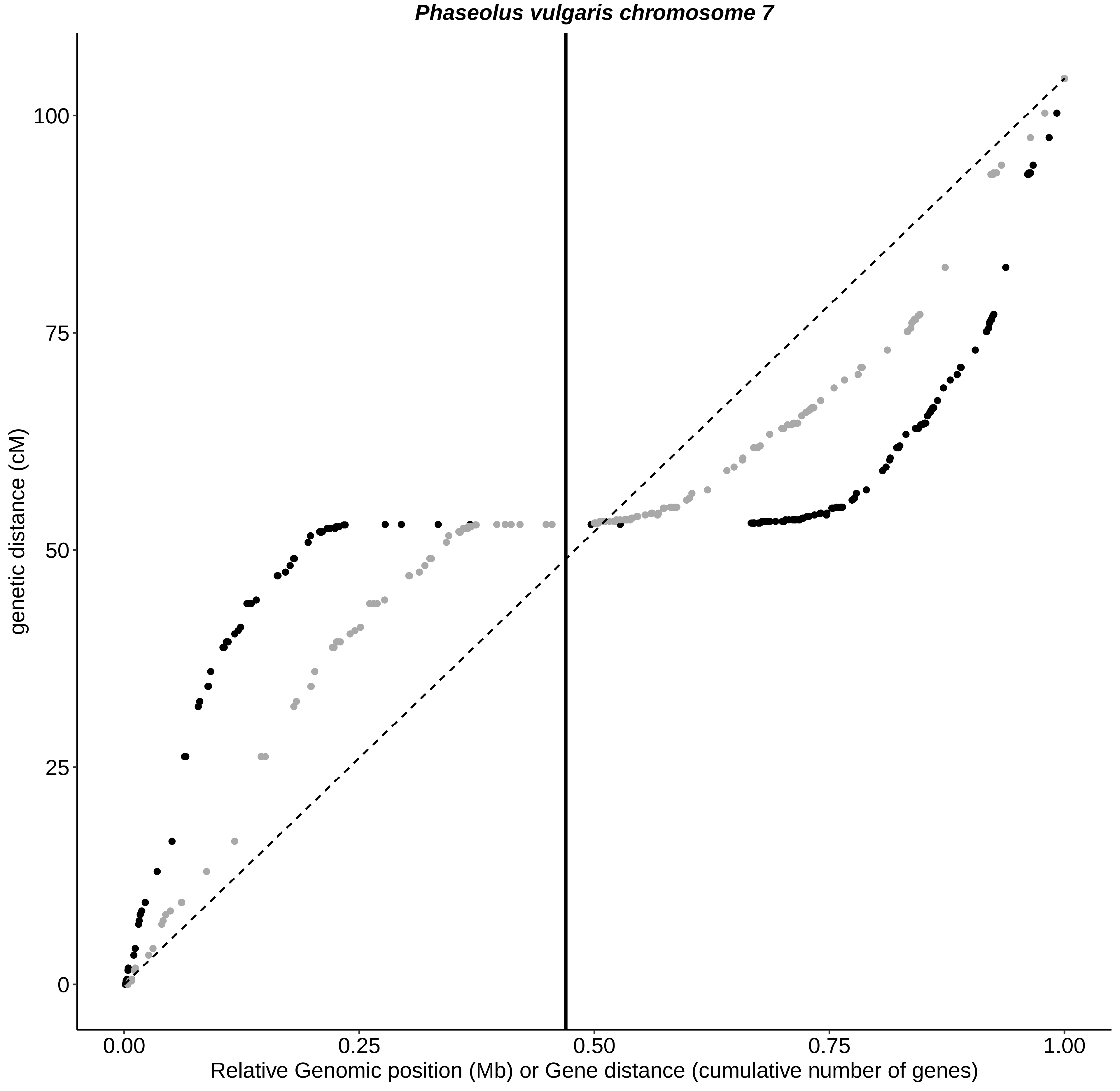
Phaseolus vulgaris chromosome 7
genetic distance (cM)
0
25
50
75
100
0.00
0.25
0.50
0.75
1.00
Relative Genomic position (Mb) or Gene distance (cumulative number of genes)

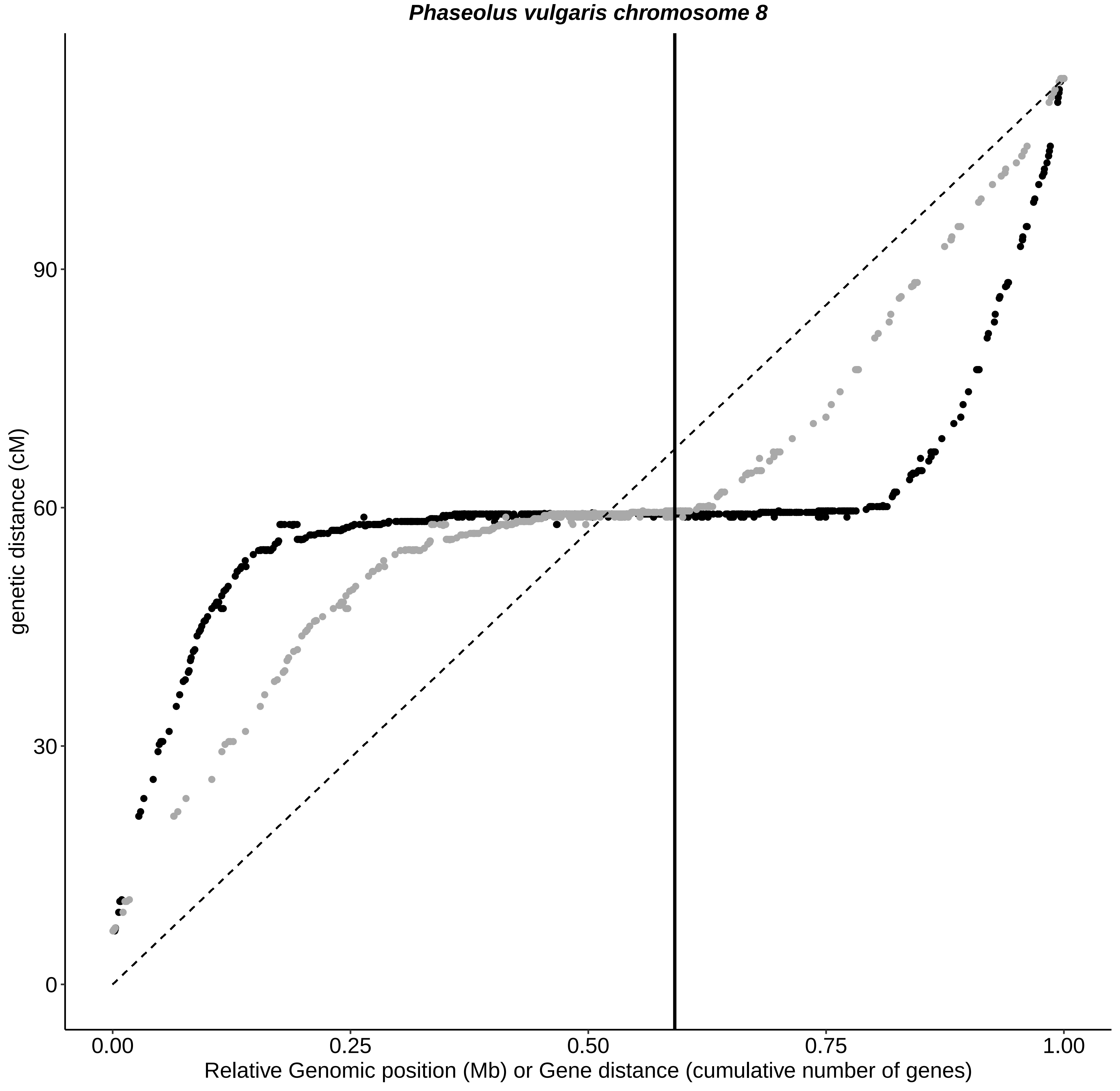

Phaseolus vulgaris chromosome 8
genetic distance (cM)
0
30
60
90
0.00
0.25
0.50
0.75
1.00
Relative Genomic position (Mb) or Gene distance (cumulative number of genes)

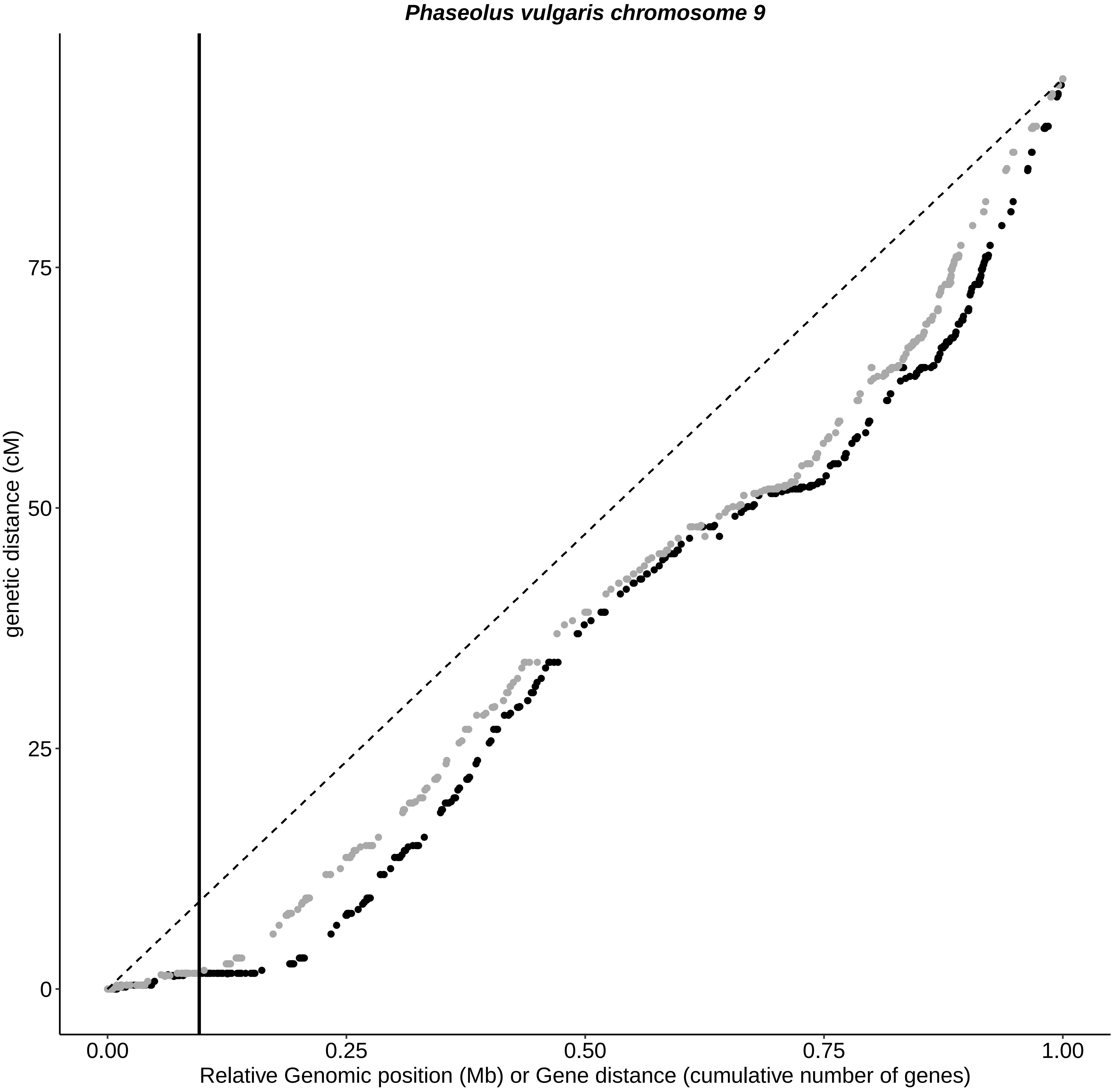
Phaseolus vulgaris chromosome 9
genetic distance (cM)
0
25
50
75
0.00
0.25
0.50
0.75
1.00
Relative Genomic position (Mb) or Gene distance (cumulative number of genes)

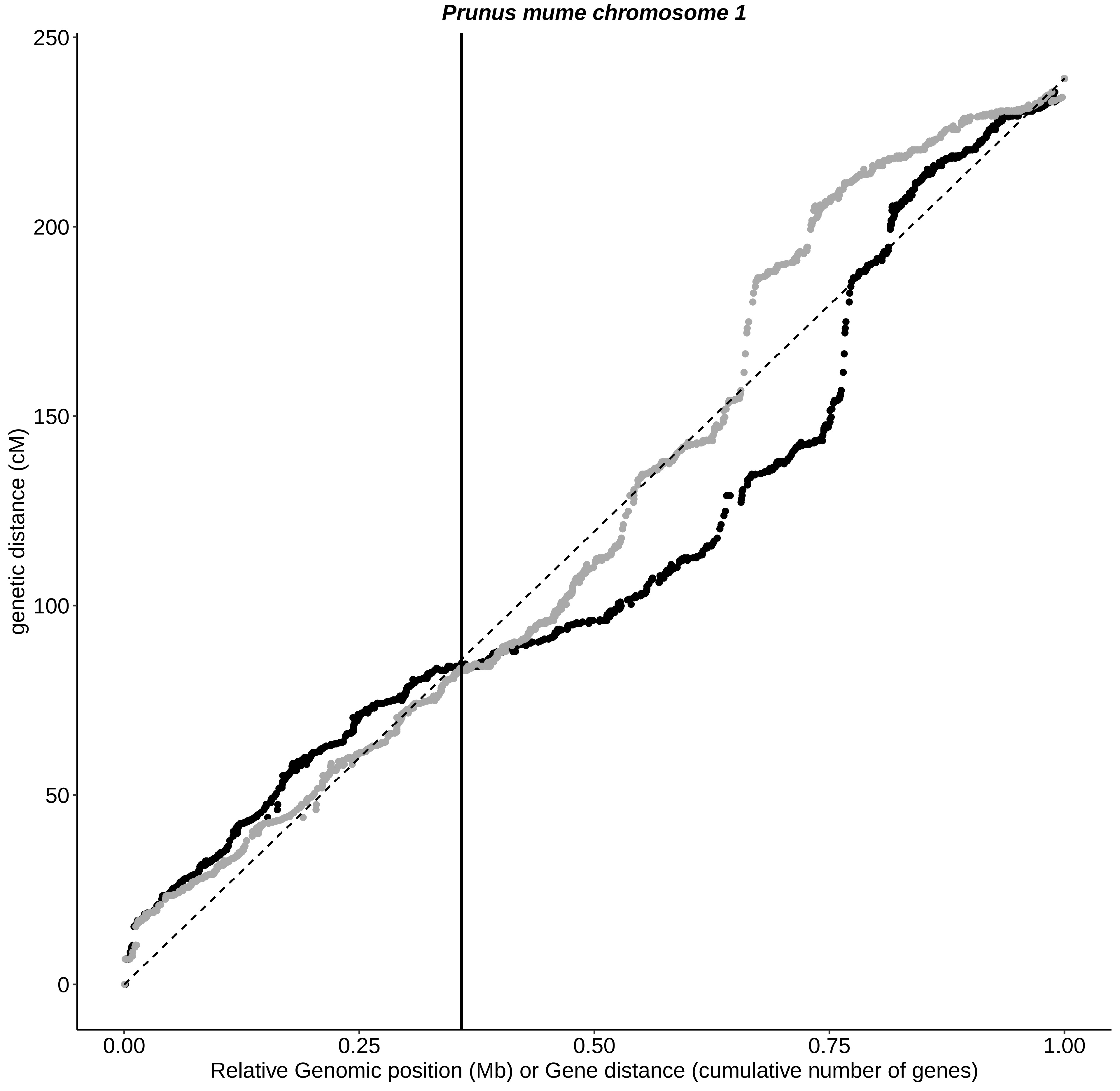
Prunus mume chromosome 1
250
200
150
100
50
0
genetic distance (cM)
0.00
0.25
0.50
0.75
1.00
Relative Genomic position (Mb) or Gene distance (cumulative number of genes)

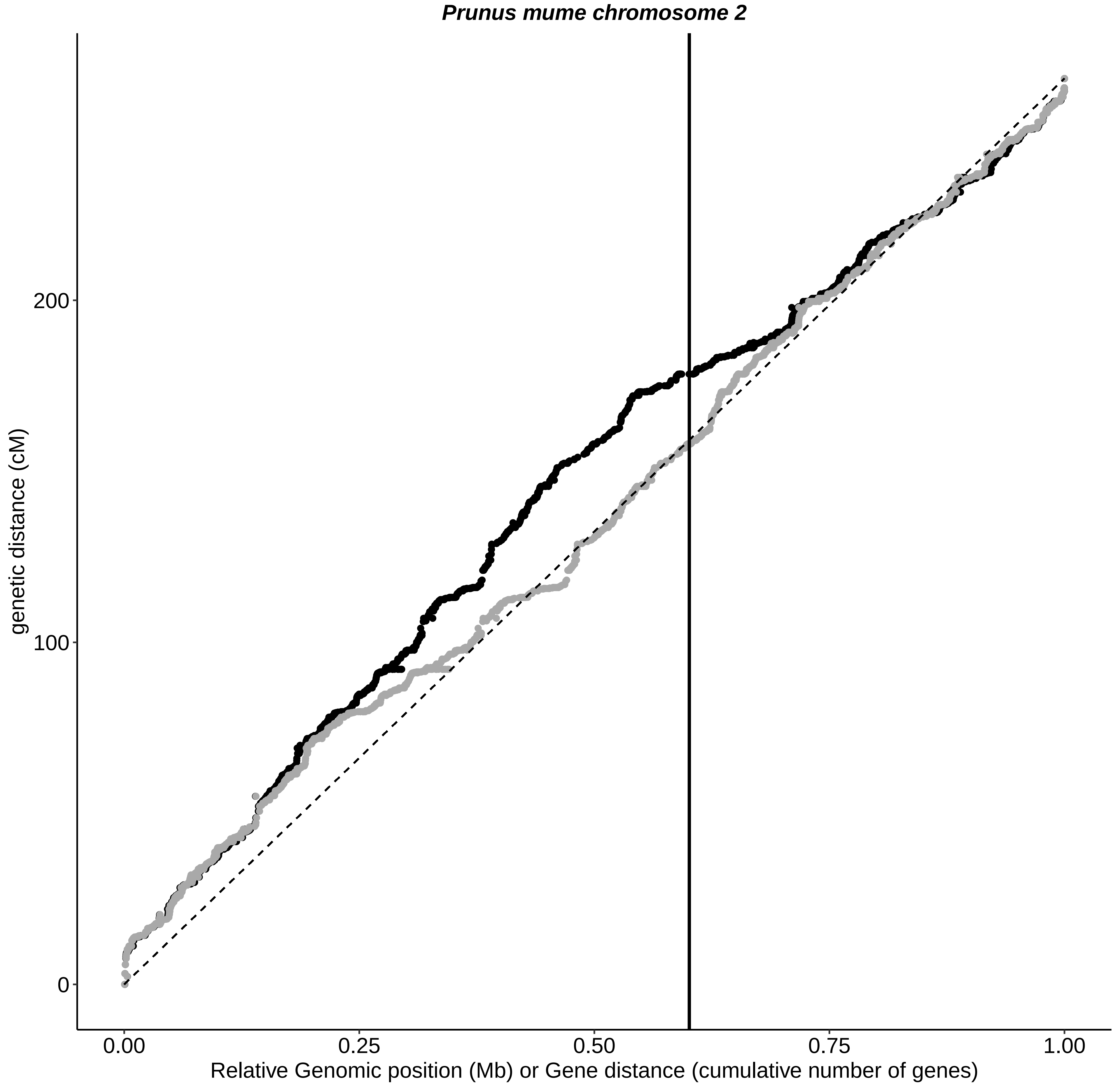
Prunus mume chromosome 2
genetic distance (cM)
0
100
200
0.00
0.25
0.50
0.75
1.00
Relative Genomic position (Mb) or Gene distance (cumulative number of genes)

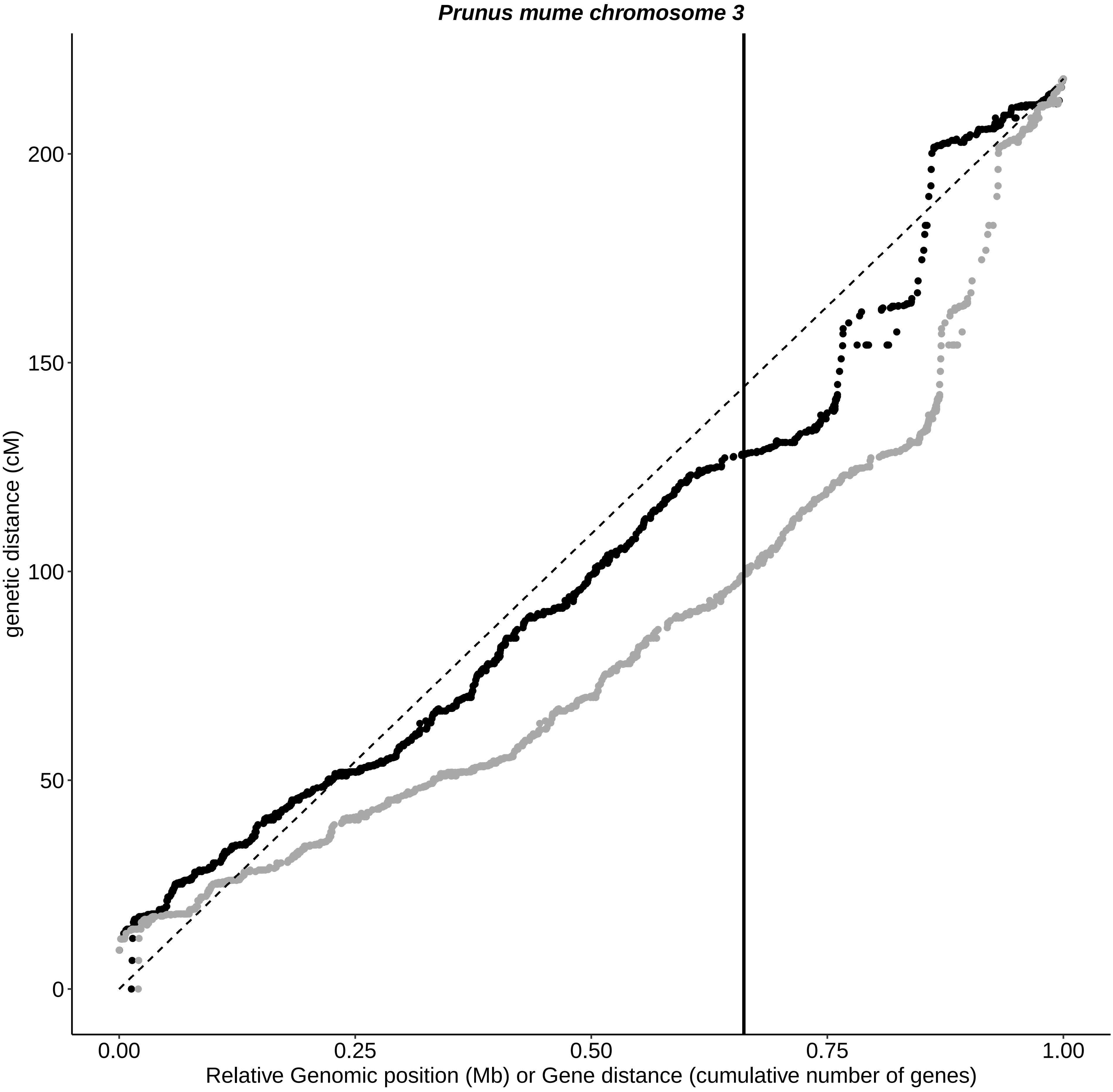
Prunus mume chromosome 3
genetic distance (cM)
200
150
100
50
0
0.00
0.25
0.50
0.75
1.00
Relative Genomic position (Mb) or Gene distance (cumulative number of genes)

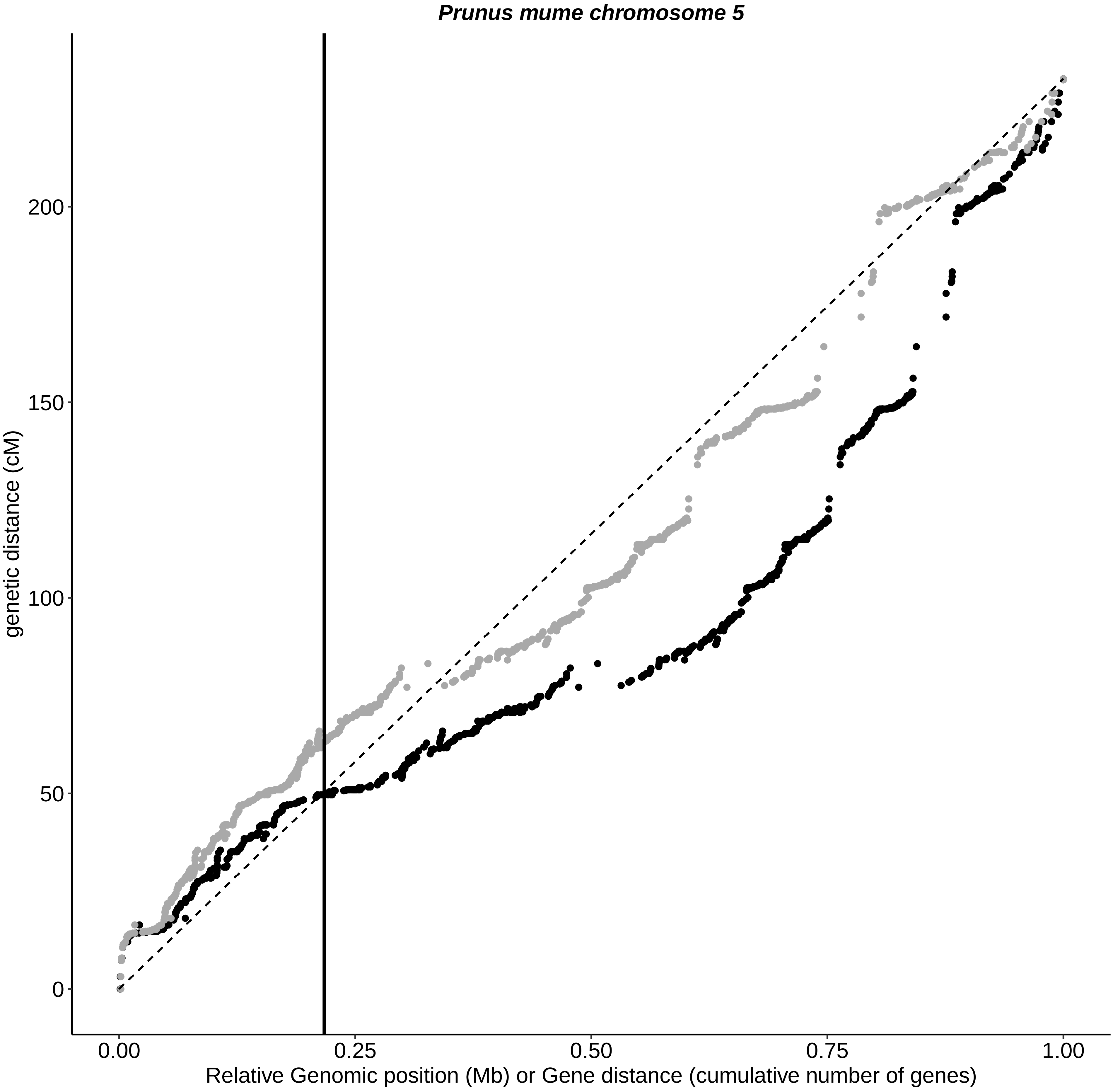
Prunus mume chromosome 5
genetic distance (cM)
0
50
100
150
200
0.00
0.25
0.50
0.75
1.00
Relative Genomic position (Mb) or Gene distance (cumulative number of genes)

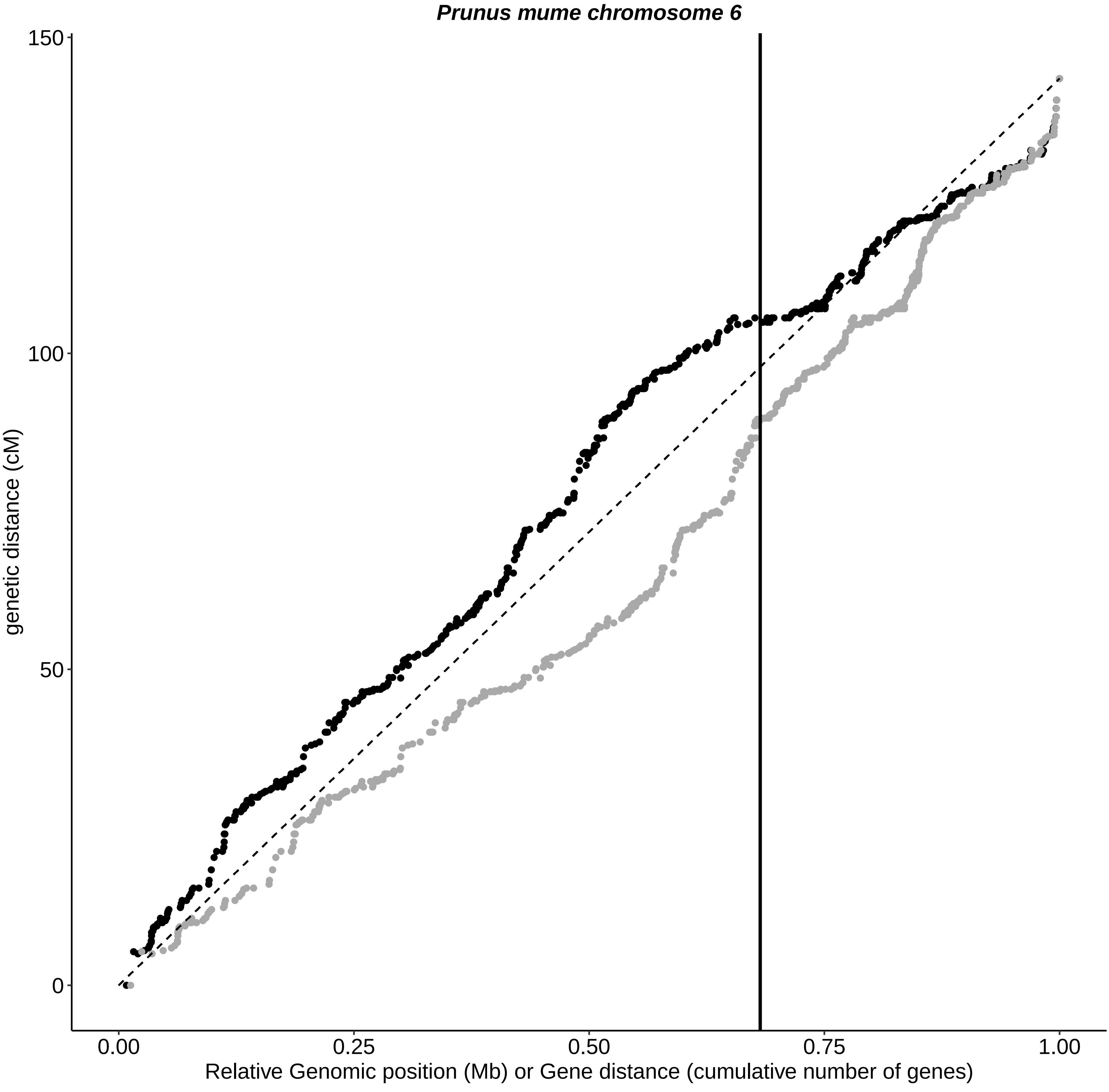

Prunus mume chromosome 6
150
100
50
0
genetic distance (cM)
0.00
0.25
0.50
0.75
1.00
Relative Genomic position (Mb) or Gene distance (cumulative number of genes)

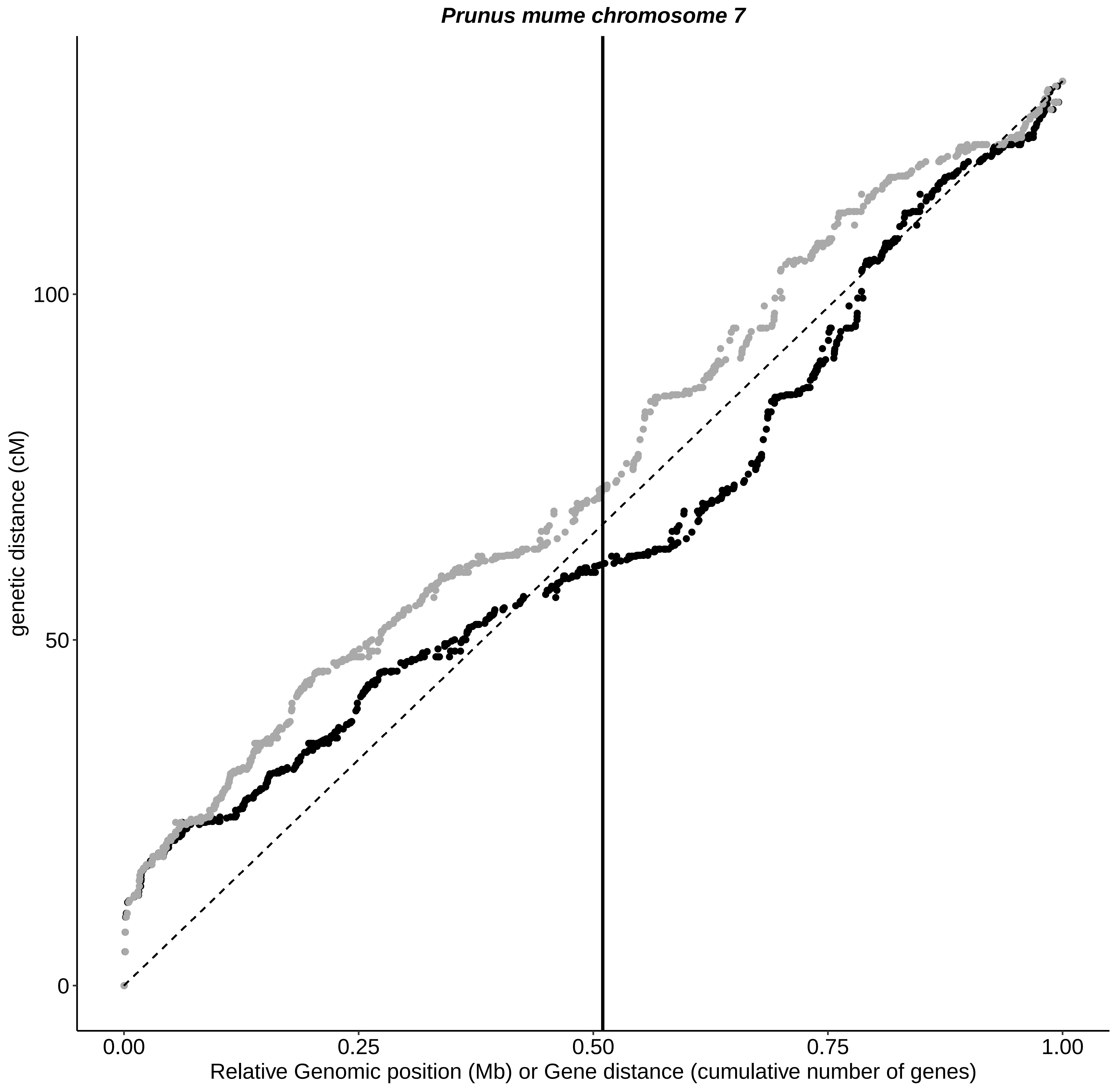
Prunus mume chromosome 7
genetic distance (cM)
0
50
100
0.00
0.25
0.50
0.75
1.00
Relative Genomic position (Mb) or Gene distance (cumulative number of genes)

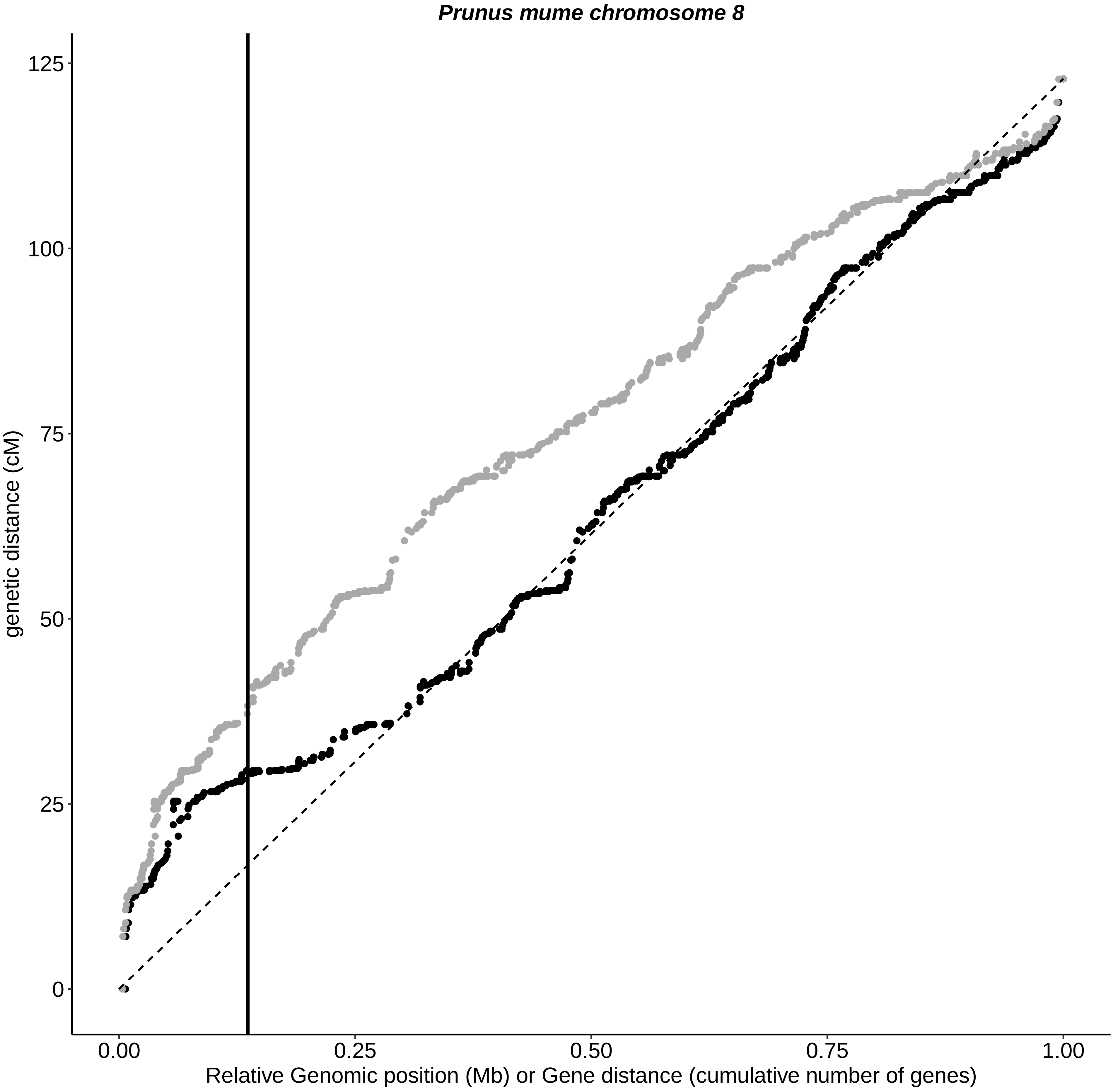
Prunus mume chromosome 8
genetic distance (cM)
125
100
75
50
25
0
0.00
0.25
0.50
0.75
1.00
Relative Genomic position (Mb) or Gene distance (cumulative number of genes)

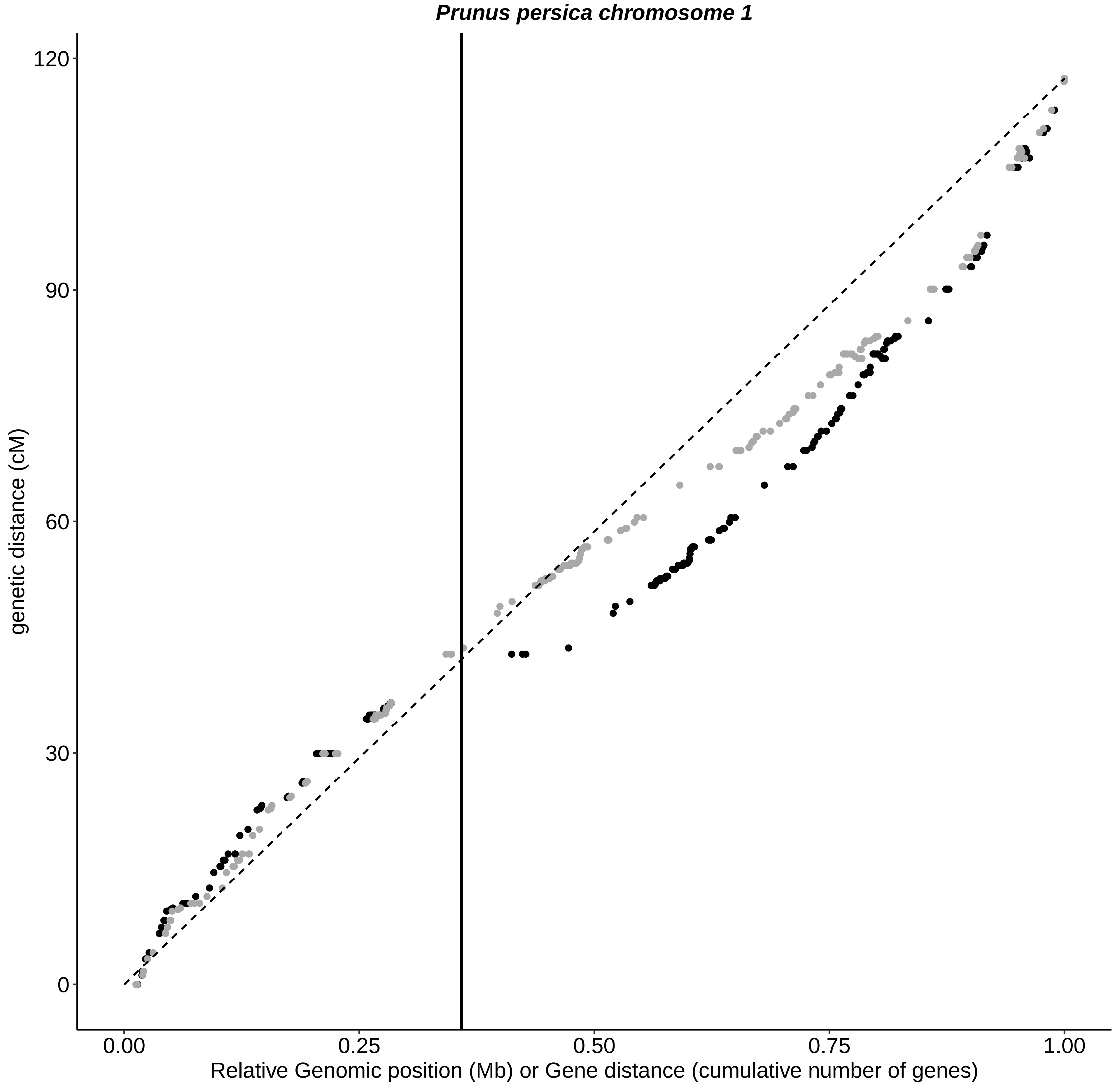
Prunus persica chromosome 1
genetic distance (cM)
120
90
60
30
0
0.00
0.25
0.50
0.75
1.00
Relative Genomic position (Mb) or Gene distance (cumulative number of genes)

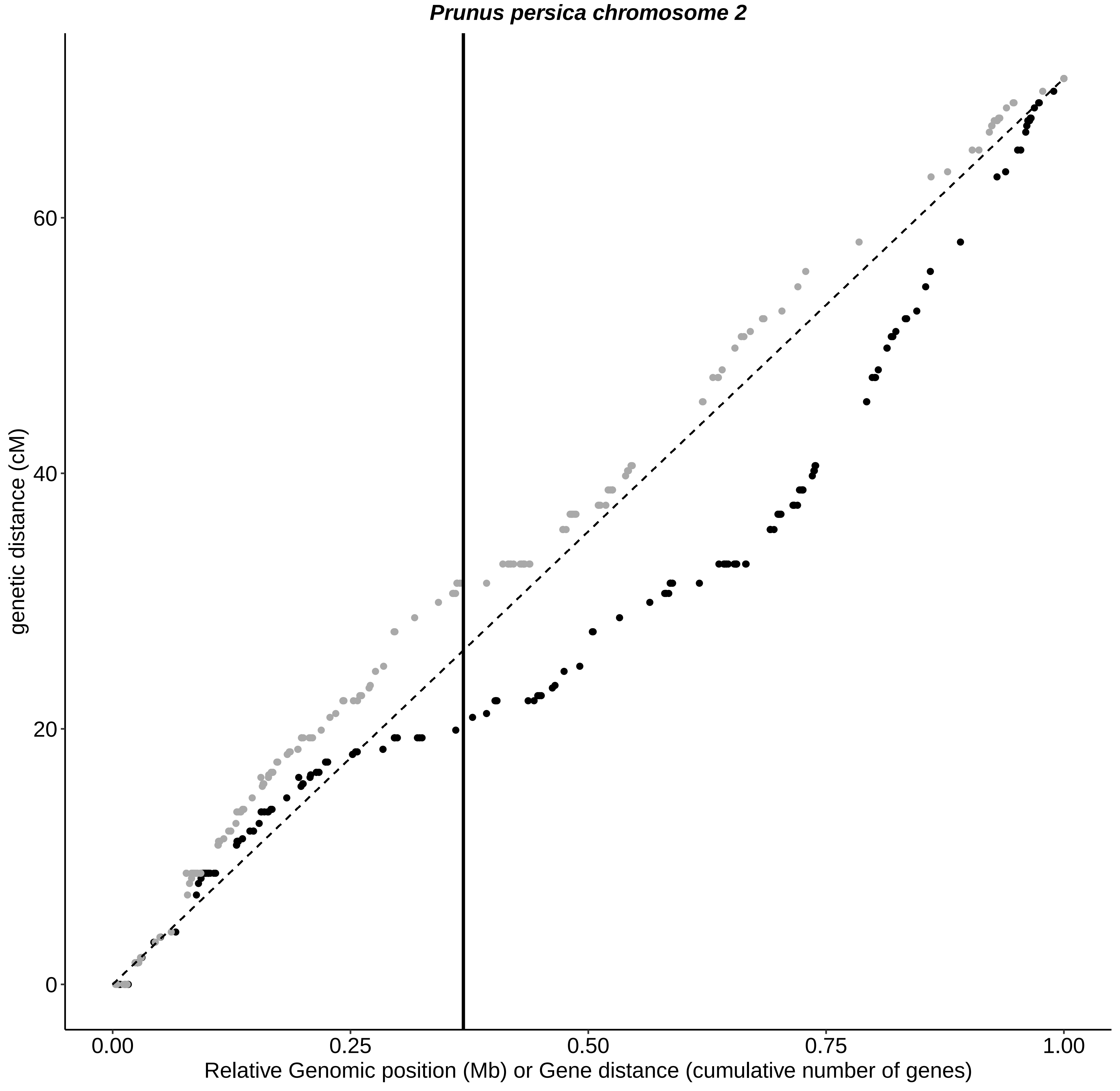
Prunus persica chromosome 2
genetic distance (cM)
0
20
40
60
0.00
0.25
0.50
0.75
1.00
Relative Genomic position (Mb) or Gene distance (cumulative number of genes)

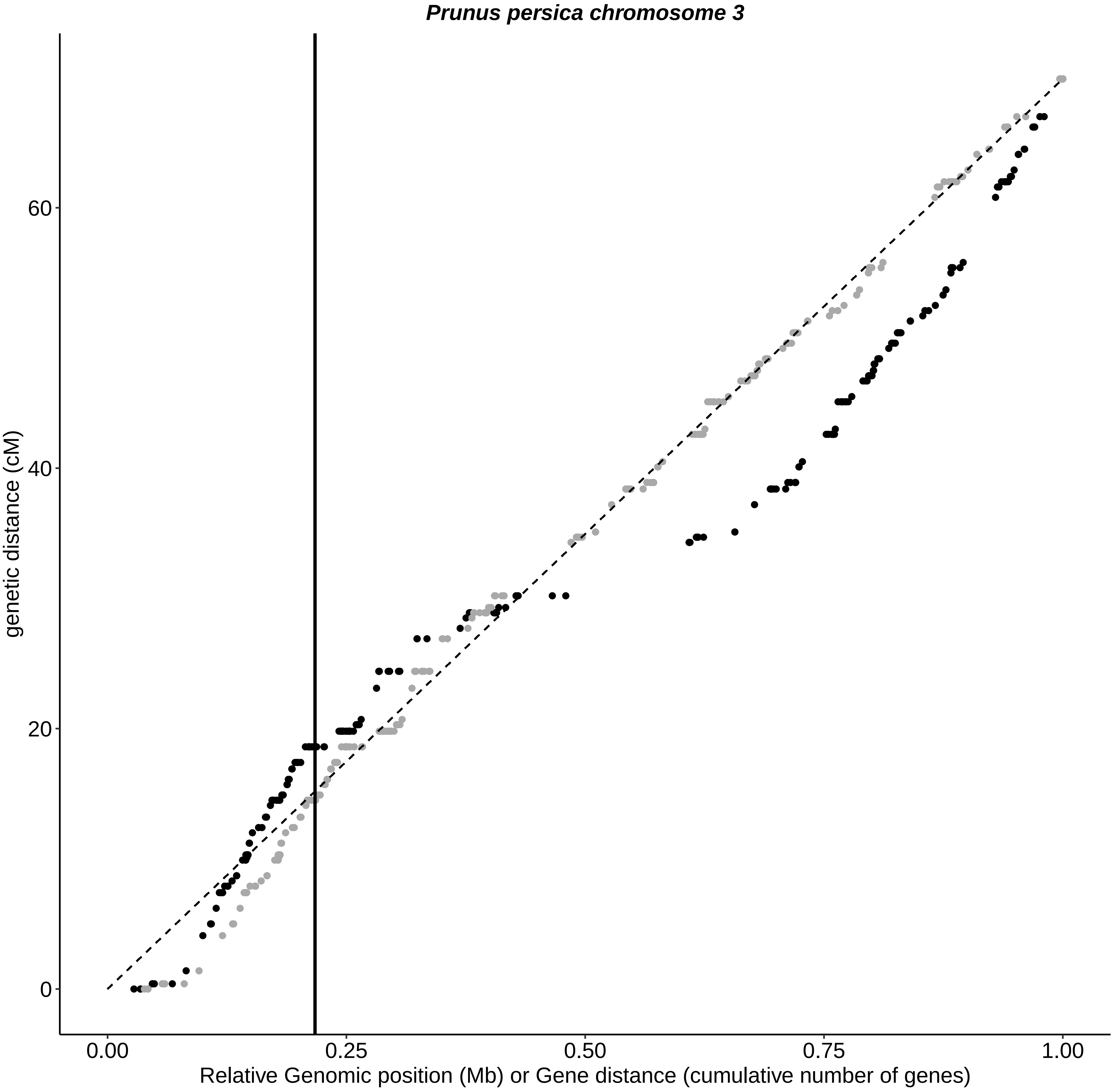
Prunus persica chromosome 3
genetic distance (cM)
0
20
40
60
0.00
0.25
0.50
0.75
1.00
Relative Genomic position (Mb) or Gene distance (cumulative number of genes)

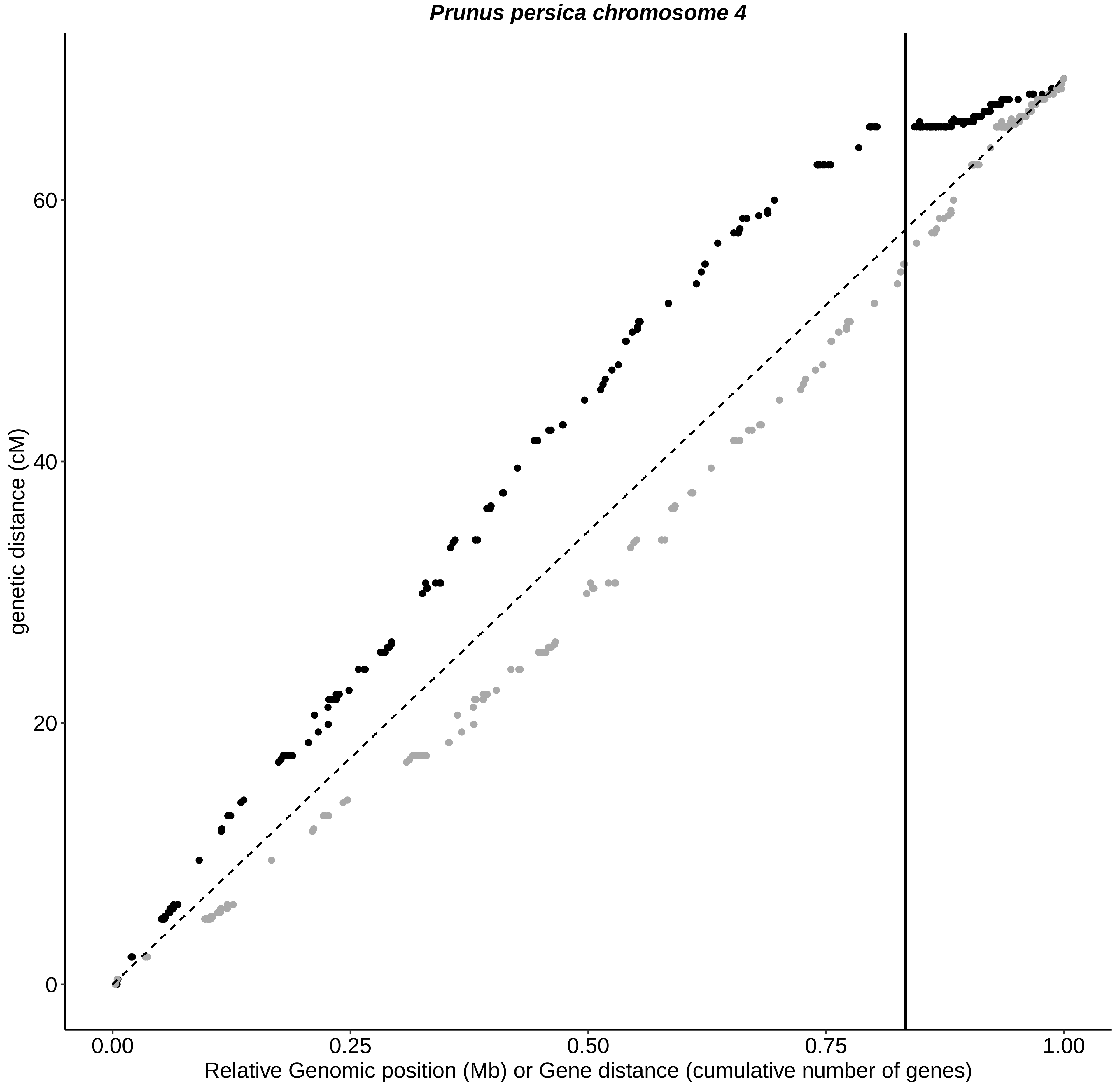
Prunus persica chromosome 4
genetic distance (cM)
0
20
40
60
0.00
0.25
0.50
0.75
1.00
Relative Genomic position (Mb) or Gene distance (cumulative number of genes)

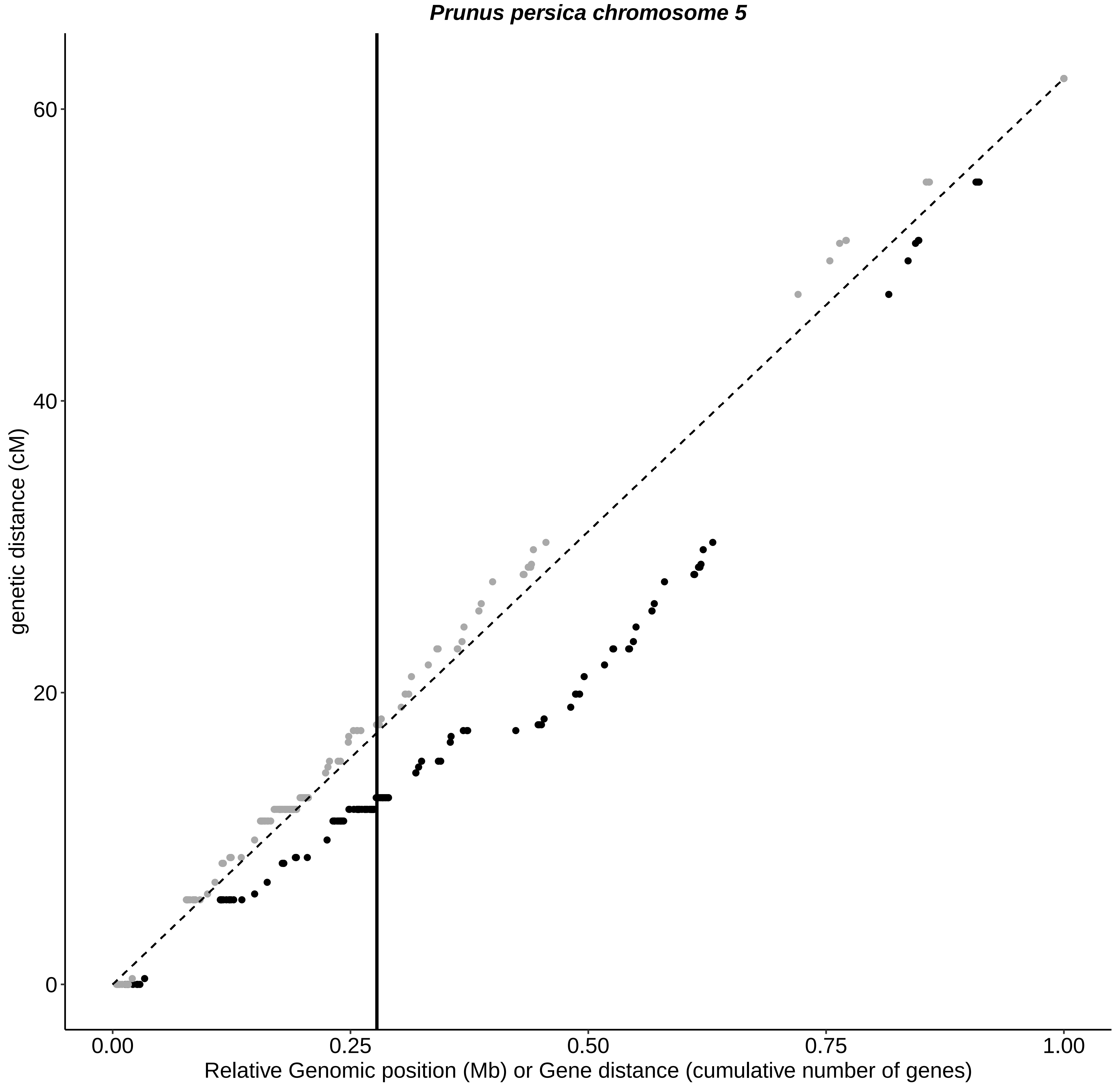
Prunus persica chromosome 5
genetic distance (cM)
60
40
20
0
0.00
0.25
0.50
0.75
1.00
Relative Genomic position (Mb) or Gene distance (cumulative number of genes)

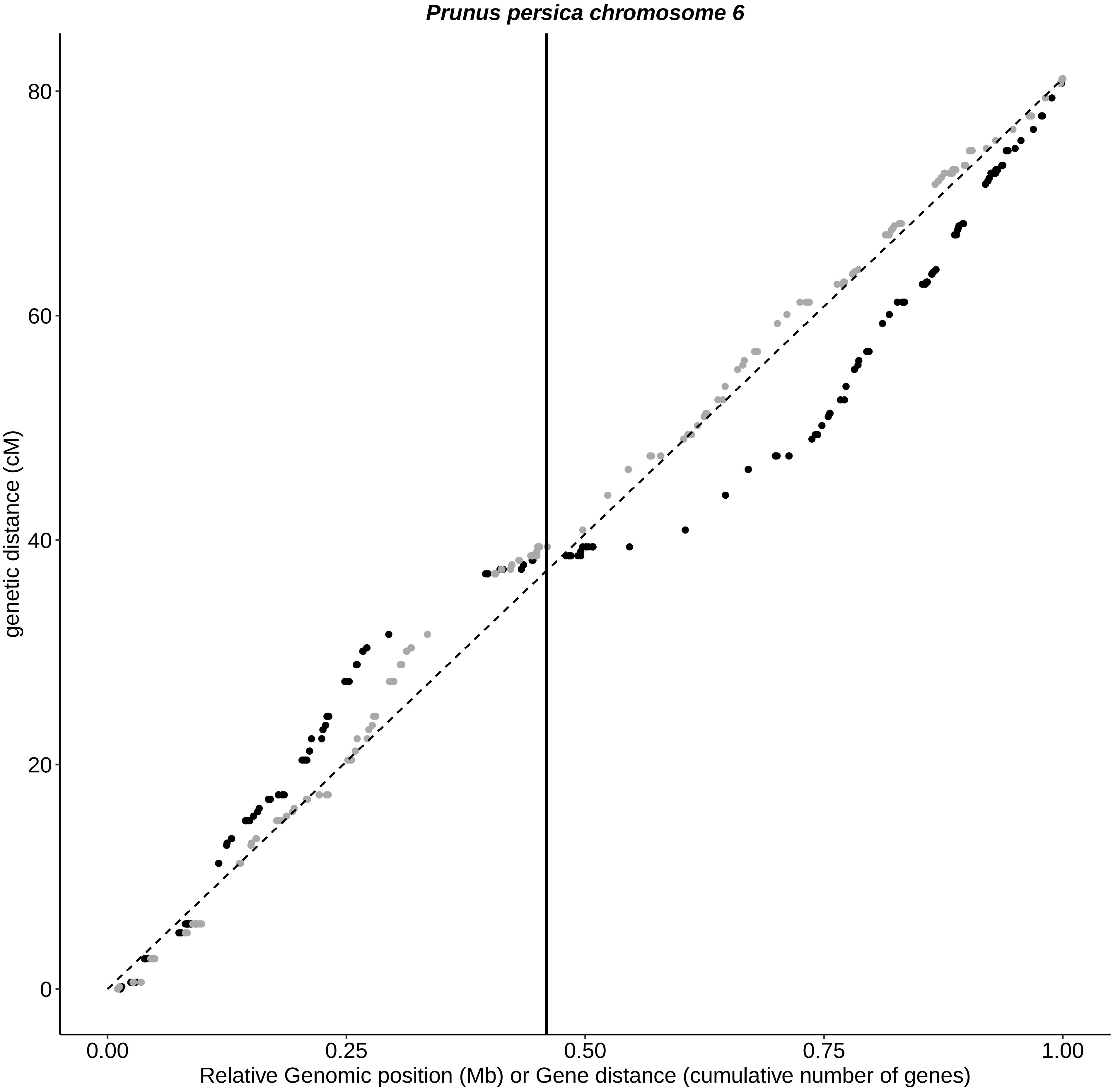
Prunus persica chromosome 6
genetic distance (cM)
0
20
40
60
80
0.00
0.25
0.50
0.75
1.00
Relative Genomic position (Mb) or Gene distance (cumulative number of genes)

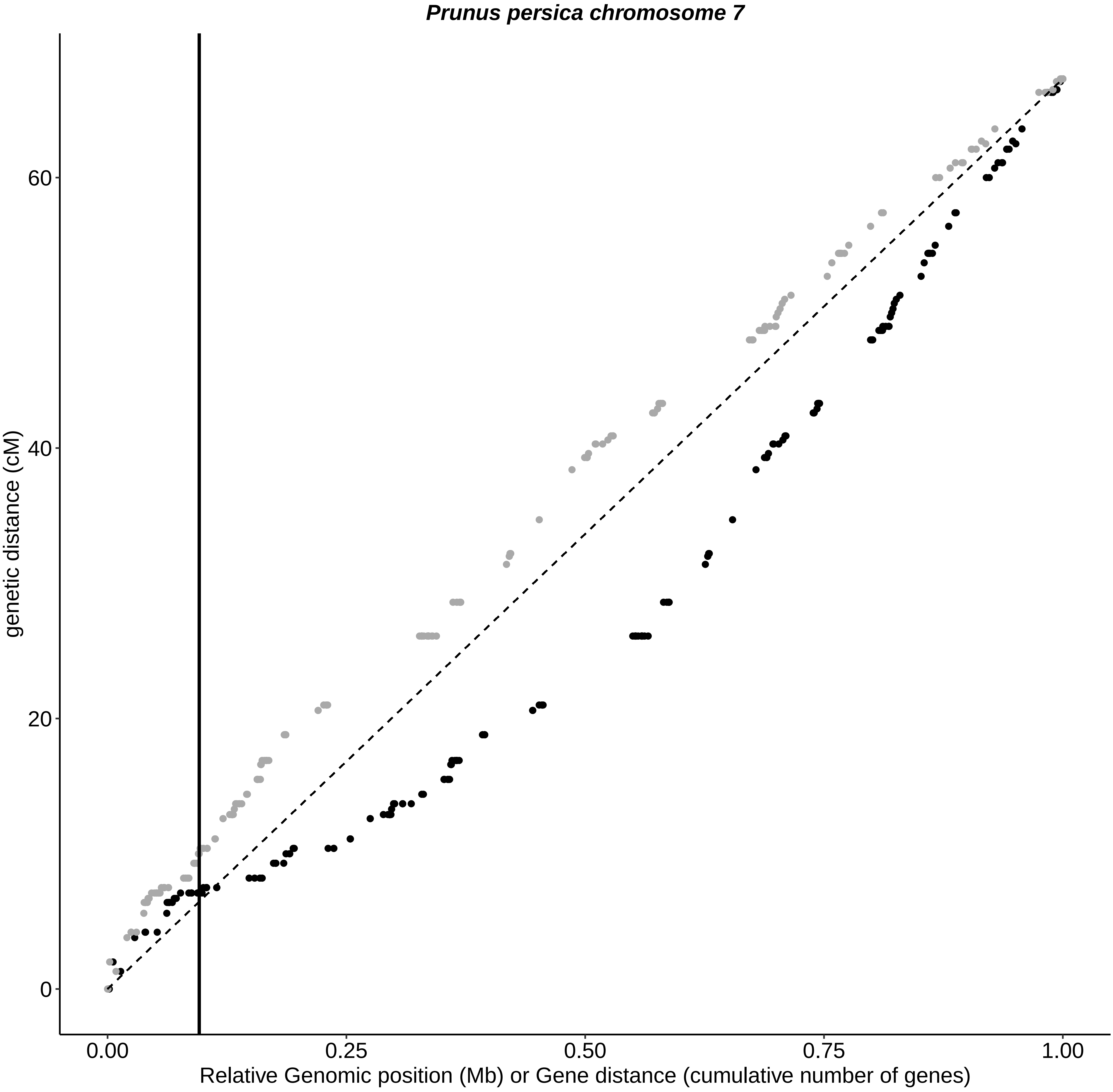

Prunus persica chromosome 7
genetic distance (cM)
0
20
40
60
0.00
0.25
0.50
0.75
1.00
Relative Genomic position (Mb) or Gene distance (cumulative number of genes)

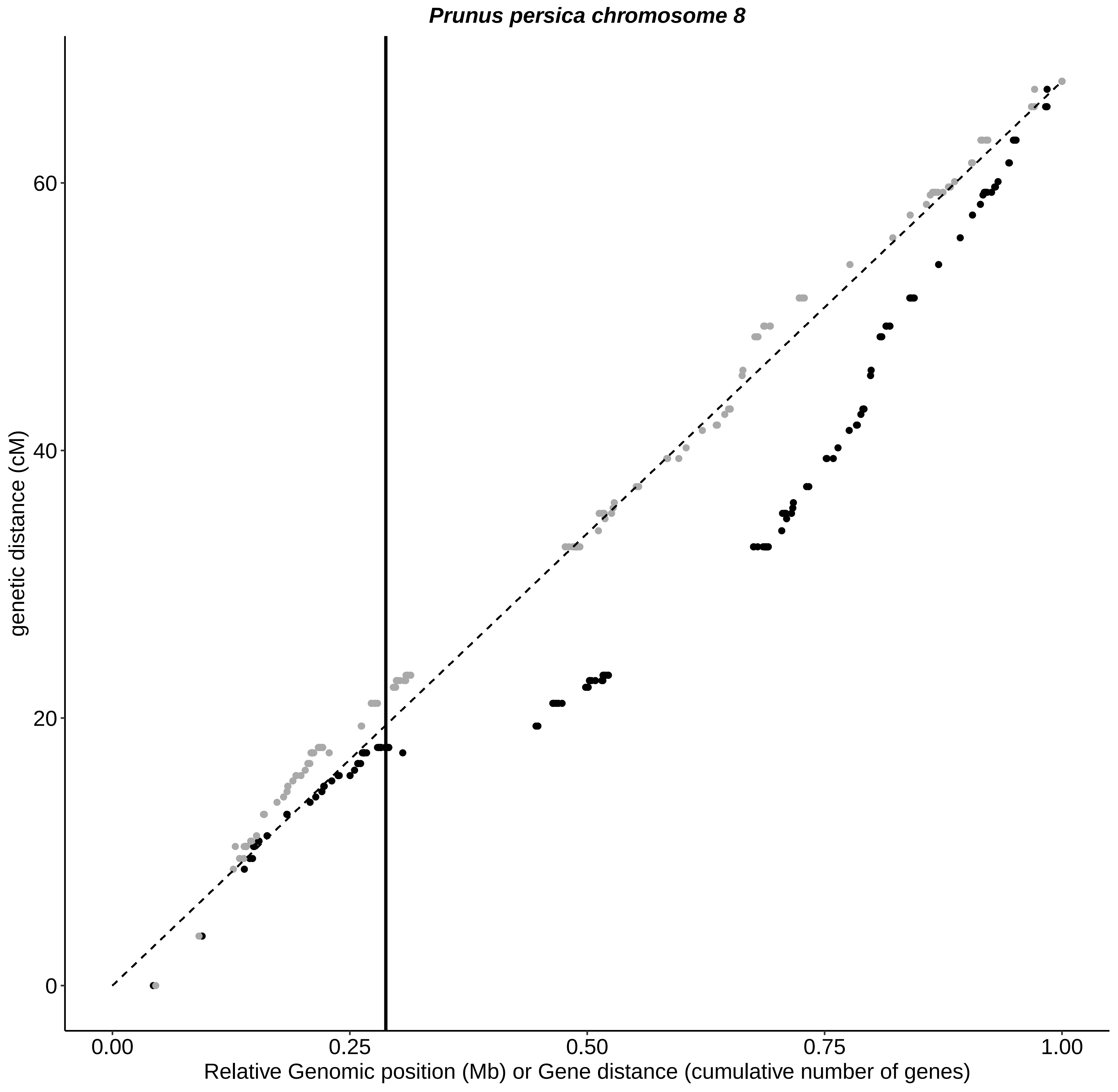
Prunus persica chromosome 8
genetic distance (cM)
0
20
40
60
0.00
0.25
0.50
0.75
1.00
Relative Genomic position (Mb) or Gene distance (cumulative number of genes)

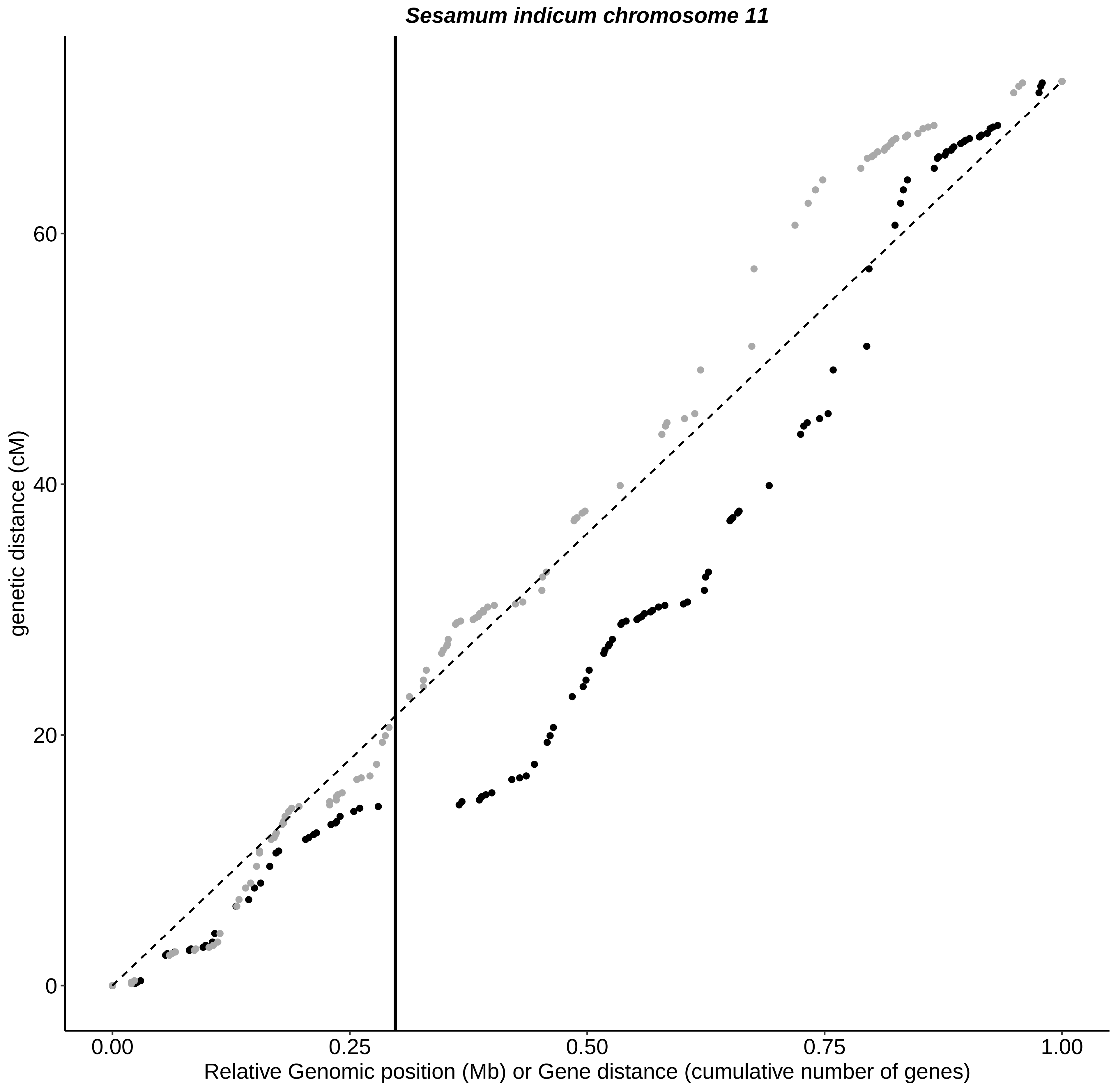
Sesamum indicum chromosome 11
genetic distance (cM)
0
20
40
60
0.00
0.25
0.50
0.75
1.00
Relative Genomic position (Mb) or Gene distance (cumulative number of genes)

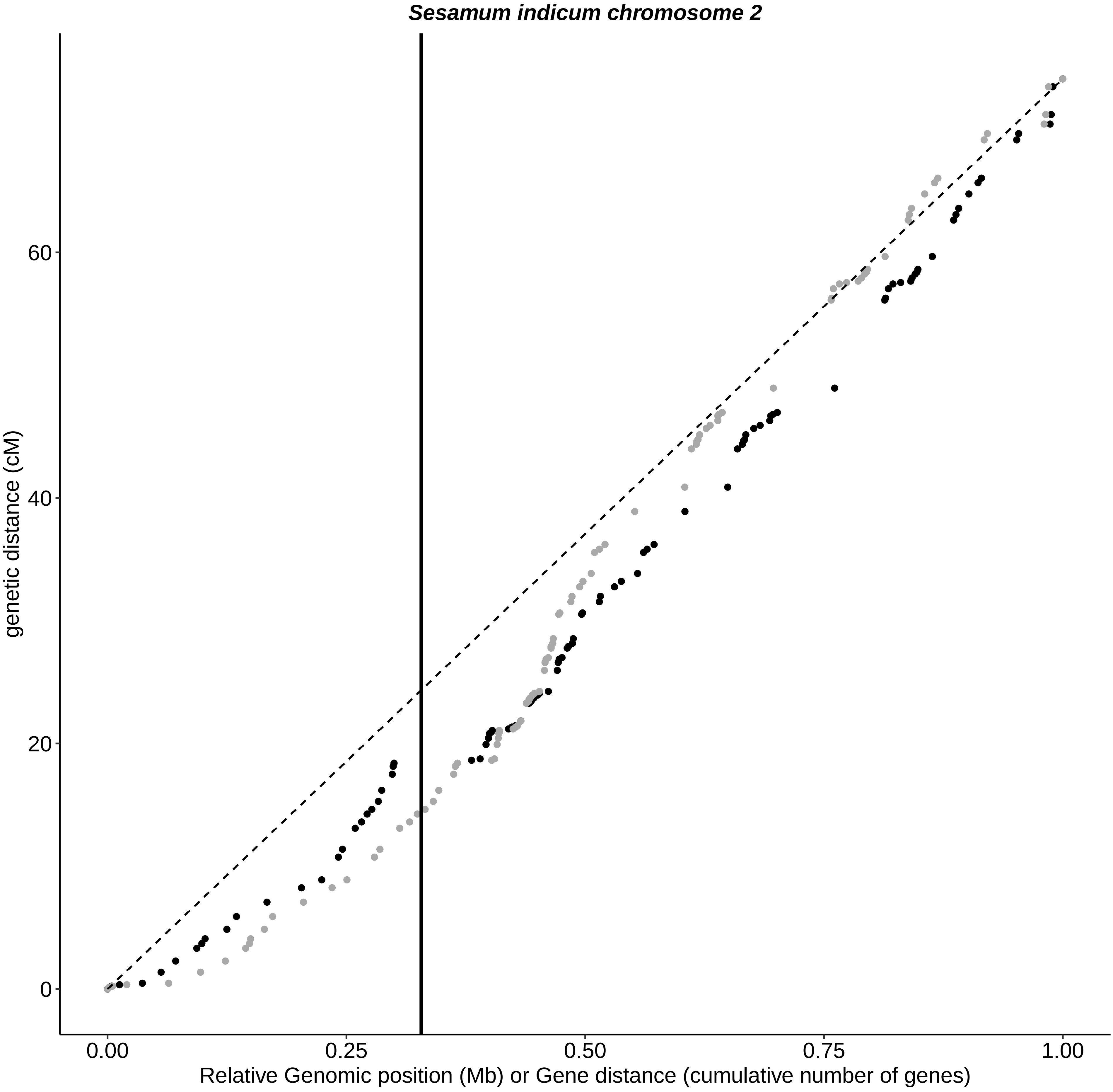

Sesamum indicum chromosome 2
genetic distance (cM)
0
20
40
60
0.00
0.25
0.50
0.75
1.00
Relative Genomic position (Mb) or Gene distance (cumulative number of genes)

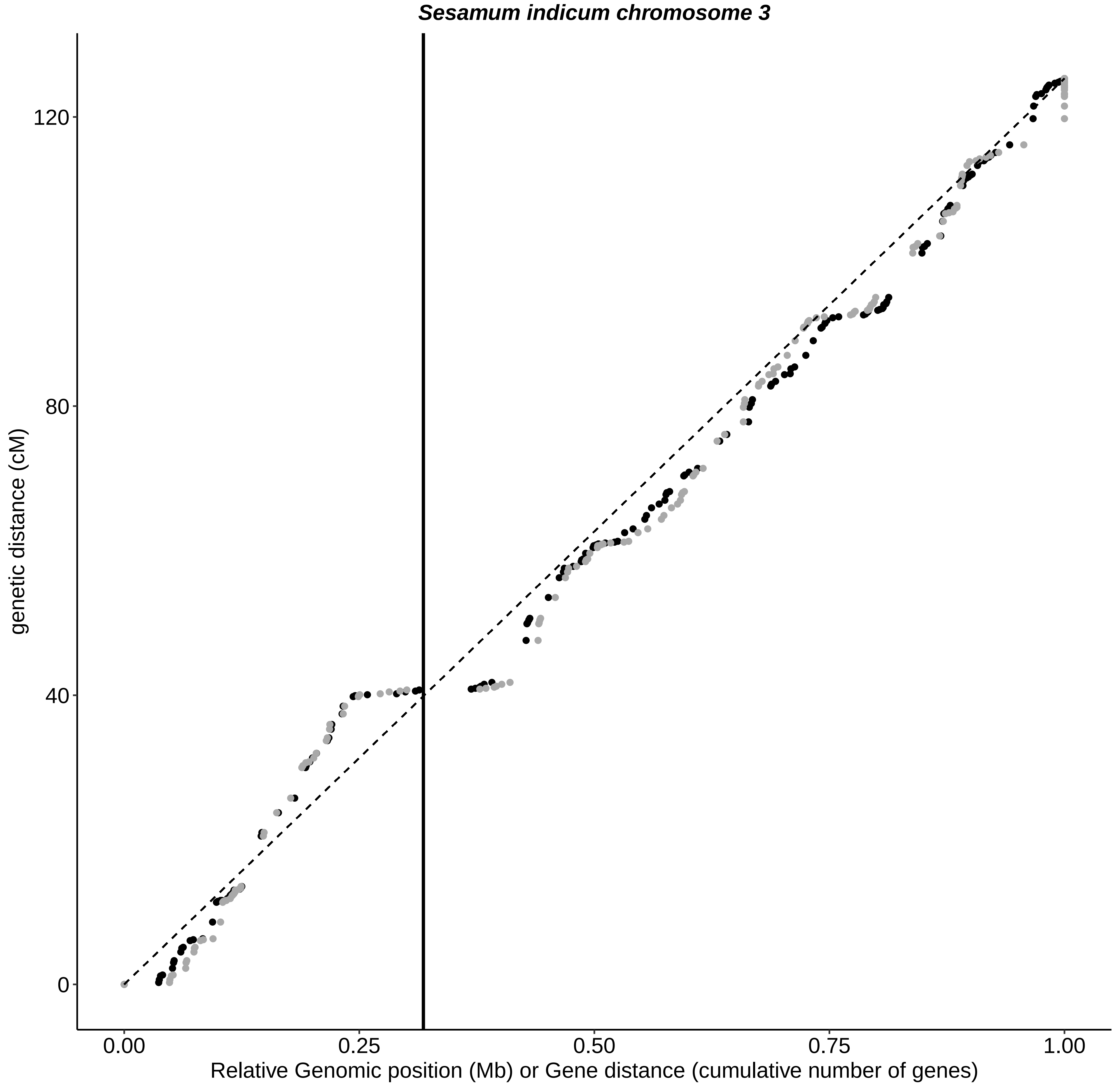
Sesamum indicum chromosome 3
genetic distance (cM)
0
40
80
120
0.00
0.25
0.50
0.75
1.00
Relative Genomic position (Mb) or Gene distance (cumulative number of genes)

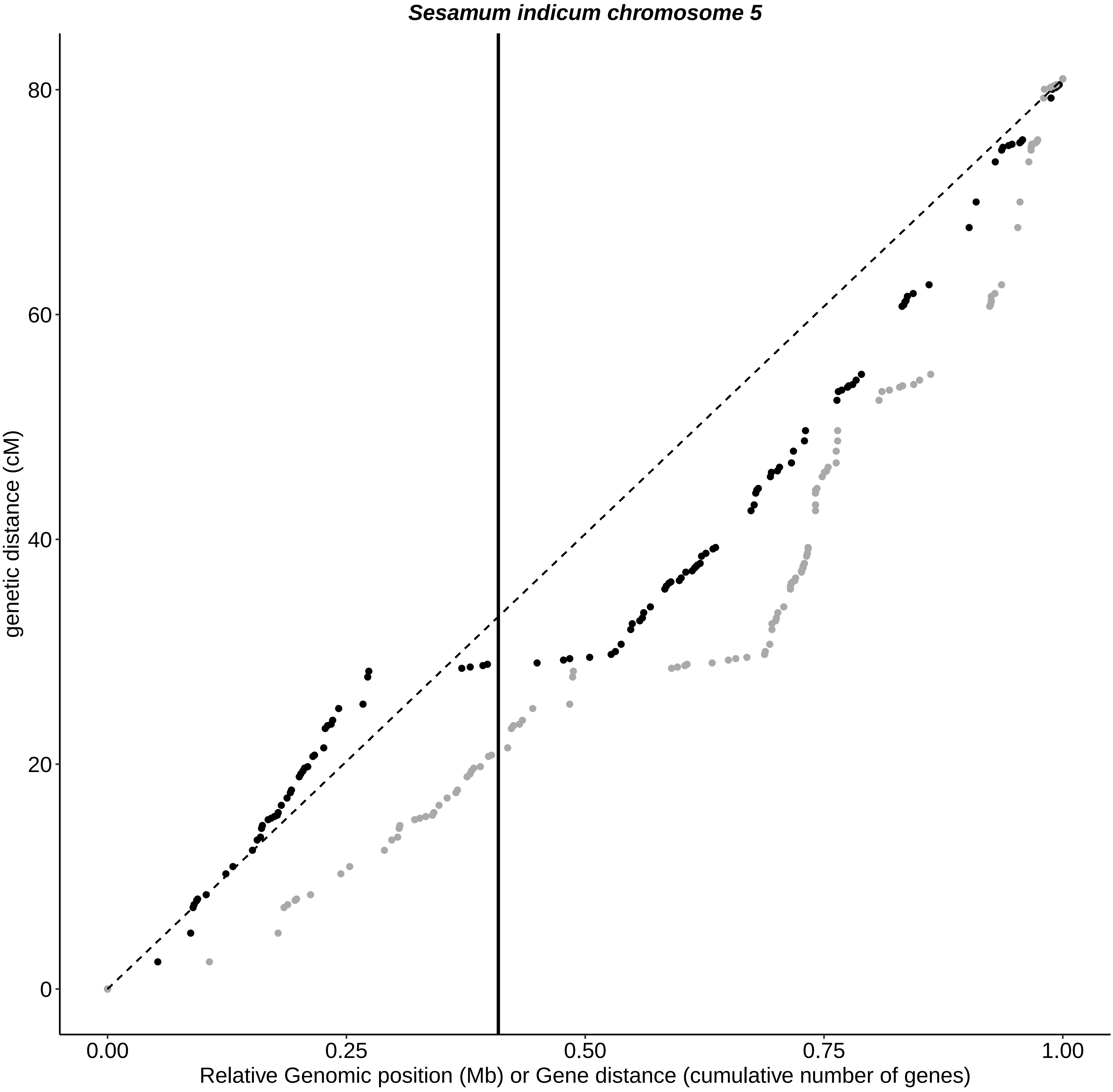
Sesamum indicum chromosome 5
genetic distance (cM)
0
20
40
60
80
0.00
0.25
0.50
0.75
1.00
Relative Genomic position (Mb) or Gene distance (cumulative number of genes)

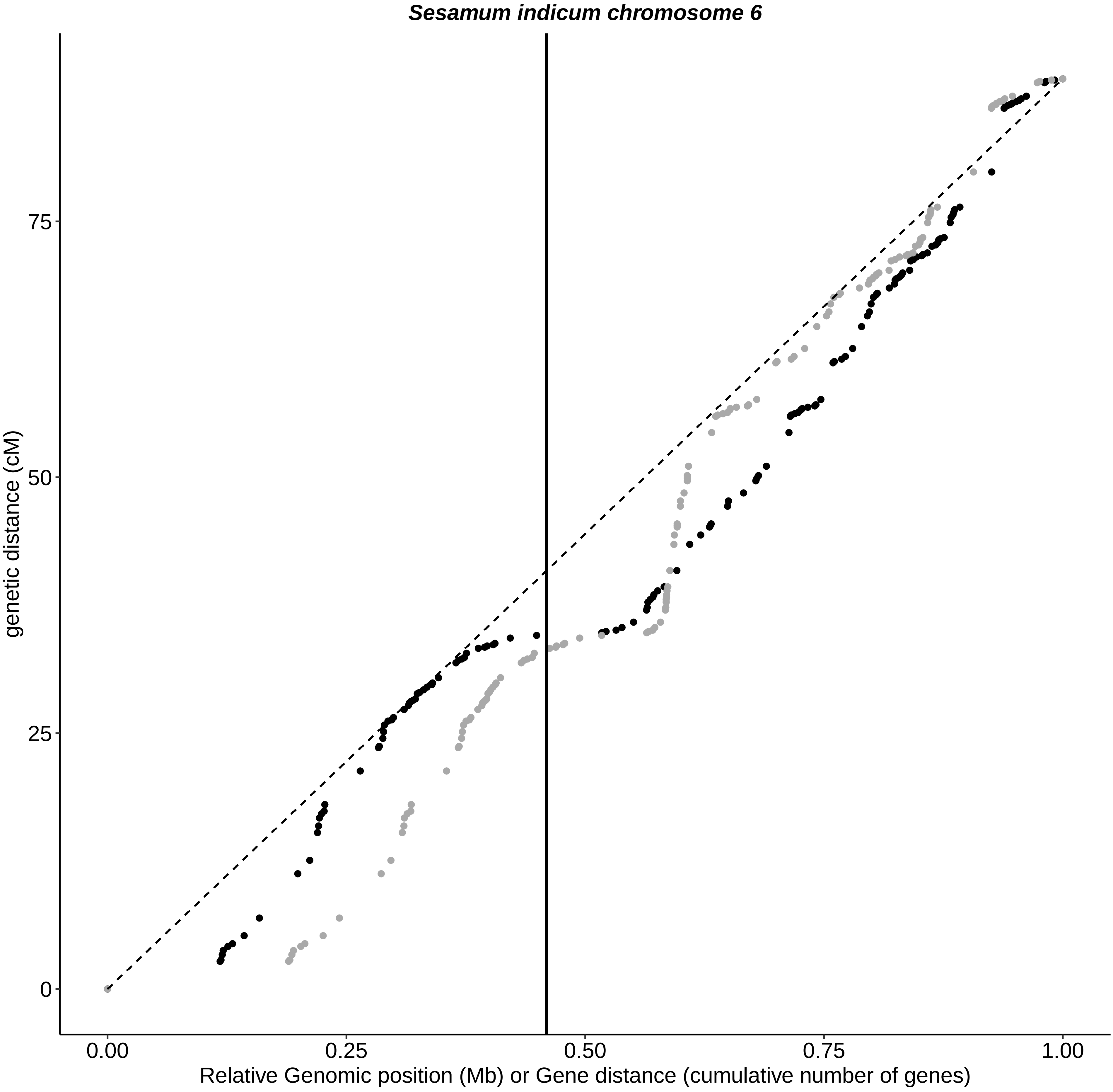
Sesamum indicum chromosome 6
genetic distance (cM)
0
25
50
75
0.00
0.25
0.50
0.75
1.00
Relative Genomic position (Mb) or Gene distance (cumulative number of genes)

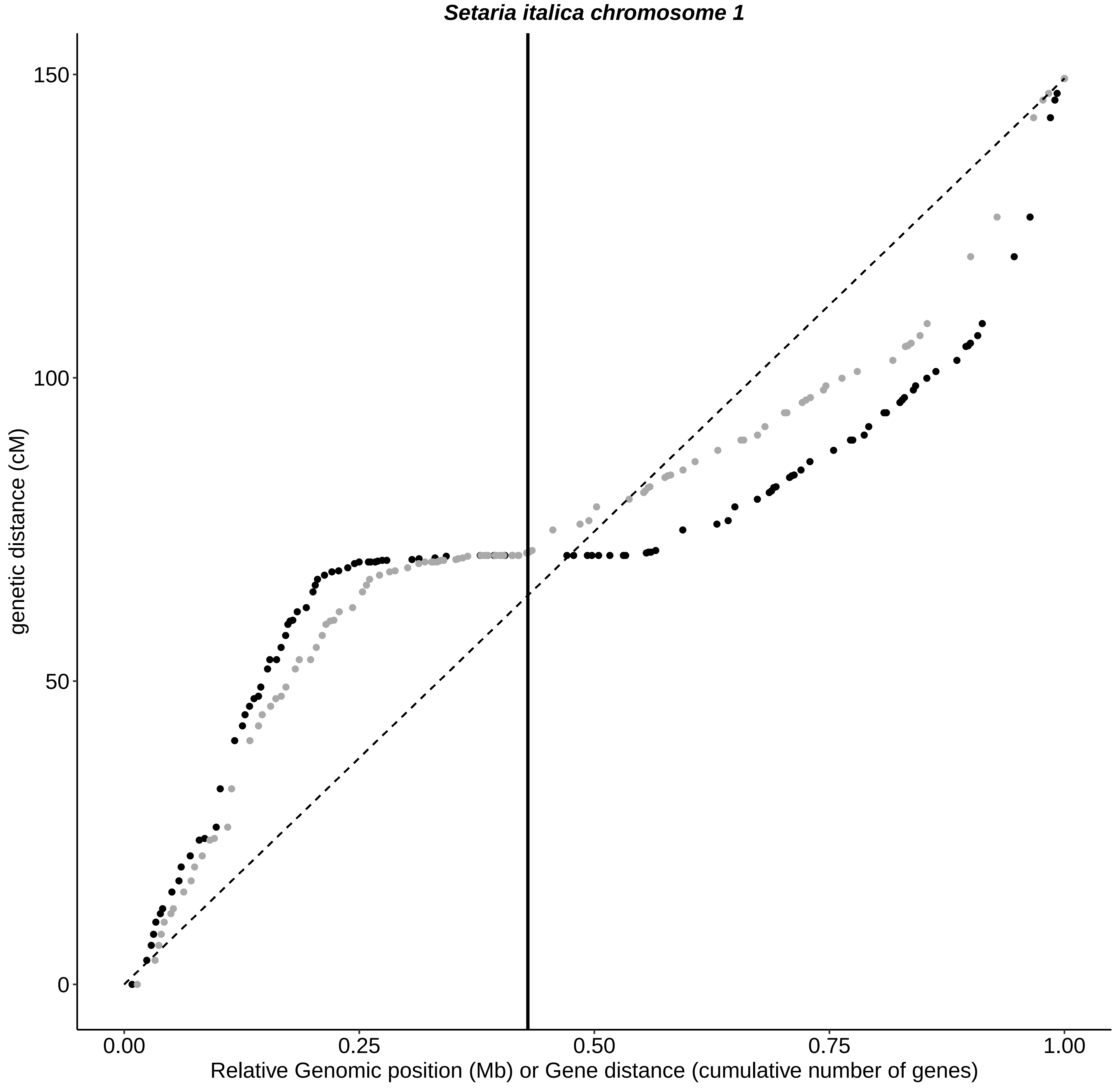
Setaria italica chromosome 1
genetic distance (cM)
150
100
50
0
0.00
0.25
0.50
0.75
1.00
Relative Genomic position (Mb) or Gene distance (cumulative number of genes)

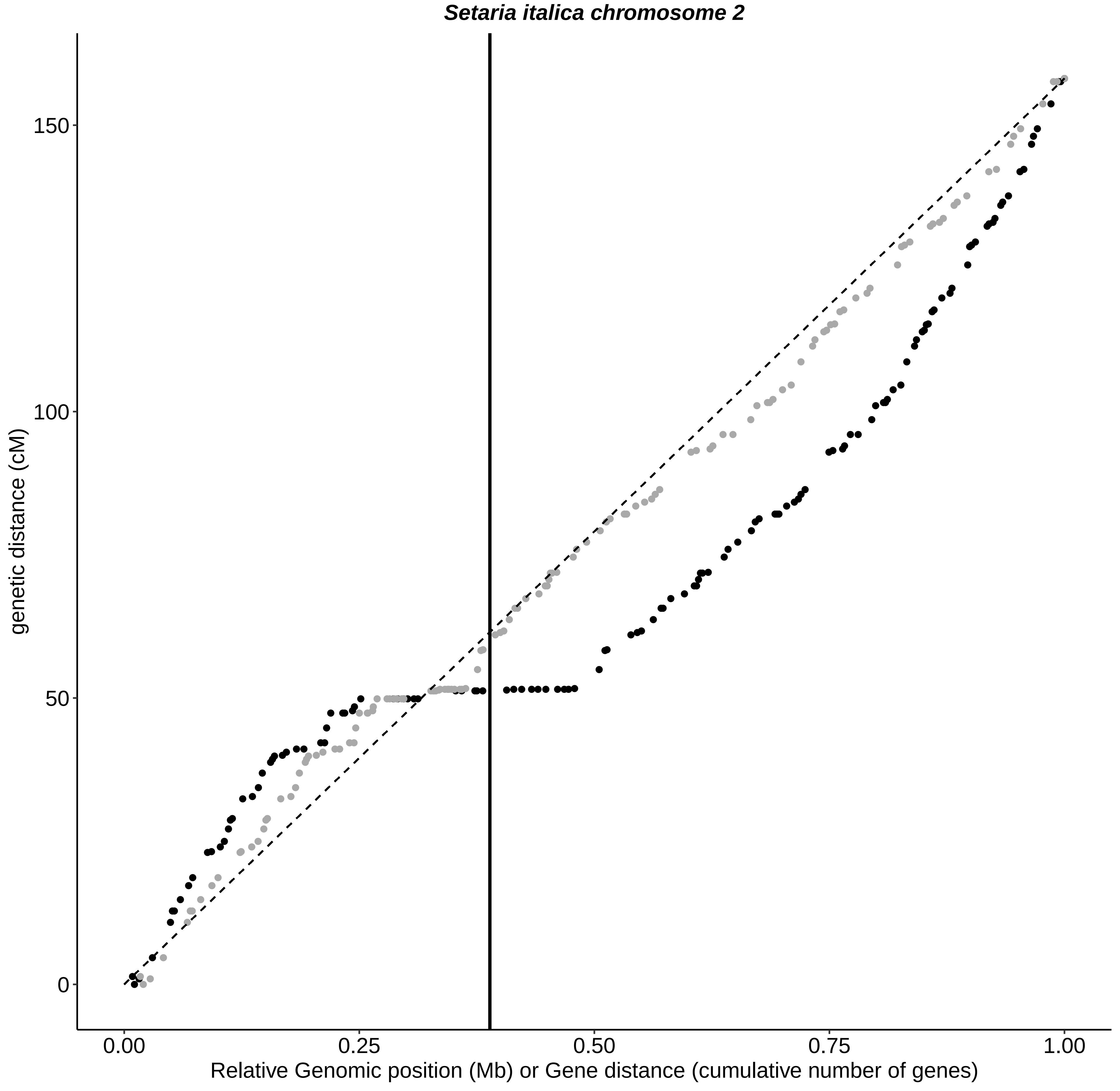
Setaria italica chromosome 2
genetic distance (cM)
150
100
50
0
0.00
0.25
0.50
0.75
1.00
Relative Genomic position (Mb) or Gene distance (cumulative number of genes)

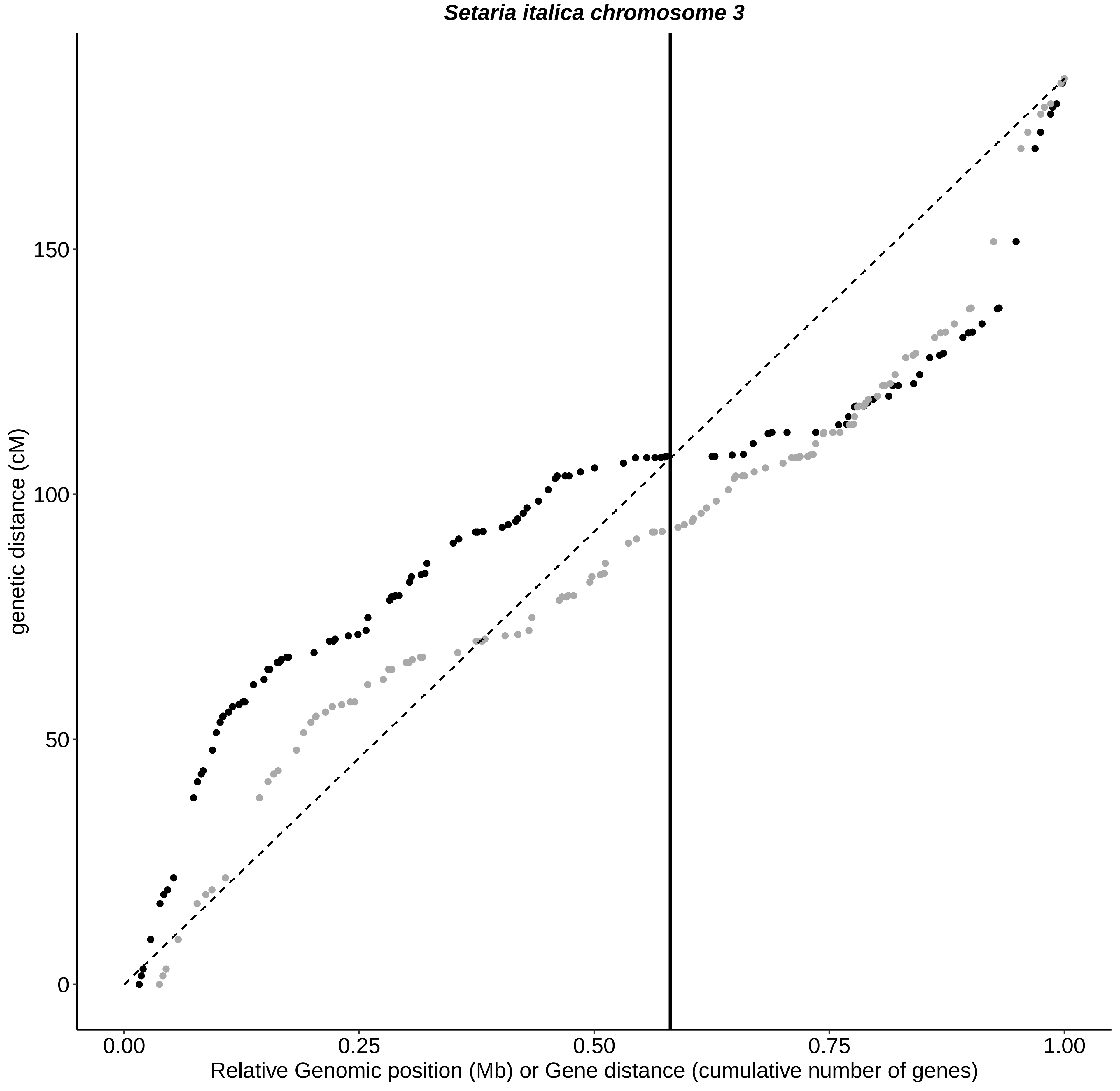

Setaria italica chromosome 3
genetic distance (cM)
150
100
50
0
0.00
0.25
0.50
0.75
1.00
Relative Genomic position (Mb) or Gene distance (cumulative number of genes)

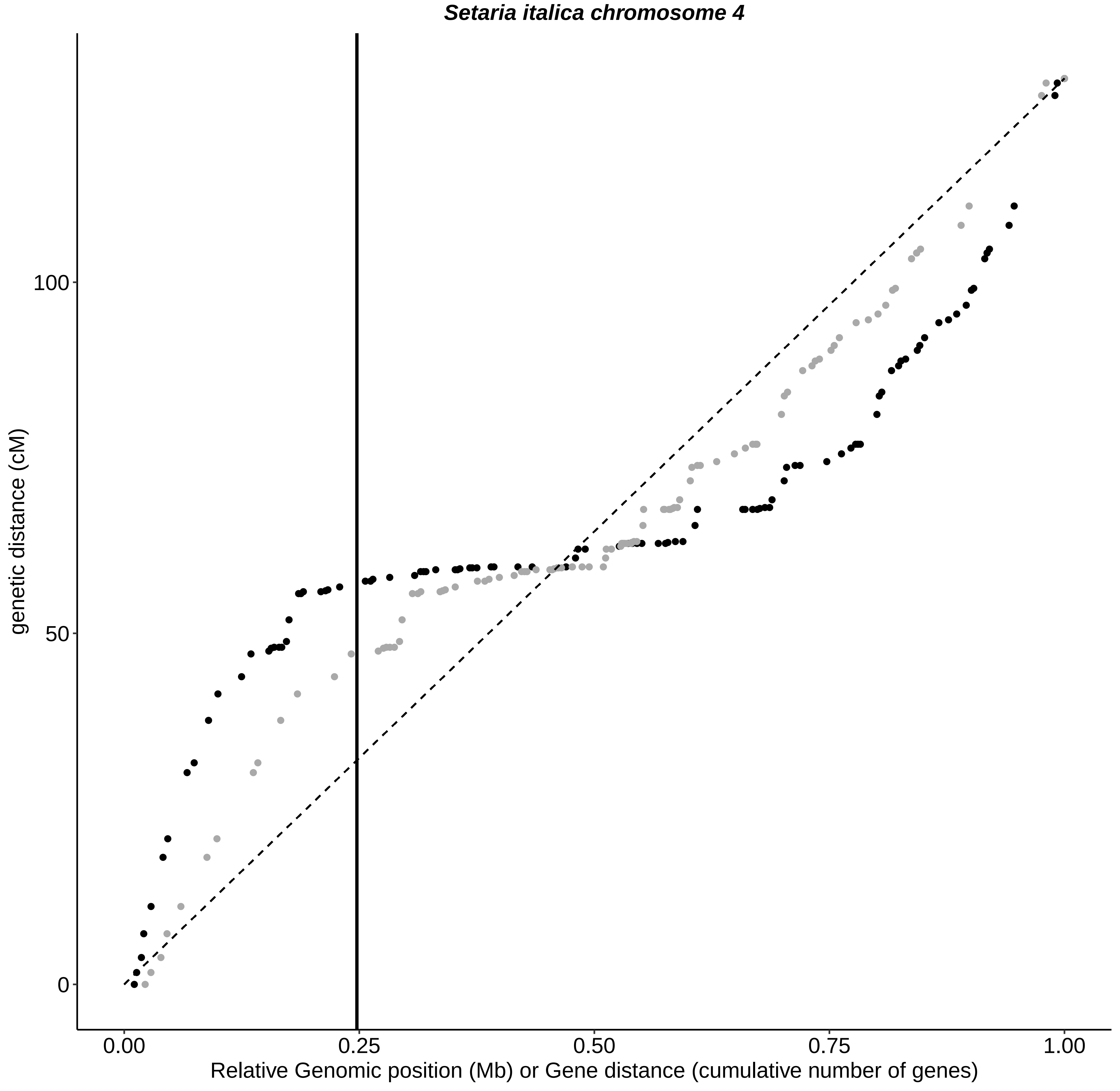
Setaria italica chromosome 4
genetic distance (cM)
0
50
100
0.00
0.25
0.50
0.75
1.00
Relative Genomic position (Mb) or Gene distance (cumulative number of genes)

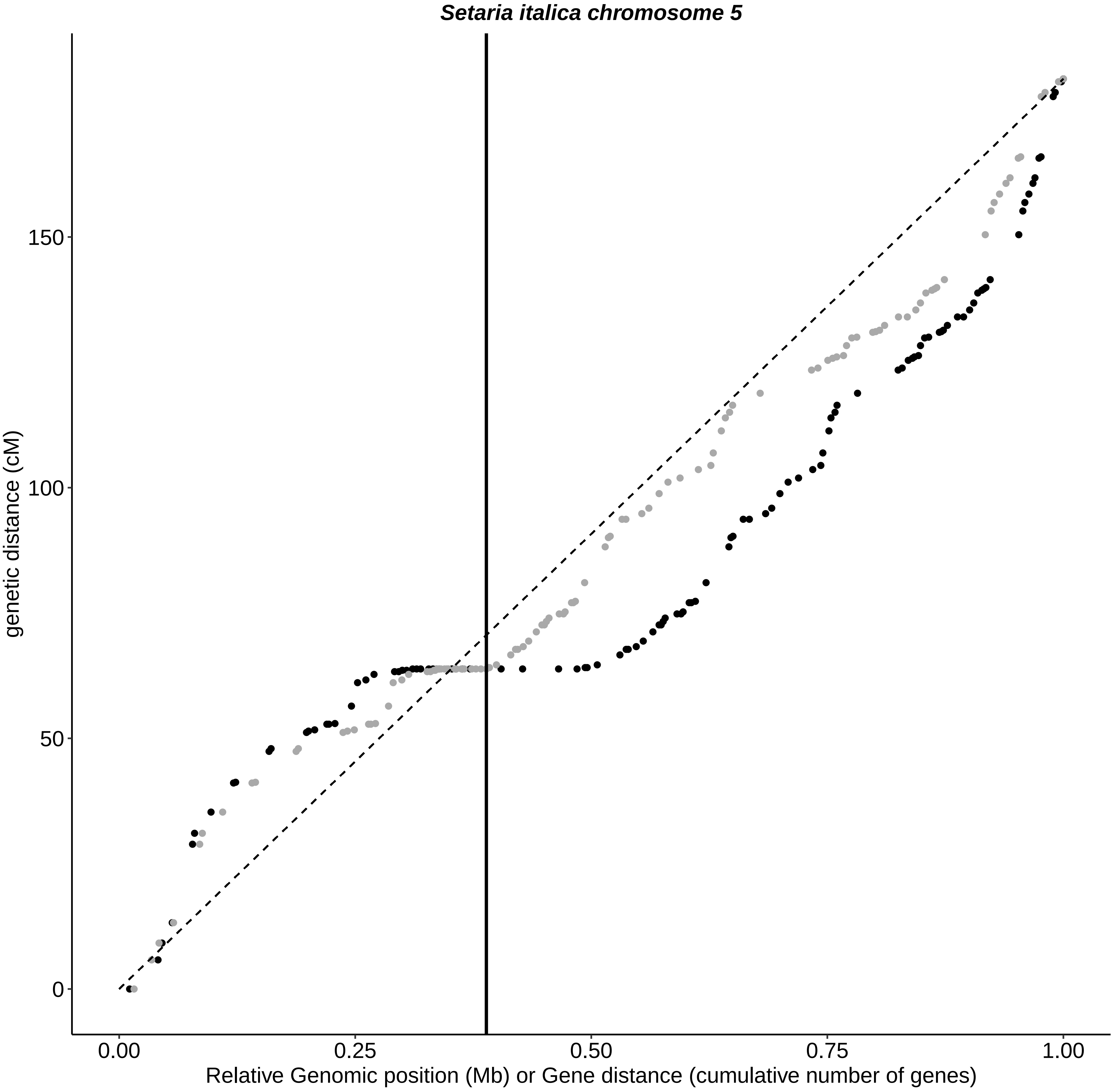
Setaria italica chromosome 5
genetic distance (cM)
150
100
50
0
0.00
0.25
0.50
0.75
1.00
Relative Genomic position (Mb) or Gene distance (cumulative number of genes)

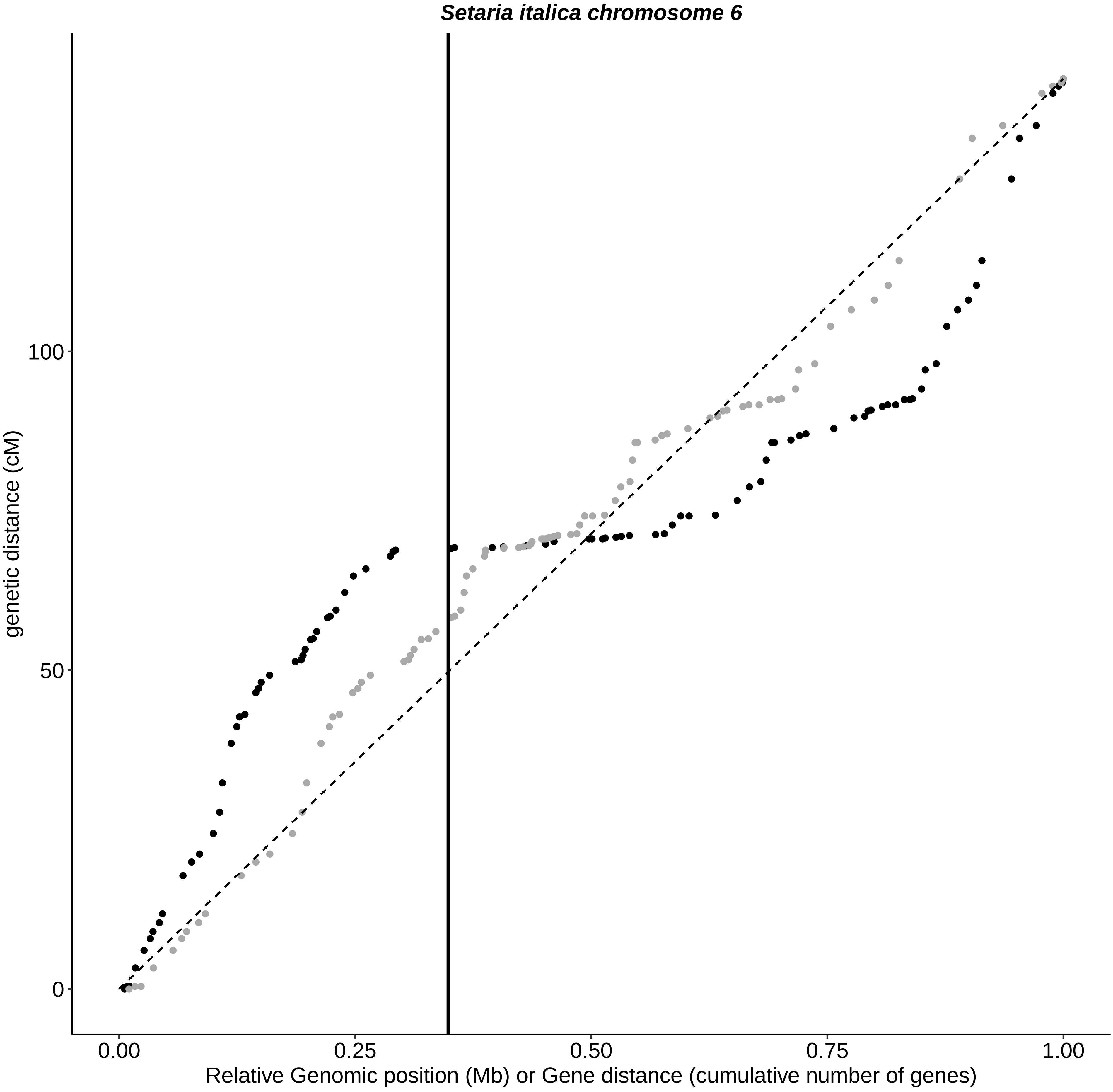
Setaria italica chromosome 6
genetic distance (cM)
0
50
100
0.00
0.25
0.50
0.75
1.00
Relative Genomic position (Mb) or Gene distance (cumulative number of genes)

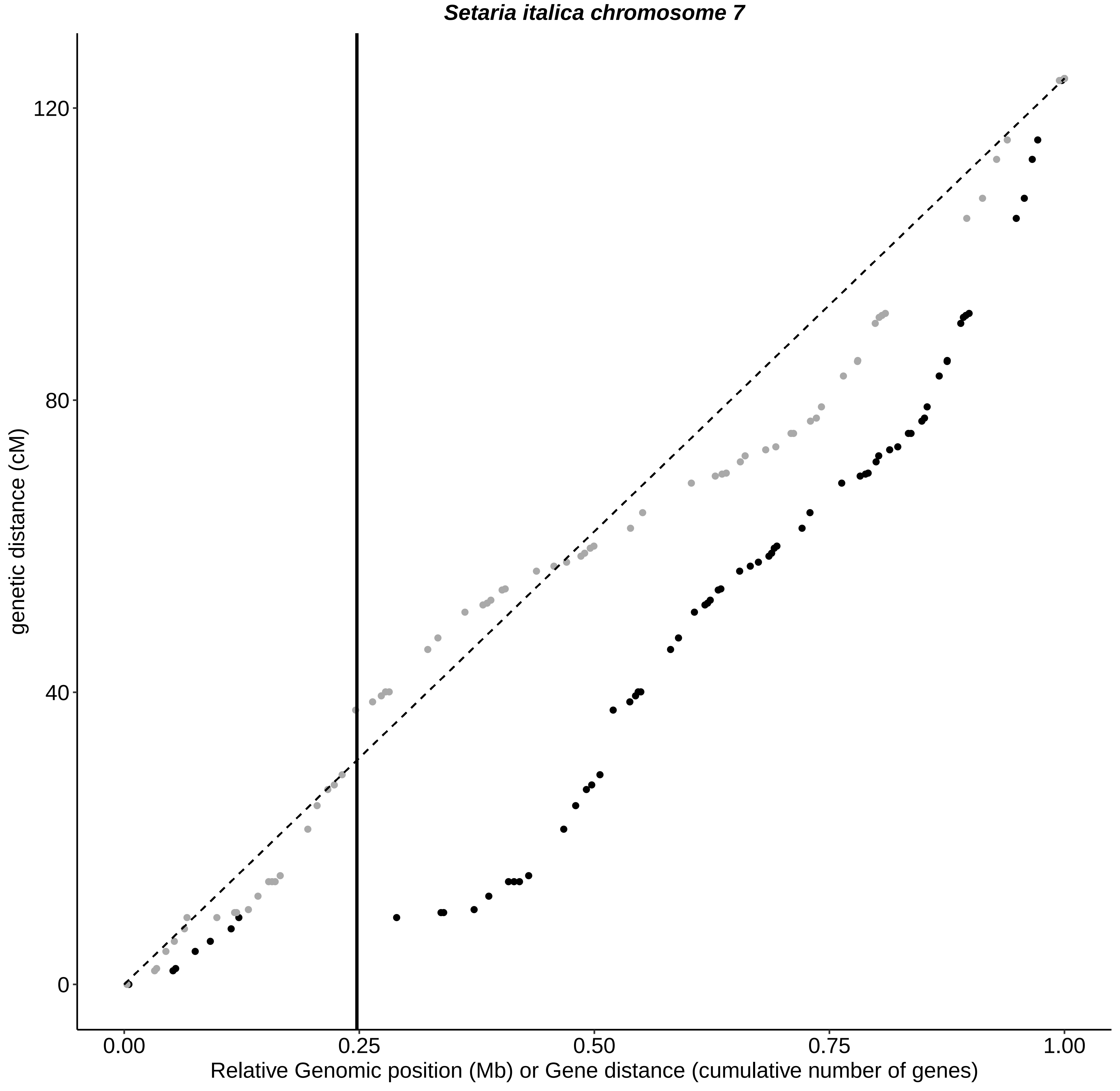
Setaria italica chromosome 7
genetic distance (cM)
0
40
80
120
0.00
0.25
0.50
0.75
1.00
Relative Genomic position (Mb) or Gene distance (cumulative number of genes)

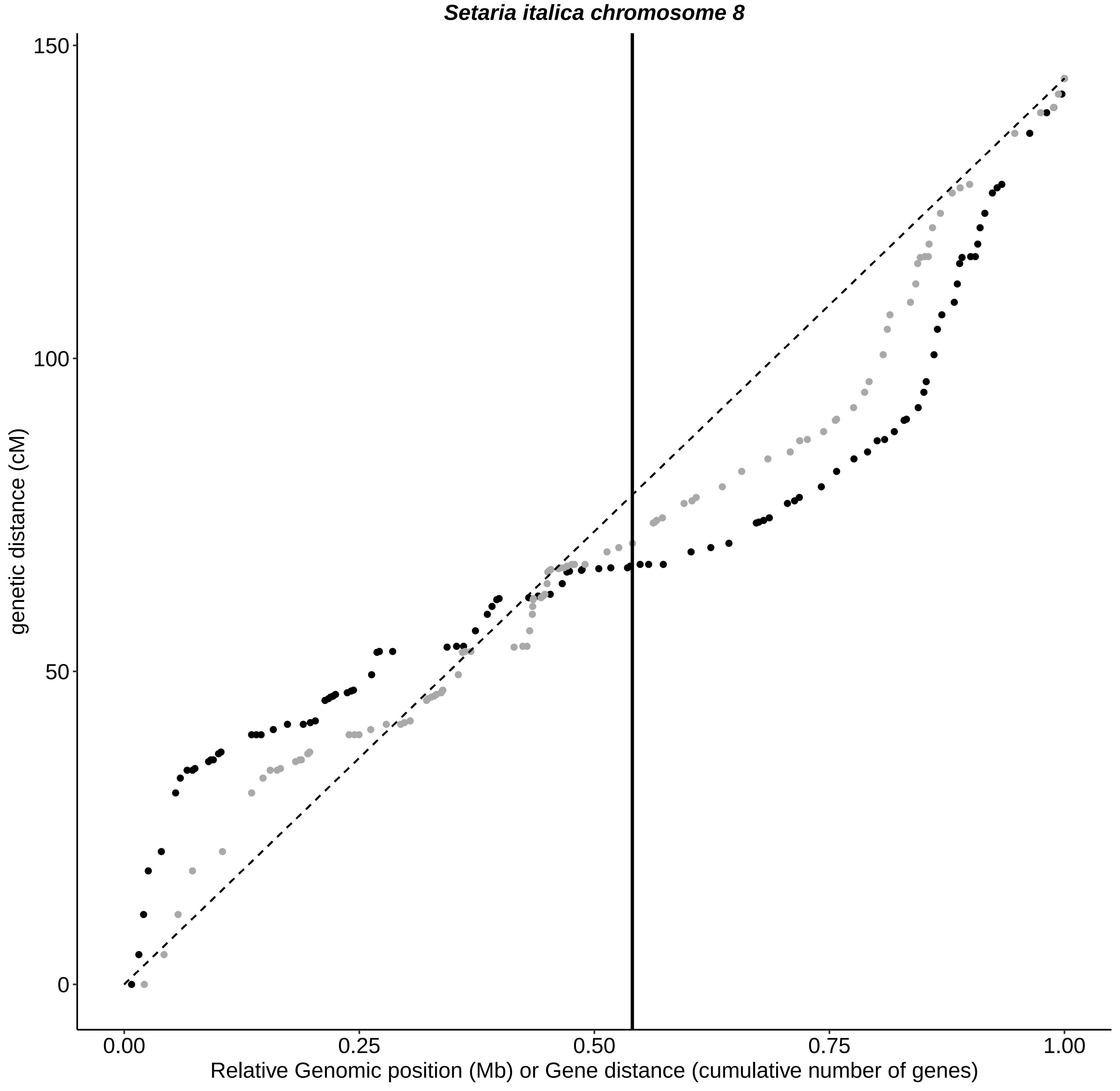
Setaria italica chromosome 8
genetic distance (cM)
150
100
50
0
0.00
0.25
0.50
0.75
1.00
Relative Genomic position (Mb) or Gene distance (cumulative number of genes)

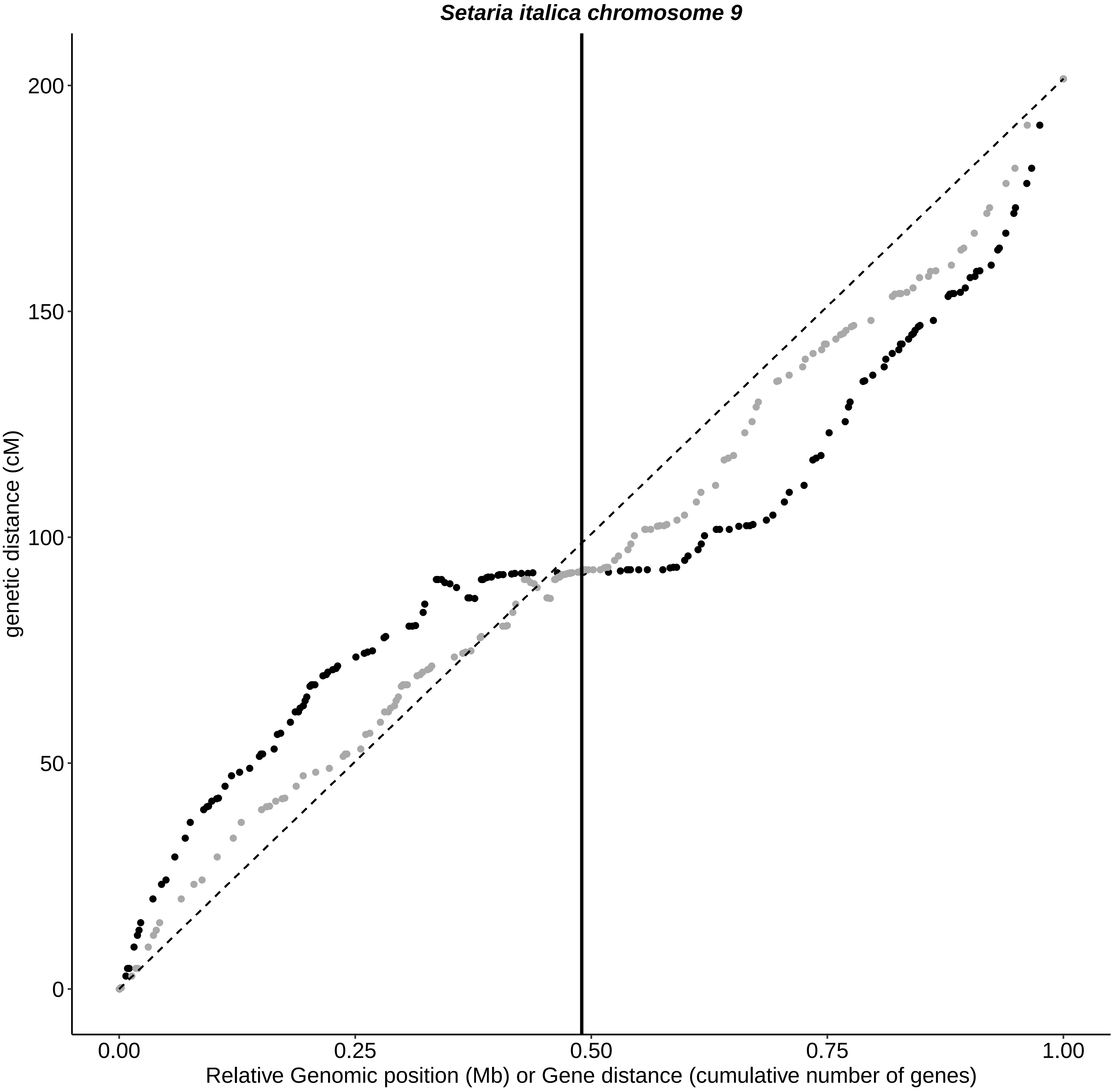
Setaria italica chromosome 9
genetic distance (cM)
0
50
100
150
200
0.00
0.25
0.50
0.75
1.00
Relative Genomic position (Mb) or Gene distance (cumulative number of genes)

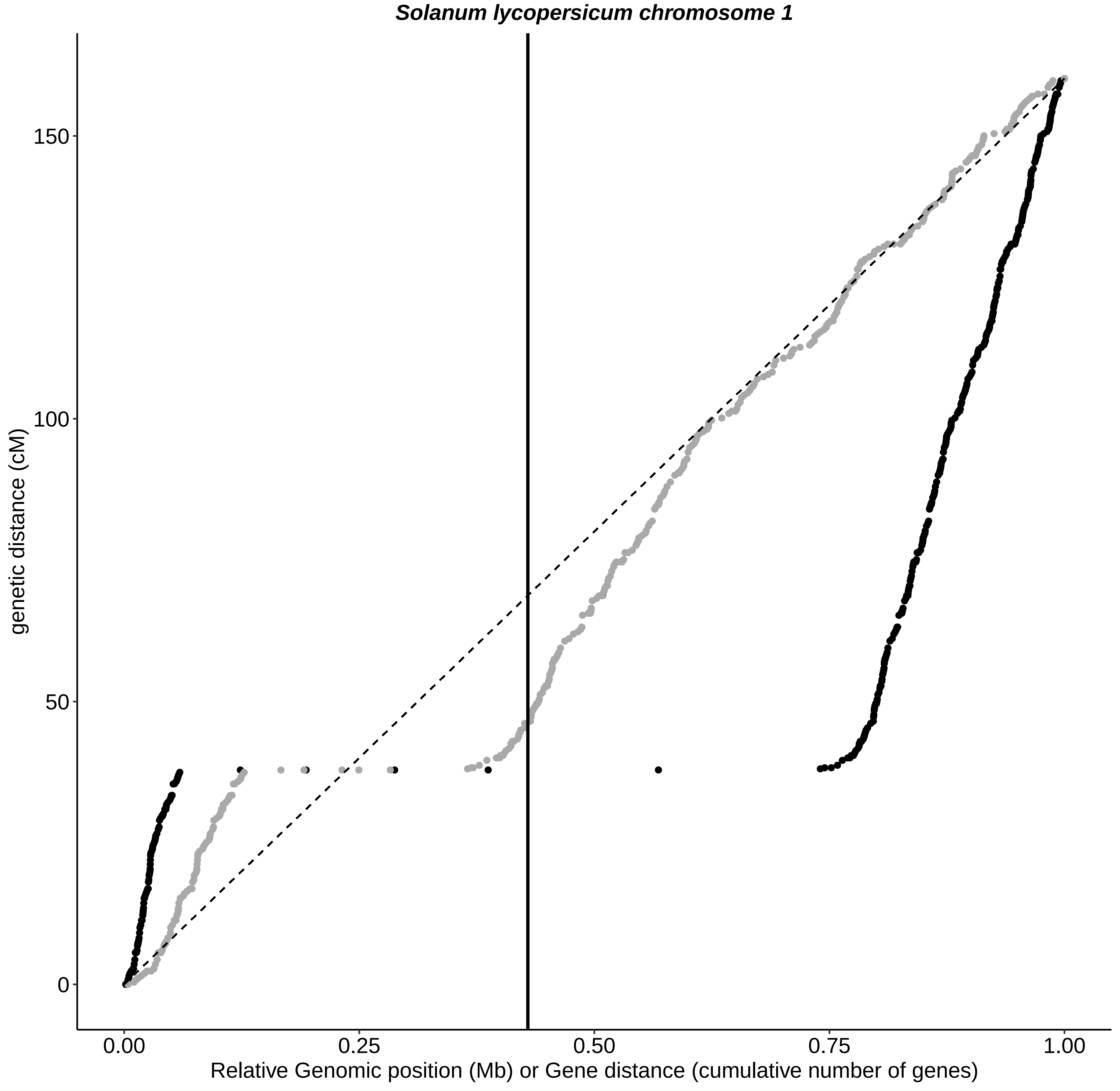

Solanum lycopersicum chromosome 1
genetic distance (cM)
0
50
100
150
0.00
0.25
0.50
0.75
1.00
Relative Genomic position (Mb) or Gene distance (cumulative number of genes)

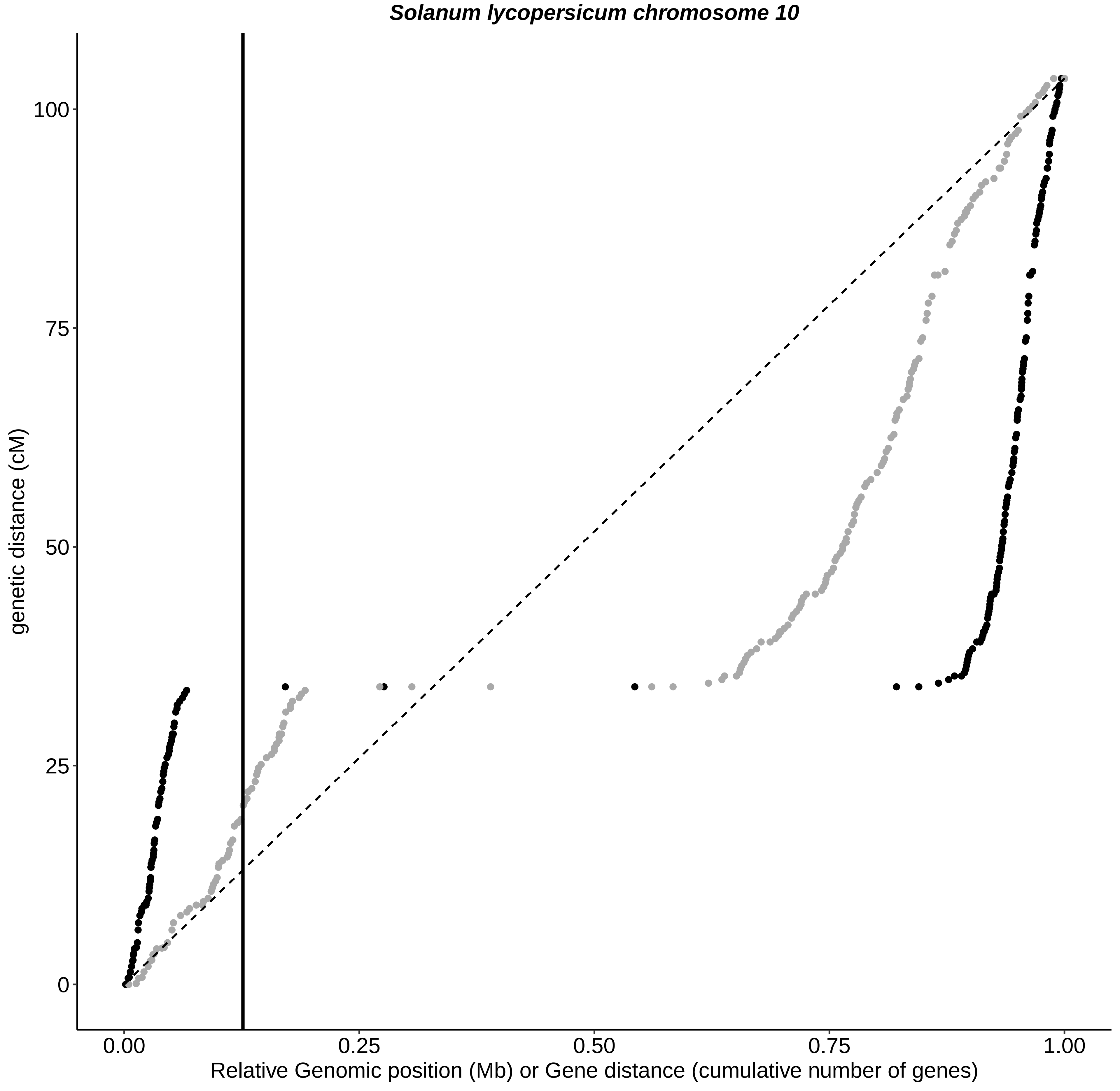
Solanum lycopersicum chromosome 10
genetic distance (cM)
100
75
50
25
0
0.00
0.25
0.50
0.75
1.00
Relative Genomic position (Mb) or Gene distance (cumulative number of genes)

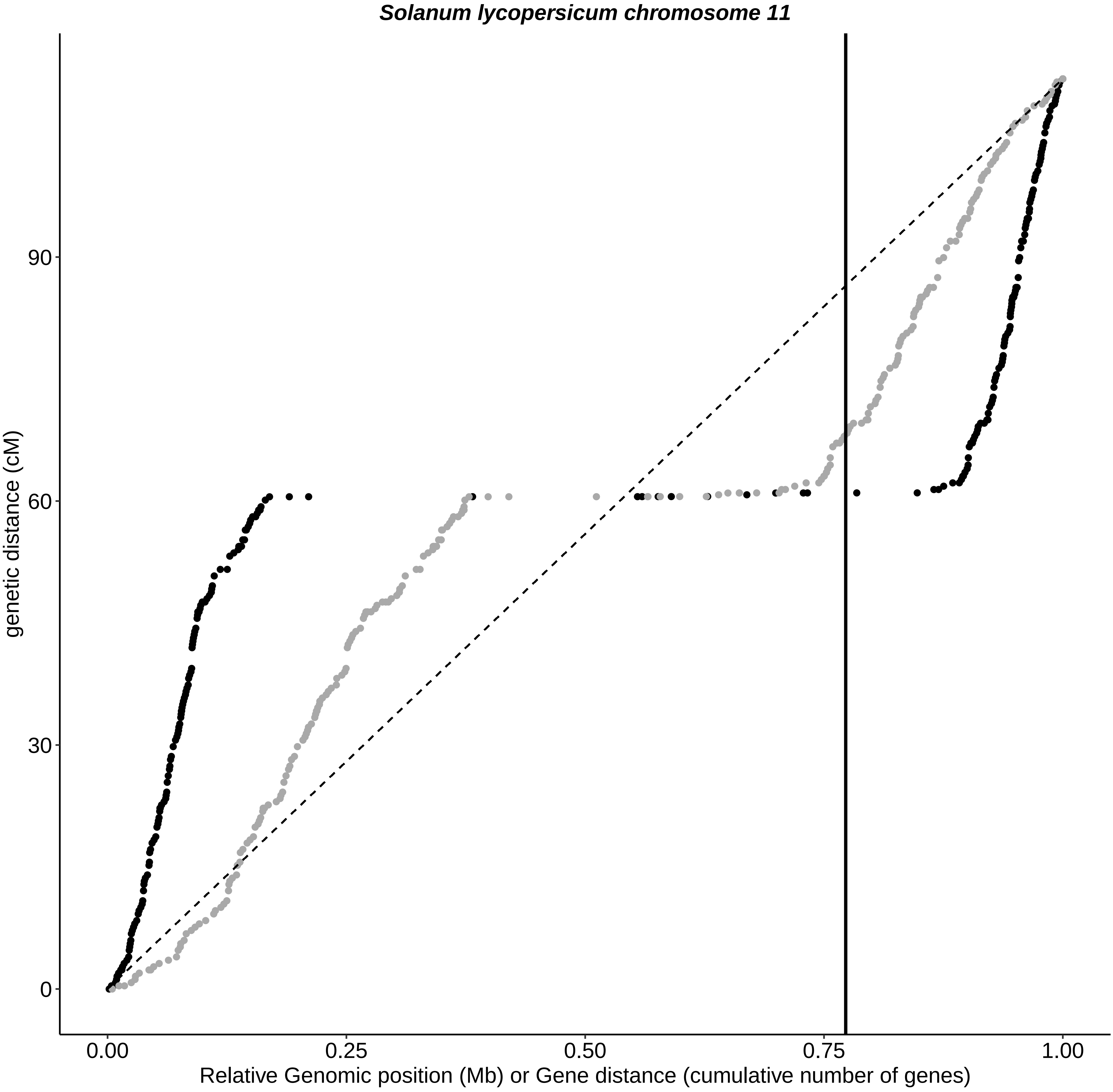
Solanum lycopersicum chromosome 11
genetic distance (cM)
0
30
60
90
0.00
0.25
0.50
0.75
1.00
Relative Genomic position (Mb) or Gene distance (cumulative number of genes)

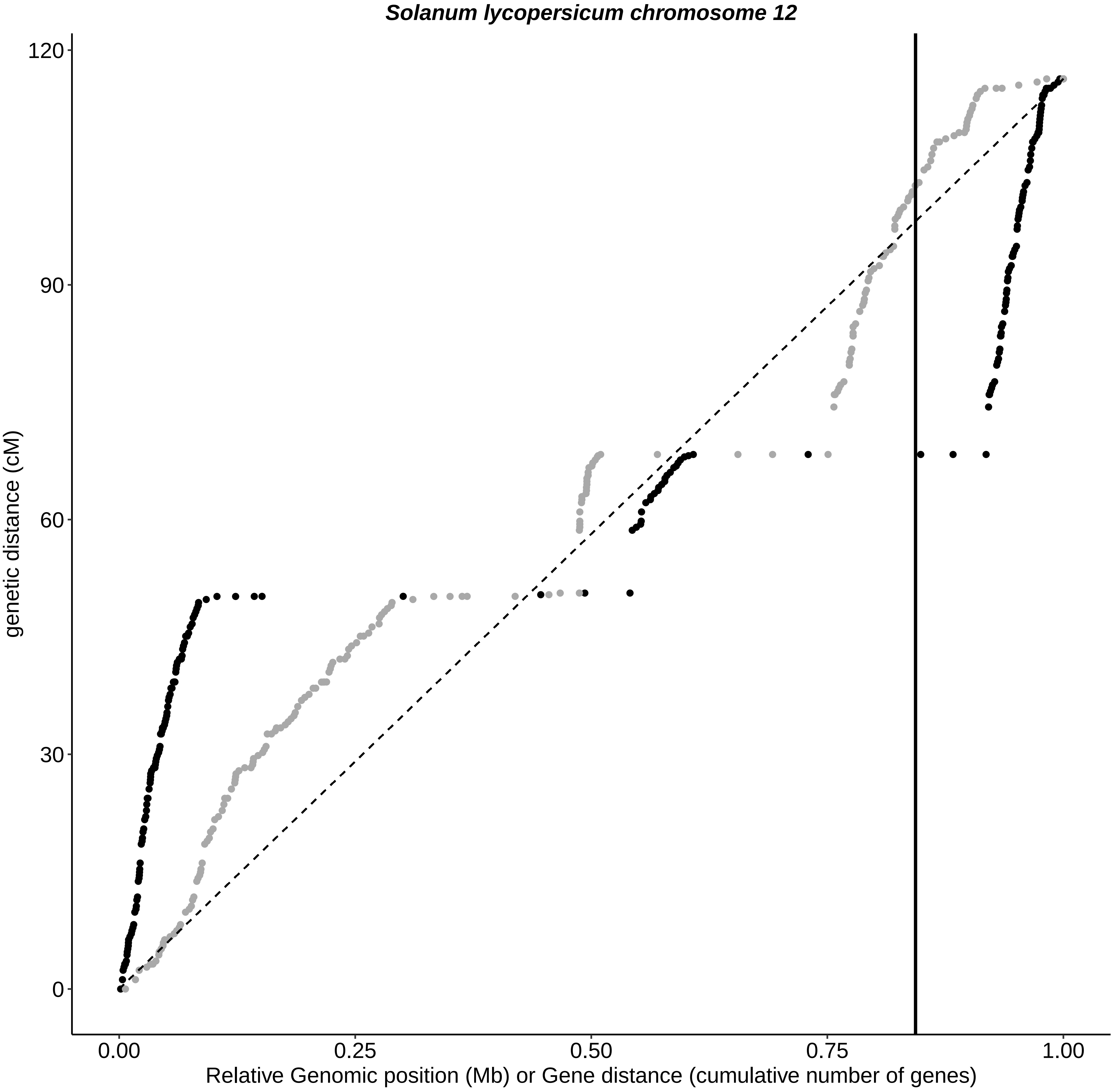
Solanum lycopersicum chromosome 12
genetic distance (cM)
120
90
60
30
0
0.00
0.25
0.50
0.75
1.00
Relative Genomic position (Mb) or Gene distance (cumulative number of genes)

***Solanum lycopersicum* chromosome 2**

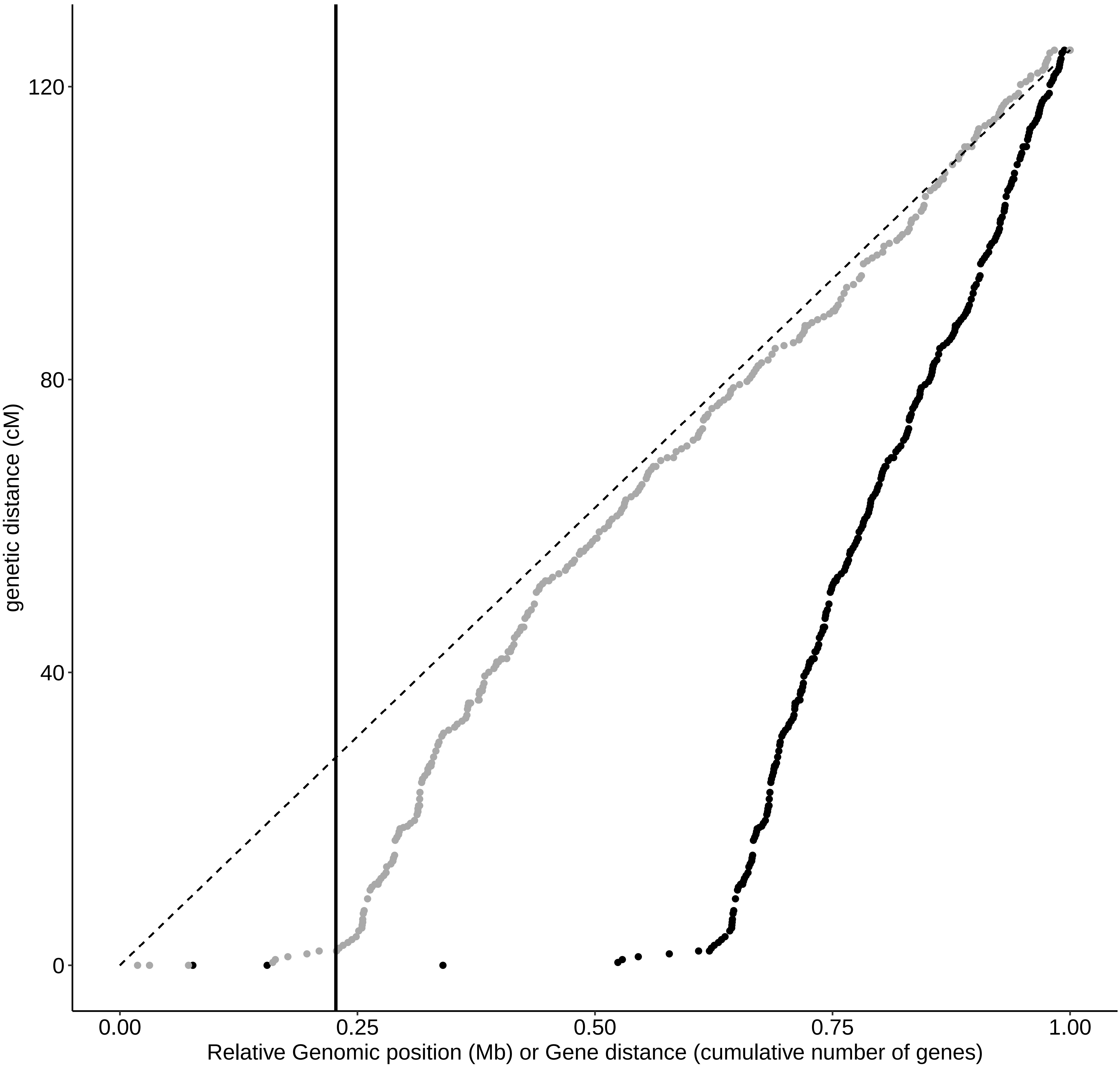

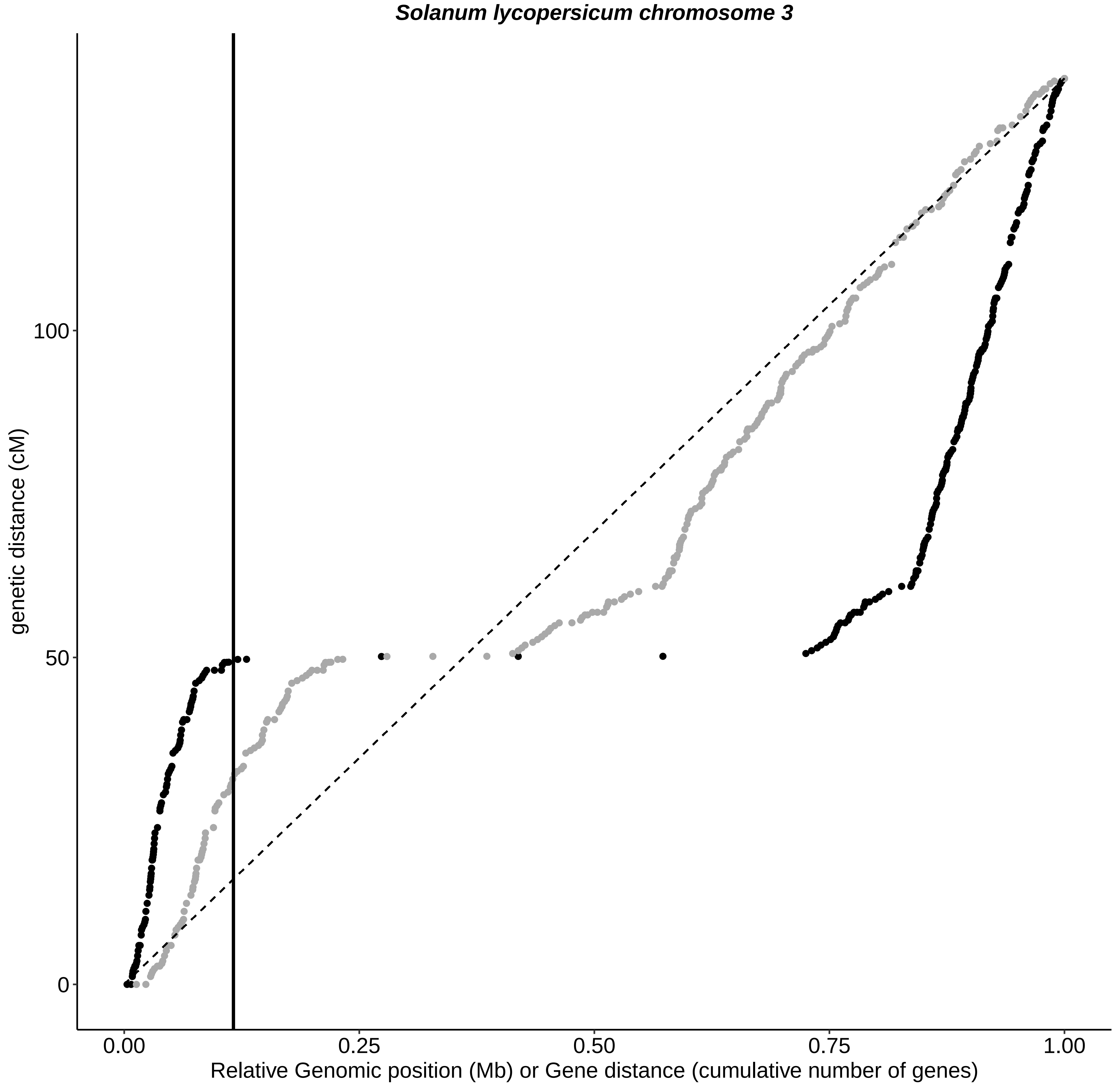

Solanum lycopersicum chromosome 3
genetic distance (cM)
0
50
100
0.00
0.25
0.50
0.75
1.00
Relative Genomic position (Mb) or Gene distance (cumulative number of genes)

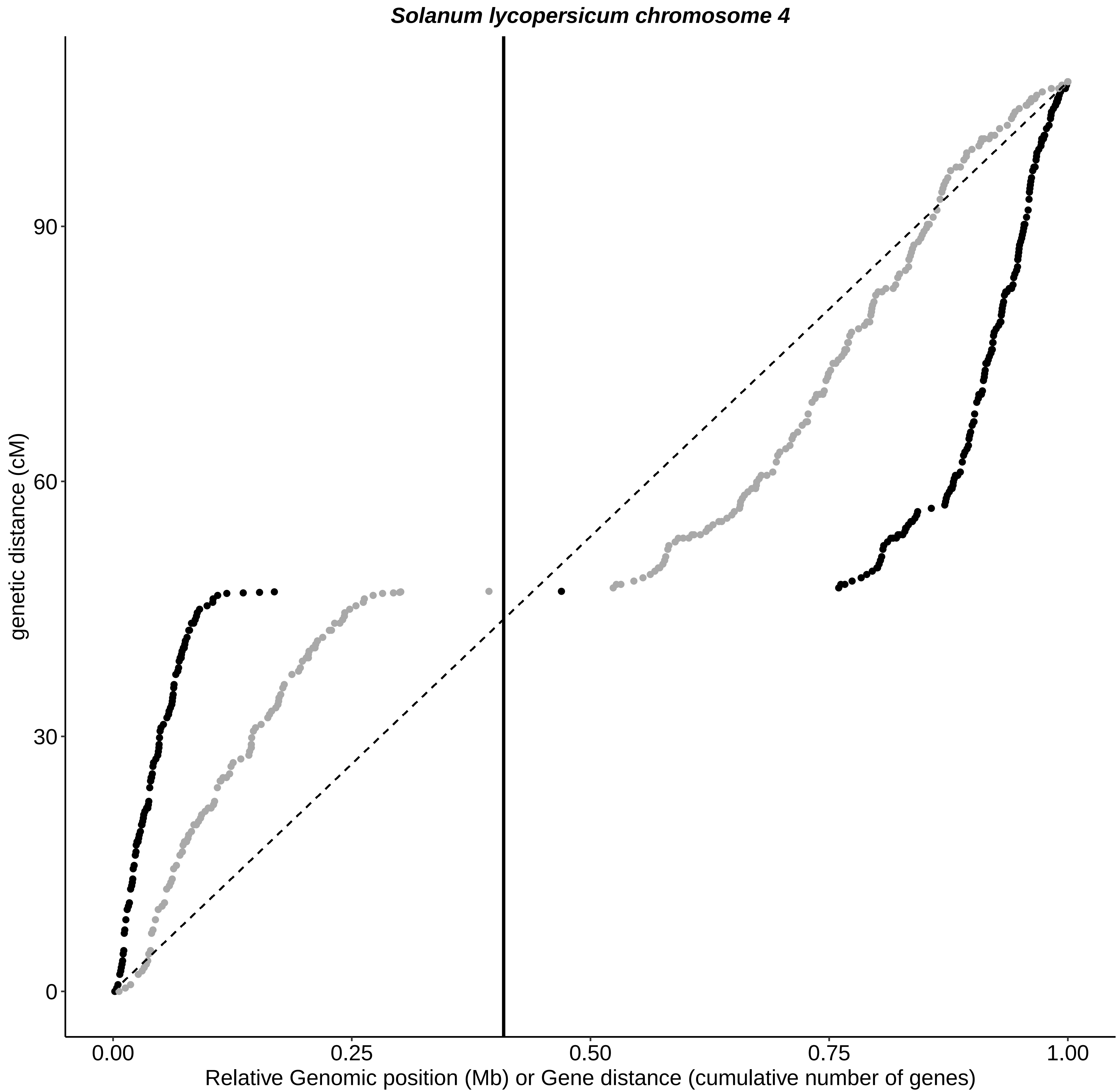
Solanum lycopersicum chromosome 4
genetic distance (cM)
0
30
60
90
0.00
0.25
0.50
0.75
1.00
Relative Genomic position (Mb) or Gene distance (cumulative number of genes)

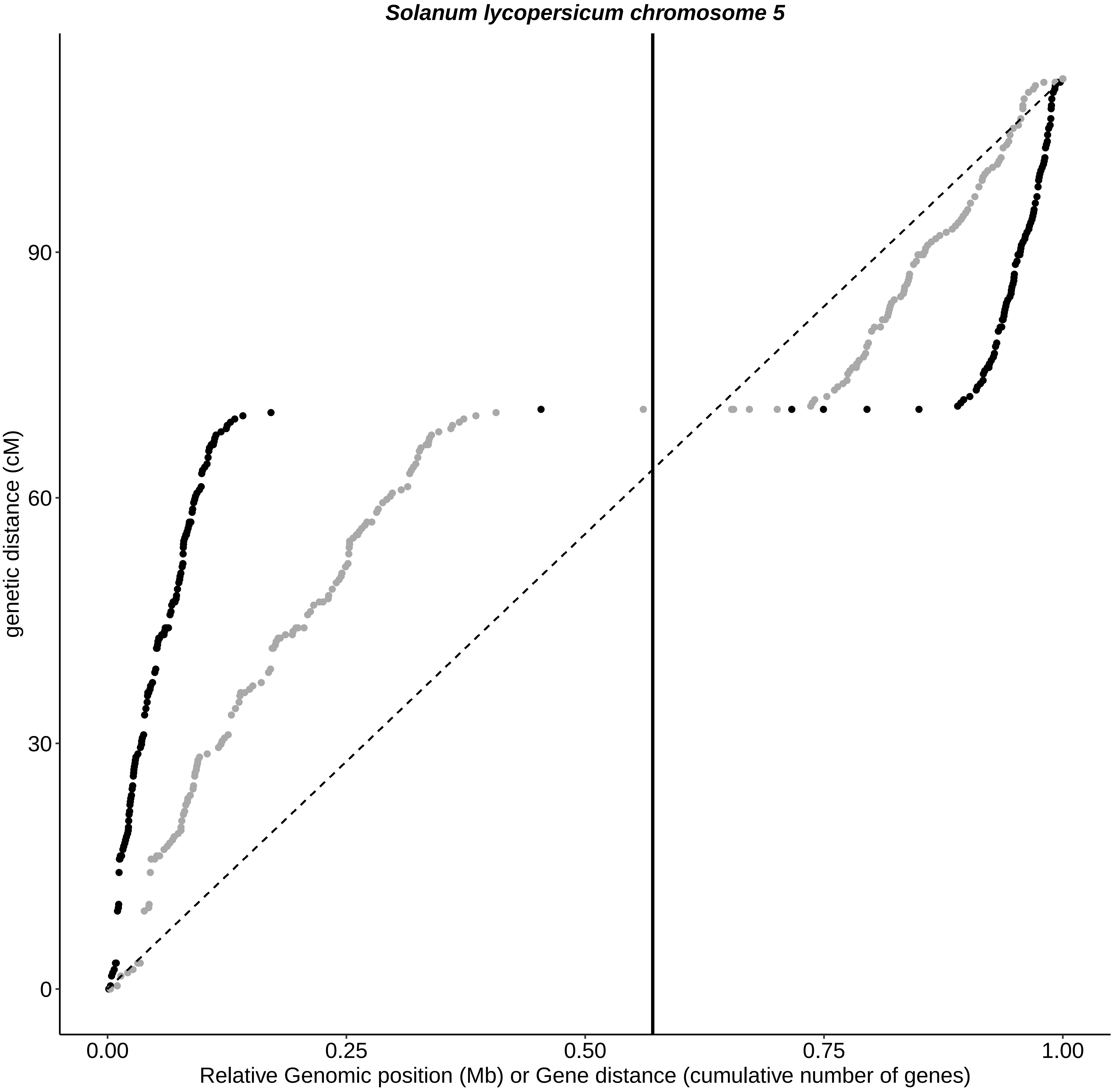
Solanum lycopersicum chromosome 5
genetic distance (cM)
0
30
60
90
0.00
0.25
0.50
0.75
1.00
Relative Genomic position (Mb) or Gene distance (cumulative number of genes)

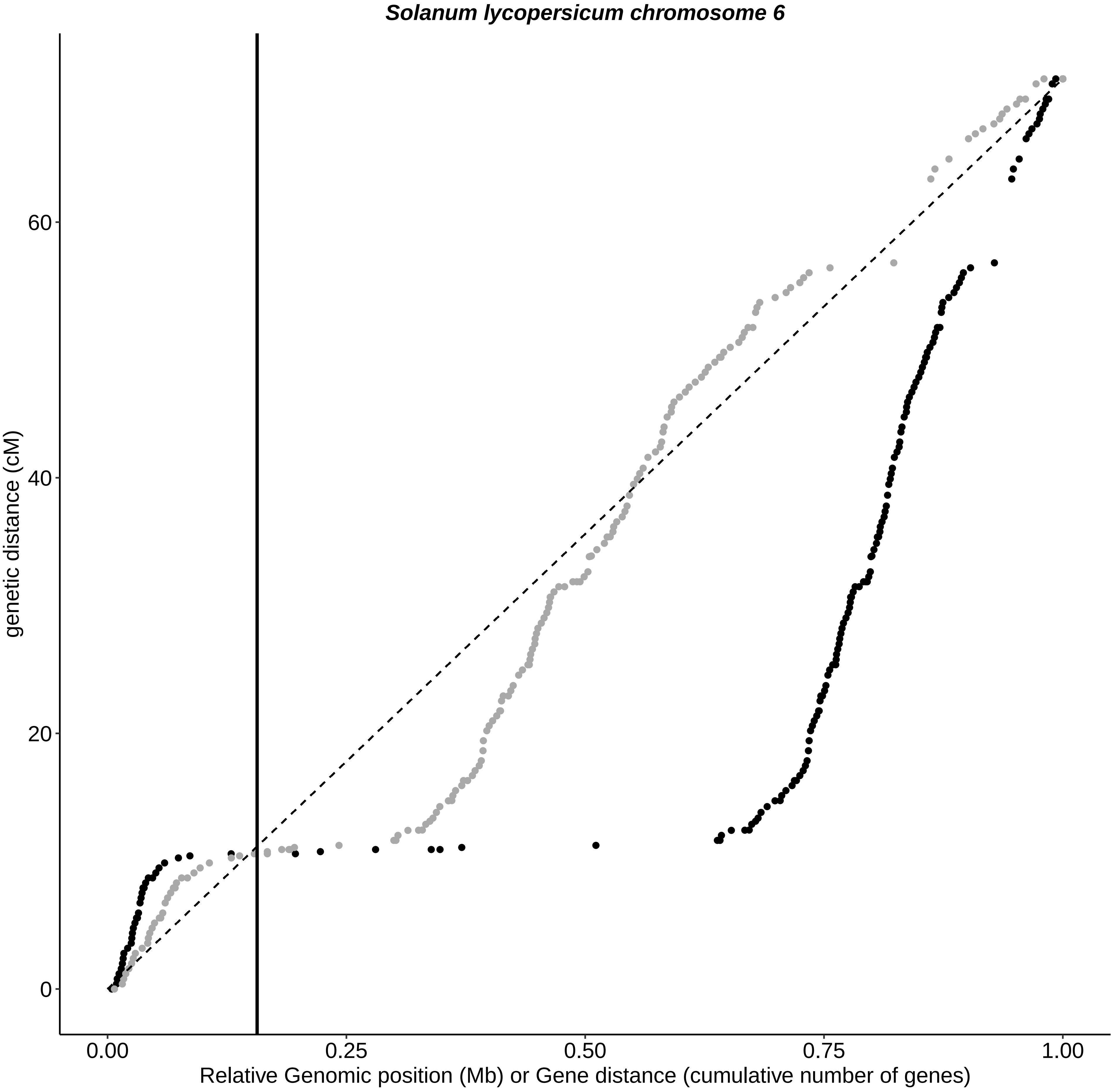

Solanum lycopersicum chromosome 6
genetic distance (cM)
0
20
40
60
0.00
0.25
0.50
0.75
1.00
Relative Genomic position (Mb) or Gene distance (cumulative number of genes)

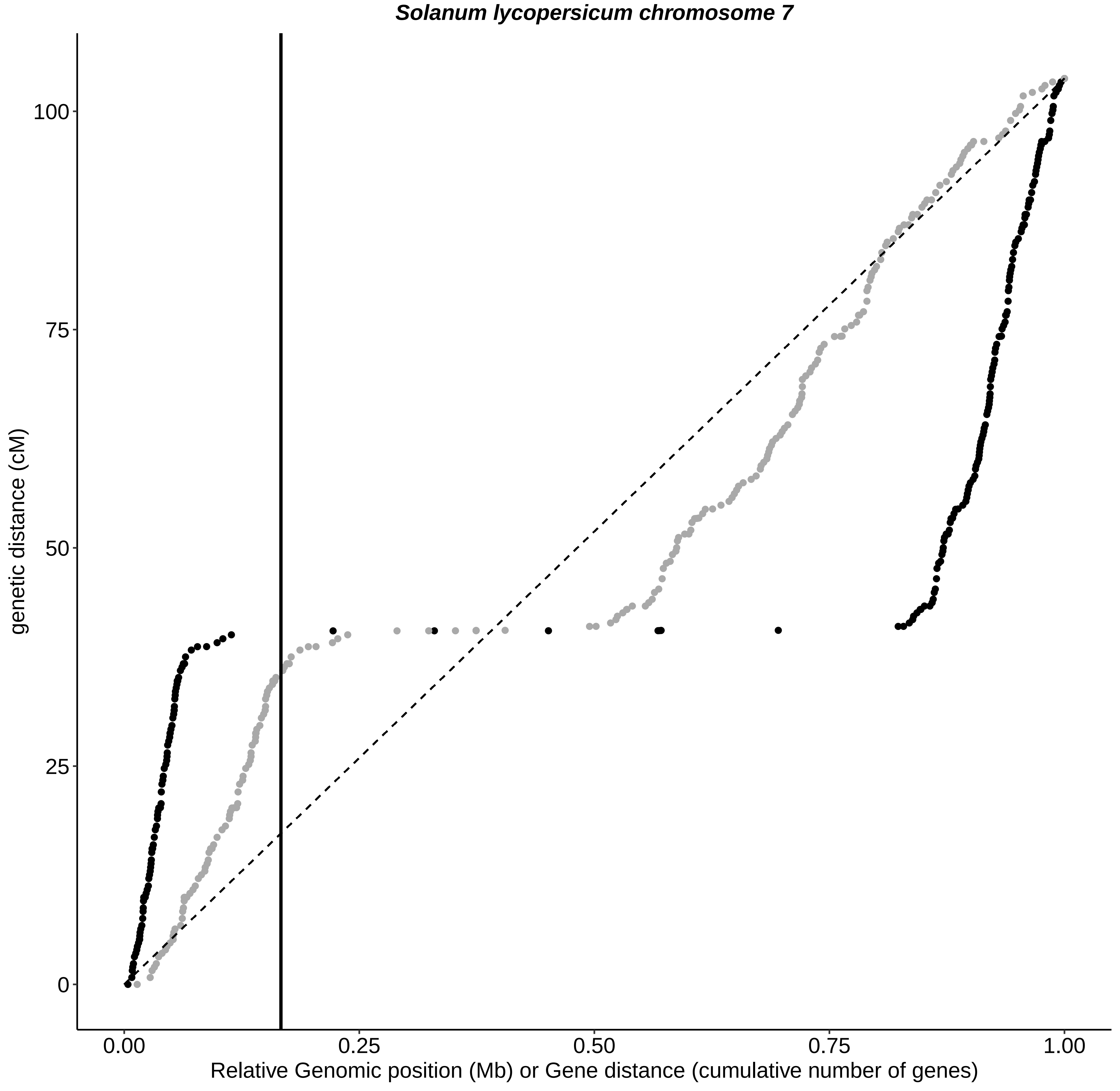
Solanum lycopersicum chromosome 7
genetic distance (cM)
100
75
50
25
0
0.00
0.25
0.50
0.75
1.00
Relative Genomic position (Mb) or Gene distance (cumulative number of genes)

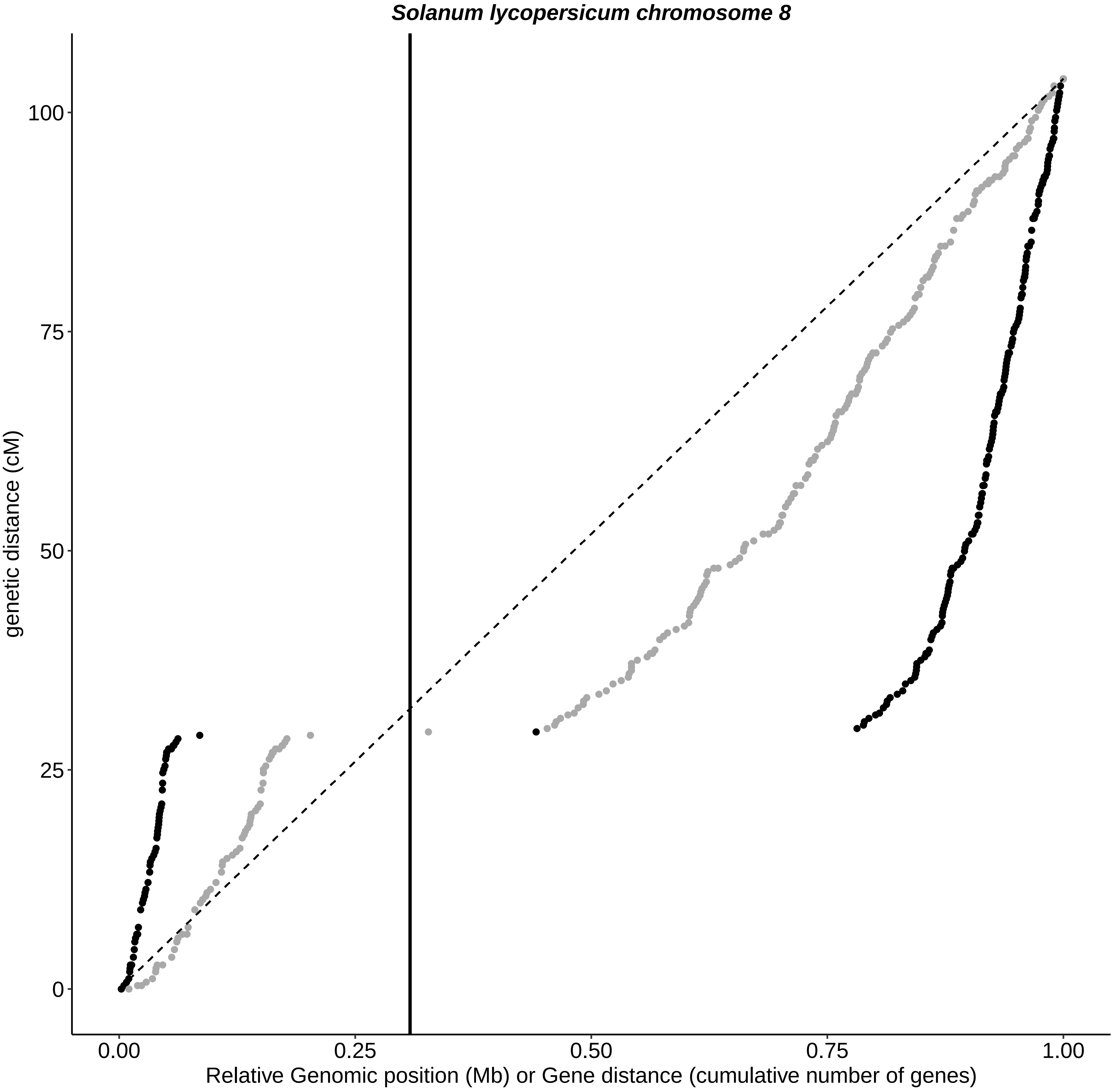

Solanum lycopersicum chromosome 8
genetic distance (cM)
100
75
50
25
0
0.00
0.25
0.50
0.75
1.00
Relative Genomic position (Mb) or Gene distance (cumulative number of genes)

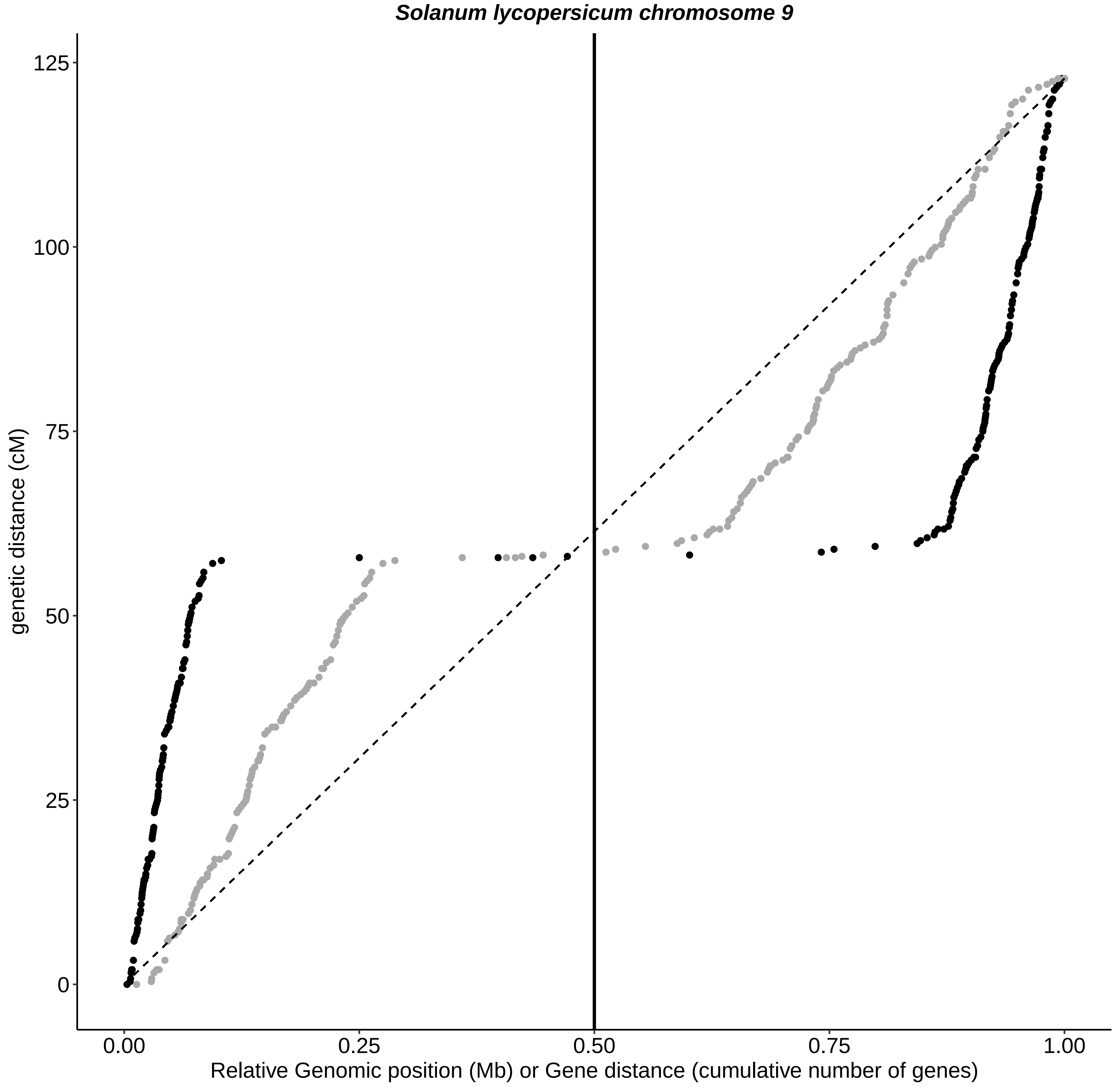
Solanum lycopersicum chromosome 9
genetic distance (cM)
125
100
75
50
25
0
0.00
0.25
0.50
0.75
1.00
Relative Genomic position (Mb) or Gene distance (cumulative number of genes)

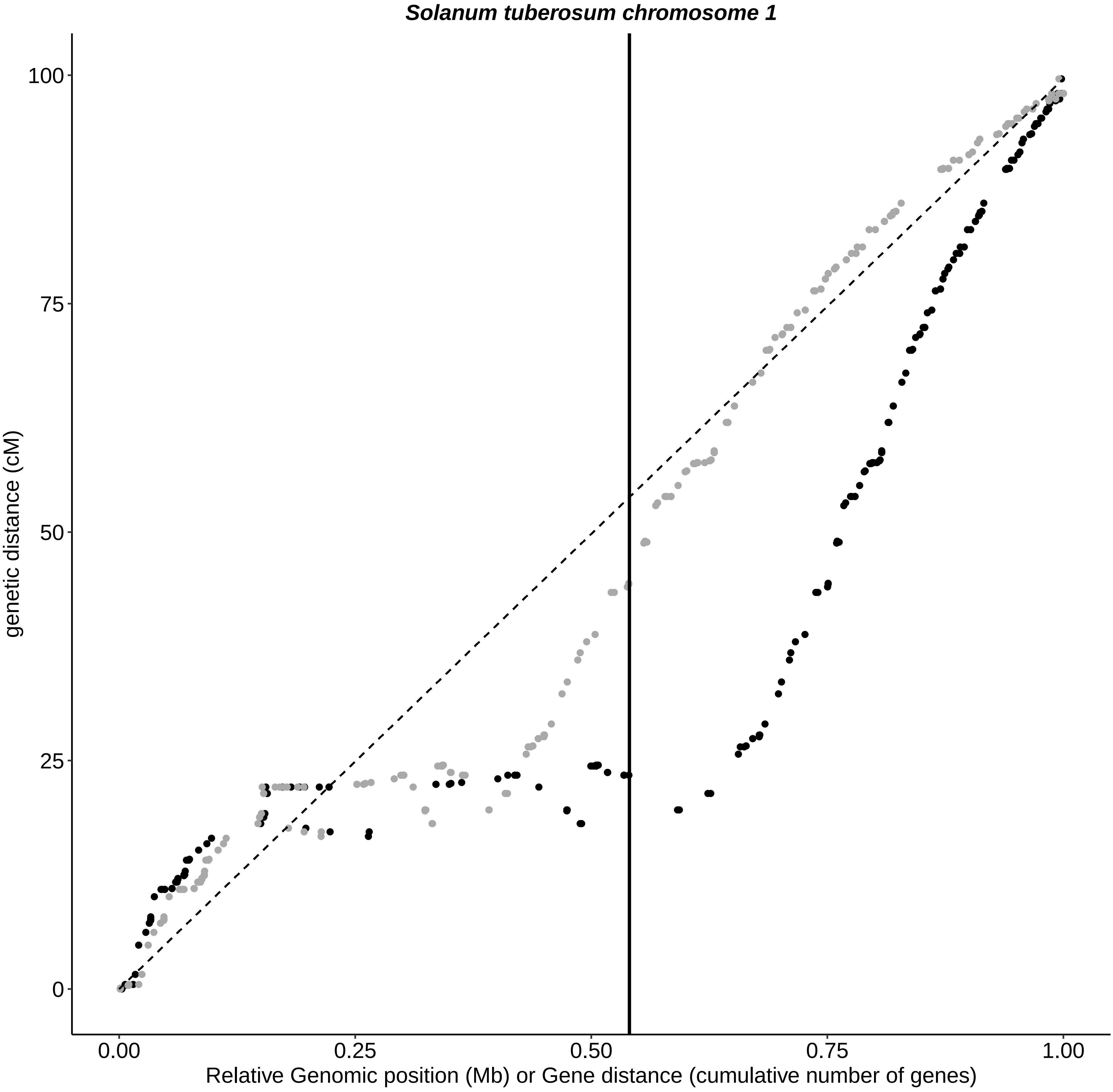

Solanum tuberosum chromosome 1
genetic distance (cM)
0
25
50
75
100
0.00
0.25
0.50
0.75
1.00
Relative Genomic position (Mb) or Gene distance (cumulative number of genes)

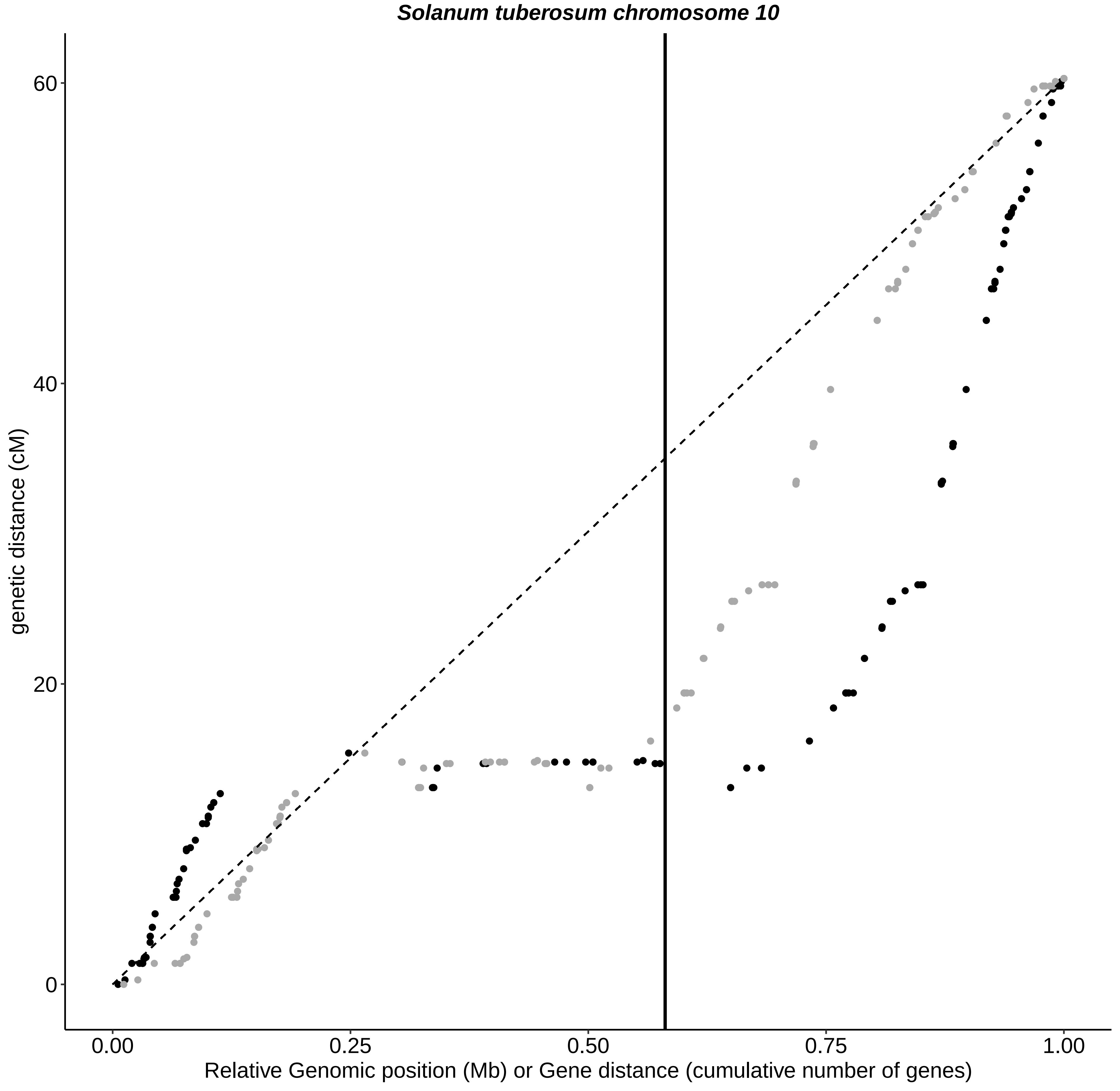
Solanum tuberosum chromosome 10
genetic distance (cM)
0
20
40
60
0.00
0.25
0.50
0.75
1.00
Relative Genomic position (Mb) or Gene distance (cumulative number of genes)

***Solanum tuberosum* chromosome 11**

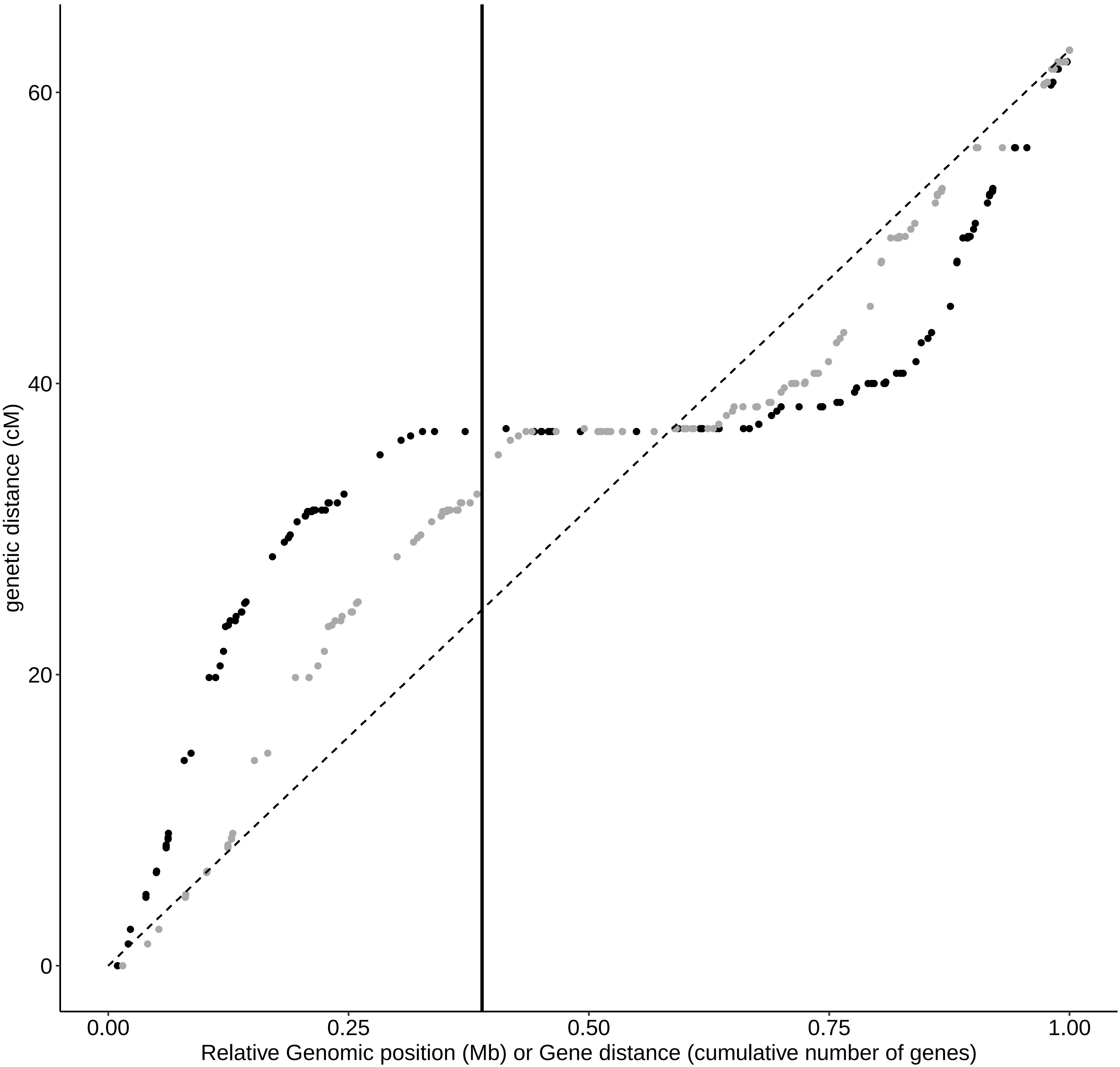

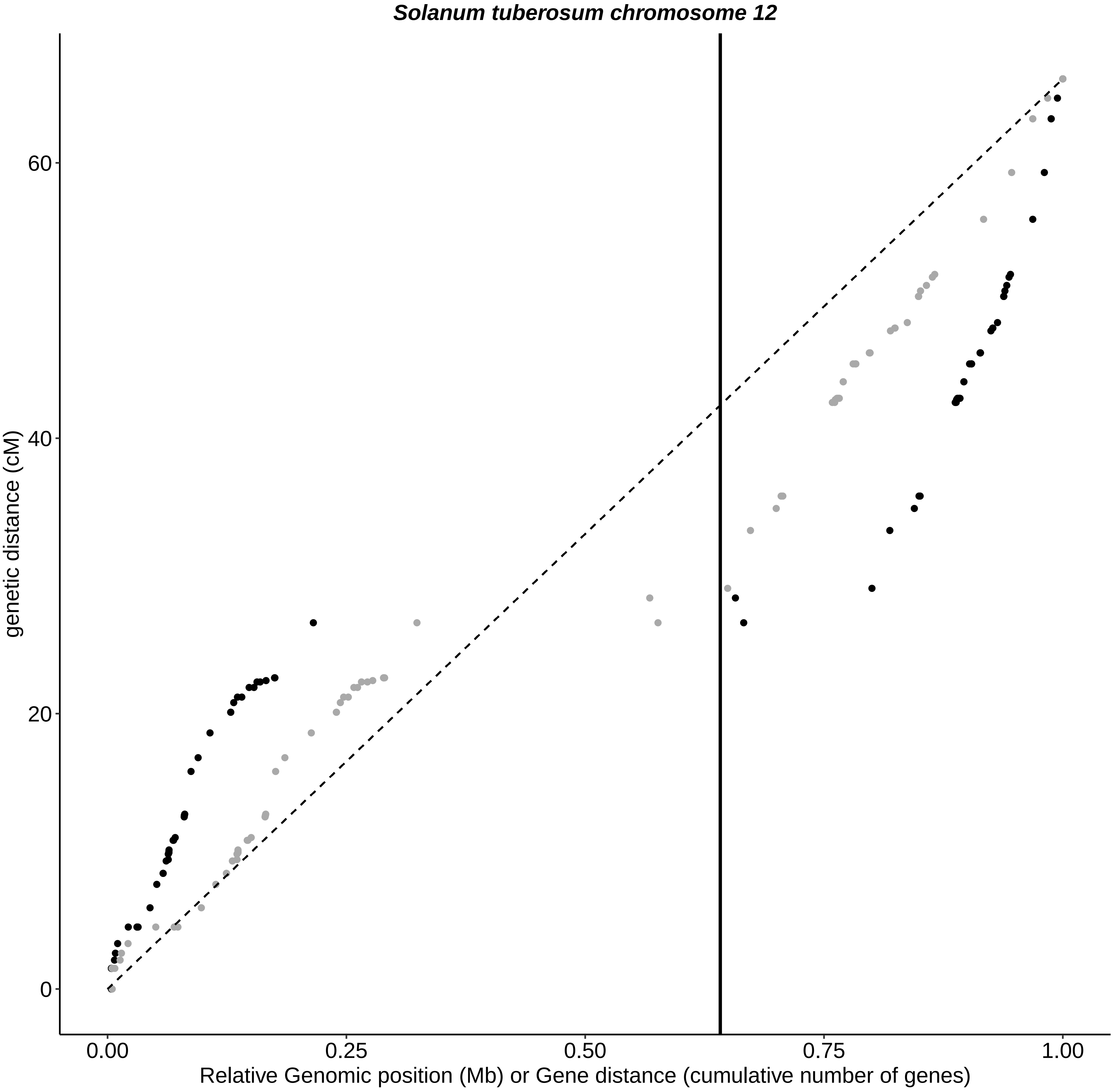
Solanum tuberosum chromosome 12
genetic distance (cM)
0
20
40
60
0.00
0.25
0.50
0.75
1.00
Relative Genomic position (Mb) or Gene distance (cumulative number of genes)

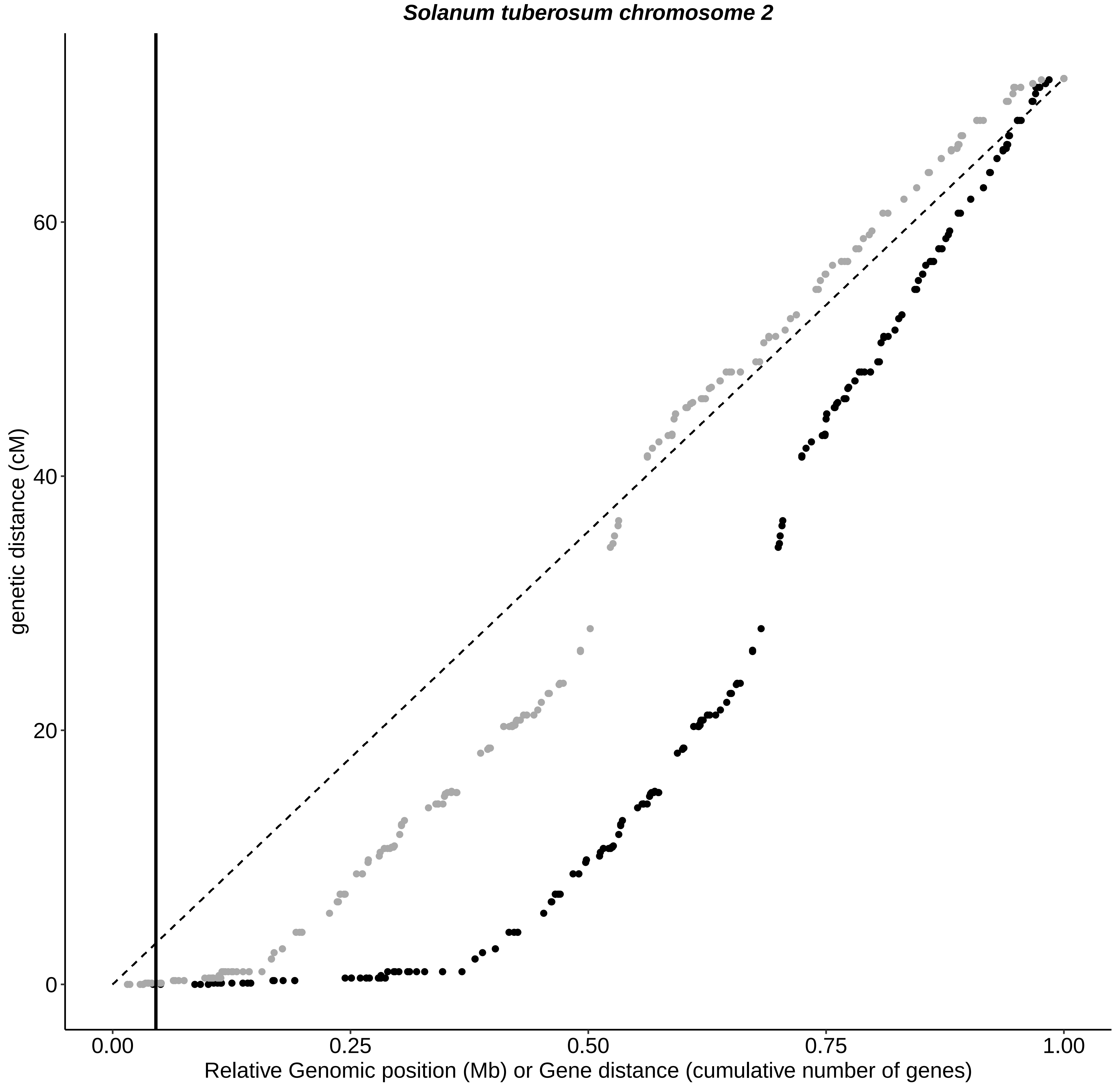
Solanum tuberosum chromosome 2
genetic distance (cM)
0
20
40
60
0.00
0.25
0.50
0.75
1.00
Relative Genomic position (Mb) or Gene distance (cumulative number of genes)

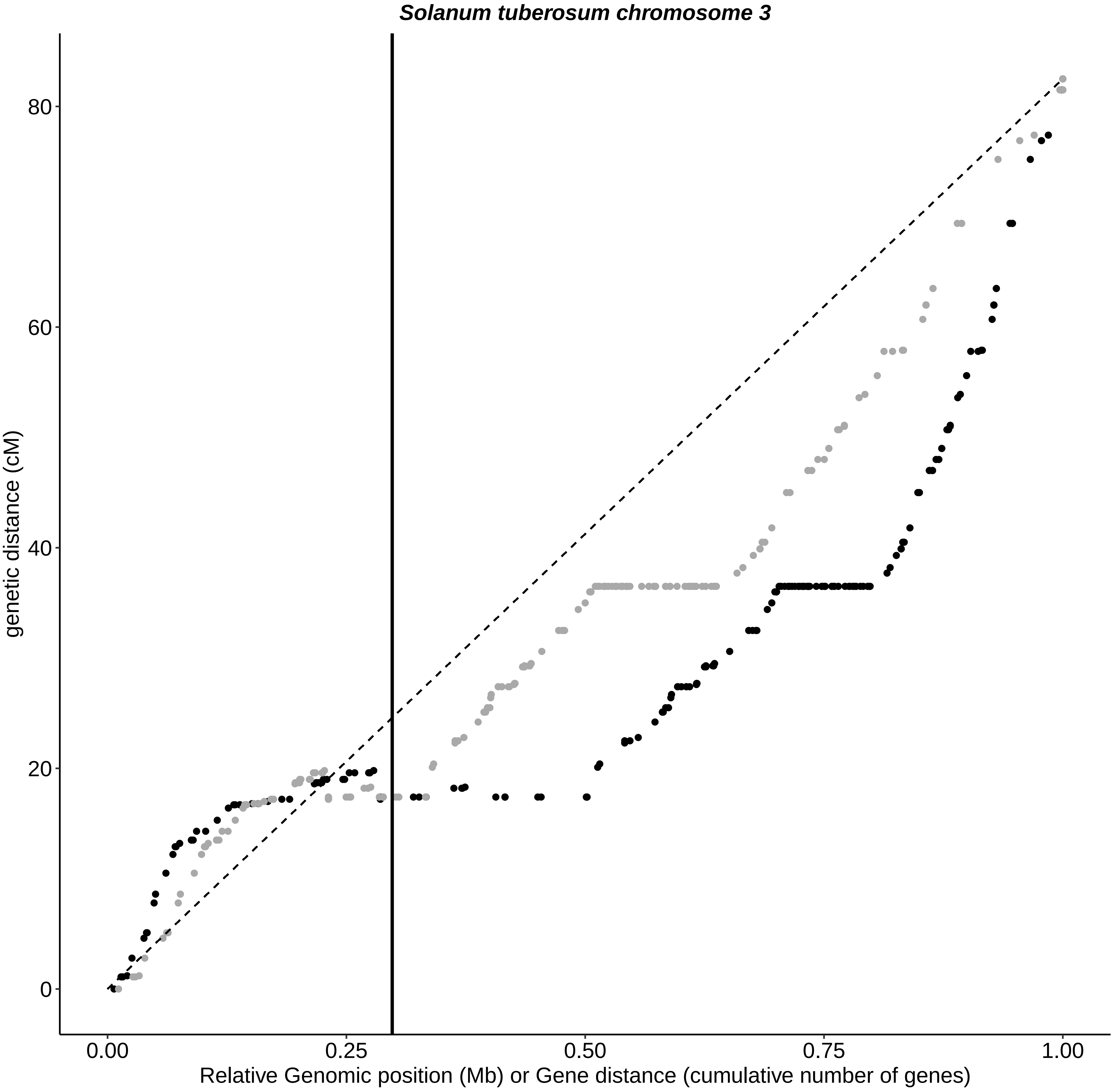
Solanum tuberosum chromosome 3
genetic distance (cM)
0
20
40
60
80
0.00
0.25
0.50
0.75
1.00
Relative Genomic position (Mb) or Gene distance (cumulative number of genes)

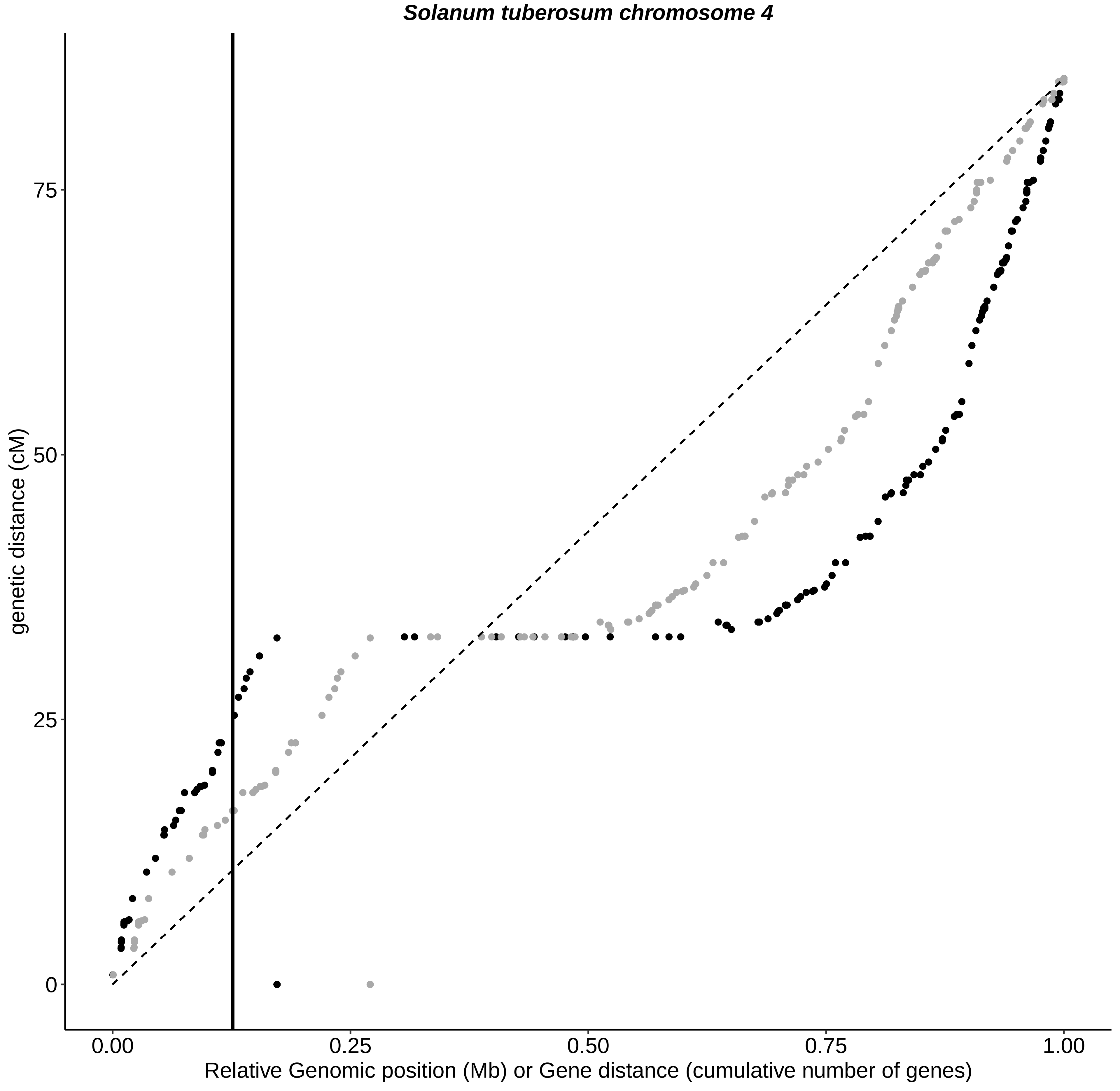
Solanum tuberosum chromosome 4
genetic distance (cM)
0
25
50
75
0.00
0.25
0.50
0.75
1.00
Relative Genomic position (Mb) or Gene distance (cumulative number of genes)

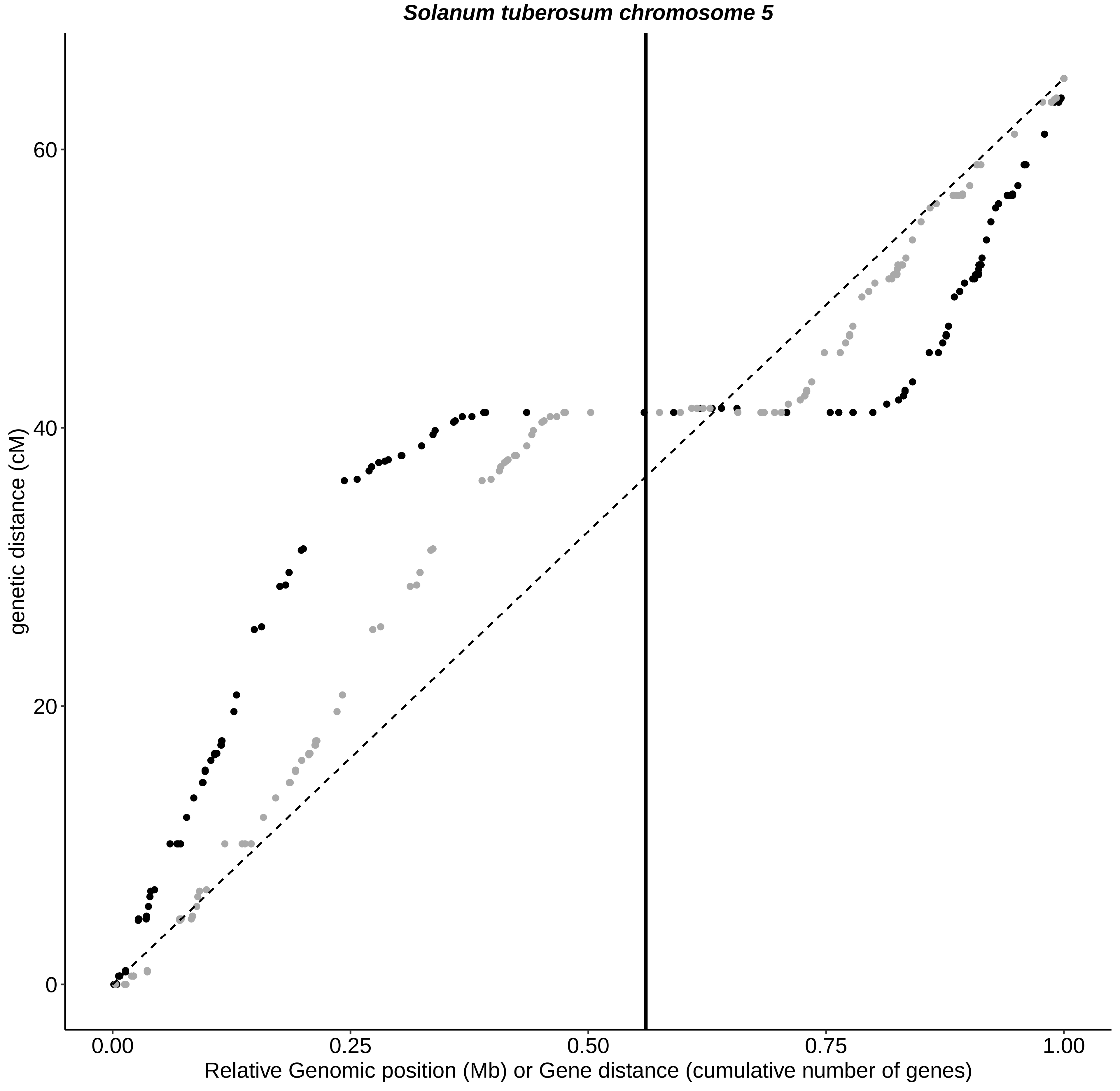
Solanum tuberosum chromosome 5
genetic distance (cM)
0
20
40
60
0.00
0.25
0.50
0.75
1.00
Relative Genomic position (Mb) or Gene distance (cumulative number of genes)

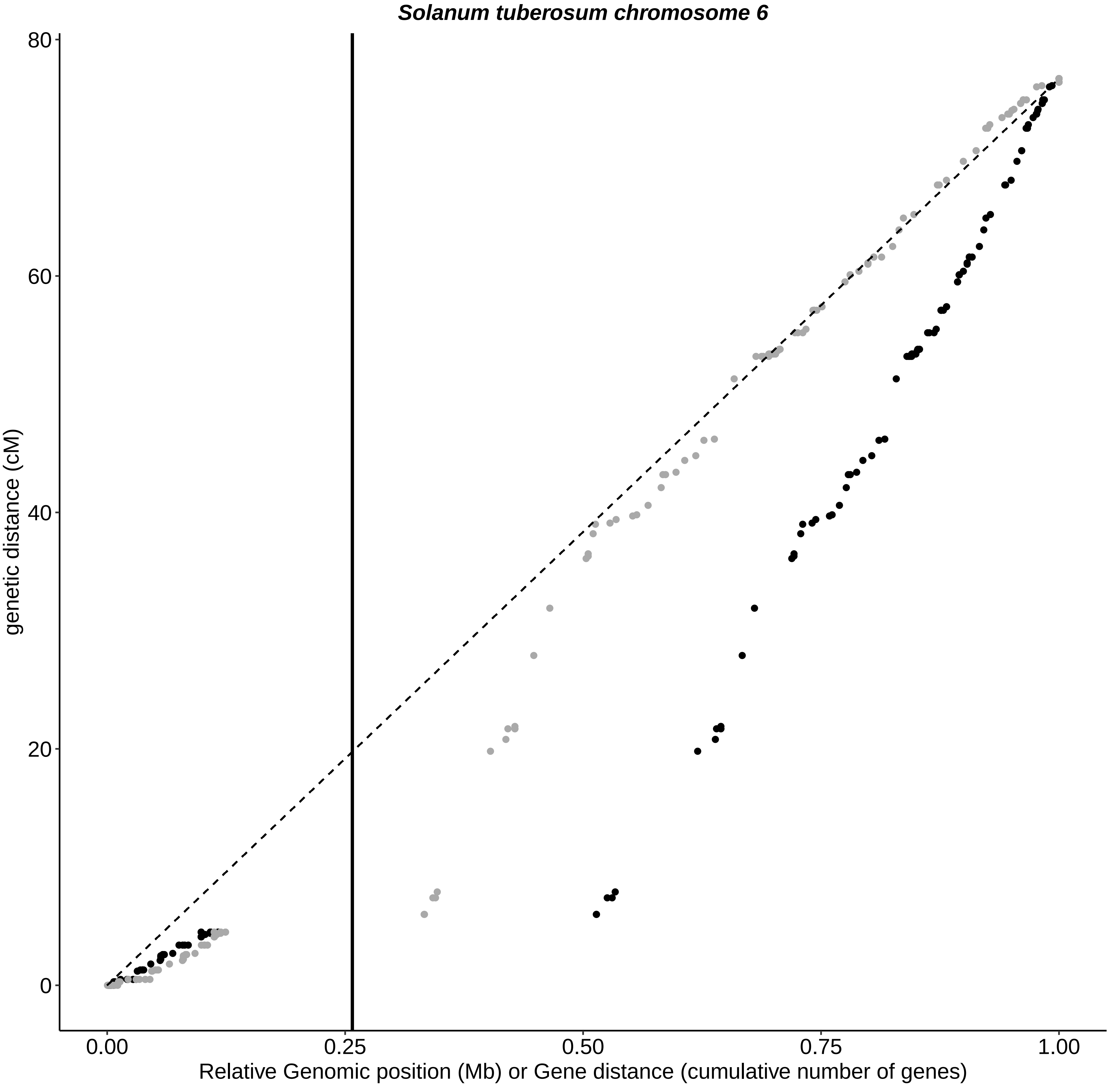
Solanum tuberosum chromosome 6
genetic distance (cM)
0
20
40
60
80
0.00
0.25
0.50
0.75
1.00
Relative Genomic position (Mb) or Gene distance (cumulative number of genes)

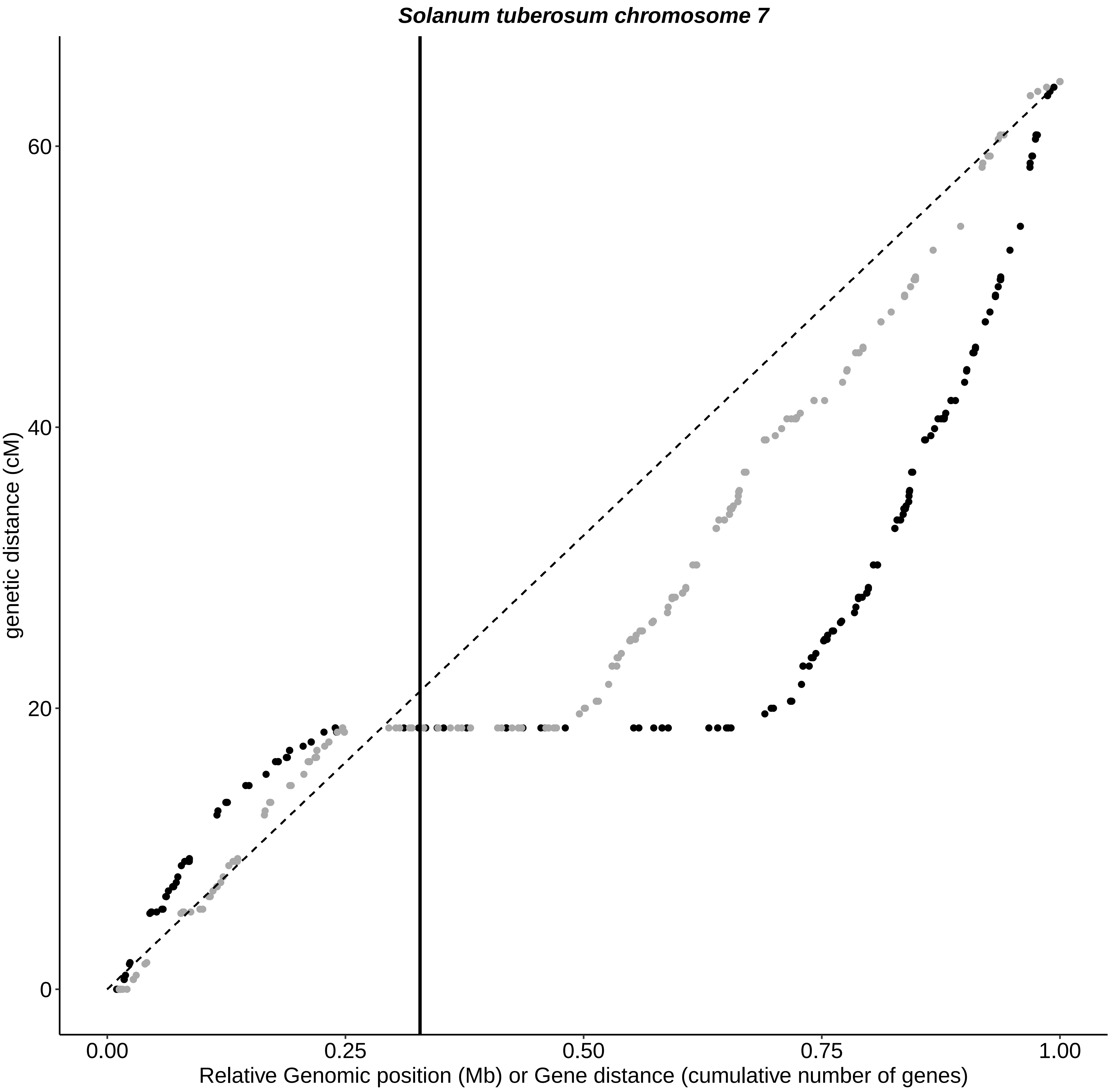
Solanum tuberosum chromosome 7
genetic distance (cM)
0
20
40
60
0.00
0.25
0.50
0.75
1.00
Relative Genomic position (Mb) or Gene distance (cumulative number of genes)

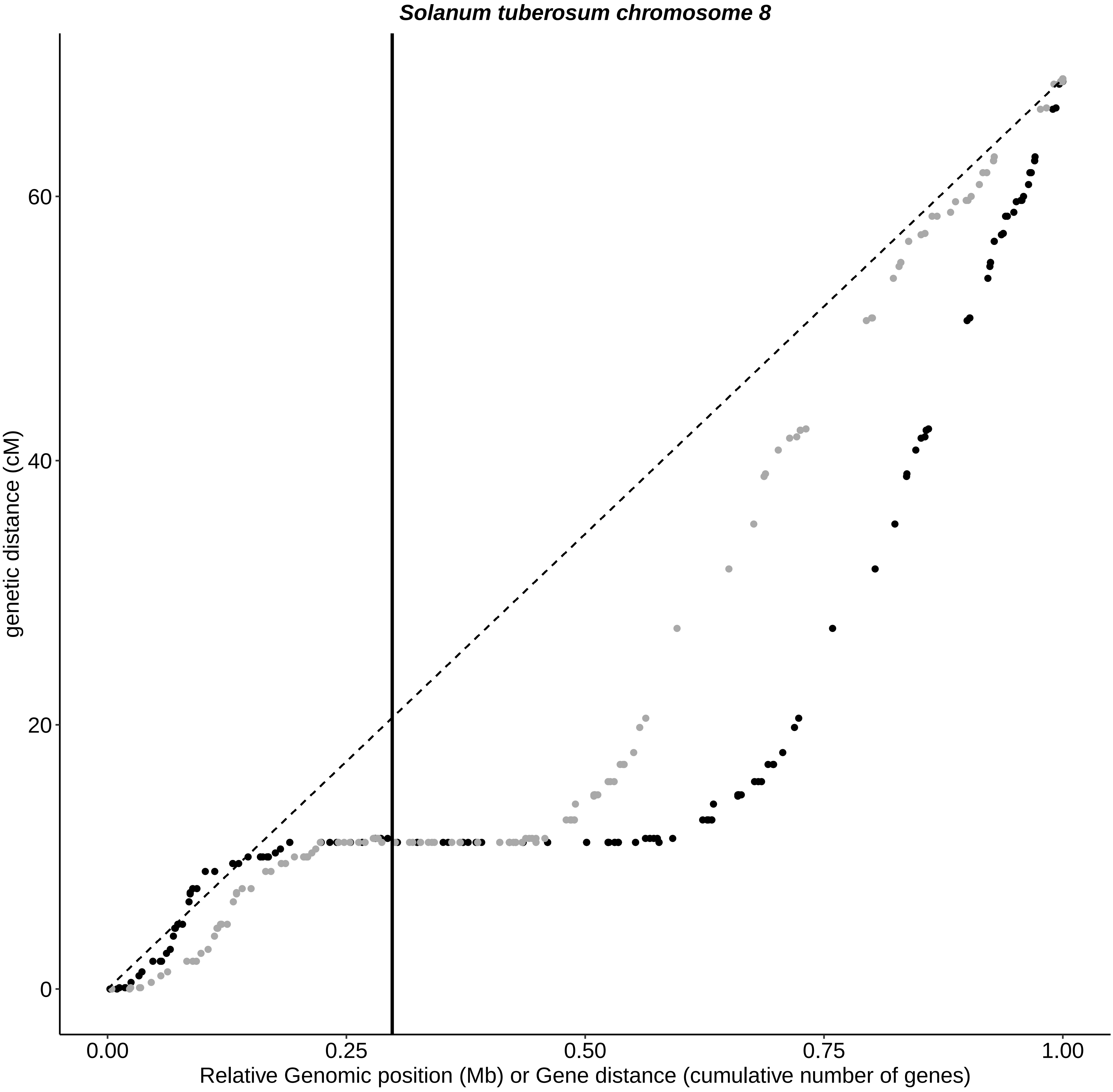
Solanum tuberosum chromosome 8
genetic distance (cM)
0
20
40
60
0.00
0.25
0.50
0.75
1.00
Relative Genomic position (Mb) or Gene distance (cumulative number of genes)

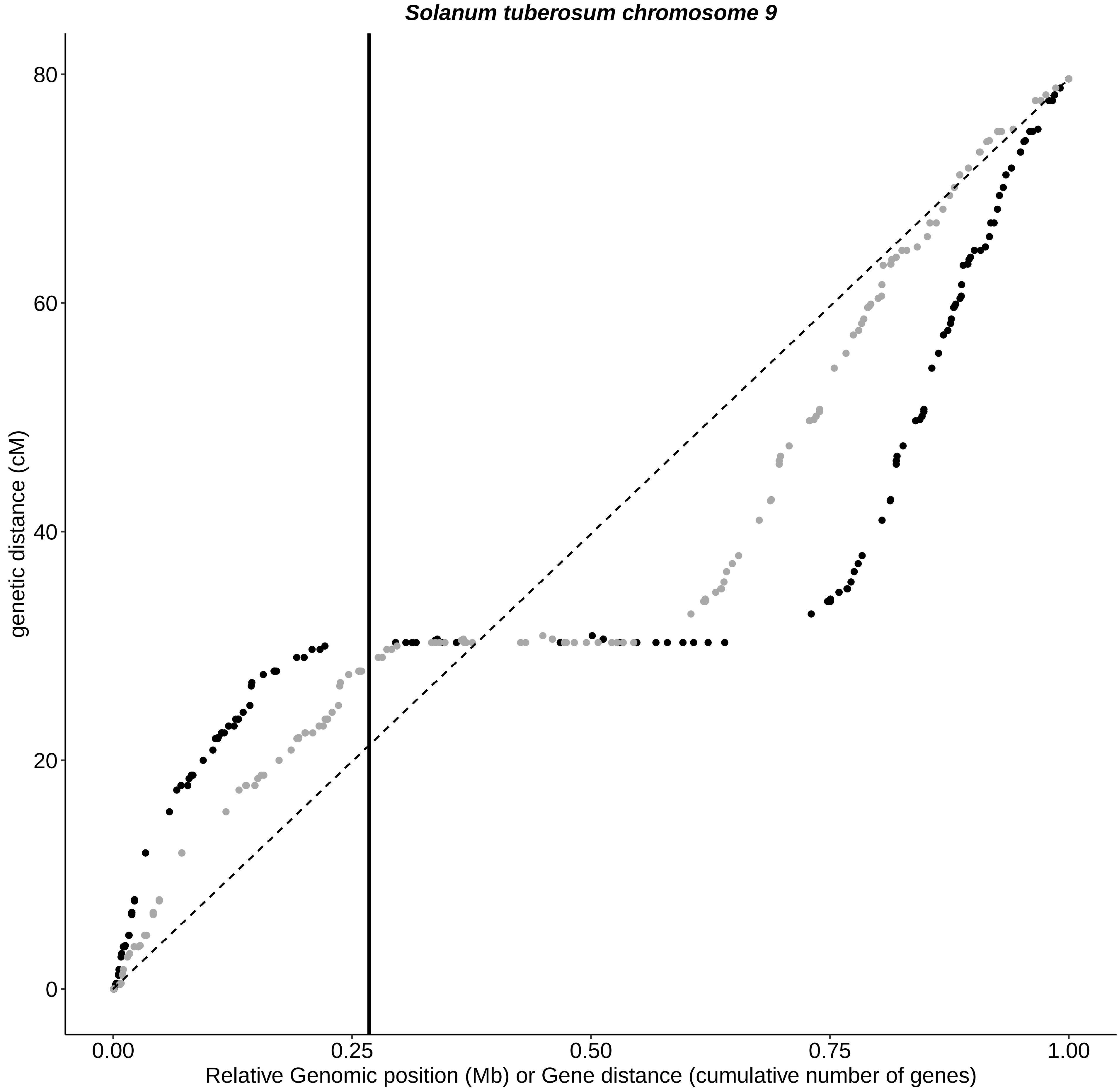
Solanum tuberosum chromosome 9
genetic distance (cM)
80
60
40
20
0
0.00
0.25
0.50
0.75
1.00
Relative Genomic position (Mb) or Gene distance (cumulative number of genes)

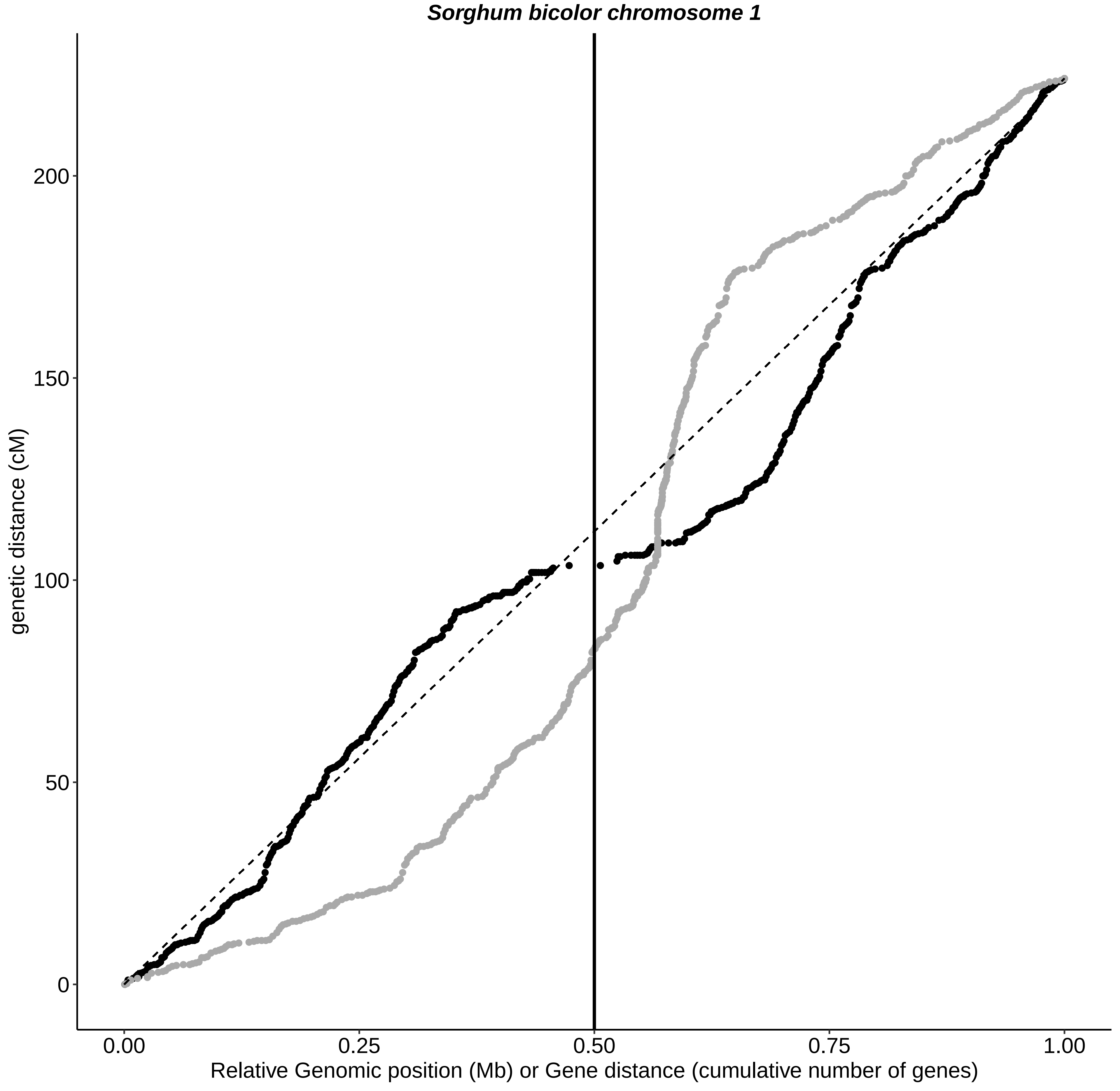
Sorghum bicolor chromosome 1
genetic distance (cM)
0
50
100
150
200
0.00
0.25
0.50
0.75
1.00
Relative Genomic position (Mb) or Gene distance (cumulative number of genes)

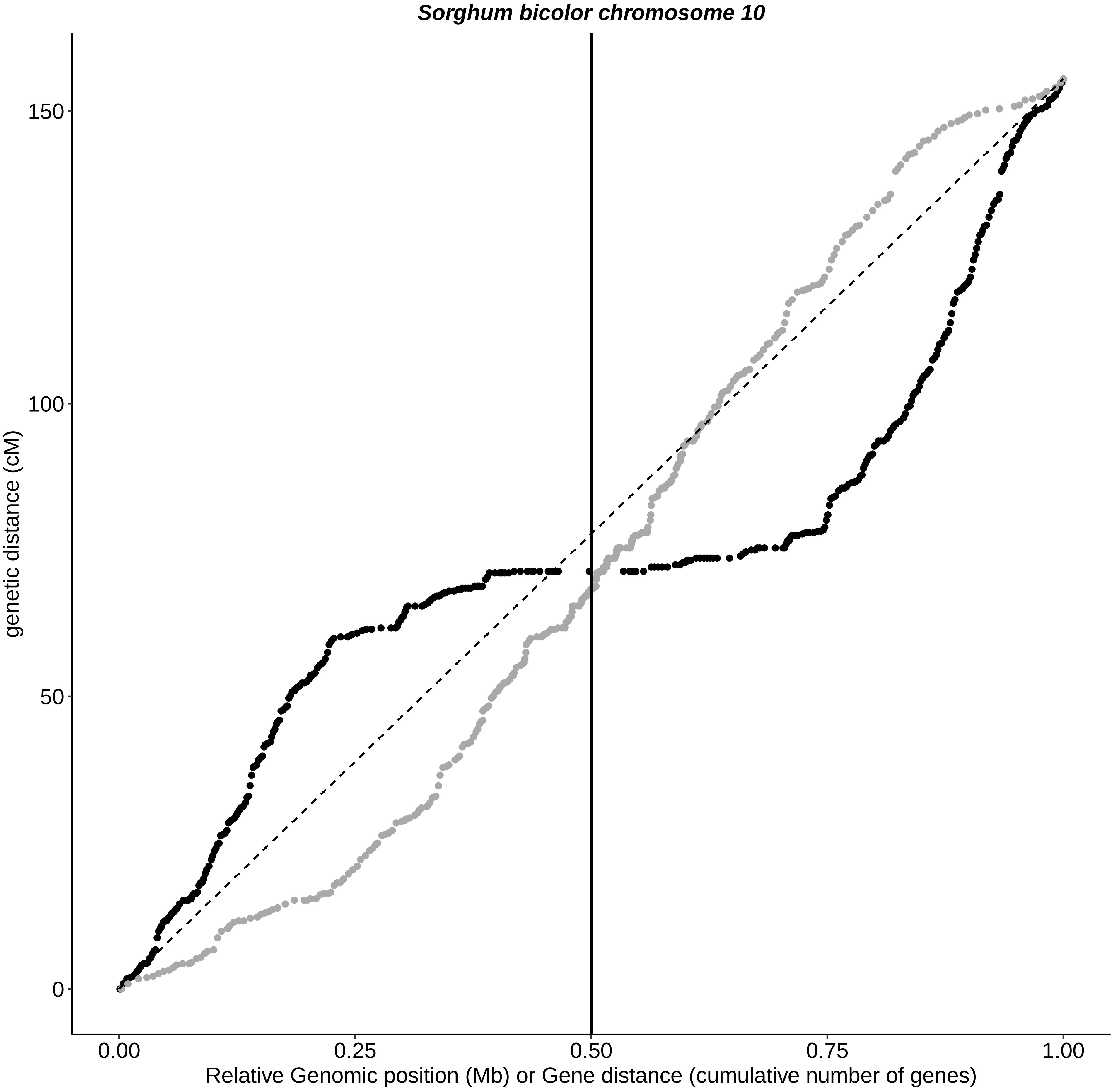
Sorghum bicolor chromosome 10
genetic distance (cM)
0
50
100
150
0.00
0.25
0.50
0.75
1.00
Relative Genomic position (Mb) or Gene distance (cumulative number of genes)

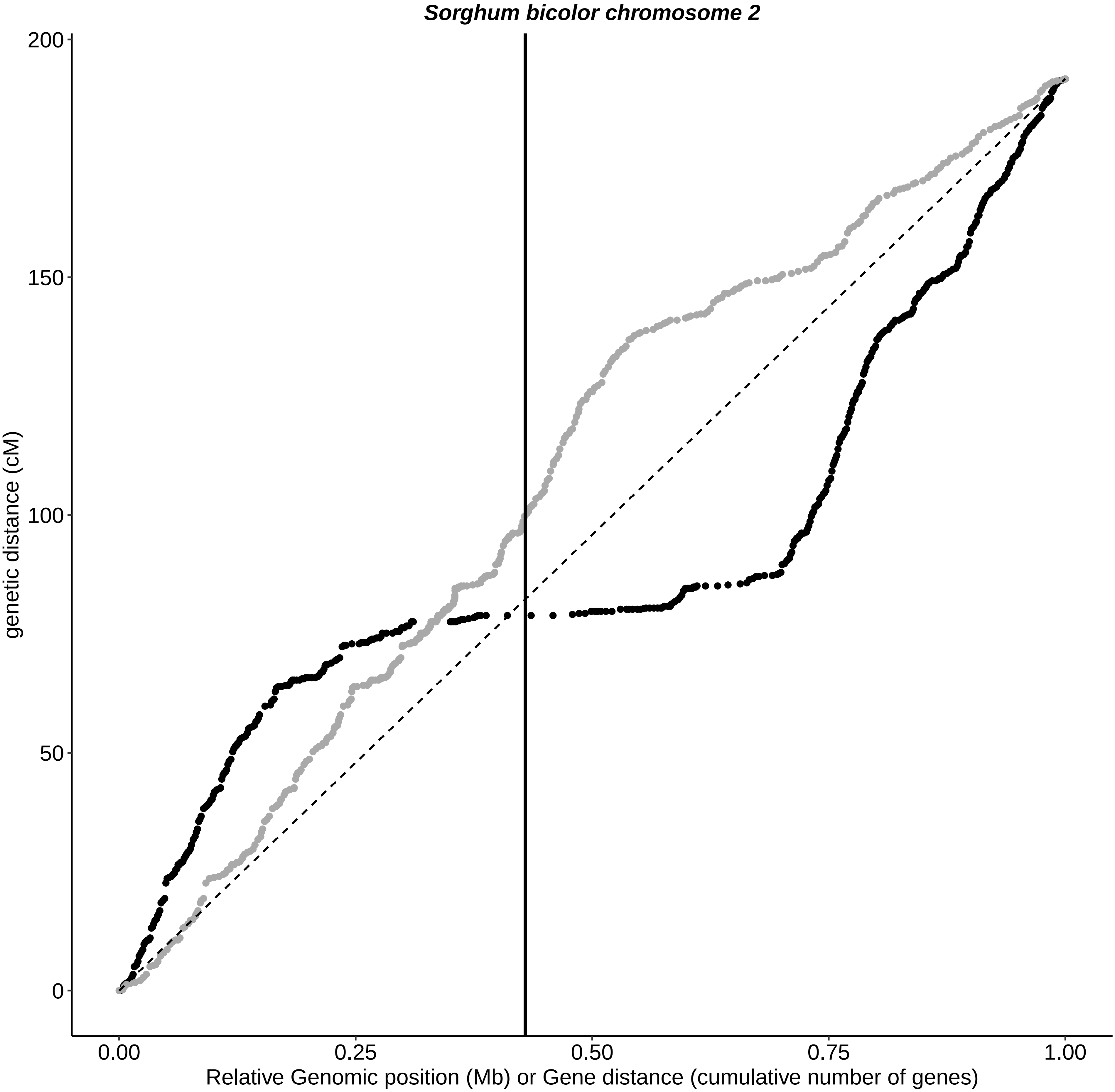
Sorghum bicolor chromosome 2
genetic distance (cM)
200
150
100
50
0
0.00
0.25
0.50
0.75
1.00
Relative Genomic position (Mb) or Gene distance (cumulative number of genes)

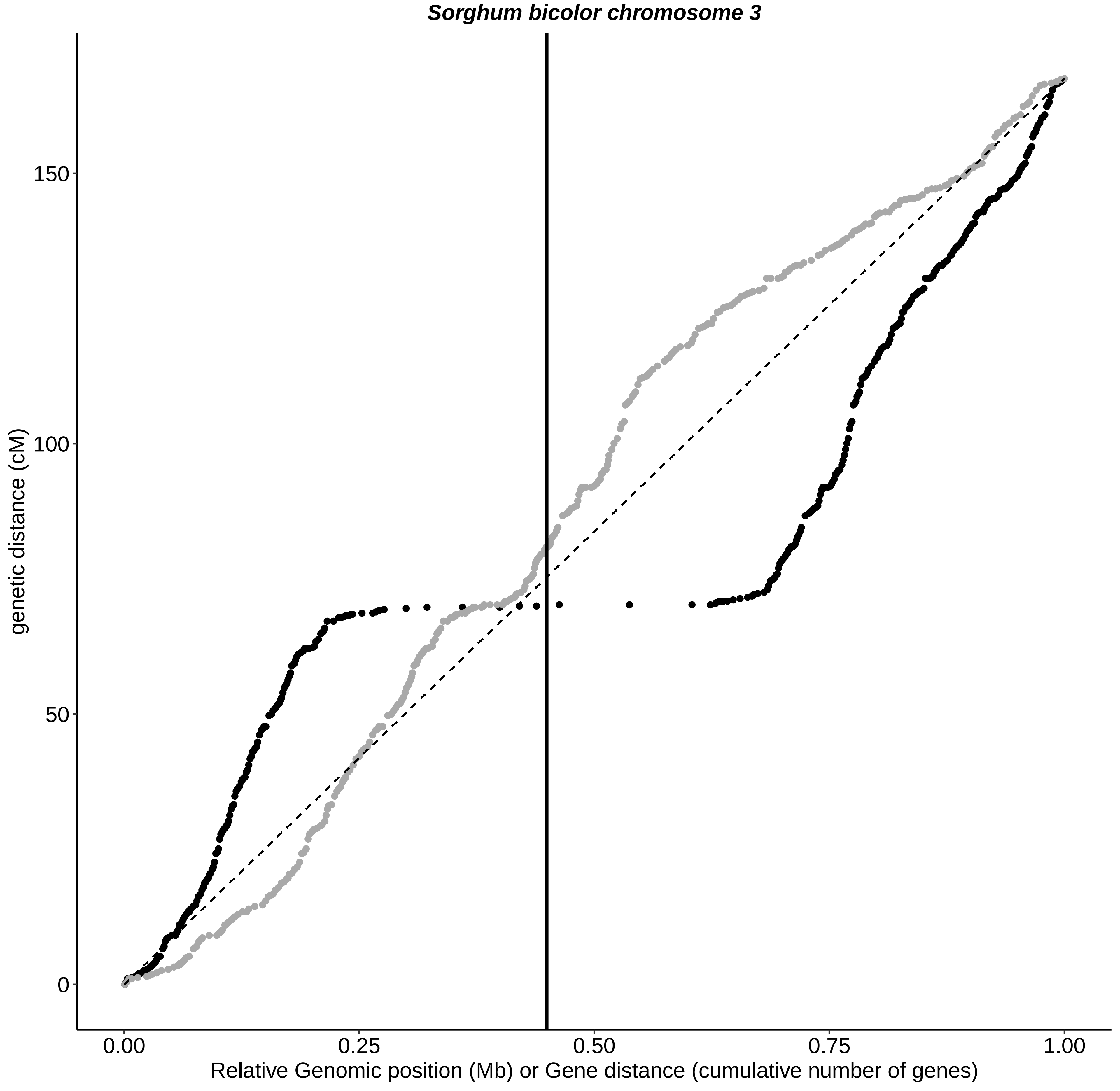
Sorghum bicolor chromosome 3
genetic distance (cM)
0
50
100
150
0.00
0.25
0.50
0.75
1.00
Relative Genomic position (Mb) or Gene distance (cumulative number of genes)

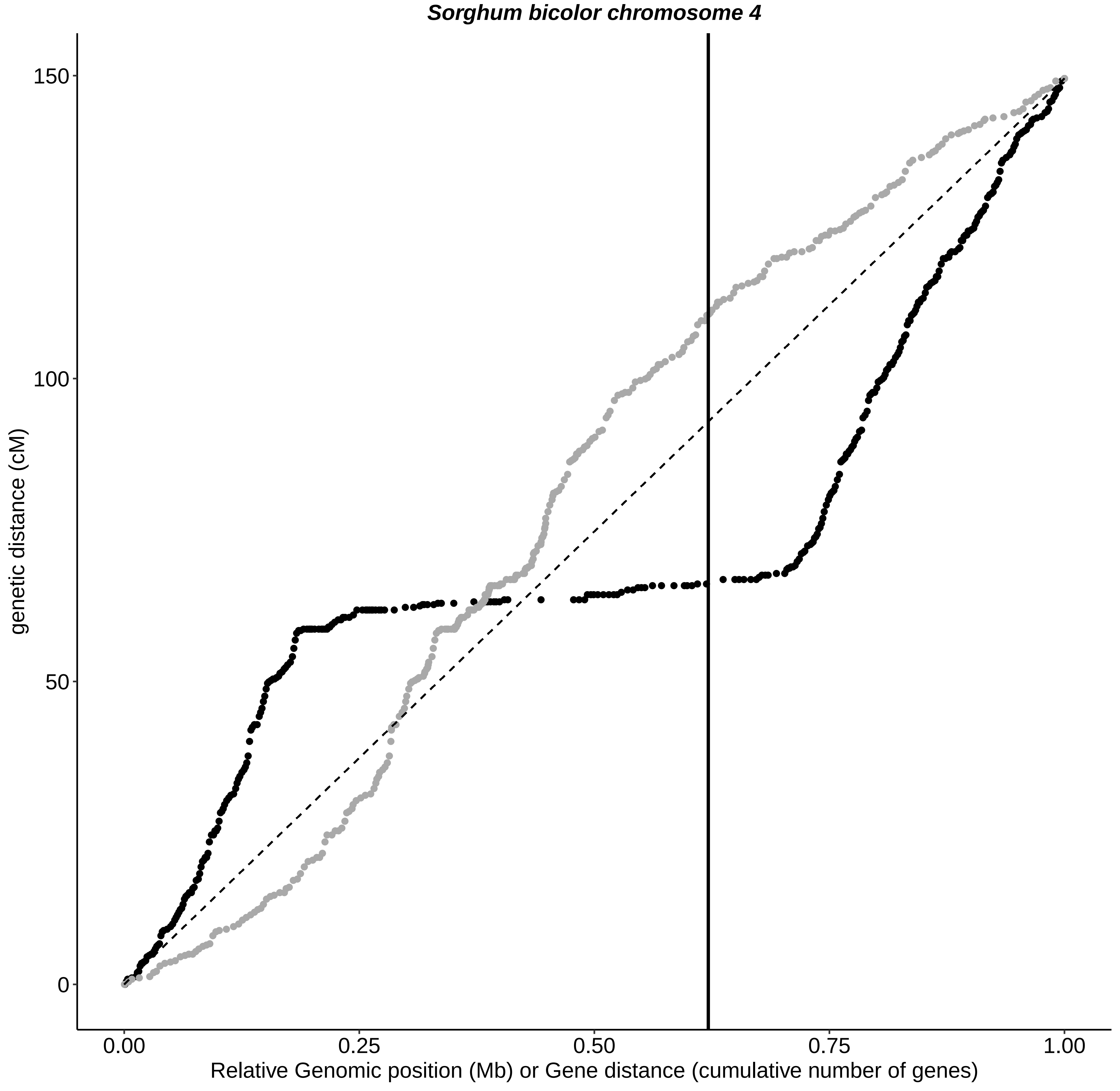

Sorghum bicolor chromosome 4
150
100
50
0
genetic distance (cM)
0.00
0.25
0.50
0.75
1.00
Relative Genomic position (Mb) or Gene distance (cumulative number of genes)

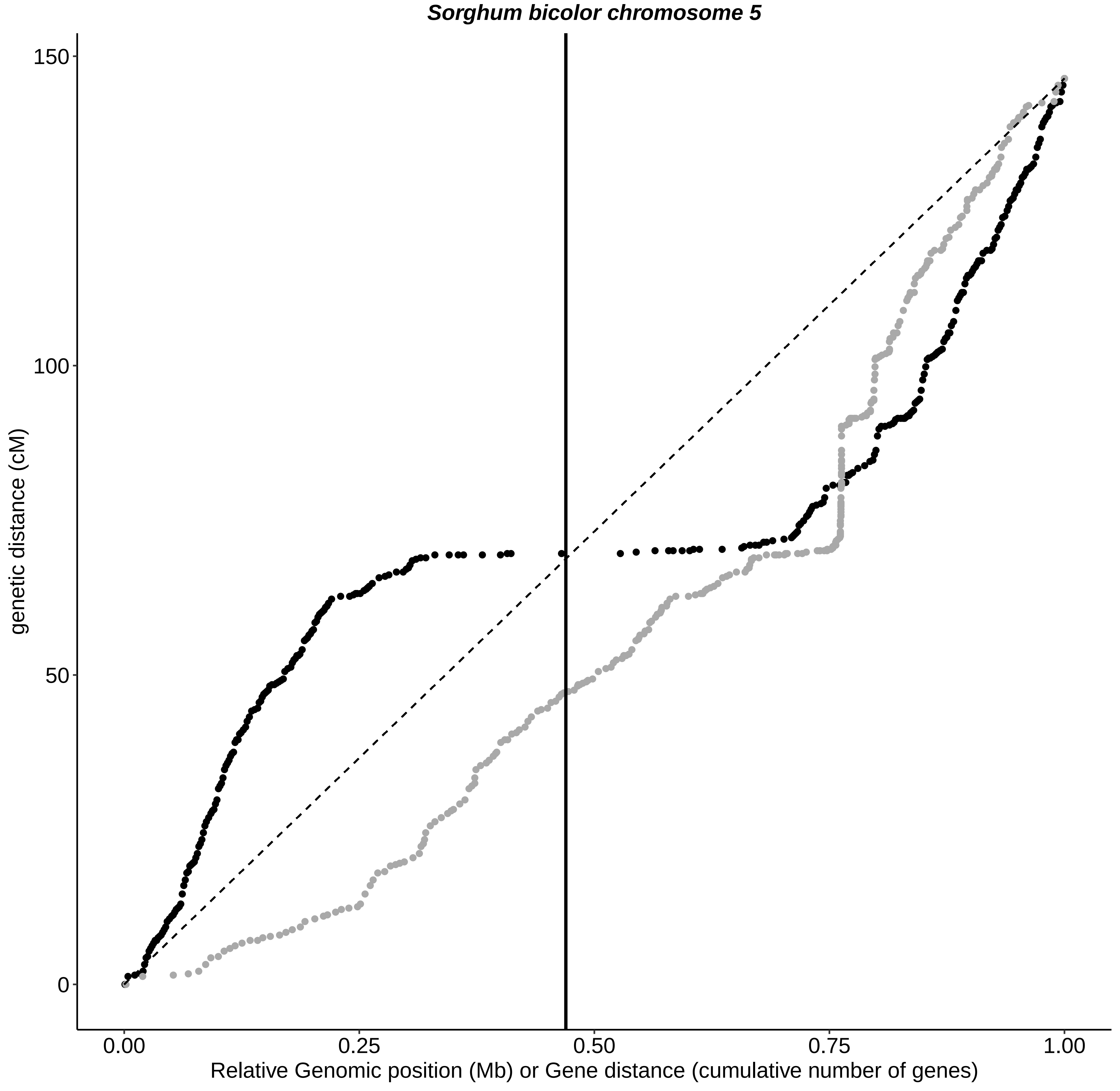
Sorghum bicolor chromosome 5
genetic distance (cM)
150
100
50
0
0.00
0.25
0.50
0.75
1.00
Relative Genomic position (Mb) or Gene distance (cumulative number of genes)

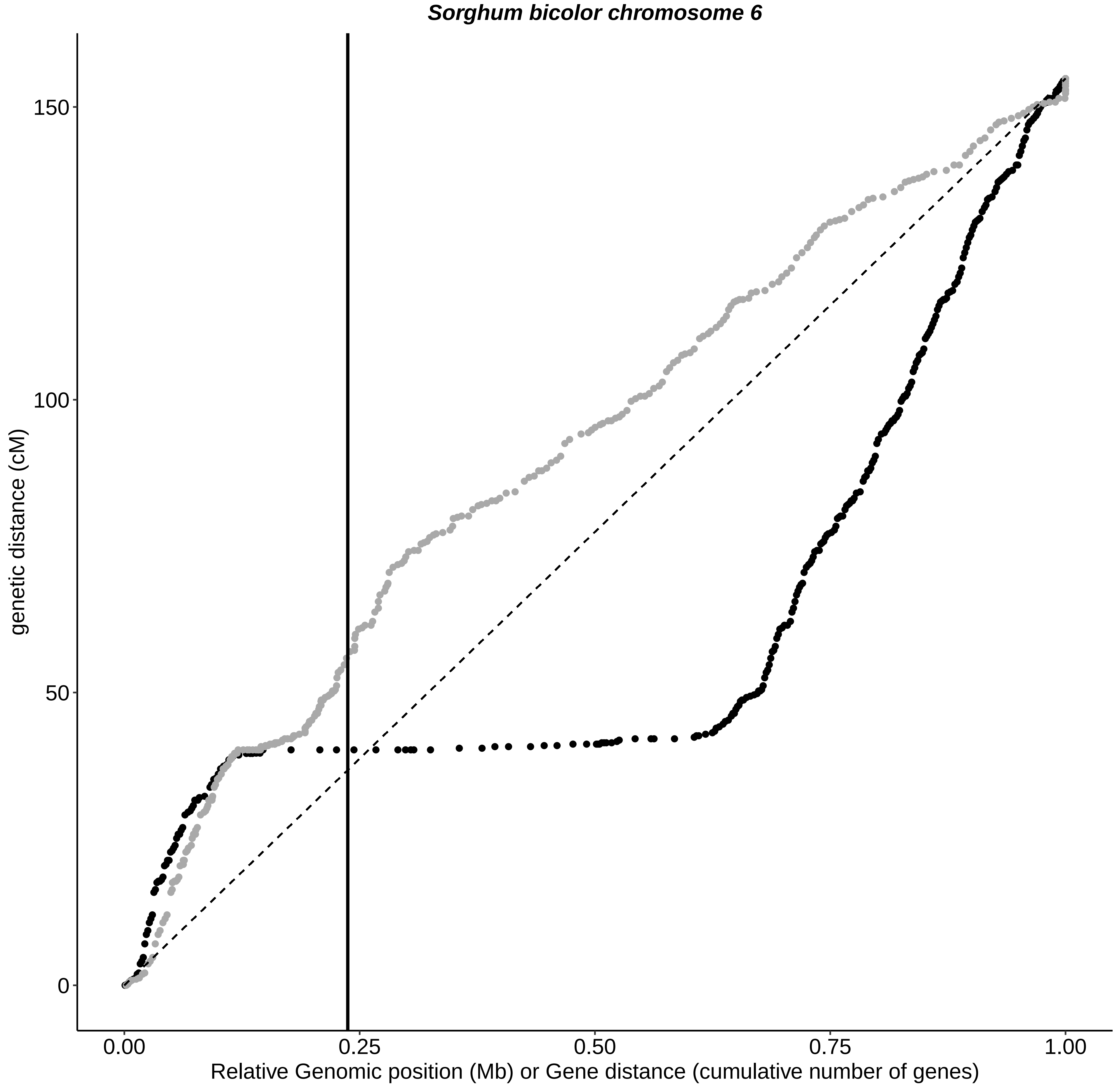
Sorghum bicolor chromosome 6
genetic distance (cM)
0
50
100
150
0.00
0.25
0.50
0.75
1.00
Relative Genomic position (Mb) or Gene distance (cumulative number of genes)

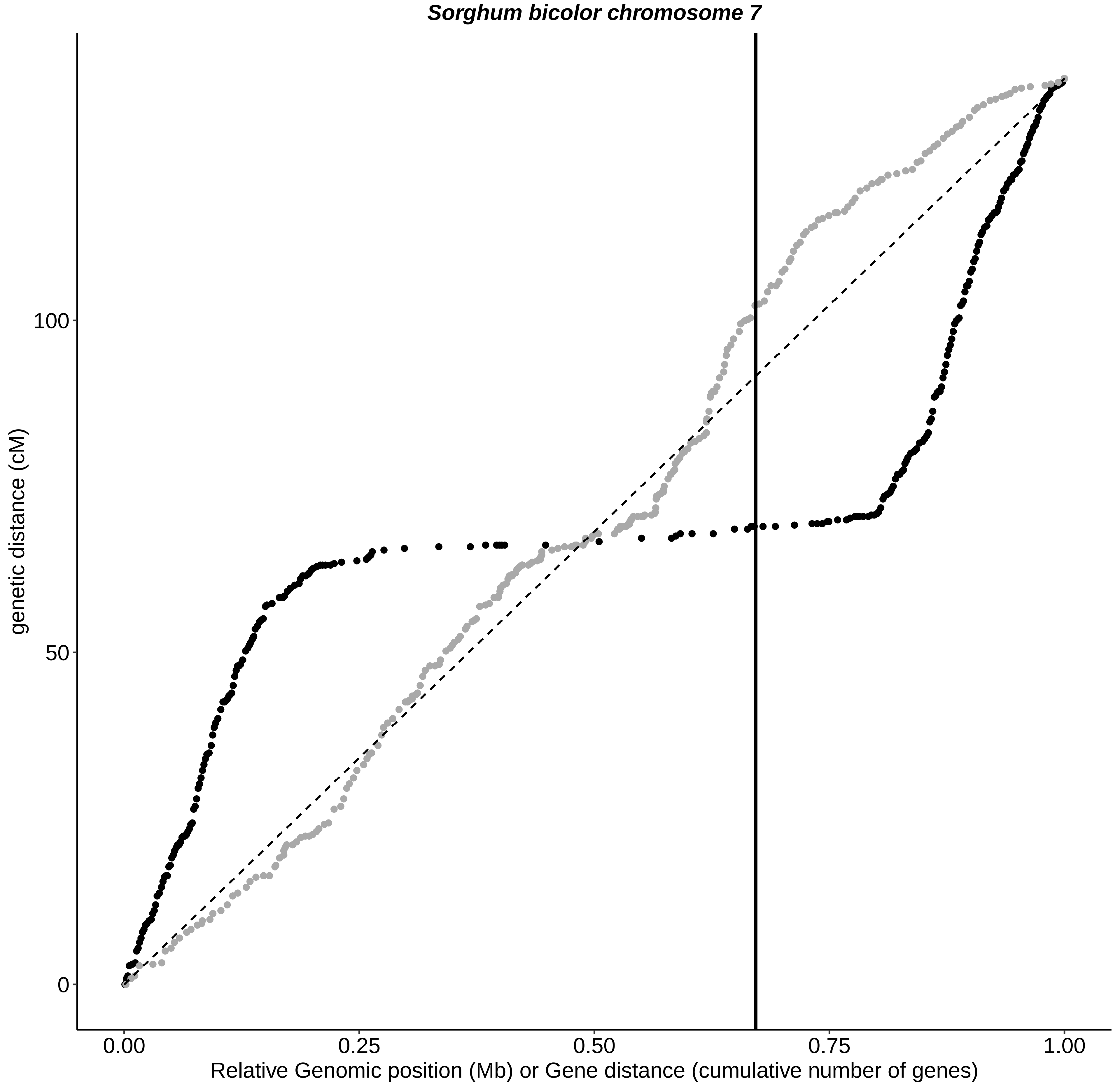
Sorghum bicolor chromosome 7
genetic distance (cM)
0
50
100
0.00
0.25
0.50
0.75
1.00
Relative Genomic position (Mb) or Gene distance (cumulative number of genes)

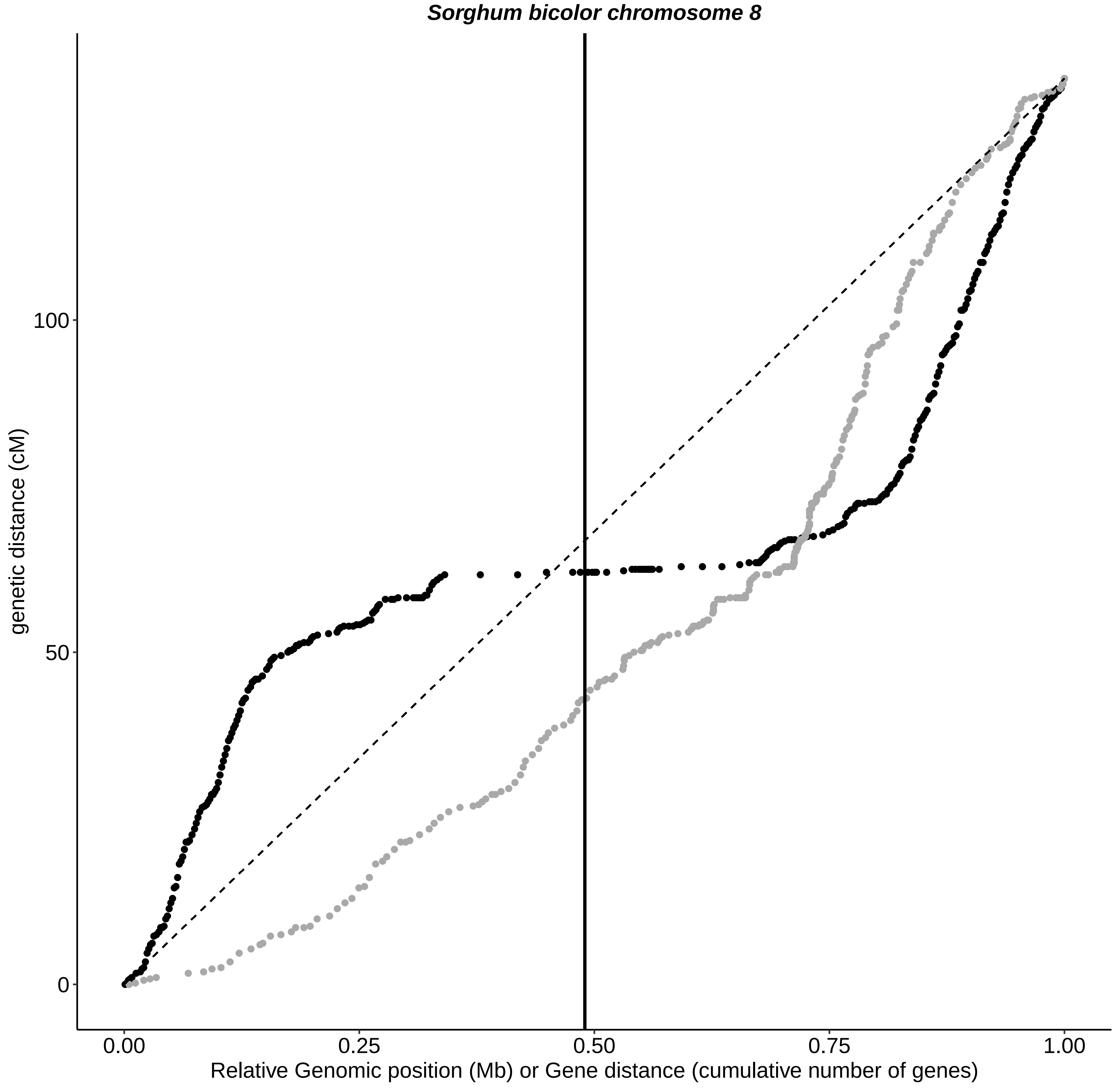
Sorghum bicolor chromosome 8
genetic distance (cM)
100
50
0
0.00
0.25
0.50
0.75
1.00
Relative Genomic position (Mb) or Gene distance (cumulative number of genes)

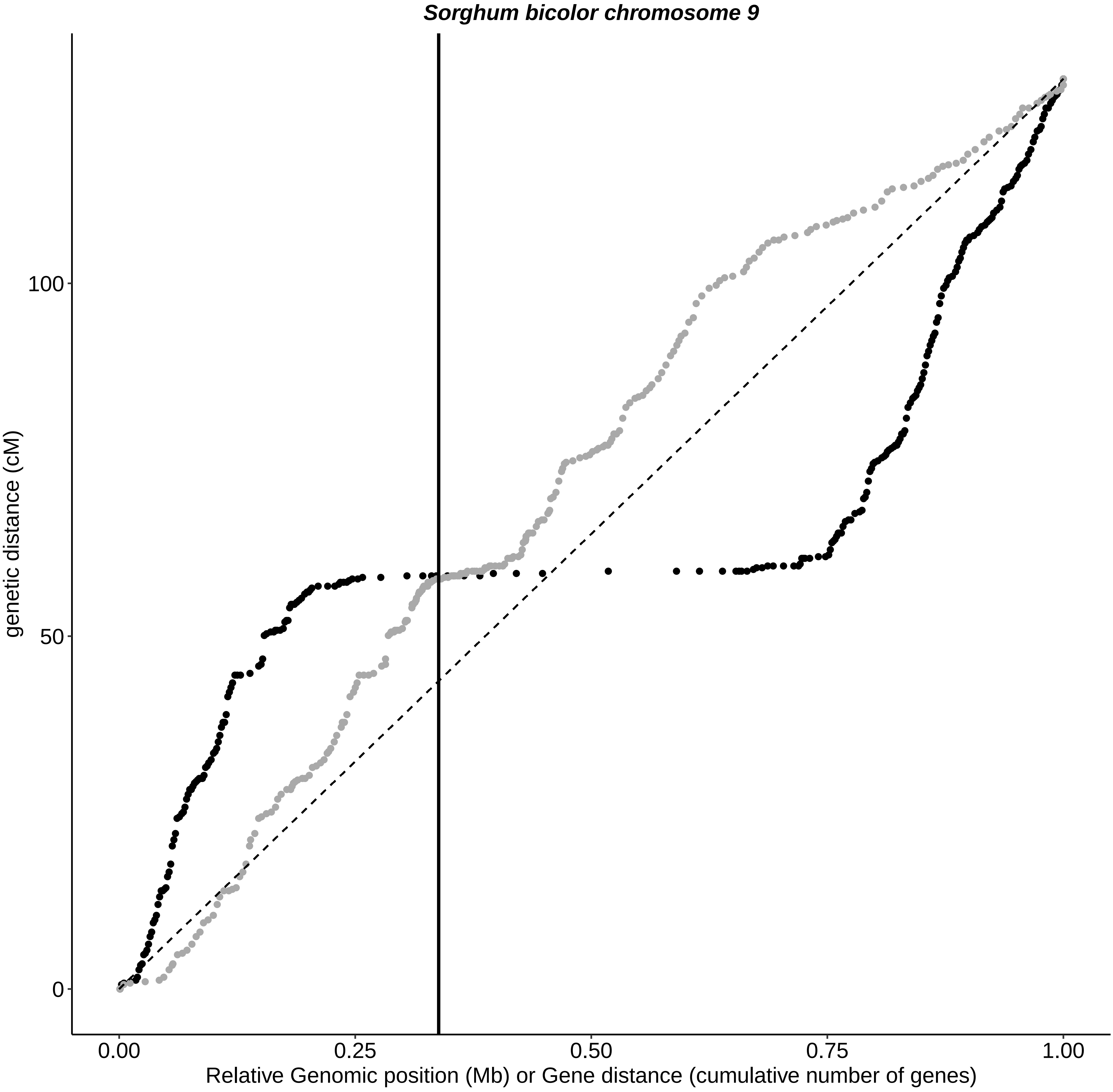
Sorghum bicolor chromosome 9
genetic distance (cM)
0
50
100
0.00
0.25
0.50
0.75
1.00
Relative Genomic position (Mb) or Gene distance (cumulative number of genes)

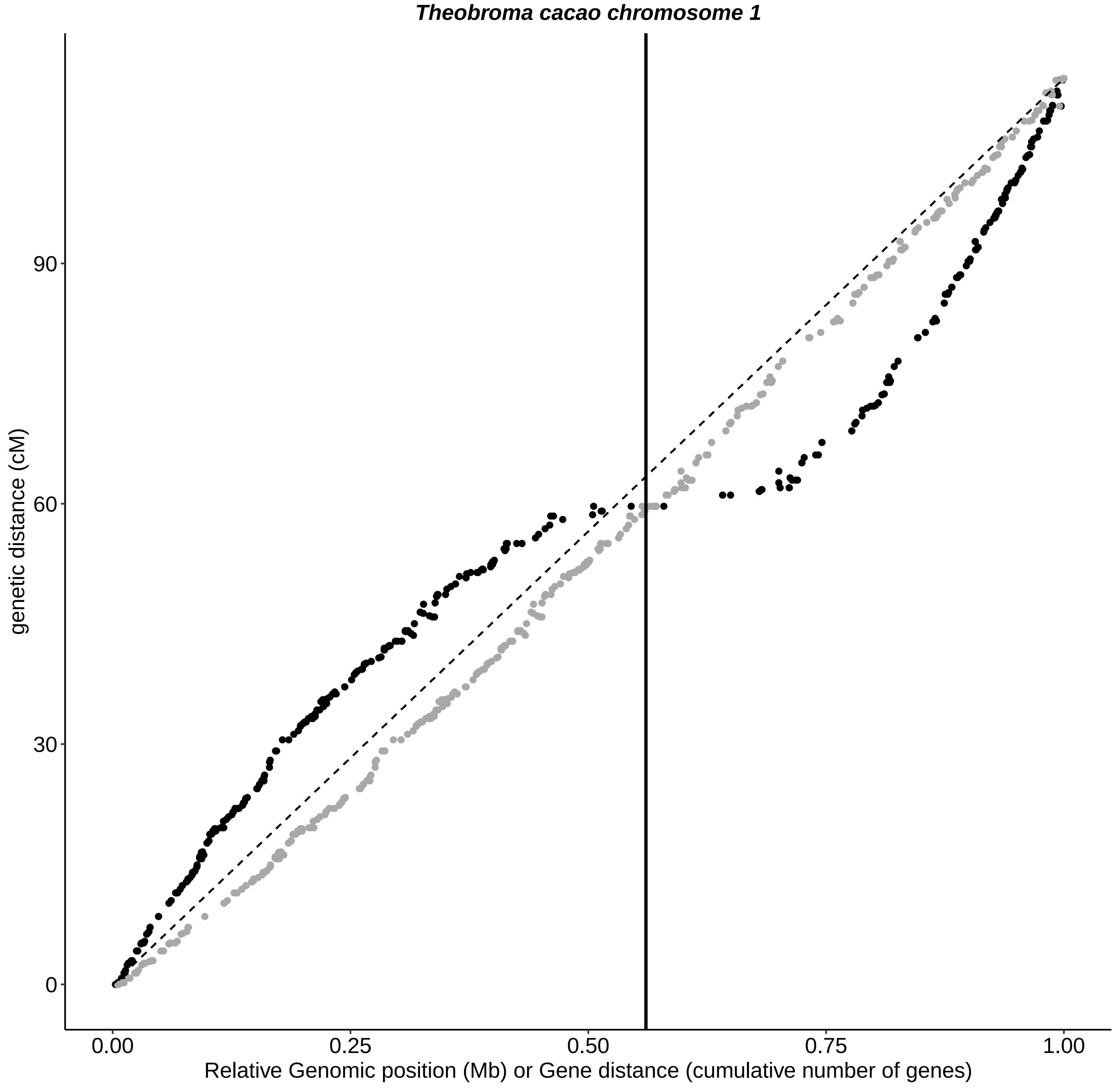
Theobroma cacao chromosome 1
genetic distance (cM)
0
30
60
90
0.00
0.25
0.50
0.75
1.00
Relative Genomic position (Mb) or Gene distance (cumulative number of genes)

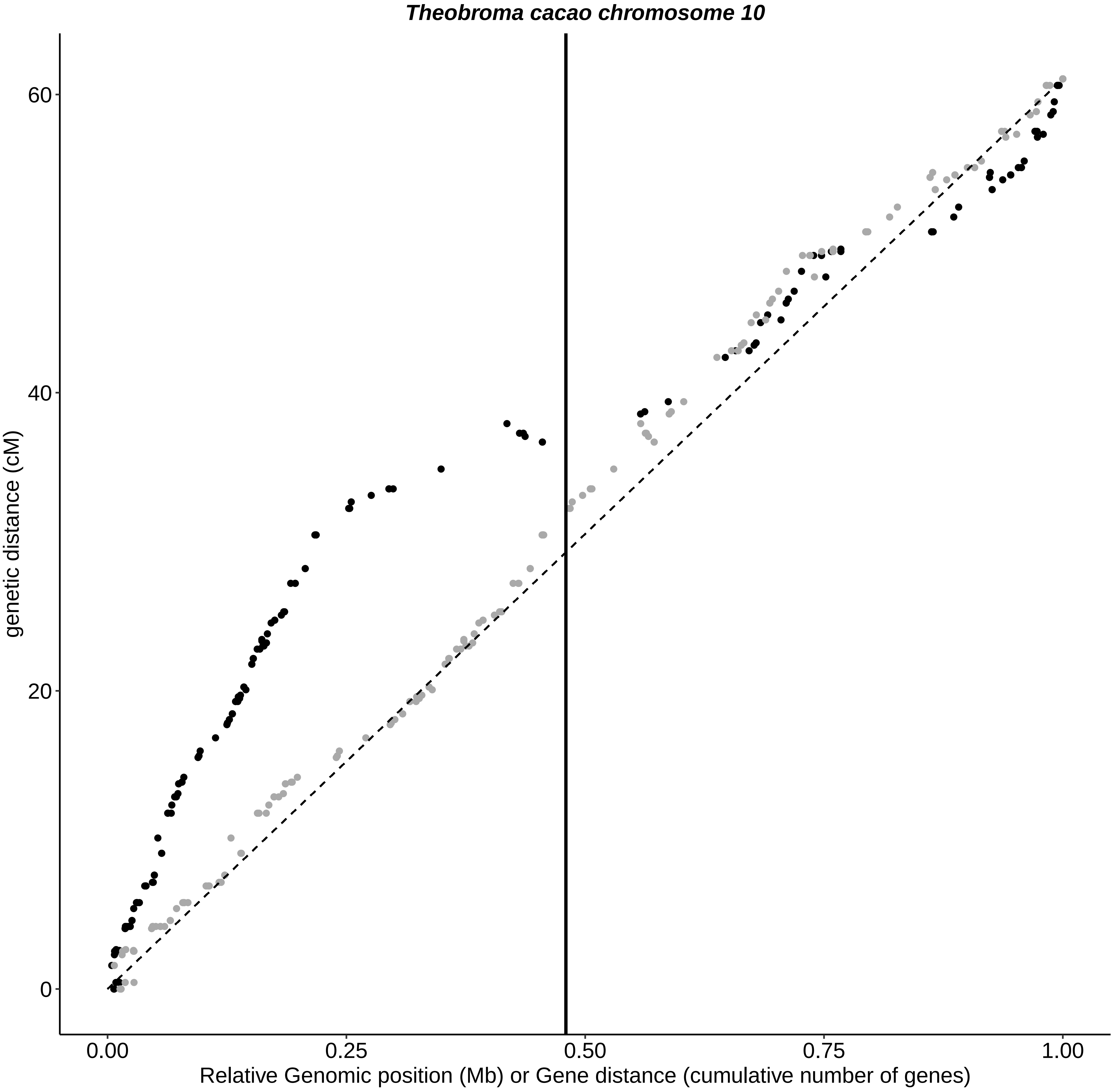
Theobroma cacao chromosome 10
genetic distance (cM)
0
20
40
60
0.00
0.25
0.50
0.75
1.00
Relative Genomic position (Mb) or Gene distance (cumulative number of genes)

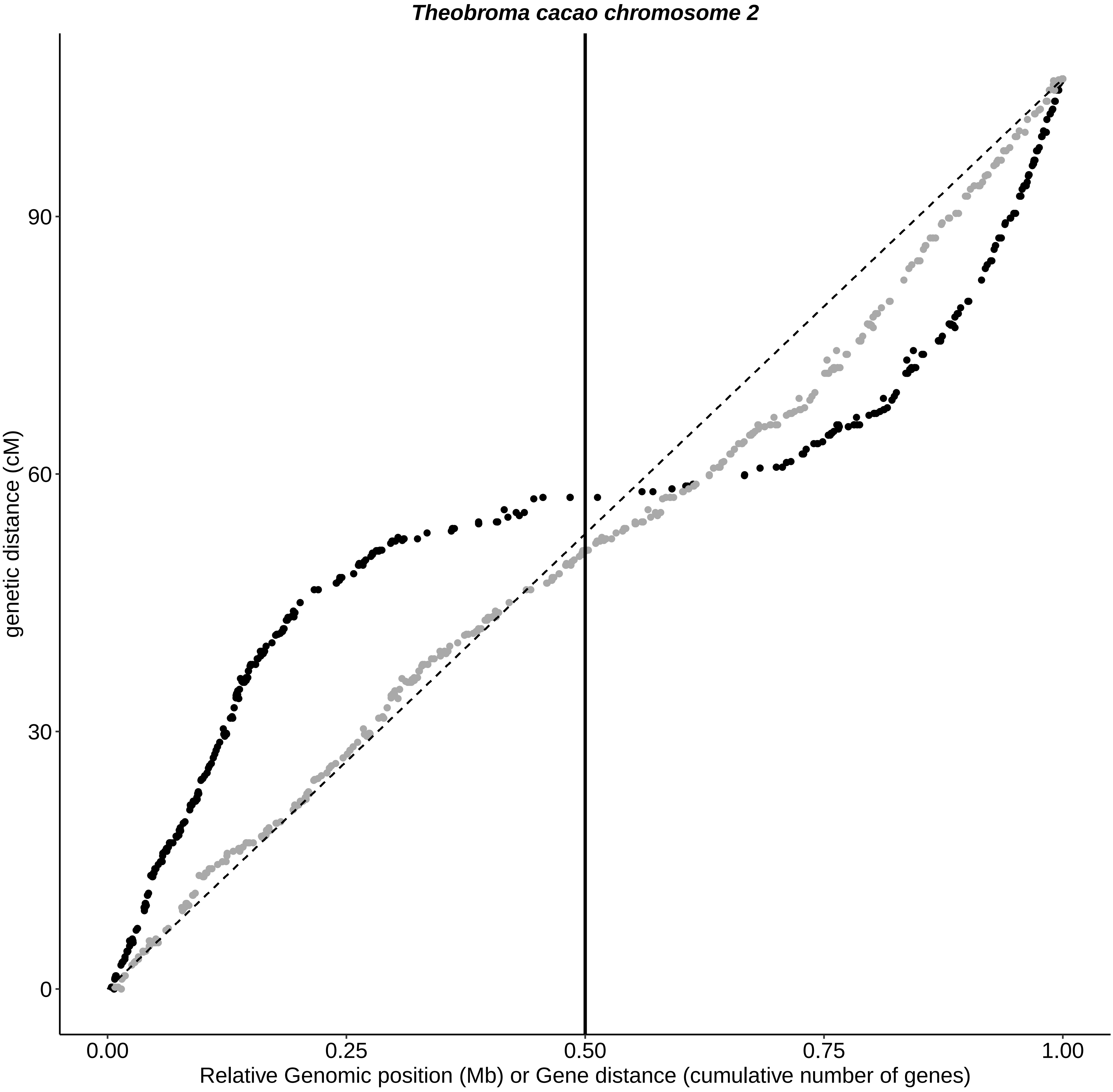
Theobroma cacao chromosome 2
genetic distance (cM)
0
30
60
90
0.00
0.25
0.50
0.75
1.00
Relative Genomic position (Mb) or Gene distance (cumulative number of genes)

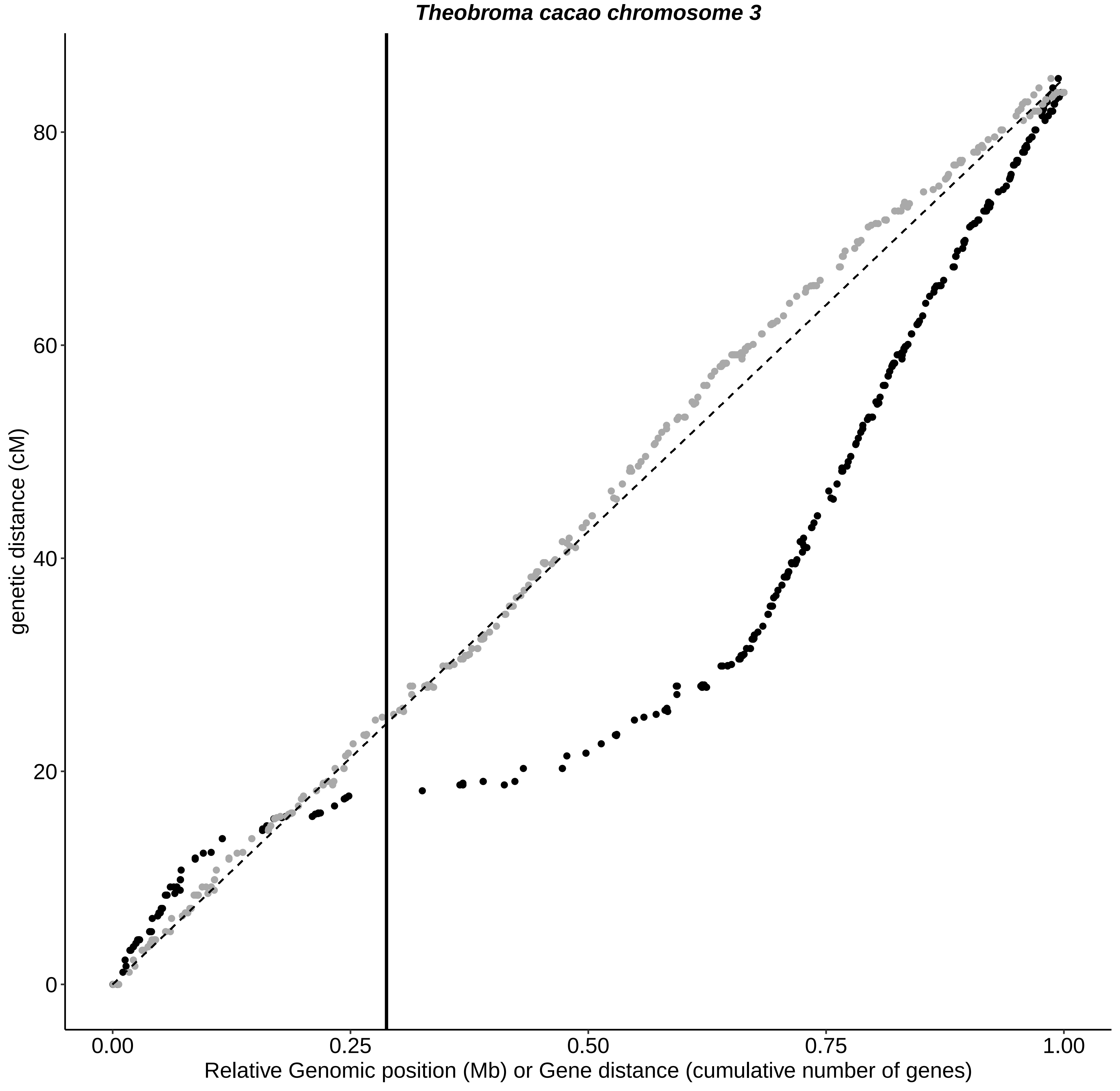
Theobroma cacao chromosome 3
genetic distance (cM)
0
20
40
60
80
0.00
0.25
0.50
0.75
1.00
Relative Genomic position (Mb) or Gene distance (cumulative number of genes)

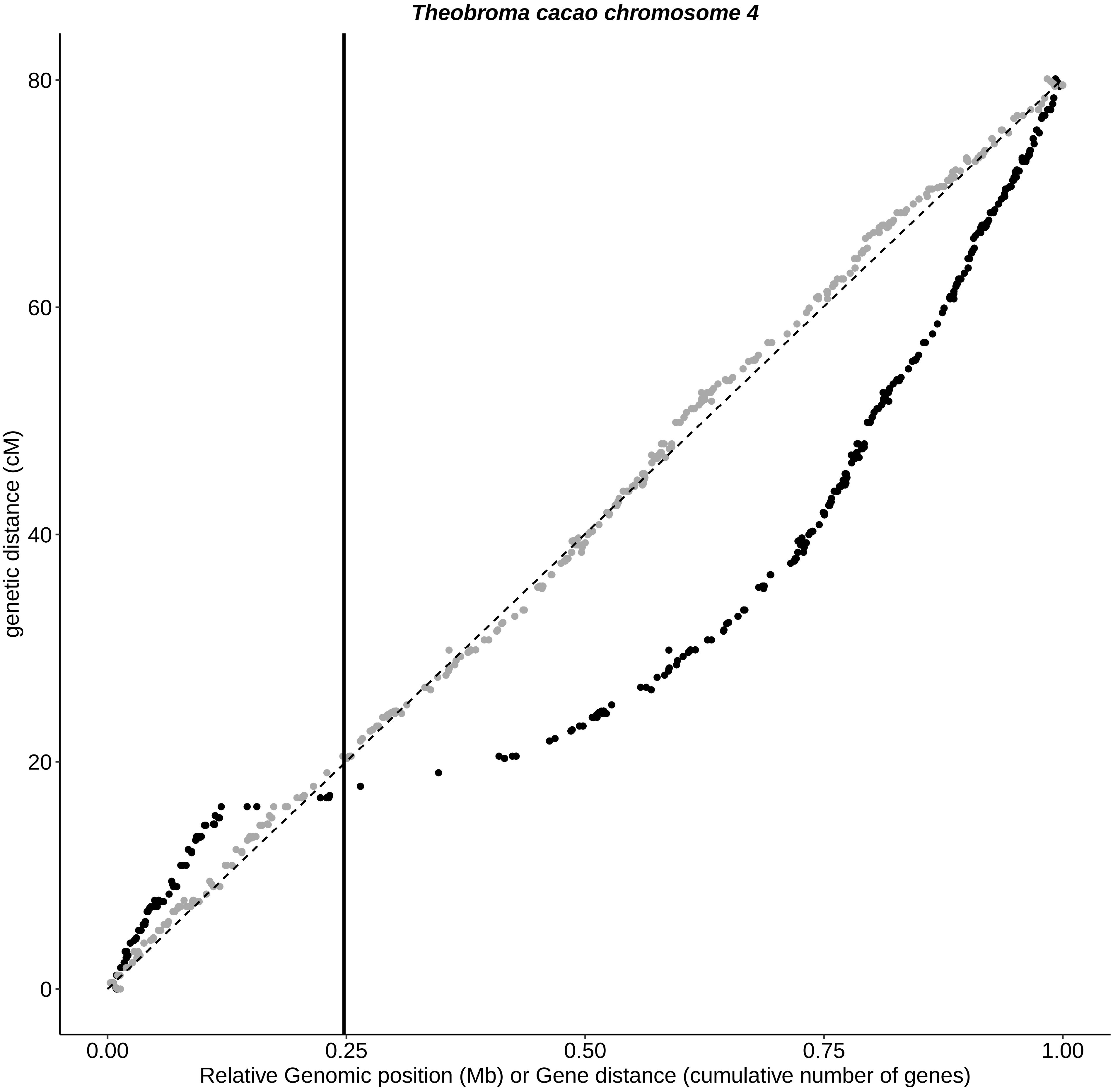
Theobroma cacao chromosome 4
genetic distance (cM)
0
20
40
60
80
0.00
0.25
0.50
0.75
1.00
Relative Genomic position (Mb) or Gene distance (cumulative number of genes)

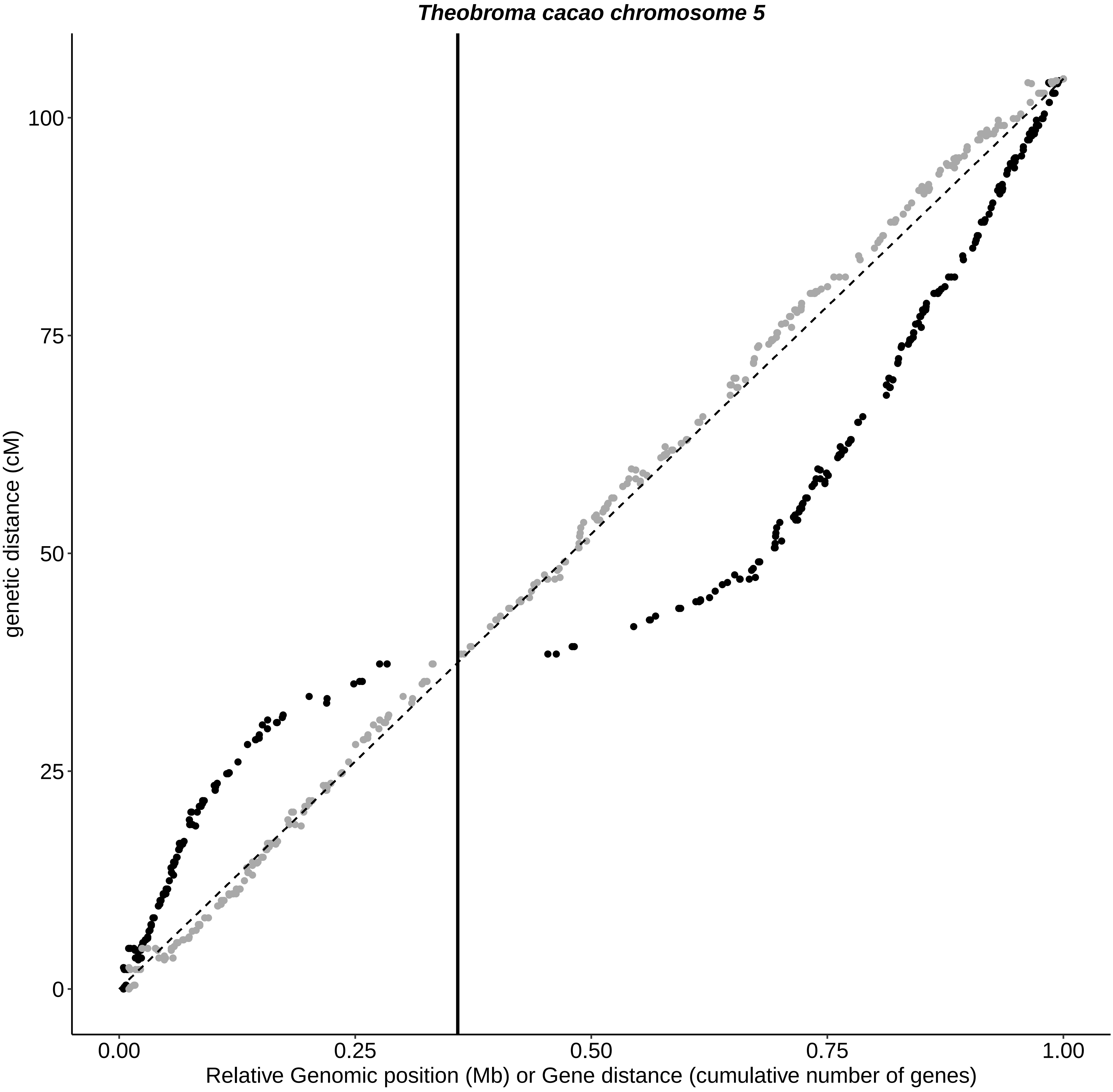
Theobroma cacao chromosome 5
genetic distance (cM)
100
75
50
25
0
0.00
0.25
0.50
0.75
1.00
Relative Genomic position (Mb) or Gene distance (cumulative number of genes)

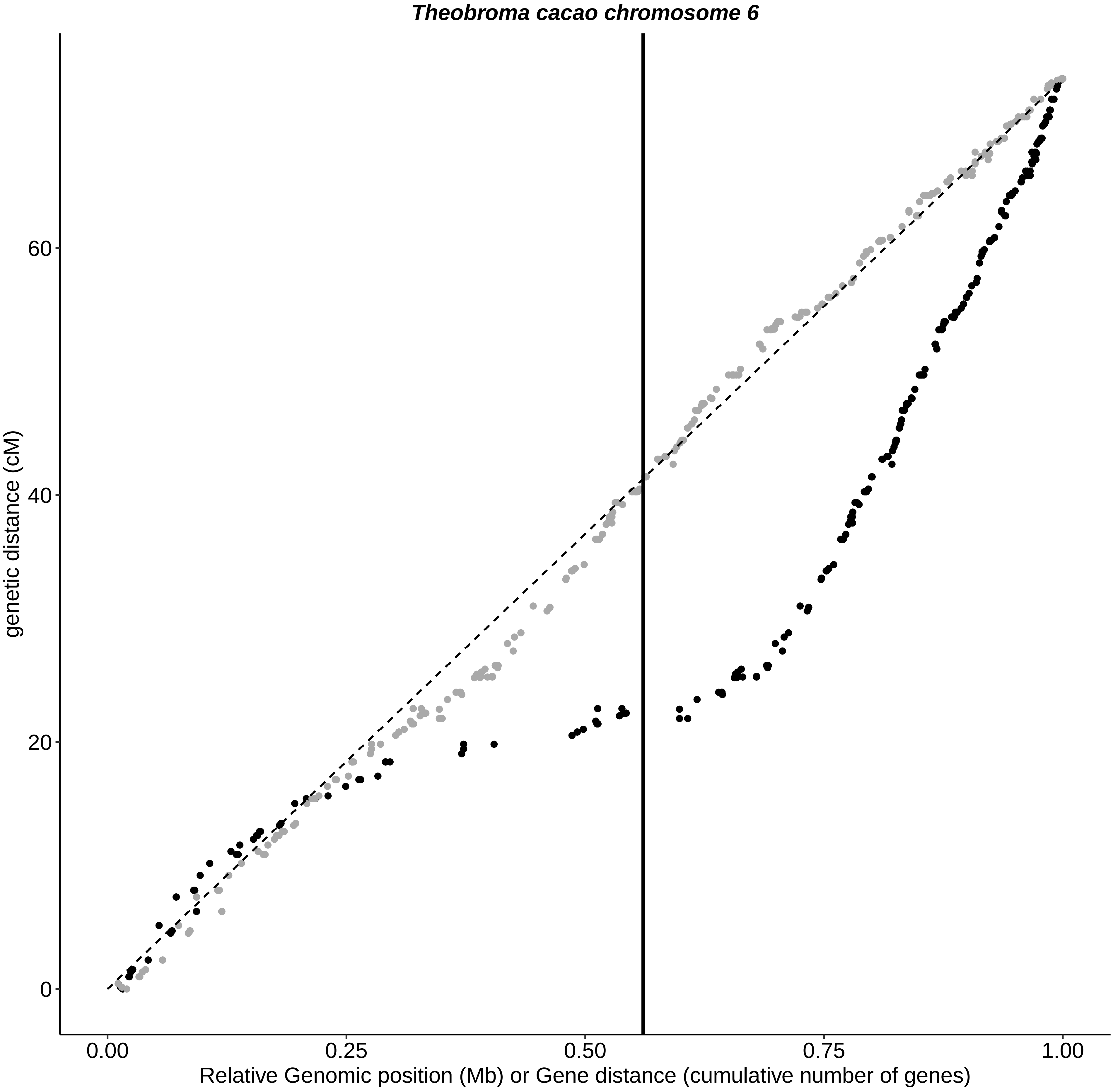
Theobroma cacao chromosome 6
genetic distance (cM)
60
40
20
0
0.00
0.25
0.50
0.75
1.00
Relative Genomic position (Mb) or Gene distance (cumulative number of genes)

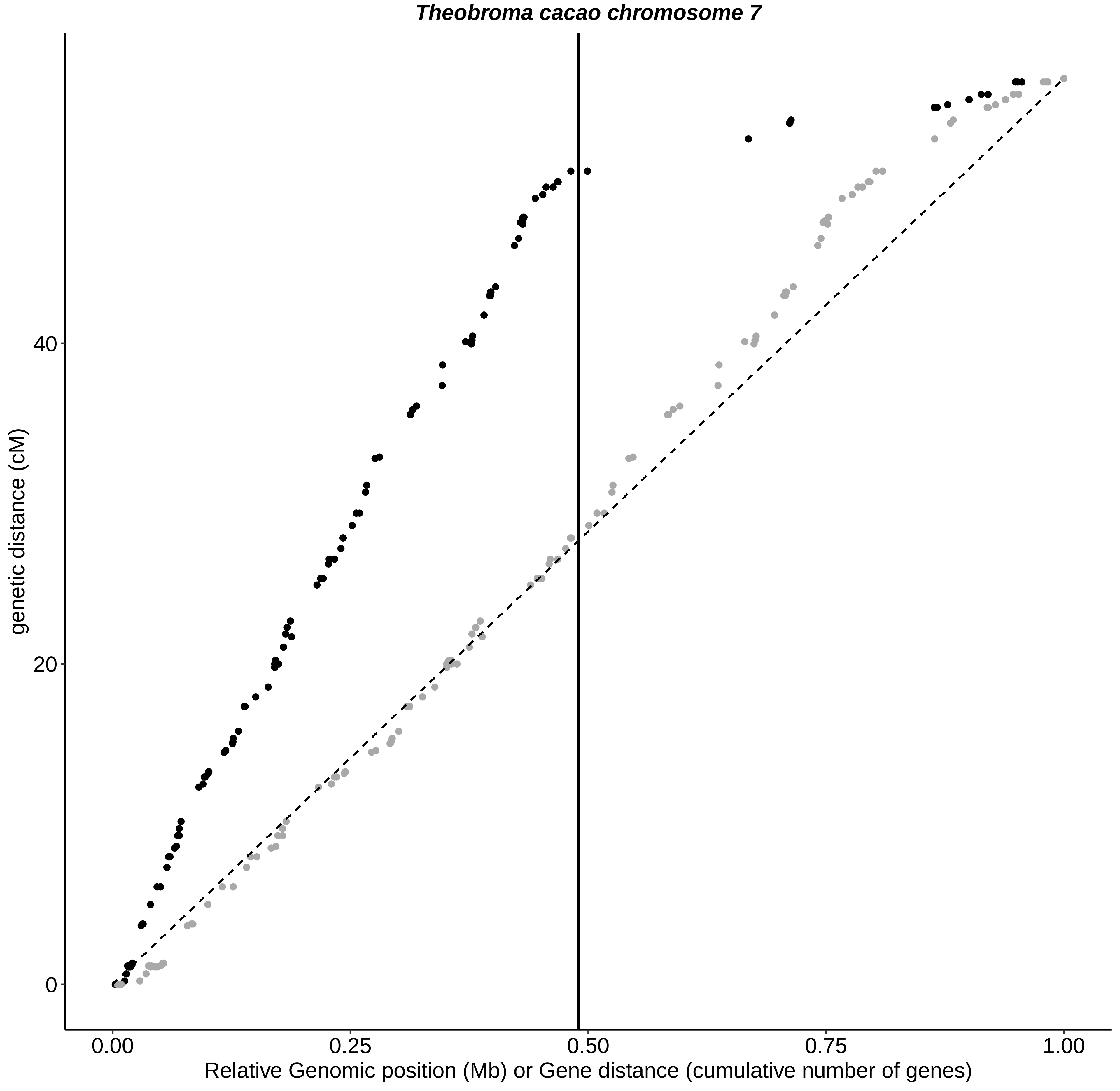
Theobroma cacao chromosome 7
genetic distance (cM)
0
20
40
0.00
0.25
0.50
0.75
1.00
Relative Genomic position (Mb) or Gene distance (cumulative number of genes)

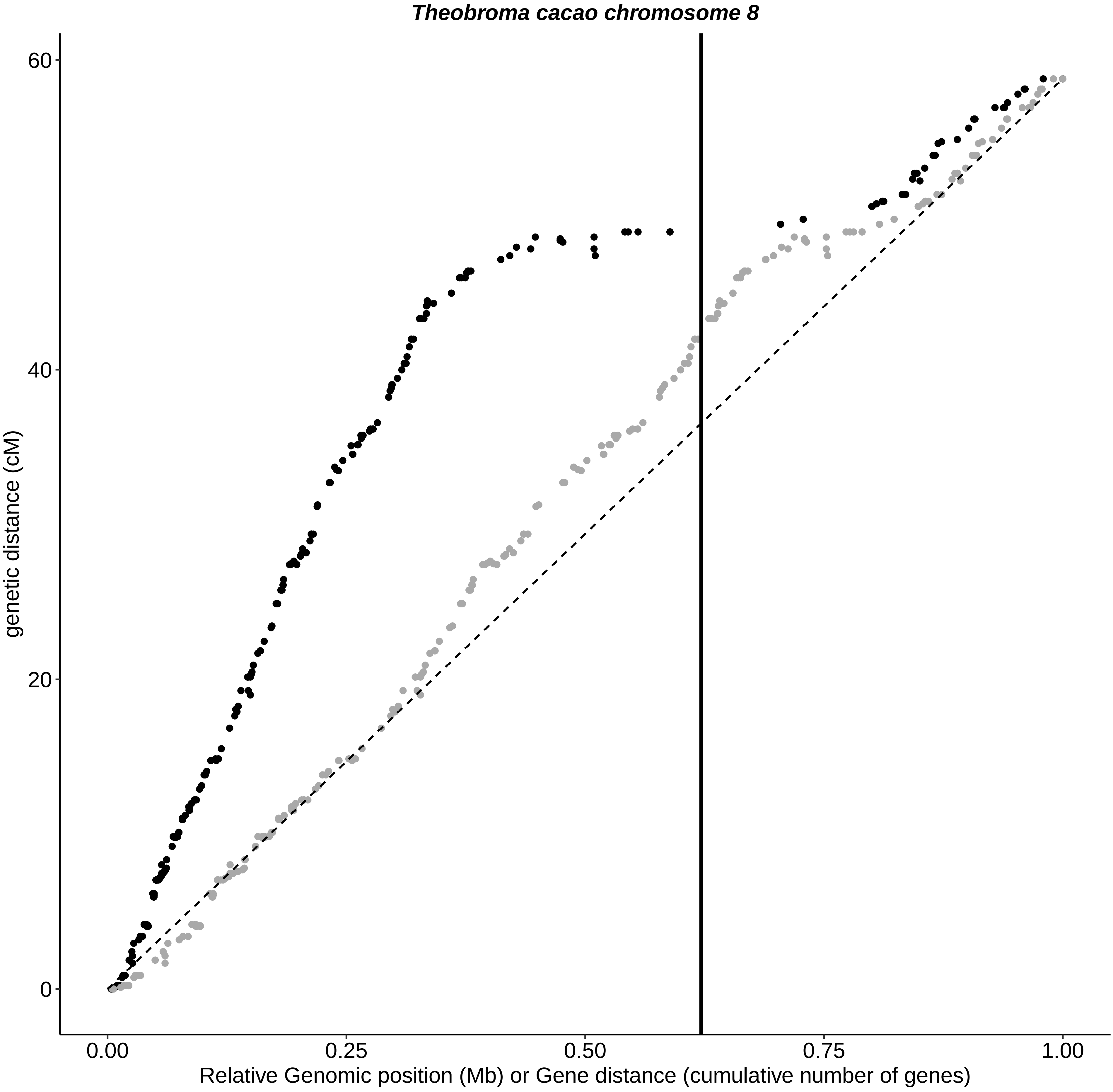

Theobroma cacao chromosome 8
genetic distance (cM)
60
40
20
0
0.00
0.25
0.50
0.75
1.00
Relative Genomic position (Mb) or Gene distance (cumulative number of genes)

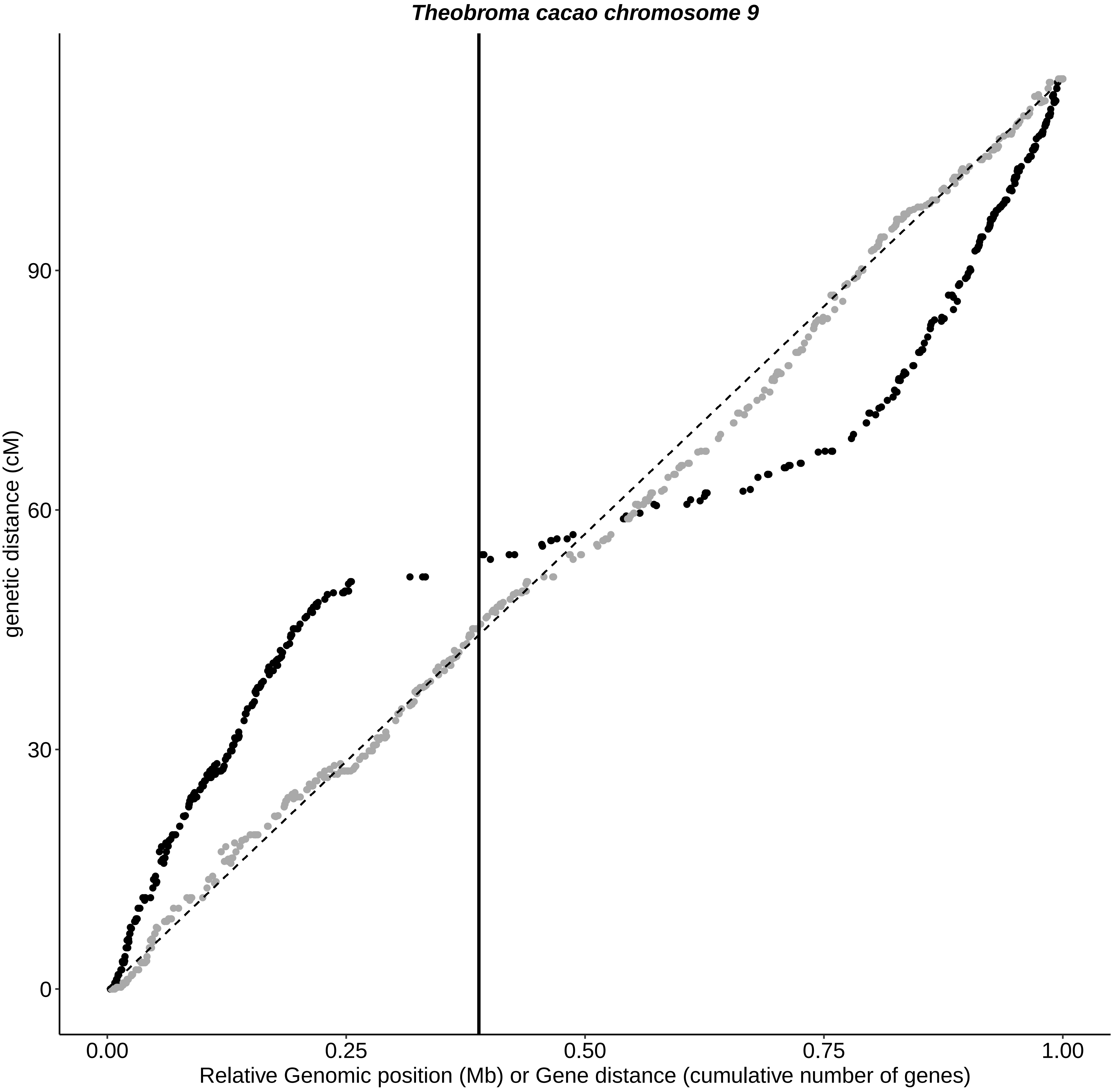

Theobroma cacao chromosome 9
genetic distance (cM)
0
30
60
90
0.00
0.25
0.50
0.75
1.00
Relative Genomic position (Mb) or Gene distance (cumulative number of genes)

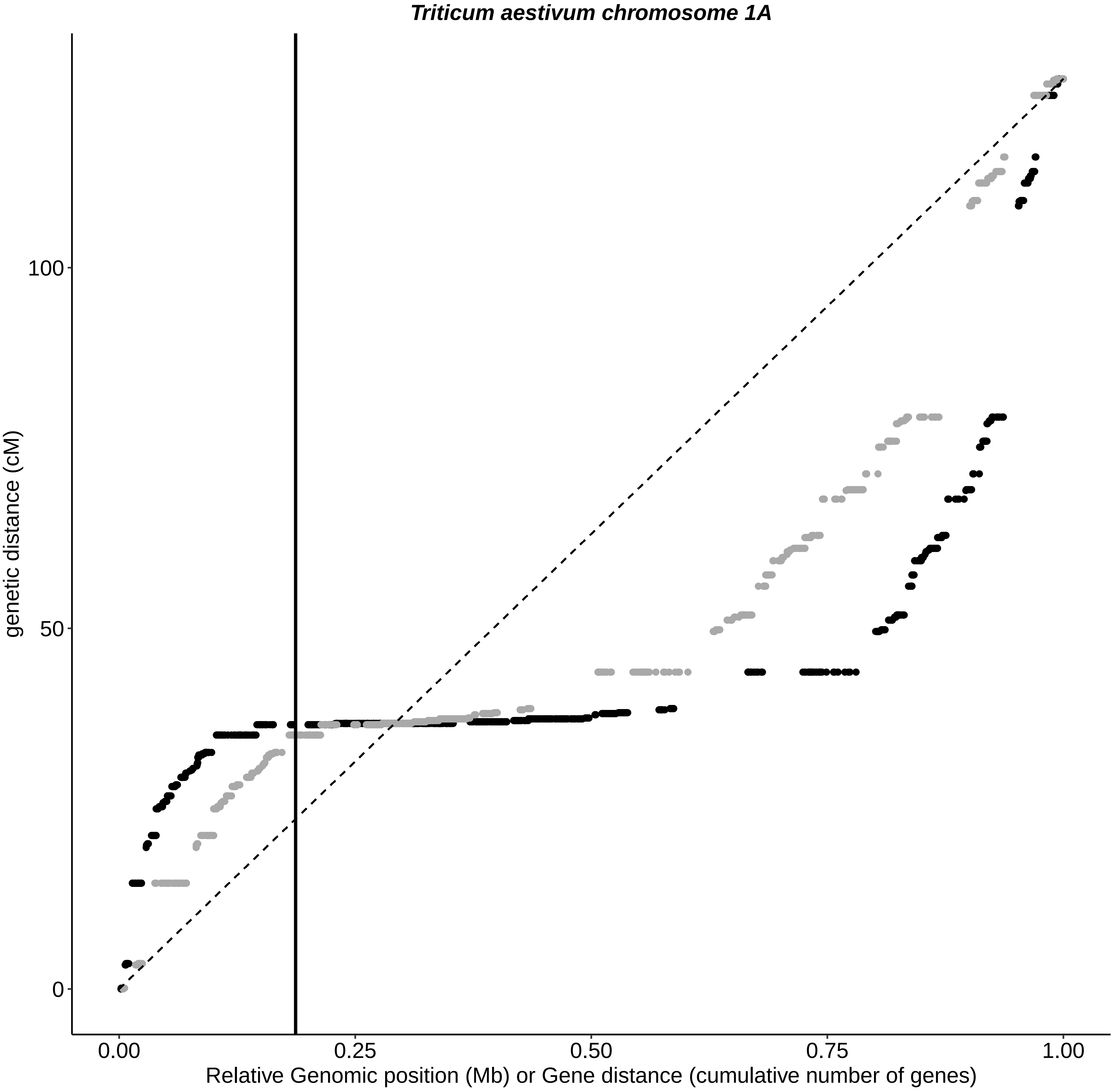
Triticum aestivum chromosome 1A
genetic distance (cM)
100
50
0
0.00
0.25
0.50
0.75
1.00
Relative Genomic position (Mb) or Gene distance (cumulative number of genes)

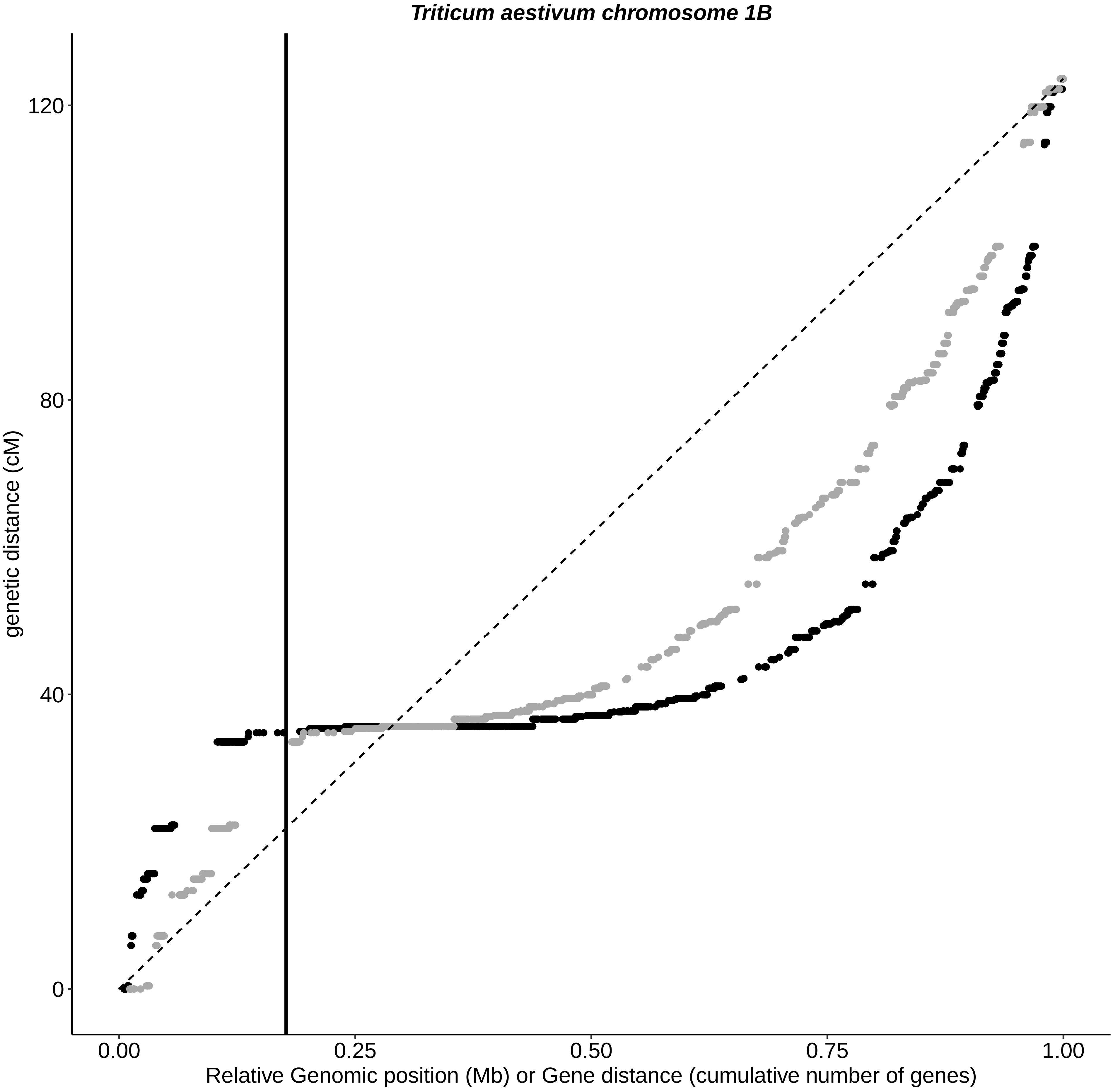
Triticum aestivum chromosome 1B
genetic distance (cM)
0
40
80
120
0.00
0.25
0.50
0.75
1.00
Relative Genomic position (Mb) or Gene distance (cumulative number of genes)

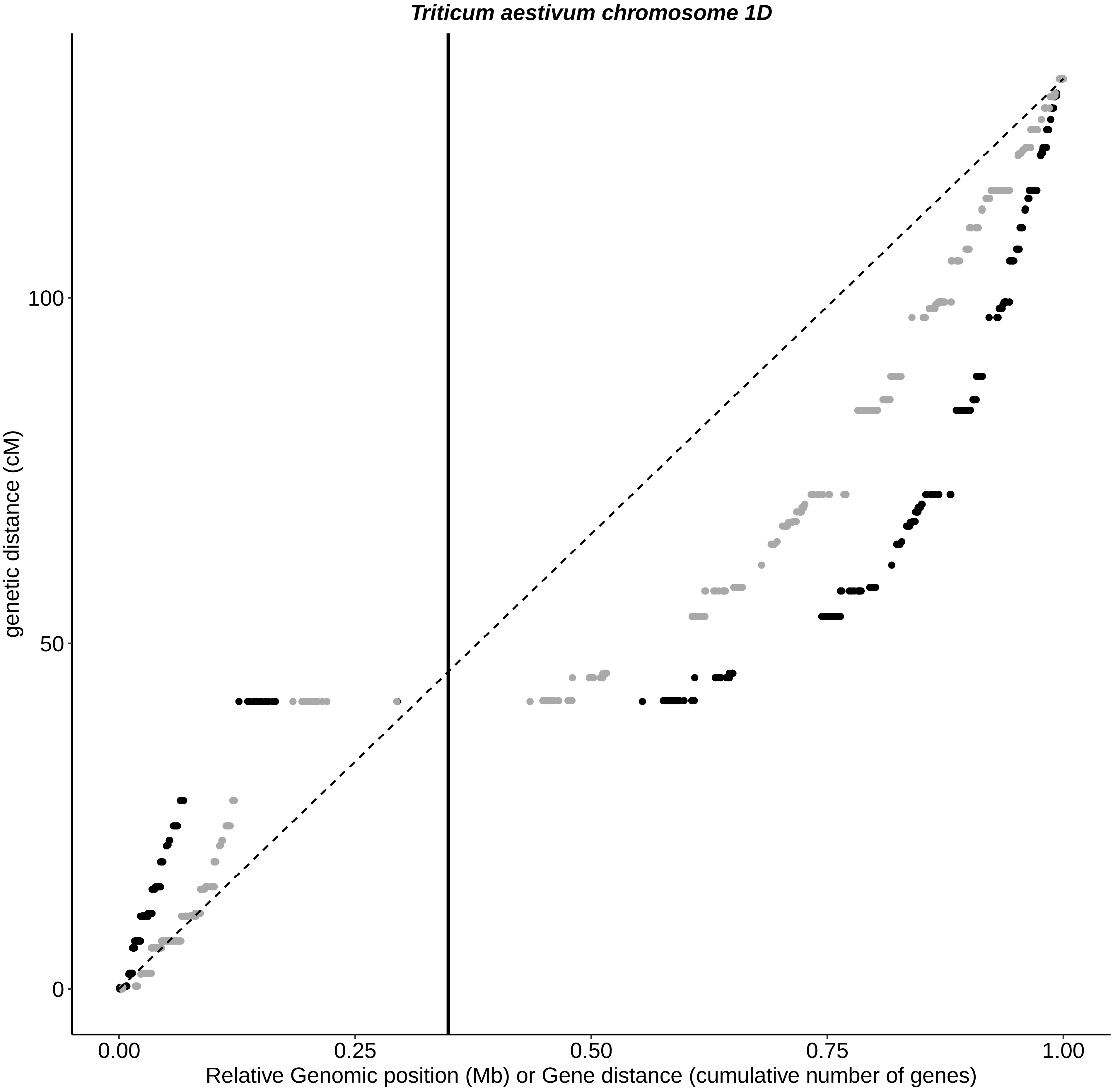
Triticum aestivum chromosome 1D
genetic distance (cM)
100
50
0
0.00
0.25
0.50
0.75
1.00
Relative Genomic position (Mb) or Gene distance (cumulative number of genes)

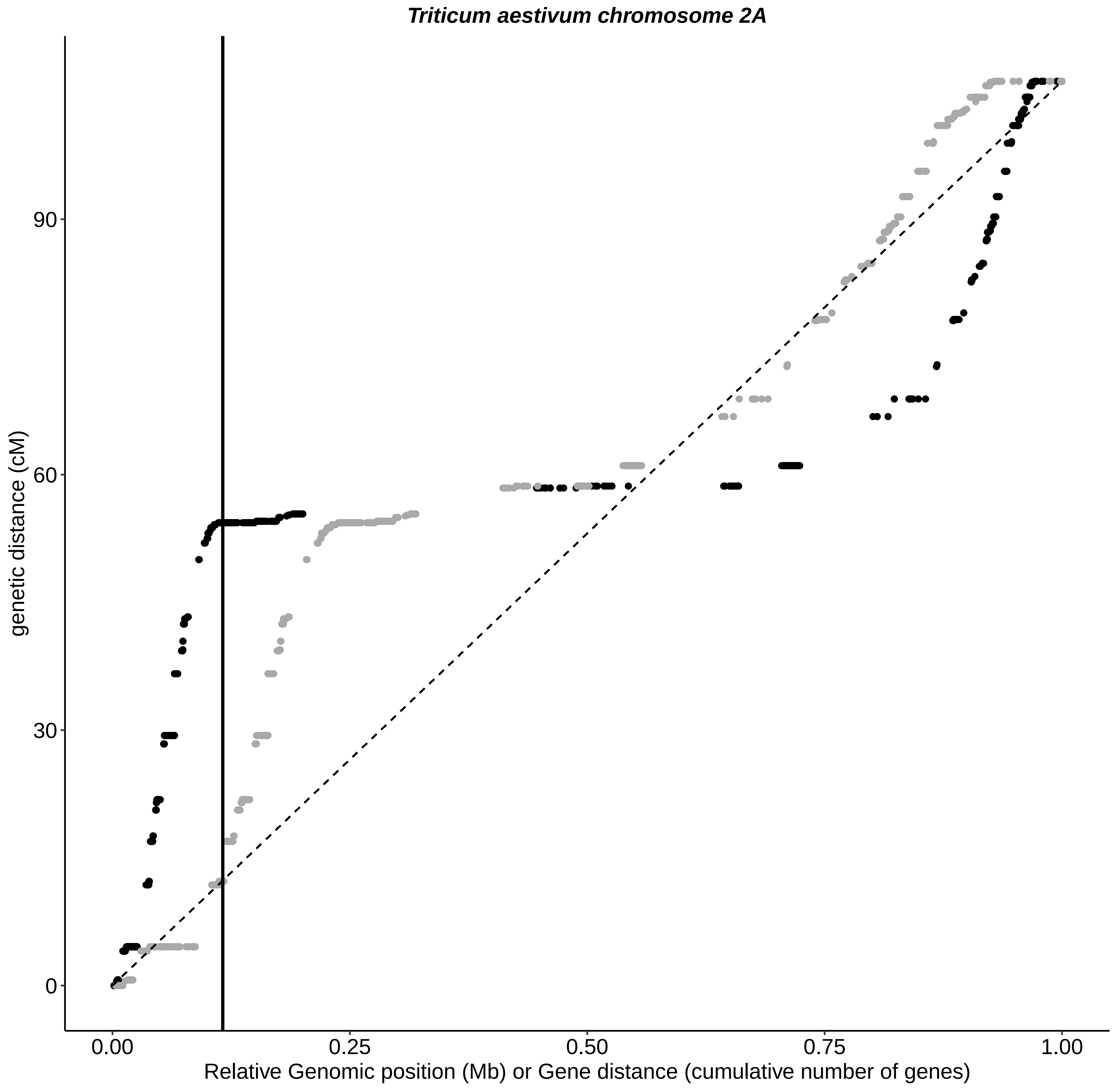
Triticum aestivum chromosome 2A
genetic distance (cM)
0
30
60
90
0.00
0.25
0.50
0.75
1.00
Relative Genomic position (Mb) or Gene distance (cumulative number of genes)

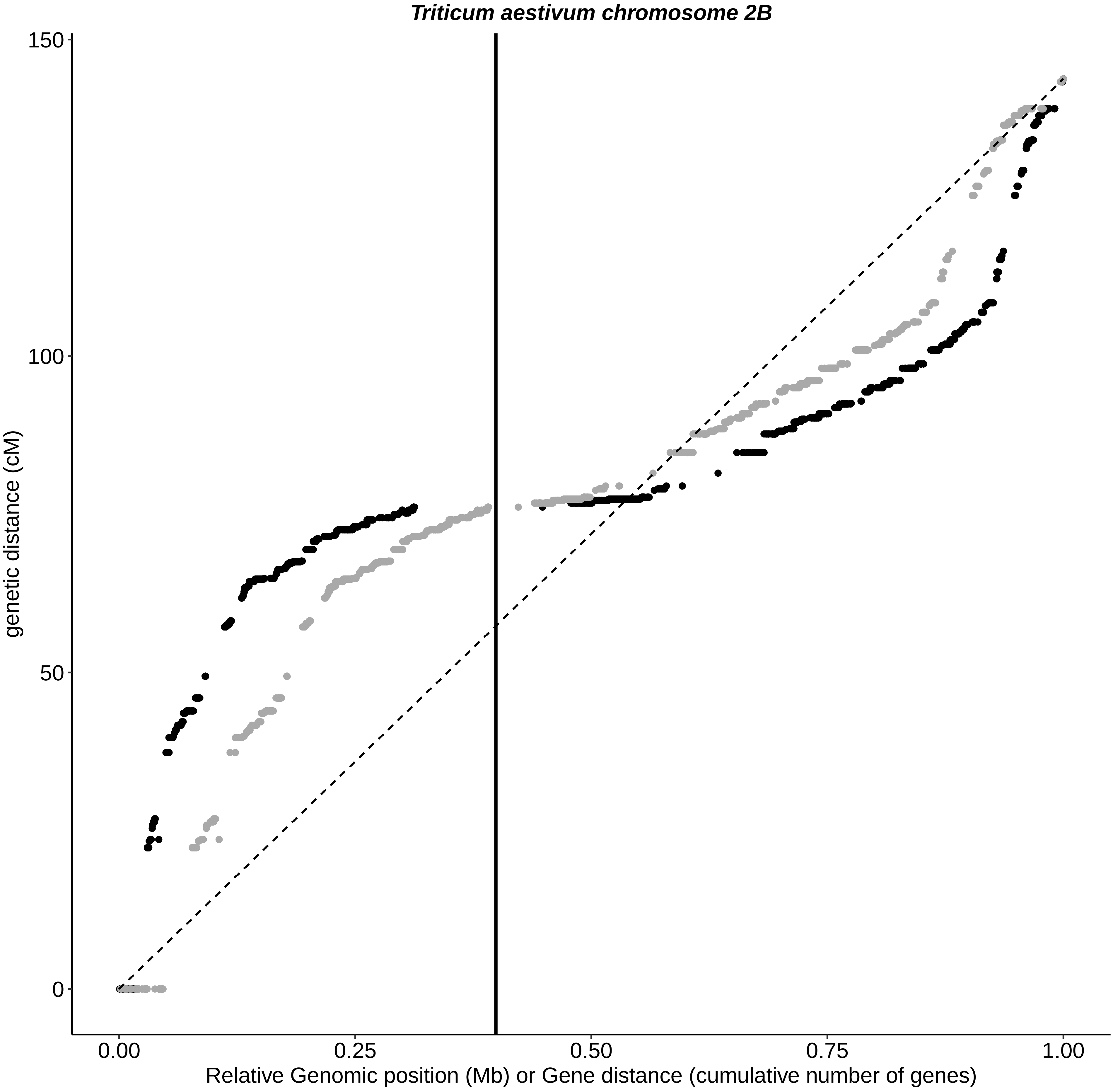

Triticum aestivum chromosome 2B
genetic distance (cM)
150
100
50
0
0.00
0.25
0.50
0.75
1.00
Relative Genomic position (Mb) or Gene distance (cumulative number of genes)

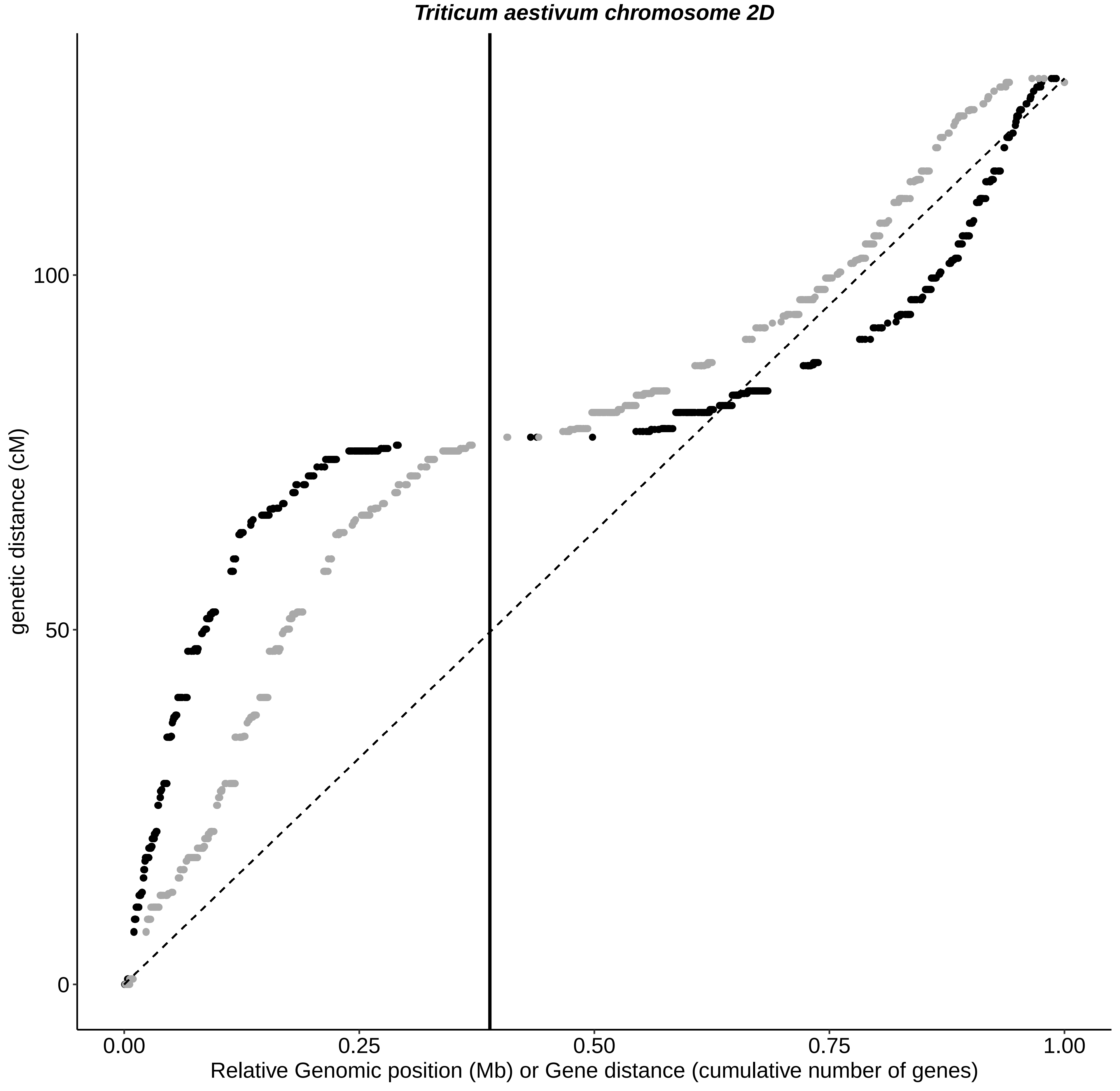

Triticum aestivum chromosome 2D
genetic distance (cM)
100
50
0
0.00
0.25
0.50
0.75
1.00
Relative Genomic position (Mb) or Gene distance (cumulative number of genes)

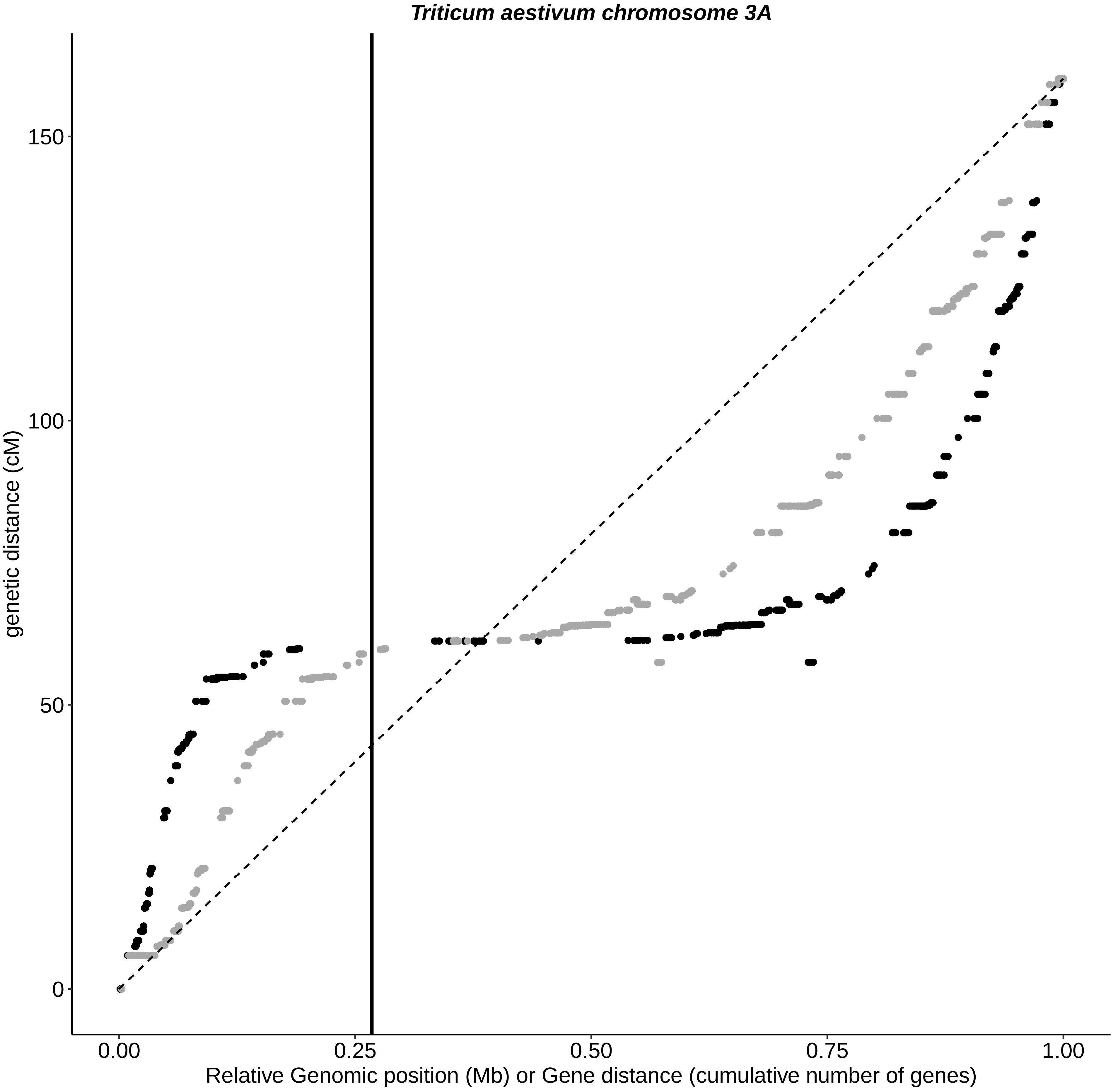
Triticum aestivum chromosome 3A
genetic distance (cM)
0
50
100
150
0.00
0.25
0.50
0.75
1.00
Relative Genomic position (Mb) or Gene distance (cumulative number of genes)

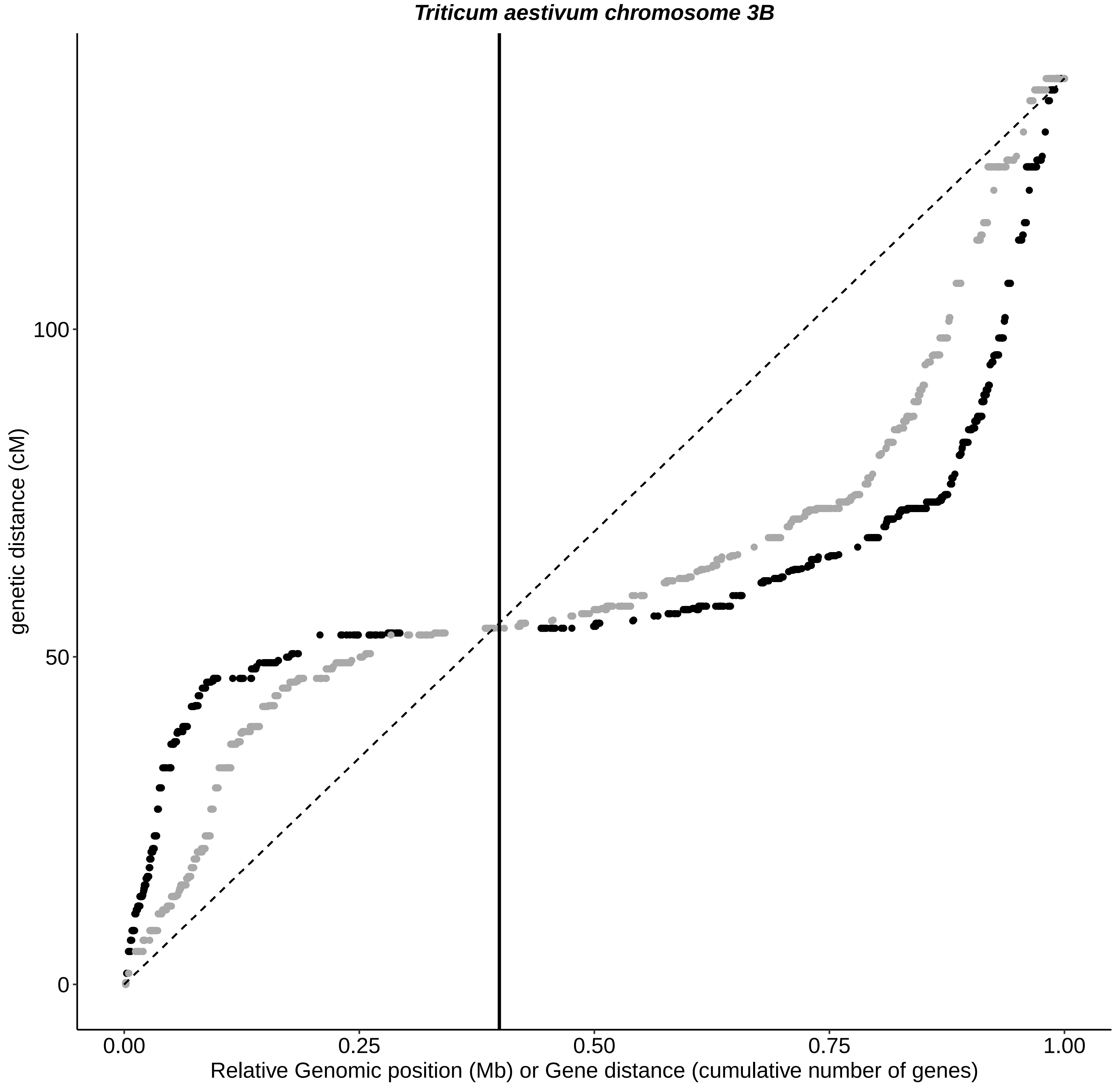
Triticum aestivum chromosome 3B
genetic distance (cM)
0
50
100
0.00
0.25
0.50
0.75
1.00
Relative Genomic position (Mb) or Gene distance (cumulative number of genes)

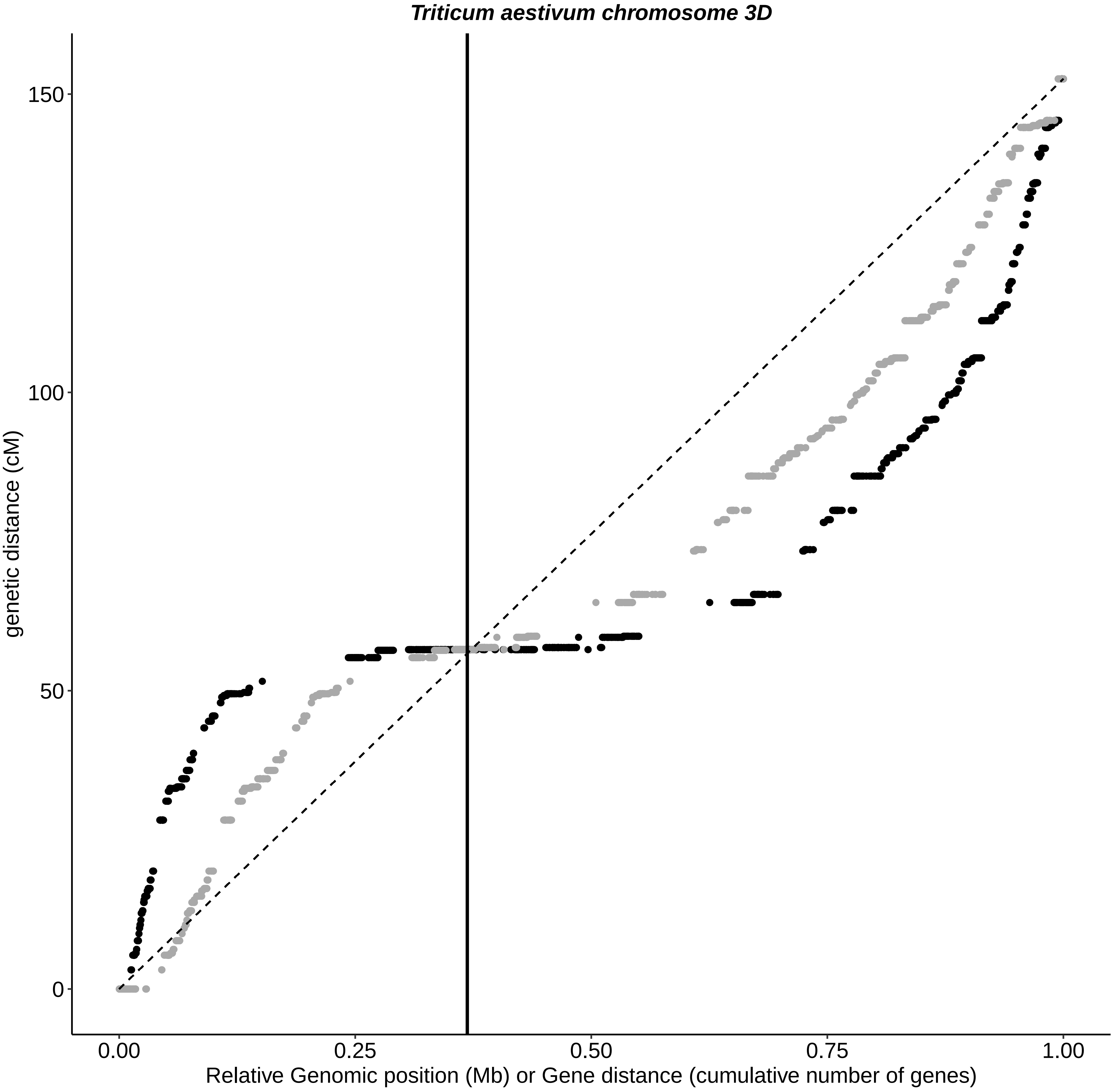
Triticum aestivum chromosome 3D
genetic distance (cM)
150
100
50
0
0.00
0.25
0.50
0.75
1.00
Relative Genomic position (Mb) or Gene distance (cumulative number of genes)

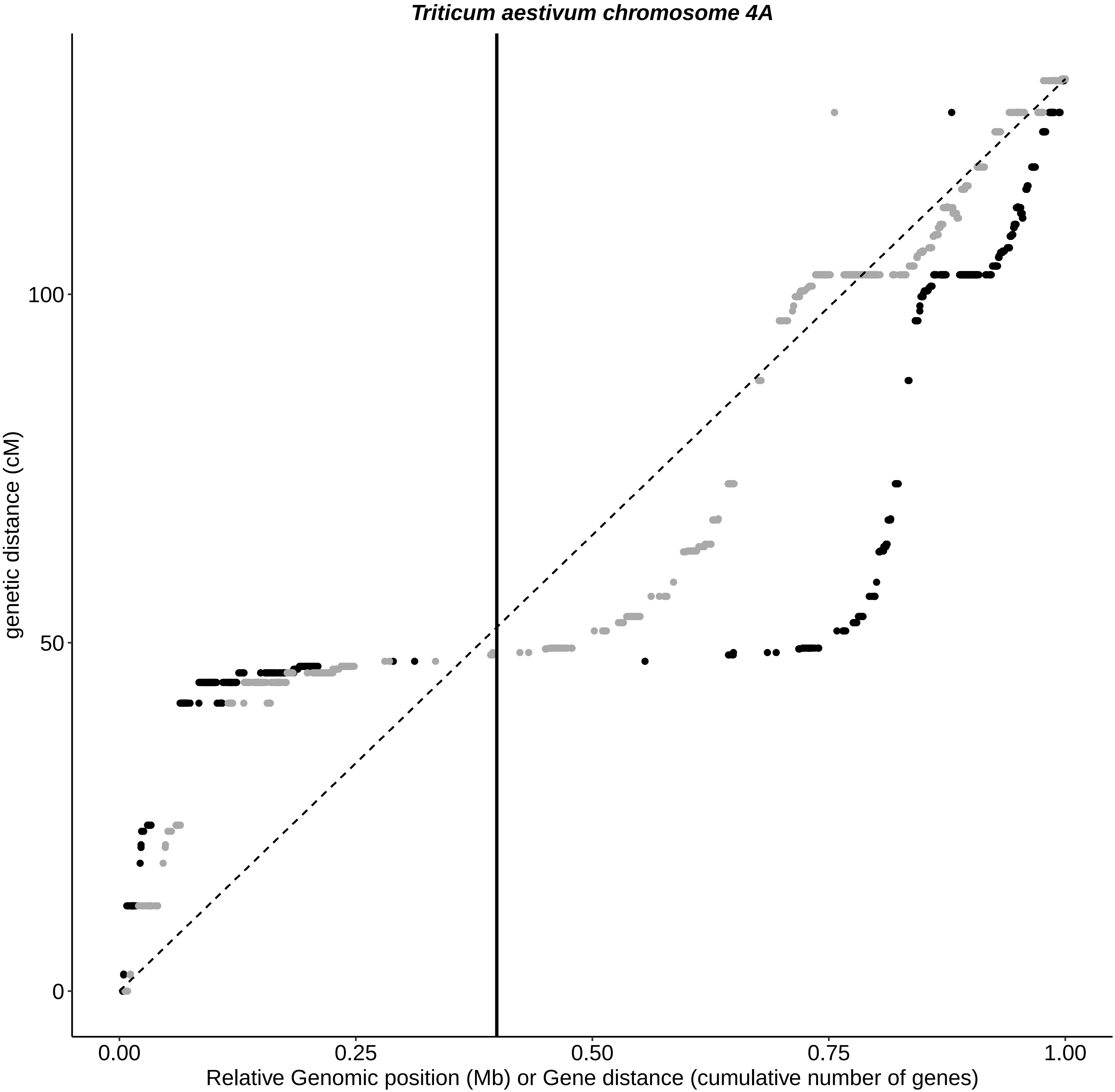
Triticum aestivum chromosome 4A
genetic distance (cM)
100
50
0
0.00
0.25
0.50
0.75
1.00
Relative Genomic position (Mb) or Gene distance (cumulative number of genes)

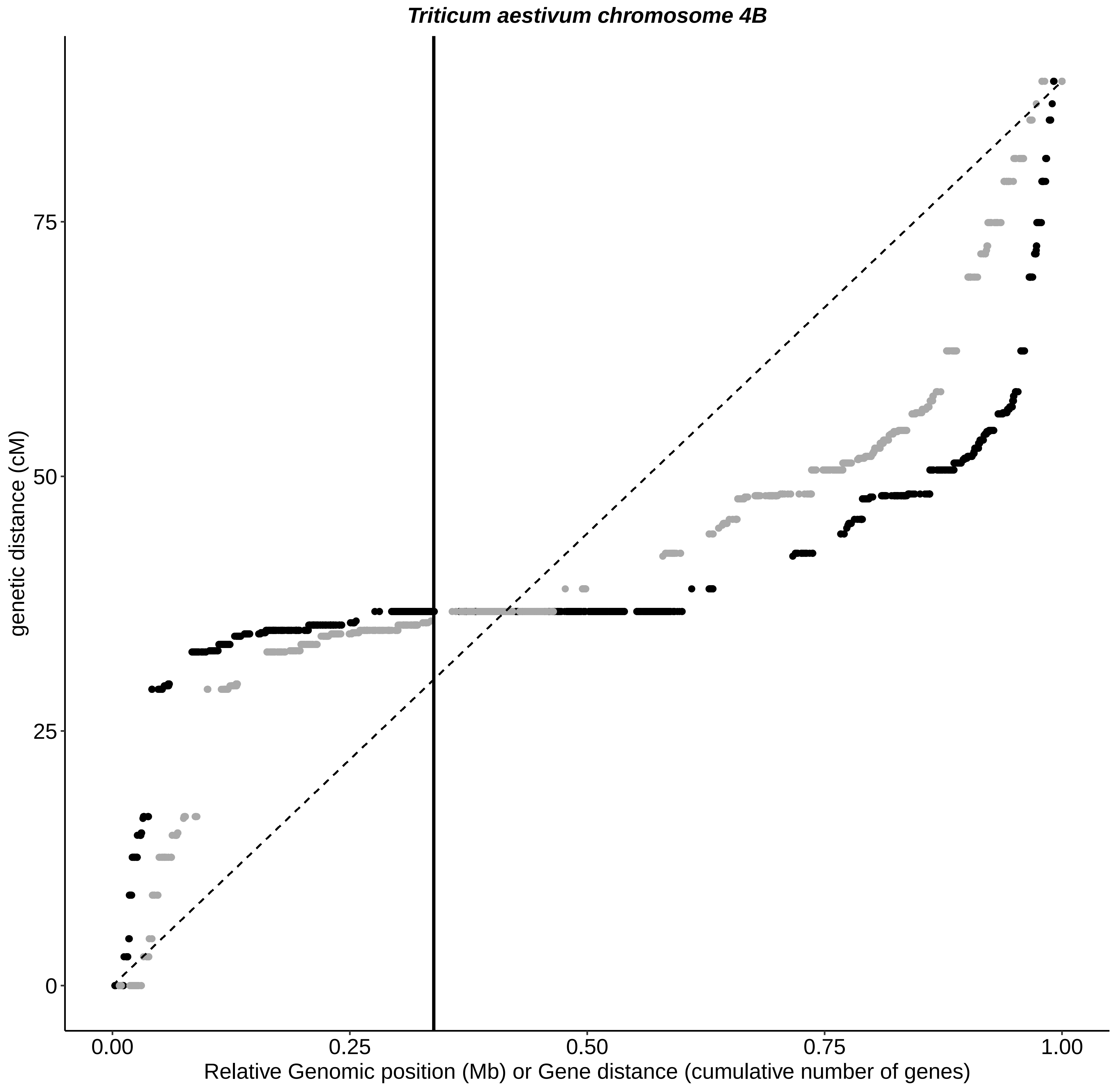

Triticum aestivum chromosome 4B
genetic distance (cM)
0
25
50
75
0.00
0.25
0.50
0.75
1.00
Relative Genomic position (Mb) or Gene distance (cumulative number of genes)

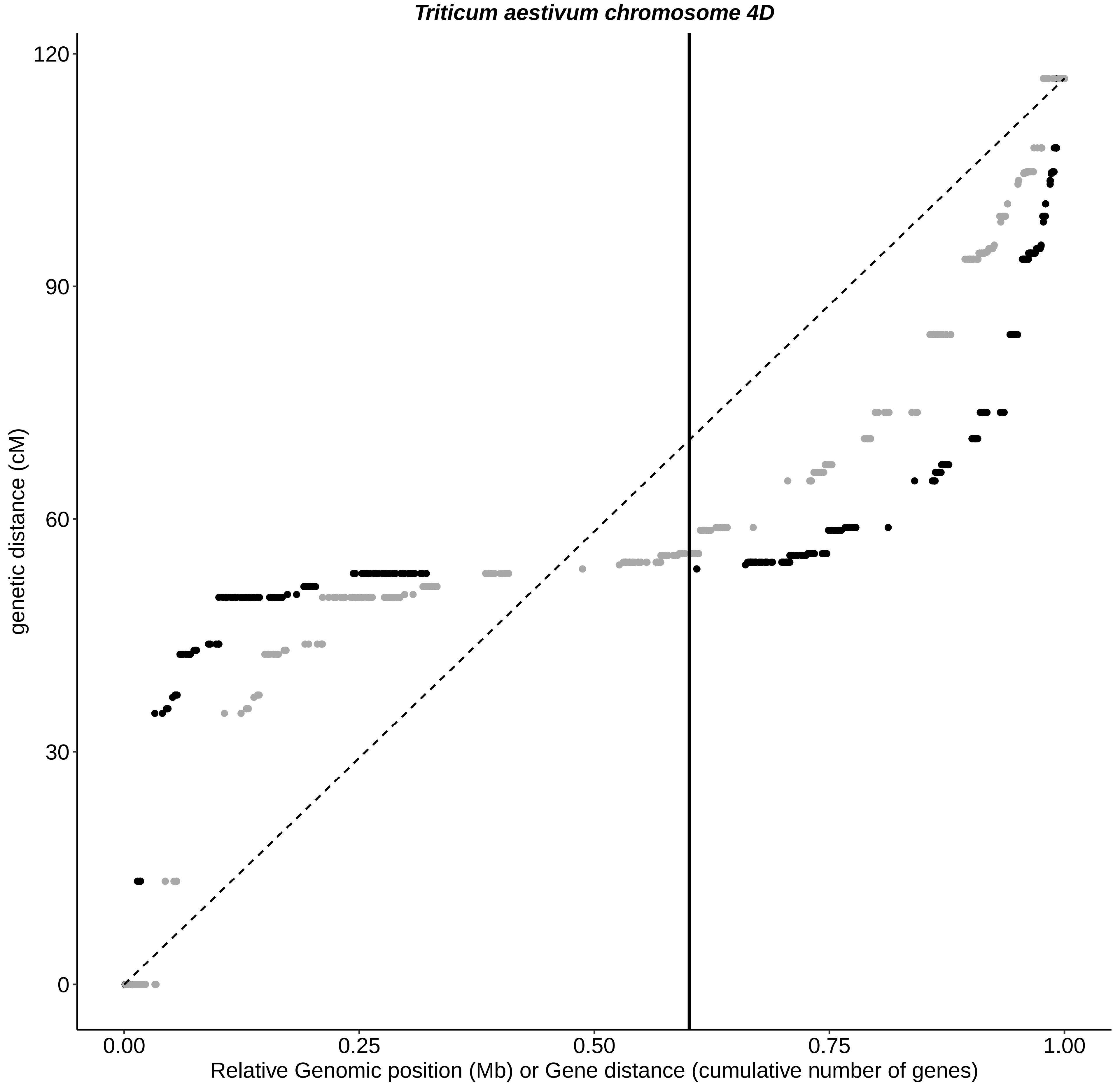
Triticum aestivum chromosome 4D
genetic distance (cM)
0
30
60
90
120
0.00
0.25
0.50
0.75
1.00
Relative Genomic position (Mb) or Gene distance (cumulative number of genes)

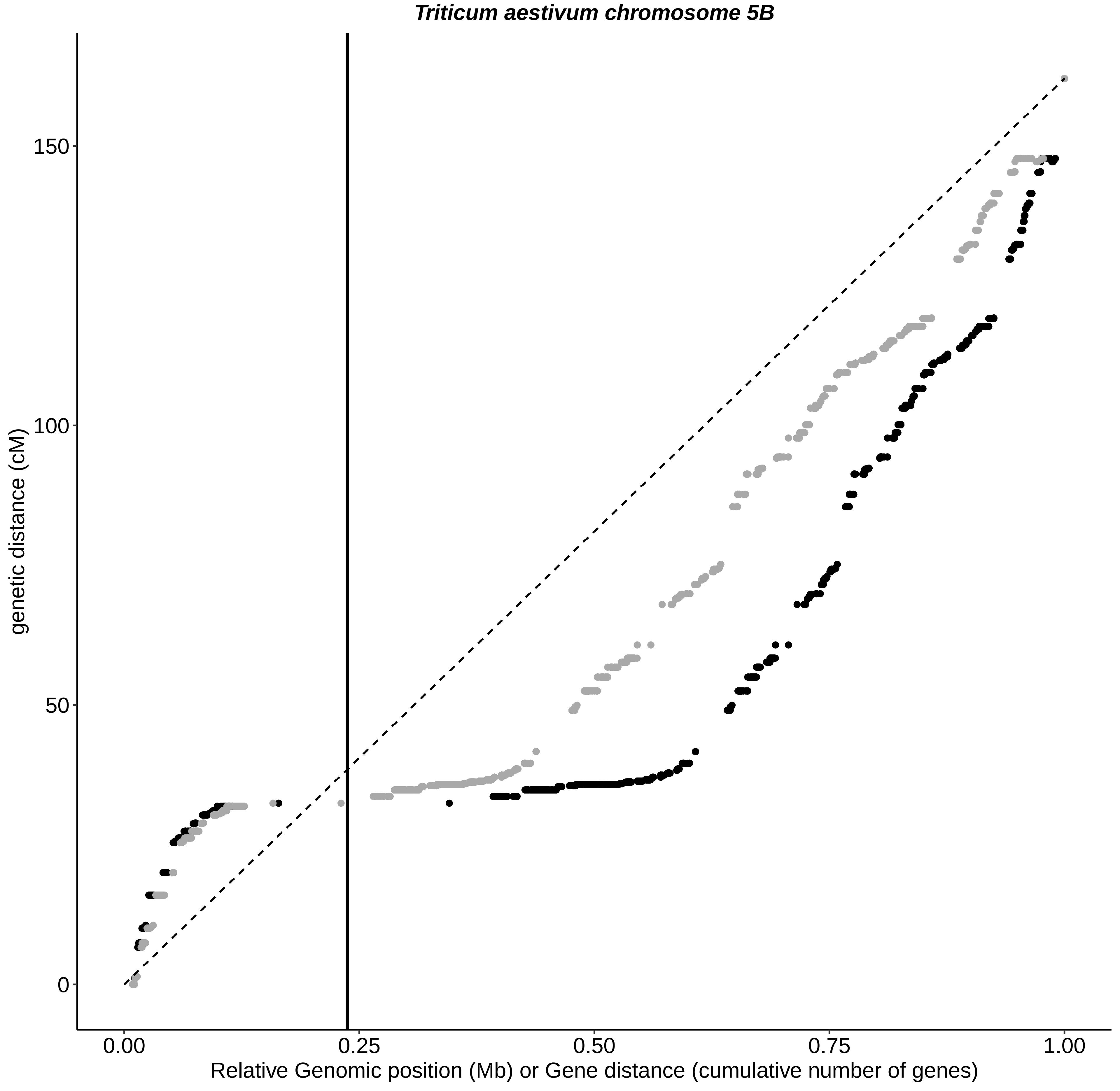
Triticum aestivum chromosome 5B
genetic distance (cM)
150
100
50
0
0.00
0.25
0.50
0.75
1.00
Relative Genomic position (Mb) or Gene distance (cumulative number of genes)

Triticum aestivum chromosome 5D

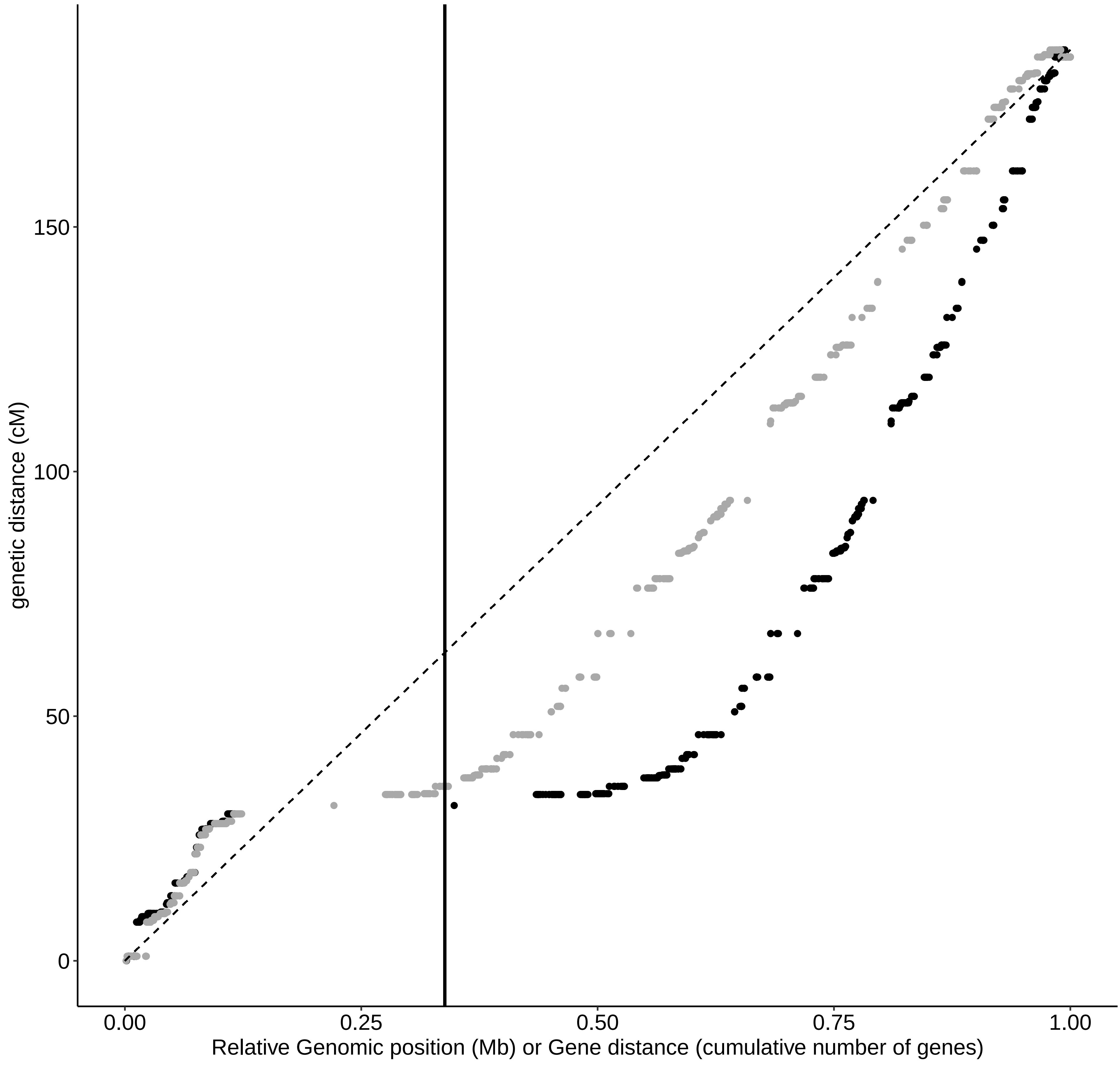

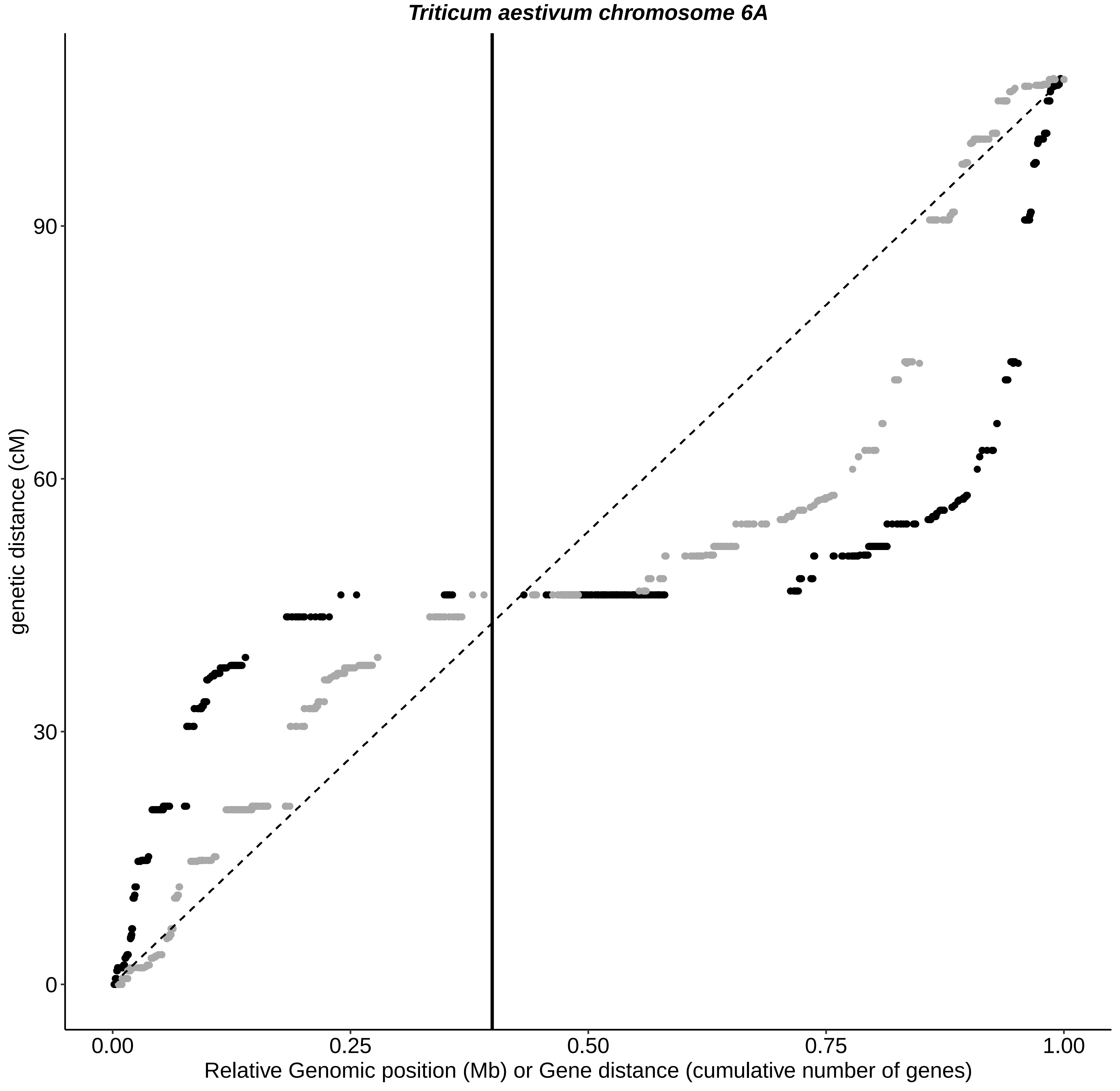
Triticum aestivum chromosome 6A
genetic distance (cM)
0
30
60
90
0.00
0.25
0.50
0.75
1.00
Relative Genomic position (Mb) or Gene distance (cumulative number of genes)

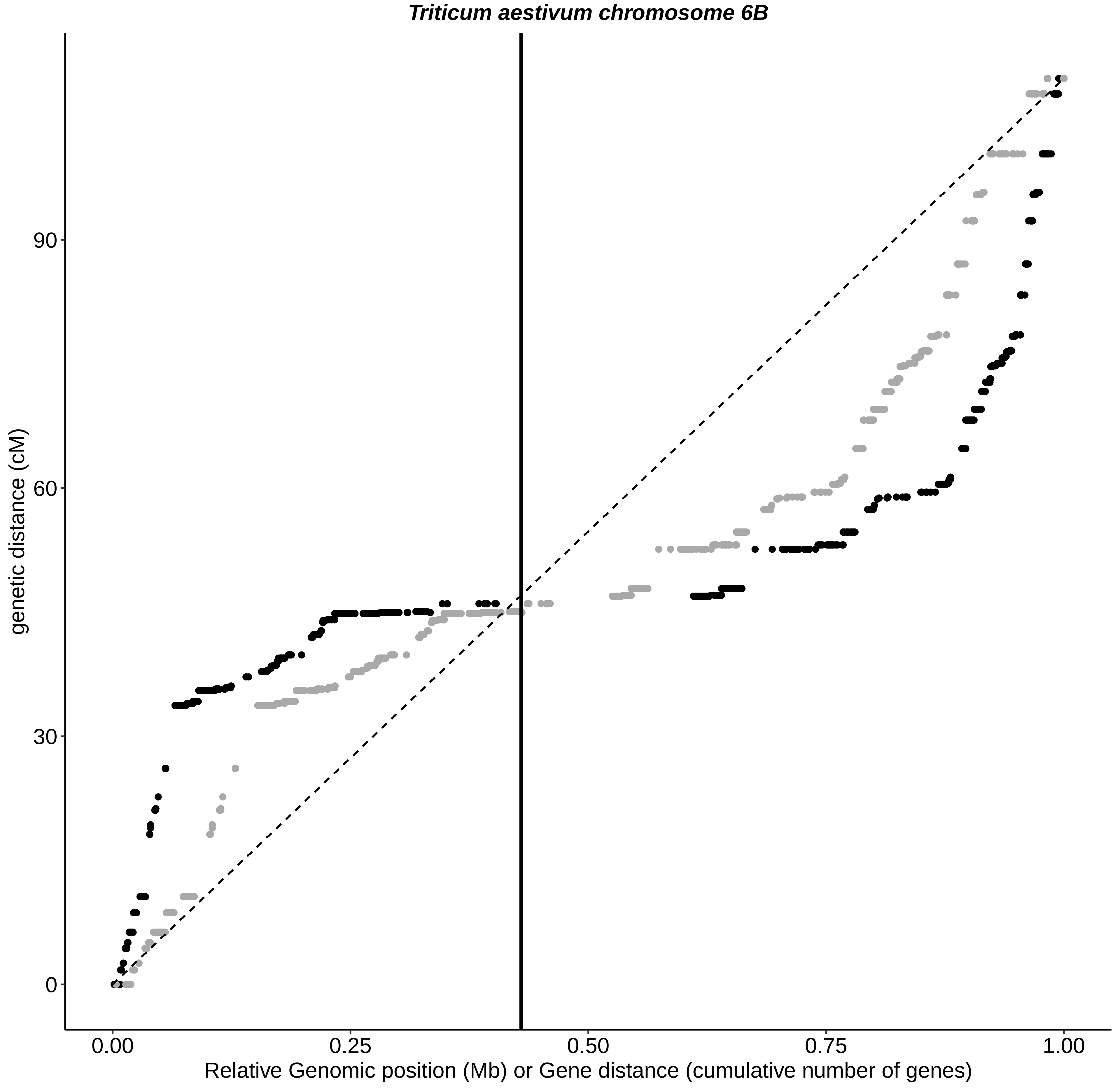
Triticum aestivum chromosome 6B
genetic distance (cM)
0
30
60
90
0.00
0.25
0.50
0.75
1.00
Relative Genomic position (Mb) or Gene distance (cumulative number of genes)

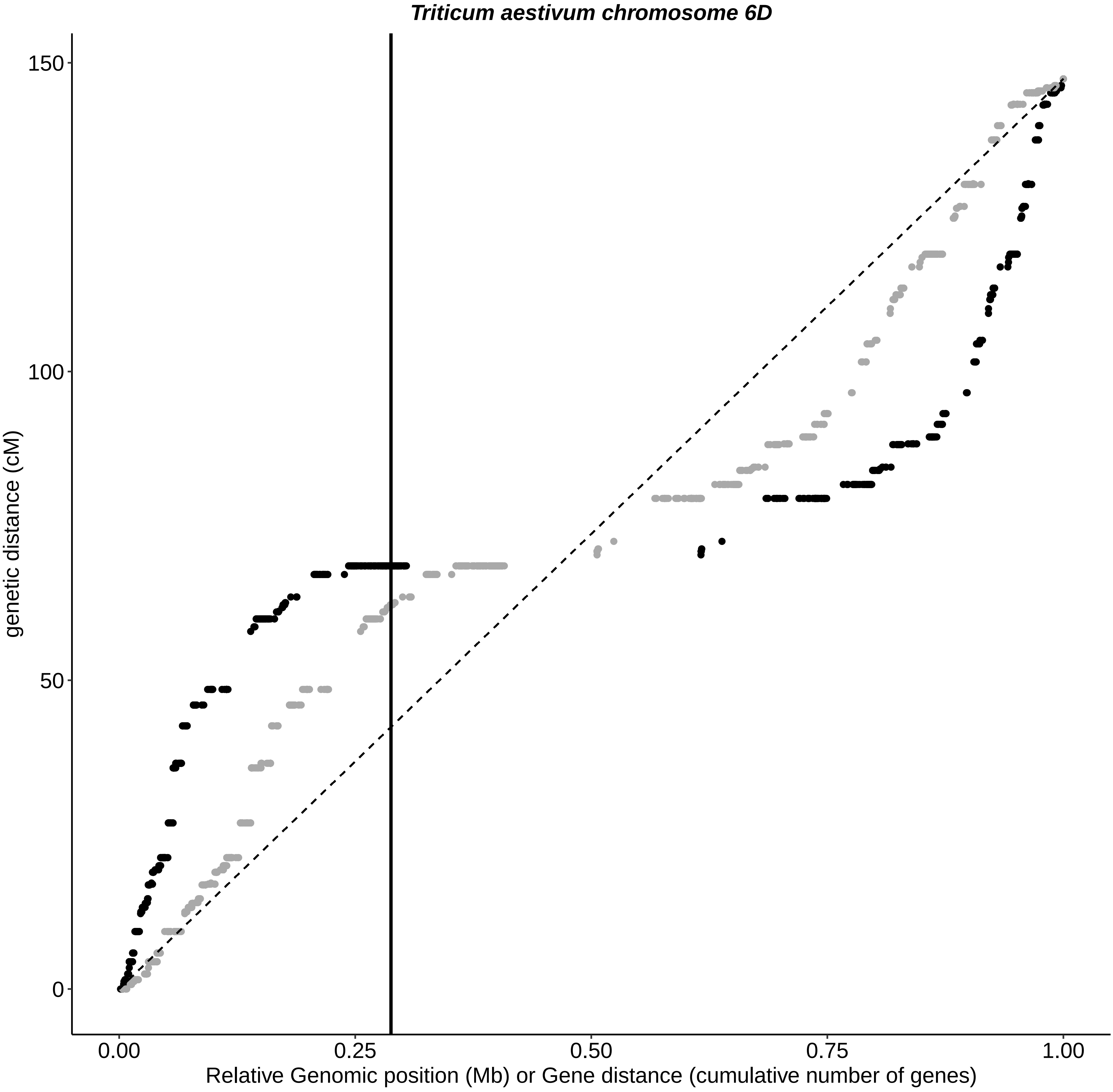
Triticum aestivum chromosome 6D
genetic distance (cM)
150
100
50
0
0.00
0.25
0.50
0.75
1.00
Relative Genomic position (Mb) or Gene distance (cumulative number of genes)

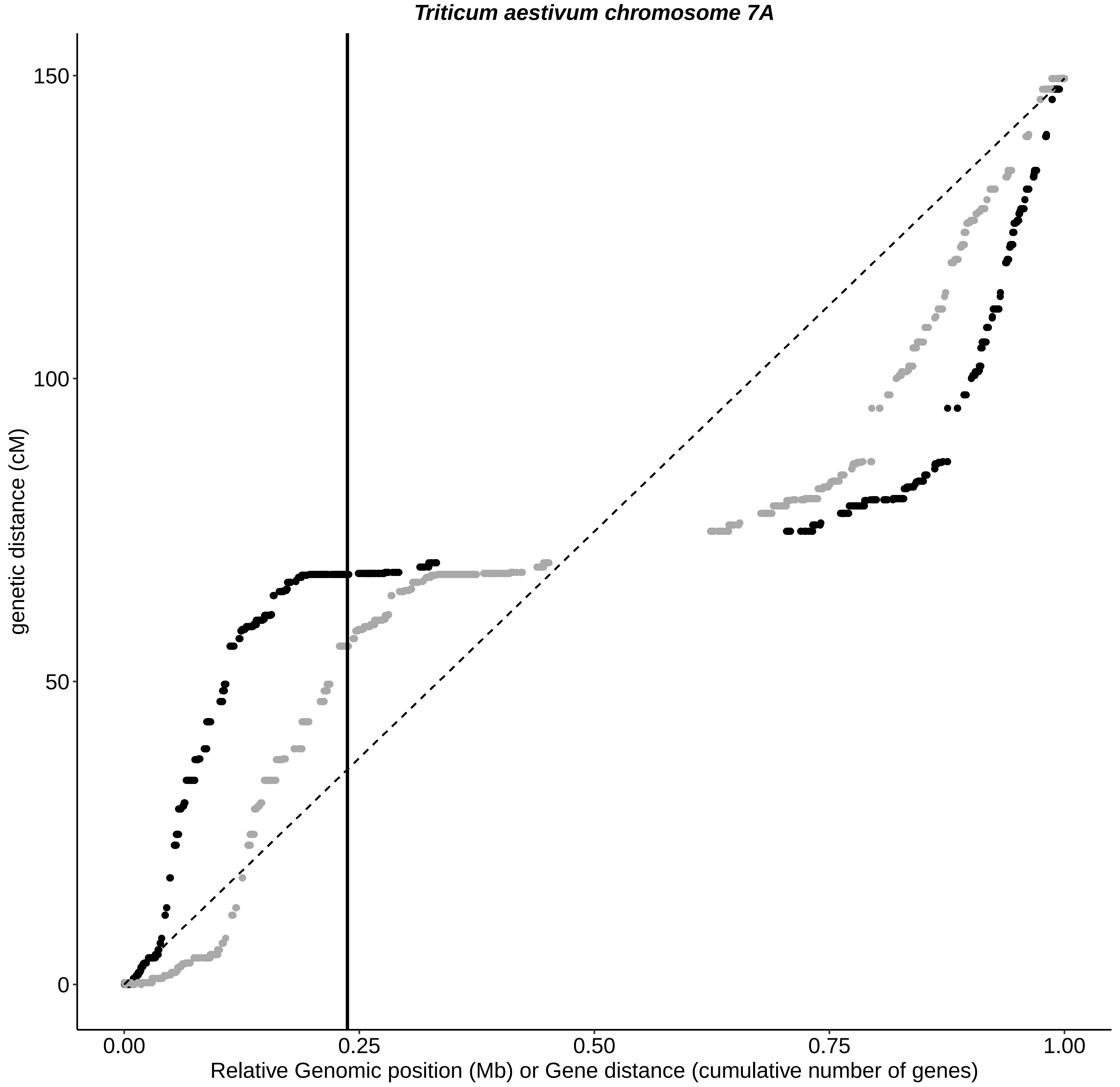

Triticum aestivum chromosome 7A
genetic distance (cM)
150
100
50
0
0.00
0.25
0.50
0.75
1.00
Relative Genomic position (Mb) or Gene distance (cumulative number of genes)

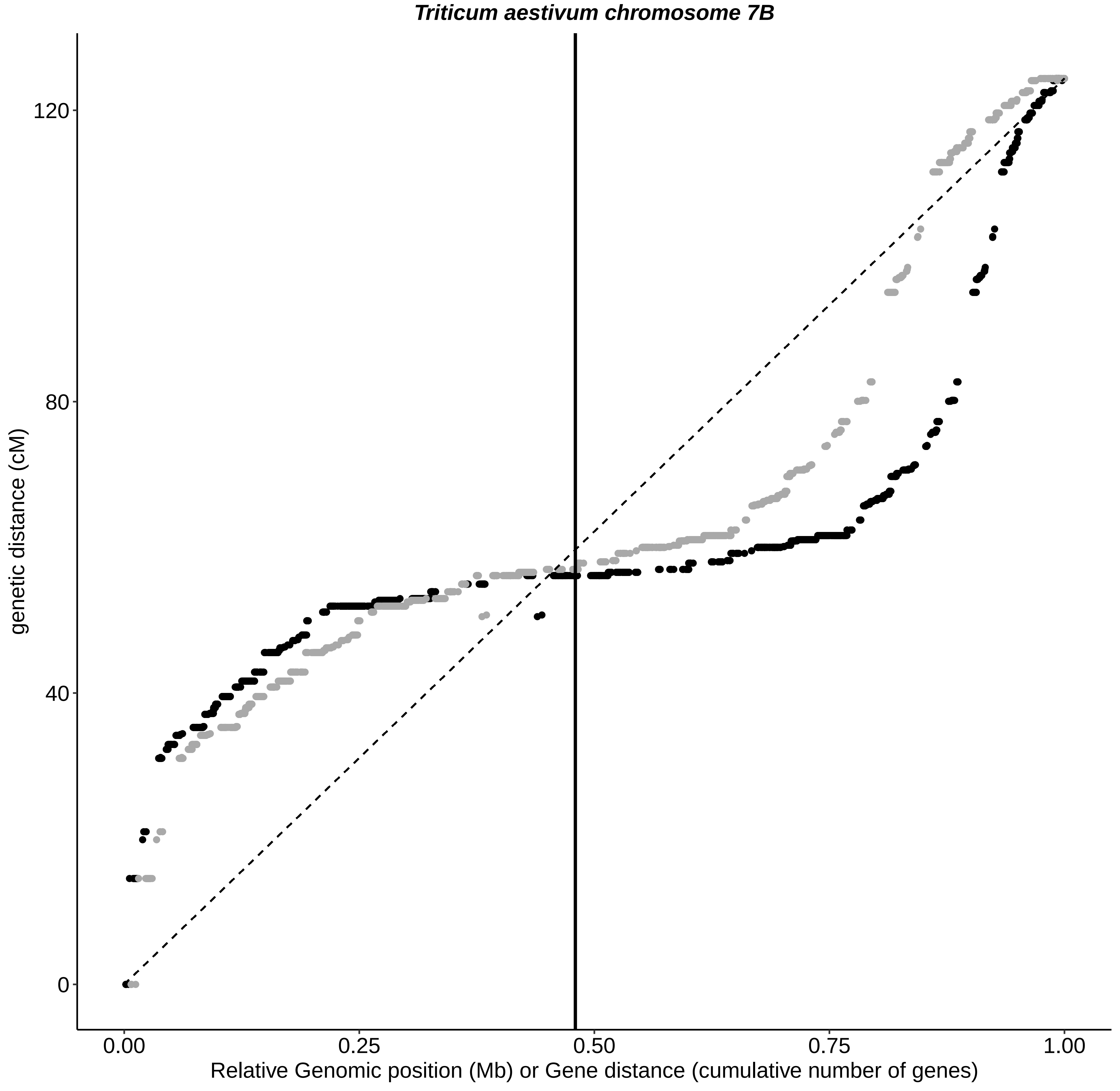
Triticum aestivum chromosome 7B
genetic distance (cM)
0
40
80
120
0.00
0.25
0.50
0.75
1.00
Relative Genomic position (Mb) or Gene distance (cumulative number of genes)

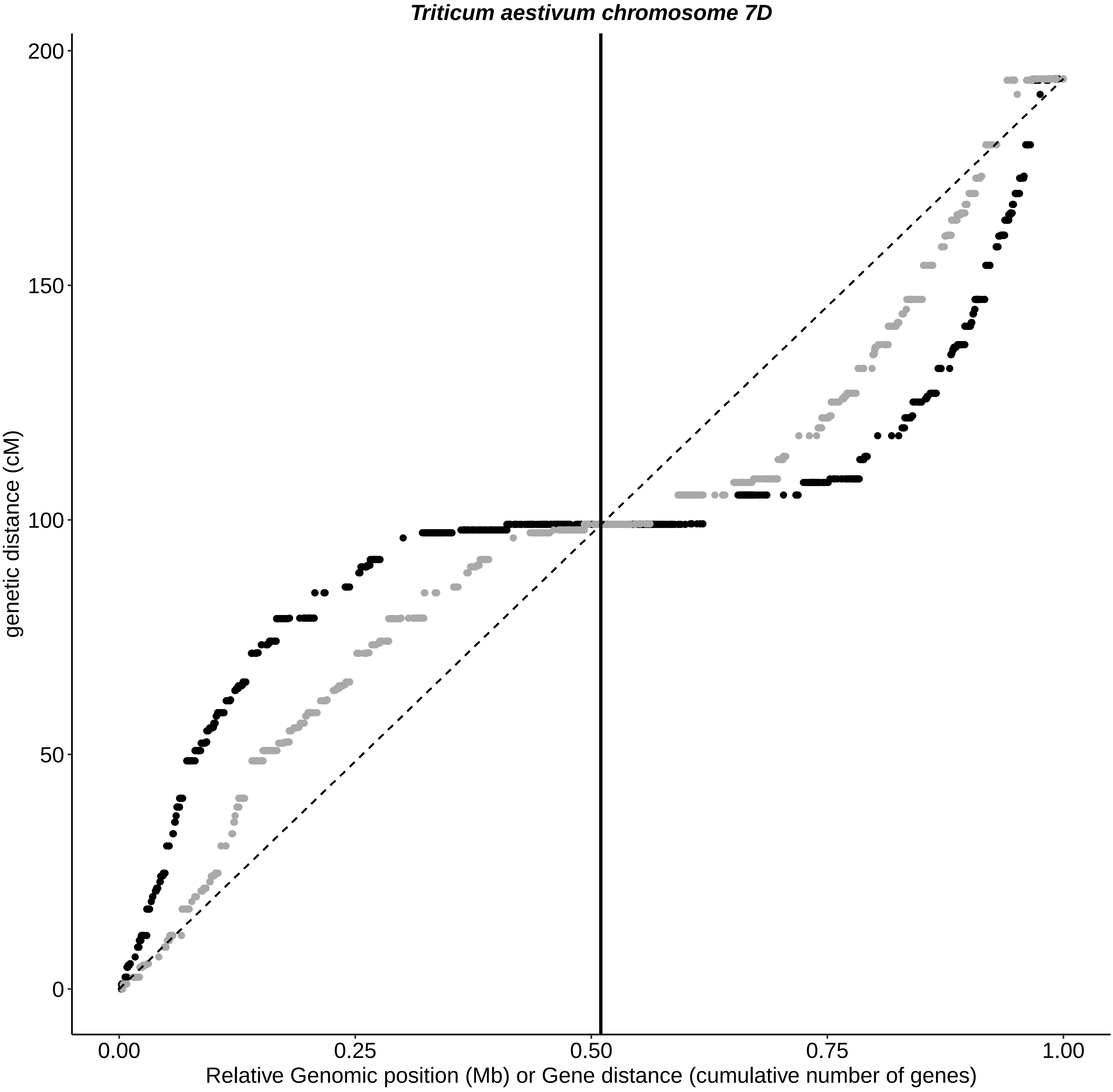
Triticum aestivum chromosome 7D
genetic distance (cM)
200
150
100
50
0
0.00
0.25
0.50
0.75
1.00
Relative Genomic position (Mb) or Gene distance (cumulative number of genes)

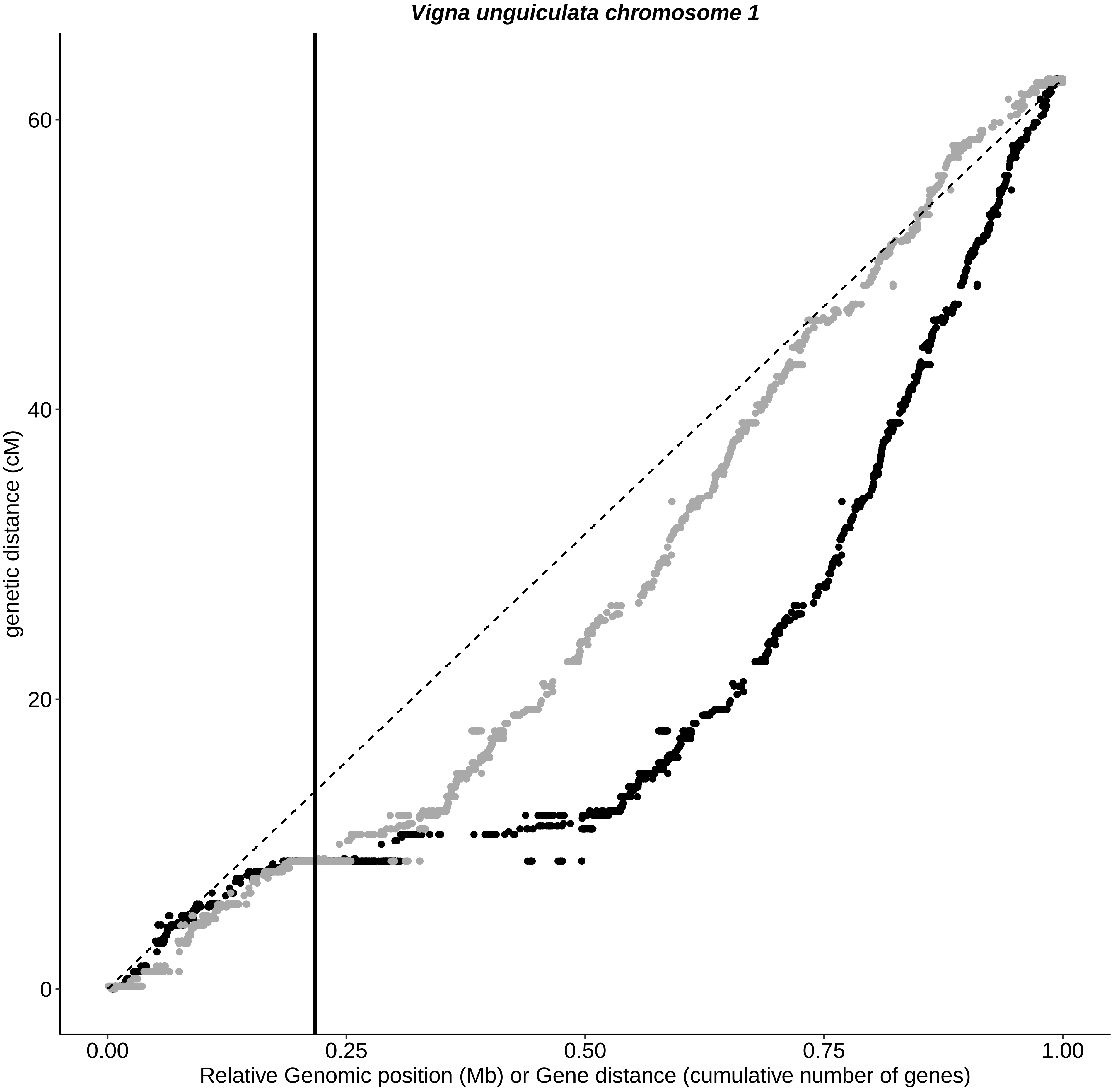
Vigna unguiculata chromosome 1
genetic distance (cM)
0
20
40
60
0.00
0.25
0.50
0.75
1.00
Relative Genomic position (Mb) or Gene distance (cumulative number of genes)

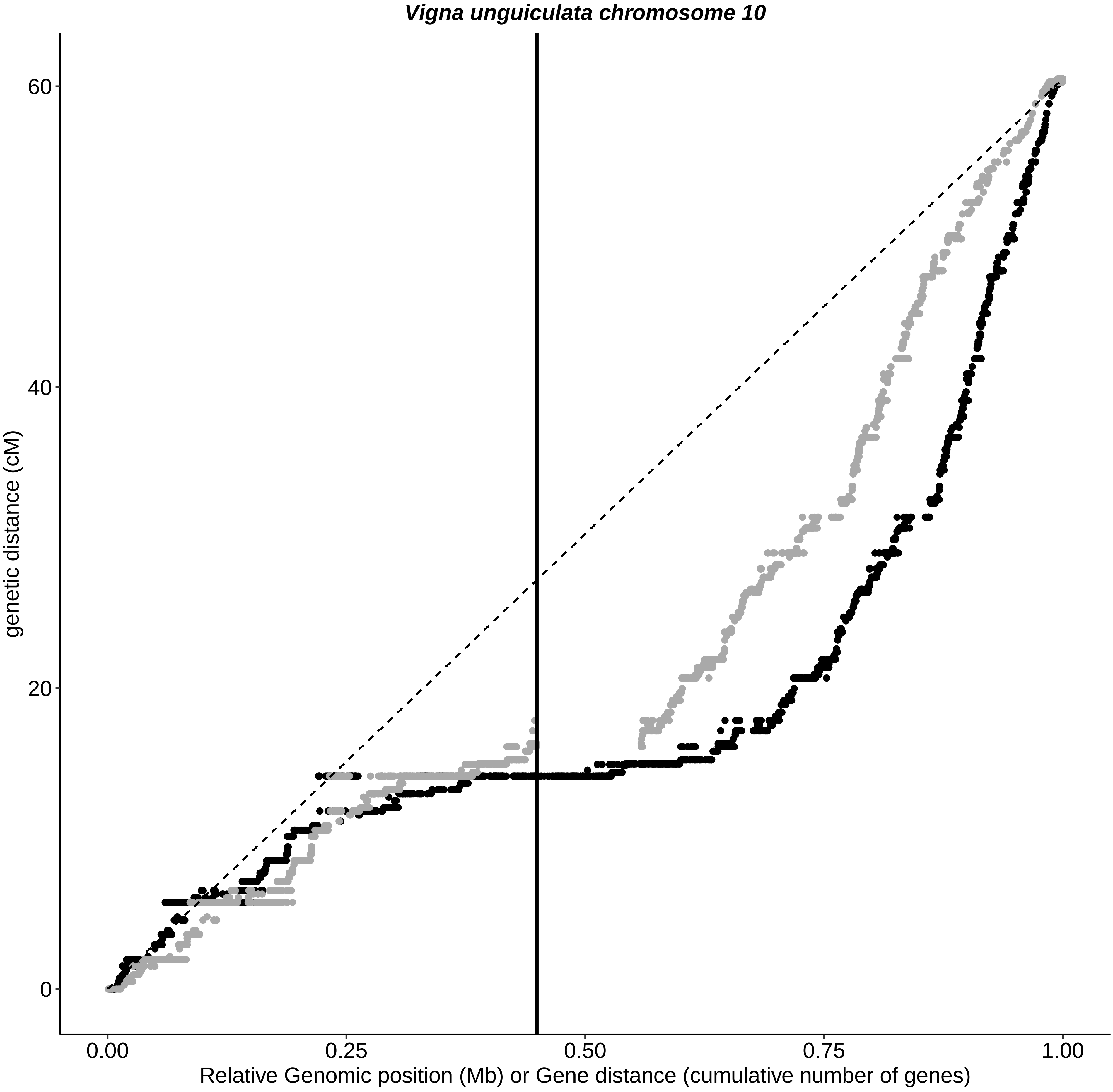
Vigna unguiculata chromosome 10
genetic distance (cM)
0
20
40
60
0.00
0.25
0.50
0.75
1.00
Relative Genomic position (Mb) or Gene distance (cumulative number of genes)

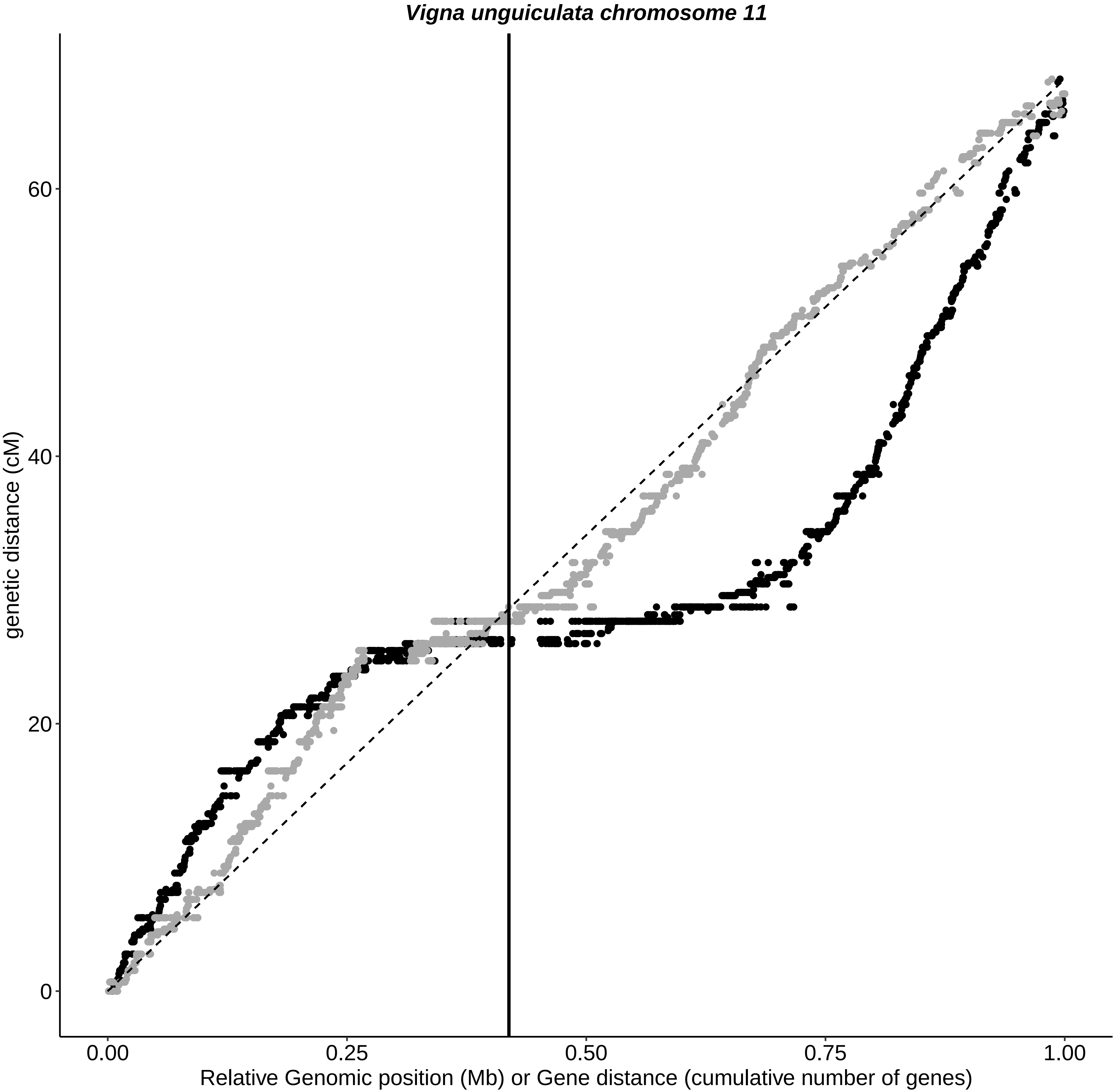
Vigna unguiculata chromosome 11
genetic distance (cM)
0
20
40
60
0.00
0.25
0.50
0.75
1.00
Relative Genomic position (Mb) or Gene distance (cumulative number of genes)

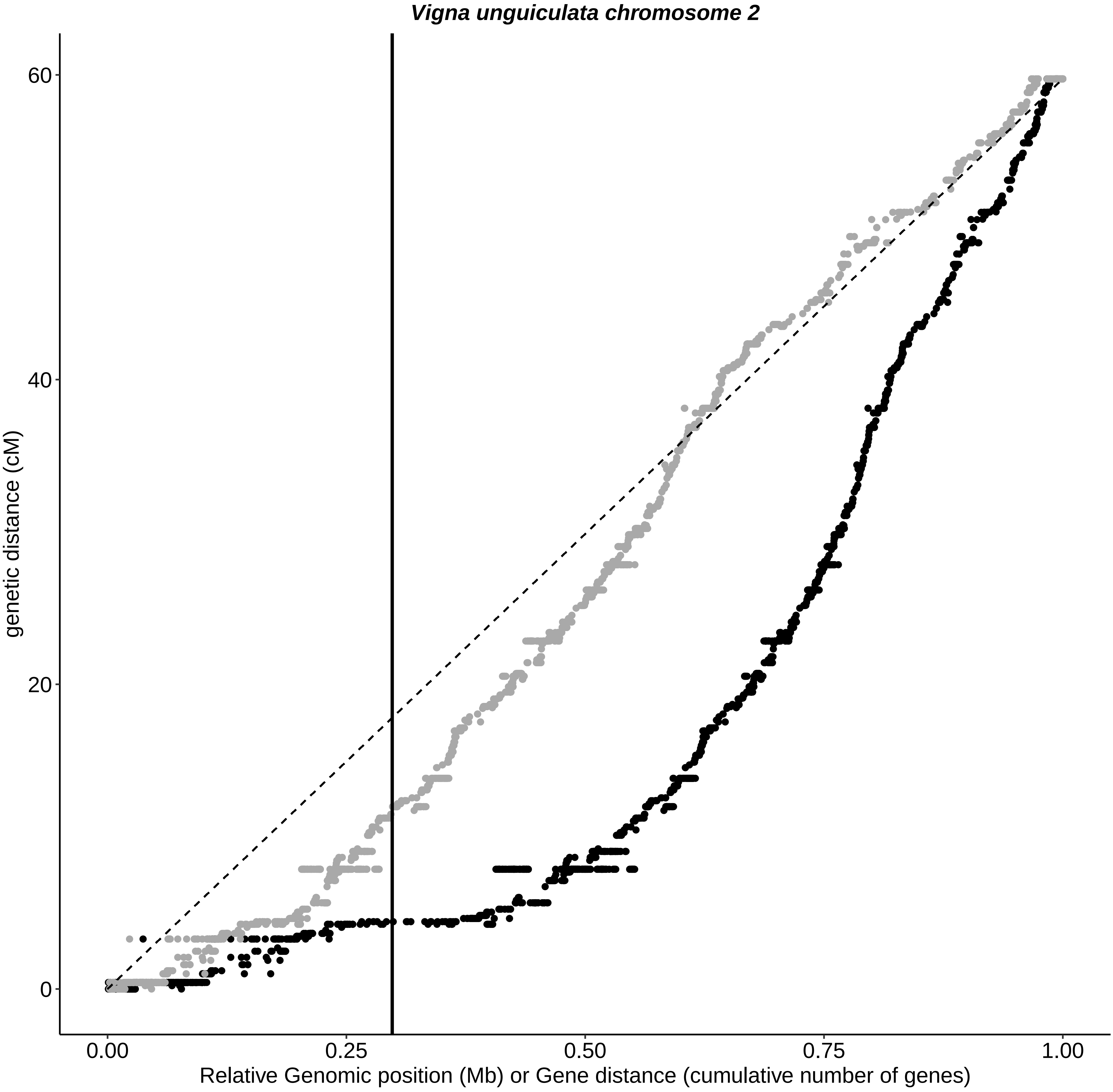
Vigna unguiculata chromosome 2
genetic distance (cM)
0
20
40
60
0.00
0.25
0.50
0.75
1.00
Relative Genomic position (Mb) or Gene distance (cumulative number of genes)

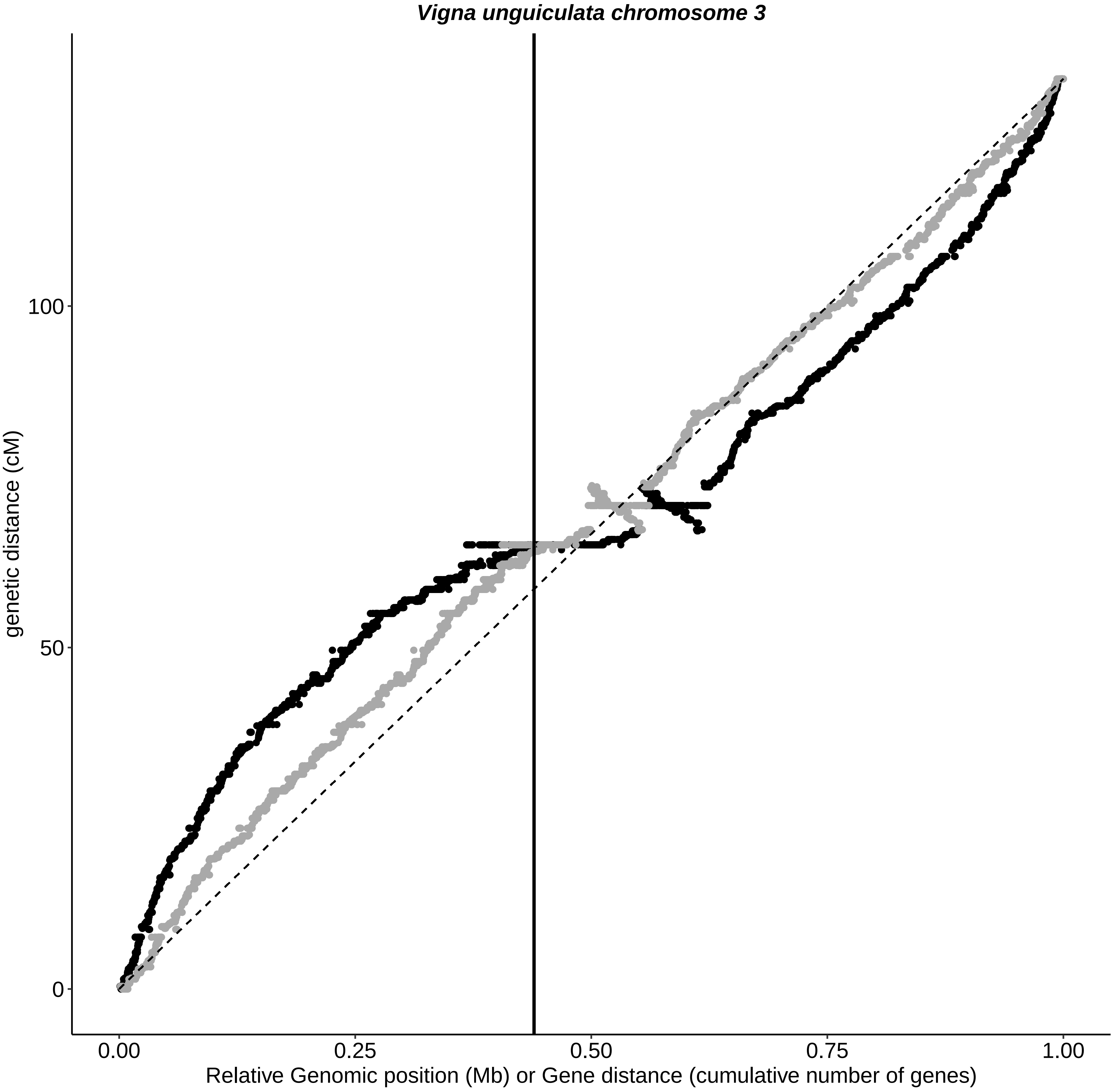
Vigna unguiculata chromosome 3
genetic distance (cM)
0
50
100
0.00
0.25
0.50
0.75
1.00
Relative Genomic position (Mb) or Gene distance (cumulative number of genes)

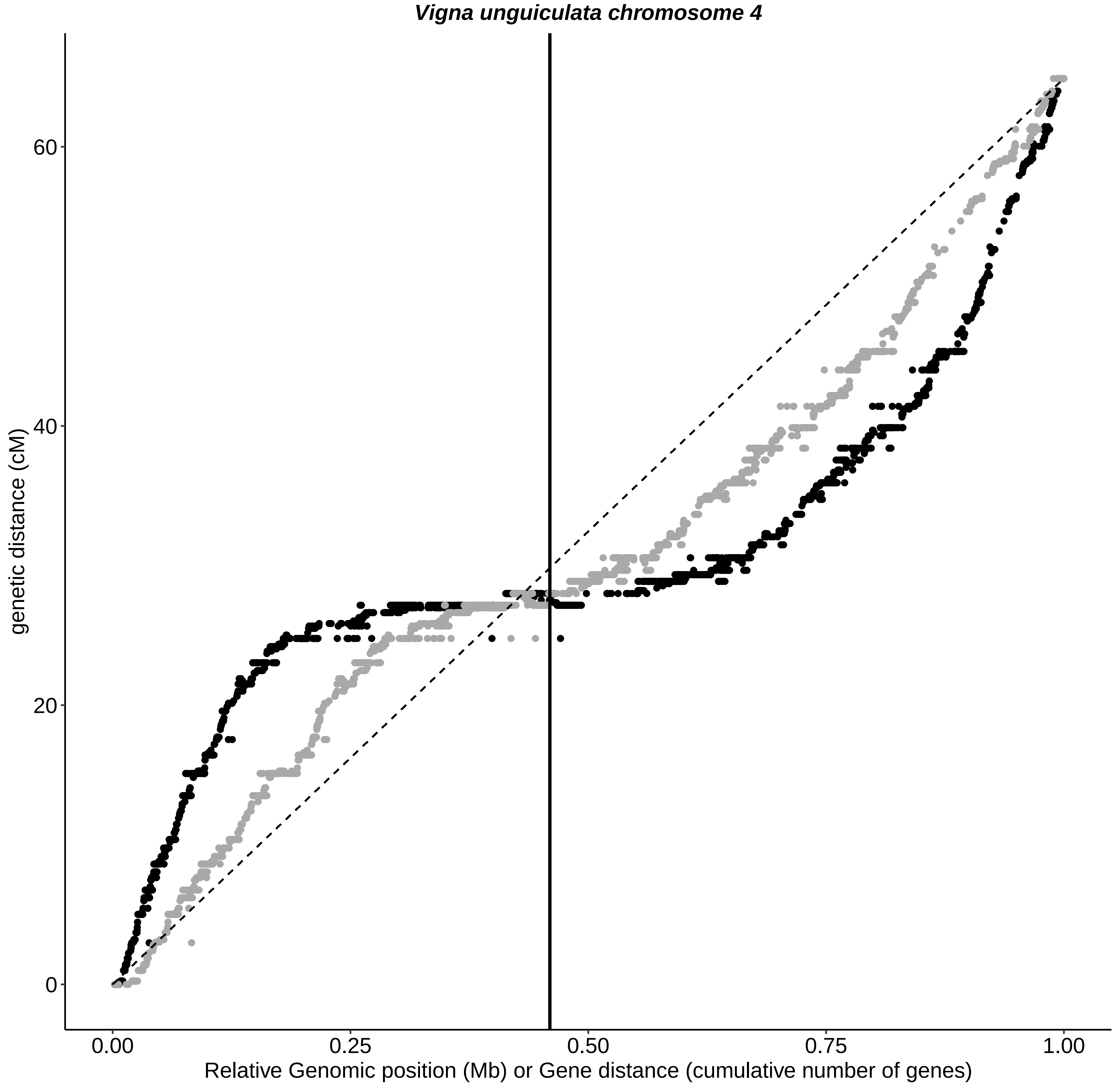
Vigna unguiculata chromosome 4
genetic distance (cM)
60
40
20
0
0.00
0.25
0.50
0.75
1.00
Relative Genomic position (Mb) or Gene distance (cumulative number of genes)

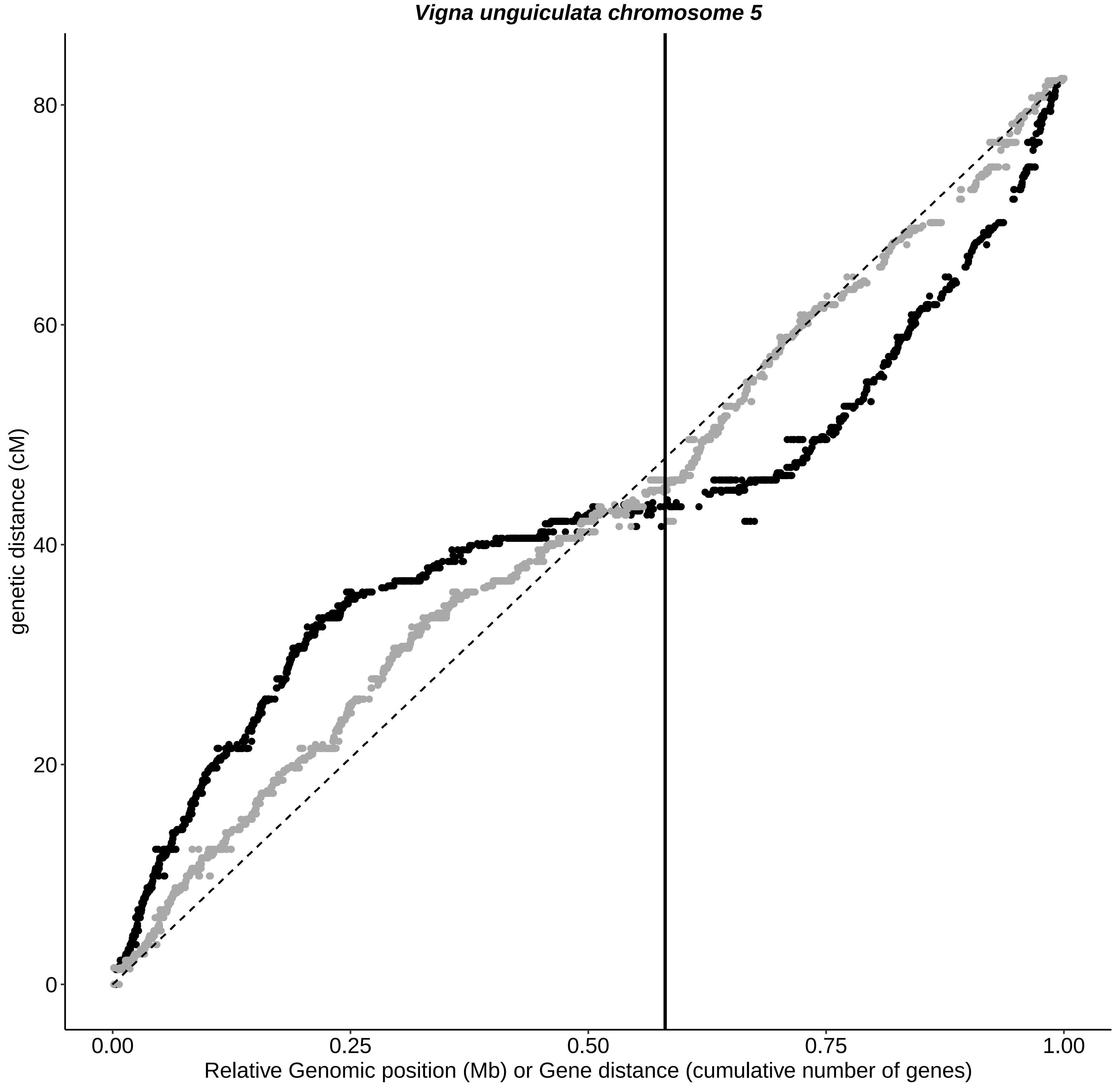
Vigna unguiculata chromosome 5
genetic distance (cM)
0
20
40
60
80
0.00
0.25
0.50
0.75
1.00
Relative Genomic position (Mb) or Gene distance (cumulative number of genes)

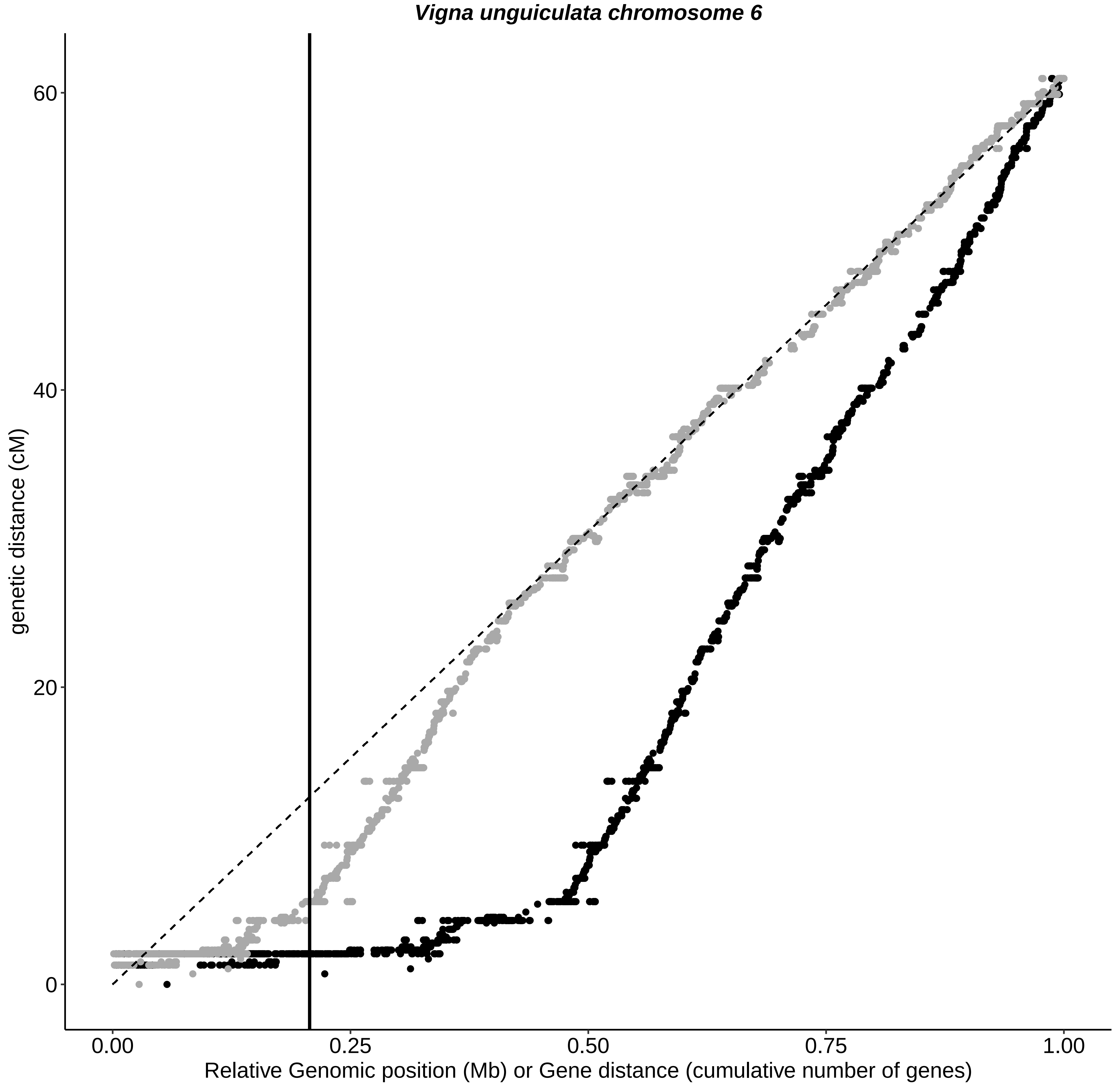
Vigna unguiculata chromosome 6
genetic distance (cM)
0
20
40
60
0.00
0.25
0.50
0.75
1.00
Relative Genomic position (Mb) or Gene distance (cumulative number of genes)

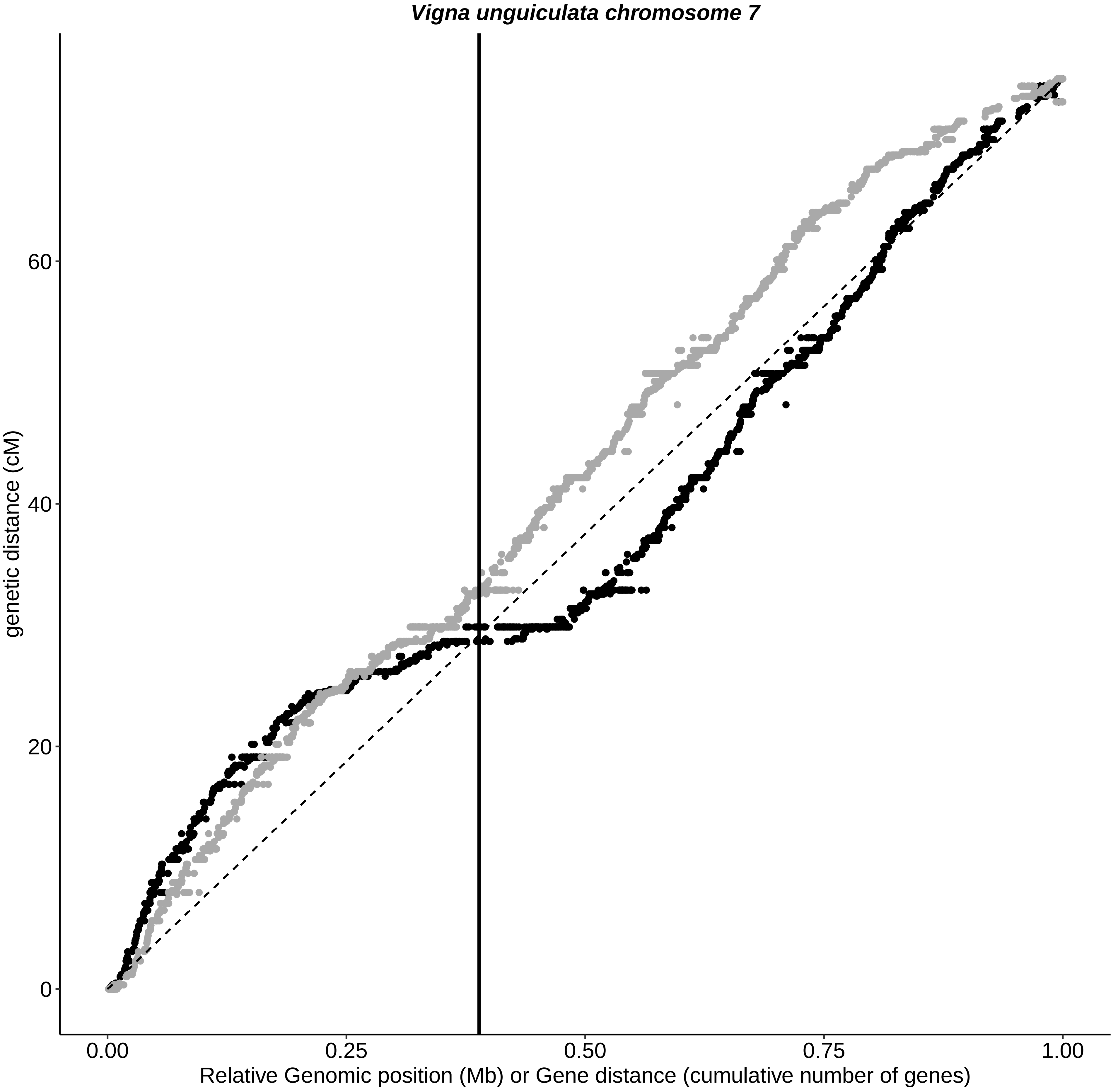
Vigna unguiculata chromosome 7
genetic distance (cM)
0
20
40
60
0.00
0.25
0.50
0.75
1.00
Relative Genomic position (Mb) or Gene distance (cumulative number of genes)

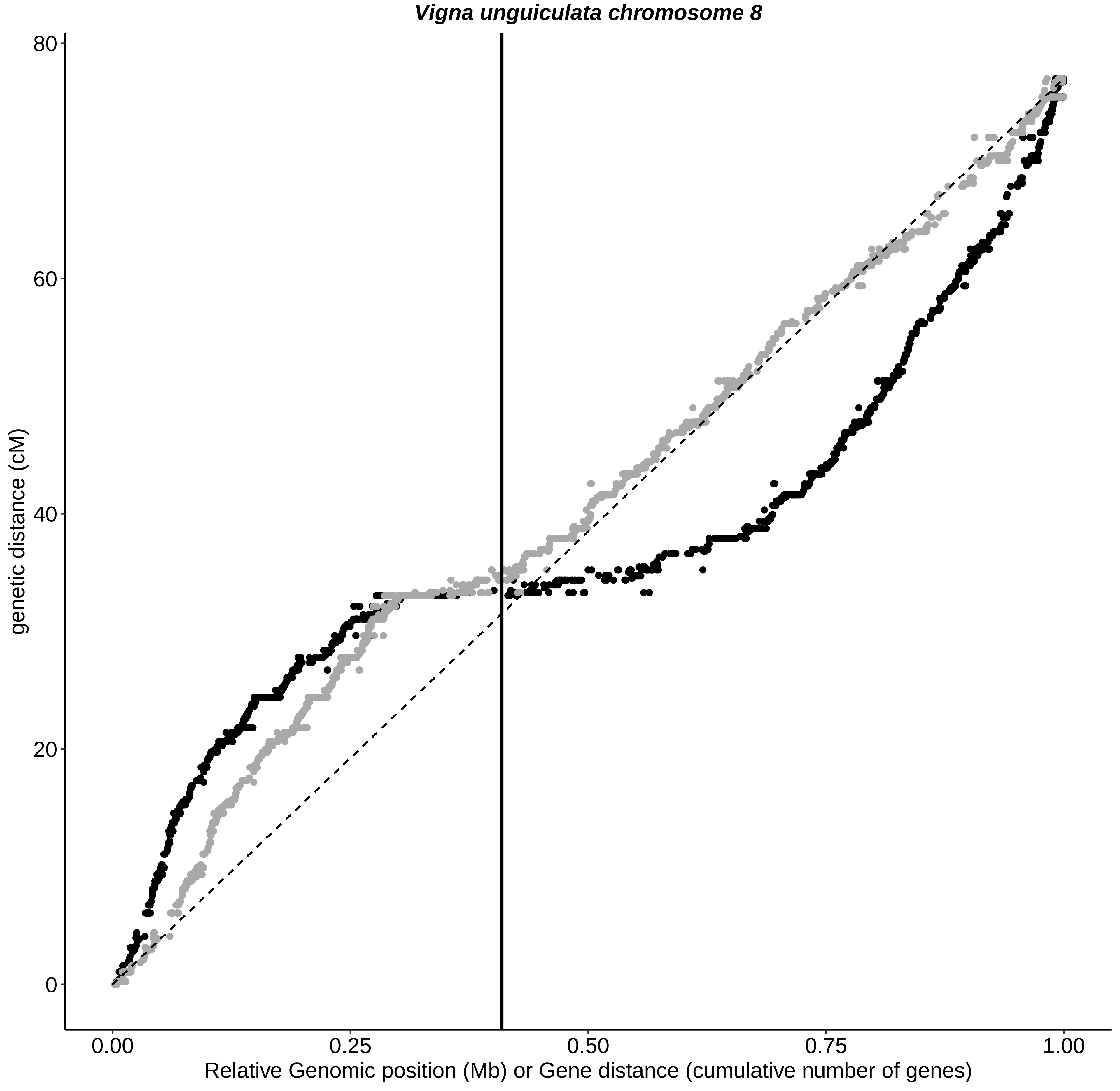
Vigna unguiculata chromosome 8
genetic distance (cM)
0
20
40
60
80
0.00
0.25
0.50
0.75
1.00
Relative Genomic position (Mb) or Gene distance (cumulative number of genes)

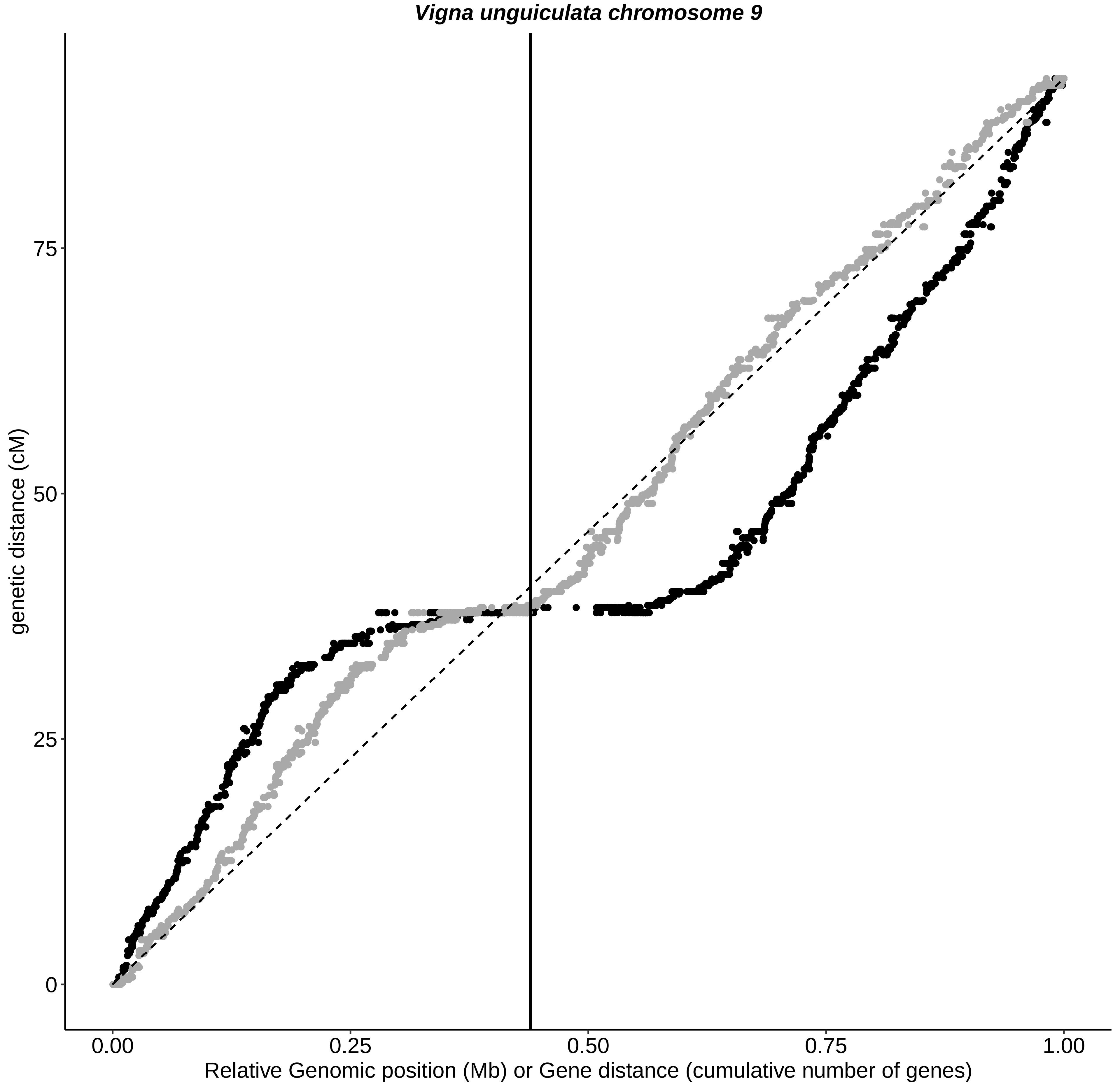
Vigna unguiculata chromosome 9
genetic distance (cM)
0
25
50
75
0.00
0.25
0.50
0.75
1.00
Relative Genomic position (Mb) or Gene distance (cumulative number of genes)

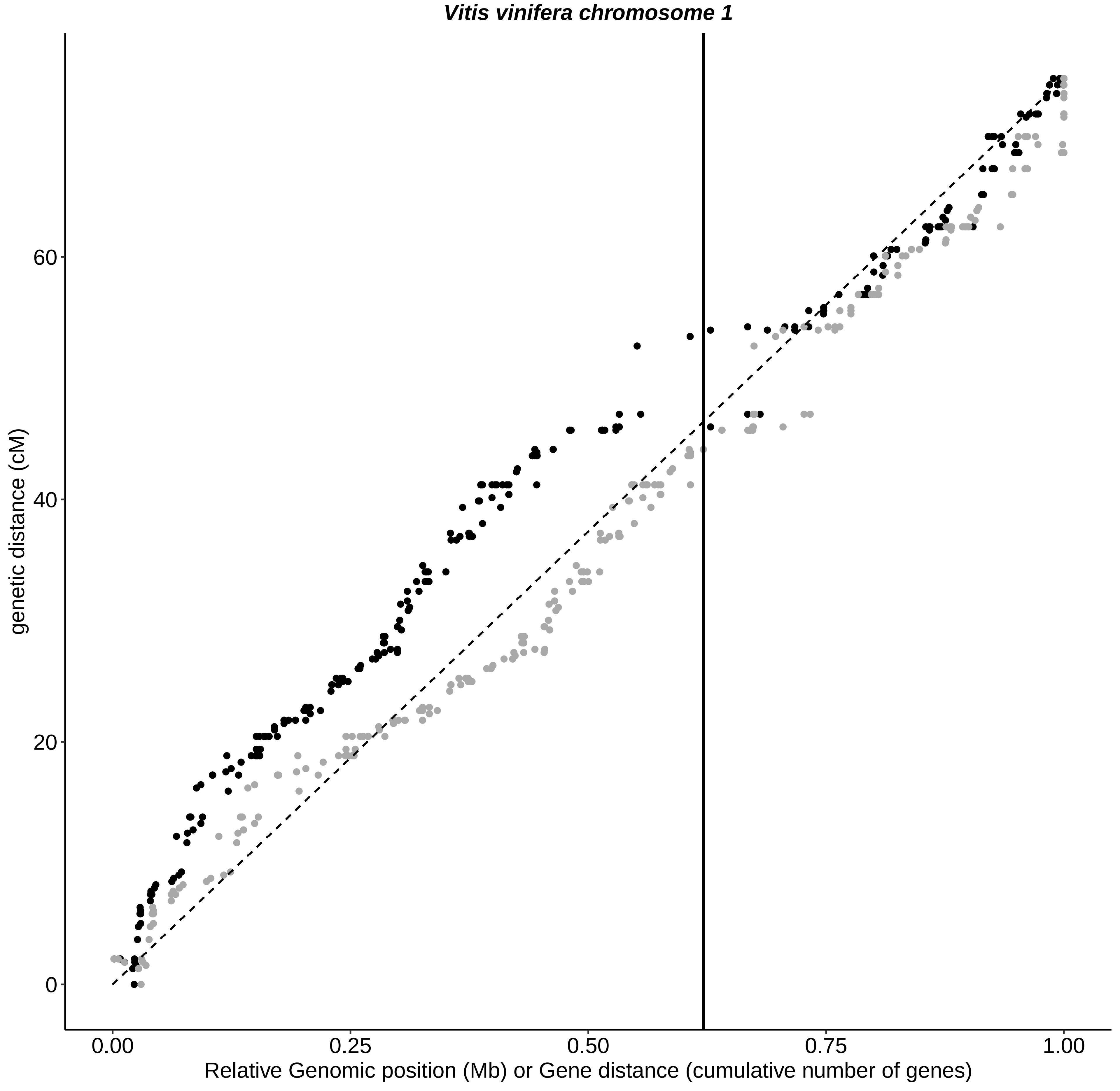
Vitis vinifera chromosome 1
genetic distance (cM)
0
20
40
60
0.00
0.25
0.50
0.75
1.00
Relative Genomic position (Mb) or Gene distance (cumulative number of genes)

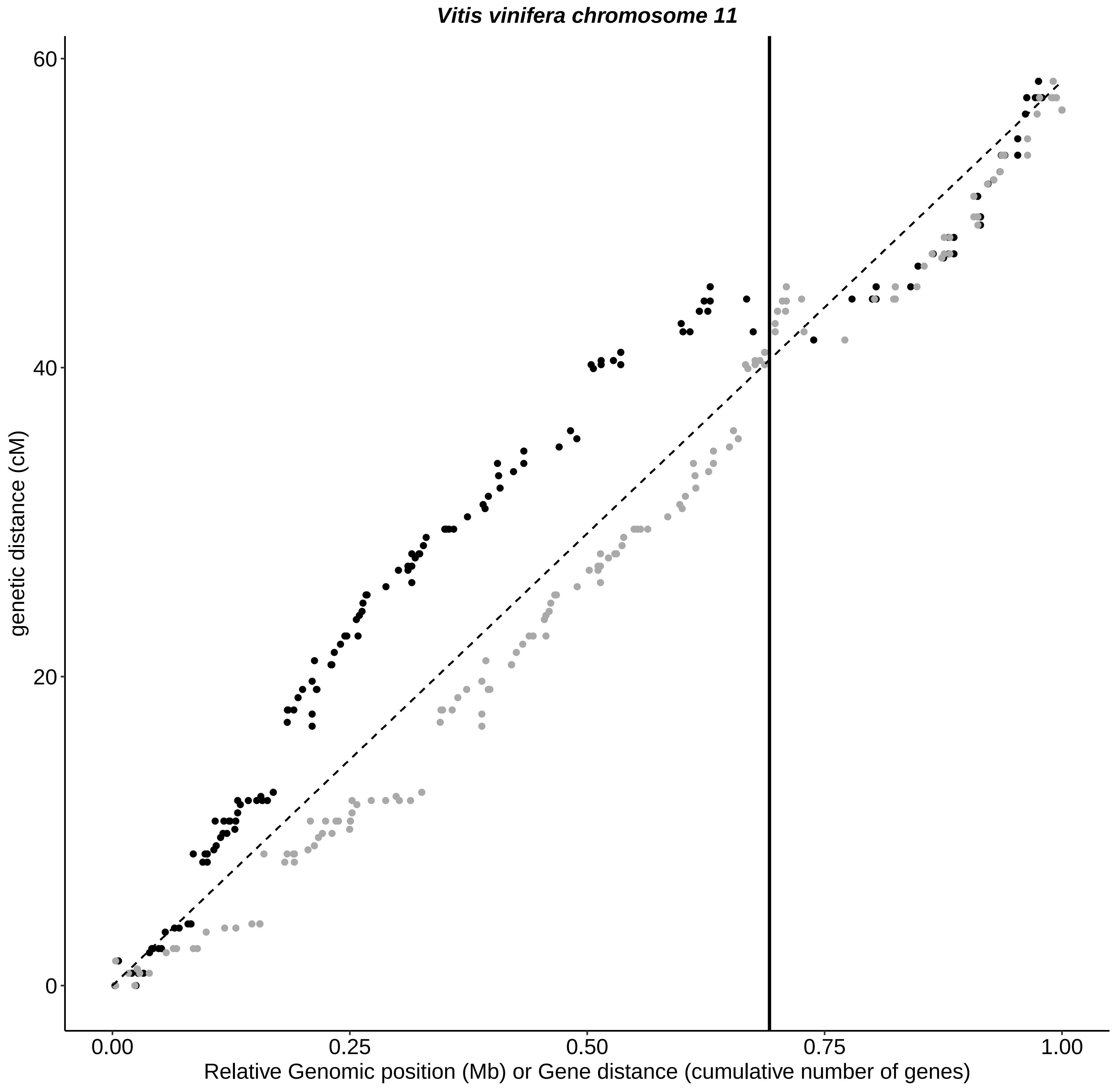
Vitis vinifera chromosome 11
genetic distance (cM)
0
20
40
60
0.00
0.25
0.50
0.75
1.00
Relative Genomic position (Mb) or Gene distance (cumulative number of genes)

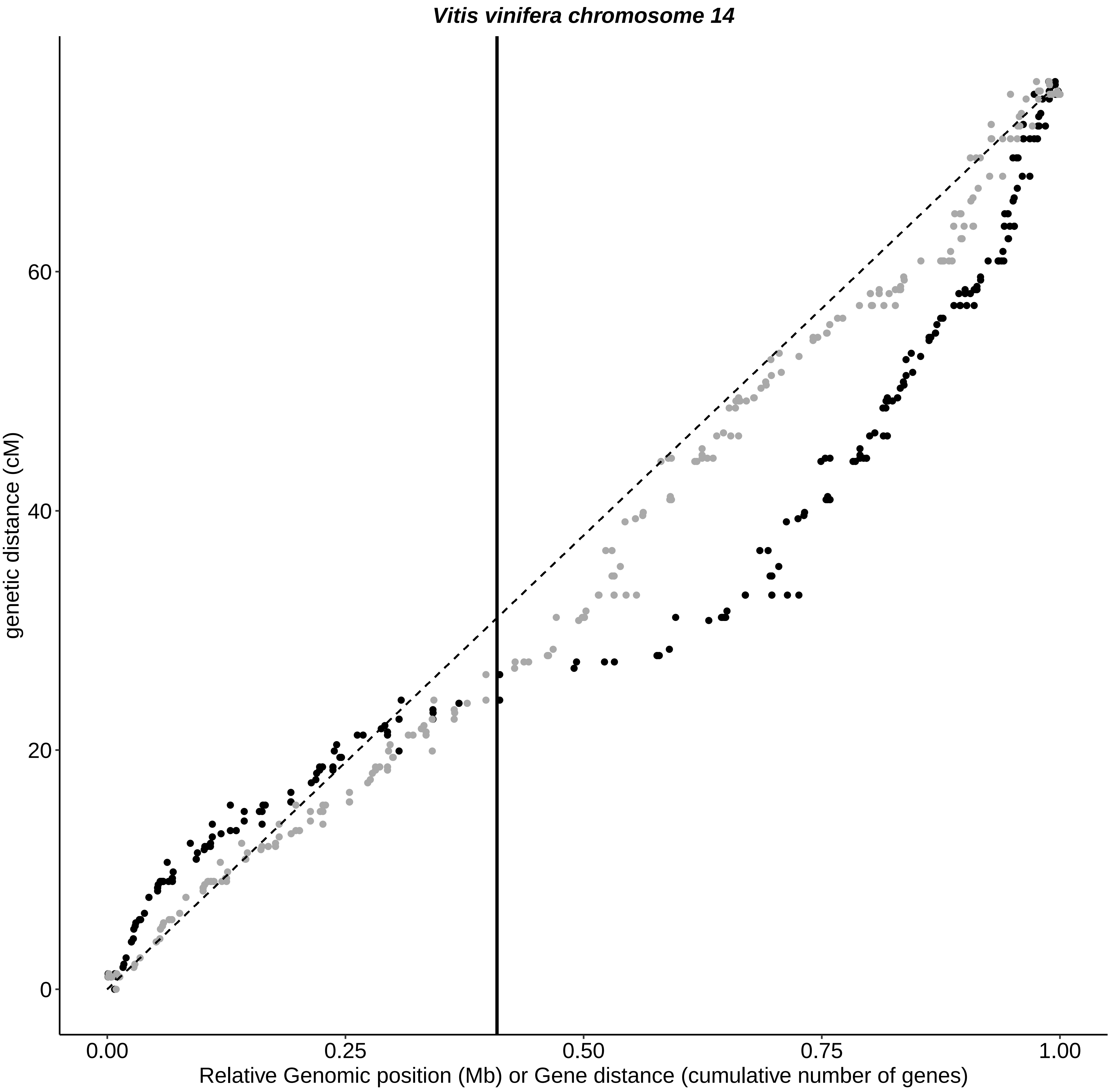
Vitis vinifera chromosome 14
genetic distance (cM)
0
20
40
60
0.00
0.25
0.50
0.75
1.00
Relative Genomic position (Mb) or Gene distance (cumulative number of genes)

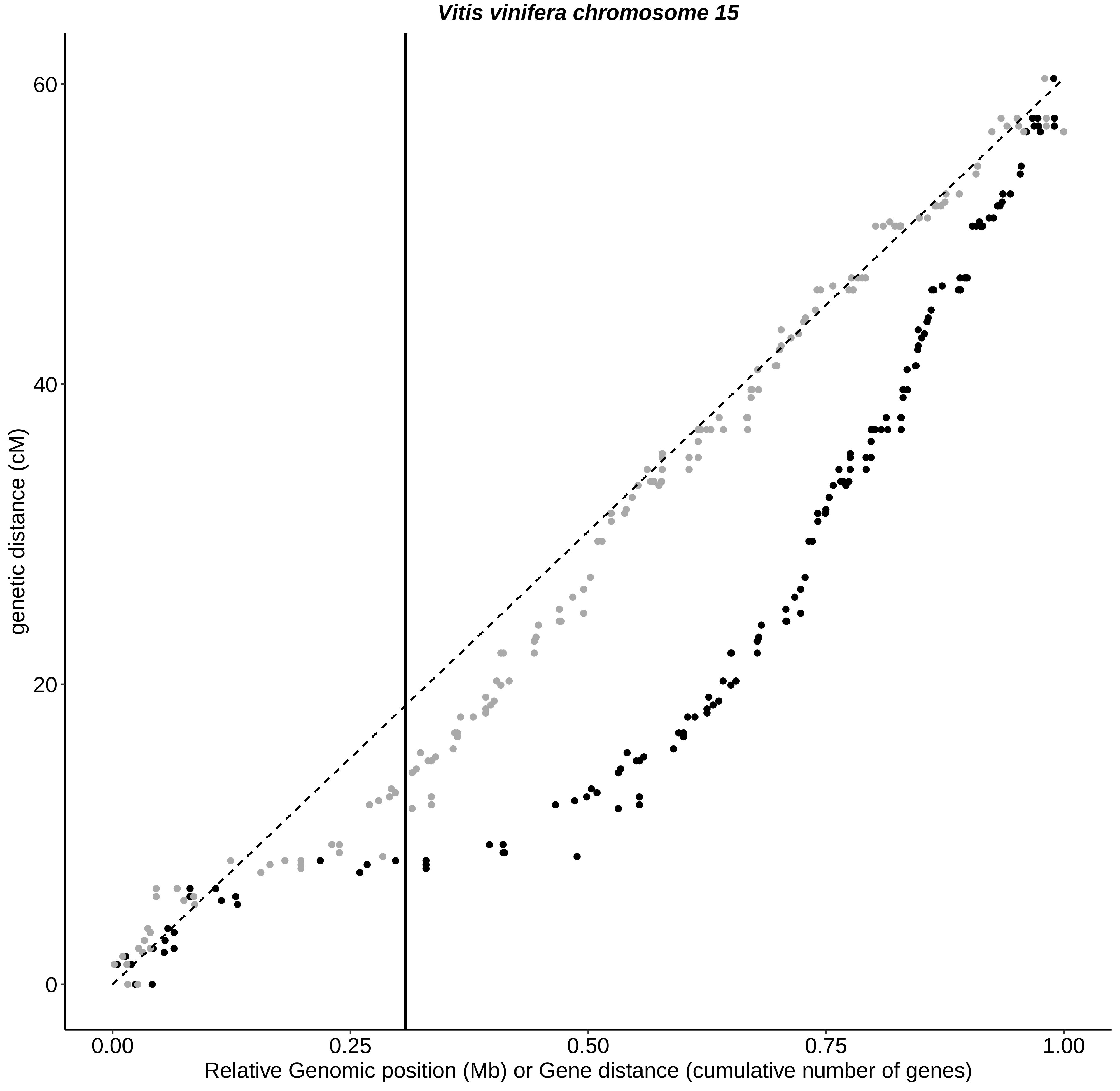

Vitis vinifera chromosome 15
genetic distance (cM)
0
20
40
60
0.00
0.25
0.50
0.75
1.00
Relative Genomic position (Mb) or Gene distance (cumulative number of genes)

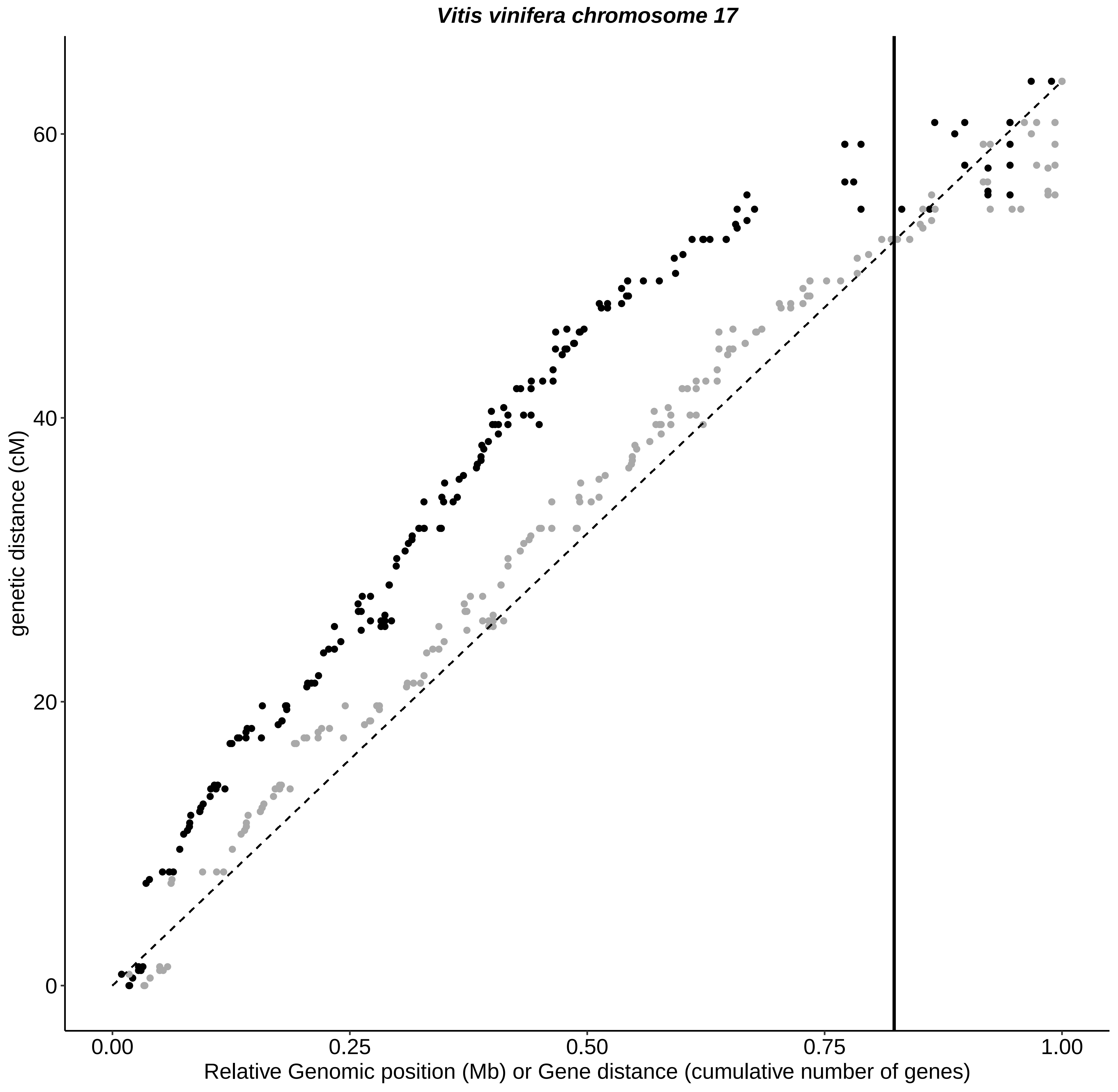
Vitis vinifera chromosome 17
genetic distance (cM)
0
20
40
60
0.00
0.25
0.50
0.75
1.00
Relative Genomic position (Mb) or Gene distance (cumulative number of genes)

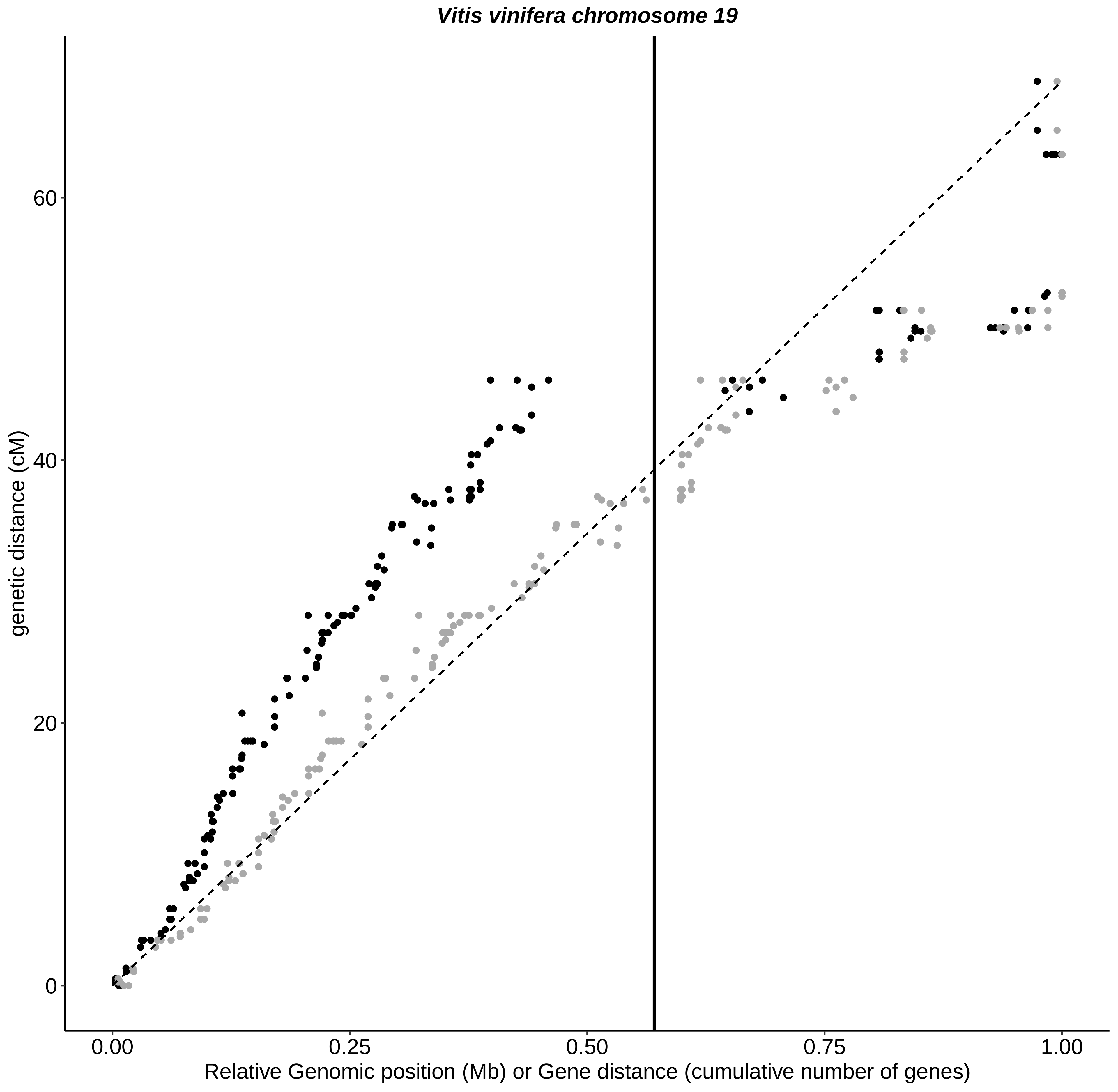
Vitis vinifera chromosome 19
genetic distance (cM)
0
20
40
60
0.00
0.25
0.50
0.75
1.00
Relative Genomic position (Mb) or Gene distance (cumulative number of genes)

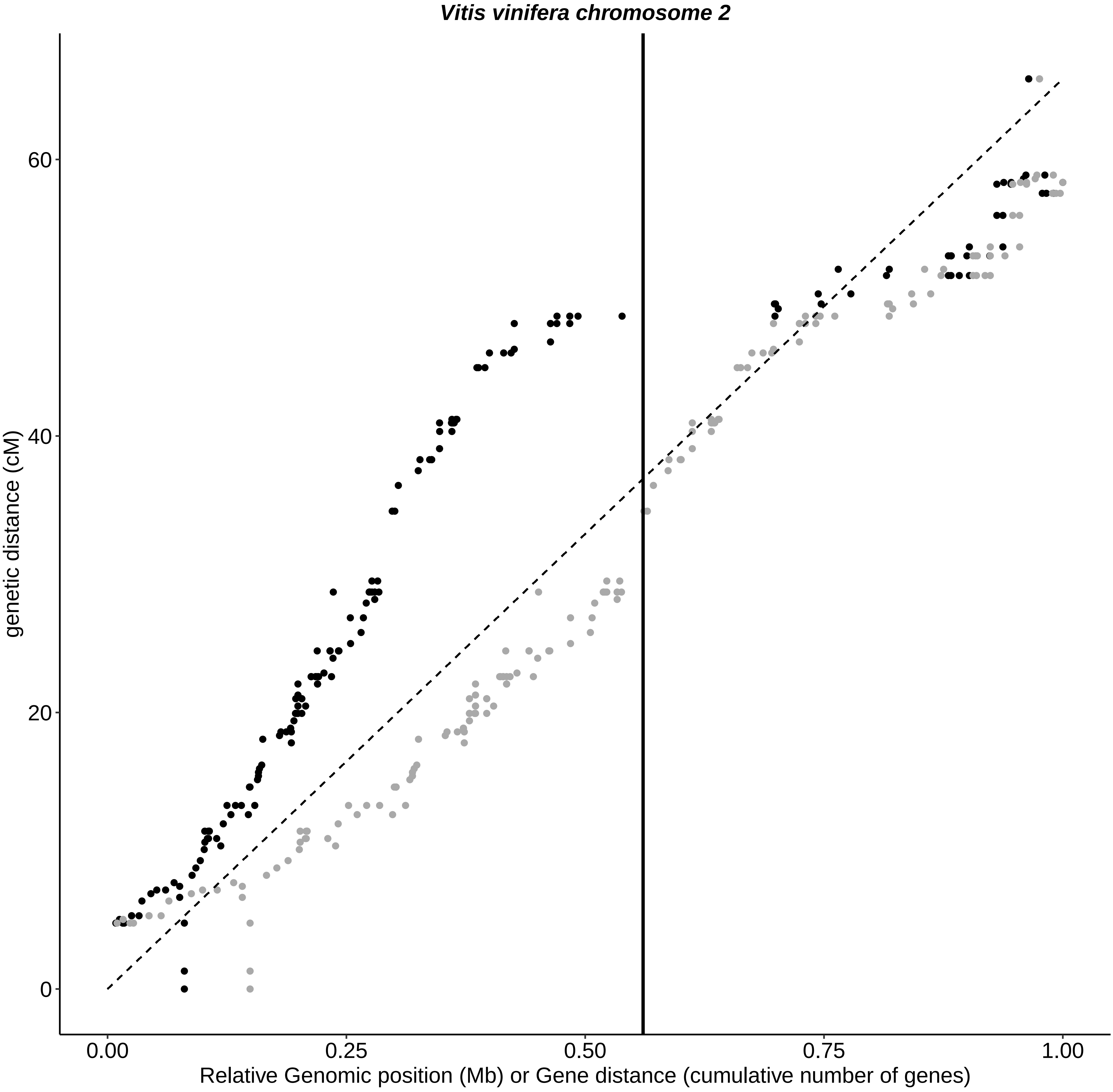

Vitis vinifera chromosome 2
genetic distance (cM)
0
20
40
60
0.00
0.25
0.50
0.75
1.00
Relative Genomic position (Mb) or Gene distance (cumulative number of genes)

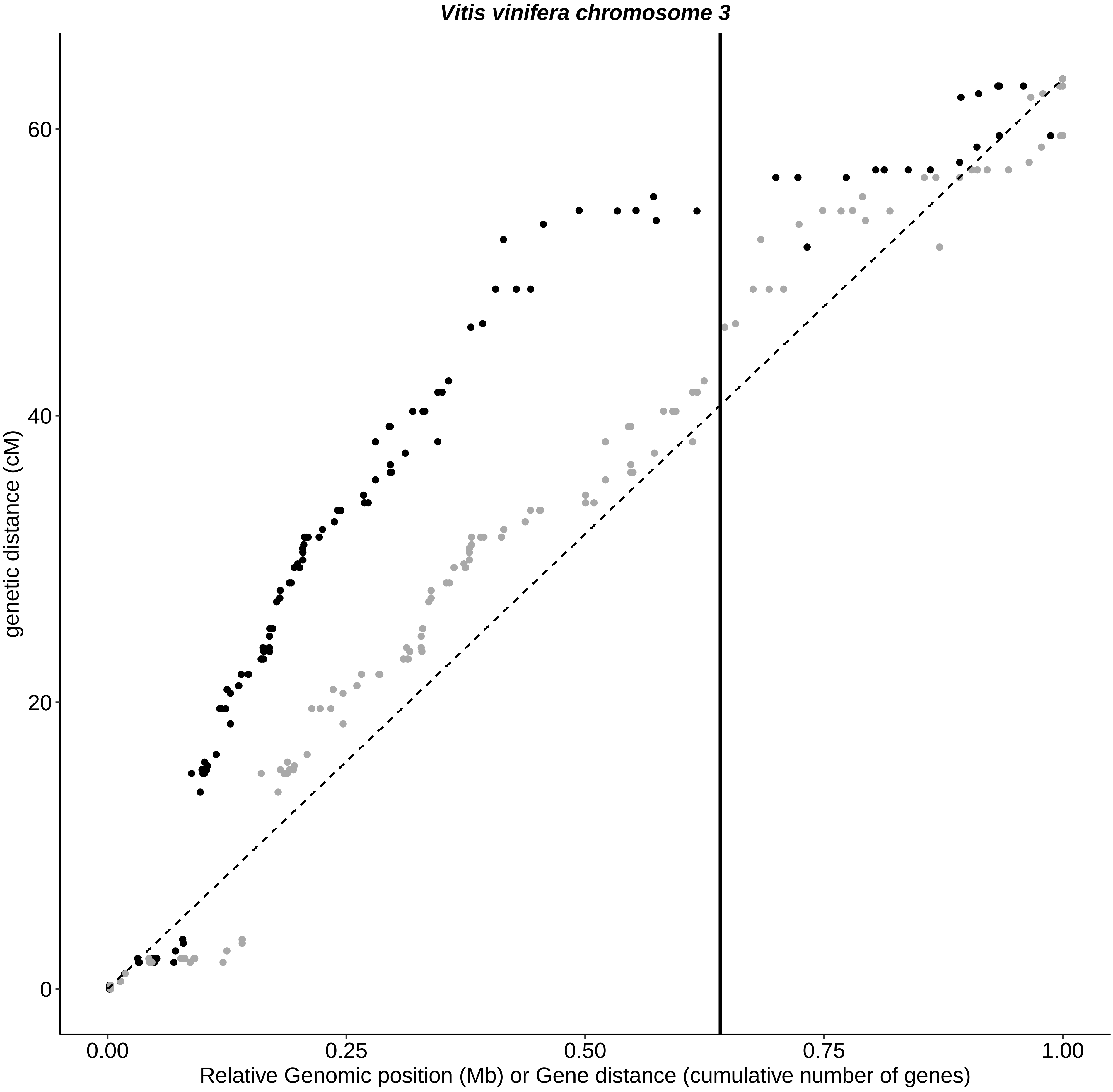
Vitis vinifera chromosome 3
genetic distance (cM)
0
20
40
60
0.00
0.25
0.50
0.75
1.00
Relative Genomic position (Mb) or Gene distance (cumulative number of genes)

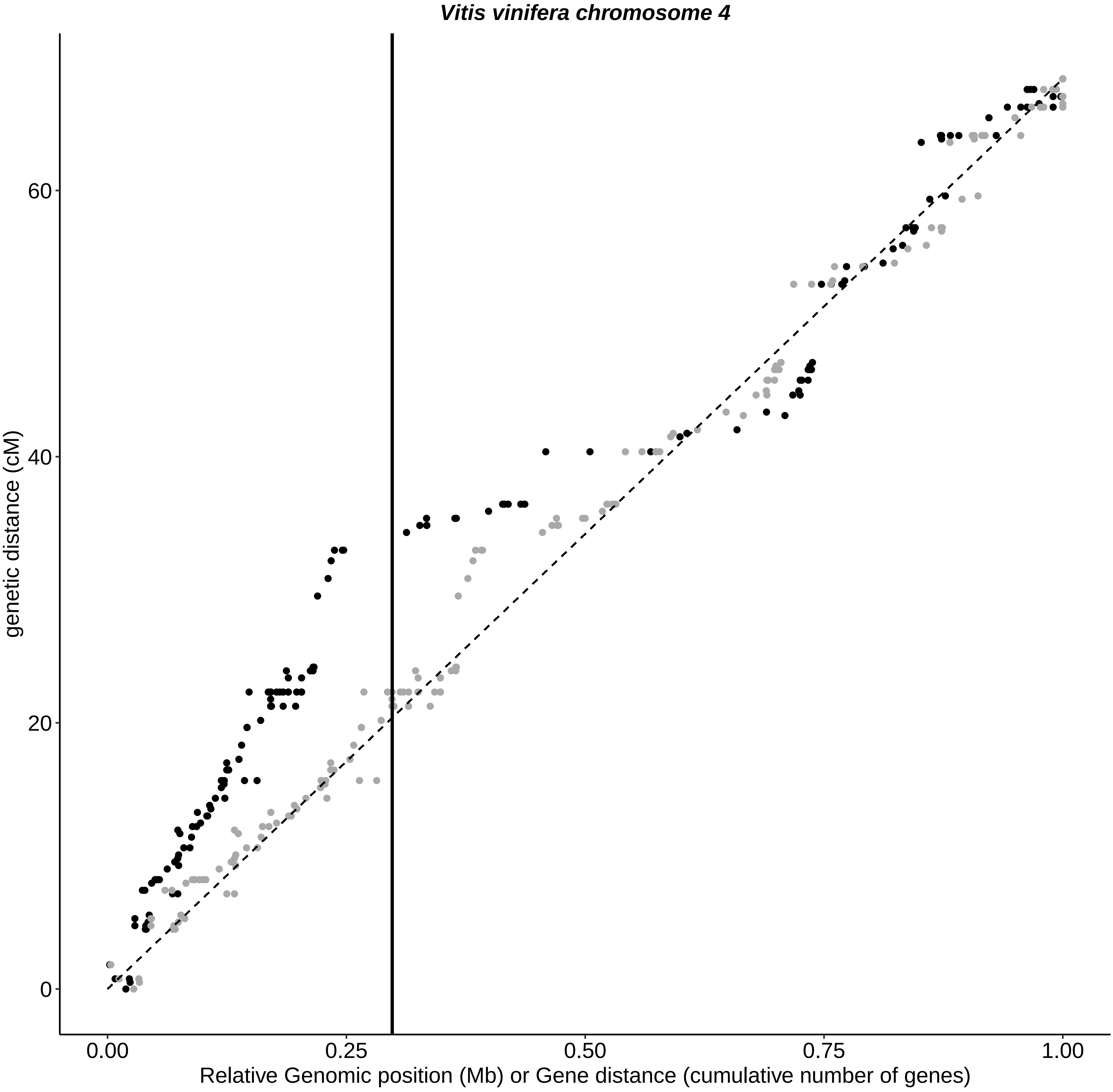
Vitis vinifera chromosome 4
genetic distance (cM)
60
40
20
0
0.00
0.25
0.50
0.75
1.00
Relative Genomic position (Mb) or Gene distance (cumulative number of genes)

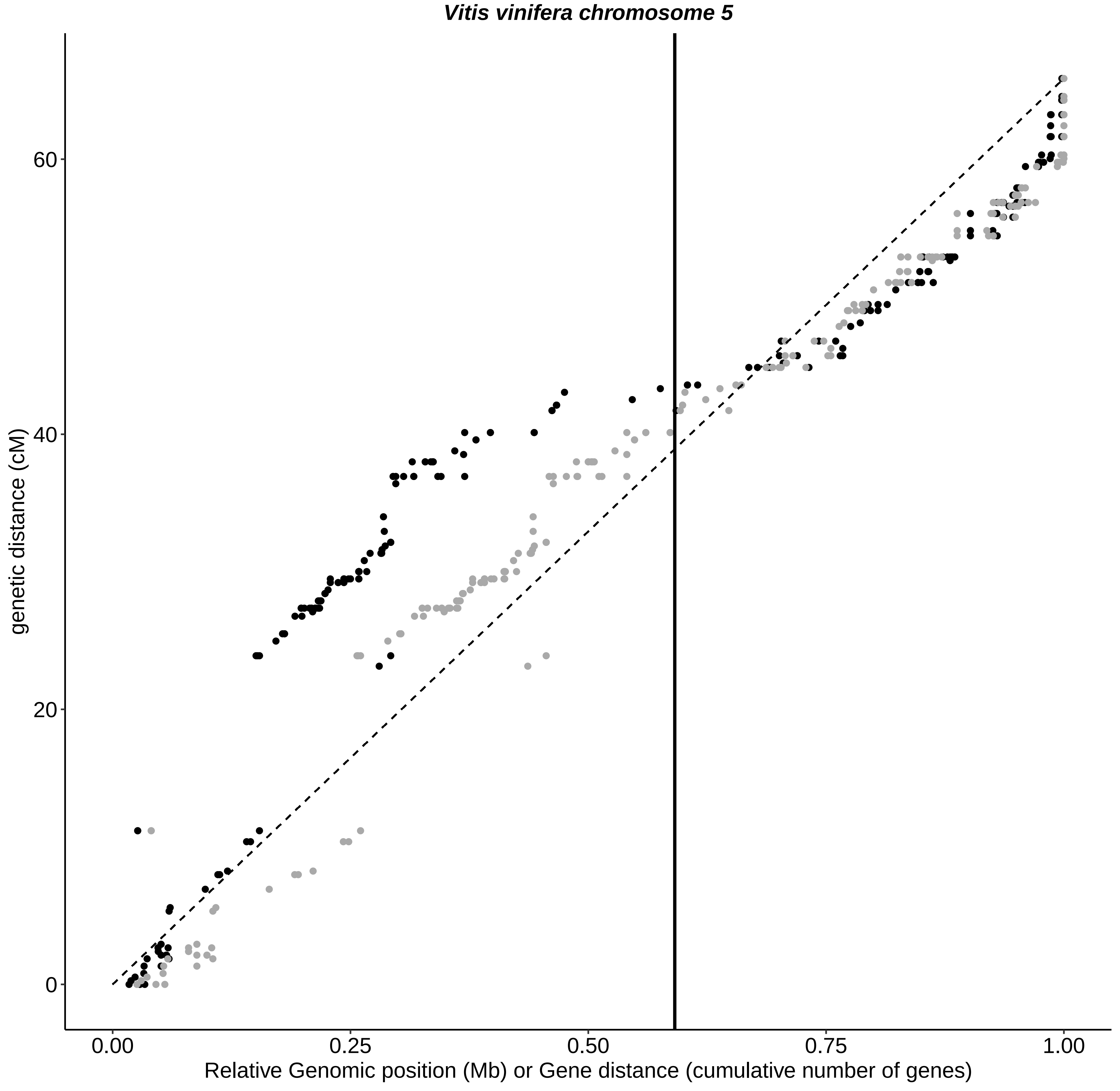
Vitis vinifera chromosome 5
genetic distance (cM)
0
20
40
60
0.00
0.25
0.50
0.75
1.00
Relative Genomic position (Mb) or Gene distance (cumulative number of genes)

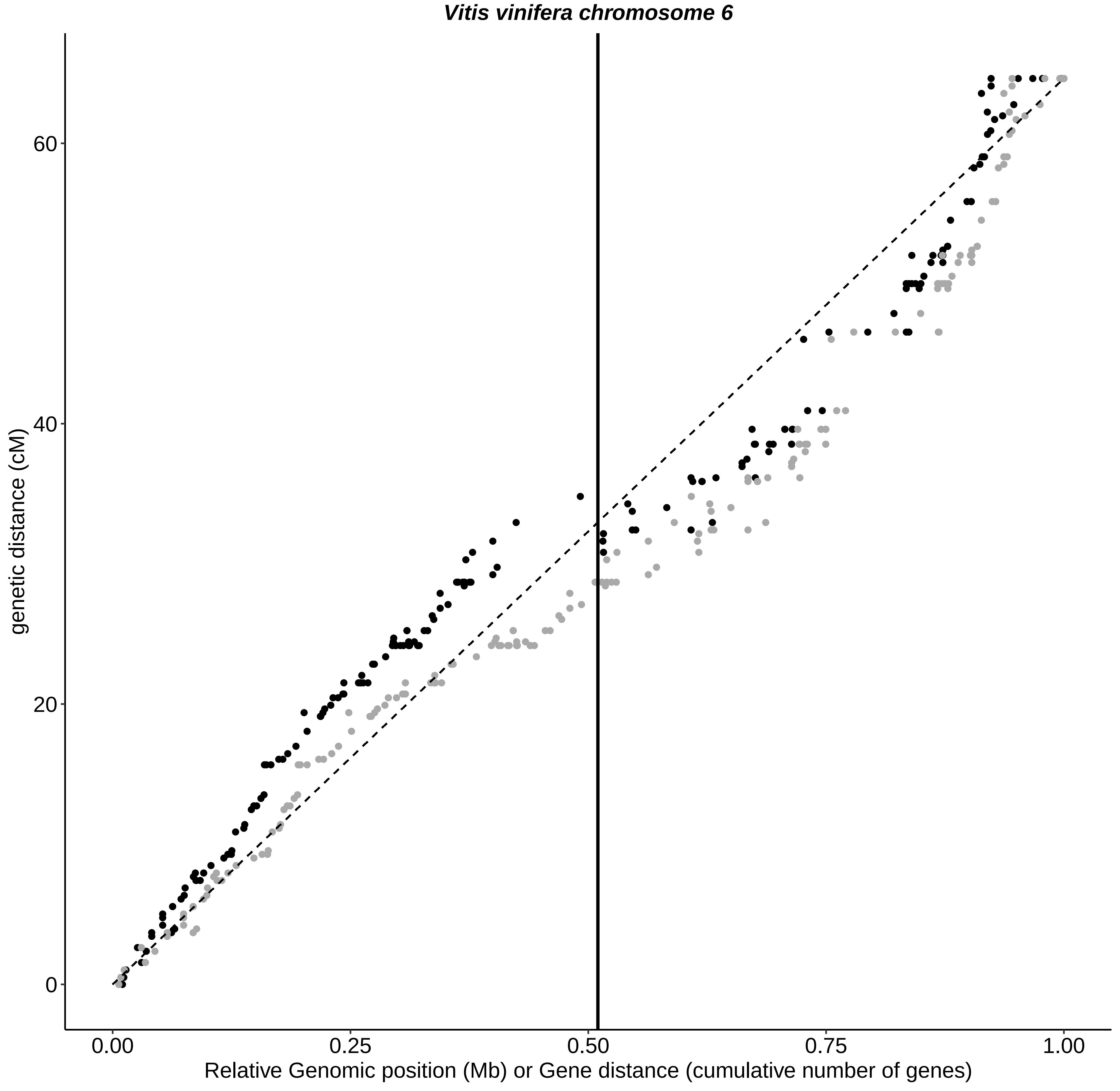
Vitis vinifera chromosome 6
genetic distance (cM)
0
20
40
60
0.00
0.25
0.50
0.75
1.00
Relative Genomic position (Mb) or Gene distance (cumulative number of genes)

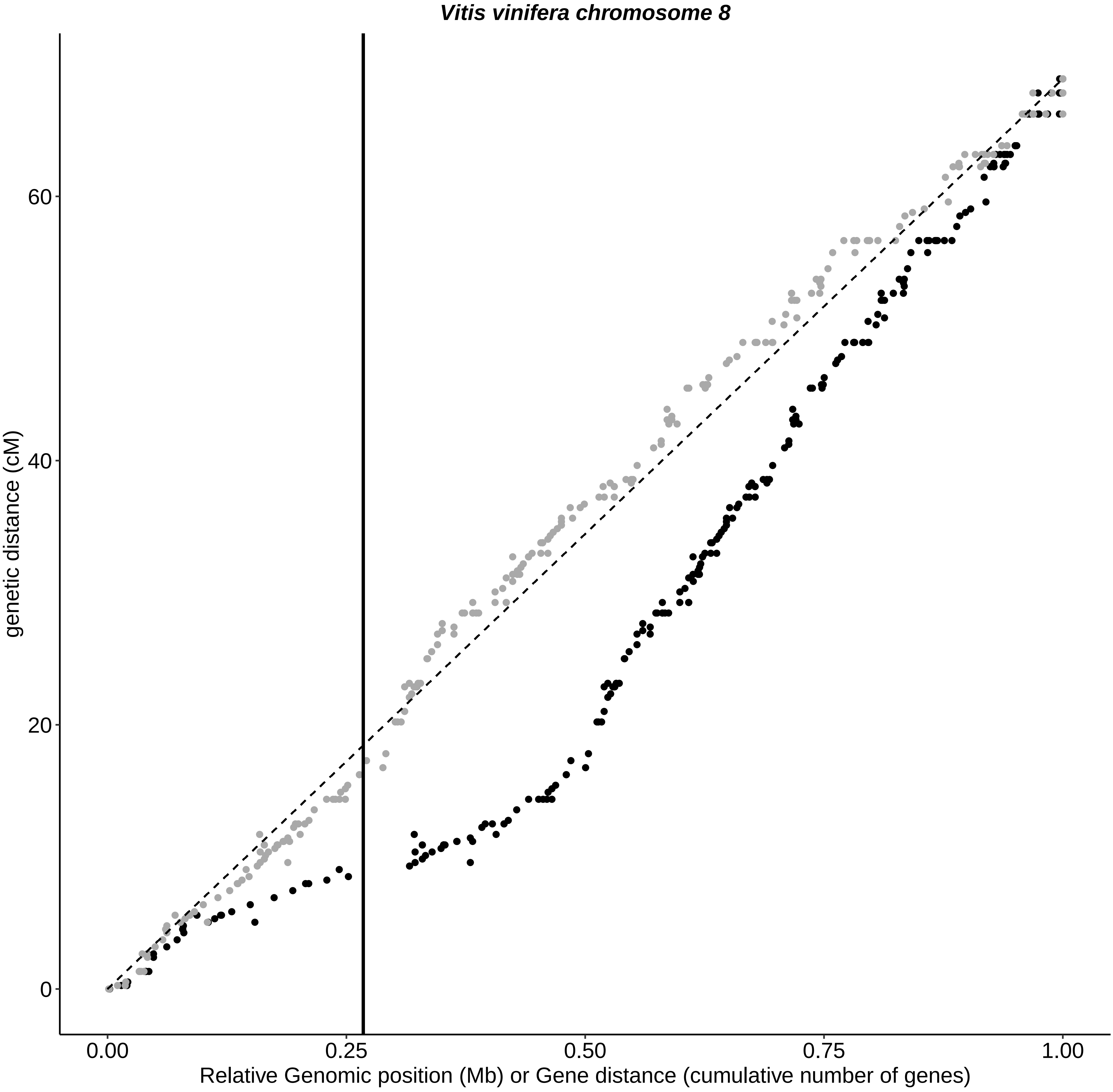
Vitis vinifera chromosome 8
genetic distance (cM)
0
20
40
60
0.00
0.25
0.50
0.75
1.00
Relative Genomic position (Mb) or Gene distance (cumulative number of genes)

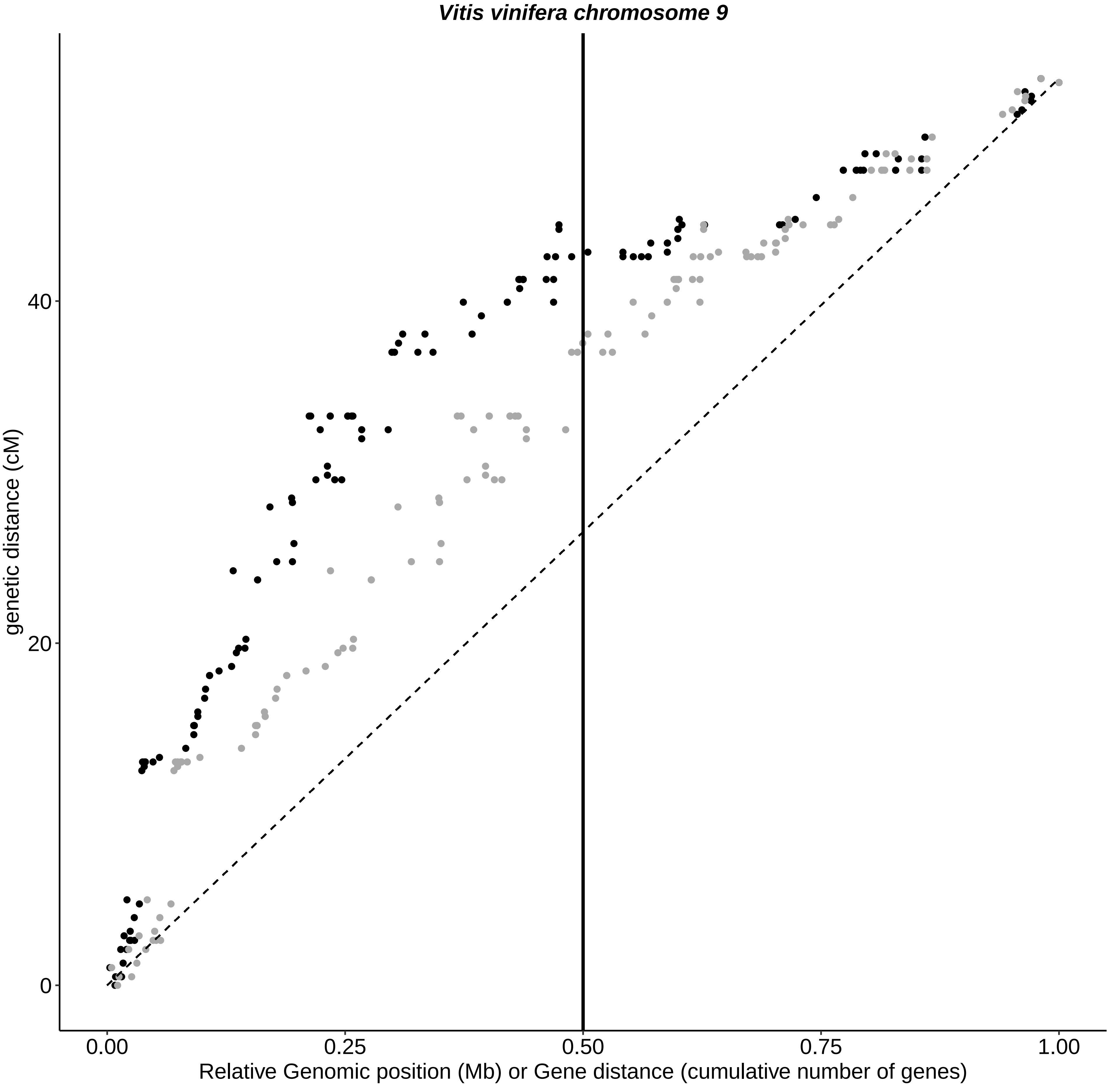
Vitis vinifera chromosome 9
genetic distance (cM)
0
20
40
0.00
0.25
0.50
0.75
1.00
Relative Genomic position (Mb) or Gene distance (cumulative number of genes)

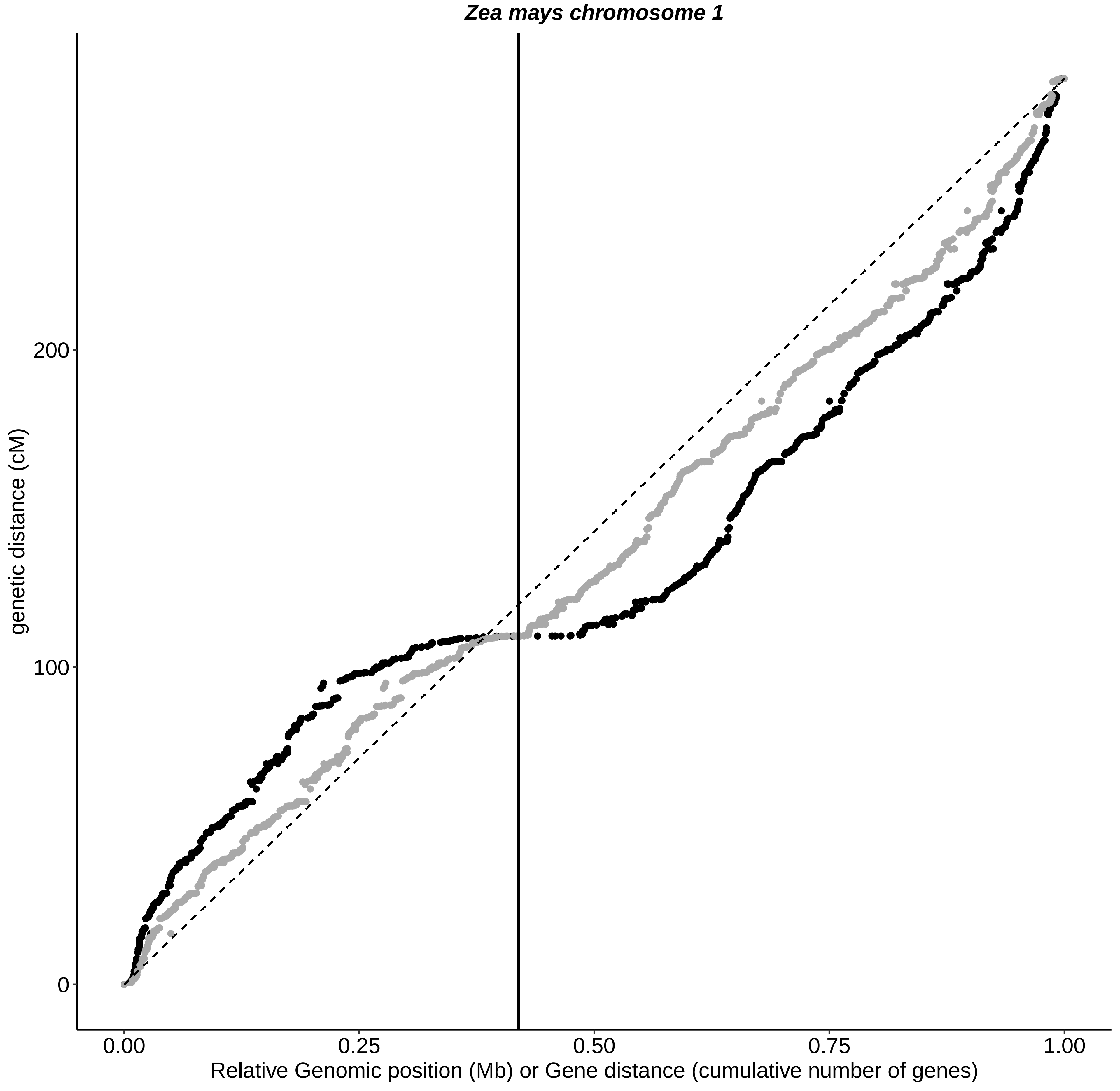
Zea mays chromosome 1
genetic distance (cM)
0
100
200
0.00
0.25
0.50
0.75
1.00
Relative Genomic position (Mb) or Gene distance (cumulative number of genes)

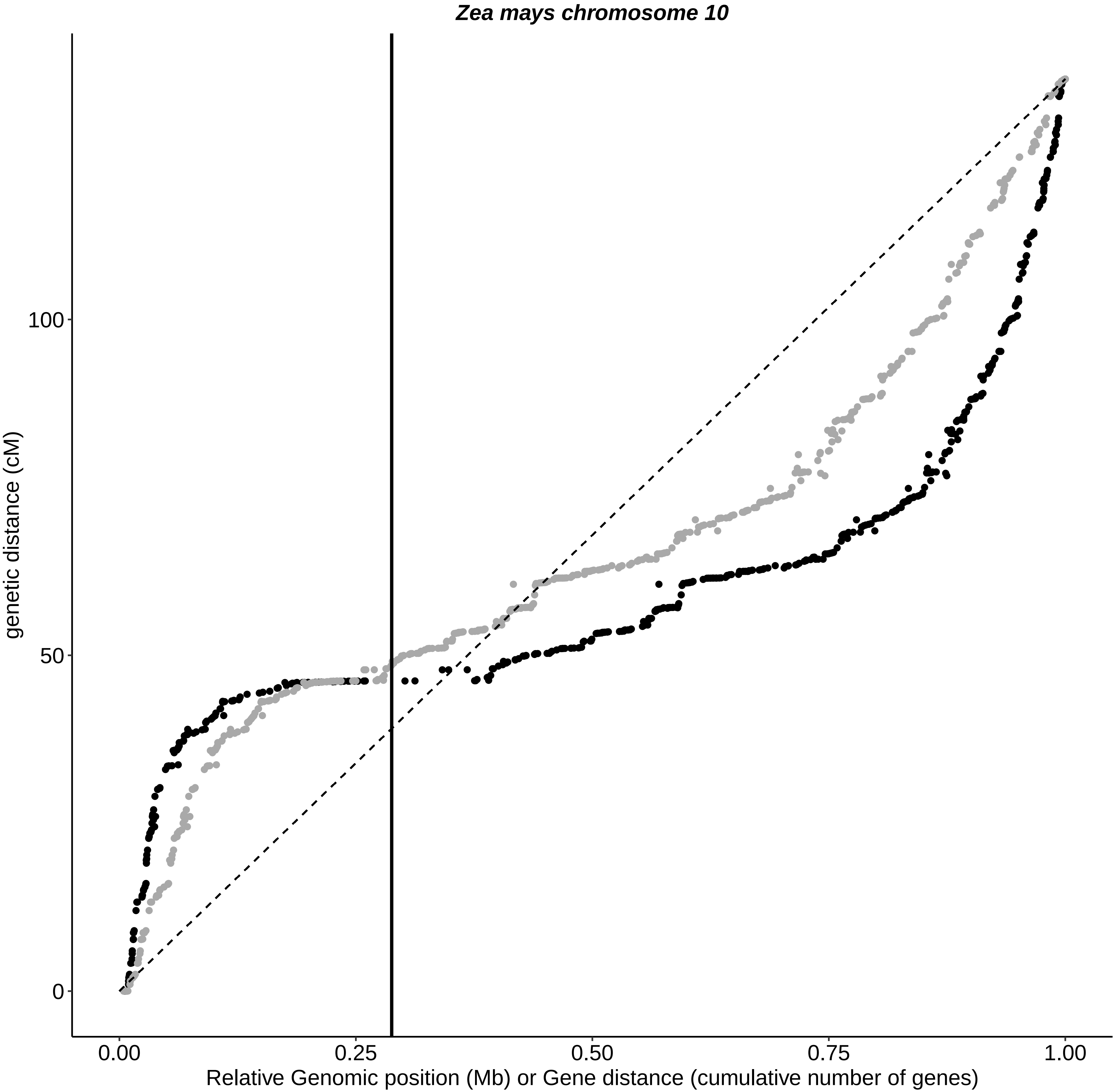
Zea mays chromosome 10
genetic distance (cM)
0
50
100
0.00
0.25
0.50
0.75
1.00
Relative Genomic position (Mb) or Gene distance (cumulative number of genes)

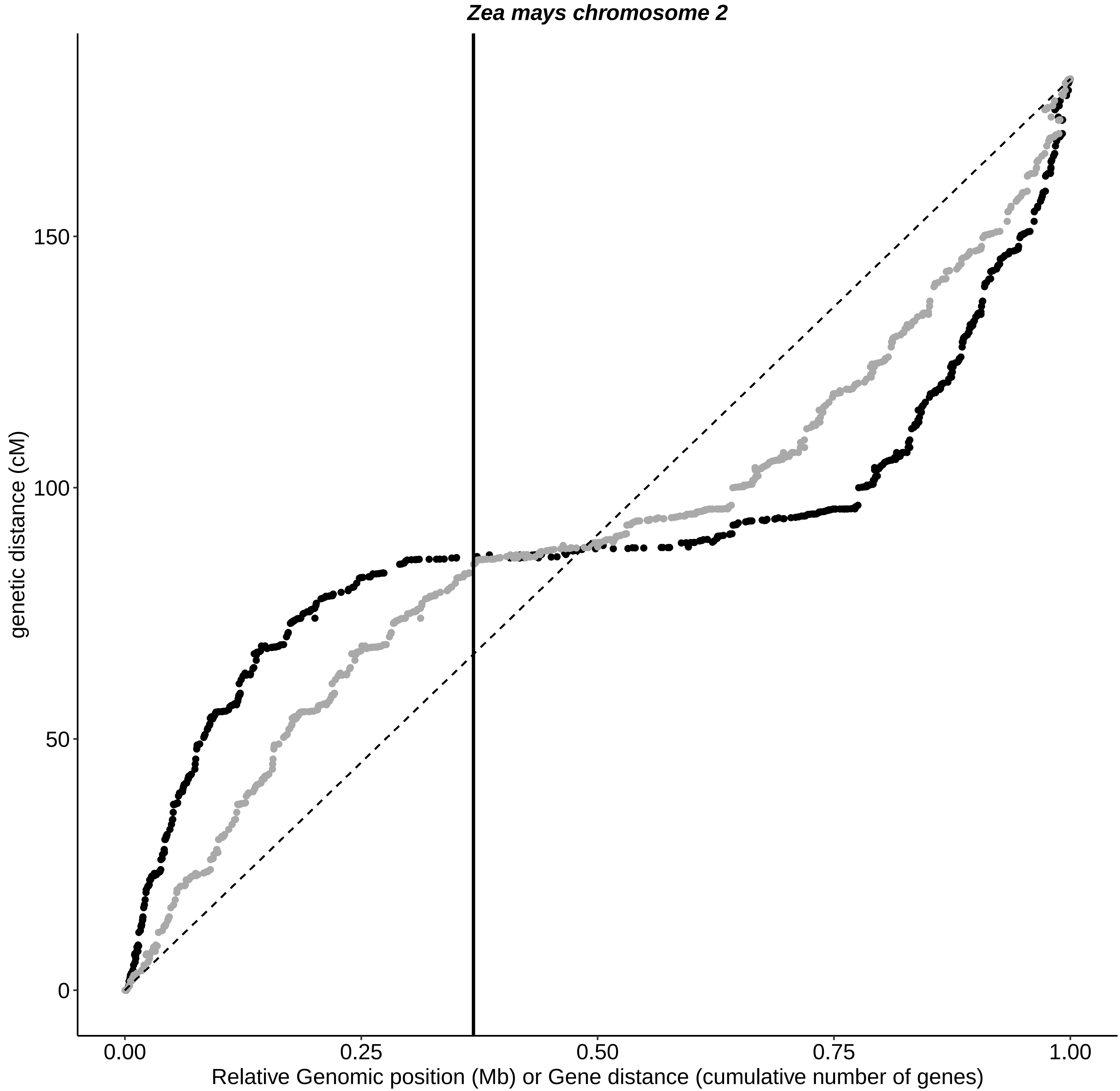
Zea mays chromosome 2
genetic distance (cM)
150
100
50
0
0.00
0.25
0.50
0.75
1.00
Relative Genomic position (Mb) or Gene distance (cumulative number of genes)

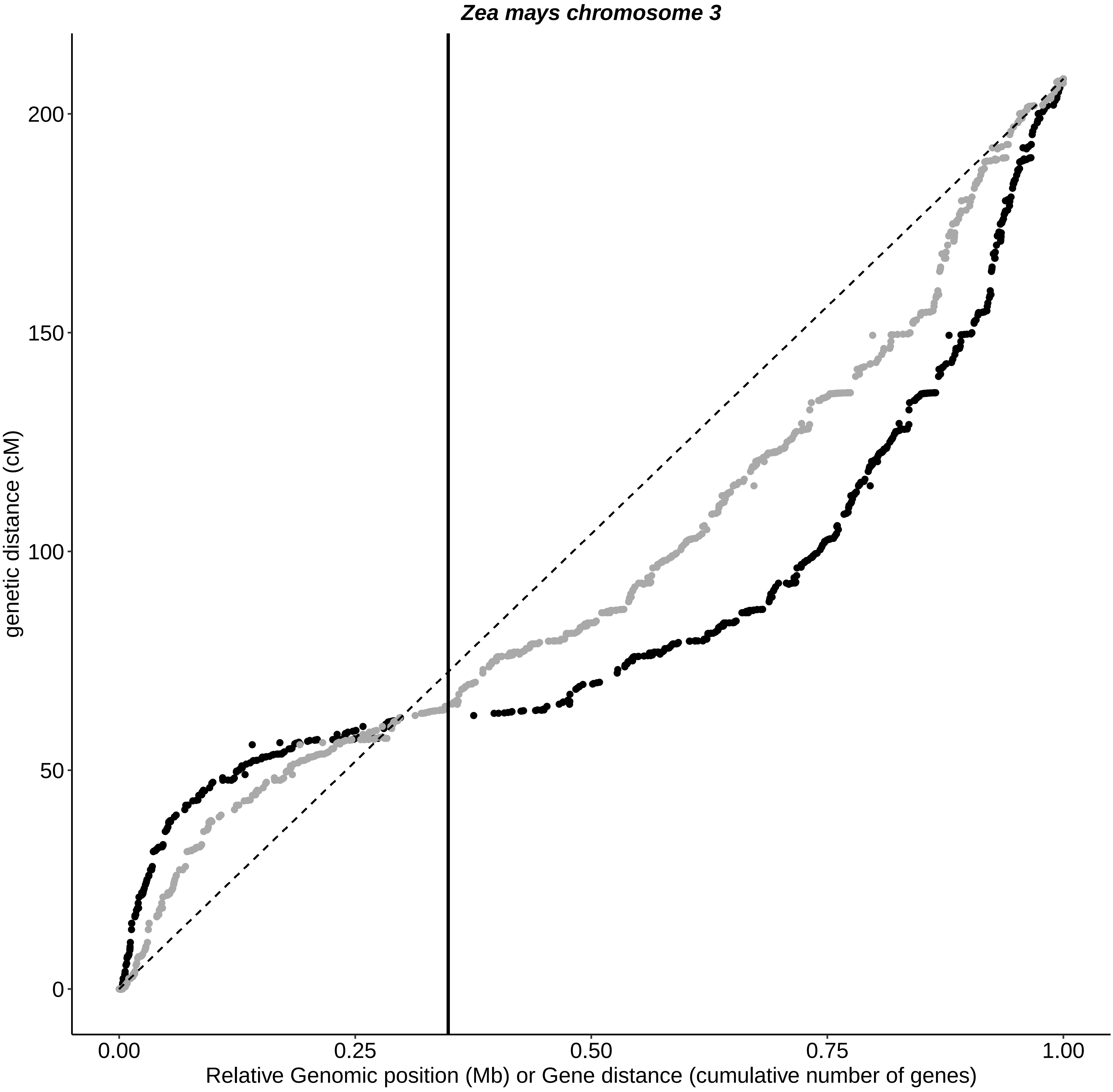
Zea mays chromosome 3
genetic distance (cM)
0
50
100
150
200
0.00
0.25
0.50
0.75
1.00
Relative Genomic position (Mb) or Gene distance (cumulative number of genes)

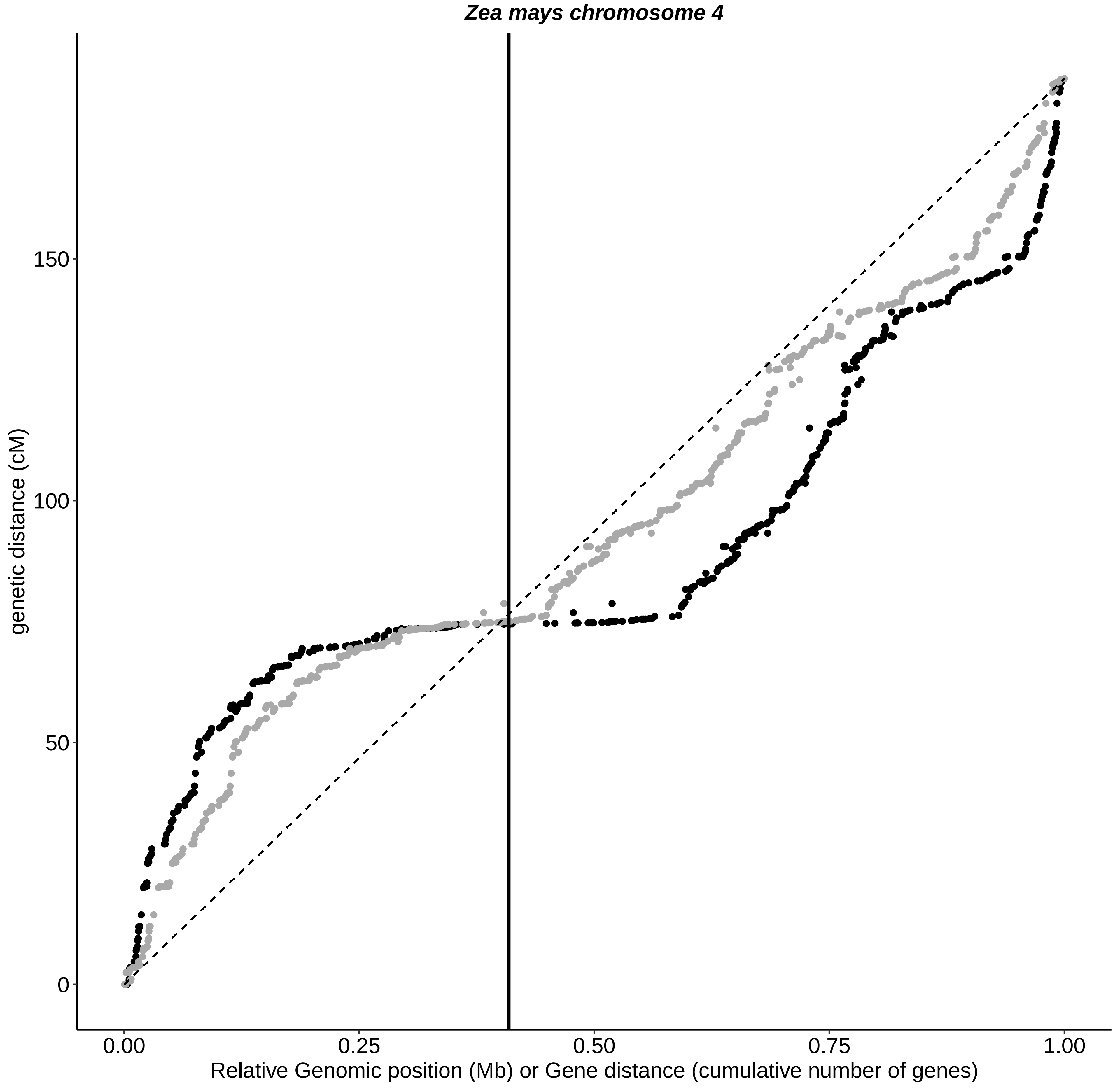
Zea mays chromosome 4
genetic distance (cM)
150
100
50
0
0.00
0.25
0.50
0.75
1.00
Relative Genomic position (Mb) or Gene distance (cumulative number of genes)

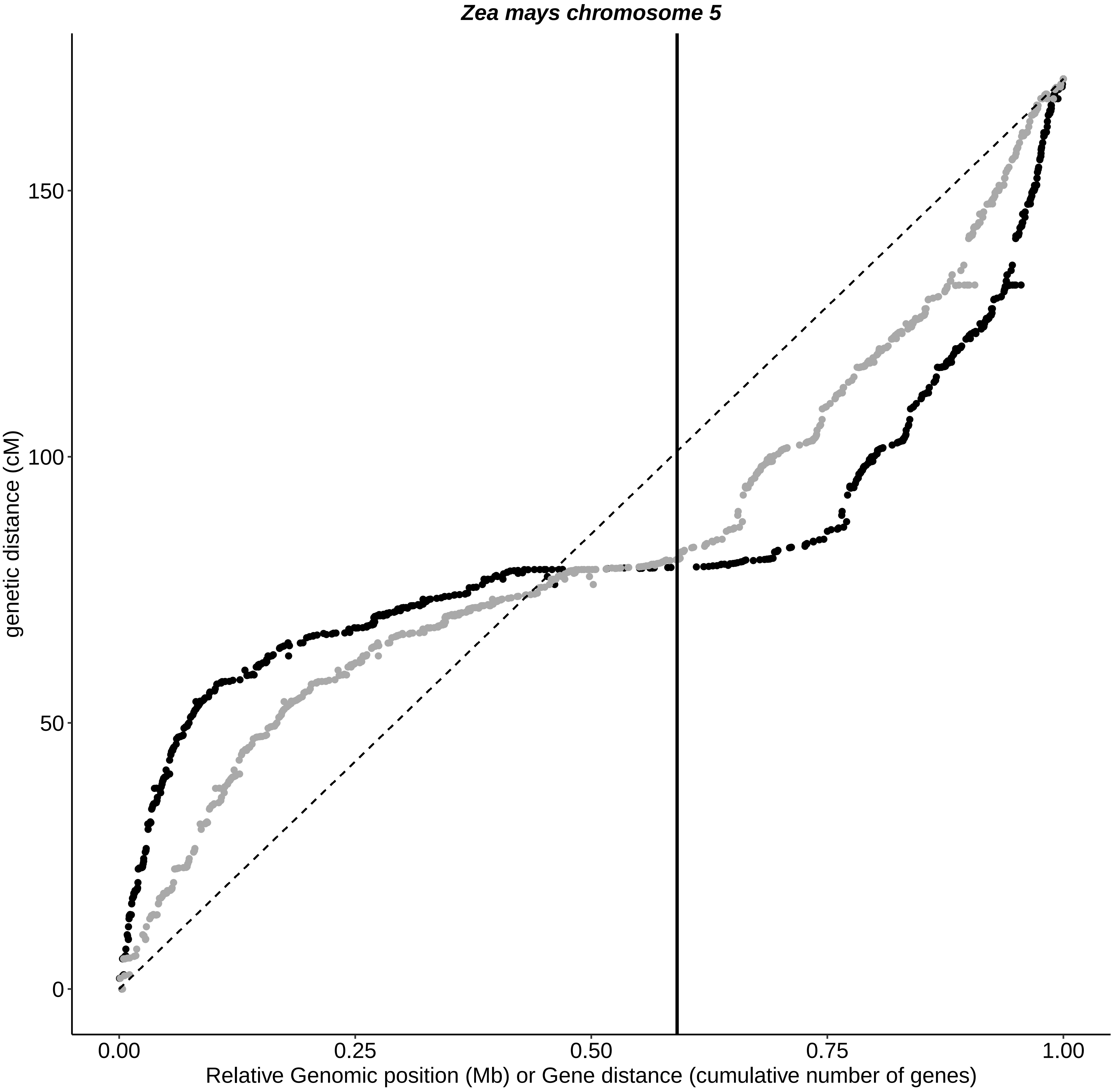
Zea mays chromosome 5
genetic distance (cM)
0
50
100
150
0.00
0.25
0.50
0.75
1.00
Relative Genomic position (Mb) or Gene distance (cumulative number of genes)

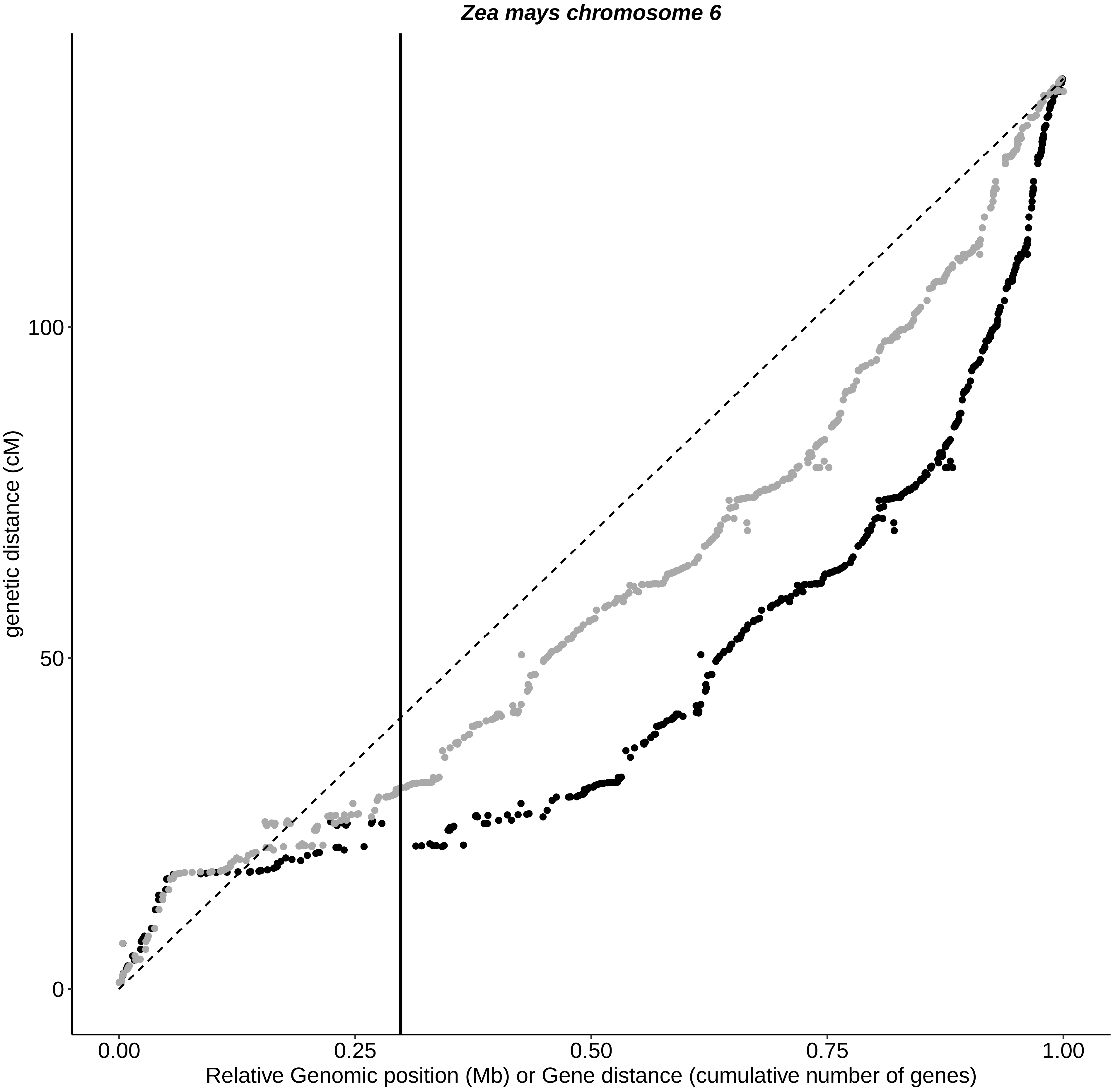
Zea mays chromosome 6
genetic distance (cM)
0
50
100
0.00
0.25
0.50
0.75
1.00
Relative Genomic position (Mb) or Gene distance (cumulative number of genes)

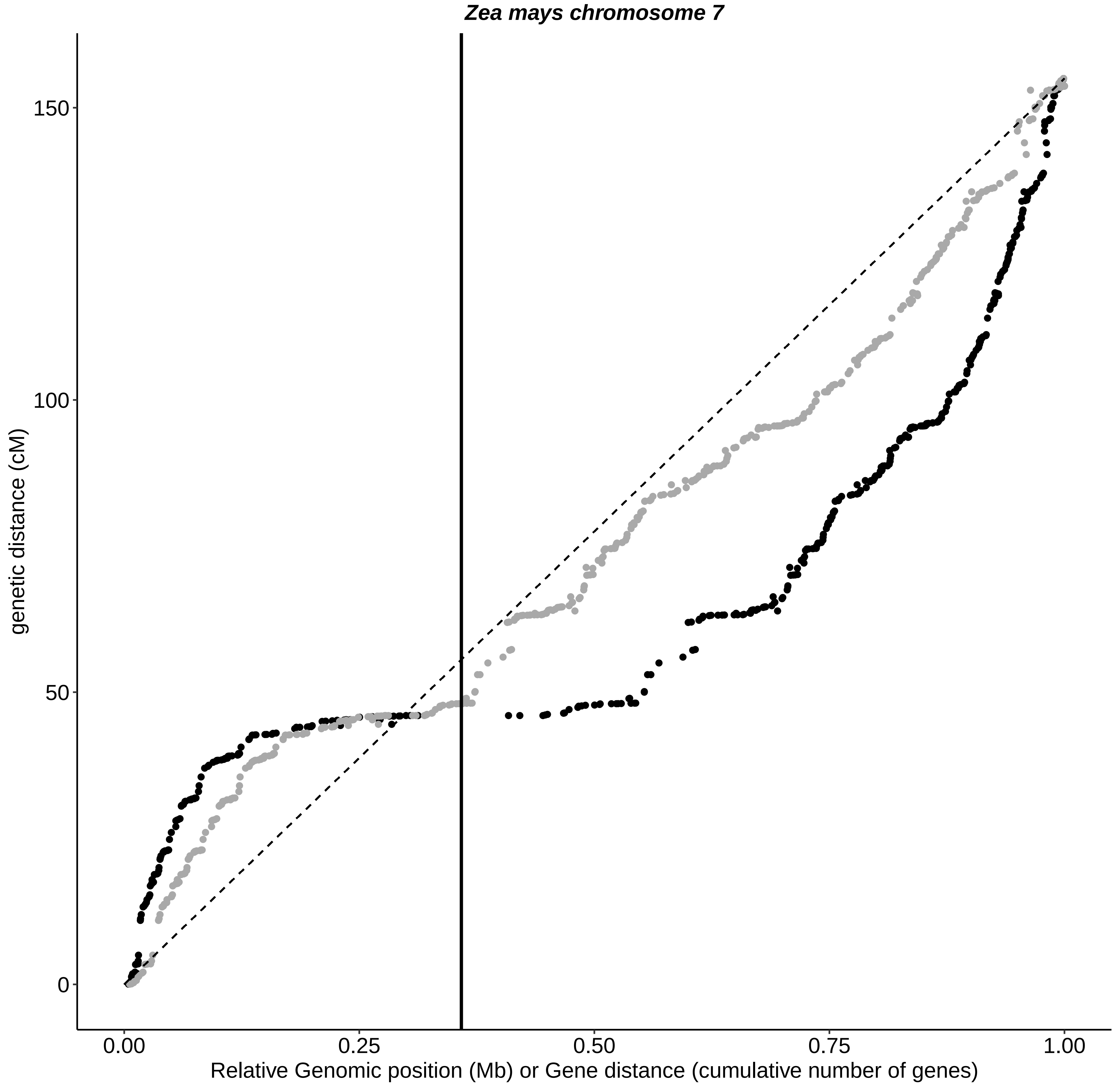
Zea mays chromosome 7
150
100
50
0
genetic distance (cM)
0.00
0.25
0.50
0.75
1.00
Relative Genomic position (Mb) or Gene distance (cumulative number of genes)

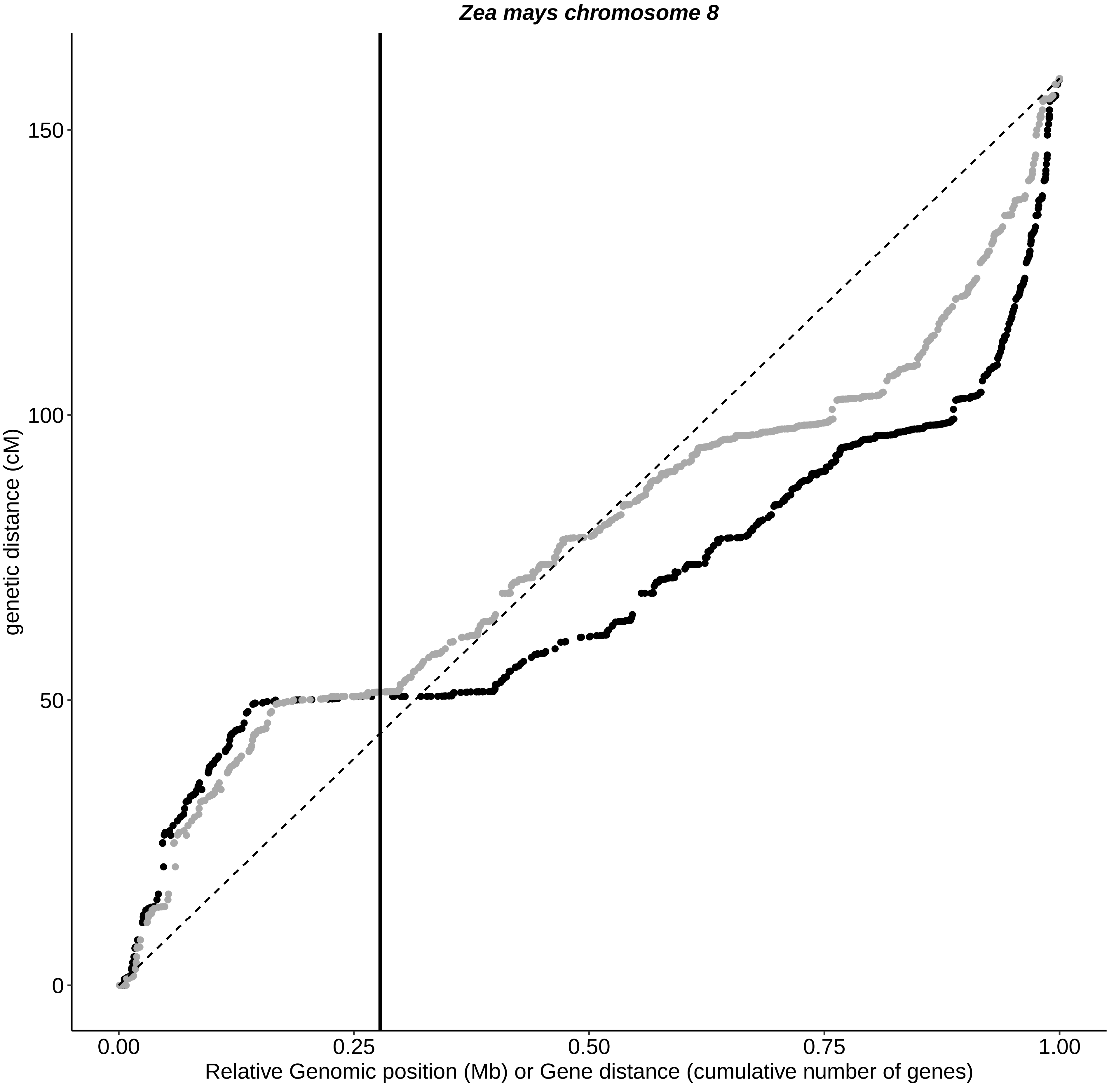

Zea mays chromosome 8
genetic distance (cM)
0
50
100
150
0.00
0.25
0.50
0.75
1.00
Relative Genomic position (Mb) or Gene distance (cumulative number of genes)

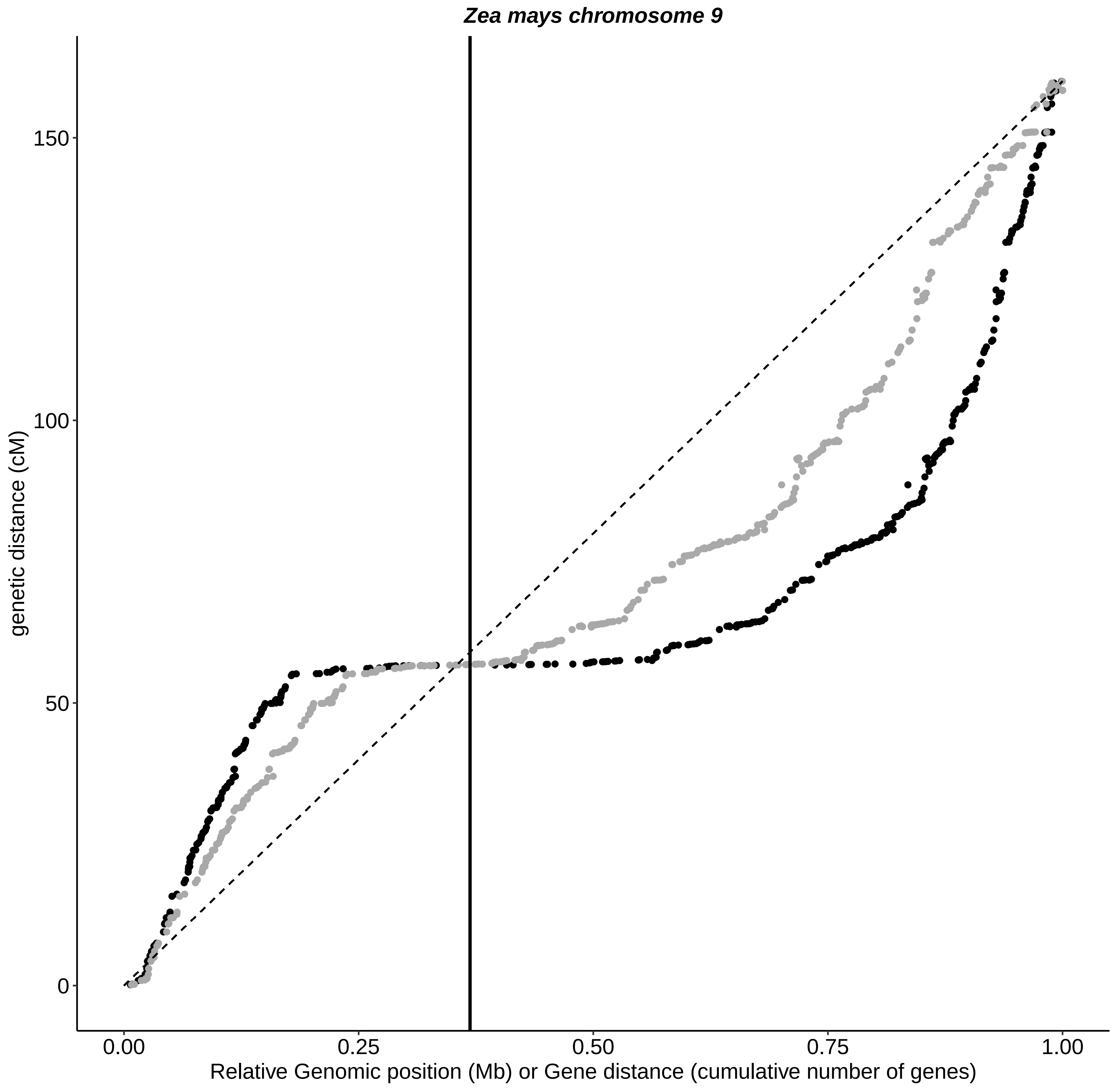
Zea mays chromosome 9
genetic distance (cM)
0
50
100
150
0.00
0.25
0.50
0.75
1.00
Relative Genomic position (Mb) or Gene distance (cumulative number of genes)
