# Supplementary material for: Diversity and determinants of recombination landscapes in flowering plants
Source: PLoS Genet. 2022 Aug 30;18(8):e1010141. doi: 10.1371/journal.pgen.1010141 (PMC9467342; doi:10.1371/journal.pgen.1010141)
Supplement: S1 Data — (PDF) [file pgen.1010141.s025.pdf]

References for dataset included in this study

Bartholomé, J., Mandrou, E., Mabiala, A., Jenkins, J., Nabihoudine, I., Klopp, C., Schmutz, J., Plomion, C., Gion, J., 2015. High-resolution genetic maps of *E ucalyptus* improve *Eucalyptus grandis* genome assembly. New Phytol 206, 1283–1296. https://doi.org/10.1111/nph.13150

Bennetzen, J.L., Schmutz, J., Wang, H., Percifield, R., Hawkins, J., Pontaroli, A.C., Estep, M., Feng, L., Vaughn, J.N., Grimwood, J., Jenkins, J., Barry, K., Lindquist, E., Hellsten, U., Deshpande, S., Wang, X., Wu, X., Mitros, T., Triplett, J., Yang, X., Ye, C.-Y., Mauro-Herrera, M., Wang, L., Li, P., Sharma, M., Sharma, R., Ronald, P.C., Panaud, O., Kellogg, E.A., Brutnell, T.P., Doust, A.N., Tuskan, G.A., Rokhsar, D., Devos, K.M., 2012. Reference genome sequence of the model plant Setaria. Nat Biotechnol 30, 555–561. https://doi.org/10.1038/nbt.2196

Bertioli, D.J., Cannon, S.B., Froenicke, L., Huang, G., Farmer, A.D., Cannon, E.K.S., Liu, X., Gao, D., Clevenger, J., Dash, S., Ren, L., Moretzsohn, M.C., Shirasawa, K., Huang, W., Vidigal, B., Abernathy, B., Chu, Y., Niederhuth, C.E., Umale, P., Araújo, A.C.G., Kozik, A., Do Kim, K., Burow, M.D., Varshney, R.K., Wang, X., Zhang, X., Barkley, N., Guimarães, P.M., Isobe, S., Guo, B., Liao, B., Stalker, H.T., Schmitz, R.J., Scheffler, B.E., Leal-Bertioli, S.C.M., Xun, X., Jackson, S.A., Michelmore, R., Ozias-Akins, P., 2016. The genome sequences of Arachis duranensis and Arachis ipaensis, the diploid ancestors of cultivated peanut. Nat Genet 48, 438–446. https://doi.org/10.1038/ng.3517

Bodénès, C., Chancerel, E., Ehrenmann, F., Kremer, A., Plomion, C., 2016. High-density linkage mapping and distribution of segregation distortion regions in the oak genome. DNA Res 23, 115–124. https://doi.org/10.1093/dnares/dsw001

Brault, C., Doligez, A., Cunff, L., Coupel-Ledru, A., Simonneau, T., Chiquet, J., This, P., Flutre, T., 2021. Harnessing multivariate, penalized regression methods for genomic prediction

and QTL detection of drought-related traits in grapevine. G3 Genes|Genomes|Genetics 11, jkab248. https://doi.org/10.1093/g3journal/jkab248

Cormier, F., Lawac, F., Maledon, E., Gravillon, M.-C., Nudol, E., Mournet, P., Vignes, H., Chaïr, H., Arnau, G., 2019. A reference high-density genetic map of greater yam (Dioscorea alata L.). Theor Appl Genet 132, 1733–1744. https://doi.org/10.1007/s00122-019-03311-6

De Leon, T.B., Linscombe, S., Subudhi, P.K., 2016. Molecular Dissection of Seedling Salinity Tolerance in Rice (Oryza sativa L.) Using a High-Density GBS-Based SNP Linkage Map. Rice 9, 52. https://doi.org/10.1186/s12284-016-0125-2

Di Pierro, E.A., Gianfranceschi, L., Di Guardo, M., Koehorst-van Putten, H.J., Kruisselbrink, J.W., Longhi, S., Troggio, M., Bianco, L., Muranty, H., Pagliarani, G., Tartarini, S., Letschka, T., Lozano Luis, L., Garkava-Gustavsson, L., Micheletti, D., Bink, M.C., Voorrips, R.E., Aziz, E., Velasco, R., Laurens, F., van de Weg, W.E., 2016. A high-density, multi-parental SNP genetic map on apple validates a new mapping approach for outcrossing species. Hortic Res 3, 16057. https://doi.org/10.1038/hortres.2016.57

Endelman, J.B., Jansky, S.H., 2016. Genetic mapping with an inbred line-derived F2 population in potato. Theor Appl Genet 129, 935–943. https://doi.org/10.1007/s00122-016-2673-7

Ganal, M.W., Durstewitz, G., Polley, A., Bérard, A., Buckler, E.S., Charcosset, A., Clarke, J.D., Graner, E.-M., Hansen, M., Joets, J., Le Paslier, M.-C., McMullen, M.D., Montalent, P., Rose, M., Schön, C.-C., Sun, Q., Walter, H., Martin, O.C., Falque, M., 2011. A Large Maize (Zea mays L.) SNP Genotyping Array: Development and Germplasm Genotyping, and Genetic Mapping to Compare with the B73 Reference Genome. PLoS ONE 6, e28334. https://doi.org/10.1371/journal.pone.0028334

Gonda, I., Ashrafi, H., Lyon, D.A., Strickler, S.R., Hulse-Kemp, A.M., Ma, Q., Sun, H., Stoffel, K., Powell, A.F., Futrell, S., Thannhauser, T.W., Fei, Z., Van Deynze, A.E., Mueller,

L.A., Giovannoni, J.J., Foolad, M.R., 2019. Sequencing-Based Bin Map Construction of a Tomato Mapping Population, Facilitating High-Resolution Quantitative Trait Loci Detection. The Plant Genome 12, 180010. https://doi.org/10.3835/plantgenome2018.02.0010

Gui, S., Peng, J., Wang, X., Wu, Z., Cao, R., Salse, J., Zhang, H., Zhu, Z., Xia, Q., Quan, Z., Shu, L., Ke, W., Ding, Y., 2018. Improving *Nelumbo nucifera* genome assemblies using high-resolution genetic maps and BioNano genome mapping reveals ancient chromosome rearrangements. Plant J 94, 721–734. https://doi.org/10.1111/tpj.13894

Gutierrez-Gonzalez, J.J., Mascher, M., Poland, J., Muehlbauer, G.J., 2019. Dense genotyping-by-sequencing linkage maps of two Synthetic W7984×Opata reference populations provide insights into wheat structural diversity. Sci Rep 9, 1793. https://doi.org/10.1038/s41598-018-38111-3

Han, K., Jeong, H.-J., Yang, H.-B., Kang, S.-M., Kwon, J.-K., Kim, S., Choi, D., Kang, B.-C., 2016. An ultra-high-density bin map facilitates high-throughput QTL mapping of horticultural traits in pepper (*Capsicum annuum*). DNA Res 23, 81–91. https://doi.org/10.1093/dnares/dsv038

Huo, N., Garvin, D.F., You, F.M., McMahon, S., Luo, M.-C., Gu, Y.Q., Lazo, G.R., Vogel, J.P., 2011. Comparison of a high-density genetic linkage map to genome features in the model grass Brachypodium distachyon. Theor Appl Genet 123, 455–464. https://doi.org/10.1007/s00122-011-1598-4

International Cassava Genetic Map Consortium (ICGMC), 2015. High-Resolution Linkage Map and Chromosome-Scale Genome Assembly for Cassava (*Manihot esculenta* Crantz) from 10 Populations. G3 Genes|Genomes|Genetics 5, 133–144. https://doi.org/10.1534/g3.114.015008

Jorgensen, C., Luo, M.-C., Ramasamy, R., Dawson, M., Gill, B.S., Korol, A.B., Distelfeld, A., Dvorak, J., 2017. A High-Density Genetic Map of Wild Emmer Wheat from the Karaca Dağ

Region Provides New Evidence on the Structure and Evolution of Wheat Chromosomes. Front. Plant Sci. 8, 1798. https://doi.org/10.3389/fpls.2017.01798

King, K., Li, H., Kang, J., Lu, C., 2019. Mapping quantitative trait loci for seed traits in Camelina sativa. Theor Appl Genet 132, 2567–2577. https://doi.org/10.1007/s00122-019-03371-8

Książkiewicz, M., Nazzicari, N., Yang, H., Nelson, M.N., Renshaw, D., Rychel, S., Ferrari, B., Carelli, M., Tomaszewska, M., Stawiński, S., Naganowska, B., Wolko, B., Annicchiarico, P., 2017. A high-density consensus linkage map of white lupin highlights synteny with narrow-leafed lupin and provides markers tagging key agronomic traits. Sci Rep 7, 15335. https://doi.org/10.1038/s41598-017-15625-w

Lee, C.-R., Wang, B., Mojica, J.P., Mandáková, T., Prasad, K.V.S.K., Goicoechea, J.L., Perera, N., Hellsten, U., Hundley, H.N., Johnson, J., Grimwood, J., Barry, K., Fairclough, S., Jenkins, J.W., Yu, Y., Kudrna, D., Zhang, J., Talag, J., Golser, W., Ghattas, K., Schranz, M.E., Wing, R., Lysak, M.A., Schmutz, J., Rokhsar, D.S., Mitchell-Olds, T., 2017. Young inversion with multiple linked QTLs under selection in a hybrid zone. Nat Ecol Evol 1, 0119. https://doi.org/10.1038/s41559-017-0119

Ling, H.-Q., Ma, B., Shi, X., Liu, H., Dong, L., Sun, H., Cao, Y., Gao, Q., Zheng, S., Li, Ye, Yu, Y., Du, H., Qi, M., Li, Yan, Lu, H., Yu, H., Cui, Y., Wang, N., Chen, C., Wu, H., Zhao, Y., Zhang, J., Li, Yiwen, Zhou, W., Zhang, B., Hu, W., van Eijk, M.J.T., Tang, J., Witsenboer, H.M.A., Zhao, S., Li, Z., Zhang, A., Wang, D., Liang, C., 2018. Genome sequence of the progenitor of wheat A subgenome Triticum urartu. Nature 557, 424–428. https://doi.org/10.1038/s41586-018-0108-0

Lonardi, S., Muñoz-Amatriaín, M., Liang, Q., Shu, S., Wanamaker, S.I., Lo, S., Tanskanen, J., Schulman, A.H., Zhu, T., Luo, M., Alhakami, H., Ounit, R., Hasan, A.Md., Verdier, J., Roberts, P.A., Santos, J.R.P., Ndeve, A., Doležel, J., Vrána, J., Hokin, S.A., Farmer, A.D.,

Cannon, S.B., Close, T.J., 2019. The genome of cowpea (*Vigna unguiculata* [L.] Walp.). Plant J 98, 767–782. https://doi.org/10.1111/tpj.14349

Lovell, J.T., Jenkins, J., Lowry, D.B., Mamidi, S., Sreedasyam, A., Weng, X., Barry, K., Bonnette, J., Campitelli, B., Daum, C., Gordon, S.P., Gould, B.A., Khasanova, A., Lipzen, A., MacQueen, A., Palacio-Mejía, J.D., Plott, C., Shakirov, E.V., Shu, S., Yoshinaga, Y., Zane, M., Kudrna, D., Talag, J.D., Rokhsar, D., Grimwood, J., Schmutz, J., Juenger, T.E., 2018. The genomic landscape of molecular responses to natural drought stress in Panicum hallii. Nat Commun 9, 5213. https://doi.org/10.1038/s41467-018-07669-x

Luo, C., Shu, B., Yao, Q., Wu, H., Xu, W., Wang, S., 2016. Construction of a High-Density Genetic Map Based on Large-Scale Marker Development in Mango Using Specific-Locus Amplified Fragment Sequencing (SLAF-seq). Front. Plant Sci. 7. https://doi.org/10.3389/fpls.2016.01310

Luo, M.-C., You, F.M., Li, P., Wang, J.-R., Zhu, T., Dandekar, A.M., Leslie, C.A., Aradhya, M., McGuire, P.E., Dvorak, J., 2015. Synteny analysis in Rosids with a walnut physical map reveals slow genome evolution in long-lived woody perennials. BMC Genomics 16, 707. https://doi.org/10.1186/s12864-015-1906-5

Ma, X., Fu, Y., Zhao, X., Jiang, L., Zhu, Z., Gu, P., Xu, W., Su, Z., Sun, C., Tan, L., 2016. Genomic structure analysis of a set of Oryza nivara introgression lines and identification of yield-associated QTLs using whole-genome resequencing. Sci Rep 6, 27425. https://doi.org/10.1038/srep27425

Markelz, R.J.C., Covington, M.F., Brock, M.T., Devisetty, U.K., Kliebenstein, D.J., Weinig, C., Maloof, J.N., 2017. Using RNA-Seq for Genomic Scaffold Placement, Correcting Assemblies, and Genetic Map Creation in a Common *Brassica rapa* Mapping Population. G3 Genes|Genomes|Genetics 7, 2259–2270. https://doi.org/10.1534/g3.117.043000

Matsumura, H., Hsiao, M.-C., Lin, Y.-P., Toyoda, A., Taniai, N., Tarora, K., Urasaki, N., Anand, S.S., Dhillon, N.P.S., Schafleitner, R., Lee, C.-R., 2020. Long-read bitter gourd (*Momordica charantia*) genome and the genomic architecture of nonclassic domestication. Proc. Natl. Acad. Sci. U.S.A. 117, 14543–14551. https://doi.org/10.1073/pnas.1921016117

Merot-L'anthoene, V., Tournebize, R., Darracq, O., Rattina, V., Lepelley, M., Bellanger, L., Tranchant-Dubreuil, C., Coulée, M., Pégard, M., Metairon, S., Fournier, C., Stoffelen, P., Janssens, S.B., Kiwuka, C., Musoli, P., Sumirat, U., Legnaté, H., Kambale, J., Ferreira da Costa Neto, J., Revel, C., de Kochko, A., Descombes, P., Crouzillat, D., Poncet, V., 2019. Development and evaluation of a genome-wide Coffee 8.5K SNP array and its application for high-density genetic mapping and for investigating the origin of *Coffea arabica* L. Plant Biotechnol J 17, 1418–1430. https://doi.org/10.1111/pbi.13066

Montero-Pau, J., Blanca, J., Esteras, C., Martínez-Pérez, E.Ma., Gómez, P., Monforte, A.J., Cañizares, J., Picó, B., 2017. An SNP-based saturated genetic map and QTL analysis of fruit-related traits in Zucchini using Genotyping-by-sequencing. BMC Genomics 18, 94. https://doi.org/10.1186/s12864-016-3439-y

Mun, J.-H., Chung, H., Chung, W.-H., Oh, M., Jeong, Y.-M., Kim, N., Ahn, B.O., Park, B.-S., Park, S., Lim, K.-B., Hwang, Y.-J., Yu, H.-J., 2015. Construction of a reference genetic map of Raphanus sativus based on genotyping by whole-genome resequencing. Theor Appl Genet 128, 259–272. https://doi.org/10.1007/s00122-014-2426-4

Muñoz-Amatriaín, M., Cuesta-Marcos, A., Endelman, J.B., Comadran, J., Bonman, J.M., Bockelman, H.E., Chao, S., Russell, J., Waugh, R., Hayes, P.M., Muehlbauer, G.J., 2014. The USDA Barley Core Collection: Genetic Diversity, Population Structure, and Potential for Genome-Wide Association Studies. PLoS ONE 9, e94688. https://doi.org/10.1371/journal.pone.0094688

Nowak, M.D., Birkeland, S., Mandáková, T., Roy Choudhury, R., Guo, X., Gustafsson, A.L.S., Gizaw, A., Schrøder-Nielsen, A., Fracassetti, M., Brysting, A.K., Rieseberg, L., Slotte,

T., Parisod, C., Lysak, M.A., Brochmann, C., 2021. The genome of *Draba nivalis* shows signatures of adaptation to the extreme environmental stresses of the Arctic. Mol Ecol Resour 21, 661–676. https://doi.org/10.1111/1755-0998.13280

Pereira, L., Ruggieri, V., Pérez, S., Alexiou, K.G., Fernández, M., Jahrmann, T., Pujol, M., Garcia-Mas, J., 2018. QTL mapping of melon fruit quality traits using a high-density GBS-based genetic map. BMC Plant Biol 18, 324. https://doi.org/10.1186/s12870-018-1537-5

Pucher, A., Hash, C.T., Wallace, J.G., Han, S., Leiser, W.L., Haussmann, B.I.G., 2018. Mapping a male-fertility restoration locus for the A4 cytoplasmic-genic male-sterility system in pearl millet using a genotyping-by-sequencing-based linkage map. BMC Plant Biol 18, 65. https://doi.org/10.1186/s12870-018-1267-8

Ren, R., Ray, R., Li, P., Xu, J., Zhang, M., Liu, G., Yao, X., Kilian, A., Yang, X., 2015a. Construction of a high-density DArTseq SNP-based genetic map and identification of genomic regions with segregation distortion in a genetic population derived from a cross between feral and cultivated-type watermelon. Mol Genet Genomics 290, 1457–1470. https://doi.org/10.1007/s00438-015-0997-7

Ren, R., Ray, R., Li, P., Xu, J., Zhang, M., Liu, G., Yao, X., Kilian, A., Yang, X., 2015b. Construction of a high-density DArTseq SNP-based genetic map and identification of genomic regions with segregation distortion in a genetic population derived from a cross between feral and cultivated-type watermelon. Mol Genet Genomics 290, 1457–1470. https://doi.org/10.1007/s00438-015-0997-7

Royaert, S., Jansen, J., da Silva, D.V., de Jesus Branco, S.M., Livingstone, D.S., Mustiga, G., Marelli, J.-P., Araújo, I.S., Corrêa, R.X., Motamayor, J.C., 2016. Identification of candidate genes involved in Witches' broom disease resistance in a segregating mapping population of Theobroma cacao L. in Brazil. BMC Genomics 17, 107. https://doi.org/10.1186/s12864-016-2415-x

Serin, E.A.R., Snoek, L.B., Nijveen, H., Willems, L.A.J., Jiménez-Gómez, J.M., Hilhorst, H.W.M., Ligterink, W., 2017. Construction of a High-Density Genetic Map from RNA-Seq Data for an Arabidopsis Bay-0 × Shahdara RIL Population. Front. Genet. 8, 201. https://doi.org/10.3389/fgene.2017.00201

Slotte, T., Hazzouri, K.M., Ågren, J.A., Koenig, D., Maumus, F., Guo, Y.-L., Steige, K., Platts, A.E., Escobar, J.S., Newman, L.K., Wang, W., Mandáková, T., Vello, E., Smith, L.M., Henz, S.R., Steffen, J., Takuno, S., Brandvain, Y., Coop, G., Andolfatto, P., Hu, T.T., Blanchette, M., Clark, R.M., Quesneville, H., Nordborg, M., Gaut, B.S., Lysak, M.A., Jenkins, J., Grimwood, J., Chapman, J., Prochnik, S., Shu, S., Rokhsar, D., Schmutz, J., Weigel, D., Wright, S.I., 2013. The Capsella rubella genome and the genomic consequences of rapid mating system evolution. Nat Genet 45, 831–835. https://doi.org/10.1038/ng.2669

Song, Q., Jia, G., Hyten, D.L., Jenkins, J., Hwang, E.-Y., Schroeder, S.G., Osorno, J.M., Schmutz, J., Jackson, S.A., McClean, P.E., Cregan, P.B., 2015. SNP Assay Development for Linkage Map Construction, Anchoring Whole-Genome Sequence, and Other Genetic and Genomic Applications in Common Bean. G3 Genes|Genomes|Genetics 5, 2285–2290. https://doi.org/10.1534/g3.115.020594

Talukder, Z.I., Gong, L., Hulke, B.S., Pegadaraju, V., Song, Q., Schultz, Q., Qi, L., 2014. A High-Density SNP Map of Sunflower Derived from RAD-Sequencing Facilitating Fine-Mapping of the Rust Resistance Gene R12. PLoS ONE 9, e98628. https://doi.org/10.1371/journal.pone.0098628

Verde, I., Jenkins, J., Dondini, L., Micali, S., Pagliarani, G., Vendramin, E., Paris, R., Aramini, V., Gazza, L., Rossini, L., Bassi, D., Troggio, M., Shu, S., Grimwood, J., Tartarini, S., Dettori, M.T., Schmutz, J., 2017. The Peach v2.0 release: high-resolution linkage mapping and deep resequencing improve chromosome-scale assembly and contiguity. BMC Genomics 18, 225. https://doi.org/10.1186/s12864-017-3606-9

Wang, L., Xia, Q., Zhang, Y., Zhu, Xiaodong, Zhu, Xiaofeng, Li, D., Ni, X., Gao, Y., Xiang, H., Wei, X., Yu, J., Quan, Z., Zhang, X., 2016. Updated sesame genome assembly and fine mapping of plant height and seed coat color QTLs using a new high-density genetic map. BMC Genomics 17, 31. https://doi.org/10.1186/s12864-015-2316-4

Wang, Y., Wang, C., Han, H., Luo, Y., Wang, Z., Yan, C., Xu, W., Qu, S., 2020. Construction of a High-Density Genetic Map and Analysis of Seed-Related Traits Using Specific Length Amplified Fragment Sequencing for Cucurbita maxima. Front. Plant Sci. 10, 1782. https://doi.org/10.3389/fpls.2019.01782

Wang, Z., Zhang, D., Wang, X., Tan, X., Guo, H., Paterson, A.H., 2013. A Whole-Genome DNA Marker Map for Cotton Based on the D-Genome Sequence of *Gossypium raimondii* L. G3 Genes|Genomes|Genetics 3, 1759–1767. https://doi.org/10.1534/g3.113.006890

Watanabe, S., Shimizu, T., Machita, K., Tsubokura, Y., Xia, Z., Yamada, T., Hajika, M., Ishimoto, M., Katayose, Y., Harada, K., Kaga, A., 2018. Development of a high-density linkage map and chromosome segment substitution lines for Japanese soybean cultivar Enrei. DNA Research 25, 123–136. https://doi.org/10.1093/dnares/dsx043

Xu, L.-Y., Wang, L.-Y., Wei, K., Tan, L.-Q., Su, J.-J., Cheng, H., 2018. High-density SNP linkage map construction and QTL mapping for flavonoid-related traits in a tea plant (Camellia sinensis) using 2b-RAD sequencing. BMC Genomics 19, 955. https://doi.org/10.1186/s12864-018-5291-8

Yaakub, Z., Kamaruddin, K., Singh, R., Mustafa, S., Marjuni, M., Ting, N.-C., Amiruddin, M.D., Leslie, L.E.-T., Cheng-Li, O.L., Sritha, K., Nookiah, R., Jansen, J., Abdullah, M.O., 2020. An Integrated Linkage Map of Interspecific Backcross 2 (BC2) Populations Reveals QTLs Associated With Fatty Acid Composition and Vegetative Parameters Influencing Compactness in Oil Palm (preprint). In Review. https://doi.org/10.21203/rs.2.12850/v3

Yang, Y., Shen, Y., Li, S., Ge, X., Li, Z., 2017. High Density Linkage Map Construction and QTL Detection for Three Silique-Related Traits in Orychophragmus violaceus Derived Brassica napus Population. Front. Plant Sci. 8, 1512. https://doi.org/10.3389/fpls.2017.01512

Zhang, J., Zhang, Q., Cheng, T., Yang, W., Pan, H., Zhong, J., Huang, L., Liu, E., 2015. High-density genetic map construction and identification of a locus controlling weeping trait in an ornamental woody plant (Prunus mume Sieb. et Zucc). DNA Research 22, 183–191. https://doi.org/10.1093/dnares/dsv003

Zhang, K., Kuraparthy, V., Fang, H., Zhu, L., Sood, S., Jones, D.C., 2019. High-density linkage map construction and QTL analyses for fiber quality, yield and morphological traits using CottonSNP63K array in upland cotton (Gossypium hirsutum L.). BMC Genomics 20, 889. https://doi.org/10.1186/s12864-019-6214-z

Zhang, M., Zhang, W., Zhu, X., Sun, Q., Yan, C., Xu, S.S., Fiedler, J., Cai, X., 2020. Dissection and physical mapping of wheat chromosome 7B by inducing meiotic recombination with its homoeologues in Aegilops speltoides and Thinopyrum elongatum. Theor Appl Genet 133, 3455–3467. https://doi.org/10.1007/s00122-020-03680-3

Zhang, W., Zhu, X., Zhang, M., Shi, G., Liu, Z., Cai, X., 2019. Chromosome engineering-mediated introgression and molecular mapping of novel Aegilops speltoides-derived resistance genes for tan spot and Septoria nodorum blotch diseases in wheat. Theor Appl Genet 132, 2605–2614. https://doi.org/10.1007/s00122-019-03374-5

Zhou, G., Jian, J., Wang, P., Li, C., Tao, Y., Li, X., Renshaw, D., Clements, J., Sweetingham, M., Yang, H., 2018. Construction of an ultra-high density consensus genetic map, and enhancement of the physical map from genome sequencing in Lupinus angustifolius. Theor Appl Genet 131, 209–223. https://doi.org/10.1007/s00122-017-2997-y

Zhu, W.-Y., Huang, L., Chen, L., Yang, J.-T., Wu, J.-N., Qu, M.-L., Yao, D.-Q., Guo, C.-L., Lian, H.-L., He, H.-L., Pan, J.-S., Cai, R., 2016. A High-Density Genetic Linkage Map for

Cucumber (Cucumis sativus L.): Based on Specific Length Amplified Fragment (SLAF) Sequencing and QTL Analysis of Fruit Traits in Cucumber. Front. Plant Sci. 7. https://doi.org/10.3389/fpls.2016.00437

Zhuang, W., Chen, H., Yang, M., Wang, Jianping, Pandey, M.K., Zhang, C., Chang, W.-C., Zhang, L., Zhang, Xingtan, Tang, R., Garg, V., Wang, Xingjun, Tang, H., Chow, C.-N., Wang, Jinpeng, Deng, Y., Wang, D., Khan, A.W., Yang, Q., Cai, T., Bajaj, P., Wu, K., Guo, B., Zhang, Xinyou, Li, J., Liang, F., Hu, J., Liao, B., Liu, S., Chitikineni, A., Yan, H., Zheng, Y., Shan, S., Liu, Q., Xie, D., Wang, Z., Khan, S.A., Ali, N., Zhao, C., Li, X., Luo, Z., Zhang, S., Zhuang, R., Peng, Z., Wang, S., Mamadou, G., Zhuang, Y., Zhao, Z., Yu, W., Xiong, F., Quan, W., Yuan, M., Li, Y., Zou, H., Xia, H., Zha, L., Fan, J., Yu, J., Xie, W., Yuan, J., Chen, K., Zhao, S., Chu, W., Chen, Y., Sun, P., Meng, F., Zhuo, T., Zhao, Yuhao, Li, C., He, G., Zhao, Yongli, Wang, C., Kavikishor, P.B., Pan, R.-L., Paterson, A.H., Wang, Xiyin, Ming, R., Varshney, R.K., 2019. The genome of cultivated peanut provides insight into legume karyotypes, polyploid evolution and crop domestication. Nat Genet 51, 865–876. https://doi.org/10.1038/s41588-019-0402-2

Zou, G., Zhai, G., Feng, Q., Yan, S., Wang, A., Zhao, Q., Shao, J., Zhang, Z., Zou, J., Han, B., Tao, Y., 2012. Identification of QTLs for eight agronomically important traits using an ultra-high-density map based on SNPs generated from high-throughput sequencing in sorghum under contrasting photoperiods. Journal of Experimental Botany 63, 5451–5462. https://doi.org/10.1093/jxb/ers205
