# Supplementary material for: Diversity and determinants of recombination landscapes in flowering plants
Source: PLoS Genet. 2022 Aug 30;18(8):e1010141. doi: 10.1371/journal.pgen.1010141 (PMC9467342; doi:10.1371/journal.pgen.1010141)
Supplement: Attachment — Submitted filename: Response to reviewers.pdf [file pgen.1010141.s026.pdf]

Response to reviewers

Dear editor,

We thank you and the three anonymous reviewers for giving us the opportunity to improve and resubmit our manuscript and we are pleased to submit a revised version of our manuscript.

The reviewers have contrasted views. Reviewers 2 and 3 are very positive, and we thank them for their positive feedback. In contrast, reviewer 1 doesn't call into question the validity of our results but has concerns about the general interest and originality of the work, and the lack of clear questions addressed in the manuscript. Although we disagree with her/him about the lack of novelty and interest (see also reviewers 2 and 3), we acknowledge that we didn't stress and explain it sufficiently and that the work was framed in a somewhat too loose way. Most of reviewer 1's concerns were already addressed or partly addressed in the manuscript but not clearly enough for the reader. In this revised version we thus pay particular attention to be more explicit on hypotheses, to clearly stress the novelty of the dataset, approach, and results, and to sharpen and streamline the text.

About the novelty of work, we would like to insist on the originality and the quality of the dataset compared to what was done so far. First, we almost doubled the number of species compared to Haenel et al. 2018 (which was the largest dataset in plants before). Second, we started from raw data (genetic and physical distance between markers) to rebuild Marey maps and inferred recombination maps with the same standardised pipeline. So our work is a new analysis using published datasets but not a meta-analysis as in Haenel et al. 2018, where heterogeneous information was collected and combined, and mostly inferred from figures (*"For the vast majority of species (>90%), CO information suitable to this study was available in graphical form only."*, p2483). Third, by an extensive literature survey, we also gathered cytological information to more precisely locate centromeres. This allowed us to perform detailed analyses that were not carried out before (ex: identifying patterns, testing the different models, measuring genetic shuffling). We briefly stress this in the introduction: *"So far, the number of studied species remained limited and, as plant genomes are highly diverse in many ways (Pellicer et al., 2018; Soltis et al., 2015), the expected diversity in recombination landscapes may have been overlooked (Gaut et al., 2007). In addition, previous studies where meta-analyses combining heterogeneous datasets (ex: mix of inferred data from graphics, final processed data and only a few raw datasets in Haenel et al. 2018) without a standard way to infer recombination maps, which prevented detailed comparison among many species."*

In this revised version, we also address the reproducibility issues raised by reviewer 3 and consider all specific comments of the three reviewers, especially to

improve the clarity of the method and results sections and the aesthetics and readability of figures.

With this revised version we hope that we have addressed all comments and issues raised by the three reviewers and that the manuscript is now suitable for publication in PLoS Genetics. Below we provide detailed answers to reviewers' comments.

Thomas Brazier and Sylvain Glémin

Reviewer #1

It is now possible to analyse genetic and physical maps of organisms, as the literature now contains suitable data from multiple species. This manuscript analyses such data from larger numbers of plant species than previous studies (55 species, 5-26 chromosomes per species), and describes results for the broad-scale recombination landscapes. However, it does not actually use the analyses to ask interesting questions, which I had hope to see. Some questions might include the following.

We respectfully disagree with the general comments that we didn't address some of the interesting questions listed below. However we acknowledge that we didn't clearly stress them and framed the work with sufficiently clearly identified questions. We have extensively rewritten the introduction to make them appear clearly and below we answer each specific question.

Do chromosome arms have an obligate crossover? How often do chromosome arms have multiple crossovers, versus a single one (as I believe is the case in C. elegans)?

We already answered these questions in the previous version in figure 1A, figure 2B and through the formal model selection (Figure 6; Table 1). We also discussed them in the discussion about the species basal recombination rate, that the smallest chromosome of the genome had between one and two CO, independently of its genomic size. However we agree that it could have appeared diluted among other results. In the current version, (i) we directly ask the question in the introduction: *"What is the range of COs per chromosome observed in plants?"*, (ii) we annotated the number of COs on isolines in

Figure 1A, and (iii) we more explicitly describe the results *"Less than 2% of chromosomes had less than one CO (n = 11). 234 chromosomes had between one and two COs, suggesting that a single CO per chromosome is sufficient, though 419 chromosomes had more than two COs."*

Do related species differ (this is an important question, as it relates to the question of whether genetic recombination is sometimes selectively favoured, leading to higher crossover numbers than required for correct segregation, and for repair mechanisms to occur). This question is discussed near the very end of the text, but is not mentioned as a question earlier, making it appear that the ms is entirely descriptive. The ms does not seem to mention that some of the species studied are close relatives, and that this can be helpful in studying such questions.

We discussed the potential selective advantage of recombination by raising the question of the potential advantage to recombine more in gene-rich regions and what would be the optimal distribution of COs along the chromosome. However, we preferred not to introduce it as a main question of the article as the dataset and the approach is not appropriate to study it properly. We have too few closely related species to properly test for the evolution of recombination rates. At the angiosperms scale, we showed that the phylogenetic effect is mainly due to the differences of chromosome size between species (the phylogenetic model did not perform better than the mixed effect model with a species random effect without phylogenetic structure). We precise it in the results section "*the introduction of phylogenetic covariance did not improve the mixed model <u>thus we did not retain a phylogenetic effect</u>*". In general, closely related species do not differ in recombination rates but they do not differ in chromosome size either.

How large are pericentromeric regions with low recombination rates in plants, and how much do they differ between related species?

We answered the first question in figure 4 and figure S2. And as said above, we do not have an appropriate sampling to properly analyse differences among related species.

Do selfers have higher recombination rates per physical length of chromosome than closely related outcrossers?

As for previous questions, our dataset is not appropriate for this specific question. We would need either more pairs of closely related selfing and outcrossing species or a larger dataset to perform an angiosperm wide phylogenetic regression analysis. Actually, out of curiosity we had already done this analysis before and the effect was not significant. As it was a side analysis and because we already had many results we decided not to present it.

Are recombination rates the same in male versus female meiosis? This is finally mentioned in line 574, but it is not made clear until then that the data analysed are sex-averaged rates.

It is indeed a really interesting question. Unfortunately, as discussed line 596 (first draft version), we do not have enough sex-specific data to study it (in general, only sex-averaged genetic maps are provided). We indicated more clearly in the introduction and results that we have only sex-averaged genetic maps: "*we have estimated the sex-averaged rate of COs along chromosomes. (…) We retrieved publicly available data for sex-averaged linkage maps*"

Instead, the ms presents rather dull statistical analyses. The results have value, but they appear mainly to confirm findings that were already well established, and the ms does not make very clear what new findings now emerge, or show what we can now understand from the results that was not already known. More than once in the text "new insights" are claimed, but it is difficult to find them, partly because of the length of the text, which is also long-winded and repetitive in several places. These problems could be ameliorated by outlining in the Introduction what questions the authors set out to study. As written, this section gives the impression that their aims were purely descriptive, which is not an encouragement to read the text. The ms also tries to interest the reader by making claims to novelty, rather than describing some interesting questions. For example, I feel that it is too strong to say that "the broad diversity of recombination landscapes among plants has rarely been investigated… and the diversity of the resulting landscapes among species and chromosomes still need[s] to be assessed", although a formal comparative genomic approach may be new and valuable. A further value from analysing more species is that exceptions to accepted generalisations may be detected, and this study did produce a few examples of such exceptions. Overall, I doubt that readers need a length Introduction to tell them that recombination patterns are interesting in relation to evolution, including evolution of patterns in genomes, such as regions with different repetitive sequence density, and consequently gene density, and with differences in GC content. A shorter Introduction could give a better idea of what is new from this study.

As mentioned above, we agree that the novelty and the strength of this work could have been diluted in the manuscript. So we streamlined the introduction (for ex we removed some general statements) and now stress the main questions addressed in this study: *"Thanks to this dataset we addressed the following questions. What is the range of COs per chromosome observed in plants? Is the distribution of COs shaped by genome structure (i.e. chromosome size, telomeres, centromeres) and if so is there a universal pattern? Since recombination hotspots have been found in gene regulatory sequences so far, are recombination landscapes generally associated with gene density? What are the consequences of recombination heterogeneity on the extent of genetic shuffling? Overall, we found that recombination*

landscapes in plants are more diverse and more complex than initially thought." **We also tried to be clearer on what is novel and what is confirmatory.**

At least several of the conclusions are just confirmations of what was already known. The following examples illustrate this problem, and my comments also include some other issues for some of them (a recurring problem throughout the text is poor writing, including long-winded writing that makes the meaning hard to understand, and I provide some examples in my 'Minor comments' below, but these are still important comments that require revisions of the text, …

We extensively revised the text to simplify the writing and we hope it is clearer now.

…including a suggestion that some species may have too little information to be used. It would be helpful to show the numbers or markers mapped in Figure 4. In addition, if the numbers are small, presumably the total genetic map lengths are unreliable, and it is not explained prominently whether any attempt was made to check for this problem.

During dataset assembly, we checked the coverage of linkage maps with the difference between raw total linkage map length and the corrected total linkage map length. We estimated the corrected total linkage map length with two different methods: Chakravarti et al. (1991) and Hall and Willis (2005) and chose to report only the more sophisticated method of Hall and Willis (2005) in the manuscript. We added our conclusions to the Results: ***"Corrected linkage map length didn't change the total linkage map length (mean difference = 1.19 cM, max difference = 5.62 cM), giving confidence in the coverage of the linkage map"*****.**

Moreover, we used different metrics to assess the quality of Marey maps during the filtering step (e.g. marker density, mean interval between markers, largest gap, percentage of the total chromosome size covered by the linkage map, genome coverage, i.e. the difference between map length in Mb and chromosome size from the fasta file) and they were useful to automatically reject a large proportion of dataset (57 dataset retained among 120 dataset gathered; 52 % of dataset discarded). We did not keep every chromosome of a dataset and discarded 17 chromosomes among the retained dataset. The quantitative criteria were adjusted by iterative trials until we found a consistent dataset, and finally their values were:

- **Exclude chromosomes with less than 50 markers**

- **At least one marker every 4 cM on average**

- Exclude maps with gaps larger than 20 cM, as they could be falsely detected as recombination peaks (the value seems large, but smaller values were extremely stringent)

- Final visual assessment to exclude undetected suspicious maps (11 chromosomes among 6 species) or to make the choice to keep maps outside criteria (19 chromosomes among 9 species).

Moreover, it is important to note the strong qualitative improvement of our dataset compared to Haenel et al. (2018). They kept chromosomes with at least 20 markers (50 for us with a few exceptions at 30, so 50% to 150% higher) . As explained above, they mixed Marey maps with recombination landscapes, hence mixing different methods of estimation. Many of their linkage maps or recombination maps were figures that were digitised to interpolate an approximate markers' positions or recombination rates in segments, potentially limiting their spatial resolution (in both cM and Mb). They mixed sex-averaged maps with sex-specific ones (or an average of them). On the contrary, we made the effort to search only for tabular data to get the exact genetic and physical position of markers, and we had to ask many authors to provide their maps (as they were not always in supplementary materials). We did the effort of mapping markers on reference genomes as much as possible (if physical positions were not provided or if a more recent assembly version was available, when we had the markers' sequences). We mapped markers' positions on a reference genome for 14 datasets. We implemented a pipeline for the Marey map approach to get as much as possible consistent and reproducible estimates (e.g. automatic tuning of smoothing, bootstrapped confidence interval) so they could be compared between species, despite the inherent heterogeneity of our dataset.

References
Hall, M. C. & Willis, J. H. Transmission Ratio Distortion in Intraspecific Hybrids of Mimulus guttatus. Genetics 170, 375–386 (2005).
Chakravarti, A. A graphical representation of genetic and physical maps: The Marey map. Genomics 11, 219–222 (1991).

1. "We observed that the bias towards the periphery was not ubiquitous across species" and "Only a subset of species, especially those with larger chromosomes, exhibited a clear bias". These conclusions are quite similar to that of Haenel et al. (2018) that a distal bias is "universal for chromosomes larger than 30 Mb" (note the incorrect English "concluded to a distal bias"). The main advance seems to be that this study finds that Nelumbo nucifera and Camellia sinensis are exceptions to this pattern, with the highest recombination rates found in the middle of their chromosomes.

We corrected the English "*concluded*" by "*assessed*".

Though we get similar conclusions, we did more than identifying two exceptions. We identified a second pattern that Haenel et al. didn't detect. Haenel et al. (2018) described and conceptualised only the distal pattern (34 species in our study) but they didn't suggest anything for species not following this pattern. Thus we identified 22 species as exceptions to the Haenel et al. model (16 species sub-distal and 7 species as exceptions). We classified *N. nucifera* and *C. sinensis* in the sub-distal instead of distal, despite their large chromosome size, thus suggesting that the Haenel model is not so universal, as they claimed. We also stated that a fraction of species do not follow any of the two patterns we described (7 species, e.g. *A. thaliana* or *C. rubella*), suggesting that the diversity of patterns may be more important than we thought (and there is room for a more extended sampling in the future).

The result is described in a rather unhelpful manner, without taking chromosomes morphology into account. The text states that, for larger chromosomes, crossovers tend to occur (not "accumulate") at the ends of chromosome, while the central regions have less. However, this would be correct only for metacentrics, and the centres of chromosome presumably means centromeric and pericentromeric regions, but this is not made clear. It is also not made clear that these are completely recombination-free regions.

We changed "*accumulate*" by "*occur*".

Most chromosomes in our dataset are metacentric or sub-metacentric, which make it difficult to discern a difference between the physical centre (midpoint) and the centromere. When we used the term centre/central, we meant the midpoint of the chromosome. Otherwise we used centromeric/pericentromeric where appropriate.

We stated in the results that the centromere was a recombination free region (line 384 in the original draft). *"When the centromere position was known, we qualitatively observed that the centromeres had an almost universal local suppressor effect (Figure 3). In small and medium-sized chromosomes, the recombination was often suppressed in short restricted centromeric regions (several Mb) displaying drastic drops in the recombination rates, whereas the rest of the map did not seem to be affected. In larger chromosomes, the suppression of recombination extends to large regions upstream and downstream of the physical centre of the chromosome (approximately 80-90% of the chromosome; Figure 4)."*

We discussed more the limits of having mostly metacentric chromosomes: *"this work suggests that centromeres do not only have just a local effect but they also influence the symmetry of recombination landscapes over long distances, though a large proportion of our sample is metacentric, which might*

limit the detection of an effect. (…) However, how centromeres (especially non-metacentric ones) may affect CO distribution at larger scales still needs to be determined."

The extent of a larger pericentromeric region (meaning, the extent of the wider region surrounding or adjacent to the centromere) is known to vary greatly between species, but it is not well described in the ms, and only examples are shown, with rather subjective criteria to define the different regions. It would, in principle, be possible to define them less subjectively, though this might not be easy. At least, it would be good to mention whether this was attempted. A further problem is that regions are shown in figures, rather than tables giving estimates of genome region sizes and recombination rates, and as relative sizes are often used, it is difficult to understand what sizes of pericentromeric regions (for example) are found in plants.

We mainly provide figures in the main text as we think it is clearer for the reader to get the main results. However, we also provide many quantitative data in tabular form in supplementary material. We also provide scripts and all Marey maps that will be available on the MareyMap online website. So it should be easy to retrieve quantitative information if needed.

It is also not a new discovery that low recombination regions tend to have low gene density. The Discussion acknowledges this, but it is strange to first describe this as if it is a new result, only to later mention that it is not. If the Introduction had laid out some questions, this could be avoided. Problems like this also make the text longer than necessary.

We hope we now go more directly to the point. In the introduction we ask: *"Since recombination hotspots have been found in gene regulatory sequences so far, are recombination landscapes generally associated with gene density?"* We also mention in the results section that we follow previous studies: *"At a fine scale, it has been shown in a few species that COs preferentially occur in gene promoters. The scale of 100 kb used here is too large to directly test whether this is a common pattern shared among angiosperms. Instead, like in Haenel et al. (2018), we assessed whether recombination increased with gene density."*

2. Recombination is unevenly distributed in genomes. Therefore one should not write that "We showed that" this is the case. Once can write "We confirmed that" (or something similar). This text also uses vague terminology "how genetic variation is shuffled during meiosis", but the word recombination already exists, so it would be better to be precise. If at some point the meaning is gene conversion, this should be used. However, I think that the text mentions conversion only in passing, and it is not considered seriously.

We changed "*showed*" by "*confirmed*".

We don't clearly see the point here. We think that it is pretty clear that we are studying crossover rates, and we have no data to analyse gene conversion. In addition gene conversion cannot shuffle genes except in the close vicinity to recombination points. Following Veller et al. 2019 we measured the effect of a CO on gene shuffling at the whole chromosome scale. However, we are open to suggestions to improve the message if we have misunderstood this comment.

In line 538, I am not sure why thw word "prediction" is used (In addition to the role of centromeres, we also observed a departure from the prediction that recombination rates should decrease with the distance to the tip of the chromosome, showing that the distal model is not generally found among plants). Is this really a prediction, or are you trying to say that you did not confirm the view that this pattern is shared by all plants? If so, references are needed to assertions that all plants share this pattern.

We removed the word "*prediction*" and used a more clear statement: *"In addition to the role of centromeres, we also observed that recombination rates <u>do not always</u> decrease <u>monotonically</u> with the distance to the tip of the chromosome…"*

The Discussion section need not repeat so much of the results. It might also mention that recombination rates vary between individuals of the same species, including from the effects of rearrangements, especially inversions, so it would be good to mention that the data are currently often from just a single maternal and paternal parental individual of each species (for selfers, perhaps just a single parental individual). Hotspots should also be mentioned, if only to make clear that this study did not attempt to detect them.

474 It is proposed that in angiosperms crossovers may be initiated in gene regulatory sequences, and it is suggested that this "sheds new light on the evolution of recombination landscapes", but without saying what new light is shed, other than this suggestion. The suggestion is not evaluated further, and I did not understand if it is a speculation, based on the correlation between recombination and gene density mentioned in this paragraph (or on some other observations). However, based on later text (line 613), I suspect that the intended meaning is that the results are consistent with such a proposal that was already published by others.

We rewrote the sentence in a more explicit way: *"This sheds new light on the evolution of recombination landscapes and whether the distribution of COs is optimal for the efficacy of genetic shuffling."* We hope it is clearer now. [Note that the complete sentence was *"This sheds new light on the efficacy of genetic shuffling and the evolution of recombination landscapes."* so we already mentioned the efficacy of genetic shuffling]

However, as the correlation must be strongly affected by the lower gene densities in genome regions with low recombination rates, which lead to accumulation of transposable elements and other repetitive sequences, it would seem difficult to disentangle this from the suggested mechanism. Line 628 states that "The positive association of COs and gene regulatory sequences, including fine-scale correlations, appears more robust", which is too vague. It seems unlikely that the effect is stronger than the very marked and consistent effect of low recombination rates on repetitive sequence density (although of course different elements are involved in different cases).

We removed this statement. "~~The positive association of COs and gene regulatory sequences, including fine-scale correlations, appears more robust (Choi et al., 2013; He et al., 2017; Marand et al., 2019), but c~~*Causality mechanisms of these multiple interactions still need to be clarified*."

Regions with high recombination rates may, however, allow patterns in crossover localisation to be detectable, and I believe that this has been studied, for example in maize (e.g. papers by Dooner and colleagues) and also in Mimulus guttatus (see the paper by Hellsten et al. cited above). Line 621 finally mentions the problem of other correlated factors. I think that the authors should revise their text so that it does not first set up an untestable idea and then mention that it is untestable. Instead, it will be preferable to set up some interesting questions early in the text, tell readers what is currently known, and then describe analyses that help understand things better than before.

Dooner, H., & He, L. (2008). Maize genome structure variation: Interplay between retrotransposon polymorphisms and genic recombination. Plant Cell, 20(2), 249-258. doi:10.1105/tpc.107.057596

Fengler, K., Allen, S. M., Li, B., & Rafalski, A. (2007). Distribution of genes, recombination, and repetitive elements in the maize genome. Crop Science, 47(Supplement), S-83-S-95.

Yao, H., Zhou, Q., Li, J., Smith, H., Yandeau, M., Nikolau, B. J., & Schnable, P. S. (2002). Molecular characterization of meiotic recombination across the 140-kb multigenic a1-sh2 interval of maize. Proceedings of the National Academy of Sciences of the USA, 99, 6157-6162.

Tenaillon, M. I., Sawkins, M. C., Anderson, L. K., Stack, S. M., Doebley, J. F., & Gaut, B. S. (2002). Patterns of diversity and recombination along chromosome 1 of maize (Zea mays ssp. mays L.). Genetics, 162, 1401-1413.

We think that even in regions with high recombination rate it can be difficult to identify the precise locations of CO with the Marey map approach, especially in large genomes as in maize, unless the size of the genetic mapping population

is very large as in the recent study in A. thaliana. Otherwise LD maps should be more appropriate.
To be more constructive and less vague on this point, we now add that the use of recombination maps at finer scale will help resolving the role of genic regions in shaping recombination landscapes: "*Causality mechanisms of these multiple interactions still need to be clarified. The use of fine scale recombination maps (using very large mapping populations or LD maps) should help identifying the respective role of genic regions (especially the role of promoters) and transposable elements (or other genomic features)".*

Another comment that applies throughout the text is that recent papers are cited for concepta and understanding that are not new. In such cases, the text should make clear that the citation is to a review paper. For example, the text gives the impression that Marand et al. (2019) discovered that gene density and recombination rates are both correlated with transposable elements (meaning densities of transposable elements). This has been known for a long time, and was reviewed in 1994 by Charlesworth et al. (Nature, 371, 215-220. doi:10.1038/371215a0).

Citations have been changed accordingly.

In first mentioning heterochiasmy, it seems strange not to mention whether the papers cited refer to plants or just to studies in animal species. It is explained later that Melamed-Bessudo et al. (2016) showed that it is not universal in plants, but the text does not explain what the term might mean in plants, and that hermaphrodites may have different crossover patterns in male and female meiosis, so readers may be puzzled.

We rephrased to indicate when we were talking specifically of plants, and when we were referring to a more universal pattern shared by plants and animals. We also define heterochiasmy when we first used the word.

3. Similarly, I was surprised to read that "We were intrigued to notice that [within species] the chromosome-wide recombination rate is proportional to the relative size of the chromosome". I was under the impression that this was already known.

We changed the wording: "*Chromosome length drives the basal recombination rate for each species, though ~~but we were intrigued to notice that~~ within species the chromosome-wide recombination rate was proportional to the relative size of the chromosome*".

However, we think it was not already known that the size effect was species specific (this is why it's the relative size effect that seems to be general). We obtained this result because we gathered a dataset of chromosome-scale recombination landscapes, analysed them on a per chromosome level and modelled a species random effect; it has not been done before to our knowledge. In the two previous meta-analyses, Stapley et al. (2017) data were restricted to genome-wide recombination rates, and Haenel et al. (2018)

averaged their chromosome-wide recombination rates to a genome-wide level in similar analyses (one point per chromosome).

It is illustrated in Figure 2D, which shows the new results, which are potentially interesting, as they relate to the question of how often arms have multiple crossovers. This figure analyses the excess of crossovers, defined as the linkage map length minus the 50 cM expected if one crossover per arm is obligate), and shows that it correlates positively with the chromosome' physical sizes divided by the average chromosome size for the species, which they term the "relative chromosome size". Such an effect is not a new result.

As explained above, what we think is novel is that there is no single relationship between absolute chromosome size and CO but possibly a single and general one with the relative chromosome size. So the qualitative pattern was known but we think that its quantification was not. We added this precision in the corresponding result part: *"More concretely, it means that two chromosomes having the same ratio of size will have the same ratio of excess of recombination rate, whatever the species and the genome size"*.

In the discussion, we also tried to better explain this point: *"However, there is no universal relationship between the absolute size of a chromosome and its mean recombination rate. Although the average recombination rate of a species is well predicted by its average chromosome size, the recombination rates of each chromosome separately are not well predicted by their absolute chromosome size. Instead, variation within species is much better explained by the relative chromosome size, and surprisingly, this relationship seems to be roughly the same among species (see Figures 1 and 2)."*

However, as I understand it, an obligate crossover is expected on each arm. If so, the number of excess crossovers, in addition to this one, should be analysed per arm. Even if my recollection about this is incorrect, the text should make clear what is known from previous studies, and why the present study uses chromosome, not arm, lengths. Line 136 mentions that the centromeric index was known for the chromosomes of 37 species, but then it remains unclear how these data were used, and also whether results can be used from the species where no such data were available. Line 285 mentions that recombination rates were negatively correlated with the distance to the nearest telomere, which seems to suggest that metacentrics may have been analysed as such, but I could not see this clearly explained.

We explained earlier in the main text that one CO seems to be sufficient (at least for some species) [*"Less than 2% of chromosomes had less than one CO (n = 11). 234 chromosomes had between one and two COs, suggesting that a single CO per chromosome is sufficient, though 419 chromosomes had more than two COs"*] so we think it's clearer why we used linkage map length minus 50 cM. In addition we showed later that the model with one mandatory CO per arm is not well supported statistically.

Line 300 states that that (in my wording) the centromere regions almost universally showed low recombination rates, but this is not completely clear in Figure 4, where large low recombination rate regions in several species, for example Vigna unguiculata, appear not to overlap the centromeres. If this is a real biological observation, the statement seems incorrect.

We completed the statement with new results quantifying this effect "*Ninety percent of chromosomes (388 chromosomes) had significantly less recombination than the chromosome average at the centromeric index (n = 425, resampling test, 1,000 bootstraps, 95 % confidence interval). 81 chromosomes (19 %) were completely recombination-free in the centromere. However, the transposition of centromere position from cytological data to genomic data may be imprecise or wrongly oriented for some chromosomes. After orienting chromosomes to map the centromeric index, 16 % of chromosomes (70 over 425) had a recombination rate higher in the inferred centromere position than on the opposite side, thus a centromere potentially mapped on the wrong side. Of these 70 chromosomes, the difference between inferred and opposite centromere position was less than 1 cM/Mb for 64 % of them (n = 45).* "

Given these possible problems with the data, I was not convinced of the value of the formal modelling analysis of the effect of the centromere in suppressing recombination, and the comparison with less simple models that suggest that telomeres may also affect patterns. Such effects are plausible, but I feel that some of these plant data do not add valuable and solid support.

We feel that this criticism is somewhat unfair. The initial simple telomere-led model has been proposed on the basis of visual observations based on species average and on the correlation between periphery-bias ratio and chromosome length on 16 animal and 11 plant species. This is a very useful starting point but it was clearly proposed as a conceptual model by the authors (their Figures 3 and 4). Here we think we go beyond this by formally testing the model and proposing alternative ones with a much larger dataset and taking individual chromosome patterns into account. We are aware of the possible noise in the data but if some centromeres are not correctly located (opposite size for example) this should go against model 3 and removing those chromosomes didn't change the results. So we think our results are robust.

Another weakness is the lack of any mention of differences between male and female meiosis, and another is the lack of any mention of outcrossing rates.

In the first version we already discussed the difference between male and female meiosis. We made it clearer that we have only sex-averaged genetic maps and that we didn't analysed sex-specific maps. Note that in the recent paper of Sardell and Kirkpatrick (Am Nat 2020, sex differences in the recombination landscapes), only five plant species are available. However we

kept the idea in the discussion as it is indeed an interesting hypothesis to explain the observed patterns.

As explained above, the dataset is not appropriate to test for the effect of the mating system.

I wondered why these papers were not cited, or other papers about Arabidopsis lyrate or helleri, which may have genetic map information.

Hellsten, U., Wright, K. M., Jenkins, J., Shu, S., Yuan, Y., Wessler, S. R., . . . Rokhsar, D. S. (2013). Fine-scale variation in meiotic recombination in Mimulus inferred from population shotgun sequencing. Proceedings of the National Academy of Sciences of the United States of America, 110(48), 19478–19482. doi:10.1073/pnas.1319032110

Kawabe, A., Hansson, B., Forrest, A., Hagenblad, J., & Charlesworth, D. (2006). Comparative gene mapping in Arabidopsis lyrata chromosomes 6 and 7 and A. thaliana chromosome IV: evolutionary history, rearrangements and local recombination rates. Genetical Research, 88, 45-56.

Hansson, B., Kawabe, A., Preuss, S., Kuittinen, H., & Charlesworth, D. (2006). Comparative gene mapping in Arabidopsis lyrata chromosomes 1 and 2 and the corresponding A. thaliana chromosome 1: recombination rates, rearrangements and centromere location. Genetical Research, 87(2), 75-85. doi:10.1017/S0016672306008287

They are interesting papers. However the first one focuses on fine-scale crossover patterns and CO hotspots, especially around genic regions and does not provide a chromosome scale map (actually not possible with the method they used). The others could have been used but they did not match our filtering criteria.

Minor problems with the English, or vague wording or unclear statements

1. In English, it should be “correlated with” (not “to”).

Corrected.

2. The word ‘drive’ should be avoided, as it is very vague. For example, the meaning is not clear in the phrase “Chromosome length drives the basal recombination rate for each species”

We changed to “*Chromosome length constrains the basal recombination rate for each species*”

3. In line 182, it should read “regression lines for species with at least 5 chromosomes mapped, 5-26 chromosomes per species, 55 species).

Corrected according to the reviewer's comment.

4. Line 232 Genomic distances (Mb) were scaled between 0 and 1 (divided by chromosome size) to compare chromosomes with different sizes.

We are sorry but we don't understand what is the issue here.

5. It is difficult to make out the meaning of the text starting in line 247. I think it means the following: "Each chromosome was divided in (it should read "into") ten bins, each one 10th of the chromosome's total physical size." The relative recombination rate is the log-transformed ratio of the expected relative genetic length (one tenth, presumably of the total genetic length) divided by the observed relative genetic length of the bin (presumably meaning the proportion of the total genetic length represented by the physical region in question. Values below zero correspond to recombination rates lower than expected under a random distribution of crossovers across the physical chromosome. Also difficult to understand "Chromosome sizes (Mb) on the left correspond to each broken stick chromosome" — maybe it means "each chromosome". Also (in line 244) "Relative recombination rates along the chromosome were estimated in ten bins using the broken stick model.

To avoid confusion, we used another representation by dividing chromosomes in ten bins of equal genomic size, computing the average recombination rate in each bin and dividing by the mean recombination rate to get a relative measure. We also reordered the species as a function of chromosome size instead of recombination heterogeneity.

6. In English, one needs to say "divided into" (not "in"). Also "pooled into" (although this reads awkwardly in English, and line 140 might be better as "the Spearman rank correlation coefficient correlation between the values for 1 Mb windows and those for the 100 kb windows within them was ….".

Corrected according to the reviewer's comment.

7. The work "linkage" in genetics means that the variants are linked. It should be distinguished from "linkage disequilibrium" (LD), which refers to associations between two or more liked variants. Line 57 should be corrected, as the text refers to the latter, but uses the former ("Recombination…. breaking the linkage between neighbouring sites and creating new genetic combinations"). The sites remain linked, but not in LD. The sentence is also confusing by adding "upon which selection can act", because selection acts on single variants, and the authors are trying to say that new genetic combinations might be more (or less) favoured by selection than the non-recombinant combinations (in other words, the different variants may interact in their effect on fitness).

We changed "*breaking the linkage*" into "*breaking linkage disequilibrium*".

We also write now: "*making selection more efficiently*" to avoid possible confusion.

8. It is a sweeping statement to say that "Plant genomes contain large regions with suppressed recombination", depending strongly on how many plants have good data on physical and genetic maps, so line 92 ought to mention the number on which this is based, and give readers at least a rough idea of what is meant by "large". There is no need to add the obvious remark that this impacts genomic averages (in addition "impact" is the wrong word, as the meaning is that it affects the average — of course the average depends on the values in all genome regions that are included in the data, so it is not worth saying explicitly).

As genome sizes vary over many orders of magnitude in plants (10 to > 1000 Mb), "large" is very species dependent. It varies from a few Mb in smaller genomes (genomes between 10 and 30 Mb) to hundreds of Mb in larger genomes (500-1000 Mb), and the proportion of genome without recombination ranges from a few percent to 80%. We clarified the sentence (added the range of values and proportions) *"Plant genomes contain large regions with suppressed recombination in various proportions (from a few Mb to hundreds of Mb, > 1-75 %),…".*

We suppressed the statement about the average recombination rate.

9. Phrases that are unnecessary (such as "it seems that" in line 93, should be pruned out, so that the text is easier to read. There are quite a few such instances, and I do not comment on all of them. The beginning of the Results section, for example, could be written more briefly and clearly.

We retrieved publicly available data for linkage maps and genome assemblies, to obtain genetic map distances and physical distances. We used linkage maps with marker positions in chromosome-level genome assemblies (except for Capsella rubella, which had a high-quality scaffold-level assembly of pseudo-chromosomes). After filtering based on the marker numbers, densities, and genome coverage, and after filtering out the outlying markers (maybe meaning outlier markers by a criterion that needs to be explained), we produced 665 Marey maps (reference needed) for 57 species (2-26 chromosomes per species); marker numbers per chromosome (or perhaps the authors mean per species, in which case perhaps some species have too little information to be used) ranged from 31 to 49,483.

We thank you for this example of paragraph and we tried to follow this advice throughout the manuscript. Note that the minimum of markers is 30 per chromosome and not per species as mentioned above.

Reviewer #2

In this paper, the authors seek to decipher genomic patterns of recombination across a large (57 species) dataset of sequenced plant genomes coupled with genetic maps. Their meta-analyses lead to several novel observations.

I thoroughly enjoyed reading this manuscript, and I congratulate the authors on a really fine paper. It will be, in my view, a very welcome addition to the literature. In the surest sign of flattery, I'm a jealous that I did not think of doing such a neat analysis.

We thank reviewer 2 for this very positive comment.

Accordingly, I have only minor comments that the authors may wish to address in revision. Most of the comments are very minor, indeed. They are offered both as an attempt to clarify the few areas of the text that I found difficult to digest and probably out of an abundance of enthusiasm for this work. I leave it to the authors to decide if my suggestions offer improvements or are better ignored…

Minor Comments:

- Line 48 – Unlike most of the rest of the paper, I found this sentence hard to read and digest. Reword, rework or shorten? Btw I'd use "in" instead of "to" ("in the production")

We splitted the sentence for clarity.

- Line 79 – This last sentence of the paragraph is really indirect and therefore pretty tough to read. I'm not really sure what manipulations are being considered here… Rewrite?

We rewrote the sentence: *"The characterization of recombination landscapes also has practical interests as variation in meiotic genes could be used to experimentally manipulated CO patterns for advantageous purposes, such as redirecting recombination towards regions of interest for crop breeding (Kuo et al., 2021)"*.

- Line 100 – as a reader, I found that a better link between the two sentences on this line could have been helpful. Maybe something as simple as "Haenel et al. considered chrosomome length, found blah blah blah and suggested a simpler telomere-led model. That model included a universal bias…"

We rewrote it more directly: *"They found that larger chromosomes have low crossover rates in their centre and suggested a simple telomere-led model with a universal bias of COs towards the periphery of the chromosome, positively driven by chromosome length."*

- Line 118 – I'd use "about" instead of "on"

Changed for "*about*"

- Line 125 – If this is reasonable, I'd love to see the filter characteristics hinted at here, even though there is a good description in the methods. That is something like "… marker density (at least 50 per chromosome), genome coverage (blah blah)"

To avoid weighting the main text too much we preferred referring to the method part for details. However, we can add more details here if needed.

- Line 701 – I'm a bit confused by the what was done when marker sequences were not available and also how many species fell into this category. I'm not concerned at all – this is a careful study – but it'd be nice understand better.

When marker sequences were not available for mapping on the most recent genome assembly, genomic positions were those of the original publication (precisions now added to the M&M). "*We remapped markers on the reference genome for 14 species for which genomic positions were not known or were mapped to an older assembly*".

- Figure 2 – It might be helpful to have X-axis say "Mean chromosome size" where appropriate (e.g., Figure 2C and B). The legend is very clear, though.

The X-axis has been changed accordingly.

- I love Figure 3. It blows my mind how consistent the patterns are between the dashed lines (genome wide) and an individual chromosome. It is bizarre and neat and thought provoking. It might be nice to report mean chromosome size (in the legend or in the figure), given that the species are ordered in that matter. It just makes me curious…

Mean chromosome size has been added to the title of each figure.

- Figure 4, since patterns seem to correlated with mean chromosome size, would it be worth adding that value after each species name? As a reader, it would help me to see the pattern and better digest the text from ~lines 213 to 227 and figure 5a, etc).

We added the mean chromosome size after species name and colored each species as the function of three patterns identified later. We also reordered species as a function of mean chromosome size.

- Figure 5A – this may make the graph too crowded, but it'd be nice to be able to compare dots in 5A to figure 4. So, it'd be nice to have the dots labelled. If that is too much, the authors might want to consider labelling a few species (e.g., the six in figure 3 or some of the species mentioned in lines 220 to 227). Personally, I'd love to know what the outliers are in this graph!

Figure 5A has been annotated with a subset of species (the 6 in figure 3 and a few species mentioned in lines 220 to 227 or seen as outliers).

- Lines 284 and following. It'd be nice to cite Figure 6A and Figure 6B separately after the word descriptions of the patterns.

We added references to Figure 6A and Figure 6B in the text.

- Line 296. I could not figure out what the "species correlation" referred to. Sorry if I missed this, but it's worth another look to be sure it is clear.

We changed the formulation: "*the correlation between recombination and the distance to the nearest telomere was significantly higher for species with larger chromosomes*".

- I'm kind of shocked that M3 is favored over M2, as isn't one CO per arm necessary for mechanism? Hence, I'd a priori predict M2 > M3. I don't think this contrast is explicitly discussed in the Discussion (e.g., lines 523 to 536), but I think it should be.

To our knowledge, it was not very clear if one CO per arm or per chromosome was necessary. It seems that CO assurance imposes one CO per chromosome, not per arm, in many species. For example, a recent study by Dukic and Bomblies in *Arabidopsis arenosa* observed a large proportion of chromosomes undergoing a single CO, while only 25/30% of bivalents contained at least two COs. Indeed, we observed less than two COs in many chromosomes, especially the smaller chromosomes within species (Figure 1). Besides, even if M2 fit well for a bunch of chromosomes (7 species over 37 species support M2), the model is not applicable for many other chromosomes just because at least one arm has less than 1 CO (length < 50 cM). To mitigate this effect, we subset only species with chromosomes having at least one CO per arm (26 species over 37) and M3 was still a better model than M2. However we must say that we do not have the perfect sampling to categorically exclude M2 or M3, because most of our chromosomes are metacentric, thus a low power to distinguish M2-M3.

- Figure 8: It'd be nice if the legend clearly stated which graph is which. I think Figure 8b is the distal recombination pattern, but I'm not 100% sure. It'd be great to have sample sizes on the graph too (n = 34 or 16 species, I think).

We now give an explicit legend; (a) distal pattern vs (b) sub-distal pattern. We also added to the figure sample sizes and an annotation for distal/sub-distal patterns

- It's pretty clear in the M&M, but on line 419, it might be nice to mention that rintra is a single value per chromosome. On my first reading, I was thinking it was some sort of transformation of cM between genes…

We now explicitly mention that r_{intra} is a single value per chromosome: *"The r_{intra} gives, <u>for a chromosome</u>, a measure of the probability of a random pair of loci to be shuffled by a crossover."*

- The analysis of gene distances is very thought provoking!

Thanks!

- Line 488 – What the heck is going on with fungi and animals! It’s certainly not necessary, but can the authors provide a quick description or explanation. They have piqued my curiousity.

Indeed this statement was not very clear; we removed this comparison with animals and fungi as it was not important to discuss our results. For your information, we were referring to the fact that linkage map length does not depend on the absolute chromosome size (Figure 2A). Stapley et al. (2017) showed that genome-wide linkage map length does not depend on total genome size in plants, in contrast to fungi and animals. It is possible that CO interference is stronger in plants than in animals and fungi, as it would explain why linkage map length remain stable across a large span of genome sizes, though it is not very clear to make the link with our results because Stapley et al. (2017) relies on genome-averaged values.

Again, I do not consider any of my comments to be critical for publication, and I want to again congratulate the authors on a thorough and interesting study.

Reviewer #3

This manuscript by Brazier and Glémin uses a comparative approach to investigate variation in recombination landscapes in flowering plants. Their study used genetic map data from 665 chromosomes in 57 species of angiosperms. At the whole chromosomal level, they found a negative correlation between chromosome size and recombination rate (cM/Mb) with a strong species-specific effect. They also found that CO excess on chromosomes was more correlated with their relative size to other chromosomes in the genome rather than their absolute size, and that this effect was consistent across species. When investigating crossover landscapes, they found that landscapes were similar within species but strongly varied between species. CO rates were not uniform across chromosomes and were often more likely to occur at the distal ends of the chromosomes, with larger chromosomes tending to have a higher "periphery bias" of COs. However (as with most things in nature), this general pattern did have a number of exceptions. The authors then investigated the joint effect of telomeres and centromeres on CO distribution, finding the strongest support for a model that incorporated the effects of the telomere, centromere and one CO per chromosome. The authors found that recombination rate increased with gene density. Finally, the authors showed that genetic shuffling was positively correlated with linkage map length, and that there was a small negative effect of the periphery-bias ratio. These effects were slightly higher when modelling genetic shuffling in terms of gene distances. Whilst the investigations here are largely correlative rather than revealing mechanisms, this study provides a useful foundation for further investigation of broad drivers of recombination rate and landscape variation across a wide range of taxa.

This paper is the most comprehensive and well analysed that I have read on this topic, and generally it is well-written and well structured, particularly the introduction and discussion. I'm impressed by the sheer breadth of analyses.

We thank reviewer 3 for his/her very positive comment.

Nevertheless, **there are parts of the methods & results that lack clarity**, which in turn leads to issues with reproducibility. In particular, a lot of the statistical models are not well described – model structures should be made explicit in the methods and/or results, rather than providing a general text for statistical analyses at the end of the methods. I would emphasise that providing code and data (where possible) would improve these issues.

Thank you for pointing this out. We addressed this issue by providing more information in the results and M&M sections. The chosen statistical models are explicitly written. Besides, the R code used for statistical analysis will be freely available in a public github repository. Marey maps will be available in the

same github repository and in an updated version of the MareyMap Online database.

I had many comments and suggestions - those marked ** should be addressed by the authors in a revised version.

ABSTRACT/INTRODUCTION

Lines 32-33: The authors should be clearer what they mean by “relative size” here (i.e. relative to the rest of the genome) and why this result is interesting.

We substantially modified this part of the abstract and hope it is clearer now: *“We found that the number of crossing-over per chromosome spans a limited range (between one to five/six) whatever the genome size, and that there is no single relationship across species between genetic map length and chromosome size. Instead, we found a general relationship between the relative size of chromosomes and recombination rate, while the absolute length constrains the basal recombination rate for each species”*. We also substantially rewrote the rest of the abstract and hope it clarifies and sharpens the message.

Lines 48-52: In the first sentence, I would add the term “crossing-over” or “crossover” here to set up the rest of the introduction. In the second sentence, I would briefly define landscape (i.e. variation in recombination rate along the chromosomes)

Crossover and the definition of recombination landscapes have been added to the first sentences.

Lines 61 – 63: Indicate that you are defining assurance in this sentence.

Crossover assurance has been added to the sentence. “*at least one CO per chromosome is mandatory (i.e. crossover assurance) to achieve proper segregation and to avoid deleterious consequences of nondisjunction*”

Line 73: Can the authors briefly mention how recombination landscapes shape the distribution of TEs?

We mentioned “*the accumulation of TEs in regions of low recombination*”

**Lines 98 – 100: I found this statement confusing, as I can’t understand how independence between linkage map length and genome size means that recombination rates will be higher in smaller genomes. I also can’t make the link between this statement and the Stapley paper – I think they found that linkage map lengths were smaller in smaller genomes, but also that chromosome number explained more variation (i.e. increased chromosome number lead to longer maps

due to a higher minimum bound of recombination due to crossover assurance). Perhaps I am wrong, but regardless, it might be worth double-checking this statement and explaining it more clearly.

"*Recombination rates are supposed to be higher in smaller genomes because the linkage map length is independent of genome size*". Since the total linkage map length do not depend on the total genome size, we can assume that smaller genomes will have roughly the same linkage map length as larger genomes, thus the same number of COs is distributed among a smaller genomic size (Mb) and recombination rate is higher (cM.Mb^{-1}). Stapley et al. (2017) made a similar statement in Box 1. "*Following the observation that linkage map length was similar across eukaryotes despite large variation in genome size, it was proposed that larger genomes have several orders of magnitude lower recombination rates.*" In their Figure 3 Stapley et al. show that linkage map length increases linearly with genome size, though in plants the best fit was a quadratic function. Accordingly, in plants, larger genomes have roughly the same linkage map length than smaller ones. Indeed, they also stated that chromosome number explained more variation than genome size.

We rephrased the statement accordingly to the reviewer's comment: assertion is true in plants, not other eukaryotes, and the number of chromosomes is more important than genome size:

"In plants, contrary to other eukaryotes, recombination rates are supposed to be higher in smaller genomes because the linkage map length is independent of genome size and the number of chromosomes explain more variation than genome size (Stapley et al., 2017)."

Line 100: on recombination rate, or landscape? Or both?

Actually, both. We added to the phrase: *"existence of a major broad-scale determinant of CO distribution and frequency"* to be more explicit.

**Line 102: Based on your argument here, it is not clear how chromosome length links to biases of CO towards the peripheries – please clarify.

We clarified the argument. *"Haenel et al. (2018) found that larger chromosomes have low crossover rates in their centre and suggested a simple telomere-led model with a universal bias of COs towards the periphery of the chromosome, positively driven by chromosome length"*

Line 117: Briefly define genetic shuffling and why it's interesting – could even be mentioned earlier e.g. around lines 56 – 58.

We added precision on the expected effect of CO patterns on genetic shuffling in line 117. *"how CO patterns affect the extent of genetic shuffling"*

We modified lines 56-58 to define genetic shuffling. ***"creating new genetic combinations transmitted to the next generation upon which selection can act, <u>i.e. genetic shuffling</u>"***

We added explicit precisions in results. ***"In most cases, genetic shuffling were slightly higher when gene distances were used instead of base pairs (Figure 10; mean = 0.22 for base pairs; mean = 0.26 for gene distances; Wilcoxon rank sum test with continuity correction, p < 0.001), <u>implying that the genetic shuffling was more efficient among coding regions than than among regions randomly sampled in the genome</u>"***

RESULTS

**Lines 130 – 132: I don't think this is described in the methods. Is there information on the number of progeny? I was curious about this but couldn't find the information in the supplementary tables.

We added a new column with the number of progeny (retrieved in the original publication of the genetic map) in supplementary table S1 Dataset Metadata.

Line 143: This header could be interpreted that smaller chromosomes have more crossover events rather than more crossovers per unit length. Perhaps "Smaller chromosomes have higher recombination rates than larger ones"?

The header was modified accordingly.

**Lines 153 – 169: Where are the methods for this LMER and what is the model structure? Is this what is being described in lines 831 – 850 of the methods? Throughout the paper, it needs to be clearer what models were run and what their fixed & random effect structures were in order to better interpret them.

Models' equations are now explicitly mentioned in the results section for each model presented.

Line 155: Does this mean that there is no/low phylogenetic signal of recombination rate?

We do not have a proper sampling to study the evolution of recombination rates along the phylogeny, with uneven sampling amongst angiosperms (many close relatives in monocots, sister species in *Brassica* sp., *Oryza* sp. or *Arachis* sp.). Though the phylogenetic mixed model (pglmm, phyr package, see method for details) assessed a phylogenetic signal (34% of the variance explained by random effects was due to a phylogenetic signal), we suspect it was mainly attributed to differences of chromosome size between monocots and eudicots (monocots with mainly large genome and eudicots with a higher

proportion of small genomes) and uneven sampling. When we add both phylogenetic effect and chromosome size, we saw that the phylogenetic mixed model did not perform better than a more parsimonious linear mixed model with a species effect without phylogenetic covariation (Table S5).

Figure 1: This figure is busy. A suggestion for panel A: perhaps the dashed lines could be fit from axis to axis, to visually demarcate the 1 – 4CO expectations a bit better? For panel B, since this is the same data plotted twice, perhaps only the regression lines need to be visualised here rather than all of the points.

We modified the figure to improve its readability. We hope it is better now:

Figure 1A. Dashed lines are now solid thin lines, going axis to axis and more easily identified. Lines were identified by the expected number of CO directly on the figure. Regions with less than one CO or more than four COs were labelled.

Figure 1B. We removed points, according to the reviewer's comment, for better visualisation of regression lines.

**Figure 2: I found this figure confusing. Some suggested edits:

Panel A could be wider to allow discerning of the slopes. I also struggled to understand what the isolines on the graph are showing even after reading several times. When using isolines, perhaps there is a need to define their values (as in Figure 1) – or perhaps they can be removed if making things too busy.

Panel B: I cannot interpret what this is showing – are there really lines in panel A that have intercepts of less than zero?

Panels B & C: I am very curious to see the error on these estimates.

Panels B & C: maybe these panels might be better suited in the supplementary?

Panel D: Again, very busy. Perhaps use of transparency of points or lines could make things clearer.

We understand the confusion on figure 2. Panel B has been removed since it did not support important findings. Panel C has been moved to supplementary materials (Figure S4). Panel A is now wider. We added transparency to points to make regression lines more distinguishable.

The genome wide recombination rate (cM/Mb) has been annotated on isolines. They tend to show that within species recombination rates are roughly similar among chromosomes despite differences in absolute chromosome size (because regression lines are parallel to their closest isoline).

**Figure 4: Accessibility issue for colour-blindness - the red dots may not be visible on the green background. The visual scale for the chromosome size is a unclear,

particularly as appears to be log – could there be line traces instead of colours here? Also – perhaps I have misunderstood – but if the chromosomes were split into ten bins, then why does the resolution of recombination rate estimation look to be much higher than 1/10th of the chromosome on the horizontal lines?

Thank you for pointing this out. Centromere position is now more visible, represented by black and white diamonds (higher contrast).

The visual scale of chromosome size is now clearer, with the height of bar plots corresponding to chromosome size. The scale is now linear instead of log. We also ordered species by mean chromosome size. Following reviewer 1's suggestion we also used another way to visualise the results to avoid possible confusion. Chromosomes are now splitted into ten bins of equal genomic sizes, not genetic length. We hope it eases the reading.

**Line 282 – 298: It seems that chromosome size was a strong correlate of recombination pattern, but I was curious if the authors tested other factors to rule out potential artefacts (e.g. differences in marker density) or to identify other biological correlates, such as ploidy? Was there a phylogenetic signal of this distal vs subdistal pattern?

We tested several factors, such as number of markers, marker density (cM & Mb) or number of progeny, that had no effect. Out of curiosity we also previously checked for possible effects of ploidy, lifespan and mating systems. None was significant. Note that our sampling is not appropriate to properly test these effects (see answer to reviewer 1). If required we can add such analyses in supplementary material.

Figure 6: There is a lot of text to wade through in the legend - it would help the reader to put annotations, sample sizes, key on the figures to allow for faster interpretation. For example, putting A: Distal pattern, N = XX, B: Subdistal pattern, N = XX on the panels make it easier to interpret. The dashed lines for the unclassified patterns are very distracting – why not include this as another panel, or put it in the supplementary material? Panel C is tiny and needs a key, or at least x-axis labels. I like the schematics of the crossover distributions but it's so tiny – perhaps include this as its own figure as it explains the model really well.

Annotations were added to the figure to reduce the legend (i.e. pattern, sample size). Unclassified patterns were removed, since they are specific patterns and they are also directly accessible in supplementary Figure S6.

X-axis labels were added to panel C.

Figure 6 was splitted. Figure 6D was moved to a new figure.

Figure 7: A & B. There needs to be a higher contrast between the colours as it's difficult to see the differences between blue and black. C. What do the colours

represent here? Adjusting the point transparency and slight x jitter may improve the visualisation here.

Figure 7A & B. The contrast was increased.

Figure 7C. We removed colours because they are not informative on this graph and do not contribute to the main message. Colours represented species (each species had its own relationship recombination rate ~ gene count) but it was difficult to distinguish them. The distribution of specific relationship recombination rate ~ gene count is already presented in Figure 7A. We added a slight jitter.

Figure 8: Same as Figure 6 – annotating the panels would be helpful.

We annotated the panels with the pattern name and the sample size (number of species). We removed the dashed lines for exceptions.

**Line 414 – I think it's important for the authors to briefly define what genetic shuffling is and why it's interesting to look at from an evolutionary perspective.

We added a short definition of genetic shuffling. *"<u>Genetic shuffling participates to the random reassortment of genes between parental homologous chromosomes.</u> To quantify how much the genetic shuffling depends on the distribution of COs, we estimated its intrachromosomal component, r_{intra} , as described in equation 10 in Veller et al. (2019)."*

Line 419: On the same chromosome

Same answer as for reviewer 2. We explicitly mentioned that r_{intra} is a single value per chromosome. *"The r_{intra} gives, <u>for a chromosome</u>, a measure of the probability of a random pair of loci to be shuffled by a crossover."*

Line 422: less efficient = resulted in less genomic shuffling?

The sentence was modified according to the reviewer's suggestion, to be more explicit on the effect of COs. *"COs clustered in distal regions are supposed <u>to generate less genetic shuffling</u> than COs evenly distributed in the chromosome."*

Figure 9: see comments on Figure 7.

We guess the comment applies better to Figure 10. The contrast was increased by changing the blue colour.

DISCUSSION

Lines 477 – 488: I'm a little puzzled by some of the statements here, so perhaps clarification is needed. I think it could be mentioned that crossover assurance will give a basal rate per chromosome of 50cM regardless of size, and then the authors can expand how the findings outlined here add to this established fact. Furthermore, I believe that in animals, larger chromosomes do have lower recombination rates within species… if I have misinterpreted this, perhaps the authors need to clarify their point better.

Indeed this statement was not very clear (see the same comment above); we removed this comparison with animals and fungi as it was not important to discuss our results.

Line 519: clarify what "association" means here… does chromosome pairing begin at the telomeres?

Yes it does. The phrase was changed to be more explicit (association replaced by chromosome pairing). ***"the early <u>chromosome pairing beginning in telomeres</u> is thought to favour distal COs "***

Line 570: put "beam-film" in inverted commas and indicate that you are about to describe it.

We put 'beam-film' in inverted commas. We indicated that we describe the conclusions of Zhang et al. (2014), we do not have results about the 'beam-film'. ***"<u>Zhang et al. (2014) assessed that the 'beam-film' model</u> is able (…) <u>If clamping is assumed, the model predicts</u> that mechanical stress culminates (…)"***

Line 582 – 583: It depends on the number of gametes measured and how many were male and female, which is easily done in dioecious species. I think authors should specify here "in angiosperms" and iterate here why heterochiasmy is difficult to investigate for the less plant-literate reader.

We are not sure to clearly understand this suggestion. The offspring generation corresponds to an equal number of males and females meiosis, even if the parents produced different numbers of gametes.

METHODS

**Lines 700 – 704: Indicate that this was from cytogenetic data. How is this information orientated correctly to the linkage map/genome sequence?

We removed the sentence about centromeric indexes, since it was better explained later in methods, in the paragraph "Testing centromere or telomere effects" : ***"We searched the literature for centromeric indices (ratio of the short arm length divided by the total chromosome length) established by cytological measures. When we had no information about the correct orientation of the chromosome (short arm/long arm), the centromeric index was oriented to***

match the region with the lowest recombination rate of the whole chromosome (i.e. putative centromere). "

**Lines 709 – 723: I think this paragraph requires a few improvements in reproducibility. Was this all done in the MareyMap package in the next paragraph? What was a ballpark criteria/example for anything that was outside the global trend?

At the beginning of the paragraph: *"We selected genetic and genomic maps after stringent filtering and corrections, using custom scripts provided in a public Github repository (https://github.com/ThomasBrazier/diversity-determinants-recombination-landscapes-flowering-plants.git). "*

We specified a qualitative criterion for map selection and quality assessment: "We assumed that markers must follow a monotone increasing function when plotting genetic distances as a function of genomic distances in a chromosome (i.e. the Marey map) and collinearity between the genetic map and the reference genome was required to keep a Marey map."

More details about the correction: *"If necessary, genetic maps were reoriented so that the Marey map function is increasing (i.e. genetic distances read in the opposite direction)."*

We specified criteria for outlier filtering and precisions about the processing: *"Markers clearly outside the global trend of the Marey map (e.g. large genetic/genomic distance from the global cloud of markers or from the interpolated Marey function, no other marker in a close neighbourhood) were visually filtered out, and multiple iterations of filtering/interpolation helped to refine outlier removal. "* We previously tried different strategies of automation for this step, as in Mansour et al. (2021), but after many trials, we concluded that it was neither efficient nor reliable for handling heterogeneous datasets. Though it is easy to distinguish clear outliers for human eyes, it is not a trivial task to set an automatic filtering for outliers. Because the noise is highly dataset dependent, one cannot use a constant threshold to exclude outliers. A threshold adjusted for the best map might be too stringent for another dataset, while a threshold set for the worst map might fail to detect the few outliers in almost perfect Marey maps. We tried automatic filtering of outliers based on the distribution of inter-marker distances (between adjacent markers), but user confirmation is required, as for many data-specific tasks. Setting a given threshold of distance or a given distribution statistic (e.g. remove 5% with the greater genetic distance than the maximum extreme value) do not take into consideration the heterogeneity between dataset. Thus, when comparing different dataset relying on very different distributions, we suspect it could introduce a kind of noise-dependent bias. The more noise there is in data, the less stringent filtering will be. Finally, visual assessment and an iterative procedure of filtering/interpolation seemed to be the more robust approach, though we lose in reproducibility. Please note that all outlier markers removed

are still present in Marey maps provided in supplementary, though they are labelled as 'not valid' and not used in subsequent analyses. This is also a possibility proposed in the MareyMap package in R.

In the next paragraph: *"Local recombination rates along the chromosome were estimated <u>with custom scripts following the Marey map approach, as described in the MareyMap R package (Rezvoy et al., 2007).</u>"*

References

Mansour, Y., Chateau, A. & Fiston-Lavier, A.-S. BREC: an R package/Shiny app for automatically identifying heterochromatin boundaries and estimating local recombination rates along chromosomes. BMC Bioinformatics 22, 396 (2021).

**Lines 724 – 737: Related to the previous comment, when looking at the plotted Marey maps (Figure S1), are the methods/results affected in any way by the "jitter" of Mb vs cM distances? I imagine that if the markers were not in the correct order in the linkage map (if the local linkage order is ABC, but the real genomic order is ACB), then the cM length of the chromosome may be overestimated, meaning that recombination rates would be consistently inflated. For example, in Camellia sinensis, the maps seems to be messier and therefore may accumulate local overestimations in recombination rate that will lead to a longer cM map than the true one, compared to Arabidopsis where the orders appear to be highly conserved between the genome and the linkage map. The potential impact of this should be discussed.

This issue was addressed by automatically adjusting the smoothing parameter of the loess regression to the data, and subsequently by evaluating the performance of interpolation using a bootstrap procedure (random resampling of markers). Due to higher levels of noise in some Marey maps, we certainly lose resolution and miss local variations for those maps, but we are confident that our method did not lead to longer genetic maps (cM).

To check for a bias in our estimates (e.g. inflating the chromosome recombination rate), we assessed the differences between the genome wide recombination rate (obtained by dividing the genetic map length by the genome length) and the averaged estimate per chromosome (the mean of recombination rates in windows of 100 kb). Both values are extremely correlated (Spearman's Rho = 0.99, p < 0.001) and the figure below shows that our mean estimates are not biased.

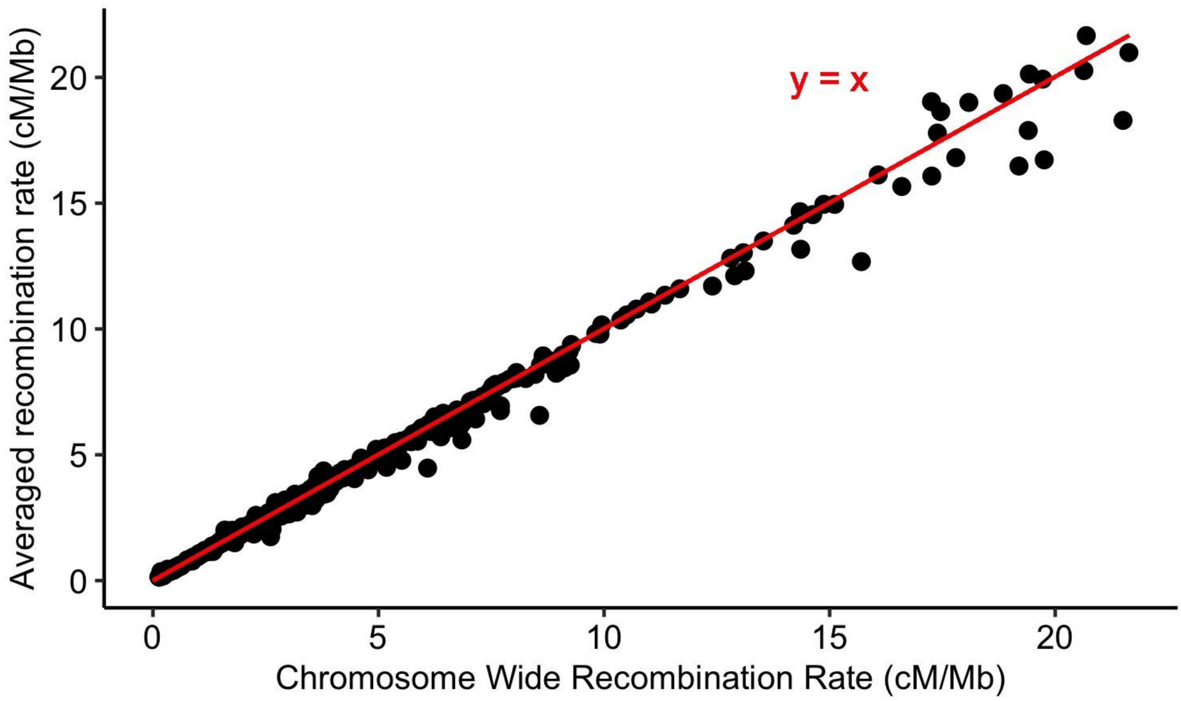

However, we cannot exclude that locally, along the chromosome, some values may reach high values that are not biologically reasonable, false peaks of recombination (i.e. data overfitting). On the other hand, the messier maps, due to stronger smoothing, should lead to a lower resolution and be more limited in the detection of local variation. That is why we implemented a K-fold cross validation procedure to automatically adjust the smoothing to avoid overfitting & underfitting issues. In addition, a bootstrap procedure (1,000 iterations), with random resampling of markers (with replacement), was used to evaluate the sensitivity of our estimates to outliers and noisy data. Recombination landscapes with large confidence intervals, indicating low data quality and/or poor performance of interpolation (precision and reliability), were discarded.

**Lines 738 – 749: Please clarify here how the relative recombination rate is calculated – is this done for each segment? i.e. if the chromosome is 100cM and 80Mb, and the first segment is e.g. 10cM & 20Mb, then how would the value be calculated? The verbal argument is unclear.

The representation of the broken stick model was not intuitive and we modified figure 4. The relative recombination rate is now estimated in 10 bins of constant genomic size and the relative recombination rate is the ratio expected genetic size divided by observed genetic size. In the legend: "*Relative recombination rates along the chromosome were estimated in ten bins of equal genomic size as the observed genetic length divided by the expected*

genetic length (one tenth of total genetic size) of the bin (log-transformed). Values below (above) zero are recombination rates that are lower (higher) than expected under a random distribution*". The verbal argument was made clearer, and the equation was added. ***"The relative recombination rate <u>in the segment i</u> was estimated by the log-ratio of the <u>observed</u> genetic size divided by the <u>expected</u> <u>genetic</u> size <u>(i.e. fixed to total genetic size / k by the model), as in the following equation.</u>

$$\text{relative recombination rate} = \log_{10}(genetic_i / (genetic_{total} / k))$$"

In the case suggested by the reviewer, it means that the chromosome is cut into 10 bins of genomic size 8 Mb (80/10). If the first bin (8 Mb) is 20 cM long, the relative recombination rate is:

$$\log_{10}(20 / (100/10)) = \log_{10}(2)$$

**Lines 831 – 850: The way this is written, it isn't connected to any specific models. It is important to describe what was modelled here and to be explicit about the model structures to ensure reproducibility.

We specified more explicitly models in the result section (model formulas are also given in Supplementary Table S5 of model selection). We compared models:
LM response variable ~ explanatory variable
LMER response variable ~ explanatory variable + (1|Species)
LMER response variable ~ explanatory variable + (response variable|Species)
PGLMM response variable ~ explanatory variable + (1|Species__); where Species__ indicates that phylogenetic structure is modelled

After model selection, we chose for all regressions at a chromosome level (samples are chromosomes) the model
LMER response variable ~ explanatory variable + (1|Species)

Since we didn't detect a phylogenetic effect, we chose the model
LM response variable ~ explanatory variable for all regressions at a species level (one point per species, chromosomes pooled).
