# Supplementary material for: Diversity and determinants of recombination landscapes in flowering plants
Source: PLoS Genet. 2022 Aug 30;18(8):e1010141. doi: 10.1371/journal.pgen.1010141 (PMC9467342; doi:10.1371/journal.pgen.1010141)
Supplement: Attachment — Submitted filename: PGENETICS-D-22-00286_R1_reviewer.pdf [file pgen.1010141.s027.pdf]

PLOS Genetics

Diversity and determinants of recombination landscapes in flowering plants

--Manuscript Draft--

**Manuscript Number:**	PGENETICS-D-22-00286R1
**Full Title:**	Diversity and determinants of recombination landscapes in flowering plants
**Short Title:**	Recombination landscapes in angiosperms
**Article Type:**	Research Article
**Section/Category:**	Evolution
**Keywords:**	meiotic recombination; crossover pattern; Marey map; genetic shuffling; comparative genomics
**Abstract:**	During meiosis, crossover rates are not randomly distributed along the chromosome and their location may have a strong impact on the functioning and evolution of the genome. To date, the broad diversity of recombination landscapes among plants has rarely been investigated and a formal comparative genomic approach is still needed to characterized and assess the determinants of recombination landscapes among species and chromosomes. We gathered genetic maps and genomes for 57 flowering plant species, corresponding to 665 chromosomes, for which we estimated large-scale recombination landscapes. We found that the number of crossing-over per chromosome spans a limited range (between one to five/six) whatever the genome size, and that there is no single relationship across species between genetic map length and chromosome size. Instead, we found a general relationship between the relative size of chromosomes and recombination rate, while the absolute length constrains the basal recombination rate for each species. At the chromosome level, we identified two main patterns (with a few exceptions) and we proposed a conceptual model explaining the broad-scale distribution of crossovers where both telomeres and centromeres play a role. These patterns globally correspond to the underlying gene distribution, which affects how efficiently genes are shuffled at meiosis. These results raised new questions not only on the evolution of recombination rates but also on their distribution along chromosomes.
**Additional Information:**	
**Question**	**Response**
**Financial Disclosure**<br><br>Enter a financial disclosure statement that describes the sources of funding for the work included in this submission. Review the submission guidelines for detailed requirements. View published research articles from *PLOS Genetics* for specific examples.<br><br>This statement is required for submission and **will appear in the published article** if the submission is accepted. Please make sure it is accurate.	SG received fundings from the Agence Nationale de la Recherche (ANR HotRec ANR-19-CE12-0019-04). The funders had no role in study design, data collection and analysis, decision to publish, or preparation of the manuscript.

Powered by Editorial Manager® and ProduXion Manager® from Aries Systems Corporation

**Unfunded studies**<br><br>Enter: *The author(s) received no specific funding for this work.*<br><br>**Funded studies**<br><br>Enter a statement with the following details:<br>• Initials of the authors who received each award<br>• Grant numbers awarded to each author<br>• The full name of each funder<br>• URL of each funder website<br>• Did the sponsors or funders play any role in the study design, data collection and analysis, decision to publish, or preparation of the manuscript?<br>• **NO** - Include this sentence at the end of your statement: *The funders had no role in study design, data collection and analysis, decision to publish, or preparation of the manuscript.*<br>• **YES** - Specify the role(s) played.<br><br>* typeset	
**Competing Interests**<br><br>Use the instructions below to enter a competing interest statement for this submission. On behalf of all authors, disclose any competing interests that could be perceived to bias this work—acknowledging all financial support and any other relevant financial or non-financial competing interests.<br><br>This statement **will appear in the published article** if the submission is accepted. Please make sure it is accurate. View published research articles from *PLOS Genetics* for specific examples.	The authors have declared that no competing interests exist.

**NO authors have competing interests**<br><br>Enter: *The authors have declared that no competing interests exist.*<br><br>**Authors with competing interests**<br><br>Enter competing interest details beginning with this statement:<br><br>*I have read the journal's policy and the authors of this manuscript have the following competing interests: [insert competing interests here]*<br><br>* typeset<br><br>This statement is required for submission and **will appear in the published article** if the submission is accepted. Please make sure it is accurate and that any funding sources listed in your Funding Information later in the submission form are also declared in your Financial Disclosure statement.	
**Data Availability**<br><br>Authors are required to make all data underlying the findings described fully available, without restriction, and from the time of publication. PLOS allows rare exceptions to address legal and ethical concerns. See the PLOS Data Policy and FAQ for detailed information.<br><br>A Data Availability Statement describing where the data can be found is required at submission. Your answers to this question constitute the Data Availability Statement and will be published in the article, if accepted.	Yes - all data are fully available without restriction

**Important:** Stating 'data available on request from the author' is not sufficient. If your data are only available upon request, select 'No' for the first question and explain your exceptional situation in the text box.<br><br>Do the authors confirm that all data underlying the findings described in their manuscript are fully available without restriction?	
**Describe where the data may be found in full sentences. If you are copying our sample text, replace any instances of XXX with the appropriate details.**<br><br>• If the data are **held or will be held in a public repository**, include URLs, accession numbers or DOIs. If this information will only be available after acceptance, indicate this by ticking the box below. For example: *All XXX files are available from the XXX database (accession number(s) XXX, XXX.*).<br>• If the data are all contained **within the manuscript and/or Supporting Information files**, enter the following: *All relevant data are within the manuscript and its Supporting Information files.*<br>• If neither of these applies but you are able to provide **details of access elsewhere**, with or without limitations, please do so. For example:<br><br>*Data cannot be shared publicly because of [XXX]. Data are available from the XXX Institutional Data Access / Ethics Committee (contact via XXX) for researchers who meet the criteria for access to confidential data.*<br><br>*The data underlying the results presented in the study are available from (include the name of the third party and contact information or URL).*<br>• This text is appropriate if the data are owned by a third party and authors do not have permission to share the data.	All data available to reproduce the results presented in the paper will be available in a public data repository (https://github.com/ThomasBrazier/diversity-determinants-recombination-landscapes-flowering-plants.git).

Powered by Editorial Manager® and ProduXion Manager® from Aries Systems Corporation

* typeset	
Additional data availability information:	Tick here if the URLs/accession numbers/DOIs will be available only after acceptance of the manuscript for publication so that we can ensure their inclusion before publication.

Powered by Editorial Manager® and ProduXion Manager® from Aries Systems Corporation

Response to reviewers

Dear editor,

We thank you and the three anonymous reviewers for giving us the opportunity to improve and resubmit our manuscript and we are pleased to submit a revised version of our manuscript.

The reviewers have contrasted views. Reviewers 2 and 3 are very positive, and we thank them for their positive feedback. In contrast, reviewer 1 doesn't call into question the validity of our results but has concerns about the general interest and originality of the work, and the lack of clear questions addressed in the manuscript. Although we disagree with her/him about the lack of novelty and interest (see also reviewers 2 and 3), we acknowledge that we didn't stress and explain it sufficiently and that the work was framed in a somewhat too loose way. Most of reviewer 1's concerns were already addressed or partly addressed in the manuscript but not clearly enough for the reader. In this revised version we thus pay particular attention to be more explicit on hypotheses, to clearly stress the novelty of the dataset, approach, and results, and to sharpen and streamline the text.

About the novelty of work, we would like to insist on the originality and the quality of the dataset compared to what was done so far. First, we almost doubled the number of species compared to Haenel et al. 2018 (which was the largest dataset in plants before). Second, we started from raw data (genetic and physical distance between markers) to rebuild Marey maps and inferred recombination maps with the same standardised pipeline. So our work is a new analysis using published datasets but not a meta-analysis as in Haenel et al. 2018, where heterogeneous information was collected and combined, and mostly inferred from figures (*"For the vast majority of species (>90%), CO information suitable to this study was available in graphical form only."*, p2483). Third, by an extensive literature survey, we also gathered cytological information to more precisely locate centromeres. This allowed us to perform detailed analyses that were not carried out before (ex: identifying patterns, testing the different models, measuring genetic shuffling). We briefly stress this in the introduction: *"So far, the number of studied species remained limited and, as plant genomes are highly diverse in many ways (Pellicer et al., 2018; Soltis et al., 2015), the expected diversity in recombination landscapes may have been overlooked (Gaut et al., 2007). In addition, previous studies where meta-analyses combining heterogeneous datasets (ex: mix of inferred data from graphics, final processed data and only a few raw datasets in Haenel et al. 2018) without a standard way to infer recombination maps, which prevented detailed comparison among many species."*

In this revised version, we also address the reproducibility issues raised by reviewer 3 and consider all specific comments of the three reviewers, especially to

improve the clarity of the method and results sections and the aesthetics and readability of figures.

With this revised version we hope that we have addressed all comments and issues raised by the three reviewers and that the manuscript is now suitable for publication in PLoS Genetics. Below we provide detailed answers to reviewers' comments.

Thomas Brazier and Sylvain Glémin

Reviewer #1

It is now possible to analyse genetic and physical maps of organisms, as the literature now contains suitable data from multiple species. This manuscript analyses such data from larger numbers of plant species than previous studies (55 species, 5-26 chromosomes per species), and describes results for the broad-scale recombination landscapes. However, it does not actually use the analyses to ask interesting questions, which I had hope to see. Some questions might include the following.

We respectfully disagree with the general comments that we didn't address some of the interesting questions listed below. However we acknowledge that we didn't clearly stress them and framed the work with sufficiently clearly identified questions. We have extensively rewritten the introduction to make them appear clearly and below we answer each specific question.

Do chromosome arms have an obligate crossover? How often do chromosome arms have multiple crossovers, versus a single one (as I believe is the case in C. elegans)?

We already answered these questions in the previous version in figure 1A, figure 2B and through the formal model selection (Figure 6; Table 1). We also discussed them in the discussion about the species basal recombination rate, that the smallest chromosome of the genome had between one and two CO, independently of its genomic size. However we agree that it could have appeared diluted among other results. In the current version, (i) we directly ask the question in the introduction: *"What is the range of COs per chromosome observed in plants?"*, (ii) we annotated the number of COs on isolines in

Figure 1A, and (iii) we more explicitly describe the results *"Less than 2% of chromosomes had less than one CO (n = 11). 234 chromosomes had between one and two COs, suggesting that a single CO per chromosome is sufficient, though 419 chromosomes had more than two COs."*

Do related species differ (this is an important question, as it relates to the question of whether genetic recombination is sometimes selectively favoured, leading to higher crossover numbers than required for correct segregation, and for repair mechanisms to occur). This question is discussed near the very end of the text, but is not mentioned as a question earlier, making it appear that the ms is entirely descriptive. The ms does not seem to mention that some of the species studied are close relatives, and that this can be helpful in studying such questions.

We discussed the potential selective advantage of recombination by raising the question of the potential advantage to recombine more in gene-rich regions and what would be the optimal distribution of COs along the chromosome. However, we preferred not to introduce it as a main question of the article as the dataset and the approach is not appropriate to study it properly. We have too few closely related species to properly test for the evolution of recombination rates. At the angiosperms scale, we showed that the phylogenetic effect is mainly due to the differences of chromosome size between species (the phylogenetic model did not perform better than the mixed effect model with a species random effect without phylogenetic structure). We precise it in the results section "*the introduction of phylogenetic covariance did not improve the mixed model thus we did not retain a phylogenetic effect*". In general, closely related species do not differ in recombination rates but they do not differ in chromosome size either.

How large are pericentromeric regions with low recombination rates in plants, and how much do they differ between related species?

We answered the first question in figure 4 and figure S2. And as said above, we do not have an appropriate sampling to properly analyse differences among related species.

Do selfers have higher recombination rates per physical length of chromosome than closely related outcrossers?

As for previous questions, our dataset is not appropriate for this specific question. We would need either more pairs of closely related selfing and outcrossing species or a larger dataset to perform an angiosperm wide phylogenetic regression analysis. Actually, out of curiosity we had already done this analysis before and the effect was not significant. As it was a side analysis and because we already had many results we decided not to present it.

Are recombination rates the same in male versus female meiosis? This is finally mentioned in line 574, but it is not made clear until then that the data analysed are sex-averaged rates.

It is indeed a really interesting question. Unfortunately, as discussed line 596 (first draft version), we do not have enough sex-specific data to study it (in general, only sex-averaged genetic maps are provided). We indicated more clearly in the introduction and results that we have only sex-averaged genetic maps: "*we have estimated the sex-averaged rate of COs along chromosomes. (…) We retrieved publicly available data for sex-averaged linkage maps*"

Instead, the ms presents rather dull statistical analyses. The results have value, but they appear mainly to confirm findings that were already well established, and the ms does not make very clear what new findings now emerge, or show what we can now understand from the results that was not already known. More than once in the text "new insights" are claimed, but it is difficult to find them, partly because of the length of the text, which is also long-winded and repetitive in several places. These problems could be ameliorated by outlining in the Introduction what questions the authors set out to study. As written, this section gives the impression that their aims were purely descriptive, which is not an encouragement to read the text. The ms also tries to interest the reader by making claims to novelty, rather than describing some interesting questions. For example, I feel that it is too strong to say that "the broad diversity of recombination landscapes among plants has rarely been investigated… and the diversity of the resulting landscapes among species and chromosomes still need[s] to be assessed", although a formal comparative genomic approach may be new and valuable. A further value from analysing more species is that exceptions to accepted generalisations may be detected, and this study did produce a few examples of such exceptions. Overall, I doubt that readers need a length Introduction to tell them that recombination patterns are interesting in relation to evolution, including evolution of patterns in genomes, such as regions with different repetitive sequence density, and consequently gene density, and with differences in GC content. A shorter Introduction could give a better idea of what is new from this study.

As mentioned above, we agree that the novelty and the strength of this work could have been diluted in the manuscript. So we streamlined the introduction (for ex we removed some general statements) and now stress the main questions addressed in this study: *"Thanks to this dataset we addressed the following questions. What is the range of COs per chromosome observed in plants? Is the distribution of COs shaped by genome structure (i.e. chromosome size, telomeres, centromeres) and if so is there a universal pattern? Since recombination hotspots have been found in gene regulatory sequences so far, are recombination landscapes generally associated with gene density? What are the consequences of recombination heterogeneity on the extent of genetic shuffling? Overall, we found that recombination*

landscapes in plants are more diverse and more complex than initially thought." **We also tried to be clearer on what is novel and what is confirmatory.**

At least several of the conclusions are just confirmations of what was already known. The following examples illustrate this problem, and my comments also include some other issues for some of them (a recurring problem throughout the text is poor writing, including long-winded writing that makes the meaning hard to understand, and I provide some examples in my 'Minor comments' below, but these are still important comments that require revisions of the text, …

We extensively revised the text to simplify the writing and we hope it is clearer now.

…including a suggestion that some species may have too little information to be used. It would be helpful to show the numbers or markers mapped in Figure 4. In addition, if the numbers are small, presumably the total genetic map lengths are unreliable, and it is not explained prominently whether any attempt was made to check for this problem.

During dataset assembly, we checked the coverage of linkage maps with the difference between raw total linkage map length and the corrected total linkage map length. We estimated the corrected total linkage map length with two different methods: Chakravarti et al. (1991) and Hall and Willis (2005) and chose to report only the more sophisticated method of Hall and Willis (2005) in the manuscript. We added our conclusions to the Results: ***"Corrected linkage map length didn't change the total linkage map length (mean difference = 1.19 cM, max difference = 5.62 cM), giving confidence in the coverage of the linkage map"*****.**

Moreover, we used different metrics to assess the quality of Marey maps during the filtering step (e.g. marker density, mean interval between markers, largest gap, percentage of the total chromosome size covered by the linkage map, genome coverage, i.e. the difference between map length in Mb and chromosome size from the fasta file) and they were useful to automatically reject a large proportion of dataset (57 dataset retained among 120 dataset gathered; 52 % of dataset discarded). We did not keep every chromosome of a dataset and discarded 17 chromosomes among the retained dataset. The quantitative criteria were adjusted by iterative trials until we found a consistent dataset, and finally their values were:

- Exclude chromosomes with less than 50 markers

- At least one marker every 4 cM on average

- Exclude maps with gaps larger than 20 cM, as they could be falsely detected as recombination peaks (the value seems large, but smaller values were extremely stringent)

- Final visual assessment to exclude undetected suspicious maps (11 chromosomes among 6 species) or to make the choice to keep maps outside criteria (19 chromosomes among 9 species).

Moreover, it is important to note the strong qualitative improvement of our dataset compared to Haenel et al. (2018). They kept chromosomes with at least 20 markers (50 for us with a few exceptions at 30, so 50% to 150% higher) . As explained above, they mixed Marey maps with recombination landscapes, hence mixing different methods of estimation. Many of their linkage maps or recombination maps were figures that were digitised to interpolate an approximate markers' positions or recombination rates in segments, potentially limiting their spatial resolution (in both cM and Mb). They mixed sex-averaged maps with sex-specific ones (or an average of them). On the contrary, we made the effort to search only for tabular data to get the exact genetic and physical position of markers, and we had to ask many authors to provide their maps (as they were not always in supplementary materials). We did the effort of mapping markers on reference genomes as much as possible (if physical positions were not provided or if a more recent assembly version was available, when we had the markers' sequences). We mapped markers' positions on a reference genome for 14 datasets. We implemented a pipeline for the Marey map approach to get as much as possible consistent and reproducible estimates (e.g. automatic tuning of smoothing, bootstrapped confidence interval) so they could be compared between species, despite the inherent heterogeneity of our dataset.

References

Hall, M. C. & Willis, J. H. Transmission Ratio Distortion in Intraspecific Hybrids of Mimulus guttatus. Genetics 170, 375–386 (2005).
Chakravarti, A. A graphical representation of genetic and physical maps: The Marey map. Genomics 11, 219–222 (1991).

1. "We observed that the bias towards the periphery was not ubiquitous across species" and "Only a subset of species, especially those with larger chromosomes, exhibited a clear bias". These conclusions are quite similar to that of Haenel et al. (2018) that a distal bias is "universal for chromosomes larger than 30 Mb" (note the incorrect English "concluded to a distal bias"). The main advance seems to be that this study finds that Nelumbo nucifera and Camellia sinensis are exceptions to this pattern, with the highest recombination rates found in the middle of their chromosomes.

We corrected the English "*concluded*" by "*assessed*".

Though we get similar conclusions, we did more than identifying two exceptions. We identified a second pattern that Haenel et al. didn't detect. Haenel et al. (2018) described and conceptualised only the distal pattern (34 species in our study) but they didn't suggest anything for species not following this pattern. Thus we identified 22 species as exceptions to the Haenel et al. model (16 species sub-distal and 7 species as exceptions). We classified *N. nucifera* and *C. sinensis* in the sub-distal instead of distal, despite their large chromosome size, thus suggesting that the Haenel model is not so universal, as they claimed. We also stated that a fraction of species do not follow any of the two patterns we described (7 species, e.g. *A. thaliana* or *C. rubella*), suggesting that the diversity of patterns may be more important than we thought (and there is room for a more extended sampling in the future).

The result is described in a rather unhelpful manner, without taking chromosomes morphology into account. The text states that, for larger chromosomes, crossovers tend to occur (not "accumulate") at the ends of chromosome, while the central regions have less. However, this would be correct only for metacentrics, and the centres of chromosome presumably means centromeric and pericentromeric regions, but this is not made clear. It is also not made clear that these are completely recombination-free regions.

We changed "*accumulate*" by "*occur*".

Most chromosomes in our dataset are metacentric or sub-metacentric, which make it difficult to discern a difference between the physical centre (midpoint) and the centromere. When we used the term centre/central, we meant the midpoint of the chromosome. Otherwise we used centromeric/pericentromeric where appropriate.

We stated in the results that the centromere was a recombination free region (line 384 in the original draft). *"When the centromere position was known, we qualitatively observed that the centromeres had an almost universal local suppressor effect (Figure 3). In small and medium-sized chromosomes, the recombination was often suppressed in short restricted centromeric regions (several Mb) displaying drastic drops in the recombination rates, whereas the rest of the map did not seem to be affected. In larger chromosomes, the suppression of recombination extends to large regions upstream and downstream of the physical centre of the chromosome (approximately 80-90% of the chromosome; Figure 4)."*

We discussed more the limits of having mostly metacentric chromosomes: *"this work suggests that centromeres do not only have just a local effect but they also influence the symmetry of recombination landscapes over long distances, <u>though a large proportion of our sample is metacentric, which might</u>*

limit the detection of an effect. (…) However, how centromeres (especially non-metacentric ones) may affect CO distribution at larger scales still needs to be determined.”

The extent of a larger pericentromeric region (meaning, the extent of the wider region surrounding or adjacent to the centromere) is known to vary greatly between species, but it is not well described in the ms, and only examples are shown, with rather subjective criteria to define the different regions. It would, in principle, be possible to define them less subjectively, though this might not be easy. At least, it would be good to mention whether this was attempted. A further problem is that regions are shown in figures, rather than tables giving estimates of genome region sizes and recombination rates, and as relative sizes are often used, it is difficult to understand what sizes of pericentromeric regions (for example) are found in plants.

We mainly provide figures in the main text as we think it is clearer for the reader to get the main results. However, we also provide many quantitative data in tabular form in supplementary material. We also provide scripts and all Marey maps that will be available on the MareyMap online website. So it should be easy to retrieve quantitative information if needed.

It is also not a new discovery that low recombination regions tend to have low gene density. The Discussion acknowledges this, but it is strange to first describe this as if it is a new result, only to later mention that it is not. If the Introduction had laid out some questions, this could be avoided. Problems like this also make the text longer than necessary.

We hope we now go more directly to the point. In the introduction we ask: *“Since recombination hotspots have been found in gene regulatory sequences so far, are recombination landscapes generally associated with gene density?”* We also mention in the results section that we follow previous studies: *“At a fine scale, it has been shown in a few species that COs preferentially occur in gene promoters. The scale of 100 kb used here is too large to directly test whether this is a common pattern shared among angiosperms. Instead, like in Haenel et al. (2018), we assessed whether recombination increased with gene density.”*

2. Recombination is unevenly distributed in genomes. Therefore one should not write that “We showed that” this is the case. Once can write “We confirmed that” (or something similar). This text also uses vague terminology “how genetic variation is shuffled during meiosis”, but the word recombination already exists, so it would be better to be precise. If at some point the meaning is gene conversion, this should be used. However, I think that the text mentions conversion only in passing, and it is not considered seriously.

We changed “*showed*” by “*confirmed*”.

We don't clearly see the point here. We think that it is pretty clear that we are studying crossover rates, and we have no data to analyse gene conversion. In addition gene conversion cannot shuffle genes except in the close vicinity to recombination points. Following Veller et al. 2019 we measured the effect of a CO on gene shuffling at the whole chromosome scale. However, we are open to suggestions to improve the message if we have misunderstood this comment.

In line 538, I am not sure why thw word "prediction" is used (In addition to the role of centromeres, we also observed a departure from the prediction that recombination rates should decrease with the distance to the tip of the chromosome, showing that the distal model is not generally found among plants). Is this really a prediction, or are you trying to say that you did not confirm the view that this pattern is shared by all plants? If so, references are needed to assertions that all plants share this pattern.

We removed the word "*prediction*" and used a more clear statement: *"In addition to the role of centromeres, we also observed that recombination rates <u>do not always</u> decrease <u>monotonically</u> with the distance to the tip of the chromosome…"*

The Discussion section need not repeat so much of the results. It might also mention that recombination rates vary between individuals of the same species, including from the effects of rearrangements, especially inversions, so it would be good to mention that the data are currently often from just a single maternal and paternal parental individual of each species (for selfers, perhaps just a single parental individual). Hotspots should also be mentioned, if only to make clear that this study did not attempt to detect them.

474 It is proposed that in angiosperms crossovers may be initiated in gene regulatory sequences, and it is suggested that this "sheds new light on the evolution of recombination landscapes", but without saying what new light is shed, other than this suggestion. The suggestion is not evaluated further, and I did not understand if it is a speculation, based on the correlation between recombination and gene density mentioned in this paragraph (or on some other observations). However, based on later text (line 613), I suspect that the intended meaning is that the results are consistent with such a proposal that was already published by others.

We rewrote the sentence in a more explicit way: *"This sheds new light on the evolution of recombination landscapes and whether the distribution of COs is optimal for the efficacy of genetic shuffling."* We hope it is clearer now. [Note that the complete sentence was *"This sheds new light on the efficacy of genetic shuffling and the evolution of recombination landscapes."* so we already mentioned the efficacy of genetic shuffling]

However, as the correlation must be strongly affected by the lower gene densities in genome regions with low recombination rates, which lead to accumulation of transposable elements and other repetitive sequences, it would seem difficult to disentangle this from the suggested mechanism. Line 628 states that “The positive association of COs and gene regulatory sequences, including fine-scale correlations, appears more robust”, which is too vague. It seems unlikely that the effect is stronger than the very marked and consistent effect of low recombination rates on repetitive sequence density (although of course different elements are involved in different cases).

We removed this statement. “~~The positive association of COs and gene regulatory sequences, including fine-scale correlations, appears more robust (Choi et al., 2013; He et al., 2017; Marand et al., 2019), but c~~*Causality mechanisms of these multiple interactions still need to be clarified*.”

Regions with high recombination rates may, however, allow patterns in crossover localisation to be detectable, and I believe that this has been studied, for example in maize (e.g. papers by Dooner and colleagues) and also in Mimulus guttatus (see the paper by Hellsten et al. cited above). Line 621 finally mentions the problem of other correlated factors. I think that the authors should revise their text so that it does not first set up an untestable idea and then mention that it is untestable. Instead, it will be preferable to set up some interesting questions early in the text, tell readers what is currently known, and then describe analyses that help understand things better than before.

Dooner, H., & He, L. (2008). Maize genome structure variation: Interplay between retrotransposon polymorphisms and genic recombination. Plant Cell, 20(2), 249-258. doi:10.1105/tpc.107.057596

Fengler, K., Allen, S. M., Li, B., & Rafalski, A. (2007). Distribution of genes, recombination, and repetitive elements in the maize genome. Crop Science, 47(Supplement), S-83-S-95.

Yao, H., Zhou, Q., Li, J., Smith, H., Yandeau, M., Nikolau, B. J., & Schnable, P. S. (2002). Molecular characterization of meiotic recombination across the 140-kb multigenic a1-sh2 interval of maize. Proceedings of the National Academy of Sciences of the USA, 99, 6157-6162.

Tenaillon, M. I., Sawkins, M. C., Anderson, L. K., Stack, S. M., Doebley, J. F., & Gaut, B. S. (2002). Patterns of diversity and recombination along chromosome 1 of maize (Zea mays ssp. mays L.). Genetics, 162, 1401-1413.

We think that even in regions with high recombination rate it can be difficult to identify the precise locations of CO with the Marey map approach, especially in large genomes as in maize, unless the size of the genetic mapping population

is very large as in the recent study in A. thaliana. Otherwise LD maps should be more appropriate.
To be more constructive and less vague on this point, we now add that the use of recombination maps at finer scale will help resolving the role of genic regions in shaping recombination landscapes: "*Causality mechanisms of these multiple interactions still need to be clarified. The use of fine scale recombination maps (using very large mapping populations or LD maps) should help identifying the respective role of genic regions (especially the role of promoters) and transposable elements (or other genomic features)".*

Another comment that applies throughout the text is that recent papers are cited for concepta and understanding that are not new. In such cases, the text should make clear that the citation is to a review paper. For example, the text gives the impression that Marand et al. (2019) discovered that gene density and recombination rates are both correlated with transposable elements (meaning densities of transposable elements). This has been known for a long time, and was reviewed in 1994 by Charlesworth et al. (Nature, 371, 215-220. doi:10.1038/371215a0).

Citations have been changed accordingly.

In first mentioning heterochiasmy, it seems strange not to mention whether the papers cited refer to plants or just to studies in animal species. It is explained later that Melamed-Bessudo et al. (2016) showed that it is not universal in plants, but the text does not explain what the term might mean in plants, and that hermaphrodites may have different crossover patterns in male and female meiosis, so readers may be puzzled.

We rephrased to indicate when we were talking specifically of plants, and when we were referring to a more universal pattern shared by plants and animals. We also define heterochiasmy when we first used the word.

3. Similarly, I was surprised to read that "We were intrigued to notice that [within species] the chromosome-wide recombination rate is proportional to the relative size of the chromosome". I was under the impression that this was already known.

We changed the wording: "*Chromosome length drives the basal recombination rate for each species, though ~~but we were intrigued to notice that~~ within species the chromosome-wide recombination rate was proportional to the relative size of the chromosome*".

However, we think it was not already known that the size effect was species specific (this is why it's the relative size effect that seems to be general). We obtained this result because we gathered a dataset of chromosome-scale recombination landscapes, analysed them on a per chromosome level and modelled a species random effect; it has not been done before to our knowledge. In the two previous meta-analyses, Stapley et al. (2017) data were restricted to genome-wide recombination rates, and Haenel et al. (2018)

averaged their chromosome-wide recombination rates to a genome-wide level in similar analyses (one point per chromosome).

It is illustrated in Figure 2D, which shows the new results, which are potentially interesting, as they relate to the question of how often arms have multiple crossovers. This figure analyses the excess of crossovers, defined as the linkage map length minus the 50 cM expected if one crossover per arm is obligate), and shows that it correlates positively with the chromosome' physical sizes divided by the average chromosome size for the species, which they term the "relative chromosome size". Such an effect is not a new result.

As explained above, what we think is novel is that there is no single relationship between absolute chromosome size and CO but possibly a single and general one with the relative chromosome size. So the qualitative pattern was known but we think that its quantification was not. We added this precision in the corresponding result part: *"More concretely, it means that two chromosomes having the same ratio of size will have the same ratio of excess of recombination rate, whatever the species and the genome size"*.

In the discussion, we also tried to better explain this point: *"However, there is no universal relationship between the absolute size of a chromosome and its mean recombination rate. Although the average recombination rate of a species is well predicted by its average chromosome size, the recombination rates of each chromosome separately are not well predicted by their absolute chromosome size. Instead, variation within species is much better explained by the relative chromosome size, and surprisingly, this relationship seems to be roughly the same among species (see Figures 1 and 2)."*

However, as I understand it, an obligate crossover is expected on each arm. If so, the number of excess crossovers, in addition to this one, should be analysed per arm. Even if my recollection about this is incorrect, the text should make clear what is known from previous studies, and why the present study uses chromosome, not arm, lengths. Line 136 mentions that the centromeric index was known for the chromosomes of 37 species, but then it remains unclear how these data were used, and also whether results can be used from the species where no such data were available. Line 285 mentions that recombination rates were negatively correlated with the distance to the nearest telomere, which seems to suggest that metacentrics may have been analysed as such, but I could not see this clearly explained.

We explained earlier in the main text that one CO seems to be sufficient (at least for some species) [*"Less than 2% of chromosomes had less than one CO (n = 11). 234 chromosomes had between one and two COs, suggesting that a single CO per chromosome is sufficient, though 419 chromosomes had more than two COs"*] so we think it's clearer why we used linkage map length minus 50 cM. In addition we showed later that the model with one mandatory CO per arm is not well supported statistically.

Line 300 states that that (in my wording) the centromere regions almost universally showed low recombination rates, but this is not completely clear in Figure 4, where large low recombination rate regions in several species, for example Vigna unguiculata, appear not to overlap the centromeres. If this is a real biological observation, the statement seems incorrect.

We completed the statement with new results quantifying this effect "*Ninety percent of chromosomes (388 chromosomes) had significantly less recombination than the chromosome average at the centromeric index (n = 425, resampling test, 1,000 bootstraps, 95 % confidence interval). 81 chromosomes (19 %) were completely recombination-free in the centromere. However, the transposition of centromere position from cytological data to genomic data may be imprecise or wrongly oriented for some chromosomes. After orienting chromosomes to map the centromeric index, 16 % of chromosomes (70 over 425) had a recombination rate higher in the inferred centromere position than on the opposite side, thus a centromere potentially mapped on the wrong side. Of these 70 chromosomes, the difference between inferred and opposite centromere position was less than 1 cM/Mb for 64 % of them (n = 45).* "

Given these possible problems with the data, I was not convinced of the value of the formal modelling analysis of the effect of the centromere in suppressing recombination, and the comparison with less simple models that suggest that telomeres may also affect patterns. Such effects are plausible, but I feel that some of these plant data do not add valuable and solid support.

We feel that this criticism is somewhat unfair. The initial simple telomere-led model has been proposed on the basis of visual observations based on species average and on the correlation between periphery-bias ratio and chromosome length on 16 animal and 11 plant species. This is a very useful starting point but it was clearly proposed as a conceptual model by the authors (their Figures 3 and 4). Here we think we go beyond this by formally testing the model and proposing alternative ones with a much larger dataset and taking individual chromosome patterns into account. We are aware of the possible noise in the data but if some centromeres are not correctly located (opposite size for example) this should go against model 3 and removing those chromosomes didn't change the results. So we think our results are robust.

Another weakness is the lack of any mention of differences between male and female meiosis, and another is the lack of any mention of outcrossing rates.

In the first version we already discussed the difference between male and female meiosis. We made it clearer that we have only sex-averaged genetic maps and that we didn't analysed sex-specific maps. Note that in the recent paper of Sardell and Kirkpatrick (Am Nat 2020, sex differences in the recombination landscapes), only five plant species are available. However we

kept the idea in the discussion as it is indeed an interesting hypothesis to explain the observed patterns.

As explained above, the dataset is not appropriate to test for the effect of the mating system.

I wondered why these papers were not cited, or other papers about Arabidopsis lyrate or helleri, which may have genetic map information.

Hellsten, U., Wright, K. M., Jenkins, J., Shu, S., Yuan, Y., Wessler, S. R., . . . Rokhsar, D. S. (2013). Fine-scale variation in meiotic recombination in Mimulus inferred from population shotgun sequencing. Proceedings of the National Academy of Sciences of the United States of America, 110(48), 19478–19482. doi:10.1073/pnas.1319032110

Kawabe, A., Hansson, B., Forrest, A., Hagenblad, J., & Charlesworth, D. (2006). Comparative gene mapping in Arabidopsis lyrata chromosomes 6 and 7 and A. thaliana chromosome IV: evolutionary history, rearrangements and local recombination rates. Genetical Research, 88, 45-56.

Hansson, B., Kawabe, A., Preuss, S., Kuittinen, H., & Charlesworth, D. (2006). Comparative gene mapping in Arabidopsis lyrata chromosomes 1 and 2 and the corresponding A. thaliana chromosome 1: recombination rates, rearrangements and centromere location. Genetical Research, 87(2), 75-85. doi:10.1017/S0016672306008287

They are interesting papers. However the first one focuses on fine-scale crossover patterns and CO hotspots, especially around genic regions and does not provide a chromosome scale map (actually not possible with the method they used). The others could have been used but they did not match our filtering criteria.

Minor problems with the English, or vague wording or unclear statements

1. In English, it should be “correlated with” (not “to”).

Corrected.

2. The word ‘drive’ should be avoided, as it is very vague. For example, the meaning is not clear in the phrase “Chromosome length drives the basal recombination rate for each species”

We changed to “*Chromosome length constrains the basal recombination rate for each species*”

3. In line 182, it should read “regression lines for species with at least 5 chromosomes mapped, 5-26 chromosomes per species, 55 species).

Corrected according to the reviewer's comment.

4. Line 232 Genomic distances (Mb) were scaled between 0 and 1 (divided by chromosome size) to compare chromosomes with different sizes.

We are sorry but we don't understand what is the issue here.

5. It is difficult to make out the meaning of the text starting in line 247. I think it means the following: "Each chromosome was divided in (it should read "into") ten bins, each one 10th of the chromosome's total physical size." The relative recombination rate is the log-transformed ratio of the expected relative genetic length (one tenth, presumably of the total genetic length) divided by the observed relative genetic length of the bin (presumably meaning the proportion of the total genetic length represented by the physical region in question. Values below zero correspond to recombination rates lower than expected under a random distribution of crossovers across the physical chromosome. Also difficult to understand "Chromosome sizes (Mb) on the left correspond to each broken stick chromosome" — maybe it means "each chromosome". Also (in line 244) "Relative recombination rates along the chromosome were estimated in ten bins using the broken stick model.

To avoid confusion, we used another representation by dividing chromosomes in ten bins of equal genomic size, computing the average recombination rate in each bin and dividing by the mean recombination rate to get a relative measure. We also reordered the species as a function of chromosome size instead of recombination heterogeneity.

6. In English, one needs to say "divided into" (not "in"). Also "pooled into" (although this reads awkwardly in English, and line 140 might be better as "the Spearman rank correlation coefficient correlation between the values for 1 Mb windows and those for the 100 kb windows within them was ….".

Corrected according to the reviewer's comment.

7. The work "linkage" in genetics means that the variants are linked. It should be distinguished from "linkage disequilibrium" (LD), which refers to associations between two or more liked variants. Line 57 should be corrected, as the text refers to the latter, but uses the former ("Recombination…. breaking the linkage between neighbouring sites and creating new genetic combinations"). The sites remain linked, but not in LD. The sentence is also confusing by adding "upon which selection can act", because selection acts on single variants, and the authors are trying to say that new genetic combinations might be more (or less) favoured by selection than the non-recombinant combinations (in other words, the different variants may interact in their effect on fitness).

We changed "*breaking the linkage*" into <u>"*breaking linkage disequilibrium*"</u>.

We also write now: "*making selection more efficiently*" to avoid possible confusion.

8. It is a sweeping statement to say that "Plant genomes contain large regions with suppressed recombination", depending strongly on how many plants have good data on physical and genetic maps, so line 92 ought to mention the number on which this is based, and give readers at least a rough idea of what is meant by "large". There is no need to add the obvious remark that this impacts genomic averages (in addition "impact" is the wrong word, as the meaning is that it affects the average — of course the average depends on the values in all genome regions that are included in the data, so it is not worth saying explicitly).

As genome sizes vary over many orders of magnitude in plants (10 to > 1000 Mb), "large" is very species dependent. It varies from a few Mb in smaller genomes (genomes between 10 and 30 Mb) to hundreds of Mb in larger genomes (500-1000 Mb), and the proportion of genome without recombination ranges from a few percent to 80%. We clarified the sentence (added the range of values and proportions) *"Plant genomes contain large regions with suppressed recombination* <u>*in various proportions (from a few Mb to hundreds of Mb, > 1-75 %)*</u>*,…".*

We suppressed the statement about the average recombination rate.

9. Phrases that are unnecessary (such as "it seems that" in line 93, should be pruned out, so that the text is easier to read. There are quite a few such instances, and I do not comment on all of them. The beginning of the Results section, for example, could be written more briefly and clearly.

We retrieved publicly available data for linkage maps and genome assemblies, to obtain genetic map distances and physical distances. We used linkage maps with marker positions in chromosome-level genome assemblies (except for Capsella rubella, which had a high-quality scaffold-level assembly of pseudo-chromosomes). After filtering based on the marker numbers, densities, and genome coverage, and after filtering out the outlying markers (maybe meaning outlier markers by a criterion that needs to be explained), we produced 665 Marey maps (reference needed) for 57 species (2-26 chromosomes per species); marker numbers per chromosome (or perhaps the authors mean per species, in which case perhaps some species have too little information to be used) ranged from 31 to 49,483.

We thank you for this example of paragraph and we tried to follow this advice throughout the manuscript. Note that the minimum of markers is 30 per chromosome and not per species as mentioned above.

Reviewer #2

In this paper, the authors seek to decipher genomic patterns of recombination across a large (57 species) dataset of sequenced plant genomes coupled with genetic maps. Their meta-analyses lead to several novel observations.

I thoroughly enjoyed reading this manuscript, and I congratulate the authors on a really fine paper. It will be, in my view, a very welcome addition to the literature. In the surest sign of flattery, I'm a jealous that I did not think of doing such a neat analysis.

We thank reviewer 2 for this very positive comment.

Accordingly, I have only minor comments that the authors may wish to address in revision. Most of the comments are very minor, indeed. They are offered both as an attempt to clarify the few areas of the text that I found difficult to digest and probably out of an abundance of enthusiasm for this work. I leave it to the authors to decide if my suggestions offer improvements or are better ignored…

Minor Comments:

- Line 48 – Unlike most of the rest of the paper, I found this sentence hard to read and digest. Reword, rework or shorten? Btw I'd use "in" instead of "to" ("in the production")

We splitted the sentence for clarity.

- Line 79 – This last sentence of the paragraph is really indirect and therefore pretty tough to read. I'm not really sure what manipulations are being considered here… Rewrite?

We rewrote the sentence: *"The characterization of recombination landscapes also has practical interests as variation in meiotic genes could be used to experimentally manipulated CO patterns for advantageous purposes, such as redirecting recombination towards regions of interest for crop breeding (Kuo et al., 2021)"*.

- Line 100 – as a reader, I found that a better link between the two sentences on this line could have been helpful. Maybe something as simple as "Haenel et al. considered chrosomome length, found blah blah blah and suggested a simpler telomere-led model. That model included a universal bias…"

We rewrote it more directly: *"They found that larger chromosomes have low crossover rates in their centre and suggested a simple telomere-led model with a universal bias of COs towards the periphery of the chromosome, positively driven by chromosome length."*

- Line 118 – I'd use "about" instead of "on"

Changed for "*about*"

- Line 125 – If this is reasonable, I'd love to see the filter characteristics hinted at here, even though there is a good description in the methods. That is something like "… marker density (at least 50 per chromosome), genome coverage (blah blah)"

To avoid weighting the main text too much we preferred referring to the method part for details. However, we can add more details here if needed.

- Line 701 – I'm a bit confused by the what was done when marker sequences were not available and also how many species fell into this category. I'm not concerned at all – this is a careful study – but it'd be nice understand better.

When marker sequences were not available for mapping on the most recent genome assembly, genomic positions were those of the original publication (precisions now added to the M&M). "*We remapped markers on the reference genome for 14 species for which genomic positions were not known or were mapped to an older assembly*".

- Figure 2 – It might be helpful to have X-axis say "Mean chromosome size" where appropriate (e.g., Figure 2C and B). The legend is very clear, though.

The X-axis has been changed accordingly.

- I love Figure 3. It blows my mind how consistent the patterns are between the dashed lines (genome wide) and an individual chromosome. It is bizarre and neat and thought provoking. It might be nice to report mean chromosome size (in the legend or in the figure), given that the species are ordered in that matter. It just makes me curious…

Mean chromosome size has been added to the title of each figure.

- Figure 4, since patterns seem to correlated with mean chromosome size, would it be worth adding that value after each species name? As a reader, it would help me to see the pattern and better digest the text from ~lines 213 to 227 and figure 5a, etc).

We added the mean chromosome size after species name and colored each species as the function of three patterns identified later. We also reordered species as a function of mean chromosome size.

- Figure 5A – this may make the graph too crowded, but it'd be nice to be able to compare dots in 5A to figure 4. So, it'd be nice to have the dots labelled. If that is too much, the authors might want to consider labelling a few species (e.g., the six in figure 3 or some of the species mentioned in lines 220 to 227). Personally, I'd love to know what the outliers are in this graph!

Figure 5A has been annotated with a subset of species (the 6 in figure 3 and a few species mentioned in lines 220 to 227 or seen as outliers).

- Lines 284 and following. It'd be nice to cite Figure 6A and Figure 6B separately after the word descriptions of the patterns.

We added references to Figure 6A and Figure 6B in the text.

- Line 296. I could not figure out what the "species correlation" referred to. Sorry if I missed this, but it's worth another look to be sure it is clear.

We changed the formulation: "*the correlation between recombination and the distance to the nearest telomere was significantly higher for species with larger chromosomes*".

- I'm kind of shocked that M3 is favored over M2, as isn't one CO per arm necessary for mechanism? Hence, I'd a priori predict M2 > M3. I don't think this contrast is explicitly discussed in the Discussion (e.g., lines 523 to 536), but I think it should be.

To our knowledge, it was not very clear if one CO per arm or per chromosome was necessary. It seems that CO assurance imposes one CO per chromosome, not per arm, in many species. For example, a recent study by Dukic and Bomblies in *Arabidopsis arenosa* observed a large proportion of chromosomes undergoing a single CO, while only 25/30% of bivalents contained at least two COs. Indeed, we observed less than two COs in many chromosomes, especially the smaller chromosomes within species (Figure 1). Besides, even if M2 fit well for a bunch of chromosomes (7 species over 37 species support M2), the model is not applicable for many other chromosomes just because at least one arm has less than 1 CO (length < 50 cM). To mitigate this effect, we subset only species with chromosomes having at least one CO per arm (26 species over 37) and M3 was still a better model than M2. However we must say that we do not have the perfect sampling to categorically exclude M2 or M3, because most of our chromosomes are metacentric, thus a low power to distinguish M2-M3.

- Figure 8: It'd be nice if the legend clearly stated which graph is which. I think Figure 8b is the distal recombination pattern, but I'm not 100% sure. It'd be great to have sample sizes on the graph too (n = 34 or 16 species, I think).

We now give an explicit legend; (a) distal pattern vs (b) sub-distal pattern. We also added to the figure sample sizes and an annotation for distal/sub-distal patterns

- It's pretty clear in the M&M, but on line 419, it might be nice to mention that rintra is a single value per chromosome. On my first reading, I was thinking it was some sort of transformation of cM between genes…

We now explicitly mention that r_{intra} is a single value per chromosome: *"The r_{intra} gives, <u>for a chromosome</u>, a measure of the probability of a random pair of loci to be shuffled by a crossover."*

- The analysis of gene distances is very thought provoking!

Thanks!

- Line 488 – What the heck is going on with fungi and animals! It's certainly not necessary, but can the authors provide a quick description or explanation. They have piqued my curiousity.

Indeed this statement was not very clear; we removed this comparison with animals and fungi as it was not important to discuss our results. For your information, we were referring to the fact that linkage map length does not depend on the absolute chromosome size (Figure 2A). Stapley et al. (2017) showed that genome-wide linkage map length does not depend on total genome size in plants, in contrast to fungi and animals. It is possible that CO interference is stronger in plants than in animals and fungi, as it would explain why linkage map length remain stable across a large span of genome sizes, though it is not very clear to make the link with our results because Stapley et al. (2017) relies on genome-averaged values.

Again, I do not consider any of my comments to be critical for publication, and I want to again congratulate the authors on a thorough and interesting study.

Reviewer #3

This manuscript by Brazier and Glémin uses a comparative approach to investigate variation in recombination landscapes in flowering plants. Their study used genetic map data from 665 chromosomes in 57 species of angiosperms. At the whole chromosomal level, they found a negative correlation between chromosome size and recombination rate (cM/Mb) with a strong species-specific effect. They also found that CO excess on chromosomes was more correlated with their relative size to other chromosomes in the genome rather than their absolute size, and that this effect was consistent across species. When investigating crossover landscapes, they found that landscapes were similar within species but strongly varied between species. CO rates were not uniform across chromosomes and were often more likely to occur at the distal ends of the chromosomes, with larger chromosomes tending to have a higher "periphery bias" of COs. However (as with most things in nature), this general pattern did have a number of exceptions. The authors then investigated the joint effect of telomeres and centromeres on CO distribution, finding the strongest support for a model that incorporated the effects of the telomere, centromere and one CO per chromosome. The authors found that recombination rate increased with gene density. Finally, the authors showed that genetic shuffling was positively correlated with linkage map length, and that there was a small negative effect of the periphery-bias ratio. These effects were slightly higher when modelling genetic shuffling in terms of gene distances. Whilst the investigations here are largely correlative rather than revealing mechanisms, this study provides a useful foundation for further investigation of broad drivers of recombination rate and landscape variation across a wide range of taxa.

This paper is the most comprehensive and well analysed that I have read on this topic, and generally it is well-written and well structured, particularly the introduction and discussion. I'm impressed by the sheer breadth of analyses.

We thank reviewer 3 for his/her very positive comment.

Nevertheless, **there are parts of the methods & results that lack clarity**, which in turn leads to issues with reproducibility. In particular, a lot of the statistical models are not well described – model structures should be made explicit in the methods and/or results, rather than providing a general text for statistical analyses at the end of the methods. I would emphasise that providing code and data (where possible) would improve these issues.

Thank you for pointing this out. We addressed this issue by providing more information in the results and M&M sections. The chosen statistical models are explicitly written. Besides, the R code used for statistical analysis will be freely available in a public github repository. Marey maps will be available in the

same github repository and in an updated version of the MareyMap Online database.

I had many comments and suggestions - those marked ** should be addressed by the authors in a revised version.

ABSTRACT/INTRODUCTION

Lines 32-33: The authors should be clearer what they mean by “relative size” here (i.e. relative to the rest of the genome) and why this result is interesting.

We substantially modified this part of the abstract and hope it is clearer now: *“We found that the number of crossing-over per chromosome spans a limited range (between one to five/six) whatever the genome size, and that there is no single relationship across species between genetic map length and chromosome size. Instead, we found a general relationship between the relative size of chromosomes and recombination rate, while the absolute length constrains the basal recombination rate for each species”*. We also substantially rewrote the rest of the abstract and hope it clarifies and sharpens the message.

Lines 48-52: In the first sentence, I would add the term “crossing-over” or “crossover” here to set up the rest of the introduction. In the second sentence, I would briefly define landscape (i.e. variation in recombination rate along the chromosomes)

Crossover and the definition of recombination landscapes have been added to the first sentences.

Lines 61 – 63: Indicate that you are defining assurance in this sentence.

Crossover assurance has been added to the sentence. “*at least one CO per chromosome is mandatory (i.e. crossover assurance) to achieve proper segregation and to avoid deleterious consequences of nondisjunction”*

Line 73: Can the authors briefly mention how recombination landscapes shape the distribution of TEs?

We mentioned “*the accumulation of TEs in regions of low recombination*”

**Lines 98 – 100: I found this statement confusing, as I can’t understand how independence between linkage map length and genome size means that recombination rates will be higher in smaller genomes. I also can’t make the link between this statement and the Stapley paper – I think they found that linkage map lengths were smaller in smaller genomes, but also that chromosome number explained more variation (i.e. increased chromosome number lead to longer maps

due to a higher minimum bound of recombination due to crossover assurance). Perhaps I am wrong, but regardless, it might be worth double-checking this statement and explaining it more clearly.

"*Recombination rates are supposed to be higher in smaller genomes because the linkage map length is independent of genome size*". Since the total linkage map length do not depend on the total genome size, we can assume that smaller genomes will have roughly the same linkage map length as larger genomes, thus the same number of COs is distributed among a smaller genomic size (Mb) and recombination rate is higher (cM.Mb^{-1}). Stapley et al. (2017) made a similar statement in Box 1. "*Following the observation that linkage map length was similar across eukaryotes despite large variation in genome size, it was proposed that larger genomes have several orders of magnitude lower recombination rates.*" In their Figure 3 Stapley et al. show that linkage map length increases linearly with genome size, though in plants the best fit was a quadratic function. Accordingly, in plants, larger genomes have roughly the same linkage map length than smaller ones. Indeed, they also stated that chromosome number explained more variation than genome size.

We rephrased the statement accordingly to the reviewer's comment: assertion is true in plants, not other eukaryotes, and the number of chromosomes is more important than genome size:

"In plants, contrary to other eukaryotes, recombination rates are supposed to be higher in smaller genomes because the linkage map length is independent of genome size and the number of chromosomes explain more variation than genome size (Stapley et al., 2017)."

Line 100: on recombination rate, or landscape? Or both?

Actually, both. We added to the phrase: *"<u>existence of a major broad-scale determinant of CO distribution and frequency</u>"* to be more explicit.

**Line 102: Based on your argument here, it is not clear how chromosome length links to biases of CO towards the peripheries – please clarify.

We clarified the argument. *"Haenel et al. (2018) <u>found that larger chromosomes have low crossover rates in their centre and </u>suggested a simple telomere-led model with a universal bias of COs towards the periphery of the chromosome, <u>positively</u> driven by chromosome length"*

Line 117: Briefly define genetic shuffling and why it's interesting – could even be mentioned earlier e.g. around lines 56 – 58.

We added precision on the expected effect of CO patterns on genetic shuffling in line 117. *"how CO patterns affect <u>the extent of</u> genetic shuffling"*

We modified lines 56-58 to define genetic shuffling. ***"creating new genetic combinations transmitted to the next generation upon which selection can act, i.e. genetic shuffling"***

We added explicit precisions in results. ***"In most cases, genetic shuffling were slightly higher when gene distances were used instead of base pairs (Figure 10; mean = 0.22 for base pairs; mean = 0.26 for gene distances; Wilcoxon rank sum test with continuity correction, p < 0.001), implying that the genetic shuffling was more efficient among coding regions than than among regions randomly sampled in the genome"***

RESULTS

**Lines 130 – 132: I don't think this is described in the methods. Is there information on the number of progeny? I was curious about this but couldn't find the information in the supplementary tables.

We added a new column with the number of progeny (retrieved in the original publication of the genetic map) in supplementary table S1 Dataset Metadata.

Line 143: This header could be interpreted that smaller chromosomes have more crossover events rather than more crossovers per unit length. Perhaps "Smaller chromosomes have higher recombination rates than larger ones"?

The header was modified accordingly.

**Lines 153 – 169: Where are the methods for this LMER and what is the model structure? Is this what is being described in lines 831 – 850 of the methods? Throughout the paper, it needs to be clearer what models were run and what their fixed & random effect structures were in order to better interpret them.

Models' equations are now explicitly mentioned in the results section for each model presented.

Line 155: Does this mean that there is no/low phylogenetic signal of recombination rate?

We do not have a proper sampling to study the evolution of recombination rates along the phylogeny, with uneven sampling amongst angiosperms (many close relatives in monocots, sister species in *Brassica* sp., *Oryza* sp. or *Arachis* sp.). Though the phylogenetic mixed model (pglmm, phyr package, see method for details) assessed a phylogenetic signal (34% of the variance explained by random effects was due to a phylogenetic signal), we suspect it was mainly attributed to differences of chromosome size between monocots and eudicots (monocots with mainly large genome and eudicots with a higher

proportion of small genomes) and uneven sampling. When we add both phylogenetic effect and chromosome size, we saw that the phylogenetic mixed model did not perform better than a more parsimonious linear mixed model with a species effect without phylogenetic covariation (Table S5).

Figure 1: This figure is busy. A suggestion for panel A: perhaps the dashed lines could be fit from axis to axis, to visually demarcate the 1 – 4CO expectations a bit better? For panel B, since this is the same data plotted twice, perhaps only the regression lines need to be visualised here rather than all of the points.

We modified the figure to improve its readability. We hope it is better now:

Figure 1A. Dashed lines are now solid thin lines, going axis to axis and more easily identified. Lines were identified by the expected number of CO directly on the figure. Regions with less than one CO or more than four COs were labelled.

Figure 1B. We removed points, according to the reviewer's comment, for better visualisation of regression lines.

**Figure 2: I found this figure confusing. Some suggested edits:

Panel A could be wider to allow discerning of the slopes. I also struggled to understand what the isolines on the graph are showing even after reading several times. When using isolines, perhaps there is a need to define their values (as in Figure 1) – or perhaps they can be removed if making things too busy.

Panel B: I cannot interpret what this is showing – are there really lines in panel A that have intercepts of less than zero?

Panels B & C: I am very curious to see the error on these estimates.

Panels B & C: maybe these panels might be better suited in the supplementary?

Panel D: Again, very busy. Perhaps use of transparency of points or lines could make things clearer.

We understand the confusion on figure 2. Panel B has been removed since it did not support important findings. Panel C has been moved to supplementary materials (Figure S4). Panel A is now wider. We added transparency to points to make regression lines more distinguishable.

The genome wide recombination rate (cM/Mb) has been annotated on isolines. They tend to show that within species recombination rates are roughly similar among chromosomes despite differences in absolute chromosome size (because regression lines are parallel to their closest isoline).

**Figure 4: Accessibility issue for colour-blindness - the red dots may not be visible on the green background. The visual scale for the chromosome size is a unclear,

particularly as appears to be log – could there be line traces instead of colours here? Also – perhaps I have misunderstood – but if the chromosomes were split into ten bins, then why does the resolution of recombination rate estimation look to be much higher than 1/10th of the chromosome on the horizontal lines?

Thank you for pointing this out. Centromere position is now more visible, represented by black and white diamonds (higher contrast).

The visual scale of chromosome size is now clearer, with the height of bar plots corresponding to chromosome size. The scale is now linear instead of log. We also ordered species by mean chromosome size. Following reviewer 1's suggestion we also used another way to visualise the results to avoid possible confusion. Chromosomes are now splitted into ten bins of equal genomic sizes, not genetic length. We hope it eases the reading.

**Line 282 – 298: It seems that chromosome size was a strong correlate of recombination pattern, but I was curious if the authors tested other factors to rule out potential artefacts (e.g. differences in marker density) or to identify other biological correlates, such as ploidy? Was there a phylogenetic signal of this distal vs subdistal pattern?

We tested several factors, such as number of markers, marker density (cM & Mb) or number of progeny, that had no effect. Out of curiosity we also previously checked for possible effects of ploidy, lifespan and mating systems. None was significant. Note that our sampling is not appropriate to properly test these effects (see answer to reviewer 1). If required we can add such analyses in supplementary material.

Figure 6: There is a lot of text to wade through in the legend - it would help the reader to put annotations, sample sizes, key on the figures to allow for faster interpretation. For example, putting A: Distal pattern, N = XX, B: Subdistal pattern, N = XX on the panels make it easier to interpret. The dashed lines for the unclassified patterns are very distracting – why not include this as another panel, or put it in the supplementary material? Panel C is tiny and needs a key, or at least x-axis labels. I like the schematics of the crossover distributions but it's so tiny – perhaps include this as its own figure as it explains the model really well.

Annotations were added to the figure to reduce the legend (i.e. pattern, sample size). Unclassified patterns were removed, since they are specific patterns and they are also directly accessible in supplementary Figure S6.

X-axis labels were added to panel C.

Figure 6 was splitted. Figure 6D was moved to a new figure.

Figure 7: A & B. There needs to be a higher contrast between the colours as it's difficult to see the differences between blue and black. C. What do the colours

represent here? Adjusting the point transparency and slight x jitter may improve the visualisation here.

Figure 7A & B. The contrast was increased.

Figure 7C. We removed colours because they are not informative on this graph and do not contribute to the main message. Colours represented species (each species had its own relationship recombination rate ~ gene count) but it was difficult to distinguish them. The distribution of specific relationship recombination rate ~ gene count is already presented in Figure 7A. We added a slight jitter.

Figure 8: Same as Figure 6 – annotating the panels would be helpful.

We annotated the panels with the pattern name and the sample size (number of species). We removed the dashed lines for exceptions.

**Line 414 – I think it's important for the authors to briefly define what genetic shuffling is and why it's interesting to look at from an evolutionary perspective.

We added a short definition of genetic shuffling. *"<u>Genetic shuffling participates to the random reassortment of genes between parental homologous chromosomes.</u> To quantify how much the genetic shuffling depends on the distribution of COs, we estimated its intrachromosomal component, r_{intra}, as described in equation 10 in Veller et al. (2019)."*

Line 419: On the same chromosome

Same answer as for reviewer 2. We explicitly mentioned that r_{intra} is a single value per chromosome. *"The r_{intra} gives, <u>for a chromosome</u>, a measure of the probability of a random pair of loci to be shuffled by a crossover."*

Line 422: less efficient = resulted in less genomic shuffling?

The sentence was modified according to the reviewer's suggestion, to be more explicit on the effect of COs. *"COs clustered in distal regions are supposed <u>to generate less genetic shuffling</u> than COs evenly distributed in the chromosome."*

Figure 9: see comments on Figure 7.

We guess the comment applies better to Figure 10. The contrast was increased by changing the blue colour.

DISCUSSION

Lines 477 – 488: I'm a little puzzled by some of the statements here, so perhaps clarification is needed. I think it could be mentioned that crossover assurance will give a basal rate per chromosome of 50cM regardless of size, and then the authors can expand how the findings outlined here add to this established fact. Furthermore, I believe that in animals, larger chromosomes do have lower recombination rates within species… if I have misinterpreted this, perhaps the authors need to clarify their point better.

Indeed this statement was not very clear (see the same comment above); we removed this comparison with animals and fungi as it was not important to discuss our results.

Line 519: clarify what "association" means here… does chromosome pairing begin at the telomeres?

Yes it does. The phrase was changed to be more explicit (association replaced by chromosome pairing). ***"the early <u>chromosome pairing beginning in telomeres</u> is thought to favour distal COs "***

Line 570: put "beam-film" in inverted commas and indicate that you are about to describe it.

We put 'beam-film' in inverted commas. We indicated that we describe the conclusions of Zhang et al. (2014), we do not have results about the 'beam-film'. ***"<u>Zhang et al. (2014) assessed that the 'beam-film' model</u> is able (…) <u>If clamping is assumed, the model predicts</u> that mechanical stress culminates (…)"***

Line 582 – 583: It depends on the number of gametes measured and how many were male and female, which is easily done in dioecious species. I think authors should specify here "in angiosperms" and iterate here why heterochiasmy is difficult to investigate for the less plant-literate reader.

We are not sure to clearly understand this suggestion. The offspring generation corresponds to an equal number of males and females meiosis, even if the parents produced different numbers of gametes.

METHODS

**Lines 700 – 704: Indicate that this was from cytogenetic data. How is this information orientated correctly to the linkage map/genome sequence?

We removed the sentence about centromeric indexes, since it was better explained later in methods, in the paragraph "Testing centromere or telomere effects" : ***"We searched the literature for centromeric indices (ratio of the short arm length divided by the total chromosome length) established by cytological measures. When we had no information about the correct orientation of the chromosome (short arm/long arm), the centromeric index was oriented to***

match the region with the lowest recombination rate of the whole chromosome (i.e. putative centromere). "

**Lines 709 – 723: I think this paragraph requires a few improvements in reproducibility. Was this all done in the MareyMap package in the next paragraph? What was a ballpark criteria/example for anything that was outside the global trend?

At the beginning of the paragraph: *"We selected genetic and genomic maps after stringent filtering and corrections, using custom scripts provided in a public Github repository (https://github.com/ThomasBrazier/diversity-determinants-recombination-landscapes-flowering-plants.git). "*

We specified a qualitative criterion for map selection and quality assessment: "We assumed that markers must follow a monotone increasing function when plotting genetic distances as a function of genomic distances in a chromosome (i.e. the Marey map) and collinearity between the genetic map and the reference genome was required to keep a Marey map."

More details about the correction: *"If necessary, genetic maps were reoriented so that the Marey map function is increasing (i.e. genetic distances read in the opposite direction)."*

We specified criteria for outlier filtering and precisions about the processing: *"Markers clearly outside the global trend of the Marey map (e.g. large genetic/genomic distance from the global cloud of markers or from the interpolated Marey function, no other marker in a close neighbourhood) were visually filtered out, and multiple iterations of filtering/interpolation helped to refine outlier removal. "* We previously tried different strategies of automation for this step, as in Mansour et al. (2021), but after many trials, we concluded that it was neither efficient nor reliable for handling heterogeneous datasets. Though it is easy to distinguish clear outliers for human eyes, it is not a trivial task to set an automatic filtering for outliers. Because the noise is highly dataset dependent, one cannot use a constant threshold to exclude outliers. A threshold adjusted for the best map might be too stringent for another dataset, while a threshold set for the worst map might fail to detect the few outliers in almost perfect Marey maps. We tried automatic filtering of outliers based on the distribution of inter-marker distances (between adjacent markers), but user confirmation is required, as for many data-specific tasks. Setting a given threshold of distance or a given distribution statistic (e.g. remove 5% with the greater genetic distance than the maximum extreme value) do not take into consideration the heterogeneity between dataset. Thus, when comparing different dataset relying on very different distributions, we suspect it could introduce a kind of noise-dependent bias. The more noise there is in data, the less stringent filtering will be. Finally, visual assessment and an iterative procedure of filtering/interpolation seemed to be the more robust approach, though we lose in reproducibility. Please note that all outlier markers removed

are still present in Marey maps provided in supplementary, though they are labelled as 'not valid' and not used in subsequent analyses. This is also a possibility proposed in the MareyMap package in R.

In the next paragraph: *"Local recombination rates along the chromosome were estimated <u>with custom scripts following the Marey map approach, as described in the MareyMap R package (Rezvoy et al., 2007).</u>"*

References

Mansour, Y., Chateau, A. & Fiston-Lavier, A.-S. BREC: an R package/Shiny app for automatically identifying heterochromatin boundaries and estimating local recombination rates along chromosomes. BMC Bioinformatics 22, 396 (2021).

**Lines 724 – 737: Related to the previous comment, when looking at the plotted Marey maps (Figure S1), are the methods/results affected in any way by the "jitter" of Mb vs cM distances? I imagine that if the markers were not in the correct order in the linkage map (if the local linkage order is ABC, but the real genomic order is ACB), then the cM length of the chromosome may be overestimated, meaning that recombination rates would be consistently inflated. For example, in Camellia sinensis, the maps seems to be messier and therefore may accumulate local overestimations in recombination rate that will lead to a longer cM map than the true one, compared to Arabidopsis where the orders appear to be highly conserved between the genome and the linkage map. The potential impact of this should be discussed.

This issue was addressed by automatically adjusting the smoothing parameter of the loess regression to the data, and subsequently by evaluating the performance of interpolation using a bootstrap procedure (random resampling of markers). Due to higher levels of noise in some Marey maps, we certainly lose resolution and miss local variations for those maps, but we are confident that our method did not lead to longer genetic maps (cM).

To check for a bias in our estimates (e.g. inflating the chromosome recombination rate), we assessed the differences between the genome wide recombination rate (obtained by dividing the genetic map length by the genome length) and the averaged estimate per chromosome (the mean of recombination rates in windows of 100 kb). Both values are extremely correlated (Spearman's Rho = 0.99, p < 0.001) and the figure below shows that our mean estimates are not biased.

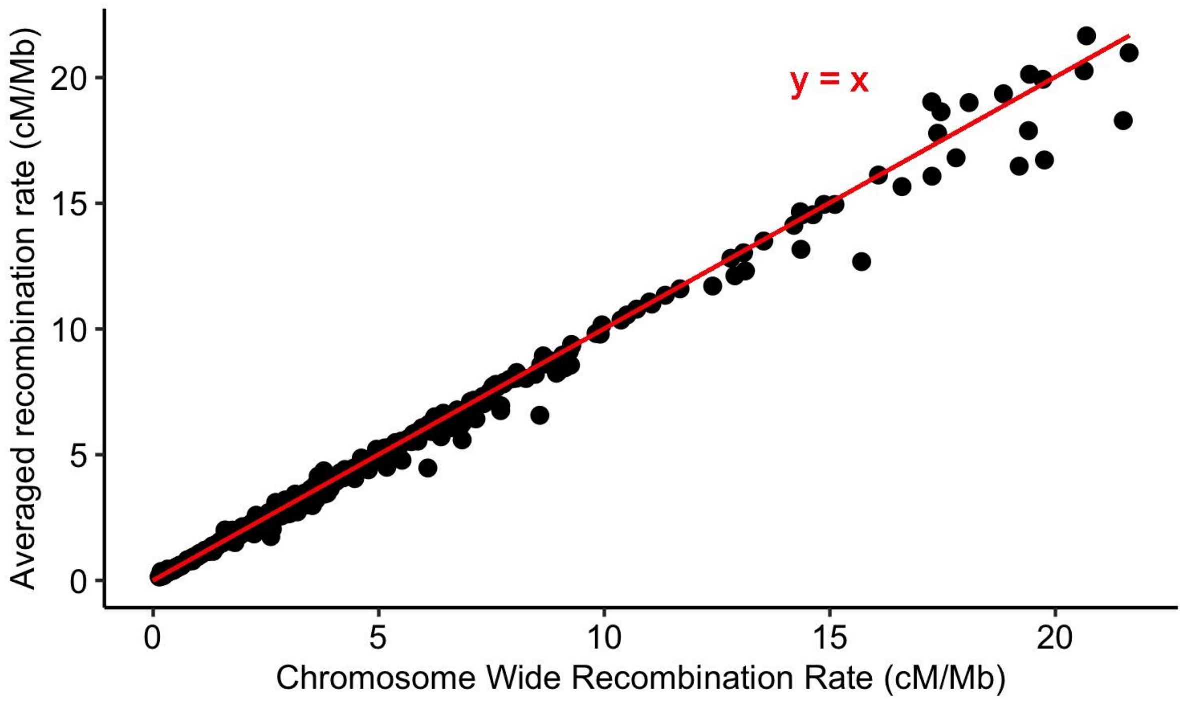

However, we cannot exclude that locally, along the chromosome, some values may reach high values that are not biologically reasonable, false peaks of recombination (i.e. data overfitting). On the other hand, the messier maps, due to stronger smoothing, should lead to a lower resolution and be more limited in the detection of local variation. That is why we implemented a K-fold cross validation procedure to automatically adjust the smoothing to avoid overfitting & underfitting issues. In addition, a bootstrap procedure (1,000 iterations), with random resampling of markers (with replacement), was used to evaluate the sensitivity of our estimates to outliers and noisy data. Recombination landscapes with large confidence intervals, indicating low data quality and/or poor performance of interpolation (precision and reliability), were discarded.

**Lines 738 – 749: Please clarify here how the relative recombination rate is calculated – is this done for each segment? i.e. if the chromosome is 100cM and 80Mb, and the first segment is e.g. 10cM & 20Mb, then how would the value be calculated? The verbal argument is unclear.

The representation of the broken stick model was not intuitive and we modified figure 4. The relative recombination rate is now estimated in 10 bins of constant genomic size and the relative recombination rate is the ratio expected genetic size divided by observed genetic size. In the legend: "*Relative recombination rates along the chromosome were estimated in ten bins of equal genomic size as the observed genetic length divided by the expected*

genetic length (one tenth of total genetic size) of the bin (log-transformed). Values below (above) zero are recombination rates that are lower (higher) than expected under a random distribution*". The verbal argument was made clearer, and the equation was added. ***"The relative recombination rate <u>in the segment i</u> was estimated by the log-ratio of the <u>observed</u> genetic size divided by the <u>expected</u> <u>genetic</u> size <u>(i.e. fixed to total genetic size / k by the model), as in the following equation.</u>

relative recombination rate = $\log_{10}$ (genetic$_i$ / (genetic$_{total}$ / k))"

In the case suggested by the reviewer, it means that the chromosome is cut into 10 bins of genomic size 8 Mb (80/10). If the first bin (8 Mb) is 20 cM long, the relative recombination rate is:

$$\log_{10}(20 / (100/10)) = \log_{10}(2)$$

**Lines 831 – 850: The way this is written, it isn't connected to any specific models. It is important to describe what was modelled here and to be explicit about the model structures to ensure reproducibility.

We specified more explicitly models in the result section (model formulas are also given in Supplementary Table S5 of model selection). We compared models:
LM response variable ~ explanatory variable
LMER response variable ~ explanatory variable + (1|Species)
LMER response variable ~ explanatory variable + (response variable|Species)
PGLMM response variable ~ explanatory variable + (1|Species__); where Species__ indicates that phylogenetic structure is modelled

After model selection, we chose for all regressions at a chromosome level (samples are chromosomes) the model
LMER response variable ~ explanatory variable + (1|Species)

Since we didn't detect a phylogenetic effect, we chose the model
LM response variable ~ explanatory variable for all regressions at a species level (one point per species, chromosomes pooled).

Manuscript

Click here to access/download;Manuscript;renamed_b7fd9.docx

Diversity and determinants of recombination landscapes in flowering plants

Short title: Recombination landscapes in angiosperms

Authors: Thomas Brazier[1], Sylvain Glémin[1, 2*]

[1] University of Rennes, CNRS, ECOBIO (Ecosystems, Biodiversity, Evolution) - UMR 6553, Rennes, France

[2] Department of Ecology and Genetics, Evolutionary Biology Center and Science for Life Laboratory, Uppsala University, Uppsala, Sweden

* Corresponding author.

Present address: Sylvain Glémin, University of Rennes, CNRS, ECOBIO (Ecosystems, Biodiversity, Evolution) - UMR 6553, Rennes, France; email: sylvain.glemin@univ-rennes1.fr

ORCIDs:

Brazier: https://orcid.org/0000-0001-5990-7545

Glémin: https://orcid.org/0000-0001-7260-4573

Abstract

During meiosis, crossover rates are not randomly distributed along the chromosome and their location may have a strong impact on the functioning and evolution of the genome. To date, the broad diversity of recombination landscapes among plants has rarely been investigated and a formal comparative genomic approach is still needed to characterized and assess the determinants of recombination landscapes among species and chromosomes. We gathered genetic maps and genomes for 57 flowering plant species, corresponding to 665 chromosomes, for which we estimated large-scale recombination landscapes. We found that the number of crossing-over per chromosome spans a limited range (between one to five/six) whatever the genome size, and that there is no single relationship across species between genetic map length and chromosome size. Instead, we found a general relationship between the relative size of chromosomes and recombination rate, while the absolute length constrains the basal recombination rate for each species. At the chromosome level, we identified two main patterns (with a few exceptions) and we proposed a conceptual model explaining the broad-scale distribution of crossovers where both telomeres and centromeres play a role. These patterns globally correspond to the underlying gene distribution, which affects how efficiently genes are shuffled at meiosis. These results raised new questions not only on the evolution of recombination rates but also on their distribution along chromosomes.

KEYWORDS: meiotic recombination, crossover pattern, Marey map, genetic shuffling, comparative genomics

Author summary

Meiotic recombination is a universal feature of sexually reproducing species. During meiosis, cross~~ing~~-overs play a fundamental role for the proper segregation of chromosomes during meiosis and ~~for~~ reshuffl~~ing~~es alleles among ~~between~~ chromosomes~~, which increases genetic diversity and the adaptive potential of a species~~. How much variation in recombination is expected within a genome and among different species remains a central question for understanding ~~to understand~~ the evolution of recombination. We characterized and compared recombination landscapes in a large set of plant species with ~~that represent~~ a wide range of genomic characteristics. We found that the number of cross~~ing-~~overs varied little among species, from one mandatory to no more than five or six crossing-overs per chromosomes, whatever the genome size. However, ~~recombination can strongly vary within a genome and~~ we identified two main patterns of variation along chromosomes (with a few exceptions) that can be explained by a new conceptual model where chromosome length, chromosome structure and gene density play a role. The strong association between gene density and was already well known, but recombination raised new questions not only about ~~on~~ the evolution of recombination rates but also on their distribution along chromosomes.

Introduction

Meiotic recombination is a universal feature of sexually reproducing species. ~~Through~~ crossovers~~, new haplotypes are passed on to offspring by the~~ reciprocal[ly] exchange ~~of~~ DNA between maternal and paternal chromosomes. However, recombination ~~landscapes — the variation in recombination~~ rate[s] ~~along the chromosome —~~ are not homogeneous across the genome and vary among species (de Massy, 2013; Haenel et al., 2018; Mézard et al., 2015; Stapley et al., 2017). Meiotic recombination ~~plays a fundamental functional role by forming~~ involves chiasmata at specific pairing sites between homologous chromosomes to ensure the physical tension needed for the proper disjunction of homologs (de Massy, 2013; Mézard et al., 2015; Zickler and Kleckner, 2015). Recombination also plays an evolutionary role by breaking linkage disequilibrium between neighbouring sites and creating new genetic combinations transmitted to the next generation ~~(i.e. genetic shuffling)~~, making selection on individual genetic variants more efficient (Barton, 1995; Charlesworth and Jensen, 2021; Otto, 2009). The number and location of crossovers (COs) along the chromosome are finely regulated through mechanisms of crossover assurance, interference and homeostasis (Otto and Payseur, 2019; Pazhayam et al., 2021). In most species, crossover assurance is necessary to achieve proper segregation and to avoid deleterious consequences of nondisjunction, though it is not very clear whether ~~if it is~~ at least one CO per chromosome or per arm is required, ~~that is mandatory~~. Additional COs are also usually regulated through interference, ensuring that they are not too numerous and not too close to each other (Pazhayam et al., 2021; Wang et al., 2015). In addition to regulation on a large scale (Cooper et al., 2016; Zelkowski et al., 2019), recombination is also finely tuned on a small scale. Generally, crossovers are concentrated in very short genomic regions (typically a few kb), i.e. recombination hotspots. In plants studied so far, CO hotspots have been found in gene regulatory sequences, and mostly in promoters (Choi et al., 2018; He et al., 2017; Marand et al., 2019).

In addition to meiosis ~~functioning~~ their functions in, variations in recombination rates ~~have a strong impact on~~ affect genome structure, functioning and evolution (Gaut et al., 2007; Haenel et al., 2018; Stapley et al., 2017; Tiley and Burleigh, 2015) and it has become a ~~key~~ challenge to integrate recombination rate variation in population genomics in the age of 'genomic landscapes' (Booker et al., 2020; Comeron, 2017). The characterization of recombination landscapes also has practical interests as variation in meiotic genes could be used to experimentally manipulate CO patterns for ~~advantageous~~ purposes~~,~~ such as redirecting recombination towards regions of interest for crop breeding (Kuo et al., 2021).

In plants, recombination rates are ~~supposed~~ believed to be species with higher in smaller genomes because the linkage map length is independent of genome size and the number of chromosomes explain more variation than genome size (Stapley et al., 2017). Several broad-scale determinants have recently been identified, such as chromosome length (Tiley and Burleigh, 2015), distance to the telomere or centromere (Blitzblau et al., 2007) and genomic and epigenetic features (Apuli et al., 2020; Marand et al., 2019; Yelina et al., 2012). Plant genomes also contain large regions with suppressed recombination in various proportions (from a few Mb to hundreds of Mb, 1 to 75 % of the genome). However, ~~despite these recent advances,~~ the diversity of recombination landscapes in plants still remain to be properly quantified.

Recently, a meta-analysis explored large-scale recombination landscapes among eukaryotes and paved the way for identifying general patterns (Haenel et al., 2018). They found that larger chromosomes have low crossover rates in their centre and suggested a simple telomere-led model with a universal bias of COs towards the periphery of the chromosome, positively driven by chromosome length. They also proposed that chromosome length played the main role in crossover patterning while position of the centromere had almost no effect (except ~~a~~ local~~one~~ ly). Alternatively, it has also been proposed that both telomeres and centromeres shape recombination landscapes (Wang and Copenhaver, 2018) and ~~the universality of~~ a universal pattern among plants has been questioned (Zelkowski et al., 2019). ~~So far, the~~ As only a limited number of ~~studied~~ species ~~remained limited~~ have been studied

and, as plant genomes are highly diverse in many ways (Pellicer et al., 2018; Soltis et al., 2015), the expected diversity in recombination landscapes may have been overlooked (Gaut et al., 2007). In addition, previous studies were meta-analyses combining heterogeneous datasets (ex: mix of inferred data from graphics, final processed data and only a few raw datasets in Haenel et al. 2018) without a standard way to infer recombination maps, which prevented detailed comparison among many species.

To overcome these limitations we gathered, to our knowledge, the largest recombination landscape dataset in flowering plants. We started from raw data by combining genetic mapping from pedigree data and genome assembly up to the chromosome scale, from which we estimated recombination maps – more precisely the sex-averaged rate of COs along chromosomes – using the same standardised method. Thanks to this dataset we addressed the following questions. What is the range of COs per chromosome observed in plants? Is the distribution of COs shaped by genome structure (i.e. chromosome size, telomeres, centromeres) and if so is there a universal pattern? Since recombination hotspots have been found in gene regulatory sequences so far, are recombination landscapes generally associated with gene density? What are the consequences of recombination heterogeneity on the extent of genetic shuffling? Overall, we found that recombination landscapes in plants are more diverse and more complex than initially thought. We identified two main patterns that are parallel to, and which may emerge from, the gene density distribution. We showed that this globally improves the genetic shuffling of coding regions, which raises new questions about the evolution of recombination.

Results

Dataset and recombination maps

We retrieved publicly available data for sex-averaged linkage maps and genome assemblies to obtain genetic and physical distances. We selected linkage maps for which the markers had genomic positions on a chromosome-level genome assembly (except for *Capsella rubella*, which had a high-quality scaffold-level assembly of pseudo-chromosomes). We remapped markers on the reference genome for 14 species for which genomic positions were not known or were mapped to an older assembly. After making a selection based on the number of markers, marker density, and genome coverage, and after filtering out the outlying markers (see methods), we produced 665 chromosome-scale Marey maps (plot of the genetic vs genomic distance, cM vs Mb) for 57 species (2-26 chromosomes per species, Table S1, S2, Fig S1, S2). The number of markers per chromosome ranged from 31 to 49,483, with a mean of 956 markers. Corrected linkage map length (Hall & Willis's method) did not change the total linkage map length (mean difference = 1.19 cM, max difference = 5.62 cM), giving confidence in the coverage of the linkage map (Hall & Willis, 2005). We verified that neither the number of markers, marker density nor the number of progenies had a significant effect on the analyses. We also retrieved gene annotations for 41 genomes. The angiosperm phylogeny was well represented in our sampling (Fig S3), with a basal angiosperm species (*Nelumbo nucifera*), 15 monocot species and 41 eudicots. From literature, we also obtained data on the centromeric index for 37 species, defined as the ratio of the short arm length divided by the total chromosome length (Table S3).

From the Marey maps, we estimated local recombination rates along the chromosomes on non-overlapping 100 kb windows with a 95% confidence interval (1,000 bootstraps). Estimates at a scale of 1 Mb yielded very similar results (the Spearman rank correlation coefficient correlation between the two estimates was Rho = 0.99, $p < 0.001$, Table S4) therefore only 100 kb landscapes were analysed in the subsequent analyses.

Smaller chromosomes have higher recombination rates than larger ones

In agreement with previous studies (Haenel et al., 2018; Stapley et al., 2017), we found a significant negative correlation between chromosome size (Mb) and the mean chromosomal recombination rate (Spearman rank correlation coefficient Rho = -0.84, $p < 0.001$; log-log Linear Model, adjusted $R^2 = 0.83$, $p < 0.001$). ~~For~~ most species~~, there were~~ had between one and four COs per chromosome ~~even~~ al though the genome sizes span almost two orders of magnitude. ~~Less~~ Fewer than 2% of chromosomes had ~~less than one~~ no CO (n = 11). 234 chromosomes had between one and two COs, suggesting that a single CO per chromosome is sufficient, though 419 chromosomes had more than two COs.

Using a Linear Mixed Model we found a significant species random effect ($\log_{10}$(recombination rate) ~ $\log_{10}$(chromosome size) + (1 | species), marginal $R^2 = 0.17$, conditional $R^2 = 0.96$, $p < 0.001$). Adding phylogenetic covariance did not improve the mixed model ~~thus~~ , so we did not retain a phylogenetic effect (Table S5). Interestingly, the (log-log) relationship between the recombination rate and ~~the~~ chromosome size was not the same within and between species, suggesting that absolute chromosome size does not have ~~a general~~ the same effect ~~among~~ in all species (Fig 1B). Similarly, the relationship between linkage map length (cM) and chromosome size (Mb) was highly species - specific (linkage map length ~ log10(chromosome size) + (1 | species), marginal $R^2 = 0.49$, conditional $R^2 = 0.99$, $p < 0.001$) (Fig 2A), with species slopes decreasing with the mean chromosome size ~~in a log-log relationship. It indicates~~ in a log-log relationship, indicating that species slopes are roughly proportional to the inverse of the mean chromosome size (Fig S4). ~~As a~~ consequen~~ce,~~tly the excess of COs on a chromosome (i.e. the linkage map length minus 50 cM) was ~~not~~ correlated with ~~the absolute chromosome~~ its relative size ~~but with the relative one (i.e. chromosome size divided by the mean chromosome size of the species; Fig 2B)~~ (i.e. chromosome size divided by the mean chromosome size of the species; Fig 2B), not the absolute size. Moreover, in contrast to the relationship between recombination rate and absolute size, we did not observe any difference between the linear model and the fixed regression of the mixed linear model, suggesting that this relationship is similar across

species (Fig 2B). ~~More concretely, it means that~~ In other words, any two chromosomes ~~having~~ with the same ratio of sizes will have the same ratio of excess of recombination rate, whatever the species and the genome size.

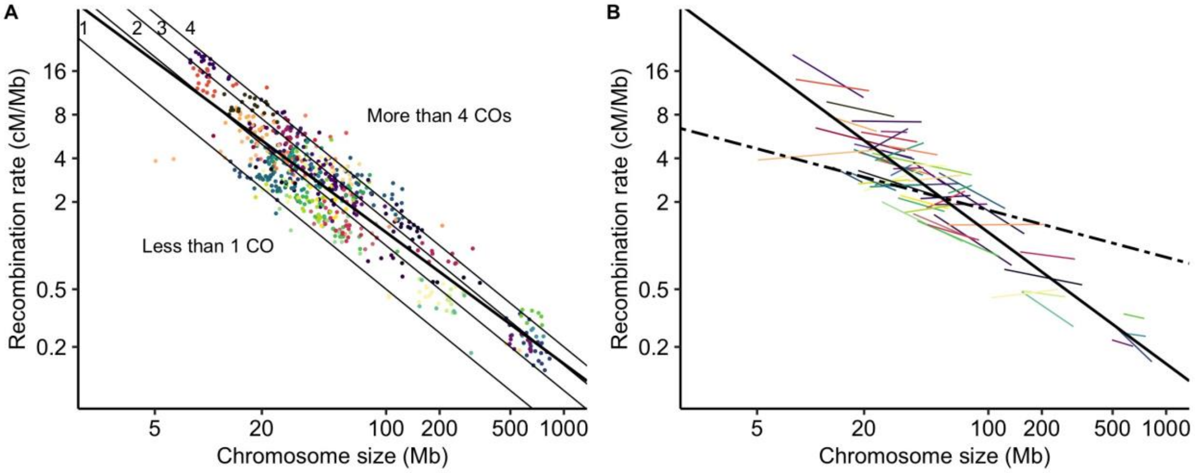

Fig 1. Mean recombination rates per chromosome (cM/Mb~~, log scale~~) are negatively correlated with chromosome physical ~~genomic~~ sizes (Mb, log scale). Each point represents a chromosome (n = 665). Species are presented in different colours (57 species). (A) The bold solid line represents the linear regression line fitted to the data. The thin ~~solid~~ lines correspond to the expectation of one, two, three or four COs per chromosome ~~respectively~~. (B) Correlations between recombination rates and chromosome size within each species with at least 5 chromosomes (coloured lines, 55 species) and the overall between-species correlation controlled for a species effect (black dashed line , n = 57 species). Solid bold line as in (A).

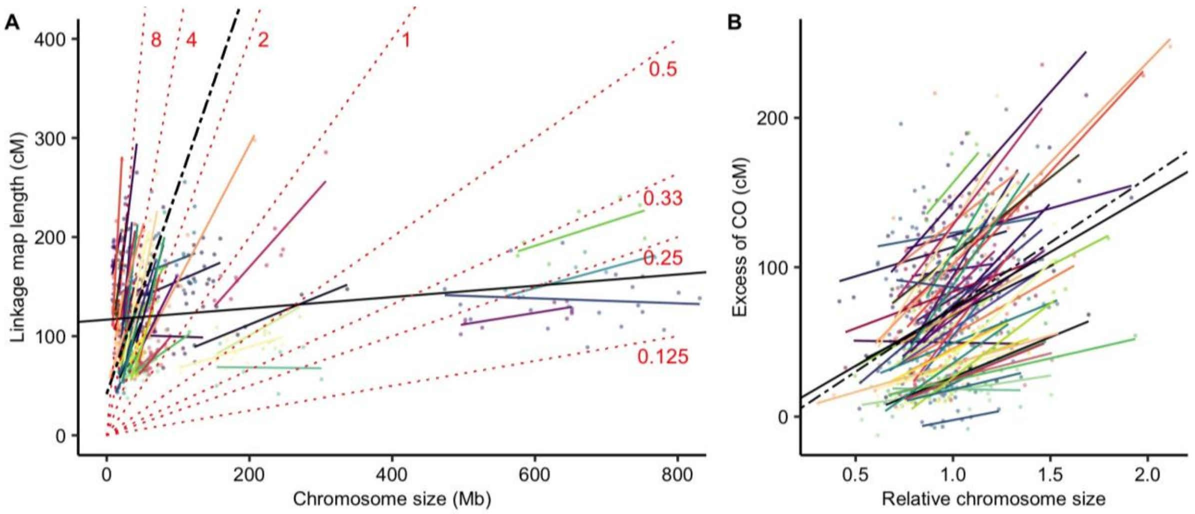

Fig 2. Linkage map length (cM) is positively correlated with genomic chromosome size (Mb). (A) Correlation between chromosome genomic size (Mb) and linkage map length (cM). Each point represents a chromosome (n = 665). Species are presented in different colours (57 species).

The black solid line represents the simple linear regression (linkage map length ~ $\log_{10}$(chromosome size), adjusted R^2 = 0.036, $p < 0.001$) and the black dashed line the fixed effect of the mixed model (linkage map length ~ $\log_{10}$(chromosome size) + (1 | species), marginal R^2 = 0.49, conditional R^2 = 0.99, $p < 0.001$). Species random slopes are shown in colours. Isolines of recombination rates are plotted for different values (indicated cM/Mb) as dotted red lines to represent regions with equal recombination. (B) The excess of COs (linkage map length minus 50 cM for the obligate CO) is positively correlated with the relative chromosome size (size / average size of the species). The black solid line is the linear regression across species (excess of CO ~ relative chromosome size, adjusted R^2 = 0.13, $p < 0.001$) and the black dashed line the fixed effect of the mixed model (excess of CO ~ relative chromosome size + (1 | species), marginal R^2 = 0.14, conditional R^2 = 0.86, $p < 0.001$). Coloured solid lines represent individual regression lines for species with at least 5 chromosomes (55 species).

Diversity of CO patterns among flowering plants

Recombination landscapes along chromosomes appeared to be qualitatively very similar within species but strongly varied between species (Fig 3, Fig S2). In the text below, we have used the terms proximal and distal regions, respectively, to avoid confusion with the molecular composition and specific position defining telomeric and centromeric regions *stricto sensu*. Some landscapes were homogeneous along chromosomes whereas others were extremely structured with recombination concentrated in the short distal parts of the genome, and wide variations between these two extremes (Fig 3). Representing relative recombination rates in ~~on~~ ten bins of equal physical ~~chromosome~~ length (see Materials and Methods for details), ~~we observed that the~~ bias towards the periphery was not ubiquitous across species (Fig 4), whereas ~~unlike~~ Haenel et al. (2018) ~~who~~ suggested that the distal bias could be universal for chromosomes larger than 30 Mb. Only a subset of species, especially those with very large chromosomes (> 100 Mb), exhibited a clear bias (Fig 4). Despite large chromosome sizes (mean chromosome sizes = 101 Mb and 198 Mb, respectively), *Nelumbo nucifera* and *Camellia sinensis* are noticeable exceptions to this pattern, with the highest recombination rates found in the middle of the chromosomes (*Nelumbo nucifera* illustrated in Fig 3E, other species in Fig S2). For small to medium-sized chromosomes, the pattern is less clear. Most species did not show any clear structure along the chromosome but a few of them (e.g. *Capsella rubella*, *Dioscorea alata, Mangifera indica, Manihot esculenta*) showed a drop in

recombination rates in the distal regions and high recombination rates in the proximal regions (*Capsella rubella* illustrated in Fig 3A).

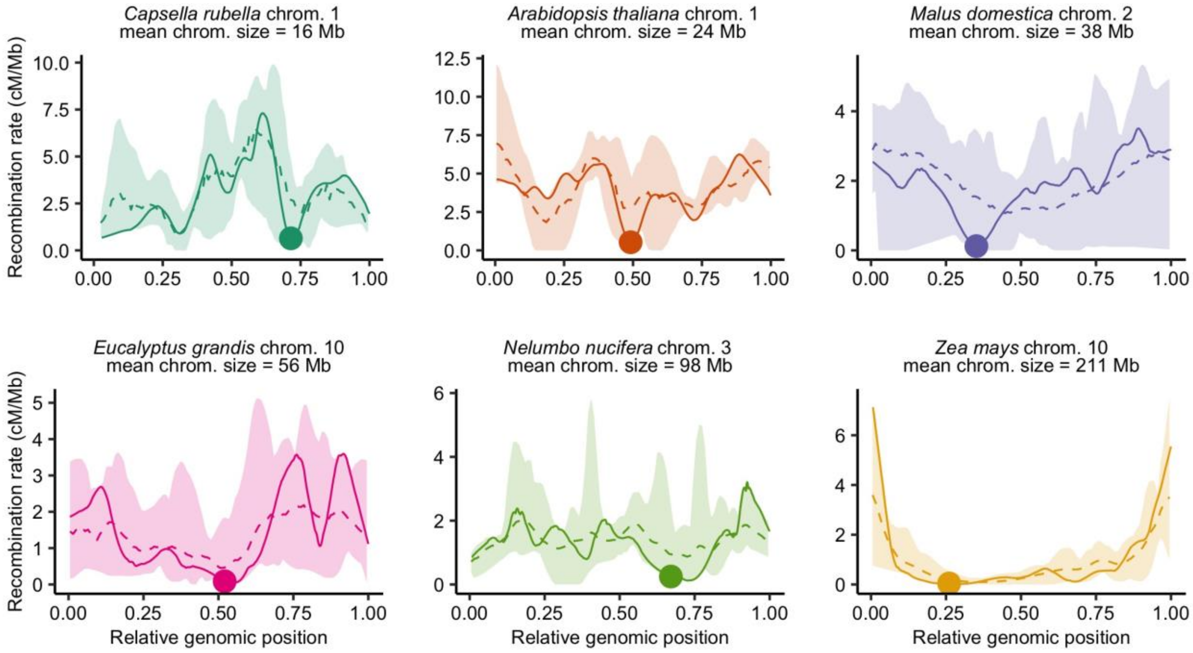

Fig 3. Diversity of recombination landscapes exemplified by six different species. Recombination landscapes are similar within species (the dashed line is the average landscape for pooled chromosomes, all recombination landscapes of the species are contained within the colour ribbon). Genomic distances (Mb) were scaled between 0 and 1 to compare chromosomes with different sizes. Estimates of the recombination rates were obtained by 1,000 bootstraps over loci in windows of 100 kb with loess regression and automatic span calibration. One chromosome per species is represented in a solid line, with the genomic position of the centromere demarcated by a dot. The six species are ordered by ascending mean chromosome size (Mb).

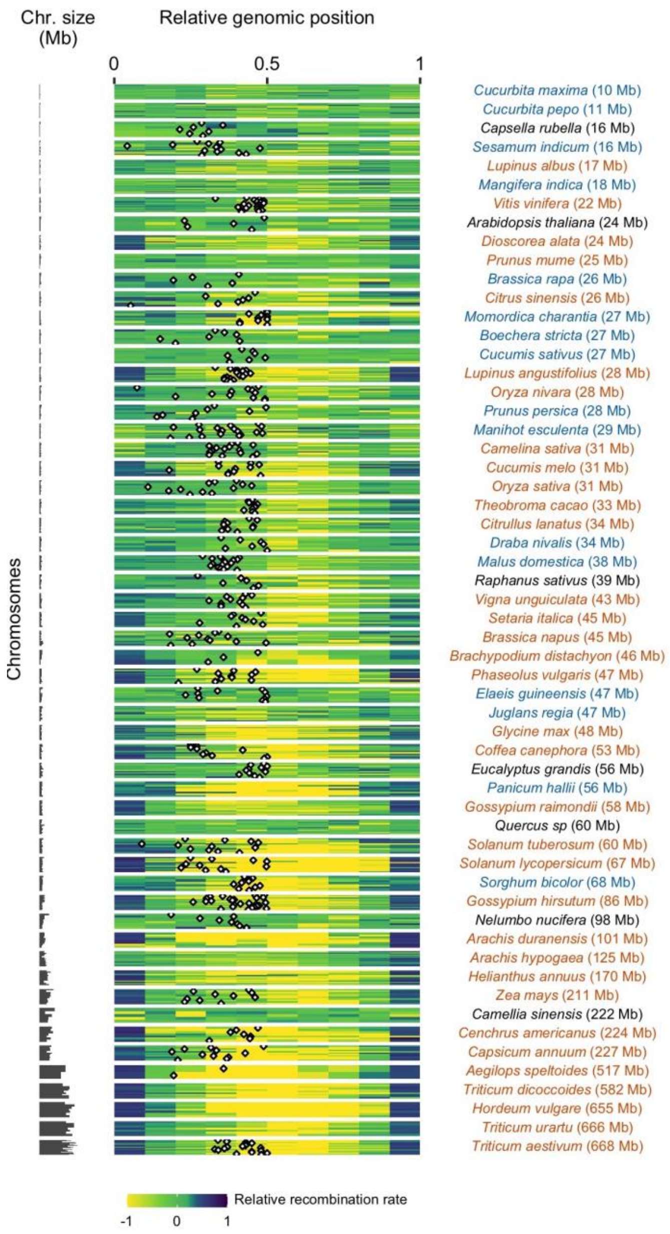

Fig 4. Patterns of recombination within chromosomes (n = 665). Relative recombination rates along the chromosome were estimated in ten bins of equal ratios of ~~genomic size as~~ the observed genetic length divided by the expected genetic length (one tenth of total size) of the bin (log-transformed). Values below (above) zero are recombination rates that are lower (higher) than expected under a random distribution. The 57 Species are ordered by ascending genome size ~~(57 species)~~. Each horizontal bar plot represents one chromosome. When available, the centromere position is mapped as a black and white diamond.

Following Haenel et al. (2018), we calculated the periphery-bias ratio as the recombination rate in ~~the tips of the chromosome (~~10% at each extremity~~)~~ of each chromosome divided by the mean recombination rate. A ratio higher than 1 indicates a higher recombination rate in the tips than the whole chromosome. By pooling chromosomes ~~per~~ within species, we detected a

significant positive effect of chromosome length on the periphery-bias ratio across species (Linear Model, adjusted R^2 = 0.44, p < 0.001; Fig 5A) with some exceptions (ex on Fig 3A and 3E). Across all species the mean periphery-bias ratio is significantly higher than 1 (95% bootstrapped confidence interval = [2.06;2.32]) and skewed towards values higher than 1 but the correlation with chromosome length within species was not clear (Fig 5B, 5C, Table S6).

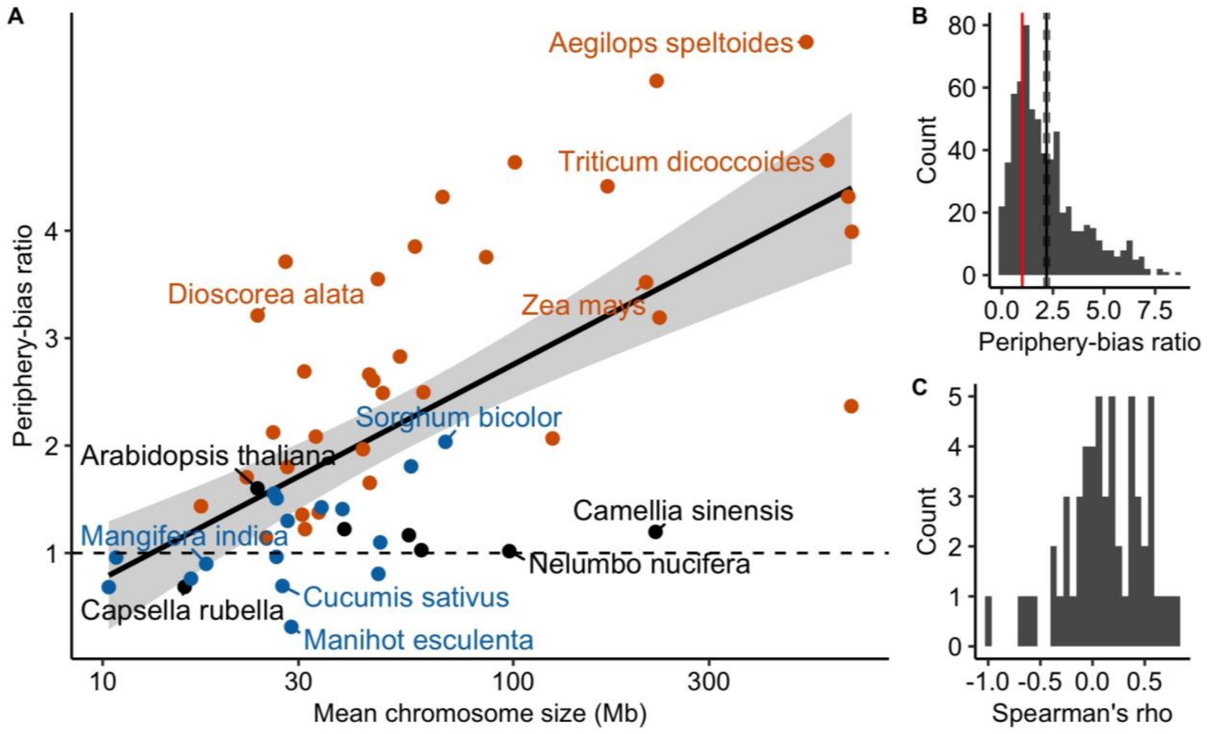

Fig 5. The periphery-bias ratio is positively correlated with chromosome genomic size. (A) Linear regression between the species mean periphery-bias ratio and the mean chromosome size (log scale) across species (n = 57 species; adjusted R^2 = 0.44, p < 0.001). Points are coloured according to the classification of the CO patterns described below (orange = distal, blue = sub-distal, black = unclassified). (B) Distribution of periphery-bias ratios (n = 665 chromosomes). The mean periphery-bias ratio and its 95% confidence interval (black solid and dashed lines) were estimated by 1,000 bootstrap replicates. The red vertical line corresponds to a ratio of one. (C) Distribution of Spearman's correlation coefficients between the periphery-bias ratio and chromosome genomic size (Mb) within species (n = 57 species).

Joint effect of telomeres and centromeres on crossover distribution along chromosomes

Globally, recombination rates were negatively correlated with the distance to the nearest telomere (Fig S5, Table S7, Table S8). However, two qualitative ~~different~~ patterns ~~qualitatively~~ emerged (Fig 6, Fig S6, Table S8). In 34 species, recombination decreased from the telomere and

reached a plateau ~~after a relative genomic distance of~~ at approximately 20% of the ~~whole~~ length (or do you mean arm length)? chromosome (the distal ~~model~~ pattern, Fig 6A), in agreement with the model suggested by Haenel et al. (2018). Sixteen species ~~presented~~ exhibited a sharp decrease in the most distal regions and a peak of recombination in the sub-distal regions (relative genomic distance between 0.1-0.2) followed by a slow decrease towards the centre of the chromosome (the sub-distal pattern, Fig 6B). There were a few exceptions to these two patterns (six species), e.g. *Capsella rubella* consistently showed higher recombination rates in the middle of the chromosome (Fig 3A). Interestingly, chromosomes from species classified as having a distal pattern were significantly larger than chromosomes with a sub-distal pattern (Wilcox rank sum test, $p < 0.001$, Fig 6C). Furthermore, the correlation between recombination and the distance to the nearest telomere was significantly higher for species with larger chromosomes (Spearman rank correlation coefficient Rho = -0.51, $p < 0.001$; Fig S5).

When the centromere position was known, we observed that the centromeres had an almost universal local suppressor effect (Fig 3, 4). In small and medium-sized chromosomes, the recombination was often suppressed only in short restricted centromeric regions (several Mb, 1-5 % of the map) displaying drastic drops in the recombination rates~~,~~ ~~whereas the rest of the map did not seem to be affected~~. In larger chromosomes, the suppression of recombination extends to large regions upstream and downstream of the physical centre of the chromosome (approximately 80-90% of the chromosome; Fig 4). Ninety percent of chromosomes (388 chromosomes) had significantly less recombination than the chromosome average at the centromeric index (n = 425, resampling test, 1,000 bootstraps, 95 % confidence interval). 81 chromosomes (19 %) were completely recombination-free in the centromere. However, the transposition of centromere position from cytological data to genomic data may be imprecise or wrongly oriented for some chromosomes. After orienting chromosomes to map the centromeric index, 16 % of chromosomes (70 over 425) had a recombination rate slightly higher in the inferred

centromere position than on the opposite side, thus a centromere potentially mapped on the wrong side.

To understand the patterns observed ~~go~~ further, we ~~formally~~ compared three models ~~that could explain the broad-scale crossover patterns we observed~~ (Fig 7). Under the strict distal model proposed by Haenel et al. (2018) (M1), the centromere ~~does not~~ plays no ~~any~~ role beyond its local suppressor effect, which predicts ~~and therefore we expect~~ an equal distribution of crossovers on both sides of the chromosome, independently of centromere position: $\frac{d(1/2)}{d(1)} = 0.5$, where $d(1/2)$ is the genetic distance (cM) to the physical? middle of the chromosome and $d(1)$ is the total genetic distance (cM). ~~In addition,~~ also we tested two alternative models with ~~adding~~ a centromere effect. We assumed that the position of the centromere, $d(c)$, affects ~~has an effect on~~ the distribution of crossovers along the chromosome. Models M2 'telomere + centromere + one CO per arm' and M3 'telomere + centromere + one CO per chromosome'~~;~~ both assume that the relative genetic distance of a chromosome arm is proportional to its relative genomic size. However, they differ in the number and distribution of mandatory COs. In M2, At least one CO in each chromosome arm (50 cM) is mandatory , ~~in M2~~ whereas only one CO is mandatory for the entire chromosome in M3. For species whose centromere position was known (37 species, 425 chromosomes) we regressed the observed values against the theoretical predictions of the three models and compared them using goodness-of-fit criteria (adjusted R^2, AIC, BIC). M1 was not supported by any species, and Model M2 was generally rejected since 22% of chromosomes had genetic maps smaller ~~showed less~~ than 50 cM in at least one arm, ~~even~~ though it was supported in a handful of species (Table 1)~~, and model M1 was not supported by any species~~. ~~Model~~ M3 was the best supported model (30 out of 37 species), with good predictive power (Spearman rank correlation between predicted and observed values: Rho = 0.72, p < 0.001; Tables 1, S9, S10). Given that ~~some chromosomes had one~~ chromosome genetic maps arm shorter than 50 cM, are ~~which is~~ incompatible with ~~one mandatory CO per arm in model~~ M2, we also compared the three models on a subset of chromosomes with at least 50 cM on each chromosome arm (n = 36 species, 333 chromosomes) which confirmed that model M3 was the best model. Similarly, we reran the model ~~without~~

chromosomes ~~with uncertainty on the~~ centromere position (n = 37 species, 355 chromosomes) and found the same results.

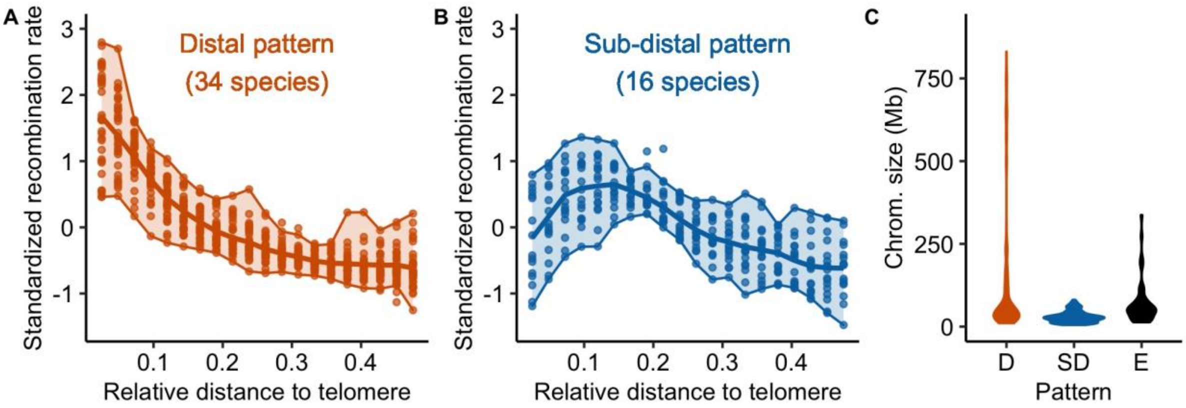

Fig 6. Distribution of crossover: main patterns. (A and B) Standardized recombination rates for species (chromosomes pooled per species, n = 57 species) are expressed as a function of the relative genomic distance from the telomere in 20 bins representing the two main patterns (orange = distal, blue = sub-distal). The seven unclassified species are not shown. Chromosomes were split in half and 0.5 corresponds to the centre of the chromosome. In each plot, the solid line represents the mean recombination rate estimated in a bin (20 bins) and each dot per bin represents the average of a species. Upper and lower boundaries of the ribbon represent the maximum and minimum values. (C) Distribution of chromosome genomic sizes (Mb) for each pattern.

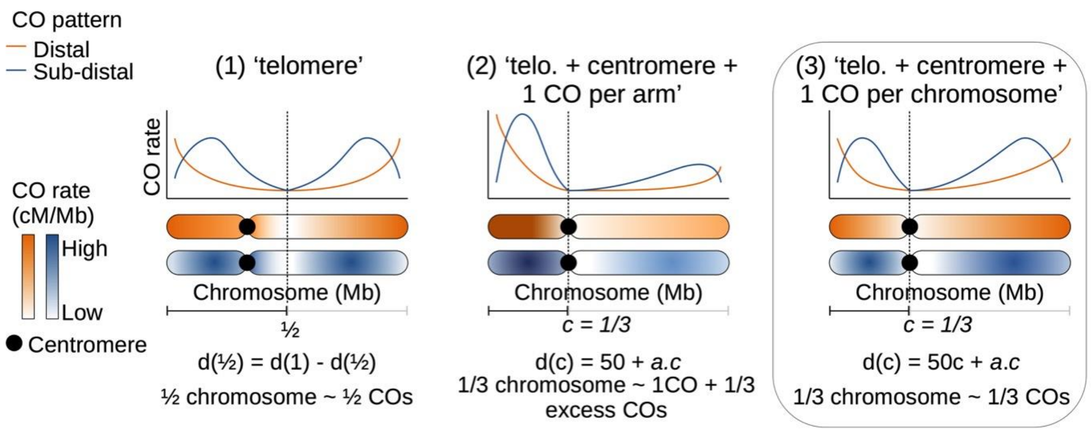

Fig 7. Possible models of crossover patterns. Schematic representation of the three competing models for the two main patterns, with an example of a centromere position at 1/3 of the chromosome. Model 3 is the best model (box).

Table 1. Model selection for the telomere/centromere effect (n = 37 species with a centromere position, 425 chromosomes). Three competing models were compared based on the adjusted R^2, p-value and AIC-BIC criteria among chromosomes (the best supported model is in bold characters). The number of species supporting each model was calculated based on the adjusted R^2 within species, for all species with at least five chromosomes. (1) ‘telomere’ model. (2) ‘telomere + centromere + one CO per arm’ model. (3) ‘telomere + centromere + one CO per chromosome’ model. $d(c)$ is the genetic distance to the centromere. $d(1)$ is the total genetic distance. A second model selection was done on a subset of chromosomes with at least 50 cM on each chromosome arm (n = 36 species, 333 chromosomes).

#	Model	Expected	Adjusted R^2	p	AIC	BIC	Species
	Full dataset (37 species, 425 chromosomes)						
1	Telomere	d(1/2) / d(1) = 0.5	0.22	< 0.001	-477.8	-465.7	0
2	Tel. + Cent. + CO per arm	(d(c) – 50) / (d(1) – 100) = c	-	0.72	3098.2	3110.4	7
3	Tel. + Cent. + CO per chr.	d(c) / d(1) = c	0.51	< 0.001	-476.6	-464.5	30
	Subset (36 species, 333 chromosomes)						
1	Telomere	d(1/2) / d(1) = 0.5	0.18	< 0.001	-407.5	-396.1	0
2	Tel. + Cent. + CO per arm	(d(c) – 50) / (d(1) – 100) = c	-0.001	0.42	1939.1	1950.5	10
3	Tel. + Cent. + CO per chr.	d(c) / d(1) = c	0.50	< 0.001	-396	-384.6	26

Recombination rates are positively correlated with gene density

~~At a fine scale,~~ it has been shown in a few species that COs preferentially occur in gene (see the Introduction) promoters. The scale of 100 kb used here is too large to ~~directly~~ test whether this ~~is a common~~ is pattern shared among angiosperms. Instead, ~~like~~ as in Haenel et al. (2018), we assessed whether recombination increased with gene density. Forty-one genomes were annotated with gene positions. Across chromosomes, the distribution of chromosomal correlations between gene count and recombination rate was clearly skewed towards positive values, independently of the previously described CO patterns (mean Spearman’s rank correlation = 0.46 [0.43; 0.49]; Fig 8A). Ninety-one percent of 483 chromosomes (41 species) showed a significant correlation between the number of genes and recombination rate at a 100 kb scale. ~~Yet~~ the strength of the relationship greatly varied across species and did not correlate with usual predictors such as the chromosome length or the genome-wide recombination rate (Fig 8B). Overall, standardized recombination rates (subtracting the mean and dividing by the standard deviation to allow comparison among species)

consistently increased with the number of genes in most species (linear quadratic regression, adjusted R^2 = 0.62, $p < 0.001$; Fig 8C).

As for recombination patterns, we classified patterns of gene density along chromosomes in three categories: distal, sub-distal and exceptions (Fig S7). Most species (30 out of 41) were classified in the same gene density and recombination pattern (Table S11). Moreover, we observed the same qualitative pattern for gene density and recombination for species with either major recombination pattern ~~when we classified species as a function of recombination patterns, we qualitatively observed the same pattern for gene density and recombination~~ (Fig 9)~~, suggesting that recombination and gene density share the same non-random distribution along the genome~~.

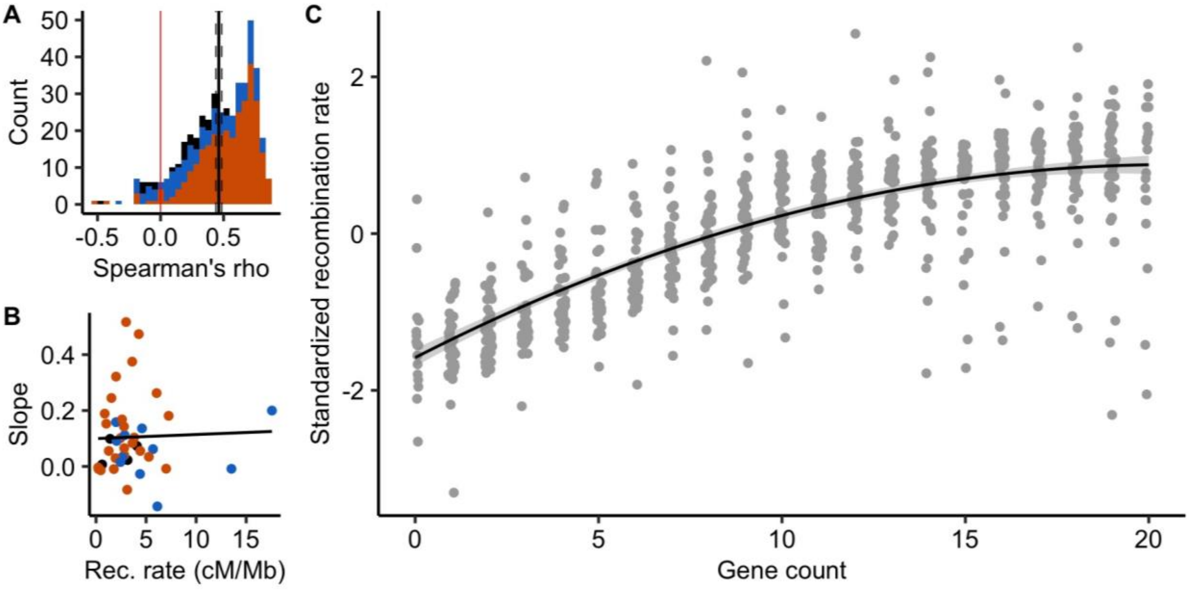

Fig 8. Recombination rates are positively correlated with gene density (n = 483 chromosomes, 41 species). (A) Distribution of chromosome Spearman's rank correlations between the number of genes and the recombination rate in 100 kb windows. The black vertical line is the mean correlation with a 95% confidence interval (dashed lines) estimated by 1,000 bootstrap replicates. Colours correspond to CO patterns (orange = distal, blue = sub-distal, black = exception). (B) Slopes of the species linear regression between gene count and recombination rates are independent of the species averaged recombination rate (Linear Model, adjusted R^2 = -0.02, p = 0.83). (C) Standardized recombination rates for each number of genes in a 100 kb window (centred-reduced, chromosomes pooled per species, one colour per species) estimated by 1,000 bootstraps and standardized within species. The gene count was estimated by counting the number of gene starting positions within each 100 kb window. The black line with a grey ribbon is the quadratic regression estimated by linear regression with a 95% parametric confidence interval (Linear Model, adjusted R^2 = 0.62, $p < 0.001$).

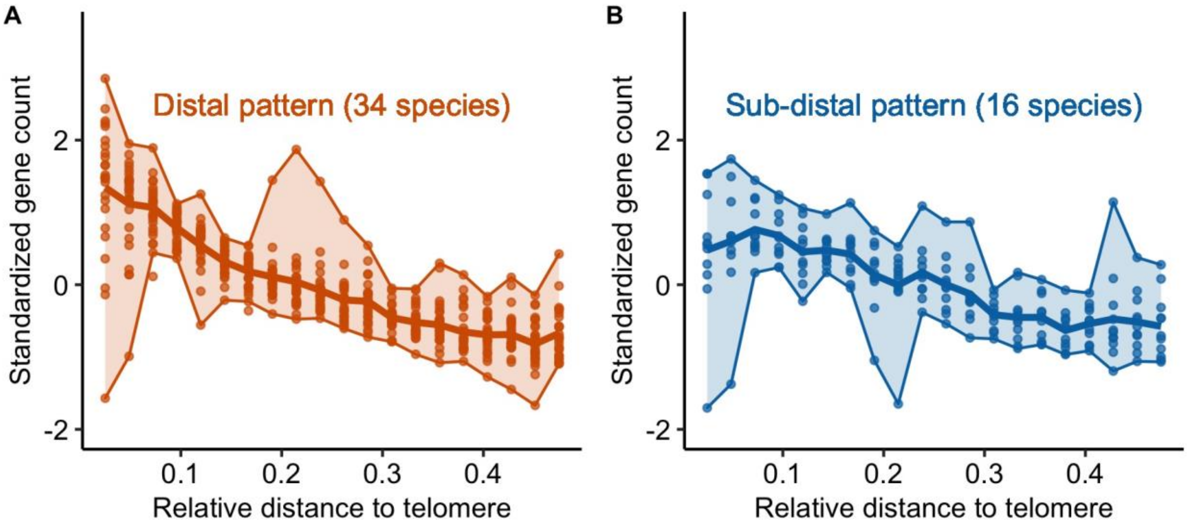

Fig 9. Gene counts patterns along the chromosome are correlated with CO patterns (n = 41 species). Standardized gene count (centred-reduced) as a function of the relative distance from the tip to the middle of the chromosome (genomic distances distributed in 20 bins). We used the same groups as identified for the CO pattern in Fig 6; (a) distal pattern vs (b) sub-distal pattern. Same legend as Fig 6.

Genetic shuffling

We confirmed that crossovers are ~~recombination is~~ unevenly distributed in genomes, which must ~~should~~ affect how genetic variation is ~~shuffled during meiosis. Genetic shuffling participates to the random~~ recombined ~~reassortment of genes~~ between parental homologous chromosomes. To quantify recombination within and between chromosomes ~~how much genetic shuffling depends on the distribution of COs~~, we estimated its intrachromosomal component, $\bar{r}_{intra}$, as described in equation 10 in Veller et al. (2019). The $\bar{r}_{intra}$ gives, for a chromosome, a measure of the probability for a random pair of loci to be recombined ~~shuffled~~ by a crossover. As expected, this ~~genetic shuffling~~ was positively ~~and significantly~~ correlated with linkage map length ($\bar{r}_{intra}$ ~ linkage map length + (1 | species), marginal $R^2 = 0.43$, conditional $R^2 = 0.88$, $p < 0.001$, Fig S8). A pattern in which COs are physically clustered in distal regions of the chromosome is thought ~~are supposed~~ to generate less recombination ~~genetic shuffling~~ than one with COs evenly distributed across ~~in~~ the chromosome. References needed recombination At a chromosomal level, the periphery-bias ratio consistently (across species) has ~~as~~ a low but significant effect on recombination ~~genetic shuffling, consistent among species~~ ($\bar{r}_{intra}$ ~ periphery-bias ratio + (1 | species), marginal $R^2 = 0.05$, conditional $R^2 = 0.68$, $p < 0.001$, Fig S9). The more COs are clustered in the tips of

within-chromosome recombination [is this your meaning?]

the chromosome, the lower the chromosomal genetic shuffling. These results verify the analytical predictions of Veller et al. (2019), although the strength of the effect remains weak.

However, the distributions of COs and genes are both non-random and often correlated (Fig 8 and S10). Genomic distances measured in base pairs may not be the most appropriate measure of genetic shuffling among functional genomic components. Thus, we also measured genomic distances in gene distances (i.e. the cumulative number of genes along the chromosome) instead of base pairs. Marey maps most often appeared more homogeneous when scaled on gene distances instead of base pair distances, with 70% (316 over 450) of Marey maps showing a smaller departure from a random distribution (Fig 10, S11, Table S11). Globally, a subset of 30 species has more homogeneous Marey maps with gene distances whereas 11 others are quantitatively more heterogeneous (notably *Capsella rubella* and *Arabidopsis thaliana*), although this could be due to low quality annotations making it difficult to precisely estimate the gene distances for some of them (e.g. *Sesamum indicum*). In most cases, genetic shuffling recombination rates? were slightly higher when gene distances were used instead of base pairs (Fig 11; mean = 0.22 for base pairs; mean = 0.26 for gene distances; Wilcoxon rank sum test with continuity correction, $p < 0.001$), implying that the genetic shuffling was more recombination efficient among coding regions than among regions randomly sampled in the genome. Interestingly, the increase in genetic shuffling calculated in gene distances compared to genomic distance was more pronounced for longer chromosomes — which are often the most heterogeneous ones, characterized by a distal pattern — whereas we saw little effect on smaller chromosomes characterized by a sub-distal pattern (difference in $\bar{r}_{intra}$ ~ log10(chromosome size) + (1 | species), marginal $R^2 = 0.21$, conditional $R^2 = 0.87$, $p < 0.001$, Fig 11).

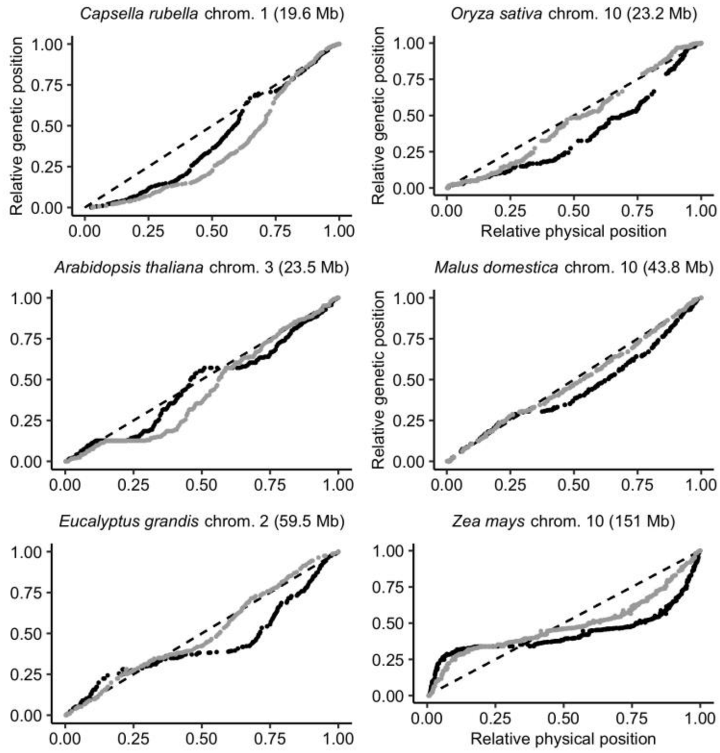

Fig 10. Marey maps of six chromosomes with the relative physical distance expressed in genomic distances (black dots, position in the genome in Mb) or in gene distances (grey dots, position measured as the cumulative number of genes along the chromosome. Marey maps are ordered by ascending chromosome size (Mb). The diagonal dashed line represents a theoretical random distribution of COs along the chromosome.

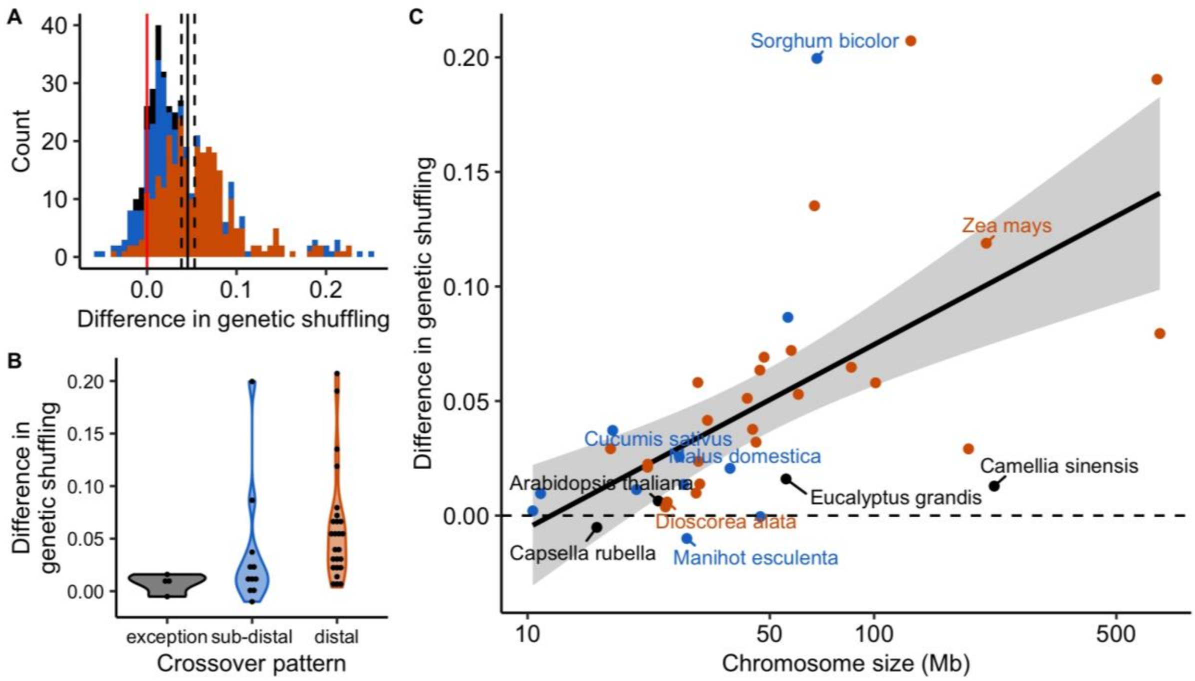

Fig 11. Differences in genetic shuffling between estimates based on genomic distances (Mb) and gene distances (cumulative number of genes). The difference is the genetic shuffling in gene

distances minus the genetic shuffling in genomic distances. Colours correspond to CO patterns (orange = distal, blue = sub-distal, black = exception). (A) Distribution of the chromosome differences in the genetic shuffling (n = 444 chromosomes). (B) Distributions of the species difference in the genetic shuffling (n = 41 species, chromosomes pooled). (C) Species differences in the genetic shuffling are positively correlated with the averaged chromosome size (Linear Model, adjusted $R^2 = 0.20$, $p = 0.002$, n = 41, 95% parametric confidence interval).

Discussion

This has already been stated, and it is unnecessary to repeat it

~~Based on a large and curated dataset, we provided, to our knowledge, the largest description of recombination landscapes among flowering plants.~~ In addition to confirming that both the chromosome-wide recombination rate and the heterogeneity of recombination landscapes vary according to chromosome length, ~~we~~ our new analyses identified two distinct CO patterns and ~~we~~ propose~~d~~ a new model that extended the strict telomere model recently proposed by Haenel et al. (2018). Moreover, the consistent correlation between recombination and gene density may have implications for the evolution of recombination landscapes and whether the distribution of COs is optimal for the efficacy of genetic shuffling.

Chromosome size and recombination rate

Some unnecessary repetition of results here can be removed — shorter will be clearer

We showed that, for most species, the smallest chromosome had roughly one or two COs, independently of chromosome size. This is in agreement with the idea that CO assurance is a ubiquitous regulation process among angiosperms (Pazhayam et al., 2021). Moreover, this constraint imposes a kind of basal recombination rate for each species, on the order of $50/Sc$ cM/Mb, where Sc is the size of the lowest chromosome in Mb. Regardless of the genome size (which ranges three orders of magnitude or more), the number of COs remains relatively stable amongst species, most probably under the joint influence of CO assurance, interference and homeostasis (Otto and Payseur, 2019; Stapley et al., 2017; Wang et al., 2015). As a result, averaged recombination rates are negatively correlated with chromosome lengths, as already known in plants (Haenel et al., 2018; Tiley and Burleigh, 2015).

Surely this is already known, for example from differences between closely related species with different rates, including selfer-outcrtosser differences

However, there is no universal relationship between the absolute size of a chromosome and its mean recombination rate. Although the average recombination rate of a species is well predicted by its average chromosome size, the recombination rates of each chromosome separately are not well predicted by their absolute chromosome size. Instead, variation within species is much better explained by the relative chromosome size, and surprisingly, this relationship seems to be roughly the same among species (see Fig 1 and 2). This suggests that CO interference is proportional to the relative size of the chromosome, as has been empirically observed in some plants (Ferreira et al., 2021). Although it is not clear yet which interference distance unit is the most relevant, genomic distances (in Mb) are excluded in most models of interference in favour of genetic distances (cM) (Foss et al., 1993) or, more likely, the length of the synaptonemal complex in micrometres (Capilla-Pérez et al., 2021; Kleckner et al., 2004; Lloyd and Jenczewski, 2019; Zickler and Kleckner, 2015). Both scales match our observation of a relative size effect. Within species, genetic maps increase with chromosome size, but among species they are uncorrelated and far less variable than genome sizes, which makes the relative chromosome size the main determinant of recombination rate variations among species. Similarly, physical sizes (in micrometres) at meiosis do not seem to scale with genome size, as chromosomal organization (nucleosomes, chromatin loops) strongly reduces the variation that could be expected given the genome size (Otto and Payseur, 2019).

Recombination patterns along chromosomes

We observed a global trend towards higher recombination rates in sub-distal regions (Gaut et al., 2007; Haenel et al., 2018). The distal bias increased with chromosome length, in agreement with the conclusions of Haenel et al. (2018), although our methods differ in resolution. We analysed species and chromosomes separately whereas Haenel et al. (2018) used averages over the different patterns, thereby masking chromosome- and species-specific particularities. For example, they did not detect the sub-distal pattern neither unclassified exceptions, whereas they seem common among species (16 and 7 species

respectively). So far, little is known about the mechanisms that could explain the link between the distal bias and chromosome length. Even if models of CO interference yield similar patterns (Falque et al., 2007; Zhang et al., 2014), the conceptual model of Haenel et al. (2018) is still the only one to explicitly consider chromosome length. The telomere effect is thought to act at a broad chromosome scale over long genomic distance. The decision of double strand breaks (DSBs) to engage in the CO pathway is made early on during meiosis and the early chromosome pairing beginning in telomeres is thought to favour distal COs (Bishop and Zickler, 2004; Higgins et al., 2012; Hinch et al., 2019). In barley, when the relative timing of the first stages of the meiotic program was shortened, COs were redistributed towards proximal regions (Higgins et al., 2012), as later observed in wheat (Osman et al., 2021).

Some unnecessary repetition of results here can be removed

Haenel et al. (2018) proposed that distance to the telomere is driving CO positioning, and therefore it should produce a symmetrical U-shaped pattern along chromosomes. However, a formal test showed that this model was too simple and that centromeres also played a role in the distribution of COs between chromosome arms. The best model (M3: ‘telomere + centromere + one CO per chromosome’) that we have proposed suggests that centromeres do not only have a local effect but also influence the symmetry of recombination landscapes over long distance, though a large proportion of our sample is metacentric, which might limit the detection of an effect. The local suppression of COs in centromeric regions is well known and largely conserved among species and seems a strong constitutive feature restricted to a short centromeric region, basically the kinetochore (Ellermeier et al., 2010; Fernandes et al., 2019). But the extent of the pericentromeric region varies drastically, most probably under the influence of DNA methylation, chromatin accessibility or RNA interference (Choi et al., 2018; Ellermeier et al., 2010; Hartmann et al., 2019; Pan et al., 2011). However, how centromeres (especially non-metacentric ones) may affect CO distribution at larger scales still needs to be determined.

Diversity of patterns among species

In addition to the role of centromeres, we also observed that recombination rates do not always decrease monotonically with the distance to the tip of the chromosome, showing that the distal model is not generally found among plants. We observed at least two different crossover patterns among plant species. Only 34 out of 57 species support a process starting at the tips (distal model), and 16 present the highest recombination rates in sub-distal regions, while seven species remain unclassified, which is at the limit of our visual classification. Globally, the distal pattern and distal bias seem to occur more often in larger chromosomes, but our data lack species with giant genomes. Giant genomes are not rare in plants, and we cannot extrapolate our conclusions to the upper range of the genome size variation (Pellicer et al., 2018). Astonishingly, a low-density genetic map in *Allium* showed higher recombination rates in the proximal regions, which is opposite to the major trend we found (Khrustaleva et al., 2005). Genera with giant genomes such as *Lilium* or *Allium* would have been valuable assets in our dataset, but the actual genomic and linkage data are relatively incomplete (Jo et al., 2017; Shahin et al., 2011).

The occurrence of various recombination patterns is in agreement with what is known of the timing of meiosis and heterochiasmy (the fact that male and female meiosis have different CO patterns). Despite the strong conservation of the main meiotic mechanism in plants, differences in the balance between key components may produce distinct CO patterns (de Massy, 2013; Higgins et al., 2012; Kuo et al., 2021; Zelkowski et al., 2019). For example, the ZYP1 and ASY1 proteins have antagonistic effects on the formation of the synaptonemal complex in plants (Lambing et al., 2020). In barley and wheat, linearization of the chromosome axis triggered by ZYP1 is gradual along the chromosome and initiated in distal regions, forming the telomere bouquet where early DSBs form (Higgins et al., 2012; Osman et al., 2021). In contrast, chromosome axes are formed at a similar time in *Arabidopsis thaliana* and chromosomes are gradually enriched in ASY1 from the telomeres to the centromeres; a gene-dosage component favours synapsis and ultimately COs towards

the proximal regions (Lambing et al., 2020). It appears that the timing of the meiotic programme is important for the distal bias, as it involves changes in the relative contribution of each meiotic component that could explain the re-localization of COs (Higgins et al., 2012; Lambing et al., 2020). Therefore, the different patterns we observed may be explained by the different balance and timing of the expression of shared key regulators of CO patterning such as ZYP1 and ASY1 (Kuo et al., 2021). It is interesting to note that this is also true for mechanistic models of interference. Zhang et al. (2014) assessed that the 'beam-film' model is able to fit both CO patterns, regardless whether the tips of the chromosomes have an effect on interference or not, i.e. clamping. If clamping is assumed, the model predicts that mechanical stress culminates in the extremities of the chromosome leading to high CO rates at the periphery where it is released first. In contrast, when clamping is limited, mechanical stress is released in the tips of the chromosome and COs occur further from the tips, until a threshold of mechanical stress is reached. The observed sub-distal pattern fits these predictions.

The two patterns of recombination we described here can also be observed in opposite sexes within the same plant species (Capilla-Pérez et al., 2021; Dukić and Bomblies, 2022; Sardell and Kirkpatrick, 2019). Marked heterochiasmy variations between species, a feature shared among plants and animals, could influence the resulting sex-averaged recombination landscape (Sardell and Kirkpatrick, 2019). The sex-averaged telomere effect can be thought of as the product of two independent sex-specific landscapes although it is not clear how sex-specific maps ultimately contribute to the sex-averaged one (Johnston et al., 2016; Lenormand et al., 2016). Recombination is usually biased towards the tips of the chromosome in male recombination maps, but is more evenly distributed in female maps in the few plant species with available data (Sardell and Kirkpatrick, 2019). In *Arabidopsis thaliana*, male meiosis has higher CO rates within the tips of the chromosome, as it has been observed in other species with large chromosomes, whereas female meiosis is more homogeneously distributed, with the lowest rates found in the distal regions (Capilla-Pérez et

al., 2021). Shorter chromosome axes in *A. thaliana* female meiosis could induce fewer DSBs and class II non-interfering COs (Lloyd and Jenczewski, 2019). Conversely, in maize, the distal bias is similar in both sexes, despite higher CO rates for females (Kianian et al., 2018). Heterochiasmy is not universal in plants (Melamed-Bessudo et al., 2016), and we suggest that the variation in recombination landscapes could also result from variation in heterochiasmy among species, as it has been suggested for broad-scale differences in recombination landscapes between *A. thaliana* and its relative *A. arenosa* (Dukić and Bomblies, 2022). This hypothesis should be tested further as more sex-specific genetic maps become available.

Recombination landscapes, gene density and genetic shuffling

We observed a strong convergence between CO patterns and gene density patterns. This correlation is consistent in our dataset despite possible errors in genome annotation and we also observed two different gene density patterns globally corresponding to similar CO patterns, emphasizing the close link between recombination and gene density. Interestingly, we found the same correlation in species with atypical chromosomes. For example, *Camellia sinensis* and *Nelumbo nucifera* have large genomes with homogenous recombination landscapes, and a recent annotation of the *Nelumbo nucifera* genome showed that genes are also evenly distributed along chromosomes at a broad scale (Shi et al., 2020), similar to *Camellia sinensis* (Wei et al., 2018). In wheat and rye, the analysis of the effect of chromosome rearrangement on recombination also suggests that CO localization is more locus-specific than location-specific: after inversions of distal and interstitial segments, COs were relocated to the new position on the distal segment (Lukaszewski, 2008; Lukaszewski et al., 2012). Overall, the parallel between gene density and recombination landscapes, confirmed by these two exceptions, is in agreement with the preferential occurrence of COs in gene regulatory sequences (Choi et al., 2018; He et al., 2017; Marand et al., 2019), and suggests that this may be a general pattern shared among angiosperms. Thus, gene distribution along chromosomes could be a main driver of recombination landscapes simply

by determining where COs may preferentially occur. It should be noted that since the gene number is usually positively correlated with chromosome size within a species but is roughly independent of genome size among species, this hypothesis also matches with the relative-size effect discussed above.

However, gene density and recombination rates are both correlated with many other genomic features, such as transposable elements (Charlesworth et al., 1994; Kent et al., 2017). The accumulation of transposable elements in low recombining regions would progressively decrease gene density in the region, and would eventually result in a positive correlation between gene density and recombination. However, the correlation of recombination rates with transposable elements is not always clear and different TE families have opposite correlations (Kent et al., 2017; Underwood and Choi, 2019). Causality mechanisms of these multiple interactions still need to be clarified. The use of fine scale recombination maps (using very large mapping populations or LD maps) should help identifying the respective role of genic regions (especially the role of promoters) and transposable elements (or other genomic features).

Irrespective of the underlying mechanism, our finding implies that the CO distribution ultimately scales with the gene distribution. Therefore, in most species, COs have a more even distribution between genes than between random genomic locations (Fig 10). The redistribution of COs towards functional regions could be a simple consequence of COs occurring within gene regulatory sequences, but it has important evolutionary implications such as increasing the genetic shuffling and homogenizing the probability of two random genes to recombine, especially for large genomes that exhibit the strongest difference in genetic shuffling between genes and between genomic locations (Fig 11). Therefore, CO patterning (and not only the global CO rate) could be under selection not only for its direct effect on the functioning of meiosis but also for its indirect effects on selection efficacy (Otto and Payseur, 2019). Recombination decreases linkage disequilibrium and negative interferences between adjacent loci (e.g. Hill-Robertson Interference), and thus locally

increases the efficacy of selection. Functional sites are targets for selection (Nachman and Payseur, 2012) and we found higher recombination rates in functional regions, meaning that only a few genes are ultimately excluded from the benefits of recombination, even under the most pronounced distal bias.

Higher recombination rates in gene-rich regions could provide a satisfying explanation as to why the distal bias is maintained among species despite its theoretical lack of efficacy for genetic shuffling (Veller et al., 2019). The association between CO hotspots and gene regulatory sequences is mechanistically driven by chromatin accessibility, but it does not exclude the evolution of the mechanism itself towards the benefits of recombining more in gene-rich regions (Lenormand et al., 2016). However, slight variations in genetic shuffling caused by the non-random distribution of COs are less likely to be under strong selection compared to stabilizing selection on molecular constraints for chromosome pairing and segregation (Ritz et al., 2017), although interference is sometimes likely to evolve towards relaxed physical constraints (Otto and Payseur, 2019). In addition, the intra-chromosomal component of the genetic shuffling is a small contributor to the genome-wide shuffling rate, as a major part is due to independent assortment among chromosomes (Veller et al., 2019). Our estimates for the chromosomal genetic shuffling do not reach the theoretical optimal value of 0.5. The pattern is not absolute, and a fraction of genes remains in low recombining regions. In grass species, up to 30% of genes are found in recombination deserts and are not subject to efficient selection (e.g. Mayer et al., 2011). Finally, it is still an open question as to whether this global distribution of COs in gene regulatory sequences is advantageous for the genetic diversity and adaptive potential of a species (Pan et al., 2016).

Conclusion

Our comparative study only demonstrates correlations, and not mechanisms, but helps to understand the diversity and determinants of recombination landscapes in flowering plants. Our results partly confirm previous studies based on fewer species (Haenel et al., 2018; Stapley et al., 2017; Tiley and Burleigh, 2015) while bringing new insights that alter previous

conclusions thanks to a detailed analysis at the species and chromosome levels. Two main and distinct CO patterns emerge across a large set of flowering plant species; it seems likely that chromosome structure (length, centromere) and gene densities are the major drivers of these patterns, and the interactions between them raise questions about the evolution of complex genomic patterns at the chromosome scale (Gaut et al., 2007; Nam and Ellegren, 2012). The new large and curated dataset we provide in the present work should be useful for addressing such questions and testing future evolutionary hypotheses regarding the role of recombination in genome architecture.

Materials and Methods

Data preparation

To build recombination maps, we combined genetic and genomic maps in angiosperms that had already been published in the literature. We conducted a literature search to collect sex-averaged genetic maps estimated on pedigree data – with markers positions in centiMorgans (cM). The keywords used were ‘genetic map’, ‘linkage map’, ‘genome assembly’, ‘plants’ and ‘angiosperms’, combined with 'high-density' or 'saturated' in order to target genetic maps with a large number of markers and progenies. Additionally, we carried out searches within public genomic databases to find publicly available genetic maps. Only species with a reference genome assembly at a chromosome level were included in our study (a complete list of genetic maps with the associated metadata is given in Tables S1, S2). As much as possible, genomic positions along the chromosome (Mb) were estimated by blasting marker sequences on the most recent genome assembly (otherwise genomic positions were those of the original publication). Genome assemblies with annotation files at a chromosome-scale were downloaded from NCBI (https://www.ncbi.nlm.nih.gov/) or public databases. Marker sequences were blasted with ‘blastn’ and a 90% identity cutoff. Markers were anchored to the genomic position of the best hit. When the sequence was a pair of primers, the mapped genomic position was the best hit between pairs of positions showing a

short distance between the forward and reverse primer (< 200 bp). In a few exceptions (see Table S1), genomic positions were mapped on a close congeneric species genome and the genomic map was kept if there was good collinearity between the genetic and genomic positions. Chromosomes were numbered as per the reference genome assembly. When marker sequences were not available, we kept the genomic positions published with the genetic map. The total genomic length was estimated by the length of the chromosome sequence in the genome assembly. The total genetic length was corrected using Hall and Willis's method (Hall and Willis, 2005) which accounts for undetected events of recombination in distal regions by adding *2s* to the length of each linkage group (where *s* is the average marker spacing in the group).

We selected genetic and genomic maps after stringent filtering and corrections, using custom scripts available in a public Github repository (https://github.com/ThomasBrazier/diversity-determinants-recombination-landscapes-flowering-plants.git). We assumed that markers must follow a monotone increasing function when plotting genetic distances as a function of genomic distances in a chromosome (i.e. the Marey map) and collinearity between the genetic map and the reference genome was required to keep a Marey map. If necessary, genetic maps were reoriented so that the Marey map function is increasing (i.e. genetic distances read in the opposite direction). In a first step, Marey maps with fewer than 50 markers per chromosome were removed, although a few exceptions were visually validated (maps with ~30 markers). Marey maps with more than 10% of the total genomic map length missing at one end of the chromosome were removed. Marey maps with obvious artefacts and assembly mismatches (e.g. lack of collinearity, large inversions, large gaps) were removed. Markers clearly outside the global trend of the Marey map (e.g. large genetic/genomic distance from the global cloud of markers or from the interpolated Marey function, no other marker in a close neighbourhood) were visually filtered out, and multiple iterations of filtering/interpolation helped to refine outlier removal. The Marey map approach is a graphical method, so figures were

systematically produced at each step as a way to evaluate the results of the filtering and corrections. Finally, when multiple datasets were available for the same species, we selected the dataset with the highest marker density – in addition to visual validation – to maintain a balanced sampling and avoid pseudo-replicates of the same chromosome.

Estimates of local recombination rates

Local recombination rates along the chromosome were estimated with custom scripts following the Marey map approach, as described in the MareyMap R package (Rezvoy et al., 2007). The mathematical function of the Marey map was interpolated with a two-degree polynomial loess regression. Each span smoothing parameter was calibrated by 1,000 iterations of hold-out partitioning (random sampling of markers between two subsets; 2/3 for training and 1/3 for testing) with the Mean Squared Error of the loess regression as a goodness-of-fit criterion. The possible span ranged from 0.2 to 0.5 and was visually adjusted for certain maps. The local recombination rate was the derivative of the interpolated smoothed function in fixed 100 kb and 1 Mb non-overlapping windows. Negative estimates were not possible as we assumed a monotonously increasing function and negative recombination rates were set to zero. The 95% confidence intervals of the recombination rates were estimated by 1,000 bootstrap replicates of the markers and recombination landscapes with large confidence interval were discarded. The quality of the estimates was checked using the correlation between the 100 kb and 1 Mb windows.

The distribution of CO along chromosomes

The spatial structure of recombination landscapes across species and chromosomes is a major feature of recombination landscapes. We divided the Marey map in k segments of equal genomic size (Mb) and then calculated the relative genetic size (cM) of each segment. Under the null model (i.e. random recombination), one expects k segments of equal genetic size $1/k$. The relative recombination rate in the segment i was estimated by the log-ratio of

the observed genetic size (i.e. genetic size of segment *i*) divided by the expected genetic size (i.e. fixed to total genetic size / *k* by the model), as in the following equation.

$$relative\ recombination\ rate = log_{10}\frac{genetic_i}{genetic_{total}/k}$$

Given the observation that most recombination landscapes are broken down into at least three segments (White and Hill, 2020), we arbitrarily chose a number of segments *k* = 10 to reach a good resolution (a larger *k* did not show any qualitative differences).

Crossover patterns and the periphery-bias ratio

We investigated the spatial bias towards distal regions of the chromosome in the distribution of recombination by estimating recombination rates as a function of relative distances to the telomere (i.e. distance to the nearest chromosome end). Chromosomes were split by their midpoint and only one side was randomly sampled for each chromosome to avoid pseudo-replicates and the averaging of two potentially contrasting patterns on opposite arms. The relative distance to the telomere was the distance to the telomere divided by total chromosome size, then divided into 20 bins of equal relative distances. A periphery-bias ratio metric similar to the one presented in Haenel et al. (2018) was estimated to measure the strength of the distal bias. We divided the recombination rates in the tip of the chromosome (10% on each side of the chromosome, and one randomly sampled tip) by the mean recombination rate of the whole chromosome. We investigated the sensitivity of this periphery-bias ratio to the sampling scale by calculating the ratio for many distal region sizes (Fig S12).

Testing centromere or telomere effects

We searched the literature for centromeric indices (ratio of the short arm length divided by the total chromosome length) established by cytological measures. When we had no information about the correct orientation of the chromosome (short arm/long arm), the centromeric index was oriented to match the region with the lowest recombination rate of the

whole chromosome (i.e. putative centromere). To determine if telomeres and centromeres play a significant role in CO patterning, we fitted empirical CO distributions to three theoretical models of CO distribution. In the following equations, *d(x)* is the relative genetic distance at the relative genomic position *x*, and *a* is a coefficient corresponding to the excess of COs per genomic distance. Under the strict 'telomere' model (1), we assumed that only telomeres played a role in CO distribution, i.e. an equal distribution of COs on both sides of the chromosome (i.e. $d(1/2) = d(1) - d(1/2)$, such that $\frac{d(1/2)}{d(1)} = 0.5$. The 'telomere + centromere + one mandatory CO per arm' model (2) assumed at least one CO per chromosome arm and a relative genetic distance of each chromosome arm proportional to its relative genomic size, corresponding to the role of centromere position, denoted *d(c)*. We have $d(c) = 50 + a \times c$ and $d(1) - d(c) = 50 + a \times (1 - c)$, such that $\frac{d(c)-50}{d(1)-100} = c$. Lastly, the 'telomere + centromere + one CO per chromosome' model (3) assumed at least one CO per chromosome and a relative genetic distance within the chromosome proportional to its relative genomic distance. We have $d(c) = c \times 50 + a \times c$ and $d(1) - d(c) = (1 - c) \times 50 + a \times (1 - c)$, such that $\frac{d(c)}{d(1)} = c$. The three competing models were compared with a linear regression between empirical and theoretical values, based on the adjusted R^2 and AIC-BIC criteria among chromosomes. The number of species supporting each model was calculated based on the adjusted R^2 within species, for all species with at least five chromosomes.

Gene density

We retrieved genome annotations ('gff' files) for genes, coding sequences and exon positions, preferentially from NCBI and otherwise from public databases (41 species). We estimated gene counts in 100 kb windows for recombination maps by counting the number of genes with a starting position falling inside the window. For each gene count, we estimated the species mean recombination rate and its confidence interval at 95% by 1,000 bootstrap replicates (chromosomes pooled per species). Most species had rarely more than 20 genes over a 100 kb span and variance dramatically increased in the upper range of the

gene counts, and therefore we pruned gene counts over 20 for graphical representation and statistical analyses.

Genetic shuffling

To assess the efficiency of the recombination between chromosomes and species, we calculated the measure of intra-chromosomal genetic shuffling described by Veller et al. (2019). To have even sampling along the chromosome, genetic positions (cM) of 1,000 pseudo-markers evenly distributed along genomic distances (Mb) were interpolated using a loess regression on each Marey map, following the same smoothing and interpolation procedure as for the estimation of the recombination rates. The chromosomal genetic shuffling $\bar{r}_{intra}$ were calculated as per the intra-chromosomal component of the equation 10 presented in Veller et al. (2019). For a single chromosome,

$$\bar{r}_{intra} = \sum_{i<j} (r_{ij} / \binom{\Lambda}{2})$$

where Λ is the total number of loci, $\binom{\Lambda}{2} = \Lambda(\Lambda - 1)/2$ and r_{ij} is the rate of shuffling for the locus pair *(i, j)*. For the intra-chromosomal component $\bar{r}_{intra}$, the pairwise shuffling rate was only calculated for linked sites, i.e. loci on the same chromosome. This pairwise shuffling rate was estimated by the recombination fraction between loci *i* and *j*. Recombination fractions were directly calculated from Haldane or Kosambi genetic distances between loci by applying a reverse Haldane function (1) or reverse Kosambi function (2), depending on the mapping function originally used for the given genetic map.

$$r_{ij} = \frac{1}{2}(1 - e^{-2d_{ij}/100}) \qquad (1)$$

$$r_{ij} = tanh\frac{1}{2}tanh(2d_{ij}/100) \qquad (2)$$

We also estimated marker positions in gene distances instead of genomic distances (Mb) to investigate the influence of the non-random distribution of genes on the recombination

landscape. Gene distances were the cumulative number of genes along the chromosome at a given marker's position. Splicing variants and overlapping genes were counted as a single gene. The genetic shuffling was re-estimated with gene distances instead of genomic distances to consider a genetic shuffling based on the gene distribution, as suggested by Veller et al. (2019). To compare the departure from a random distribution along the chromosome among both types of distances (i.e. genomic and genes), we calculated the Root Mean Square Error (RMSE) of each Marey map and for both distances. To assess if the distribution of genes influenced the heterogeneity of recombination landscapes, the type of distance with the lower RMSE was considered as the more homogeneous landscape. However, this measure for gene distances is sensitive to annotation errors and artefacts. False negatives are therefore expected (when Marey maps were assessed as more homogeneous in genomic distances while the inverse is true) and this classification remains conservative.

Statistical analyses

All statistical analyses were performed with R version 4.0.4 (R Core Team, 2019). We assessed statistical relationships with the non-parametric Spearman's rank correlation and regression models. Linear Models were used for regressions with species data since we did not detect a phylogenetic effect. The structure in the chromosome dataset was accounted for by Linear Mixed Models (LMER) implemented in the 'lme4' R package (Bates et al., 2015, p. 4) and the phylogenetic structure was tested by fitting the Phylogenetic Generalized Linear Mixed Model (PGLMM) of the 'phyr' R package (Ives et al., 2019). The phylogenetic time-calibrated supertree used for the covariance matrix was retrieved from the publicly available phylogeny constructed by Smith and Brown (Smith and Brown, 2018). Marginal and conditional R^2 values for LMER were estimated with the 'MuMIn' R package (Bartoń, 2020). Significance of the model parameters was tested with the 'lmerTest' R package (Kuznetsova et al., 2017). We selected the model based on AIC/BIC criteria and diagnostic plots. Reliability and stability of the various models were assessed by checking quantile-quantile

plots for the normality of residuals and residuals plotted as a function of fitted values for homoscedasticity. Model quality was checked by the comparison of predicted and observed values. Given the skewed nature of some distributions, we used logarithm (base 10) transformations when appropriate. For comparison between species, statistics were standardized (i.e. by subtracting the mean and dividing by standard deviation). Mean statistics and 95% confidence intervals were estimated by 1,000 bootstrap replicates.

Acknowledgements

We thank Eric Janczewski, Laurent Duret, Anne-Marie Chèvre, Eric Petit and Armel Salmon, as well as three anonymous reviewers, for precious comments on the results and manuscript. We thank all the people that provided us genetic data that were not published yet.

Author contributions

SG conceptualised and supervised the study. TB produced and analysed data. Both authors contributed to writing the paper.

References

Apuli, R.-P., Bernhardsson, C., Schiffthaler, B., Robinson, K.M., Jansson, S., Street, N.R., Ingvarsson, P.K., 2020. Inferring the Genomic Landscape of Recombination Rate Variation in European Aspen (*Populus tremula*). G3 GenesGenomesGenetics 10, 299–309. https://doi.org/10.1534/g3.119.400504

Bartoń, K., 2020. MuMIn: Multi-model inference (manual).

Barton, N.H., 1995. A general model for the evolution of recombination. Genet. Res. 65, 123–144. https://doi.org/10.1017/S0016672300033140

Bates, D., Mächler, M., Bolker, B., Walker, S., 2015. Fitting linear mixed-effects models using lme4. J. Stat. Softw. 67, 1–48. https://doi.org/10.18637/jss.v067.i01

Bishop, D.K., Zickler, D., 2004. Early decision; meiotic crossover interference prior to stable strand exchange and synapsis. Cell 117, 9–15. https://doi.org/10.1016/S0092-8674(04)00297-1

Blitzblau, H.G., Bell, G.W., Rodriguez, J., Bell, S.P., Hochwagen, A., 2007. Mapping of Meiotic Single-Stranded DNA Reveals Double-Strand-Break Hotspots near Centromeres and Telomeres. Curr. Biol. 17, 2003–2012. https://doi.org/10.1016/j.cub.2007.10.066

Booker, T.R., Yeaman, S., Whitlock, M.C., 2020. Variation in recombination rate affects detection of outliers in genome scans under neutrality. Mol. Ecol. mec.15501. https://doi.org/10.1111/mec.15501

Capilla-Pérez, L., Durand, S., Hurel, A., Lian, Q., Chambon, A., Taochy, C., Solier, V., Grelon, M., Mercier, R., 2021. The synaptonemal complex imposes crossover interference and heterochiasmy in *Arabidopsis*. Proc. Natl. Acad. Sci. 118, e2023613118. https://doi.org/10.1073/pnas.2023613118

Charlesworth, B., Sniegowski, P. & Stephan, W., 1994. The evolutionary dynamics of repetitive DNA in eukaryotes. Nature 371, 215–220.

Charlesworth, B., Jensen, J.D., 2021. Effects of Selection at Linked Sites on Patterns of Genetic Variability. Annu. Rev. Ecol. Evol. Syst. 52, 177–197. https://doi.org/10.1146/annurev-ecolsys-010621-044528

Choi, K., Zhao, X., Kelly, K.A., Venn, O., Higgins, J.D., Yelina, N.E., Hardcastle, T.J., Ziolkowski, P.A., Copenhaver, G.P., Franklin, F.C.H., McVean, G., Henderson, I.R., 2013. Arabidopsis meiotic crossover hot spots overlap with H2A.Z nucleosomes at gene promoters. Nat. Genet. 45, 1327–1336. https://doi.org/10.1038/ng.2766

Choi, K., Zhao, X., Tock, A.J., Lambing, C., Underwood, C.J., Hardcastle, T.J., Serra, H., Kim, Juhyun, Cho, H.S., Kim, Jaeil, Ziolkowski, P.A., Yelina, N.E., Hwang, I., Martienssen, R.A., Henderson, I.R., 2018. Nucleosomes and DNA methylation shape meiotic DSB frequency in *Arabidopsis thaliana* transposons and gene regulatory regions. Genome Res. 28, 532–546. https://doi.org/10.1101/gr.225599.117

Comeron, J.M., 2017. Background selection as null hypothesis in population genomics: insights and challenges from *Drosophila* studies. Philos. Trans. R. Soc. B Biol. Sci. 372, 20160471. https://doi.org/10.1098/rstb.2016.0471

Cooper, T.J., Garcia, V., Neale, M.J., 2016. Meiotic DSB patterning: A multifaceted process. Cell Cycle 15, 13–21. https://doi.org/10.1080/15384101.2015.1093709

Corbett-Detig, R.B., Hartl, D.L., Sackton, T.B., 2015. Natural Selection Constrains Neutral Diversity across A Wide Range of Species. PLOS Biol. 13, e1002112. https://doi.org/10.1371/journal.pbio.1002112

de Massy, B., 2013. Initiation of Meiotic Recombination: How and Where? Conservation and Specificities Among Eukaryotes. Annu. Rev. Genet. 47, 563–599. https://doi.org/10.1146/annurev-genet-110711-155423

Dukić, M., Bomblies, K., 2022. Male and female recombination landscapes of diploid *Arabidopsis arenosa*. Genetics iyab236. https://doi.org/10.1093/genetics/iyab236

Ellermeier, C., Higuchi, E.C., Phadnis, N., Holm, L., Geelhood, J.L., Thon, G., Smith, G.R., 2010. RNAi and heterochromatin repress centromeric meiotic recombination. Proc. Natl. Acad. Sci. 107, 8701–8705. https://doi.org/10.1073/pnas.0914160107

Falque, M., Mercier, R., Mézard, C., de Vienne, D., Martin, O.C., 2007. Patterns of Recombination and MLH1 Foci Density Along Mouse Chromosomes: Modeling Effects of Interference and Obligate Chiasma. Genetics 176, 1453–1467. https://doi.org/10.1534/genetics.106.070235

Fernandes, J.B., Wlodzimierz, P., Henderson, I.R., 2019. Meiotic recombination within plant centromeres. Curr. Opin. Plant Biol. 48, 26–35. https://doi.org/10.1016/j.pbi.2019.02.008

Ferreira, M.T.M., Glombik, M., Perničková, K., Duchoslav, M., Scholten, O., Karafiátová, M., Techio, V.H., Doležel, J., Lukaszewski, A.J., Kopecký, D., 2021. Direct evidence for crossover and chromatid interference in meiosis of two plant hybrids (*Lolium multiflorum*×*Festuca pratensis* and *Allium cepa*×*A. roylei*). J. Exp. Bot. 72, 254–267. https://doi.org/10.1093/jxb/eraa455

Foss, E., Lande, R., Stahl, F., Steinberg, C., 1993. Chiasma interference as a function of genetic distance. Genetics 133, 681–691.

Galtier, N., Roux, C., Rousselle, M., Romiguier, J., Figuet, E., Glémin, S., Bierne, N., Duret, L., 2018. Codon Usage Bias in Animals: Disentangling the Effects of Natural Selection, Effective Population Size, and GC-Biased Gene Conversion. Mol. Biol. Evol. 35, 1092–1103. https://doi.org/10.1093/molbev/msy015

Gaut, B.S., Wright, S.I., Rizzon, C., Dvorak, J., Anderson, L.K., 2007. Recombination: an underappreciated factor in the evolution of plant genomes. Nat. Rev. Genet. 8, 77–84. https://doi.org/10.1038/nrg1970

Glémin, S., Clément, Y., David, J., Ressayre, A., 2014. GC content evolution in coding regions of angiosperm genomes: a unifying hypothesis. Trends Genet. 30, 263–270. https://doi.org/10.1016/j.tig.2014.05.002

Haenel, Q., Laurentino, T.G., Roesti, M., Berner, D., 2018. Meta-analysis of chromosome-scale crossover rate variation in eukaryotes and its significance to evolutionary genomics. Mol. Ecol. 27, 2477–2497. https://doi.org/10.1111/mec.14699

Hall, M.C., Willis, J.H., 2005. Transmission Ratio Distortion in Intraspecific Hybrids of *Mimulus guttatus*: Implications for Genomic Divergence. Genetics 170, 375–386. https://doi.org/10.1534/genetics.104.038653

Hartmann, M., Umbanhowar, J., Sekelsky, J., 2019. Centromere-Proximal Meiotic Crossovers in *Drosophila melanogaster* Are Suppressed by Both Highly Repetitive Heterochromatin and Proximity to the Centromere. Genetics 213, 113–125. https://doi.org/10.1534/genetics.119.302509

He, Y., Wang, M., Dukowic-Schulze, S., Zhou, A., Tiang, C.-L., Shilo, S., Sidhu, G.K., Eichten, S., Bradbury, P., Springer, N.M., Buckler, E.S., Levy, A.A., Sun, Q., Pillardy, J., Kianian, P.M.A., Kianian, S.F., Chen, C., Pawlowski, W.P., 2017. Genomic features shaping the landscape of meiotic double-strand-break hotspots in maize. Proc. Natl. Acad. Sci. 114, 12231–12236. https://doi.org/10.1073/pnas.1713225114

Higgins, J.D., Perry, R.M., Barakate, A., Ramsay, L., Waugh, R., Halpin, C., Armstrong, S.J., Franklin, F.C.H., 2012. Spatiotemporal Asymmetry of the Meiotic Program Underlies the Predominantly Distal Distribution of Meiotic Crossovers in Barley. Plant Cell 24, 4096–4109. https://doi.org/10.1105/tpc.112.102483

Hinch, A.G., Zhang, G., Becker, P.W., Moralli, D., Hinch, R., Davies, B., Bowden, R., Donnelly, P., 2019. Factors influencing meiotic recombination revealed by whole-genome sequencing of single sperm. Science 363, eaau8861. https://doi.org/10.1126/science.aau8861

Ives, A., Dinnage, R., Nell, L.A., Helmus, M., Li, D., 2019. phyr: Model based phylogenetic analysis (manual).

Jo, J., Purushotham, P.M., Han, K., Lee, H.-R., Nah, G., Kang, B.-C., 2017. Development of a Genetic Map for Onion (*Allium cepa* L.) Using Reference-Free Genotyping-by-

Sequencing and SNP Assays. Front. Plant Sci. 8, 1606. https://doi.org/10.3389/fpls.2017.01606

Johnston, S.E., Bérénos, C., Slate, J., Pemberton, J.M., 2016. Conserved Genetic Architecture Underlying Individual Recombination Rate Variation in a Wild Population of Soay Sheep (*Ovis aries*). Genetics 203, 583–598. https://doi.org/10.1534/genetics.115.185553

Kent, T.V., Uzunović, J., Wright, S.I., 2017. Coevolution between transposable elements and recombination. Philos. Trans. R. Soc. B Biol. Sci. 372, 20160458. https://doi.org/10.1098/rstb.2016.0458

Khrustaleva, L.I., de Melo, P.E., van Heusden, A.W., Kik, C., 2005. The Integration of Recombination and Physical Maps in a Large-Genome Monocot Using Haploid Genome Analysis in a Trihybrid Allium Population. Genetics 169, 1673–1685. https://doi.org/10.1534/genetics.104.038687

Kianian, P.M.A., Wang, M., Simons, K., Ghavami, F., He, Y., Dukowic-Schulze, S., Sundararajan, A., Sun, Q., Pillardy, J., Mudge, J., Chen, C., Kianian, S.F., Pawlowski, W.P., 2018. High-resolution crossover mapping reveals similarities and differences of male and female recombination in maize. Nat. Commun. 9, 2370. https://doi.org/10.1038/s41467-018-04562-5

Kleckner, N., Zickler, D., Jones, G.H., Dekker, J., Padmore, R., Henle, J., Hutchinson, J., 2004. A mechanical basis for chromosome function. Proc. Natl. Acad. Sci. 101, 12592–12597. https://doi.org/10.1073/pnas.0402724101

Kuo, P., Da Ines, O., Lambing, C., 2021. Rewiring Meiosis for Crop Improvement. Front. Plant Sci. 12, 708948. https://doi.org/10.3389/fpls.2021.708948

Kuznetsova, A., Brockhoff, P.B., Christensen, R.H.B., 2017. lmerTest package: Tests in linear mixed effects models. J. Stat. Softw. 82, 1–26. https://doi.org/10.18637/jss.v082.i13

Lambing, C., Kuo, P.C., Tock, A.J., Topp, S.D., Henderson, I.R., 2020. ASY1 acts as a dosage-dependent antagonist of telomere-led recombination and mediates crossover interference in *Arabidopsis*. Proc. Natl. Acad. Sci. 117, 13647–13658. https://doi.org/10.1073/pnas.1921055117

Lenormand, T., Engelstädter, J., Johnston, S.E., Wijnker, E., Haag, C.R., 2016. Evolutionary mysteries in meiosis. Philos. Trans. R. Soc. B Biol. Sci. 371, 20160001. https://doi.org/10.1098/rstb.2016.0001

Lloyd, A., Jenczewski, E., 2019. Modelling Sex-Specific Crossover Patterning in *Arabidopsis*. Genetics 211, 847–859. https://doi.org/10.1534/genetics.118.301838

Lukaszewski, A.J., 2008. Unexpected behavior of an inverted rye chromosome arm in wheat. Chromosoma 117, 569–578. https://doi.org/10.1007/s00412-008-0174-4

Lukaszewski, A.J., Kopecky, D., Linc, G., 2012. Inversions of chromosome arms 4AL and 2BS in wheat invert the patterns of chiasma distribution. Chromosoma 121, 201–208. https://doi.org/10.1007/s00412-011-0354-5

Marand, A.P., Zhao, H., Zhang, W., Zeng, Z., Fang, C., Jiang, J., 2019. Historical Meiotic Crossover Hotspots Fueled Patterns of Evolutionary Divergence in Rice. Plant Cell 31, 645–662. https://doi.org/10.1105/tpc.18.00750

Mayer, K.F.X., Martis, M., Hedley, P.E., Šimková, H., Liu, H., Morris, J.A., Steuernagel, B., Taudien, S., Roessner, S., Gundlach, H., Kubaláková, M., Suchánková, P., Murat, F., Felder, M., Nussbaumer, T., Graner, A., Salse, J., Endo, T., Sakai, H., Tanaka, T., Itoh, T., Sato, K., Platzer, M., Matsumoto, T., Scholz, U., Doležel, J., Waugh, R., Stein, N., 2011. Unlocking the Barley Genome by Chromosomal and Comparative Genomics. Plant Cell 23, 1249–1263. https://doi.org/10.1105/tpc.110.082537

Melamed-Bessudo, C., Shilo, S., Levy, A.A., 2016. Meiotic recombination and genome evolution in plants. Curr. Opin. Plant Biol. 30, 82–87. https://doi.org/10.1016/j.pbi.2016.02.003

Mézard, C., Tagliaro Jahns, M., Grelon, M., 2015. Where to cross? New insights into the location of meiotic crossovers. Trends Genet. 31, 393–401. https://doi.org/10.1016/j.tig.2015.03.008

Nachman, M.W., Payseur, B.A., 2012. Recombination rate variation and speciation: theoretical predictions and empirical results from rabbits and mice. Philos. Trans. R. Soc. B Biol. Sci. 367, 409–421. https://doi.org/10.1098/rstb.2011.0249

Nam, K., Ellegren, H., 2012. Recombination Drives Vertebrate Genome Contraction. PLoS Genet. 8, e1002680. https://doi.org/10.1371/journal.pgen.1002680

Osman, K., Algopishi, U., Higgins, J.D., Henderson, I.R., Edwards, K.J., Franklin, F.C.H., Sanchez-Moran, E., 2021. Distal Bias of Meiotic Crossovers in Hexaploid Bread Wheat Reflects Spatio-Temporal Asymmetry of the Meiotic Program. Front. Plant Sci. 12, 631323. https://doi.org/10.3389/fpls.2021.631323

Otto, S.P., 2009. The Evolutionary Enigma of Sex. Am. Nat. 174, S1–S14. https://doi.org/10.1086/599084

Otto, S.P., Payseur, B.A., 2019. Crossover Interference: Shedding Light on the Evolution of Recombination. Annu. Rev. Genet. 53, 19–44. https://doi.org/10.1146/annurev-genet-040119-093957

Pan, J., Sasaki, M., Kniewel, R., Murakami, H., Blitzblau, H.G., Tischfield, S.E., Zhu, X., Neale, M.J., Jasin, M., Socci, N.D., Hochwagen, A., Keeney, S., 2011. A Hierarchical Combination of Factors Shapes the Genome-wide Topography of Yeast Meiotic Recombination Initiation. Cell 144, 719–731. https://doi.org/10.1016/j.cell.2011.02.009

Pan, Q., Li, L., Yang, X., Tong, H., Xu, S., Li, Z., Li, W., Muehlbauer, G.J., Li, J., Yan, J., 2016. Genome-wide recombination dynamics are associated with phenotypic variation in maize. New Phytol. 210, 1083–1094. https://doi.org/10.1111/nph.13810

Pazhayam, N.M., Turcotte, C.A., Sekelsky, J., 2021. Meiotic Crossover Patterning. Front. Cell Dev. Biol. 9, 681123. https://doi.org/10.3389/fcell.2021.681123

Pellicer, J., Hidalgo, O., Dodsworth, S., Leitch, I., 2018. Genome Size Diversity and Its Impact on the Evolution of Land Plants. Genes 9, 88. https://doi.org/10.3390/genes9020088

R Core Team, 2019. R: A Language and Environment for Statistical Computing. R Foundation for Statistical Computing, Vienna, Austria.

Rezvoy, C., Charif, D., Gueguen, L., Marais, G.A.B., 2007. MareyMap: an R-based tool with graphical interface for estimating recombination rates. Bioinformatics 23, 2188–2189. https://doi.org/10.1093/bioinformatics/btm315

Ritz, K.R., Noor, M.A.F., Singh, N.D., 2017. Variation in Recombination Rate: Adaptive or Not? Trends Genet. 33, 364–374. https://doi.org/10.1016/j.tig.2017.03.003

Sandhu, D., Gill, K.S., 2002. Gene-Containing Regions of Wheat and the Other Grass Genomes. Plant Physiol. 128, 803–811. https://doi.org/10.1104/pp.010745

Sardell, J.M., Kirkpatrick, M., 2019. Sex differences in the recombination landscape. Am. Nat. 704943. https://doi.org/10.1086/704943

Shahin, A., Arens, P., Van Heusden, A.W., Van Der Linden, G., Van Kaauwen, M., Khan, N., Schouten, H.J., Van De Weg, W.E., Visser, R.G.F., Van Tuyl, J.M., 2011. Genetic mapping in *Lilium*: mapping of major genes and quantitative trait loci for several ornamental traits and disease resistances. Plant Breed. 130, 372–382. https://doi.org/10.1111/j.1439-0523.2010.01812.x

Shi, T., Rahmani, R.S., Gugger, P.F., Wang, M., Li, H., Zhang, Y., Li, Z., Wang, Q., Van de Peer, Y., Marchal, K., Chen, J., 2020. Distinct Expression and Methylation Patterns for Genes with Different Fates following a Single Whole-Genome Duplication in Flowering Plants. Mol. Biol. Evol. 37, 2394–2413. https://doi.org/10.1093/molbev/msaa105

Smith, S.A., Brown, J.W., 2018. Constructing a broadly inclusive seed plant phylogeny. Am. J. Bot. 105, 302–314. https://doi.org/10.1002/ajb2.1019

Soltis, P.S., Marchant, D.B., Van de Peer, Y., Soltis, D.E., 2015. Polyploidy and genome evolution in plants. Curr. Opin. Genet. Dev. 35, 119–125. https://doi.org/10.1016/j.gde.2015.11.003

Stapley, J., Feulner, P.G.D., Johnston, S.E., Santure, A.W., Smadja, C.M., 2017. Variation in recombination frequency and distribution across eukaryotes: patterns and processes. Philos. Trans. R. Soc. B Biol. Sci. 372, 20160455. https://doi.org/10.1098/rstb.2016.0455

Tiley, G.P., Burleigh, J.G., 2015. The relationship of recombination rate, genome structure, and patterns of molecular evolution across angiosperms. BMC Evol. Biol. 15, 194. https://doi.org/10.1186/s12862-015-0473-3

Underwood, C.J., Choi, K., 2019. Heterogeneous transposable elements as silencers, enhancers and targets of meiotic recombination. Chromosoma 128, 279–296. https://doi.org/10.1007/s00412-019-00718-4

Veller, C., Kleckner, N., Nowak, M.A., 2019. A rigorous measure of genome-wide genetic shuffling that takes into account crossover positions and Mendel’s second law. Proc. Natl. Acad. Sci. 116, 1659–1668. https://doi.org/10.1073/pnas.1817482116

Wang, S., Zickler, D., Kleckner, N., Zhang, L., 2015. Meiotic crossover patterns: Obligatory crossover, interference and homeostasis in a single process. Cell Cycle 14, 305–314. https://doi.org/10.4161/15384101.2014.991185

Wang, Y., Copenhaver, G.P., 2018. Meiotic Recombination: Mixing It Up in Plants. Annu. Rev. Plant Biol. 69, 577–609. https://doi.org/10.1146/annurev-arplant-042817-040431

Wei, C., Yang, H., Wang, S., Zhao, J., Liu, C., Gao, L., Xia, E., Lu, Y., Tai, Y., She, G., Sun, J., Cao, H., Tong, W., Gao, Q., Li, Y., Deng, W., Jiang, X., Wang, W., Chen, Q., Zhang, S., Li, H., Wu, J., Wang, P., Li, P., Shi, C., Zheng, F., Jian, J., Huang, B., Shan, D., Shi, M., Fang, C., Yue, Y., Li, F., Li, D., Wei, S., Han, B., Jiang, C., Yin, Y., Xia, T., Zhang, Z., Bennetzen, J.L., Zhao, S., Wan, X., 2018. Draft genome sequence of *Camellia sinensis* var. *sinensis* provides insights into the evolution of the tea genome and tea quality. Proc. Natl. Acad. Sci. 115, E4151–E4158. https://doi.org/10.1073/pnas.1719622115

White, I.M.S., Hill, W.G., 2020. Effect of heterogeneity in recombination rate on variation in realised relationship. Heredity 124, 28–36. https://doi.org/10.1038/s41437-019-0241-z

Yelina, N.E., Choi, K., Chelysheva, L., Macaulay, M., de Snoo, B., Wijnker, E., Miller, N., Drouaud, J., Grelon, M., Copenhaver, G.P., Mezard, C., Kelly, K.A., Henderson, I.R., 2012. Epigenetic Remodeling of Meiotic Crossover Frequency in *Arabidopsis thaliana* DNA Methyltransferase Mutants. PLoS Genet. 8, e1002844. https://doi.org/10.1371/journal.pgen.1002844

Zelkowski, M., Olson, M.A., Wang, M., Pawlowski, W., 2019. Diversity and Determinants of Meiotic Recombination Landscapes. Trends Genet. 35, 359–370. https://doi.org/10.1016/j.tig.2019.02.002

Zhang, L., Liang, Z., Hutchinson, J., Kleckner, N., 2014. Crossover Patterning by the Beam-Film Model: Analysis and Implications. PLoS Genet. 10, e1004042. https://doi.org/10.1371/journal.pgen.1004042

Zickler, D., Kleckner, N., 2015. Recombination, Pairing, and Synapsis of Homologs during Meiosis. Cold Spring Harb. Perspect. Biol. 7, a016626. https://doi.org/10.1101/cshperspect.a016626

Supporting information

Fig S1. Markers positions in genetic distance (cM) as a function of genomic distance (Mb), namely Mary maps, for each chromosome included in the dataset (n = 665 chromosomes). The black vertical line is the centromere position estimated by cytological measures, when available in the literature.

Fig S2. Recombination landscapes for each chromosome included in the dataset (n = 665 chromosomes). Recombination rate (cM/Mb) estimated in windows of 100kb along genomic distances (Mb). Confidence interval at 95% (grey ribbon) estimated by 1,000 bootstraps of loci. The black vertical line is the centromere position estimated by cytological measures, when available in the literature.

Fig S3. Phylogenetic tree of species in our dataset (n = 57), annotated with mean recombination rate (cM/Mb) and mean chromosome size (Mb). The supertree was retrieved from the publicly available phylogeny constructed by Smith and Brown (Smith & Brown, 2018).

Fig S4. Slopes of the linear regression within species (linkage map length ~ chromosome size) as a function of the species mean genomic chromosome size (Mb).

Fig S5. The negative correlation (Spearman’s Rho coefficient) between recombination rates (cM/Mb) and the distance to the nearest telomere is stronger for species with a larger chromosome size (n = 57). The linear regression line and its parametric 95% confidence interval were estimated in ggplot2. The inset presents the distribution of Spearman’s Rho coefficients for chromosomes (n = 665 chromosomes). The mean correlation and its 95% confidence interval (black solid and dashed lines) were estimated by 1,000 bootstraps. The red vertical line is for a null correlation.

Fig S6. Standardized recombination rate (cM/Mb) as a function of the relative distance (Mb) from the telomere along the chromosome (physical distances expressed in 20 bins).

Chromosomes were split in halves, a relative distance of 0.5 being the centre of the chromosome, and only one side was randomly sampled to avoid averaging patterns. Then, chromosomes were pooled per species. Each colour is a species. A loess regression was estimated for each species. Species presented in four plots for clarity.

Fig S7. Standardized gene count as a function of the relative distance (Mb) from the telomere along the chromosome (physical distances expressed in 20 bins). Chromosomes were split in halves, a relative distance of 0.5 being the centre of the chromosome, and only one side was randomly sampled to avoid averaging patterns. Then, chromosomes were pooled per species. Each colour is a species. A loess regression was estimated for each species. Species presented in four plots for clarity.

Fig S8. The genetic shuffling $\bar{r}_{intra}$ increases with the size of the genetic map (cM). Linear mixed regression with a species random effect and its 95% confidence interval estimated by ggplot2 (black line and grey ribbon). Each colour is a species. A linear regression was estimated for each species.

Fig S9. The genetic shuffling $\bar{r}_{intra}$ decreases with the periphery-bias ratio. Linear mixed regression with a species random effect and its 95% confidence interval estimated by ggplot2 (black line and grey ribbon). Each colour is a species. A linear regression was estimated for each species.

FigS10. Gene count in windows of 100kb along genomic distances (Mb) for each chromosome with gene annotations (n = 480 chromosomes). Recombination rate (cM/Mb) estimated in windows of 100kb . Loess regression of gene count along the chromosome in blue line with parametric confidence interval at 95% in grey.

Fig S11. Marey maps with genomic distances (black points) and gene distances (gray points). Markers positions in genetic distance (cM) as a function of the relative physical distance (either Mb of cumulative number of genes) for each chromosome with gene annotations (n = 480 chromosomes). The black dashed line is a theoretical uniform

distribution of markers. The black vertical line is the centromere position estimated by cytological measures, when available in the literature.

Fig S12. Sensitivity of the periphery-bias ratio to the size of the sampled distal region (i.e. number of bins sampled at the tips). The periphery-bias ratio was estimated for different numbers of bins sampled and always divided by the mean chromosomal recombination rate. Linear regression (black line) shows a decrease of the periphery-bias ratio as the number of bins increases, towards a ratio value of 1 (dashed line).

Table S1. Metadata for 665 recombination landscapes, with name of the dataset collected and literal name of the chromosome used in our study, chromosome name in annotation (gff), size of the genetic map (cM, raw and corrected by methods of Chakravarti et al. (1991) or Hal & Willis (2005)), size of the genomic sequence in genome assembly (Mb), number of markers, density of markers in cM and bp, mean interval between markers in cM and bp, span parameter of the loess function, type of mapping function (Haldane, Kosambi or none), accession of the reference genome used for markers genomic positions, link to data repository and doi reference of the study in which the genetic map was published.

Table S2. Flowering plant species included in the study, with authors, year and doi reference of the genetic map publication, and accession of the reference genome.

Table S3. Centromeric indexes estimated in cytological studies, with unit of measurement, mean and standard error of long and short chromosome arms, centromeric index (ratio of short arm length divided by total chromosome length), and doi reference to the original study.

Table S4. Correlation between recombination landscapes estimated at two different genomic scales (1Mb and 100kb). Spearman's Rho coefficient was estimated for each chromosome between recombination rates estimated directly in windows of 1Mb and the mean recombination rate of 100kb windows pooled together in 1Mb windows. Mean of the Spearman's Rho coefficient among chromosomes and proportion of significant p-values given for each species.

Table S5. Selection of the regression model between LM, LMER and PGLMM which explains best the relationship between the mean recombination rate (cM/Mb) and the chromosome size (Mb), based on AIC and BIC criteria.

Table S6. Species averaged correlation between the averaged chromosome size (Mb) and the averaged periphery-bias ratio. Mean of the Spearman's Rho coefficient among

correlations at chromosome scale and proportion of significant p-values given for each species.

Table S7. Chromosome correlation between the recombination rate (cM/Mb) and the relative distance to the telomere, with Spearman's Rho coefficient and p-value of the test per chromosome.

Table S8. Species averaged correlation between the recombination rate (cM/Mb) and the relative distance to the telomere. Mean of the Spearman's Rho coefficient among correlations at chromosome scale and proportion of significant p-values given for each species.

Table S9. Selection of the best model of crossover distribution for each species, based on Adjusted R-Squared between observed values and theoretical values predicted by the model. The best model selected for each species is the one maximizing the Adjusted R-Squared.

Table S10. Selection of the best model of crossover distribution for each species in a subset of chromosomes with at least 50cM on each chromosome arm, based on Adjusted R-Squared between observed values and theoretical values predicted by model. The best model selected for each species is the one maximizing the Adjusted R-Squared.

Table S11. Convergence between crossover patterns and gene patterns at a species scale. For each species is given the type of crossover pattern, the type of gene count pattern, the difference RMSE(gene pattern) - RMSE(crossover pattern) which indicates how gene patterns are more/less homogeneous than crossover patterns, the homogenization effect of gene patterns (more/less), the difference genetic shuffling(gene pattern) - genetic shuffling(crossover pattern) and the averaged chromosome size (Mb).

Data S1. References for linkage map data included in this study.

Figure

Click here to access/download;Figure;Fig1.tif

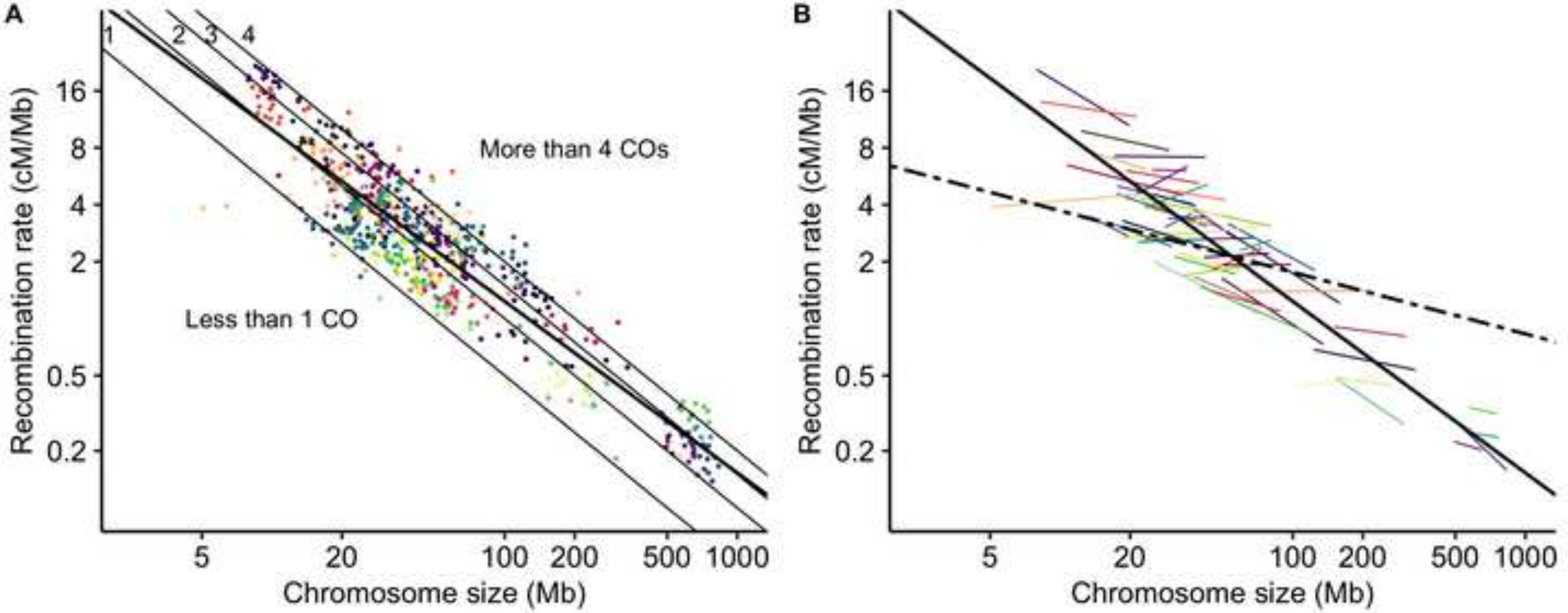

Figure

Click here to access/download;Figure;Fig2.tif

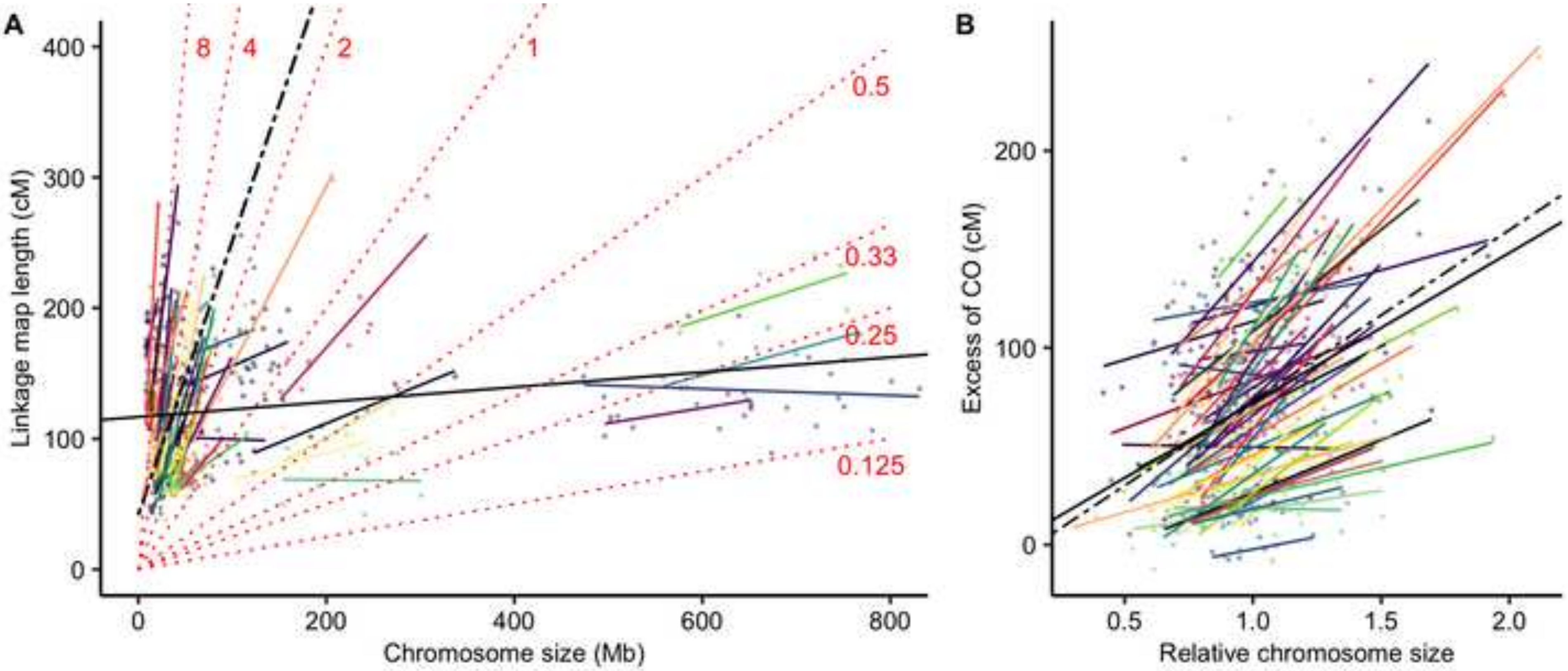

Figure

Click here to access/download;Figure;Fig3.tif

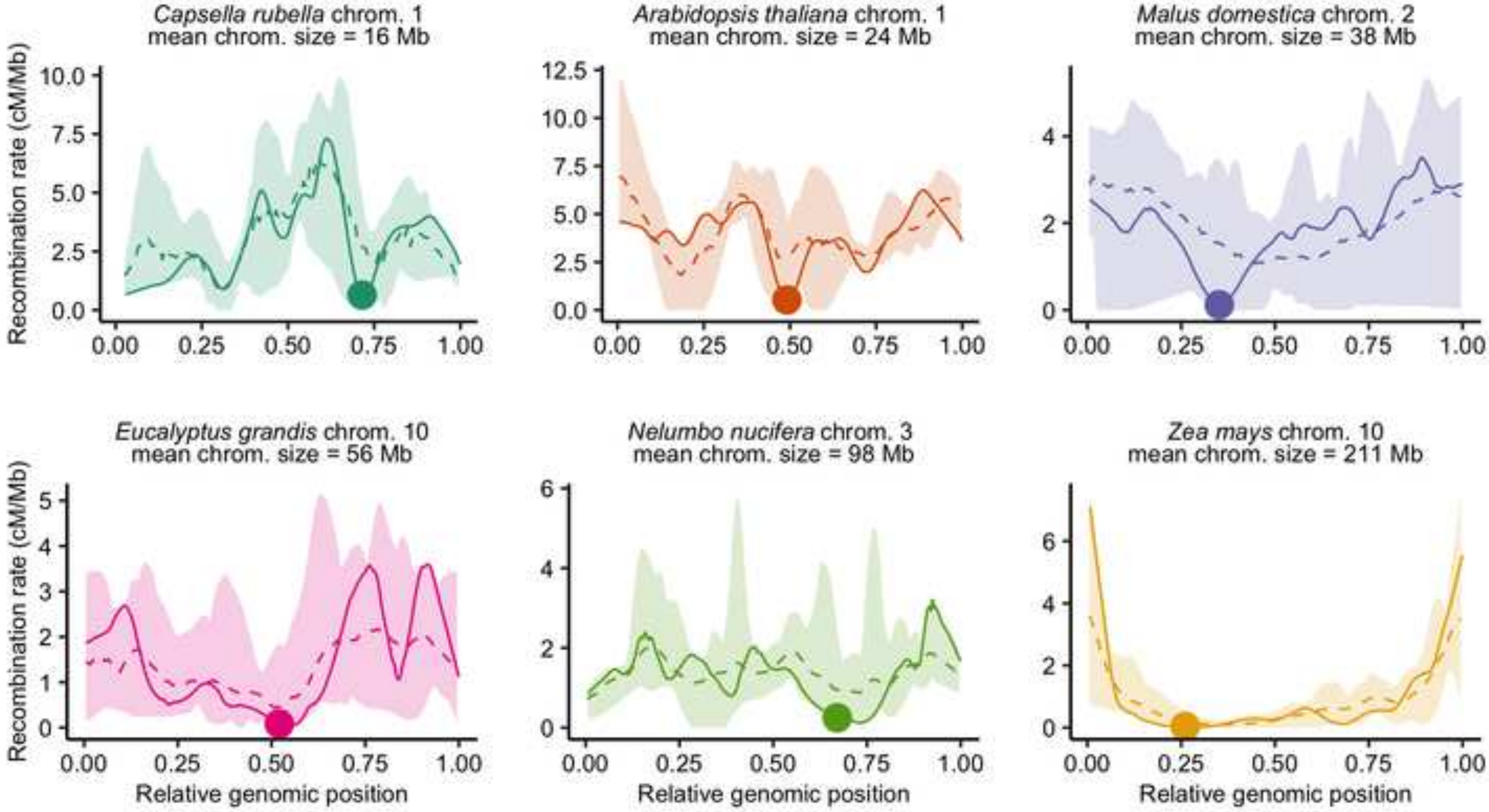

Figure

Click here to access/download;Figure;Fig4.tif

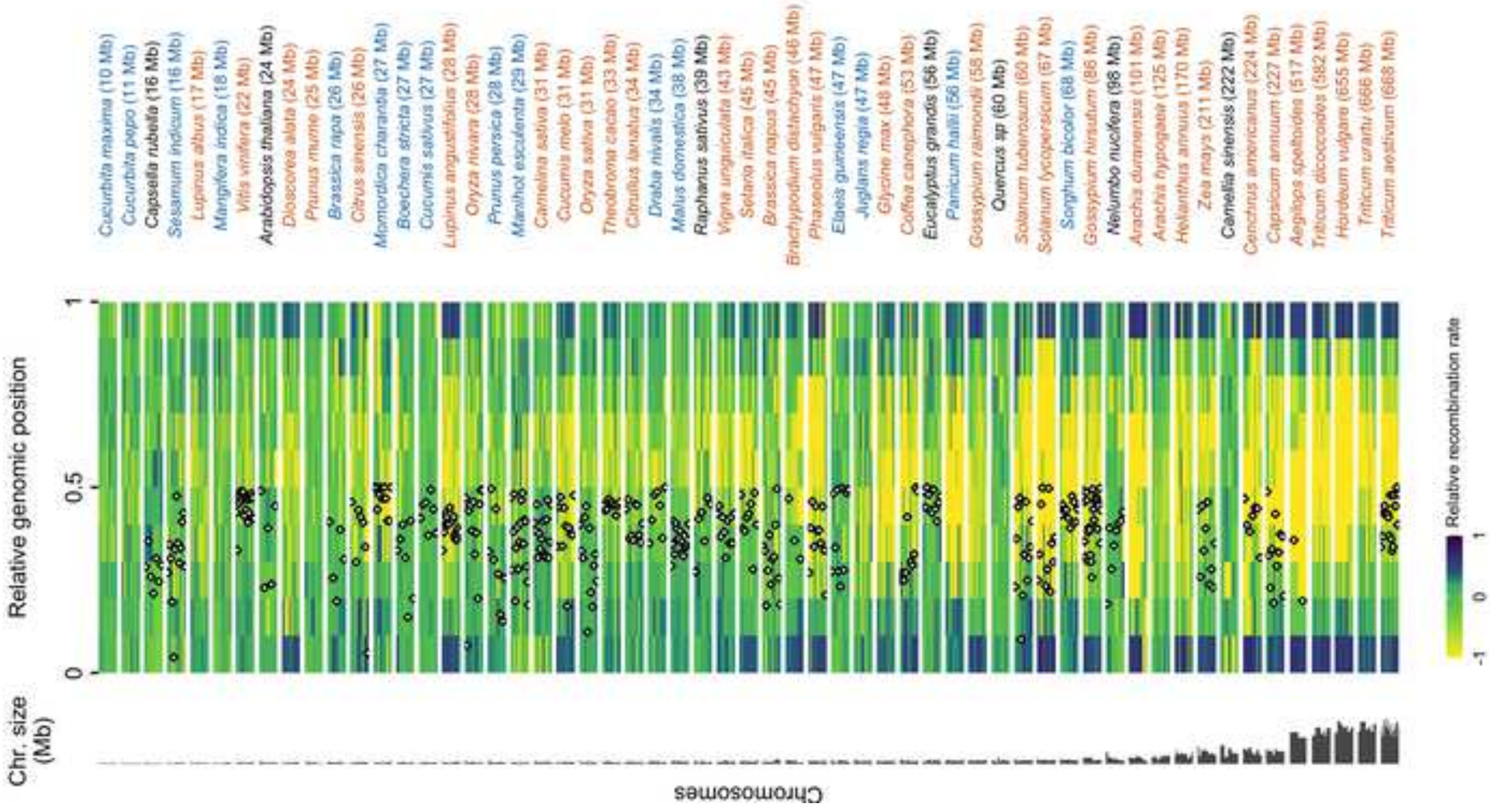

Figure

Click here to access/download;Figure;Fig5.tif

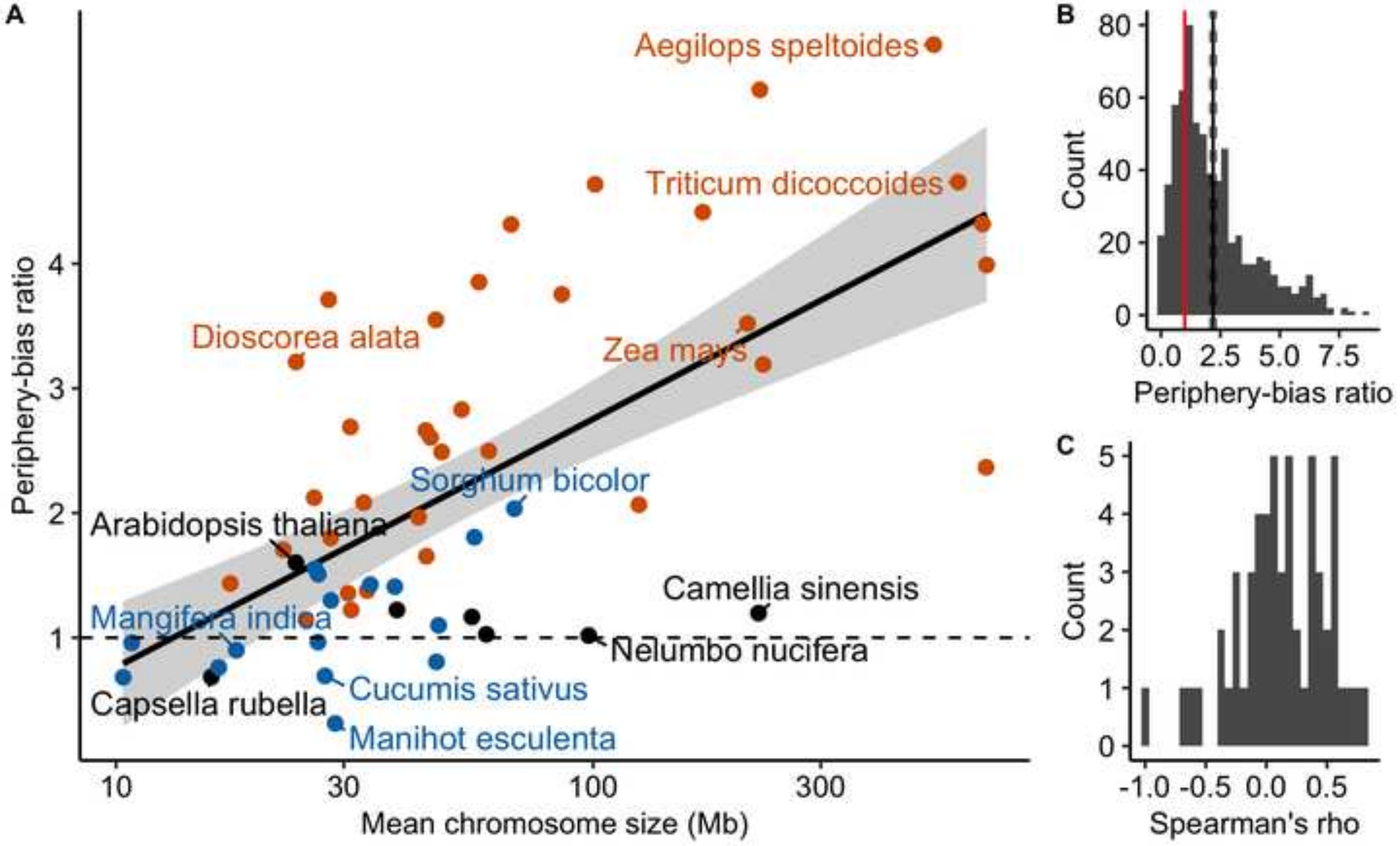

Click here to access/download;Figure;Fig6.tif

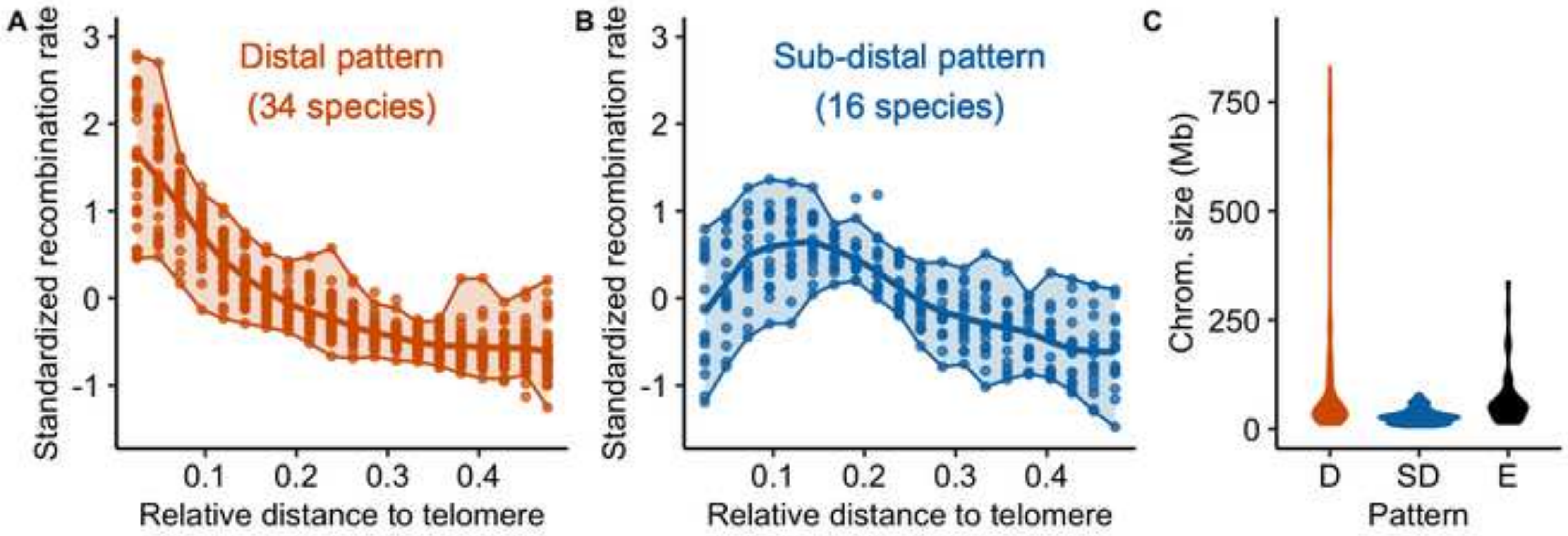

Figure Click here to access/download;Figure;Fig7.tif

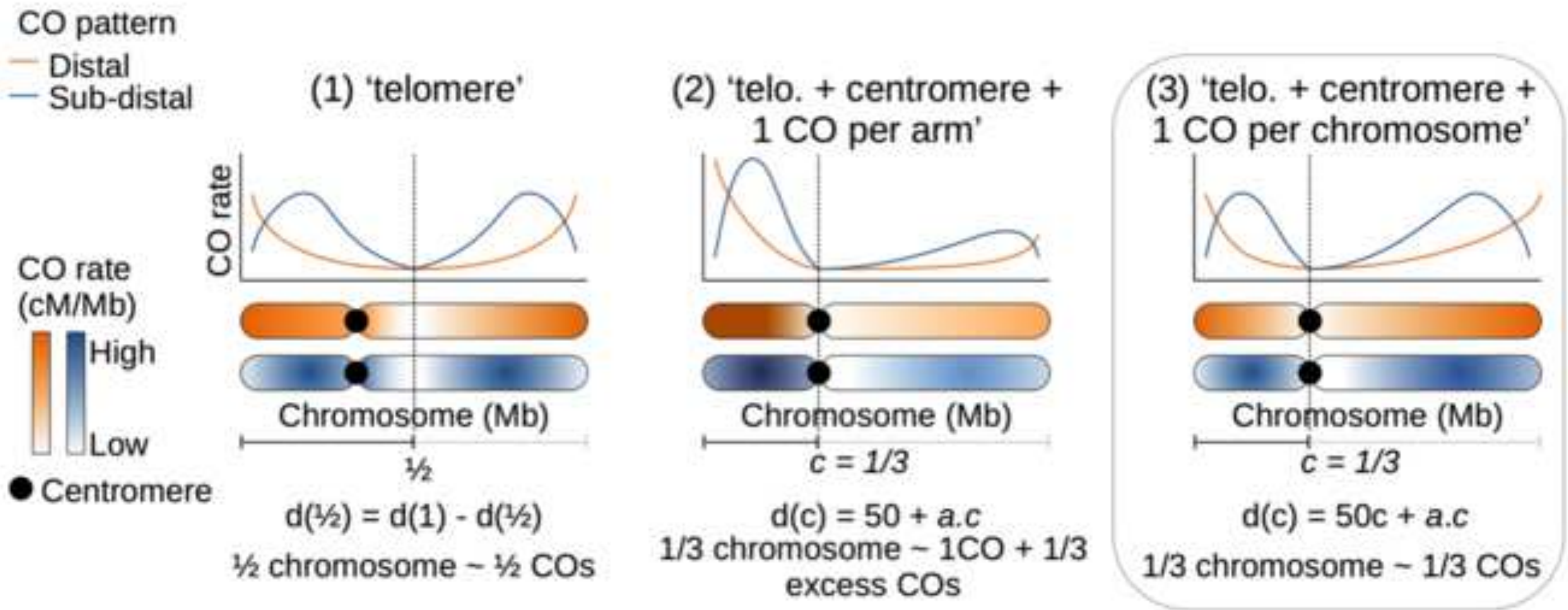

 Click here to access/download;Figure;Fig8.tif

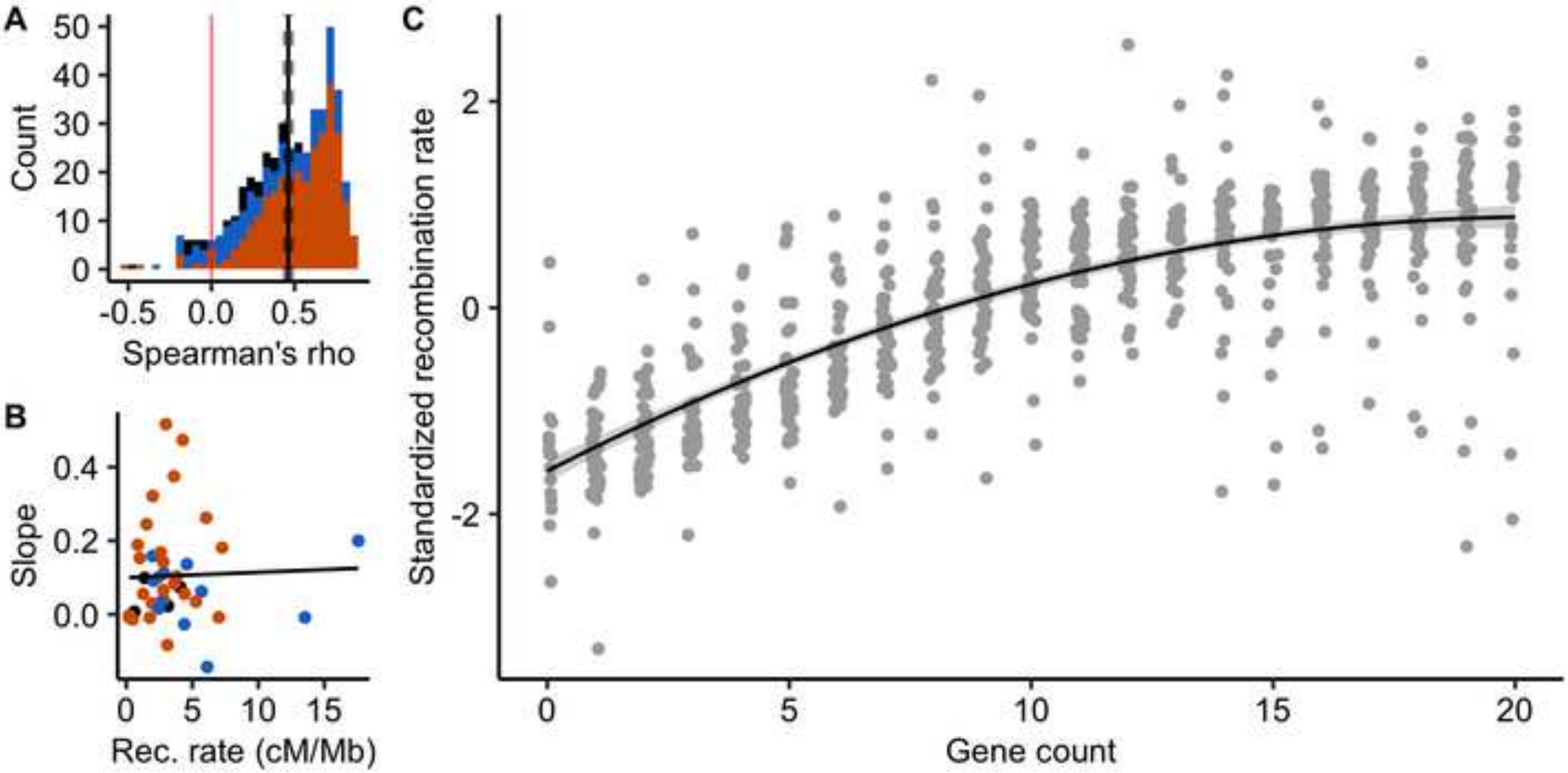

Figure

Click here to access/download;Figure;Fig9.tif

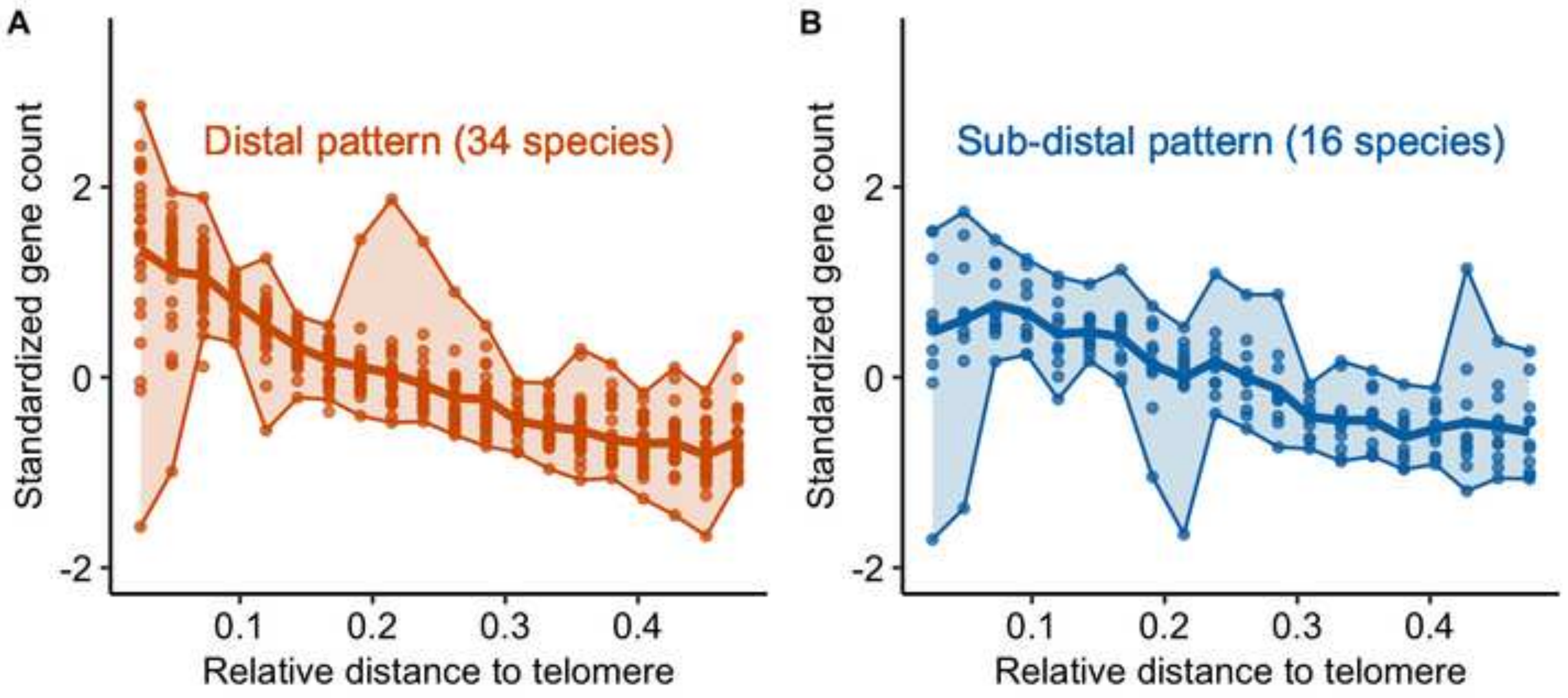

Figure
Click here to access/download;Figure;Fig10.tif

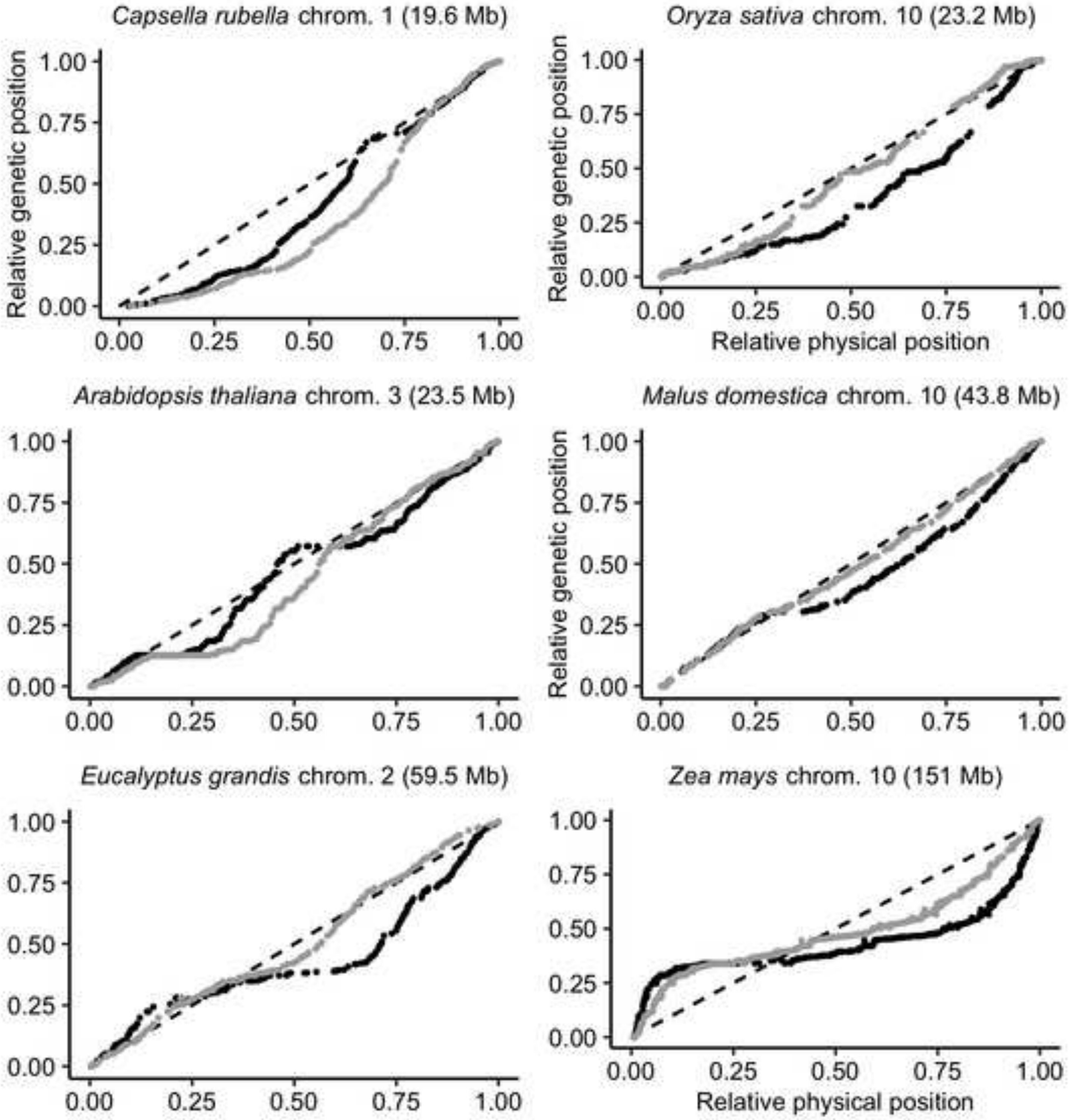

Figure

Click here to access/download;Figure;Fig11.tif

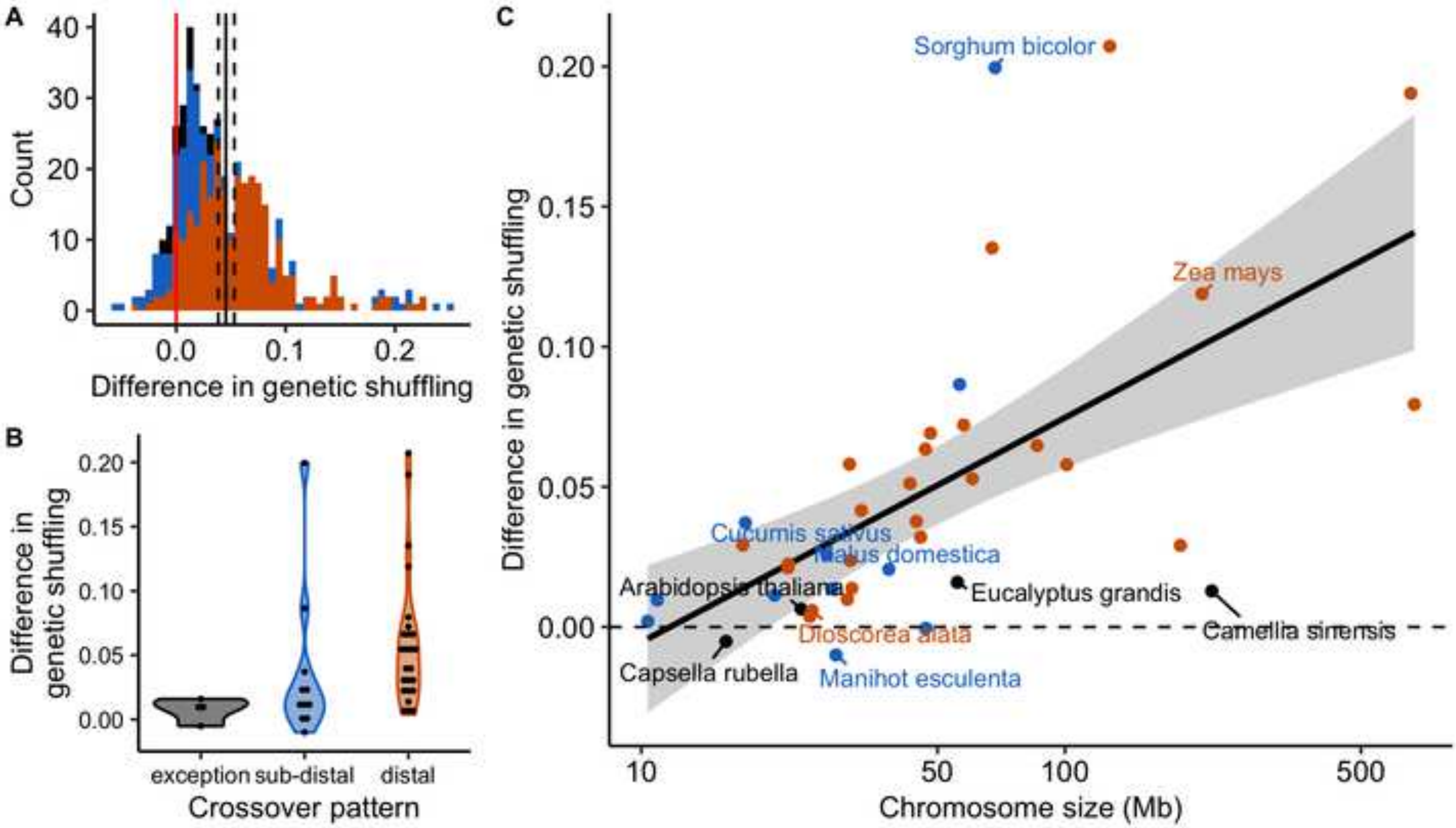

Figure

Click here to access/download
Supporting Information
FigS1.pdf

Figure

Click here to access/download
Supporting Information
FigS2.pdf

Figure

Click here to access/download
Supporting Information
FigS3.tif

Figure

Click here to access/download
Supporting Information
FigS4.tif

Figure

Click here to access/download
Supporting Information
FigS5.tif

Figure

Click here to access/download
Supporting Information
FigS6.tif

Figure

Click here to access/download
Supporting Information
FigS7.tif

Figure

Click here to access/download
Supporting Information
FigS8.tif

Figure

Click here to access/download
Supporting Information
FigS9.tif

Figure

Click here to access/download
Supporting Information
FigS10.pdf

Figure

Click here to access/download

Supporting Information

FigS11.pdf

Figure

Click here to access/download
Supporting Information
FigS12.tif

Click here to access/download
Supporting Information
Table S1.xlsx

Click here to access/download
Supporting Information
Table S2.xlsx

Click here to access/download
Supporting Information
Table S3.xlsx

Click here to access/download
Supporting Information
Table S4.xlsx

Click here to access/download
Supporting Information
Table S5.xlsx

Click here to access/download
Supporting Information
Table S6.xlsx

Click here to access/download
Supporting Information
Table S7.xlsx

Click here to access/download
Supporting Information
Table S8.xlsx

Click here to access/download
Supporting Information
Table S9.xlsx

Click here to access/download
Supporting Information
Table S10.xlsx

Click here to access/download
Supporting Information
Table S11.xlsx

Click here to access/download
Supporting Information
Data S1.pdf

Diversity and determinants of recombination landscapes in flowering plants

Short title: Recombination landscapes in angiosperms

Authors: Thomas Brazier[1], Sylvain Glémin[1, 2*]

[1] University of Rennes, CNRS, ECOBIO (Ecosystems, Biodiversity, Evolution) - UMR 6553, Rennes, France

[2] Department of Ecology and Genetics, Evolutionary Biology Center and Science for Life Laboratory, Uppsala University, Uppsala, Sweden

* Corresponding author.

Present address: Sylvain Glémin, University of Rennes, CNRS, ECOBIO (Ecosystems, Biodiversity, Evolution) - UMR 6553, Rennes, France; email: sylvain.glemin@univ-rennes1.fr

ORCIDs:

Brazier: https://orcid.org/0000-0001-5990-7545

Glémin: https://orcid.org/0000-0001-7260-4573

Formatted: English (United States)

Formatted: Normal (Web)

Formatted: English (United States)

Formatted: Normal (Web)

Abstract

During meiosis, crossover rates are not randomly distributed along the chromosome and ~~therefore they locally influence the creation of novel genotypes and the efficacy of selection~~their location may have a st~~o~~ron~~nr~~g impact on the functioning and evolution of the genome. To date, the broad diversity of recombination landscapes among plants has rarely been investigated and~~,~~ a~~undermining the overall understanding of the constraints driving the evolution of crossover frequency and distribution.~~ ~~A~~ formal comparative genomic approach is still needed to characterized and assess ~~could be valuable to assess~~ t~~T~~he determinants ~~that shape the local crossover rate and the diversity of the resulting~~of recombination landscapes among species and chromosomes~~ still need to be assessed in a formal comparative genomic approach~~. We gathered genetic maps and genomes for 57 flowering plant species, corresponding to 665 chromosomes, for which we estimated large-scale recombination landscapes. We found that the number of crossing-over per chromosome spans a limited range (between one to five/six) whatever the genome size, and that there is no single relationship across species between genetic map length and ~~Chromosome~~ chromosome size. Instead, we found a general relationship between the relative size of chromosomes and recombination rate, while the absolute length ~~drives~~ constrains the basal recombination rate for each species~~, but though within species we were intrigued to notice that the chromosome-wide recombination rate is was proportional to the relative size of the chromosome~~. ~~Moreover, for larger chromosomes, crossovers tend to accumulate occur at the ends of the chromosome leaving the central regions as recombination-free regions.~~At the chromosome level, we identified two main patterns (with a few exceptions) and~~ Based on identified crossover patterns and testable predictions,~~ we proposed a conceptual model explaining the broad-scale distribution of crossovers where both telomeres and centromeres ~~are important~~play a role. ~~Finally, we qualitatively identified two recurrent crossover patterns among species and highlighted that t~~These patterns globally correspond to the underlying gene distribution, which affects how efficiently genes are shuffled at meiosis. ~~In addition to~~

the positive correlation between recombination and gene density, we argue that crossover patterns are essential for the efficiency of chromosomal genetic shuffling, even though the ultimate evolutionary potential forged by the diversity of recombination landscapes remains an open question.These results raised new questions not only on the evolution of recombination rates but also on their distribution along chromosomes.

KEYWORDS: meiotic recombination, crossover pattern, Marey map, genetic shuffling, comparative genomics

Author summary

Meiotic recombination is a universal feature of sexually reproducing species. During meiosis, crossing-overs play a fundamental role for the proper segregation of chromosomes during meiosis and for reshuffling alleles among between chromosomes, which increases genetic diversity and the adaptive potential of a species. How much variation in recombination is expected within a genome and among different species remains a central question to understand the evolution of recombination. We characterized and compared recombination landscapes in a large set of plant species that represent a wide range of genomic characteristics. We found that the number of crossing-overs varied little among species, from one mandatory to no more than five or six crossing-overs per chromosomes, whatever the genome size. However, recombination can strongly vary within a genome and we identified two main patterns of variation along chromosomes (with a few exceptions) that can be explained by a new conceptual model where chromosome length, chromosome structure and gene density play a role. The strong association between gene density and recombination raised new questions not only on the evolution of recombination rates but also on their distribution along chromosomes.

Formatted: Indent: First line: 0", Line spacing: Double

Introduction

Meiotic r~~R~~ecombination is a universal feature of sexually reproducing species. Through crossovers,~~that participates to the production of~~ new haplotypes are passed on to offspring by the reciprocal exchange of DNA between maternal and paternal chromosomes~~ during meiosis~~. However, recombination landscapes — the variation in recombination rate along the chromosome — are not homogeneous ~~along the chromosome~~across the genome and vary among species (de Massy, 2013; Haenel et al., 2018; Mézard et al., 2015; Stapley et al., 2017). Meiotic recombination plays a fundamental functional role by forming chiasmata at specific pairing sites between homologous chromosomes to ensure the physical tension needed for the proper disjunction of homologs (de Massy, 2013; Mézard et al., 2015; Zickler and Kleckner, 2015). Recombination also plays an evolutionary role by breaking ~~the~~ linkage disequilibrium between neighbouring sites and creating new genetic combinations transmitted to the next generation (i.e. genetic shuffling), ~~- upon which~~making selection ~~can act~~ more efficient~~ly~~ ~~-~~(Barton, 1995; Charlesworth and Jensen, 2021; Otto, 2009). ~~However,~~ ~~t~~The number and location of crossovers (COs) along the chromosome are finely regulated through mechanisms of crossover assurance, interference and homeostasis (Otto and Payseur, 2019; Pazhayam et al., 2021). In most species, crossover assurance is necessary to achieve proper segregation and to avoid deleterious consequences of nondisjunction, though it is not very clear if it is at least~~at least~~ one CO per chromosome or per arm that is mandatory~~ to achieve proper segregation and to avoid deleterious consequences of nondisjunction~~. Additional COs are also usually regulated through interference, ensuring that they are not too numerous and not too close to each other (Pazhayam et al., 2021; Wang et al., 2015). In addition to regulation on a large scale (Cooper et al., 2016; Zelkowski et al., 2019), recombination is also finely tuned on a small scale. Generally, crossovers are concentrated in very short genomic regions (typically a few kb), i.e. recombination hotspots. In plants studied so far, CO hotspots ~~are usually~~have been found in gene regulatory sequences, and mostly in promoters (Choi et al., 2018; He et al., 2017; Marand et al., 2019).

In addition to meiosis functioning, variations in recombination rates have a strong impact on genome structure, functioning and evolution (Gaut et al., 2007; Haenel et al., 2018; Stapley et al., 2017; Tiley and Burleigh, 2015) and it ~~. For example, recombination landscapes are thought to shape genetic diversity and the distribution of transposable elements (TEs) along chromosomes, through the indirect effect of recombination in modulating the extent of linked selection~~ and the accumulation of TEs in regions of low recombination ~~(Corbett-Detig et al., 2015; Kent et al., 2017). Recombination can also shape nucleotide landscapes through the effect of GC-biased gene conversion (Galtier et al., 2018; Glémin et al., 2014). As a consequence, it~~ has become a key challenge to integrate recombination rate variation in population genomics in the age of ‘genomic landscapes’ (Booker et al., 2020; Comeron, 2017). The characterization of recombination landscapes also has practical interests ~~since it is likely that~~ as variation in meiotic genes cou~~b~~ld be used to~~changes in CO patterns is to be~~ experimentally manipulate~~d~~ CO patterns ~~–~~for ~~an~~ advantageous purpos~~ese~~, such as redirecting recombination towards regions of interest for crop breeding~~creating specific genetic combinations or directly influencing genetic diversity and the adaptive potential of a species~~ (Kuo et al., 2021).

In plants, ~~contrary to other eukaryotes,~~ recombination rates are supposed to be higher in smaller genomes because the linkage map length is independent of genome size and the number of chromosomes explain more variation than genome size (Stapley et al., 2017). Several broad-scale determinants have recently been identified, such as chromosome length (Tiley and Burleigh, 2015), distance to the telomere or centromere (Blitzblau et al., 2007) and genomic and epigenetic features (Apuli et al., 2020; Marand et al., 2019; Yelina et al., 2012).

~~To date, the diversity of recombination landscapes in plants has not been properly quantified; it is often limited to genome-wide recombination rates (Stapley et al., 2017), even though it could be used as a lever to identify the major determinants shaping crossover patterns across species (Gaut et al., 2007). Recently, a meta-analysis explored large-scale~~

recombination landscapes among eukaryotes and concluded that chromosome length plays a major role in crossover patterns, but this analysis only included a limited number of plant species (Haenel et al., 2018). As plant genomes are highly diverse in many ways (Pellicer et al., 2018; Soltis et al., 2015), the expected diversity in recombination landscapes has been overlooked (Gaut et al., 2007). Plant genomes also contain large regions with suppressed recombination in various proportions (from a few Mb to hundreds of Mb, 1 to 75 % of the genome), which impact affect genomic averages (Gaut et al., 2007; Haenel et al., 2018)), and it seems that the physical location of COs along the chromosome is species-specific (Wang and Copenhaver, 2018). However, despite these recent advances, the diversity of recombination landscapes in plants still remain to be properly quantified. Nonetheless, several broad-scale determinants have recently been identified, such as chromosome length (Haenel et al., 2018; Tiley and Burleigh, 2015), distance to the telomere or centromere (Blitzblau et al., 2007; Haenel et al., 2018) and genomic and epigenetic features (Apuli et al., 2020; Marand et al., 2019; Yelina et al., 2012). In plants, contrary to other eukaryotes, recombination rates are supposed to be higher in smaller genomes because the linkage map length is independent of genome size and the number of chromosomes explain more variation than genome size (Stapley et al., 2017).

Recently, a meta-analysis explored large-scale recombination landscapes among eukaryotes and paved the way for identifying general patterns (Haenel et al., 2018). They found that larger chromosomes have low crossover rates in their centre and suggested a simple telomere-led model with a universal bias of COs towards the periphery of the chromosome, positively driven by chromosome length. They also proposed that chromosome length played the main role in crossover patterning while position of the centromere had almost no effect (except a local one). Alternatively, it has also been proposed that both telomeres and centromeres shape recombination landscapes (Wang and Copenhaver, 2018) and the universality of a universal pattern among plants has been questioned (Zelkowski et al., 2019). So far, the number of studied species remained limited

and, as plant genomes are highly diverse in many ways (Pellicer et al., 2018; Soltis et al., 2015), the expected diversity in recombination landscapes may have been overlooked (Gaut et al., 2007). In addition, previous studies where meta-analyses combining heterogeneous datasets (ex: mix of inferred data from graphics, final processed data and only a few raw datasets in Haenel et al. 2018) without a standard way to infer recombination maps, which prevented detailed comparison among many species. and tThe existence of a major broad-scale determinant of CO distribution and frequency, such as chromosome size, needs to be tested. Haenel et al. (2018) found that larger chromosomes have low crossover rates in their centre and suggested a simple telomere-led model with a universal bias of COs towards the periphery of the chromosome, positively driven by chromosome length; this new conceptual model still needs to be tested on data. However, there is little evidence supporting a universal pattern among plants (Zelkowski et al., 2019); and Iit has been proposed that both telomeres and centromeres shape recombination landscapes, although this is not yet fully understood (Wang and Copenhaver, 2018).

To overcome these limitations Since recombination hotspots are supposedly found in gene regulatory sequences, gene density could also be a universal driver of recombination rates among plant species, leading to the emergence of crossover patternsbut this still needs to be tested.

By combining genetic mapping from pedigree data and genome assembly up to the chromosome scale, we have gathered, to the best of our knowledge, the largest recombination landscape dataset in flowering plants. We started from raw data by combining genetic mapping from pedigree data and genome assembly up to the chromosome scale, from which we estimated recombination maps – more precisely the sex-averaged rate of COs along chromosomes – using the same standardiszed method. More precisely, we have estimated the sex-averaged rate of COs along chromosomes. Thanks to this dataset we addressed the following questions. What is the range of COs per chromosome observed in plants? Is the distribution of COs shaped by genome structure (i.e. chromosome size,

telomeres, centromeres) and if so is there a universal pattern? Since recombination hotspots have been found in gene regulatory sequences so far, are recombination landscapes generally associated with gene density? What are the consequences of recombination heterogeneity on the extent of genetic shuffling? Overall, we found that recombination landscapes in plants are more diverse and more complex than initially thought. We tested the relationship between recombination and chromosome length and assessed if the distribution of COs could be shaped by genome structure (i.e. chromosome size, telomeres, centromeres) or genome features (i.e. gene density). We identified two main patterns of recombination that are parallel to, and which may emerge from, the gene density distribution. We showed . Finally, we discussed the possible evolutionary implications of the heterogeneity of recombination landscapes by quantifying how CO patterns affect the extent of genetic shuffling. We assessed that COs patterns were improvingthat this globally improves the genetic shuffling of coding regions, which raises new questions on about the evolution of recombination.

Formatted: English (United States)

Results

Dataset and recombination maps

We retrieved publicly available data for sex-averaged linkage maps and genome assemblies, to obtain genetic map distances and physical distances. We selected linkage maps for which the markers had genomic positions on a chromosome-level genome assembly (except for *Capsella rubella*, which had a high-quality scaffold-level assembly, i.e. of pseudo-chromosomes). We remapped markers on the reference genome for 14 species for which genomic positions were not known or were mapped to an older assembly. After making a selection based on the number of markers, marker density, and genome coverage,

and after filtering out the outlying markers (see methods), ~~we adopted a qualitative visual validation. Recombination landscapes with large confidence intervals were discarded. In the end, ~~we ~~retained ~~produced 665 chromosome-scale Marey maps (plot of the genetic vs genomic distance, cM vs Mb) for 57 species (2-26 chromosomes per species,~~),~~ ~~from which we successfully inferred recombination landscapes (~~Table S1, S2, Fig~~ures~~ S1, S2). ~~After removing the outliers, t~~The number of markers per chromosome ~~map ~~ranged from 31 to 49,483, with a mean of 956 markers. Corrected linkage map length (Hall & Willis's method) did not~~n't~~ change the total linkage map length (mean difference = 1.19 cM, max difference = 5.62 cM), giving confidence in the coverage of the linkage map (Hall & Willis, 2005)~~ per map~~. We ~~used a linear regression on the models' residuals to verify~~verified that neither the number of markers, marker density nor the number of progenies had a significant effect on the analyses. We also retrieved gene annotations for 41 genomes. The angiosperm phylogeny was well represented in our sampling (~~Figure~~Fig S3), with a basal angiosperm species (*Nelumbo nucifera*), 15 monocot species and 41 eudicots. From literature, w~~W~~e also ~~searched ~~obtained ~~the literature for ~~data on the centromeric index for 37 species, defined as the ratio of the short arm length divided by the total chromosome length (Table S3).

From the Marey maps, we estimated local recombination rates along the chromosomes on non-overlapping 100 kb windows with a 95% confidence interval (1,000 bootstraps). Estimates at a scale of 1 Mb yielded very similar results (the Spearman rank correlation coefficient correlation between the ~~values for 1 Mb windows and those for the 100 kb windows within them~~two estimates was~~correlation between 1 Mb windows and 100 kb windows pooled in 1 Mb windows, Spearman rank correlation coefficient~~ Rho = 0.99, p < 0.001, Table S4) therefore only 100 kb landscapes were analysed in the subsequent analyses.

Smaller chromosomes ~~recombine ~~have higher recombination rates~~more often~~ than larger ones

Formatted: Font: 11 pt, Font color: Auto, English (United Kingdom)

Formatted: Font: 11 pt, Font color: Auto, English (United Kingdom)

Formatted: Font: 11 pt, Font color: Auto, English (United Kingdom)

~~Our results are i~~In agreement with previous studies ~~showing that smaller chromosomes have a higher recombination rate per Mb than larger ones~~ (Haenel et al., 2018; Stapley et al., 2017), ~~and our sampling suggests a~~w~~ consistent pattern across species (Figure 1A). W~~e found a significant negative correlation between chromosome size (Mb) and the mean chromosomal recombination rate (Spearman rank correlation coefficient Rho = -0.84, p < 0.001; log-log Linear Model, adjusted R^2 = 0.83, p < 0.001). For most species, there were between one and four COs per chromosome~~, which suggests that the number of COs per chromosome remains stable across species~~ even though the genome sizes span almost two orders of magnitude. Less than 2% of chromosomes had less than one CO (n = 11), 234 chromosomes had between one and two COs, suggesting that a single CO per chromosome is sufficient, though 419 chromosomes had more than two COs.

Using a Linear Mixed Model~~ (LMER)~~ we found a significant species random effect ~~for both the intercept and the slopes~~ (~~the best model was log-log LMER~~ log_{10}(recombination rate) ~ log_{10}(chromosome size) + (1 | species), marginal R^2 = 0.17, conditional R^2 = 0.96, p < 0.001). ~~;~~ ~~the introduction of~~Adding phylogenetic covariance did not improve the mixed model thus we did not retain a phylogenetic effect (Table S5). Interestingly, ~~the LMER results showed that~~ the (log-log) relationship between the recombination rate and the chromosome size was not the same within and between species, suggesting that absolute chromosome size does not have a general effect among species (~~Figure~~Fig 1B). Similarly, the relationship between linkage map length (cM) and chromosome size (Mb) was highly species specific (linkage map length ~ log10(chromosome size) + (1 | species)~~log-log linear mixed model~~, marginal R^2 = 0.49, conditional R^2 = 0.99, p < 0.001) (~~Figure~~Fig 2A), with species slopes decreasing with the mean chromosome size in a log-log relationship. It~~,~~ indicates~~ing~~ that species slopes are roughly proportional to the inverse of the mean chromosome size (~~Figure~~Fig S4~~2C~~). As a consequence, the excess of COs on a chromosome (i.e. the linkage map length minus 50 cM) was not ~~correlated to~~ correlated with the absolute chromosome size but ~~to~~ with the relative one (i.e. chromosome size divided by the mean chromosome

Formatted: Font: 11 pt, English (United Kingdom)

Formatted: Subscript

Formatted: Subscript

Formatted: Superscript

Formatted: Superscript

size of the species; Fig 2B). Moreover, in contrast to the relationship between recombination rate and absolute size, we did not observe any difference between the linear model and the fixed regression of the mixed linear model, suggesting that this relationship is similar across species (~~Figure~~Fig 2B~~D~~). More concretely, it means that two chromosomes having the same ratio of size will have the same ratio of excess of recombination rate, whatever the species and the genome size.

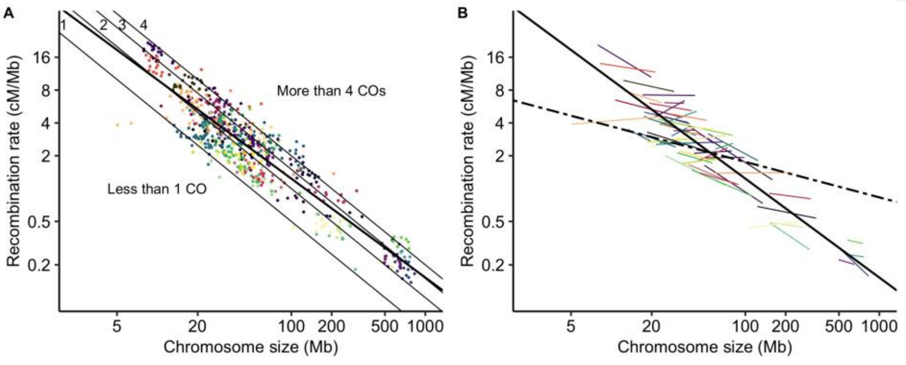

~~Figure~~Fig 1. Mean ~~Recombination~~ recombination rates per chromosome (cM/Mb, log scale) are negatively correlated ~~to~~ with chromosome genomic size (Mb, log scale). ~~Recombination rates were estimated with the loess regression function in windows of 100 kb and averaged per chromosome.~~ Each point represents a chromosome (n = 665). Species are presented in different colours (57 species). (A) The ~~large black~~bold solid line represents the l~~L~~inear ~~Model~~ regression line fitted to the data. ~~(LM log10(recombination rate) ~ log10(chromosome size)log-log Linear Model, adjusted R^2 = 0.83, p < 0.001).~~ The ~~lower~~ thin solid~~long dashed~~ lines correspond~~s~~ to the expectation of one, ~~CO~~ two, three or four COs per chromosome respectively. ~~, and the upper dashed lines correspond to two, three or four COs respectively (ascending order).~~ (B) Correlations between recombination rates and chromosome size within each species with at least 5 chromosomes (coloured lines, 55 species) and the~~a~~ overall between-species correlation controlled for a species effect (black dashed line , n = 57 species). Solid bold line as in (A). ~~The black dashed line represents the selected Linear Mixed Model with a species effect (LMER log10(recombination rate) ~ log10(chromosome size) + (1 | species)log-log LMER, marginal R^2 = 0.17, conditional R^2 = 0.96, p < 0.001). Coloured lines show the random regressions for species (55 species regression lines for species with at least 5 chromosomes~~ mapped~~, 5-26 chromosomes per species~~, 55 species~~)~~.

Formatted: Normal

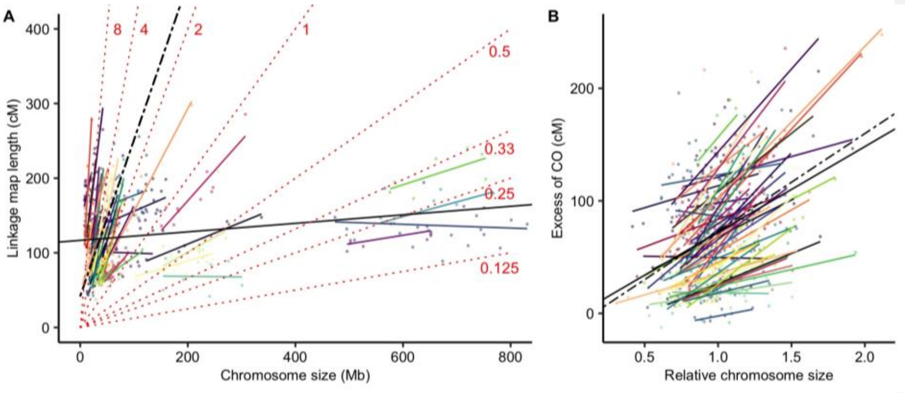

FigureFig 2. Linkage map length (cM) is positively correlated to with genomic chromosome size (Mb). (A) Correlation between chromosome genomic size (Mb) and linkage map length (cM). Each point represents a chromosome (n = 665). Species are presented in different colours (57 species). The black sThe linear regression is the solid black line represents the simple linear regression (LM linkage map length ~ log_{10}(chromosome size)Linear Model, adjusted R^2 = 0.036, p < 0.001) and the . The fixed regression of the Linear Mixed Model is the black dashed line the fixed effect of the mixed model (LMER linkage map length ~ log_{10}(chromosome size) + (1 | species)LMER, marginal R^2 = 0.49, conditional R^2 = 0.99, p < 0.001). Species random slopes are shown in colours. Isolines of the Genome-wide rRecombination rRates (GwRR)are were plotted for different values (indicated cM/Mb) as dotted red lines to represent regions with equal recombination rates and GwRR (cM.Mb^{-1}) were annotated. (B) Random intercepts for species as a function of the species mean genomic chromosome size (Mb). (C) Random slopes for species as a function of the species mean genomic chromosome size (Mb). (BD) The excess of COs (i.e. linkage map length minus 50 cM for the obligate CO) is consistently positively correlated to with the relative chromosome size (i.e. chromosome size divided by the averaged chromosome size of the species)(size / average size of the species). Each point represents a chromosome (n = 665). Species are presented in different colours (57 species). The black solid line is the linear regression across species (LM excess of CO ~ relative chromosome sizeLinear Model, adjusted R^2 = 0.13, p < 0.001) and . The the black dashed line is the linear mixed regression with a random species effectthe fixed effect of the mixed model (LMER excess of CO ~ relative chromosome size + (1 | species)LMER, marginal R^2 = 0.14, conditional R^2 = 0.86, p < 0.001). Coloured solid lines represent individual regression lines (Linear Model) for species with at least 5 chromosomes (55 species, 5-26 chromosomes per species).

Formatted: Subscript

Formatted: Subscript

Formatted: Superscript

Formatted: Subtitle

Diversity of CO patterns among flowering plants

Recombination landscapes along chromosomes appeared to be qualitatively very similar within species but strongly varied between species (FigureFig 3, FigureFig S2). COs were not evenly distributed between the centre and extremities of the chromosomes. In the text below, we have used the terms proximal and distal regions, respectively, to avoid confusion

with the molecular composition and specific position defining telomeric and centromeric regions *stricto sensu*. Some landscapes were homogeneous along chromosomes whereas others were extremely structured with ~~the~~ recombination concentrated in the short distal parts of the genome, ~~with~~ and wide ~~pattern~~ variations between these two extremes (~~Figure~~Fig 3). ~~Using a broken stick representation where~~Representing— relative recombination rates ~~were presented~~ on ten bins of equal chromosome length (see Materials and Methods for details), we observed that the bias towards the periphery was not ubiquitous across species (~~Figure~~Fig 4), unlike ~~;~~ Haenel et al. (2018) ~~concluded to assesseda a~~who suggested that the distal bias could be universal for chromosomes larger than 30 Mb. Only a subset of species, especially those with very large~~r~~ chromosomes (> 100 Mb), exhibited a clear bias~~, with COs clustered in the distal regions and with recombination rates that were lower than expected at the centre~~ (~~Figure~~Fig 4). Despite large chromosome sizes (mean chromosome sizes = 101 Mb and 198 Mb, respectively), *Nelumbo nucifera* and *Camellia sinensis* are noticeable exceptions to this pattern, with the highest recombination rates found in the middle of the chromosomes (*Nelumbo nucifera* illustrated in ~~Figure~~Fig 3E, other species in ~~Figure~~Fig S2). For small to medium-sized chromosomes, the pattern is less clear. Most species did not show any clear structure along the chromosome but a few of them (e.g. *Capsella rubella*, *Dioscorea alata, Mangifera indica, Manihot esculenta*) showed a drop in recombination rates in the distal regions and high recombination rates in the proximal regions (*Capsella rubella* illustrated in ~~Figure~~Fig 3A).

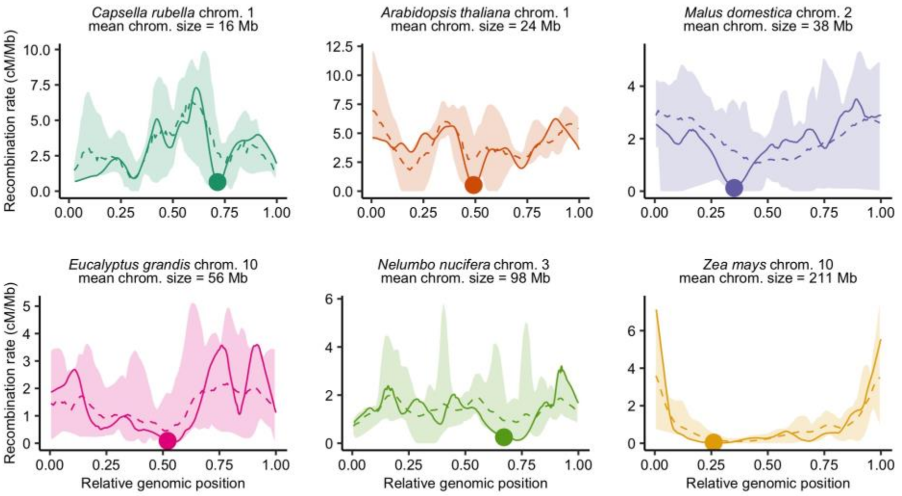

~~Figure~~Fig 3. ~~The d~~Diversity of recombination landscapes ~~in angiosperms is~~ exemplified by six different ~~emblematic~~ species. Recombination landscapes are similar within species (the dashed line is the average landscape for pooled chromosomes, all recombination landscapes of the species are contained within the colour ribbon). Genomic distances (Mb) were scaled between 0 and 1 ~~(divided by chromosome size)~~ to compare chromosomes with different sizes. Estimates of the recombination rates were obtained by 1,000 bootstraps ~~replicates of~~over loci in windows of 100 kb with loess regression and automatic span calibration. ~~The averaged species recombination landscape (dashed line) was estimated by calculating the mean recombination rate in 100 bins along the chromosome axis, all chromosomes pooled. Similarly, the lower and upper boundaries (pale ribbon) were estimated by taking the minimum and maximum recombination rates in 100 bins.~~ One chromosome per species is represented in a solid line, with the genomic position of the centromere demarcated by a dot. The six species are ordered by ascending mean chromosome size (Mb).

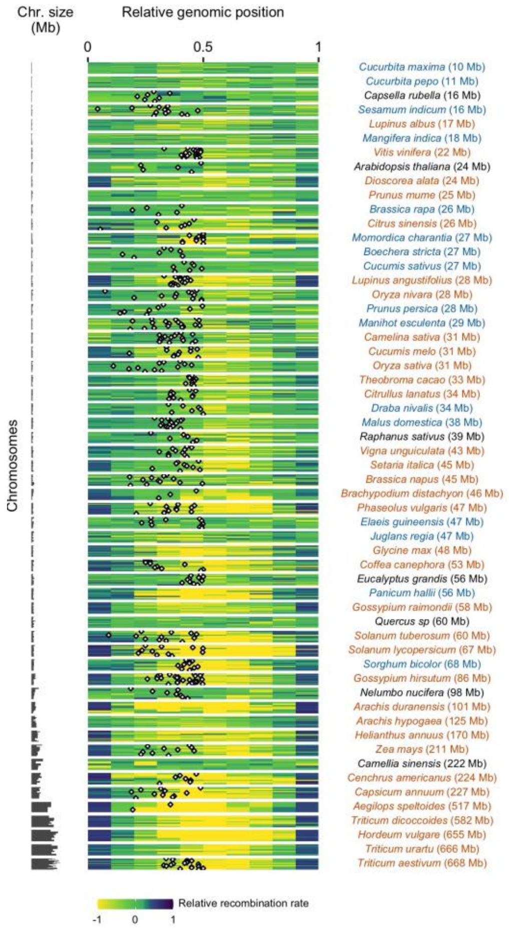

~~Figure~~Fig 4. Patterns of recombination within chromosomes (n = 665). Relative recombination rates along the chromosome were estimated in ten bins ~~using the broken stick model~~of equal genomic size as the ~~expected~~observed ~~relative~~ genetic length ~~(one tenth)~~ divided by the ~~observed~~expected ~~relative~~ genetic length (one tenth of total size) of the bin (~~log-transformed~~log-transformed). ~~).~~ Values below (above) zero are recombination rates that are lower (higher) than expected under a random distribution.~~.~~ ~~–~~Species are ordered by ascending ~~(top to bottom) variation in the relative recombination rates~~genome size (57 species). Each horizontal bar plot represents ~~the spatial distribution of recombination along a~~one chromosome~~. Each chromosome was divided into ten bins of equal genomic genetic size, i.e. 1/10 of the total genomic genetic mapsize (cMMb).~~. ~~The relative recombination rate is the log-transformed ratio of the expected relative genetic genomic length (one tenth) divided by the observed relative genetic genomic length of the bin (Mb). It means that values below zero are recombination rates that are lower than expected under a random distribution of COs whereas values above zero are recombination rates that are higher than expected. Whenever~~When available, the

centromere position on the chromosome is available, this information is mapped as a red black and white diamonddot and chromosomes are oriented with the longer arm on the right. Chromosome sizes (Mb) on the left correspond to each broken stick chromosome.

Following Haenel et al. (2018), we calculated the periphery-bias ratio as the recombination rate in the tips of the chromosome (10% at each extremity) divided by the mean recombination rate. A ratio higher than 1 indicates a higher recombination rate in the tips than the whole chromosome. By pooling chromosomes per species, we detected a significant positive effect of chromosome length on the periphery-bias ratio across species (Spearman rank correlation coefficient Rho = 0.60, p < 0.001; Linear Model, adjusted R^2 = 0.44, p < 0.001;) (FigureFig 5A) with some exceptions (ex on FiguresFig 3A and 3E).. At the chromosome level,Across all species the mean periphery-bias ratio is significantly higher than 1 (95% bootstrapped confidence interval = [2.06;2.32]) and skewed towards values higher than 1 but the correlation with chromosome length within species was not clear (FigureFig 5B, 5C, Table S6). Although we do find some ratios below 1 (Figure 5B), the distribution of the periphery-bias ratios is clearly skewed towards values higher than 1, suggesting that spatial clustering in the tips of the chromosome is a common feature among angiosperms, however with many exceptions (Figure 3A, 3E).

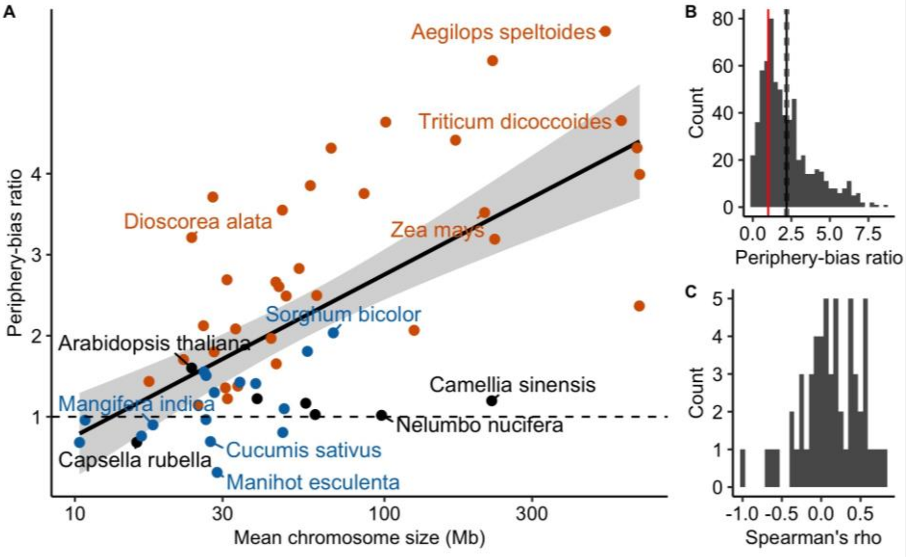

~~Figure~~Fig 5. The periphery-bias ratio is positively correlated ~~to~~ with chromosome genomic size. (A) ~~The~~ Linear regression between the species mean periphery-bias ratio ~~depends on~~and the mean chromosome size (~~Mb,~~ log scale) across species (n = 57 species;)~~. The linear regression line and its parametric 95% confidence interval were estimated in ggplot2 (Linear Model, chromosome size log-transformed,~~ adjusted R^2 = 0.44, p < 0.001). ~~A periphery-bias ratio above 1 (dashed horizontal line) indicates that recombination rates in the tips of the chromosome are higher than the mean chromosome recombination rate.~~ Points are coloured according to the classification of the CO patterns described below (orange = distal, blue = sub-distal, black = unclassified). (B) Distribution of periphery-bias ratios (n = 665 chromosomes). The mean periphery-bias ratio and its 95% confidence interval (black solid and dashed lines) were estimated by 1,000 bootstrap replicates. The red vertical line corresponds to a ratio of one ~~shows the theoretical value for an equal recombination in the tips compared to the rest of the chromosome (periphery-bias ratio = 1)~~. (C) Distribution of Spearman's correlation coefficients between the periphery-bias ratio and chromosome genomic size (Mb) within species (n = 57 species).

Joint effect of telomeres and centromeres on crossover distribution along chromosomes

Globally, recombination rates were negatively correlated ~~to~~ with the distance to the nearest telomere (~~Figure~~Fig S5~~4~~, Table S7, Table S8). However, two different patterns qualitatively emerged (~~Figure~~Fig 6, ~~Figure~~Fig S6~~5~~, Table S8). In 34 species, recombination decreased from the telomere and reached a plateau after a relative genomic distance of approximately 20% of the whole chromosome (the distal model, Fig 6A), in agreement with the model suggested by Haenel et al. (2018). Sixteen species presented a sharp decrease in the most distal regions and a peak of recombination in the sub-distal regions (relative genomic distance between 0.1-0.2) followed by a slow decrease towards the centre of the chromosome (the sub-distal pattern, Fig 6B). There were ~~very~~ a few exceptions to these two patterns (six species), e.g. *Capsella rubella* consistently showed higher recombination rates in the middle of the chromosome (~~Figure~~Fig 3A). Interestingly, chromosomes from species classified as having a distal pattern were significantly larger than chromosomes with a sub-distal pattern (Wilcox rank sum test, p < 0.001, ~~Figure~~Fig 6C). Furthermore, the ~~the species~~ correlation between recombination and the distance to the nearest telomere was significantly higher ~~was significantly negatively correlated to with~~for species with larger chromosomes

the mean chromosome length (Spearman rank correlation coefficient Rho = -0.51, p < 0.001; FigureFig S54).

When the centromere position was known, we qualitatively observed that the centromeres had an almost universal local suppressor effect (FigureFig 3, 4)). In small and medium-sized chromosomes, the recombination was often suppressed in short restricted centromeric regions (several Mb, 1-5 % of the map) displaying drastic drops in the recombination rates, whereas the rest of the map did not seem to be affected. In larger chromosomes, the suppression of recombination extends to large regions upstream and downstream of the physical centre of the chromosome (approximately 80-90% of the chromosome; FigureFig 4). Ninety percent of chromosomes (388 chromosomes) had significantly less recombination than the chromosome average at the centromeric index (n = 425, resampling test, 1,000 bootstraps, 95 % confidence interval). 81 chromosomes (19 %) were completely recombination-free in the centromere. However, the transposition of centromere position from cytological data to genomic data may be imprecise or wrongly oriented for some chromosomes. After orienting chromosomes to map the centromeric index, 16 % of chromosomes (70 over 425) had a recombination rate slightly higher in the inferred centromere position than on the opposite side, thus a centromere potentially mapped on the wrong side.

To go further, we formally compared three models that could explain the broad-scale crossover patterns we observed (FigureFig 76D). Under the strict distal model proposed by Haenel et al. (2018) (M1), the centromere does not play any role beyond its local suppressor effect, and therefore we expect an equal distribution of crossovers on both sides of the chromosome is expected, independently of centromere position: . In other words, we should expect $\frac{d(1/2)}{d(1)} = 0.5$, where $d(1/2)$ is the genetic distance (cM) to the middle of the chromosome and $d(1)$ is the total genetic distance (cM). In addition, , to this model (M1), we tested two nested alternative models adding a centromere effect. We assumed that the

position of the centromere, $d(c)$, has an effect on the distribution of crossovers along the chromosome. Models M2 'telomere + centromere + one CO per arm' and M3 'telomere + centromere + one CO per chromosome'; both assume that the relative genetic distance of a chromosome arm is proportional to its relative genomic size. However, ~~models M2 and M3~~they differ in the number and distribution of mandatory COs. At least one CO in each chromosome arm (50 cM) is mandatory in M2 whereas only one CO is mandatory for the entire chromosome in M3. For species whose centromere position was known (37 species, 425 chromosomes) we regressed the observed values against the theoretical predictions of the three models and compared them ~~Based on~~using goodness-of-fit criteria (adjusted R^2, AIC, BIC).~~,~~ ~~we used linear regression to compare the theoretical predictions of the three competing models to the observed values for Marey maps in which the centromere position was known (37 species, 425 chromosomes).~~ Model M2 was generally rejected since 22% of chromosomes showed less than 50 cM in at least one arm, even though it was supported in a handful of species (Table 1), and model M1 was not supported by any species. Model M3 was the best supported model (30 out of 37 species), with good predictive power (Spearman rank correlation between predicted and observed values: Rho = 0.72, p < 0.001; Tables 1, S9, S10). Given that some chromosomes had one chromosome arm shorter than 50 cM, which is incompatible with one mandatory CO per arm in model M2, we also ~~performed a second model selection~~compared the three models on a subset of chromosomes with at least 50 cM on each chromosome arm (n = 36 species, 333 chromosomes) which confirmed that model M3 was the best model. Similarly, we reran the model without chromosomes with uncertainty on the centromere position (n = 37 species, 355 chromosomes) and found the same results.

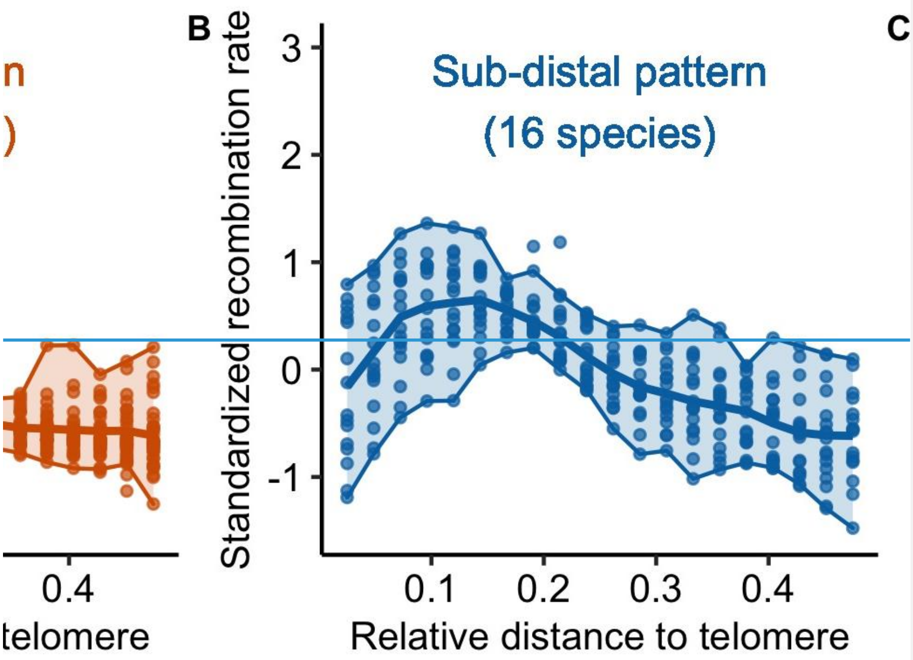

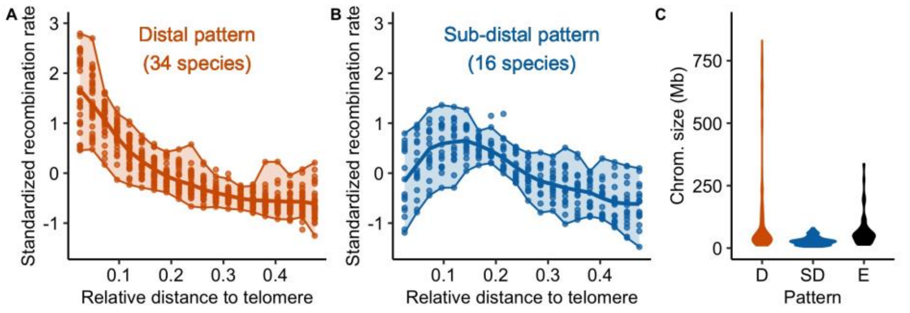

~~Figure~~Fig 6. Distribution of c~~C~~rossover: main patterns~~. and possible models.~~ (A and B) ~~can be classified in two different patterns, where recombination rates are higher in distal regions and lower near the centre of the chromosome.~~ Standardized recombination rates for species (~~centred-reduced cM/Mb,~~ chromosomes pooled per species, n = 57 species) are expressed as a function of the relative genomic distance from the telomere in 20 bins representing the two main patterns (orange = distal, blue = sub-distal). ~~Two patterns were identified and species were pooled accordingly, with 7~~The seven unclassified species ~~(orange = distal, blue = sub-distal, black = unclassified).~~are not shown. ~~(A) In the distal pattern (34 species), recombination rates decreased immediately from the tip of the chromosome (left plot, orange line and ribbon). (B) In the sub-distal pattern (16 species), recombination rates were reduced in the distal regions and the peak of recombination was in the sub-distal region (right plot, blue line and ribbon).~~ Chromosomes were split in half and 0.5 corresponds to the centre of the chromosome. In each plot, the solid line represents the mean recombination rate estimated in a bin (20 bins)

and each dot per bin represents the average of a species.. Chromosomes were split in half, where a distance of 0.5 is the centre of the chromosome. Then, chromosomes were pooled per species (each point is the mean recombination rate of all chromosomes in a species, for a distance bin to the tip of the chromosome). Upper and lower boundaries of the ribbon represent the maximum and minimum values attained for a particular pattern. Patterns that were not classified (7 species) were represented by individual loess regressions using black dashed lines. (C) Distribution of chromosome genomic sizes (Mb) in for each pattern. (D) Schematic representation of the Three three competing models for the two main patterns, compatible with the distal and sub-distal crossover patterns were tested using formal tests on predictions. Models are compatible with the distal and sub-distal crossover patterns we identified.with an exemple of a centromere position at 1/3 of the chromosome. Predictions are made on the distribution of crossovers between both chromosome sides (separated by the middle of the chromosome, model 1) or chromosome arms (separated by the centromere, models 2 and 3). Symbolic chromosomes illustrate the distribution of crossovers expected under each model and for each pattern. Model 3 is the best model (box).

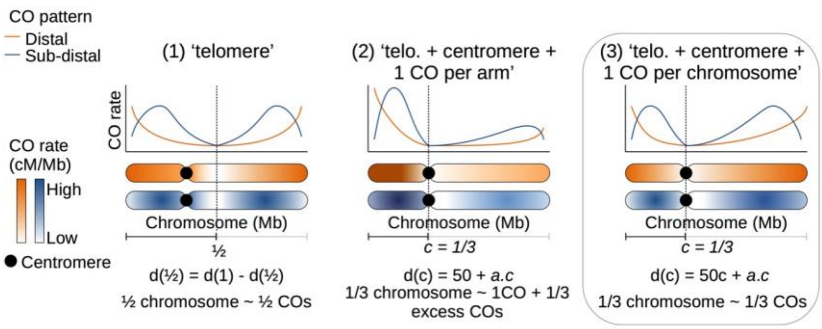

Formatted: Normal

Fig 7. Possible models of crossover patterns. Schematic representation of the three competing models for the two main patterns, with an example of a centromere position at 1/3 of the chromosome. Model 3 is the best model (box).

Formatted: Normal

Table 1. Model selection for the telomere/centromere effect (n = 37 species with a centromere position, 425 chromosomes). Three competing models were compared based on the adjusted R^2~~-~~, p-value and AIC-BIC criteria among chromosomes (the best supported model is in bold characters). The number of species supporting each model was calculated based on the adjusted R^2 within species, for all species with at least five chromosomes. (1) ‘telomere’ model. (2) ‘telomere + centromere + one CO per arm’ model. (3) ‘telomere + centromere + one CO per chromosome’ model. $d(c)$ is the genetic distance to the centromere. $d(1)$ is the total genetic distance. A second model selection was done on a subset of chromosomes with at least 50 cM on each chromosome arm (n = 36 species, 333 chromosomes).

#	Model	Expected	Adjusted R^2	p	AIC	BIC	Species
		Full dataset (37 species, 425 chromosomes)					
1	Telomere	d(1/2) / d(1) = 0.5	0.22	< 0.001	-477.8	-465.7	0
2	Tel. + Cent. + CO per arm	(d(c) – 50) / (d(1) – 100) = c	-	0.72	3098.2	3110.4	7
3	Tel. + Cent. + CO per chr.	d(c) / d(1) = c	0.51	< 0.001	-476.6	-464.5	30
		Subset (36 species, 333 chromosomes)					
1	Telomere	d(1/2) / d(1) = 0.5	0.18	< 0.001	-407.5	-396.1	0
2	Tel. + Cent. + CO per arm	(d(c) – 50) / (d(1) – 100) = c	-0.001	0.42	1939.1	1950.5	10
3	Tel. + Cent. + CO per chr.	d(c) / d(1) = c	0.50	< 0.001	-396	-384.6	26

Recombination rates are positively correlated ~~to~~ with gene density

At a fine scale, it has been shown in a few species that COs preferentially occur in gene promoters. The scale of 100 kb used here is too large to directly test whether this is a common pattern shared among angiosperms. Instead, like in Haenel et al. (2018), we assessed whether recombination increased with gene density. Forty-one genomes were annotated with gene positions. Across chromosomes, the distribution of chromosomal correlations between gene count and recombination rate was clearly skewed towards positive values, independently of the previously described CO patterns (mean Spearman’s rank correlation = 0.46 [0.43; 0.49]; ~~Figure~~Fig 8~~7~~A). Ninety-one percent ~~(91%)~~ of 483 chromosomes (41 species) showed a significant correlation between the number of genes and recombination rate at a 100 kb scale. Yet the strength of the relationship greatly varied across species and did not correlate with usual predictors such as the chromosome length or the genome-wide recombination rate (~~Figure~~Fig 8~~7~~B). Overall, standardized recombination rates (subtracting the mean and dividing by the standard deviation to allow comparison

among species) consistently increased with the number of genes in most species (linear quadratic regression, adjusted R^2 = 0.62, p < 0.001; FigureFig 87C).

Formatted: Superscript

As for recombination patterns, we classified patterns of gene density along chromosomes in three categories: distal, sub-distal and exceptions (FigureFig S76). Most species (30 out of 41) were classified in the same gene density and recombination pattern (Table S11). Moreover, when we classified species as a function of recombination patterns, we qualitatively observed the same pattern for gene density and recombination (FigureFig 98), suggesting that recombination and gene density share the same non-random distribution along the genome.

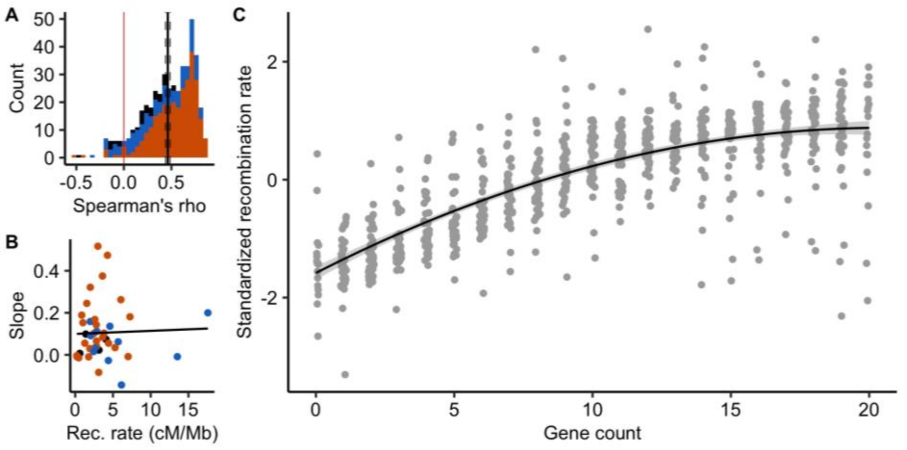

FigureFig 87. Recombination rates are positively correlated to with gene density (n = 483 chromosomes, 41 species). (A) Distribution of chromosome Spearman's rank correlations between the number of genes and the recombination rate in 100 kb windows. The black vertical line is the mean correlation with a 95% confidence interval (dashed lines) estimated by 1,000 bootstrap replicates. Colours correspond to CO patterns (orange = distal, blue = sub-distal, black = exception). (B) Slopes of the species linear regression between gene count and recombination rates are independent of the species averaged recombination rate (Linear Model, adjusted R^2 = -0.02, p = 0.83). (C) Standardized recombination rates for each number of genes in a 100 kb window (centred-reduced, chromosomes pooled per species, one colour per species) estimated by 1,000 bootstraps and standardized within species. The gene count was estimated by counting the number of gene starting positions within each 100 kb window. The black line with a grey ribbon is the quadratic regression estimated by linear regression with a 95% parametric confidence interval (Linear Model, adjusted R^2 = 0.62, p < 0.001).

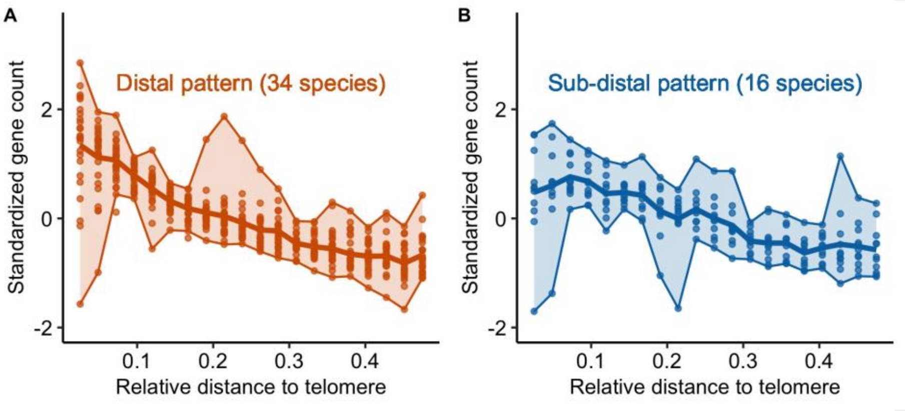

FigureFig 98. Gene counts patterns along the chromosome are correlated to with CO patterns (n = 41 species). Standardized gene count (centred-reduced) as a function of the relative distance from the tip to the middle of the chromosome (genomic distances distributed in 20 bins). We used the same groups as identified for the CO pattern in FigureFig 6; (a) distal pattern vs (b) sub-distal pattern.) and observed the same patterns along the chromosome. The solid line represents the mean gene count estimated in a bin and the upper and lower boundaries of the ribbon represent the maximum and minimum values in a bin. Patterns that were not classified (4 species with a gene annotation) were represented by loess regression in grey dashed lines. To estimate gene counts in bins of relative distances, chromosomes were split in half, where a distance of 0.5 is the centre of the chromosome. Chromosomes were pooled per species (n = 483 chromosomes).Same legend as FigureFig 6.

Genetic shuffling

We showed confirmed that recombination is unevenly distributed in genomes, which should affect how genetic variation is shuffled during meiosis. Genetic shuffling participates to the random reassortment of genes between parental homologous chromosomes. To quantify how much the genetic shuffling depends on the distribution of COs, we estimated the its intrachromosomal component, of the genetic shuffling $\bar{r}_{intra}$, as described in equation 10 provided byin Veller et al. (2019). The $\bar{r}_{intra}$ gives, for a chromosome, a measure of the probability of for a random pair of loci to be shuffled by a crossover. As expected, genetic shuffling was positively and significantly correlated with towith linkage map length (LMER $\bar{r}_{intra}$ ~ linkage map length + (1 | species)Linear Mixed Model, marginal R^2 = 0.43, conditional R^2 = 0.88, p < 0.001, FigureFig S87). COs clustered in distal regions are supposedly less efficientto generate less genetic shuffling than COs evenly distributed in the

chromosome. At a chromosomal level, ~~linear mixed regression (controlling for a species effect) revealed a low but significant negative effect of~~ the periphery-bias ratio as a low but significant effect on ~~the~~ genetic shuffling, consistent among species (~~LMER~~ $\bar{r}_{intra}$ ~ periphery-bias ratio + (1 | species), marginal $R^2 = 0.05$, conditional $R^2 = 0.68$, $p < 0.001$, ~~Figure~~Fig S9~~8~~). The more COs are clustered in the tips of the chromosome, the lower the chromosomal genetic shuffling. These results verify the analytical predictions of Veller et al. (2019), although the strength of ~~this~~ the effect remains weak.

However, the distributions of COs and genes are both non-random and often correlated (~~Figures~~Fig 8~~7~~ and S10~~9~~). Genomic distances measured in base pairs may not be the most appropriate measure of genetic shuffling among functional genomic components. Thus, we measured genomic distances in gene distances (i.e. the cumulative number of genes along the chromosome) instead of base pairs. Marey maps most often appeared more homogeneous when scaled on gene distances instead of base pair distances, with 70% (316 over 450) of Marey maps showing a smaller ~~scale~~ departure from a random distribution (~~Figures~~Fig 10~~9~~, S11~~0~~, Table S11). Globally, a subset of 30 species ~~have~~ has more homogeneous Marey maps with gene distances whereas 11 others are quantitatively more heterogeneous (notably *Capsella rubella* and *Arabidopsis thaliana*), although this could be due to low quality annotations making it difficult to precisely estimate the gene distances for some of them (e.g. *Sesamum indicum*). In most cases, genetic shuffling were slightly higher when gene distances were used instead of base pairs (~~Figure~~Fig 11~~0~~; mean = 0.22 for base pairs; mean = 0.26 for gene distances; Wilcoxon rank sum test with continuity correction, $p < 0.001$), implying that the genetic shuffling was more efficient among coding regions than ~~the whole genome~~among regions randomly sampled in the genome~~ with a distribution slightly skewed towards higher values for gene distances~~. Interestingly, the increase in genetic shuffling ~~when scaled with~~calculated in gene distances compared to genomic distance was more pronounced for longer chromosomes — which are often the most heterogeneous ones, characterized by a distal pattern — whereas we saw little effect on smaller chromosomes

characterized by a sub-distal pattern (~~LMER~~ difference in $\bar{r}_{intra}$ ~ log10(chromosome size) + (1 | species)~~Linear Mixed Model~~, marginal R^2 = 0.21~~16~~, conditional R^2 = 0.87~~3~~, $p < 0.001$, ~~Figure~~Fig 11~~0~~).

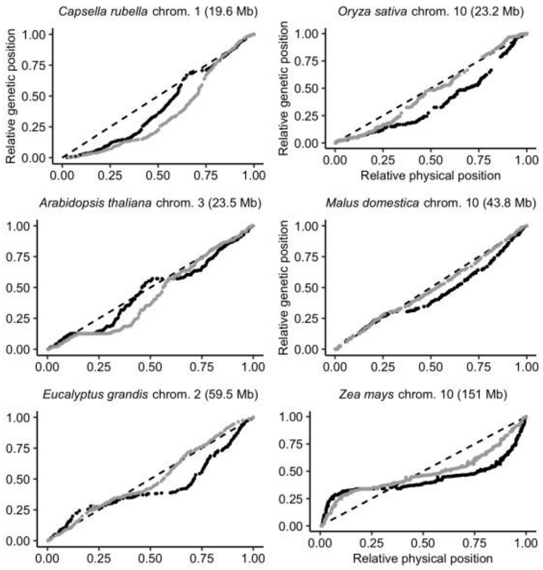

~~Figure~~Fig 10~~9~~. Marey maps of six chromosomes with the relative physical distance expressed in genomic distances (black dots, position in the genome in Mb) or in gene distances (grey dots, position measured as the cumulative number of genes along the chromosome. ~~).~~ Marey maps are ordered by ascending chromosome size (Mb). ~~The relative genetic position is the position of the marker on the linkage map.~~ The diagonal dashed line represents a theoretical random distribution of COs along the chromosome.

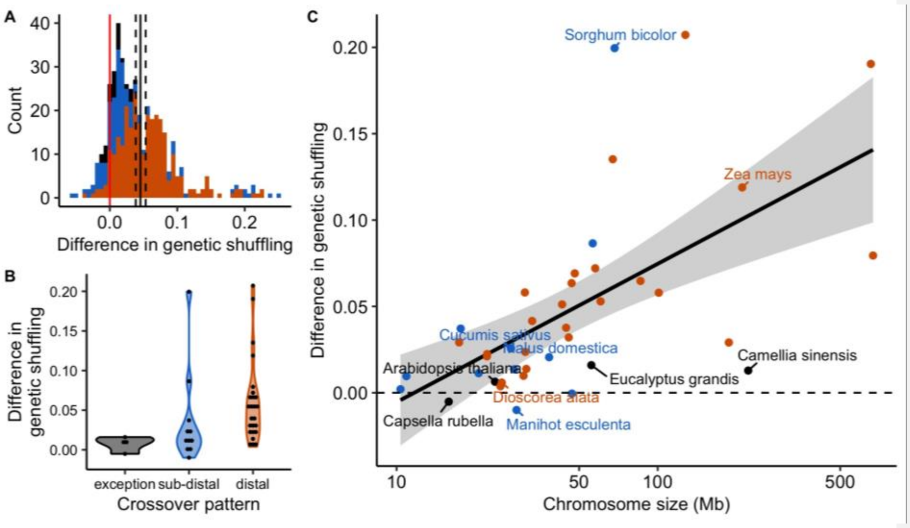

~~Figure~~Fig 11~~0~~. Differences in genetic shuffling between estimates based on genomic distances (Mb) and gene distances (cumulative number of genes). The difference is the genetic shuffling in ~~(~~gene distances~~)~~ minus the genetic shuffling in ~~(~~genomic distances~~), thus positive values indicate an increase in the genetic shuffling based on gene distances compared to genomic distances~~. Colours correspond to CO patterns (orange = distal, blue = sub-distal, black = exception). (A) Distribution of the chromosome differences in the genetic shuffling (n = 444 chromosomes). (B) Distributions of the species difference in the genetic shuffling (n = 41 species, chromosomes pooled). (C) Species differences in the genetic shuffling are positively correlated ~~to~~ with the averaged chromosome size (Linear Model, adjusted R^2 = 0.20, p = 0.002, n = 41, 95% parametric confidence interval).

Discussion

Based on a large and~~,~~ curated dataset, we provided ~~here~~, to ~~the best of~~ our knowledge, the largest description of recombination landscapes among flowering plants. In addition to confirming that both the chromosome-wide recombination rate and the heterogeneity of recombination landscapes vary according to chromosome length, we identified two distinct CO patterns and we proposed a new model that ~~builds on~~extended the strict telomere model recently ~~suggested~~ proposed by Haenel et al. (2018). Moreover, the consistent correlation between recombination and gene density ~~that we observed suggests that crossover initiation in gene regulatory sequences could be shared among angiosperms. This sheds new light on~~may have implications for the evolution of recombination landscapes and whether the

distribution of COs is optimal for the efficacy of genetic shuffling~~ and the evolution of recombination landscapes~~.

Chromosome size and recombination rate

We showed that, for most species, the smallest chromosome had roughly one or two COs, independently of chromosome size. This is in agreement with the idea that CO assurance is a ubiquitous regulation process among angiosperms (Pazhayam et al., 2021). Moreover, ~~it seems that~~ this constraint imposes a kind of basal recombination rate for each species, on the order of $50/Sc$ cM/Mb, where Sc is the size of the lowest chromosome in Mb. Regardless of the genome size (which ranges three orders of magnitude or more), the number of COs remains relatively stable amongst species, most probably under the joint influence of CO assurance, interference and homeostasis (Otto and Payseur, 2019; Stapley et al., 2017; Wang et al., 2015). As a result, averaged recombination rates are negatively correlated ~~to~~ with chromosome lengths, as already known in plants (Haenel et al., 2018; Tiley and Burleigh, 2015). ~~and in contrast to fungi and animals (Stapley et al., 2017).~~

~~Surprisingly, we found that,~~ However, there is no universal relationship between the absolute size of a chromosome and its mean recombination rate. Although the average recombination rate of a species is well predicted by its average chromosome size, the recombination rates of each chromosome separately are not well predicted by their absolute chromosome size. Instead, variation within species is much better explained by the ~~within a species,~~ relative chromosome size~~.~~, and surprisingly, this relationship seems to be roughly the same among species (see ~~Figures~~Fig 1 and 2). ~~was a stronger determinant of the genetic map than absolute chromosome size.~~ This suggests that CO interference is proportional to the relative size of the chromosome, as has been empirically observed in some plants (Ferreira et al., 2021). Although it is not clear yet which interference distance unit is the most relevant, genomic distances (in Mb) are excluded in most models of interference in favour of genetic distances (cM) (Foss et al., 1993) or, more likely, the length

of the synaptonemal complex in micrometres (Capilla-Pérez et al., 2021; Kleckner et al., 2004; Lloyd and Jenczewski, 2019; Zickler and Kleckner, 2015). Both scales~~ (in genetic distances or in size) of the synaptonemal complex, in micrometres,~~ match our observation of a relative size effect. Within species, genetic maps increase with chromosome size, but among species they are uncorrelated and far less variable than genome sizes, which makes the relative chromosome size the main determinant of recombination rate variations among species. Similarly, physical sizes (in micrometres) at meiosis do not seem to scale with genome size, as chromosomal organization (nucleosomes, chromatin loops) strongly reduces the variation that could be expected given the genome size (Otto and Payseur, 2019).

Recombination patterns along chromosomes

We observed a global trend towards higher recombination rates in sub-distal regions (Gaut et al., 2007; Haenel et al., 2018). The distal bias increased with chromosome length, in agreement with the conclusions of Haenel et al. (2018), although our methods differ in resolution. We analysed species and chromosomes separately whereas Haenel et al. (2018) used averages over the different patterns, thereby masking chromosome- and species-specific particularities. For example, they did not detect the sub-distal pattern ~~and ~~neither unclassified exceptions, whereas they seem common among species (1~~4~~6 and 7 species respectively). So far, little is known about the mechanisms that could explain the link between the distal bias and chromosome length. Even if models of CO interference yield similar patterns (Falque et al., 2007; Zhang et al., 2014), the conceptual model of Haenel et al. (2018) is still the only one to explicitly consider chromosome length. The telomere effect is thought to act at a broad chromosome scale over long genomic distance~~s~~. The decision of double strand breaks (DSBs) to engage in the CO pathway is made early on during meiosis and the early ~~association ~~chromosome pairing beginning in~~of~~ telomeres is thought to favour distal COs (Bishop and Zickler, 2004; Higgins et al., 2012; Hinch et al., 2019). In barley, when the relative timing of the first stages of the meiotic program was shortened, COs were

redistributed towards proximal regions (Higgins et al., 2012), as later observed in wheat (Osman et al., 2021).

Haenel et al. (2018) proposed that distance to the telomere is driving CO positioning, and therefore it should produce a symmetrical U-shaped pattern along chromosomes. However, a formal test showed that this model was too simple and that centromeres also played a role in the distribution of COs between chromosome arms. The best model (M3: ‘telomere + centromere + one CO per chromosome’) that we have proposed ~~in this work~~ suggests that centromeres do not only have ~~just~~ a local effect but ~~they~~ also influence the symmetry of recombination landscapes over long distance~~s~~, though a large proportion of our sample is metacentric, which might limit the detection of an effect. The local suppression of COs in centromeric regions is well known and largely conserved among species and seems a strong constitutive feature restricted to a short centromeric region, basically the kinetochore (Ellermeier et al., 2010; Fernandes et al., 2019). But the extent of ~~a larger~~the pericentromeric region varies drastically, most probably under the influence of DNA methylation, chromatin accessibility or RNA interference (Choi et al., 2018; Ellermeier et al., 2010; Hartmann et al., 2019; Pan et al., 2011). However, how centromeres (especially non-metacentric ones) may affect CO distribution at larger scales still needs to be determined.

Diversity of patterns among species

In addition to the role of centromeres, we also observed ~~a departure from the prediction~~ that recombination rates ~~should~~ do not always decrease ~~linearly~~monotonically with the distance to the tip of the chromosome, showing that the distal model is not generally found among plants. We observed at least two different crossover patterns among plant species. Only 34 out of 57 species support a process starting ~~in~~ at the tips (distal model), and 16 present the highest recombination rates in sub-distal regions, while seven species remain unclassified, which is at the limit of our visual classification. Globally, the distal pattern and distal bias seem to occur more often in larger chromosomes, but our data lack species with giant genomes. Giant genomes are not rare in plants, and we cannot extrapolate our

conclusions to the upper range of the genome size variation (Pellicer et al., 2018). Astonishingly, a low-density genetic map in *Allium* showed higher recombination rates in the proximal regions, which is opposite to the major trend we found (Khrustaleva et al., 2005). Genera with giant genomes such as *Lilium* or *Allium* would have been valuable assets in our dataset, but the actual genomic and linkage data are relatively incomplete (Jo et al., 2017; Shahin et al., 2011).

~~Contrary to the single pattern described by Haenel et al. (2018), this pattern diversity is more~~The occurrence of various recombination patterns is in agreement with what is known of the timing of meiosis and heterochiasmy (the fact that male and female meiosis have different CO patterns). Despite the strong conservation of the main meiotic mechanism in plants, differences in the balance between key components may produce distinct CO patterns (de Massy, 2013; Higgins et al., 2012; Kuo et al., 2021; Zelkowski et al., 2019). For example, the ZYP1 ~~telomere-led recombination~~ and ~~the~~ ASY1 proteins ~~are two~~have antagonistic ~~forces acting~~effects on the formation of the synaptonemal complex in plants (Lambing et al., 2020). In barley and wheat, linearization of the chromosome axis triggered by ZYP1 is gradual along the chromosome and~~,~~ initiated in distal regions, ~~whereas early DSBs form in the~~forming the telomere bouquet where early DSBs form (Higgins et al., 2012; Osman et al., 2021). In contrast, chromosome axes are formed at a similar time in *Arabidopsis thaliana* and chromosomes are gradually enriched in ASY1 from the telomeres to the centromeres; a gene-dosage component favours synapsis and ultimately COs towards the proximal regions (Lambing et al., 2020). It appears that the timing of the meiotic programme is important for the distal bias, as it involves changes in the relative contribution of each meiotic component that could explain the re-localization of COs (Higgins et al., 2012; Lambing et al., 2020). Therefore, the different patterns we observed may be explained by the different balance and timing of the expression of shared key regulators of CO patterning such as ZYP1 and ASY1 (Kuo et al., 2021). It is interesting to note that this is also true for mechanistic models of interference. Zhang et al. (2014) assessed that t~~T~~he 'beam-film'

model is able to fit both CO patterns, regardless ~~if~~ whether the tips of the chromosomes have an effect on interference or not, i.e. clamping ~~(Zhang et al., 2014)~~. If ~~there is~~ clamping is assumed, the model predicts that mechanical stress culminates in the extremities of the chromosome leading to high CO rates at the periphery where it is released first. In contrast, when clamping is limited, mechanical stress is released in the tips of the chromosome and COs occur further from the tips, until a threshold of mechanical stress is reached. The observed sub-distal pattern fits these predictions.

The two patterns of recombination we described here can also be observed in opposite sexes within the same plant species~~, i.e. heterochiasmy~~ (Capilla-Pérez et al., 2021; Dukić and Bomblies, 2022; Sardell and Kirkpatrick, 2019). Marked heterochiasmy variations between species, a feature shared among plants and animals, could influence the resulting sex-averaged recombination landscape (Sardell and Kirkpatrick, 2019). The sex-averaged telomere effect can be thought of as the product of two independent sex-specific landscapes although it is not clear how sex-specific maps ultimately contribute to the sex-averaged one (Johnston et al., 2016; Lenormand et al., 2016). Recombination is usually biased towards the tips of the chromosome in male recombination maps, but is more evenly distributed in female maps in ~~most~~ the few plant species with available data~~s~~ (Sardell and Kirkpatrick, 2019). In *Arabidopsis thaliana*, male meiosis has higher CO rates within the tips of the chromosome, as it has been observed in other species with large chromosomes, whereas female meiosis is more homogeneously distributed, with the lowest rates found in the distal regions (Capilla-Pérez et al., 2021). Shorter chromosome axes in *A. thaliana* female meiosis could induce fewer DSBs and class II non-interfering COs (Lloyd and Jenczewski, 2019). Conversely, in maize, the distal bias is similar in both sexes, despite higher CO rates for females (Kianian et al., 2018). Heterochiasmy is not universal in plants (Melamed-Bessudo et al., 2016), and we suggest that the variation in recombination landscapes could also result from variation in heterochiasmy among species, as it has been suggested for broad-scale differences in recombination landscapes between *A. thaliana* and its relative *A. arenosa*

(Dukić and Bomblies, 2022). This hypothesis should be tested further as more sex-specific genetic maps become available.

Recombination landscapes, gene density and genetic shuffling

We observed a strong convergence between CO patterns and gene density patterns. This correlation is consistent in our dataset despite possible errors in genome annotation and we also observed two different gene density patterns globally corresponding to similar CO patterns, emphasizing the close link between recombination and gene density. Interestingly, ~~gene density also had a strong correlation with the recombination rate~~we found the same correlation in species with atypical chromosomes. For example, *Camellia sinensis* and *Nelumbo nucifera* have large genomes with homogenous recombination landscapes, ~~whereas large genomes are usually associated with a distal pattern.~~and ~~A~~ a recent annotation of ~~a new genome assembly of~~the *Nelumbo nucifera* genome showed that genes are also evenly distributed along chromosomes at a broad scale (Shi et al., 2020), similar to *Camellia sinensis* (Wei et al., 2018). In wheat and rye, the analysis of the effect of chromosome rearrangement on recombination also suggests that CO localization is more locus-specific than location-specific: after inversions of distal and interstitial segments, COs were relocated to the new position on the distal segment (Lukaszewski, 2008; Lukaszewski et al., 2012). Overall, the parallel between gene density and recombination landscapes, confirmed by these two exceptions, is in agreement with the preferential occurrence of COs in gene regulatory sequences (Choi et al., 2018; He et al., 2017; Marand et al., 2019), and suggests that this may be a general pattern shared among angiosperms. Thus, gene distribution along chromosomes could be a main driver of recombination landscapes simply by determining where COs may preferentially occur. It should be noted that since the gene number is usually positively correlated ~~with~~ ~~to~~with chromosome size within a species but is roughly independent of genome size among species, this hypothesis also matches with the relative-size effect discussed above.

However, gene density and recombination rates are both correlated ~~to~~ with many other genomic features, such as transposable elements ~~(Marand et al., 2019)~~(Charlesworth et al., 1994; Kent et al., 2017). The accumulation of transposable elements in low recombining regions would progressively decrease gene density in the region, ~~-~~and would eventually result in a positive correlation between gene density and recombination ~~(Kent et al., 2017)~~. However, the correlation of recombination rates with transposable elements is not always clear and different TE families have opposite correlations (Kent et al., 2017; Underwood and Choi, 2019). ~~The positive association of COs and gene regulatory sequences, including fine-scale correlations, appears more robust (Choi et al., 2013; He et al., 2017; Marand et al., 2019), but c~~Causality mechanisms of these multiple interactions still need to be clarified. The use of fine scale recombination maps (using very large mapping populations or LD maps) should help identifying the respective role of genic regions (especially the role of promoters) and transposable elements (or other genomic features). ~~.~~

Irrespective of the underlying mechanism, our finding implies that the CO distribution ultimately scales with the gene distribution. Therefore, in most species, COs have a more even distribution between genes than between random genomic locations (~~Figure~~Fig 10~~9~~). The redistribution of COs towards functional regions could be a simple consequence of COs occurring within gene regulatory sequences, but it has important evolutionary implications such as increasing the genetic shuffling and homogenizing the probability of two random genes to recombine, especially for large genomes that exhibit the strongest difference in genetic shuffling between genes and between genomic locations (~~Figure~~Fig 1~~1~~0). Therefore, CO patterning (and not only the global CO rate) could be under selection not only for its direct effect on the functioning of meiosis but also for its indirect effects on selection efficacy (Otto and Payseur, 2019). Recombination decreases linkage disequilibrium and negative interferences between adjacent loci (e.g. Hill-Robertson Interference), and thus locally increases the efficacy of selection. Functional sites are targets for selection (Nachman and Payseur, 2012) and we found higher recombination rates in functional regions, meaning that

only a few genes are ultimately excluded from the benefits of recombination, even under the most pronounced distal bias.

Higher recombination rates in gene-rich regions could provide a satisfying explanation as to why the distal bias is maintained among species despite its theoretical lack of efficacy for genetic shuffling (Veller et al., 2019). The association between CO hotspots and gene regulatory sequences is mechanistically driven by chromatin accessibility, but it does not exclude the evolution of the mechanism itself towards the benefits of recombining more in gene-rich regions (Lenormand et al., 2016). However, slight variations in genetic shuffling caused by the non-random distribution of COs are less likely to be under strong selection compared to stabilizing selection on molecular constraints for chromosome pairing and segregation (Ritz et al., 2017), although interference is sometimes likely to evolve towards relaxed physical constraints (Otto and Payseur, 2019). In addition, the intra-chromosomal component of the genetic shuffling is a small contributor to the genome-wide shuffling rate, as a major part is due to independent assortment among chromosomes (Veller et al., 2019) ~~even though there may be significant selective pressure towards more recombination between genes within chromosomes~~. Our estimates for the chromosomal genetic shuffling do not reach the theoretical optimal value of 0.5. The pattern is not absolute, and a fraction of genes remains in low recombining regions. In grass species, up to 30% of genes are found in recombination deserts and are not subject to efficient selection (e.g. Mayer et al., 2011). Finally, it is still an open question as to whether this global distribution of COs in gene regulatory sequences is advantageous for the genetic diversity and adaptive potential of a species (Pan et al., 2016).

Conclusion

Our comparative study only demonstrates correlations, and not mechanisms, but helps to understand the diversity and determinants of recombination landscapes in flowering plants. Our results partly confirm previous studies based on fewer species (Haenel et al., 2018; Stapley et al., 2017; Tiley and Burleigh, 2015) while bringing new insights that alter previous

conclusions thanks to a detailed analysis at the species and chromosome levels. Two main and distinct CO patterns emerge across a large set of flowering plant species; it seems likely that chromosome structure (length, centromere) and gene densities are the major drivers of these patterns, and the interactions between them raise questions about the evolution of complex genomic patterns at the chromosome scale (Gaut et al., 2007; Nam and Ellegren, 2012). The new large and curated dataset we provide in the present work should be useful for addressing such questions and testing future evolutionary hypotheses regarding the role of recombination in genome architecture.

Materials and Methods

Data preparation

To build recombination maps, we combined genetic and genomic maps in angiosperms that had already been published in the literature. We conducted a literature search to collect sex-averaged genetic maps estimated on pedigree data – with markers positions in centiMorgans (cM). The keywords used were ‘genetic map’, ‘linkage map’, ‘genome assembly’, ‘plants’ and ‘angiosperms’, combined with 'high-density' or 'saturated' in order to target genetic maps with a large number of markers and progenies. Additionally, we carried out searches within public genomic databases to find publicly available genetic maps. Only species with a reference genome assembly at a chromosome level were included in our study (a complete list of genetic maps with the associated metadata is given in Tables S1, S2). As much as possible, genomic positions along the chromosome (Mb) were estimated by blasting marker sequences on the most recent genome assembly (otherwise genomic positions were those of the original publication). Genome assemblies with annotation files at a chromosome-scale were downloaded from NCBI (https://www.ncbi.nlm.nih.gov/) or public databases. Marker sequences were blasted with ‘blastn’ and a 90% identity cutoff. Markers were anchored to the genomic position of the best hit. When the sequence was a pair of primers, the mapped genomic position was the best hit between pairs of positions showing a

short distance between the forward and reverse primer (< 200 bp). In a few exceptions (see Table S1), genomic positions were mapped on a close congeneric species genome and the genomic map was kept if there was good collinearity between the genetic and genomic positions. Chromosomes were numbered as per the reference genome assembly. When marker sequences were not available, we kept the genomic positions published with the genetic map. ~~The centromere position was retrieved from the literature (i.e. the centromeric index, the ratio of the short arm length versus the total chromosome length).~~ The total genomic length was estimated by the length of the chromosome sequence in the genome assembly. The total genetic length was corrected using Hall and Willis's method (Hall and Willis, 2005) which accounts for undetected events of recombination in distal regions by adding *2s* to the length of each linkage group (where *s* is the average marker spacing in the group).

We selected genetic and genomic maps after stringent filtering and corrections, using custom scripts available in a public Github repository (https://github.com/ThomasBrazier/diversity-determinants-recombination-landscapes-flowering-plants.git). We assumed that markers must follow a monotone increasing function when plotting genetic distances as a function of genomic distances in a chromosome (i.e. the Marey map) and ~~to guarantee~~ collinearity between the genetic map and the reference genome was required to keep a Marey map. If necessary, genetic maps were reoriented so that the Marey map function is increasing (i.e. genetic distances read in the opposite direction). In a first step, Marey maps with fewer than 50 markers per chromosome were removed, although a few exceptions were visually validated (maps with ~30 markers). Marey maps with more than 10% of the total genomic map length missing at one end of the chromosome were removed. Marey maps with obvious artefacts and assembly mismatches (e.g. lack of collinearity, large inversions, large gaps) were removed. Markers clearly outside the global trend of the Marey map (e.g. large genetic/genomic distance from the global cloud of markers or from the interpolated Marey function, no other marker in a close

neighbourhood) were visually filtered out, and multiple iterations of filtering/interpolation helped to refine outlier removal. The Marey map approach is a graphical method, so figures were systematically produced at each step as a way to evaluate the results of the filtering and corrections. Finally, when multiple datasets were available for the same species, we selected the dataset with the highest marker density – in addition to visual validation – to maintain a balanced sampling and avoid pseudo-replicates of the same chromosome.

Estimates of local recombination rates

Local recombination rates along the chromosome were estimated with custom scripts following the Marey map approach, as described in the MareyMap R package (Rezvoy et al., 2007)~~ with the Marey map approach described in the MareyMap R package (Rezvoy et al., 2007)~~. The mathematical function of the Marey map was interpolated with a two-degree polynomial loess regression. Each span smoothing parameter was calibrated by 1,000 iterations of hold-out partitioning (random sampling of markers between two subsets; 2/3 for training and 1/3 for testing) with the Mean Squared Error of the loess regression as a goodness-of-fit criterion. The possible span ranged from 0.2 to 0.5 and was visually adjusted for certain maps. The local recombination rate was the derivative of the interpolated smoothed function in fixed 100 kb and 1 Mb non-overlapping windows. Negative estimates were not possible as we assumed a monotonously increasing function and negative recombination rates were set to zero. The 95% confidence intervals of the recombination rates were estimated by 1,000 bootstrap replicates of the markers and recombination landscapes with large confidence interval were discarded. The quality of the estimates was checked using the correlation between the 100 kb and 1 Mb windows.

The distribution of CO along chromosomes~~broken stick model~~

The spatial structure of recombination landscapes across species and chromosomes is a major feature of recombination landscapes. ~~Applied to the distribution of recombination in Marey maps, our implementation of the broken stick model seemed effective to visualize the~~

~~broad-scale variation of recombination rates (White and Hill, 2020).~~ We divided the Marey map in *k* segments of equal ~~genetic~~ genomic size (Mb) and then calculated the relative ~~genomic~~ genetic size (cM) of each segment. Under the null model (i.e. random recombination), one expects *k* segments of equal ~~genomic~~ genetic size 1/*k*. The relative recombination rate in the segment *i* was estimated by the log-ratio of the ~~relative~~ observed genetic size (i.e. genetic size of segment *i*) divided by the ~~relative~~ expected ~~genomic~~ genetic size (i.e. fixed to total genetic size / *k* by the model), as in the following equation.

$$relative\ recombination\ rate = log_{10}\frac{genetic_i}{genetic_{total}/k}$$

Given the observation that most recombination landscapes are broken down into at least three segments (White and Hill, 2020), we arbitrarily chose a number of segments *k* = 10 to reach a good resolution (a larger *k* did not show any qualitative differences).

Crossover patterns and the periphery-bias ratio

We investigated the spatial bias towards distal regions of the chromosome in the distribution of recombination by estimating recombination rates as a function of relative distances to the telomere (i.e. distance to the nearest chromosome end). Chromosomes were split by their midpoint and only one side was randomly sampled for each chromosome to avoid pseudo-replicates and the averaging of two potentially contrasting patterns on opposite arms. The relative distance to the telomere was the distance to the telomere divided by total chromosome size, then divided into 20 bins of equal relative distances. A periphery-bias ratio metric similar to the one presented in Haenel et al. (2018) was estimated to measure the strength of the distal bias. We divided the recombination rates in the tip of the chromosome (10% on each side of the chromosome, and one randomly sampled tip) by the mean recombination rate of the whole chromosome. We investigated the sensitivity of this periphery-bias ratio to the sampling scale by calculating the ratio for many distal region sizes (~~Figure~~Fig S12~~1~~).

Formatted: Font: Italic

Testing centromere or telomere effects

We searched the literature for centromeric indices (ratio of the short arm length divided by the total chromosome length) established by cytological measures. When we had no information about the correct orientation of the chromosome (short arm/long arm), the centromeric index was oriented to match the region with the lowest recombination rate of the whole chromosome (i.e. putative centromere). To determine if telomeres and centromeres play a significant role in CO patterning, we fitted empirical CO distributions to three theoretical models of CO distribution. In the following equations, *d(x)* is the relative genetic distance at the relative genomic position *x*, and *a* is a coefficient corresponding to the excess of COs per genomic distance. Under the strict 'telomere' model (1), we assumed that only telomeres played a role in CO distribution, i.e. an equal distribution of COs on both sides of the chromosome (i.e. $d(1/2) = d(1) - d(1/2)$, such that $\frac{d(1/2)}{d(1)} = 0.5$. The 'telomere + centromere + one mandatory CO per arm' model (2) assumed at least one CO per chromosome arm and a relative genetic distance of each chromosome arm proportional to its relative genomic size, corresponding to the role of centromere position, denoted *d(c)*. We have $d(c) = 50 + a \times c$ and $d(1) - d(c) = 50 + a \times (1 - c)$, such that $\frac{d(c)-50}{d(1)-100} = c$. Lastly, the 'telomere + centromere + one CO per chromosome' model (3) assumed at least one CO per chromosome and a relative genetic distance within the chromosome proportional to its relative genomic distance. We have $d(c) = c \times 50 + a \times c$ and $d(1) - d(c) = (1 - c) \times 50 + a \times (1 - c)$, such that $\frac{d(c)}{d(1)} = c$. The three competing models were compared with a linear regression between empirical and theoretical values, based on the adjusted R^2 and AIC-BIC criteria among chromosomes. The number of species supporting each model was calculated based on the adjusted R^2 within species, for all species with at least five chromosomes.

Gene density

We retrieved genome annotations ('gff' files) for genes, coding sequences and exon positions, preferentially from NCBI and otherwise from public databases (41 species). We

estimated gene counts in 100 kb windows for recombination maps by counting the number of genes with a starting position falling inside the window. For each gene count, we estimated the species mean recombination rate and its confidence interval at 95% by 1,000 bootstrap replicates (chromosomes pooled per species). Most species had rarely more than 20 genes over a 100 kb span and variance dramatically increased in the upper range of the gene counts, and therefore we pruned gene counts over 20 for graphical representation and statistical analyses.

Genetic shuffling

To assess the efficiency of the recombination between chromosomes and species, we calculated the measure of intra-chromosomal genetic shuffling described by Veller et al. (2019). To have even sampling along the chromosome, genetic positions (cM) of 1,000 pseudo-markers evenly distributed along genomic distances (Mb) were interpolated using a loess regression on each Marey map, following the same smoothing and interpolation procedure as for the estimation of the recombination rates. The chromosomal genetic shuffling $\bar{r}_{intra}$ were calculated as per the intra-chromosomal component of the equation 10 presented in Veller et al. (2019). For a single chromosome,

$$\bar{r}_{intra} = \sum_{i<j} (r_{ij} / \binom{\Lambda}{2})$$

where Λ is the total number of loci, $\binom{\Lambda}{2} = \Lambda(\Lambda - 1)/2$ and r_{ij} is the rate of shuffling for the locus pair *(i, j)*. For the intra-chromosomal component $\bar{r}_{intra}$, the pairwise shuffling rate was only calculated for linked sites, i.e. loci on the same chromosome. This pairwise shuffling rate was estimated by the recombination fraction between loci *i* and *j*. Recombination fractions were directly calculated from Haldane or Kosambi genetic distances between loci by applying a reverse Haldane function (1) or reverse Kosambi function (2), depending on the mapping function originally used for the given genetic map.

$$r_{ij} = \frac{1}{2}(1 - e^{-2d_{ij}/100}) \qquad (1)$$

$$r_{ij} = tanh\frac{1}{2}tanh(2d_{ij}/100) \qquad (2)$$

We also estimated marker positions in gene distances instead of genomic distances (Mb) to investigate the influence of the non-random distribution of genes on the recombination landscape. Gene distances were the cumulative number of genes along the chromosome at a given marker's position. Splicing variants and overlapping genes were counted as a single gene. The genetic shuffling was re-estimated with gene distances instead of genomic distances to consider a genetic shuffling based on the gene distribution, as suggested by Veller et al. (2019). To compare the departure from a random distribution along the chromosome among both types of distances (i.e. genomic and genes), we calculated the Root Mean Square Error (RMSE) of each Marey map and for both distances. To assess if the distribution of genes influenced the heterogeneity of recombination landscapes, the type of distance with the lower RMSE was considered as the more homogeneous landscape. However, this measure for gene distances is sensitive to annotation errors and artefacts. False negatives are therefore expected (when Marey maps were assessed as more homogeneous in genomic distances while the inverse is true) and this classification remains conservative.

Statistical analyses

All statistical analyses were performed with R version 4.0.4 (R Core Team, 2019). We assessed statistical relationships with the non-parametric Spearman's rank correlation and regression models. Linear Models were used for regressions with species data since we did not detect a phylogenetic effect. The structure in the chromosome dataset was accounted for by Linear Mixed Models (LMER) implemented in the 'lme4' R package (Bates et al., 2015, p. 4) and the phylogenetic structure was tested by fitting the Phylogenetic Generalized Linear Mixed Model (PGLMM) of the 'phyr' R package (Ives et al., 2019). The phylogenetic time-calibrated supertree used for the covariance matrix was retrieved from the publicly available

phylogeny constructed by Smith and Brown (Smith and Brown, 2018). Marginal and conditional R^2 values for LMER were estimated with the 'MuMIn' R package (Bartoń, 2020). Significance of the model parameters was tested with the 'lmerTest' R package (Kuznetsova et al., 2017). We selected the model based on AIC/BIC criteria and diagnostic plots. Reliability and stability of the various models were assessed by checking quantile-quantile plots for the normality of residuals and residuals plotted as a function of fitted values for homoscedasticity. Model quality was checked by the comparison of predicted and observed values. Given the skewed nature of some distributions, we used logarithm (base 10) transformations when appropriate. For comparison between species, statistics were standardized (i.e. by subtracting the mean and dividing by standard deviation). Mean statistics and 95% confidence intervals were estimated by 1,000 bootstrap replicates.

Acknowledgements

We thank Eric Janczewski, Laurent Duret, Anne-Marie Chèvre, Eric Petit and Armel Salmon, as well as three anonymous reviewers, for precious comments on the results and manuscript. We thank all the people that provided us genetic data that were not published yet.

Formatted: Normal

Author contributions

SG conceptualised and supervised the study. TB produced and analysed data. Both authors contributed to writing the paper.

Formatted: Indent: First line: 0"

References

Apuli, R.-P., Bernhardsson, C., Schiffthaler, B., Robinson, K.M., Jansson, S., Street, N.R., Ingvarsson, P.K., 2020. Inferring the Genomic Landscape of Recombination Rate Variation

in European Aspen (*Populus tremula*). G3 GenesGenomesGenetics 10, 299–309. https://doi.org/10.1534/g3.119.400504

Bartoń, K., 2020. MuMIn: Multi-model inference (manual).

Barton, N.H., 1995. A general model for the evolution of recombination. Genet. Res. 65, 123–144. https://doi.org/10.1017/S0016672300033140

Bates, D., Mächler, M., Bolker, B., Walker, S., 2015. Fitting linear mixed-effects models using lme4. J. Stat. Softw. 67, 1–48. https://doi.org/10.18637/jss.v067.i01

Bishop, D.K., Zickler, D., 2004. Early decision; meiotic crossover interference prior to stable strand exchange and synapsis. Cell 117, 9–15. https://doi.org/10.1016/S0092-8674(04)00297-1

Blitzblau, H.G., Bell, G.W., Rodriguez, J., Bell, S.P., Hochwagen, A., 2007. Mapping of Meiotic Single-Stranded DNA Reveals Double-Strand-Break Hotspots near Centromeres and Telomeres. Curr. Biol. 17, 2003–2012. https://doi.org/10.1016/j.cub.2007.10.066

Booker, T.R., Yeaman, S., Whitlock, M.C., 2020. Variation in recombination rate affects detection of outliers in genome scans under neutrality. Mol. Ecol. mec.15501. https://doi.org/10.1111/mec.15501

Capilla-Pérez, L., Durand, S., Hurel, A., Lian, Q., Chambon, A., Taochy, C., Solier, V., Grelon, M., Mercier, R., 2021. The synaptonemal complex imposes crossover interference and heterochiasmy in *Arabidopsis*. Proc. Natl. Acad. Sci. 118, e2023613118. https://doi.org/10.1073/pnas.2023613118

Charlesworth, B., Sniegowski, P. & Stephan, W., 1994. The evolutionary dynamics of repetitive DNA in eukaryotes. Nature 371, 215–220.

Formatted: Swedish (Sweden)

Formatted: Font: (Default) Arial, 11 pt, English (United Kingdom)

Formatted: Font: (Default) Arial, 11 pt, English (United Kingdom)

Formatted: Indent: First line: 0.2"

Formatted: Font: (Default) Arial, 11 pt, Not Italic, English (United Kingdom)

Formatted: Font: (Default) Arial, 11 pt, English (United Kingdom)

Formatted: Font: (Default) Arial, 11 pt, Not Bold, English (United Kingdom)

Formatted: Font: (Default) Arial, 11 pt, English (United Kingdom)

Charlesworth, B., Jensen, J.D., 2021. Effects of Selection at Linked Sites on Patterns of Genetic Variability. Annu. Rev. Ecol. Evol. Syst. 52, 177–197. https://doi.org/10.1146/annurev-ecolsys-010621-044528

Choi, K., Zhao, X., Kelly, K.A., Venn, O., Higgins, J.D., Yelina, N.E., Hardcastle, T.J., Ziolkowski, P.A., Copenhaver, G.P., Franklin, F.C.H., McVean, G., Henderson, I.R., 2013. Arabidopsis meiotic crossover hot spots overlap with H2A.Z nucleosomes at gene promoters. Nat. Genet. 45, 1327–1336. https://doi.org/10.1038/ng.2766

Choi, K., Zhao, X., Tock, A.J., Lambing, C., Underwood, C.J., Hardcastle, T.J., Serra, H., Kim, Juhyun, Cho, H.S., Kim, Jaeil, Ziolkowski, P.A., Yelina, N.E., Hwang, I., Martienssen, R.A., Henderson, I.R., 2018. Nucleosomes and DNA methylation shape meiotic DSB frequency in *Arabidopsis thaliana* transposons and gene regulatory regions. Genome Res. 28, 532–546. https://doi.org/10.1101/gr.225599.117

Comeron, J.M., 2017. Background selection as null hypothesis in population genomics: insights and challenges from *Drosophila* studies. Philos. Trans. R. Soc. B Biol. Sci. 372, 20160471. https://doi.org/10.1098/rstb.2016.0471

Cooper, T.J., Garcia, V., Neale, M.J., 2016. Meiotic DSB patterning: A multifaceted process. Cell Cycle 15, 13–21. https://doi.org/10.1080/15384101.2015.1093709

Corbett-Detig, R.B., Hartl, D.L., Sackton, T.B., 2015. Natural Selection Constrains Neutral Diversity across A Wide Range of Species. PLOS Biol. 13, e1002112. https://doi.org/10.1371/journal.pbio.1002112

de Massy, B., 2013. Initiation of Meiotic Recombination: How and Where? Conservation and Specificities Among Eukaryotes. Annu. Rev. Genet. 47, 563–599. https://doi.org/10.1146/annurev-genet-110711-155423

Dukić, M., Bomblies, K., 2022. Male and female recombination landscapes of diploid *Arabidopsis arenosa*. Genetics iyab236. https://doi.org/10.1093/genetics/iyab236

Ellermeier, C., Higuchi, E.C., Phadnis, N., Holm, L., Geelhood, J.L., Thon, G., Smith, G.R., 2010. RNAi and heterochromatin repress centromeric meiotic recombination. Proc. Natl. Acad. Sci. 107, 8701–8705. https://doi.org/10.1073/pnas.0914160107

Falque, M., Mercier, R., Mézard, C., de Vienne, D., Martin, O.C., 2007. Patterns of Recombination and MLH1 Foci Density Along Mouse Chromosomes: Modeling Effects of Interference and Obligate Chiasma. Genetics 176, 1453–1467. https://doi.org/10.1534/genetics.106.070235

Fernandes, J.B., Wlodzimierz, P., Henderson, I.R., 2019. Meiotic recombination within plant centromeres. Curr. Opin. Plant Biol. 48, 26–35. https://doi.org/10.1016/j.pbi.2019.02.008

Ferreira, M.T.M., Glombik, M., Perničková, K., Duchoslav, M., Scholten, O., Karafiátová, M., Techio, V.H., Doležel, J., Lukaszewski, A.J., Kopecký, D., 2021. Direct evidence for crossover and chromatid interference in meiosis of two plant hybrids (*Lolium multiflorum×Festuca pratensis* and *Allium cepa×A. roylei*). J. Exp. Bot. 72, 254–267. https://doi.org/10.1093/jxb/eraa455

Foss, E., Lande, R., Stahl, F., Steinberg, C., 1993. Chiasma interference as a function of genetic distance. Genetics 133, 681–691.

Galtier, N., Roux, C., Rousselle, M., Romiguier, J., Figuet, E., Glémin, S., Bierne, N., Duret, L., 2018. Codon Usage Bias in Animals: Disentangling the Effects of Natural Selection, Effective Population Size, and GC-Biased Gene Conversion. Mol. Biol. Evol. 35, 1092–1103. https://doi.org/10.1093/molbev/msy015

Gaut, B.S., Wright, S.I., Rizzon, C., Dvorak, J., Anderson, L.K., 2007. Recombination: an underappreciated factor in the evolution of plant genomes. Nat. Rev. Genet. 8, 77–84. https://doi.org/10.1038/nrg1970

Formatted: English (United States)

Formatted: Swedish (Sweden)

Glémin, S., Clément, Y., David, J., Ressayre, A., 2014. GC content evolution in coding regions of angiosperm genomes: a unifying hypothesis. Trends Genet. 30, 263–270. https://doi.org/10.1016/j.tig.2014.05.002

Haenel, Q., Laurentino, T.G., Roesti, M., Berner, D., 2018. Meta-analysis of chromosome-scale crossover rate variation in eukaryotes and its significance to evolutionary genomics. Mol. Ecol. 27, 2477–2497. https://doi.org/10.1111/mec.14699

Hall, M.C., Willis, J.H., 2005. Transmission Ratio Distortion in Intraspecific Hybrids of *Mimulus guttatus*: Implications for Genomic Divergence. Genetics 170, 375–386. https://doi.org/10.1534/genetics.104.038653

Hartmann, M., Umbanhowar, J., Sekelsky, J., 2019. Centromere-Proximal Meiotic Crossovers in *Drosophila melanogaster* Are Suppressed by Both Highly Repetitive Heterochromatin and Proximity to the Centromere. Genetics 213, 113–125. https://doi.org/10.1534/genetics.119.302509

He, Y., Wang, M., Dukowic-Schulze, S., Zhou, A., Tiang, C.-L., Shilo, S., Sidhu, G.K., Eichten, S., Bradbury, P., Springer, N.M., Buckler, E.S., Levy, A.A., Sun, Q., Pillardy, J., Kianian, P.M.A., Kianian, S.F., Chen, C., Pawlowski, W.P., 2017. Genomic features shaping the landscape of meiotic double-strand-break hotspots in maize. Proc. Natl. Acad. Sci. 114, 12231–12236. https://doi.org/10.1073/pnas.1713225114

Higgins, J.D., Perry, R.M., Barakate, A., Ramsay, L., Waugh, R., Halpin, C., Armstrong, S.J., Franklin, F.C.H., 2012. Spatiotemporal Asymmetry of the Meiotic Program Underlies the Predominantly Distal Distribution of Meiotic Crossovers in Barley. Plant Cell 24, 4096–4109. https://doi.org/10.1105/tpc.112.102483

Hinch, A.G., Zhang, G., Becker, P.W., Moralli, D., Hinch, R., Davies, B., Bowden, R., Donnelly, P., 2019. Factors influencing meiotic recombination revealed by whole-genome

sequencing of single sperm. Science 363, eaau8861. https://doi.org/10.1126/science.aau8861

Ives, A., Dinnage, R., Nell, L.A., Helmus, M., Li, D., 2019. phyr: Model based phylogenetic analysis (manual).

Jo, J., Purushotham, P.M., Han, K., Lee, H.-R., Nah, G., Kang, B.-C., 2017. Development of a Genetic Map for Onion (*Allium cepa* L.) Using Reference-Free Genotyping-by-Sequencing and SNP Assays. Front. Plant Sci. 8, 1606. https://doi.org/10.3389/fpls.2017.01606

Johnston, S.E., Bérénos, C., Slate, J., Pemberton, J.M., 2016. Conserved Genetic Architecture Underlying Individual Recombination Rate Variation in a Wild Population of Soay Sheep (*Ovis aries*). Genetics 203, 583–598. https://doi.org/10.1534/genetics.115.185553

Kent, T.V., Uzunović, J., Wright, S.I., 2017. Coevolution between transposable elements and recombination. Philos. Trans. R. Soc. B Biol. Sci. 372, 20160458. https://doi.org/10.1098/rstb.2016.0458

Khrustaleva, L.I., de Melo, P.E., van Heusden, A.W., Kik, C., 2005. The Integration of Recombination and Physical Maps in a Large-Genome Monocot Using Haploid Genome Analysis in a Trihybrid Allium Population. Genetics 169, 1673–1685. https://doi.org/10.1534/genetics.104.038687

Kianian, P.M.A., Wang, M., Simons, K., Ghavami, F., He, Y., Dukowic-Schulze, S., Sundararajan, A., Sun, Q., Pillardy, J., Mudge, J., Chen, C., Kianian, S.F., Pawlowski, W.P., 2018. High-resolution crossover mapping reveals similarities and differences of male and female recombination in maize. Nat. Commun. 9, 2370. https://doi.org/10.1038/s41467-018-04562-5

Formatted: Swedish (Sweden)

Formatted: Swedish (Sweden)

Kleckner, N., Zickler, D., Jones, G.H., Dekker, J., Padmore, R., Henle, J., Hutchinson, J., 2004. A mechanical basis for chromosome function. Proc. Natl. Acad. Sci. 101, 12592–12597. https://doi.org/10.1073/pnas.0402724101

Kuo, P., Da Ines, O., Lambing, C., 2021. Rewiring Meiosis for Crop Improvement. Front. Plant Sci. 12, 708948. https://doi.org/10.3389/fpls.2021.708948

Kuznetsova, A., Brockhoff, P.B., Christensen, R.H.B., 2017. lmerTest package: Tests in linear mixed effects models. J. Stat. Softw. 82, 1–26. https://doi.org/10.18637/jss.v082.i13

Lambing, C., Kuo, P.C., Tock, A.J., Topp, S.D., Henderson, I.R., 2020. ASY1 acts as a dosage-dependent antagonist of telomere-led recombination and mediates crossover interference in *Arabidopsis*. Proc. Natl. Acad. Sci. 117, 13647–13658. https://doi.org/10.1073/pnas.1921055117

Lenormand, T., Engelstädter, J., Johnston, S.E., Wijnker, E., Haag, C.R., 2016. Evolutionary mysteries in meiosis. Philos. Trans. R. Soc. B Biol. Sci. 371, 20160001. https://doi.org/10.1098/rstb.2016.0001

Lloyd, A., Jenczewski, E., 2019. Modelling Sex-Specific Crossover Patterning in *Arabidopsis*. Genetics 211, 847–859. https://doi.org/10.1534/genetics.118.301838

Lukaszewski, A.J., 2008. Unexpected behavior of an inverted rye chromosome arm in wheat. Chromosoma 117, 569–578. https://doi.org/10.1007/s00412-008-0174-4

Lukaszewski, A.J., Kopecky, D., Linc, G., 2012. Inversions of chromosome arms 4AL and 2BS in wheat invert the patterns of chiasma distribution. Chromosoma 121, 201–208. https://doi.org/10.1007/s00412-011-0354-5

Marand, A.P., Zhao, H., Zhang, W., Zeng, Z., Fang, C., Jiang, J., 2019. Historical Meiotic Crossover Hotspots Fueled Patterns of Evolutionary Divergence in Rice. Plant Cell 31, 645–662. https://doi.org/10.1105/tpc.18.00750

Formatted: Swedish (Sweden)

Formatted: Swedish (Sweden)

Mayer, K.F.X., Martis, M., Hedley, P.E., Šimková, H., Liu, H., Morris, J.A., Steuernagel, B., Taudien, S., Roessner, S., Gundlach, H., Kubaláková, M., Suchánková, P., Murat, F., Felder, M., Nussbaumer, T., Graner, A., Salse, J., Endo, T., Sakai, H., Tanaka, T., Itoh, T., Sato, K., Platzer, M., Matsumoto, T., Scholz, U., Doležel, J., Waugh, R., Stein, N., 2011. Unlocking the Barley Genome by Chromosomal and Comparative Genomics. Plant Cell 23, 1249–1263. https://doi.org/10.1105/tpc.110.082537

Melamed-Bessudo, C., Shilo, S., Levy, A.A., 2016. Meiotic recombination and genome evolution in plants. Curr. Opin. Plant Biol. 30, 82–87. https://doi.org/10.1016/j.pbi.2016.02.003

Mézard, C., Tagliaro Jahns, M., Grelon, M., 2015. Where to cross? New insights into the location of meiotic crossovers. Trends Genet. 31, 393–401. https://doi.org/10.1016/j.tig.2015.03.008

Nachman, M.W., Payseur, B.A., 2012. Recombination rate variation and speciation: theoretical predictions and empirical results from rabbits and mice. Philos. Trans. R. Soc. B Biol. Sci. 367, 409–421. https://doi.org/10.1098/rstb.2011.0249

Nam, K., Ellegren, H., 2012. Recombination Drives Vertebrate Genome Contraction. PLoS Genet. 8, e1002680. https://doi.org/10.1371/journal.pgen.1002680

Osman, K., Algopishi, U., Higgins, J.D., Henderson, I.R., Edwards, K.J., Franklin, F.C.H., Sanchez-Moran, E., 2021. Distal Bias of Meiotic Crossovers in Hexaploid Bread Wheat Reflects Spatio-Temporal Asymmetry of the Meiotic Program. Front. Plant Sci. 12, 631323. https://doi.org/10.3389/fpls.2021.631323

Otto, S.P., 2009. The Evolutionary Enigma of Sex. Am. Nat. 174, S1–S14. https://doi.org/10.1086/599084

Otto, S.P., Payseur, B.A., 2019. Crossover Interference: Shedding Light on the Evolution of Recombination. Annu. Rev. Genet. 53, 19–44. https://doi.org/10.1146/annurev-genet-040119-093957

Pan, J., Sasaki, M., Kniewel, R., Murakami, H., Blitzblau, H.G., Tischfield, S.E., Zhu, X., Neale, M.J., Jasin, M., Socci, N.D., Hochwagen, A., Keeney, S., 2011. A Hierarchical Combination of Factors Shapes the Genome-wide Topography of Yeast Meiotic Recombination Initiation. Cell 144, 719–731. https://doi.org/10.1016/j.cell.2011.02.009

Pan, Q., Li, L., Yang, X., Tong, H., Xu, S., Li, Z., Li, W., Muehlbauer, G.J., Li, J., Yan, J., 2016. Genome-wide recombination dynamics are associated with phenotypic variation in maize. New Phytol. 210, 1083–1094. https://doi.org/10.1111/nph.13810

Pazhayam, N.M., Turcotte, C.A., Sekelsky, J., 2021. Meiotic Crossover Patterning. Front. Cell Dev. Biol. 9, 681123. https://doi.org/10.3389/fcell.2021.681123

Pellicer, J., Hidalgo, O., Dodsworth, S., Leitch, I., 2018. Genome Size Diversity and Its Impact on the Evolution of Land Plants. Genes 9, 88. https://doi.org/10.3390/genes9020088

R Core Team, 2019. R: A Language and Environment for Statistical Computing. R Foundation for Statistical Computing, Vienna, Austria.

Rezvoy, C., Charif, D., Gueguen, L., Marais, G.A.B., 2007. MareyMap: an R-based tool with graphical interface for estimating recombination rates. Bioinformatics 23, 2188–2189. https://doi.org/10.1093/bioinformatics/btm315

Ritz, K.R., Noor, M.A.F., Singh, N.D., 2017. Variation in Recombination Rate: Adaptive or Not? Trends Genet. 33, 364–374. https://doi.org/10.1016/j.tig.2017.03.003

Sandhu, D., Gill, K.S., 2002. Gene-Containing Regions of Wheat and the Other Grass Genomes. Plant Physiol. 128, 803–811. https://doi.org/10.1104/pp.010745

Formatted: Swedish (Sweden)

Sardell, J.M., Kirkpatrick, M., 2019. Sex differences in the recombination landscape. Am. Nat. 704943. https://doi.org/10.1086/704943

Shahin, A., Arens, P., Van Heusden, A.W., Van Der Linden, G., Van Kaauwen, M., Khan, N., Schouten, H.J., Van De Weg, W.E., Visser, R.G.F., Van Tuyl, J.M., 2011. Genetic mapping in *Lilium*: mapping of major genes and quantitative trait loci for several ornamental traits and disease resistances. Plant Breed. 130, 372–382. https://doi.org/10.1111/j.1439-0523.2010.01812.x

Shi, T., Rahmani, R.S., Gugger, P.F., Wang, M., Li, H., Zhang, Y., Li, Z., Wang, Q., Van de Peer, Y., Marchal, K., Chen, J., 2020. Distinct Expression and Methylation Patterns for Genes with Different Fates following a Single Whole-Genome Duplication in Flowering Plants. Mol. Biol. Evol. 37, 2394–2413. https://doi.org/10.1093/molbev/msaa105

Smith, S.A., Brown, J.W., 2018. Constructing a broadly inclusive seed plant phylogeny. Am. J. Bot. 105, 302–314. https://doi.org/10.1002/ajb2.1019

Soltis, P.S., Marchant, D.B., Van de Peer, Y., Soltis, D.E., 2015. Polyploidy and genome evolution in plants. Curr. Opin. Genet. Dev. 35, 119–125. https://doi.org/10.1016/j.gde.2015.11.003

Stapley, J., Feulner, P.G.D., Johnston, S.E., Santure, A.W., Smadja, C.M., 2017. Variation in recombination frequency and distribution across eukaryotes: patterns and processes. Philos. Trans. R. Soc. B Biol. Sci. 372, 20160455. https://doi.org/10.1098/rstb.2016.0455

Tiley, G.P., Burleigh, J.G., 2015. The relationship of recombination rate, genome structure, and patterns of molecular evolution across angiosperms. BMC Evol. Biol. 15, 194. https://doi.org/10.1186/s12862-015-0473-3

Formatted: Swedish (Sweden)

Underwood, C.J., Choi, K., 2019. Heterogeneous transposable elements as silencers, enhancers and targets of meiotic recombination. Chromosoma 128, 279–296. https://doi.org/10.1007/s00412-019-00718-4

Veller, C., Kleckner, N., Nowak, M.A., 2019. A rigorous measure of genome-wide genetic shuffling that takes into account crossover positions and Mendel’s second law. Proc. Natl. Acad. Sci. 116, 1659–1668. https://doi.org/10.1073/pnas.1817482116

Wang, S., Zickler, D., Kleckner, N., Zhang, L., 2015. Meiotic crossover patterns: Obligatory crossover, interference and homeostasis in a single process. Cell Cycle 14, 305–314. https://doi.org/10.4161/15384101.2014.991185

Wang, Y., Copenhaver, G.P., 2018. Meiotic Recombination: Mixing It Up in Plants. Annu. Rev. Plant Biol. 69, 577–609. https://doi.org/10.1146/annurev-arplant-042817-040431

Wei, C., Yang, H., Wang, S., Zhao, J., Liu, C., Gao, L., Xia, E., Lu, Y., Tai, Y., She, G., Sun, J., Cao, H., Tong, W., Gao, Q., Li, Y., Deng, W., Jiang, X., Wang, W., Chen, Q., Zhang, S., Li, H., Wu, J., Wang, P., Li, P., Shi, C., Zheng, F., Jian, J., Huang, B., Shan, D., Shi, M., Fang, C., Yue, Y., Li, F., Li, D., Wei, S., Han, B., Jiang, C., Yin, Y., Xia, T., Zhang, Z., Bennetzen, J.L., Zhao, S., Wan, X., 2018. Draft genome sequence of *Camellia sinensis* var. *sinensis* provides insights into the evolution of the tea genome and tea quality. Proc. Natl. Acad. Sci. 115, E4151–E4158. https://doi.org/10.1073/pnas.1719622115

White, I.M.S., Hill, W.G., 2020. Effect of heterogeneity in recombination rate on variation in realised relationship. Heredity 124, 28–36. https://doi.org/10.1038/s41437-019-0241-z

Yelina, N.E., Choi, K., Chelysheva, L., Macaulay, M., de Snoo, B., Wijnker, E., Miller, N., Drouaud, J., Grelon, M., Copenhaver, G.P., Mezard, C., Kelly, K.A., Henderson, I.R., 2012. Epigenetic Remodeling of Meiotic Crossover Frequency in *Arabidopsis thaliana* DNA Methyltransferase Mutants. PLoS Genet. 8, e1002844. https://doi.org/10.1371/journal.pgen.1002844

Formatted: Swedish (Sweden)

Zelkowski, M., Olson, M.A., Wang, M., Pawlowski, W., 2019. Diversity and Determinants of Meiotic Recombination Landscapes. Trends Genet. 35, 359–370. https://doi.org/10.1016/j.tig.2019.02.002

Zhang, L., Liang, Z., Hutchinson, J., Kleckner, N., 2014. Crossover Patterning by the Beam-Film Model: Analysis and Implications. PLoS Genet. 10, e1004042. https://doi.org/10.1371/journal.pgen.1004042

Zickler, D., Kleckner, N., 2015. Recombination, Pairing, and Synapsis of Homologs during Meiosis. Cold Spring Harb. Perspect. Biol. 7, a016626. https://doi.org/10.1101/cshperspect.a016626

Supporting information

FigS1. Markers positions in genetic distance (cM) as a function of genomic distance (Mb), namely Mary maps, for each chromosome included in the dataset (n = 665 chromosomes). The black vertical line is the centromere position estimated by cytological measures, when available in the literature.

FigS2. Recombination landscapes for each chromosome included in the dataset (n = 665 chromosomes). Recombination rate (cM/Mb) estimated in windows of 100kb along genomic distances (Mb). Confidence interval at 95% (grey ribbon) estimated by 1,000 bootstraps of loci. The black vertical line is the centromere position estimated by cytological measures, when available in the literature.

FigS3. Phylogenetic tree of species in our dataset (n = 57), annotated with mean recombination rate (cM/Mb) and mean chromosome size (Mb). The supertree was retrieved from the publicly available phylogeny constructed by Smith and Brown (Smith & Brown, 2018).

Formatted: Swedish (Sweden)

Formatted: Swedish (Sweden)

Formatted: Heading 1, Left

Formatted: Justified

Formatted: Justified, Indent: First line: 0.2", Space After: 10 pt, Pattern: Clear

Formatted: Not Highlight

FigS4. Slopes of the linear regression within species (linkage map length ~ chromosome size) as a function of the species mean genomic chromosome size (Mb).

FigS5. The negative correlation (Spearman's Rho coefficient) between recombination rates (cM/Mb) and the distance to the nearest telomere is stronger for species with a larger chromosome size (n = 57). The linear regression line and its parametric 95% confidence interval were estimated in ggplot2. The inset presents the distribution of Spearman's Rho coefficients for chromosomes (n = 665 chromosomes). The mean correlation and its 95% confidence interval (black solid and dashed lines) were estimated by 1,000 bootstraps. The red vertical line is for a null correlation.

Formatted: Font: 10.5 pt, Not Bold, Font color: Gray-80%, Highlight

FigS6. Standardized recombination rate (cM/Mb) as a function of the relative distance (Mb) from the telomere along the chromosome (physical distances expressed in 20 bins). Chromosomes were split in halves, a relative distance of 0.5 being the centre of the chromosome, and only one side was randomly sampled to avoid averaging patterns. Then, chromosomes were pooled per species. Each colour is a species. A loess regression was estimated for each species. Species presented in four plots for clarity.

Formatted: Font: 11 pt, Bold, Font color: Red, Not Highlight

FigS7. Standardized gene count as a function of the relative distance (Mb) from the telomere along the chromosome (physical distances expressed in 20 bins). Chromosomes were split in halves, a relative distance of 0.5 being the centre of the chromosome, and only one side was randomly sampled to avoid averaging patterns. Then, chromosomes were pooled per species. Each colour is a species. A loess regression was estimated for each species. Species presented in four plots for clarity.

Formatted: Justified, Indent: First line: 0.2", Space After: 10 pt, Pattern: Clear

Formatted: Font: 11 pt, Not Bold, Font color: Auto

FigS8. The genetic shuffling $\bar{r}_{intra}$ increases with the size of the genetic map (cM). Linear mixed regression with a species random effect and its 95% confidence interval estimated by ggplot2 (black line and grey ribbon). Each colour is a species. A linear regression was estimated for each species.

Formatted: Font: 10.5 pt, Bold, Font color: Gray-80%, Highlight

FigS9. The genetic shuffling $\bar{r}_{intra}$ decreases with the periphery-bias ratio. Linear mixed regression with a species random effect and its 95% confidence interval estimated by ggplot2 (black line and grey ribbon). Each colour is a species. A linear regression was estimated for each species.

FigS10. Gene count in windows of 100kb along genomic distances (Mb) for each chromosome with gene annotations (n = 480 chromosomes). Recombination rate (cM/Mb) estimated in windows of 100kb . Loess regression of gene count along the chromosome in blue line with parametric confidence interval at 95% in grey.

FigS11. Marey maps with genomic distances (black points) and gene distances (gray points). Markers positions in genetic distance (cM) as a function of the relative physical distance (either Mb of cumulative number of genes) for each chromosome with gene annotations (n = 480 chromosomes). The black dashed line is a theoretical uniform distribution of markers. The black vertical line is the centromere position estimated by cytological measures, when available in the literature.

Formatted: Justified

FigS12. Sensitivity of the periphery-bias ratio to the size of the sampled distal region (i.e. number of bins sampled at the tips). The periphery-bias ratio was estimated for different numbers of bins sampled and always divided by the mean chromosomal recombination rate. Linear regression (black line) shows a decrease of the periphery-bias ratio as the number of bins increases, towards a ratio value of 1 (dashed line).

Table S1. Metadata for 665 recombination landscapes, with name of the dataset collected and literal name of the chromosome used in our study, chromosome name in annotation (gff), size of the genetic map (cM, raw and corrected by methods of Chakravarti et al. (1991) or Hal & Willis (2005)), size of the genomic sequence in genome assembly (Mb), number of markers, density of markers in cM and bp, mean interval between markers in cM and bp, span parameter of the loess function, type of mapping function (Haldane, Kosambi or none), accession of the reference genome used for markers genomic positions, link to data repository and doi reference of the study in which the genetic map was published.

Table S2. Flowering plant species included in the study, with authors, year and doi reference of the genetic map publication, and accession of the reference genome.

Formatted: Font: (Default) Arial, 11 pt, Not Bold, Font color: Auto, Not Highlight

Table S3. Centromeric indexes estimated in cytological studies, with unit of measurement, mean and standard error of long and short chromosome arms, centromeric index (ratio of short arm length divided by total chromosome length), and doi reference to the original study.

Table S4. Correlation between recombination landscapes estimated at two different genomic scales (1Mb and 100kb). Spearman's Rho coefficient was estimated for each chromosome between recombination rates estimated directly in windows of 1Mb and the mean recombination rate of 100kb windows pooled together in 1Mb windows. Mean of the Spearman's Rho coefficient among chromosomes and proportion of significant p-values given for each species.

Table S5. Selection of the regression model between LM, LMER and PGLMM which explains best the relationship between the mean recombination rate (cM/Mb) and the chromosome size (Mb), based on AIC and BIC criteria.

Table S6. Species averaged correlation between the averaged chromosome size (Mb) and the averaged periphery-bias ratio. Mean of the Spearman's Rho coefficient among

correlations at chromosome scale and proportion of significant p-values given for each species.

Table S7. Chromosome correlation between the recombination rate (cM/Mb) and the relative distance to the telomere, with Spearman's Rho coefficient and p-value of the test per chromosome.

Table S8. Species averaged correlation between the recombination rate (cM/Mb) and the relative distance to the telomere. Mean of the Spearman's Rho coefficient among correlations at chromosome scale and proportion of significant p-values given for each species.

Table S9. Selection of the best model of crossover distribution for each species, based on Adjusted R-Squared between observed values and theoretical values predicted by the model. The best model selected for each species is the one maximizing the Adjusted R-Squared.

Table S10. Selection of the best model of crossover distribution for each species in a subset of chromosomes with at least 50cM on each chromosome arm, based on Adjusted R-Squared between observed values and theoretical values predicted by model. The best model selected for each species is the one maximizing the Adjusted R-Squared.

Table S11. Convergence between crossover patterns and gene patterns at a species scale. For each species is given the type of crossover pattern, the type of gene count pattern, the difference RMSE(gene pattern) - RMSE(crossover pattern) which indicates how gene patterns are more/less homogeneous than crossover patterns, the homogenization effect of gene patterns (more/less), the difference genetic shuffling(gene pattern) - genetic shuffling(crossover pattern) and the averaged chromosome size (Mb).

Data S1. References for linkage map data included in this study.

Formatted: Font: Not Bold

Formatted: Font: Not Bold
