# Supplementary material for: Diversity and determinants of recombination landscapes in flowering plants
Source: PLoS Genet. 2022 Aug 30;18(8):e1010141. doi: 10.1371/journal.pgen.1010141 (PMC9467342; doi:10.1371/journal.pgen.1010141)
Supplement: Attachment — Submitted filename: AnswerToReviewers_V2.docx [file pgen.1010141.s028.docx]

Dear Editors,

We are pleased to submit a revised version of our manuscript following the revision plan we proposed you. Below we detail the answers to reviewers. We noted in bold the additions or changes compared to previous version. We thank again the reviewers and yourself for the work on our manuscript that clearly improved it, and we hope it is now suitable for publication in PLoS Genetics.

Sincerely,

Thomas Brazier and Sylvain Glémin

## Answer to Reviewer #1

The revised manuscript still requires extensive revision of the English, which is frequently hard to understand (I attach an annotated pdf file as well as comments below).

*We greatly thank the reviewer for the time spent to edit our manuscript. We took most suggestions into account. Note that we kept the term genetic shuffling at some places but added genetic shuffling measure, to be explicit on the fact that it is a quantification of the process. When it was not necessary, we replaced it by recombination as proposed by the reviewer.*

The Discussion also seems unduly long (7 pages) and could be shorter and clearer if unnecessary repetition of results were removed.

*We partly streamlined the discussion but we preferred not to reduce it too much as the two other reviewers clearly appreciated this new version (see rev 3 for ex).*

Another general comment is that the text frequently ignores the state of the art in the field. When a concept is already well-established, I feel that, if one cites a recent paper, the citation should make clear that this is a review of the topic. As written, without doing this, or citing the early literature, the manuscript misleadingly gives the impression that these things are new discoveries. My comments on Lines 56 and 127 below are in a category that I consider “not so minor”.

*We think it’s unfair to say that we often ignored the state of the art in the field. It is rather standard to cite a recent reference to summarize well-established results. We are aware that recombination has been studied for a long time but we didn’t aim to do a historical survey here. Also, when we wrote that we observed a pattern in the data it didn’t mean that we discovered it. We tried to be more explicit, if possible, to precise when it was already fully known or only known in a few species and that we provided a broader characterization.*

The chief question remains whether the present study represents an advance sufficient for acceptance by PLoS Genetics, as opposed to being better suited for a journal like G3 or Heredity. In other words, what new discovery does it provide?
It is correct that the size of the dataset is an improvement on previous analyses, and I consider it improvements in quality to describe recombination patterns, not just total map lengths, and to describe centromere locations (the value of such information was stressed in this recent paper, which should be cited: Yoshida, K., and J. Kitano, 2021 Tempo and mode in karyotype evolution revealed by a probabilistic model incorporating both chromosome number and morphology. PLoS Genetics 17: e1009502. doi: 10.1371/journal.pgen.1009502). Clearly, these points should be emphasised. My criticisms did not dispute the value of such improvements, or of re-analysing the data to make results comparable between species.
The authors’ responses make clear that several of the most interesting questions could not be studied, and it would be helpful to readers to mention these explicitly, perhaps in a brief “Future directions” section at the end. This would help readers understand how difficult it is to reach conclusions about these questions, and that the right kind of data are now obtainable, albeit with considerable effort.

*We partly disagree with that. The reviewer listed a series of “interesting questions” in the first review. In the revised version we more clearly stressed these questions and we answered to two out of the five she/he proposed. Following her/his suggestion, we added a few sentences at the end to propose future directions, especially about comparison between close relatives and sex differences in recombination maps: “****we hope that many new species will be added, especially thanks to the increasing number of fully-sequenced plant genomes. We also encourage experimentalists to quantify separately male and female recombination landscapes, as potential sex differences could bring important insight on the evolution of recombination patterns (Sardell and Kirkpatrick, 2019). Comparing recombination maps among closely related species, or even among population of a same species, would also help understanding how recombination landscapes evolve****”.*

The issue of family sizes appears not to be mentioned, but surely the ability to detect recombinants will depend on the family size, and, in small families, none may be detected in regions with infrequent crossing over even if crossovers can occasionally occur. At present, the genetic maps are taken as facts, not estimates.

*This is a new criticism that was not raised in the first round and we are a bit surprised because we tested for the possible effect of dataset characteristics, including family size, which had no significant effect (see previous answer to reviewer 3): l150-152: ”* *We verified that neither the number of markers, marker density nor the number of progenies had a significant effect on the analyses.” However, we agree that we didn’t give the detail. We added these results in supplementary material* ***(Fig S3).***

*We are clearly aware that genetic maps are estimates and not fact! That’s the reason why we did different tests such as comparing the raw maps with the maps that used mapping functions corrections, comparing the mean recombination rates from the genome wide estimates and from averaging along the Marey maps, bootstrapping markers, test for the effect of the number of markers, progeny size…. So, we think that we did more controls and more advanced statistics than previous studies. Overall, we think our results are globally robust, especially the clear patterns we observed can hardly be explained by potential bias due to family size.*

This problem extends beyond the issue of family sizes, and I believe that it also affects the analysis of the models. As crossovers happen in the 4-strand stage, the genetic map distances don’t directly tell us the number of crossovers per bivalent, and so it is difficult to infer anything clear about the number that is required for correct segregation. It might be nice to know whether the evidence supports a requirement for a crossover per chromosome, or whether this requirement is enforced for each chromosome arm. However, it is well-established that, despite some such requirement, crossover numbers show a distribution that includes zero events, yet correct segregation is possible.

*We agree that recombination maps inform about average processes and don’t give access to the distribution of crossovers within a meiosis. We added “on average” when presenting the number of chromosomes and we also added two sentences to recall the limits of genetic maps: “However, as genetic maps are based on the average of several meiosis, they do not give access to the distribution of COs per meiosis. Thus, it is worth noting that chromosome genetic maps higher than 50 cM do not imply that all chromosome always exhibit at least one CO.”*

*R: We note that the reviewer wanted us to address this question in the first round of review (“Do chromosome arms have an obligate crossover? How often do chromosome arms have multiple crossovers, versus a single one?”), which is a bit contradictory if she/he thinks that recombination maps cannot help.*

However, my major criticism of the manuscript is still that the main new conclusion (line 434 onwards) does not seem justified. In my wording, this is that recombination between coding regions occurs more (“was more efficient”) than among regions randomly sampled from the genome, especially for longer chromosomes, which tend to have distal crossover localisation, and consequently the most heterogeneous recombination rates. The reasoning used to reach the conclusion that this is an important evolutionary observation seems to overlook an “elephant in the room” — the accumulation of repetitive sequences in low recombination regions of genomes. A direct outcome of this tendency is that regions with low recombination rates tend also have low gene densities. I don’t think that this has (in my edited wording) “implications for the evolution of crossover landscapes and whether the distribution of COs is optimal for the efficacy of genetic recombination”.
Following Haenel et al. (2018), the authors show that recombination rates correlate positively with gene density. This is unsurprising, as gene density goers down as repetitive content goes up, and the latter increases in genome regions with low crossover rates. This has been established in many species, including plants.

*We disagree on this comment for several reasons: we think that it is not our main new conclusion. We also bring new information about the diversity of recombination landscape, on the absolute vs relative effect of chromosome size, on the possible role of centromere position, which was not clearly known before, even if it may raise new questions rather than bring definitive answers. We also think that the effect on genetic shuffling is new and interesting, we didn’t claim that the relationship was not known before but we tested it in a systematic way.*

*We agree that accumulation of TEs is a possible explanation and we already discussed it in the manuscript. We have re-written the manuscript at several places to present the two, not mutually exclusive, mechanisms more equally:*

*In introduction:*

*“Several broad-scale determinants have recently been identified, such as chromosome length (Tiley and Burleigh, 2015), distance to the telomere or centromere (Blitzblau et al., 2007) and genomic and epigenetic features (Apuli et al., 2020; Marand et al., 2019; Yelina et al., 2012),* ***notably the density of transposable elements (TEs), which is usually negatively correlated with recombination rates (Kent et al, 2017)****.”*

*“Since recombination* ***is negatively associated with TEs and*** *recombination hotspots have been found in gene regulatory, are recombination landscapes* ***always*** *associated with gene density?”*

*In Results:*

*“It has been shown in a few species that COs preferentially occur in gene promoters. The scale of 100 kb used here is too large to test whether this pattern is shared among angiosperms. Instead, like in Haenel et al. (2018), we assessed whether recombination increased with gene density.* ***This pattern is also predicted if there is a negative association between TEs and recombination****.”*

*In the discussion:*

*“****On the one hand, gene density could directly determine recombination landscapes, leading to the accumulation of TEs in gene-poor regions, which would be amplified by a positive feedback loop. On the other hand, recombination could be targeted to gene-rich regions to avoid the deleterious effects of ectopic recombination between TEs (Kent et al. 2017). Recombination, gene density and TEs could thus co-evolve*** *and causal mechanisms of these multiple interactions still need to be clarified* ***(Kent et al. 2017).”***

*However, whatever the underlying mechanism, it can have an impact on the efficacy of recombination and we just suggested that it MAY have implications… So, we don’t think it is a strong claim. In addition, if the driver of the pattern is that recombination is targeted to genic region (rather than a passive consequence of TE accumulation in non-recombining regions) it seems reasonable to raise the question whether it can be adaptive or not (But clearly we don’t want and we don’t mean to answer it in this study)*

Line 373 onwards says “the strength of the relationship greatly varied across species and did not correlate with usual predictors such as the chromosome length or the genome-wide recombination rate”. But surely the usual predictors of gene density are not chromosome length or the genome-wide recombination rate, but local recombination rates, with clear effects of proximity to centromeres. This is because these effects are pronounced only in genome regions with pretty low recombination rates, and cannot be detected from correlations using minor or local recombination rate differences.

*We suppressed “with usual predictors” that brought some confusion.*

By writing that recombination rates “consistently increased with the number of genes” in 100 kb windows, the text suggests in the readers mind that the causative factor is the number of genes (even though clearly a correlation does not imply such a causation, and of course I don’t think that the authors intended to create this misleading impression).

*This is an unfair criticism. It’s just the presentation of a result. We can write “is positively correlated” instead but we don’t see the difference. And we used consistently because the same relation is observed in all species.*

Marey maps most often appeared more homogeneous when the x axis measures distances in terms of cumulative number of genes along the chromosome instead of base pair distances. When genes are sparse, a small value of this “gene distance” corresponds to a large physical distance, so the inhomogeneity of the maps is diminished, as a direct arithmetical consequence (provided that the annotation of the genes is accurate). This has no special biological significance.
If I have misunderstood the authors’ reasoning, I am happy to be corrected. Certainly, the writing needs to make the meaning clearer if what I have understood them to be saying is not their meaning.

*This is a misunderstanding. All these analyses are on relative scales. If genes are homogenously distributed along chromosomes, then the Marey maps (on a relative scale) are the same, so with the same heterogeneity. Note that if it were just an arithmetic property, we couldn’t find the reverse effect (higher heterogeneity at the gene scale) for some species. We though that the rationale was quite straightforward but we added this sentence to help understanding: “****This is not an automatic effect of changing scale as we compared the two maps on relative scales.****”*

Not so minor

Lines 56 and 127: The text should not give the impression that an association between gene density and recombination is a new observation. Line 127 says “are recombination landscapes generally associated with gene density?” (meaning “are recombination RATEs generally associated with gene density?”), but such a relationship is already extremely well established, because it is known that repetitive sequences accumulate in low recombination regions of genomes. If this is to be mentioned in the Introduction, it needs to be changed to make clear that this well-known pattern is confirmed by these new analyses. The fact that recombination hotspots have been found in gene regulatory sequences is a minor contributor to such a pattern.

*The question was clearly to test whether the pattern is general so rephrase it with “always” instead of “generally” and we also refer to TEs in the question (see comment above): “****Since recombination is negatively associated with TEs*** *and recombination hotspots have been found in gene regulatory, are recombination landscapes* ***always*** *associated with gene density?”*

Line 66: It is strange to cite de Massy, 2013 and later papers for the discovery that recombination rates are not homogeneous across the genome and vary among species, as this has been known for a very long time. Centromeric and pericentromeric regions with low rates were known for plants at least since work on maize in the 1930s, and work on tomatoes shortly afterwards, showing the heterochromatic regions physically surrounding centromeres are recombinationally inert.

*We think it is rather classical to cite a rather recent review that summarizes the literature. We don’t see the point of doing another review by citing all pioneer works in the introduction.*

Similarly, in line 294, it reads strangely that “we observed that the centromeres had an almost universal local suppressor effect”, as this is such a well-known phenomenon (since Beadle’s work in Drosophila in 1932), and is known to act in addition to the effect of heterochromatin. It would be better to write that thewell-known phenomenon of centromeric low crossover rates is confirmed. In this paragraph, a caveat should be expressed to the statement about “completely recombination-free in the centromere” (and also about the short genetic maps of some arms in line 331), as surely the ability to detect recombinants will depend on the family size, and none may be detected in small families, even if crossovers occasionally occur.

*We also found this criticism unfair. It’s in the part where we present the results so we don’t know why it’s strange to say “we observed”. We didn’t say “we discovered”. We replaced observed by confirmed. We also added a statement about family size: “****In*** *81 chromosomes (19 %)* ***estimated recombination was null*** *in the centromere* ***(although it can be non-zero but lower than the detection threshold given the number of individuals used for genetic maps).****”*

Line 192: Figure 1 shows that most of the 57 plant species analysed have estimated cM/Mb rates >1. This is a valuable result, but the text doesn’t mention it. I would have liked to see the value of 1 indicated by a horizontal line, as it is not shown on the y axis. From the figure, it appears that 4 species consistently have values < 0.5. Their names should be mentioned, as such low values are unusual, and these results should be examined to find out whether they are trustworthy, of if terminal markers may be lacking. Figure 2A seems to show that these same species may have very long chromosomes. This too seems worthy of explicit remark, as many readers will be interested to learn that plant chromosomes can be larger than 500 Mb (again, the species should be named, as perhaps these are species already known to have highly repetitive, and physically large, genomes). As presented, the reader does not even know whether these are related species (looking at Figure 4, I wonder if these are the 4 grass species at the bottom of that Figure?).

*We don’t understand why 1 cM/Mb should be an important threshold. Yes, it corresponds to species with very large chromosomes so this not surprising and we think it is well known that some species, like wheat, can have very large chromosomes. We don’t see the point to comment more here. On figure 1 we didn’t add the names of the species because it would make it unreadable. At the beginning of the second part of results we added this sentence:* ***“Mean genome-wide recombination rates spanned two orders of magnitude, ranging from 0.2 cM/Mb in bread wheat (Triticum aestivum) to 16.9 cM/Mb in squash (Cucurbita maxima).”***

This comment exemplifies my previous concern that only relationships between quantities are studied, when in fact some of the values are of interest in themselves. Omitting to even mention them makes the findings hard to understand, as it is easy to “miss the wood for the trees”, or understand clearly what the complicated relationships might mean.

*This is also unfair as we discuss some values, like the number of average crossover and the total length of genetic maps. On Figure 3 we also give examples with a range of chromosome size. We added values for the periphery-bias ratio, see below.*

Line 222: the text is still not clear (“used the terms proximal and distal regions, respectively, to avoid confusion with the molecular composition and specific position defining telomeric and centromeric regions stricto sensu”). Do “proximal and distal regions” mean centromere-proximal versus distal positions (on both arms for metacentrics), respectively? (and I think that the correct phrase is “sensu strictu”). And does “middle” or “centre” refer to the middle of an arm in a metacentric, or to the centromeric region and its neighbourhood (whereas, for an acrocentric, it would refer to a region distant from the centromere). Clarification is still needed.

*We have corrected the text as follows: “****In the text below, to avoid confusion with the molecular composition and specific position defining telomeric and centromeric regions stricto sensu, we have used instead the terms distal regions for the extremities of the chromosomes and proximal regions for the central part of the chromosomes around the centromere. Note that in the species we surveyed none had acrocentric chromosome.****” We hope it is clearer now. About the use of the latin expression stricto sensu vs sensu stricto, the correct term is stricto sensu even if both can be used.*

I also did not understand which landscapes were homogeneous along chromosomes — it would be helpful to name explicitly the species in Figure 3 that fall into the different categories, and mention the test for homogeneity that was used. The differences are not obvious to me.

*We rephrase it as “appeared rather homogeneous” and refer to Fig 4 instead of Fig 3. Fig 4 gives a better visual idea of homogeneity vs heterogeneity of the different landscapes. However, we didn’t perform statistical test for homogeneity. Nonetheless, we estimated the Gini index as a measure of heterogeneity (Table S1) and added the range of the Gini index in the text.* ***"The Gini index is a measure of heterogeneity bounded between 0 (perfect homogeneity) and 1 (maximal heterogeneity). The range of the Gini index estimated on recombination landscapes was between 0.1 and 0.9 (Table S1)."***

Line 265 states that the confidence interval for the periphery bias is 2.06 - 2.32., indicating that recombination rates are highest in the tips of chromosomes., though the differences are not usually extreme. Confusingly, the text earlier in this section strongly emphasised that a bias towards the periphery was not ubiquitous across species. These things are not necessarily contradictory, but they give the impression of being so. In fact, if I have understood correctly, it appears from Figure 5 that Haenel et al. were correct in thinking that this is a common pattern. If so, this should be clearly stated in the text, as it is an important conclusion, and readers should not be left with the impression that they were wrong (even if there are a few exceptions to this pattern — in fact, the exceptions could be of interest).

*Here the confidence interval is on the mean so we don’t think it is a problem to say that it’s not ubiquitous. It’s obvious on Figure 5 that the bias is on average higher than 1 but that several species have bias close to one or even negative. Despite this global bias, 16 species don’t follow the model proposed by Haenel and six species appear atypical. We think it’s clear from Fig 6 and from the text where we proposed two main patterns, with Haenel’s pattern being the most frequent. We now give more quantitative values and we tried to better explain the results: “By pooling chromosomes within species,* ***the periphery-bias ratio ranged from 0.31 to 5.76. Most species exhibited a ratio higher than one but the ratio was lower than one for nine species and just above one (<1.2) for six species (see Sup Table S5).*** *We detected a significant positive effect of chromosome length on the periphery-bias ratio across species (Linear Model, adjusted R^2^ = 0.44, p < 0.001; Fig 5A) with some exceptions (ex on Fig 3A and 3E). Analysing chromosomes independently across all species the mean periphery-bias ratio is significantly higher than 1 (95% bootstrapped confidence interval* ***of the mean*** *= [2.06;2.32]) and skewed towards values higher than 1 but the correlation with chromosome length within each species was not clear (Fig 5B, 5C, Table S6). ”*

Minor comments

A minor, but general, issue is the failure to distinguish between physical positions in the chromosome assemblies and genetic map positions. Although the text makes use of both these measures, it is sometimes unclear which one is meant, so that the meaning is not clear (e.g. lines 313 to 315).

*We corrected this, thanks to the annotated file.*

It is simplistic to write that recombination “increases genetic diversity and the adaptive potential of a species”. Most readers will know that genetic recombination is interesting and worthy of study.

*We removed this sentence as suggested by the reviewer. Note, however, that it was in the “author summary”, which is supposed to be for a broader less specialist readership.*

Line 51: what genomic characteristics? It would be better to say “genome size and ….” specifying the other characteristics.

*Corrected*

Line 83: It is too sweeping to say that recombination hotspots are general. Some organisms don’t have them, so please write more cautiously. In addition, please make clear what definitions were used for the plant hotspots cited, as many authors use this term extremely loosely, to mean any region where they detect higher recombination than other regions, even if the regions are large, unlike the original definition of hotspots.

*We have replaced “generally” by “in plants studied so far”*

Line 87: it is too vague to just say that recombination affects genome structure, functioning and evolution. Please be explicit. For example, recombination events can affect genome structure by causing ectopic recombination between similar sequences, including repeats, in different locations. As written, readers will not understand what you have in mind.

*We are now more explicit: “In addition to their function in meiosis, variations in recombination rates affect genome structure, functioning and evolution* ***through direct effects – such as mutagenic effects, bias-conversion, and ectopic exchanges – and indirect effects by modulating the efficacy of selection (Webster and Hurst 2011)****, and it has become a challenge to integrate recombination rate variation in population genomics in the age of ‘genomic landscapes’ (Booker et al., 2020; Comeron, 2017).”*

Line 107: the meaning is unclear of “positively driven by chromosome length”

*Changed to “correlated with”*

Line 119: the phrase “to our knowledge” is in the wrong place in the sentence.

*Moved at the end of the sentence*

Line 128: the question “What are the consequences of recombination heterogeneity on the extent of genetic shuffling?” is hard to understand, as “genetic shuffling” is usually just “baby language” for recombination (in my opinion, it should not be used in a scientific paper). Is this an attempt to mention that recombination can involve both crossing over and gene conversion? If so, it is certainly not clear enough. I would advise a different term for the sub-heading in line 405.

*We found this comment somewhat condescending. We took specifically the term genetic shuffling from Veller et al. who proposed a formal quantification and although we focused on recombination along chromosome, genetic shuffling is not, in general, synonymous to recombination as it also includes re-association of alleles among different chromosomes. The term shuffling or reshuffling are also used in other articles on recombination. Here are examples taken at random in recent literature: Roze, 2021. A simple expression for the strength of selection on recombination generated by interference among mutations. PNAS. Mieulet et al. 2018. Unleashing meiotic crossovers in crops. Nature plants. Peñalba et al. 2020. From molecules to populations: appreciating and estimating recombination rate variation. Nature Genetics.*

*At some places in the manuscript, we removed shuffling as suggested by the reviewer. For the question in the introduction, we chose to keep this term and for the sub-heading we added “****Quantification of*** *genetic shuffling” to be clearer on the fact that we focused on a quantitative measure.*

Line 169: Are the crossover numbers means? It is difficult to understand what is meant by “234 chromosomes had between one and two COs”, as a chromosome has a definite number. This, and the preceding sentence should be revised to make the meaning clear.

*We added “on average” or “average number” to be clearer.*

Line 173: The meaning of a “species random effect” is not clear enough.

*We think the meaning is clear here because we directly refer to the mixed linear model and give the equation of the model in parenthesis. So, we kept the phrasing here.*

Line 263: what does “ex on Fig 3A and 3E” mean?

*Replaced by “see Capsella rubella and Nelumbo nucifera on Fig 3”.*

Line 289: the meaning is unclear. The text reads “chromosomes from species classified as having a distal pattern were significantly larger than chromosomes with a sub-distal pattern”. Does this mean “species classified as having the distal pattern had significantly larger chromosomes than those of species assigned to the set with the sub-distal pattern”.

*We rephrased it as follows: “Interestingly,* ***species classified as having a distal pattern had significantly larger chromosomes than species classified as having a sub-distal pattern****”*

Line 313: The meaning is unclear for “equal distribution of crossovers on both sides of the
chromosome”,
*We changed it to: “equal distribution of crossovers on both sides* ***of the centre of*** *chromosomes”. We hope it makes it clearer.*

Line 321: This reads “At least one CO in each chromosome arm (50 cM) is mandatory in M2 whereas only one CO is mandatory for the entire chromosome in M3”, which is difficult to understand. Does it mean “In M2, at least one CO in each chromosome arm (50 cM) is mandatory, whereas in M3 only a single CO is mandatory for the entire chromosome, even if it has two arms”?

*Yes. Corrected accordingly. “****In M2****, at least one CO in each chromosome arm (50 cM) is mandatory whereas only one CO is mandatory for the entire chromosome in M3,* ***even if it has two arms (which always the case in our dataset)****”*

## Answer to Reviewer #3

## • Throughout the Abstract/Summary/Intro, I would use “crossover” rather than “crossing-over” when referring to the process as a noun e.g. number of crossovers vs. during crossing-over (verb). (I realise I may not have been clear enough in my original review)

## *Thank you for pointing it. It is corrected now.*

## • Line 24: characterize

## *Corrected*

## • Line 35: correspond globally

## *Corrected*

## • Lines 63 – 64: might make more sense to say something like “New haplotypes are passed on to offspring by the reciprocal exchange of DNA between maternal and paternal chromosomes, known as crossovers (COs)” and then remove “(COs)” on line 74

*Thanks for the suggestion. Corrected.*

## • Line 68: specific pairing sites implies that recombination always takes place in the same spot

## *We removed “specific”*

## • Lines 84-85: naïve question for you to check – is it in regulatory regions rather that sequence? Promotor regions?

## *We replaced sequence by regions*

## • Lines 172 – 174: can you get at how much of the variance species explains?

## *We added the value:* ***82 % of the variance explained by the species random effect (marginal R^2^ = 0.17, conditional R^2^ = 0.96).***

## • Figure 6: Define pattern “E” in the legend

## *Added in the legend*

## • For my comment about the local linkage order potentially inflating map length, I thought your response was good – could it be included in the methods in case another reader had the same concern?

## *We added a part of this response in the methods*

## Formatting changes

All figures have been processed through the PACE automated figure assessment.

Citations have been formatted according to the PLoS Genetics citation style.
